# Simulation-based estimates and projections of global, regional and country-level maternal mortality by cause, 1990–2050

**DOI:** 10.1038/s41591-023-02310-x

**Published:** 2023-04-20

**Authors:** Zachary J. Ward, Rifat Atun, Gary King, Brenda Sequeira Dmello, Sue J. Goldie

**Affiliations:** 1 Center for Health Decision Science, Harvard T.H. Chan School of Public Health, Harvard University, Boston, MA USA; 2 Department of Global Health and Population, Harvard T.H. Chan School of Public Health, Harvard University, Boston, MA USA; 3 Department of Health Policy and Management, Harvard T.H. Chan School of Public Health, Harvard University, Boston, MA USA; 4 Department of Global Health and Social Medicine, Harvard Medical School, Harvard University, Boston, MA USA; 5 Institute for Quantitative Social Science, Harvard University, Cambridge, MA USA; 6 Maternal and Newborn Healthcare, Comprehensive Community Based Rehabilitation in Tanzania (CCBRT), Dar Es Salaam, Tanzania; 7 Global Health Education and Learning Incubator, Harvard University, Cambridge, MA USA

**Keywords:** Reproductive disorders, Health care

## Abstract

Maternal mortality is a major global health challenge. Although progress has been made globally in reducing maternal deaths, measurement remains challenging given the many causes and frequent underreporting of maternal deaths. We developed the Global Maternal Health microsimulation model for women in 200 countries and territories, accounting for individual fertility preferences and clinical histories. Demographic, epidemiologic, clinical and health system data were synthesized from multiple sources, including the medical literature, Civil Registration Vital Statistics systems and Demographic and Health Survey data. We calibrated the model to empirical data from 1990 to 2015 and assessed the predictive accuracy of our model using indicators from 2016 to 2020. We projected maternal health indicators from 1990 to 2050 for each country and estimate that between 1990 and 2020 annual global maternal deaths declined by over 40% from 587,500 (95% uncertainty intervals (UI) 520,600–714,000) to 337,600 (95% UI 307,900–364,100), and are projected to decrease to 327,400 (95% UI 287,800–360,700) in 2030 and 320,200 (95% UI 267,100–374,600) in 2050. The global maternal mortality ratio is projected to decline to 167 (95% UI 142–188) in 2030, with 58 countries above 140, suggesting that on current trends, maternal mortality Sustainable Development Goal targets are unlikely to be met. Building on the development of our structural model, future research can identify context-specific policy interventions that could allow countries to accelerate reductions in maternal deaths.

## Abstract (web summary)

Modeling analyses of maternal mortality data for 200 countries and territories reveal that based on current trends, the maternal mortality Sustainable Development Goal targets for 2030 are unlikely to be met.

## Main

Maternal mortality is a major global health challenge, with the risk of maternal death still higher in the poorest countries today than it was more than a century ago in the wealthiest nations^1^. Although progress has been made with the Millennium Development Goals, the maternal mortality targets were not met^2^. Although highly cost-effective interventions exist to address pregnancy-related complications and reduce maternal mortality^3^, critical gaps in knowledge remain about how to adapt and implement strategies in various contexts^4^ (for example, specific countries, urban or rural location and so on).

Evaluating the real-world effectiveness of strategies and monitoring the comparative progress of countries is complicated, because the measurement of maternal mortality is difficult. Compared with the frequency of pregnancy, maternal mortality is a relatively rare event; extremely large samples or complete enumeration are therefore needed to calculate stable estimates^5^. However, many of the countries with the highest burden of maternal mortality have inadequate data collection and incomplete vital registration systems^6^. Maternal deaths are thus underreported across the pregnancy continuum, especially in early pregnancy and from complications of induced abortion and indirect causes (for example, HIV, malaria)^1^. Even when recorded, maternal mortality is often misclassified because it is not a single diagnosis but a composite of many distinct conditions, each with their own pathophysiology^7^.

Despite these challenges, tracking global progress does require reliable quantitative measures to inform funding, policy and practice. The United Nations (UN) Sustainable Development Goals (SDGs) includes a target (3.1) to reduce the global maternal mortality ratio (MMR) to fewer than 70 maternal deaths per 100,000 live births by 2030, with no individual country exceeding 140 deaths^8^. Statistical methods have been used by the UN Maternal Mortality Estimation Inter-agency Group^9,10^ and the Institute for Health Metrics and Evaluation^11,12^ to produce estimates of maternal mortality based on data that are available. These approaches estimate the cross-sectional associations between aggregate country-level factors and levels of maternal mortality, with gross domestic product the largest driver of trends^13^. Although these regression models provide insight into global progress, they may not adequately capture within-country longitudinal trends in maternal deaths, estimates of which are crucial to inform country-specific planning^14^.

Given the multifactorial nature of maternal mortality, a single indicator provides limited information to assess the comparative impact of strategies aimed at improving maternal health. For example, although the MMR (maternal deaths per 100,000 live births) is used to assess global progress, it reflects obstetrical risks for a single point in time and is not sensitive to changes in fertility^15^. Other indicators, such as the lifetime risk of maternal mortality, are needed to reflect the impact of changes in both fertility and obstetrical risk. In addition, compared with the MMR, process indicators and intermediate outcomes (for example, number of cesarean section (C-section) deliveries) may be observed with more certainty, and can provide insight into specific health system barriers to be targeted.

In contrast to aggregate models, which are based solely on previous trends in the outcome of interest, a structural model of women’s reproductive life cycles, based on a defined system of causal components and their relationships, can synthesize evidence on various factors from multiple sources and can offer more robust predictions for complex systems^16^. Such a structural approach is also better-suited to exploring counterfactual scenarios to estimate the potential impact of various strategies to improve maternal health in different contexts. Indeed, microsimulation modeling is increasingly recognized in epidemiology as another approach for causal inference, because it utilizes the robust foundations of graphical causal models and can explore the effects of complex interventions that occur over prolonged periods^17^.

**Fig. 1:**
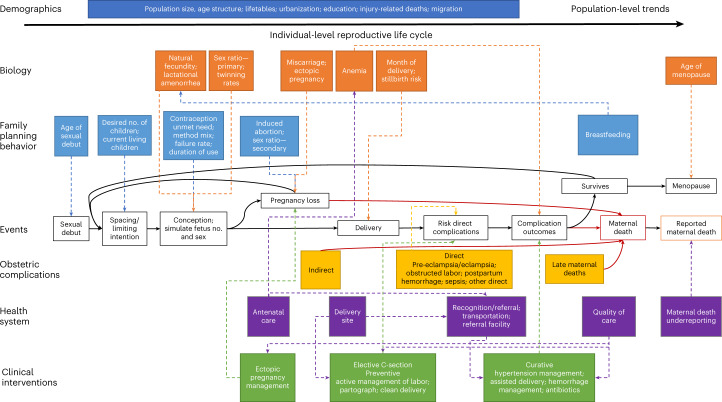
GMatH model conceptual framework. The GMatH microsimulation model simulates the reproductive histories of individual women in 200 countries and territories, accounting for heterogeneity in education and urban or rural location, family planning preferences and history of maternal complications. The GMatH model accounts for population-level demographic and secular trends, and simulates various individual-level processes related to maternal health, including biological processes, family planning behaviors, clinical practice and health system factors.

In this study, we describe the development and calibration of the Global Maternal Health (GMatH) microsimulation model (Fig. 1 and Appendix A), and provide estimates and projections for six maternal mortality indicators (as well as cause-specific estimates of maternal deaths and various other fertility and process indicators) for 200 countries and territories from 1990 to 2050. Table 1 summarizes our findings and policy implications.

**Table 1:** Policy summary

Background	Maternal mortality is a major global health problem. Although progress has been made globally in reducing maternal deaths, measurement remains challenging given the many causes and frequent underreporting of maternal deaths.
Main findings and limitations	We developed the GMatH microsimulation model to simulate the reproductive life courses of women in 200 countries and territories, accounting for individual fertility preferences and clinical histories. We estimate that between 1990 and 2020, annual global maternal deaths declined by more than 40% from 587,500 (95% UI 520,600–714,000) to 337,600 (95% UI 307,900–364,100), and on current trends are projected to decrease to 327,400 (95% UI 287,800–360,700) in 2030 and 320,200 (95% UI 267,100–374,600) in 2050. The global MMR is projected to decline to 167 (95% UI 142–188) in 2030, with 58 countries projected to have an MMR above 140, suggesting that the maternal mortality SDG targets are unlikely to be met. Our estimates of global maternal deaths and MMR are similar to those from the UN, but are substantially higher than estimates by the GBD study, with large country-level differences. Structural modeling of women’s reproductive life cycles allows for synthesis of data all along the reproductive pathway from multiple sources, leveraging data which may be observed with more certainty to infer parameters that are more uncertain, or unobserved. Such an approach can improve the robustness of results and identify potential reasons for large differences in country-level estimates, as well as provide insight into specific health system barriers that can be targeted to improve maternal health outcomes in various contexts. However, our approach is not without limitations, including the computationally intensive nature of the model, and data limitations for many model parameters. Although we leveraged empirical data when available, we were not able to set informative priors for some parameters when calibrating the model. However, as more data become available we can refine our model assumptions and estimates.
Policy implications	Structural modeling is a feasible approach to produce robust global and country-specific estimates of maternal mortality. Although maternal deaths have declined in recent years, many countries are not on track to achieve the SDG targets. Building on the development of our structural model, future research can identify context-specific policy interventions that could allow countries to accelerate reductions in maternal deaths.

## Results

### Model performance

Synthesizing estimates from various sources (Table 2), we calibrated our model to empirical estimates for a range of maternal health indicators (Appendix A.3). Posterior predictive checks of our calibration training set (used to fit the model) found that our coverage probability was 88.5% for all targets in the model, and 96.0% across the various maternal mortality indicators, with a mean absolute error by cause of 10.6 deaths on average. The coverage probability for estimates in our testing set (not used for calibration) was 91.4% overall and 96.0% for maternal mortality indicators, with a mean absolute error by cause of 10.5 deaths on average. A full list of model performance indicators is reported in Appendix A.3.4.

**Table 2:** Summary of model inputs

	Parameter type	Variability (within-country)	Data source	Appendix A
**Demographics**				
Countries/territories	NA	NA	UN World Population Prospects 2022	1.1.1
Population size projections; Urbanization projections	NA	Age, year; year	UN World Population Prospects 2022; UN World Urbanization Prospects 2018	1.1.2
Lifetables	Annual mortality rate	Age, year	UN World Population Prospects 2022	1.1.3
Proportion of deaths due to injury	Logistic regression	Year	GBD 2019	1.1.4
Education projections	Multinomial logistic regression	Year, urban/rural	DHS, UNESCO	1.1.5
Migration	Cycle-specific weights estimated via raking	Year, subgroup	UN World Population Prospects 2022; UN World Urbanization Prospects 2018	1.1.6
**Biological parameters**				
Natural fecundity/fertility	Splines	Age	Medical literature	1.2.1
Sex ratio—primary	Ratio	Overall	Medical literature	1.2.2
Twinning rates	Monozygotic: rate; dizygotic: splines	Monozygotic: overall; dizygotic: age	Medical literature	1.2.3
Miscarriage	Splines; relative risk	Age; history of miscarriage	Medical literature	1.2.4
Ectopic pregnancy	Naïve incidence: splines; recurrence, mortality: probabilities	Naïve incidence: age; recurrence, mortality: overall	Medical literature	1.2.5
Stillbirths	Splines; male relative risk	Age; overall	Medical literature	1.2.6
Month of delivery	Fetal lifetable	Overall	Medical literature	1.2.7
Lactational amenorrhea	Exclusive breastfeeding: failure rate; nonexclusive breastfeeding: relative rate	Overall	Medical literature	1.2.8
Menopause	Normal distribution	Overall	Medical literature	1.2.9
Anemia (hemoglobin distribution)	Normal distribution	Year, subgroup	DHS data, WHO GHO database	1.2.10
**Family planning parameters**				
Age of sexual debut	Log-normal distribution	Subgroup	DHS data	1.3.1
Number of living children	Poisson distribution	Year, age, subgroup	DHS data	1.3.2
Desired number of children	Inflated Poisson distribution	Year, subgroup	DHS data	1.3.3
Unmet need	Logistic regression	Year, age, subgroup	DHS data	1.3.4
Contraception method mix	Multinomial logistic regression	Year, subgroup	DHS data	1.3.5
Contraception failure rates	Annual failure probability	Subgroup	DHS data, medical literature	1.3.6
Method duration of use	Duration: exponential distribution; switch method: probability	Subgroup	DHS data	1.3.7
Abortion	Incidence, safe proportion: logistic regression; mortality: probability; infertility: probabilities	Incidence, safe proportion: year, subgroup; mortality: anemia status; infertility: overall	Medical literature	1.3.8
Sex ratio—secondary	Ratio	Year, subgroup	UN World Population Prospects 2022	1.3.9
Breastfeeding	Logistic regression	Year, subgroup	UNICEF data	1.3.10
**Health system parameters**				
Antenatal care	Number of visits: two-part Poisson model; hemoglobin, complication recognition increase: coefficient per visit	Number of visits: year, subgroup; hemoglobin, complication recognition increase: subgroup	DHS data; medical literature	1.4.1
Starting delivery site	Multinomial logistic regression	Year, subgroup	DHS data, WHO GHO database, medical literature	1.4.2
Recognition and referral	Probability	Complication type/severity, delivery site, subgroup	Medical literature	1.4.3
Transportation	Probability	Delivery site, subgroup	Medical literature	1.4.4
Referral facility	Probabilities	Delivery site, subgroup	Medical literature	1.4.5
Quality of care	Logistic regression	Year, delivery site	Prior probabilities set by income group	1.4.6
Maternal death underreporting	Logistic regression	Year, delivery site	Medical literature	1.4.7
**Obstetric complications**				
Pre-eclampsia/eclampsia	Probabilities	Age, anemia, multiple gestation, history of pre-eclampsia/eclampsia	Medical literature	1.5.1
Obstructed labor	Probabilities	Age	Medical literature	1.5.2
Postpartum hemorrhage	Probabilities	Anemia	Medical literature	1.5.3
Sepsis	Probabilities	Anemia, C-section	Medical literature	1.5.4
Other direct	Probabilities	Incidence: overall; mortality*:* delivery site	Medical literature, WHO Mortality Database	1.5.5
Late maternal deaths	Probabilities	Incidence: overall; mortality: treatment site	Medical literature, WHO Mortality Database	1.5.6
Indirect maternal deaths	Logistic regression	Year	WHO Mortality Database	1.5.7
**Clinical interventions**				
Elective C-section	Logistic regression	Year, subgroup	DHS data, UNICEF data	1.6.1
Preventive				
Active management of the third stage of labor	Availability: logistic regression; efficacy: relative incidence reduction	Availability: year, delivery site; efficacy: overall	Medical literature	1.6.2
Partograph	Availability: logistic regression; efficacy: relative incidence reduction	Availability: year, delivery site; efficacy: overall	Medical literature	1.6.3
Clean delivery	Availability: logistic regression; efficacy: relative incidence reduction	Availability: year, delivery site; efficacy: overall	Medical literature	1.6.4
Curative				
Ectopic pregnancy management	Availability: logistic regression; efficacy: relative risk	Availability: year, treatment site; efficacy: overall	Medical literature	1.6.5
Hypertension management	Availability: logistic regression; efficacy: relative risk	Availability: year, delivery site; efficacy: overall	Medical literature	1.6.6
Assisted delivery	Availability: logistic regression; efficacy: relative risk	Availability: year, delivery site; efficacy: overall	Medical literature	1.6.7
Hemorrhage management	Availability: logistic regression; efficacy: relative risk	Availability: year, delivery site; efficacy: overall	Medical literature	1.6.8
Antibiotics	Availability: logistic regression; efficacy: relative risk	Availability: year, delivery site; efficacy: overall	Medical literature	1.6.9

NA, not applicable; UNESCO, United Nations Education, Scientific and Cultural Organization; UNICEF, United Nations Children’s Fund; WHO GHO, World Health Organization Global Health Observatory database.

Subgroup corresponds to education level + urban/rural location.

### Historical and current estimates

**Extended Data Fig. 1:**
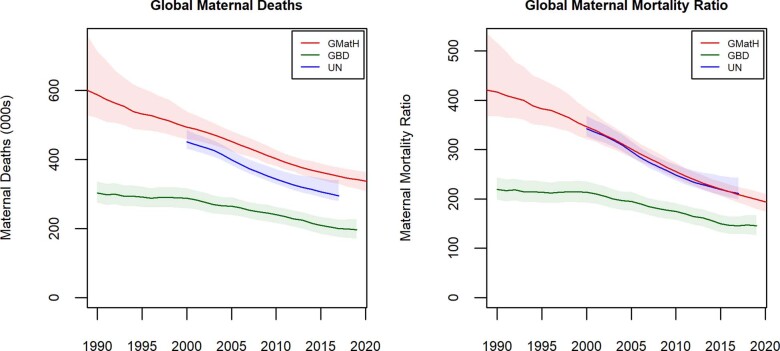
Comparison of estimated global maternal deaths and maternal mortality ratio across GMatH, GBD, and UN models: 1990–2020. GMatH: Global Maternal Health microsimulation model. GBD: Global Burden of Disease – 2019 estimates^18^. UN: United Nations – 2019 estimates^10^. Lines represent means. Shaded areas represent 95% uncertainty intervals for the GMatH and GBD estimates, and 80% uncertainty intervals for the UN estimates.

We estimate that between 1990 and 2020, global maternal deaths decreased from 587,500 (95% UI 520,600–714,000) to 337,600 (95% UI 307,900–364,100), and the global MMR decreased from 416 per 100,000 live births (95% UI 368–516) to 194 (95% UI 174–210). These global estimates are similar to those from the UN^10^, but are substantially higher than estimates from the Global Burden of Disease (GBD)^18^ (Extended Data Fig. 1). As described above, the UN and GBD estimates are based on aggregate-level regression models, in contrast to our individual-level structural simulation model.

We estimate that in 2020, the vast majority of maternal deaths (98.7%; 333,300 of 337,600) occurred in low- and middle-income countries (LMICs), with the top 10 countries by number of maternal deaths accounting for 57.7% and the top 20 accounting for 73.3% of global maternal deaths. Country-level estimates of maternal deaths and MMR for 2017 (the most recent year available across all three models) diverge between the GMatH, UN and GBD models, with substantial differences for some countries, such as Nigeria, Ethiopia and Afghanistan (Table 3; Appendix B has results for all countries).

**Table 3:** Comparison of top 20 countries by maternal deaths and MMR across GMatH, GBD and UN models in 2017

A. Maternal deaths						
	GMatH		UN		GBD	
No.	Country	Maternal deaths	Country	Maternal deaths	Country	Maternal deaths
1	Nigeria	46,529 (37,102–54,632)	Nigeria	67,000 (48,000–96,000)	India	40,309 (33,398–47,641)
2	India	39,083 (27,338–52,535)	India	35,000 (28,000–43,000)	Nigeria	18,156 (11,708–28,562)
3	Ethiopia	35,368 (24,252–42,253)	Democratic Republic of the Congo	16,000 (12,000–24,000)	Pakistan	16,851 (12,620–21,957)
4	Democratic Republic of the Congo	18,251 (12,542–26,460)	Ethiopia	14,000 (10,000–20,000)	Democratic Republic of the Congo	10,767 (8,026–13,521)
5	Afghanistan	16,222 (11,699–21,755)	United Republic of Tanzania	11,000 (8,100–14,000)	Ethiopia	7,651 (5,681–10,144)
6	Pakistan	12,308 (8,077–17,376)	Indonesia	8,600 (6,200–12,000)	Bangladesh	6,913 (5,345–8,833)
7	United Republic of Tanzania	9,546 (6,607–12,451)	Pakistan	8,300 (5,000–14,000)	Indonesia	5,826 (4,614–7,410)
8	Bangladesh	8,336 (5,772–10,660)	Afghanistan	7,700 (5,100–12,000)	United Republic of Tanzania	5,538 (3,889–7,525)
9	Chad	8,194 (6,107–10,572)	Chad	7,300 (5,400–10,000)	Afghanistan	4,123 (3,040–5,476)
10	China	7,694 (2,550–19,881)	Uganda	6,000 (4,500–8,400)	Kenya	3,757 (2,770–4,924)
11	Kenya	6,439 (3,594–10,462)	Côte d’Ivoire	5,400 (3,800–7,900)	Niger	3,394 (2,316–4,590)
12	Uganda	6,305 (4,427–8,526)	Bangladesh	5,100 (3,900–6,900)	Mali	3,379 (2,332–4,548)
13	Indonesia	6,200 (3,371–9,160)	Niger	5,100 (3,700–7,300)	Chad	3,130 (2,233–4,011)
14	Niger	5,788 (4,187–7,850)	Somalia	5,100 (2,400–9,800)	Cameroon	2,996 (1,822–4,225)
15	South Sudan	5,482 (3,242–8,375)	Kenya	5,000 (3,700–7,000)	Somalia	2,931 (1,957–4,159)
16	Yemen	5,463 (3,700–7,991)	China	4,900 (3,700–6,000)	Sudan	2,553 (1,466–3,930)
17	Mozambique	5,204 (3,774–6,558)	Cameroon	4,700 (3,300–7,000)	Madagascar	2,420 (1,793–3,080)
18	Côte d’Ivoire	5,116 (3,199–7,817)	South Sudan	4,500 (3,000–6,600)	Côte d’Ivoire	2,357 (1,562–3,245)
19	Angola	5,070 (3,321–7,127)	Mali	4,400 (3,300–6,100)	Guinea	2,275 (1,689–2,940)
20	Madagascar	4,846 (3,125–6,864)	Sudan	3,900 (2,800–5,400)	Brazil	2,161 (2,079–2,239)

B. MMR						
	GMatH		UN		GBD	
No.	Country	MMR	Country	MMR	Country	MMR
1	Afghanistan	1,295 (861–1,800)	South Sudan	1,150 (789–1,710)	Liberia	544 (384–720)
2	South Sudan	1,247 (807–1,764)	Chad	1,140 (847–1,590)	Sierra Leone	512 (359–670)
3	Chad	1,000 (737–1,285)	Sierra Leone	1,120 (808–1,620)	Mauritania	494 (337–683)
4	Guinea	794 (505–1,107)	Nigeria	917 (658–1,320)	Guinea	494 (367–639)
5	Liberia	779 (423–1,193)	Central African Republic	829 (463–1,470)	Haiti	491 (351–661)
6	Haiti	770 (546–1,037)	Somalia	829 (385–1,590)	Central African Republic	449 (302–633)
7	Somalia	757 (234–1,166)	Mauritania	766 (528–1,140)	Eritrea	445 (310–642)
8	Guinea-Bissau	730 (505–1,048)	Guinea-Bissau	667 (457–995)	Chad	426 (304–547)
9	Ethiopia	724 (510–849)	Liberia	661 (481–943)	Gambia	423 (313–550)
10	Central African Republic	690 (341–1,192)	Afghanistan	638 (427–1,010)	Senegal	406 (286–526)
11	Sierra Leone	637 (418–958)	Côte d’Ivoire	617 (426–896)	Mali	377 (260–507)
12	Lesotho	614 (357–917)	Gambia	597 (440–808)	Democratic Republic of the Congo	372 (278–468)
13	Niger	545 (389–747)	Guinea	576 (437–779)	Djibouti	372 (200–563)
14	Nigeria	527 (433–606)	Mali	562 (419–784)	Somalia	359 (240–509)
15	Côte d’Ivoire	522 (332–736)	Burundi	548 (413–728)	Cameroon	338 (206–477)
16	Madagascar	501 (339–685)	Lesotho	544 (391–788)	Lesotho	334 (201–509)
17	Eritrea	482 (243–813)	Cameroon	529 (376–790)	Niger	321 (219–434)
18	Yemen	456 (324–653)	United Republic of Tanzania	524 (399–712)	Nepal	301 (212–399)
19	Cameroon	433 (293–585)	Niger	509 (368–724)	Zimbabwe	292 (211–395)
20	Mali	424 (310–525)	Eritrea	480 (327–718)	Afghanistan	291 (215–387)

UN 2019 estimates^10^.

GBD 2019 estimates^18^.

Data shown are means (95% UI) for the GMatH and GBD estimates, and means (80% UI) for the UN estimates.

Table 4 illustrates a range of maternal mortality indicators estimated for 2022 by country income group and region. We find that whereas the estimated MMR varies about 25 times between low- and high-income countries (480 versus 17), the lifetime risk of maternal death varies nearly 75 times (2.19% versus 0.03%), highlighting the impact of differences in repeated pregnancy exposure as a result of higher fertility on cumulative mortality risk.

**Table 4:** Estimated maternal mortality indicators dashboard by region in 2022

	Total maternal deaths	MMR	Pregnancy mortality ratio	Proportional mortality ratio (%)	Maternal mortality rate	Lifetime risk of maternal death (%)
**Global**	**338,962 (305,457–367,774)**	**190 (167–208)**	**199 (174–220)**	**36.43 (30.63–41.49)**	**15 (14–17)**	**0.48 (0.43–0.53)**
Low income	164,182 (139,895–182,994)	480 (411–539)	486 (416–546)	60.60 (50.62–68.51)	75 (63–85)	2.19 (1.86–2.46)
Lower middle income	148,319 (126,057–167,132)	174 (150–197)	189 (162–215)	29.59 (21.48–34.76)	15 (13–17)	0.48 (0.41–0.55)
Upper middle income	22,101 (17,041–35,306)	46 (32–78)	49 (35–81)	16.10 (10.91–23.95)	3 (2–5)	0.09 (0.06–0.15)
High income	4,360 (3,478–5,392)	17 (13–22)	20 (15–26)	8.88 (6.13–12.58)	1 (1–1)	0.03 (0.02–0.04)
**Africa**	**223,037 (194,249–242,847)**	**387 (333–423)**	**404 (352–441)**	**37.96 (34.07–41.54)**	**60 (52–66)**	**1.78 (1.53–1.95)**
Eastern Africa	84,911 (62,918–97,057)	441 (338–518)	449 (346–525)	46.64 (34.21–56.35)	68 (50–78)	1.98 (1.50–2.31)
Middle Africa	41,320 (33,184–50,057)	412 (327–505)	424 (338–529)	49.61 (36.02–61.13)	86 (68–106)	2.47 (1.94–3.09)
Northern Africa	5,640 (3,712–8,209)	69 (43–101)	73 (45–106)	32.76 (16.76–59.86)	7 (4–10)	0.22 (0.13–0.33)
Southern Africa	2,442 (1,487–3,861)	129 (66–241)	131 (68–242)	15.28 (8.22–29.34)	12 (7–20)	0.38 (0.21–0.63)
Western Africa	88,724 (76,026–99,789)	457 (396–515)	492 (429–555)	31.54 (27.61–35.89)	83 (71–94)	2.49 (2.12–2.84)
**Asia**	**97,054 (79,794–115,999)**	**98 (79–122)**	**103 (84–132)**	**51.19 (25.32–67.89)**	**6 (5–8)**	**0.21 (0.17–0.27)**
Central Asia	1,659 (897–2,591)	83 (39–147)	85 (41–151)	46.03 (16.61–76.02)	7 (3–12)	0.22 (0.10–0.38)
Eastern Asia	6,856 (2,598–18,536)	26 (5–82)	27 (7–85)	44.69 (10.01–81.36)	1 (0–4)	0.04 (0.01–0.14)
South-Eastern Asia	13,828 (9,446–18,626)	96 (65–132)	97 (66–132)	70.75 (59.18–81.10)	6 (4–9)	0.20 (0.13–0.28)
Southern Asia	67,293 (53,241–84,338)	132 (103–167)	141 (106–186)	49.24 (20.26–70.62)	10 (8–13)	0.33 (0.25–0.42)
Western Asia	7,417 (5,057–10,639)	83 (52–127)	84 (52–128)	75.74 (60.94–85.22)	8 (5–12)	0.24 (0.15–0.38)
**Europe**	**3,594 (2,679–4,709)**	**31 (20–45)**	**35 (23–55)**	**9.89 (4.44–15.77)**	**1 (1–2)**	**0.05 (0.03–0.07)**
Eastern Europe	2,187 (1,335–3,273)	54 (29–89)	60 (31–109)	17.73 (3.86–39.01)	2 (1–4)	0.07 (0.04–0.12)
Northern Europe	350 (177–566)	12 (5–23)	15 (6–27)	7.88 (2.60–16.35)	1 (0–1)	0.02 (0.01–0.04)
Southern Europe	510 (315–730)	24 (12–40)	31 (17–49)	3.94 (1.97–6.28)	1 (0–2)	0.03 (0.02–0.05)
Western Europe	547 (373–756)	13 (6–21)	14 (7–23)	10.56 (4.71–18.97)	1 (0–1)	0.02 (0.01–0.04)
**Latin America and the Caribbean**	**12,965 (10,685–15,424)**	**91 (70–121)**	**101 (78–131)**	**13.57 (10.31–18.19)**	**6 (5–8)**	**0.21 (0.17–0.26)**
Caribbean	2,630 (1,765–3,704)	313 (217–424)	331 (231–445)	29.40 (20.10–47.33)	23 (15–32)	0.73 (0.49–1.04)
Central America	4,043 (2,904–5,396)	95 (56–140)	107 (64–159)	12.81 (8.54–20.07)	7 (5–10)	0.23 (0.15–0.33)
South America	6,292 (4,808–8,206)	68 (47–99)	76 (53–108)	11.74 (7.66–17.69)	5 (3–6)	0.15 (0.11–0.21)
**Northern America**	**1,289 (622–2,117)**	**11 (4–20)**	**11 (4–21)**	**48.25 (22.17–74.42)**	**1 (0–1)**	**0.03 (0.01–0.05)**
**Oceania**	**1,022 (436–1,892)**	**115 (46–215)**	**128 (54–229)**	**29.38 (9.64–58.19)**	**8 (3–16)**	**0.28 (0.11–0.53)**
Australia/New Zealand	131 (0–285)	31 (0–70)	53 (12–105)	7.24 (0.00–21.62)	2 (0–4)	0.05 (0.00–0.13)
Melanesia	862 (282–1,718)	193 (62–383)	196 (64–384)	75.63 (32.46–96.21)	24 (7–51)	0.77 (0.23–1.62)
Micronesia	15 (5–28)	168 (55–330)	203 (79–374)	17.39 (6.60–33.16)	16 (5–32)	0.52 (0.16–1.06)
Polynesia	14 (5–27)	135 (40–260)	193 (76–327)	10.17 (3.19–18.82)	17 (5–33)	0.58 (0.17–1.11)

Values are shown as mean (95% UI).

Total maternal deaths is the number of maternal deaths + the number of late maternal deaths. MMR is the maternal mortality ratio, defined as the number of maternal deaths per 100,000 live births.

The pregnancy mortality ratio is the number of pregnancy-related deaths per 100,000 live births.

The proportional mortality ratio is the ratio of maternal deaths to all deaths among women aged 15–49.

Maternal mortality rate is the number of maternal deaths per 100,000 women aged 15–49.

The lifetime risk of maternal death is the probability that a 15-year-old female will eventually die from a maternal cause, assuming fertility and mortality risks do not change in the future. Estimated as the sum of age-specific maternal mortality rates from ages 15 to 49.

Regional totals are shown in bold.

### Maternal mortality projections

**Extended Data Fig. 2:**
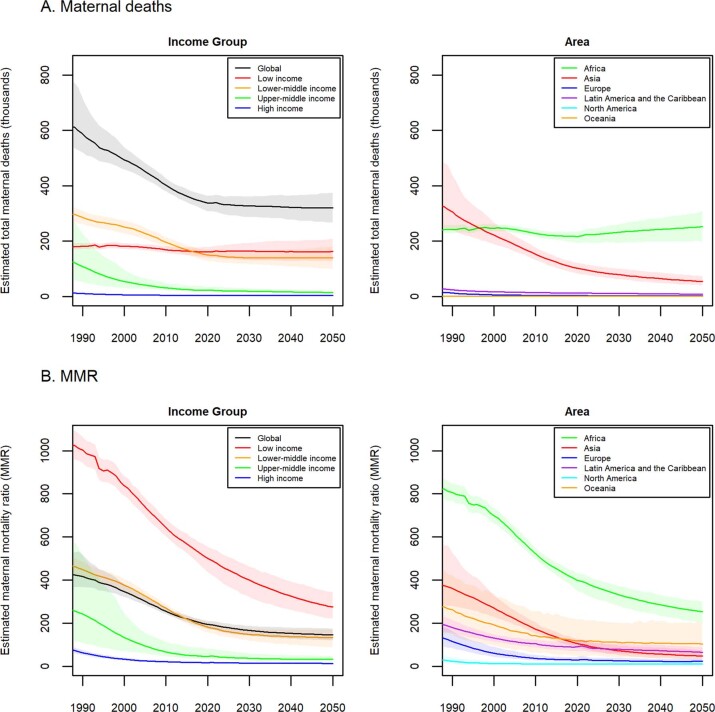
Estimated maternal deaths and MMR by area and country income group, 1990–2050. **A**. Maternal Deaths. **B**. Maternal mortality ratio (maternal deaths per 100,000 live births). Lines represent means. Shaded areas indicate 95% uncertainty intervals.

On current trends, global maternal deaths are projected to decrease from 339,000 (95% UI 305,500–367,800) in 2022 to 327,400 (95% UI 287,800–360,700) in 2030, and to 320,200 (95% UI 267,100–374,600) in 2050. This decline is largely due to decreases in Asia, with most of the projected future maternal deaths occurring in Africa (Extended Data Fig. 2a). The global MMR is projected to decline from 190 (95% UI 167–208) in 2022 to 167 (95% UI 142–188) in 2030, and to 146 (95% UI 120–174) in 2050 (Extended Data Fig. 2b).

**Fig. 2:**
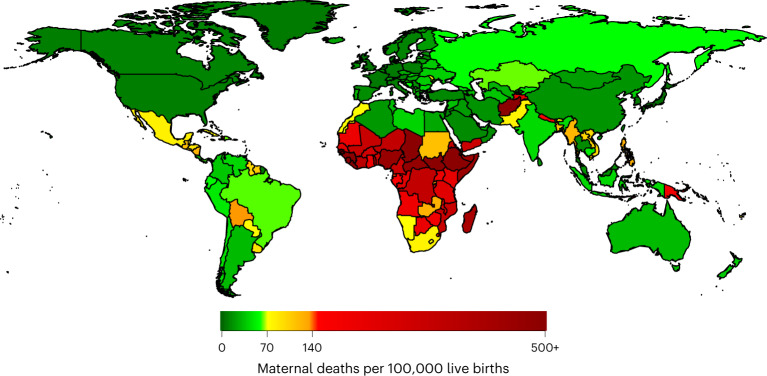
Projected MMR by country in 2030. Mean projected MMR (maternal deaths per 100,000 live births) by country in 2030.

We find that in 2030, 105 countries are projected to have a MMR below 70, and 142 will have a MMR below 140, meaning that 58 countries, mainly in sub-Saharan Africa, are projected to not meet the SDG target of MMR <140 by 2030 (Fig. 2). Country-specific results are available in Appendix B and also in a public data repository. Model posterior parameters are also presented in Appendix C.

### Causes of maternal death

**Extended Data Fig. 3:**
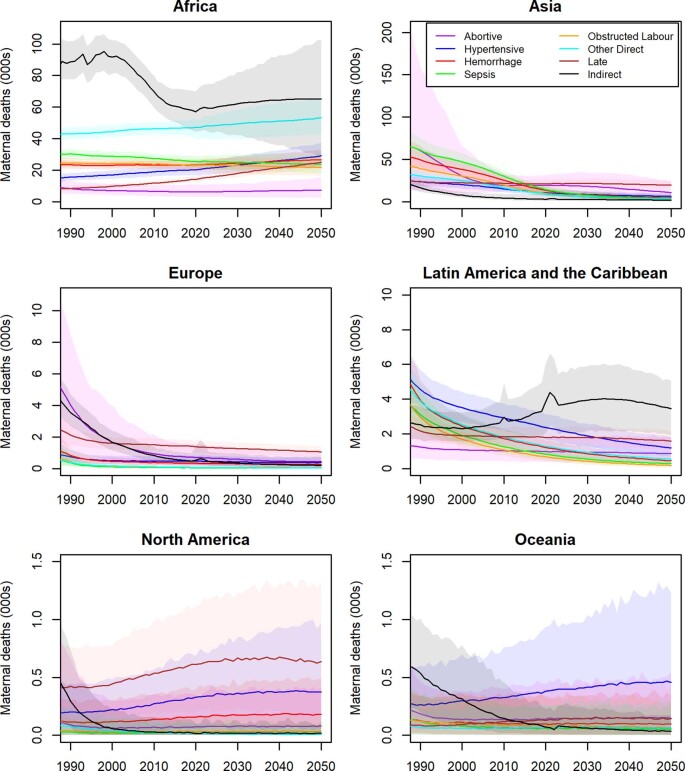
Estimated maternal deaths by cause 1990–2050, mean and 95% UI. Causes of maternal death: Abortive (abortion, ectopic pregnancy, miscarriage), Hypertensive disorders, Hemorrhage, Sepsis and other infections, Obstructed labour, Other direct, Late maternal deaths, and Indirect maternal deaths. Lines represent means. Shaded areas indicate 95% uncertainty intervals.

We find that the main causes of maternal death vary by setting and have changed over time (Extended Data Fig. 3). As an example, in Africa, indirect deaths (for example, due to HIV, malaria) are estimated to have declined over time but remain the leading cause of maternal death, whereas other direct maternal deaths (from various causes, such as obstetric embolism or complications of anesthesia) have slowly increased over time. In Asia, direct causes such as sepsis and hemorrhage have declined, whereas late maternal deaths and deaths from abortive causes are now estimated to be the leading causes of maternal death. In Latin America and the Caribbean, indirect maternal deaths may have increased over time, whereas in Oceania deaths from hypertensive disorders are estimated to be the leading cause of maternal death, with late maternal deaths estimated to be the leading cause in Europe and North America.

## Discussion

Although measurement of maternal mortality remains challenging, reliable quantitative estimates are needed to track progress over time and evaluate the impact of policies. We developed a microsimulation model of global maternal health, synthesizing demographic, epidemiologic, clinical and health system data from many sources, including the medical literature, Civil Registration Vital Statistics (CRVS) data and individual-level Demographic and Health Survey (DHS) data for more than 4.6 million women, and accounting for heterogeneity both across and within countries. We calibrated the model to multiple sources of empirical primary data on fertility, process and mortality indicators (Appendix A.3.2), and find that our model has a high degree of predictive accuracy compared with our testing set (external data not used to calibrate the model) and is consistent with multiple outcomes across a range of indicators. We find that although the global MMR and annual maternal deaths are projected to decline, based on current trends the projected decreases will not be enough to achieve the SDG Target 3.1 of a global MMR of 70 by 2030—a finding consistent with a 2019 UN assessment^10^.

Nevertheless, we find that progress has been made. Our model estimates that between 1990 and 2020 global maternal deaths per year declined by more than 40%. Our annual global estimates are similar to those of the UN^10^, but much higher than the GBD estimates^18^, with large country-level differences across all three models. These large country-level differences have implications for both local and global planning and resource allocation, particularly as the top 20 countries are estimated to account for nearly 75% of global maternal deaths. Given the large uncertainty around many aspects of maternal health, our use of a fundamentally different modeling approach may help to shed light on potential reasons for such divergent estimates. Further research on the impact of some common inputs, such as the use of World Health Organization lifetables for both the UN and GMatH models (which only impact competing mortality risks and indirect maternal deaths in the GMatH model), could also help to identify areas for future model development.

We also explicitly include underreporting of maternal deaths as part of the modeled data-generating process, allowing us to estimate underreporting parameters that vary by setting and period, and yield results consistent with the number of reported maternal deaths from various sources, such as CRVS systems and survey-based estimates (Appendix A.3.2). Including underreporting as part of the data-generating process in the model is more consistent with a Bayesian modeling framework in which parameters are random and empirical data are fixed, as opposed to making ex ante adjustments to the data used to the fit the model, which is the approach taken by the GBD and UN^9,12^. To promote model transparency, we provide extensive documentation for all model parameters, including data sources, assumptions and model implementation details as supplementary appendices, as well as online (www.gmath-model.org).

Our model projects that many countries will have an MMR above 140 in 2030, mostly in sub-Saharan Africa, with Africa estimated to have overtaken Asia since 2000 as the continent with the highest number of maternal deaths. Although the MMR is projected to decrease in all continents, because of population growth in Africa the number of maternal deaths is projected to remain relatively constant over time, highlighting the need to consider multiple indicators of maternal mortality when evaluating progress. For example, policy interventions or socioeconomic trends that impact fertility may yield little change in the MMR, while having a substantial impact on other indicators such as the number of maternal deaths and the lifetime risk of maternal death^15^.

Providing estimates for multiple indicators is thus important, both for improving the robustness of the results, and to provide insight into different health system barriers that can be targeted to improve maternal health. Structural modeling of women’s reproductive life cycles allows for synthesis of data along the reproductive pathway from multiple sources, leveraging data that may be observed with more certainty to infer parameters which are more uncertain, or unobserved. Such an approach can improve the robustness of results and identify potential reasons for large differences in country-level estimates, as well as provide insights into specific health system barriers that can be targeted to improve maternal health outcomes in various contexts (for example, specific countries, urban or rural location and so on).

Although computationally intensive, the development of our structural model offers two major benefits over existing methods. First, the specification of causal relationships makes explicit all model assumptions, and provides the potential for more robust, detailed estimates of maternal health outcomes, because we can incorporate data for multiple indicators along the reproductive pathway that may be more accurately/frequently observed than maternal mortality. This approach is therefore less sensitive to individual model inputs, such as lifetable estimates, because we model each cause of death separately, rather than modeling maternal mortality as a proportion of estimated all-cause female mortality. Our model also explicitly considers individual-level heterogeneity, as well as trends in demographic composition (urban or rural and education level) and how these trends impact various aspects of maternal health. In future work we plan to estimate trends in maternal mortality by subgroup, providing more detailed information on disparities in maternal mortality, both globally and within countries. Second, the use of a structural model allows realistic policy interventions (that is, counterfactual scenarios) to be simulated. Current estimates of maternal mortality are based on associative models (with aggregate predictors not amenable to policy intervention, such as gross domestic product)—although they can provide an estimate of the burden of maternal mortality, they cannot then be used to model interventions to provide actionable guidance on how the burden can be reduced.

In addition to the uncertainty around underreporting, we faced data limitations for other model parameters. Although we leveraged empirical data when available, we were not able to set informative priors for some parameters (for example, quality of care) when calibrating the model. Additional research could therefore help to refine our assumptions and improve the precision of our estimates. For example, specific empirical indicators of quality of care would be especially useful because we fit these parameters solely via calibration owing to a lack of data. Estimates of the extent to which surveys may underestimate maternal mortality would be useful for the same reason. We account for uncertainty around all model parameters, and report uncertainty intervals for all model outcomes, but recognize that because these are conditional on the model structure there are likely other sources of uncertainty (for example, structural assumptions) that are not reflected in our reported measures of uncertainty.

Although we account for indirect maternal deaths in the model as a proportion of competing (that is, nondirect maternal) mortality risks, we do not disaggregate indirect maternal deaths by cause (for example, HIV versus malaria), potentially limiting the utility of the model in evaluating interventions to address specific causes of indirect maternal deaths. Further model developments to incorporate the country-specific prevalence of diseases such as HIV and malaria, and their contribution to indirect maternal deaths by setting would be needed to refine this area of the model. We also did not account for the potential impact of the COVID-19 pandemic on maternal health outcomes (except via 2022 UN lifetable estimates, which impact indirect maternal deaths). However, as more data become available we can refine our model assumptions and estimates.

Structural modeling is a feasible approach to produce global and country-specific estimates of maternal mortality. On current trends, we find that many countries are not on track to achieve an MMR below 140 by 2030 and find large differences for country-specific estimates between the UN, GBD and GMatH models. Building on the development of our structural model, future research can evaluate counterfactual scenarios to identify realistic policy interventions in different contexts that could allow countries to make substantial progress toward improving maternal health.

## Methods

### Simulation model overview

Building on a previous conceptual model^19^, we developed the GMatH microsimulation model to simulate the reproductive histories of individual women in 200 countries and territories, accounting for heterogeneity in education and urban or rural location, family planning preferences and history of maternal complications (Fig. 1). The model progresses in monthly cycles and follows an open population in which new women enter each cycle, allowing population-level trends to be estimated by calendar year.

The probability of pregnancy is based on age, contraceptive use and breastfeeding status, with the number of fetuses based on age-specific twinning rates. Women may initiate, switch or discontinue contraception to space or limit the number of children given their desired family size. Pregnant women may experience ectopic pregnancy or miscarriage based on maternal and gestational age-specific probabilities, or may elect to terminate an unwanted pregnancy.

The incidence and case fatality rates of complications associated with pregnancy and childbirth are based on complication severity, individual-level risk factors (for example, anemia) and access to appropriate clinical interventions. In addition to death from pregnancy-related complications, women face risks of age-specific competing mortality from other causes.

### Model development

We followed several guiding principles when developing the model structure and selecting the data sources used to inform the model parameters. For example, we used empirical data whenever possible to set prior probability distributions for parameters, including individual-level DHS data for more than 4.6 million women from 322 surveys in 83 countries (Appendix A.2.1). We also relied on empirical data when developing the model structure and defining allowable relationships between parameters, aiming to balance model parsimony while still accounting for important mediating factors (for example, the impact of anemia on maternal health outcomes).

When empirical data were not available, we relied on expert opinion and general medical knowledge to inform the model development. We also accounted for uncertainty around all model inputs, using a hierarchical modeling approach with up to five levels (global, country income group, area (continent), region and country) to set prior probability distributions for all model parameters. We then calibrated the model (that is, fitted all model parameters) to empirical data for a range of maternal health outcomes.

Following this approach, we synthesized the best available epidemiologic and clinical evidence from multiple sources (Table 2), including randomized clinical trials, observational studies, meta-analyses, expert opinion, census data and primary survey data, as described below. Comprehensive supplemental appendices are provided with additional details for each model parameter, as well as an accompanying website (www.gmath-model.org).

### Demographics

We simulate individual women in 200 countries and territories, derived from an exhaustive list of areas (Appendix A.1.1.1). We obtained country-specific population projections from the UN, and used annual, age-specific estimates of the female population in each country from 1985 to 2021, and probabilistic projections to estimate population trends from 2022 to 2050 (Appendix A.1.1.2). The urban proportion of each country’s population was based on the UN Urbanization Prospects (Appendix A.1.1.2). Country-specific lifetables (all-cause annual mortality rates) for 1950–2100 were obtained from the UN (Appendix A.1.1.3). Because maternal deaths do not include deaths due to accidental or incidental causes, we model the proportion of deaths due to injuries, based on estimates from the GBD 2019 (Appendix A.1.1.4).

To account for heterogeneity within each country, we model each woman’s urban/rural location and level of education (low (less than primary), middle (less than secondary) or high (completed secondary or higher)), based on data from the DHS and the United Nations Educational, Scientific and Cultural Organization (Appendix A.1.1.5).

To account for international and internal (for example, rural to urban) migration, we estimated poststratification weights (via raking) by cycle, allowing us to re-weight our simulated estimates to reflect population trends not already included in the model (for example, migration, differential background mortality). Cycle-specific weights were calculated for six demographic subgroups within each country (urban/rural location by three education levels) (Appendix A.1.1.6).

### Biological parameters

Sexually active women of childbearing age face underlying age-specific probabilities of pregnancy in the absence of contraceptive use or breastfeeding (‘natural fertility/fecundity’) (Appendix A.1.2.1). At conception, the number and sex of each fetus is based on the primary sex ratio and twinning rate (Appendix A.1.2.2, A.1.2.3). Risks of miscarriage (spontaneous abortion) or ectopic pregnancy depend on maternal age and history of pregnancy loss (Appendices A.1.2.4 and A.1.2.5). Risks of antepartum stillbirth are based on maternal age and fetus sex, whereas intrapartum stillbirths may result from obstetric complications (Appendix A.1.2.6). The month of delivery is simulated based on fetal lifetable data (Appendix A.1.2.7). The impact of lactational amenorrhea on fecundity is modeled up to nine months after delivery for breastfeeding women (Appendix A.1.2.8). Age of menopause is drawn from a distribution for each woman (Appendix A.1.2.9). Anemia status is based on the underlying hemoglobin distribution in each country, with time trends estimated by subgroup (Appendix A.1.2.10).

### Family planning parameters

We simulate each woman’s family planning preferences and behaviors, based on country-specific individual-level empirical data. Age of sexual debut signals the beginning of a woman’s reproductive life cycle (Appendix A.1.3.1). At model initialization each woman is assigned a number of living children given her age, history of sexual activity and subgroup (Appendix A.1.3.2). We also draw a desired number of children (ideal family size) for each woman (Appendix A.1.3.3). Women who have met or exceeded this number are assumed to be ‘limiting’ their family size and are otherwise assumed to be ‘spacing’ births. Given her fertility preferences (limiting, spacing or desires birth soon), we model the probability that each woman’s need for contraception is met (Appendix A.1.3.4), and a method is assigned given spacing/limiting intention (Appendix A.1.3.5). Each method has a modeled failure rate (Appendix A.1.3.6) and duration of use for women who are spacing (Appendix A.1.3.7). Women may switch methods, or discontinue all contraception if they desire birth soon. Unintended pregnancies face a risk of induced abortion, a proportion of which may be ‘unsafe’ (for example, conducted by untrained personnel) and associated with higher morbidity and mortality (Appendix A.1.3.8). The risk of abortion for female fetuses is modified by a country’s secondary sex ratio (Appendix A.1.3.9) to account for sex-selective abortions. Breastfeeding duration and exclusive/nonexclusive status are simulated at delivery to inform the impact of lactational amenorrhea on fecundity (Appendix A.1.3.10).

### Health system parameters

The number of antenatal care visits is modeled for each pregnancy, and is assumed to impact anemia status and recognition of pregnancy complications (Appendix A.1.4.1). Five delivery sites are modeled, accounting for the emergency obstetric care (EmOC) status of health facilities: Home, Home-SBA (skilled birth attendant), nonEmOC (no EmOC) facility, BEmOC (basic EmOC) facility and CEmOC (comprehensive EmOC) facility^20^. The starting site is modeled for each delivery given a woman’s subgroup and year of delivery (Appendix A.1.4.2).

We model probabilities of recognition/referral for incident complications (that is, the ‘first delay’)^21^, and assume that recognition/referral improves with delivery site, with severe complications more likely to be recognized (Appendix A.1.4.3). We simulate whether timely transportation is available for each referral (Appendix A.1.4.4), and the target referral facility, accounting for possibilities of ‘horizontal transfer’ (transfer to a facility of the same EmOC status) and facility preference (bypassing lower-level facilities) (Appendix A.1.4.5). We also model the quality of care at each delivery site to capture health system and facility-level factors that account for residual differences in maternal mortality not explained by delivery site and availability of clinical interventions (Appendix A.1.4.6).

Because we fit the model to reported estimates of maternal mortality, we also include site-specific parameters to account for underreporting of maternal deaths (Appendix A.1.4.7). We differentiate between estimates from CRVS (for example, passive reporting) and survey-based estimates (for example, active investigation/case finding) of maternal mortality when fitting to specific targets.

### Obstetrical complications

For each delivery, we include the risk of major direct obstetrical complications associated with labor and childbirth: pre-eclampsia/eclampsia (Appendix A.1.5.1), obstructed labor (Appendix A.1.5.2), postpartum hemorrhage (Appendix A.1.5.3) and sepsis (Appendix A.1.5.4), accounting for individual-level risk factors and complication severity. We also include mortality from other direct maternal causes (Appendix A.1.5.5), late maternal mortality in the year following delivery (Appendix A.1.5.6) and indirect maternal deaths, which are not due to direct obstetric causes but are aggravated by pregnancy, such as malaria and HIV-related maternal deaths (Appendix A.1.5.7)^22^.

### Clinical interventions

Some interventions are routinely used to reduce the incidence of complications, such as active management of the third stage of labor, which reduces the risk of postpartum hemorrhage (Appendix A.1.6.2), partograph monitoring, which provides early detection of obstructed labor (Appendix A.1.6.3), and clean delivery, which reduces the risk of sepsis (Appendix A.1.6.4). We also model elective C-sections, which are nonmedically indicated but commonly used in some settings (Appendix A.1.6.1). Other interventions are applied once a complication is recognized, such as management of ectopic pregnancy (Appendix A.1.6.5), hypertension management (Appendix A.1.6.6), assisted delivery (Appendix A.1.6.7), hemorrhage management (Appendix A.1.6.8) and antibiotic use (Appendix A.1.6.9). We assume that the impact of postpartum care is captured by site-specific probabilities of complication incidence and morbidity/mortality.

For each intervention, we model the ‘availability’ (probability it can be used at a particular site), ‘efficacy’ (maximum clinical effectiveness) and real-world ‘effectiveness’ (actual impact on complication outcomes, accounting for site-specific quality of care), all of which depend on a woman’s delivery site.

### Model outcomes

Model outcomes include annual estimates of maternal deaths (total and by cause), live births, MMR, pregnancy mortality ratio, maternal death rate, lifetime risk of maternal death and proportional mortality ratio, as well as other fertility- and process-related indicators (Appendix A.2.4). We report the mean and 95% UI for each outcome, calculated as the 2.5 and 97.5 percentiles of the simulation results, which account for both first-order (individual-level stochastic) and second-order (parameter) uncertainty. We start the model in 1985 to allow for a ‘burn-in’ period, and report estimates starting in 1990. The GMatH model was developed in Java (v.1.8.0), and statistical analyses were performed in R (v.3.6.1).

### Statistical analysis

Model calibration involves comparing the model predictions with empirical data to identify parameter sets that provide a good fit (Appendix A.3.1). We fitted the model to primary data (not modeled estimates) for a range of maternal health indicators (Appendix A.3.2). Because of the complex interactions between model events and outcomes, we calibrated all model parameters simultaneously, sampling from the priors specified for each parameter (described above).

For calibration targets, we estimated reported maternal deaths from CRVS data, both total and by eight grouped causes (see Appendix A.2.3. for International Classification of Diseases codes). We calibrated to data from 1990 to 2015 (training set), reserving estimates from 2016 to 2020 as a testing set to assess the predictive accuracy of our model. We had 22,495 targets in the training set and 1,525 estimates in the testing set.

We used a Bayesian approach in which the empirical data are considered fixed and the model parameters are random variables. We used a stochastic optimization algorithm (simulated annealing) to identify good-fitting parameter sets. A goodness-of-fit score for each proposed parameter set was calculated as the sum of the distanced-squared between the model predictions and empirical estimates. We sampled from the final 100 best-fitting parameter sets to simulate 1,000 iterations of the model, thus accounting for both first-order (stochastic) and second-order (parameter) uncertainty.

As a posterior predictive check of the calibrated model, we compared our model predictions with the observed data in our training set (1990–2015). To evaluate the predictive accuracy of our model, we compared our predictions with the test set of estimates (2016–2020) not used in model calibration. We calculated how often (that is, the proportion of estimates) our prediction intervals (95% UI) contained the reported (empirical) point estimate (coverage probability), the mean absolute error and the mean error.

### Model projections

Using the calibrated model, we made projections for each country from 1990 to 2050. Projected indicators are driven both by trends in demographic composition and parameter-specific secular trends. We model demographic trends (including uncertainty) within each country, accounting for factors such as age structure, urbanization and educational attainment, and many model parameters are conditional on these demographic factors (see Table 2 for parameters that vary by ‘age’ or ‘subgroup’). In addition, some parameters are modeled with an independent coefficient to account for time trends (see Table 2 for parameters that vary by ‘year’). For these parameters, predicted values vary over time as a function of the calibrated coefficients for the trend term. To account for potential nonlinear trends in these parameter values, we also include an exponential trend modifier (*α* ≤ 1) to allow for (weakly) monotonic trends while helping to guard against extrapolating to unreasonable levels (Appendix A.2.2.2).

### Ethics and inclusion statement

All data for this study, including from LMICs, were obtained from publicly available sources. One colleague (B.S.D.) is from an LMIC and the corresponding author (Z.J.W.) is originally from an LMIC and is now based in a high-income country. We fully endorse the Nature Portfolio journals’ guidance on LMIC authorship and inclusion. Because this work builds on previous modeling work, authorship was based, in part, on prior participation and collaboration. However, we are strongly committed to collaboration with researchers from LMICs in future work, especially for analyses focused on specific contexts or countries.

This research is locally relevant to all countries included because we report findings by country, providing local policy-makers with important data on maternal health outcomes.

Because our modeling approach employed only publicly available data, as well as published data from the medical literature for each country, ethics review was not required. The data collection and analysis techniques employed raised no risks pertaining to stigmatization, incrimination, discrimination, animal welfare, the environment, health, safety, security or other personal risks. No biological materials, cultural artifacts or associated traditional knowledge has been transferred out of any country. In preparing the manuscript, the authors reviewed relevant studies from all countries for which data were available, as described in Appendix A.

### Reporting summary

Further information on research design is available in the Nature Portfolio Reporting Summary linked to this article.

## Online content

Any methods, additional references, Nature Portfolio reporting summaries, source data, extended data, supplementary information, acknowledgements, peer review information; details of author contributions and competing interests; and statements of data and code availability are available at 10.1038/s41591-023-02310-x.

## Supplementary information

Supplementary Information Appendix A: Model inputs, datasets and definitions, and model calibration. Appendix B: Country profiles. Appendix C: Calibrated parameters.

Reporting Summary

## Data Availability

Simulation results are available in a public data repository: 10.7910/DVN/UBGY9P. We also provide documentation for all model parameters, including data sources, assumptions and model implementation details as supplementary appendices, as well as online (www.gmath-model.org).

## Code availability

No software was used for any primary data collection in this study. The GMatH simulation model was developed in Java (v.1.8.0), and statistical analyses were performed in R (v.3.6.1). Pseudocode describing the simulation algorithm is available in Appendix A.1.7. Requests for more code details should be addressed to Z.J.W.
