# Supplementary material for: Simulation-based estimates and projections of global, regional and country-level maternal mortality by cause, 1990–2050
Source: Nat Med. 2023 Apr 20;29(5):1253–61. doi: 10.1038/s41591-023-02310-x (PMC10202807; doi:10.1038/s41591-023-02310-x)
Supplement: Supplementary file 2 — Reporting Summary [file 41591_2023_2310_MOESM2_ESM.pdf]

nature portfolio

Corresponding author(s): Zachary J. Ward

Last updated by author(s): Mar 9, 2023

Reporting Summary

Nature Portfolio wishes to improve the reproducibility of the work that we publish. This form provides structure for consistency and transparency in reporting. For further information on Nature Portfolio policies, see our Editorial Policies and the Editorial Policy Checklist.

Statistics

For all statistical analyses, confirm that the following items are present in the figure legend, table legend, main text, or Methods section.

n/a	Confirmed	
☐	☒	The exact sample size (n) for each experimental group/condition, given as a discrete number and unit of measurement
☐	☒	A statement on whether measurements were taken from distinct samples or whether the same sample was measured repeatedly
☒	☐	The statistical test(s) used AND whether they are one- or two-sided<br>*Only common tests should be described solely by name; describe more complex techniques in the Methods section.*
☒	☐	A description of all covariates tested
☐	☒	A description of any assumptions or corrections, such as tests of normality and adjustment for multiple comparisons
☐	☒	A full description of the statistical parameters including central tendency (e.g. means) or other basic estimates (e.g. regression coefficient) AND variation (e.g. standard deviation) or associated estimates of uncertainty (e.g. confidence intervals)
☒	☐	For null hypothesis testing, the test statistic (e.g. F, t, r) with confidence intervals, effect sizes, degrees of freedom and P value noted<br>*Give P values as exact values whenever suitable.*
☐	☒	For Bayesian analysis, information on the choice of priors and Markov chain Monte Carlo settings
☐	☒	For hierarchical and complex designs, identification of the appropriate level for tests and full reporting of outcomes
☒	☐	Estimates of effect sizes (e.g. Cohen's d, Pearson's r), indicating how they were calculated

Our web collection on statistics for biologists contains articles on many of the points above.

Software and code

Policy information about availability of computer code

Data collection	No software was used for any primary data collection in this study.
Data analysis	The GMatH simulation model was developed in Java (v1.8.0), and statistical analyses were performed in R (v3.6.1). Pseudocode describing the simulation algorithm is available in Appendix A.1.7. Requests for more code details should be addressed to Zachary J Ward.

For manuscripts utilizing custom algorithms or software that are central to the research but not yet described in published literature, software must be made available to editors and reviewers. We strongly encourage code deposition in a community repository (e.g. GitHub). See the Nature Portfolio guidelines for submitting code & software for further information.

Data

Policy information about availability of data

All manuscripts must include a data availability statement. This statement should provide the following information, where applicable:

- Accession codes, unique identifiers, or web links for publicly available datasets
- A description of any restrictions on data availability
- For clinical datasets or third party data, please ensure that the statement adheres to our policy

Simulation results are available in a public data repository: https://doi.org/10.7910/DVN/UBGY9P. We also provide documentation for all model parameters, including data sources, assumptions, and model implementation details as supplementary appendices, as well as online (www.gmath-model.org).

March 2021

Human research participants

Policy information about studies involving human research participants and Sex and Gender in Research.

Reporting on sex and gender	As our study focuses on maternal mortality, only females are considered in this analysis.
Population characteristics	See "Sample size" description below.
Recruitment	We simulated nationally-representative populations of individual women in 200 countries and territories.
Ethics oversight	As our modelling approach employed only publicly available data, as well as published data from the medical literature for each country, ethics review was not required.

Note that full information on the approval of the study protocol must also be provided in the manuscript.

Field-specific reporting

Please select the one below that is the best fit for your research. If you are not sure, read the appropriate sections before making your selection.

☒ Life sciences ☐ Behavioural & social sciences ☐ Ecological, evolutionary & environmental sciences

For a reference copy of the document with all sections, see nature.com/documents/nr-reporting-summary-flat.pdf

Life sciences study design

All studies must disclose on these points even when the disclosure is negative.

Sample size	We simulated nationally-representative populations of individual women in 200 countries and territories, with the number of women based on UN population projections. To account for international and internal (e.g. rural to urban) migration, we estimated post-stratification weights (via raking) by cycle, allowing us to re-weight our simulated estimates to reflect population trends not already included in the model (e.g., migration, differential background mortality, etc.).
Data exclusions	No data were excluded, but some empirical data were withheld from model fitting as a testing set to assess the predictive accuracy of the model - see the next point below.
Replication	We calibrated the simulation model to empirical data on a range of maternal health indicators from 1990-2015 (i.e. training set), reserving estimates from 2016-2020 as a testing set to assess the predictive accuracy of our model. We had 22,495 targets in the training set, and 1,525 estimates in the testing set. The coverage probability for estimates in our testing set (not used for calibration) was 91.4% overall, and was 96.0% for maternal mortality indicators, with a mean absolute error by cause of 10.5 deaths on average.
Randomization	As this is a modelling study no experimental randomization was performed. Covariates such as age, year, educational attainment, urban/rural location, etc., were controlled for, as described for each model parameter in Table 2 and Appendix A.
Blinding	A testing set of data was withheld from model calibration, as described above.

Reporting for specific materials, systems and methods

We require information from authors about some types of materials, experimental systems and methods used in many studies. Here, indicate whether each material, system or method listed is relevant to your study. If you are not sure if a list item applies to your research, read the appropriate section before selecting a response.

Materials & experimental systems

n/a	Involved in the study
☒	☐ Antibodies
☒	☐ Eukaryotic cell lines
☒	☐ Palaeontology and archaeology
☒	☐ Animals and other organisms
☒	☐ Clinical data
☒	☐ Dual use research of concern

Methods

n/a	Involved in the study
☒	☐ ChIP-seq
☒	☐ Flow cytometry
☒	☐ MRI-based neuroimaging
