# Supplementary material for: Simulation-based estimates and projections of global, regional and country-level maternal mortality by cause, 1990–2050
Source: Nat Med. 2023 Apr 20;29(5):1253–61. doi: 10.1038/s41591-023-02310-x (PMC10202807; doi:10.1038/s41591-023-02310-x)
Supplement: Supplementary file 1 — Appendix A: Model inputs, datasets and definitions, and model calibration. Appendix B: Country profiles. Appendix C: Calibrated parameters. [file 41591_2023_2310_MOESM1_ESM.pdf]

nature medicine

Article

https://doi.org/10.1038/s41591-023-02310-x

Simulation-based estimates and projections of global, regional and country-level maternal mortality by cause, 1990–2050

In the format provided by the authors and unedited

Contents

A Appendix A: Model Inputs, Datasets and Definitions, Model Calibration 9

A.1 Model Inputs . . . 9
A.1.1 Demographic Data . . . 9
A.1.1.1 Countries/Territories . . . 9
A.1.1.2 Population Projections . . . 11
A.1.1.3 Lifetables . . . 12
A.1.1.4 Proportion of Deaths due to Injury . . . 13
A.1.1.5 Education Projections . . . 17
A.1.1.6 Migration . . . 19
A.1.2 Biological Parameters . . . 20
A.1.2.1 Natural Fecundity/Fertility . . . 20
A.1.2.2 Sex Ratio - Primary . . . 23
A.1.2.3 Twinning Rates . . . 25
A.1.2.4 Miscarriage . . . 29
A.1.2.5 Ectopic Pregnancy . . . 32
A.1.2.6 Stillbirths . . . 37
A.1.2.7 Month of Delivery . . . 41
A.1.2.8 Lactational Amenorrhea (LAM) . . . 42
A.1.2.9 Menopause . . . 46
A.1.2.10 Anemia . . . 49
A.1.3 Family Planning . . . 56
A.1.3.1 Age of Sexual Debut . . . 56
A.1.3.2 Number of Living Children . . . 65
A.1.3.3 Desired Number of Children . . . 74
A.1.3.4 Unmet Need . . . 87
A.1.3.5 Contraception Method Mix . . . 121
A.1.3.6 Contraception Failure Rates . . . 244
A.1.3.7 Method Duration of Use . . . 247
A.1.3.8 Abortion . . . 257
A.1.3.9 Sex Ratio - Secondary . . . 271
A.1.3.10 Breastfeeding . . . 277
A.1.4 Health System Parameters . . . 281
A.1.4.1 Antenatal Care . . . 281
A.1.4.2 Starting Delivery Site . . . 294
A.1.4.3 Recognition/Referral . . . 314
A.1.4.4 Transportation . . . 323
A.1.4.5 Referral Facility . . . 326
A.1.4.6 Quality of Care . . . 330
A.1.4.7 Maternal Death Underreporting . . . 332
A.1.5 Obstetric Complications . . . 339
A.1.5.1 Preeclampsia/Eclampsia . . . 340
A.1.5.2 Obstructed Labor . . . 347
A.1.5.3 Postpartum Hemorrhage . . . 351
A.1.5.4 Sepsis . . . 358
A.1.5.5 Other Direct Complications . . . 362
A.1.5.6 Late Maternal Deaths . . . 366
A.1.5.7 Indirect Maternal Deaths . . . 372
A.1.6 Clinical Interventions . . . 376
A.1.6.1 Elective Caesarean Section . . . 377
A.1.6.2 Active Management of Labor . . . 385
A.1.6.3 Partograph . . . 387
A.1.6.4 Clean delivery . . . 389

A.1.6.5 Management of Ectopic Pregnancy . . . 392
A.1.6.6 Hypertension Management . . . 395
A.1.6.7 Assisted Delivery . . . 400
A.1.6.8 Hemorrhage Treatment . . . 404
A.1.6.9 Antibiotics . . . 406
A.1.7 GMatH Pseudocode . . . 408
A.2 Datasets and Definitions . . . 414
A.2.1 DHS Datasets . . . 414
A.2.2 Parameter Types . . . 416
A.2.2.1 Splines . . . 416
A.2.2.2 Trend Modifiers . . . 416
A.2.3 Maternal Death ICD Codes . . . 417
A.2.3.1 ICD-8 . . . 417
A.2.3.2 ICD-9 . . . 417
A.2.3.3 ICD-10 . . . 417
A.2.4 Maternal Health Indicators . . . 421
A.3 Model Calibration . . . 423
A.3.1 Calibration Overview . . . 423
A.3.2 Calibration Targets . . . 423
A.3.3 Scores . . . 428
A.3.4 Posterior Predictive Checks . . . 430

B Appendix B: Country Profiles **433**
Afghanistan . . . 435
Albania . . . 436
Algeria . . . 437
Andorra . . . 438
Angola . . . 439
Antigua and Barbuda . . . 440
Argentina . . . 441
Armenia . . . 442
Australia . . . 443
Austria . . . 444
Azerbaijan . . . 445
Bahamas . . . 446
Bahrain . . . 447
Bangladesh . . . 448
Barbados . . . 449
Belarus . . . 450
Belgium . . . 451
Belize . . . 452
Benin . . . 453
Bermuda . . . 454
Bhutan . . . 455
Bolivia (Plurinational State of) . . . 456
Bosnia and Herzegovina . . . 457
Botswana . . . 458
Brazil . . . 459
Brunei Darussalam . . . 460
Bulgaria . . . 461
Burkina Faso . . . 462
Burundi . . . 463
Cabo Verde . . . 464
Cambodia . . . 465

Cameroon . . . 466
Canada . . . 467
Cayman Islands . . . 468
Central African Republic . . . 469
Chad . . . 470
Chile . . . 471
China . . . 472
Colombia . . . 473
Comoros . . . 474
Congo . . . 475
Costa Rica . . . 476
Côte d'Ivoire . . . 477
Croatia . . . 478
Cuba . . . 479
Cyprus . . . 480
Czechia . . . 481
Dem. People's Republic of Korea . . . 482
Democratic Republic of the Congo . . . 483
Denmark . . . 484
Djibouti . . . 485
Dominica . . . 486
Dominican Republic . . . 487
Ecuador . . . 488
Egypt . . . 489
El Salvador . . . 490
Equatorial Guinea . . . 491
Eritrea . . . 492
Estonia . . . 493
Eswatini . . . 494
Ethiopia . . . 495
Faroe Islands . . . 496
Fiji . . . 497
Finland . . . 498
France . . . 499
Gabon . . . 500
Gambia . . . 501
Georgia . . . 502
Germany . . . 503
Ghana . . . 504
Greece . . . 505
Greenland . . . 506
Grenada . . . 507
Guatemala . . . 508
Guinea . . . 509
Guinea-Bissau . . . 510
Guyana . . . 511
Haiti . . . 512
Honduras . . . 513
Hungary . . . 514
Iceland . . . 515
India . . . 516
Indonesia . . . 517
Iran (Islamic Republic of) . . . 518
Iraq . . . 519

Ireland . . . 520
Israel . . . 521
Italy . . . 522
Jamaica . . . 523
Japan . . . 524
Jordan . . . 525
Kazakhstan . . . 526
Kenya . . . 527
Kiribati . . . 528
Kuwait . . . 529
Kyrgyzstan . . . 530
Lao People's Democratic Republic . . . 531
Latvia . . . 532
Lebanon . . . 533
Lesotho . . . 534
Liberia . . . 535
Libya . . . 536
Liechtenstein . . . 537
Lithuania . . . 538
Luxembourg . . . 539
Madagascar . . . 540
Malawi . . . 541
Malaysia . . . 542
Maldives . . . 543
Mali . . . 544
Malta . . . 545
Marshall Islands . . . 546
Mauritania . . . 547
Mauritius . . . 548
Mexico . . . 549
Micronesia (Fed. States of) . . . 550
Monaco . . . 551
Mongolia . . . 552
Montenegro . . . 553
Morocco . . . 554
Mozambique . . . 555
Myanmar . . . 556
Namibia . . . 557
Nauru . . . 558
Nepal . . . 559
Netherlands . . . 560
New Zealand . . . 561
Nicaragua . . . 562
Niger . . . 563
Nigeria . . . 564
North Macedonia . . . 565
Norway . . . 566
Oman . . . 567
Pakistan . . . 568
Palau . . . 569
Panama . . . 570
Papua New Guinea . . . 571
Paraguay . . . 572
Peru . . . 573

Philippines . . . 574
Poland . . . 575
Portugal . . . 576
Puerto Rico . . . 577
Qatar . . . 578
Republic of Korea . . . 579
Republic of Moldova . . . 580
Romania . . . 581
Russian Federation . . . 582
Rwanda . . . 583
Saint Kitts and Nevis . . . 584
Saint Lucia . . . 585
Saint Vincent and the Grenadines . . . 586
Samoa . . . 587
San Marino . . . 588
Sao Tome and Principe . . . 589
Saudi Arabia . . . 590
Senegal . . . 591
Serbia . . . 592
Seychelles . . . 593
Sierra Leone . . . 594
Singapore . . . 595
Slovakia . . . 596
Slovenia . . . 597
Solomon Islands . . . 598
Somalia . . . 599
South Africa . . . 600
South Sudan . . . 601
Spain . . . 602
Sri Lanka . . . 603
State of Palestine . . . 604
Sudan . . . 605
Suriname . . . 606
Sweden . . . 607
Switzerland . . . 608
Syrian Arab Republic . . . 609
Taiwan . . . 610
Tajikistan . . . 611
Thailand . . . 612
Timor-Leste . . . 613
Togo . . . 614
Tonga . . . 615
Trinidad and Tobago . . . 616
Tunisia . . . 617
Türkiye . . . 618
Turkmenistan . . . 619
Tuvalu . . . 620
Uganda . . . 621
Ukraine . . . 622
United Arab Emirates . . . 623
United Kingdom . . . 624
United Republic of Tanzania . . . 625
United States of America . . . 626
Uruguay . . . 627

Uzbekistan 628
Vanuatu 629
Venezuela (Bolivarian Republic of) 630
Viet Nam 631
Yemen 632
Zambia 633
Zimbabwe 634

C Appendix C: Calibrated Parameters 636
C.1 Demographic Parameters 636
C.1.1 Proportion of Deaths Due to Injury 636
C.2 Biological Parameters 638
C.2.1 Natural Fecundity/Fertility 638
C.2.2 Sex Ratio - Primary 640
C.2.3 Twinning Rates 641
C.2.3.1 Monozygotic 641
C.2.3.2 Dizygotic 641
C.2.4 Miscarriage 643
C.2.4.1 Incidence 643
C.2.4.2 Relative Risk | Previous Miscarriage 644
C.2.5 Ectopic Pregnancy 646
C.2.5.1 Incidence 646
C.2.5.2 Recurrence 647
C.2.5.3 Rupture 648
C.2.5.4 CFR 648
C.2.6 Stillbirths 650
C.2.6.1 Incidence 650
C.2.6.2 Male RR 651
C.2.7 Lactational Amenorrhea 653
C.2.8 Menopause 655
C.2.9 Anemia 656
C.3 Family Planning 659
C.3.1 Age of Sexual Debut 659
C.3.2 Number of Living Children 661
C.3.3 Desired Number of Children 664
C.3.4 Unmet Need 670
C.3.4.1 No Need 670
C.3.4.2 Spacing 673
C.3.4.3 Limiting 676
C.3.5 Contraception Method Mix 680
C.3.5.1 Pill 680
C.3.5.2 IUD 682
C.3.5.3 Injections 685
C.3.5.4 Condom 688
C.3.5.5 Female sterilization 691
C.3.5.6 Male sterilization 693
C.3.5.7 Periodic abstinence 695
C.3.5.8 Withdrawal 698
C.3.5.9 Other 701
C.3.5.10 Implants 704
C.3.6 Contraception Failure Rates 708
C.3.6.1 Pill 708
C.3.6.2 IUD 709
C.3.6.3 Injections 710

C.3.6.4 Condom . . . 711
C.3.6.5 Female sterilization . . . 712
C.3.6.6 Male sterilization . . . 713
C.3.6.7 Periodic abstinence . . . 714
C.3.6.8 Withdrawal . . . 715
C.3.6.9 Other . . . 717
C.3.6.10 Implants . . . 718
C.3.7 Method Duration of Use . . . 719
C.3.7.1 Duration of Use . . . 719
C.3.7.2 Discontinue All Methods . . . 728
C.3.8 Abortion . . . 737
C.3.8.1 p(Abortion|Unintended Pregnancy) . . . 737
C.3.8.2 p(Safe Abortion|Abortion) . . . 738
C.3.8.3 p(Mortality) . . . 740
C.3.8.4 p(Infertility) . . . 740
C.3.8.5 Anemia RR - Mortality . . . 741
C.3.9 Sex Ratio - Secondary . . . 743
C.3.10 Breastfeeding . . . 744
C.3.10.1 Any Breastfeeding . . . 744
C.3.10.2 Exclusive Breastfeeding . . . 745
C.3.10.3 6 Months | Any Breastfeeding . . . 747
C.4 Health System Parameters . . . 749
C.4.1 Antenatal Care . . . 749
C.4.1.1 Any ANC . . . 749
C.4.1.2 Number of Visits . . . 751
C.4.1.3 Hb Increase . . . 753
C.4.1.4 Recognition/Referral Increase . . . 754
C.4.2 Starting Delivery Site . . . 756
C.4.2.1 Facility Delivery . . . 756
C.4.2.2 SBA|Home . . . 757
C.4.2.3 non-EmOC|Facility . . . 759
C.4.2.4 BEmOC|Facility . . . 761
C.4.2.5 CEmOC|Facility . . . 763
C.4.3 Recognition/Referral . . . 766
C.4.3.1 Home . . . 766
C.4.3.2 Home-SBA . . . 773
C.4.3.3 non-EmOC . . . 780
C.4.3.4 BEmOC . . . 787
C.4.3.5 CEmOC . . . 794
C.4.4 Transportation . . . 802
C.4.4.1 Home . . . 802
C.4.4.2 Home-SBA . . . 803
C.4.4.3 non-EmOC . . . 804
C.4.4.4 BEmOC . . . 805
C.4.5 Referral Facility . . . 807
C.4.5.1 Home . . . 807
C.4.5.2 Home-SBA . . . 808
C.4.5.3 non-EmOC . . . 810
C.4.5.4 BEmOC . . . 812
C.4.6 Under-reporting . . . 815
C.4.6.1 CRVS . . . 815
C.4.6.2 Survey . . . 819
C.4.7 Quality of Care . . . 825
C.5 Obstetric Complications . . . 830

C.5.1 Pre-Eclampsia/Eclampsia . . . 830
C.5.1.1 Incidence . . . 830
C.5.1.2 Risk Factors . . . 831
C.5.1.3 Outcomes . . . 832
C.5.2 Obstructed Labour . . . 836
C.5.2.1 Incidence . . . 836
C.5.2.2 Risk Factors . . . 837
C.5.2.3 Outcomes . . . 837
C.5.3 Postpartum Hemorrhage . . . 841
C.5.3.1 Incidence . . . 841
C.5.3.2 Morbidity/Mortality . . . 842
C.5.3.3 Anemia Relative Risks/Outcomes . . . 844
C.5.4 Sepsis . . . 847
C.5.4.1 Incidence . . . 847
C.5.4.2 Risk Factors . . . 848
C.5.4.3 Mortality/Morbidity . . . 849
C.5.4.4 Anemia Relative Risks . . . 850
C.5.5 Other Direct . . . 852
C.5.5.1 Incidence . . . 852
C.5.5.2 Mortality . . . 852
C.5.6 Late Maternal Deaths . . . 855
C.5.6.1 Incidence . . . 855
C.5.6.2 Mortality . . . 855
C.5.7 Indirect Maternal Deaths . . . 858
C.6 Clinical Interventions . . . 860
C.6.1 Elective Caesarean Section . . . 860
C.6.2 Active Management of Labour . . . 863
C.6.2.1 Use . . . 863
C.6.2.2 Efficacy . . . 866
C.6.3 Partograph . . . 868
C.6.3.1 Use . . . 868
C.6.3.2 Efficacy . . . 871
C.6.4 Clean Delivery . . . 873
C.6.4.1 Use . . . 873
C.6.4.2 Efficacy . . . 877
C.6.5 Management of Ectopic Pregnancy . . . 879
C.6.5.1 Availability . . . 879
C.6.5.2 Efficacy . . . 882
C.6.6 Hypertension Management . . . 884
C.6.6.1 Availability . . . 884
C.6.6.2 Efficacy . . . 887
C.6.6.3 C-Section|EmOC . . . 888
C.6.7 Assisted Delivery . . . 889
C.6.7.1 Availability . . . 889
C.6.7.2 Efficacy . . . 892
C.6.7.3 C-Section|EmOC, Moderate OL . . . 893
C.6.8 Hemorrhage Treatment . . . 894
C.6.8.1 Availability . . . 894
C.6.8.2 Efficacy . . . 897
C.6.9 Antibiotics . . . 899
C.6.9.1 Availability . . . 899
C.6.9.2 Efficacy . . . 902

A Appendix A: Model Inputs, Datasets and Definitions, Model Calibration

A.1 Model Inputs

A.1.1 Demographic Data

A.1.1.1 Countries/Territories

We obtained a list of 237 countries/territories from the UN Population Projections 2022. We excluded the following areas, mainly due to political affiliations with other countries. Our final dataset contained 200 countries/territories.

Excluded Area	Political Status
American Samoa	United States Territory
Anguilla	British Overseas Territory
Aruba	Kingdom of the Netherlands
Bonaire, Sint Eustatius and Saba	Caribbean Netherlands (special municipalities)
British Virgin Islands	British Overseas Territory
Cook Islands	Free association with New Zealand
Curaçao	Kingdom of the Netherlands
Falkland Islands (Malvinas)	British Overseas Territory
French Guiana	French Overseas Department
French Polynesia	French Overseas Collectivity
Gibraltar	British Overseas Territory
Guadeloupe	French Overseas Department
Guam	United States Territory
Guernsey	British Crown dependency
Holy See	City-state
Hong Kong	Chinese special administrative region
Isle of Man	British Crown dependency
Jersey	British Crown dependency
Kosovo	Partially recognised state
Macao	Chinese special administrative region
Martinique	French Overseas Department
Mayotte	French Overseas Department
Montserrat	British Overseas Territory
New Caledonia	French Overseas Collectivity
Niue	Free association with New Zealand
Northern Mariana Islands	United States Commonwealth
Réunion	French Overseas Department
Saint-Barthélemy	French Overseas Collectivity
Saint Helena	British Overseas Territory
Saint Martin	French Overseas Collectivity
Saint Pierre and Miquelon	French Overseas Collectivity
Sint Maarten	Kingdom of the Netherlands
Tokelau	Dependent territory of New Zealand
Turks and Caicos Islands	British Overseas Territory
United States Virgin Islands	United States Territory
Wallis and Futuna Islands	French Overseas Collectivity
Western Sahara	Disputed territory

Geographical Group

We used the UN geography groupings to assign each country/territory to a geographic Area (continent) and Region. These groupings were used to define various hierarchical models when estimating model parameters.

Area	Region	# Countries
Africa	Eastern Africa	18
	Middle Africa	9
	Northern Africa	6
	Southern Africa	5
	Western Africa	16
Asia	Central Asia	5
	Eastern Asia	6
	South-Eastern Asia	11
	Southern Asia	9
	Western Asia	18
Europe	Eastern Europe	10
	Northern Europe	11
	Southern Europe	14
	Western Europe	9
Latin America and the Caribbean	Caribbean	15
	Central America	8
	South America	12
Northern America	Northern America	4
Oceania	Australia/New Zealand	2
	Melanesia	4
	Micronesia	5
	Polynesia	3

Income Group
Countries/territories were also assigned to an income group based on the 2018 World Bank Income Groups.

Income Group	# Countries
Low income	34
Lower middle income	46
Upper middle income	55
High income	65

A.1.1.2 Population Projections

Population Size

We obtained country-specific population projections from the 2022 UN World Population Prospects. We used annual, age-specific estimates of the female population to model each country's population from 1985 to 2021. To project forward to 2050 we used the probabilistic population projections (PPP) to estimate the number of women in the population from 2022 onwards. Annual age-specific projections from the deterministic Medium Fertility scenario were used to construct the projected age structure (by single ages).

For computational efficiency we do not simulate the entire population of women in each country. Instead we specify a scalar and simulate a proportion of the female population (sampled at random) and re-weight our modeled estimates back to the total population size. To improve the stability of modeled estimates for small countries we specify a minimum population size of 10,000 women to simulate in a country and adjust the country-specific population scalar accordingly.

Urbanicity

We obtained projections of the urban proportion of each country's population from the UN Urbanization Prospects 2018. Due to lack of data we assumed that these projections were representative of males and females of all ages. This approach may thus overestimate the urban proportion at older ages in countries where urbanization is driven by younger people. However, given that the population of interest in our model is women of reproductive age this assumption is unlikely to substantially impact our estimates.

References

United Nations, Department of Economic and Social Affairs, Population Division (2022). World Population Prospects 2022, Online Edition. https://population.un.org/wpp/Download/Standard/CSV/

United Nations, Department of Economic and Social Affairs, Population Division (2018). World Urbanization Prospects: The 2018 Revision, Online Edition. https://population.un.org/wup/Download/

A.1.1.3 Lifetables

We obtained country-specific estimated lifetables (annual mortality rates) for 1985-1950 from the UN World Population Prospects 2022 by single age and year. Annual mortality rates were converted to monthly probabilities for use in the model.

References

United Nations, Department of Economic and Social Affairs, Population Division (2022). World Population Prospects 2022, Online Edition.

A.1.1.4 Proportion of Deaths due to Injury

Overview

The World Health Organization (WHO) defines a maternal death as "the death of a woman while pregnant or within 42 days of termination of pregnancy, irrespective of the duration and site of the pregnancy, from any cause related to or aggravated by the pregnancy or its management but not from accidental or incidental causes". (https://www.who.int/healthinfo/statistics/indmaternalmortality/en/)

In addition to deaths from direct obstetric causes explicitly included in the model, we therefore also include maternal deaths from indirect causes when calculating maternal indicators. To estimate the risk of death from these indirect causes we use background mortality rates for women ages 15-49 from the WHO lifetables, and remove a proportion of deaths due to accidental/incidental causes (e.g. traffic accidents, other injuries, etc).

Data

We use estimates of cause of death from the Global Burden of Disease (GBD) 2019 to model the proportion of deaths due to these causes. We obtained estimates of cause of death among women ages 15-49 for 197 countries included in the model from 1990 to 2019. Based on the mean GBD estimates, we calculated the proportion of all deaths due to injuries (code C. Injuries).

Parameters

We used a hierarchical logistic regression model to estimate how the proportion of deaths due to injuries changes over time.

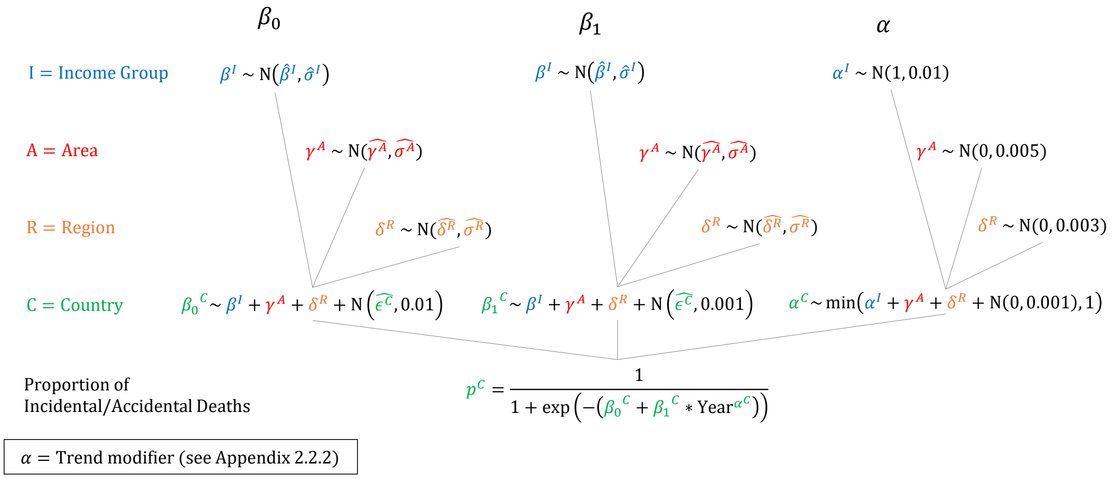

Priors

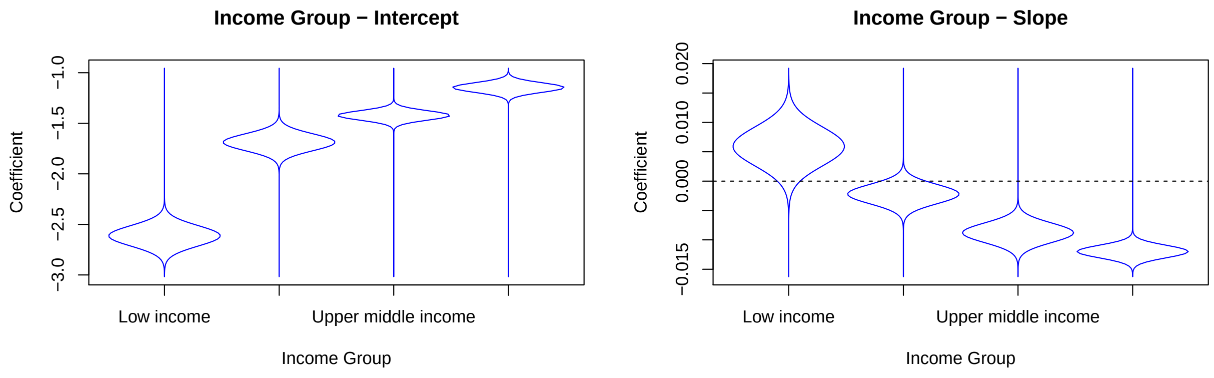

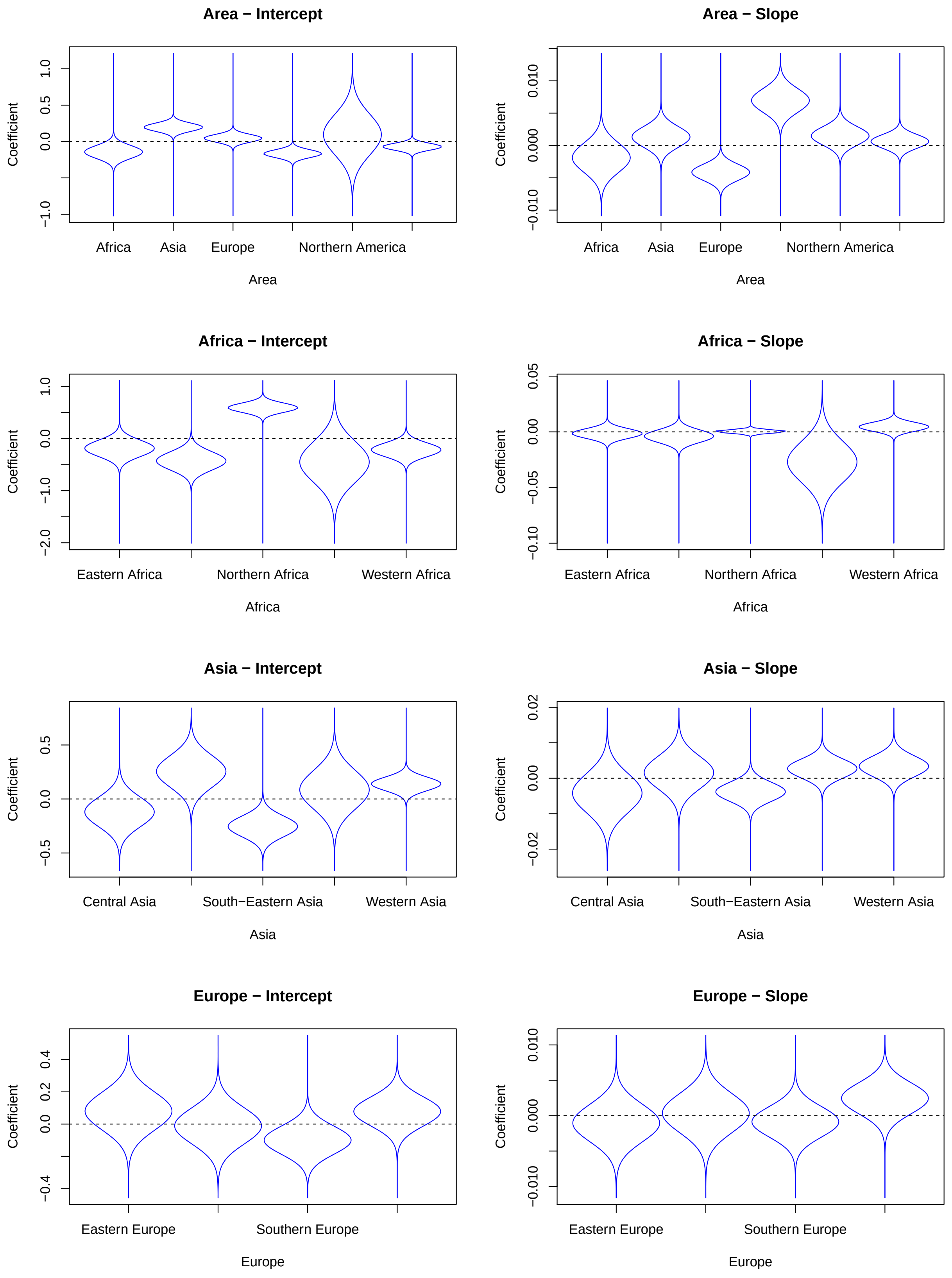
Area − Intercept
Coefficient
1.0
0.5
0.0
−1.0
Africa
Asia
Europe
Northern America
Area
Area − Slope
Coefficient
0.010
0.000
−0.010
Africa
Asia
Europe
Northern America
Area
Africa − Intercept
Coefficient
1.0
0.0
−1.0
−2.0
Eastern Africa
Northern Africa
Western Africa
Africa
Africa − Slope
Coefficient
0.05
0.00
−0.05
−0.10
Eastern Africa
Northern Africa
Western Africa
Africa
Asia − Intercept
Coefficient
0.5
0.0
−0.5
Central Asia
South−Eastern Asia
Western Asia
Asia
Asia − Slope
Coefficient
0.02
0.00
−0.02
Central Asia
South−Eastern Asia
Western Asia
Asia
Europe − Intercept
Coefficient
0.4
0.2
0.0
−0.4
Eastern Europe
Southern Europe
Europe
Europe − Slope
Coefficient
0.010
0.000
−0.010
Eastern Europe
Southern Europe
Europe

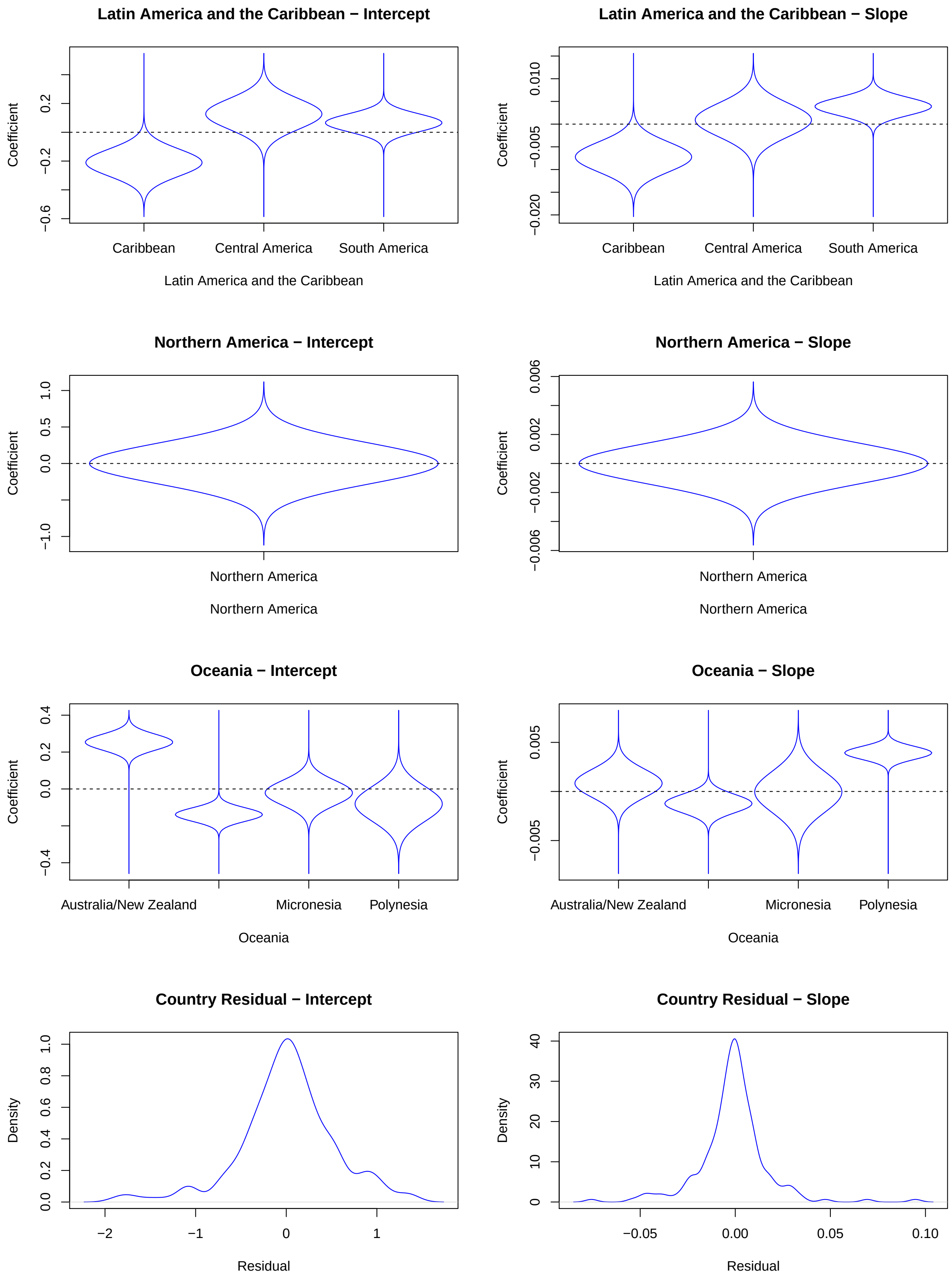

Model Implementation

Background mortality deaths (i.e. deaths from the lifetable) that occur in the model during pregnancy will be classified as 'maternal' deaths if they are not due to incidental/accidental causes, as controlled by the estimated proportions over time.

References

Global Burden of Disease Study 2019 (GBD 2019) Results. Seattle, United States: Institute for Health Metrics and Evaluation (IHME), 2020. Available from https://vizhub.healthdata.org/gbd-results/.

World Health Organization. Maternal mortality ratio (per 100 000 live births). Available at https://www.who.int/healthinfo/statistics/indmaternalmortality/en/.

A.1.1.5 Education Projections

Women's education is associated with family planning preferences/behaviours and maternal health outcomes. To capture heterogeneity we therefore model each woman's level of education and urban/rural location. We model 3 levels of education: low (less than primary), middle (less than secondary), and high (completed secondary or higher).

Country-level UNESCO data on educational attainment for females aged 25 years or older was available for 178 countries from 1950 to 2018 - a total of 1214 estimates were available.

To supplement these estimates, and to estimate educational attainment by urban/rural residence, we analyzed DHS data (see DHS datasets). Taking the complex survey design of each DHS survey into account, we estimated the proportion of women with each level of educational attainment aged 25 years and older (n=2,871,974) for consistency with the UN data. We estimated educational attainment overall, and by urban/rural location.

Combining both UNESCO and DHS data we had 1508 observations for 186 countries.

Education Level	DHS Levels (v149)	UNESCO Levels
Low (less than primary)	No education	No schooling
	Incomplete primary	Incomplete primary
Middle (less than secondary)	Complete primary	Primary (ISCED 1)
	Incomplete secondary	Lower secondary (ISCED 2)
High (completed secondary or higher)	Complete secondary	Upper secondary (ISCED 3)
	Higher	Post-secondary non-tertiary (ISCED 4)
		Short-cycle tertiary (ISCED 5)
		Bachelor's or equivalent (ISCED 6)
		Master's or equivalent (ISCED 7)
		Doctoral or equivalent (ISCED 8)

To estimate trends in educational attainment over time we fit Bayesian hierarchical logistic regression models.

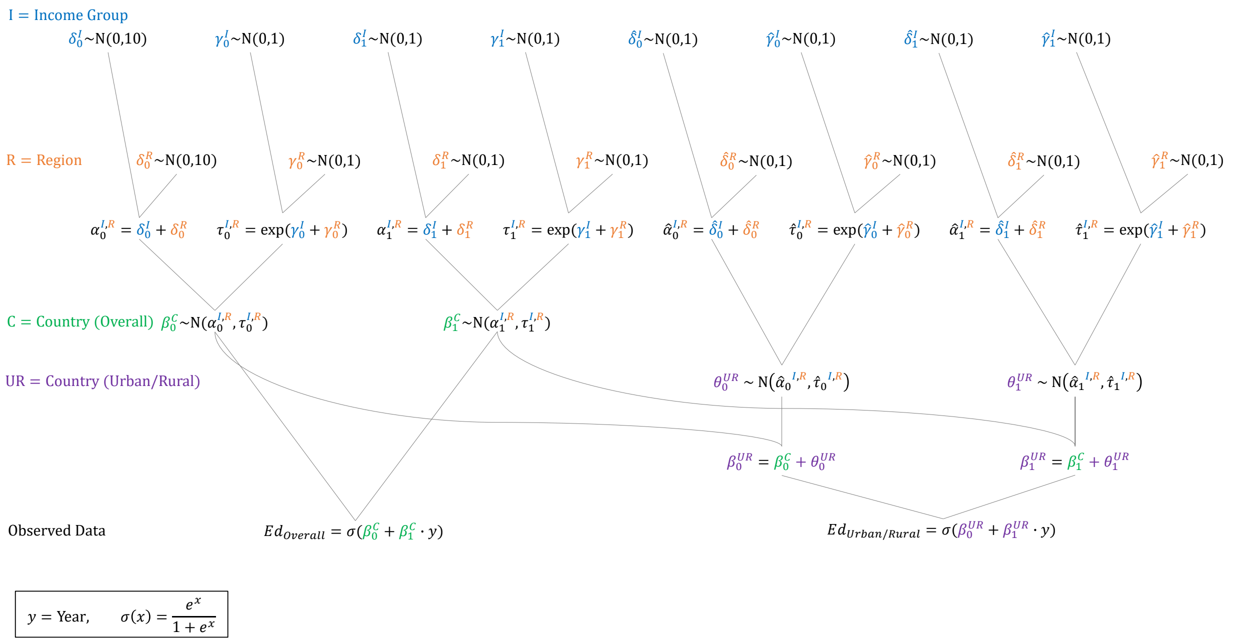

Separate models were fit for each level of educational attainment (i.e. low/middle/high) and the predictions from each model were re-normalized:

$$P(Ed^i) = \frac{\sigma(\beta_0^i + \beta_1^i \cdot year)}{\sum_{k=1}^{3} \sigma(\beta_0^k + \beta_1^k \cdot year)}$$

where

$$\sigma(x) = \frac{e^x}{1+e^x}$$

We obtained 1,000 posterior samples from each model. Sampling was performed using Stan (version 2.19.2) with 4 chains, 1,000 warmup iterations, step size of 0.8, and maximum tree depth of 20.

We evaluated the model fit using posterior predictive checks (i.e. comparing our model posterior predictions to the observed estimates) for estimates from 1990 onwards. These checks revealed a mean absolute error of 0.044 and coverage probabilities (i.e. proportion of times the observed value fell within the model 95% UIs) of 80.65%. See Country Profiles for country-specific education projections.

In each iteration of the simulation model we sample a parameter set from the education posteriors.

Due to lack of data, we used overall country estimates for both urban and rural women in high income countries, and for the following countries with sparse data: Algeria, Belarus, Belize, Bosnia and Herzegovina, Botswana, Bulgaria, China, Costa Rica, Cuba, North Korea, Dominica, Fiji, Grenada, Iran, Iraq, Jamaica, Kiribati, Lebanon, Libya, Malaysia, Marshall Islands, Mexico, Micronesia, Mongolia, Montenegro, Nauru, North Macedonia, Romania, Russia, Saint Lucia, Saint Vincent and the Grenadines, Samoa, Serbia, Solomon Islands, Thailand, Tonga, Turkmenistan, Tuvalu, Vanuatu, Venezuela.

For Dominican Republic, Ecuador, Equatorial Guinea, and Mauritius, the urban projections were unstable so we used overall trends for urban women for these countries.

References

UNESCO Institute for Statistics (UIS). Share of population by educational attainment, population 25 years and older. February 2019 Release. http://uis.unesco.org/sites/default/files/datacentre/Educational_attainment_-_Niveau_d'%C3%A9ducation_atteint.xlsx

ICF. Demographic and Health Surveys (various) [Datasets]. Funded by USAID. Rockville, Maryland: ICF [Distributor].

A.1.1.6 Migration

To account for international and internal (e.g. rural to urban) migration, we estimated post-stratification weights (w) via raking by cycle. This approach allows us to re-weight our simulated estimates (x) over time to reflect changes in the population not already explicitly accounted for in the model (e.g., migration, differential background mortality, etc). Cycle-specific weights were calculated (iteratively until convergence: $\hat{\pi} \rightarrow \pi^*$) for 6 demographic groups within each country: Urban/Rural location (i) by 3 Education Levels j:

$$\hat{\pi}_i^{(t)} = \sum_j x_{ij} * w_{ij}^{(t)}, \hat{\pi}_j^{(t)} = \sum_i x_{ij} * w_{ij}^{(t)}$$

$$w_{ij}^{(t+1)} = w_{ij}^{(t)} * \frac{\pi_i^*}{\hat{\pi}_i^{(t)}}, \text{if } t \text{ is even}$$

$$w_{ij}^{(t+1)} = w_{ij}^{(t)} * \frac{\pi_j^*}{\hat{\pi}_j^{(t)}}, \text{if } t \text{ is odd}$$

Raking was performed among women aged 25-49 to account for the educational attainment projections. The weights were then re-scaled to adjust the population of women aged 15-49 to match the sampled UN population projections.

A.1.2 Biological Parameters

A.1.2.1 Natural Fecundity/Fertility

Overview

Fecundity is defined as the biological capacity to reproduce, a theoretical potential that differs from fertility, which is the actual realization of a live birth. Fecundity is therefore more difficult to measure, but has been estimated by examining populations which do not use deliberate family planning - so called "natural fertility" populations that likely reflect natural age-related physiological changes in reproductive capacity and natural sterility (Wilson 1988, O'Connor 1998).

Natural fertility

Natural fertility was first estimated by Henry 1961, based on empirical data from 13 populations of married women who did not use deliberate birth control. While the absolute level of fertility varied among populations, it was observed that relative fertility rates followed the same characteristic age profile. In later work, Coale and Trussell (1974, 1978) averaged 10 of these 13 natural fertility populations to obtain more refined estimates after discarding those suspected to be biased by age misreporting. More recent analyses have focused on fitting statistical models to the empirical data (Xie 1990,1991,1992, Wilson 1998).

Age patterns of natural fertility may vary somewhat between populations, with patterns determined by context-specific reproductive behaviors (Wilson 1988). However, after adjusting each population to the same fertility rate at age 20, the age-related decline in fertility is almost identical in all populations, suggesting a common pattern of female reproductive aging (O'Connor 1998). Evidence also suggests that even very different marriage patterns have only a minor effect on the overall fertility pattern (Wilson 1988).

Natural fecundity

However, note that "natural fertility" is based on observed live births, and therefore underestimates total pregnancies since it does not take into account pregnancy loss (e.g. spontaneous or elective abortion). Since the model does simulate the risk of pregnancy loss, we need to estimate the probability of natural fecundity (i.e. risk of conception), rather than natural fertility. This estimation is complicated by the fact that risk of miscarriage varies substantially by maternal age. To address the inherent uncertainty in this parameter we fit quadratic splines to estimated rates of pregnancy after adjusting for age-specific risks of miscarriage.

Data

For baseline natural fertility estimates, we averaged age group data from Coale and Trussell (1974) and Xie (1990) - estimates were similar from both sources. This provided the relative shape of fertility by age. To adjust for pregnancy loss, we used estimates of miscarriage from Andersen 2000. We further inflated the priors by 25% to better approximate other data from Trussell 2007, which estimated that women not using contraception faced an annual probability of pregnancy of 0.85. However, the population of women under study was desiring pregnancy, and the denominator also only included women at risk of pregnancy (i.e. not already pregnant or breastfeeding). Thus we would expect the actual probability to be lower in the general population.

	Natural Fertility (Annual probability of live birth)			Risk of Miscarriage
Age Group	Coale and Trussell (1974)	Xie (1990)	Average	Andersen (2000)
20-24	0.46	0.46	0.46	0.089
25-29	0.431	0.436	0.4335	0.095
30-34	0.395	0.392	0.3935	0.12
35-39	0.322	0.333	0.3275	0.197
40-44	0.167	0.199	0.183	0.408
45-49	0.024	0.043	0.0335	0.747

Parameters

We fit quadratic B-splines to the midpoints of the natural fecundity age groups. Spline Width: 30

Spline #	Knot Location	Height
1	20	0.66

Spline #	Knot Location	Height
2	30	0.58
3	40	0.5

We used a hierarchical model with 4 levels (global/area/region/country) to account for potential differences in factors that affect fecundity (e.g. biological and cultural factors).

G = Global $\beta_i^G \sim N(\hat{\alpha}_i, 0.05)$

A = Area $\gamma_i^A \sim N(\beta_i^G, 0.01)$ $\sigma_i^A \sim \text{HalfNorm}(0.01)$

R = Region $\delta_i^R \sim N(\gamma_i^A, \sigma_i^A)$ $\sigma_i^R \sim \text{HalfNorm}(0.01)$

C = Country $\alpha_i^C \sim \max\left(N\left(\delta_i^R, \sigma_i^R\right), 0\right)$

B−Splines $f(x) = \sum_{i=1}^{3} \alpha_i^C B_i(\hat{x})$

Priors

We enforced declining α by age when sampling spline heights.

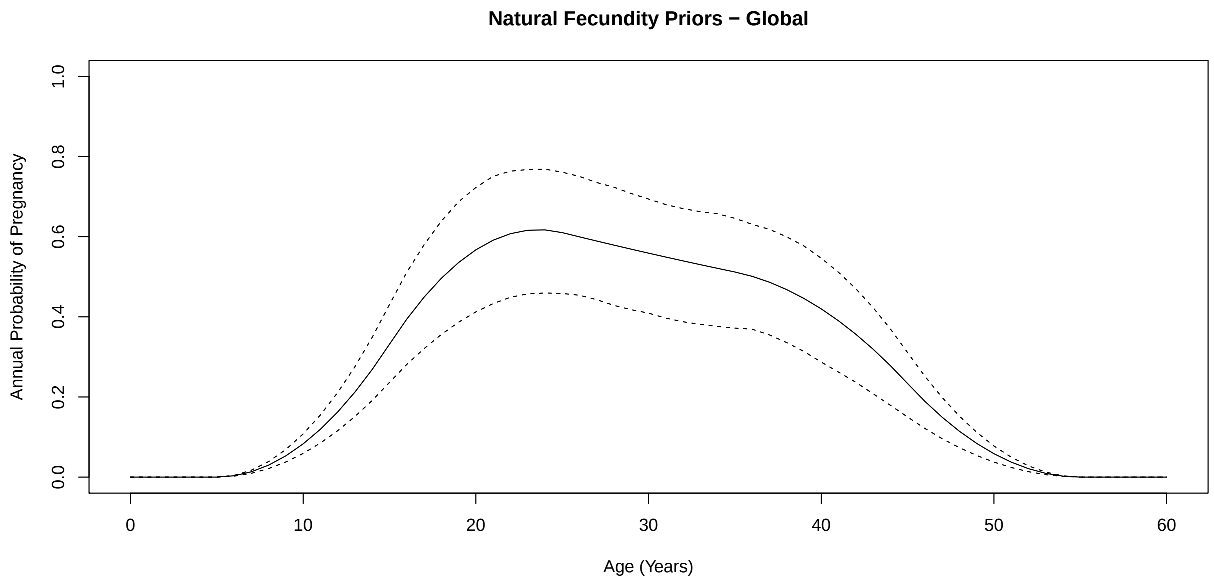

Model Implementation

The annual probabilities of pregnancy are converted to monthly probabilities. Age-specific natural fertility is used in the model to determine the probability of pregnancy in the absence of any contraception (or LAM). Note that as we do not explicitly account for marital status or frequency of sexual intercourse, the calibrated

age-specific natural fertility parameters implicitly reflect both the frequency of sexual intercourse and the probability of pregnancy given intercourse.

References

Andersen AM, Wohlfahrt J, Christens P, Olsen J, Melbye M. Maternal age and fetal loss: population based register linkage study. BMJ 2000; 320: 1708-12.

Coale AJ, Trussell TJ. Model Fertility Schedules: Variations in The Age Structure of Childbearing in Human Populations. Popul Index 1974; 40(2): 185-258.

Coale AJ, Trussell JT. Technical Note: Finding the Two Parameters That Specify a Model Schedule of Marital Fertility. Popul Index 1978; 44: 203-13.

Henry L. Some data on natural fertility. Eugen Q 1961; 8: 81-91.

O'Connor KA, Holman DJ, Wood JW. Declining fecundity and ovarian ageing in natural fertility populations. Maturitas 1998; 30(2): 127-136.

Trussell J. Contraceptive efficacy. In Hatcher RA, Trussell J, Nelson AL, Cates W, Stewart FH, Kowal D. Contraceptive Technology: Nineteenth Revised Edition. New York NY: Ardent Media, 2007.

Wilson C, Oeppen J, Pardoe M. What is natural fertility? The modelling of a Concept. Popul Index 1988; 54(1): 4-20.

Xie Y. What is natural fertility? The remodeling of a Concept. Popul Index 1990; 56(4): 656-663.

Xie Y. Model Fertility Schedules Revisited: The Log-Multiplicative Model Approach. Soc Sci Res 1991; 20: 355-368.

Xie Y, Pimental EE. Age patterns of marital fertility: revising the Coale-Trussell method. J Am Stat Assoc 1992; 87(420): 977-984.

A.1.2.2 Sex Ratio - Primary

Overview

The primary sex ratio is a measure of how many males are conceived compared to females. This ratio is used to simulate the sex of fetuses at conception.

Data

In humans, the natural primary sex ratio has been estimated at about 1.05 to 1.06 (Grech 2002), although it has been shown to vary. A study of trends in the United States over many years (Mathews 2005) found that the sex ratio at birth was 1.06-1.07 for mothers having their first baby, and that it decreased with each additional birth towards 1.03. Recent research also claims that the sex ratio at conception is actually 1.00, and thus not male-biased as is commonly thought (Orzack 2015). Instead, the authors show that total female mortality during pregnancy exceeds total male mortality, leading to a sex ratio above 1.0 as gestation progresses. However, we do not model differential risks of miscarriage by fetal sex, and so we assume that the sex ratio is constant throughout gestation, and by birth order.

Parameters

For simplicity, we assume that the primary sex ratio remains constant over time. We constructed a hierarchical model (global/area/region/country) to allow for potential differences due to genetics or environmental exposures.

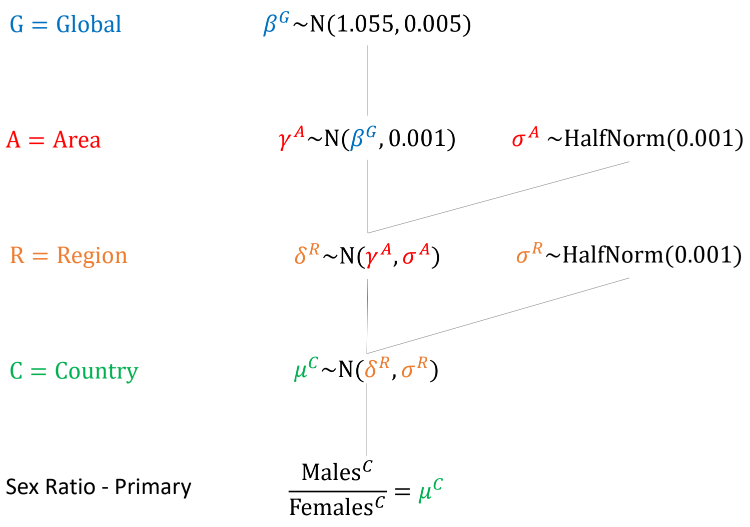

Priors

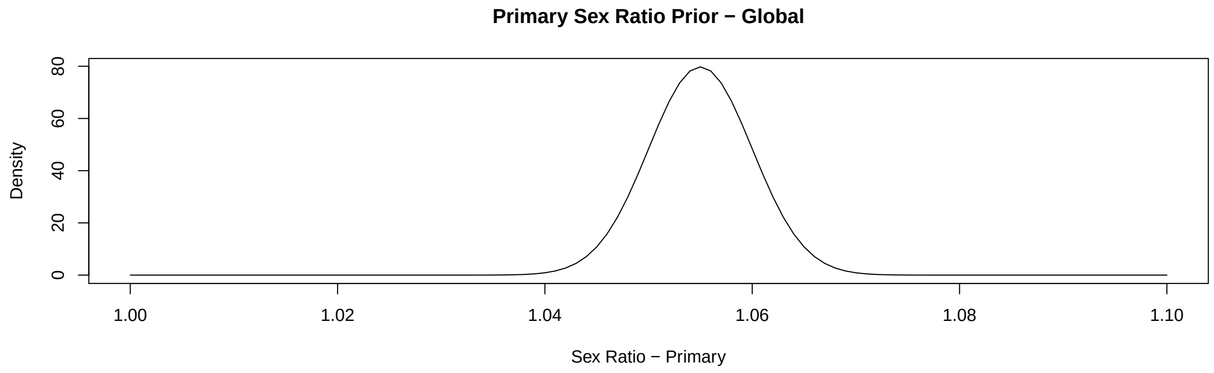

Model Implementation

We use the sex ratio to simulate fetal sex at conception. We also compare the primary sex ratio to the secondary sex ratio in order to simulate the probability of sex selective abortion.

References

Grech V, Savona-Ventura C, Vassallo-Agius P. Unexplained differences in sex ratios at birth in Europe and North America. BMJ 2002; 324(7344): 1010-1.

Mathews TJ, Hamilton BE. Trend Analysis of the Sex Ratio at Birth in the United States. National Vital Statistics Reports 2005; 53: 20.

Orzack SH, Stubblefield JW, Akmaev VR, et al. The human sex ratio from conception to birth. Proc Natl Acad Sci USA 2015; 112(16): E2102-11.

A.1.2.3 Twinning Rates

Overview

Twins are simulated in the model to more accurately represent the number of births in the population, and to account for increased risks of delivery complications that may occur with multiple fetuses. We do not model triplets or higher order multiples due to their very low incidence (Fellman 2009).

Data

Monozygotic twinning is thought to occur at a relatively constant rate of 3.5–4 per 1000 births across human populations (Hall 2003, Smits 2011). However, the rate of dizygotic twinning varies greatly around the world, leading to large differences in overall twinning rates, which range from about 6 per 1000 births in Asia, to 10-20 per 1000 births in Europe and the USA, to about 40 per 1000 births in parts of Africa (Hall 2003).

A study based on 150 Demographic and Health Surveys, conducted between 1987 and 2010 in 75 low and middle income countries (Smits 2011) found similar differences in twinning rates, with variation caused by dizygotic twinning rates. In developed countries, twinning rates have been increasing (Pison 2015). A study of trends in the US found that only about 1/3 of the increase was due to increasing maternal age, with the rest likely explained by increased use of infertility treatments (Martin 2012).

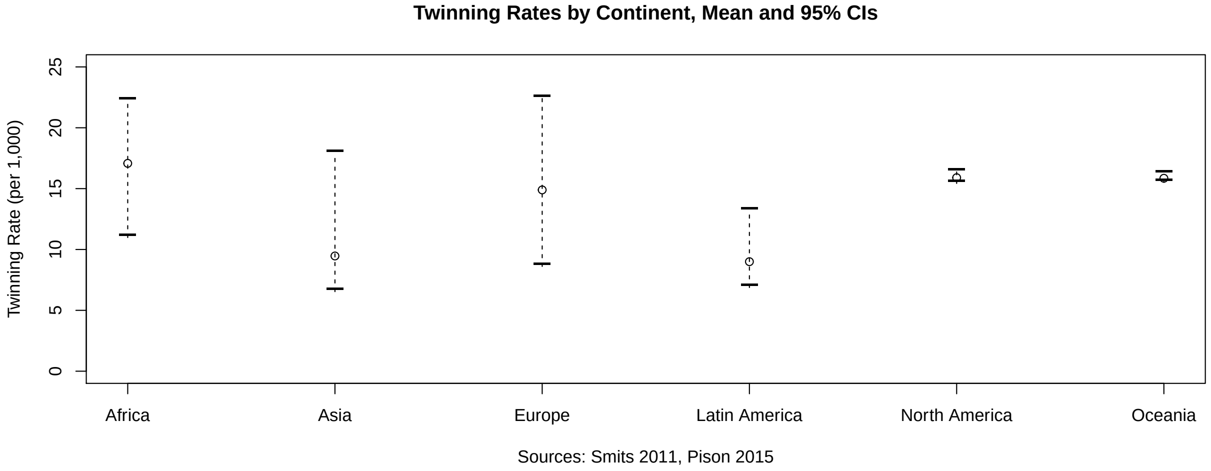

The number of twin pregnancies increases substantially with maternal age (Hoekstra 2008, Smits 2011), with estimated twinning rates increasing more than 4-fold between the ages 15 and 37 years with parity held constant (Bulmer 1970, Bortolus 1999).

Several studies have reported twinning rates (per 1,000) by maternal age:

Maternal Age	Monozygotic	Dizygotic	Country	Source
<20	5.4	3.6	Israel	Harlap 1979
20-24	5.1	5.8		
25-29	5.0	11.3		
30-34	5.1	8.3		
35-39	7.3	17.3		
$\geq$ 40	7.4	—		
$\leq$ 29	4.2	32.3	Nigeria	Nylander 1981
$\geq$ 30	4.7	72.8		
<25	3.7	1.9	India	Rao 1983
25-29	4.6	5.9		
30-34	1.1	10.7		

Maternal Age	Monozygotic	Dizygotic	Country	Source
≥ 35	1.0	12.0		
<19	3.6	2.5	Italy	Parazzini 1991
20-24	3.6	4.2		
25-29	4.2	5.4		
30-34	4.1	7.3		
35-39	4.5	8.1		
40-44	3.8	6.0		
≥ 45	4.7	—		
<20	0.0	2.26	India	Satija 2008
20-24	3.98	3.98		
25-29	3.19	11.46		
30-34	2.51	20.1		
<15	3.2	0.3	Brazil	Otta 2016
15-19	3.6	2.1		
20-24	3.9	4.2		
25-29	4.7	6.6		
30-34	4.9	10.1		
35-39	5.0	13.0		
40-44	4.2	12.5		
45+	9.1	35.1		

Parameters
We assumed that monozygotic twin rates are constant by maternal age. To model age-related dizygotic rates we used quadratic B-splines fit to the midpoint of the maternal age groups. The estimated dizygotic twinning rate at age 38 was used as the value for later maternal ages.

We set area-specific dizygotic priors for 'low' and 'high' twinning countries.

Spline Width: 30

Spline #	Knot Location	Africa	Asia	Europe	Latin America	North America	Oceania
1	20	$N(10,5)$	$N(2.5,1)$	$N(10,5)$	$N(2.5,1)$	$N(10,5)$	$N(10,5)$
2	30	$N(25,5)$	$N(10,3)$	$N(25,5)$	$N(10,3)$	$N(25,5)$	$N(25,5)$
3	40	$N(40,5)$	$N(20,3)$	$N(40,5)$	$N(20,3)$	$N(40,5)$	$N(40,5)$

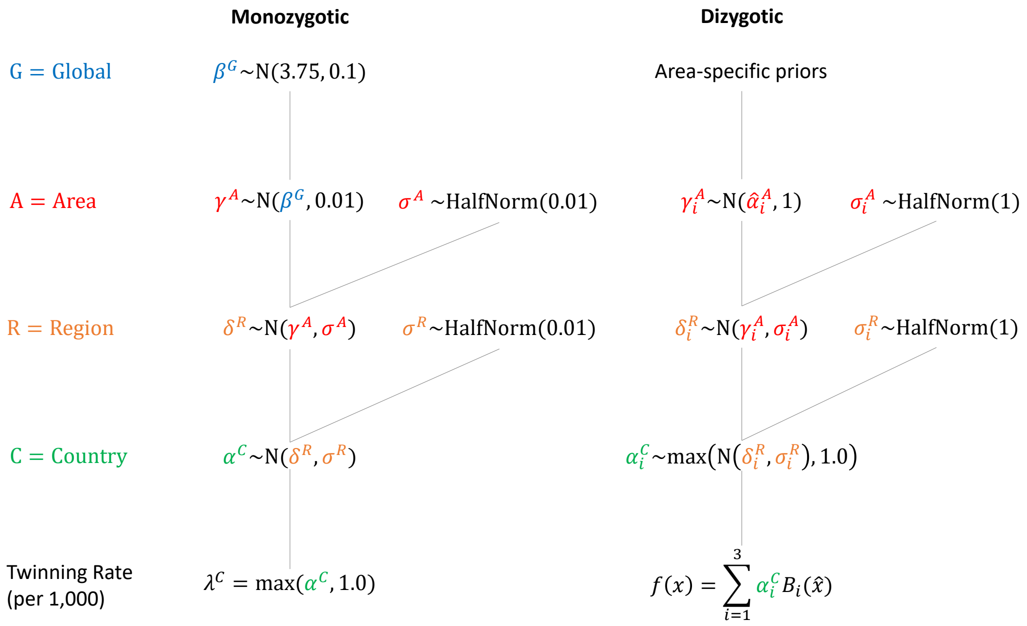

Priors

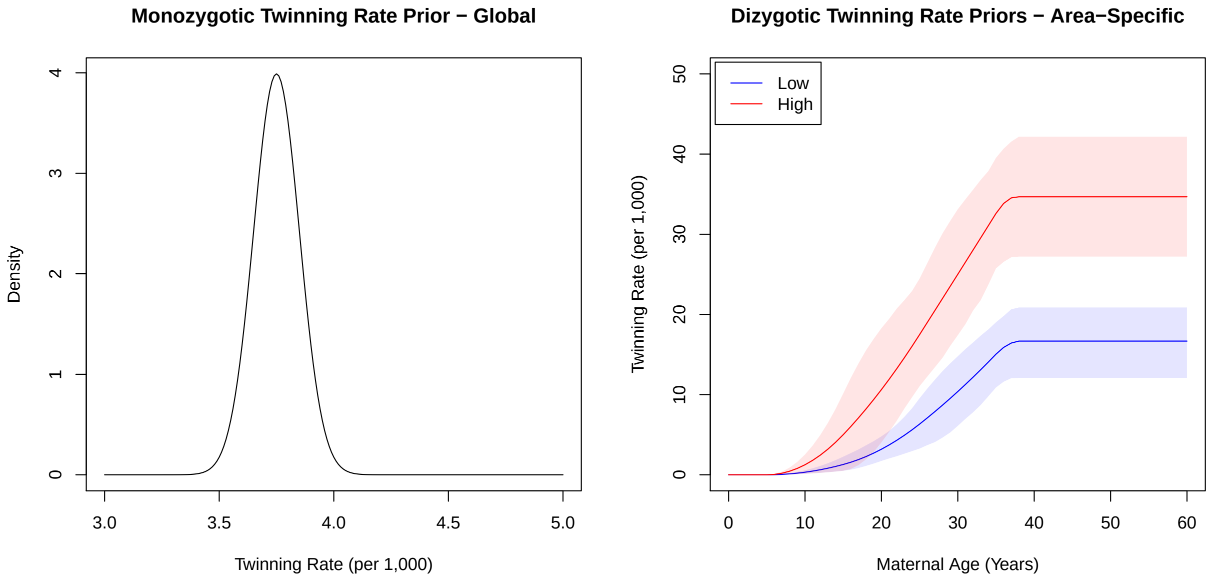

Model Implementation

At conception, the model simulates whether the pregnancy is a singleton or twins. If monozygotic twins are simulated, then a single sex is simulated based on the sex ratio and applied to both fetuses. If dizygotic twins are simulated then a sex is simulated for each fetus. We assume that sex-selective abortion does not apply to twins. Country-specific twinning rate estimates are included as calibration targets to help fit the model.

References

Bortolus R, Parazzini F, Chatenoud L, Benzi G, Bianchi MM, Marini A. The epidemiology of multiple births. Hum Reprod Update 1999; 5(2): 179-187.

Bulmer MG. The Biology of Twinning in Man. Oxford: Clarendon Press, 1970.

Fellman F, Eriksson AW. Statistical analyses of Hellin's law. Twin Res Hum Genet 2009; 12(2): 191-200.

Hall JG. Twinning. Lancet 2003; 362: 735–43.

Harlap S. Multiple births in former oral contraceptive users. Br J Obstet Gynaecol 1979; 86(7): 557-62.

Hoekstra C, Zhao ZZ, Lambalk CB, et al. Dizygotic twinning. Hum Reprod Update 2008; 14(1): 37-47.

Martin JA, Hamilton BE, Osterman MJK. Three Decades of Twin Births in the United States, 1980-2009. NCHS Data Brief 2012, No. 80. Available at: https://www.cdc.gov/nchs/data/databriefs/db80.pdf

Nylander PPS. Causes of high twinning frequencies in Nigeria. In: Nance WE, Allen G, Parisi P (eds). Twin Research. Part B: Biology and Epidemiology. New York: Alan R. Liss, 1978.

Otta E, Fernandes ES, Acquaviva TG, et al. Twinning and Multiple Birth Rates According to Maternal Age in the City of São Paulo, Brazil: 2003-2014. Twin Res Hum Genet 2016; 19(6): 679-686.

Parazzini F, Tozzi L, Mezzanotte G, et al. Trends in multiple births in Italy: 1955-1983. Br J Obstet Gynaecol 1991; 98(6): 535-9.

Pison G, Monden C, Smits J. Twinning Rates in Developed Countries: Trends and Explanations. Popul Dev Rev 2015; 41(4): 629-649.

Rao PS, Inbaraj SG, Muthurathnam S. Twinning rates in Tamilnadu. J Epidemiol Community Health 1983; 37(2): 117-20.

Smits J, Monden C Twinning across the Developing World. PLoS One 2011; 6(9): e25239.

Satija M, Sharma S, Soni RK, Sachar RK, Singh GP. Twinning and its correlates: community-based study in a rural area of India. Hum Biol 2008; 80(6): 611-621.

A.1.2.4 Miscarriage

Overview

The term 'miscarriage' in the model refers to spontaneous abortion. Ectopic pregnancy is modeled separately as a high risk condition with risks of morbidity/mortality. In contrast, we assume that spontaneous abortion poses no physical risks to the mother.

Data

Maternal age is a strong predictor of miscarriage. Andersen (2000) presents rates of spontaneous abortion by maternal age group from a population-based register linkage study in Denmark. After adjusting the denominator by subtracting all induced abortions from the number of pregnancies, the adjusted risk of miscarriage was calculated by age group. These estimates are thus conditional on no induced abortion.

The risk of miscarriage given previous miscarriages is also modeled, to take into account within-women clustering/high-risk women. Estimates were modeled using data from Risch 1988, which estimated a Relative Risk of miscarriage based on the number of previous miscarriages (NPM). The equation is: $(1 + NPM)^{\alpha}$, where α was estimated to be 1.01 (95% CI 0.83-1.18). Some of the estimated increased risk is likely due to older age, so we use calibration to estimate the overall risk of miscarriage, taking into account maternal age and previous number of miscarriages.

A systematic review by Avalos et al. (2012) reported risk of miscarriage by gestational age up to 20 weeks gestation. The distribution of gestational age at miscarriage across the 4 sources (Taylor 1970, Harlap 1980, Goldhaber and Fireman 1991, Li 2002) was averaged in order to estimate the distribution of gestational ages at which miscarriages occur. All miscarriages are assumed to occur by 6 months gestation. The midpoint (in weeks) of month of gestation was used and the following distribution is assumed in the model:

Month of Gestation	Cumulative Probability of Miscarriage | Miscarriage
2	0.2262
3	0.5866
4	0.8824
5	0.9684
6	1.0

Estimates of miscarriage in early pregnancy are especially difficult to ascertain as women may not know that they are pregnant. We therefore begin modeling the risk of miscarriage in the second month of gestation.

Parameters

We fit quadratic B-splines to the midpoints of the age groups. Spline Width: 40

Spline #	Knot Location	Height
1	8	$N(0.15, 0.02)$
2	22 $^1/_3$	$N(0.09, 0.02)$
3	37 $^1/_3$	$N(0.14, 0.02)$
4	52	$N(1.15, 0.08)$

We used the estimated probability of miscarriage at age 50 for older ages.

To model increased risk given a history of miscarriage, we base our prior on Risch 1988, which estimated α to be 1.01 (95% CI 0.83-1.18), which translates to $\alpha \sim N(1.01, 0.089)$. A maximum of 5 previous miscarriages is used when estimating increased risks of miscarriage.

	p(Spontaneous Abortion | Maternal Age)		NPM Relative Risk	
G = Global	$\beta_i^G \sim \mathrm{N}(\hat{\alpha}_i, \hat{\sigma}_i)$		$\beta^G \sim \mathrm{N}(1.01, 0.089)$	
A = Area	$\gamma_i^A \sim \mathrm{N}(\beta_i^G, 0.01)$	$\sigma_i^A \sim \mathrm{HalfNorm}(0.01)$	$\gamma^A \sim \mathrm{N}(\beta^G, 0.01)$	$\sigma^A \sim \mathrm{HalfNorm}(0.01)$
R = Region	$\delta_i^R \sim \mathrm{N}(\gamma_i^A, \sigma_i^A)$	$\sigma_i^R \sim \mathrm{HalfNorm}(0.01)$	$\delta^R \sim \mathrm{N}(\gamma^A, \sigma^A)$	$\sigma^R \sim \mathrm{HalfNorm}(0.01)$
C = Country	$\alpha_i^C \sim \max(\mathrm{N}(\delta_i^R, \sigma_i^R), 0)$		$\alpha^C \sim \mathrm{N}(\delta^R, \sigma^R)$	
B-Splines/RR	$f(x) = \sum_{i=1}^{4} \alpha_i^C B_i(\hat{x})$		$RR = (1 + \min(NPM, 5))^{\alpha^C}$	

Priors

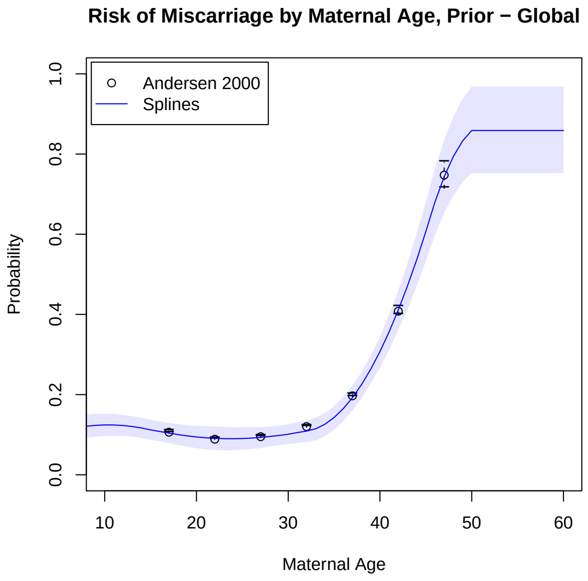

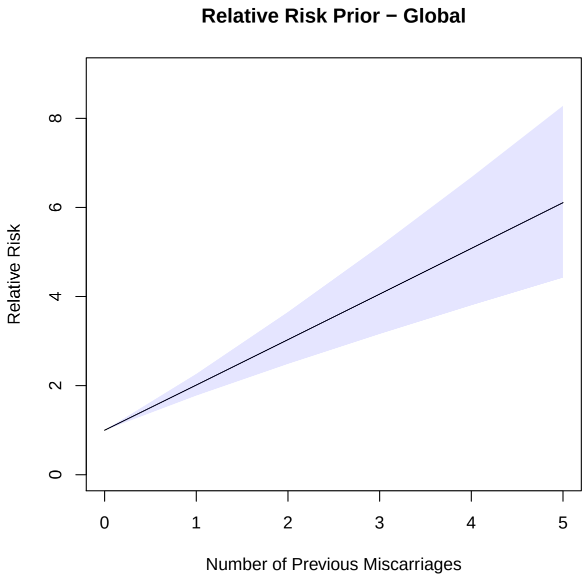

Model Implementation

Following conception in the model, the risk of miscarriage is simulated based on a woman's age and her history of previous miscarriages. If a miscarriage is simulated to happen, the gestational age at which it occurs is drawn from the distribution of months of gestation. We assume that there is no risk of maternal morbidity or mortality from miscarriage.

References

Andersen AM, Wohlfahrt J, Christens P, Olsen J, Melbye M. Maternal age and fetal loss: population based register linkage study. BMJ 2000; 320(7251): 1708-12.

Avalos LA, Galindo C, Li DK. A systematic review to calculate background miscarriage rates using life table analysis. Birth Defects Res A Clin Mol Teratol 2012; 94(6): 417-432.

Goldhaber MK, Fireman BH. The fetal life table revisited: spontaneous abortion rates in three Kaiser Permanente cohorts. Epidemiology 1991; 2(1): 33-39.

Harlap S, Shiono PH, Ramcharan S. A life table of spontaneous abortions and the effects of age, parity and other variables. In: Porter IH, Hook EB, editors. Human Embryonic and Fetal Death. New York: Academic Press, 1980.

Li DK, Odouli R, Wi S, et al. A population-based prospective cohort study of personal exposure to magnetic fields during pregnancy and the risk of miscarriage. Epidemiology 2002; 13(1): 9-20.

Risch HA, Weiss NS, Clarke EA, Miller AB. Risk factors for spontaneous abortion and its recurrence. Am J Epidemiol. 1988; 128(2): 420-30.

Taylor WF. The probability of fetal death. In: Fraser FC, McKusick VA, editors. Congenital malformations: proceedings of the Third International Conference, The Hague, The Netherlands, Sept 7-13, 1969. 2nd ed. Amsterdam: Excepta Medica, 1970.

A.1.2.5 Ectopic Pregnancy

Overview

Ectopic pregnancy is a pregnancy in which the fertilized ovum implants outside the intrauterine cavity, with more than 95% occurring in the fallopian tubes (Tenore 2000). Because these sites can not accommodate placental attachment or embryo growth, the potential for rupture and hemorrhage make ectopic pregnancy a high risk condition and the leading cause of maternal mortality in the first trimester (Tenore 2000, Lozeau 2005).

Prompt ultrasound evaluation is key in diagnosing ectopic pregnancy (Lozeau 2005). Appropriate treatment for patients with nonruptured ectopic pregnancy may include expectant management, medical management with methotrexate, or surgery. Surgical treatment is appropriate if ruptured ectopic pregnancy is suspected (Lozeau 2005).

Data

Incidence

Ectopic pregnancies occur in around 2% of pregnancies, with increasing risk for older women (Stulberg 2013, Jacob 2017, Ranji 2018). Andersen (2000) presents rates of ectopic pregnancy by maternal age group from a population-based register linkage study in Denmark. After adjusting the denominator by subtracting all induced abortions from the number of pregnancies, the adjusted risk of ectopic pregnancy was calculated by age group. A population-based study in France found similar effect sizes for increased risk of ectopic pregnancy by maternal age (Bouyer 2003).

Prior ectopic pregnancy is a strong risk factor for recurrent ectopic pregnancy, with recurrence rates reported from 10% to 25% (Panelli 2015, Chouinard 2019), or 10 times the risk of the general population. For example, Bhattacharya 2012 reports an adjusted hazard rate of 13.0 (95% CI 11.63-16.86) and Jacob 2017 reports an adjusted odds ratio of 8.17 (95% CI 4.87-13.69).

Although many women diagnosed with ectopic pregnancy have no identifiable risk factors, a number of other risk factors have been associated with ectopic pregnancy, including smoking, alcohol, tubal surgery or tubal damage from sexually transmitted infections, prior pelvic infection, IUD use, and pregnancy conceived by assisted reproduction (Li 2015, Panelli 2015, Jacob 2017, Assouni Mindjah 2018, Bronson 2018, Gaskins 2018, Asah-Opoku 2019). Some of these risk factors may be associated with maternal age. For example, increased risk of ectopic pregnancies in teenage women is most likely caused by pelvic inflammatory disease (Andersen 2000).

We model risk by maternal age for first-time ectopic pregnancy. For women with a previous ectopic pregnancy we model a risk of recurrence. Using a hierarchical model with country-specific effects allows us to account for different underlying risks not explicitly modeled, such as STI prevalence and assisted reproduction.

Morbidity/Mortality

Tubal pregnancies may either diminish in size and spontaneously resolve, or continue to grow and eventually lead to tubal rupture. However, there are no reliable clinical, sonographic, or biological markers (e.g. serum beta hCH or serum progesterone) that can predict rupture of a tubal ectopic pregnancy (Kumar 2015). In addition, the time from conception to tubal rupture is short, often occurring within 6-8 weeks of conception (Bronson 2018).

Reported tubal rupture rates range from population-based reports of 18% (Job-Spira 1999), to clinic/physician office-based reports of 32% (Bickell 2004), to hospital-based reports of 80% and higher (Singh 1992). For example, an analysis of 80 cases of ectopic pregnancy in Pakistan found a rupture rate over 90% (Ashfaq 2017), likely due to selection bias of ruptured cases presenting at hospital.

In contrast, a study of women on Medicaid in the US found that ectopic pregnancy-associated complications occurred in only 11% of cases, with a mortality ratio of 0.48 per 100,000 live births (Stulberg 2016). Another study found that the ectopic pregnancy mortality ratio in the US declined from 1.15 to 0.50 deaths per 100,000 live births between 1980-1984 and 2003-2007 (Creanga 2011). In a review of African developing countries, a majority of hospital-based studies reported ectopic pregnancy case fatality rates (CFR) of 1-3%

(10 times higher than reported in developed countries), with late diagnosis and emergency surgical treatment likely accounting for the high fatality rates (Goyaux 2003).

Because CFR estimates are based on diagnosed cases, we condition CFR on rupture in the model. We assume that the probability of rupture is around 10-20%, based on the population-based studies (Job-Spira 1999, Stulberg 2016). We assume that given rupture, the CFR is around 3-5% in the absence of treatment (i.e. natural history). Although tubal rupture seriously affects the current health of the mother, it seems to have no effect on subsequent fertility (Job-Spira 1999). We therefore assume that women who survive a ruptured ectopic pregnancy have no continuing morbidity, besides the increased risk of recurrent ectopic pregnancy in the future.

Parameters

Incidence

We fit quadratic B-splines to the midpoints of the age groups, based on data from Andersen 2000. Spline Width: 40

Spline #	Knot Location	Height
1	8	$N(0.03, 0.002)$
2	$22\ ^1/_3$	$N(0.01, 0.002)$
3	$37\ ^1/_3$	$N(0.05, 0.002)$
4	52	$N(0.11, 0.015)$

We extended the probability from age 50 to older ages.

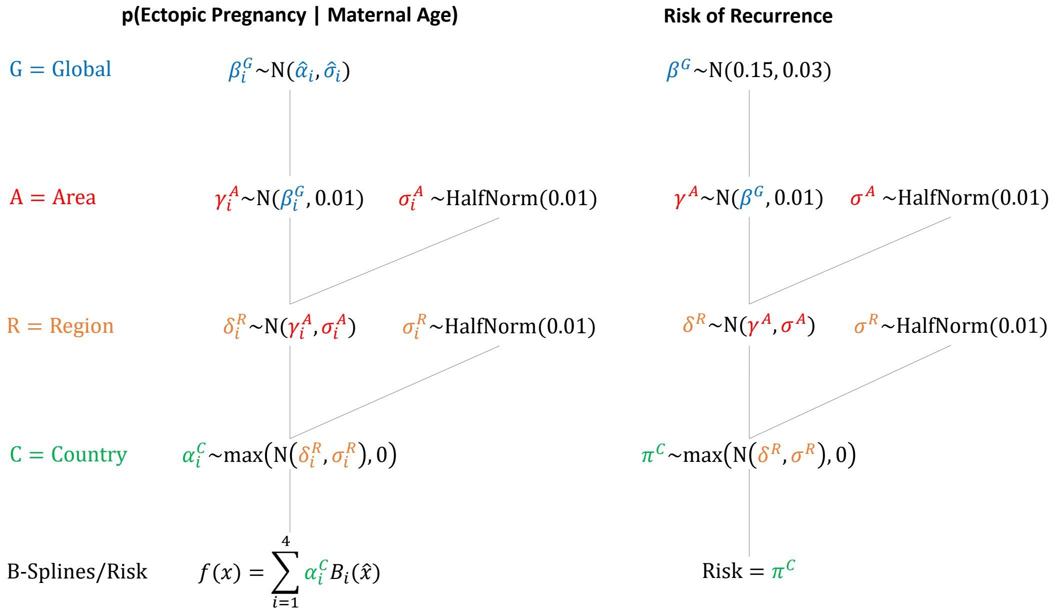

Morbidity/Mortality

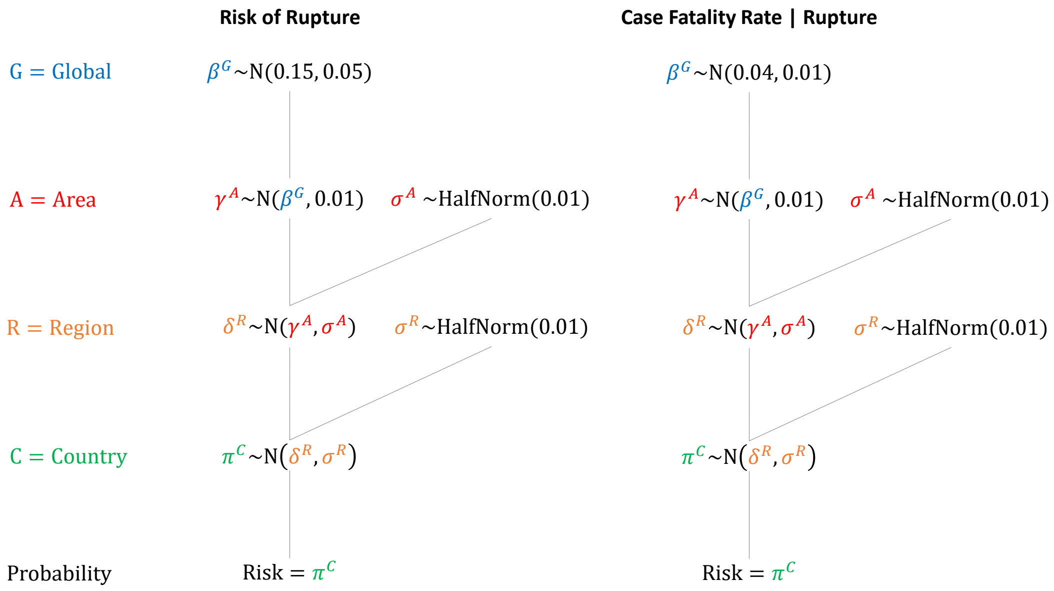

Priors

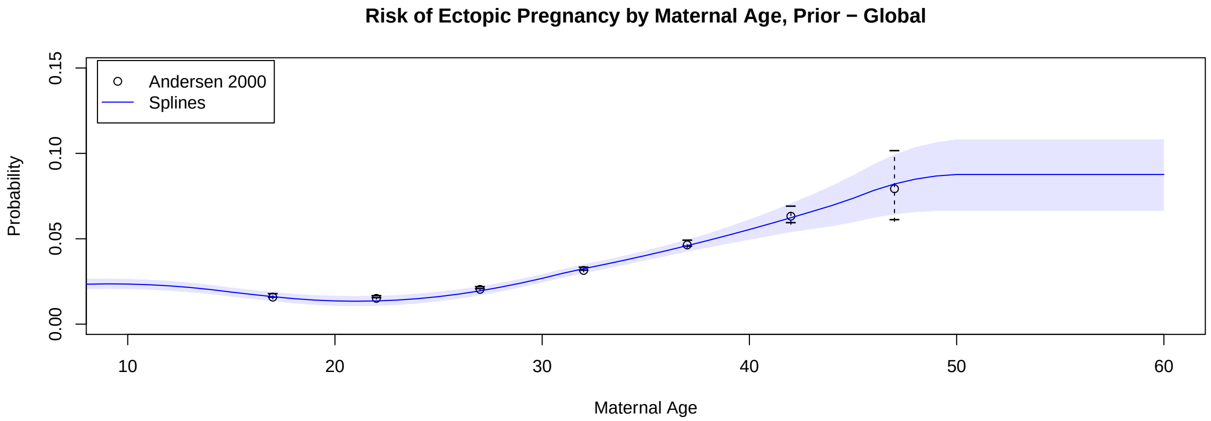

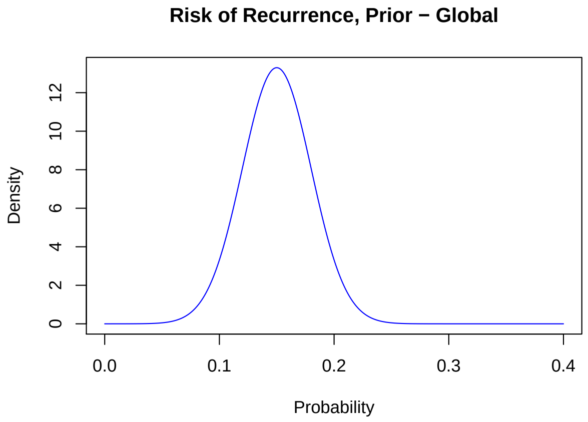

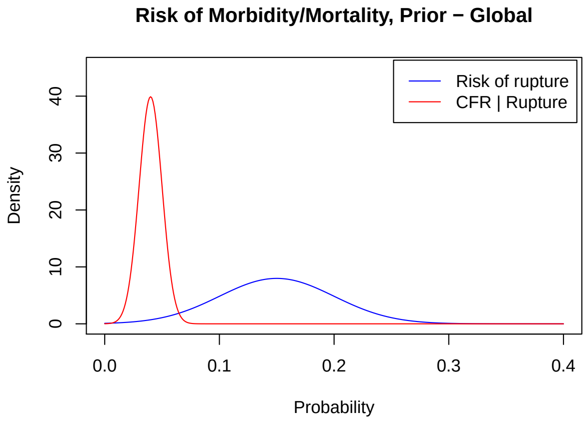

Model Implementation

Following conception in the model, the risk of ectopic pregnancy is simulated based on a woman's age and her history of previous ectopic pregnancy. We assume that ectopic pregnancies happen during the first trimester (i.e. month 3) in the model.

References

Andersen AM, Wohlfahrt J, Christens P, Olsen J, Melbye M. Maternal age and fetal loss: population based register linkage study. BMJ 2000; 320(7251): 1708-12.

Asah-Opoku K, Oppong SA, Ameme DK, et al. Risk factors for ectopic pregnancy among pregnant women attending a tertiary healthcare facility in Accra, Ghana. Int J Gynaecol Obstet 2019; doi: 10.1002/ijgo.12928.

Ashfaq S, Sultan S, Aziz S, Irfan MM, Hasan M, Siddique A. Ectopic Pregnancy With Tubal Rupture: An Analysis Of 80 Cases. J Ayub Med Coll Abbottabad 2017; 29(2): 254-257.

Assouni Mindjah YA, Essiben F, Foumane P, Dohbit JS, Mboudou ET. Risk factors for ectopic pregnancy in a population of Cameroonian women: A case-control study. PLoS One 2018; 13(12): e0207699.

Bhattacharya S, McLernon DJ, Lee AJ, Bhattacharya S. Reproductive outcomes following ectopic pregnancy: register-based retrospective cohort study. PLoS Med 2012; 9(6): e1001243.

Bickell NA, Bodian C, Anderson RM, Kase N. Time and the risk of ruptured tubal pregnancy. Obstet Gynecol 2004; 104(4): 789-94.

Bouyer J, Coste J, Shojaei T, et al. Risk factors for ectopic pregnancy: a comprehensive analysis based on a large case-control, population-based study in France. Am J Epidemiol 2003; 157(3): 185-94.

Bronson R. Ectopic pregnancy - still a challenge. Fertil Steril 2018; 110(7): 1265-1266.

Chouinard M, Mayrand MH, Ayoub A, Healy-Profitós J, Auger N. Ectopic pregnancy and outcomes of future intrauterine pregnancy. Fertil Steril 2019; 112(1): 112-119.

Creanga AA, Shapiro-Mendoza CK, Bish CL, Zane S, Berg CJ, Callaghan WM. Trends in ectopic pregnancy mortality in the United States: 1980-2007. Obstet Gynecol 2011; 117(4): 837-43.

Gaskins AJ, Missmer SA, Rich-Edwards JW, Williams PL, Souter I, Chavarro JE. Demographic, lifestyle, and reproductive risk factors for ectopic pregnancy. Fertil Steril 2018; 110(7): 1328-1337.

Goyaux N, Leke R, Keita N, Thonneau P. Ectopic pregnancy in African developing countries. Acta Obstet Gynecol Scand 2003; 82(4): 305-12.

Jacob L, Kalder M, Kostev K. Risk factors for ectopic pregnancy in Germany: a retrospective study of 100,197 patients. Ger Med Sci 2017; 15: Doc19.

Job-Spira N, Fernandez H, Bouyer J, Pouly JL, Germain E, Coste J. Ruptured tubal ectopic pregnancy: risk factors and reproductive outcome. Results of a population-based study in France. Am J Obstet Gynecol 1999; 180: 938–44.

Kumar V, Gupta J. Tubal ectopic pregnancy. BMJ Clin Evid 2015; 11: 1406.

Li C, Zhao WH, Zhu Q, et al. Risk factors for ectopic pregnancy: a multi-center case-control study. BMC Pregnancy Childbirth 2015; 15: 187.

Lozeau AM, Potter B. Diagnosis and management of ectopic pregnancy. Am Fam Physician 2005; 72(9): 1707-14.

Panelli DM, Phillips CH, Brady PC. Incidence, diagnosis and management of tubal and nontubal ectopic pregnancies: a review. Fertil Res Pract 2015; 1: 15.

Ranji GG, Usha Rani G, Varshini S. Ectopic Pregnancy: Risk Factors, Clinical Presentation and Management. J Obstet Gynaecol India 2018; 68(6): 487-492.

Singh KB, Poole CA, Otterson WN, Dunnihoo DR, Bairnsfather LE, Long DC. Characteristics of indigent women with ruptured and unruptured tubal pregnancies. J Reprod Med 1992; 37: 745–8.

Stulberg DB, Cain LR, Dahlquist I, Lauderdale DS. Ectopic pregnancy rates in the Medicaid population. Am J Obstet Gynecol 2013; 208(4): 274.e1-7.

Stulberg DB, Cain L, Dahlquist IH, Lauderdale DS. Ectopic pregnancy morbidity and mortality in low-income women, 2004-2008. Hum Reprod 2016; 31(3): 666-71.

Tenore JL. Ectopic pregnancy. Am Fam Physician 2000; 61(4): 1080-8.

A.1.2.6 Stillbirths

Overview

The WHO recommends that stillbirth be defined as a baby born with no signs of life at or after 28 weeks' gestation (WHO website). Other definitions are based on birthweight. However, it is argued that a gestational age threshold is more appropriate as it is a better predictor of maturity (and hence viability), with many fetuses at risk of stillbirth having preceding fetal growth restriction (Mohangoo 2013, Lawn 2016). Intrapartum stillbirth is a death which occurs after the onset of labour but before birth.

Data

The estimated global stillbirth rate in 2015 was 18.4 per 1000 births, down from 24.7 in 2000, with an estimated 2.6 million stillbirths per year (Blencowe 2016). It is estimated that 98% of all stillbirths occur in low-income and middle-income countries, with 77% in south Asia and sub-Saharan Africa (Blencowe 2016). Stillbirth rates are estimated to range from 3.4 per 1000 births in developed regions to 28.7 in sub-Saharan Africa, with intrapartum stillbirths estimated to account for 50% of stillbirths globally, ranging from 10% in developed countries to 60% in Southern Asia (Lawn 2016).

An analysis of 18 countries with reliable data found that congenital abnormalities accounted for a median of only 7.4% of stillbirths (Lawn 2016). Thus, disorders associated with many stillbirths are potentially modifiable and often coexist, such as maternal infections, non-communicable diseases, and nutrition and lifestyle factors (Lawn 2016).

Maternal age (older than 35 years, or younger than 16 years) is also associated with increased risk of stillbirth (Lawn 2016). Andersen (2000) presents rates of stillbirth by maternal age group from a population-based register linkage study in Denmark. After adjusting the denominator by subtracting all induced abortions from the number of pregnancies, the adjusted risk of stillbirth was calculated by age group, resulting in rates around 3-4 per 1,000. Due to the high quality of obstetric and neonatal care in Denmark, we assume that intrapartum stillbirths are rare in this population, (Lawn 2016) and use these estimates as priors for rates of antepartum stillbirth.

Other risk factors for stillbirth include infections during pregnancy, such as malaria and syphilis (Lawn 2016). Non-communicable diseases, especially hypertension and diabetes also have an impact, with about 10% of stillbirths globally attributed to these disorders (Lawn 2016). Pregnancy lasting longer than 42 weeks is associated with increased risk, accounting for an estimated 14% of stillbirths worldwide (Lawn 2016). We do not model these risk factors explicitly, but by using a hierarchical modeling approach we can implicitly account for these country-specific factors.

A systematic review of over 30 million birth outcomes found that male babies are at a higher risk of stillbirth, with a relative risk of 1.10 (95% CI: 1.07-1.13) (Mondal 2014). Probable explanations include X-linked congenital conditions, increased risk of preterm labour, and poor fetal growth for male babies (Lawn 2016).

For intrapartum stillbirths, we model the risk related to obstructed labor and pre-eclampsia/eclampsia. For example, a review of studies found that prolonged deliveries which caused obstetric fistula also resulted in stillbirth for 78% to 96% of patients (Tebeu 2012). Another systematic review found the reported frequency of stillbirth associated with obstetric fistula ranged from 32.3% to 100%, with estimates from the largest studies around 92% (Cowgill 2015). Pre-eclampsia and eclampsia may contribute to poor fetal growth and have an estimated population attributable risk for stillbirths of about 5% (Lawn 2016). As maternal age may be associated with these pregnancy complications, these increased risks may explain some of the association between maternal age and stillbirths.

In summary, we model the risk of stillbirth from 3 general causes:

Stillbirth Type	Modeled Cause
Antepartum	Hierarchical spline model accounts for congenital risk, maternal age, and other country-specific risk factors such as infections and NCDs
	Relative risk for male fetuses
Intrapartum	Prolonged/obstructed labour

Stillbirth Type	Modeled Cause
Antepartum/ Intrapartum	Pre-eclampsia/eclampsia

We include country-specific estimates of stillbirths as calibration targets.

Parameters

We fit quadratic B-splines to the midpoints of maternal age groups, based on stillbirth data from Andersen 2000. Using a hierarchical model allows us to adjust these estimates to account for other country-specific risk factors.

Spline Width: 40

Spline #	Knot Location	Height
1	8	$N(6, 0.75)$
2	22 $^1/_3$	$N(4, 0.1)$
3	37 $^1/_3$	$N(4.5, 0.3)$
4	52	$N(3.8, 2)$

We extended the probability from age 50 to older ages.

We model a relative risk for males based on the estimated relative risk from Mondal 2014: RR = 1.10 (95% CI: 1.07-1.13).

We used hierarchical models to account for risk of stillbirths from OL and HTN (see delivery complications…) We assume no excess risk for males from these intrapartum causes.

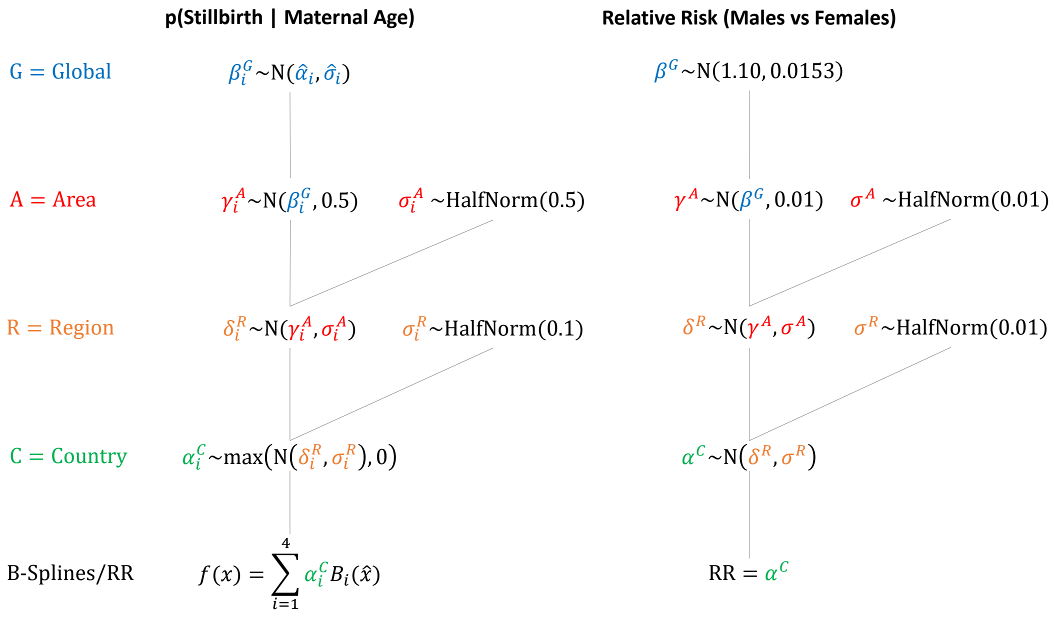

Priors

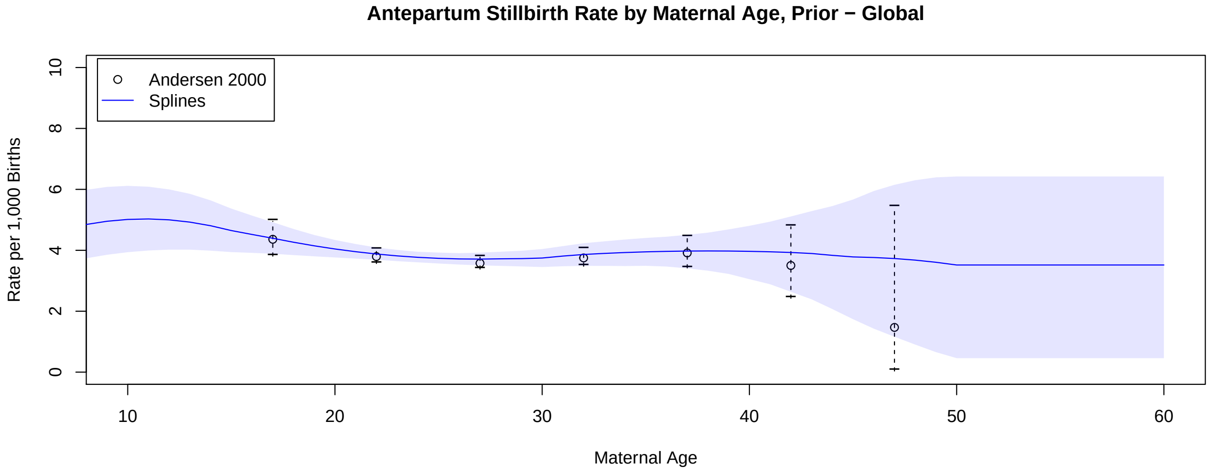

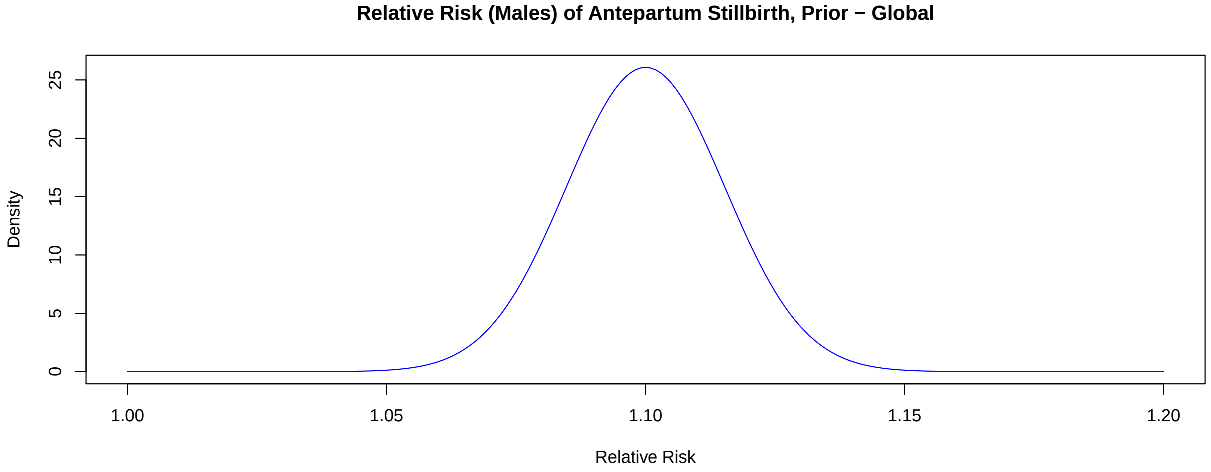

Model Implementation

Antepartum stillbirths are simulated based on country-specific risks modified by maternal age and fetus sex. Intrapartum stillbirths are simulated as a result of delivery complications (i.e. prolonged labour and pre-eclampsia/eclampsia). We assume that the risks of other delivery complications (i.e. post-partum hemorrhage and sepsis) are the same for both stillbirths and live births.

References

Andersen AM, Wohlfahrt J, Christens P, Olsen J, Melbye M. Maternal age and fetal loss: population based register linkage study. BMJ 2000; 320(7251): 1708-12.

Blencowe H, Cousens S, Jassir FB, Say L, Chou D, Mathers C, et al. National, regional, and worldwide estimates of stillbirth rates in 2015, with trends from 2000: a systematic analysis. Lancet Glob Health 2016; 4(2): e98-e108.

Cowgill KD, Bishop J, Norgaard AK, Rubens C, Gravett MG. Obstetric fistula in low-resource countries: an under-valued and under-studied problem–systematic review of its incidence, prevalence, and association with stillbirth. BMC Pregnancy Childbirth 2015; 15: 193.

Lawn JE, Blencowe H, Waswa P, Amouzou A, Mathers C, Hogan D, et al. Stillbirths: rates, risk factors, and acceleration towards 2030. Lancet 2016; 387(10018): 587-603.

Mohangoo AD, Blondel B, Gissler M, Velebil P, Macfarlane A, Zeitlin J. International comparisons of fetal

and neonatal mortality rates in high-income countries: should exclusion thresholds be based on birth weight or gestational age? PLoS One 2013; 8: e64869.

Mondal D, Galloway TS, Bailey TC, Mathews F. Elevated risk of stillbirth in males: systematic review and meta-analysis of more than 30 million births. BMC Med 2014; 12: 220.

Tebeu PM, Fomulu JN, Khaddaj S, de Bernis L, Delvaux T, Rochat CH. Risk factors for obstetric fistula: a clinical review. Int Urogynecol J 2012; 23: 387–94.

World Health Organization. Stillbirths. https://www.who.int/maternal_child_adolescent/epidemiology/stillbirth/en/

A.1.2.7 Month of Delivery

Overview
This parameter refers to the gestational age at delivery (in months).

Data
Data are based on the fetal lifetable by Goldhaber and Fireman (1991). Estimates of live births from Table 2 were converted into cumulative probabilities of birth by gestational month:

Gestational Month	Cumulative Prob. Of Delivery
2	0
3	0
4	0
5	0.0005
6	0.0018
7	0.0044
8	0.0263
9	0.5999
10	1.0

Parameters
For simplicity, we do not explore any uncertainty surrounding this parameter and assume that the probabilities are fixed.

Model Implementation
If a pregnancy is simulated to end in a delivery (i.e. live birth or stillbirth), the month of delivery is drawn from the table of probabilities. We currently do not differentiate between delivery times for singleton and twins, although twins are generally born earlier.

References

Goldhaber MK, Fireman BH. The fetal life table revisited: spontaneous abortion rates in three Kaiser Permanente cohorts. Epidemiology 1991; 2: 33-39.

A.1.2.8 Lactational Amenorrhea (LAM)

Overview

Breastfeeding can play an important role in improving maternal and child health by increasing birth intervals due to the temporary contraceptive effect from lactation. This method, called the lactational amenorrhea method (LAM), is defined in the 1988 Bellagio Consensus as the informed use of breastfeeding as a contraceptive by a woman who is still amenorrheic and who does not feed her baby with supplements for up to 6 months after delivery (Kennedy 1989). Correct use of LAM means adherence to the three LAM criteria of 1) no resumption of menses; 2) not supplementing regularly or allowing long periods without breastfeeding, day or night; and 3) baby not aged older than 6 months, with some monthly deviation allowed from the feeding criteria (Labbok 1997). Incorrect LAM use is defined as continued reliance on LAM when any of these three criteria are no longer met.

Virtually all women, in a short period of time postpartum, will be amenorrheic, regardless of their breastfeeding status (Bracher 1992). The high efficacy is due in part to the fact that most women, whether contracepting or not, will be anovulatory and cannot become pregnant in the first 6-12 weeks postpartum (Bracher 1992; Labbok 1997). However, this protection declines with time, and the risk of return of menses and ovulation, and subsequent risk of pregnancy, differs based on breastfeeding status. Breastfeeding women have longer durations of amenorrhea than non-breastfeeders, and breastfeeding women who delay supplementation and/or nurse more frequently have the longest duration of amenorrhea. Among those breastfeeding, women who are fully breastfeeding have ovulation delayed longer than women who have begun supplementing (Bender 1998).

Several studies show that up to 50% of women ovulated before resumption of menses (Bender 1998), so women could be experiencing amenorrhea yet still be at risk for pregnancy, though this risk is small. Once menstruation has returned, breastfeeding can no longer be relied upon to protect the nursing mother against pregnancy, as mothers who are menstruating during breastfeeding have a chance of contraception that is less than normally cycling non-lactating women, but high enough to require alternative forms of contraception (Short 1991, Howie 1993, Trussell 2007).

The impact of the timing of supplemental feeding on the efficacy of lactational amenorrhea and return of menses for the mother is debated. Two studies suggest that LAM can be relied upon as the sole method of highly effective contraception up to six months, irrespective of when supplements are introduced to the infant's diet (Short 1991, Kennedy 1992). However, while partial breastfeeding still suppresses fertility, supplementation reduces the child's need to suckle, which decreases breast stimulus and quickens menstruation (Kennedy 1992). Although supplementation with other foods decreases protective effects of breastfeeding against pregnancy, women who continue to breastfeed beyond 6 months may still experience subfertility, albeit at a rate of pregnancy higher than that experienced at 6 months (Kennedy 1996). Two studies find that after 9 months postpartum, there is no protective effect of breastfeeding (Howie 1993, Dihidar 2002).

Data

LAM is highly effective against ovulation and pregnancy, with the maximum benefit for spacing and greatest protection against ovulation and pregnancy amongst women who are exclusively or fully breastfeeding (Kennedy 1992, Bender 1998, Van der Wijden 2015). Several studies find that with correct use among amenorrheic women who are exclusively or fully breastfeeding, efficacy rates of LAM as a contraceptive method are 98%. A Cochrane review conducted by Van der Wijden et al. (2015) reported life table pregnancy rates of LAM users ranging from 0.45%-2.45% at 6 months. LAM is equally effective in a variety of healthcare settings, both in developed and developing countries (Labbok 1997). Kennedy (1992) found cumulative pregnancy rates of 2.9 and 5.9 per 100 women at 6 and 12 months, respectively, compared with 0.7 among LAM users at 6 months. Other estimates find that the efficacy for correct use of LAM is 98.5% at 6 months and 92.2% at 12 months (Labbok 1997). Another study in India of 5,161 households found that among women who were breastfeeding compared to not breastfeeding, the relative risk of return to menses at time t was 0.47 (SE: 0.15) for $0 < t < 6$ months and 0.93 (SE: 0.36) for $6 < t < 9$ months (Dihidar 2002).

Study	6-month efficacy	9-month efficacy	12-month efficacy
Bellagio consensus 1988	More than 98% for fully or nearly fully breastfeeding	—	—
Labbok 1997	98.5% for correct use	—	—
	98.3% for incorrect use	—	92.2%
	98.0% for lactational amenorrhea	—	91.2%
Kennedy 1992	99.3% for ‘correct use’	—	—
	97.1% for lactational amenorrhea	—	94.1%
	92.4% for only baby less than 6 mos	—	82.8%
Ramos 1996	99.0% for correct use	—	—
	97.5% for incorrect use	—	—
	98.5% for all women during correct or incorrect use	—	—
	99.1% for lactational amenorrhea	97.4%	97.4%
Short 1991	98.3%	—	93.0%
Van der Wijden 2015	97.55, 99.55% in two controlled studies of LAM	—	—
	92.5-100% in six uncontrolled studies of LAM	—	—
	98.8-99.12% among fully breastfeeding women who were amenorrheic who were not aware of LAM as a contraceptive method	—	—

Parameters

As a conservative assumption, we assume no protective efficacy of LAM after 9 months. Based on estimates from the literature, we assume a LAM efficacy for exclusive breastfeeding of around 98% up to 6 months post-partum (i.e. failure rate of 2%), and around 97% up to 9 months post-partum. For women who are non-exclusively breastfeeding we model a relative rate of pregnancy at 6 months and 9 months based on estimates from Dihidar 2002.

When drawing parameters, we constrain the failure rate/relative rate for 0-6 months to be lower than for 6-9 months.

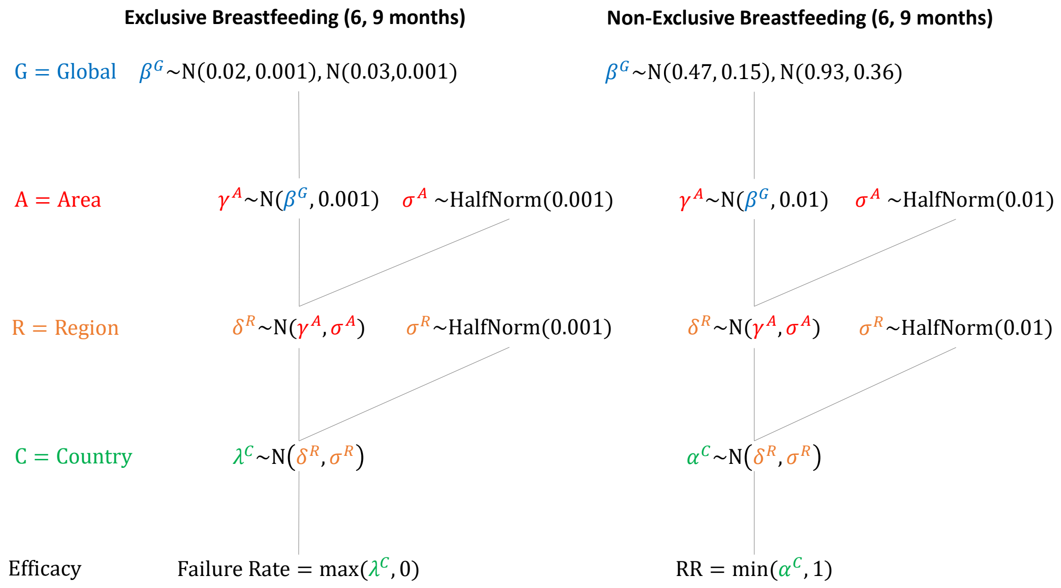

Priors

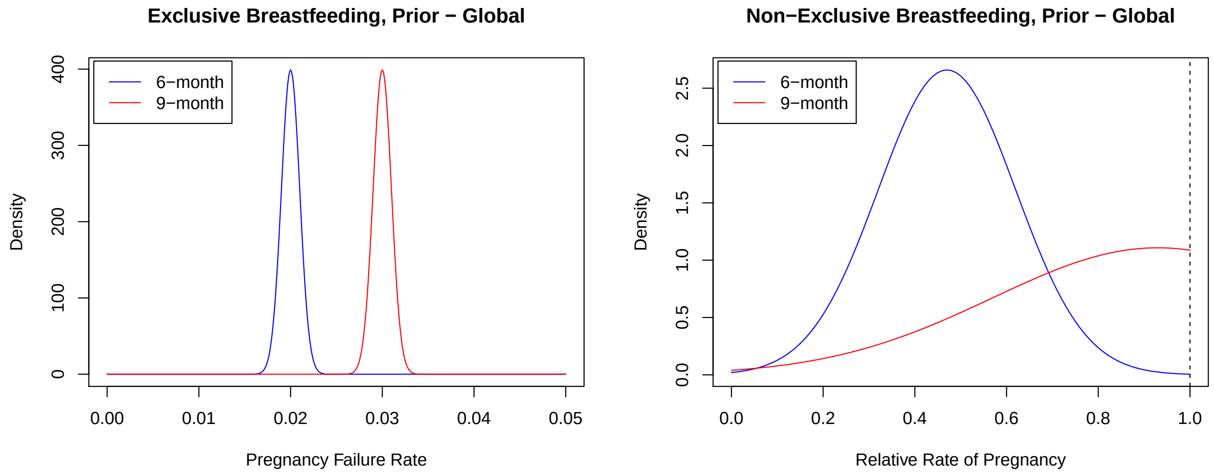

Model Implementation

At delivery, a woman's breastfeeding status (exclusive/non-exclusive) and duration (months) are simulated (see Breastfeeding). If the woman breastfeeds exclusively, the failure rate of LAM is used, converted to monthly probabilities assuming a constant failure rate over the relevant period (6 months or 9 months). If a woman breastfeeds non-exclusively, the associated relative rate of pregnancy is applied to the age-specific pregnancy rate. We assume that the efficacy of LAM and contraceptive methods are independent.

References

Bender DE, Dusch E, McCann MF. From efficacy to effectiveness: selecting indicators for a community-based lactational amenorrhea method promotion programme. J Biosoc Sci 1998; 30(2): 193-225.

Bracher M. Breastfeeding, lactational infecundity, contraception and the spacing of births: implications of the Bellagio Consensus Statement. Health Transit Rev 1992; 2(1): 19-47.

Dihidar S, Bhattacharya BN, Mukherjee KL. Description of breastfeeding practices among poorer sections in Calcutta Metropolitan Area and its impact on postpartum infecundity. J Child Health Care 2002; 6(3): 203-19.

Howie PW. Natural regulation of fertility. Br Med Bull 1993; 49(1): 182-199.

Kennedy KI, Visness CM. Contraceptive efficacy of lactational amenorrhoea. Lancet 1992; 339: 227-30.

Kennedy KI, Labbok MH, Van Look PF. Lactational amenorrhea method for family planning. Int J Gynaecol Obstet 1996; 54(1): 55-57.

Kennedy KI, Rivera R, McNeilly AS. Consensus statement on the use of breastfeeding as a family planning method. Contraception 1989; 39(5): 477-96.

Labbok MH, Hight-Laukaran V, Peterson AE, Fletcher V, von Hertzen H, Van Look PF. Multicenter study of the Lactational Amenorrhea Method (LAM): I. Efficacy, duration, and implications for clinical application. Contraception 1997; 55(6): 327-336.

Ramos R, Kennedy KI, Visness CM. Effectiveness of lactational amenorrhoea in prevention of pregnancy in Manila, the Philippines: non-comparative prospective trial. BMJ 1996; 313: 909-12.

Short RV, Lewis PR, Renfree MB, Shaw G. Contraceptive effects of extended lactational amenorrhoea: beyond the Bellagio Consensus. Lancet 1991; 337: 715-7.

Trussell J. Contraceptive efficacy. In Hatcher RA, Trussell J, Nelson AL, Cates W, Stewart FH, Kowal D. Contraceptive Technology: Nineteenth Revised Edition. New York NY: Ardent Media, 2007.

Van der Wijden C, Manion C. Lactational amenorrhoea method for family planning. Cochrane Database Syst Rev 2015; CD001329.

World Health Organization Task Force on Methods for the Natural Regulation of Fertility. The World Health Organization multinational study of breast-feeding and lactational amenorrhea. III. Pregnancy during breast-feeding. Fertil Steril 1999; 72(3): 431-40.

A.1.2.9 Menopause

Overview
The transition into natural menopause typically begins in a woman's mid-40s, when the function of the ovaries becomes inconsistent (Treloar 1974, MicKinlay 1992). While age at natural menopause is a complex trait with high heritability (Shi 2016), older age at menopause typically reflects good overall health, whereas early menopause - generally defined as occurring before age 40 - can reflect poorer health (Schmidt 2017).

Data
A study of over 3,000 women from five racial/ethnic groups in the United States found that the median age of natural menopause was 52.5 years, and that women with higher education levels and good overall health tended to have a later age of menopause (Gold 2013). The study also found that menopausal age did not vary by race/ethnicity after controlling for behavioral and health variables (Gold 2013). For example, smoking is most consistently associated with earlier menopausal age, with studies showing that women who smoke stop menstruating an average of 1-2 years earlier than comparable nonsmokers (Gold 2011, Butts 2014).

A systematic review and meta-analysis of 46 studies across 24 countries found a mean age of menopause of 48.8 years (95% CI 48.3-49.2), with lower ages in African, Latin American, Asian, and Middle Eastern countries, and higher ages in Europe, Australia, and the US (Schoenaker 2014). Higher education was associated with later age at menopause, with a weighted mean difference of 0.64 years (95% CI 0.26-1.02) between women with high vs low education (Schoenaker 2014).

Here we present the mean ages by continent as reported in Figure 2, Schoenaker 2014:

Continent	Mean Age (95% CI)
Africa	48.38 (48.12 to 48.65)
Asia	48.75 (48.08 to 49.42)
Australia	51.25 (49.75 to 52.76)
Europe	50.54 (50.04 to 51.05)
Latin America	47.24 (45.88 to 48.60)
Middle East	47.37 (46.91 to 47.84)
USA	49.11 (48.78 to 49.43)

Parameters
To account for country-specific factors we use a hierarchical model with the following area-specific priors:

Area	Prior
Africa	$N(48.38, 0.135)$
Asia	$N(48.75, 0.342)$
Europe	$N(50.54, 0.258)$
Latin America	$N(47.24, 0.694)$
North America	$N(49.11, 0.166)$
Oceania	$N(51.25, 0.768)$

Given the relatively small effect of education on age of menopause we do not include parameters to account for the impact of education in the interest of parsimony.

Gold 2013 plots estimated cumulative distributions of final menstrual period by age, showing a sigmoid curve from ages 42 to 60, implying a standard deviation of around 3. While age at menopause is generally skewed to the left (i.e. towards earlier ages), for simplicity we use a normal distribution. We impose a ceiling of age 60 on sampled age at menopause.

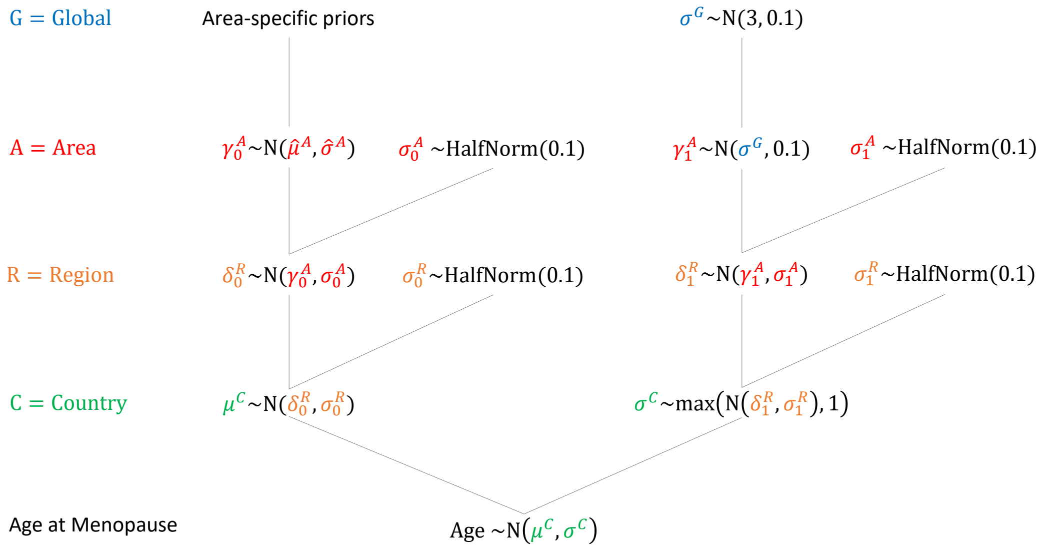

Priors

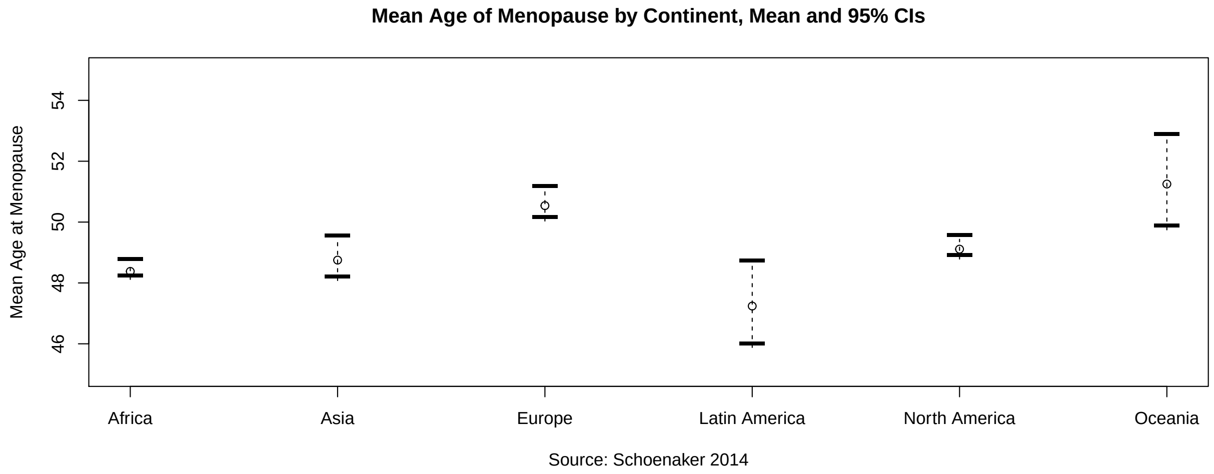

References

Butts SF, Sammel MD, Greer C, Rebbeck TR, Boorman DW, Freeman EW. Cigarettes, genetic background, and menopausal timing: the presence of single nucleotide polymorphisms in cytochrome P450 genes is associated with increased risk of natural menopause in European-American smokers. Menopause 2014; 21(7):694-701.

Gold EB. The timing of the age at which natural menopause occurs. Obstet Gynecol Clin North Am 2011; 38(3):425-40.

Gold EB, Crawford SL, Avis NE, et al. Factors related to age at natural menopause: longitudinal analyses from SWAN. Am J Epidemiol 2013; 178(1):70-83.

McKinlay SM, Brambilla DJ, Posner JG. The normal menopause transition. Maturitas 1992; 14(2):103-15.

Schmidt CW. Age at Menopause: Do Chemical Exposures Play A Role? Environ Health Perspect 2017; 125(6): 062001.

Schoenaker DA, Jackson CA, Rowlands JV, Mishra GD. Socioeconomic position, lifestyle factors and age at natural menopause: a systematic review and meta-analyses of studies across six continents. Int J Epidemiol 2014; 43(5): 1542-62.

Shi J, Zhang B, Choi JY, et al. Age at menarche and age at natural menopause in East Asian women: a genome-wide association study. Age (Dordr) 2016; 38(5-6): 513-523.

Treloar AE. Menarche, menopause, and intervening fecundability. Hum Biol 1974; 46(1):89-107.

A.1.2.10 Anemia

Overview

Anemia is a leading cause or risk factor for maternal deaths and adverse pregnancy outcomes, especially in developing countries (Kassebaum 2016, Rahman 2016). For example, the relative risk of death from maternal complications has been estimated to be 3.5 times greater with severe anemia and 1.35 times greater with moderate anemia, compared to a woman without anemia (Brabin 2001). Anemia is thus an important risk factor to include in the model.

The main causes of anemia are poor nutrition (e.g. iron and vitamin deficiencies), infectious diseases (e.g. malaria), and untreated genetic hemoglobin disorders (Rahman 2016).

Anemia status is determined by hemoglobin levels, as defined by the WHO for different populations (e.g. children of different ages, pregnant women, etc). Here we present the WHO definitions for anemia among pregnant women (WHO 2011).

Status	Hemoglobin level at sea level (g/l)
No anemia	110 or higher
Mild	100 to 109
Moderate	70 to 99
Severe	Lower than 70

Note: While it is a common term, 'mild' anemia is misleading, as iron deficiency is already advanced by the time anemia is detected, which may have consequences even before anemia is clinically apparent (WHO 2011).

As residential elevation above sea level and smoking are known to increase hemoglobin concentrations, anemia definitions must be adjusted for altitude and smoking so that the prevalence is not underestimated. The WHO recommends the following adjustments (WHO 2011):

Altitude (meters above sea level)	Measured hemoglobin adjustment (g/l)
<1000	0
1000	-2
1500	-5
2000	-8
2500	-13
3000	-19
3500	-27
4000	-35
4500	-45

Smoking status	Measured hemoglobin adjustment (g/l)
Non-smoker	0
Smoker (all)	-3
0.5-1 packet/day	-3
1-2 packets/day	-5
2+ packets/day	-7

Data

In high income countries, it was estimated that 16% of women and 22% of pregnant women had anemia in 2011, with the highest rates of anemia occurring in low income countries, especially Central and West Africa

(56% of pregnant women) and South Asia (52% of pregnant women) (Stevens 2013). Overall, it is estimated that developing countries account for 90% of the anemia burden (Kassebaum 2016).

Modeled estimates of overall anemia prevalence among pregnant women by country were available from the WHO GHO database (WHO 2017). Estimates were available for 186 countries for 1990-2016. We supplemented these data with an analysis of individual-level hemoglobin data from pregnant women in 111 DHS surveys (n=97,613), stratified by urban/rural location and level of education. Here we plot the empirical distributions of hemoglobin estimated from DHS data.

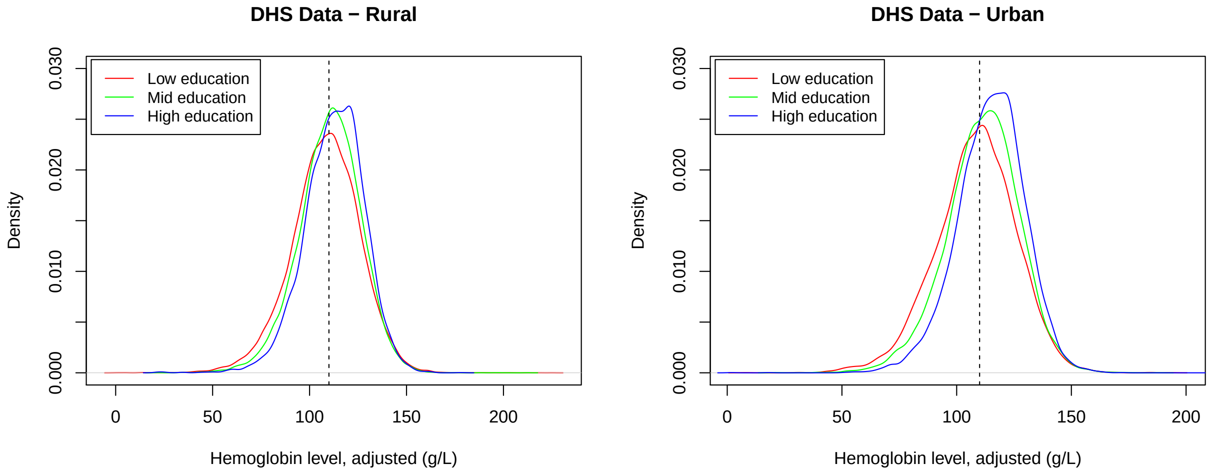

Parameters

We model the underlying distribution of hemoglobin to better model the prevalence of anemia severity. We assume that hemoglobin levels are normally distributed - an assumption borne out by the empirical data (see above). While hemoglobin may be slightly skewed to the left, because we are only interested in anemia prevalence, we only need to accurately model the left side of the distribution.

Anemia is defined as hemoglobin less than 110 g/L, so we use the normal CDF to estimate underlying parameter values of μ and σ for each country that give rise to the estimated anemia prevalence over time.

$$\text{p(Anemia)} = \frac{1}{2}\left[1 + \text{erf}\left(\frac{110-\mu}{\sigma\sqrt{2}}\right)\right]$$
$$2 \cdot \text{p(Anemia)} - 1 = \text{erf}\left(\frac{110-\mu}{\sigma\sqrt{2}}\right)$$
$$\text{erf}^{-1}\left(2 \cdot \text{p(Anemia)} - 1\right) = \frac{110-\mu}{\sigma\sqrt{2}}$$
$$\mu = 110 - \sigma\sqrt{2} \cdot \text{erf}^{-1}\left(2 \cdot \text{p(Anemia)} - 1\right)$$

However, because we only have 1 number (prevalence) at each timepoint, and 2 parameters to fit, we have to fix one parameter in order to estimate the other given the data. We estimated the empirical SDs of hemoglobin distribution in each DHS dataset and found they had a mean of 15.81 and SD of 1.93. We sampled a value for σ from this distribution and held it fixed across countries, then estimated μ using the inverse normal CDF.

We then fit linear regressions (weighted by the inverse of the prevalence CIs) to estimate how μ has changed over time. We repeated this 100 times to estimate a set of regression coefficients for different values of σ. We then estimated priors for a hierarchical model based on these estimated coefficients.

To estimate SDs we fit linear regressions to DHS estimates of hemoglobin SDs over time. We bootstrapped the DHS estimates 1000 times to estimate the variance for these estimates. We used these estimates as global

priors.

We estimated the empirical means and SDs of the subgroup-specific (urban/rural and education) hemoglobin distributions in DHS. Below we plot the differences of means and SDs for each subgroup compared to the country overall. These subgroup-specific offsets were used to offset the estimated overall priors by shifting the income intercept.

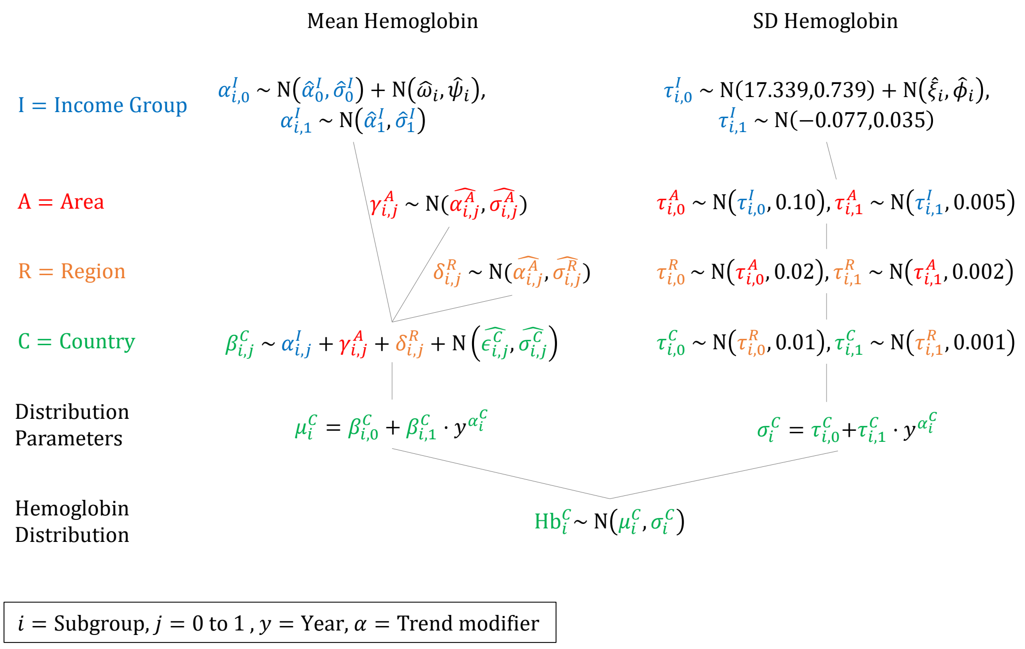

Priors

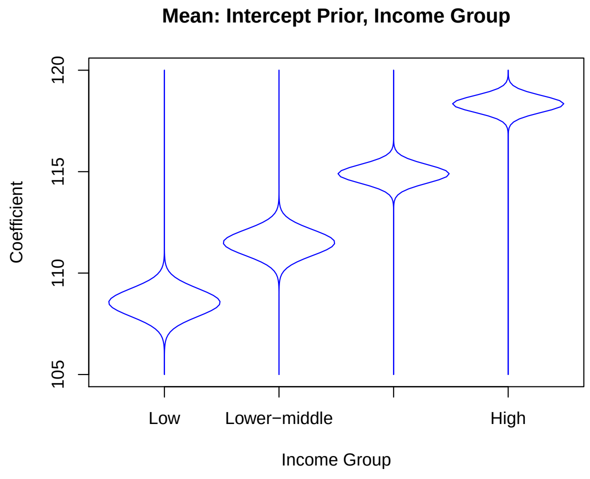

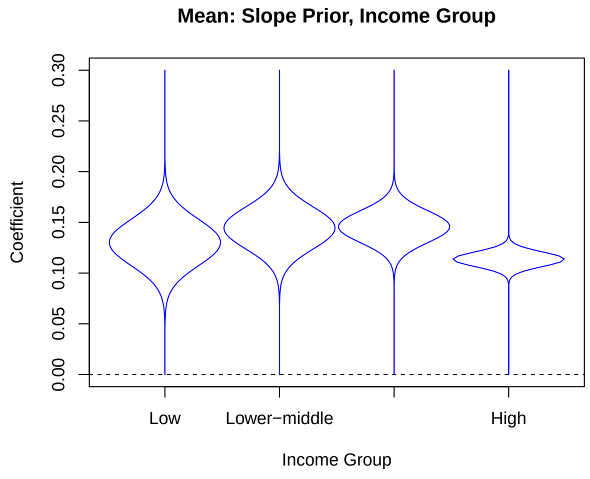

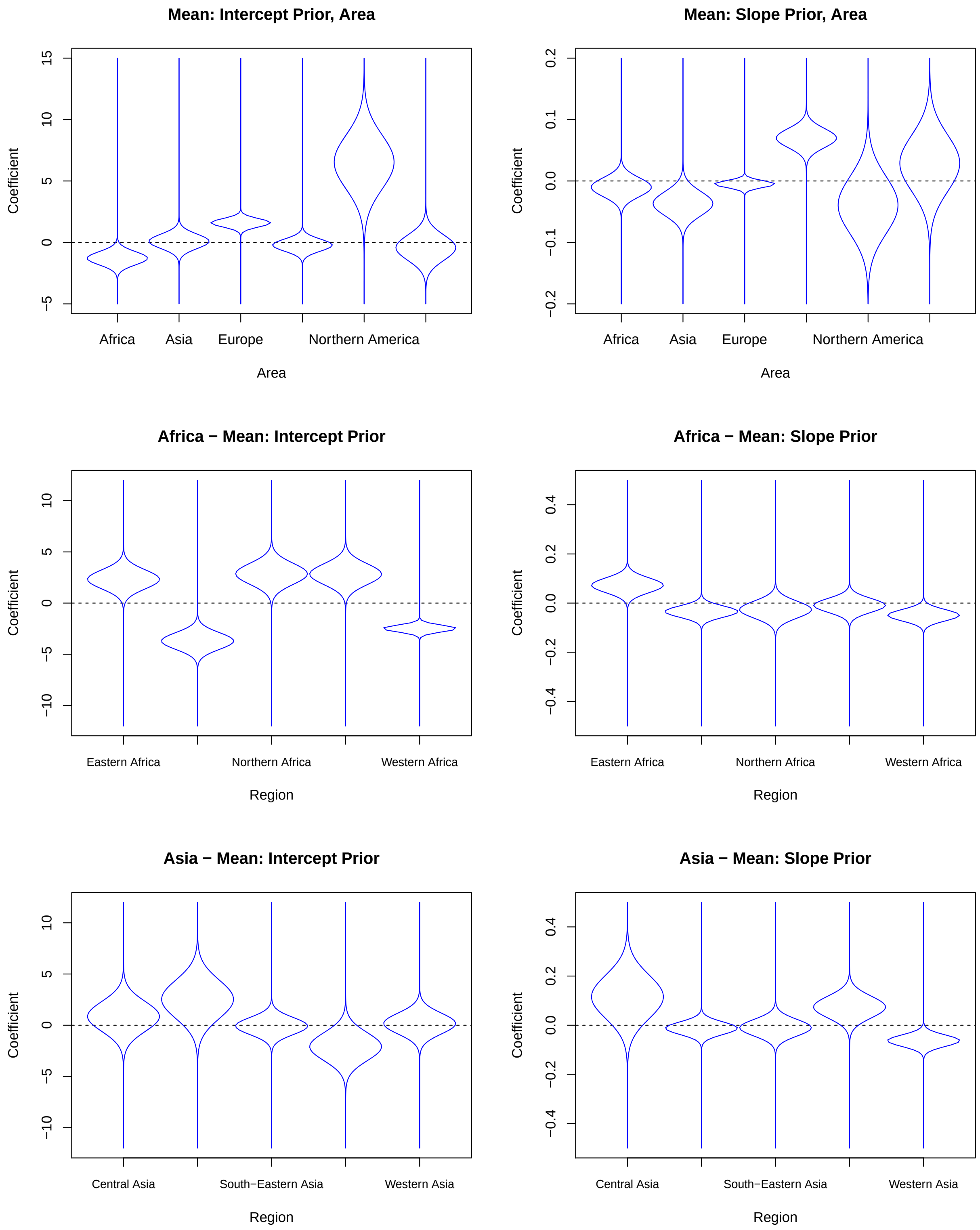
Mean: Intercept Prior, Area
Coefficient
Africa
Asia
Europe
Northern America
Area
Mean: Slope Prior, Area
Coefficient
Africa
Asia
Europe
Northern America
Area
Africa − Mean: Intercept Prior
Coefficient
Eastern Africa
Northern Africa
Western Africa
Region
Africa − Mean: Slope Prior
Coefficient
Eastern Africa
Northern Africa
Western Africa
Region
Asia − Mean: Intercept Prior
Coefficient
Central Asia
South−Eastern Asia
Western Asia
Region
Asia − Mean: Slope Prior
Coefficient
Central Asia
South−Eastern Asia
Western Asia
Region

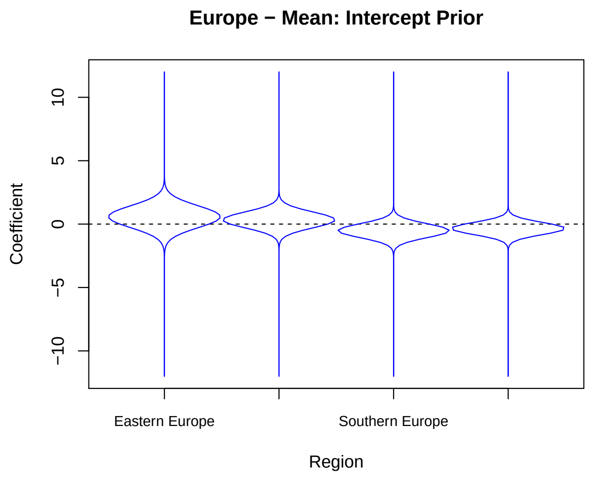
Europe − Mean: Intercept Prior
Coefficient
10
5
0
−5
−10
Eastern Europe
Southern Europe
Region

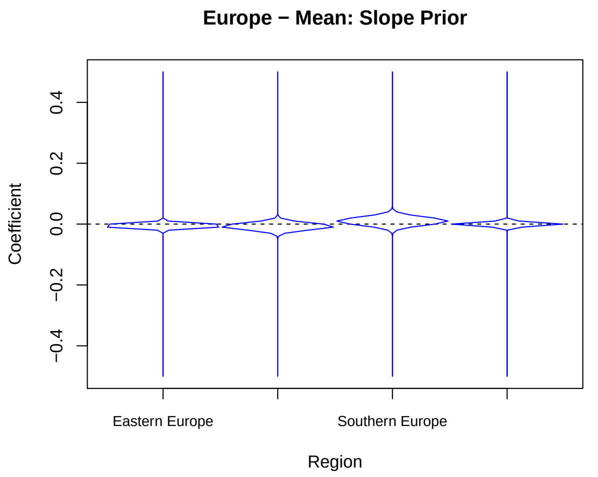
Europe − Mean: Slope Prior
Coefficient
0.4
0.2
0.0
−0.2
−0.4
Eastern Europe
Southern Europe
Region

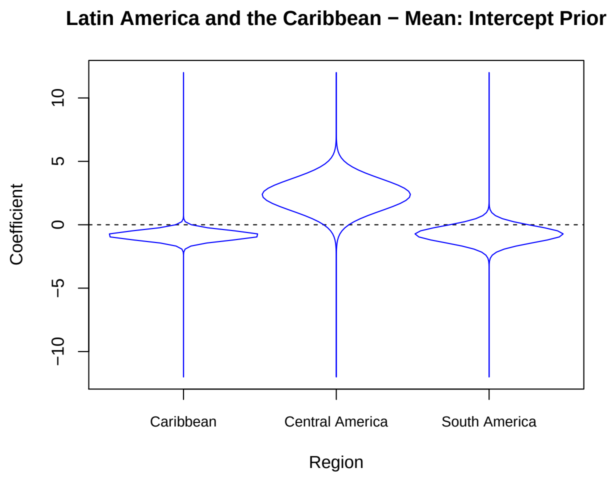
Latin America and the Caribbean − Mean: Intercept Prior
Coefficient
10
5
0
−5
−10
Caribbean
Central America
South America
Region

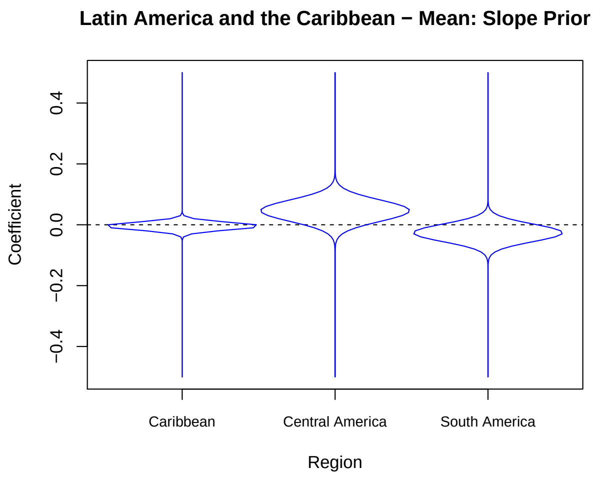
Latin America and the Caribbean − Mean: Slope Prior
Coefficient
0.4
0.2
0.0
−0.2
−0.4
Caribbean
Central America
South America
Region

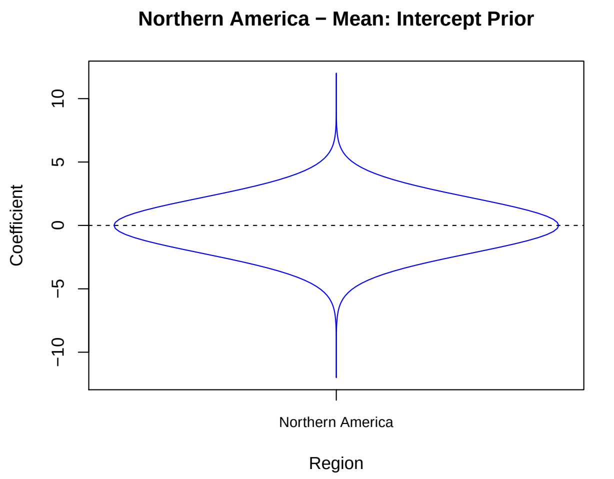
Northern America − Mean: Intercept Prior
Coefficient
10
5
0
−5
−10
Northern America
Region

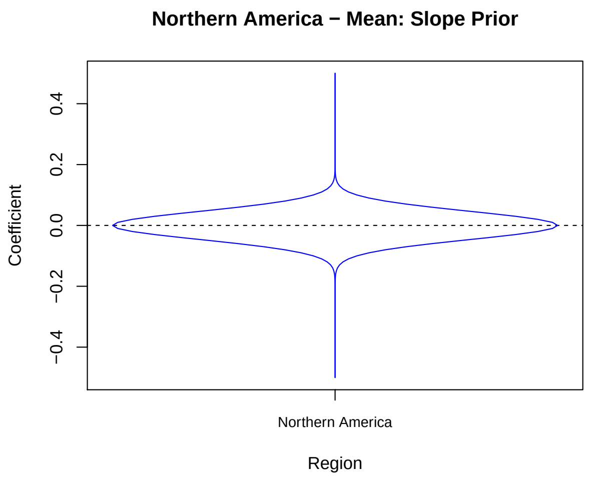
Northern America − Mean: Slope Prior
Coefficient
0.4
0.2
0.0
−0.2
−0.4
Northern America
Region

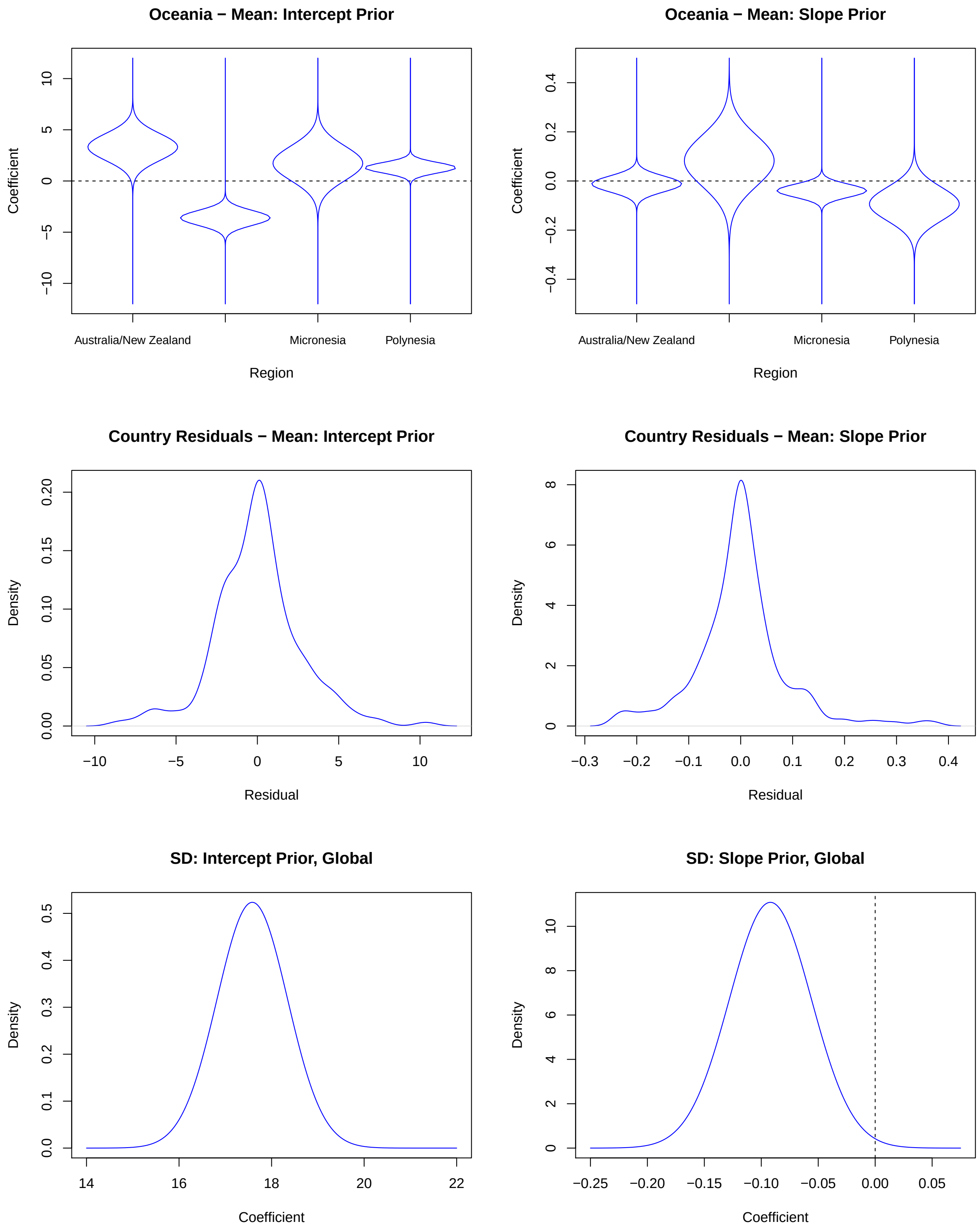
Oceania − Mean: Intercept Prior
Coefficient
10
5
0
−5
−10
Australia/New Zealand
Micronesia
Polynesia
Region
Oceania − Mean: Slope Prior
Coefficient
0.4
0.2
0.0
−0.2
−0.4
Australia/New Zealand
Micronesia
Polynesia
Region
Country Residuals − Mean: Intercept Prior
Density
0.20
0.15
0.10
0.05
0.00
−10
−5
0
5
10
Residual
Country Residuals − Mean: Slope Prior
Density
8
6
4
2
0
−0.3
−0.2
−0.1
0.0
0.1
0.2
0.3
0.4
Residual
SD: Intercept Prior, Global
Density
0.5
0.4
0.3
0.2
0.1
0.0
14
16
18
20
22
Coefficient
SD: Slope Prior, Global
Density
10
8
6
4
2
0
−0.25
−0.20
−0.15
−0.10
−0.05
0.00
0.05
Coefficient

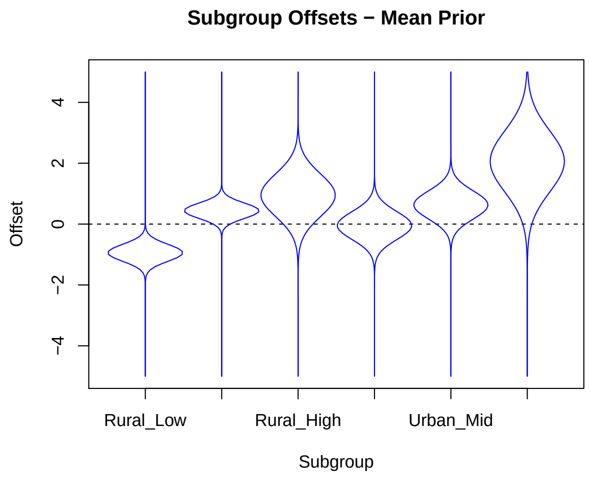

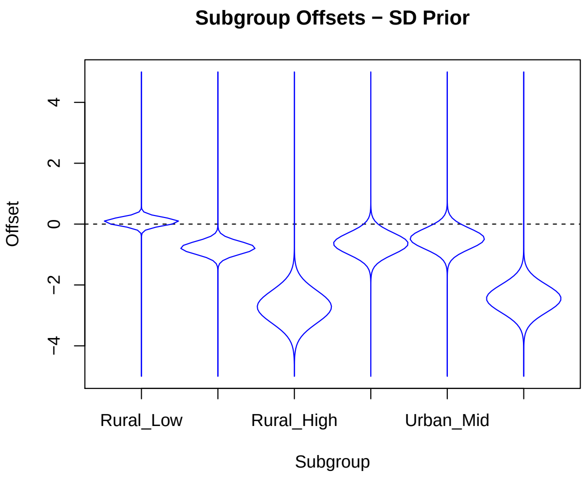

Model Implementation

We simulate a hemoglobin value (and thus anemia status) at the beginning of each pregnancy. We simulate a latent variable for each woman which tracks her relative position in the distribution, while the distribution itself is updated over time to reflect secular trends in anemia prevalence. We model an improvement in hemoglobin levels for women who attend antenatal care, and a decrease in hemoglobin for women who experience postpartum hemorrhage.

References

Brabin BJ, Hakimi M, Pelletier D. An analysis of anemia and pregnancy-related maternal mortality. J Nutr 2001; 131(2S-2): 604S-614S.

Kassebaum NJ, GBD 2013 Anemia Collaborators. The Global Burden of Anemia. Hematol Oncol Clin North Am 2016; 30(2):247-308.

Rahman MM, Abe SK, Rahman MS, et al. Maternal anemia and risk of adverse birth and health outcomes in low- and middle-income countries: systematic review and meta-analysis. Am J Clin Nutr 2016; 103(2): 495-504.

Stevens GA, Finucane MM, De-Regil LM, et al. Global, regional, and national trends in haemoglobin concentration and prevalence of total and severe anaemia in children and pregnant and non-pregnant women for 1995–2011: a systematic analysis of population-representative data. Lancet Glob Health 2013; 1: e16–25.

WHO. Haemoglobin concentrations for the diagnosis of anaemia and assessment of severity. Geneva: World Health Organization, 2011. Available at: https://www.who.int/vmnis/indicators/haemoglobin/en/

WHO. Prevalence of anaemia in pregnant women - Estimates by country. Global Health Observatory. Last updated 2017-08-30. Available at: http://apps.who.int/gho/data/view.main.ANAEMIAWOMENPWv

A.1.3 Family Planning

A.1.3.1 Age of Sexual Debut

Overview

Age of sexual debut is used in the model to simulate the beginning of a woman's reproductive life-cycle. We do not model frequency of sexual behavior, but assume that once a woman becomes sexually active she faces age-specific rates of fecundity which may be modified by the use of contraception.

Data

We analyzed DHS data (v531) from 1,150,813 women aged 35 and over from 230 surveys in 71 countries. We restricted the dataset to women aged 35 and over to limit the impact of censoring - women who have not yet experienced sexual debut cannot report the age at which they started, leading to biased estimates among observations at younger ages. Among women aged 35 and over, 1.5% reported not having had sex. This small group of potentially censored ages has limited impact on our estimates, so for simplicity we removed them from the dataset. Here we plot the distribution of reported ages of sexual debut by location (urban/rural) and level of education.

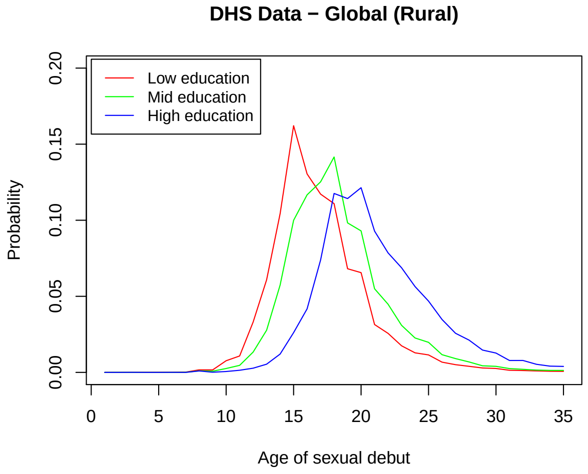

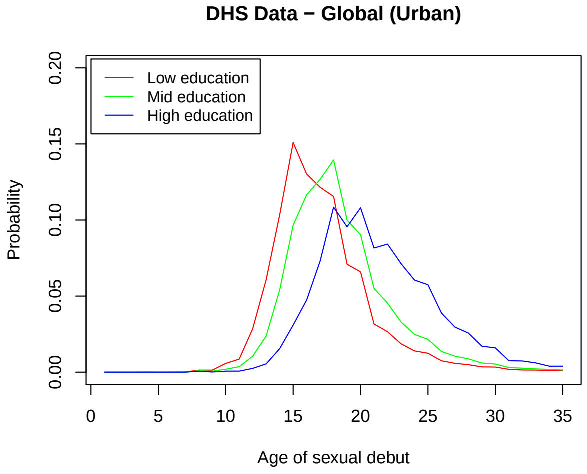

To explore potential secular trends we estimated the mean and variance of age of sexual debut by year for each urban/rural location and education group. We see that they are fairly stable across years, so for simplicity we assume that the distribution within these demographic groups is stationary over time.

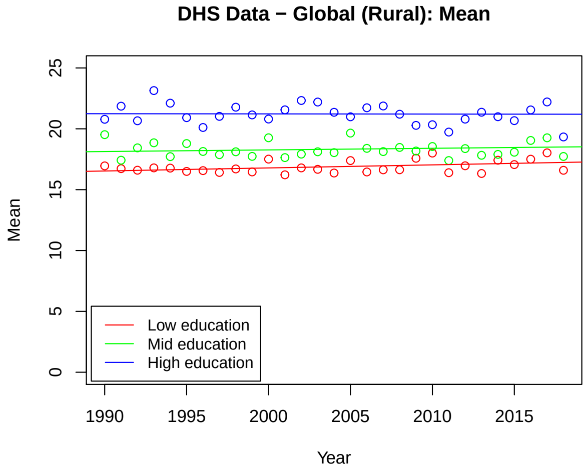

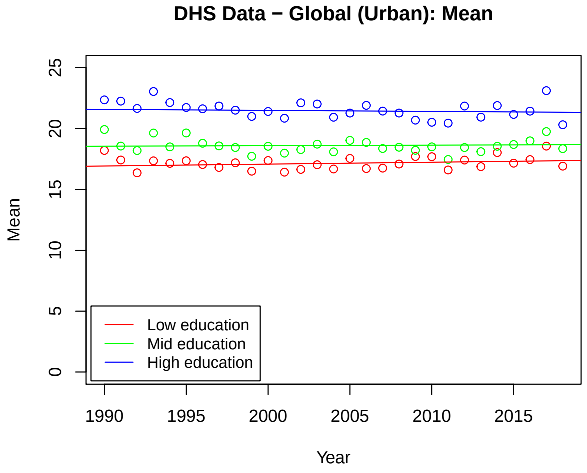

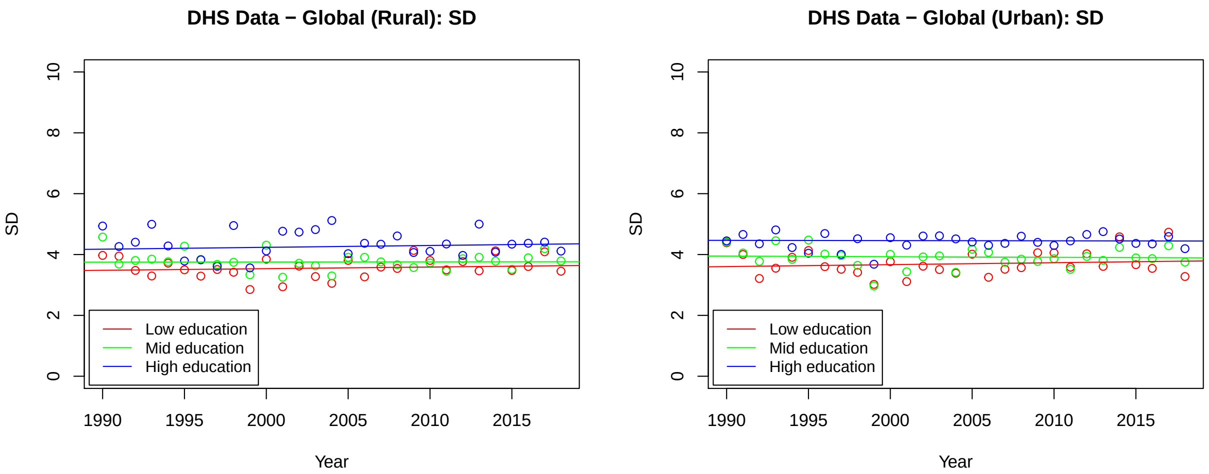

Parameters

We assumed that age of sexual debut follows a log-normal distribution, and used hierarchical models to estimate the mean and variance for each country. Because no high income countries are available in the DHS data we used the priors estimated for upper-middle income countries to set priors for high income countries.

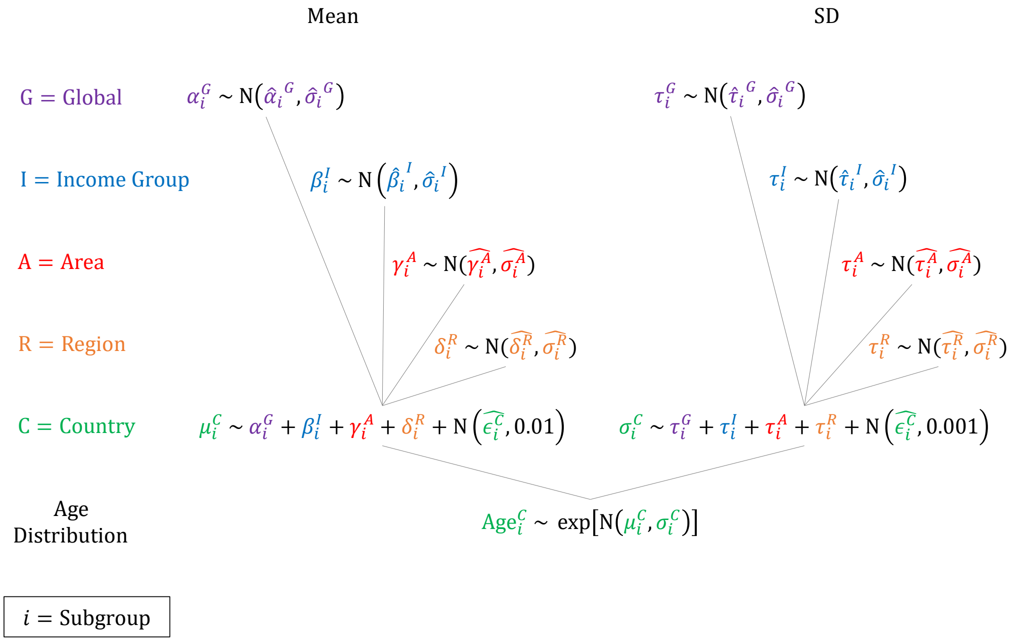

Priors

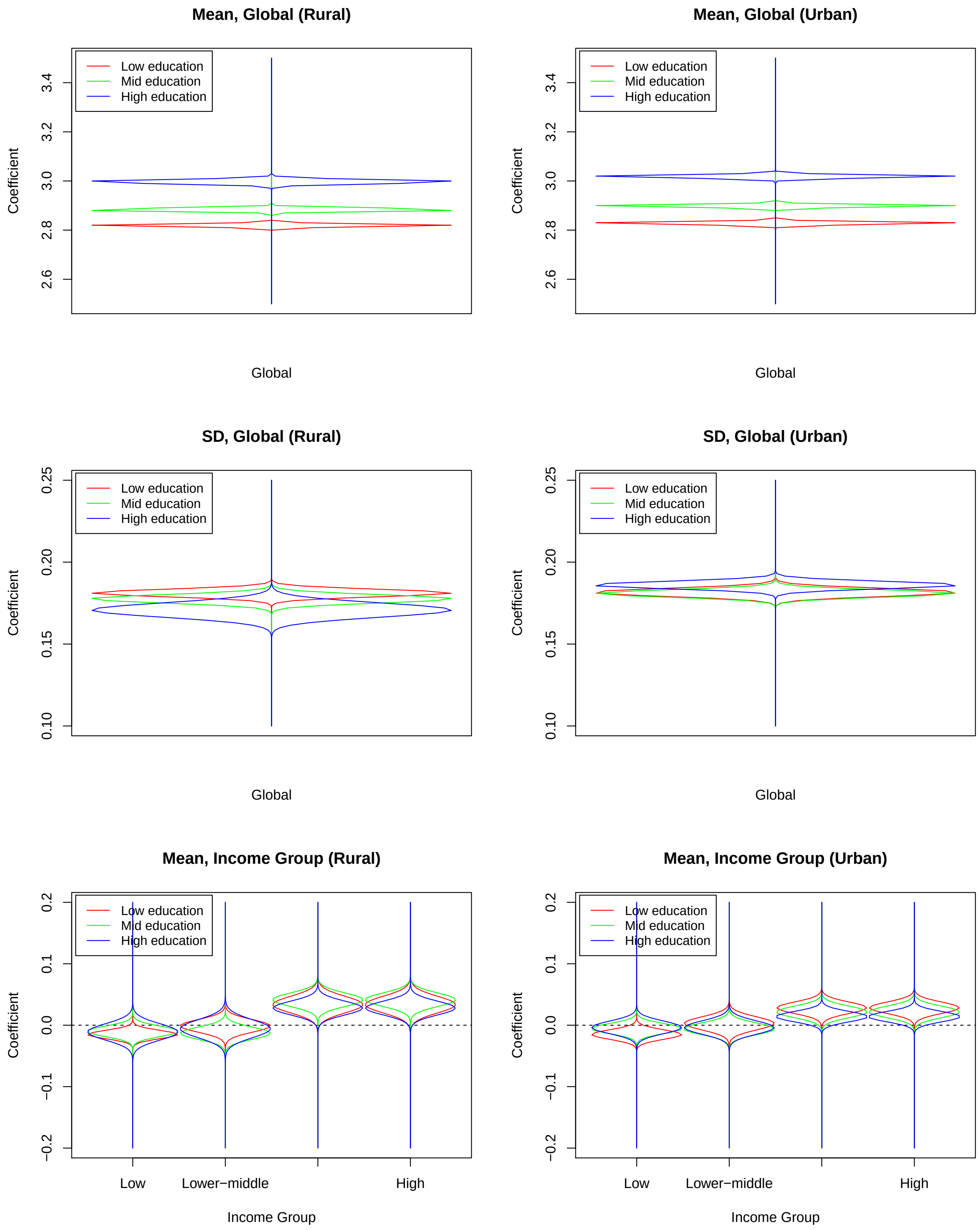
Mean, Global (Rural)
Mean, Global (Urban)
SD, Global (Rural)
SD, Global (Urban)
Mean, Income Group (Rural)
Mean, Income Group (Urban)
Low education
Mid education
High education
Coefficient
Global
Low
Lower−middle
High
Income Group
3.4
3.2
3.0
2.8
2.6
0.25
0.20
0.15
0.10
0.2
0.1
0.0
−0.1
−0.2

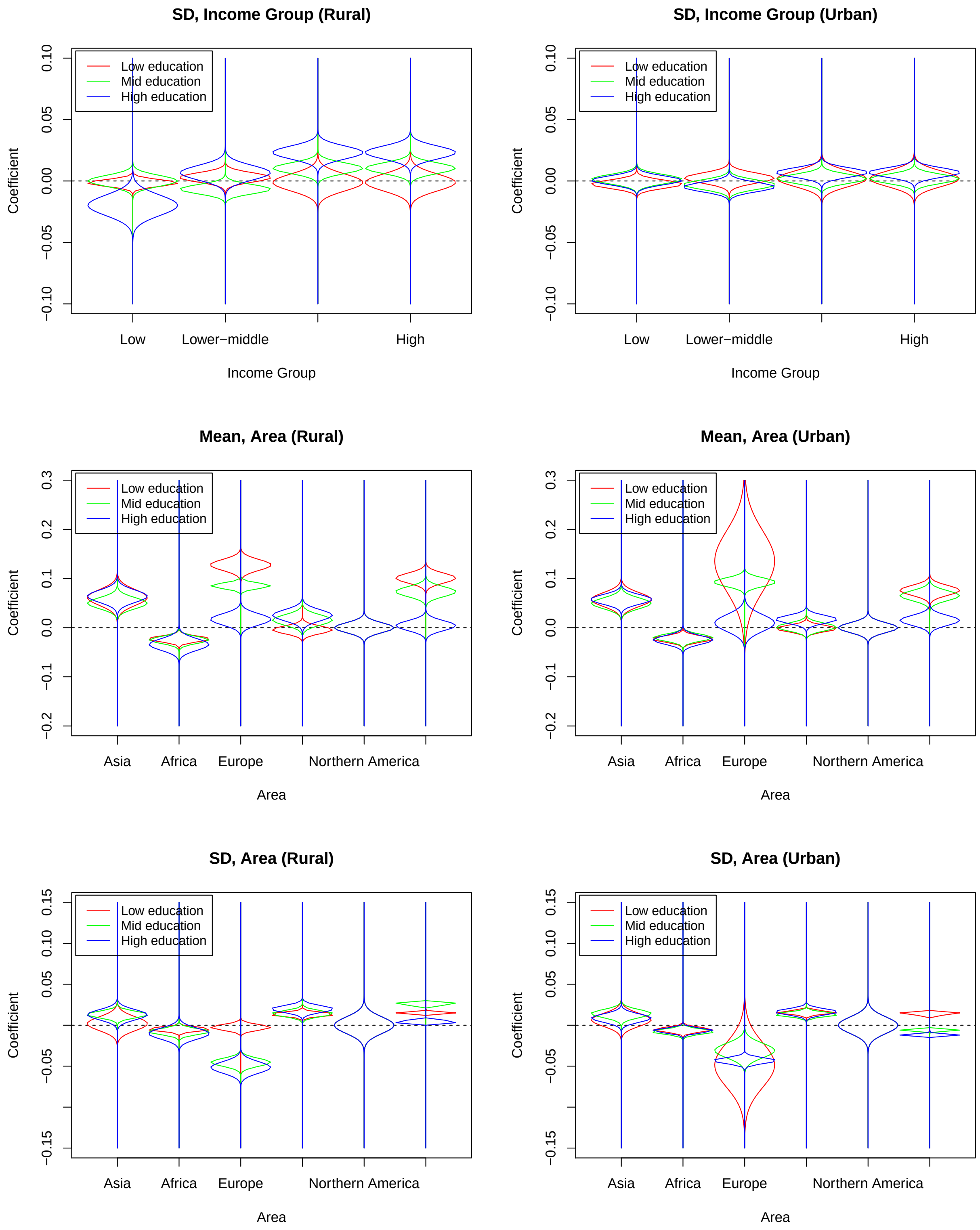
SD, Income Group (Rural)
SD, Income Group (Urban)
Low education
Mid education
High education
Coefficient
Low
Lower−middle
High
Income Group
Mean, Area (Rural)
Mean, Area (Urban)
Asia
Africa
Europe
Northern America
Area
SD, Area (Rural)
SD, Area (Urban)

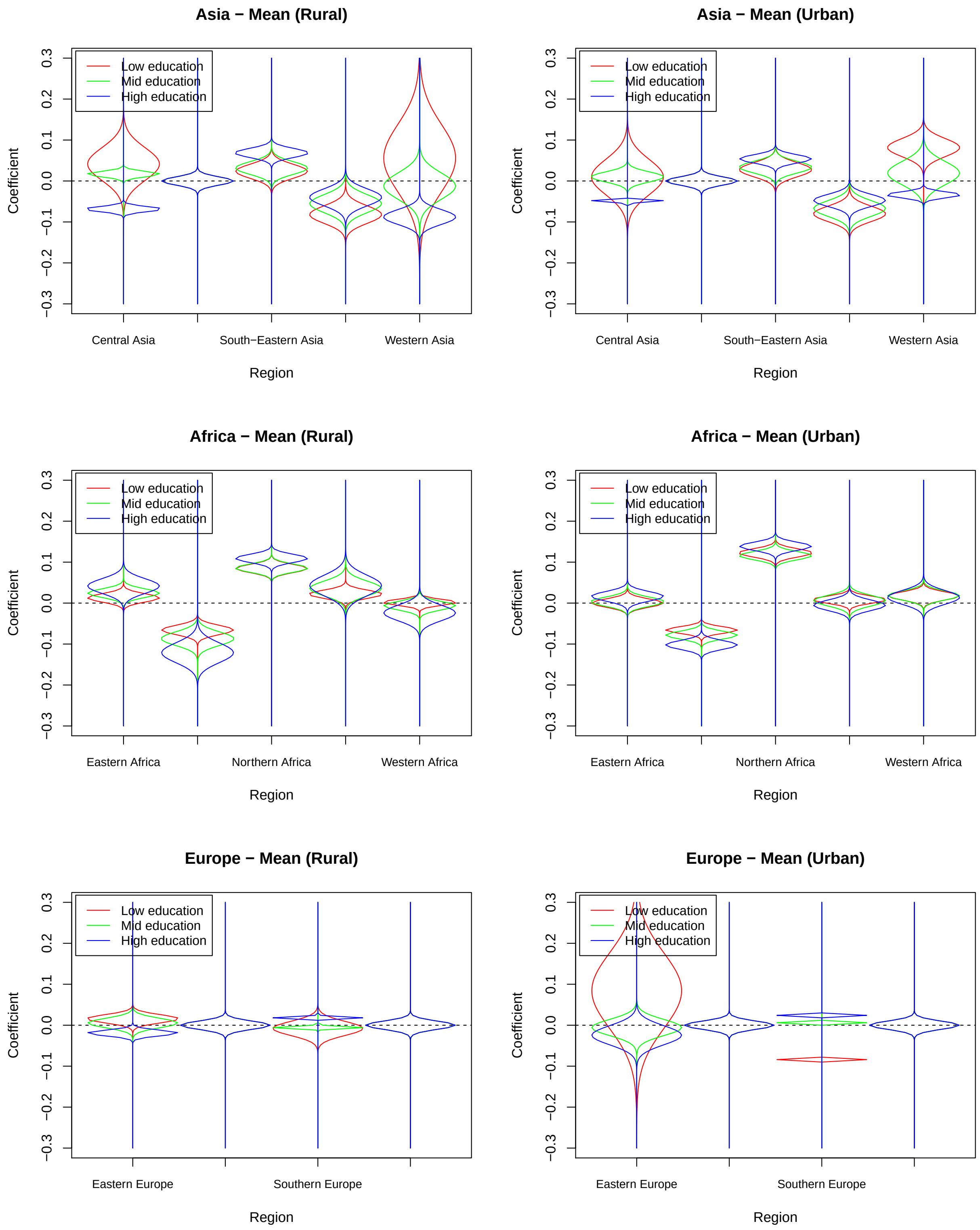
Asia − Mean (Rural)
Asia − Mean (Urban)
Africa − Mean (Rural)
Africa − Mean (Urban)
Europe − Mean (Rural)
Europe − Mean (Urban)
Low education
Mid education
High education
Coefficient
Region
0.3
0.2
0.1
0.0
−0.1
−0.2
−0.3
Central Asia
South−Eastern Asia
Western Asia
Eastern Africa
Northern Africa
Western Africa
Eastern Europe
Southern Europe

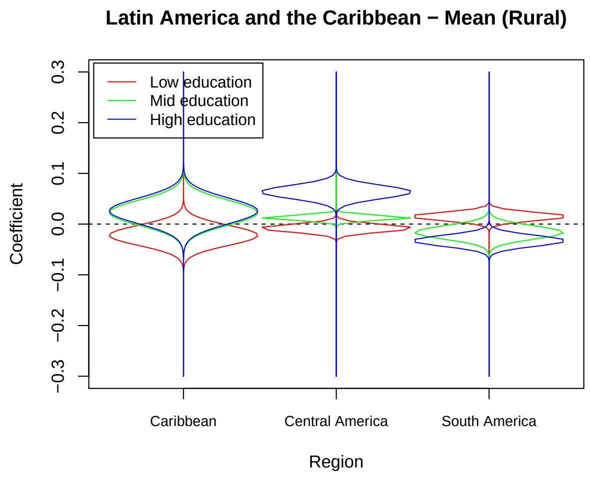
Latin America and the Caribbean − Mean (Rural)
Low education
Mid education
High education
Coefficient
0.3
0.2
0.1
0.0
−0.1
−0.2
−0.3
Caribbean
Central America
South America
Region

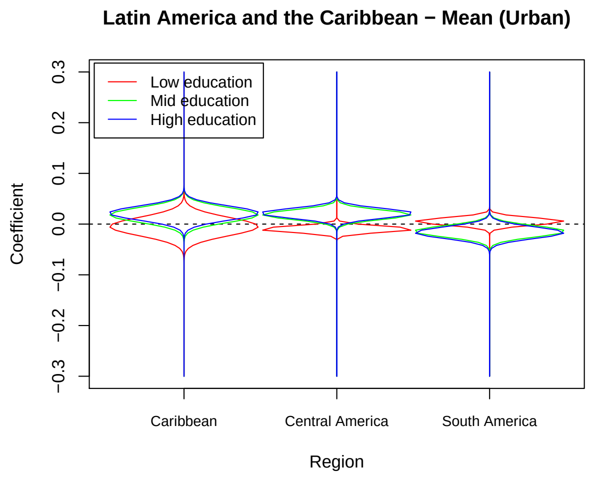
Latin America and the Caribbean − Mean (Urban)
Low education
Mid education
High education
Coefficient
0.3
0.2
0.1
0.0
−0.1
−0.2
−0.3
Caribbean
Central America
South America
Region

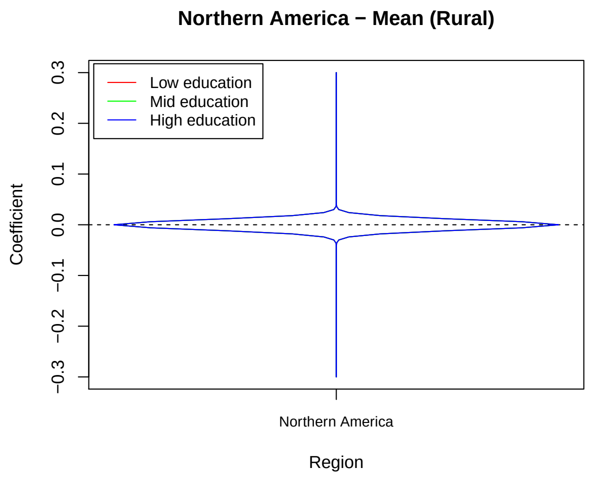
Northern America − Mean (Rural)
Low education
Mid education
High education
Coefficient
0.3
0.2
0.1
0.0
−0.1
−0.2
−0.3
Northern America
Region

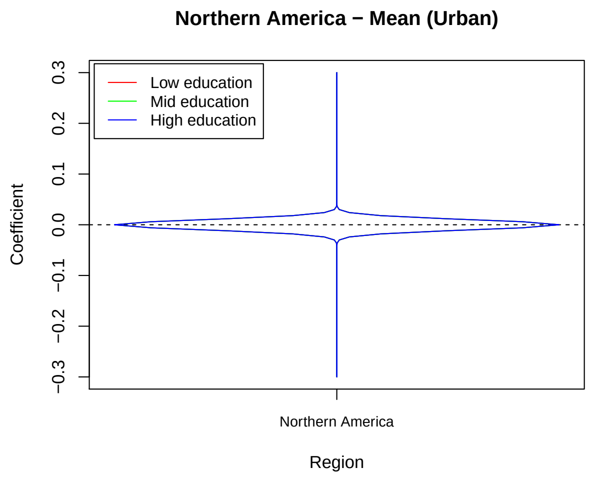
Northern America − Mean (Urban)
Low education
Mid education
High education
Coefficient
0.3
0.2
0.1
0.0
−0.1
−0.2
−0.3
Northern America
Region

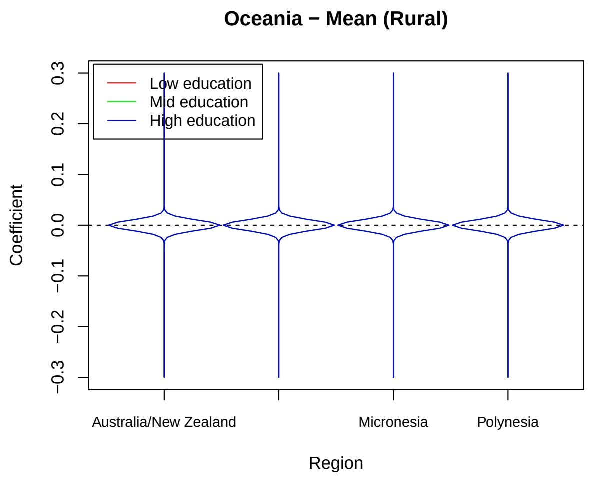
Oceania − Mean (Rural)
Low education
Mid education
High education
Coefficient
0.3
0.2
0.1
0.0
−0.1
−0.2
−0.3
Australia/New Zealand
Micronesia
Polynesia
Region

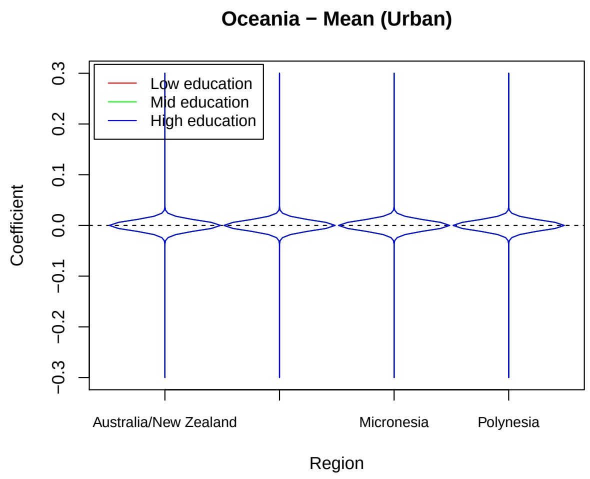
Oceania − Mean (Urban)
Low education
Mid education
High education
Coefficient
0.3
0.2
0.1
0.0
−0.1
−0.2
−0.3
Australia/New Zealand
Micronesia
Polynesia
Region

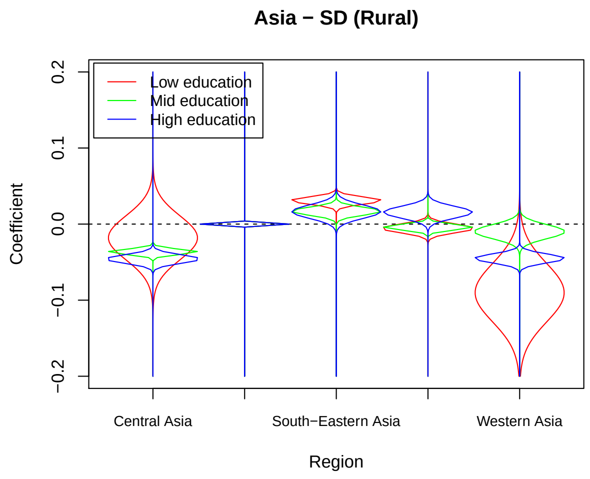
Asia − SD (Rural)
Low education
Mid education
High education
Coefficient
0.2
0.1
0.0
−0.1
−0.2
Central Asia
South−Eastern Asia
Western Asia
Region

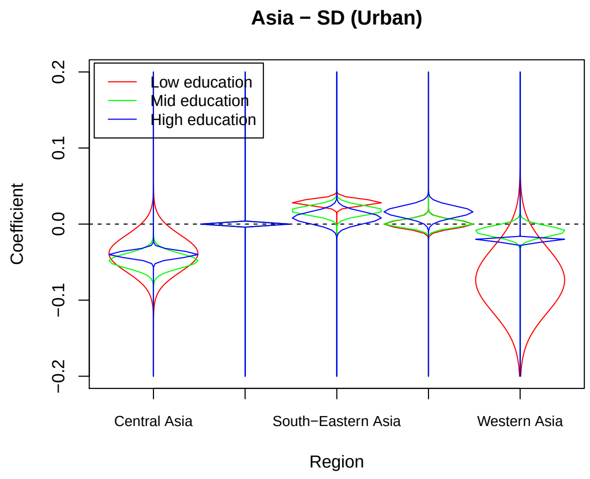
Asia − SD (Urban)
Low education
Mid education
High education
Coefficient
0.2
0.1
0.0
−0.1
−0.2
Central Asia
South−Eastern Asia
Western Asia
Region

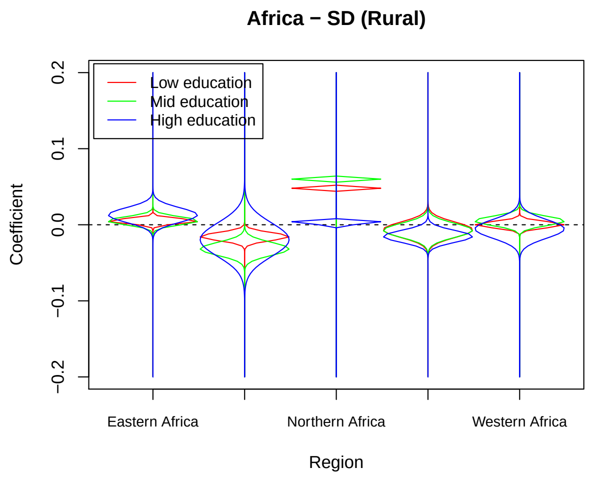
Africa − SD (Rural)
Low education
Mid education
High education
Coefficient
0.2
0.1
0.0
−0.1
−0.2
Eastern Africa
Northern Africa
Western Africa
Region

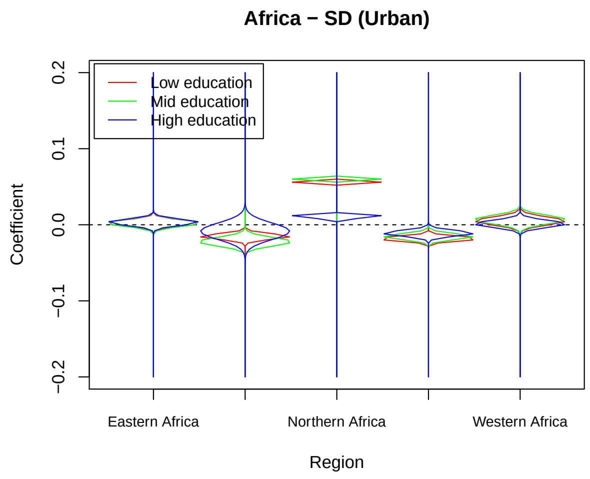
Africa − SD (Urban)
Low education
Mid education
High education
Coefficient
0.2
0.1
0.0
−0.1
−0.2
Eastern Africa
Northern Africa
Western Africa
Region

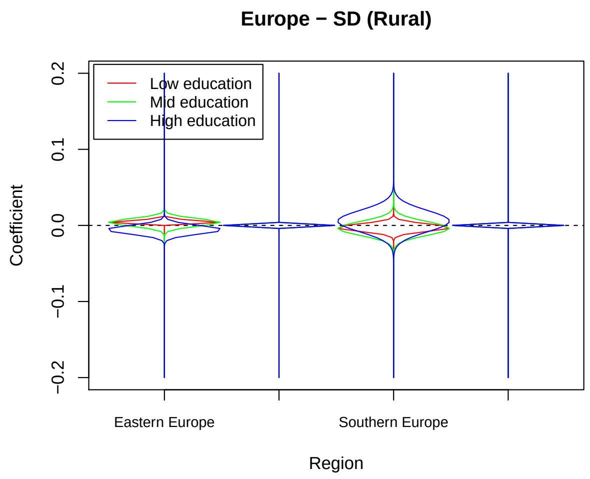
Europe − SD (Rural)
Low education
Mid education
High education
Coefficient
0.2
0.1
0.0
−0.1
−0.2
Eastern Europe
Southern Europe
Region

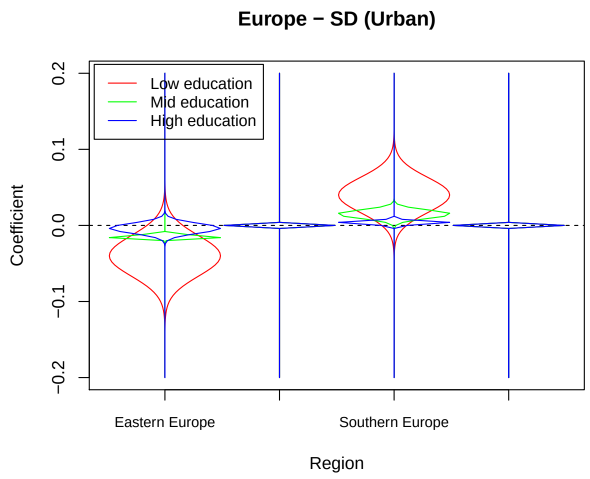
Europe − SD (Urban)
Low education
Mid education
High education
Coefficient
0.2
0.1
0.0
−0.1
−0.2
Eastern Europe
Southern Europe
Region

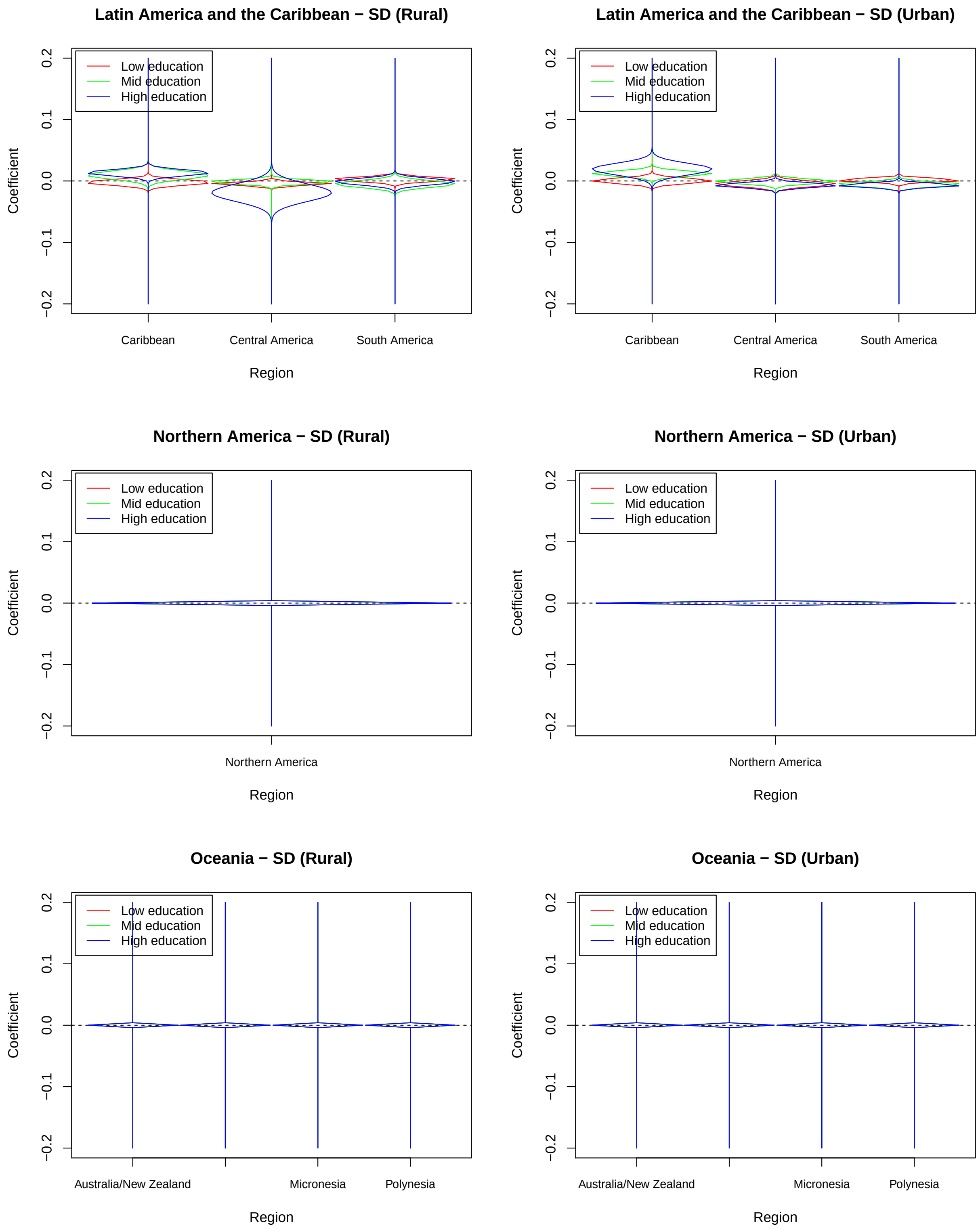

Latin America and the Caribbean − SD (Rural)
Latin America and the Caribbean − SD (Urban)
Northern America − SD (Rural)
Northern America − SD (Urban)
Oceania − SD (Rural)
Oceania − SD (Urban)
Low education
Mid education
High education
Coefficient
Region
Caribbean
Central America
South America
Northern America
Australia/New Zealand
Micronesia
Polynesia
0.2
0.1
0.0
−0.1
−0.2

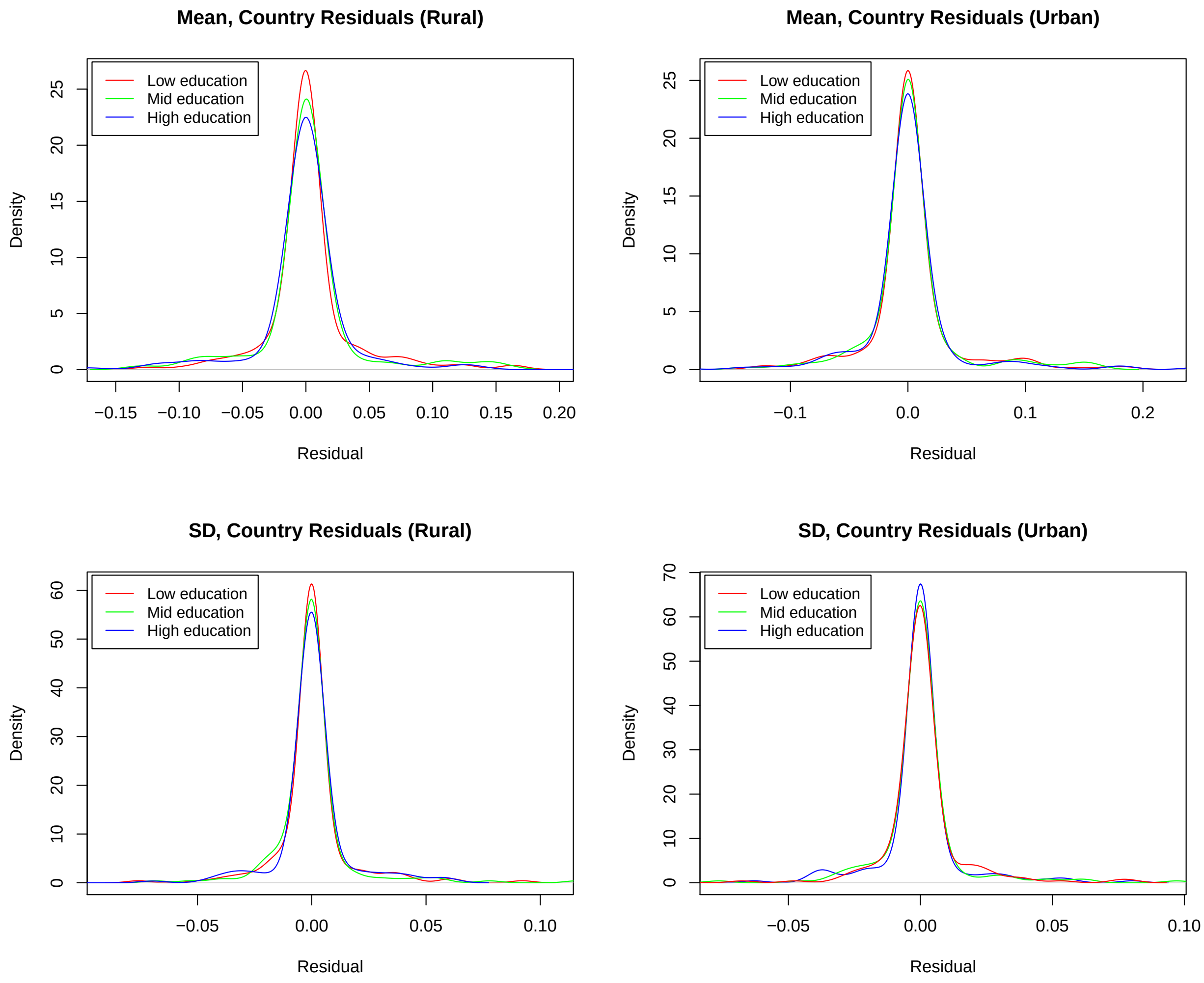

Model Implementation

Age of sexual debut is drawn for each woman at model initiation. Once she reaches this age she begins to face monthly probabilities of pregnancy. As a simplifying assumption, all women are given an age of sexual debut, although in reality a small percentage report never having sex.

A.1.3.2 Number of Living Children

Overview
At the beginning of the model, women are initialized with a number of living children who were born before the analytic timeframe begins. Modelling the number of existing children that women have at the start of the model allows for family planning preferences for 'limiting' or 'spacing' to be more appropriately captured.

Data
We estimated the number of living children based on DHS data. We restricted our analysis to women who were sexually active (i.e. past age of sexual debut). Data were available for 3,046,522 women from 230 surveys in 71 countries.

Parameters
We assumed that the number of living children follows a Poisson distribution. We fit hierarchical models to estimate the mean number of children by age and year, stratified by subgroup (urban/rural and level of education). We used upper middle income priors for high income countries due to lack of DHS data in high income countries.

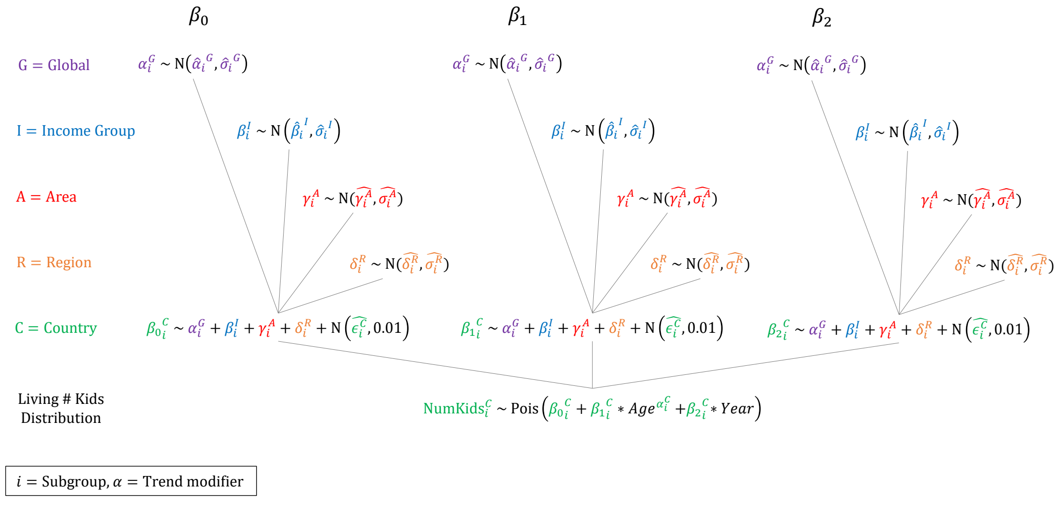

Priors

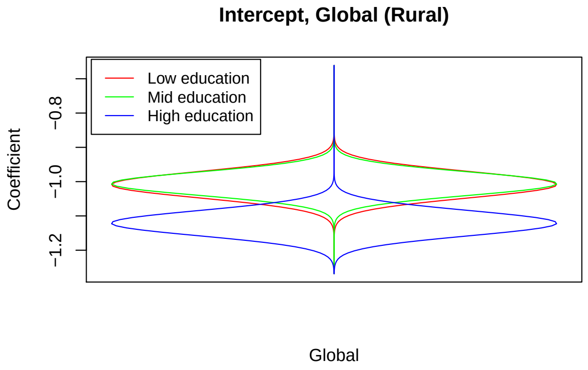

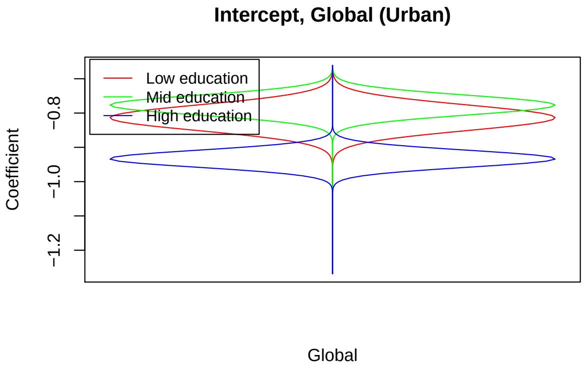

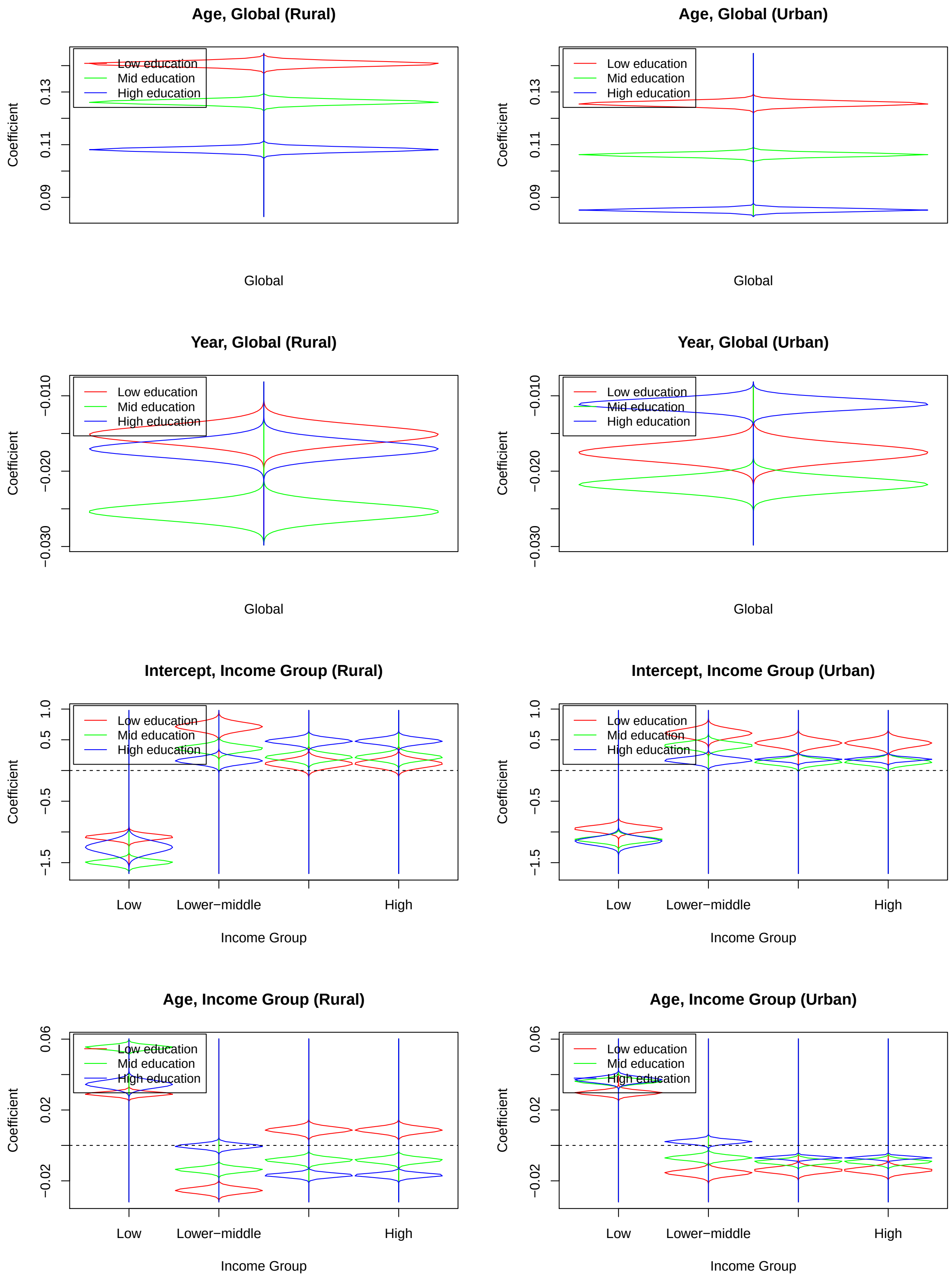
Age, Global (Rural)
Age, Global (Urban)
Year, Global (Rural)
Year, Global (Urban)
Intercept, Income Group (Rural)
Intercept, Income Group (Urban)
Age, Income Group (Rural)
Age, Income Group (Urban)
Low education
Mid education
High education
Coefficient
Global
Income Group
Low
Lower−middle
High
0.09
0.11
0.13
−0.010
−0.020
−0.030
1.0
0.5
−0.5
−1.5
0.06
0.02
−0.02

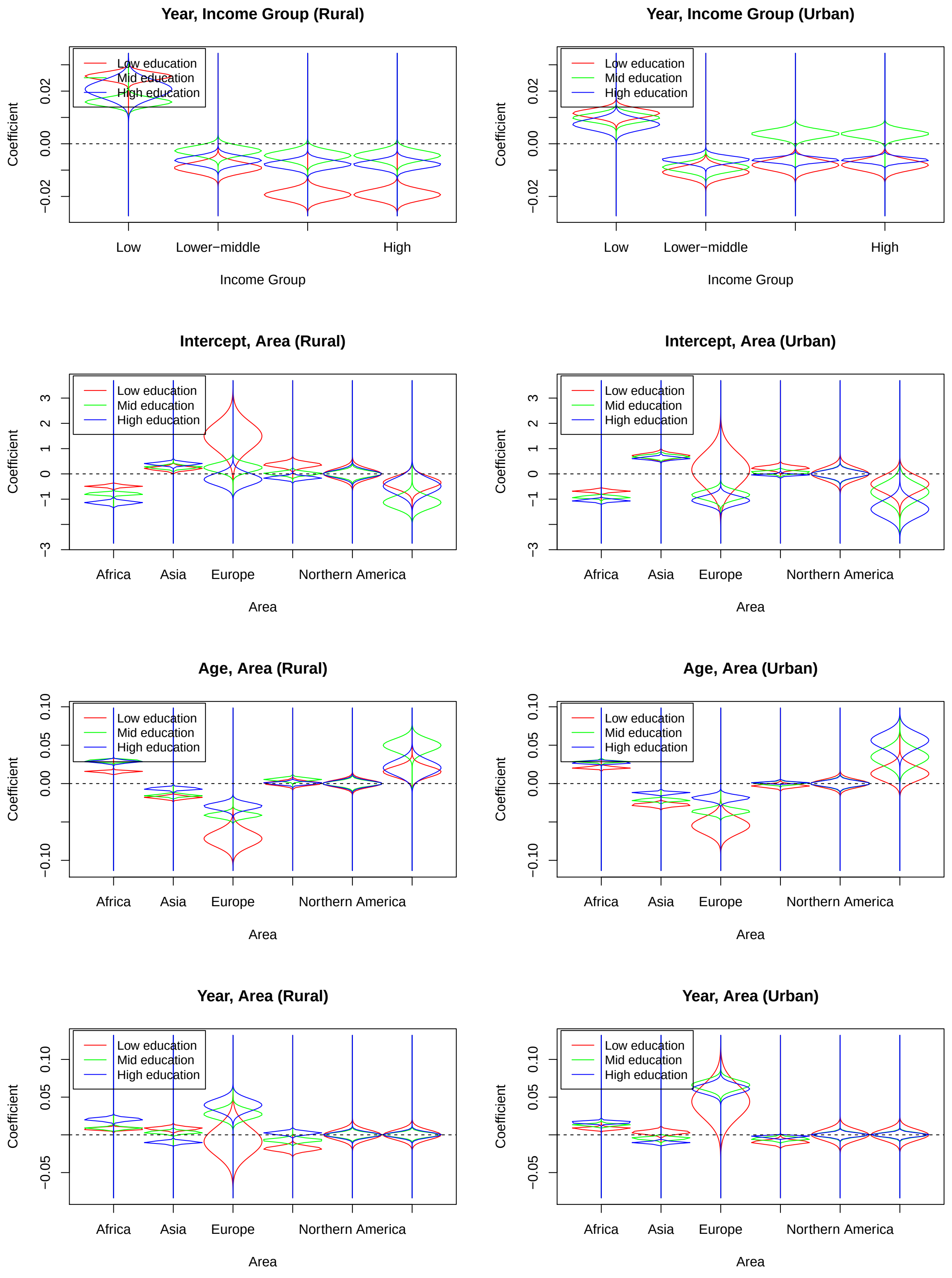
Year, Income Group (Rural)
Year, Income Group (Urban)
Low education
Mid education
High education
Coefficient
0.02
0.00
−0.02
Low
Lower−middle
High
Income Group
Intercept, Area (Rural)
Intercept, Area (Urban)
3
2
1
0
−1
−3
Africa
Asia
Europe
Northern America
Area
Age, Area (Rural)
Age, Area (Urban)
0.10
0.05
0.00
−0.10
Year, Area (Rural)
Year, Area (Urban)
0.10
0.05
−0.05

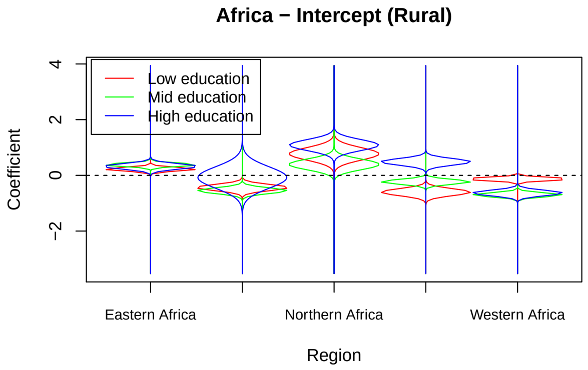
Africa − Intercept (Rural)
Low education
Mid education
High education
Coefficient
4
2
0
−2
Eastern Africa
Northern Africa
Western Africa
Region

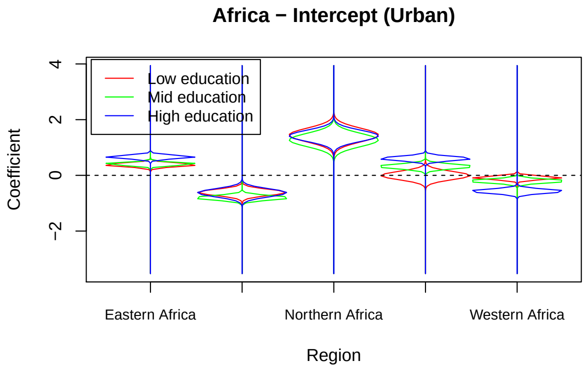
Africa − Intercept (Urban)
Low education
Mid education
High education
Coefficient
4
2
0
−2
Eastern Africa
Northern Africa
Western Africa
Region

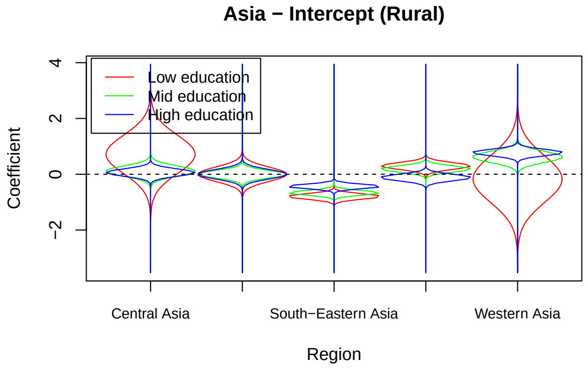
Asia − Intercept (Rural)
Low education
Mid education
High education
Coefficient
4
2
0
−2
Central Asia
South−Eastern Asia
Western Asia
Region

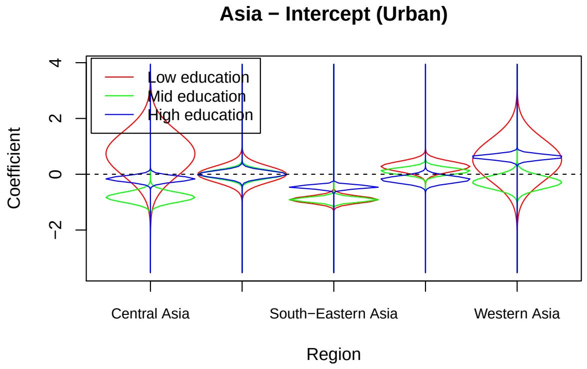
Asia − Intercept (Urban)
Low education
Mid education
High education
Coefficient
4
2
0
−2
Central Asia
South−Eastern Asia
Western Asia
Region

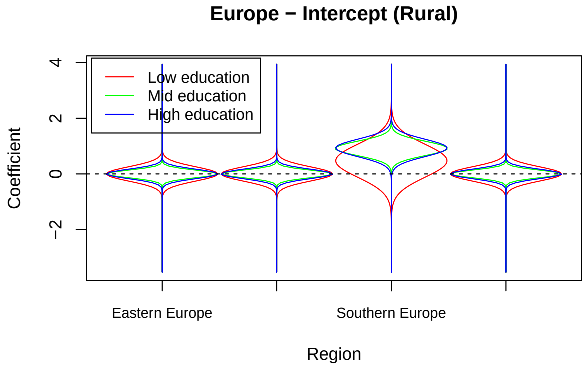
Europe − Intercept (Rural)
Low education
Mid education
High education
Coefficient
4
2
0
−2
Eastern Europe
Southern Europe
Region

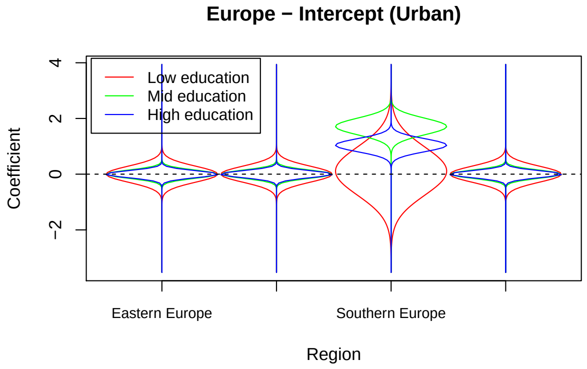
Europe − Intercept (Urban)
Low education
Mid education
High education
Coefficient
4
2
0
−2
Eastern Europe
Southern Europe
Region

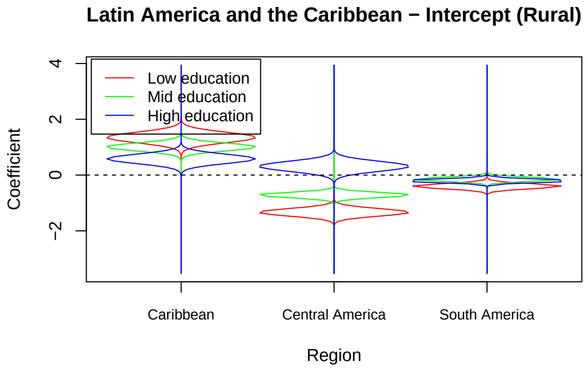
Latin America and the Caribbean − Intercept (Rural)
Low education
Mid education
High education
Coefficient
4
2
0
−2
Caribbean
Central America
South America
Region

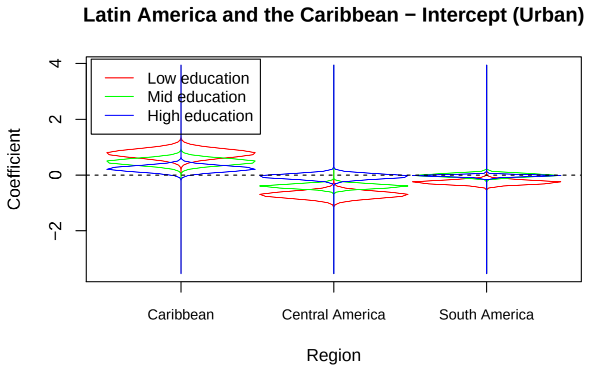
Latin America and the Caribbean − Intercept (Urban)
Low education
Mid education
High education
Coefficient
4
2
0
−2
Caribbean
Central America
South America
Region

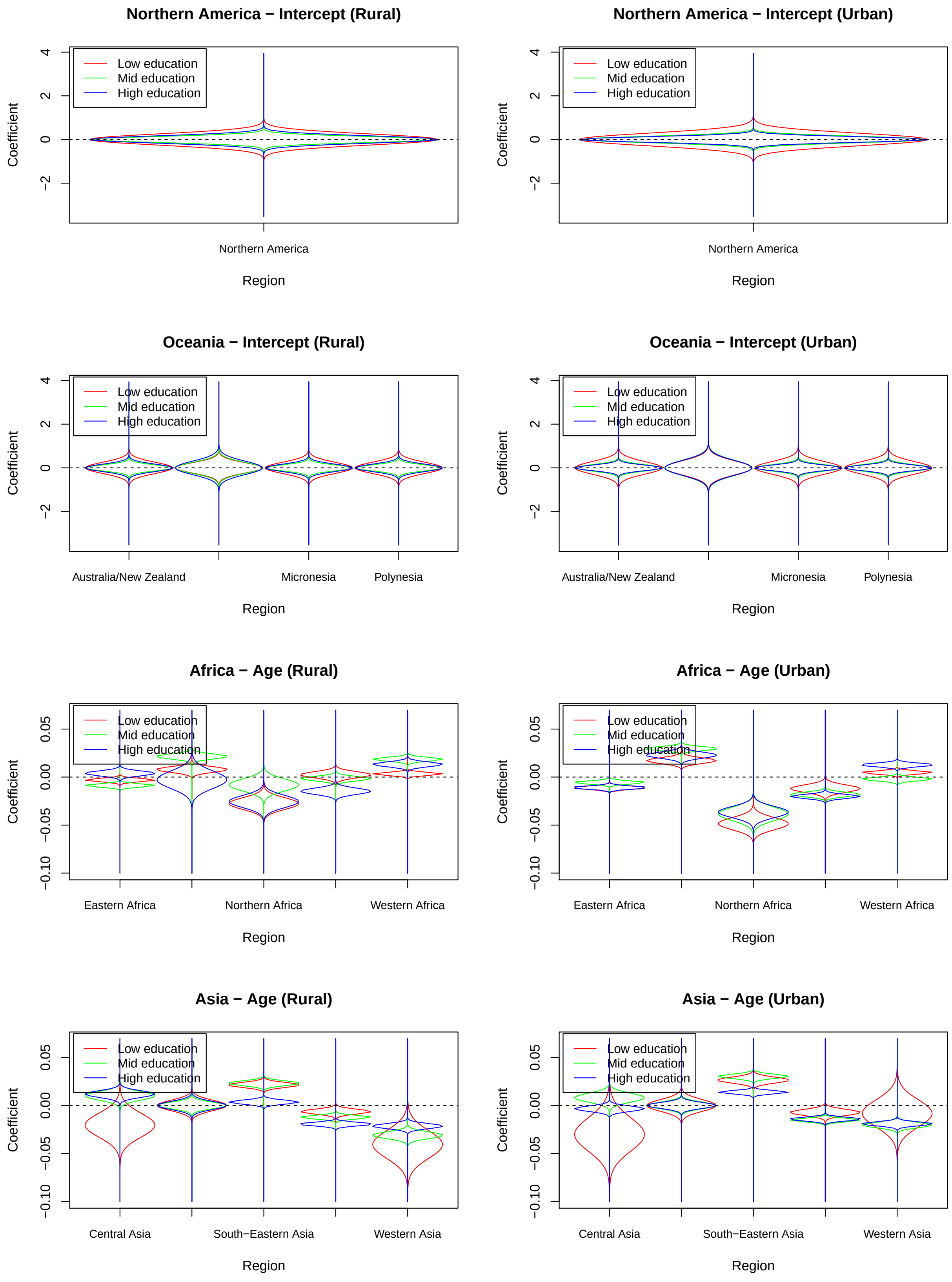

Northern America − Intercept (Rural)
Northern America − Intercept (Urban)
Oceania − Intercept (Rural)
Oceania − Intercept (Urban)
Africa − Age (Rural)
Africa − Age (Urban)
Asia − Age (Rural)
Asia − Age (Urban)
Low education
Mid education
High education
Coefficient
Region
Northern America
Australia/New Zealand
Micronesia
Polynesia
Eastern Africa
Northern Africa
Western Africa
Central Asia
South−Eastern Asia
Western Asia

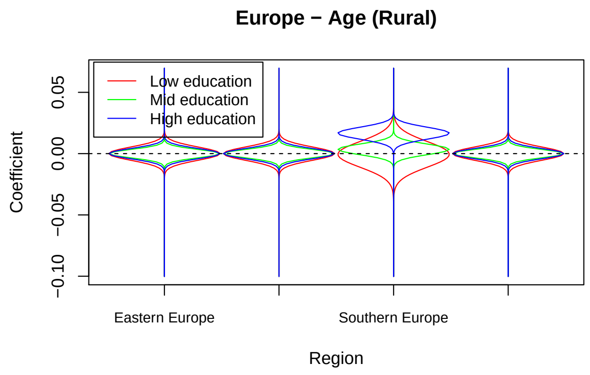
Europe − Age (Rural)
Low education
Mid education
High education
Coefficient
0.05
0.00
−0.05
−0.10
Eastern Europe
Southern Europe
Region

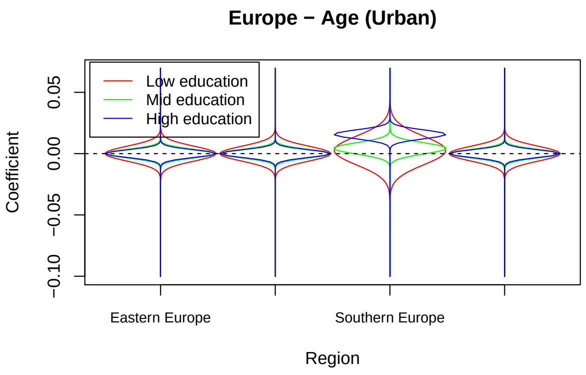
Europe − Age (Urban)
Low education
Mid education
High education
Coefficient
0.05
0.00
−0.05
−0.10
Eastern Europe
Southern Europe
Region

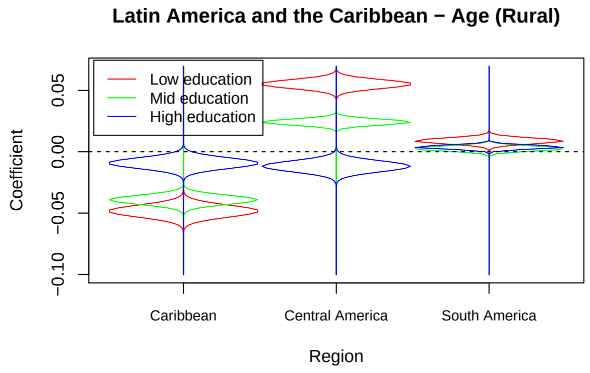
Latin America and the Caribbean − Age (Rural)
Low education
Mid education
High education
Coefficient
0.05
0.00
−0.05
−0.10
Caribbean
Central America
South America
Region

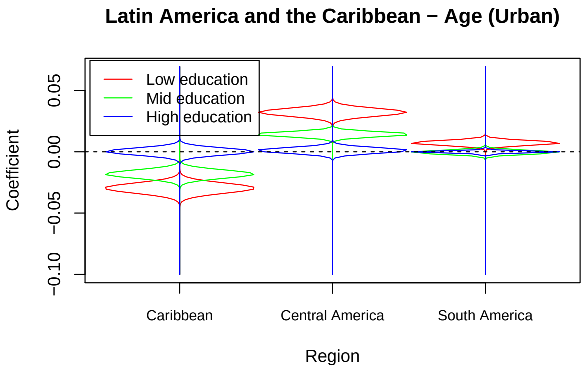
Latin America and the Caribbean − Age (Urban)
Low education
Mid education
High education
Coefficient
0.05
0.00
−0.05
−0.10
Caribbean
Central America
South America
Region

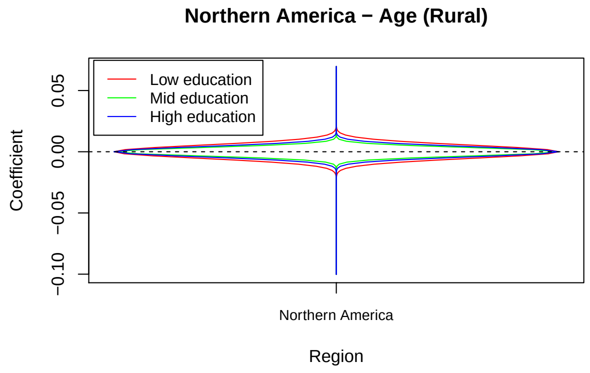
Northern America − Age (Rural)
Low education
Mid education
High education
Coefficient
0.05
0.00
−0.05
−0.10
Northern America
Region

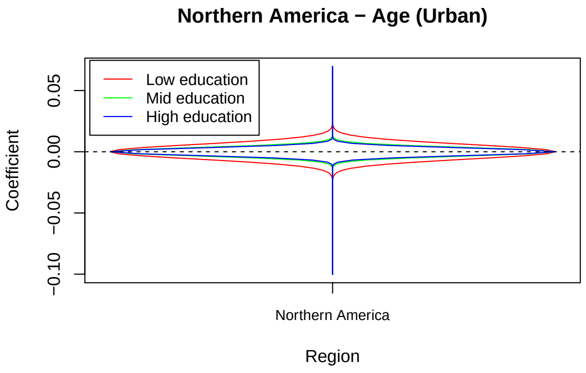
Northern America − Age (Urban)
Low education
Mid education
High education
Coefficient
0.05
0.00
−0.05
−0.10
Northern America
Region

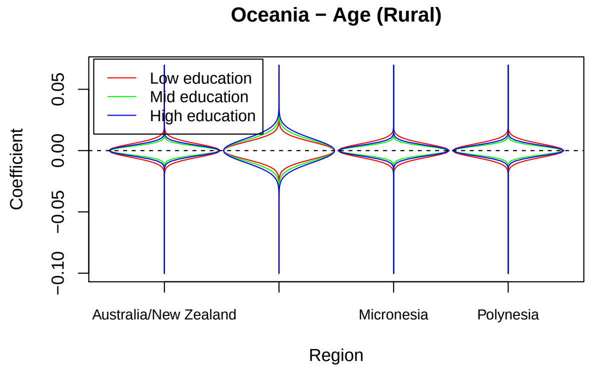
Oceania − Age (Rural)
Low education
Mid education
High education
Coefficient
0.05
0.00
−0.05
−0.10
Australia/New Zealand
Micronesia
Polynesia
Region

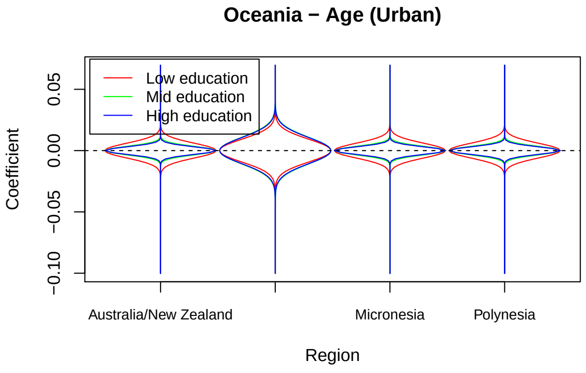
Oceania − Age (Urban)
Low education
Mid education
High education
Coefficient
0.05
0.00
−0.05
−0.10
Australia/New Zealand
Micronesia
Polynesia
Region

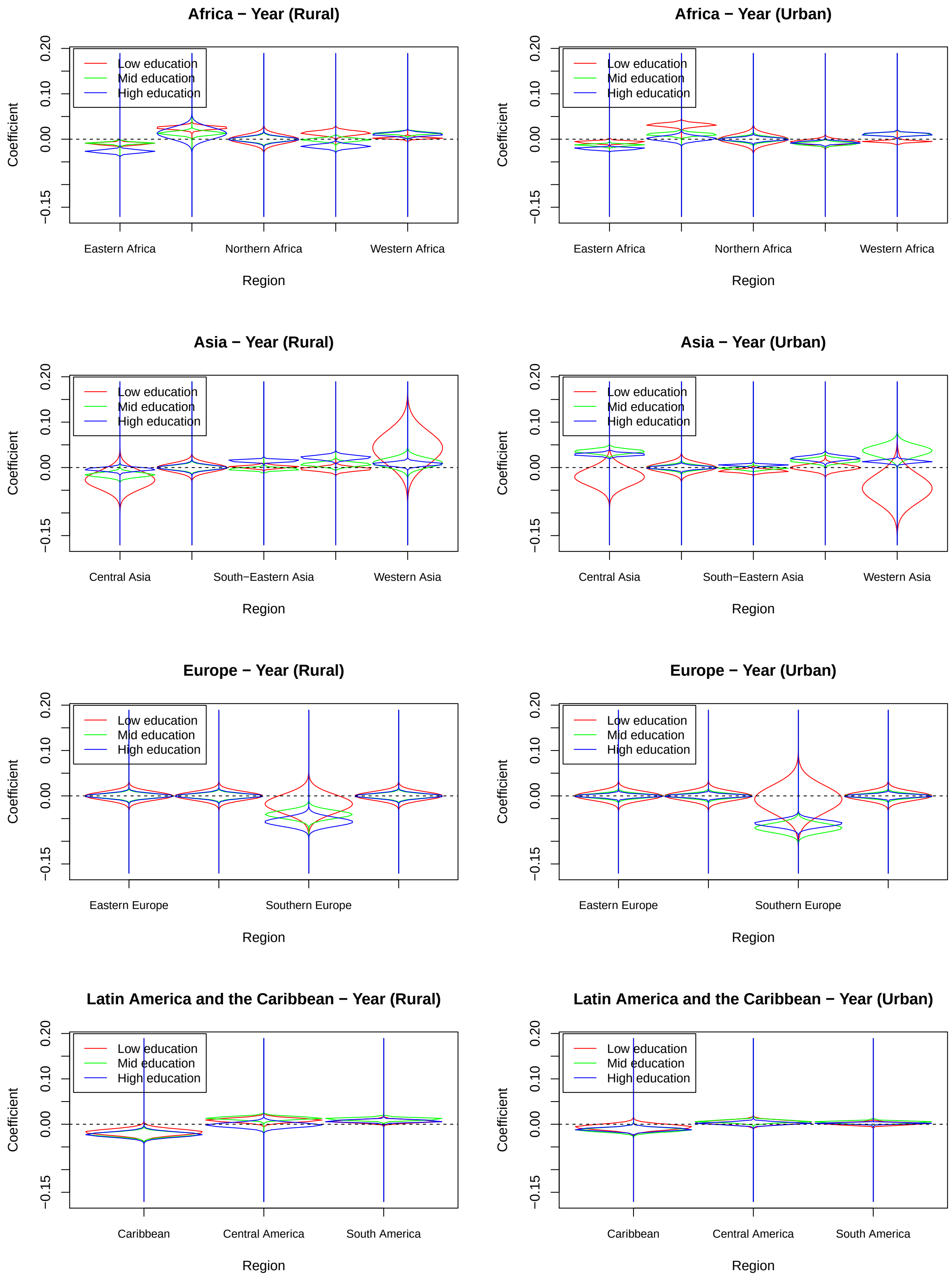
Africa − Year (Rural)
Africa − Year (Urban)
Asia − Year (Rural)
Asia − Year (Urban)
Europe − Year (Rural)
Europe − Year (Urban)
Latin America and the Caribbean − Year (Rural)
Latin America and the Caribbean − Year (Urban)
Low education
Mid education
High education
Coefficient
0.20
0.10
0.00
−0.15
Region
Eastern Africa
Northern Africa
Western Africa
Central Asia
South−Eastern Asia
Western Asia
Eastern Europe
Southern Europe
Caribbean
Central America
South America

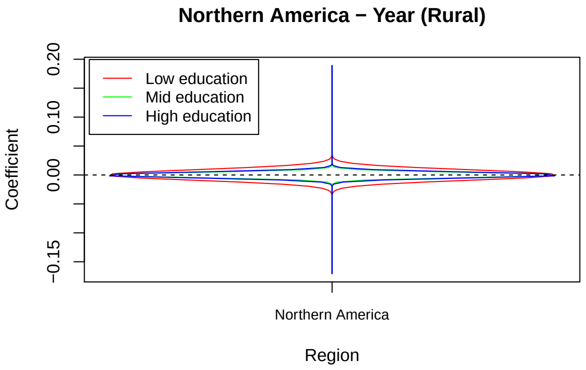
Northern America − Year (Rural)
Low education
Mid education
High education
Coefficient
−0.15
0.00
0.10
0.20
Northern America
Region

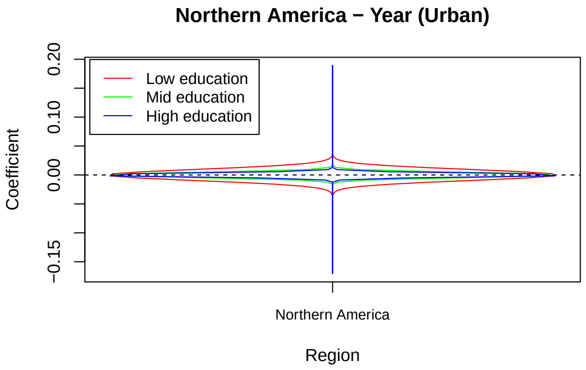
Northern America − Year (Urban)
Low education
Mid education
High education
Coefficient
−0.15
0.00
0.10
0.20
Northern America
Region

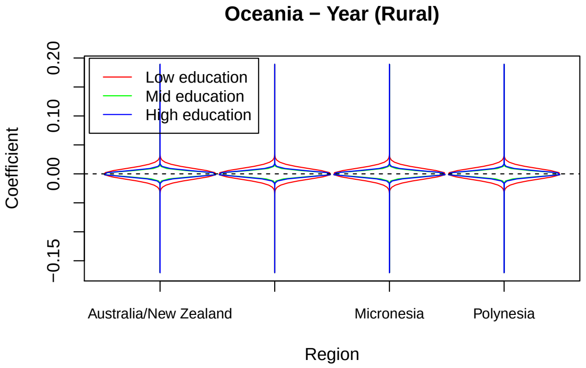
Oceania − Year (Rural)
Low education
Mid education
High education
Coefficient
−0.15
0.00
0.10
0.20
Australia/New Zealand
Micronesia
Polynesia
Region

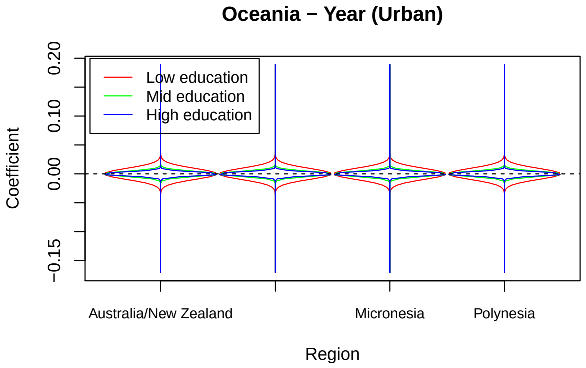
Oceania − Year (Urban)
Low education
Mid education
High education
Coefficient
−0.15
0.00
0.10
0.20
Australia/New Zealand
Micronesia
Polynesia
Region

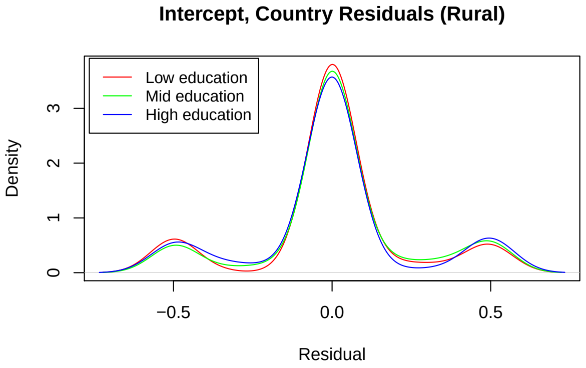
Intercept, Country Residuals (Rural)
Low education
Mid education
High education
Density
0
1
2
3
−0.5
0.0
0.5
Residual

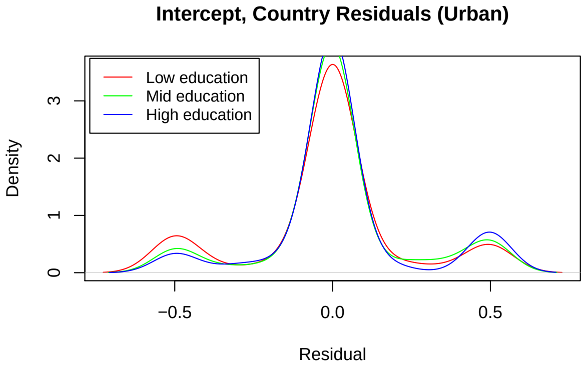
Intercept, Country Residuals (Urban)
Low education
Mid education
High education
Density
0
1
2
3
−0.5
0.0
0.5
Residual

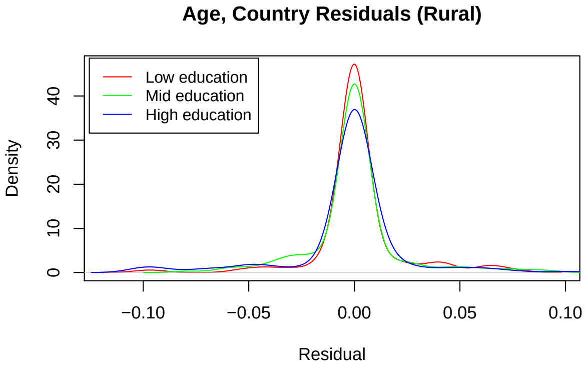
Age, Country Residuals (Rural)
Low education
Mid education
High education
Density
0
10
20
30
40
−0.10
−0.05
0.00
0.05
0.10
Residual

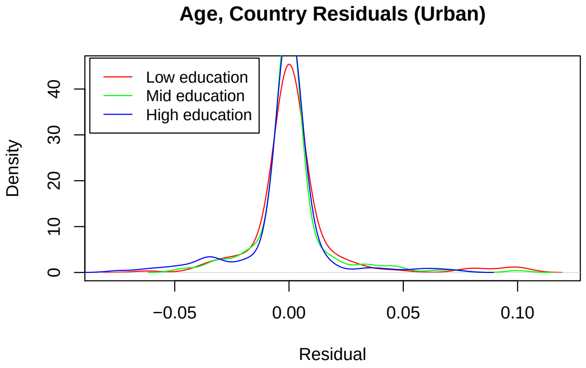
Age, Country Residuals (Urban)
Low education
Mid education
High education
Density
0
10
20
30
40
−0.05
0.00
0.05
0.10
Residual

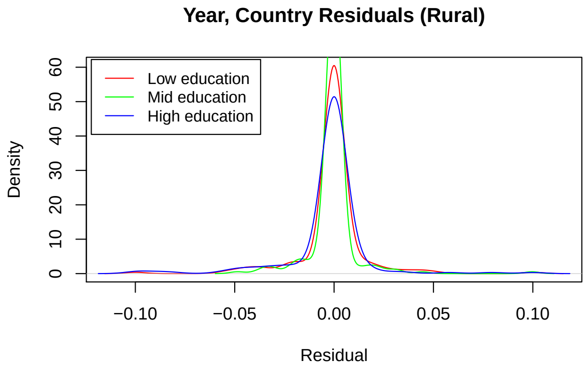

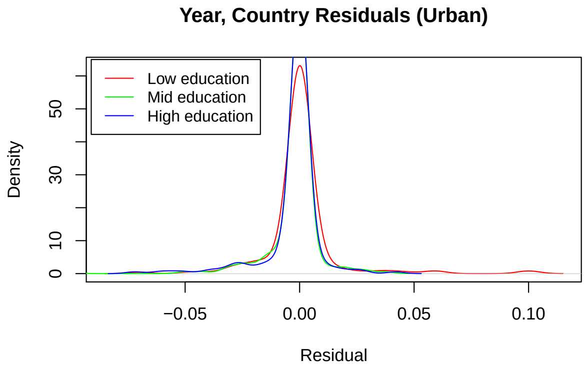

Model Implementation

Given the parameters described above (intercept, age, year), the mean number of living children λ_w is predicted for each woman who has been sexually active for at least 9 months in 1985 (cycle 0). Her number of children is then drawn from a Poisson distribution: $\text{Pois}(\lambda_w)$. The sex of each child is assigned randomly (50% male), and the current age of each child is drawn uniformly at random based on the interval between the mother's age at sexual debut (plus 9 months) and her current age.

A.1.3.3 Desired Number of Children

Overview

We assume that each woman in the model has certain fertility preferences, operationalized by her stated ideal family size, or desired number of children. This variable determines what 'mentality' of family planning a woman is currently operating under: 'spacing' or 'limiting'. While women have less than their desired number of children they are considered to be 'spacing'. Once they have met (or exceeded) this number they are considered to be 'limiting'.

Data

We assume that a woman's desired number of children remains constant over her reproductive life-cycle. We did not find any studies that looked at changes in desired number of children over the course of a woman's life. However, we did find four related studies that examined consistency in reporting fertility preferences over time, ranging from five months to four years. Two longitudinal studies from India and Morocco specifically assessed consistency of women's reported desired number of children (Bankole 1998, Roy 2008), and two studies from rural Ghana assessed women's desire to stop childbearing over time (Kodzi 2010, 2012). A review of this literature did not reveal any consistent trends in how desired fertility may change throughout a woman's life.

We analyzed data on the reported ideal number of children from the DHS (v613). We removed missing or "Don't know" responses. Data were available for 4,102,452 women from 254 surveys in 74 countries. We assumed that non-numeric responses indicated fatalistic attitudes towards family size (i.e. no preference). These responses (n=212,933) occurred on average for 5.2% of women, ranging from near 0% in Lesotho and Vietnam to around 20% in Afghanistan and Chad and nearly 30% in Yemen. We re-coded these responses to 10 desired children, and top-coded the number of specified desired children at 20 (500 responses were top-coded). Here we plot the global distribution of desired number of children (by subgroup) and explore potential secular trends in the mean and variance of this distribution.

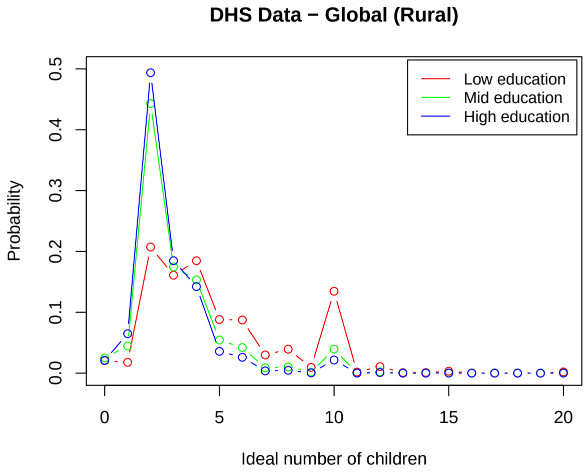

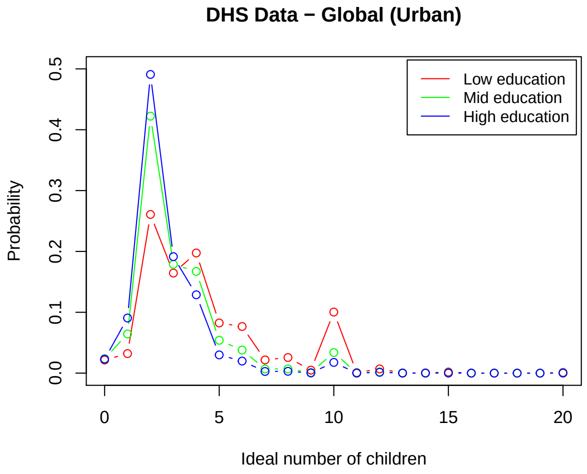

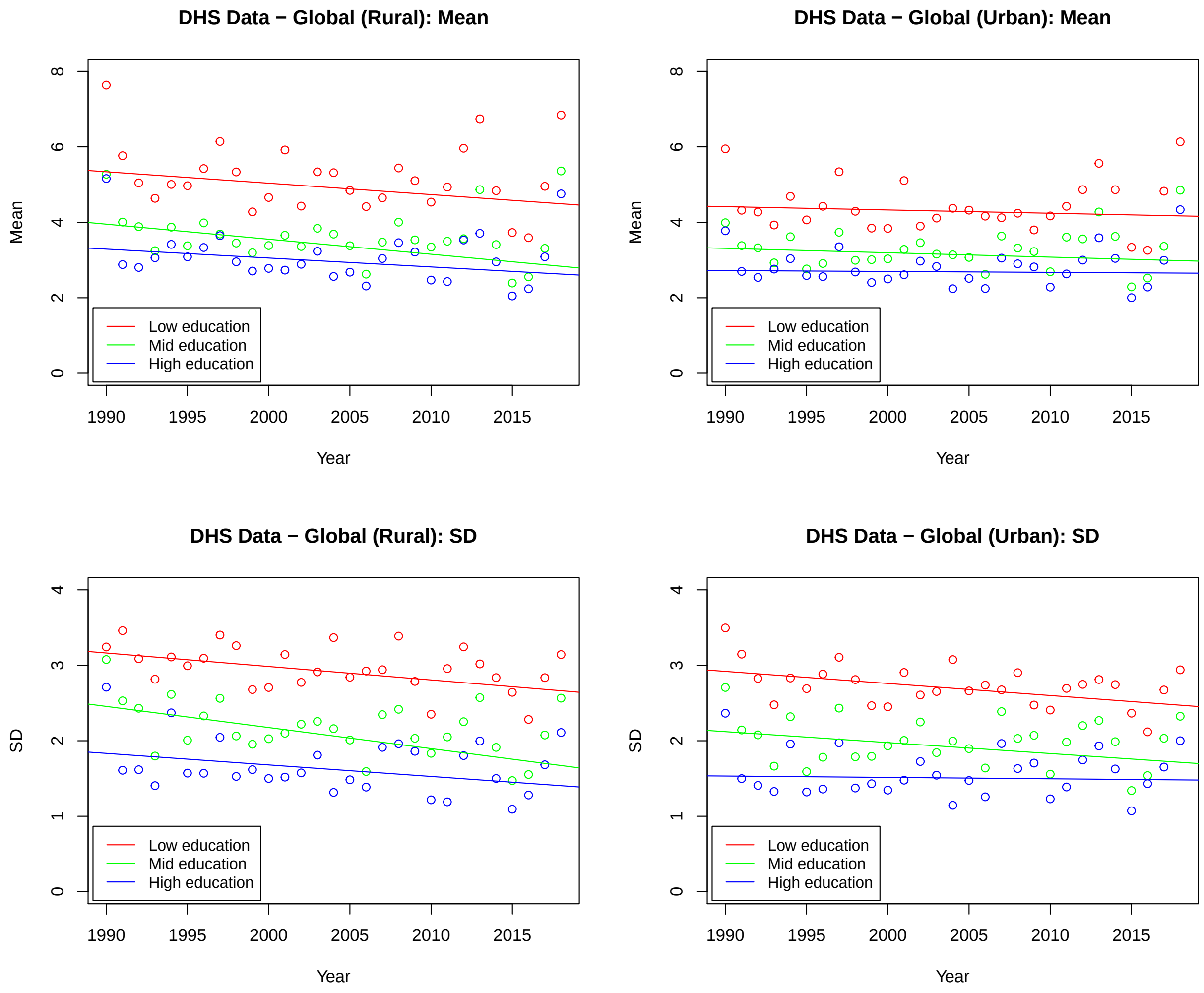

Parameters

We assume that the distribution of number of desired children follows a Poisson distribution (Pois(λ)), with an additional point mass (π) at p. This allows us to account for high concentrations of particular answers, for example, a large spike at 2 desired children:

$$P(K = k) = (1 - \pi)\frac{\lambda^k e^{-\lambda}}{k!} + \mathbb{I}(k = p)\pi$$

We fit hierarchical models to estimate priors for these parameters, stratified by subgroup (urban/rural and level of education). We used upper middle income priors for high income countries due to lack of DHS data in high income countries.

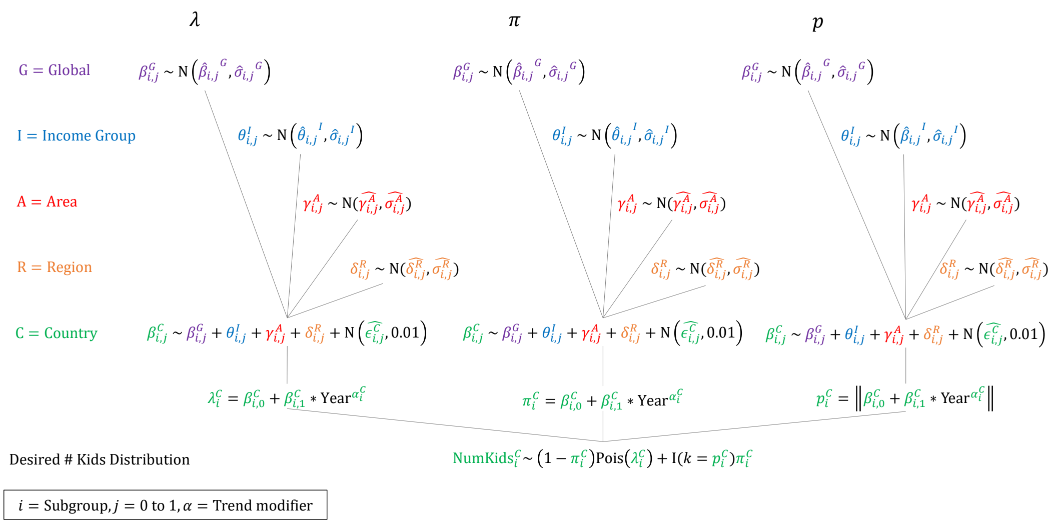
λ
π
p
G = Global
I = Income Group
A = Area
R = Region
C = Country
Desired # Kids Distribution
$\beta^G_{i,j} \sim \mathrm{N}\left(\hat{\beta}_{i,j}{}^G, \hat{\sigma}_{i,j}{}^G\right)$
$\theta^I_{i,j} \sim \mathrm{N}\left(\hat{\theta}_{i,j}{}^I, \hat{\sigma}_{i,j}{}^I\right)$
$\theta^I_{i,j} \sim \mathrm{N}\left(\hat{\beta}_{i,j}{}^I, \hat{\sigma}_{i,j}{}^I\right)$
$\gamma^A_{i,j} \sim \mathrm{N}(\widehat{\gamma^A_{i,j}}, \widehat{\sigma^A_{i,j}})$
$\delta^R_{i,j} \sim \mathrm{N}(\widehat{\delta^R_{i,j}}, \widehat{\sigma^R_{i,j}})$
$\beta^C_{i,j} \sim \beta^G_{i,j} + \theta^I_{i,j} + \gamma^A_{i,j} + \delta^R_{i,j} + \mathrm{N}\left(\widehat{\epsilon^C_{i,j}}, 0.01\right)$
$\lambda^C_i = \beta^C_{i,0} + \beta^C_{i,1} * \mathrm{Year}^{\alpha^C_i}$
$\pi^C_i = \beta^C_{i,0} + \beta^C_{i,1} * \mathrm{Year}^{\alpha^C_i}$
$p^C_i = \left\| \beta^C_{i,0} + \beta^C_{i,1} * \mathrm{Year}^{\alpha^C_i} \right\|$
$\mathrm{NumKids}^C_i \sim (1 - \pi^C_i)\mathrm{Pois}(\lambda^C_i) + \mathrm{I}(k = p^C_i)\pi^C_i$
i = Subgroup, j = 0 to 1, α = Trend modifier

Priors

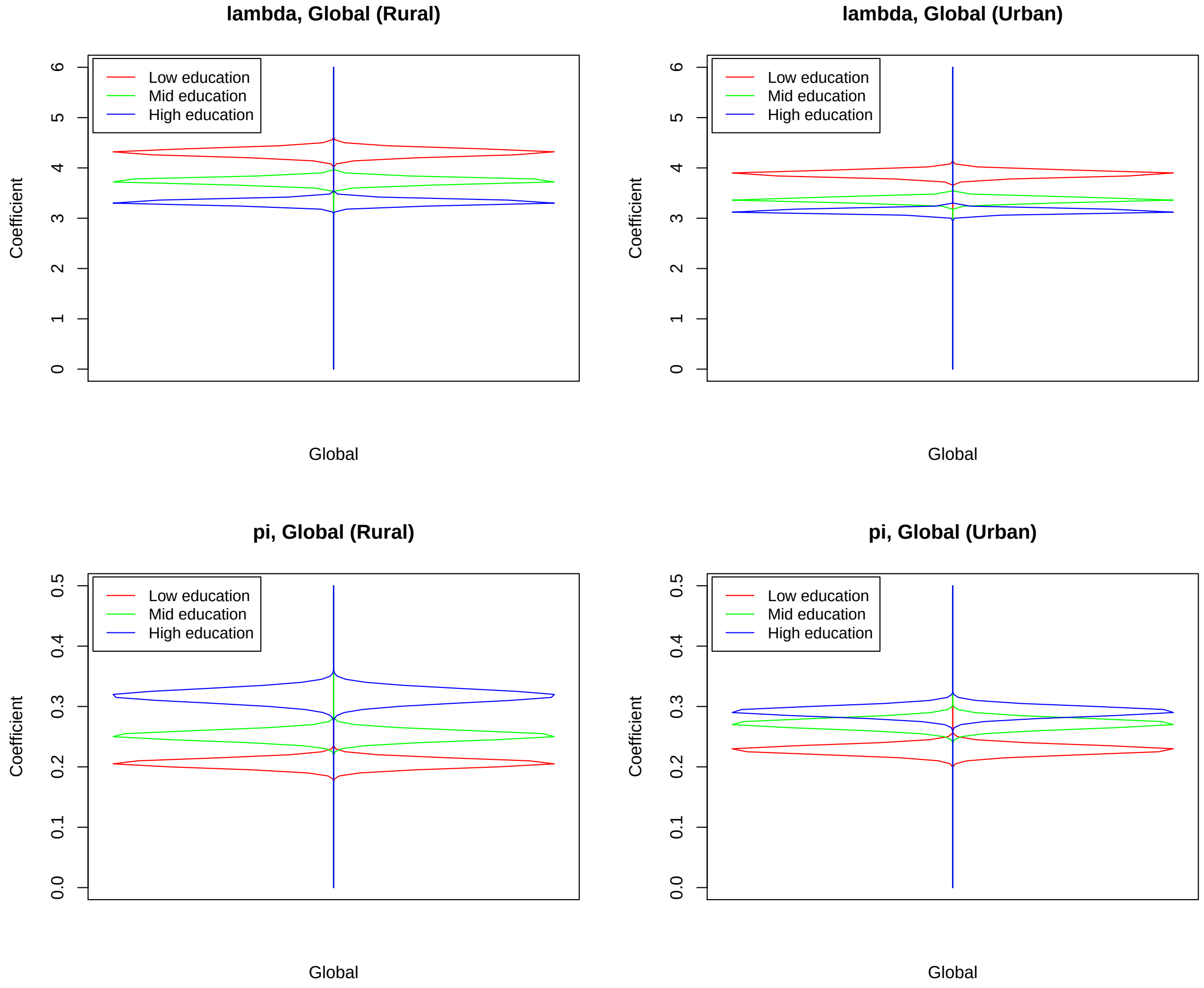
lambda, Global (Rural)
lambda, Global (Urban)
pi, Global (Rural)
pi, Global (Urban)
Low education
Mid education
High education
Coefficient
Global
0
1
2
3
4
5
6
0.0
0.1
0.2
0.3
0.4
0.5

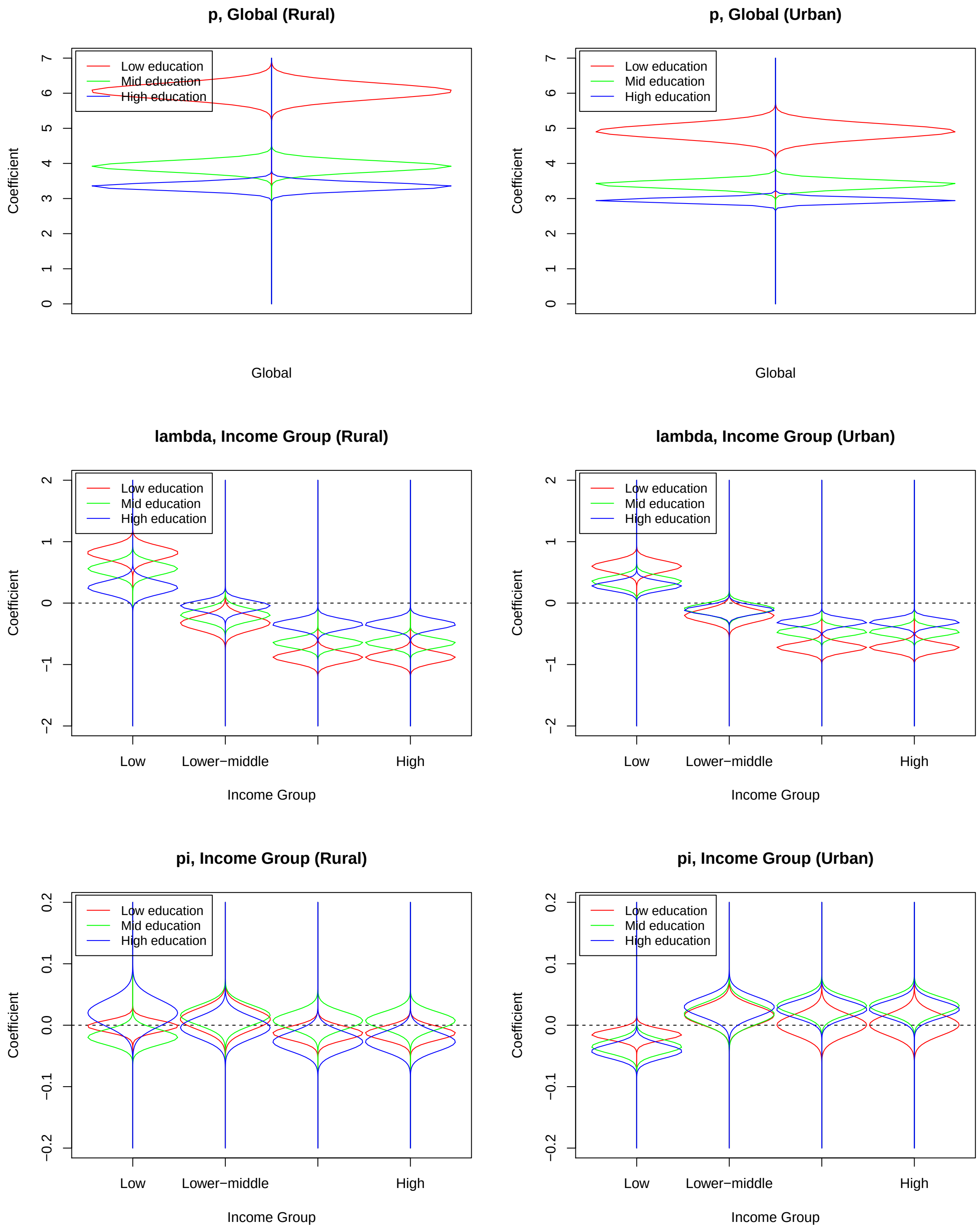
p, Global (Rural)
p, Global (Urban)
Low education
Mid education
High education
Coefficient
Global
lambda, Income Group (Rural)
lambda, Income Group (Urban)
Low
Lower−middle
High
Income Group
pi, Income Group (Rural)
pi, Income Group (Urban)

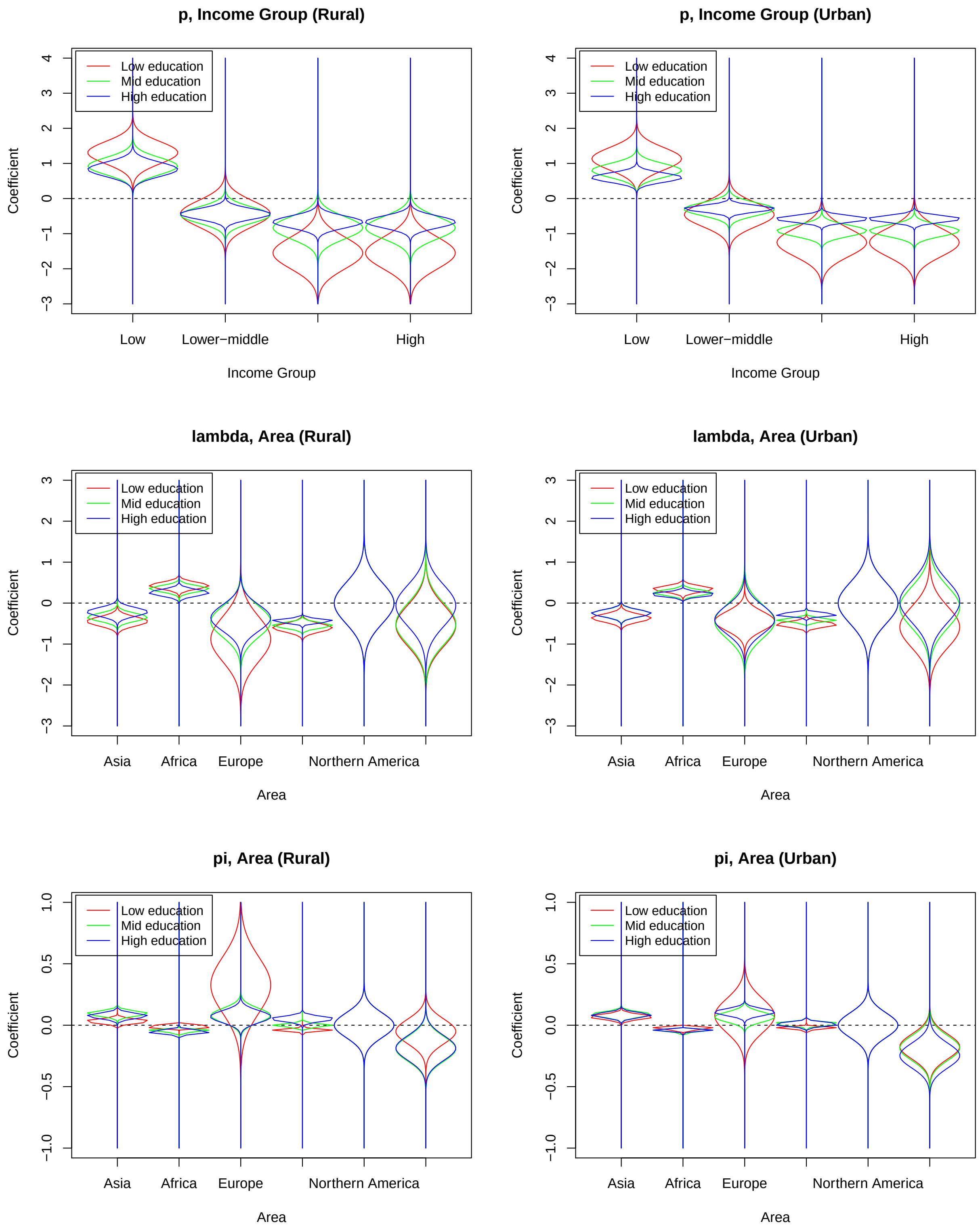
p, Income Group (Rural)
Low education
Mid education
High education
Coefficient
Low
Lower−middle
High
Income Group
p, Income Group (Urban)
Low education
Mid education
High education
Coefficient
Low
Lower−middle
High
Income Group
lambda, Area (Rural)
Low education
Mid education
High education
Coefficient
Asia
Africa
Europe
Northern America
Area
lambda, Area (Urban)
Low education
Mid education
High education
Coefficient
Asia
Africa
Europe
Northern America
Area
pi, Area (Rural)
Low education
Mid education
High education
Coefficient
Asia
Africa
Europe
Northern America
Area
pi, Area (Urban)
Low education
Mid education
High education
Coefficient
Asia
Africa
Europe
Northern America
Area

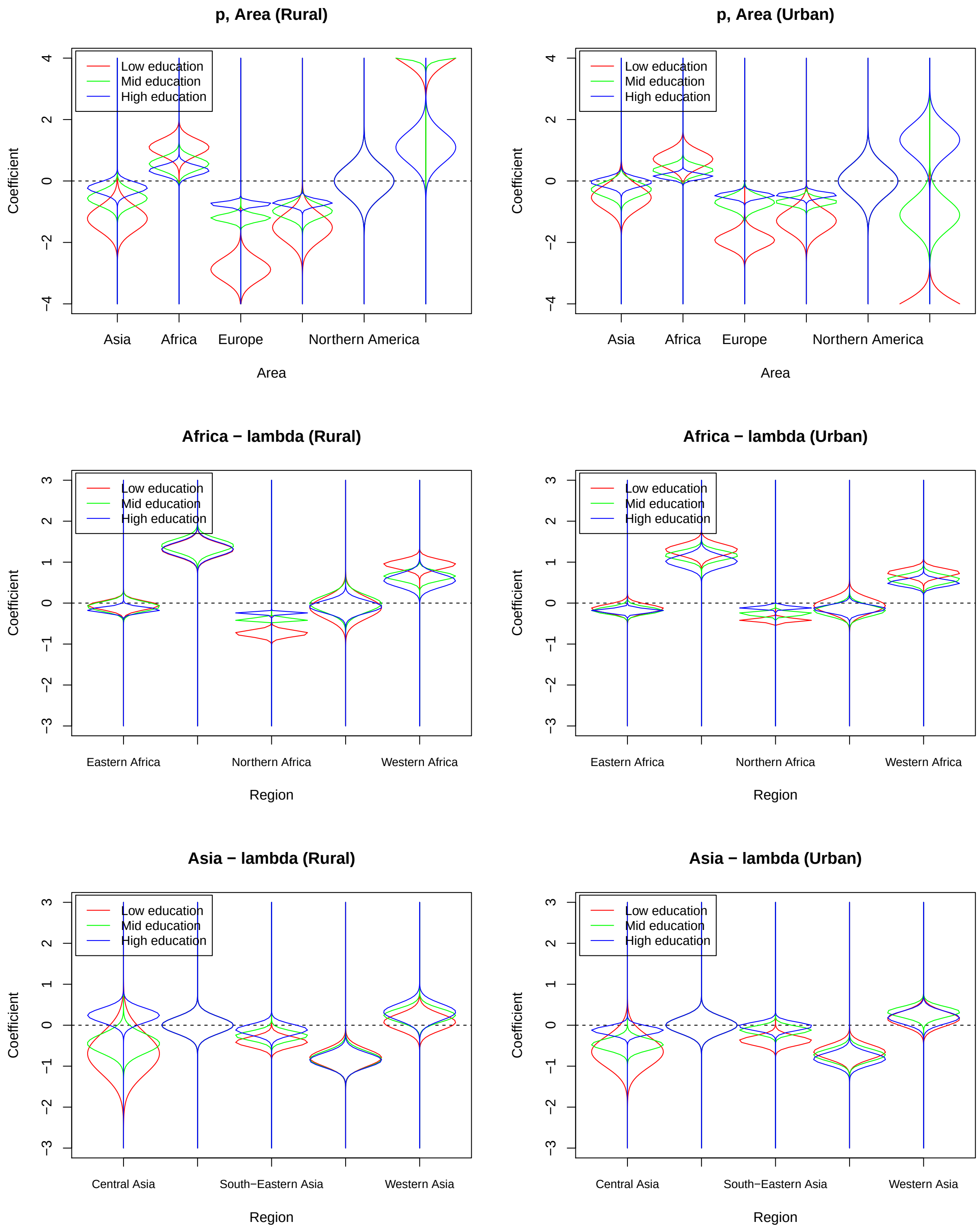

p, Area (Rural)
p, Area (Urban)
Low education
Mid education
High education
Coefficient
Asia
Africa
Europe
Northern America
Area
Africa − lambda (Rural)
Africa − lambda (Urban)
Eastern Africa
Northern Africa
Western Africa
Region
Asia − lambda (Rural)
Asia − lambda (Urban)
Central Asia
South−Eastern Asia
Western Asia

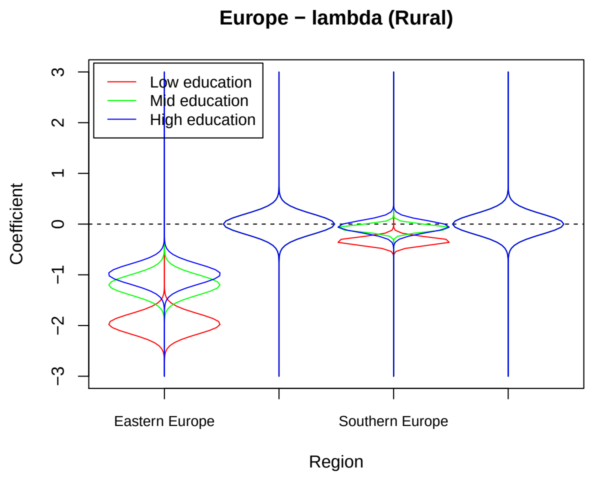
Europe − lambda (Rural)
Low education
Mid education
High education
Coefficient
3
2
1
0
−1
−2
−3
Eastern Europe
Southern Europe
Region

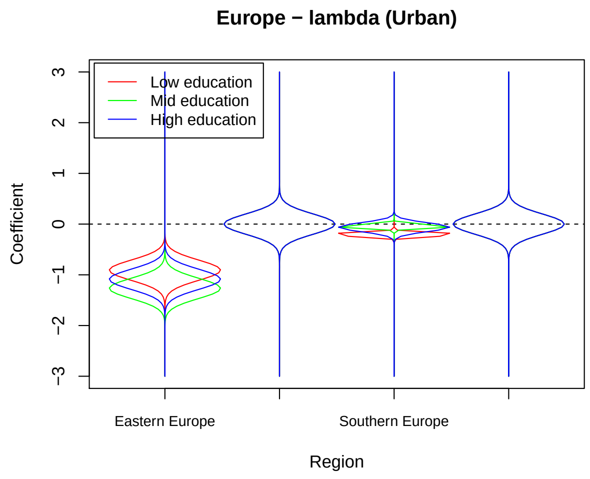
Europe − lambda (Urban)
Low education
Mid education
High education
Coefficient
3
2
1
0
−1
−2
−3
Eastern Europe
Southern Europe
Region

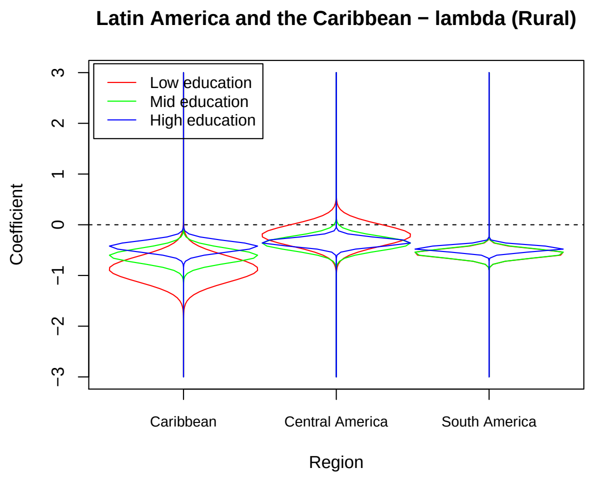
Latin America and the Caribbean − lambda (Rural)
Low education
Mid education
High education
Coefficient
3
2
1
0
−1
−2
−3
Caribbean
Central America
South America
Region

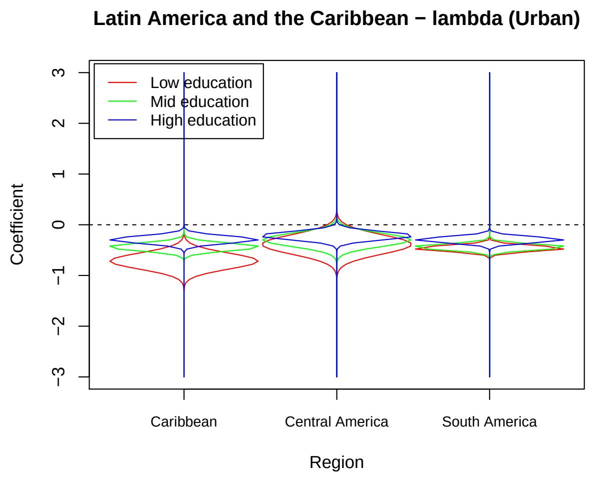
Latin America and the Caribbean − lambda (Urban)
Low education
Mid education
High education
Coefficient
3
2
1
0
−1
−2
−3
Caribbean
Central America
South America
Region

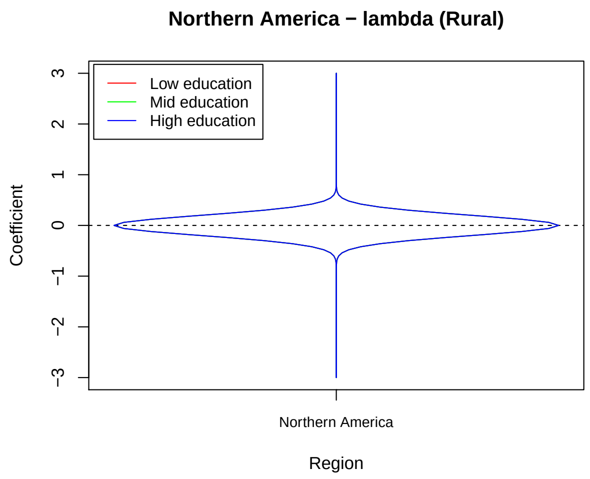
Northern America − lambda (Rural)
Low education
Mid education
High education
Coefficient
3
2
1
0
−1
−2
−3
Northern America
Region

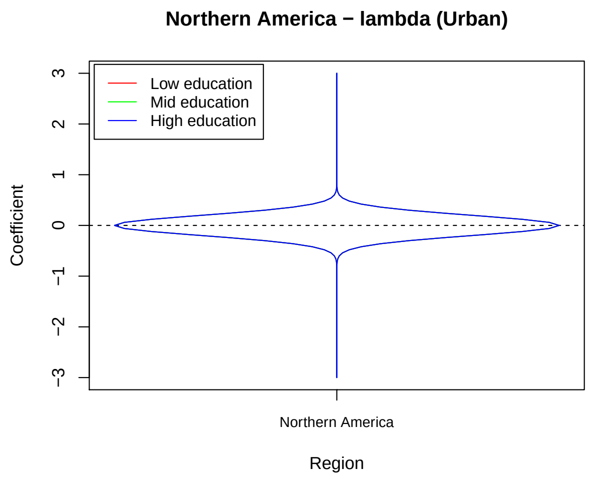
Northern America − lambda (Urban)
Low education
Mid education
High education
Coefficient
3
2
1
0
−1
−2
−3
Northern America
Region

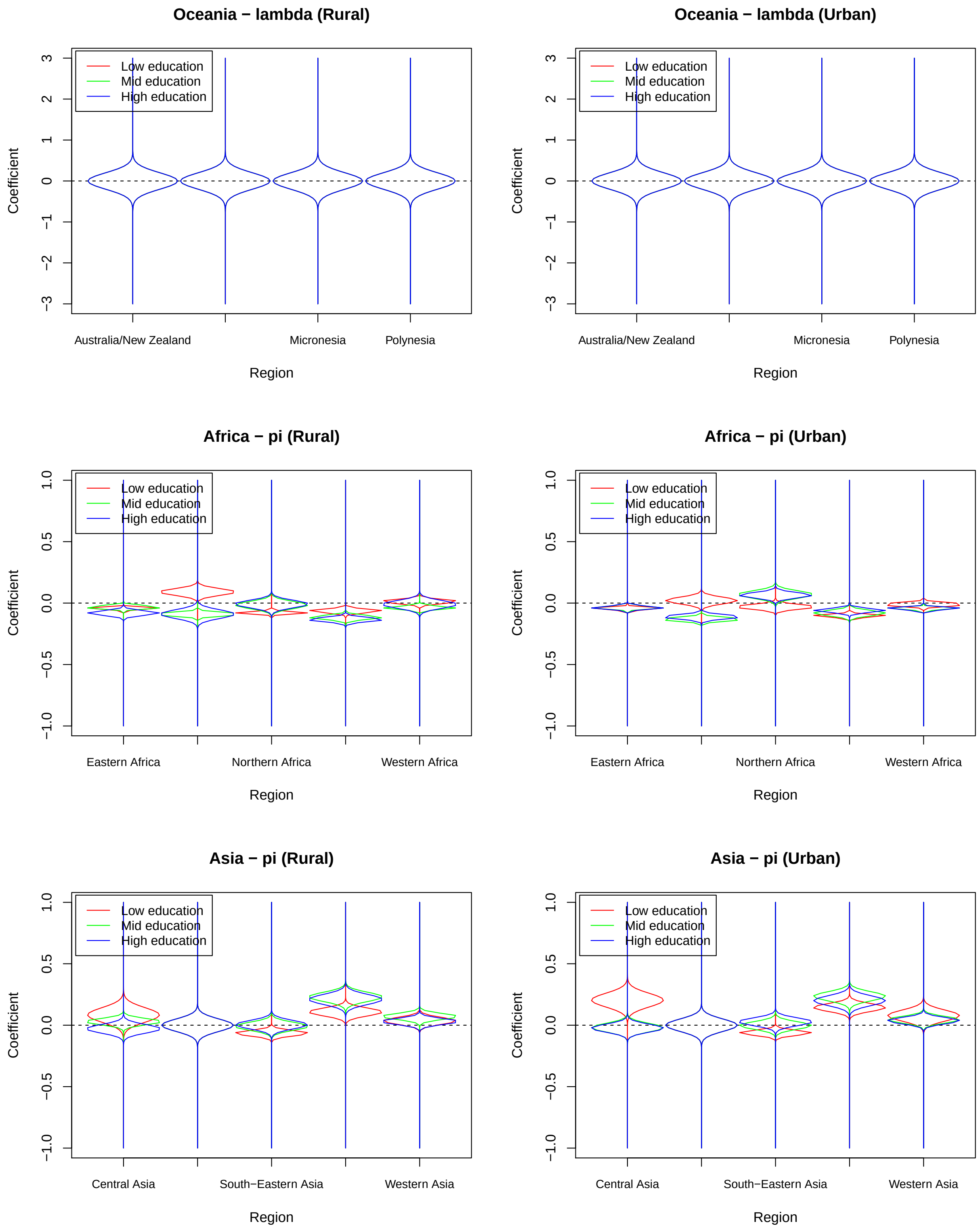

Oceania − lambda (Rural)
Low education
Mid education
High education
Coefficient
Australia/New Zealand
Micronesia
Polynesia
Region
Oceania − lambda (Urban)
Africa − pi (Rural)
Eastern Africa
Northern Africa
Western Africa
Africa − pi (Urban)
Asia − pi (Rural)
Central Asia
South−Eastern Asia
Western Asia
Asia − pi (Urban)

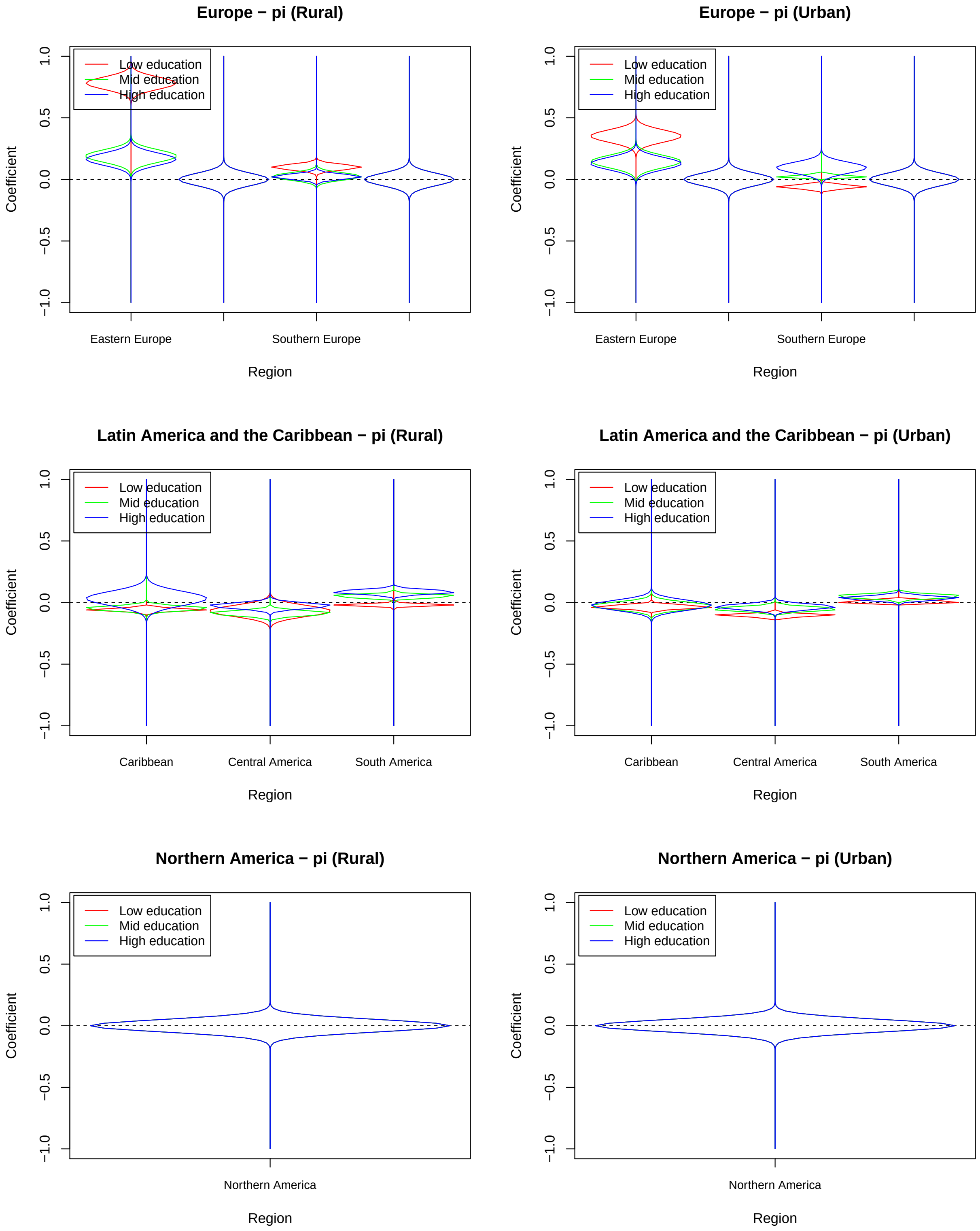
Europe − pi (Rural)
Europe − pi (Urban)
Latin America and the Caribbean − pi (Rural)
Latin America and the Caribbean − pi (Urban)
Northern America − pi (Rural)
Northern America − pi (Urban)
Low education
Mid education
High education
Coefficient
1.0
0.5
0.0
−0.5
−1.0
Eastern Europe
Southern Europe
Caribbean
Central America
South America
Northern America
Region

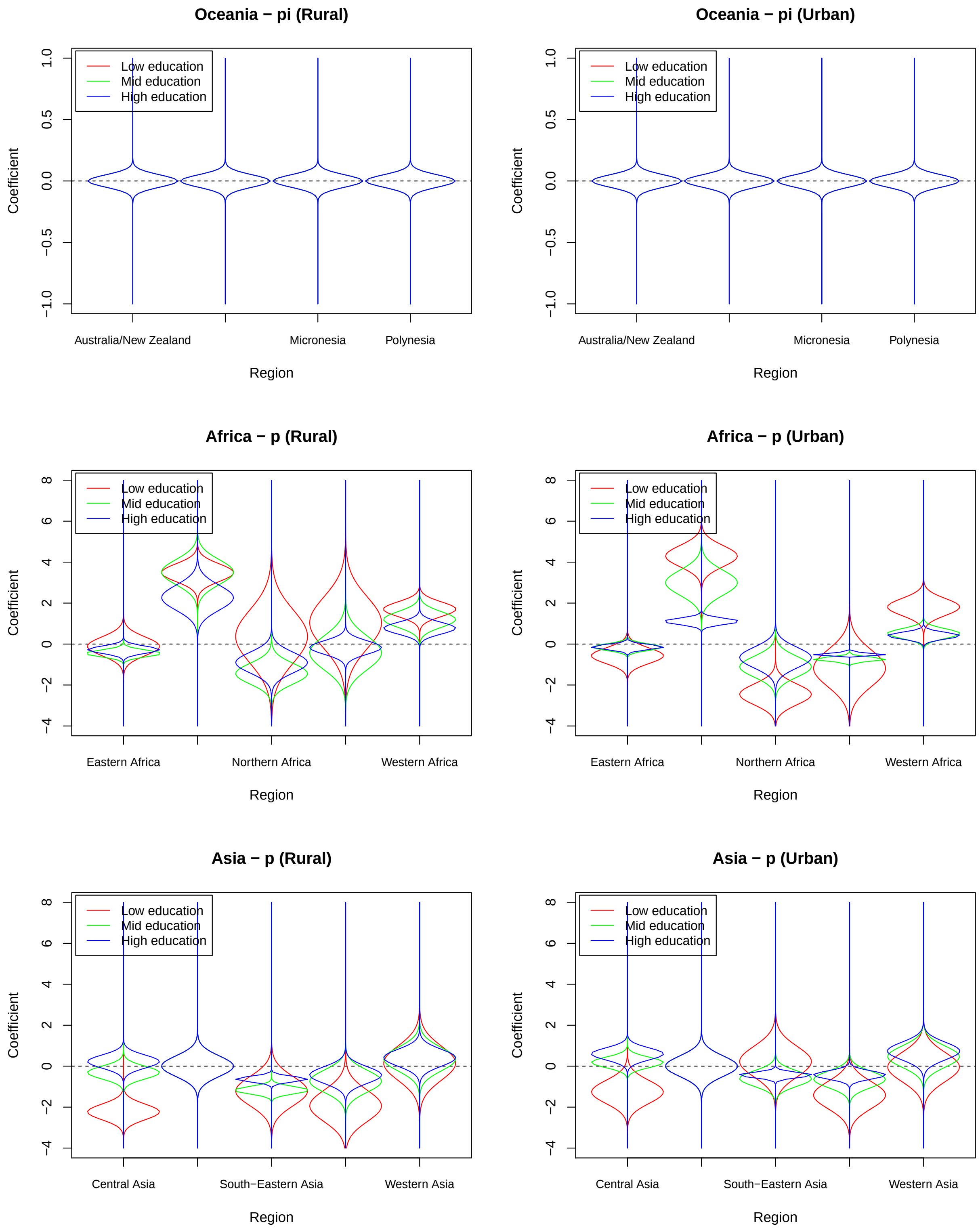
Oceania − pi (Rural)
Oceania − pi (Urban)
Africa − p (Rural)
Africa − p (Urban)
Asia − p (Rural)
Asia − p (Urban)
Low education
Mid education
High education
Coefficient
Region
Australia/New Zealand
Micronesia
Polynesia
Eastern Africa
Northern Africa
Western Africa
Central Asia
South−Eastern Asia
Western Asia

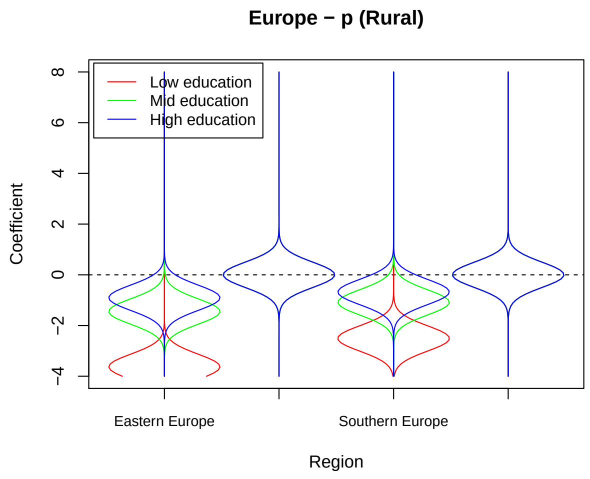
Europe − p (Rural)
Low education
Mid education
High education
Coefficient
8
6
4
2
0
−2
−4
Eastern Europe
Southern Europe
Region

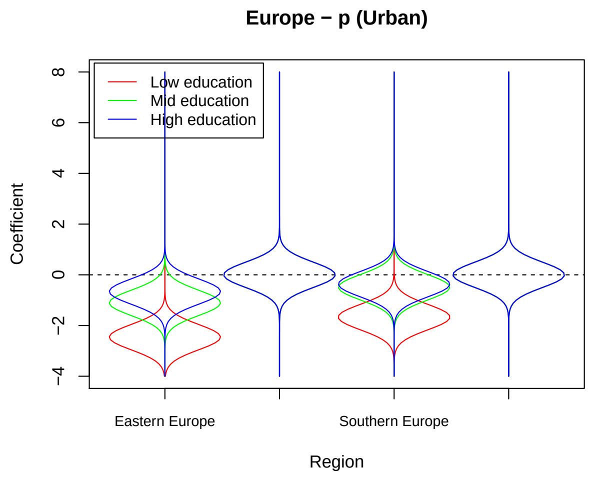
Europe − p (Urban)
Low education
Mid education
High education
Coefficient
8
6
4
2
0
−2
−4
Eastern Europe
Southern Europe
Region

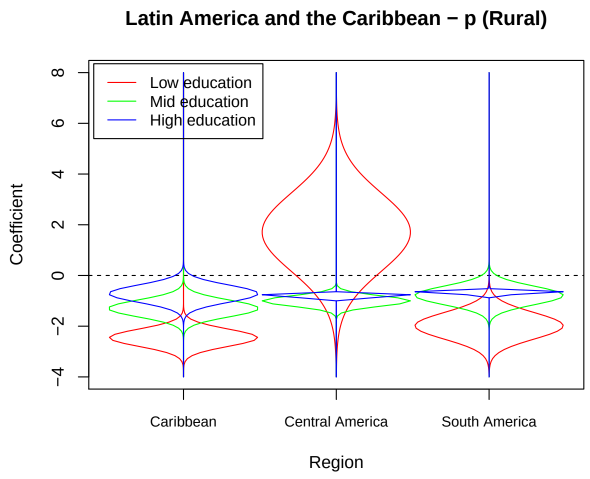
Latin America and the Caribbean − p (Rural)
Low education
Mid education
High education
Coefficient
8
6
4
2
0
−2
−4
Caribbean
Central America
South America
Region

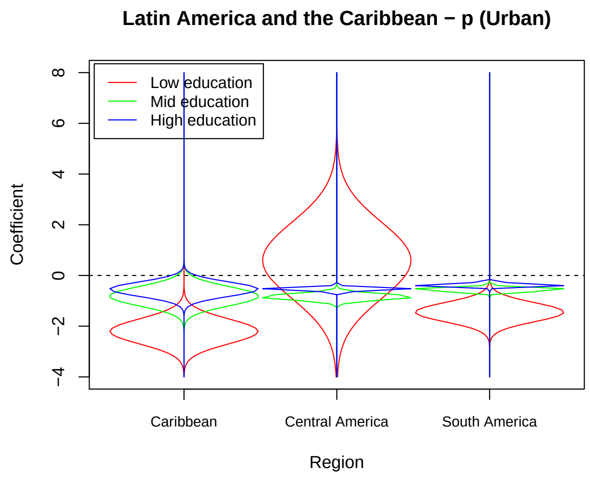
Latin America and the Caribbean − p (Urban)
Low education
Mid education
High education
Coefficient
8
6
4
2
0
−2
−4
Caribbean
Central America
South America
Region

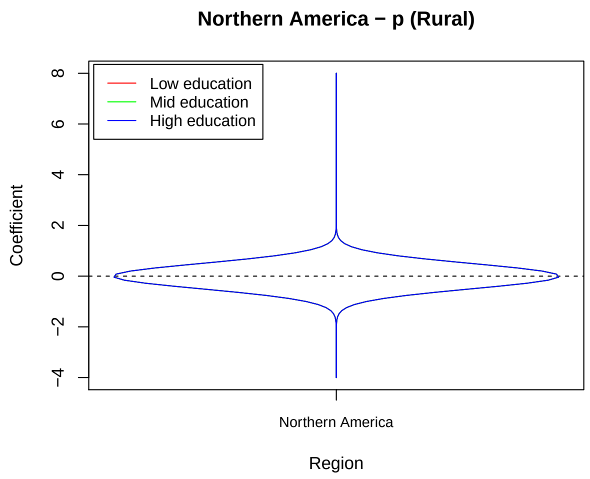
Northern America − p (Rural)
Low education
Mid education
High education
Coefficient
8
6
4
2
0
−2
−4
Northern America
Region

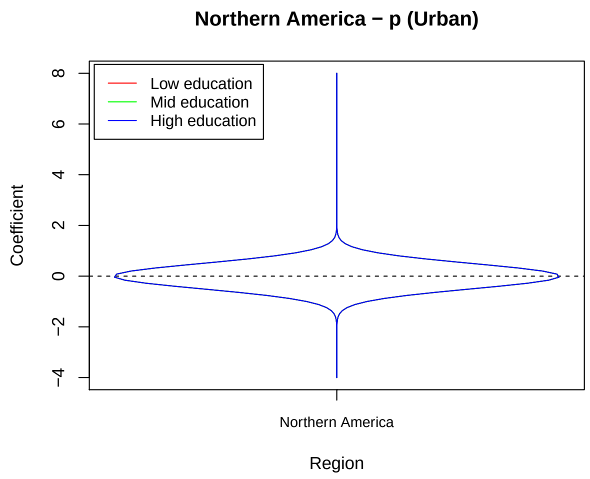
Northern America − p (Urban)
Low education
Mid education
High education
Coefficient
8
6
4
2
0
−2
−4
Northern America
Region

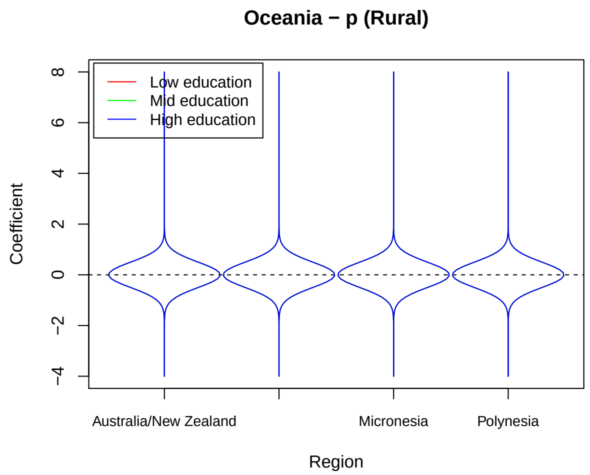
Oceania − p (Rural)
Low education
Mid education
High education
Coefficient
Australia/New Zealand
Micronesia
Polynesia
Region

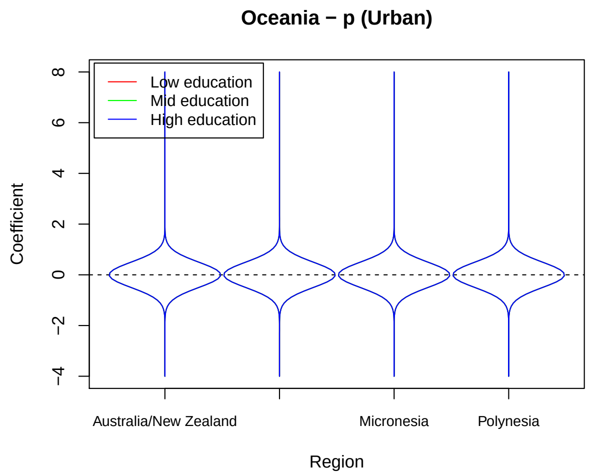
Oceania − p (Urban)
Low education
Mid education
High education
Coefficient
Australia/New Zealand
Micronesia
Polynesia
Region

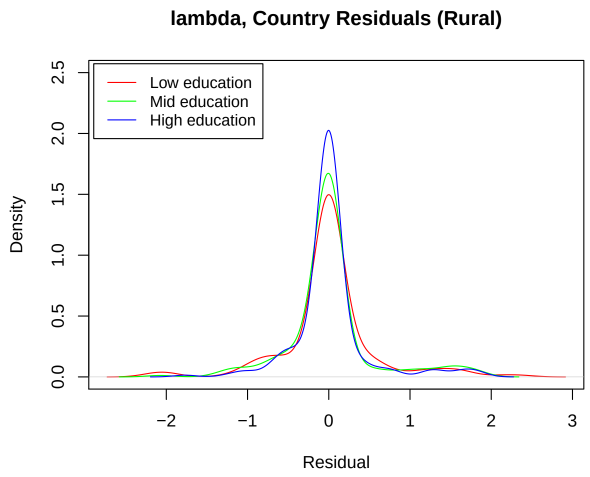
lambda, Country Residuals (Rural)
Low education
Mid education
High education
Density
Residual

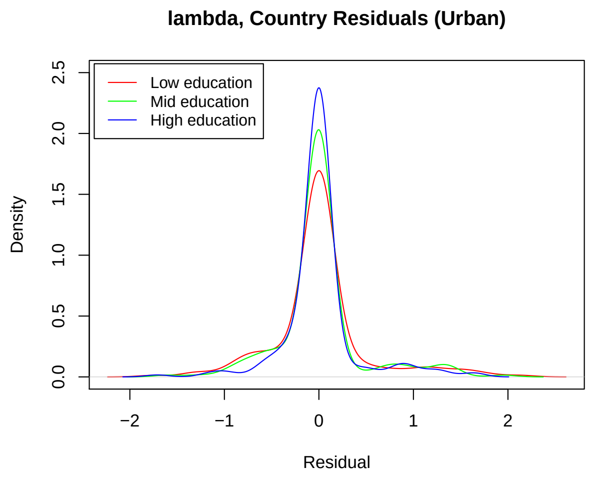
lambda, Country Residuals (Urban)
Low education
Mid education
High education
Density
Residual

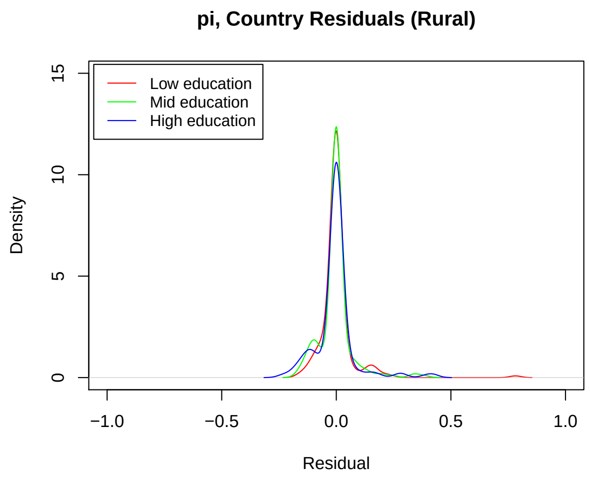
pi, Country Residuals (Rural)
Low education
Mid education
High education
Density
Residual

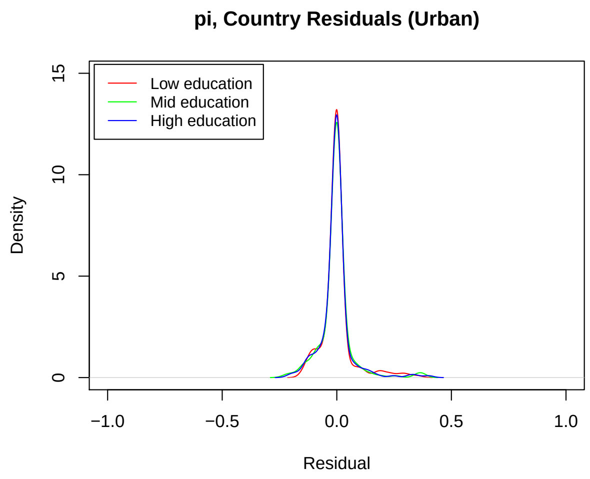
pi, Country Residuals (Urban)
Low education
Mid education
High education
Density
Residual

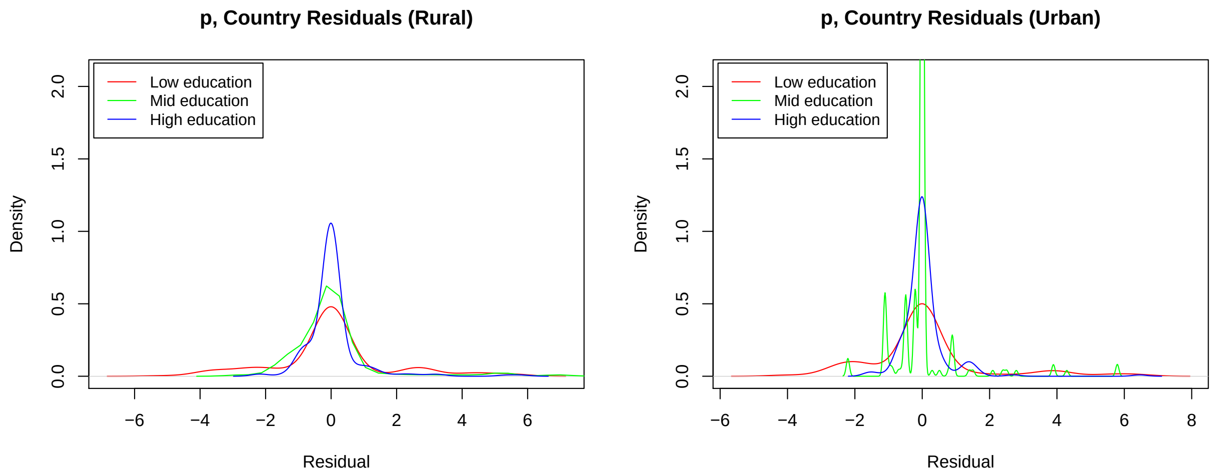

Model Implementation

A desired number of children is drawn for each woman at model initialization. Women who have less living children than the desired number are assumed to be ‘spacing’, while women who have met (or exceeded) this number are assumed to be ‘limiting’. If the death of a child in the model reduces a woman’s total number of children below her desired family size, we assume that she reverts to a spacing ‘mentality’.

References

Bankole A, Westoff CF. The consistency and validity of reproductive attitudes: evidence from Morocco. J Biosoc Sci 1998;30:439-455.

Kodzi IA, Casterline JB, Aglobitse P. The time dynamics of individual fertility preferences among rural Ghanaian women. Stud Fam Plann 2010;41(1):45-54.

Kodzi IA, Johnson DR, Casterline JB. To have or not to have another child: life cycle, health and cost considerations of Ghanaian women. Soc Sci Med 2012;74:966-972.

Roy TK, Sinha RK, Koenig M, Mohanty SK, Patel SK. Consistency and predictive ability of fertility preference indicators: longitudinal evidence from rural India. Int Fam Plann Perspect 2008;34(3):138-145.

A.1.3.4 Unmet Need

Overview

Unmet need for family planning is defined as "women who do not want to become pregnant but are not using contraception" (DHS website). We model unmet need for 'spacing' for women who have not met their desired family size yet, and unmet need for 'limiting' for women who do not want to have any more children.

Data

We used DHS data (variable v626) to estimate trends in unmet need by age and year among women who were sexually active and pre-menopausal. Data were available for 2,644,156 women from 254 surveys in 74 countries. Some women do not have any need for contraception because they desire pregnancy soon (v626==7). These women are excluded from our unmet need calculations, and unmet need for spacing (v626==1) is then calculated among the remaining women. Total spacing is comprised of women who responded: "Unmet need to space" (v626==1), "Using to space" (v626==3), "Spacing failure" (v626==5), and "Desire birth" (v626==7). Unmet need for limiting (v626==2) is more straightforward, and is calculated among the total limiting population: "Unmet need to limit" (v626==2), "Using to limit" (v626==4), and "Limiting failure" (v626==6).

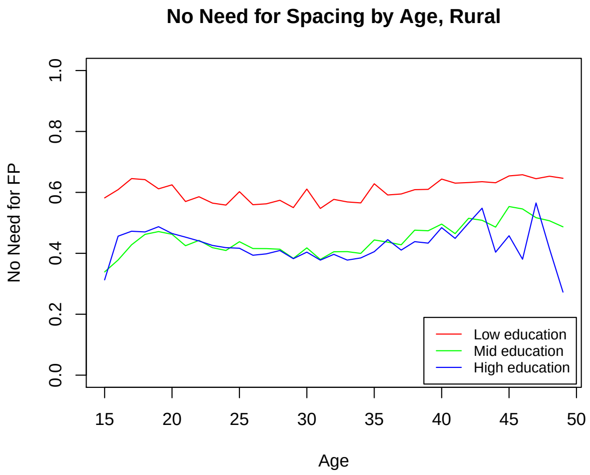

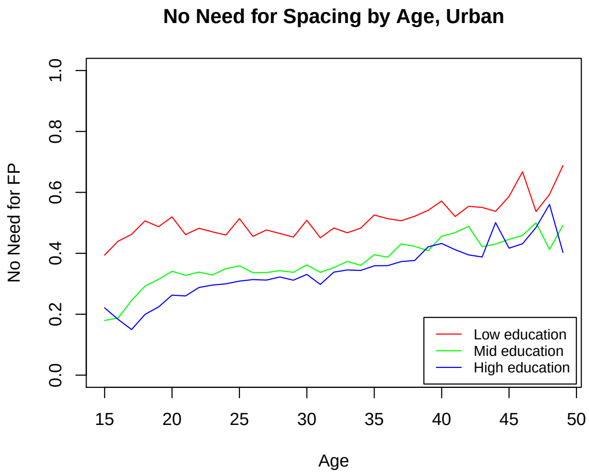

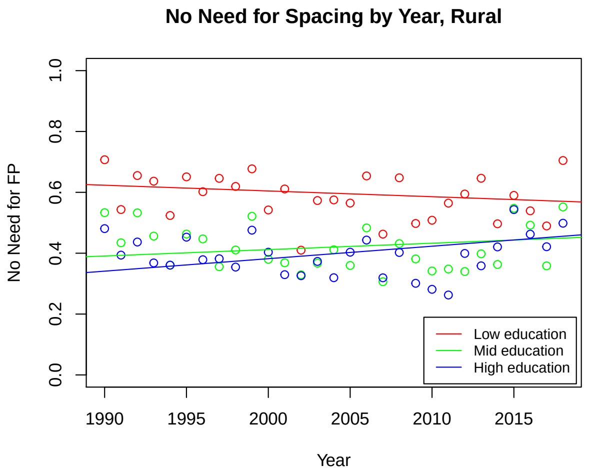

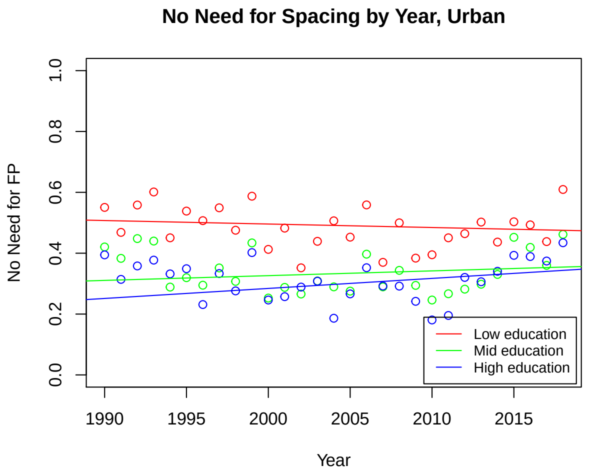

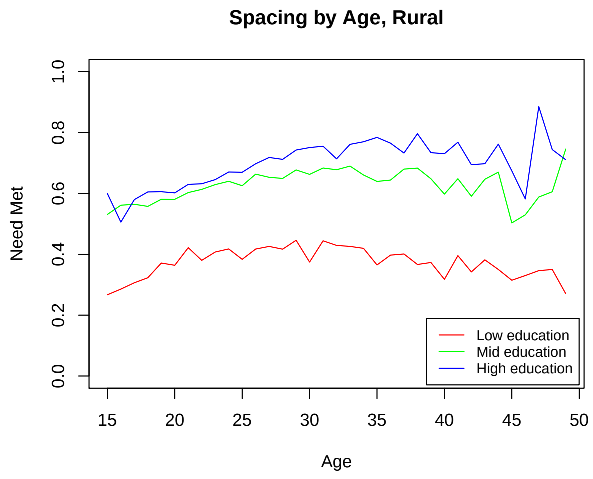
Spacing by Age, Rural
Need Met
0.0
0.2
0.4
0.6
0.8
1.0
15
20
25
30
35
40
45
50
Age
Low education
Mid education
High education

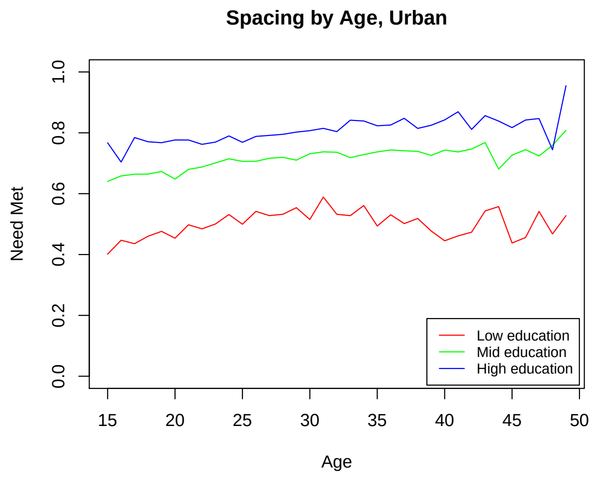
Spacing by Age, Urban
Need Met
0.0
0.2
0.4
0.6
0.8
1.0
15
20
25
30
35
40
45
50
Age
Low education
Mid education
High education

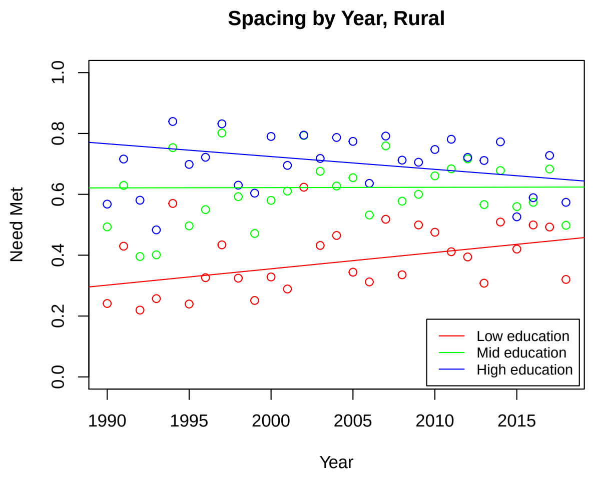
Spacing by Year, Rural
Need Met
0.0
0.2
0.4
0.6
0.8
1.0
1990
1995
2000
2005
2010
2015
Year
Low education
Mid education
High education

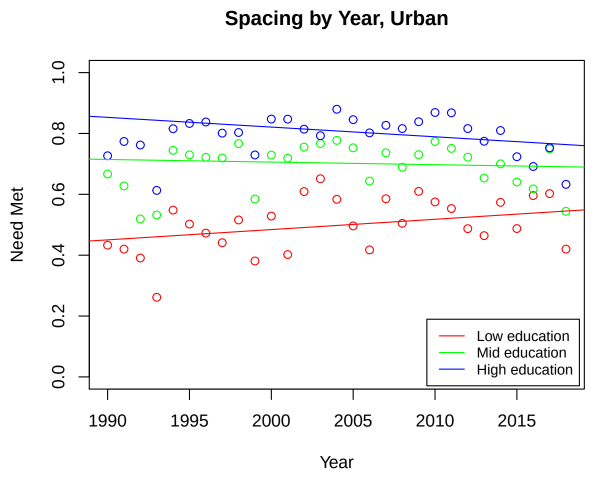
Spacing by Year, Urban
Need Met
0.0
0.2
0.4
0.6
0.8
1.0
1990
1995
2000
2005
2010
2015
Year
Low education
Mid education
High education

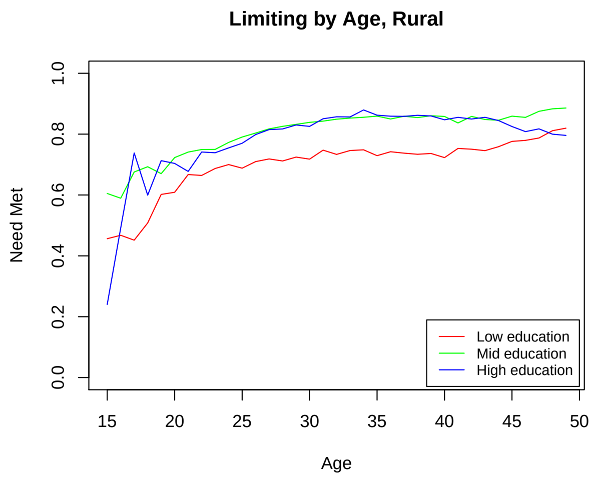
Limiting by Age, Rural
Need Met
0.0
0.2
0.4
0.6
0.8
1.0
15
20
25
30
35
40
45
50
Age
Low education
Mid education
High education

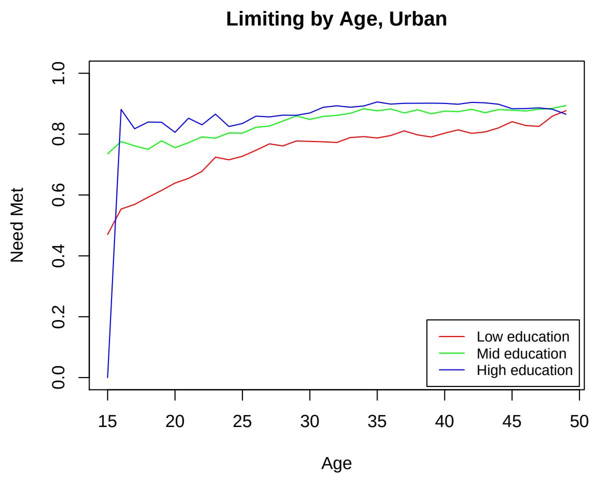
Limiting by Age, Urban
Need Met
0.0
0.2
0.4
0.6
0.8
1.0
15
20
25
30
35
40
45
50
Age
Low education
Mid education
High education

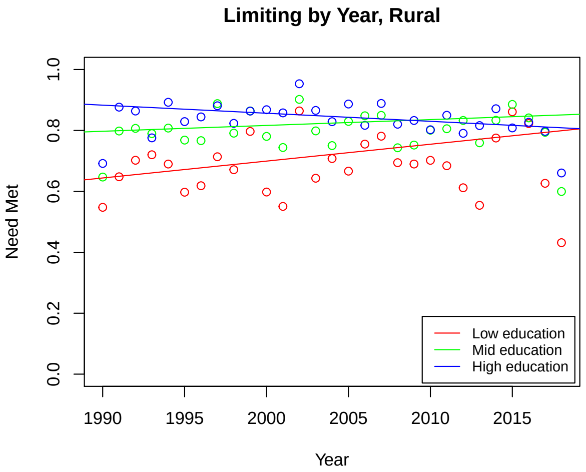

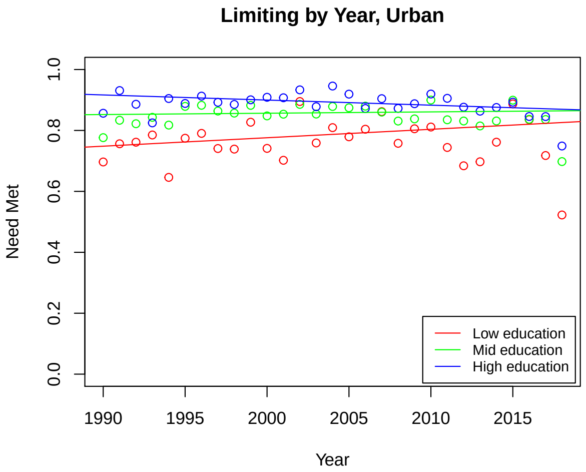

Parameters

We estimated hierarchical logistic regression models for each outcome (desired birth, unmet need for spacing, and unmet need for limiting) by subgroup (urban/rural and education level). We used upper middle income priors for high income countries due to lack of DHS data in high income countries.

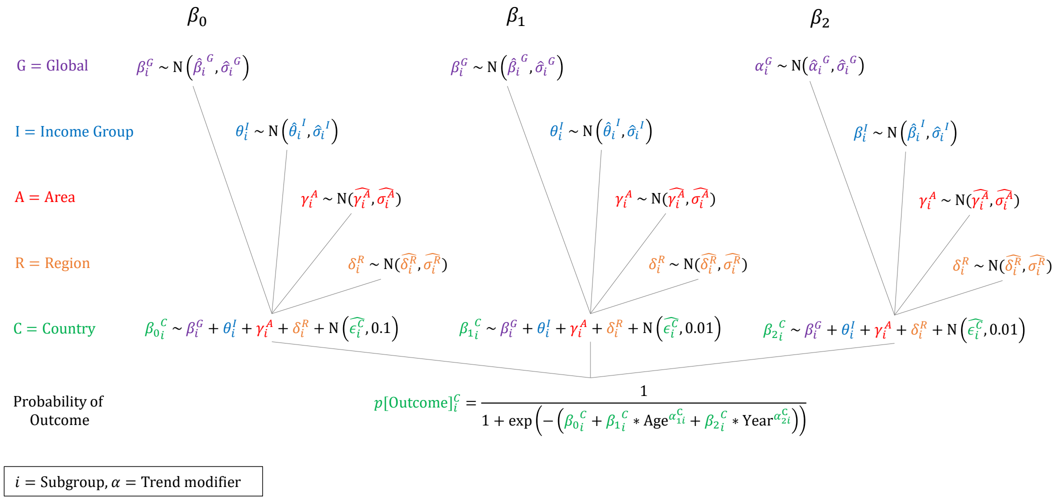

Priors

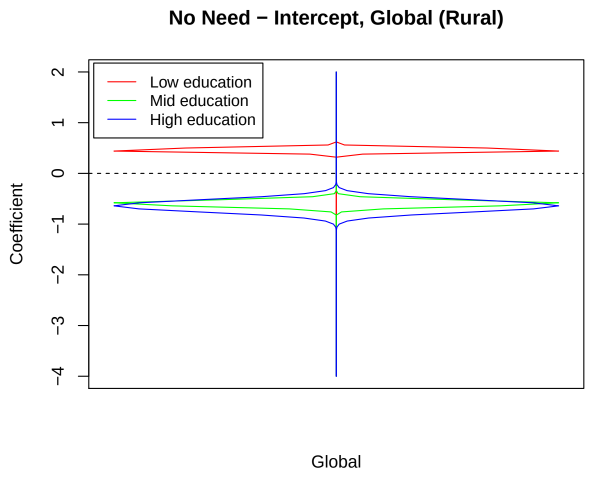
No Need − Intercept, Global (Rural)
Low education
Mid education
High education
Coefficient
2
1
0
−1
−2
−3
−4
Global

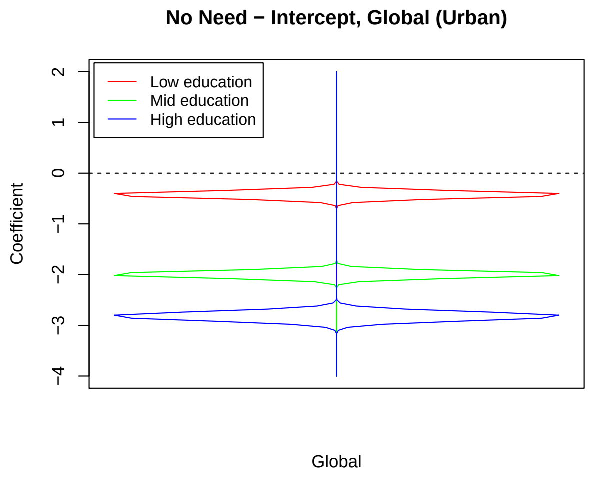
No Need − Intercept, Global (Urban)
Low education
Mid education
High education
Coefficient
2
1
0
−1
−2
−3
−4
Global

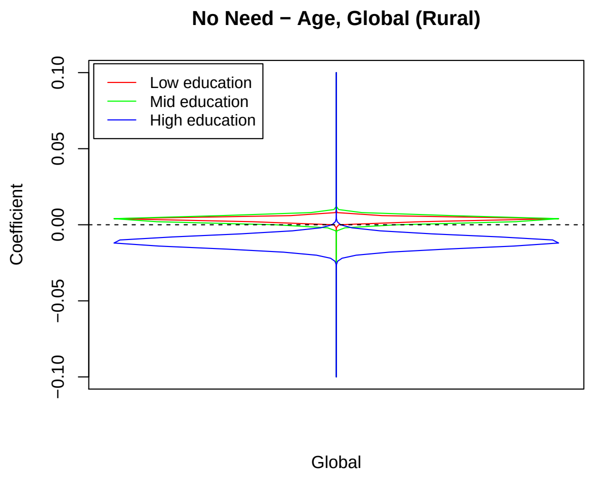
No Need − Age, Global (Rural)
Low education
Mid education
High education
Coefficient
0.10
0.05
0.00
−0.05
−0.10
Global

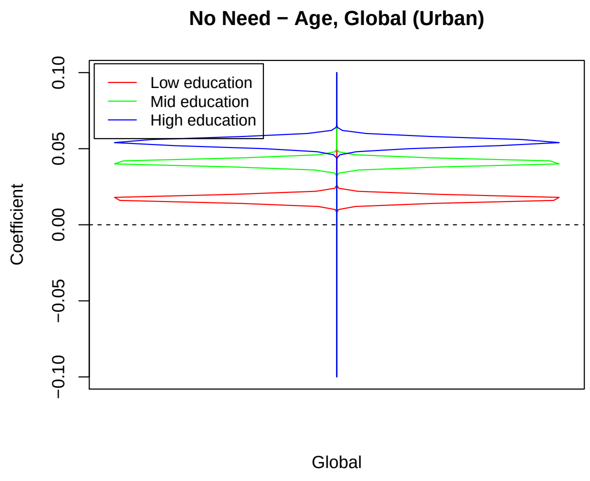
No Need − Age, Global (Urban)
Low education
Mid education
High education
Coefficient
0.10
0.05
0.00
−0.05
−0.10
Global

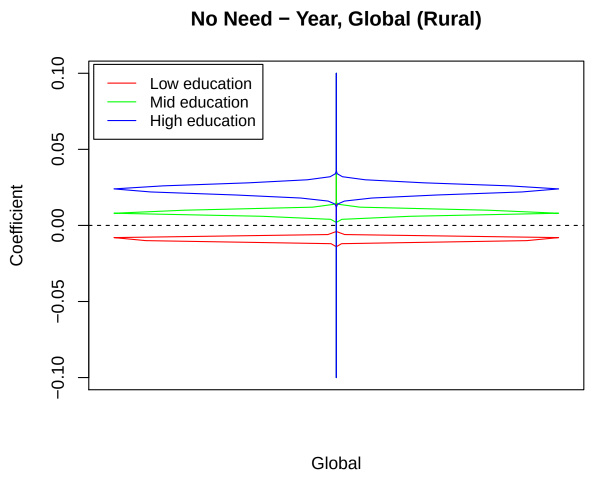
No Need − Year, Global (Rural)
Low education
Mid education
High education
Coefficient
0.10
0.05
0.00
−0.05
−0.10
Global

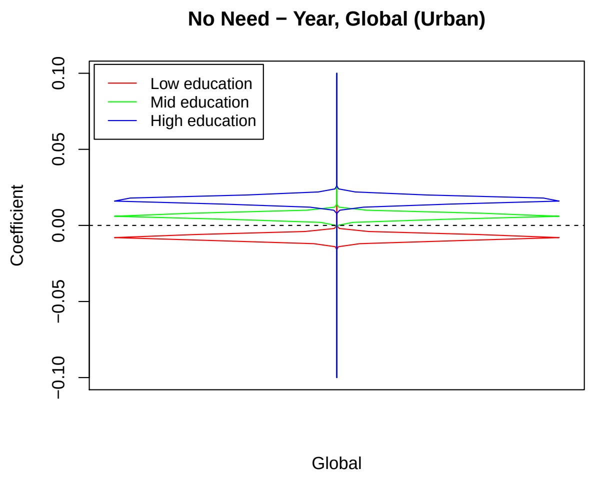
No Need − Year, Global (Urban)
Low education
Mid education
High education
Coefficient
0.10
0.05
0.00
−0.05
−0.10
Global

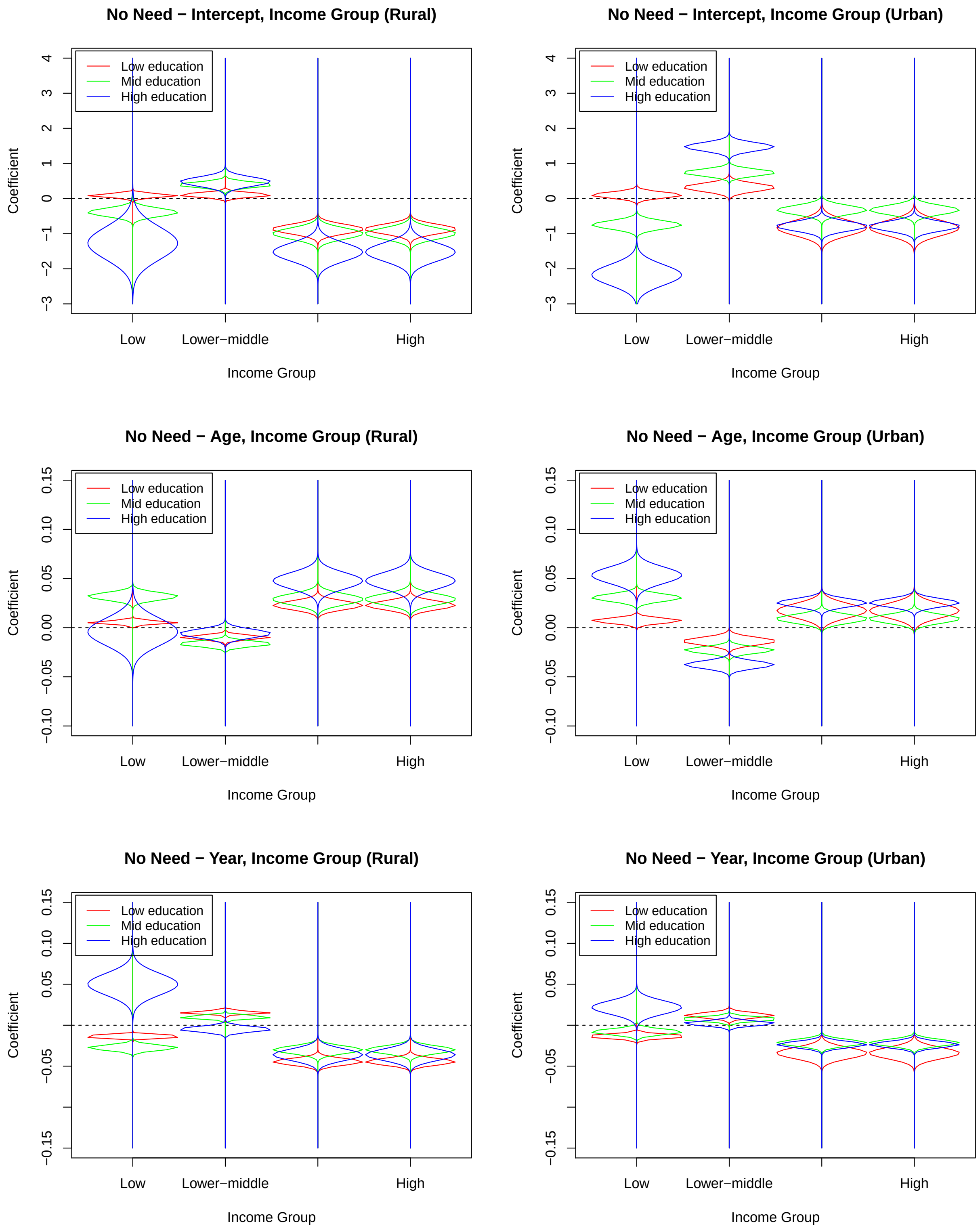
No Need − Intercept, Income Group (Rural)
No Need − Intercept, Income Group (Urban)
No Need − Age, Income Group (Rural)
No Need − Age, Income Group (Urban)
No Need − Year, Income Group (Rural)
No Need − Year, Income Group (Urban)
Low education
Mid education
High education
Coefficient
Income Group
Low
Lower−middle
High
4
3
2
1
0
−1
−2
−3
0.15
0.10
0.05
0.00
−0.05
−0.10
−0.15

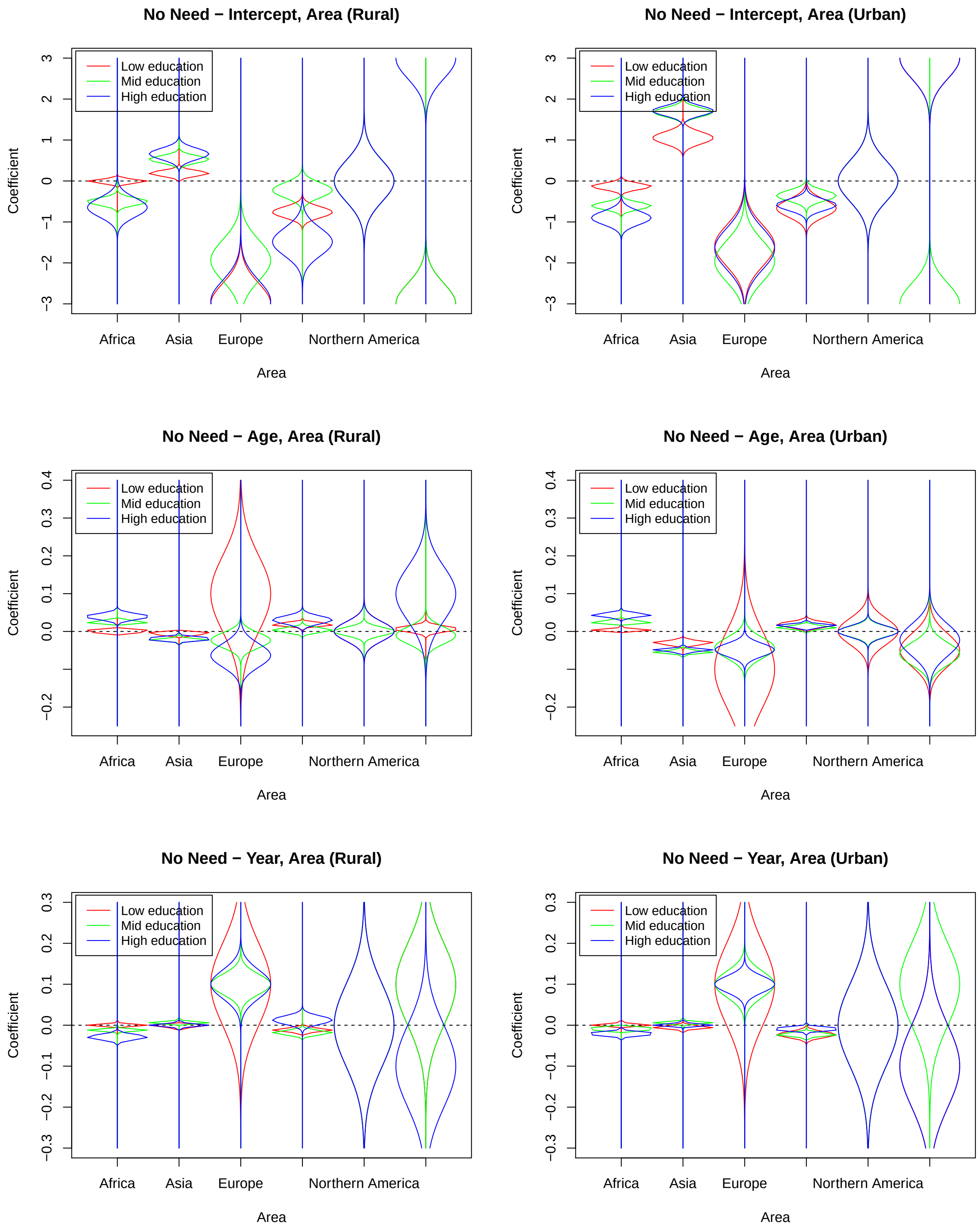
No Need − Intercept, Area (Rural)
No Need − Intercept, Area (Urban)
No Need − Age, Area (Rural)
No Need − Age, Area (Urban)
No Need − Year, Area (Rural)
No Need − Year, Area (Urban)
Low education
Mid education
High education
Coefficient
Area
Africa
Asia
Europe
Northern America

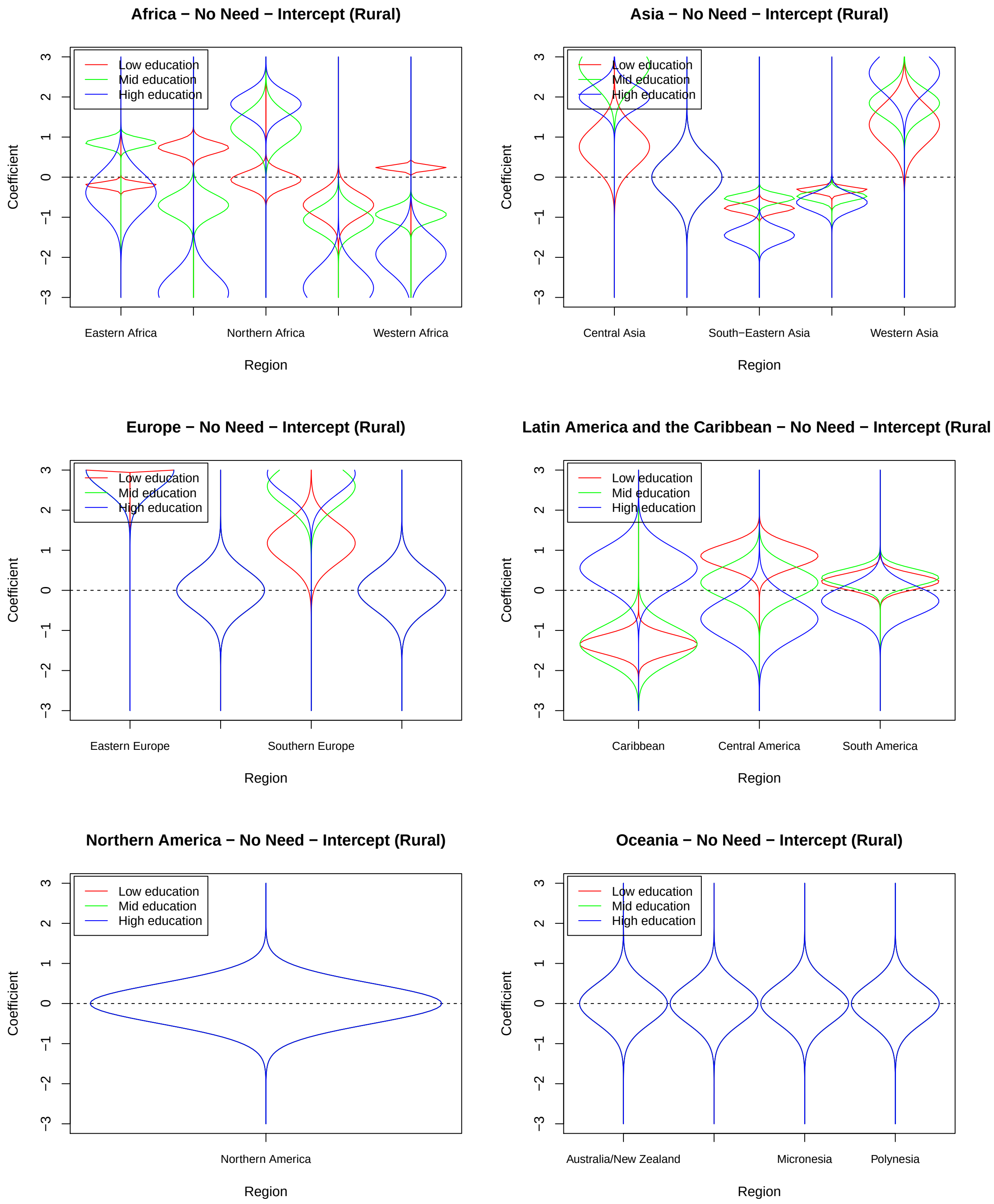
Africa − No Need − Intercept (Rural)
Low education
Mid education
High education
Coefficient
Eastern Africa
Northern Africa
Western Africa
Region
Asia − No Need − Intercept (Rural)
Central Asia
South−Eastern Asia
Western Asia
Europe − No Need − Intercept (Rural)
Eastern Europe
Southern Europe
Latin America and the Caribbean − No Need − Intercept (Rural
Caribbean
Central America
South America
Northern America − No Need − Intercept (Rural)
Northern America
Oceania − No Need − Intercept (Rural)
Australia/New Zealand
Micronesia
Polynesia

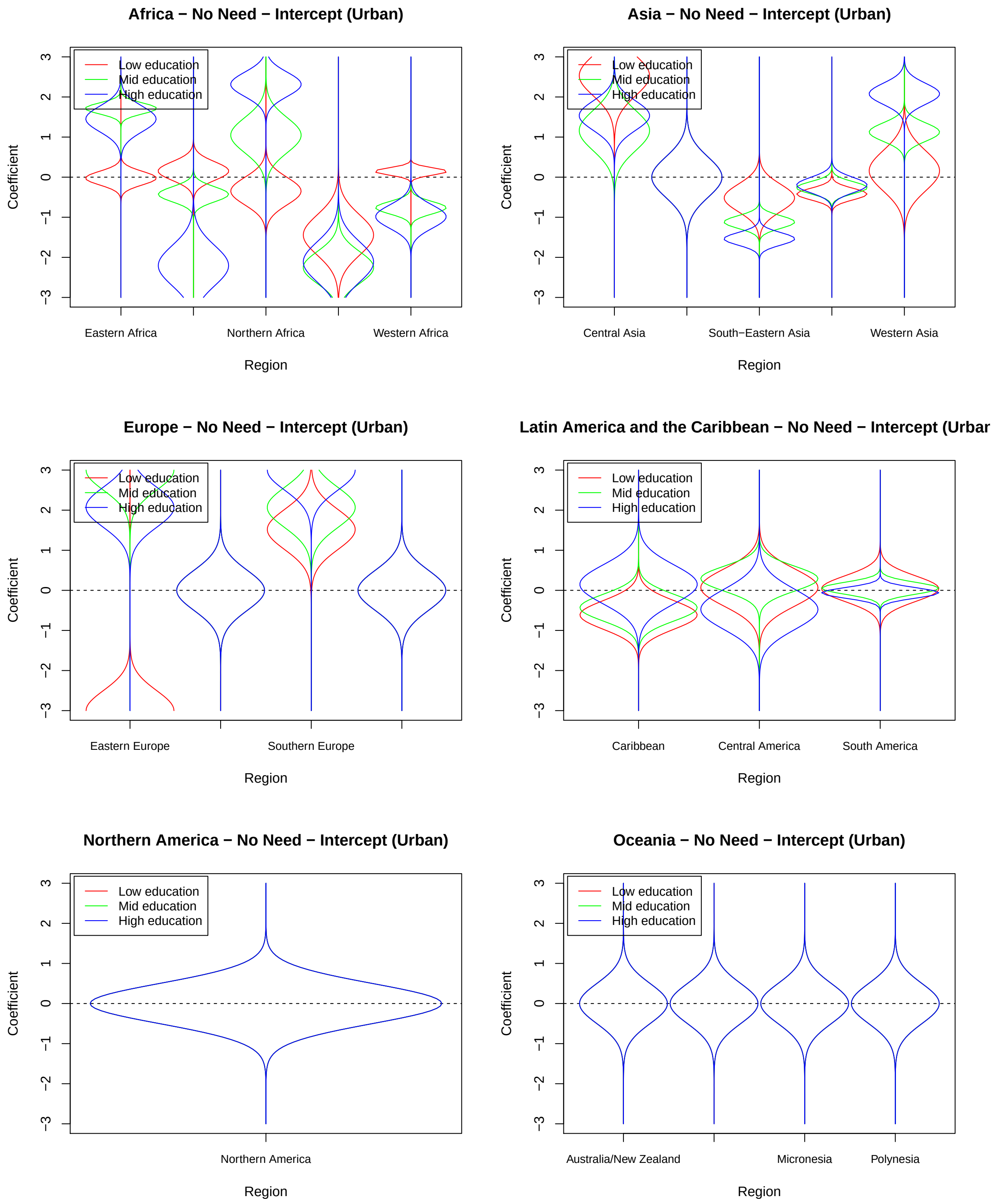
Africa − No Need − Intercept (Urban)
Low education
Mid education
High education
Coefficient
3
2
1
0
−1
−2
−3
Eastern Africa
Northern Africa
Western Africa
Region
Asia − No Need − Intercept (Urban)
Low education
Mid education
High education
Coefficient
Central Asia
South−Eastern Asia
Western Asia
Region
Europe − No Need − Intercept (Urban)
Low education
Mid education
High education
Coefficient
Eastern Europe
Southern Europe
Region
Latin America and the Caribbean − No Need − Intercept (Urbar
Low education
Mid education
High education
Coefficient
Caribbean
Central America
South America
Region
Northern America − No Need − Intercept (Urban)
Low education
Mid education
High education
Coefficient
Northern America
Region
Oceania − No Need − Intercept (Urban)
Low education
Mid education
High education
Coefficient
Australia/New Zealand
Micronesia
Polynesia
Region

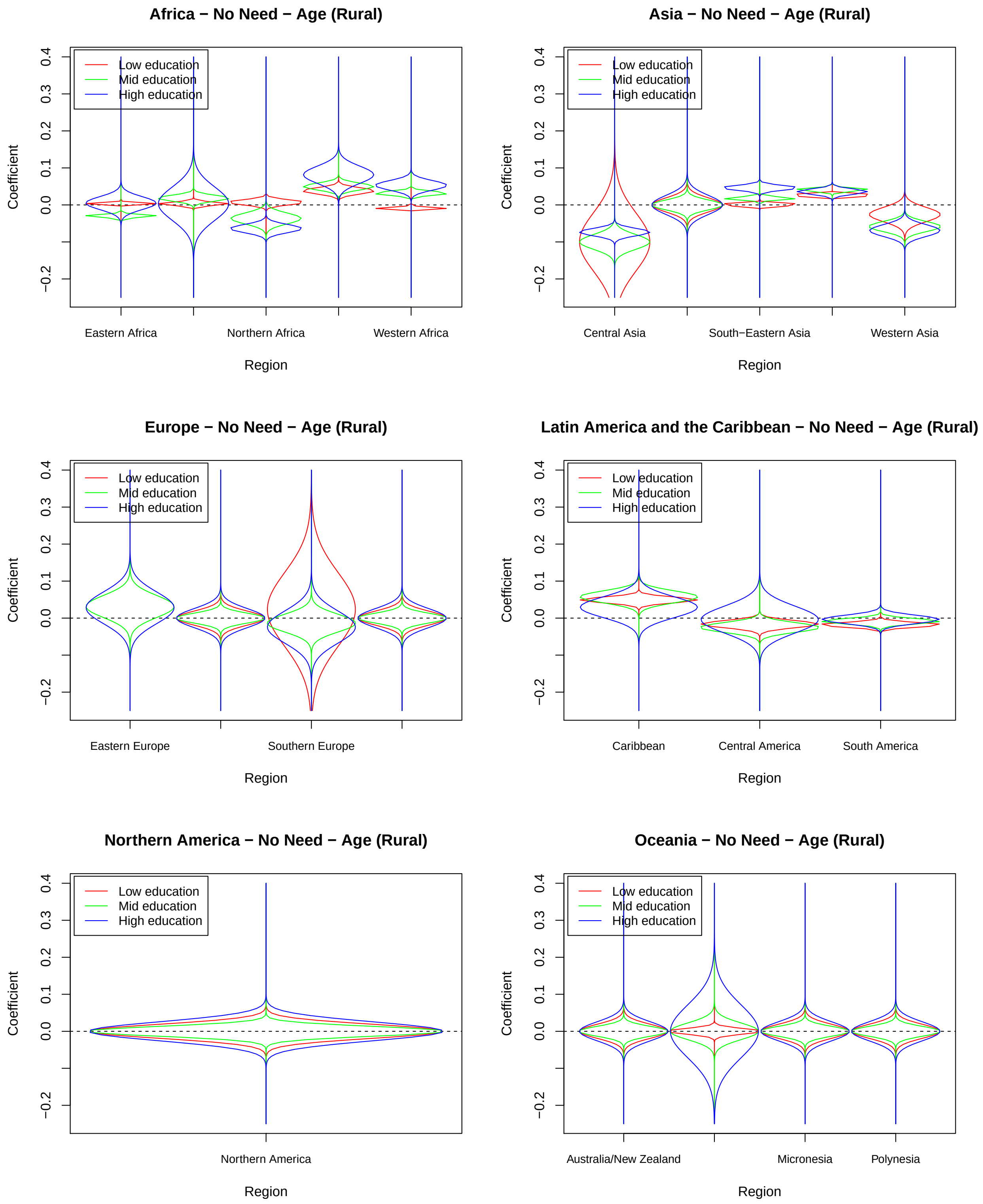

Africa − No Need − Age (Rural)
Asia − No Need − Age (Rural)
Europe − No Need − Age (Rural)
Latin America and the Caribbean − No Need − Age (Rural)
Northern America − No Need − Age (Rural)
Oceania − No Need − Age (Rural)
Low education
Mid education
High education
Coefficient
Region
Eastern Africa
Northern Africa
Western Africa
Central Asia
South−Eastern Asia
Western Asia
Eastern Europe
Southern Europe
Caribbean
Central America
South America
Northern America
Australia/New Zealand
Micronesia
Polynesia

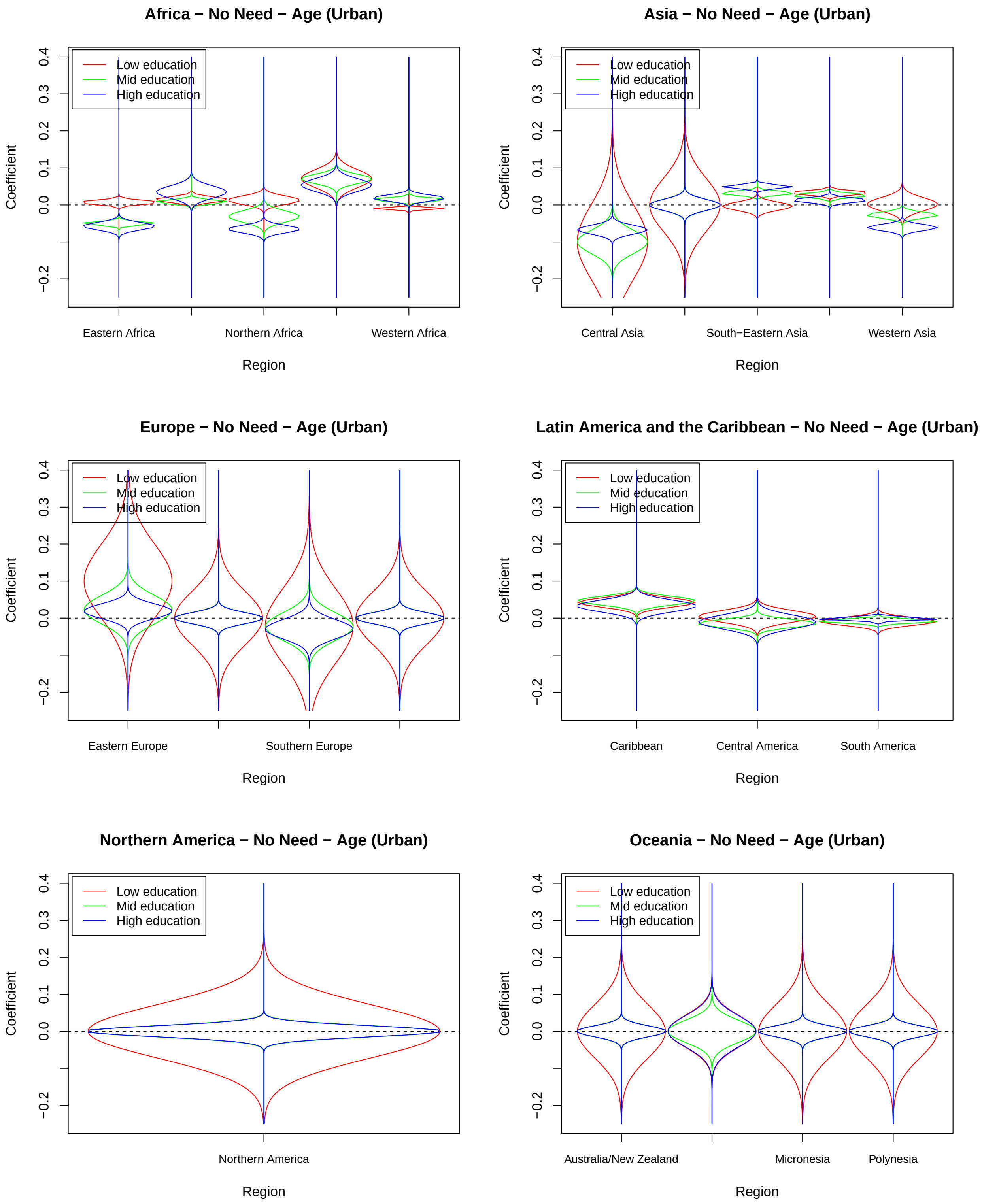
Africa − No Need − Age (Urban)
Low education
Mid education
High education
Coefficient
Eastern Africa
Northern Africa
Western Africa
Region
Asia − No Need − Age (Urban)
Central Asia
South−Eastern Asia
Western Asia
Europe − No Need − Age (Urban)
Eastern Europe
Southern Europe
Latin America and the Caribbean − No Need − Age (Urban)
Caribbean
Central America
South America
Northern America − No Need − Age (Urban)
Northern America
Oceania − No Need − Age (Urban)
Australia/New Zealand
Micronesia
Polynesia
0.4
0.3
0.2
0.1
0.0
−0.2

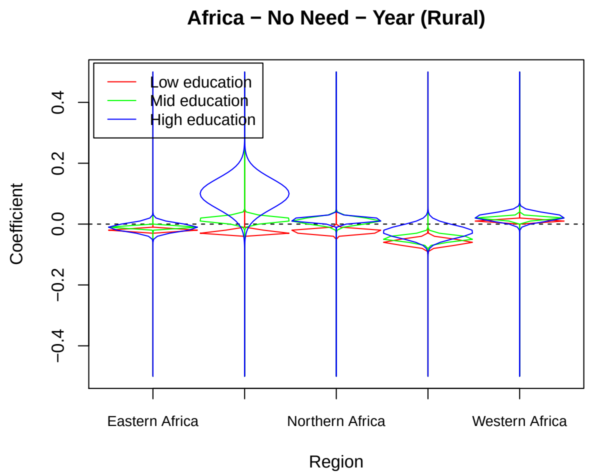
Africa − No Need − Year (Rural)
Low education
Mid education
High education
Coefficient
0.4
0.2
0.0
−0.2
−0.4
Eastern Africa
Northern Africa
Western Africa
Region

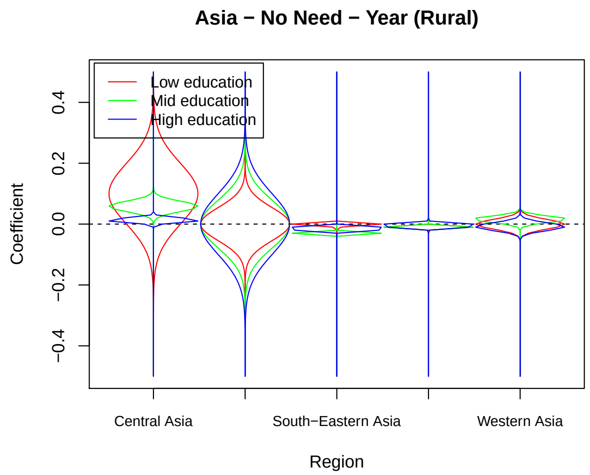
Asia − No Need − Year (Rural)
Low education
Mid education
High education
Coefficient
0.4
0.2
0.0
−0.2
−0.4
Central Asia
South−Eastern Asia
Western Asia
Region

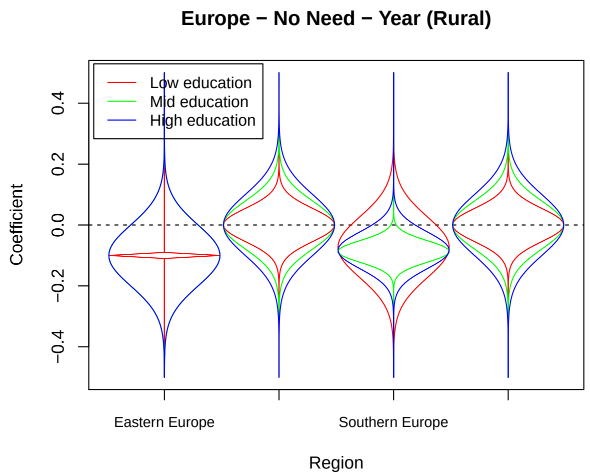
Europe − No Need − Year (Rural)
Low education
Mid education
High education
Coefficient
0.4
0.2
0.0
−0.2
−0.4
Eastern Europe
Southern Europe
Region

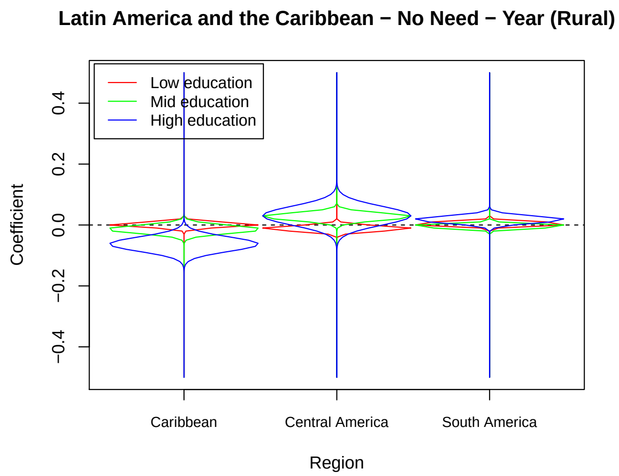
Latin America and the Caribbean − No Need − Year (Rural)
Low education
Mid education
High education
Coefficient
0.4
0.2
0.0
−0.2
−0.4
Caribbean
Central America
South America
Region

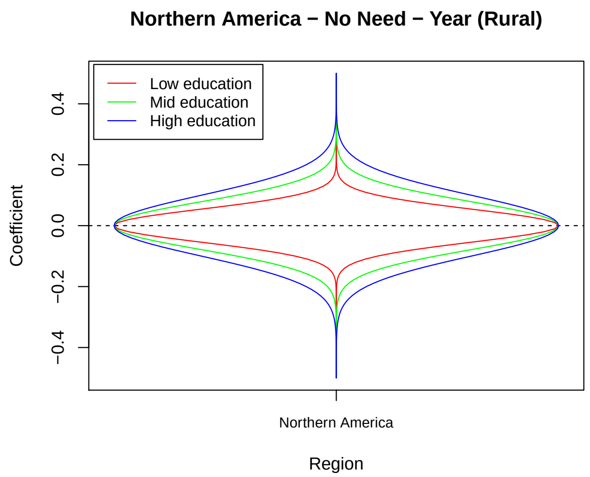
Northern America − No Need − Year (Rural)
Low education
Mid education
High education
Coefficient
0.4
0.2
0.0
−0.2
−0.4
Northern America
Region

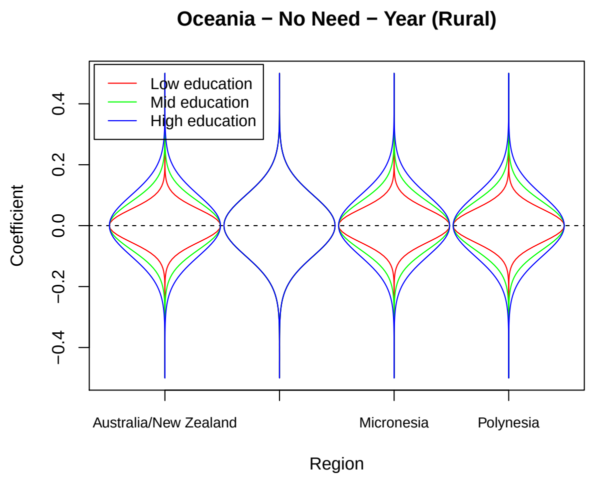
Oceania − No Need − Year (Rural)
Low education
Mid education
High education
Coefficient
0.4
0.2
0.0
−0.2
−0.4
Australia/New Zealand
Micronesia
Polynesia
Region

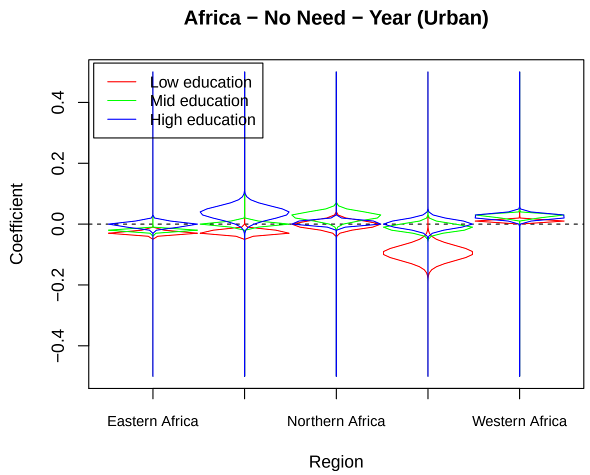
Africa − No Need − Year (Urban)
Low education
Mid education
High education
Coefficient
0.4
0.2
0.0
−0.2
−0.4
Eastern Africa
Northern Africa
Western Africa
Region

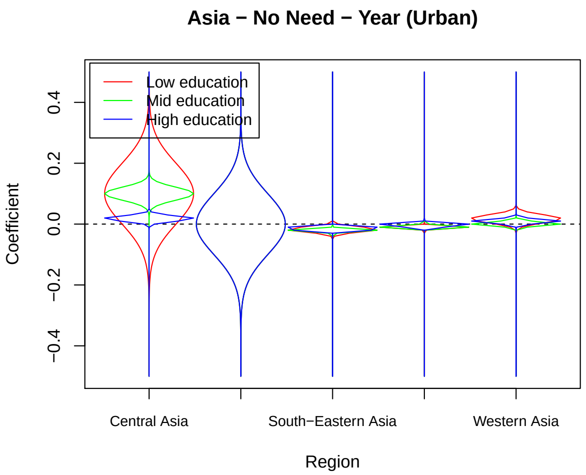
Asia − No Need − Year (Urban)
Low education
Mid education
High education
Coefficient
0.4
0.2
0.0
−0.2
−0.4
Central Asia
South−Eastern Asia
Western Asia
Region

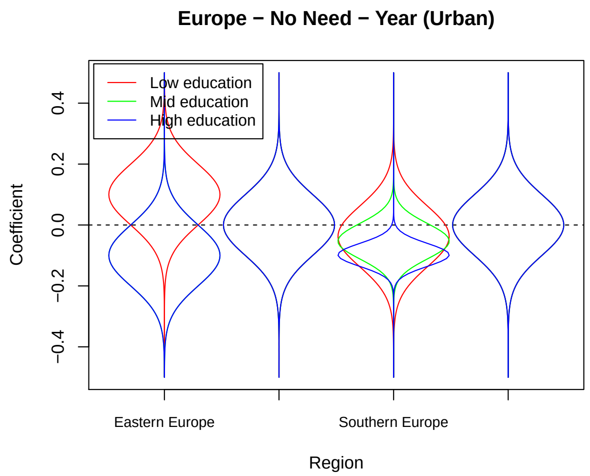
Europe − No Need − Year (Urban)
Low education
Mid education
High education
Coefficient
0.4
0.2
0.0
−0.2
−0.4
Eastern Europe
Southern Europe
Region

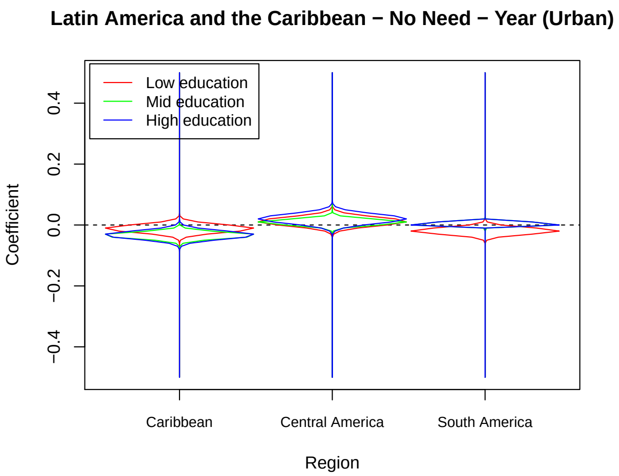
Latin America and the Caribbean − No Need − Year (Urban)
Low education
Mid education
High education
Coefficient
0.4
0.2
0.0
−0.2
−0.4
Caribbean
Central America
South America
Region

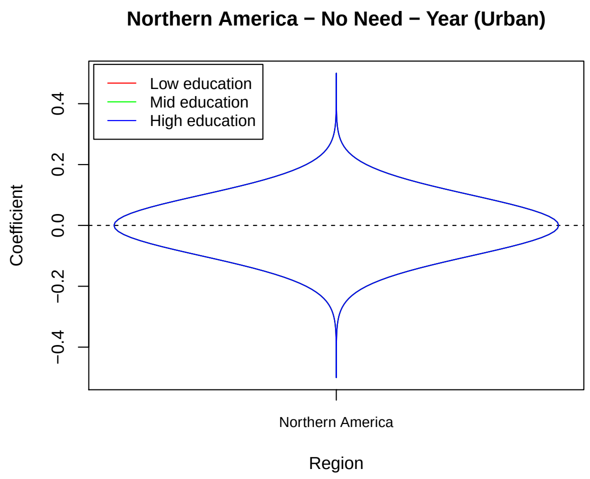
Northern America − No Need − Year (Urban)
Low education
Mid education
High education
Coefficient
0.4
0.2
0.0
−0.2
−0.4
Northern America
Region

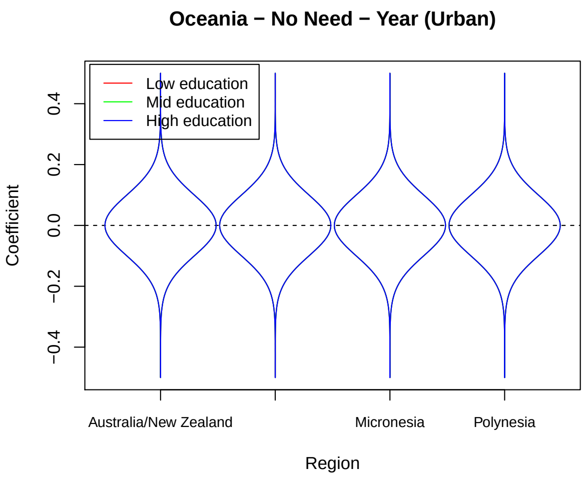
Oceania − No Need − Year (Urban)
Low education
Mid education
High education
Coefficient
0.4
0.2
0.0
−0.2
−0.4
Australia/New Zealand
Micronesia
Polynesia
Region

No Need − Intercept − Country Residuals (Rural)

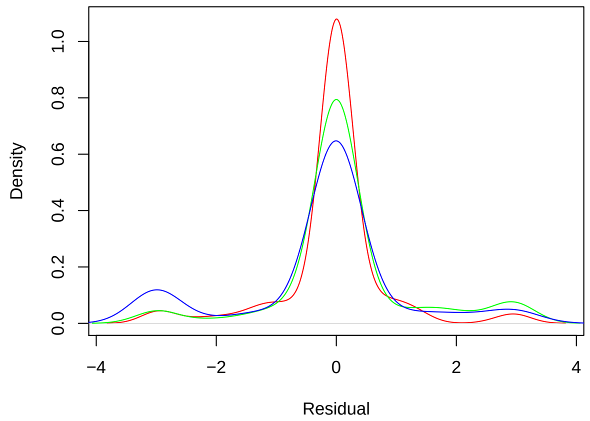

No Need − Intercept − Country Residuals (Urban)

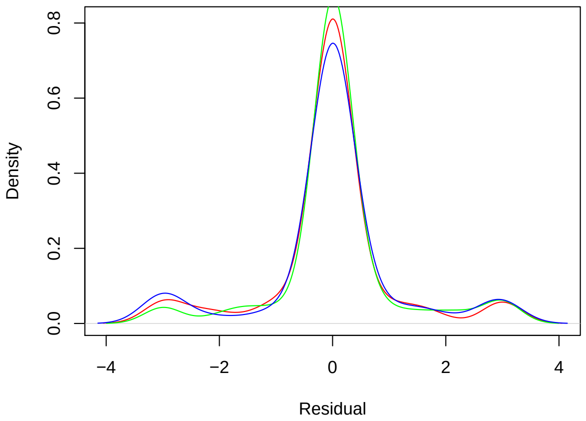

No Need − Age − Country Residuals (Rural)

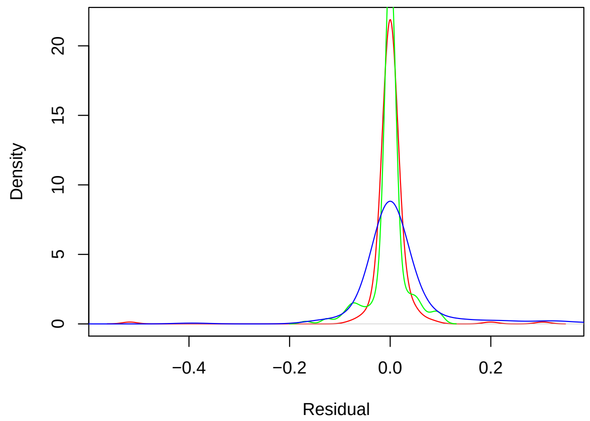

No Need − Age − Country Residuals (Urban)

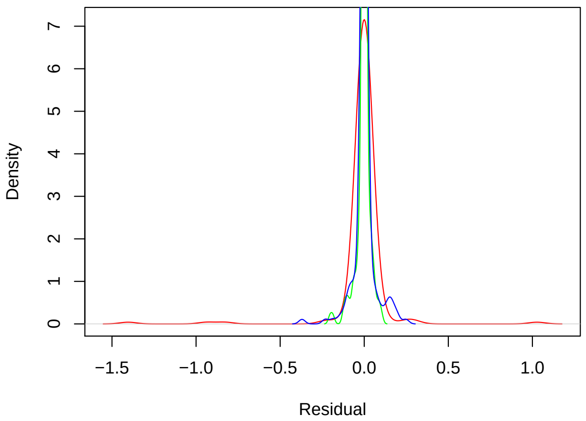

No Need − Year − Country Residuals (Rural)

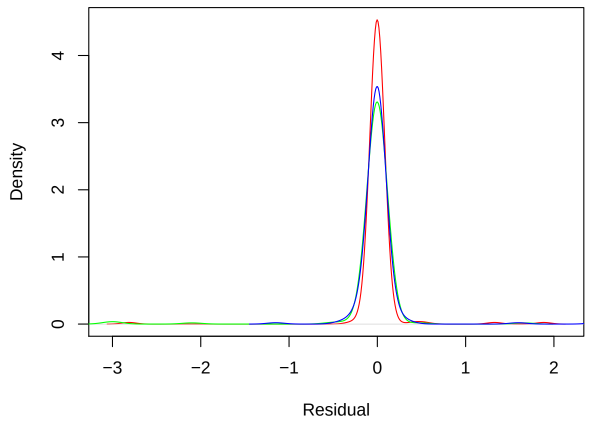

No Need − Year − Country Residuals (Urban)

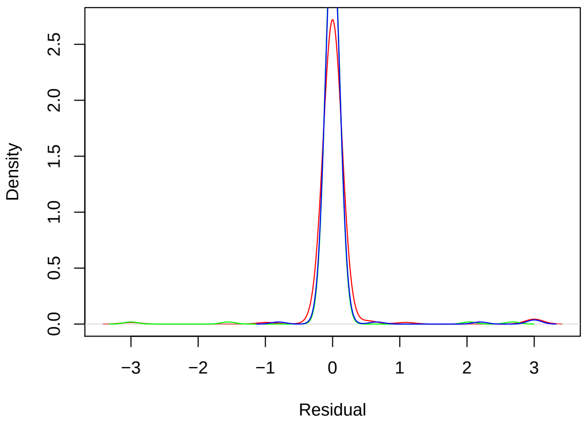

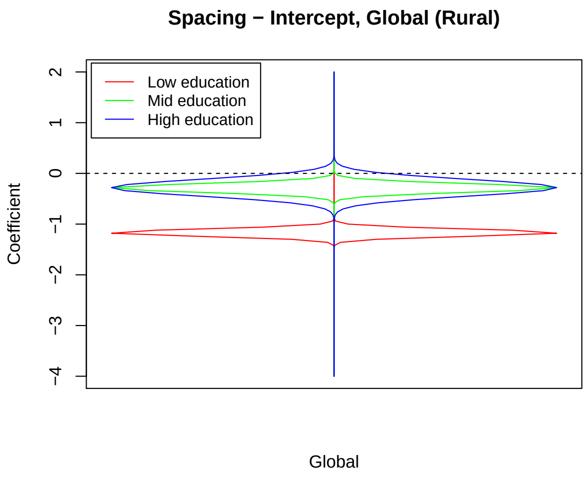
Spacing − Intercept, Global (Rural)
Low education
Mid education
High education
Coefficient
2
1
0
−1
−2
−3
−4
Global

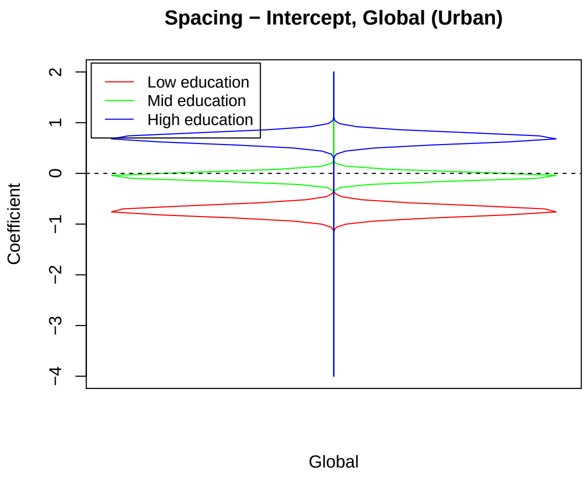
Spacing − Intercept, Global (Urban)
Low education
Mid education
High education
Coefficient
2
1
0
−1
−2
−3
−4
Global

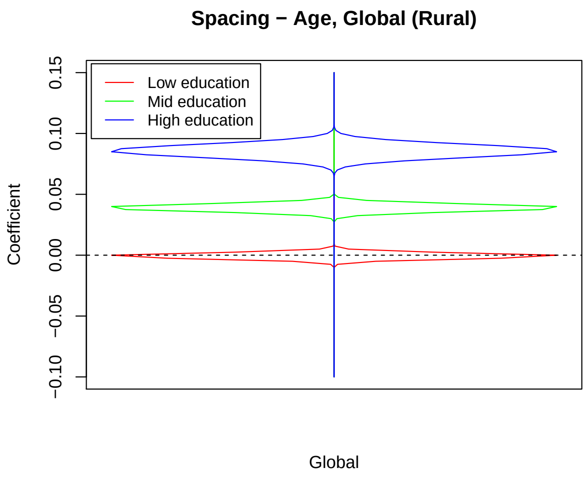
Spacing − Age, Global (Rural)
Low education
Mid education
High education
Coefficient
0.15
0.10
0.05
0.00
−0.05
−0.10
Global

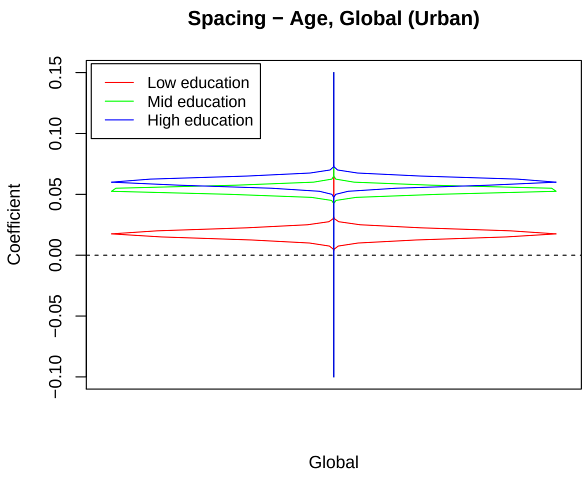
Spacing − Age, Global (Urban)
Low education
Mid education
High education
Coefficient
0.15
0.10
0.05
0.00
−0.05
−0.10
Global

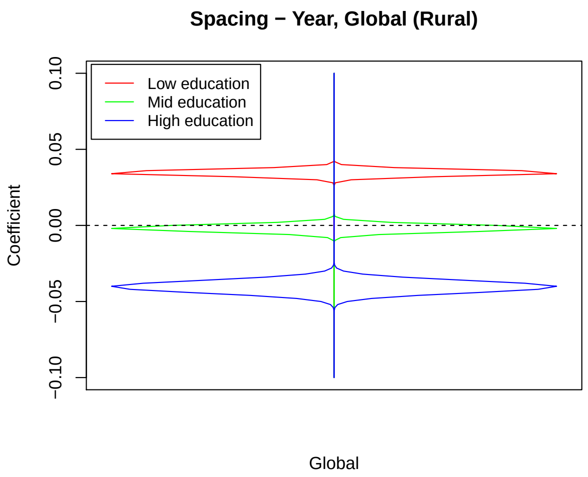
Spacing − Year, Global (Rural)
Low education
Mid education
High education
Coefficient
0.10
0.05
0.00
−0.05
−0.10
Global

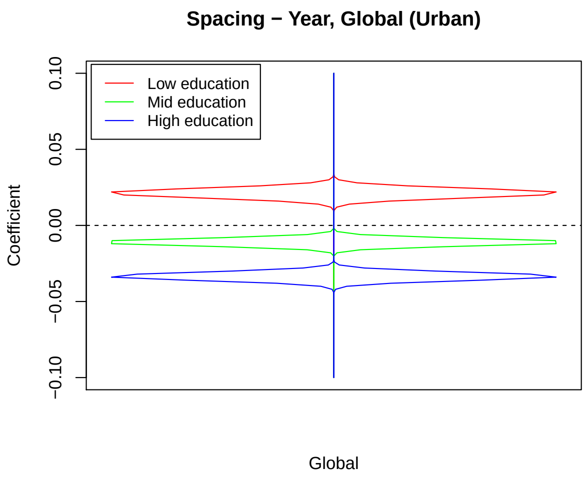
Spacing − Year, Global (Urban)
Low education
Mid education
High education
Coefficient
0.10
0.05
0.00
−0.05
−0.10
Global

Spacing − Intercept, Income Group (Rural)

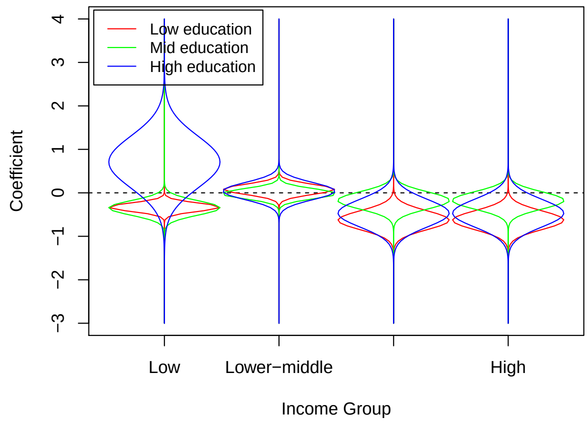

Spacing − Intercept, Income Group (Urban)

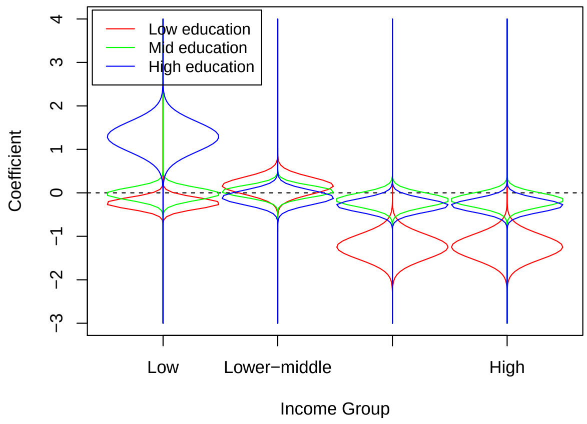

Spacing − Age, Income Group (Rural)

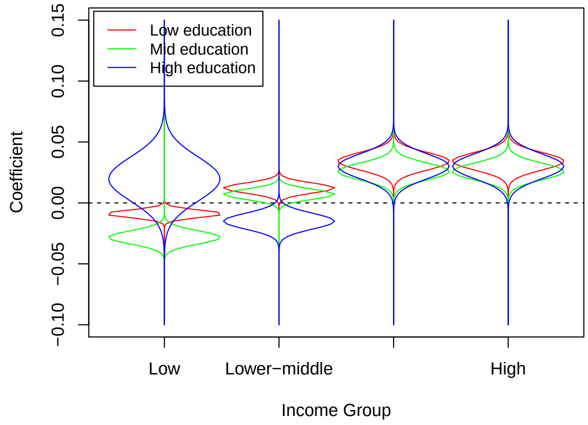

Spacing − Age, Income Group (Urban)

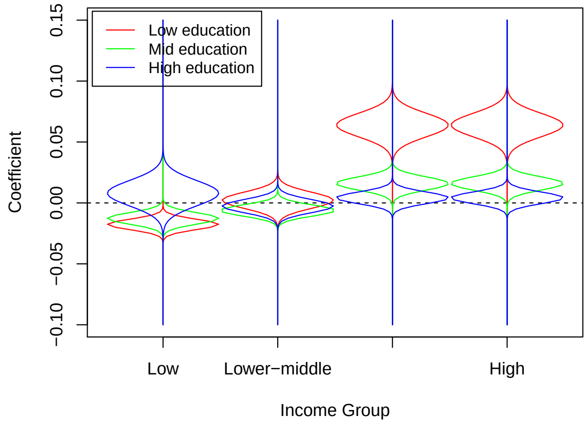

Spacing − Year, Income Group (Rural)

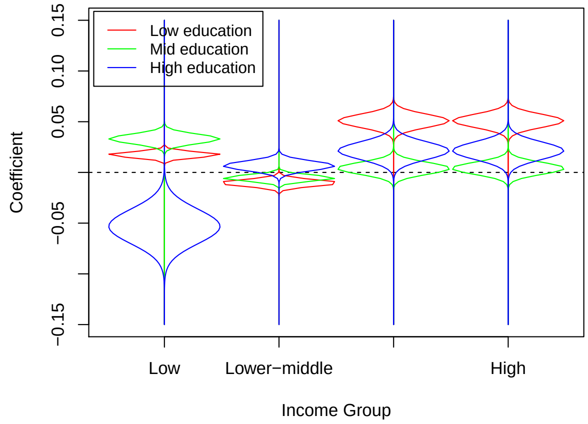

Spacing − Year, Income Group (Urban)

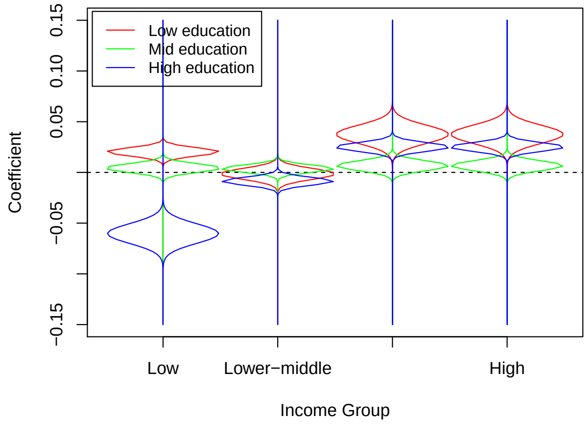

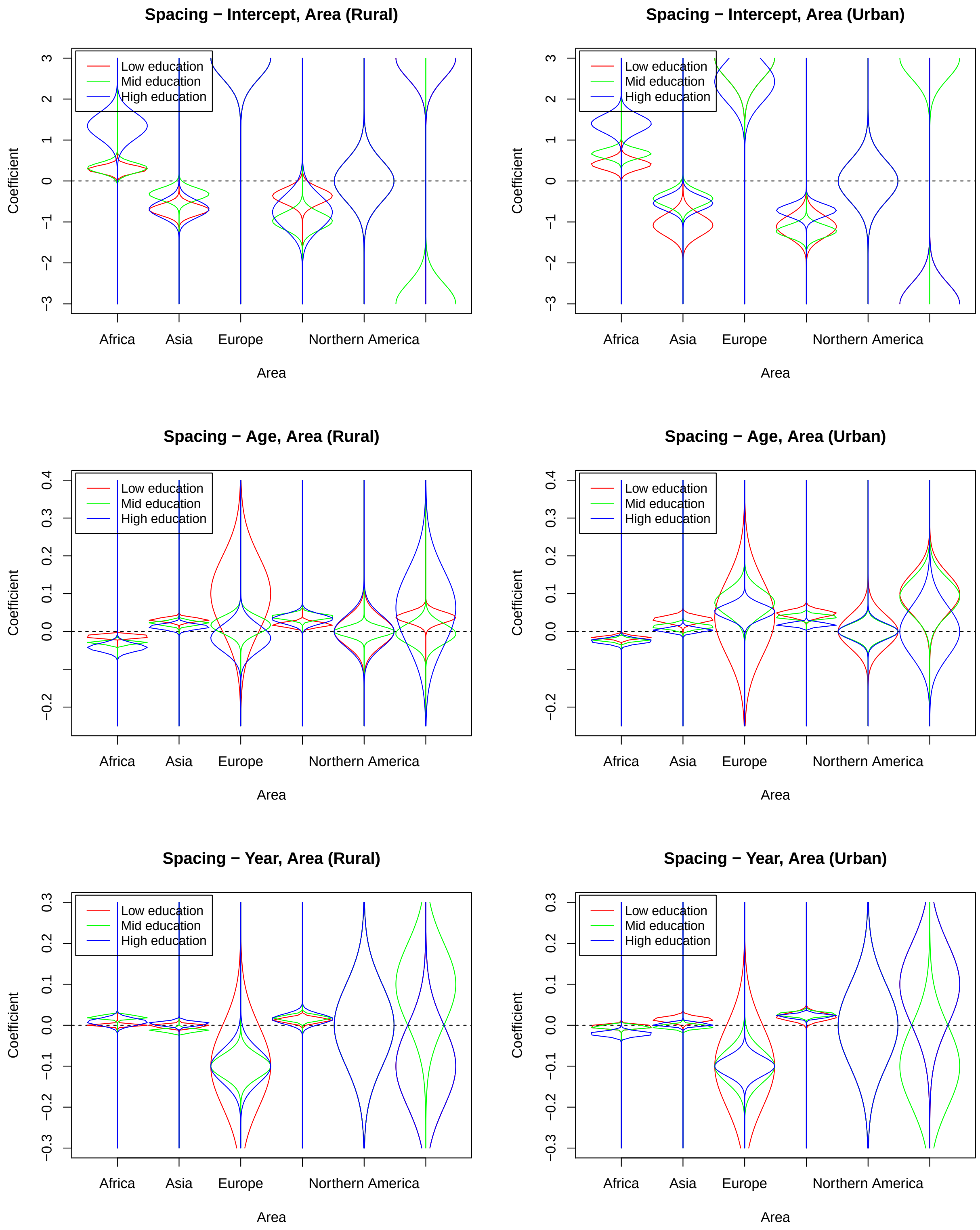
Spacing − Intercept, Area (Rural)
Low education
Mid education
High education
Coefficient
3
2
1
0
−1
−2
−3
Africa
Asia
Europe
Northern America
Area
Spacing − Intercept, Area (Urban)
Low education
Mid education
High education
Coefficient
3
2
1
0
−1
−2
−3
Africa
Asia
Europe
Northern America
Area
Spacing − Age, Area (Rural)
Low education
Mid education
High education
Coefficient
0.4
0.3
0.2
0.1
0.0
−0.2
Africa
Asia
Europe
Northern America
Area
Spacing − Age, Area (Urban)
Low education
Mid education
High education
Coefficient
0.4
0.3
0.2
0.1
0.0
−0.2
Africa
Asia
Europe
Northern America
Area
Spacing − Year, Area (Rural)
Low education
Mid education
High education
Coefficient
0.3
0.2
0.1
0.0
−0.1
−0.2
−0.3
Africa
Asia
Europe
Northern America
Area
Spacing − Year, Area (Urban)
Low education
Mid education
High education
Coefficient
0.3
0.2
0.1
0.0
−0.1
−0.2
−0.3
Africa
Asia
Europe
Northern America
Area

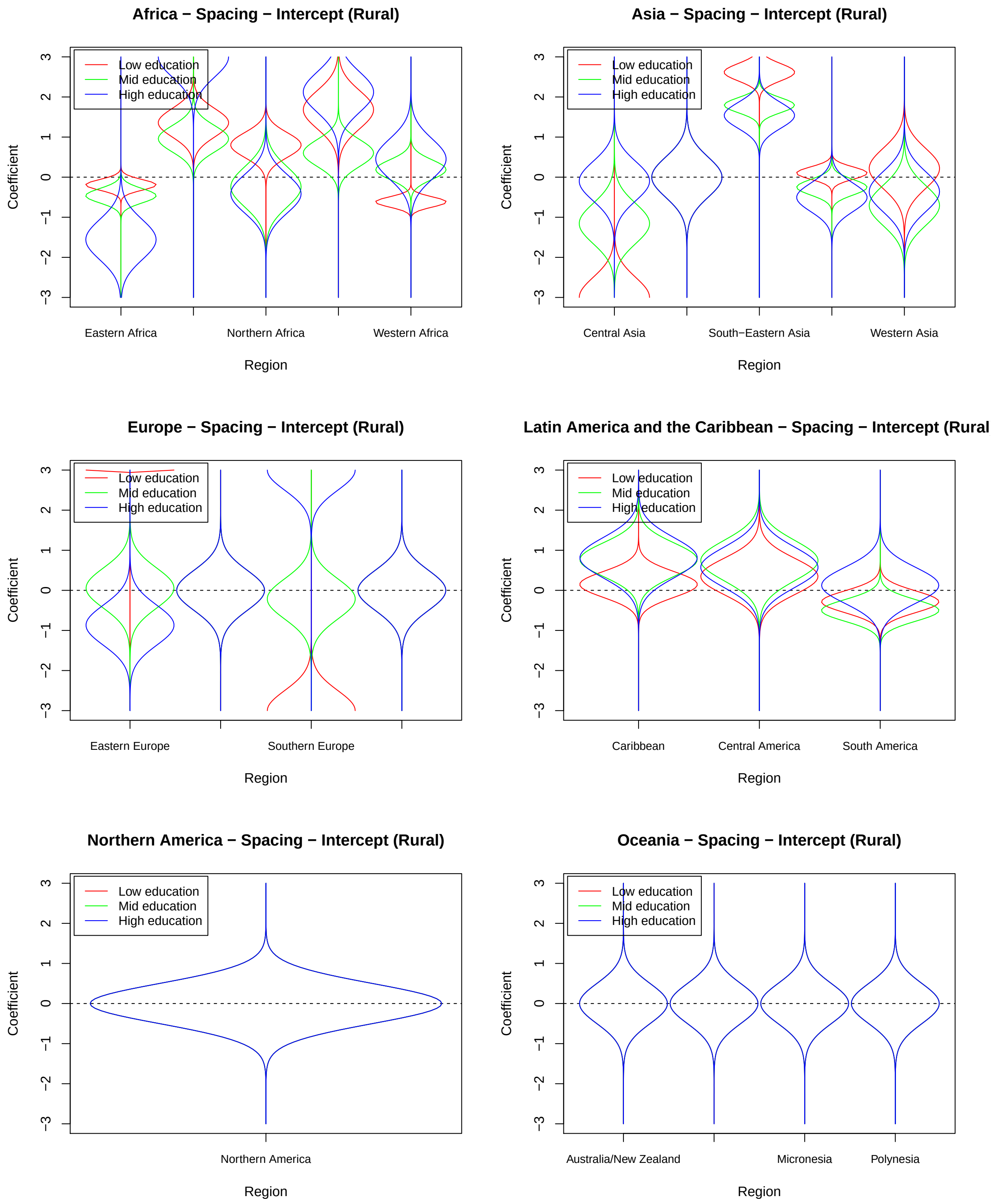
Africa − Spacing − Intercept (Rural)
Low education
Mid education
High education
Coefficient
Eastern Africa
Northern Africa
Western Africa
Region
Asia − Spacing − Intercept (Rural)
Low education
Mid education
High education
Coefficient
Central Asia
South−Eastern Asia
Western Asia
Region
Europe − Spacing − Intercept (Rural)
Low education
Mid education
High education
Coefficient
Eastern Europe
Southern Europe
Region
Latin America and the Caribbean − Spacing − Intercept (Rural
Low education
Mid education
High education
Coefficient
Caribbean
Central America
South America
Region
Northern America − Spacing − Intercept (Rural)
Low education
Mid education
High education
Coefficient
Northern America
Region
Oceania − Spacing − Intercept (Rural)
Low education
Mid education
High education
Coefficient
Australia/New Zealand
Micronesia
Polynesia
Region

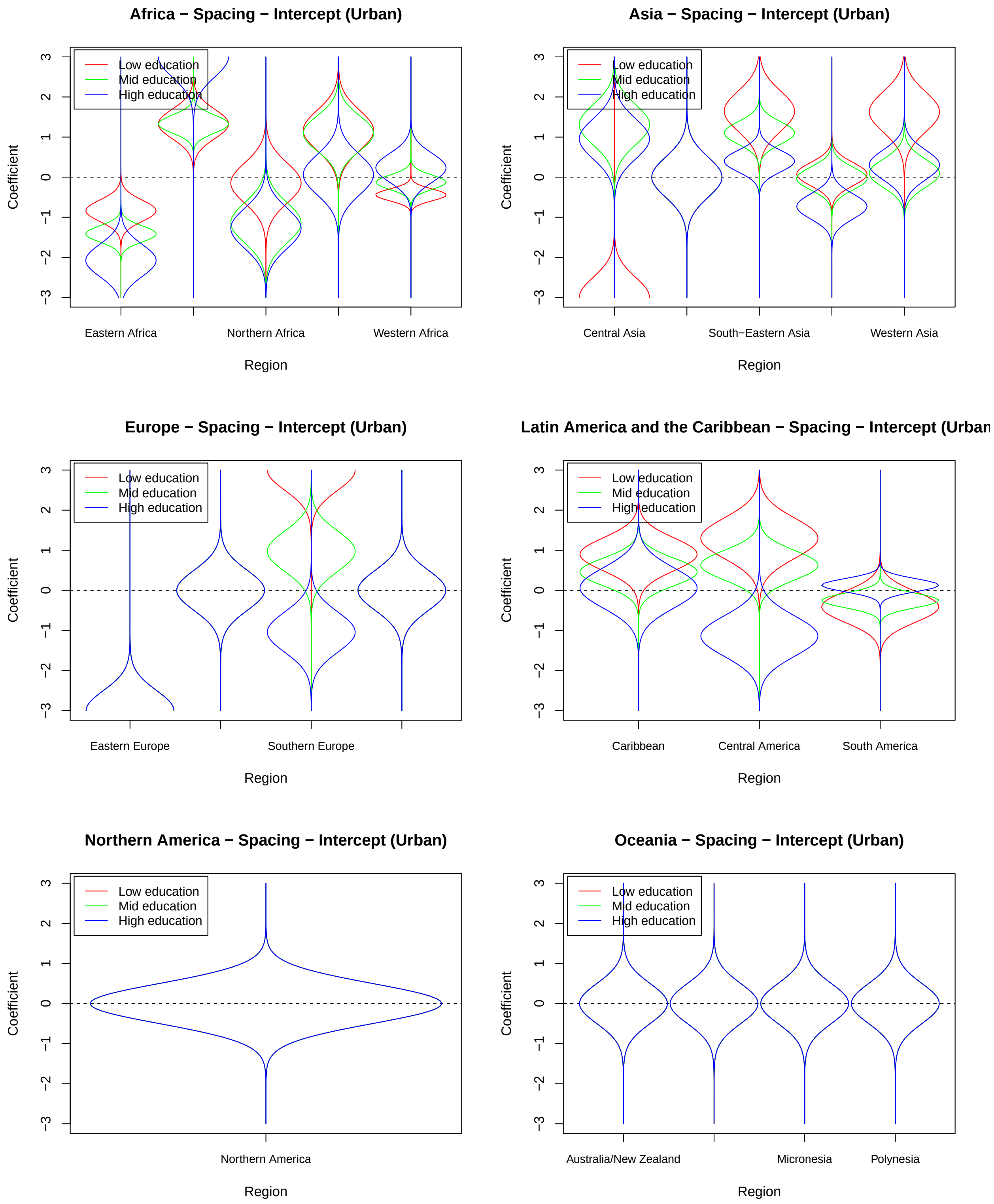
Africa − Spacing − Intercept (Urban)
Low education
Mid education
High education
Coefficient
Eastern Africa
Northern Africa
Western Africa
Region
Asia − Spacing − Intercept (Urban)
Central Asia
South−Eastern Asia
Western Asia
Europe − Spacing − Intercept (Urban)
Eastern Europe
Southern Europe
Latin America and the Caribbean − Spacing − Intercept (Urban
Caribbean
Central America
South America
Northern America − Spacing − Intercept (Urban)
Northern America
Oceania − Spacing − Intercept (Urban)
Australia/New Zealand
Micronesia
Polynesia

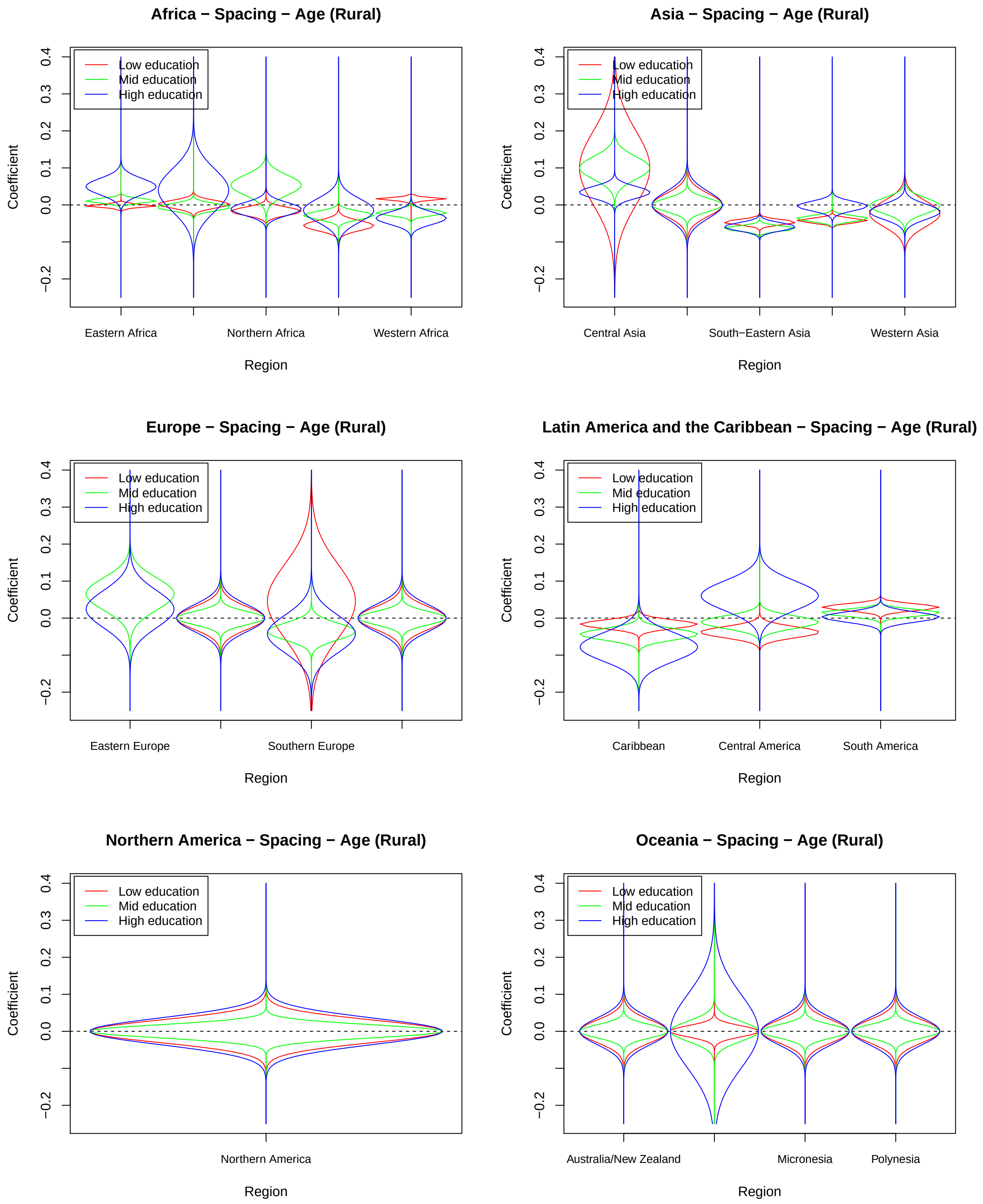

Africa − Spacing − Age (Rural)
Low education
Mid education
High education
Coefficient
Eastern Africa
Northern Africa
Western Africa
Region
Asia − Spacing − Age (Rural)
Central Asia
South−Eastern Asia
Western Asia
Europe − Spacing − Age (Rural)
Eastern Europe
Southern Europe
Latin America and the Caribbean − Spacing − Age (Rural)
Caribbean
Central America
South America
Northern America − Spacing − Age (Rural)
Northern America
Oceania − Spacing − Age (Rural)
Australia/New Zealand
Micronesia
Polynesia

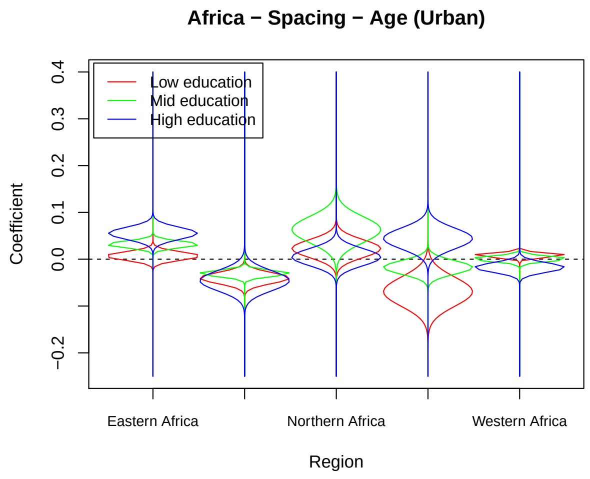
Africa − Spacing − Age (Urban)
Low education
Mid education
High education
Coefficient
0.4
0.3
0.2
0.1
0.0
−0.2
Eastern Africa
Northern Africa
Western Africa
Region

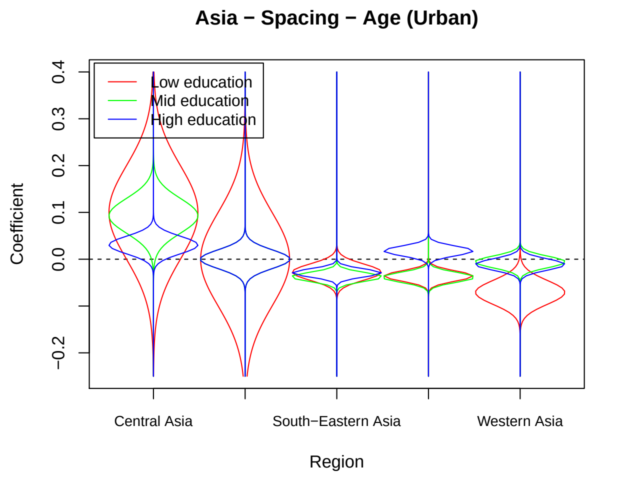
Asia − Spacing − Age (Urban)
Low education
Mid education
High education
Coefficient
0.4
0.3
0.2
0.1
0.0
−0.2
Central Asia
South−Eastern Asia
Western Asia
Region

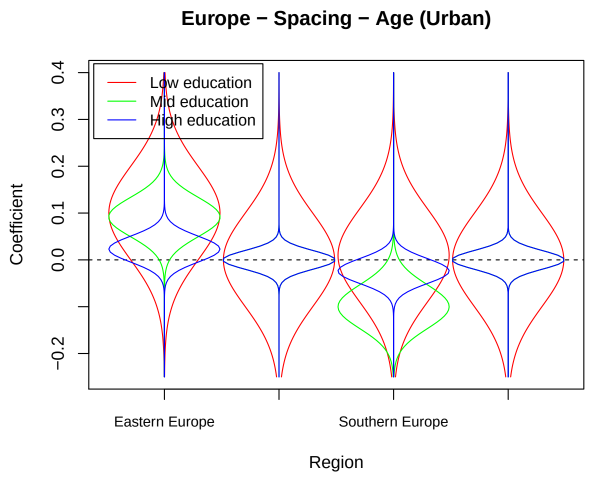
Europe − Spacing − Age (Urban)
Low education
Mid education
High education
Coefficient
0.4
0.3
0.2
0.1
0.0
−0.2
Eastern Europe
Southern Europe
Region

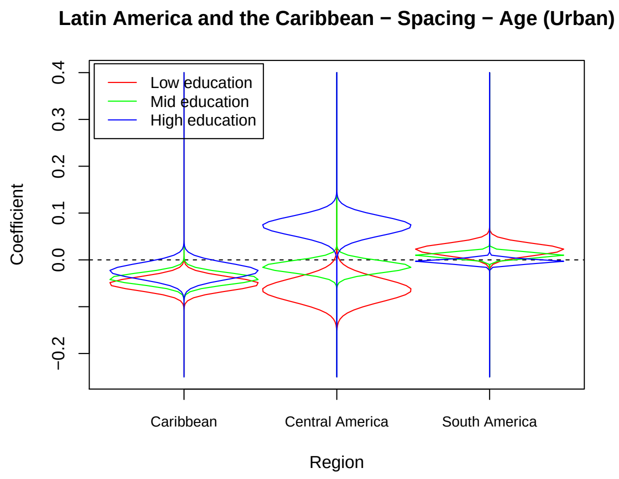
Latin America and the Caribbean − Spacing − Age (Urban)
Low education
Mid education
High education
Coefficient
0.4
0.3
0.2
0.1
0.0
−0.2
Caribbean
Central America
South America
Region

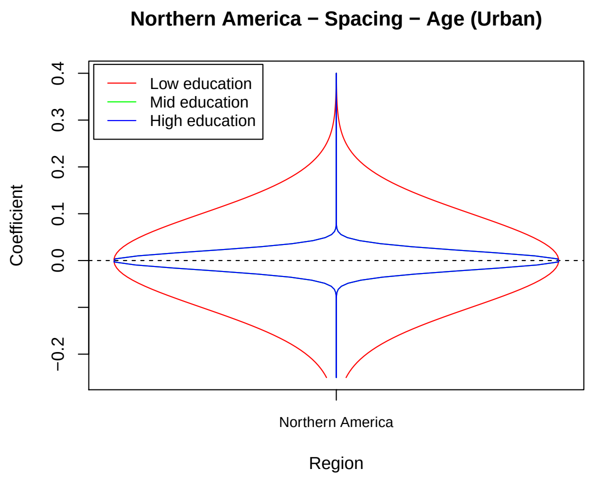
Northern America − Spacing − Age (Urban)
Low education
Mid education
High education
Coefficient
0.4
0.3
0.2
0.1
0.0
−0.2
Northern America
Region

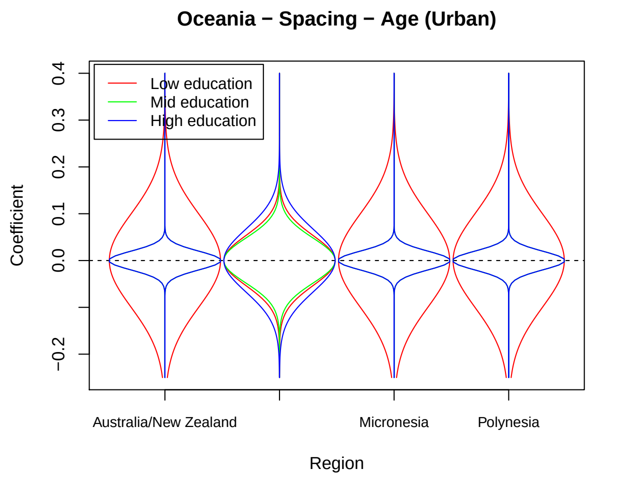
Oceania − Spacing − Age (Urban)
Low education
Mid education
High education
Coefficient
0.4
0.3
0.2
0.1
0.0
−0.2
Australia/New Zealand
Micronesia
Polynesia
Region

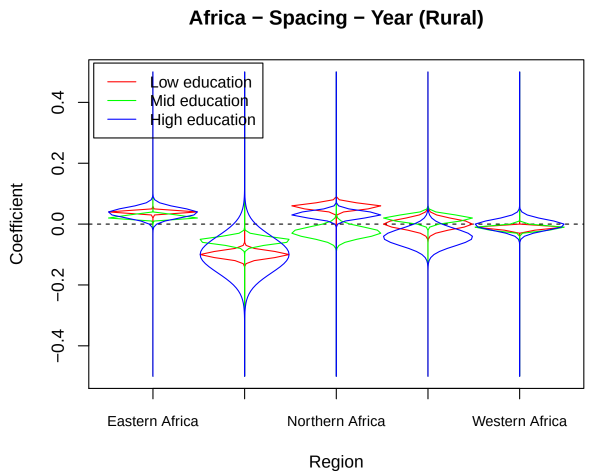
Africa − Spacing − Year (Rural)
Low education
Mid education
High education
Coefficient
−0.4
−0.2
0.0
0.2
0.4
Eastern Africa
Northern Africa
Western Africa
Region

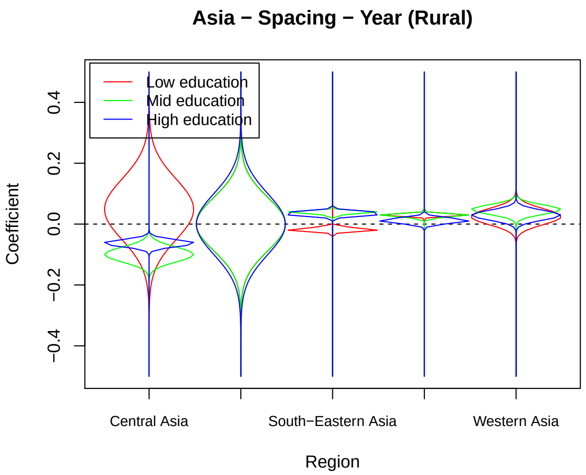
Asia − Spacing − Year (Rural)
Low education
Mid education
High education
Coefficient
−0.4
−0.2
0.0
0.2
0.4
Central Asia
South−Eastern Asia
Western Asia
Region

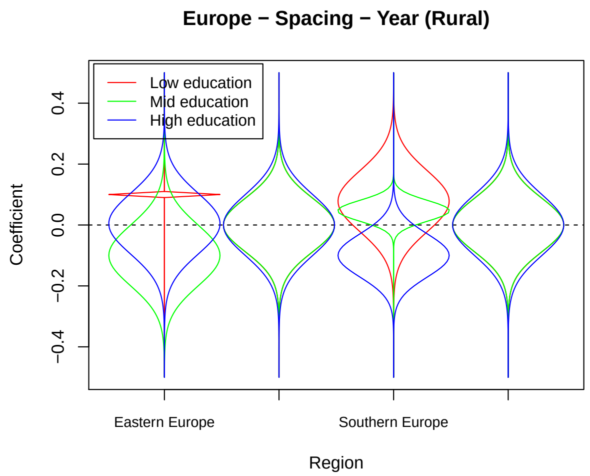
Europe − Spacing − Year (Rural)
Low education
Mid education
High education
Coefficient
−0.4
−0.2
0.0
0.2
0.4
Eastern Europe
Southern Europe
Region

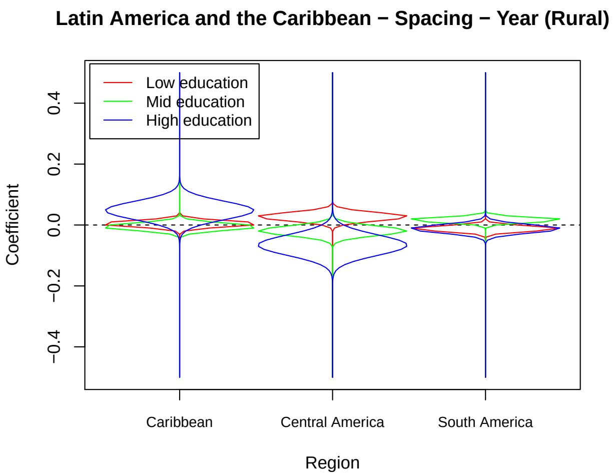
Latin America and the Caribbean − Spacing − Year (Rural)
Low education
Mid education
High education
Coefficient
−0.4
−0.2
0.0
0.2
0.4
Caribbean
Central America
South America
Region

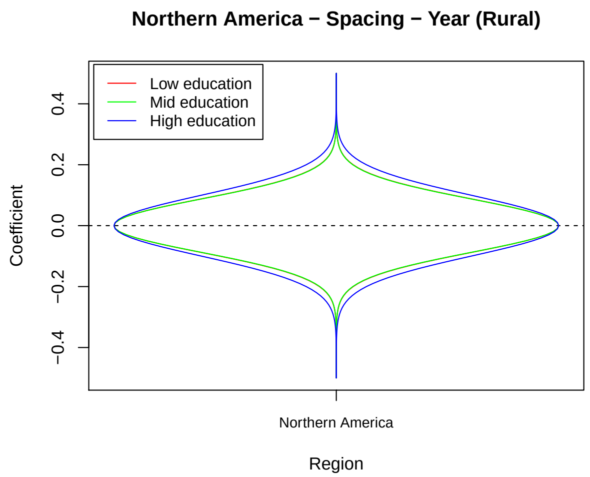
Northern America − Spacing − Year (Rural)
Low education
Mid education
High education
Coefficient
−0.4
−0.2
0.0
0.2
0.4
Northern America
Region

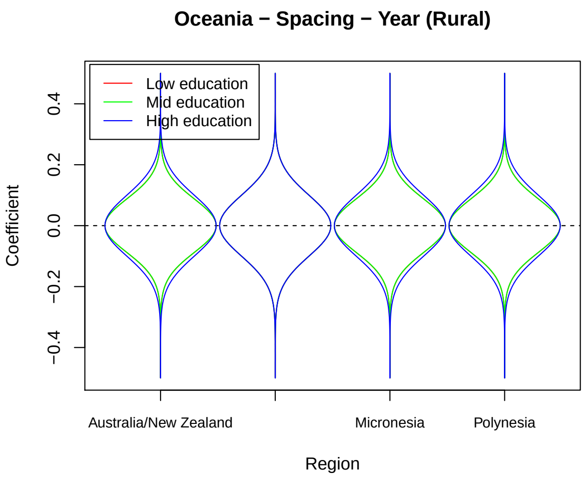
Oceania − Spacing − Year (Rural)
Low education
Mid education
High education
Coefficient
−0.4
−0.2
0.0
0.2
0.4
Australia/New Zealand
Micronesia
Polynesia
Region

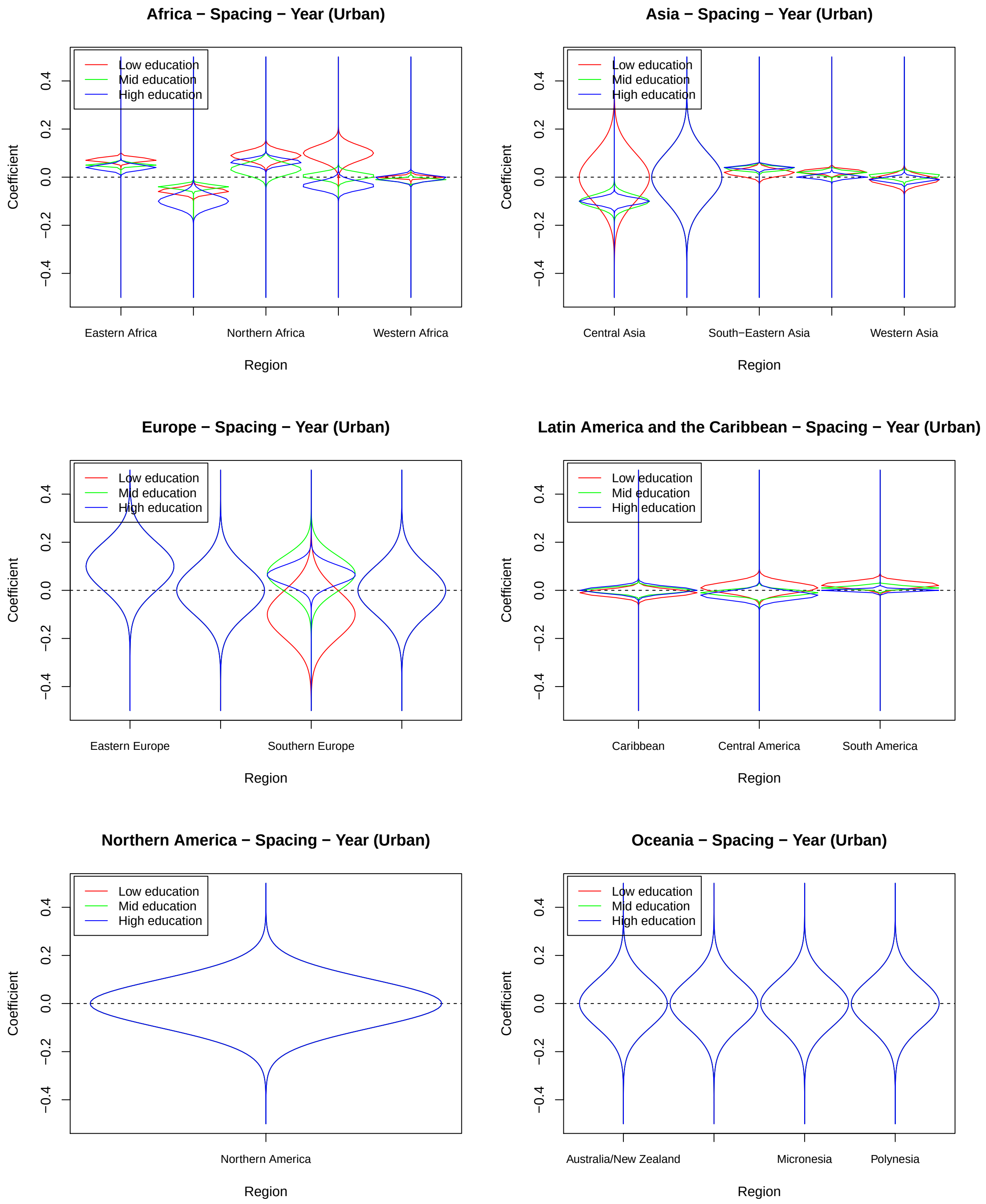
Africa − Spacing − Year (Urban)
Low education
Mid education
High education
Coefficient
Eastern Africa
Northern Africa
Western Africa
Region
Asia − Spacing − Year (Urban)
Central Asia
South−Eastern Asia
Western Asia
Europe − Spacing − Year (Urban)
Eastern Europe
Southern Europe
Latin America and the Caribbean − Spacing − Year (Urban)
Caribbean
Central America
South America
Northern America − Spacing − Year (Urban)
Northern America
Oceania − Spacing − Year (Urban)
Australia/New Zealand
Micronesia
Polynesia

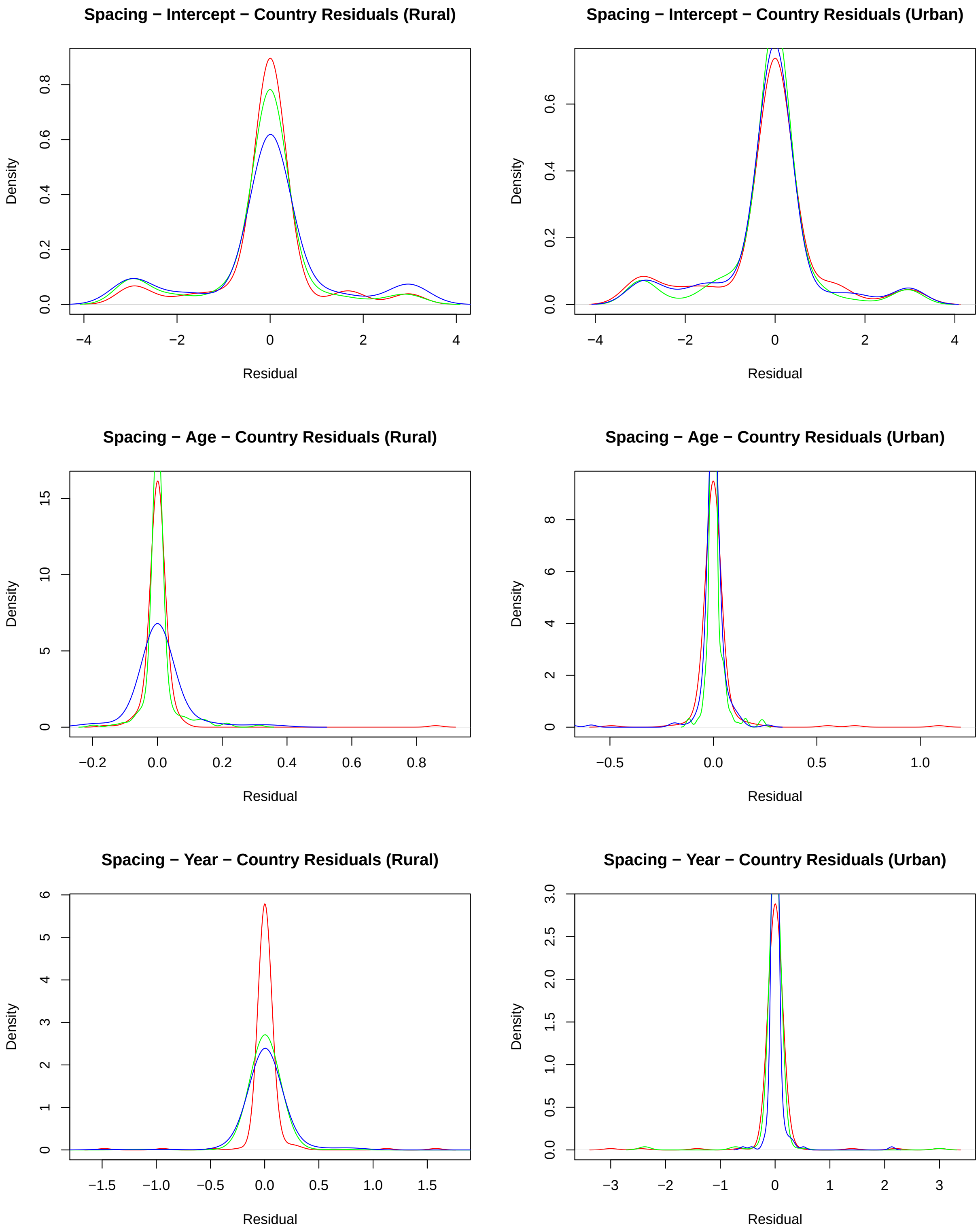

Spacing − Intercept − Country Residuals (Rural)
Spacing − Intercept − Country Residuals (Urban)
Spacing − Age − Country Residuals (Rural)
Spacing − Age − Country Residuals (Urban)
Spacing − Year − Country Residuals (Rural)
Spacing − Year − Country Residuals (Urban)
Density
Residual

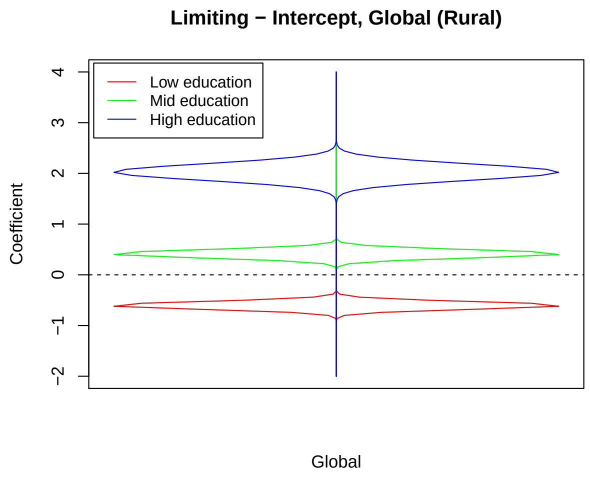
Limiting − Intercept, Global (Rural)
Low education
Mid education
High education
Coefficient
Global

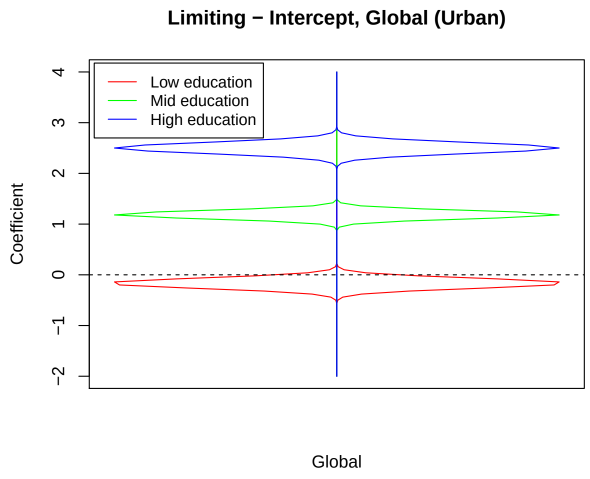
Limiting − Intercept, Global (Urban)
Low education
Mid education
High education
Coefficient
Global

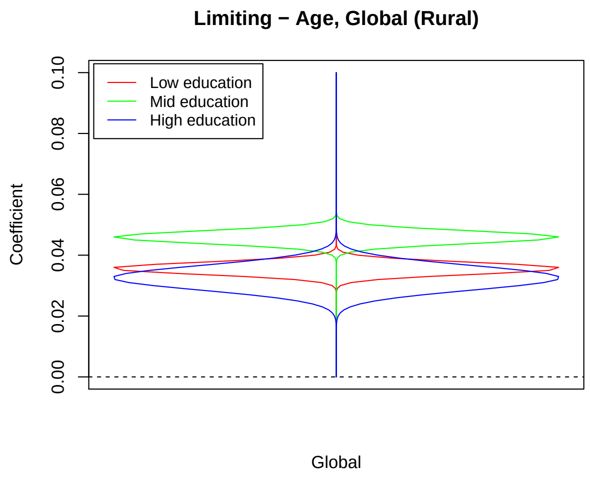
Limiting − Age, Global (Rural)
Low education
Mid education
High education
Coefficient
Global

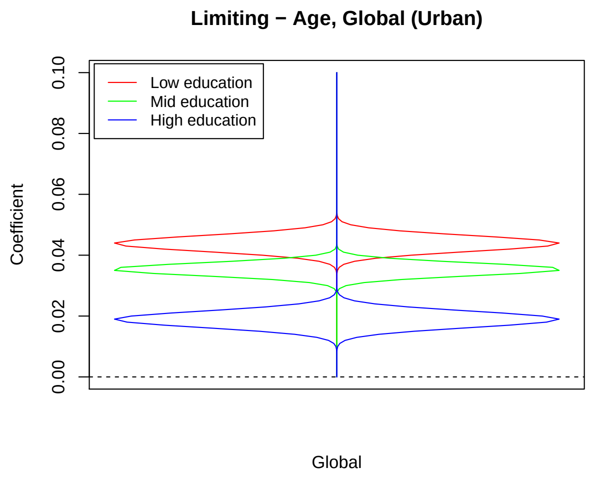
Limiting − Age, Global (Urban)
Low education
Mid education
High education
Coefficient
Global

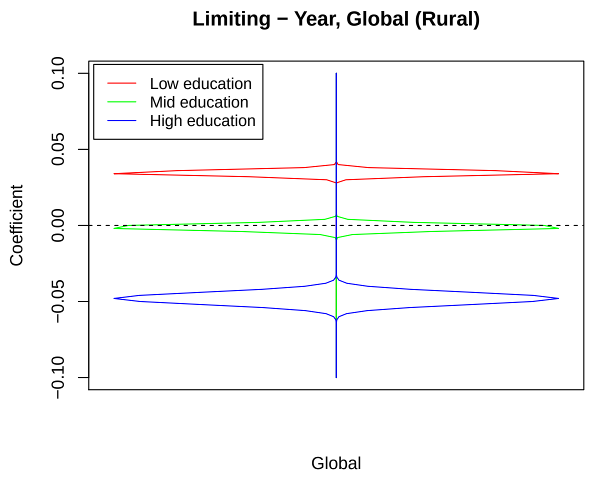
Limiting − Year, Global (Rural)
Low education
Mid education
High education
Coefficient
Global

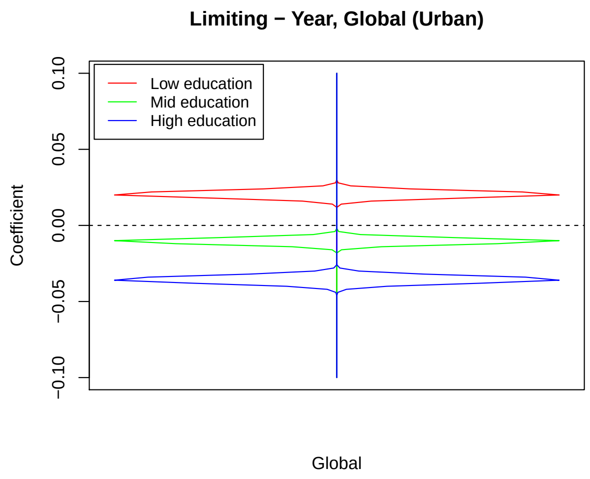
Limiting − Year, Global (Urban)
Low education
Mid education
High education
Coefficient
Global

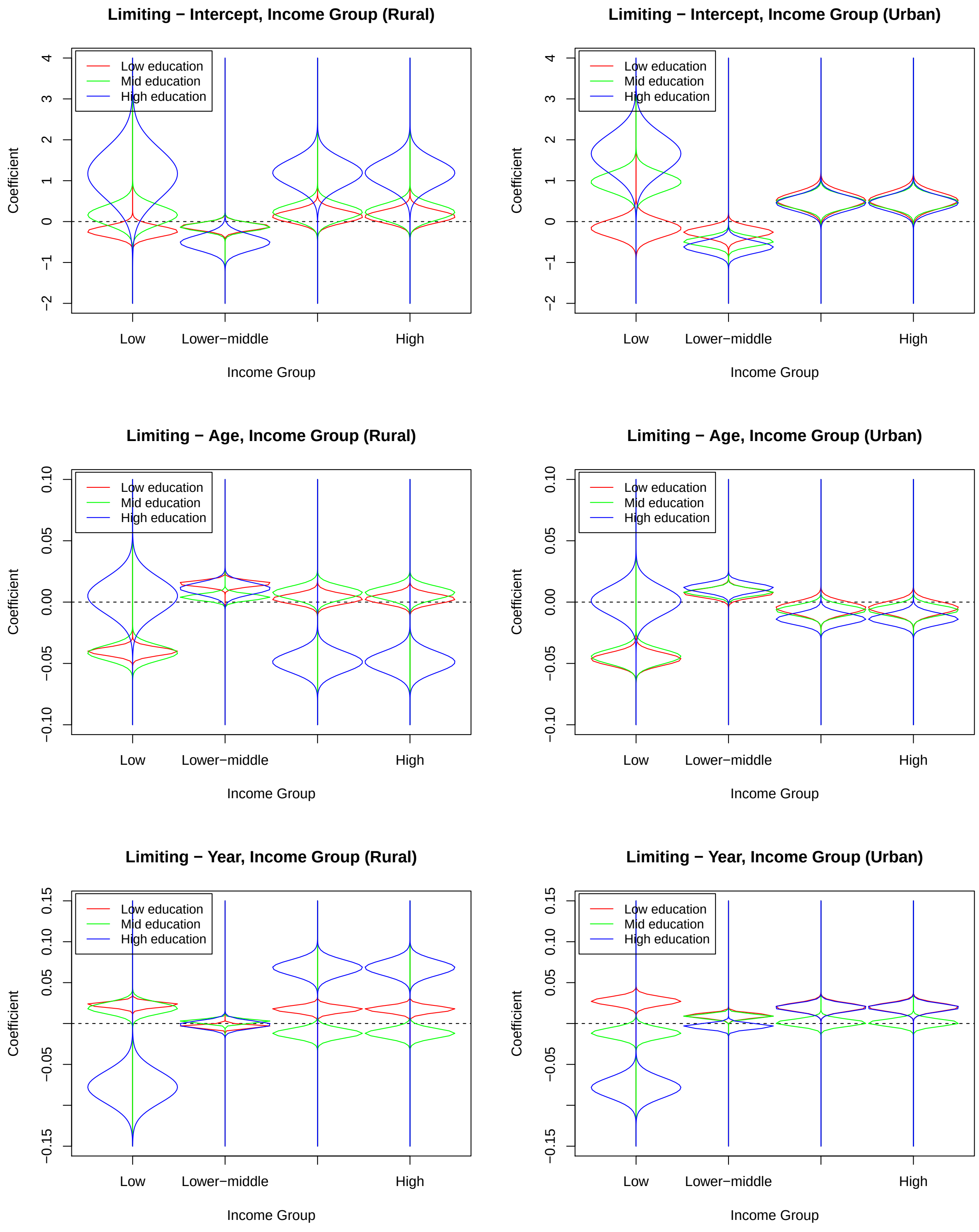

Limiting − Intercept, Income Group (Rural)
Limiting − Intercept, Income Group (Urban)
Limiting − Age, Income Group (Rural)
Limiting − Age, Income Group (Urban)
Limiting − Year, Income Group (Rural)
Limiting − Year, Income Group (Urban)
Low education
Mid education
High education
Coefficient
Income Group
Low
Lower−middle
High
−2
−1
0
1
2
3
4
−0.10
−0.05
0.00
0.05
0.10
−0.15
0.15

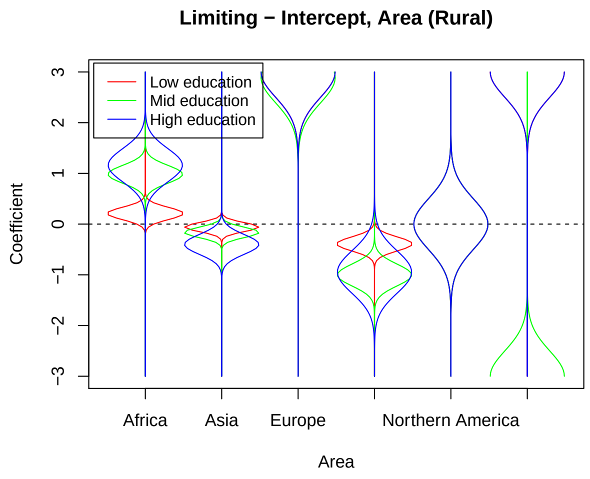
Limiting − Intercept, Area (Rural)
Low education
Mid education
High education
Coefficient
Africa
Asia
Europe
Northern America
Area

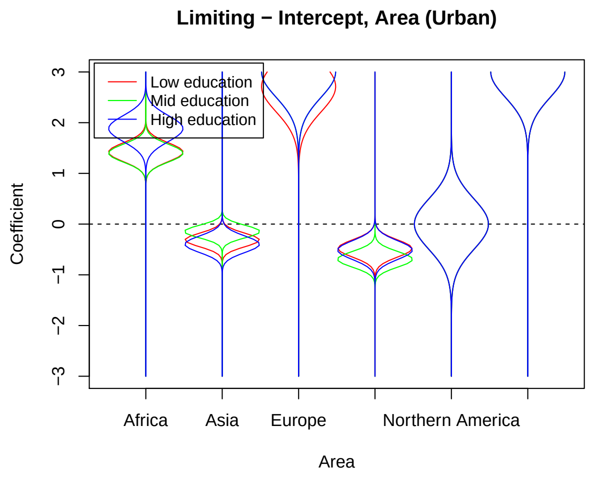
Limiting − Intercept, Area (Urban)
Low education
Mid education
High education
Coefficient
Africa
Asia
Europe
Northern America
Area

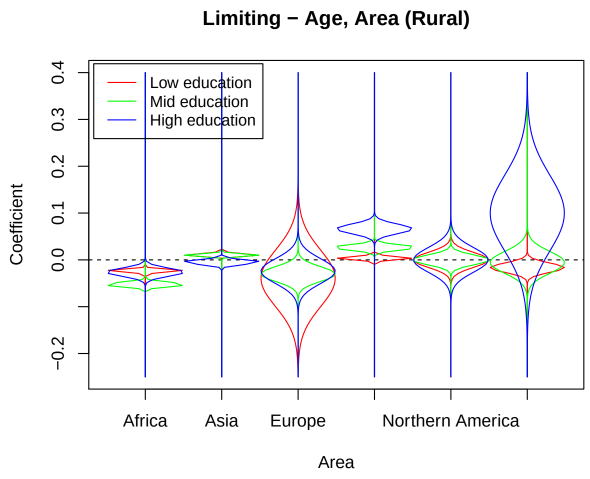
Limiting − Age, Area (Rural)
Low education
Mid education
High education
Coefficient
Africa
Asia
Europe
Northern America
Area

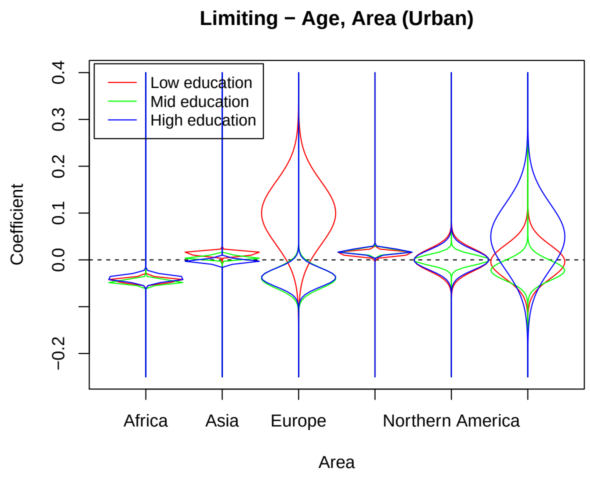
Limiting − Age, Area (Urban)
Low education
Mid education
High education
Coefficient
Africa
Asia
Europe
Northern America
Area

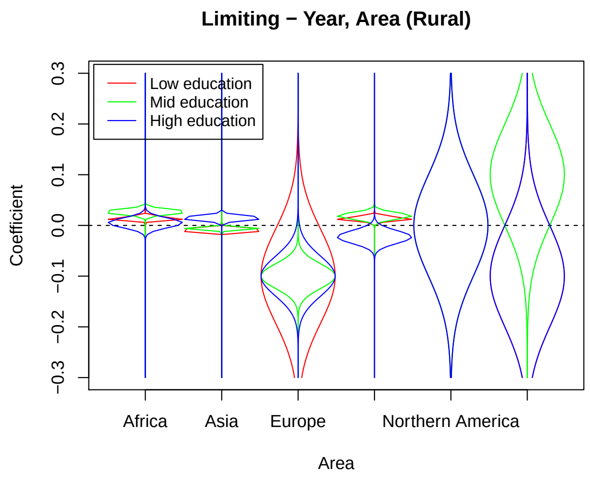
Limiting − Year, Area (Rural)
Low education
Mid education
High education
Coefficient
Africa
Asia
Europe
Northern America
Area

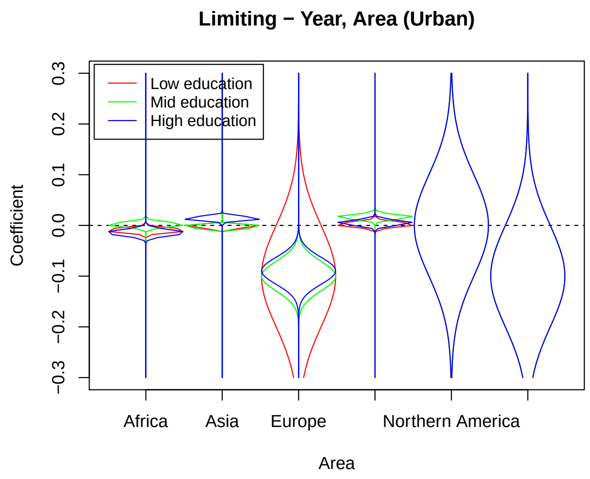
Limiting − Year, Area (Urban)
Low education
Mid education
High education
Coefficient
Africa
Asia
Europe
Northern America
Area

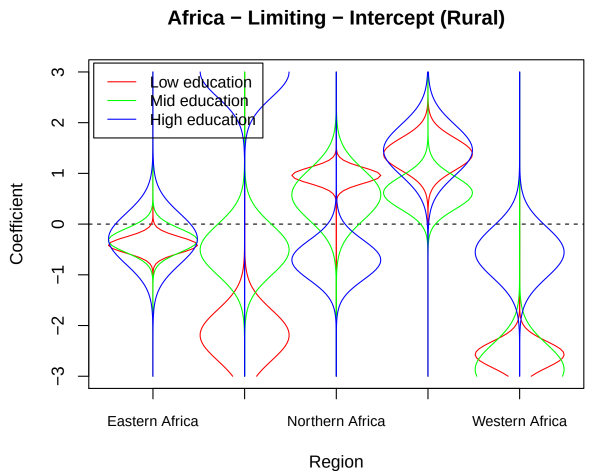
Africa − Limiting − Intercept (Rural)
Low education
Mid education
High education
Coefficient
3
2
1
0
−1
−2
−3
Eastern Africa
Northern Africa
Western Africa
Region

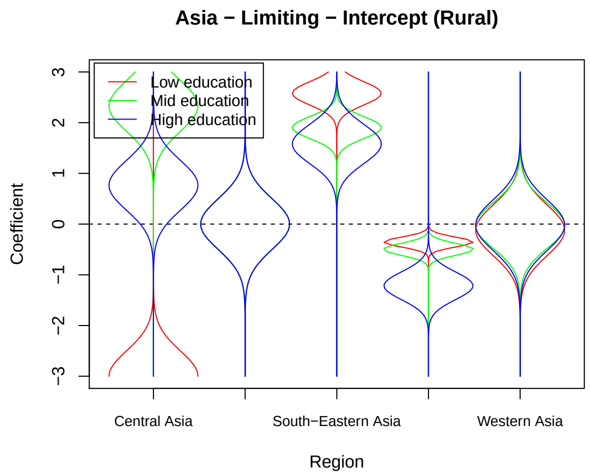
Asia − Limiting − Intercept (Rural)
Low education
Mid education
High education
Coefficient
3
2
1
0
−1
−2
−3
Central Asia
South−Eastern Asia
Western Asia
Region

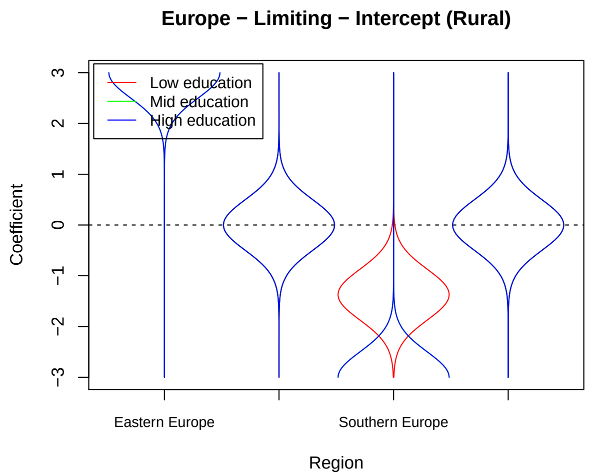
Europe − Limiting − Intercept (Rural)
Low education
Mid education
High education
Coefficient
3
2
1
0
−1
−2
−3
Eastern Europe
Southern Europe
Region

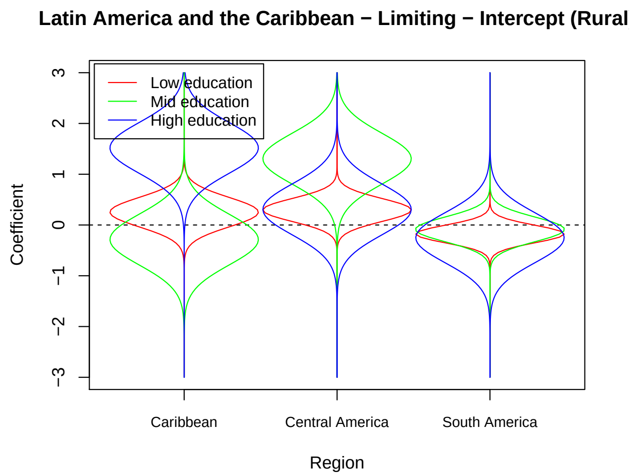
Latin America and the Caribbean − Limiting − Intercept (Rural
Low education
Mid education
High education
Coefficient
3
2
1
0
−1
−2
−3
Caribbean
Central America
South America
Region

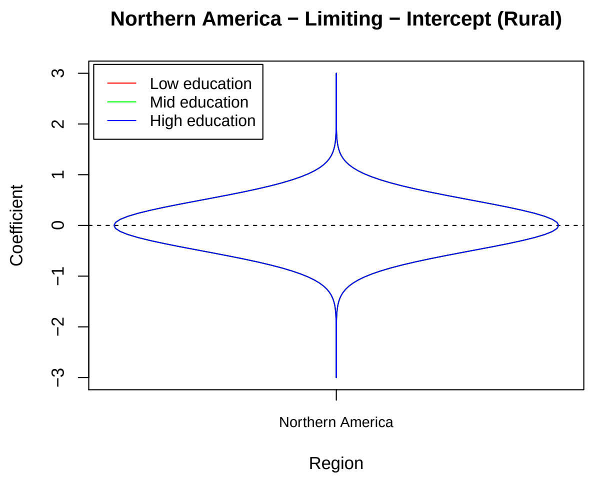
Northern America − Limiting − Intercept (Rural)
Low education
Mid education
High education
Coefficient
3
2
1
0
−1
−2
−3
Northern America
Region

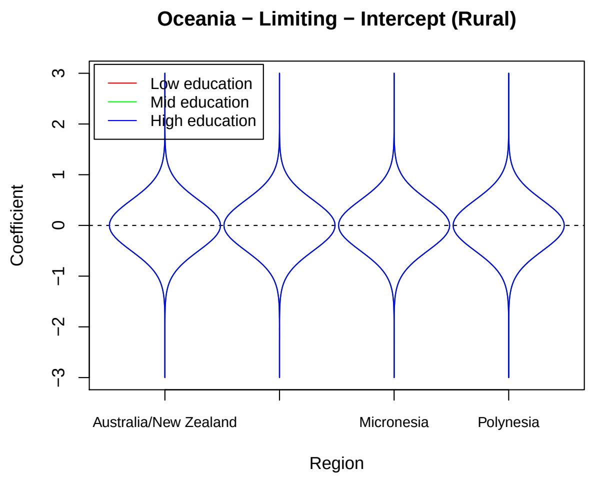
Oceania − Limiting − Intercept (Rural)
Low education
Mid education
High education
Coefficient
3
2
1
0
−1
−2
−3
Australia/New Zealand
Micronesia
Polynesia
Region

Africa − Limiting − Intercept (Urban)

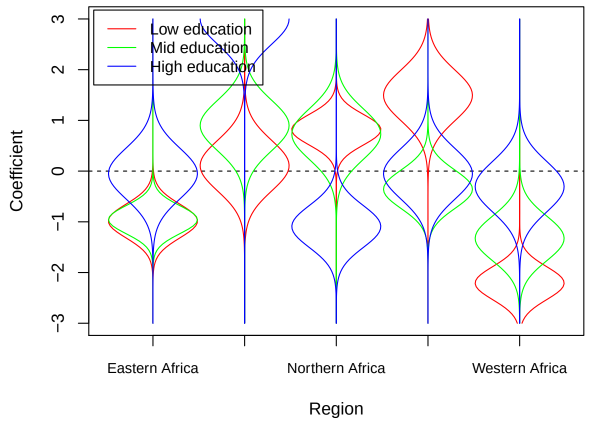

Asia − Limiting − Intercept (Urban)

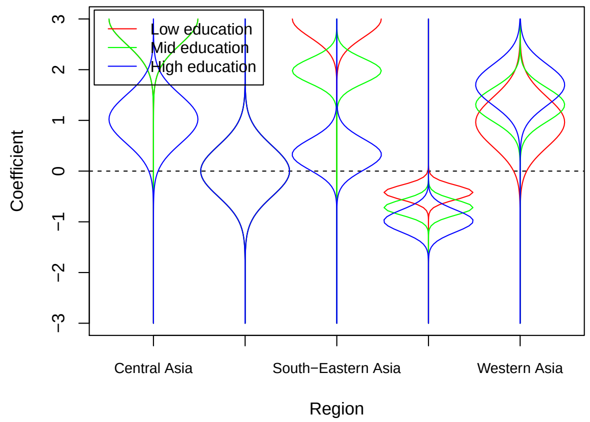

Europe − Limiting − Intercept (Urban)

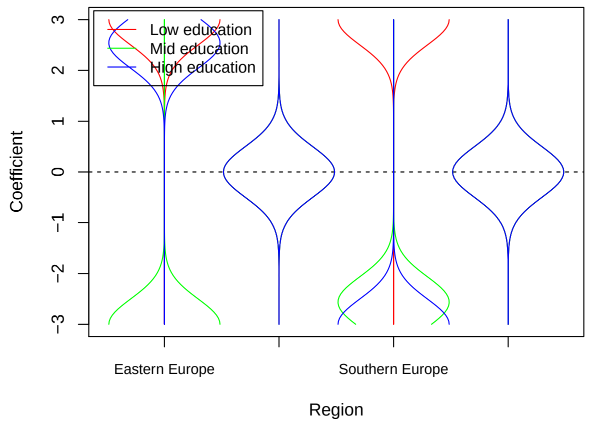

Latin America and the Caribbean − Limiting − Intercept (Urban

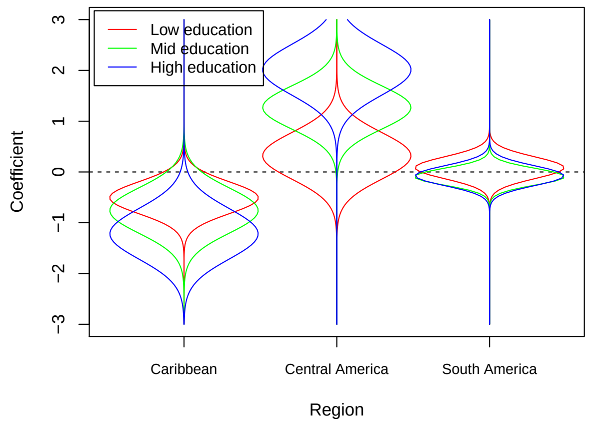

Northern America − Limiting − Intercept (Urban)

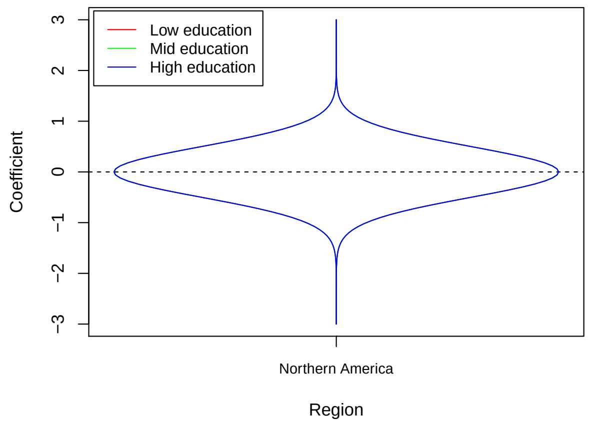

Oceania − Limiting − Intercept (Urban)

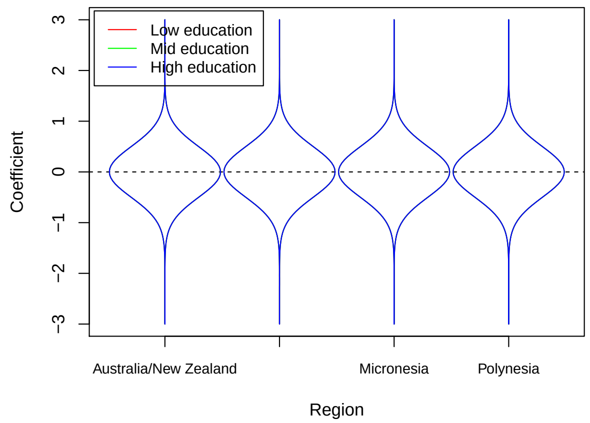

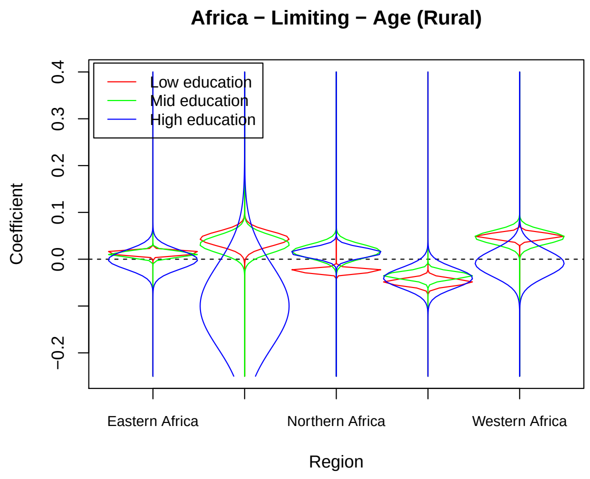
Africa − Limiting − Age (Rural)
Low education
Mid education
High education
Coefficient
Eastern Africa
Northern Africa
Western Africa
Region

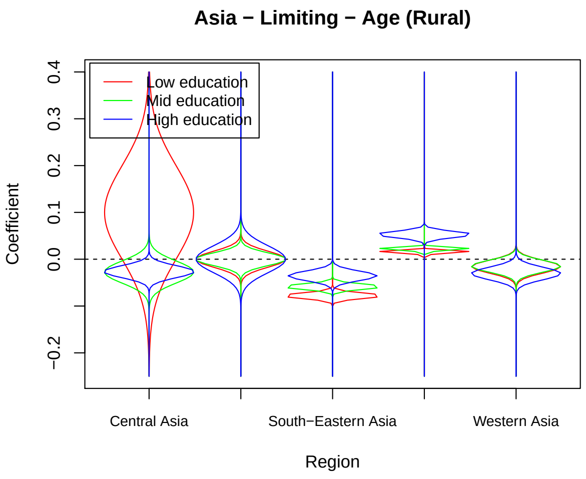
Asia − Limiting − Age (Rural)
Low education
Mid education
High education
Coefficient
Central Asia
South−Eastern Asia
Western Asia
Region

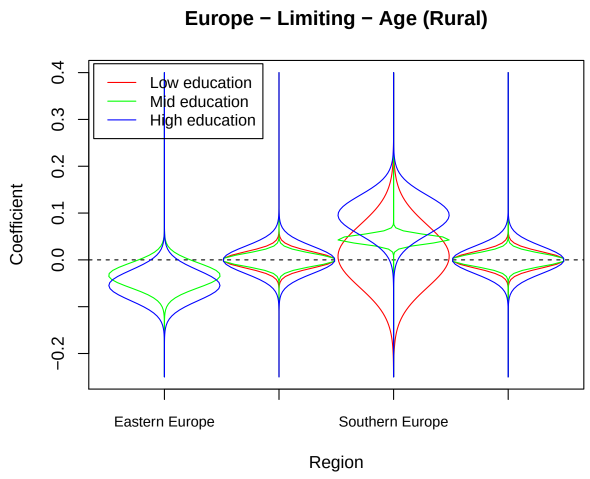
Europe − Limiting − Age (Rural)
Low education
Mid education
High education
Coefficient
Eastern Europe
Southern Europe
Region

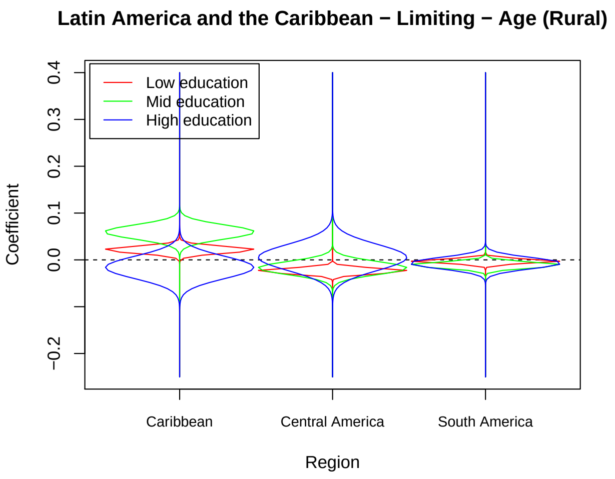
Latin America and the Caribbean − Limiting − Age (Rural)
Low education
Mid education
High education
Coefficient
Caribbean
Central America
South America
Region

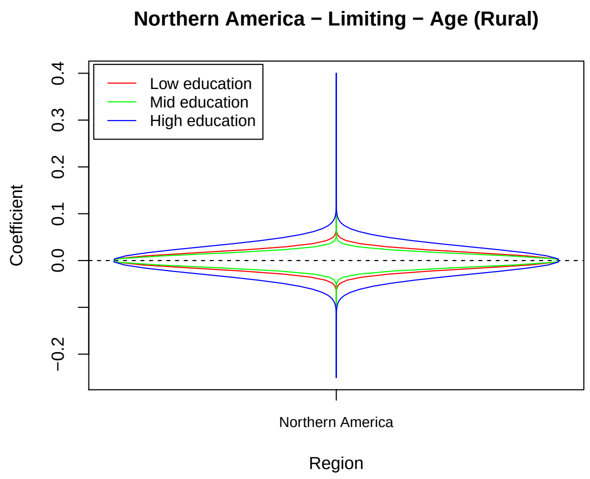
Northern America − Limiting − Age (Rural)
Low education
Mid education
High education
Coefficient
Northern America
Region

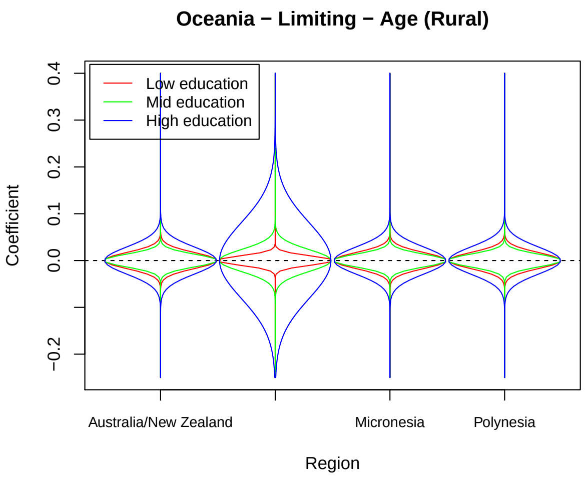
Oceania − Limiting − Age (Rural)
Low education
Mid education
High education
Coefficient
Australia/New Zealand
Micronesia
Polynesia
Region

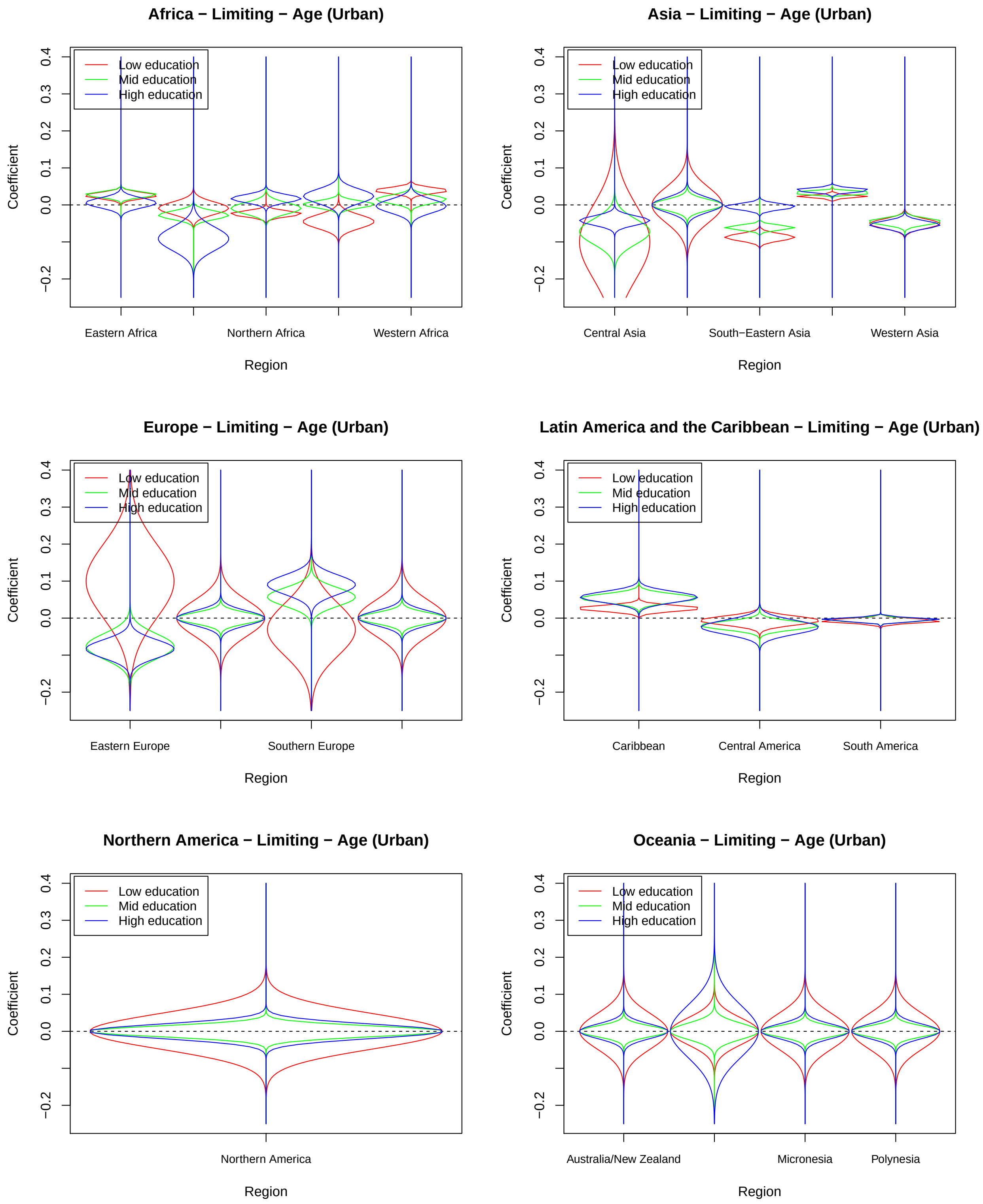

Africa − Limiting − Age (Urban)
Low education
Mid education
High education
Coefficient
Eastern Africa
Northern Africa
Western Africa
Region
Asia − Limiting − Age (Urban)
Central Asia
South−Eastern Asia
Western Asia
Europe − Limiting − Age (Urban)
Eastern Europe
Southern Europe
Latin America and the Caribbean − Limiting − Age (Urban)
Caribbean
Central America
South America
Northern America − Limiting − Age (Urban)
Northern America
Oceania − Limiting − Age (Urban)
Australia/New Zealand
Micronesia
Polynesia

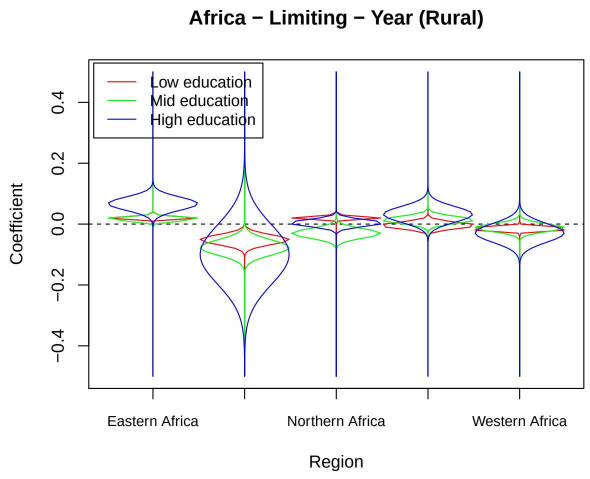
Africa − Limiting − Year (Rural)
Low education
Mid education
High education
Coefficient
−0.4
−0.2
0.0
0.2
0.4
Eastern Africa
Northern Africa
Western Africa
Region

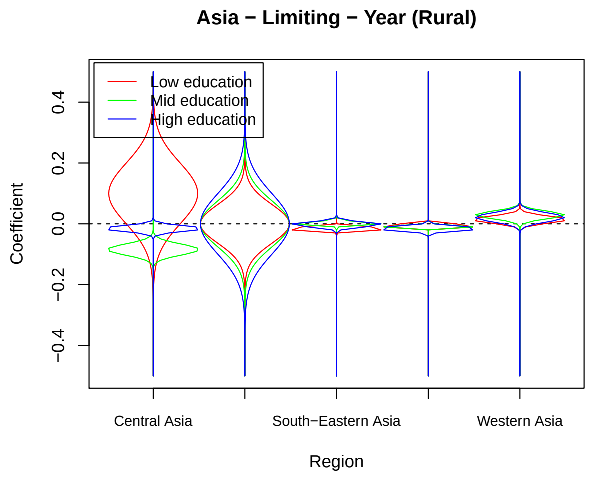
Asia − Limiting − Year (Rural)
Low education
Mid education
High education
Coefficient
−0.4
−0.2
0.0
0.2
0.4
Central Asia
South−Eastern Asia
Western Asia
Region

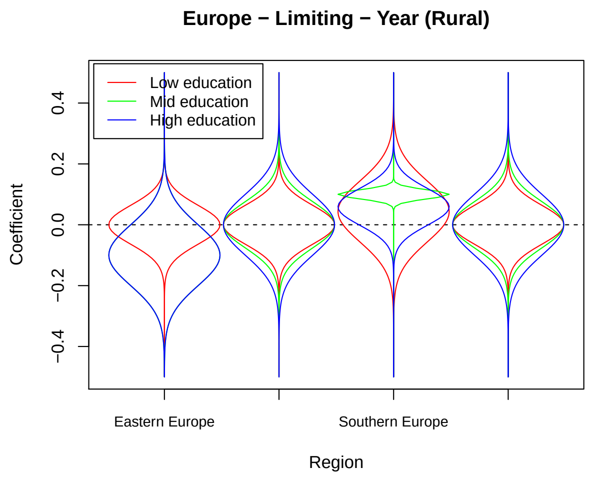
Europe − Limiting − Year (Rural)
Low education
Mid education
High education
Coefficient
−0.4
−0.2
0.0
0.2
0.4
Eastern Europe
Southern Europe
Region

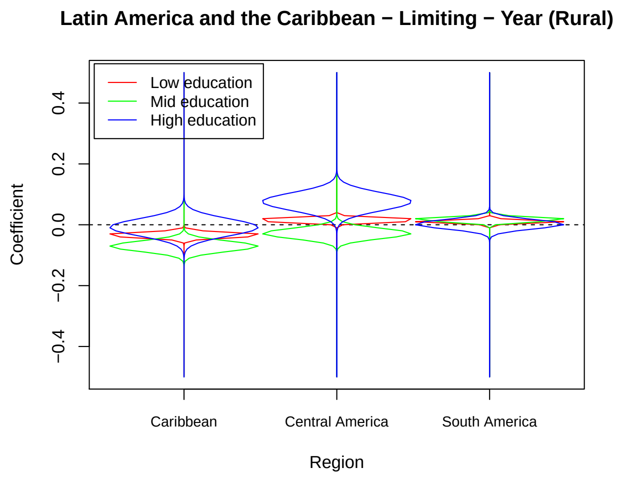
Latin America and the Caribbean − Limiting − Year (Rural)
Low education
Mid education
High education
Coefficient
−0.4
−0.2
0.0
0.2
0.4
Caribbean
Central America
South America
Region

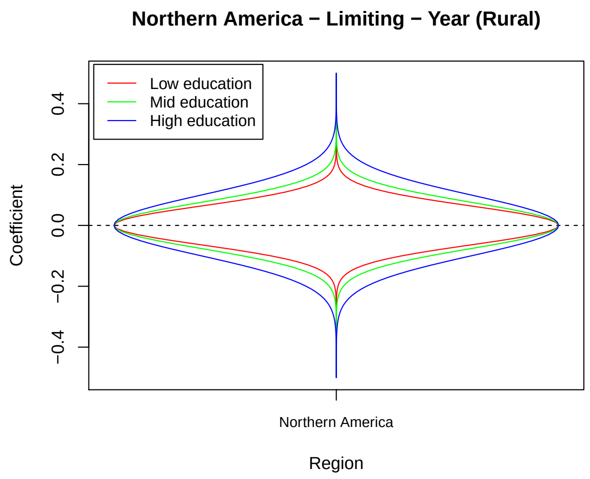
Northern America − Limiting − Year (Rural)
Low education
Mid education
High education
Coefficient
−0.4
−0.2
0.0
0.2
0.4
Northern America
Region

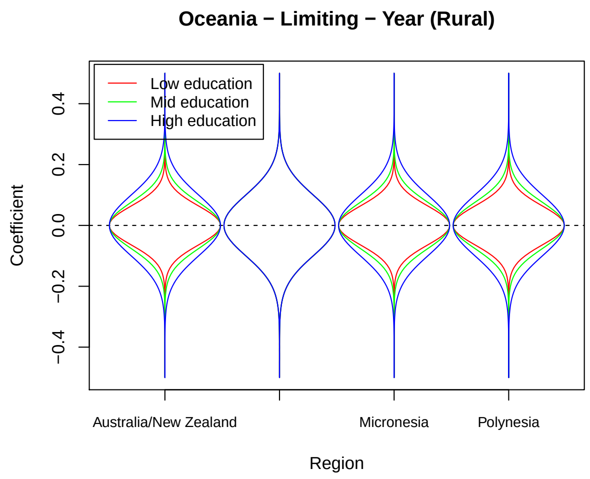
Oceania − Limiting − Year (Rural)
Low education
Mid education
High education
Coefficient
−0.4
−0.2
0.0
0.2
0.4
Australia/New Zealand
Micronesia
Polynesia
Region

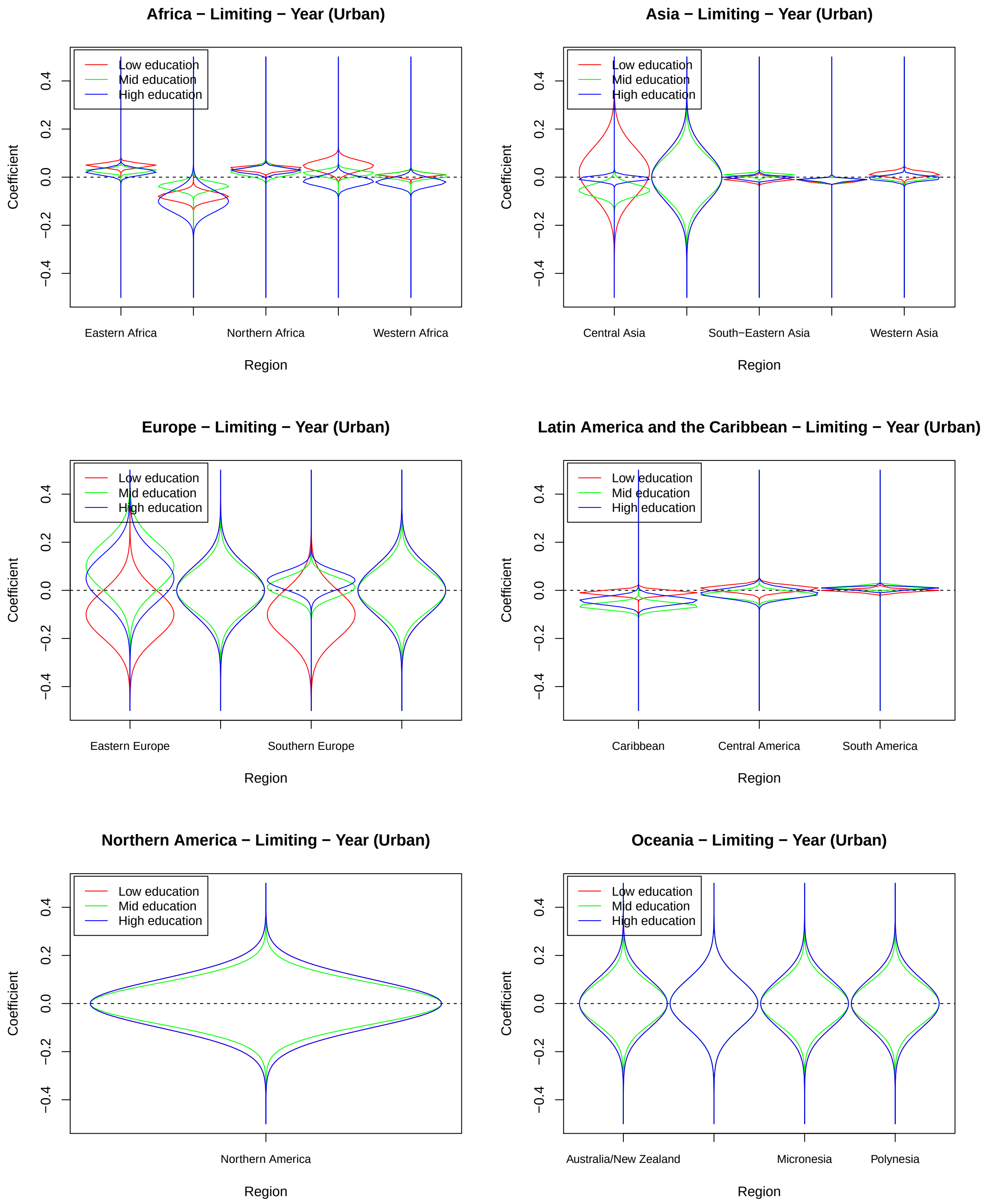
Africa − Limiting − Year (Urban)
Low education
Mid education
High education
Coefficient
0.4
0.2
0.0
−0.2
−0.4
Eastern Africa
Northern Africa
Western Africa
Region
Asia − Limiting − Year (Urban)
Low education
Mid education
High education
Coefficient
0.4
0.2
0.0
−0.2
−0.4
Central Asia
South−Eastern Asia
Western Asia
Region
Europe − Limiting − Year (Urban)
Low education
Mid education
High education
Coefficient
0.4
0.2
0.0
−0.2
−0.4
Eastern Europe
Southern Europe
Region
Latin America and the Caribbean − Limiting − Year (Urban)
Low education
Mid education
High education
Coefficient
0.4
0.2
0.0
−0.2
−0.4
Caribbean
Central America
South America
Region
Northern America − Limiting − Year (Urban)
Low education
Mid education
High education
Coefficient
0.4
0.2
0.0
−0.2
−0.4
Northern America
Region
Oceania − Limiting − Year (Urban)
Low education
Mid education
High education
Coefficient
0.4
0.2
0.0
−0.2
−0.4
Australia/New Zealand
Micronesia
Polynesia
Region

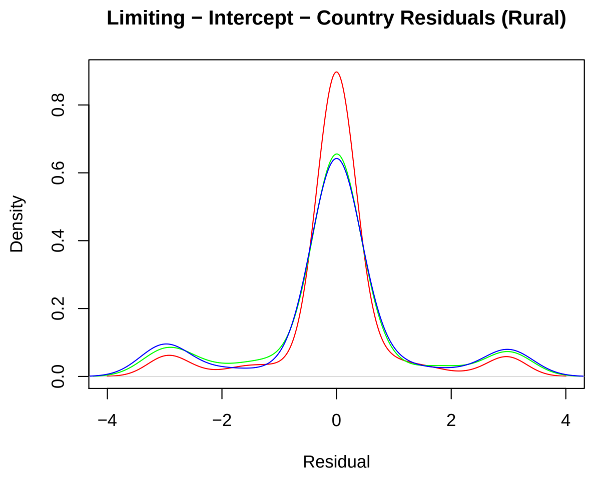

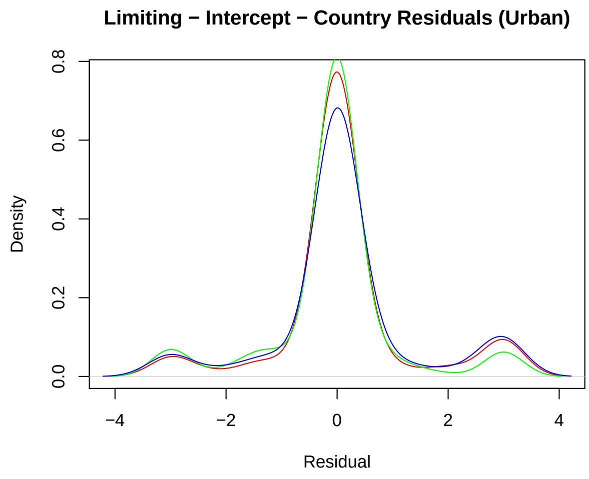

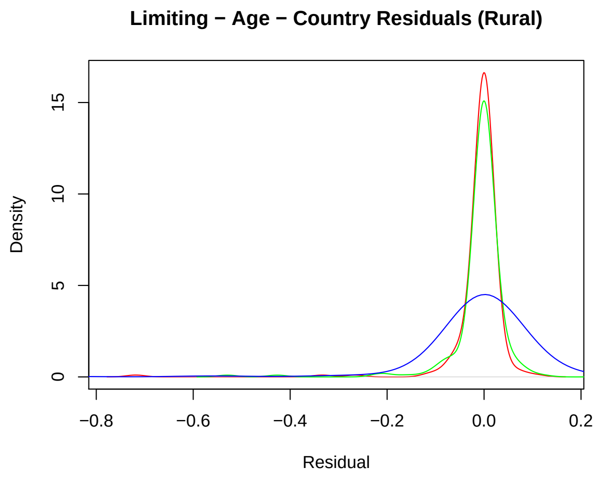

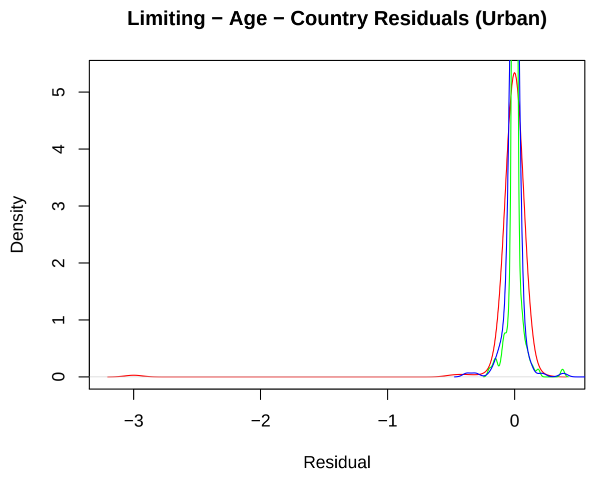

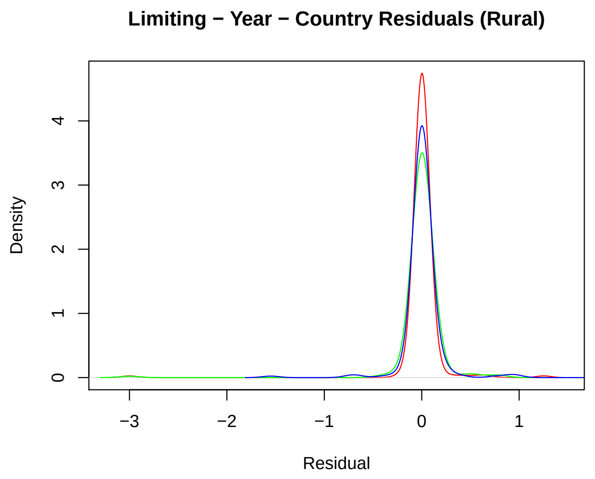

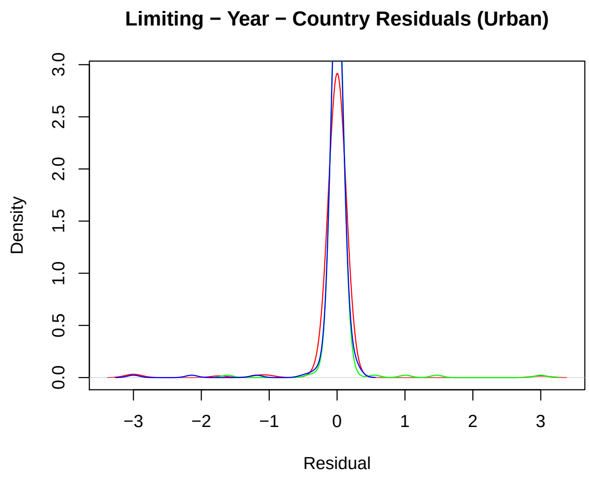

Model Implementation

Because unmet need tends to decrease with age, women who experience unmet need are checked annually in the model to simulate whether their need becomes met.

References

Demographic and Health Survey Documentation. Unmet need. Available: http://dhsprogram.com/topics/Unmet-Need.cfm.

A.1.3.5 Contraception Method Mix

Overview

The mix of contraceptive methods that women may use is simulated in the model based on demographic characteristics and age. Women may switch between methods in the model for reasons such as side-effects or method failure.

Data

Method of contraception is based on data from the DHS (variable v312), conditional on whether a woman is spacing or limiting. We estimated age and time trends based on DHS data for 1,409,587 women from 254 surveys in 74 countries.

We model the use of 10 contraception methods: Pill, IUD, Injections, Condom, Female sterilization, Male sterilization, Periodic abstinence, Withdrawal, Implants, Other. Due to the small number of responses for certain methods we combined 'Diaphragm' with 'Condom', and 'Prolonged abstinence' with 'Periodic abstinence'. We re-coded other methods not listed above as 'Other'. Here we plot time trends in method mix for spacing and limiting by income group, based on DHS data. (Note: No high income countries are available in the DHS.)

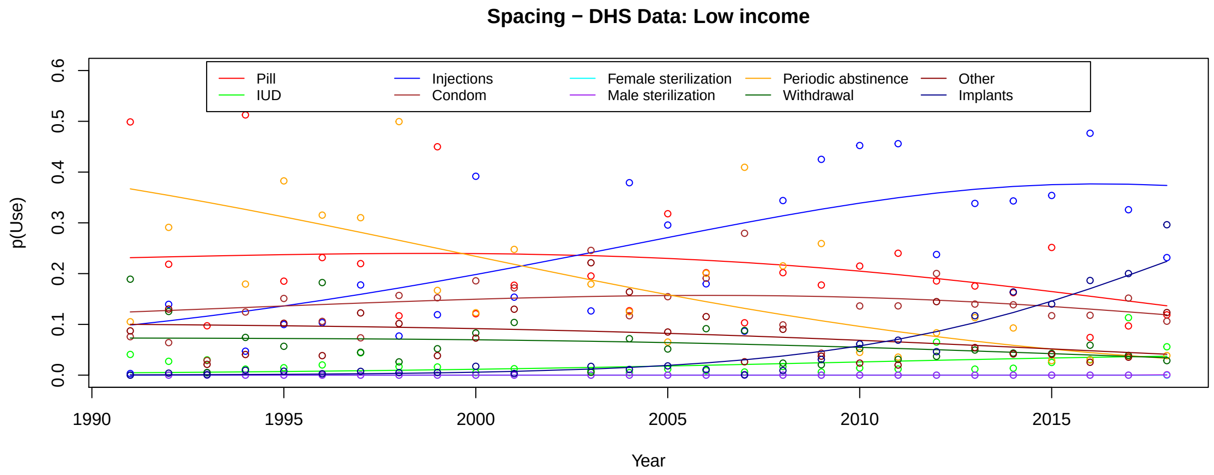

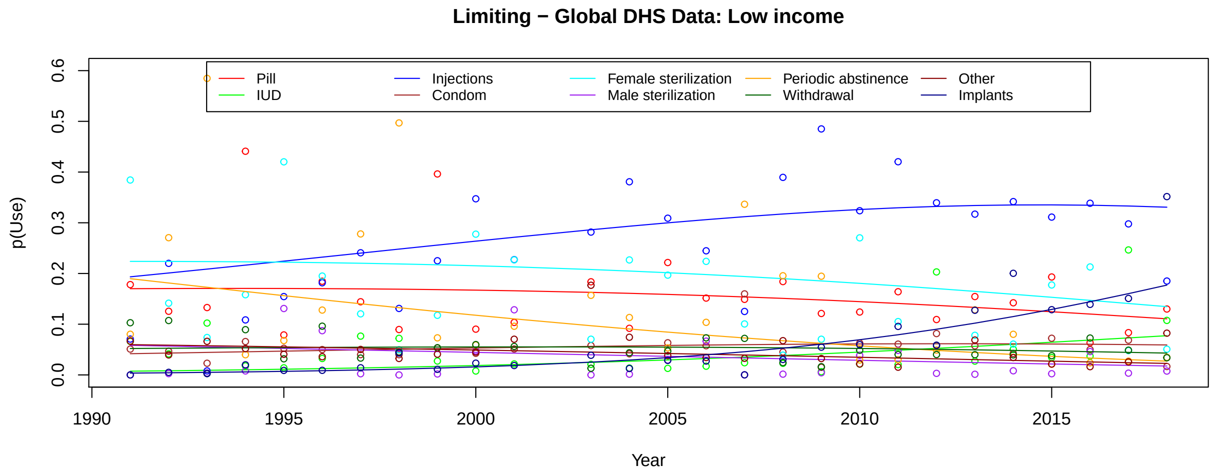

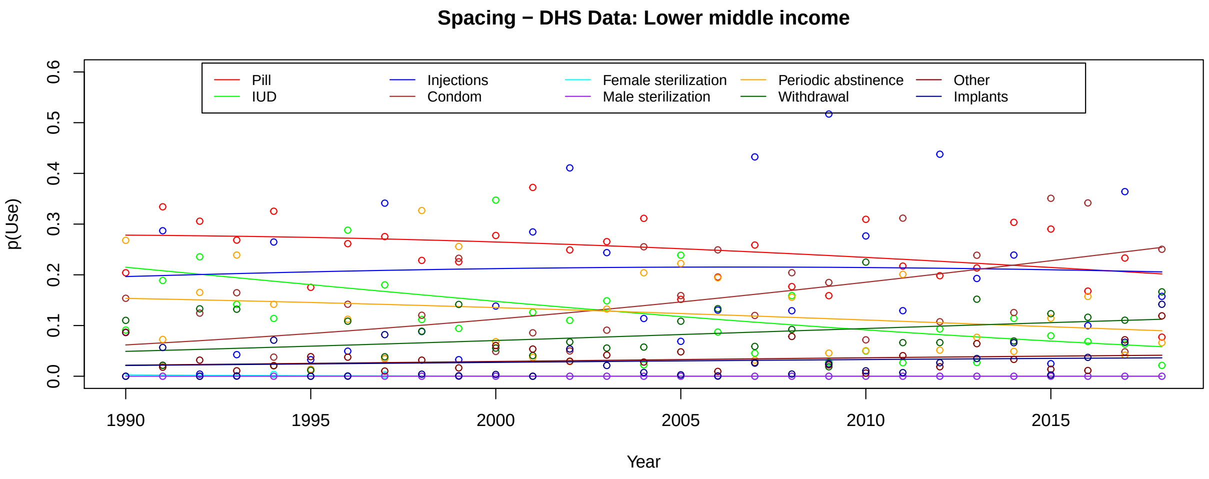
Spacing − DHS Data: Lower middle income
Pill
IUD
Injections
Condom
Female sterilization
Male sterilization
Periodic abstinence
Withdrawal
Other
Implants
p(Use)
Year

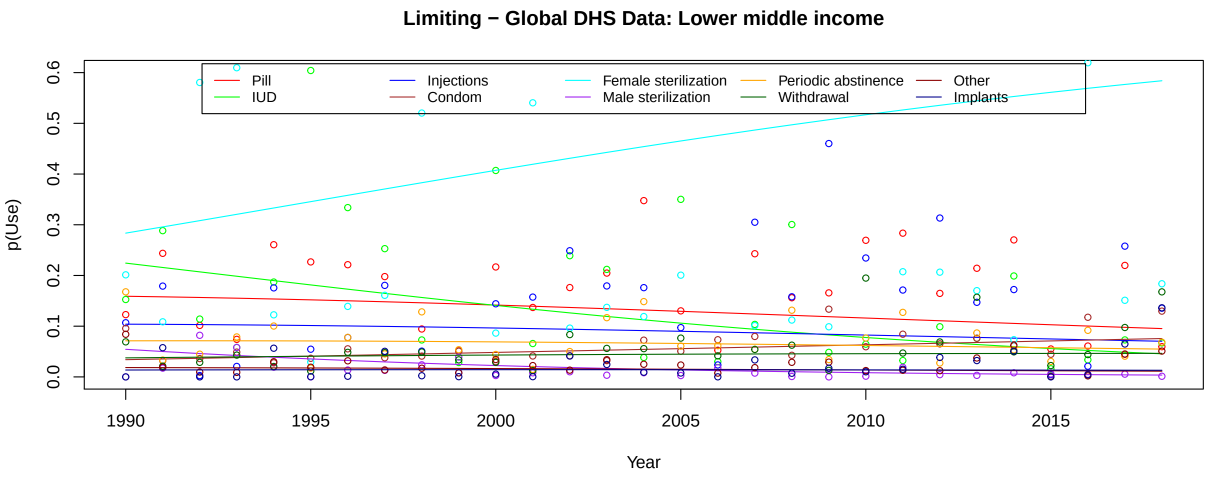
Limiting − Global DHS Data: Lower middle income
Pill
IUD
Injections
Condom
Female sterilization
Male sterilization
Periodic abstinence
Withdrawal
Other
Implants
p(Use)
Year

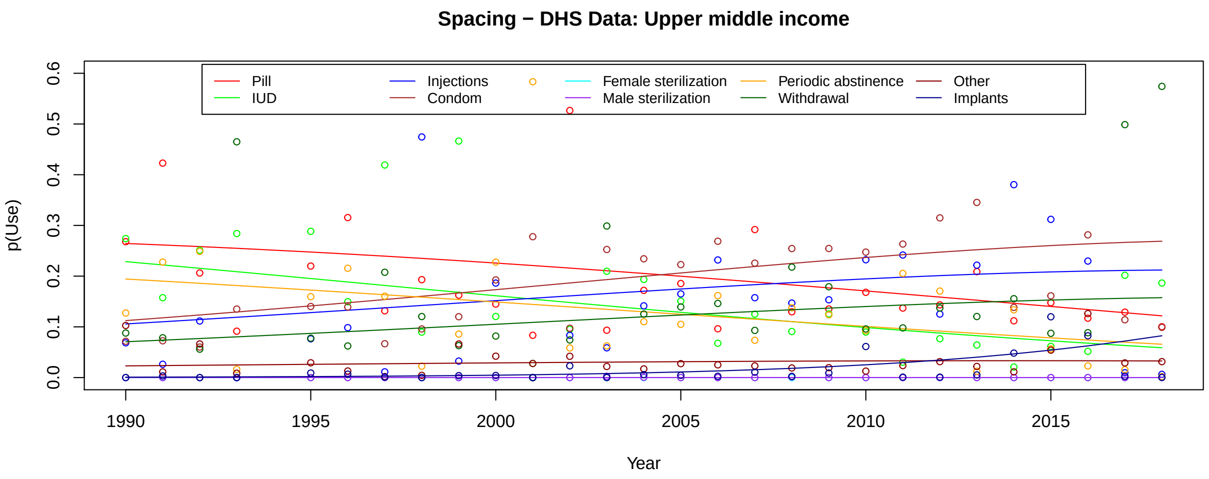
Spacing − DHS Data: Upper middle income
Pill
IUD
Injections
Condom
Female sterilization
Male sterilization
Periodic abstinence
Withdrawal
Other
Implants
p(Use)
Year

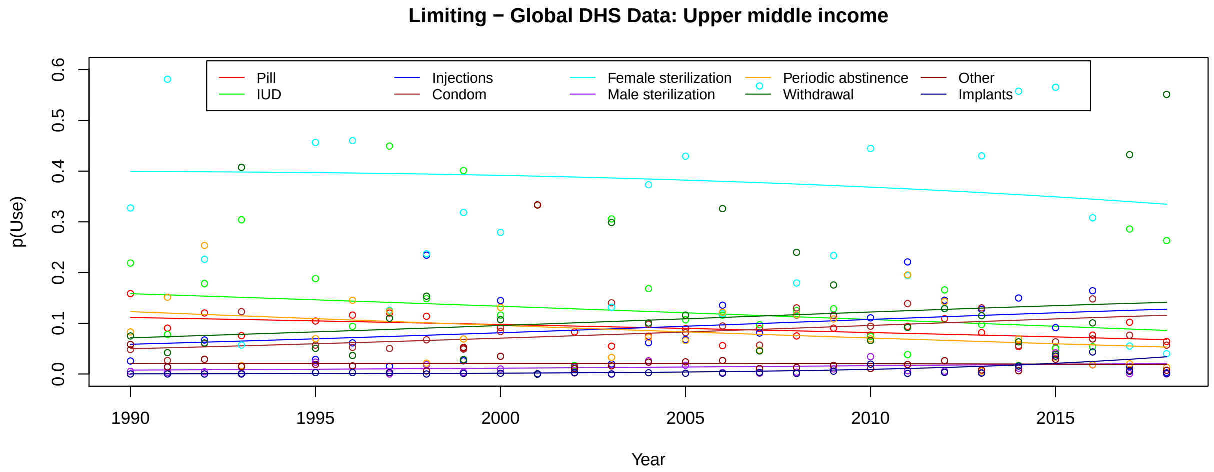

Parameters

We estimated hierarchical logistic regression models for each method, stratified by subgroup. Separate models were estimated for spacing and limiting. The predicted probabilities of each method were re-normalized to ensure that the probabilities sum to 1.0. We used upper middle income priors for high income countries due to lack of DHS data in high income countries.

β_0 | β_1

I = Income Group

$$\beta^{I}_{m,i,j} \sim N\left(\hat{\beta}_{m,i,j}{}^{I}, \hat{\sigma}_{m,i,j}{}^{I}\right) \qquad \beta^{I}_{i} \sim N\left(\hat{\beta}_{m,i,j}{}^{I}, \hat{\sigma}_{m,i,j}{}^{I}\right)$$

A = Area

$$\gamma^{A}_{m,i,j} \sim N(\widehat{\gamma^{A}_{m,i,j}}, \widehat{\sigma^{A}_{m,i,j}}) \qquad \gamma^{A}_{m,i,j} \sim N(\widehat{\gamma^{A}_{m,i,j}}, \widehat{\sigma^{A}_{m,i,j}})$$

R = Region

$$\delta^{R}_{m,i,j} \sim N(\widehat{\delta^{R}_{m,i,j}}, \widehat{\sigma^{R}_{m,i,j}}) \qquad \delta^{R}_{m,i,j} \sim N(\widehat{\delta^{R}_{m,i,j}}, \widehat{\sigma^{R}_{m,i,j}})$$

C = Country

$$\beta_0{}^{C}_{m,i,j} \sim \beta^{I}_{m,i,j} + \gamma^{A}_{m,i,j} + \delta^{R}_{m,i,j} + N\left(\widehat{\epsilon^{C}_{m,i,j}}, 0.01\right) \qquad \beta_1{}^{C}_{m,i,j} \sim \beta^{I}_{m,i,j} + \gamma^{A}_{m,i,j} + \delta^{R}_{m,i,j} + N\left(\widehat{\epsilon^{C}_{m,i,j}}, 0.01\right)$$

$$\pi^{C}_{m,i,j} = \frac{1}{1 + \exp\left(-\left(\beta_0{}^{C}_{m,i,j} + \beta_1{}^{C}_{m,i,j} * \text{Year}^{\alpha^{C}_{m,i,j}}\right)\right)}$$

Probability of Using Method

$$p[\text{Method} = m]^{C}_{i,j} = \frac{\pi^{C}_{m,i,j}}{\sum_{k=1}^{M} \pi^{C}_{k,i,j}}$$

m = Method
i = Subgroup
j = Spacing/Limiting
α = Trend modifier

Priors

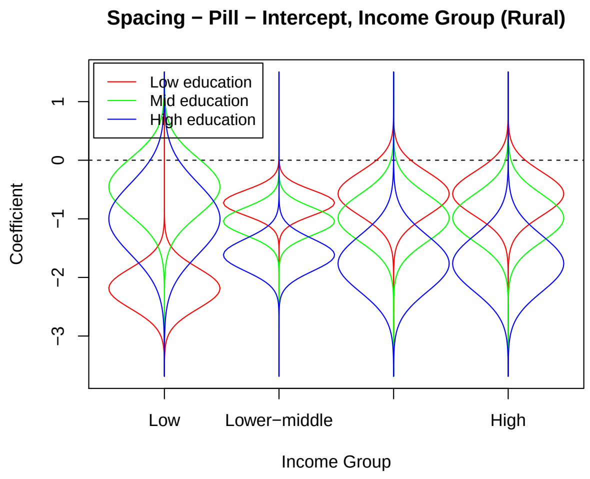
Spacing − Pill − Intercept, Income Group (Rural)
Low education
Mid education
High education
Coefficient
1
0
−1
−2
−3
Low
Lower−middle
High
Income Group

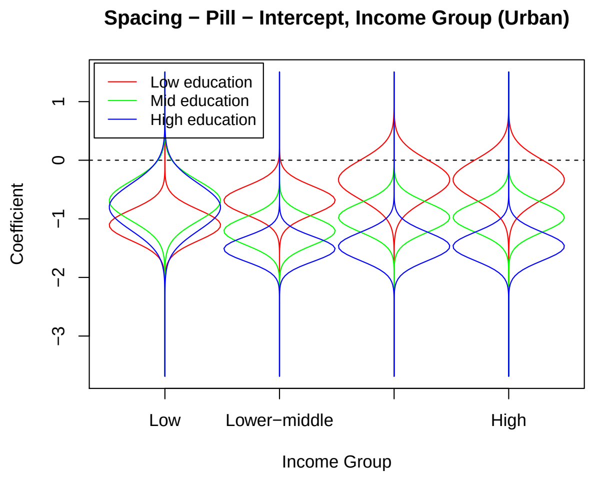
Spacing − Pill − Intercept, Income Group (Urban)
Low education
Mid education
High education
Coefficient
1
0
−1
−2
−3
Low
Lower−middle
High
Income Group

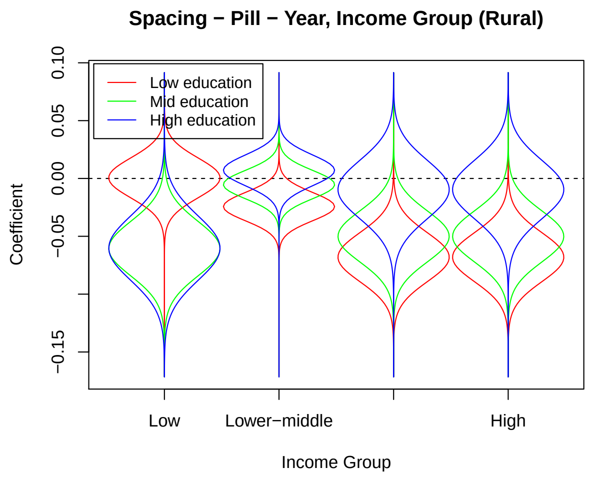
Spacing − Pill − Year, Income Group (Rural)
Low education
Mid education
High education
Coefficient
0.10
0.05
0.00
−0.05
−0.15
Low
Lower−middle
High
Income Group

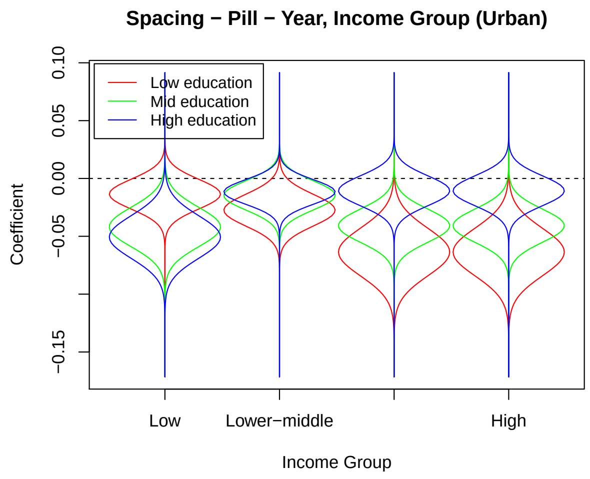
Spacing − Pill − Year, Income Group (Urban)
Low education
Mid education
High education
Coefficient
0.10
0.05
0.00
−0.05
−0.15
Low
Lower−middle
High
Income Group

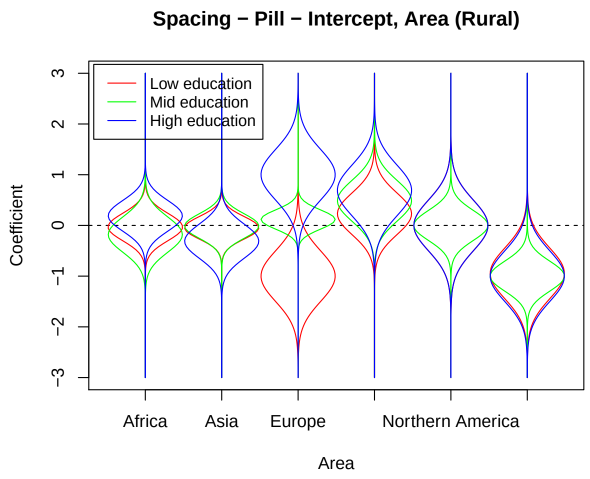
Spacing − Pill − Intercept, Area (Rural)
Low education
Mid education
High education
Coefficient
3
2
1
0
−1
−2
−3
Africa
Asia
Europe
Northern America
Area

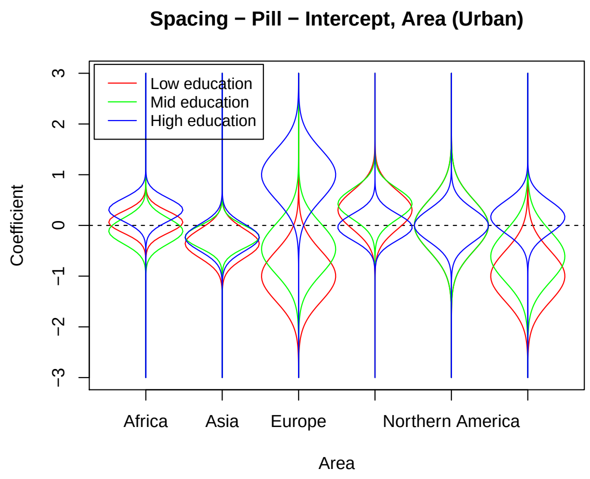
Spacing − Pill − Intercept, Area (Urban)
Low education
Mid education
High education
Coefficient
3
2
1
0
−1
−2
−3
Africa
Asia
Europe
Northern America
Area

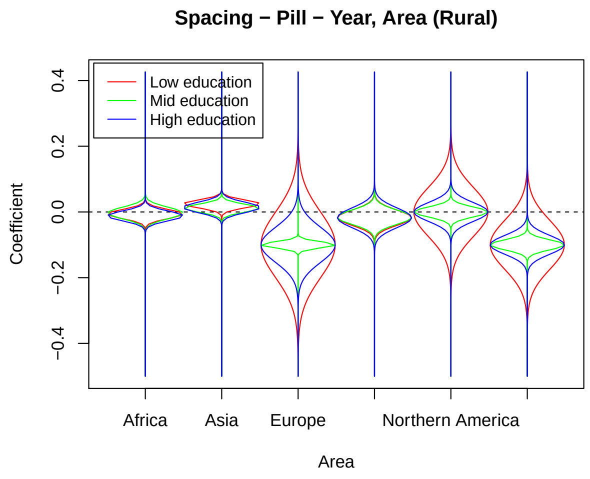
Spacing − Pill − Year, Area (Rural)
Low education
Mid education
High education
Coefficient
0.4
0.2
0.0
−0.2
−0.4
Africa
Asia
Europe
Northern America
Area

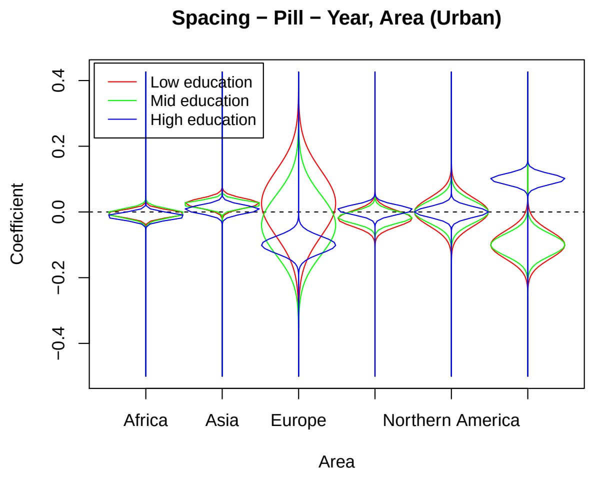
Spacing − Pill − Year, Area (Urban)
Low education
Mid education
High education
Coefficient
0.4
0.2
0.0
−0.2
−0.4
Africa
Asia
Europe
Northern America
Area

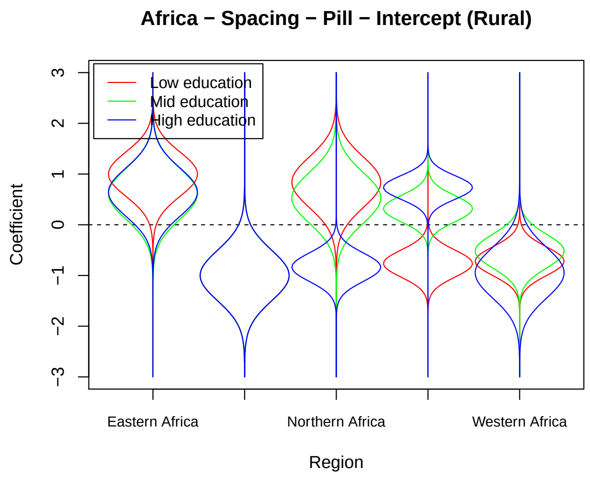
Africa − Spacing − Pill − Intercept (Rural)
Low education
Mid education
High education
Coefficient
3
2
1
0
−1
−2
−3
Eastern Africa
Northern Africa
Western Africa
Region

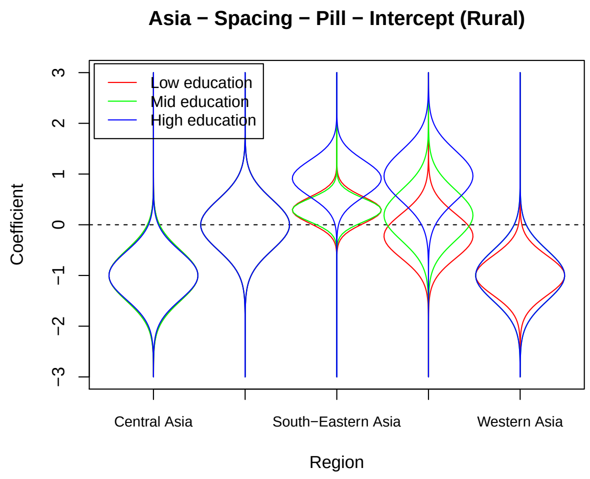
Asia − Spacing − Pill − Intercept (Rural)
Low education
Mid education
High education
Coefficient
3
2
1
0
−1
−2
−3
Central Asia
South−Eastern Asia
Western Asia
Region

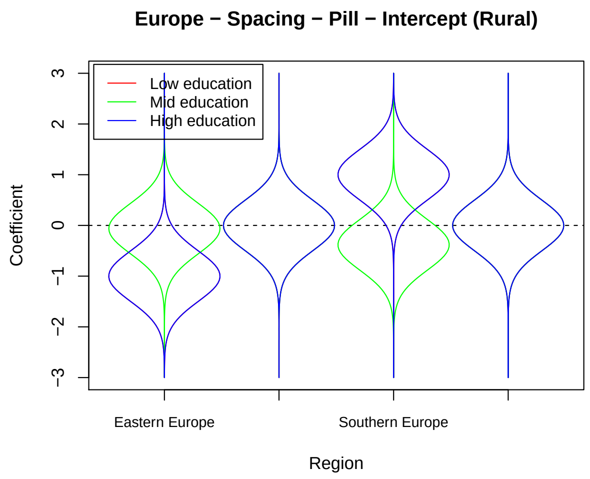
Europe − Spacing − Pill − Intercept (Rural)
Low education
Mid education
High education
Coefficient
3
2
1
0
−1
−2
−3
Eastern Europe
Southern Europe
Region

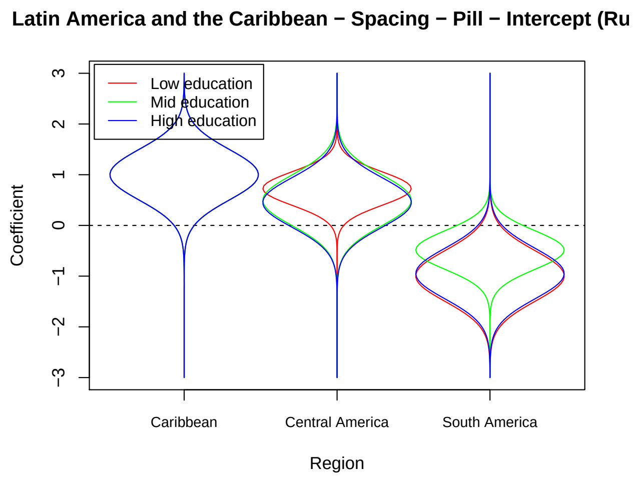
Latin America and the Caribbean − Spacing − Pill − Intercept (Ru
Low education
Mid education
High education
Coefficient
3
2
1
0
−1
−2
−3
Caribbean
Central America
South America
Region

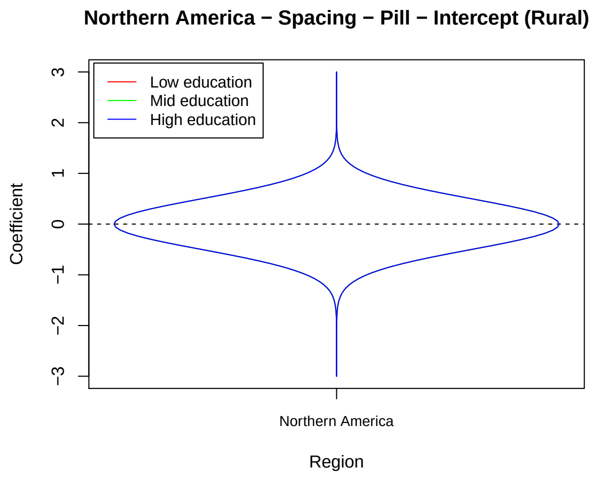
Northern America − Spacing − Pill − Intercept (Rural)
Low education
Mid education
High education
Coefficient
Northern America
Region

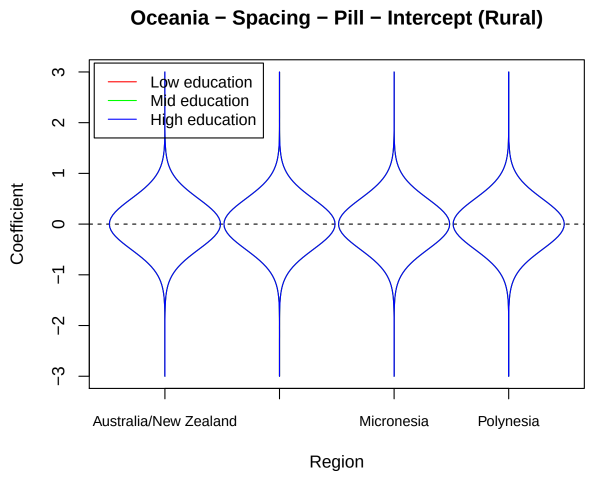
Oceania − Spacing − Pill − Intercept (Rural)
Low education
Mid education
High education
Coefficient
Australia/New Zealand
Micronesia
Polynesia
Region

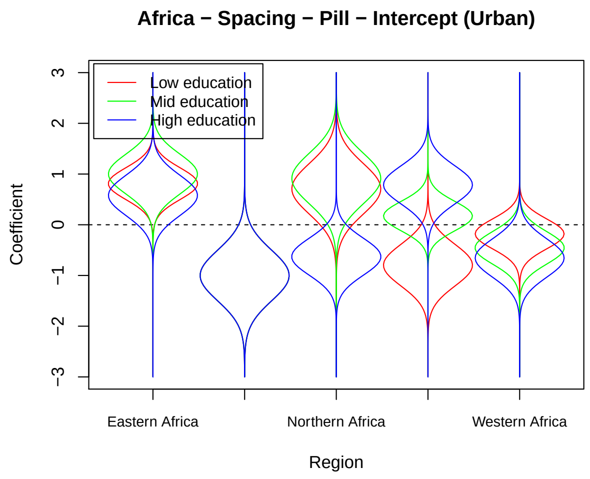
Africa − Spacing − Pill − Intercept (Urban)
Low education
Mid education
High education
Coefficient
Eastern Africa
Northern Africa
Western Africa
Region

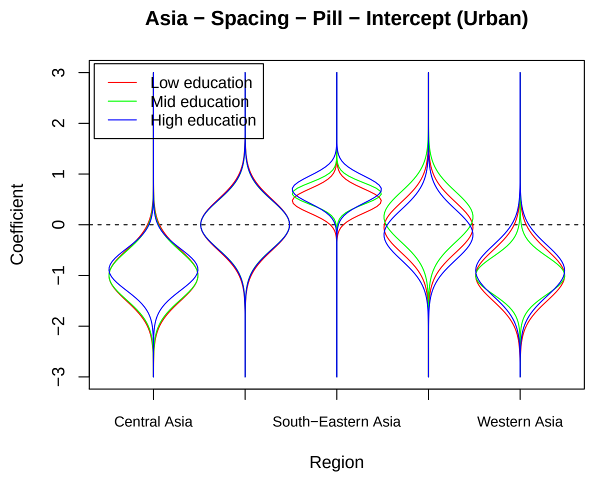
Asia − Spacing − Pill − Intercept (Urban)
Low education
Mid education
High education
Coefficient
Central Asia
South−Eastern Asia
Western Asia
Region

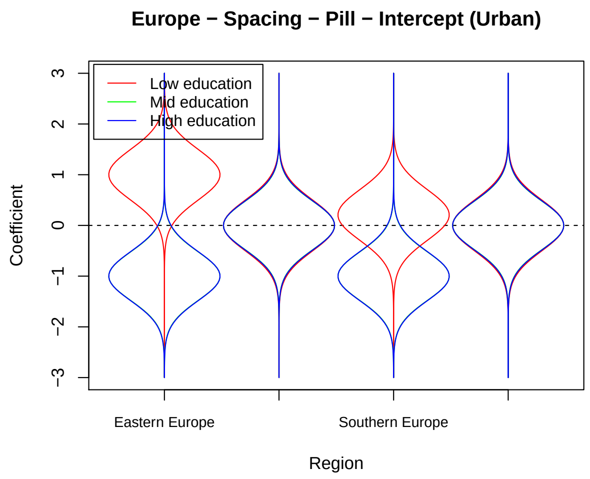
Europe − Spacing − Pill − Intercept (Urban)
Low education
Mid education
High education
Coefficient
Eastern Europe
Southern Europe
Region

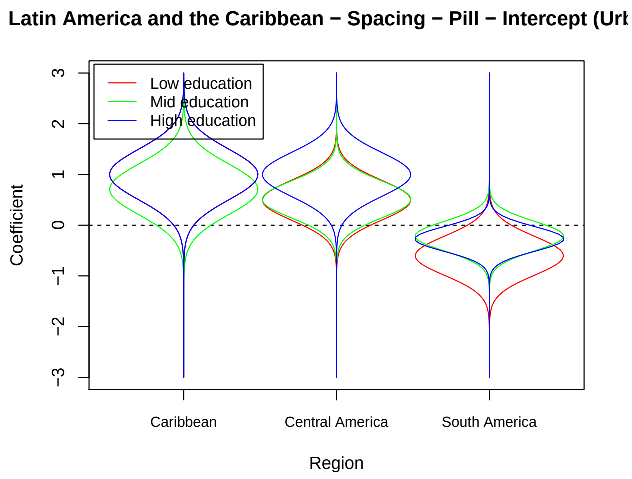
Latin America and the Caribbean − Spacing − Pill − Intercept (Urb
Low education
Mid education
High education
Coefficient
Caribbean
Central America
South America
Region

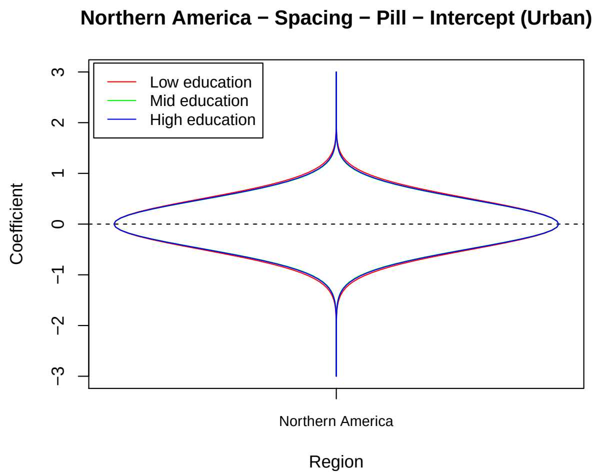
Northern America − Spacing − Pill − Intercept (Urban)
Low education
Mid education
High education
Coefficient
Northern America
Region

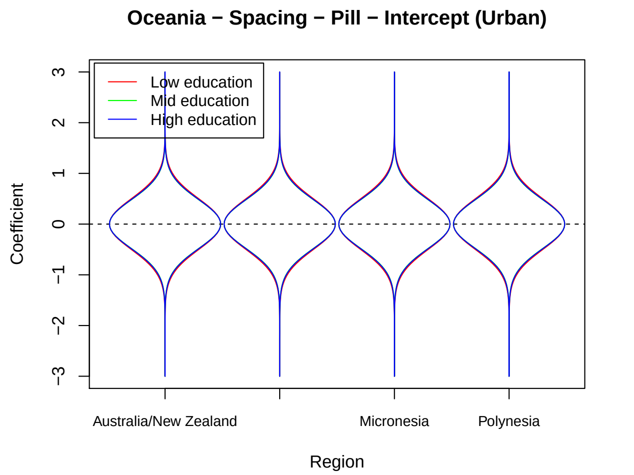
Oceania − Spacing − Pill − Intercept (Urban)
Low education
Mid education
High education
Coefficient
Australia/New Zealand
Micronesia
Polynesia
Region

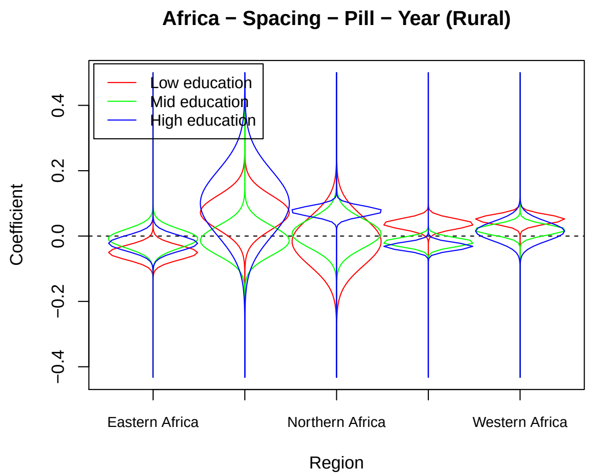
Africa − Spacing − Pill − Year (Rural)
Low education
Mid education
High education
Coefficient
Eastern Africa
Northern Africa
Western Africa
Region

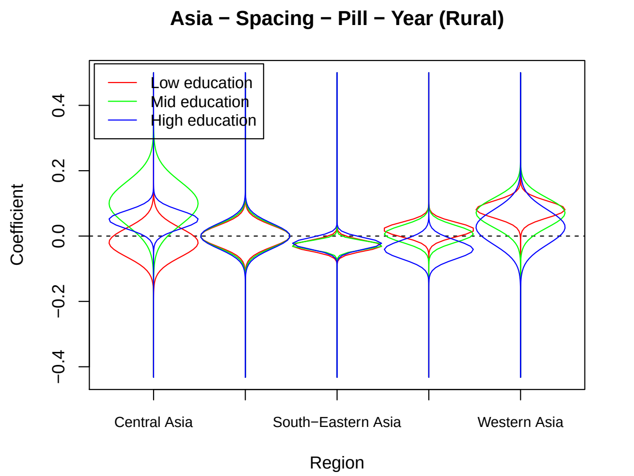
Asia − Spacing − Pill − Year (Rural)
Low education
Mid education
High education
Coefficient
Central Asia
South−Eastern Asia
Western Asia
Region

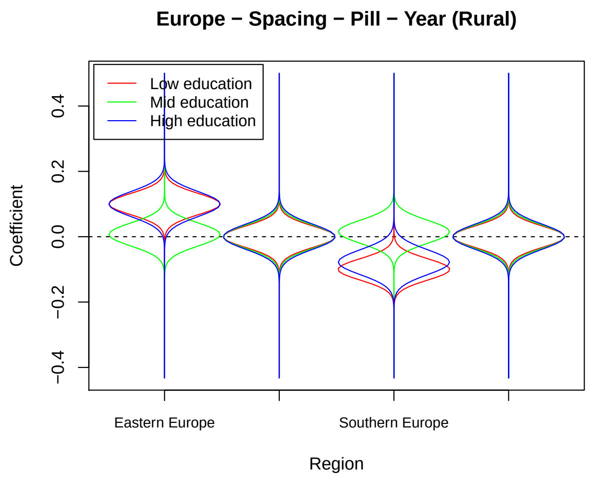
Europe − Spacing − Pill − Year (Rural)
Low education
Mid education
High education
Coefficient
Eastern Europe
Southern Europe
Region

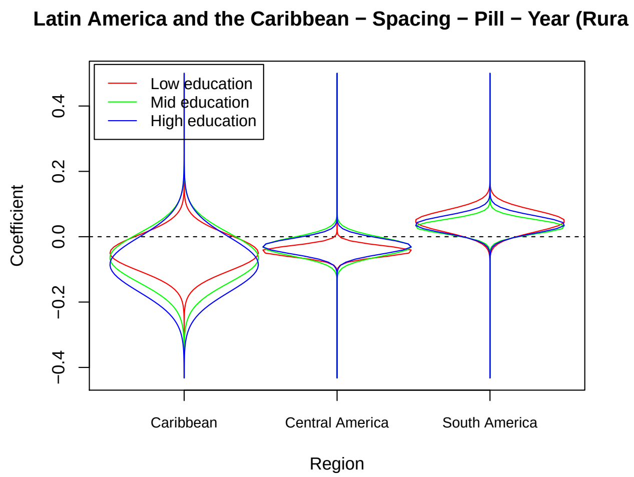
Latin America and the Caribbean − Spacing − Pill − Year (Rura
Low education
Mid education
High education
Coefficient
Caribbean
Central America
South America
Region

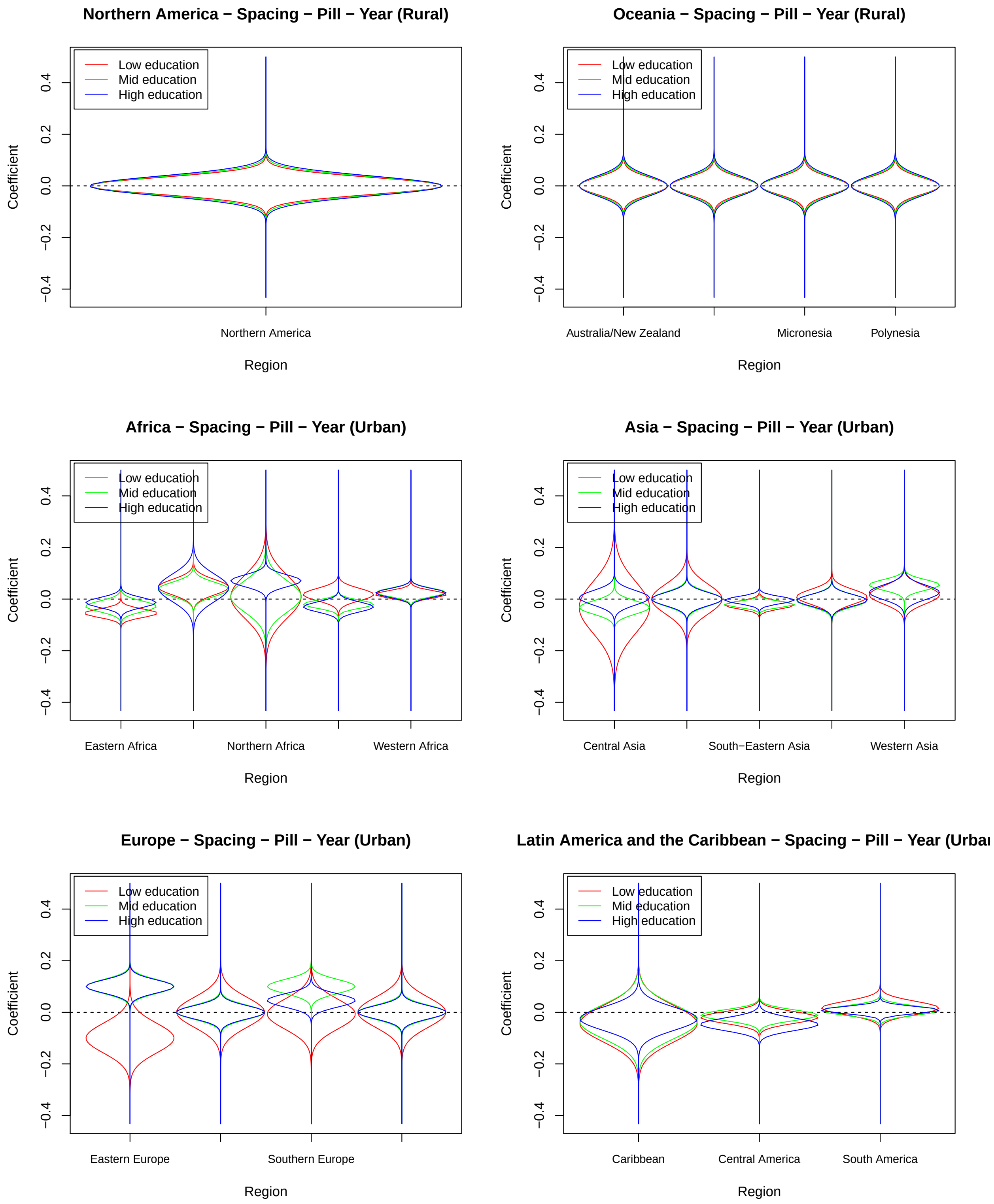
Northern America − Spacing − Pill − Year (Rural)
Low education
Mid education
High education
Coefficient
Northern America
Region
Oceania − Spacing − Pill − Year (Rural)
Australia/New Zealand
Micronesia
Polynesia
Africa − Spacing − Pill − Year (Urban)
Eastern Africa
Northern Africa
Western Africa
Asia − Spacing − Pill − Year (Urban)
Central Asia
South−Eastern Asia
Western Asia
Europe − Spacing − Pill − Year (Urban)
Eastern Europe
Southern Europe
Latin America and the Caribbean − Spacing − Pill − Year (Urba
Caribbean
Central America
South America
−0.4
−0.2
0.0
0.2
0.4

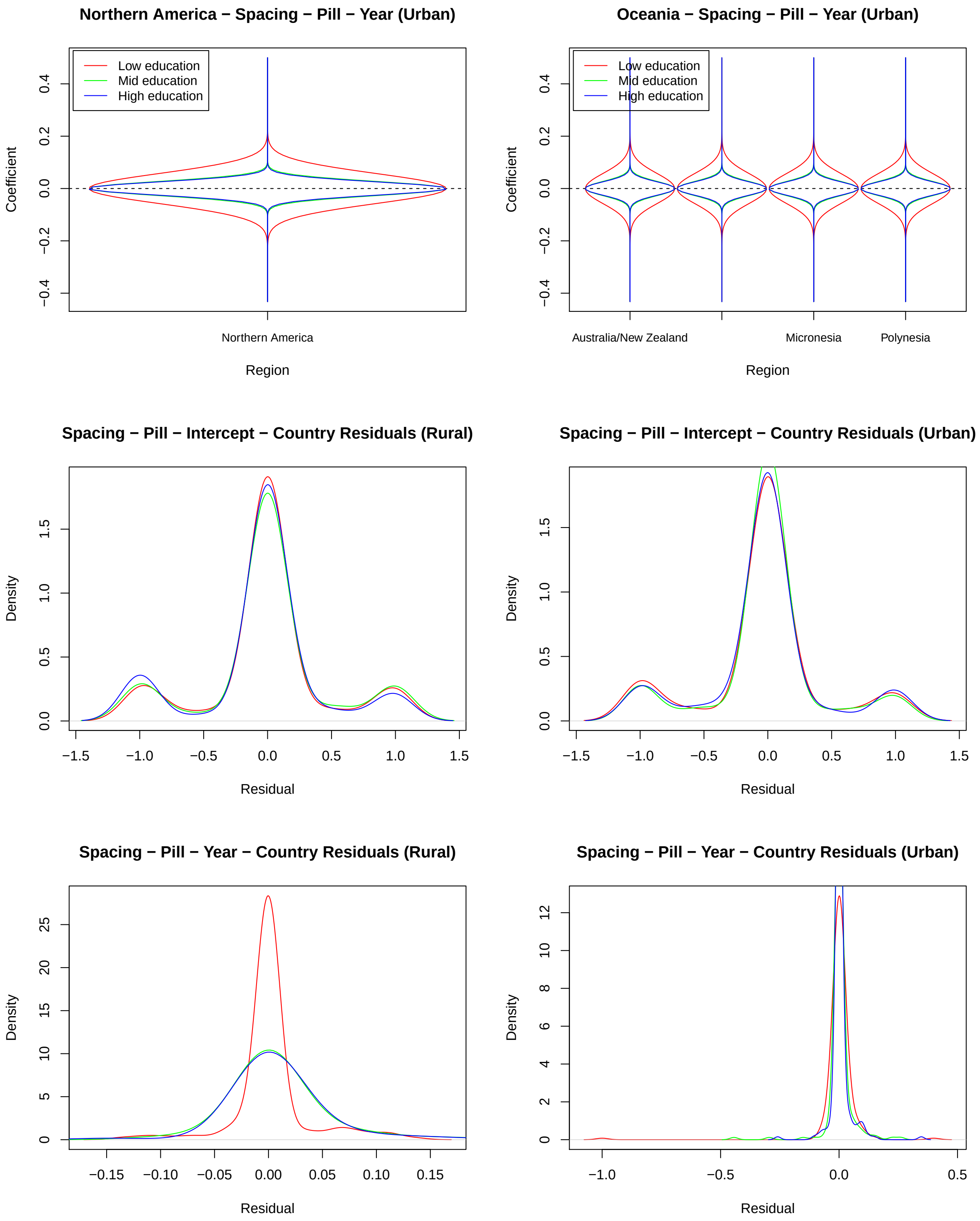
Northern America − Spacing − Pill − Year (Urban)
Low education
Mid education
High education
Coefficient
0.4
0.2
0.0
−0.2
−0.4
Northern America
Region
Oceania − Spacing − Pill − Year (Urban)
Low education
Mid education
High education
Coefficient
Australia/New Zealand
Micronesia
Polynesia
Region
Spacing − Pill − Intercept − Country Residuals (Rural)
Density
1.5
1.0
0.5
0.0
−1.5
−1.0
−0.5
0.0
0.5
1.0
1.5
Residual
Spacing − Pill − Intercept − Country Residuals (Urban)
Density
Residual
Spacing − Pill − Year − Country Residuals (Rural)
Density
25
20
15
10
5
0
−0.15
−0.10
−0.05
0.00
0.05
0.10
0.15
Residual
Spacing − Pill − Year − Country Residuals (Urban)
Density
12
10
8
6
4
2
0
−1.0
−0.5
0.0
0.5
Residual

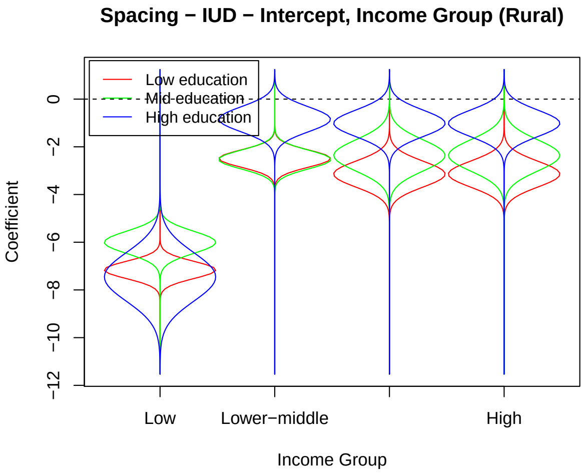
Spacing − IUD − Intercept, Income Group (Rural)
Low education
Mid education
High education
Coefficient
Low
Lower−middle
High
Income Group

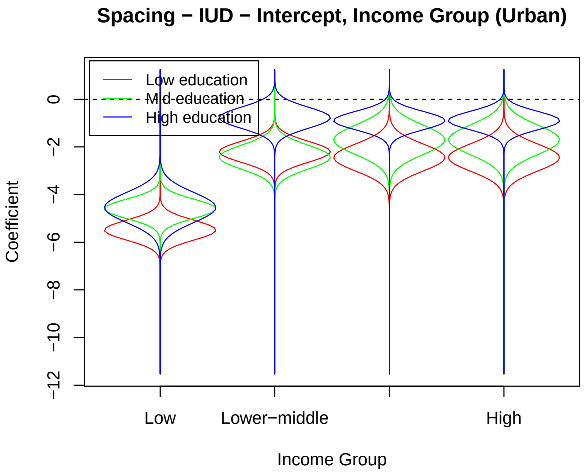
Spacing − IUD − Intercept, Income Group (Urban)
Low education
Mid education
High education
Coefficient
Low
Lower−middle
High
Income Group

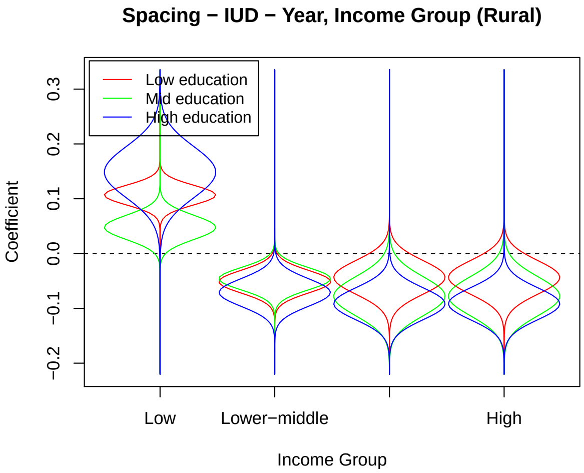
Spacing − IUD − Year, Income Group (Rural)
Low education
Mid education
High education
Coefficient
Low
Lower−middle
High
Income Group

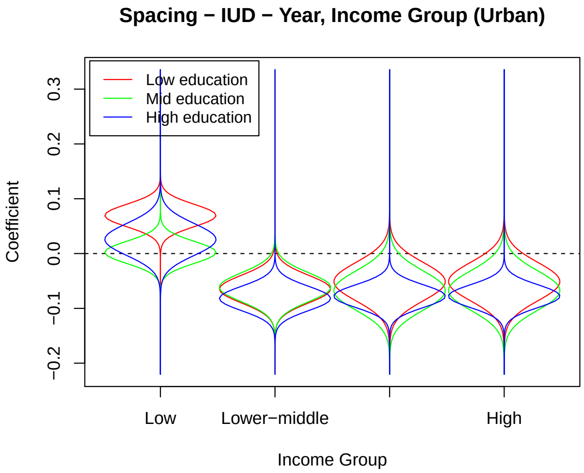
Spacing − IUD − Year, Income Group (Urban)
Low education
Mid education
High education
Coefficient
Low
Lower−middle
High
Income Group

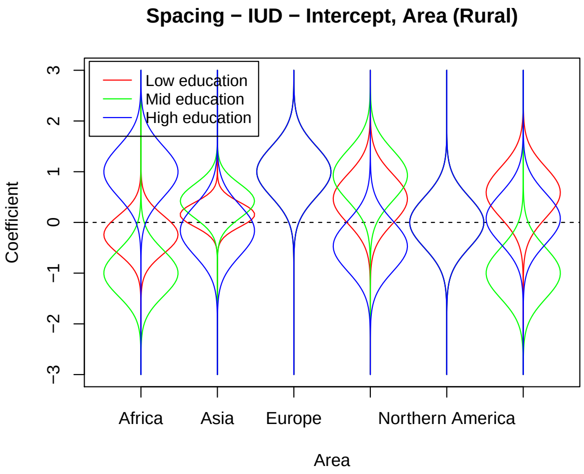
Spacing − IUD − Intercept, Area (Rural)
Low education
Mid education
High education
Coefficient
Africa
Asia
Europe
Northern America
Area

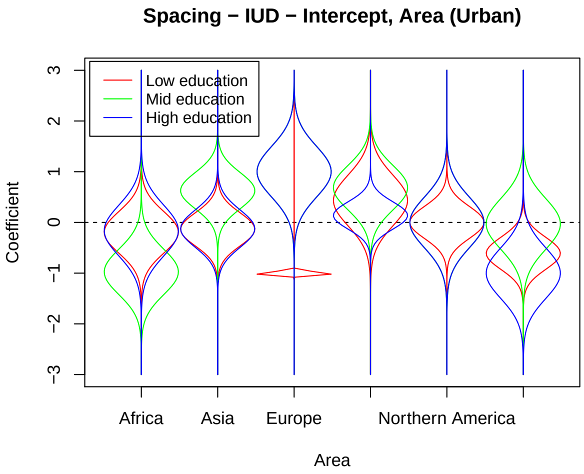
Spacing − IUD − Intercept, Area (Urban)
Low education
Mid education
High education
Coefficient
Africa
Asia
Europe
Northern America
Area

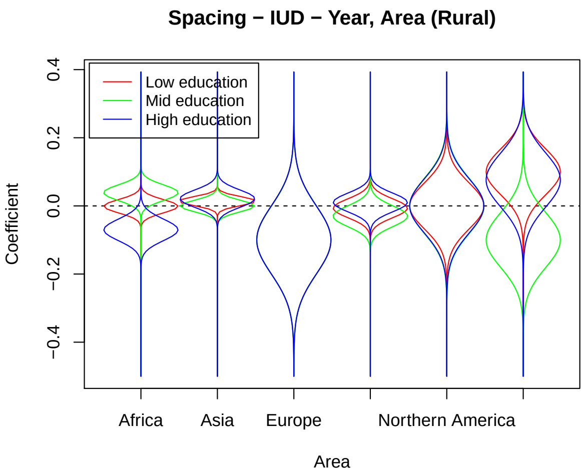
Spacing − IUD − Year, Area (Rural)
Low education
Mid education
High education
Coefficient
0.4
0.2
0.0
−0.2
−0.4
Africa
Asia
Europe
Northern America
Area

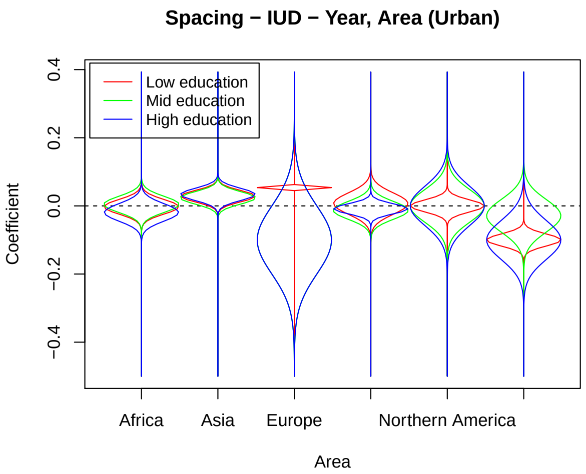
Spacing − IUD − Year, Area (Urban)
Low education
Mid education
High education
Coefficient
0.4
0.2
0.0
−0.2
−0.4
Africa
Asia
Europe
Northern America
Area

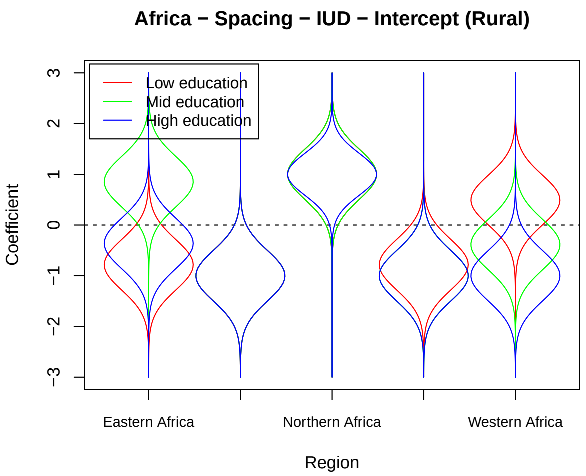
Africa − Spacing − IUD − Intercept (Rural)
Low education
Mid education
High education
Coefficient
3
2
1
0
−1
−2
−3
Eastern Africa
Northern Africa
Western Africa
Region

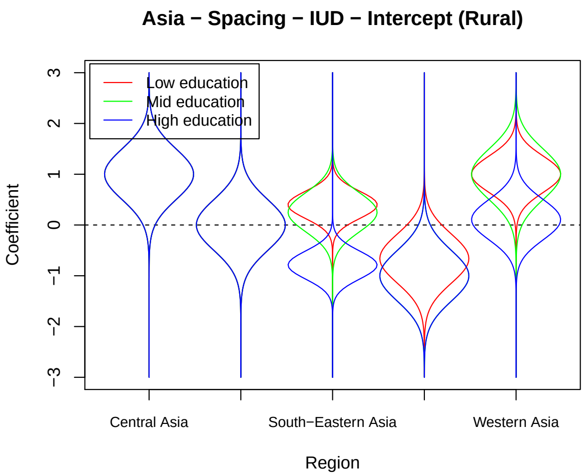
Asia − Spacing − IUD − Intercept (Rural)
Low education
Mid education
High education
Coefficient
3
2
1
0
−1
−2
−3
Central Asia
South−Eastern Asia
Western Asia
Region

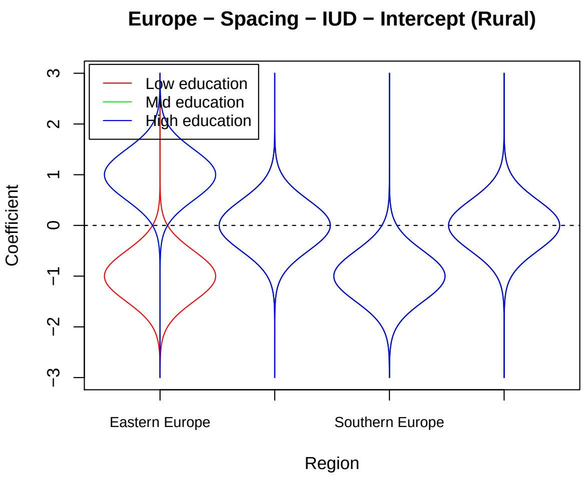
Europe − Spacing − IUD − Intercept (Rural)
Low education
Mid education
High education
Coefficient
3
2
1
0
−1
−2
−3
Eastern Europe
Southern Europe
Region

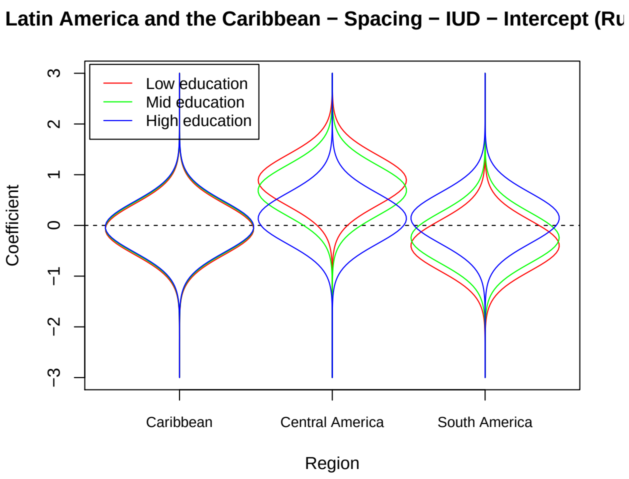
Latin America and the Caribbean − Spacing − IUD − Intercept (Ru
Low education
Mid education
High education
Coefficient
3
2
1
0
−1
−2
−3
Caribbean
Central America
South America
Region

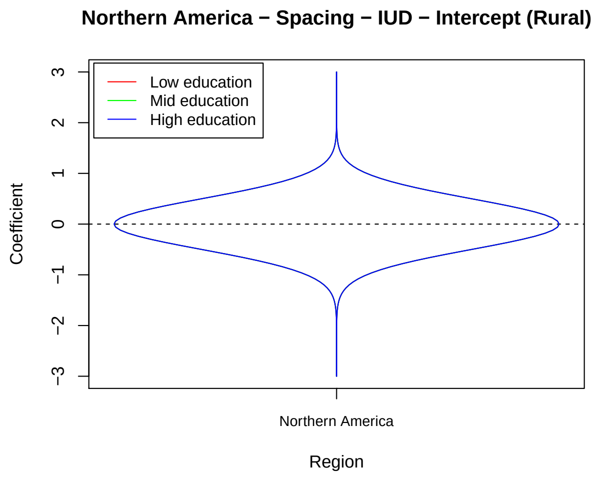
Northern America − Spacing − IUD − Intercept (Rural)
Low education
Mid education
High education
Coefficient
3
2
1
0
−1
−2
−3
Northern America
Region

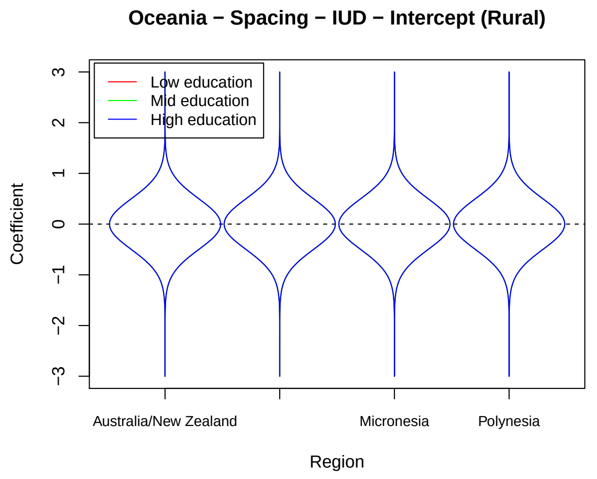
Oceania − Spacing − IUD − Intercept (Rural)
Low education
Mid education
High education
Coefficient
3
2
1
0
−1
−2
−3
Australia/New Zealand
Micronesia
Polynesia
Region

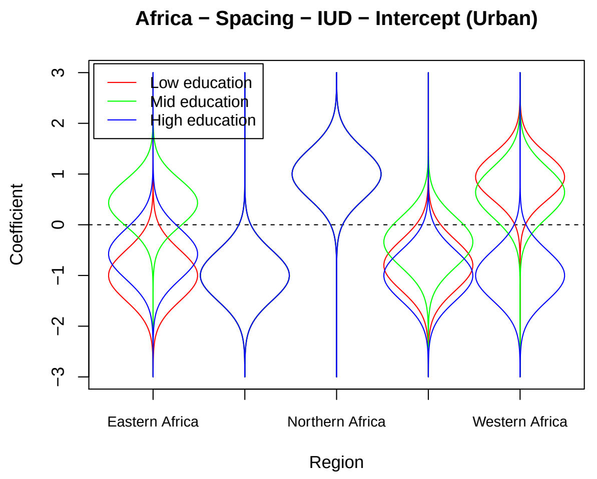
Africa − Spacing − IUD − Intercept (Urban)
Low education
Mid education
High education
Coefficient
3
2
1
0
−1
−2
−3
Eastern Africa
Northern Africa
Western Africa
Region

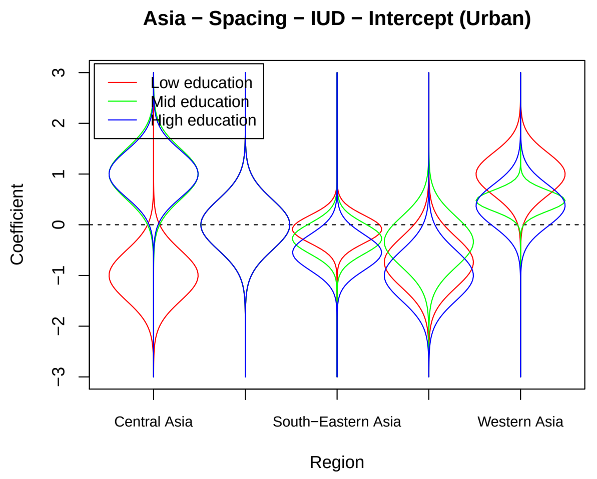
Asia − Spacing − IUD − Intercept (Urban)
Low education
Mid education
High education
Coefficient
3
2
1
0
−1
−2
−3
Central Asia
South−Eastern Asia
Western Asia
Region

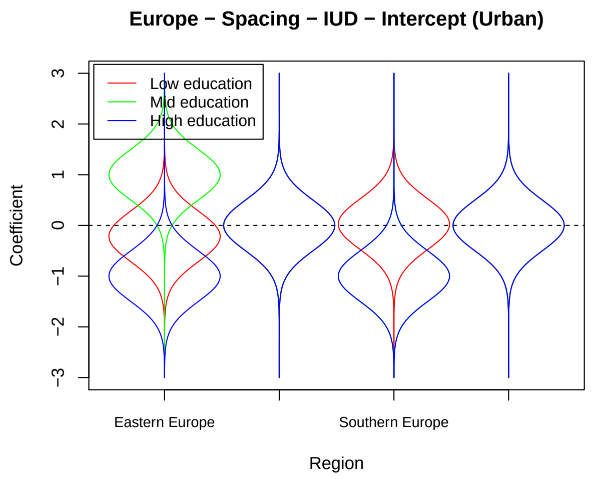
Europe − Spacing − IUD − Intercept (Urban)
Low education
Mid education
High education
Coefficient
3
2
1
0
−1
−2
−3
Eastern Europe
Southern Europe
Region

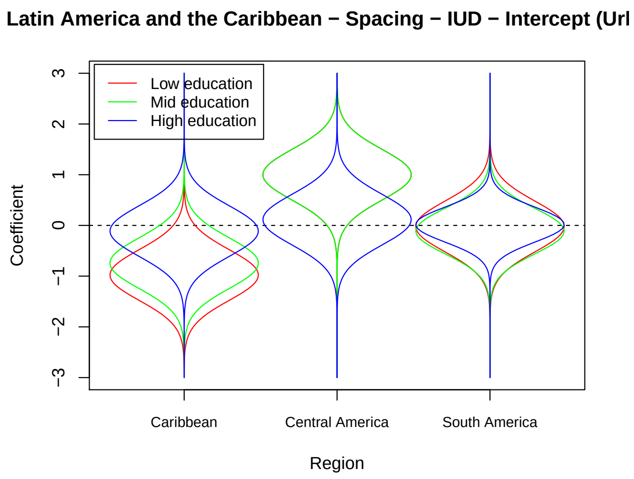
Latin America and the Caribbean − Spacing − IUD − Intercept (Url
Low education
Mid education
High education
Coefficient
3
2
1
0
−1
−2
−3
Caribbean
Central America
South America
Region

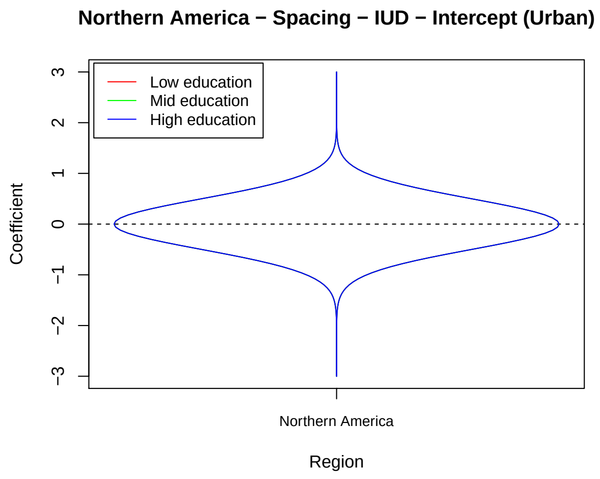
Northern America − Spacing − IUD − Intercept (Urban)
Low education
Mid education
High education
Coefficient
Northern America
Region

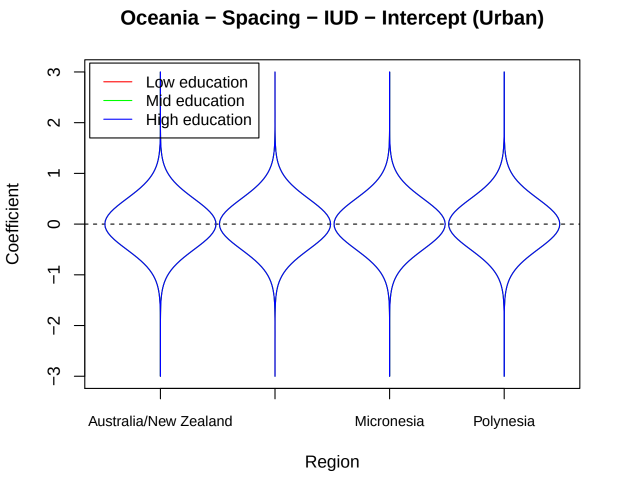
Oceania − Spacing − IUD − Intercept (Urban)
Low education
Mid education
High education
Coefficient
Australia/New Zealand
Micronesia
Polynesia
Region

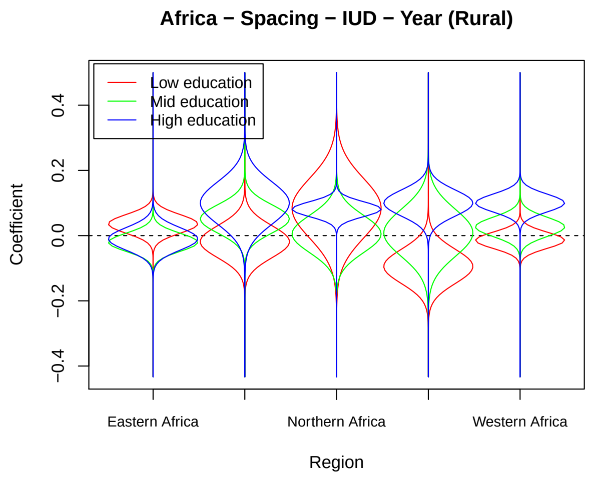
Africa − Spacing − IUD − Year (Rural)
Low education
Mid education
High education
Coefficient
Eastern Africa
Northern Africa
Western Africa
Region

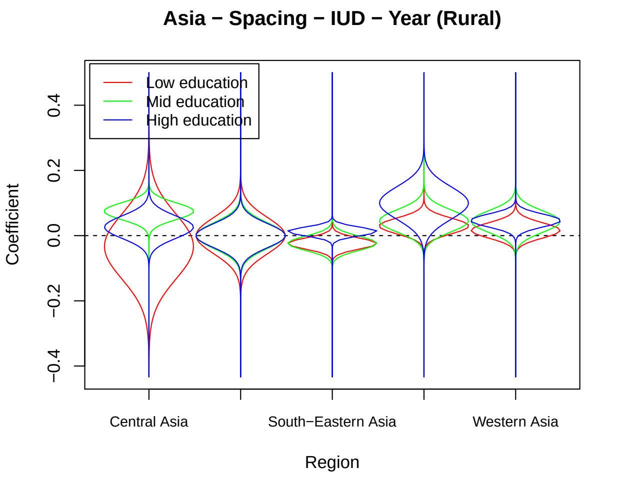
Asia − Spacing − IUD − Year (Rural)
Low education
Mid education
High education
Coefficient
Central Asia
South−Eastern Asia
Western Asia
Region

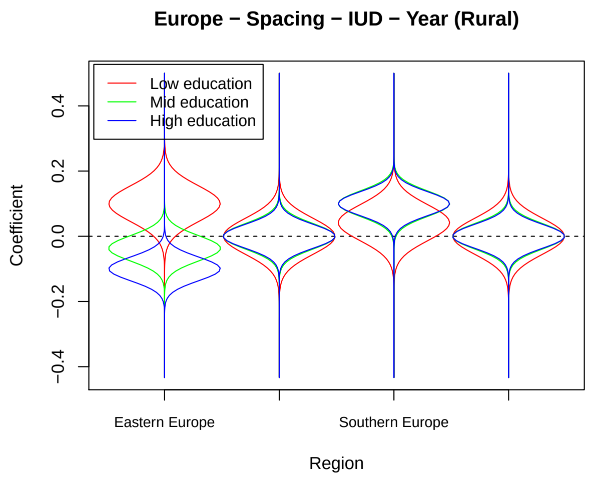
Europe − Spacing − IUD − Year (Rural)
Low education
Mid education
High education
Coefficient
Eastern Europe
Southern Europe
Region

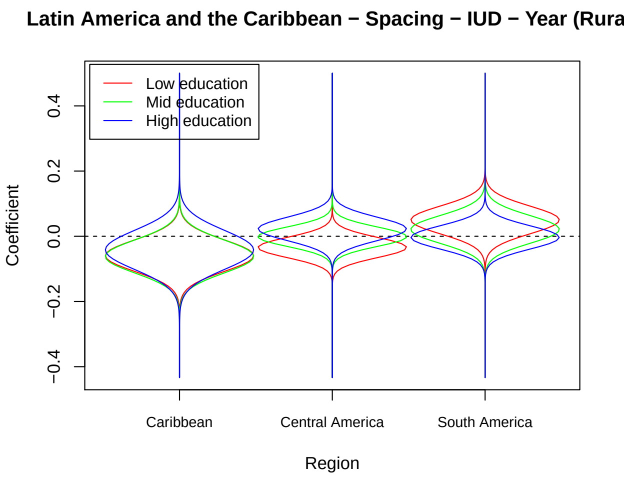
Latin America and the Caribbean − Spacing − IUD − Year (Rura
Low education
Mid education
High education
Coefficient
Caribbean
Central America
South America
Region

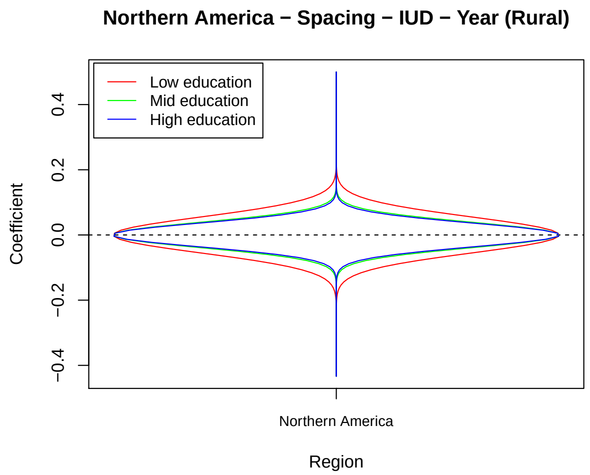
Northern America − Spacing − IUD − Year (Rural)
Low education
Mid education
High education
Coefficient
−0.4
−0.2
0.0
0.2
0.4
Northern America
Region

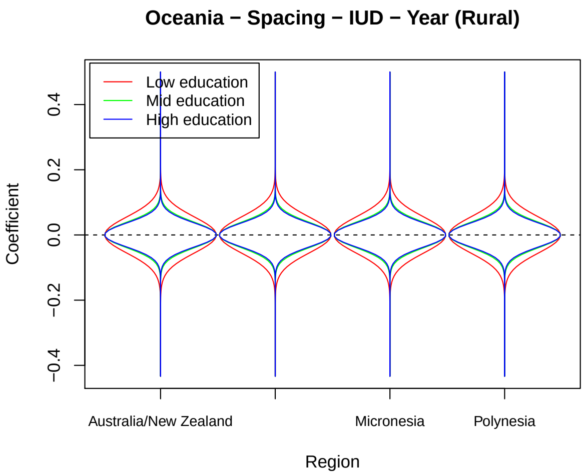
Oceania − Spacing − IUD − Year (Rural)
Low education
Mid education
High education
Coefficient
−0.4
−0.2
0.0
0.2
0.4
Australia/New Zealand
Micronesia
Polynesia
Region

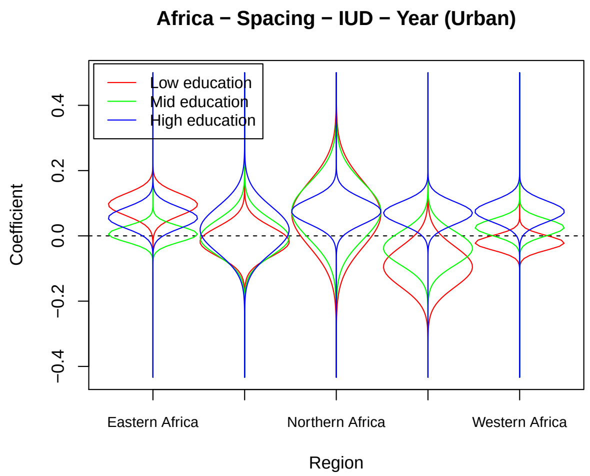
Africa − Spacing − IUD − Year (Urban)
Low education
Mid education
High education
Coefficient
−0.4
−0.2
0.0
0.2
0.4
Eastern Africa
Northern Africa
Western Africa
Region

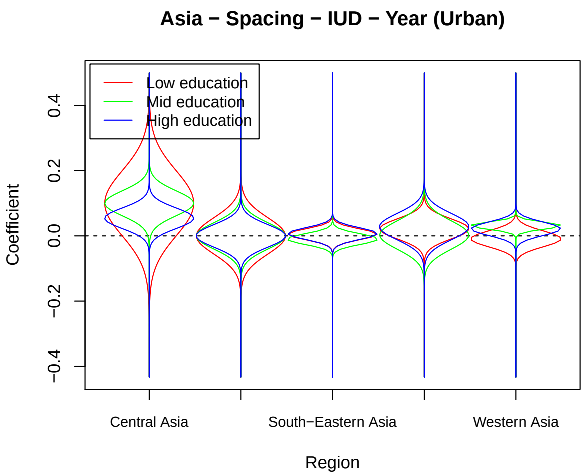
Asia − Spacing − IUD − Year (Urban)
Low education
Mid education
High education
Coefficient
−0.4
−0.2
0.0
0.2
0.4
Central Asia
South−Eastern Asia
Western Asia
Region

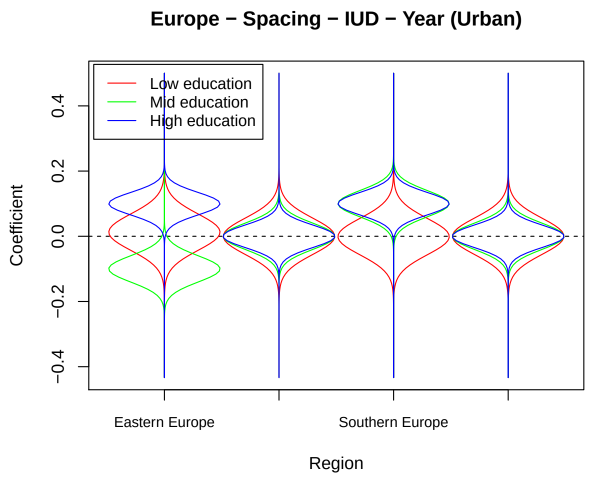
Europe − Spacing − IUD − Year (Urban)
Low education
Mid education
High education
Coefficient
−0.4
−0.2
0.0
0.2
0.4
Eastern Europe
Southern Europe
Region

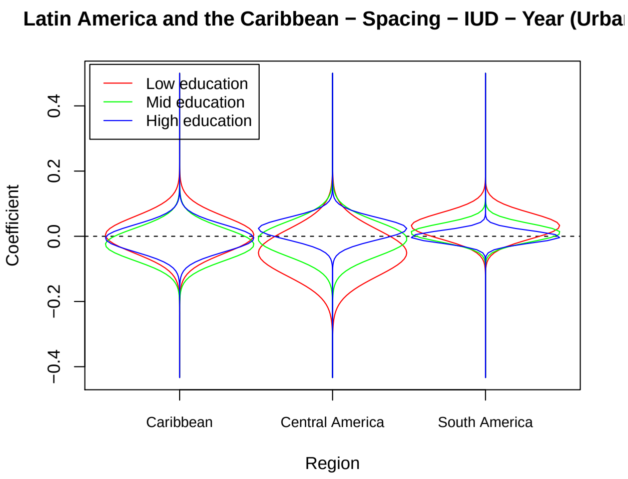
Latin America and the Caribbean − Spacing − IUD − Year (Urba
Low education
Mid education
High education
Coefficient
−0.4
−0.2
0.0
0.2
0.4
Caribbean
Central America
South America
Region

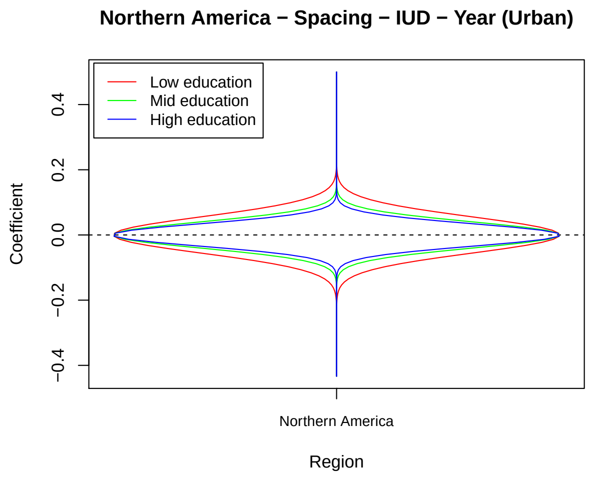
Northern America − Spacing − IUD − Year (Urban)
Low education
Mid education
High education
Coefficient
−0.4
−0.2
0.0
0.2
0.4
Northern America
Region

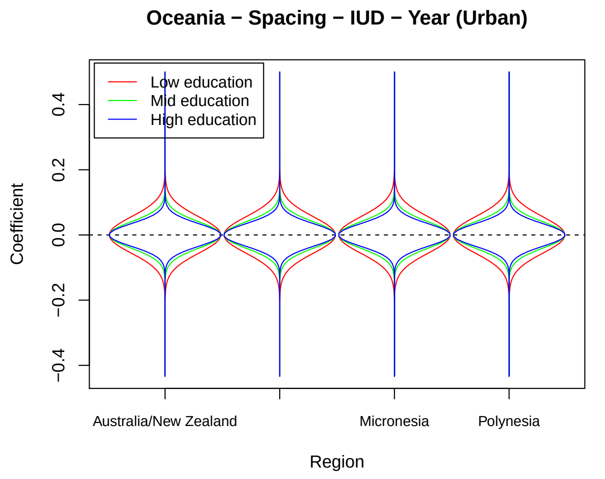
Oceania − Spacing − IUD − Year (Urban)
Low education
Mid education
High education
Coefficient
−0.4
−0.2
0.0
0.2
0.4
Australia/New Zealand
Micronesia
Polynesia
Region

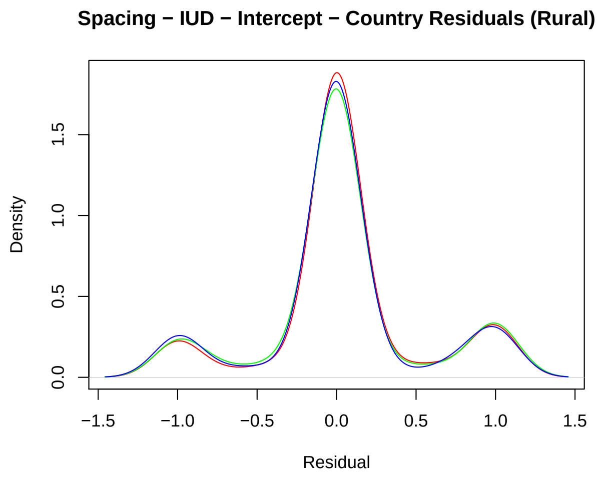
Spacing − IUD − Intercept − Country Residuals (Rural)
Density
0.0
0.5
1.0
1.5
−1.5
−1.0
−0.5
0.0
0.5
1.0
1.5
Residual

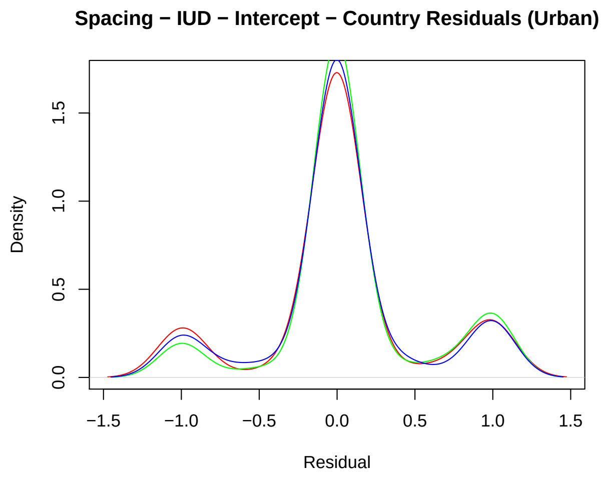
Spacing − IUD − Intercept − Country Residuals (Urban)
Density
0.0
0.5
1.0
1.5
−1.5
−1.0
−0.5
0.0
0.5
1.0
1.5
Residual

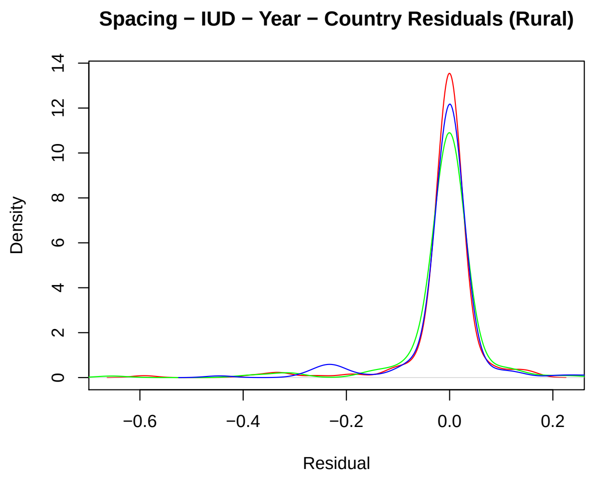
Spacing − IUD − Year − Country Residuals (Rural)
Density
0
2
4
6
8
10
12
14
−0.6
−0.4
−0.2
0.0
0.2
Residual

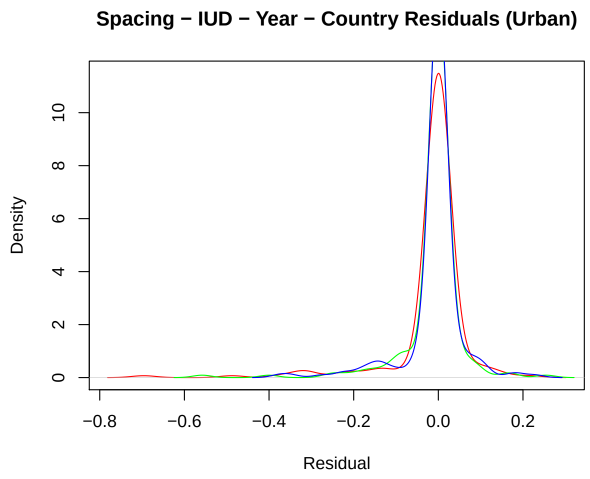
Spacing − IUD − Year − Country Residuals (Urban)
Density
0
2
4
6
8
10
−0.8
−0.6
−0.4
−0.2
0.0
0.2
Residual

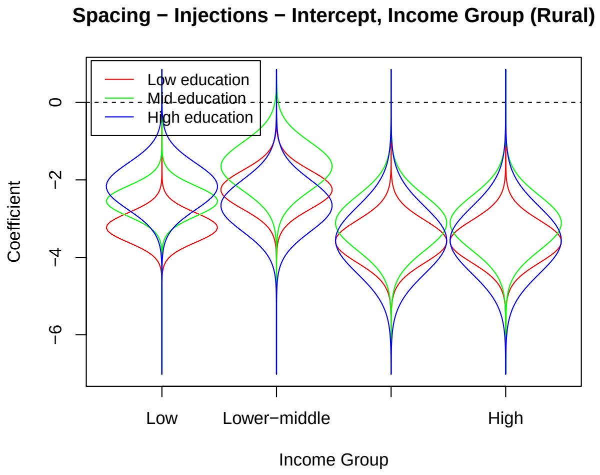
Spacing − Injections − Intercept, Income Group (Rural)
Low education
Mid education
High education
Coefficient
0
−2
−4
−6
Low
Lower−middle
High
Income Group

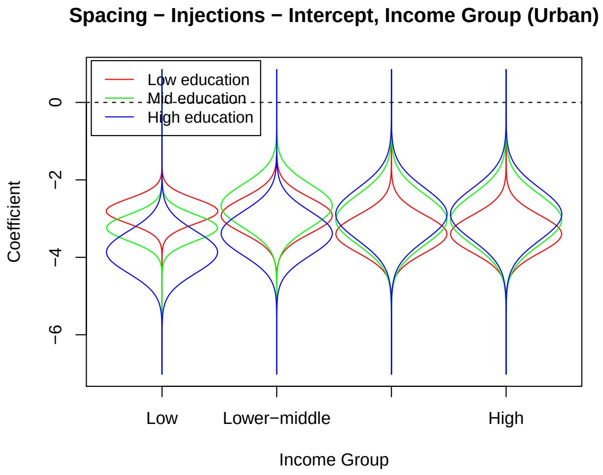
Spacing − Injections − Intercept, Income Group (Urban)
Low education
Mid education
High education
Coefficient
0
−2
−4
−6
Low
Lower−middle
High
Income Group

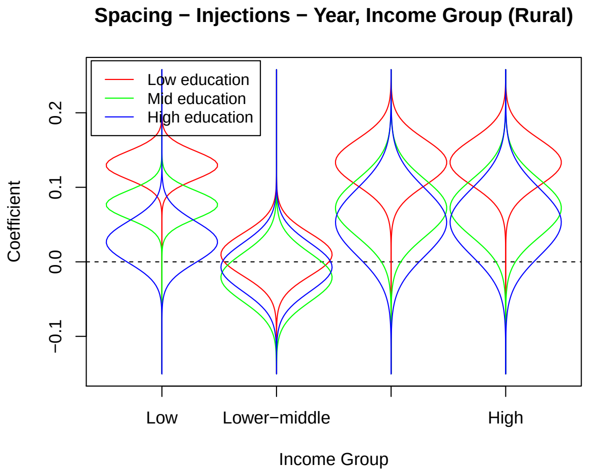
Spacing − Injections − Year, Income Group (Rural)
Low education
Mid education
High education
Coefficient
0.2
0.1
0.0
−0.1
Low
Lower−middle
High
Income Group

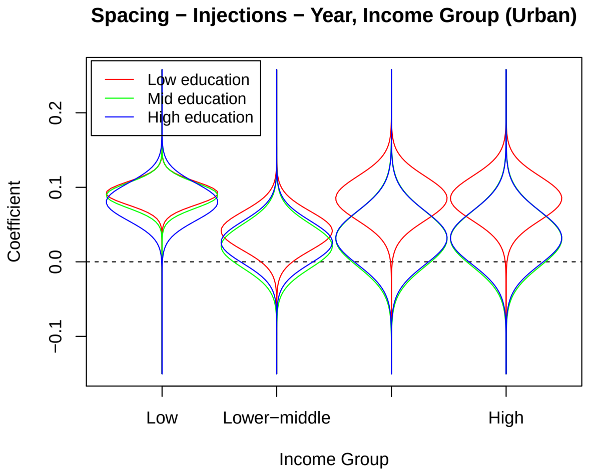
Spacing − Injections − Year, Income Group (Urban)
Low education
Mid education
High education
Coefficient
0.2
0.1
0.0
−0.1
Low
Lower−middle
High
Income Group

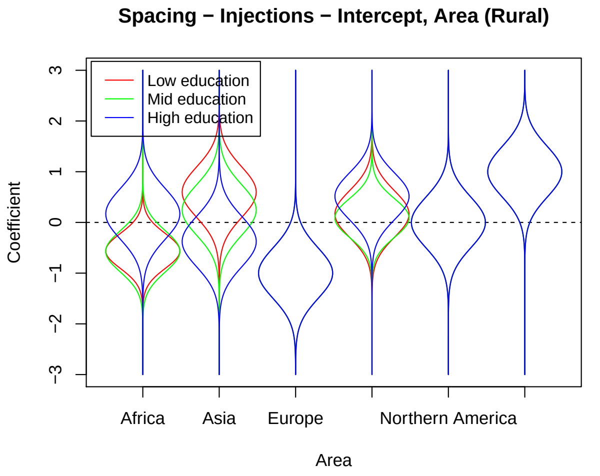
Spacing − Injections − Intercept, Area (Rural)
Low education
Mid education
High education
Coefficient
3
2
1
0
−1
−2
−3
Africa
Asia
Europe
Northern America
Area

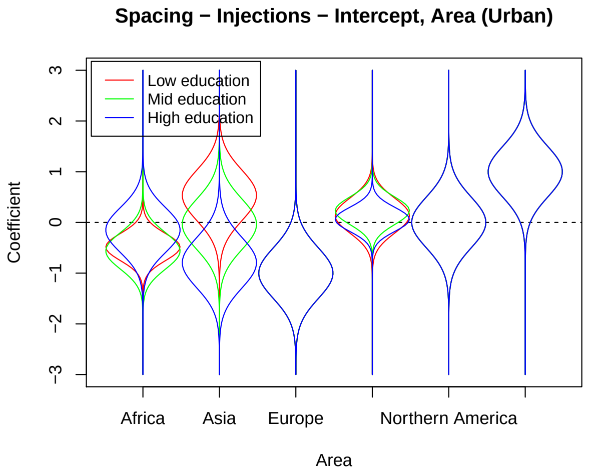
Spacing − Injections − Intercept, Area (Urban)
Low education
Mid education
High education
Coefficient
3
2
1
0
−1
−2
−3
Africa
Asia
Europe
Northern America
Area

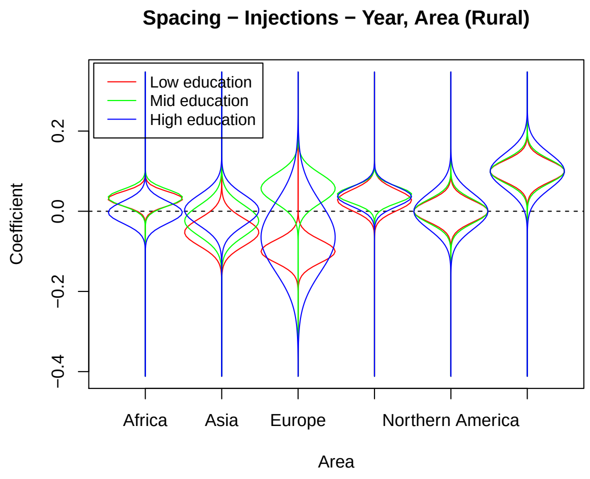
Spacing − Injections − Year, Area (Rural)
Low education
Mid education
High education
Coefficient
0.2
0.0
−0.2
−0.4
Africa
Asia
Europe
Northern America
Area

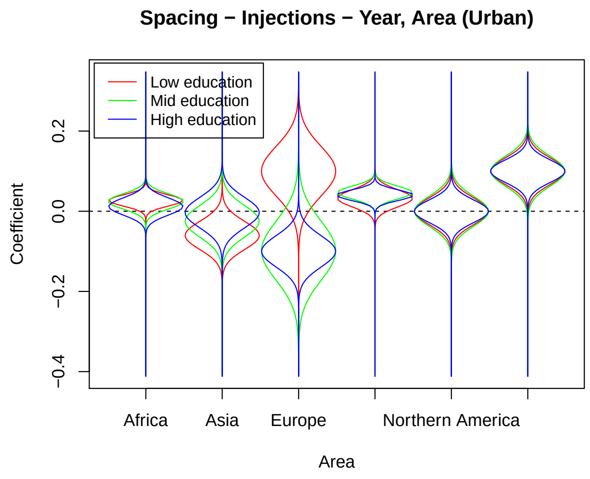
Spacing − Injections − Year, Area (Urban)
Low education
Mid education
High education
Coefficient
0.2
0.0
−0.2
−0.4
Africa
Asia
Europe
Northern America
Area

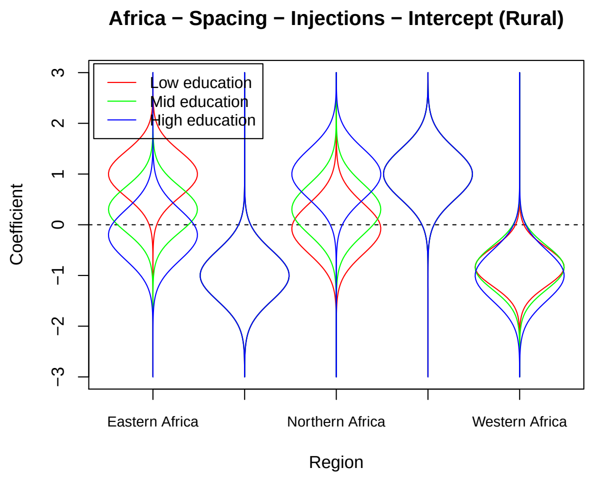
Africa − Spacing − Injections − Intercept (Rural)
Low education
Mid education
High education
Coefficient
3
2
1
0
−1
−2
−3
Eastern Africa
Northern Africa
Western Africa
Region

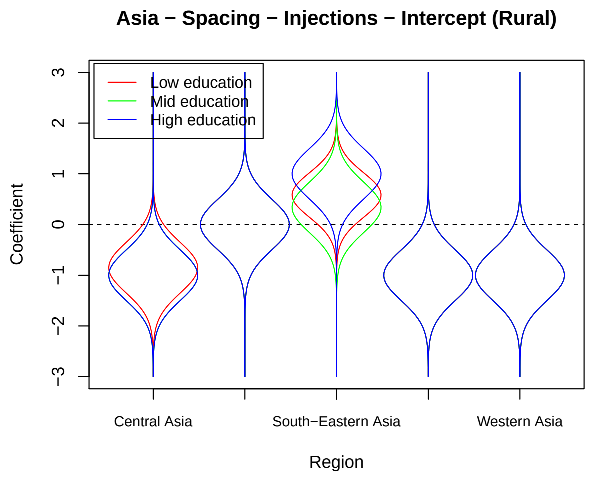
Asia − Spacing − Injections − Intercept (Rural)
Low education
Mid education
High education
Coefficient
3
2
1
0
−1
−2
−3
Central Asia
South−Eastern Asia
Western Asia
Region

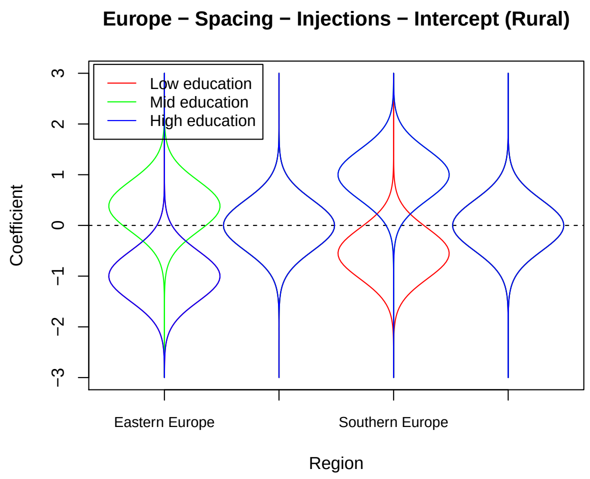
Europe − Spacing − Injections − Intercept (Rural)
Low education
Mid education
High education
Coefficient
3
2
1
0
−1
−2
−3
Eastern Europe
Southern Europe
Region

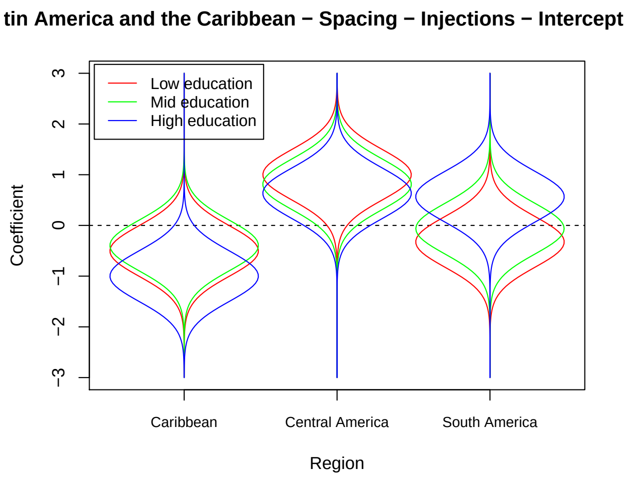
tin America and the Caribbean − Spacing − Injections − Intercept
Low education
Mid education
High education
Coefficient
3
2
1
0
−1
−2
−3
Caribbean
Central America
South America
Region

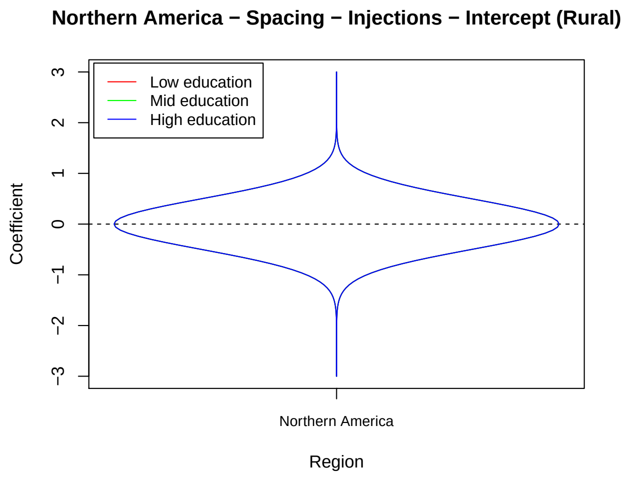
Northern America − Spacing − Injections − Intercept (Rural)
Low education
Mid education
High education
Coefficient
3
2
1
0
−1
−2
−3
Northern America
Region

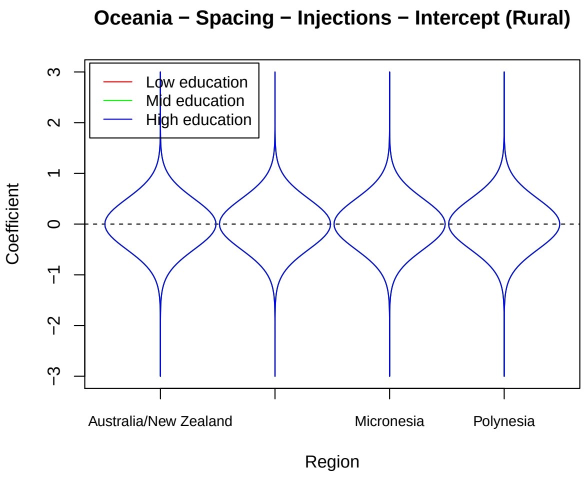
Oceania − Spacing − Injections − Intercept (Rural)
Low education
Mid education
High education
Coefficient
3
2
1
0
−1
−2
−3
Australia/New Zealand
Micronesia
Polynesia
Region

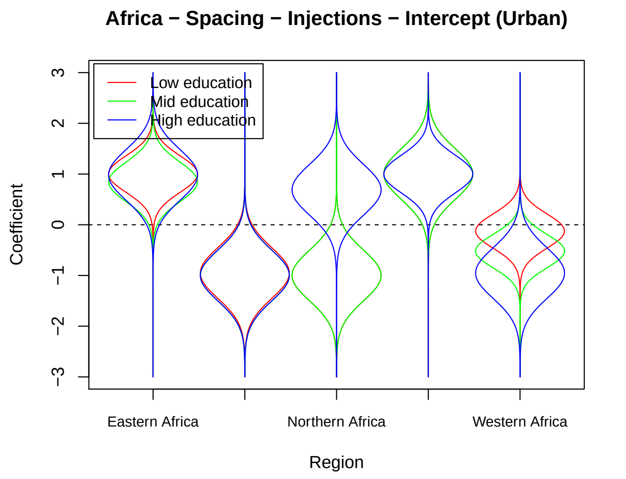
Africa − Spacing − Injections − Intercept (Urban)
Low education
Mid education
High education
Coefficient
3
2
1
0
−1
−2
−3
Eastern Africa
Northern Africa
Western Africa
Region

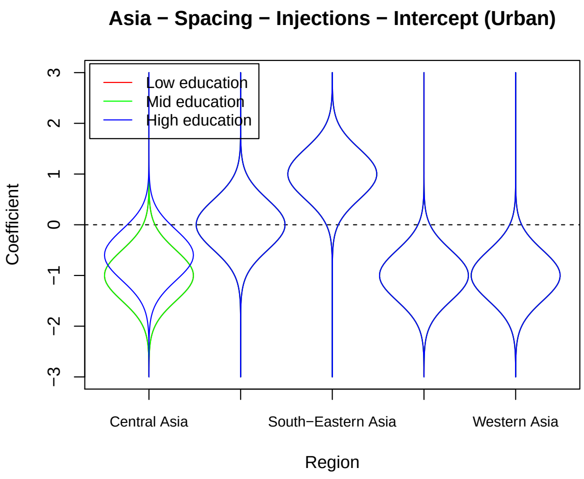
Asia − Spacing − Injections − Intercept (Urban)
Low education
Mid education
High education
Coefficient
3
2
1
0
−1
−2
−3
Central Asia
South−Eastern Asia
Western Asia
Region

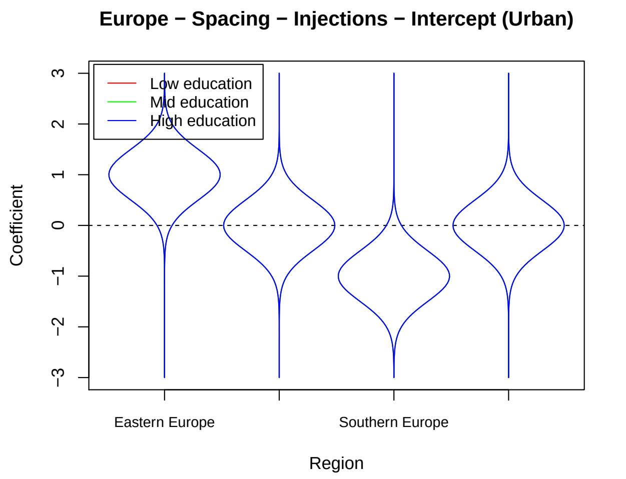
Europe − Spacing − Injections − Intercept (Urban)
Low education
Mid education
High education
Coefficient
3
2
1
0
−1
−2
−3
Eastern Europe
Southern Europe
Region

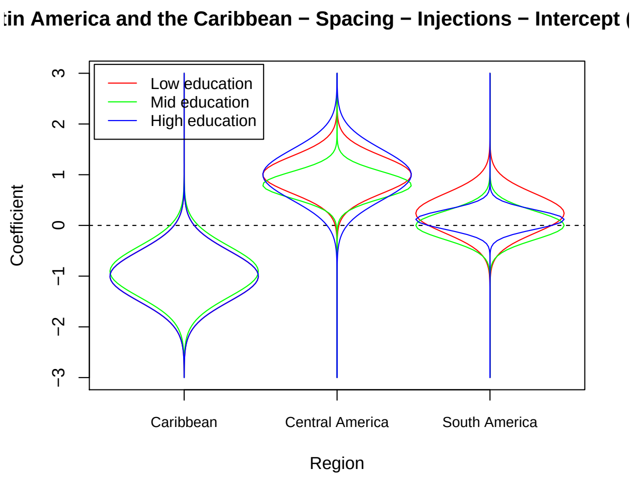
tin America and the Caribbean − Spacing − Injections − Intercept (
Low education
Mid education
High education
Coefficient
3
2
1
0
−1
−2
−3
Caribbean
Central America
South America
Region

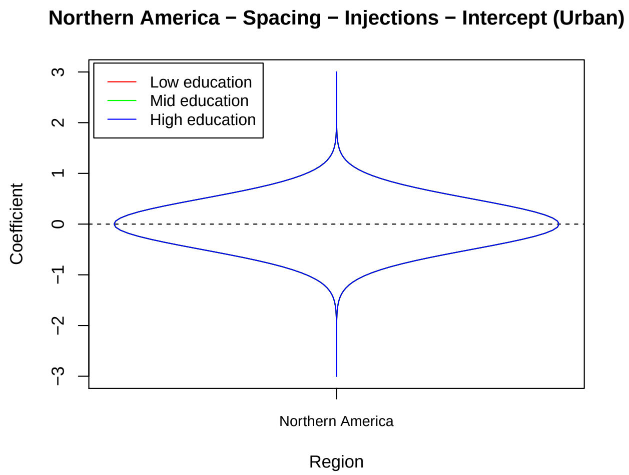
Northern America − Spacing − Injections − Intercept (Urban)
Low education
Mid education
High education
Coefficient
3
2
1
0
−1
−2
−3
Northern America
Region

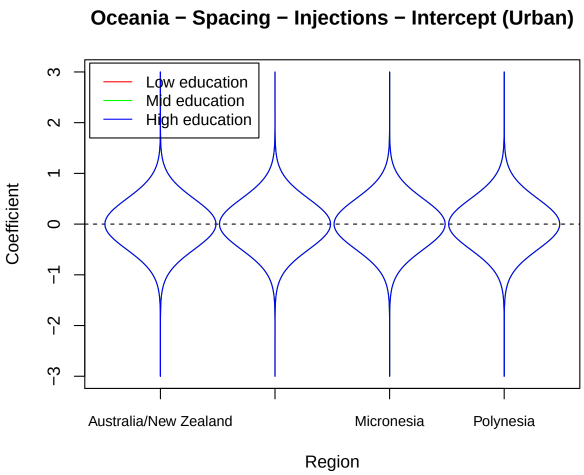
Oceania − Spacing − Injections − Intercept (Urban)
Low education
Mid education
High education
Coefficient
3
2
1
0
−1
−2
−3
Australia/New Zealand
Micronesia
Polynesia
Region

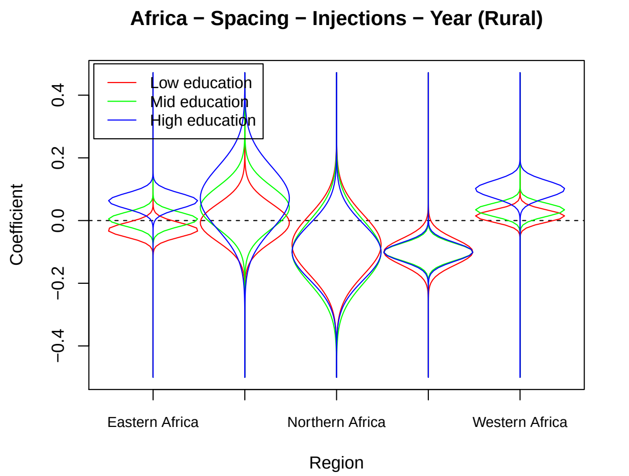
Africa − Spacing − Injections − Year (Rural)
Low education
Mid education
High education
Coefficient
0.4
0.2
0.0
−0.2
−0.4
Eastern Africa
Northern Africa
Western Africa
Region

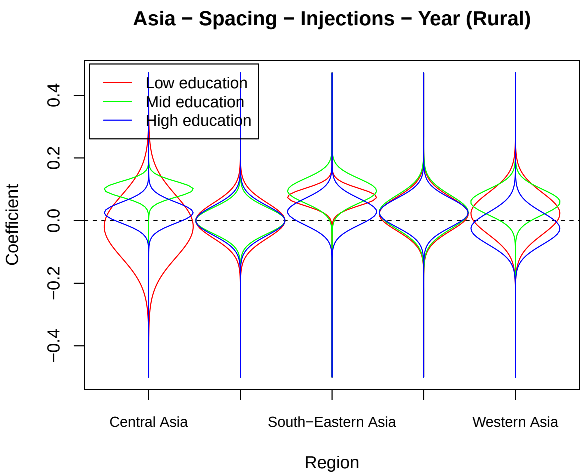
Asia − Spacing − Injections − Year (Rural)
Low education
Mid education
High education
Coefficient
0.4
0.2
0.0
−0.2
−0.4
Central Asia
South−Eastern Asia
Western Asia
Region

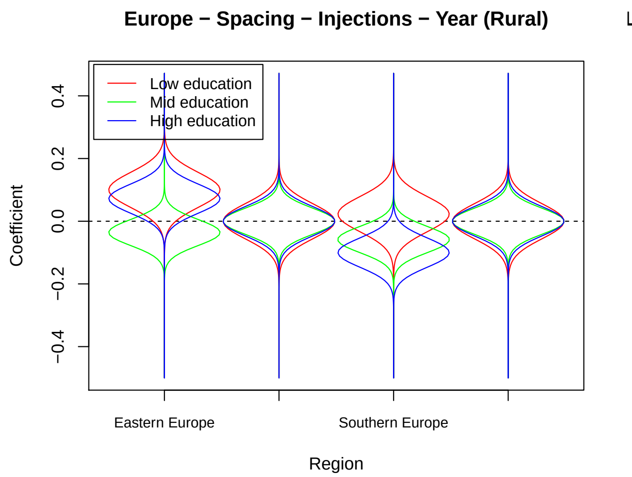
Europe − Spacing − Injections − Year (Rural)
Low education
Mid education
High education
Coefficient
0.4
0.2
0.0
−0.2
−0.4
Eastern Europe
Southern Europe
Region

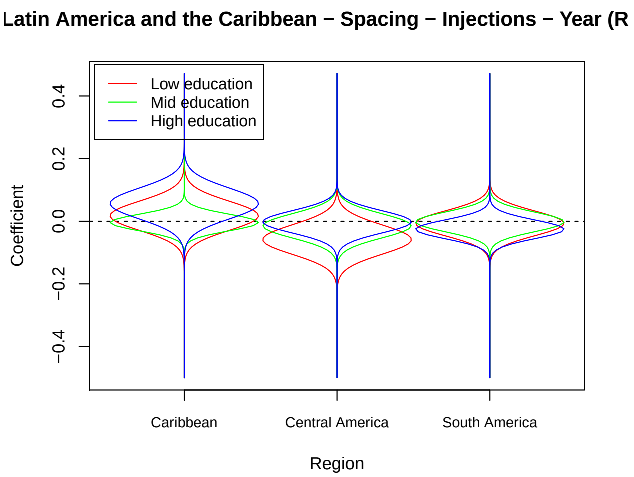
Latin America and the Caribbean − Spacing − Injections − Year (R
Low education
Mid education
High education
Coefficient
0.4
0.2
0.0
−0.2
−0.4
Caribbean
Central America
South America
Region

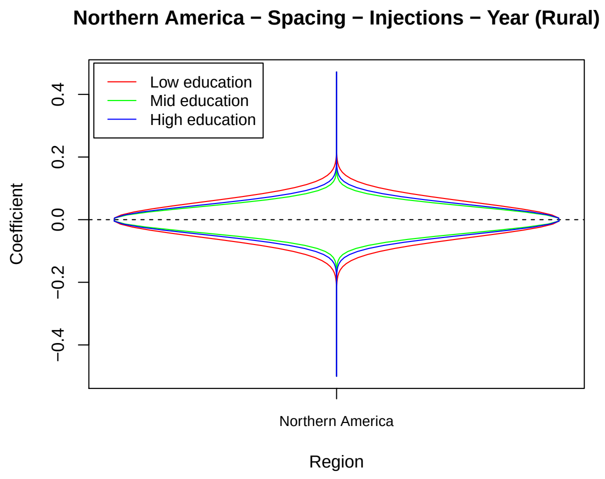
Northern America − Spacing − Injections − Year (Rural)
Low education
Mid education
High education
Coefficient
0.4
0.2
0.0
−0.2
−0.4
Northern America
Region

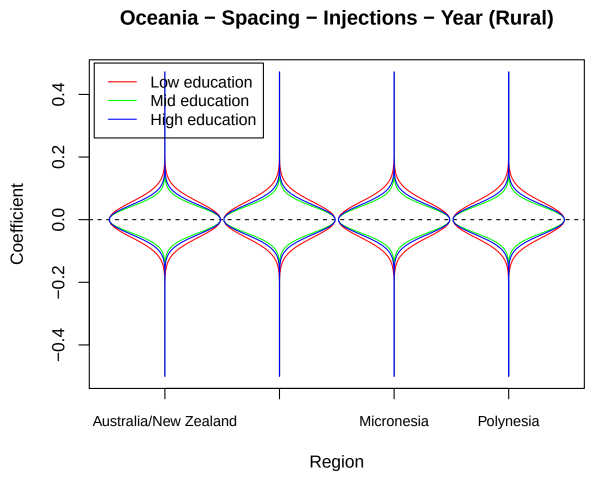
Oceania − Spacing − Injections − Year (Rural)
Low education
Mid education
High education
Coefficient
0.4
0.2
0.0
−0.2
−0.4
Australia/New Zealand
Micronesia
Polynesia
Region

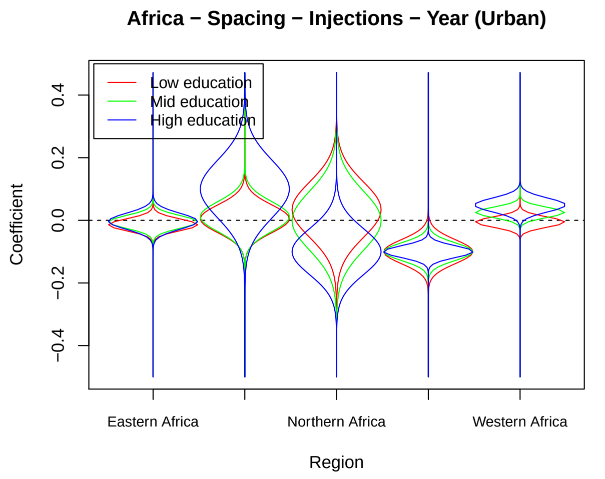
Africa − Spacing − Injections − Year (Urban)
Low education
Mid education
High education
Coefficient
0.4
0.2
0.0
−0.2
−0.4
Eastern Africa
Northern Africa
Western Africa
Region

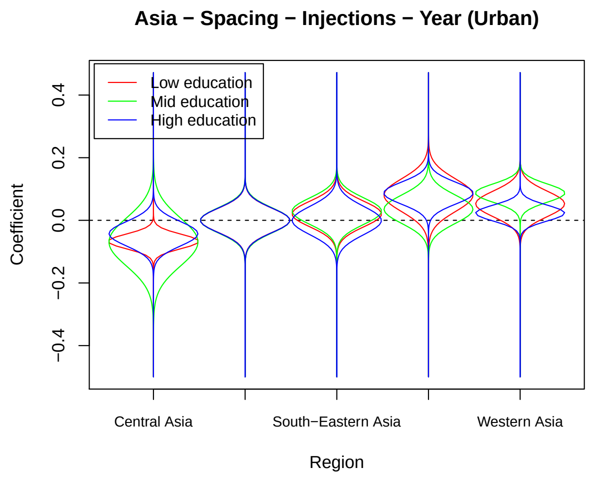
Asia − Spacing − Injections − Year (Urban)
Low education
Mid education
High education
Coefficient
0.4
0.2
0.0
−0.2
−0.4
Central Asia
South−Eastern Asia
Western Asia
Region

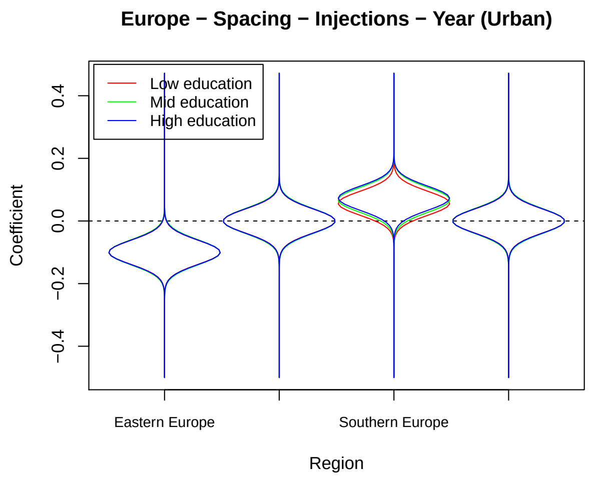
Europe − Spacing − Injections − Year (Urban)
Low education
Mid education
High education
Coefficient
0.4
0.2
0.0
−0.2
−0.4
Eastern Europe
Southern Europe
Region

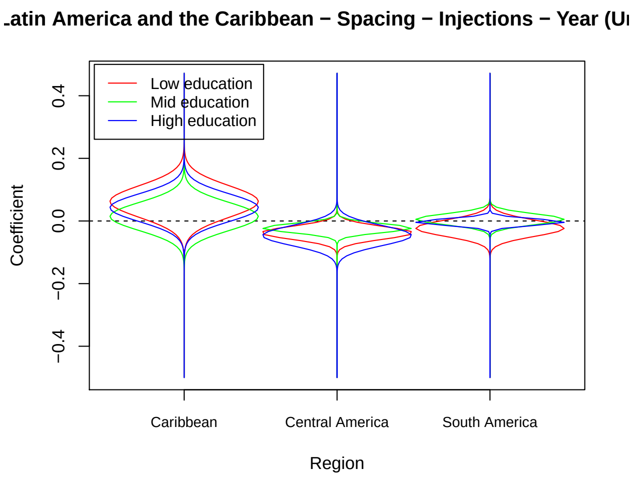
.atin America and the Caribbean − Spacing − Injections − Year (Ui
Low education
Mid education
High education
Coefficient
0.4
0.2
0.0
−0.2
−0.4
Caribbean
Central America
South America
Region

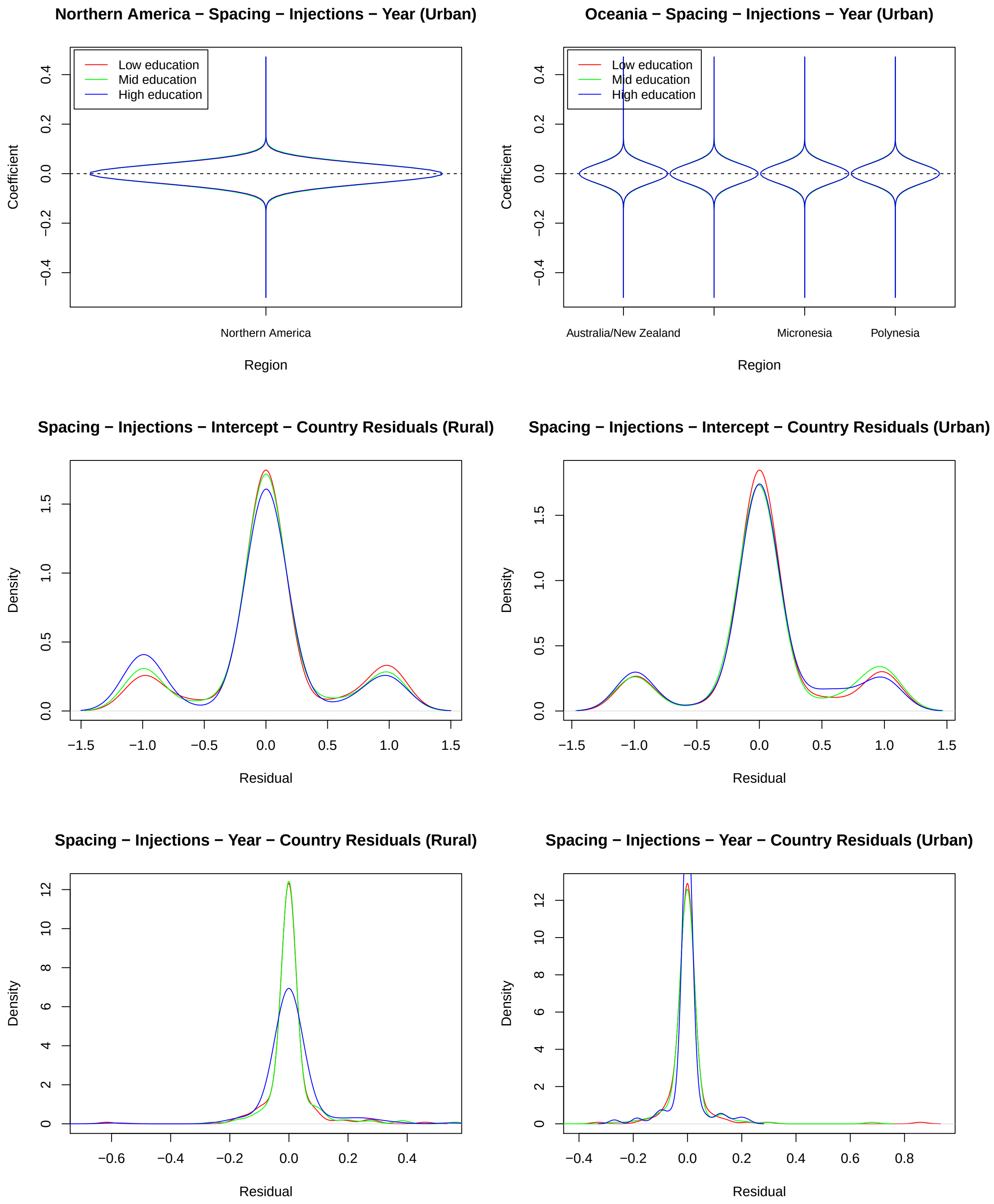
Northern America − Spacing − Injections − Year (Urban)
Low education
Mid education
High education
Coefficient
−0.4
−0.2
0.0
0.2
0.4
Northern America
Region
Oceania − Spacing − Injections − Year (Urban)
Low education
Mid education
High education
Coefficient
Australia/New Zealand
Micronesia
Polynesia
Region
Spacing − Injections − Intercept − Country Residuals (Rural)
Density
0.0
0.5
1.0
1.5
−1.5
−1.0
−0.5
0.0
0.5
1.0
1.5
Residual
Spacing − Injections − Intercept − Country Residuals (Urban)
Density
Residual
Spacing − Injections − Year − Country Residuals (Rural)
Density
0
2
4
6
8
10
12
−0.6
−0.4
−0.2
0.0
0.2
0.4
Residual
Spacing − Injections − Year − Country Residuals (Urban)
Density
−0.4
−0.2
0.0
0.2
0.4
0.6
0.8
Residual

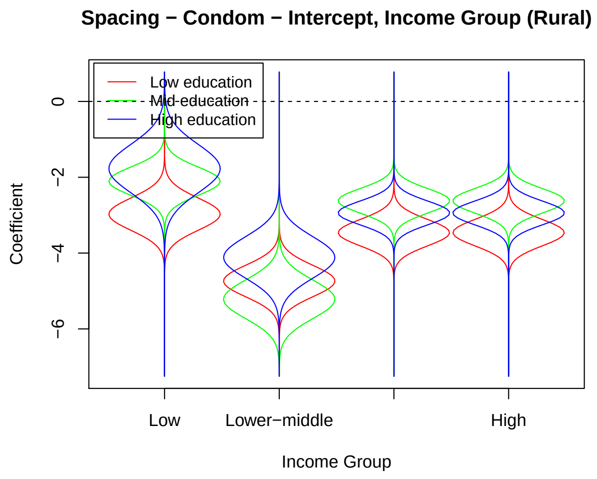
Spacing − Condom − Intercept, Income Group (Rural)
Low education
Mid education
High education
Coefficient
0
−2
−4
−6
Low
Lower−middle
High
Income Group

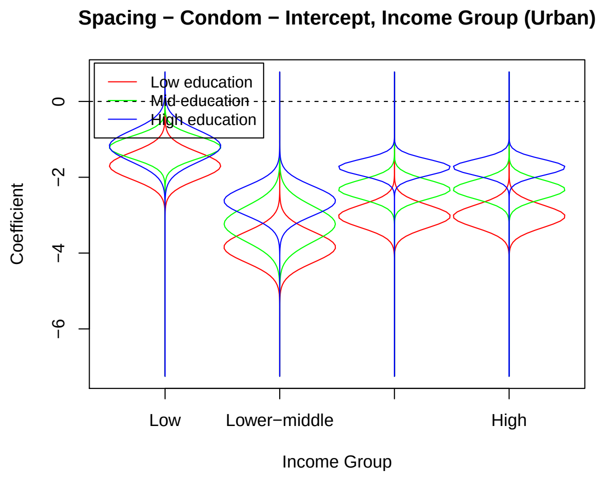
Spacing − Condom − Intercept, Income Group (Urban)
Low education
Mid education
High education
Coefficient
0
−2
−4
−6
Low
Lower−middle
High
Income Group

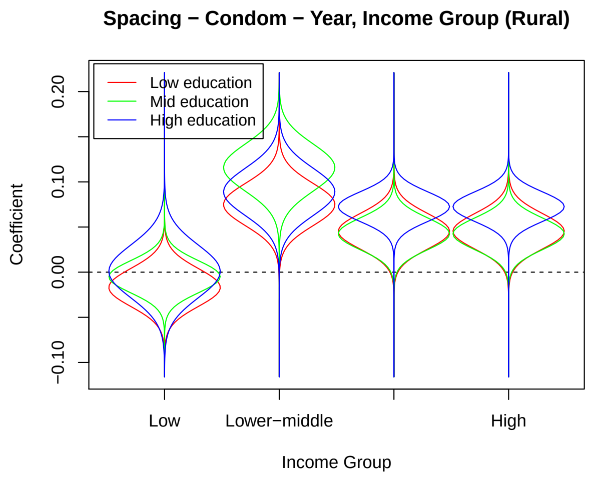
Spacing − Condom − Year, Income Group (Rural)
Low education
Mid education
High education
Coefficient
0.20
0.10
0.00
−0.10
Low
Lower−middle
High
Income Group

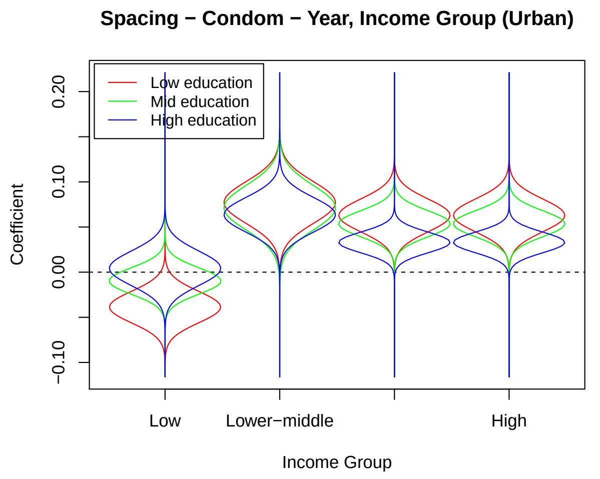
Spacing − Condom − Year, Income Group (Urban)
Low education
Mid education
High education
Coefficient
0.20
0.10
0.00
−0.10
Low
Lower−middle
High
Income Group

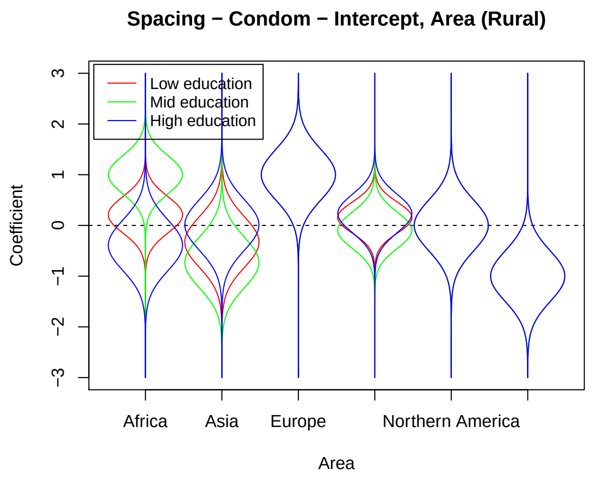
Spacing − Condom − Intercept, Area (Rural)
Low education
Mid education
High education
Coefficient
3
2
1
0
−1
−2
−3
Africa
Asia
Europe
Northern America
Area

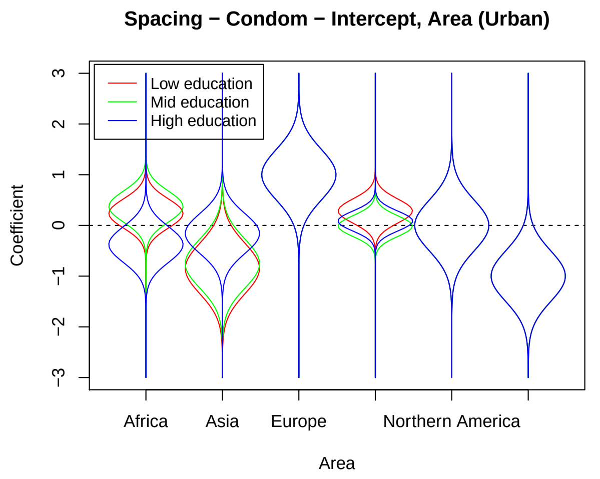
Spacing − Condom − Intercept, Area (Urban)
Low education
Mid education
High education
Coefficient
3
2
1
0
−1
−2
−3
Africa
Asia
Europe
Northern America
Area

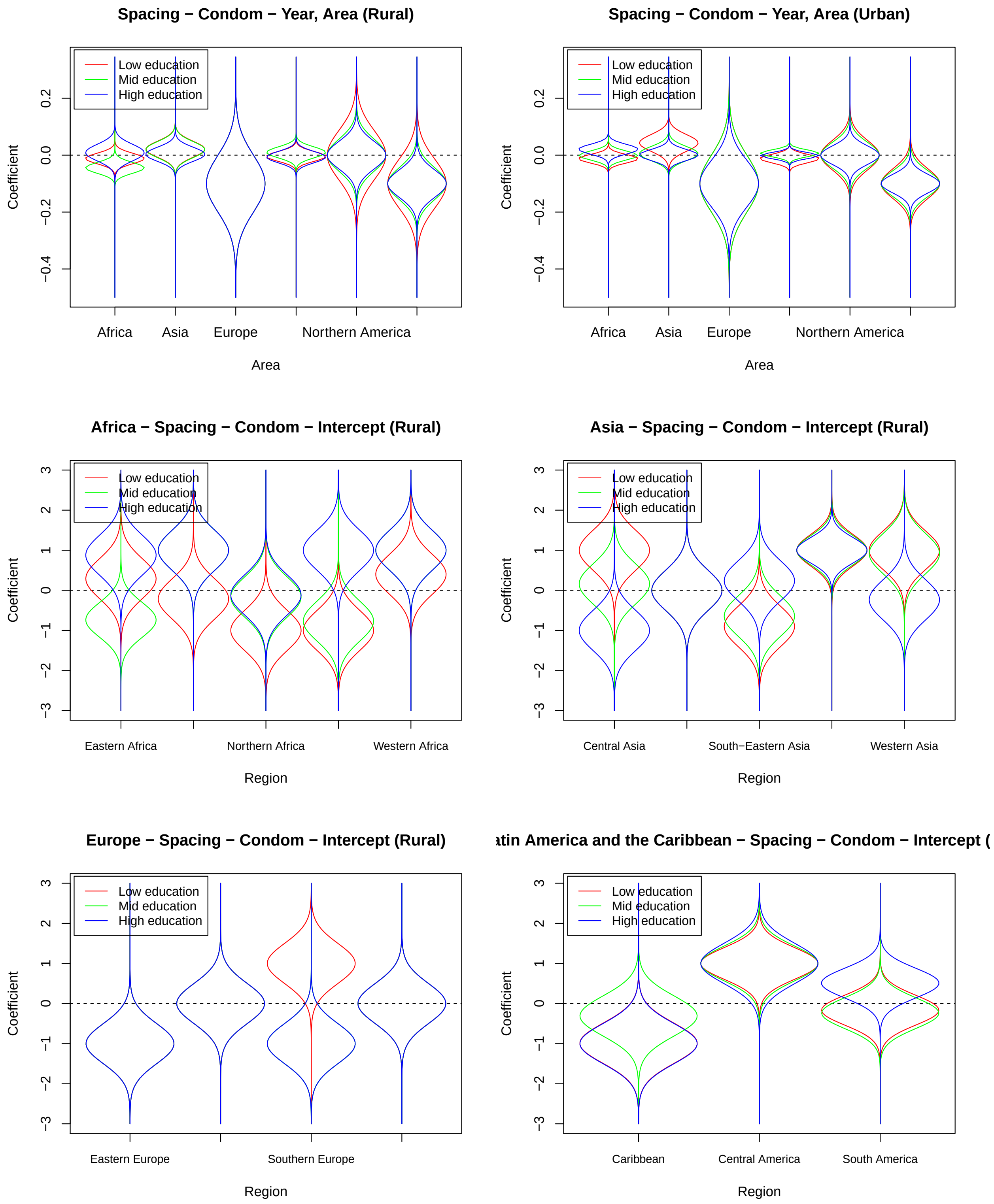
Spacing − Condom − Year, Area (Rural)
Low education
Mid education
High education
Coefficient
0.2
0.0
−0.2
−0.4
Africa
Asia
Europe
Northern America
Area
Spacing − Condom − Year, Area (Urban)
Low education
Mid education
High education
Coefficient
0.2
0.0
−0.2
−0.4
Africa
Asia
Europe
Northern America
Area
Africa − Spacing − Condom − Intercept (Rural)
Low education
Mid education
High education
Coefficient
3
2
1
0
−1
−2
−3
Eastern Africa
Northern Africa
Western Africa
Region
Asia − Spacing − Condom − Intercept (Rural)
Low education
Mid education
High education
Coefficient
3
2
1
0
−1
−2
−3
Central Asia
South−Eastern Asia
Western Asia
Region
Europe − Spacing − Condom − Intercept (Rural)
Low education
Mid education
High education
Coefficient
3
2
1
0
−1
−2
−3
Eastern Europe
Southern Europe
Region
atin America and the Caribbean − Spacing − Condom − Intercept (
Low education
Mid education
High education
Coefficient
3
2
1
0
−1
−2
−3
Caribbean
Central America
South America
Region

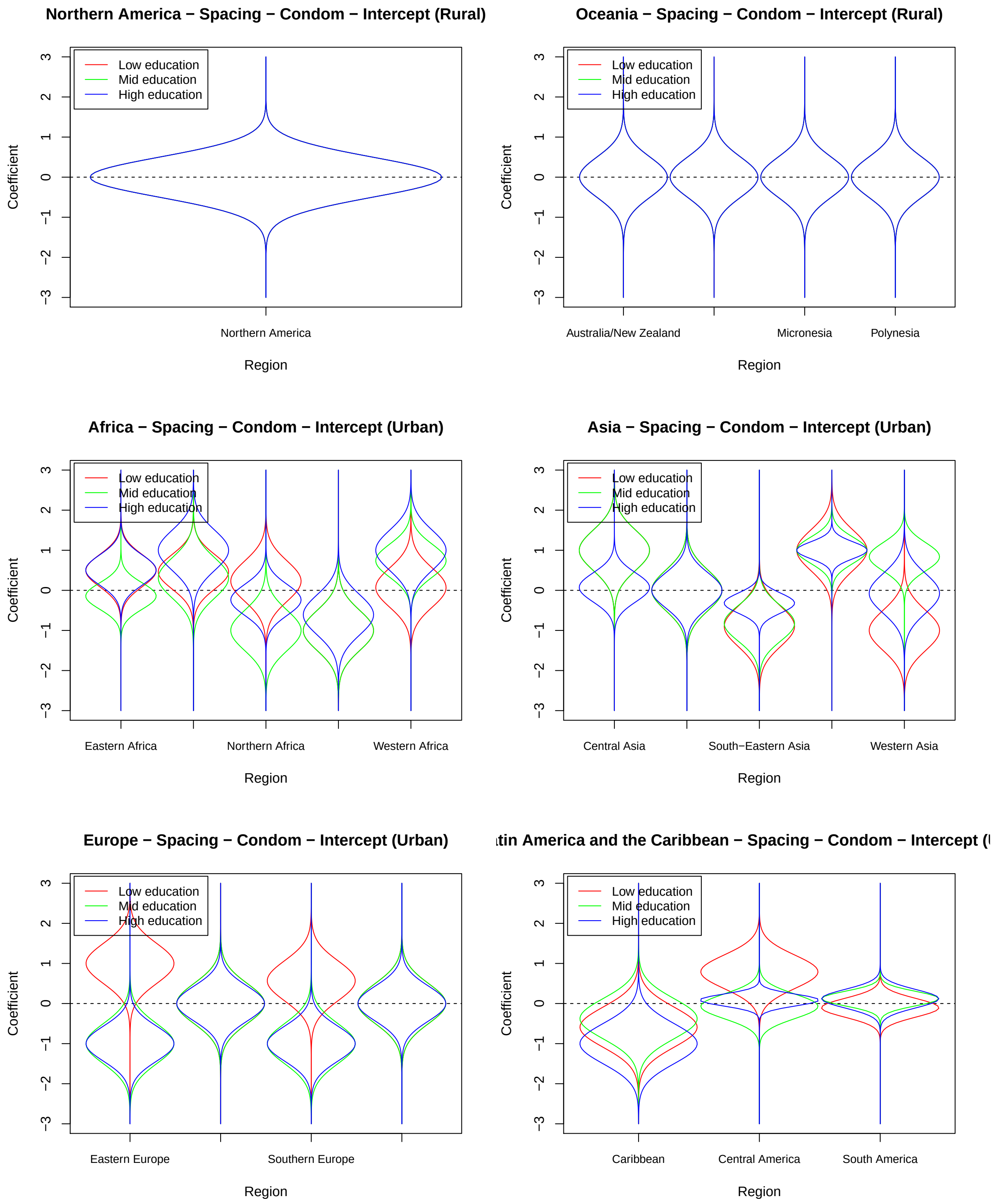
Northern America − Spacing − Condom − Intercept (Rural)
Oceania − Spacing − Condom − Intercept (Rural)
Africa − Spacing − Condom − Intercept (Urban)
Asia − Spacing − Condom − Intercept (Urban)
Europe − Spacing − Condom − Intercept (Urban)
tin America and the Caribbean − Spacing − Condom − Intercept (
Low education
Mid education
High education
Coefficient
Region
Northern America
Australia/New Zealand
Micronesia
Polynesia
Eastern Africa
Northern Africa
Western Africa
Central Asia
South−Eastern Asia
Western Asia
Eastern Europe
Southern Europe
Caribbean
Central America
South America

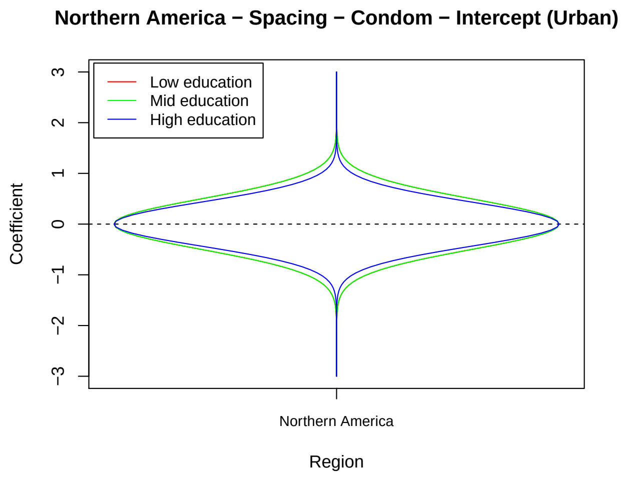
Northern America − Spacing − Condom − Intercept (Urban)
Low education
Mid education
High education
Coefficient
3
2
1
0
−1
−2
−3
Northern America
Region

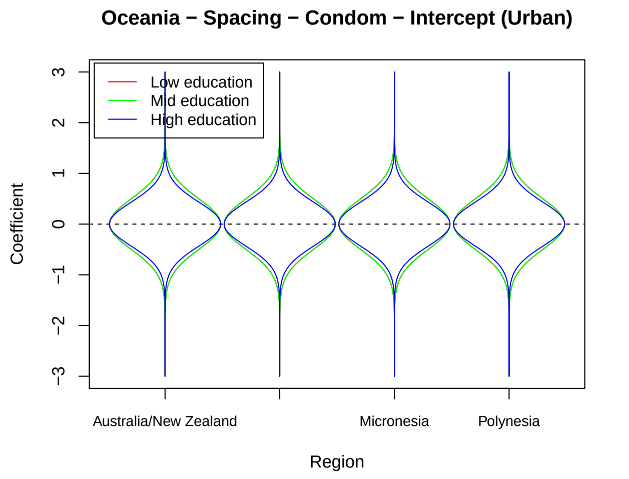
Oceania − Spacing − Condom − Intercept (Urban)
Low education
Mid education
High education
Coefficient
3
2
1
0
−1
−2
−3
Australia/New Zealand
Micronesia
Polynesia
Region

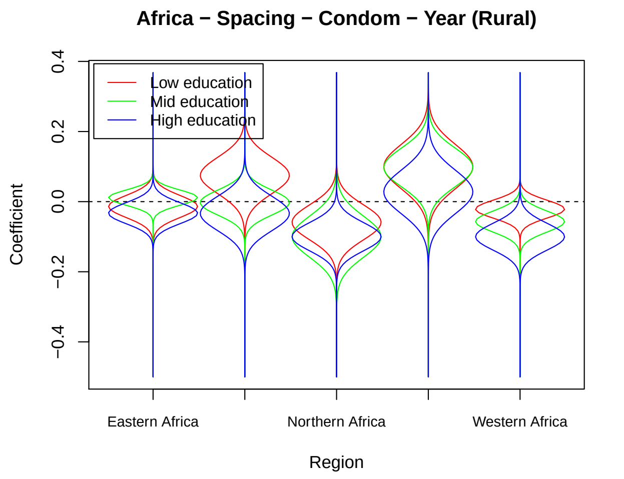
Africa − Spacing − Condom − Year (Rural)
Low education
Mid education
High education
Coefficient
0.4
0.2
0.0
−0.2
−0.4
Eastern Africa
Northern Africa
Western Africa
Region

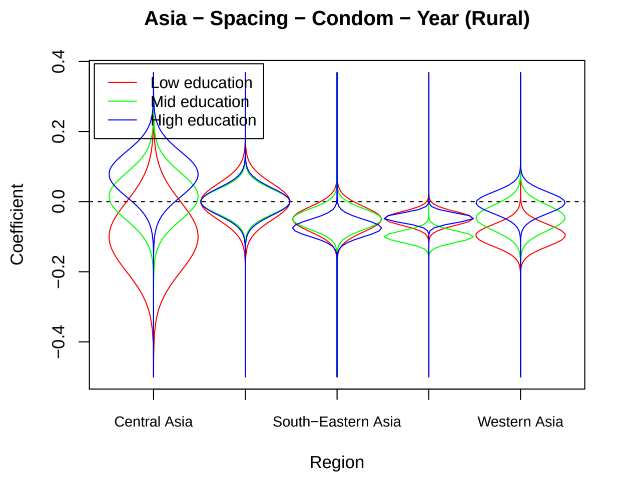
Asia − Spacing − Condom − Year (Rural)
Low education
Mid education
High education
Coefficient
0.4
0.2
0.0
−0.2
−0.4
Central Asia
South−Eastern Asia
Western Asia
Region

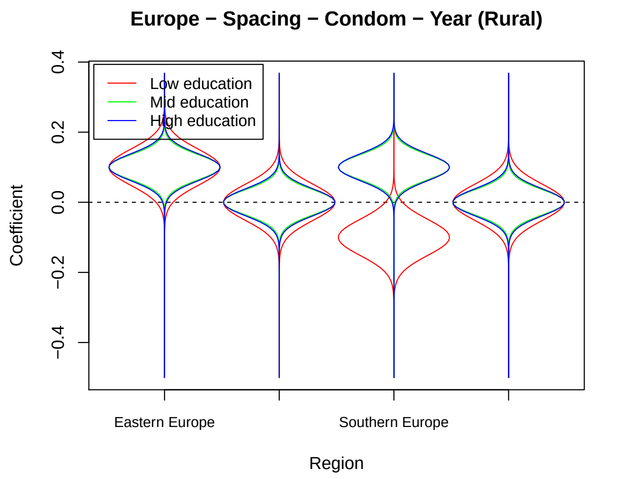
Europe − Spacing − Condom − Year (Rural)
Low education
Mid education
High education
Coefficient
0.4
0.2
0.0
−0.2
−0.4
Eastern Europe
Southern Europe
Region

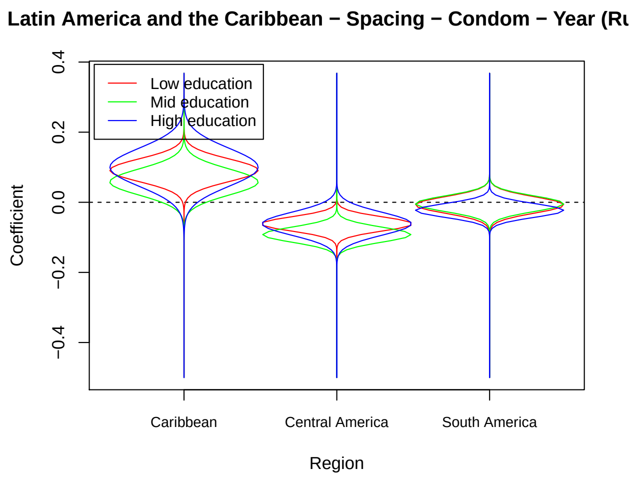
Latin America and the Caribbean − Spacing − Condom − Year (Ru
Low education
Mid education
High education
Coefficient
0.4
0.2
0.0
−0.2
−0.4
Caribbean
Central America
South America
Region

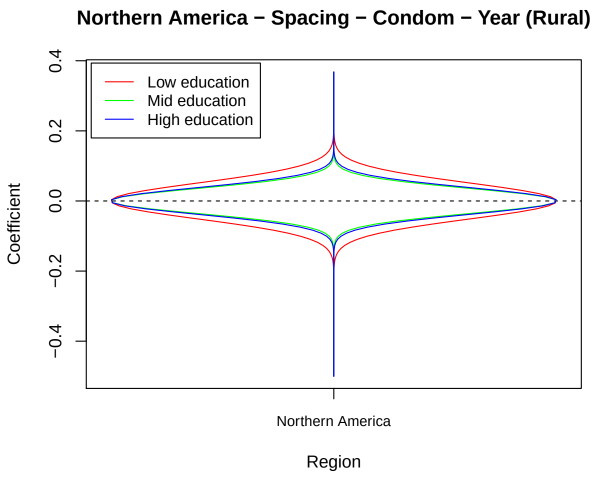
Northern America − Spacing − Condom − Year (Rural)
Low education
Mid education
High education
Coefficient
0.4
0.2
0.0
−0.2
−0.4
Northern America
Region

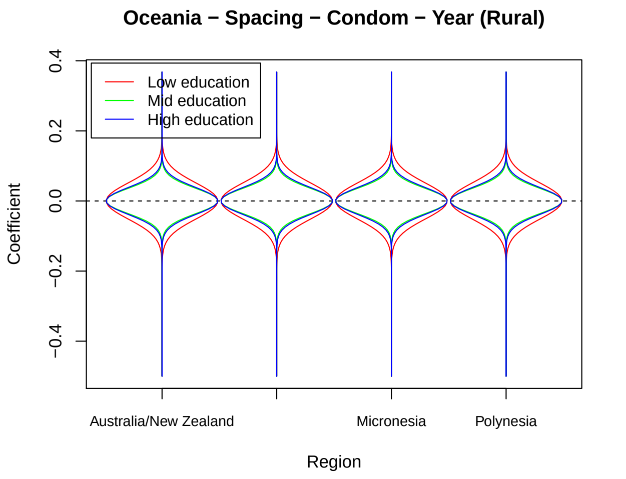
Oceania − Spacing − Condom − Year (Rural)
Low education
Mid education
High education
Coefficient
0.4
0.2
0.0
−0.2
−0.4
Australia/New Zealand
Micronesia
Polynesia
Region

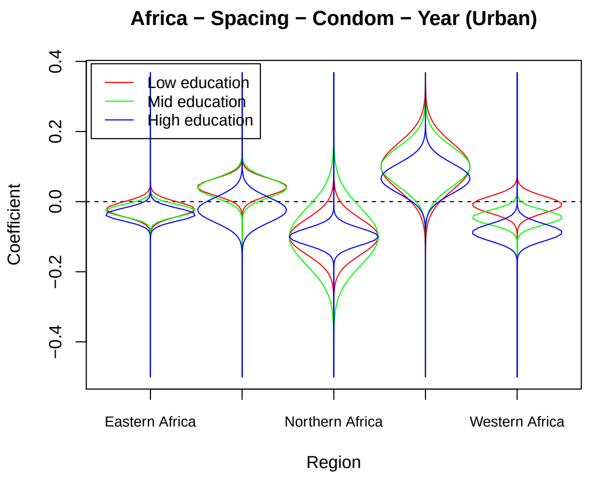
Africa − Spacing − Condom − Year (Urban)
Low education
Mid education
High education
Coefficient
0.4
0.2
0.0
−0.2
−0.4
Eastern Africa
Northern Africa
Western Africa
Region

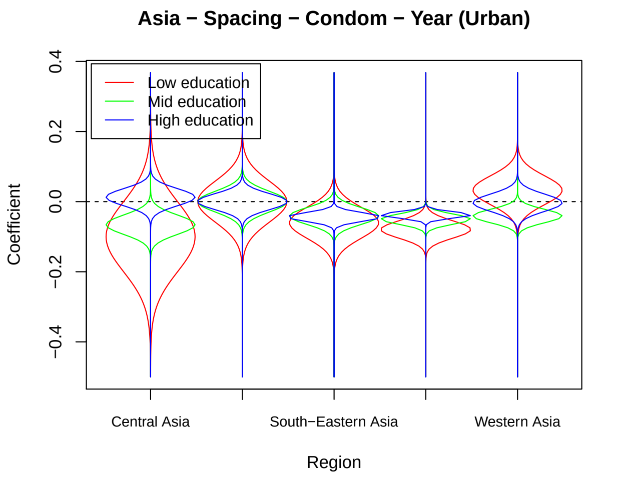
Asia − Spacing − Condom − Year (Urban)
Low education
Mid education
High education
Coefficient
0.4
0.2
0.0
−0.2
−0.4
Central Asia
South−Eastern Asia
Western Asia
Region

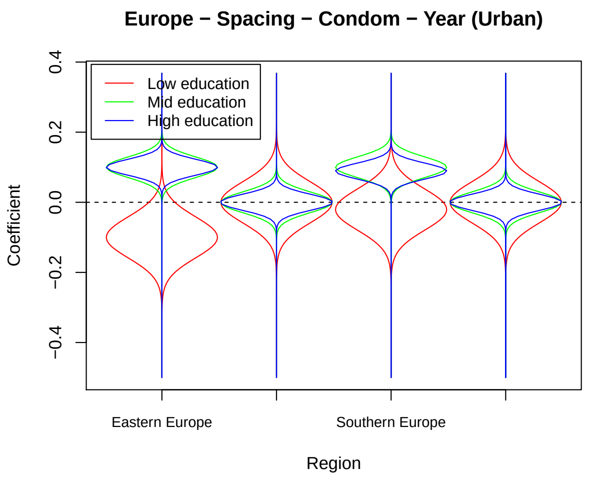
Europe − Spacing − Condom − Year (Urban)
Low education
Mid education
High education
Coefficient
0.4
0.2
0.0
−0.2
−0.4
Eastern Europe
Southern Europe
Region

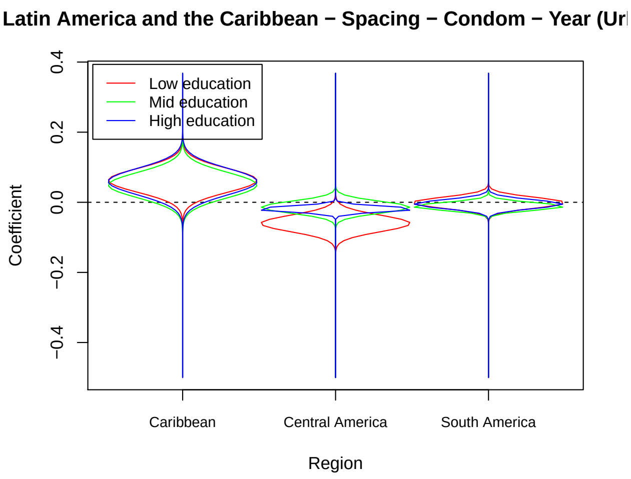
Latin America and the Caribbean − Spacing − Condom − Year (Ur
Low education
Mid education
High education
Coefficient
0.4
0.2
0.0
−0.2
−0.4
Caribbean
Central America
South America
Region

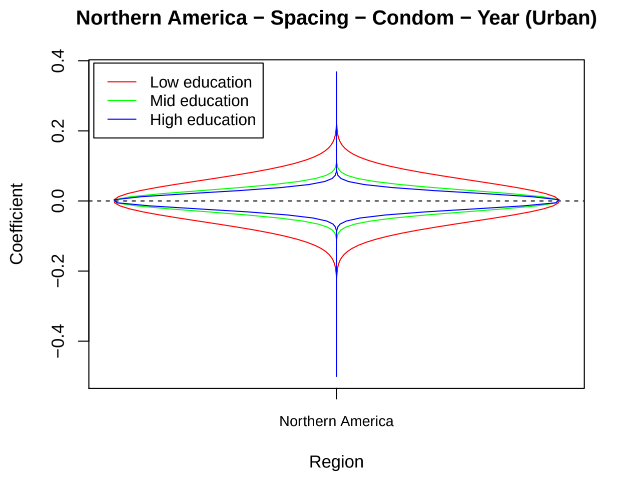
Northern America − Spacing − Condom − Year (Urban)
Low education
Mid education
High education
Coefficient
0.4
0.2
0.0
−0.2
−0.4
Northern America
Region

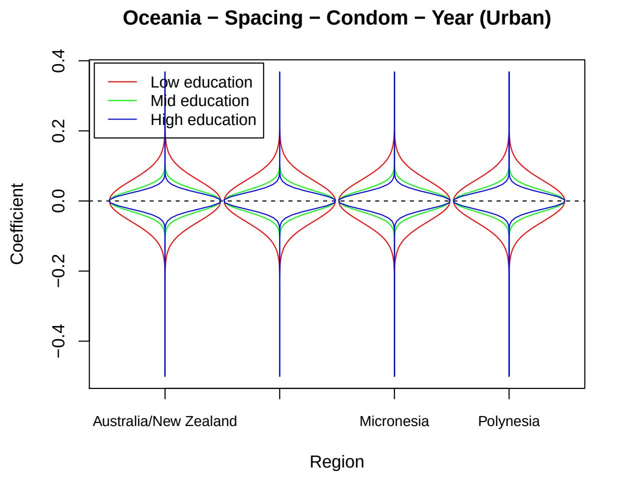
Oceania − Spacing − Condom − Year (Urban)
Low education
Mid education
High education
Coefficient
0.4
0.2
0.0
−0.2
−0.4
Australia/New Zealand
Micronesia
Polynesia
Region

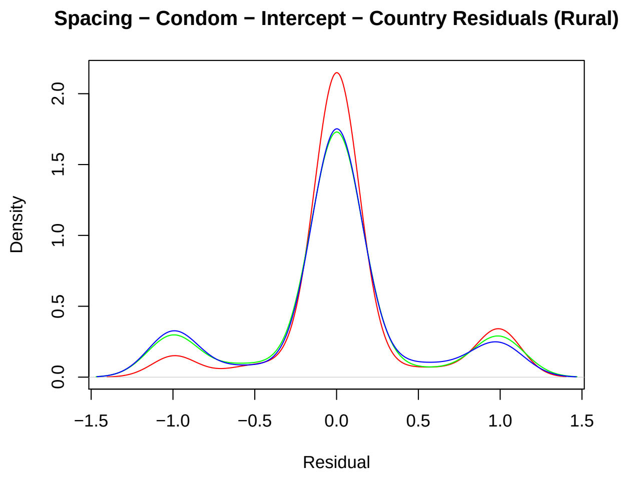
Spacing − Condom − Intercept − Country Residuals (Rural)
Density
2.0
1.5
1.0
0.5
0.0
−1.5
−1.0
−0.5
0.0
0.5
1.0
1.5
Residual

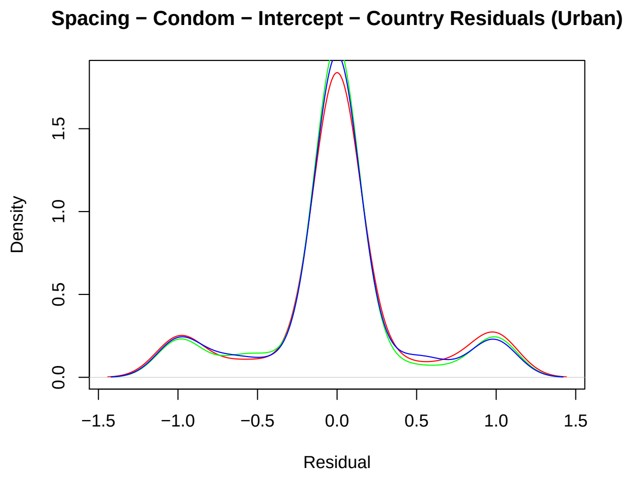
Spacing − Condom − Intercept − Country Residuals (Urban)
Density
1.5
1.0
0.5
0.0
−1.5
−1.0
−0.5
0.0
0.5
1.0
1.5
Residual

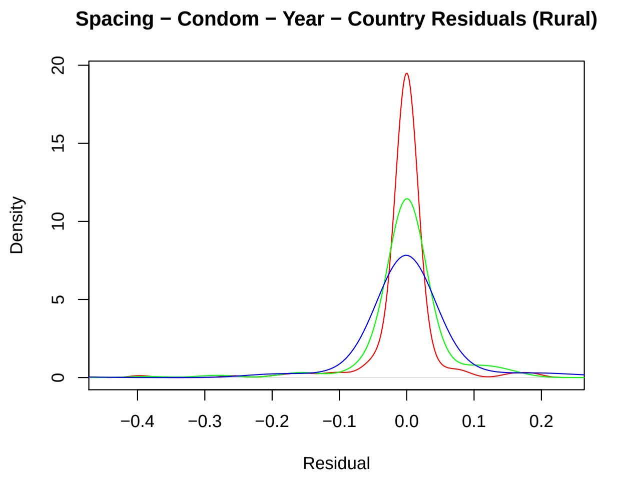
Spacing − Condom − Year − Country Residuals (Rural)
Density
20
15
10
5
0
−0.4
−0.3
−0.2
−0.1
0.0
0.1
0.2
Residual

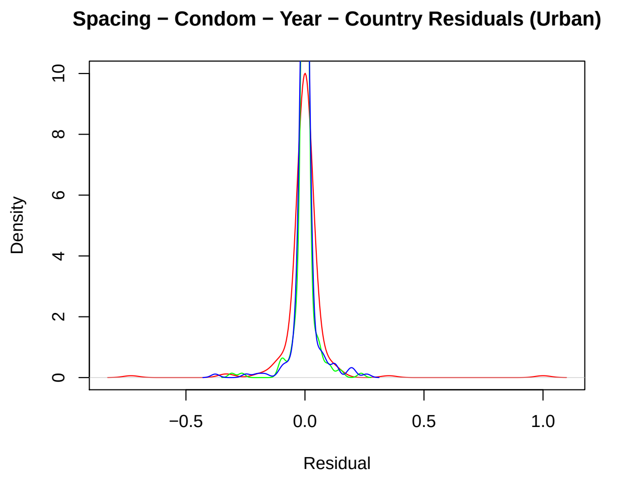
Spacing − Condom − Year − Country Residuals (Urban)
Density
10
8
6
4
2
0
−0.5
0.0
0.5
1.0
Residual

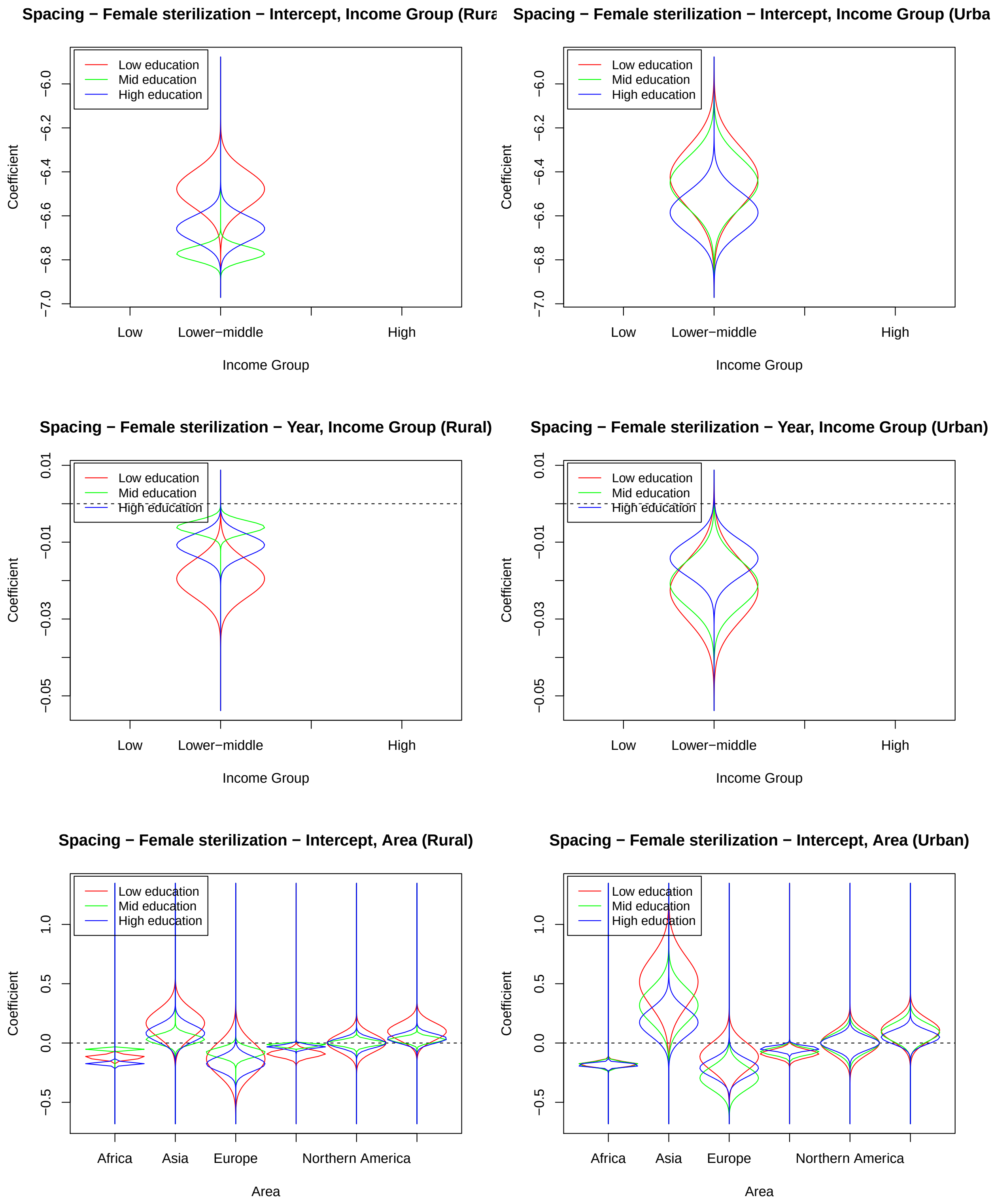

Spacing − Female sterilization − Intercept, Income Group (Rura
Spacing − Female sterilization − Intercept, Income Group (Urba
Spacing − Female sterilization − Year, Income Group (Rural)
Spacing − Female sterilization − Year, Income Group (Urban)
Spacing − Female sterilization − Intercept, Area (Rural)
Spacing − Female sterilization − Intercept, Area (Urban)
Low education
Mid education
High education
Coefficient
Income Group
Low
Lower−middle
High
Area
Africa
Asia
Europe
Northern America

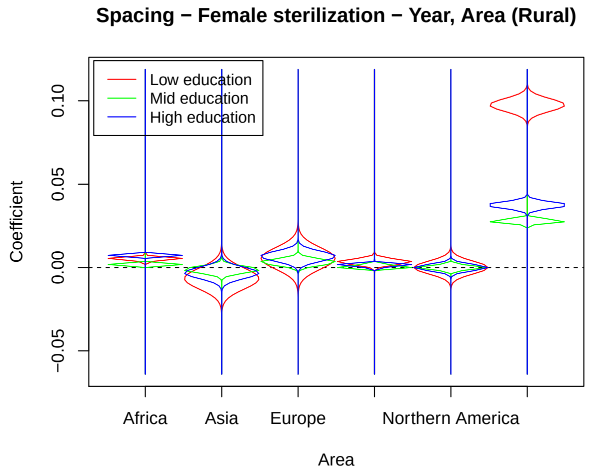
Spacing − Female sterilization − Year, Area (Rural)
Low education
Mid education
High education
Coefficient
0.10
0.05
0.00
−0.05
Africa
Asia
Europe
Northern America
Area

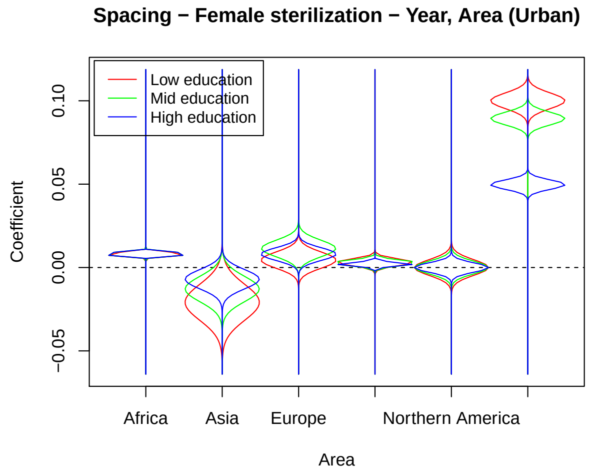
Spacing − Female sterilization − Year, Area (Urban)
Low education
Mid education
High education
Coefficient
0.10
0.05
0.00
−0.05
Africa
Asia
Europe
Northern America
Area

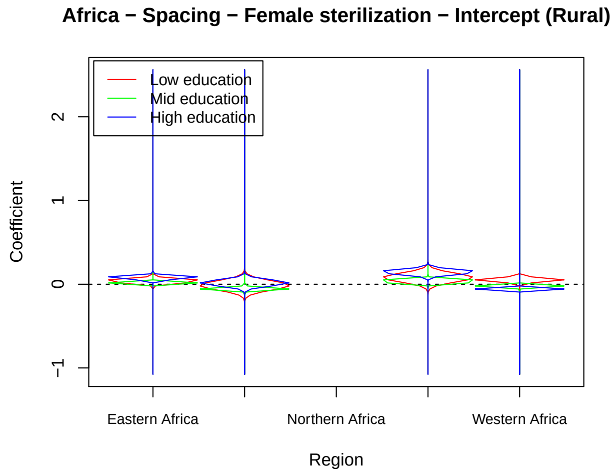
Africa − Spacing − Female sterilization − Intercept (Rural)
Low education
Mid education
High education
Coefficient
2
1
0
−1
Eastern Africa
Northern Africa
Western Africa
Region

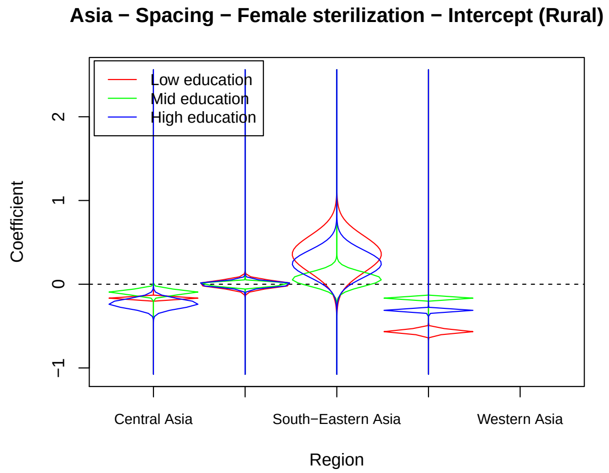
Asia − Spacing − Female sterilization − Intercept (Rural)
Low education
Mid education
High education
Coefficient
2
1
0
−1
Central Asia
South−Eastern Asia
Western Asia
Region

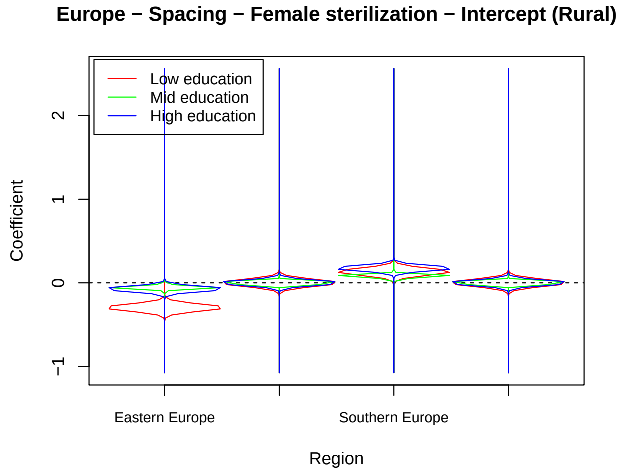
Europe − Spacing − Female sterilization − Intercept (Rural)
Low education
Mid education
High education
Coefficient
2
1
0
−1
Eastern Europe
Southern Europe
Region

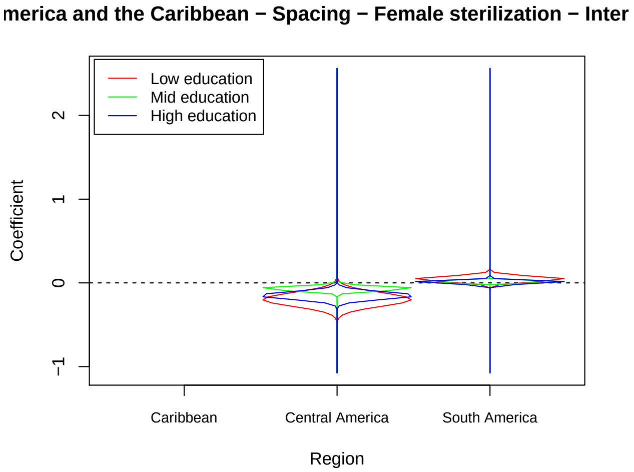
merica and the Caribbean − Spacing − Female sterilization − Inter
Low education
Mid education
High education
Coefficient
2
1
0
−1
Caribbean
Central America
South America
Region

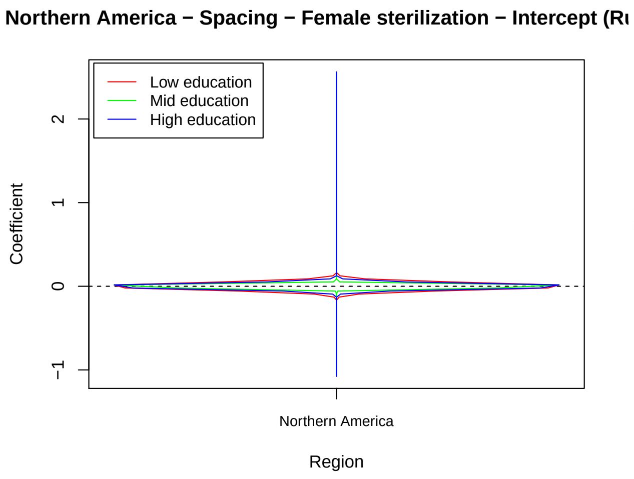
Northern America − Spacing − Female sterilization − Intercept (Ru
Low education
Mid education
High education
Coefficient
−1
0
1
2
Northern America
Region

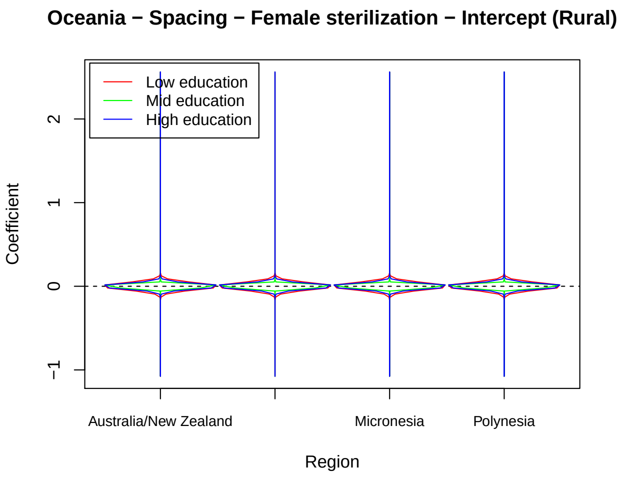
Oceania − Spacing − Female sterilization − Intercept (Rural)
Low education
Mid education
High education
Coefficient
−1
0
1
2
Australia/New Zealand
Micronesia
Polynesia
Region

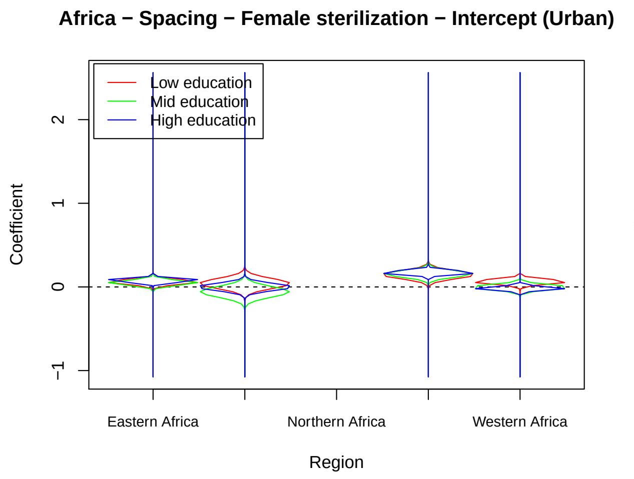
Africa − Spacing − Female sterilization − Intercept (Urban)
Low education
Mid education
High education
Coefficient
−1
0
1
2
Eastern Africa
Northern Africa
Western Africa
Region

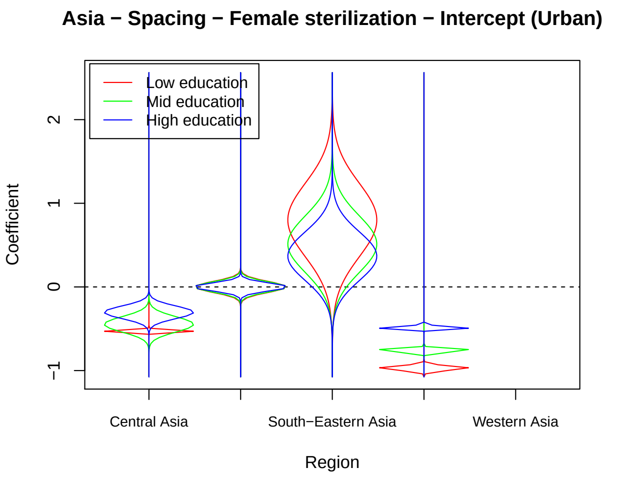
Asia − Spacing − Female sterilization − Intercept (Urban)
Low education
Mid education
High education
Coefficient
−1
0
1
2
Central Asia
South−Eastern Asia
Western Asia
Region

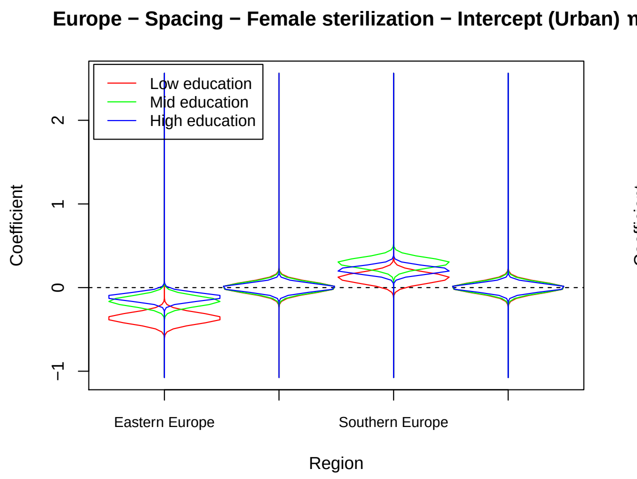
Europe − Spacing − Female sterilization − Intercept (Urban)
Low education
Mid education
High education
Coefficient
−1
0
1
2
Eastern Europe
Southern Europe
Region

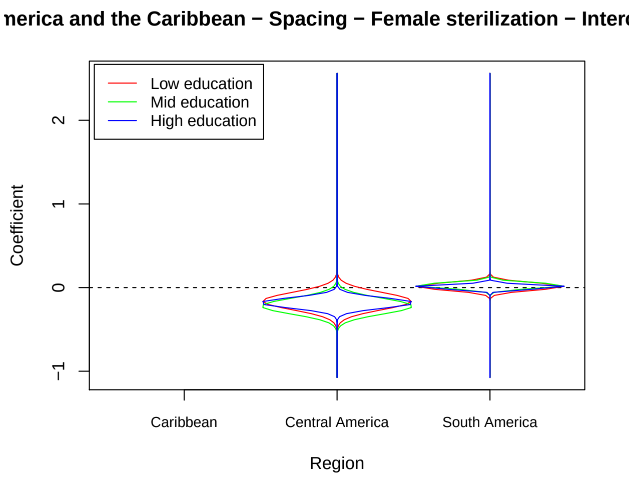
nerica and the Caribbean − Spacing − Female sterilization − Interc
Low education
Mid education
High education
Coefficient
−1
0
1
2
Caribbean
Central America
South America
Region

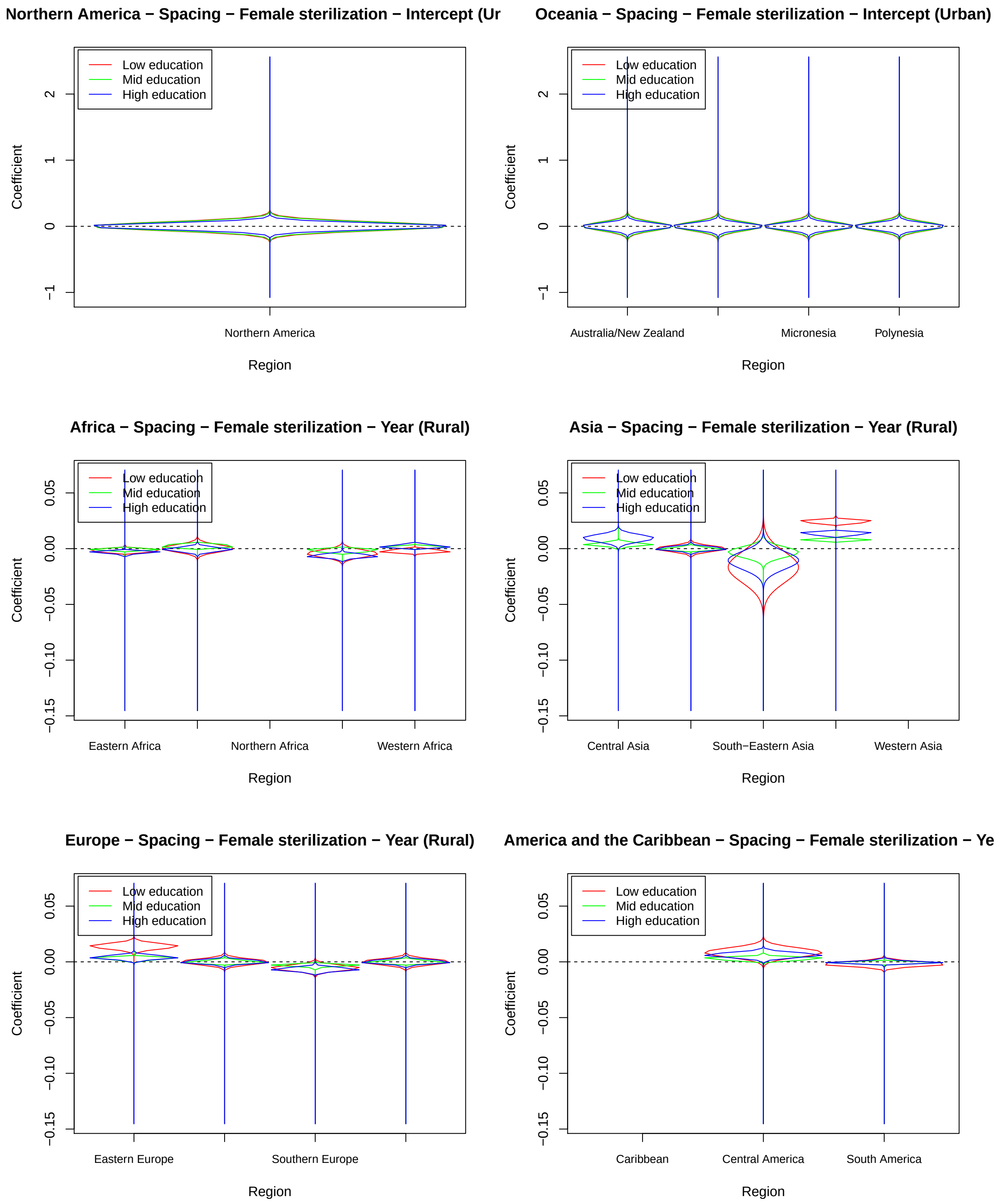
Northern America − Spacing − Female sterilization − Intercept (Ur
Low education
Mid education
High education
Coefficient
Region
Northern America
−1
0
1
2
Oceania − Spacing − Female sterilization − Intercept (Urban)
Australia/New Zealand
Micronesia
Polynesia
Africa − Spacing − Female sterilization − Year (Rural)
Eastern Africa
Northern Africa
Western Africa
−0.15
−0.10
−0.05
0.00
0.05
Asia − Spacing − Female sterilization − Year (Rural)
Central Asia
South−Eastern Asia
Western Asia
Europe − Spacing − Female sterilization − Year (Rural)
Eastern Europe
Southern Europe
America and the Caribbean − Spacing − Female sterilization − Ye
Caribbean
Central America
South America

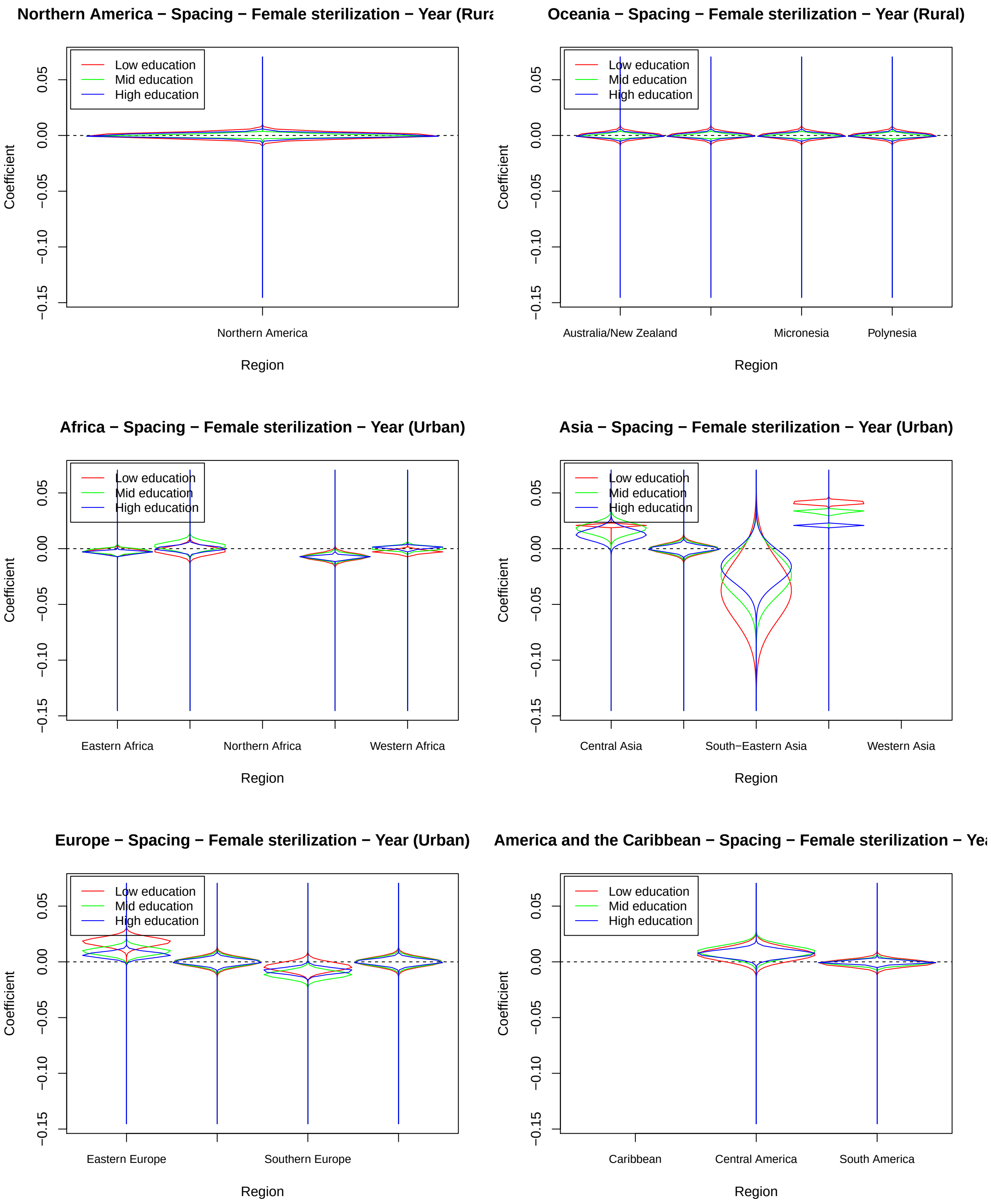

Northern America − Spacing − Female sterilization − Year (Rura
Low education
Mid education
High education
Coefficient
0.05
0.00
−0.05
−0.10
−0.15
Northern America
Region
Oceania − Spacing − Female sterilization − Year (Rural)
Low education
Mid education
High education
Coefficient
Australia/New Zealand
Micronesia
Polynesia
Region
Africa − Spacing − Female sterilization − Year (Urban)
Low education
Mid education
High education
Coefficient
Eastern Africa
Northern Africa
Western Africa
Region
Asia − Spacing − Female sterilization − Year (Urban)
Low education
Mid education
High education
Coefficient
Central Asia
South−Eastern Asia
Western Asia
Region
Europe − Spacing − Female sterilization − Year (Urban)
Low education
Mid education
High education
Coefficient
Eastern Europe
Southern Europe
Region
America and the Caribbean − Spacing − Female sterilization − Yea
Low education
Mid education
High education
Coefficient
Caribbean
Central America
South America
Region

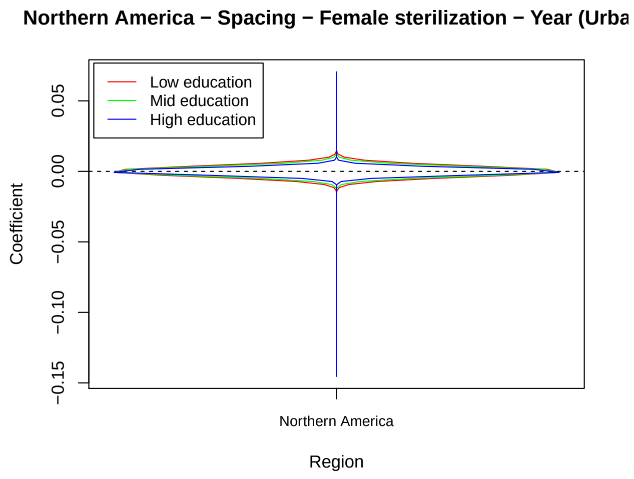
Northern America − Spacing − Female sterilization − Year (Urba
Low education
Mid education
High education
Coefficient
0.05
0.00
−0.05
−0.10
−0.15
Northern America
Region

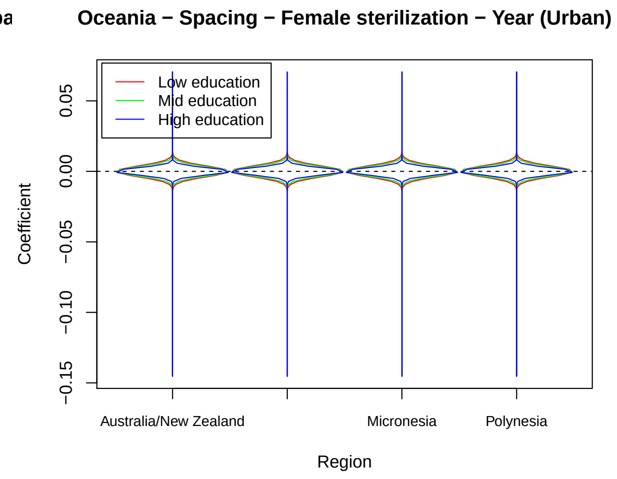
Oceania − Spacing − Female sterilization − Year (Urban)
Low education
Mid education
High education
Coefficient
0.05
0.00
−0.05
−0.10
−0.15
Australia/New Zealand
Micronesia
Polynesia
Region

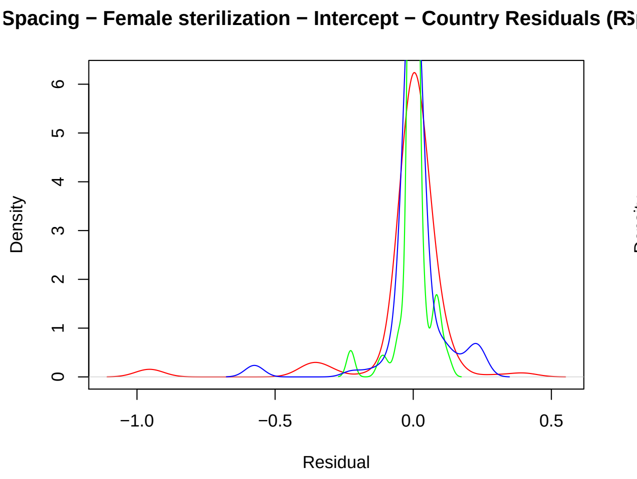
Spacing − Female sterilization − Intercept − Country Residuals (R
Density
0
1
2
3
4
5
6
−1.0
−0.5
0.0
0.5
Residual

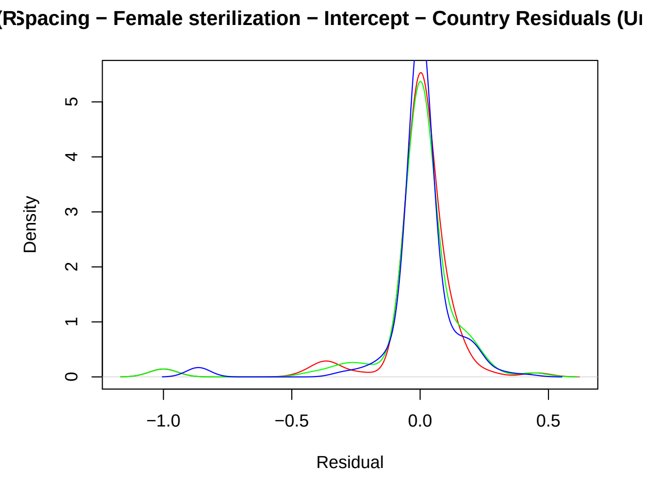
Spacing − Female sterilization − Intercept − Country Residuals (U
Density
0
1
2
3
4
5
−1.0
−0.5
0.0
0.5
Residual

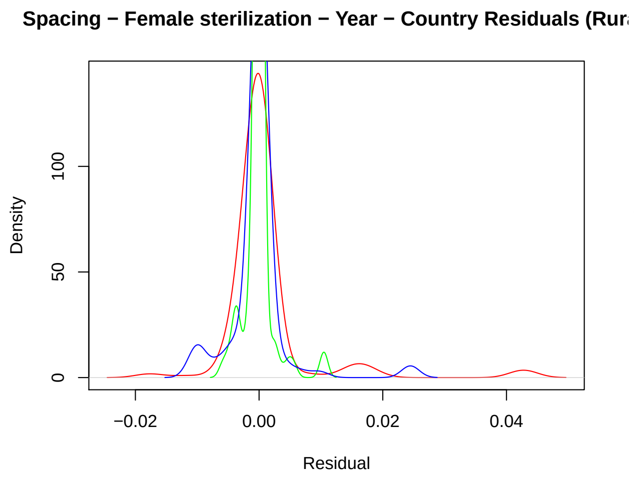
Spacing − Female sterilization − Year − Country Residuals (Rur
Density
0
50
100
−0.02
0.00
0.02
0.04
Residual

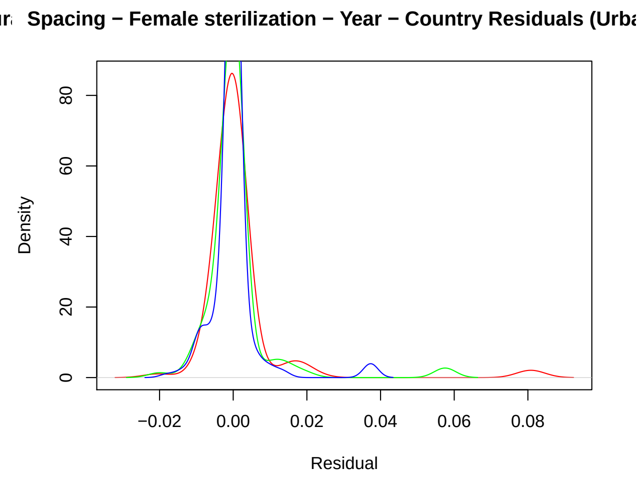
Spacing − Female sterilization − Year − Country Residuals (Urba
Density
0
20
40
60
80
−0.02
0.00
0.02
0.04
0.06
0.08
Residual

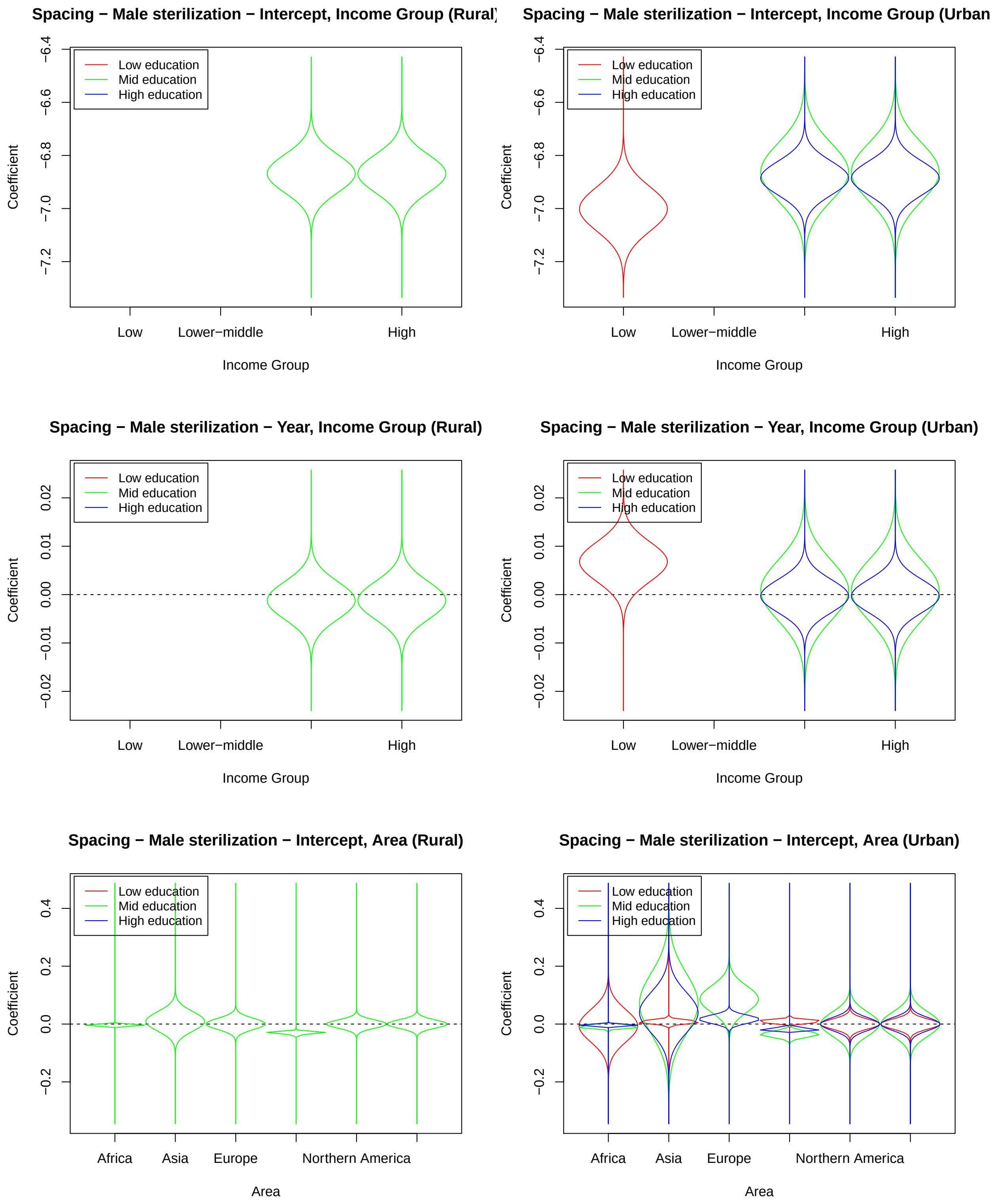
Spacing − Male sterilization − Intercept, Income Group (Rural)
Low education
Mid education
High education
Coefficient
−6.4
−6.6
−6.8
−7.0
−7.2
Low
Lower−middle
High
Income Group
Spacing − Male sterilization − Intercept, Income Group (Urban
Low education
Mid education
High education
Coefficient
−6.4
−6.6
−6.8
−7.0
−7.2
Low
Lower−middle
High
Income Group
Spacing − Male sterilization − Year, Income Group (Rural)
Low education
Mid education
High education
Coefficient
0.02
0.01
0.00
−0.01
−0.02
Low
Lower−middle
High
Income Group
Spacing − Male sterilization − Year, Income Group (Urban)
Low education
Mid education
High education
Coefficient
0.02
0.01
0.00
−0.01
−0.02
Low
Lower−middle
High
Income Group
Spacing − Male sterilization − Intercept, Area (Rural)
Low education
Mid education
High education
Coefficient
0.4
0.2
0.0
−0.2
Africa
Asia
Europe
Northern America
Area
Spacing − Male sterilization − Intercept, Area (Urban)
Low education
Mid education
High education
Coefficient
0.4
0.2
0.0
−0.2
Africa
Asia
Europe
Northern America
Area

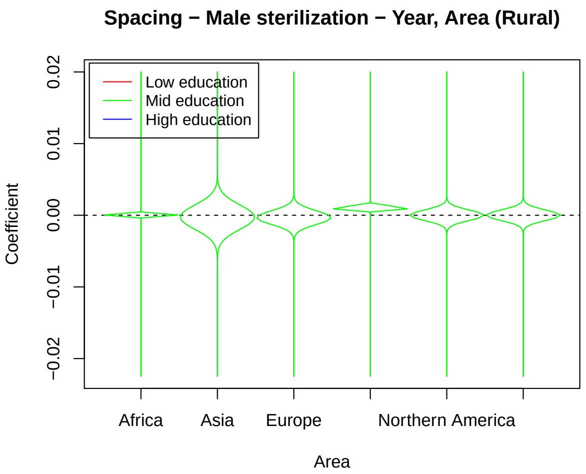
Spacing − Male sterilization − Year, Area (Rural)
Low education
Mid education
High education
Coefficient
0.02
0.01
0.00
−0.01
−0.02
Africa
Asia
Europe
Northern America
Area

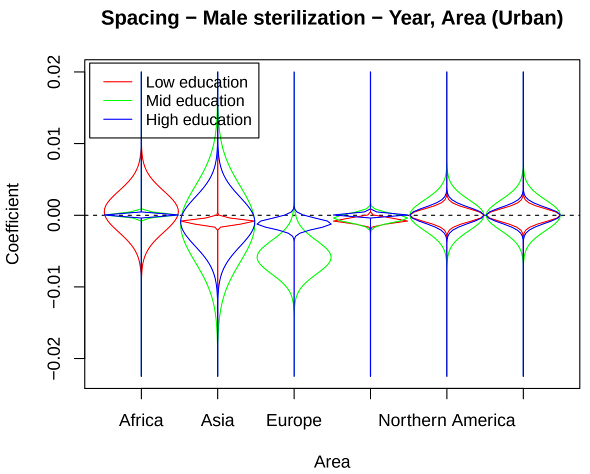
Spacing − Male sterilization − Year, Area (Urban)
Low education
Mid education
High education
Coefficient
0.02
0.01
0.00
−0.01
−0.02
Africa
Asia
Europe
Northern America
Area

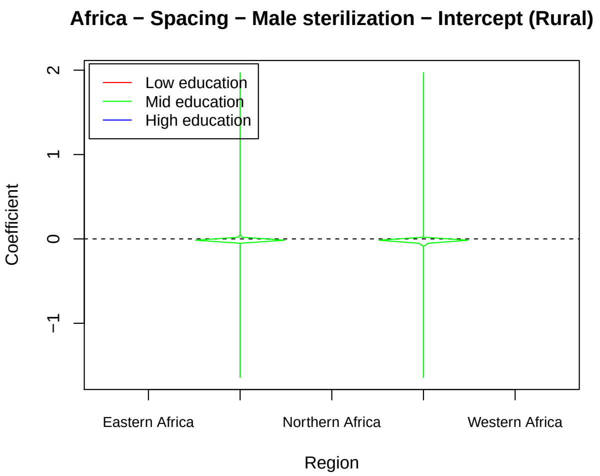
Africa − Spacing − Male sterilization − Intercept (Rural)
Low education
Mid education
High education
Coefficient
2
1
0
−1
Eastern Africa
Northern Africa
Western Africa
Region

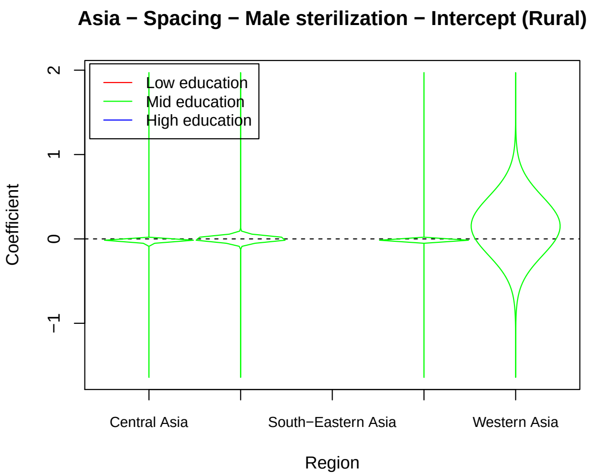
Asia − Spacing − Male sterilization − Intercept (Rural)
Low education
Mid education
High education
Coefficient
2
1
0
−1
Central Asia
South−Eastern Asia
Western Asia
Region

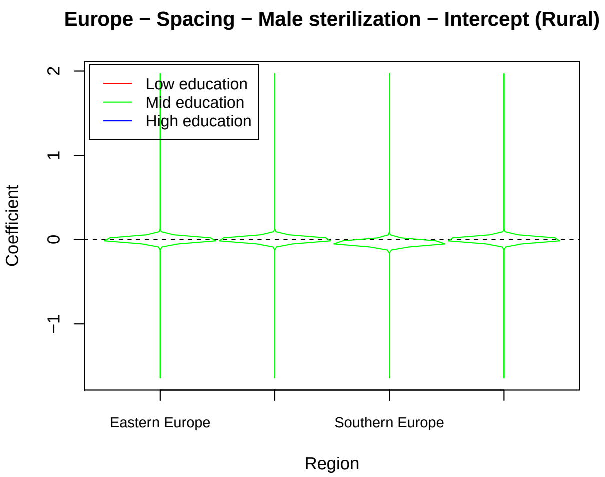
Europe − Spacing − Male sterilization − Intercept (Rural)
Low education
Mid education
High education
Coefficient
2
1
0
−1
Eastern Europe
Southern Europe
Region

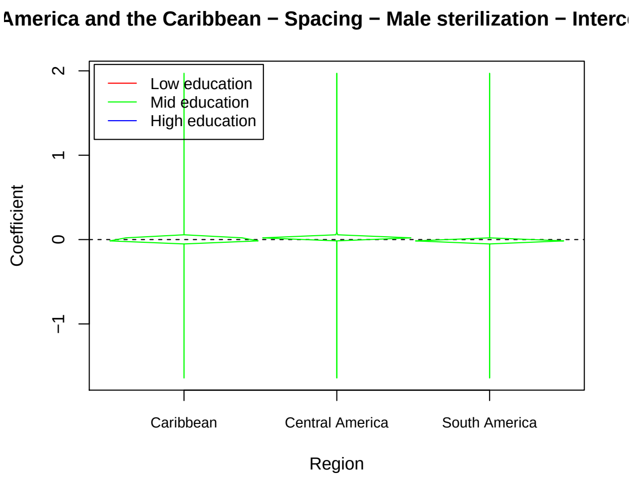
America and the Caribbean − Spacing − Male sterilization − Interc
Low education
Mid education
High education
Coefficient
2
1
0
−1
Caribbean
Central America
South America
Region

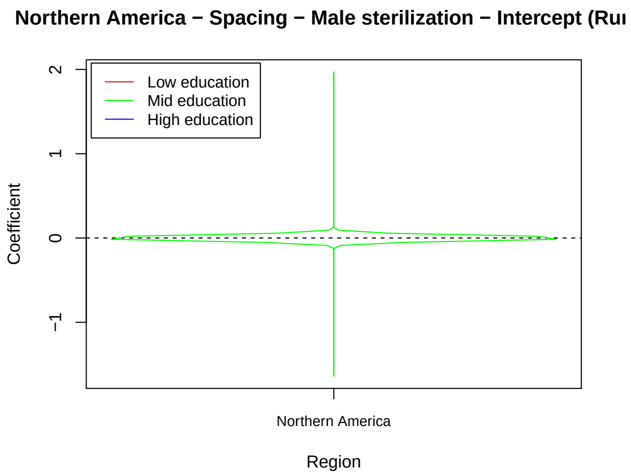
Northern America − Spacing − Male sterilization − Intercept (Ru
Low education
Mid education
High education
Coefficient
2
1
0
−1
Northern America
Region

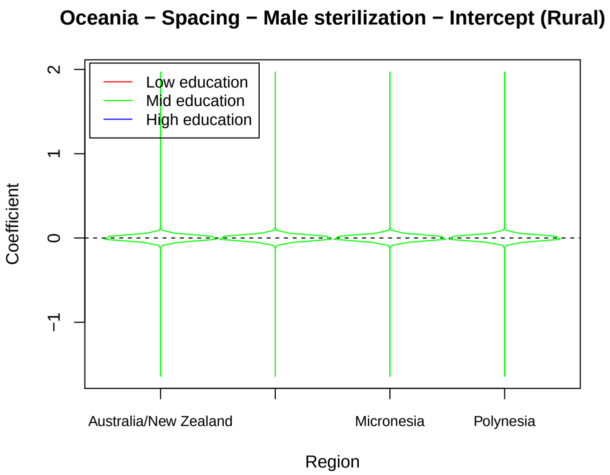
Oceania − Spacing − Male sterilization − Intercept (Rural)
Low education
Mid education
High education
Coefficient
2
1
0
−1
Australia/New Zealand
Micronesia
Polynesia
Region

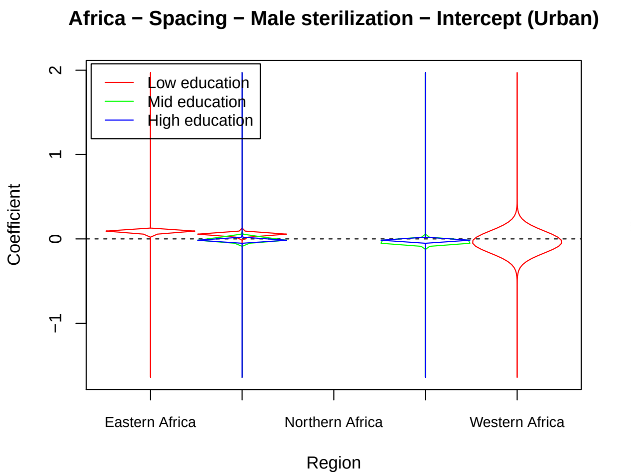
Africa − Spacing − Male sterilization − Intercept (Urban)
Low education
Mid education
High education
Coefficient
2
1
0
−1
Eastern Africa
Northern Africa
Western Africa
Region

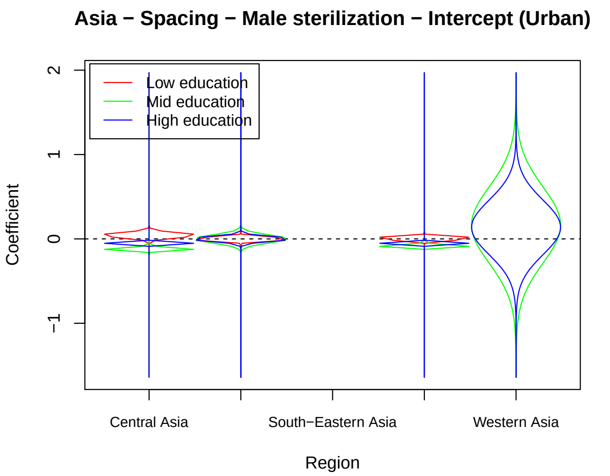
Asia − Spacing − Male sterilization − Intercept (Urban)
Low education
Mid education
High education
Coefficient
2
1
0
−1
Central Asia
South−Eastern Asia
Western Asia
Region

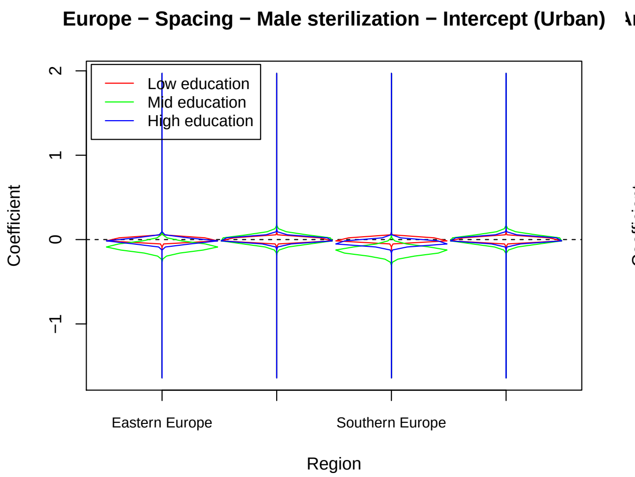
Europe − Spacing − Male sterilization − Intercept (Urban)
Low education
Mid education
High education
Coefficient
2
1
0
−1
Eastern Europe
Southern Europe
Region

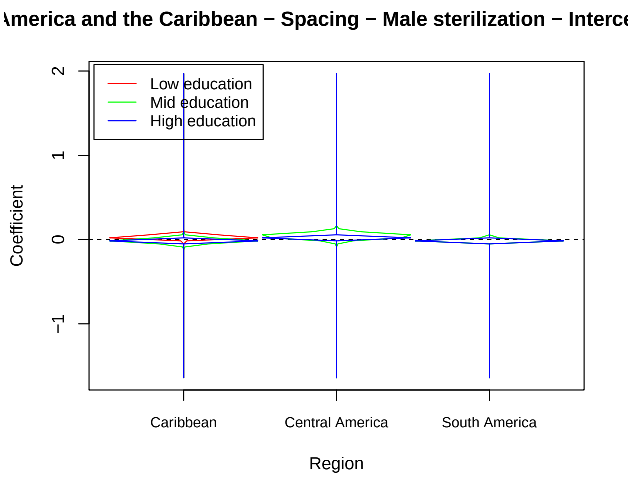
America and the Caribbean − Spacing − Male sterilization − Interce
Low education
Mid education
High education
Coefficient
2
1
0
−1
Caribbean
Central America
South America
Region

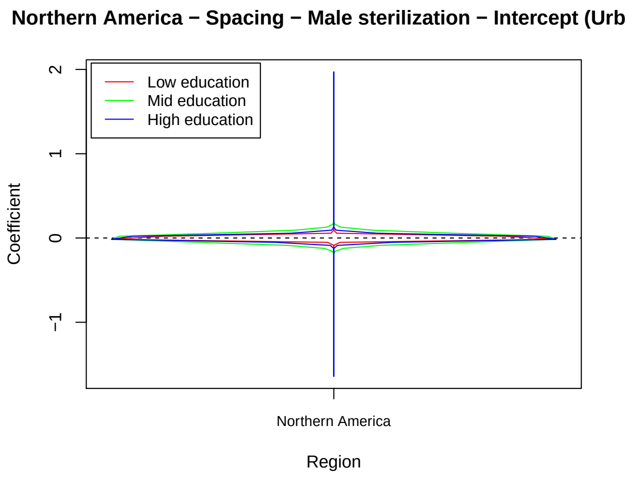
Northern America − Spacing − Male sterilization − Intercept (Urb
Low education
Mid education
High education
Coefficient
Northern America
Region

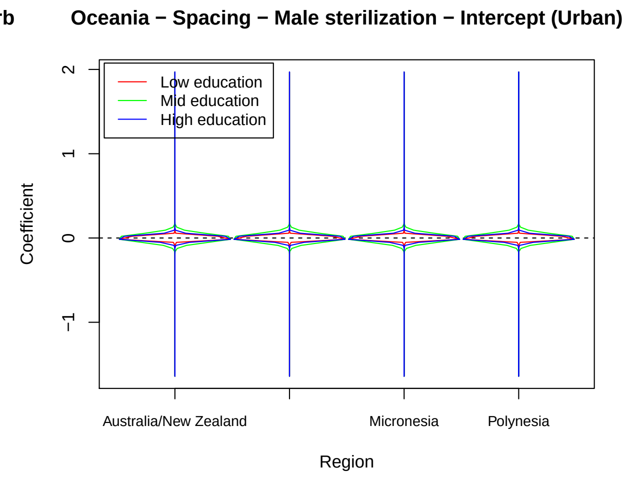
Oceania − Spacing − Male sterilization − Intercept (Urban)
Low education
Mid education
High education
Coefficient
Australia/New Zealand
Micronesia
Polynesia
Region

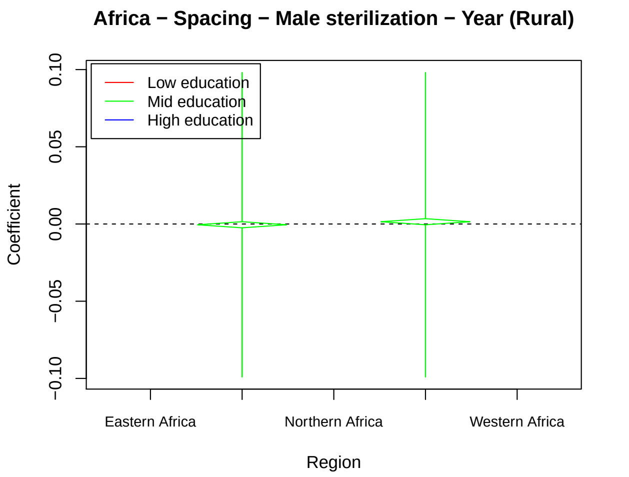
Africa − Spacing − Male sterilization − Year (Rural)
Low education
Mid education
High education
Coefficient
Eastern Africa
Northern Africa
Western Africa
Region

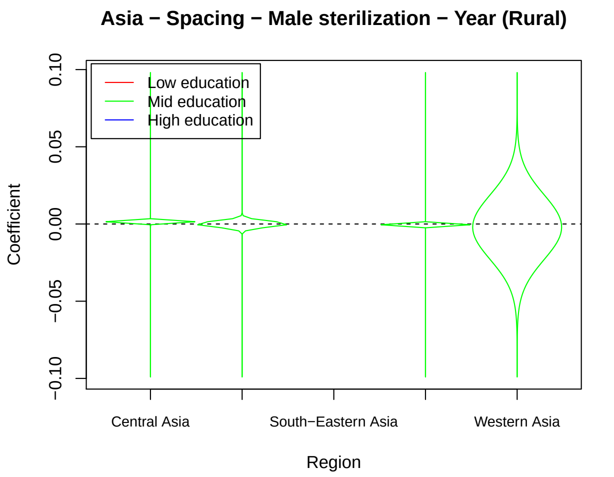
Asia − Spacing − Male sterilization − Year (Rural)
Low education
Mid education
High education
Coefficient
Central Asia
South−Eastern Asia
Western Asia
Region

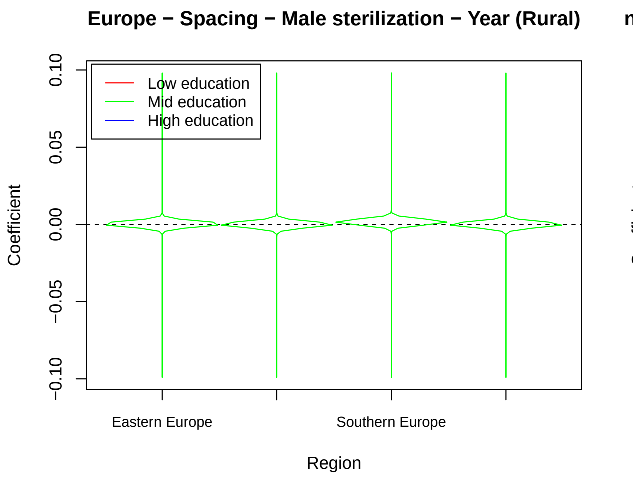
Europe − Spacing − Male sterilization − Year (Rural)
Low education
Mid education
High education
Coefficient
Eastern Europe
Southern Europe
Region

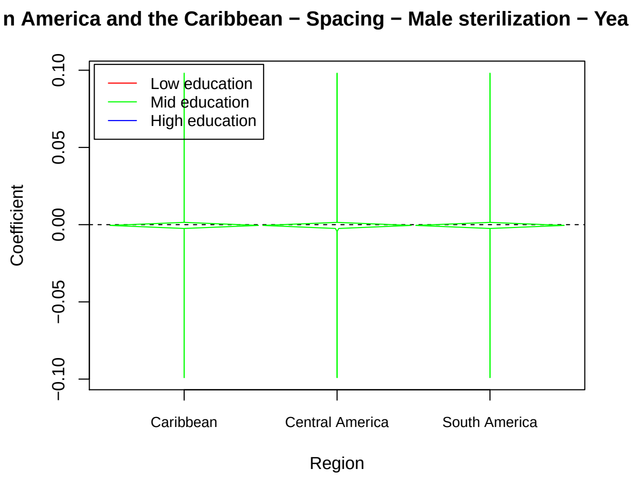
n America and the Caribbean − Spacing − Male sterilization − Yea
Low education
Mid education
High education
Coefficient
Caribbean
Central America
South America
Region

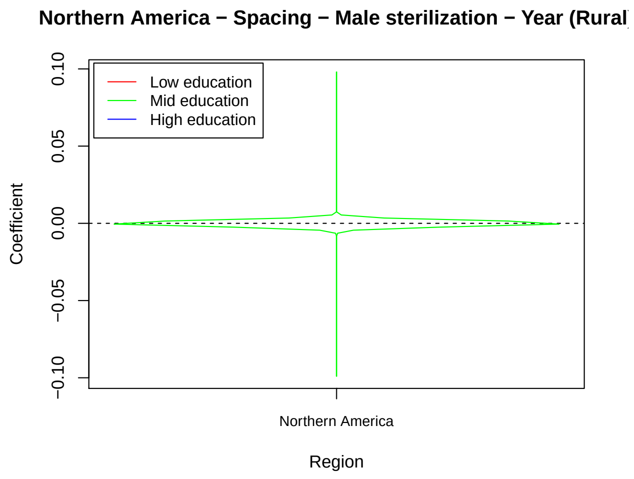
Northern America − Spacing − Male sterilization − Year (Rural
Low education
Mid education
High education
Coefficient
0.10
0.05
0.00
−0.05
−0.10
Northern America
Region

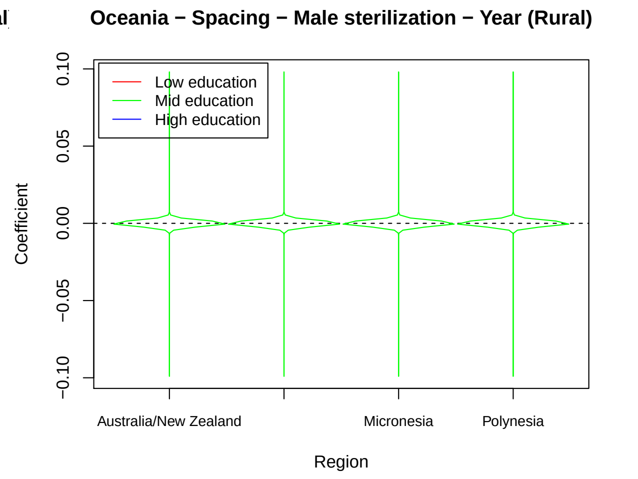
Oceania − Spacing − Male sterilization − Year (Rural)
Low education
Mid education
High education
Coefficient
0.10
0.05
0.00
−0.05
−0.10
Australia/New Zealand
Micronesia
Polynesia
Region

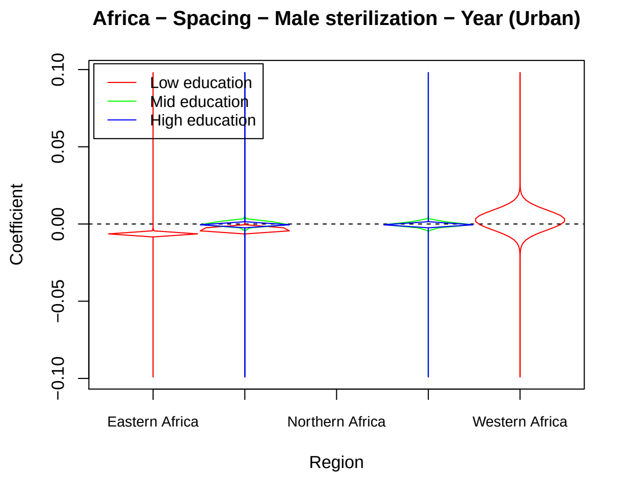
Africa − Spacing − Male sterilization − Year (Urban)
Low education
Mid education
High education
Coefficient
0.10
0.05
0.00
−0.05
−0.10
Eastern Africa
Northern Africa
Western Africa
Region

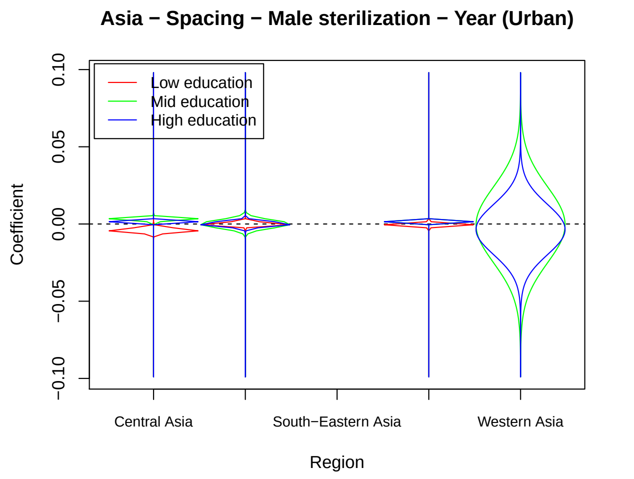
Asia − Spacing − Male sterilization − Year (Urban)
Low education
Mid education
High education
Coefficient
0.10
0.05
0.00
−0.05
−0.10
Central Asia
South−Eastern Asia
Western Asia
Region

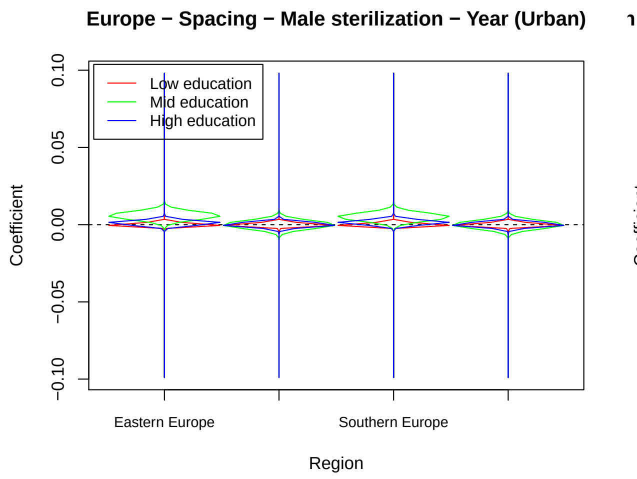
Europe − Spacing − Male sterilization − Year (Urban)
Low education
Mid education
High education
Coefficient
0.10
0.05
0.00
−0.05
−0.10
Eastern Europe
Southern Europe
Region

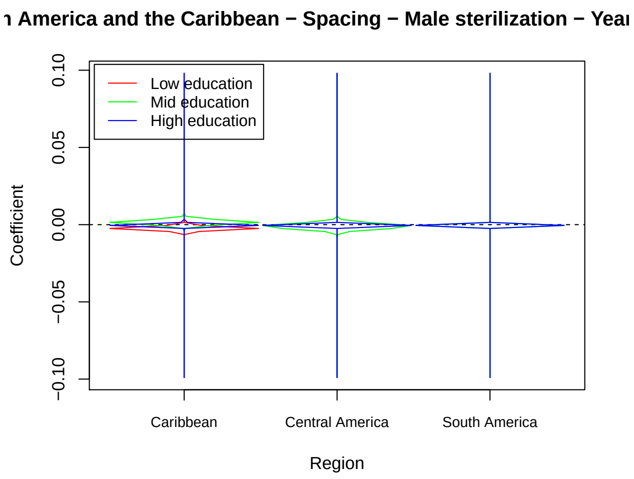
n America and the Caribbean − Spacing − Male sterilization − Yea
Low education
Mid education
High education
Coefficient
0.10
0.05
0.00
−0.05
−0.10
Caribbean
Central America
South America
Region

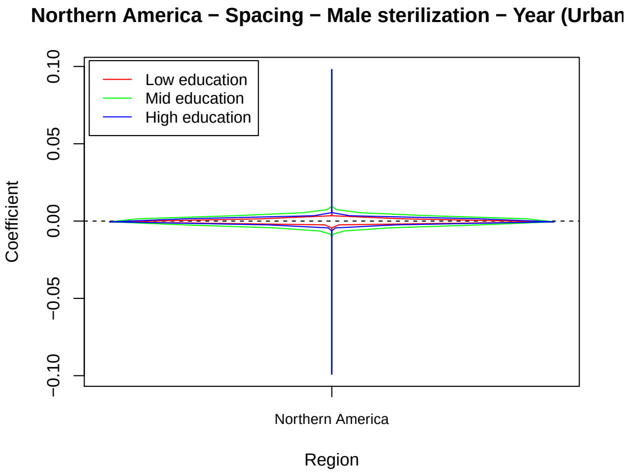
Northern America − Spacing − Male sterilization − Year (Urban
Low education
Mid education
High education
Coefficient
0.10
0.05
0.00
−0.05
−0.10
Northern America
Region

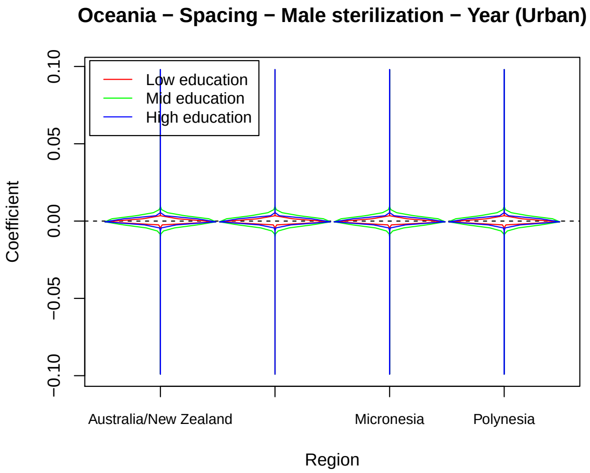
Oceania − Spacing − Male sterilization − Year (Urban)
Low education
Mid education
High education
Coefficient
0.10
0.05
0.00
−0.05
−0.10
Australia/New Zealand
Micronesia
Polynesia
Region

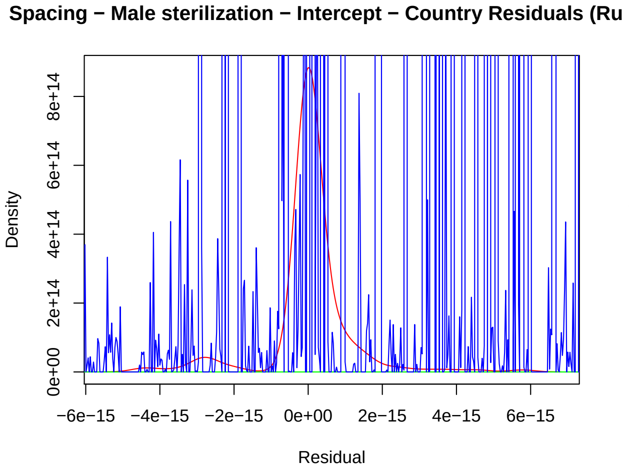
Spacing − Male sterilization − Intercept − Country Residuals (Ru
Density
0e+00
2e+14
4e+14
6e+14
8e+14
−6e−15
−4e−15
−2e−15
0e+00
2e−15
4e−15
6e−15
Residual

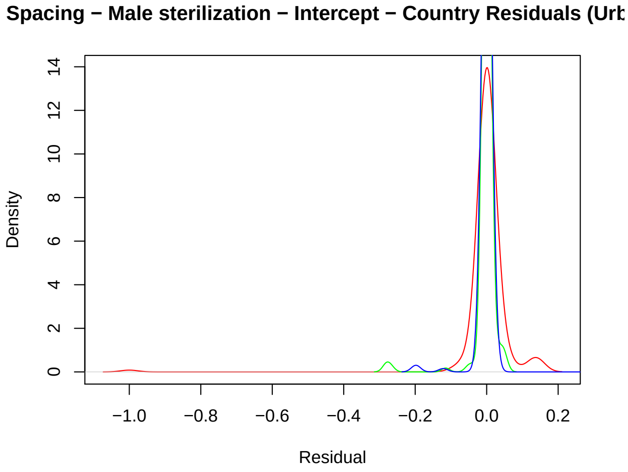
Spacing − Male sterilization − Intercept − Country Residuals (Urb
Density
0
2
4
6
8
10
12
14
−1.0
−0.8
−0.6
−0.4
−0.2
0.0
0.2
Residual

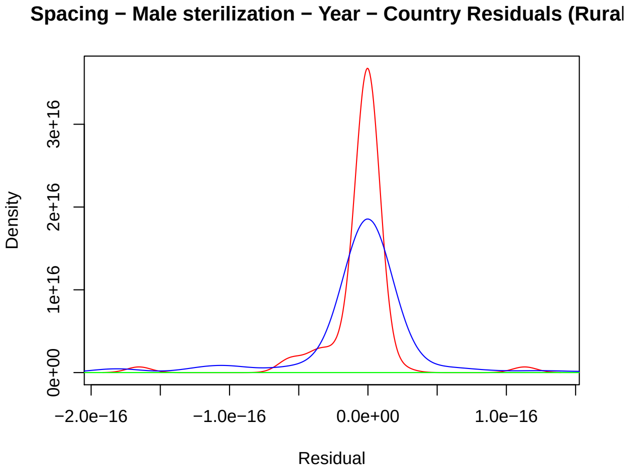
Spacing − Male sterilization − Year − Country Residuals (Rural
Density
0e+00
1e+16
2e+16
3e+16
−2.0e−16
−1.0e−16
0.0e+00
1.0e−16
Residual

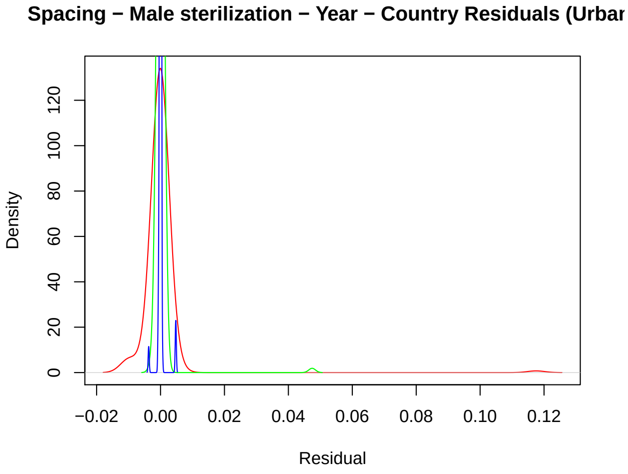
Spacing − Male sterilization − Year − Country Residuals (Urbar
Density
0
20
40
60
80
100
120
−0.02
0.00
0.02
0.04
0.06
0.08
0.10
0.12
Residual

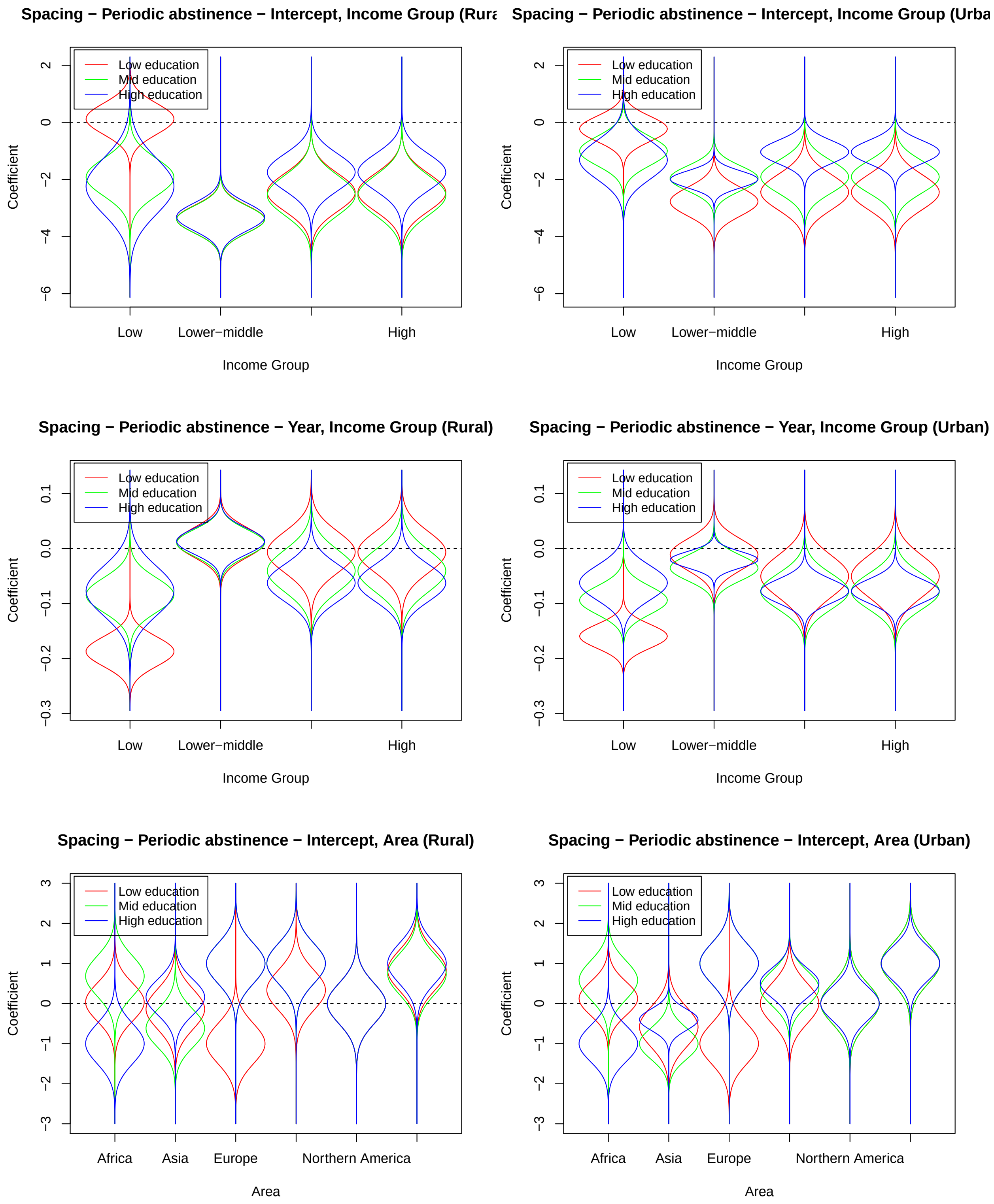
Spacing − Periodic abstinence − Intercept, Income Group (Rura
Spacing − Periodic abstinence − Intercept, Income Group (Urba
Spacing − Periodic abstinence − Year, Income Group (Rural)
Spacing − Periodic abstinence − Year, Income Group (Urban)
Spacing − Periodic abstinence − Intercept, Area (Rural)
Spacing − Periodic abstinence − Intercept, Area (Urban)
Low education
Mid education
High education
Coefficient
Income Group
Low
Lower−middle
High
Area
Africa
Asia
Europe
Northern America

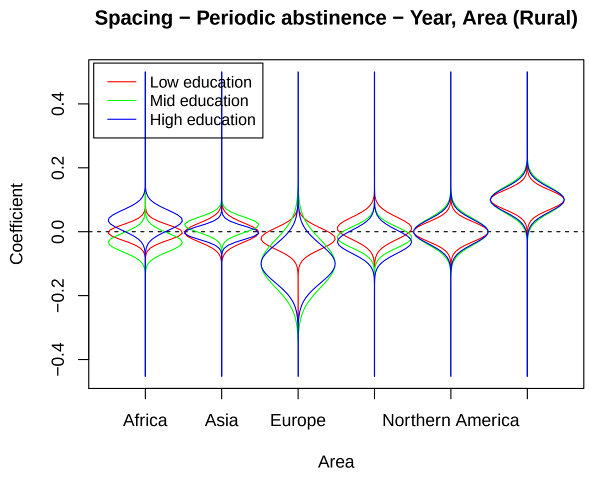
Spacing − Periodic abstinence − Year, Area (Rural)
Low education
Mid education
High education
Coefficient
0.4
0.2
0.0
−0.2
−0.4
Africa
Asia
Europe
Northern America
Area

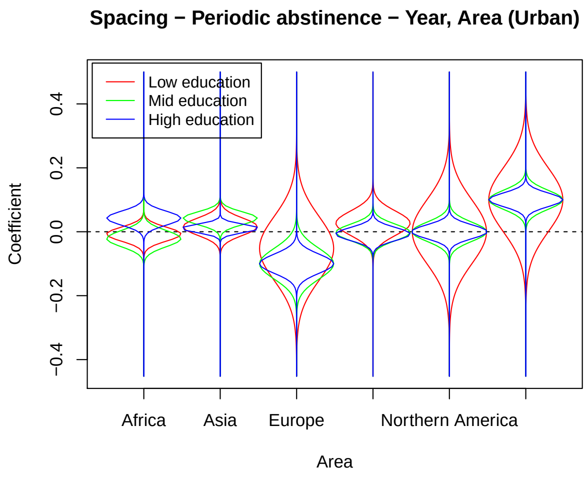
Spacing − Periodic abstinence − Year, Area (Urban)
Low education
Mid education
High education
Coefficient
0.4
0.2
0.0
−0.2
−0.4
Africa
Asia
Europe
Northern America
Area

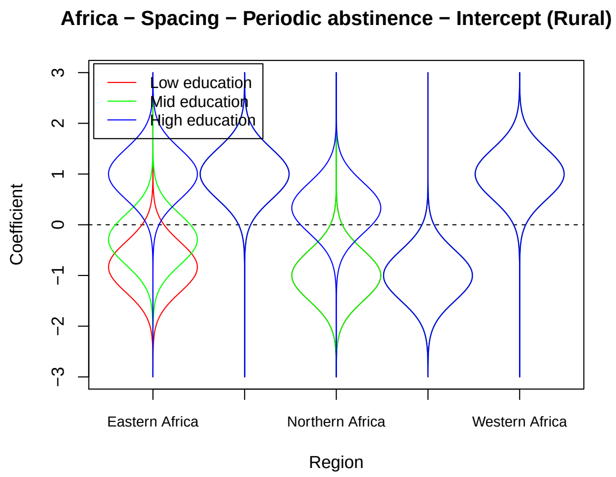
Africa − Spacing − Periodic abstinence − Intercept (Rural)
Low education
Mid education
High education
Coefficient
3
2
1
0
−1
−2
−3
Eastern Africa
Northern Africa
Western Africa
Region

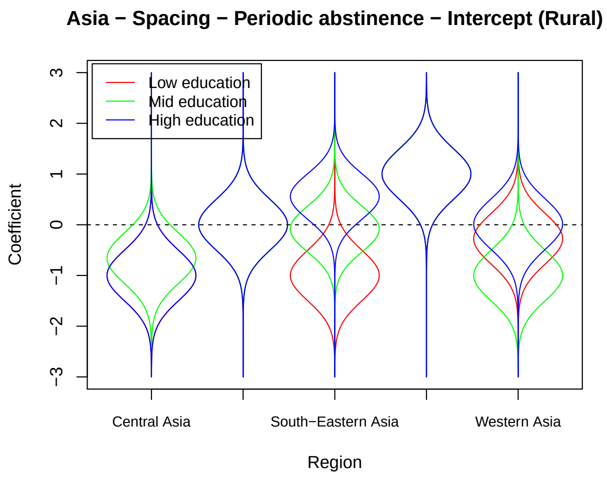
Asia − Spacing − Periodic abstinence − Intercept (Rural)
Low education
Mid education
High education
Coefficient
3
2
1
0
−1
−2
−3
Central Asia
South−Eastern Asia
Western Asia
Region

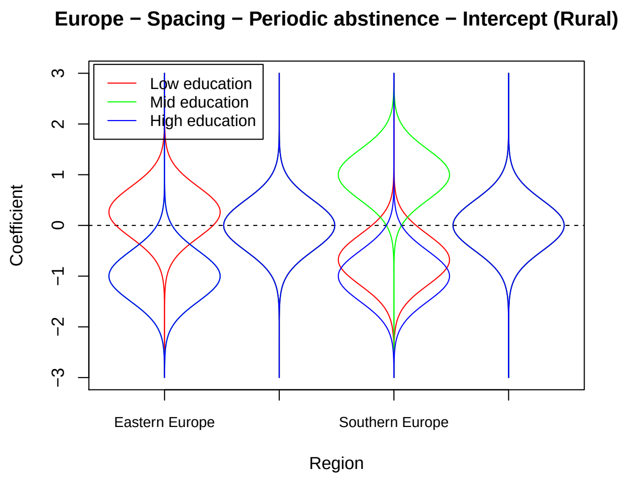
Europe − Spacing − Periodic abstinence − Intercept (Rural)
Low education
Mid education
High education
Coefficient
3
2
1
0
−1
−2
−3
Eastern Europe
Southern Europe
Region

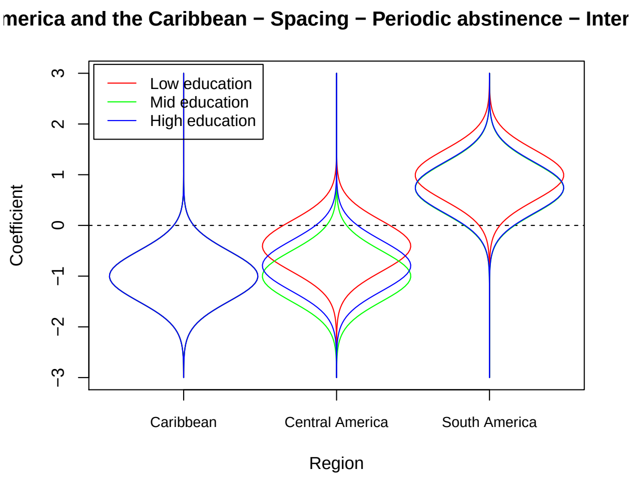
merica and the Caribbean − Spacing − Periodic abstinence − Inter
Low education
Mid education
High education
Coefficient
3
2
1
0
−1
−2
−3
Caribbean
Central America
South America
Region

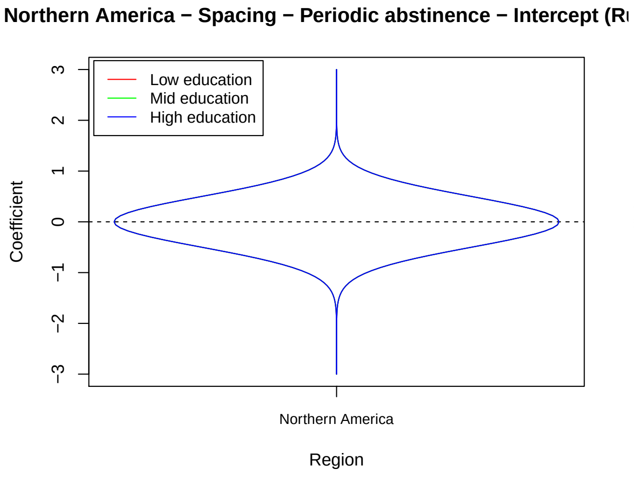
Northern America − Spacing − Periodic abstinence − Intercept (Ru
Low education
Mid education
High education
Coefficient
Northern America
Region

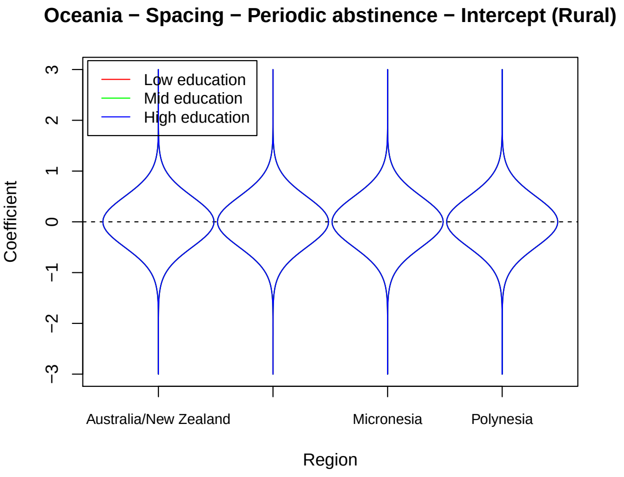
Oceania − Spacing − Periodic abstinence − Intercept (Rural)
Low education
Mid education
High education
Coefficient
Australia/New Zealand
Micronesia
Polynesia
Region

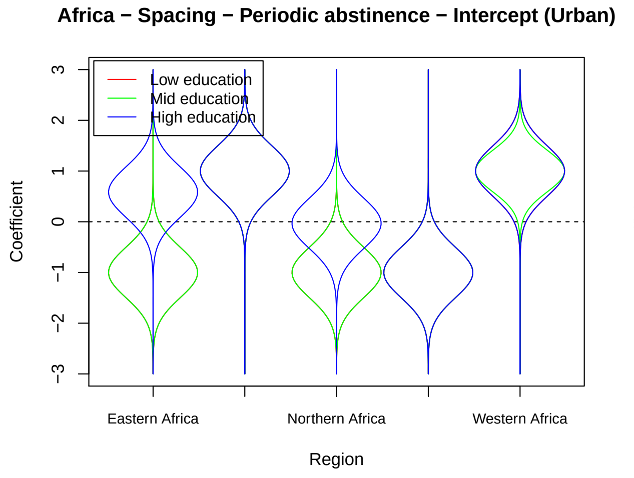
Africa − Spacing − Periodic abstinence − Intercept (Urban)
Low education
Mid education
High education
Coefficient
Eastern Africa
Northern Africa
Western Africa
Region

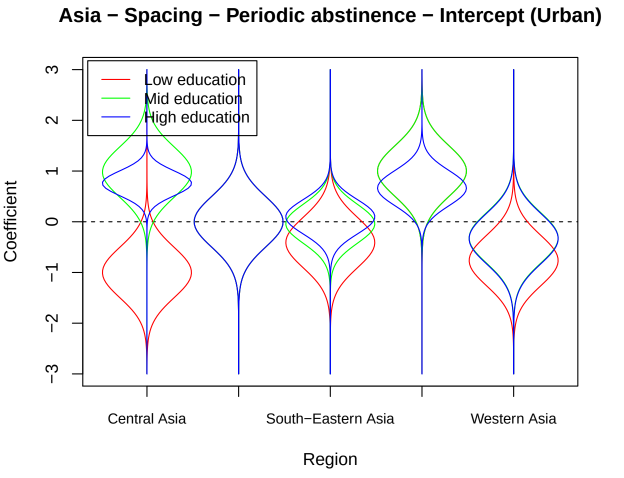
Asia − Spacing − Periodic abstinence − Intercept (Urban)
Low education
Mid education
High education
Coefficient
Central Asia
South−Eastern Asia
Western Asia
Region

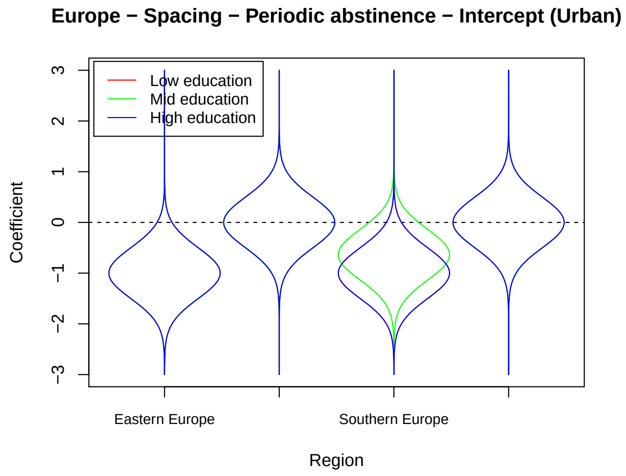
Europe − Spacing − Periodic abstinence − Intercept (Urban)
Low education
Mid education
High education
Coefficient
Eastern Europe
Southern Europe
Region

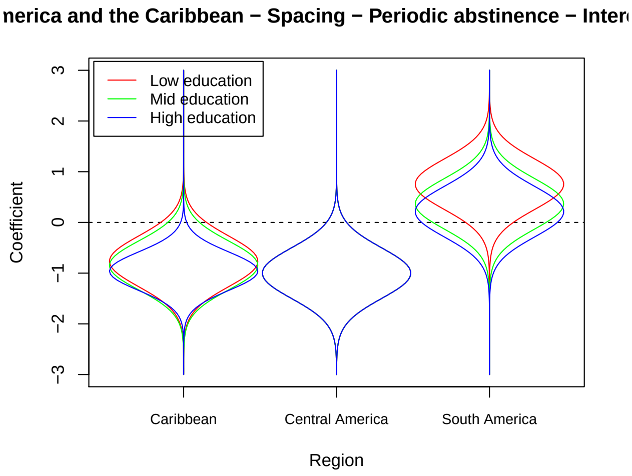
nerica and the Caribbean − Spacing − Periodic abstinence − Inter
Low education
Mid education
High education
Coefficient
Caribbean
Central America
South America
Region

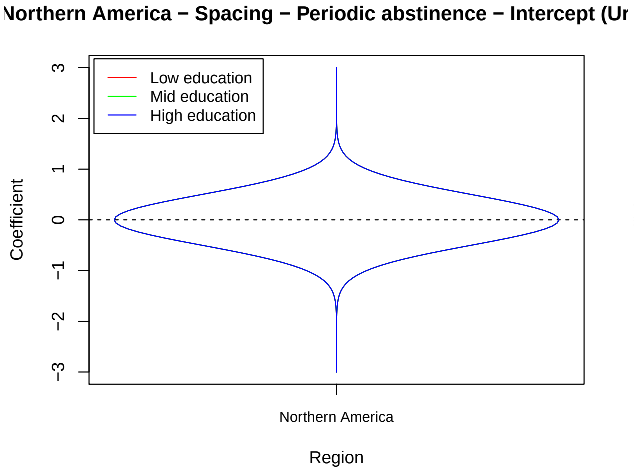
Northern America − Spacing − Periodic abstinence − Intercept (Ur
Low education
Mid education
High education
Coefficient
Northern America
Region

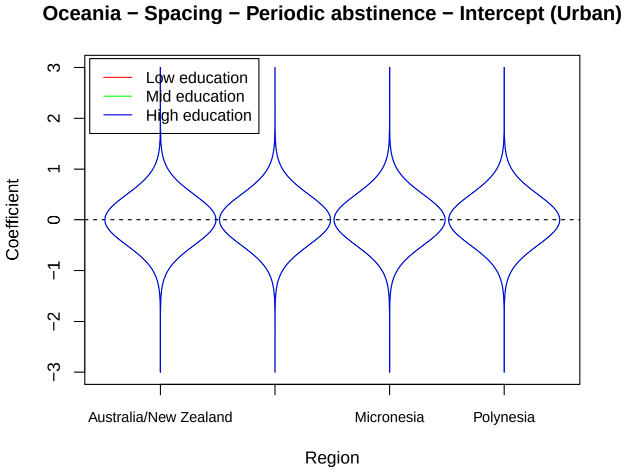
Oceania − Spacing − Periodic abstinence − Intercept (Urban)
Low education
Mid education
High education
Coefficient
Australia/New Zealand
Micronesia
Polynesia
Region

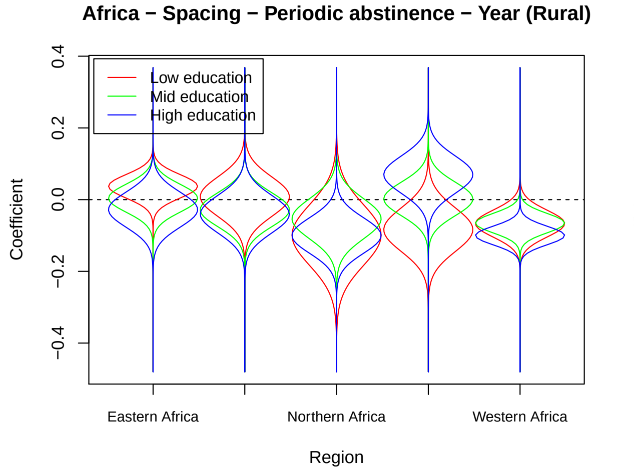
Africa − Spacing − Periodic abstinence − Year (Rural)
Low education
Mid education
High education
Coefficient
Eastern Africa
Northern Africa
Western Africa
Region

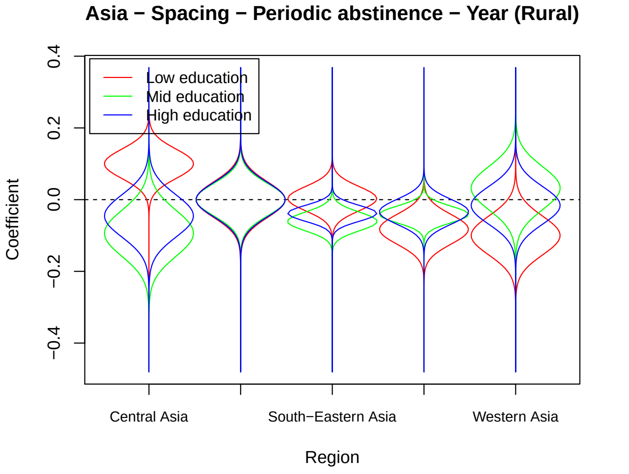
Asia − Spacing − Periodic abstinence − Year (Rural)
Low education
Mid education
High education
Coefficient
Central Asia
South−Eastern Asia
Western Asia
Region

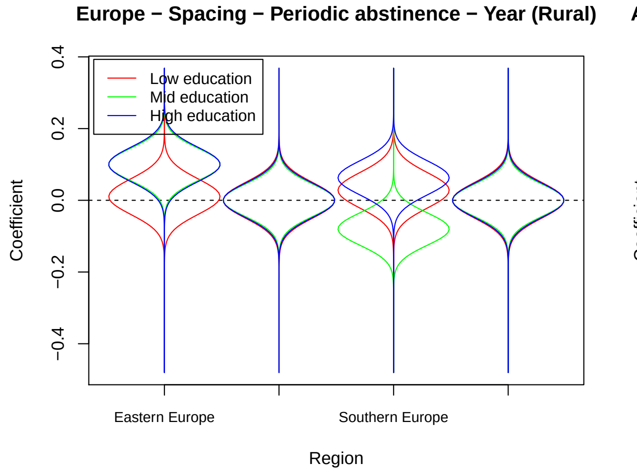
Europe − Spacing − Periodic abstinence − Year (Rural)
Low education
Mid education
High education
Coefficient
Eastern Europe
Southern Europe
Region

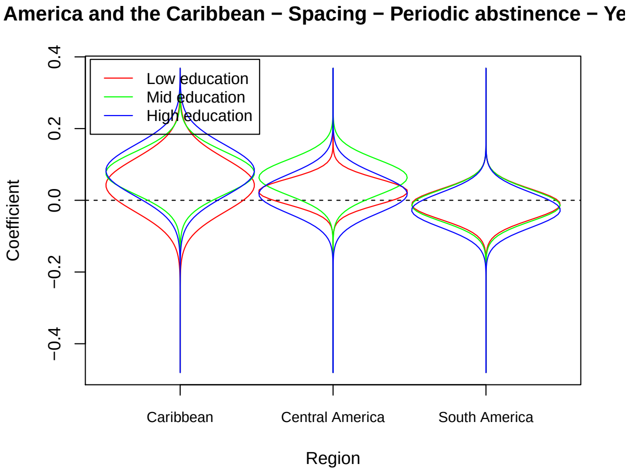
America and the Caribbean − Spacing − Periodic abstinence − Ye
Low education
Mid education
High education
Coefficient
Caribbean
Central America
South America
Region

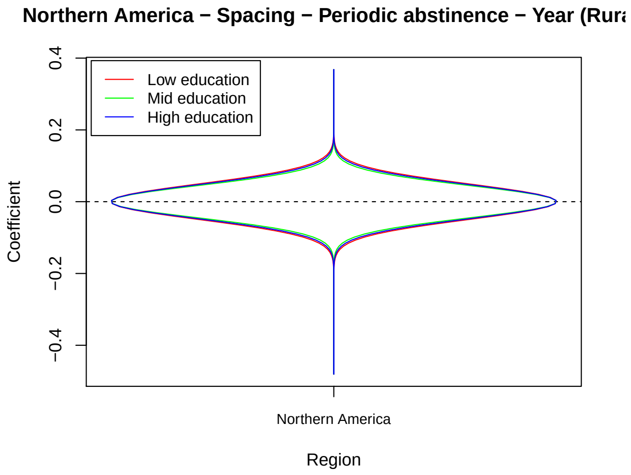

Northern America − Spacing − Periodic abstinence − Year (Rura
Low education
Mid education
High education
Coefficient
0.4
0.2
0.0
−0.2
−0.4
Northern America
Region

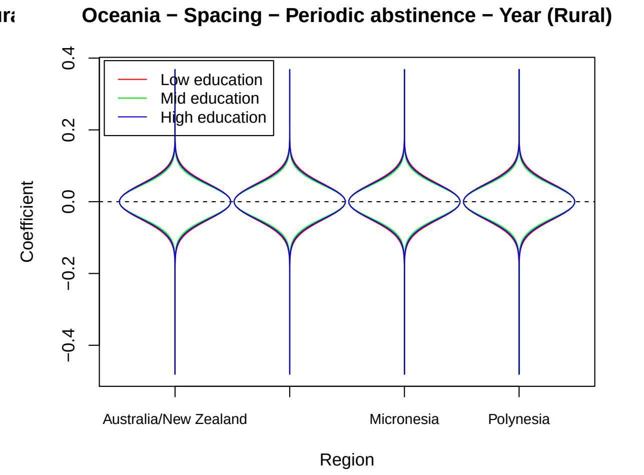

Oceania − Spacing − Periodic abstinence − Year (Rural)
Low education
Mid education
High education
Coefficient
0.4
0.2
0.0
−0.2
−0.4
Australia/New Zealand
Micronesia
Polynesia
Region

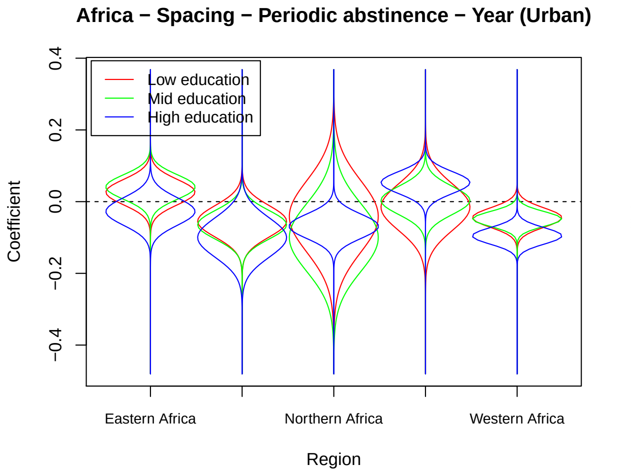

Africa − Spacing − Periodic abstinence − Year (Urban)
Low education
Mid education
High education
Coefficient
0.4
0.2
0.0
−0.2
−0.4
Eastern Africa
Northern Africa
Western Africa
Region

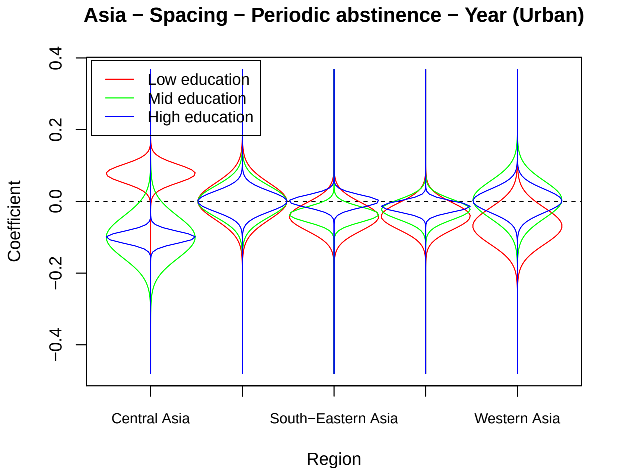

Asia − Spacing − Periodic abstinence − Year (Urban)
Low education
Mid education
High education
Coefficient
0.4
0.2
0.0
−0.2
−0.4
Central Asia
South−Eastern Asia
Western Asia
Region

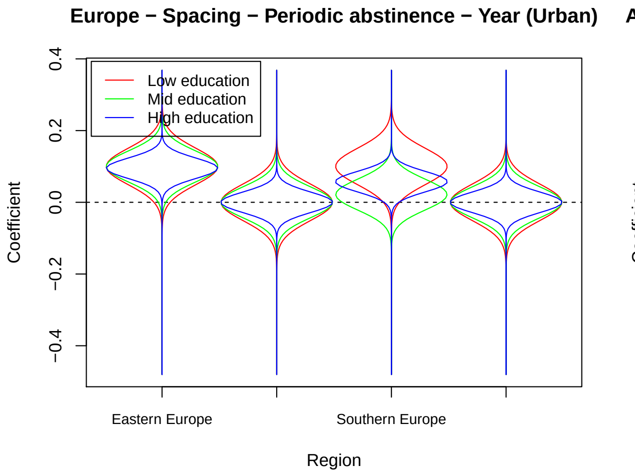

Europe − Spacing − Periodic abstinence − Year (Urban)
Low education
Mid education
High education
Coefficient
0.4
0.2
0.0
−0.2
−0.4
Eastern Europe
Southern Europe
Region

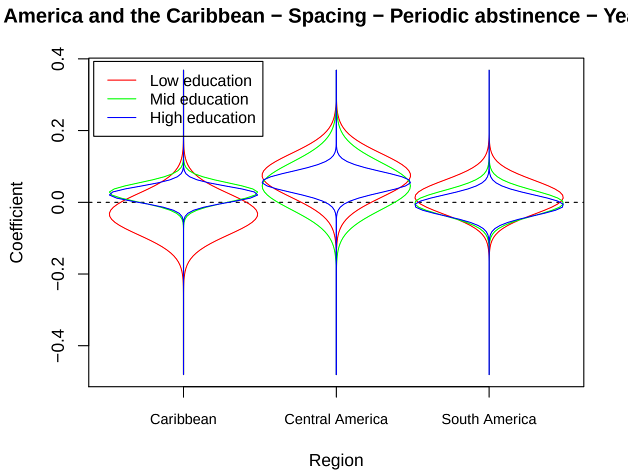

America and the Caribbean − Spacing − Periodic abstinence − Ye
Low education
Mid education
High education
Coefficient
0.4
0.2
0.0
−0.2
−0.4
Caribbean
Central America
South America
Region

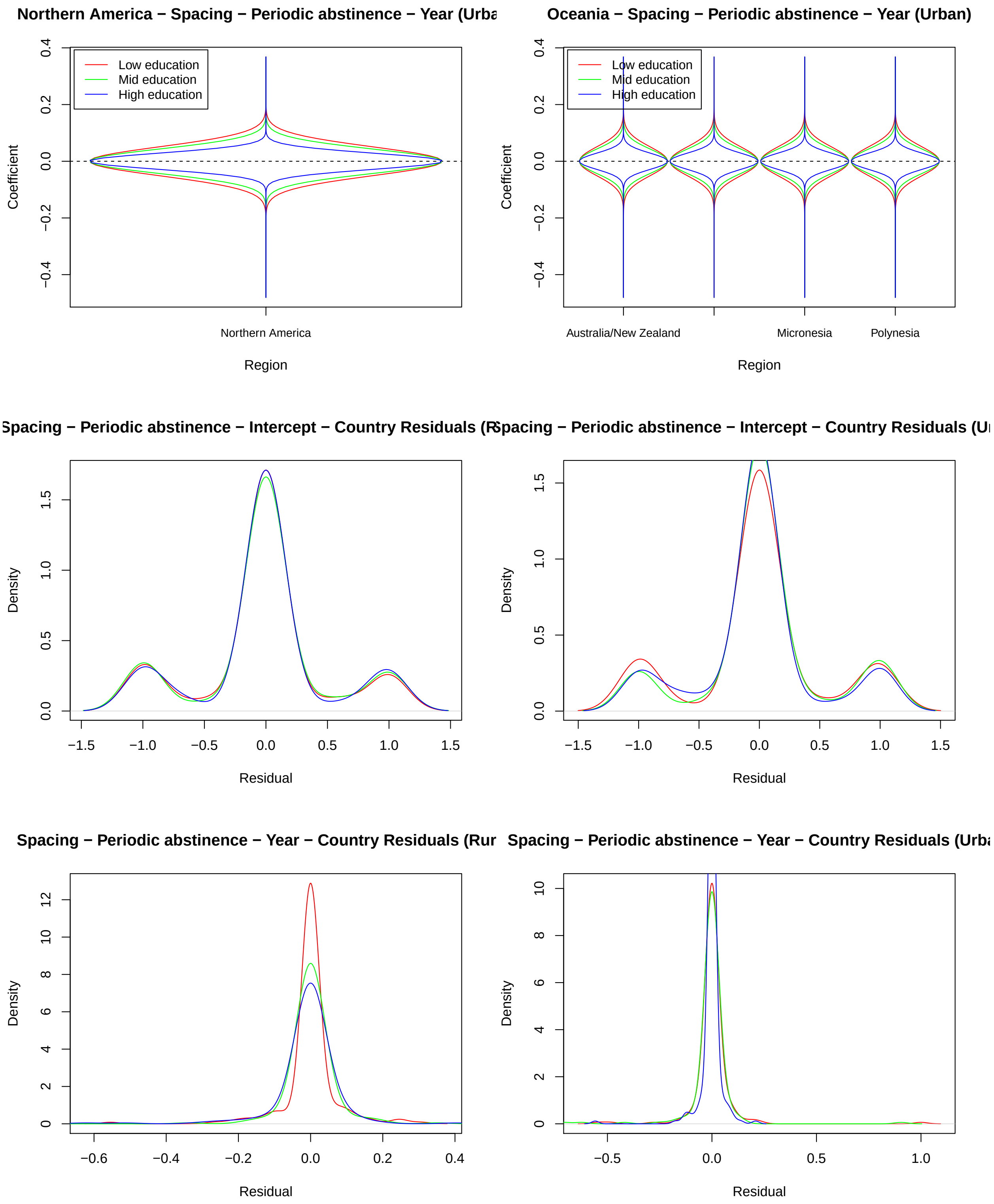
Northern America − Spacing − Periodic abstinence − Year (Urba
Oceania − Spacing − Periodic abstinence − Year (Urban)
Low education
Mid education
High education
Coefficient
Northern America
Australia/New Zealand
Micronesia
Polynesia
Region
Spacing − Periodic abstinence − Intercept − Country Residuals (R
Spacing − Periodic abstinence − Intercept − Country Residuals (U
Density
Residual
Spacing − Periodic abstinence − Year − Country Residuals (Rur
Spacing − Periodic abstinence − Year − Country Residuals (Urba

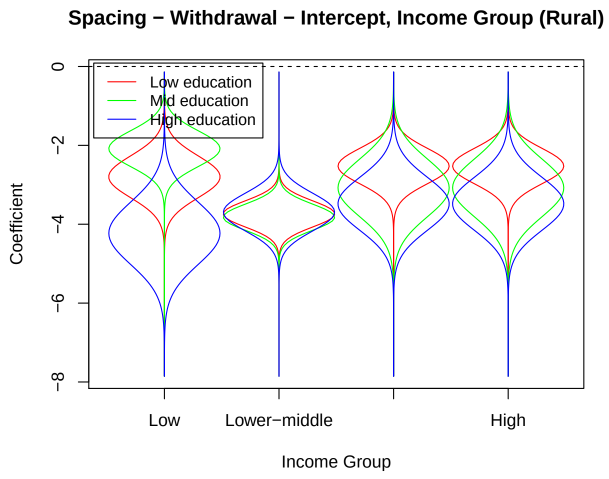
Spacing − Withdrawal − Intercept, Income Group (Rural)
Low education
Mid education
High education
Coefficient
0
−2
−4
−6
−8
Low
Lower−middle
High
Income Group

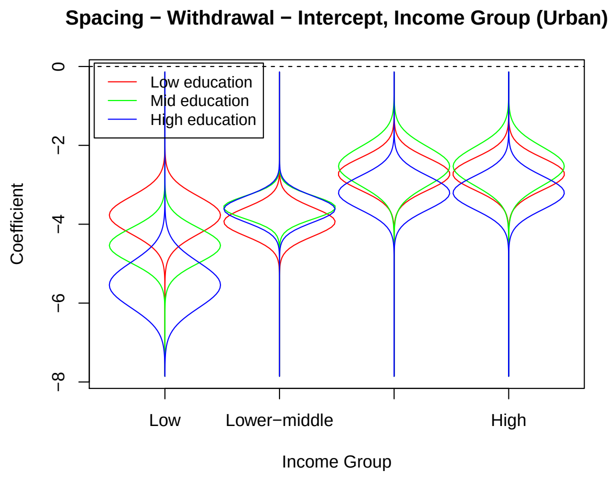
Spacing − Withdrawal − Intercept, Income Group (Urban)
Low education
Mid education
High education
Coefficient
0
−2
−4
−6
−8
Low
Lower−middle
High
Income Group

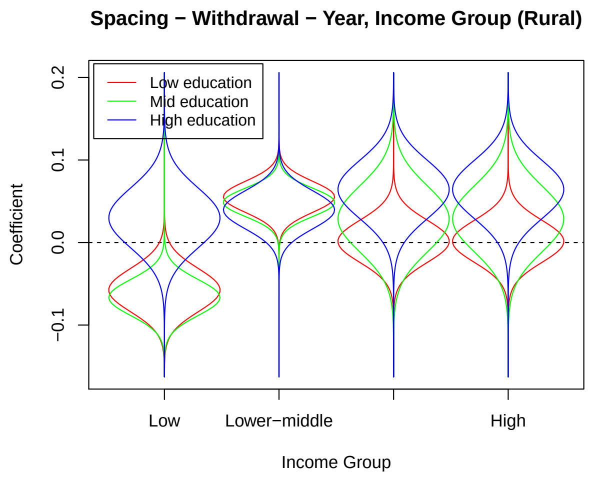
Spacing − Withdrawal − Year, Income Group (Rural)
Low education
Mid education
High education
Coefficient
0.2
0.1
0.0
−0.1
Low
Lower−middle
High
Income Group

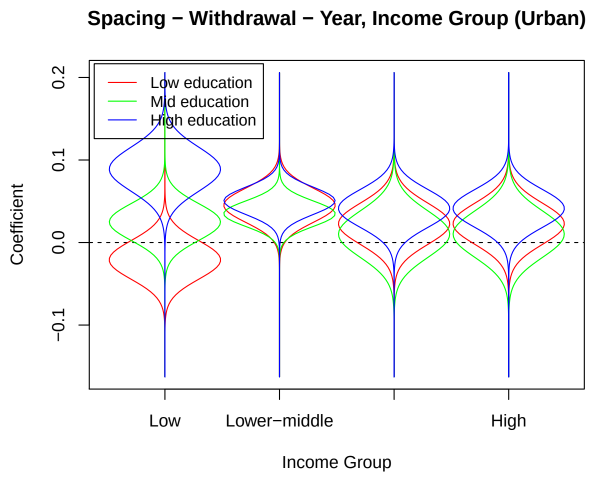
Spacing − Withdrawal − Year, Income Group (Urban)
Low education
Mid education
High education
Coefficient
0.2
0.1
0.0
−0.1
Low
Lower−middle
High
Income Group

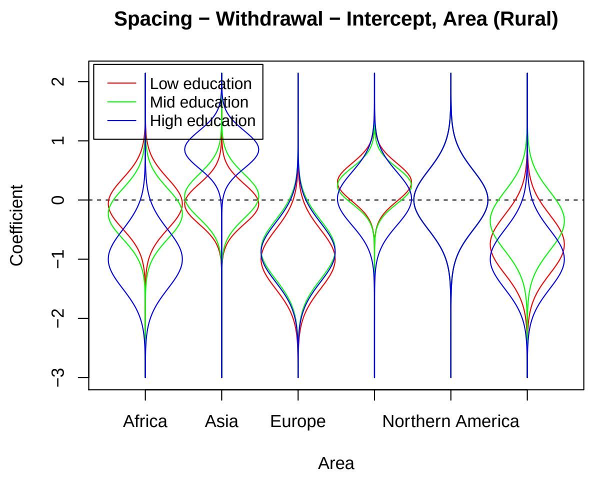
Spacing − Withdrawal − Intercept, Area (Rural)
Low education
Mid education
High education
Coefficient
2
1
0
−1
−2
−3
Africa
Asia
Europe
Northern America
Area

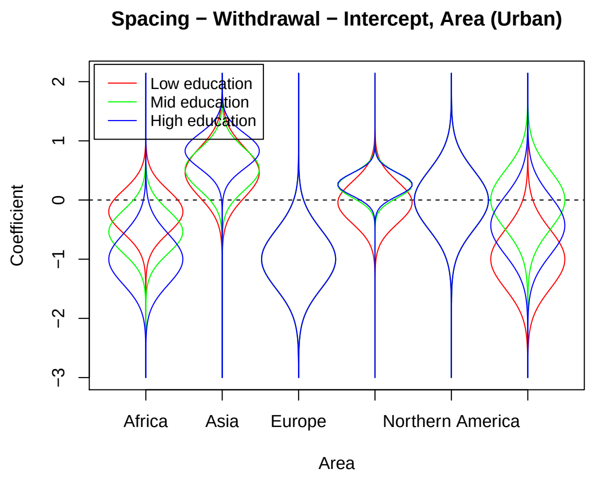
Spacing − Withdrawal − Intercept, Area (Urban)
Low education
Mid education
High education
Coefficient
2
1
0
−1
−2
−3
Africa
Asia
Europe
Northern America
Area

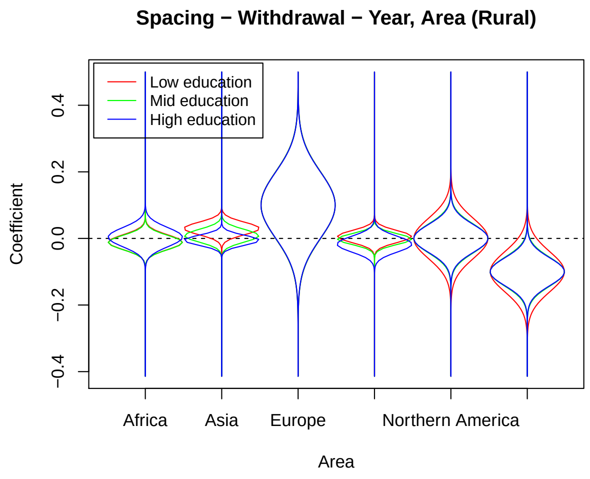
Spacing − Withdrawal − Year, Area (Rural)
Low education
Mid education
High education
Coefficient
0.4
0.2
0.0
−0.2
−0.4
Africa
Asia
Europe
Northern America
Area

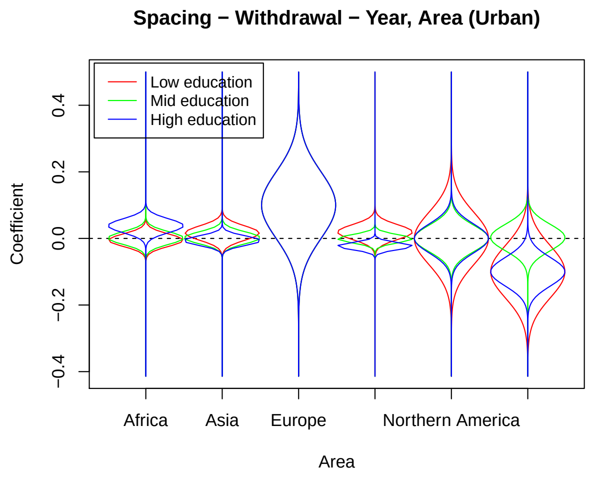
Spacing − Withdrawal − Year, Area (Urban)
Low education
Mid education
High education
Coefficient
0.4
0.2
0.0
−0.2
−0.4
Africa
Asia
Europe
Northern America
Area

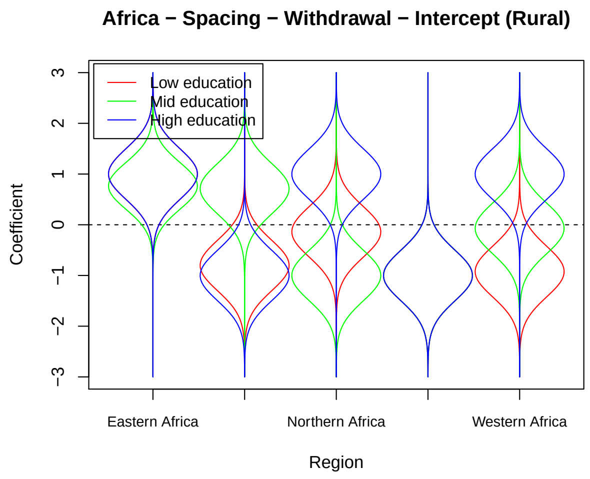
Africa − Spacing − Withdrawal − Intercept (Rural)
Low education
Mid education
High education
Coefficient
3
2
1
0
−1
−2
−3
Eastern Africa
Northern Africa
Western Africa
Region

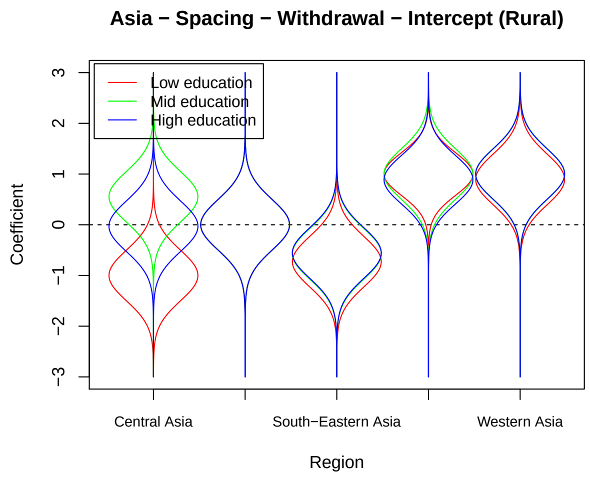
Asia − Spacing − Withdrawal − Intercept (Rural)
Low education
Mid education
High education
Coefficient
3
2
1
0
−1
−2
−3
Central Asia
South−Eastern Asia
Western Asia
Region

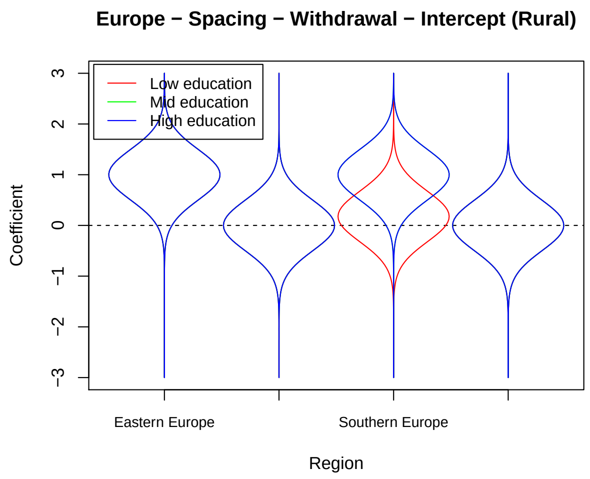
Europe − Spacing − Withdrawal − Intercept (Rural)
Low education
Mid education
High education
Coefficient
3
2
1
0
−1
−2
−3
Eastern Europe
Southern Europe
Region

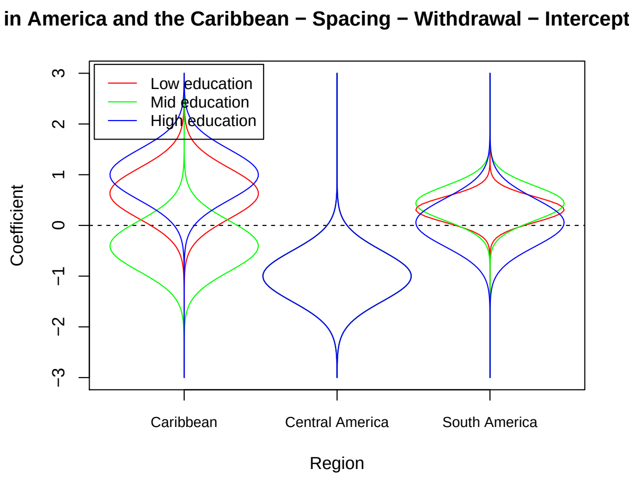
in America and the Caribbean − Spacing − Withdrawal − Intercept
Low education
Mid education
High education
Coefficient
3
2
1
0
−1
−2
−3
Caribbean
Central America
South America
Region

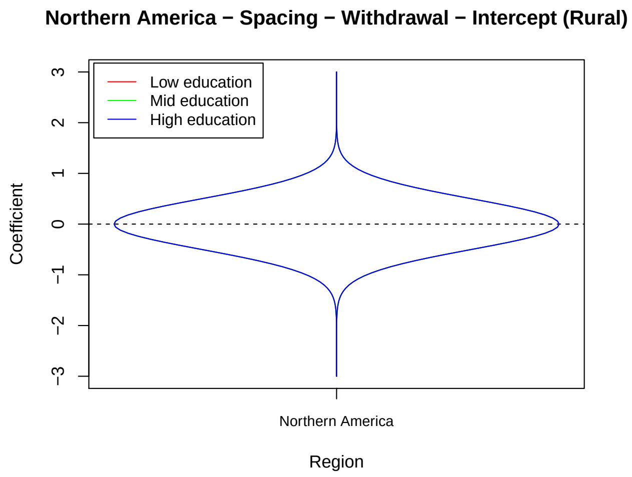
Northern America − Spacing − Withdrawal − Intercept (Rural)
Low education
Mid education
High education
Coefficient
Northern America
Region

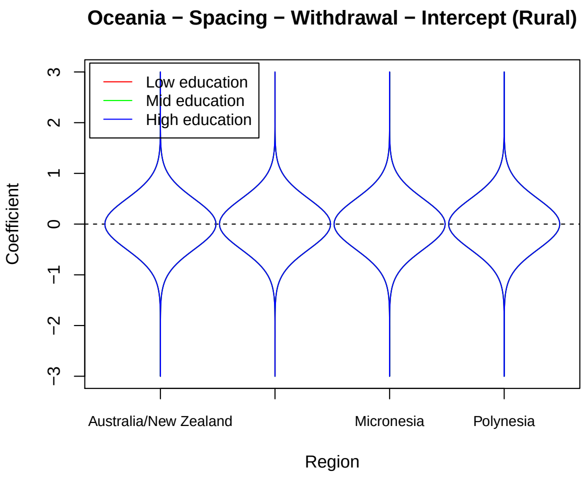
Oceania − Spacing − Withdrawal − Intercept (Rural)
Low education
Mid education
High education
Coefficient
Australia/New Zealand
Micronesia
Polynesia
Region

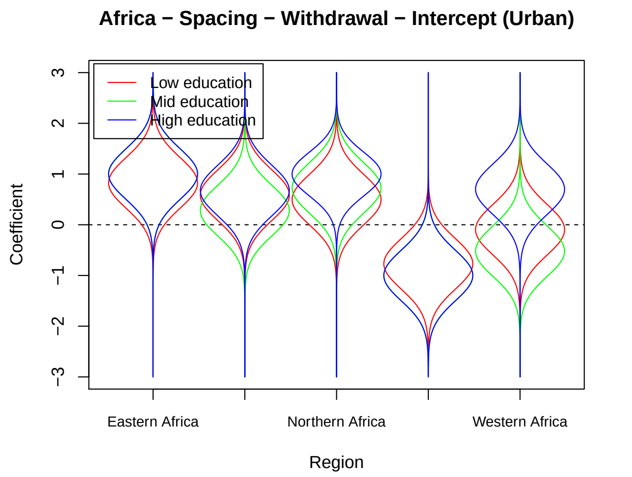
Africa − Spacing − Withdrawal − Intercept (Urban)
Low education
Mid education
High education
Coefficient
Eastern Africa
Northern Africa
Western Africa
Region

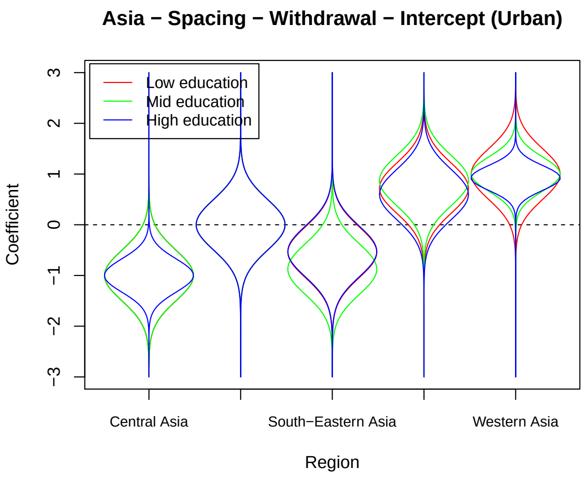
Asia − Spacing − Withdrawal − Intercept (Urban)
Low education
Mid education
High education
Coefficient
Central Asia
South−Eastern Asia
Western Asia
Region

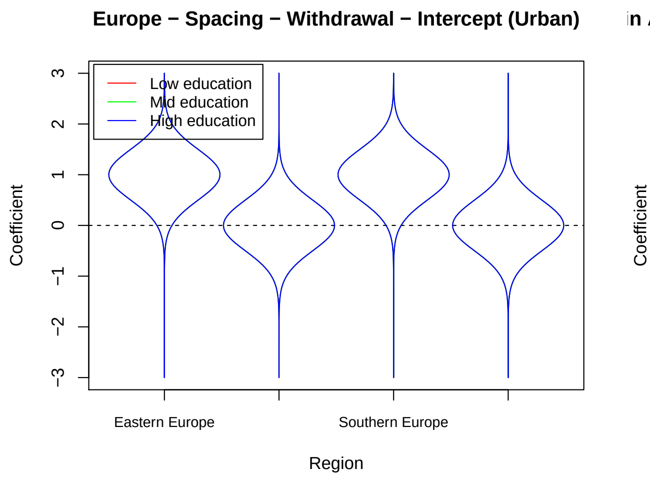
Europe − Spacing − Withdrawal − Intercept (Urban)
Low education
Mid education
High education
Coefficient
Eastern Europe
Southern Europe
Region

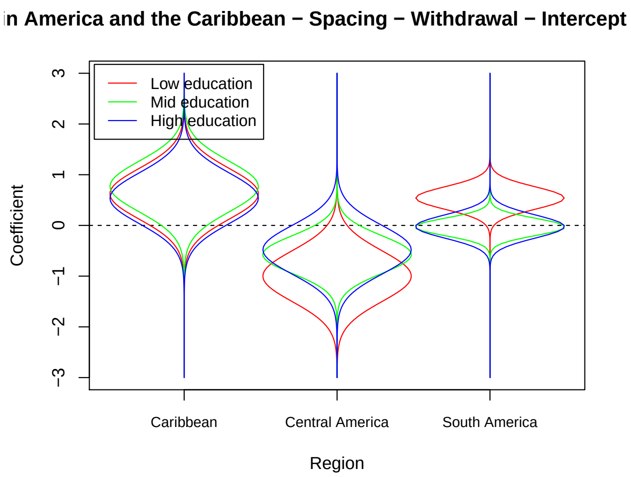
in America and the Caribbean − Spacing − Withdrawal − Intercept
Low education
Mid education
High education
Coefficient
Caribbean
Central America
South America
Region

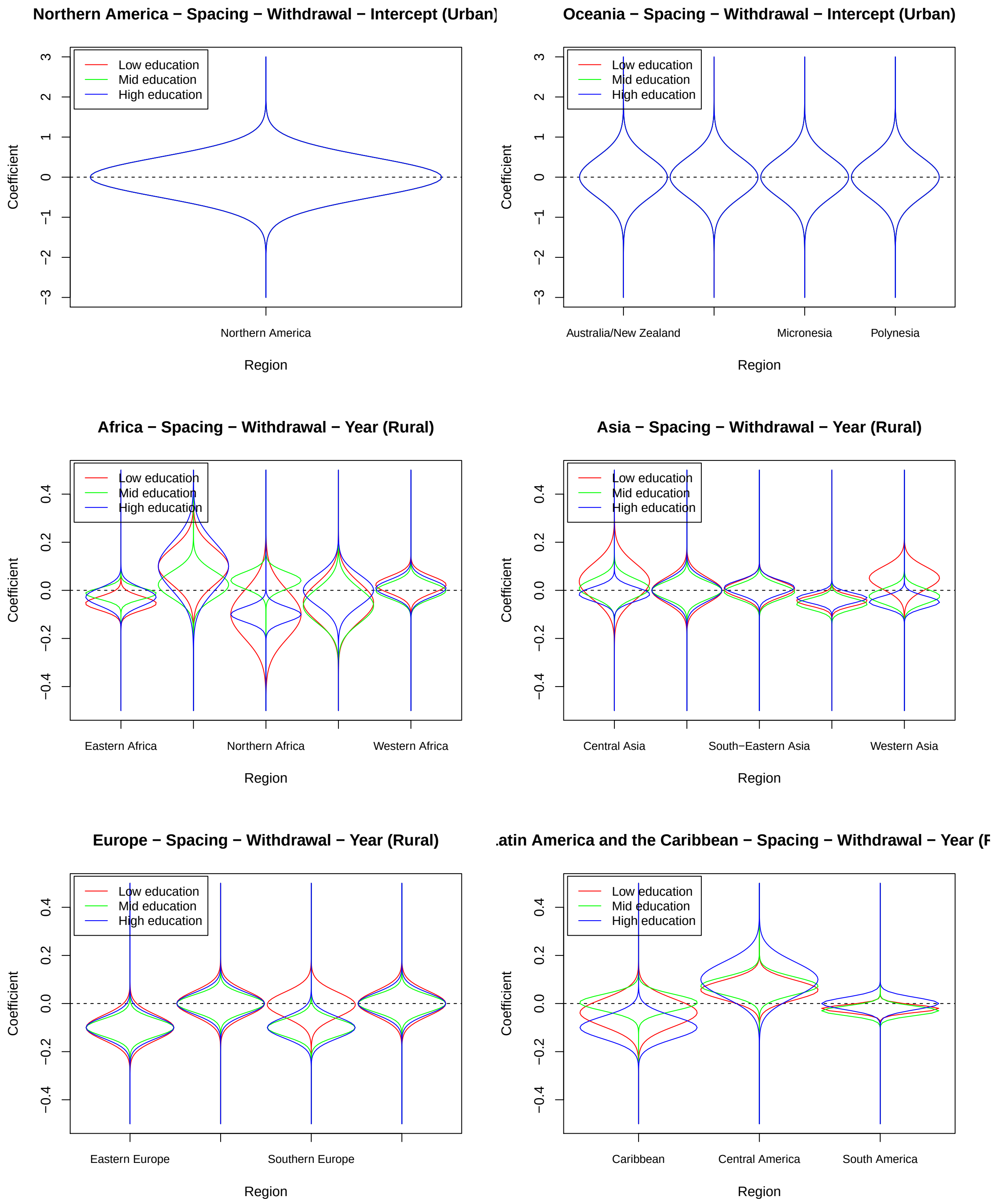
Northern America − Spacing − Withdrawal − Intercept (Urban)
Oceania − Spacing − Withdrawal − Intercept (Urban)
Africa − Spacing − Withdrawal − Year (Rural)
Asia − Spacing − Withdrawal − Year (Rural)
Europe − Spacing − Withdrawal − Year (Rural)
.atin America and the Caribbean − Spacing − Withdrawal − Year (F
Low education
Mid education
High education
Coefficient
Region
Northern America
Australia/New Zealand
Micronesia
Polynesia
Eastern Africa
Northern Africa
Western Africa
Central Asia
South−Eastern Asia
Western Asia
Eastern Europe
Southern Europe
Caribbean
Central America
South America

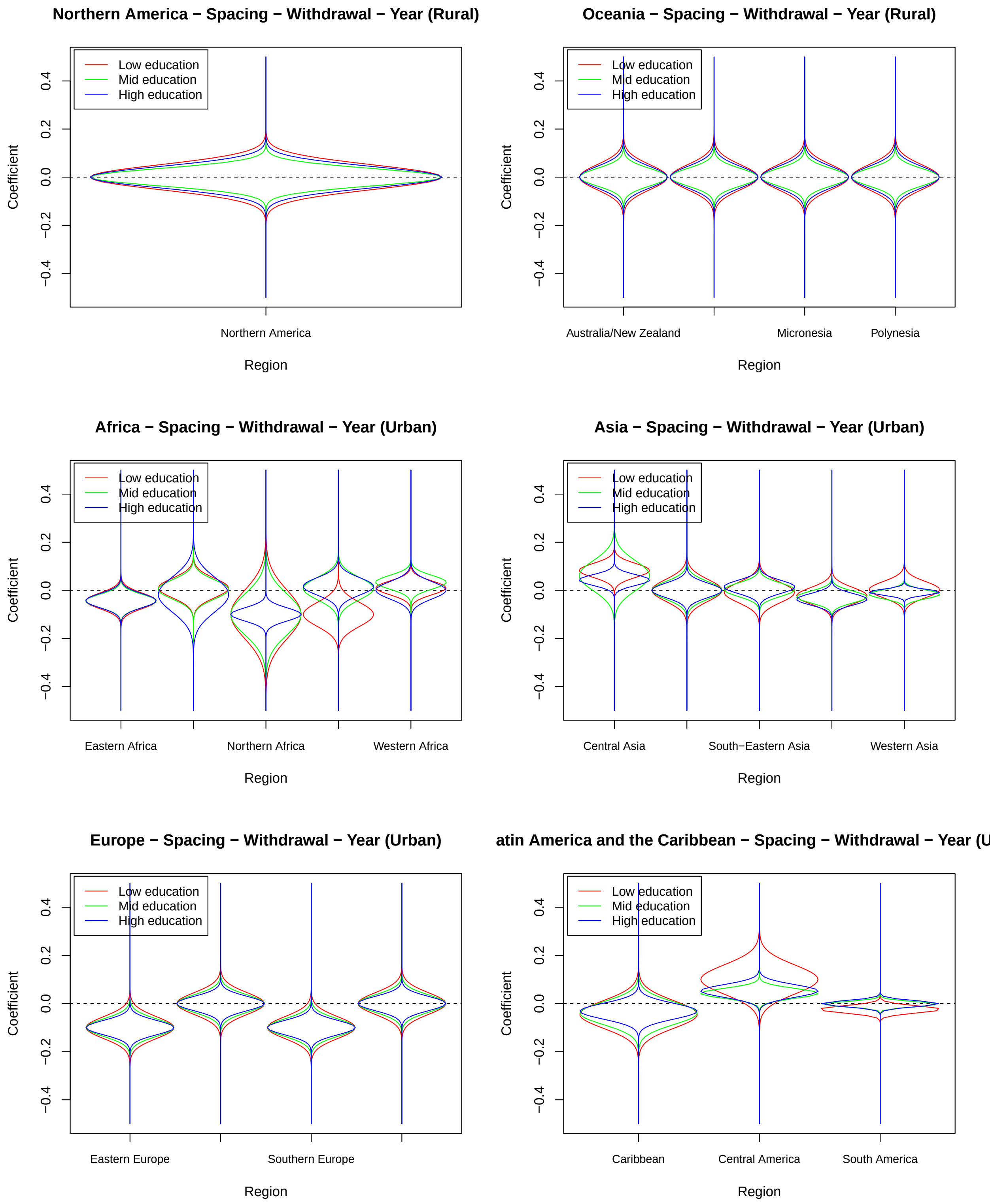
Northern America − Spacing − Withdrawal − Year (Rural)
Oceania − Spacing − Withdrawal − Year (Rural)
Africa − Spacing − Withdrawal − Year (Urban)
Asia − Spacing − Withdrawal − Year (Urban)
Europe − Spacing − Withdrawal − Year (Urban)
atin America and the Caribbean − Spacing − Withdrawal − Year (U
Low education
Mid education
High education
Coefficient
Region
−0.4
−0.2
0.0
0.2
0.4
Northern America
Australia/New Zealand
Micronesia
Polynesia
Eastern Africa
Northern Africa
Western Africa
Central Asia
South−Eastern Asia
Western Asia
Eastern Europe
Southern Europe
Caribbean
Central America
South America

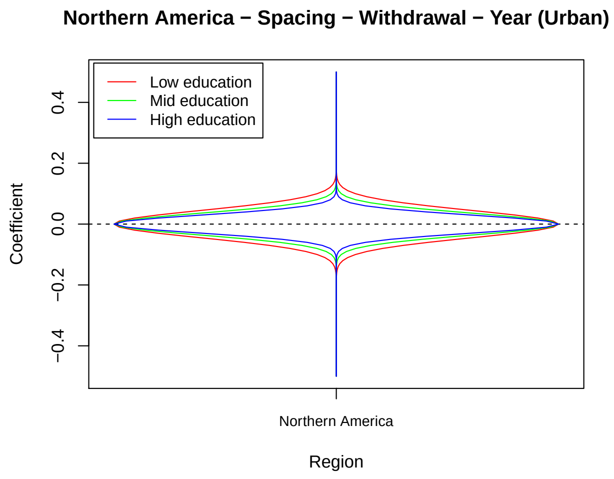
Northern America − Spacing − Withdrawal − Year (Urban)
Low education
Mid education
High education
Coefficient
Northern America
Region

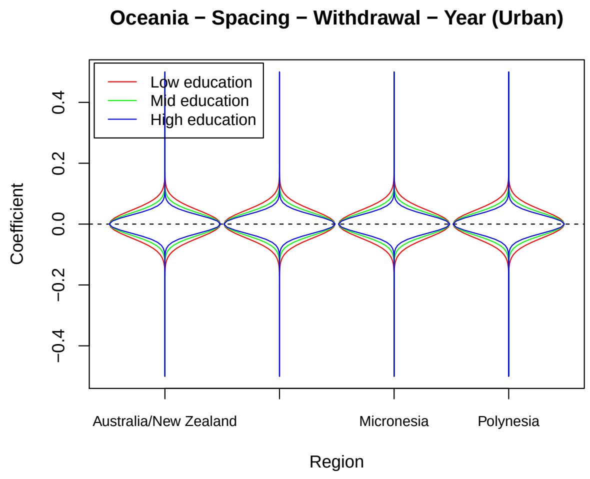
Oceania − Spacing − Withdrawal − Year (Urban)
Low education
Mid education
High education
Coefficient
Australia/New Zealand
Micronesia
Polynesia
Region

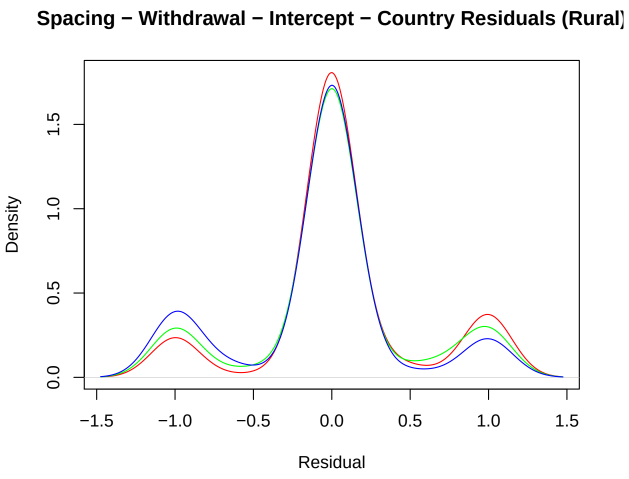
Spacing − Withdrawal − Intercept − Country Residuals (Rural)
Density
Residual

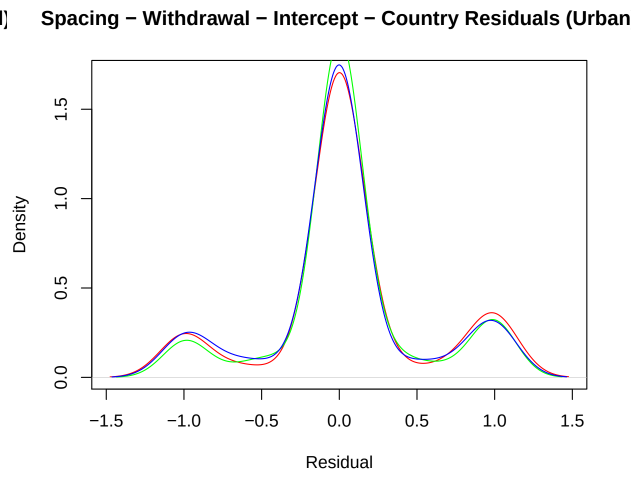
Spacing − Withdrawal − Intercept − Country Residuals (Urban
Density
Residual

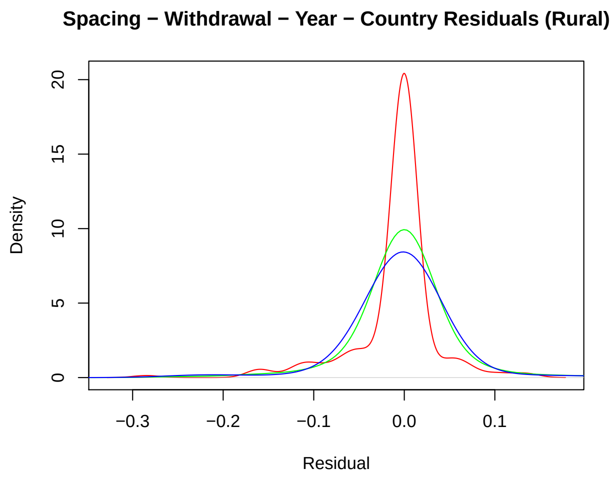
Spacing − Withdrawal − Year − Country Residuals (Rural)
Density
Residual

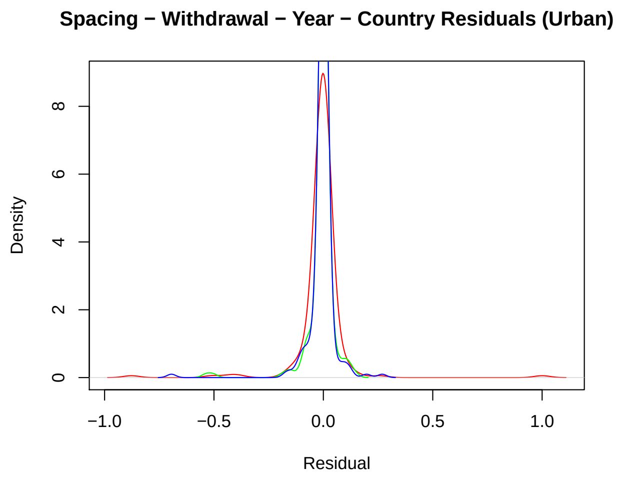
Spacing − Withdrawal − Year − Country Residuals (Urban)
Density
Residual

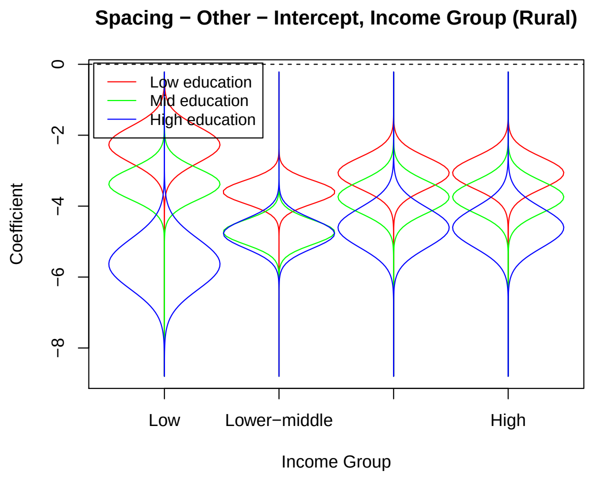
Spacing − Other − Intercept, Income Group (Rural)
Low education
Mid education
High education
Coefficient
0
−2
−4
−6
−8
Low
Lower−middle
High
Income Group

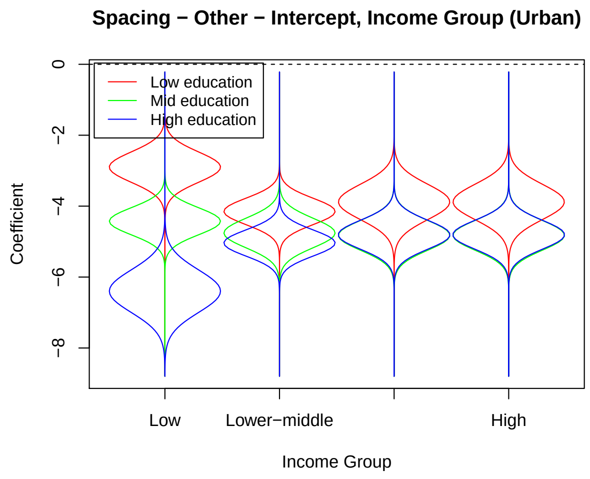
Spacing − Other − Intercept, Income Group (Urban)
Low education
Mid education
High education
Coefficient
0
−2
−4
−6
−8
Low
Lower−middle
High
Income Group

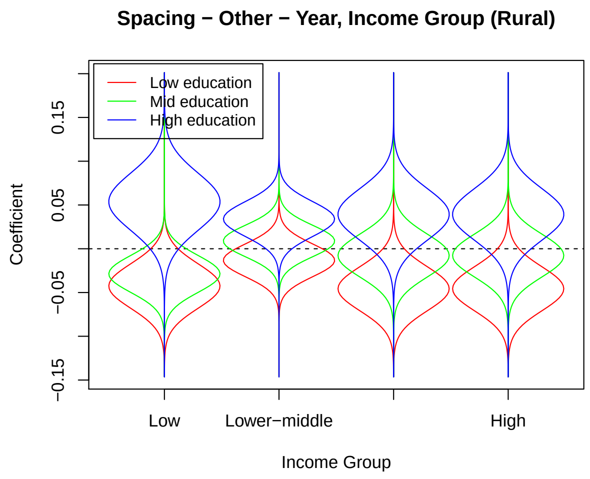
Spacing − Other − Year, Income Group (Rural)
Low education
Mid education
High education
Coefficient
0.15
0.05
−0.05
−0.15
Low
Lower−middle
High
Income Group

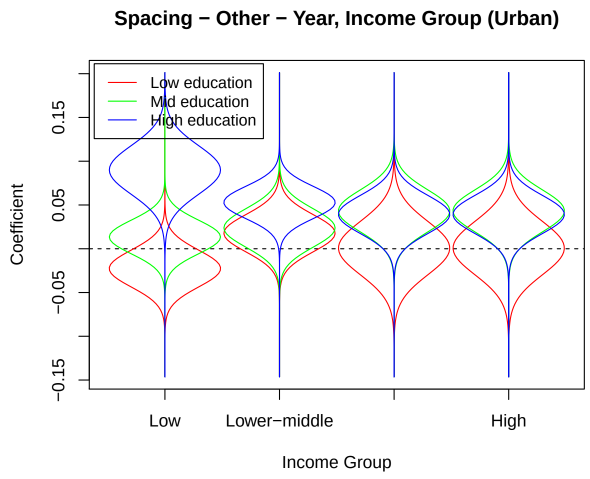
Spacing − Other − Year, Income Group (Urban)
Low education
Mid education
High education
Coefficient
0.15
0.05
−0.05
−0.15
Low
Lower−middle
High
Income Group

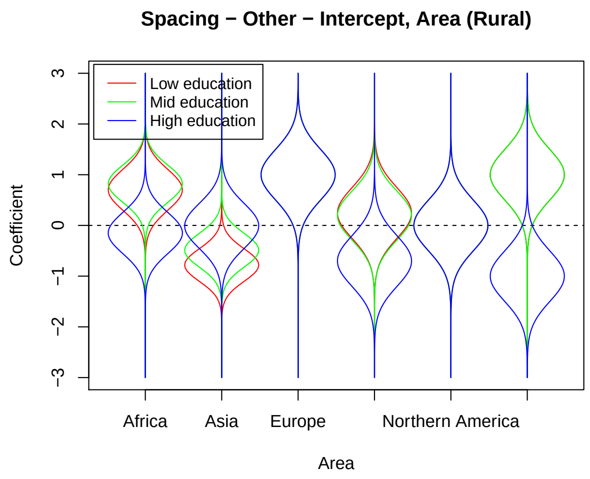
Spacing − Other − Intercept, Area (Rural)
Low education
Mid education
High education
Coefficient
3
2
1
0
−1
−2
−3
Africa
Asia
Europe
Northern America
Area

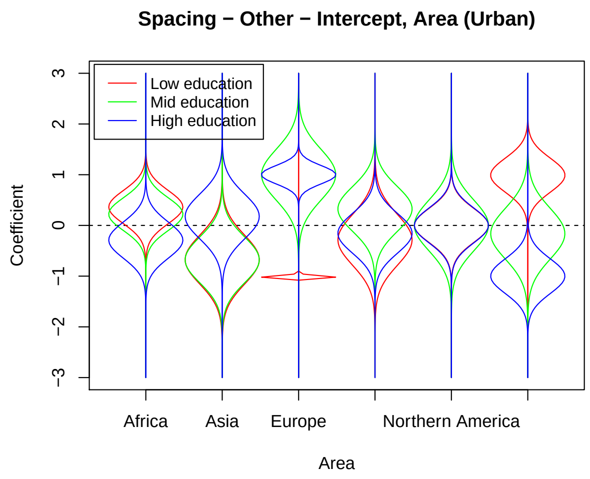
Spacing − Other − Intercept, Area (Urban)
Low education
Mid education
High education
Coefficient
3
2
1
0
−1
−2
−3
Africa
Asia
Europe
Northern America
Area

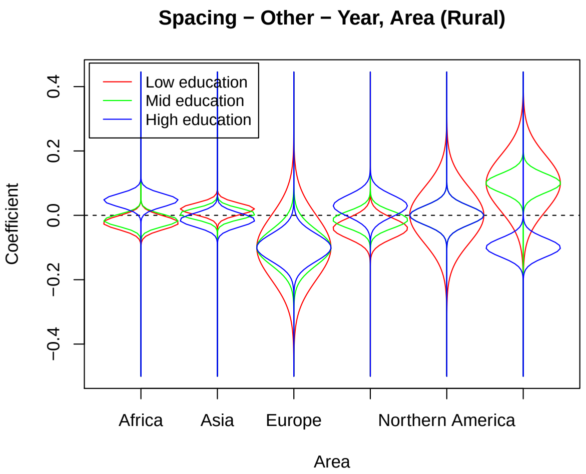
Spacing − Other − Year, Area (Rural)
Low education
Mid education
High education
Coefficient
0.4
0.2
0.0
−0.2
−0.4
Africa
Asia
Europe
Northern America
Area

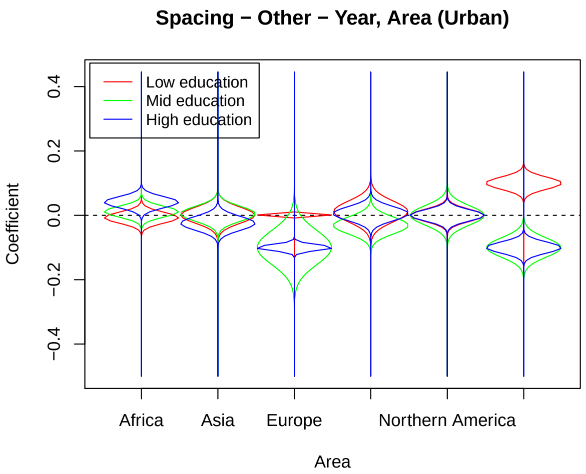
Spacing − Other − Year, Area (Urban)
Low education
Mid education
High education
Coefficient
0.4
0.2
0.0
−0.2
−0.4
Africa
Asia
Europe
Northern America
Area

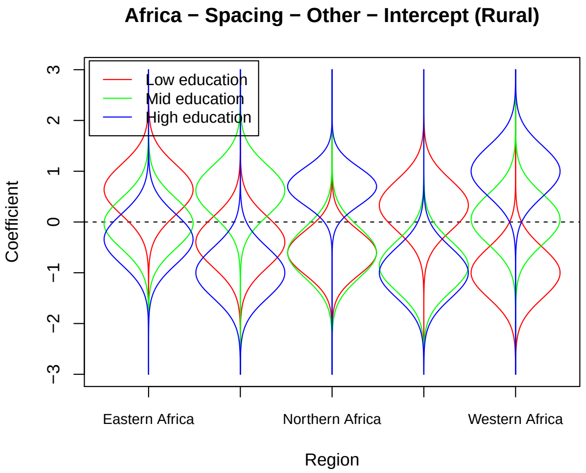
Africa − Spacing − Other − Intercept (Rural)
Low education
Mid education
High education
Coefficient
3
2
1
0
−1
−2
−3
Eastern Africa
Northern Africa
Western Africa
Region

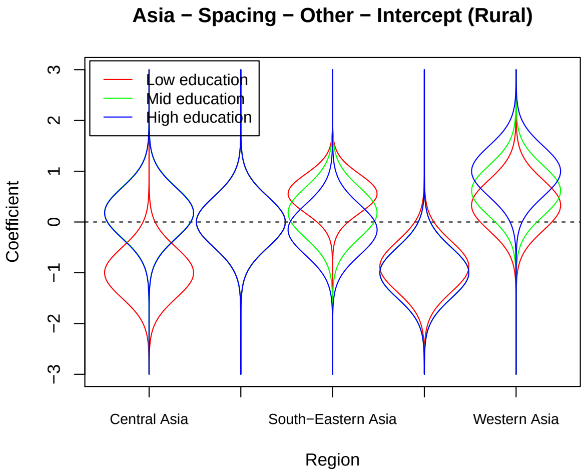
Asia − Spacing − Other − Intercept (Rural)
Low education
Mid education
High education
Coefficient
3
2
1
0
−1
−2
−3
Central Asia
South−Eastern Asia
Western Asia
Region

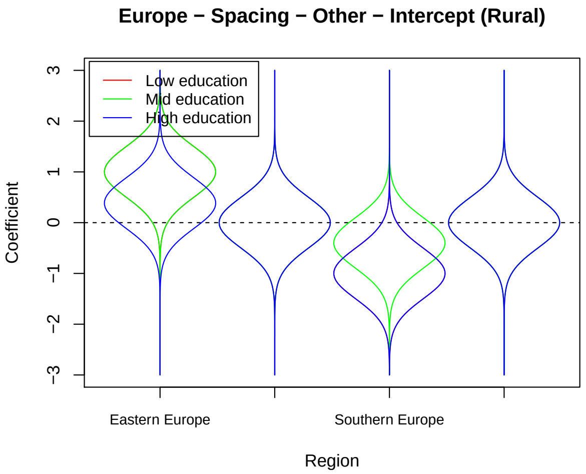
Europe − Spacing − Other − Intercept (Rural)
Low education
Mid education
High education
Coefficient
3
2
1
0
−1
−2
−3
Eastern Europe
Southern Europe
Region

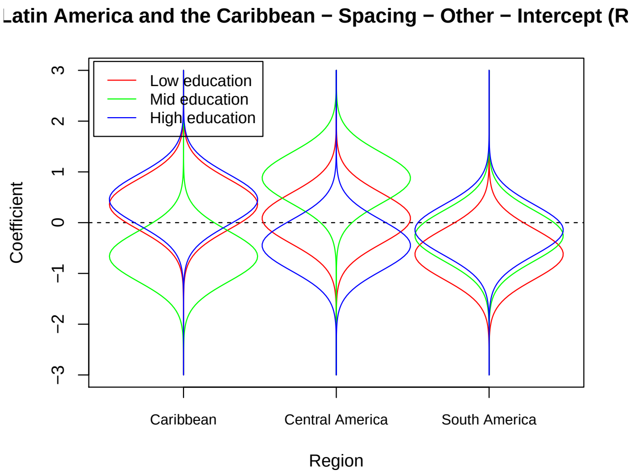
Latin America and the Caribbean − Spacing − Other − Intercept (R
Low education
Mid education
High education
Coefficient
3
2
1
0
−1
−2
−3
Caribbean
Central America
South America
Region

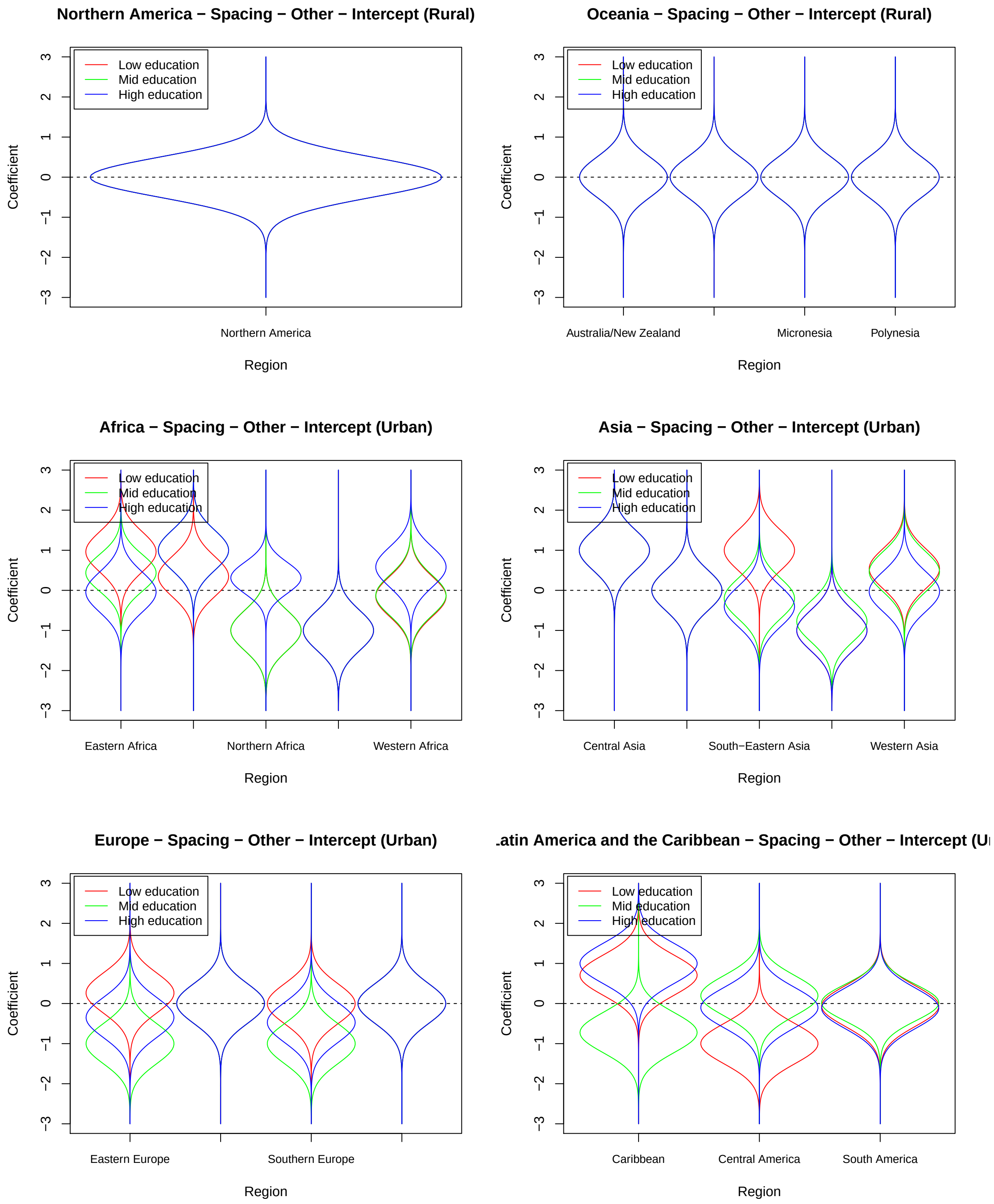
Northern America − Spacing − Other − Intercept (Rural)
Low education
Mid education
High education
Coefficient
3
2
1
0
−1
−2
−3
Northern America
Region
Oceania − Spacing − Other − Intercept (Rural)
Low education
Mid education
High education
Coefficient
Australia/New Zealand
Micronesia
Polynesia
Region
Africa − Spacing − Other − Intercept (Urban)
Low education
Mid education
High education
Coefficient
Eastern Africa
Northern Africa
Western Africa
Region
Asia − Spacing − Other − Intercept (Urban)
Low education
Mid education
High education
Coefficient
Central Asia
South−Eastern Asia
Western Asia
Region
Europe − Spacing − Other − Intercept (Urban)
Low education
Mid education
High education
Coefficient
Eastern Europe
Southern Europe
Region
.atin America and the Caribbean − Spacing − Other − Intercept (U
Low education
Mid education
High education
Coefficient
Caribbean
Central America
South America
Region

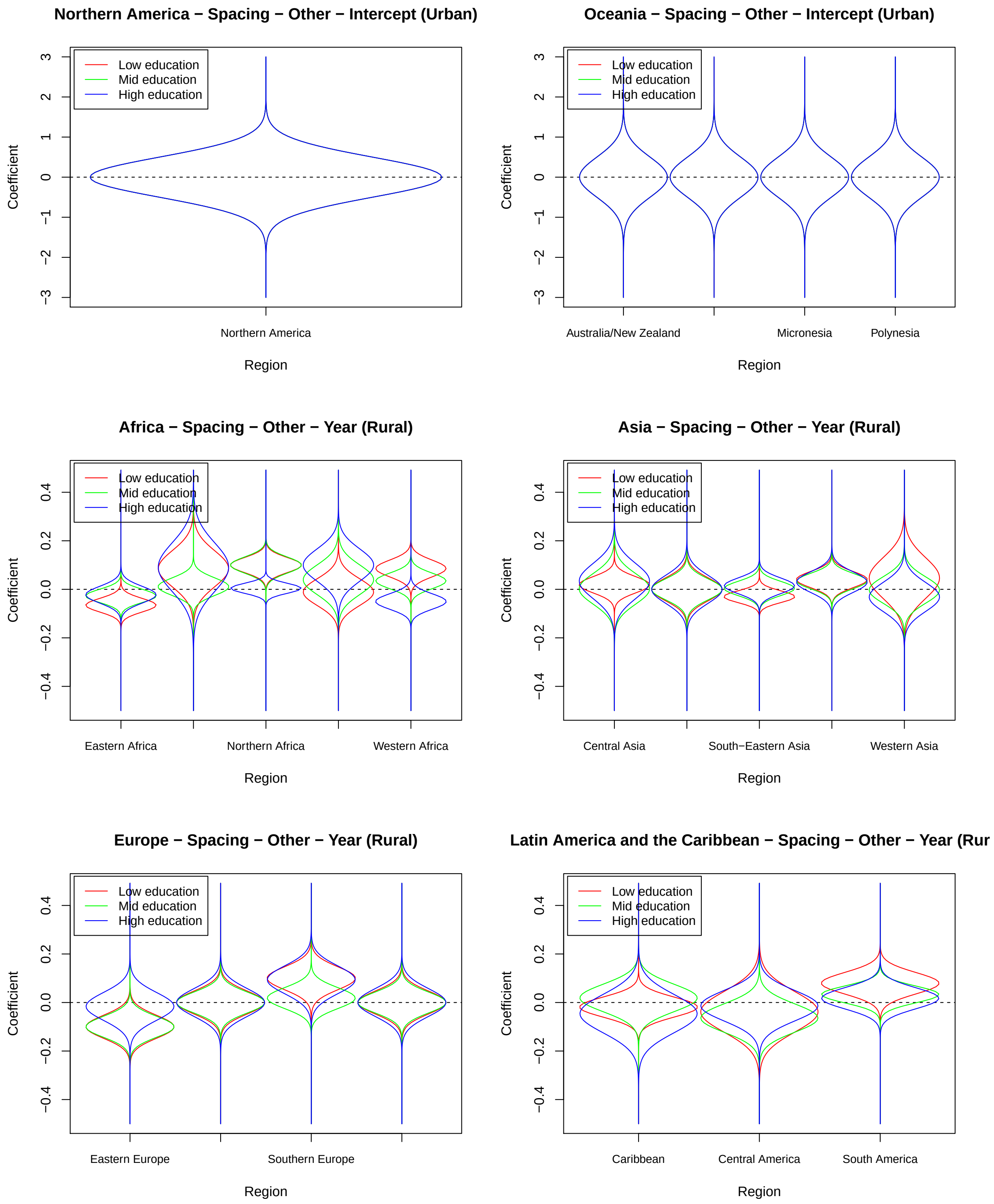
Northern America − Spacing − Other − Intercept (Urban)
Low education
Mid education
High education
Coefficient
Northern America
Region
Oceania − Spacing − Other − Intercept (Urban)
Australia/New Zealand
Micronesia
Polynesia
Africa − Spacing − Other − Year (Rural)
Eastern Africa
Northern Africa
Western Africa
Asia − Spacing − Other − Year (Rural)
Central Asia
South−Eastern Asia
Western Asia
Europe − Spacing − Other − Year (Rural)
Eastern Europe
Southern Europe
Latin America and the Caribbean − Spacing − Other − Year (Rur
Caribbean
Central America
South America

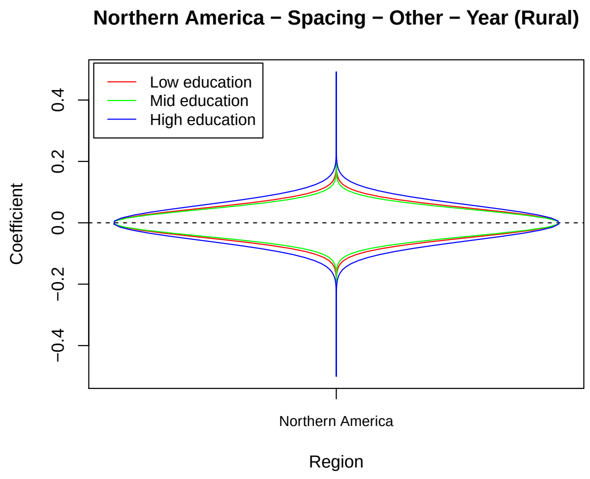
Northern America − Spacing − Other − Year (Rural)
Low education
Mid education
High education
Coefficient
Northern America
Region

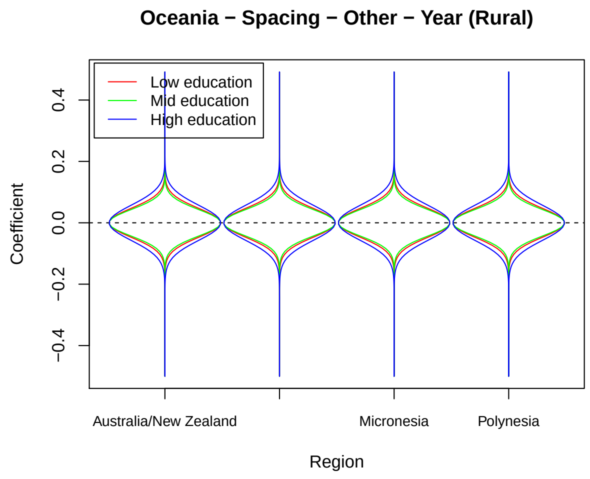
Oceania − Spacing − Other − Year (Rural)
Low education
Mid education
High education
Coefficient
Australia/New Zealand
Micronesia
Polynesia
Region

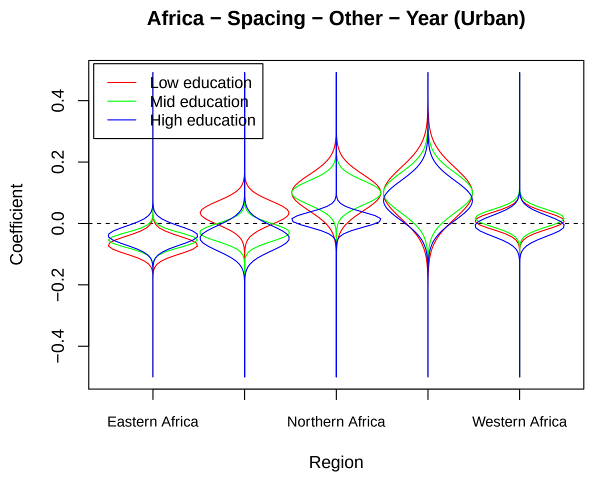
Africa − Spacing − Other − Year (Urban)
Low education
Mid education
High education
Coefficient
Eastern Africa
Northern Africa
Western Africa
Region

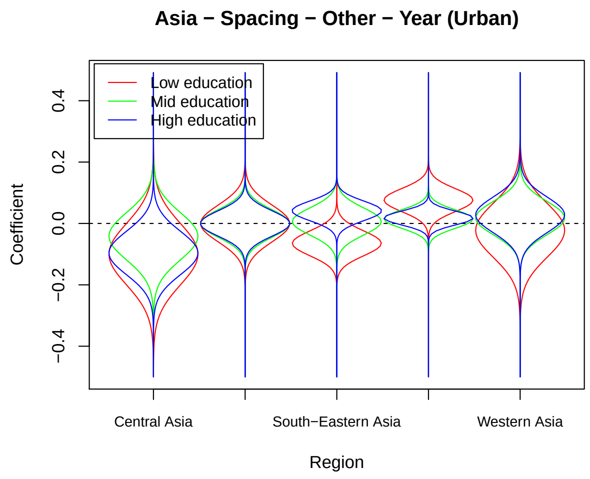
Asia − Spacing − Other − Year (Urban)
Low education
Mid education
High education
Coefficient
Central Asia
South−Eastern Asia
Western Asia
Region

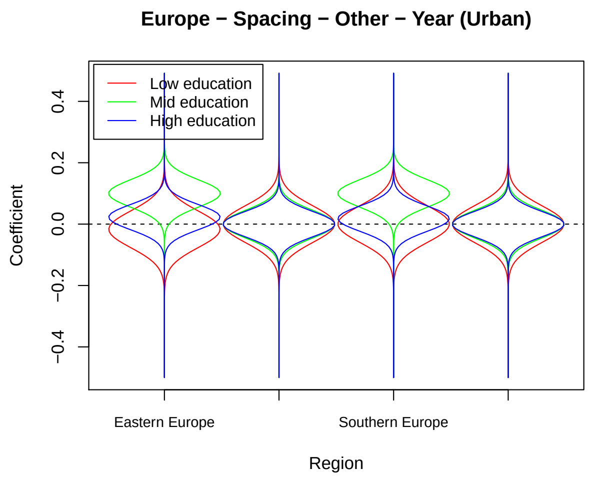
Europe − Spacing − Other − Year (Urban)
Low education
Mid education
High education
Coefficient
Eastern Europe
Southern Europe
Region

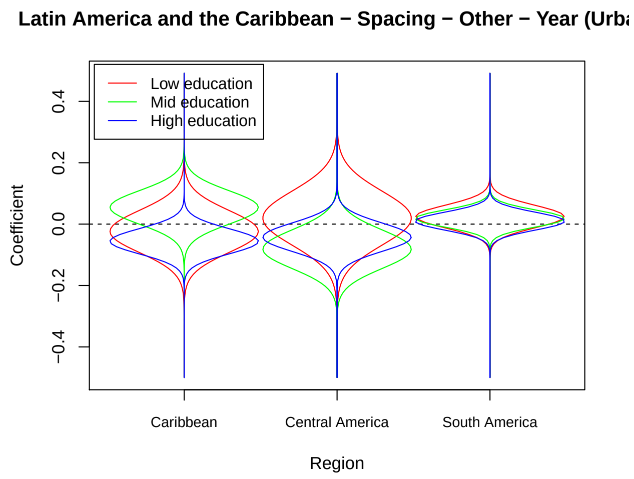
Latin America and the Caribbean − Spacing − Other − Year (Urba
Low education
Mid education
High education
Coefficient
Caribbean
Central America
South America
Region

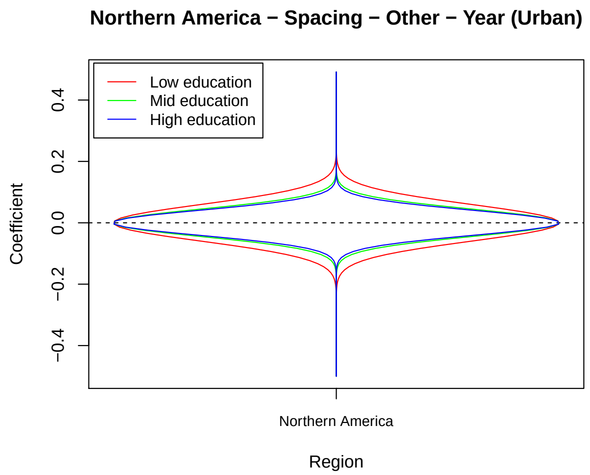
Northern America − Spacing − Other − Year (Urban)
Low education
Mid education
High education
Coefficient
0.4
0.2
0.0
−0.2
−0.4
Northern America
Region

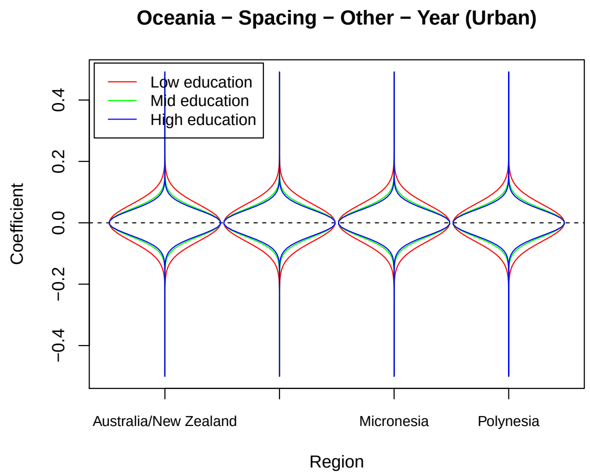
Oceania − Spacing − Other − Year (Urban)
Low education
Mid education
High education
Coefficient
0.4
0.2
0.0
−0.2
−0.4
Australia/New Zealand
Micronesia
Polynesia
Region

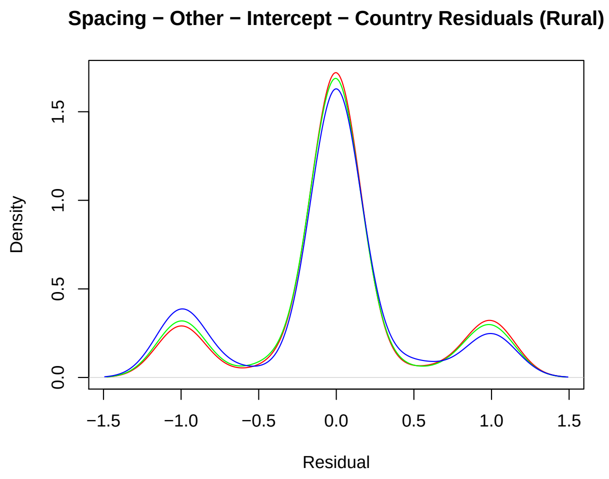
Spacing − Other − Intercept − Country Residuals (Rural)
Density
0.0
0.5
1.0
1.5
−1.5
−1.0
−0.5
0.0
0.5
1.0
1.5
Residual

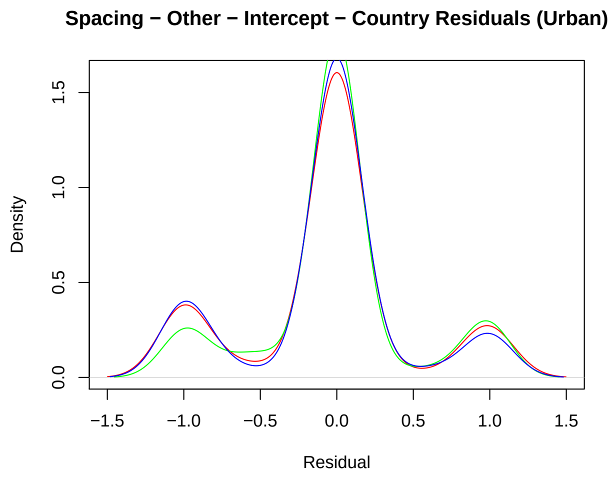
Spacing − Other − Intercept − Country Residuals (Urban)
Density
0.0
0.5
1.0
1.5
−1.5
−1.0
−0.5
0.0
0.5
1.0
1.5
Residual

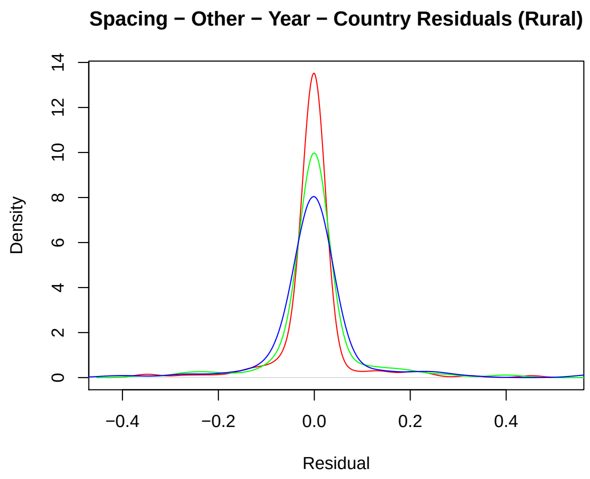
Spacing − Other − Year − Country Residuals (Rural)
Density
0
2
4
6
8
10
12
14
−0.4
−0.2
0.0
0.2
0.4
Residual

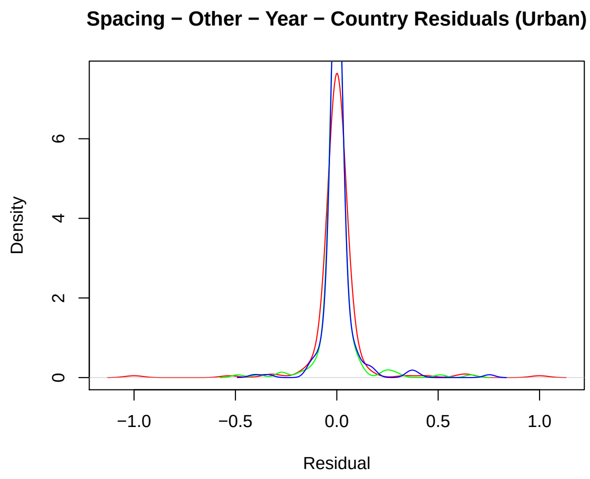
Spacing − Other − Year − Country Residuals (Urban)
Density
0
2
4
6
−1.0
−0.5
0.0
0.5
1.0
Residual

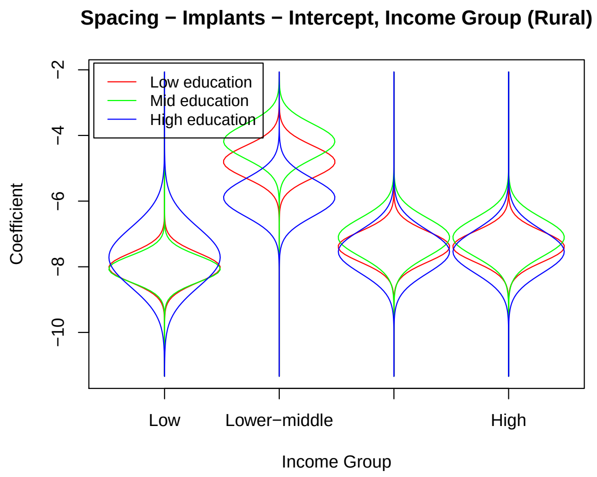
Spacing − Implants − Intercept, Income Group (Rural)
Low education
Mid education
High education
Coefficient
−2
−4
−6
−8
−10
Low
Lower−middle
High
Income Group

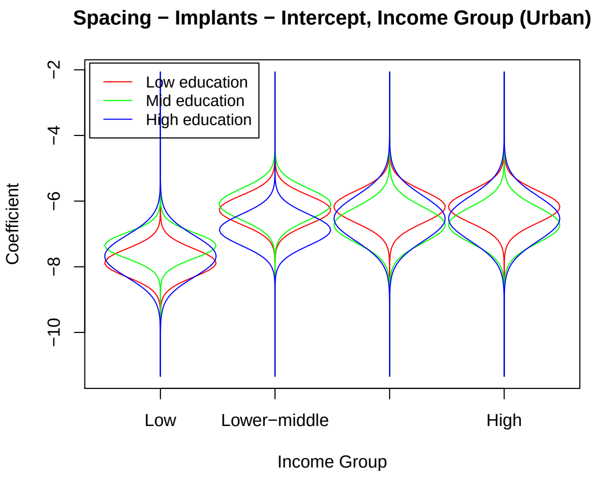
Spacing − Implants − Intercept, Income Group (Urban)
Low education
Mid education
High education
Coefficient
−2
−4
−6
−8
−10
Low
Lower−middle
High
Income Group

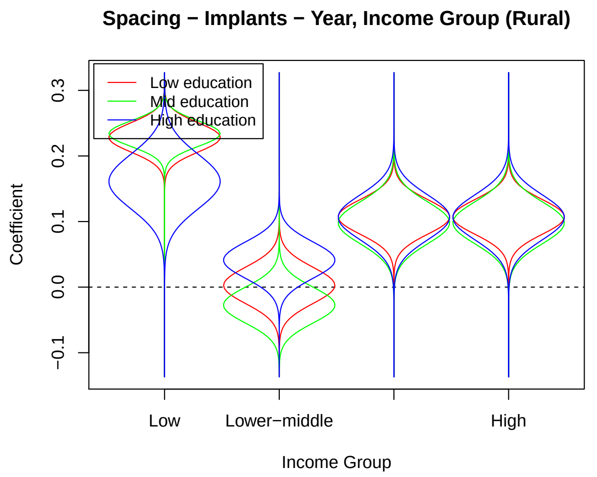
Spacing − Implants − Year, Income Group (Rural)
Low education
Mid education
High education
Coefficient
0.3
0.2
0.1
0.0
−0.1
Low
Lower−middle
High
Income Group

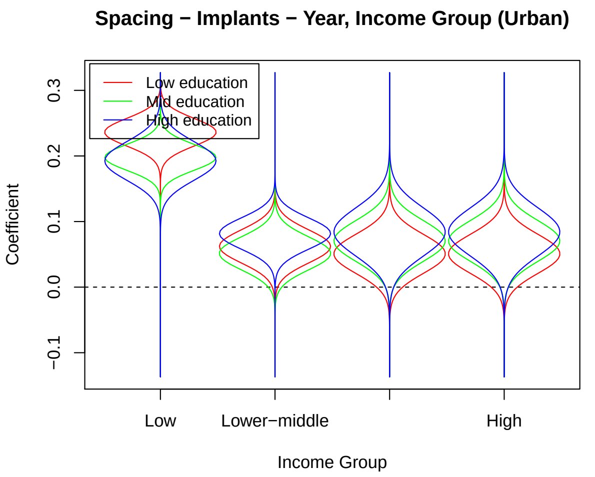
Spacing − Implants − Year, Income Group (Urban)
Low education
Mid education
High education
Coefficient
0.3
0.2
0.1
0.0
−0.1
Low
Lower−middle
High
Income Group

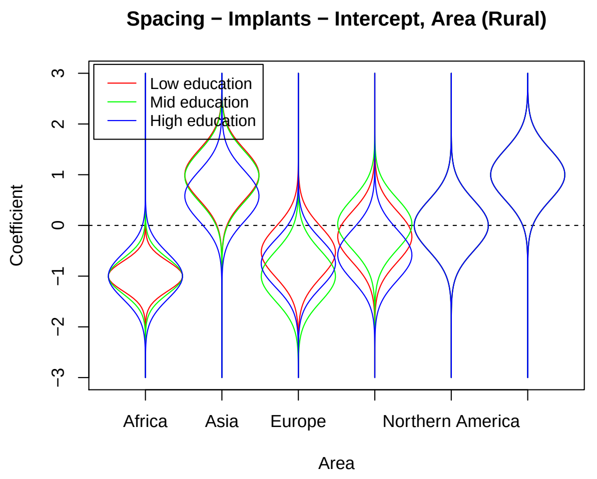
Spacing − Implants − Intercept, Area (Rural)
Low education
Mid education
High education
Coefficient
3
2
1
0
−1
−2
−3
Africa
Asia
Europe
Northern America
Area

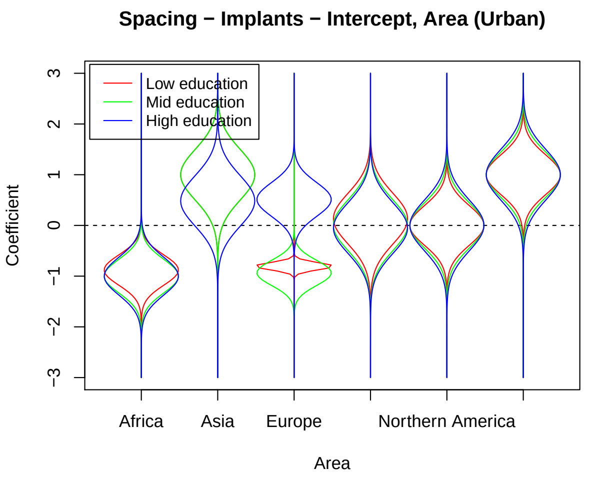
Spacing − Implants − Intercept, Area (Urban)
Low education
Mid education
High education
Coefficient
3
2
1
0
−1
−2
−3
Africa
Asia
Europe
Northern America
Area

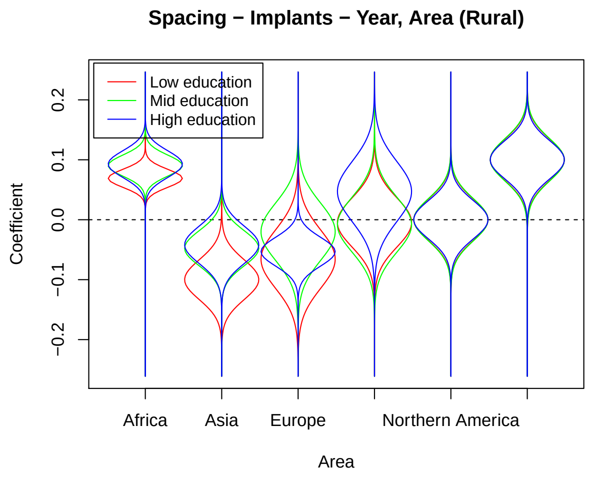
Spacing − Implants − Year, Area (Rural)
Low education
Mid education
High education
Coefficient
0.2
0.1
0.0
−0.1
−0.2
Africa
Asia
Europe
Northern America
Area

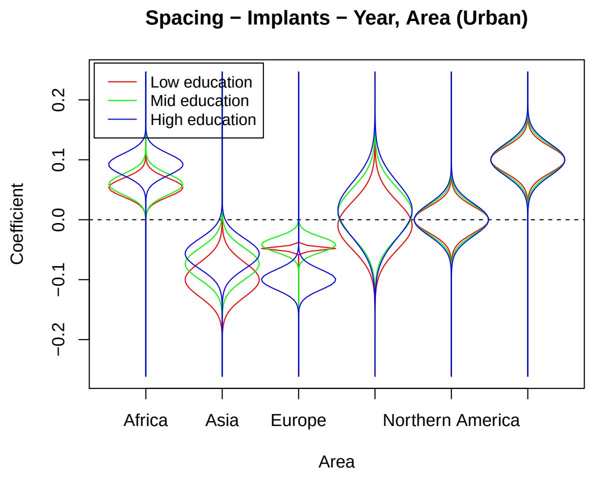
Spacing − Implants − Year, Area (Urban)
Low education
Mid education
High education
Coefficient
0.2
0.1
0.0
−0.1
−0.2
Africa
Asia
Europe
Northern America
Area

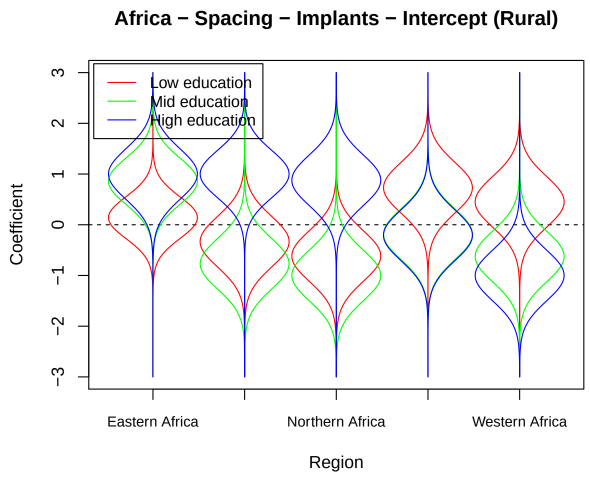
Africa − Spacing − Implants − Intercept (Rural)
Low education
Mid education
High education
Coefficient
3
2
1
0
−1
−2
−3
Eastern Africa
Northern Africa
Western Africa
Region

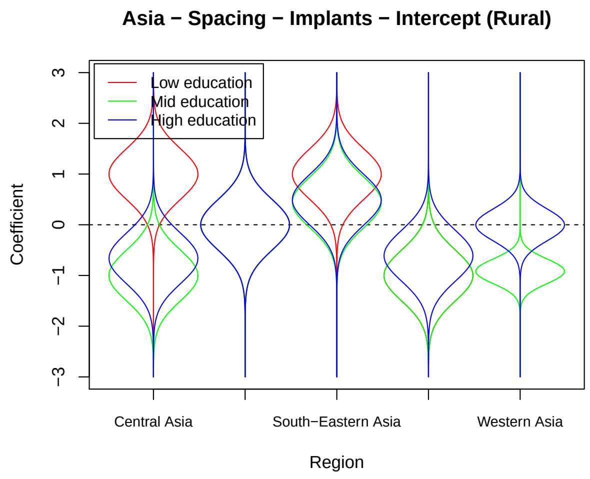
Asia − Spacing − Implants − Intercept (Rural)
Low education
Mid education
High education
Coefficient
3
2
1
0
−1
−2
−3
Central Asia
South−Eastern Asia
Western Asia
Region

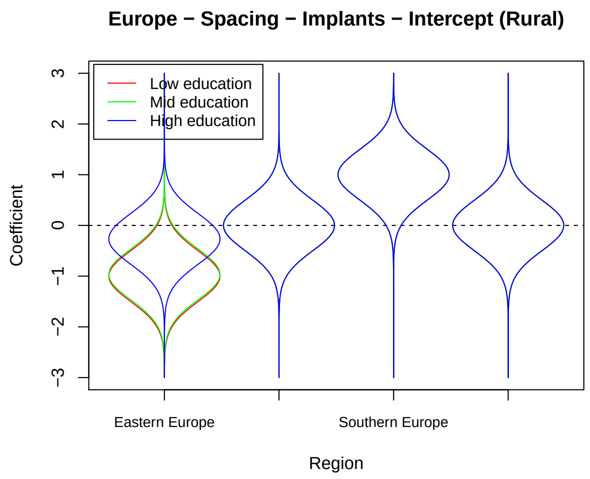
Europe − Spacing − Implants − Intercept (Rural)
Low education
Mid education
High education
Coefficient
3
2
1
0
−1
−2
−3
Eastern Europe
Southern Europe
Region

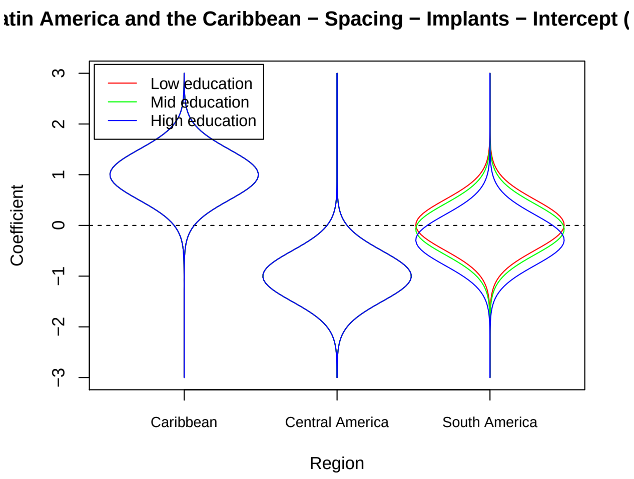
atin America and the Caribbean − Spacing − Implants − Intercept (
Low education
Mid education
High education
Coefficient
3
2
1
0
−1
−2
−3
Caribbean
Central America
South America
Region

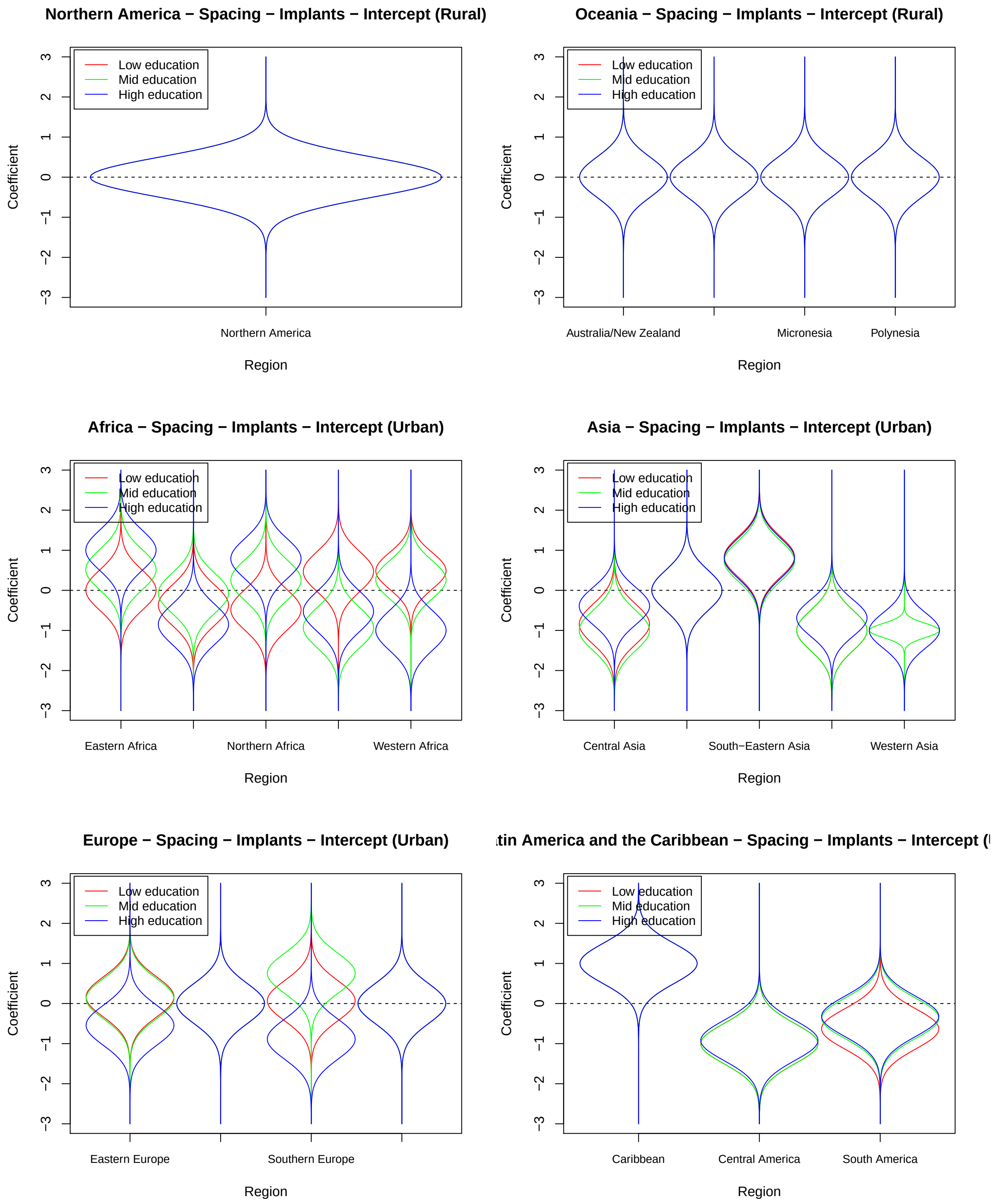
Northern America − Spacing − Implants − Intercept (Rural)
Low education
Mid education
High education
Coefficient
Northern America
Region
Oceania − Spacing − Implants − Intercept (Rural)
Australia/New Zealand
Micronesia
Polynesia
Africa − Spacing − Implants − Intercept (Urban)
Eastern Africa
Northern Africa
Western Africa
Asia − Spacing − Implants − Intercept (Urban)
Central Asia
South−Eastern Asia
Western Asia
Europe − Spacing − Implants − Intercept (Urban)
Eastern Europe
Southern Europe
tin America and the Caribbean − Spacing − Implants − Intercept (
Caribbean
Central America
South America

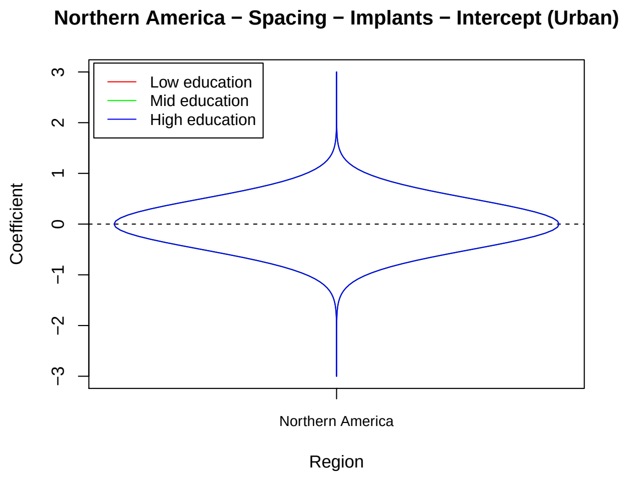
Northern America − Spacing − Implants − Intercept (Urban)
Low education
Mid education
High education
Coefficient
3
2
1
0
−1
−2
−3
Northern America
Region

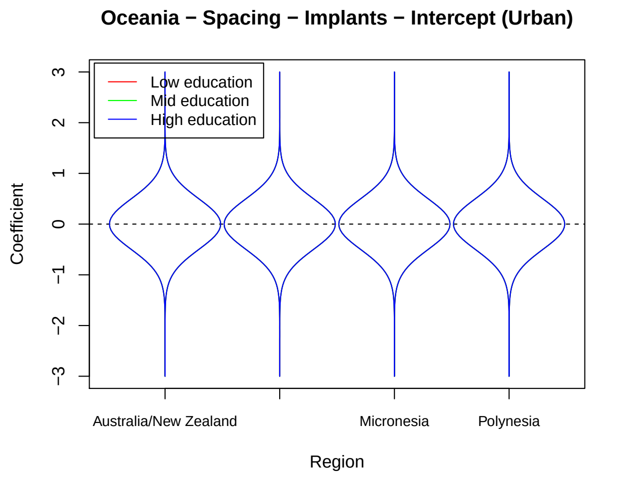
Oceania − Spacing − Implants − Intercept (Urban)
Low education
Mid education
High education
Coefficient
3
2
1
0
−1
−2
−3
Australia/New Zealand
Micronesia
Polynesia
Region

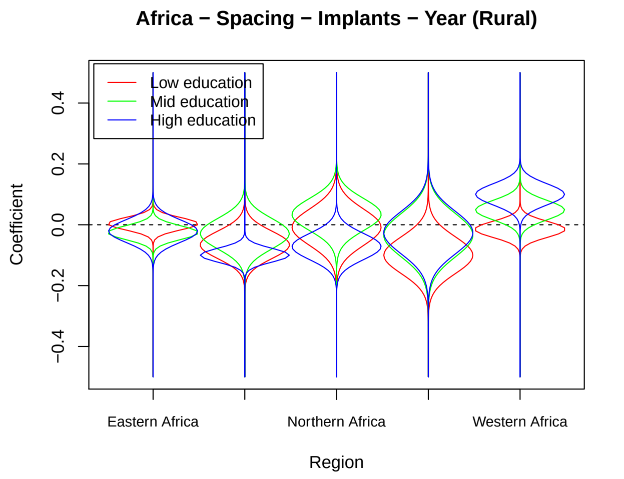
Africa − Spacing − Implants − Year (Rural)
Low education
Mid education
High education
Coefficient
0.4
0.2
0.0
−0.2
−0.4
Eastern Africa
Northern Africa
Western Africa
Region

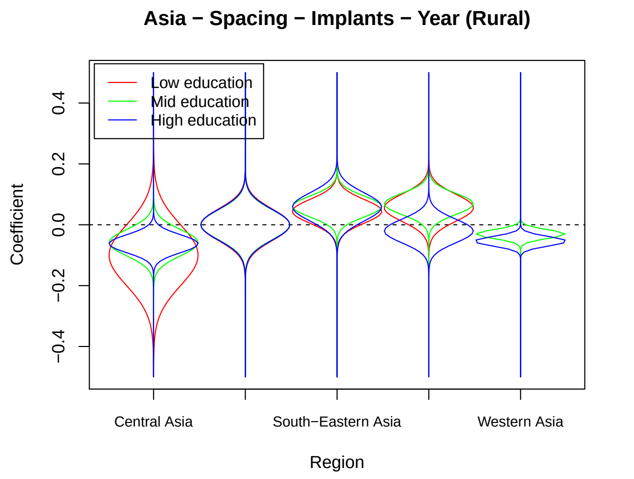
Asia − Spacing − Implants − Year (Rural)
Low education
Mid education
High education
Coefficient
0.4
0.2
0.0
−0.2
−0.4
Central Asia
South−Eastern Asia
Western Asia
Region

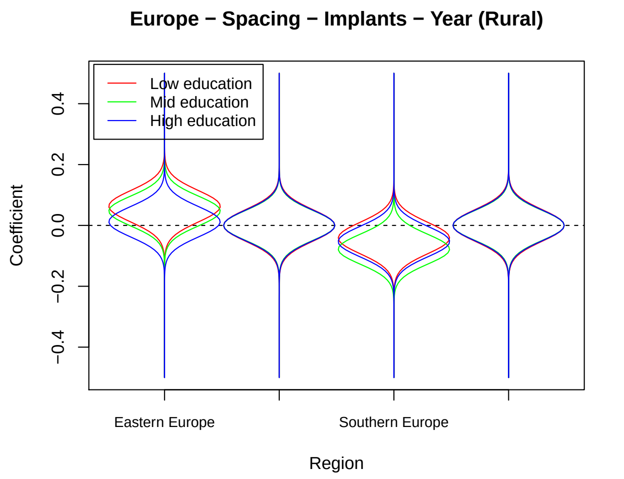
Europe − Spacing − Implants − Year (Rural)
Low education
Mid education
High education
Coefficient
0.4
0.2
0.0
−0.2
−0.4
Eastern Europe
Southern Europe
Region

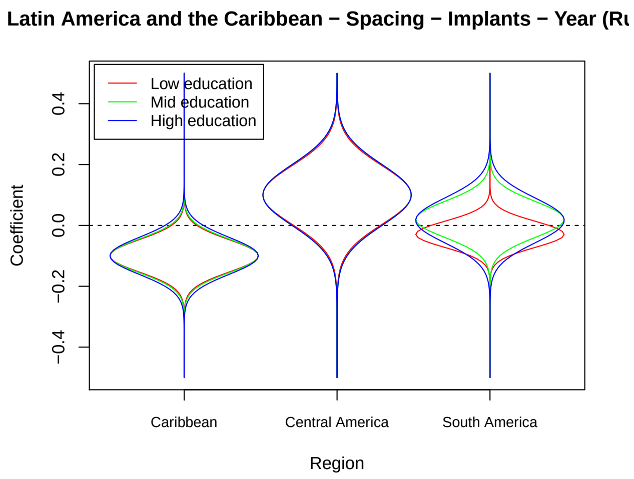
Latin America and the Caribbean − Spacing − Implants − Year (Ru
Low education
Mid education
High education
Coefficient
0.4
0.2
0.0
−0.2
−0.4
Caribbean
Central America
South America
Region

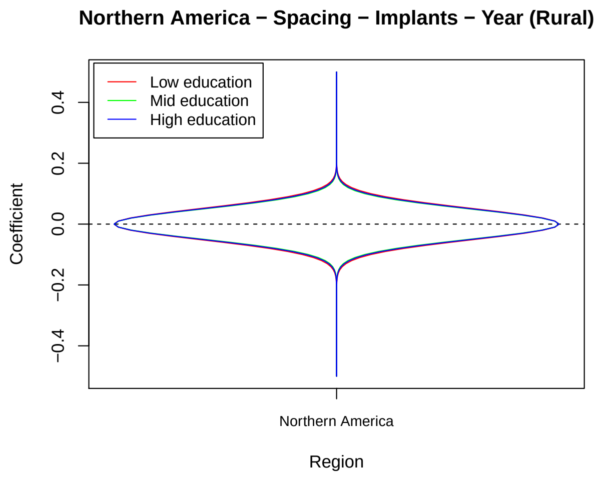
Northern America − Spacing − Implants − Year (Rural)
Low education
Mid education
High education
Coefficient
0.4
0.2
0.0
−0.2
−0.4
Northern America
Region

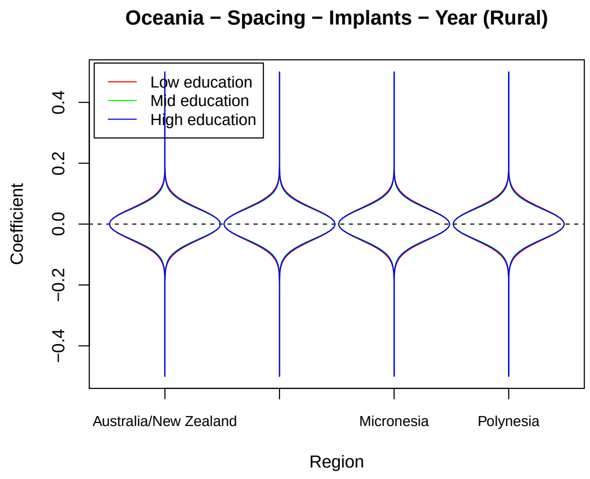
Oceania − Spacing − Implants − Year (Rural)
Low education
Mid education
High education
Coefficient
0.4
0.2
0.0
−0.2
−0.4
Australia/New Zealand
Micronesia
Polynesia
Region

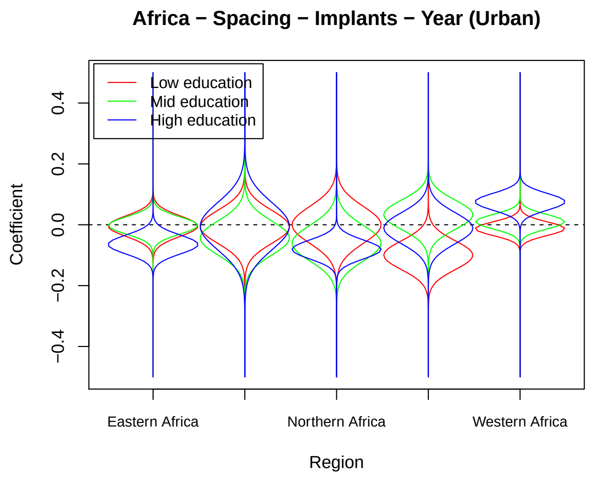
Africa − Spacing − Implants − Year (Urban)
Low education
Mid education
High education
Coefficient
0.4
0.2
0.0
−0.2
−0.4
Eastern Africa
Northern Africa
Western Africa
Region

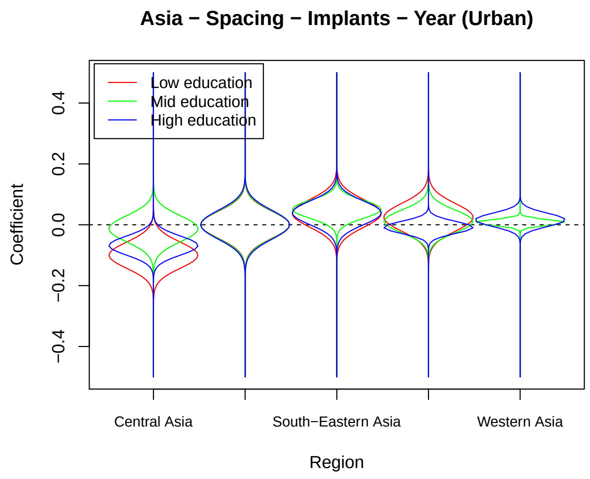
Asia − Spacing − Implants − Year (Urban)
Low education
Mid education
High education
Coefficient
0.4
0.2
0.0
−0.2
−0.4
Central Asia
South−Eastern Asia
Western Asia
Region

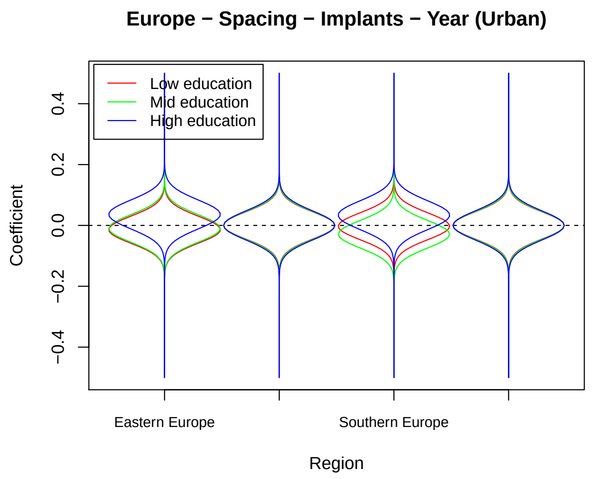
Europe − Spacing − Implants − Year (Urban)
Low education
Mid education
High education
Coefficient
0.4
0.2
0.0
−0.2
−0.4
Eastern Europe
Southern Europe
Region

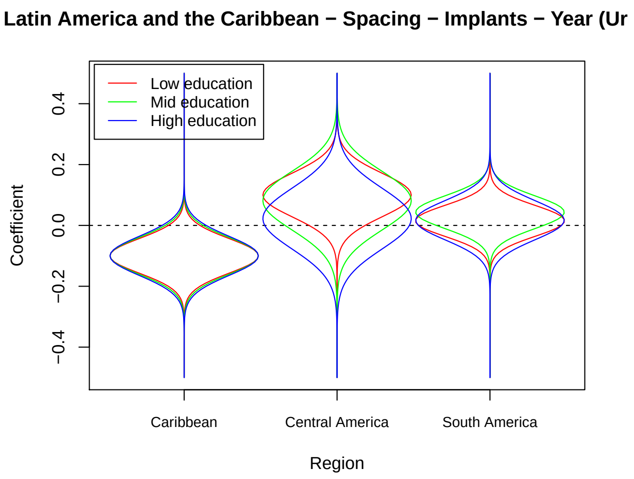
Latin America and the Caribbean − Spacing − Implants − Year (Ur
Low education
Mid education
High education
Coefficient
0.4
0.2
0.0
−0.2
−0.4
Caribbean
Central America
South America
Region

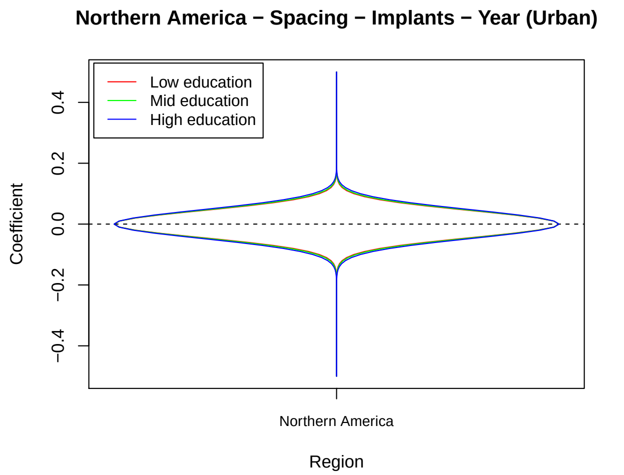
Northern America − Spacing − Implants − Year (Urban)
Low education
Mid education
High education
Coefficient
Northern America
Region

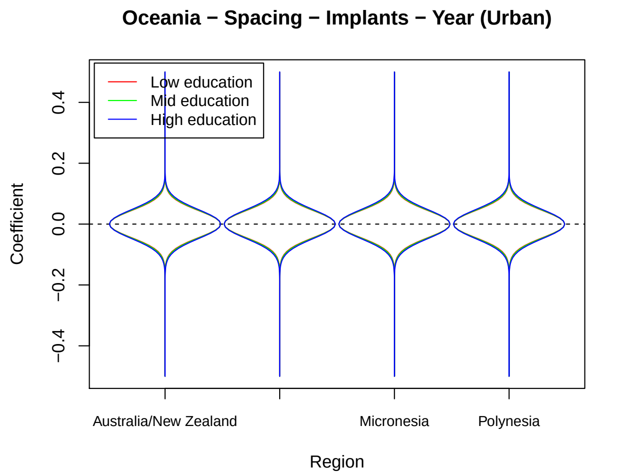
Oceania − Spacing − Implants − Year (Urban)
Low education
Mid education
High education
Coefficient
Australia/New Zealand
Micronesia
Polynesia
Region

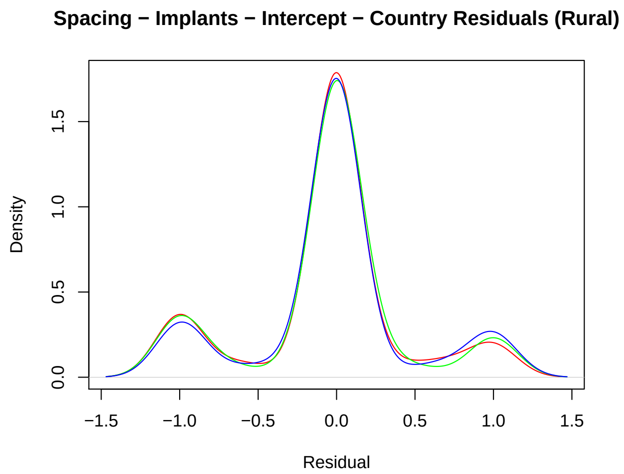
Spacing − Implants − Intercept − Country Residuals (Rural)
Density
Residual

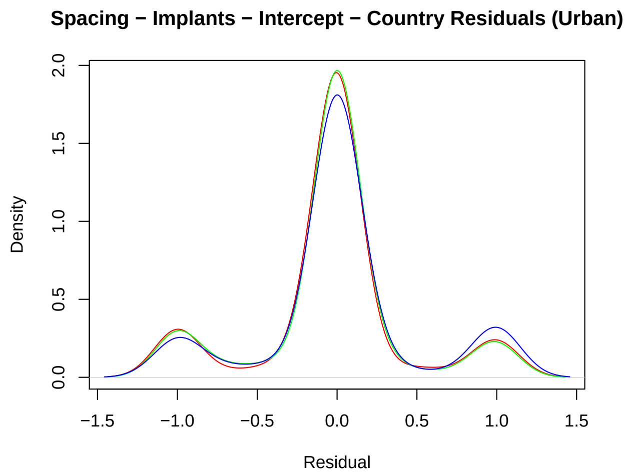
Spacing − Implants − Intercept − Country Residuals (Urban)
Density
Residual

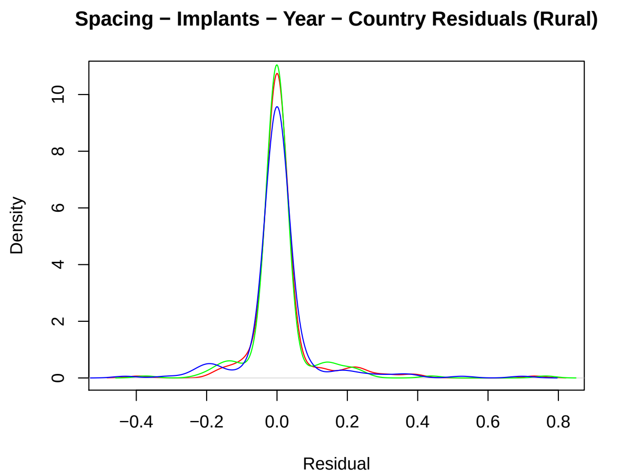
Spacing − Implants − Year − Country Residuals (Rural)
Density
Residual

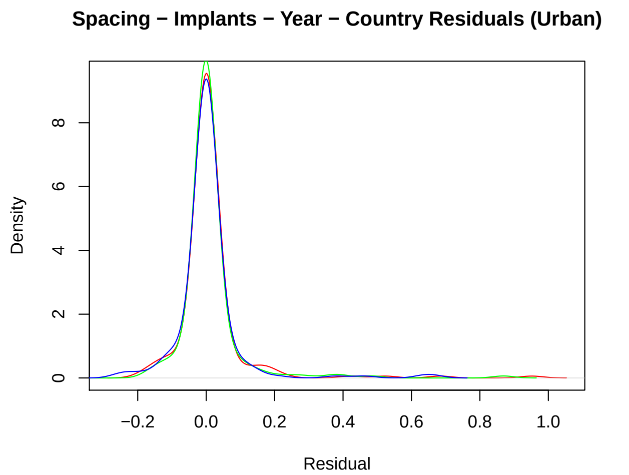
Spacing − Implants − Year − Country Residuals (Urban)
Density
Residual

Limiting − Pill − Intercept, Income Group (Rural)

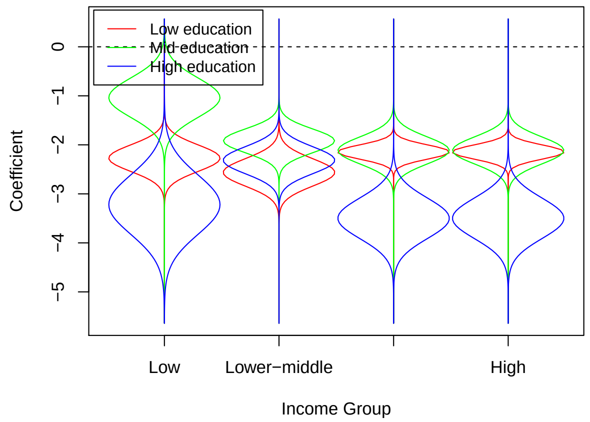

Limiting − Pill − Intercept, Income Group (Urban)

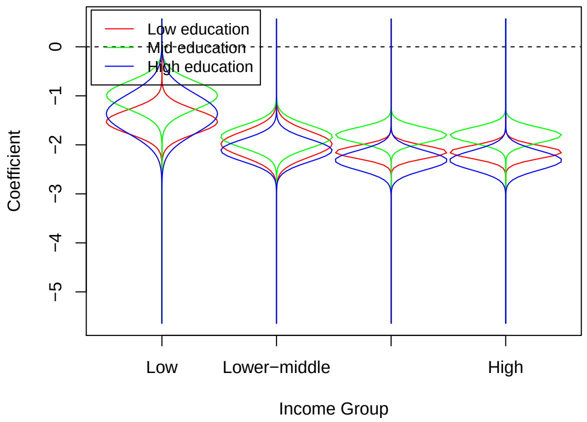

Limiting − Pill − Year, Income Group (Rural)

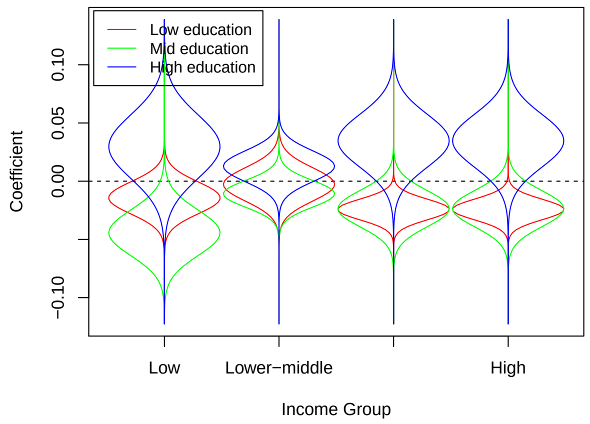

Limiting − Pill − Year, Income Group (Urban)

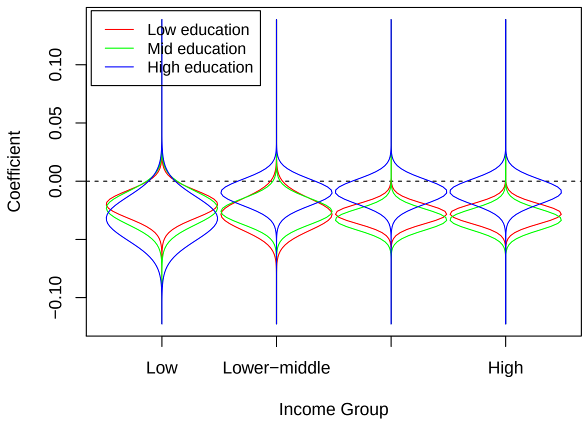

Limiting − Pill − Intercept, Area (Rural)

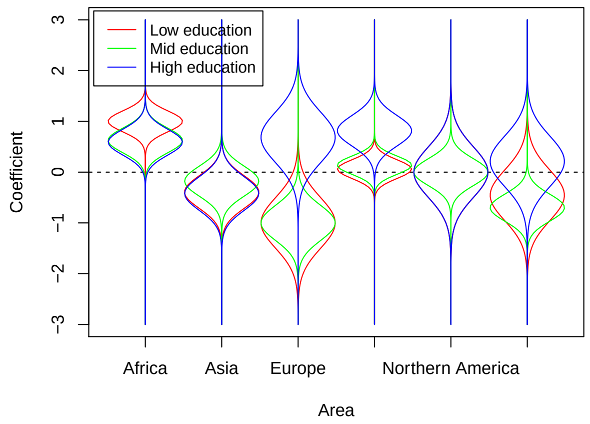

Limiting − Pill − Intercept, Area (Urban)

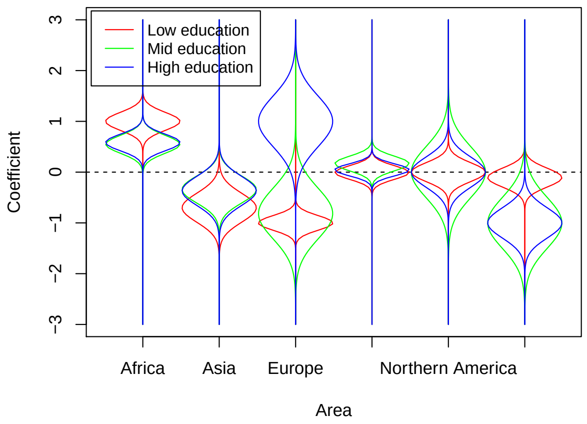

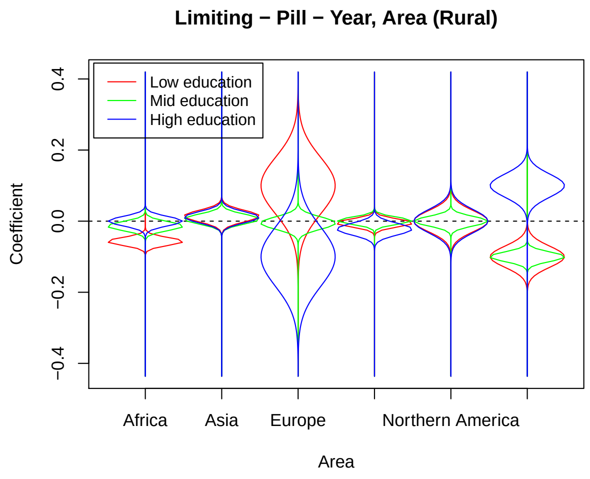
Limiting − Pill − Year, Area (Rural)
Low education
Mid education
High education
Coefficient
0.4
0.2
0.0
−0.2
−0.4
Africa
Asia
Europe
Northern America
Area

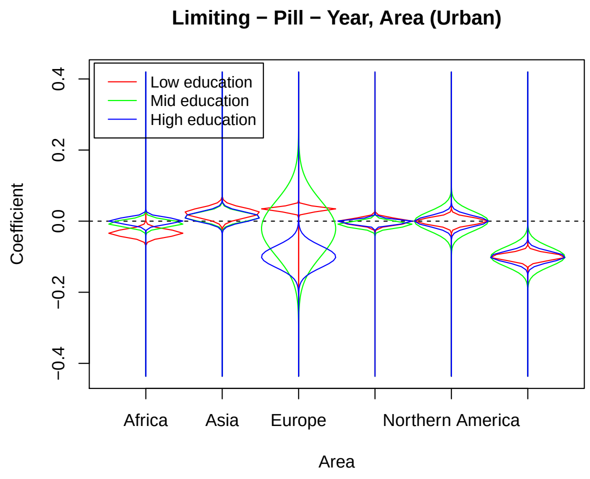
Limiting − Pill − Year, Area (Urban)
Low education
Mid education
High education
Coefficient
0.4
0.2
0.0
−0.2
−0.4
Africa
Asia
Europe
Northern America
Area

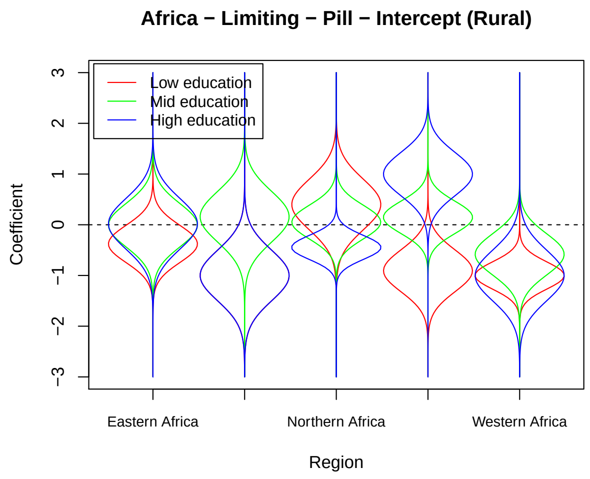
Africa − Limiting − Pill − Intercept (Rural)
Low education
Mid education
High education
Coefficient
3
2
1
0
−1
−2
−3
Eastern Africa
Northern Africa
Western Africa
Region

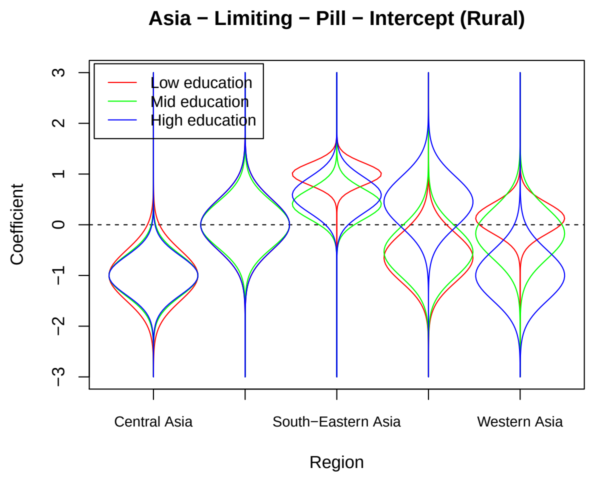
Asia − Limiting − Pill − Intercept (Rural)
Low education
Mid education
High education
Coefficient
3
2
1
0
−1
−2
−3
Central Asia
South−Eastern Asia
Western Asia
Region

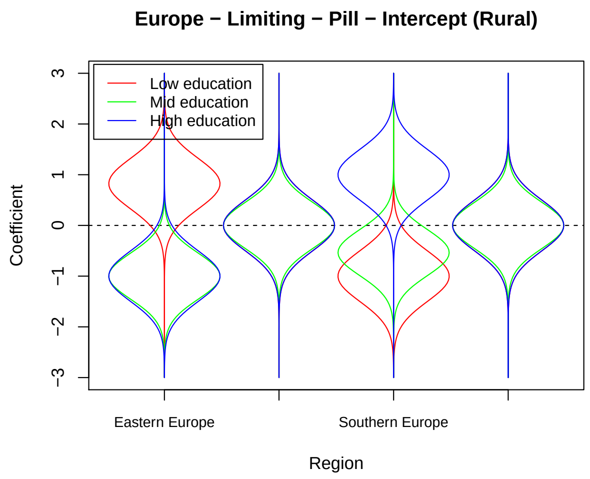
Europe − Limiting − Pill − Intercept (Rural)
Low education
Mid education
High education
Coefficient
3
2
1
0
−1
−2
−3
Eastern Europe
Southern Europe
Region

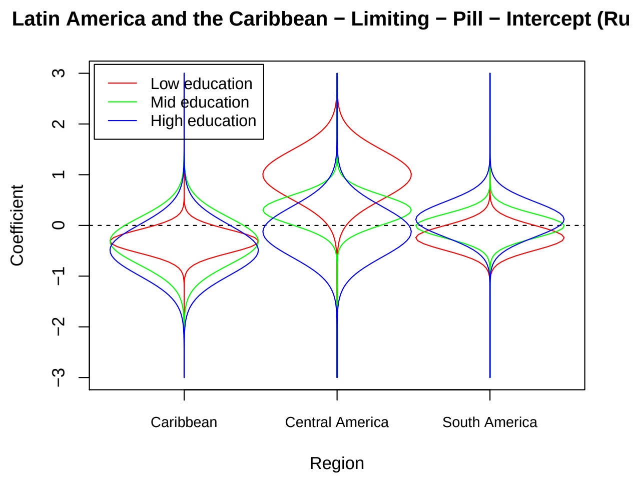
Latin America and the Caribbean − Limiting − Pill − Intercept (Ru
Low education
Mid education
High education
Coefficient
3
2
1
0
−1
−2
−3
Caribbean
Central America
South America
Region

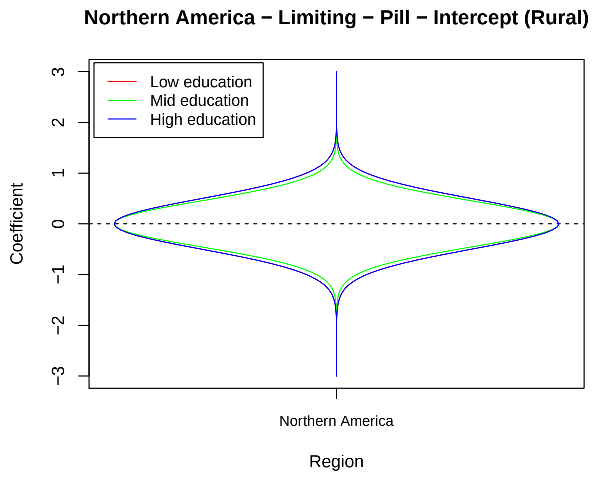
Northern America − Limiting − Pill − Intercept (Rural)
Low education
Mid education
High education
Coefficient
3
2
1
0
−1
−2
−3
Northern America
Region

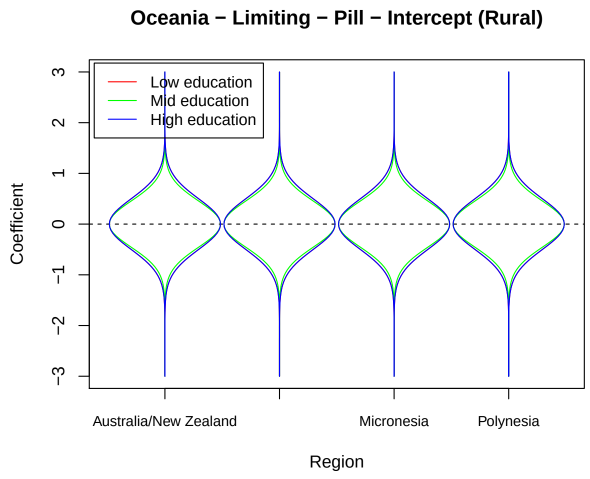
Oceania − Limiting − Pill − Intercept (Rural)
Low education
Mid education
High education
Coefficient
3
2
1
0
−1
−2
−3
Australia/New Zealand
Micronesia
Polynesia
Region

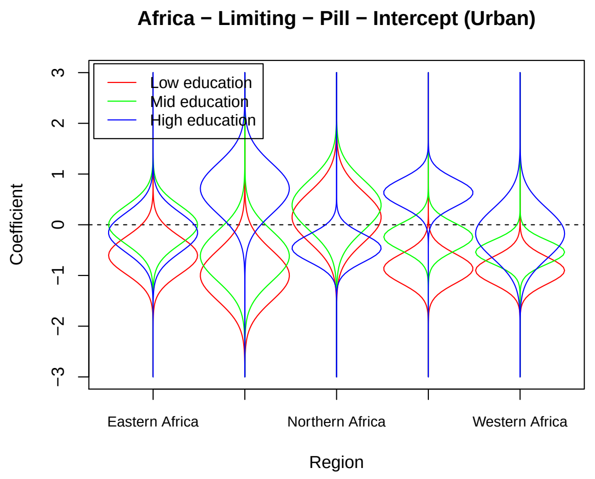
Africa − Limiting − Pill − Intercept (Urban)
Low education
Mid education
High education
Coefficient
3
2
1
0
−1
−2
−3
Eastern Africa
Northern Africa
Western Africa
Region

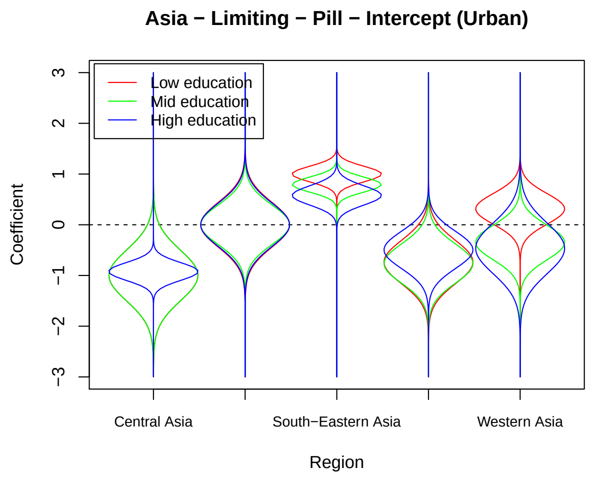
Asia − Limiting − Pill − Intercept (Urban)
Low education
Mid education
High education
Coefficient
3
2
1
0
−1
−2
−3
Central Asia
South−Eastern Asia
Western Asia
Region

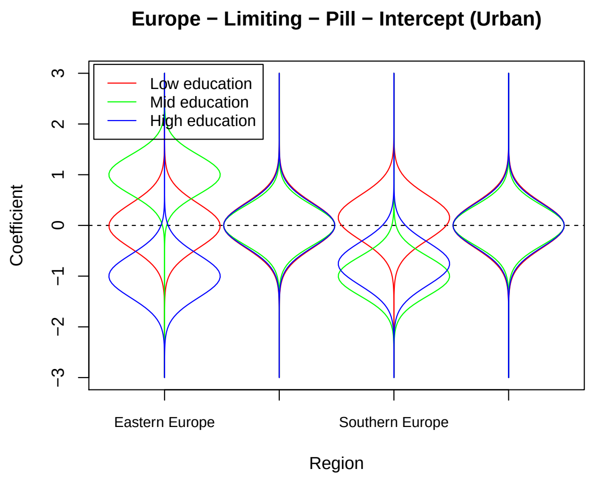
Europe − Limiting − Pill − Intercept (Urban)
Low education
Mid education
High education
Coefficient
3
2
1
0
−1
−2
−3
Eastern Europe
Southern Europe
Region

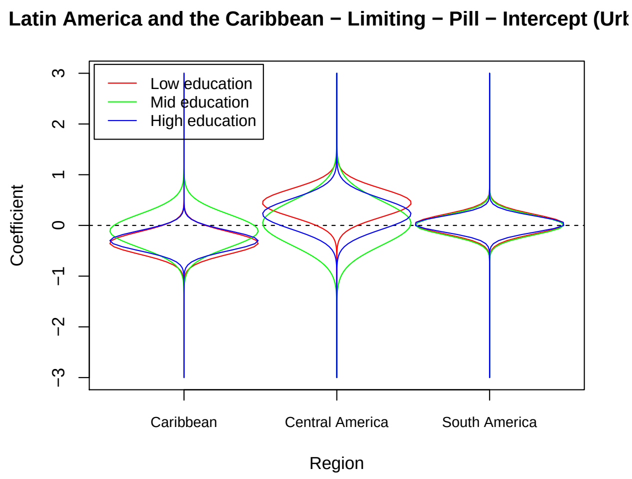
Latin America and the Caribbean − Limiting − Pill − Intercept (Urb
Low education
Mid education
High education
Coefficient
3
2
1
0
−1
−2
−3
Caribbean
Central America
South America
Region

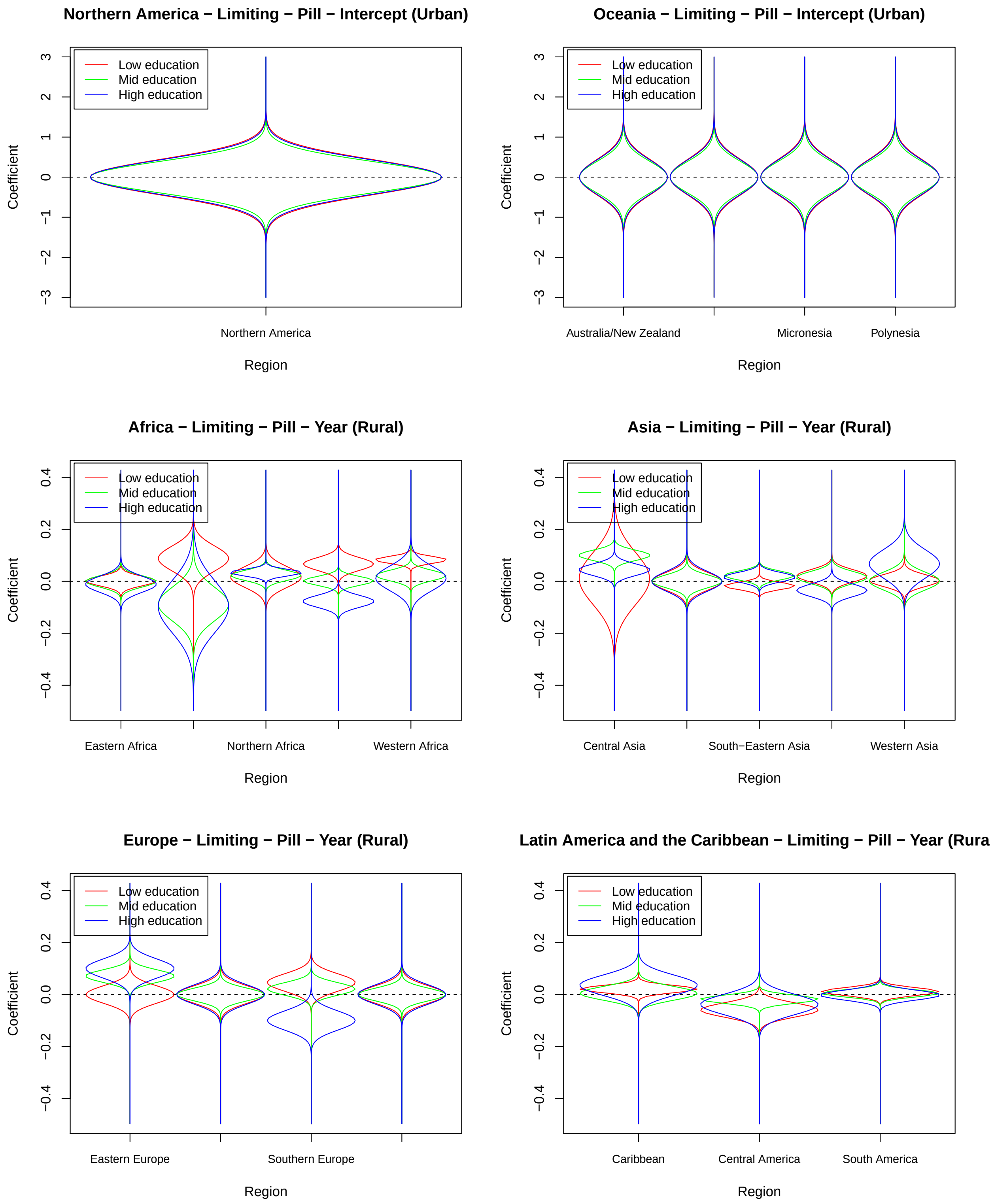
Northern America − Limiting − Pill − Intercept (Urban)
Low education
Mid education
High education
Coefficient
Northern America
Region
Oceania − Limiting − Pill − Intercept (Urban)
Australia/New Zealand
Micronesia
Polynesia
Africa − Limiting − Pill − Year (Rural)
Eastern Africa
Northern Africa
Western Africa
Asia − Limiting − Pill − Year (Rural)
Central Asia
South−Eastern Asia
Western Asia
Europe − Limiting − Pill − Year (Rural)
Eastern Europe
Southern Europe
Latin America and the Caribbean − Limiting − Pill − Year (Rura
Caribbean
Central America
South America

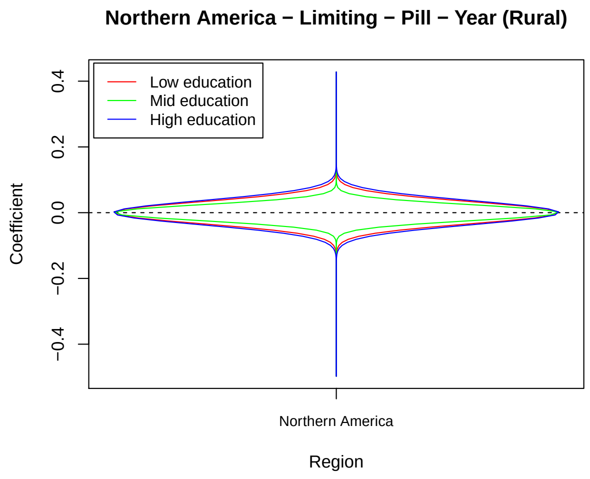
Northern America − Limiting − Pill − Year (Rural)
Low education
Mid education
High education
Coefficient
0.4
0.2
0.0
−0.2
−0.4
Northern America
Region

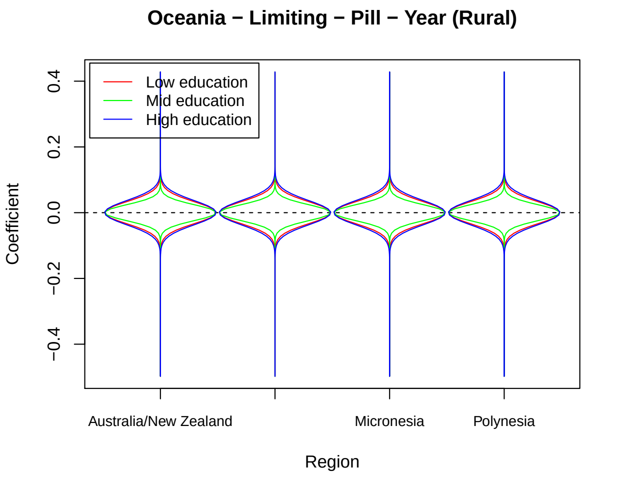
Oceania − Limiting − Pill − Year (Rural)
Low education
Mid education
High education
Coefficient
0.4
0.2
0.0
−0.2
−0.4
Australia/New Zealand
Micronesia
Polynesia
Region

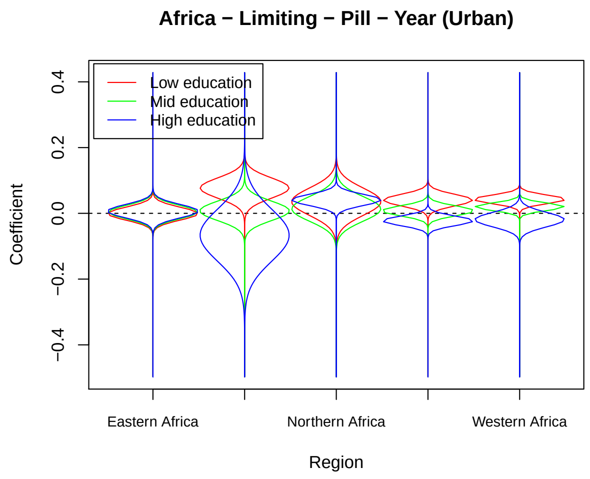
Africa − Limiting − Pill − Year (Urban)
Low education
Mid education
High education
Coefficient
0.4
0.2
0.0
−0.2
−0.4
Eastern Africa
Northern Africa
Western Africa
Region

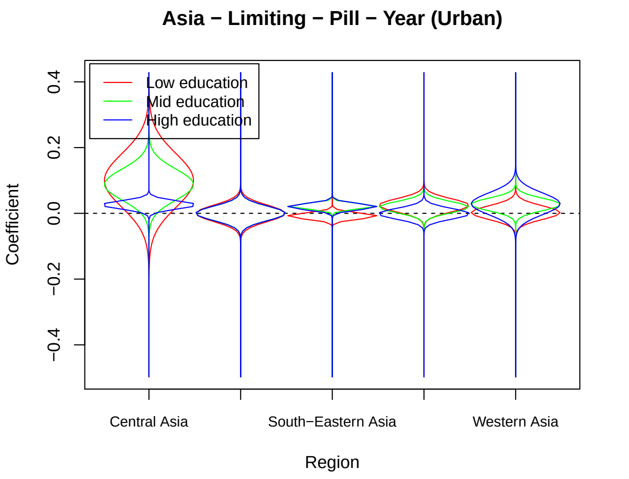
Asia − Limiting − Pill − Year (Urban)
Low education
Mid education
High education
Coefficient
0.4
0.2
0.0
−0.2
−0.4
Central Asia
South−Eastern Asia
Western Asia
Region

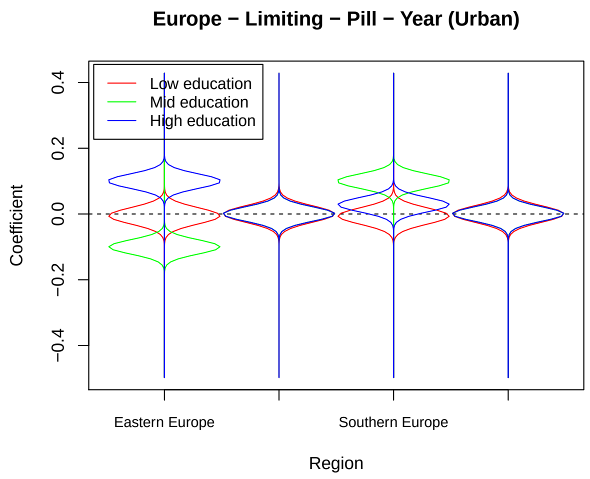
Europe − Limiting − Pill − Year (Urban)
Low education
Mid education
High education
Coefficient
0.4
0.2
0.0
−0.2
−0.4
Eastern Europe
Southern Europe
Region

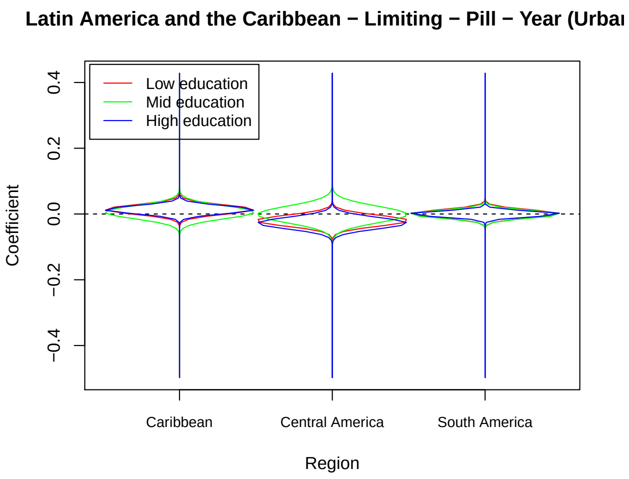
Latin America and the Caribbean − Limiting − Pill − Year (Urba
Low education
Mid education
High education
Coefficient
0.4
0.2
0.0
−0.2
−0.4
Caribbean
Central America
South America
Region

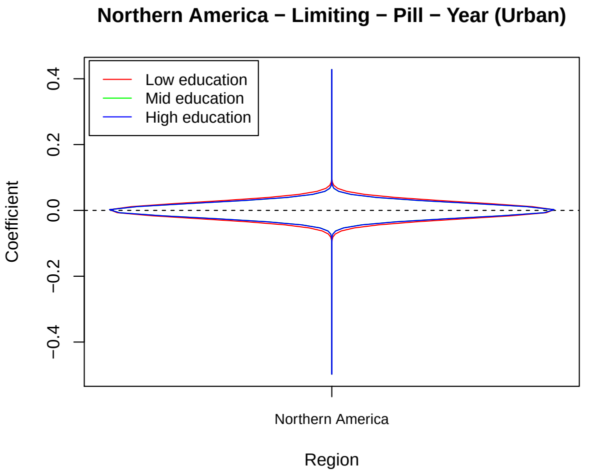
Northern America − Limiting − Pill − Year (Urban)
Low education
Mid education
High education
Coefficient
0.4
0.2
0.0
−0.2
−0.4
Northern America
Region

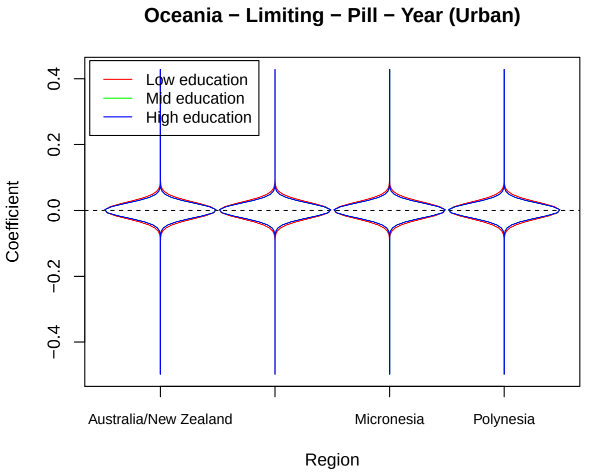
Oceania − Limiting − Pill − Year (Urban)
Low education
Mid education
High education
Coefficient
0.4
0.2
0.0
−0.2
−0.4
Australia/New Zealand
Micronesia
Polynesia
Region

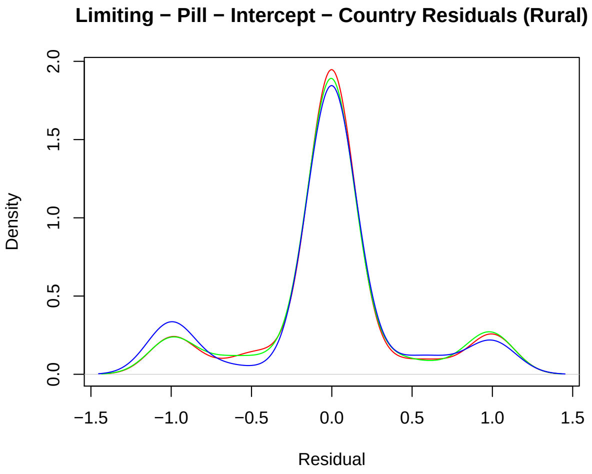
Limiting − Pill − Intercept − Country Residuals (Rural)
Density
0.0
0.5
1.0
1.5
2.0
−1.5
−1.0
−0.5
0.0
0.5
1.0
1.5
Residual

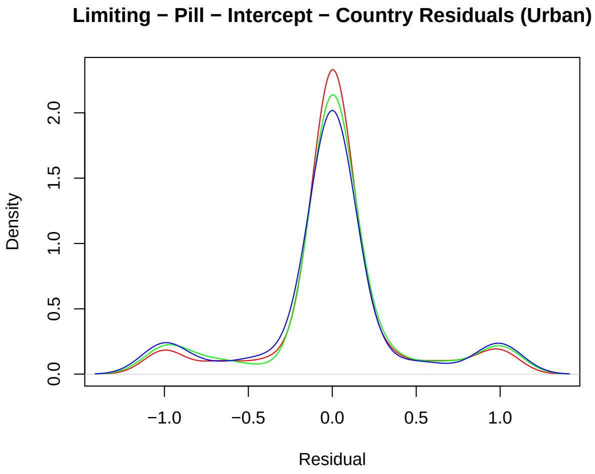
Limiting − Pill − Intercept − Country Residuals (Urban)
Density
0.0
0.5
1.0
1.5
2.0
−1.0
−0.5
0.0
0.5
1.0
Residual

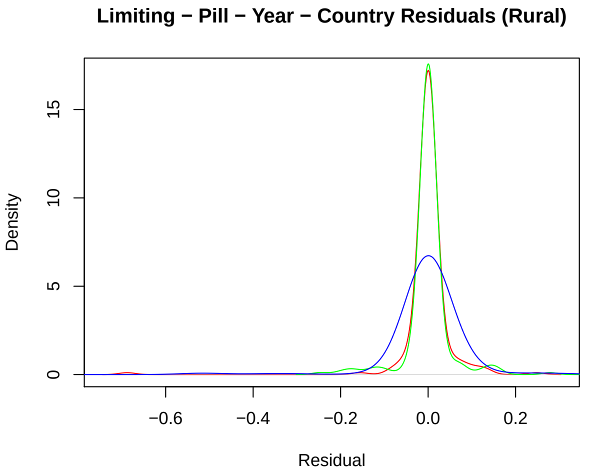
Limiting − Pill − Year − Country Residuals (Rural)
Density
0
5
10
15
−0.6
−0.4
−0.2
0.0
0.2
Residual

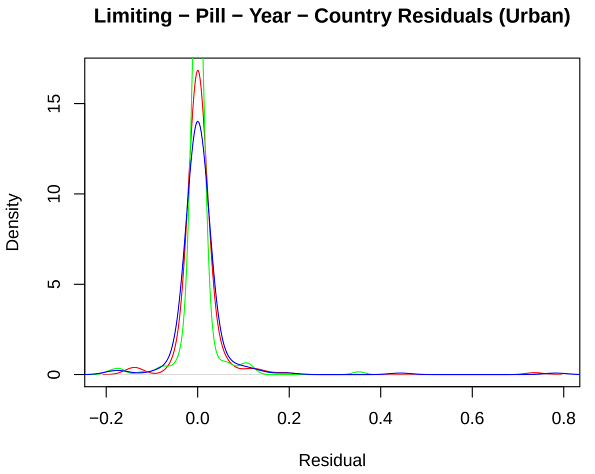
Limiting − Pill − Year − Country Residuals (Urban)
Density
0
5
10
15
−0.2
0.0
0.2
0.4
0.6
0.8
Residual

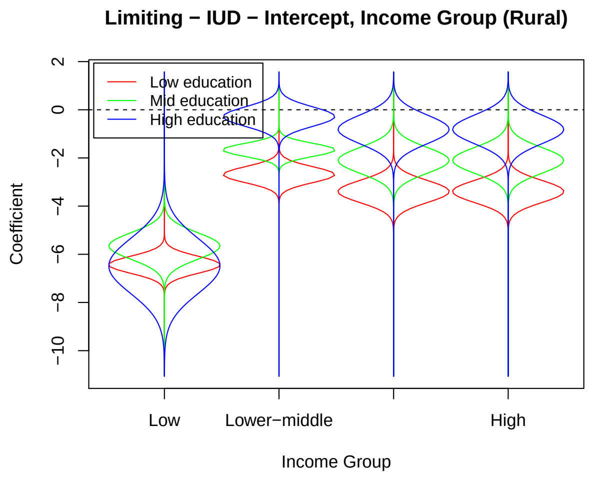
Limiting − IUD − Intercept, Income Group (Rural)
Low education
Mid education
High education
Coefficient
2
0
−2
−4
−6
−8
−10
Low
Lower−middle
High
Income Group

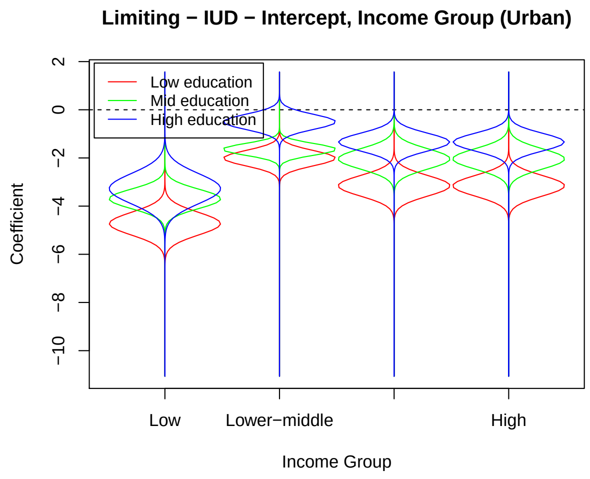
Limiting − IUD − Intercept, Income Group (Urban)
Low education
Mid education
High education
Coefficient
2
0
−2
−4
−6
−8
−10
Low
Lower−middle
High
Income Group

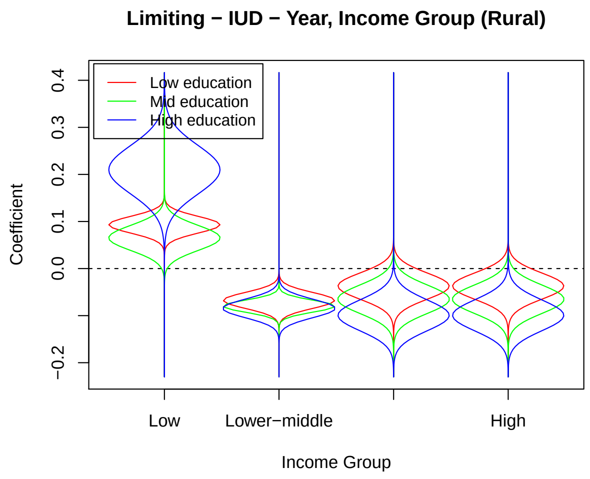
Limiting − IUD − Year, Income Group (Rural)
Low education
Mid education
High education
Coefficient
0.4
0.3
0.2
0.1
0.0
−0.2
Low
Lower−middle
High
Income Group

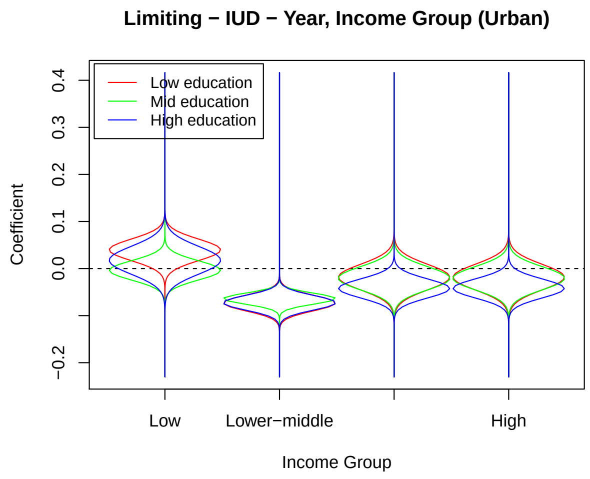
Limiting − IUD − Year, Income Group (Urban)
Low education
Mid education
High education
Coefficient
0.4
0.3
0.2
0.1
0.0
−0.2
Low
Lower−middle
High
Income Group

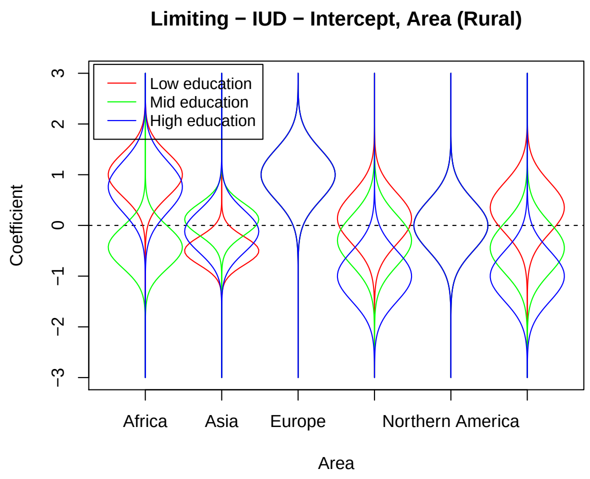
Limiting − IUD − Intercept, Area (Rural)
Low education
Mid education
High education
Coefficient
3
2
1
0
−1
−2
−3
Africa
Asia
Europe
Northern America
Area

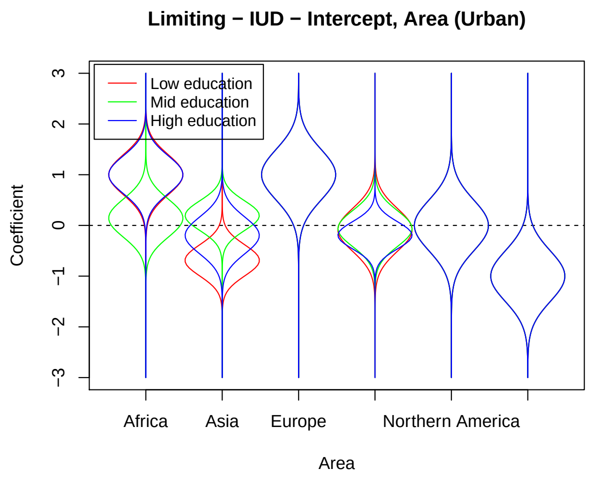
Limiting − IUD − Intercept, Area (Urban)
Low education
Mid education
High education
Coefficient
3
2
1
0
−1
−2
−3
Africa
Asia
Europe
Northern America
Area

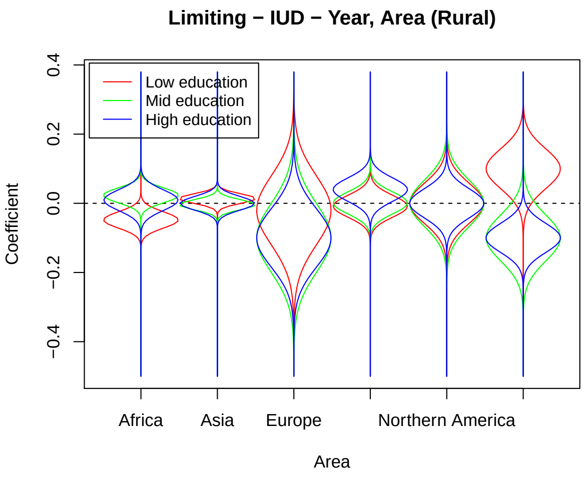
Limiting − IUD − Year, Area (Rural)
Low education
Mid education
High education
Coefficient
0.4
0.2
0.0
−0.2
−0.4
Africa
Asia
Europe
Northern America
Area

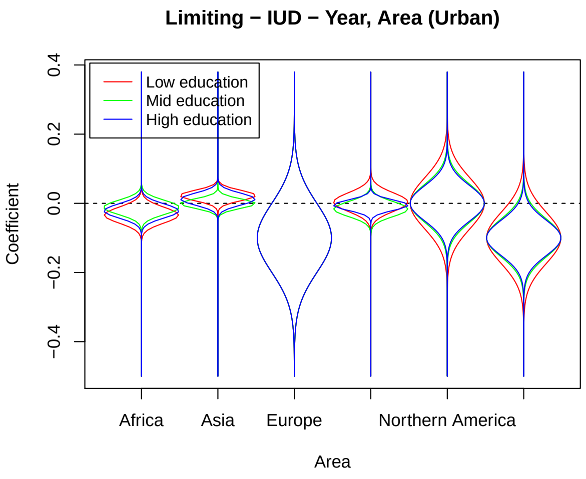
Limiting − IUD − Year, Area (Urban)
Low education
Mid education
High education
Coefficient
0.4
0.2
0.0
−0.2
−0.4
Africa
Asia
Europe
Northern America
Area

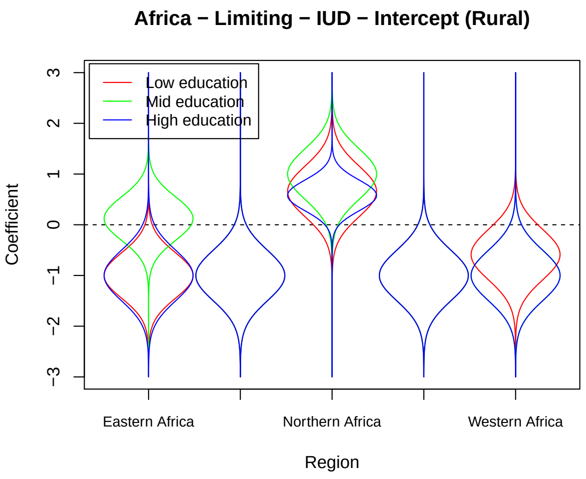
Africa − Limiting − IUD − Intercept (Rural)
Low education
Mid education
High education
Coefficient
3
2
1
0
−1
−2
−3
Eastern Africa
Northern Africa
Western Africa
Region

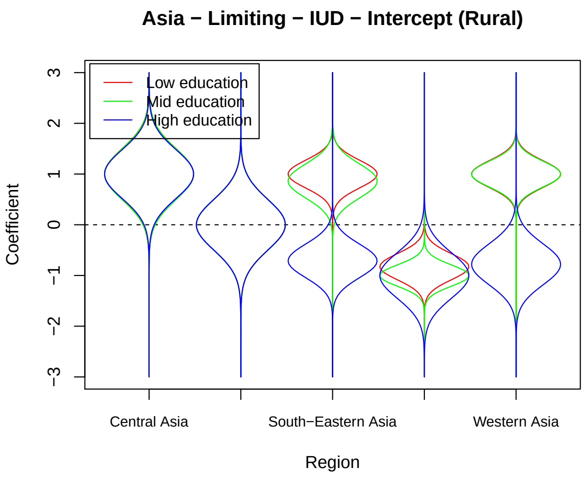
Asia − Limiting − IUD − Intercept (Rural)
Low education
Mid education
High education
Coefficient
3
2
1
0
−1
−2
−3
Central Asia
South−Eastern Asia
Western Asia
Region

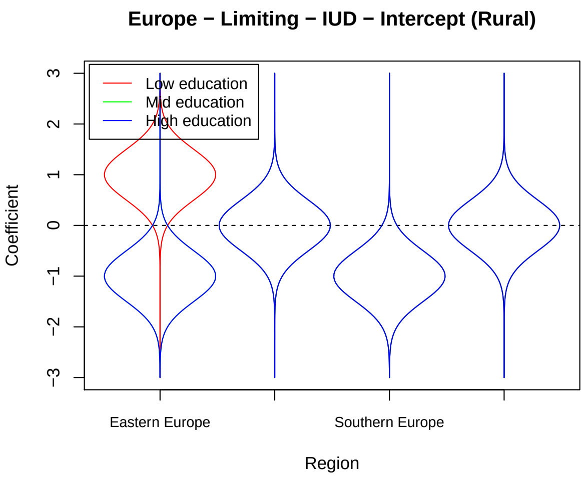
Europe − Limiting − IUD − Intercept (Rural)
Low education
Mid education
High education
Coefficient
3
2
1
0
−1
−2
−3
Eastern Europe
Southern Europe
Region

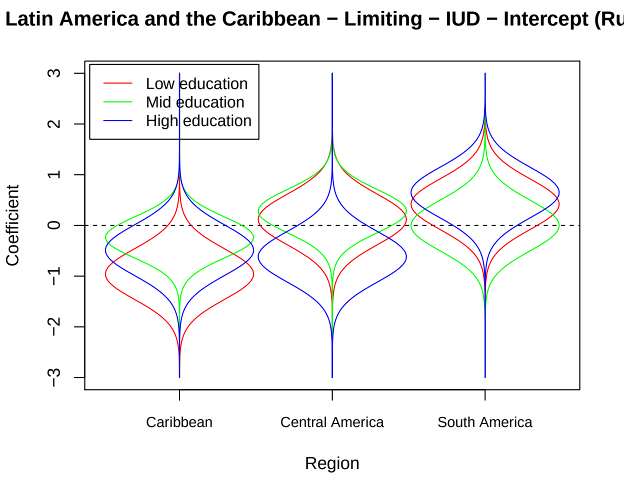
Latin America and the Caribbean − Limiting − IUD − Intercept (Ru
Low education
Mid education
High education
Coefficient
3
2
1
0
−1
−2
−3
Caribbean
Central America
South America
Region

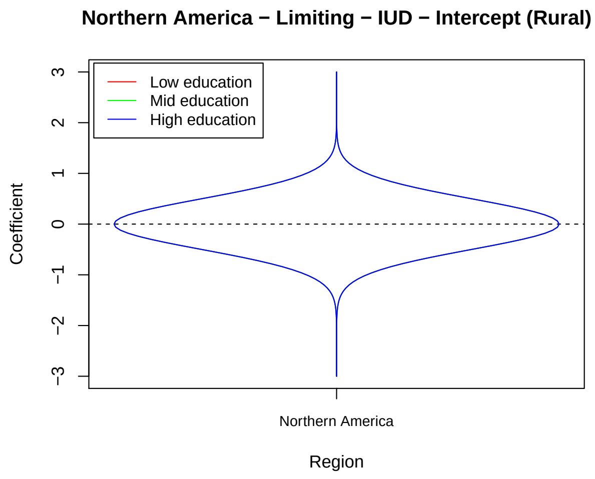
Northern America − Limiting − IUD − Intercept (Rural)
Low education
Mid education
High education
Coefficient
3
2
1
0
−1
−2
−3
Northern America
Region

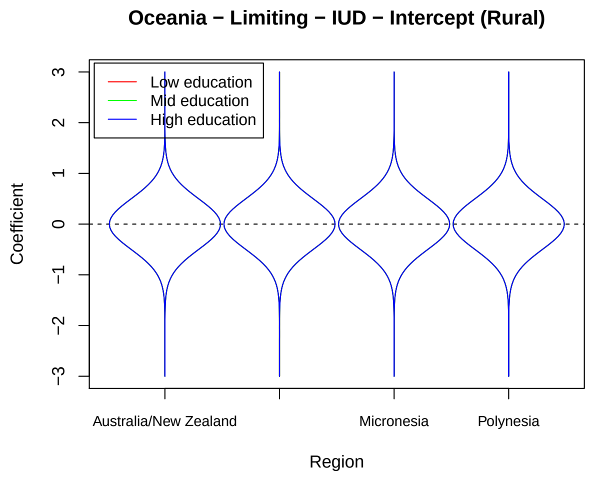
Oceania − Limiting − IUD − Intercept (Rural)
Low education
Mid education
High education
Coefficient
3
2
1
0
−1
−2
−3
Australia/New Zealand
Micronesia
Polynesia
Region

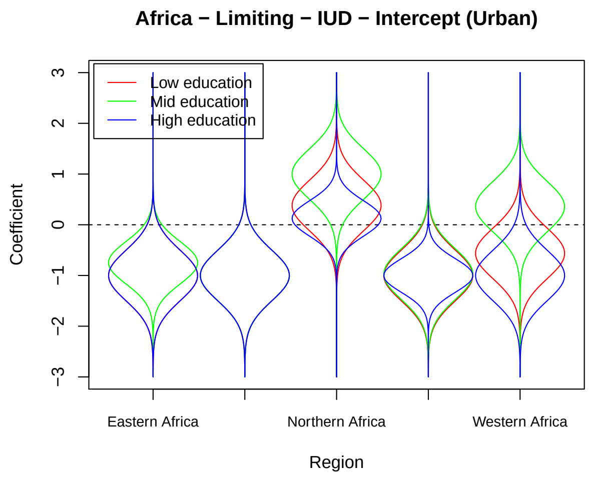
Africa − Limiting − IUD − Intercept (Urban)
Low education
Mid education
High education
Coefficient
3
2
1
0
−1
−2
−3
Eastern Africa
Northern Africa
Western Africa
Region

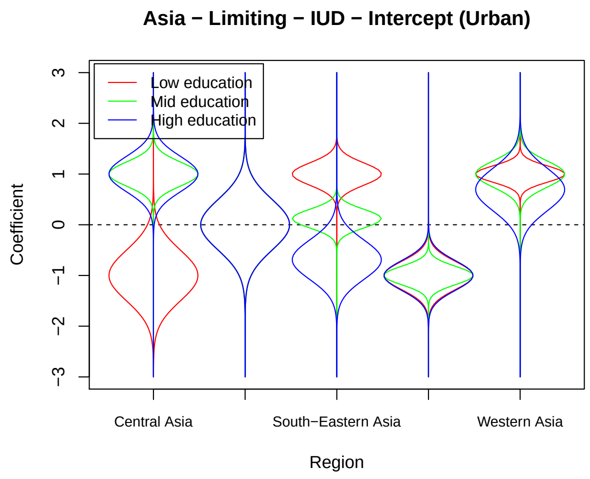
Asia − Limiting − IUD − Intercept (Urban)
Low education
Mid education
High education
Coefficient
3
2
1
0
−1
−2
−3
Central Asia
South−Eastern Asia
Western Asia
Region

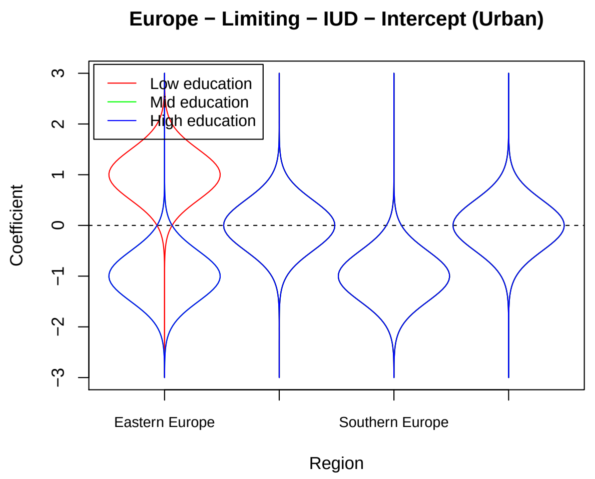
Europe − Limiting − IUD − Intercept (Urban)
Low education
Mid education
High education
Coefficient
3
2
1
0
−1
−2
−3
Eastern Europe
Southern Europe
Region

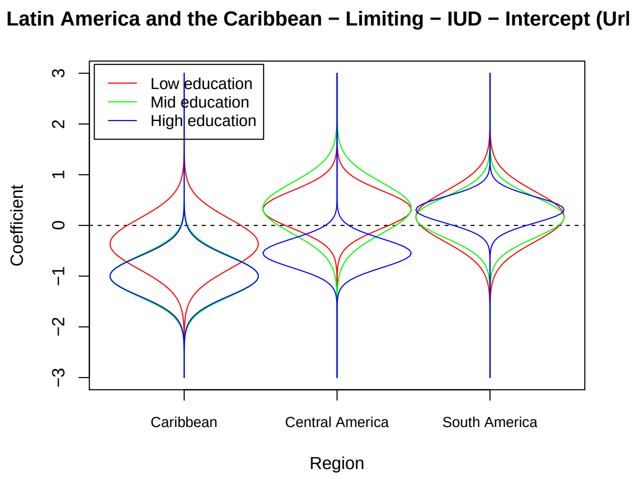
Latin America and the Caribbean − Limiting − IUD − Intercept (Url
Low education
Mid education
High education
Coefficient
3
2
1
0
−1
−2
−3
Caribbean
Central America
South America
Region

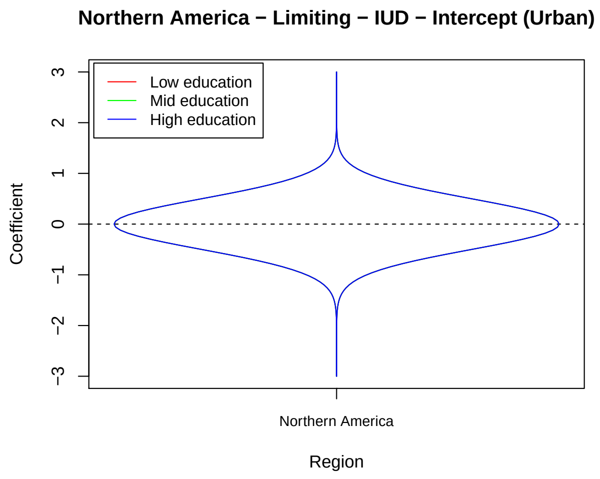
Northern America − Limiting − IUD − Intercept (Urban)
Low education
Mid education
High education
Coefficient
3
2
1
0
−1
−2
−3
Northern America
Region

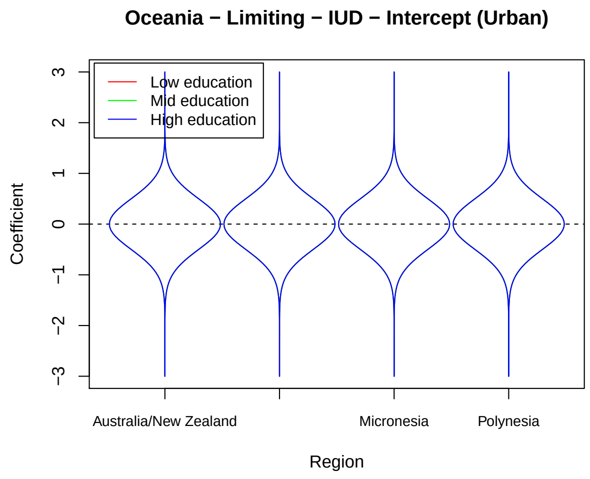
Oceania − Limiting − IUD − Intercept (Urban)
Low education
Mid education
High education
Coefficient
3
2
1
0
−1
−2
−3
Australia/New Zealand
Micronesia
Polynesia
Region

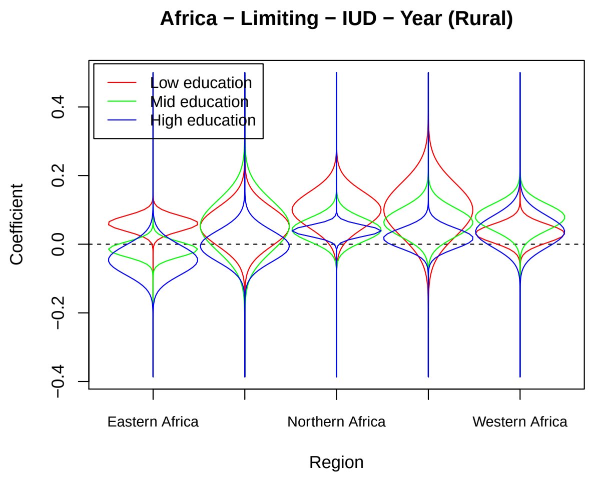
Africa − Limiting − IUD − Year (Rural)
Low education
Mid education
High education
Coefficient
0.4
0.2
0.0
−0.2
−0.4
Eastern Africa
Northern Africa
Western Africa
Region

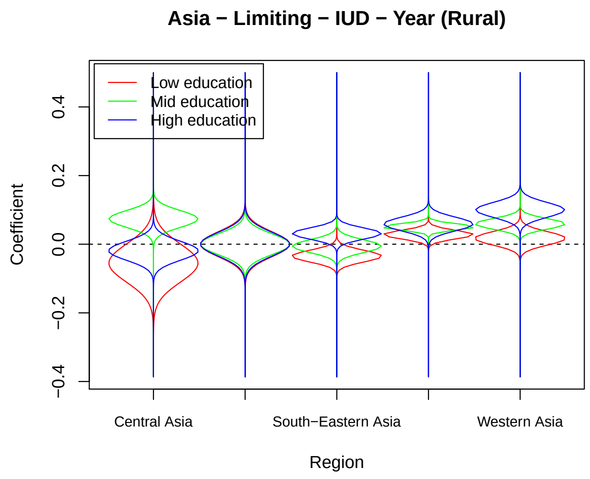
Asia − Limiting − IUD − Year (Rural)
Low education
Mid education
High education
Coefficient
0.4
0.2
0.0
−0.2
−0.4
Central Asia
South−Eastern Asia
Western Asia
Region

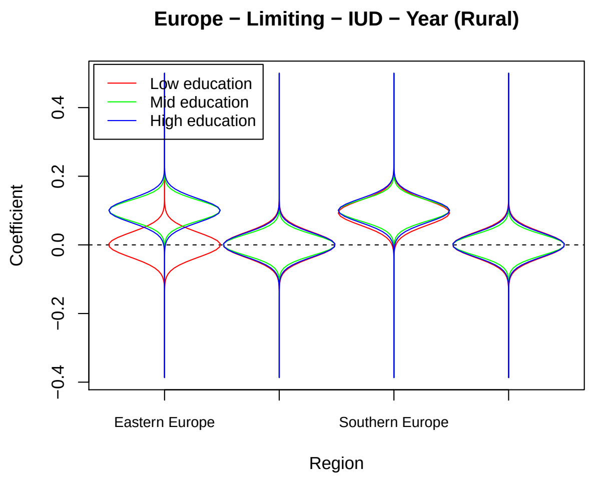
Europe − Limiting − IUD − Year (Rural)
Low education
Mid education
High education
Coefficient
0.4
0.2
0.0
−0.2
−0.4
Eastern Europe
Southern Europe
Region

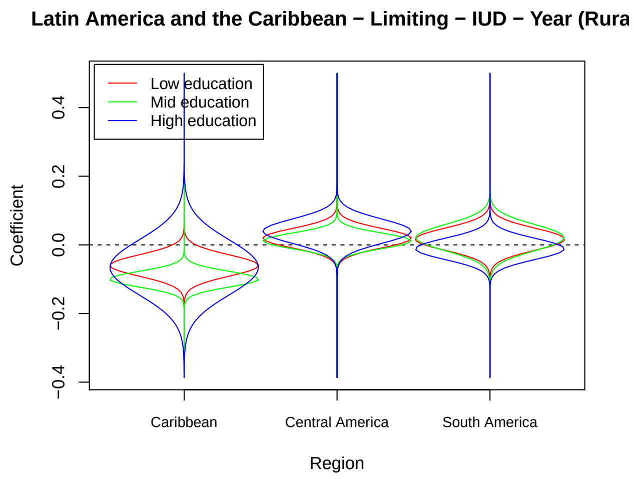
Latin America and the Caribbean − Limiting − IUD − Year (Rura
Low education
Mid education
High education
Coefficient
0.4
0.2
0.0
−0.2
−0.4
Caribbean
Central America
South America
Region

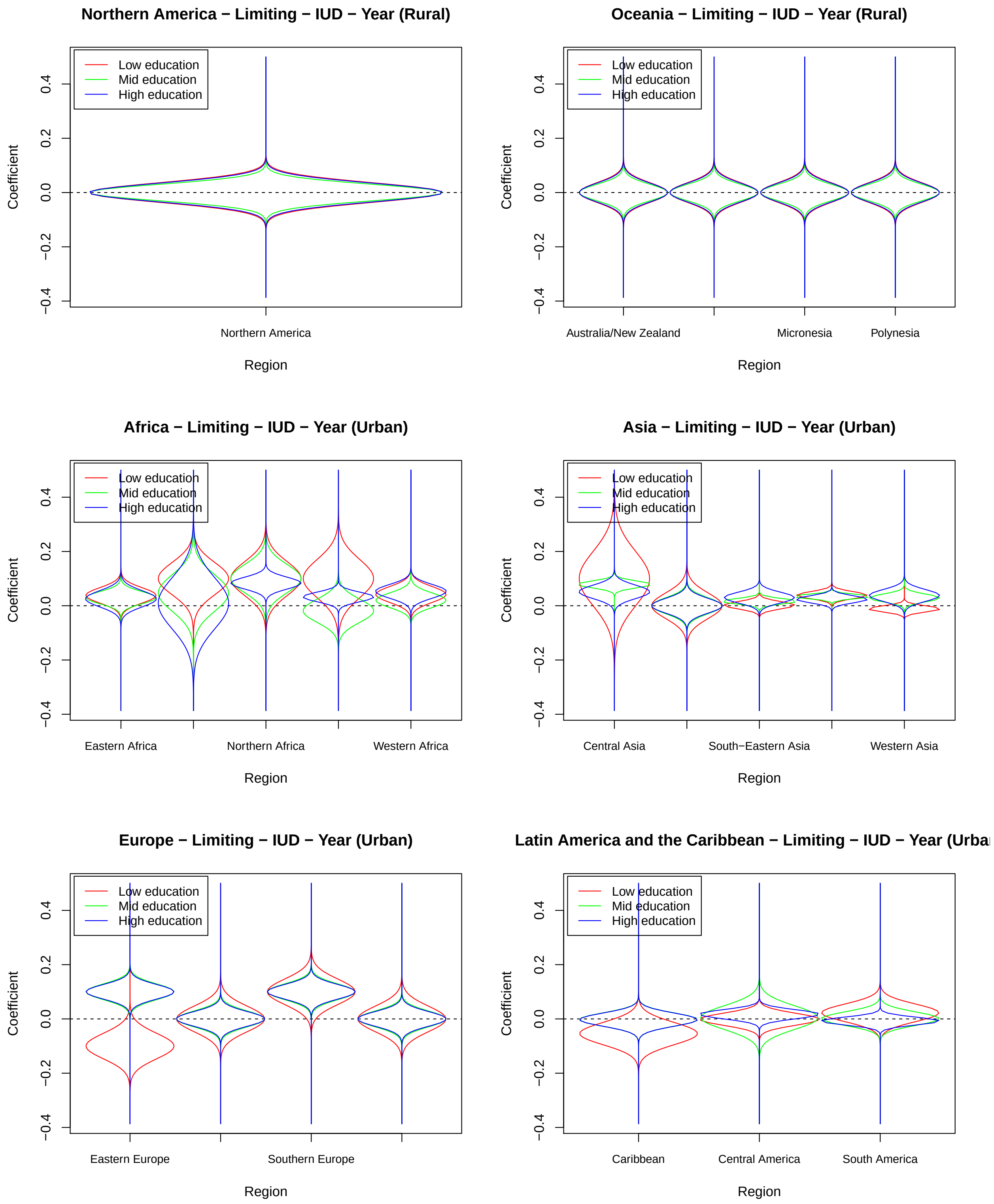
Northern America − Limiting − IUD − Year (Rural)
Low education
Mid education
High education
Coefficient
0.4
0.2
0.0
−0.2
−0.4
Northern America
Region
Oceania − Limiting − IUD − Year (Rural)
Australia/New Zealand
Micronesia
Polynesia
Africa − Limiting − IUD − Year (Urban)
Eastern Africa
Northern Africa
Western Africa
Asia − Limiting − IUD − Year (Urban)
Central Asia
South−Eastern Asia
Western Asia
Europe − Limiting − IUD − Year (Urban)
Eastern Europe
Southern Europe
Latin America and the Caribbean − Limiting − IUD − Year (Urba
Caribbean
Central America
South America

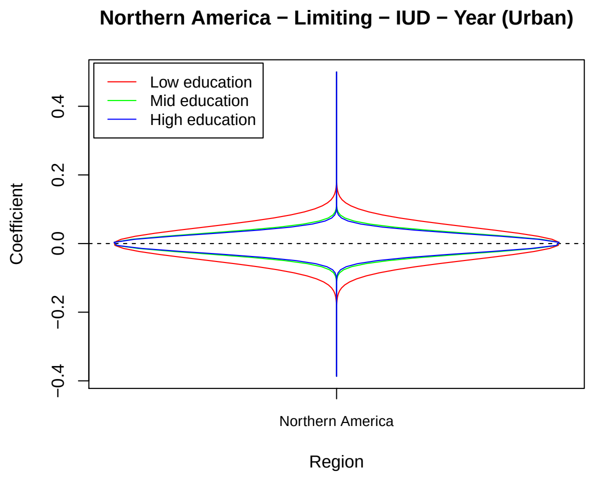
Northern America − Limiting − IUD − Year (Urban)
Low education
Mid education
High education
Coefficient
−0.4
−0.2
0.0
0.2
0.4
Northern America
Region

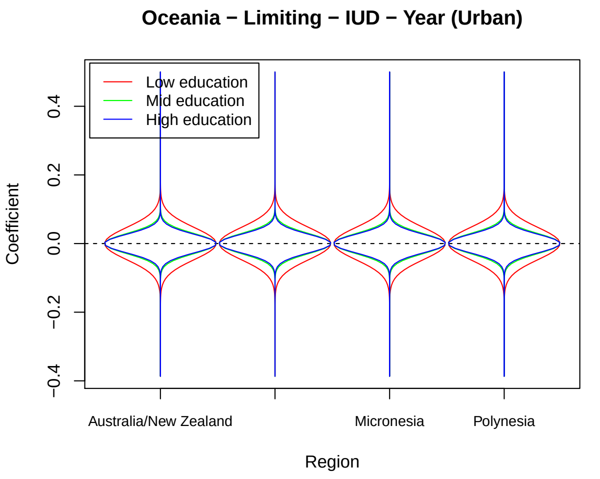
Oceania − Limiting − IUD − Year (Urban)
Low education
Mid education
High education
Coefficient
−0.4
−0.2
0.0
0.2
0.4
Australia/New Zealand
Micronesia
Polynesia
Region

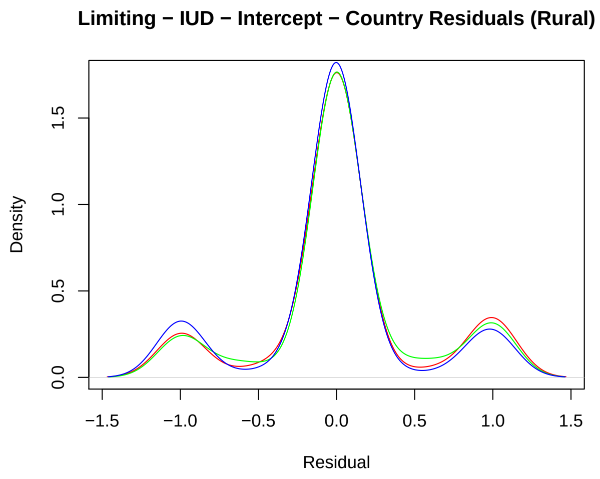
Limiting − IUD − Intercept − Country Residuals (Rural)
Density
0.0
0.5
1.0
1.5
−1.5
−1.0
−0.5
0.0
0.5
1.0
1.5
Residual

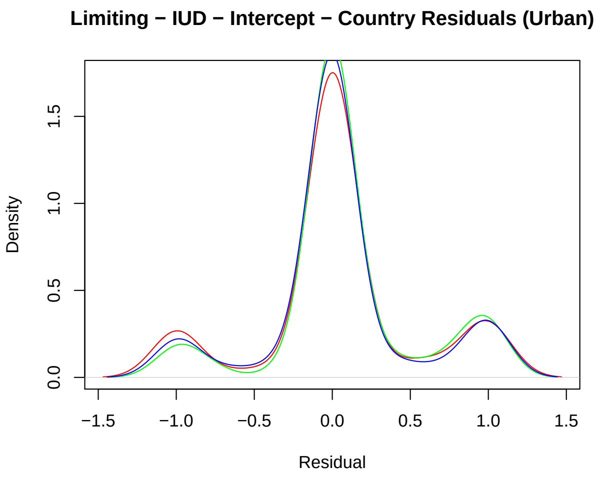
Limiting − IUD − Intercept − Country Residuals (Urban)
Density
0.0
0.5
1.0
1.5
−1.5
−1.0
−0.5
0.0
0.5
1.0
1.5
Residual

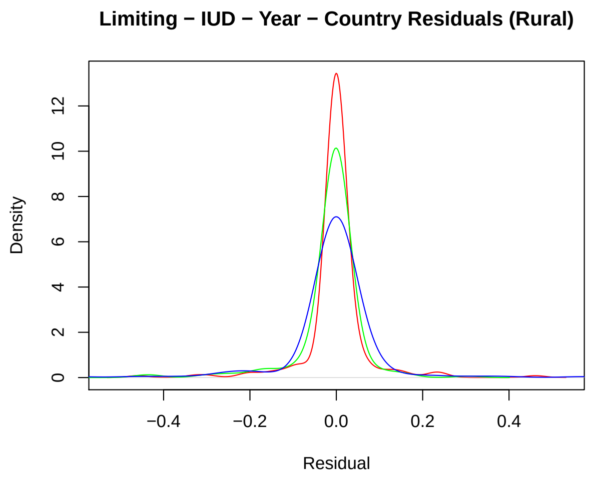
Limiting − IUD − Year − Country Residuals (Rural)
Density
0
2
4
6
8
10
12
−0.4
−0.2
0.0
0.2
0.4
Residual

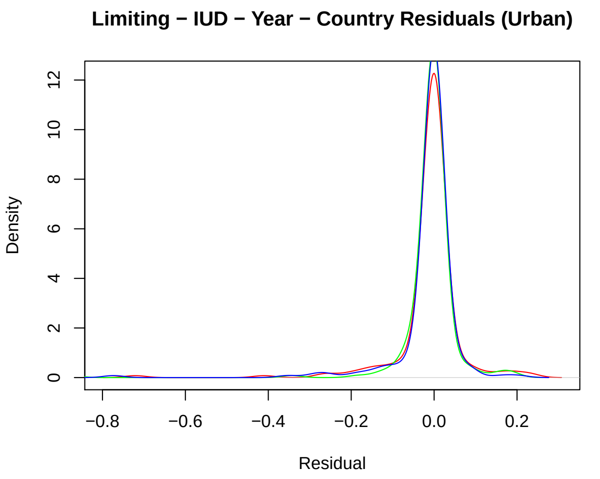
Limiting − IUD − Year − Country Residuals (Urban)
Density
0
2
4
6
8
10
12
−0.8
−0.6
−0.4
−0.2
0.0
0.2
Residual

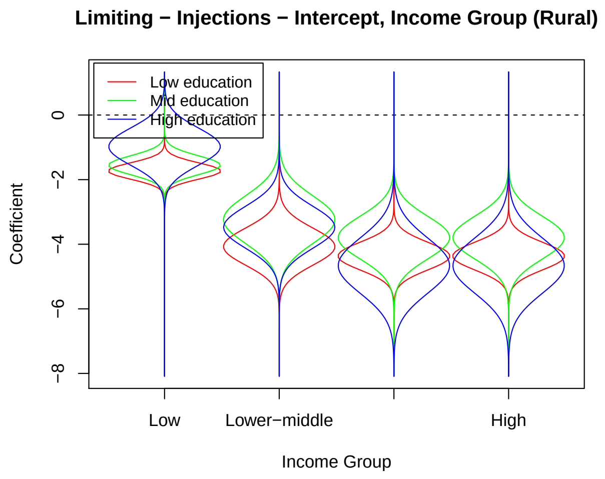
Limiting − Injections − Intercept, Income Group (Rural)
Low education
Mid education
High education
Coefficient
0
−2
−4
−6
−8
Low
Lower−middle
High
Income Group

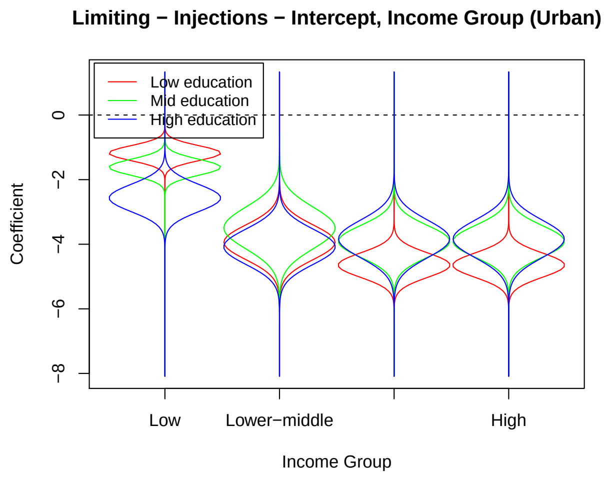
Limiting − Injections − Intercept, Income Group (Urban)
Low education
Mid education
High education
Coefficient
0
−2
−4
−6
−8
Low
Lower−middle
High
Income Group

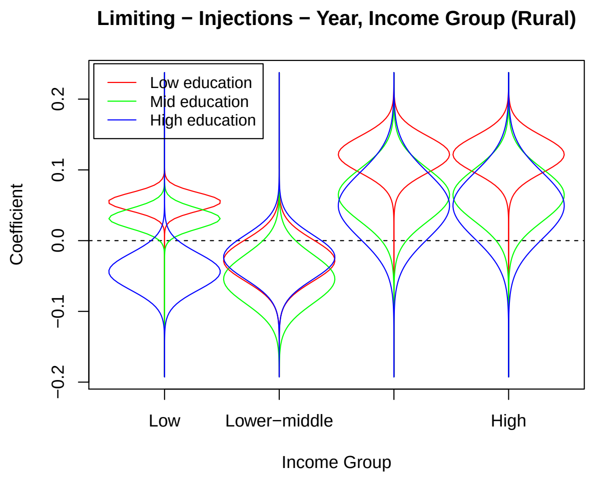
Limiting − Injections − Year, Income Group (Rural)
Low education
Mid education
High education
Coefficient
0.2
0.1
0.0
−0.1
−0.2
Low
Lower−middle
High
Income Group

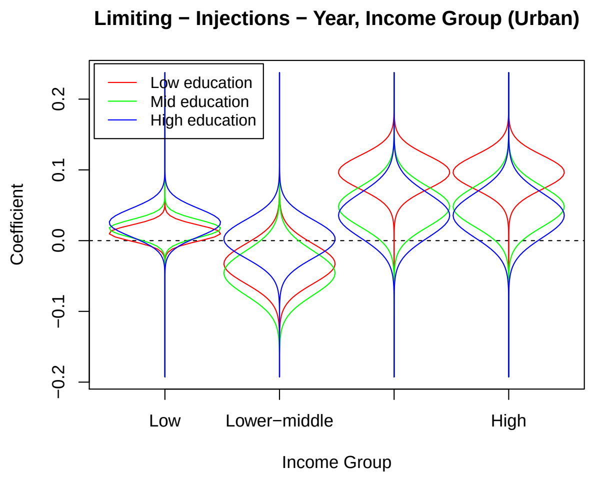
Limiting − Injections − Year, Income Group (Urban)
Low education
Mid education
High education
Coefficient
0.2
0.1
0.0
−0.1
−0.2
Low
Lower−middle
High
Income Group

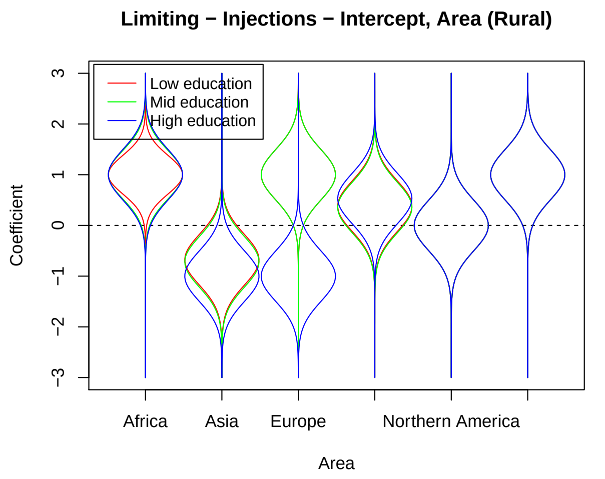
Limiting − Injections − Intercept, Area (Rural)
Low education
Mid education
High education
Coefficient
3
2
1
0
−1
−2
−3
Africa
Asia
Europe
Northern America
Area

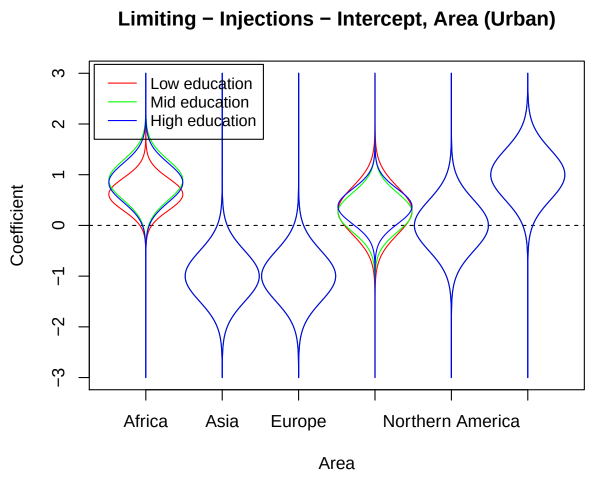
Limiting − Injections − Intercept, Area (Urban)
Low education
Mid education
High education
Coefficient
3
2
1
0
−1
−2
−3
Africa
Asia
Europe
Northern America
Area

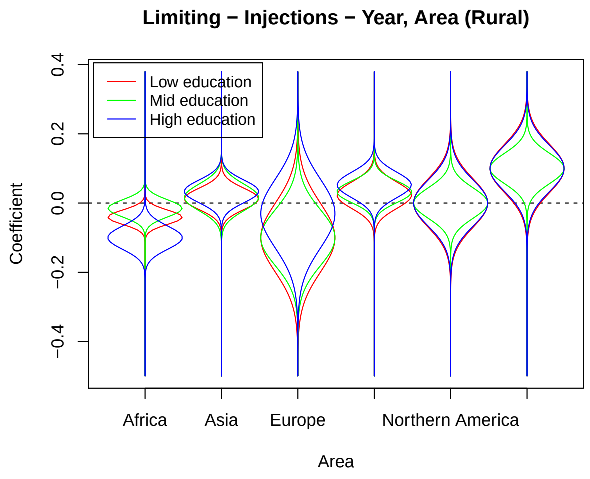
Limiting − Injections − Year, Area (Rural)
Low education
Mid education
High education
Coefficient
0.4
0.2
0.0
−0.2
−0.4
Africa
Asia
Europe
Northern America
Area

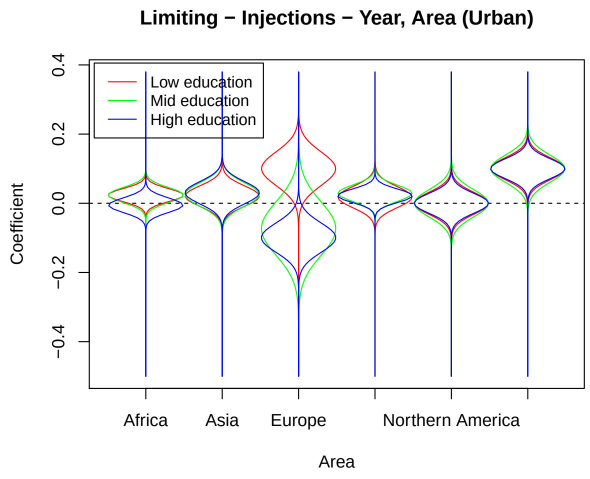
Limiting − Injections − Year, Area (Urban)
Low education
Mid education
High education
Coefficient
0.4
0.2
0.0
−0.2
−0.4
Africa
Asia
Europe
Northern America
Area

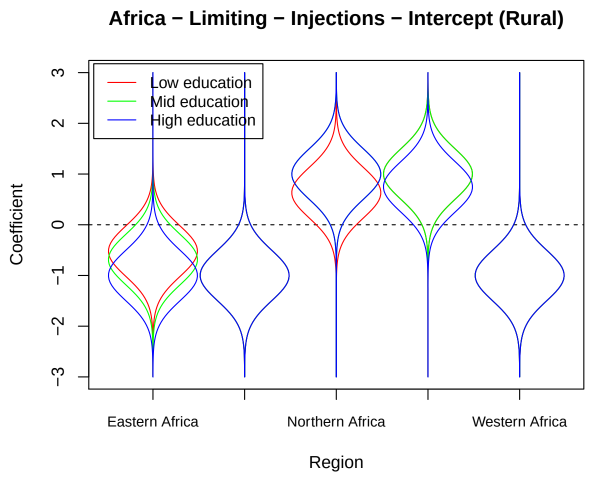
Africa − Limiting − Injections − Intercept (Rural)
Low education
Mid education
High education
Coefficient
3
2
1
0
−1
−2
−3
Eastern Africa
Northern Africa
Western Africa
Region

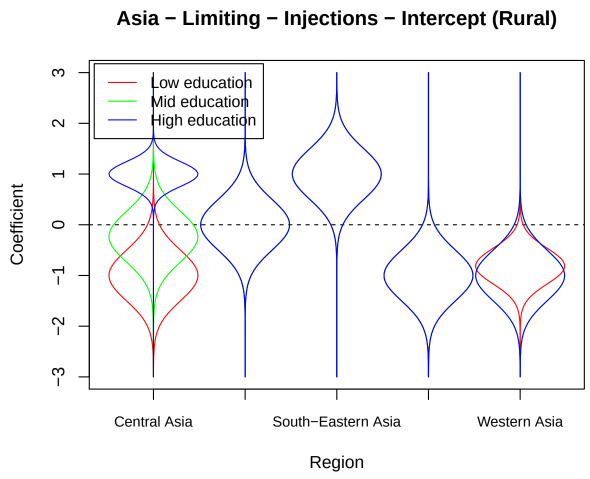
Asia − Limiting − Injections − Intercept (Rural)
Low education
Mid education
High education
Coefficient
3
2
1
0
−1
−2
−3
Central Asia
South−Eastern Asia
Western Asia
Region

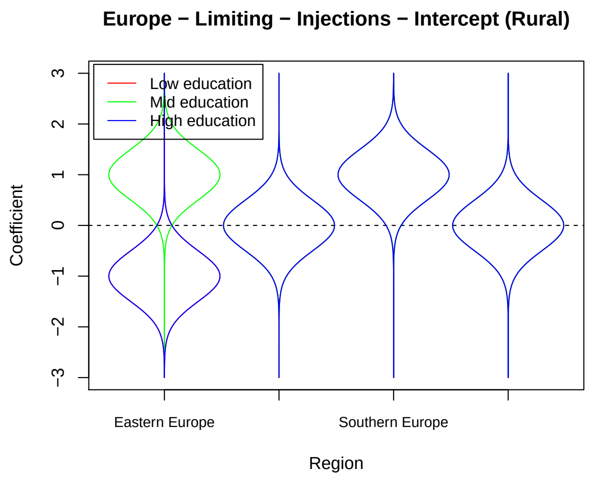
Europe − Limiting − Injections − Intercept (Rural)
Low education
Mid education
High education
Coefficient
3
2
1
0
−1
−2
−3
Eastern Europe
Southern Europe
Region

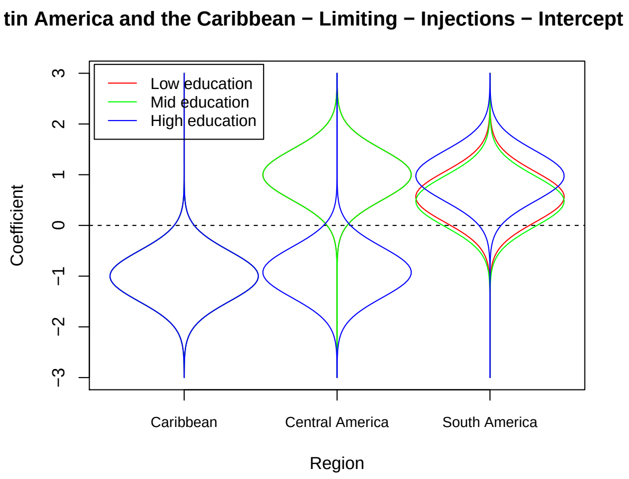
tin America and the Caribbean − Limiting − Injections − Intercept
Low education
Mid education
High education
Coefficient
3
2
1
0
−1
−2
−3
Caribbean
Central America
South America
Region

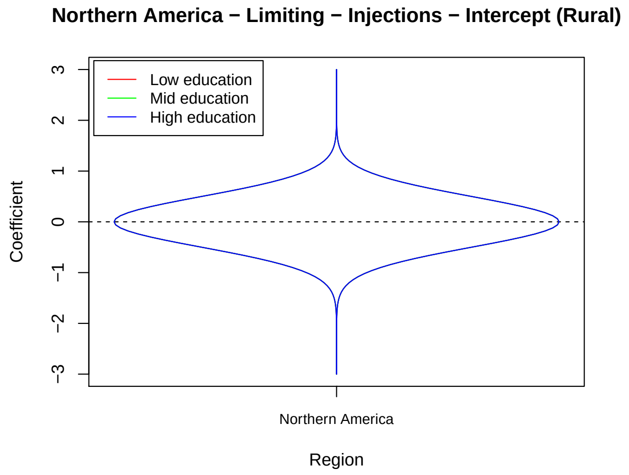
Northern America − Limiting − Injections − Intercept (Rural)
Low education
Mid education
High education
Coefficient
Northern America
Region

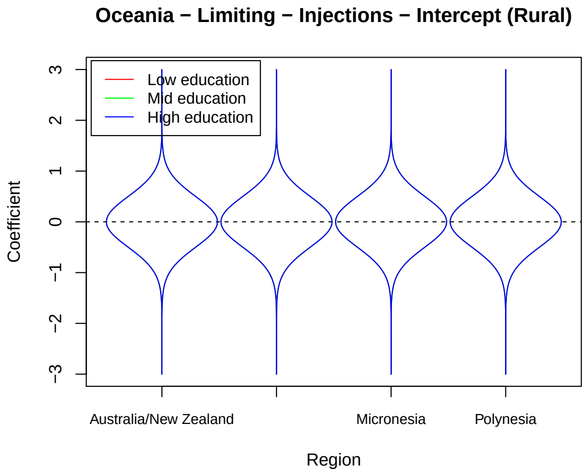
Oceania − Limiting − Injections − Intercept (Rural)
Low education
Mid education
High education
Coefficient
Australia/New Zealand
Micronesia
Polynesia
Region

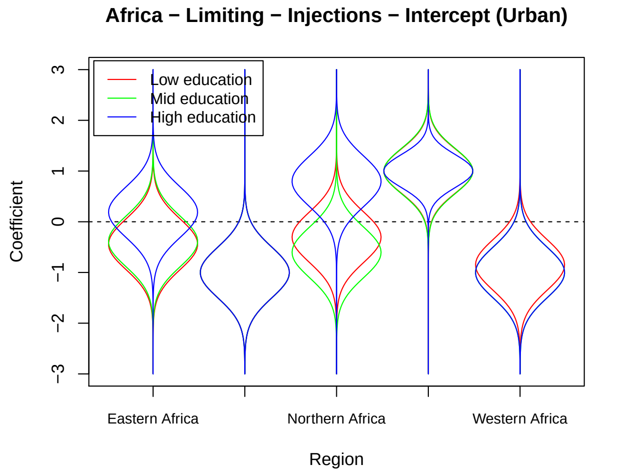
Africa − Limiting − Injections − Intercept (Urban)
Low education
Mid education
High education
Coefficient
Eastern Africa
Northern Africa
Western Africa
Region

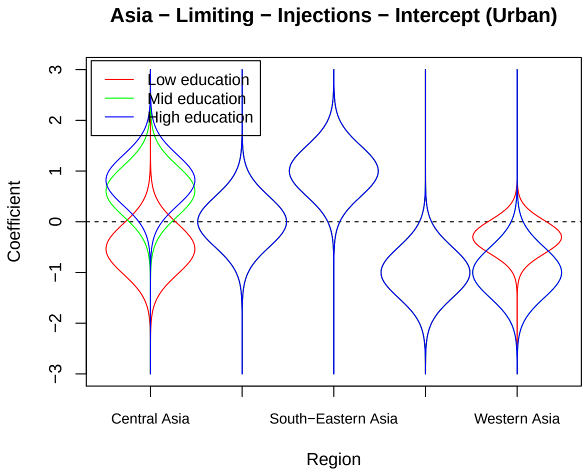
Asia − Limiting − Injections − Intercept (Urban)
Low education
Mid education
High education
Coefficient
Central Asia
South−Eastern Asia
Western Asia
Region

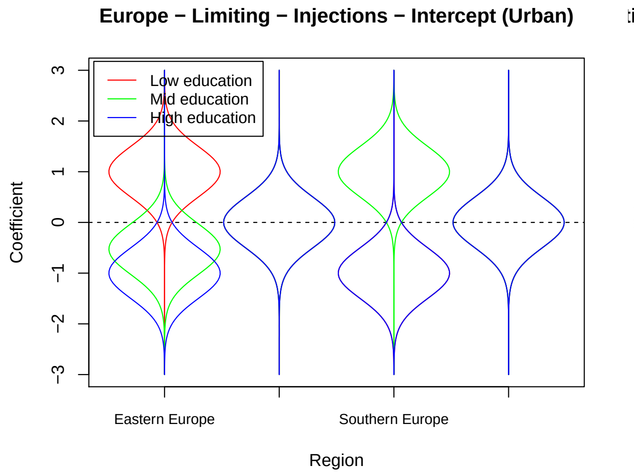
Europe − Limiting − Injections − Intercept (Urban)
Low education
Mid education
High education
Coefficient
Eastern Europe
Southern Europe
Region

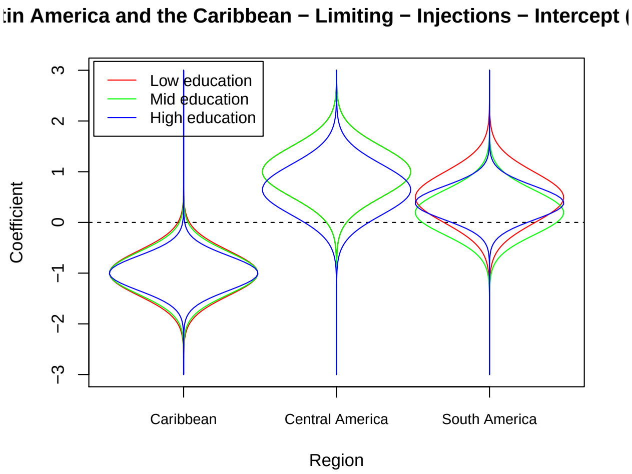
tin America and the Caribbean − Limiting − Injections − Intercept (
Low education
Mid education
High education
Coefficient
Caribbean
Central America
South America
Region

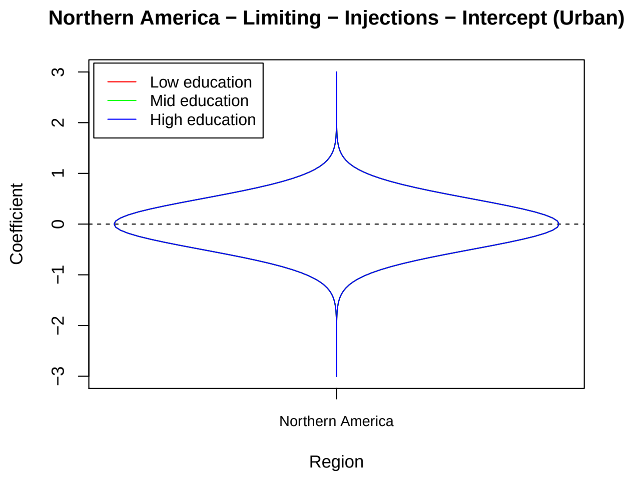
Northern America − Limiting − Injections − Intercept (Urban)
Low education
Mid education
High education
Coefficient
Northern America
Region

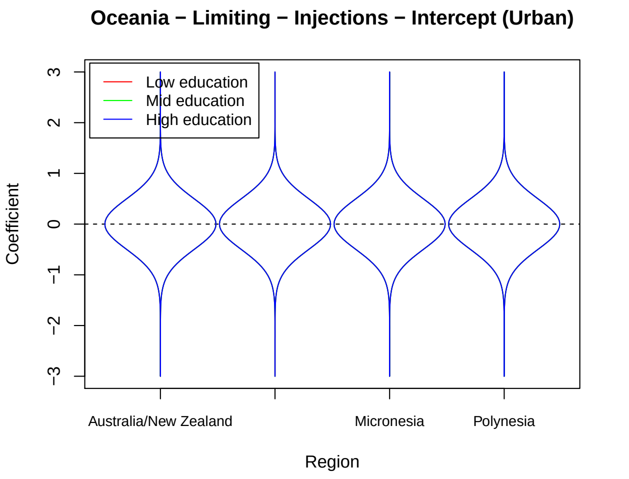
Oceania − Limiting − Injections − Intercept (Urban)
Low education
Mid education
High education
Coefficient
Australia/New Zealand
Micronesia
Polynesia
Region

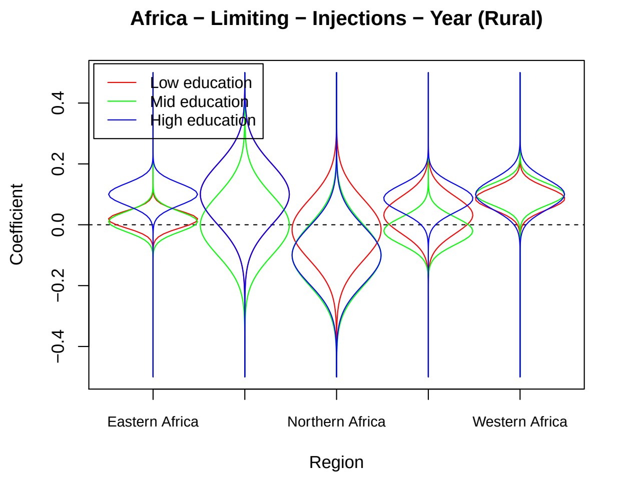
Africa − Limiting − Injections − Year (Rural)
Low education
Mid education
High education
Coefficient
Eastern Africa
Northern Africa
Western Africa
Region

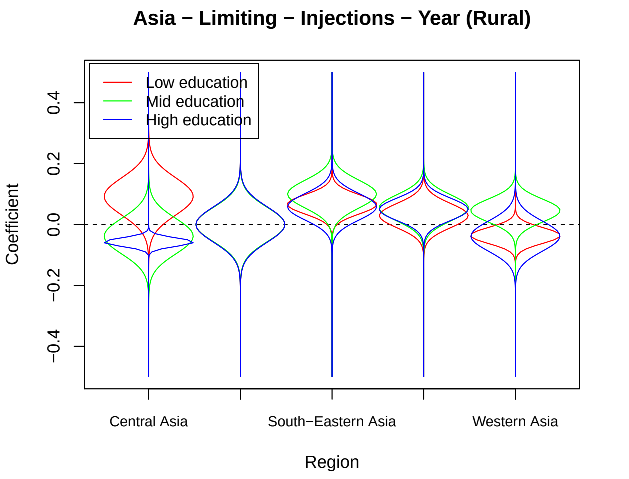
Asia − Limiting − Injections − Year (Rural)
Low education
Mid education
High education
Coefficient
Central Asia
South−Eastern Asia
Western Asia
Region

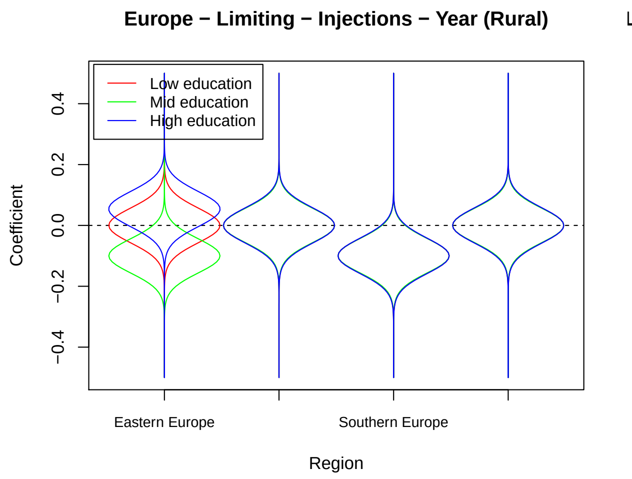
Europe − Limiting − Injections − Year (Rural)
Low education
Mid education
High education
Coefficient
Eastern Europe
Southern Europe
Region

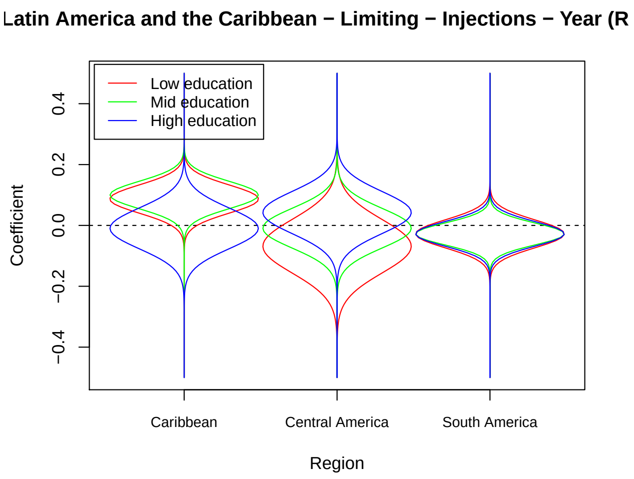
Latin America and the Caribbean − Limiting − Injections − Year (R
Low education
Mid education
High education
Coefficient
Caribbean
Central America
South America
Region

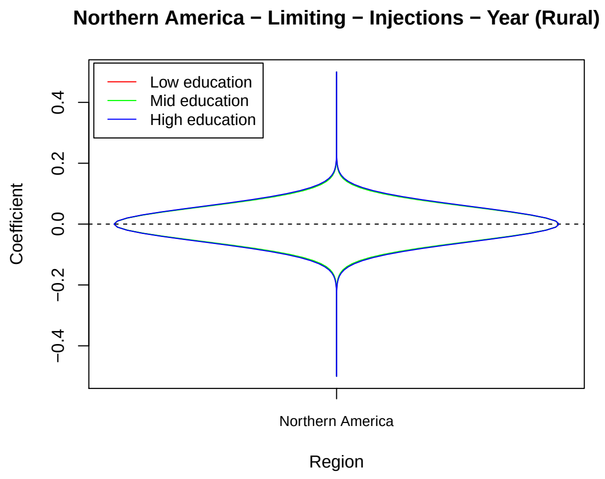
Northern America − Limiting − Injections − Year (Rural)
Low education
Mid education
High education
Coefficient
0.4
0.2
0.0
−0.2
−0.4
Northern America
Region

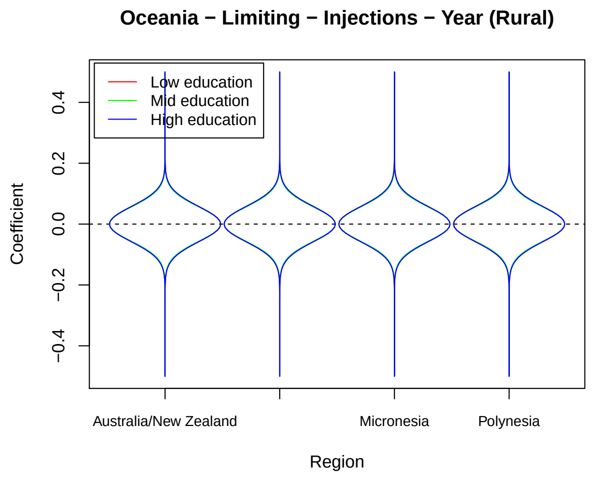
Oceania − Limiting − Injections − Year (Rural)
Low education
Mid education
High education
Coefficient
0.4
0.2
0.0
−0.2
−0.4
Australia/New Zealand
Micronesia
Polynesia
Region

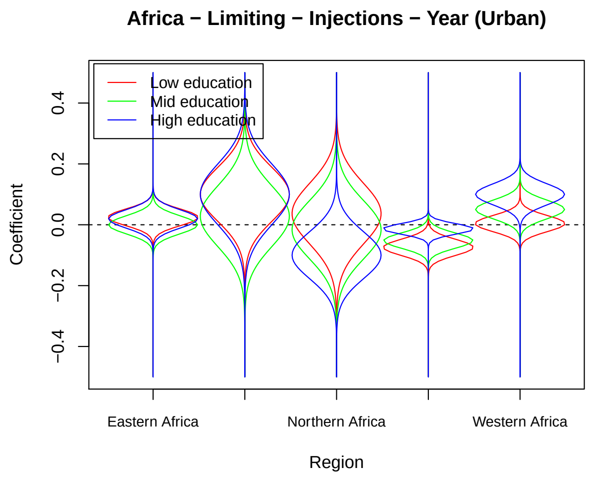
Africa − Limiting − Injections − Year (Urban)
Low education
Mid education
High education
Coefficient
0.4
0.2
0.0
−0.2
−0.4
Eastern Africa
Northern Africa
Western Africa
Region

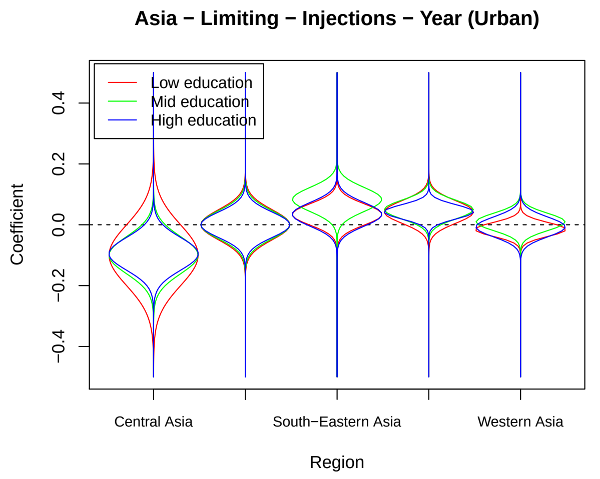
Asia − Limiting − Injections − Year (Urban)
Low education
Mid education
High education
Coefficient
0.4
0.2
0.0
−0.2
−0.4
Central Asia
South−Eastern Asia
Western Asia
Region

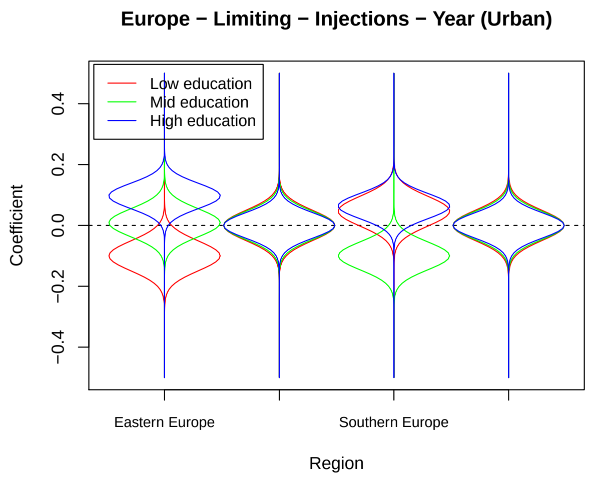
Europe − Limiting − Injections − Year (Urban)
Low education
Mid education
High education
Coefficient
0.4
0.2
0.0
−0.2
−0.4
Eastern Europe
Southern Europe
Region

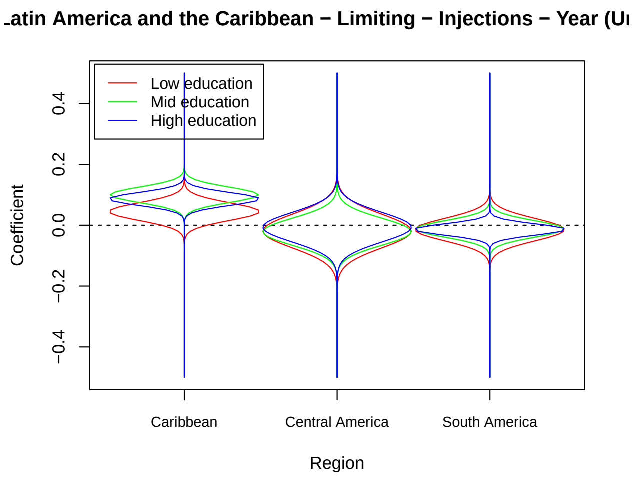
atin America and the Caribbean − Limiting − Injections − Year (U
Low education
Mid education
High education
Coefficient
0.4
0.2
0.0
−0.2
−0.4
Caribbean
Central America
South America
Region

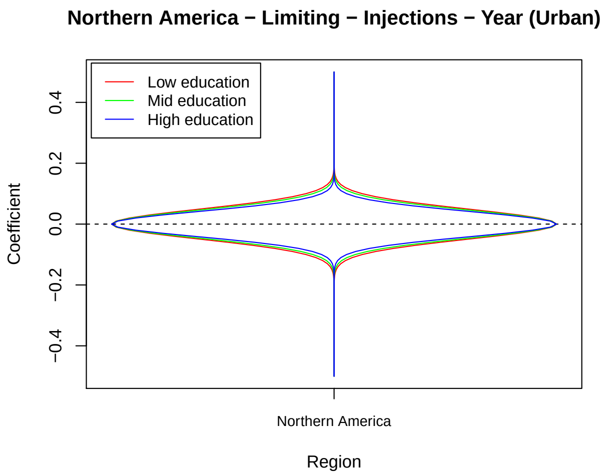
Northern America − Limiting − Injections − Year (Urban)
Low education
Mid education
High education
Coefficient
Northern America
Region

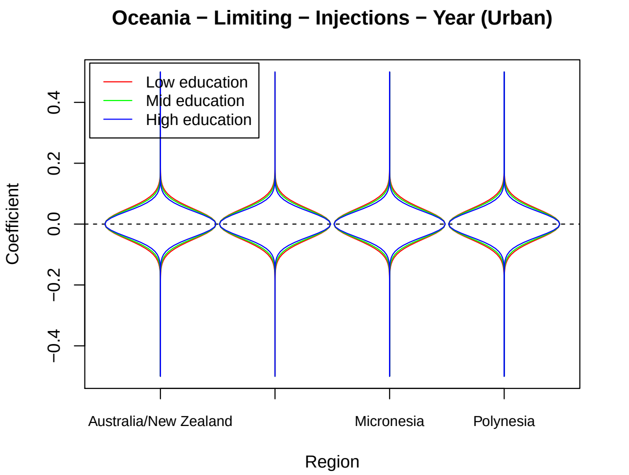
Oceania − Limiting − Injections − Year (Urban)
Low education
Mid education
High education
Coefficient
Australia/New Zealand
Micronesia
Polynesia
Region

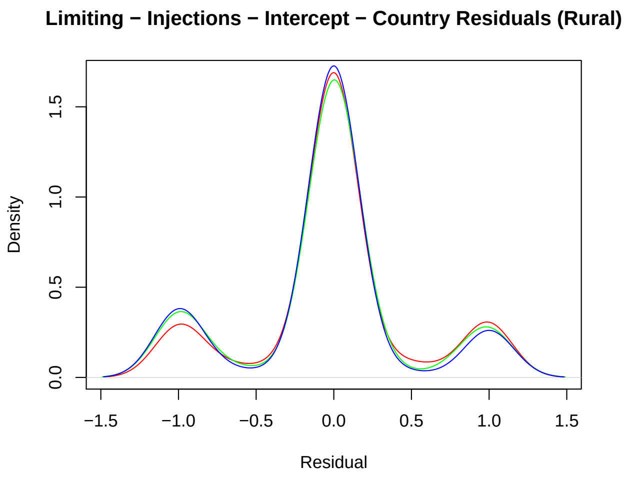
Limiting − Injections − Intercept − Country Residuals (Rural)
Density
Residual

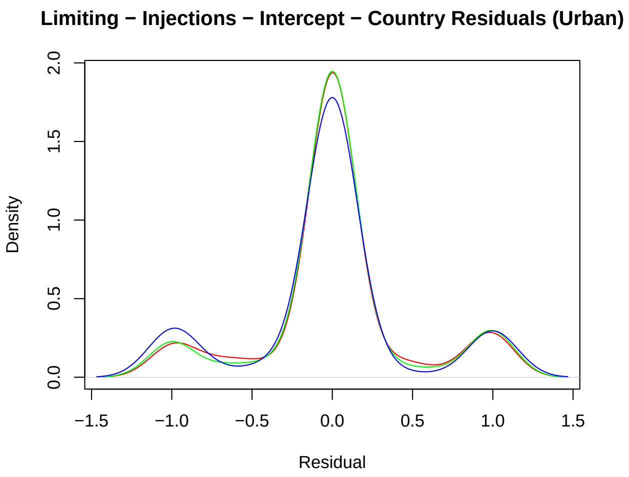
Limiting − Injections − Intercept − Country Residuals (Urban)
Density
Residual

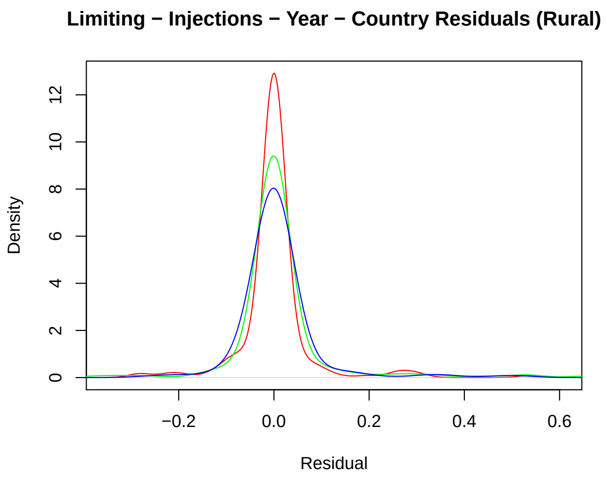
Limiting − Injections − Year − Country Residuals (Rural)
Density
Residual

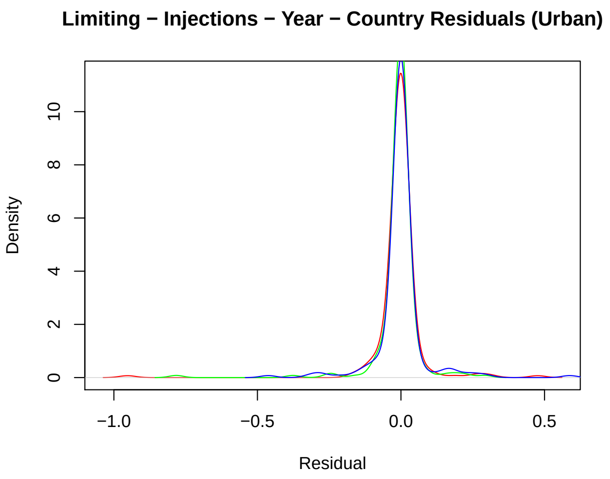
Limiting − Injections − Year − Country Residuals (Urban)
Density
Residual

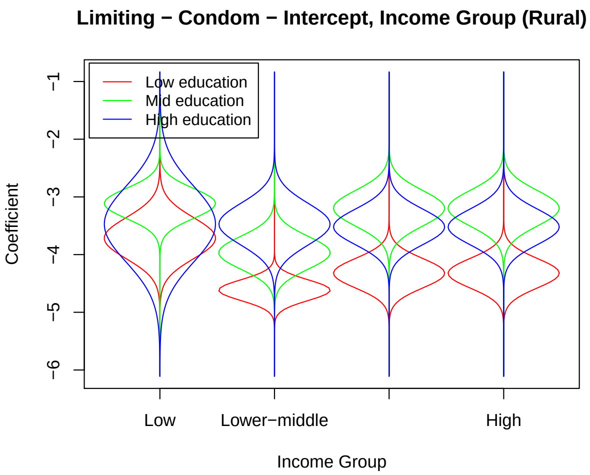
Limiting − Condom − Intercept, Income Group (Rural)
Low education
Mid education
High education
Coefficient
−1
−2
−3
−4
−5
−6
Low
Lower−middle
High
Income Group

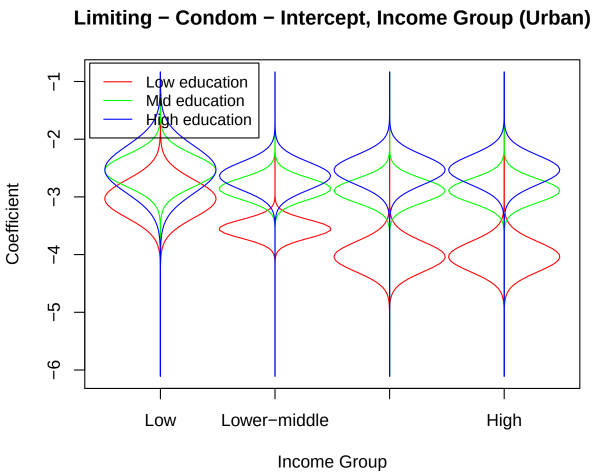
Limiting − Condom − Intercept, Income Group (Urban)
Low education
Mid education
High education
Coefficient
−1
−2
−3
−4
−5
−6
Low
Lower−middle
High
Income Group

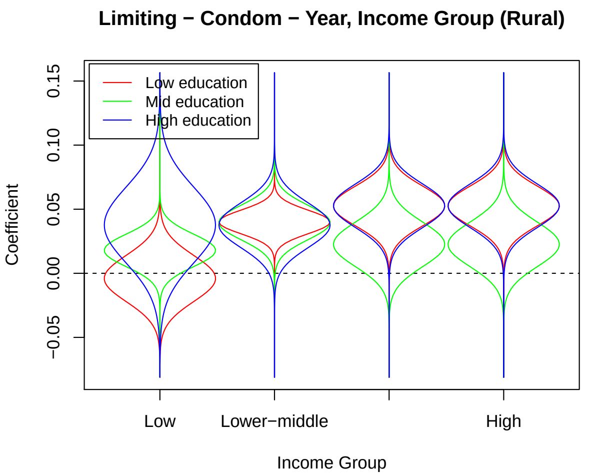
Limiting − Condom − Year, Income Group (Rural)
Low education
Mid education
High education
Coefficient
0.15
0.10
0.05
0.00
−0.05
Low
Lower−middle
High
Income Group

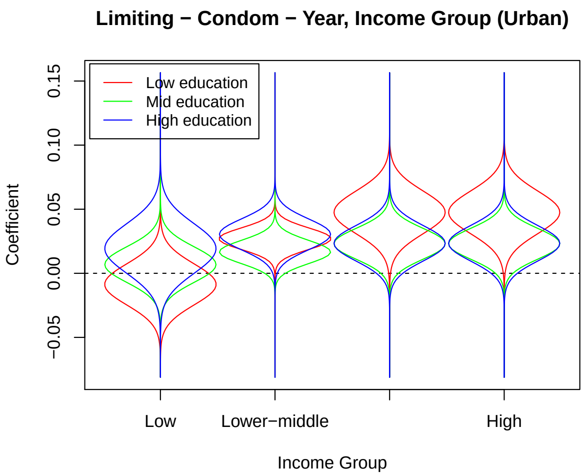
Limiting − Condom − Year, Income Group (Urban)
Low education
Mid education
High education
Coefficient
0.15
0.10
0.05
0.00
−0.05
Low
Lower−middle
High
Income Group

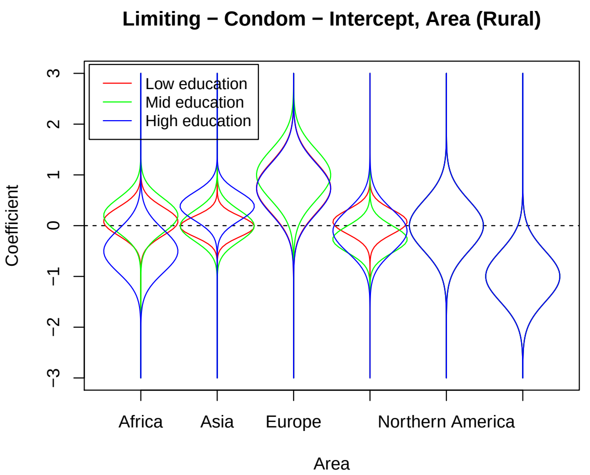
Limiting − Condom − Intercept, Area (Rural)
Low education
Mid education
High education
Coefficient
3
2
1
0
−1
−2
−3
Africa
Asia
Europe
Northern America
Area

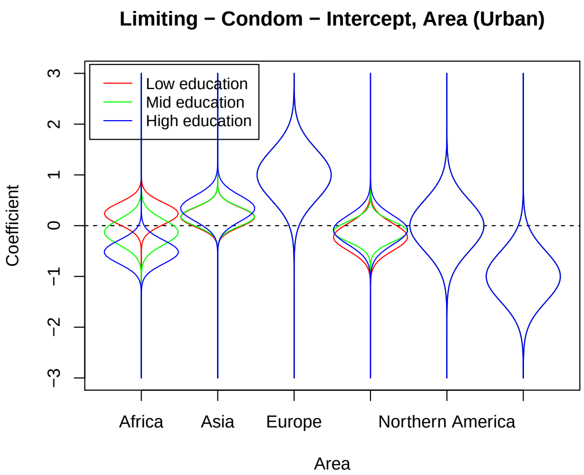
Limiting − Condom − Intercept, Area (Urban)
Low education
Mid education
High education
Coefficient
3
2
1
0
−1
−2
−3
Africa
Asia
Europe
Northern America
Area

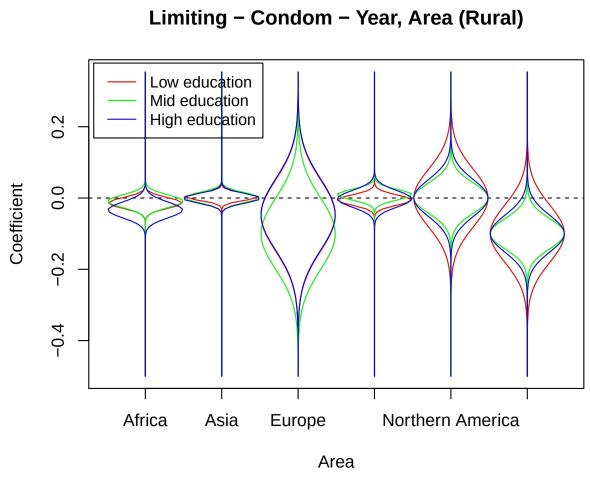
Limiting − Condom − Year, Area (Rural)
Low education
Mid education
High education
Coefficient
0.2
0.0
−0.2
−0.4
Africa
Asia
Europe
Northern America
Area

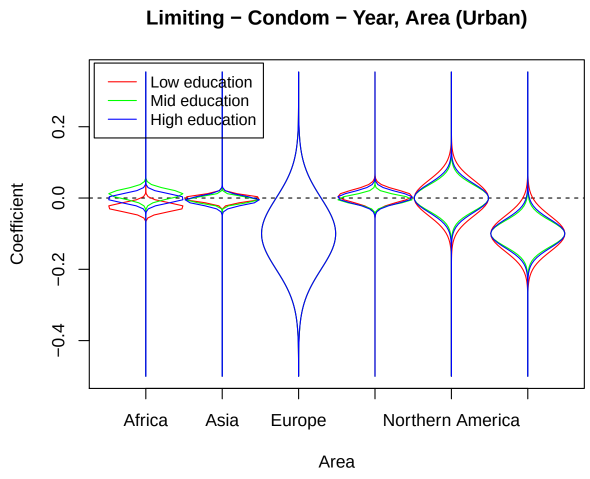
Limiting − Condom − Year, Area (Urban)
Low education
Mid education
High education
Coefficient
0.2
0.0
−0.2
−0.4
Africa
Asia
Europe
Northern America
Area

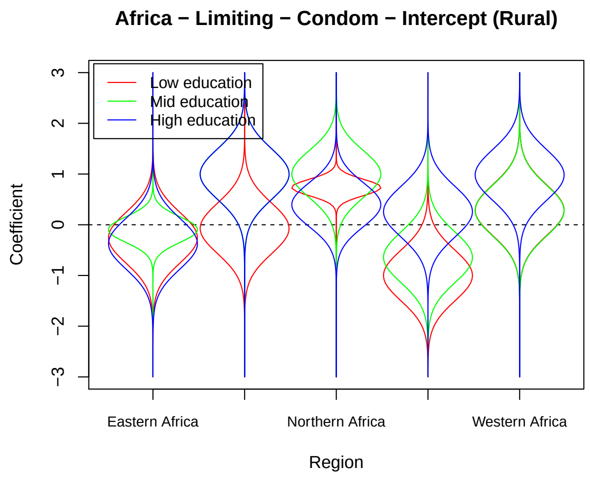
Africa − Limiting − Condom − Intercept (Rural)
Low education
Mid education
High education
Coefficient
3
2
1
0
−1
−2
−3
Eastern Africa
Northern Africa
Western Africa
Region

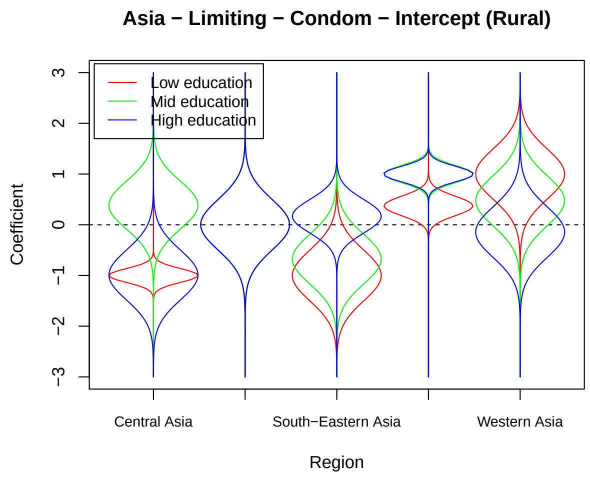
Asia − Limiting − Condom − Intercept (Rural)
Low education
Mid education
High education
Coefficient
3
2
1
0
−1
−2
−3
Central Asia
South−Eastern Asia
Western Asia
Region

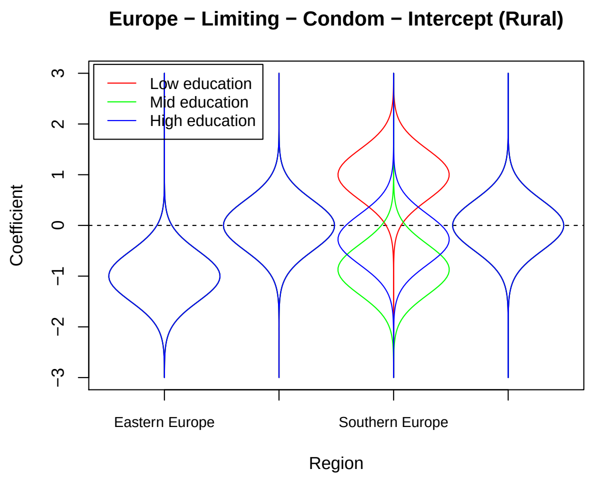
Europe − Limiting − Condom − Intercept (Rural)
Low education
Mid education
High education
Coefficient
3
2
1
0
−1
−2
−3
Eastern Europe
Southern Europe
Region

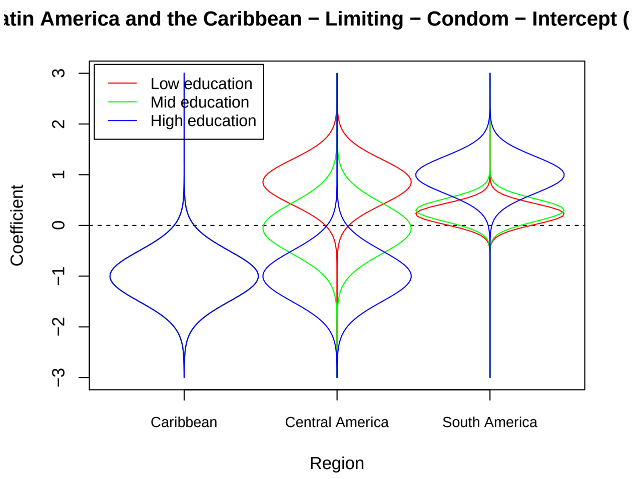
atin America and the Caribbean − Limiting − Condom − Intercept (
Low education
Mid education
High education
Coefficient
3
2
1
0
−1
−2
−3
Caribbean
Central America
South America
Region

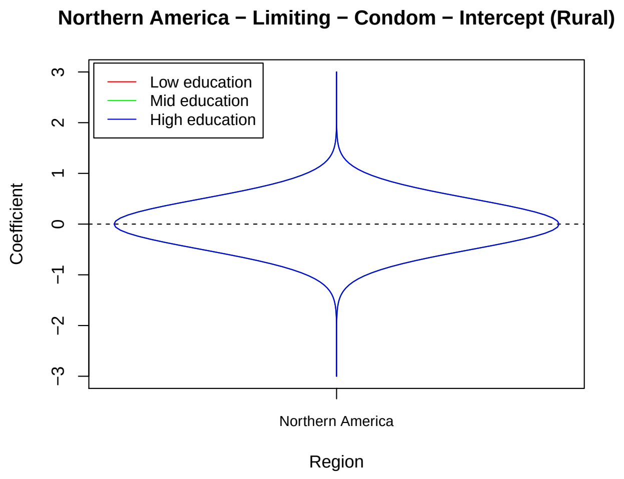
Northern America − Limiting − Condom − Intercept (Rural)
Low education
Mid education
High education
Coefficient
Northern America
Region

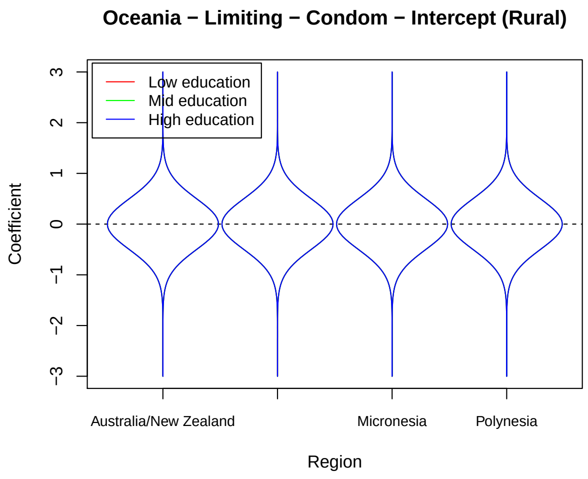
Oceania − Limiting − Condom − Intercept (Rural)
Low education
Mid education
High education
Coefficient
Australia/New Zealand
Micronesia
Polynesia
Region

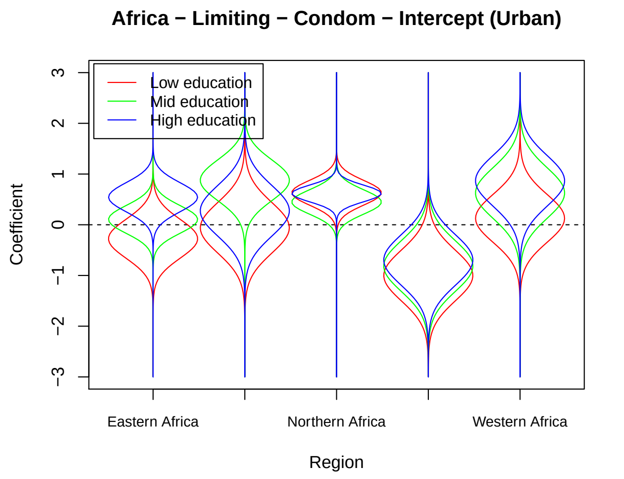
Africa − Limiting − Condom − Intercept (Urban)
Low education
Mid education
High education
Coefficient
Eastern Africa
Northern Africa
Western Africa
Region

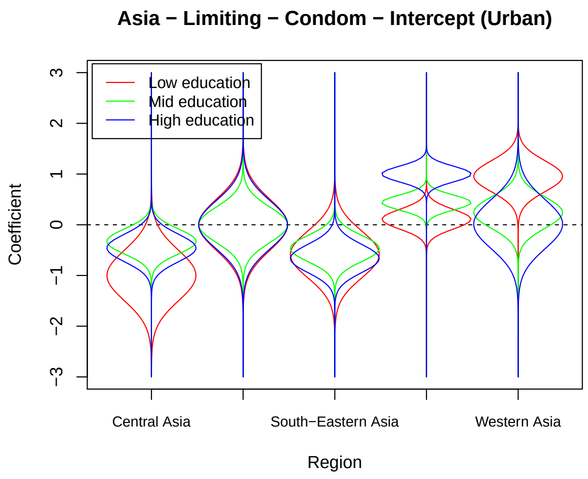
Asia − Limiting − Condom − Intercept (Urban)
Low education
Mid education
High education
Coefficient
Central Asia
South−Eastern Asia
Western Asia
Region

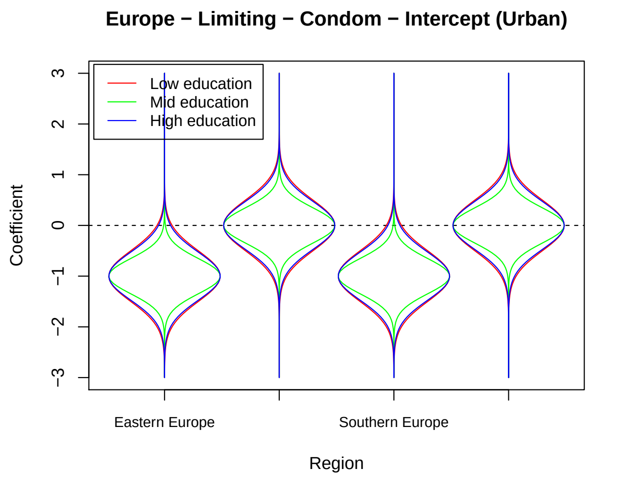
Europe − Limiting − Condom − Intercept (Urban)
Low education
Mid education
High education
Coefficient
Eastern Europe
Southern Europe
Region

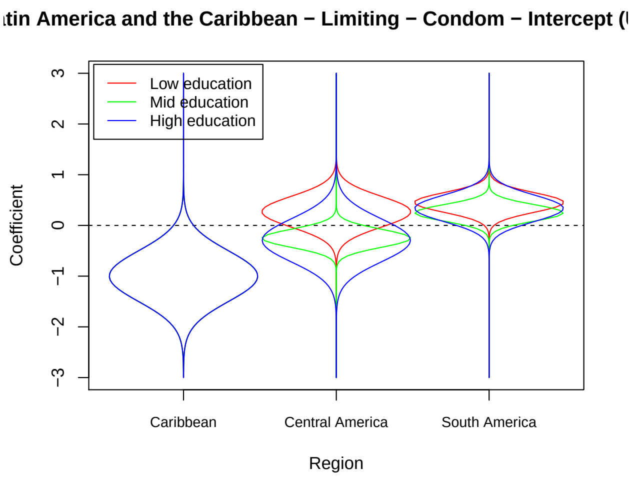
tin America and the Caribbean − Limiting − Condom − Intercept (
Low education
Mid education
High education
Coefficient
Caribbean
Central America
South America
Region

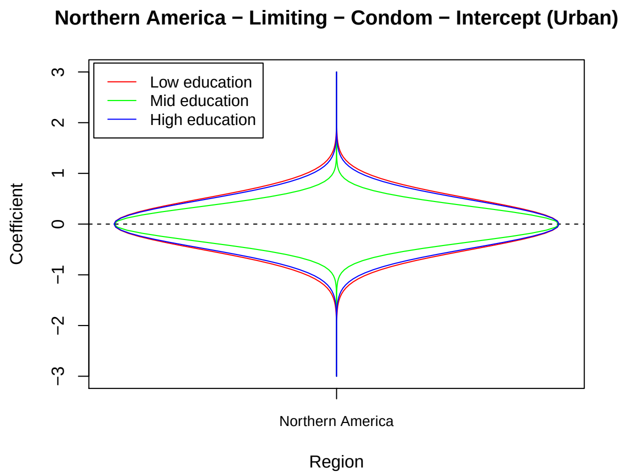
Northern America − Limiting − Condom − Intercept (Urban)
Low education
Mid education
High education
Coefficient
3
2
1
0
−1
−2
−3
Northern America
Region

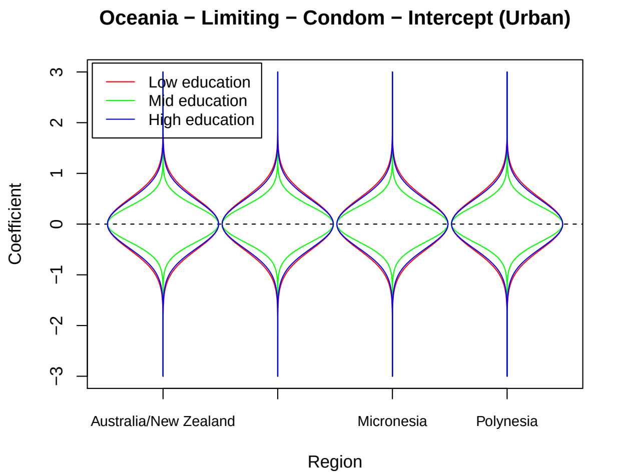
Oceania − Limiting − Condom − Intercept (Urban)
Low education
Mid education
High education
Coefficient
3
2
1
0
−1
−2
−3
Australia/New Zealand
Micronesia
Polynesia
Region

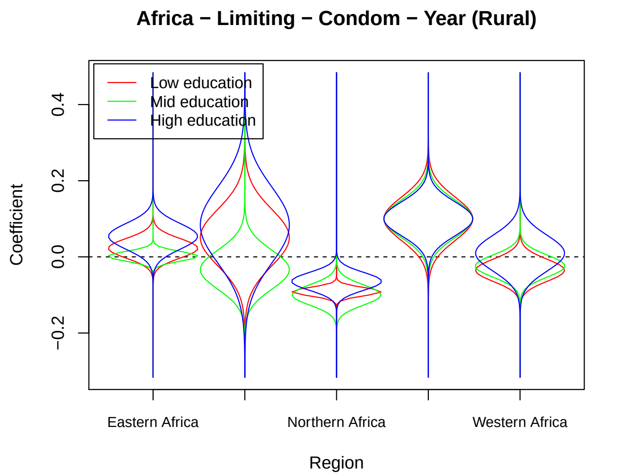
Africa − Limiting − Condom − Year (Rural)
Low education
Mid education
High education
Coefficient
0.4
0.2
0.0
−0.2
Eastern Africa
Northern Africa
Western Africa
Region

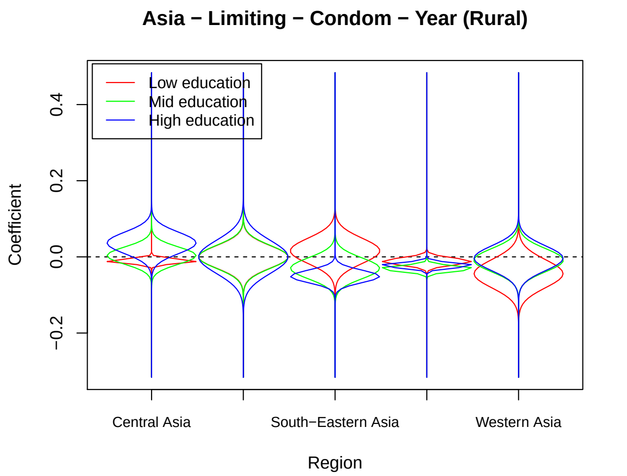
Asia − Limiting − Condom − Year (Rural)
Low education
Mid education
High education
Coefficient
0.4
0.2
0.0
−0.2
Central Asia
South−Eastern Asia
Western Asia
Region

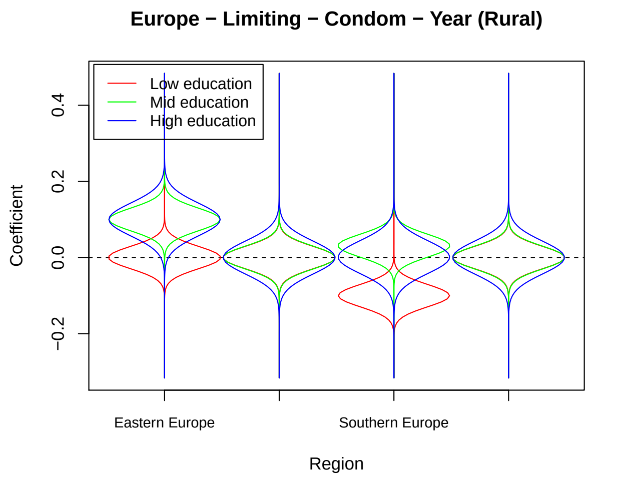
Europe − Limiting − Condom − Year (Rural)
Low education
Mid education
High education
Coefficient
0.4
0.2
0.0
−0.2
Eastern Europe
Southern Europe
Region

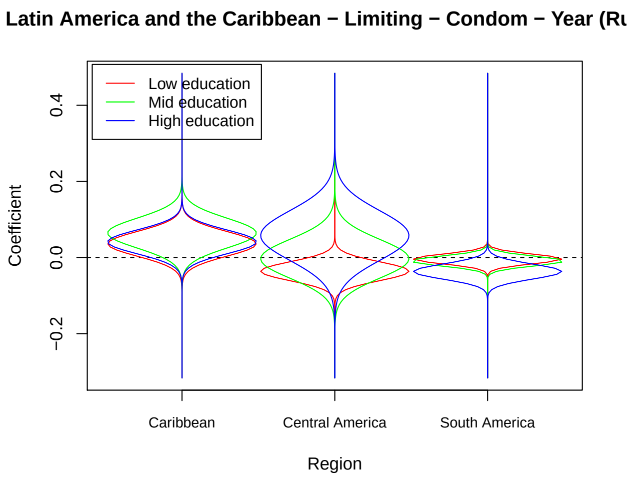
Latin America and the Caribbean − Limiting − Condom − Year (Ru
Low education
Mid education
High education
Coefficient
0.4
0.2
0.0
−0.2
Caribbean
Central America
South America
Region

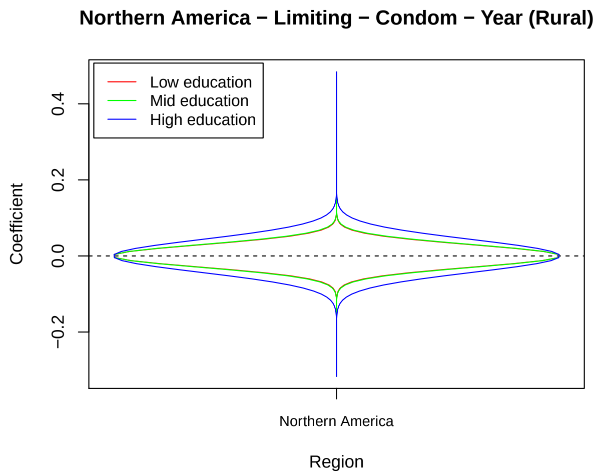
Northern America − Limiting − Condom − Year (Rural)
Low education
Mid education
High education
Coefficient
Northern America
Region

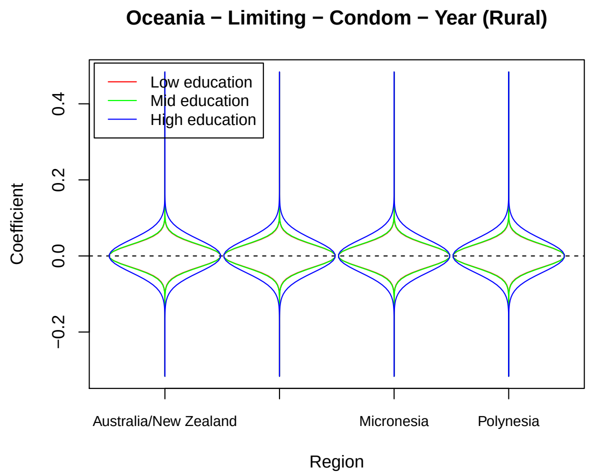
Oceania − Limiting − Condom − Year (Rural)
Low education
Mid education
High education
Coefficient
Australia/New Zealand
Micronesia
Polynesia
Region

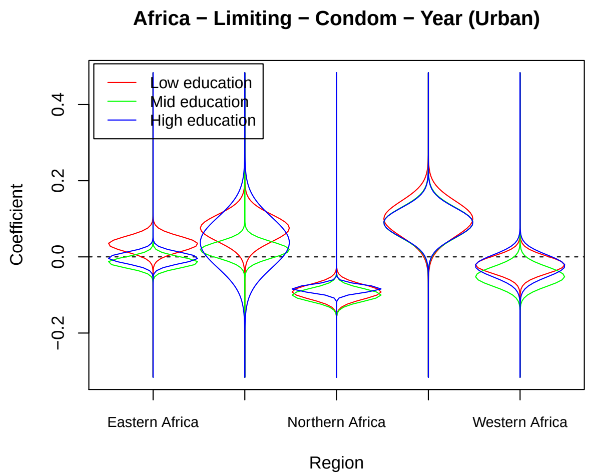
Africa − Limiting − Condom − Year (Urban)
Low education
Mid education
High education
Coefficient
Eastern Africa
Northern Africa
Western Africa
Region

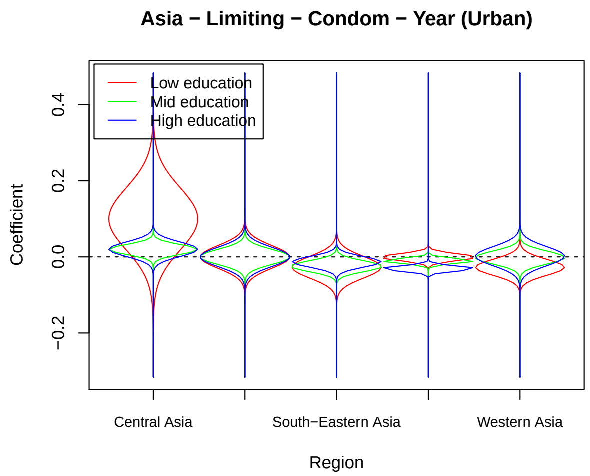
Asia − Limiting − Condom − Year (Urban)
Low education
Mid education
High education
Coefficient
Central Asia
South−Eastern Asia
Western Asia
Region

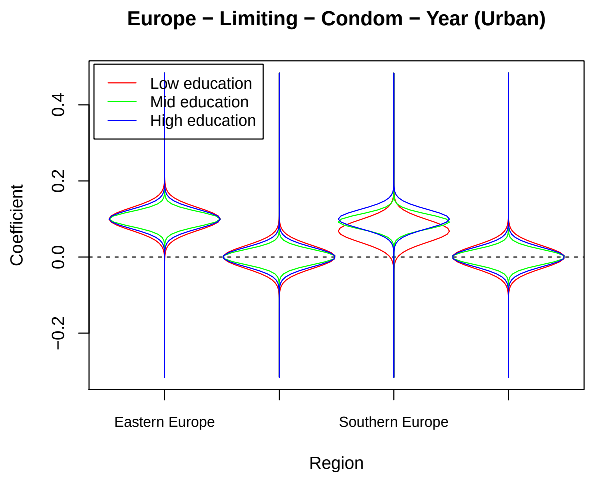
Europe − Limiting − Condom − Year (Urban)
Low education
Mid education
High education
Coefficient
Eastern Europe
Southern Europe
Region

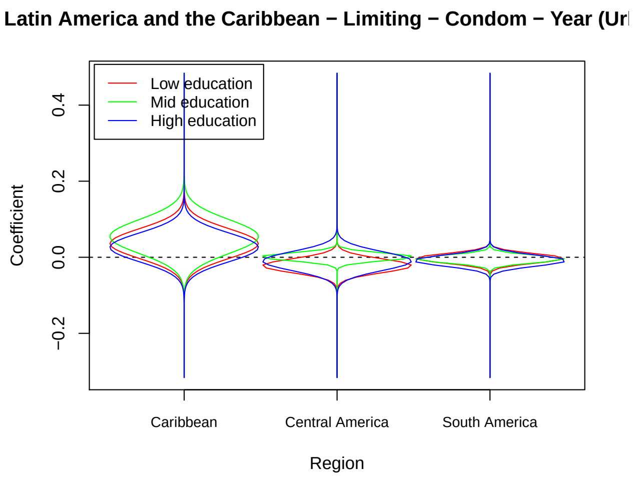
Latin America and the Caribbean − Limiting − Condom − Year (Ur
Low education
Mid education
High education
Coefficient
Caribbean
Central America
South America
Region

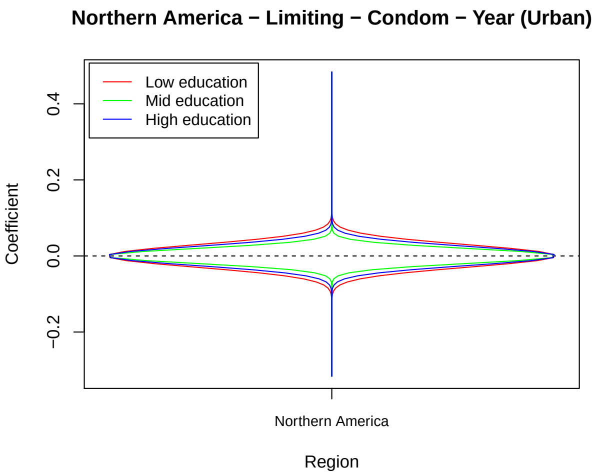
Northern America − Limiting − Condom − Year (Urban)
Low education
Mid education
High education
Coefficient
−0.2
0.0
0.2
0.4
Northern America
Region

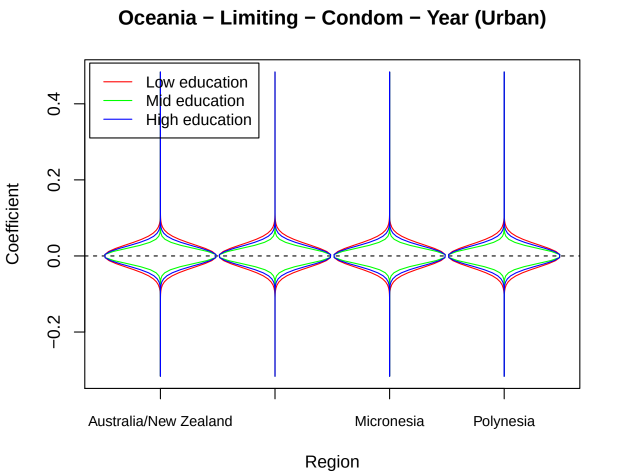
Oceania − Limiting − Condom − Year (Urban)
Low education
Mid education
High education
Coefficient
−0.2
0.0
0.2
0.4
Australia/New Zealand
Micronesia
Polynesia
Region

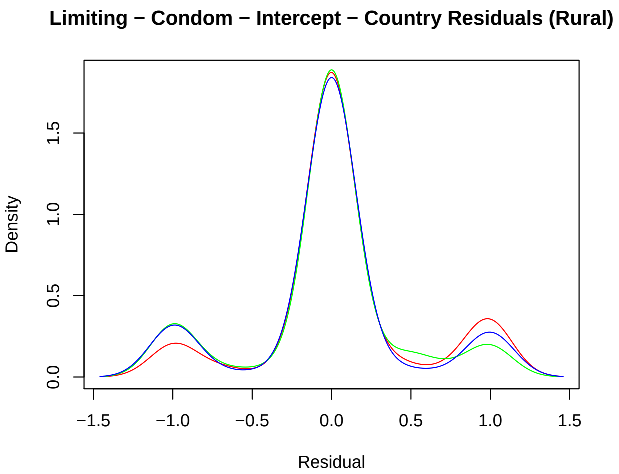
Limiting − Condom − Intercept − Country Residuals (Rural)
Density
0.0
0.5
1.0
1.5
−1.5
−1.0
−0.5
0.0
0.5
1.0
1.5
Residual

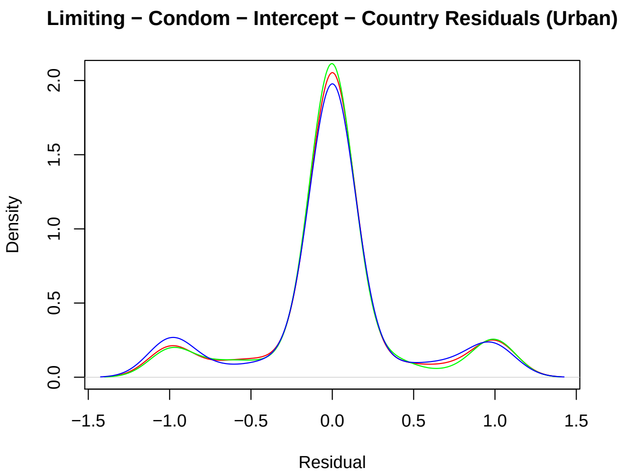
Limiting − Condom − Intercept − Country Residuals (Urban)
Density
0.0
0.5
1.0
1.5
2.0
−1.5
−1.0
−0.5
0.0
0.5
1.0
1.5
Residual

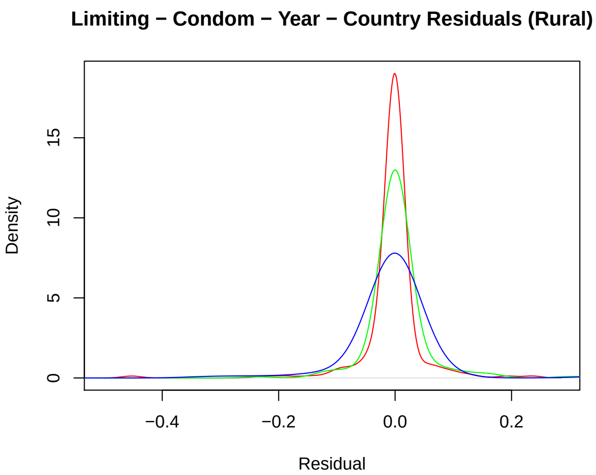
Limiting − Condom − Year − Country Residuals (Rural)
Density
0
5
10
15
−0.4
−0.2
0.0
0.2
Residual

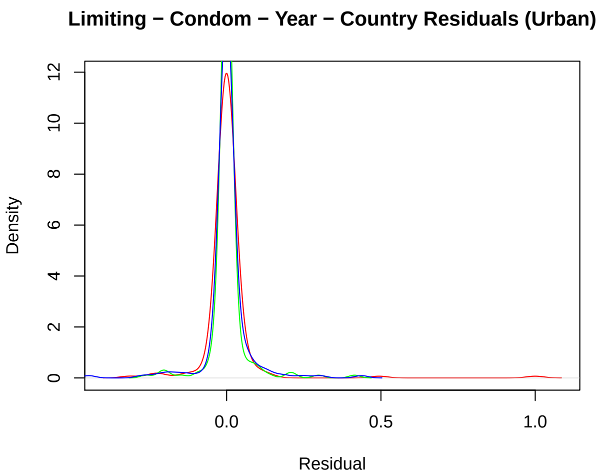
Limiting − Condom − Year − Country Residuals (Urban)
Density
0
2
4
6
8
10
12
0.0
0.5
1.0
Residual

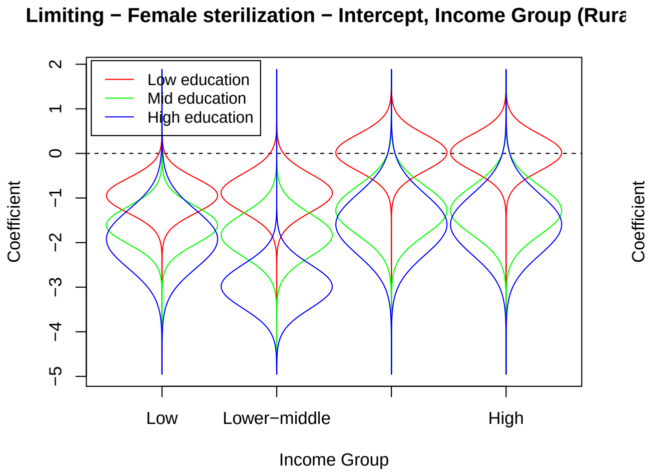
Limiting − Female sterilization − Intercept, Income Group (Rura
Low education
Mid education
High education
Coefficient
2
1
0
−1
−2
−3
−4
−5
Low
Lower−middle
High
Income Group

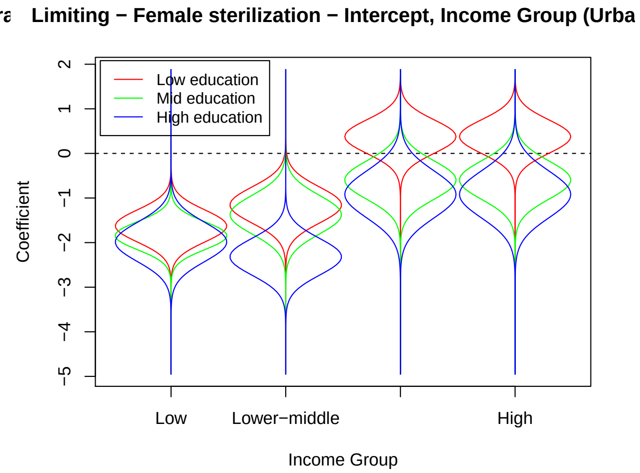
Limiting − Female sterilization − Intercept, Income Group (Urba
Low education
Mid education
High education
Coefficient
2
1
0
−1
−2
−3
−4
−5
Low
Lower−middle
High
Income Group

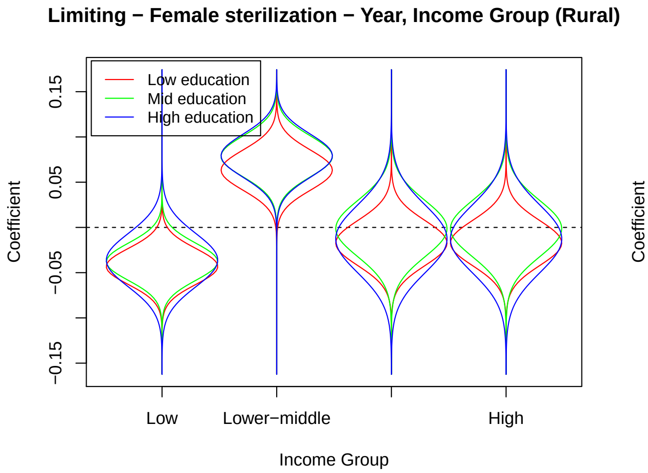
Limiting − Female sterilization − Year, Income Group (Rural)
Low education
Mid education
High education
Coefficient
0.15
0.05
−0.05
−0.15
Low
Lower−middle
High
Income Group

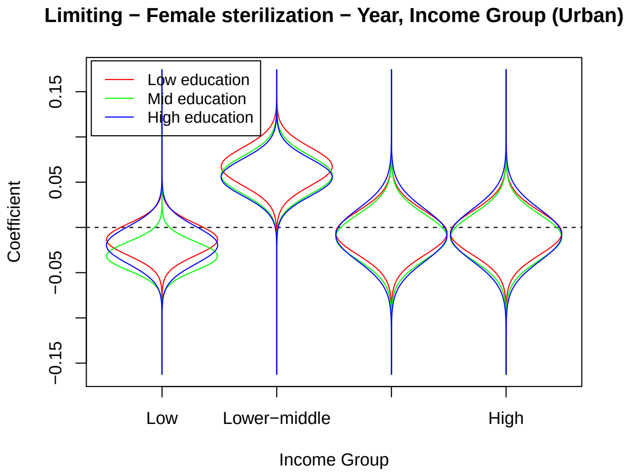
Limiting − Female sterilization − Year, Income Group (Urban)
Low education
Mid education
High education
Coefficient
0.15
0.05
−0.05
−0.15
Low
Lower−middle
High
Income Group

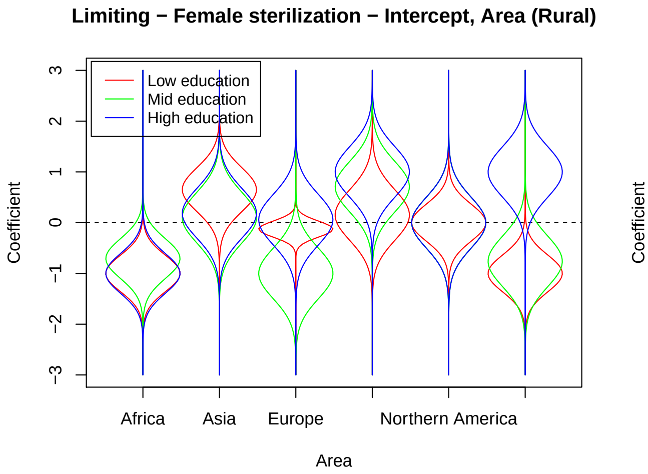
Limiting − Female sterilization − Intercept, Area (Rural)
Low education
Mid education
High education
Coefficient
3
2
1
0
−1
−2
−3
Africa
Asia
Europe
Northern America
Area

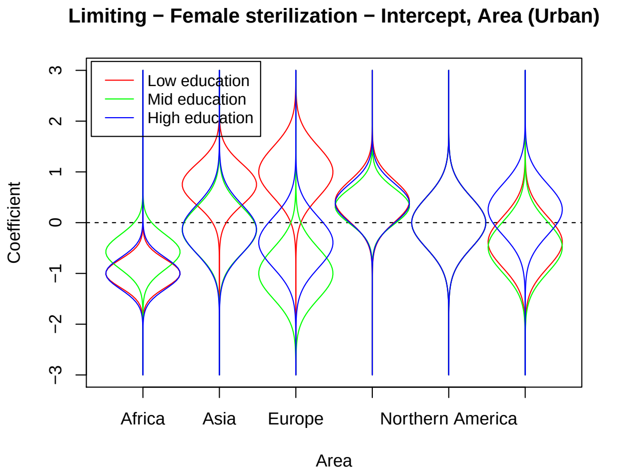
Limiting − Female sterilization − Intercept, Area (Urban)
Low education
Mid education
High education
Coefficient
3
2
1
0
−1
−2
−3
Africa
Asia
Europe
Northern America
Area

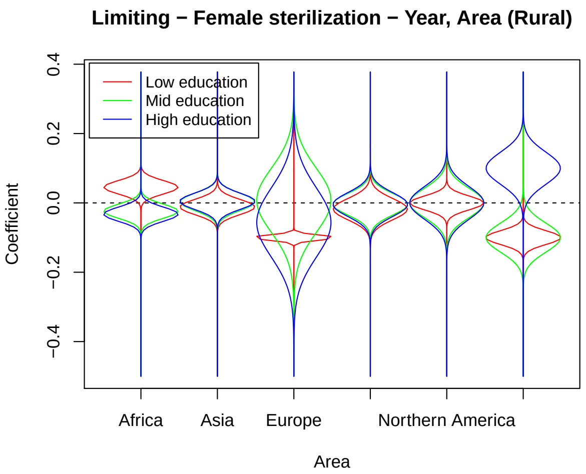
Limiting − Female sterilization − Year, Area (Rural)
Low education
Mid education
High education
Coefficient
0.4
0.2
0.0
−0.2
−0.4
Africa
Asia
Europe
Northern America
Area

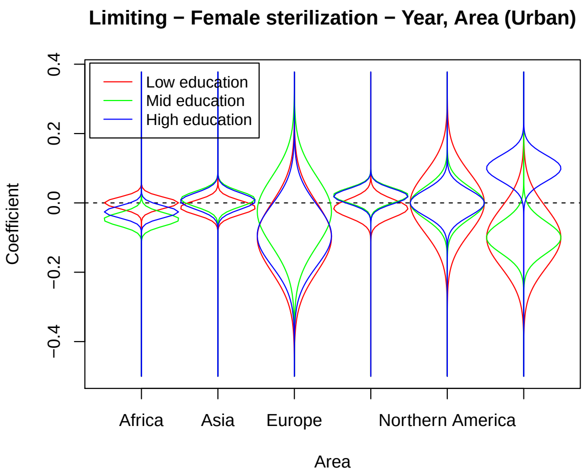
Limiting − Female sterilization − Year, Area (Urban)
Low education
Mid education
High education
Coefficient
0.4
0.2
0.0
−0.2
−0.4
Africa
Asia
Europe
Northern America
Area

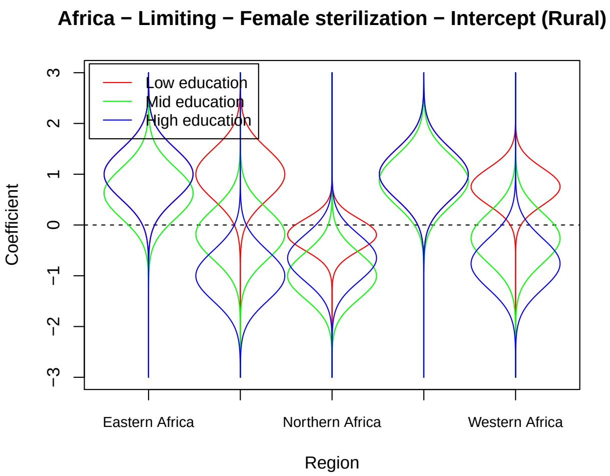
Africa − Limiting − Female sterilization − Intercept (Rural)
Low education
Mid education
High education
Coefficient
3
2
1
0
−1
−2
−3
Eastern Africa
Northern Africa
Western Africa
Region

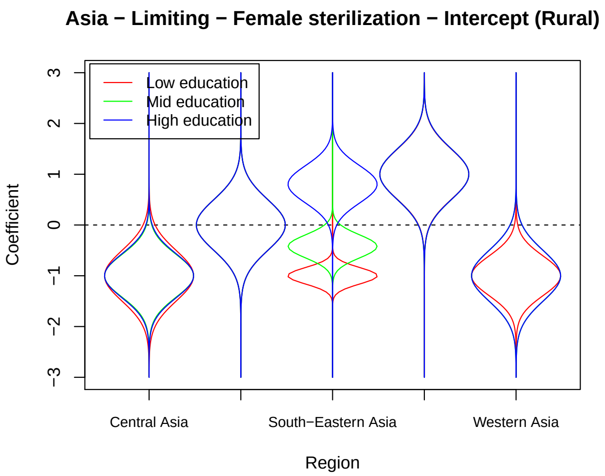
Asia − Limiting − Female sterilization − Intercept (Rural)
Low education
Mid education
High education
Coefficient
3
2
1
0
−1
−2
−3
Central Asia
South−Eastern Asia
Western Asia
Region

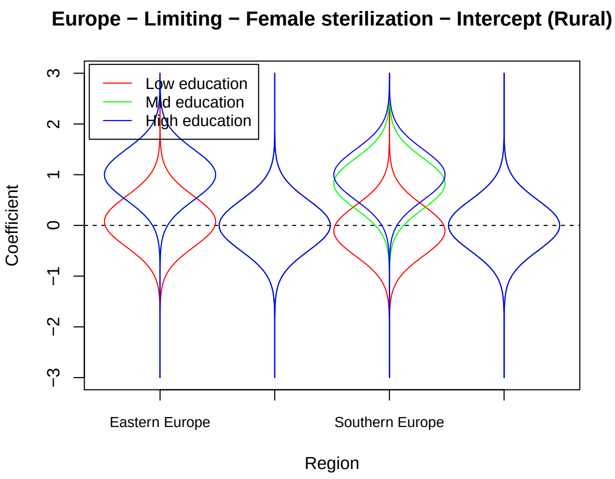
Europe − Limiting − Female sterilization − Intercept (Rural)
Low education
Mid education
High education
Coefficient
3
2
1
0
−1
−2
−3
Eastern Europe
Southern Europe
Region

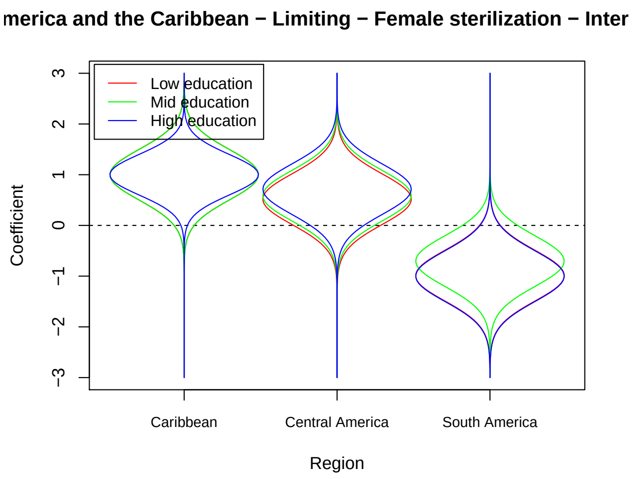
merica and the Caribbean − Limiting − Female sterilization − Inter
Low education
Mid education
High education
Coefficient
3
2
1
0
−1
−2
−3
Caribbean
Central America
South America
Region

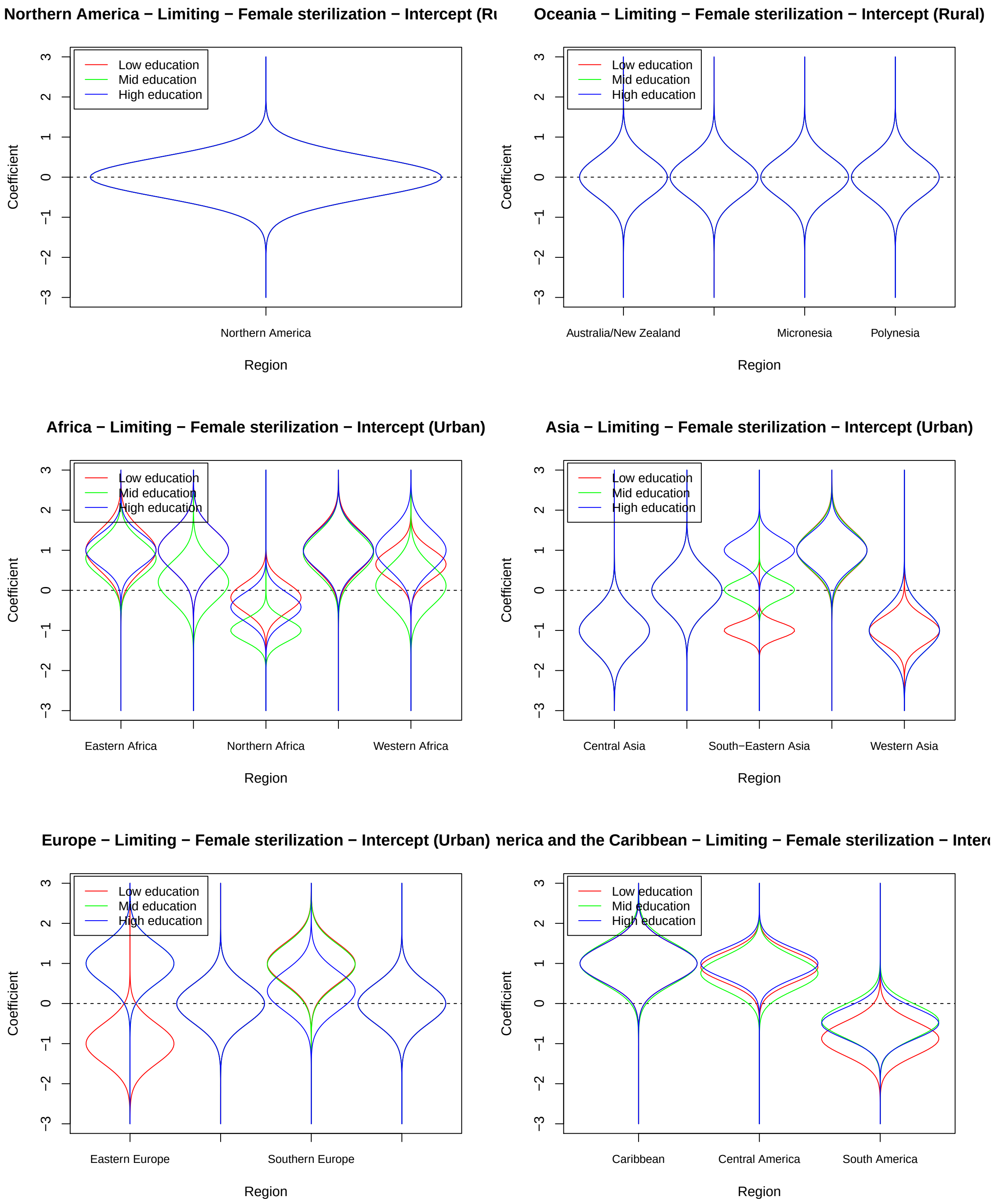
Northern America − Limiting − Female sterilization − Intercept (Ru
Oceania − Limiting − Female sterilization − Intercept (Rural)
Africa − Limiting − Female sterilization − Intercept (Urban)
Asia − Limiting − Female sterilization − Intercept (Urban)
Europe − Limiting − Female sterilization − Intercept (Urban)
nerica and the Caribbean − Limiting − Female sterilization − Interc
Low education
Mid education
High education
Coefficient
Region
Northern America
Australia/New Zealand
Micronesia
Polynesia
Eastern Africa
Northern Africa
Western Africa
Central Asia
South−Eastern Asia
Western Asia
Eastern Europe
Southern Europe
Caribbean
Central America
South America
3
2
1
0
−1
−2
−3

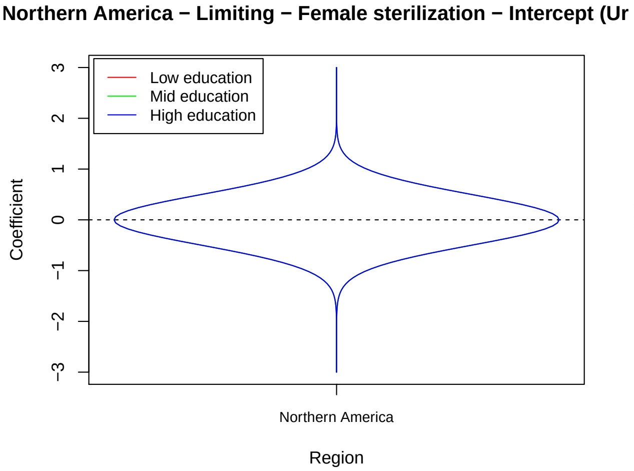
Northern America − Limiting − Female sterilization − Intercept (Ur
Low education
Mid education
High education
Coefficient
3
2
1
0
−1
−2
−3
Northern America
Region

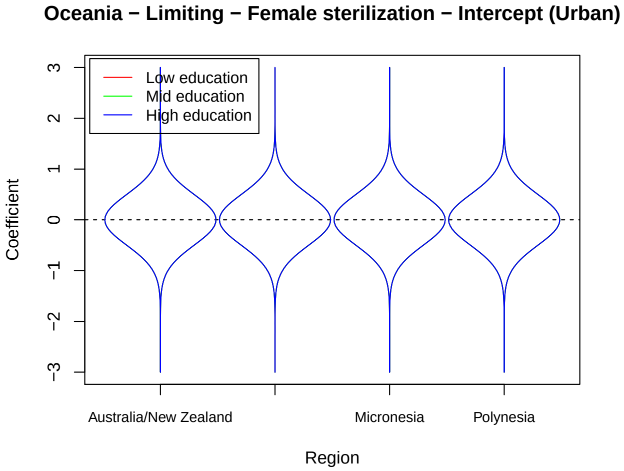
Oceania − Limiting − Female sterilization − Intercept (Urban)
Low education
Mid education
High education
Coefficient
3
2
1
0
−1
−2
−3
Australia/New Zealand
Micronesia
Polynesia
Region

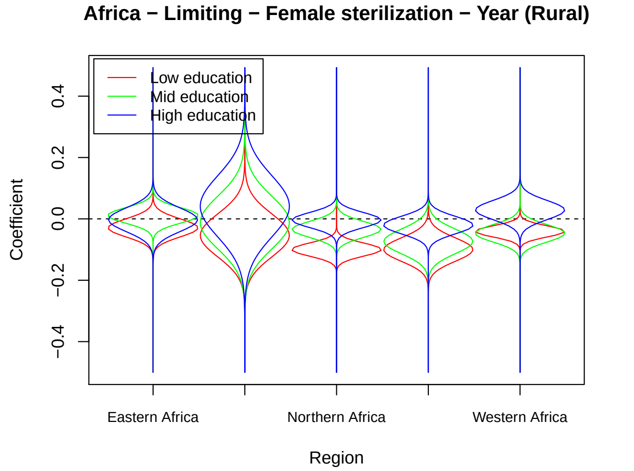
Africa − Limiting − Female sterilization − Year (Rural)
Low education
Mid education
High education
Coefficient
0.4
0.2
0.0
−0.2
−0.4
Eastern Africa
Northern Africa
Western Africa
Region

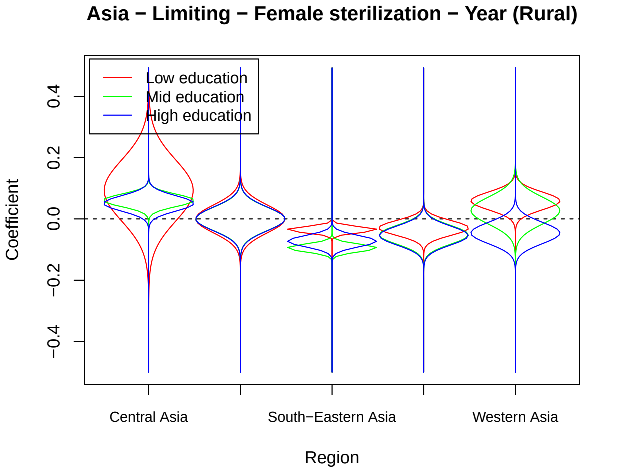
Asia − Limiting − Female sterilization − Year (Rural)
Low education
Mid education
High education
Coefficient
0.4
0.2
0.0
−0.2
−0.4
Central Asia
South−Eastern Asia
Western Asia
Region

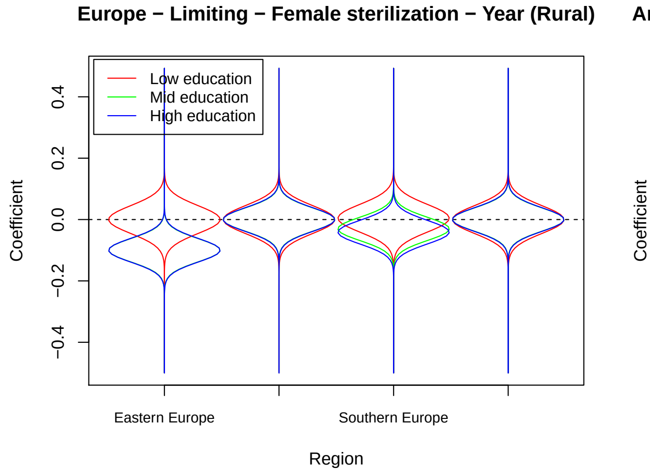
Europe − Limiting − Female sterilization − Year (Rural)
Low education
Mid education
High education
Coefficient
0.4
0.2
0.0
−0.2
−0.4
Eastern Europe
Southern Europe
Region

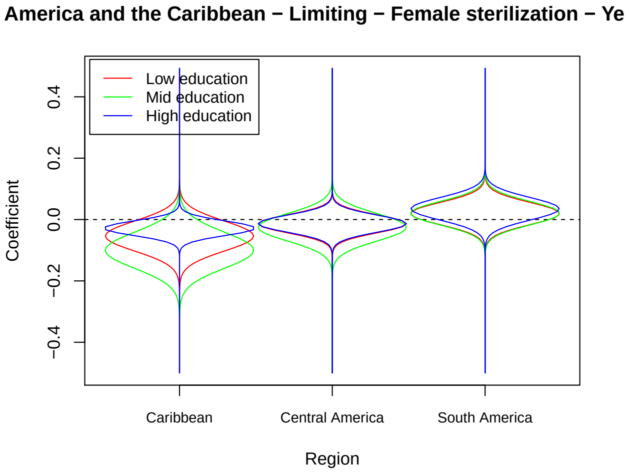
America and the Caribbean − Limiting − Female sterilization − Ye
Low education
Mid education
High education
Coefficient
0.4
0.2
0.0
−0.2
−0.4
Caribbean
Central America
South America
Region

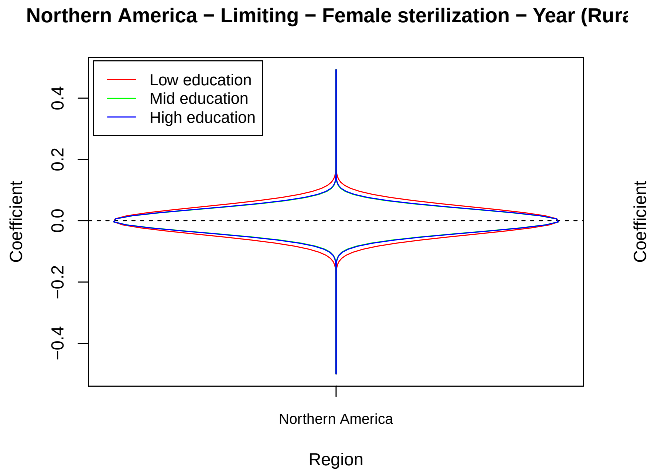
Northern America − Limiting − Female sterilization − Year (Rura
Low education
Mid education
High education
Coefficient
0.4
0.2
0.0
−0.2
−0.4
Northern America
Region

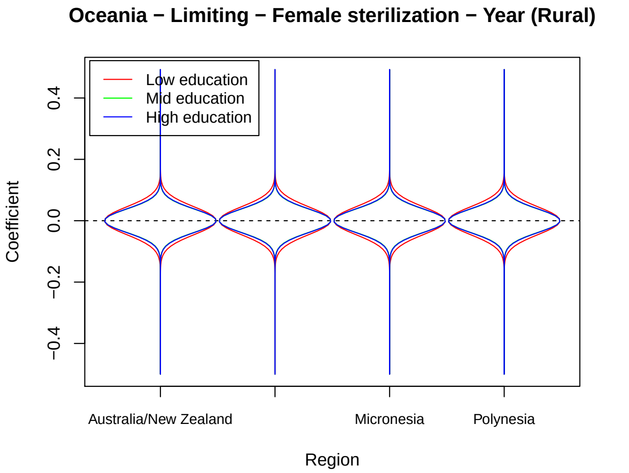
Oceania − Limiting − Female sterilization − Year (Rural)
Low education
Mid education
High education
Coefficient
0.4
0.2
0.0
−0.2
−0.4
Australia/New Zealand
Micronesia
Polynesia
Region

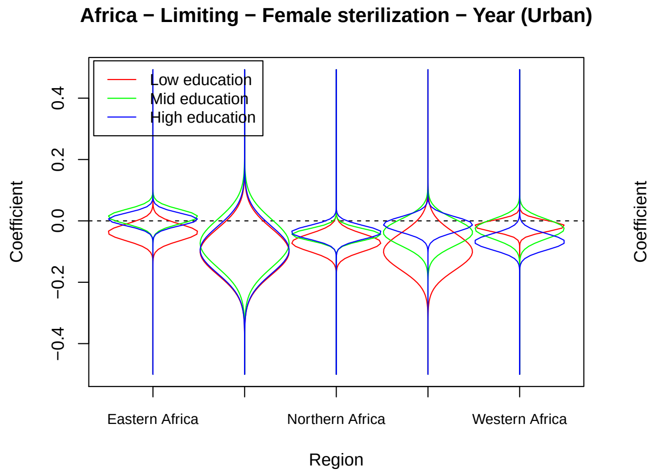
Africa − Limiting − Female sterilization − Year (Urban)
Low education
Mid education
High education
Coefficient
0.4
0.2
0.0
−0.2
−0.4
Eastern Africa
Northern Africa
Western Africa
Region

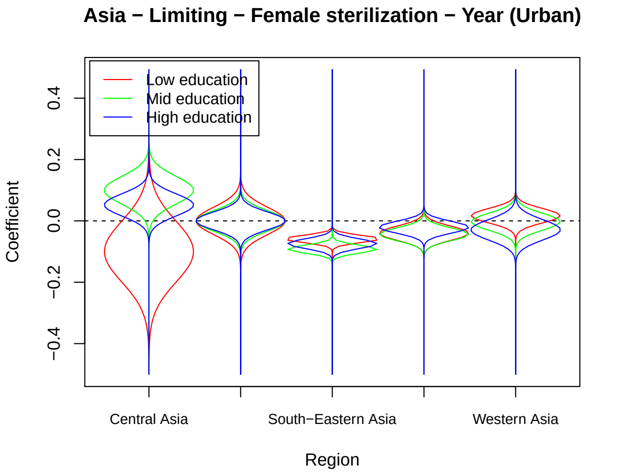
Asia − Limiting − Female sterilization − Year (Urban)
Low education
Mid education
High education
Coefficient
0.4
0.2
0.0
−0.2
−0.4
Central Asia
South−Eastern Asia
Western Asia
Region

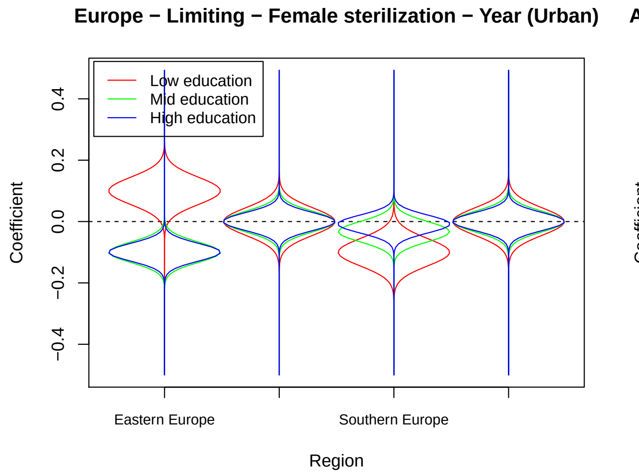
Europe − Limiting − Female sterilization − Year (Urban)
Low education
Mid education
High education
Coefficient
0.4
0.2
0.0
−0.2
−0.4
Eastern Europe
Southern Europe
Region

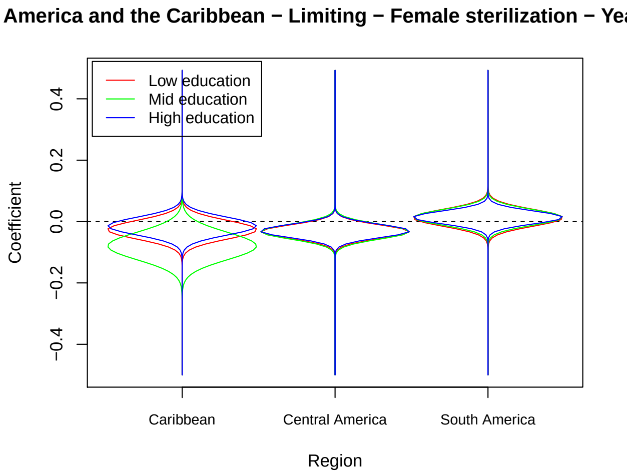
America and the Caribbean − Limiting − Female sterilization − Yea
Low education
Mid education
High education
Coefficient
0.4
0.2
0.0
−0.2
−0.4
Caribbean
Central America
South America
Region

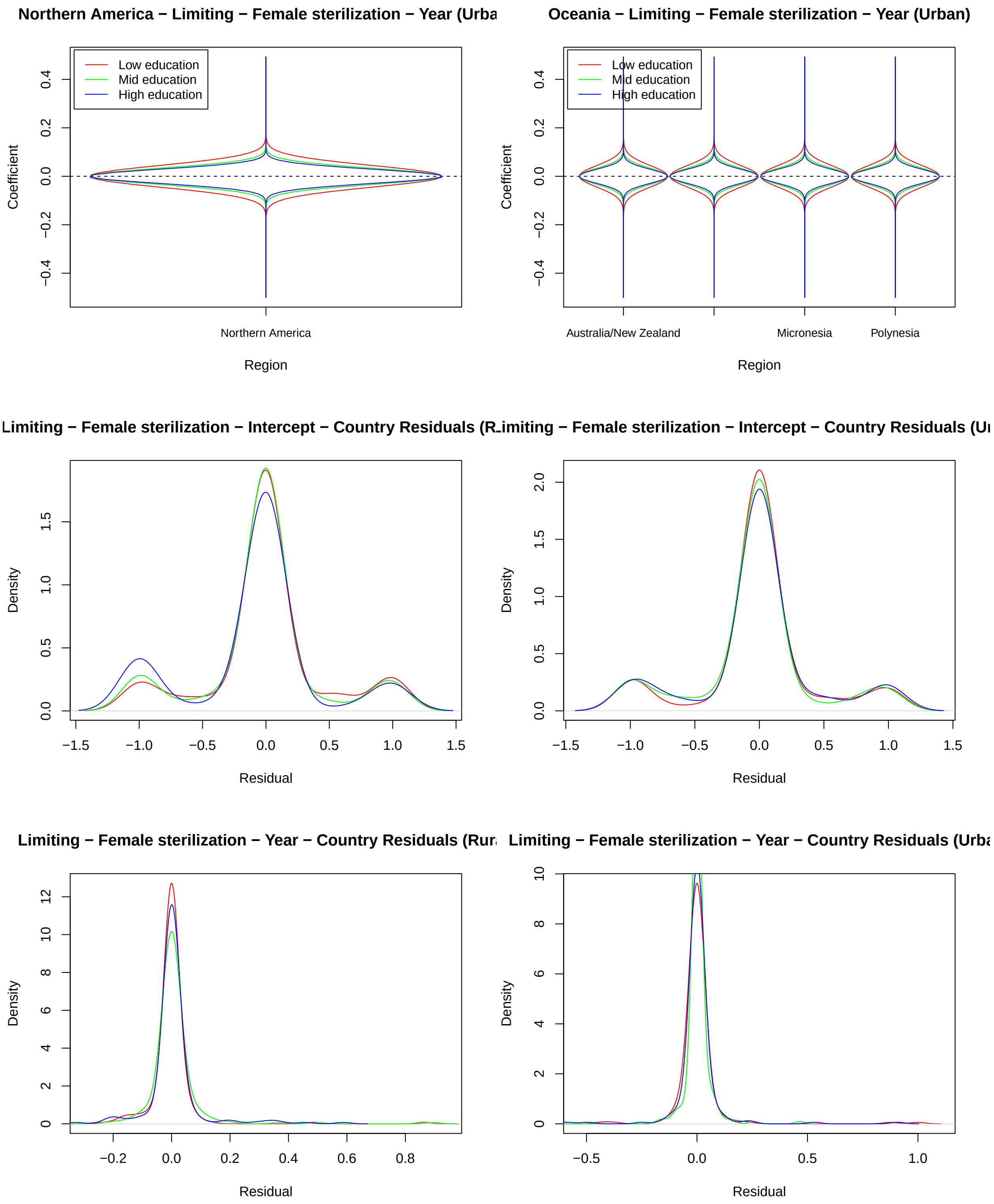

Northern America − Limiting − Female sterilization − Year (Urba
Oceania − Limiting − Female sterilization − Year (Urban)
Low education
Mid education
High education
Coefficient
Region
Northern America
Australia/New Zealand
Micronesia
Polynesia
Limiting − Female sterilization − Intercept − Country Residuals (R
Limiting − Female sterilization − Intercept − Country Residuals (U
Limiting − Female sterilization − Year − Country Residuals (Rur
Limiting − Female sterilization − Year − Country Residuals (Urba
Density
Residual

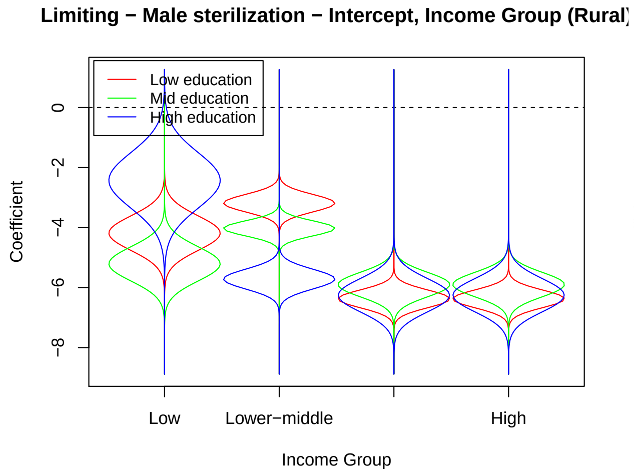
Limiting − Male sterilization − Intercept, Income Group (Rural)
Low education
Mid education
High education
0
−2
−4
−6
−8
Coefficient
Low
Lower−middle
High
Income Group

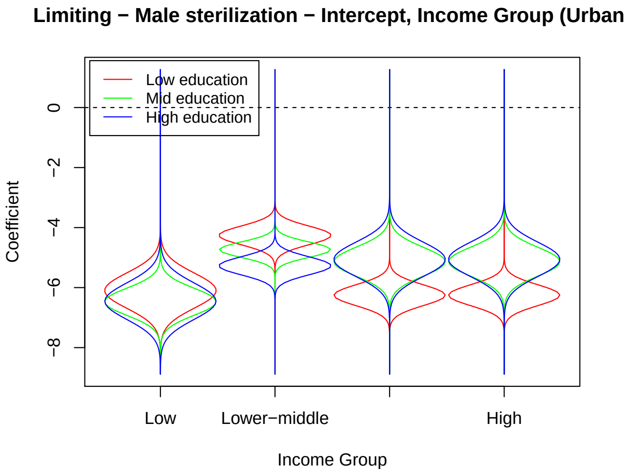
Limiting − Male sterilization − Intercept, Income Group (Urban
Low education
Mid education
High education
0
−2
−4
−6
−8
Coefficient
Low
Lower−middle
High
Income Group

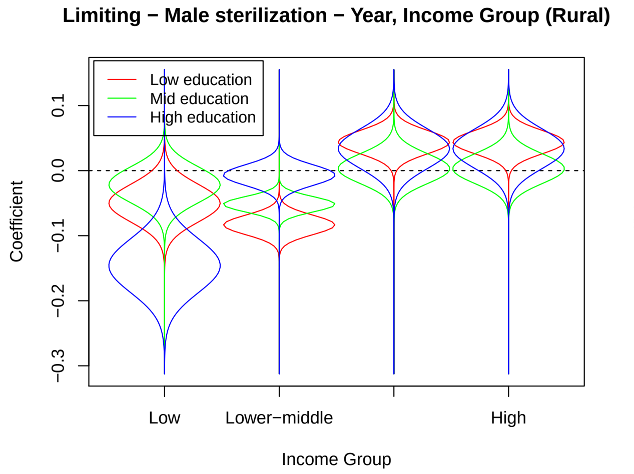
Limiting − Male sterilization − Year, Income Group (Rural)
Low education
Mid education
High education
0.1
0.0
−0.1
−0.2
−0.3
Coefficient
Low
Lower−middle
High
Income Group

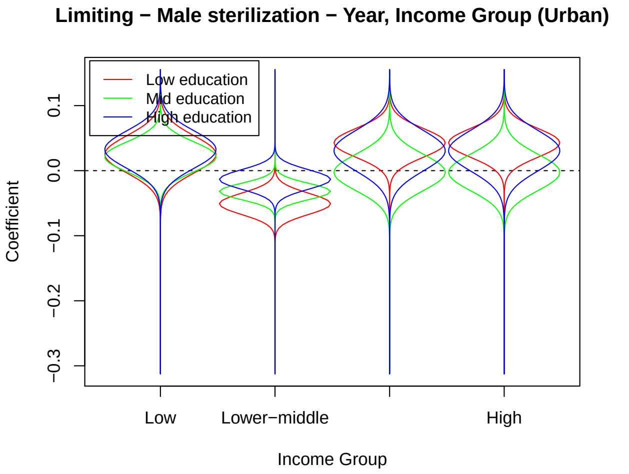
Limiting − Male sterilization − Year, Income Group (Urban)
Low education
Mid education
High education
0.1
0.0
−0.1
−0.2
−0.3
Coefficient
Low
Lower−middle
High
Income Group

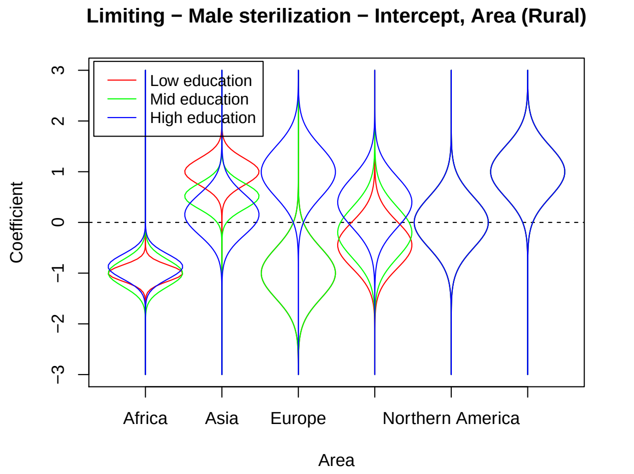
Limiting − Male sterilization − Intercept, Area (Rural)
Low education
Mid education
High education
3
2
1
0
−1
−2
−3
Coefficient
Africa
Asia
Europe
Northern America
Area

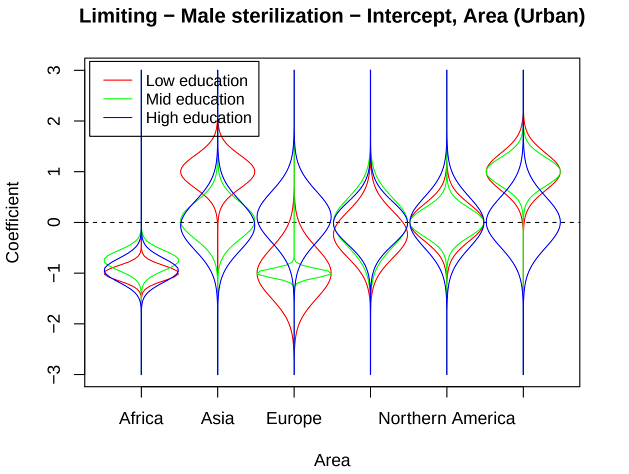
Limiting − Male sterilization − Intercept, Area (Urban)
Low education
Mid education
High education
3
2
1
0
−1
−2
−3
Coefficient
Africa
Asia
Europe
Northern America
Area

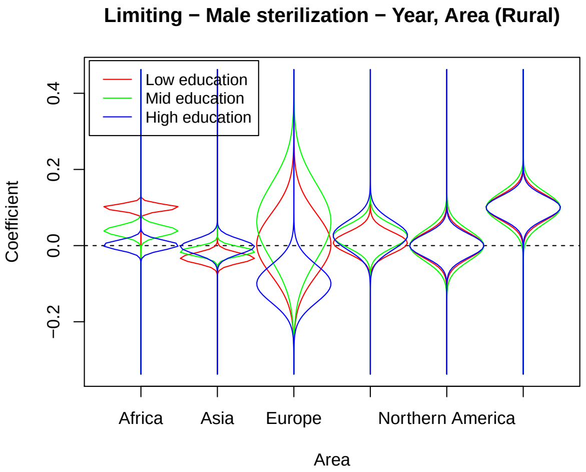
Limiting − Male sterilization − Year, Area (Rural)
Low education
Mid education
High education
Coefficient
−0.2
0.0
0.2
0.4
Africa
Asia
Europe
Northern America
Area

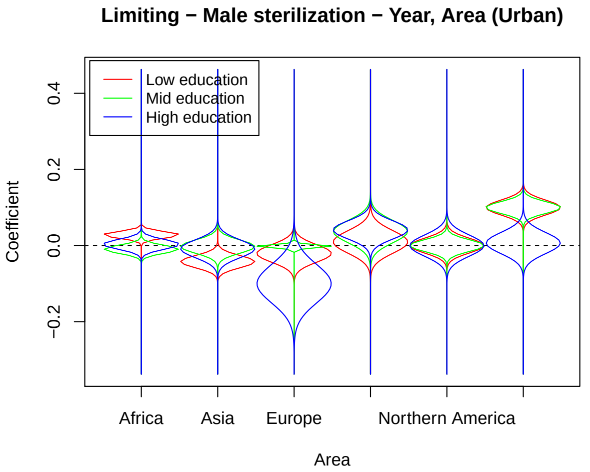
Limiting − Male sterilization − Year, Area (Urban)
Low education
Mid education
High education
Coefficient
−0.2
0.0
0.2
0.4
Africa
Asia
Europe
Northern America
Area

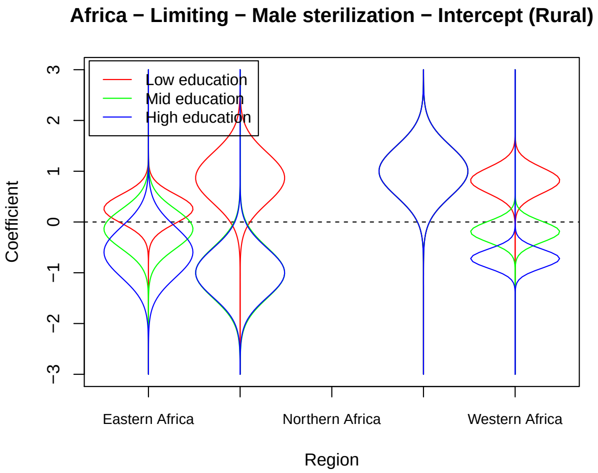
Africa − Limiting − Male sterilization − Intercept (Rural)
Low education
Mid education
High education
Coefficient
−3
−2
−1
0
1
2
3
Eastern Africa
Northern Africa
Western Africa
Region

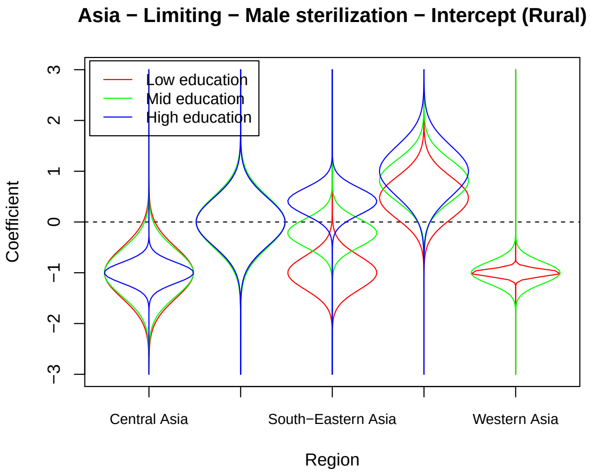
Asia − Limiting − Male sterilization − Intercept (Rural)
Low education
Mid education
High education
Coefficient
−3
−2
−1
0
1
2
3
Central Asia
South−Eastern Asia
Western Asia
Region

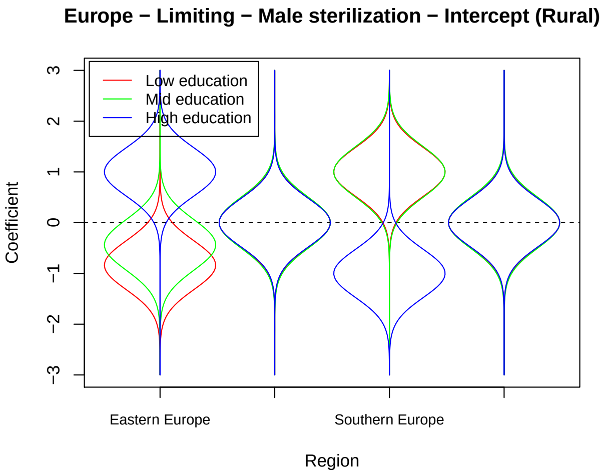
Europe − Limiting − Male sterilization − Intercept (Rural)
Low education
Mid education
High education
Coefficient
−3
−2
−1
0
1
2
3
Eastern Europe
Southern Europe
Region

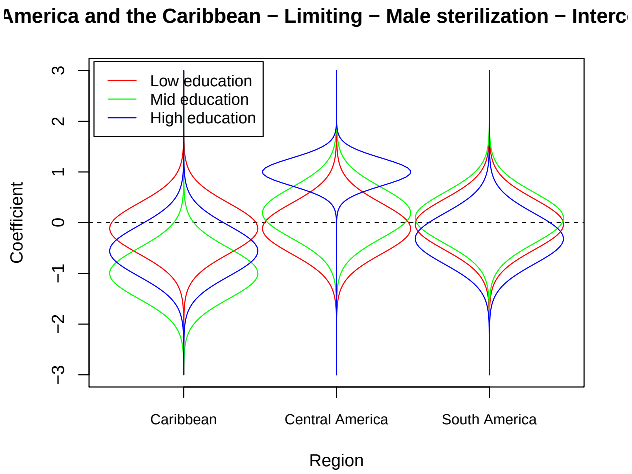
America and the Caribbean − Limiting − Male sterilization − Interc
Low education
Mid education
High education
Coefficient
−3
−2
−1
0
1
2
3
Caribbean
Central America
South America
Region

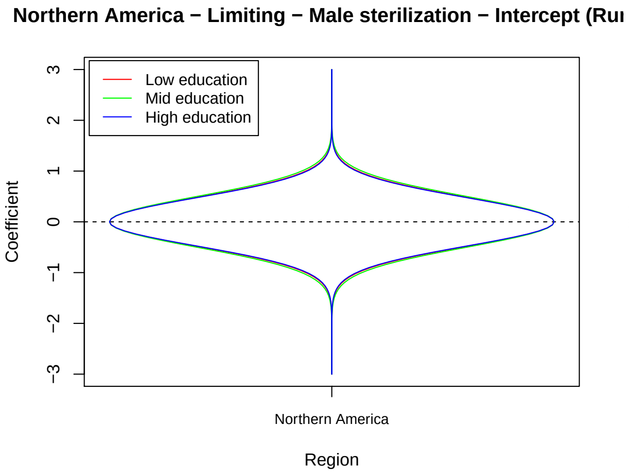
Northern America − Limiting − Male sterilization − Intercept (Rur
Low education
Mid education
High education
Coefficient
3
2
1
0
−1
−2
−3
Northern America
Region

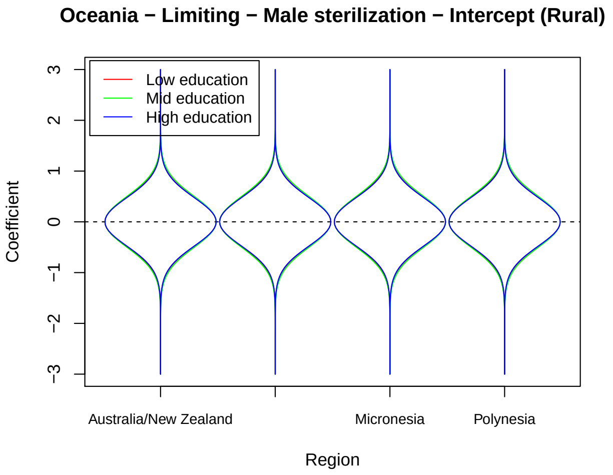
Oceania − Limiting − Male sterilization − Intercept (Rural)
Low education
Mid education
High education
Coefficient
3
2
1
0
−1
−2
−3
Australia/New Zealand
Micronesia
Polynesia
Region

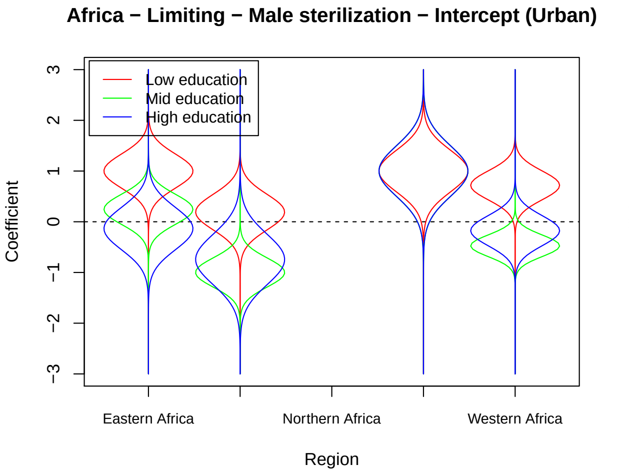
Africa − Limiting − Male sterilization − Intercept (Urban)
Low education
Mid education
High education
Coefficient
3
2
1
0
−1
−2
−3
Eastern Africa
Northern Africa
Western Africa
Region

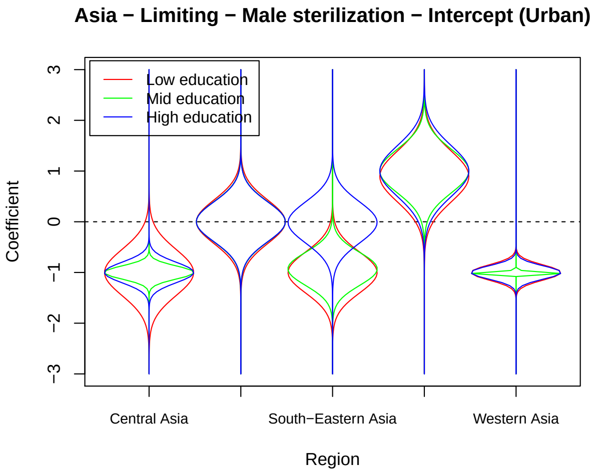
Asia − Limiting − Male sterilization − Intercept (Urban)
Low education
Mid education
High education
Coefficient
3
2
1
0
−1
−2
−3
Central Asia
South−Eastern Asia
Western Asia
Region

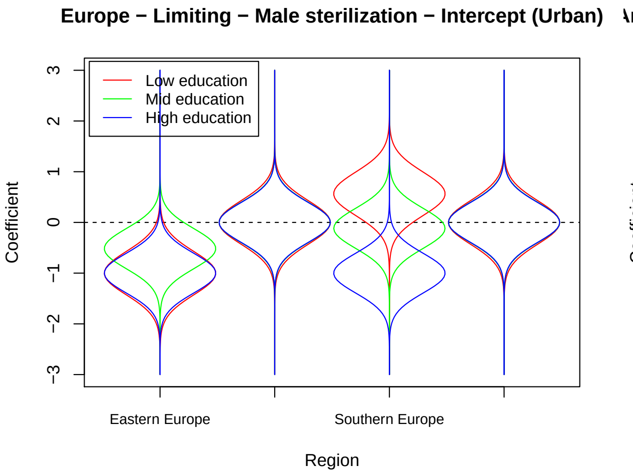
Europe − Limiting − Male sterilization − Intercept (Urban)
Low education
Mid education
High education
Coefficient
3
2
1
0
−1
−2
−3
Eastern Europe
Southern Europe
Region

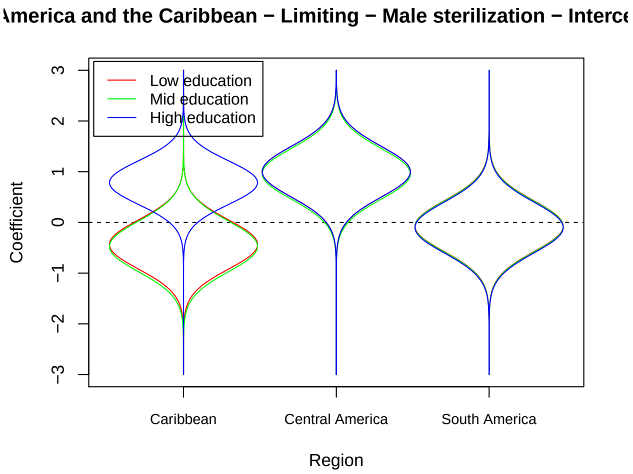
America and the Caribbean − Limiting − Male sterilization − Interce
Low education
Mid education
High education
Coefficient
3
2
1
0
−1
−2
−3
Caribbean
Central America
South America
Region

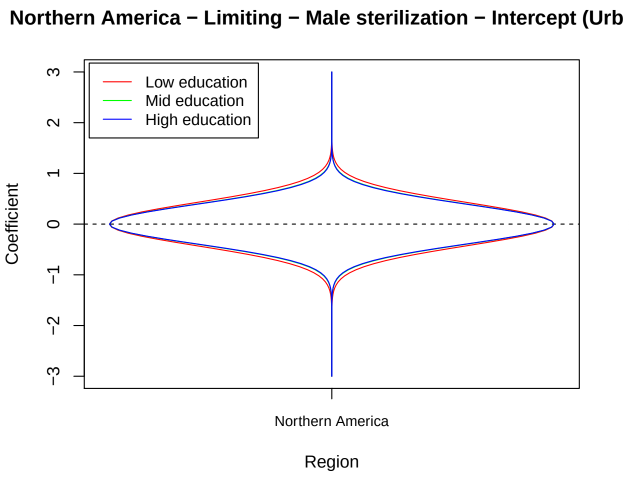
Northern America − Limiting − Male sterilization − Intercept (Urb
Low education
Mid education
High education
Coefficient
3
2
1
0
−1
−2
−3
Northern America
Region

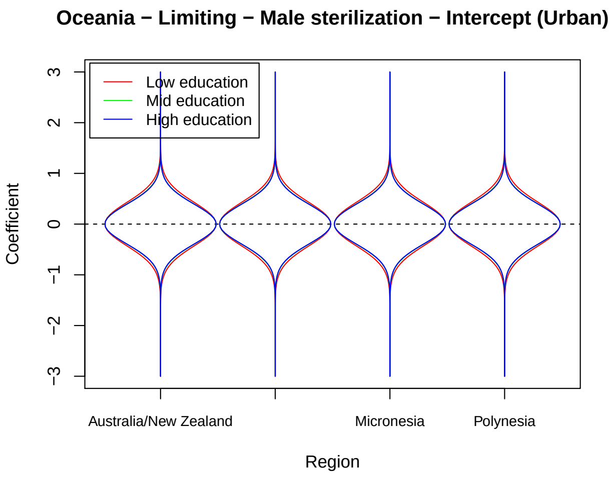
Oceania − Limiting − Male sterilization − Intercept (Urban)
Low education
Mid education
High education
Coefficient
3
2
1
0
−1
−2
−3
Australia/New Zealand
Micronesia
Polynesia
Region

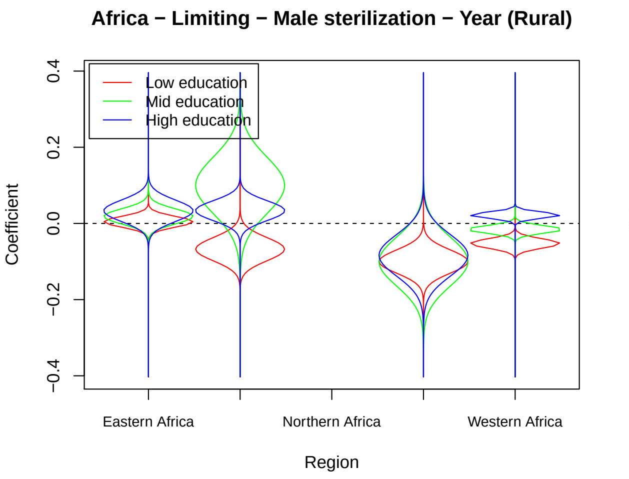
Africa − Limiting − Male sterilization − Year (Rural)
Low education
Mid education
High education
Coefficient
0.4
0.2
0.0
−0.2
−0.4
Eastern Africa
Northern Africa
Western Africa
Region

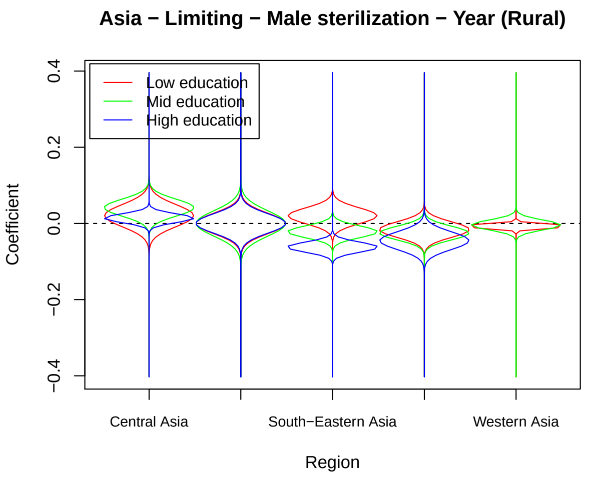
Asia − Limiting − Male sterilization − Year (Rural)
Low education
Mid education
High education
Coefficient
0.4
0.2
0.0
−0.2
−0.4
Central Asia
South−Eastern Asia
Western Asia
Region

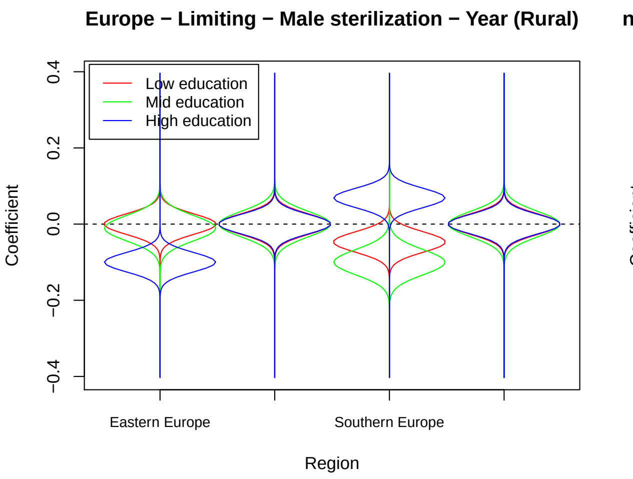
Europe − Limiting − Male sterilization − Year (Rural)
Low education
Mid education
High education
Coefficient
0.4
0.2
0.0
−0.2
−0.4
Eastern Europe
Southern Europe
Region

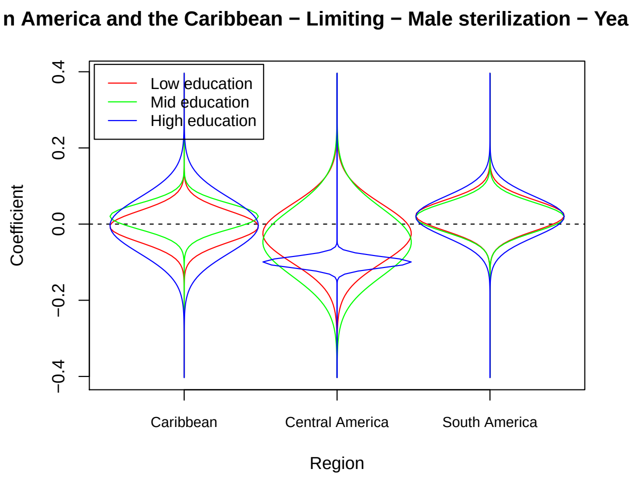
n America and the Caribbean − Limiting − Male sterilization − Yea
Low education
Mid education
High education
Coefficient
0.4
0.2
0.0
−0.2
−0.4
Caribbean
Central America
South America
Region

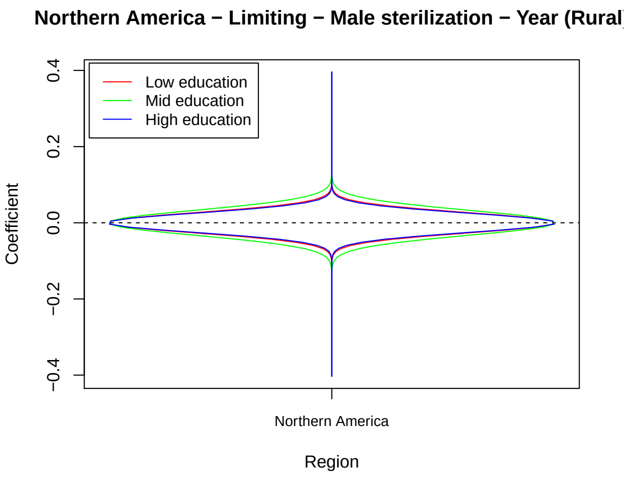
Northern America − Limiting − Male sterilization − Year (Rural
Low education
Mid education
High education
Coefficient
0.4
0.2
0.0
−0.2
−0.4
Northern America
Region

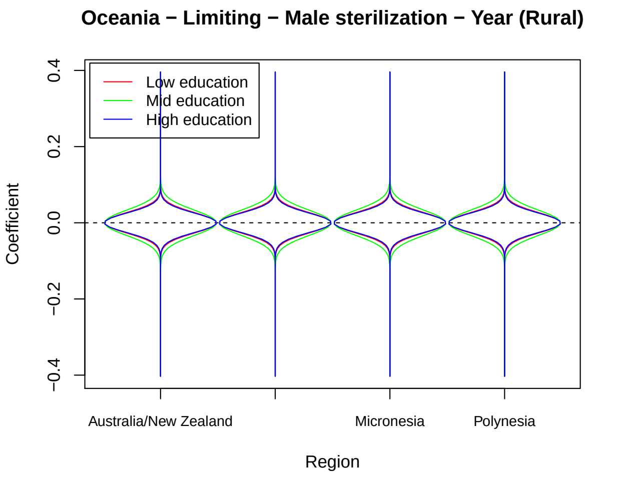
Oceania − Limiting − Male sterilization − Year (Rural)
Low education
Mid education
High education
Coefficient
0.4
0.2
0.0
−0.2
−0.4
Australia/New Zealand
Micronesia
Polynesia
Region

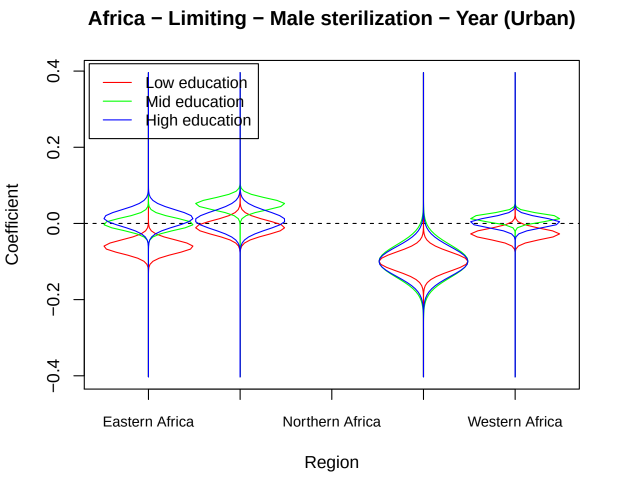
Africa − Limiting − Male sterilization − Year (Urban)
Low education
Mid education
High education
Coefficient
0.4
0.2
0.0
−0.2
−0.4
Eastern Africa
Northern Africa
Western Africa
Region

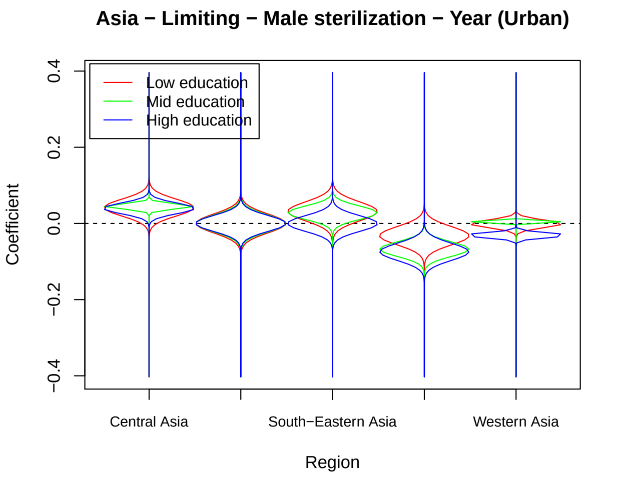
Asia − Limiting − Male sterilization − Year (Urban)
Low education
Mid education
High education
Coefficient
0.4
0.2
0.0
−0.2
−0.4
Central Asia
South−Eastern Asia
Western Asia
Region

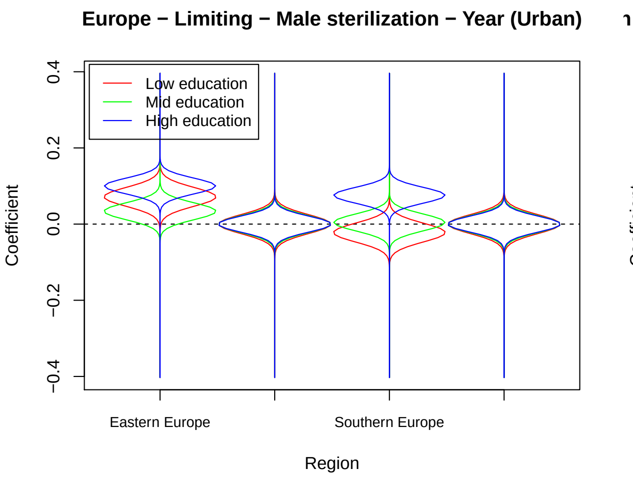
Europe − Limiting − Male sterilization − Year (Urban)
Low education
Mid education
High education
Coefficient
0.4
0.2
0.0
−0.2
−0.4
Eastern Europe
Southern Europe
Region

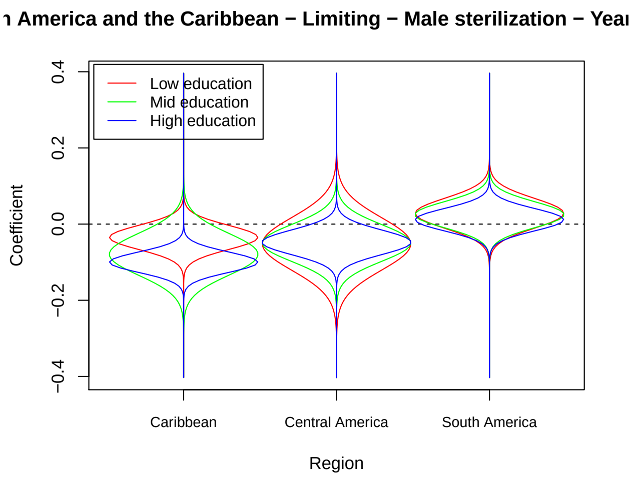
America and the Caribbean − Limiting − Male sterilization − Yea
Low education
Mid education
High education
Coefficient
0.4
0.2
0.0
−0.2
−0.4
Caribbean
Central America
South America
Region

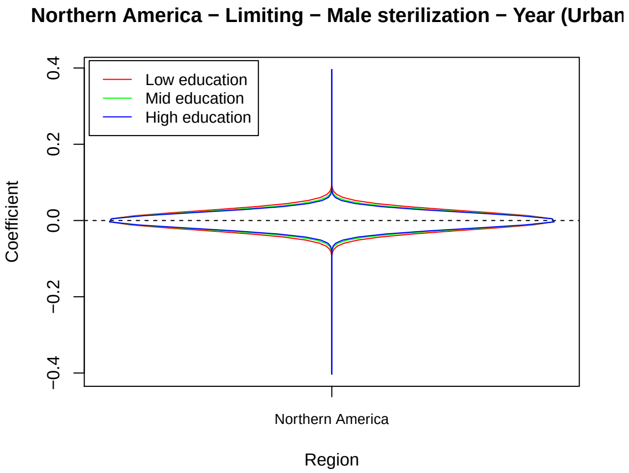
Northern America − Limiting − Male sterilization − Year (Urban
Low education
Mid education
High education
Coefficient
0.4
0.2
0.0
−0.2
−0.4
Northern America
Region

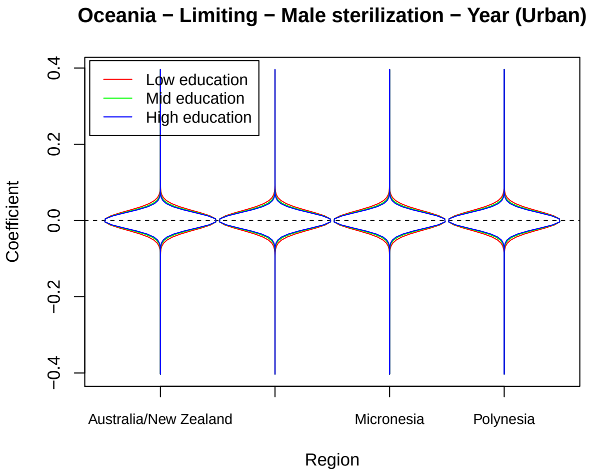
Oceania − Limiting − Male sterilization − Year (Urban)
Low education
Mid education
High education
Coefficient
0.4
0.2
0.0
−0.2
−0.4
Australia/New Zealand
Micronesia
Polynesia
Region

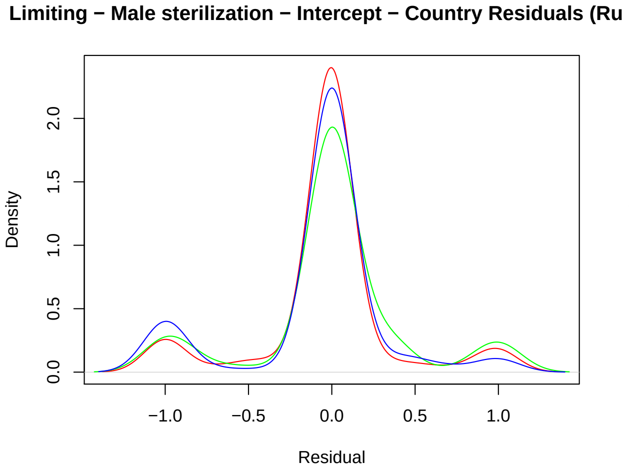
Limiting − Male sterilization − Intercept − Country Residuals (Ru
Density
2.0
1.5
1.0
0.5
0.0
−1.0
−0.5
0.0
0.5
1.0
Residual

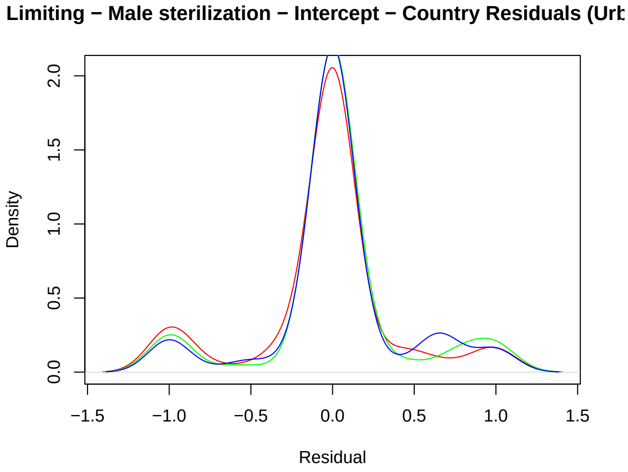
Limiting − Male sterilization − Intercept − Country Residuals (Urb
Density
2.0
1.5
1.0
0.5
0.0
−1.5
−1.0
−0.5
0.0
0.5
1.0
1.5
Residual

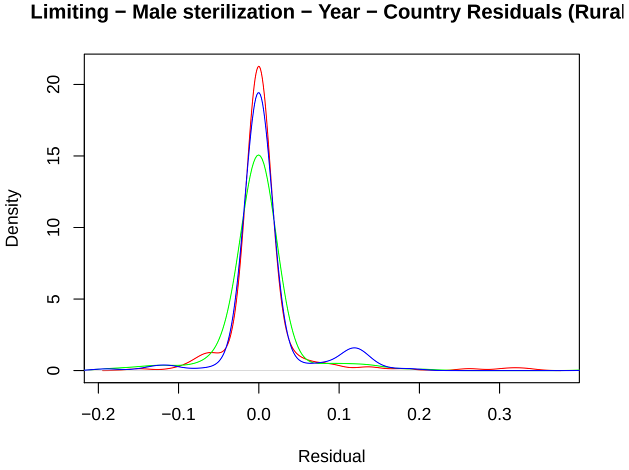
Limiting − Male sterilization − Year − Country Residuals (Rural
Density
20
15
10
5
0
−0.2
−0.1
0.0
0.1
0.2
0.3
Residual

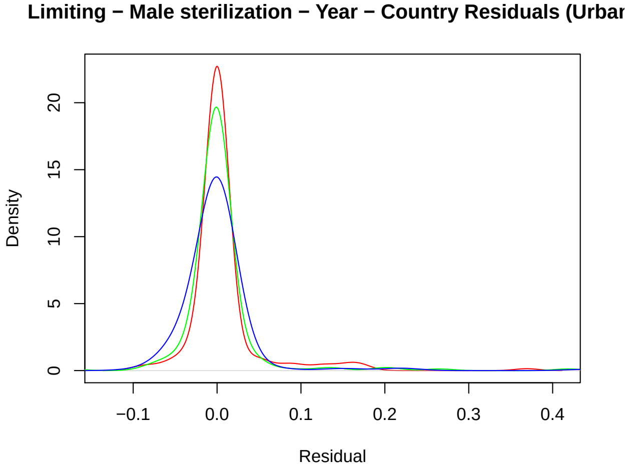
Limiting − Male sterilization − Year − Country Residuals (Urbar
Density
20
15
10
5
0
−0.1
0.0
0.1
0.2
0.3
0.4
Residual

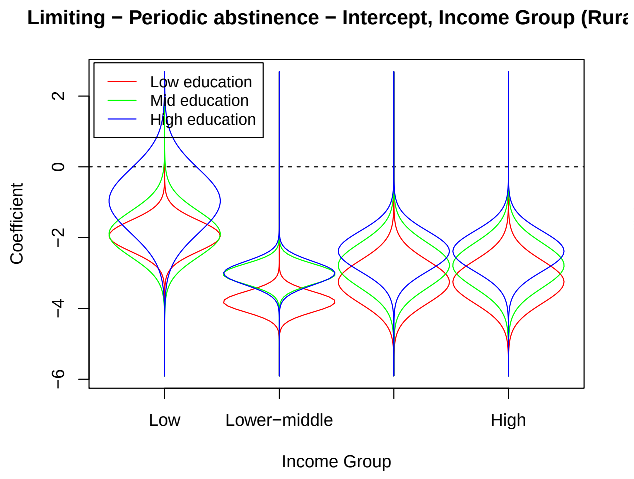
Limiting − Periodic abstinence − Intercept, Income Group (Rura
Low education
Mid education
High education
Coefficient
2
0
−2
−4
−6
Low
Lower−middle
High
Income Group

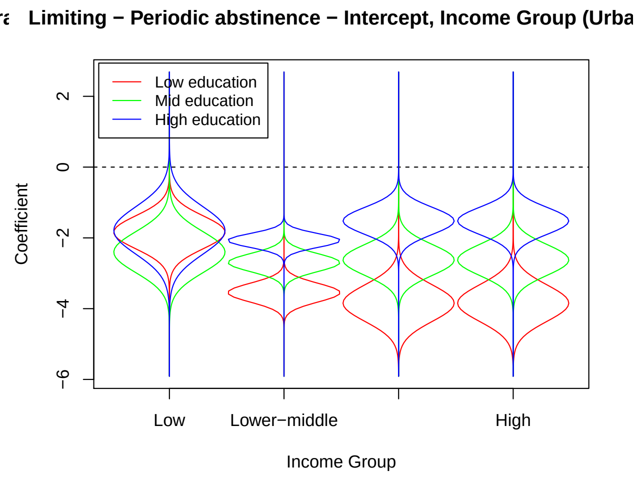
Limiting − Periodic abstinence − Intercept, Income Group (Urba
Low education
Mid education
High education
Coefficient
2
0
−2
−4
−6
Low
Lower−middle
High
Income Group

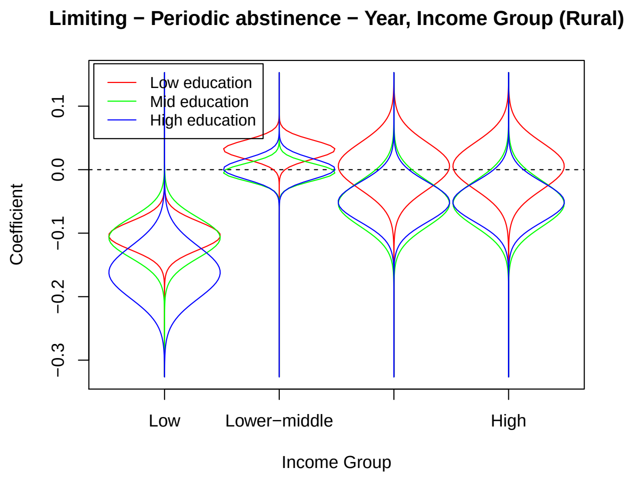
Limiting − Periodic abstinence − Year, Income Group (Rural)
Low education
Mid education
High education
Coefficient
0.1
0.0
−0.1
−0.2
−0.3
Low
Lower−middle
High
Income Group

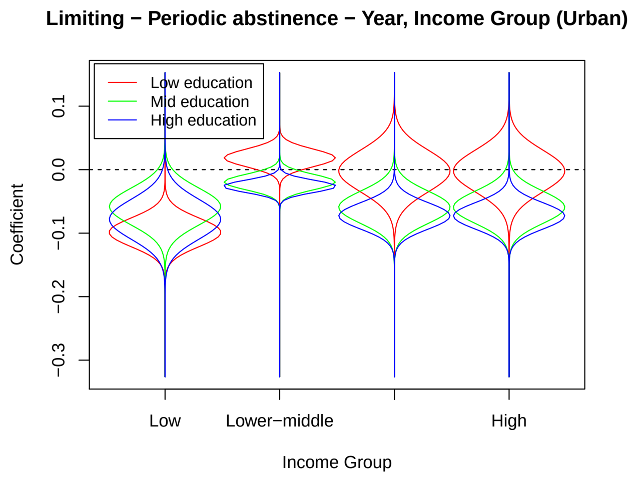
Limiting − Periodic abstinence − Year, Income Group (Urban)
Low education
Mid education
High education
Coefficient
0.1
0.0
−0.1
−0.2
−0.3
Low
Lower−middle
High
Income Group

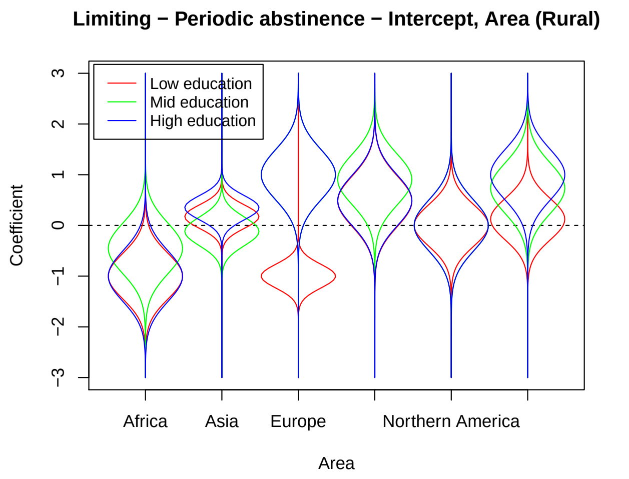
Limiting − Periodic abstinence − Intercept, Area (Rural)
Low education
Mid education
High education
Coefficient
3
2
1
0
−1
−2
−3
Africa
Asia
Europe
Northern America
Area

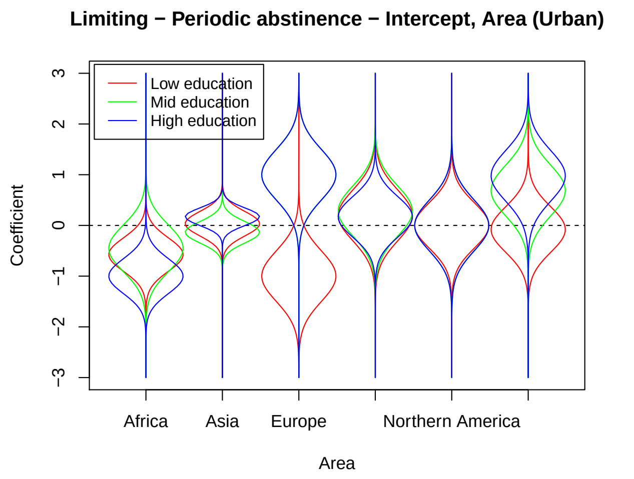
Limiting − Periodic abstinence − Intercept, Area (Urban)
Low education
Mid education
High education
Coefficient
3
2
1
0
−1
−2
−3
Africa
Asia
Europe
Northern America
Area

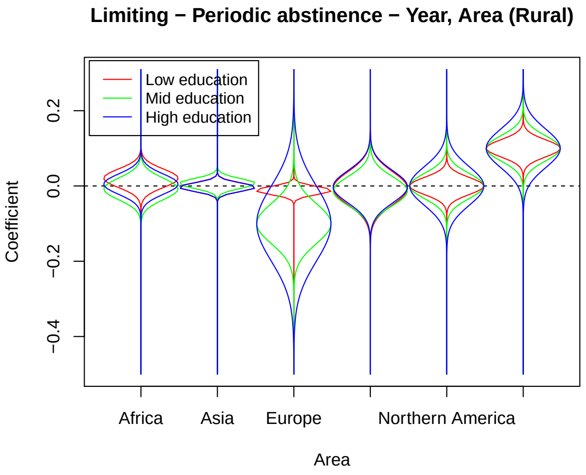
Limiting − Periodic abstinence − Year, Area (Rural)
Low education
Mid education
High education
Coefficient
Africa
Asia
Europe
Northern America
Area

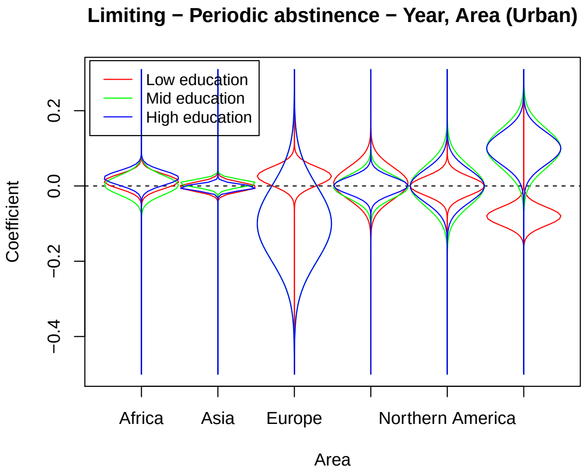
Limiting − Periodic abstinence − Year, Area (Urban)
Low education
Mid education
High education
Coefficient
Africa
Asia
Europe
Northern America
Area

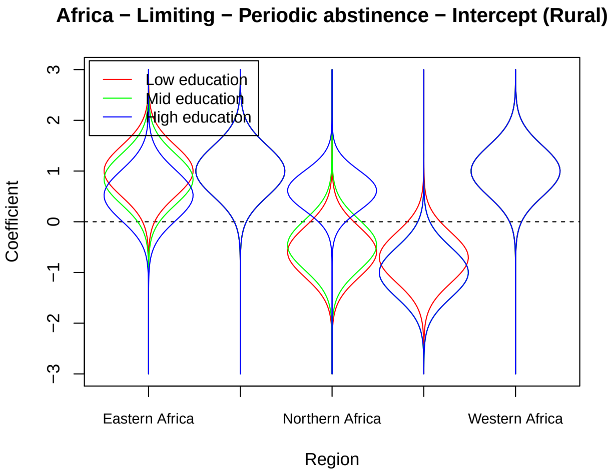
Africa − Limiting − Periodic abstinence − Intercept (Rural)
Low education
Mid education
High education
Coefficient
Eastern Africa
Northern Africa
Western Africa
Region

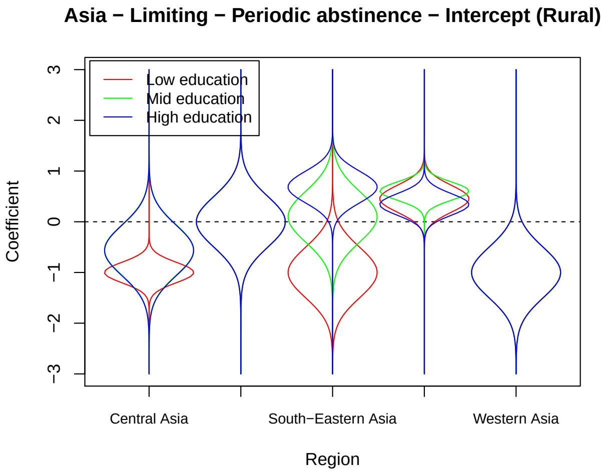
Asia − Limiting − Periodic abstinence − Intercept (Rural)
Low education
Mid education
High education
Coefficient
Central Asia
South−Eastern Asia
Western Asia
Region

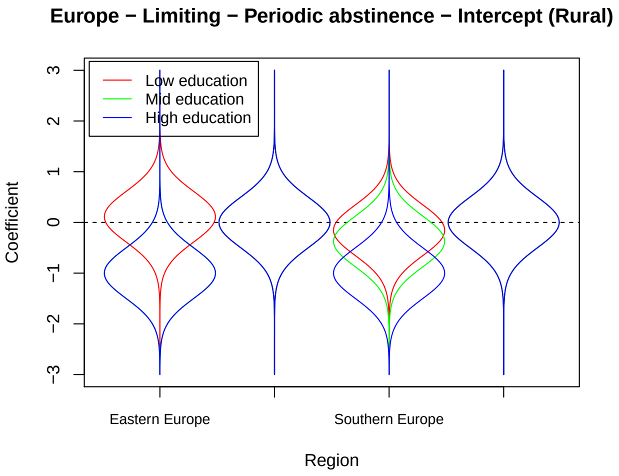
Europe − Limiting − Periodic abstinence − Intercept (Rural)
Low education
Mid education
High education
Coefficient
Eastern Europe
Southern Europe
Region

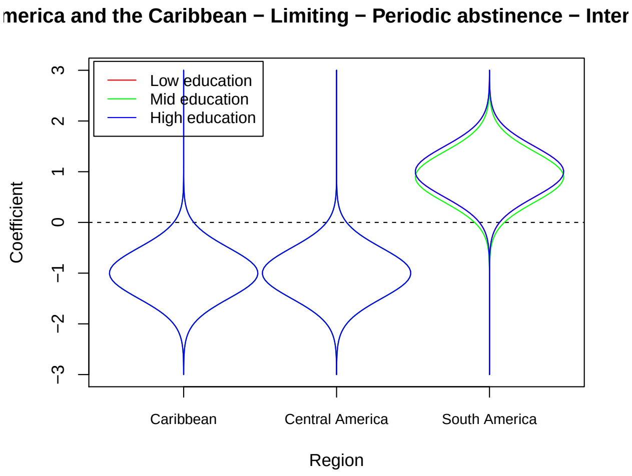
merica and the Caribbean − Limiting − Periodic abstinence − Inter
Low education
Mid education
High education
Coefficient
Caribbean
Central America
South America
Region

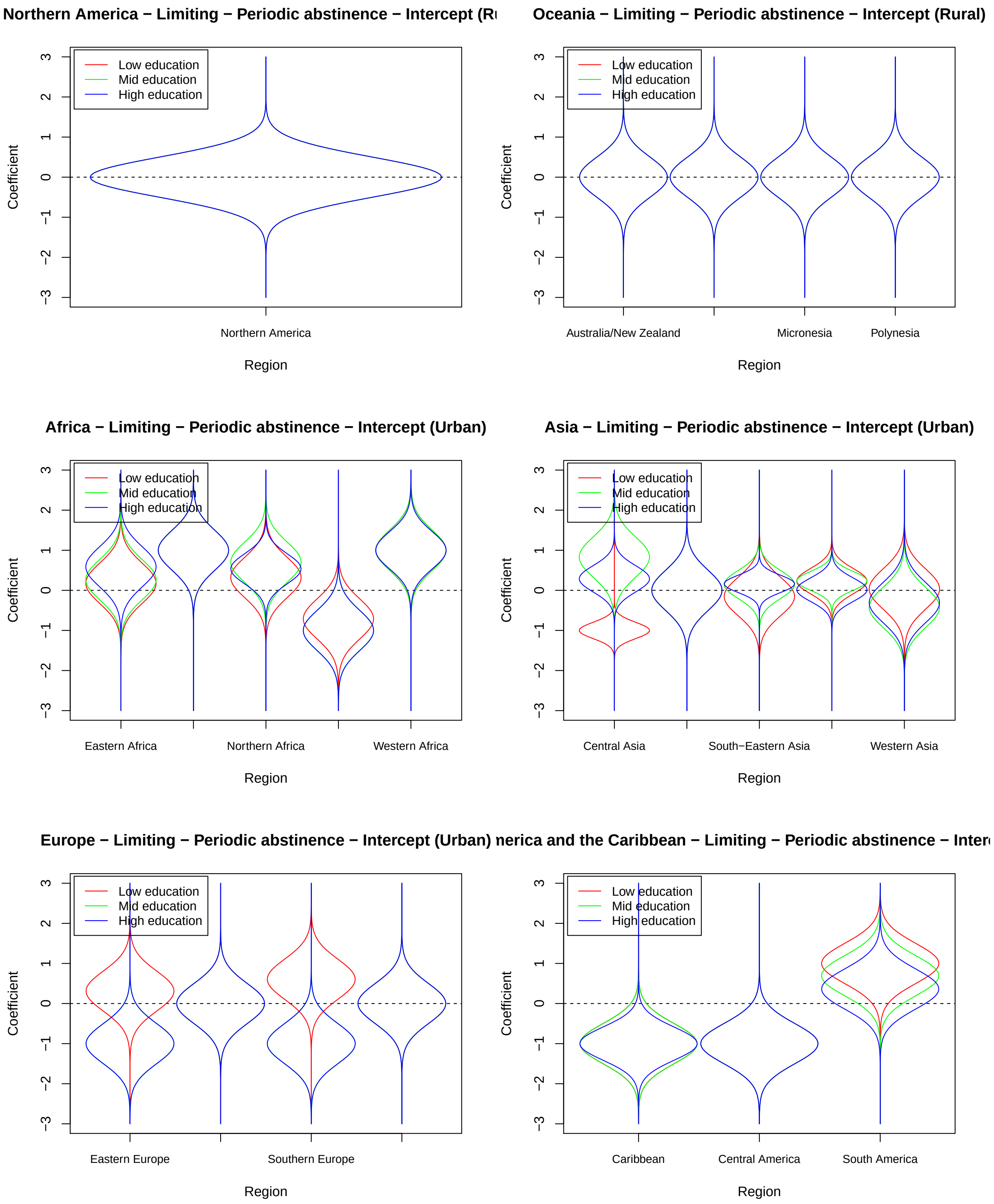
Northern America − Limiting − Periodic abstinence − Intercept (Ru
Oceania − Limiting − Periodic abstinence − Intercept (Rural)
Africa − Limiting − Periodic abstinence − Intercept (Urban)
Asia − Limiting − Periodic abstinence − Intercept (Urban)
Europe − Limiting − Periodic abstinence − Intercept (Urban)
nerica and the Caribbean − Limiting − Periodic abstinence − Interc
Low education
Mid education
High education
Coefficient
Region
Northern America
Australia/New Zealand
Micronesia
Polynesia
Eastern Africa
Northern Africa
Western Africa
Central Asia
South−Eastern Asia
Western Asia
Eastern Europe
Southern Europe
Caribbean
Central America
South America

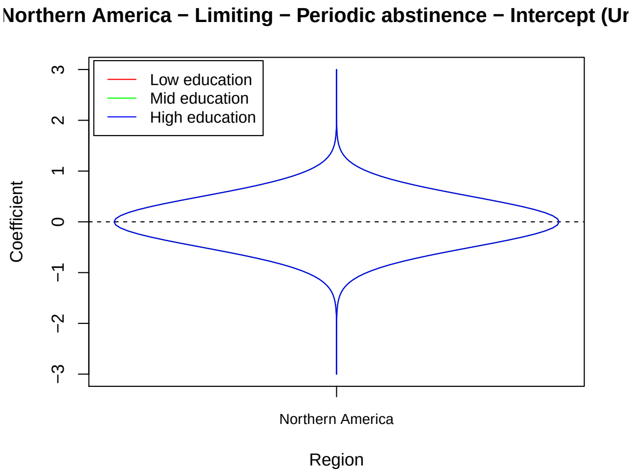
Northern America − Limiting − Periodic abstinence − Intercept (Ur
Low education
Mid education
High education
Coefficient
3
2
1
0
−1
−2
−3
Northern America
Region

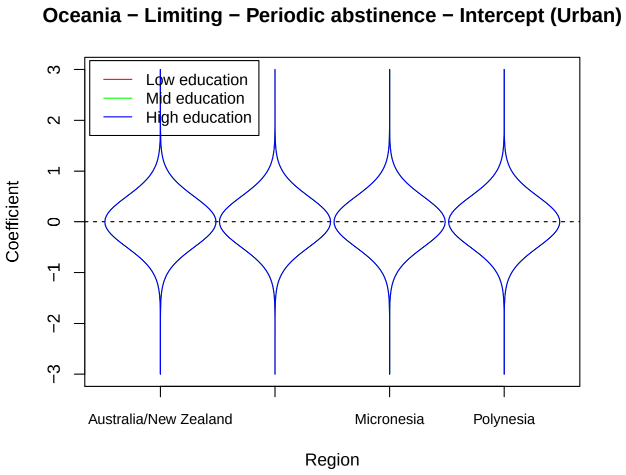
Oceania − Limiting − Periodic abstinence − Intercept (Urban)
Low education
Mid education
High education
Coefficient
3
2
1
0
−1
−2
−3
Australia/New Zealand
Micronesia
Polynesia
Region

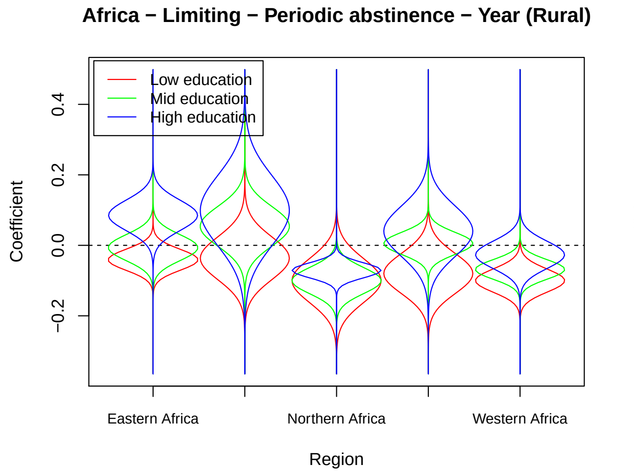
Africa − Limiting − Periodic abstinence − Year (Rural)
Low education
Mid education
High education
Coefficient
0.4
0.2
0.0
−0.2
Eastern Africa
Northern Africa
Western Africa
Region

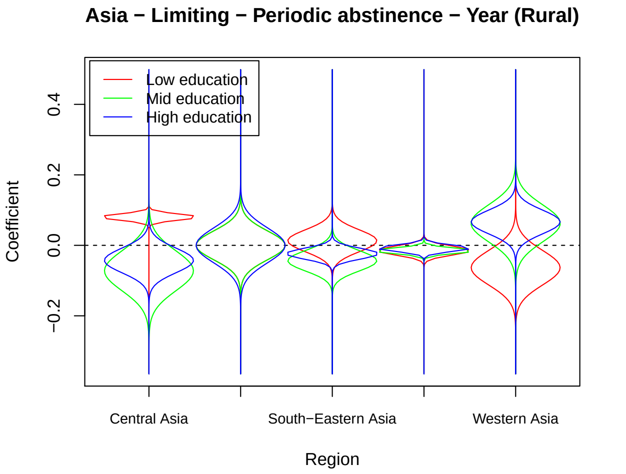
Asia − Limiting − Periodic abstinence − Year (Rural)
Low education
Mid education
High education
Coefficient
0.4
0.2
0.0
−0.2
Central Asia
South−Eastern Asia
Western Asia
Region

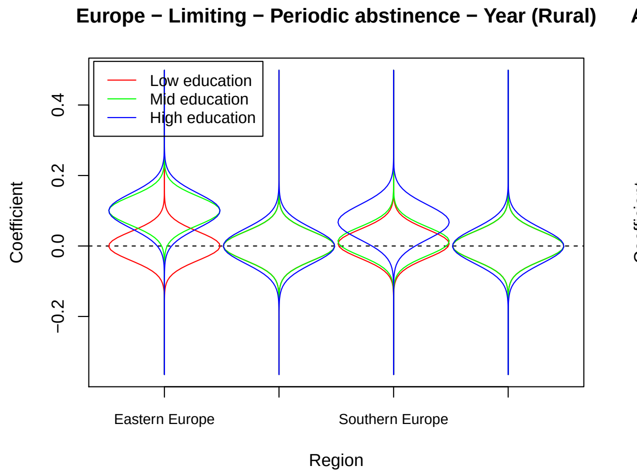
Europe − Limiting − Periodic abstinence − Year (Rural)
Low education
Mid education
High education
Coefficient
0.4
0.2
0.0
−0.2
Eastern Europe
Southern Europe
Region

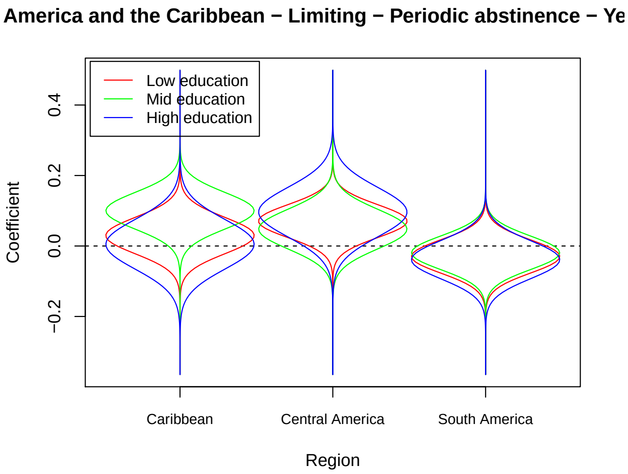
America and the Caribbean − Limiting − Periodic abstinence − Ye
Low education
Mid education
High education
Coefficient
0.4
0.2
0.0
−0.2
Caribbean
Central America
South America
Region

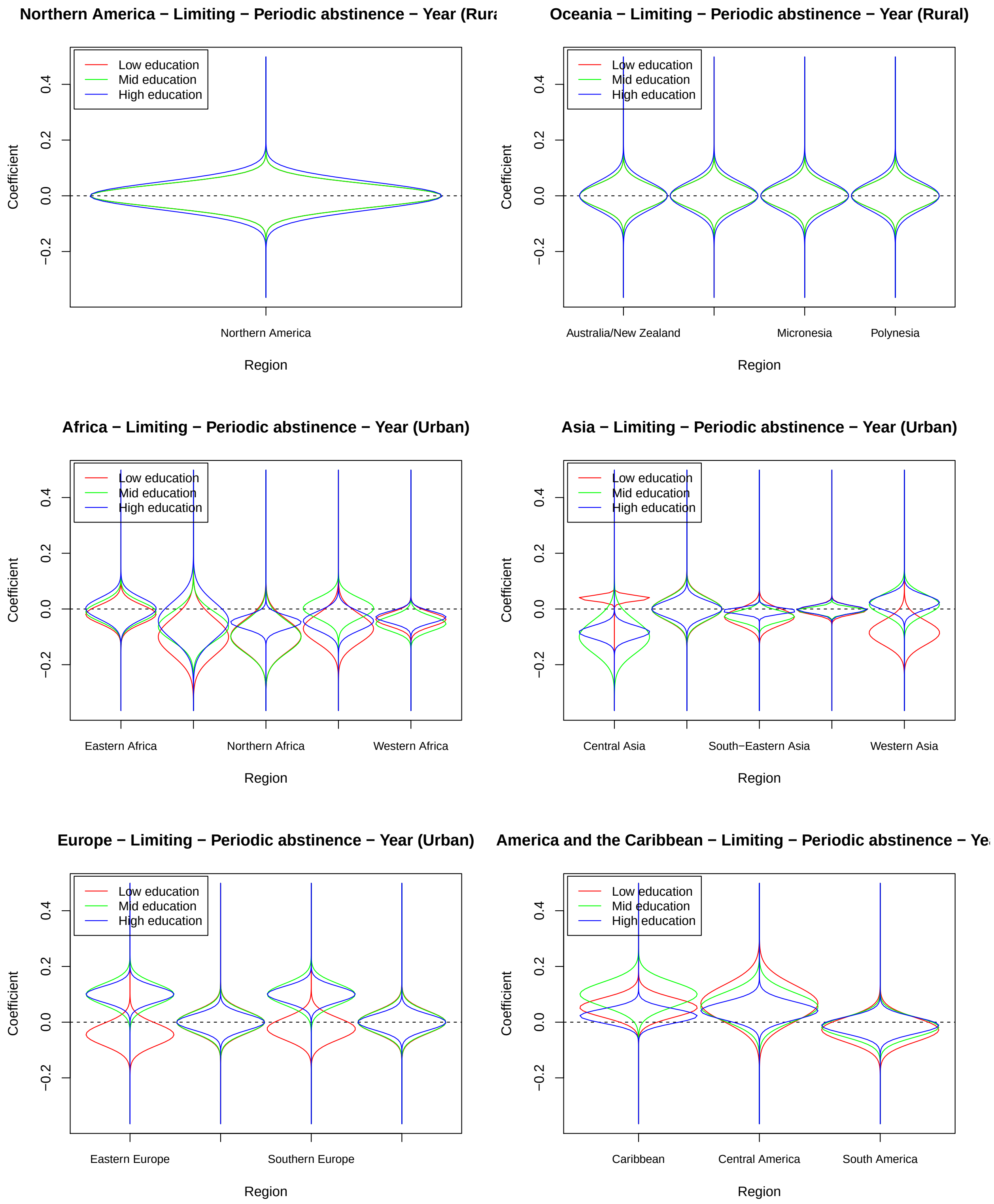
Northern America − Limiting − Periodic abstinence − Year (Rura
Oceania − Limiting − Periodic abstinence − Year (Rural)
Africa − Limiting − Periodic abstinence − Year (Urban)
Asia − Limiting − Periodic abstinence − Year (Urban)
Europe − Limiting − Periodic abstinence − Year (Urban)
America and the Caribbean − Limiting − Periodic abstinence − Ye
Low education
Mid education
High education
Coefficient
Region
Northern America
Australia/New Zealand
Micronesia
Polynesia
Eastern Africa
Northern Africa
Western Africa
Central Asia
South−Eastern Asia
Western Asia
Eastern Europe
Southern Europe
Caribbean
Central America
South America
−0.2
0.0
0.2
0.4

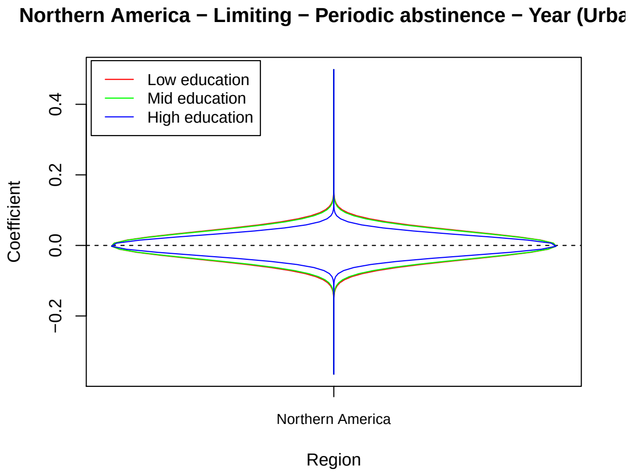
Northern America − Limiting − Periodic abstinence − Year (Urba
Low education
Mid education
High education
Coefficient
Northern America
Region

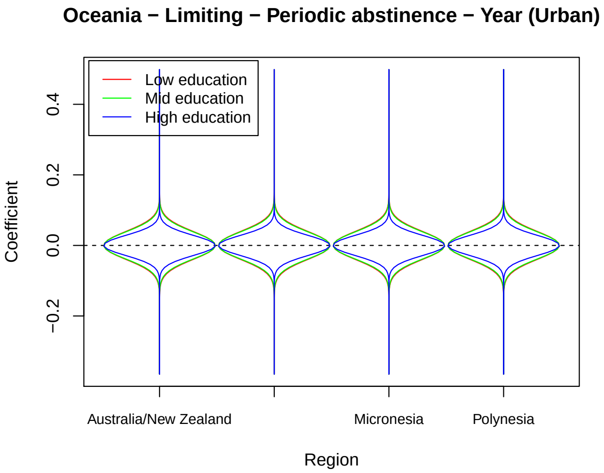
Oceania − Limiting − Periodic abstinence − Year (Urban)
Low education
Mid education
High education
Coefficient
Australia/New Zealand
Micronesia
Polynesia
Region

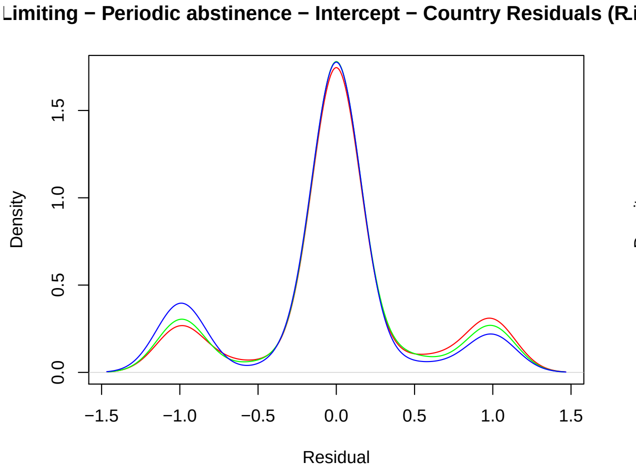
Limiting − Periodic abstinence − Intercept − Country Residuals (R
Density
Residual

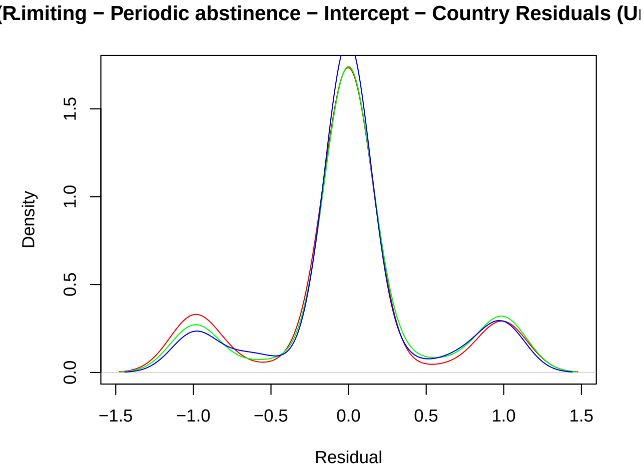
Limiting − Periodic abstinence − Intercept − Country Residuals (Ur
Density
Residual

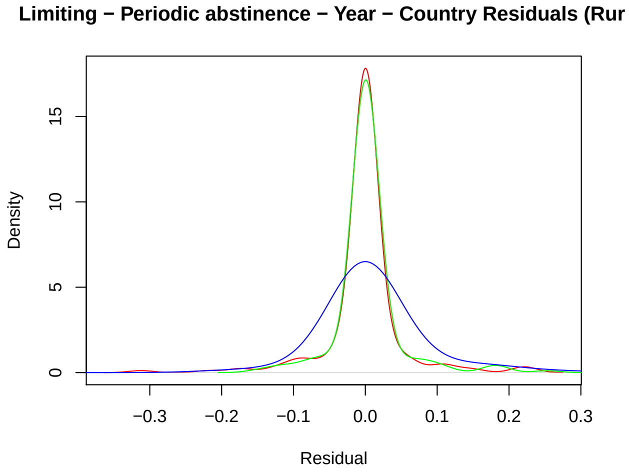
Limiting − Periodic abstinence − Year − Country Residuals (Rur
Density
Residual

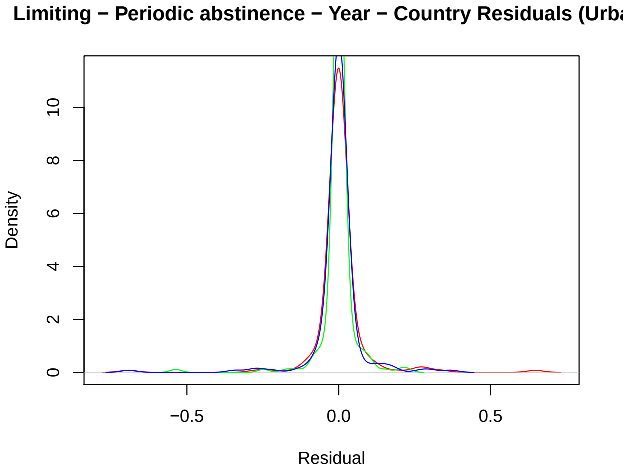
Limiting − Periodic abstinence − Year − Country Residuals (Urba
Density
Residual

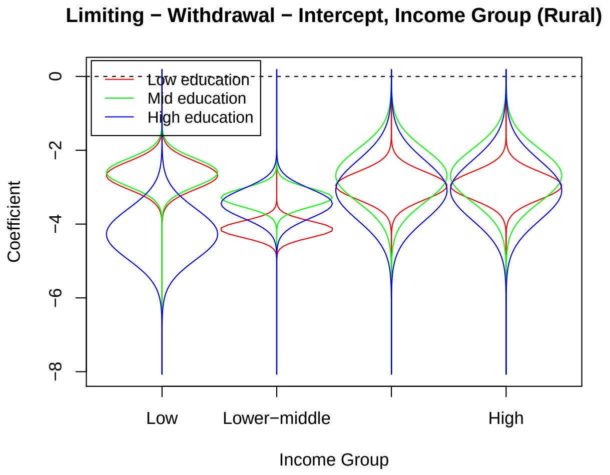
Limiting − Withdrawal − Intercept, Income Group (Rural)
Low education
Mid education
High education
Coefficient
0
−2
−4
−6
−8
Low
Lower−middle
High
Income Group

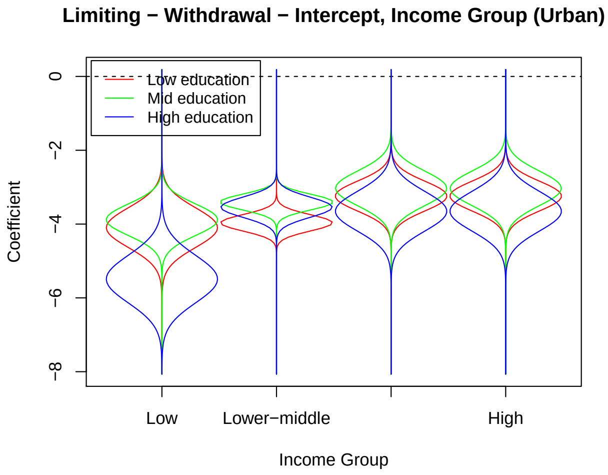
Limiting − Withdrawal − Intercept, Income Group (Urban)
Low education
Mid education
High education
Coefficient
0
−2
−4
−6
−8
Low
Lower−middle
High
Income Group

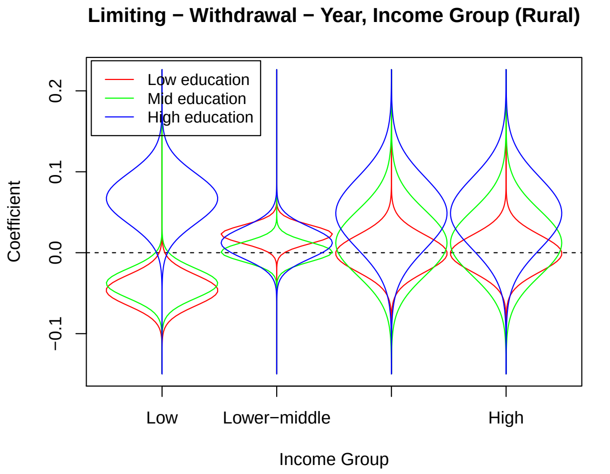
Limiting − Withdrawal − Year, Income Group (Rural)
Low education
Mid education
High education
Coefficient
0.2
0.1
0.0
−0.1
Low
Lower−middle
High
Income Group

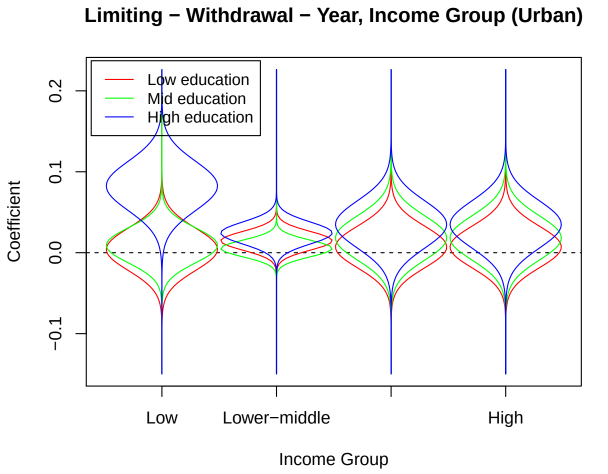
Limiting − Withdrawal − Year, Income Group (Urban)
Low education
Mid education
High education
Coefficient
0.2
0.1
0.0
−0.1
Low
Lower−middle
High
Income Group

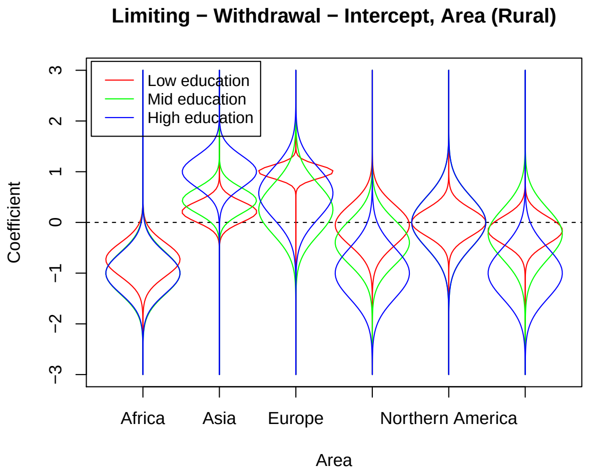
Limiting − Withdrawal − Intercept, Area (Rural)
Low education
Mid education
High education
Coefficient
3
2
1
0
−1
−2
−3
Africa
Asia
Europe
Northern America
Area

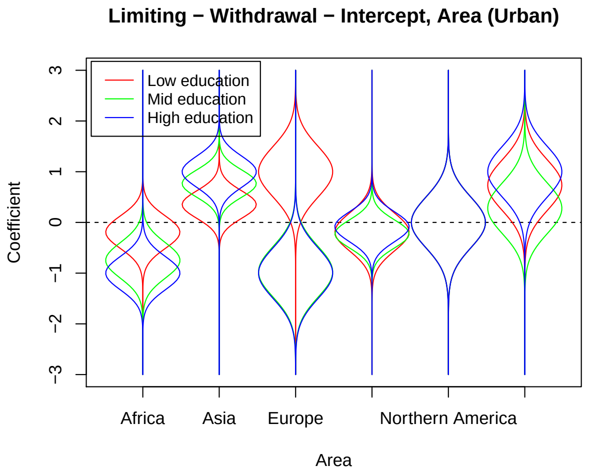
Limiting − Withdrawal − Intercept, Area (Urban)
Low education
Mid education
High education
Coefficient
3
2
1
0
−1
−2
−3
Africa
Asia
Europe
Northern America
Area

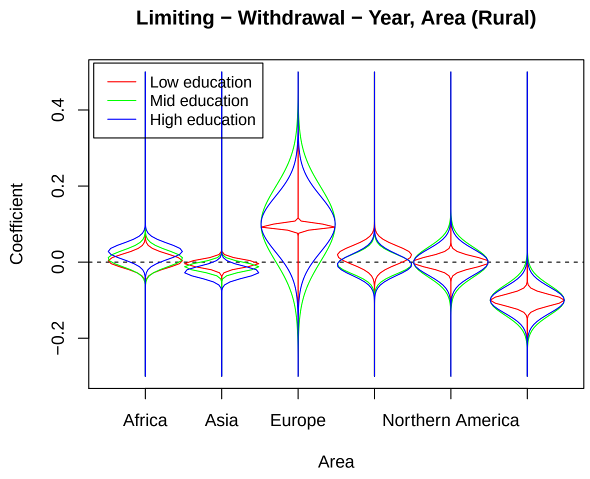
Limiting − Withdrawal − Year, Area (Rural)
Low education
Mid education
High education
Coefficient
−0.2
0.0
0.2
0.4
Africa
Asia
Europe
Northern America
Area

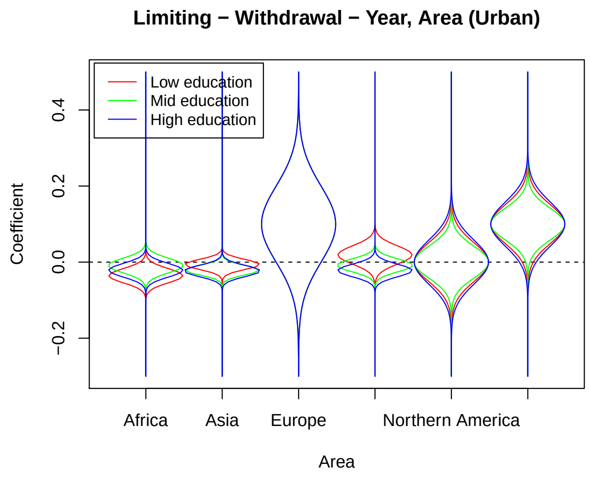
Limiting − Withdrawal − Year, Area (Urban)
Low education
Mid education
High education
Coefficient
−0.2
0.0
0.2
0.4
Africa
Asia
Europe
Northern America
Area

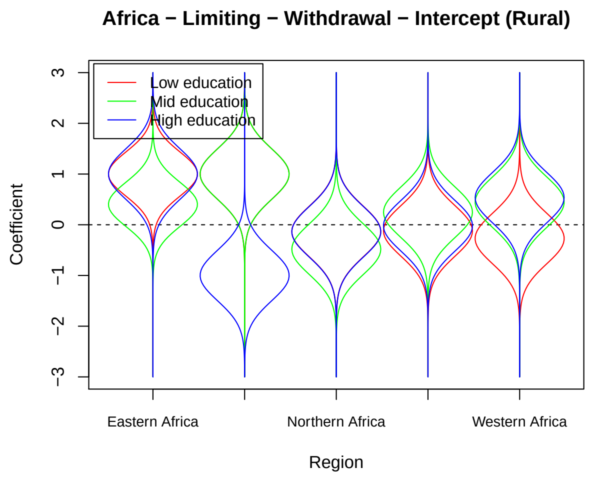
Africa − Limiting − Withdrawal − Intercept (Rural)
Low education
Mid education
High education
Coefficient
−3
−2
−1
0
1
2
3
Eastern Africa
Northern Africa
Western Africa
Region

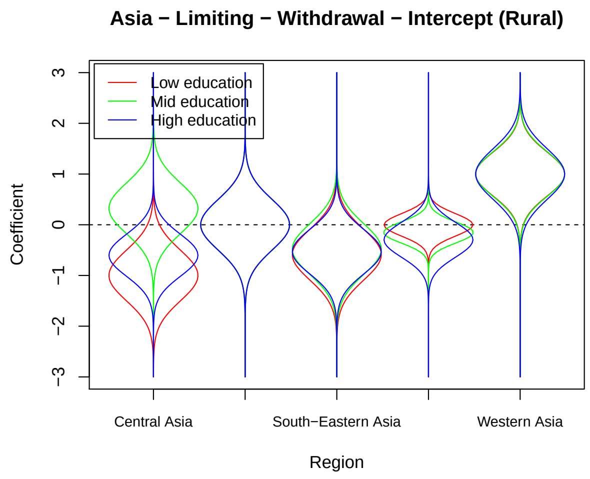
Asia − Limiting − Withdrawal − Intercept (Rural)
Low education
Mid education
High education
Coefficient
−3
−2
−1
0
1
2
3
Central Asia
South−Eastern Asia
Western Asia
Region

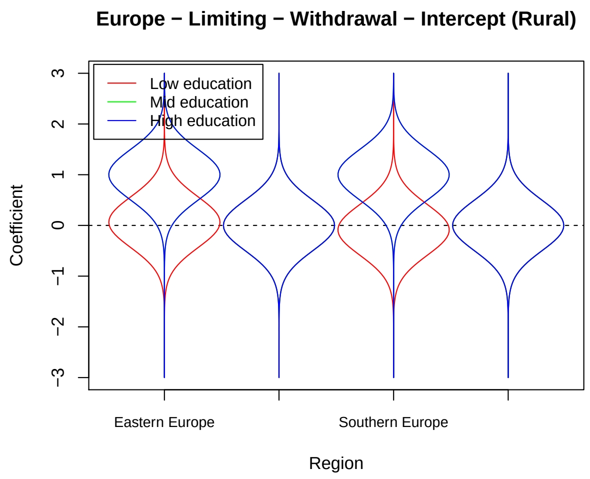
Europe − Limiting − Withdrawal − Intercept (Rural)
Low education
Mid education
High education
Coefficient
−3
−2
−1
0
1
2
3
Eastern Europe
Southern Europe
Region

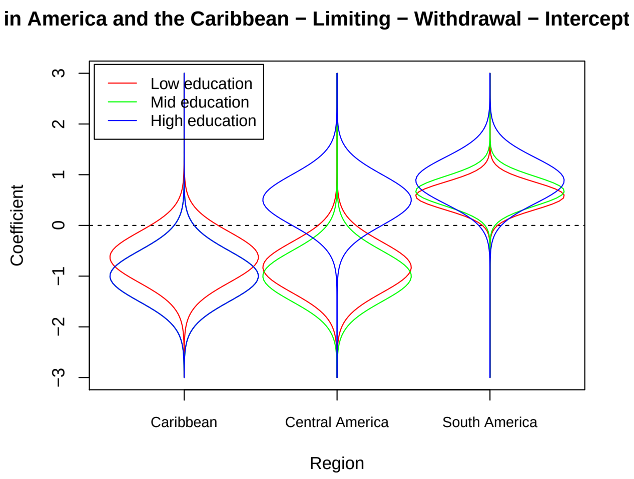
in America and the Caribbean − Limiting − Withdrawal − Intercept
Low education
Mid education
High education
Coefficient
−3
−2
−1
0
1
2
3
Caribbean
Central America
South America
Region

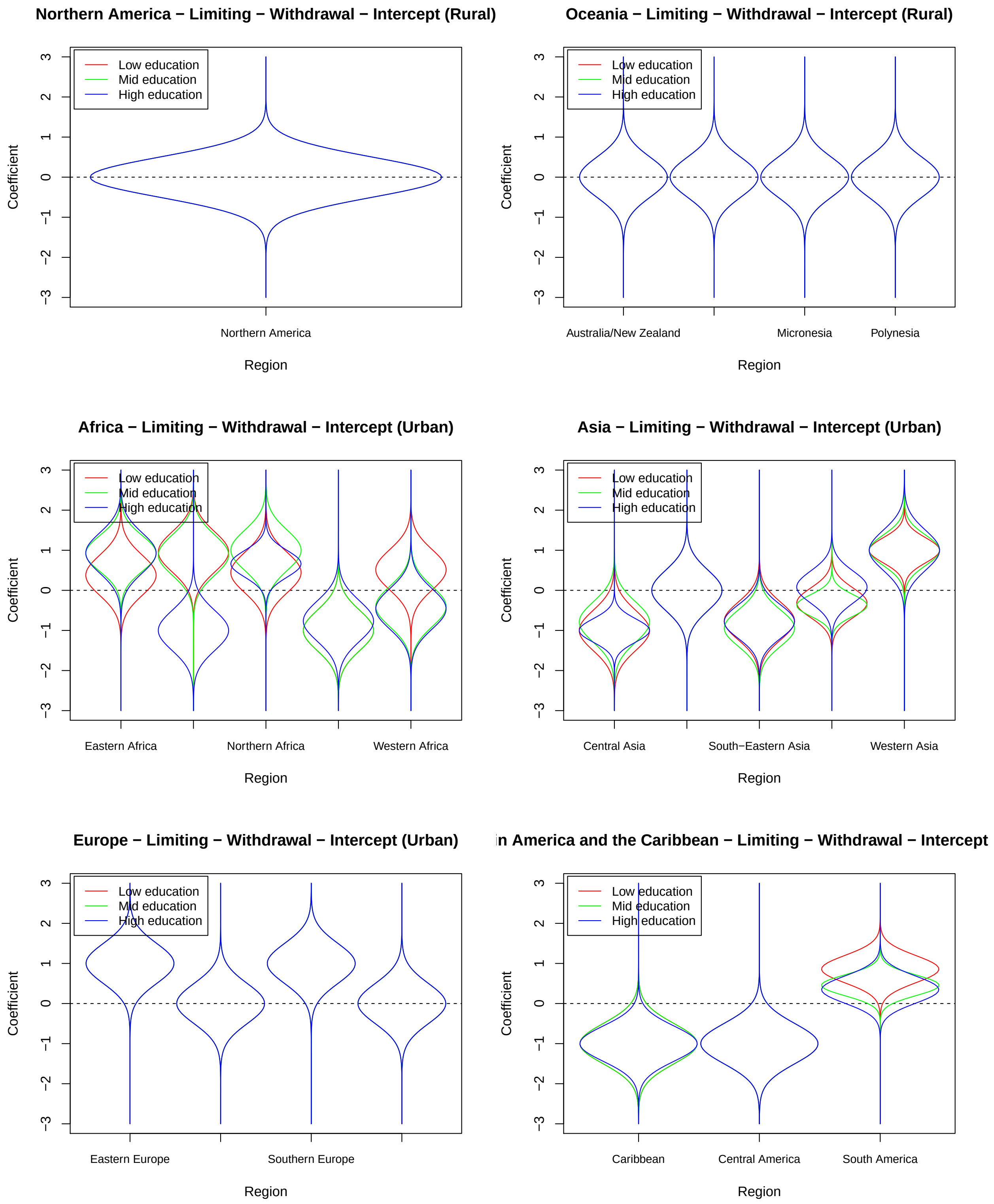
Northern America − Limiting − Withdrawal − Intercept (Rural)
Oceania − Limiting − Withdrawal − Intercept (Rural)
Africa − Limiting − Withdrawal − Intercept (Urban)
Asia − Limiting − Withdrawal − Intercept (Urban)
Europe − Limiting − Withdrawal − Intercept (Urban)
in America and the Caribbean − Limiting − Withdrawal − Intercept
Low education
Mid education
High education
Coefficient
Region
Northern America
Australia/New Zealand
Micronesia
Polynesia
Eastern Africa
Northern Africa
Western Africa
Central Asia
South−Eastern Asia
Western Asia
Eastern Europe
Southern Europe
Caribbean
Central America
South America

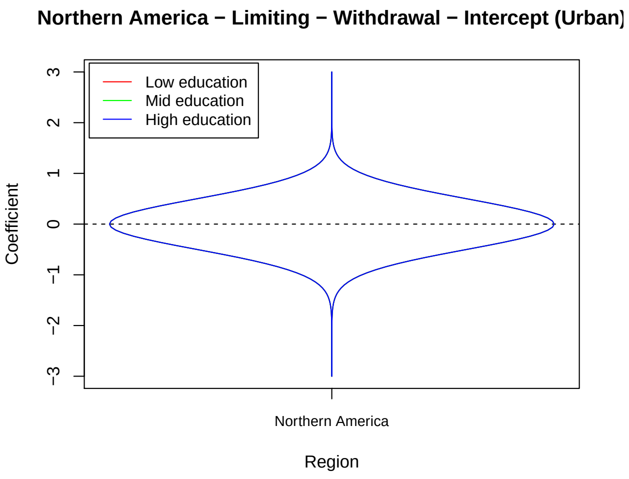
Northern America − Limiting − Withdrawal − Intercept (Urban)
Low education
Mid education
High education
Coefficient
3
2
1
0
−1
−2
−3
Northern America
Region

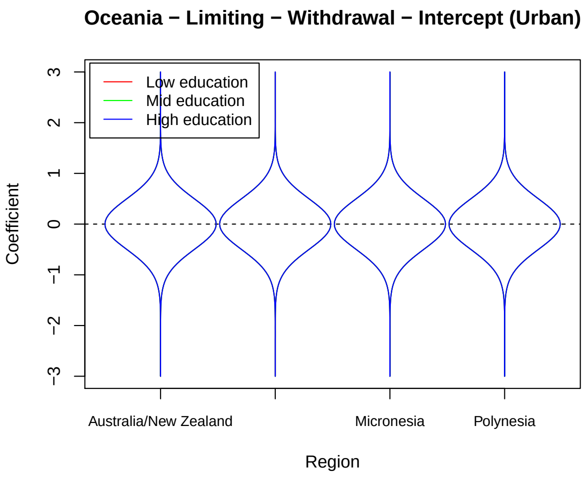
Oceania − Limiting − Withdrawal − Intercept (Urban)
Low education
Mid education
High education
Coefficient
3
2
1
0
−1
−2
−3
Australia/New Zealand
Micronesia
Polynesia
Region

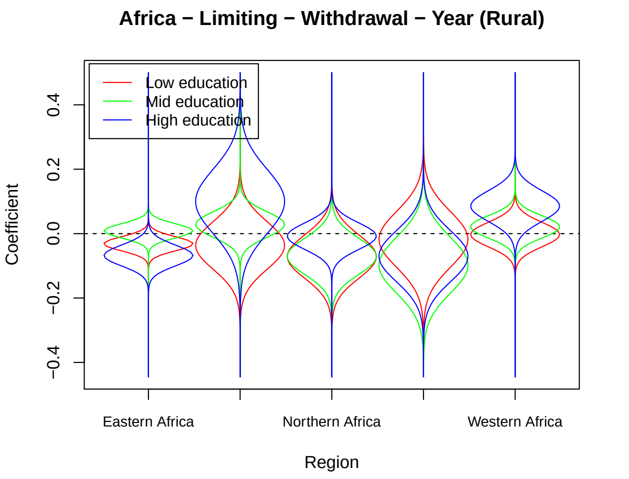
Africa − Limiting − Withdrawal − Year (Rural)
Low education
Mid education
High education
Coefficient
0.4
0.2
0.0
−0.2
−0.4
Eastern Africa
Northern Africa
Western Africa
Region

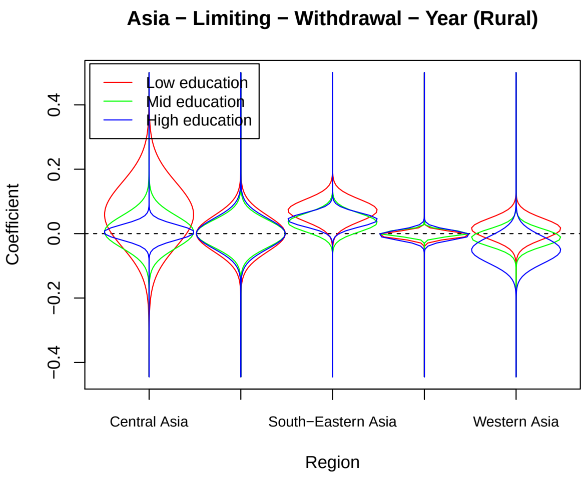
Asia − Limiting − Withdrawal − Year (Rural)
Low education
Mid education
High education
Coefficient
0.4
0.2
0.0
−0.2
−0.4
Central Asia
South−Eastern Asia
Western Asia
Region

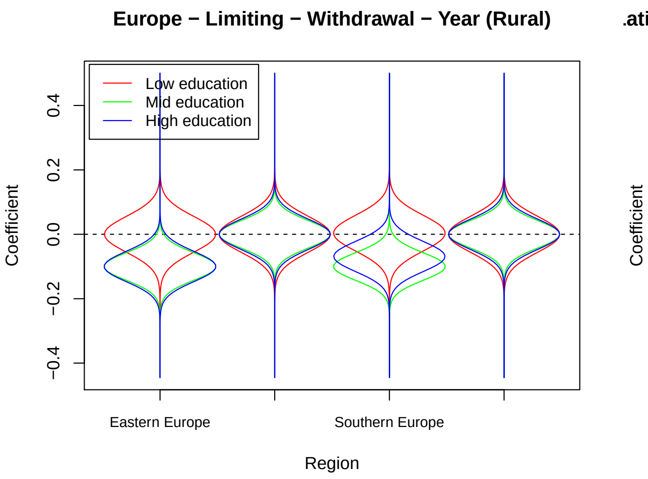
Europe − Limiting − Withdrawal − Year (Rural)
Low education
Mid education
High education
Coefficient
0.4
0.2
0.0
−0.2
−0.4
Eastern Europe
Southern Europe
Region

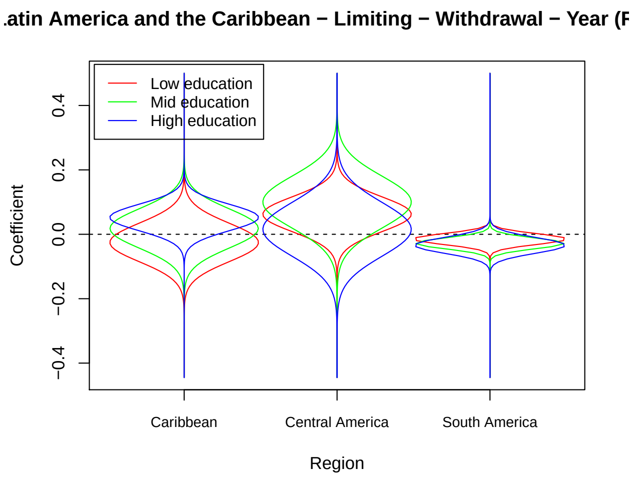
.atin America and the Caribbean − Limiting − Withdrawal − Year (F
Low education
Mid education
High education
Coefficient
0.4
0.2
0.0
−0.2
−0.4
Caribbean
Central America
South America
Region

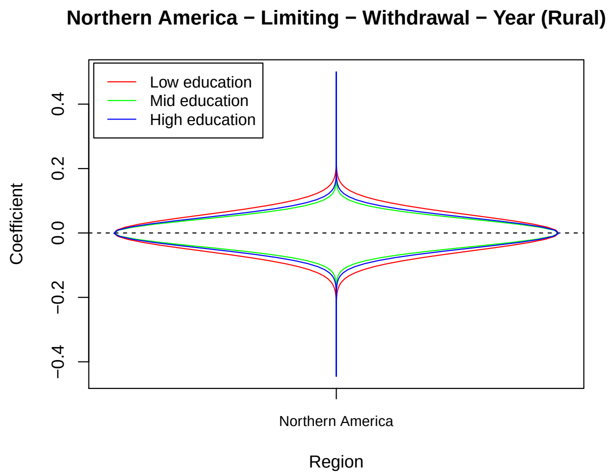
Northern America − Limiting − Withdrawal − Year (Rural)
Low education
Mid education
High education
Coefficient
Northern America
Region

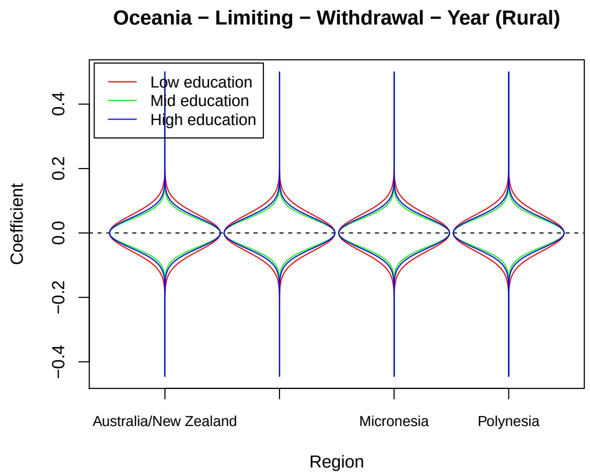
Oceania − Limiting − Withdrawal − Year (Rural)
Low education
Mid education
High education
Coefficient
Australia/New Zealand
Micronesia
Polynesia
Region

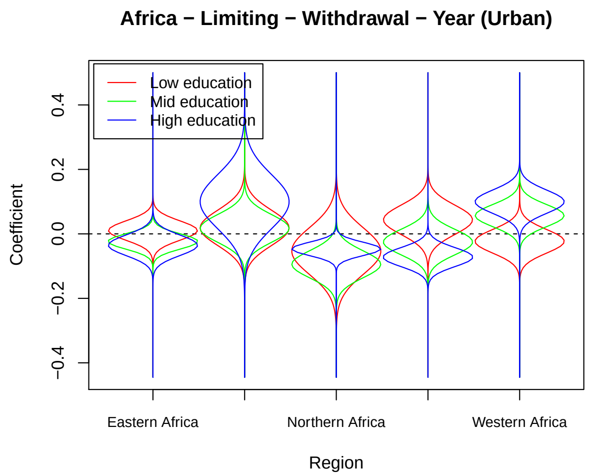
Africa − Limiting − Withdrawal − Year (Urban)
Low education
Mid education
High education
Coefficient
Eastern Africa
Northern Africa
Western Africa
Region

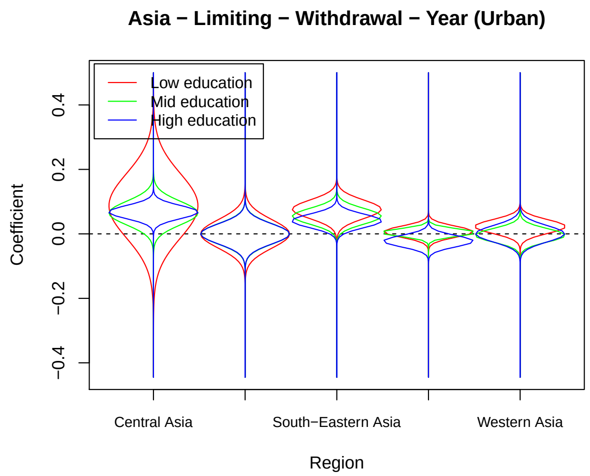
Asia − Limiting − Withdrawal − Year (Urban)
Low education
Mid education
High education
Coefficient
Central Asia
South−Eastern Asia
Western Asia
Region

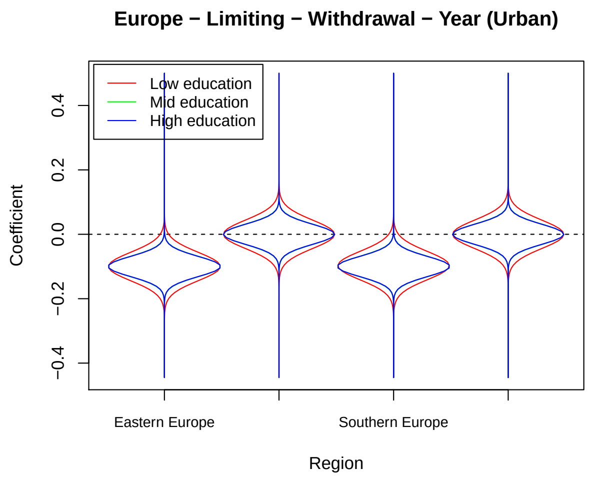
Europe − Limiting − Withdrawal − Year (Urban)
Low education
Mid education
High education
Coefficient
Eastern Europe
Southern Europe
Region

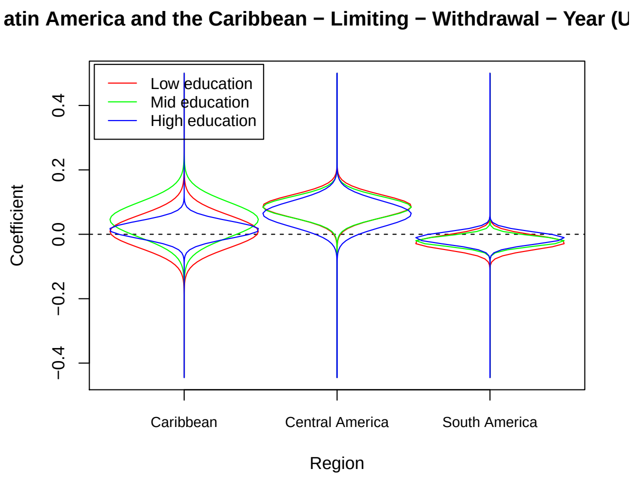
atin America and the Caribbean − Limiting − Withdrawal − Year (U
Low education
Mid education
High education
Coefficient
Caribbean
Central America
South America
Region

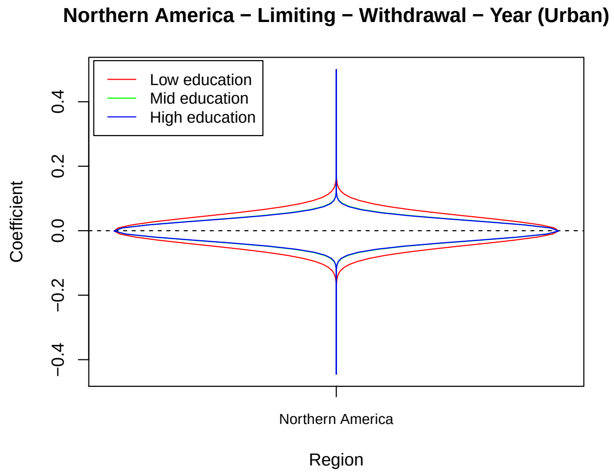
Northern America − Limiting − Withdrawal − Year (Urban)
Low education
Mid education
High education
Coefficient
Northern America
Region

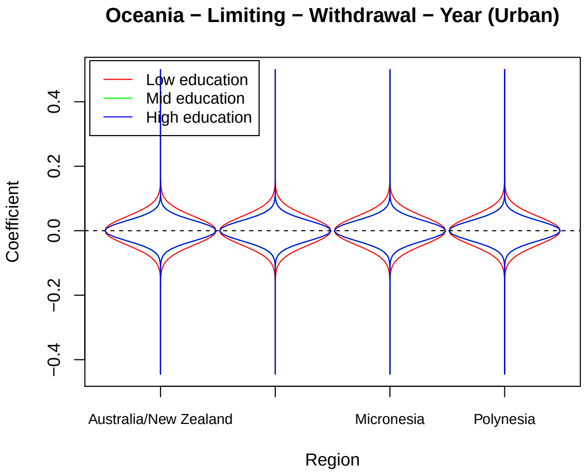
Oceania − Limiting − Withdrawal − Year (Urban)
Low education
Mid education
High education
Coefficient
Australia/New Zealand
Micronesia
Polynesia
Region

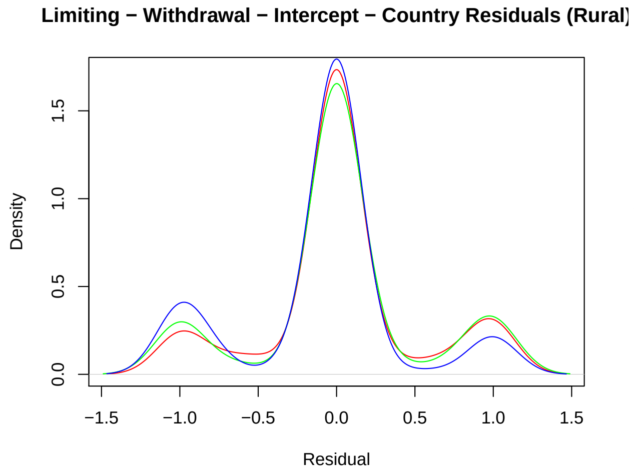
Limiting − Withdrawal − Intercept − Country Residuals (Rural)
Density
Residual

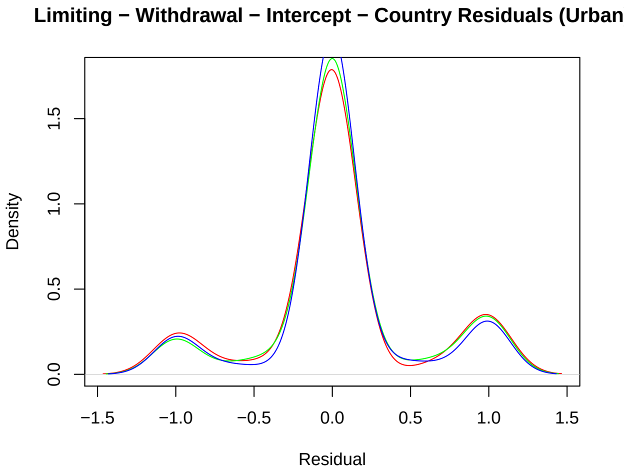
Limiting − Withdrawal − Intercept − Country Residuals (Urban
Density
Residual

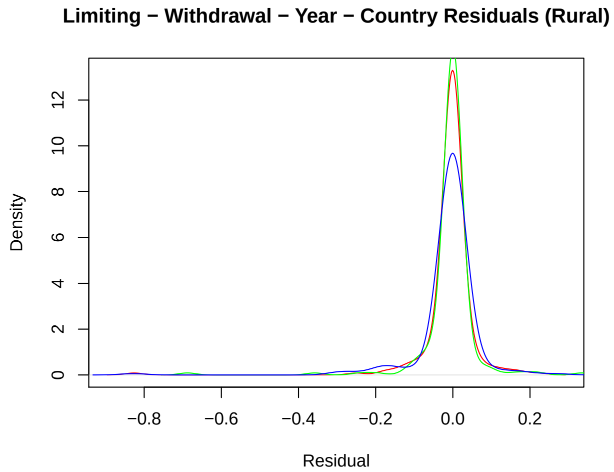
Limiting − Withdrawal − Year − Country Residuals (Rural)
Density
Residual

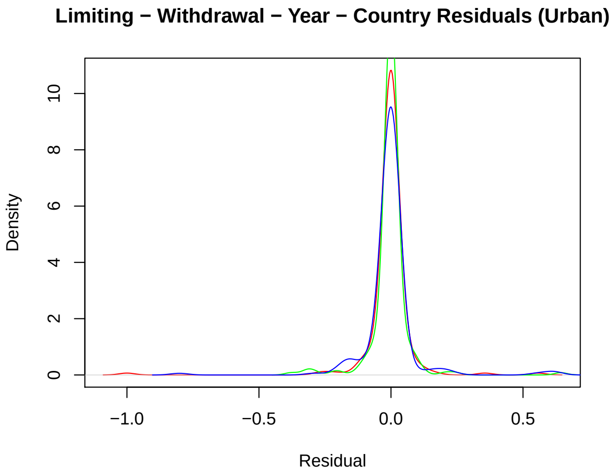
Limiting − Withdrawal − Year − Country Residuals (Urban)
Density
Residual

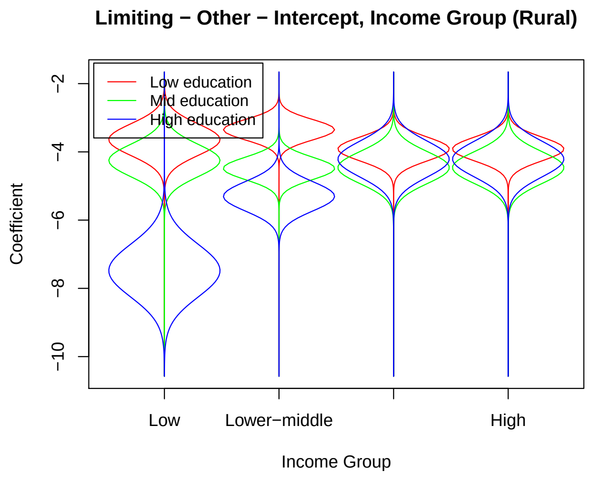
Limiting − Other − Intercept, Income Group (Rural)
Low education
Mid education
High education
Coefficient
−2
−4
−6
−8
−10
Low
Lower−middle
High
Income Group

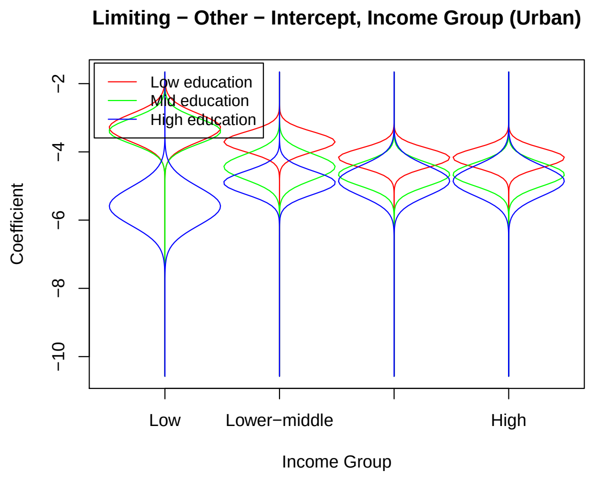
Limiting − Other − Intercept, Income Group (Urban)
Low education
Mid education
High education
Coefficient
−2
−4
−6
−8
−10
Low
Lower−middle
High
Income Group

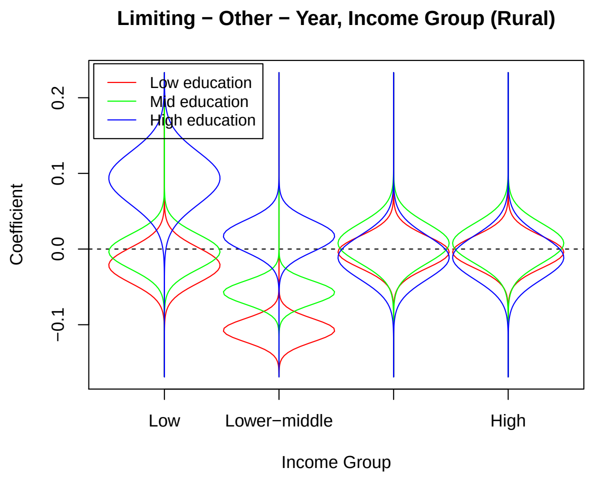
Limiting − Other − Year, Income Group (Rural)
Low education
Mid education
High education
Coefficient
0.2
0.1
0.0
−0.1
Low
Lower−middle
High
Income Group

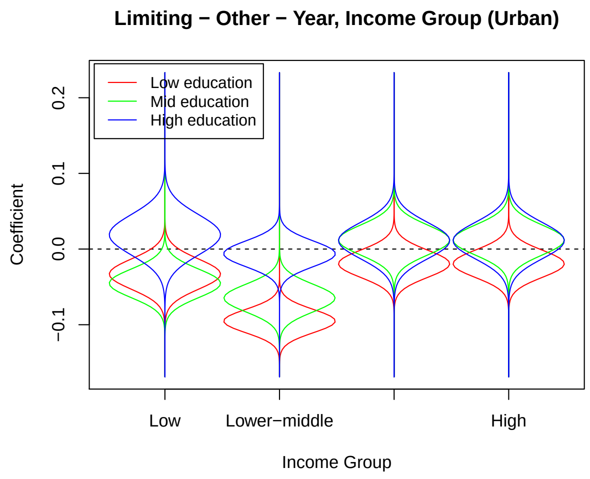
Limiting − Other − Year, Income Group (Urban)
Low education
Mid education
High education
Coefficient
0.2
0.1
0.0
−0.1
Low
Lower−middle
High
Income Group

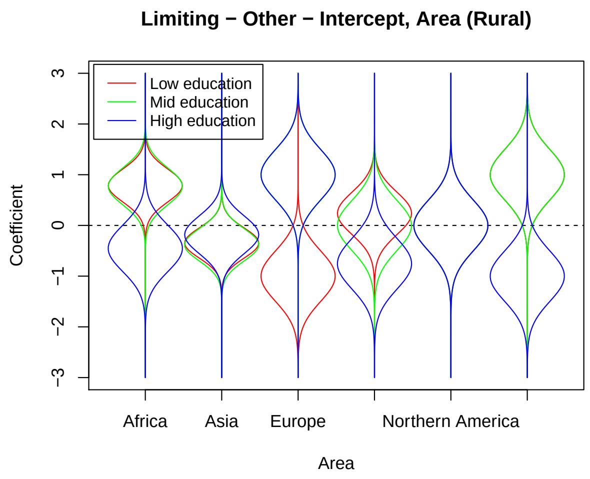
Limiting − Other − Intercept, Area (Rural)
Low education
Mid education
High education
Coefficient
3
2
1
0
−1
−2
−3
Africa
Asia
Europe
Northern America
Area

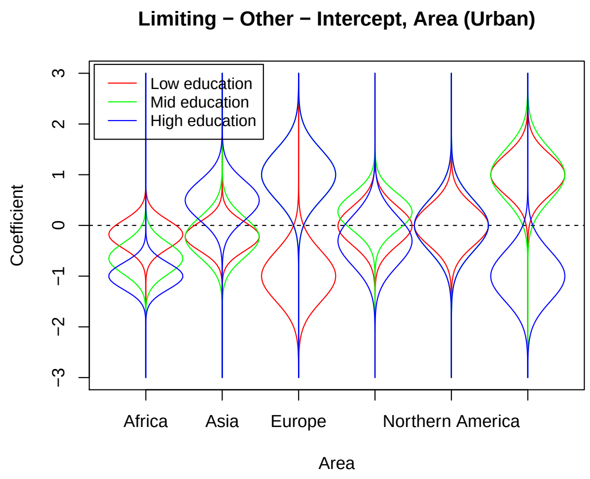
Limiting − Other − Intercept, Area (Urban)
Low education
Mid education
High education
Coefficient
3
2
1
0
−1
−2
−3
Africa
Asia
Europe
Northern America
Area

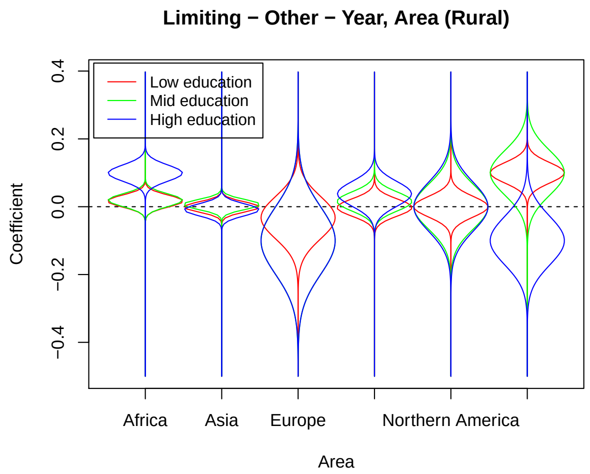
Limiting − Other − Year, Area (Rural)
Low education
Mid education
High education
Coefficient
Africa
Asia
Europe
Northern America
Area

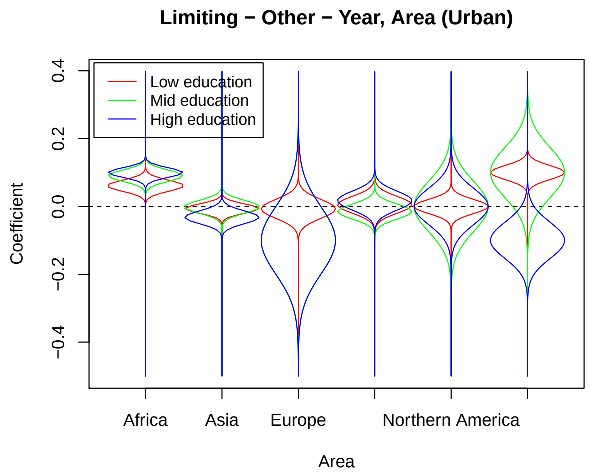
Limiting − Other − Year, Area (Urban)
Low education
Mid education
High education
Coefficient
Africa
Asia
Europe
Northern America
Area

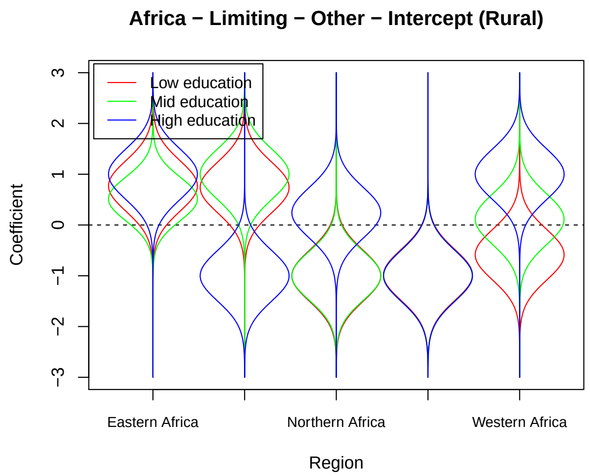
Africa − Limiting − Other − Intercept (Rural)
Low education
Mid education
High education
Coefficient
Eastern Africa
Northern Africa
Western Africa
Region

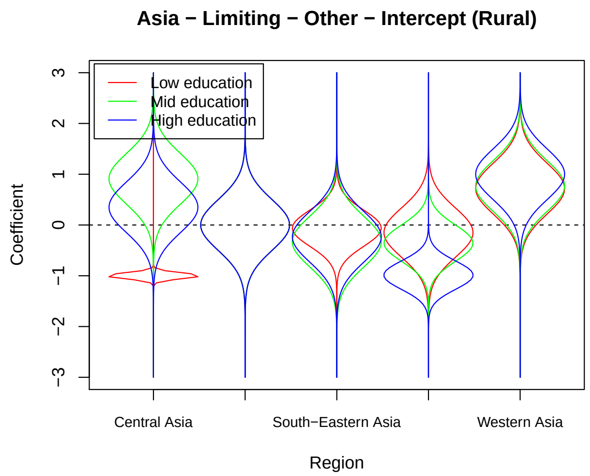
Asia − Limiting − Other − Intercept (Rural)
Low education
Mid education
High education
Coefficient
Central Asia
South−Eastern Asia
Western Asia
Region

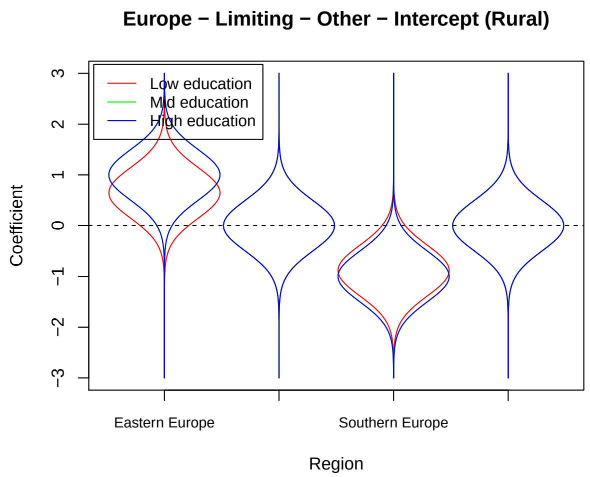
Europe − Limiting − Other − Intercept (Rural)
Low education
Mid education
High education
Coefficient
Eastern Europe
Southern Europe
Region

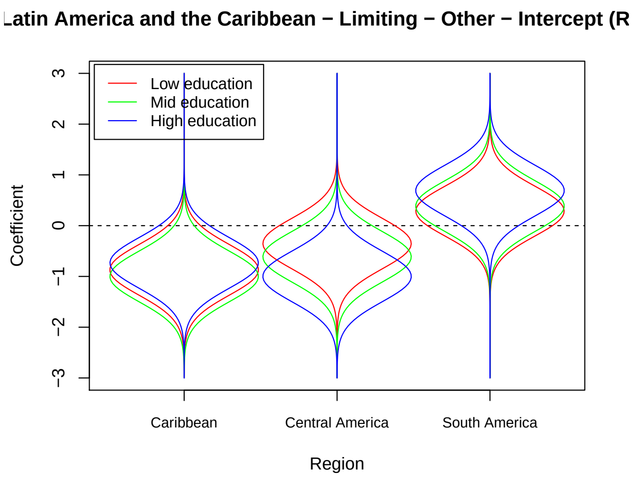
Latin America and the Caribbean − Limiting − Other − Intercept (R
Low education
Mid education
High education
Coefficient
Caribbean
Central America
South America
Region

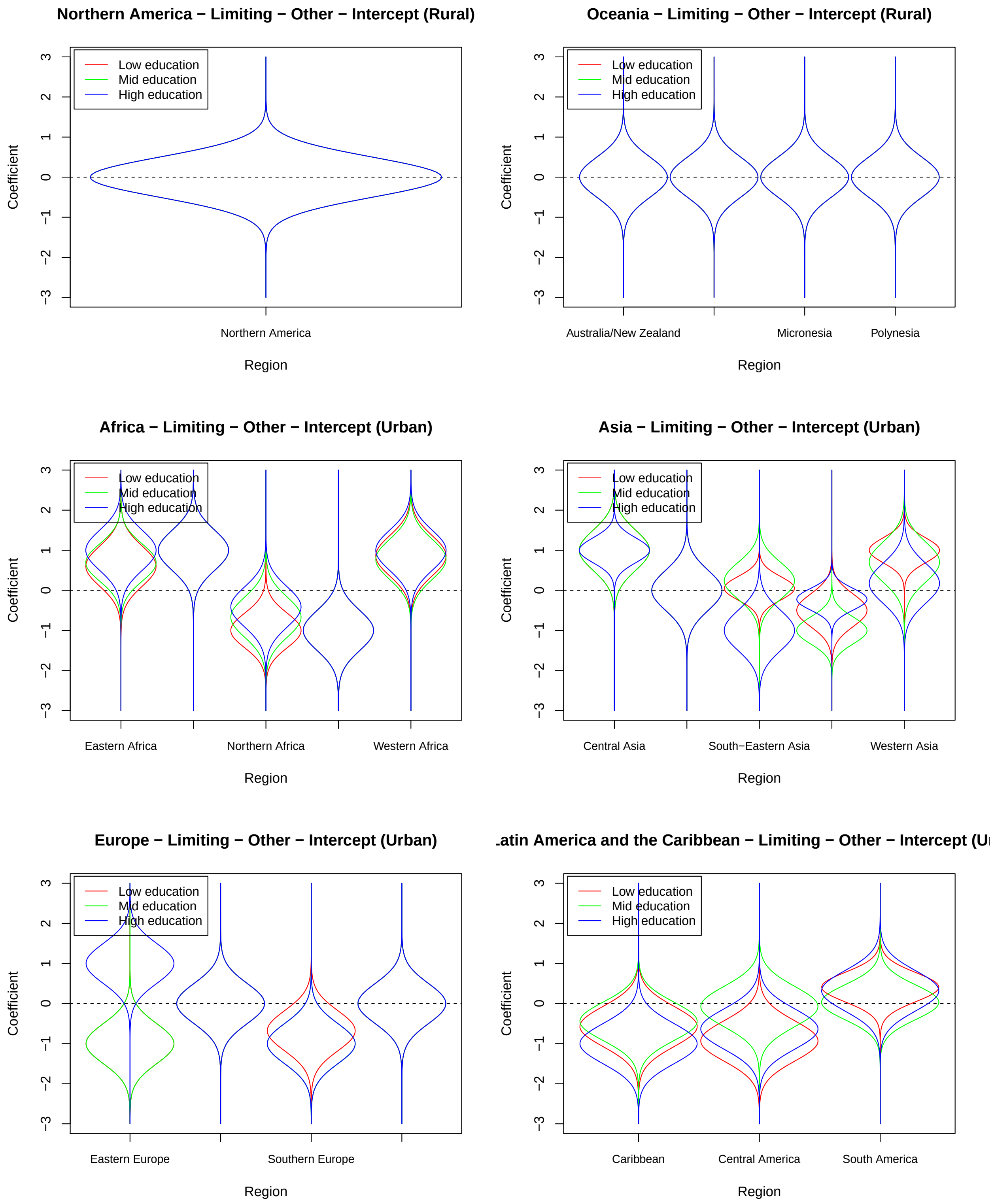
Northern America − Limiting − Other − Intercept (Rural)
Oceania − Limiting − Other − Intercept (Rural)
Africa − Limiting − Other − Intercept (Urban)
Asia − Limiting − Other − Intercept (Urban)
Europe − Limiting − Other − Intercept (Urban)
.atin America and the Caribbean − Limiting − Other − Intercept (U
Low education
Mid education
High education
Coefficient
Region
Northern America
Australia/New Zealand
Micronesia
Polynesia
Eastern Africa
Northern Africa
Western Africa
Central Asia
South−Eastern Asia
Western Asia
Eastern Europe
Southern Europe
Caribbean
Central America
South America

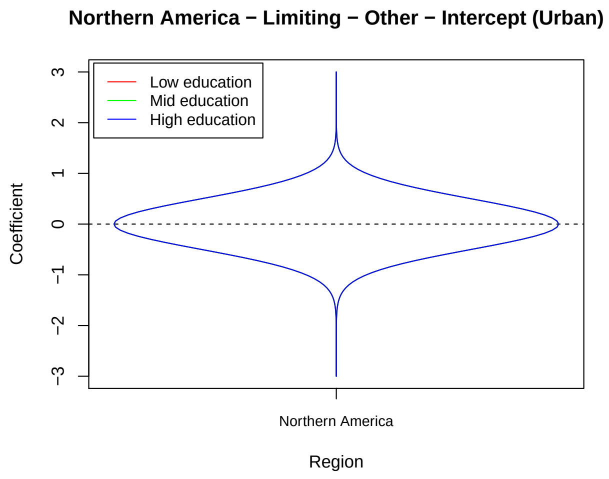
Northern America − Limiting − Other − Intercept (Urban)
Low education
Mid education
High education
Coefficient
3
2
1
0
−1
−2
−3
Northern America
Region

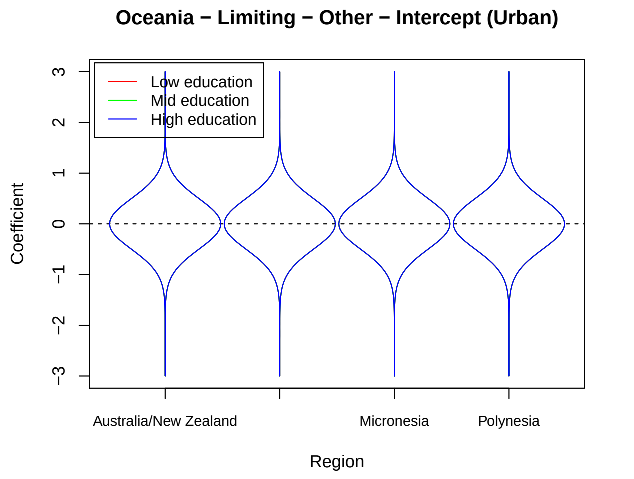
Oceania − Limiting − Other − Intercept (Urban)
Low education
Mid education
High education
Coefficient
Australia/New Zealand
Micronesia
Polynesia
Region

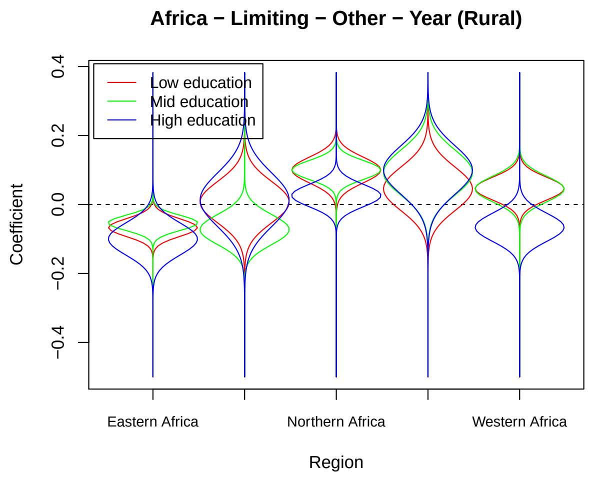
Africa − Limiting − Other − Year (Rural)
Low education
Mid education
High education
Coefficient
0.4
0.2
0.0
−0.2
−0.4
Eastern Africa
Northern Africa
Western Africa
Region

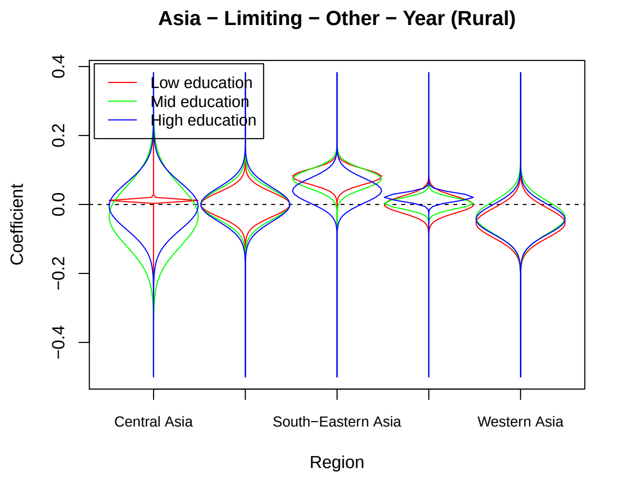
Asia − Limiting − Other − Year (Rural)
Low education
Mid education
High education
Coefficient
Central Asia
South−Eastern Asia
Western Asia
Region

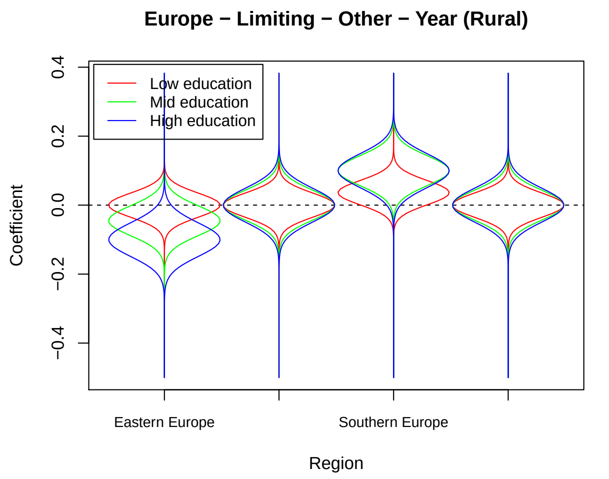
Europe − Limiting − Other − Year (Rural)
Low education
Mid education
High education
Coefficient
Eastern Europe
Southern Europe
Region

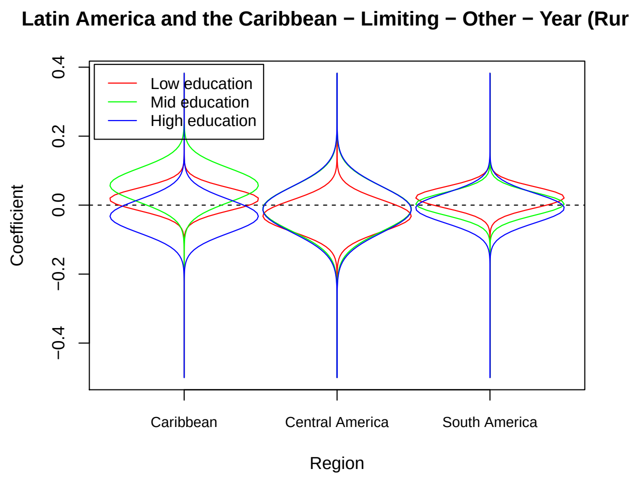
Latin America and the Caribbean − Limiting − Other − Year (Rur
Low education
Mid education
High education
Coefficient
Caribbean
Central America
South America
Region

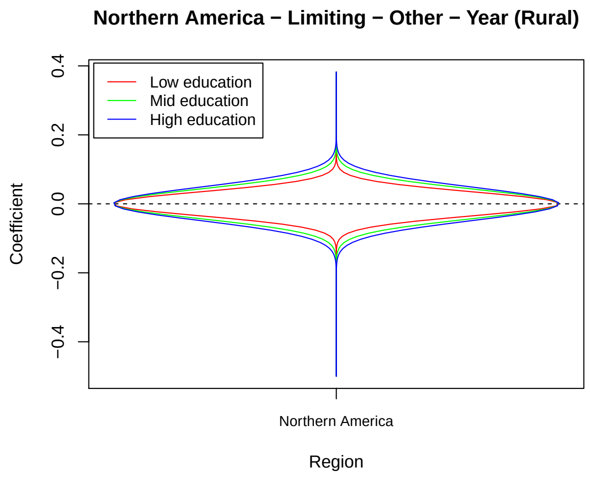
Northern America − Limiting − Other − Year (Rural)
Low education
Mid education
High education
Coefficient
0.4
0.2
0.0
−0.2
−0.4
Northern America
Region

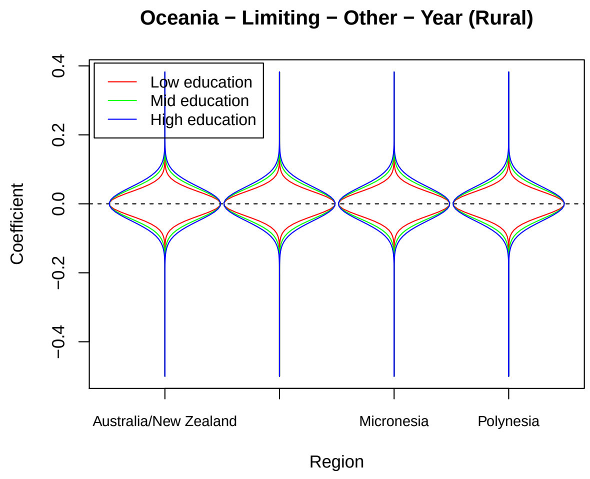
Oceania − Limiting − Other − Year (Rural)
Low education
Mid education
High education
Coefficient
0.4
0.2
0.0
−0.2
−0.4
Australia/New Zealand
Micronesia
Polynesia
Region

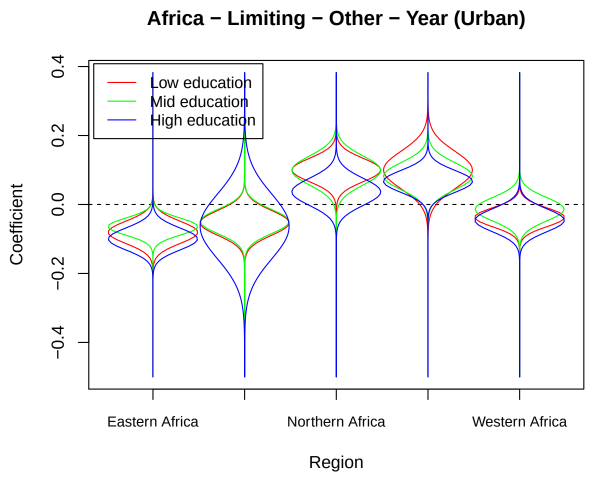
Africa − Limiting − Other − Year (Urban)
Low education
Mid education
High education
Coefficient
0.4
0.2
0.0
−0.2
−0.4
Eastern Africa
Northern Africa
Western Africa
Region

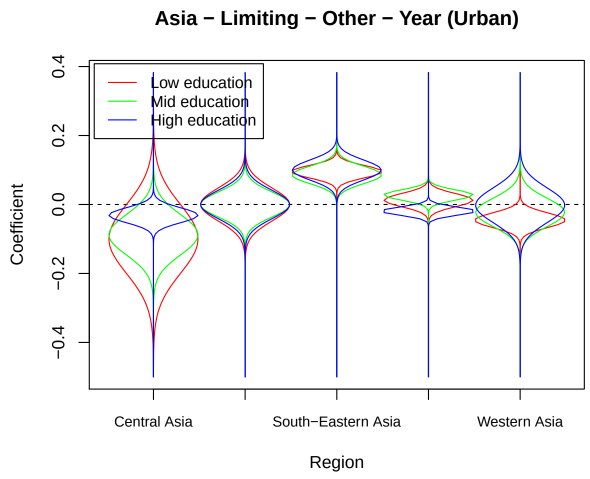
Asia − Limiting − Other − Year (Urban)
Low education
Mid education
High education
Coefficient
0.4
0.2
0.0
−0.2
−0.4
Central Asia
South−Eastern Asia
Western Asia
Region

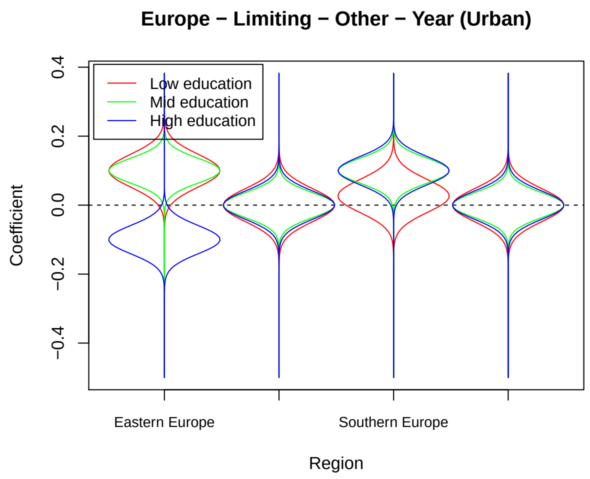
Europe − Limiting − Other − Year (Urban)
Low education
Mid education
High education
Coefficient
0.4
0.2
0.0
−0.2
−0.4
Eastern Europe
Southern Europe
Region

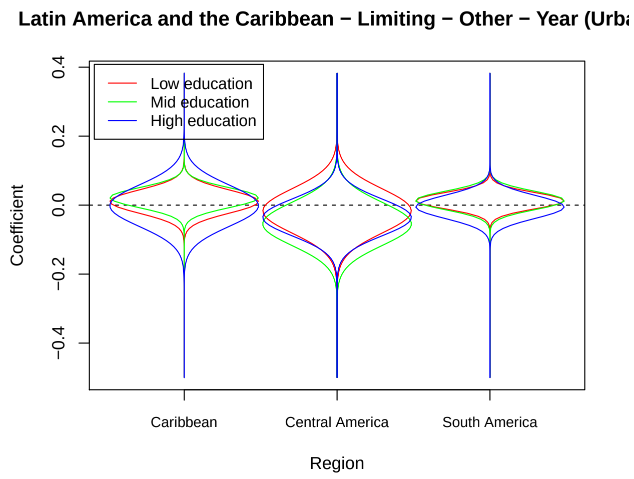
Latin America and the Caribbean − Limiting − Other − Year (Urba
Low education
Mid education
High education
Coefficient
0.4
0.2
0.0
−0.2
−0.4
Caribbean
Central America
South America
Region

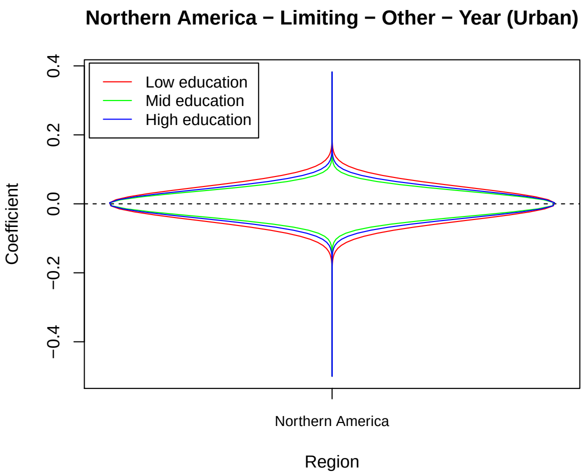
Northern America − Limiting − Other − Year (Urban)
Low education
Mid education
High education
Coefficient
0.4
0.2
0.0
−0.2
−0.4
Northern America
Region

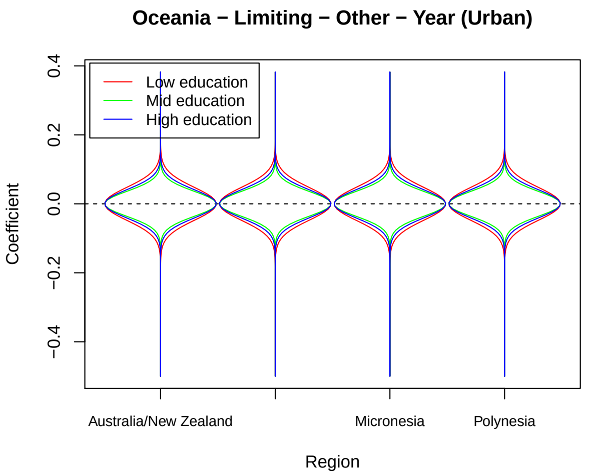
Oceania − Limiting − Other − Year (Urban)
Low education
Mid education
High education
Coefficient
0.4
0.2
0.0
−0.2
−0.4
Australia/New Zealand
Micronesia
Polynesia
Region

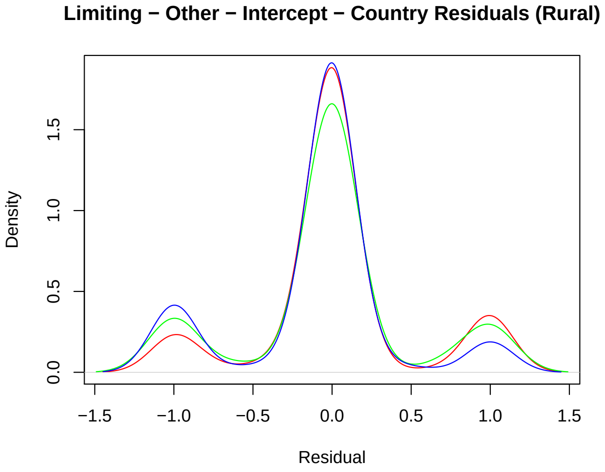
Limiting − Other − Intercept − Country Residuals (Rural)
Density
0.0
0.5
1.0
1.5
−1.5
−1.0
−0.5
0.0
0.5
1.0
1.5
Residual

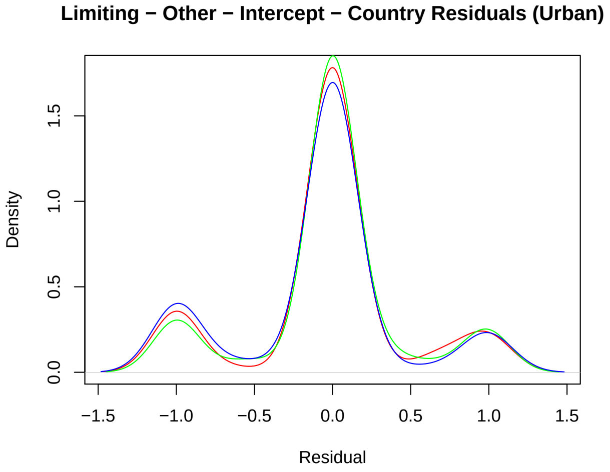
Limiting − Other − Intercept − Country Residuals (Urban)
Density
0.0
0.5
1.0
1.5
−1.5
−1.0
−0.5
0.0
0.5
1.0
1.5
Residual

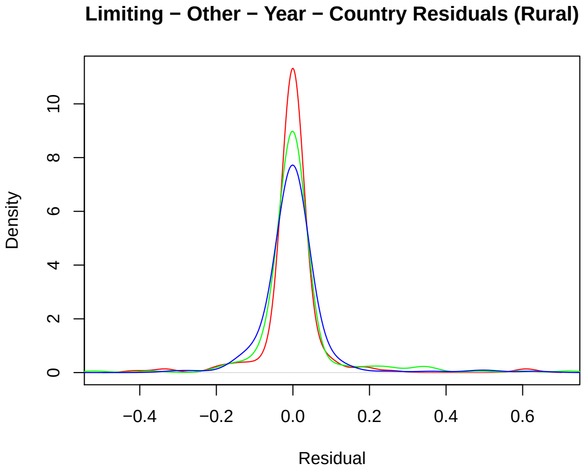
Limiting − Other − Year − Country Residuals (Rural)
Density
0
2
4
6
8
10
−0.4
−0.2
0.0
0.2
0.4
0.6
Residual

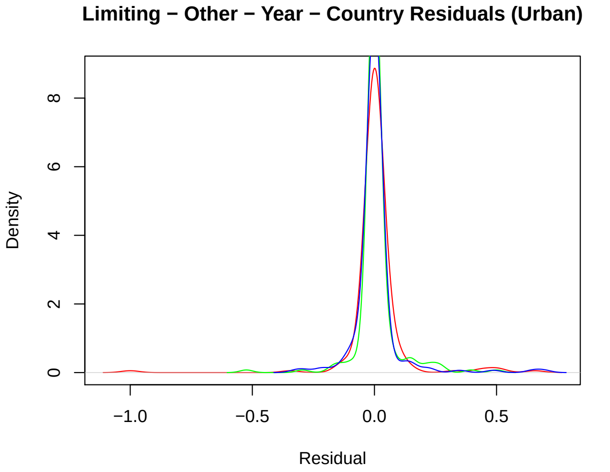
Limiting − Other − Year − Country Residuals (Urban)
Density
0
2
4
6
8
−1.0
−0.5
0.0
0.5
Residual

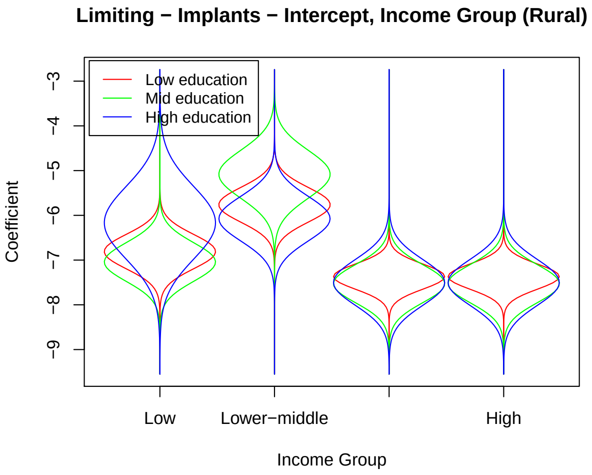
Limiting − Implants − Intercept, Income Group (Rural)
Low education
Mid education
High education
Coefficient
−3
−4
−5
−6
−7
−8
−9
Low
Lower−middle
High
Income Group

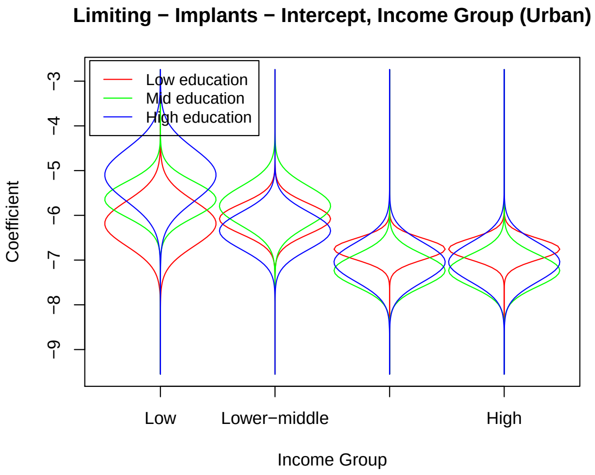
Limiting − Implants − Intercept, Income Group (Urban)
Low education
Mid education
High education
Coefficient
−3
−4
−5
−6
−7
−8
−9
Low
Lower−middle
High
Income Group

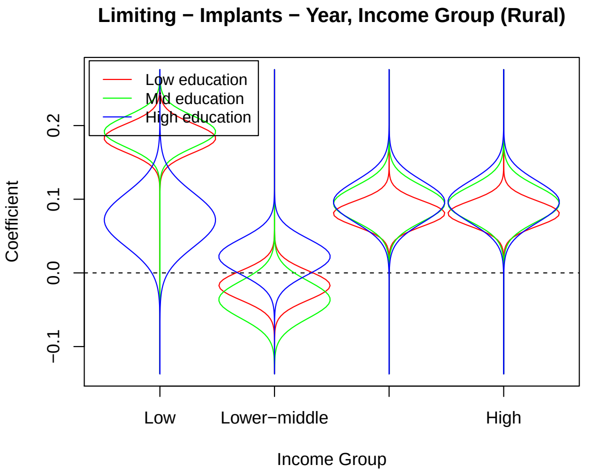
Limiting − Implants − Year, Income Group (Rural)
Low education
Mid education
High education
Coefficient
0.2
0.1
0.0
−0.1
Low
Lower−middle
High
Income Group

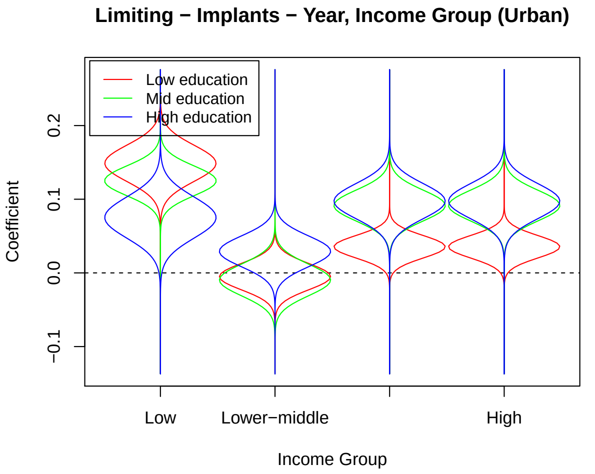
Limiting − Implants − Year, Income Group (Urban)
Low education
Mid education
High education
Coefficient
0.2
0.1
0.0
−0.1
Low
Lower−middle
High
Income Group

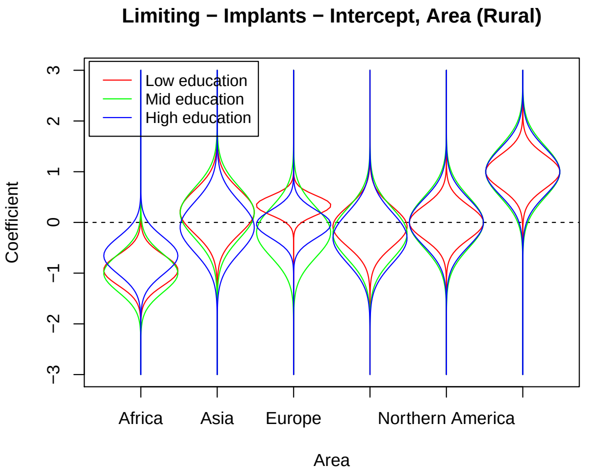
Limiting − Implants − Intercept, Area (Rural)
Low education
Mid education
High education
Coefficient
3
2
1
0
−1
−2
−3
Africa
Asia
Europe
Northern America
Area

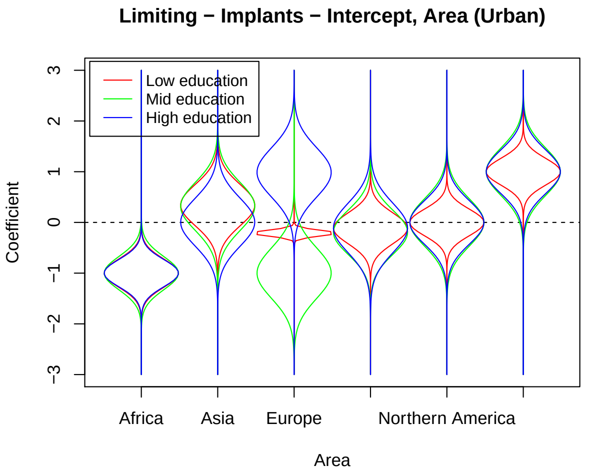
Limiting − Implants − Intercept, Area (Urban)
Low education
Mid education
High education
Coefficient
3
2
1
0
−1
−2
−3
Africa
Asia
Europe
Northern America
Area

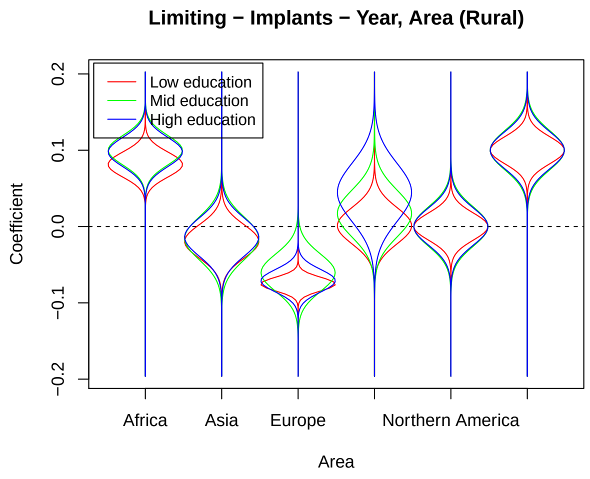
Limiting − Implants − Year, Area (Rural)
Low education
Mid education
High education
Coefficient
0.2
0.1
0.0
−0.1
−0.2
Africa
Asia
Europe
Northern America
Area

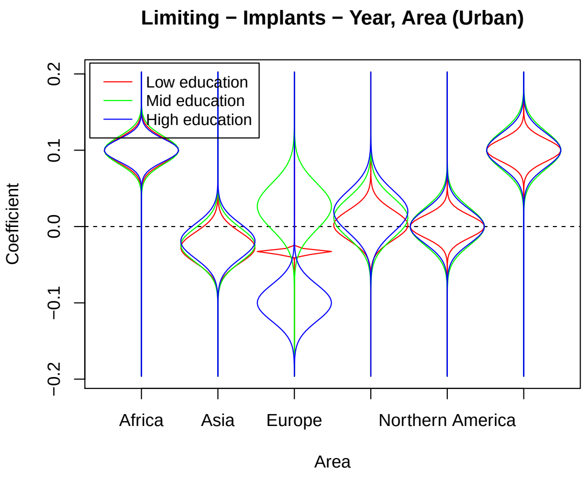
Limiting − Implants − Year, Area (Urban)
Low education
Mid education
High education
Coefficient
0.2
0.1
0.0
−0.1
−0.2
Africa
Asia
Europe
Northern America
Area

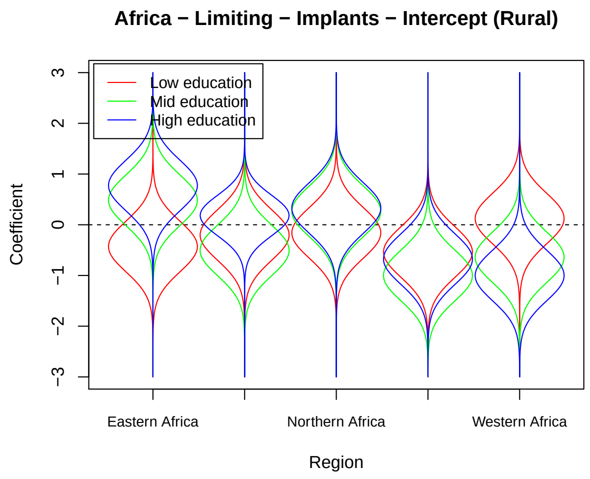
Africa − Limiting − Implants − Intercept (Rural)
Low education
Mid education
High education
Coefficient
3
2
1
0
−1
−2
−3
Eastern Africa
Northern Africa
Western Africa
Region

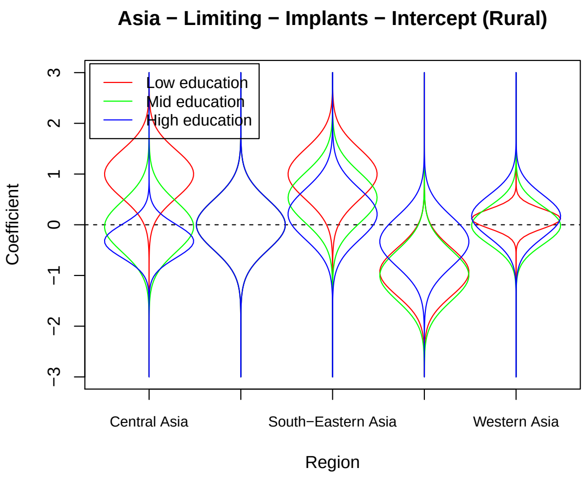
Asia − Limiting − Implants − Intercept (Rural)
Low education
Mid education
High education
Coefficient
3
2
1
0
−1
−2
−3
Central Asia
South−Eastern Asia
Western Asia
Region

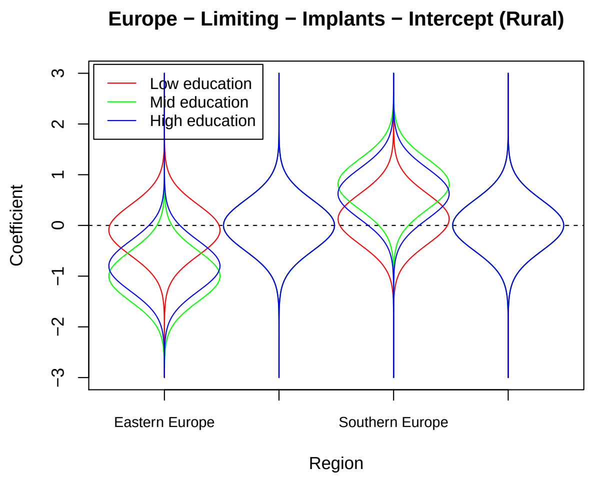
Europe − Limiting − Implants − Intercept (Rural)
Low education
Mid education
High education
Coefficient
3
2
1
0
−1
−2
−3
Eastern Europe
Southern Europe
Region

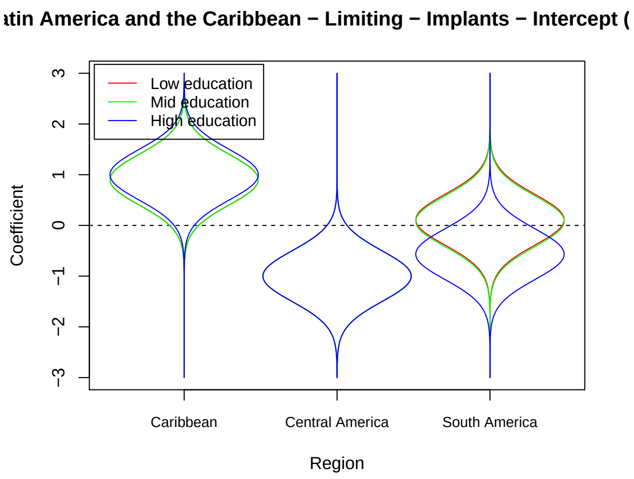
atin America and the Caribbean − Limiting − Implants − Intercept (
Low education
Mid education
High education
Coefficient
3
2
1
0
−1
−2
−3
Caribbean
Central America
South America
Region

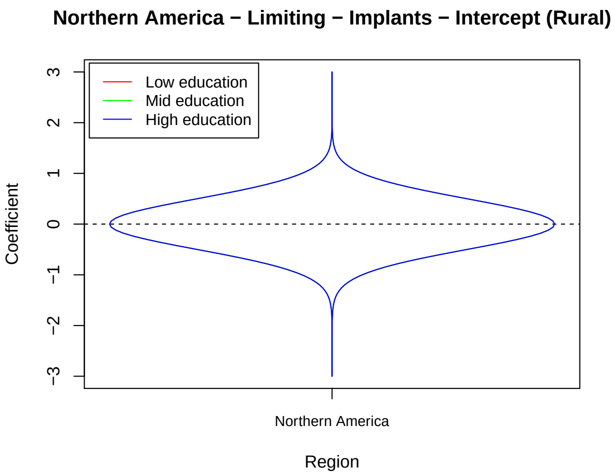
Northern America − Limiting − Implants − Intercept (Rural)
Low education
Mid education
High education
Coefficient
Northern America
Region

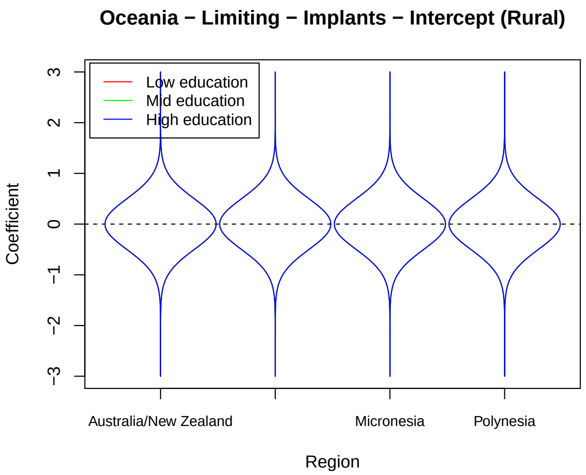
Oceania − Limiting − Implants − Intercept (Rural)
Low education
Mid education
High education
Coefficient
Australia/New Zealand
Micronesia
Polynesia
Region

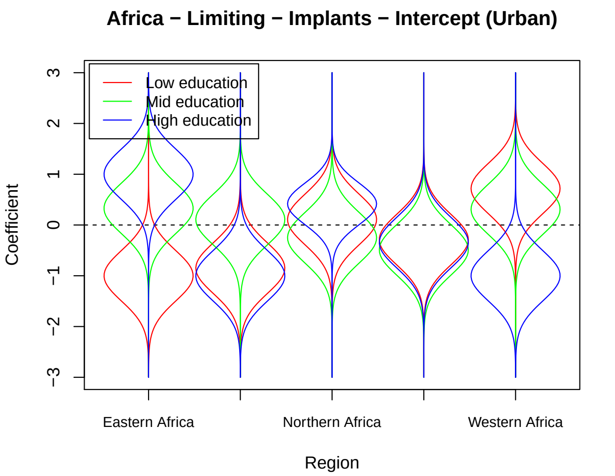
Africa − Limiting − Implants − Intercept (Urban)
Low education
Mid education
High education
Coefficient
Eastern Africa
Northern Africa
Western Africa
Region

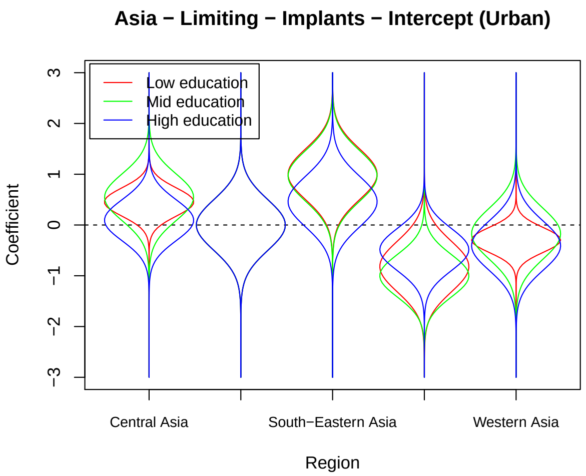
Asia − Limiting − Implants − Intercept (Urban)
Low education
Mid education
High education
Coefficient
Central Asia
South−Eastern Asia
Western Asia
Region

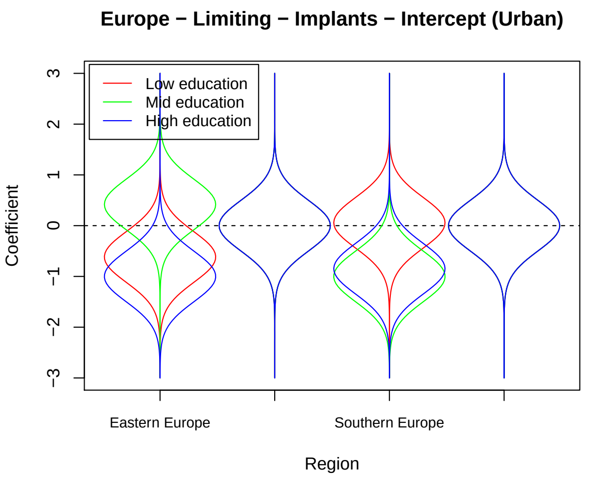
Europe − Limiting − Implants − Intercept (Urban)
Low education
Mid education
High education
Coefficient
Eastern Europe
Southern Europe
Region

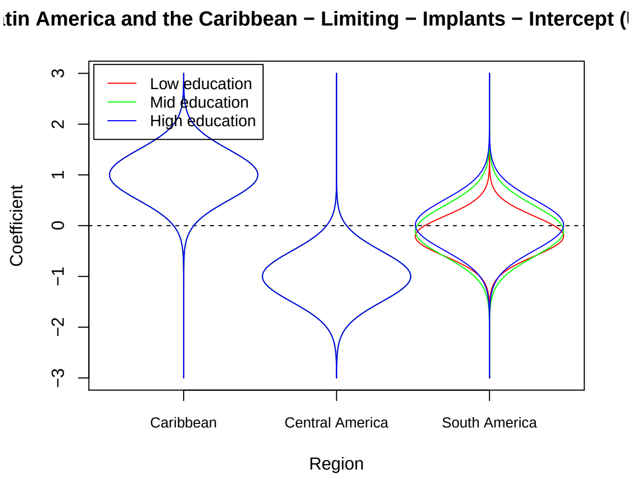
tin America and the Caribbean − Limiting − Implants − Intercept (
Low education
Mid education
High education
Coefficient
Caribbean
Central America
South America
Region

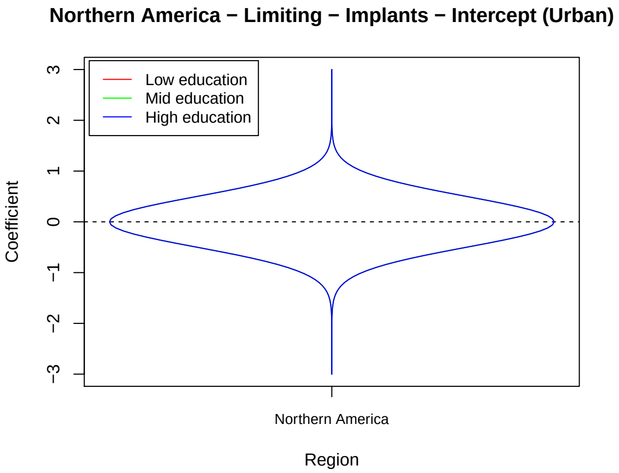
Northern America − Limiting − Implants − Intercept (Urban)
Low education
Mid education
High education
Coefficient
3
2
1
0
−1
−2
−3
Northern America
Region

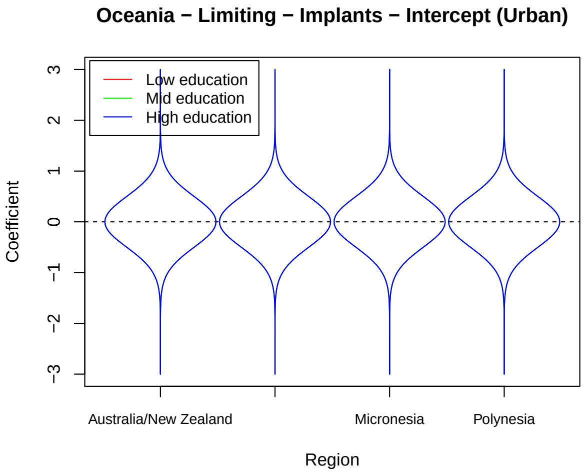
Oceania − Limiting − Implants − Intercept (Urban)
Low education
Mid education
High education
Coefficient
3
2
1
0
−1
−2
−3
Australia/New Zealand
Micronesia
Polynesia
Region

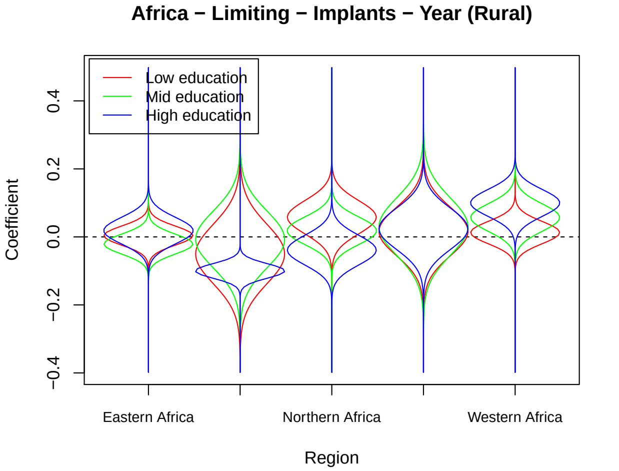
Africa − Limiting − Implants − Year (Rural)
Low education
Mid education
High education
Coefficient
0.4
0.2
0.0
−0.2
−0.4
Eastern Africa
Northern Africa
Western Africa
Region

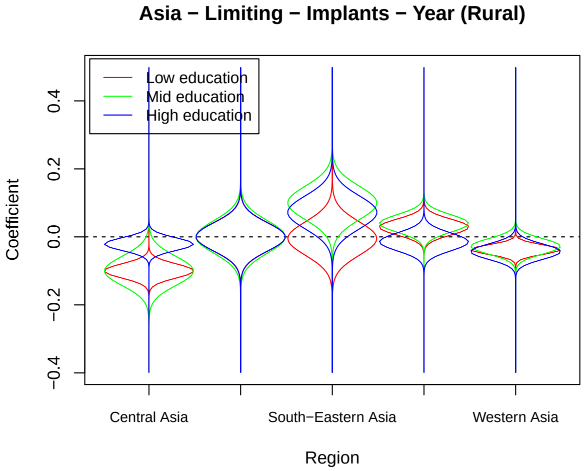
Asia − Limiting − Implants − Year (Rural)
Low education
Mid education
High education
Coefficient
0.4
0.2
0.0
−0.2
−0.4
Central Asia
South−Eastern Asia
Western Asia
Region

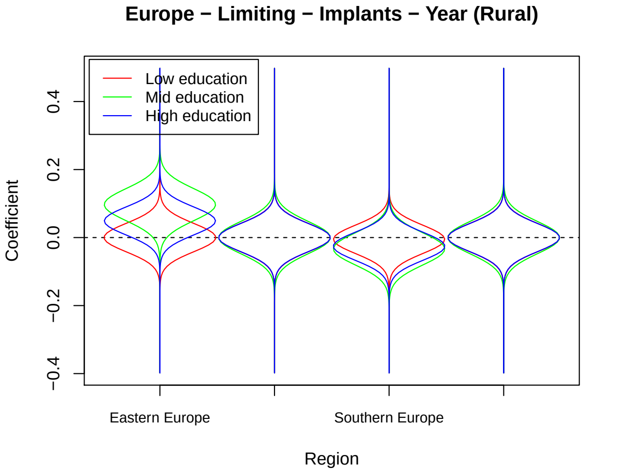
Europe − Limiting − Implants − Year (Rural)
Low education
Mid education
High education
Coefficient
0.4
0.2
0.0
−0.2
−0.4
Eastern Europe
Southern Europe
Region

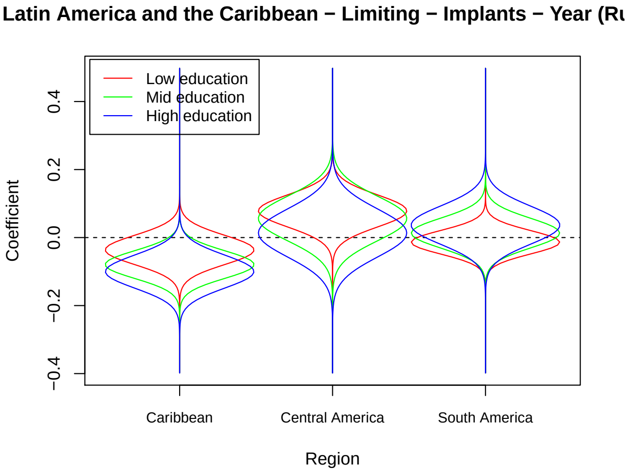
Latin America and the Caribbean − Limiting − Implants − Year (Ru
Low education
Mid education
High education
Coefficient
0.4
0.2
0.0
−0.2
−0.4
Caribbean
Central America
South America
Region

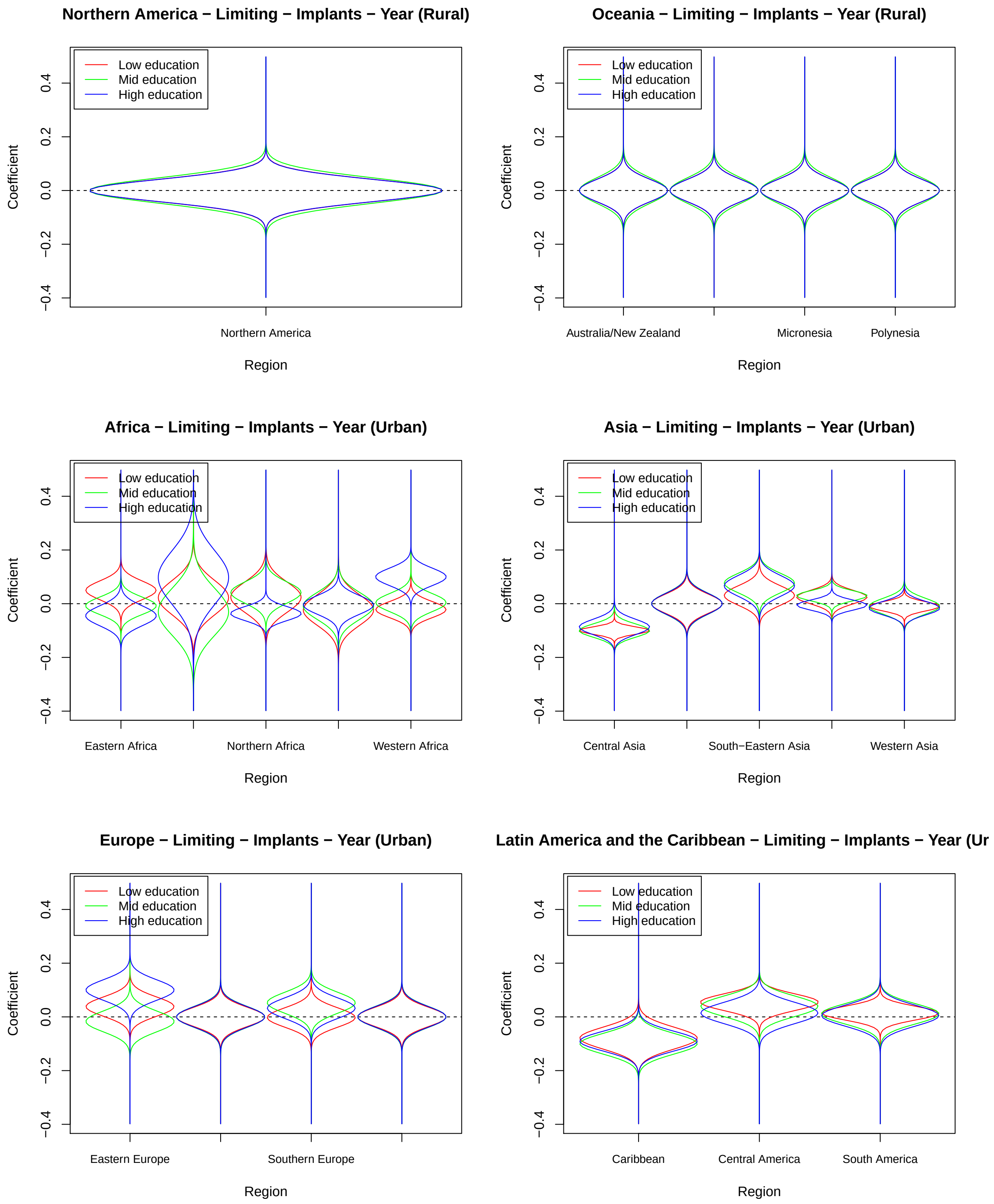
Northern America − Limiting − Implants − Year (Rural)
Low education
Mid education
High education
Coefficient
0.4
0.2
0.0
−0.2
−0.4
Northern America
Region
Oceania − Limiting − Implants − Year (Rural)
Australia/New Zealand
Micronesia
Polynesia
Africa − Limiting − Implants − Year (Urban)
Eastern Africa
Northern Africa
Western Africa
Asia − Limiting − Implants − Year (Urban)
Central Asia
South−Eastern Asia
Western Asia
Europe − Limiting − Implants − Year (Urban)
Eastern Europe
Southern Europe
Latin America and the Caribbean − Limiting − Implants − Year (Ur
Caribbean
Central America
South America

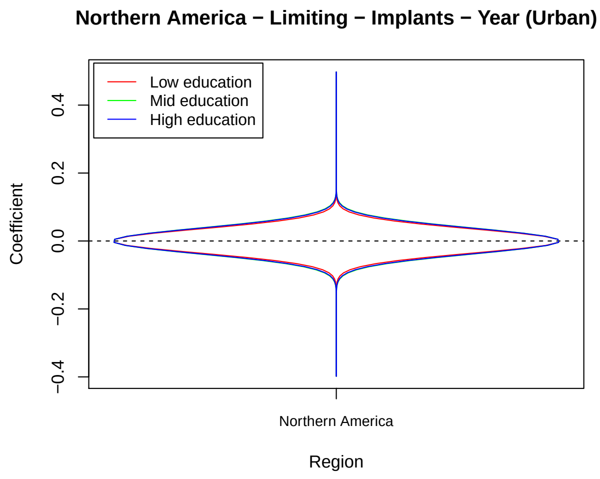

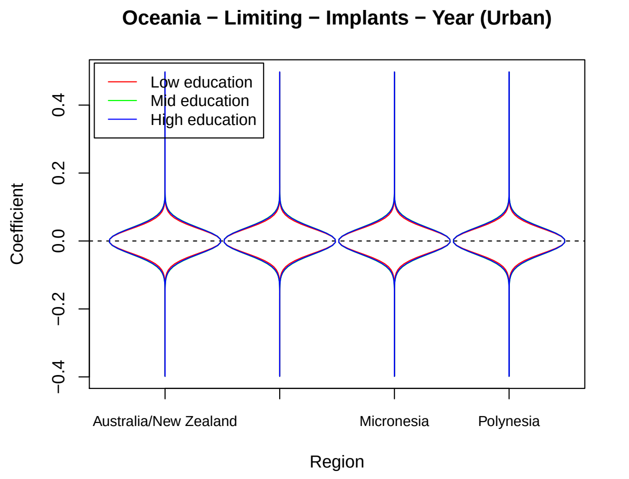

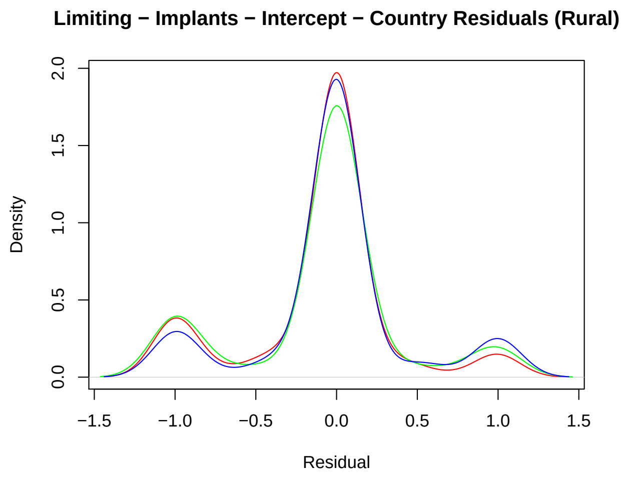

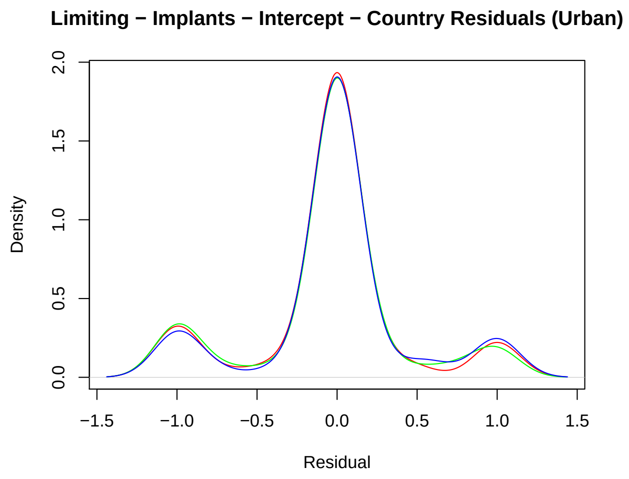

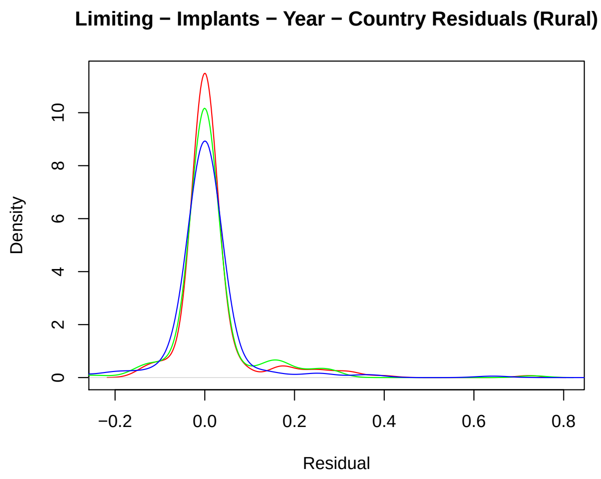

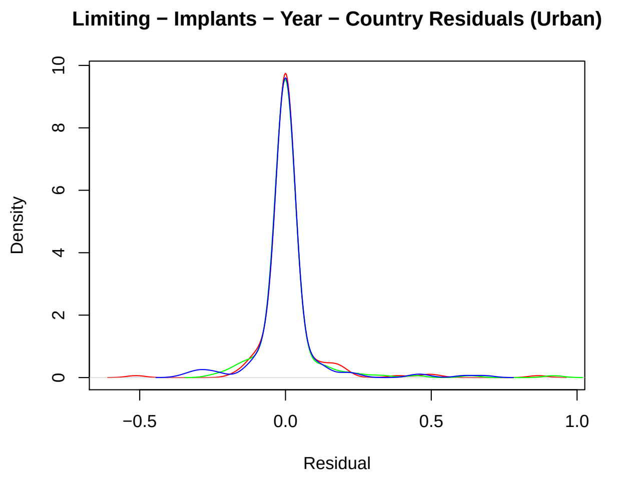

Model Implementation

A method of contraception is assigned to women if their need for spacing or limiting is met. Methods can be assigned whenever a woman experiences a fertility event, such as sexual debut, at the end of pregnancy (due to delivery or termination), or at the end of her simulated duration of use.

A.1.3.6 Contraception Failure Rates

Overview
Every method of contraception has some failure rate - that is, the chance that the user will become pregnant. Failure rates are typically calculated as the risk of becoming pregnant within 1 year under 'perfect' use or 'typical' use. Perfect use implies that the method is properly used in every instance, and so represents the maximum efficacy of a given method. Typical use refers to the general practice of usage. These two rates may be quite different if people fail to use methods properly and consistently. In the model we use estimates of typical use, as these better represent what is actually happening in the general population.

Data
Here we compare estimated failure rates from different sources. Trussell 2018 and Sundaram 2017 are based on data from the US, Moreau 2007 is based on data from France, and Bradley 2019 and Polis 2016 are based on analyses of DHS data from multiple countries.

Failure probability (within first year of typical use):

Method	Trussell 2018	Moreau 2007	Sundaram 2017	Bradley 2019	Polis 2016
No method	85%	—	—	—	—
Pill	7%	2.4% (1.7%-3.2%)	7.2% (0.68)	6.3% (5.9%-6.8%)	5.5% (3.5%-7.3%)
IUD	0.1%-0.8%	1.1% (0.5%-2.2%)	1.4% (0.68)	1.2% (0.9%-1.5%)	1.4% (0.0%-2.4%)
Injections	4% (Depo-Provera)	—	4.0% (1.06)	2.0% (1.7%-2.3%)	1.7% (0.6%-2.9%)
Condom	13%	3.3% (2.4%-4.7%)	12.6% (1.11)	8.6% (7.6%-9.6%)	5.4% (2.3%-8.7%)
Female sterilization	0.5%	—	—	—	—
Male sterilization	0.15%	—	—	—	—
Periodic abstinence	15%	7.7% (2.6%-21.5%)	—	19.0% (17.4%-20.6%)	13.9% (9.2%-19.3%)
Withdrawal	20%	10.1% (4.3%-22.5%)	19.9% (2.03)	17.3% (15.9%-18.7%)	13.4% (9.1%-17.1%)
Implants	0.1% (Nexplanon)	—	—	0.3% (0.1%-0.9%)	0.6% (0.0%-2.4%)
Other	—	—	—	—	—

To estimate priors we pooled data on failure rates for 43 DHS countries from Polis 2016, and added estimates for the US (Sundaram 2017) and France (Moreau 2007).

Within countries, failure rates vary by socioeconomic status, with the poorest and youngest women at highest risk of experiencing unintended pregnancy (Bradley 2019). A similar gradient of failure rates by education level was found in France (Moreau 2007).

Mortality Risk As an invasive procedure, female sterilization has been associated with a risk of death, especially in developing countries (Aubert 1980). Recent reports of mass deaths in sterilization camps in India highlight the concerns over poor quality of medical care when performing such procedures (Das 2014, Pulla 2014). Mortality from sterilization has previously been estimated at between 2.5 and 10.0 per 100,000 procedures (Smith 1985). For calibration purposes, we do not currently incorporate any risk of morbidity/mortality from contraceptive methods, but this can be included in the model for future analyses.

Parameters
We estimated hierarchical logistic regression models for each method. We set the same priors for each subgroup within a country (urban/rural & education level), but constrained the sampled failure rates to be

non-decreasing by increasing education level to account for the socioeconomic gradient in failure rates. We used the same global priors from ‘Withdrawal’ for ‘Other’ due to lack of data.

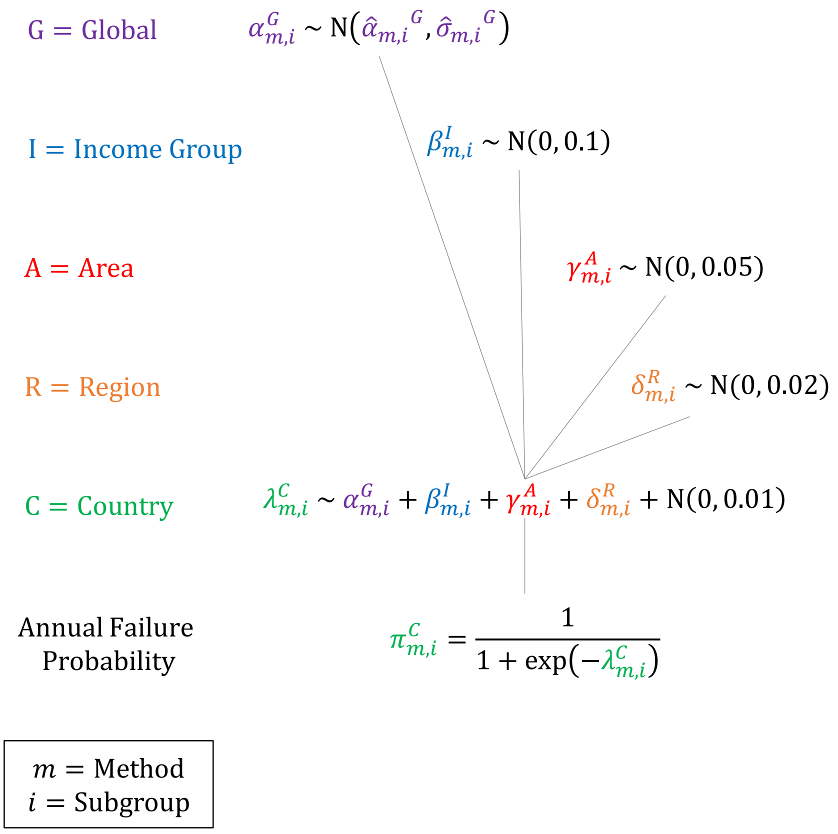

Priors

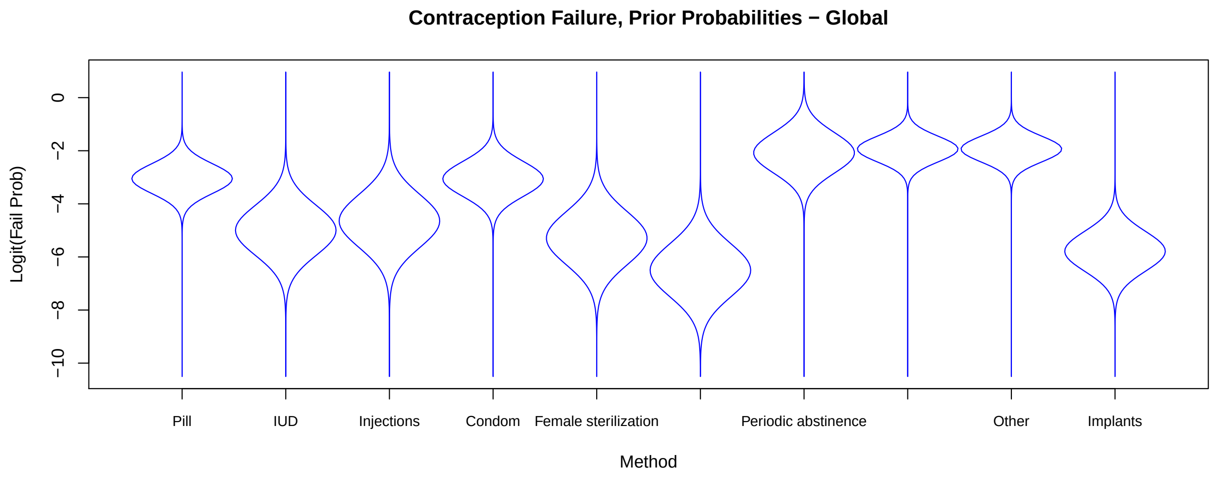

Model Implementation

To estimate the protective efficacy of each method we converted the annual failure probabilities to monthly rates and estimated relative rates compared to ‘No method’. The relative rates of average protective efficacy

were then applied to the underlying age-specific natural fertility rates. We assume that the protective effects of LAM and contraception methods are independent. We also assume that the failure rate of each method is the same regardless of fertility intent (i.e. spacing or limiting).

References

Aubert JM, Lubell I, Schima M. Mortality risk associated with female sterilization. Int J Gynaecol Obstet 1980;18(6):406-10.

Bradley SEK, Polis CB, Bankole A, Croft T. Global Contraceptive Failure Rates: Who Is Most at Risk? Stud Fam Plann 2019; 50(1): 3-24.

Das A, Contractor S. India's latest sterilisation camp massacre. BMJ 2014;349:g7282.

Moreau C, Trussell J, Rodriguez G, Bajos N, Bouyer J. Contraceptive failure rates in France: results from a population-based survey. Human Reprod 2007; 22(9): 2422-2427.

Polis CB, Bradley SE, Bankole A, Onda T, Croft T, Singh S. Typical-use contraceptive failure rates in 43 countries with Demographic and Health Survey data: summary of a detailed report. Contraception 2016; 94(1): 11-7.

Polis C, Bradley SEK, Bankole A, et al. Contraceptive Failure Rates in the Developing World: An Analysis of Demographic and Health Survey Data in 43 Countries. Guttmacher Institute 2016. Available at: https://www.guttmacher.org/report/contraceptive-failure-rates-in-developing-world

Pulla P. Why are women dying in India's sterilisation camps? BMJ 2014;349:g7509.

Smith GL, Taylor GP, Smith KF. Comparative risks and costs of male and female sterilization. Am J Public Health 1985;75:370-374.

Sundaram A, Vaughan B, Kost K, Bankole A, Finer L, Singh S, Trussell J. Contraceptive Failure in the United States: Estimates from the 2006-2010 National Survey of Family Growth. Perspect Sex Reprod Health 2017; 49(1): 7-16.

Trussell J, Aiken ARA, Micks E, Guthrie KA. Efficacy, safety, and personal considerations. In: Hatcher RA, Nelson AL, Trussell J, Cwiak C, Cason P, Policar MS, Edelman A, Aiken ARA, Marrazzo J, Kowal D, eds. Contraceptive technology. 21st ed. New York, NY: Ayer Company Publishers, Inc., 2018. Table available at: http://www.contraceptivetechnology.org/wp-content/uploads/2013/09/Contraceptive-Failure-Rates.pdf

A.1.3.7 Method Duration of Use

Overview
Duration of method use is simulated for women using contraception for spacing. We assume that women who are limiting use a method indefinitely until menopause or method failure. Women who are spacing can either switch to another method, or stop using any method of contraception.

Data
We used data on length of current contraceptive use from the DHS. Data on duration of method use were available for 405,932 women from 248 surveys in 75 countries. We also analyzed the reasons given for method discontinuation. We assumed that women who gave any of the following reasons would discontinue use of any contraceptive method:

- Wanted to become pregnant
- Husband disapproved
- Infrequent sex, husband away
- Fatalistic
- Difficult pregnancy, menopause
- Marital dissolution
- Other

For other reasons such as side effects, costs, access, etc. we assumed that the woman switched to a different method.

Here we plot the estimated probabilities that women would discontinue all contraceptive use conditional on the last method they reported using.

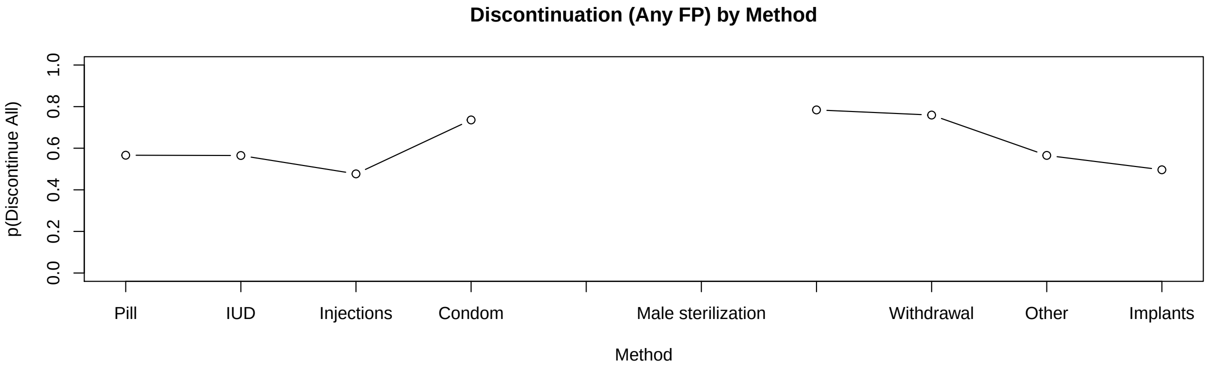

Parameters
We fit hierarchical models to the DHS data to estimate priors for the duration of use for each method and the probability of discontinuing use of all contraception at the end of the simulated duration of use. For simplicity, we assumed that duration of use follows an exponential distribution, and used logistic regression models to simulate the probability of discontinuing all methods. We used upper middle income priors for high income countries due to lack of DHS data in high income countries.

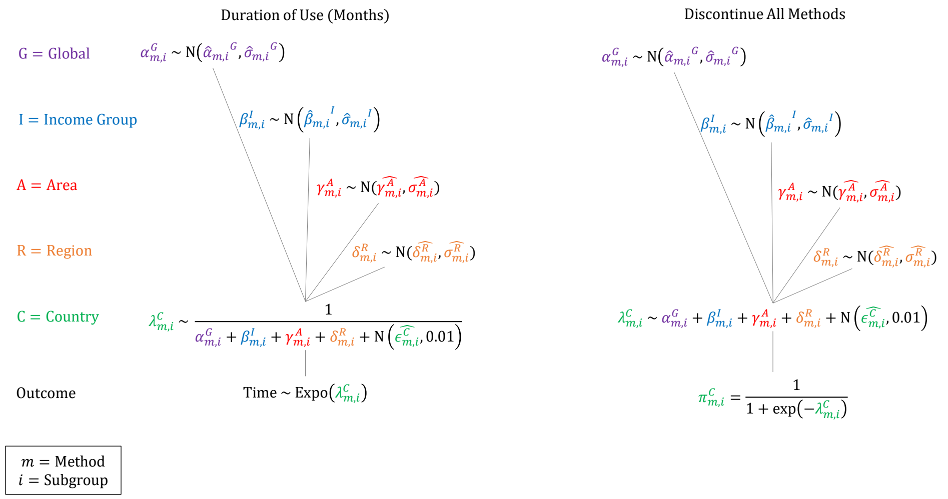
Duration of Use (Months)
Discontinue All Methods
G = Global
I = Income Group
A = Area
R = Region
C = Country
Outcome
$\alpha^G_{m,i} \sim N(\hat{\alpha}_{m,i}{}^G, \hat{\sigma}_{m,i}{}^G)$
$\beta^I_{m,i} \sim N(\hat{\beta}_{m,i}{}^I, \hat{\sigma}_{m,i}{}^I)$
$\gamma^A_{m,i} \sim N(\widehat{\gamma^A_{m,i}}, \widehat{\sigma^A_{m,i}})$
$\delta^R_{m,i} \sim N(\widehat{\delta^R_{m,i}}, \widehat{\sigma^R_{m,i}})$
$\lambda^C_{m,i} \sim \frac{1}{\alpha^G_{m,i} + \beta^I_{m,i} + \gamma^A_{m,i} + \delta^R_{m,i} + N(\widehat{\epsilon^C_{m,i}}, 0.01)}$
Time ~ Expo$(\lambda^C_{m,i})$
$\lambda^C_{m,i} \sim \alpha^G_{m,i} + \beta^I_{m,i} + \gamma^A_{m,i} + \delta^R_{m,i} + N(\widehat{\epsilon^C_{m,i}}, 0.01)$
$\pi^C_{m,i} = \frac{1}{1 + \exp(-\lambda^C_{m,i})}$
m = Method
i = Subgroup

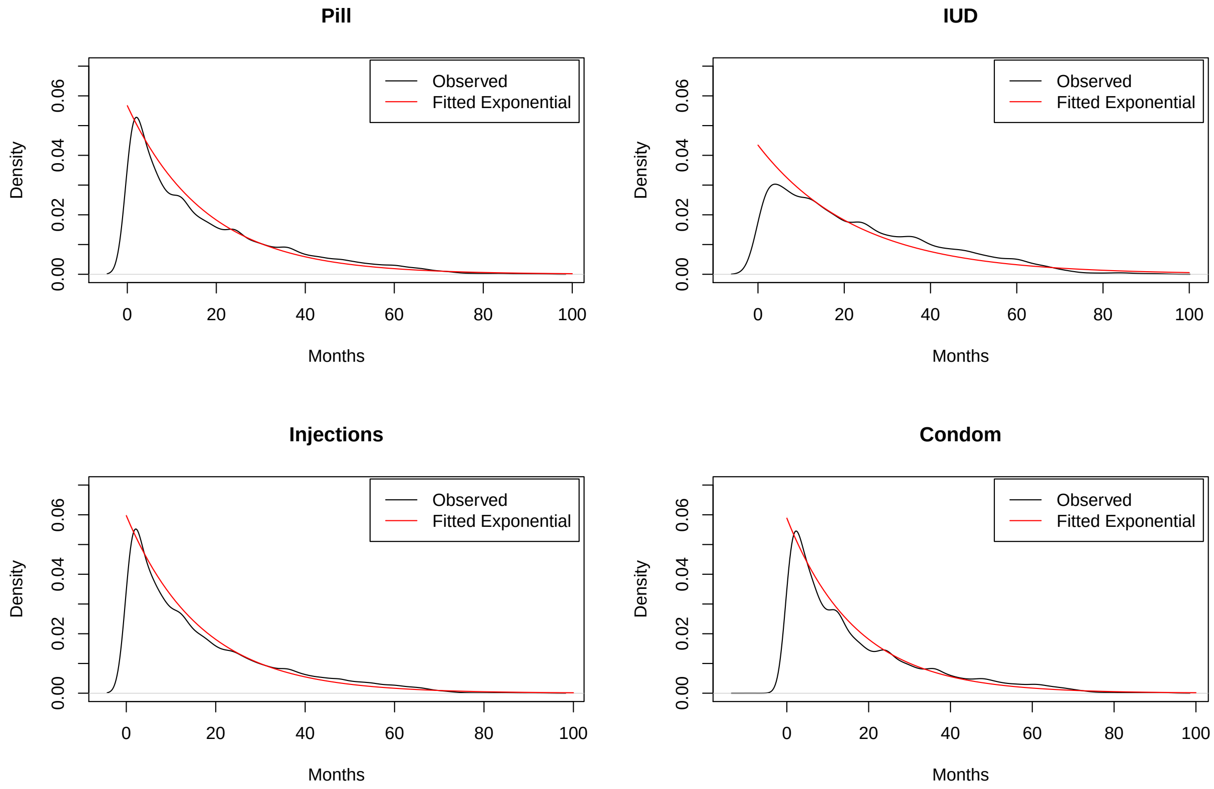
Pill
IUD
Injections
Condom
Observed
Fitted Exponential
Density
Months
0.00
0.02
0.04
0.06
0
20
40
60
80
100

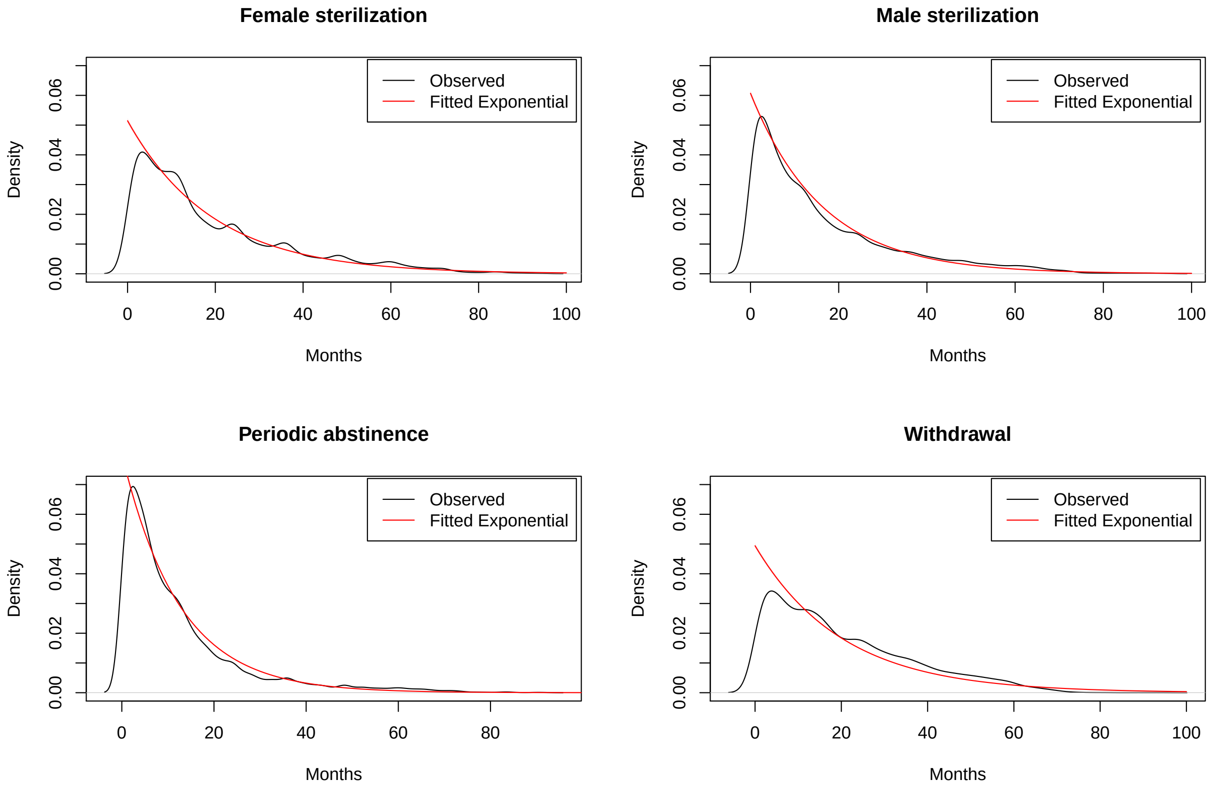
Female sterilization
Male sterilization
Periodic abstinence
Withdrawal
Observed
Fitted Exponential
Density
Months
0.00
0.02
0.04
0.06
0
20
40
60
80
100

Priors

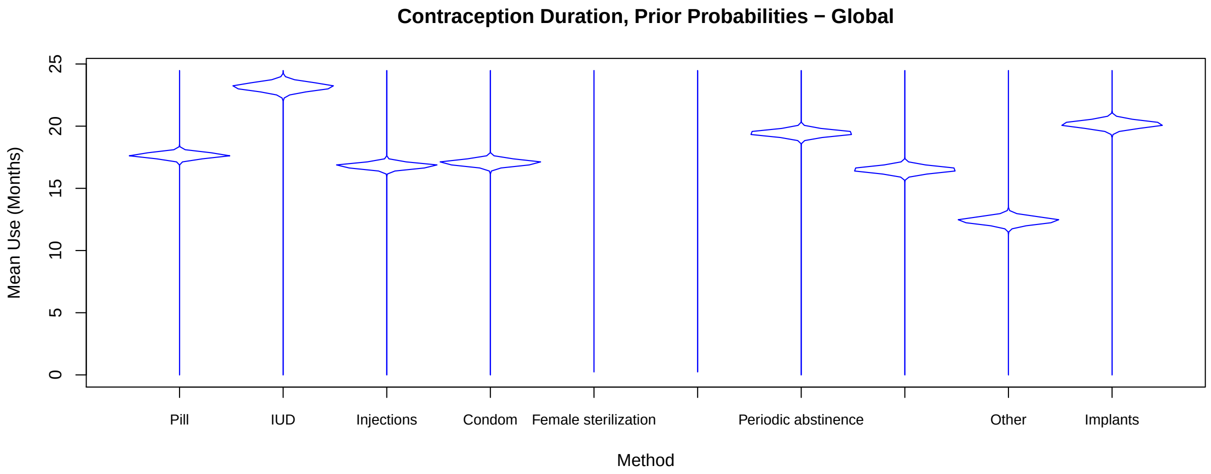
Contraception Duration, Prior Probabilities − Global
Mean Use (Months)
0
5
10
15
20
25
Pill
IUD
Injections
Condom
Female sterilization
Periodic abstinence
Other
Implants
Method

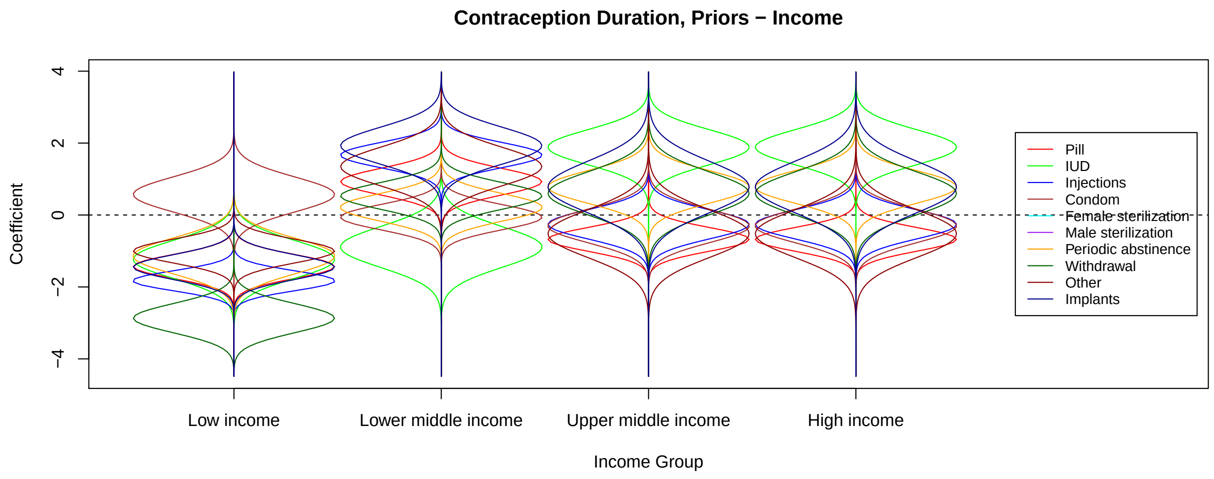
Contraception Duration, Priors − Income
Coefficient
4
2
0
−2
−4
Low income
Lower middle income
Upper middle income
High income
Income Group
Pill
IUD
Injections
Condom
Female sterilization
Male sterilization
Periodic abstinence
Withdrawal
Other
Implants

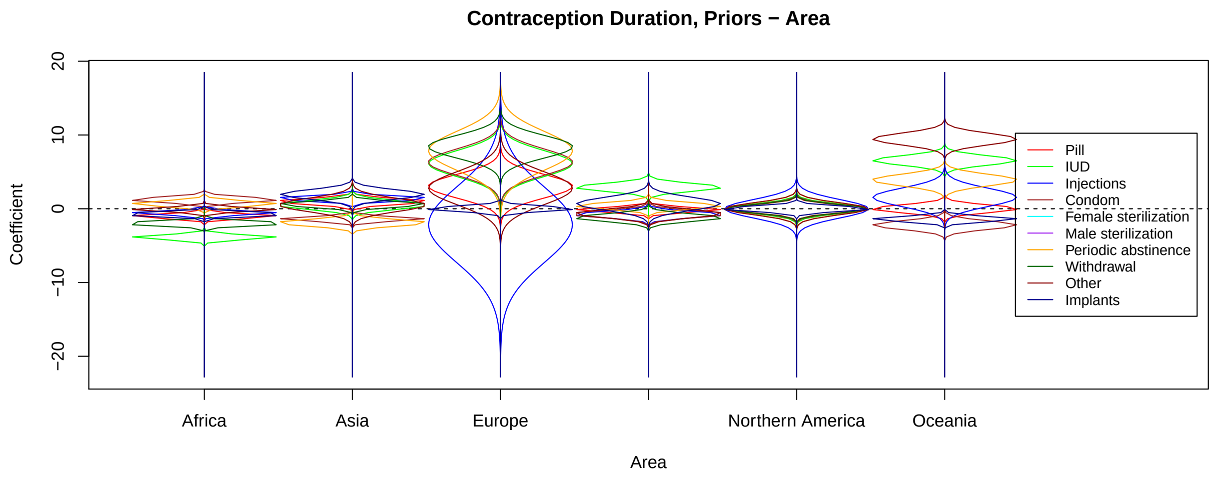
Contraception Duration, Priors − Area
Coefficient
20
10
0
−10
−20
Africa
Asia
Europe
Northern America
Oceania
Area
Pill
IUD
Injections
Condom
Female sterilization
Male sterilization
Periodic abstinence
Withdrawal
Other
Implants

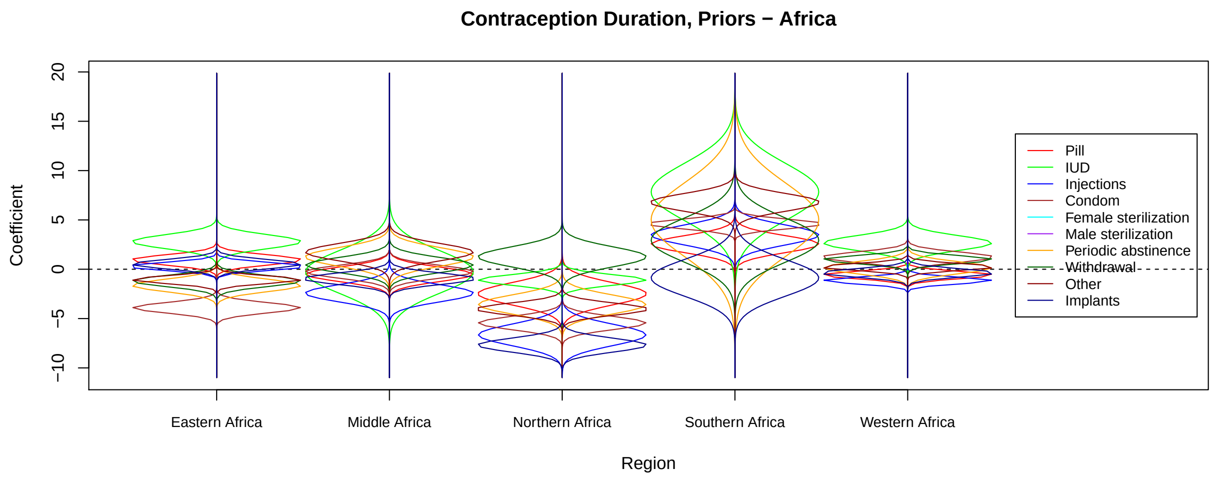
Contraception Duration, Priors − Africa
Coefficient
20
15
10
5
0
−5
−10
Eastern Africa
Middle Africa
Northern Africa
Southern Africa
Western Africa
Region
Pill
IUD
Injections
Condom
Female sterilization
Male sterilization
Periodic abstinence
Withdrawal
Other
Implants

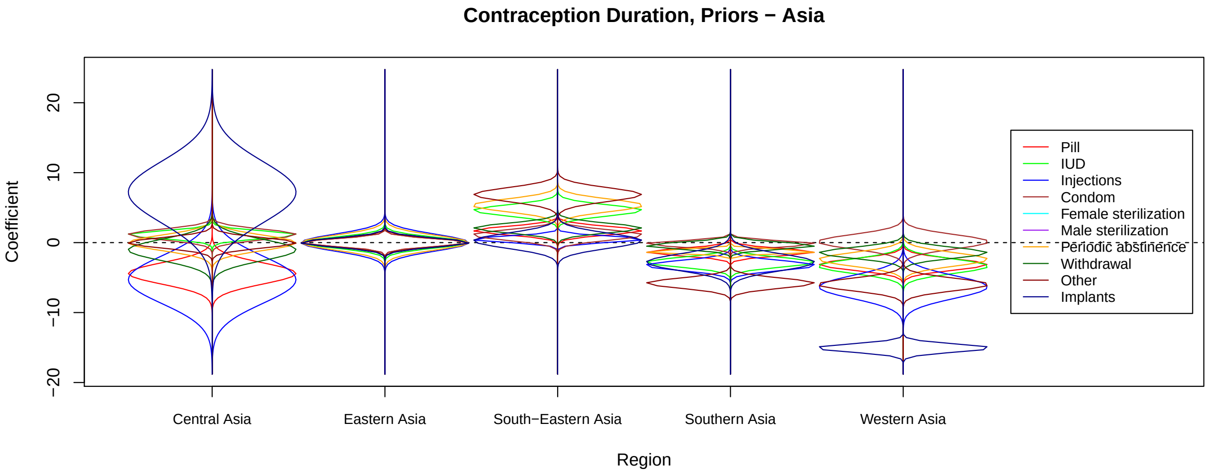
Contraception Duration, Priors − Asia
Coefficient
20
10
0
−10
−20
Central Asia
Eastern Asia
South−Eastern Asia
Southern Asia
Western Asia
Region
Pill
IUD
Injections
Condom
Female sterilization
Male sterilization
Periodic abstinence
Withdrawal
Other
Implants

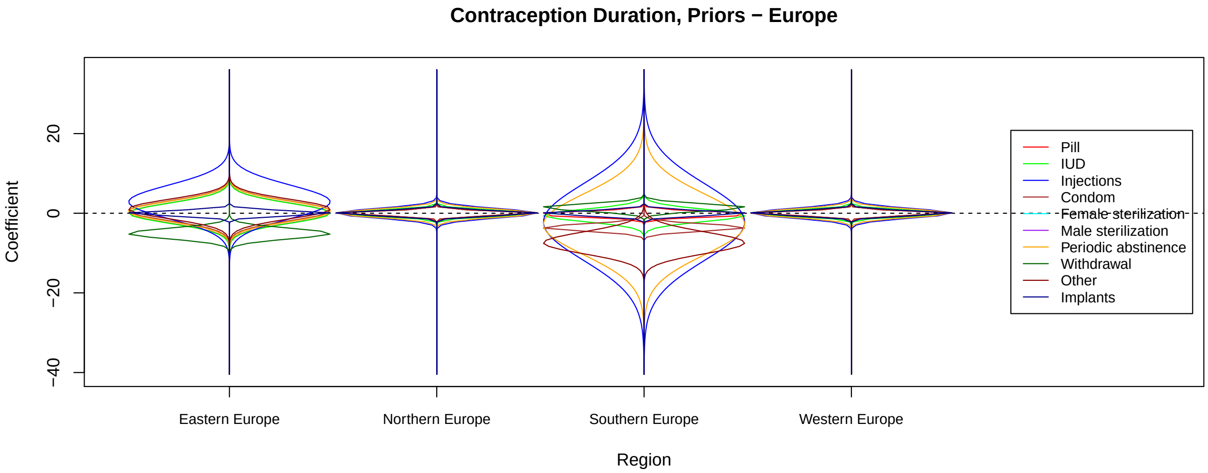
Contraception Duration, Priors − Europe
Coefficient
20
0
−20
−40
Eastern Europe
Northern Europe
Southern Europe
Western Europe
Region
Pill
IUD
Injections
Condom
Female sterilization
Male sterilization
Periodic abstinence
Withdrawal
Other
Implants

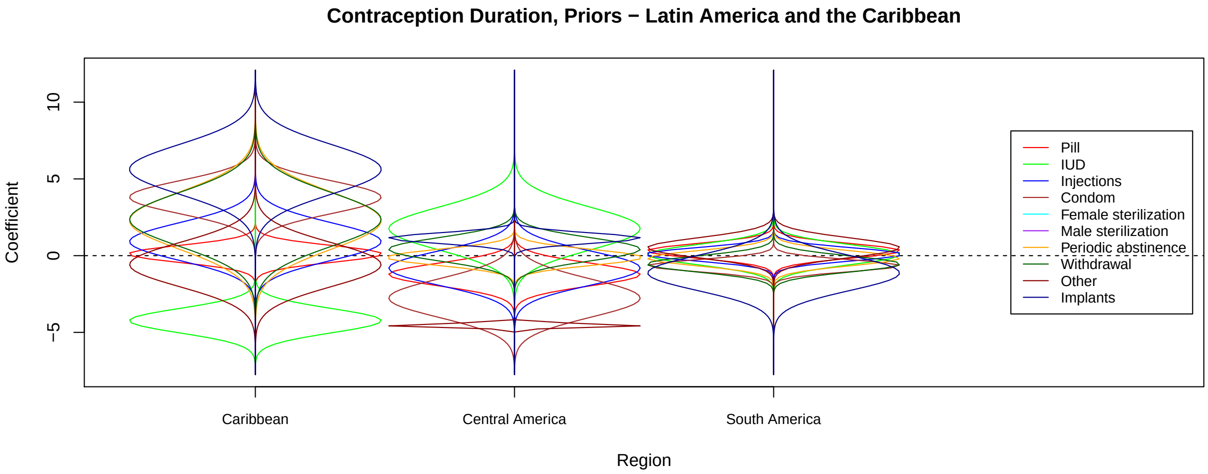
Contraception Duration, Priors − Latin America and the Caribbean
Coefficient
10
5
0
−5
Caribbean
Central America
South America
Region
Pill
IUD
Injections
Condom
Female sterilization
Male sterilization
Periodic abstinence
Withdrawal
Other
Implants

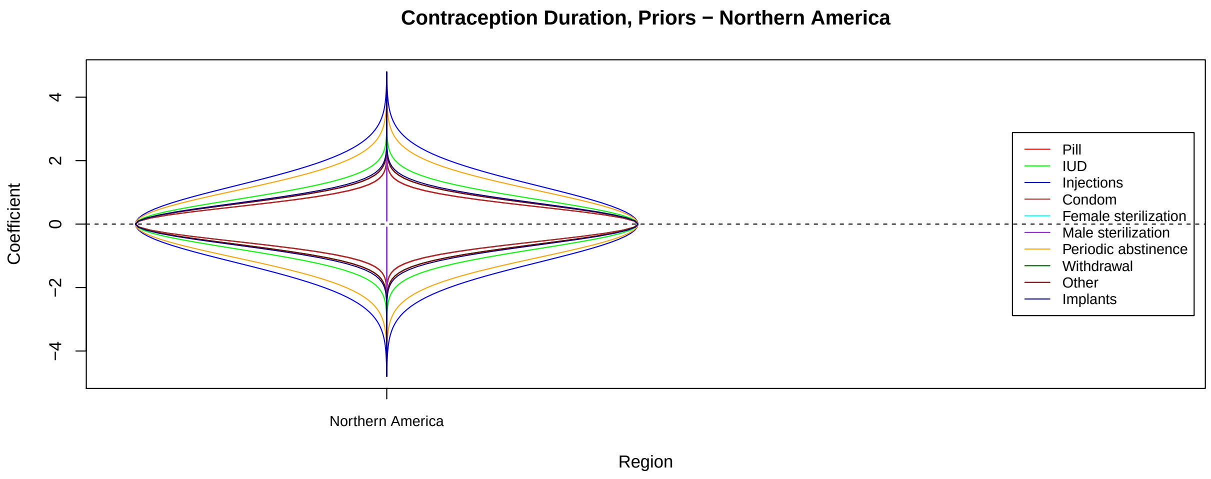
Contraception Duration, Priors − Northern America
Coefficient
4
2
0
−2
−4
Northern America
Region
Pill
IUD
Injections
Condom
Female sterilization
Male sterilization
Periodic abstinence
Withdrawal
Other
Implants

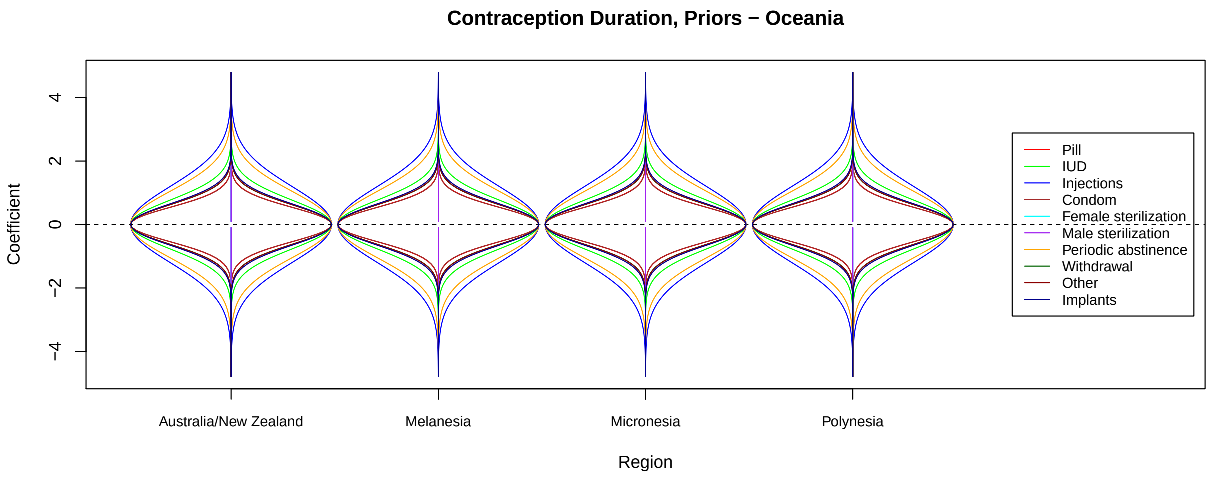
Contraception Duration, Priors − Oceania
Coefficient
4
2
0
−2
−4
Australia/New Zealand
Melanesia
Micronesia
Polynesia
Region
Pill
IUD
Injections
Condom
Female sterilization
Male sterilization
Periodic abstinence
Withdrawal
Other
Implants

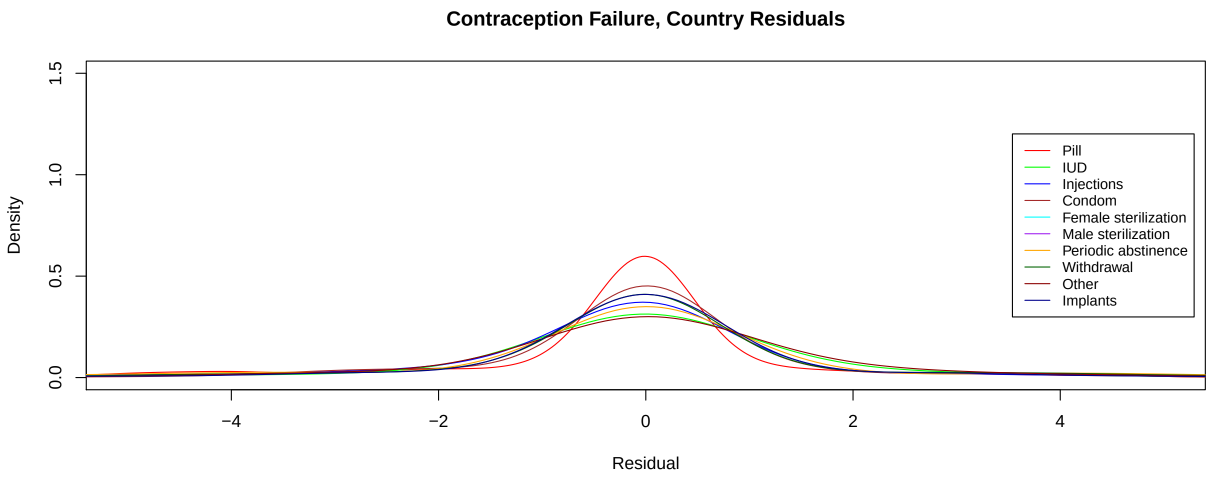
Contraception Failure, Country Residuals
Density
1.5
1.0
0.5
0.0
−4
−2
0
2
4
Residual
Pill
IUD
Injections
Condom
Female sterilization
Male sterilization
Periodic abstinence
Withdrawal
Other
Implants

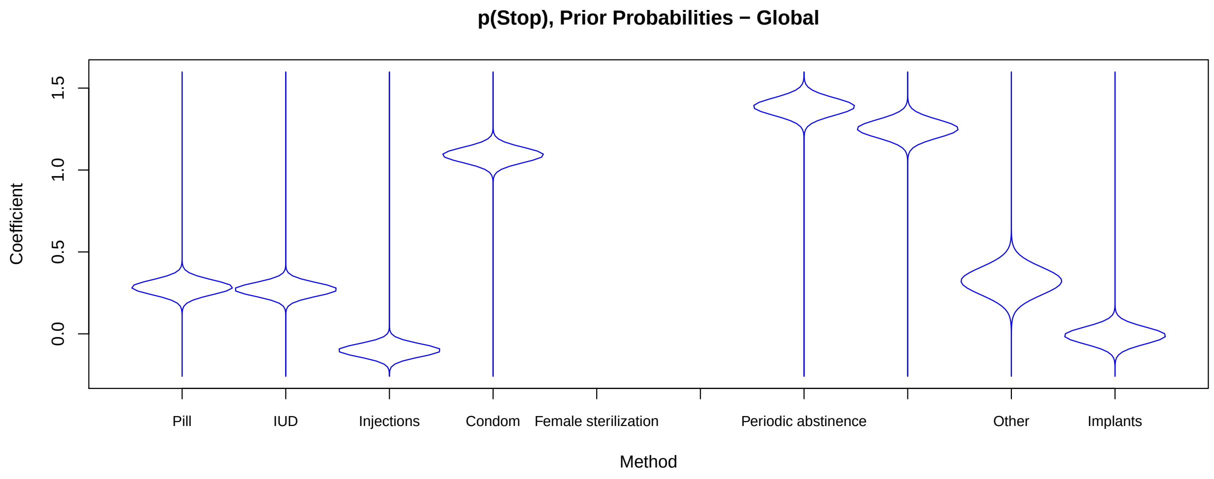
p(Stop), Prior Probabilities − Global
Coefficient
1.5
1.0
0.5
0.0
Pill
IUD
Injections
Condom
Female sterilization
Periodic abstinence
Other
Implants
Method

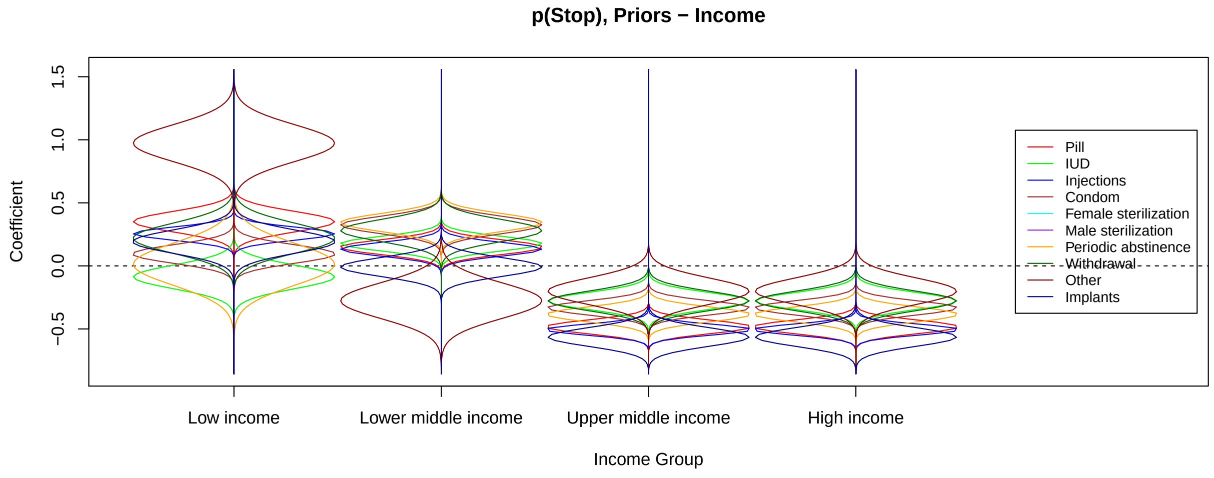
p(Stop), Priors − Income
Coefficient
1.5
1.0
0.5
0.0
−0.5
Low income
Lower middle income
Upper middle income
High income
Income Group
Pill
IUD
Injections
Condom
Female sterilization
Male sterilization
Periodic abstinence
Withdrawal
Other
Implants

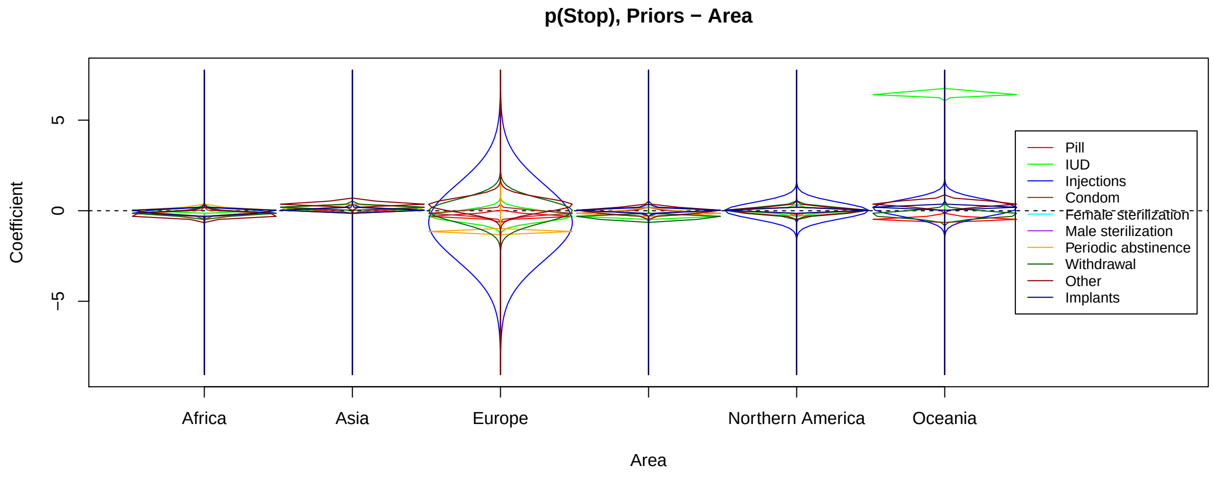
p(Stop), Priors − Area
Coefficient
5
0
−5
Africa
Asia
Europe
Northern America
Oceania
Area
Pill
IUD
Injections
Condom
Female sterilization
Male sterilization
Periodic abstinence
Withdrawal
Other
Implants

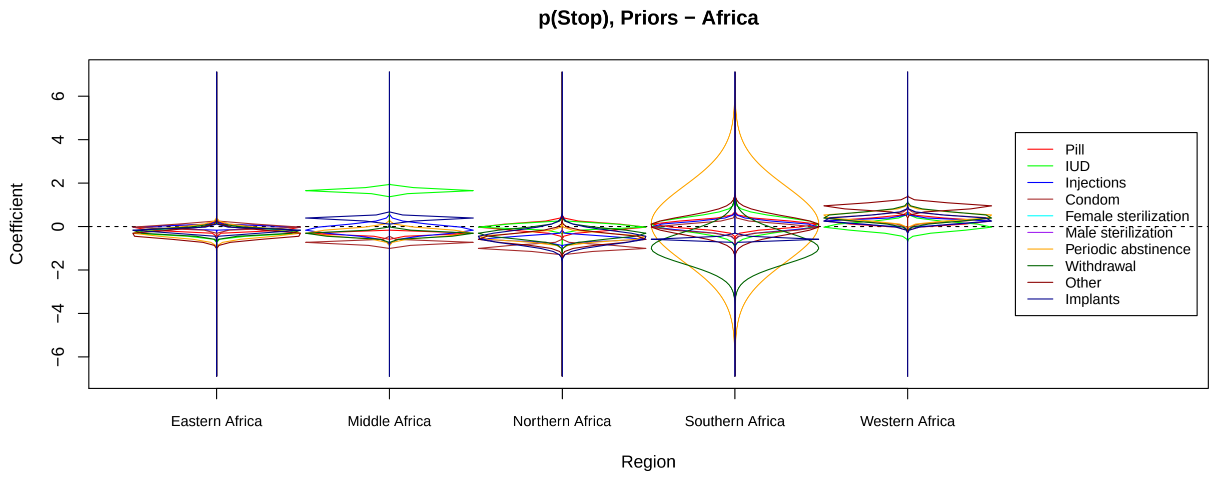
p(Stop), Priors − Africa
Coefficient
6
4
2
0
−2
−4
−6
Pill
IUD
Injections
Condom
Female sterilization
Male sterilization
Periodic abstinence
Withdrawal
Other
Implants
Eastern Africa
Middle Africa
Northern Africa
Southern Africa
Western Africa
Region

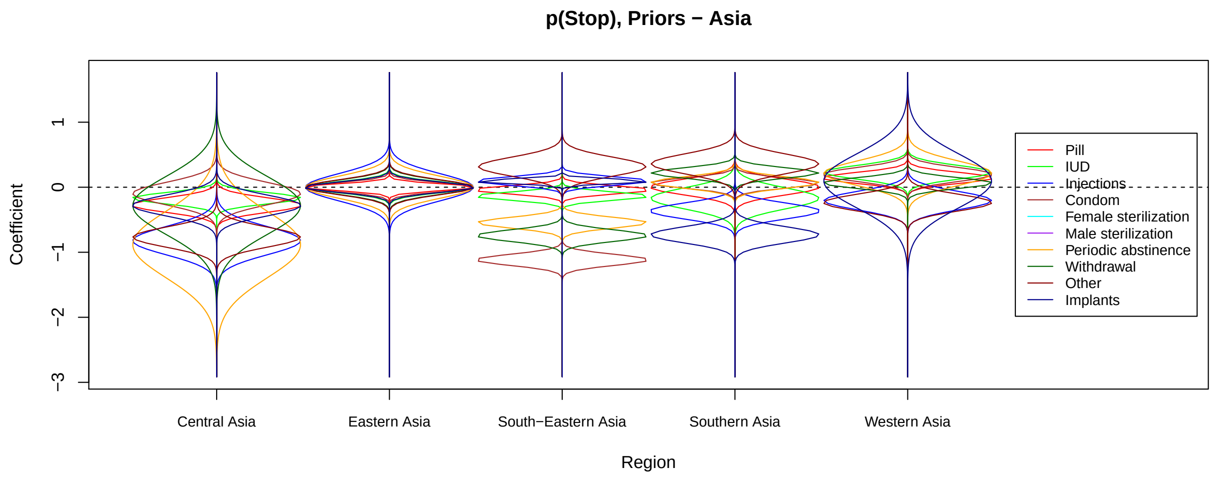
p(Stop), Priors − Asia
Coefficient
1
0
−1
−2
−3
Pill
IUD
Injections
Condom
Female sterilization
Male sterilization
Periodic abstinence
Withdrawal
Other
Implants
Central Asia
Eastern Asia
South−Eastern Asia
Southern Asia
Western Asia
Region

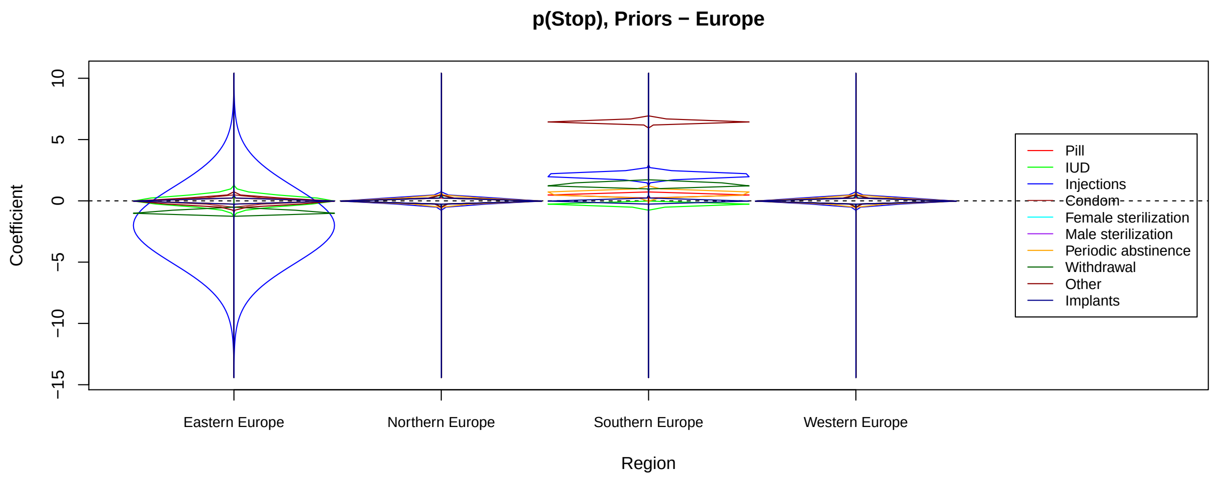
p(Stop), Priors − Europe
Coefficient
10
5
0
−5
−10
−15
Pill
IUD
Injections
Condom
Female sterilization
Male sterilization
Periodic abstinence
Withdrawal
Other
Implants
Eastern Europe
Northern Europe
Southern Europe
Western Europe
Region

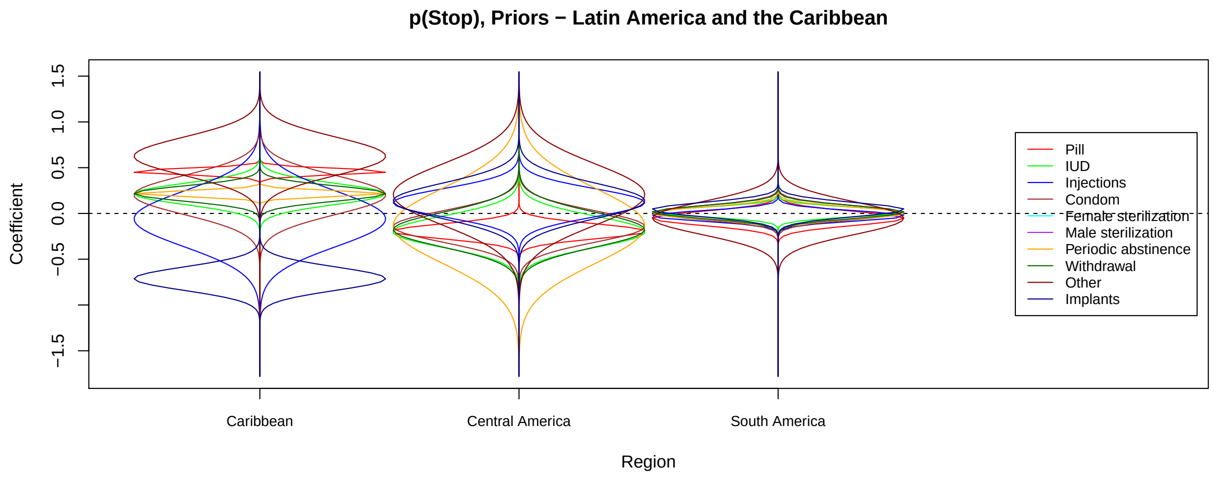
p(Stop), Priors − Latin America and the Caribbean
Coefficient
1.5
1.0
0.5
0.0
−0.5
−1.5
Caribbean
Central America
South America
Region
Pill
IUD
Injections
Condom
Female sterilization
Male sterilization
Periodic abstinence
Withdrawal
Other
Implants

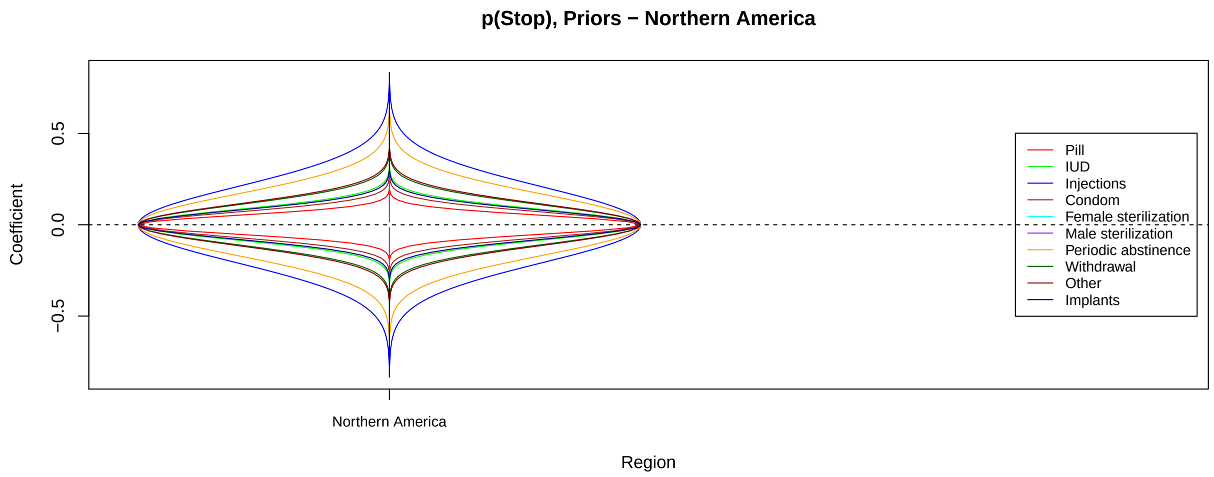
p(Stop), Priors − Northern America
Coefficient
0.5
0.0
−0.5
Northern America
Region
Pill
IUD
Injections
Condom
Female sterilization
Male sterilization
Periodic abstinence
Withdrawal
Other
Implants

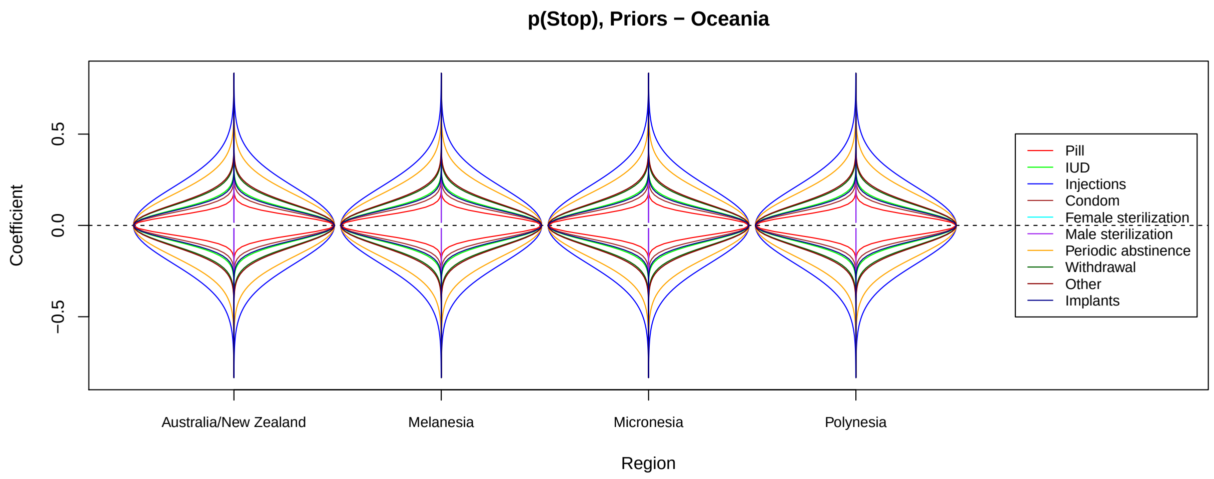
p(Stop), Priors − Oceania
Coefficient
0.5
0.0
−0.5
Australia/New Zealand
Melanesia
Micronesia
Polynesia
Region
Pill
IUD
Injections
Condom
Female sterilization
Male sterilization
Periodic abstinence
Withdrawal
Other
Implants

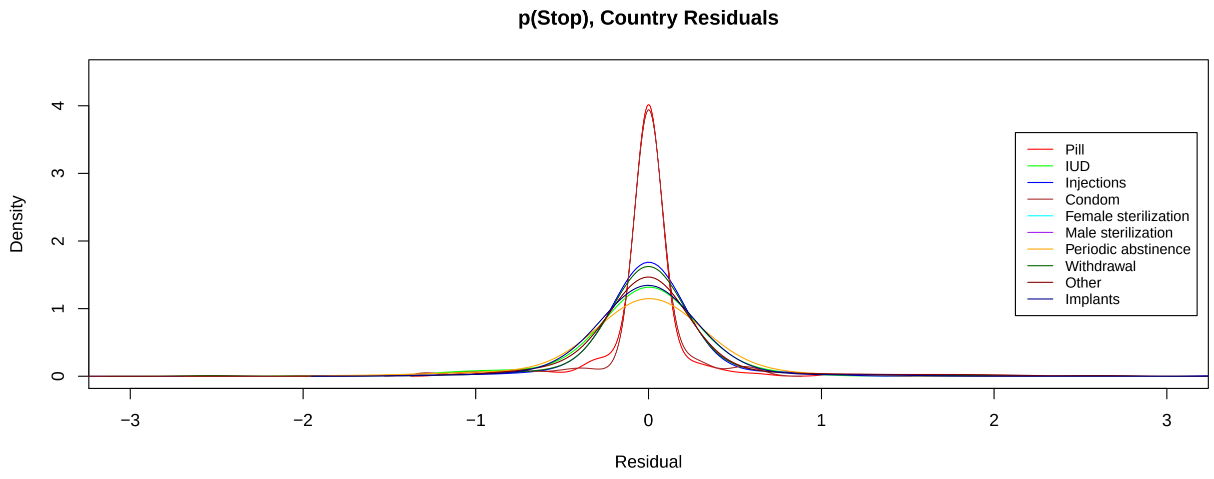

Model Implementation

When a spacing method is assigned, a duration of use (in months) is drawn from the exponential distribution. Duration of use is renormalized so that the maximum possible length is 10 years. If a woman completes the duration (i.e. the method does not fail), the reason for discontinuation is simulated.

A.1.3.8 Abortion

Overview

Induced abortion is usually classified as either 'safe' (eg: medical abortion with misoprostol) or 'unsafe' (eg: traditional methods). While medical abortions still pose some level of health risk to the mother, unsafe abortion has a much higher risk of maternal mortality/morbidity, depending on the quality of the facilities and skill of the abortion provider, the intervention method used, and the general health of the mother and stage of her pregnancy (WHO 2011). Unsafe abortion may be induced by the woman herself, a nonmedical person under unhygienic conditions, or by a health worker outside of the prescribed facilities (WHO 2011). The outcome of unsafe abortion also depends on a woman's willingness to turn to medical services in the event of a complication, and the readiness of medical staff to deal promptly with the complications (WHO 2011). Deaths due to unsafe abortion are mainly caused by bleeding or severe infections caused by the abortion procedure, or due to organ damage. Women may also suffer long-term health consequences such as infertility, as well as shorter-term effects (WHO 2011).

Data

Incidence

To estimate the probability of abortion given unintended pregnancy we use estimates from a global model of unintended pregnancy and its outcomes (Bearak 2020). Here we plot estimates of the proportion of unintended pregnancies which end in abortion by region over time.

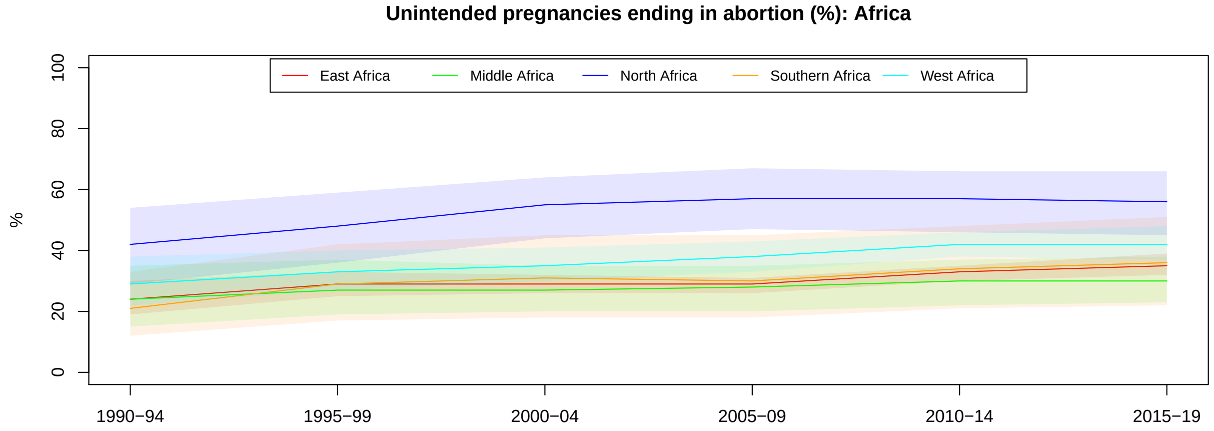

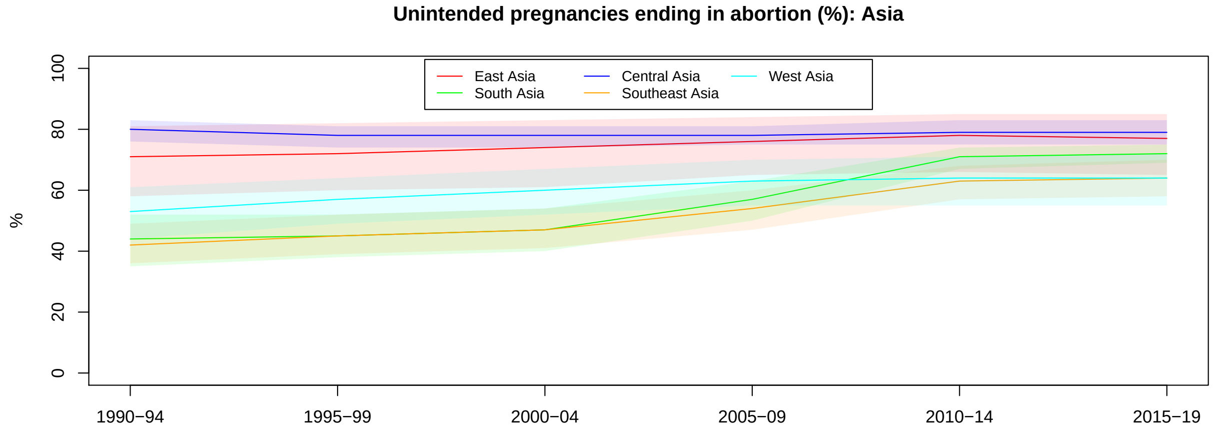

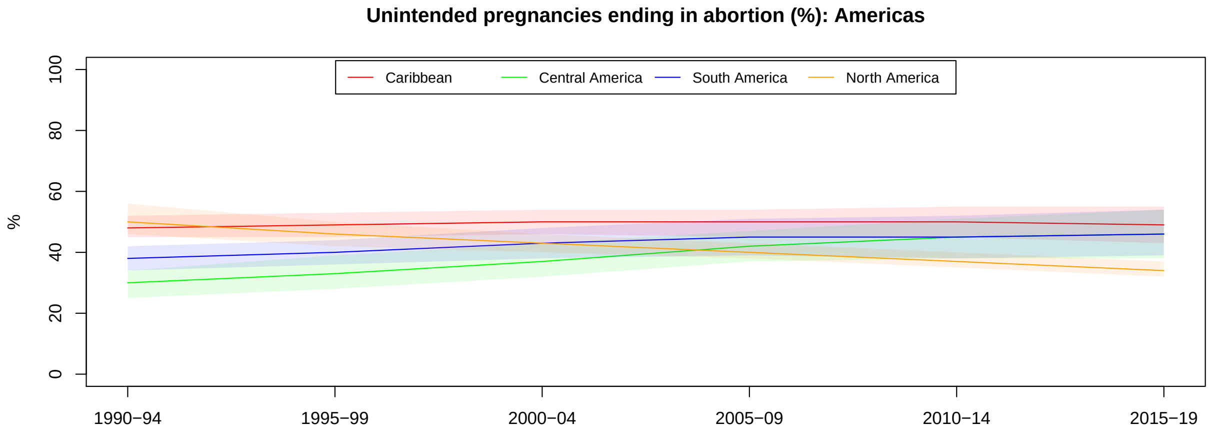

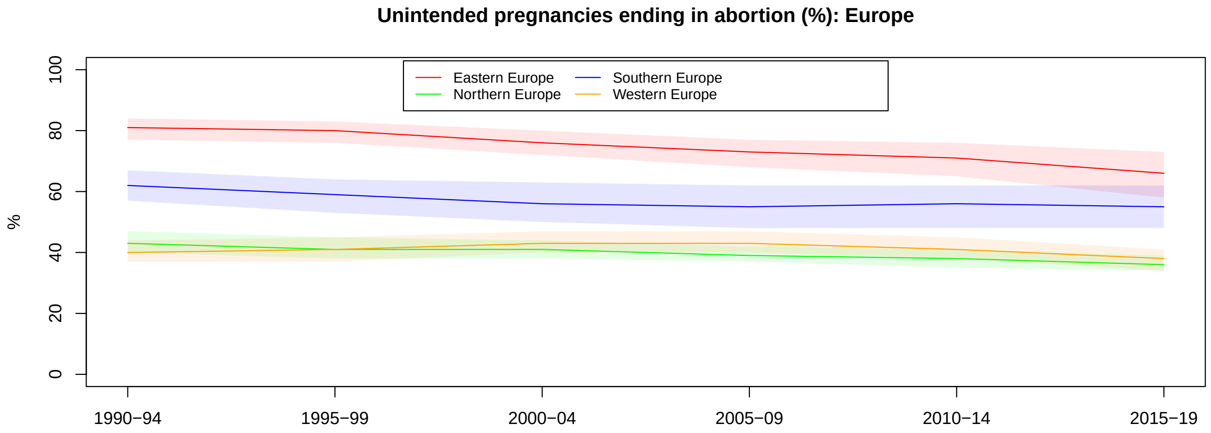

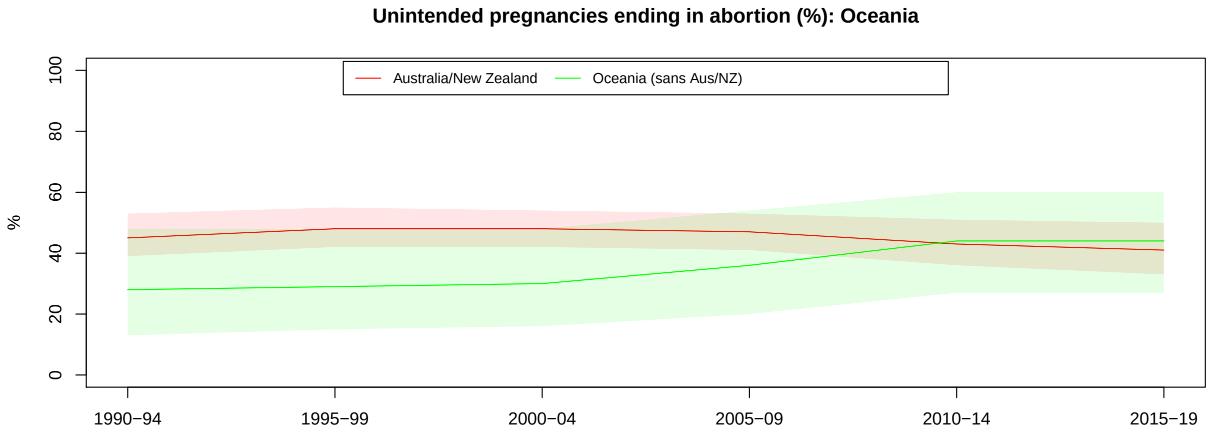

To estimate the proportion of abortions that are 'safe' we used regional estimates from a model of global abortions (Ganatra 2017):

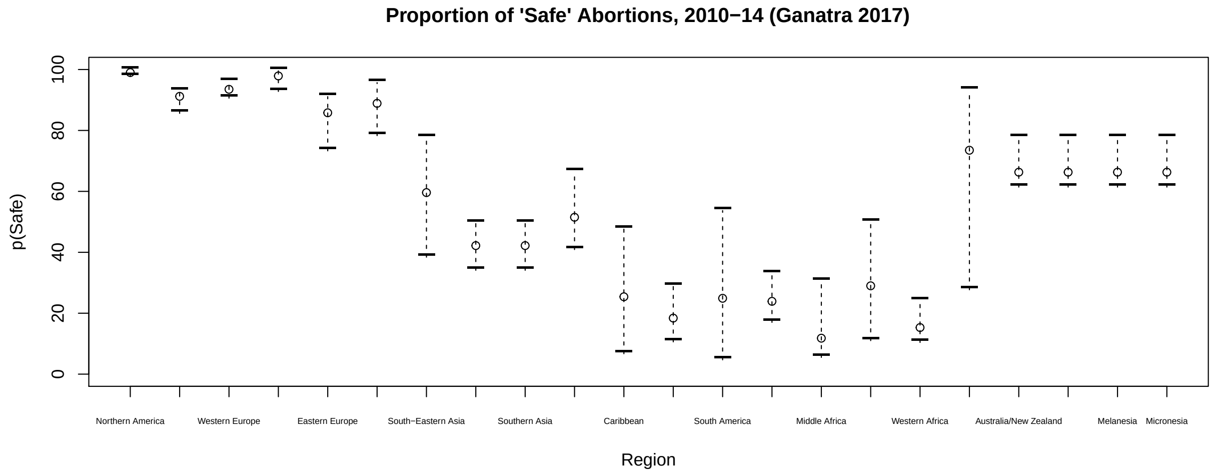

Mortality

Estimates of abortion mortality vary by location. Estimates of abortion-related mortality in the US in 2000-2009 find a rate of 0.7 per 100,000 abortions (Raymond 2014, Zane 2015). A study in India found that out of 89,194 abortions in 2012-2013, there were 253 maternal deaths, with 67% of abortions classified as ‘unsafe’, indicating an overall CFR of 0.3% (Yokoe 2019). A study in Zimbabwe of 1002 women presenting with abortion complications found that 0.2% died, with 19% presenting with ‘severe’ complications, and 3% with ‘near miss’ complications (Madziyire 2018). A study in Kinshasa of women who were admitted to receive obstetric and gynecological emergency care services in 2014, the mortality rate related to induced abortion was 5.6% (95% CI: 2.3–11.3%) (Ishoso 2018). A systematic review of 70 studies from 28 countries between 1988 and 2014 found that at least 9% of abortion-related hospital admissions have a near-miss event and approximately 1.5% end in death, with hemorrhage the most common complication reported (Calvert 2018). The WHO estimates that the global case-fatality rate associated with unsafe abortion (220 per 100,000) is 350 times higher than the rate associated with legal induced abortions in the US, with rates more than 800 times higher in sub-Saharan Africa - even in developed countries, the case-fatality rate for unsafe abortion is 40 times higher than for legal induced abortion (WHO 2011). Here we report the WHO estimates of unsafe abortion CFRs by region (adapted from Table 7, WHO 2011):

Region	Estimated number of deaths per 100,000 unsafe abortions
WORLD	220
Developed regions	30
Developing regions	220
AFRICA	460
Eastern Africa	520
Middle Africa	470
Northern Africa	170
Southern Africa	370
Western Africa	540
ASIA	160
South-Central Asia	200
South-Eastern Asia	70
Western Asia	70
EUROPE	30
Eastern Europe	30
LATIN AMERICA AND THE CARIBBEAN	30
Caribbean	80
Central America	20
South America	20

Region	Estimated number of deaths per 100,000 unsafe abortions
OCEANIA	400

Morbidity
A nationwide register study of induced abortion in Finland (which we assume are virtually all 'safe') found that the proportion of women with IVF treatment after induced abortion in the whole cohort was 0.4% (Mannisto 2019). Factors associated with induced abortion such as gestational age at abortion, method of abortion, and complications following abortion were not associated with the use of IVF treatment later. The WHO estimates that 20-30% of unsafe abortions result in reproductive tract infections, of which 20-40% result in upper-genital-tract infection and infertility (Abouzahr 1998). Global Burden of Disease (2000) estimates of the probability of secondary infertility from unsafe abortion ranged from 3% to 12% by region (Ahman 2000).

Parameters
We based our priors for abortion incidence on estimates by Bearak 2020. To estimate priors we simulated data by drawing samples from the reported posteriors. We drew 100 points for each time period and fit logistic regressions to the simulated data for each region, using the estimated coefficients and standard errors as priors. To estimate priors for the proportion of abortions that are 'safe', we logit transformed the Ganatra 2017 estimates. Because estimates were only available for the period 2010-14 we also set a prior for a 'year' coefficient to allow for time trends in safe abortion.

For mortality, we based region priors for safe abortion CFR on US estimates of abortion CFR, as nearly all abortions in North America are estimated to be 'safe'. For unsafe abortion CFR, we based the priors on regional estimates from the WHO (WHO 2011), and set σ as 10% of the estimated CFR. To account for the relative risk of mortality from anemia, we set priors based on data from Brabin 2001. Given that estimates of 'near miss' events are around 10 times higher than mortality, we set priors for infertility from safe abortion 10 times higher than the mortality estimates. For unsafe abortion we set priors based on the WHO estimates of 4%-12%.

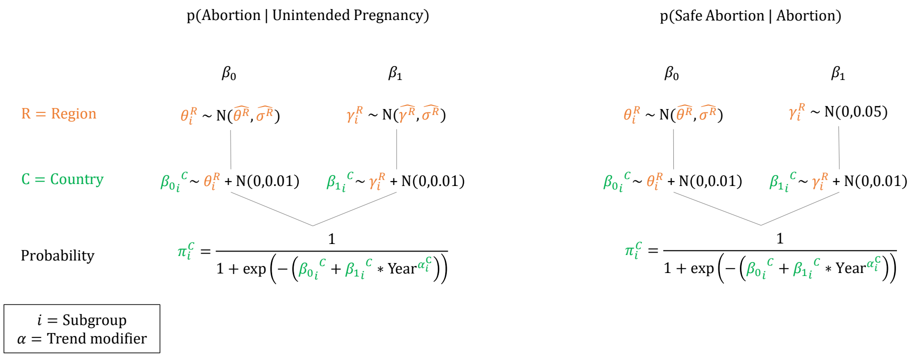

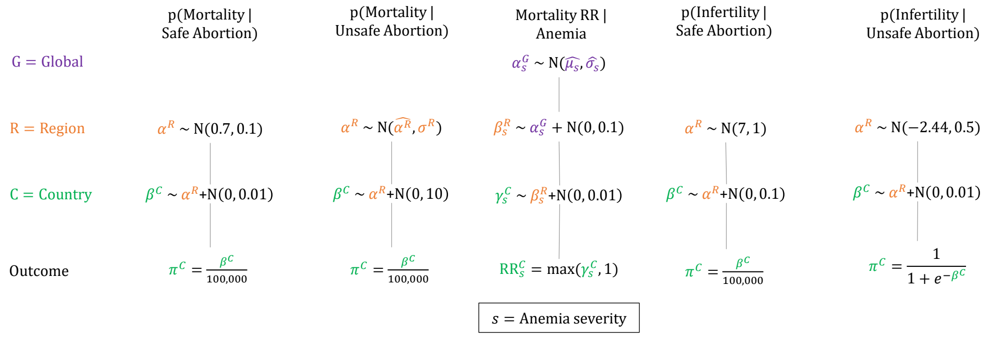

Priors

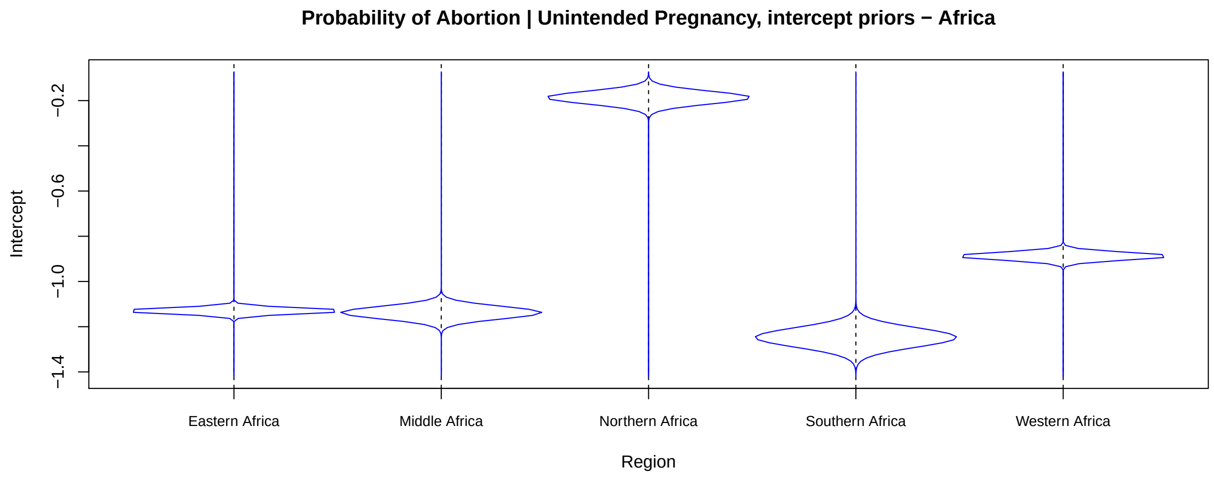
Probability of Abortion | Unintended Pregnancy, intercept priors − Africa
Intercept
−0.2
−0.6
−1.0
−1.4
Eastern Africa
Middle Africa
Northern Africa
Southern Africa
Western Africa
Region

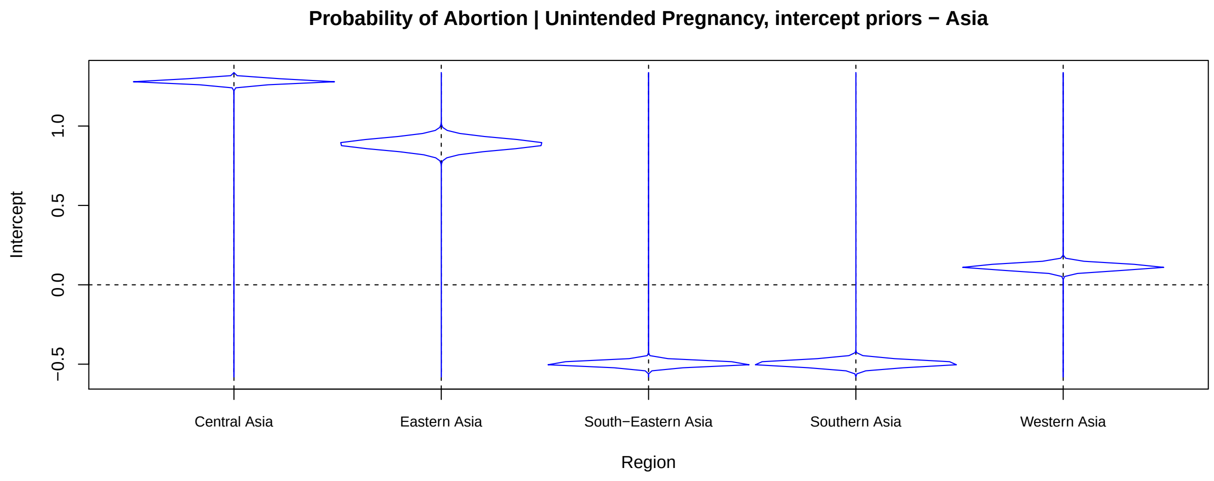
Probability of Abortion | Unintended Pregnancy, intercept priors − Asia
Intercept
1.0
0.5
0.0
−0.5
Central Asia
Eastern Asia
South−Eastern Asia
Southern Asia
Western Asia
Region

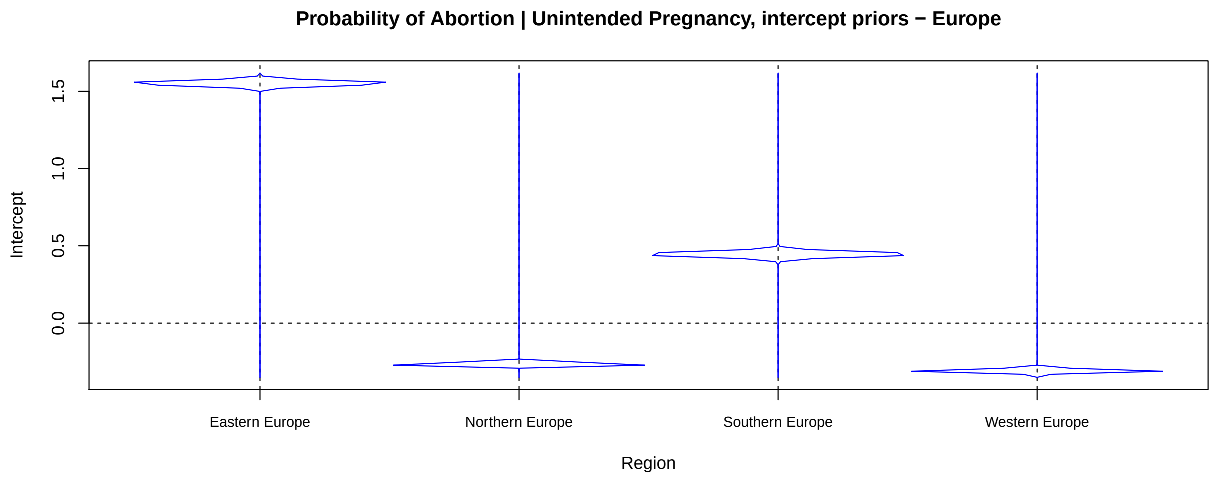
Probability of Abortion | Unintended Pregnancy, intercept priors − Europe
Intercept
1.5
1.0
0.5
0.0
Eastern Europe
Northern Europe
Southern Europe
Western Europe
Region

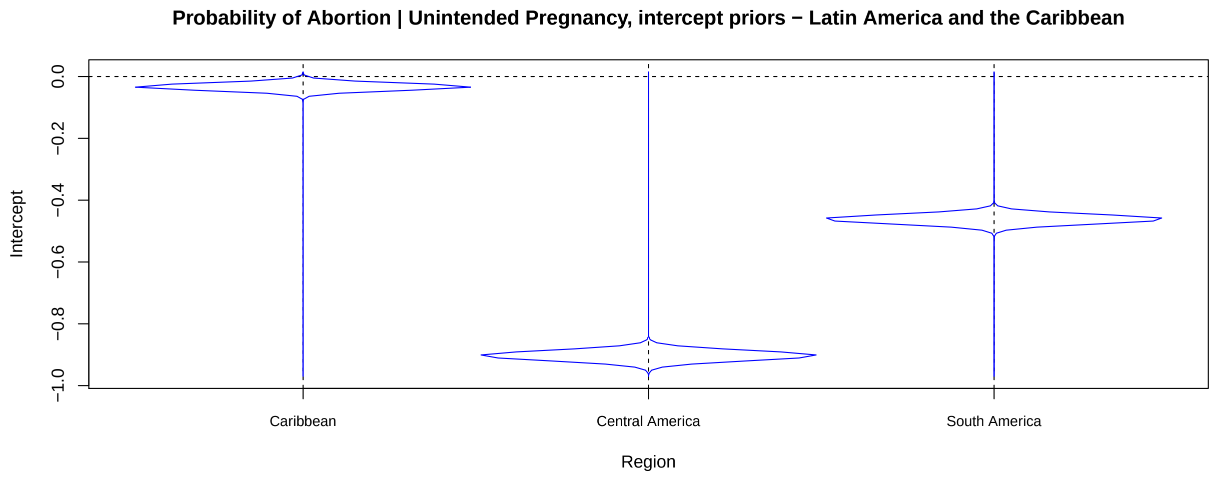

Probability of Abortion | Unintended Pregnancy, intercept priors − Latin America and the Caribbean
Intercept
0.0
−0.2
−0.4
−0.6
−0.8
−1.0
Caribbean
Central America
South America
Region

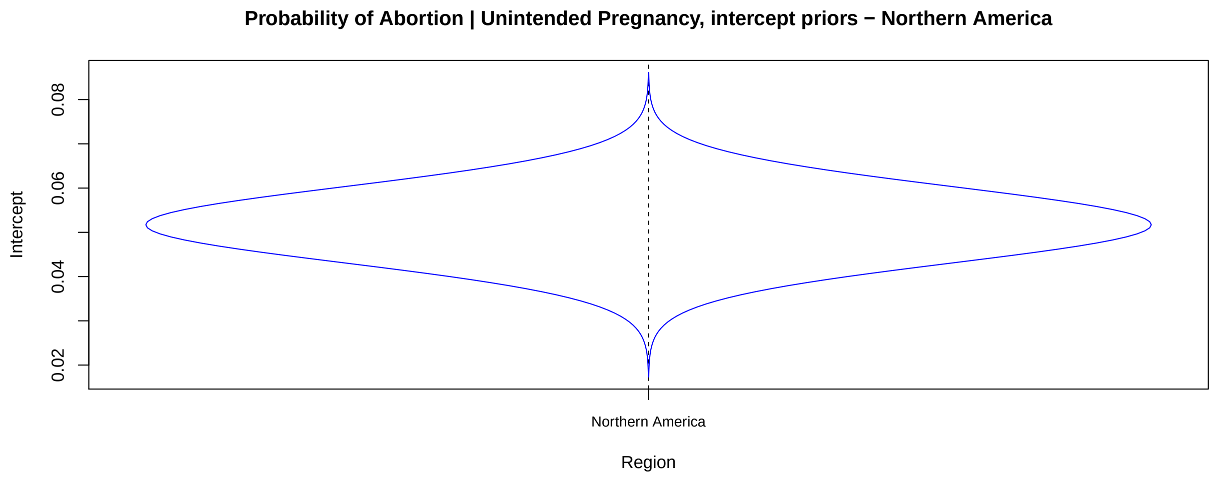

Probability of Abortion | Unintended Pregnancy, intercept priors − Northern America
Intercept
0.08
0.06
0.04
0.02
Northern America
Region

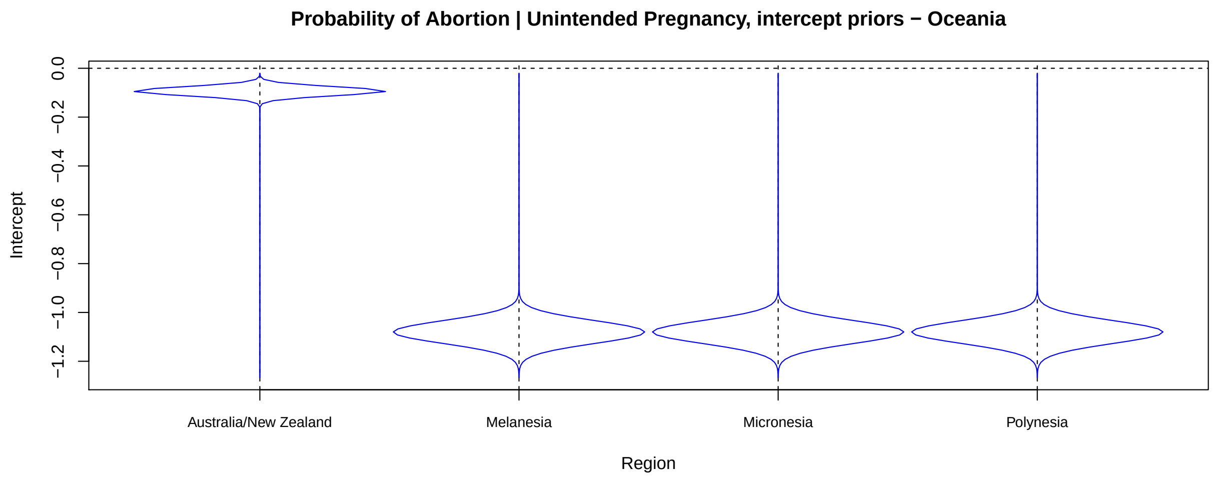

Probability of Abortion | Unintended Pregnancy, intercept priors − Oceania
Intercept
0.0
−0.2
−0.4
−0.6
−0.8
−1.0
−1.2
Australia/New Zealand
Melanesia
Micronesia
Polynesia
Region

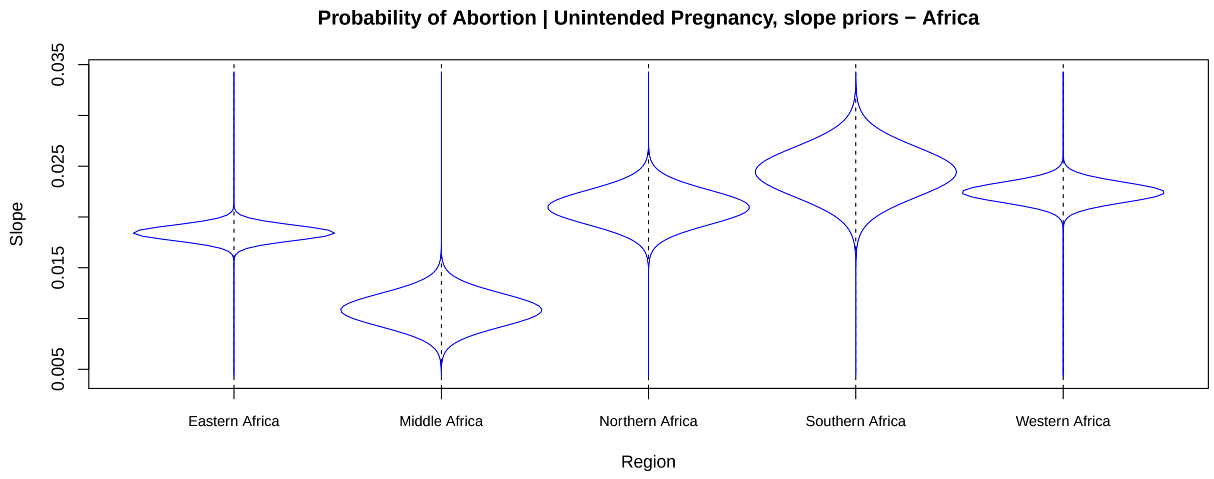
Probability of Abortion | Unintended Pregnancy, slope priors − Africa
Slope
0.005
0.015
0.025
0.035
Eastern Africa
Middle Africa
Northern Africa
Southern Africa
Western Africa
Region

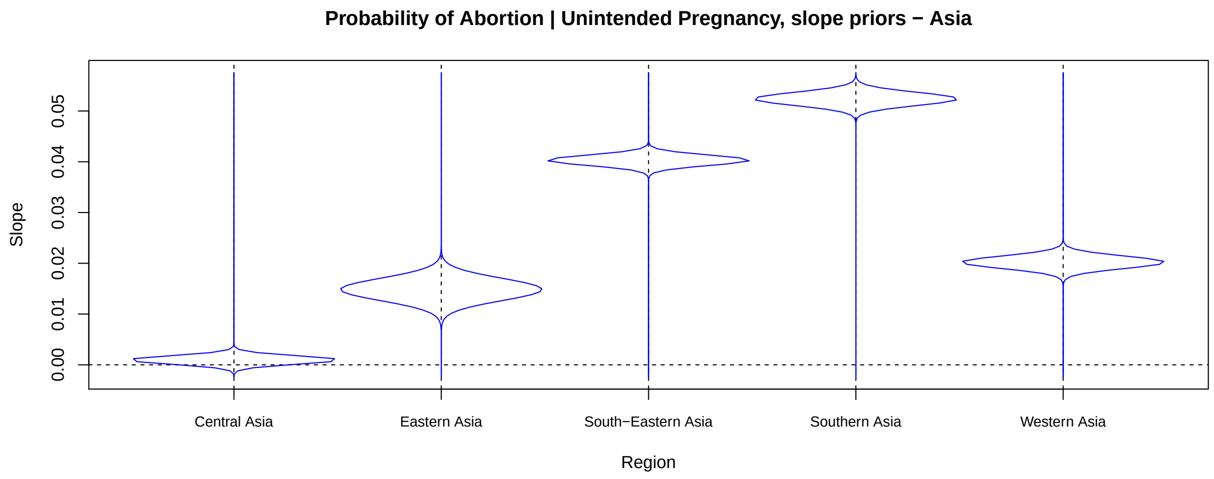
Probability of Abortion | Unintended Pregnancy, slope priors − Asia
Slope
0.00
0.01
0.02
0.03
0.04
0.05
Central Asia
Eastern Asia
South−Eastern Asia
Southern Asia
Western Asia
Region

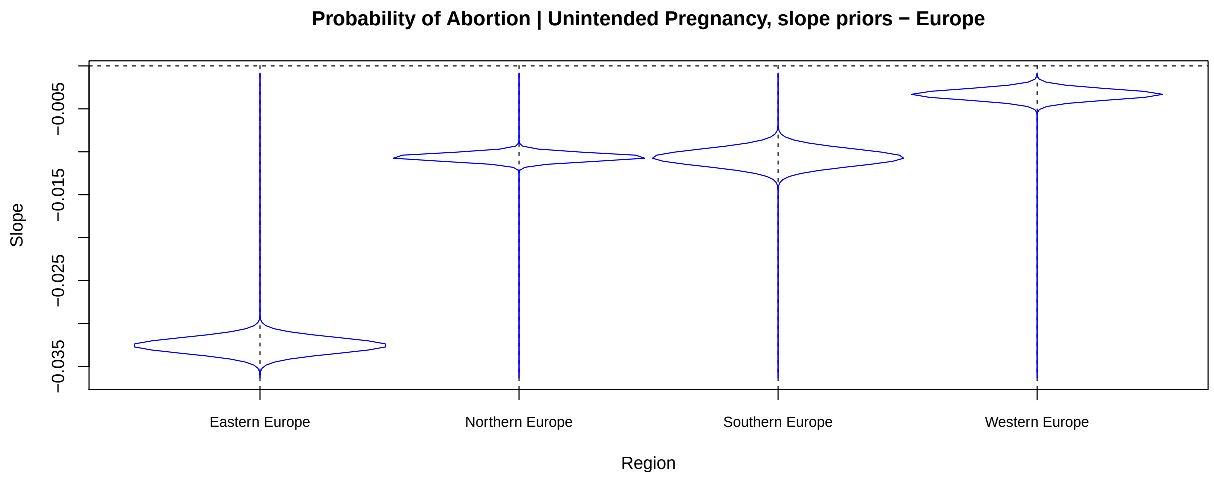
Probability of Abortion | Unintended Pregnancy, slope priors − Europe
Slope
−0.035
−0.025
−0.015
−0.005
Eastern Europe
Northern Europe
Southern Europe
Western Europe
Region

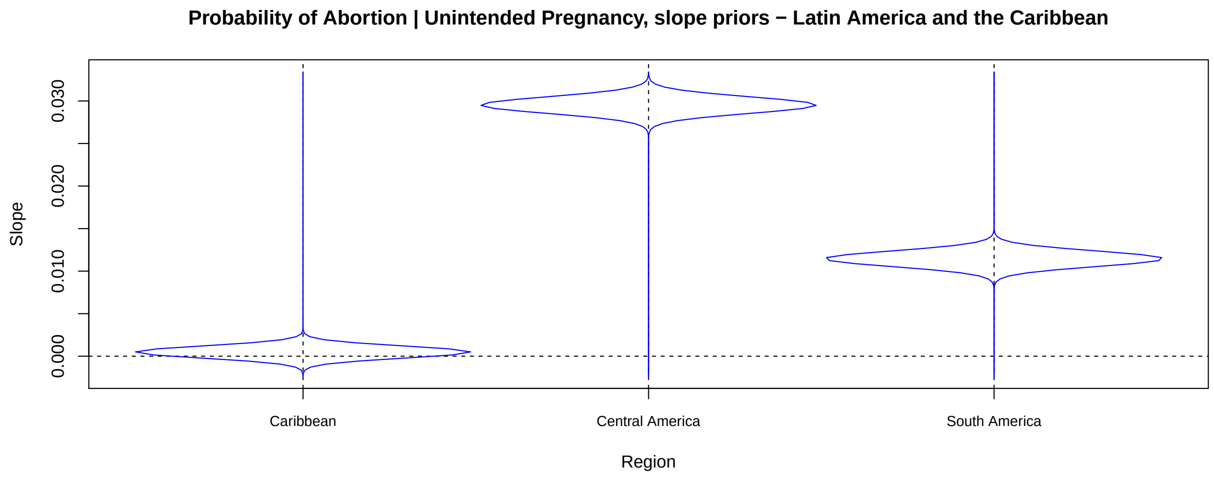
Probability of Abortion | Unintended Pregnancy, slope priors − Latin America and the Caribbean
Slope
0.000
0.010
0.020
0.030
Caribbean
Central America
South America
Region

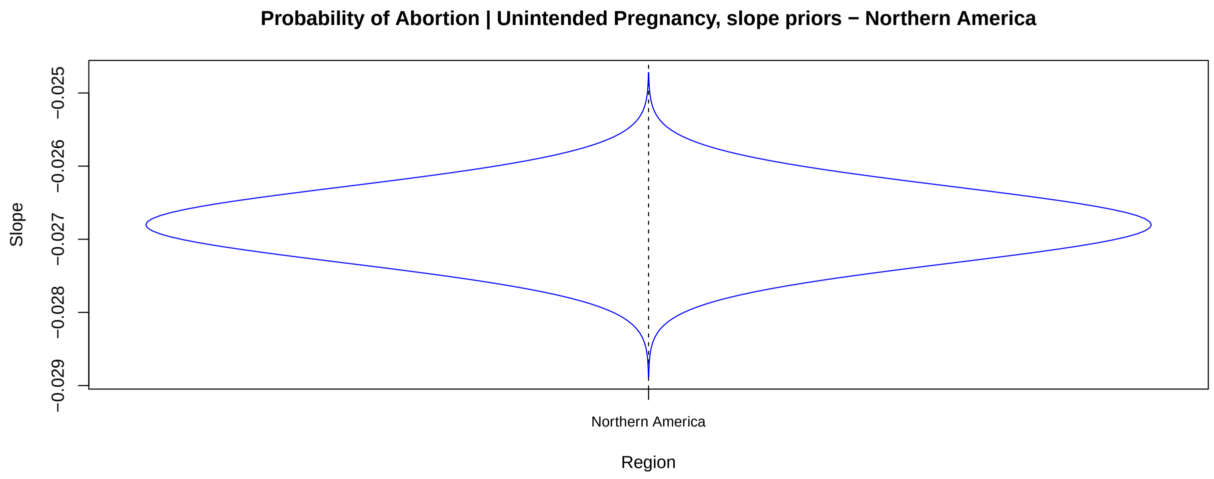
Probability of Abortion | Unintended Pregnancy, slope priors − Northern America
Slope
−0.029
−0.028
−0.027
−0.026
−0.025
Northern America
Region

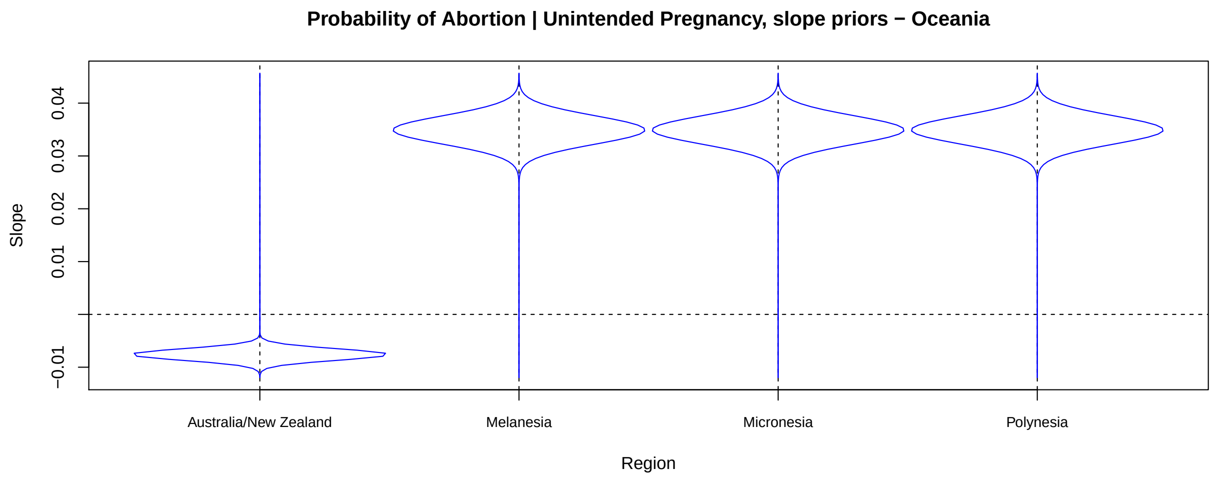
Probability of Abortion | Unintended Pregnancy, slope priors − Oceania
Slope
−0.01
0.01
0.02
0.03
0.04
Australia/New Zealand
Melanesia
Micronesia
Polynesia
Region

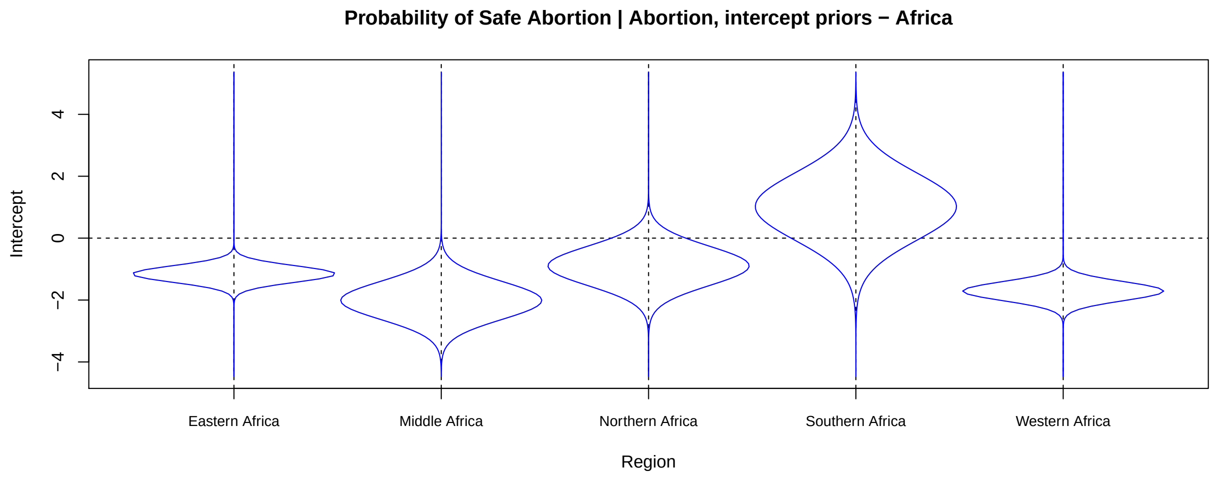
Probability of Safe Abortion | Abortion, intercept priors − Africa
Intercept
−4
−2
0
2
4
Eastern Africa
Middle Africa
Northern Africa
Southern Africa
Western Africa
Region

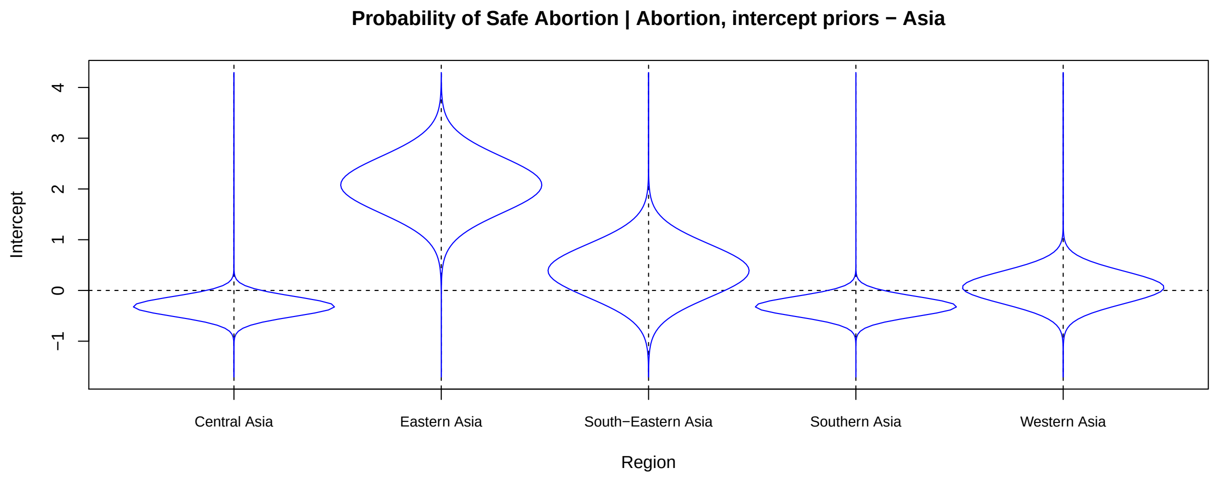
Probability of Safe Abortion | Abortion, intercept priors − Asia
Intercept
−1
0
1
2
3
4
Central Asia
Eastern Asia
South−Eastern Asia
Southern Asia
Western Asia
Region

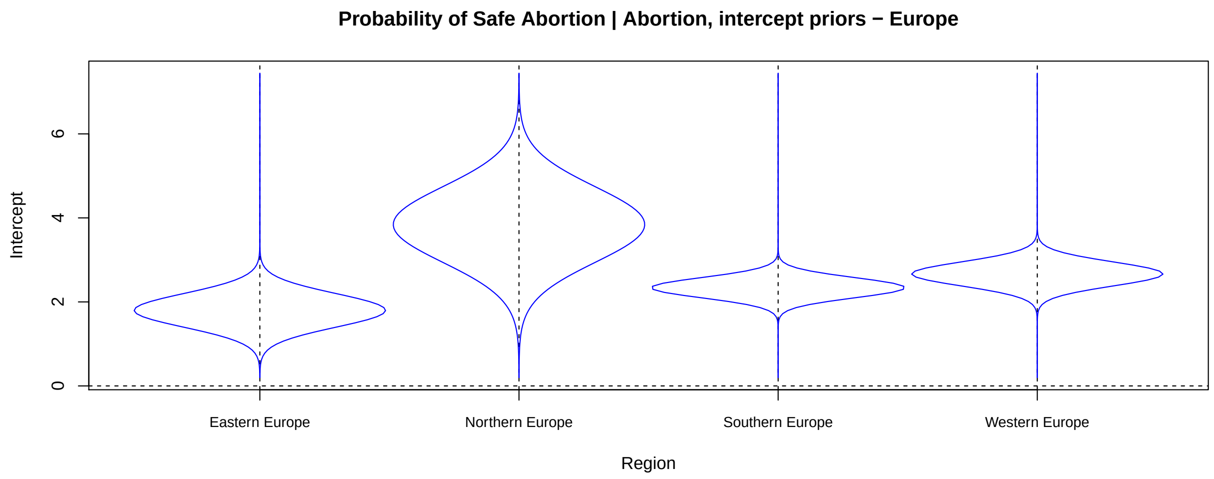
Probability of Safe Abortion | Abortion, intercept priors − Europe
Intercept
0
2
4
6
Eastern Europe
Northern Europe
Southern Europe
Western Europe
Region

Probability of Safe Abortion | Abortion, intercept priors − Latin America and the Caribbean

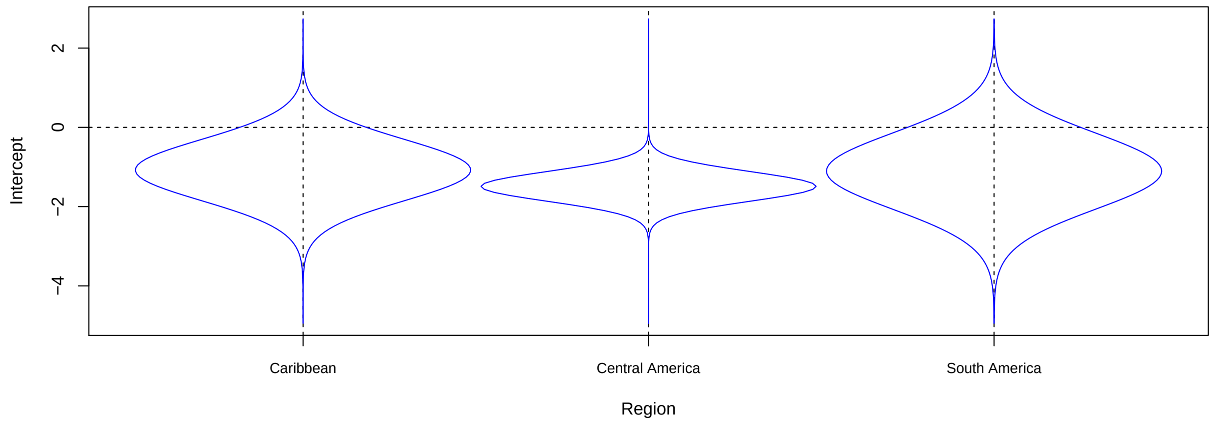

Probability of Safe Abortion | Abortion, intercept priors − Northern America

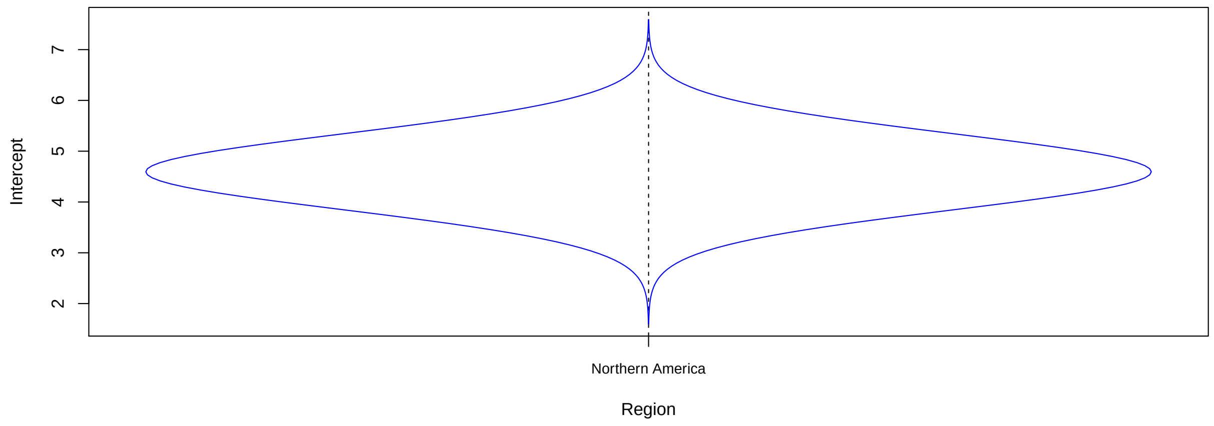

Probability of Safe Abortion | Abortion, intercept priors − Oceania

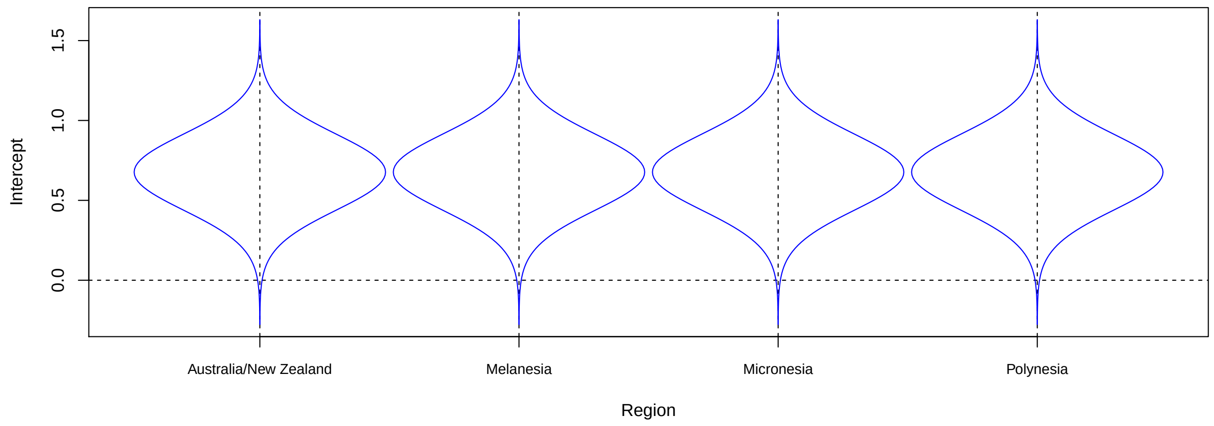

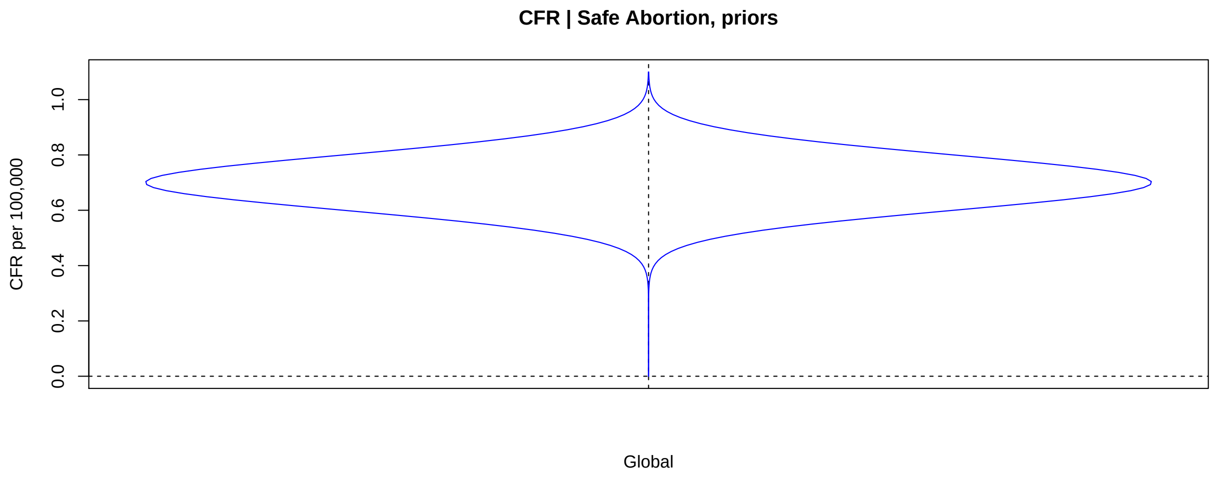
CFR | Safe Abortion, priors
CFR per 100,000
0.0
0.2
0.4
0.6
0.8
1.0
Global

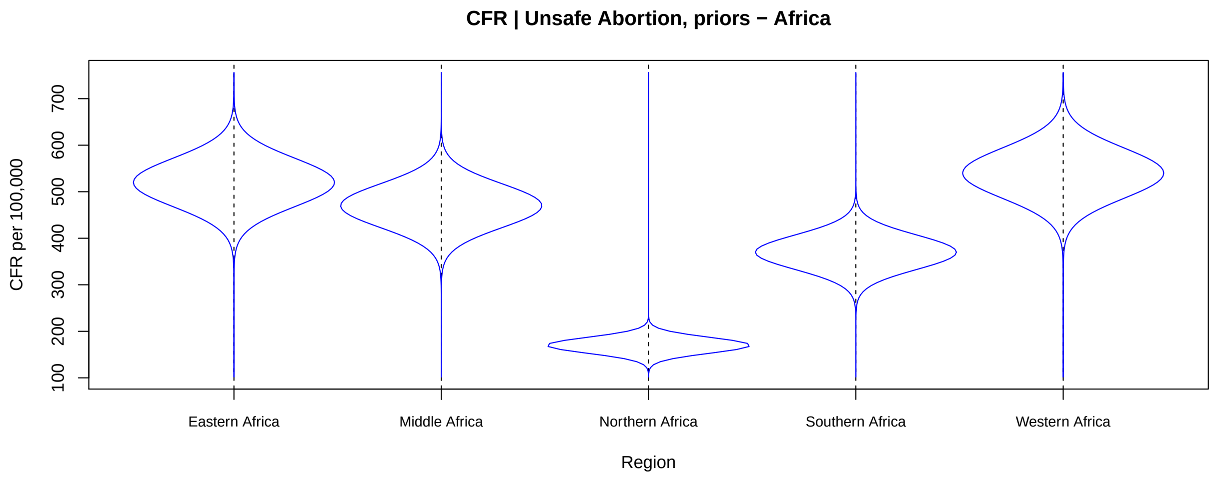
CFR | Unsafe Abortion, priors − Africa
CFR per 100,000
100
200
300
400
500
600
700
Eastern Africa
Middle Africa
Northern Africa
Southern Africa
Western Africa
Region

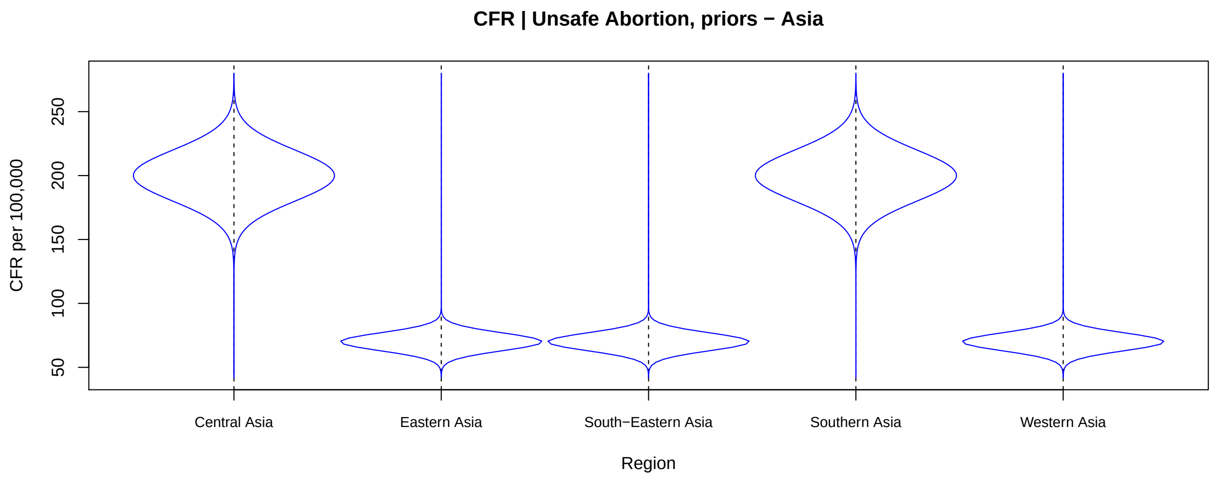
CFR | Unsafe Abortion, priors − Asia
CFR per 100,000
50
100
150
200
250
Central Asia
Eastern Asia
South−Eastern Asia
Southern Asia
Western Asia
Region

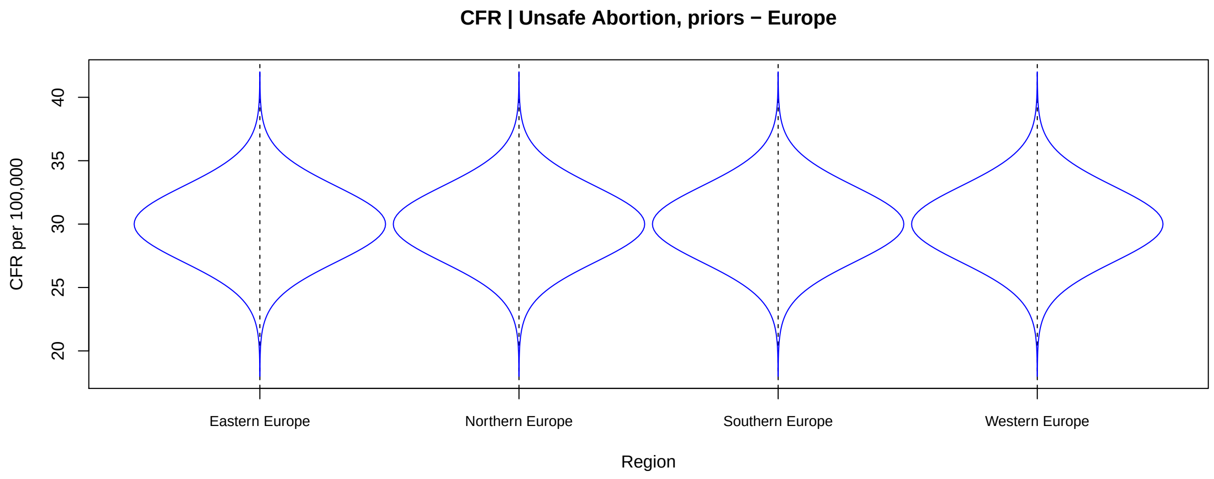
CFR | Unsafe Abortion, priors − Europe
CFR per 100,000
20
25
30
35
40
Eastern Europe
Northern Europe
Southern Europe
Western Europe
Region

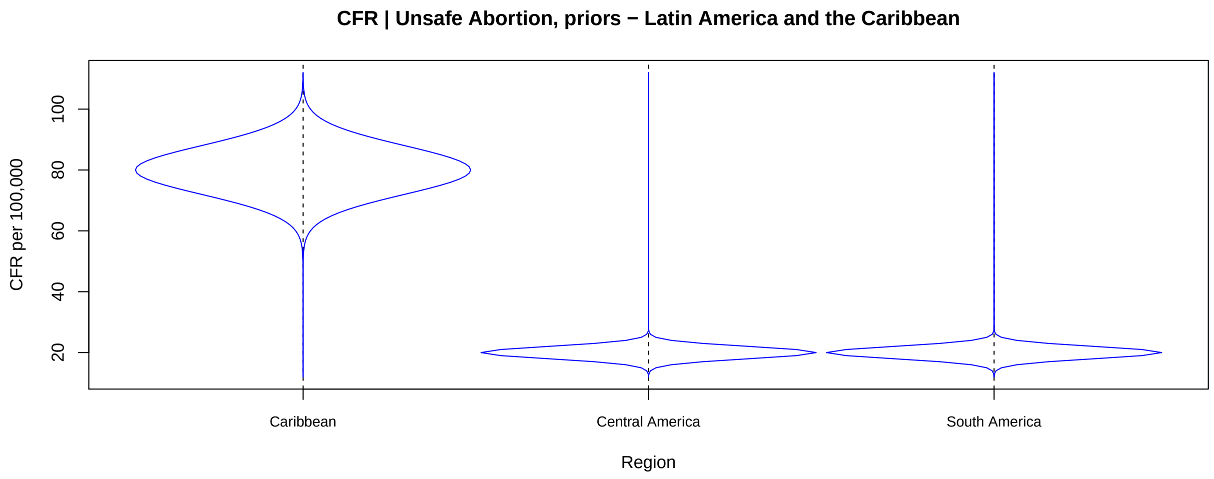
CFR | Unsafe Abortion, priors − Latin America and the Caribbean
CFR per 100,000
20
40
60
80
100
Caribbean
Central America
South America
Region

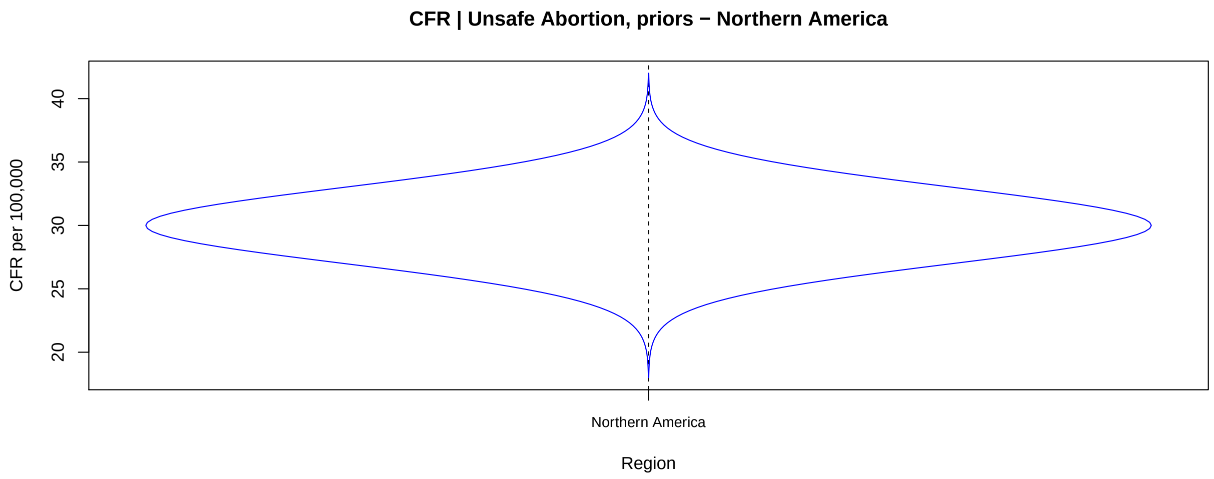
CFR | Unsafe Abortion, priors − Northern America
CFR per 100,000
20
25
30
35
40
Northern America
Region

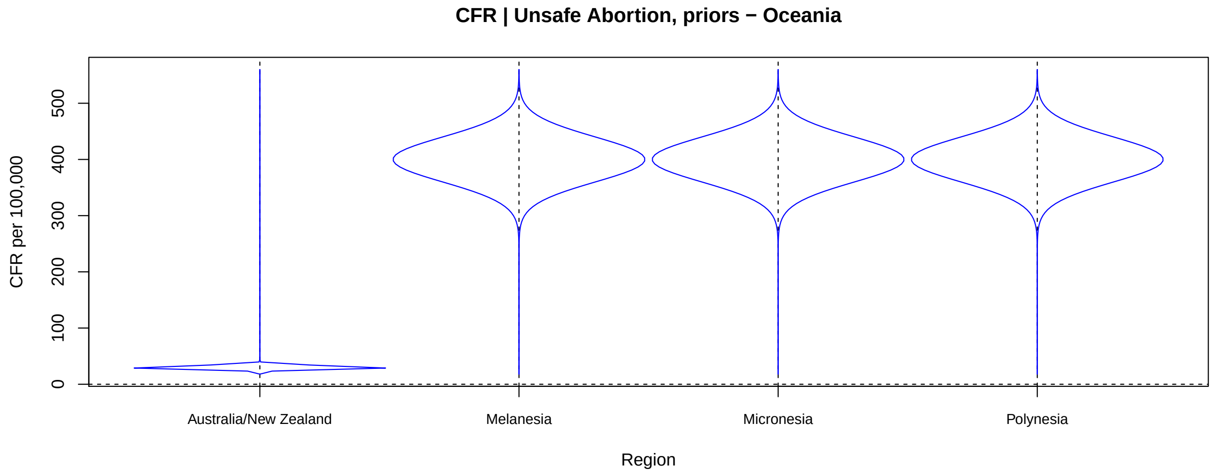

Model Implementation

The chance of abortion is only simulated for women who do not want to become pregnant at the time of conception. The probability of abortion is adjusted if the fetus is a (singleton) female, based on the secondary sex ratio. If an abortion is simulated to occur, the gestational age is then drawn. We make the simplifying assumption that the distribution of gestational age at induced abortion follows that of spontaneous abortion (see Miscarriage). At the time of termination, the abortion is simulated to be either safe or unsafe. Risks of mortality are adjusted by a woman's anemia severity. When sampling anemia RRs we ensured that they were at least 1.0, and non-decreasing by severity.

References

AbouZahr C, Åhman E. Unsafe abortion and ectopic pregnancy. In: Murray CJL, Lopez AD, eds. Health dimensions of sex and reproduction: the global burden of sexually transmitted diseases, HIV, maternal conditions, perinatal disorders, and congenital anomalies. Cambridge, MA, Harvard University Press, 1998.

Åhman E, Dolea C, Shah I. The global burden of unsafe abortion in the year 2000. Global Burden of Disease 2000. Available at: https://www.who.int/healthinfo/statistics/bod_abortions.pdf

Bearak J, Popinchalk A, Ganatra B, et al. Unintended pregnancy and abortion by income, region, and the legal status of abortion: estimates from a comprehensive model for 1990-2019. Lancet Glob Health 2020; 8(9): e1152-e1161.

Brabin BJ, Hakimi M, Pelletier D. An analysis of anemia and pregnancy-related maternal mortality. J Nutr 2001; 131(2S-2): 604S-614S.

Calvert C, Owolabi OO, Yeung F. The magnitude and severity of abortion-related morbidity in settings with limited access to abortion services: a systematic review and meta-regression. BMJ Glob Health 2018; 3(3): e000692.

Ganatra B, Gerdts C, Rossier C, et al. Global, regional, and subregional classification of abortions by safety, 2010-14: estimates from a Bayesian hierarchical model. Lancet 2017; 390(10110): 2372-2381.

Ishoso DK, Tshefu AK, Coppieters Y. Analysis of induced abortion-related complications in women admitted to referral health facilities in Kinshasa, Democratic Republic of the Congo. PLoS One 2018; 13(8): e0203186.

Madziyire MG, Polis CB, Riley T, Sully EA, Owolabi O, Chipato T. Severity and management of postabortion complications among women in Zimbabwe, 2016: a cross-sectional study. BMJ Open 2018; 8(2): e019658.

Männistö J, Mentula M, Bloigu A, Gissler M, Heikinheimo O, Niinimäki M. Induced abortion and future use of IVF treatment; A nationwide register study. PLoS One 2019; 14(11): e0225162.

Raymond EG, Grossman D, Weaver MA, Toti S, Winikoff B. Mortality of induced abortion, other outpatient surgical procedures and common activities in the United States. Contraception 2014; 90(5): 476–9.

Sedgh G, Bearak J, Singh S, et al. Abortion incidence between 1990 and 2014: global, regional, and subregional levels and trends. Lancet 2016; 388(10041): 258-67.

WHO. Unsafe abortion. Global and regional estimates of incidence of unsafe abortion and associated mortality in 2008. 6th edn. Geneva: World Health Organization, 2011. Available: https://www.who.int/reproductive health/publications/unsafe_abortion/9789241501118/en/.

Yokoe R, Rowe R, Choudhury SS, Rani A, Zahir F, Nair M. Unsafe abortion and abortion-related death among 1.8 million women in India. BMJ Glob Health 2019; 4(3): e001491.

Zane S, Creanga AA, Berg CJ, et al. Abortion-related mortality in the United States: 1998–2010. Obstet Gynecol 2015; 126(2): 258–65.

A.1.3.9 Sex Ratio - Secondary

Overview

The secondary sex ratio is the ratio at birth (males/females), which may be higher than the primary sex ratio due to sex-selective abortion, driven by factors such as son preference, readily available technology of prenatal sex determination, and fertility decline (Chao 2019, Chao 2021). In the model, the difference between the secondary and primary sex ratio in each country is used to modify the probability of abortion for female fetuses.

Data

The UN World Population Prospects 2022 includes estimates of sex ratio at birth for each country over time (UN 2022). Estimates range from 1.0 to nearly 1.2 in countries such as China and Azerbaijan where sex-selective abortion may be more prevalent.

Parameters

To estimate priors we fitted hierarchical models by region to trends in the estimated secondary sex ratios between 1990 and 2020.

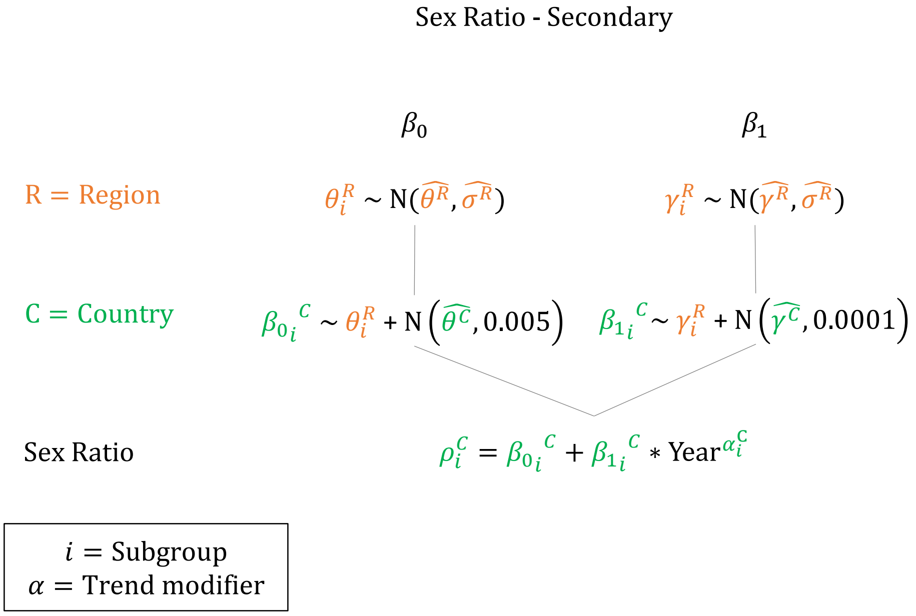

Priors

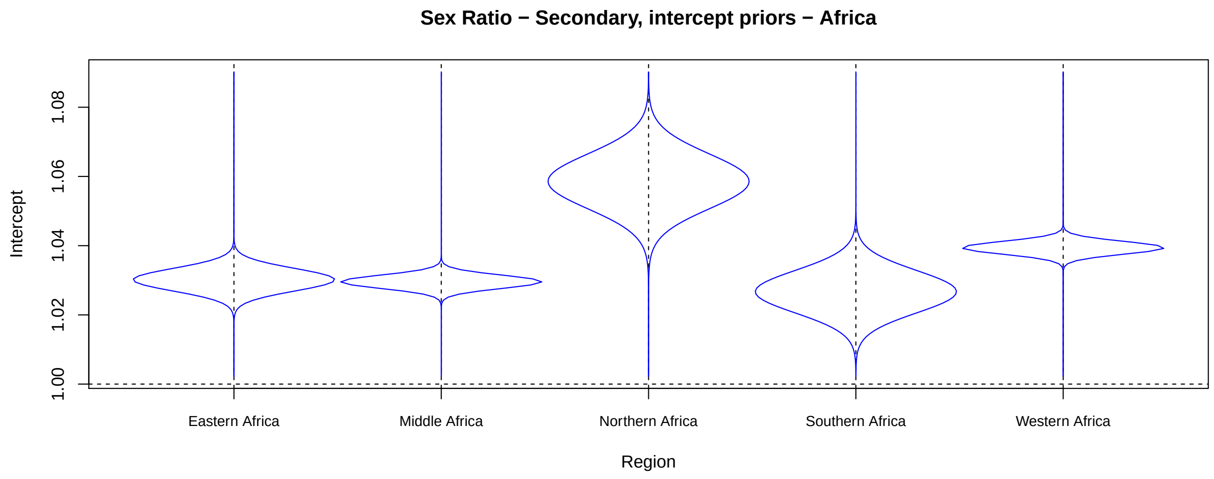
Sex Ratio − Secondary, intercept priors − Africa
Intercept
1.00
1.02
1.04
1.06
1.08
Eastern Africa
Middle Africa
Northern Africa
Southern Africa
Western Africa
Region

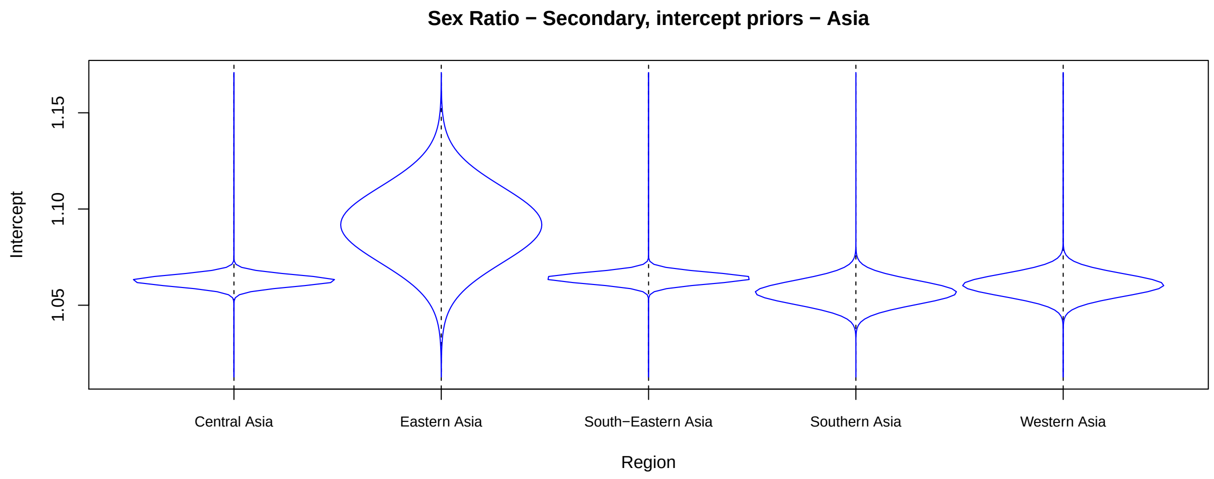
Sex Ratio − Secondary, intercept priors − Asia
Intercept
1.05
1.10
1.15
Central Asia
Eastern Asia
South−Eastern Asia
Southern Asia
Western Asia
Region

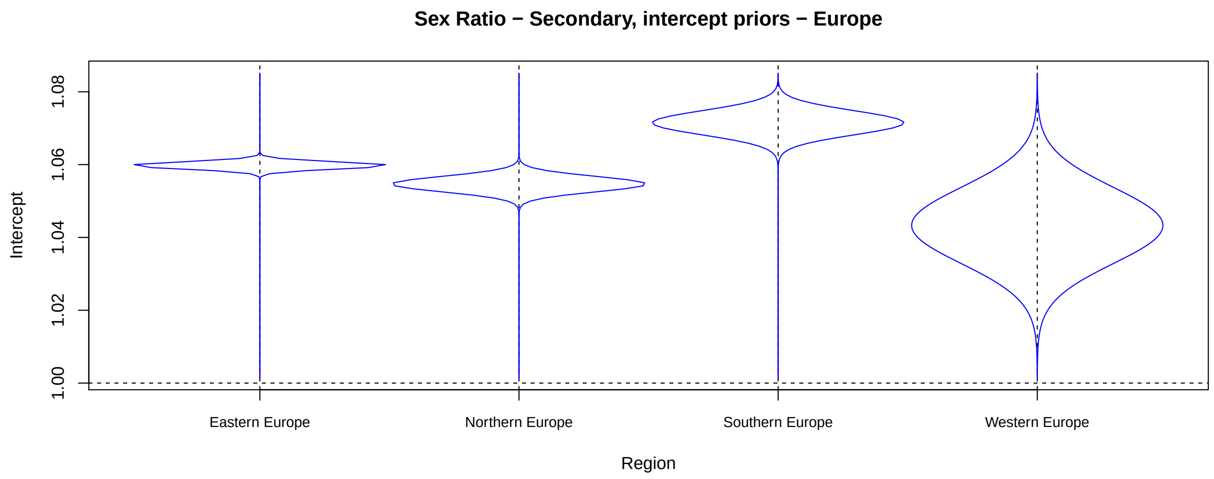
Sex Ratio − Secondary, intercept priors − Europe
Intercept
1.00
1.02
1.04
1.06
1.08
Eastern Europe
Northern Europe
Southern Europe
Western Europe
Region

Sex Ratio − Secondary, intercept priors − Latin America and the Caribbean

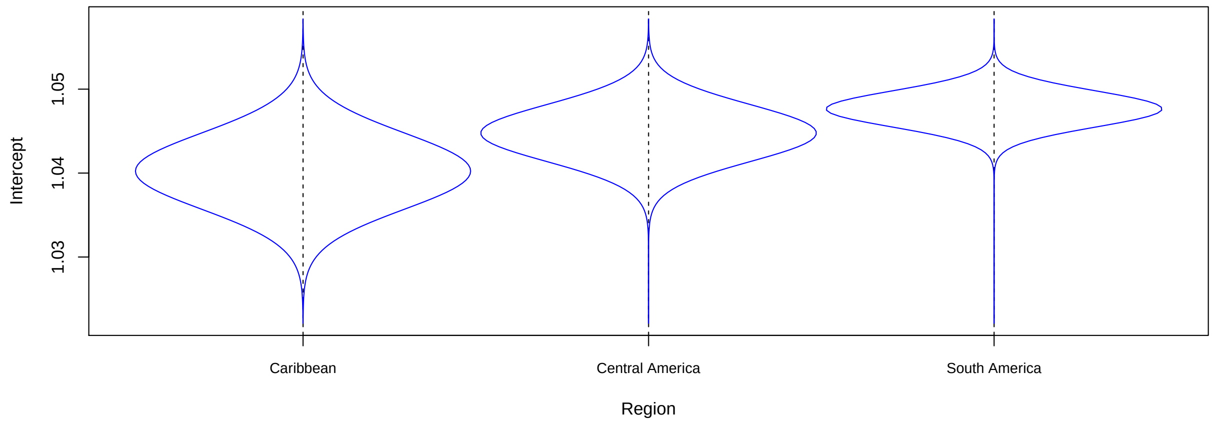

Sex Ratio − Secondary, intercept priors − Northern America

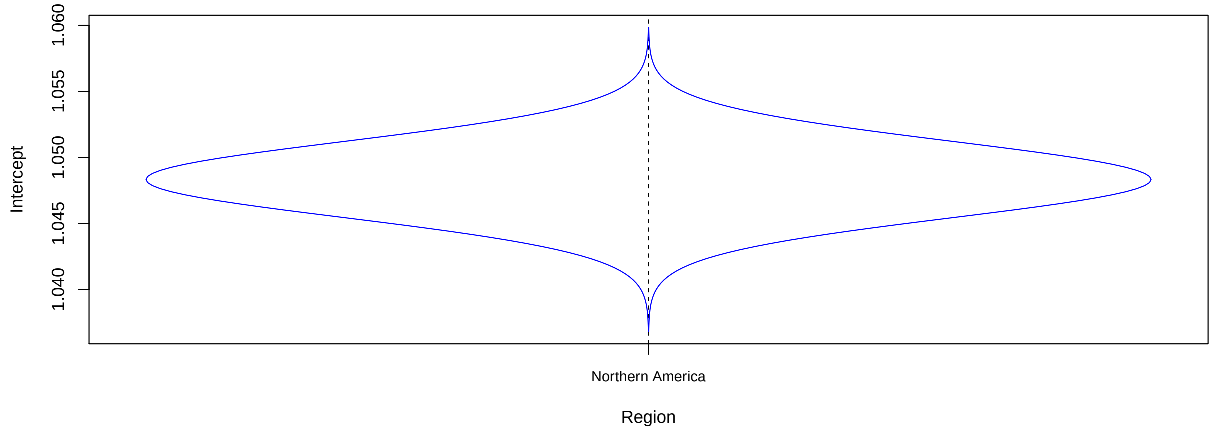

Sex Ratio − Secondary, intercept priors − Oceania

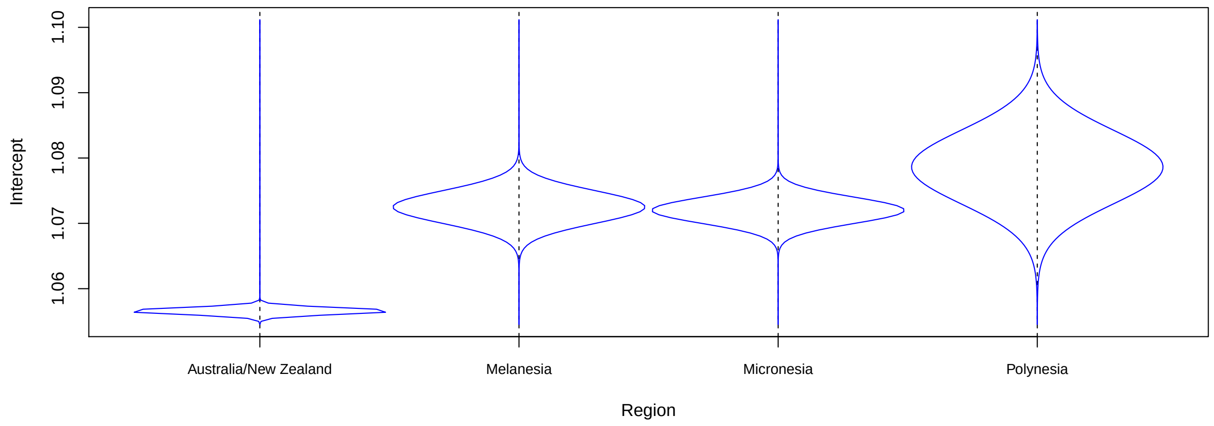

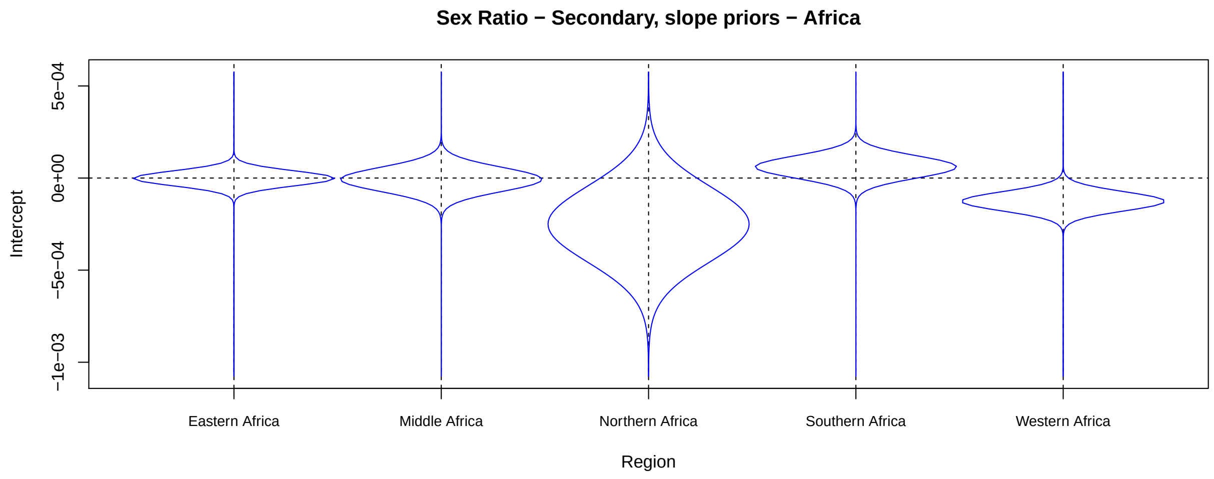
Sex Ratio − Secondary, slope priors − Africa
Intercept
5e−04
0e+00
−5e−04
−1e−03
Eastern Africa
Middle Africa
Northern Africa
Southern Africa
Western Africa
Region

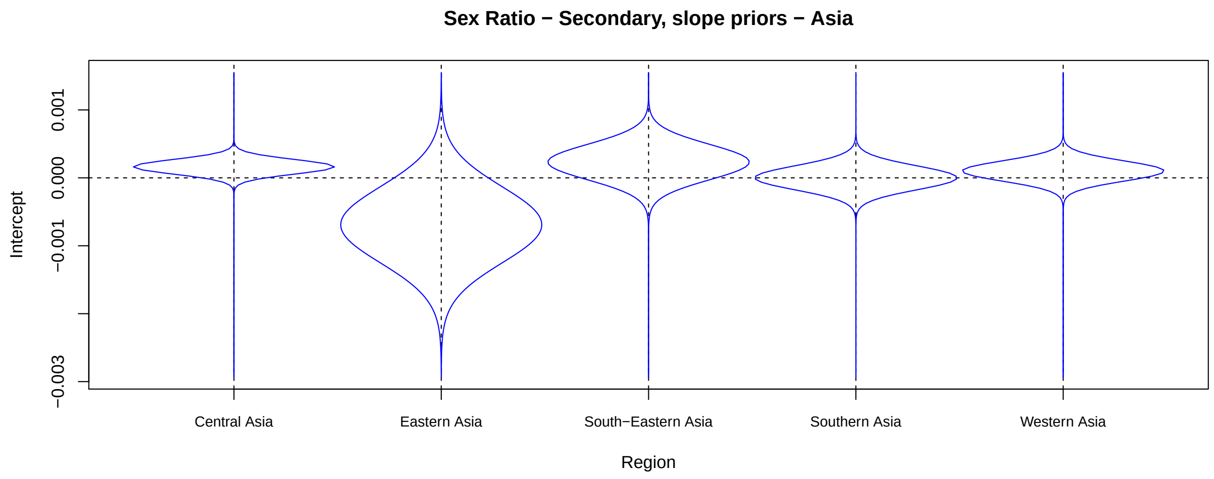
Sex Ratio − Secondary, slope priors − Asia
Intercept
0.001
0.000
−0.001
−0.003
Central Asia
Eastern Asia
South−Eastern Asia
Southern Asia
Western Asia
Region

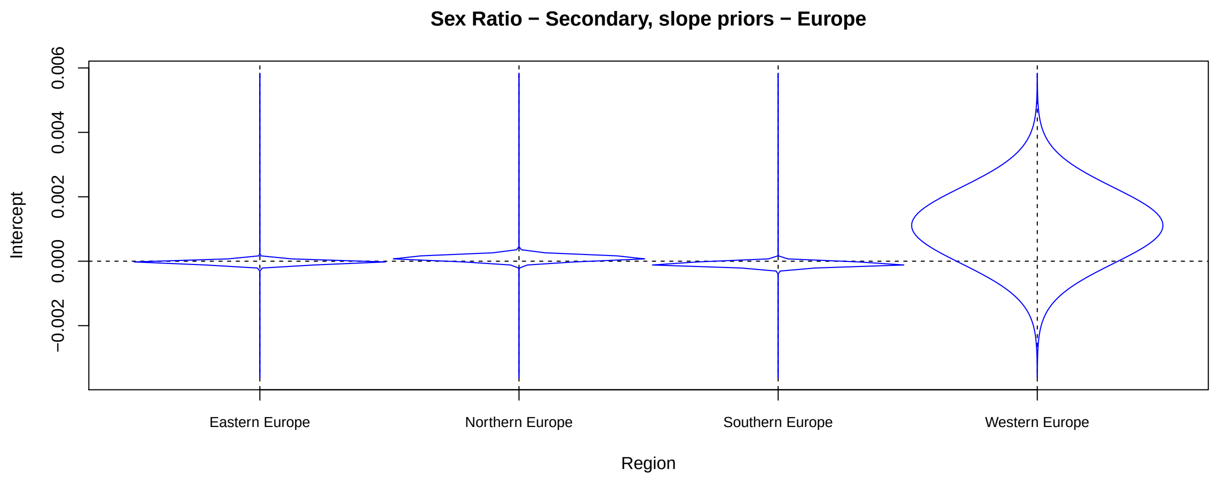
Sex Ratio − Secondary, slope priors − Europe
Intercept
0.006
0.004
0.002
0.000
−0.002
Eastern Europe
Northern Europe
Southern Europe
Western Europe
Region

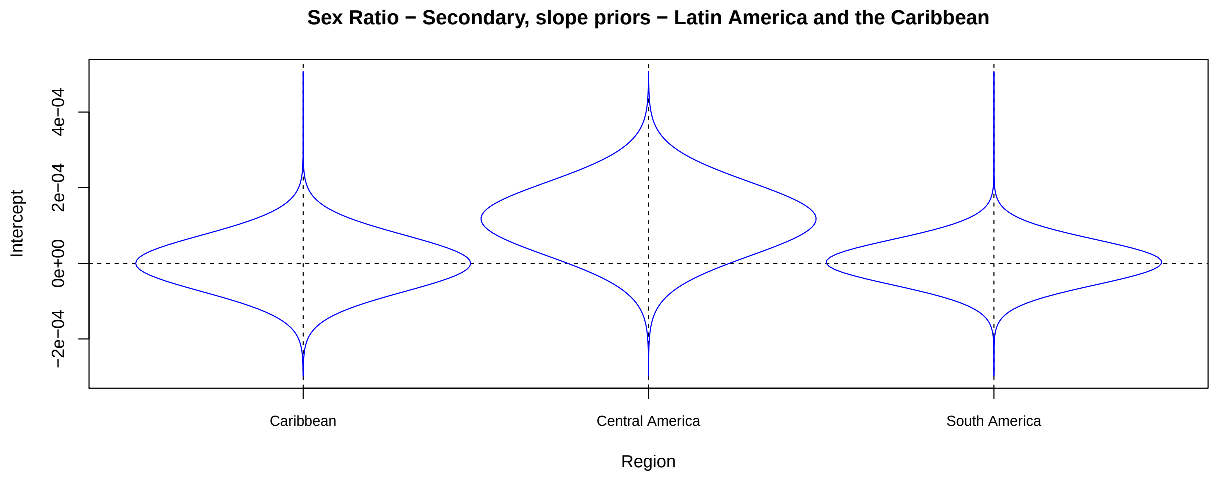

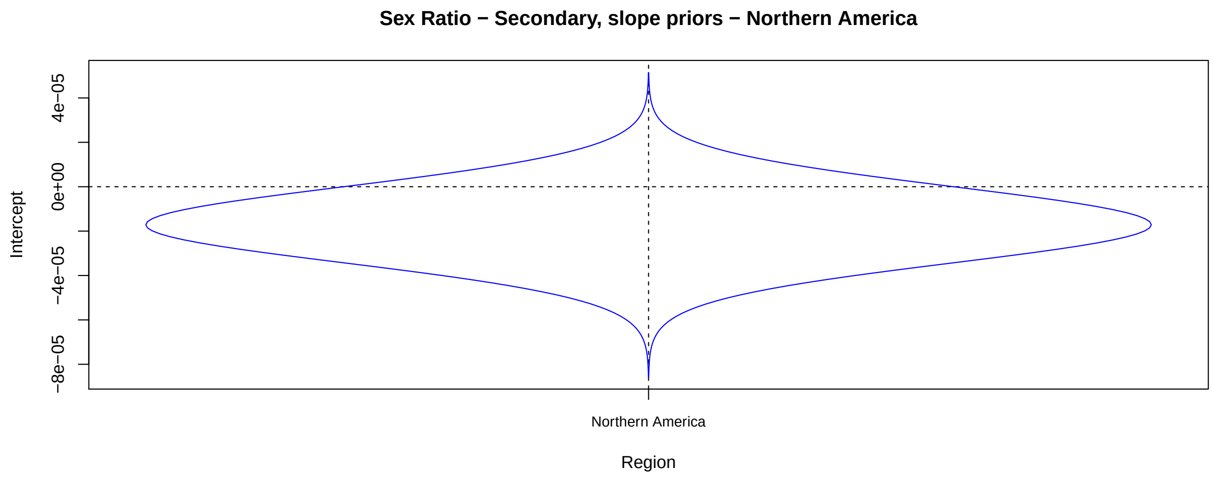

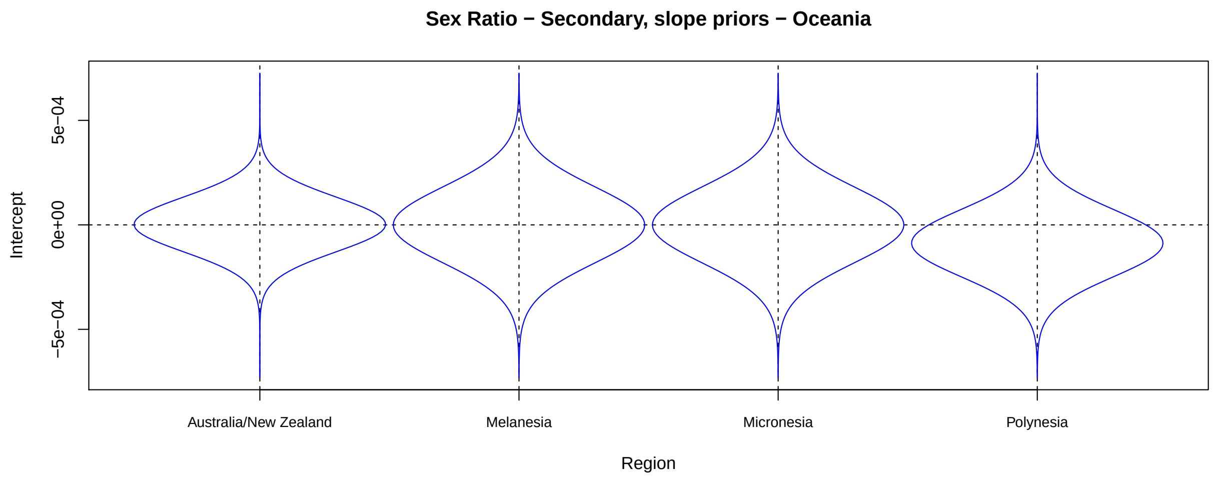

Model Implementation

The difference between the primary and secondary sex ratio is used to modify the risk of abortion for female fetuses. (Note: This only applies to singleton pregnancies in the model.) To estimate the relative risk of sex-selective abortion based on these ratios, let ρ_1 by the primary sex ratio $\frac{m_1}{f_1}$, and ρ_2 by the secondary

sex ratio $\frac{m_2}{f_2}$. Let π by the probability of abortion for males. We then have $\rho_2 = \frac{m_1 * (1-\pi)}{f_1 * (1-\pi*\alpha)}$, where α is the relative risk of abortion for females. Solving for α we have

$$\alpha = \frac{\pi + r - 1}{r\pi}$$

where $r = \frac{\rho_2}{\rho_1}$.

We enforced a floor of 1.0 for the relative risk of sex-selective abortion (α).

References

Chao F, Gerland P, Cook AR, Alkema L. Systematic assessment of the sex ratio at birth for all countries and estimation of national imbalances and regional reference levels. Proc Natl Acad Sci U S A 2019; 116(19): 9303-9311.

Chao F, Gerland P, Cook AR, Alkema L. Global estimation and scenario-based projections of sex ratio at birth and missing female births using a Bayesian hierarchical time series mixture model. Ann Appl Stat 2021; 15(3): 1499-1528.

United Nations, Department of Economic and Social Affairs, Population Division (2022). World Population Prospects 2022, Online Edition. https://population.un.org/wpp/Download/Standard/CSV/

A.1.3.10 Breastfeeding

Overview

Breastfeeding status and duration are simulated in the model after delivery, and are used to determine if women experience protective effects of lactational amenorrhea on risk of pregnancy (see LAM section).

Data

We obtained data compiled by UNICEF on 'any breastfeeding' for 131 countries, and 'exclusive breastfeeding' of infants 0-5 months for 134 countries (UNICEF). Globally, it is estimated that 42% of infants 0-5 months of age were exclusively breastfed in 2018, with variation by region.

Region	Exclusive Breastfeeding (%)
Eastern and Southern Africa	55
South Asia	54
Latin America and the Caribbean	38
North America	35
West and Central Africa	34
Eastern Europe and Central Asia	33
East Asia and the Pacific	30
Middle East and North Africa	30

Parameters

We estimated hierarchical logistic regression models for the probability of any breastfeeding, and of exclusive breastfeeding for infants 0-5 months based on the compiled UNICEF data. We also set priors for the probability of breastfeeding for 6 months (as opposed to 9 months, the maximum duration of LAM efficacy we model) conditional on any breastfeeding. We allowed the sampled parameters to vary by subgroup in the model.

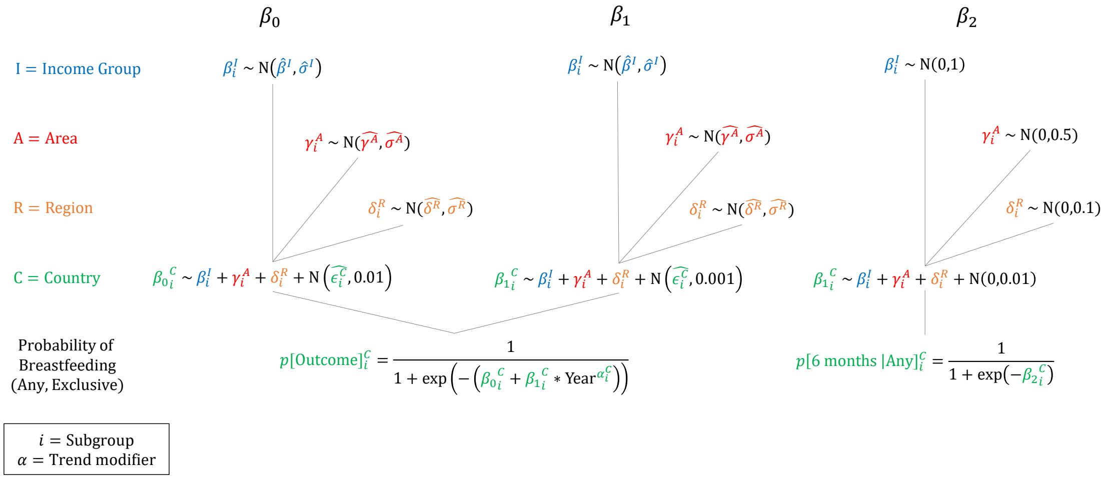

Priors

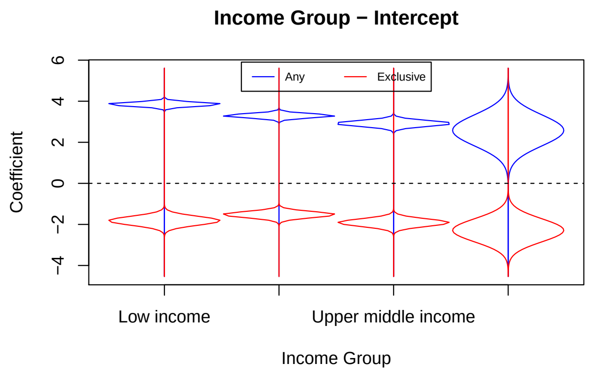
Income Group − Intercept
Any
Exclusive
Coefficient
6
4
2
0
−2
−4
Low income
Upper middle income
Income Group

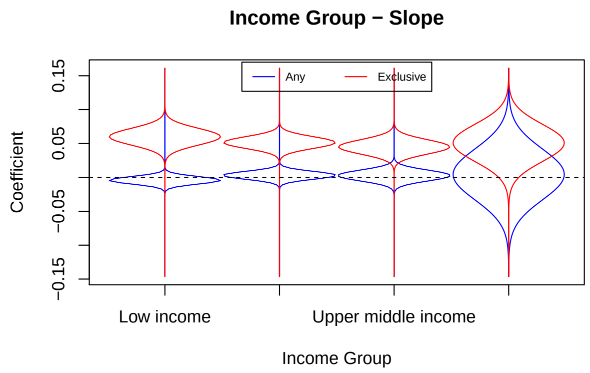
Income Group − Slope
Any
Exclusive
Coefficient
0.15
0.05
−0.05
−0.15
Low income
Upper middle income
Income Group

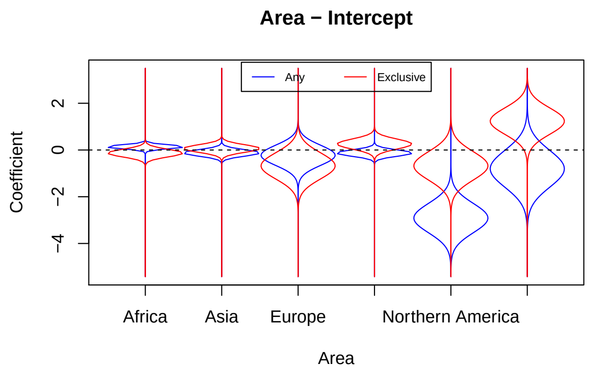
Area − Intercept
Any
Exclusive
Coefficient
2
0
−2
−4
Africa
Asia
Europe
Northern America
Area

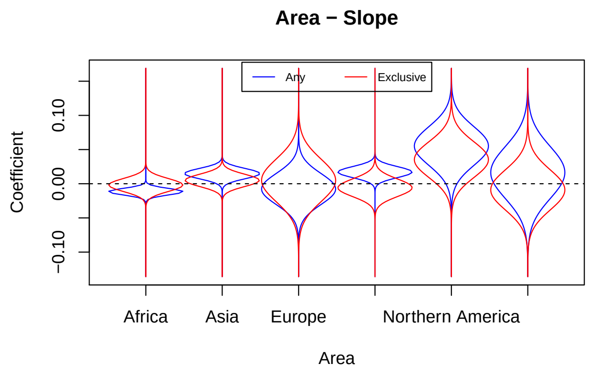
Area − Slope
Any
Exclusive
Coefficient
0.10
0.00
−0.10
Africa
Asia
Europe
Northern America
Area

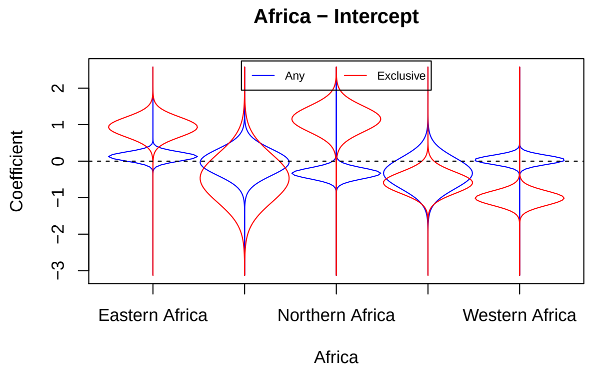
Africa − Intercept
Any
Exclusive
Coefficient
2
1
0
−1
−2
−3
Eastern Africa
Northern Africa
Western Africa
Africa

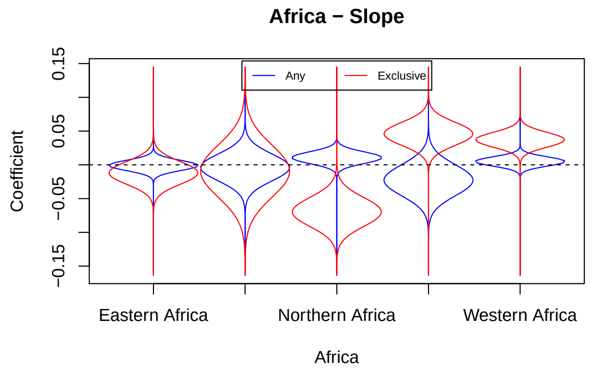
Africa − Slope
Any
Exclusive
Coefficient
0.15
0.05
−0.05
−0.15
Eastern Africa
Northern Africa
Western Africa
Africa

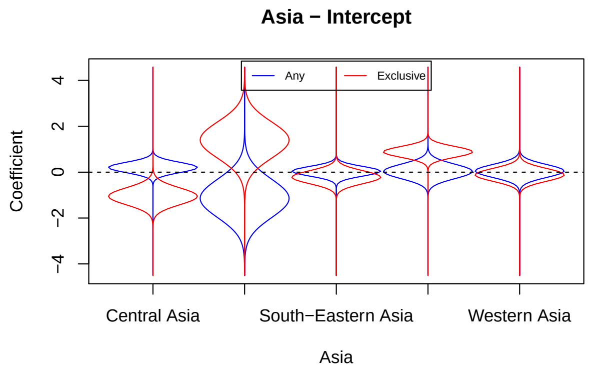
Asia − Intercept
Any
Exclusive
Coefficient
4
2
0
−2
−4
Central Asia
South−Eastern Asia
Western Asia
Asia

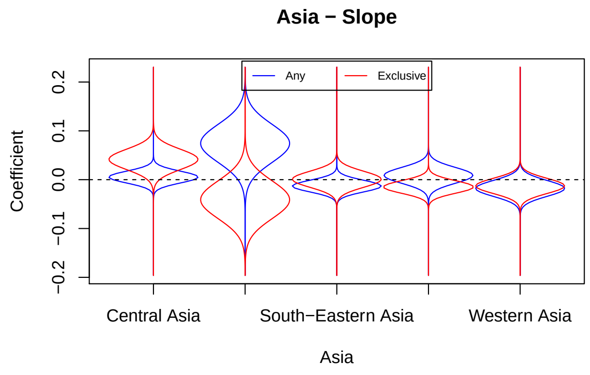
Asia − Slope
Any
Exclusive
Coefficient
0.2
0.1
0.0
−0.1
−0.2
Central Asia
South−Eastern Asia
Western Asia
Asia

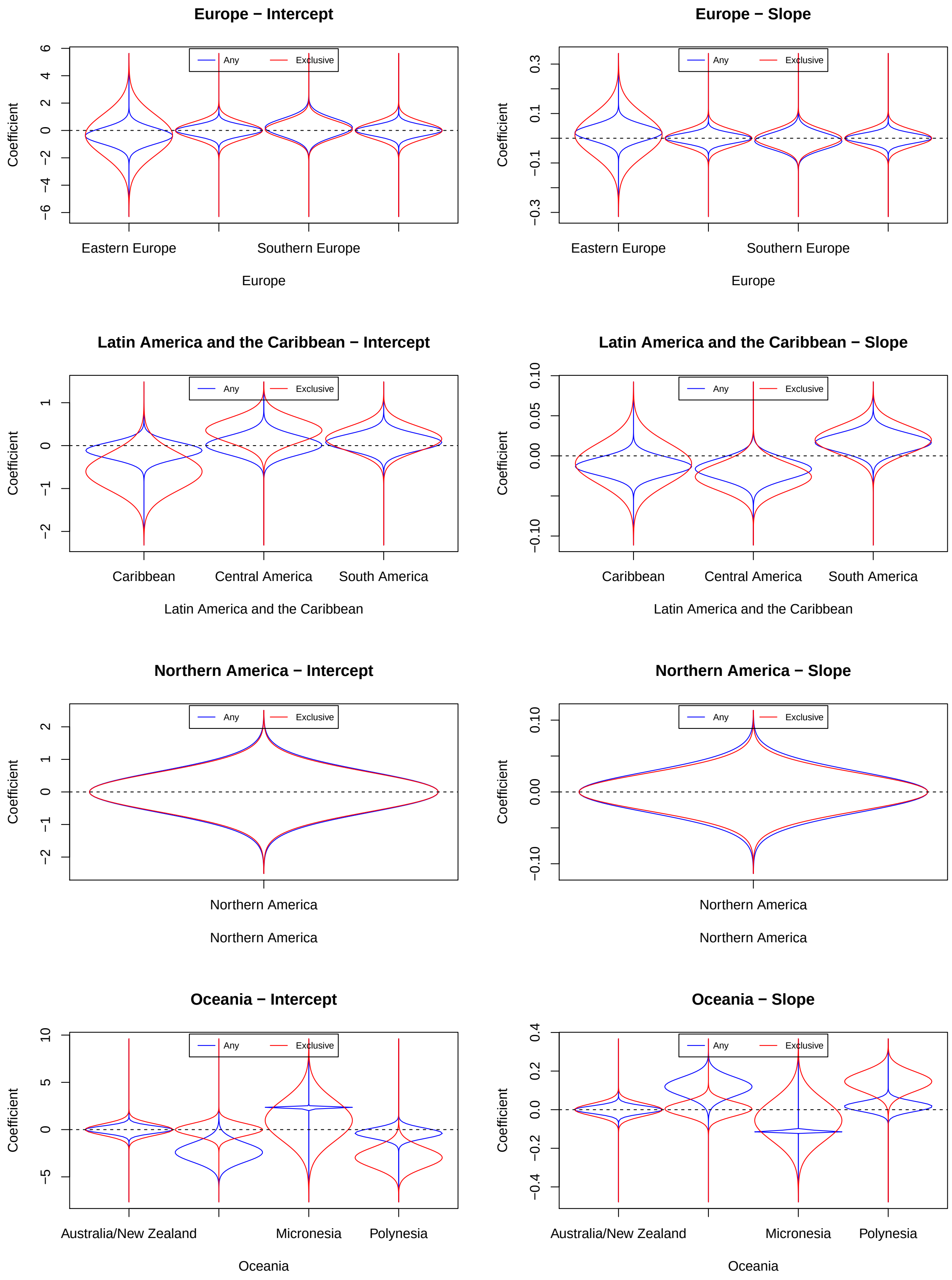
Europe − Intercept
Europe − Slope
Latin America and the Caribbean − Intercept
Latin America and the Caribbean − Slope
Northern America − Intercept
Northern America − Slope
Oceania − Intercept
Oceania − Slope
Any
Exclusive
Coefficient
Eastern Europe
Southern Europe
Europe
Caribbean
Central America
South America
Latin America and the Caribbean
Northern America
Australia/New Zealand
Micronesia
Polynesia
Oceania

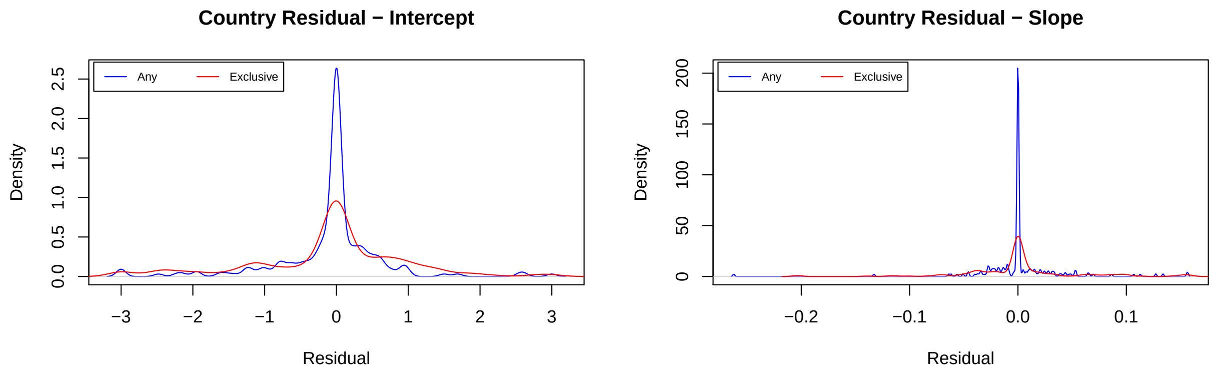

Model Implementation

Breastfeeding status is simulated after live birth delivery in the model. Conditional on any breastfeeding, a status (exclusive/non-exclusive) and duration are assigned. We assume that women who breastfeed exclusively for any duration will then continue to breastfeed non-exclusively for an additional 3 months. If a breastfeeding child dies, we assume that the mother stops breastfeeding and no longer experiences any protective efficacy from LAM. (Note: If a mother had twins, then LAM protective efficacy continues if at least 1 breastfeeding child is still alive.)

References

United Nations Children's Fund, Division of Data, Analysis, Planning and Monitoring (2019). Global UNICEF Global Databases: Infant and Young Child Feeding: Early initiation of breastfeeding, Ever Breastfed, New York, June 2019. Available at: https://data.unicef.org/topic/nutrition/infant-and-young-child-feeding/

United Nations Children's Fund, Division of Data, Analysis, Planning and Monitoring (2019). Global UNICEF Global Databases: Infant and Young Child Feeding: Exclusive breastfeeding, Predominant breastfeeding, New York, June 2019. Available at: https://data.unicef.org/topic/nutrition/infant-and-young-child-feeding/

A.1.4 Health System Parameters

A.1.4.1 Antenatal Care

Overview

Antenatal care (ANC) can be defined as care provided by skilled health-care professionals during pregnancy to ensure the best health conditions for both mother and baby, including: risk identification, prevention and management of pregnancy-related or concurrent diseases, and health education and health promotion (WHO 2016). ANC can reduce maternal (and perinatal) morbidity and mortality directly, through detection and treatment of pregnancy-related complications, and indirectly, through identification of pregnancies at higher risk of developing complications. In addition, ANC also provides an important opportunity to prevent and manage concurrent diseases which may contribute to indirect causes of maternal morbidity and mortality, such as HIV and malaria infections (WHO 2016). Previously, a minimum of four ANC visits was recommended. However, the WHO now recommends a minimum of eight ANC visits (or 'contacts') as the four-visit focused ANC model does not offer women adequate contact with health-care practitioners (WHO 2018).

Data

We analyzed DHS data on ANC attendance from 1,713,875 women from 264 surveys in 75 countries. Here we plot the mean number of ANC visits by year for each subgroup.

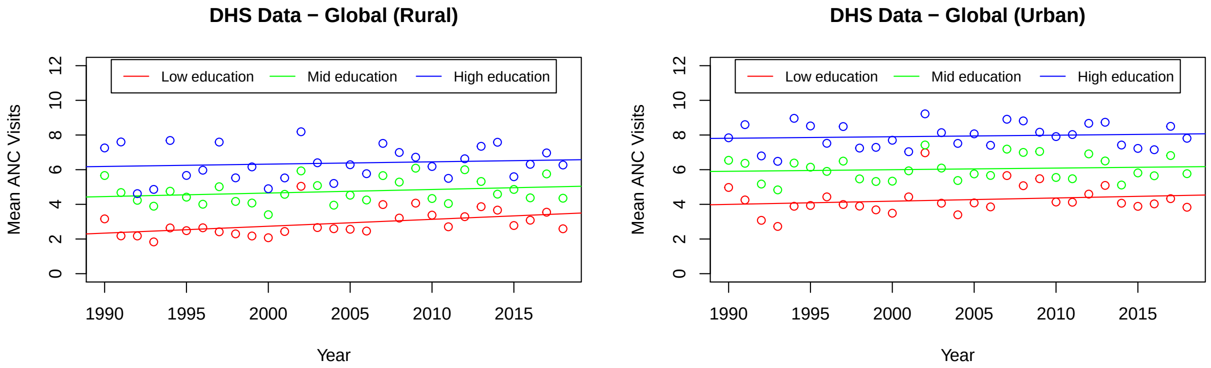

Anemia It is estimated that about half of anemia in pregnant women is amenable to iron supplementation (WHO 2015), however, this may be quite variable and is likely much lower in malaria-endemic areas. The WHO recommends daily oral iron and folic acid supplementation with 30 mg to 60 mg of elemental iron and 400 μg folic acid (WHO 2016). In settings where anemia in pregnant women is a severe public health problem, a daily dose of 60 mg of elemental iron is preferred (WHO 2016). If a woman is diagnosed with anemia during pregnancy, her daily elemental iron should be increased to 120 mg until her Hb concentration rises to normal (WHO 2016). A WHO review of the literature finds that low-certainty evidence shows that daily iron supplementation may reduce the risk of anemia at term (Hb <100g/L at 37 weeks of gestation or later) (14 trials, 2199 women; RR: 0.30, 95% CI: 0.19-0.46) and severe postpartum anemia (Hb <80g/L) (8 trials, 1339 women; RR: 0.04, 95% CI: 0.01-0.28) (WHO 2016). Low-certainty evidence also shows that daily iron supplementation may increase maternal Hb concentration at or near term (34 weeks of gestation or more) (19 trials, 3704 women; MD: 8.88 g/L higher, 95% CI: 6.96-10.8 g/L) (WHO 2016).

Complication recognition ANC provides a unique opportunity to educate women on how to recognize complications during pregnancy, delivery, and postpartum, and a number of studies have found a positive association between ANC visits and knowledge of obstetric danger signs. A study in Madagascar found that receiving information about danger signs at ANC was associated with better knowledge of danger signs during delivery, with an adjusted odds ratio of 1.50 (95% CI 0.90-2.49) (Salem 2018). A study in Bangladesh found that the odds of being "well prepared" was six times (OR 5.98, 95% CI: 3.85-9.28) higher for women with four or more ANC visits, compared to women with none or one ANC visit (Pervin 2018). A study in Ghana found that pregnant women learn about danger signs from a range of providers, and regular contact with formal providers typically coincided with increased knowledge of danger signs (Aborigo 2014). A study in India found that adequate prenatal care was associated with better knowledge regarding the danger signs

of pregnancy, with an odds ratio of 2.72 (95% CI 1.03-7.16) (Gopalakrishnan 2019). Another study assessing birth preparedness and complication readiness found an odds ratio for 4+ ANC visits of 2.4 (95% CI 0.83-6.96) (Akshaya 2017). In Tanzania, studies controlling for confounding and clustering effects found an AOR of 4+ ANC visits of 1.94 (95% CI 1.17-3.21) for birth preparedness and complication readiness (Bintabara 2015), and 1.4 (95% CI 1.1-1.9) for awareness of danger signs of obstetric complications (Pembe 2009).

Parameters

We used a two-part hierarchical Poisson model to estimate the number of ANC visits based on DHS data. We used upper middle income priors for high income countries due to lack of DHS data in high income countries. We also model the impact of ANC visits on anemia prevalence via an improvement in Hb concentration. We set priors based on WHO estimates that daily iron supplementation increases Hb concentration by 8.88 g/L (95% CI 6.96-10.80), assuming a linear relationship between number of ANC visits and and probability/efficacy of iron supplementation. Given that the mean number of ANC visits reported in the DHS data was 5.4, we re-scaled the Hb increase to be per ANC visit when sampling in the model. To model the impact of ANC visits on the probability of recognizing a complication, we set priors for a coefficient per ANC visit that is added to the hierarchical logistic regression model of recognition/referral. We assume that ANC only improves recognition/referral for women delivering at Home.

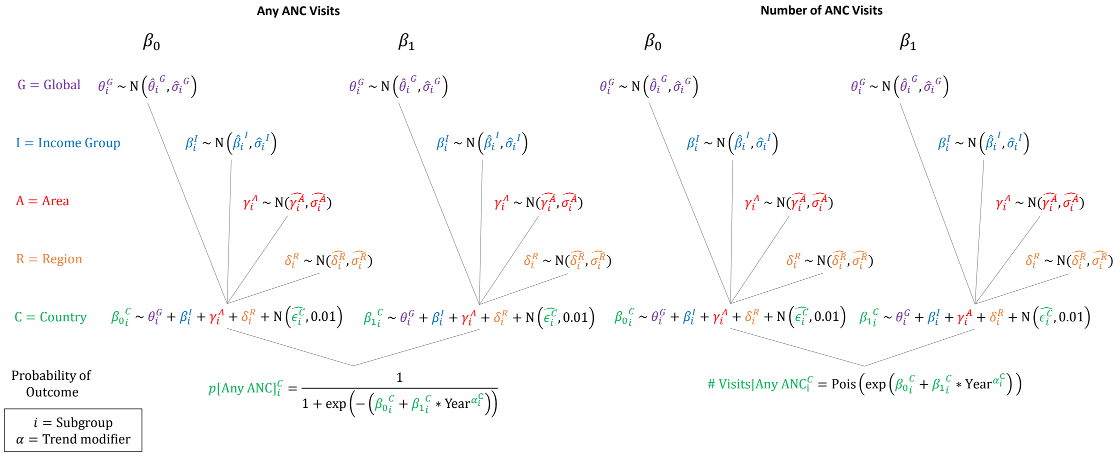

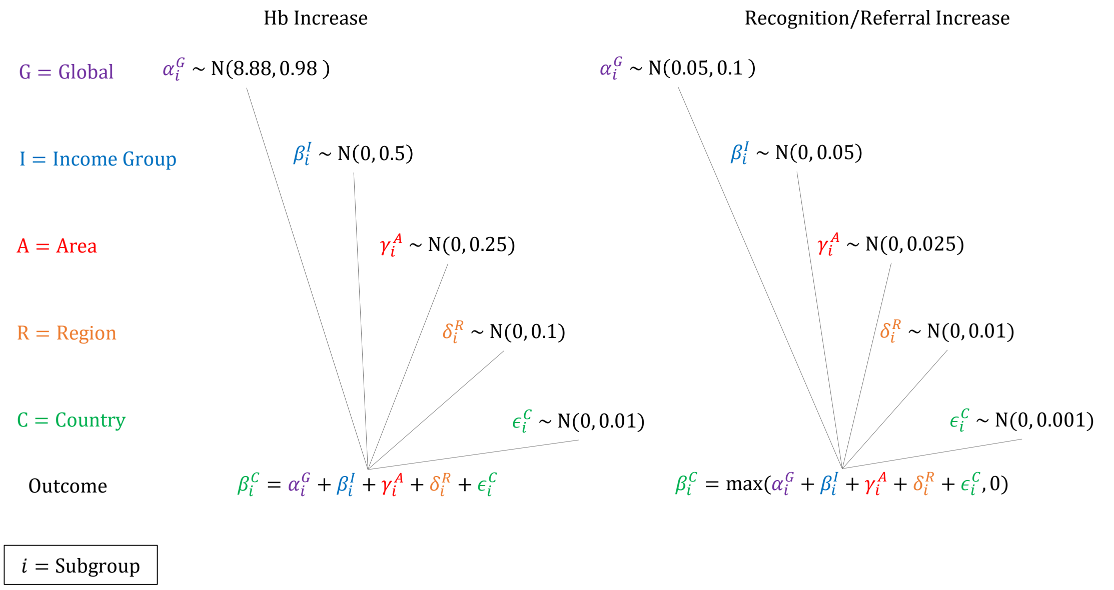
Hb Increase
Recognition/Referral Increase
G = Global
$\alpha_i^G \sim N(8.88, 0.98)$
$\alpha_i^G \sim N(0.05, 0.1)$
I = Income Group
$\beta_i^I \sim N(0, 0.5)$
$\beta_i^I \sim N(0, 0.05)$
A = Area
$\gamma_i^A \sim N(0, 0.25)$
$\gamma_i^A \sim N(0, 0.025)$
R = Region
$\delta_i^R \sim N(0, 0.1)$
$\delta_i^R \sim N(0, 0.01)$
C = Country
$\epsilon_i^C \sim N(0, 0.01)$
$\epsilon_i^C \sim N(0, 0.001)$
Outcome
$\beta_i^C = \alpha_i^G + \beta_i^I + \gamma_i^A + \delta_i^R + \epsilon_i^C$
$\beta_i^C = \max(\alpha_i^G + \beta_i^I + \gamma_i^A + \delta_i^R + \epsilon_i^C, 0)$
i = Subgroup

Priors

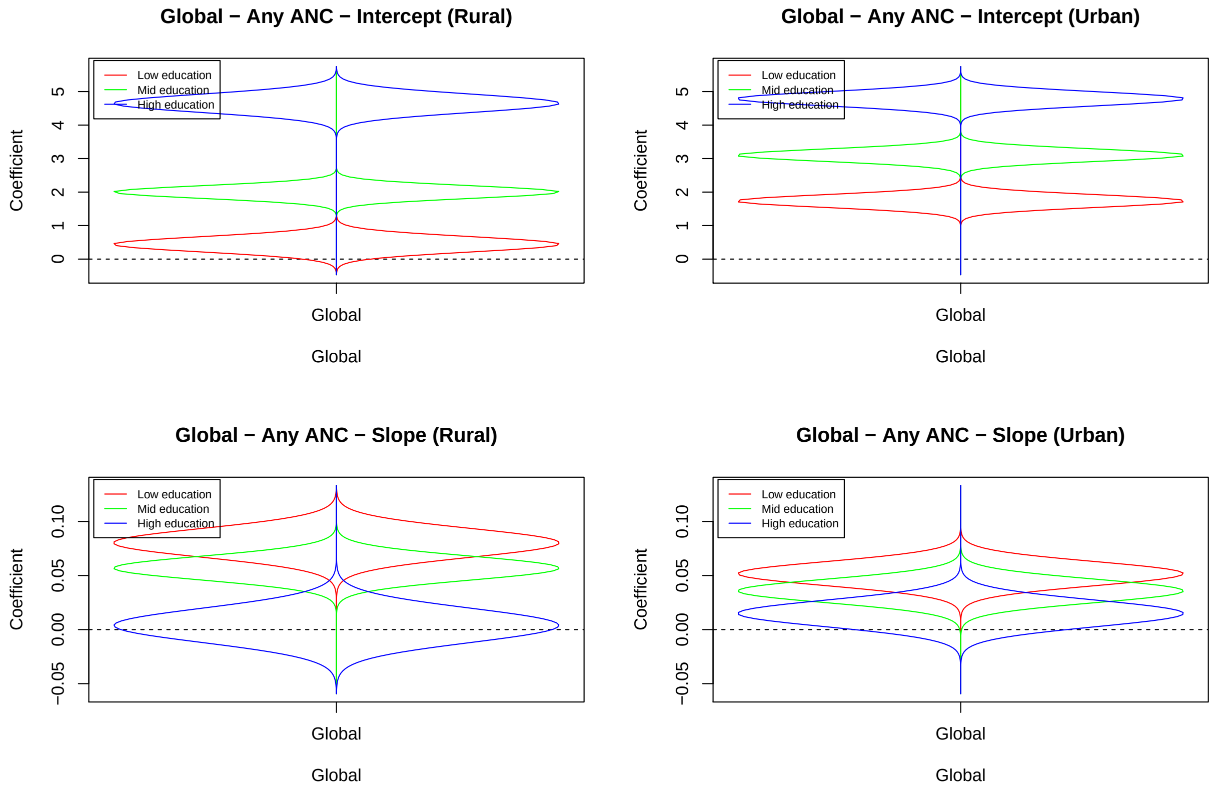
Global − Any ANC − Intercept (Rural)
Global − Any ANC − Intercept (Urban)
Global − Any ANC − Slope (Rural)
Global − Any ANC − Slope (Urban)
Low education
Mid education
High education
Coefficient
Global
Global

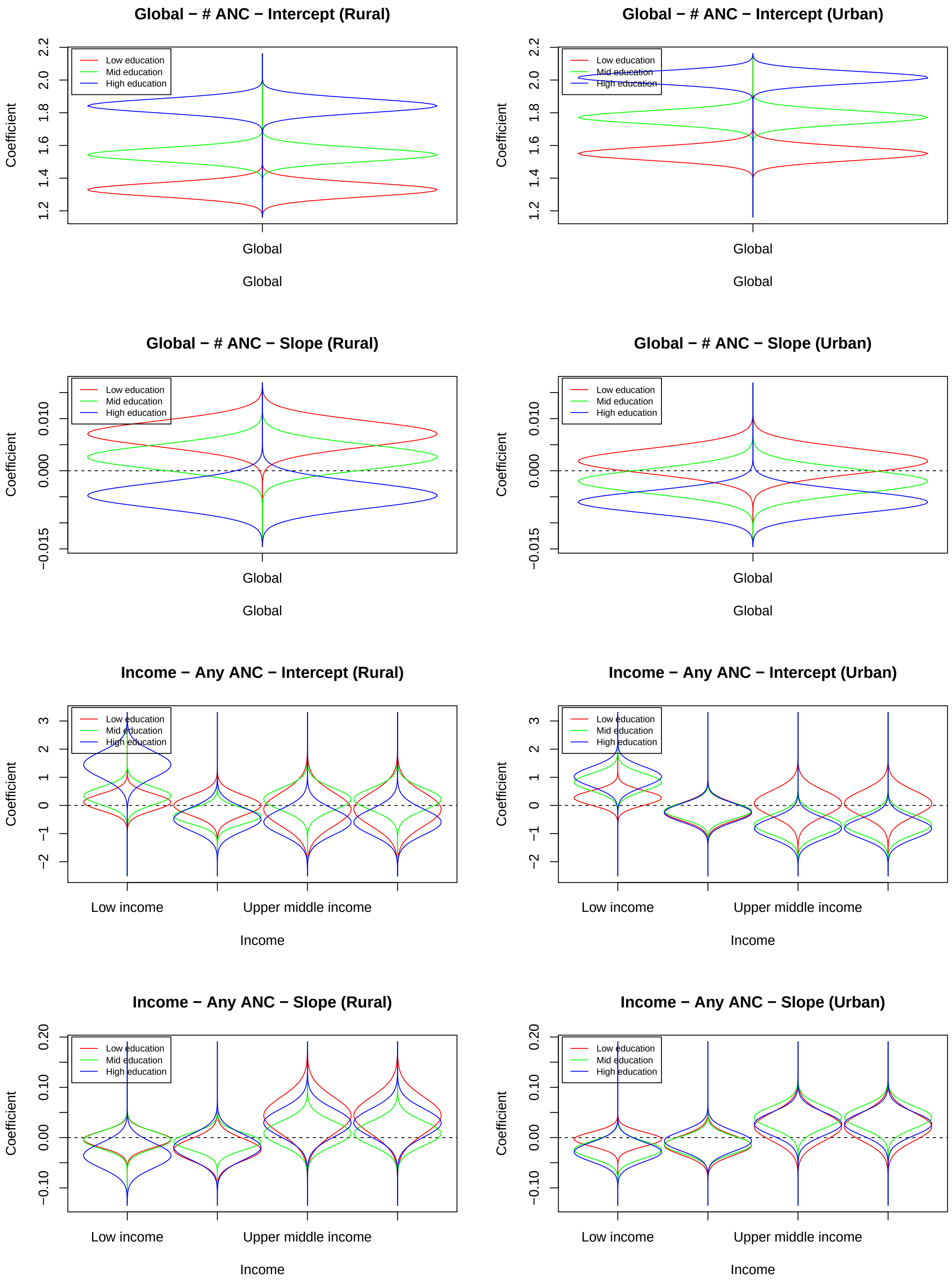
Global − # ANC − Intercept (Rural)
Global − # ANC − Intercept (Urban)
Global − # ANC − Slope (Rural)
Global − # ANC − Slope (Urban)
Income − Any ANC − Intercept (Rural)
Income − Any ANC − Intercept (Urban)
Income − Any ANC − Slope (Rural)
Income − Any ANC − Slope (Urban)
Low education
Mid education
High education
Coefficient
Global
Low income
Upper middle income
Income

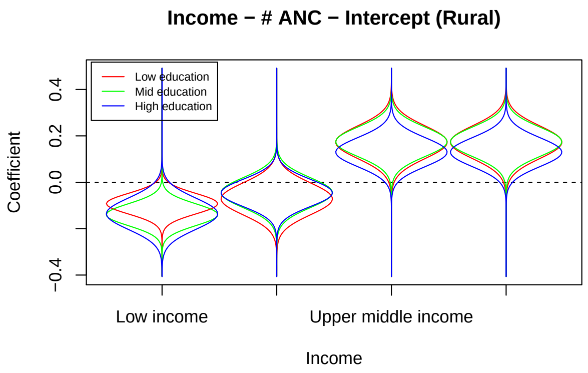
Income − # ANC − Intercept (Rural)
Low education
Mid education
High education
Coefficient
−0.4
0.0
0.2
0.4
Low income
Upper middle income
Income

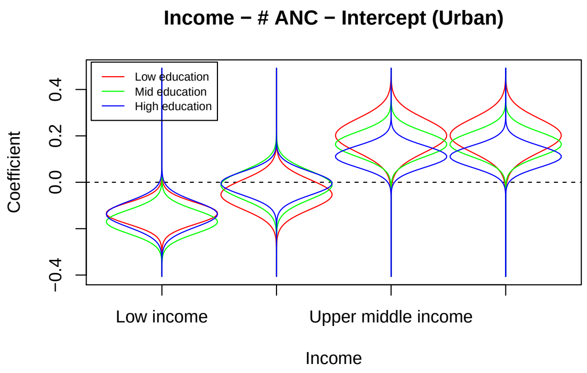
Income − # ANC − Intercept (Urban)
Low education
Mid education
High education
Coefficient
−0.4
0.0
0.2
0.4
Low income
Upper middle income
Income

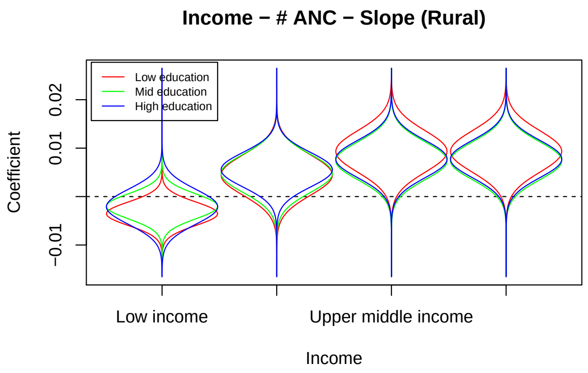
Income − # ANC − Slope (Rural)
Low education
Mid education
High education
Coefficient
−0.01
0.01
0.02
Low income
Upper middle income
Income

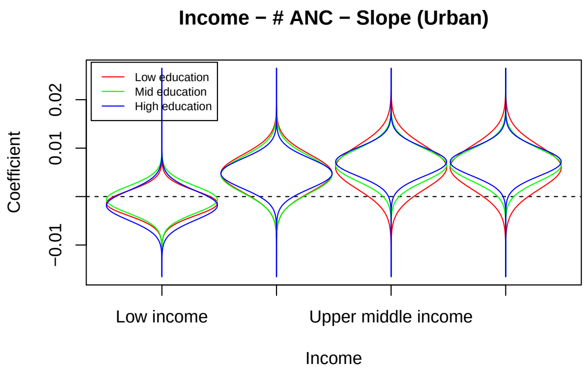
Income − # ANC − Slope (Urban)
Low education
Mid education
High education
Coefficient
−0.01
0.01
0.02
Low income
Upper middle income
Income

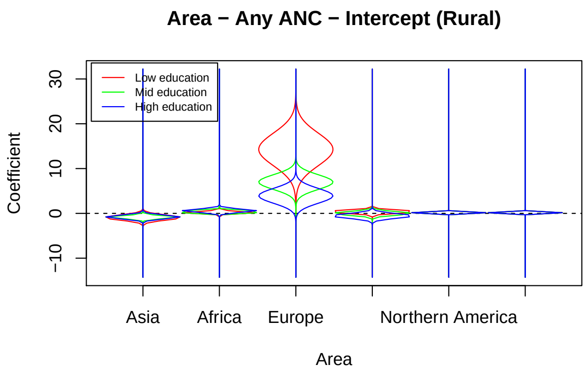
Area − Any ANC − Intercept (Rural)
Low education
Mid education
High education
Coefficient
−10
0
10
20
30
Asia
Africa
Europe
Northern America
Area

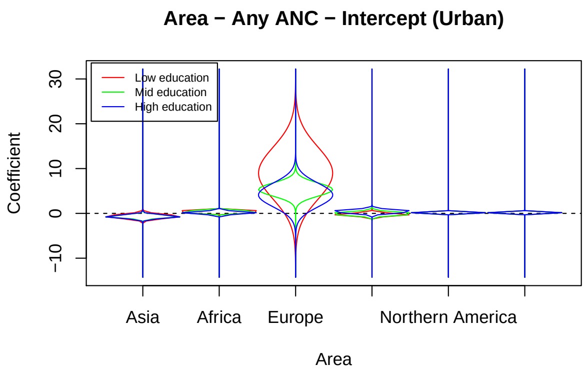
Area − Any ANC − Intercept (Urban)
Low education
Mid education
High education
Coefficient
−10
0
10
20
30
Asia
Africa
Europe
Northern America
Area

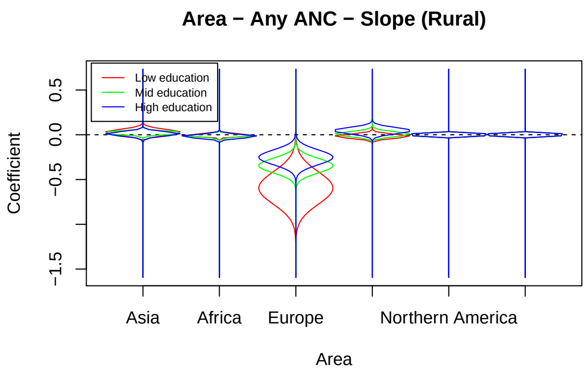
Area − Any ANC − Slope (Rural)
Low education
Mid education
High education
Coefficient
−1.5
−0.5
0.0
0.5
Asia
Africa
Europe
Northern America
Area

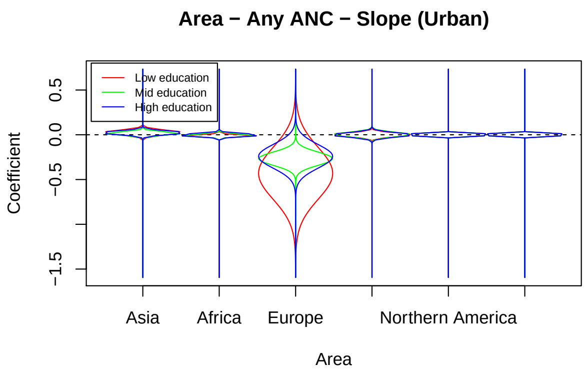
Area − Any ANC − Slope (Urban)
Low education
Mid education
High education
Coefficient
−1.5
−0.5
0.0
0.5
Asia
Africa
Europe
Northern America
Area

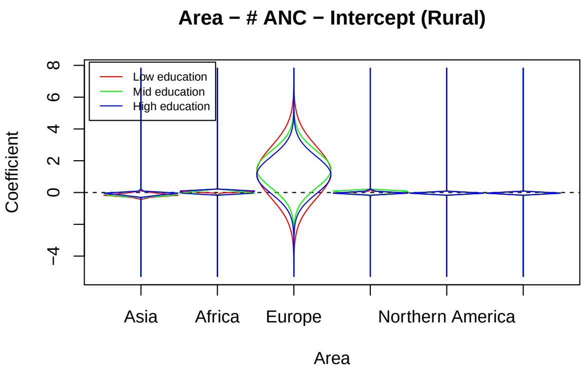
Area − # ANC − Intercept (Rural)
Low education
Mid education
High education
Coefficient
8
6
4
2
0
−4
Asia
Africa
Europe
Northern America
Area

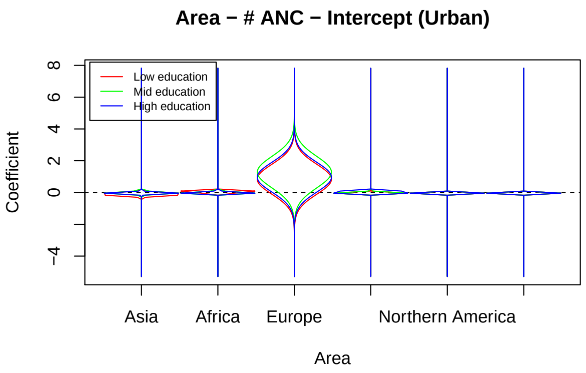
Area − # ANC − Intercept (Urban)
Low education
Mid education
High education
Coefficient
8
6
4
2
0
−4
Asia
Africa
Europe
Northern America
Area

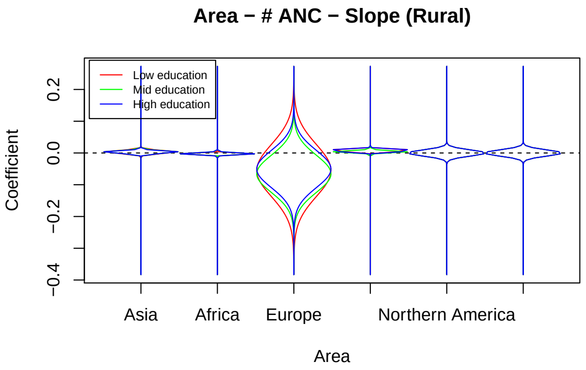
Area − # ANC − Slope (Rural)
Low education
Mid education
High education
Coefficient
0.2
0.0
−0.2
−0.4
Asia
Africa
Europe
Northern America
Area

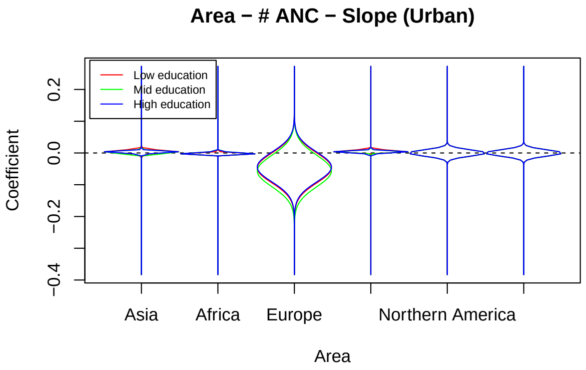
Area − # ANC − Slope (Urban)
Low education
Mid education
High education
Coefficient
0.2
0.0
−0.2
−0.4
Asia
Africa
Europe
Northern America
Area

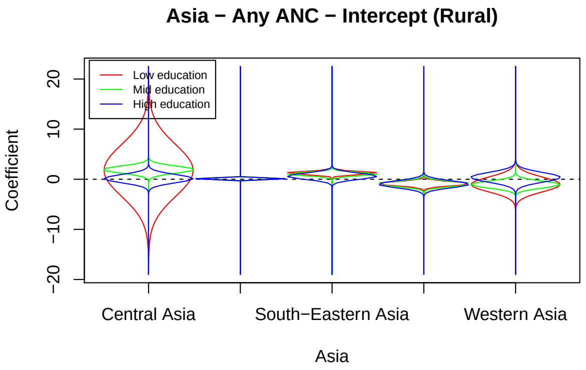
Asia − Any ANC − Intercept (Rural)
Low education
Mid education
High education
Coefficient
20
10
0
−10
−20
Central Asia
South−Eastern Asia
Western Asia
Asia

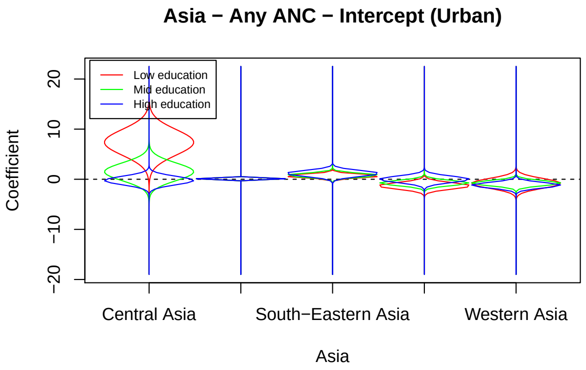
Asia − Any ANC − Intercept (Urban)
Low education
Mid education
High education
Coefficient
20
10
0
−10
−20
Central Asia
South−Eastern Asia
Western Asia
Asia

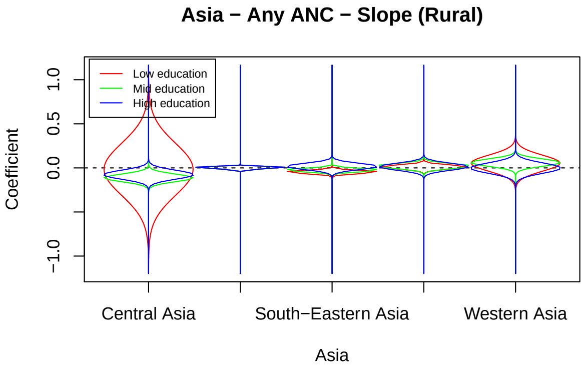
Asia − Any ANC − Slope (Rural)
Low education
Mid education
High education
Coefficient
1.0
0.5
0.0
−1.0
Central Asia
South−Eastern Asia
Western Asia
Asia

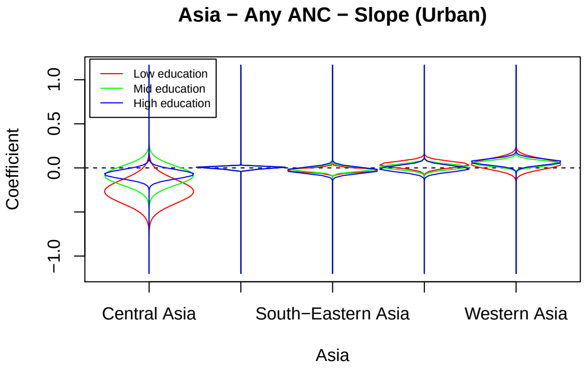
Asia − Any ANC − Slope (Urban)
Low education
Mid education
High education
Coefficient
1.0
0.5
0.0
−1.0
Central Asia
South−Eastern Asia
Western Asia
Asia

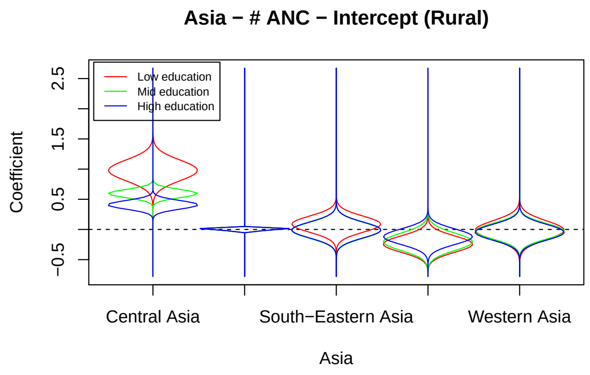
Asia − # ANC − Intercept (Rural)
Low education
Mid education
High education
Coefficient
−0.5
0.5
1.5
2.5
Central Asia
South−Eastern Asia
Western Asia
Asia

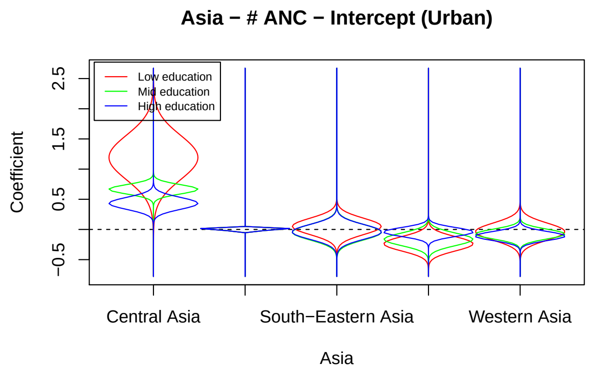
Asia − # ANC − Intercept (Urban)
Low education
Mid education
High education
Coefficient
−0.5
0.5
1.5
2.5
Central Asia
South−Eastern Asia
Western Asia
Asia

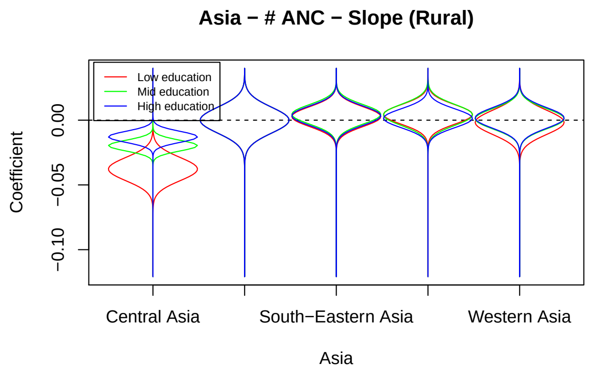
Asia − # ANC − Slope (Rural)
Low education
Mid education
High education
Coefficient
−0.10
−0.05
0.00
Central Asia
South−Eastern Asia
Western Asia
Asia

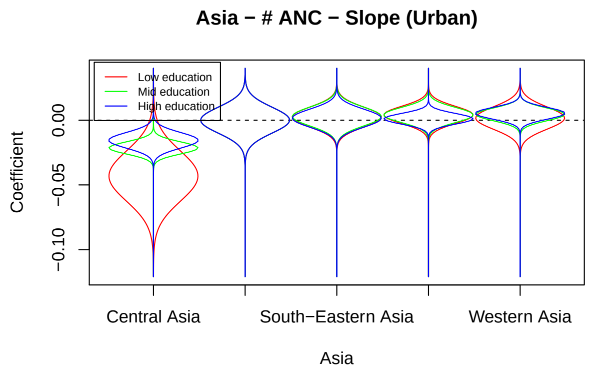
Asia − # ANC − Slope (Urban)
Low education
Mid education
High education
Coefficient
−0.10
−0.05
0.00
Central Asia
South−Eastern Asia
Western Asia
Asia

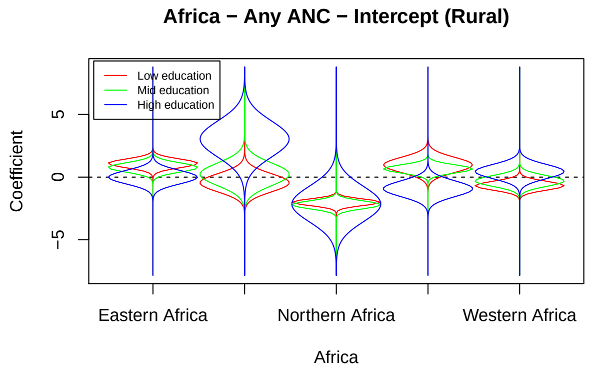
Africa − Any ANC − Intercept (Rural)
Low education
Mid education
High education
Coefficient
−5
0
5
Eastern Africa
Northern Africa
Western Africa
Africa

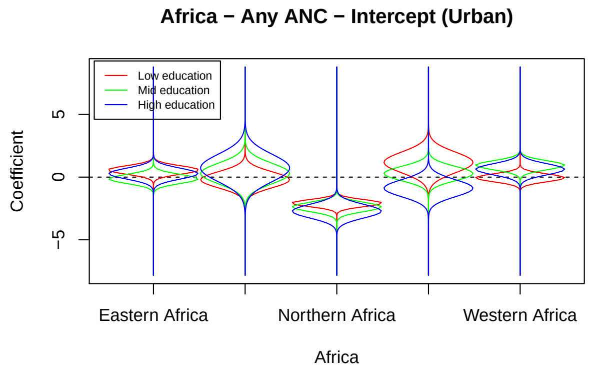
Africa − Any ANC − Intercept (Urban)
Low education
Mid education
High education
Coefficient
−5
0
5
Eastern Africa
Northern Africa
Western Africa
Africa

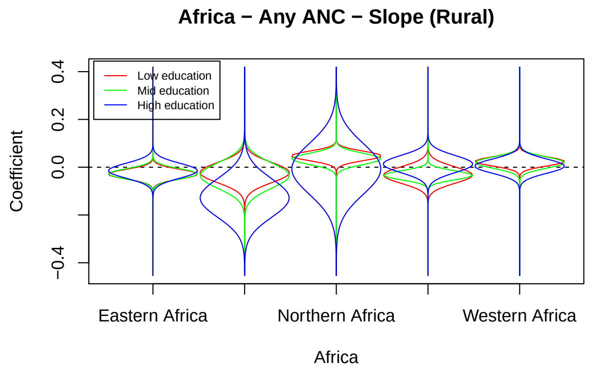
Africa − Any ANC − Slope (Rural)
Low education
Mid education
High education
Coefficient
−0.4
0.0
0.2
0.4
Eastern Africa
Northern Africa
Western Africa
Africa

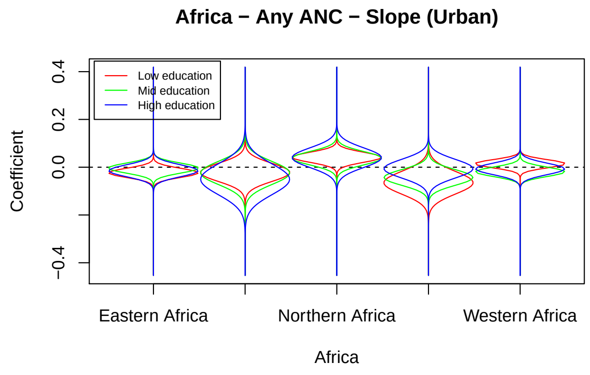
Africa − Any ANC − Slope (Urban)
Low education
Mid education
High education
Coefficient
−0.4
0.0
0.2
0.4
Eastern Africa
Northern Africa
Western Africa
Africa

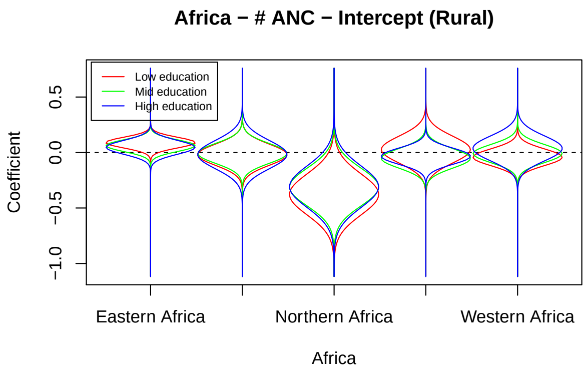
Africa − # ANC − Intercept (Rural)
Low education
Mid education
High education
Coefficient
0.5
0.0
−0.5
−1.0
Eastern Africa
Northern Africa
Western Africa
Africa

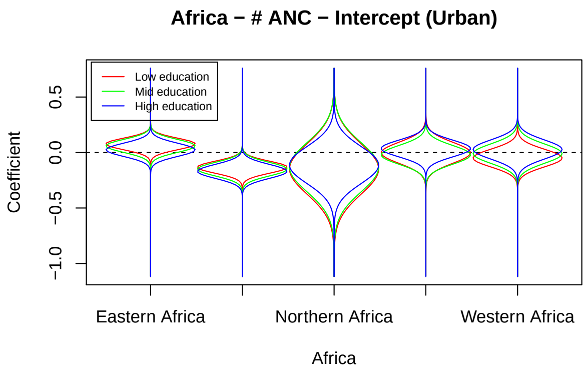
Africa − # ANC − Intercept (Urban)
Low education
Mid education
High education
Coefficient
0.5
0.0
−0.5
−1.0
Eastern Africa
Northern Africa
Western Africa
Africa

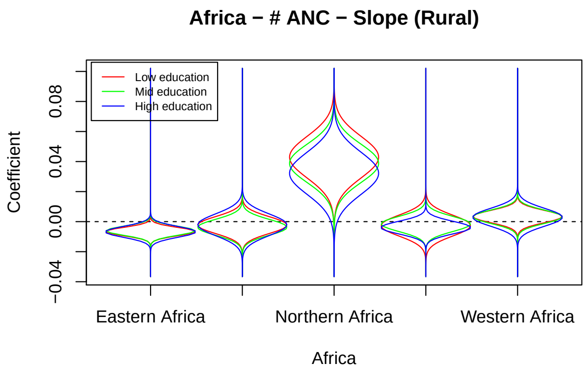
Africa − # ANC − Slope (Rural)
Low education
Mid education
High education
Coefficient
0.08
0.04
0.00
−0.04
Eastern Africa
Northern Africa
Western Africa
Africa

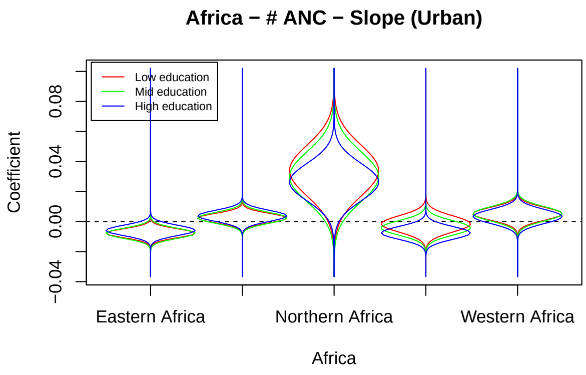
Africa − # ANC − Slope (Urban)
Low education
Mid education
High education
Coefficient
0.08
0.04
0.00
−0.04
Eastern Africa
Northern Africa
Western Africa
Africa

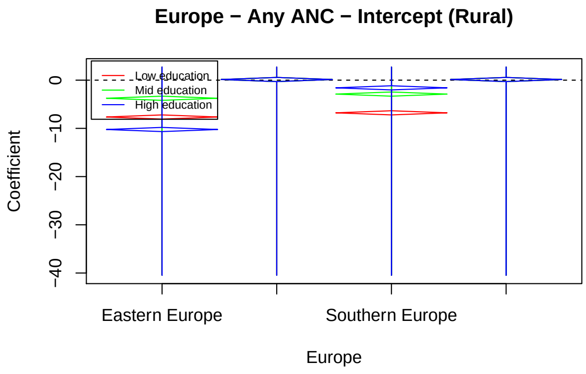
Europe − Any ANC − Intercept (Rural)
Low education
Mid education
High education
Coefficient
0
−10
−20
−30
−40
Eastern Europe
Southern Europe
Europe

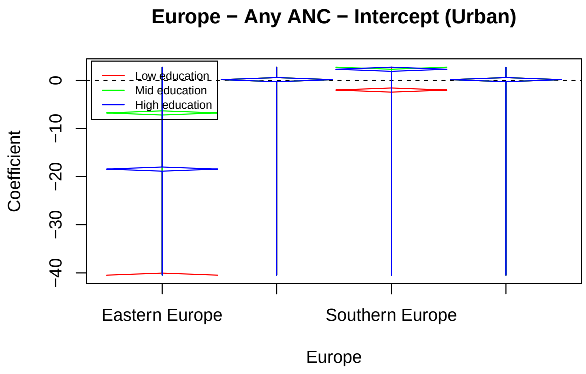
Europe − Any ANC − Intercept (Urban)
Low education
Mid education
High education
Coefficient
0
−10
−20
−30
−40
Eastern Europe
Southern Europe
Europe

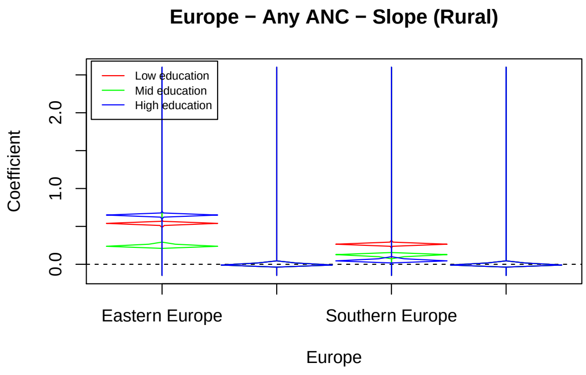
Europe − Any ANC − Slope (Rural)
Low education
Mid education
High education
Coefficient
2.0
1.0
0.0
Eastern Europe
Southern Europe
Europe

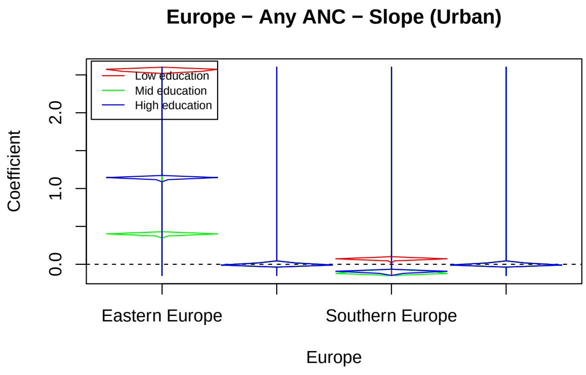
Europe − Any ANC − Slope (Urban)
Low education
Mid education
High education
Coefficient
2.0
1.0
0.0
Eastern Europe
Southern Europe
Europe

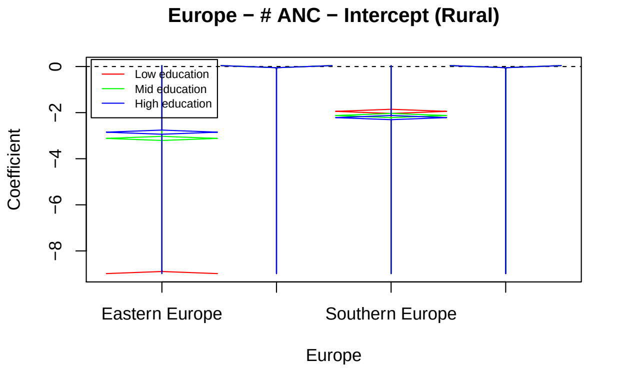
Europe − # ANC − Intercept (Rural)
Low education
Mid education
High education
Coefficient
0
−2
−4
−6
−8
Eastern Europe
Southern Europe
Europe

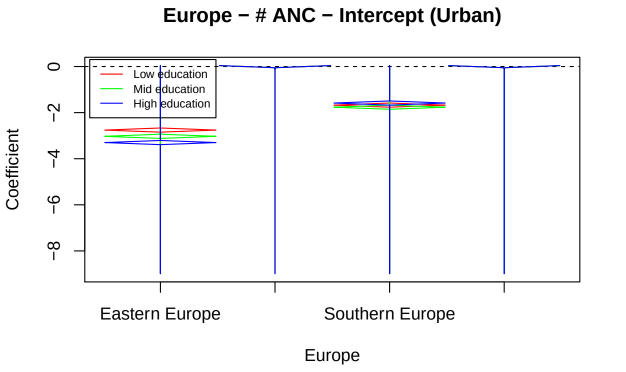
Europe − # ANC − Intercept (Urban)
Low education
Mid education
High education
Coefficient
0
−2
−4
−6
−8
Eastern Europe
Southern Europe
Europe

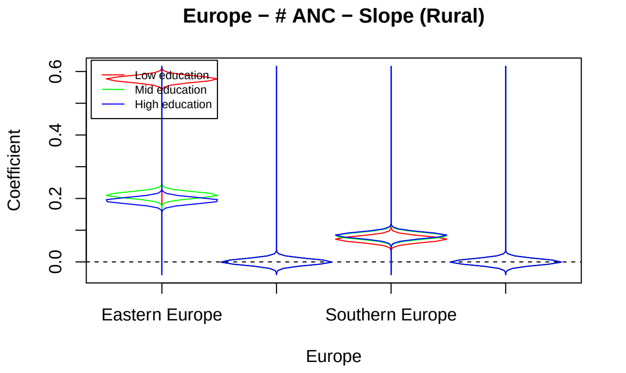
Europe − # ANC − Slope (Rural)
Low education
Mid education
High education
Coefficient
0.0
0.2
0.4
0.6
Eastern Europe
Southern Europe
Europe

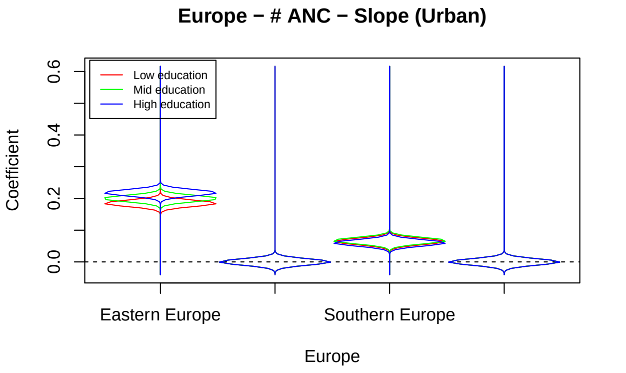
Europe − # ANC − Slope (Urban)
Low education
Mid education
High education
Coefficient
0.0
0.2
0.4
0.6
Eastern Europe
Southern Europe
Europe

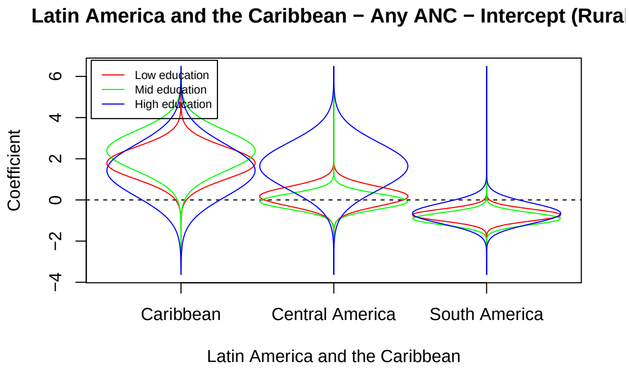
Latin America and the Caribbean − Any ANC − Intercept (Rura
Low education
Mid education
High education
Coefficient
−4
−2
0
2
4
6
Caribbean
Central America
South America
Latin America and the Caribbean

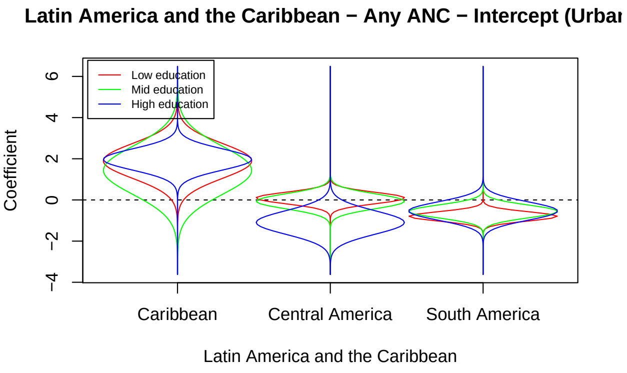
Latin America and the Caribbean − Any ANC − Intercept (Urbai
Low education
Mid education
High education
Coefficient
−4
−2
0
2
4
6
Caribbean
Central America
South America
Latin America and the Caribbean

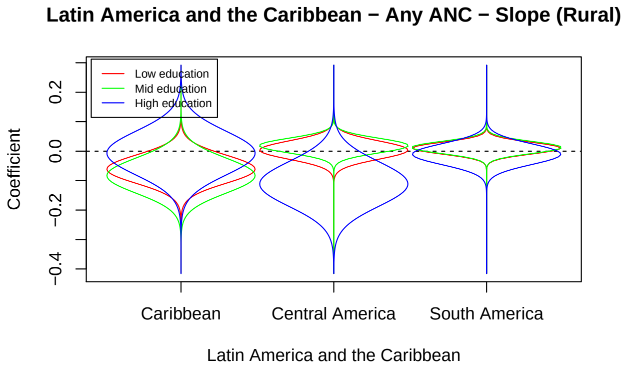
Latin America and the Caribbean − Any ANC − Slope (Rural)
Low education
Mid education
High education
Coefficient
−0.4
−0.2
0.0
0.2
Caribbean
Central America
South America
Latin America and the Caribbean

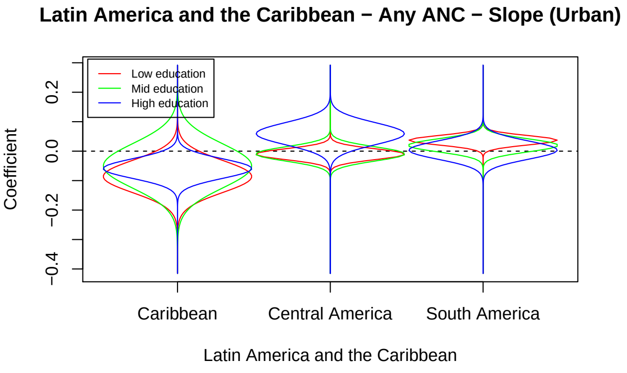
Latin America and the Caribbean − Any ANC − Slope (Urban)
Low education
Mid education
High education
Coefficient
−0.4
−0.2
0.0
0.2
Caribbean
Central America
South America
Latin America and the Caribbean

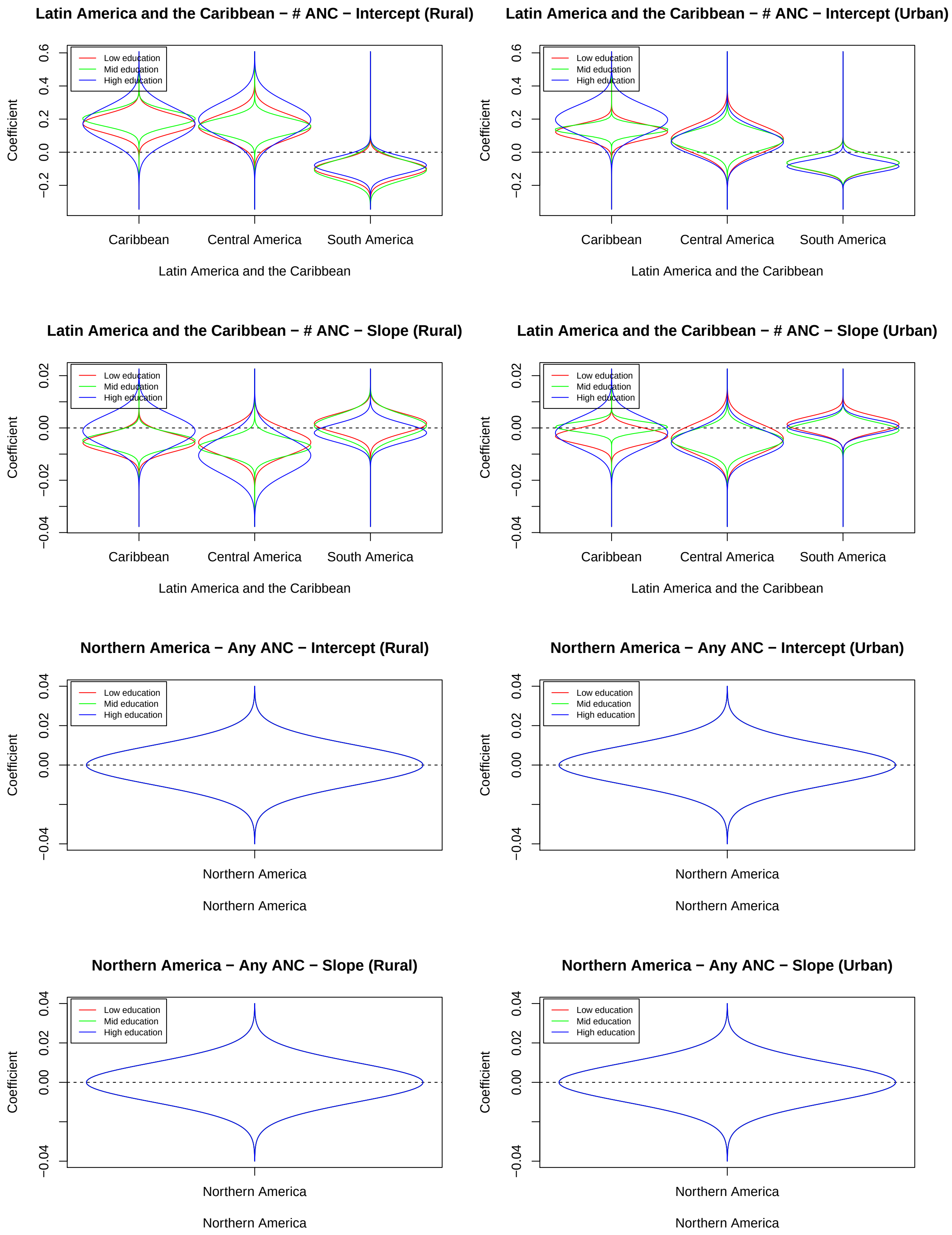
Latin America and the Caribbean − # ANC − Intercept (Rural)
Latin America and the Caribbean − # ANC − Intercept (Urban)
Latin America and the Caribbean − # ANC − Slope (Rural)
Latin America and the Caribbean − # ANC − Slope (Urban)
Northern America − Any ANC − Intercept (Rural)
Northern America − Any ANC − Intercept (Urban)
Northern America − Any ANC − Slope (Rural)
Northern America − Any ANC − Slope (Urban)
Low education
Mid education
High education
Coefficient
Caribbean
Central America
South America
Latin America and the Caribbean
Northern America

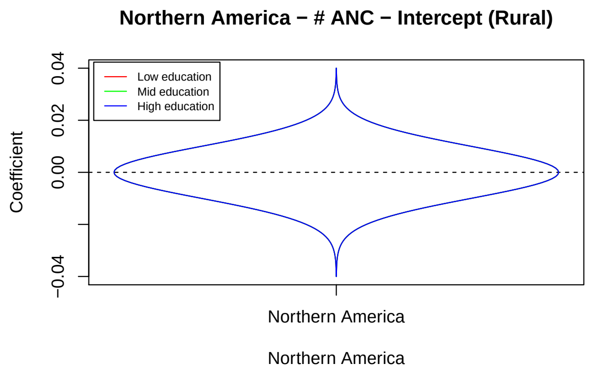
Northern America − # ANC − Intercept (Rural)
Low education
Mid education
High education
Coefficient
0.04
0.02
0.00
−0.04
Northern America
Northern America

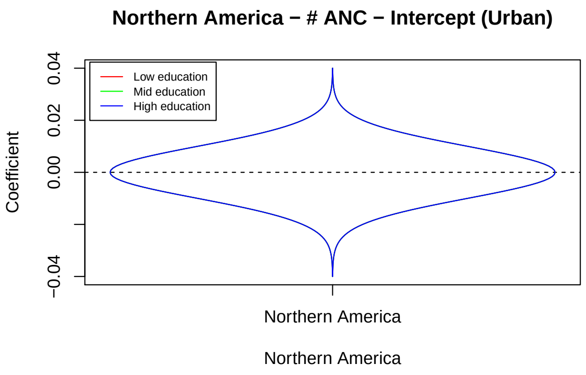
Northern America − # ANC − Intercept (Urban)
Low education
Mid education
High education
Coefficient
0.04
0.02
0.00
−0.04
Northern America
Northern America

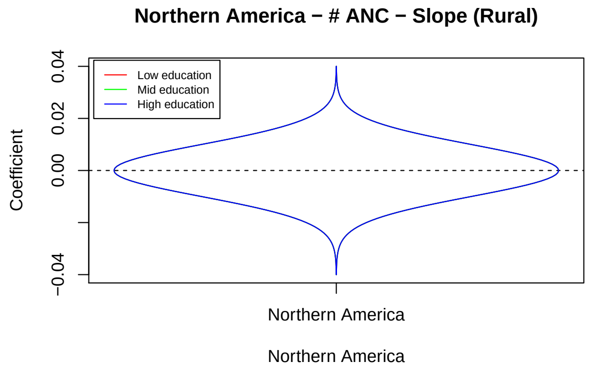
Northern America − # ANC − Slope (Rural)
Low education
Mid education
High education
Coefficient
0.04
0.02
0.00
−0.04
Northern America
Northern America

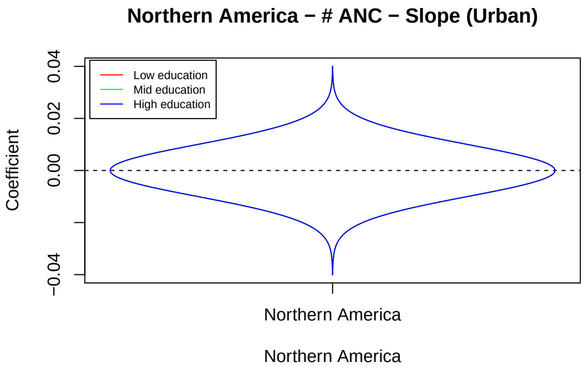
Northern America − # ANC − Slope (Urban)
Low education
Mid education
High education
Coefficient
0.04
0.02
0.00
−0.04
Northern America
Northern America

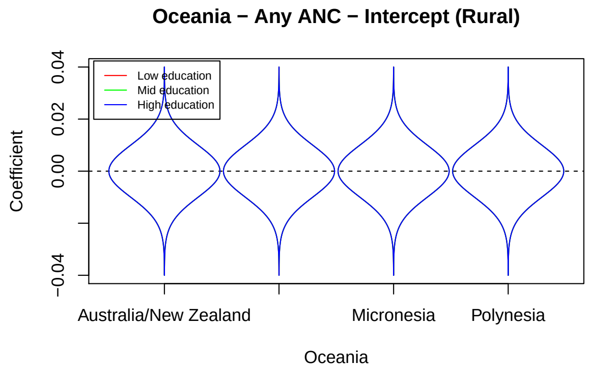
Oceania − Any ANC − Intercept (Rural)
Low education
Mid education
High education
Coefficient
0.04
0.02
0.00
−0.04
Australia/New Zealand
Micronesia
Polynesia
Oceania

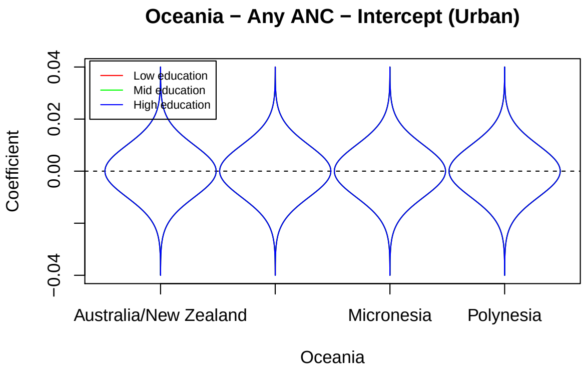
Oceania − Any ANC − Intercept (Urban)
Low education
Mid education
High education
Coefficient
0.04
0.02
0.00
−0.04
Australia/New Zealand
Micronesia
Polynesia
Oceania

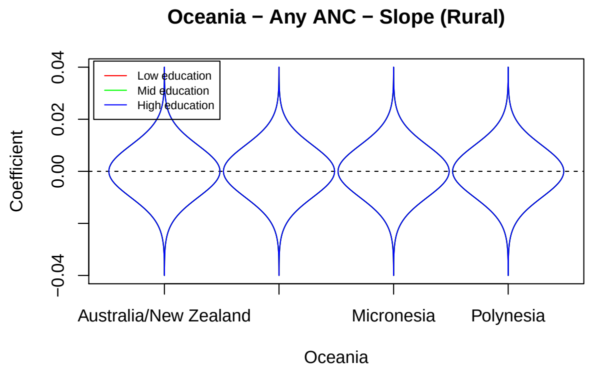
Oceania − Any ANC − Slope (Rural)
Low education
Mid education
High education
Coefficient
0.04
0.02
0.00
−0.04
Australia/New Zealand
Micronesia
Polynesia
Oceania

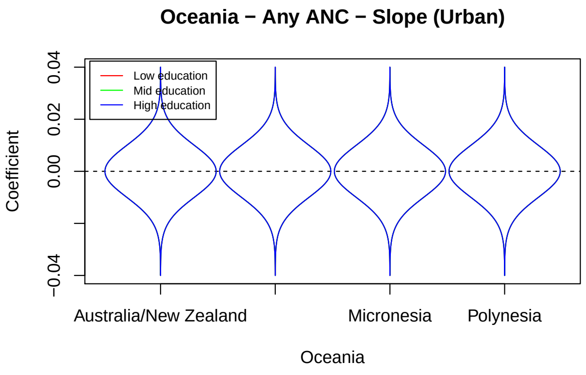
Oceania − Any ANC − Slope (Urban)
Low education
Mid education
High education
Coefficient
0.04
0.02
0.00
−0.04
Australia/New Zealand
Micronesia
Polynesia
Oceania

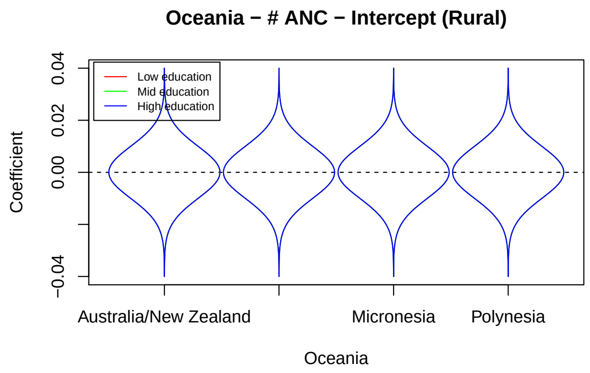

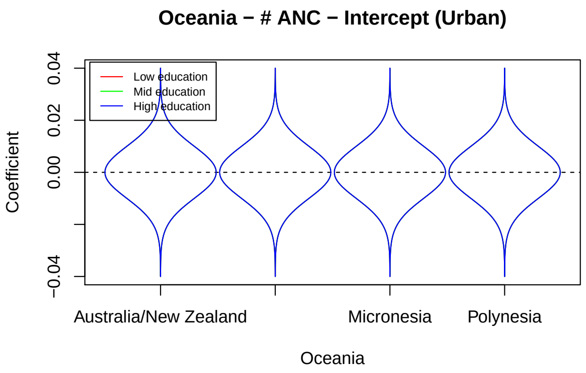

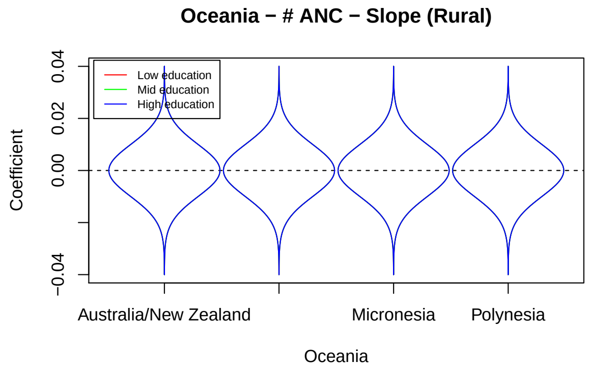

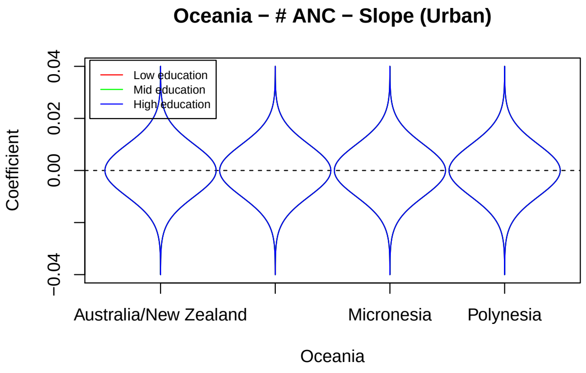

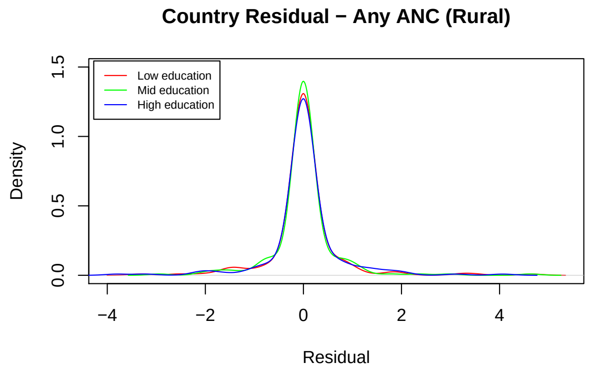

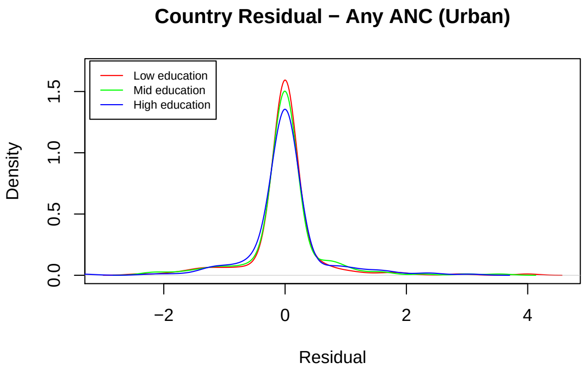

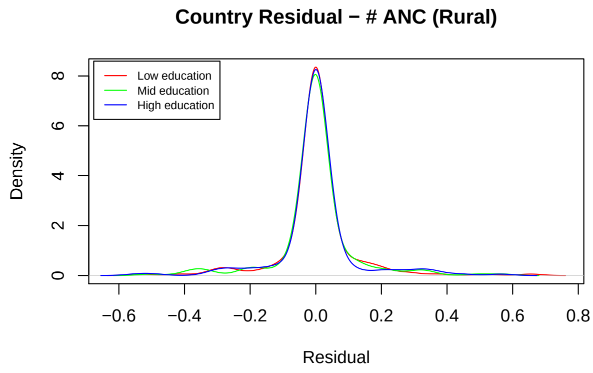

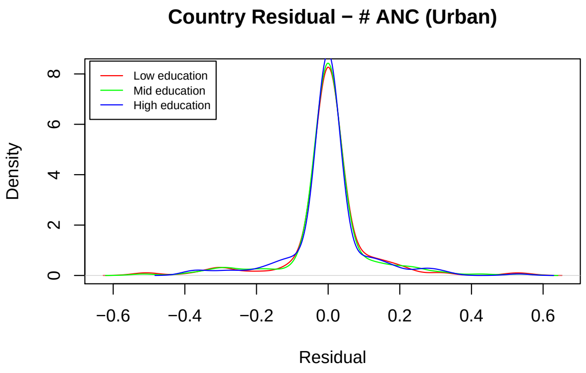

Model Implementation

The number of ANC visits is sampled at the beginning of each woman's pregnancy. Based on her number of ANC visits, her hemoglobin concentration is adjusted and anemia status re-evaluated. Because the priors for anemia status are based on DHS data that include women who attended ANC, model calibration is used to lower the 'natural history' (i.e. without ANC) hemoglobin concentrations for women so that the distribution of anemia prevalence after accounting for current ANC coverage is similar to the observed DHS data.

References

Aborigo RA, Moyer CA, Gupta M, et al. Obstetric danger signs and factors affecting health seeking behaviour among the Kassena-Nankani of Northern Ghana: a qualitative study. Afr J Reprod Health 2014; 18(3): 78-86. PMID: https://pubmed.ncbi.nlm.nih.gov/25438512/

Akshaya KM, Shivalli S. Birth preparedness and complication readiness among the women beneficiaries of selected rural primary health centers of Dakshina Kannada district, Karnataka, India. PLoS One 2017; 12(8): e0183739. DOI: https://doi.org/10.1371/journal.pone.0183739

Bintabara D, Mohamed MA, Mghamba J, Wasswa P, Mpembeni RNM. Birth preparedness and complication readiness among recently delivered women in chamwino district, central Tanzania: a cross sectional study. Reprod Health 2015; 12: 44. DOI: https://doi.org/10.1186/s12978-015-0041-8

Gopalakrishnan S, Eashwar VMA, Muthulakshmi M. Health-seeking behaviour among antenatal and postnatal rural women in Kancheepuram District of Tamil Nadu: A cross-sectional Study. J Family Med Prim Care 2019; 8(3): 1035-1042. DOI: https://doi.org/10.4103/jfmpc.jfmpc_323_18

Pembe AB, Urassa DP, Carlstedt A, Lindmark G, Nyström L, Darj E. Rural Tanzanian women's awareness of danger signs of obstetric complications. BMC Pregnancy Childbirth 2009; 9: 12. DOI: https://doi.org/10.1186/1471-2393-9-12

Pervin J, Nu UT, Rahman AMQ, et al. Level and determinants of birth preparedness and complication readiness among pregnant women: A cross sectional study in a rural area in Bangladesh. PLoS One 2018; 13(12): e0209076. DOI: https://doi.org/10.1371/journal.pone.0209076

Salem A, Lacour O, Scaringella S, et al. Cross-sectional survey of knowledge of obstetric danger signs among women in rural Madagascar. BMC Pregnancy Childbirth 2018; 18(1): 46. DOI: https://doi.org/10.1186/s12884-018-1664-x

World Health Organization. The global prevalence of anaemia in 2011. 2015. Available at: http://apps.who.int/iris/bitstream/10665/177094/1/9789241564960_eng.pdf

World Health Organization. WHO recommendations on antenatal care for a positive pregnancy experience. 2016. Available at: http://apps.who.int/iris/bitstream/10665/250796/1/9789241549912-eng.pdf

World Health Organization. WHO recommendation on antenatal care contact schedules. 2018. Available at: https://extranet.who.int/rhl/topics/improving-health-system-performance/who-recommendation-antenatal-care-contact-schedules

A.1.4.2 Starting Delivery Site

Overview

Women may deliver either at home or in a facility. Medical facilities are classified by the level of emergency obstetric care (EmOC) they can provide. Basic EmOC (BEmOC) is comprised of six signal functions which include administration of injectable antibiotics, oxytocics, and sedatives/anti-convulsants, performance of manual removal of placenta, removal of retained products, and assisted vaginal delivery. In addition to these six signal functions, comprehensive EmOC (CEmOC) offers blood transfusion, surgical capability, and management of shock (WHO 2009). The WHO recommends a minimum of 5 EmOC (combination of BEmOC and CEmOC) facilities per 500,000 population, where at least 1 is comprehensive (WHO 2009). Delivery sites are split into 5 categories in the model: Home, Home - Skilled Birth Attendant (SBA), non-EmOC, BEmOC, and CEmOC. The starting site determines what preventative care a woman may receive (if any), thus potentially reducing her risk of delivery complications, and influences the probabilities that a complication may be recognized, referred, and transported.

Data

Facility Delivery

We analyzed delivery site (home vs facility) from DHS data (variable m15) for 1,717,203 women in 75 countries from 261 surveys. We supplemented this with country-level data from the Global Health Observatory on births attended by skilled health personnel for 108 countries not available in the DHS data (GHO database). We use the data on skilled birth personnel as a proxy for facility delivery. We used the same priors for all subgroups in these (non-DHS) countries. From both sources of data we obtained estimates for 183 countries between 1990 and 2018. For "Home SBA", we considered SBA as reported in DHS as a doctor or nurse/midwife. Overall, few women delivered at home with SBA (about 3.5% globally). We estimated the probability of SBA given home birth among 684,977 women in 75 countries from 257 DHS surveys. Due to lack of DHS data for high income countries we used the priors for upper middle income countries.

Facility Type

To estimate the distribution of EmOC facilities we undertook a targeted literature review, summarized here by continent.

Africa:

- Angola: A 2019 report in focus provinces estimated that 50% of municipalities could provide BEmOC and 10% could provide CEmOC (UNFPA 2019).

- Benin: A study of services provided in 2002 found 26 CEmOC facilities and 19 BEmOC facilities, and that 72% of deliveries occurred in any facility while 13% occurred in EmOC facilities (AMDD 2004).

- Burkina Faso: A comparison of two EmOC surveys in 2010 and 2014 found that 71.7% of births occurred in facilities and 3.4% occurred in CEmOC in 2010, and that 42.6% of births occurred in facilities and 4.5% occurred in CEmOC in 2014 (Kouanda 2016).

- Burundi: A 2018 UNFPA document reported that there were 7 BEmOC and 23 and CEmOC facilities (UNFPA 2018).

- Cameroon: A 2000-2001 study in 5/10 provinces found 5 BEmOC and 21 CEmOC and estimated that 5.9% of births occurred in all facilities (UNFPA 2003).

- Chad: A study of services provided in 2002 found 24 CEmOC facilities and 7 BEmOC facilities and that 9% of deliveries occurred in any facility, with nearly all occurring in EmOC facilities (28153/29226) (AMDD 2004).

- Côte d'Ivoire: A 2000-2001 study in 16/46 districts found 104 BEmOC and 15 CEmOC facilities, and estimated 45.1% of deliveries occurred in all facilities and that 31.3% occurred in EmOC facilities (UNFPA 2003).

- DRC: A 2012 study in three provinces found that out of 266 facilities 5 were BEmOC and 4 were CEmOC, and estimated that 0.8% of births occurred in an EmOC facility, and that 25.4% occurred in any facility (UNFPA 2012).

- Ethiopia: A census of almost all health centers and hospitals in the country (795 facilities) in 2008 found that there were 58 CEmOC, 25 BEmOC, 712 'partially functioning' or non-EmOC facilities (Admasu 2011). The study estimated that 7% of expected births occurred in a facility, and 3% of births occurred in an EmOC facility (Admasu 2011).

- Gabon: A needs assessment of services provided in 2001 found that of 77 facilities 11 were CEmOC and 5 were BEmOC (Fauveau 2007). 74% of births occurred in any facility, and 14.5% occurred in EmOC facilities.

- Gambia: A study of services provided in 2002 in 47 health facilities found that 4 were CEmOC and 8 were BEmOC, and that 30% of deliveries occurred in EmOC facilities (Fauveau 2007).

- Ghana: A nationwide EmOC needs assessment in 2010 found that 57.7% of expected births occurred in facilities, and 20.7% of births occurred in EmOC facilities (Bosomprah 2016).

- Guinea: A national EmOC needs assessment was conducted in 2012, including all public, private, and faith-based health facilities that performed at least one delivery during the period of study. Out of 502 health facilities survey, 421 health centers were expected to perform BEmOC signal functions, but none was actually a fully functioning BEmOC facility due to low performance in terms of assisted vaginal delivery and neonatal resuscitation - all 15 EmOC facilities were CEmOC, and only 2 were in rural areas (Baguiya 2016).

- Guinea-Bissau: A nationwide survey of 107 health facilities in 2002 found that 3 were CEmOC and 11 were BEmOC, and that 29% of deliveries occurred in EmOC facilities (Fauveau 2007).

- Kenya: The 2010 Service Provision Assessment of 129 facilities offering delivery services found that 9% of facilities reported the ability to carry out BEmOC, and 7% reported the ability to carry out CEmOC (NCAPD 2011). A 2003 study in North-Eastern Province estimated that 8.8% of deliveries took place in EmOC facilities (Pearson 2005).

- Malawi: A 2006 study of three districts in central Malawi found that 40.6% of deliveries took place in facilities, and that 22.5% occurred in EmOC facilities (Kongnyuy 2009).

- Mali: A study in 2001-2004 in two districts (Bougouni and Yanfolila) found that the proportion of births in EmOC facilities was 5-6% on average (Otchere 2007).

- Mauritania: A 2000-2001 study of all facilities found 1 BEmOC and 7 CEmOC facilities, and estimated that 35% of deliveries occurred in all facilities (UNFPA 2003).

- Morocco: A study of services provided in 2000-2002 of 510 government maternities and birthing centers found 137 BEmOC and 61 CEmOC facilities, and estimated that 49.4% of birth occurred in all facilities, with 40.5% of births occurring in EmOC facilities (AMDD 2003). Private hospitals were not surveyed however.

- Mozambique: A study of availability of EmOC facilities in Mozambique found that the institutional delivery rate at EmOC facilities increased from 17% in 2007 (57% all facilities) to 19% in 2012 (67% all facilities). Nationally there were 78 EmOC facilities (33 CEmOC) in 2007, and 67 EmOC facilities (32 CEmOC) in 2012 (Augusto 2018). A needs assessment in Sofala province in 2000 found 1 BEmOC and 4 CEmOC facilities, and estimated that 34.2% of births occurred in any facility, and 12% occurred in

EmOC facilities (Bailey 2002).

- Namibia: The 2016 national emergency obstetric and newborn care needs assessment estimated that 94% of births occurred in hospitals expected to provide CEmOC, however, only 28.3% of them occurred in fully functional EmOC facilities. The report estimated that in 2016 95.7% of births occurred in health facilities - 94% in hospitals, 5% at health centers, and 1% in clinics. In 2005, 70.9% of births occurred in health facilities, of which 95% occurred in hospitals, with 23% of all births delivering in EmOC facilities (MHSS 2016).

- Niger: A study of services provided in 2000 found 54 BEmOC facilities and 22 CEmOC facilities, and that 11% of deliveries occurred in any facility - however, the number of deliveries in EmOC facilities was not available (AMDD 2003).

- Nigeria: A study of facilities in Bauchi State between 2012 and 2015 found that BEmOC facilities increased from 3/59 to 8/59, and CEmOC increased from 3/59 to 13/59, and that facility deliveries increased from 28,455 to 35,818, of which 8,483 and 19,383 deliveries were in EmOC facilities, an increase of 30% to 55% of facility deliveries (Kabo 2019). Another study in Kwara state in 2013 found that 38.7% of all deliveries took place in health facilities, and 13.6% of all births occurred at EmOC facilities (Saidu 2013).

- Rwanda: A study in 2004 in 4 districts estimated that 7.2% of deliveries occurred in EmOC facilities (Pearson 2005).

- Senegal: A national needs assessment of 172 facilities in 2001 found 5 BEmOC and 33 CEmOC facilities, and estimated that 28.6% of deliveries occurred in all facilities surveyed, and 9.7% occurred in EmOC facilities (Bailey 2002).

- Sierra Leone: Sierra Leone is divided in 13 districts, and each district has one healthcare facility designated to provided CEmOC and five or six designated to provide BEmOC. In total, 13 facilities were designed to provided CEmOC and 67 facilities were designated to provide BEmOC (Jones 2016). A previous study in 2008 found that 9.6% of births took place in a health facility, and 2.3% of births occurred in an EmOC facility (Oyerinde 2011).

- South Sudan: A study in 2003 found that the proportion of deliveries in EmOC facilities was 0.6% in Yambio and 1.5% in Rumbek (Pearson 2005).

- Tanzania: A health facility survey in Zanzibar in 2012 found that 47% of all births occurred in EmOC health facilities, and of 79 sampled facilities 9% were CEmOC, 7.6% were BEmOC, 27.9% were 'partially BEmOC', and 55.7% were non-EmOC (Fakih 2016). A needs assessment for 1999-2000 in 2 districts in Mwanza Region estimated that 43% of births occurred in a facility and that 14% occurred in EmOC facilities (AMDD 2003).
- Uganda: A study in 2003 estimated that nationally 5% of deliveries occurred in EmOC facilities (Pearson 2005).

Asia:

- Afghanistan: An assessment in secure areas of Afghanistan in late 2009 to early 2010 found that 53 of the 78 facilities (68%) performed all basic EmOC signal functions, and 44 of the 78 (56%) performed all comprehensive EmOC signal functions (Young-Mi 2012).

- Bangladesh: A study of a sample of 14 public and private health facilities in rural northwest Bangladesh in October 2011-January 2012 found that 3 offered BEmOC and 11 offered CEmOC. All private clinics (7/7) provided CEmOC, while 4/7 public facilities provided CEmOC, however, only 3,217 deliveries occurred in private facilities, compared with 10,377 in public facilities (Sikder 2015). A national health facility assessment performed between November 2007 and July 2008 of all public EmOC facilities and

private facilities providing obstetric services in the 64 districts of Bangladesh found that of 2376 facilities assessed, 316 had all CEmOC signal functions and 359 had all BEmOC signal functions (Alam 2015).

- India: A study in 2001 in four districts of West Bengal of 408 facilities found 17 CEmOC and 48 BEmOC facilities, and estimated that 26.2% of births occurred in EmOC (Biswas 2005). A study in Gujarat in 2012-2013 of 159 facilities found 23 CEmOC and 3 BEmOC facilities, and estimated that among the facility births 24.1% occurred in CEmOC, 1.8% occurred in BEmOC, 46.8% occurred in less-than-BEmOC but with capability of C-section, and 27.3% occurred in less-than-BEmOC (Salazar 2016). A needs assessment in 2010 in Karnataka found that of 466 facilities there were 136 CEmOC and 42 BEmOC facilities (Mony 2013).

- Nepal: A study in rural Nepal in 2018 found that only 1/16 facilities were capable of CEmOC, and at most 2 had all BEmOC signal functions (Banstola 2020). A needs assessment of 157 facilities in Eastern, Western and Mid-western regions in 2000 found 5 BEmOC and 18 CEmOC facilities, and estimated that 5.2% of births occurred in EmOC facilities (Bailey 2002).

- Pakistan: A study of 2 districts in Pakistan (Haripur and Nowshera) in 2016 found that 22% of all births take place in public health facilities in both districts, of which 41% were conducted at health facilities that did not meet BEmOC criteria (Brückmann 2019). A needs assessment in 3 districts in Sindh Province in 1999 found 16 BEmOC and 13 CEmOC facilities, and estimated that 7.9% of births occurred in EmOC facilities (AMDD 2002).

- Sri Lanka: A needs assessment of services provided in 1999 estimated that 72.9% of births occurred in CEmOC facilities, with 3.8% in BEmOC and 9.6% in BEmOC-2 (missing AVD and removal of retained products) (AMDD 2003).

- Thailand: A study in the five southernmost provinces of Thailand in 2004-2005 of 56 facilities found 12 CEmOC and and 43 BEmOC facilities, and estimated that 89.5% of births occurred in hospitals (Liabsuetrakul 2007).

- Timor-Leste: A national EmOC assessment in 2015 estimated that 47.8% of all expected live births occurred in facilities, and that 24.6% of birth occurred in EmOC facilities (MOH Timor-Leste 2015). Out of 75 facilities they found 2 BEmOC and 6 CEmOC.

Latin America:

- Bolivia: A needs assessment in 2003 found that 33% of births occurred in all facilities, and 24% occurred in EmOC and EmOC-1 facilities (performance of other signal functions but the absence of assisted vaginal delivery) (Bailey 2005). Out of 85 selected facilities in the study 7 were BEmOC-1, 20 were CEmOC, and 13 were CEmOC-1.

- El Salvador: A needs assessment in 2002 found that 46% of births occurred in facilities, and nearly all occurred in EmOC-1 facilities (Bailey 2005). Out of 33 surveyed facilities 28 were CEmOC-1 (no AVD) and none were BEmOC.

- Honduras: A needs assessment in 2002 found that 42% of births occurred in facilities, and 34% occurred in EmOC and EmOC-1 facilities (no AVD) (Bailey 2005). Out of 27 surveyed facilities 6 were CEmOC, 16 were CEmOC-1, and none were BEmOC.

- Nicaragua: A needs assessment of services provided in 1999-2000 in 112 facilities found 9 BEmOC-1 and 10 CEmOC-1 facilities (i.e. no AVD), and estimated that 44.5% of deliveries occurred in all facilities, and 28.7% occurred in EmOC facilities (AMDD 2003).
- Peru: A needs assessment of services in 31 facilities in the six most northern provinces of Ayacucho in 1999-2000 found 2 CEmOC-1 (no AVD) facilities and no BEmOC, and estimated that 54.6% of births occurred in any facility and 25.9% occurred in the CEmOC facilities (AMDD 2002).

North America:

- USA: In the US in 2012, 1.36% of US births occurred outside a hospital - 66% of out-of-hospital births occurred at home, and another 29% occurred in a freestanding birthing center, with the remaining 5% of out-of-hospital births occurring in a clinic or doctor's office or other location (MacDorman 2014).

Parameters

We used hierarchical logistic regression models to estimate priors for facility delivery and home-SBA based on the DHS data, and for EmOC status based on estimates from the literature. Modelled EmOC status probabilities are renormalized so that they sum to 1.0. While needs assessments often report the number of types of facilities, these counts do not account for the volume of deliveries by facility. When the percentage of deliveries by EmOC vs non-EmOC was reported, we used data on facility breakdown to set priors for the relative breakdown of deliveries by EmOC status. When only facility counts were available we assumed that CEmOCs have 2x the delivery volume of BEmOCs, and that BEmOCs have 2.5x the delivery volume of non-EmOCs when setting priors.

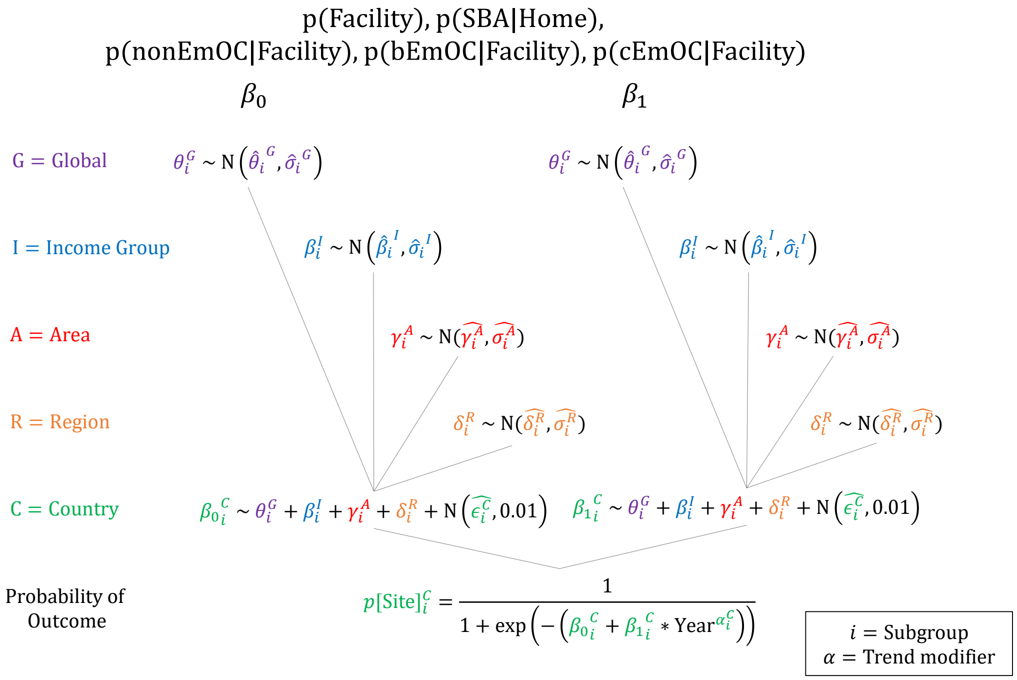

Priors

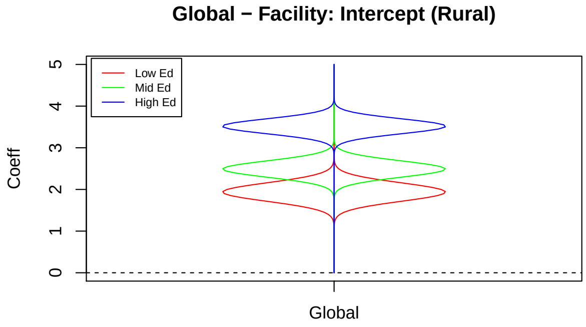

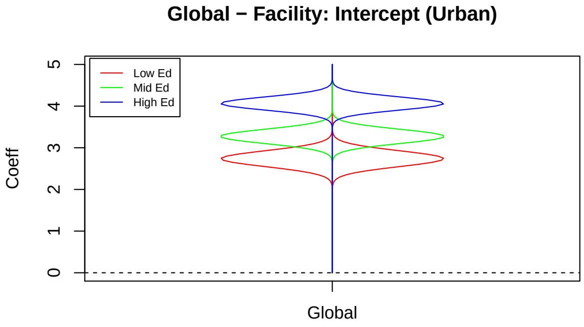

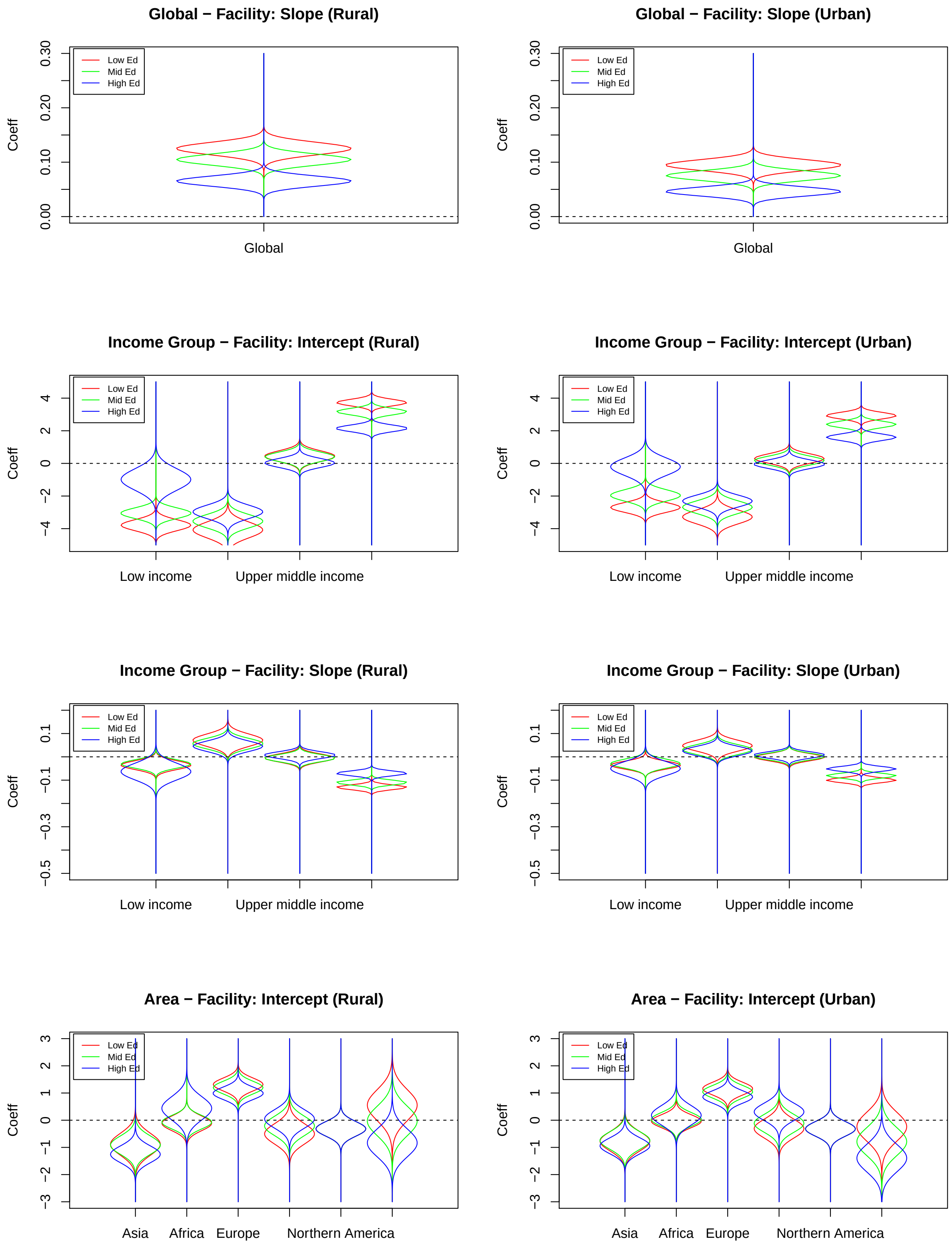
Global − Facility: Slope (Rural)
Global − Facility: Slope (Urban)
Income Group − Facility: Intercept (Rural)
Income Group − Facility: Intercept (Urban)
Income Group − Facility: Slope (Rural)
Income Group − Facility: Slope (Urban)
Area − Facility: Intercept (Rural)
Area − Facility: Intercept (Urban)
Low Ed
Mid Ed
High Ed
Coeff
Global
Low income
Upper middle income
Asia
Africa
Europe
Northern America

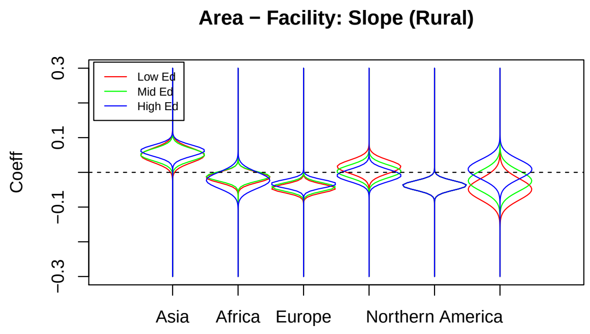
Area − Facility: Slope (Rural)
Coeff
0.3
0.1
−0.1
−0.3
Low Ed
Mid Ed
High Ed
Asia
Africa
Europe
Northern America

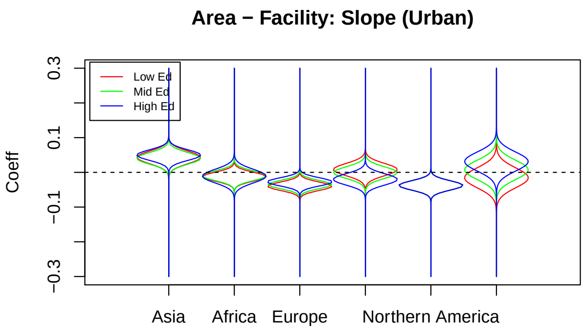
Area − Facility: Slope (Urban)
Coeff
0.3
0.1
−0.1
−0.3
Low Ed
Mid Ed
High Ed
Asia
Africa
Europe
Northern America

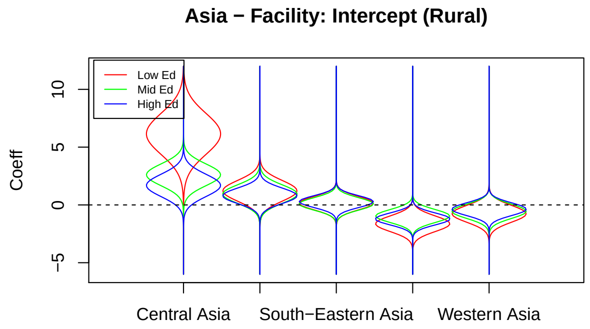
Asia − Facility: Intercept (Rural)
Coeff
10
5
0
−5
Low Ed
Mid Ed
High Ed
Central Asia
South−Eastern Asia
Western Asia

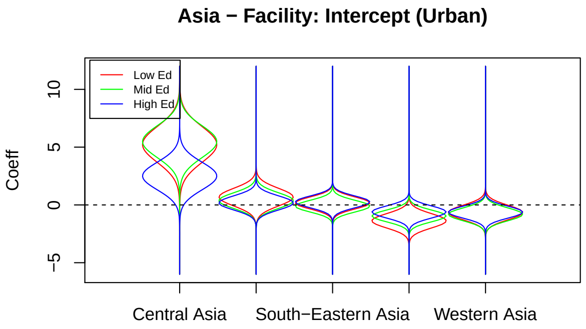
Asia − Facility: Intercept (Urban)
Coeff
10
5
0
−5
Low Ed
Mid Ed
High Ed
Central Asia
South−Eastern Asia
Western Asia

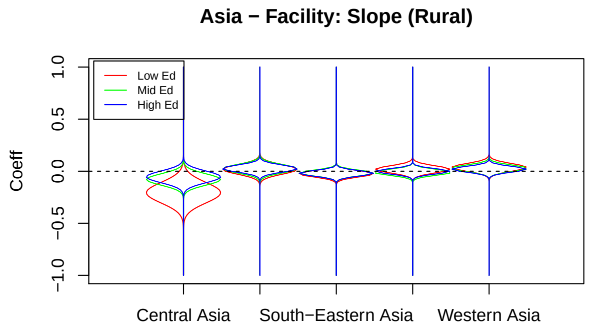
Asia − Facility: Slope (Rural)
Coeff
1.0
0.5
0.0
−0.5
−1.0
Low Ed
Mid Ed
High Ed
Central Asia
South−Eastern Asia
Western Asia

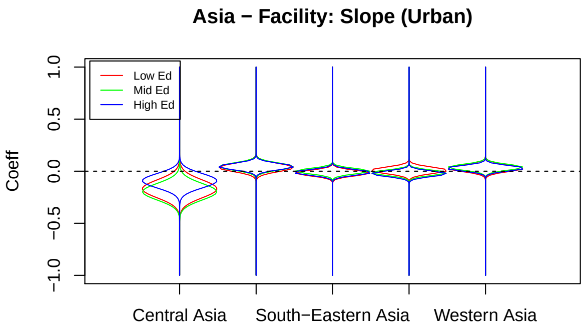
Asia − Facility: Slope (Urban)
Coeff
1.0
0.5
0.0
−0.5
−1.0
Low Ed
Mid Ed
High Ed
Central Asia
South−Eastern Asia
Western Asia

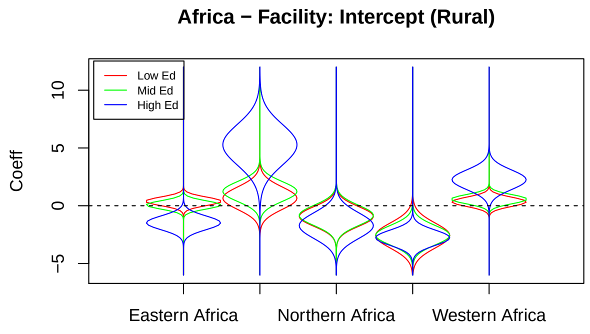
Africa − Facility: Intercept (Rural)
Coeff
10
5
0
−5
Low Ed
Mid Ed
High Ed
Eastern Africa
Northern Africa
Western Africa

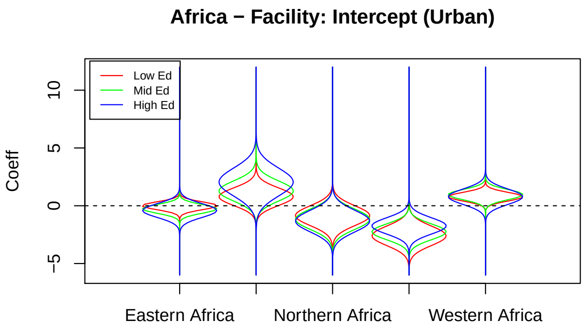
Africa − Facility: Intercept (Urban)
Coeff
10
5
0
−5
Low Ed
Mid Ed
High Ed
Eastern Africa
Northern Africa
Western Africa

Africa − Facility: Slope (Rural)

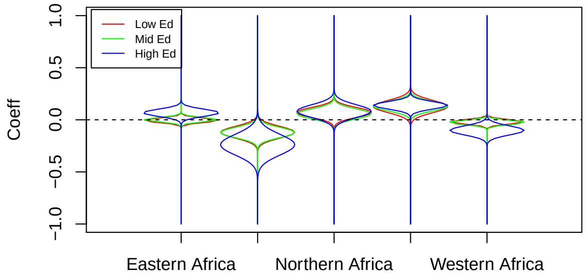

Africa − Facility: Slope (Urban)

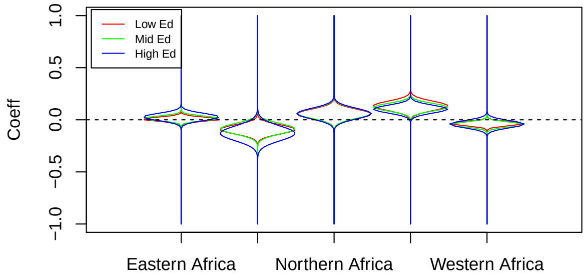

Europe − Facility: Intercept (Rural)

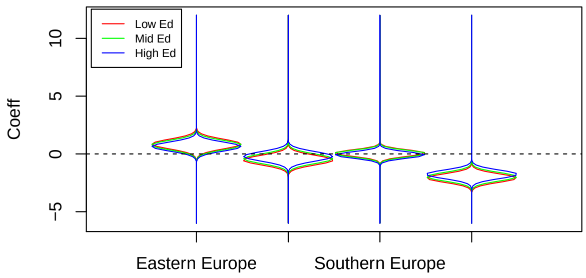

Europe − Facility: Intercept (Urban)

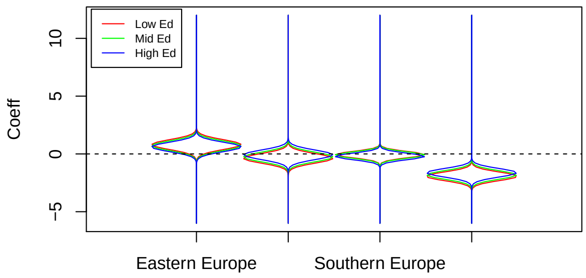

Europe − Facility: Slope (Rural)

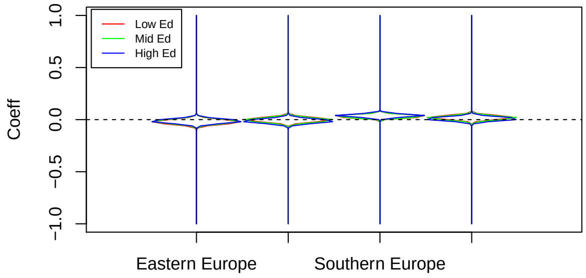

Europe − Facility: Slope (Urban)

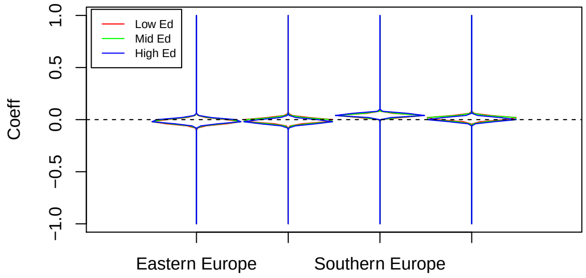

Latin America and the Caribbean − Facility: Intercept (Rural)

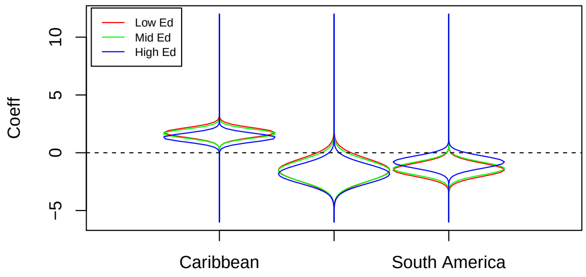

Latin America and the Caribbean − Facility: Intercept (Urban)

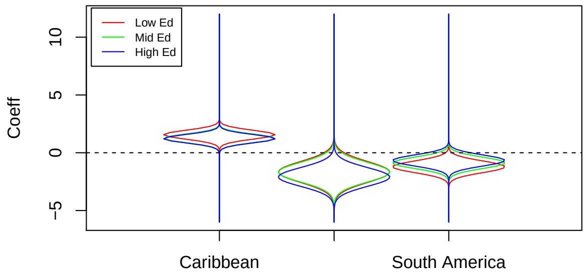

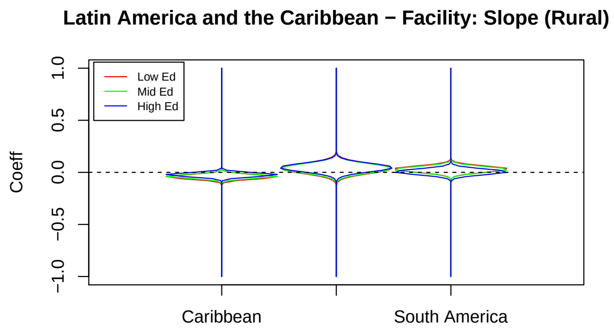
Latin America and the Caribbean − Facility: Slope (Rural)
Low Ed
Mid Ed
High Ed
Coeff
1.0
0.5
0.0
−0.5
−1.0
Caribbean
South America

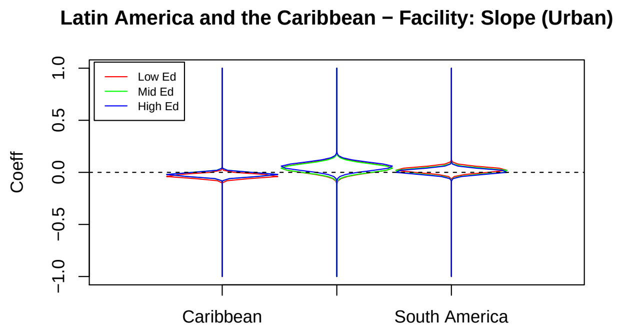
Latin America and the Caribbean − Facility: Slope (Urban)
Low Ed
Mid Ed
High Ed
Coeff
1.0
0.5
0.0
−0.5
−1.0
Caribbean
South America

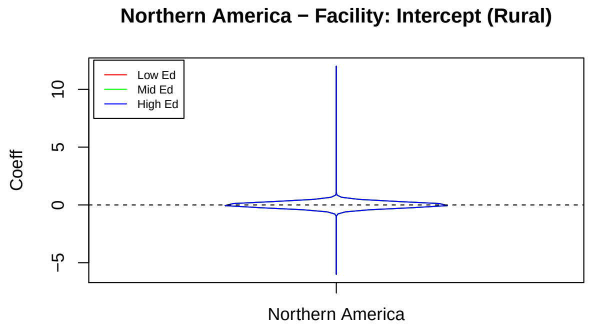
Northern America − Facility: Intercept (Rural)
Low Ed
Mid Ed
High Ed
Coeff
10
5
0
−5
Northern America

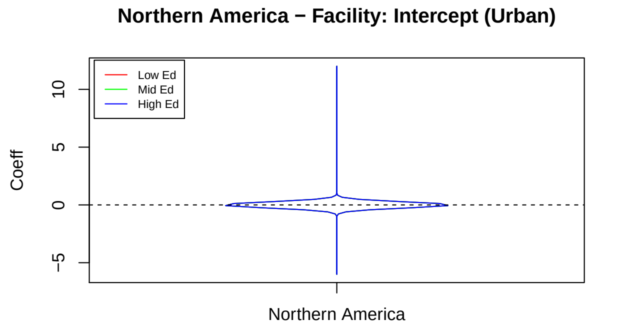
Northern America − Facility: Intercept (Urban)
Low Ed
Mid Ed
High Ed
Coeff
10
5
0
−5
Northern America

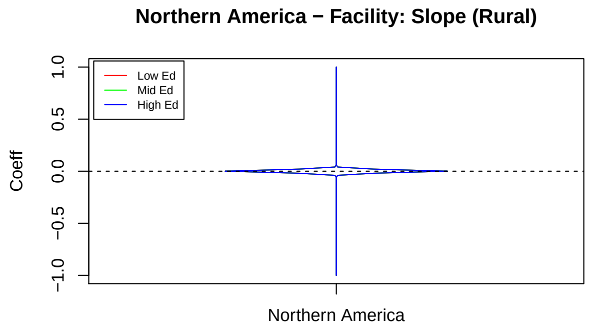
Northern America − Facility: Slope (Rural)
Low Ed
Mid Ed
High Ed
Coeff
1.0
0.5
0.0
−0.5
−1.0
Northern America

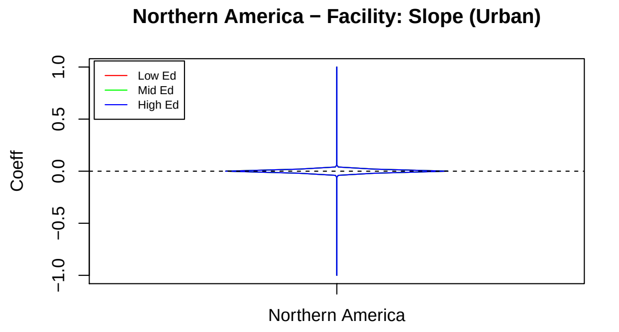
Northern America − Facility: Slope (Urban)
Low Ed
Mid Ed
High Ed
Coeff
1.0
0.5
0.0
−0.5
−1.0
Northern America

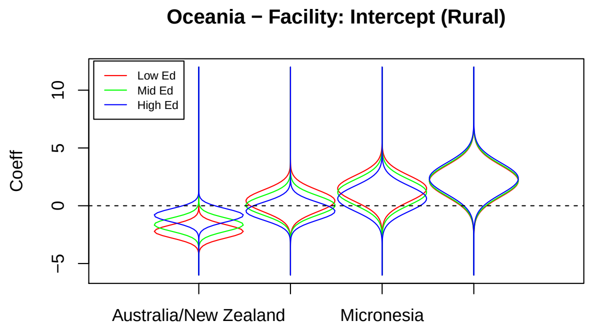
Oceania − Facility: Intercept (Rural)
Low Ed
Mid Ed
High Ed
Coeff
10
5
0
−5
Australia/New Zealand
Micronesia

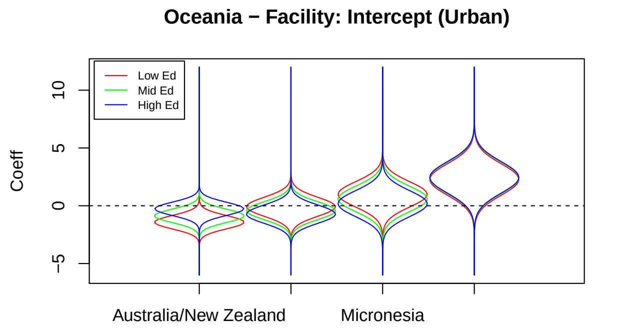
Oceania − Facility: Intercept (Urban)
Low Ed
Mid Ed
High Ed
Coeff
10
5
0
−5
Australia/New Zealand
Micronesia

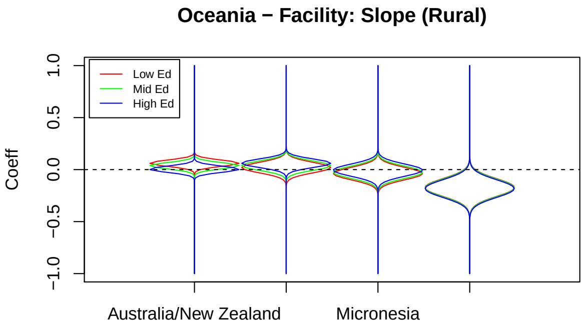
Oceania − Facility: Slope (Rural)
Low Ed
Mid Ed
High Ed
Coeff
−1.0
−0.5
0.0
0.5
1.0
Australia/New Zealand
Micronesia

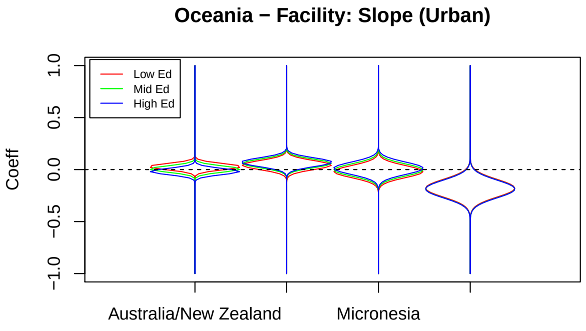
Oceania − Facility: Slope (Urban)
Low Ed
Mid Ed
High Ed
Coeff
−1.0
−0.5
0.0
0.5
1.0
Australia/New Zealand
Micronesia

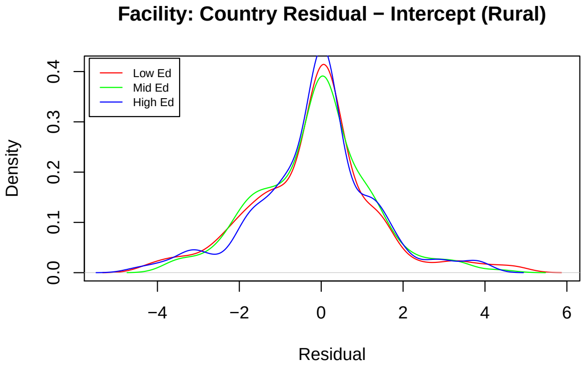
Facility: Country Residual − Intercept (Rural)
Low Ed
Mid Ed
High Ed
Density
0.0
0.1
0.2
0.3
0.4
−4
−2
0
2
4
6
Residual

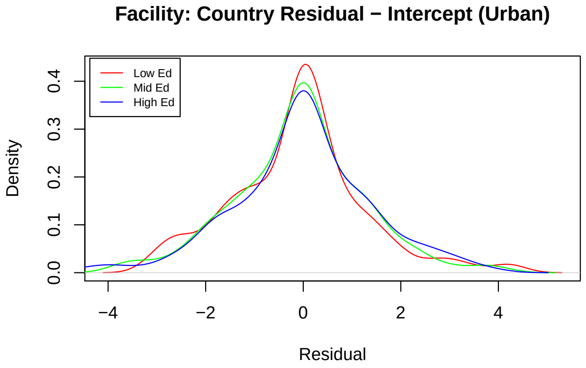
Facility: Country Residual − Intercept (Urban)
Low Ed
Mid Ed
High Ed
Density
0.0
0.1
0.2
0.3
0.4
−4
−2
0
2
4
Residual

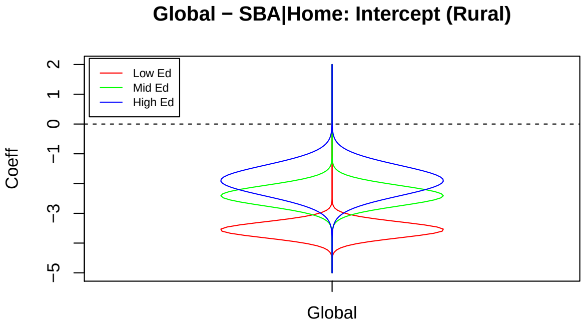
Global − SBA|Home: Intercept (Rural)
Low Ed
Mid Ed
High Ed
Coeff
−5
−3
−1
0
1
2
Global

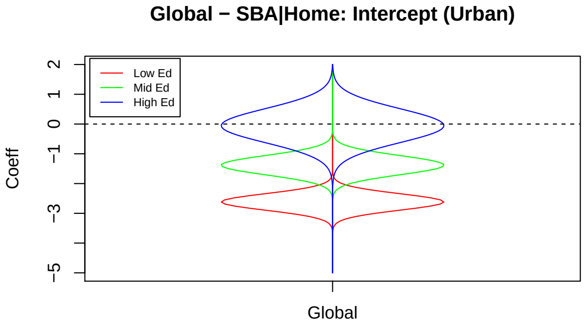
Global − SBA|Home: Intercept (Urban)
Low Ed
Mid Ed
High Ed
Coeff
−5
−3
−1
0
1
2
Global

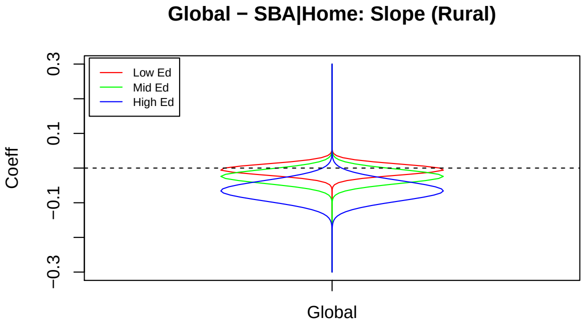
Global − SBA|Home: Slope (Rural)
Low Ed
Mid Ed
High Ed
Coeff
−0.3
−0.1
0.1
0.3
Global

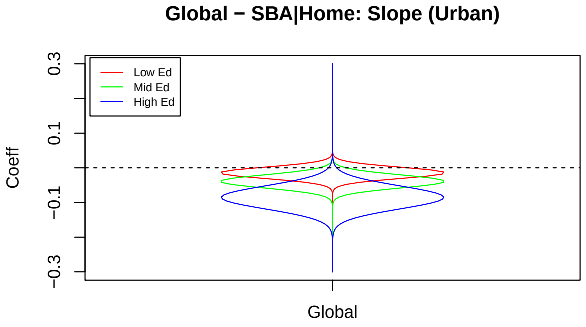
Global − SBA|Home: Slope (Urban)
Low Ed
Mid Ed
High Ed
Coeff
−0.3
−0.1
0.1
0.3
Global

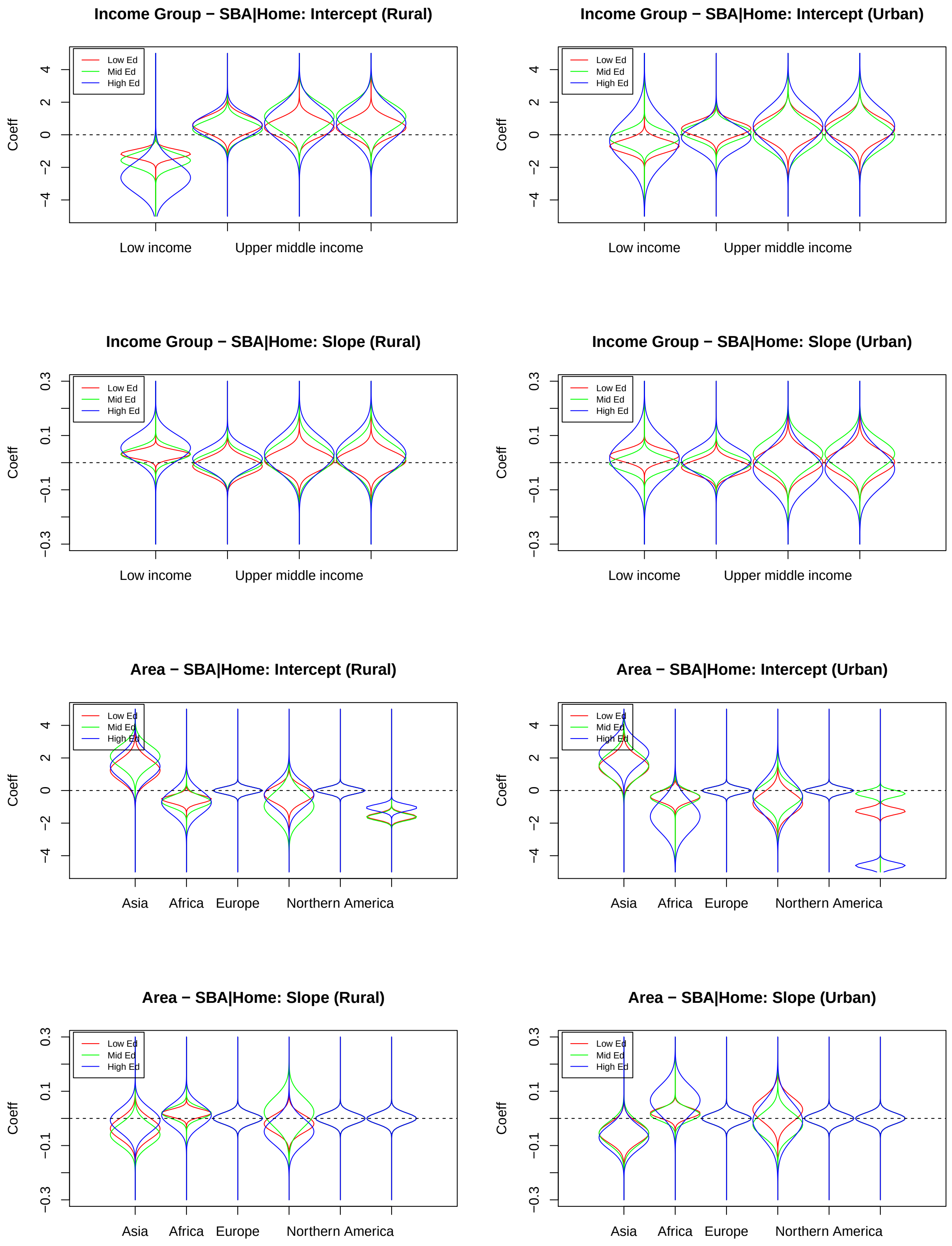
Income Group − SBA|Home: Intercept (Rural)
Low Ed
Mid Ed
High Ed
Coeff
4
2
0
−2
−4
Low income
Upper middle income
Income Group − SBA|Home: Intercept (Urban)
Low Ed
Mid Ed
High Ed
Coeff
Low income
Upper middle income
Income Group − SBA|Home: Slope (Rural)
Low Ed
Mid Ed
High Ed
Coeff
0.3
0.1
−0.1
−0.3
Low income
Upper middle income
Income Group − SBA|Home: Slope (Urban)
Low Ed
Mid Ed
High Ed
Coeff
Low income
Upper middle income
Area − SBA|Home: Intercept (Rural)
Low Ed
Mid Ed
High Ed
Coeff
Asia
Africa
Europe
Northern America
Area − SBA|Home: Intercept (Urban)
Low Ed
Mid Ed
High Ed
Coeff
Asia
Africa
Europe
Northern America
Area − SBA|Home: Slope (Rural)
Low Ed
Mid Ed
High Ed
Coeff
Asia
Africa
Europe
Northern America
Area − SBA|Home: Slope (Urban)
Low Ed
Mid Ed
High Ed
Coeff
Asia
Africa
Europe
Northern America

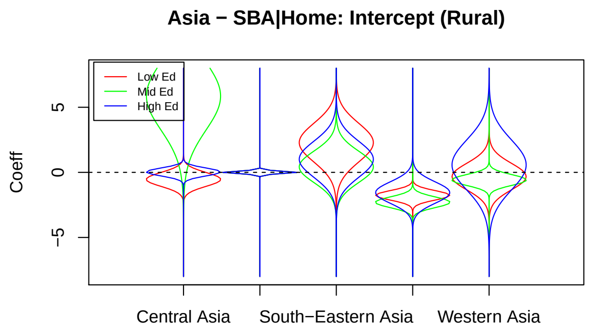
Asia − SBA|Home: Intercept (Rural)
Low Ed
Mid Ed
High Ed
Coeff
5
0
−5
Central Asia
South−Eastern Asia
Western Asia

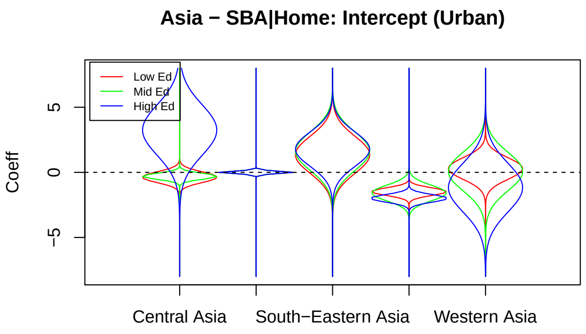
Asia − SBA|Home: Intercept (Urban)
Low Ed
Mid Ed
High Ed
Coeff
5
0
−5
Central Asia
South−Eastern Asia
Western Asia

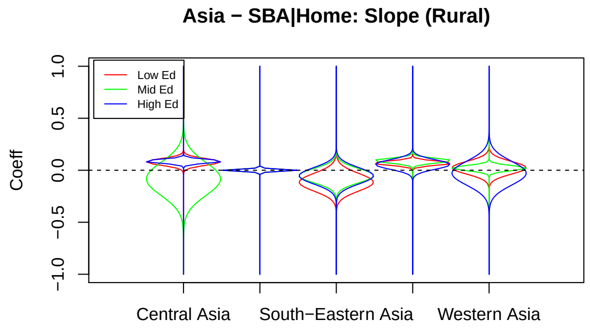
Asia − SBA|Home: Slope (Rural)
Low Ed
Mid Ed
High Ed
Coeff
1.0
0.5
0.0
−0.5
−1.0
Central Asia
South−Eastern Asia
Western Asia

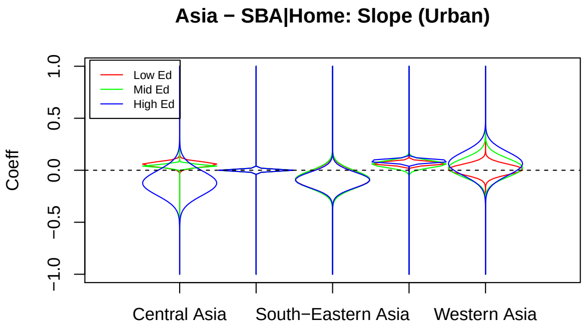
Asia − SBA|Home: Slope (Urban)
Low Ed
Mid Ed
High Ed
Coeff
1.0
0.5
0.0
−0.5
−1.0
Central Asia
South−Eastern Asia
Western Asia

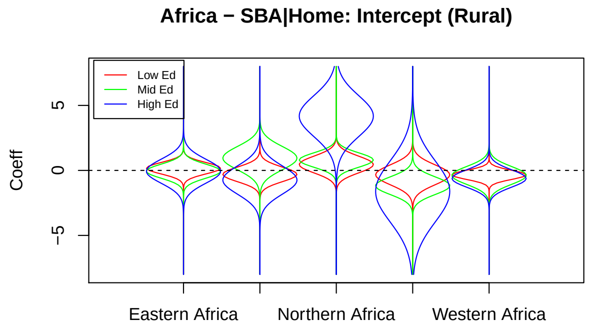
Africa − SBA|Home: Intercept (Rural)
Low Ed
Mid Ed
High Ed
Coeff
5
0
−5
Eastern Africa
Northern Africa
Western Africa

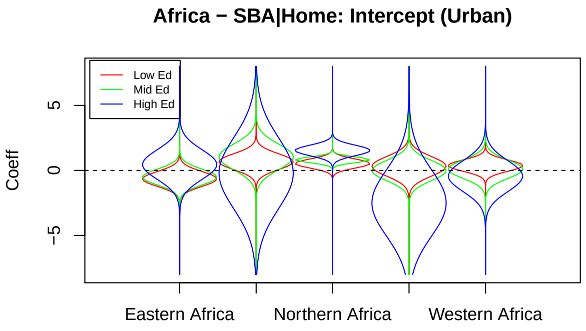
Africa − SBA|Home: Intercept (Urban)
Low Ed
Mid Ed
High Ed
Coeff
5
0
−5
Eastern Africa
Northern Africa
Western Africa

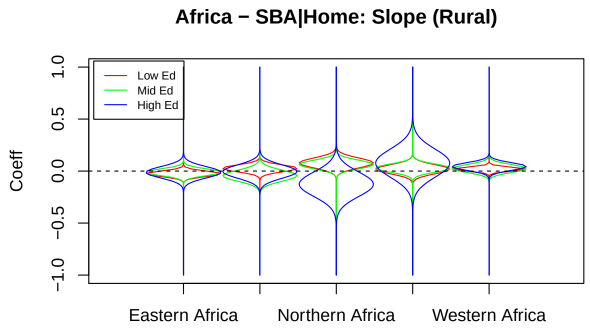
Africa − SBA|Home: Slope (Rural)
Low Ed
Mid Ed
High Ed
Coeff
1.0
0.5
0.0
−0.5
−1.0
Eastern Africa
Northern Africa
Western Africa

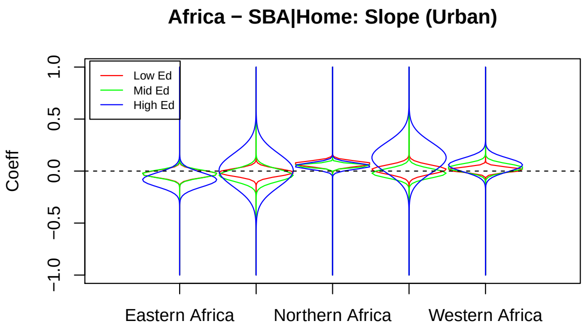
Africa − SBA|Home: Slope (Urban)
Low Ed
Mid Ed
High Ed
Coeff
1.0
0.5
0.0
−0.5
−1.0
Eastern Africa
Northern Africa
Western Africa

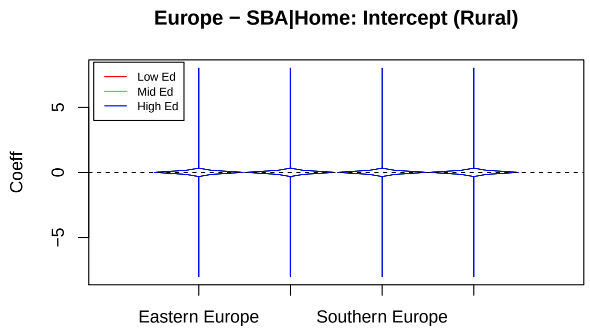
Europe − SBA|Home: Intercept (Rural)
Low Ed
Mid Ed
High Ed
Coeff
5
0
−5
Eastern Europe
Southern Europe

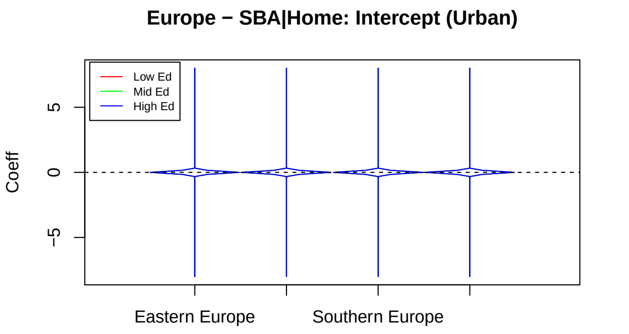
Europe − SBA|Home: Intercept (Urban)
Low Ed
Mid Ed
High Ed
Coeff
5
0
−5
Eastern Europe
Southern Europe

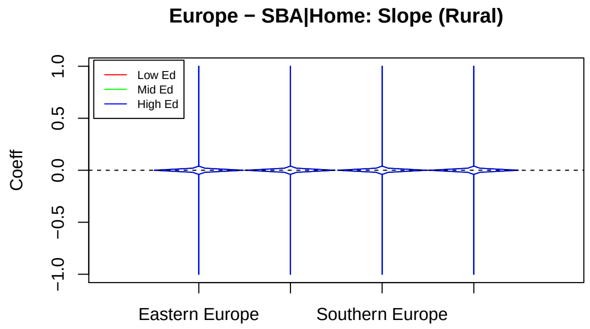
Europe − SBA|Home: Slope (Rural)
Low Ed
Mid Ed
High Ed
Coeff
1.0
0.5
0.0
−0.5
−1.0
Eastern Europe
Southern Europe

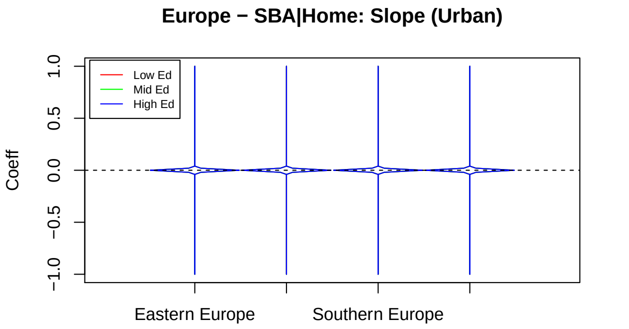
Europe − SBA|Home: Slope (Urban)
Low Ed
Mid Ed
High Ed
Coeff
1.0
0.5
0.0
−0.5
−1.0
Eastern Europe
Southern Europe

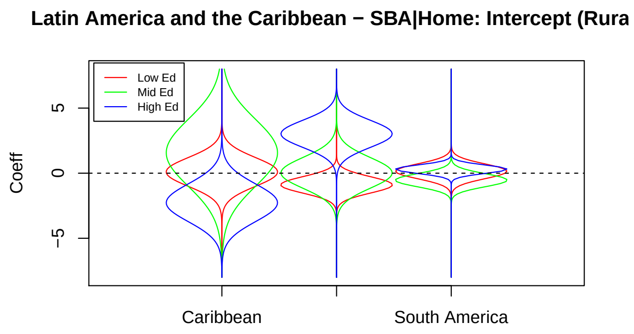
Latin America and the Caribbean − SBA|Home: Intercept (Rura
Low Ed
Mid Ed
High Ed
Coeff
5
0
−5
Caribbean
South America

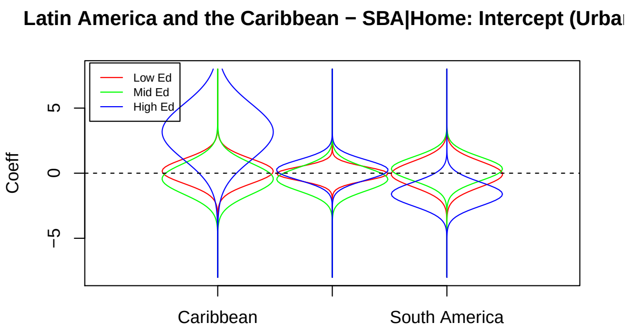
Latin America and the Caribbean − SBA|Home: Intercept (Urba
Low Ed
Mid Ed
High Ed
Coeff
5
0
−5
Caribbean
South America

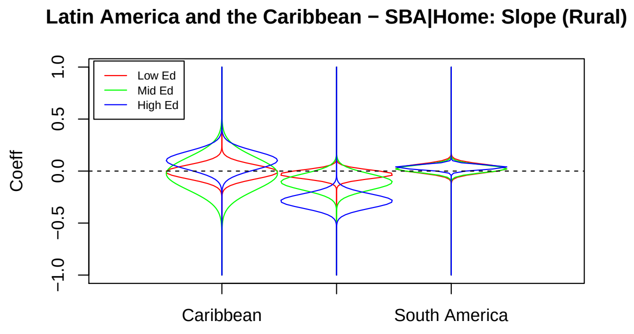
Latin America and the Caribbean − SBA|Home: Slope (Rural)
Low Ed
Mid Ed
High Ed
Coeff
1.0
0.5
0.0
−0.5
−1.0
Caribbean
South America

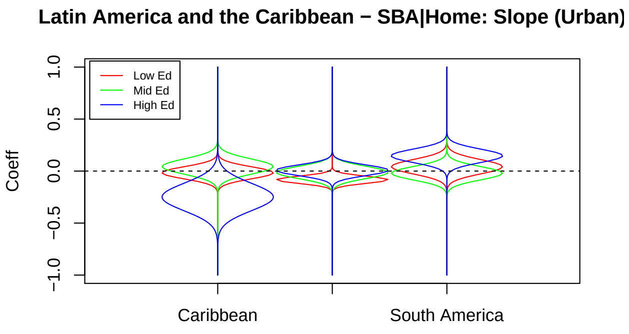
Latin America and the Caribbean − SBA|Home: Slope (Urban)
Low Ed
Mid Ed
High Ed
Coeff
1.0
0.5
0.0
−0.5
−1.0
Caribbean
South America

Northern America − SBA|Home: Intercept (Rural)

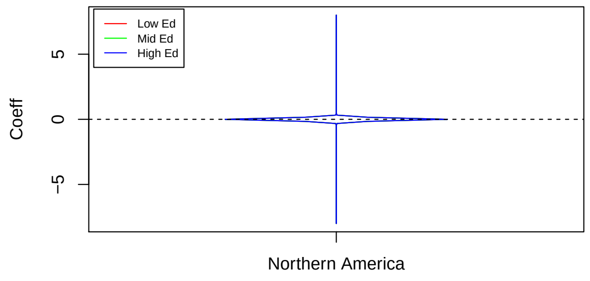

Northern America − SBA|Home: Intercept (Urban)

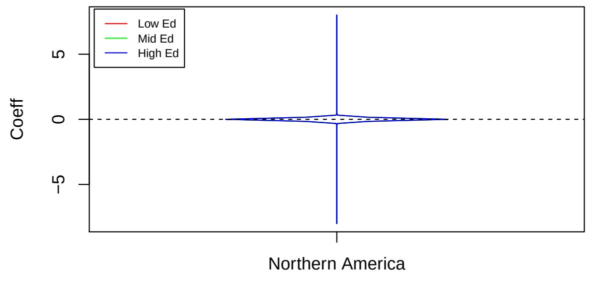

Northern America − SBA|Home: Slope (Rural)

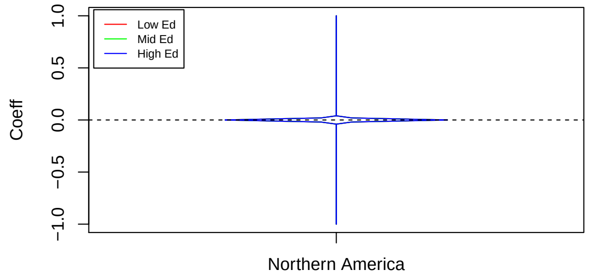

Northern America − SBA|Home: Slope (Urban)

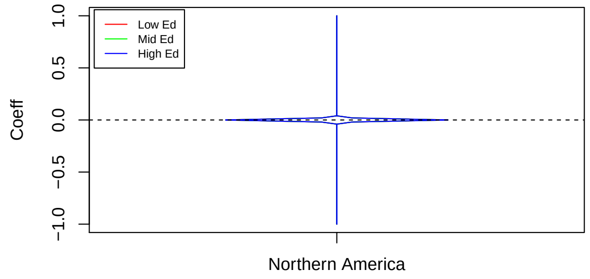

Oceania − SBA|Home: Intercept (Rural)

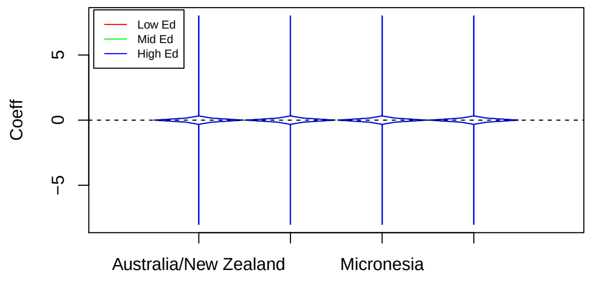

Oceania − SBA|Home: Intercept (Urban)

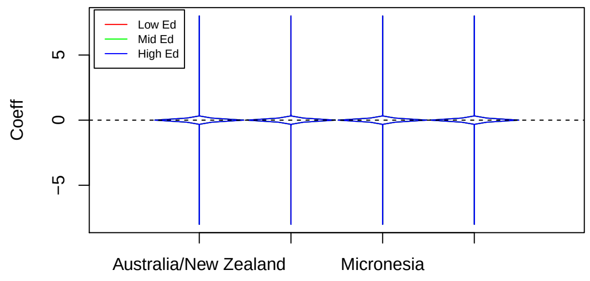

Oceania − SBA|Home: Slope (Rural)

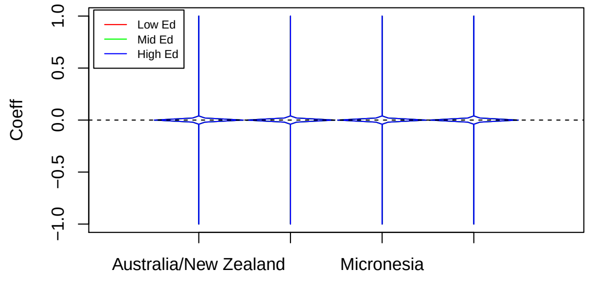

Oceania − SBA|Home: Slope (Urban)

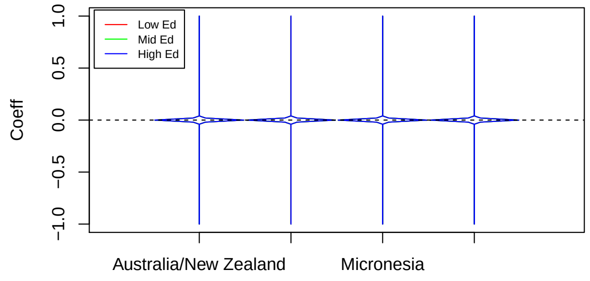

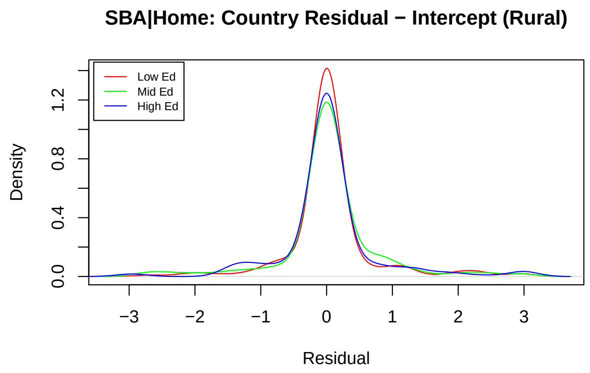
SBA|Home: Country Residual − Intercept (Rural)
Low Ed
Mid Ed
High Ed
Density
0.0
0.4
0.8
1.2
−3
−2
−1
0
1
2
3
Residual

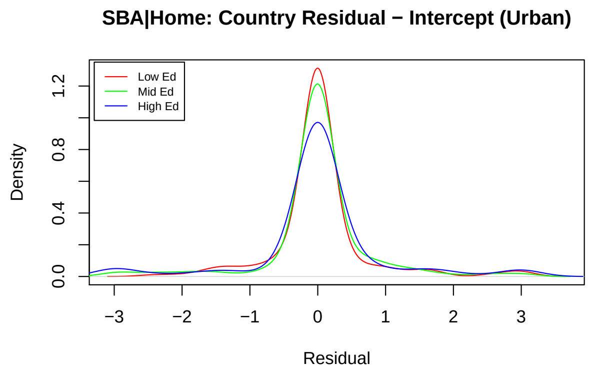
SBA|Home: Country Residual − Intercept (Urban)
Low Ed
Mid Ed
High Ed
Density
0.0
0.4
0.8
1.2
−3
−2
−1
0
1
2
3
Residual

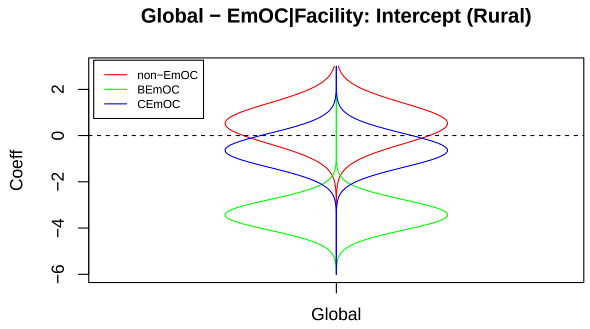
Global − EmOC|Facility: Intercept (Rural)
non−EmOC
BEmOC
CEmOC
Coeff
−6
−4
−2
0
2
Global

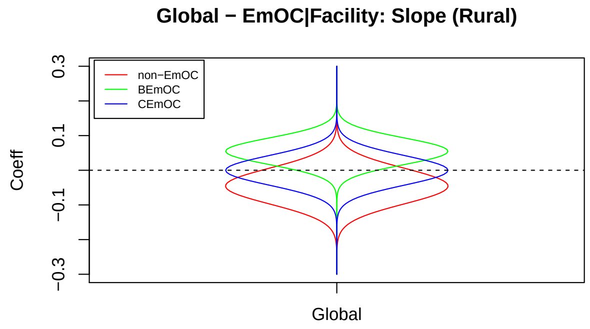
Global − EmOC|Facility: Slope (Rural)
non−EmOC
BEmOC
CEmOC
Coeff
−0.3
−0.1
0.1
0.3
Global

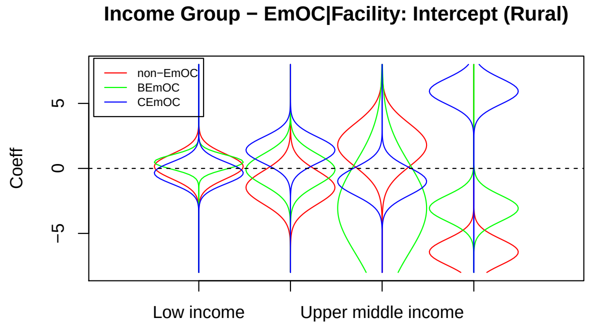
Income Group − EmOC|Facility: Intercept (Rural)
non−EmOC
BEmOC
CEmOC
Coeff
−5
0
5
Low income
Upper middle income

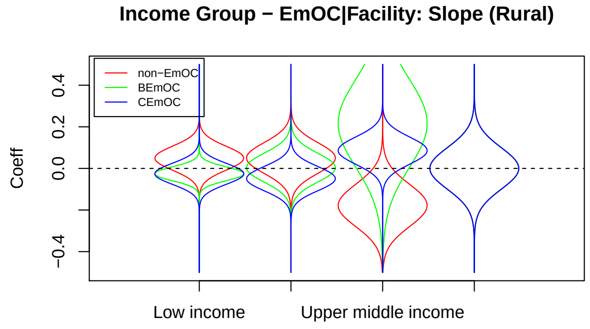
Income Group − EmOC|Facility: Slope (Rural)
non−EmOC
BEmOC
CEmOC
Coeff
−0.4
0.0
0.2
0.4
Low income
Upper middle income

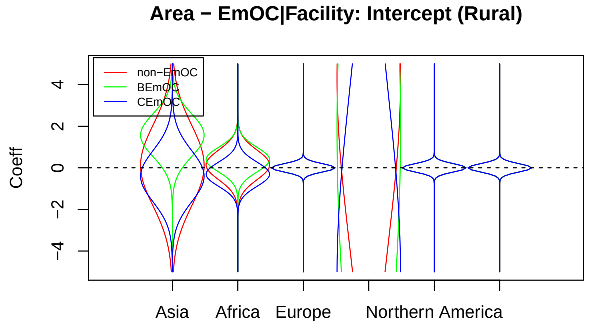
Area − EmOC|Facility: Intercept (Rural)
non−EmOC
BEmOC
CEmOC
Coeff
−4
−2
0
2
4
Asia
Africa
Europe
Northern America

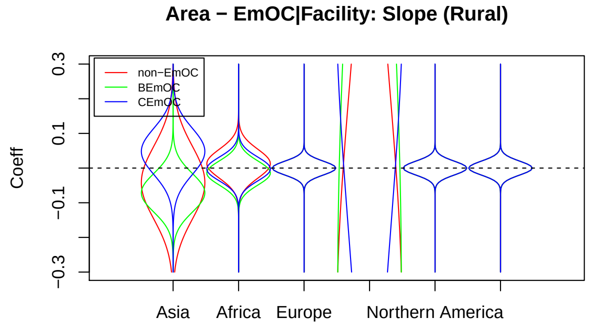
Area − EmOC|Facility: Slope (Rural)
non−EmOC
BEmOC
CEmOC
Coeff
−0.3
−0.1
0.1
0.3
Asia
Africa
Europe
Northern America

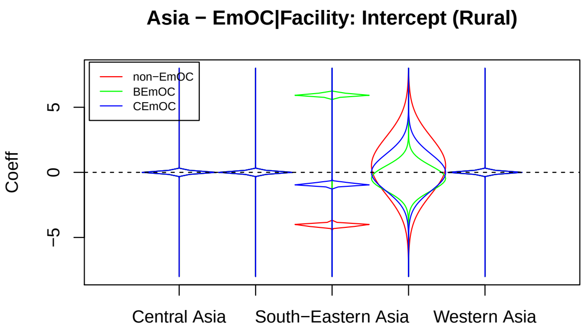
Asia − EmOC|Facility: Intercept (Rural)
non−EmOC
BEmOC
CEmOC
Coeff
5
0
−5
Central Asia
South−Eastern Asia
Western Asia

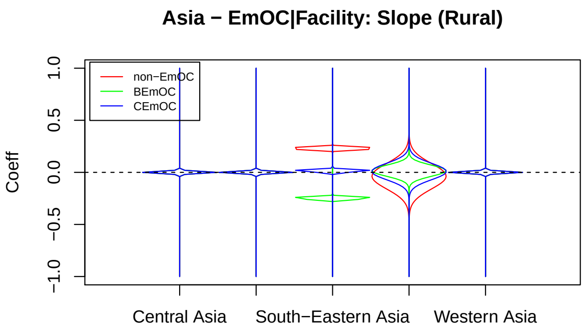
Asia − EmOC|Facility: Slope (Rural)
non−EmOC
BEmOC
CEmOC
Coeff
1.0
0.5
0.0
−0.5
−1.0
Central Asia
South−Eastern Asia
Western Asia

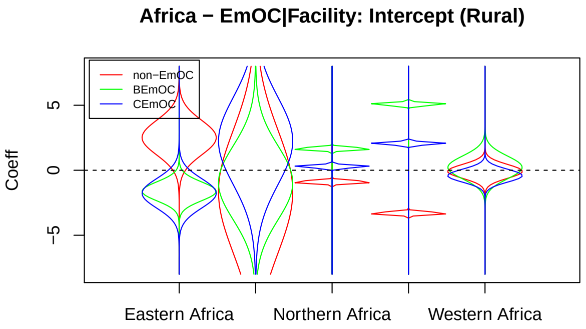
Africa − EmOC|Facility: Intercept (Rural)
non−EmOC
BEmOC
CEmOC
Coeff
5
0
−5
Eastern Africa
Northern Africa
Western Africa

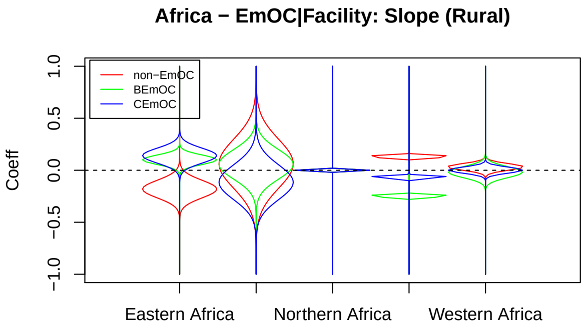
Africa − EmOC|Facility: Slope (Rural)
non−EmOC
BEmOC
CEmOC
Coeff
1.0
0.5
0.0
−0.5
−1.0
Eastern Africa
Northern Africa
Western Africa

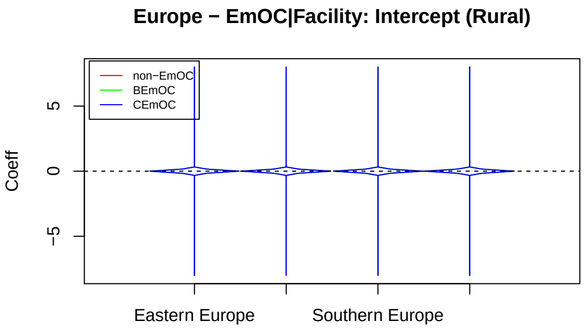
Europe − EmOC|Facility: Intercept (Rural)
non−EmOC
BEmOC
CEmOC
Coeff
5
0
−5
Eastern Europe
Southern Europe

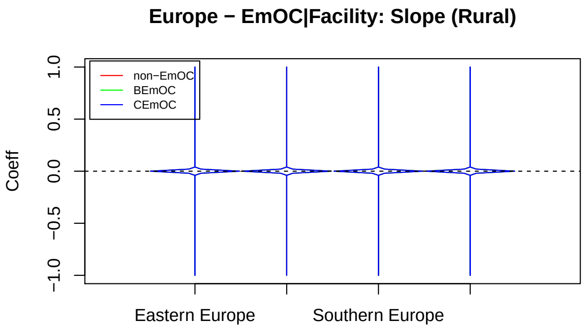
Europe − EmOC|Facility: Slope (Rural)
non−EmOC
BEmOC
CEmOC
Coeff
1.0
0.5
0.0
−0.5
−1.0
Eastern Europe
Southern Europe

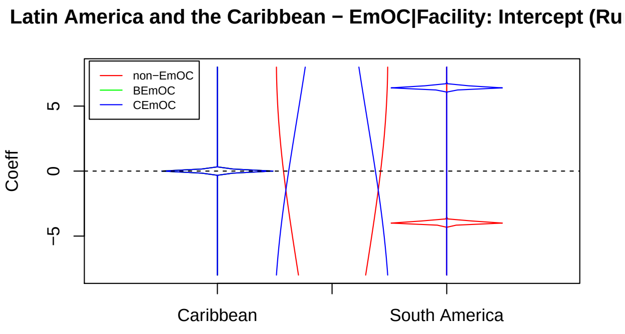
Latin America and the Caribbean − EmOC|Facility: Intercept (Ru
non−EmOC
BEmOC
CEmOC
Coeff
5
0
−5
Caribbean
South America

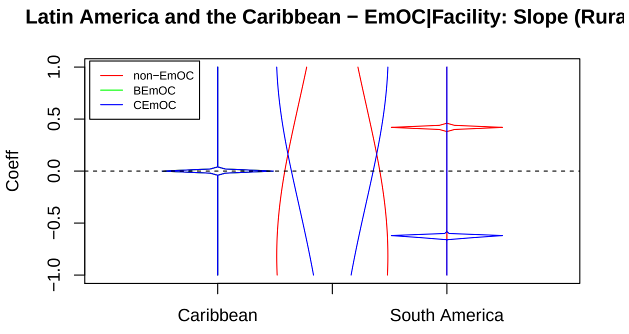
Latin America and the Caribbean − EmOC|Facility: Slope (Rura
non−EmOC
BEmOC
CEmOC
Coeff
1.0
0.5
0.0
−0.5
−1.0
Caribbean
South America

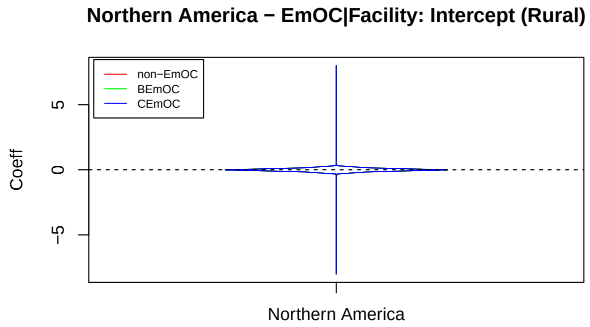

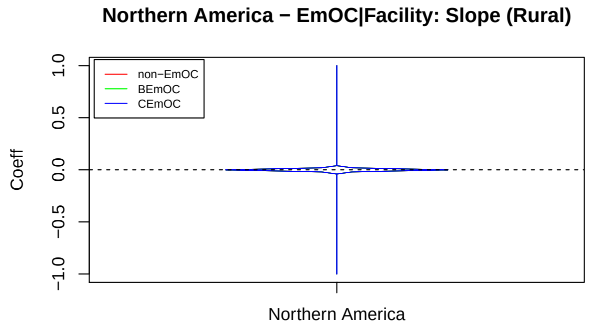

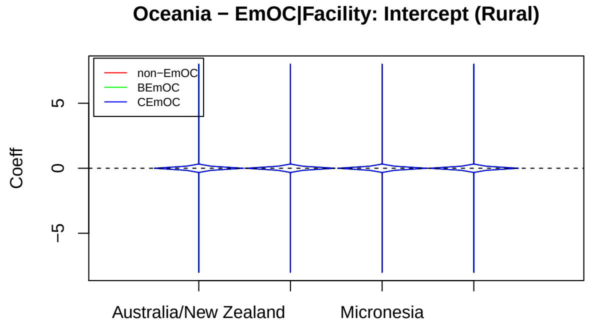

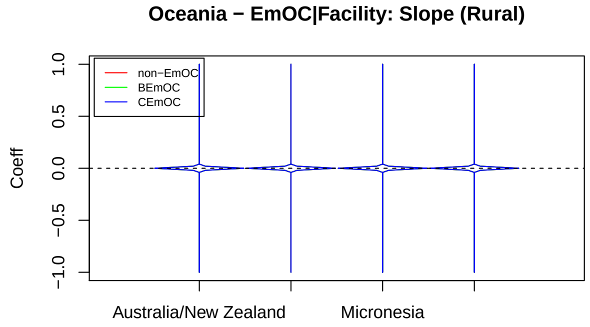

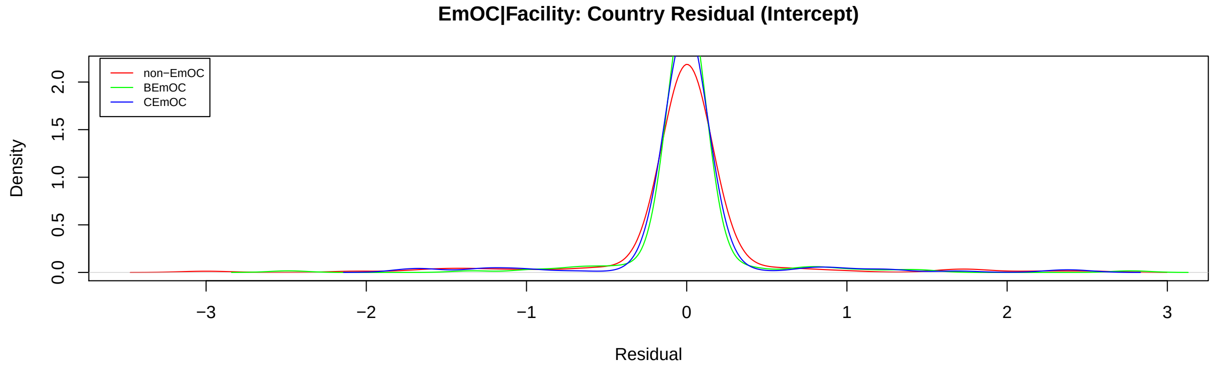

Model Implementation

At the time of delivery, the model simulates whether the woman will deliver at Home, Home-SBA, or at a Facility. Conditional on delivering at an institution, the type of facility is then drawn: non-EmOC, BEmOC, or CEmOC. Site-specific probabilities of preventive care and complication referral are then simulated.

References

Admasu K, Haile-Mariam A, Bailey P. Indicators for availability, utilization, and quality of emergency obstetric care in Ethiopia, 2008. Int J Gynaecol Obstet 2011; 115(1): 101-5. DOI: https://doi.org/10.1016/j.ijgo.2011.07.010

Alam B, Mridha MK, Biswas TK, Roy L, Rahman M, Chowdhury ME. Coverage of emergency obstetric care and availability of services in public and private health facilities in Bangladesh. Int J Gynaecol Obstet 2015; 131(1): 63-9. DOI: https://doi.org/10.1016/j.ijgo.2015.04.041

AMDD Working Group on Indicators. Averting Maternal Death and Disability. Program Note: Using

UN process indicators to assess needs in emergency obstetric services: Pakistan, Peru and Vietnam. Int J Gynaecol Obstet 2002; 78(3): 275-82. DOI: https://doi.org/10.1016/s0020-7292(02)00190-x

AMDD Working Group on Indicators. Program Note: Using UN process indicators to assess needs in emergency obstetric services: Morocco, Nicaragua and Sri Lanka. Int J Gynaecol Obstet 2003; 80(2): 222-30. DOI: https://doi.org/10.1016/s0020-7292(02)00390-9

AMDD Working Group on Indicators. Program Note: Using UN process indicators to assess needs in emergency obstetric services: Niger, Rwanda and Tanzania. Int J Gynaecol Obstet 2003; 83(1): 112-20. DOI: https://doi.org/10.1016/s0020-7292(03)00187-5

AMDD Working Group on Indicators. Program Note: Using UN process indicators to assess needs in emergency obstetric services: Benin and Chad. Int J Gynaecol Obstet 2004; 86(1): 110-20. DOI: https://doi.org/10.1016/j.ijgo.2004.04.034

Augusto O, Keyes EE, Madede T, et al. Progress in Mozambique: Changes in the availability, use, and quality of emergency obstetric and newborn care between 2007 and 2012. PLoS One 2018; 13(7): e0199883. DOI: https://doi.org/10.1371/journal.pone.0199883

Baguiya A, Meda IB, Millogo T, Kourouma M, Mouniri H, Kouanda S. Availability and utilization of obstetric and newborn care in Guinea: a national needs assessment. Int J Gynaecol Obstet 2016; 135 Suppl 1: S2-S6. DOI: https://doi.org/10.1016/j.ijgo.2016.09.004

Bailey PE, Paxton A. Program Note: Using UN process indicators to assess needs in emergency obstetric services. Int J Gynaecol Obstet 2002; 76(3): 299-305. DOI: https://doi.org/10.1016/s0020-7292(01)00592-6

Bailey P, AMDD Working Group on Indicators. Program Note: Using UN process indicators to assess needs in emergency obstetric services: Bolivia, El Salvador and Honduras. Int J Gynaecol Obstet 2005; 89(2): 221-30. DOI: https://doi.org/10.1016/j.ijgo.2004.12.045

Banstola A, Simkhada P, van Teijlingen E, et al. The availability of emergency obstetric care in birthing centres in rural Nepal: a cross-sectional survey. Matern Child Health J 2020; 24(6): 806-816. DOI: https://doi.org/10.1007/s10995-019-02832-2

Biswas AB, Das DK, Misra R, Roy RN, Ghosh D, Mitra K. Availability and use of emergency obstetric care services in four districts of West Bengal, India. J Health Popul Nutr 2005; 23(3): 266-74. PMID: https://pubmed.ncbi.nlm.nih.gov/16262024/

Bosomprah S, Tatem AJ, Dotse-Gborgbortsi W, Aboagye P, Matthews Z. Spatial distribution of emergency obstetric and newborn care services in Ghana: using the evidence to plan interventions. Int J Gynaecol Obstet 2016; 132(1): 130-4. DOI: https://doi.org/10.1016/j.ijgo.2015.11.004

Brückmann P, Hashmi A, Kuch M, Kuhnt J, Monfared I, Vollmer S. Public provision of emergency obstetric care: a case study in two districts of Pakistan. BMJ Open 2019; 9(5): e027187. DOI: https://doi.org/10.1136/bmjopen-2018-027187

Fakih B, Nofly AAS, Ali AO, et al. The status of maternal and newborn health care services in Zanzibar. BMC Pregnancy Childbirth 2016; 16(1): 134. DOI: https://doi.org/10.1186/s12884-016-0928-6

Fauveau V, UNFPA-AMDD Working Group on Indicators. Program Note: Using UN process indicators to assess needs in emergency obstetric services: Gabon, Guinea-Bissau, and The Gambia. Int J Gynaecol Obstet 2007; 96(3): 233-40. DOI: https://doi.org/10.1016/j.ijgo.2006.12.004

Global Health Observatory data repository. Births attended by skilled health personnel. Available at: https://apps.who.int/gho/data/node.main.SKILLEDBIRTHATTENDANTS?lang=en. Last updated: 2020-04-08

Jones SA, Gopalakrishnan S, Ameh CA, White S, van den Broek NR. 'Women and babies are dying but not of Ebola': the effect of the Ebola virus epidemic on the availability, uptake and outcomes of maternal and newborn health services in Sierra Leone. BMJ Glob Health 2016; 1(3): e000065. DOI: https://doi.org/10.1136/bmjgh-2016-000065

Kabo I, Orobaton N, Abdulkarim M, et al. Strengthening and monitoring health system's capacity to improve availability, utilization and quality of emergency obstetric care in northern Nigeria. PLoS One 2019; 14(2): e0211858. DOI: https://doi.org/10.1371/journal.pone.0211858

Kongnyuy EJ, Hofman J, Mlava G, Mhango C, van den Broek N. Availability, utilisation and quality of basic and comprehensive emergency obstetric care services in Malawi. Matern Child Health J 2009; 13(5): 687-94. DOI: https://doi.org/10.1007/s10995-008-0380-y

Kouanda S, Ouédraogo AM, Ouédraogo GH, Sanon D, Belemviré S, Ouédraogo L. Emergency obstetric and neonatal care needs assessment: Results of the 2010 and 2014 surveys in Burkina Faso. Int J Gynaecol Obstet 2016; 135 Suppl 1: S11-S15. DOI: https://doi.org/10.1016/j.ijgo.2016.08.008

Liabsuetrakul T, Peeyananjarassri K, Tassee S, Sanguanchua S, Chaipinitpan S. Emergency obstetric care in the southernmost provinces of Thailand. Int J Qual Health Care 2007; 19(4): 250-6. DOI: https://doi.org/10.1093/intqhc/mzm023

MacDorman MF, Matthews TJ, Declercq E. Trends in out-of-hospital births in the United States, 1990-2012. NCHS Data Brief 2014; 144: 1-8. Available at: https://www.cdc.gov/nchs/data/databriefs/db144.pdf

Ministry of Health and Social Services, Namibia. Report on emergency obstetric and newborn care needs assessment. 2016. Available at: https://cmnh.lstmed.ac.uk/sites/default/files/content/centre-news-articles/attachments/203264-Full%20report_Namibia%20EmONC%20Assessment.pdf

Ministry of Health, Timor-Leste. Emergency obstetric and newborn care needs assessment - Timor-Leste 2015. Available at: https://reliefweb.int/report/timor-leste/emergency-obstetric-and-newborn-care-needs-assessment-timor-leste-2015

Mony PK, Krishnamurthy J, Thomas A, et al. Availability and distribution of emergency obstetric care services in Karnataka State, South India: access and equity considerations. PLoS One 2013; 8(5): e64126. DOI: https://doi.org/10.1371/journal.pone.0064126

National Coordinating Agency for Population and Development (NCAPD), Ministry of Medical Services (MOMS), Ministry of Public Health and Sanitation (MOPHS), Kenya National Bureau of Statistics (KNBS), ICF Macro. 2011. Kenya Service Provision Assessment Survey 2010. Nairobi, Kenya. Available at: https://dhsprogram.com/pubs/pdf/SPA17/SPA17.pdf

Otchere SA, Kayo A. The challenges of improving emergency obstetric care in two rural districts in Mali. Int J Gynaecol Obstet 2007; 99(2): 173-82. DOI: https://doi.org/10.1016/j.ijgo.2007.07.004

Oyerinde K, Harding Y, Amara P, Kanu R, Shoo R, Daoh K. The status of maternal and newborn care services in Sierra Leone 8 years after ceasefire. Int J Gynaecol Obstet 2011; 114(2): 168-73. DOI: https://doi.org/10.1016/j.ijgo.2011.05.006

Pearson L, Shoo R. Availability and use of emergency obstetric services: Kenya, Rwanda, Southern Sudan, and Uganda. Int J Gynaecol Obstet 2005; 88(2): 208-15. DOI: https://doi.org/10.1016/j.ijgo.2004.09.027

Saidu R, August EM, Alio AP, Salihu HM, Saka MJ, Jimoh AAG. An assessment of essential maternal health services in Kwara State, Nigeria. Afr J Reprod Health 2013; 17(1): 41-8. PMID: https://pubmed.ncbi.nlm.nih.gov/24069733/

Salazar M, Vora K, De Costa A. The dominance of the private sector in the provision of emergency obstetric care: studies from Gujarat, India. BMC Health Serv Res 2016; 16: 225. DOI: https://doi.org/10.1186/s12913-016-1473-8

Sikder SS, Labrique AB, Ali H, et al. Availability of emergency obstetric care (EmOC) among public and private health facilities in rural northwest Bangladesh. BMC Public Health 2015; 15:36. DOI: https://doi.org/10.1186/s12889-015-1405-2

UNFPA, AMDD. Making safe motherhood a reality in West Africa: Using indicators to programme for results. 2003. Available at: https://www.unfpa.org/publications/making-safe-motherhood-reality-west-africa

UNFPA. Evaluation des besoins en soins obstetricaux et neonatals d'urgence dans trois provinces de la République Démocratique du Congo. 2012. Available at: https://drc.unfpa.org/sites/default/files/pub-pdf/SONU_RAPPORT_ENQUETE_FINAL_DU_7_12_2012.pdf

UNFPA. Country programme document for Burundi. DP/FPA/CPD/BDI/8. 2018. Available at: https://www.unfpa.org/sites/default/files/portal-document/DPFPACPDBDI8_EN.pdf

UNFPA. Country programme document for Angola. DP/FPA/CPD/AGO/8. 2019. Available at: https://www.unfpa.org/sites/default/files/event-pdf/DP.FPA_.CPD_.AGO_.8_-_Angola_CPD_2020-2022_-_DRAFT_Final_-14Jun19.pdf

World Health Organization, UNFPA, UNICEF, AMDD. Monitoring emergency obstetric care: a handbook. Geneva: World Health Organization, 2009. Available at: http://whqlibdoc.who.int/publications/2009/9789241547734_eng.pdf

Young-Mi K, Zainullah P, Mungia J, Tappis H, Bartlett L, Zaka N. Availability and quality of emergency obstetric and neonatal care services in Afghanistan. Int J Gynaecol Obstet 2012; 116(3): 192-6. DOI: https://doi.org/10.1016/j.ijgo.2011.10.017

A.1.4.3 Recognition/Referral

Overview
In order to be treated, a complication must first be recognized, and perhaps referred to an appropriate facility. Recognition/referral of a complication is conceptualized as the "first delay" in the framework developed by Thaddeus and Maine (1994). We model different probabilities of recognition/referral by starting delivery site and complication type/severity.

The broader concept of recognition/referral encapsulates different aspects of complication management and health-seeking behavior such as: technical knowledge of complication danger signs, cultural acceptability of referral (ex: whether the husband is present and gives permission for the wife to go), financial barriers, childcare arrangements, etc. A systematic review found five main sub-themes related to the first delay: socio-economic factors, community perceptions about obstetric complications, women's autonomy and insufficient male partner involvement in the management of obstetric emergency, maternal obstetric history and health service utilization, and women's knowledge about obstetric danger signs. These factors contributed to delays at home to seeking appropriate obstetric care in a timely manner when needed (Geleto 2018).

Data
We undertook a targeted literature review of recognition/referral pathways and women's knowledge of obstetric danger signs, summarized by country below.

Bangladesh:
A study describing the process of recognition and response to symptoms during potential prolonged labour found that the most frequent and usually first action was to seek care from untrained attendants at home, followed from professional attendants outside the home, with care-seeking outside the home occurring a median of 19 hours after perceived labor onset (Head 2011). Another study in rural Bangladesh of women who reported a 'serious or very serious' complication during their last pregnancy/delivery found that most women sought care for 'serious' complications (86%), with 42% seeking multiple sources of care (Moran 2007). A community-based cross-sectional survey in Matlab of women who delivered in 2014 found that 26% of women could recognize three or more pregnancy danger signs, and 23% could recognize three or more delivery danger signs (Pervin 2018).

Ethiopia:
A systematic review and meta-analysis found that 32% (95% CI: 25.6-38.5) of pregnant women were prepared for birth and its complications, and only 26.33% of pregnant women were aware of danger signs during pregnancy (Berhe 2018). Another systematic review and meta-analysis found that the level of women's knowledge about obstetric danger signs during pregnancy, delivery, and postpartum was 48%, 43%, and 32%, respectively. Maternal age, education, income, health service use, distance from facility, and women's autonomy were reported in several studies as determinants of women's knowledge of obstetric danger signs (Geleto 2019).

Ghana:
A household survey in East Mamprusi District found that 51.1%, 29.4%, and 34.6% of women knew at least three key danger signs during pregnancy, delivery, and postpartum period, respectively (Saaka 2017).

Haiti, Malawi, Senegal:
A study of ANC clients based on service provision and assessment (SPA) surveys in Haiti, Malawi, and Senegal found that in all three countries more than half of clients knew at least one danger sign, but most clients could report only one, while almost no clients knew all seven (Assaf 2018).

India:
A study of pregnant women attending the outpatient department of an urban tertiary care hospital for the first time found that 20%, 15.8%, and 12% had knowledge of at least 3 danger signs of pregnancy, labor, and severe illness in newborns, respectively (Kamineni 2017). A cross-sectional study of antenatal and postnatal mothers registered in a Rural Health Training Center in Kancheepruam district, Tamil Nadu found that 21% of women had adequate knowledge (correctly answering at least 6/12 questions) regarding the danger signs of pregnancy, with maternal literacy and adequate prenatal care major determinants of respondent knowledge (Gopalakrishnan 2019). Another study in Dakshina Kannada district, Karnataka found that 53.8% of women

were aware of at least 6 danger signs (Akshaya 2017). A study in a rural block of Haryana found that >8 danger signs were known to only 9.5% of participants (Sharma 2019). Studies have found that knowledge of danger signs is likely somewhat higher among trained birth attendants. A study of Accredited Social Health Activists (ASHAs) in a rural district in Karnataka found that 1%, 4.8%, and 7.2% were aware of all key danger signs for labor and child birth, postpartum period, and pregnancy period, with a majority (71%) scoring 4-7 out of 8 (Kochukuttan 2013). A study in three states (Maharashtra, Rajasthan, and Odisha) exploring the adequacy of knowledge and clinical skills of ayurvedic and homeopathic practitioners engaged by the state governments to provide maternity care services found that nearly 76% had adequate theoretical knowledge of essential obstetric care and identification and management of complications of pregnancy (Chandhiok 2018). Looking at actual responses to complications (not just general knowledge of danger signs), studies have found higher proportions of women who seek care. Using a broader definition of "complication", the Coverage Evaluation Survey (CES) 2009-10 found that 32.8% of women who experienced a complication did not consult anybody, implying that the remaining ~70% of women did seek some form of care. Similar findings were observed by Montgomery (2014): a study based on a nationally representative survey found that two-thirds of women died seeking some form of healthcare, with most seeking care in a critical medical condition - it also found that rural areas of poorer states had proportionately lower access to and utilization of healthcare services than in urban areas, but that this rural-urban disparity was not seen in richer states. Another study found that out of 153 maternal deaths identified through community workers, verbal autopsies indicated that 23% occurred at home and that 30% occurred on the way to a health facility (Raj 2013).

Indonesia:
A study in Jayawijaya district, Papua province in eastern Indonesia, found that the decision to seek care lies predominantly with the husband. There was no significant lag time between the decision-making stage and the process of seeking care for the maternal cases. On average, the interval between symptom recognition and the decision to seek care in the home was less than 6 hours. Rapid decision-making usually occurred when visible symptoms of excessive bleeding began to appear (Rosales 2017).

Kenya:
A study of women attending antenatal care at Kenyatta National hospital found that 67% of respondents knew at least one danger sign in pregnancy, while only 6.9% knew of three or more danger signs, with level of education positively influencing birth preparedness (Mutiso 2008). A CEmOC facility-based qualitative study of 30 women who experience obstetric "near miss" in Malindi found that 16/30 women experienced delays in making the decision to seek care, compared to 8 women who experienced delays in reaching the care facility, and 6 experienced delays in receiving care at the facility (Echoka 2014). A community-based cross-sectional survey of community health workers/volunteers in North and East Kamagambo, Migori County found that 60% of participants in North Kamagambo knew 3 or more danger signs in 3 or more categories, compared to 24% of participants in East Kamagambo, revealing varying levels of knowledge among the two populations of lay health workers (Heerboth 2020).

Madagascar:
A convenience sample of 372 women in their first year postpartum between April-October 2015 found that knowledge of at least one danger sign varied from 80.9% of women knowing danger signs in pregnancy, to 51.9% at delivery and 50.8% postpartum, with women with higher education more likely to know danger signs. (Salem 2018)

Malawi:
A study of primigravidae women in a rural health center in Blantyre found that 82% (95% CI 67-96) of women had some knowledge and could make an informed decision to go to a health facility with pregnancy complications, with 61% (95% CI 42-79) able to do so with complications after delivery (Kumbani 2002). A following study of primigravidae women in an urban health center found that 60% of the participants were knowledgeable about obstetric complications in pregnancy, however, 73% and 82.2% did not know of any problems that could occur during and after delivery, respectively (Kumbani 2006). Participants had limited knowledge of complications that may need immediate treatment during all three periods: 58% (95% CI 43-73) had some knowledge and could make an informed decision to go to a health facility with pregnancy complications, however only 24% (95% CI 11-38) could do the same for complications after delivery (Kumbani 2006).

Morocco & Netherlands:
A qualitative study to describe knowledge of hypertensive disorders in pregnancy among pregnant Moroccan women in Morocco and the Netherlands found that half of them had never heard about hypertension in pregnancy and had no knowledge of symptoms or alarm signals (Ouasmani 2018).

Myanmar:
A cross-sectional survey of 262 auxiliary midwives from July 2015 to July 2016 found that only 8% were able to identify at least 80% of 20 critical danger signs, and 57.6% knew three or more critical danger signs during pregnancy, and 54.2% knew four or more critical signs during birth and postpartum (Than 2017).

Nepal:
A study of group antenatal care in six villages in Achham found that among the control cohort, 8-10% of women could identify danger signs during pregnancy, labor and childbirth, or postpartum period (Thapa 2019).

Nicaragua:
A study examining the impact of a radio-education intervention on knowledge of pregnancy danger signs found that the total number of signs identified by study participants increased by 53.8%, and found that urban setting and more than sixth-grade education were factors making it significantly more likely to score higher on post-test related to knowledge of danger signs (Radoff 2013).

Nigeria:
A community-based survey in Osogbo metropolis found that 70.8% of women were aware of danger signs in pregnancy, with vaginal bleeding the commonest danger sign mentioned (Sabageh 2017). A cross-sectional study at the postnatal ward at Federal Teaching Hospital in Ebonyi found that most of the women knew about birth preparedness (87.7%) and complication readiness (79.5%) (Onoh 2020). A cross-sectional study of recently delivered women at selected health facilities in Ikenne found that 34.6% of women had knowledge of 5+ danger signs of pregnancy (Imaralu 2020). A cross-sectional study of pregnant women attending the prenatal clinic at a tertiary hospital found that 81.5% were "well prepared" for birth and its complications, however, only 27.4% knew key danger signs during labor/delivery and 24.9% knew those in the first 2 days after delivery (Aduloju 2017). A study of women in Zaria found that 18.31%, 9.89%, and 9.24% knew at least four danger signs during pregnancy, labor and delivery, and postpartum period, respectively (George 2014). A study evaluating the Maternal and Child Health Integrated Program (MCHIP) in Kano and Zamfara states found that mothers' knowledge of birth preparedness/complication readiness was 32.2% among those who received counseling compared with 11.2% among those who did not (Ishola 2017).

Pakistan:
A study in three rural areas around Islamabad found that the majority of women (75%) were unaware of key obstetric danger signs and symptoms, with traditional practices and home remedies commonly used to manage complications in pregnancy before seeking medical care, which was only done when the condition became unmanageable at home (Salam 2018).

Rwanda:
A study in 2015 at University Teach Hospital of Kigali of all admitted pregnancy-related complications found that the majority of first delays resulted from mothers failing to recognize the problem (22.3%). Women with hypertensive disorders represented 9.1% who failed to recognized signs of severity, followed by women who experienced abortive outcomes (abortion or ectopic pregnancy) (Benimana 2018). A cross-sectional study among pregnant women who were referred to Ruhengeri hospital between July and November 2015, found that out of 350 women, 296 (84.6%), 271 (77.4%) and 288 (82.3%) could mention at least one key danger sign during pregnancy, labor and postpartum respectively, but only 23 (6.6%) could mention three or more key danger signs during all three periods (Smeele 2018).

South Africa:
A study of 340 pregnant women in KwaZulu-Natal found that although most of the study population (92%) attended health care facilities, only half (52%) of them knew about some of the "danger signs" of pregnancy, and 39% of them knew about their HIV status (Hoque 2011).

Tanzania:

A community-based cross-sectional study of women who delivered in the previous two years in Chamwino found that 58.2% of respondents were considered to be prepared for birth and its complications, with maternal education a significant determinant of preparedness (Bintabara 2015). However, 68.6% of women knew 0 danger signs, with 6.1% knowing 1-4 and 25.2$ knowing 5+ (Bintabara 2015). A follow-up study in Chamwino District found that 25.2% of respondents could mention at least five danger signs in any of the three phases of childbirth (pregnancy, childbirth, postpartum) with at least one in each phase, while 68.7% did not mention any danger signs (Bintabara 2017). A study of women who became pregnant or gave birth in the two preceding years in Mpwapwa found that 14.8% of women knew three or more obstetric danger signs, with the most commonly known signs including vaginal bleeding during pregnancy (19%), foul smelling vaginal discharge (15%), and baby stops moving (14.3%) (Urassa 2012). A study to evaluate the effectiveness of Home Based Life Saving Skills education found that the proportion of women who knew three or more danger signs improved substantially: 15.2% vs 48.1% during pregnancy, 15.3% vs 43.1% during childbirth, and 8.8% vs 19.8% in the postpartum period (August 2016). A study of women how had been pregnant in the past two years found that 51.1% knew at least one obstetric danger sign, with 26% knowing at least one danger sign during pregnancy, 23% during delivery, and 40% after delivery. However, few women knew three or more danger signs. Having secondary education was found to increase the likelihood of awareness of obstetric danger signs six-fold (OR=5.8, 95% CI 1.8-19.0) in comparison with no education at all (Pembe 2009). A study of randomly selected pregnant women attending antenatal care in Dodoma municipality found that only 46.0% of women in the intervention and 44.7% of participants had knowledge on obstetric and newborn danger signs, rising to 77.3% after the intervention, compared to 48.0% in the control group (Masoi 2019). A study in Kinondoni Municipality, Dar es Salam found that 57.8% of women were able to spontaneously mention only one to three danger signs, 31% had correct knowledge of at least four signs, and 2.7% were not able to mention any item (Mwilike 2018). However, among women who reported actually experiencing danger signs during their pregnancy (17.4% of respondents), 91% visited a healthcare facility (Mwilike 2018). The most commonly known pregnancy signs were vaginal bleeding (81%), swelling of the fingers, face, and legs (46%), and severe headache (44%), and older women were more likely to have knowledge of danger signs than young women (OR 1.61, 95% CI 1.05-2.46) (Mwilike 2018).

Turkey:
A study of 125 pregnant women who had been admitted to the emergency department for antenatal bleeding found that advanced age, high level of education, lack of health insurance, receiving antenatal care, nuclear family structure, and knowledge of the danger signs during pregnancy were found to affect the use of emergency obstetric care services (Koşum 2013).

Uganda:
A study of 810 women admitted in the antepartum period to Mulago hospital, found that only about 1 in 3 women were able to mention at least three of the five basic components of birth preparedness/complication readiness, and 1 in every 4 women could not mention any of the five components (Mbalinda 2014). A study of 764 recently delivered women from 112 villages in Mbarara district found that 52% of women knew at least one key danger sign during pregnancy, 72% during delivery, and 72% during postpartum. Only 19% had knowledge of 3 or more key danger signs during the three periods. Overall 35% of the respondents were birth prepared. Young age and high levels of education had a synergistic effect on the relationship between knowledge and birth preparedness (Kabakyenga 2011). A study 0f 80 pregnant women attending antenatal clinic in Adjumani found that 76.25% of the respondents mentioned vaginal bleeding and 62.5% vomiting as danger signs in pregnancy, while 12.5% did not know any danger sign in pregnancy (Florence 2019).

Parameters
We used hierarchical logistic regression models to model the probability of recognition and referral. Based on findings in the literature, we assume that women with higher education are more likely to recognize and have the autonomy and necessary support to be referred. We also assume that more severe complications are more likely to be recognized and referred, and that recognition/referral probabilities increase with more advanced delivery site, with Home being the lowest, followed by Home-SBA, non-EmOC, BEmOC, and CEmOC. These constraints were enforced when sampling probabilities in the model. Based on the literature, we also assume that ANC visits improve the probability of recognizing complications for women delivering at Home (see ANC).

Recognition/Referral

G = Global — $\alpha^{G}_{i,k} \sim N([0,1,2,3,3],1)$ — $s = [\text{Home}, \text{Home} - \text{SBA}, \text{nonEmOC}, \text{bEmOC}, \text{cEmOC}]$

I = Income Group — $\beta^{I}_{i,k,s} \sim N(0,0.2)$

A = Area — $\gamma^{A}_{i,k,s} \sim N(0,0.1)$

R = Region — $\delta^{R}_{i,k,s} \sim N(0,0.05)$

C = Country — $\phi^{C}_{i,k,s} \sim \alpha^{G}_{i,k,s} + \beta^{I}_{i,k,s} + \gamma^{A}_{i,k,s} + \delta^{R}_{i,k,s} + N(0,0.01)$

Probability — $p^{C}_{i,k,s} = \dfrac{1}{1+\exp\left(-\phi^{C}_{i,k,s}\right)}$

i = Subgroup
k = Complication type/severity
s = Current delivery site

Priors

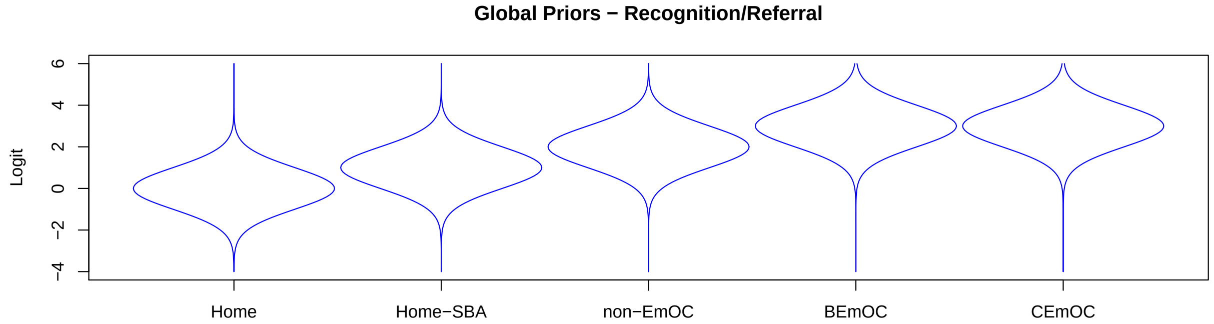

Model Implementation

If a delivery complication occurs, the probability of recognition/referral is drawn. A complication has to be recognized in order to be treated in the model, and depending on the woman's current delivery site and the severity of the complication, she may be referred to another facility. Specifically, we assume that any complication that occurs at Home or non-EmOC will be referred, and any 'severe' complication that occurs at a BEmOC will be referred. Recognized complications that occur at a CEmOC facility are not referred. We currently assume that no false positives occur (i.e. 100% specificity).

References

Aduloju OP, Akintayo AA, Aduloju T, Akin-Akintayo OO. Birth preparedness and complication readiness

among prenatal attendees in a teaching hospital in South West Nigeria. Int J Gynaecol Obstet 2017; 139(2): 202-210. DOI: https://doi.org/10.1002/ijgo.12271

Akshaya KM, Shivalli S. Birth preparedness and complication readiness among the women beneficiaries of selected rural primary health centers of Dakshina Kannada district, Karnataka, India. PLoS One 2017; 12(8): e0183739. DOI: https://doi.org/10.1371/journal.pone.0183739

Assaf S. Counseling and Knowledge of Danger Signs of Pregnancy Complications in Haiti, Malawi, and Senegal. Matern Child Health J. 2018; 22(11): 1659-1667. DOI: https://doi.org/10.1007/s10995-018-2563-5

August F, Pembe AB, Mpembeni R, Axemo P, Darj E. Effectiveness of the Home Based Life Saving Skills training by community health workers on knowledge of danger signs, birth preparedness, complication readiness and facility delivery, among women in Rural Tanzania. BMC Pregnancy Childbirth 2016; 16(1): 129. DOI: https://doi.org/10.1186/s12884-016-0916-x

Benimana C, Small M, Rulisa S. Preventability of maternal near miss and mortality in Rwanda: A case series from the University Teaching Hospital of Kigali (CHUK). PloS One 2018; 13(6): e0195711. DOI: https://doi.org/10.1371/journal.pone.0195711

Berhe AK, Muche AA, Fekadu GA, Kassa GM. Birth preparedness and complication readiness among pregnant women in Ethiopia: a systematic review and Meta-analysis. Reprod Health 2018; 15(1): 182. DOI: https://doi.org/10.1186/s12978-018-0624-2

Bintabara D, Mohamed MA, Mghamba J, Wasswa P, Mpembeni RNM. Birth preparedness and complication readiness among recently delivered women in chamwino district, central Tanzania: a cross sectional study. Reprod Health 2015; 12: 44. DOI: https://doi.org/10.1186/s12978-015-0041-8

Bintabara D, Mpembeni RNM, Mohamed AA. Knowledge of obstetric danger signs among recently-delivered women in Chamwino district, Tanzania: a cross-sectional study. BMC Pregnancy Childbirth 2017; 17(1): 276. DOI: https://doi.org/10.1186/s12884-017-1469-3

Chandhiok N, Singh S, Chaudhury N, Shrotri A. Knowledge and Skills of Ayurvedic and Homeopathic Practitioners to Provide Skilled Birth Attendance in India: An Observational Study. Indian J Community Med 2018; 43(3): 175-179. DOI: https://doi.org/10.4103/ijcm.ijcm_280_17

Echoka E, Makokha A, Dubourg D, Kombe Y, Nyandieka L, Byskov J. Barriers to emergency obstetric care services: accounts of survivors of life threatening obstetric complications in Malindi District, Kenya. Pan Afr Med J 2014; 17 Suppl 1(Suppl 1): 4. DOI: https://doi.org/10.11694/pamj.supp.2014.17.1.3042

Florence M, Atuhaire C, Nkfusai CN, Shirinde J, Cumber SN. Knowledge and practice of birth preparedness and complication readiness among pregnant women attending antenatal clinic in Openzinzi Hciii, Adjumani District, Uganda. Pan Afr Med J 2019; 34: 46. DOI: https://doi.org/10.11604/pamj.2019.34.46.16869

Geleto A, Chojenta C, Musa A, Loxton D. Barriers to access and utilization of emergency obstetric care at health facilities in sub-Saharan Africa: a systematic review of literature. Syst Rev 2018; 7(1): 183. DOI: https://doi.org/10.1186/s13643-018-0842-2

Geleto A, Chojenta C, Musa A, Loxton D. WOMEN's Knowledge of Obstetric Danger signs in Ethiopia (WOMEN's KODE):a systematic review and meta-analysis. Syst Rev 2019; 8(1): 63. DOI: https://doi.org/10.1186/s13643-019-0979-7

George SO, Yisa IO, Alawode G. Knowledge of obstetric danger signs amongst women of reproductive age in PATHS2 Zaria cluster, Kaduna Nigeria. Niger J Med 2014; 23(1): 26-32. PMID: https://pubmed.ncbi.nlm.nih.gov/24946451/

Gopalakrishnan S, Eashwar VMA, Muthulakshmi M. Health-seeking behaviour among antenatal and postnatal rural women in Kancheepuram District of Tamil Nadu: A cross-sectional Study. J Family Med Prim Care 2019; 8(3): 1035-1042. DOI: https://doi.org/10.4103/jfmpc.jfmpc_323_18

Head SK, Yount KM, Sibley LM. Delays in recognition of and care-seeking response to prolonged labor in Bangladesh. Soc Sci Med 2011; 72(7): 1157-68. DOI: https://doi.org/10.1016/j.socscimed.2011.01.030

Heerboth SA, Hennessey C, Omondi B, et al. Knowledge of Obstetric and Neonatal Danger Signs among Community Health Workers in the Rongo Sub-County of Migori County, Kenya: Results of a Community-based Cross-Sectional Survey. Afr J Reprod Health 2020; 24(1): 121-132. DOI: https://doi.org/10.29063/ajrh2020/v24i1.13

Hoque M, Hoque ME. Knowledge of danger signs for major obstetric complications among pregnant KwaZulu-Natal women: implications for health education. Asia Pac J Public Health 2011; 23(6): 946-56. DOI: https://doi.org/10.1177/1010539511428698

Imaralu JO, Ani IF, Olaleye AO, Jaiyesimi E, Afolabi-Imaralu A, Odugbemi OO. Maternal and perinatal outcomes of birth preparedness and complication readiness in recently delivered women of a Southwestern Nigerian Town. Ann Afr Med 2020; 19(1): 60-67. DOI: https://doi.org/10.4103/aam.aam_29_19

India Coverage Evaluation Survey 2009-2010. Ministry of Health and Family Welfare (India), ORG Centre for Social Research (ORG CSR), United Nations Children's Fund (UNICEF). Available: http://ghdx.healthdata.org/record/india-coverage-evaluation-survey-2009-2010.

Ishola G, Fayehun F, Isiugo-Abanihe, et al. Effect of Volunteer Household Counseling in Improving Knowledge of Birth Preparedness and Complication Readiness of Pregnant Women in Northwest Nigeria. Afr J Reprod Health 2017; 21(1): 39-48. DOI: https://doi.org/10.29063/ajrh2017/v21i1.3

Kabakyenga JK, Östergren PO, Turyakira E, Pettersson KO. Knowledge of obstetric danger signs and birth preparedness practices among women in rural Uganda. Reprod Health 2011; 8: 33. DOI: https://doi.org/10.1186/1742-4755-8-33

Kamineni V, Murki AD, Kota VL. Birth preparedness and complication readiness in pregnant women attending urban tertiary care hospital. J Family Med Prim Care 2017; 6(2): 297-300. DOI: https://doi.org/10.4103/2249-4863.220006

Kochukuttan S, Ravindran TS, Krishnan S. Evaluating Birth Preparedness and Pregnancy Complications Readiness Knowledge and Skills of Accredited Social Health Activists in India. Int J MCH AIDS 2013; 2(1): 121-8. DOI: https://doi.org/10.21106/ijma.17

Koşum Z, Yurdakul M. Factors affecting the use of emergency obstetric care among pregnant women with antenatal bleeding. Midwifery 2013; 29(5): 440-6. DOI: https://doi.org/10.1016/j.midw.2012.02.008

Kumbani L, McInerney P. The knowledge of obstetric complications among primigravidae in a rural health centre in the district of Blantyre, Malawi. Curationis 2002; 25(3): 43-54. DOI: https://doi.org/10.4102/curationis.v25i3.786

Kumbani LC, McInerney P. Primigravidae's knowledge about obstetric complications in an urban health centre in Malawi. Curationis 2006; 29(3): 41-9. DOI: https://doi.org/10.4102/curationis.v29i3.1092

Masoi TJ, Kibusi SM. Improving pregnant women's knowledge on danger signs and birth preparedness practices using an interactive mobile messaging alert system in Dodoma region, Tanzania: a controlled quasi experimental study. Reprod Health 2019; 16(1): 177. DOI: https://doi.org/10.1186/s12978-019-0838-y

Mbalinda SN, Nakimuli A, Kakaire O, Osinde MO, Kakande N, Kaye DK. Does knowledge of danger signs of pregnancy predict birth preparedness? A critique of the evidence from women admitted with pregnancy complications. Health Res Policy Syst 2014; 12: 60. DOI: https://doi.org/10.1186/1478-4505-12-60

Montgomery AL, Ram U, Kumar R, Jha P, Million Death Study Collaborators. Maternal mortality in India: causes and healthcare service use based on a nationally representative survey. PLoS One 2014; 9(1): e83331. DOI: https://doi.org/10.1371/journal.pone.0083331

Moran AC, Winch PJ, Sultana N, et al. Patterns of maternal care seeking behaviours in rural Bangladesh. Trop Med Int Health 2007; 12(7): 823-32. DOI: https://doi.org/10.1111/j.1365-3156.2007.01852.x

Mutiso SM, Qureshi Z, Kinuthia J. Birth preparedness among antenatal clients. East Afr Med J 2008; 85(6): 275-83. DOI: https://doi.org/10.4314/eamj.v85i6.9625

Mwilike B, Nalwadda G, Kagawa M, Malima K, Mselle L, Horiuchi S. Knowledge of danger signs during pregnancy and subsequent healthcare seeking actions among women in Urban Tanzania: a cross-sectional study. BMC Pregnancy Childbirth 2018; 18(1): 4. DOI: https://doi.org/10.1186/s12884-017-1628-6

Onoh RC, Egede JO, Lawani LO, Ekwedigwe KC, Aja LO, Anozie BO. Birth preparedness and complication readiness among women of reproductive age group in Abakaliki, Southeast Nigeria. Niger J Clin Pract 2020; 23(3): 362-370. DOI: https://doi.org/10.4103/njcp.njcp_670_18

Ouasmani F, Engeltjes B, Raho BH, Belayachi O, Verhoeven C. Knowledge of hypertensive disorders in pregnancy of Moroccan women in Morocco and in the Netherlands: a qualitative interview study. BMC Pregnancy Childbirth 2018; 18(1): 344. DOI: https://doi.org/10.1186/s12884-018-1980-1

Pembe AB, Urassa DP, Carlstedt A, Lindmark G, Nyström L, Darj E. Rural Tanzanian women's awareness of danger signs of obstetric complications. BMC Pregnancy Childbirth 2009; 9: 12. DOI: https://doi.org/10.1186/1471-2393-9-12

Pervin J, Nu UT, Rahman AMQ, et al. Level and determinants of birth preparedness and complication readiness among pregnant women: A cross sectional study in a rural area in Bangladesh. PLoS One 2018; 13(12): e0209076. DOI: https://doi.org/10.1371/journal.pone.0209076

Radoff KA, Levi AJ, Thompson LM. A radio-education intervention to improve maternal knowledge of obstetric danger signs. Rev Panam Salud Publica 2013; 34(4): 213-9. PMID: https://pubmed.ncbi.nlm.nih.gov/24301731/

Raj SS, Maine D, Sahoo PK, Manthri S, Chauhan K. Meeting the community halfway to reduce maternal deaths? Evidence from a community-based maternal death review in Uttar Pradesh, India. Glob Health Sci Pract 2013; 1(1): 84-96. DOI: https://doi.org/10.9745/ghsp-d-12-00049

Rosales A, Sulistyo S, Miko O, et al. Recognition of and care-seeking for maternal and newborn complications in Jayawijaya district, Papua province, Indonesia: a qualitative study. J Health Popul Nutr 2017; 36(Suppl1): 44. DOI: https://doi.org/10.1186/s41043-017-0122-0

Saaka M, Aryee P, Kuganab-Lem R, Ali M, Masahudu AR. The effect of social behavior change communication package on maternal knowledge in obstetric danger signs among mothers in East Mamprusi District of Ghana. Global Health 2017; 13(1): 19. DOI: https://doi.org/10.1186/s12992-017-0243-7

Sabageh AO, Adeoye OA, Adeomi AA, Sabageh D, Adejimi AA. Birth preparedness and complication readiness among pregnant women in Osogbo Metropolis, Southwest Nigeria. Pan Afr Med J 2017; 27: 74. DOI: https://doi.org/10.11604/pamj.2017.27.74.7266

Salam FT, Sarfraz M. Perceptions and practices among married women of child bearing age regarding obstetric danger signs in rural Islamabad: A qualitative study. J Pak Med Assoc 2018; 68(10): 1496-1501. Available at: https://jpma.org.pk/article-details/8892?article_id=8892

Salem A, Lacour O, Scaringella S, et al. Cross-sectional survey of knowledge of obstetric danger signs among women in rural Madagascar. BMC Pregnancy Childbirth 2018; 18(1): 46. DOI: https://doi.org/10.1186/s12884-018-1664-x

Sharma N, Kumar N, Singh S, Malik JS, Jangra A. Status and determinants of birth preparedness and complication readiness in a rural block of Haryana. J Family Med Prim Care 2019; 8(2): 482-486. DOI: https://doi.org/10.4103/jfmpc.jfmpc_372_18

Smeele P, Kalisa R, van Elteren M, van Roosmalen J, van den Akker T. Birth preparedness and complication readiness among pregnant women admitted in a rural hospital in Rwanda. BMC Pregnancy Childbirth 2018; 18(1): 190. DOI: https://doi.org/10.1186/s12884-018-1818-x

Thaddeus S, Maine D. Too far to walk: maternal mortality in context. Soc Sci Med 1994; 38(8): 1091-110. DOI: https://doi.org/10.1016/0277-9536(94)90226-7

Thapa P, Bangura AH, Nirola I, et al. The power of peers: an effectiveness evaluation of a cluster-controlled trial of group antenatal care in rural Nepal. Reprod Health 2019; 16(1): 150. DOI: https://doi.org/10.1186/s12978-

019-0820-8

Than KK, Morgan A, Pham MD, Beeson JG, Luchters S. Determinants of knowledge of critical danger signs, safe childbirth and immediate newborn care practices among auxiliary midwives: a cross sectional survey in Myanmar. BMJ Open 2017; 7(6): e017180. DOI: https://doi.org/10.1136/bmjopen-2017-017180

Urassa DP, Pembe AB, Mganga F. Birth preparedness and complication readiness among women in Mpwapwa district, Tanzania. Tanzan J Health Res 2012; 14(1): 42-7. DOI: https://doi.org/10.4314/thrb.v14i1.8

A.1.4.4 Transportation

Overview

Once a complication has been recognized/referred, the woman needs some way to get to a facility in time to receive care. A systematic review found three main themes related to this ‘second delay’: availability of transportation infrastructure, distance from the health facility, and lack of finance for transportation (Geleto 2018). These barriers resulted in delays on the way to a health facility even if the decision to seek care was made in a timely manner, with the majority of delays due to a shortage of vehicles, poor road infrastructure, and shortage of ambulances (Geleto 2018). This probability in the model encapsulates both the availability and timeliness of transport.

Data

A systematic review found it was usually difficult for women with lower household income to afford transportation fees, and distance from a health facility was also found to a major barrier (Geleto 2018). However, a study in Rwanda found that lack of transportation and the quality of roads were not identified as major barriers to care, with only 0.8% of maternal near misses and deaths considered preventable due to difficulty reaching a tertiary care facility (Benimana 2017). In India, all women in the Coverage Evaluation Survey 2009-10 who had institutional deliveries were asked about the mode of transport used to reach the health facility and the costs incurred. Around 6.5% of women used an ambulance and 30.9% used a jeep/car to reach the health facility, while 7.5% travelled by motorcycle or scooter. However, many women reportedly used other modes of transport such as bus/train (6.6%), and tempo/auto/tractor (34.1%). Nearly 5% of women reportedly reached the health facility on foot. Women in rural areas travelled an average of 11.2 km, taking an average time of 39.2 minutes, compared to those in urban areas who travelled 4.9 km, taking 24.3 minutes. The mean transport cost was 192 Rupees (Rural: 243; Urban: 140), although about 21% of women did not incur any transportation cost to reach the facility. Overall, only 10.4% of women who delivered at home said they did not deliver at an institution because it was too far, or that they lacked transport. However, the availability of emergency transportation for complications may be quite different than for planned deliveries in a facility. According to the District Level Household Survey (2007-08), only 55% of villages had referral services for complications. Successfully transferring a woman from a site of complication to a definite level of care requires stabilization and transfer protocols, communications technology, and transportation and cost arrangements, with reliable transportation often a missing link to timely, accessible, and affordable emergency care (Raj 2015).

Transport availability can also be lacking for women who are already at a facility. In a review of public health facilities in Uttar Pradesh, India, it was found that there were only 10 ambulances available for 15 facilities, compared to the 19 required by the Indian Public Health Standards (Raj 2015). Furthermore, only 8 of 15 facilities had ambulances in working order with at least 1 driver and funds available for fuel and maintenance. No facility was offering regular, round-the-clock referral transport, including the district hospital, and it was estimated that 27% of maternal deaths occurred en route to a facility, highlighting the importance of timely transportation (Raj 2015). Another study by Chaturvedi (2014) found that the average inter-facility travel time was 1.25 hours, while Sabde (2014) found that the median time needed to reach a facility after making the decision to seek care was nearly 2 hours, with only 12% of women reaching a facility within 1 hour. However, improvements are being made in some areas. A study by Prinja (2014) of the National Ambulance Service (NAS) in Haryana found that once a call was made, the NAS ambulance took an average of 18.8 min to reach the site of emergency and 21.7 min to transport the patient to the hospital from site of emergency. In nearly 90% of the cases, a NAS ambulance reached the site of emergency within 30 min of call, while in 80% of the cases the patient reached a facility within 1 hour of calling. A 2008 study in Sierra Leone found that only 10% of health facilities had an ambulance, while 22% had an unequipped ambulance (Oyerinde 2011). A study in the Lake and Western zones in Tanzania found that 25% of hospitals and health centers reported that they were not able to access an ambulance (Maswanya 2018).

Parameters

We used hierarchical logistic regression models to model the probability of timely transportation. We assume that women with higher education are more likely to have the resources to obtain transport, and that women in urban areas have shorter distances to travel. We also assume that transport probabilities increase with more advanced delivery site. These constraints were enforced when sampling probabilities in the model.

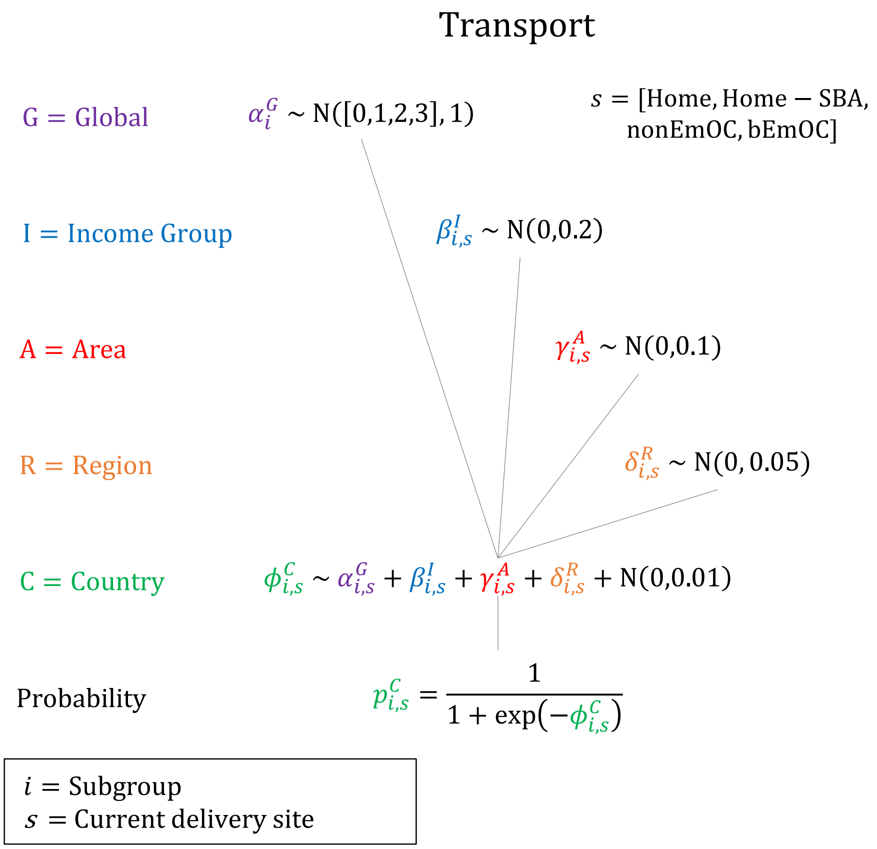

Priors

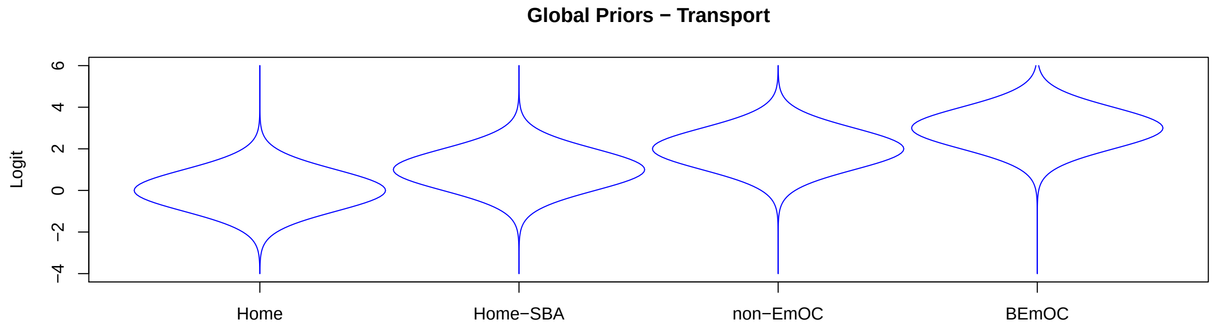

Model Implementation

Conditional on successful recognition and the need for referral, the probabilities of successful (and timely) transport are simulated based on a woman's current site of delivery and subgroup.

References

Benimana C, Small M, Rulisa S. Preventability of maternal near miss and mortality in Rwanda: A case series from the University Teaching Hospital of Kigali (CHUK). PLoS One 2018; 13(6): e0195711. DOI: https://doi.org/10.1371/journal.pone.0195711

Chaturvedi S, Randive B, Diwan V, De Costa A. Quality of obstetric referral services in India's JSY cash

transfer programme for institutional births: a study from Madhya Pradesh province. PLoS One 2014; 9(5): e96773. DOI: https://doi.org/10.1371/journal.pone.0096773

Geleto A, Chojenta C, Musa A, Loxton D. Barriers to access and utilization of emergency obstetric care at health facilities in sub-Saharan Africa: a systematic review of literature. Syst Rev 2018; 7(1): 183. DOI: https://doi.org/10.1186/s13643-018-0842-2

India Coverage Evaluation Survey 2009-2010. Ministry of Health and Family Welfare (India), ORG Centre for Social Research (ORG CSR), United Nations Children's Fund (UNICEF). Available: http://ghdx.healthdata.org/record/india-coverage-evaluation-survey-2009-2010.

International Institute for Population Sciences. India District Level Household Survey 2007-2008. Mumbai, India: International Institute for Population Sciences; 2010. Available: http://rchiips.org/PRCH-3.html.

Maswanya E, Muganyizi P, Kilima S, Mogella D, Massaga J. Practice of emergency obstetric care signal functions and reasons for non-provision among health centers and hospitals in Lake and Western zones of Tanzania. BMC Health Serv Res 2018; 18(1): 944. DOI: https://doi.org/10.1186/s12913-018-3685-6

Oyerinde K, Harding Y, Amara P, Kanu R, Shoo R, Daoh K. The status of maternal and newborn care services in Sierra Leone 8 years after ceasefire. Int J Gynaecol Obstet 2011; 114(2): 168-73. DOI: https://doi.org/10.1016/j.ijgo.2011.05.006

Prinja S, Jeet G, Kaur M, Aggarwal AK, Manchanda N, Kumar R. Impact of referral transport system on institutional deliveries in Haryana, India. Indian J Med Res 2014; 139(6): 883-91. PMID: https://pubmed.ncbi.nlm.nih.gov/25109723/

Raj SS, Manthri S, Sahoo PK. Emergency referral transport for maternal complication: lessons from the community based maternal deaths audits in Unnao district, Uttar Pradesh, India. Int J Health Policy Manag. 2015; 4(2): 99-106. DOI: https://doi.org/10.15171/ijhpm.2015.14

Sabde Y, De Costa A, Diwan V. A spatial analysis to study access to emergency obstetric transport services under the public private "Janani Express Yojana" program in two districts of Madhya Pradesh, India. Reprod Health 2014; 11: 57. DOI: https://doi.org/10.1186/1742-4755-11-57

A.1.4.5 Referral Facility

Overview

Once transportation is arranged, a target referral facility must also be chosen. When referral networks are well-established, or in cases with good communication links with facilities, women can be taken to a facility that is able to provide the appropriate level of care. However, sometimes women are taken to multiple places in an attempt to find a facility capable of treating them. The selection of an inappropriate referral facility can be another barrier to receiving the care that is needed.

Data

A study in Malindi, Kenya, found that a majority of the women from rural areas seeking care first went to a dispensary or health center, from which they were referred to the district hospital (Echoka 2014). In India, women who reported an obstetric complication in the Coverage Evaluation Survey (2009-10) were asked about the source of treatment they received. Most women consulted private doctors (54%), followed by government doctors (29%) and PHC (8%). A study of over 1,000 mothers at 96 facilities found that most came directly from home, while 9.4% were referred from another facility (Chaturvedi 2014). Of the mothers who arrived at the study facilities, 6% were referred on for higher care. Tertiary level facilities were found to receive the most referrals, while secondary level facilities received very few referrals at all. In all, 12% of mothers who had been referred once required a further referral, half of whom went on to a private facility. In general, secondary level facilities were the common senders and tertiary level facilities were the common receivers. About half of referrals from primary level were sent directly to the tertiary level. Another study (Sabde 2014) revealed that some women bypassed non-functional BEmOCs to seek care in higher-level facilities offering caesarean section services. Bypassing primary care facilities to seek care in higher level facilities indicates that perceived poor quality of care may be an important determinant for bypassing behavior. If already at a facility, women can also experience 'horizontal transfer', meaning that they are referred to another facility that is similar to their current site. For example, in a study of maternal deaths, Raj (2015) found that 25% of women referred were taken to 1 facility, 32% to 2 facilities, and 25% taken to three facilities. (The remaining 19% did not reach any facility before death.) The mean time to make arrangements for travel from home to the first facility was 3.1 hours, from facility 1 to 2 was 9.9 hours, and from facility 2 to facility 3 was 3.1 hours (Raj 2015).

Parameters

Given the preference for quality of care as seen by women bypassing lower-level facilities, we assume that referrals to CEmOC facilities are more common when setting priors.

Referral Facility

G = Global $\quad \alpha^{G}_{i,s,t} \sim \mathrm{N}\left(\widehat{\alpha}_{i,s,t}, 1\right)$

$$\widehat{\alpha}_i = \mathrm{logit}\begin{bmatrix} 0 & 0 & 0.3 & 0.2 & 0.5 \\ 0 & 0 & 0.2 & 0.2 & 0.6 \\ 0 & 0 & 0.1 & 0.2 & 0.7 \\ 0 & 0 & 0 & 0.2 & 0.8 \\ 0 & 0 & 0 & 0 & 1 \end{bmatrix} s \quad (\text{columns: } t)$$

Site=[Home, Home-SBA, nonEmOC, bEmOC, cEMOC]

I = Income Group $\quad \beta^{I}_{i,s,t} \sim \mathrm{N}(0,0.2)$

A = Area $\quad \gamma^{A}_{i,s,t} \sim \mathrm{N}(0,0.1)$

R = Region $\quad \delta^{R}_{i,s,t} \sim \mathrm{N}(0, 0.05)$

C = Country $\quad \phi^{C}_{i,s,t} \sim \alpha^{G}_{i,s,t} + \beta^{I}_{i,s,t} + \gamma^{A}_{i,s,t} + \delta^{R}_{i,s,t} + \mathrm{N}(0,0.01)$

$$\pi^{C}_{i,s,t} = \frac{1}{1 + \exp\left(-\phi^{C}_{i,s,t}\right)}$$

Probability $\quad p[\mathrm{Site} = t]^{\,C}_{i,s} = \dfrac{\pi^{C}_{i,s,t}}{\sum_{k=1}^{T} \pi^{C}_{i,s,k}}$

i = Subgroup<br>s = Current delivery site<br>t = New delivery site

Priors

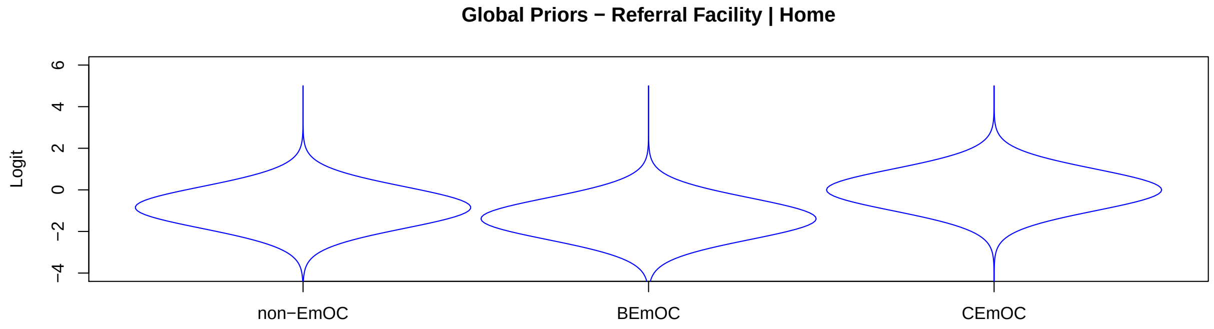

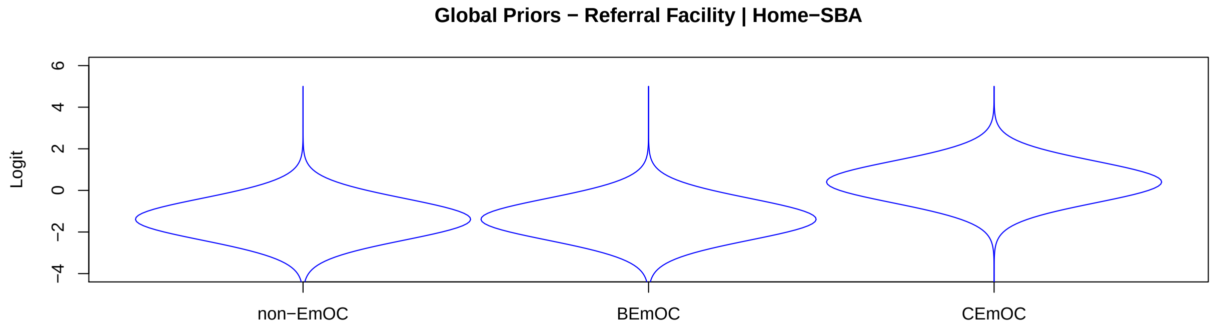

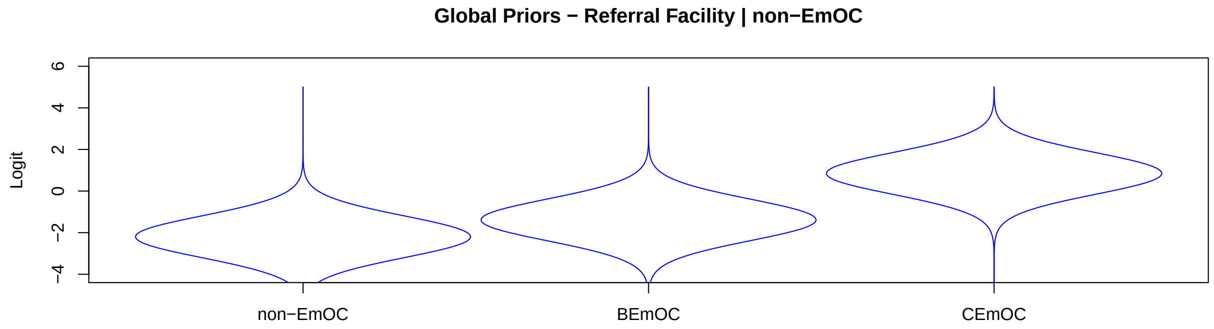

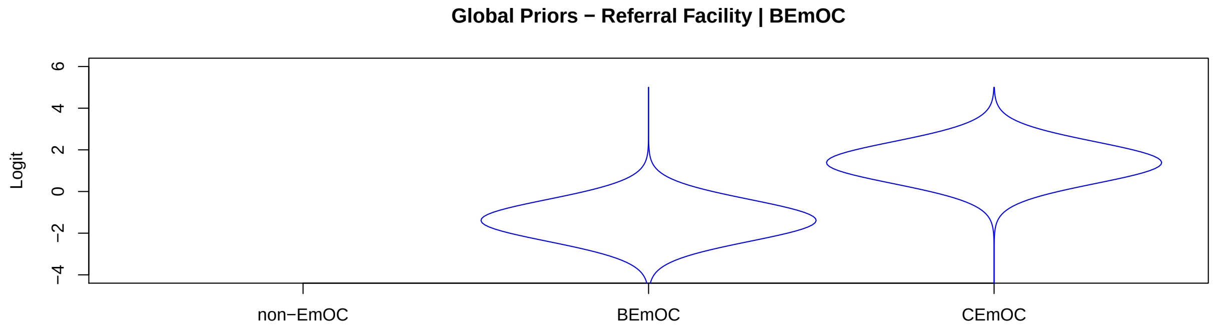

Model Implementation

If transport is available, the final referral facility is simulated conditional on the current site. We assume that women in BEmOC are not referred to non-EmOC facilities.

References

Chaturvedi S, Randive B, Diwan V, De Costa A. Quality of obstetric referral services in India's JSY cash transfer programme for institutional births: a study from Madhya Pradesh province. PLoS One 2014; 9(5): e96773.

Echoka E, Makokha A, Dubourg D, Kombe Y, Nyandieka L, Byskov J. Barriers to emergency obstetric care services: accounts of survivors of life threatening obstetric complications in Malindi District, Kenya. Pan Afr Med J 2014; 17 Suppl 1(Suppl 1): 4. DOI: https://doi.org/10.11694/pamj.supp.2014.17.1.3042

India Coverage Evaluation Survey 2009-2010. Ministry of Health and Family Welfare (India), ORG Centre for Social Research (ORG CSR), United Nations Children's Fund (UNICEF). Available: http://ghdx.healthd

ata.org/record/india-coverage-evaluation-survey-2009-2010.

Raj SS, Manthri S, Sahoo PK. Emergency referral transport for maternal complication: lessons from the community based maternal deaths audits in Unnao district, Uttar Pradesh, India. Int J Health Policy Manag 2015; 4(2): 99-106.

Sabde Y, De Costa A, Diwan V. A spatial analysis to study access to emergency obstetric transport services under the public private “Janani Express Yojana” program in two districts of Madhya Pradesh, India. Reprod Health 2014; 11: 57.

A.1.4.6 Quality of Care

Overview

To account for health-system and facility-level factors not explicitly included in the model we include a parameter for 'quality of care', which has been defined as the "degree to which health services for individuals and populations increase the likelihood of desired health outcomes and are consistent with current professional knowledge" (IOM 1990).

Real-world maternal health interventions have highlighted quality of care as an important determinant of outcomes, independent of facility delivery and complication referrals. For example, Janani Suraksha Yojana (JSY), a conditional cash-transfer program to incentivize facility delivery in India, succeeded in substantially increasing facility births, but had no significant effect on mortality due to lack of focus on the quality of facilities (Randive 2013, Scott 2014).

We therefore include this parameter in the model to account for residual differences in maternal mortality not explained by differences in the incidence of obstetric complications and availability or efficacy of clinical interventions. Although it is a broad concept, the Institute of Medicine has described 'quality of care' as having six key features: care that is safe, effective, patient-centered, efficient, timely, and equitable. Although all these features are important, there are often particularly substantial deficiencies regarding patient safety (e.g., iatrogenic harm), effectiveness (e.g., providers may fail to provide the appropriate treatment even if it is available), and patient-centeredness (e.g., patients may opt out of or not adhere to treatment if they lack confidence in the health system) (Scott 2014).

Parameters

We assume that quality improves by delivery site (Home $<$ Home SBA $<$ non-EmOC $<$ BEmOC $<$ CEmOC) and by income group. For example, we assume that higher-level facilities will generally have better-trained staff, such that interventions available in both BEmOC and CEmOC settings are expected to be delivered with higher quality in CEmOC facilities. We also assume that quality improves over time, and constrain the slope parameter to be non-negative when sampling.

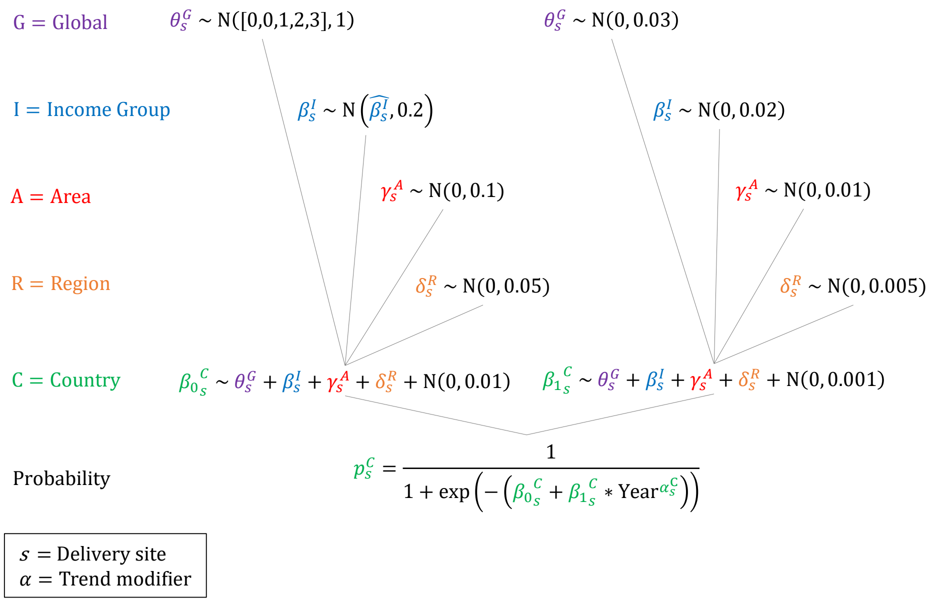

Priors

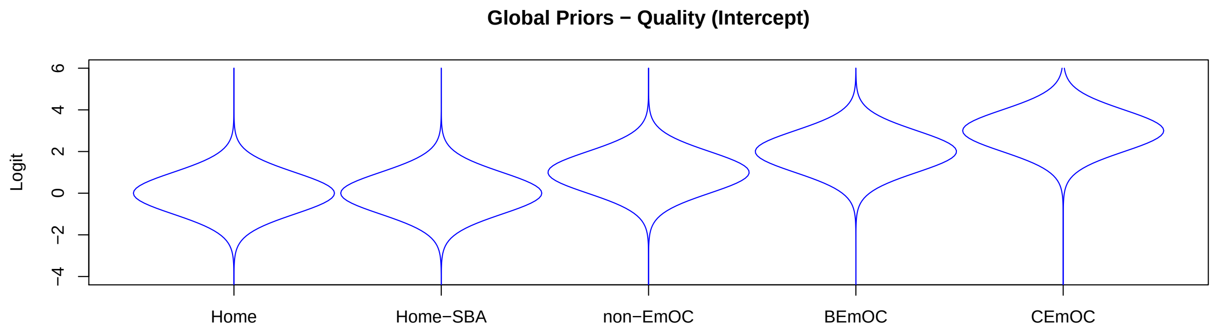

Model Implementation

The estimated quality of care at each delivery site is used to modify the efficacy of clinical interventions that may be delivered.

References

Institute of Medicine. Medicare: a strategy for quality assurance. Washington, DC: National Academy Press, 1990.

Randive B, Diwan V, De Costa A. India's conditional cash transfer program (the JSY) to promote institutional birth: is there an association between institutional birth proportion and maternal mortality? PLoS One 2013; 8(6): e67452.

Scott KW, Jha AK. Putting quality on the global health agenda. N Engl J Med 2014; 371: 1.

A.1.4.7 Maternal Death Underreporting

Overview

Empirical estimates of maternal mortality notoriously suffer from underreporting due to misclassification or omission altogether of maternal deaths. Civil registration vital statistics (CRVS) and health information systems in most developing countries are weak, and even estimates derived from complete CRVS systems in high income countries suffer from misclassification and underreporting of maternal deaths (WHO). However, the amount of underestimation varies by setting and may change over time.

The accuracy of CRVS systems can be assessed by comparing the recorded mortality rates to those obtained from specialized studies which are rigorous assessments of maternal mortality. Previous work comparing study-based mortality to CRVS-based mortality found that study-based estimates of maternal mortality were around 150% higher than CRVS-based estimates, suggesting that CRVS systems do not adequately capture all maternal deaths (Wilmoth 2012).

A recent analysis which informs the UN-MMEIG model found that the sensitivity of CRVS data for maternal deaths is poor, at 0.586 (80% CI 0.511-0.656), while specificity is good (0.9993, 80% CI 0.9990-0.9996) (Peterson 2019). The Global Burden of Disease also address underreporting of maternal mortality, and found that the net effect of data processing steps on vital registration data to correct for underreporting increased maternal deaths by 168% (Kassebaum 2016).

However, underreporting is likely even higher in lower income settings with weaker or non-existent CRVS systems. Furthermore, adjusting the reported estimates of maternal mortality directly is a crude approach which does not account for the uncertainty around levels of underreporting, and is inconsistent with a fundamental tenet of Bayesian analysis: that data are fixed and parameters are random variables. We therefore include underreporting of maternal deaths as part of our modeled data generating process which gives rise to the empirical (though biased) maternal mortality estimates used to fit the model. This approach allows us to correct for underreporting in a Bayesian framework while also accounting for the (often substantial) uncertainty around levels and trends in underreporting of maternal deaths. To account for differential levels of maternal death underreporting in data from CRVS systems (based on passive reporting) and surveys (based on active case finding), we model two levels of underreporting (CRVS vs survey), and assume that survey-based (i.e. specialized study) estimates of maternal deaths are more complete than CRVS-based data. This is consistent with empirical data as specialized survey studies are often used to estimate the magnitude of underreporting present in CRVS data.

Data

We undertook a literature review to obtain estimates of maternal mortality underreporting by country, where available.

Argentina: A retrospective review of medical records of deaths of women of childbearing age in 2014 estimated that maternal cause of death was omitted in 14.2% of maternal deaths and 33.3% of late maternal deaths (Abalos 2019).

Austria: A study of maternal mortality in 1980-98 found 119 maternal deaths registered in the official statistics, while confidential inquiries found 191 maternal deaths, suggesting under-reporting of 38% (95% CI 31-45), with more indirect deaths under-reported (81%, 95% CI 64-92) than direct obstetrical deaths (28%, 95% CI 21-36) (Karimian-Teherani 2002).

Brazil: A study estimating maternal death underreporting in 1988 in eight public maternity clinics in Rio de Janeiro found that the official death rate, based on maternal deaths as declared in death certificates, underreported by 60% compared to the corrected death rate (Silva 1996). A study of maternal deaths Paraná State in 1991-2005 based on Reproductive Age Mortality Surveys found that maternal mortality decreased by 26% over the 15 years, but that the level of underreporting remained the same over the period, ranging between 35.5% to 45.3% (Soares 2008).

Georgia: A national Reproductive Age Mortality Survey (RAMOS) conducted to examine maternal mortality in 2012 identified 23 maternal deaths, of which 14 (60.9%) were officially recognized by the Georgian CRVS, while all of the 36 pregnancy-related deaths identified were recorded in the CRVS (Berdzuli 2020).

Ghana: A Reproductive Age Mortality Survey (RAMOS) conducted in Accra identified 179 maternal deaths

in 2002, of which 101 (56%) were reported in the official statistics (Zakariah 2009).

India: A study of deaths among women of reproductive age in Vellore between 1999 and 2004 identified 28 maternal deaths, of which 14 were recorded in the official statistics, and only 10 were classified as maternal deaths, yielding a maternal mortality reporting rate of 36% (Kim 2009).

Indonesia: A study of 155 maternal deaths during 2003-2004 identified in the district hospitals of Pandeglang and Serang, in Banten Province, found that only 67 maternal deaths were recorded in the hospitals' routine reports, suggesting that only 43% of maternal deaths were captured (Qomariyah 2009). A later study in Banten Province of maternal deaths in 2016 estimated that 57% were captured by the district health office system (Qomariyah 2020).

Italy: A study linking death registry and hospital discharge registry in 10 regions covering 77% of total national births from 2006 to 2012 identified 277 maternal deaths, and estimated that the official MMR underreports maternal mortality by 60.3% (Donati 2018).

Jamaica: A cross-sectional study to examine all maternal deaths occurring in 2008 from multiple data sources identified 50 maternal deaths, of which 10 (20%) were unregistered (McCaw-Binns 2015).

Malawi: A prospective Reproductive Age Mortality Survey (RAMOS) conducted in Mangochi District in 2011-2012 identified 151 maternal deaths, of which 86 (57%) had been recorded in the official registration system, all of which were facility-based deaths (Mgawadere 2016).

Mexico: An analysis of six studies examining underreporting of maternal deaths in Yucatan estimated that underreporting was 19% during 1997-2001, with rates varying between 10.5% and 28.6% (Rodríguez-Angulo 2018).

Morocco: A retrospective survey in Gharb Chrarda Bni Hssen region of deaths of women of reproductive age between January 1, 2013 and September 30, 2014 identified 69 maternal deaths, of which only 29 were registered in the maternal deaths surveillance system (Abouchadi 2018).

Netherlands: A nationwide, retrospective study of maternal deaths during 1983-1992 estimated that the level of maternal mortality underreporting in The Netherlands was 26% (Schuitemaker 1997).

Norway: A study linking birth and cause of death registries supplemented with data from maternity clinics identified 26 maternal deaths in 2005-2009, of which only 14 (53.8%) were registered in the the official statistics (Vangen 2014).

Papua New Guinea: A retrospective review of maternal deaths at Modilon General Hospital, Madang found that during 2008-2011 there was underreporting to the provincial health information office of maternal deaths (19/60, 31.7%) and also live births (2148/9098, 23.6%) (Bolnga 2014).

Portugal: A study using multiple-capture methods from different data sources to examine maternal deaths between 2001 and 2006 estimated that maternal mortality was underestimated by 9%-26% in the official statistics (Gomes 2012).

Sri Lanka: A study carried out in 1994-95 estimated that maternal deaths in the Western Province were 24% underreported (Bandhutilaka 1996).

Suriname: A reproductive age mortality survey conducted between 2010 and 2014 estimated that 26% were underreported in vital registration data (Kodan 2017).

Sweden: A national register-based study of deaths of women of reproductive age in 1988-2007 estimated that maternal mortality was 64% higher than reported to the World Health Organization (Esscher 2013).

Switzerland: A study of Federal Statistical Office death certificates between 1995 and 2004 compared with maternal mortality cases from the archive of the Institute of Legal Medicine estimated that MMR was underreported by 20% (Fässler 2010).

Taiwan: A census of all registered deaths among women of reproductive age in 1984-1988 found that only 53% of maternal deaths were correctly classified (Kao 1997). A nationwide data linkage study examining

maternal deaths in 2004-2011 identified 236 maternal deaths, of which only 102 were reported in officially published mortality data, suggesting an underreporting rate of 57% (Wu 2015).

Tanzania: A study of the medical birth registry at Kilimanjaro Christian Medical Center in Moshi found that in 2000-2004 18 maternal deaths were recorded, however, hospital case records were found for 62 maternal deaths during the same period, thus only 29% of maternal deaths were entered into the registry (Bergsjø 2010). A study carried out in 1995 in the main catchment area of Haydom Lutheran Hospital in rural Arusha identified 26 maternal deaths, of which 22 were recorded by the hospital, but only 2 were included in the official district statistics (Olsen 2002).

Thailand: A study combining data from civil registration and inpatient diagnoses to identify maternal deaths in 2007-2014 estimated that the maternal mortality ratio (MMR) varied between 30.7-42.5, compared to the official MMR estimates of 8.9-23.3 (Chandoevwit 2016).

USA: In the US, the National Vital Statistics System (NVSS) is the source of official maternal mortality estimates. However, it has been unable to supply accurate estimates of maternal mortality in the US due to problems in reporting and coding of data (MacDorman 2018). A study of maternal deaths occurring in Maryland during the years 1993 through 2000 found that 38% of maternal deaths were unreported on death certificates - half or more deaths were unreported for women who were undelivered at the time of death, experienced a fetal death or therapeutic abortion, died more than a week after delivery, or died as a result of a cardiovascular disorder (Horon 2005).

Zambia: A study comprising of hospital data and a sisterhood method survey in Kalabo District, rural western Zambia in 1994, found that the official number of maternal deaths was 13, but that further investigation revealed 28 maternal deaths in total, yielding a reporting rate of 46% (Vork 1997).

Parameters

We used hierarchical logistic regression models to estimate the probability that maternal deaths are correctly classified and reported. We assume that reporting of maternal mortality is generally improving over time, so we constrain the year coefficient to be non-negative when sampling. Given the underreporting observed in institutions where record-keeping is theoretically in place, it would be expected that maternal deaths which occur in the community would be underreported to an even greater extent (Cross 2010). We therefore model differential probabilities of underreporting by delivery site and assume that the probability of a maternal death being accurately reported increases by site: Home < Home-SBA < non-EmOC < BEmOC < CEmOC. We also assume that survey data are more likely to report maternal deaths as surveys may be more representative of the population, and active case finding would be expected to yield more accurate estimates than passive death reporting. We therefore ensure that survey-based reporting probabilities are at least as high as the CRVS-based reporting probabilities.

p(Maternal death reported)

	β_0	β_1
G = Global	$\alpha^G_{s,m} \sim \mathrm{N}\left(\widehat{\alpha_m}^G, \widehat{\sigma_m}^G\right)$	$\alpha^G_{s,m} \sim \mathrm{N}\left(\widehat{\alpha_m}^G, \widehat{\sigma_m}^G\right)$
I = Income Group	$\beta^I_{s,m} \sim \mathrm{N}\left(\widehat{\beta_m}^I, \widehat{\sigma_m}^I\right)$	$\beta^I_{s,m} \sim \mathrm{N}\left(\widehat{\beta_m}^I, \widehat{\sigma_m}^I\right)$
A = Area	$\gamma^A_{s,m} \sim \mathrm{N}(\widehat{\gamma^A_m}, \widehat{\sigma^A_m})$	$\gamma^A_{s,m} \sim \mathrm{N}(\widehat{\gamma^A_m}, \widehat{\sigma^A_m})$
R = Region	$\delta^R_{s,m} \sim \mathrm{N}(0,0.1)$	$\delta^R_{s,m} \sim \mathrm{N}(0,0.01)$
C = Country	${\beta_{s,m_0}}^C = \alpha^G_{s,m} + \beta^I_{s,m} + \gamma^A_{s,m} + \delta^R_{s,m} + \mathrm{N}(0,0.01)$	${\beta_{s,m_1}}^C = \alpha^G_{s,m} + \beta^I_{s,m} + \gamma^A_{s,m} + \delta^R_{s,m} + \mathrm{N}(0,0.001)$

Probability

$$p[\text{Reported}]_{s,m}{}^C = \frac{1}{1 + \exp\left(-\left({\beta_{s,m_0}}^C + {\beta_{s,m_1}}^C * \text{Year}^{\alpha^C_{s,m}}\right)\right)}$$

s = Delivery site
m = Mode (CRVS vs Survey)
α = Trend modifier

Priors

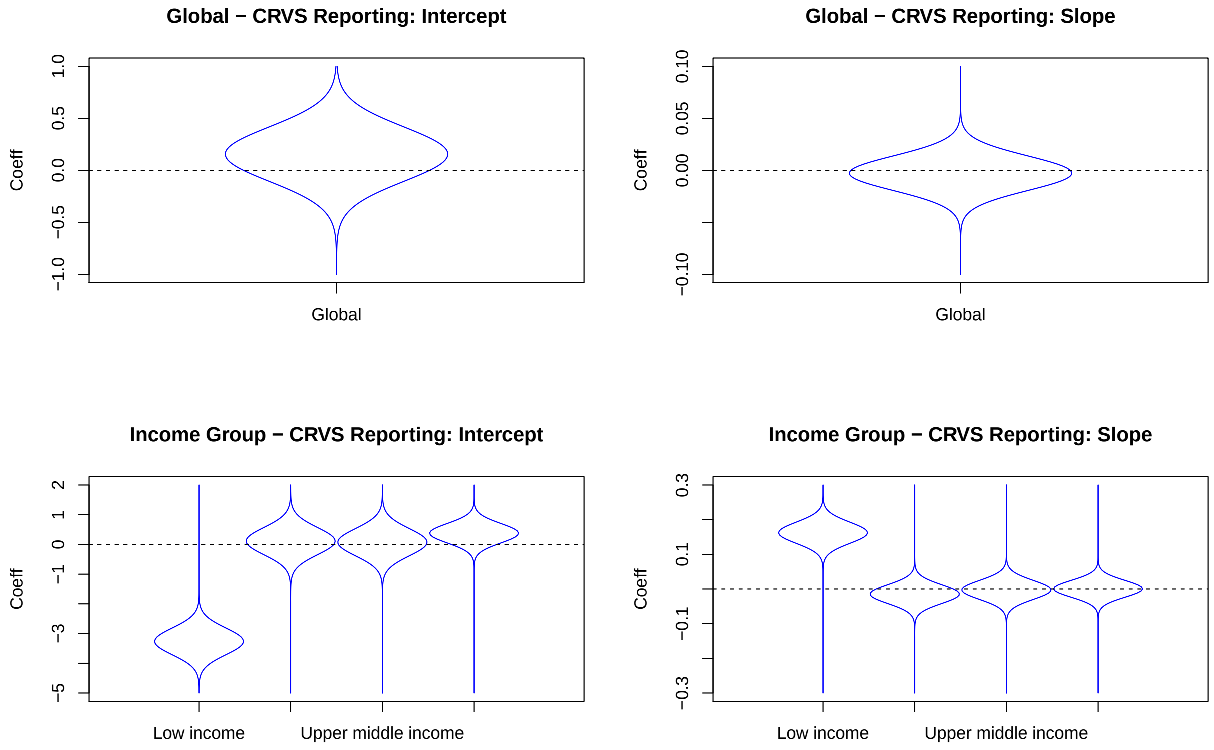

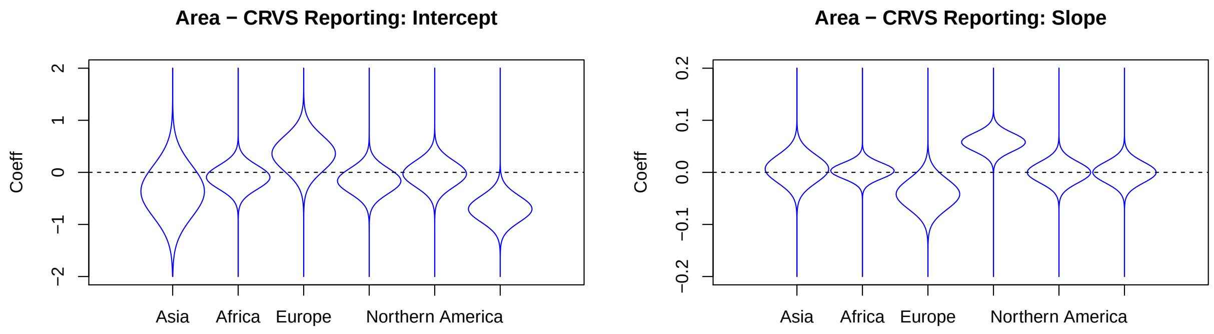

Model Implementation

The (time-dependent) probabilities of underreporting are used to simulate whether each maternal death in the model is appropriately classified and recorded. Only recorded deaths in the model are scored against the calibration targets for maternal mortality, accounting for the reporting mode (CRVS vs survey) of each empirical estimate. For simplicity, we currently assume that underreporting of maternal deaths within each country varies by delivery site and reporting mode, but is non-differential by cause of death. However, this assumption could be relaxed in the future if more data on differential underreporting by cause of death become available.

References

Abalos E, Duhau M, Escobar P, et al. [Omission of causes of maternal death in death certificates in Argentina: nationwide observational study Omissão do registro de causas maternas de morte na Argentina: estudo observacional de alcance nacional]. Rev Panam Salud Publica 2019; 43: e13. DOI: https://doi.org/10.26633/rpsp.2019.13

Abouchadi S, Zhang WH, De Brouwere V. Underreporting of deaths in the maternal deaths surveillance system in one region of Morocco. PLoS One 2018; 13(1): e0188070. DOI: https://doi.org/10.1371/journal.pone.0188070

Bandhutilaka THC. Epidemiology of Maternal Mortality in Sri Lanka. 1996. MD thesis. Colombo: Post Graduate Institute of Medicine, University of Colombo. http://librepository.pgim.cmb.ac.lk/handle/1/968

Berdzuli N, Lomia N, Staff AC, Kereselidze M, Lazdane G, Jacobsen AF. Maternal Mortality in Georgia: Incidence, Causes and Level of Underreporting: A National Reproductive Age Mortality Study 2014. Int J Womens Health 2020; 12: 277-286. DOI: https://doi.org/10.2147/ijwh.s227349

Bergsjø P, Vangen S, Lie RT, Lyatuu R, Lie-Nielsen E, Oneko O. Recording of maternal deaths in an East African university hospital. Acta Obstet Gynecol Scand. 2010; 89(6): 789-793. DOI: https://doi.org/10.3109/00016341003801664

Bolnga JW, Hamura NN, Umbers AJ, Rogerson SJ, Unger HW. Insights into maternal mortality in Madang Province, Papua New Guinea. Int J Gynaecol Obstet 2014; 124(2): 123-7. DOI: https://doi.org/10.1016/j.ijgo.2013.08.012

Chandoevwit W, Phatchana P, Sirigomon K, Ieawsuwan K, Thungthong J, Ruangdej S. Improving the measurement of maternal mortality in Thailand using multiple data sources. Popul Health Metr 2016; 14: 16. DOI: https://doi.org/10.1186/s12963-016-0087-z

Cross S, Bell JS, Graham WJ. What you count is what you target: the implications of maternal death classification for tracking progress towards reducing maternal mortality in developing countries. Bull World Health Organ 2010; 88(2): 147–153. DOI: https://dx.doi.org/10.2471%2FBLT.09.063537

Donati S, Maraschini A, Lega I, et al. Maternal mortality in Italy: Results and perspectives of record-linkage analysis. Acta Obstet Gynecol Scand 2018; 97(11): 1317-1324. DOI: https://doi.org/10.1111/aogs.13415

Esscher A, Högberg U, Haglund B, Essën B. Maternal mortality in Sweden 1988-2007: more deaths than officially reported. Acta Obstet Gynecol Scand 2013; 92(1): 40-46. DOI: https://doi.org/10.1111/aogs.12037

Fässler M, Zimmermann R, QuackLötscher KC. Maternal mortality in Switzerland 1995-2004. Swiss Med Wkly 2010; 140(1-2): 25-30. Available at: https://www.zora.uzh.ch/id/eprint/44439/1/Faessler_smw-12768.pdf

Gomes MC, Ventura MT, Nunes RS. How many maternal deaths are there in Portugal? J Matern Fetal Neonatal Med 2012; 25(10): 1975-9. DOI: https://doi.org/10.3109/14767058.2012.668587

Horon IL. Underreporting of Maternal Deaths on Death Certificates and the Magnitude of the Problem of Maternal Mortality. Am J Public Health 2005; 95(3): 478-482. DOI: https://dx.doi.org/10.2105%2FAJPH.2004.040063

Kao S, Chen LM, Shi L, Weinrich MC. Underreporting and misclassification of maternal mortality in Taiwan. Acta Obstet Gynecol Scand 1997; 76(7): 629-636. DOI: https://doi.org/10.3109/00016349709024602

Karimian-Teherani D, Haidinger G, Waldhoer T, Beck A, Vutuc C. Under-reporting of direct and indirect obstetrical deaths in Austria, 1980-98. Acta Obstet Gynecol Scand 2002; 81(4): 323-327. DOI: https://doi.org/10.1034/j.1600-0412.2002.810408.x

Kassebaum NJ, Barber RM, Bhutta ZA, et al. Global, regional, and national levels of maternal mortality, 1990-2015: a systematic analysis for the Global Burden of Disease Study 2015. Lancet 2016; 388(10053): 1775-1812. DOI: https://doi.org/10.1016/s0140-6736(16)31470-2

Kim SY, Rochat R, Rajaratnam A, Digirolamo A. Evaluating completeness of maternal mortality reporting in a rural health and social affairs unit in Vellore, India, 2004. J Biosoc Sci 2009; 41(2): 195-205. DOI: https://doi.org/10.1017/s0021932008003064

Kodan LR, Verschueren KJC, van Roosmalen J, Kanhai HHH, Bloemenkamp KWM. Maternal mortality audit in Suriname between 2010 and 2014, a reproductive age mortality survey. BMC Pregnancy Childbirth 2017; 17(1): 275. DOI: https://doi.org/10.1186/s12884-017-1466-6

MacDorman MF, Declercq E. The failure of United States maternal mortality reporting and its impact on women's lives. Birth 2018; 45(2): 105-108. DOI: https://doi.org/10.1111/birt.12333

McCaw-Binns AM, Mullings JA, Yvette Holder. Vital registration and under-reporting of maternal mortality in Jamaica. Int J Gynaecol Obstet 2015; 128(1): 62-7. DOI: https://doi.org/10.1016/j.ijgo.2014.07.023

Mgawadere F, Unkels R, Adegoke A, van den Broek N. Measuring maternal mortality using a Reproductive Age Mortality Study (RAMOS). BMC Pregnancy Childbirth 2016; 16(1): 291. DOI: https://doi.org/10.1186/s12884-016-1084-8

Olsen BE, Hinderaker SG, Lie RT, Bergsjø P, Gasheka P, Kvåle G. Maternal mortality in northern rural Tanzania: assessing the completeness of various information sources. Acta Obstet Gynecol Scand 2002; 81(4): 301-307. DOI: https://doi.org/10.1034/j.1600-0412.2002.810404.x

Peterson E, Chou D, Moller AB, Gemmill A, Say L, Alkema L. Estimating maternal mortality using data from national civil registration vital statistics systems: A Bayesian hierarchical bivariate random walk model to estimate sensitivity and specificity of reporting. arxiv 2019: arXiv:1909.08578v1 https://arxiv.org/pdf/1909.08578.pdf

Qomariyah SN, Bell JS, Pambudi ES, et al. A practical approach to identifying maternal deaths missed from routine hospital reports: lessons from Indonesia. Glob Health Action 2009; 2. DOI: https://doi.org/10.3402/gha.v2i0.1905

Qomariyah SN, Sethi R, Izati YN, et al. No one data source captures all: A nested case-control study of the completeness of maternal death reporting in Banten Province, Indonesia. PLoS One 2020; 15(5): e0232080. DOI: https://doi.org/10.1371/journal.pone.0232080

Rodríguez-Angulo EM, Hoil-Santos JJ, Ojeda-Rodríguez R. Problems with Epidemiological Surveillance and Under-Reporting of Maternal Deaths in Yucatan, Mexico: An Analysis of Six Studies. In: Schwartz D. (eds) Maternal Death and Pregnancy-Related Morbidity Among Indigenous Women of Mexico and Central

America. Global Maternal and Child Health (Medical, Anthropological, and Public Health Perspectives). 2018. Springer, Cham. DOI: https://doi.org/10.1007/978-3-319-71538-4_15

Schuitemaker N, Van Roosmalen J, Dekker G, Van Dongen P, Van Geijn H, Gravenhorst JB. Underreporting of maternal mortality in The Netherlands. Obstet Gynecol 1997; 90(1): 78-82. DOI: https://doi.org/10.1016/s0029-7844(97)00128-2

Silva LK, Russomano FB. [Underreporting of maternal mortality in Rio de Janeiro, Brazil: comparison of 2 information systems]. Bol Oficina Sanit Panam 1996; 120(1): 36-43. PMID: https://pubmed.ncbi.nlm.nih.gov/8850128/

Soares VM, de Azevedo EM, Watanabe TL. [Underreporting of maternal deaths in Paraná State, Brazil: 1991-2005]. Cad Saude Publica 2008; 24(10): 2418-2426. DOI: https://doi.org/10.1590/s0102-311x2008001000022

United Nations Maternal Mortality Estimation Inter-Agency Group. Trends in maternal mortality: 2000 to 2017. Estimates by WHO, UNICEF, UNFPA, The World Bank and the United Nations Population Division. Geneva, World Health Organization, 2019. Available at: www.who.int/reproductivehealth/publications/maternal-mortality-2017/en/

Vangen S, Ellingsen L, Andersgaard AB, et al. [Maternal deaths in Norway 2005-2009]. Tidsskr Nor Laegeforen 2014; 134: 836-9. DOI: https://doi.org/10.4045/tidsskr.13.0203

Vork FC, Kyanamina S, van Roosmalen J. Maternal mortality in rural Zambia. Acta Obstet Gynecol Scand 1997; 76(7): 646-650. DOI: https://doi.org/10.3109/00016349709024604

Wilmoth JR, Mizoguchi N, Oestergaard MZ, et al. A New Method for Deriving Global Estimates of Maternal Mortality. Stat Politics Policy 2012; 3(2): 2151-7509. DOI: https://doi.org/10.1515/2151-7509.1038

World Health Organization. Maternal mortality ratio (per 100 000 live births). The Global Health Observatory. https://www.who.int/data/gho/indicator-metadata-registry/imr-details/26

Wu TP, Liang FW, Huang YL, Chen LH, Lu TH. Maternal Mortality in Taiwan: A Nationwide Data Linkage Study. PLoS One 2015; 10(8): e0132547. DOI: https://dx.doi.org/10.1371%2Fjournal.pone.0132547

Zakariah AY, Alexander S, van Roosmalen J, Buekens P, Kwawukume EY, Frimpong P. Reproductive age mortality survey (RAMOS) in Accra, Ghana. Reprod Health 2009; 6: 7. DOI: https://doi.org/10.1186/1742-4755-6-7

A.1.5 Obstetric Complications

The underlying probabilities of obstetric complications incidence and morbidity/mortality are modeled under "natural history" assumptions (i.e. the absence of any intervention). Interventions that occur tacitly at facilities (e.g., active management of labor, clean delivery, etc.) therefore need to be explicitly modeled.

Estimating the incidence of complications under "natural history" conditions is challenging since data based on observed cases in the population include a proportion of women with access to preventive and/or treatment measures. Most estimates have made assumptions about the regional variation in available coverage with maternal health services. These estimates may not be used for the natural history variables in the model without adjustment that accounts for treatment, but they can provide insight into the magnitude of the global burden and also provide a face validity check when we run the model with reported coverage rates (e.g. SBA or proportion of facility births) by country.

We assume that some of the variation reported in the literature is attributable to the heterogeneity in severity, and estimates based on small sample sizes. Case fatality rates are thus adjusted in the model based on complication severity (e.g., life threatening complications requiring CEmOC). We assume that life-threatening complications requiring CEmOC-level services are associated with a higher case fatality rate (in the absence of treatment) than that of non-life-threatening complications.

The following obsetric complications are included in the model: - Preeclampsia/Eclampsia
- Obstructed Labor
- Postpartum Hemmorhage
- Sepsis
- Other Direct Complications
- Late Maternal Deaths
- Indirect Maternal Deaths

A.1.5.1 Preeclampsia/Eclampsia

Overview

Hypertensive disorders of pregnancy refer to a range of conditions associated with high blood pressure, proteinuria and, rarely, seizures. Severe preeclampsia and eclampsia have the highest case fatality rates of the hypertensive disorders of pregnancy, and can lead to placental abruption, disseminated intravascular coagulopathy (DIC), adult respiratory distress syndrome (ARDS), cerebral hemorrhage, seizures, and death (Duley 2009). While medication can alleviate the symptoms and their negative effects, the only cure for the pregnant woman is expedited delivery (Filippi 2016).

The etiology of the condition remains unclear. It is thought to arise from the placenta and is associated with malfunction of the lining of blood vessels. The clinical spectrum of disease in preeclampsia varies, ranging from mild, asymptomatic disease, often occurring close to term, to severe, uncontrolled hypertension typically developing remote from term (less than 34 weeks) (Gülmezoglu 2016). Eclampsia and preeclampsia tend to occur more frequently in the second half of pregnancy, and can also occur postpartum, with up to 26% of eclamptic seizures occurring beyond 48 hours and as late as six weeks following delivery (Al-Safi 2011, Rawlins 2018). Preeclampsia and eclampsia are associated with perinatal deaths, abruption placenta, and cardio-vascular disease later in life (Filippi 2016).

The initial assessment of a woman presenting with symptoms suggestive of preeclampsia/eclampsia includes assessment of blood pressure, testing of urine for proteins, observation for the presence of convulsions, and taking the fetal heart rate. A woman with severe preeclampsia presents with high blood pressure (>160/110 mm Hg) and proteinurea; in addition, eclampsia is associated with convulsions, and needs to be managed with oxygen, intravenous fluids, anti-hypertensives and anticonvulsants. Emergency transportation has to be arranged to a facility that can offer follow-up care after initial management of preeclampsia/eclampsia, and which has facilities for induction of labor or operative delivery if needed. During follow up care, blood pressure and fluid intake have to be closely monitored, the patient needs to be monitored for convulsions and symptoms related to magnesium toxicity, and the option of inducing labor or operative delivery should be considered (Jayanna 2014).

Data

Incidence

Preeclampsia/eclampsia (PE/E) is a relatively frequent pregnancy complication, with reported incidence estimates of 2%-8% (Duley 2009). However, geographic, social, economic, and racial differences are thought to be responsible for incidence rates up to 3 times higher in some populations (Ghulmiyyah 2012). Generalized seizures (eclampsia) occur in up to 8% of women with preeclampsia in developing countries, a rate 10-30 times higher than in developed countries (Steegers 2010).

A systematic review covering 39 million women in 40 countries estimated an incidence of 4.6% (95% CI 2.7-8.2) for preeclampsia among all deliveries, and 1.4% (95% CI 1.0-2.0) for eclampsia (Abalos 2013), while a secondary analysis of WHO data found the combined prevalence of PE/E to be 4% (Bilano 2014). There was high regional variation, with sub-Saharan Africa estimated to have the highest prevalence (Abalos 2013). A separate secondary analysis of the WHO Multicountry Survey on Maternal and Newborn Health (WHOMCS) found incidences of 2.16% for pre-eclampsia, 0.28% for eclampsia, and 0.29% for chronic hypertension (Abalos 2014). This is similar to previous figures by the WHO which estimated that preeclampsia occurred in 3.2% of live births, and eclampsia occurred in 0.5% (AbouZahr 2004). A WHO multi-country survey on maternal and newborn health estimated that preeclampsia is associated with over 25 percent of severe maternal outcomes and is the direct cause of 20 percent of reported maternal deaths (Souza 2013).

A analysis of DHS data on 55,384 live deliveries from six surveys during 2005-2012 in Colombia, Bangladesh, Indonesia, Mali, Niger, and Peru found that indications of eclampsia were recorded for 1.2% (95% CI 1.0–1.3), 1.7% (95% CI 1.5–2.1), and 1.7% (95% CI 1.5–2.1) of deliveries reported from the American, South East Asian, and African regions, respectively (Bellizzi 2017). A prospective cohort study in the US estimated a prevalence of preeclampsia of 9.0% (Paré 2014), while a nationally representative study in the US using data from the National Hospital Discharge Survey from 1988 to 1997 of approximately 300,000 deliveries estimated that eclampsia was reported at 1.0 per 1,000 deliveries (95% CI 0.9-1.2), and that the overall

incidence of hypertensive disorders in pregnancy was 5.9% (95% CI 5.2-6.5) (Zhang 2003). A cohort study of all women giving birth in 1998-2000 in Scandinavia estimated that the incidence of eclampsia was 5.0/10,000 deliveries (95% CI 4.3-5.7), with 86% being diagnosed with preeclampsia before the seizure, and 90% of seizures occurring after admission to hospital (Andersgaard 2006). A national surveillance study in the UK in 1992 recorded an incidence of 4.9/10,000 deliveries, while an updated population-based study using the UK Obstetric Surveillance System (UKOSS) for all women delivering in the UK in 2005-2006 found the incidence of eclampsia was 2.7 cases per 10,000 births (95% CI 2.4–3.1) (Knight 2007).

Several risk factors for PE/E have been identified, including increasing maternal age (age 30-34 AOR: 1.40 [95% CI 1.31-1.51], age 35+ AOR: 1.95 [95% CI 1.80-2.12]), severe anemia (AOR: 2.38 [95% CI 1.86-3.05]), and multiple gestation (AOR: 2.96 [95% CI 1.74-5.03]) (Paré 2014, Bilano 2014). Other risk factors include high body mass index, chronic hypertension, and gestational diabetes. In clinical practice, although these factors predict just 30% of women who develop PE/E (Mol 2016), women with a history of PE/E in a previous pregnancy have an increased risk (AOR: 3.63 [95% CI 2.29-5.73]) of developing it again (Paré 2014).

Morbidity/Mortality

Eclampsia has a high case fatality rate, which varies among regions of the world, presumably as a function of the access to and quality of health care (Dolea 2003). Although eclampsia is associated with an increased risk of maternal death in developed countries (0%-1.8%), the mortality rate is as high as 15% in developing countries (Ghulmiyyah 2012). A retrospective study of PE/E-related deaths found that access to and delay in seeking care was a major determinant of mortality, with 37.7% in grade IV coma and 54% with recurrent convulsions prior to admission (Sawhney 2000). Concurrent eclampsia with HELLP Syndrome was found to be linked in 5-6 out of every 10 deaths associated with either condition (Vigil-De Gracia 2009).

Graham 2006 reports an overall case fatality rate of 1.7% for PE/E, while a study in India of 864 eclampsia patients reported a case fatality rate of 7.29% (Biswas 2005). These women were reported to all have been treated with magnesium sulphate, and nearly half had caesarean sections, implying that this CFR may be low compared to ‘natural history’ outcomes. An earlier study showed that severe PE/E required treatment with intravenous hydralazine and magnesium sulphate and in addition, approximately 10% of all cases were assumed to require emergency cesarean section (Cahuana-Hurtado 2004). A study of eclampsia in University of Benin Teaching Hospital, Nigeria during 1995-2002 found 11 maternal deaths occurred among 103 cases of eclampsia, with a case fatality rate of 10.7% (Onuh 2004). A similar study of eclampsia in Dar es Salaam, Tanzania found a case fatality rate of 5.0% for women who delivered at Muhimibili National Hospital, and 16% for those referred to the hospital after delivering elsewhere (Urassa 2006).

Hypertensive disorders of pregnancy are also associated with increased maternal morbidity, predominantly because of maternal stroke (Martin 2005, Folk 2018). While the general incidence of stroke during pregnancy ranges from 10 to 34 per 100,000 deliveries (Bushnell 2011), women with a history of preeclampsia have approximately double the risk for fatal and nonfatal stroke (Bellamy 2007, McDonald 2008).

In addition to maternal morbidity/mortality, PE/E increases the risk of stillbirth and neonatal mortality. A population-based study in the UK in 2005-2006 found that 8/222 infants delivered by mothers with eclampsia were stillborn and 5 died in the neonatal period (Knight 2007). PE/E may contribute to poor fetal growth and have an estimated population attributable risk for stillbirths of about 5% (Lawn 2016). The risk of fetal demise for women with preeclampsia has been estimated at 5.2 per 1,000 pregnancies (Harmon 2015), and an analysis of DHS data found that eclampsia was associated with a two-fold increase (95% CI 1.4–3.2) in the risk of early neonatal mortality (Bellizzi 2017).

Parameters

We used a hierarchical logistic regression model to model incidence of PE/E, based on priors estimated on data from Abalos 2013 and Bilano 2014. In addition, we account for the following individual-level risk factors: maternal age (30-34, 35+), severe anemia, multiple gestation, and history of PE/E. We enforce non-decreasing RRs by increasing maternal age when sampling. Conditional on developing PE/E we model risks of mortality, neurological sequelae, and stillbirth, with higher risks for eclampsia vs preeclampsia.

Baseline Incidence

G = Global: $\alpha_s^G \sim N(\hat{\alpha}_s{}^G, \hat{\sigma}_s{}^G)$

I = Income Group: $\beta_s^I \sim N\left(\hat{\beta}_s{}^I, \hat{\sigma}_s{}^I\right)$

A = Area: $\gamma_s^A \sim N(\hat{\gamma}_s^A, \hat{\sigma}_s^A)$

R = Region: $\delta_s^R \sim N(0,0.1)$

C = Country: $\beta_s{}^C \sim \alpha_s^G + \beta_s^I + \gamma_s^A + \delta_s^R + N(0,0.01)$

Probability: $p[\text{Incidence}]_s{}^C = \dfrac{1}{1 + \exp(-\beta_s{}^C)}$

Risk Factors

$\alpha_r^G \sim N(\hat{\alpha}_r{}^G, \hat{\sigma}_r{}^G)$

$\gamma_r^A \sim N(0,0.05)$

$\delta_r^R \sim N(0,0.01)$

$\pi_r^C \sim \alpha_r^G + \gamma_r^A + \delta_r^R + N(0,0.001)$

$RR_r^C = \max(\pi_r^C, 1)$

s = Severity<br>r = Risk factor

Morbidity/Mortality Outcomes

G = Global: $\alpha_s^G \sim N(\hat{\alpha}_s{}^G, \hat{\sigma}_s{}^G)$

I = Income Group: $\beta_s^I \sim N(0,0.1)$

A = Area: $\gamma_s^A \sim N(0,0.05)$

R = Region: $\delta_s^R \sim N(0,0.01)$

C = Country: $\beta_s{}^C \sim \alpha_s^G + \beta_s^I + \gamma_s^A + \delta_s^R + N(0,0.001)$

Probability: $p[\text{Outcome}]_s{}^C = \dfrac{1}{1 + \exp(-\beta_s{}^C)}$

s = Severity

Priors

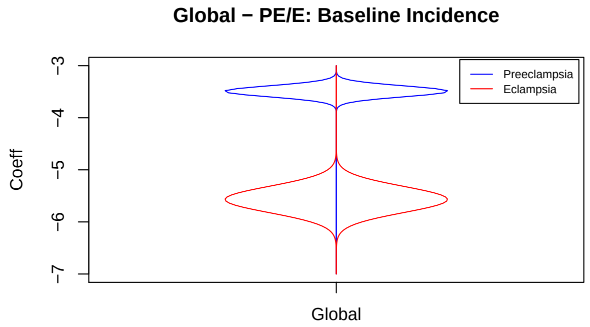

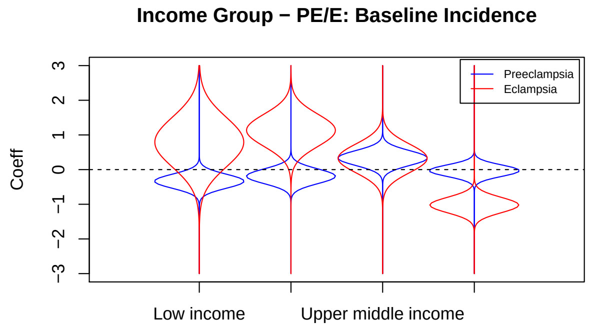

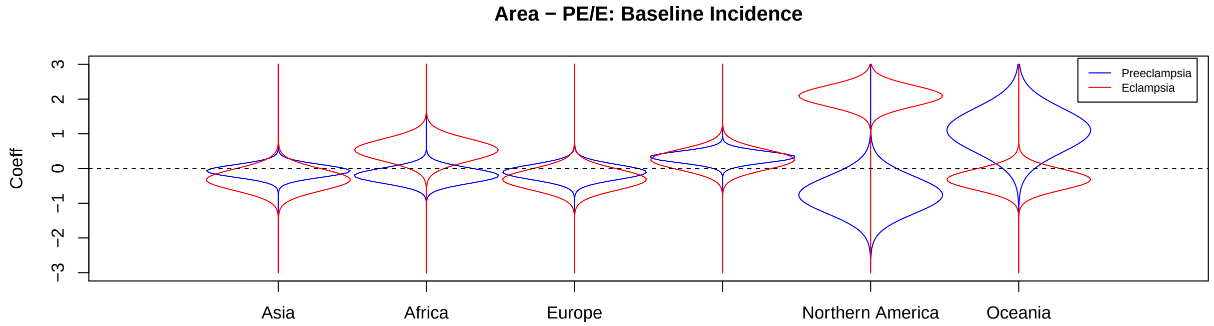

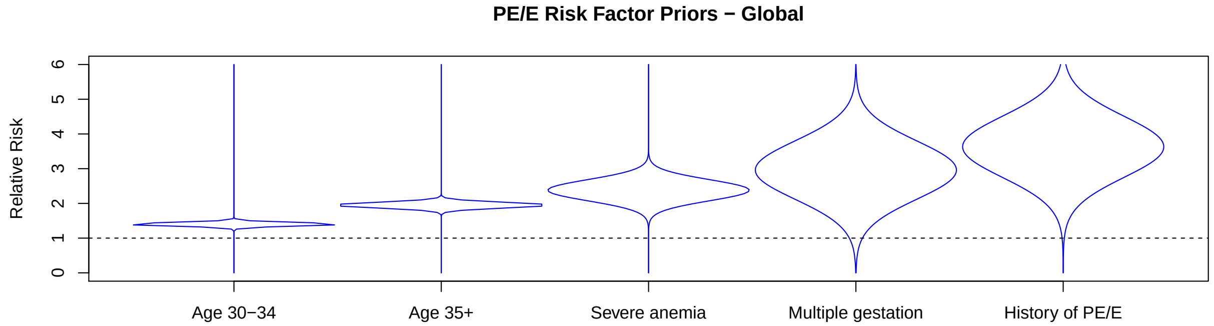

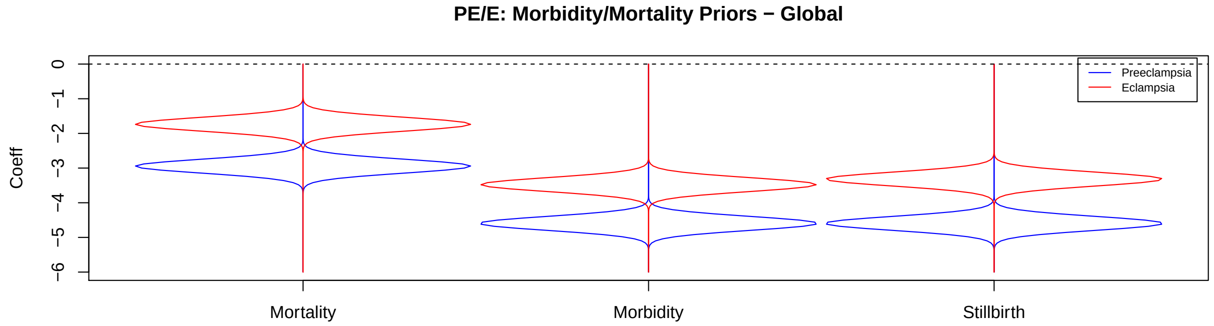

Model Implementation

At the time of delivery, the incidence and outcomes of PE/E are simulated, together with the availability and efficacy of interventions that modify the 'natural history' probabilities. We assume that the risk factors that affect incidence are independent in the model. While we model the risk of stillbirths for women who develop PE/E, we do not currently take into account neonatal outcomes in the model.

References

Andersgaard AB, Herbst A, Johansen M, et al. Eclampsia in Scandinavia: Incidence, substandard care, and potentially preventable cases. Acta Obstet Gynecol Scand 2006; 85(8): 929–936. DOI: https://doi.org/10.1080/00016340600607149

Abalos E, Cristina C, Grosso AL, Chou D, Say L. Global and regional estimates of preeclampsia and eclampsia: a systematic review. European Journal of Obstetrics and Gynecology and Reproductive Biology 2013; 170(1): 1-7. DOI: https://doi.org/10.1016/j.ejogrb.2013.05.005

Abalos E, Cuesta C, Carroli G, et al. Pre-eclampsia, eclampsia and adverse maternal and perinatal outcomes: a secondary analysis of the World Health Organization Multicountry Survey on Maternal and Newborn Health. BJOG 2014; 121(S1): 14-24. DOI: https://doi.org/10.1111/1471-0528.12629

AbouZahr C, Wardlaw T. Maternal mortality in 2000: estimates developed by WHO, UNICEF and UNFPA. Department of Reproductive Health and Research, World Health Organization: Geneva, 2004. Available at: https://apps.who.int/iris/handle/10665/42930

Al-Safi Z, Imudia AN, Filetti LC, Hobson DT, Bahado-Singh RO, Awonuga AO. Delayed postpartum preeclampsia and eclampsia: demographics, clinical course, and complications. Obstet Gynecol 2011; 118(5): 1102-7. DOI: https://doi.org/10.1097/aog.0b013e318231934c

Bellamy L, Casas JP, Hingorani AD, Williams DJ. Pre-eclampsia and risk of cardiovascular disease and cancer in later life: systematic review and meta-analysis. BMJ 2007; 335(7627): 974. DOI: https://doi.org/10.1136/bmj.39335.385301.be

Bellizzi S, Sobel HL, Ali MM. Signs of eclampsia during singleton deliveries and early neonatal mortality in low- and middle-income countries from three WHO regions. Int J Gynaecol Obstet 2017; 139(1): 50-54. DOI: https://doi.org/10.1002/ijgo.12262

Bilano VL, Ota E, Ganchimeg T, Mori R, Souza JP. Risk factors of pre-eclampsia/eclampsia and its adverse outcomes in low- and middle-income countries: a WHO secondary analysis. PLoS ONE 2014; 9(3): e91198. DOI: https://doi.org/10.1371/journal.pone.0091198

Biswas A, Modak R, Baksi S, Biswas S. Epidemiological study of eclampsia in a referral teaching hospital. J Indian Med Associ 2005; 103(6): 323-4, 326. PMID: https://pubmed.ncbi.nlm.nih.gov/16225158/

Bushnell C, Chireau M. Preeclampsia and Stroke: Risks during and after Pregnancy. Stroke Res Treat 2011; 2011: 858134. DOI: https://dx.doi.org/10.4061%2F2011%2F858134

Cahuana-Hurtado L, Sosa-Rubi S, Bertozzi S. Cost of mother-child care in Morelos State. Salud Publica Mex 2004; 46(4): 316-25. DOI: https://doi.org/10.1590/s0036-36342004000400006

Dolea C, AbouZahr C. Global Burden of Hypertensive Disorders in Pregnancy in the Year 2000. Evidence and information for policy (EIP). Global Burden of Disease Working Paper, 2003. Available at: https://www.who.int/healthinfo/statistics/bod_hypertensivedisordersofpregnancy.pdf

Duley L. The global impact of pre-eclampsia and eclampsia. Semin Perinatol 2009; 33(3): 130-7. DOI: https://doi.org/10.1053/j.semperi.2009.02.010

Filippi V, Chou D, Ronsmans C, Graham W, Say L. Chapter 3. Levels and causes of maternal mortality and morbidity. In: Black RE, Laxminarayan L, Walker N, Temmerman M, eds. Disease control priorities 3rd edn. Volume 2. Reproductive, maternal, newborn and child health. Washington, DC: World Bank, 2016. Available at: https://www.ncbi.nlm.nih.gov/books/NBK361917/

Folk DM. Hypertensive Disorders of Pregnancy: Overview and Current Recommendations. J Midwifery Womens Health 2018; 63(3): 289-300. DOI: https://doi.org/10.1111/jmwh.12725

Ghulmiyyah L, Sibai B. Maternal mortality from preeclampsia/eclampsia. Semin Perinatol 2012; 36(1): 56-9. DOI: https://doi.org/10.1053/j.semperi.2011.09.011

Graham WJ, Cairns J, Bhattacharya S, et al. Maternal and Perinatal Conditions. In: Jamison DT, Breman JG, Measham AR, et al, eds. Disease Control Priorities in Developing Countries. 2nd edition. Washington (DC): World Bank; 2006. Available at: https://www.ncbi.nlm.nih.gov/books/NBK11742/

Gülmezoglu AM, Lawrie TA, Hezelgrave N, et al. Chapter 7. Interventions to Reduce Maternal and Child Morbidity and Mortality. In: Black RE, Laxminarayan L, Walker N, Temmerman M, eds. Disease control priorities 3rd edn. Volume 2. Reproductive, maternal, newborn and child health. Washington, DC: World Bank, 2016. Available at: https://www.ncbi.nlm.nih.gov/books/NBK361904/

Harmon QE, Huang L, Umbach DM, et al. Risk of fetal death with preeclampsia. Obstet Gynecol 2015; 125(3): 628-635. DOI: https://doi.org/10.1097/aog.0000000000000696

Jayanna K, Mony P, Ramesh BM, et al. Assessment of facility readiness and provider preparedness for dealing with postpartum haemorrhage and pre-eclampsia/eclampsia in public and private health facilities of northern Karnataka, India: a cross-sectional study. BMC Pregnancy Childbirth 2014; 14: 304. DOI: https://doi.org/10.1186/1471-2393-14-304

Knight M, UKOSS. Eclampsia in the United Kingdom 2005. BJOG 2007; 114(9): 1072-8. DOI: https://doi.org/10.1111/j.1471-0528.2007.01423.x

Lawn JE, Blencowe H, Wasiwa P, et al. Stillbirths: rates, risk factors, and acceleration towards 2030. Lancet 2016; 387(10018): 587-603. DOI: https://doi.org/10.1016/S0140-6736(15)00837-5

Martin JN Jr, Thigpen BD, Moore RC, Rose CH, Cushman J, May W. Stroke and severe preeclampsia and eclampsia: a paradigm shift focusing on systolic blood pressure. Obstet Gynecol 2005; 105(2): 246-254. DOI: https://doi.org/10.1097/01.aog.0000151116.84113.56

McDonald SD, Malinowski A, Zhou Q, Yusuf S, Devereaux PJ. Cardiovascular sequelae of preeclampsia/eclampsia: a systematic review and meta-analyses. Am Heart J 2008; 156(5): 918-930. DOI: https://doi.org/10.1016/j.ahj.2008.06.042

Mol BWJ, Roberts CT, Thangaratinam S, Magee LA, de Groot CJM, Hofmeyr GJ. Pre-eclampsia. Lancet 2016; 387(10022): 999-1011. DOI: https://doi.org/10.1016/s0140-6736(15)00070-7

Onuh SO, Aisien AO. Maternal and fetal outcome in eclamptic patients in Benin City, Nigeria. J Obstet Gynaecol 2004; 24(7): 765-8. DOI: https://doi.org/10.1080/01443610400009451

Paré E, Parry S, McElrath TF, Pucci D, Netwon A, Lim KH. Clinical risk factors for preeclampsia in the 21st century. Obstet Gynecol 2014; 124(4): 763-70. DOI: https://doi.org/10.1097/aog.0000000000000451

Rawlins B, Plotkin M, Rakotovao JP, et al. Screening and management of pre-eclampsia and eclampsia in antenatal and labor and delivery services: findings from cross-sectional observation studies in six sub-Saharan African countries. BMC Pregnancy Childbirth 2018; 18(1): 346. DOI: https://doi.org/10.1186/s12884-018-1972-1

Sawhney H, Aggarwal N, Biswas R, Vasishta K, Gopalan S. Maternal mortality associated with eclampsia and severe preeclampsia of pregnancy. J Obstet Gynaecol Res 2000; 26(5): 351-6. DOI: https://doi.org/10.1111/j.1447-0756.2000.tb01338.x

Souza JP, Gülmezoglu AM, Vogel J, et al. Moving beyond essential interventions for reduction of maternal mortality (the WHO Multicountry Survey on Maternal and Newborn Health): a cross-sectional study. Lancet 2013; 381(9879): 1747-55. DOI: https://doi.org/10.1016/s0140-6736(13)60686-8

Steegers EA, von Dadelszen P, Duvekot JJ, Pijnenborg R. Pre-eclampsia. Lancet 2010; 376(9741): 631-44. DOI: https://doi.org/10.1016/s0140-6736(10)60279-6

Urassa DP, Carlstedt A, Nyström L, Massawe SN, Lindmark G. Eclampsia in Dar es Salaam, Tanzania – Incidence, outcome, and the role of antenatal care. Acta Obstet Gynecol Scand 2006; 85(5): 571-8. DOI: https://doi.org/10.1080/00016340600604880

Vigil-De Gracia P. Maternal deaths due to eclampsia and HELLP syndrome. Int J Gynaecol Obstet 2009; 104(2): 90-94. DOI: https://doi.org/10.1016/j.ijgo.2008.09.014

Zhang J, Meikle S, Trumble A. Severe maternal morbidity associated with hypertensive disorders in pregnancy in the United States. Hypertens Pregnancy 2003; 22(2): 203-12. DOI: https://doi.org/10.1081/prg-120021066

A.1.5.2 Obstructed Labor

Overview

Labor is considered obstructed when the presenting part of the fetus cannot progress into the birth canal, despite strong uterine contractions (Dolea 2003). It usually arises from mechanical factors of maternal origin (e.g. small pelvis, pelvic contractures, nongynecoid pelvis, etc.) or of fetal origin (abnormal presentation and/or position, large fetus [macrosomia], abnormally large fetal parts [e.g. hydrocephalus, tumors], etc.) (Lawson 1965). Obstruction can occur at the pelvic inlet, in the pelvic cavity or at the pelvic outlet. If neglected, it can result in endometritis, obstetric fistula (rectovaginal or vesicovaginal), and ruptured uterus with consequent hemorrhage, shock or death. It also leads to significant perinatal morbidity and mortality. Women who are malnourished, marry young, or engage in childbearing at an early age before the pelvis has reached adult proportions, are at high risk for obstructed labor. However, there is no evidence to show that interventions aimed at providing adequate childhood nutrition or delayed childbearing prevents obstructed labor (Tsu 2009).

Data

Incidence

Obstructed labor is estimated to affect 4.6% of live births, although it varies considerably among different regions of the world (Dolea 2003, Khan 2006). A review of studies from 1960-2003 reports incidence estimates ranging from 1% to 7% (Wall 2012). According to Filippi 2016, an unpublished systematic review by Adler and others yielded only 16 published population-based studies of obstructed and prolonged labor worldwide since 2000. The studies could not be combined through meta-analysis to obtain a global prevalence because of high heterogeneity, which was largely attributed to differences in case definitions. However, the median prevalence was estimated to be 1.9 per 100 deliveries for obstructed labor, and 8.7 per 100 deliveries for combined obstructed and prolonged labor. There is evidence that a higher incidence of obstructed labor is associated with younger age groups - Kabakyenga 2011 estimated an odds ratio of obstructed labor of 1.43 (95% CI 1.23-1.67) for women between the ages of 15-19, compared to older women.

Morbidity/Mortality

A review conducted by the Disease Control Priorities Project (DCP2) reported an average case fatality rate of 0.7% for obstructed labor (Graham 2006), while a review of studies from 1960-2003 finds an average mortality risk of 5.5%, ranging from 0.8% to 16.6% (Wall 2012). In addition to mortality, there is also the risk of fistula from obstructed labor. As a result of the severe delay in delivery and continuous pressure of the fetal head on maternal tissues, blood flow is blocked resulting in necrosis. This leaves abnormal gaps (or communications) between the vagina and bladder or rectum, allowing urine or stool to pass continuously through the vagina (Filippi 2016). Obstetric fistula was estimated to occur in 2.15% of neglected obstructed labor cases (Dolea 2003), while a review of studies from 1960-2003 finds an average risk of fistula from obstructed labor of 3.4%, ranging from 0.4% to 6.6% (Wall 2012).

In addition, obstructed labor is linked with increased rates of stillbirth, especially for women who develop fistula as a result of obstructed labor. Estimates of the proportion of women with obstetric fistula who also experience stillbirth range from 32.3% to 100%, with estimates from the largest studies around 92% (Cowgill 2015).

Parameters

We used a hierarchical logistic regression model for the incidence of obstructed labor and model 2 levels of severity: moderate and severe obstructed labor. We also account for higher risk of obstructed labor for women less than 20 years old. Conditional on developing obstructed labor we model risks of mortality, obstetric fistula, and stillbirth, with higher risks for severe vs moderate obstructed labor. To account for the relationship between obstetric fistula and stillbirth we model a higher risk of stillbirth for women who experience fistula.

Baseline Incidence

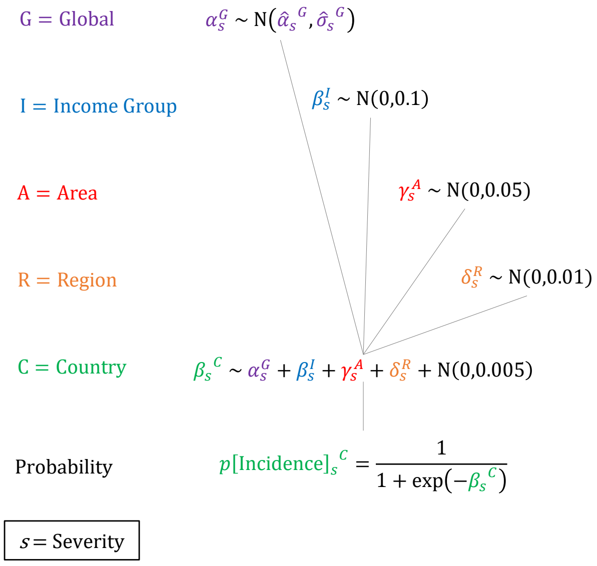

Relative Risk: Age <20

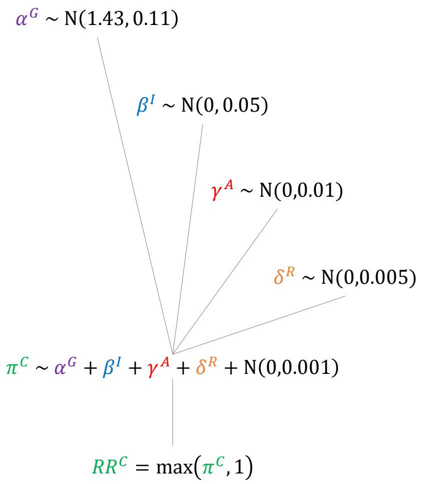

Morbidity/Mortality Outcomes

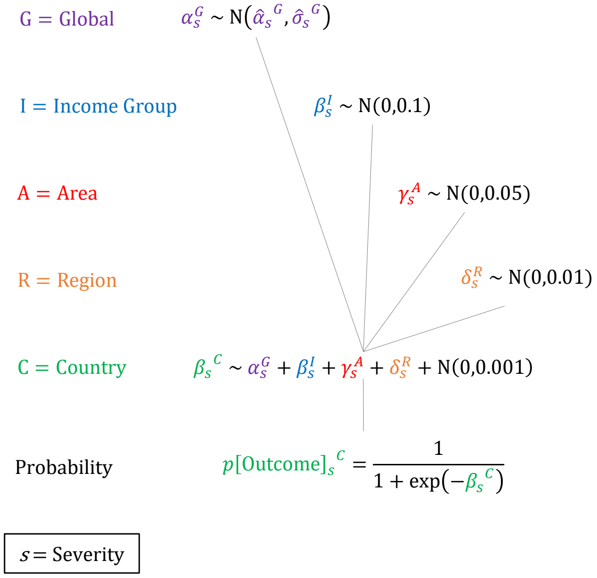

Priors

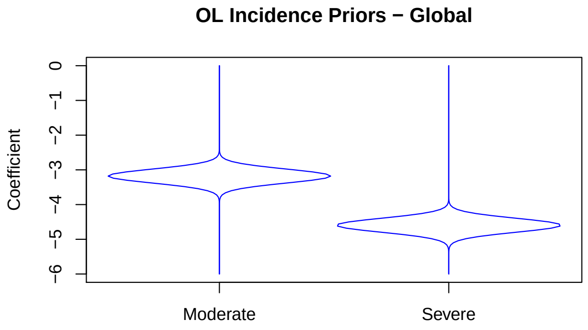

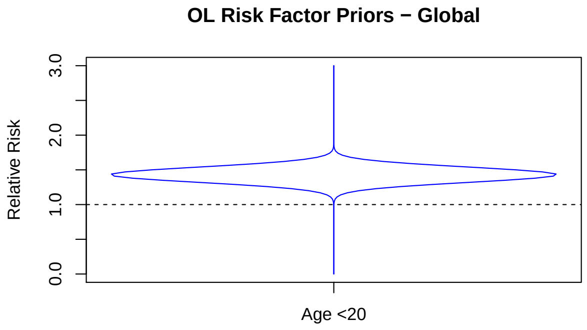

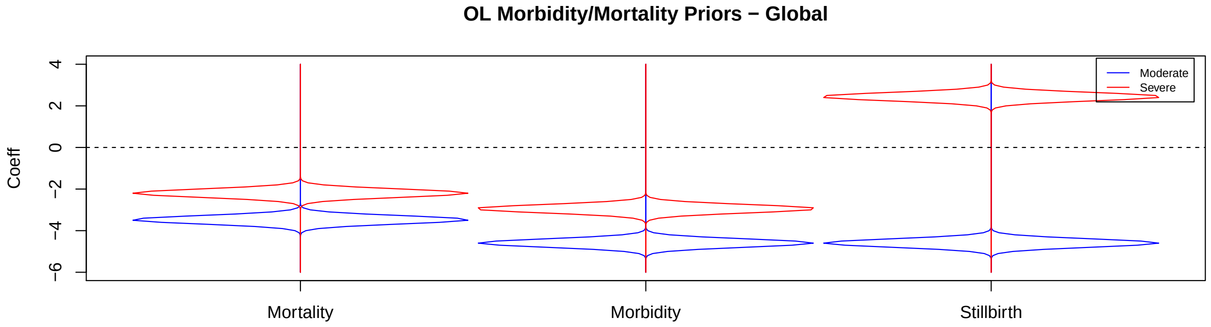

Note: For stillbirth risk, 'Severe' denotes that the woman experienced an obstetric fistula.

Model Implementation

At the time of delivery, the incidence and outcomes of obstructed labor are simulated, together with the availability and efficacy of interventions that modify the 'natural history' probabilities.

References

Cowgill KD, Bishop J, Norgaard AK, Rubens CE, Gravett MG. Obstetric fistula in low-resource countries: an under-valued and under-studied problem–systematic review of its incidence, prevalence, and association with stillbirth. BMC Pregnancy Childbirth 2015; 15: 193. DOI: https://doi.org/10.1186/s12884-015-0592-2

Dolea C, AbouZahr C. Global Burden of Obstructed Labour in the Year 2000. Evidence and information for policy (EIP). Global Burden of Disease Working Paper, 2003. Available at: https://www.who.int/healthinfo/statistics/bod_obstructedlabour.pdf

Filippi V, Chou D, Ronsmans C, Graham W, Say L. Chapter 3. Levels and causes of maternal mortality and morbidity. In: Black RE, Laxminarayan L, Walker N, Temmerman M, eds. Disease control priorities 3rd edn. Volume 2. Reproductive, maternal, newborn and child health. Washington, DC: World Bank, 2016. Available at: https://www.ncbi.nlm.nih.gov/books/NBK361917/

Graham WJ, Cairns J, Bhattacharya S, et al. Maternal and Perinatal Conditions. In: Jamison DT, Breman JG, Measham AR, et al, eds. Disease Control Priorities in Developing Countries. 2nd edition. Washington (DC): World Bank; 2006. Available at: https://www.ncbi.nlm.nih.gov/books/NBK11742/

Kabakyenga JK, Östergren PO, Turyakira E, Mukasa PE, Pettersson KO. Individual and health facility factors and the risk for obstructed labour and its adverse outcomes in south-western Uganda. BMC Pregnancy Childbirth 2011; 11: 73. DOI: https://doi.org/10.1186/1471-2393-11-73

Khan KS, Wojdyla D, Say L, Gülmezoglu AM, Van Look PF. WHO analysis of causes of maternal death: a systematic review. Lancet 2006; 367(9516): 1066-1074. DOI: https://doi.org/10.1016/s0140-6736(06)68397-9

Lawson JB. Obstructed Labour. BJOG 1965; 72(6): 877-880. DOI: https://doi.org/10.1111/j.1471-0528.1965.tb01506.x

Tsu VD, Coffey PS. New and underutilised technologies to reduce maternal mortality and morbidity: what progress have we made since Bellagio 2003? BJOG 2009; 116(2): 247-56. DOI: https://doi.org/10.1111/j.1471-0528.2008.02046.x

Wall LL. A framework for analyzing the determinants of obstetric fistula formation. Stud Fam Plann 2012; 43(4): 255-72. DOI: https://doi.org/10.1111/j.1728-4465.2012.00325.x

A.1.5.3 Postpartum Hemorrhage

Overview

Maternal hemorrhage is defined generally as severe bleeding that occurs during pregnancy (ante-partum), labor, or post-partum. Bleeding after childbirth (postpartum hemorrhage, PPH) comprises the majority of maternal hemorrhage cases. After a woman gives birth, womb muscles should contract, clamping down on the blood vessels and helping to limit bleeding when the placenta has detached. If the muscles do not contract strongly enough, very heavy bleeding can occur, which can be life threatening (Mousa 2014).

PPH is defined as blood loss from the genital tract of 500 mL or more in the first 24 hours after the delivery of the baby, and severe postpartum hemorrhage is defined as blood loss from the genital tract of 1000 mL or more in the first 24 hours after the delivery of the baby (WHO 2009). Levels of very severe PPH (2500 mL or more) have also been discussed, but no data exist for this outcome (Begley 2019). However, it is commonly acknowledged that 500 mL is an arbitrary cut-off and may not be clinically significant (Meher 2019). For example, the American College of Obstetricians and Gynecologists have revised their definition of PPH as at least 1000 mL of blood loss with signs of hypovolaemia to account for levels of blood loss more likely to correlate with maternal morbidity (Menard 2014).

Clinical signs and symptoms of blood loss (also used for diagnosis) include weakness, sweating, and tachycardia (i.e., fast heart rate), with hemodynamic collapse (i.e., loss of adequate blood pressure to get blood and oxygen to vital organs) occurring at losses of between 35% and 45% of blood (Bonnar 2000). Among the 5 major direct causes of maternal death, PPH is the most rapidly fatal with an estimated 2 hour interval from onset to death if untreated, which highlights important considerations for prevention of deaths (WHO 2005).

Common causes of PPH include failure of the uterus to contract adequately after birth (atonic PPH), which accounts for 90% of PPH in most countries; trauma to the genital tract (traumatic PPH), which accounts for about 7% of PPH; and bleeding due to retention of placental tissue and failure in the coagulation system, which accounts for the remaining 3% (Carroli 2008).

The initial management of PPH includes assessment and treatment of shock through administration of oxygen and fluids, and specific management of the cause of PPH, followed by referring to a higher facility where blood transfusion and operative delivery, if needed, are available (Jayanna 2014).

Antepartum hemorrhage Antepartum hemorrhage (APH) is defined as bleeding from the genital tract in the second half of pregnancy (but before birth) (Giordano 2010). The most important causes of APH are placenta previa or placental abruption (Fan 2017), although these are not the most common (RCOG 2011). APH complicates an estimated 2-5% of pregnancies (Sinha 2008). However, there are no consistent definitions of the severity of APH (RCOG 2011). We do not currently include antepartum hemorrhage as a separate complication in the model due to insufficient data on its epidemiology and its relatively small impact on maternal mortality.

Data

Incidence

There have been several studies, including systematic reviews, of the incidence of maternal hemorrhage under different management interventions, with some reporting severe PPH as an outcome and some reporting blood loss. There are two ways to estimate natural history incidence from these studies: (1) if there is an arm of the trial with no treatment (generally there is not), (2) if there is an arm in the trial with expectant management only (this estimate can be adjusted by applying the reduction in PPH with expectant management versus no management).

We used a number of studies to explore the total reported incidence, and the severity distribution of maternal hemorrhage. Studies vary in their definition of “severe” but most rely on blood loss >1000 ml. There is clearly heterogeneity among these “severe cases” in that some of them will be associated with rapid death from massive blood loss and very high case fatality rates. Others will still have a higher CFR compared with <500ml but not as high as the most severe cases.

In the 2000 GBD, the authors calculated an incidence of severe PPH of 10.5 per 100 live births, and 11.98 per 1000 women 15-49 for SEARO-D. This only included severe cases with >1000ml blood loss. They assumed

that in SEARO-D only 20% of women would have access to active management of labor and the births covered by SBA would be expectantly managed. They also assumed that severe PPH within 1 hour represents 90% of all severe PPH, and inflated their estimates accordingly. In this GBD study, described in Dolea 2003, the authors used clinical trial evidence from a large study of uterotonics (MISO trial). In that trial, severe PPH, defined as >1000ml of blood loss within 1 hour of delivery, occurred in 2.85% of patients in the oxytocin arm who were actively managed (Dolea 2003). To calculate the incidence that would have occurred without active management, the GBD method used findings from a Cochrane review that active management with oxytocin results in a relative risk of severe PPH of 0.33 compared to expectant management. This review has since been withdrawn due to concerns regarding the validity of these findings (Prendiville 2009). However, a newer study showed an estimate of 0.34, implying that the updated results would be similar (Begley 2019). The GBD method thus assumed the fraction of deliveries managed expectantly by SBAs would be twice as much as active management (5.7%) (Chandhiok 2006, Dolea 2003, Gülmezoglu 2001) and twice as high again with no SBA present (11.4%) (Alfirevic 2007, Derman 2006, Høj 2005, Tunçalp 2012, Walraven 2005).

In one of the largest systemic reviews covering the period 1997-2006, Carroli (2008) included 24 studies from the WHO database covering a period of 1997-2002 and an additional 166 reports assessed (from 2003-2006) with 14 included (total = 38) for a total of 224 datasets. These were stratified by those that reported PPH (n=120) and those that reported severe PPH (n=70), with severe PPH being defined as blood loss >1000 ml. Overall quality was deemed adequate for ~47% of PPH datasets and ~59% of severe PPH datasets. Overall prevalence of PPH was 6.09% (95% CI 6.06-6.11), rising to 10.55% when the blood loss was measured objectively. Overall prevalence of severe PPH was 1.86% (95% CI 1.82-1.90), rising to 3.04% when the blood loss was measured objectively. A high degree of heterogeneity was reported even in subgroups with similar characteristics. Another systematic review (Calvert 2012) found a global prevalence of blood loss equal to or greater than 500 ml of 10.8 percent in vaginal deliveries (95% CI 9.6-12.1); the prevalence of severe hemorrhage (equal to or greater than 1,000 ml) was 2.8 percent (95% CI 2.4-3.2). The review included many study settings in which active management of the third stage of labor was practiced, suggesting that the incidence of postpartum hemorrhage under natural history conditions (ex: home deliveries) is probably higher (Filippi 2016). Indeed, under expectant management, Carroli 2008 reported that PPH prevalence was 17.96% (95% CI 16.97-18.95).

Morbidity/Mortality
DCP 2 estimated a CFR for severe PPH (>1000 mL) of 1.0%, assuming that no intensive care was available (Graham 2006). However, data from India suggest that, in rural India, the case fatality rate for overall PPH is at least 4 percent (Sibley 2005), while data from a teaching hospital in Nigeria (Etuk 1997) indicate a case fatality rate of 2.2 percent. In rural areas and in non-hospital settings in Nigeria - where most births in that country occur - a far higher rate would be expected. Reports of case fatality rates ranging from 2 to 8 percent have been reported from different African settings (Brabin 2001). Experts have also estimated that 25%-50% of women would die with the most severe forms of PPH and no access to treatment, and that case fatality rates in general are higher for women with anemia and with >1000ml blood loss (Brabin 2001).

Other setting-specific studies typically find higher case fatality rates as well. One analysis estimated a CFR in sub-Saharan Africa of 15% for severe PPH (Pagel 2009). A study of hemorrhage in a North Indian tertiary care hospital found that of 178 women with early postpartum hemorrhage who either delivered in or were referred to a hospital after delivery, 94 women died (nearly 50%), with early PPH accounting for 11.7% of the deaths, and 19 women experienced "near-miss" morbidities due to early PPH (Kaul 2006). A prospective observational study in a tertiary referral hospital in Kolkata, India found a CFR of 7.5% (Shirazee 2010). A Nigerian study found that evidence-based management guidelines for PPH reduced the CFR from 13.6% to 2.5%, implying that the higher CFR would be a closer to natural history conditions (Ezugwu 2014). A retrospective review of 8 hospitals in Malawi found a CFR of 7-10% (Kongnyuy 2009), while a 10-year review of a hospital in Ethiopia found a CFR of 9.3% (Gessessew 2007). Other reported CFRs for hemorrhage range between 2.8% to 27.3% (Kaye 2011).

In addition to mortality risks, Sheehan's syndrome is a well known complication of PPH, which can present with chronic symptoms after a relatively long latent period, including failure to lactate, mild headache, fatigue, nausea, and amenorrhea (Dökmetaş 2006). While the exact incidence of Sheehan's syndrome is unknown and it rarely occurs in modern obstetric practices (Matsuzaki 2017), it is estimated to occur in 1-2% of women

who lose 1-2 L of blood with associated hypotension (Vaphiades 2003, Kelestimur 2003).

Women who survive PPH are also at risk for developing anemia. It was estimated that globally, 12% of women who survive PPH develop severe anemia (AbouZahr 2003). A systematic review of articles published between 1990 and 2009 revealed a higher prevalence of anemia in women who had experienced hemorrhage, irrespective of the timing of measurement post-partum (Wagner 2012). In high-income countries, women who had a hemorrhage were at 5.68 (95% CI 5.04-6.40) times higher risk of post-partum anemia than women who did not, while in low-income countries, the prevalence of anemia was 1.58 (95% CI 0.96-2.60) times higher (Wagner 2012).

Parameters

In order to capture a higher risk of death from extreme blood loss, we model three levels of PPH severity: Moderate, Severe, and Most Severe. We constrain incidence to decrease by severity, and risk of morbidity/mortality to increase by PPH severity when sampling parameters.

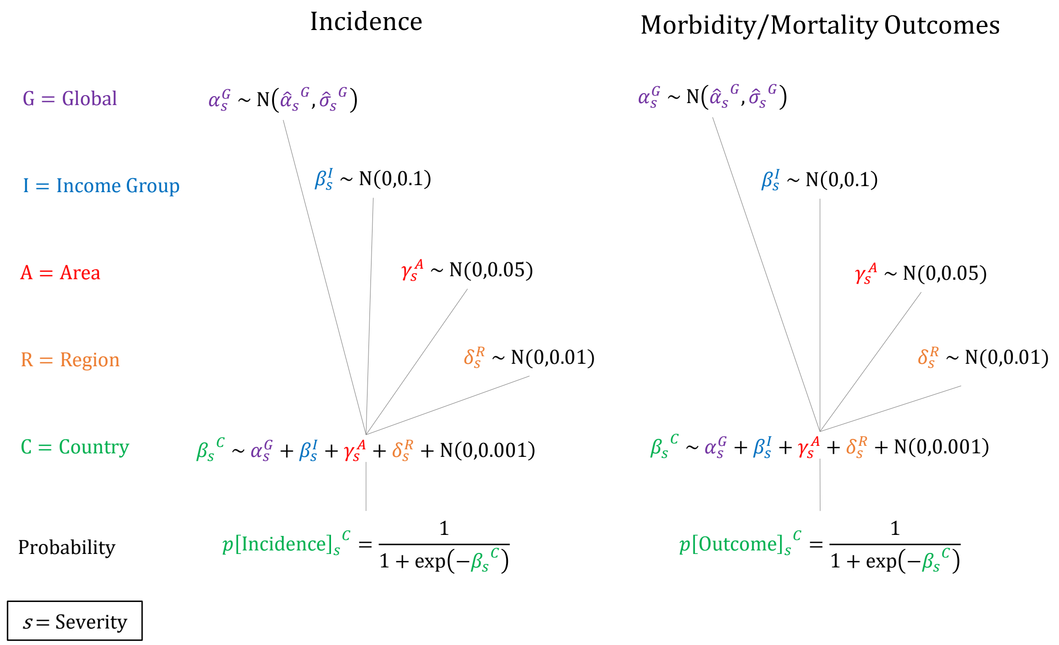

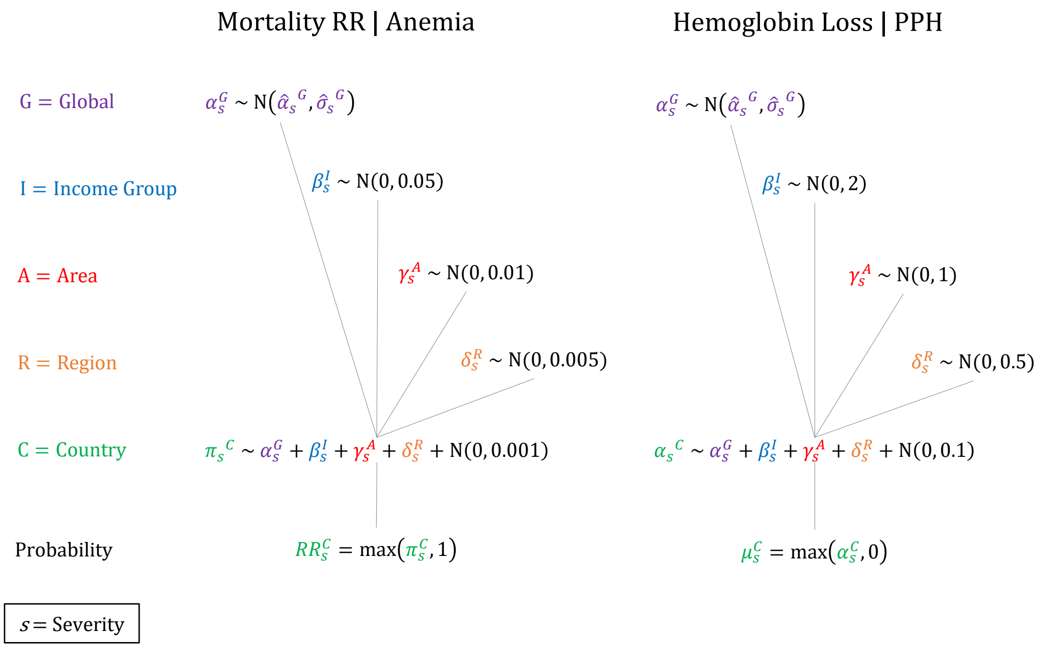

Priors

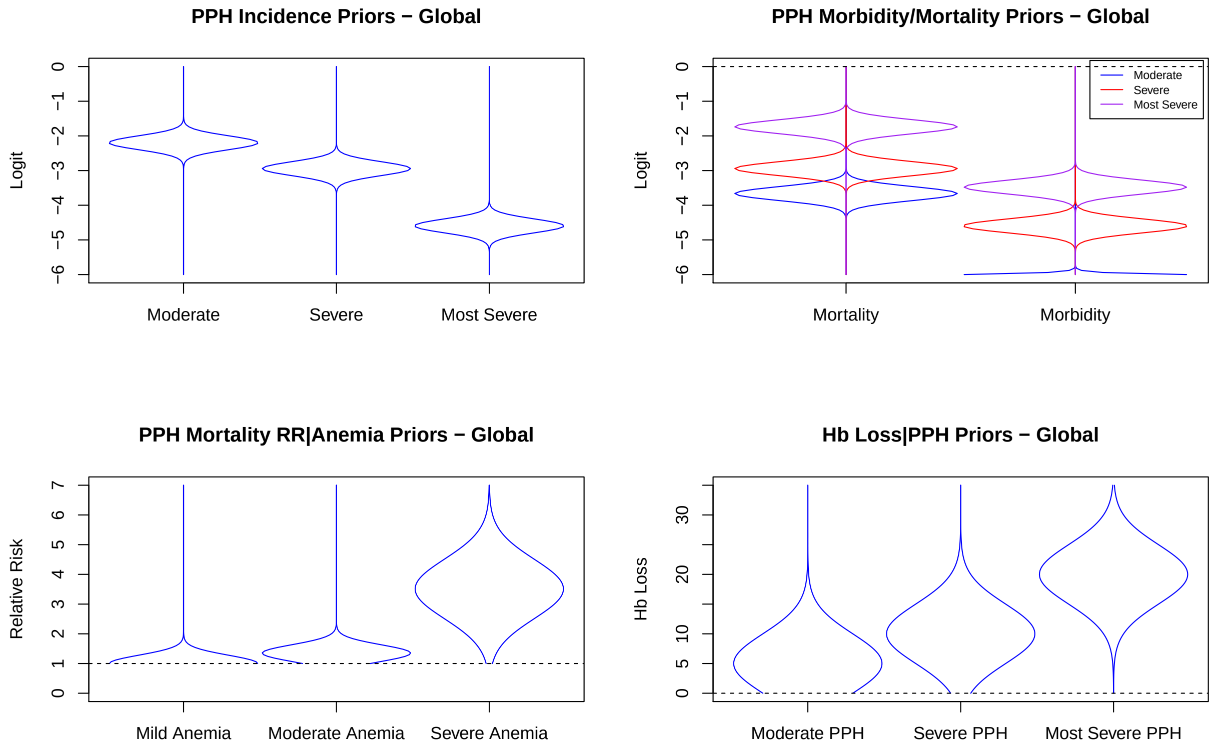

Model Implementation

At the time of delivery, the incidence and outcomes of PPH are simulated, together with the availability and efficacy of interventions that modify the 'natural history' probabilities. We assume that women who develop

Sheehan's syndrome are infertile in the model. We also include a parameter to reduce hemoglobin levels (i.e. increased risk of anemia) for women who survive PPH.

References

AbouZahr C. Global burden of maternal death and disability. Br Med Bull 2003; 67: 1-11. DOI: https://doi.org/10.1093/bmb/ldg015

Alfirevic Z, Blum J, Walraven G, Weeks A, Winikoff B. Prevention of postpartum hemorrhage with misoprostol. Int J Gynaecol Obstet 2007; 99(S2): S198-201. DOI: https://doi.org/10.1016/j.ijgo.2007.09.012

Begley CM, Gyte GM, Devane D, McGuire W, Weeks A, Biesty LM. Active versus expectant management for women in the third stage of labour. Cochrane Database Syst Rev 2019; 2(2):CD007412. DOI: https://doi.org/10.1002/14651858.cd007412.pub5

Bonnar J. Massive obstetric haemorrhage. Baillieres Best Pract Res Clin Obstet Gynaecol 2000; 14(1): 1-18. DOI: https://doi.org/10.1053/beog.1999.0060

Brabin BJ, Hakimi M, Pelletier D. An analysis of anemia and pregnancy-related maternal mortality. J Nutr 2001; 131(2S-2): 604S-614S. DOI: https://doi.org/10.1093/jn/131.2.604s

Calvert C, Thomas SL, Ronsmans C, Wagner KS, Adler AJ, Filippi V. Identifying regional variation in the prevalence of postpartum haemorrhage: a systematic review and meta-analysis. PLoS One 2012; 7(7): e41114. DOI: https://doi.org/10.1371/journal.pone.0041114

Carroli G, Cuesta C, Abalos E, Gülmezoglu AM. Epidemiology of postpartum haemorrhage: a systematic review. Best Pract Res Clin Obstet Gynaecol 2008; 22(6): 999-1012. DOI: https://doi.org/10.1016/j.bpobgyn.2008.08.004

Chandhiok N, Dhillon BS, Datey S, Mathur A, Saxena NC. Oral misoprostol for prevention of postpartum hemorrhage by paramedical workers in India. Int J Gynaecol Obstet 2006; 92(2): 170-5. DOI: https://doi.org/10.1016/j.ijgo.2005.10.019

Derman RJ, Kodkany BS, Goudar SS, et al. Oral misoprostol in preventing postpartum haemorrhage in resource-poor communities: a randomised controlled trial. Lancet 2006; 368(9543): 1248-53. DOI: https://doi.org/10.1016/s0140-6736(06)69522-6

Dolea C, AbouZahr C, Stein C. Global burden of maternal haemorrhage in the year 2000. Evidence and information for policy (EIP). Global Burden of Disease Working Paper, 2003. Available at: https://www.who.int/healthinfo/statistics/bod_maternalhaemorrhage.pdf

Dökmetaş HS, Kilicli F, Korkmaz S, Yonem O. Characteristic features of 20 patients with Sheehan's syndrome. Gynecol Endocrinol 2006; 22(5): 279–283. DOI: https://doi.org/10.1080/09513590600630504

Etuk SJ, Asuquo EE. Maternal mortality following post-partum haemorrhage in Calabar a 6-year review. West Afr J Med 1997; 16(3): 165-9. PMID: https://pubmed.ncbi.nlm.nih.gov/9329285/

Ezugwu EC, Agu PU, Nwoke MO, Ezugwu FO. Reducing maternal deaths in a low resource setting in Nigeria. Niger J Clin Pract 2014; 17(1): 62-6. DOI: https://doi.org/10.4103/1119-3077.122842

Fan D, Wu S, Liu L, et al. Prevalence of antepartum hemorrhage in women with placenta previa: a systematic review and meta-analysis. Sci Rep 2017; 7: 40320. DOI: https://doi.org/10.1038/srep40320

Filippi V, Chou D, Ronsmans C, Graham W, Say L. Chapter 3. Levels and causes of maternal mortality and morbidity. In: Black RE, Laxminarayan L, Walker N, Temmerman M, eds. Disease control priorities 3rd edn. Volume 2. Reproductive, maternal, newborn and child health. Washington, DC: World Bank, 2016. Available at: https://www.ncbi.nlm.nih.gov/books/NBK361917/

Gessessew A. Maternal complications–in a zonal hospital. Ethiop Med J 2007; 45(1): 47-54. PMID: https://pubmed.ncbi.nlm.nih.gov/17642157/

Giordano R, Cacciatore A, Cignini P, Vigna R, Romano M. Antepartum Haemorrhage. J Prenat Med 2010; 4(1): 12-16. Available at: https://www.ncbi.nlm.nih.gov/pmc/articles/PMC3263934/

Graham WJ, Cairns J, Bhattacharya S, et al. Maternal and Perinatal Conditions. In: Jamison DT, Breman JG, Measham AR, et al, eds. Disease Control Priorities in Developing Countries. 2nd edition. Washington (DC): World Bank; 2006. Available at: https://www.ncbi.nlm.nih.gov/books/NBK11742/

Gülmezoglu AM, Villar J, Ngoc NT, et al. WHO multicentre randomised trial of misoprostol in the management of the third stage of labour. Lancet 2001; 358(9283): 689-695. DOI: https://doi.org/10.1016/s0140-6736(01)05835-4

Høj L, Cardoso P, Nielsen BB, Hvidman L, Nielsen J, Aaby P. Effect of sublingual misoprostol on severe postpartum haemorrhage in a primary health centre in Guinea-Bissau: randomised double blind clinical trial. BMJ 2005; 331(7519): 723. DOI: https://doi.org/10.1136/bmj.331.7519.723

Jayanna K, Mony P, Ramesh BM, et al. Assessment of facility readiness and provider preparedness for dealing with postpartum haemorrhage and pre-eclampsia/eclampsia in public and private health facilities of northern Karnataka, India: a cross-sectional study. BMC Pregnancy Childbirth 2014; 14: 304. DOI: https://doi.org/10.1186/1471-2393-14-304

Kaul V, Bagga R, Jain V, Gopalan S. The impact of primary postpartum hemorrhage in "near-miss" morbidity and mortality in a tertiary care hospital in North India. Indian J Med Sci 2006; 60(6): 233-40. DOI: https://doi.org/10.4103/0019-5359.25905

Kaye DK, Kakaire O, Osinde MO. Systematic review of the magnitude and case fatality ratio for severe maternal morbidity in sub-Saharan Africa between 1995 and 2010. BMC Pregnancy Childbirth 2011; 11: 65. DOI: https://doi.org/10.1186/1471-2393-11-65

Keleştimur F. Sheehan's syndrome. Pituitary 2003; 6(4): 181–188. DOI: https://doi.org/10.1023/b:pitu.0000023425.20854.8e

Kongnyuy EJ, Mlava G, van den Broek N. Using criteria-based audit to improve the management of postpartum haemorrhage in resource limited countries: a case study of Malawi. Matern Child Health J 2009; 13(6): 873-8. DOI: https://doi.org/10.1007/s10995-008-0408-3

Matsuzaki S, Endo M, Ueda Y, et al. A case of acute Sheehan's syndrome and literature review: a rare but life-threatening complication of postpartum hemorrhage. BMC Pregnancy Childbirth 2017; 17: 188. DOI: https://dx.doi.org/10.1186%2Fs12884-017-1380-y

Meher S. How should we diagnose and assess the severity of PPH in clinical trials? Best Pract Res Clin Obstet Gynaecol 2019; 61: 41-54. DOI: https://doi.org/10.1016/j.bpobgyn.2019.04.003

Menard MK, Main EK, Currigan SM. Executive summary of the reVITALize initiative. Obstet Gynecol 2014; 124(1): 150-3. DOI: https://doi.org/10.1097/aog.0000000000000322

Mousa HA, Blum J, Abou El Senoun G, Shakur H, Alfirevic Z. Treatment for primary postpartum haemorrhage. Cochrane Database Syst Rev 2014; 2014(2): CD003249. DOI: https://doi.org/10.1002/14651858.cd003249.pub3

Pagel C, Lewcyka S, Colbourn T, et al. Estimation of potential effects of improved community-based drug provision, to augment health-facility strengthening, on maternal mortality due to post-partum haemorrhage and sepsis in sub-Saharan Africa: an equity-effectiveness model. Lancet 2009; 374(9699): 1441-8. DOI: https://doi.org/10.1016/s0140-6736(09)61566-x

Prendiville WJ, Elbourne D, McDonald S. [WITHDRAWN] Active versus expectant management in the third stage of labour. Cochrane Database Syst Rev 2009; 2009(3): CD000007. DOI: https://doi.org/10.1002/14651858.cd000007.pub2

Royal College of Obstetricians & Gynaecologists. Antepartum Haemorrhage. Green-top Guideline No. 63; November 2011. Available at: https://www.rcog.org.uk/globalassets/documents/guidelines/gtg_63.pdf

Shirazee HH, Saha SK, Das I, Mondal T, Samanta S, Sarkar M. Postpartum haemorrhage: a cause of maternal morbidity. J Indian Med Assoc 2010; 108(10): 663-6. PMID: https://pubmed.ncbi.nlm.nih.gov/21510550/

Sibley L, Caleb-Varkey L, Upadhyay J, et al. Recognition of and response to postpartum hemorrhage in rural northern India. J Midwifery Womens Health 2005; 50(4): 301-8. DOI: https://doi.org/10.1016/j.jmwh.2005.03.006

Sinha P, Kuruba N. Ante-partum haemorrhage: an update. J Obstet Gynaecol 2008; 28(4): 377-81. DOI: https://doi.org/10.1080/01443610802091487

Tunçalp O, Hofmeyr GJ, Gülmezoglu AM. Prostaglandins for preventing postpartum haemorrhage. Cochrane Database Syst Rev 2012; 2012(8): CD000494. DOI: https://doi.org/10.1002/14651858.cd000494.pub4

Vaphiades MS, Simmons D, Archer RL, Stringer W. Sheehan syndrome: a splinter of the mind. Surv Ophthalmol 2003; 48(2): 230–233. DOI: https://doi.org/10.1016/s0039-6257(02)00459-9

Wagner KS, Ronsmans C, Thomas SL, et al. Women who experience obstetric haemorrhage are at higher risk of anaemia, in both rich and poor countries. Trop Med Int Health 2012; 17(1): 9-22. DOI: https://doi.org/10.1111/j.1365-3156.2011.02883.x

Walraven G, Blum J, Dampha Y, et al. Misoprostol in the management of the third stage of labour in the home delivery setting in rural Gambia: a randomised controlled trial. BJOG 2005; 112(9): 1277-1283. DOI: https://doi.org/10.1111/j.1471-0528.2005.00711.x

World Health Organization. World Health Report 2005: Chapter 4 - Make Every Mother and Child Count; 61-78. Available: http://www.who.int/whr/2005/chap4-en.pdf.

World Health Organization. WHO guidelines for the management of postpartum haemorrhage and retained placenta, 2009. Available: http://apps.who.int/iris/bitstream/10665/44171/1/9789241598514_eng.pdf.

A.1.5.4 Sepsis

Overview

Puerperal (or postpartum) infection is generally defined as any bacterial infection of the female reproductive tract following childbirth or miscarriage. Signs and symptoms usually include a fever greater than 38.0°C (100.4°F), chills, lower abdominal pain, and possibly bad-smelling vaginal discharge (Cunningham 2014). Puerperal sepsis is the pregnancy-related infection that causes the greatest concern because of its severity. While various definitions of sepsis have been used in the literature (Bonet 2018), the WHO convened a group of experts to propose an up-to-date global definition for maternal sepsis, defined as "a life-threatening condition defined as organ dysfunction resulting from infection during pregnancy, childbirth, post-abortion, or postpartum period" (WHO 2017).

Data

Incidence

In the eighteenth and nineteenth centuries it is estimated that there were roughly 6-9 cases of sepsis per 1,000 deliveries in Europe, with far more occurring during epidemics of 'puerperal fever' (Loudon 2000). With modern birth practices, available data on pregnancy-related sepsis from high income countries report incidences ranging from 9 to 49 per 100,000 deliveries (Oud 2014). Scarce data from low income countries makes the incidence of sepsis difficult to determine (van Dillen 2010). According to Filippi (2016), there has been no recent published review of sepsis incidence since the work conducted for the 2000 Global Burden of Disease (Dolea 2003). That review calculated that the incidence of sepsis ranged from 2.7 and 5.2 per 100 live births according to world region. Since then, a recent community-based study in India found that the incidence of puerperal sepsis in the first week postpartum was 1.2 percent after home delivery and 1.4 percent after facility-based delivery. The incidence of fever was higher at 4 percent overall in the same Indian study (Iyengar 2012). Another study in India found a high incidence of puerperal infections at home (10 percent) and of fever (12 percent), but the study used broader definitions and followed women for 28 days (Bang 2004). Another study in Pakistan found that out of 3,316 obstetrical admissions, 3.89% had puerperal sepsis (Khaskheli 2013).

There is a growing body of evidence that the single most important risk factor for postpartum infection is caesarean section (Buddeberg 2015). Women who undergo caesarean section have a five to 20-fold greater risk for infection (and infectious morbidity) compared with a vaginal birth (Smaill 2014). A study from Denmark found that within 30 days after birth, 1.6% of women who delivered vaginally acquired an infection, compared to 7.6% of women who had a caesarean section - an almost five-fold increase in the risk of infection (Leth 2009). Women undergoing cesarean section can be divided into low- and high-risk groups for infection, with women undergoing an elective procedure at lowest risk, while women at high risk include those undergoing cesarean section after rupture of the membranes or onset of labor (ACOG 2011). A previous Cochrane systematic review on antibiotic prophylaxis for caesarean section found an average rate of endometritis in women undergoing caesarean section without antibiotic coverage of 9.2% for elective caesarean sections and 28.6% for non-elective caesarean sections, while the use of antibiotic prophylaxis reduced the incidence of endometritis by two-thirds to three-quarters for all studies combined (Smaill 2002). Antibiotic prophylaxis thus mitigates the risk of infection, but it is nevertheless higher after a caesarean section than after a spontaneous vaginal delivery (Buddeberg 2015).

Morbidity/Mortality

In historical epidemics of puerperal fever in Europe, the case fatality rate tended to be highest at the beginning of the epidemic, sometimes reaching levels of 70%-80% before declining over a period of a couple of years, while it is estimated that case fatality rates in sporadic cases were likely in the region of 25%-30% before the introduction of sulphonamides in the mid-1930s (Loudon 2000). In modern times, mortality from maternal sepsis in high income countries is estimated to be around 17 deaths per 100,000 live births, but it may be increasing (van Dillen 2010, Bauer 2013, Vaught 2018). Previous studies from low and middle income countries report case fatality rates between 4% and 50% (Bonet 2018, Dolea 2003). A review conducted by the Disease Control Priorities Project (DCP2) (Graham 2006) reported an average CFR of 1.3%, while some studies reported higher rates: 4.1% (Dare 1998), and 8.5% (Khaskheli 2013). Determining risk factors for maternal sepsis mortality is difficult as few deaths occur in countries where there are resources to accurately measure risk, rendering studies insufficiently powered to draw conclusions (Acosta 2013). However, we do

assume increased risk of death for women with anemia (Brabin 2001).

Apart from death, women who experience peripartum infections are also prone to long-term disabilities such as chronic pelvic pain, fallopian tube blockage, and secondary infertility (WHO 2015). Estimates of the risk of infertility given sepsis are scarce however. The GBD 2000 assumed that puerperal infection resulted in infertility between 5% in developed countries to 12% in developing countries (Abouzahr 1998).

Parameters

We model two levels of sepsis severity: Moderate and Severe. We constrain incidence to decrease by severity, and risk of morbidity/mortality to increase by severity when sampling parameters.

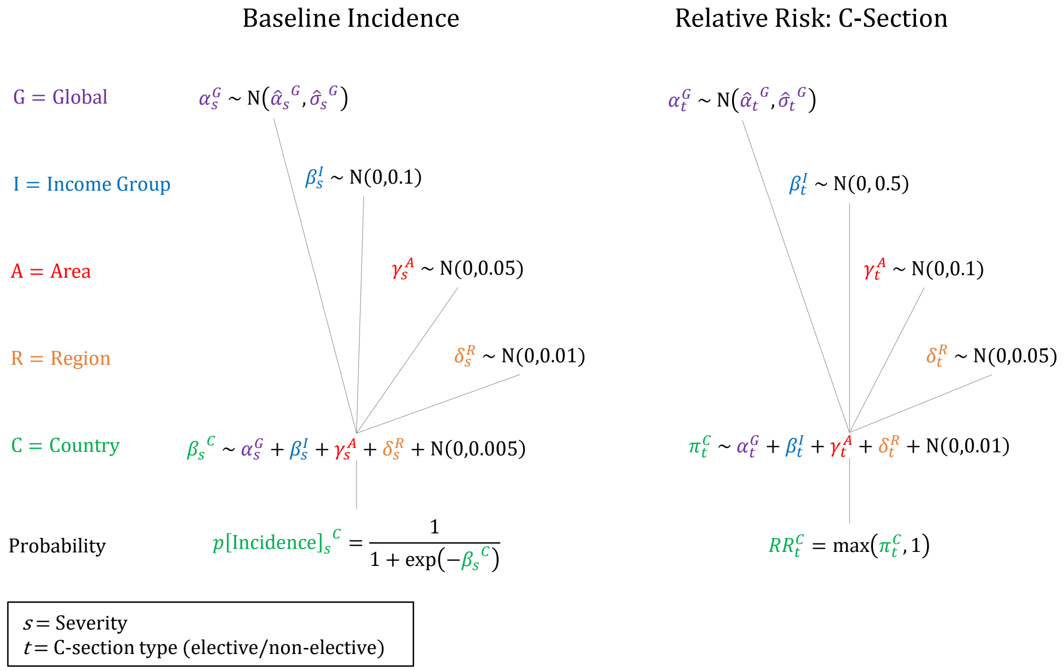

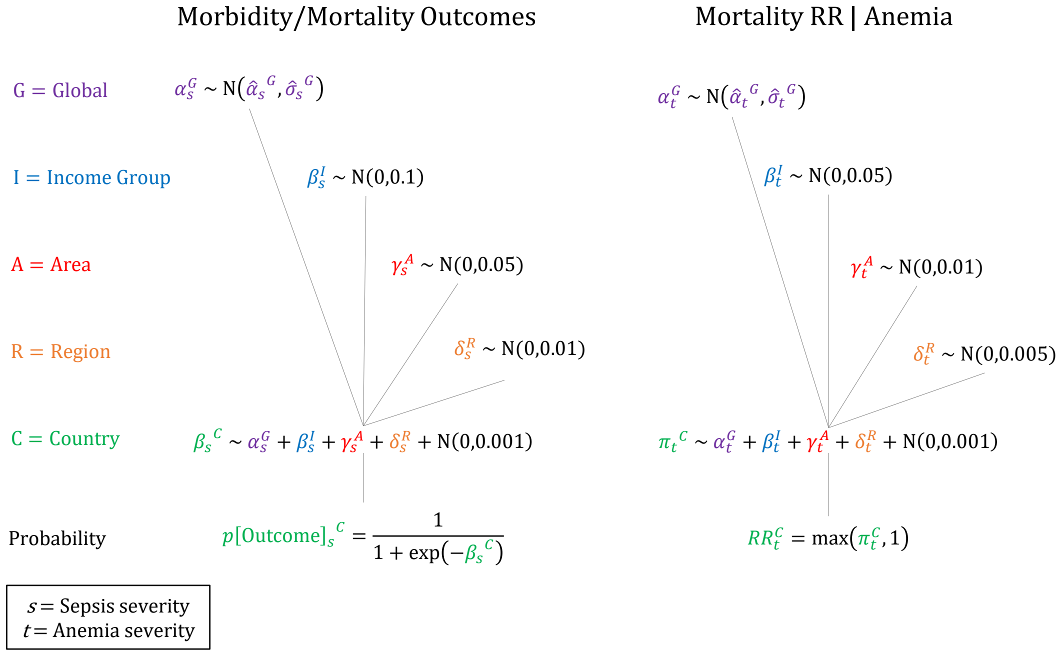

Priors

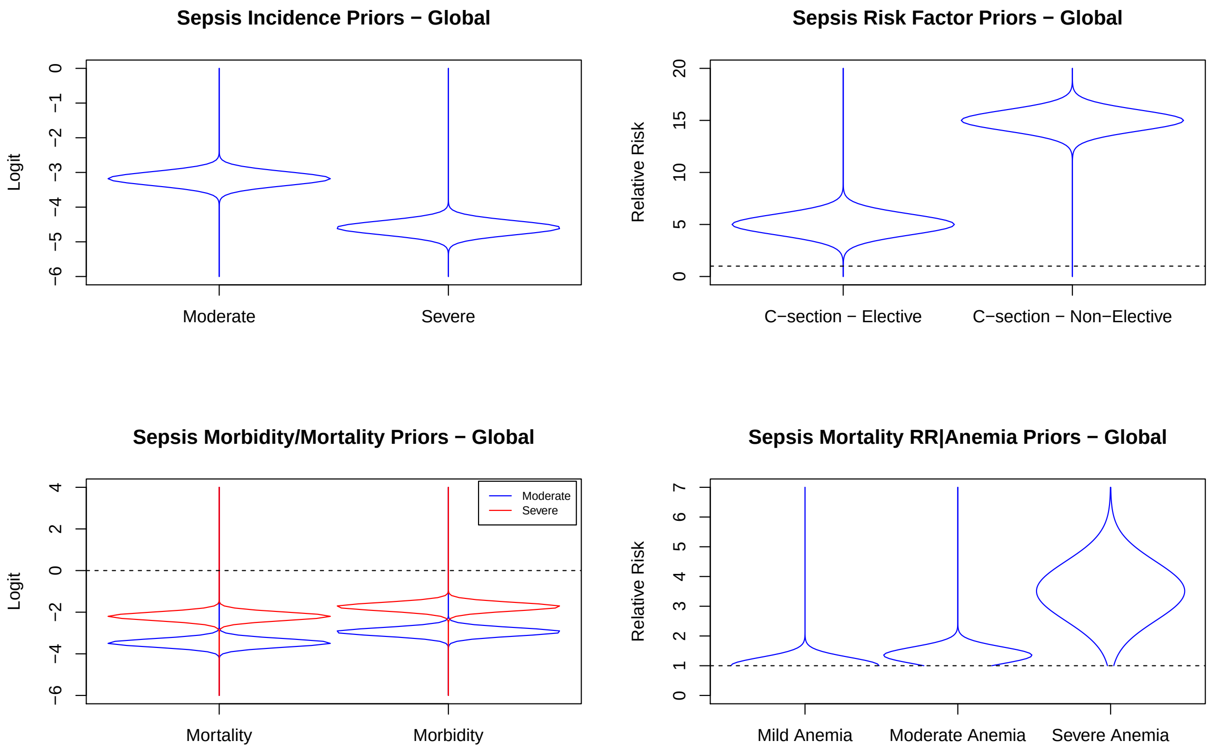

Model Implementation

At the time of delivery, the incidence and outcomes of Sepsis are simulated, together with the availability and efficacy of interventions that modify the 'natural history' probabilities.

References

Abouzahr C, Aaahman E, Guidotti R. Puerperal sepsis and other puerperal infections. In Health dimensions of sex and reproduction: the global burden of sexually transmitted diseases, maternal conditions, perinatal disorders, and congenital anomalies, eds. CJL Murray and AD Lopez, WHO 1998. https://apps.who.int/iris/handle/10665/42161

American College of Obstetricians and Gynecologists. ACOG Practice Bulletin No. 120: Use of prophylactic antibiotics in labor and delivery. Obstet Gynecol 2011; 117(6): 1472-83. DOI: https://doi.org/10.1097/aog.0b013e3182238c31

Acosta CD, Knight M. Sepsis and maternal mortality. Curr Opin Obstet Gynecol 2013; 25(2): 109-16. DOI: https://doi.org/10.1097/gco.0b013e32835e0e82

Bang RA, Bang AT, Reddy MH, Deshmukh MD, Baitule SB, Filippi V. Maternal morbidity during labour and the puerperium in rural homes and the need for medical attention: A prospective observational study in Gadchiroli, India. BJOG 2004; 111(3): 231-8. DOI: https://doi.org/10.1111/j.1471-0528.2004.00063.x

Bauer ME, Bateman BT, Bauer ST, Shanks AM, Mhyre JM. Maternal sepsis mortality and morbidity during hospitalization for delivery: temporal trends and independent associations for severe sepsis. Anesth Analg 2013; 117(4): 944–50. DOI: https://doi.org/10.1213/ane.0b013e3182a009c3

Bonet M, Souza JP, Abalos E, et al. The global maternal sepsis study and awareness campaign (GLOSS): study protocol. Reprod Health 2018; 15(1): 16. DOI: https://doi.org/10.1186/s12978-017-0437-8

Brabin BJ, Hakimi M, Pelletier D. An analysis of anemia and pregnancy-related maternal mortality. J Nutr

2001; 131(2S-2): 604S-614S. DOI: https://doi.org/10.1093/jn/131.2.604s

Buddeberg BS, Aveling W. Puerperal sepsis in the 21st century: progress, new challenges and the situation worldwide. Postgrad Med J 2015; 91(1080): 572-8. DOI: https://doi.org/10.1136/postgradmedj-2015-133475

Cunningham FG, Leveno KJ, Bloom SL et al. Chapter 37: Puerperal Complications. In: Williams Obstetrics (24th ed). McGraw-Hill Medical, New York, NY, 2014. Available at: https://accessmedicine.mhmedical.com/content.aspx?bookid=1918&sectionid=141464707

Dare FO, Bako AU, Ezechi OC. Puerperal sepsis: a preventable post-partum complication. Trop Doct 1998; 28(2): 92-5. DOI: https://doi.org/10.1177/004947559802800212

Dolea C, Stein C. Global Burden of Maternal Sepsis in the Year 2000. Evidence and information for policy (EIP). Global Burden of Disease Working Paper, 2003. Available at http://www.who.int/healthinfo/statistics/bod_maternalsepsis.pdf.

Filippi V, Chou D, Ronsmans C, Graham W, Say L. Chapter 3. Levels and causes of maternal mortality and morbidity. In: Black RE, Laxminarayan L, Walker N, Temmerman M, eds. Disease control priorities 3rd edn. Volume 2. Reproductive, maternal, newborn and child health. Washington, DC: World Bank, 2016. Available at: https://www.ncbi.nlm.nih.gov/books/NBK361917/

Graham WJ, Cairns J, Bhattacharya S, et al. Maternal and Perinatal Conditions. In: Jamison DT, Breman JG, Measham AR, et al, eds. Disease Control Priorities in Developing Countries. 2nd edition. Washington (DC): World Bank; 2006. Available at: https://www.ncbi.nlm.nih.gov/books/NBK11742/

Iyengar K. Early postpartum maternal morbidity among rural women of Rajasthan, India: a community-based study. J Health Popul Nutr 2012; 30(2): 213-25. DOI: https://doi.org/10.3329/jhpn.v30i2.11316

Khaskheli MN, Baloch S, Sheeba A. Risk factors and complications of puerperal sepsis at a tertiary healthcare centre. Pak J Med Sci 2013; 29(4): 972-6. DOI: https://doi.org/10.12669/pjms.294.3389

Leth RA, Møller JK, Thomsen RW, Uldbjerg M, Nørgaard M. Risk of selected postpartum infections after cesarean section compared with vaginal birth: a five-year cohort study of 32,468 women. Acta Obstet Gynecol Scand 2009; 88(9): 976–83. DOI: https://doi.org/10.1080/00016340903147405

Loudon I. The Tragedy of Childbed Fever. Oxford University Press, 2000. DOI: https://doi.org/10.1093/acprof:oso/9780198204992.001.0001

Oud L. Pregnancy-associated severe sepsis: contemporary state and future challenges. Infect Dis Ther 2014; 3(2): 175–89. DOI: https://doi.org/10.1007/s40121-014-0037-7

Smaill F, Hofmyer GJ. Antibiotic prophylaxis for caesarean section. Cochrane Database Syst Rev 2002; 3: CD000933. DOI: https://doi.org/10.1002/14651858.cd000933

Smaill FM, Grivell RM. Antibiotic prophylaxis versus no prophylaxis for preventing infection after cesarean section. Cochrane Database Syst Rev 2014; 10: CD007482. DOI: https://doi.org/10.1002/14651858.CD007482.pub3

van Dillen J, Zwart J, Schutte J, van Roosmalen J. Maternal sepsis: epidemiology, etiology and outcome. Curr Opin Infect Dis 2010; 23(3): 249-54. DOI: https://doi.org/10.1097/qco.0b013e328339257c

Vaught AJ. Maternal sepsis. Semin Perinatol 2018; 42(1): 9-12. DOI: https://doi.org/10.1053/j.semperi.2017.11.003

World Health Organization. WHO recommendations for prevention and treatment of maternal peripartum infections. 2015. Available at: https://www.who.int/reproductivehealth/publications/maternal_perinatal_health/peripartum-infections-guidelines/en/

World Health Organization. Statement on Maternal Sepsis. 2017. Available at: http://apps.who.int/iris/bitstream/10665/254608/1/WHO-RHR-17.02-eng.pdf?ua=1

A.1.5.5 Other Direct Complications

Overview

In addition to the major direct causes of maternal death included in the model, there also exist various other complications which pose mortality risks. While the risk of each individual cause is small, all together these 'other direct' causes form a substantial source of maternal morbidity and mortality.

Data

To examine reported maternal deaths due to other direct causes we obtained deaths by ICD-10 code from the WHO Mortality Database, a compilation of mortality data by age, sex, and cause of death, as reported annually by countries from their civil registration systems (WHO 2019).

We identified maternal deaths using the following ICD-10 codes:

Code	Description
N96	Habitual aborter
N98	Complications associated with artificial fertilization
O00-O99	Pregnancy, childbirth and the puerperium
1087	Summary code for O00-O99 Pregnancy, childbirth and the puerperium
1088	Summary code for O00-O07 Pregnancy with abortive outcome
1089	Summary code for O10-O92 Other direct obstetric deaths
1090	Summary code for O98-O99 Indirect obstetric deaths
1091	O95-O97 Remainder of pregnancy, childbirth and the puerperium

We restricted the dataset to country/year estimates between 1995-2017 with non-zero maternal deaths, totalling 1484 observations from 114 countries. According to these data, 'other direct' causes accounted for nearly 20% of reported global maternal deaths. We also calculated the proportion of maternal deaths due to 'other direct' causes by income group (not enough data were available for low income countries). We see a gradient by income group, with a higher proportion of deaths due to 'other direct' causes in high income countries, likely because the risk of death from other major obstetric complications is lower.

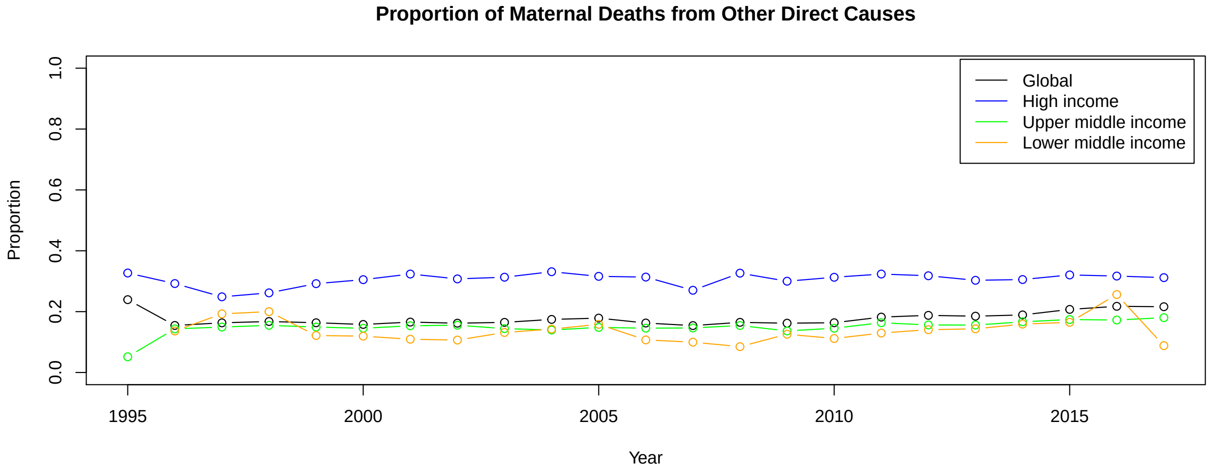

We also calculated the distribution of the reported causes of death for ICD-10 codes included in 'other direct' maternal deaths:

ICD-10	Description	Percent (%)
O88	Obstetric embolism	24.0
O26	Maternal care for other conditions predominantly related to pregnancy	14.4

ICD-10	Description	Percent (%)
O75	Other complications of labour and delivery, not elsewhere classified	14.1
O90	Complications of the puerperium, not elsewhere classified	13.2
O95	Obstetric death of unspecified cause	13.0
O36	Maternal care for other known or suspected fetal problems	3.9
O41	Other disorders of amniotic fluid and membranes	2.4
O73	Retained placenta and membranes, without haemorrhage	2.3
O22	Venous complications and haemorrhoids in pregnancy	2.0
O87	Venous complications and haemorrhoids in the puerperium	1.9
O74	Complications of anaesthesia during labour and delivery	1.5
O43	Placental disorders	1.2
O42	Premature rupture of membranes	1.0
O34	Maternal care for known or suspected abnormality of pelvic organs	0.8
O21	Excessive vomiting in pregnancy	0.6
O29	Complications of anaesthesia during pregnancy	0.5
O60	Preterm labour and delivery	0.5
O89	Complications of anaesthesia during the puerperium	0.4
O30	Multiple gestation	0.4
O68	Labour and delivery complicated by fetal stress [distress]	0.4
O70	Perineal laceration during delivery	0.3
O69	Labour and delivery complicated by umbilical cord complications	0.2
O40	Polyhydramnios	0.2
O48	Prolonged pregnancy	0.1
O35	Maternal care for known or suspected fetal abnormality and damage	0.1
O31	Complications specific to multiple gestation	0.1
O61	Failed induction of labour	0.1
O47	False labour	0.1
N98	Complications associated with artificial fertilization	0.1
O92	Other disorders of breast and lactation associated with childbirth	0.1
O28	Abnormal findings on antenatal screening of mother	<0.1

We see that nearly 25% of reported 'other direct' maternal deaths are due to obstetric embolism, 55% are due to 'other conditions' or unspecified causes, and 20% are due to various remaining causes. While it is the largest cause of 'other direct' maternal deaths, obstetric embolism is rare - a study of deliveries in the US from 1980 to 2005 estimated that it occurs in around 10 per 100,000 births (Stein 2009), with estimated case fatality rates varying between 13% to 26% (Kramer 2006, Gilbert 1999). A study of in-hospital maternal deaths in Canada found similar estimates, with obstetric embolism associated with 1.8 maternal deaths per 100,000 deliveries (Lisonkova 2011).

Parameters
Because the causes of 'other direct' maternal death are relatively rare individually, we model them as a group. Given that the largest single cause, obstetric embolism, is estimated to occur in about 1 per 10,000 deliveries, and accounts for about 25% of deaths from other direct causes, we set (fairly wide) priors for incidence of other direct causes around 4 per 10,000 births. While some causes of death may be related to clinical interventions at facilities (e.g. anaesthesia), given the relatively small number of potential iatrogenic

deaths, for simplicity we assume that the incidence of other direct causes is non-differential by delivery site. However, as many of these causes are mainly treated with supportive care, we do not model specific clinical interventions, but simulate mortality risks conditional on a woman's delivery site. We assume that case fatality rates decrease with higher level delivery sites and enforce these constraints when sampling parameters (Home > Home-SBA > non-EmOC > BEmOC > CEmOC).

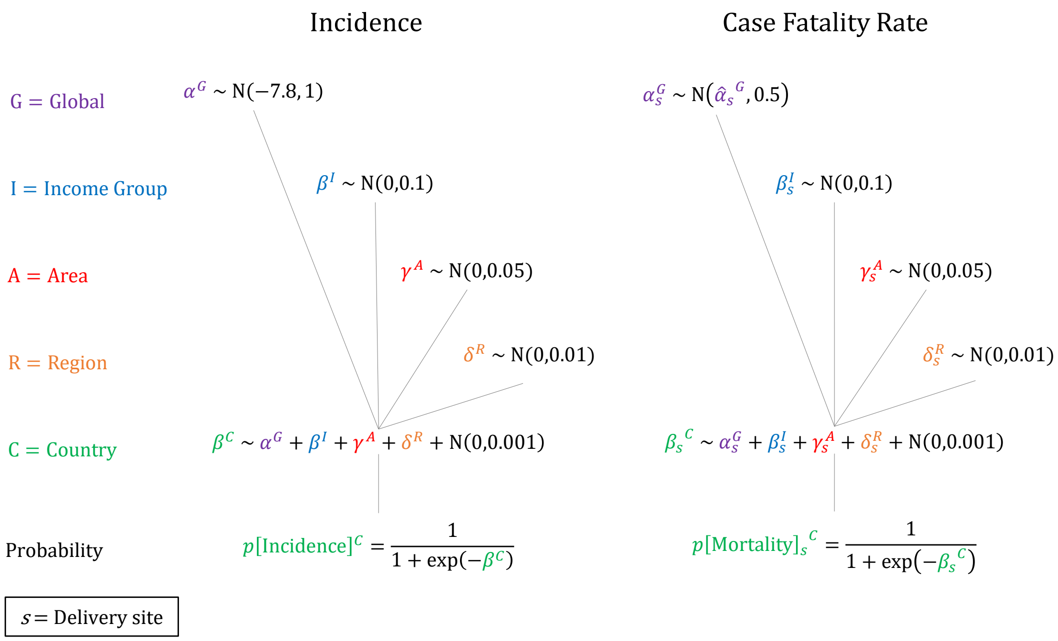

Priors

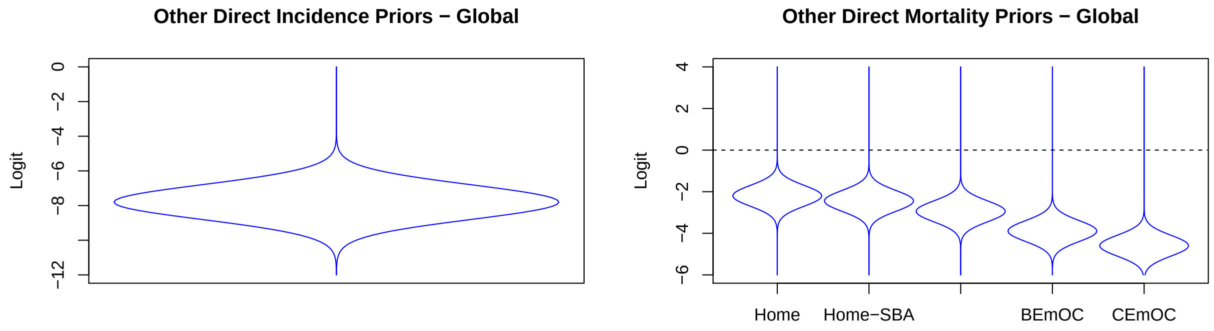

Model Implementation

Although some complications included in 'other direct' deaths may occur earlier in pregnancy, for simplicity we model the risk of these complications at the time of delivery. If a complication occurs, the risk of mortality is simulated based on a woman's delivery site (and quality of care).

References

Gilbert WM, Danielsen B. Amniotic fluid embolism: decreased mortality in a population-based study. Obstet Gynecol 1999; 93: 973–77. https://doi.org/10.1016/s0029-7844(99)00004-6

Kramer MS, Rouleau J, Baskett TF, Joseph KS, Maternal Health Study Group of the Canadian Perinatal Surveillance System. Amniotic-fluid embolism and medical induction of labour: a retrospective, population-

based cohort study. Lancet 2006; 368(9545): 1444-8. DOI: https://doi.org/10.1016/s0140-6736(06)69607-4

Lisonkova S, Liu S, Bartholomew S, Liston RM, Joseph KS, Maternal Health Study Group of the Canadian Perinatal Surveillance System. Temporal trends in maternal mortality in Canada II: estimates based on hospitalization data. J Obstet Gynaecol Can 2011; 33(10): 1020-30. DOI: https://doi.org/10.1016/s1701-2163(16)35051-4

Stein PD, Matta F, Yaekoub AY. Incidence of amniotic fluid embolism: relation to cesarean section and to age. J Womens Health (Larchmt) 2009; 18(3): 327-9. DOI: https://doi.org/10.1089/jwh.2008.0974

World Health Organization. WHO Mortality Database - Last updated: 15 December 2019. Available at: https://www.who.int/healthinfo/mortality_data/en/

A.1.5.6 Late Maternal Deaths

Overview

A late maternal death is defined as the death of a woman from direct or indirect obstetric causes, more than 42 days but less than one year after the end of pregnancy (WHO). Late maternal deaths are often classified into four main categories: cardiovascular causes, thromboembolism, cancer, and suicide (Sliwa 2016). Pregnancy can trigger or aggravate disease, or cause specific diseases, such as peripartum cardiomyopathy, which typically presents 1-3 months postpartum (Sliwa 2016). Pre-existing mental disorders, the need to cope with the demands of caring for a newborn baby, pregnancy loss, and intimate partner violence can also increase a woman's vulnerability to suicide risk (Sliwa 2016).

Data

To examine reported late maternal deaths we obtained data on deaths by ICD-10 code from the WHO Mortality Database, a compilation of mortality data by age, sex, and cause of death, as reported annually by countries from their civil registration systems (WHO 2019). We identified late maternal deaths using the ICD-10 codes O96 (Death from any obstetric cause occurring more than 42 days but less than one year after delivery) and O97 (Death from sequelae of obstetric causes). We restricted the dataset to country/year estimates between 1995-2017 with non-zero maternal deaths, totalling 1484 observations from 114 countries. According to these data, late maternal deaths accounted for an increasing proportion of reported global maternal deaths, rising to over 15% by 2017. This increase may reflect improved identification of late maternal deaths over time. We also calculated the proportion of late maternal deaths (out of all maternal deaths) by income group (not enough data were available for low income countries). We see a gradient by income group, with a higher proportion of late maternal deaths in high income countries, likely because the risk of death from other major obstetric complications is lower.

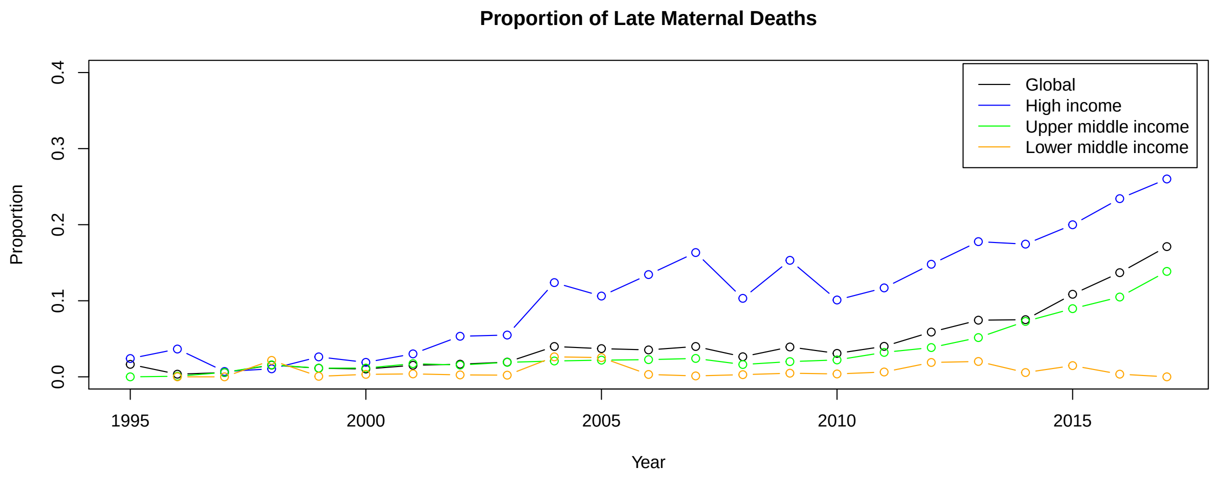

A study of seven countries in the Americas using the regional mortality database of PAHO between 1999 and 2013 found that the proportion of late maternal deaths and deaths from sequelae of obstetric causes ranged between 2.40% (CI 0.85% – 5.48%) and 18.68% (CI 17.06% – 20.47%) (de Cosio 2016).

Other reported estimates vary by setting:

Argentina: A study found higher rates of underreporting for late maternal deaths (15.4% under-registration, n=26) than for early maternal deaths (9.5% under-registration, n=95) (Ramos 2007).

Australia: A study identified 173 maternal deaths in 1994-2001, of which 97 were classified as occurring within 42 days of pregnancy ending, and 76 were classified as occurring between 43 and 365 days after pregnancy (Cliffe 2008). Most (73/76) of these deaths were classified as indirect maternal deaths with the most common causes of deaths reported as suicide (n=23), cardiac disorders (n=16) or accident/violence (n=16) (Cliffe 2008). Another study found that of 129 reported maternal deaths, 37 were early deaths and 92 occurred

late, with 67% of deceased women having a mental health diagnosis or issue related to substance abuse, and trauma (including suicide, accidental injury, vehicle accidents, and homicides) accounting for 73% of all maternal deaths (early and late) (Thornton 2013).

Brazil: A population-based study including all the late maternal deaths from the case series of the Maternal Mortality Committees in the city of São Paulo and the state of Paraná found that late maternal deaths accounted for 13.4% of all maternal deaths in São Paulo and 12.1% in Paraná, with direct obstetric causes accounting for 32.1% of late maternal deaths in São Paulo and 42.1% in Paraná (Vega 2017). Death occurred between 43 and 69 days postpartum in 44% of the cases in São Paulo and 39.5% in Paraná (Vega 2017).

Canada: A study of late maternal deaths in Canada (excluding Quebec and Manitoba) estimated that the mortality rate was 9.3 per 100,00 deliveries (95% CI 8.5-10.3), and reported the following late maternal mortality rates by cause (Lisonkova 2011):

Diagnosis	Rate per 100,000 deliveries (95% CI)
Neoplasms	2.68 (2.15-3.33)
Diseases of circulatory system	2.08 (1.63-2.67)
Diseases of nervous system	1.62 (1.22-2.14)
Diseases of respiratory system	1.59 (1.20-2.11)
Injury, poisoning, external causes of death	1.55 (1.17-2.07)
Diseases of blood and blood-forming organs	1.49 (1.11-1.99)
Endocrine, nutritional and metabolic diseases, immunity disorders	0.99 (0.69-1.42)
Infectious and parasitic diseases	0.86 (0.59-1.26)

Another study in Canada found that of 120 late maternal, 16 had direct obstetric causes (including 9 deaths by suicide), while 100 were due to indirect obstetric causes (including 46 related to malignancy), and 4 were due to undetermined causes (Boutin 2020).

Georgia: A study found 23 maternal deaths, of which 15 (65.2%) were early and 8 (34.8%) were late maternal deaths (Berdzuli 2020).

Italy: A study identified 277 early maternal deaths and 543 late maternal deaths, the majority of which (38.8%) were caused by malignant neoplasms, followed by violent deaths (suicide: 10.0%, homicide: 3.7%, other: 2.0%), road accidents (9.6%), cardiac deaths (8.9%), and neurological deaths (3.9%) (Donati 2018).

Jamaica: Of 903 pregnancy-related deaths identified in 1998-2015, 81% were early maternal deaths (n=735) and 19% were late maternal deaths (n=168), with the most common causes of deaths being malignancy, cardiovascular disease, HIV/AIDS, and cardiomyopathy (McCaw-Binns 2018).

Kenya: A study in two Nairobi slums estimated a maternal mortality rate of 706 per 100,000 live births based on 29 maternal deaths, and also identified 22 late maternal deaths, implying a late maternal mortality rate of around 530 per 100,000 live births (Ziraba 2009).

Mali: A cohort study with complete follow-up data for 4,717 women recorded 15 maternal deaths, yielding an overall MMR of 327, and identified 5 additional late maternal deaths (Etard 1999).

Mexico: A study in Mexico found that late maternal deaths are more likely to be underreported. Of 9,043 maternal deaths identified by a review process there was a 13% increase in overall identified deaths and a threefold increase in the proportion of maternal deaths classified as late maternal deaths (from 2.1% to 6.9%) (Hogan 2016).

Morocco: A study found that of 69 maternal deaths, 9 were late maternal deaths, of which 5 occurred in a health facility while 4 occurred at home (Abouchadi 2018).

South Africa: A study found that 84 women died from 'early maternal death', while 100 died from 'late maternal death' (Houle 2015).

Sweden: A study of maternal deaths in 1980-1988 identified 58 early maternal deaths and 76 late maternal deaths, predominantly caused by malignancy, stroke, heart disease, and suicide (Högberg 1994).

Tanzania: A study found that 111 women died within 42 days of childbirth, while 229 maternal deaths occurred within 365 days, suggesting that half of maternal deaths were late maternal deaths - however, these estimates exclude deaths during pregnancy (Finlay 2015).

USA: A study in Colorado found that of maternal deaths in 2011, 30% resulted from self-harm, with overdose and suicide leading causes (Metz 2016). Deaths were equally distributed throughout the first postpartum year (Metz 2016).

Although most of the estimates we identified reported the proportion of maternal deaths due to late maternal deaths, we did obtain some estimates of the incidence of late maternal deaths, which varied by setting. Here we summarize the reported (or inferred) estimates.

Estimate (per 100,000 births)	Country	Source
5.4	Canada	Lisonkova 2011
9.7	Sweden	Högberg 1994
16.6	Australia	Thornton 2013
18	Italy	Donati 2018
109	Mali	Etard 1999
530	Kenya	Ziraba 2009

Estimates of case fatality rates are scarce. However, a study in South Africa of 269 peripartum women presenting with CVD in pregnancy or within 6-months postpartum estimated a CFR of 5.9% in Group 1 (treated by a dedicated cardiac-obstetric) team, and 0.9% in Group 2 (treated with additional interventions, including early postpartum follow, referral to dedicated specialist clinics, and increased beta-blocker therapy) (Sliwa 2018). Mortality risks for women not enrolled in a trial are likely higher, and may be higher for other causes of late maternal death as well.

Parameters
We model the probability of late maternal death for all causes combined (both direct and indirect), which likely vary by country. Estimated mortality rates from late maternal deaths vary from 5.4 to 530 per 100,000 births, and estimates of CFR from CVD with specialist care ranged between 1-5%. Assuming a higher CFR of around 10% for non-specialist care, we set wide priors for incidence, centered around 1% incidence (1 per 100 births), which we model over 10 months. We assume that late maternal deaths are uniformly distributed during the year following pregnancy, consistent with data from Metz 2016. We assume that the incidence of late maternal events decreases by income group. Given an incident late maternal complication, we simulate mortality risks conditional on the site of treatment (accounting for referral probabilities) and quality of care. While there is likely substantial heterogeneity in CFR by cause (e.g. suicide attempt versus neoplasm), for simplicity we model one aggregate CFR, and set priors centered around 10% for women at Home, decreasing to around 1% for women in CEmOC facilities. We assume that case fatality rates decrease with higher level delivery sites and enforce these constraints when sampling parameters (Home > Home-SBA > non-EmOC > BEmOC > CEmOC).

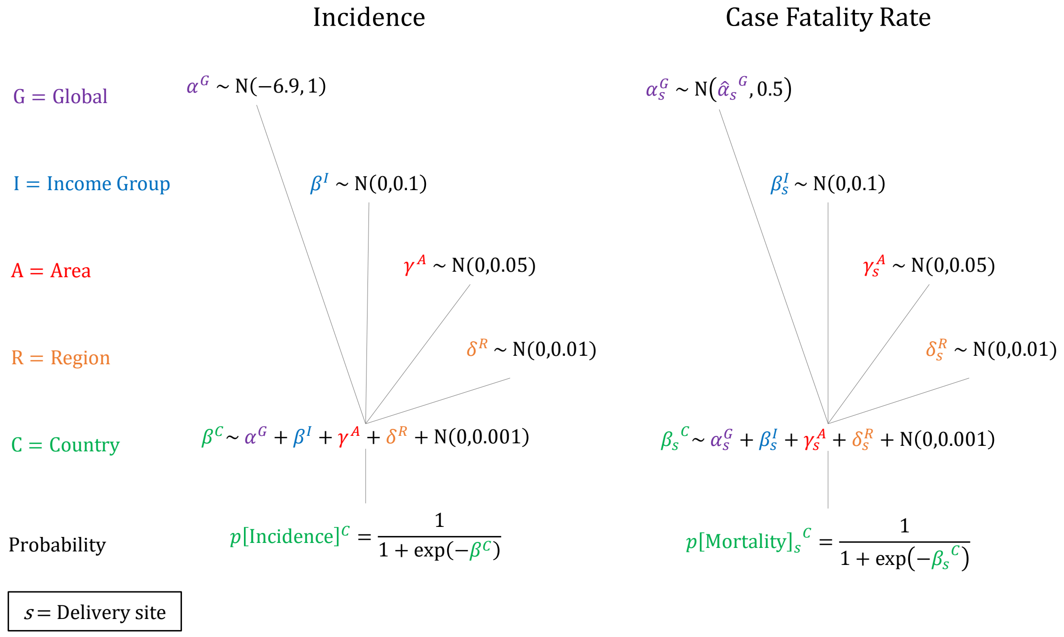

Priors

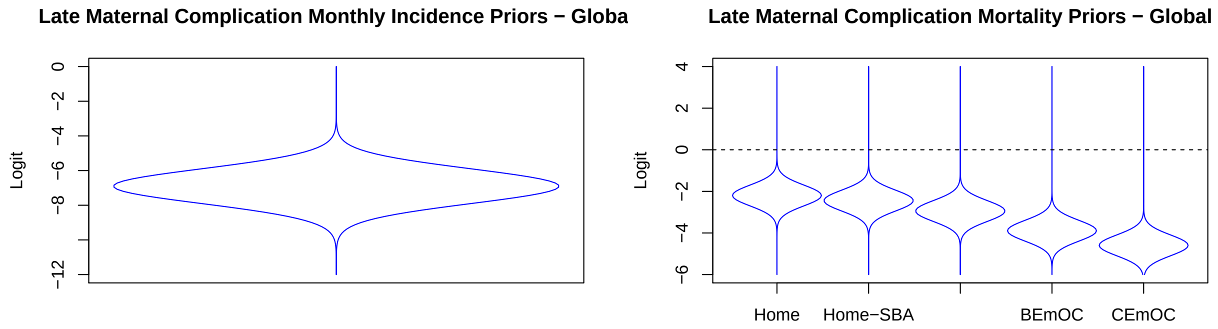

Model Implementation

We use a monthly cycle in the model, so we model 'early maternal deaths' as occurring within 2 months of delivery. For the next 10 months we simulate the risk of late maternal death, with a constant risk per month. If a late complication/event occurs, the risk of mortality is simulated based on a woman's treatment site, accounting for referral probabilities and quality of care.

References

Abouchadi S, Zhang WH, De Brouwere V. Underreporting of deaths in the maternal deaths surveillance system in one region of Morocco. PLoS One 2018; 13(1): e0188070. DOI: https://doi.org/10.1371/journal.pone.0188070

Berdzuli N, Lomia N, Staff AC, Kereselidze M, Lazdane G, Jacobsen AF. Maternal Mortality in Georgia: Incidence, Causes and Level of Underreporting: A National Reproductive Age Mortality Study 2014. Int J Womens Health 2020; 12: 277-286. DOI: https://doi.org/10.2147/ijwh.s227349

Boutin A, Cherian A, Liauw J, et al. Database Autopsy: An Efficient and Effective Confidential Enquiry into Maternal Deaths in Canada. J Obstet Gynaecol Can 2020; S1701-2163(20)30584-3. DOI: https://doi.org/10.1016/j.jogc.2020.06.026

Cliffe S, Black D, Bryant J, Sullivan E. Maternal deaths in New South Wales, Australia: a data linkage project. Aust N Z J Obstet Gynaecol 2008; 48(3): 255-60. DOI: https://doi.org/10.1111/j.1479-828x.2008.00878.x

de Cosio FG, Jiwani SS, Sanhueza A, Soliz PN, Becerra-Posada F, Espinal MA. Late Maternal Deaths and Deaths from Sequelae of Obstetric Causes in the Americas from 1999 to 2013: A Trend Analysis. PLoS One 2016; 11(9): e0160642. DOI: https://dx.doi.org/10.1371%2Fjournal.pone.0160642

Donati S, Maraschini A, Lega I, et al. Maternal mortality in Italy: Results and perspectives of record-linkage analysis. Acta Obstet Gynecol Scand 2018; 97(11): 1317-1324. DOI: https://doi.org/10.1111/aogs.13415

Etard JF, Kodio B, Traoré S. Assessment of maternal mortality and late maternal mortality among a cohort of pregnant women in Bamako, Mali. Br J Obstet Gynaecol 1999; 106(1): 60-5. DOI: https://doi.org/10.1111/j.1471-0528.1999.tb08086.x

Finlay JE, Moucheraud C, Goshev S, et al. The Effects of Maternal Mortality on Infant and Child Survival in Rural Tanzania: A Cohort Study. Matern Child Health J 2015; 19(11): 2393-402. DOI: https://doi.org/10.1007/s10995-015-1758-2

Hogan MC, Saavedra-Avendano B, Darney BG, et al. Reclassifying causes of obstetric death in Mexico: a repeated cross-sectional study. Bull World Health Organ 2016; 94(5): 362-369B. DOI: https://doi.org/10.2471/blt.15.163360

Högberg U, Innala E, Sandström A. Maternal mortality in Sweden, 1980-1988. Obstet Gynecol 1994; 84(2): 240-4. PMID: https://pubmed.ncbi.nlm.nih.gov/8041538/

Houle B, Clark SJ, Kahn K, Tollman S, Yamin A. The impacts of maternal mortality and cause of death on children's risk of dying in rural South Africa: evidence from a population based surveillance study (1992-2013). Reprod Health 2015; 12 Suppl 1(Suppl 1): S7. DOI: https://doi.org/10.1186/1742-4755-12-s1-s7

Lisonkova S, Liu S, Bartholomew S, Liston RM, Joseph KS, Maternal Health Study Group of the Canadian Perinatal Surveillance System. Temporal trends in maternal mortality in Canada II: estimates based on hospitalization data. J Obstet Gynaecol Can 2011; 33(10): 1020-30. DOI: https://doi.org/10.1016/s1701-2163(16)35051-4

McCaw-Binns AM, Campbell LV, Spence SS. The evolving contribution of non-communicable diseases to maternal mortality in Jamaica, 1998-2015: a population-based study. BJOG 2018; 125(10): 1254-1261. DOI: https://doi.org/10.1111/1471-0528.15154

Metz TD, Rovner P, Hoffman MC, Allshouse AA, Beckwith KM, Binswanger IA. Maternal Deaths From Suicide and Overdose in Colorado, 2004-2012. Obstet Gynecol 2016; 128(6): 1233-1240. DOI: https://doi.org/10.1097/aog.0000000000001695

Ramos S, Karolinski A, Romero M, Mercer R, Maternal Mortality in Argentina Study Group. A comprehensive assessment of maternal deaths in Argentina: translating multicentre collaborative research into action. Bull World Health Organ 2007; 85(8): 615-22. DOI: https://doi.org/10.2471/blt.06.032334

Sliwa K, Anthony J. Late maternal deaths: a neglected responsibility. Lancet 2016; 387(10033): 2072-2073. DOI: https://doi.org/10.1016/s0140-6736(16)30391-9

Sliwa K, Azibani F, Baard J, et al. Reducing late maternal death due to cardiovascular disease - A pragmatic pilot study. Int J Cardiol 2018; 272: 70-76. DOI: https://doi.org/10.1016/j.ijcard.2018.07.140

Thornton C, Schmied V, Dennis CL, Barnett B, Dahlen HG. Maternal deaths in NSW (2000-2006) from nonmedical causes (suicide and trauma) in the first year following birth. Biomed Res Int 2013; 2013: 623743. DOI: https://doi.org/10.1155/2013/623743

Vega CEP, Soares VMN, Lourenço Francisco Nasr AM. [Late maternal mortality: comparison of maternal mortality committees in Brazil]. Cad Saude Publica 2017; 33(3): e00197315. DOI: https://doi.org/10.1590/0102-311x00197315

World Health Organization. Maternal mortality ratio (per 100 000 live births). The Global Health Observatory. https://www.who.int/data/gho/indicator-metadata-registry/imr-details/26

World Health Organization. WHO Mortality Database - Last updated: 15 December 2019. Available at: https://www.who.int/healthinfo/mortality_data/en/

Ziraba AK, Madise N, Mills S, Kyobutungi C, Ezeh A. Maternal mortality in the informal settlements of Nairobi city: what do we know? Reprod Health 2009; 6: 6. DOI: https://doi.org/10.1186/1742-4755-6-6

A.1.5.7 Indirect Maternal Deaths

Overview

An indirect maternal death is defined as a "maternal death resulting from previous existing disease or disease that developed during pregnancy and which was not due to direct obstetric causes, but which was aggravated by physiologic effects of pregnancy" (WHO 2012). Indirect maternal deaths outnumber direct maternal deaths due to obstetric causes in many high-income countries, and have grown substantially as a proportion of all maternal deaths in low- and middle-income countries (Nair 2017). It is important to distinguish between direct and indirect maternal deaths when planning and evaluating interventions to improve maternal health, as failure to differentiate between causes may lead to unrealistic expectations about the effect of an intervention on total maternal deaths, or misallocation of resources away from major causes of death (Cross 2010).

Data

We obtained data on reported deaths from all causes for women ages 15-49 years from the WHO Mortality Database, a compilation of mortality data by age, sex, and cause of death, as reported annually by countries from their civil registration systems (WHO 2019). We restricted the data to observations where the reported causes of maternal death summed to the total number of reported maternal deaths, yielding 1730 estimates from 115 countries between 1994-2018. We used these data to set priors on the proportion of all-cause mortality due to direct maternal deaths (i.e. due to ICD-10 codes corresponding to causes of death explicitly included in the model - see Appendix 2.3 Maternal Death ICD Codes). We use these estimates to 'back out' the risk of direct maternal death for women ages 15-49 from the lifetables used in the model, leaving the risk of indirect maternal deaths as part of the background mortality rates included in the model.

Parameters

We used a hierarchical logistic regression model to model the proportion of total female mortality (15-49) from direct maternal causes included in the model. This approach allows us to account for differences in the risk of indirect maternal deaths in different settings.

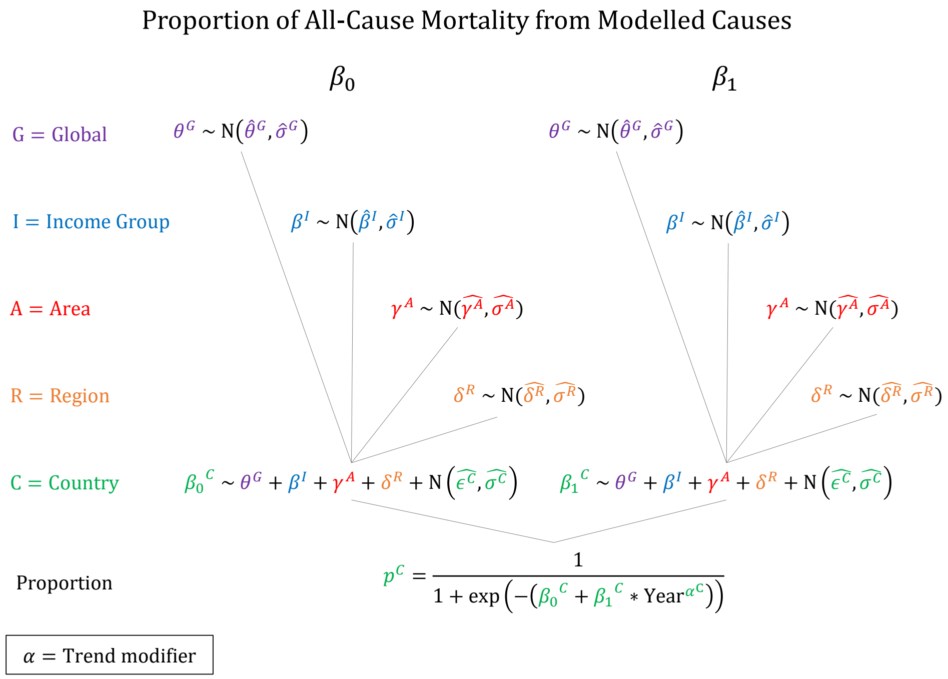

Priors

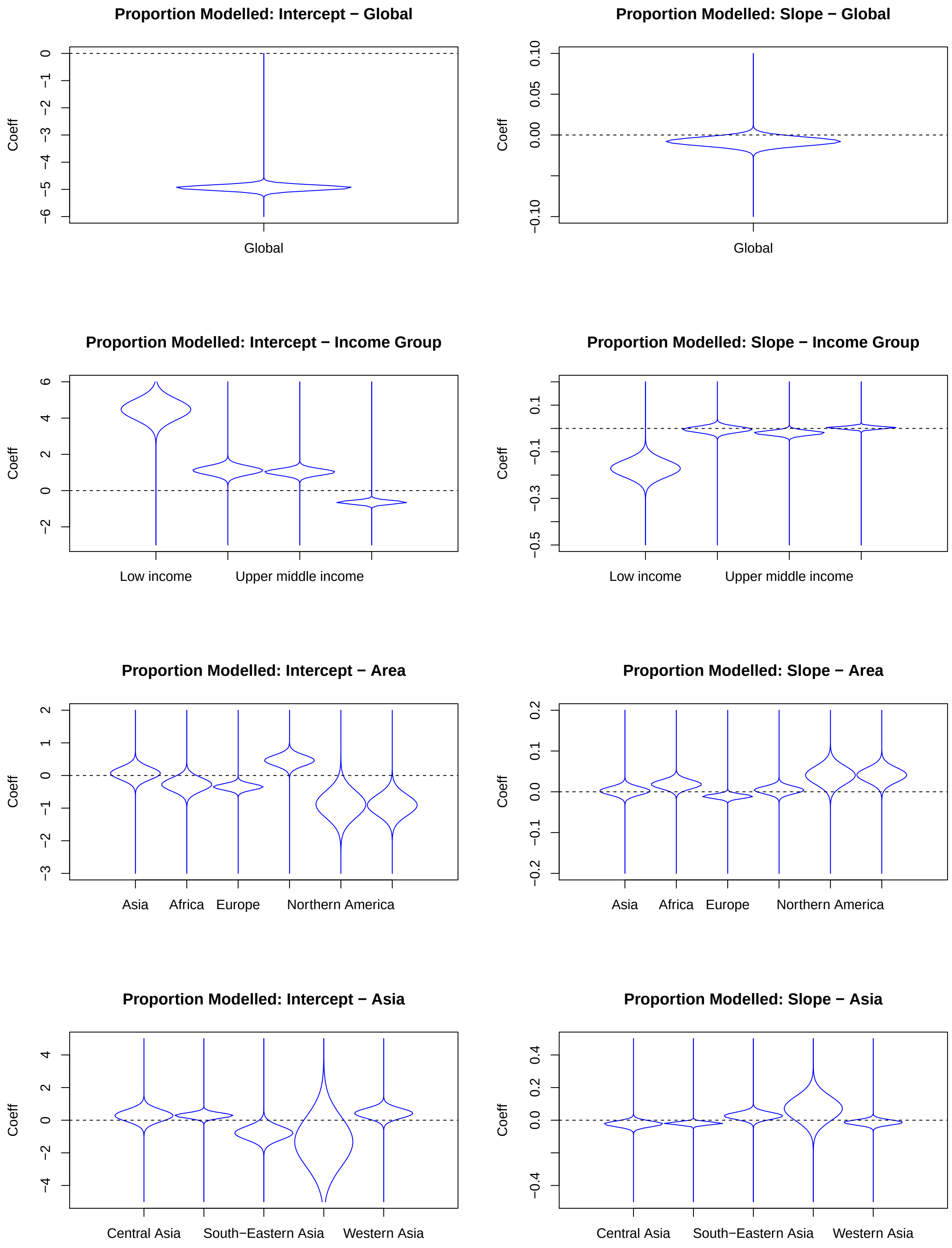
Proportion Modelled: Intercept − Global
Coeff
0
−1
−2
−3
−4
−5
−6
Global
Proportion Modelled: Slope − Global
Coeff
0.10
0.05
0.00
−0.10
Global
Proportion Modelled: Intercept − Income Group
Coeff
6
4
2
0
−2
Low income
Upper middle income
Proportion Modelled: Slope − Income Group
Coeff
0.1
−0.1
−0.3
−0.5
Low income
Upper middle income
Proportion Modelled: Intercept − Area
Coeff
2
1
0
−1
−2
−3
Asia
Africa
Europe
Northern America
Proportion Modelled: Slope − Area
Coeff
0.2
0.1
0.0
−0.1
−0.2
Asia
Africa
Europe
Northern America
Proportion Modelled: Intercept − Asia
Coeff
4
2
0
−2
−4
Central Asia
South−Eastern Asia
Western Asia
Proportion Modelled: Slope − Asia
Coeff
0.4
0.2
0.0
−0.4
Central Asia
South−Eastern Asia
Western Asia

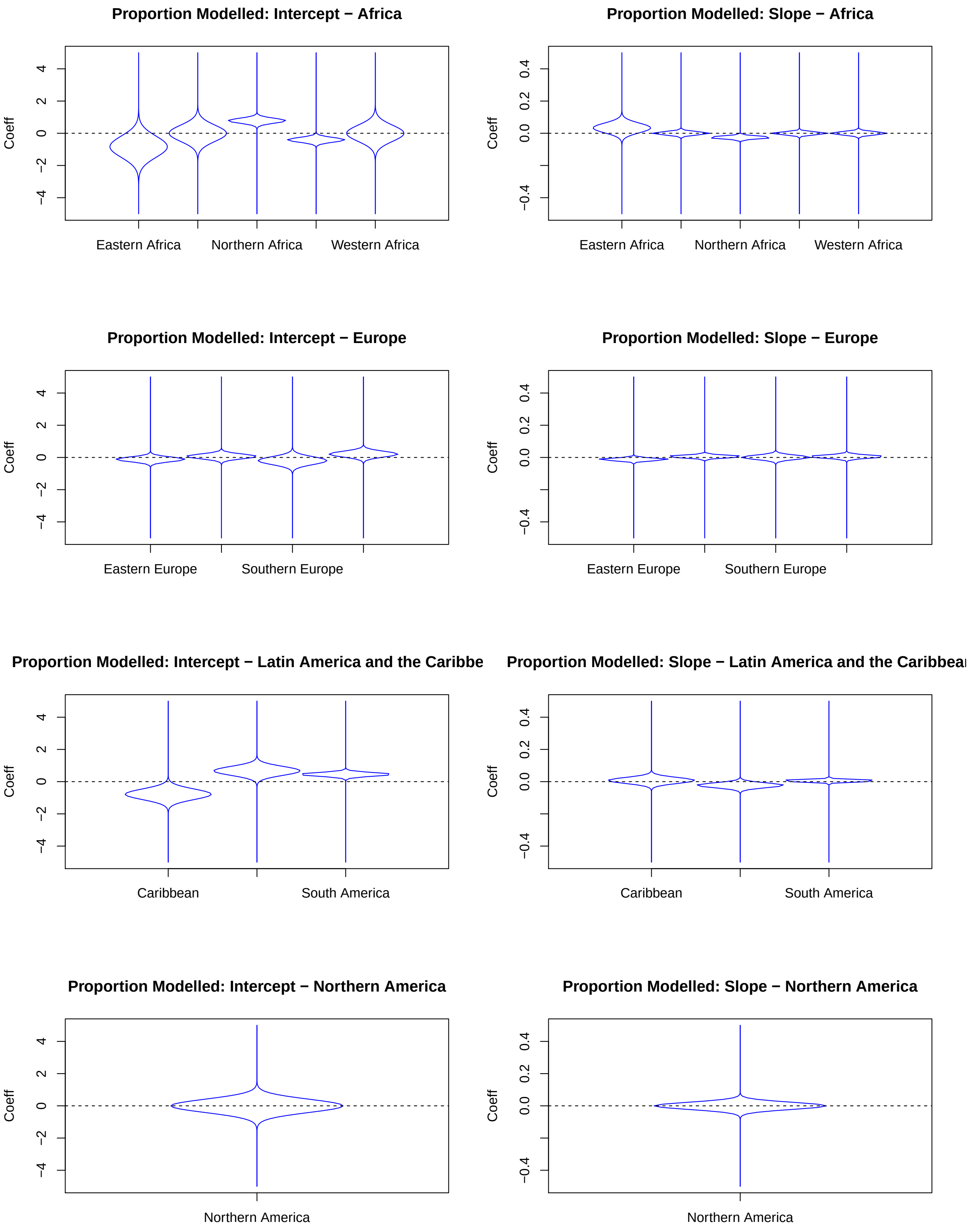
Proportion Modelled: Intercept − Africa
Proportion Modelled: Slope − Africa
Proportion Modelled: Intercept − Europe
Proportion Modelled: Slope − Europe
Proportion Modelled: Intercept − Latin America and the Caribbe
Proportion Modelled: Slope − Latin America and the Caribbea
Proportion Modelled: Intercept − Northern America
Proportion Modelled: Slope − Northern America
Coeff
−4
−2
0
2
4
−0.4
0.0
0.2
0.4
Eastern Africa
Northern Africa
Western Africa
Eastern Europe
Southern Europe
Caribbean
South America
Northern America

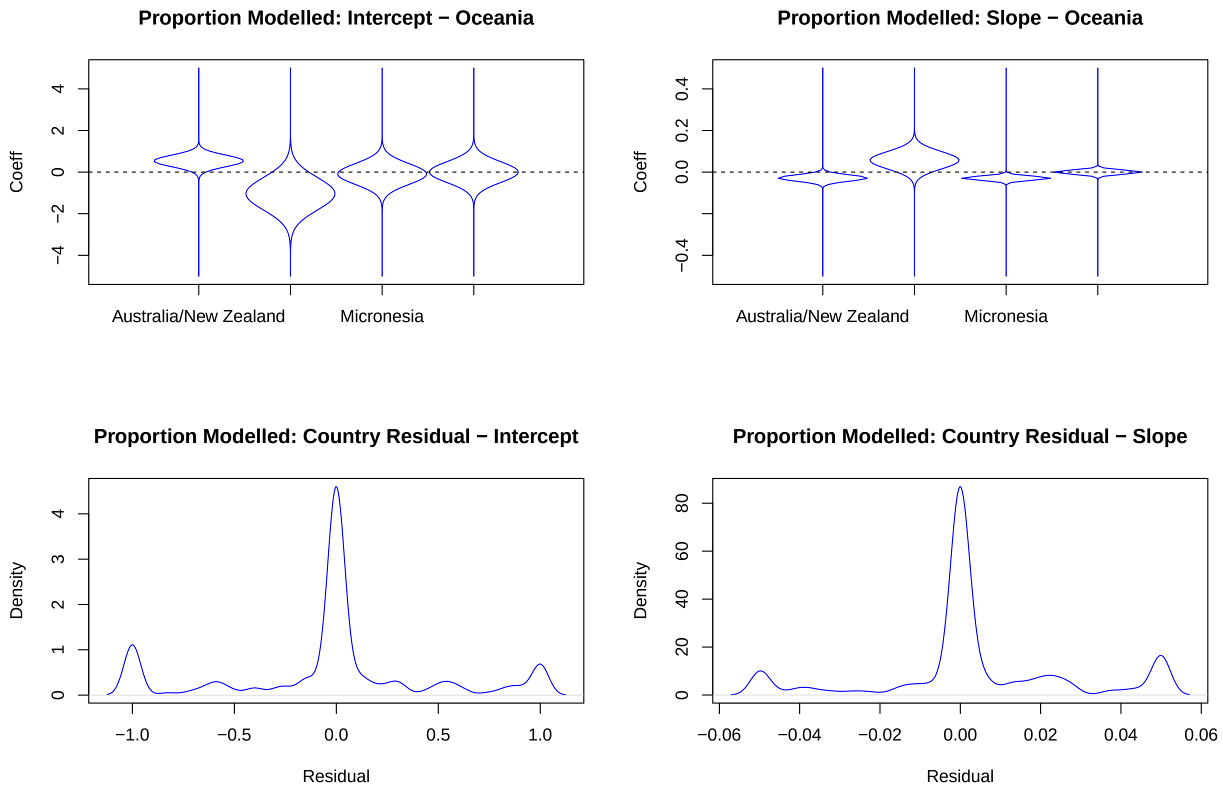

Model Implementation

We use the modelled proportion of female deaths already explicitly included in the model to proportionally lower the all-cause mortality rates from the lifetables for women ages 15-49. If a woman dies from this adjusted background mortality risk during or within 2 months of pregnancy it is considered an indirect maternal death if it is not due to incidental/accidental causes.

References

Cross S, Bell JS, Graham WJ. What you count is what you target: the implications of maternal death classification for tracking progress towards reducing maternal mortality in developing countries. Bull World Health Organ 2010; 88(2): 147-53. DOI: https://doi.org/10.2471/blt.09.063537

Nair M, Nelson-Piercy C, Knight M. Indirect maternal deaths: UK and global perspectives. Obstet Med 2017; 10(1): 10-15. DOI: https://dx.doi.org/10.1177%2F1753495X16689444

World Health Organization. The WHO application of ICD-10 to deaths during pregnancy, childbirth and the puerperium: ICD-MM, Geneva: World Health Organization, 2012. Available at: https://www.who.int/reproductivehealth/publications/monitoring/9789241548458/en/

World Health Organization. WHO Mortality Database - Last updated: 15 December 2019. Available at: https://www.who.int/healthinfo/mortality_data/en/

A.1.6 Clinical Interventions

Clinical interventions refer to actions taken during pregnancy or at the time of delivery that may change the course of a woman's natural history. The use of interventions depends on the site of delivery, and can be preventive (i.e. used if available), or curative (i.e. used in response to recognizing a complication). Preventive interventions can reduce the risk of complication incidence, while curative interventions reduce the risk of mortality/morbidity from a complication. Facilities are able to treat different severity levels of complications depending on their EmOC status, and the efficacy of interventions can also differ by site status. In the model, the probabilities of intervention availability/use are ensured to be non-decreasing by improving delivery site (e.g. we assume that CEmOC will always be at least as good as BEmOC).

We model the availability and efficacy of each intervention. Availability represents the probability that the intervention is able to be delivered at a particular site, and efficacy represents the inherent maximum clinical effectiveness of the intervention. The actual 'real-word' effectiveness of interventions is then modelled, accounting for the site-specific quality of care.

We model elective c-section and also preventive interventions and curative interventions (in response to recognized complication):

- Elective Caesarean Section
- Preventive Interventions
  - Active Management of Labor
  - Partograph
  - Clean Delivery
- Curative Interventions
  - Management of Ectopic Pregnancy
  - Hypertension Management
  - Assisted Delivery
  - Hemorrhage Treatment
  - Antibiotics

A.1.6.1 Elective Caesarean Section

Overview

Caesarean section (c-section) use has increased during the past few decades to a level often in excess of the 10-15% of deliveries for which it is though to be optimal, driven by major increases in non-medically indicated c-sections in many middle-income and high-income countries (Boerma 2018). In contrast, many low-income and middle-income countries have c-section rates of less than 10%, which is considered to reflect inadequate access to medically indicated procedures (Boerma 2018). In addition, large differences in c-section use have been observed between the poorest and richest women within many countries, (Boerma 2018) with many women in private facilities choosing to deliver by c-section without indications of complication (Ronsmans 2006). It is estimated that the global increase in c-section use between 2000 and 2015 was driven mainly by an increasing proportion of births taking place in health facilities (accounting for 66% of the increase), while an increase in c-section use within health facilities accounted for an estimated 33% of the increase (Boerma 2018).

Although c-section can be a live-saving intervention for mothers and children, it can result in short-term and long-term health consequences (Sandall 2018). The risk of postpartum infection is especially high for c-section (Buddeberg 2015). While elective c-section is less risky than non-elective c-section (ACOG 2011, Smaill 2002), there is nevertheless a higher risk of infection after a c-section than after a spontaneous vaginal delivery (Buddeberg 2015).

Data

We obtained data on national c-section rates by country from the UNICEF Maternal and Newborn Health Coverage Database. 548 estimates were available for 166 countries included in the model between 1990 to 2020. Estimates ranged from 0% in Chad to over 50% in countries such as Venezuela, Brazil, and the Dominican Republic. Here we plot the national UNICEF estimates by income group.

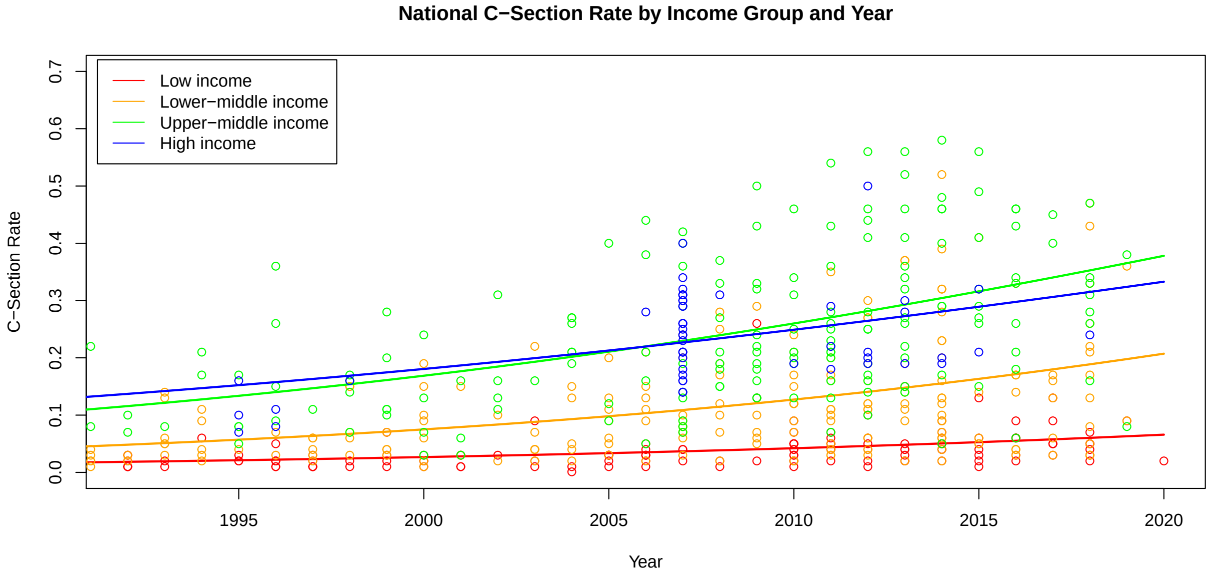

To examine trends within countries we supplemented the UNICEF data with analysis of DHS data for 1,642,046 women from 252 datasets in 75 countries. Here we plot the estimated c-section rates by subgroup (urban/rural and level of education) and year.

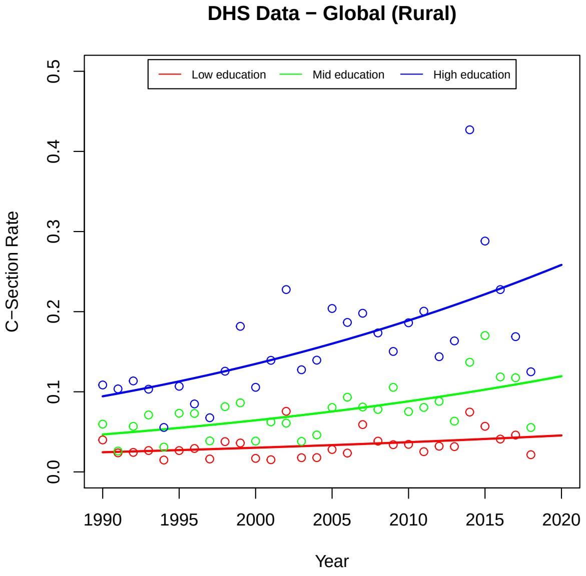

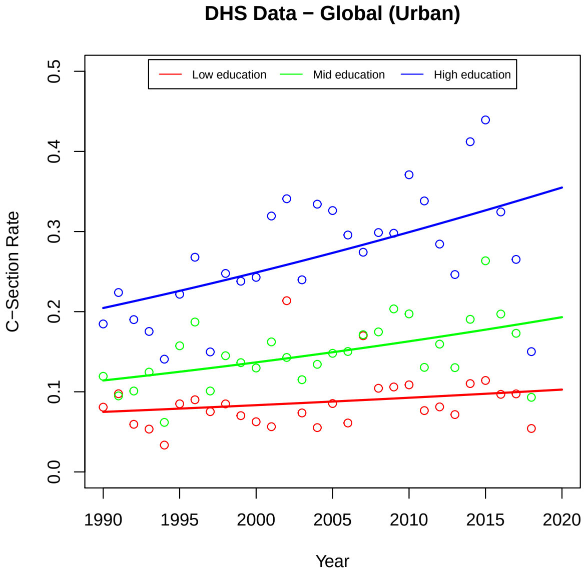

To estimate priors by subgroup we imputed subgroup-specific estimates from the national UNICEF data for countries not available in the DHS data. Note that these estimates of c-section rates include both elective and emergency c-sections. We therefore rely on calibration to total c-section rates (elective and emergency) in the model to fit probabilities of elective c-section, which are expected to be lower than the estimated priors (see calibration targets).

Parameters

We used a hierarchical logistic regression model to simulate the probability of elective c-section. We assume that elective c-section is only available in EmOC facilities.

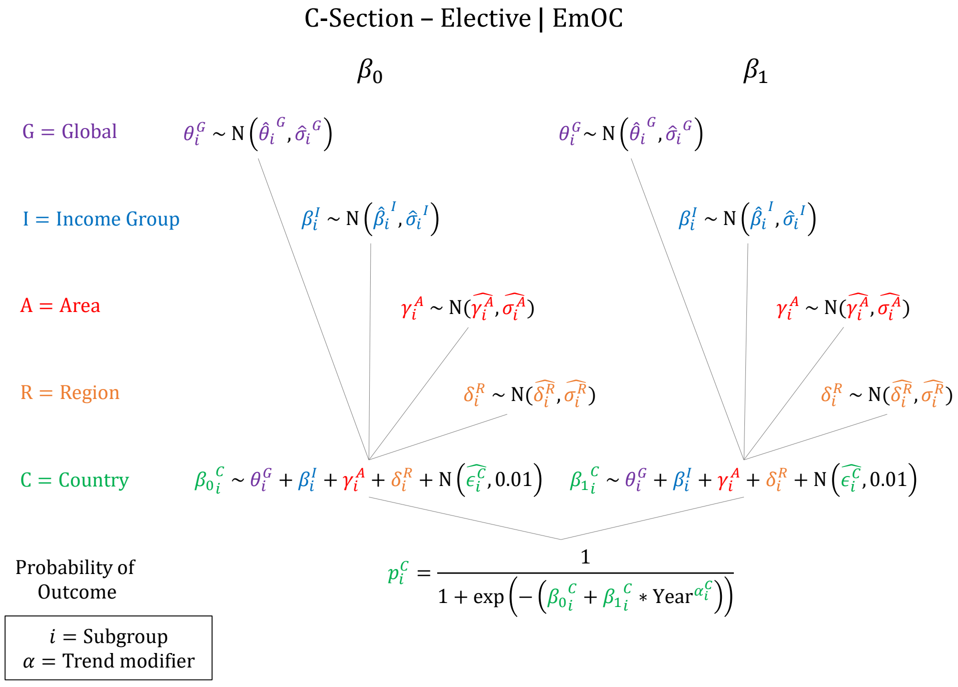

Priors

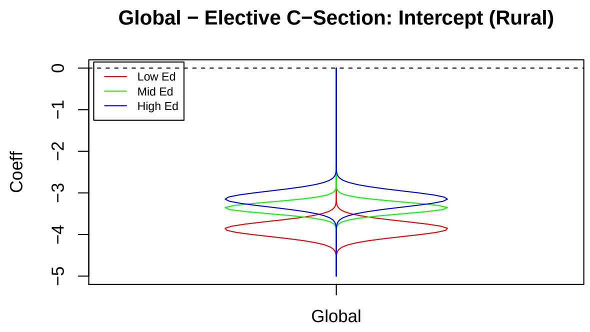
Global − Elective C−Section: Intercept (Rural)
Low Ed
Mid Ed
High Ed
Coeff
0
−1
−2
−3
−4
−5
Global

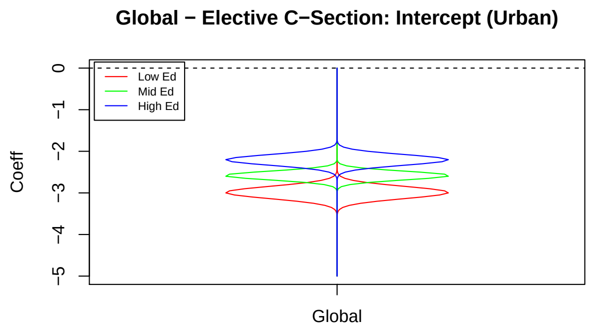
Global − Elective C−Section: Intercept (Urban)
Low Ed
Mid Ed
High Ed
Coeff
0
−1
−2
−3
−4
−5
Global

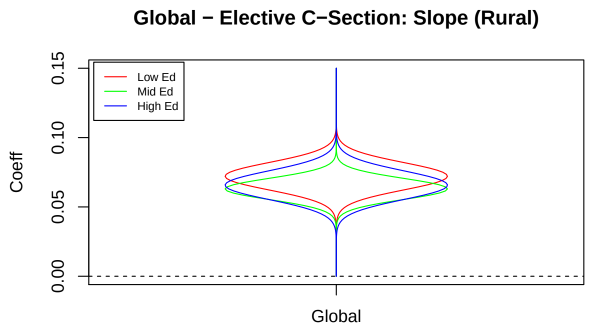
Global − Elective C−Section: Slope (Rural)
Low Ed
Mid Ed
High Ed
Coeff
0.00
0.05
0.10
0.15
Global

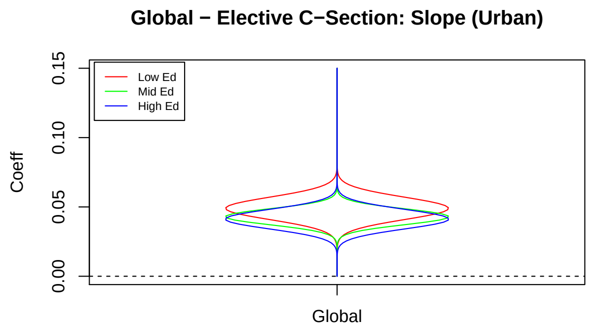
Global − Elective C−Section: Slope (Urban)
Low Ed
Mid Ed
High Ed
Coeff
0.00
0.05
0.10
0.15
Global

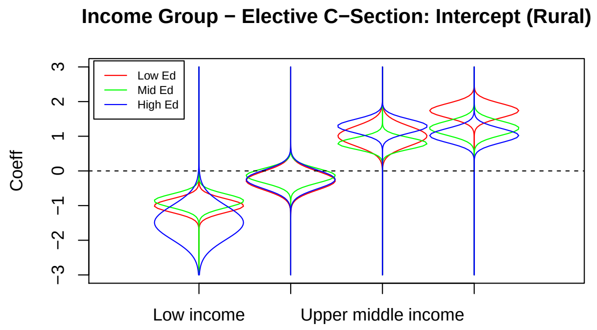
Income Group − Elective C−Section: Intercept (Rural)
Low Ed
Mid Ed
High Ed
Coeff
3
2
1
0
−1
−2
−3
Low income
Upper middle income

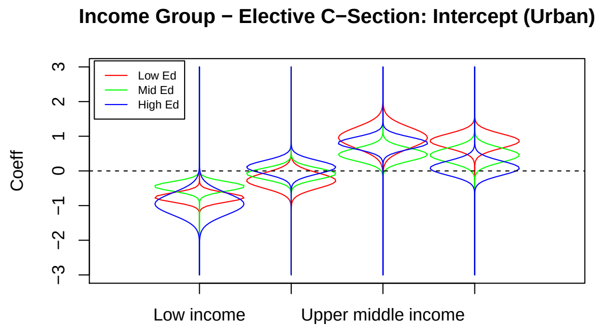
Income Group − Elective C−Section: Intercept (Urban)
Low Ed
Mid Ed
High Ed
Coeff
3
2
1
0
−1
−2
−3
Low income
Upper middle income

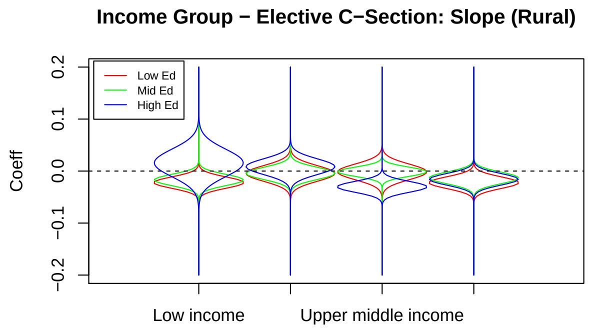
Income Group − Elective C−Section: Slope (Rural)
Low Ed
Mid Ed
High Ed
Coeff
0.2
0.1
0.0
−0.1
−0.2
Low income
Upper middle income

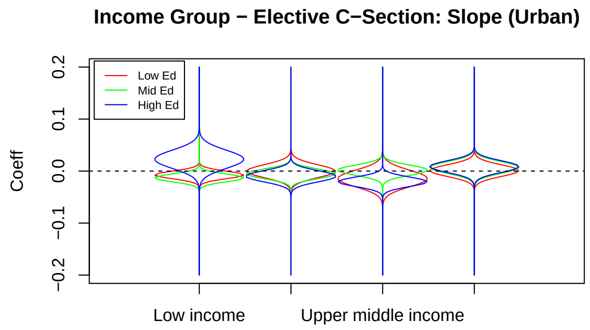
Income Group − Elective C−Section: Slope (Urban)
Low Ed
Mid Ed
High Ed
Coeff
0.2
0.1
0.0
−0.1
−0.2
Low income
Upper middle income

Area − Elective C−Section: Intercept (Rural)

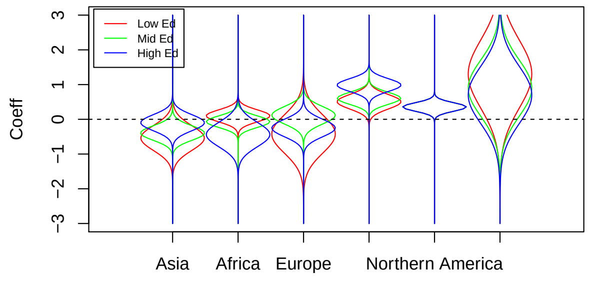

Area − Elective C−Section: Intercept (Urban)

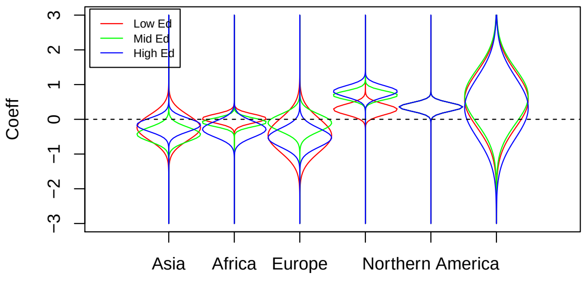

Area − Elective C−Section: Slope (Rural)

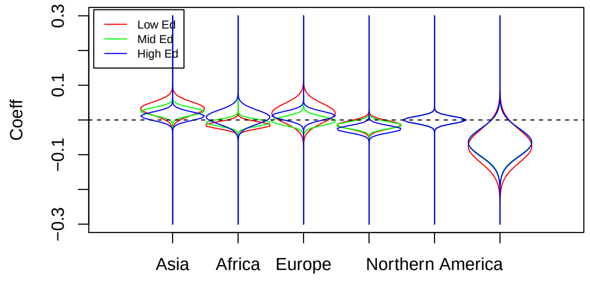

Area − Elective C−Section: Slope (Urban)

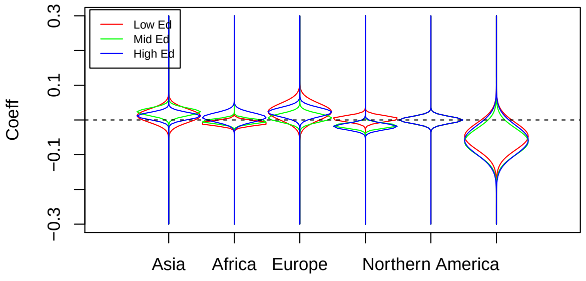

Asia − Elective C−Section: Intercept (Rural)

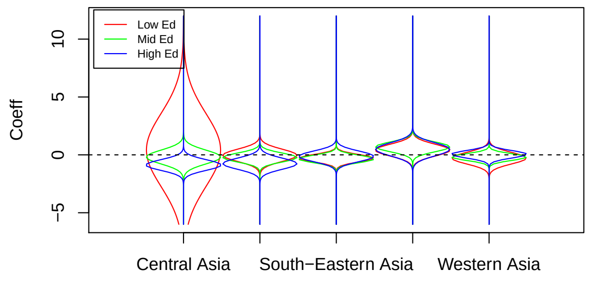

Asia − Elective C−Section: Intercept (Urban)

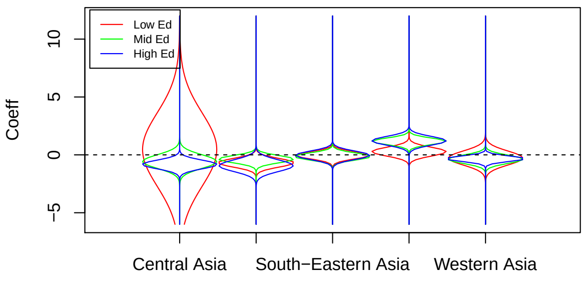

Asia − Elective C−Section: Slope (Rural)

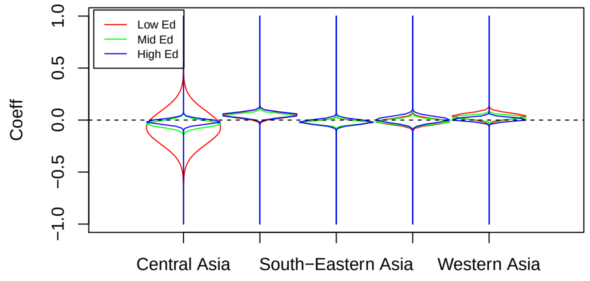

Asia − Elective C−Section: Slope (Urban)

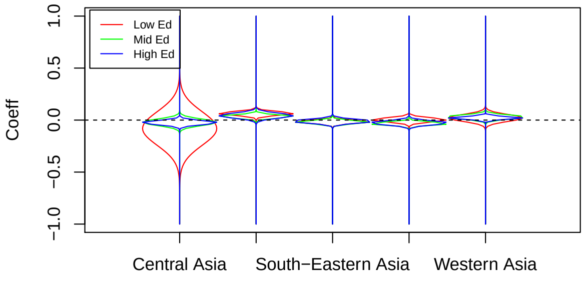

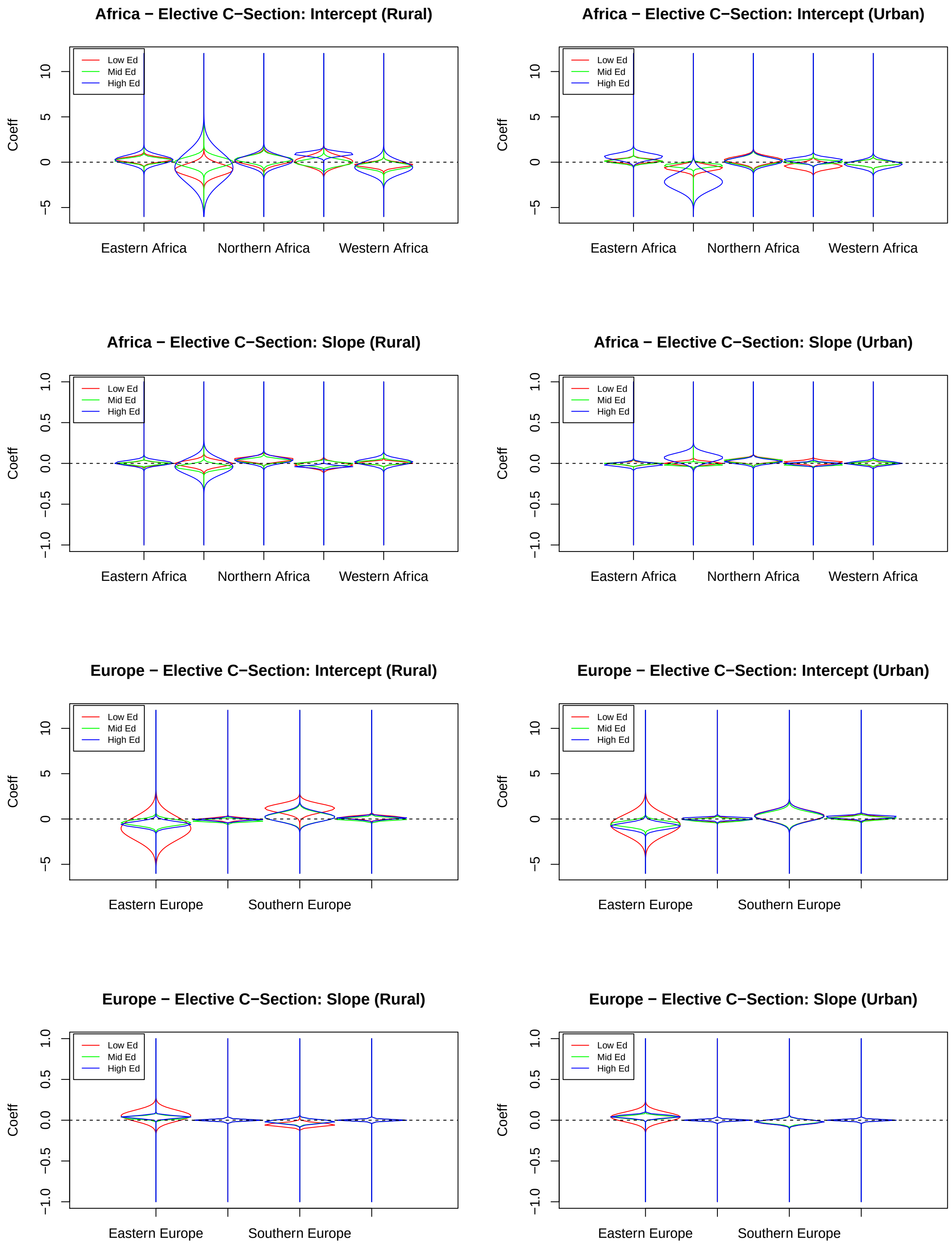
Africa − Elective C−Section: Intercept (Rural)
Africa − Elective C−Section: Intercept (Urban)
Africa − Elective C−Section: Slope (Rural)
Africa − Elective C−Section: Slope (Urban)
Europe − Elective C−Section: Intercept (Rural)
Europe − Elective C−Section: Intercept (Urban)
Europe − Elective C−Section: Slope (Rural)
Europe − Elective C−Section: Slope (Urban)
Low Ed
Mid Ed
High Ed
Coeff
Eastern Africa
Northern Africa
Western Africa
Eastern Europe
Southern Europe

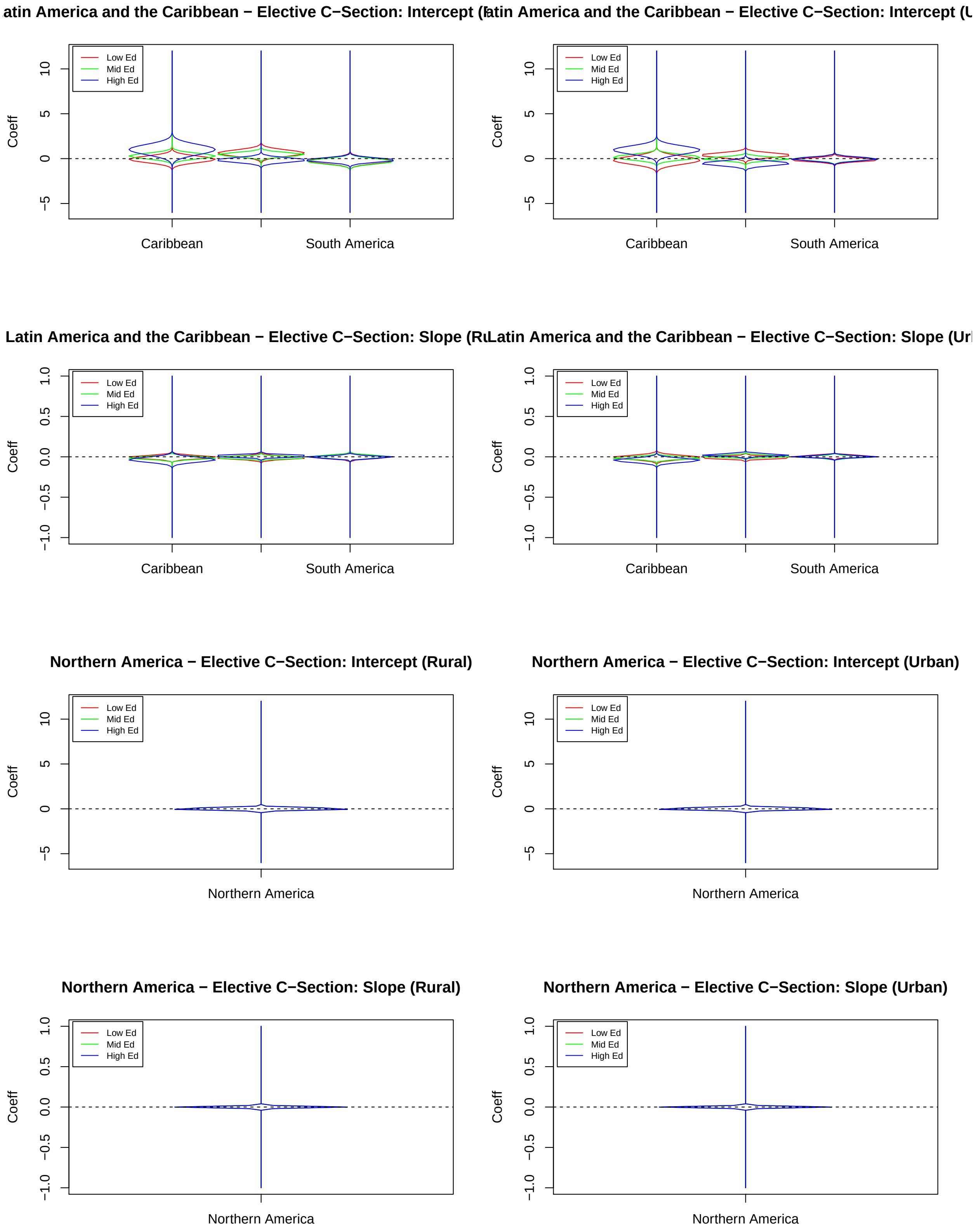
atin America and the Caribbean − Elective C−Section: Intercept (R
atin America and the Caribbean − Elective C−Section: Intercept (U
Latin America and the Caribbean − Elective C−Section: Slope (Ru
Latin America and the Caribbean − Elective C−Section: Slope (Ur
Northern America − Elective C−Section: Intercept (Rural)
Northern America − Elective C−Section: Intercept (Urban)
Northern America − Elective C−Section: Slope (Rural)
Northern America − Elective C−Section: Slope (Urban)
Low Ed
Mid Ed
High Ed
Coeff
Caribbean
South America
Northern America

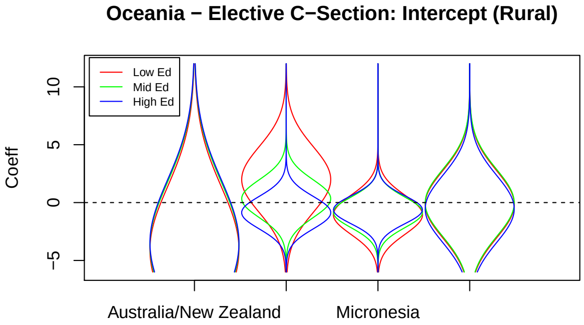

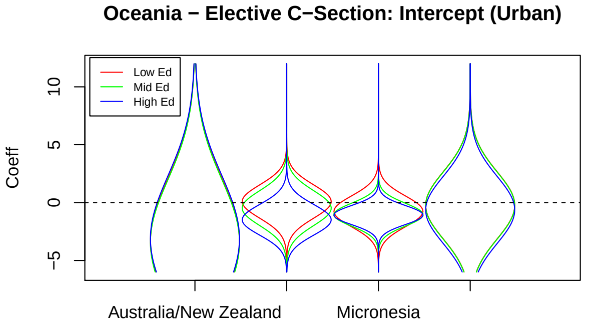

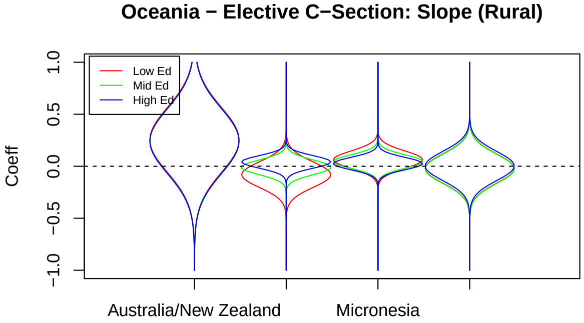

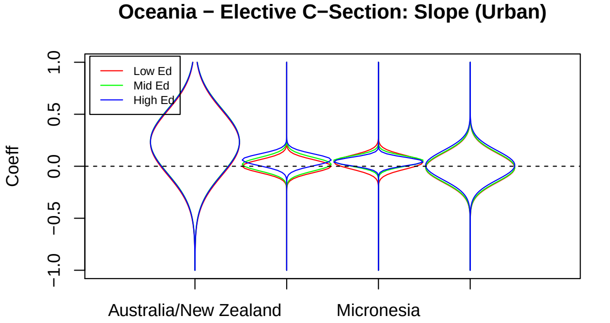

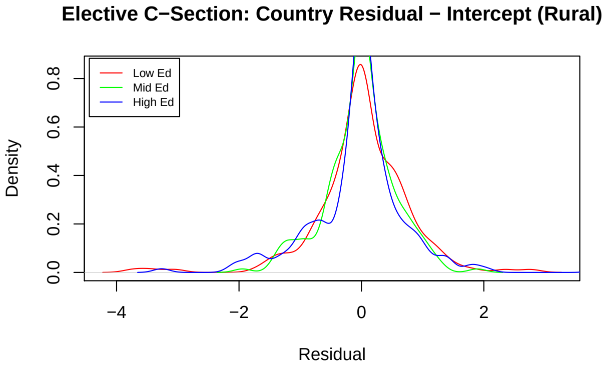

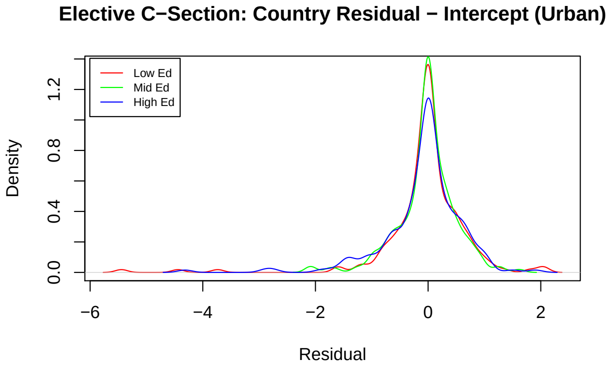

Model Implementation

At the time of delivery we simulate the probability of elective c-section for women in EmOC facilities. For simplicity, we assume that woman who undergo elective c-section are not at risk for preeclampsia/eclampsia, obstructed labour, or postpartum hemorrhage. We do model an increased risk of sepsis however, and also model the risk of other direct complications.

References

American College of Obstetricians and Gynecologists. ACOG Practice Bulletin No. 120: Use of prophylactic antibiotics in labor and delivery. Obstet Gynecol 2011; 117(6): 1472-83. DOI: https://doi.org/10.1097/aog.0b013e3182238c31

Boerma T, Ronsmans C, Melesse DY, et al. Global epidemiology of use of and disparities in caesarean sections. Lancet 2018; 392(10155): 1341-1348. DOI: https://doi.org/10.1016/s0140-6736(18)31928-7

Buddeberg BS, Aveling W. Puerperal sepsis in the 21st century: progress, new challenges and the situation

worldwide. Postgrad Med J 2015; 91(1080): 572-8. DOI: https://doi.org/10.1136/postgradmedj-2015-133475

Ronsmans C, Holtz S, Stanton C. Socioeconomic differentials in caesarean rates in developing countries: A retrospective analysis. Lancet 2006; 368(9546): 1516-1523. DOI: https://doi.org/10.1016/s0140-6736(06)69639-6

Sandall J, Tribe RM, Avery L, et al. Short-term and long-term effects of caesarean section on the health of women and children. Lancet 2018; 392(10155): 1349-1357. DOI: https://doi.org/10.1016/s0140-6736(18)31930-5

Smaill F, Hofmyer GJ. Antibiotic prophylaxis for caesarean section. Cochrane Database Syst Rev 2002; 3: CD000933. DOI: https://doi.org/10.1002/14651858.cd000933

UNICEF. Maternal and Newborn Health Coverage Database, August 2020. Available at: https://data.unicef.org/topic/maternal-health/delivery-care/

A.1.6.2 Active Management of Labor

Overview

After delivery of the baby, continued uterine contractions normally facilitate the shrinking of the uterus, separation of the placenta, and closing maternal blood vessels within the uterine wall. The purpose of management of the third stage of labor is to ensure that this happens, thus enabling the prevention/early detection of uterine atony, the most common cause of PPH (WHO 2009). Labor management involves either a "hands-off" approach (Expectant management) or a "hands-on" approach (Active management).

Expectant management (also called physiological or conservative management of labor) involves watching out for signs of placental separation and allowing the placenta to deliver spontaneously, or via the aid of gravity or nipple stimulation. On the other hand, active management involves interventions that facilitate the third stage of labor.

Active management of the third stage involves three components: 1) giving a drug (a uterotonic) to contract the uterus; 2) clamping the cord early (usually before, alongside, or immediately after giving the uterotonic), and this is before cord pulsation stops; 3) traction is applied to the cord with counter-pressure on the uterus to deliver the placenta (controlled cord traction). Specific ways the three components are used often vary. Mixed management uses some, but not all, of the three components (Begley 2019).

Data

While active management is commonly used for facility births, it is not necessarily universally practiced. A survey of members of the Royal College of Obstetricians and Gynaecologists (RCOG) and members of the Royal College of Midwives (RCM) in the UK found that the majority (94% and 71%, respectively) 'always' or 'usually' use active management, and 73% and 40% clamp the cord within 20 seconds (Farrar 2010).

A recent Cochrane review (Begley 2019) reported the effect of active management compared to expectant management. For severe PPH (1000ml+), they reported a risk ratio of 0.34 (95% CI 0.14-0.87) - however, the authors stated that they were uncertain of this finding because of the very low-quality evidence (Begley 2019). Another Cochrane review compared prophylactic oxytocin (a critical component of active management) with placebo, and found that it reduced the risk of PPH (>500mL), with a risk ratio of 0.51 (95% CI 0.37-0.72), and reduced blood loss of >1000mL with a risk ratio of 0.59 (95% CI 0.42-0.83) (Salati 2019). A systematic review of active management vs expectant management found that it reduced overall PPH incidence by 26% (17.96% [95% CI 16.97-18.95] vs 13.22% [95% CI 12.87-13.57]), and severe PPH by 22% (3.84% [95% CI 3.31-4.37] vs 2.99% [95% CI 2.80-3.18]) (Carolli 2008).

We could not find estimates of the effect of expectant management of labor compared to no management. It is also difficult to determine where expectant management is being practiced - given its low-tech approach, it may be common practice for mothers at home to use gravity/nipple stimulation. As a conservative assumption we do not explicitly model expectant management, but rather assume it is a tacit component of all natural history deliveries. Given that our estimates of the efficacy of active management are compared to expectant management, this is also necessary so that we do not bias our modeled effects of active management.

Parameters

We model the use and efficacy of active management. We assume that the use of active management increases by delivery site and income group, and that it is not available at home. We assume that use of active management increases over time and so constrain the year coefficient to be non-negative when sampling. We assume that the efficacy of active management on reducing PPH incidence is non-differential by severity.

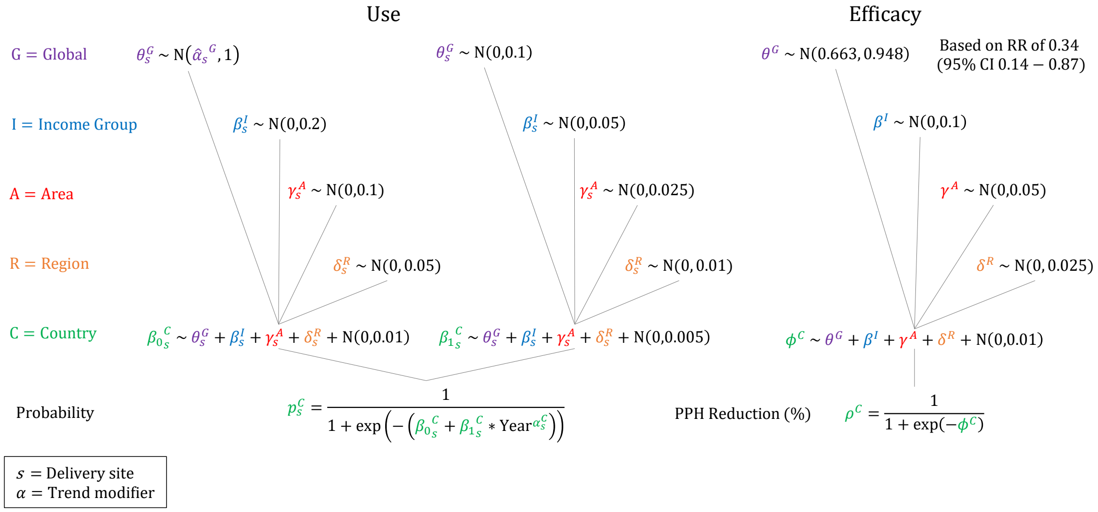

Priors

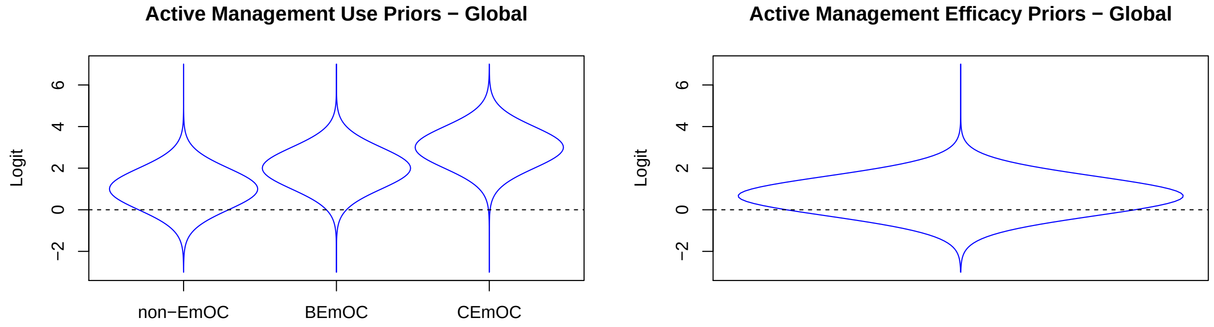

Model Implementation

Conditional on the site of delivery we simulate the probability that active management will be used and its efficacy on reducing PPH incidence.

References

Begley CM, Gyte GM, Devane D, McGuire W, Weeks A, Biesty LM. Active versus expectant management for women in the third stage of labour. Cochrane Database Syst Rev 2019; 2(2): CD007412. DOI: https://doi.org/10.1002/14651858.cd007412.pub5

Carroli G, Cuesta C, Abalos E, Gulmezoglu AM. Epidemiology of postpartum haemorrhage: a systematic review. Best Pract Res Clin Obstet Gynaecol 2008; 22(6): 999-1012. DOI: https://doi.org/10.1016/j.bpobgyn.2008.08.004

Farrar D, Tuffnell D, Airey R, Duley L. Care during the third stage of labour: a postal survey of UK midwives and obstetricians. BMC Pregnancy Childbirth 2010; 10: 23. DOI: https://doi.org/10.1186/1471-2393-10-23

Salati JA, Leathersich SJ, Williams MJ, Cuthbert A, Tolosa JE. Prophylactic oxytocin for the third stage of labour to prevent postpartum haemorrhage. Cochrane Database Syst Rev 2019; 4(4): CD001808. DOI: https://doi.org/10.1002/14651858.cd001808.pub3

World Health Organization. WHO guidelines for the management of postpartum haemorrhage and retained placenta, 2009. Available: http://apps.who.int/iris/bitstream/10665/44171/1/9789241598514_eng.pdf.

A.1.6.3 Partograph

Overview

The partograph graphically represents key events in labor and provides an early warning system. The World Health Organization partographs are the best known partographs in low resource settings. Experiences with World Health Organization and other types of partographs in low resource settings suggest that when used with defined management protocols, this inexpensive tool can effectively monitor labor and prevent obstructed labor (Mathai 2009).

Data

A WHO trial in 1987 in South-East Asia involving 35,484 women found that partograph use reduced by half both prolonged labor (from 6.4% to 3.4%), and the proportion of labors requiring augmentation (from 20.7% to 9.1%) (WHO 1994). Similar reductions were found in a prospective study of 1000 women in Karachi, Pakistan. Before introduction of the partograph only 80.8% of women delivered within 12 hours, compared to 91.6% after the partograph was introduced (Javed 2007).

However, a recent Cochrane review concluded that there was no clear evidence that partographs improve outcomes (e.g. c-section rate, oxytocin augmentation), or that one type of partograph is greatly superior to another (Lavender 2018). Nevertheless, the review acknowledges that partograph use is widespread in both low-income and high-income settings, with reported quality of care benefits (Bedwell 2017), and that evidence from non-randomized trials report potential benefits of partograph use (Bedwell 2017, Bosse 2002, Fawole 2008). Given these findings, we set wide priors for the efficacy of partographs in reducing the incidence of obstructed labor.

Parameters

We model the use and efficacy of partographs, and assume that use increases by delivery site and income group, and that it is not available at home. We assume that partograph use increases over time and so constrain the year coefficient to be non-negative when sampling. We assume that the efficacy of partographs on reducing obstructed labor incidence is non-differential by severity.

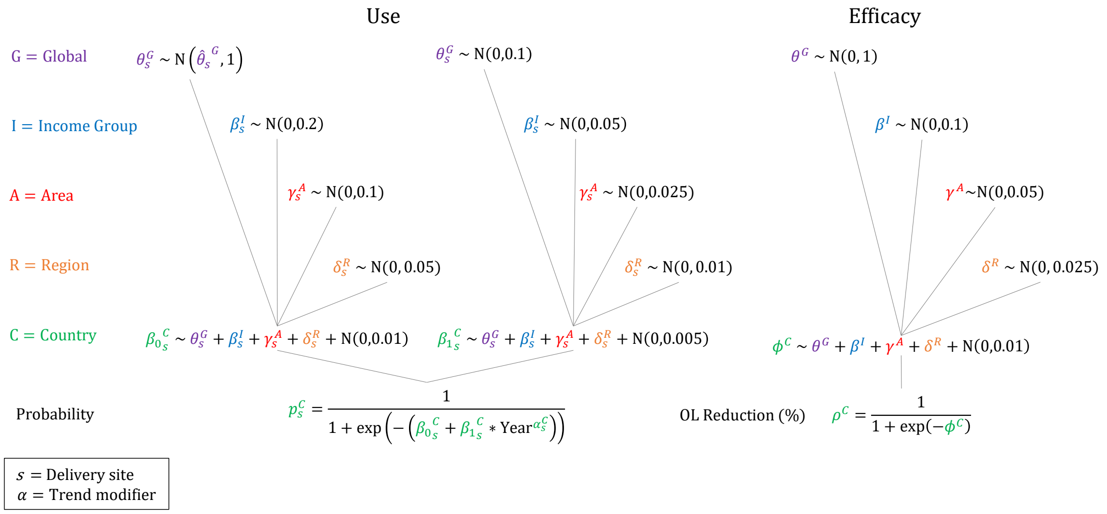

Priors

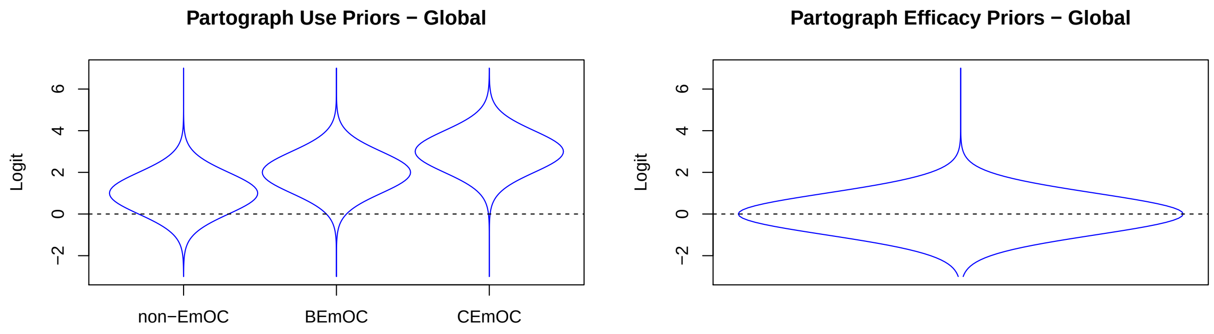

Model Implementation

Conditional on the site of delivery we simulate the probability that a partograph will be used and its efficacy on reducing the incidence of obstructed labor.

References

Bedwell C, Levin K, Pett C, Lavender T. A realist review of the partograph: when and how does it work for labour monitoring? BMC Pregnancy Childbirth 2017; 17: 31. DOI: https://doi.org/10.1186/s12884-016-1213-4

Bosse G, Massawe S, Jahn A. The partograph in daily practice: It's quality that matters. Int J Gynaecol Obstet 2002; 77(3): 243–4. DOI: https://doi.org/10.1016/s0020-7292(02)00004-8

Fawole AO, Hunyinbo KI, Adekanle DA. Knowledge and utilization of the partograph among obstetric care givers in South West Nigeria. Afr J Reprod Health 2008; 12(1): 22–9. PMID: https://pubmed.ncbi.nlm.nih.gov/20695151/

Javed I, Bhutta S, Shoaib T. Role of partogram in preventing prolonged labour. J Pak Med Assoc 2007; 57(8): 408-11. PMID: https://pubmed.ncbi.nlm.nih.gov/17902525/

Lavender T, Cuthbert A, Smyth RM. Effect of partograph use on outcomes for women in spontaneous labour at term and their babies. Cochrane Database Syst Rev 2018; 8(8): CD005461. DOI: https://doi.org/10.1002/14651858.cd005461.pub5

Mathai M. The partograph for the prevention of obstructed labor. Clin Obstet Gynecol 2009; 52(2): 256-69. DOI: https://doi.org/10.1097/grf.0b013e3181a4f163

World Health Organization Maternal Health and Safe Motherhood Programme. World Health Organization partograph in management of labour. Lancet. 1994; 343(8910): 1399-404. PMID: https://pubmed.ncbi.nlm.nih.gov/7910888/

A.1.6.4 Clean delivery

Overview
The most effective interventions for preventing maternal sepsis are the use of antiseptics and observation of stringent infection control measures to limit the spread of microorganisms, especially within hospital environments. General measures such as handwashing with soap are widely accepted practices to prevent infection transmission (Gülmezoglu 2016).

Clean birth kits (CBKs) for home use have also been developed to prevent infection, which the WHO recommends contain at a minimum: soap, plastic sheet, razor blade, cord ties, alcohol swabs, and gauze (Hundley 2012, Lassi 2020).

Data
A systematic review of CBK use found that reported usage varied widely, with estimates between 8% and 99% for Nepal alone, depending on the study (Lassi 2020). Proper use of the items in the CBK also varied substantially (Lassi 2020). For facility births, an assessment of a sample of facilities in Gujarat in 2009 found that 70% of respondents said that standard infection control procedures were followed, but a written procedure was only available in 5% of facilities (Mehta 2011). Furthermore, the study found that alcohol rubs were not used for hand cleaning, and surgical gloves were reused in over 70% of facilities, especially for vaginal examinations in the labor room.

Although there are no systematic reviews evaluating the effect of handwashing on maternal mortality from sepsis, the work of Semmelweis in the mid-19th century shed light on the epidemiological basis of puerperal sepsis and confirmed the impact of this simple and inexpensive measure on reducing maternal and morbidity (Noakes 2008, Gülmezoglu 2016).

A 2012 systematic review concluded that while providing birth kits to facilitate clean practices seems common sense, there is no evidence to indicate effects (positive or negative) independent of those achieved by a broader intervention package (Hundley 2012). A 2015 Cochrane review examined community intervention packages, in which a large component was increasing use of clean delivery kits: uptake increased by 82% while maternal mortality decreased (RR 0.80, [95% CI 0.64-1.00]), and maternal morbidity decreased (RR 0.75, [95% CI 0.61-0.92]) (Lassi 2015). However, since many things were changed (including a 20% increase in facility deliveries), and the deaths were not broken out by cause, it is difficult to ascertain what effect (if any) any single component, such as clean delivery kits, had on any particular delivery complication. A 2020 systematic review also found that the evaluation of CBKs was rarely found in isolation from intervention packages involving education programmes or service delivery, and that the components of the kits varied in each study, limiting the comparability of the studies (Lassi 2020). While the studies could not be pooled because of heterogeneity, they did find reductions in sepsis.

A cluster randomised controlled trial from Pakistan reported a reduction in puerperal sepsis (OR 0.18, 95% CI 0.14-0.22) with the introduction of community-based intervention packages including provision of CBKs (Jokhio 2005). A cross-sectional study from Egypt reported that mothers who used a CBK were less likely to develop puerperal sepsis (OR 0.11, 95% CI 0.01-1.06) (Darmstadt 2009). Two studies in Tanzania by Mosha (2005) and Winani (2007) concluded that women who bathed before delivery and women who used a clean delivery kit were 2.6 and 3.2 times less likely to develop puerperal sepsis than women who did not, respectively. A case control study from Ethiopia found that women who were given a CBK had fewer symptoms of puerperal sepsis ($p<0.00$) (Onolemhemhen 2012).

We set wide priors for availability of clean delivery, and set priors for the efficacy on reduction of sepsis centered around 95%. Note that this parameter governs the maximum possible full efficacy - the actual modelled effect is modified by site-specific quality of care parameters for each country.

Parameters
We model the use and efficacy of clean delivery practices, and assume that use increases by delivery site and income group. We assume that clean delivery practices increase over time and so constrain the year coefficient to be non-negative when sampling. We assume that the efficacy of clean delivery on reducing sepsis incidence is non-differential by severity.

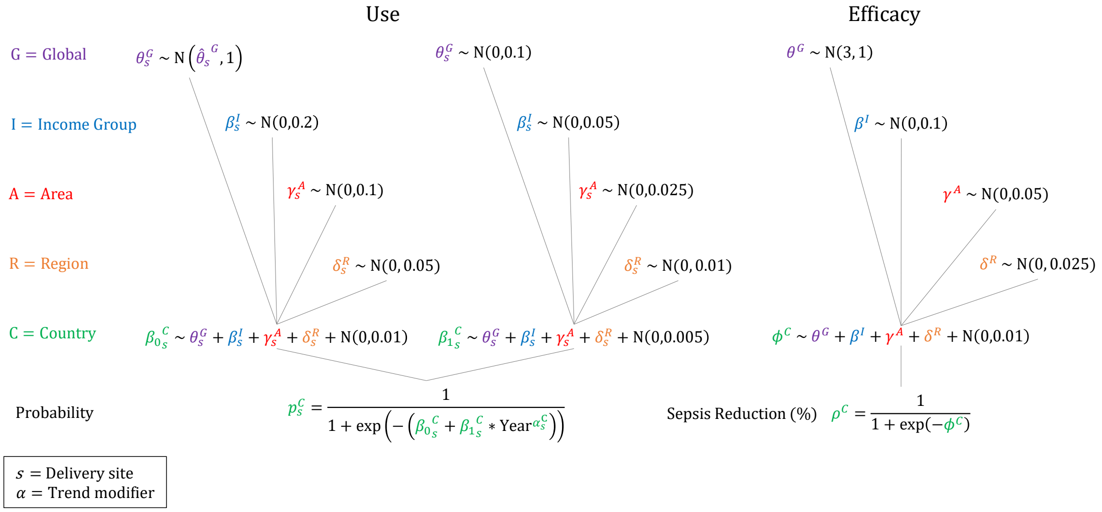

Priors

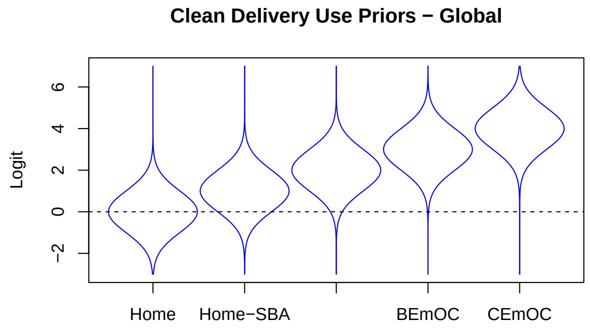

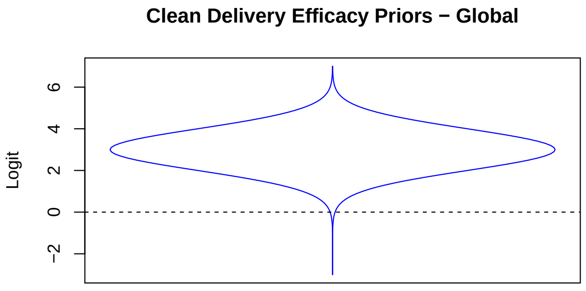

Model Implementation

Conditional on the site of delivery we simulate the probability that the delivery will be 'clean' and the impact on reducing the incidence of sepsis.

References

Darmstadt GL, Hassan M, Balsara ZP, Winch PJ, Gipson R, Santosham M. Impact of clean delivery-kit use on newborn umbilical cord and maternal puerperal infections in Egypt. J Health Popul Nutr 2009; 27(6): 746-54. DOI: https://doi.org/10.3329/jhpn.v27i6.4326

Gülmezoglu, Lawrie TA, Hezelgrave N, et al. Chapter 7. Interventions to Reduce Maternal and Child Morbidity and Mortality. In: Black RE, Laxminarayan L, Walker N, eds. Reproductive, Maternal, Newborn, and Child Health: Disease Control Priorities, Third Edition (Volume 2). Washington (DC): The International Bank for Reconstruction and Development / The World Bank; 2016. DOI: https://doi.org/10.1596/978-1-4648-0348-2_ch7

Hundley V, Avan B, Braunholtz D, Graham WJ. Are birth kits a good idea? A systematic review of the evidence. Midwifery 2012; 28(2): 204-215. DOI: https://doi.org/10.1016/j.midw.2011.03.004

Jokhio AH, Winter HR, Cheng KK. An intervention involving traditional birth attendants and perinatal and maternal mortality in Pakistan. N Engl J Med 2005; 352(20): 2091–9. DOI: https://doi.org/10.1056/nejmsa042830

Lassi ZS, Bhutta ZA. Community-based intervention packages for reducing maternal and neonatal morbidity

and mortality and improving neonatal outcomes. Cochrane Database Syst Rev 2015; 3: CD007754. DOI: https://doi.org/10.1002/14651858.cd007754.pub3

Lassi ZS, Fisher Z, Andraweera P, Cummins A, Roberts CT. Effectiveness of birthing kits for clean childbirth: a systematic review. Int Health 2020; 12(1): 3-10. DOI: https://doi.org/10.1093/inthealth/ihz022

Mehta R, Mavalankar DV, Ramani KV, Sharma S, Hussein J. Infection control in delivery care units, Gujarat state, India: a needs assessment. BMC Pregnancy Childbirth 2011; 11: 37. DOI: https://doi.org/10.1186/1471-2393-11-37

Mosha F, Winani S, Wood S, Changalucha J, Ngasalla B. Evaluation of the effectiveness of a clean delivery kit intervention in preventing cord infection and puerperal sepsis among neonates and their mothers in rural Mwanza Region, Tanzania. Tanzan Health Res Bull 2005; 7(3): 185-8. DOI: https://doi.org/10.4314/thrb.v7i3.14258

Noakes TD, Borresen J, Hew-Butler T, Lambert MI, Jordaan E. Semmelweis and the aetiology of puerperal sepsis 160 years on: an historical review. Epidemiol Infect 2008; 136(1): 1-9. DOI: https://doi.org/10.1017/s0950268807008746

Onolemhemhen D. The Ethiopian Birth Kit Project. Paper presented at: 13th World Conference on Public health 2012; Addis Abba, Ethiopia. Available at: https://www.researchgate.net/publication/268139777_The_Ethiopian_Birth_Kit_Project

Winani S, Wood S, Coffey P, Chirwa T, Mosha F, Changalucha J. Use of a clean delivery kit and factors associated with cord infection and puerperal sepsis in Mwanza, Tanzania. J Midwifery Womens Health 2007; 52(1): 37-43. DOI: https://doi.org/10.1016/j.jmwh.2006.09.004

A.1.6.5 Management of Ectopic Pregnancy

Overview

Ectopic pregnancy is a difficult diagnosis and may be missed initially. While diagnosis is often delayed due to very early presentations, it can also be missed because patients may not have the same risk factors or symptoms, such as serum B-human chorionic gonadotropin levels/trends or ultrasound findings (Robertson 2017). Most cases of tubal ectopic pregnancy that are detected early can be successfully treated with methotrexate alone or minimally invasive surgery (ACOG 2018). However, tubal ectopic pregnancy in an unstable patient requires prompt surgical intervention (ACOG 2018), and is indicated in patients with hemodynamic compromise or other clinical signs of ruptured ectopic pregnancy including pain or evidence of intra-abdominal bleeding (Panelli 2015). Laparoscopic surgery has been found to be a cost-effective treatment in the surgical management of tubal ectopic pregnancy (Hajenius 2007).

Data

Ectopic pregnancy can be associated with severe maternal morbidity, with up to a third of women being managed after the pregnancy has ruptured (McGurk 2019). While 15% to 40% of ectopic pregnancies may be suitable for non-surgical management (expected management or methotrexate treatment) (Kumar 2015), despite initial medical treatment, around 15% of women eventually require surgery (Lee 2018).

A study of 221 women with tubal pregnancy in the US found that physicians were certain of the diagnosis at the first examination for only 48% of women (Bickell 2004). Overall, 32% of women experienced tubal rupture (n = 71), and 12 women with rupture and 3 without received a blood transfusion. Ten women were treated with methotrexate only, 21 received methotrexate and subsequently underwent surgical treatment, 51 underwent salpingostomy, and 160 had salpingectomy (Bickell 2004). Another study in northeast Brazil found that 63.4% of patients presented with ruptured ectopic pregnancy and 5.9% had hemodynamic instability (Cordeiro 2018). Among the patients with ruptured ectopic pregnancy, 61% had already sought care at another center. With regard to initial treatment, 78.2% underwent surgery (27.2%, laparoscopy), 16.8% used methotrexate, and 5% underwent expectant management. However, among those who received methotrexate, 41.2% needed subsequent surgery because of elevated blood-hCG level (57.1%) and clinical signs of ruptured ectopic pregnancy (42.9%) (Cordeiro 2018).

Although complicated ectopic pregnancy was nearly universally fatal until the beginning of the nineteenth century, earlier diagnosis and improvements in technology have resulted in substantially decreased mortality in high income countries, with current mortality rates estimated at 0.35/1000 in the UK and 0.5/1000 in the USA (McGurk 2019).

Parameters

We model the availability and efficacy of ectopic pregnancy treatment. Because surgery is often needed, we assume that the availability of treatment increases by site and income group, and that treatment is not available at home. We assume that the availability of treatment increases over time and so constrain the year coefficient to be non-negative when sampling. Given the low mortality rates from ectopic pregnancy in high income countries, we assume that the efficacy of treatment is very high and set priors around 99%.

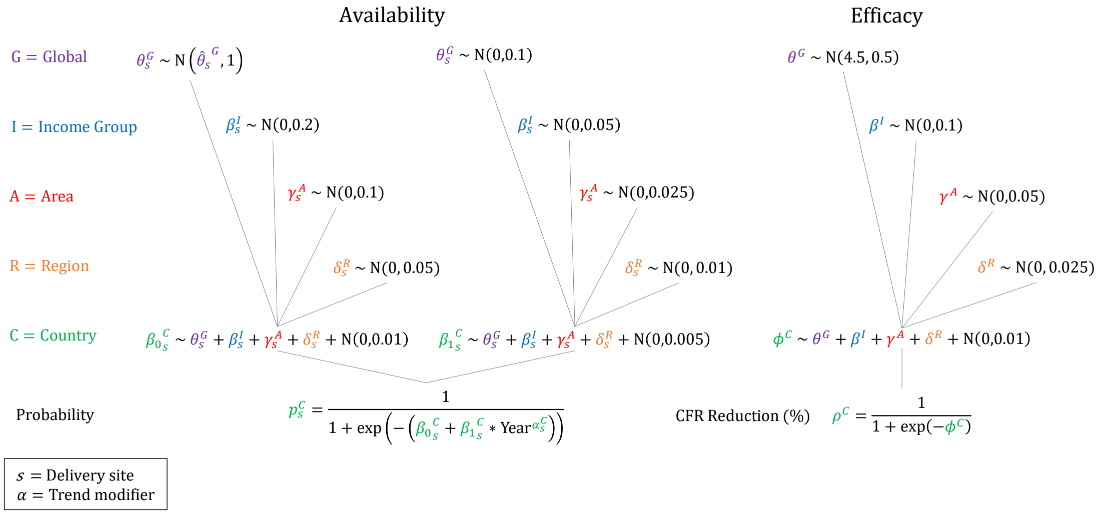

Priors

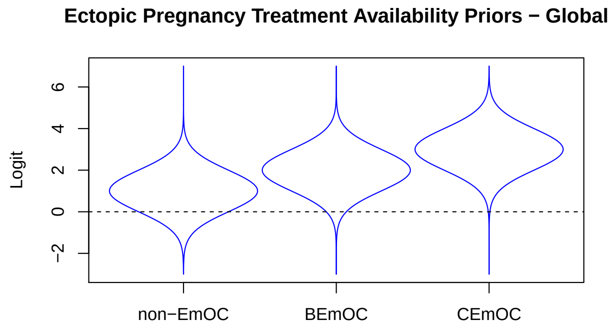

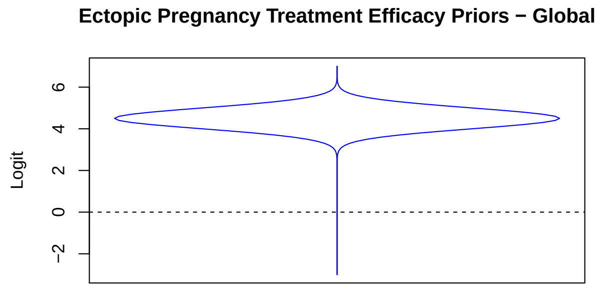

Model Implementation

We assume that women are recognized/referred for ruptured ectopic pregnancy from home. Conditional on the site of treatment we simulate the probabilities that treatment will be available and the efficacy of treatment on ectopic pregnancy mortality, accounting for quality of care.

References

ACOG Practice Bulletin No. 193 Summary: Tubal Ectopic Pregnancy. Obstet Gynecol 2018; 131(3): 613-615. DOI: https://doi.org/10.1097/aog.0000000000002559

Bickell NA, Bodian C, Anderson RM, Kase N. Time and the risk of ruptured tubal pregnancy. Obstet Gynecol 2004; 104(4): 789-94. DOI: https://doi.org/10.1097/01.aog.0000139912.65553.58

Cordeiro FDE, Alves GJA, Araujo Júnior E, Feitosa LEE. Ectopic pregnancies: a retrospective cohort analysis in a tertiary reference center in the Northeast Region of Brazil. Ceska Gynekol 2018; 83(6): 434-439. PMID: https://pubmed.ncbi.nlm.nih.gov/30848148/

Hajenius PJ, Mol F, Mol BWJ, Bossuyt PMM, Ankum WM, van der Veen F. Interventions for tubal ectopic pregnancy. Cochrane Database Syst Rev 2007; 1: CD000324. DOI: https://doi.org/10.1002/14651858.cd000324.pub2

Kumar V, Gupta J. Tubal ectopic pregnancy. BMJ Clin Evid 2015; 11: 1406. Available at: https://www.ncbi.nlm.nih.gov/pmc/articles/PMC4646159/

Lee JH, Kim S, Lee I, et al. A risk prediction model for medical treatment failure in tubal pregnancy. Eur J Obstet Gynecol Reprod Biol 2018; 225: 148-154. DOI: https://doi.org/10.1016/j.ejogrb.2018.04.020

McGurk L, Oliver R, Odejinmi F. Severe morbidity with ectopic pregnancy is associated with late presentation. J Obstet Gynaecol 2019; 39(5): 670-674. DOI: https://doi.org/10.1080/01443615.2018.1557610

Panelli DM, Phillips CH, Brady PC. Incidence, diagnosis and management of tubal and nontubal ectopic pregnancies: a review. Fertil Res Pract 2015; 1: 15. DOI: https://doi.org/10.1186/s40738-015-0008-z

Robertson JJ, Long B, Koyfman A. Emergency Medicine Myths: Ectopic Pregnancy Evaluation, Risk Factors, and Presentation. J Emerg Med 2017; 53(6): 819-828. DOI: https://doi.org/10.1016/j.jemermed.2017.08.074

A.1.6.6 Hypertension Management

Overview

A review of historical declines in PE/E mortality in high-income countries found that the the most important interventions were widespread use of prenatal care with blood pressure and urine protein measurement, and increased access to hospital care for timely induction of labor or cesarean delivery for women with severe PE/E (Goldenberg 2011). While the only definitive cure for pre-eclampsia is delivery of the baby (by induction of labor or pre-labor caesarean section), the mainstays of treatment are antihypertensive drugs for blood pressure control and magnesium sulphate (MgSO4) for eclampsia (Gülmezoglu 2016).

Data

The only interventions which have been shown to prevent pre-eclampsia are anti-platelet agents, primarily low-dose aspirin, and calcium supplementation. For example, a Cochrane review found an 18% reduction in the risk of pre-eclampsia associated with the use of antiplatelet agents (Duley 2019). Currently, professional bodies including the American College of Obstetricians and Gynecologists in the US and the National Institute for Health and Care Excellence in the UK suggest prophylactic use of aspirin for women considered at high risk for pre-eclampsia (Voutetakis 2019). A systematic review and metaanalysis found that administration of aspirin was associated with a reduction in the risk of preterm pre-eclampsia (RR 0.62, 95% CI 0.45-0.87), but that there was no significant effect on term pre-eclampsia (RR 0.92, 95% CI 0.70-1.21) (Roberge 2018). A Cochrane review found that calcium supplementation with at least 1 g of calcium could reduce high blood pressure (RR 0.65, 95% CI 0.53-0.81) as well as the risk of pre-eclampsia (RR 0.45, 95% CI 0.31-0.65) amongst pregnant women, with the greatest risk reduction occurring for women at high risk of pre-eclampsia (Hofmeyr 2018). To reduce the risk of pre-eclampsia, the WHO therefore recommends daily calcium supplementation (1.5-2.0 g oral elemental calcium) for populations with low dietary calcium intake (WHO 2016). However, except for calcium supplementation for women with low dietary calcium intake, all other preventive interventions need further assessment and are not recommended for use outside the context of clinical trials (Mol 2016). We therefore do not model specific interventions to prevent pre-eclampsia/eclampsia in the model, but do model the effect of management that can prevent pre-eclampsia from progressing to eclampsia.

While data from trials are insufficiently conclusive as to the optimal timing of delivery with pre-eclampsia, there is robust evidence that magnesium sulfate can prevent and control eclamptic seizures, and for pre-eclampsia, reduces the risk of eclampsia by more than 50% (Duley 2009, Langer 2008, Tukur 2009). A Cochrane Review of magnesium sulphate and other anticonvulsants for women with pre-eclampsia compiled evidence from 6 trials, the largest of which was the Magpie Trial Collaborative Group (Duley 2010). This review found a 59% reduction in risk of eclampsia in women with pre-eclampsia (RR 0.41, 95% CI 0.29-0.58) and a 46% reduction (RR 0.54, 95% CI 0.26-1.10) in the risk of dying in women with pre-eclampsia randomized to magnesium sulfate (Duley 2010). A review showed that magnesium sulphate was the better anticonvulsant choice when treating women with eclampsia, and substantially reduced the risk of further seizures when compared to diazepam (Duley 2009). A second, smaller, review showed that magnesium sulphate performed better than a lytic cocktail at preventing maternal deaths as well as preventing breathing problems and convulsions (Duley 2010). One goal of this study was whether induction of labor in women with pregnancy induced hypertension or pre-eclampsia at term reduced costs and improved quality of life as compared to expectant monitoring. Two studies looked only at mild pre-eclampsia and gestational diabetes, but not at cases of maternal or neonatal death or eclampsia (Koopmans 2007, 2009). A study by one collaborative group found that the use of magnesium sulphate for women with pre-eclampsia was associated with a 16% reduction in the risk of death or serious morbidity related to pre-eclampsia 2 to 3 years later (Magpie Trial 2007).

Repeated doses of nifedipine, intravenous hydralazine, or labetalol every 15–30 min have been found to achieve blood pressure control in at least 80% of women - however, oral labetalol is not widely available in low-income and middle-income countries (Mol 2016). The availability of magnesium sulphate also varies by location. For example, one assessment of facilities in Karnataka found that magnesium sulphate was available in 18% of primary health centers, 48% of higher public facilities, and 70% of private facilities (Jayanna 2014). A follow up study in Karnataka found that in public facilities, magnesium sulphate was available in 60% of primary health centres, 68% of community health centers, and 100% of sub-district and district hospitals, while 92% of private facilities reported availability of magnesium sulphate (Katageri 2018).

A Cochrane review of planned c-section versus planned vaginal birth for severe pre-eclampsia did not find any randomized clinical trials, and concludes that there are no rigorous data to assess whether c-section or vaginal birth is better for pregnant women with severe pre-eclampsia, and their babies (Amorim 2017). However, reported rates of c-section for women with eclampsia are high. For example, a study in Mali of 116 women with eclampsia reported that cesarean section was performed in 77.6% of cases, and magnesium sulphate was used in 75% of cases (Kampo 2020). A study in French Guiana in 2019 found that of 156 deliveries diagnosed with a hypertensive disorder, delivery was by cesarean in 49.7% of cases (Mhiri 2020). Another study in Zambia reported that c-section was performed for 7/8 cases of eclampsia (Miyoshi 2019). However, while commonly used for women with eclampsia, c-sections resulting from eclampsia comprise a small percentage of all c-sections - a multi-country study in sub-Saharan Africa found that PE/E accounted for only 2.4% of all c-sections (Chu 2012). Given the uncertainty around the relative efficacy of c-section vs other methods of induced labor, we do not model differential mortality/morbidity by use of c-section, but include a probability of c-section for women with eclampsia to better match reported estimates of total c-sections, and to account for the higher risks of sepsis associated with emergency c-section.

Parameters
For women with PE/E, we model the availability and efficacy of PE/E management, conditional on delivery site. We assume that the availability of treatment increases by site and income group, and that treatment is not available at home. We assume that treatment availability increases over time and so constrain the year coefficient to be non-negative when sampling. We also assume that a proportion of women with eclampsia at an EmOC facility will undergo c-section. For simplicity, we assume that the efficacy of treatment is non-differential by PE/E severity, and apply the same risk reduction for all mortality and morbidity outcomes. However, we assume that severe PE/E can only be treated at CEmOC facilities.

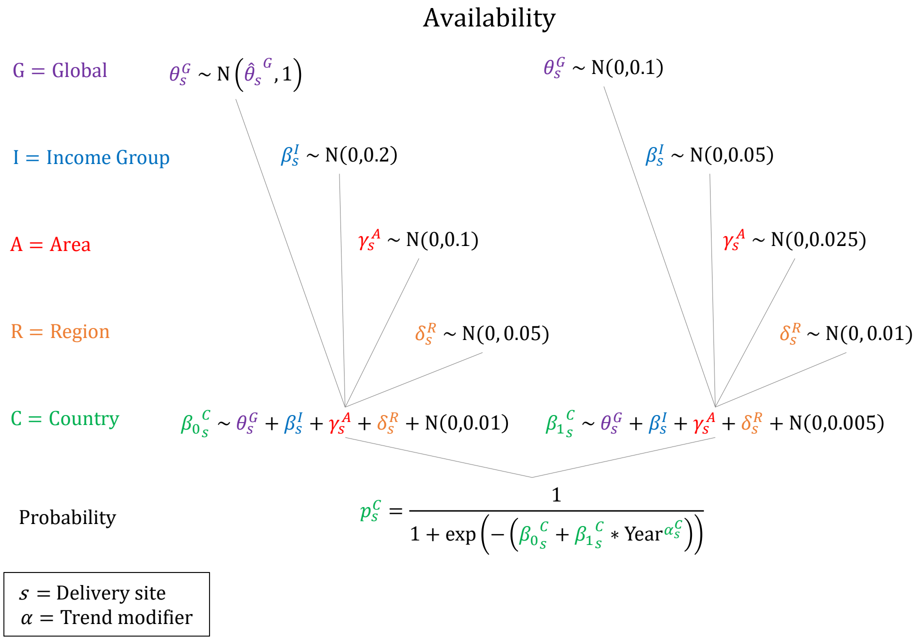

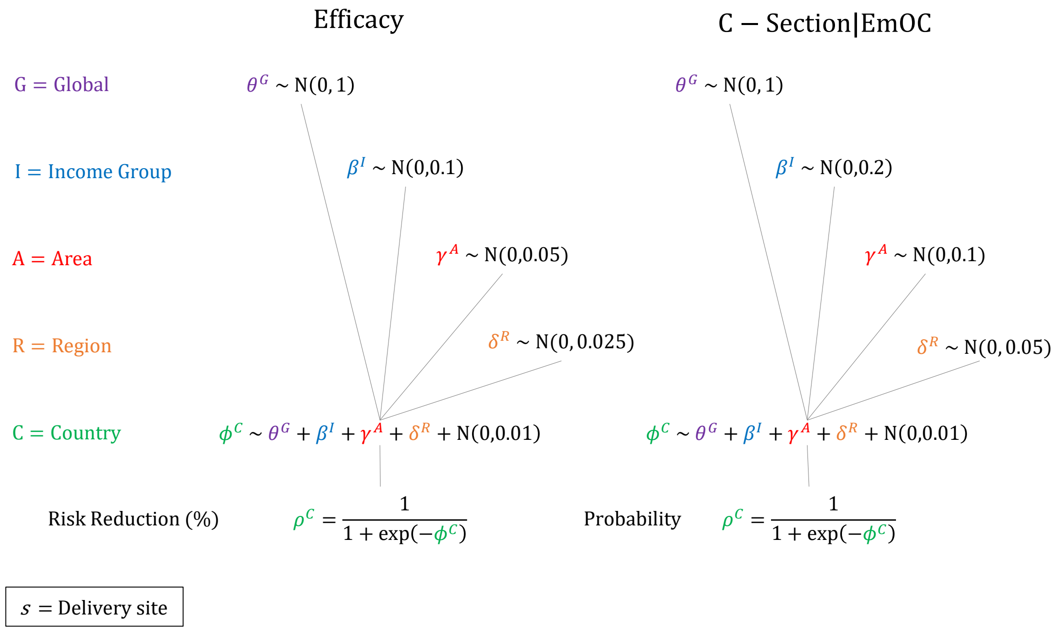

Priors

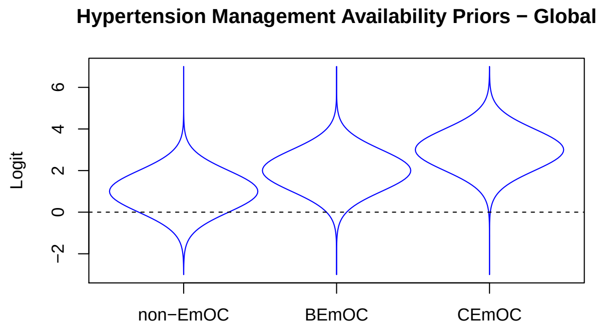

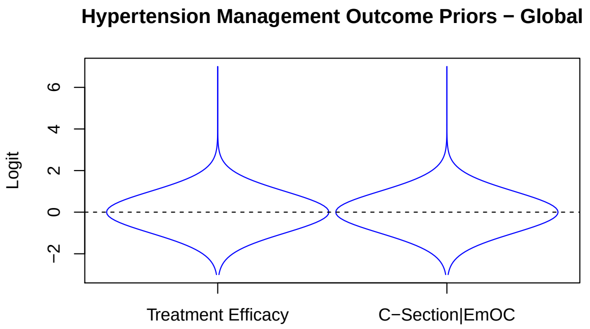

Model Implementation

Conditional on the delivery site we simulate the probabilities that treatment will be available and the efficacy of treatment on PE/E outcomes, accounting for quality of care.

References

Amorim MMR, Souza ASR, Katz L. Planned caesarean section versus planned vaginal birth for severe pre-eclampsia. Cochrane Database Syst Rev 2017; 10: CD009430. DOI: https://dx.doi.org/10.1002%2F14651858.CD009430.pub2

Chu K, Cortier H, Maldonado F, Mashant T, Ford N, Trelles M. Cesarean section rates and indications in sub-Saharan Africa: a multi-country study from Medecins sans Frontieres. PLoS One 2012; 7(9): e44484. DOI: https://doi.org/10.1371/journal.pone.0044484

Duley L. The global impact of pre-eclampsia and eclampsia. Semin Perinatol 2009; 33(3): 130-7. DOI: https://doi.org/10.1053/j.semperi.2009.02.010

Duley L, Gülmezoglu AM, Henderson-Smart DJ, Chou D. Magnesium sulphate and other anticonvulsants for women with pre-eclampsia. Cochrane Database Syst Rev 2010; 11: CD000025. DOI: https://doi.org/10.100

2/14651858.cd000025.pub2

Duley L, Meher S, Hunter KE, Seidler A, Askie LM. Antiplatelet agents for preventing pre-eclampsia and its complications. Cochrane Database Syst Rev 2019; 10: CD004659. DOI: https://doi.org/10.1002/14651858.cd004659.pub3

Goldenberg RL, McClure EM, Macguire ER, Kamath BD, Jobe AH. Lessons for low-income regions following the reduction in hypertension-related maternal mortality in high-income countries. Int J Gynaecol Obstet 2011; 113: 91-5. DOI: https://doi.org/10.1016/j.ijgo.2011.01.002

Gülmezoglu, Lawrie TA, Hezelgrave N, et al. Chapter 7. Interventions to Reduce Maternal and Child Morbidity and Mortality. In: Black RE, Laxminarayan L, Walker N, eds. Reproductive, Maternal, Newborn, and Child Health: Disease Control Priorities, Third Edition (Volume 2). Washington (DC): The International Bank for Reconstruction and Development / The World Bank; 2016. DOI: https://doi.org/10.1596/978-1-4648-0348-2_ch7

Hofmeyr GJ, Lawrie TA, Atallah ÁN, Torloni MR. Calcium supplementation during pregnancy for preventing hypertensive disorders and related problems. Cochrane Database Syst Rev 2018; 10: CD001059. DOI: https://doi.org/10.1002/14651858.cd001059.pub5

Jayanna K, Mony P, Ramesh BM, et al. Assessment of facility readiness and provider preparedness for dealing with postpartum haemorrhage and pre-eclampsia/eclampsia in public and private health facilities of northern Karnataka, India: a cross-sectional study. BMC Pregnancy Childbirth 2014; 14: 304. DOI: https://doi.org/10.1186/1471-2393-14-304

Kampo MI, Sogoba S, Kassogué D. [Maternal and perinatal prognosis of eclampsia at the Timbuktu Hospital in Mali]. Pan Afr Med J 2020; 36: 175. DOI: https://doi.org/10.11604/pamj.2020.36.175.17976

Katageri G, Charantimath U, Joshi A, et al. Availability and use of magnesium sulphate at health care facilities in two selected districts of North Karnataka, India. Reprod Health 2018; 15(Suppl 1): 91. DOI: https://doi.org/10.1186/s12978-018-0531-6

Koopmans CM, Bijlenga D, Aarnoudse JG, et al. Induction of labour versus expectant monitoring in women with pregnancy induced hypertension or mild preeclampsia at term: the HYPITAT trial. BMC Pregnancy Childbirth 2007; 7: 14. DOI: https://doi.org/10.1186/1471-2393-7-14

Koopmans CM, Bijlenga D, Groen H, et al. Induction of labour versus expectant monitoring for gestational hypertension or mild pre-eclampsia after 36 weeks' gestation (HYPITAT): a multicentre, open-label randomised controlled trial. Lancet 2009; 374(9694): 979-88. DOI: https://doi.org/10.1016/s0140-6736(09)60736-4

Langer A, Villar J, Tell K, Kim T, Kennedy S. Reducing eclampsia-related deaths–a call to action. Lancet 2008; 371(9614): 705-6. DOI: https://doi.org/10.1016/s0140-6736(08)60321-9

Magpie Trial Follow-Up Study Collaborative Group. The Magpie Trial: a randomised trial comparing magnesium sulphate with placebo for pre-eclampsia. Outcome for women at 2 years. BJOG 2007; 114(3): 300-9. DOI: https://doi.org/10.1111/j.1471-0528.2006.01166.x

Mhiri R, Mvogo A, Kamga A, et al. Epidemiology and maternal prognosis of hypertension disorders of pregnancy in French Guiana. Pregnancy Hypertens 2020; 20: 96-101. DOI: https://doi.org/10.1016/j.preghy.2020.03.010

Miyoshi Y, Matsubara K, Takata N, Oka Y. Significance of pre-hospital care to reduce the morbidity of eclampsia in rural Zambia. Pregnancy Hypertens 2019; 17: 100-103. DOI: https://doi.org/10.1016/j.preghy.2019.05.008

Mol BWJ, Roberts CT, Thangaratinam S, Magee LA, de Groot CJM, Hofmeyr GJ. Pre-eclampsia. Lancet 2016; 387(10022): 999-1011. DOI: https://doi.org/10.1016/s0140-6736(15)00070-7

Roberge S, Bujold E, Nicolaides KH. Aspirin for the prevention of preterm and term preeclampsia: systematic review and metaanalysis. Am J Obstet Gynecol 2018; 218(3): 287-293.e1. DOI: https://doi.org/10.1016/j.ajog.2017.11.561

Tukur J. The use of magnesium sulphate for the treatment of severe pre-eclampsia and eclampsia. Ann Afr Med 2009; 8(2): 76-80. DOI: https://doi.org/10.4103/1596-3519.56232

Voutetakis A, Pervanidou P, Kanaka-Gantenbein C. Aspirin for the Prevention of Preeclampsia and Potential Consequences for Fetal Brain Development. JAMA Pediatr 2019; 173(7): 619-620. DOI: https://doi.org/10.1001/jamapediatrics.2019.1260

World Health Organization. WHO recommendations on antenatal care for a positive pregnancy experience. 2016. Available at: http://apps.who.int/iris/bitstream/10665/250796/1/9789241549912-eng.pdf

A.1.6.7 Assisted Delivery

Overview
Most women give birth spontaneously, but some need assistance during the second stage of labour with instruments such as obstetric forceps or vacuum, especially in the case of obstructed or prolonged labor. A Cochrane review found that overall, forceps or metal cup appear to be the most effective at achieving a vaginal birth, but with increased risk of maternal trauma with forceps and neonatal trauma with the metal cup (O'Mahony 2010). Over the past two decades authorities have declared vacuum extraction the method of choice in modern obstetric practice due to improved safety for the fetus and less likelihood of maternal morbidity (Bailey 2017). The risks of failed delivery and trade-offs between risks of maternal and neonatal trauma thus need to be considered when choosing an instrument.

In the event that labor cannot be resolved by manipulation (to reposition the fetus) or instrumentation, Caesarean section may be required. Caesarean section (C-section) is a common abdominal operation for surgical delivery of a baby and the placenta, with techniques varying depending on the clinical situation and surgeon preferences (Dodd 2014). However, C-sections require effective anesthesia which can be regional (epidural or spinal) or a general anesthetic (Afolabi 2012).

Data
The rates of instrumental vaginal births range from 5% to 20% of births in high-income countries, with less information about the incidence in low-income countries (Majoko 2012). In general, assisted vaginal delivery (AVD) is more common in high-income countries than in low- and middle-income countries (LMICs) (Bailey 2017). A limited review of EmOC signal functions in 2006 found that AVD was generally the signal function least likely to have been performed (Bailey 2006). Other studies in sub-Saharan Africa also find that AVD was the signal function most often missing in health facilities (AMDD 2004, Pearson 2005). In Latin America, availability of AVD may be even lower - previous studies reported it to be completely missing for facilities in Nicaragua (AMDD 2003), and uncommon in Peru and no longer part of the pre-service training curriculum at some of the country's leading medical schools (AMDD 2002). A review of AVD found that it is more likely to be performed in hospitals than in health centres and clinics, and the top reported reason for non-performance was equipment-related, followed by lack of staff training (Bailey 2017). Most countries surveyed appeared to show a preference for vacuum extraction over forceps, especially at non-hospitals, which may reflect the global transition in instrument choice and a consensus that forceps is more difficult to use and less versatile (Bailey 2017).

We identified multiple studies that examined the efficacy of treating obstructed labor on reducing maternal mortality rates (Schuitemaker 1997, Adam 2005, Pasupathy 2008, Ameh 2009). The efficacy of assisted delivery for obstructed labor is high - for example, Yarrow 2004 reported a 94.1% success rate when using vacuum-assisted deliveries, and of the nine failed vacuum deliveries, four were subsequently delivered by forceps and five by cesarean section, with no maternal mortality reported. AVD is thus sometimes first attempted, with a c-section performed if needed. A multi-country study in sub-Saharan Africa found that obstructed labor accounted for 31.3% of all c-sections, followed by malpresentation (18.3%) (Chu 2012). A WHO Global survey in 2004-2008 found that operative vaginal deliveries accounted for only 2.6% of deliveries, compared to nearly 25% of deliveries by c-section with indications, suggesting that c-sections are far more commonly used than AVD (Souza 2010). We include these estimates of operative vaginal deliveries as calibration targets in the model.

Parameters
For women with obstructed labour, we model the availability and efficacy of assisted delivery, conditional on delivery site. We assume that the availability of treatment increases by site and income group, and that treatment is not available at home. We assume that treatment availability increases over time and so constrain the year coefficient to be non-negative when sampling. We assume that 'severe' OL requires c-section and can only be treated at CEmOC facilities, while 'moderate' OL can be treated with AVD if available. However, given the preference for c-section over AVD in many cases, we simulate a probability of c-section instead of AVD for women with moderate OL at EmOC as well. We assume that the efficacy of treatment is non-differential by severity (moderate/severe) or mode (AVD/c-section), and apply the same risk reduction for all mortality and morbidity outcomes. We set priors centered around 95% efficacy for risk

reduction based on the empirical data.

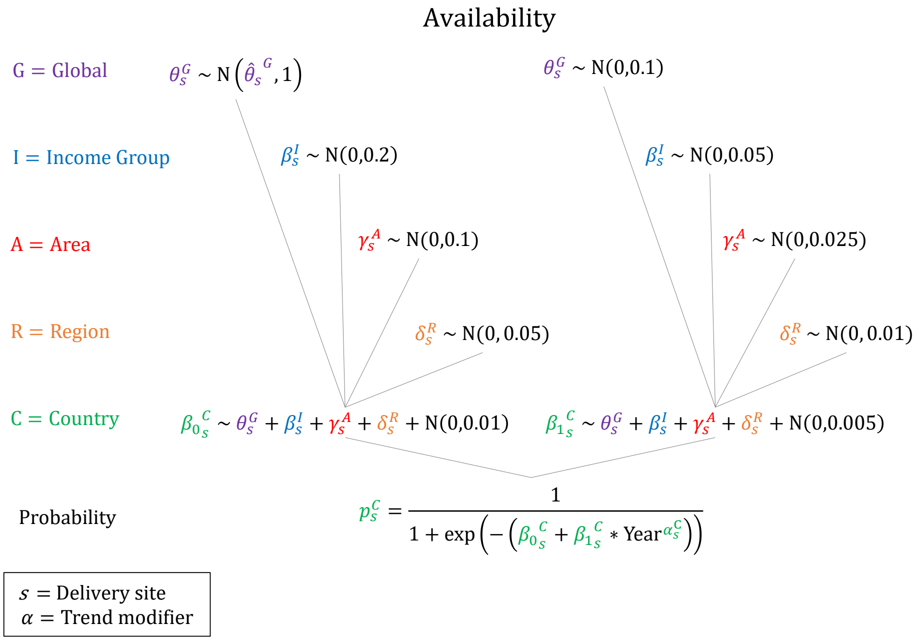

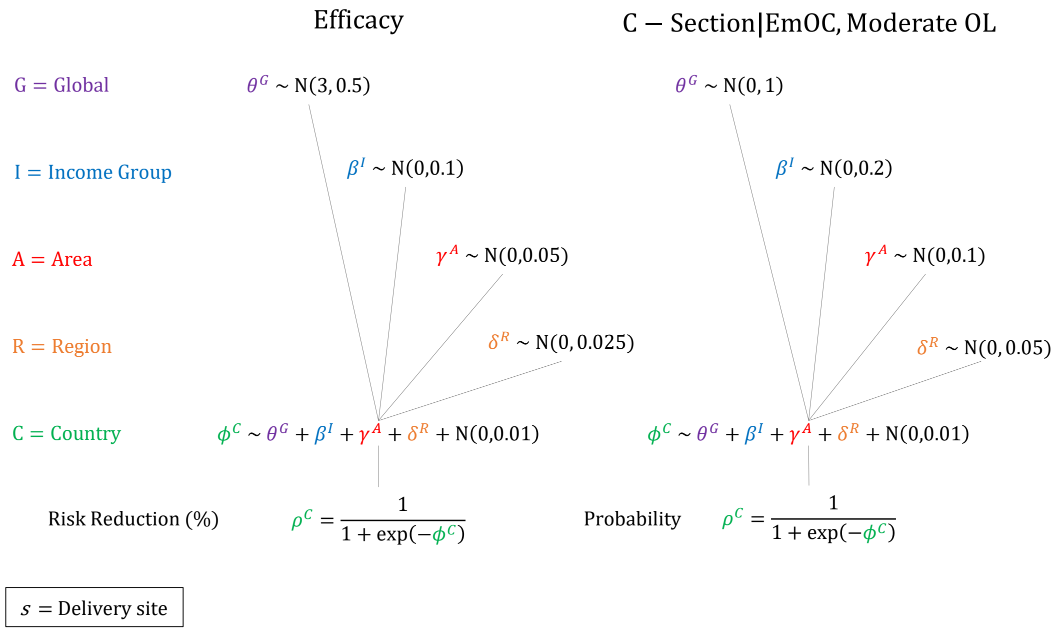

Priors

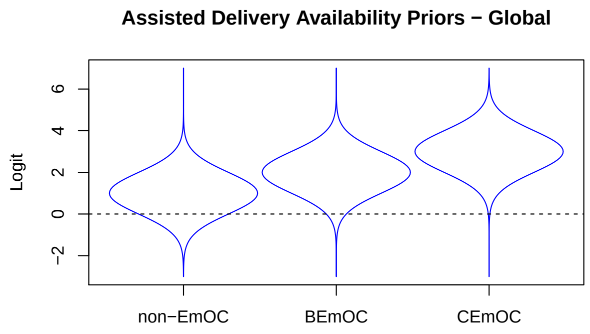

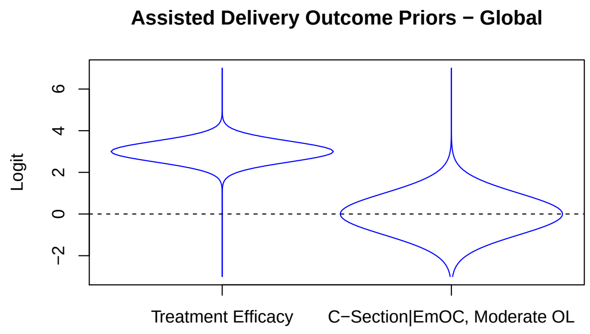

Model Implementation

Conditional on the delivery site we simulate the probabilities that treatment will be available and the efficacy of treatment on obstructed labour outcomes, accounting for quality of care.

References

Adam T, Lim SS, Mehta S, et al. Cost effectiveness analysis of strategies for maternal and neonatal health in developing countries. BMJ 2005; 331(7525): 1107. DOI: https://doi.org/10.1136/bmj.331.7525.1107

Afolabi BB, Lesi FE. Regional versus general anaesthesia for caesarean section. Cochrane Database Syst Rev 2012; 10: CD004350. DOI: https://doi.org/10.1002/14651858.cd004350.pub3

AMDD Working Group on Indicators. Averting Maternal Death and Disability. Program Note: Using UN process indicators to assess needs in emergency obstetric services: Pakistan, Peru and Vietnam. Int J Gynaecol Obstet 2002; 78(3): 275-82. DOI: https://doi.org/10.1016/s0020-7292(02)00190-x

AMDD Working Group on Indicators. Program Note: Using UN process indicators to assess needs in emergency obstetric services: Morocco, Nicaragua and Sri Lanka. Int J Gynaecol Obstet 2003; 80(2): 222-30. DOI: https://doi.org/10.1016/s0020-7292(02)00390-9

AMDD Working Group on Indicators. Program Note: Using UN process indicators to assess needs in emergency obstetric services: Benin and Chad. Int J Gynaecol Obstet 2004; 86(1): 110-20. DOI: https://doi.org/10.1016/j.ijgo.2004.04.034

Ameh CA, Weeks AD. The role of instrumental vaginal delivery in low resource settings. BJOG 2009; 116(S1): 22-25. DOI: https://doi.org/10.1111/j.1471-0528.2009.02331.x

Bailey PE, van Roosmalen J, Mola G, Evans C, de Bernis L, Dao B. Assisted vaginal delivery in low and middle income countries: an overview. BJOG 2017; 124: 1335–1344. DOI: https://doi.org/10.1111/1471-0528.14477

Bailey P, Paxton A, Lobis S, Fry D. The availability of life-saving obstetric services in developing countries: an in-depth look at the signal functions for emergency obstetric care. Int J Gynecol Obstet 2006; 93(3): 285–91. DOI: https://doi.org/10.1016/j.ijgo.2006.01.028

Chu K, Cortier H, Maldonado F, Mashant T, Ford N, Trelles M. Cesarean section rates and indications in sub-Saharan Africa: a multi-country study from Medecins sans Frontieres. PLoS One 2012; 7(9): e44484. DOI: https://doi.org/10.1371/journal.pone.0044484

Dodd JM, Anderson ER, Gates S, Grivell RM. Surgical techniques for uterine incision and uterine closure at the time of caesarean section. Cochrane Database Syst Rev 2014; 7: CD004732. DOI: https://doi.org/10.1002/14651858.cd004732.pub3

Majoko F, Gardener G. Trial of instrumental delivery in theatre versus immediate caesarean section for anticipated difficult assisted births. Cochrane Database Syst Rev 2012; 10: CD005545. DOI: https://doi.org/10.1002/14651858.cd005545.pub3

O'Mahony F, Hofmeyr GJ, Menon V. Choice of instruments for assisted vaginal delivery. Cochrane Database Syst Rev 2010; 11: CD005455. DOI: https://doi.org/10.1002/14651858.cd005455.pub2

Pasupathy D, Wood AM, Pell JP, Fleming M, Smith GCS. Time trend in the risk of delivery-related perinatal and neonatal death associated with breech presentation at term. Int J Epidemiol 2009; 38(2): 490-498. DOI: https://doi.org/10.1093/ije/dyn225

Pearson L, Shoo R. Availability and use of emergency obstetric services: Kenya, Rwanda, Southern Sudan, and Uganda. Int J Gynaecol Obstet 2005; 88(2): 208-15. DOI: https://doi.org/10.1016/j.ijgo.2004.09.027

Schuitemaker N, van Roosmalen J, Dekker G, van Dongen P, van Geijn H, Gravenhost JB. Maternal mortality after cesarean section in The Netherlands. Acta Obstet Gynecol Scand 1997; 76(4): 332-334. DOI: https://doi.org/10.1111/j.1600-0412.1997.tb07987.x

Souza JP, Gülmezoglu A, Lumbiganon P, et al. Caesarean section without medical indications is associated with an increased risk of adverse short-term maternal outcomes: the 2004–2008 WHO Global Survey on Maternal and Perinatal Health. BMC Med 2010; 8: 71. DOI: https://doi.org/10.1186/1741-7015-8-71

Yarrow C, Benoit G, Klein MC. Outcomes after vacuum-assisted deliveries. Births attended by community family practitioners. Can Fam Physician 2004; 50: 1109-14. Available at: https://www.cfp.ca/content/50/8/1109.long

A.1.6.8 Hemorrhage Treatment

Overview

Treatment options for hemorrhage include uterotonic drugs to increase muscle contractions (such as oxytocin and misoprostol) and surgical techniques (such as tying off or blocking the uterine artery) and blood transfusion (Mousa 2014). In addition, although it is not a uterotonic, tranexamic acid may reduce mortality due to bleeding from postpartum hemorrhage when given within the first three hours and may be considered as an adjuvant therapy (Evensen 2017). For women with severe, intractable hemorrhage hysterectomy provides a definitive treatment (Evensen 2017). While hysterectomy is often a last resort in high-income settings, in settings where many women are anaemic and blood supplies are limited, such as Africa and Asia, hysterectomy is often used as an early intervention to prevent death from exsanguination (WOMAN 2017).

Data

Oxytocin is the most effective treatment for PPH, even if already used for labor induction or augmentation or as part of active management (Evensen 2017), and is strongly recommended by the WHO (WHO 2012). Uterotonics can also be provided for women delivering at home. For example, a study in Indonesia found that in the majority of the PPH cases included in the study, uterotonic medication was started at home by the healthcare provider - in most cases, an IV line was established and the first dose of intramuscular oxytocin was provided before transportation to the third level of care (Rosales 2017). While uterotonics are often widely available, surgical intervention and blood transfusion are less available. For example, data from the India District Level Household Survey 2007-2008 reported that that 9.1% of community health centers have a blood storage facility (IIPS 2010).

An international randomised controlled trial of tranexamic acid found that death due to bleeding was significantly reduced in women given tranexamic acid (1.5% vs 1.9% in the placebo group, RR 0.81, 95% CI 0.65–1.00), especially for women given treatment within 3 hours of giving birth (1.2% in the tranexamic acid group vs 1.7% in the placebo group, RR 0.69, 95% CI 0.52–0.91) (WOMAN 2017).

Parameters

For women with PPH, we model the availability and efficacy of hemorrhage treatment, conditional on delivery site. We assume that the availability of treatment increases by site and income group, and that while uterotonics may be delivered at Home-SBA, surgical management and transfusion is not available at home. We therefore assume that 'severe' and 'most severe' PPH require surgical intervention and can only be treated at CEmOC facilities. We assume that treatment availability increases over time and so constrain the year coefficient to be non-negative when sampling. We assume that the efficacy of treatment is non-differential by severity, and apply the same risk reduction for all mortality and morbidity outcomes. We set efficacy priors centered near 75%.

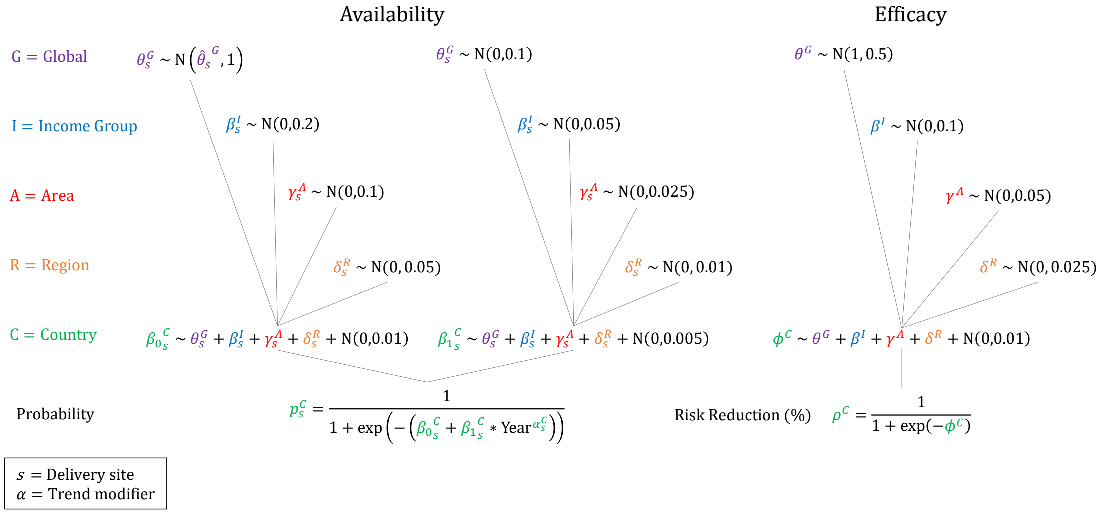

Priors

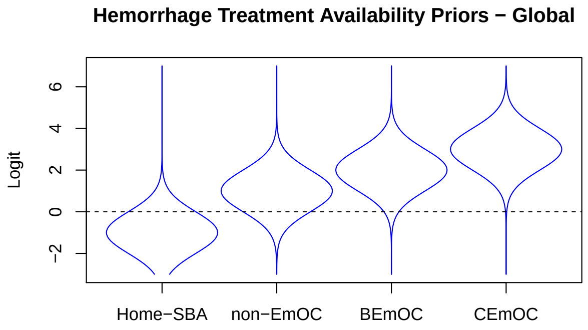

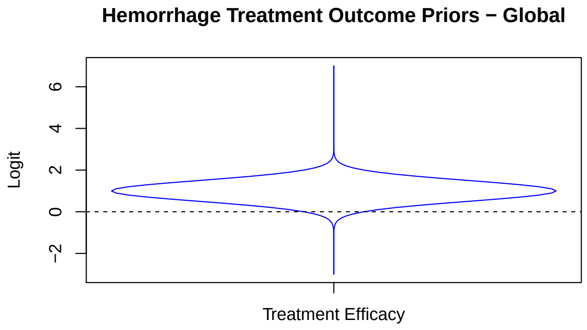

Model Implementation

Conditional on the delivery site we simulate the probabilities that treatment will be available and the efficacy of treatment on hemorrhage outcomes, accounting for quality of care. We assume that women with 'most severe' PPH can only be treated at CEmOC facilities and undergo a hysterectomy.

References

Evensen A, Anderson JM, Fontaine P. Postpartum Hemorrhage: Prevention and Treatment. Am Fam Physician 2017; 95(7): 442-449. Available at: https://www.aafp.org/afp/2017/0401/p442.html

International Institute for Population Sciences. India District Level Household Survey 2007-2008. Mumbai, India: International Institute for Population Sciences; 2010. Available: http://rchiips.org/PRCH-3.html.

Mousa HA, Blum J, Abou El Senoun G, Shakur H, Alfirevic Z. Treatment for primary postpartum haemorrhage. Cochrane Database Syst Rev 2014; 2: CD003249. DOI: https://doi.org/10.1002/14651858.cd003249.pub3

Rosales A, Sulistyo S, Miko O, et al. Recognition of and care-seeking for maternal and newborn complications in Jayawijaya district, Papua province, Indonesia: a qualitative study. J Health Popul Nutr 2017; 36(Suppl1): 44. DOI: https://doi.org/10.1186/s41043-017-0122-0

World Health Organization. WHO recommendations for the prevention and treatment of postpartum haemorrhage. 2012. Available at: https://www.who.int/reproductivehealth/publications/maternal_perinatal_health/9789241548502/en/

WOMAN Trial Collaborators. Effect of early tranexamic acid administration on mortality, hysterectomy, and other morbidities in women with post-partum haemorrhage (WOMAN): an international, randomised, double-blind, placebo-controlled trial. Lancet 2017; 389(10084): 2105-2116. DOI: https://doi.org/10.1016/s0140-6736(17)30638-4

A.1.6.9 Antibiotics

Overview

The mainstay of treating maternal sepsis is antibiotics, and we model the use of antibiotics in response to infection, rather than as a preventive measure. A Cochrane review found that while data are scarce, prophylactic antibiotics appear to be effective in reducing ill-health caused by infections in women who undergo operative vaginal delivery (Liabsuetrakul 2020). While the use of antibiotics for women undergoing caesarean section was found to reduce the occurrence of infection (Smaill 2014), we assume that they are part of routine care for C-sections, and so do not explicitly model the use of prophylactic antibiotics. With the sharp decrease in maternal morbidity observed with the introduction of the antibiotic era, it is now accepted that antibiotic treatment is warranted for postpartum infection (Mackeen 2015).

Data

A Cochrane review evaluated the comparative efficacy and side effects of different antibiotic regimens for postpartum endometritis, finding that intravenous clindamycin plus gentamicin is more effective than other antibiotics or combinations of antibiotics for treatment of womb infection after childbirth (Mackeen 2015). While we could not find empirical estimates of the impact of antibiotic treatment on maternal mortality compared to no treatment, a previous modelling study assumed a case fatality rate for sepsis of 17% without antibiotics vs 2.1% if antibiotics are taken, implying an efficacy of nearly 90% on reducing mortality, although the authors acknowledged that these parameters were the most difficult to estimate (Pagel 2009).

Parameters

For women who develop sepsis, we model the availability and efficacy of antibiotic treatment, conditional on delivery site. We assume that the availability of treatment increases by site and income group, and that treatment is not available at home. We assume that the efficacy of treatment is non-differential by severity, and apply the same risk reduction for all mortality and morbidity outcomes. We set efficacy priors centered near 90%.

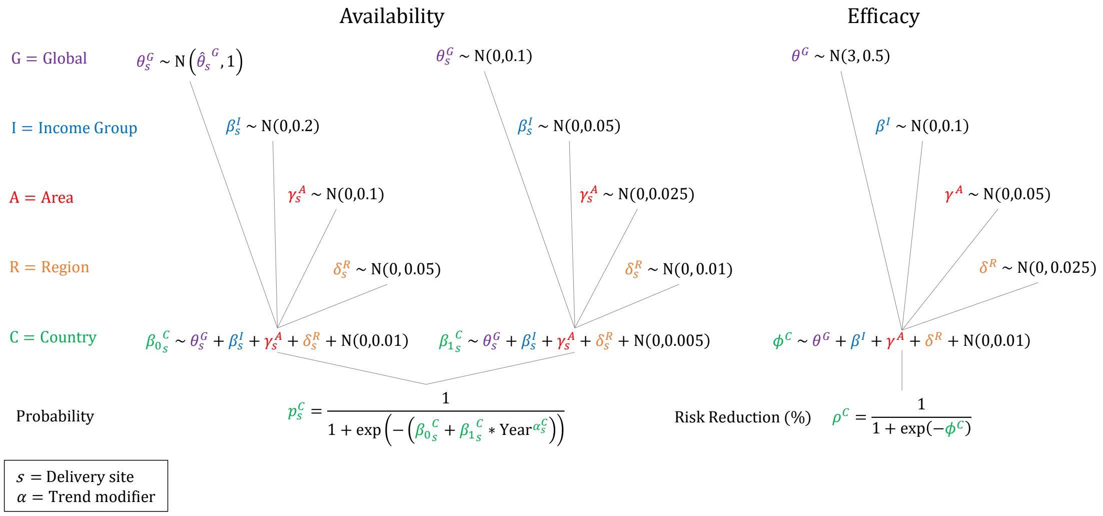

$$\theta_s^G \sim N(\hat{\theta}_s^{\,G}, 1) \quad \beta_s^I \sim N(0,0.2) \quad \gamma_s^A \sim N(0,0.1) \quad \delta_s^R \sim N(0, 0.05)$$

$${\beta_0}_s^C \sim \theta_s^G + \beta_s^I + \gamma_s^A + \delta_s^R + N(0,0.01)$$

$$\theta_s^G \sim N(0,0.1) \quad \beta_s^I \sim N(0,0.05) \quad \gamma_s^A \sim N(0,0.025) \quad \delta_s^R \sim N(0, 0.01)$$

$${\beta_1}_s^C \sim \theta_s^G + \beta_s^I + \gamma_s^A + \delta_s^R + N(0,0.005)$$

$$p_s^C = \frac{1}{1 + \exp\left(-\left({\beta_0}_s^C + {\beta_1}_s^C * \text{Year}^{\alpha_s^C}\right)\right)}$$

$$\theta^G \sim N(3, 0.5) \quad \beta^I \sim N(0,0.1) \quad \gamma^A \sim N(0,0.05) \quad \delta^R \sim N(0, 0.025)$$

$$\phi^C \sim \theta^G + \beta^I + \gamma^A + \delta^R + N(0,0.01)$$

$$\rho^C = \frac{1}{1 + \exp(-\phi^C)}$$

Priors

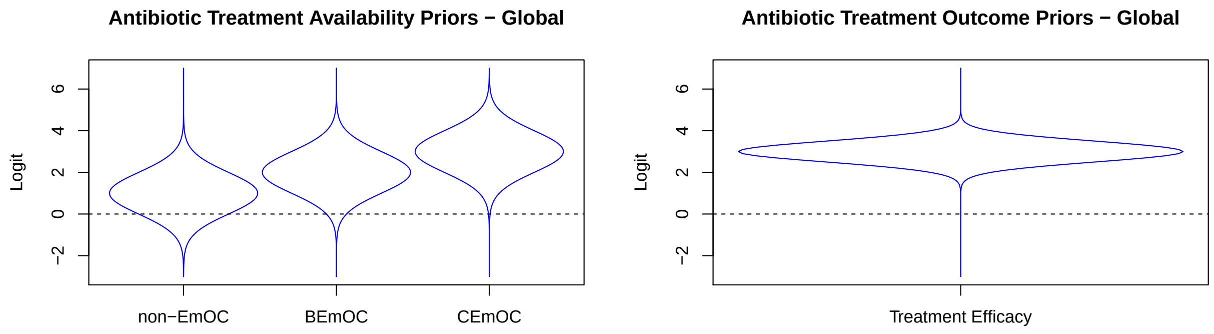

Model Implementation

Conditional on the delivery site we simulate the probabilities that treatment will be available and the efficacy of treatment on sepsis outcomes, accounting for quality of care.

References

Liabsuetrakul T, Choobun T, Peeyananjarassri K, Islam QM. Antibiotic prophylaxis for operative vaginal delivery. Cochrane Database Syst Rev 2020; 3(3): CD004455. DOI: https://doi.org/10.1002/14651858.cd004455.pub5

Mackeen AD, Packard RE, Ota E, Speer L. Antibiotic regimens for postpartum endometritis. Cochrane Database Syst Rev 2015; 2: CD001067. DOI: https://doi.org/10.1002/14651858.cd001067.pub3

Pagel C, Lewycka S, Colbourn T, et al. Estimation of potential effects of improved community-based drug provision, to augment health-facility strengthening, on maternal mortality due to post-partum haemorrhage and sepsis in sub-Saharan Africa: an equity-effectiveness model. Lancet 2009; 374(9699): 1441-8. DOI: https://doi.org/10.1016/s0140-6736(09)61566-x

Smaill FM, Grivell RM. Antibiotic prophylaxis versus no prophylaxis for preventing infection after cesarean section. Cochrane Database Syst Rev 2014; 10: CD007482. DOI: https://doi.org/10.1002/14651858.cd007482.pub3

A.1.7 GMatH Pseudocode

```
//Simulate each country - all parameters are country-specific

//Initialize population trends for each country
Initialize population projections //(see Appendix A.1.1.2: Population Projections)
Initialize education projections //(see Appendix A.1.1.5 Education Projections)

//Initialize individual-level characteristics at model startup
For each woman:
    Assign subgroup (urban/rural location + education level) | Birth year
    Assign age of sexual debut | Subgroup //(see Appendix A.1.3.1 Age of Sexual Debut)
    Assign age of menopause //(see Appendix A.1.2.9 Menopause)
    Assign hemoglobin latent variable //~Norm(0,1) (see Appendix A.1.2.10 Anemia)
    Assign desired number of children | Subgroup, Year /*(see Appendix A.1.3.3
                                                       Desired Number of Children)*/
    Initialize current living children | Subgroup, Age, Year /*(see Appendix A.1.3.2
                                                             Number of Living Children)*/
    Initialize history of previous miscarriages | Current living children
    //~Pois(0.2*numLivingKids) assume 20% risk of historic miscarriage

//Simulate population going forward
for(t=0; t<numCycles; t++){ //monthly cycle length
    Add incoming females to population //(see Appendix A.1.1.2: Population Projections)

    for(w=0; w<numWomen; w++){ //For each woman currently in the population
        Risk background mortality | Age, Year (remove risk of maternal deaths)
        //(see Appendices A.1.1.3: Lifetables, A.1.5.7 Indirect Maternal Deaths)
        Risk indirect maternal mortality (ages 15-49) | Year
          (remove accidental/incidental deaths)
        /*(see Appendices A.1.5.7 Indirect Maternal Deaths,
          A.1.1.4 Proportion of Deaths due to Injury)*/
        Risk incidence of late maternal event (if >2 months and <=1 year of last delivery)
        //(see Appendix A.1.5.6 Late Maternal Deaths)
        if(lateEvent==true){ //has late maternal event
            simulateReferral() //Simulate facility referral, see function below
            Risk late maternal death | Site, Quality, Year /*(see Appendices A.1.5.6
                                                              Late Maternal Deaths,
                                                              A.1.4.6 Quality of Care)*/
        }
        If woman died, simulate if reported | Site, Year /*(see Appendix A.1.4.7
                                                         Maternal Death Underreporting)*/

        if alive{
            Risk child mortality for each living child | Age, Sex, Year
            //(see Appendix A.1.1.3 Lifetables)

            //check for sexual debut (see Appendix 1.3.1 Age of Sexual Debut)
            if(sexuallyActive==false & curAge>=ageAtSexualDebut){
                sexuallyActive=true
                assignContraception() /*Assign contraception status + method,
                                        see function below*/
            }
```

```
//check for menopause (see Appendix A.1.2.9 Menopause)
if(menopause==false & curAge>=ageAtMenopause){
    menopause=true
}

//If fecund (sexually active, not currently pregnant, pre-menopausal)
if(pregnant==false & sexuallyActive==true & menopause==false){

    //risk pregnancy
    updateContraception() //Update contraception status, see function below
    Update breastfeeding (LAM) status | Time since delivery, # living infants
    //(see Appendix A.1.3.10 Breastfeeding)
    Risk conception | Age, LAM, Contraception method(subgroup)
    /*(see Appendices A.1.2.1 Natural Fecundity/Fertility,
      A.1.2.8 Lactational Amenorrhea (LAM),
      A.1.3.6 Contraception Failure Rates)*/
    if(conception==true){ //becomes pregnant
        Assign ANC visits | Subgroup, Year /*(see Appendix A.1.4.1
                                              Antenatal Care)*/
        Assign anemia (Hb) | Subgroup, Year, ANC /*(see Appendix A.1.2.10
                                                    Anemia*/
        Simulate number of fetuses | Maternal age /*(see Appendix A.1.2.3
                                                     Twinning Rates)*/
        Assign sex for each fetus /(see Appendix A.1.2.2 Sex Ratio - Primary)
        //Pre-draw pregnancy outcome and time to event
        Simulate risk of elective abortion if unwanted pregnancy |
          Subgroup, Year, Fetus sex, Secondary sex ratio(Subgroup,Year)
          //(see Appendices A.1.3.8 Abortion, A.1.3.9 Sex Ratio - Secondary)
        /*risk ectopic pregnancy, miscarriage, stillbirth
          (estimates are conditional on no induced abortion)*/
        if(abortion==false){
            /*Note: Ectopic pregnancy, miscarriage, and stillbirth are
          modelled as mutually exclusive events*/
            Simulate risk of ectopic pregnancy |
            Maternal age, History of ectopic pregnancy /*(see Appendix
                                                   A.1.2.5 Ectopic Pregnancy)*/
            Simulate risk of miscarriage |
            Maternal age, History of miscarriage /*(see Appendix A.1.2.4
                                                  Miscarriage)*/
            Simulate risk of antepartum stillbirth | Maternal age, Fetus sex
            //(see Appendix A.1.2.6 Stillbirths)
            Draw time to event //(see Appendix A.1.2.7 Month of Delivery)
        }
    }

    //if currently pregnant and time for pregnancy outcome
    if(pregnant==true & t==eventTime){
        //pregnancy loss
        if(outcome=='abortion'){ /*elective abortion (see Appendix A.1.3.8
                                                        Abortion)*/
            Simulate abortion type ('safe'/'unsafe') | Subgroup, Year
            Risk maternal death | Abortion type, Anemia
            If woman survives, risk infertility | Abortion type
```

```
}
else if(outcome=='miscarriage'){ /*miscarriage (see Appendix A.1.2.4
                                     Miscarriage)*/
    Update miscarriage history
}
else if(outcome=='ectopic'){ /*ectopic pregnancy (see Appendix A.1.2.5
                                     Ectopic Pregnancy)*/
    Risk rupture
    if(rupture){
        simulateReferral() /*Simulate facility referral,
                             see function below*/
        Risk maternal death |
        Site, Treatment Availability(Year)+Efficacy, Quality(Year)
        /*(see Appendices A.1.6.5 Management of Ectopic Pregnancy,
           A.1.4.6 Quality of Care)*/
    }
}

//pregnancy is carried to delivery
if(outcome=='delivery'){
    Simulate starting delivery site | Subgroup, Year
    //(see Appendix A.1.4.2 Starting Delivery Site)
    Simulate elective c-section | Subgroup, Year
      //(see Appendix A.1.6.1 Elective Caesarean Section)

    //risk delivery complications
    /*Preeclampsia/Eclampsia (see Appendix A.1.5.1
                             Preeclampsia/Eclampsia)*/
    if(electiveCSection==false){
        Risk PE/E incidence | Maternal age, Severe anemia,
                            Multiple gestation, History of PE/E
        if(hasComplication){
            simulateReferral() /*Simulate facility referral,
                                 see function below*/
            Risk maternal death | Severity, Site,
             Treatment Availability(Year)+Efficacy, Quality(Year)
          /*(see Appendices A.1.6.6 Hypertension Management,
             A.1.4.6 Quality of Care)*/
            if(alive){ //woman survives
                Risk morbidity (subsequent infertility)
                Risk intrapartum stillbirth
            }
        }
    }
    //Obstructed labour (see Appendix A.1.5.2 Obstructed Labor)
    if(alive==true & electiveCSection==false){
        Risk OL incidence | Maternal Age,
      Partograph Availability(Year)+Efficacy, Quality(Year)
      //(see Appendices A.1.6.3 Partograph, A.1.4.6 Quality of Care)
        if(hasComplication){
            simulateReferral() /*Simulate facility referral,
                                 see function below*/
            Risk maternal death | Severity, Site,
```

```
            Treatment Availability(Year)+Efficacy, Quality(Year)
            /*(see Appendices A.1.6.7 Assisted Delivery,
               A.1.4.6 Quality of Care)*/
              if(alive){ //woman survives
                  Risk morbidity (fistula, assume subsequent infertility)
                  Risk intrapartum stillbirth
              }
          }
      }
      //Other direct (see Appendix A.1.5.5 Other Direct Complications)
      if(alive==true){
          Risk other direct incidence
          if(hasComplication){
              simulateReferral() /*Simulate facility referral,
                                   see function below*/
              Risk maternal death | Site, Quality(Year)
            //(see Appendix A.1.4.6 Quality of Care)
          }
      }
      /*Postpartum hemorrhage (see Appendix A.1.5.3
                                 Postpartum Hemorrhage)*/
      if(alive==true & electiveCSection==false){
          Risk PPH incidence |
          Active Management Availability(Year)+Efficacy, Quality(Year)
          /*(see Appendices A.1.6.2 Active Management of Labor,
             A.1.4.6 Quality of Care)*/
          if(hasComplication){
              simulateReferral() /*Simulate facility referral,
                                   see function below*/
              Risk maternal death | Severity, Anemia, Site,
              Treatment Availability(Year)+Efficacy, Quality(Year)
              /*(see Appendices A.1.2.10 Anemia,
                 A.1.6.8 Hemorrhage Treatment,
                 A.1.4.6 Quality of Care)*/
              if(alive){ //woman survives
                  Risk morbidity (assume subsequent infertility)
                  Simulate hemoglobin loss
              }
          }
      }
      //Sepsis (see Appendix A.1.5.4 Sepsis)
      if(alive==true){
          Risk sepsis incidence | C-section,
          Clean Delivery Availability(Year)+Efficacy, Quality(Year)
        //(see Appendix A.1.6.4 Clean delivery, A.1.4.6 Quality of Care)
          if(hasComplication){
              simulateReferral() /*Simulate facility referral,
                                   see function below*/
              Risk maternal death | Severity, Anemia, Site,
              Treatment Availability(Year)+Efficacy, Quality(Year)
              /*(see Appendices A.1.2.10 Anemia, A.1.6.9 Antibiotics,
                 A.1.4.6 Quality of Care)*/
              if(alive){ //woman survives
```

```
                                    Risk morbidity (secondary infertility)
                                }
                            }
                        }

                        if(woman is alive & fetus(es) is/are alive){
                            Record birth(s)
                            Add new infant child(ren)
                            Simulate breastfeeding status (exclusive/non-exclusive)
                              and duration //(see Appendix A.1.3.10 Breastfeeding)
                        }
                    }

                    if(alive==false){ //woman died
                        Simulate if maternal death is reported | Site, Year
                          //(see Appendix A.1.4.7 Maternal Death Underreporting)
                    }
                    else{ //woman is alive
                        assignContraception() /*Assign contraception status + method,
                                                    see function below*/
                    }

                } //end pregnancy outcome

            } //end if fecund
        } //end if alive
    } //end woman loop
} //end cycle loop

//Simulates referral to a healthcare facility
function simulateReferral(){
    Simulate recognition | Site, Complication (type+severity), Subgroup, ANC (home only)
    //(see Appendices A.1.4.3 Recognition/Referral, A.1.4.1 Antenatal Care)
    if(recognized){
        Simulate transport availability | Site, Subgroup /*(see Appendix A.1.4.4
                                                             Transportation)*/
        if(transportAvailable){
            Simulate referral site | Current site, Subgroup /*(see Appendix A.1.4.5
                                                                 Referral Facility)*/
            Update current site
        }
    }
}

//Assigns contraception status and method
function assignContraception(){
    if(numCurrentChildren < numDesiredChildren){ //spacing
        Draw spacing intention | Subgroup, Age, Year //(see Appendix A.1.3.4 Unmet Need)
        if(desiresBirthSoon){
            No need for contraception (no method assigned)
        }
        else{ //wants to space children
            Check if spacing need is met | Subgroup, Age, Year /*(see Appendix A.1.3.4
```

```
                                                                    Unmet Need)*/
            if(needMet){
                Assign contraception method | Subgroup, Spacing, Year
                  //(see Appendix A.1.3.5 Contraception Method Mix)
                Assign intended duration of use | Method, Subgroup
                  //(see Appendix A.1.3.7 Method Duration of Use)
            }
            else{ //unmet need
                No method assigned
            }
        }
    }
    else{ //limiting (met or exceeded desired number of children)
        Check if limited need is met | Subgroup, Age, Year /*(see Appendix A.1.3.4
                                                                Unmet Need)*/
        if(needMet){
            Assign contraception method | Subgroup, Limiting, Year
              //(see Appendix A.1.3.5 Contraception Method Mix)
            Assume indefinite use of method
        }
        else{ //unmet need
            No method assigned
        }
    }
}

//Updates contraception status
function updateContraception(){
    if(usingContraception & timeUse==duration){ //check if duration of use has elapsed
        Simulate if woman discontinues all methods or switches to another method
          //(see Appendix A.1.3.7 Method Duration of Use)
        if(switchesMethods){
            Assign new contraception method | Subgroup, Spacing, Year
              //(see Appendix A.1.3.5 Contraception Method Mix)
            Assign new intended duration of use | Method, Subgroup
              //(see Appendix A.1.3.7 Method Duration of Use)
        }
        else{
            Discontinues all methods (no method assigned)
        }
    }
}
```

A.2 Datasets and Definitions

A.2.1 DHS Datasets

We pooled all Demographic and Health Survey (DHS) datasets that were available as of January 1, 2020. More information on the DHS Program is available here: https://dhsprogram.com/. We accounted for the complex survey design of each survey when performing analyses. Data were available for 4,654,705 women from 322 surveys in 83 countries.

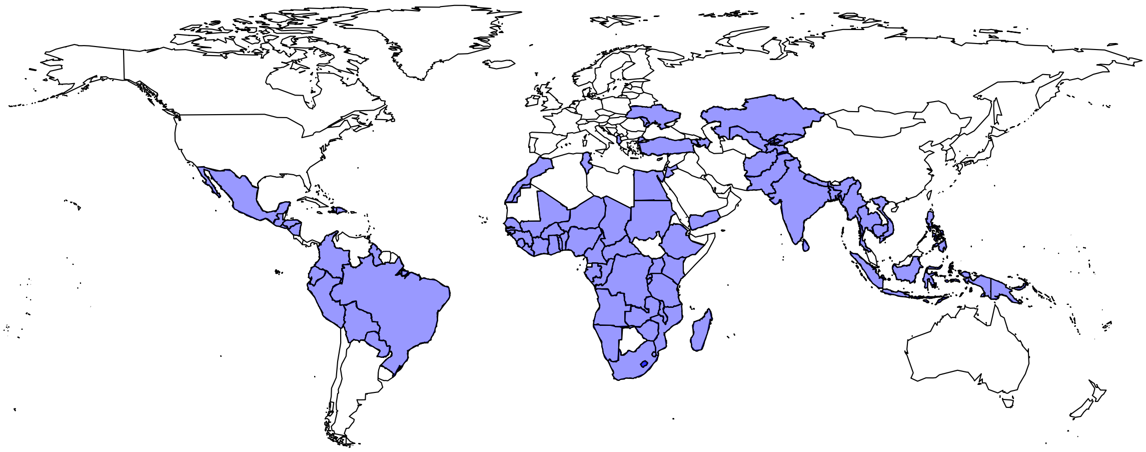

Country	DHS Years
Afghanistan	2015
Albania	2008-2009, 2017-2018
Angola	2006-2007, 2011, 2015-2016
Armenia	2000, 2005, 2010, 2015-2016
Azerbaijan	2006
Bangladesh	1993-1994, 1996-1997, 1999-2000, 2004, 2007, 2011, 2014
Benin	1996, 2001, 2006, 2011-2012, 2017-2018
Bolivia	1989, 1993-1994, 1998, 2003-2004, 2008
Brazil	1986, 1991-1992, 1996
Burkina Faso	1992-1993, 1998-1999, 2003, 2010, 2014, 2017-2018
Burundi	1987, 2010-2011, 2012-2013, 2016-2017
Cambodia	2000, 2005-2006, 2010-2011, 2014
Cameroon	1991, 1998, 2004, 2011
Central African Republic	1994-1995
Chad	1996-1997, 2004, 2014-2015
Colombia	1986, 1990, 1995, 2000, 2004-2005, 2009-2010, 2015-2016
Comoros	1996, 2012
Congo	2005, 2009, 2011-2012
Congo Democratic Republic	2007, 2013-2014
Cote d'Ivoire	1994, 1998-1999, 2005, 2011-2012
Dominican Republic	1986, 1991, 1996, 1999, 2002, 2007, 2007, 2013, 2013
Ecuador	1987
Egypt	1988-1989, 1992-1993, 1995-1996, 2000, 2003, 2005, 2008, 2014
El Salvador	1985
Ethiopia	2000, 2005, 2011, 2016
Gabon	2000-2001, 2012
Gambia	2013
Ghana	1988, 1993-1994, 1998-1999, 2003, 2008, 2014, 2016
Guatemala	1987, 1995, 1998-1999, 2014-2015
Guinea	1999, 2005, 2012, 2018
Guyana	2005, 2009
Haiti	1994-1995, 2000, 2005-2006, 2012, 2016-2017
Honduras	2005-2006, 2011-2012
India	1992-1993, 1998-2000, 2005-2006, 2015-2016
Indonesia	1987, 1991, 1994, 1997, 2002-2003, 2007, 2012, 2017
Jordan	1990, 1997, 2002, 2007, 2009, 2012, 2017-2018
Kazakhstan	1995, 1999

Country	DHS Years
Kenya	1988-1989, 1993, 1998, 2003, 2008-2009, 2014, 2015
Kyrgyz Republic	1997, 2012
Lesotho	2004-2005, 2009-2010, 2014
Liberia	1986, 2006-2007, 2008-2009, 2011, 2013, 2016
Madagascar	1992, 1997, 2003-2004, 2008-2009, 2011, 2013, 2016
Malawi	1992, 2000, 2004-2005, 2010, 2012, 2014, 2015-2016, 2017
Maldives	2009, 2016-2017
Mali	1987, 1995-1996, 2001, 2006, 2012-2013, 2015, 2018
Mexico	1987
Moldova	2005
Morocco	1987, 1992, 2003-2004
Mozambique	1997, 2003-2004, 2009, 2011, 2015, 2018
Myanmar	2015-2016
Namibia	1992, 2000, 2006-2007, 2013
Nepal	1995-1996, 2000-2001, 2005-2006, 2010-2011, 2016
Nicaragua	1997-1998, 2001
Niger	1992, 1998, 2006, 2012
Nigeria	1990, 2003, 2008, 2010, 2013, 2015, 2018
Pakistan	1990-1991, 2006-2007, 2012-2013, 2017-2018
Papua New Guinea	2016-2018
Paraguay	1990
Peru	1986, 1991-1992, 1996, 2000, 2003-2008, 2009, 2010, 2011, 2012
Philippines	1993, 1998, 2003, 2008, 2013, 2017
Rwanda	1992, 2000, 2005, 2007-2008, 2010-2011, 2013, 2014-2015, 2017
Sao Tome and Principe	2008-2009
Senegal	1986, 1992-1993, 1997, 2005, 2006, 2008-2009, 2010-2011, 2012-2013, 2012-2014, 2014, 2015, 2016, 2017
Sierra Leone	2008, 2013, 2016
South Africa	1998, 2016
Sri Lanka	1987
Sudan	1989-1990
Eswatini	2006-2007
Tajikistan	2012, 2017
Tanzania	1991-1992, 1996, 1999, 2003-2004, 2004-2005, 2007-2008, 2009-2010, 2011-2012, 2015-2016, 2017
Thailand	1987
Timor-Leste	2009-2010, 2016
Togo	1988, 1998, 2013-2014, 2017
Trinidad and Tobago	1987
Tunisia	1988
Turkey	1993, 1998, 2003-2004, 2008, 2013
Uganda	1988-1989, 1995, 2000-2001, 2006, 2009-2010, 2011, 2011, 2014-2015, 2016
Ukraine	2007
Uzbekistan	1996
Vietnam	1997, 2002, 2005
Yemen	1991-1992, 2013
Zambia	1992, 1996-1997, 2001-2002, 2007, 2013-2014
Zimbabwe	1988-1989, 1994, 1999, 2005-2006, 2010-2011, 2015

A.2.2 Parameter Types

A.2.2.1 Splines

B-splines play the role of basis functions, so that any function in the space can be expressed as a linear combination of B-splines.

$$f(x) = \sum_i \alpha_i B_i(\hat{x})$$

where α_i is the height coefficient for spline i, $\hat{x}$ is the rescaled position $\hat{x} = \frac{x - k_i - \frac{1}{2}\omega}{\omega}$ where k_i is the position of knot i and ω is the spline width. The basis spline function is given by

$$B(x) = \left\{ \begin{array}{ll} \dfrac{x^2}{2} & 0 < x \leq 1 \\ \dfrac{-2x^2 + 6x - 3}{2} & 1 < x \leq 2 \\ \dfrac{x^2 - 6x + 9}{2} & 2 < x \leq 3 \\ 0 & x \leq 0 \text{ and } x > 3 \end{array} \right\}$$

A.2.2.2 Trend Modifiers

Some model parameters include time trends (e.g. with a year variable) or age trends using logistic (or sometimes linear) regression models: $\beta_0 + \beta_1 * x$. To account for the potential of non-linear trends we include an exponent α to modify the trend variable: $\beta_0 + \beta_1 * x^\alpha$ which is fit via calibration. We constrain $\alpha \leq 1$ to ensure that the trend relationship is either linear ($\alpha = 1$) or concave ($\alpha < 1$) to model potential attenuation of trends over time (or by age). This approach maintains a monotonic trend relationship while helping to guard against extrapolating to unreasonable levels.

A.2.3 Maternal Death ICD Codes

We used the following ICD codes to identify maternal deaths in the WHO Mortality Database, a compilation of mortality data by age, sex, and cause of death, as reported annually by countries from their civil registration systems. Mortality data are available here: https://www.who.int/data/data-collection-tools/who-mortality-database.

A.2.3.1 ICD-8

Code	Description
A112	Toxaemias of pregnancy and the puerperium
A113	Haemorrhage of pregnancy and childbirth
A114	Abortion induced for legal indications
A115	Other and unspecified abortion
A116	Sepsis of childbirth and the puerperium
A117	Other complications of pregnancy, childbirth and the puerperium
A118	Delivery without mention of complication

A.2.3.2 ICD-9

Code	Subcode	Description
B38		ABORTION
	B380	Spontaneous abortion
	B381	Legally induced abortion
	B382	Illegally induced abortion
	B389	Remainder of B38
B39		DIRECT OBSTETRIC CAUSES
	B390	Haemorrhage of pregnancy and childbirth
	B391	Toxaemia of pregnancy
	B392	Infections of genitourinary tract in pregnancy
	B393	Obstructed labour
	B394	Complications of the puerperium
	B399	Remainder of B39
B40		INDIRECT OBSTETRIC CAUSES
B41		NORMAL DELIVERY

In addition to the ICD9 BTL codes listed above, additional death codes are uniquely used for Armenia, Belarus, Kazakhstan, Kyrgyzstan, Latvia, Lithuania, Russian Federation, Ukraine and the former USSR for some years: CH11 = Obstetric Complications.

A.2.3.3 ICD-10

Maternal deaths:

Code	Description
N96	Habitual aborter
N98	Complications associated with artificial fertilization
O00-O99	Pregnancy, childbirth and the puerperium
1087	Summary code for O00-O99 Pregnancy, childbirth and the puerperium
1088	Summary code for O00-O07 Pregnancy with abortive outcome
1089	Summary code for O10-O92 Other direct obstetric deaths
1090	Summary code for O98-O99 Indirect obstetric deaths

Code	Description
1091	O95-O97 Remainder of pregnancy, childbirth and the puerperium

We also categorized maternal deaths by cause, detailed below:
- Abortion, ectopic pregnancy, miscarriage
- Hypertensive disorders
- Hemorrhage
- Sepsis and other infections
- Obstructed labor
- Other direct
- Late maternal deaths
- Indirect

Abortion, ectopic pregnancy, miscarriage

ICD-10 Code	Description
N96	Habitual aborter
O00	Ectopic pregnancy
O01	Hydatidiform mole
O02	Other abnormal products of conception
O03	Spontaneous abortion
O04	Medical abortion
O05	Other abortion
O06	Unspecified abortion
O07	Failed attempted abortion

Hypertensive disorders

ICD-10 Code	Description
O10	Pre-existing hypertension complicating pregnancy, childbirth and the puerperium
O11	Pre-eclampsia superimposed on chronic hypertension
O12	Gestational [pregnancy-induced] oedema and proteinuria without hypertension
O13	Gestational [pregnancy-induced] hypertension
O14	Pre-eclampsia
O15	Eclampsia
O16	Unspecified maternal hypertension

Hemorrhage

ICD-10 Code	Description
O20	Haemorrhage in early pregnancy
O44	Placenta praevia
O45	Premature separation of placenta [abruptio placentae]
O46	Antepartum haemorrhage, not elsewhere classified
O62	Abnormalities of forces of labour
O67	Labour and delivery complicated by intrapartum haemorrhage, not elsewhere classified
O72	Postpartum haemorrhage

Sepsis and other infections

ICD-10 Code	Description
O23	Infections of genitourinary tract in pregnancy
O85	Puerperal sepsis
O86	Other puerperal infections
O91	Infections of breast associated with childbirth

Obstructed labor

ICD-10 Code	Description
O32	Maternal care for known or suspected malpresentation of fetus
O33	Maternal care for known or suspected disproportion
O63	Long labour
O64	Obstructed labour due to malposition and malpresentation of fetus
O65	Obstructed labour due to maternal pelvic abnormality
O66	Other obstructed labour
O71	Other obstetric trauma

Other direct

ICD-10 Code	Description
N98	Complications associated with artificial fertilization
O21	Excessive vomiting in pregnancy
O22	Venous complications and haemorrhoids in pregnancy
O26	Maternal care for other conditions predominantly related to pregnancy
O28	Abnormal findings on antenatal screening of mother
O29	Complications of anaesthesia during pregnancy
O30	Multiple gestation
O31	Complications specific to multiple gestation
O34	Maternal care for known or suspected abnormality of pelvic organs
O35	Maternal care for known or suspected fetal abnormality and damage
O36	Maternal care for other known or suspected fetal problems
O40	Polyhydramnios
O41	Other disorders of amniotic fluid and membranes
O42	Premature rupture of membranes
O43	Placental disorders
O47	False labour
O48	Prolonged pregnancy
O60	Preterm labour and delivery
O61	Failed induction of labour
O68	Labour and delivery complicated by fetal stress [distress]
O69	Labour and delivery complicated by umbilical cord complications
O70	Perineal laceration during delivery
O73	Retained placenta and membranes, without haemorrhage
O74	Complications of anaesthesia during labour and delivery
O75	Other complications of labour and delivery, not elsewhere classified
O87	Venous complications and haemorrhoids in the puerperium
O88	Obstetric embolism
O89	Complications of anaesthesia during the puerperium
O90	Complications of the puerperium, not elsewhere classified

ICD-10 Code	Description
O92	Other disorders of breast and lactation associated with childbirth
O95	Obstetric death of unspecified cause

Late maternal deaths

ICD-10 Code	Description
O96	Death from any obstetric cause occurring more than 42 days but less than one year after delivery
O97	Death from sequelae of obstetric causes

Indirect

ICD-10 Code	Description
O24	Diabetes mellitus in pregnancy
O25	Malnutrition in pregnancy
O98	Maternal infectious and parasitic diseases classifiable elsewhere but complicating pregnancy, childbirth and the puerperium
O99	Other maternal diseases classifiable elsewhere but complicating pregnancy, childbirth and the puerperium

A.2.4 Maternal Health Indicators

We calculate a number of maternal health indicators in the GMatH model, described below. All indicators are reported per year.

Fertility Indicators

Births: Number of live births

Total Fertility Rate (TFR): Age-standardized fertility rates, calculated among women ages 15-49. TFR = $\sum_{a=15}^{49} \frac{\text{births}_a}{\text{women}_a}$

Contraceptive Prevalence Rate (CPR): Contraceptive use (any method) among women ages 15-49

Contraceptive Method Mix: Prevalence of use of each contraceptive method among women using any method

Twinning Rate: Number of twins per 1,000 deliveries

Pregnancy Outcomes: Proportion of pregnancies that end in miscarriage, elective abortion, or ectopic pregnancy

Process Indicators

Stillbirth Rate: Number of stillbirths per 1,000 births

Anemia Prevalence: Prevalence of overall, mild, moderate, and severe anemia among pregnant women

Facility Deliveries: Percentage of deliveries which take place in a health facility (non-EmOC, BEmOC, or CEmOC)

Delivery Site: Percentage of deliveries which take place at each delivery site (Home, Home-SBA, non-EmOC, BEmOC, CEmOC)

C-Section Rate: Percentage of deliveries which take place via caesarean section (elective or emergency)

Delivery Mode: Percentage of deliveries which take place via each delivery mode (spontaneous vaginal delivery, assisted vaginal delivery, elective c-section, emergency c-section)

Mortality Indicators

Maternal Deaths: Number of maternal deaths per year. According to the WHO, a maternal death is "the death of a woman while pregnant or within 42 days of termination of pregnancy, irrespective of the duration and site of the pregnancy, from any cause related to or aggravated by the pregnancy or its management but not from accidental or incidental causes"

Maternal Mortality Ratio (MMR): Number of maternal deaths per 100,000 live births

Pregnancy Mortality Ratio: Number of pregnancy-related deaths per 100,000 live births. Pregnancy-related death is defined as the death of a woman while pregnant or within 42 days of termination of pregnancy, irrespective of the cause of death. Pregnancy-related deaths thus include all maternal deaths, and also deaths from accidental or incidental causes.

Proportional Mortality Ratio: Ratio of maternal deaths to all deaths among women ages 15-49

Maternal Mortality Rate: Number of maternal deaths per 100,000 women ages 15-49

Lifetime Risk of Maternal Death: The probability that a 15-year old female will eventually die from a maternal cause, assuming fertility and mortality risks do not change in the future. Estimated as the sum of age-specific maternal mortality rates from ages 15 to 49

Maternal Deaths by Cause: Number of maternal deaths due to 8 mutually exclusive grouped causes (Abortive, Hypertensive disorders, Hemorrhage, Sepsis and other infections, Obstructed labour, Other direct, Late, Indirect)

Note: For each mortality indicator we report both the estimated total and reported deaths, accounting for underreporting

A.3 Model Calibration

A.3.1 Calibration Overview

Many processes in a woman's reproductive life are unobserved, and those that are observed are surrounded with much uncertainty. Model calibration offers a way to systematically explore the uncertainty around each input parameter, and generate parameter sets that fit well to observed data. In this way, we can reflect the uncertainty that surrounds each model input by presenting a range of plausible (calibrated) estimates.

We calibrated all model parameters simultaneously, sampling from the priors (described above for each model parameter) to iteratively generate proposed parameter sets. Note that priors for model parameters were set using regression models of empirical data when possible (i.e. empirical Bayes/maximum marginal likelihood approach), while priors for other parameters which lacked empirical data were set based on subject matter knowledge and expert opinion. Calibrating all model parameters simultaneously to empirical data on a range of maternal health indicators allows us to: 1) revise the estimated priors from various data sources as necessary to fit the observed data, 2) estimate values for parameters that lack empirical data which yield model predictions consistent with prior knowledge and observed data, and 3) induce appropriate covariance between all parameters, which is not possible to achieve when fitting independent regression models.

We used a directed search algorithm (simulated annealing, see Kirkpatrick et al. 1983 for more details) in which we reduced the variance of the proposal distributions following an exponential cooling schedule. This allowed us to more widely explore the parameter space near the beginning of each search chain and then narrow the proposed jumps around the best set found so far, allowing us to fine-tune the calibrated parameters. Simulated annealing (with 'greedy' parameter set acceptance) was chosen as a computationally efficient approach which aims to return samples from the mode of the posterior (i.e. maximum a posteriori or MAP estimates). Our use of multiple independent search chains allows us to explore the (likely multi-modal) parameter space, generally approximating the posterior.

Parameter sets were scored based on the squared distance between the model predictions and the observed data. For targets for which confidence intervals were available, we re-weighted the targets by the inverse of the width of the 95% CIs to allow more precise estimates to have more influence in the calibration scoring.

We performed iterative rounds of search chains, using the best sets of hyperparameters found from the previous round of calibration for computational efficiency.

Reference: Kirkpatrick S, Gelatt CD, Vecchi MP. Optimization by simulated annealing. Science 1983; 220: 671–80.

A.3.2 Calibration Targets

Target Summary

We fitted the model to empirical primary data (i.e. not modeled estimates) for various maternal health indicators. We calibrated to data from 1990-2015, reserving estimates from 2016-2020 as a test set to assess the predictive accuracy of our model. We had 22,495 targets in the training set, and 1,525 estimates in the testing set.

Indicator	Training Set (1990-2015): # Countries (# Estimates)	Testing Set (2016-2020): # Countries (# Estimates)	Data Source
Fertility Indicators			
Total fertility rate (TFR) (age 15-49)	186 (6,633)	120 (249)	UN World Fertility data
Contraceptive prevalence (age 15-49	74 (235)	20 (21)	DHS data
Twinning rate	107 (107)	0 (0)	Smits 2011, Pison 2015
Process Indicators			

Indicator	Training Set (1990-2015): # Countries (# Estimates)	Testing Set (2016-2020): # Countries (# Estimates)	Data Source
Anemia prevalence (by severity)	48 (291)	16 (48)	DHS data
Stillbirth rate	93 (656)	0 (0)	Blencowe 2016
Facility delivery %	121 (286)	84 (85)	DHS data, WHO GHO database
C-section rate	161 (479)	60 (69)	UNICEF Maternal and Newborn Health Coverage Database
Spontaneous/operative vaginal delivery %	24 (48)	0 (0)	WHO Global Survey on Maternal and Perinatal Health
Mortality Indicators			
Maternal deaths (total) (CRVS data)	123 (2,311)	70 (117)	WHO Mortality Database
Maternal deaths by cause (CRVS data)	114 (11,216)	69 (928)	WHO Mortality Database
Pregnancy-related mortality ratio	56 (137)	0 (0)	DHS data
Miscellaneous (maternal deaths, maternal mortality ratio, pregnancy-related mortality ratio)	22 (96)	3 (8)	Country-specific sources

Fertility Indicators

Total Fertility Rate (TFR): We obtained estimates of total fertility rates from the UN World Fertility Data 2019. The database includes data from civil registration systems, population censuses, and sample surveys available as of August 2019 and covers the time period from 1950 to the present.

Contraceptive Prevalence Rate: We estimated contraceptive prevalence rates (i.e. proportion of women ages 15-49 using any method of contraception) based on DHS data for 4,052,949 women from 256 surveys in 75 countries.

Twinning Rate: We obtained estimates of twinning rates (per 1,000 deliveries) for 107 countries from the medical literature (Smits 2011, Pison 2015).

Process Indicators

Anemia Prevalence: We estimated the prevalence of anemia by severity (mild/moderate/severe) based on DHS data for 98,558 pregnant women from 113 surveys in 52 countries.

Stillbirth Rate: We obtained estimates of stillbirth rates from Blencowe 2016, which collated stillbirth data from national routine or registration systems, nationally representative surveys, and other data sources identified through a systematic review, web-based searches, and expert consultation. We restricted the extracted estimates to data which reported the number of stillbirths and were based on the 28 weeks definition of stillbirth. We only included estimates from national routine informations systems or nationally-representative household surveys (i.e. data types 1-3), excluding sub-national data. This yielded 656 estimates from 93 countries.

Facility Delivery %: We estimated the proportion of deliveries which occurred in a facility based on DHS data for 1,717,203 women from 261 surveys in 75 countries. We supplemented these estimates with

country-level data from the Global Health Observatory on births attended by skilled health personnel for 108 countries not available in the DHS data. We use the data on skilled birth personnel as a proxy for facility delivery.

C-Section Rate: We obtained data on national c-section rates by country from the UNICEF Maternal and Newborn Health Coverage Database. 548 estimates were available for 166 countries included in the model between 1990 to 2020.

Spontaneous and Operative Vaginal Delivery Rate: We obtained estimates of spontaneous and operative vaginal delivery rates from the World Health Organization Global Survey on Maternal and Perinatal Health (WHOGS): a multicountry, facility-based survey that collected data for all women delivering in randomly selected facilities from randomly selected countries (Souza 2010). A total of 24 countries took part in the study. Data collection took place during 2004 and 2005 in Africa and the Americas and during 2007 and 2008 in Asia. As this is a facility-based survey, we compare these estimates to the mode of delivery among facility-based births in the model.

Mortality Indicators

Maternal Deaths: We obtained estimates of total (reported) maternal deaths from the WHO Mortality Database which maintains data from Civil Registration Vital Statistics (CRVS) systems. We extracted all maternal deaths as defined by ICD-8, ICD-9, and ICD-10 codes (see Appendix 2.3), depending on the country/year.

Maternal Deaths by Cause: We obtained estimates of maternal deaths by 8 causes (grouped by ICD-10 code, see Appendix 2.3) from the WHO Mortality Database. We restricted these estimates to observations where the total reported maternal deaths equaled the sum of the specific causes, ensuring that there were no unspecified (or generally coded) maternal deaths included in these calibration targets.

Pregnancy-Related Mortality Ratio (PRMR): We obtained estimates of the pregnancy-related mortality ratio from DHS surveys, which were generally estimated using the sisterhood method to inquire about pregnancy-related deaths which occurred in the seven years preceding the survey. We therefore offset the year of the estimates to the midpoint of the seven-year period preceding the survey when calibrating the model.

Miscellaneous Maternal Mortality Estimates: We obtained various estimates of maternal mortality (maternal deaths, maternal mortality ratio [MMR], pregnancy-related mortality ratio [PRMR]) from available country-specific sources for countries for which we did not already have mortality estimates from the sources noted above. When calibrating the model we took into account the reporting mode (i.e. CRVS or survey) of each reported estimate. We summarize these other estimates of maternal mortality here.

Country	Year	Reporting Mode	Indicators	Source
Algeria	1992	Survey	MMR	National Institute of Public Health
Algeria	1999	Survey	Maternal deaths, MMR	National Institute of Public Health
Bangladesh	1993	Survey	PRMR	BMMS 2001
Bangladesh	1997	Survey	PRMR	BMMS 2001
Bangladesh	1999	Survey	MMR, PRMR	BMMS 2001
Bangladesh	2008	Survey	MMR, PRMR	BMMS 2010
Bangladesh	2014	Survey	MMR, PRMR	BMMS 2016
Bhutan	2011	Survey	PRMR	NHS 2012
Botswana	2008	CRVS	Maternal deaths, MMR	Botswana Maternal Mortality Ratio
Botswana	2009	CRVS	Maternal deaths, MMR	Botswana Maternal Mortality Ratio
Botswana	2010	CRVS	Maternal deaths, MMR	Botswana Maternal Mortality Ratio
Botswana	2011	CRVS	Maternal deaths, MMR	Botswana Maternal Mortality Ratio
Botswana	2012	CRVS	Maternal deaths, MMR	Botswana Maternal Mortality Ratio
Botswana	2013	CRVS	Maternal deaths, MMR	Botswana Maternal Mortality Ratio
Botswana	2014	CRVS	Maternal deaths, MMR	Botswana Maternal Mortality Ratio
Botswana	2015	CRVS	Maternal deaths, MMR	Botswana Maternal Mortality Ratio
Botswana	2016	CRVS	Maternal deaths, MMR	Botswana Maternal Mortality Ratio
Botswana	2017	CRVS	Maternal deaths, MMR	Botswana Maternal Mortality Ratio
China	1990	CRVS	MMR	WHO Data
China	1995	CRVS	MMR	WHO Data
China	1997	CRVS	MMR	WHO Data
China	1999	CRVS	MMR	WHO Data
China	2000	CRVS	MMR	WHO Data
China	2001	CRVS	MMR	WHO Data
China	2002	CRVS	MMR	WHO Data
China	2003	CRVS	MMR	WHO Data

Country	Year	Reporting Mode	Indicators	Source
China	2004	CRVS	MMR	WHO Data
China	2005	CRVS	MMR	WHO Data
China	2006	CRVS	MMR	WHO Data
China	2007	CRVS	MMR	WHO Data
China	2008	CRVS	MMR	WHO Data
China	2009	CRVS	MMR	WHO Data
China	2010	CRVS	MMR	WHO Data
China	2011	CRVS	MMR	WHO Data
China	2012	CRVS	MMR	WHO Data
Equatorial Guinea	2006	Survey	MMR	DHS 2011
Ghana	1995	Survey	PRMR	MHS 2007
Ghana	2000	Survey	PRMR	MHS 2007
Ghana	2005	Survey	PRMR	MHS 2007
Ghana	2009	Survey	PRMR	Census 2010
Ghana	2014	Survey	MMR	MHS 2017
Guinea-Bissau	2010	Survey	MMR	MICS 2014
India	1991	Survey	MMR	NFHS-1
India	1997	Survey	MMR	NFHS-2
India	2005	Survey	MMR	Sample Registration System
India	2008	Survey	MMR	Sample Registration System
India	2011	Survey	MMR	Sample Registration System
India	2012	Survey	MMR	Sample Registration System
India	2015	Survey	MMR	Sample Registration System
India	2016	Survey	MMR	Sample Registration System
Laos	1995	Survey	MMR	Census 1995
Laos	1996	Survey	MMR	LRHS 2000
Laos	2000	Survey	MMR	LHS 2003
Laos	2005	Survey	MMR	Census 2005
Laos	2008	Survey	MMR	MICS 2011-2012
Laos	2014	Survey	Maternal deaths	Census 2015
Lebanon	2008	Survey	MMR	RAMOS 2008
Lebanon	2011	CRVS	Maternal deaths	MOPH
Lebanon	2012	CRVS	Maternal deaths	MOPH
Lebanon	2013	CRVS	Maternal deaths	MOPH
Lebanon	2014	CRVS	Maternal deaths	MOPH
Lebanon	2015	CRVS	Maternal deaths	MOPH
Lebanon	2016	CRVS	Maternal deaths	MOPH
Lebanon	2017	CRVS	Maternal deaths	Maternal Death Surveillance And Response - Annual Report 2017
Lebanon	2018	CRVS	Maternal deaths	MOPH
Nauru	2009	CRVS	Maternal deaths, MMR	CRVS
Nauru	2012	CRVS	Maternal deaths, MMR	CRVS
North Korea	2007	Survey	Maternal deaths, MMR	Census 2008
Pakistan	1990	Survey	MMR	RHFP 2001
Pakistan	2005	Survey	MMR, PRMR	DHS 2006-07
Solomon Islands	2009	Survey	Maternal deaths, MMR	Census 2009
South Sudan	2006	Survey	MMR	SHHS 2006
Sudan	2010	Survey	MMR	MICS 2010
Taiwan	2004	CRVS	MMR	Wu et al 2015
Taiwan	2006	CRVS	MMR	Wu et al 2015
Taiwan	2008	CRVS	MMR	Wu et al 2015
Taiwan	2010	CRVS	MMR	Wu et al 2015
Tunisia	1993	CRVS	MMR	Farhat et al 2011
Tunisia	2006	CRVS	MMR	Farhat et al 2011
Tunisia	2008	Survey	MMR	RAMOS 2010
United Arab Emirates	2000	CRVS	MMR	Ghazal-Aswad et al 2011
Vanuatu	2004	CRVS	Maternal deaths, MMR	Carter et al 2016
Viet Nam	1994	CRVS	MMR	Hieu et al 1999
Viet Nam	2001	Survey	MMR	MICS 2006

Model Comparisons

We also compared our model results to estimates from other sources. These estimates were not used to calibrate the model, but allow us to assess the convergent validity of our model results with estimates from other sources.

Indicator	Source
Births	UN World Population Prospects 2022
Total fertility rate (TFR)	UN World Population Prospects 2022
Anemia prevalence (overall) among pregnant women	WHO GHO database
Contraceptive prevalence	UN World Contraceptive Use 2019

Indicator	Source
Maternal deaths	United Nations Maternal Mortality Estimation Interagency Group 2019, GBD 2019
Maternal mortality ratio (MMR)	United Nations Maternal Mortality Estimation Interagency Group 2019, GBD 2019

References

Blencowe H, Cousens S, Jassir FB, Say L, Chou D, Mathers C, et al. National, regional, and worldwide estimates of stillbirth rates in 2015, with trends from 2000: a systematic analysis. Lancet Glob Health 2016; 4(2): e98-e108. DOI: https://doi.org/10.1016/S2214-109X(15)00275-2

Global Burden of Disease Collaborative Network. Global Burden of Disease Study 2019 (GBD 2019) Results. Seattle, United States: Institute for Health Metrics and Evaluation (IHME), 2020. Accessed 2021-01-08. Available at: http://ghdx.healthdata.org/gbd-results-tool.

Global Health Observatory data repository. Births attended by skilled health personnel. Last updated: 2020-04-08. Available at: https://apps.who.int/gho/data/node.main.SKILLEDBIRTHATTENDANTS?lang=en.

Global Health Observatory data repository. Prevalence of anaemia in pregnant women. Last updated: 2017-08-30. Available at: https://www.who.int/data/gho/data/themes/topics/indicator-groups/indicator-group-details/GHO/prevalence-of-anaemia-in-pregnant-women

Pison G, Monden C, Smits J. Twinning Rates in Developed Countries: Trends and Explanations. Popul Dev Rev 2015; 41(4): 629-649. DOI: https://doi.org/10.1111/j.1728-4457.2015.00088.x

Smits J, Monden C Twinning across the Developing World. PLoS One 2011; 6(9): e25239. DOI: https://doi.org/10.1371/journal.pone.0025239

Souza JP, Gülmezoglu A, Lumbiganon P, et al. Caesarean section without medical indications is associated with an increased risk of adverse short-term maternal outcomes: the 2004–2008 WHO Global Survey on Maternal and Perinatal Health. BMC Med 2010; 8: 71. DOI: https://doi.org/10.1186/1741-7015-8-71

UNICEF. Maternal and Newborn Health Coverage Database, August 2020. Available at: https://data.unicef.org/topic/maternal-health/delivery-care/

United Nations, Department of Economic and Social Affairs, Population Division (2019). World Contraceptive Use 2019. Available at: https://www.un.org/en/development/desa/population/publications/dataset/contraception/wcu2019.asp

United Nations, Department of Economic and Social Affairs, Population Division (2019). World Fertility Data 2019. POP/DB/Fert/Rev2019. Available at: https://www.un.org/en/development/desa/population/publications/dataset/fertility/wfd2019.asp

United Nations, Department of Economic and Social Affairs, Population Division (2022). World Population Prospects 2022, Online Edition. Available at: https://population.un.org/wpp/Download/Standard/CSV/

United Nations Maternal Mortality Estimation Interagency Group 2019. Available at: https://data.unicef.org/topic/maternal-health/maternal-mortality/

World Health Organization. WHO Mortality Database. Last update: 15 December 2019. Available at: https://www.who.int/data/data-collection-tools/who-mortality-database

A.3.3 Scores

Here we plot the scores for the best 100 parameter sets, higlighted in red.

We also plot the score for each target type.

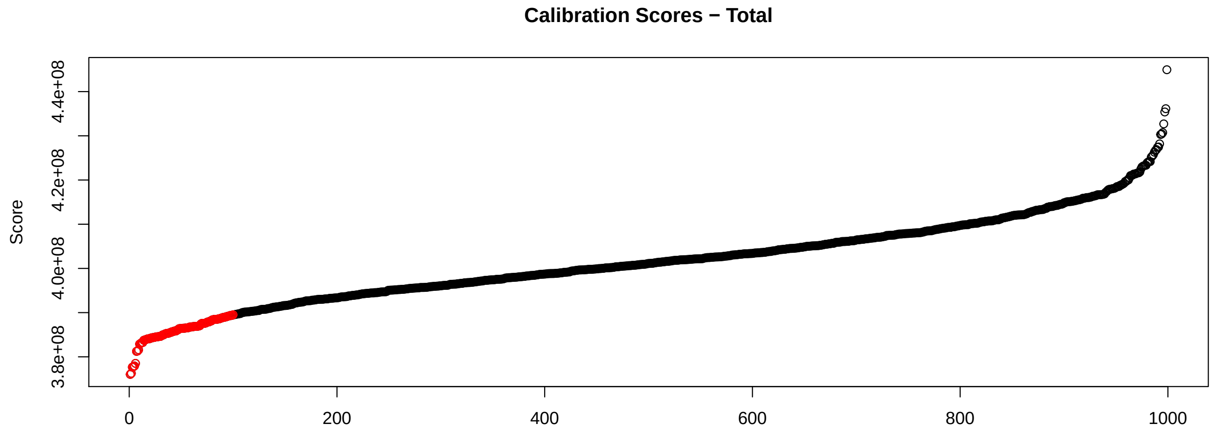

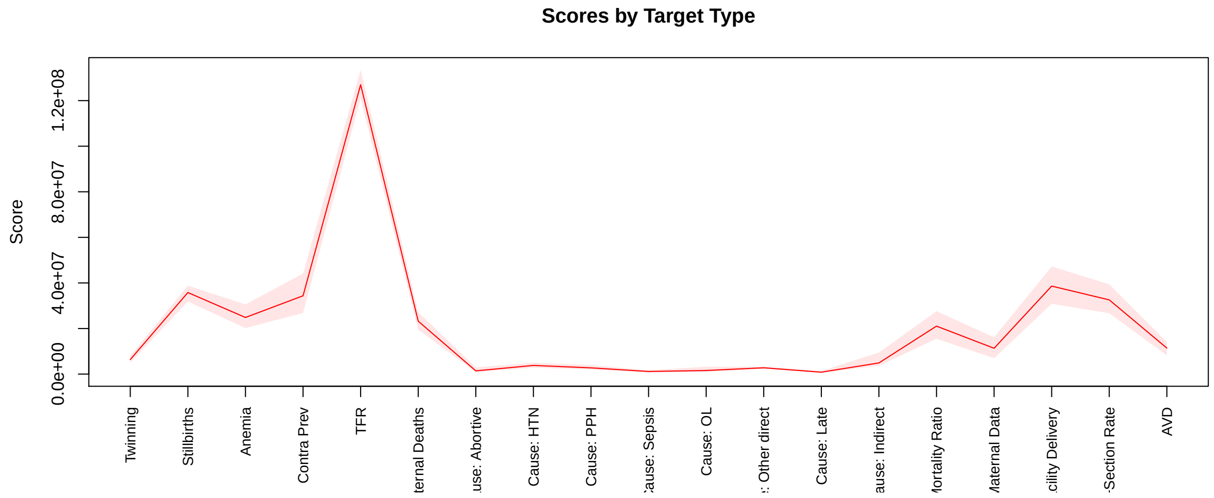

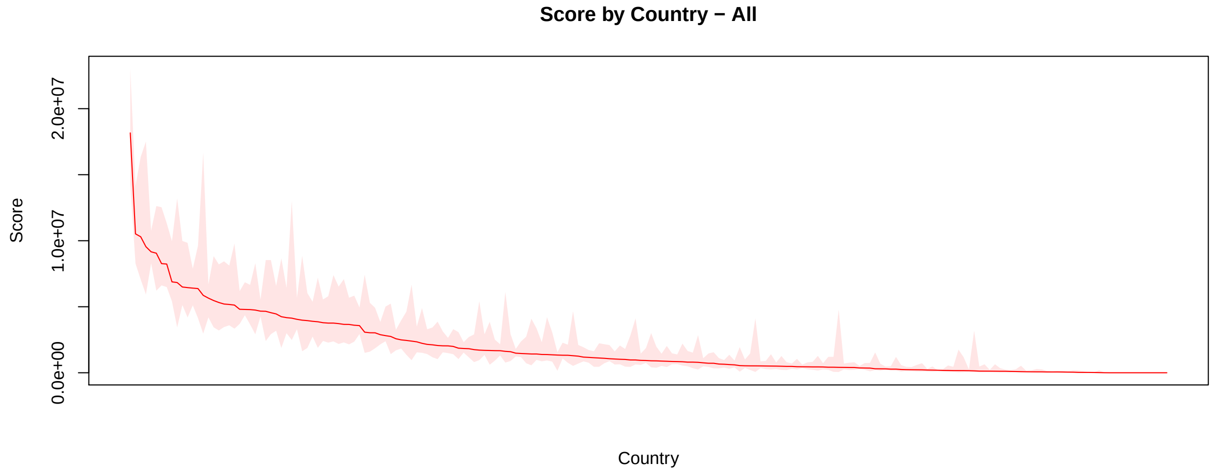

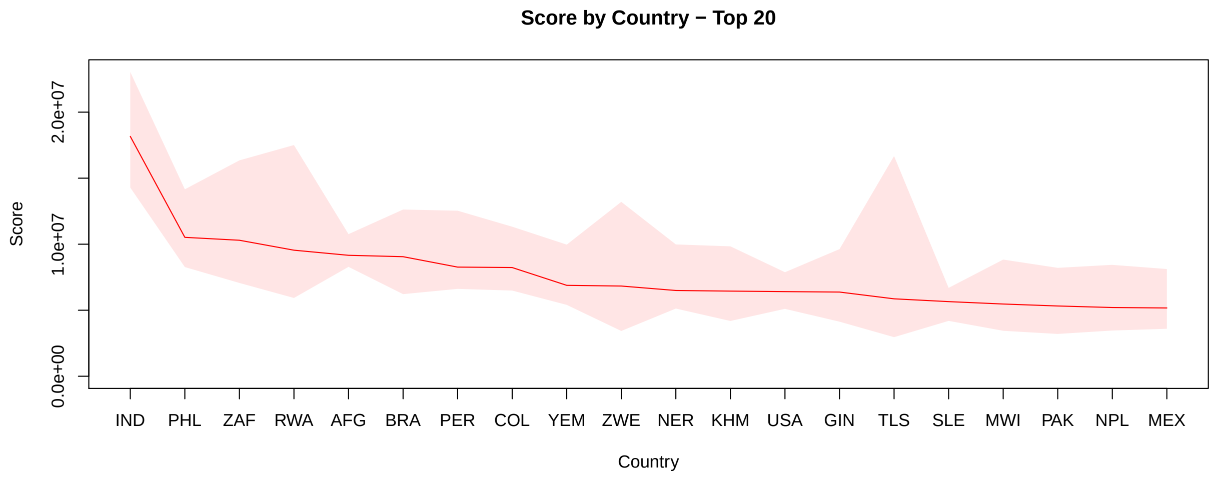
Score by Country − Top 20
Score
2.0e+07
1.0e+07
0.0e+00
IND
PHL
ZAF
RWA
AFG
BRA
PER
COL
YEM
ZWE
NER
KHM
USA
GIN
TLS
SLE
MWI
PAK
NPL
MEX
Country

A.3.4 Posterior Predictive Checks

Coverage Probabilities

Calculated as the proportion of model prediction intervals (95% uncertainty intervals [UIs]) which contain the reported point estimate

Indicator	Training Set (1990-2015)	Testing Set (2016-2020)
Fertility Indicators	*78.2%*	*90.0%*
Total fertility rate (TFR) (age 15-49)	78.0%	92.0%
Contraceptive prevalence (age 15-49)	87.2%	66.7%
Twinning rate	70.1%	NA
Process Indicators	*70.6%*	*69.3%*
Anemia prevalence (by severity)	50.9%	58.3%
Stillbirth rate	68.1%	NA
Facility delivery %	86.0%	61.2%
C-section rate	81.4%	87.0%
Spontaneous/operative vaginal delivery %	25.0%	NA
Mortality Indicators	*96.0%*	*96.0%*
Maternal deaths (total) (CRVS data)	92.3%	96.6%
Abortive deaths (CRVS data)	96.0%	100%
PE/E deaths (CRVS data)	98.9%	100%
PPH (CRVS data)	99.7%	100%
Sepsis deaths (CRVS data)	99.2%	100%
OL deaths (CRVS data)	98.9%	100%
Other direct deaths (CRVS data)	90.0%	81.0%
Late maternal deaths (CRVS data)	99.0%	96.6%
Indirect maternal deaths (CRVS data)	94.0%	89.7%
Pregnancy-related mortality ratio	84.7%	NA
Miscellaneous (maternal deaths, maternal mortality ratio, pregnancy-related mortality ratio)	87.5%	100.0%
**OVERALL**	**88.5%**	**91.4%**

Mean Absolute Error

Indicator	Training Set (1990-2015)	Testing Set (2016-2020)
Fertility Indicators		
Total fertility rate (TFR) (age 15-49)	0.24	0.19
Contraceptive prevalence (age 15-49)	2.9	6.5
Twinning rate	3.3	NA
Process Indicators		
Anemia prevalence (by severity)	5.0	7.7
Stillbirth rate	2.0	NA
Facility delivery %	3.1	4.3
C-section rate	1.6	3.1
Spontaneous/operative vaginal delivery %	4.2	NA
Mortality Indicators		
Maternal deaths (total) (CRVS data)	44.0	47.5
Abortive deaths (CRVS data)	7.6	5.3
PE/E deaths (CRVS data)	14.8	15.4
PPH deaths (CRVS data)	11.5	9.5
Sepsis deaths (CRVS data)	6.0	2.9

Indicator	Training Set (1990-2015)	Testing Set (2016-2020)
OL deaths (CRVS data)	7.7	4.3
Other direct deaths (CRVS data)	12.4	16.7
Late maternal deaths (CRVS data)	5.6	13.6
Indirect maternal deaths (CRVS data)	19.6	16.2
Pregnancy-related mortality ratio	81.1	NA
Miscellaneous (maternal deaths, maternal mortality ratio, pregnancy-related mortality ratio)	46.7	18.3

Mean Error Reported values are mean (SE)

Indicator	Training Set (1990-2015)	Testing Set (2016-2020)
Fertility Indicators		
Total fertility rate (TFR) (age 15-49)	0.018 (0.005)	-0.055 (0.017)
Contraceptive prevalence (age 15-49)	-0.9 (0.3)	-5.3 (1.5)
Twinning rate	-1.4 (0.4)	NA
Process Indicators		
Anemia prevalence (by severity)	2.9 (0.4)	4.6 (1.4)
Stillbirth rate	1.0 (0.1)	NA
Facility delivery %	-0.2 (0.3)	1.1 (0.8)
C-section rate	0.1 (0.1)	1.2 (0.5)
Spontaneous/operative vaginal delivery %	-1.4 (0.8)	NA
Mortality Indicators		
Maternal deaths (total) (CRVS data)	-11.1 (1.8)	2.6 (8.9)
Abortive deaths (CRVS data)	2.0 (0.4)	-2.7 (0.9)
PE/E deaths (CRVS data)	-4.4 (0.9)	-12.3 (3.2)
PPH deaths (CRVS data)	1.4 (0.7)	-5.7 (1.8)
Sepsis deaths (CRVS data)	-1.9 (0.5)	-0.6 (0.5)
OL deaths (CRVS data)	-7.1 (0.7)	-4.1 (0.9)
Other direct deaths (CRVS data)	9.3 (1.0)	15.7 (5.3)
Late maternal deaths (CRVS data)	-2.7 (0.4)	7.0 (3.1)
Indirect maternal deaths (CRVS data)	1.7 (1.4)	6.0 (3.8)
Pregnancy-related mortality ratio	10.7 (9.5)	NA
Miscellaneous (maternal deaths, maternal mortality ratio, pregnancy-related mortality ratio)	18.3 (9.9)	18.3 (4.6)

Coverage Probabilities by Country Grouping

Here we summarize coverage probabilities (and number of targets) by indicator type and country group

Group	Fertility: Train	Fertility: Test	Process: Train	Process: Test	Mortality: Train	Mortality: Test	Overall: Train	Overall: Test
Global	78.2% (n=6975)	90.0% (n=270)	70.6% (n=1760)	69.3% (n=202)	96.0% (n=13760)	96.0% (n=1053)	88.5% (n=22495)	91.4% (n=1525)
Low income	59.4% (n=1315)	80.0% (n=35)	67.2% (n=393)	69.2% (n=65)	91.7% (n=168)	100% (n=9)	63.9% (n=1876)	75.2% (n=109)
LM income	77.5% (n=1753)	90.9% (n=44)	66.6% (n=428)	81.4% (n=43)	97.2% (n=1636)	94.5% (n=128)	84.8% (n=3817)	91.2% (n=215)
UM income	77.6% (n=2097)	91.2% (n=80)	68.7% (n=470)	76.4% (n=55)	97.1% (n=4581)	95.9% (n=367)	89.5% (n=7148)	93.0% (n=502)

Group	Fertility: Train	Fertility: Test	Process: Train	Process: Test	Mortality: Train	Mortality: Test	Overall: Train	Overall: Test
High income	93.1% (n=1810)	91.9% (n=111)	79.1% (n=469)	46.2% (n=39)	95.2% (n=7375)	96.4% (n=549)	94.0% (n=9654)	92.8% (n=699)
Africa	73.0% (n=1651)	83.3% (n=48)	66.8% (n=588)	68.7% (n=67)	96.4% (n=806)	90.3% (n=31)	78.0% (n=3045)	78.1% (n=146)
Asia	69.7% (n=2742)	90.4% (n=83)	66.6% (n=380)	81.5% (n=65)	96.3% (n=2699)	95.5% (n=265)	81.8% (n=5821)	92.3% (n=413)
Europe	95.8% (n=1224)	94.3% (n=88)	86.6% (n=403)	46.7% (n=30)	95.8% (n=4979)	98.4% (n=370)	95.2% (n=6606)	94.5% (n=488)
Latin America	85.7% (n=1142)	90.2% (n=41)	66.1% (n=336)	74.2% (n=31)	96.6% (n=4504)	95.9% (n=315)	92.8% (n=5982)	93.5% (n=387)
North America	95.9% (n=49)	0% (n=2)	15.4% (n=13)	0% (n=2)	89.1% (n=331)	88.9% (n=36)	87.5% (n=393)	80.0% (n=40)
Oceania	82.6% (n=167)	100% (n=8)	60.0% (n=40)	57.1% (n=7)	95.2% (n=441)	88.9% (n=36)	89.8% (n=648)	86.3% (n=51)

B Appendix B: Country Profiles

We calculate a number of maternal health indicators in the GMatH model, described below. All indicators are reported per year.

Indicator	Description
Demographics	
Female Population, Age 15-49	Number of females ages 15-49 in the population
Urbanicity	Proportion of the population living in an urban area
Level of Education	Low (less than primary), Middle (less than secondary), High (completed secondary or higher)
Fertility Indicators	
Births	Number of live births
Total Fertility Rate (TFR)	Age-standardized fertility rates, calculated among women ages 15-49. $\text{TFR} = \sum_{a=15}^{49} \frac{\text{births}_a}{\text{women}_a}$
Twinning Rate	Number of twins per 1,000 deliveries
Pregnancy Outcomes	Proportion of pregnancies that end in miscarriage, elective abortion, or ectopic pregnancy
Contraceptive Prevalence	Prevalence of contraceptive use (any method) among women ages 15-49
Contraceptive Method Mix	Prevalence of use of each contraceptive method among women using any method
Process Indicators	
Stillbirth Rate	Number of stillbirths per 1,000 births
Anemia Prevalence	Prevalence of overall, mild, moderate, and severe anemia among pregnant women
Facility Deliveries	Percentage of deliveries which take place in a health facility (non-EmOC, BEmOC, or CEmOC)
Delivery Site	Percentage of deliveries which take place at each delivery site (Home, Home-SBA, non-EmOC, BEmOC, CEmOC)
C-Section Rate	Percentage of deliveries which take place via caesarean section (elective or emergency)
Delivery Mode	Percentage of deliveries which take place via each delivery mode (spontaneous vaginal delivery, assisted vaginal delivery, elective c-section, emergency c-section)
Mortality Indicators	
Maternal Deaths (including Late)	Number of maternal deaths per year, including late maternal deaths
Maternal Mortality Ratio (MMR)	Number of maternal deaths per 100,000 live births
Pregnancy Mortality Ratio	Number of pregnancy-related deaths per 100,000 live births. Pregnancy-related death is defined as the death of a woman while pregnant or within 42 days of termination of pregnancy, irrespective of the cause of death. Pregnancy-related deaths thus include all maternal deaths, and also deaths from accidental or incidental causes.
Proportional Mortality Ratio	Ratio of maternal deaths to all deaths among women ages 15-49
Maternal Mortality Rate	Number of maternal deaths per 100,000 women ages 15-49
Lifetime Risk of Maternal Death	The probability that a 15-year old female will eventually die from a maternal cause, assuming fertility and mortality risks do not change in the future. Estimated as the sum of age-specific maternal mortality rates from ages 15 to 49
Maternal Deaths: Abortive	Maternal deaths from abortion, ectopic pregnancy, or miscarriage

Indicator	Description
Maternal Deaths: HTN	Maternal deaths from hypertensive disorders (e.g. preeclampsia/eclampsia)
Maternal Deaths: Hemorrhage	Maternal deaths from hemorrhage
Maternal Deaths: Sepsis	Maternal deaths from sepsis and other infections
Maternal Deaths: OL	Maternal deaths from obstructed labour
Maternal Deaths: Other Direct	Maternal deaths from other direct causes
Maternal Deaths: Late	Maternal deaths from any obstetric cause or sequelae occurring more than 42 days but less than one year after delivery
Maternal Deaths: Indirect	Maternal deaths not due to direct obstetric causes, but which are aggravated by physiologic effects of pregnancy (e.g. malaria, HIV)

Afghanistan

ISO Code	Region	Area	Income Group
AFG	Southern Asia	Asia	Low income

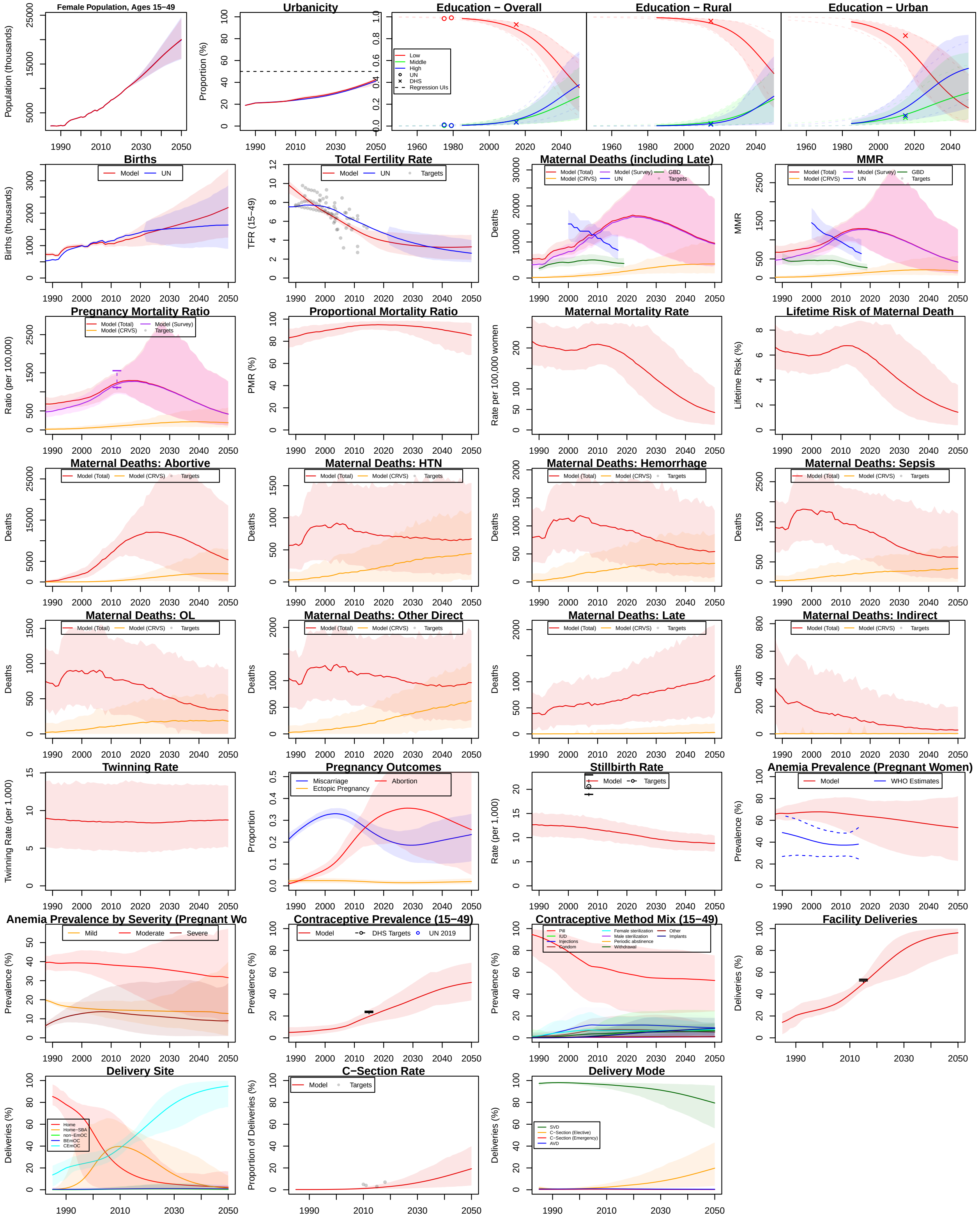

Albania

ISO Code	Region	Area	Income Group
ALB	Southern Europe	Europe	Upper middle income

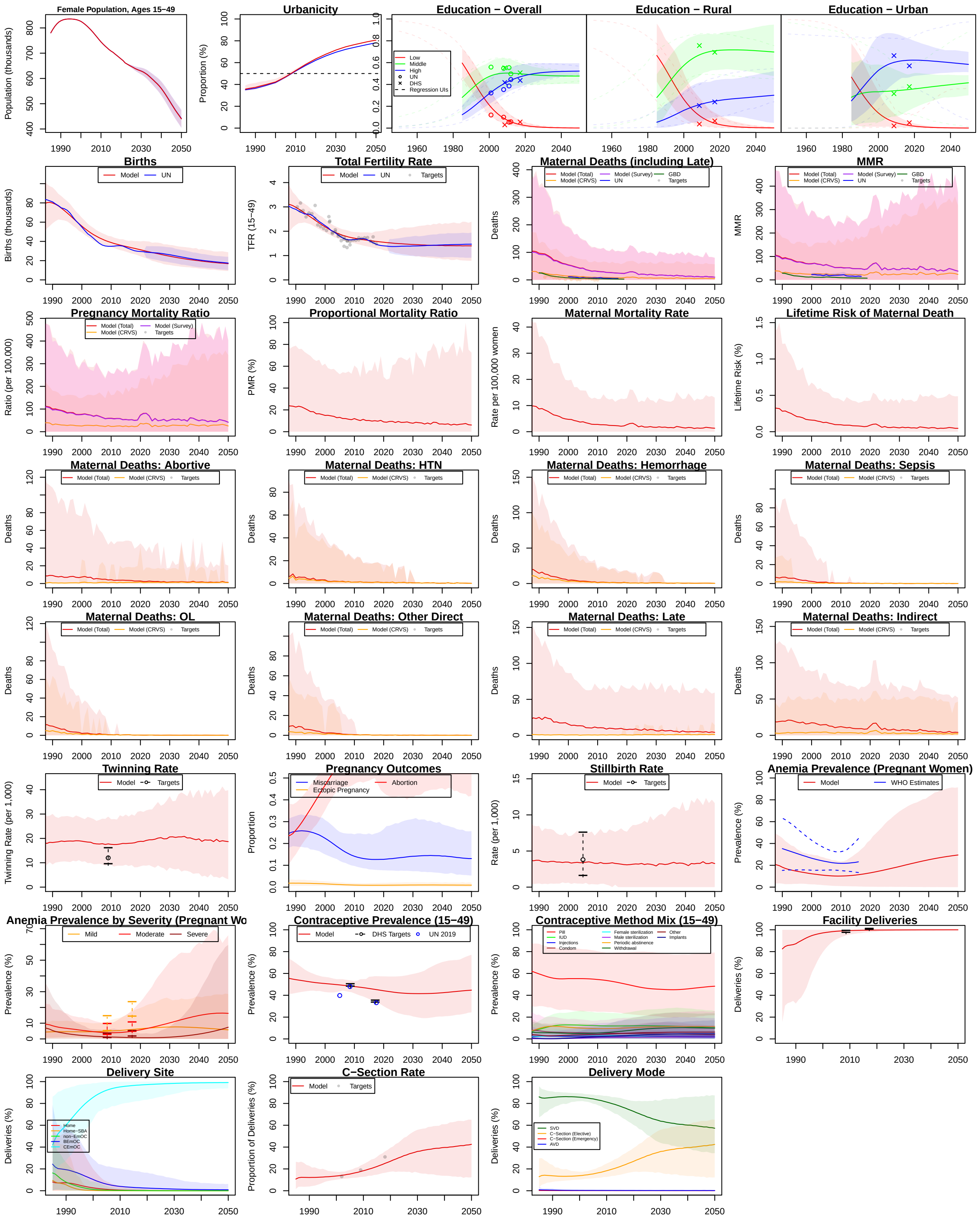

Algeria

ISO Code	Region	Area	Income Group
DZA	Northern Africa	Africa	Upper middle income

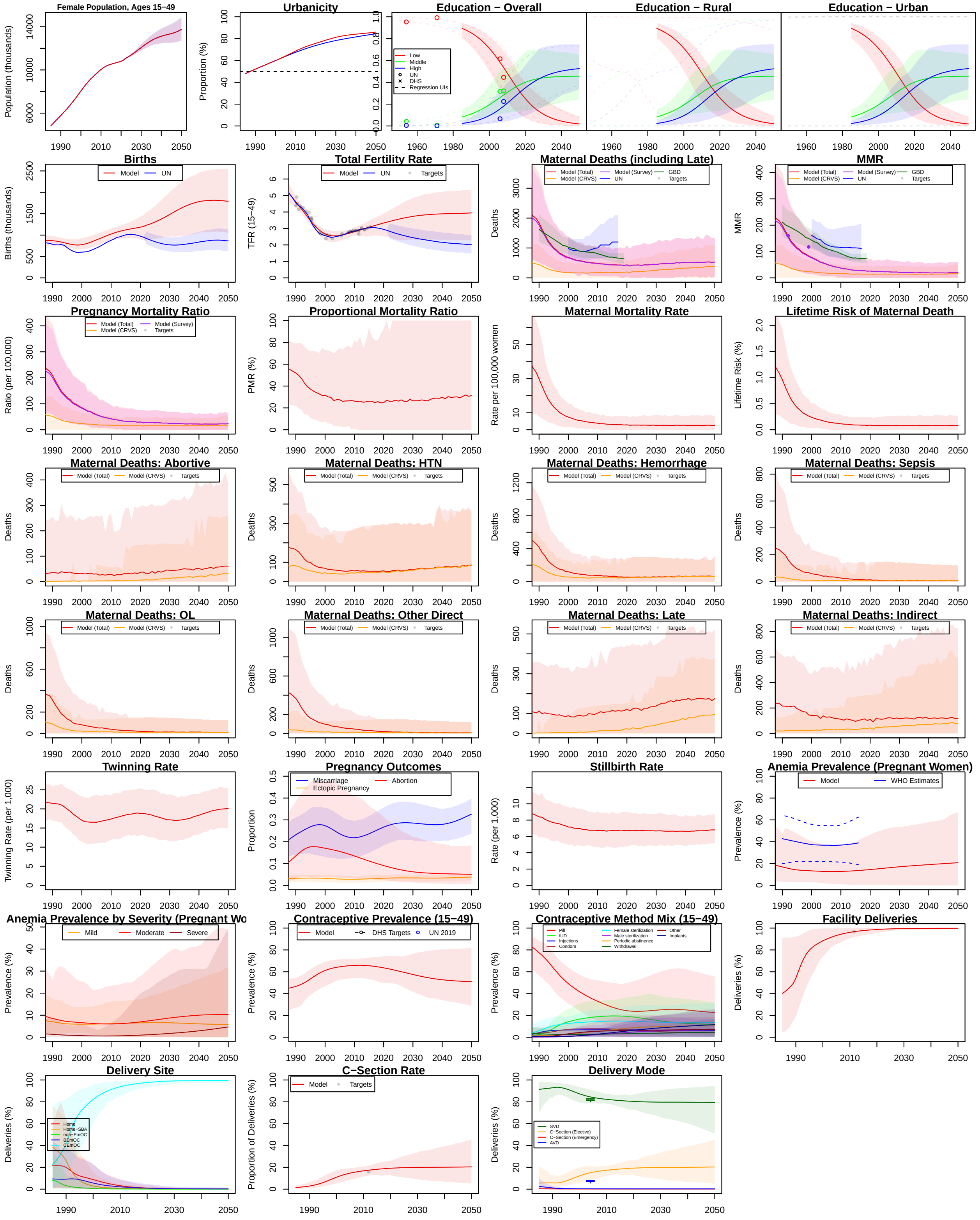

Andorra

ISO Code	Region	Area	Income Group
AND	Southern Europe	Europe	High income

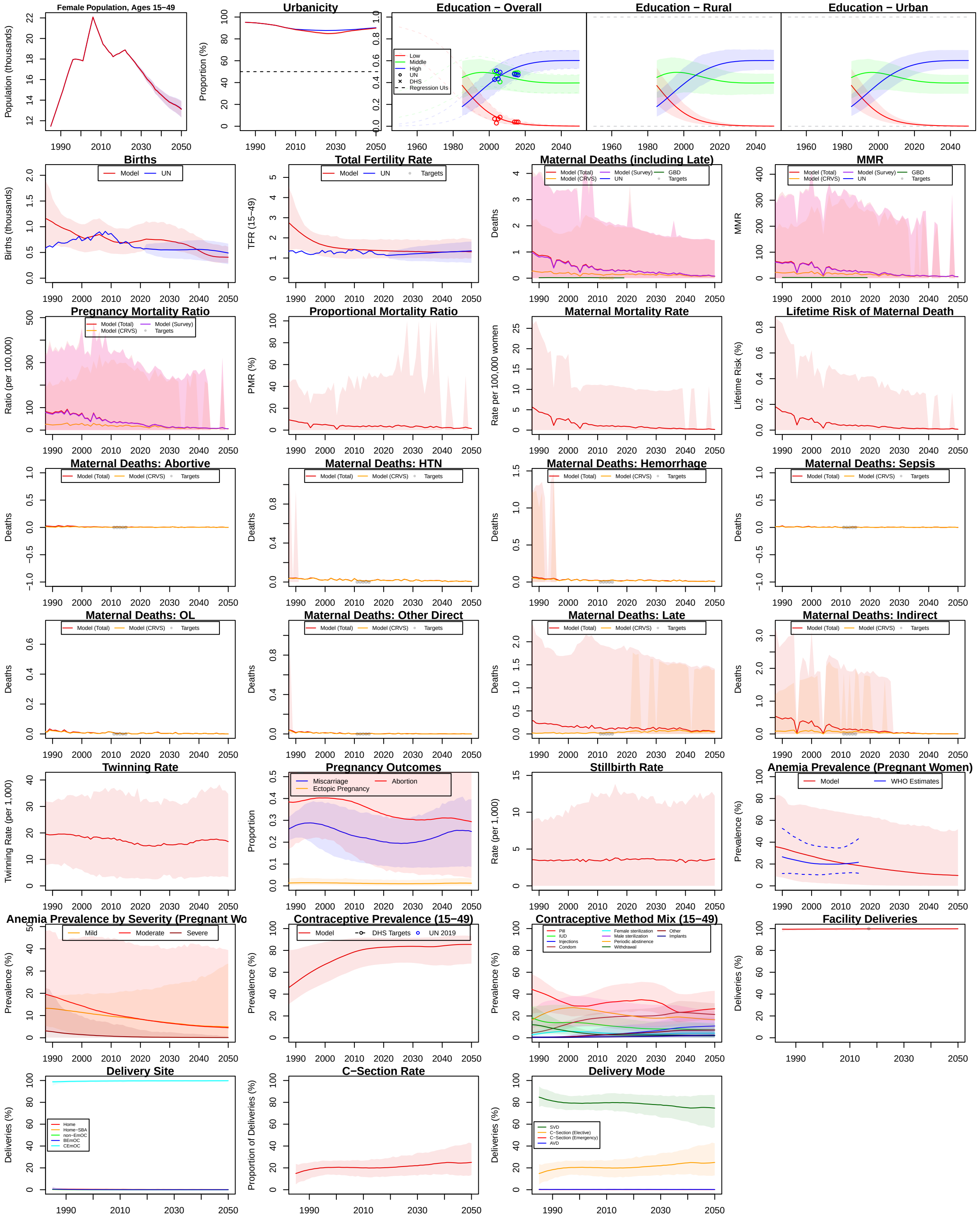

Angola

ISO Code	Region	Area	Income Group
AGO	Middle Africa	Africa	Lower middle income

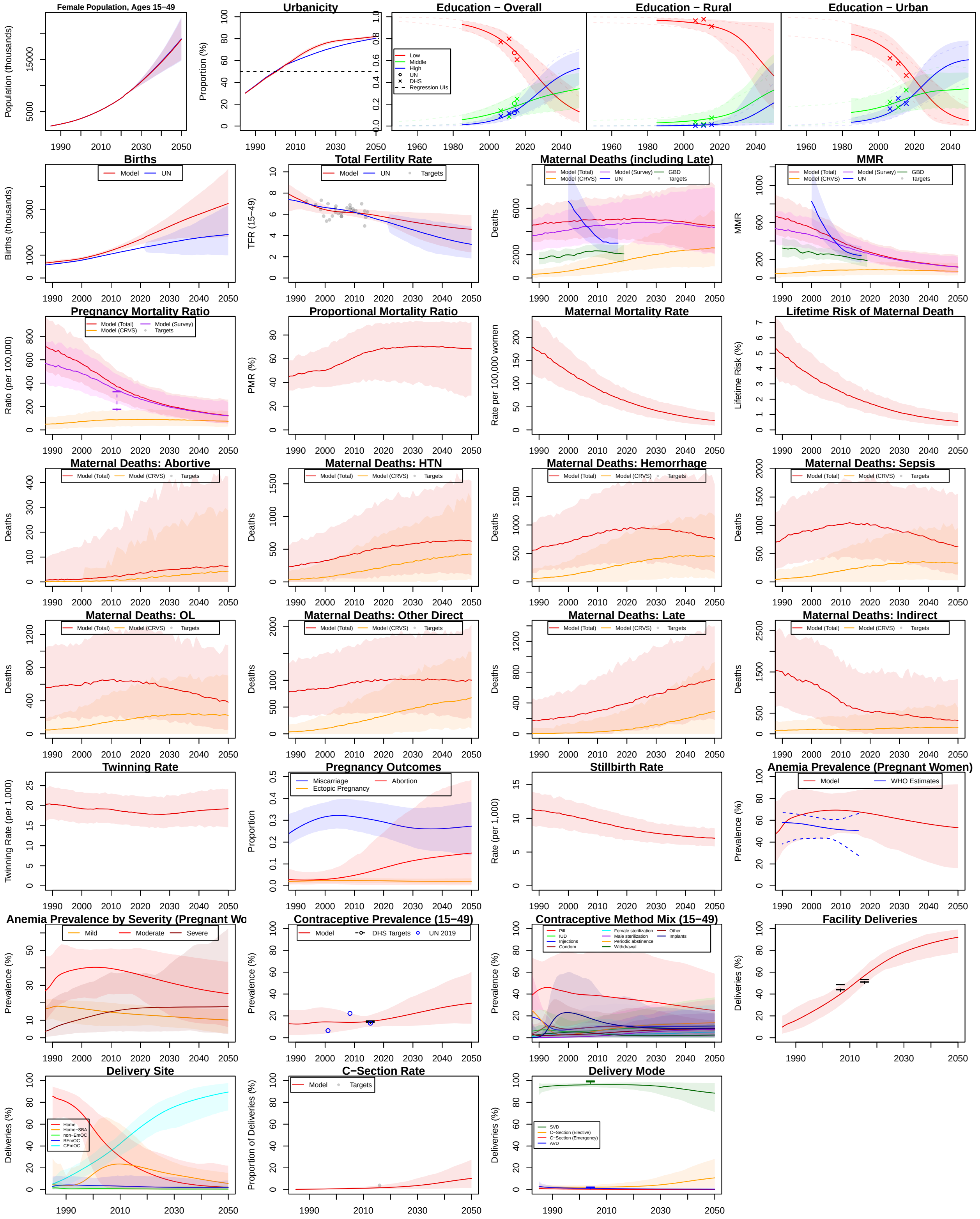

Antigua and Barbuda

ISO Code	Region	Area	Income Group
ATG	Caribbean	Latin America and the Caribbean	High income

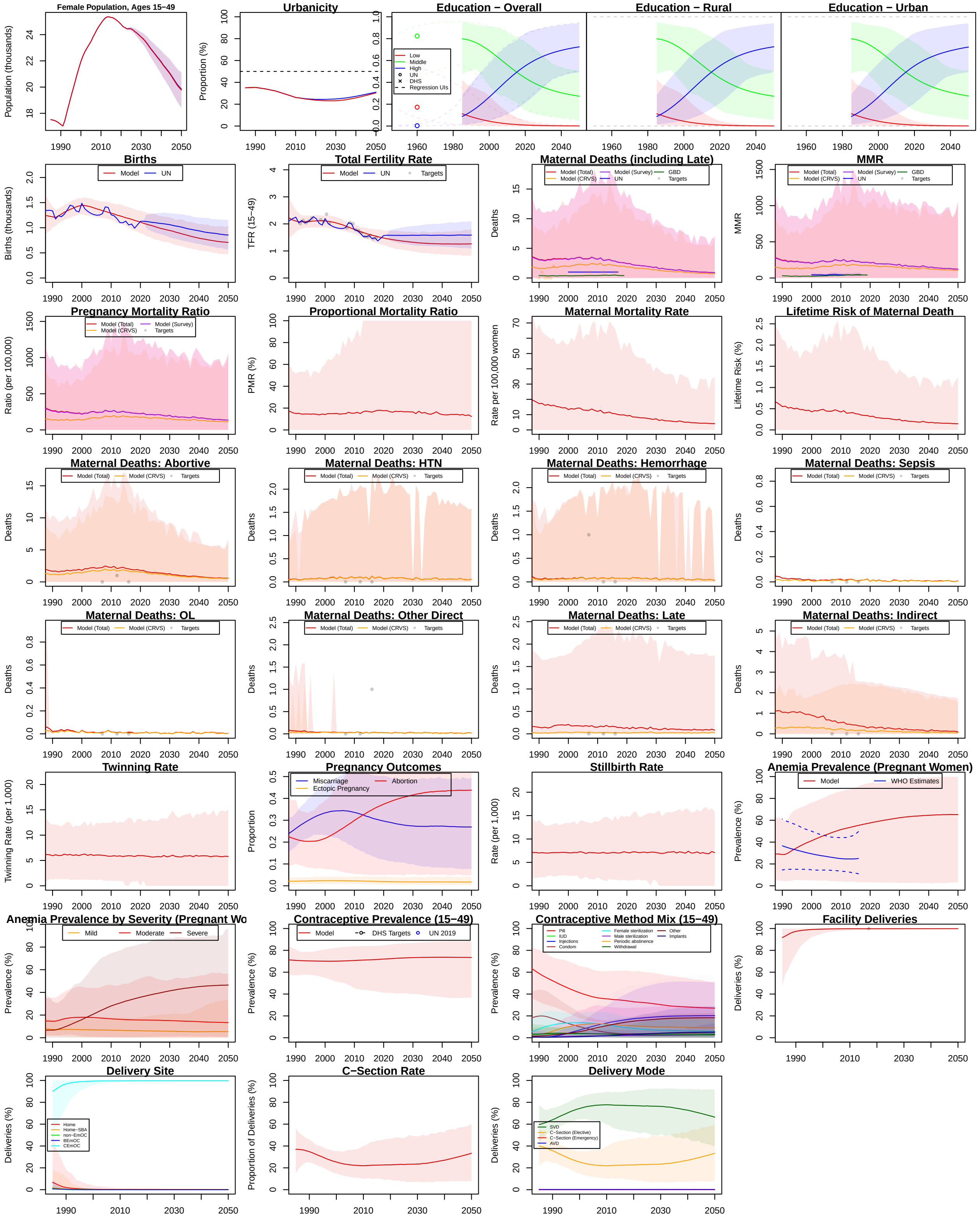

Argentina

ISO Code	Region	Area	Income Group
ARG	South America	Latin America and the Caribbean	High income

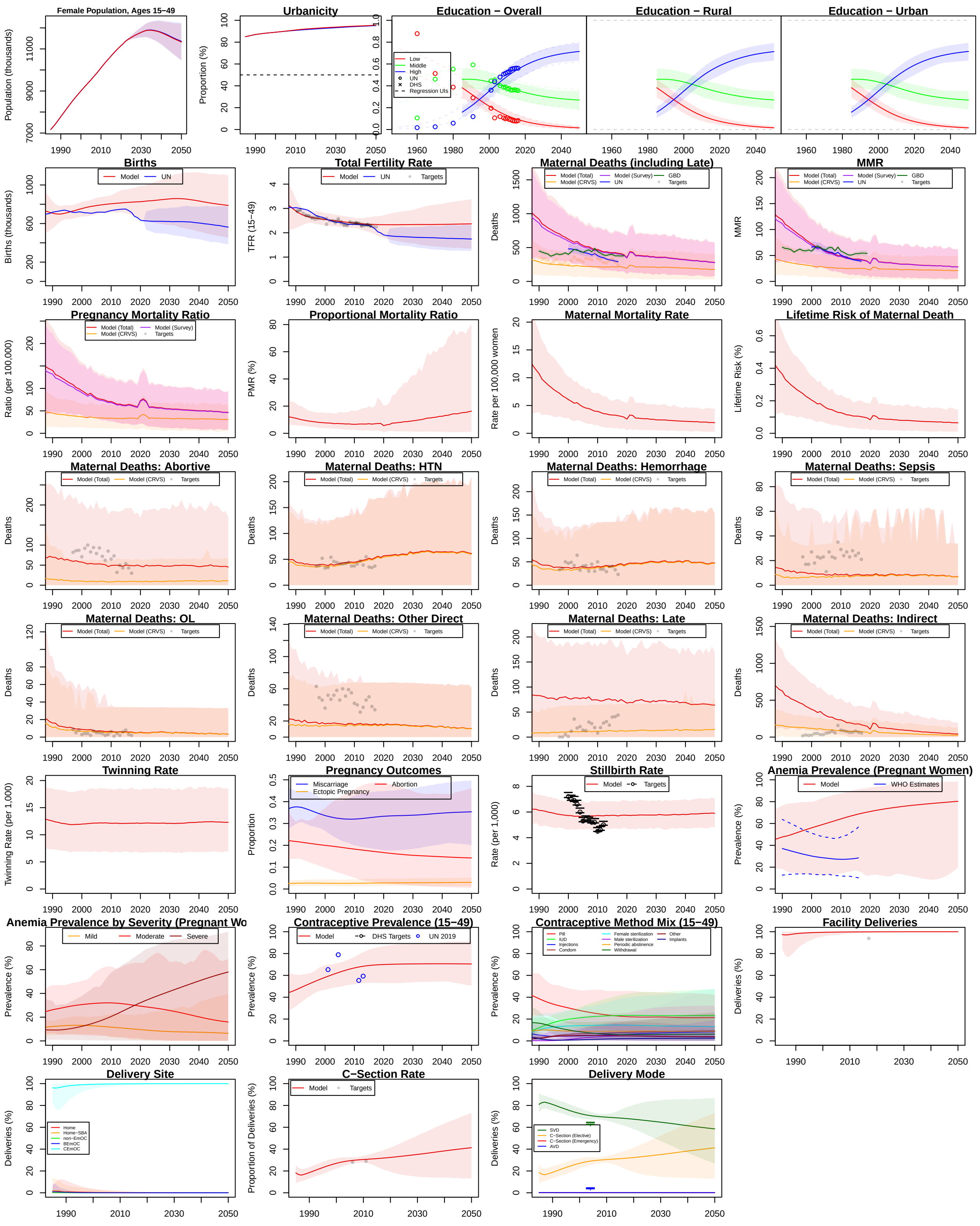

Armenia

ISO Code	Region	Area	Income Group
ARM	Western Asia	Asia	Upper middle income

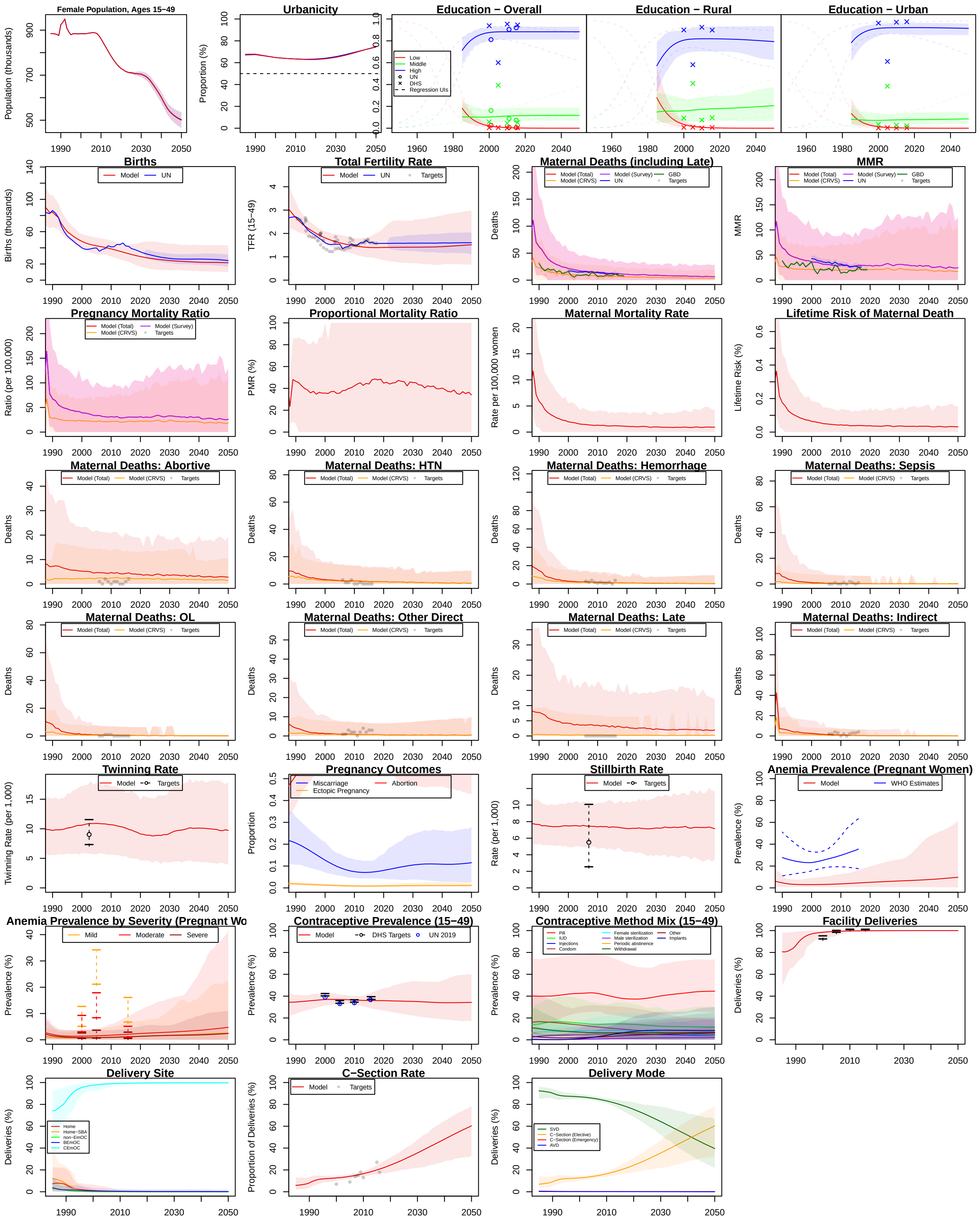

Australia

ISO Code	Region	Area	Income Group
AUS	Australia/New Zealand	Oceania	High income

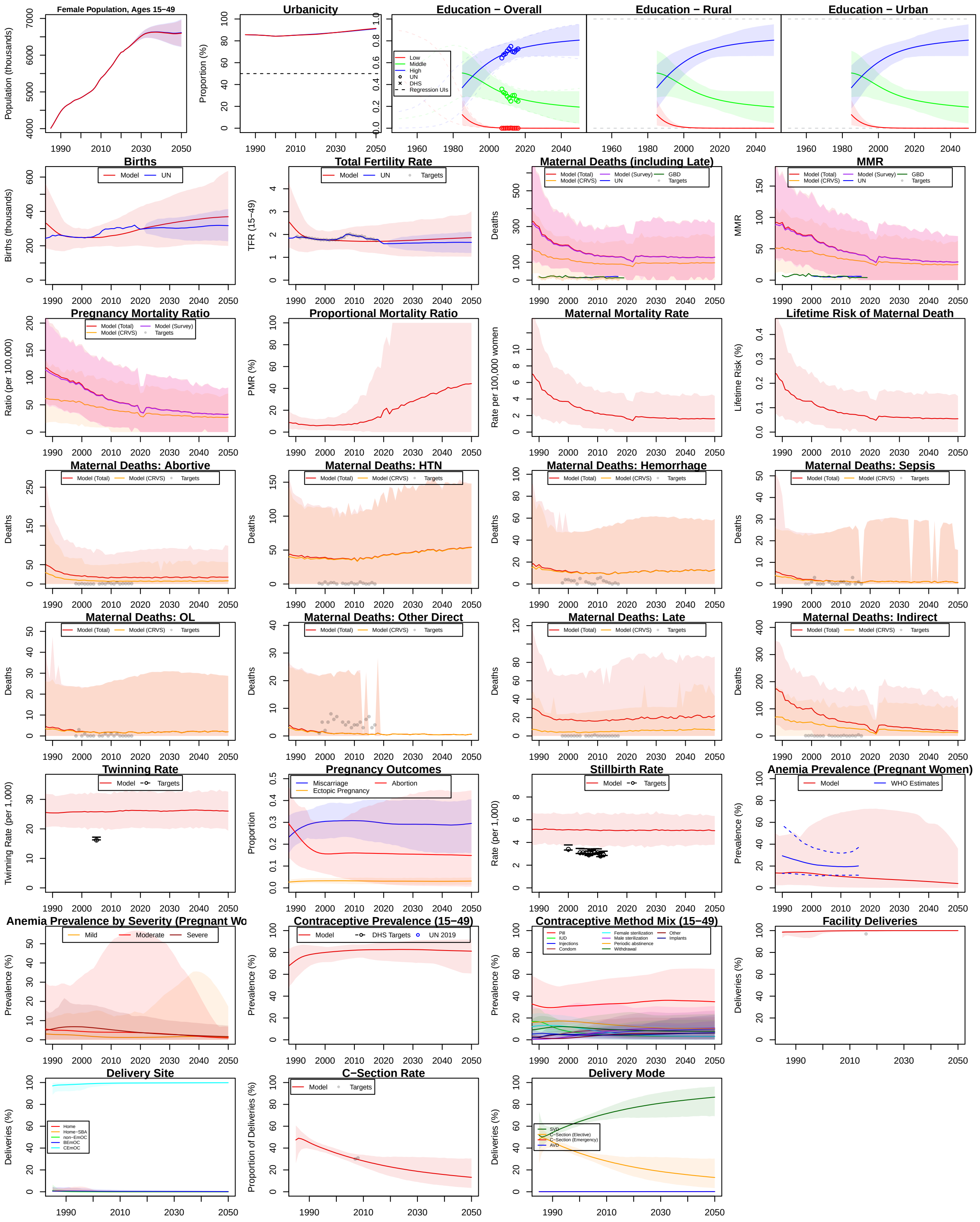

Austria

ISO Code	Region	Area	Income Group
AUT	Western Europe	Europe	High income

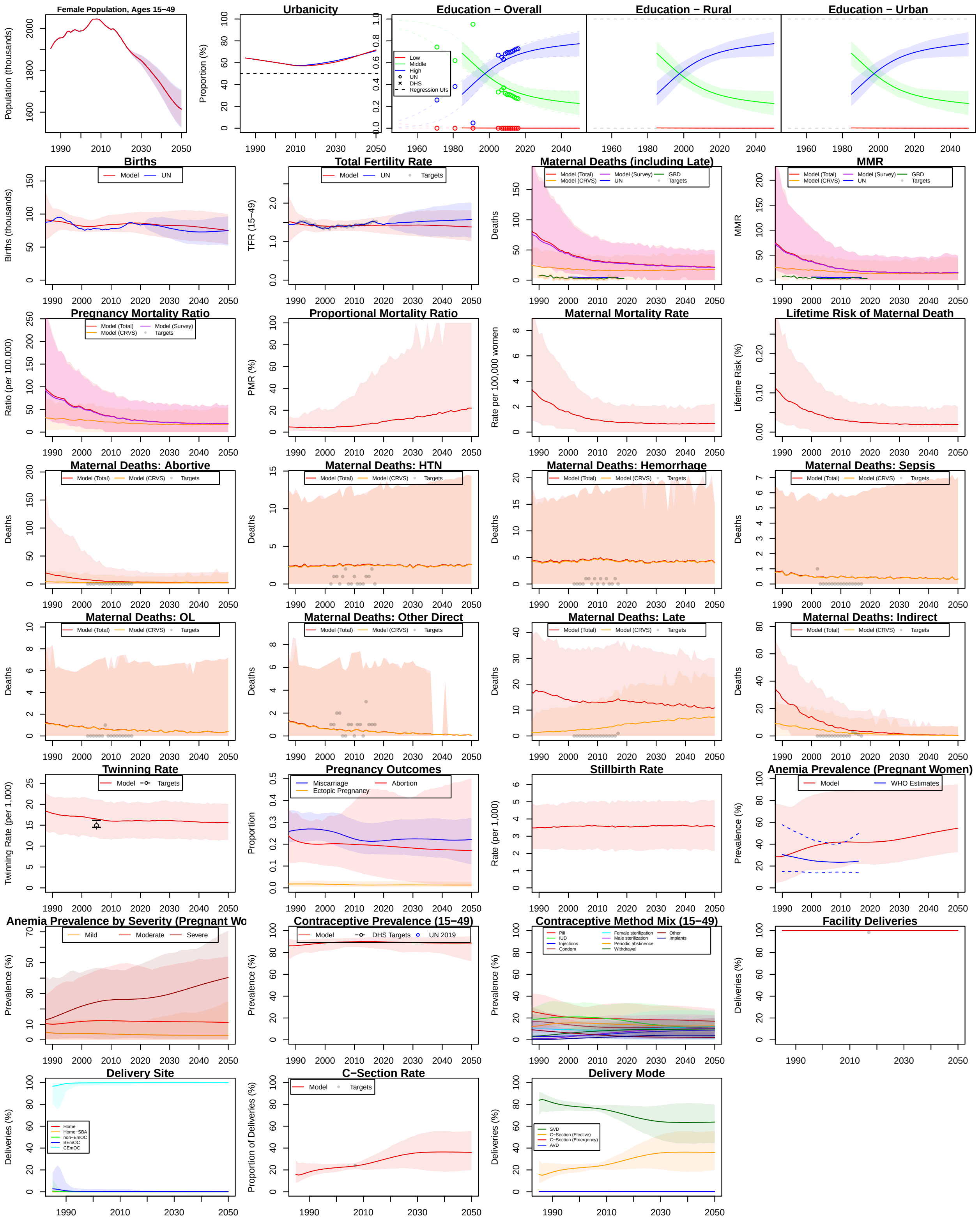

Azerbaijan

ISO Code	Region	Area	Income Group
AZE	Western Asia	Asia	Upper middle income

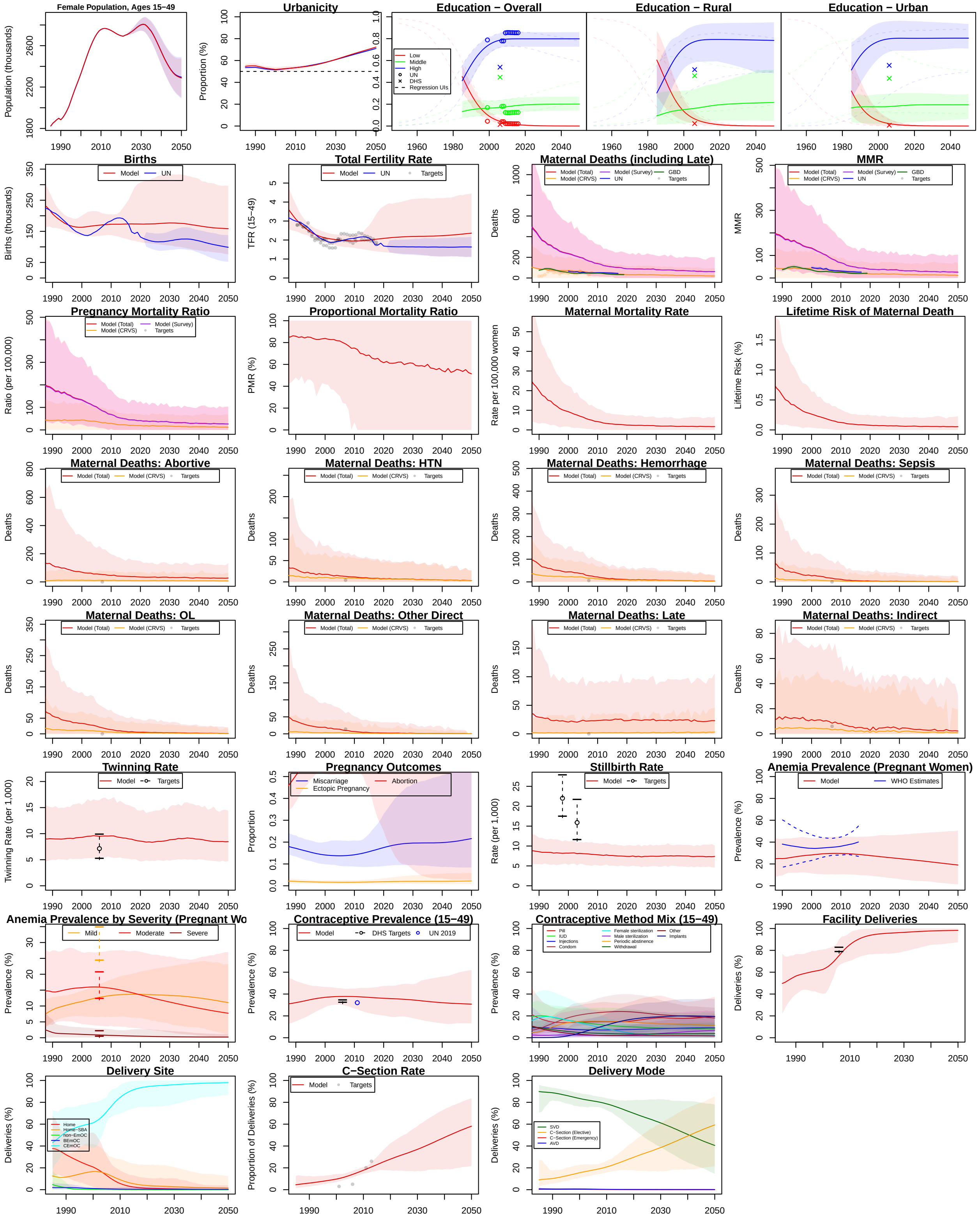

Bahamas

ISO Code	Region	Area	Income Group
BHS	Caribbean	Latin America and the Caribbean	High income

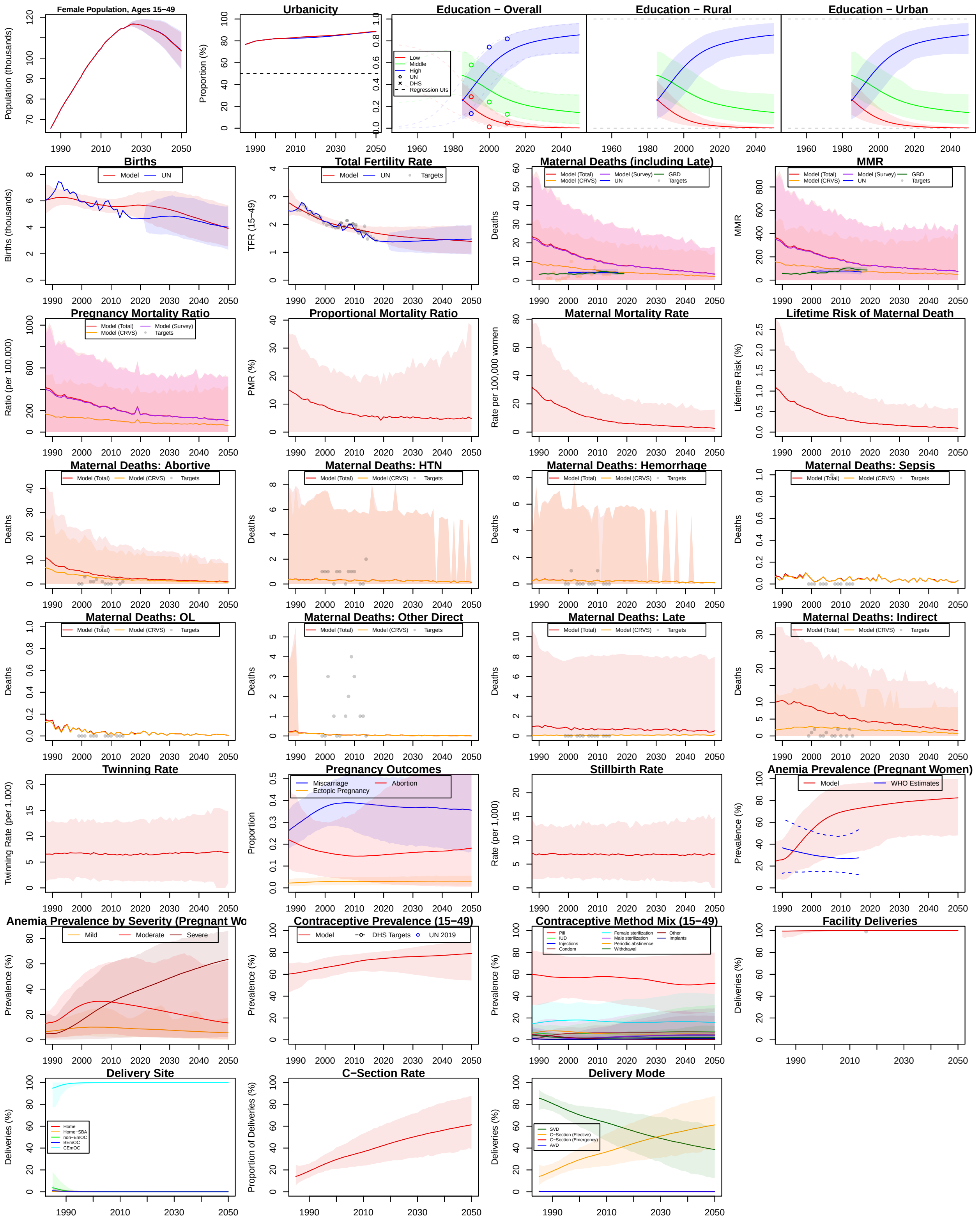

Bahrain

ISO Code	Region	Area	Income Group
BHR	Western Asia	Asia	High income

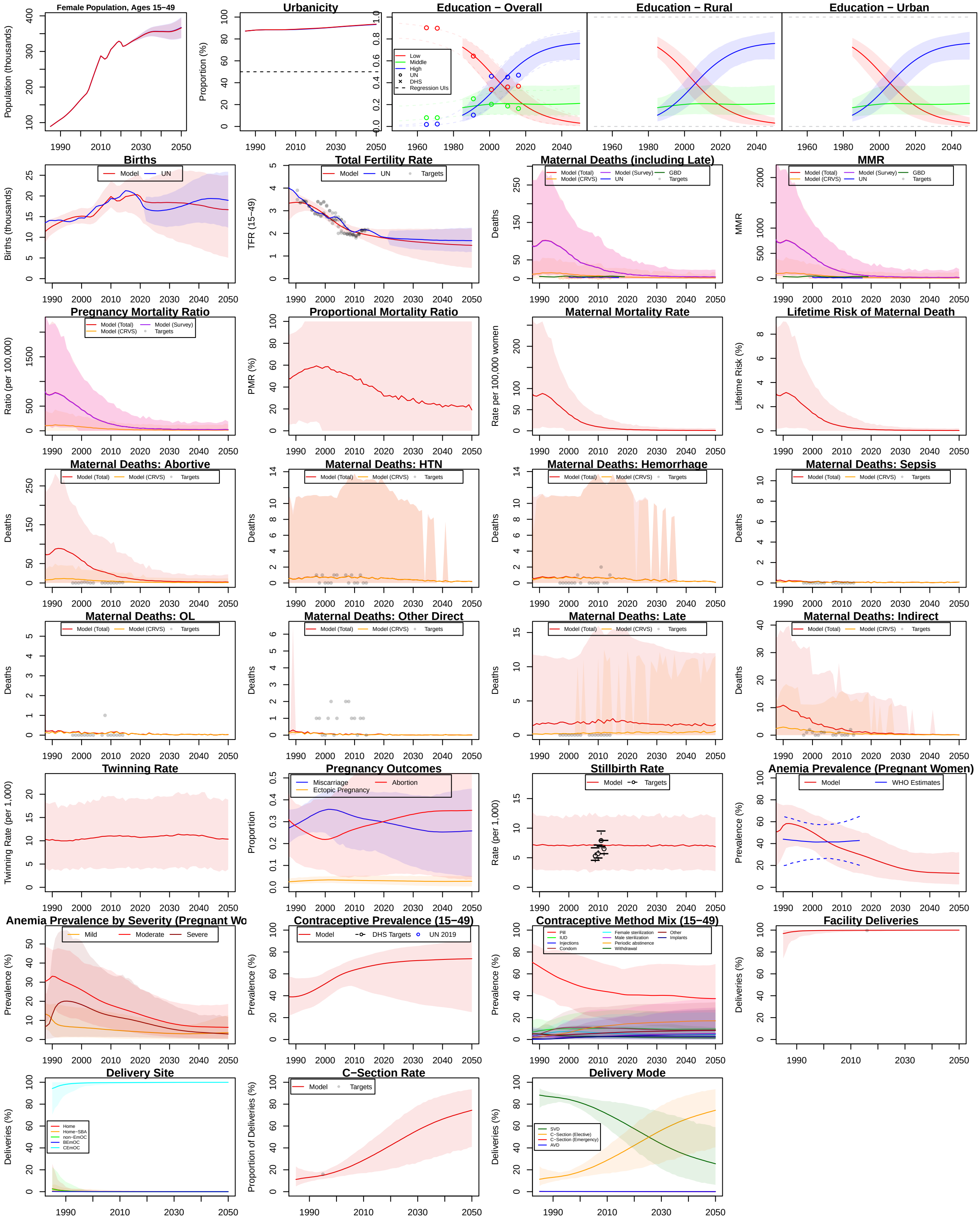

Bangladesh

ISO Code	Region	Area	Income Group
BGD	Southern Asia	Asia	Lower middle income

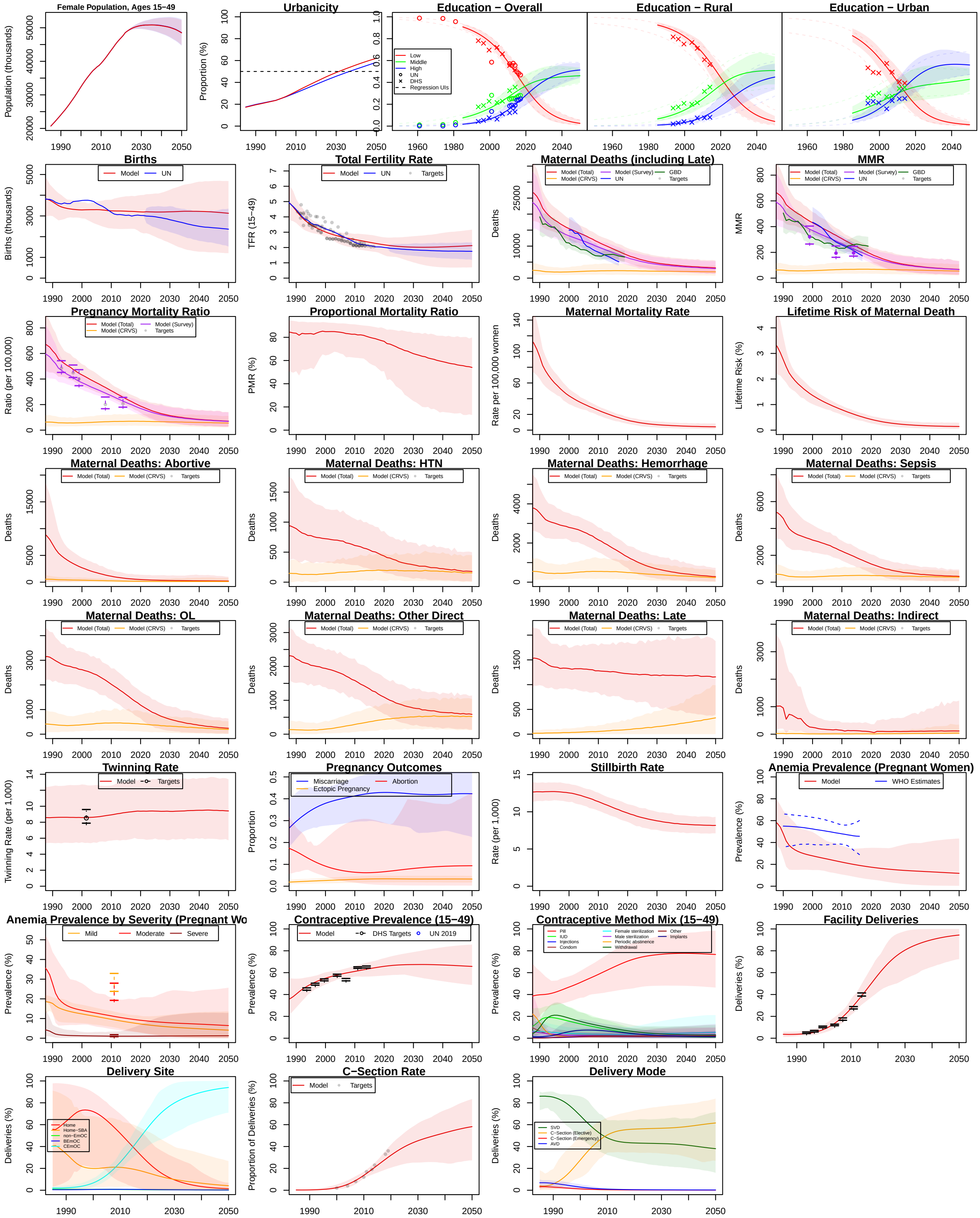

Barbados

ISO Code	Region	Area	Income Group
BRB	Caribbean	Latin America and the Caribbean	High income

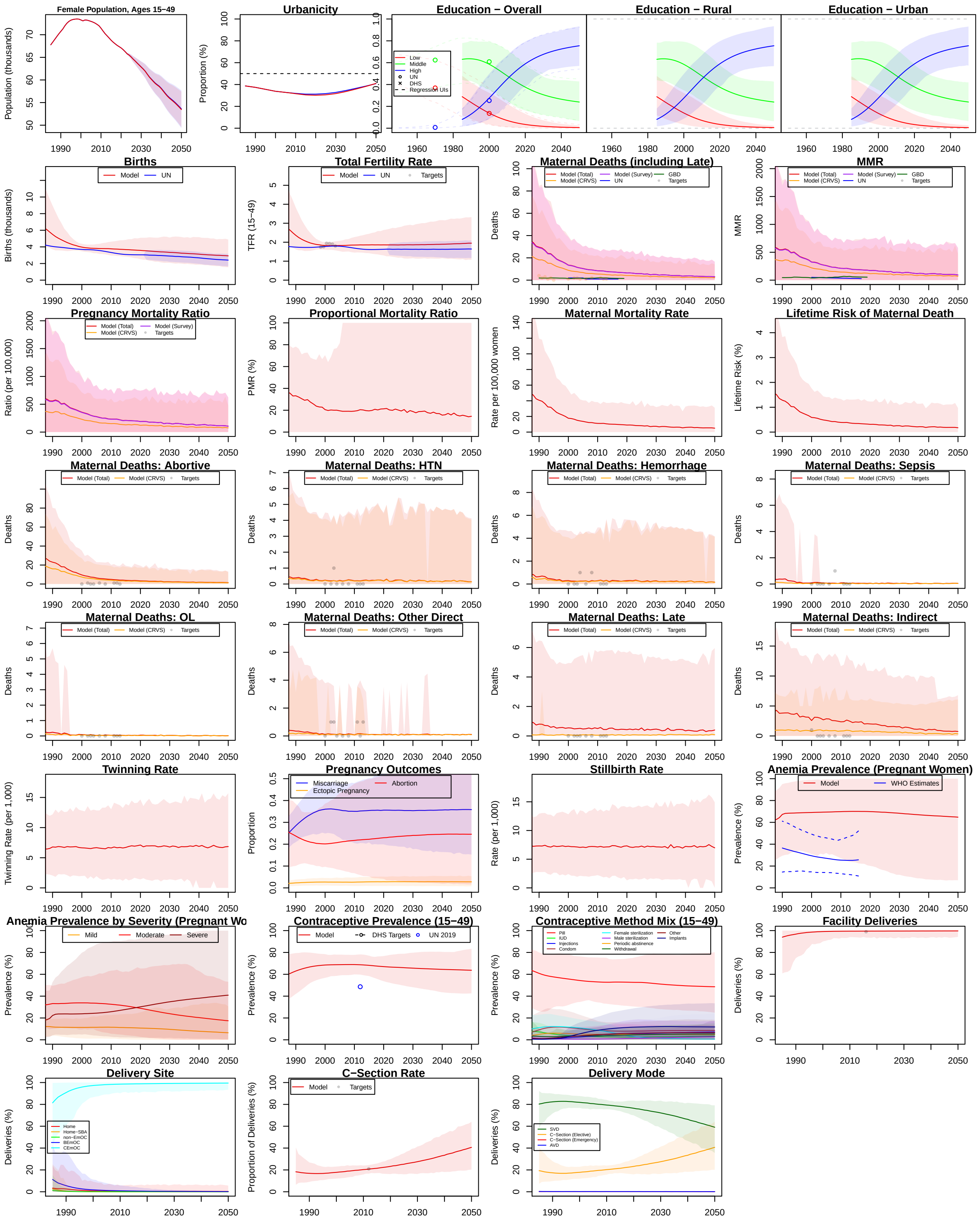

Belarus

ISO Code	Region	Area	Income Group
BLR	Eastern Europe	Europe	Upper middle income

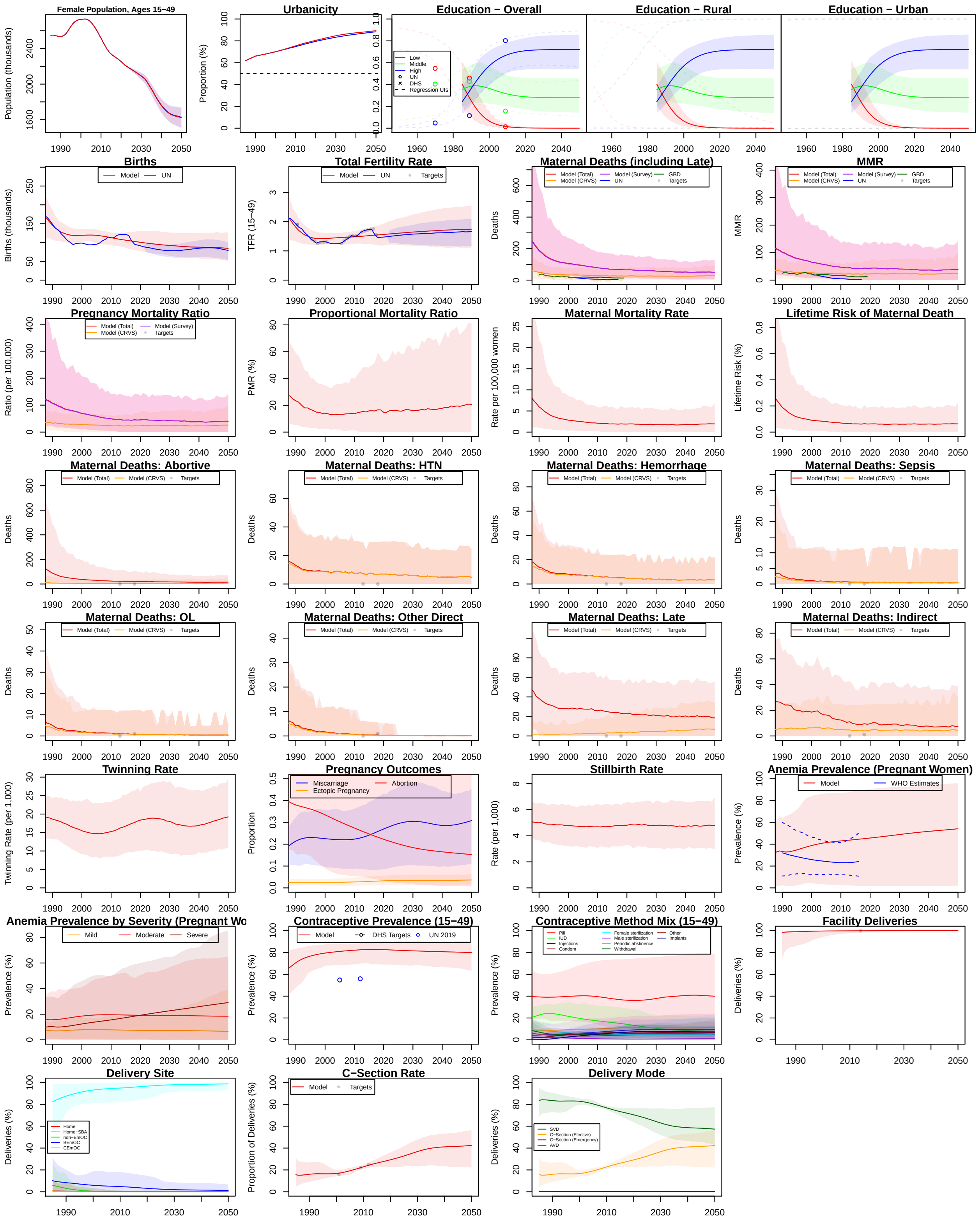

Belgium

ISO Code	Region	Area	Income Group
BEL	Western Europe	Europe	High income

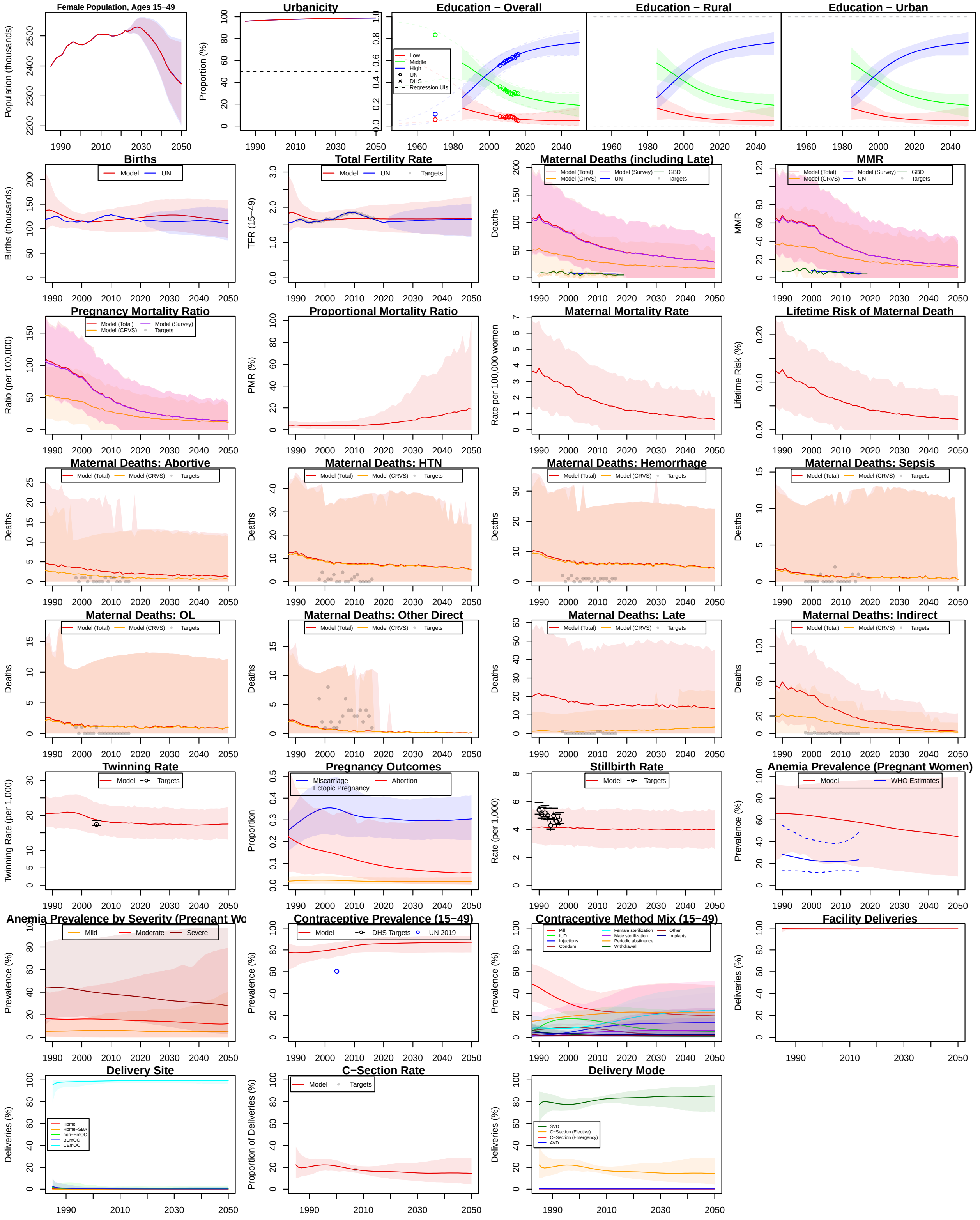

Belize

ISO Code	Region	Area	Income Group
BLZ	Central America	Latin America and the Caribbean	Upper middle income

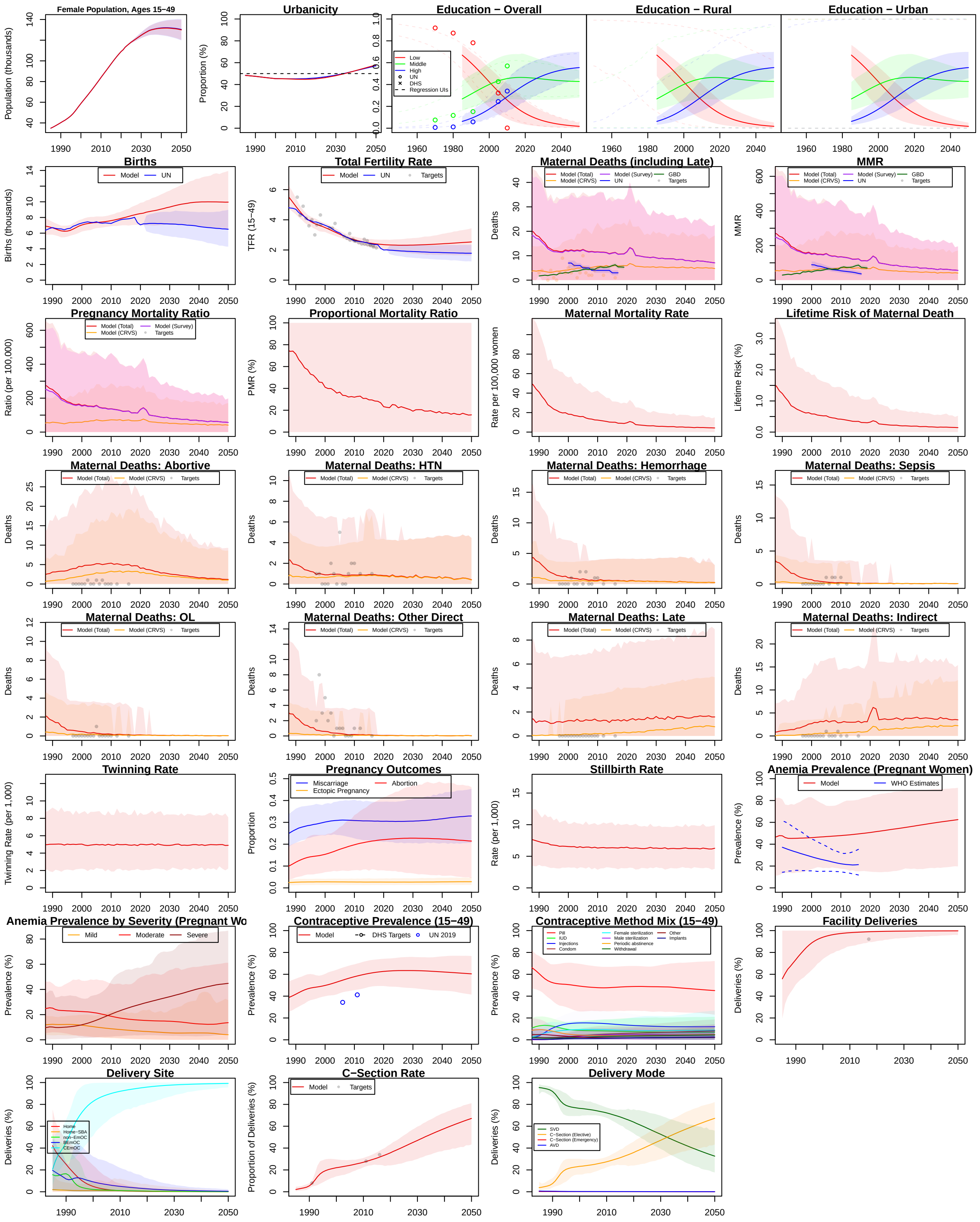

Benin

ISO Code	Region	Area	Income Group
BEN	Western Africa	Africa	Low income

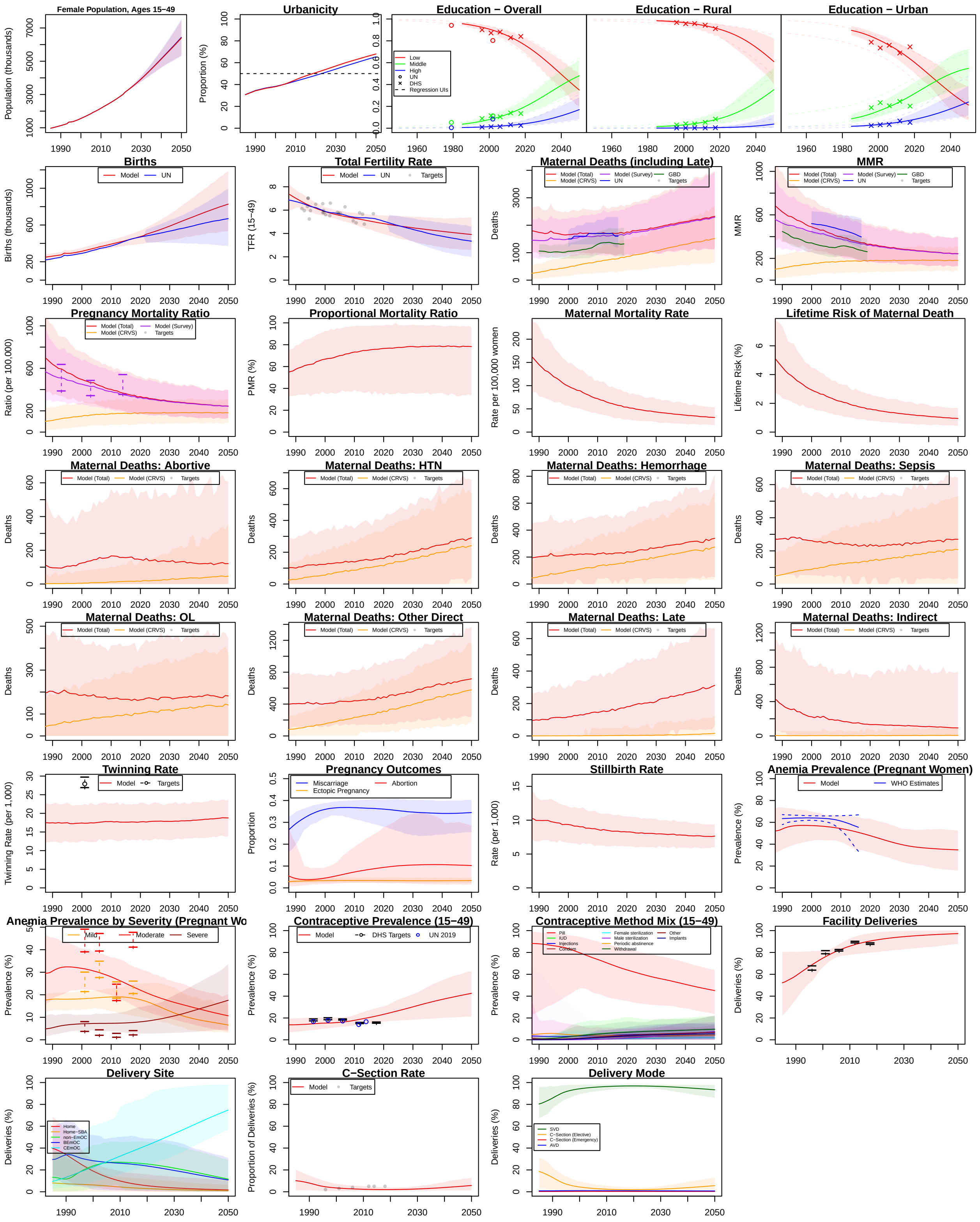

Bermuda

ISO Code	Region	Area	Income Group
BMU	Northern America	Northern America	High income

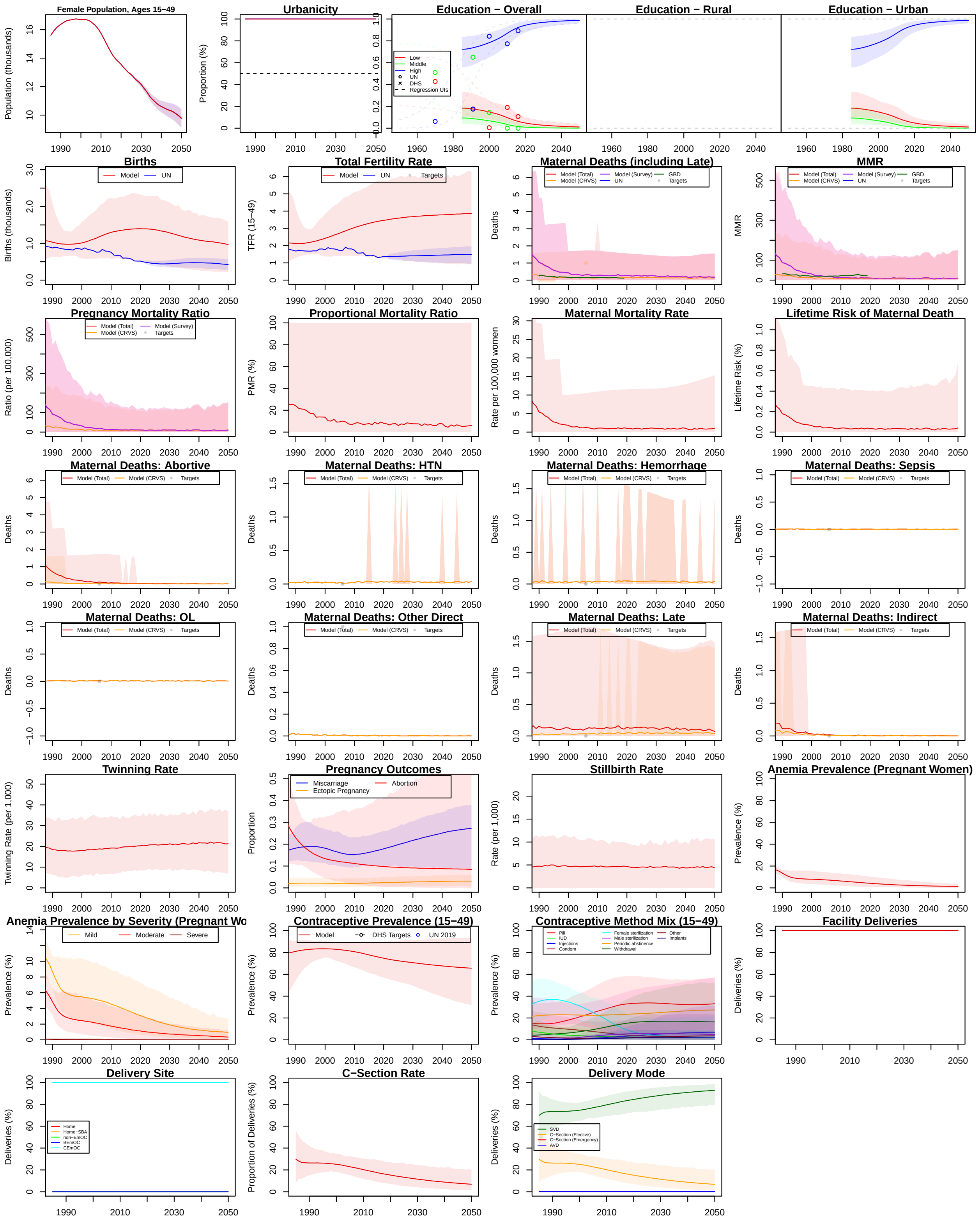

Bhutan

ISO Code	Region	Area	Income Group
BTN	Southern Asia	Asia	Lower middle income

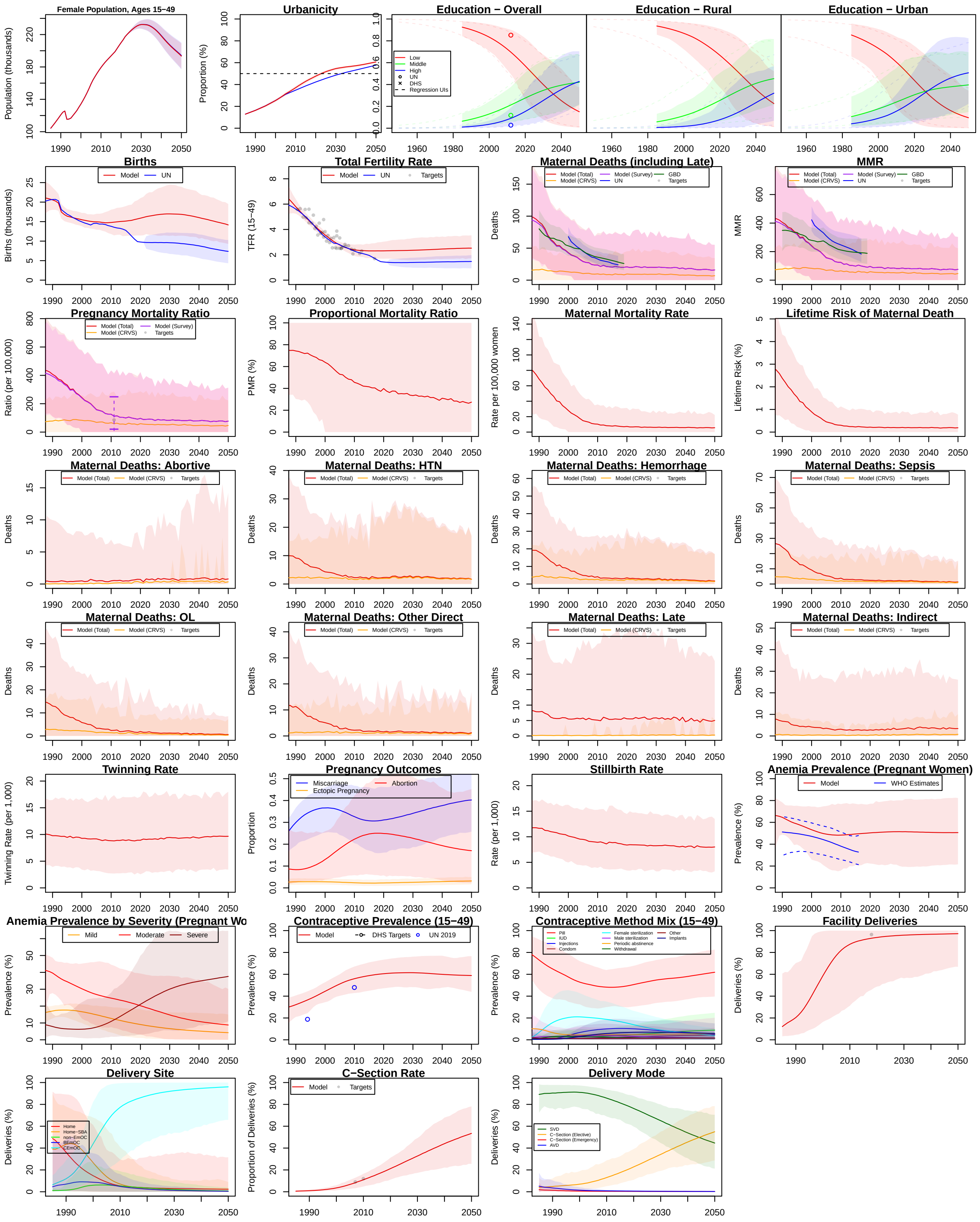

Bolivia (Plurinational State of)

ISO Code	Region	Area	Income Group
BOL	South America	Latin America and the Caribbean	Lower middle income

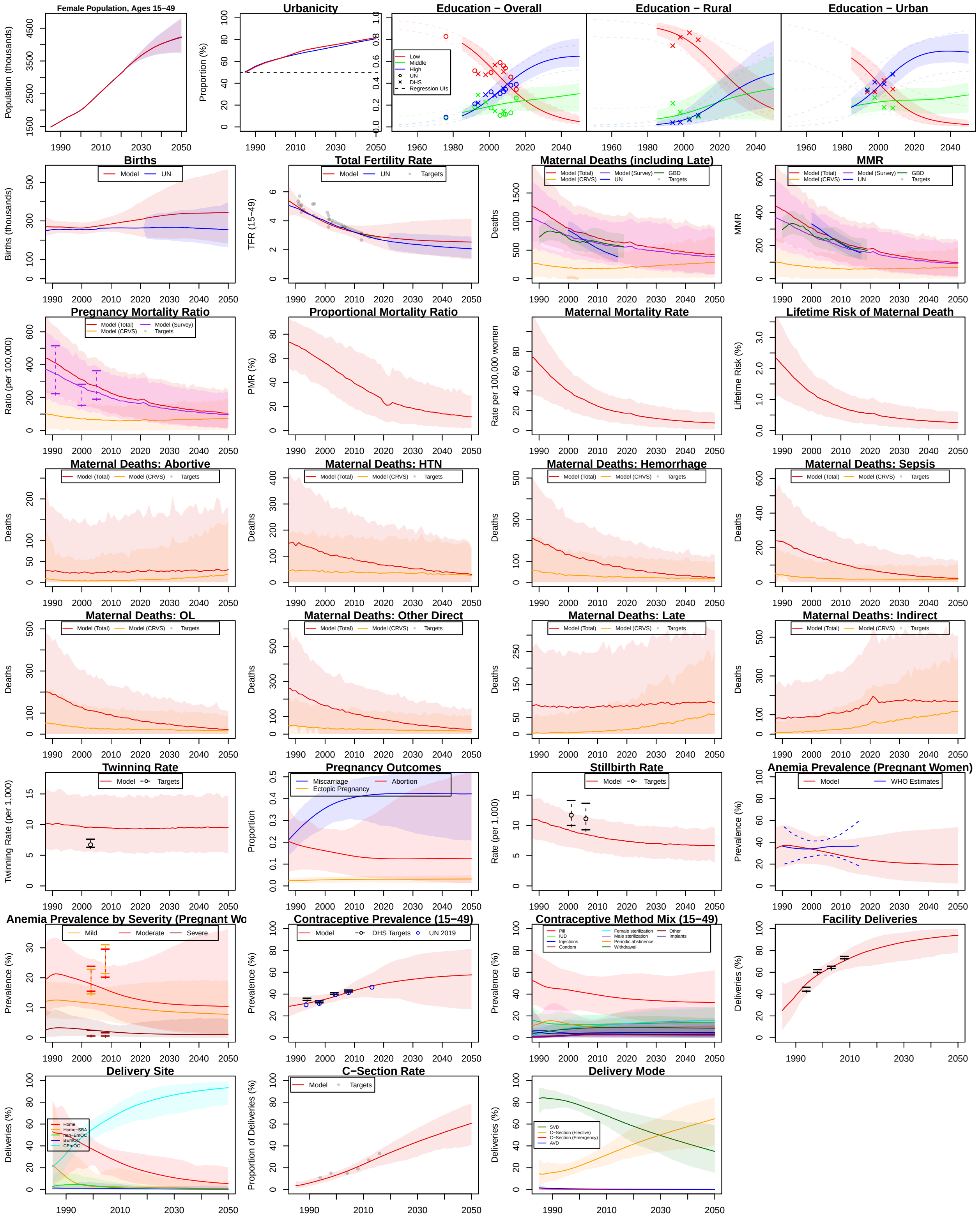

Bosnia and Herzegovina

ISO Code	Region	Area	Income Group
BIH	Southern Europe	Europe	Upper middle income

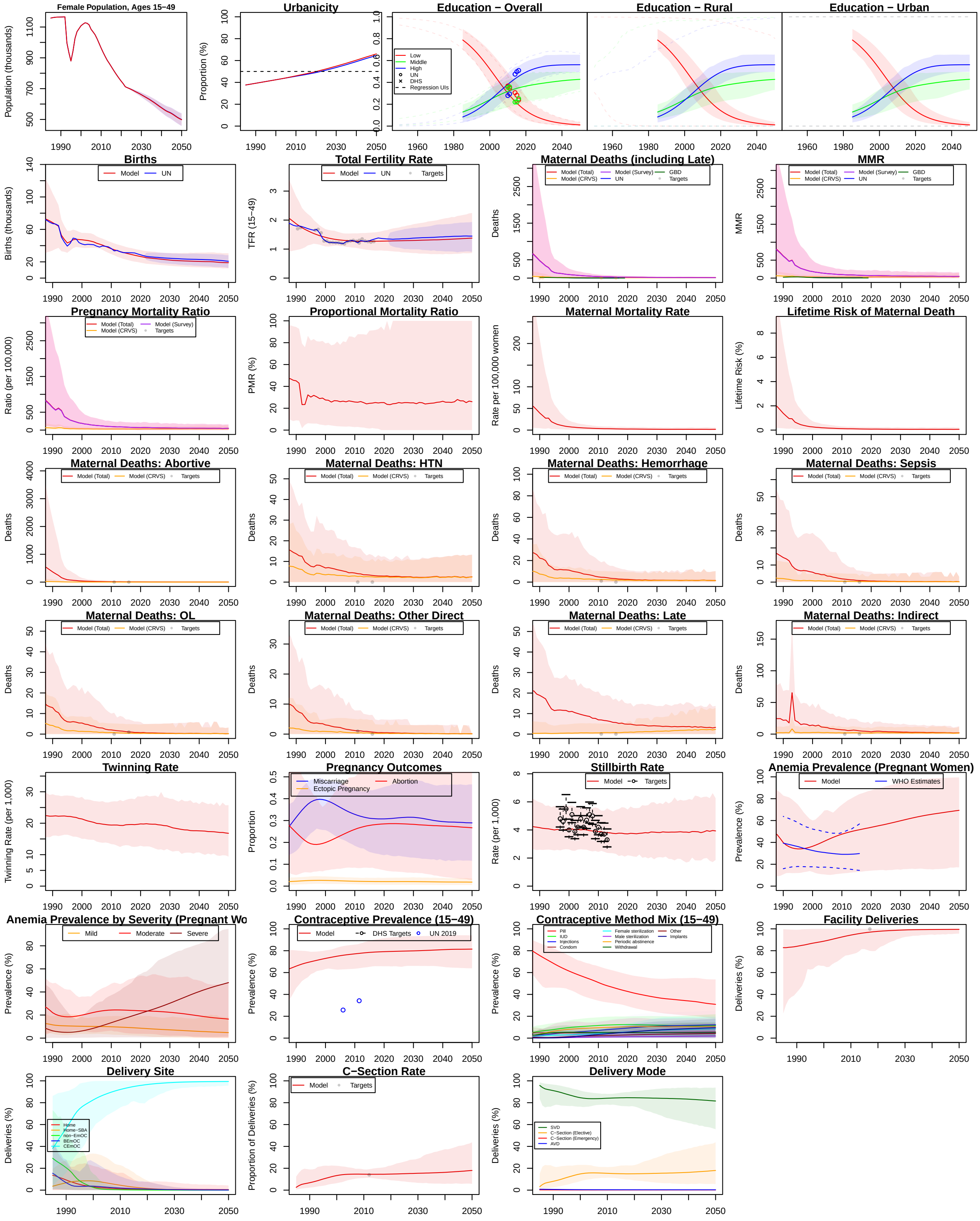

Botswana

ISO Code	Region	Area	Income Group
BWA	Southern Africa	Africa	Upper middle income

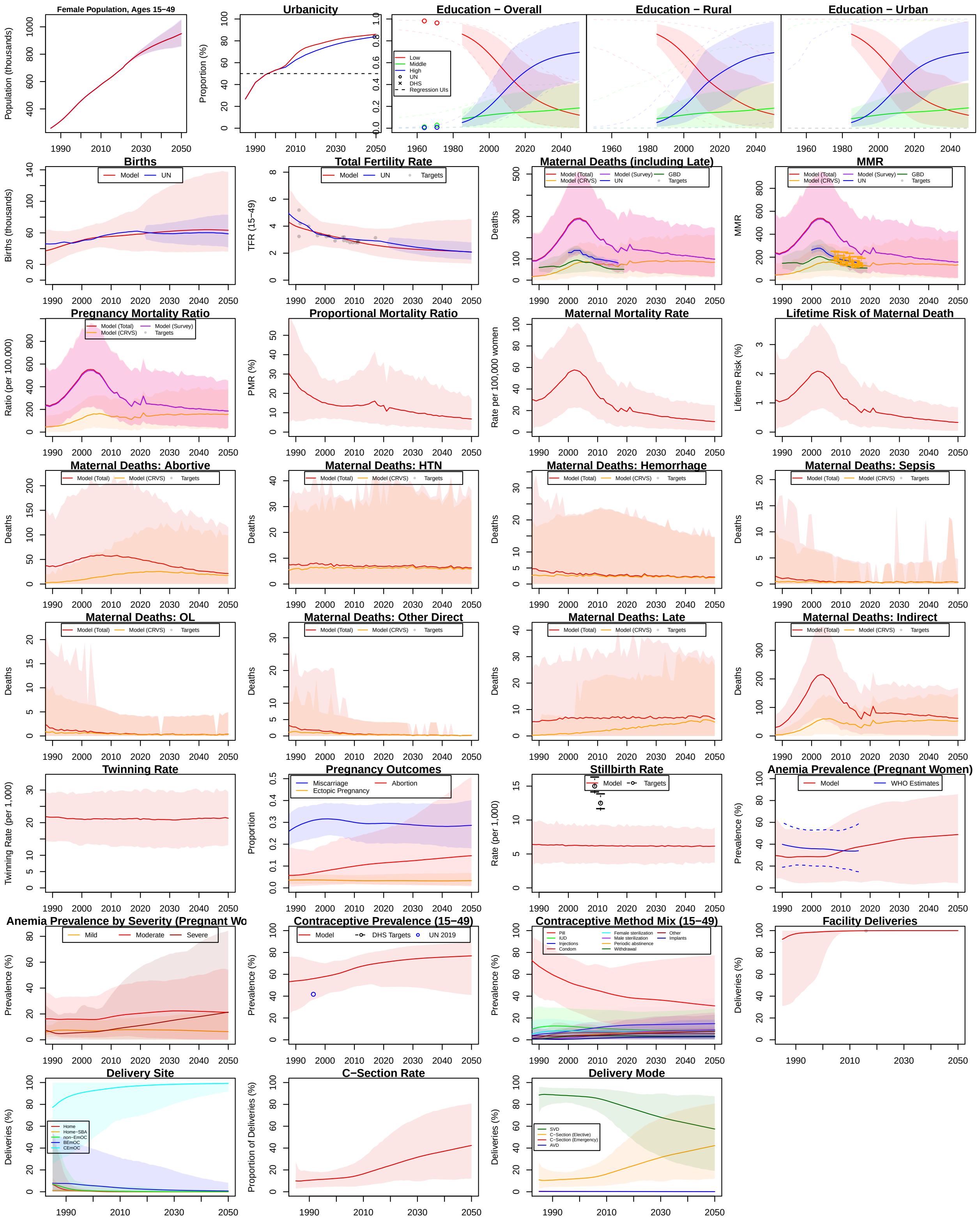

Brazil

ISO Code	Region	Area	Income Group
BRA	South America	Latin America and the Caribbean	Upper middle income

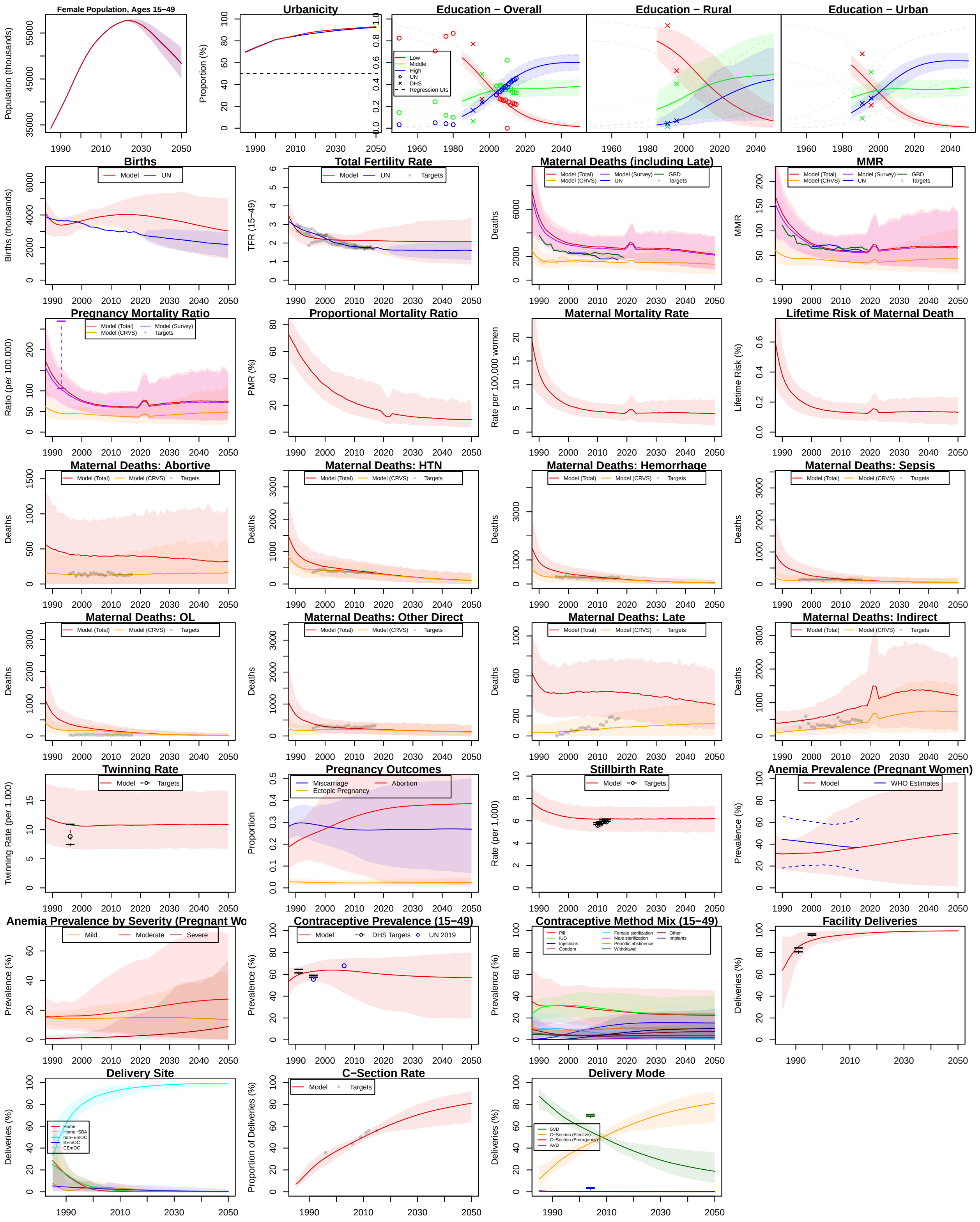

Brunei Darussalam

ISO Code	Region	Area	Income Group
BRN	South-Eastern Asia	Asia	High income

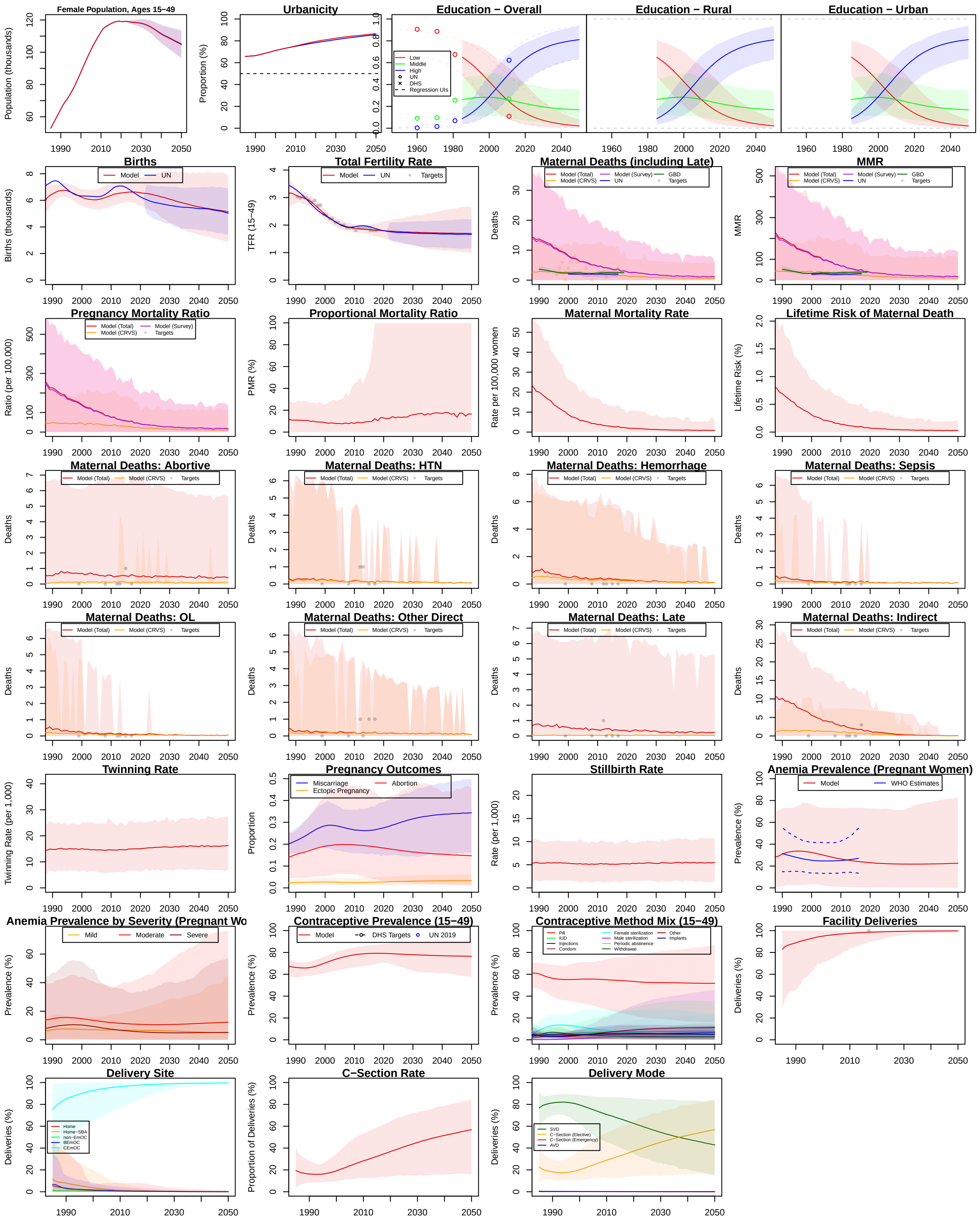

Bulgaria

ISO Code	Region	Area	Income Group
BGR	Eastern Europe	Europe	Upper middle income

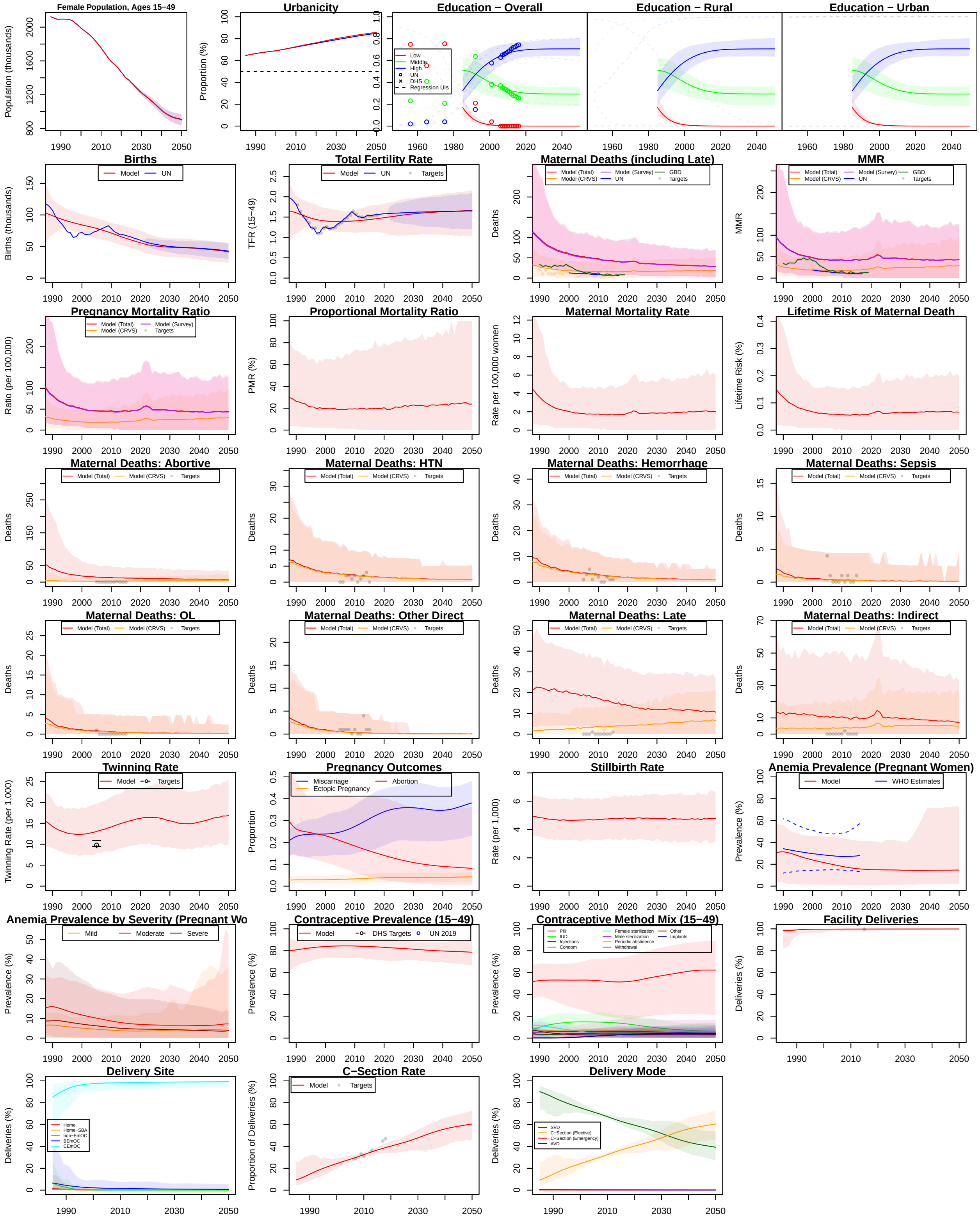

Burkina Faso

ISO Code	Region	Area	Income Group
BFA	Western Africa	Africa	Low income

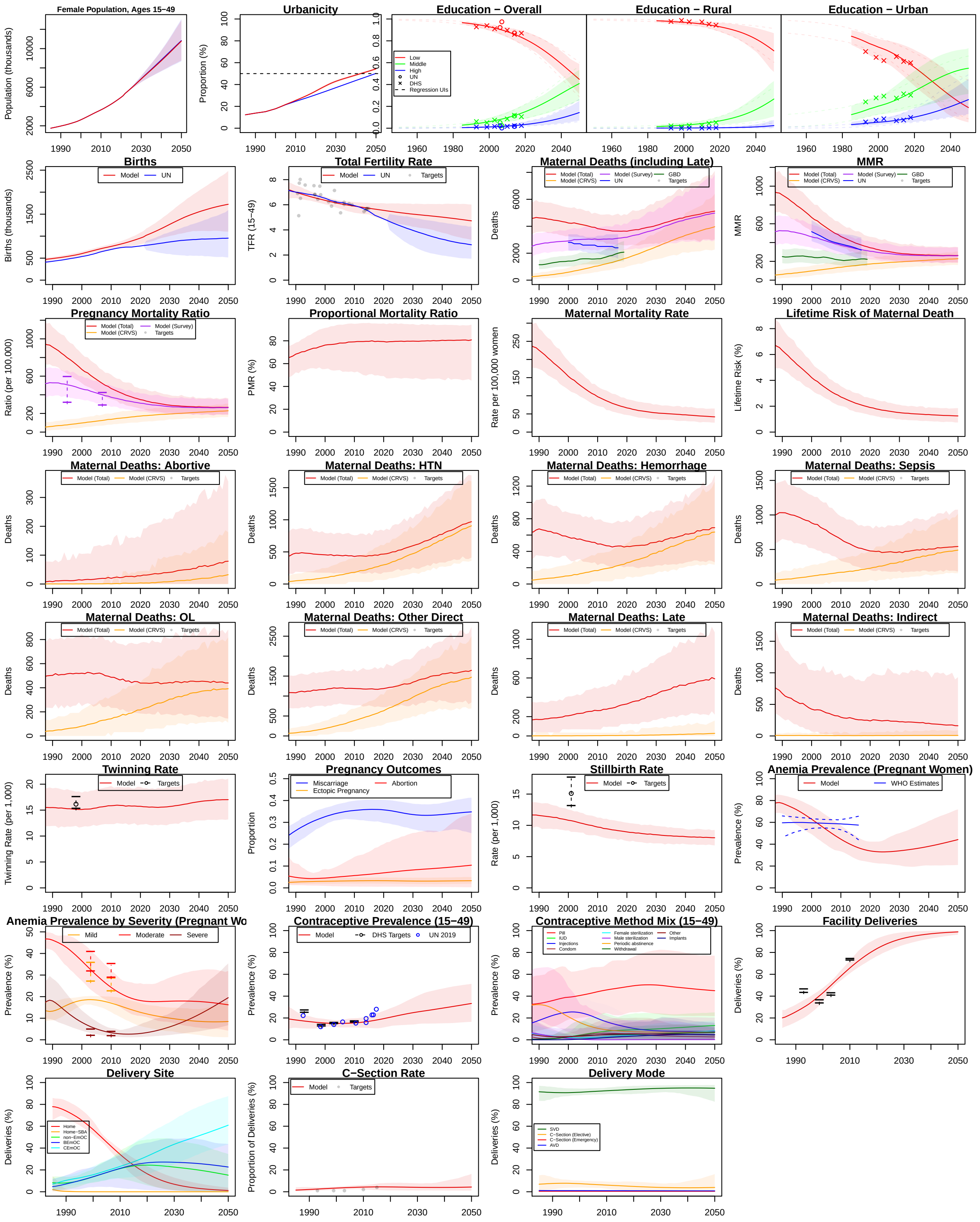

Burundi

ISO Code	Region	Area	Income Group
BDI	Eastern Africa	Africa	Low income

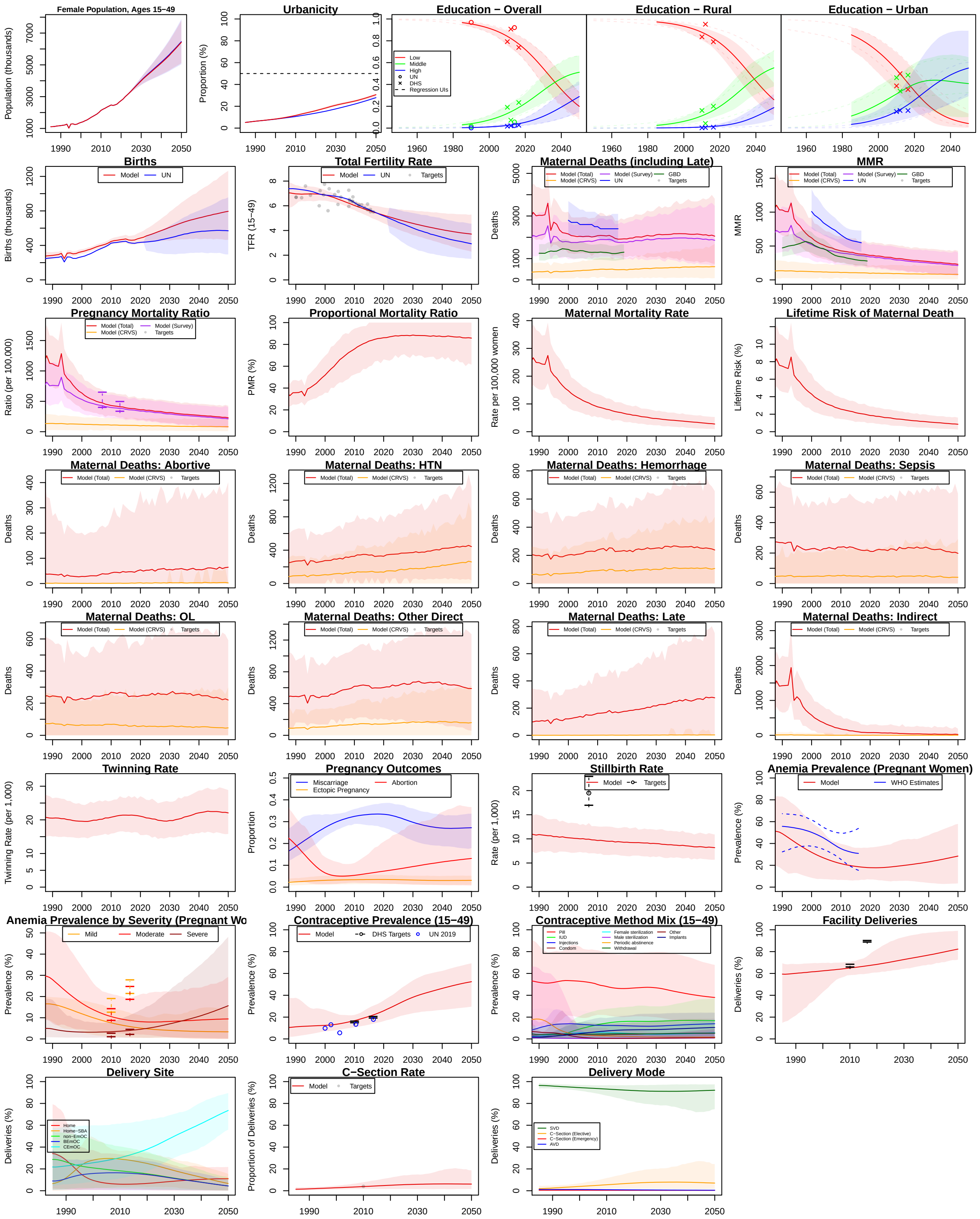

Cabo Verde

ISO Code	Region	Area	Income Group
CPV	Western Africa	Africa	Lower middle income

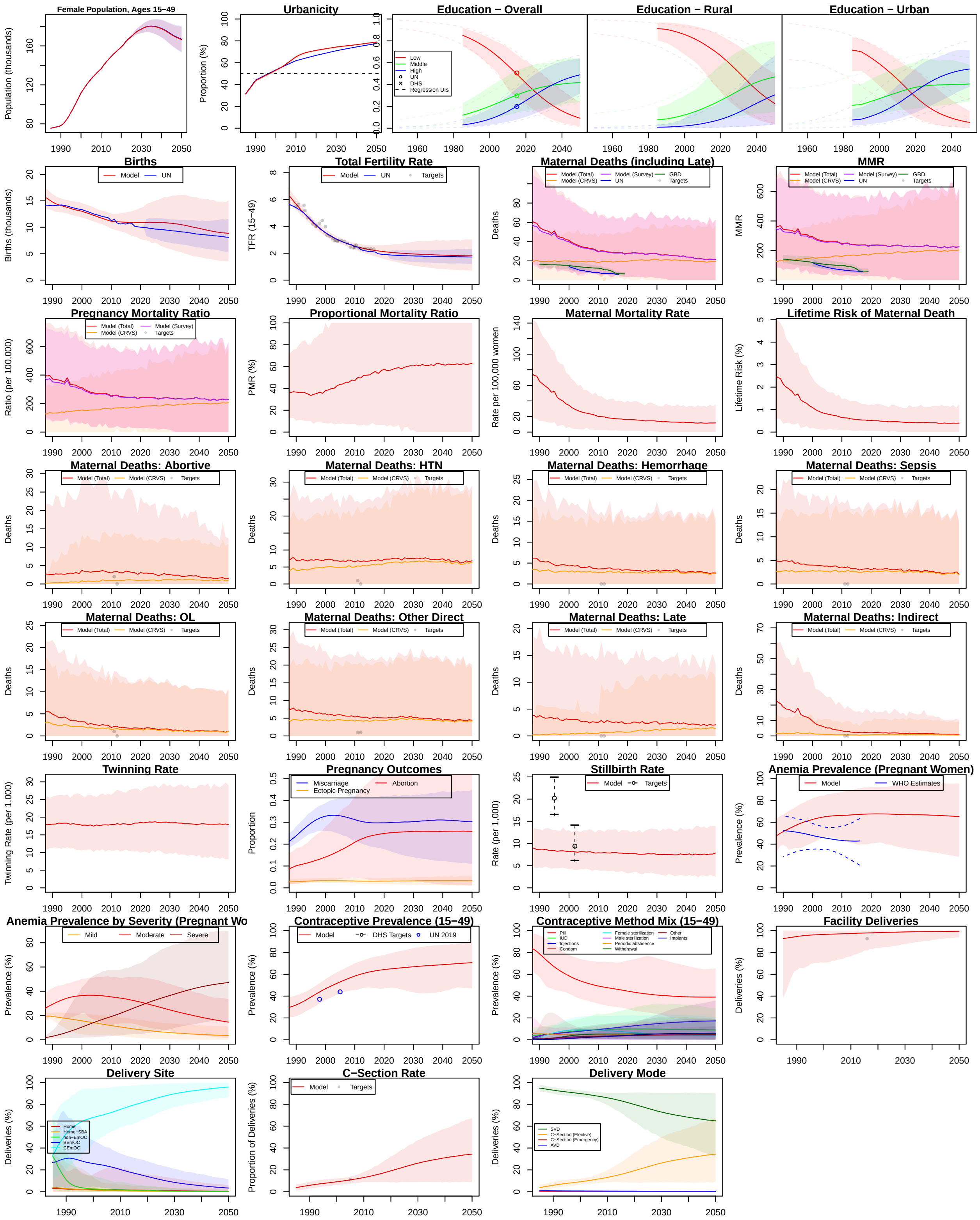

Cambodia

ISO Code	Region	Area	Income Group
KHM	South-Eastern Asia	Asia	Lower middle income

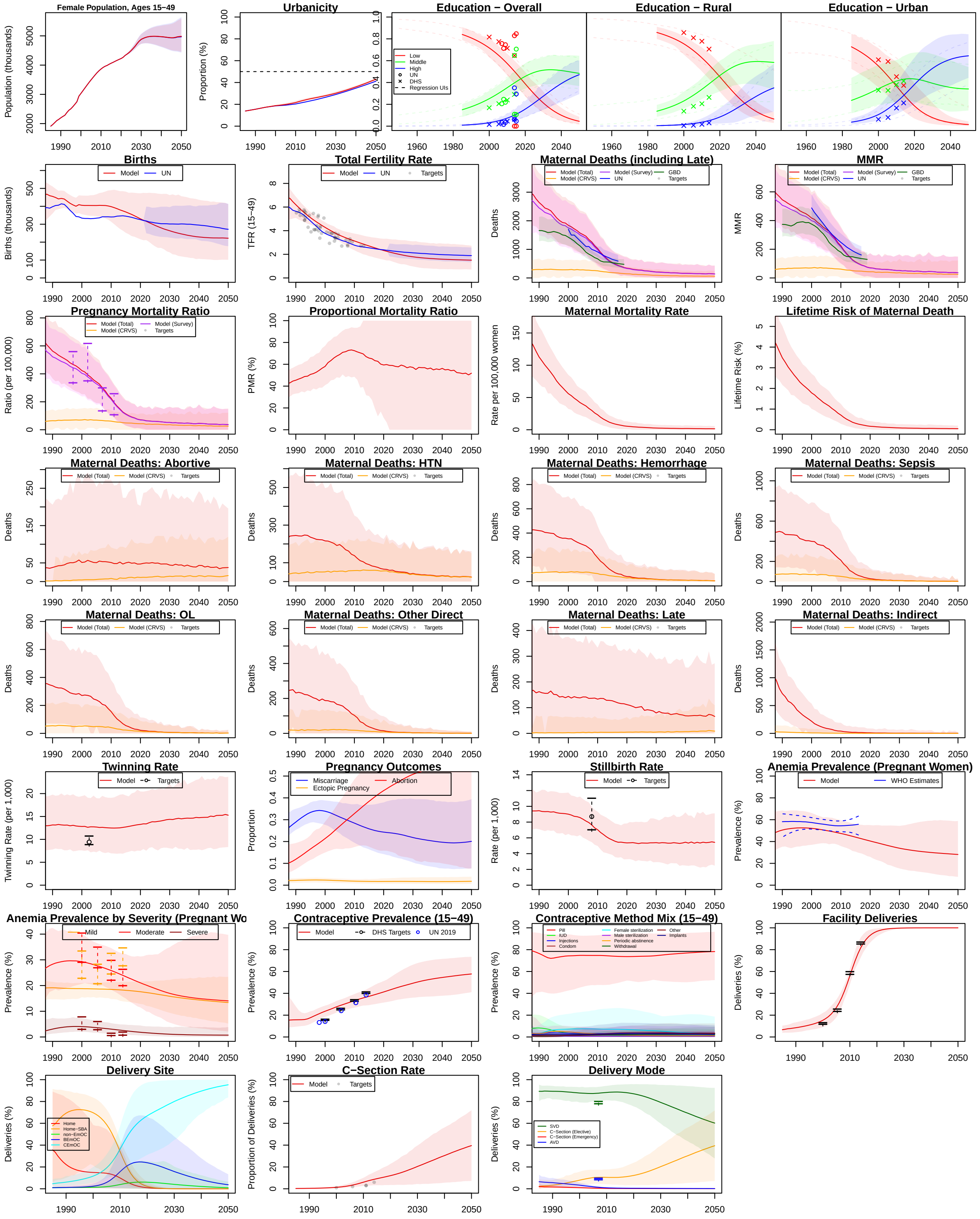

Cameroon

ISO Code	Region	Area	Income Group
CMR	Middle Africa	Africa	Lower middle income

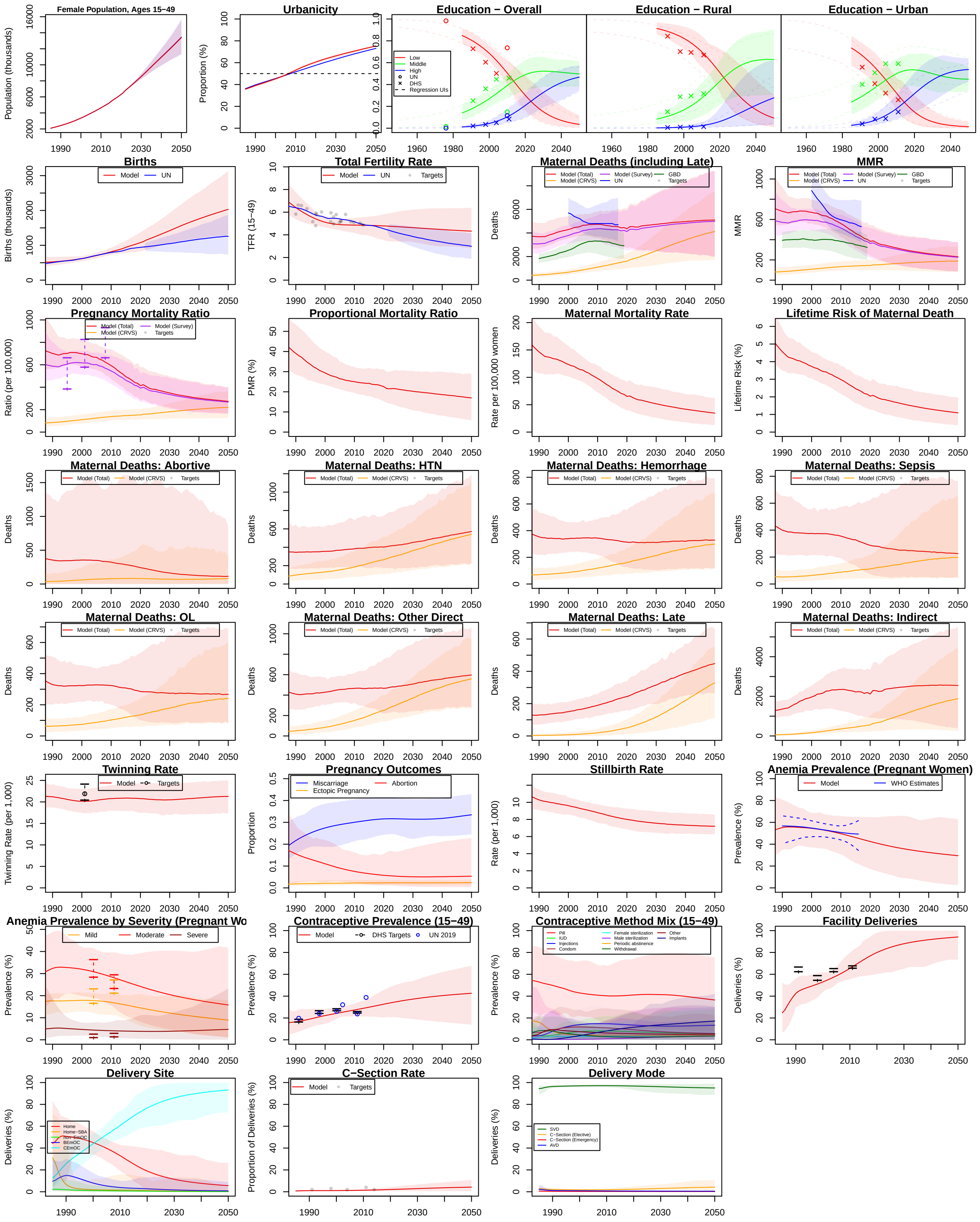

Canada

ISO Code	Region	Area	Income Group
CAN	Northern America	Northern America	High income

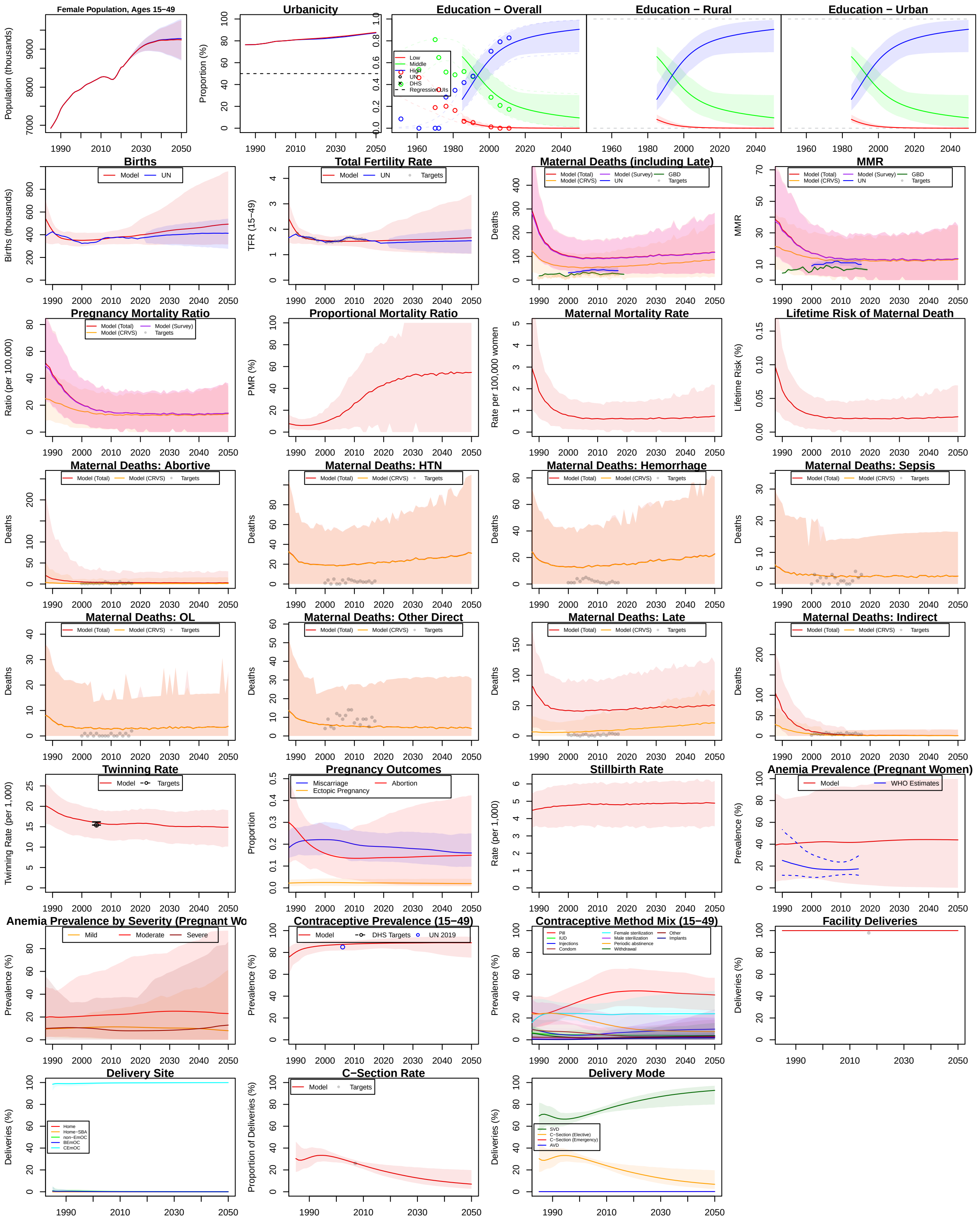

Cayman Islands

ISO Code	Region	Area	Income Group
CYM	Caribbean	Latin America and the Caribbean	High income

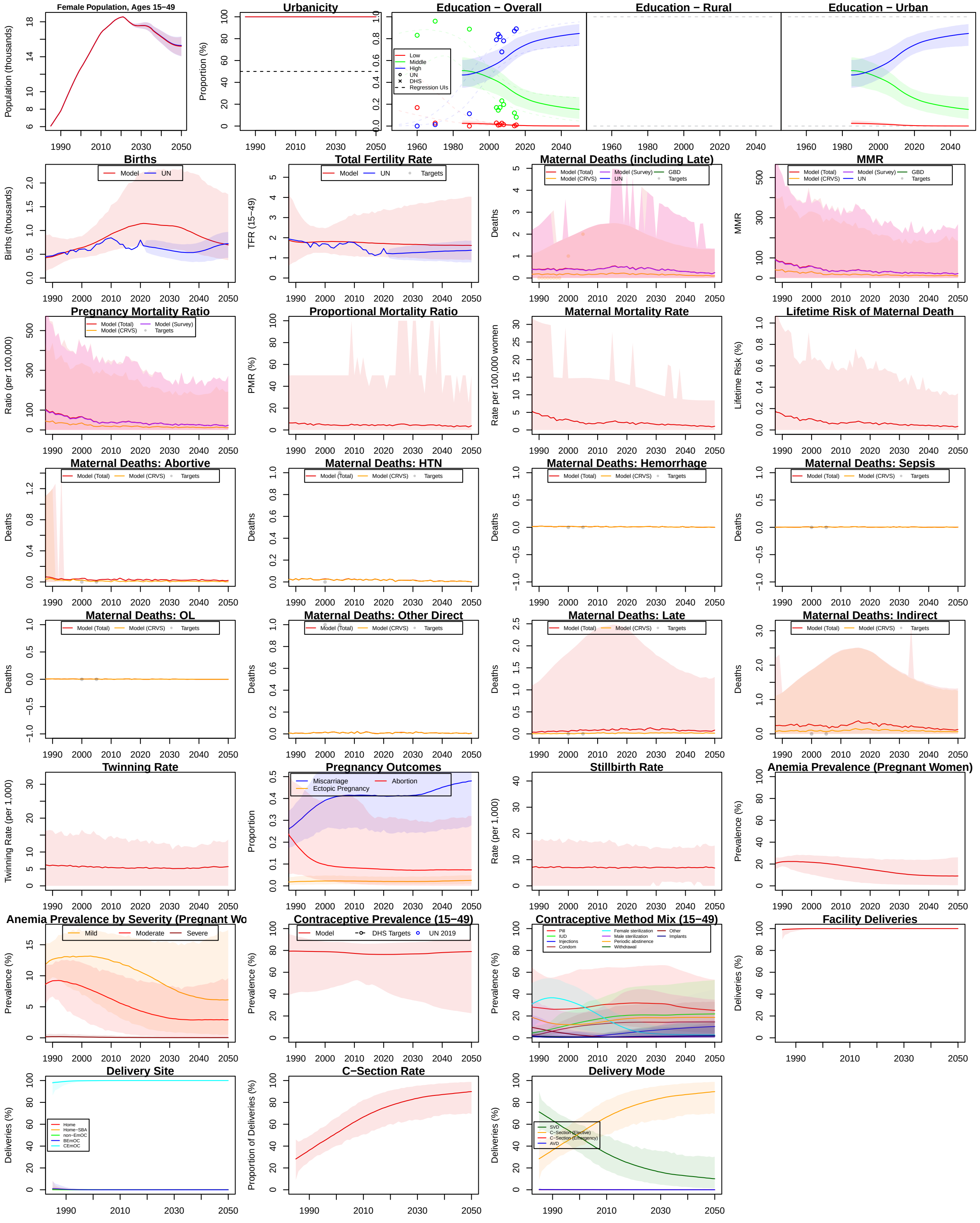

Central African Republic

ISO Code	Region	Area	Income Group
CAF	Middle Africa	Africa	Low income

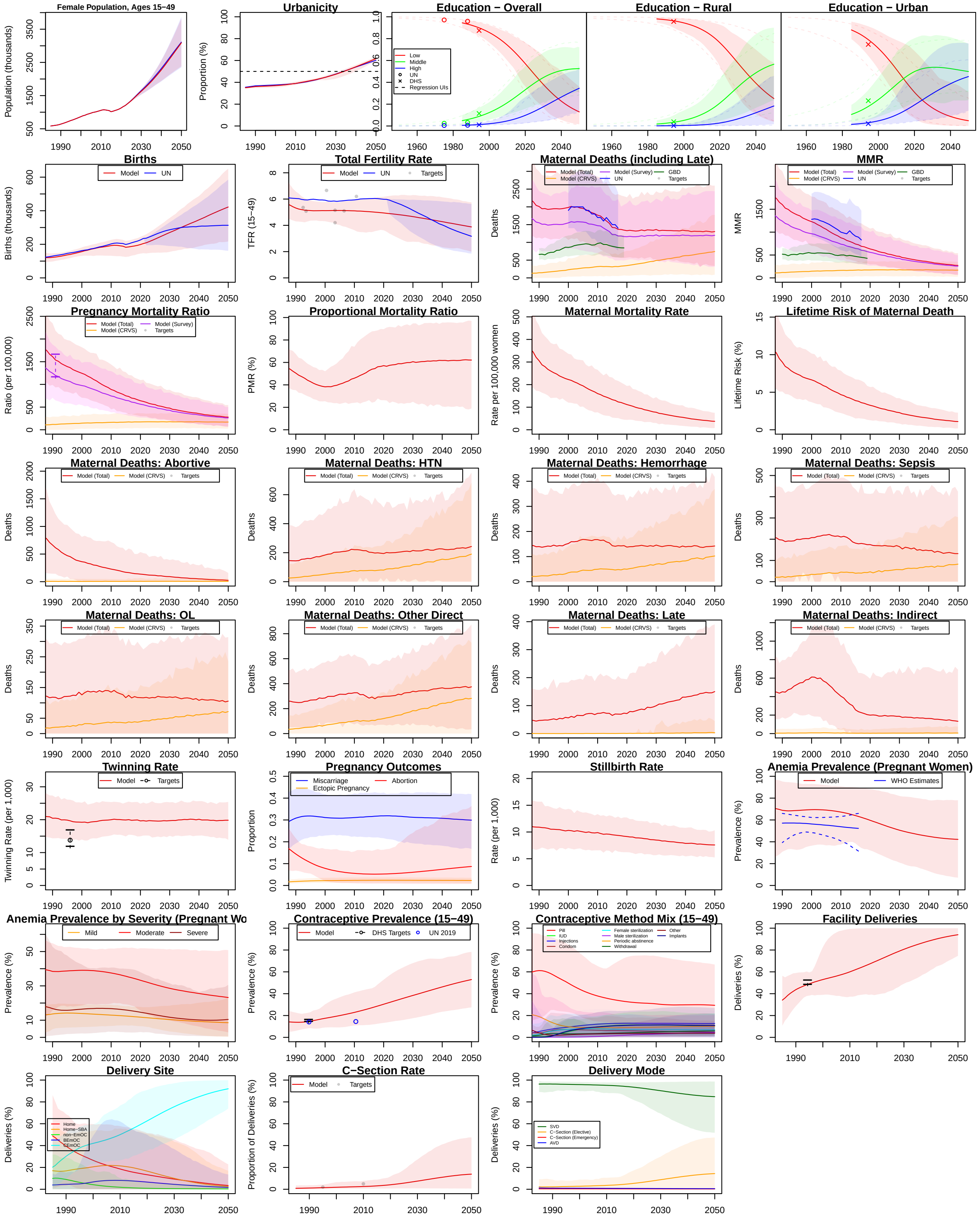

Chad

ISO Code	Region	Area	Income Group
TCD	Middle Africa	Africa	Low income

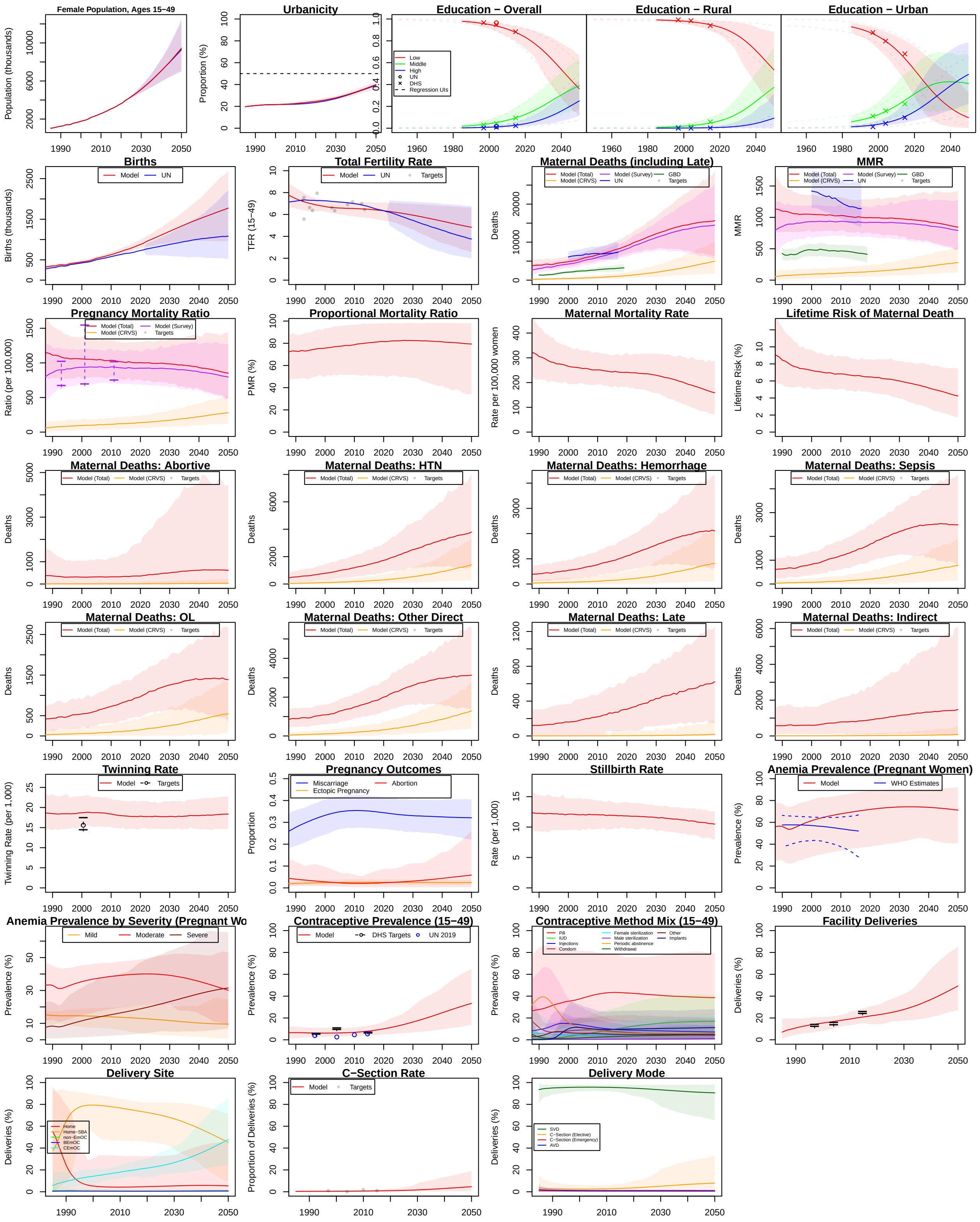

Chile

ISO Code	Region	Area	Income Group
CHL	South America	Latin America and the Caribbean	High income

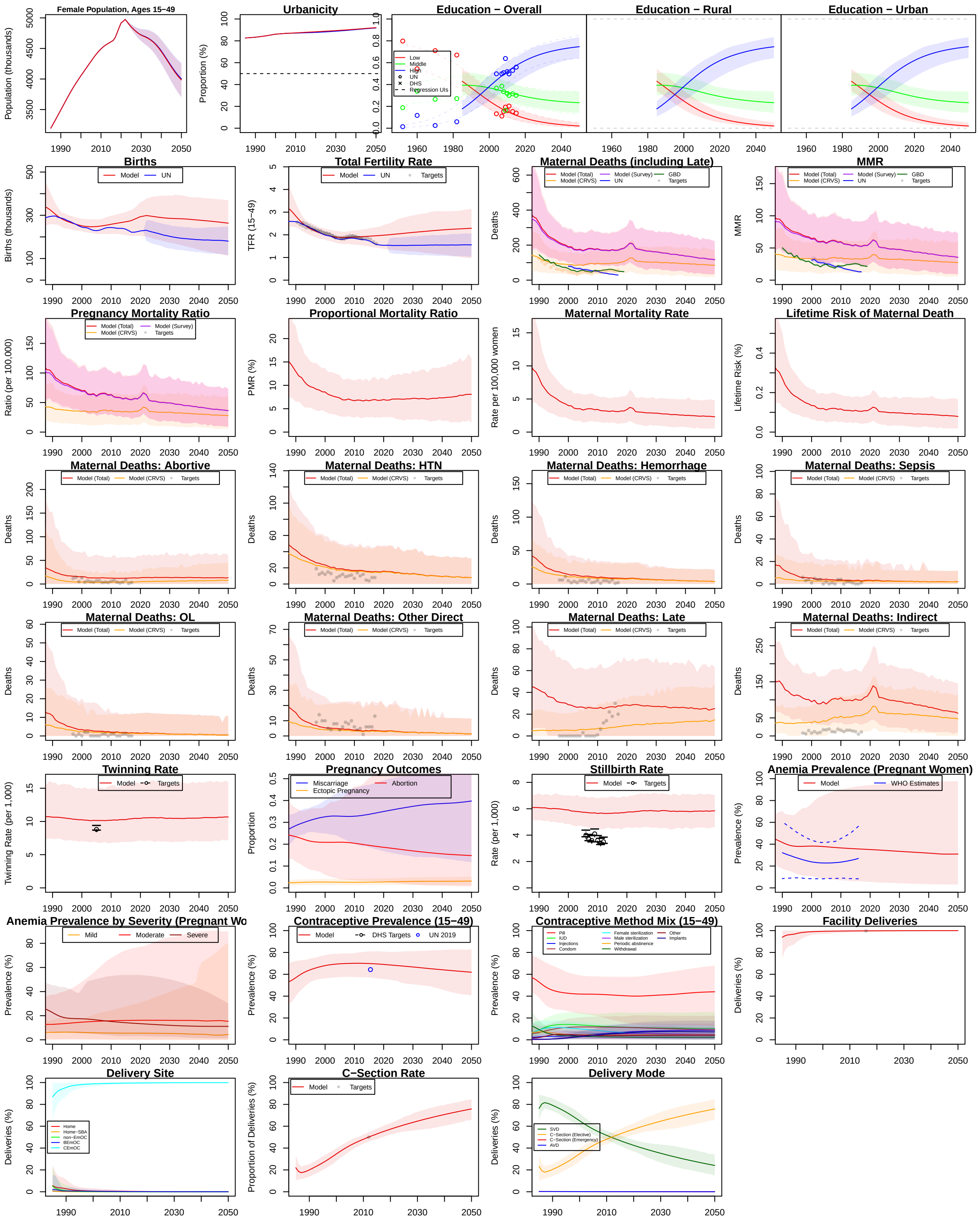

China

ISO Code	Region	Area	Income Group
CHN	Eastern Asia	Asia	Upper middle income

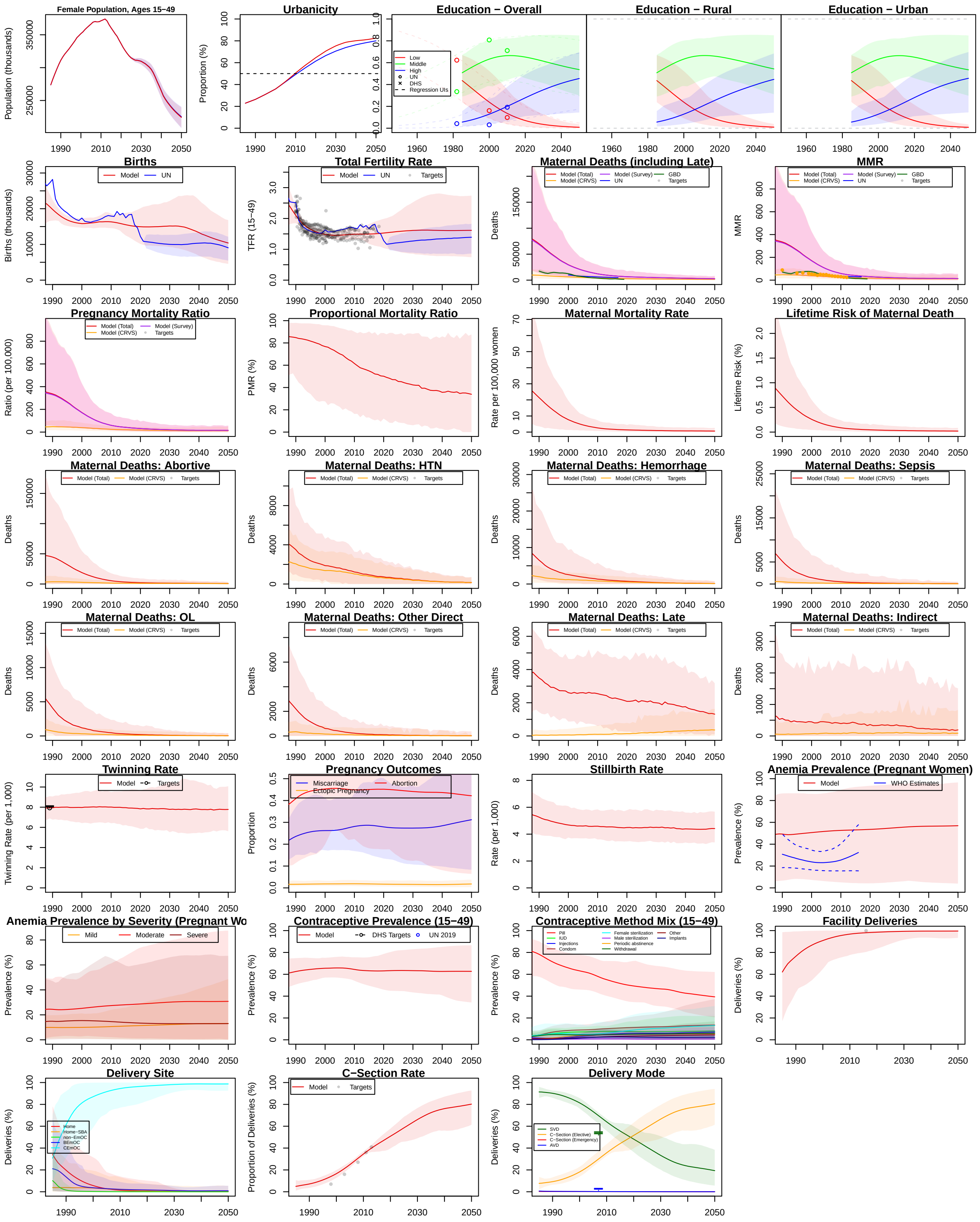

Colombia

ISO Code	Region	Area	Income Group
COL	South America	Latin America and the Caribbean	Upper middle income

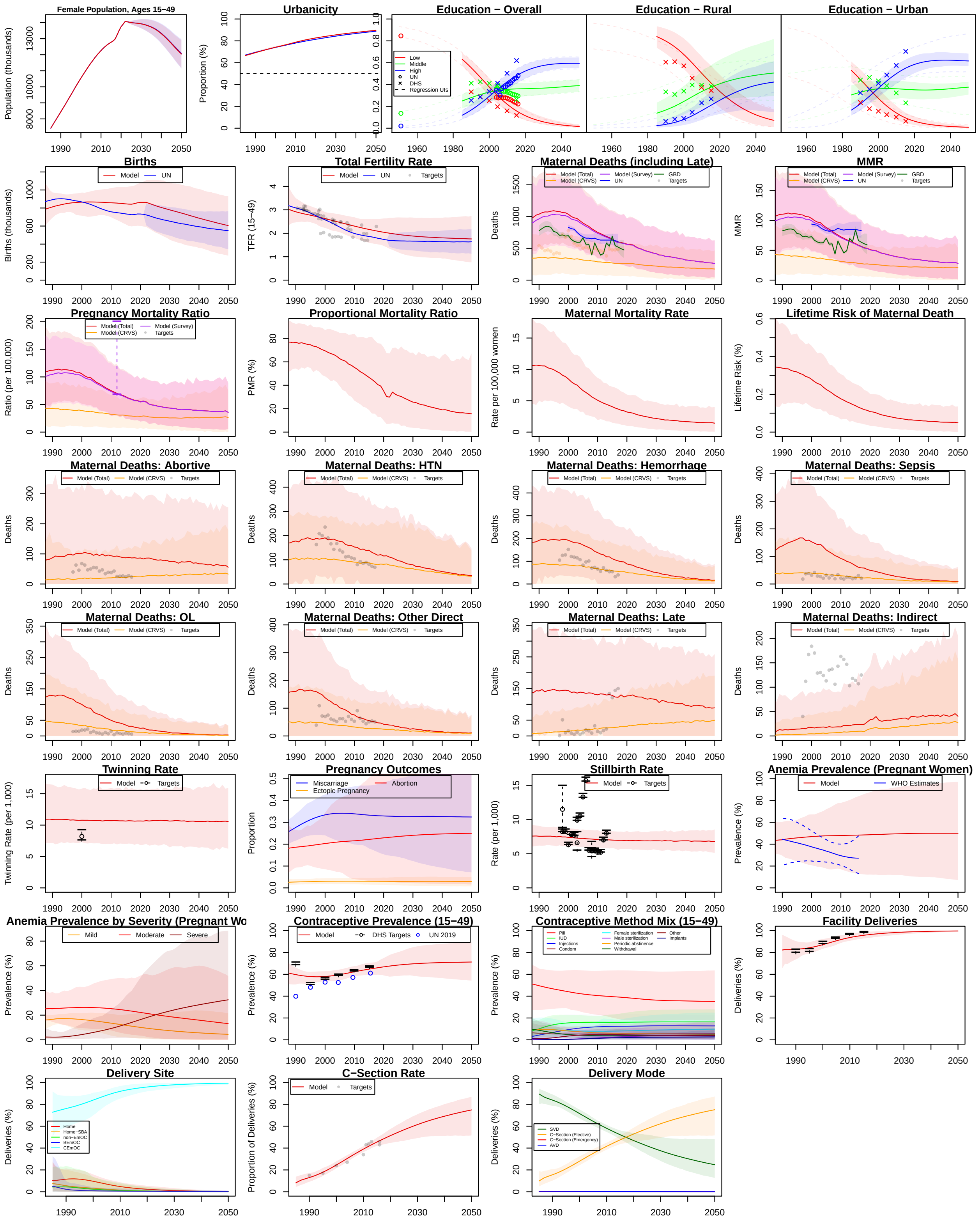

Comoros

ISO Code	Region	Area	Income Group
COM	Eastern Africa	Africa	Low income

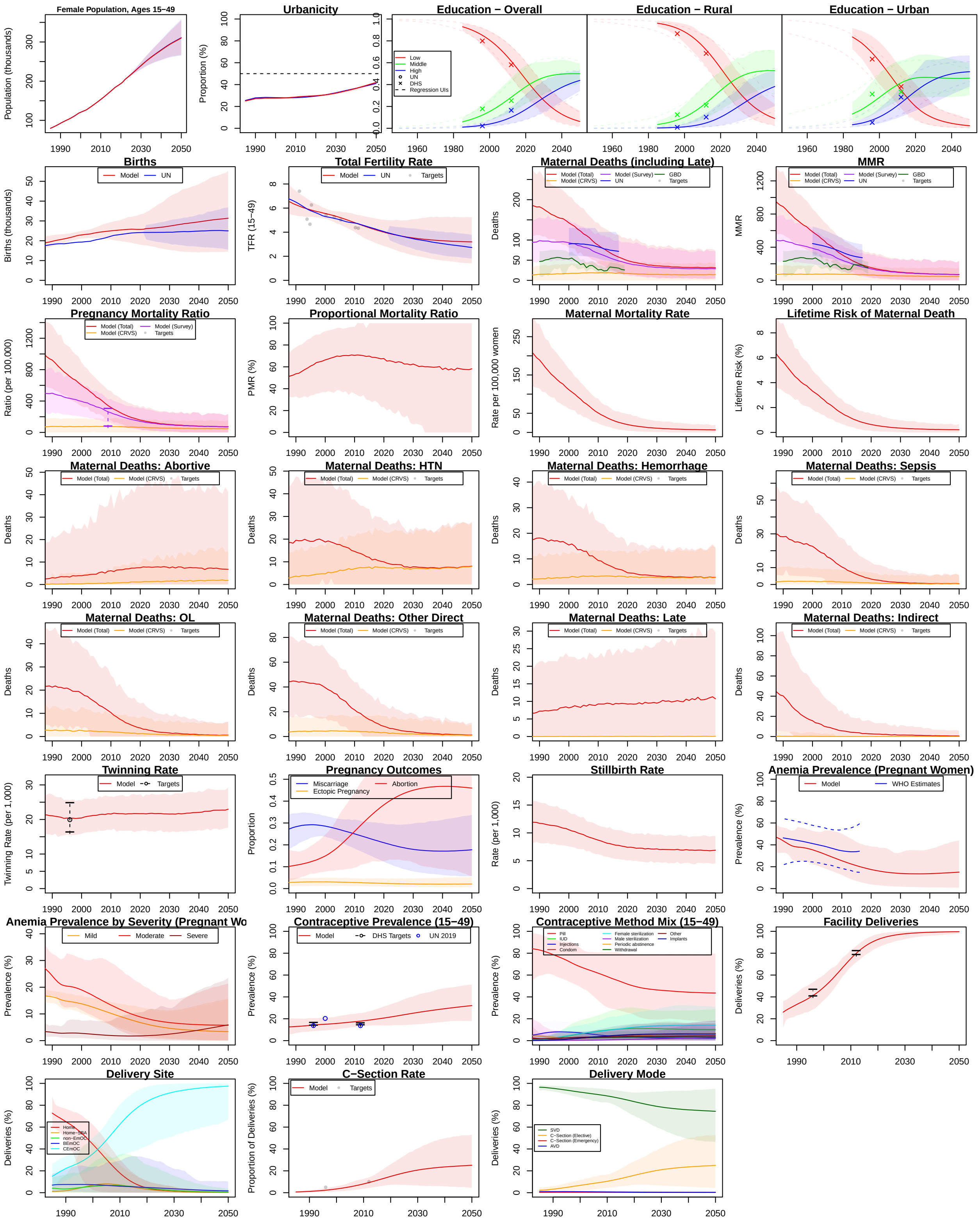

Congo

ISO Code	Region	Area	Income Group
COG	Middle Africa	Africa	Lower middle income

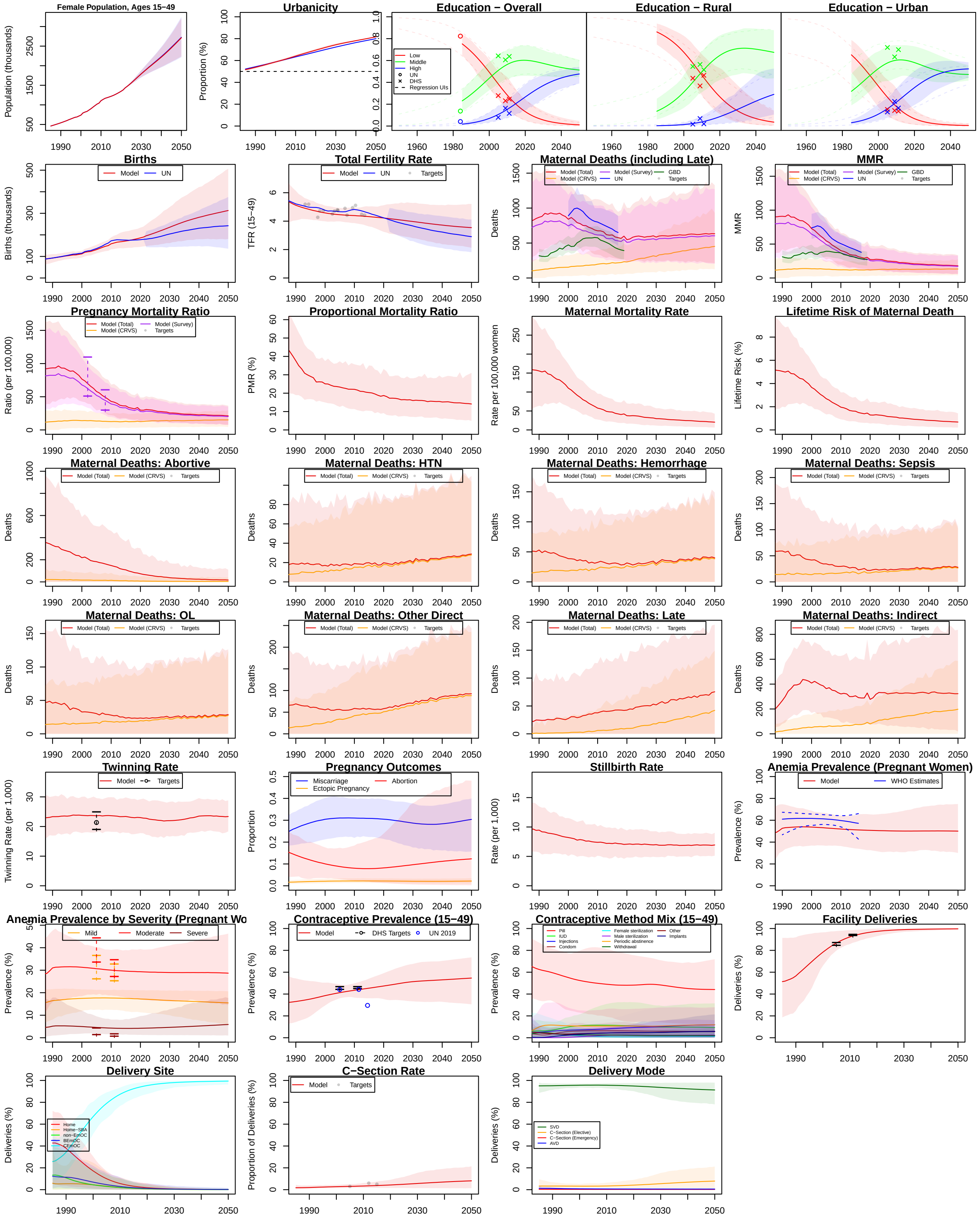

Costa Rica

ISO Code	Region	Area	Income Group
CRI	Central America	Latin America and the Caribbean	Upper middle income

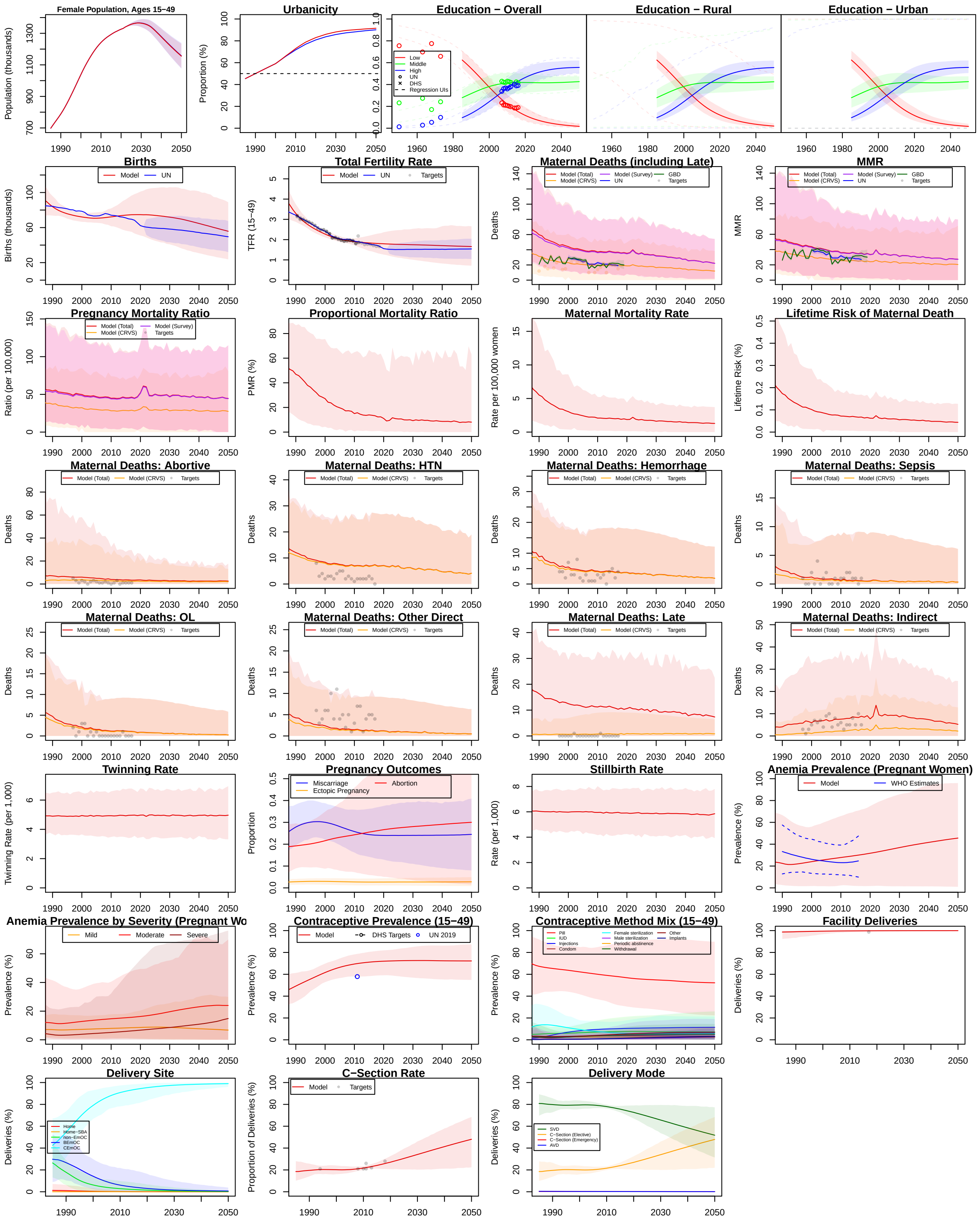

Côte d'Ivoire

ISO Code	Region	Area	Income Group
CIV	Western Africa	Africa	Lower middle income

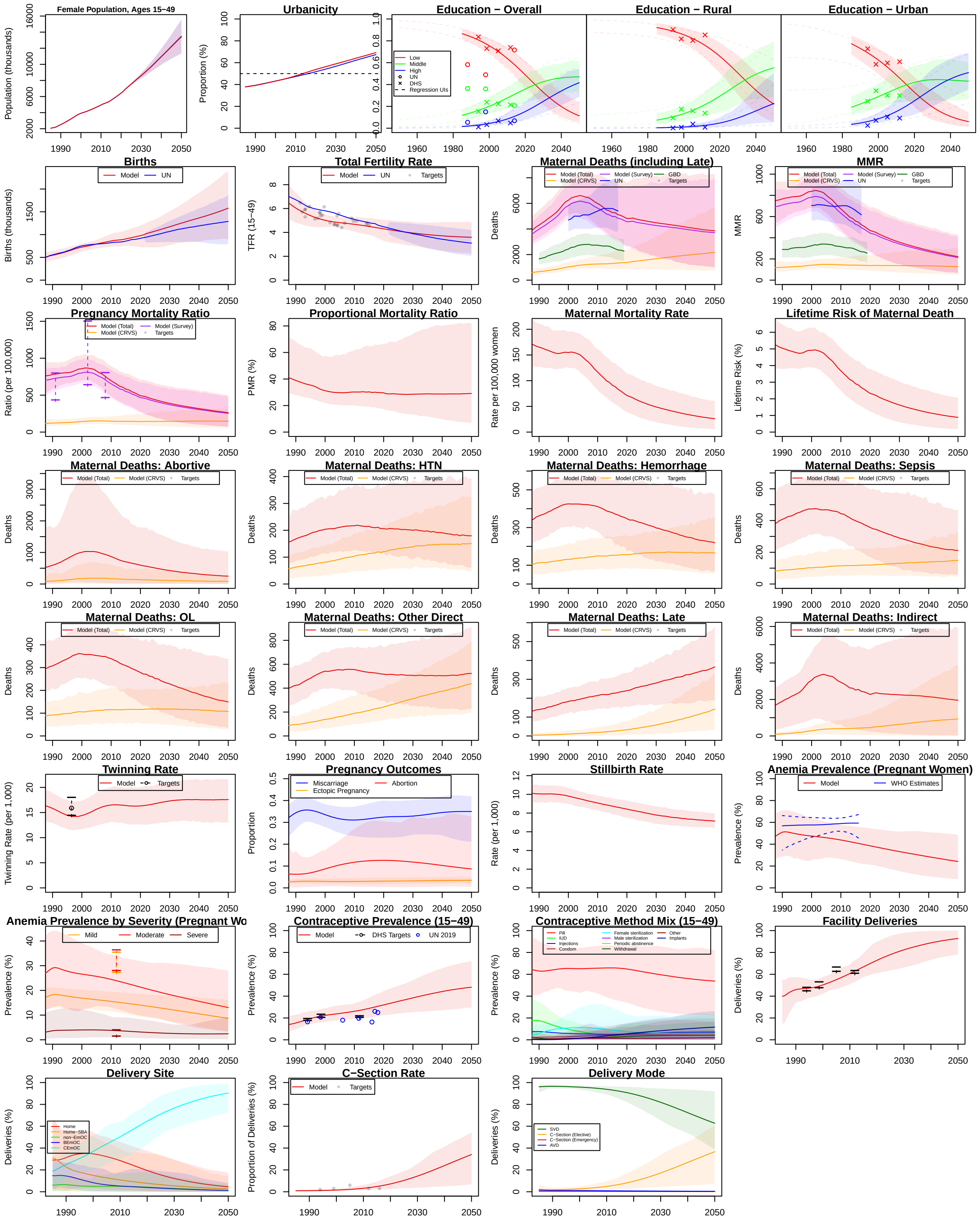

Croatia

ISO Code	Region	Area	Income Group
HRV	Southern Europe	Europe	High income

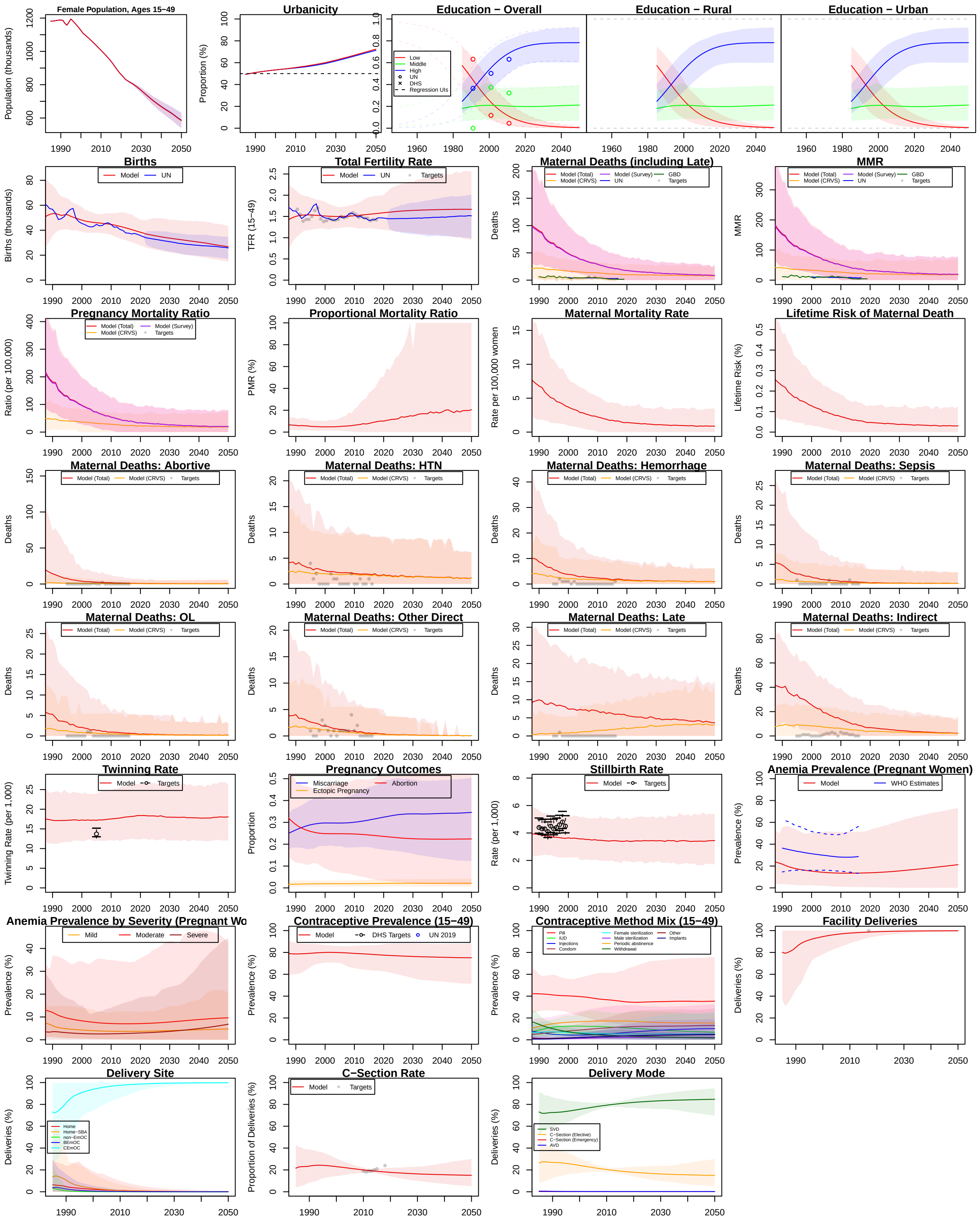

Cuba

ISO Code	Region	Area	Income Group
CUB	Caribbean	Latin America and the Caribbean	Upper middle income

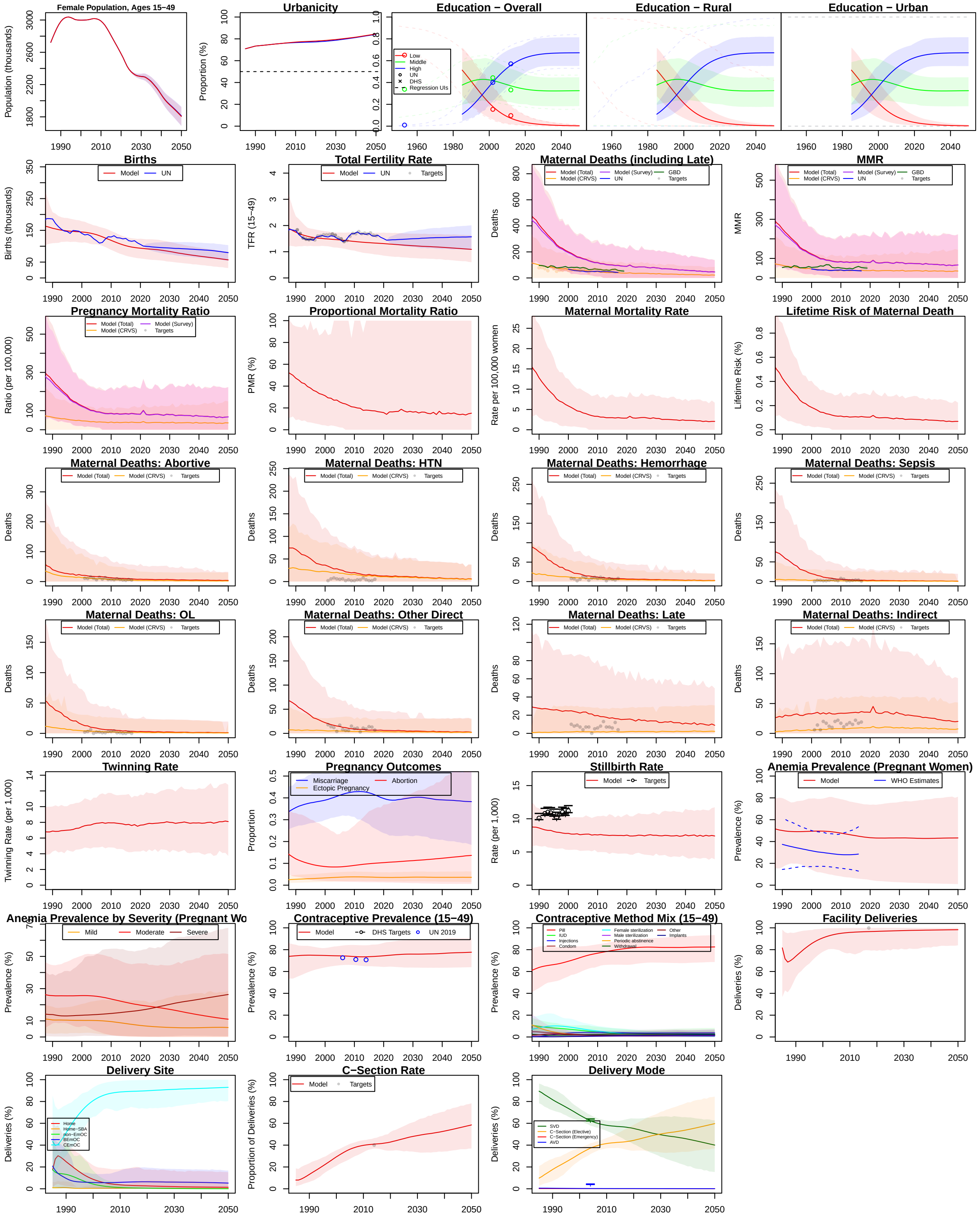

Cyprus

ISO Code	Region	Area	Income Group
CYP	Western Asia	Asia	High income

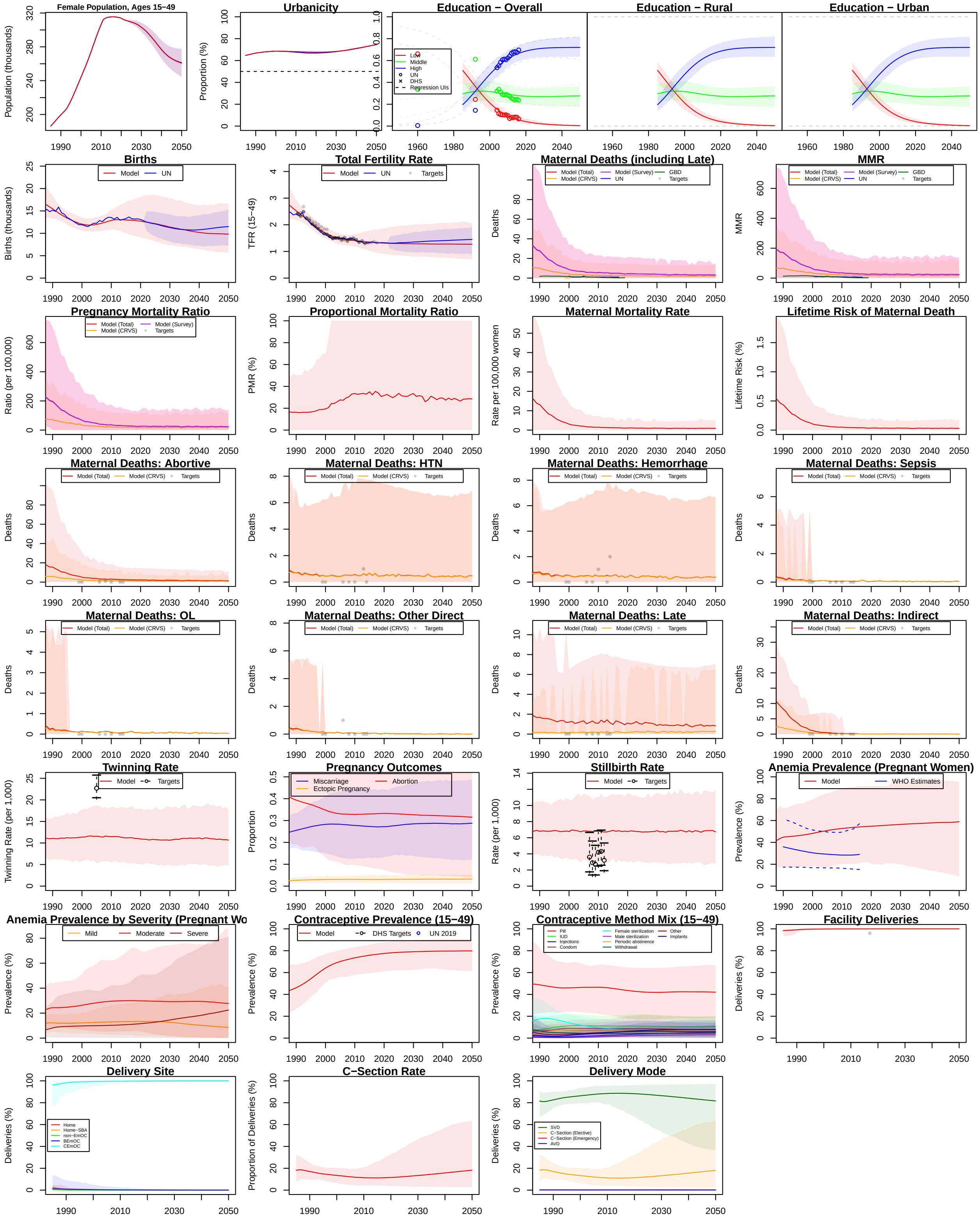

Czechia

ISO Code	Region	Area	Income Group
CZE	Eastern Europe	Europe	High income

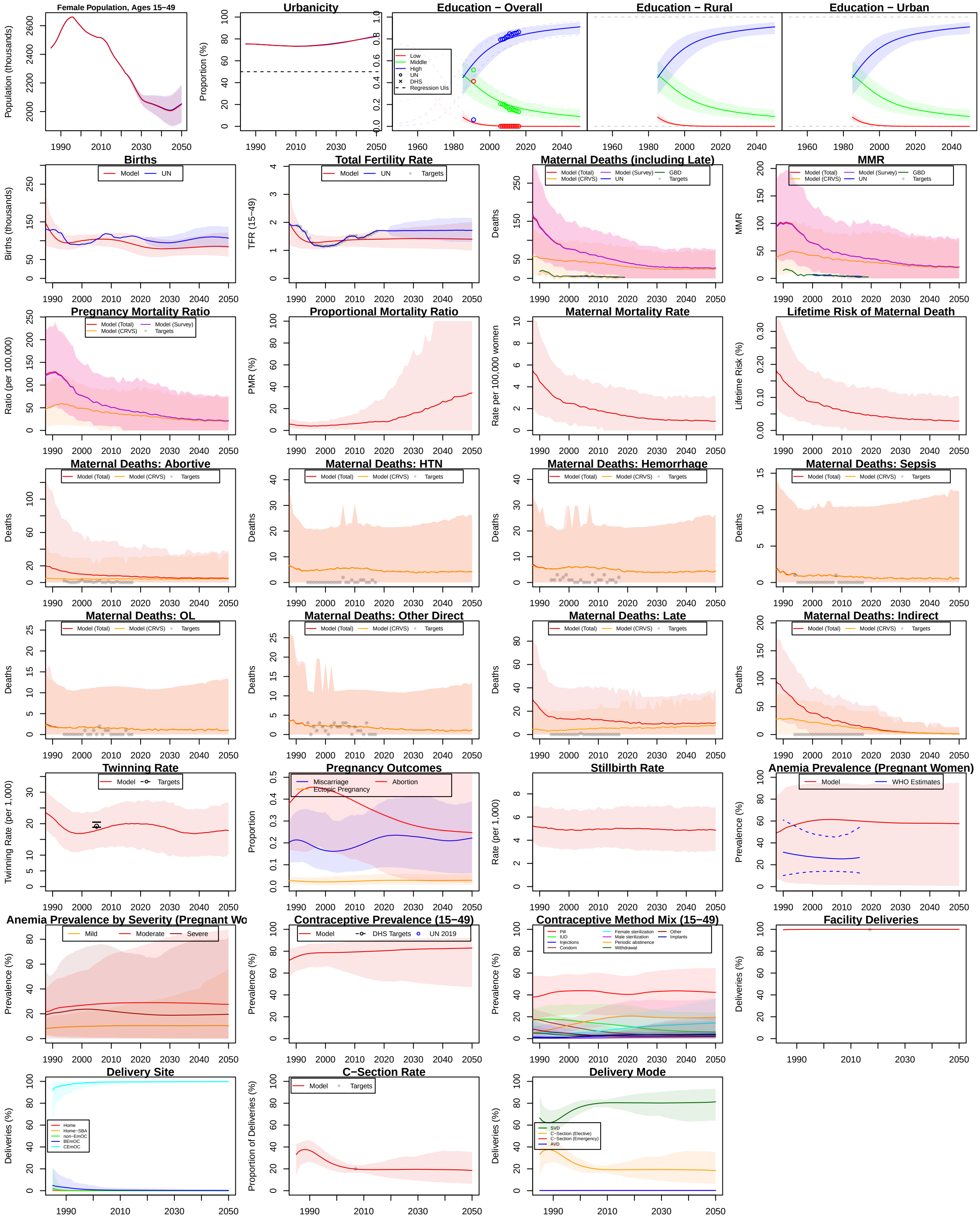

Dem. People's Republic of Korea

ISO Code	Region	Area	Income Group
PRK	Eastern Asia	Asia	Low income

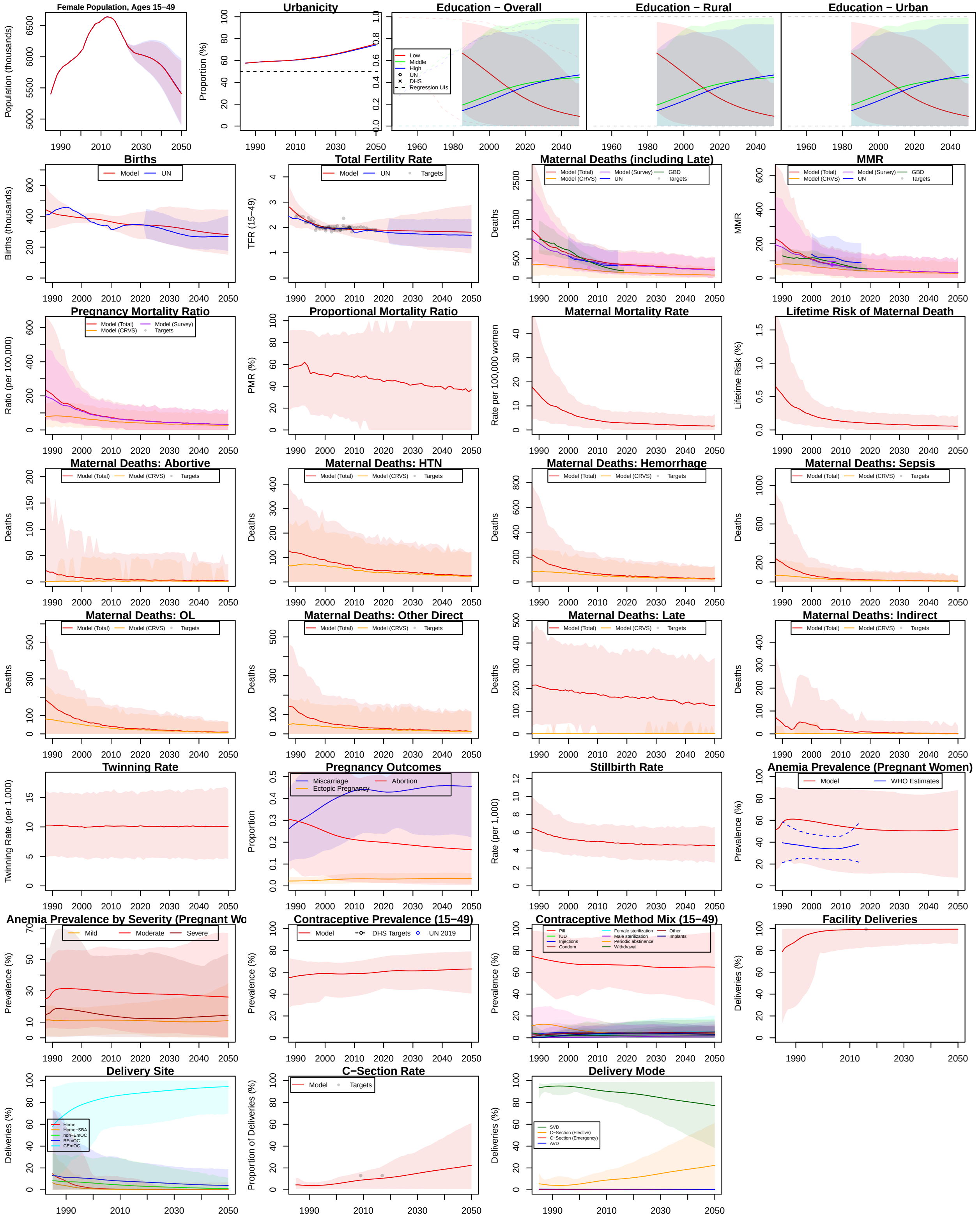

Democratic Republic of the Congo

ISO Code	Region	Area	Income Group
COD	Middle Africa	Africa	Low income

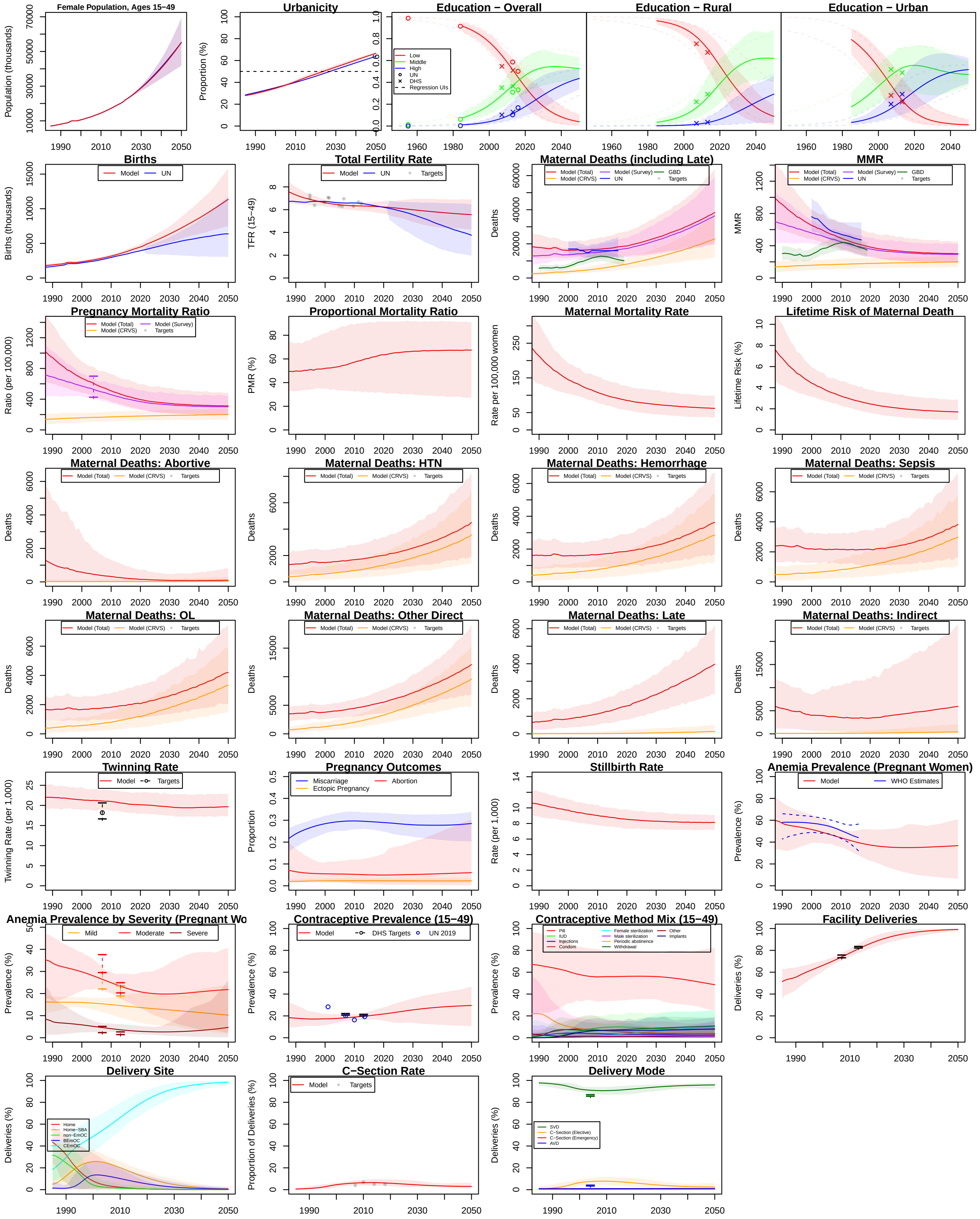

Denmark

ISO Code	Region	Area	Income Group
DNK	Northern Europe	Europe	High income

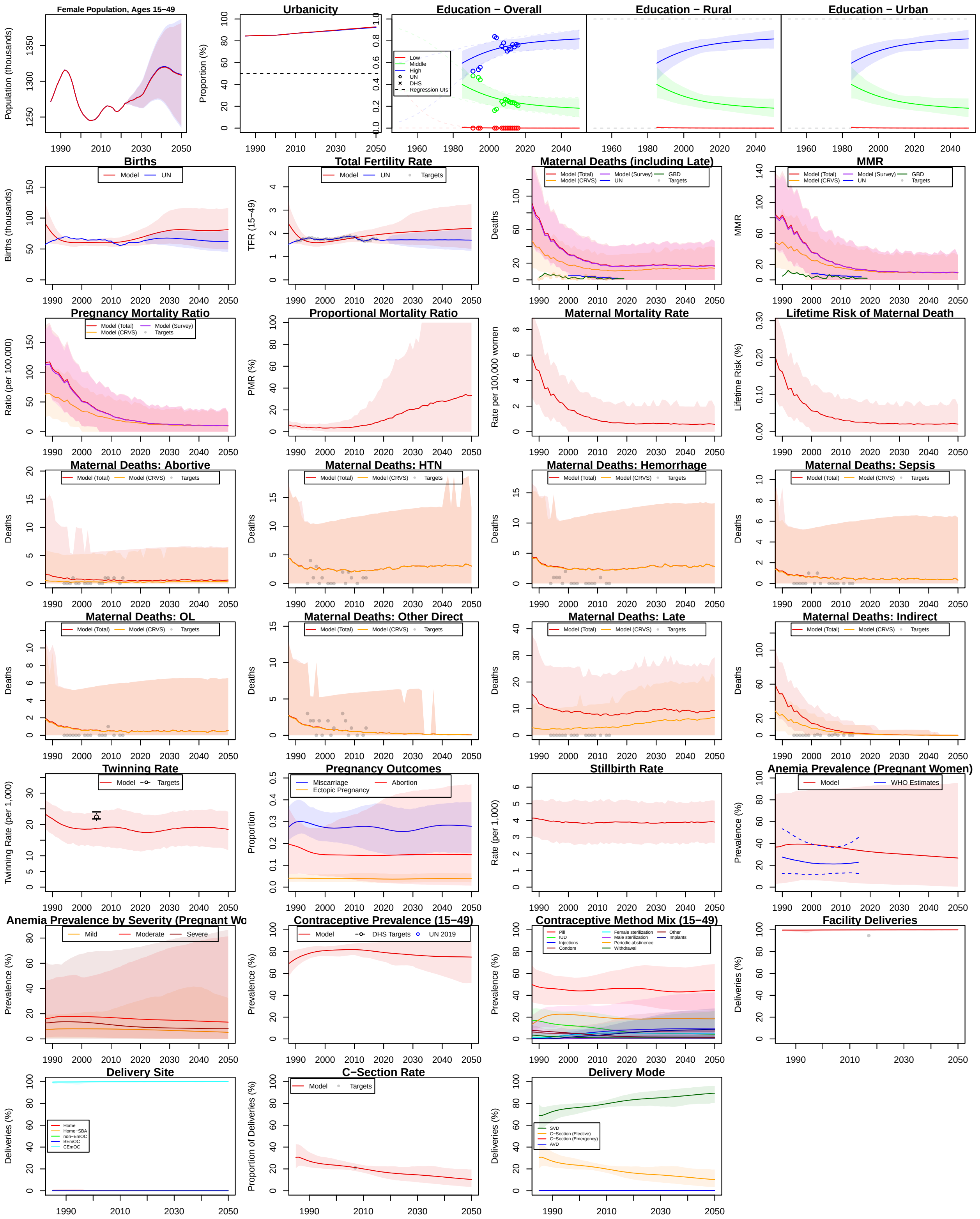

Djibouti

ISO Code	Region	Area	Income Group
DJI	Eastern Africa	Africa	Lower middle income

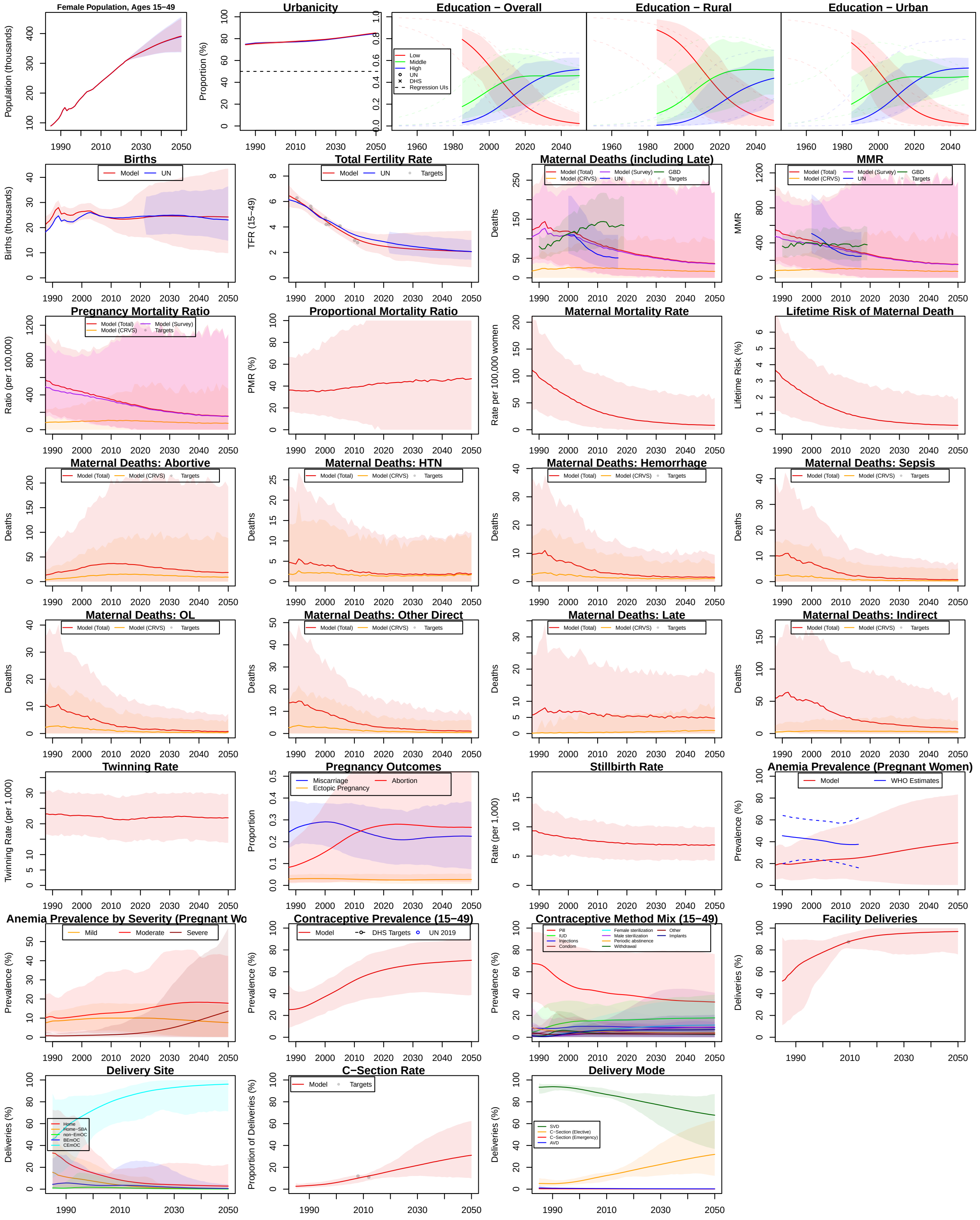

Dominica

ISO Code	Region	Area	Income Group
DMA	Caribbean	Latin America and the Caribbean	Upper middle income

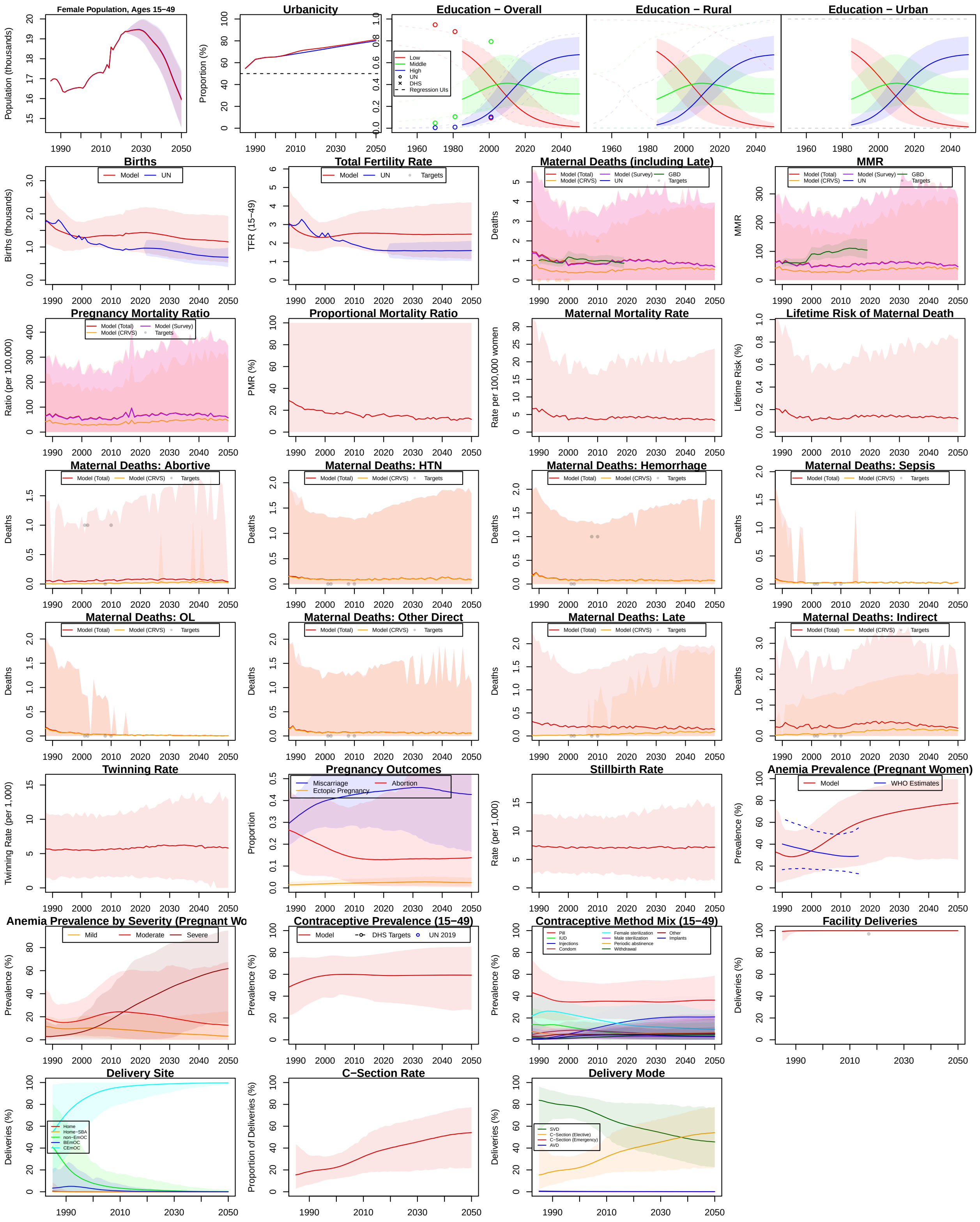

Dominican Republic

ISO Code	Region	Area	Income Group
DOM	Caribbean	Latin America and the Caribbean	Upper middle income

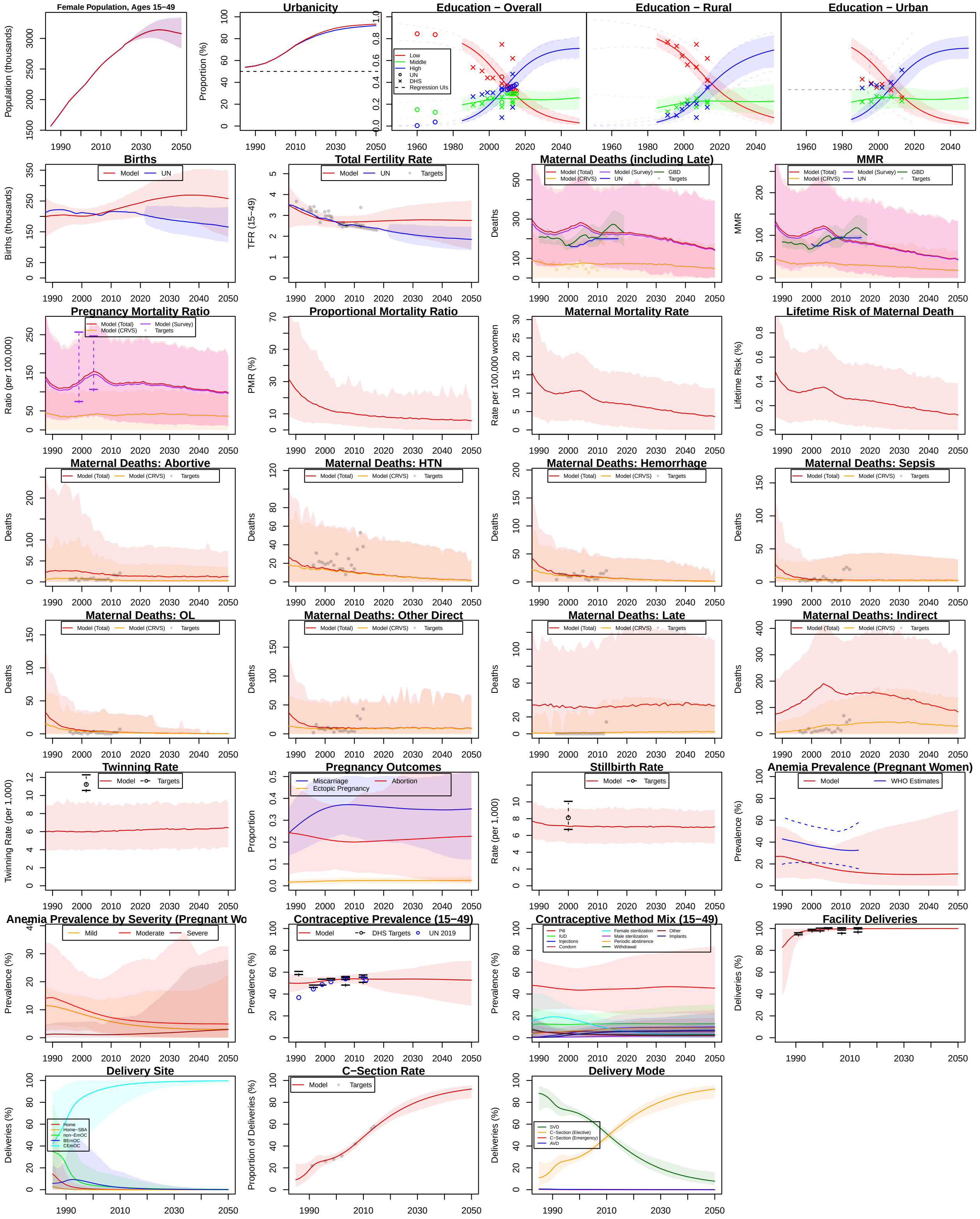

Ecuador

ISO Code	Region	Area	Income Group
ECU	South America	Latin America and the Caribbean	Upper middle income

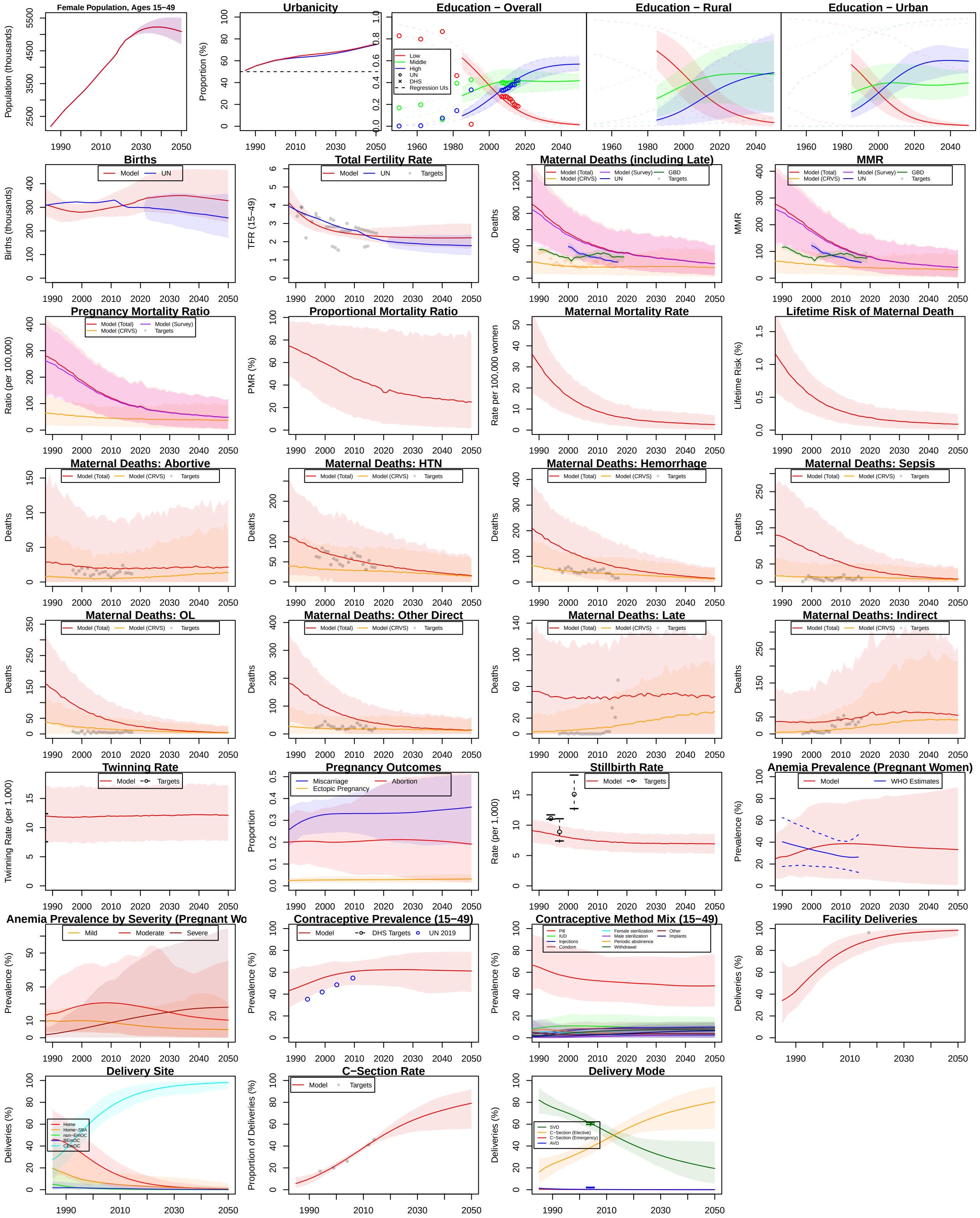

Egypt

ISO Code	Region	Area	Income Group
EGY	Northern Africa	Africa	Lower middle income

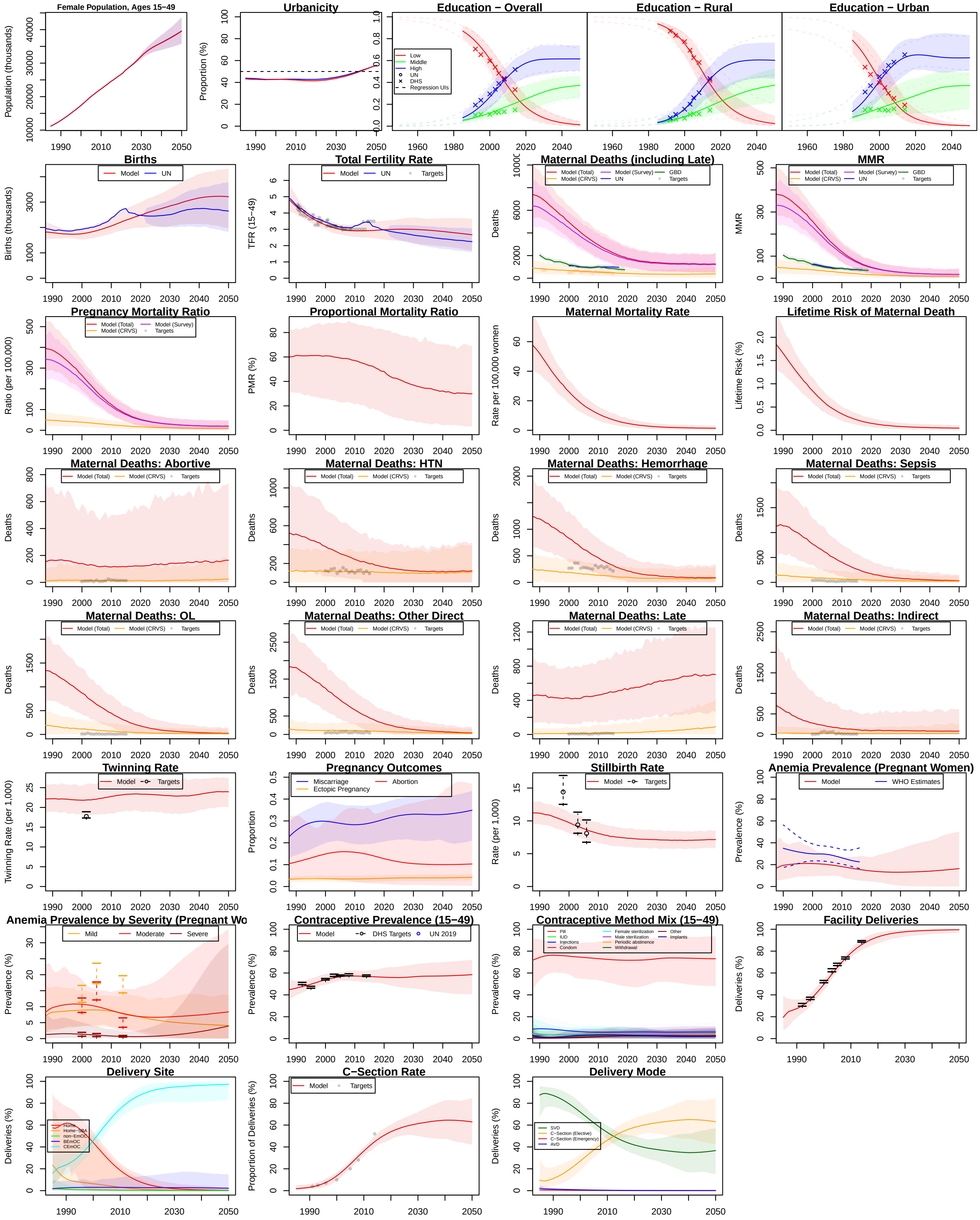

El Salvador

ISO Code	Region	Area	Income Group
SLV	Central America	Latin America and the Caribbean	Lower middle income

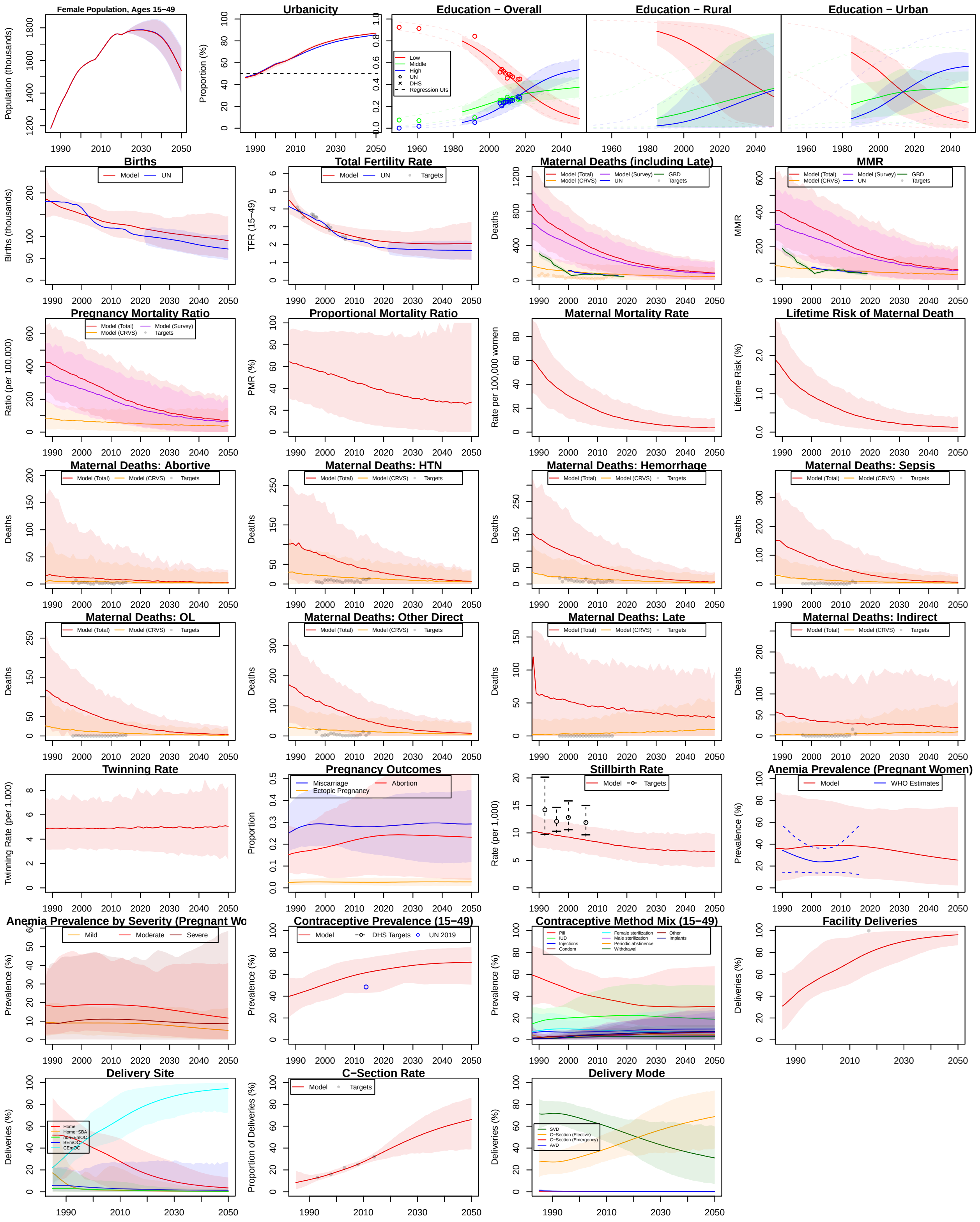

Equatorial Guinea

ISO Code	Region	Area	Income Group
GNQ	Middle Africa	Africa	Upper middle income

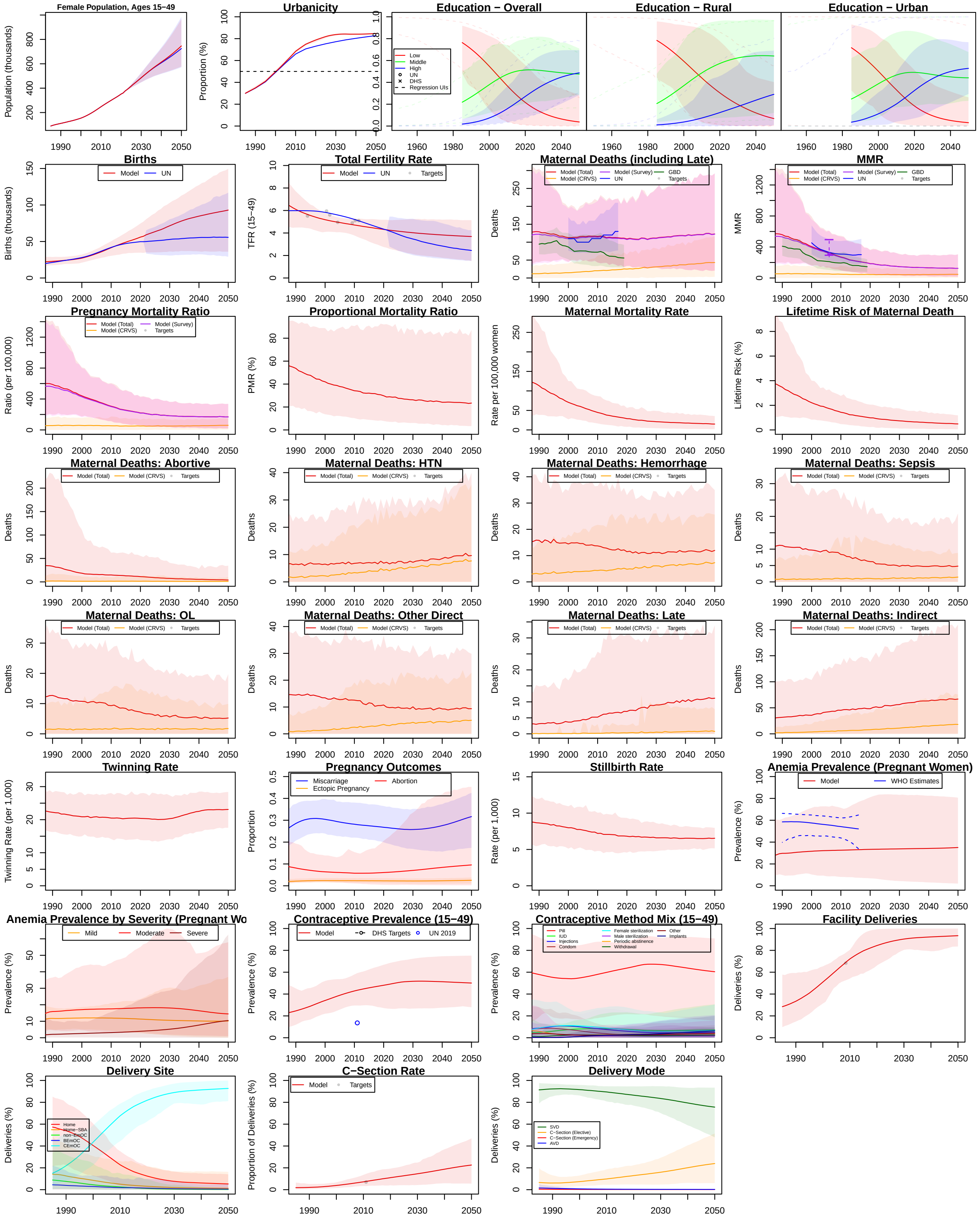

Eritrea

ISO Code	Region	Area	Income Group
ERI	Eastern Africa	Africa	Low income

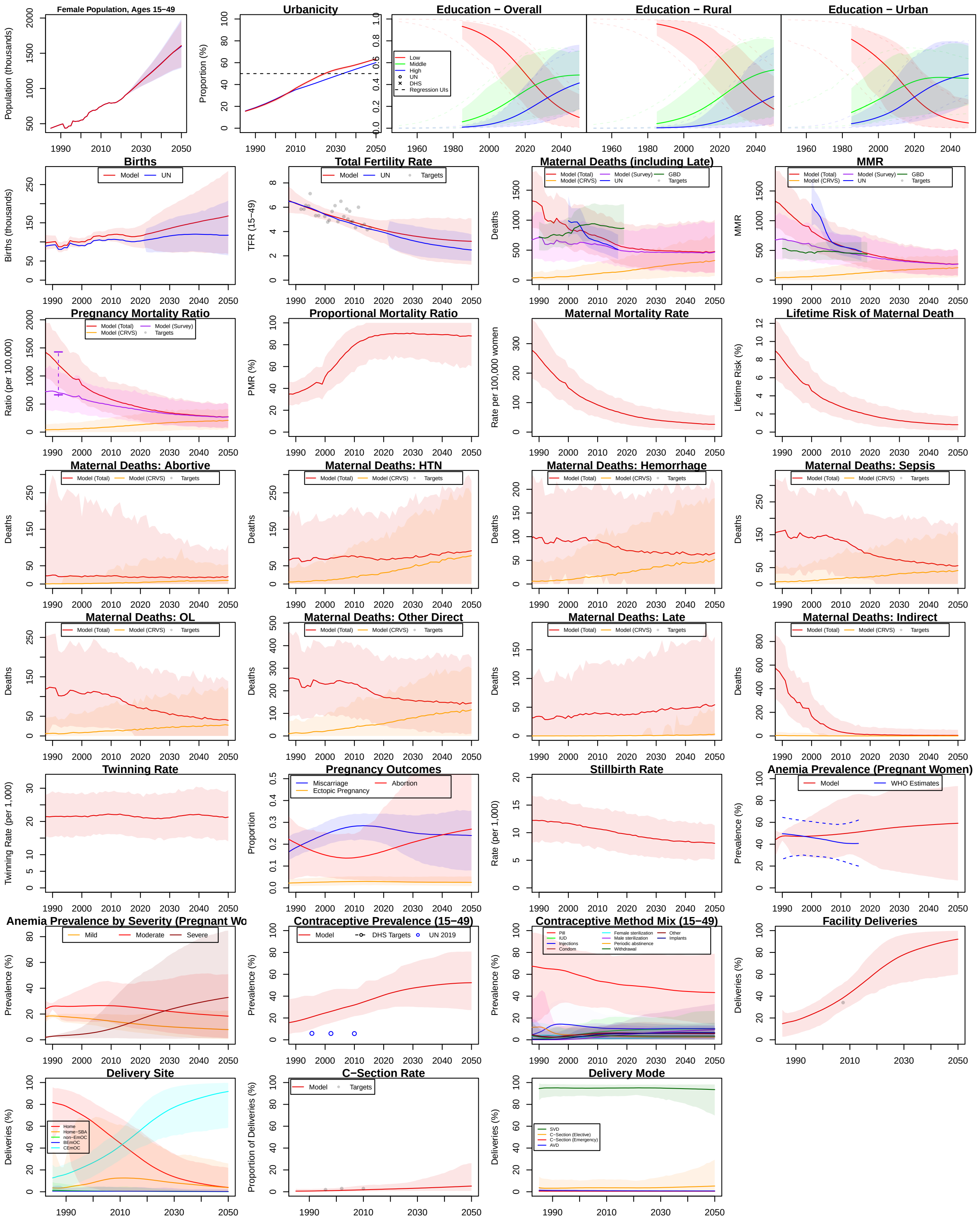

Estonia

ISO Code	Region	Area	Income Group
EST	Northern Europe	Europe	High income

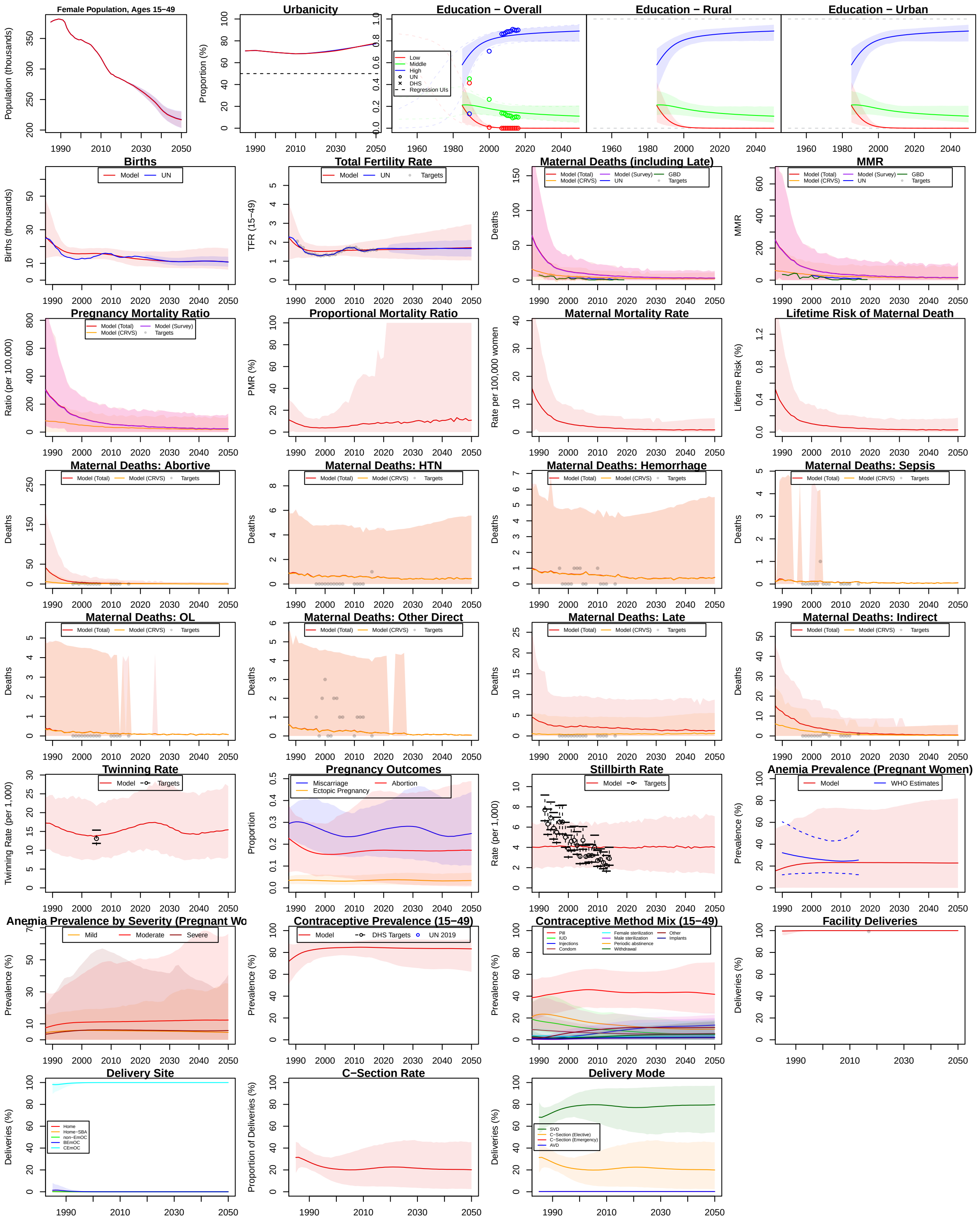

Eswatini

ISO Code	Region	Area	Income Group
SWZ	Southern Africa	Africa	Lower middle income

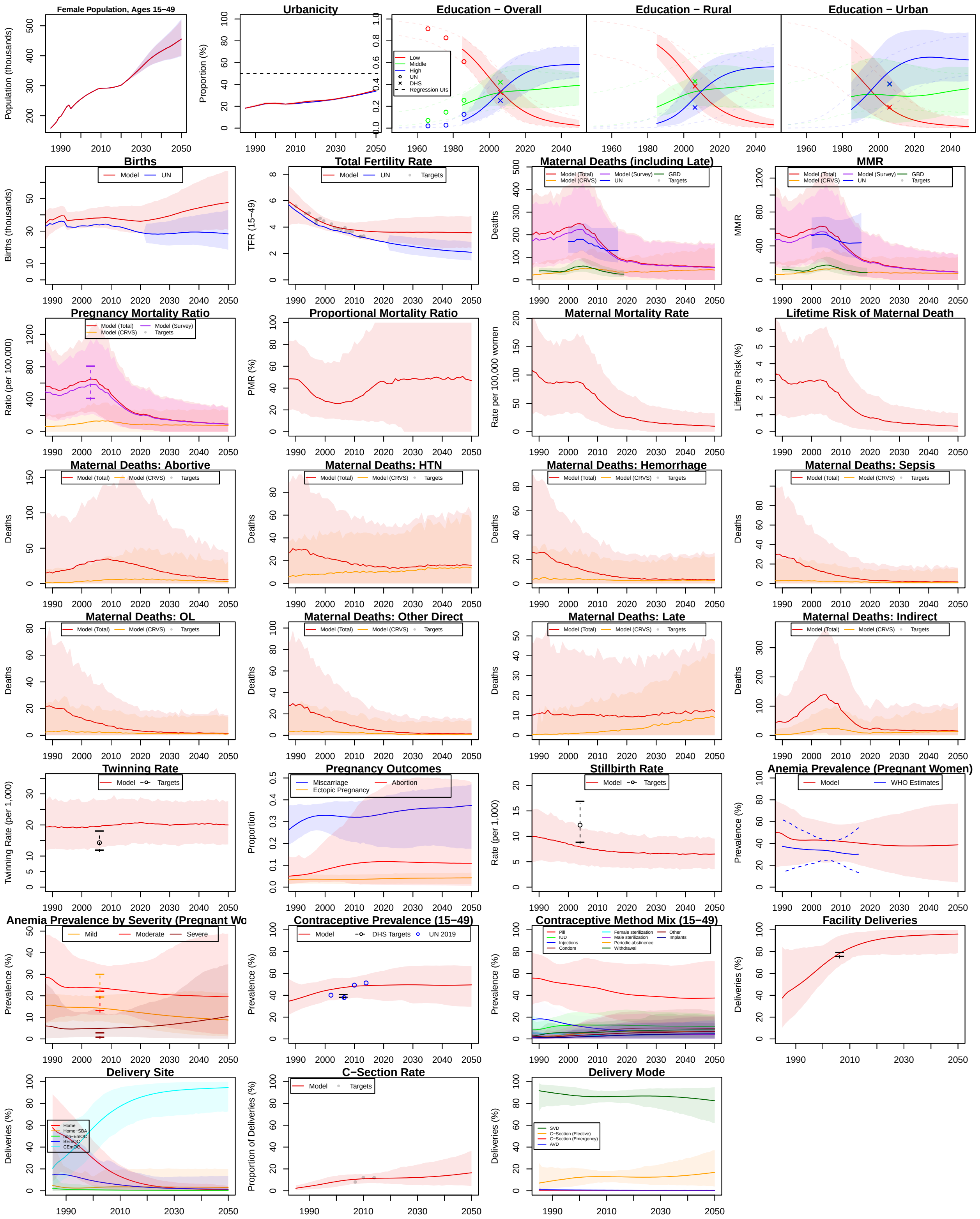

Ethiopia

ISO Code	Region	Area	Income Group
ETH	Eastern Africa	Africa	Low income

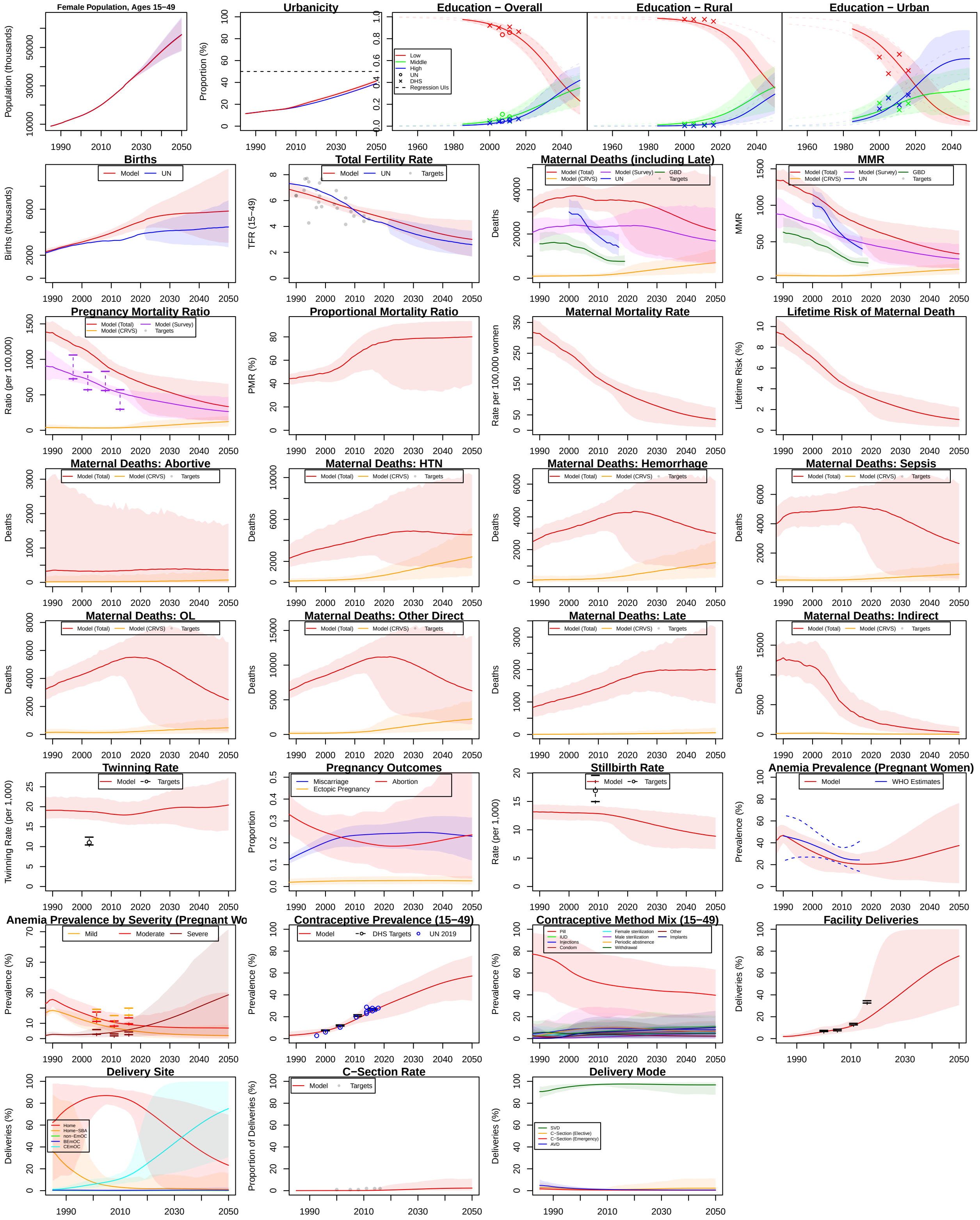

Faroe Islands

ISO Code	Region	Area	Income Group
FRO	Northern Europe	Europe	High income

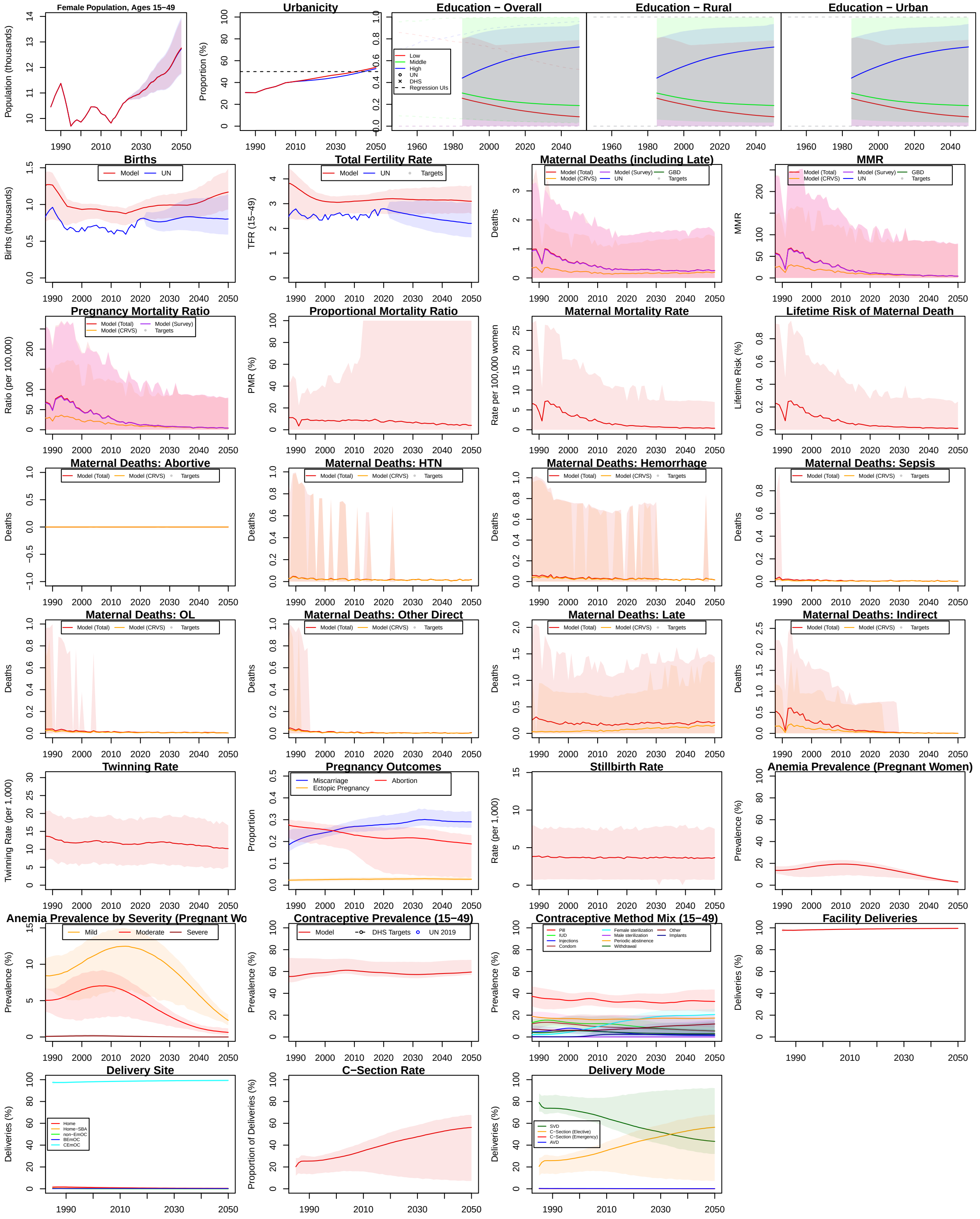

Fiji

ISO Code	Region	Area	Income Group
FJI	Melanesia	Oceania	Upper middle income

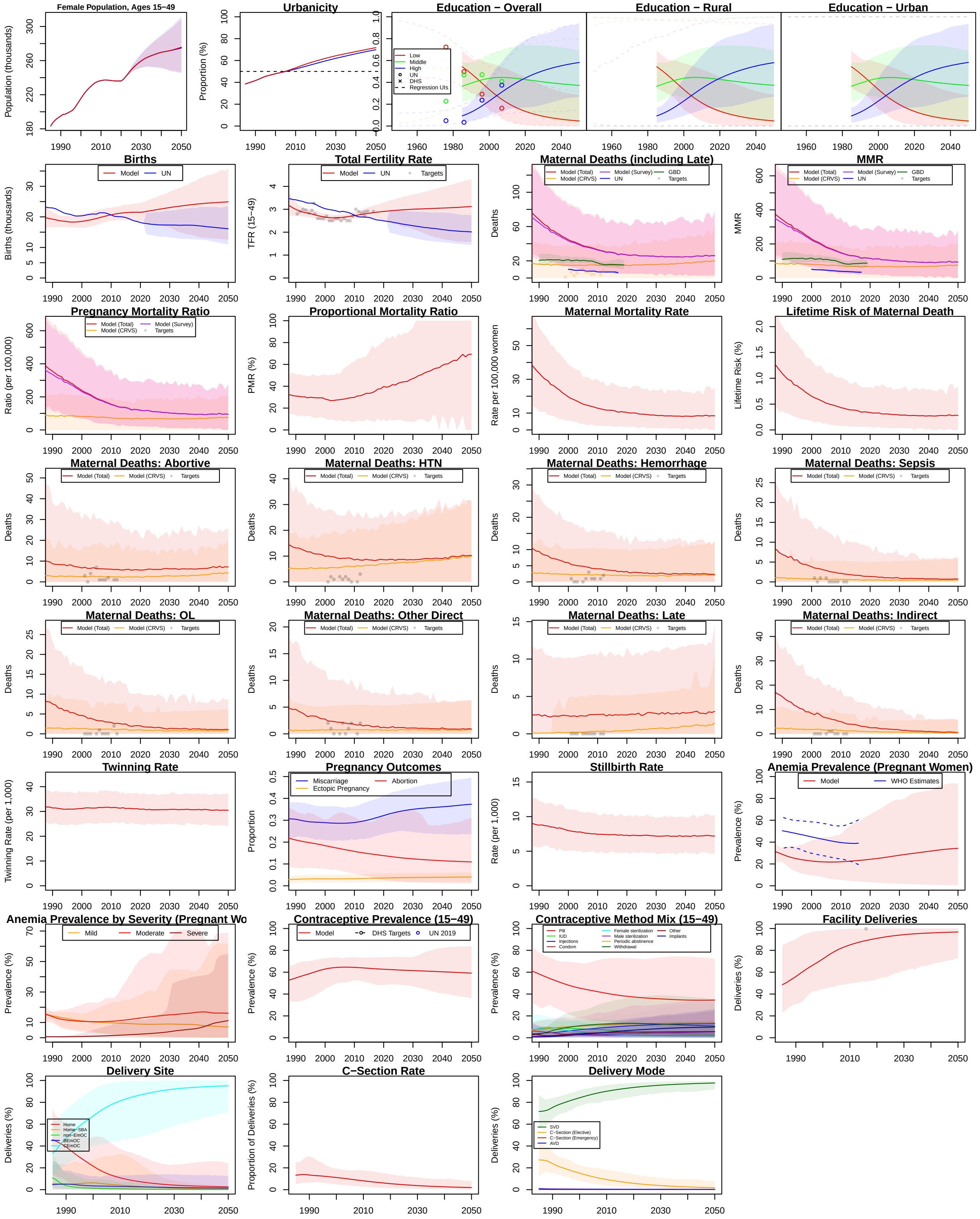

Finland

ISO Code	Region	Area	Income Group
FIN	Northern Europe	Europe	High income

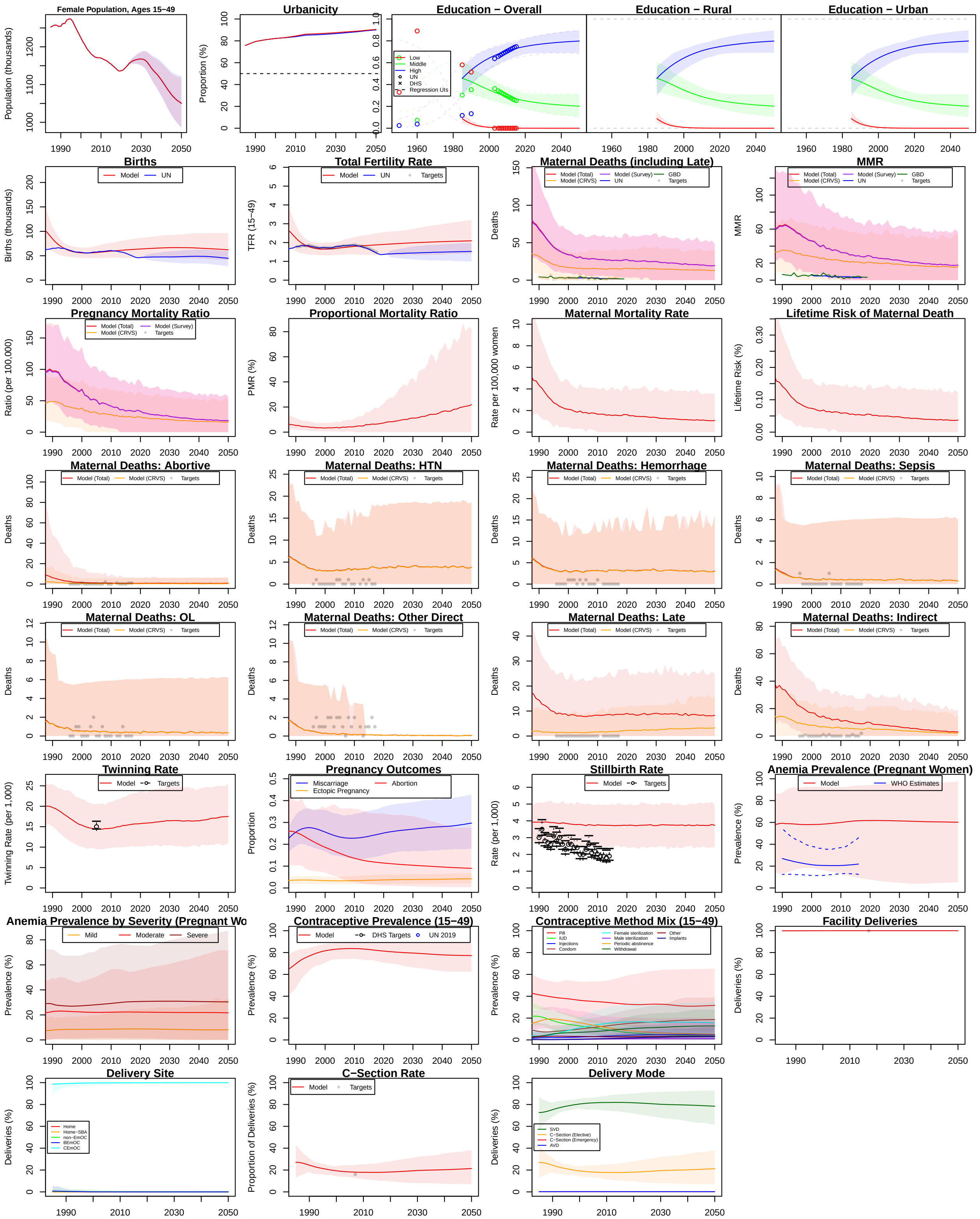

France

ISO Code	Region	Area	Income Group
FRA	Western Europe	Europe	High income

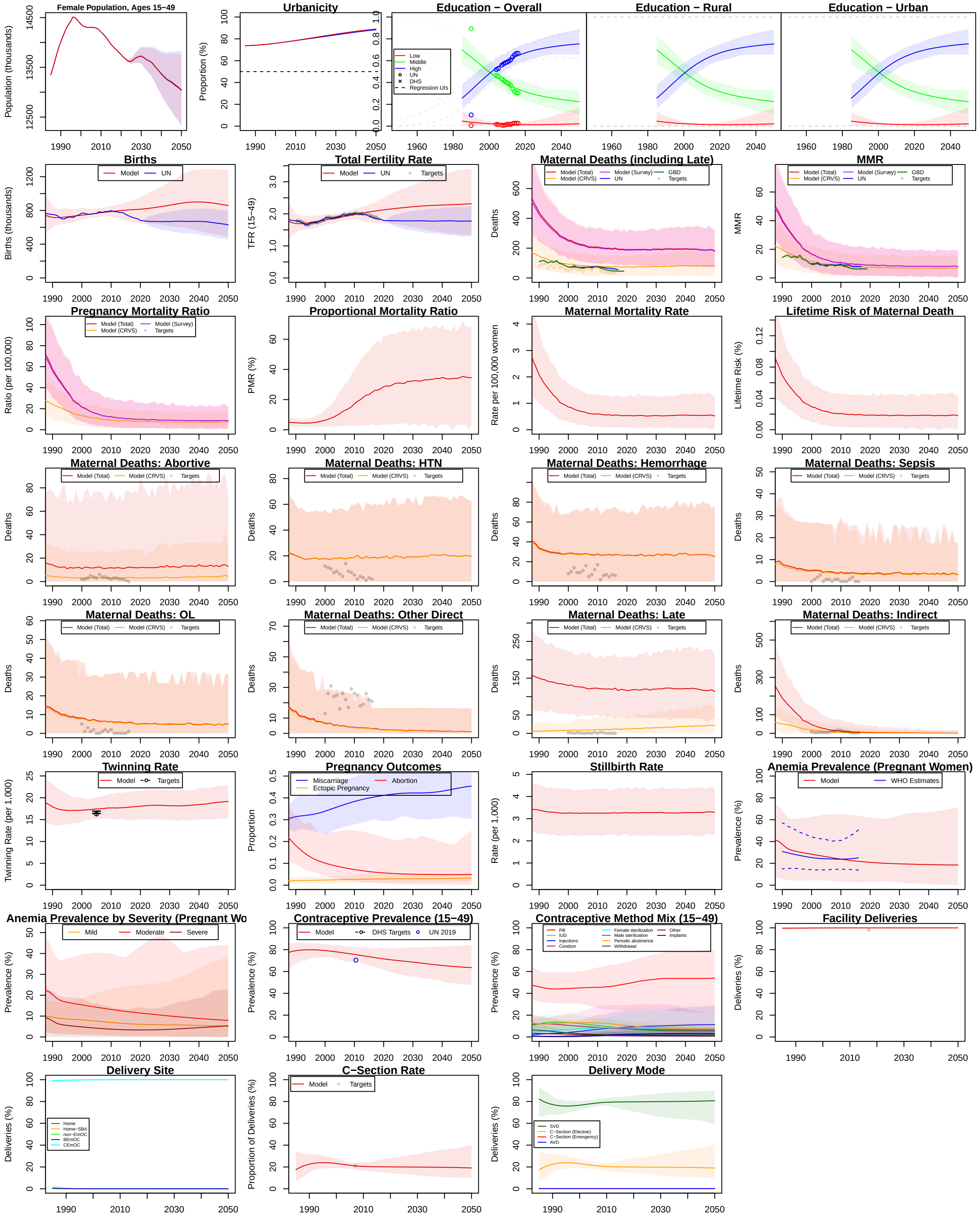

Gabon

ISO Code	Region	Area	Income Group
GAB	Middle Africa	Africa	Upper middle income

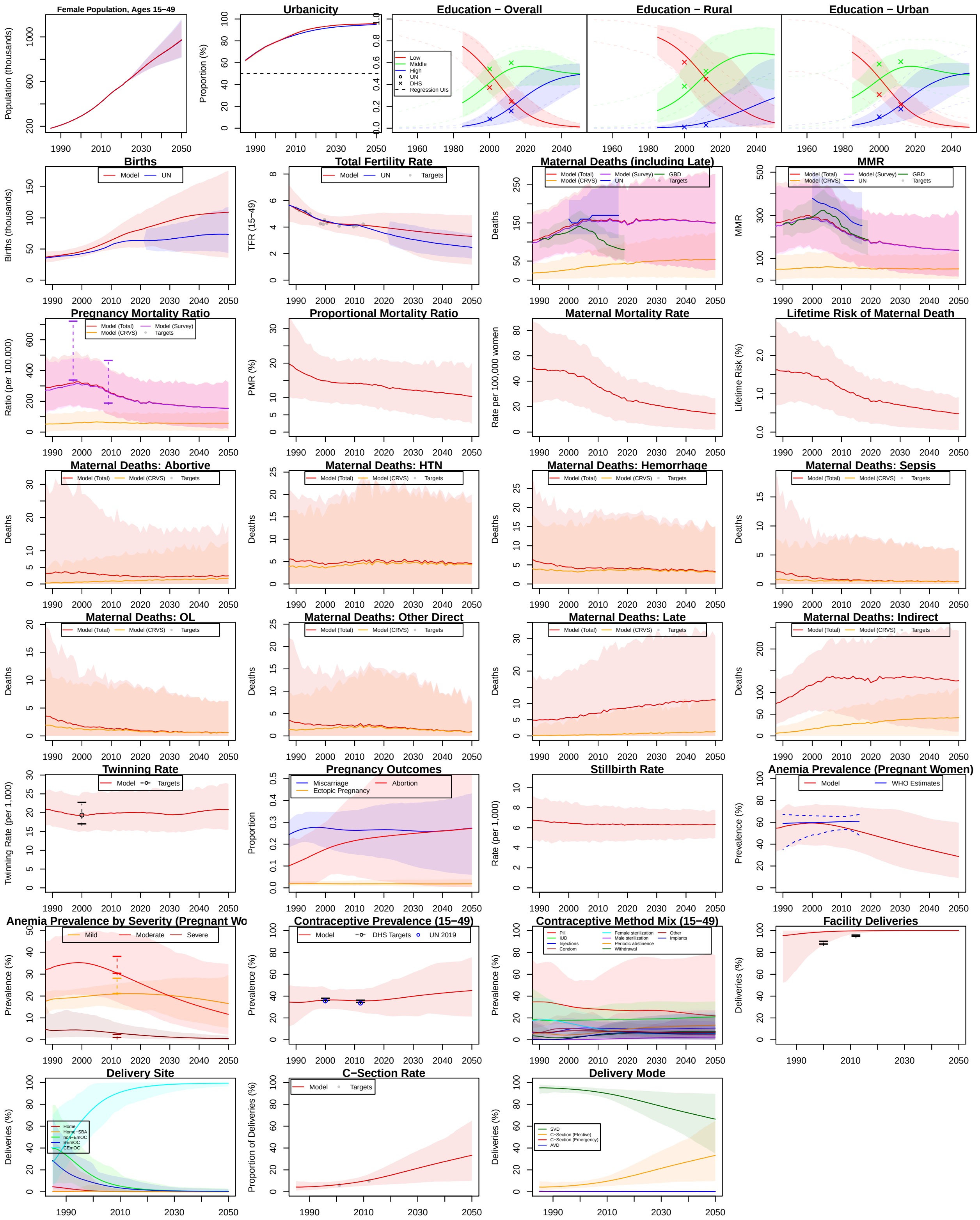

Gambia

ISO Code	Region	Area	Income Group
GMB	Western Africa	Africa	Low income

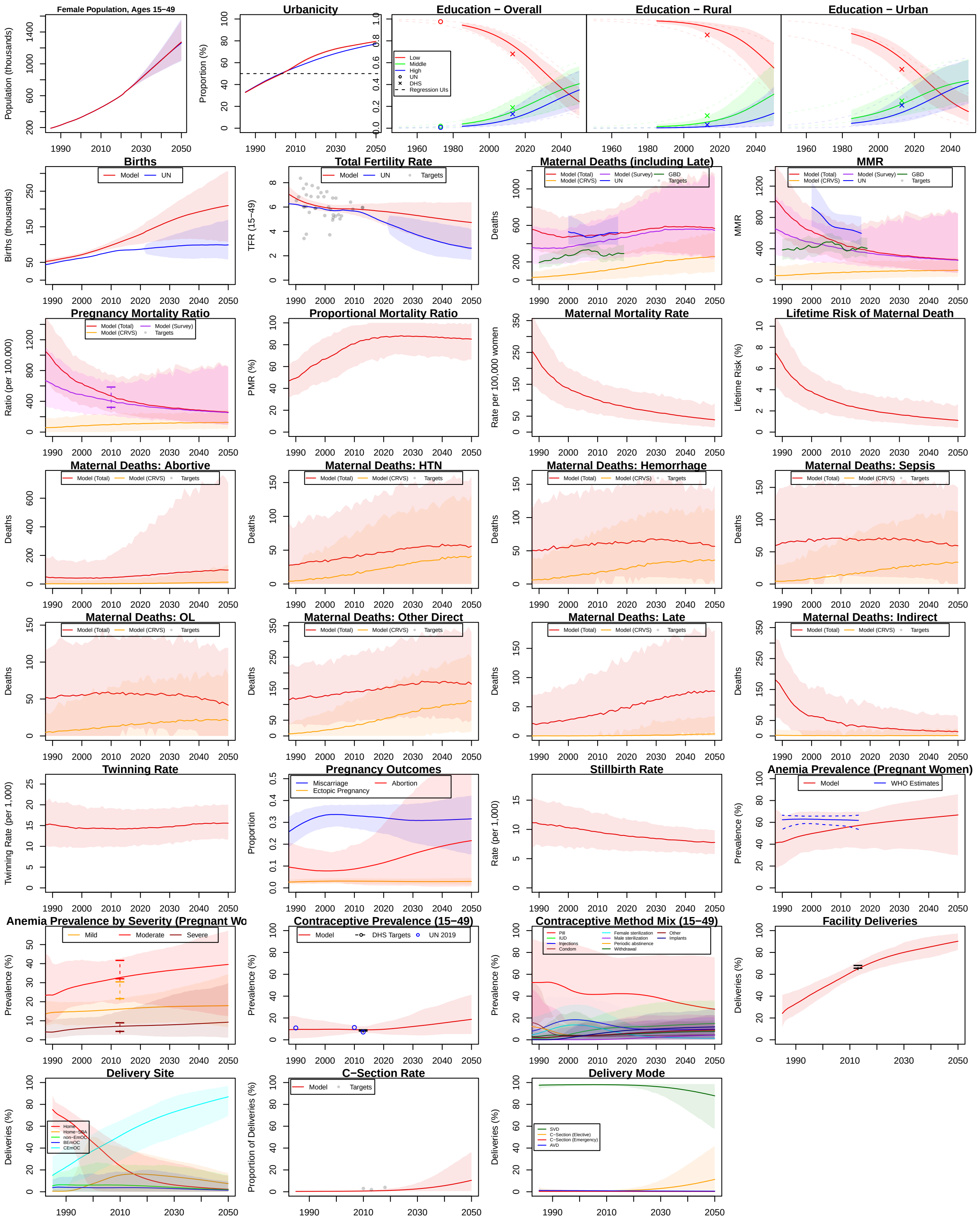

Georgia

ISO Code	Region	Area	Income Group
GEO	Western Asia	Asia	Lower middle income

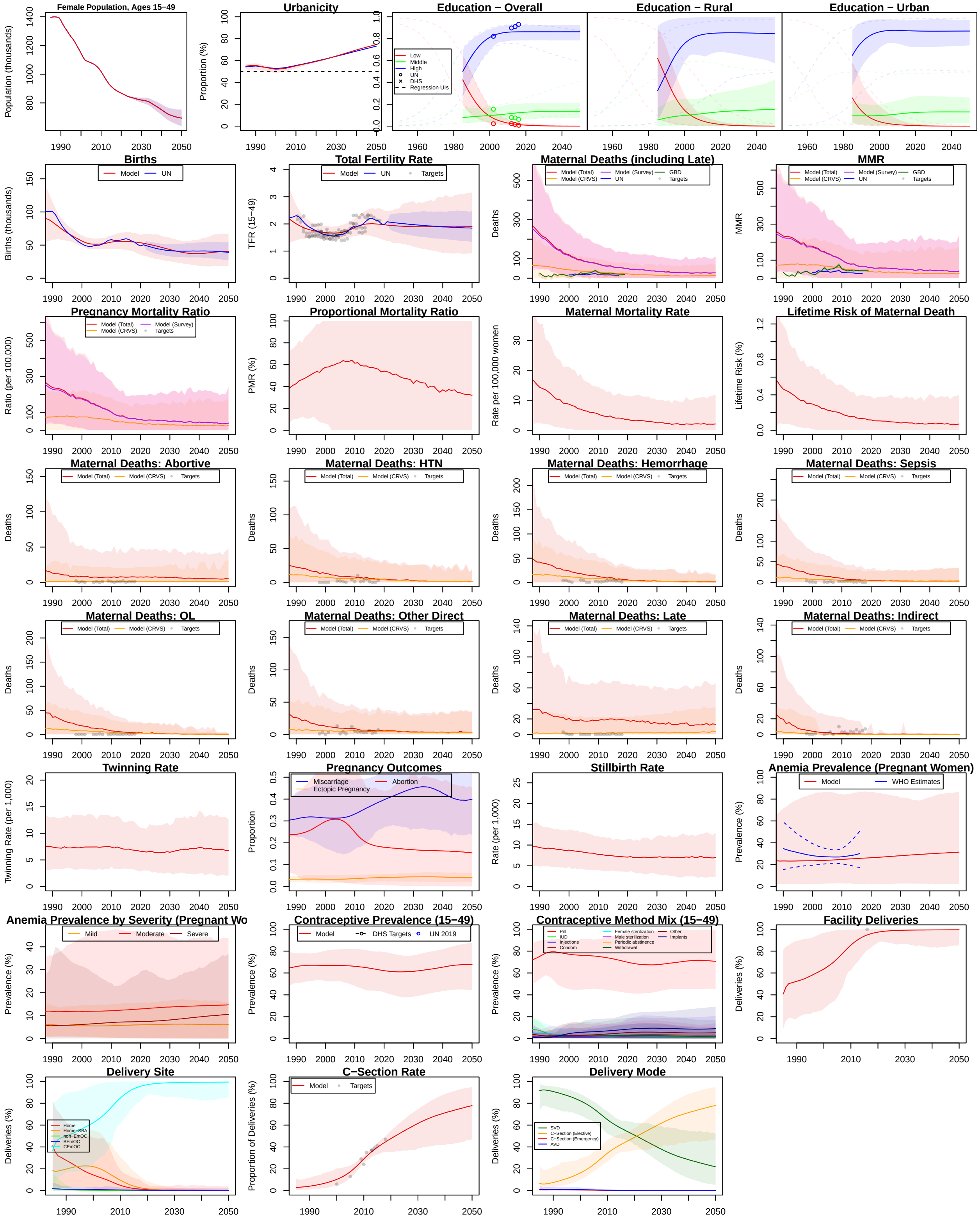

Germany

ISO Code	Region	Area	Income Group
DEU	Western Europe	Europe	High income

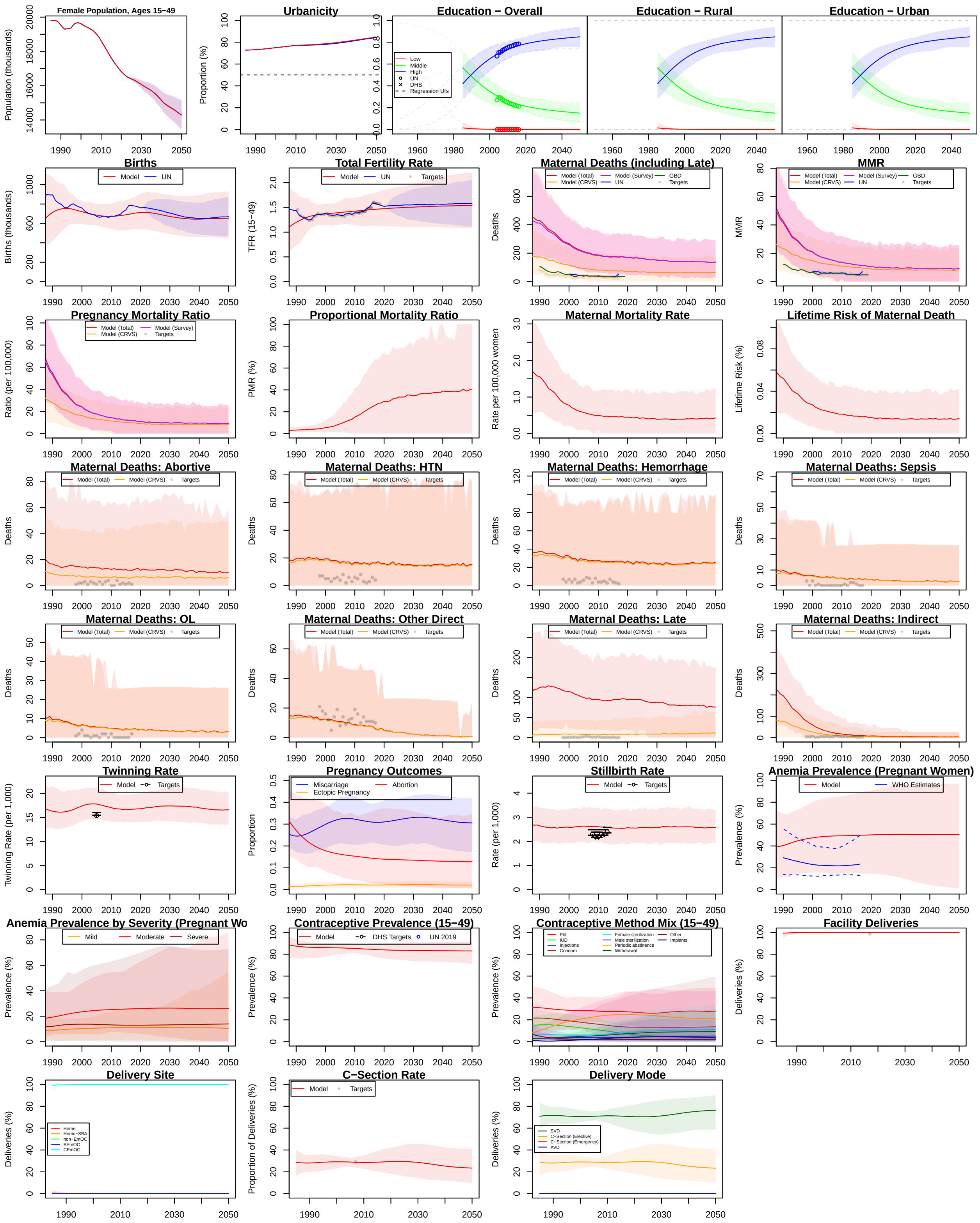

Ghana

ISO Code	Region	Area	Income Group
GHA	Western Africa	Africa	Lower middle income

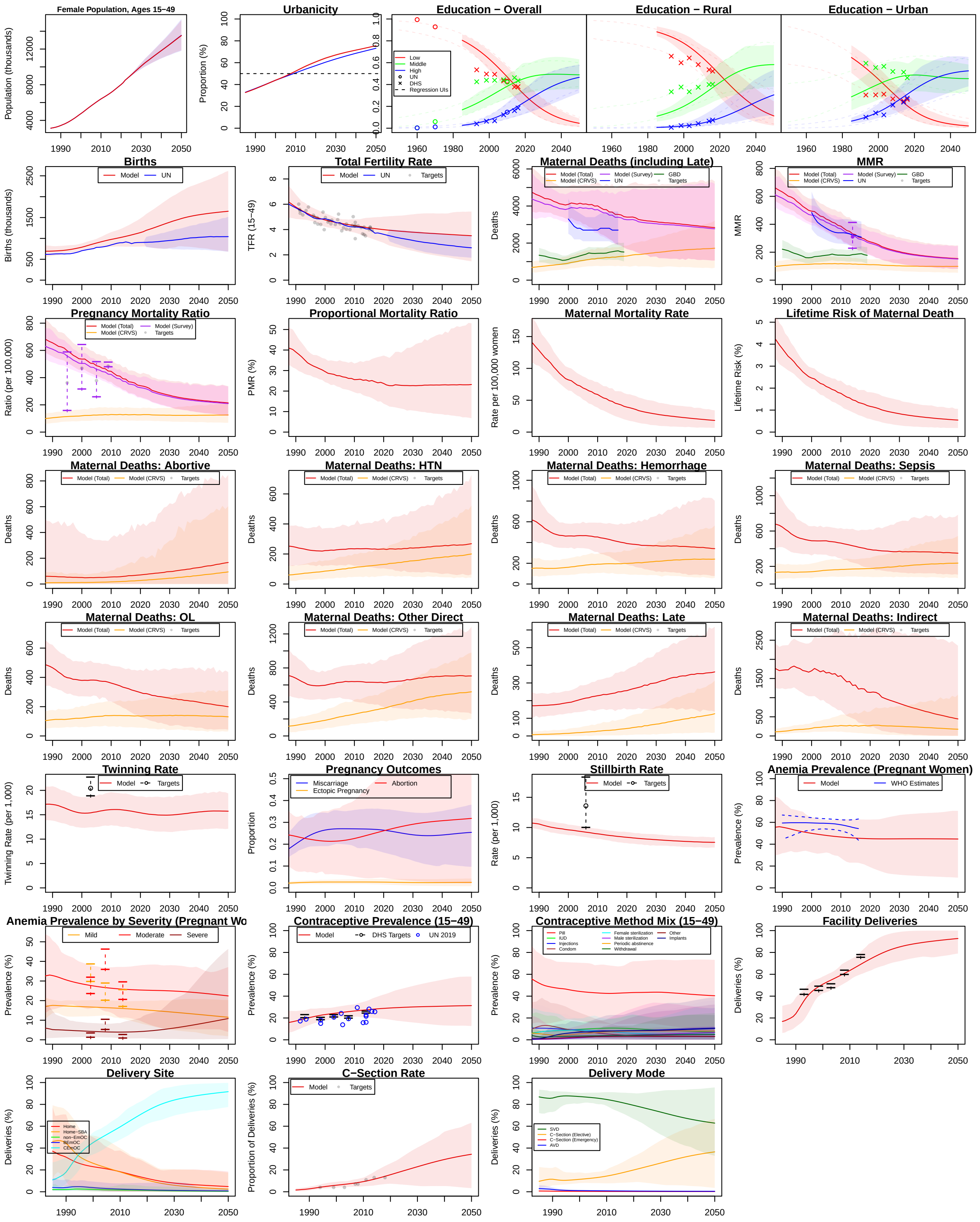

Greece

ISO Code	Region	Area	Income Group
GRC	Southern Europe	Europe	High income

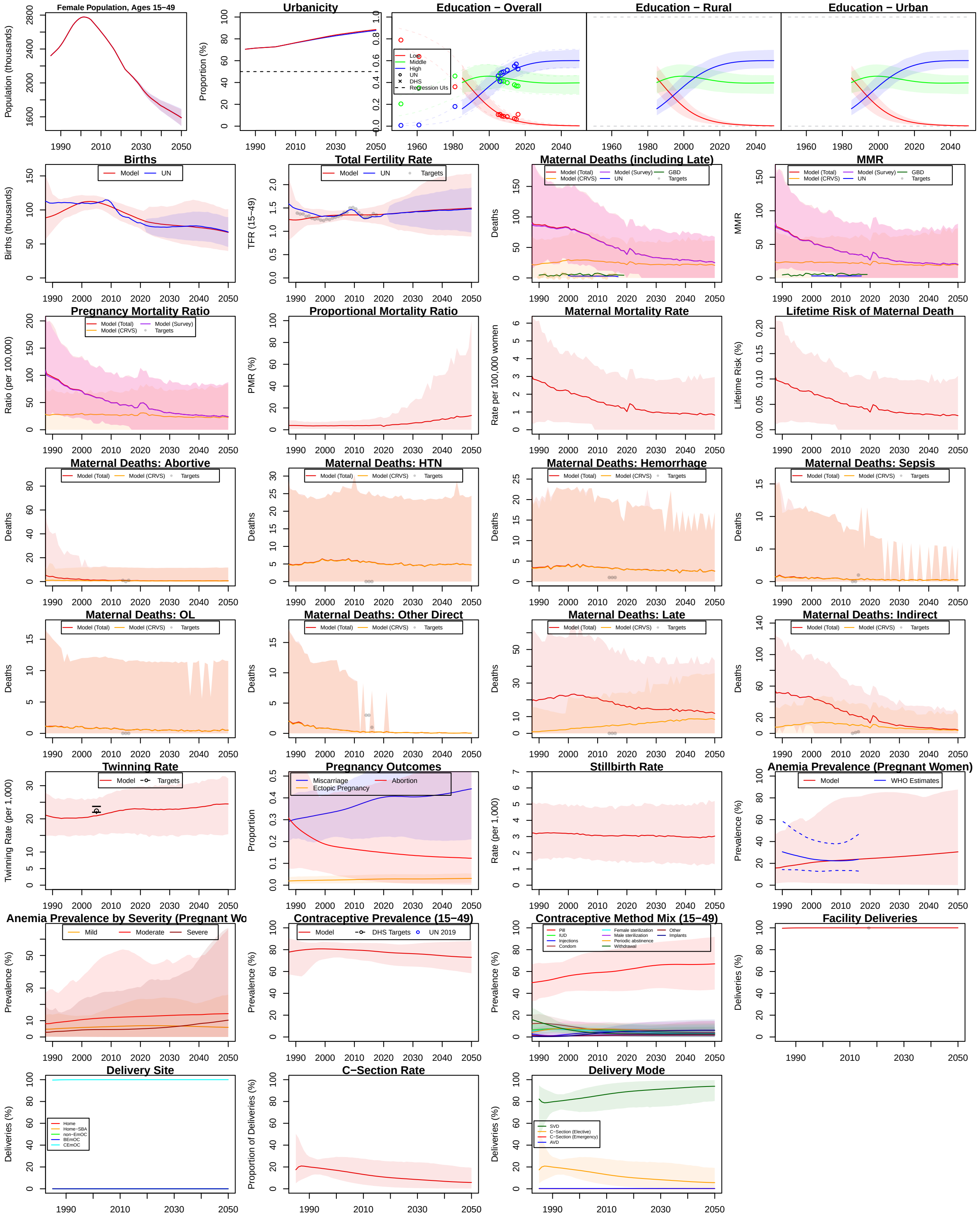

Greenland

ISO Code	Region	Area	Income Group
GRL	Northern America	Northern America	High income

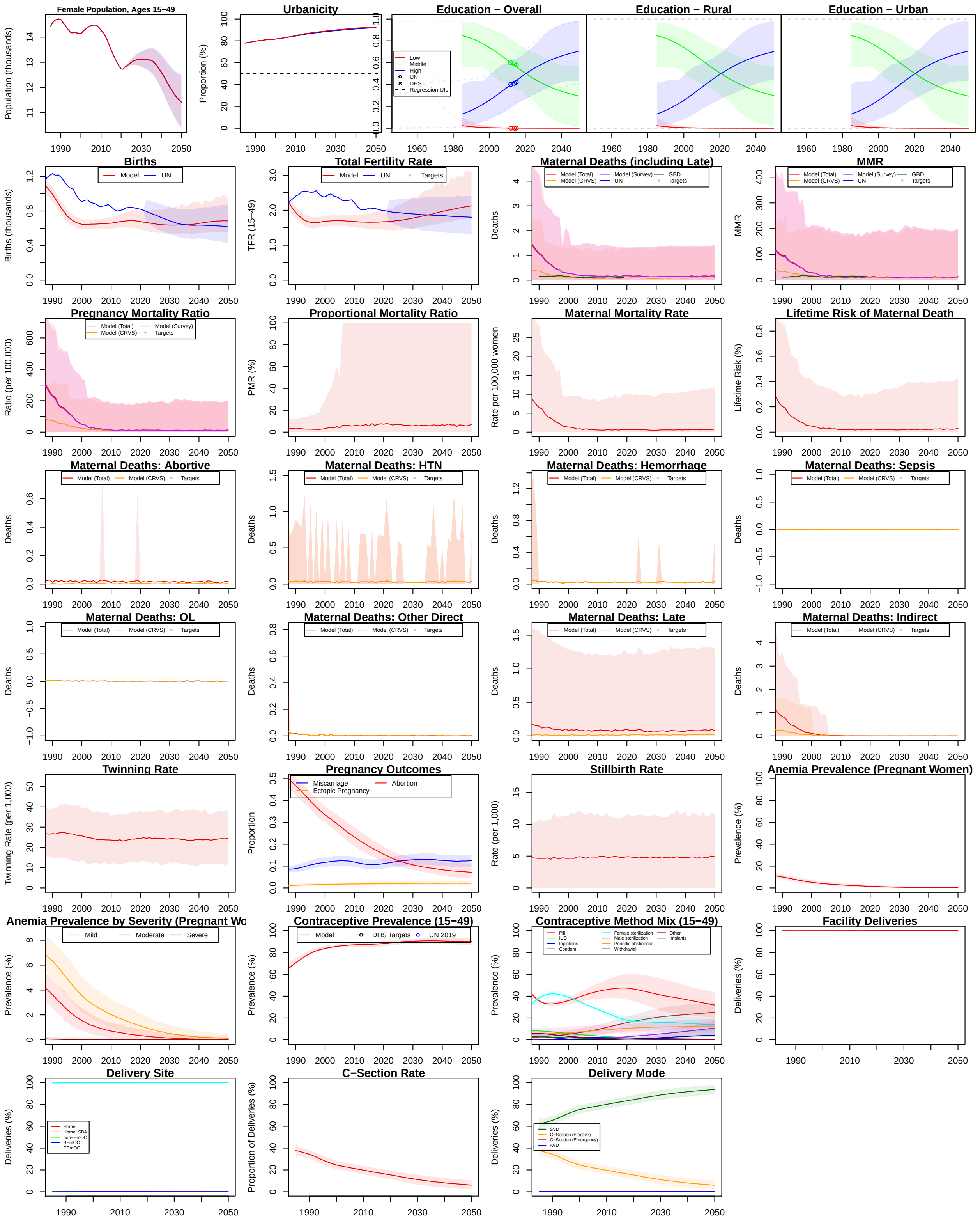

Grenada

ISO Code	Region	Area	Income Group
GRD	Caribbean	Latin America and the Caribbean	Upper middle income

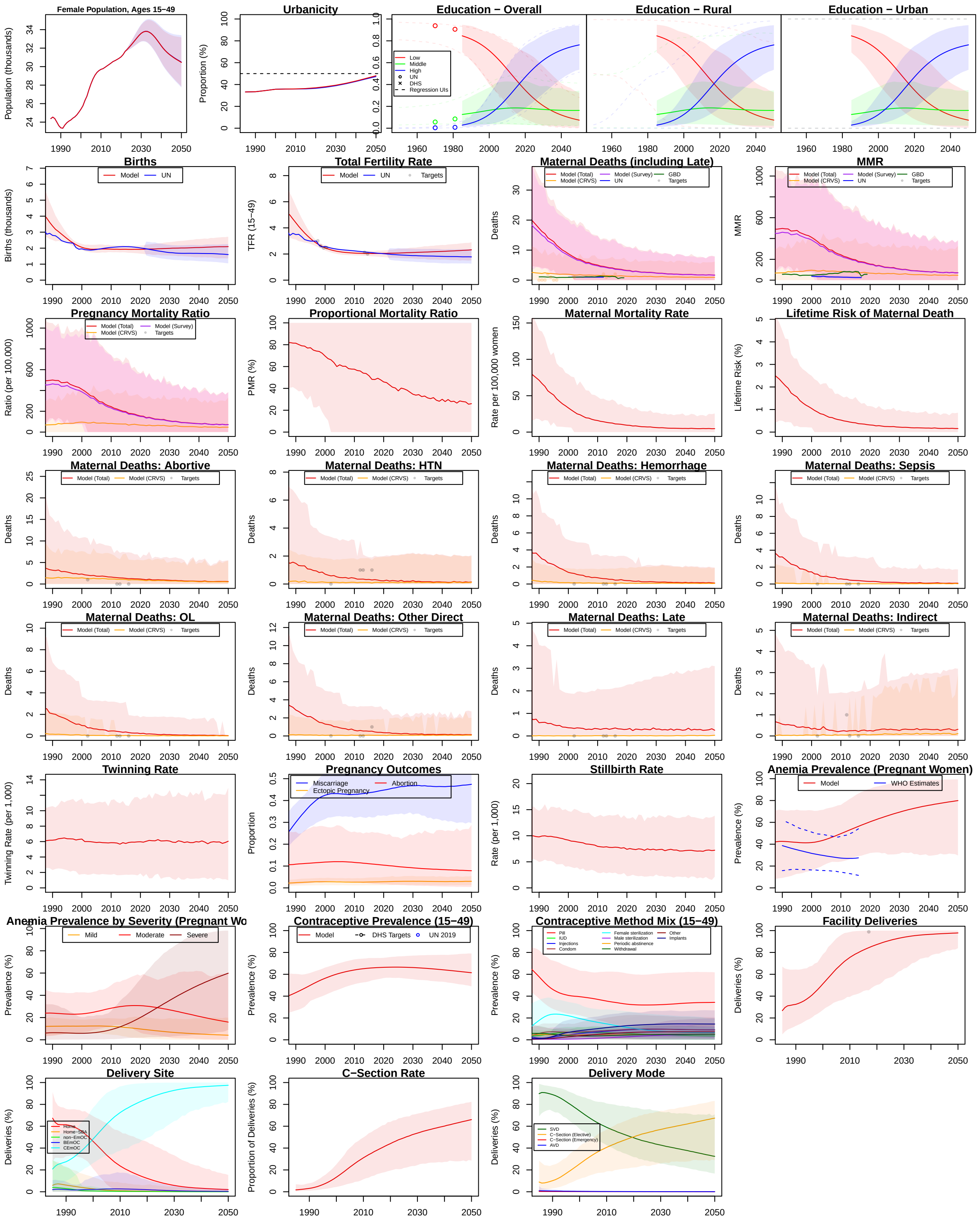

Guatemala

ISO Code	Region	Area	Income Group
GTM	Central America	Latin America and the Caribbean	Upper middle income

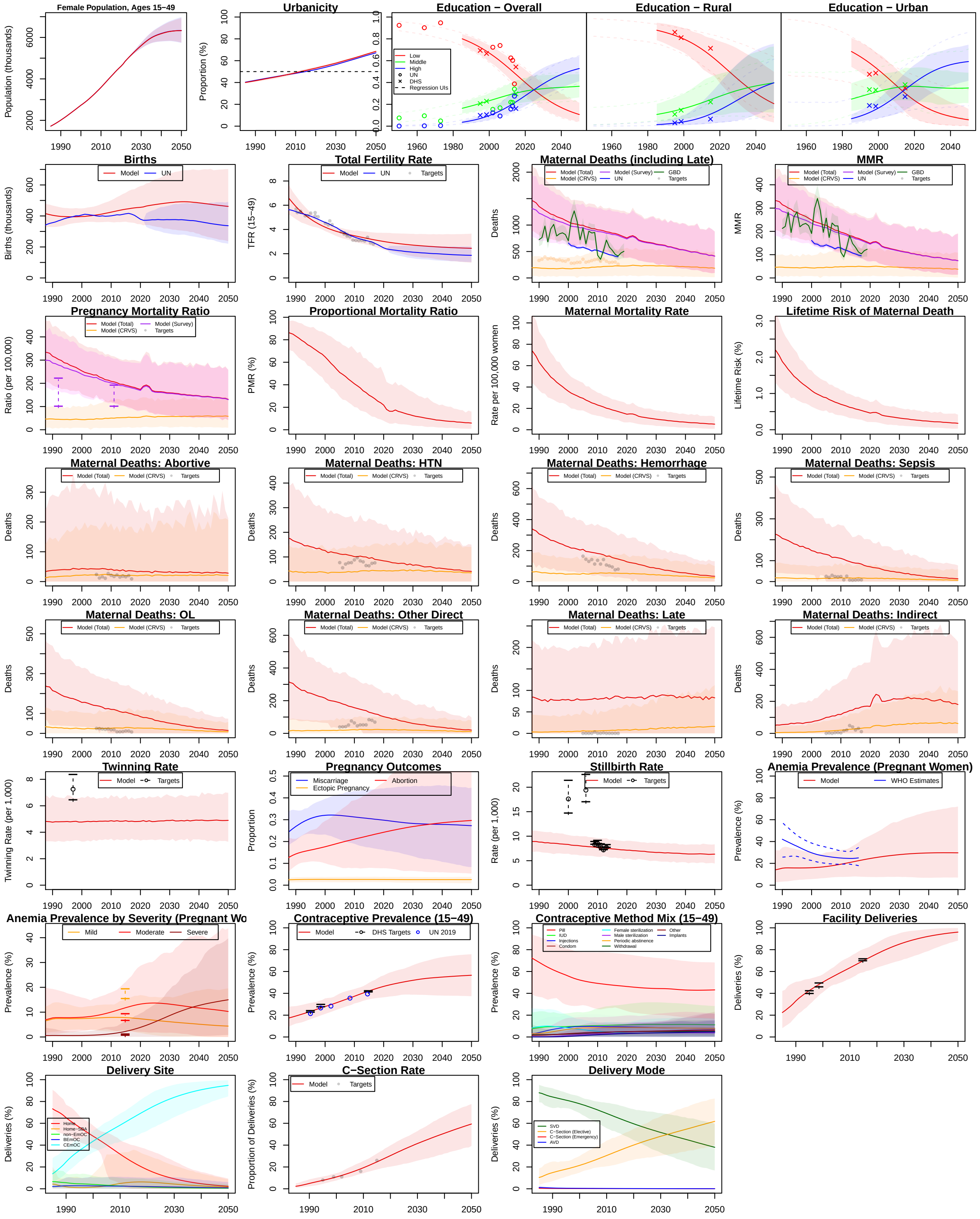

Guinea

ISO Code	Region	Area	Income Group
GIN	Western Africa	Africa	Low income

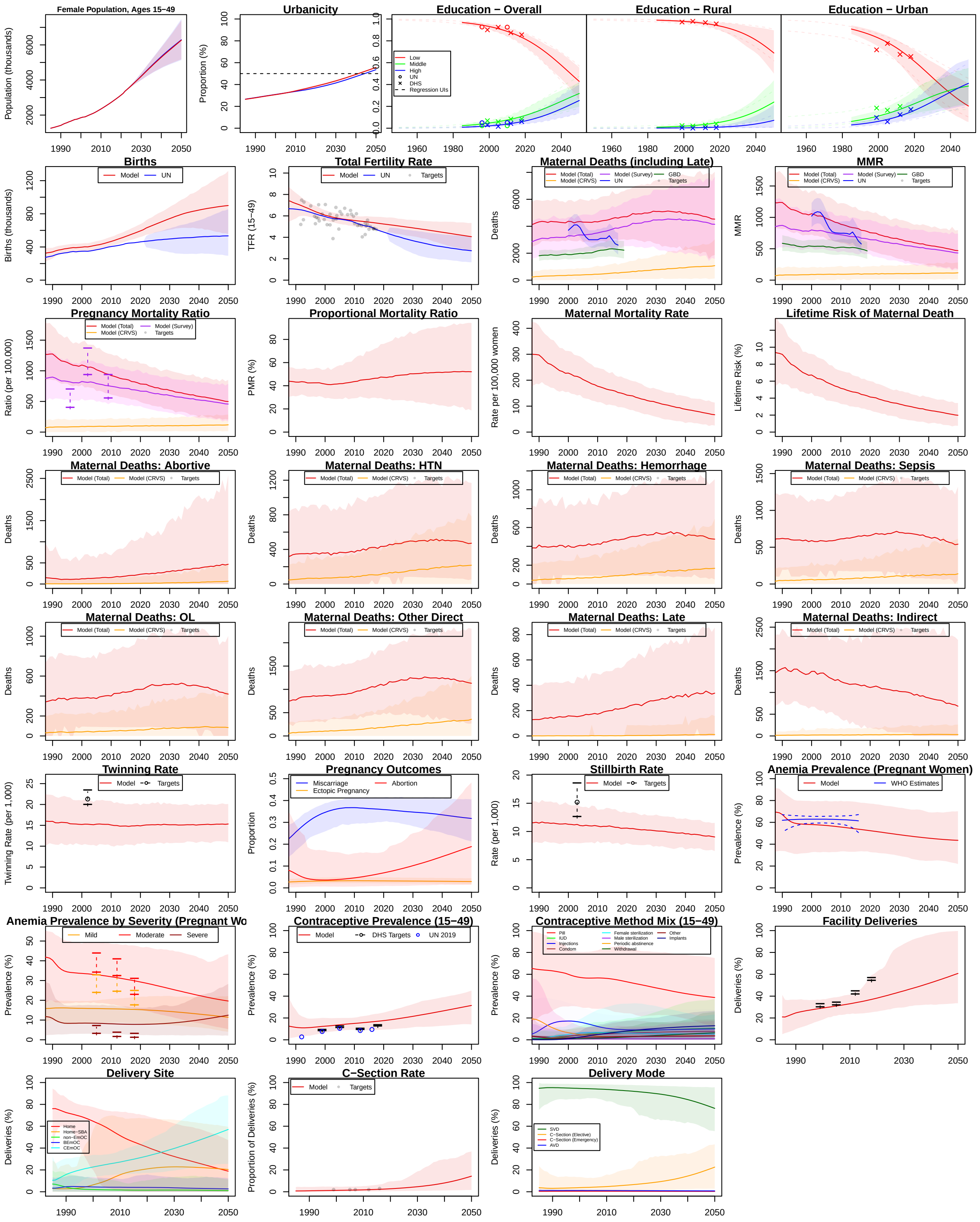

Guinea-Bissau

ISO Code	Region	Area	Income Group
GNB	Western Africa	Africa	Low income

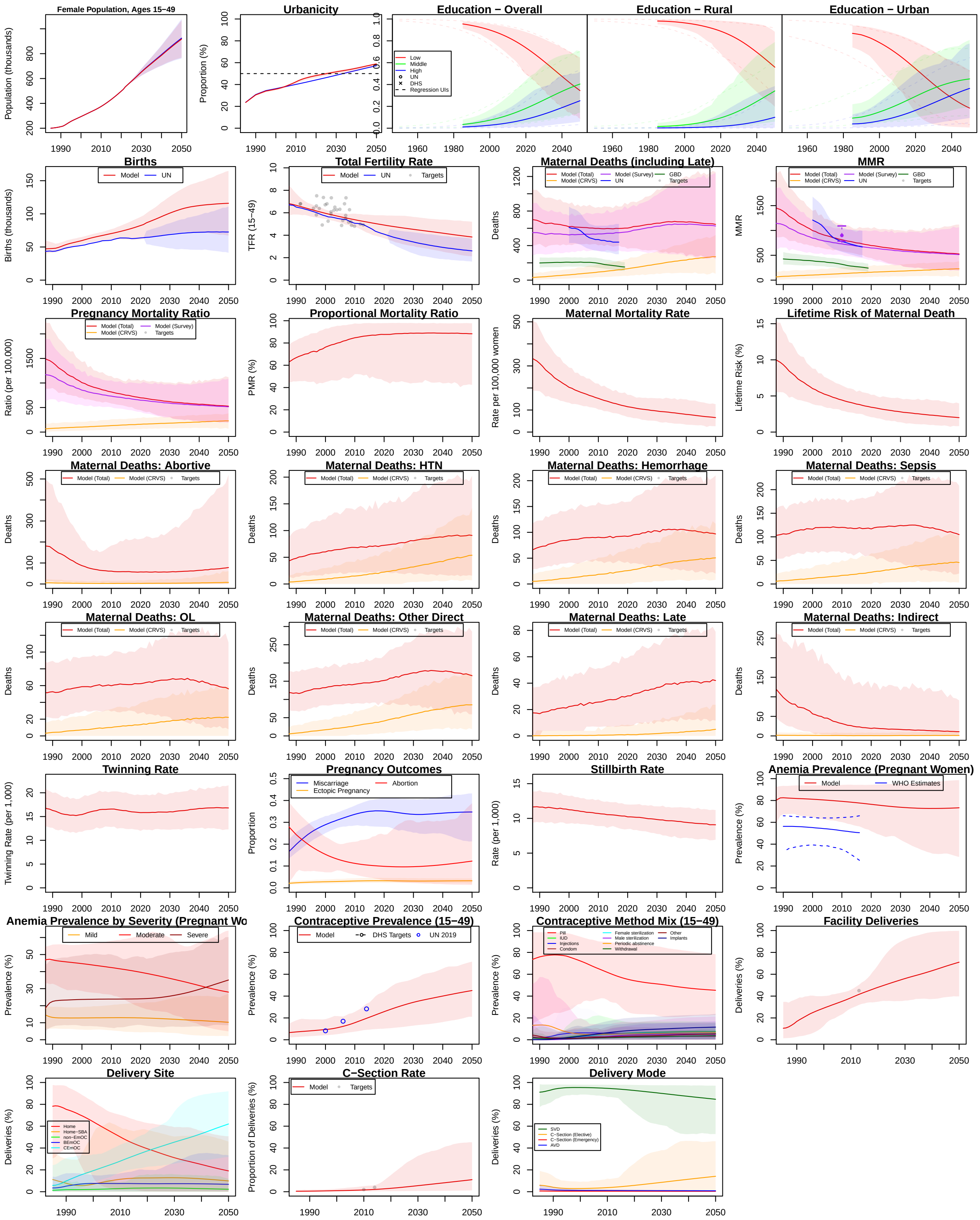

Guyana

ISO Code	Region	Area	Income Group
GUY	South America	Latin America and the Caribbean	Upper middle income

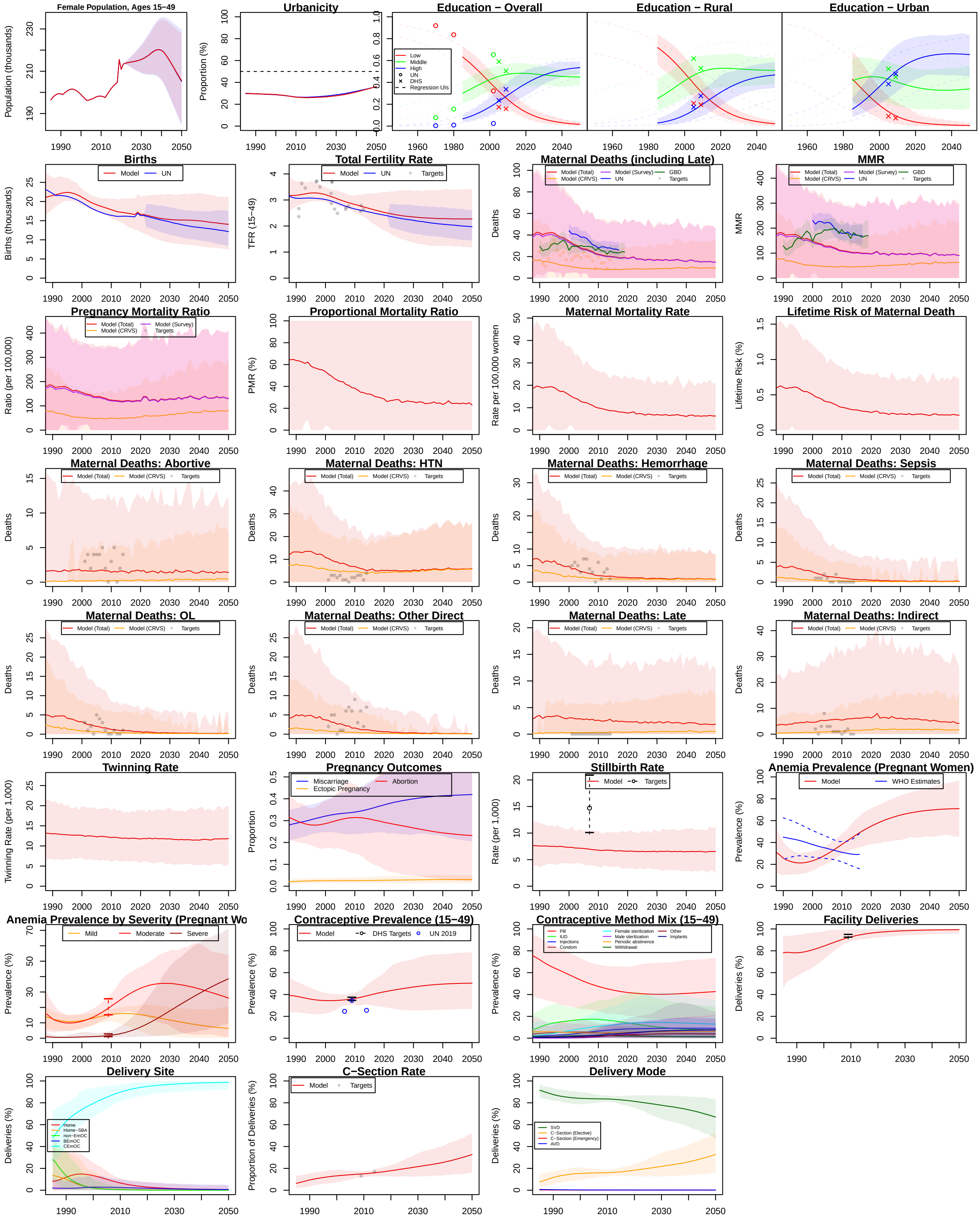

Haiti

ISO Code	Region	Area	Income Group
HTI	Caribbean	Latin America and the Caribbean	Low income

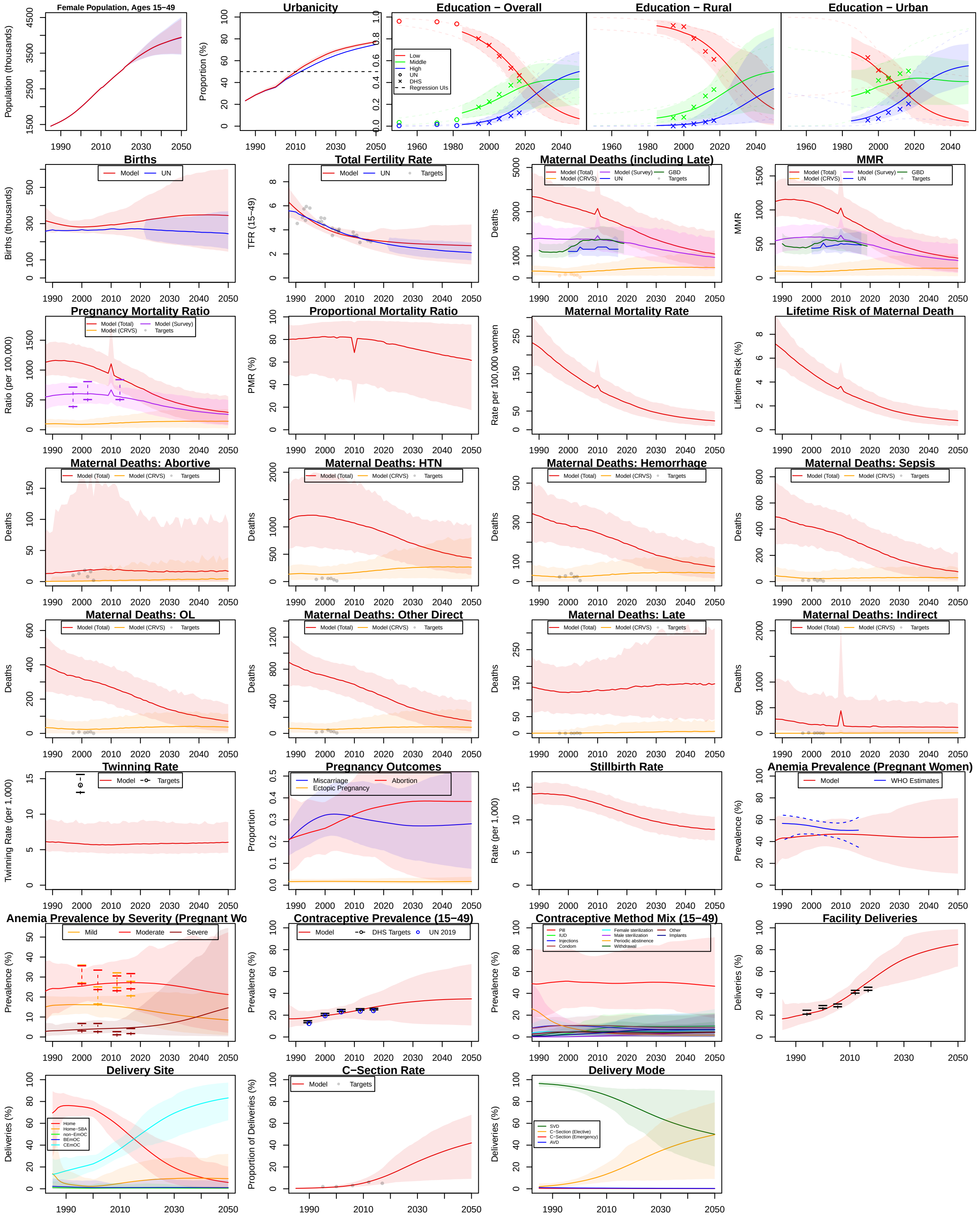

Honduras

ISO Code	Region	Area	Income Group
HND	Central America	Latin America and the Caribbean	Lower middle income

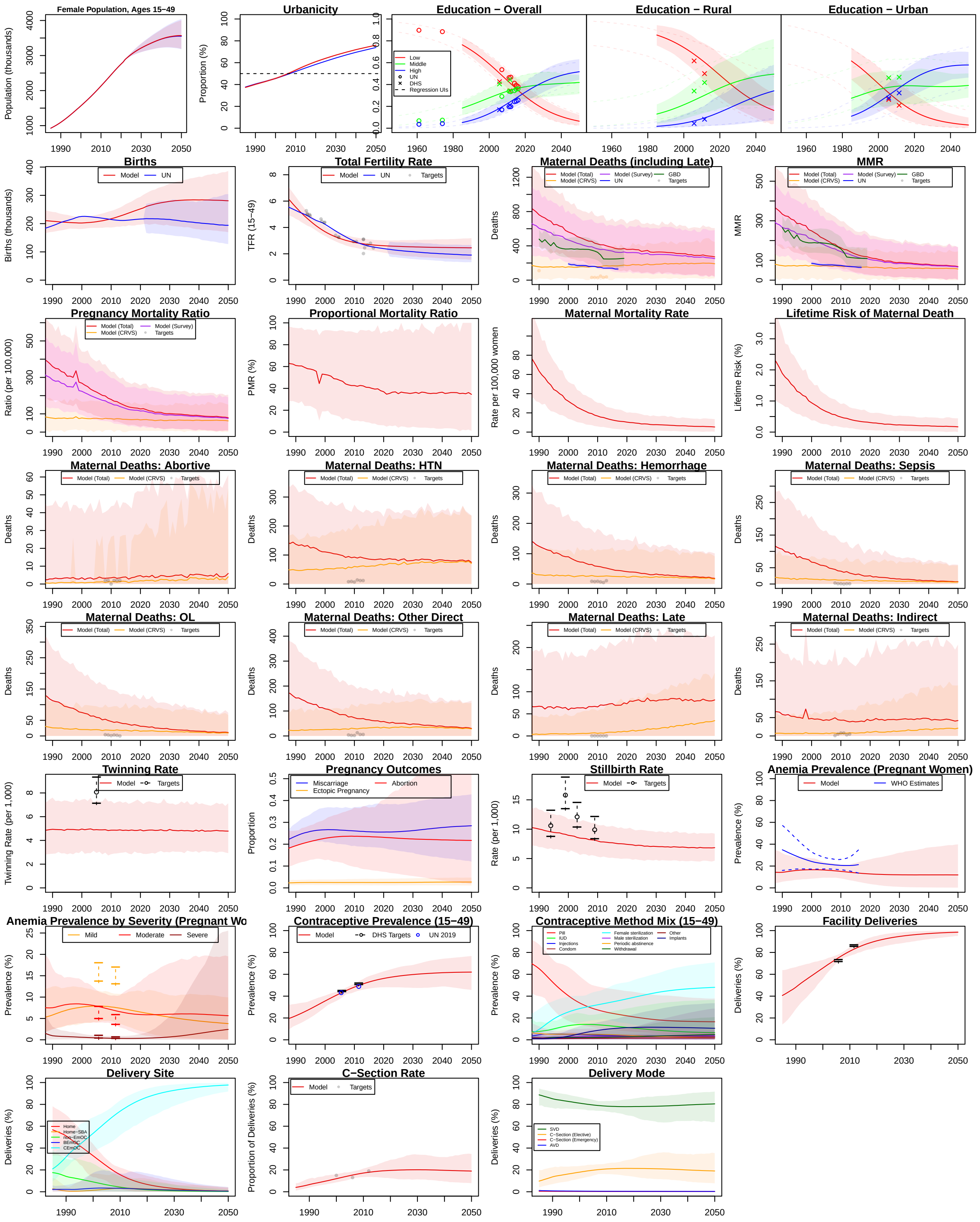

Hungary

ISO Code	Region	Area	Income Group
HUN	Eastern Europe	Europe	High income

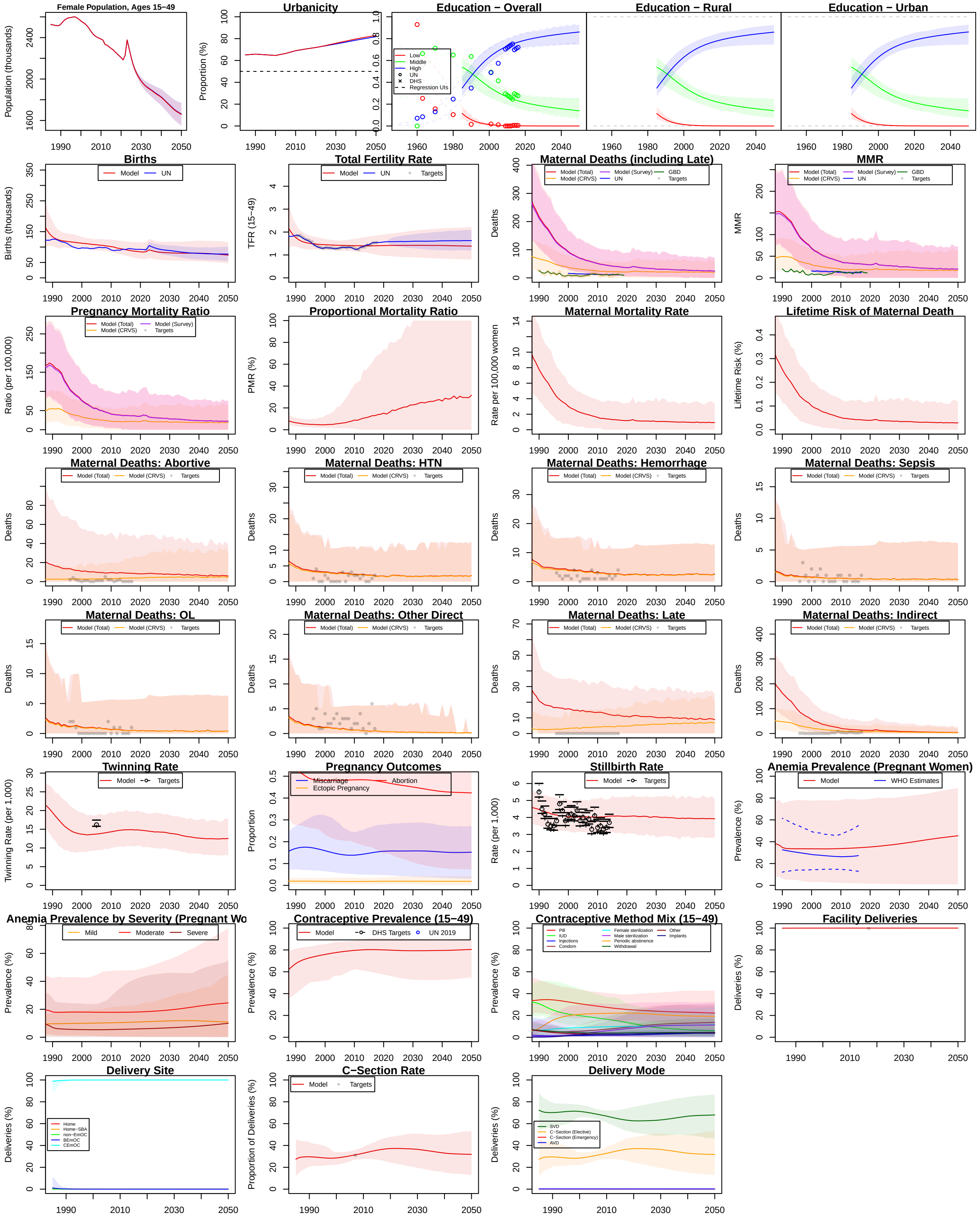

Iceland

ISO Code	Region	Area	Income Group
ISL	Northern Europe	Europe	High income

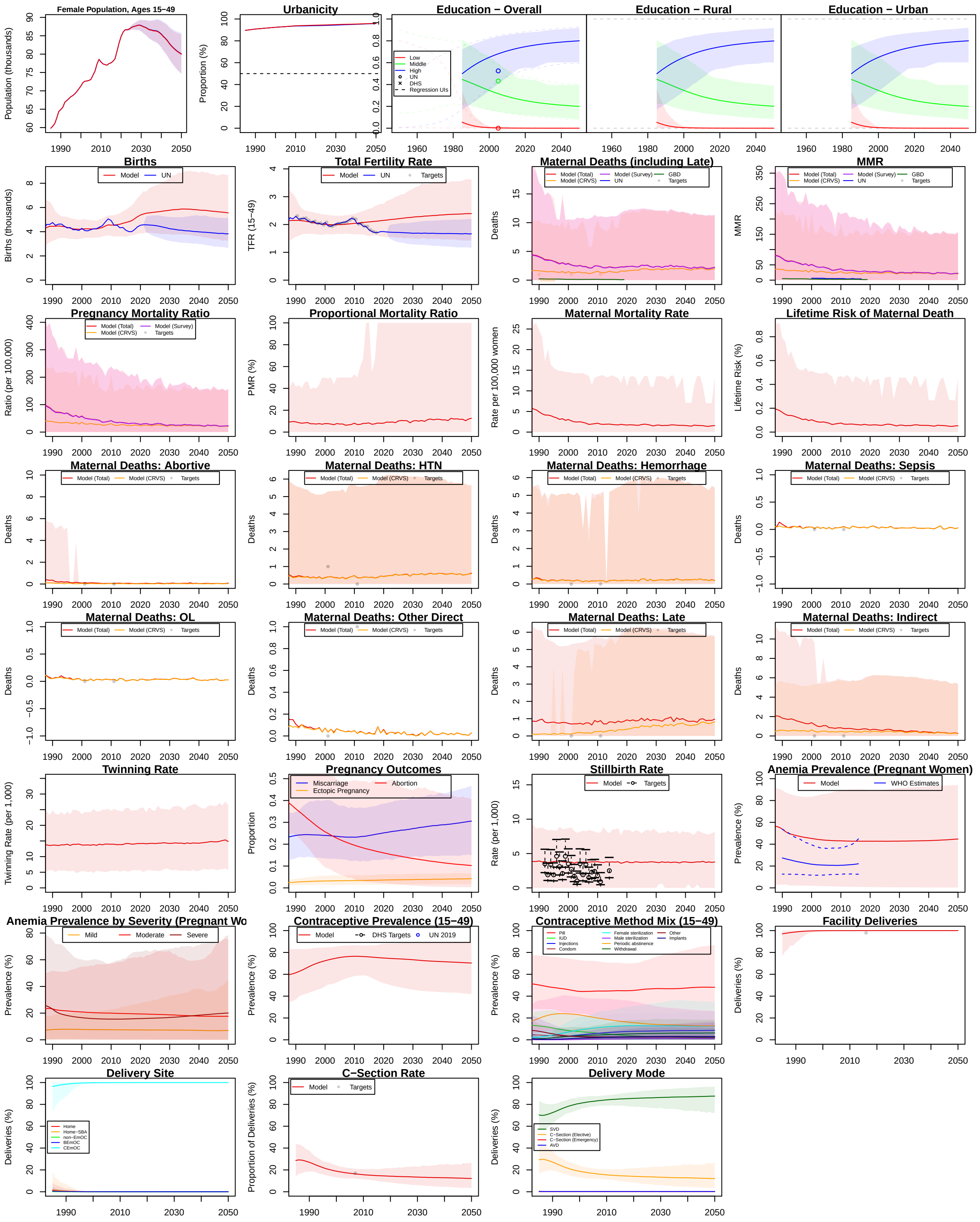

India

ISO Code	Region	Area	Income Group
IND	Southern Asia	Asia	Lower middle income

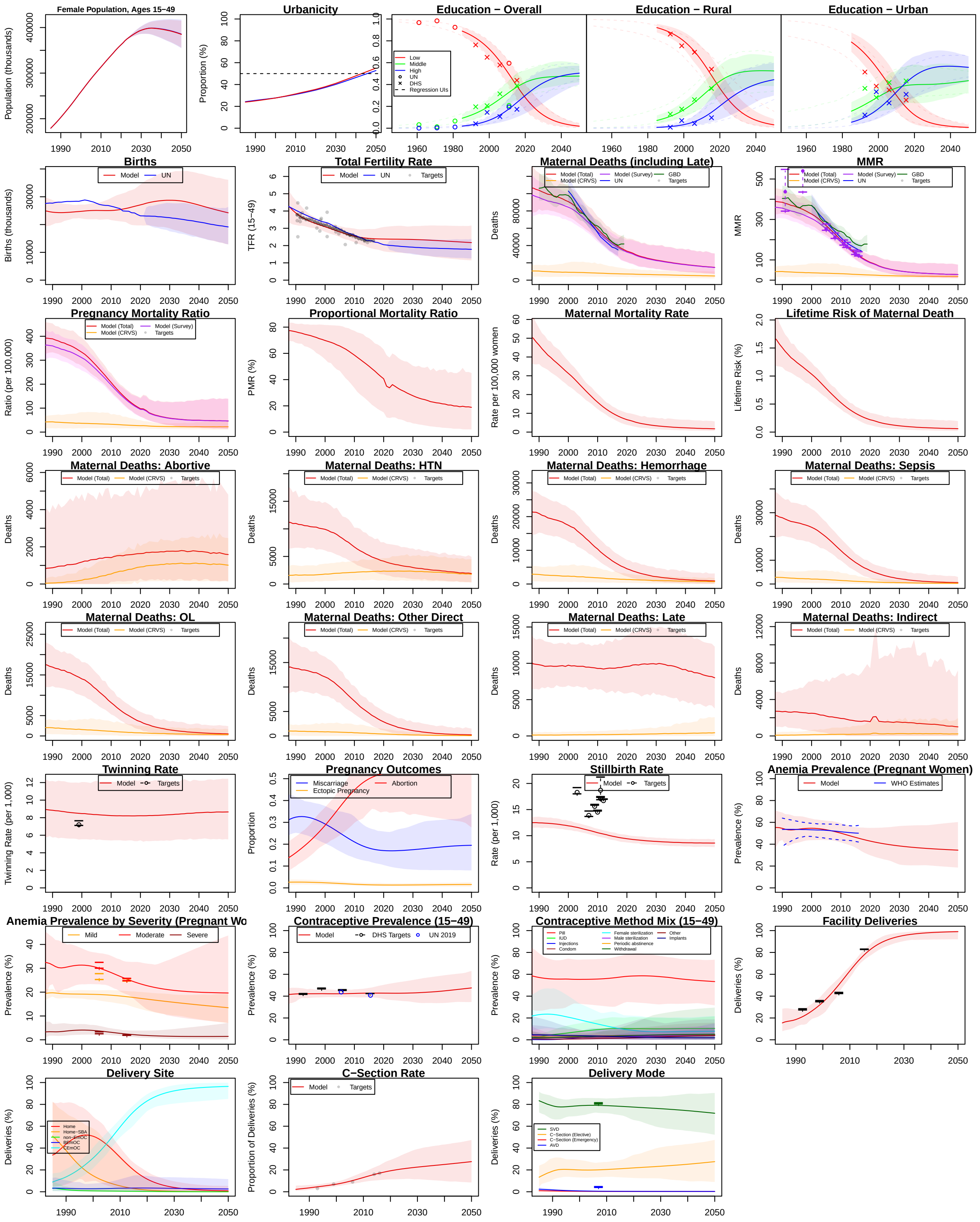

Indonesia

ISO Code	Region	Area	Income Group
IDN	South-Eastern Asia	Asia	Lower middle income

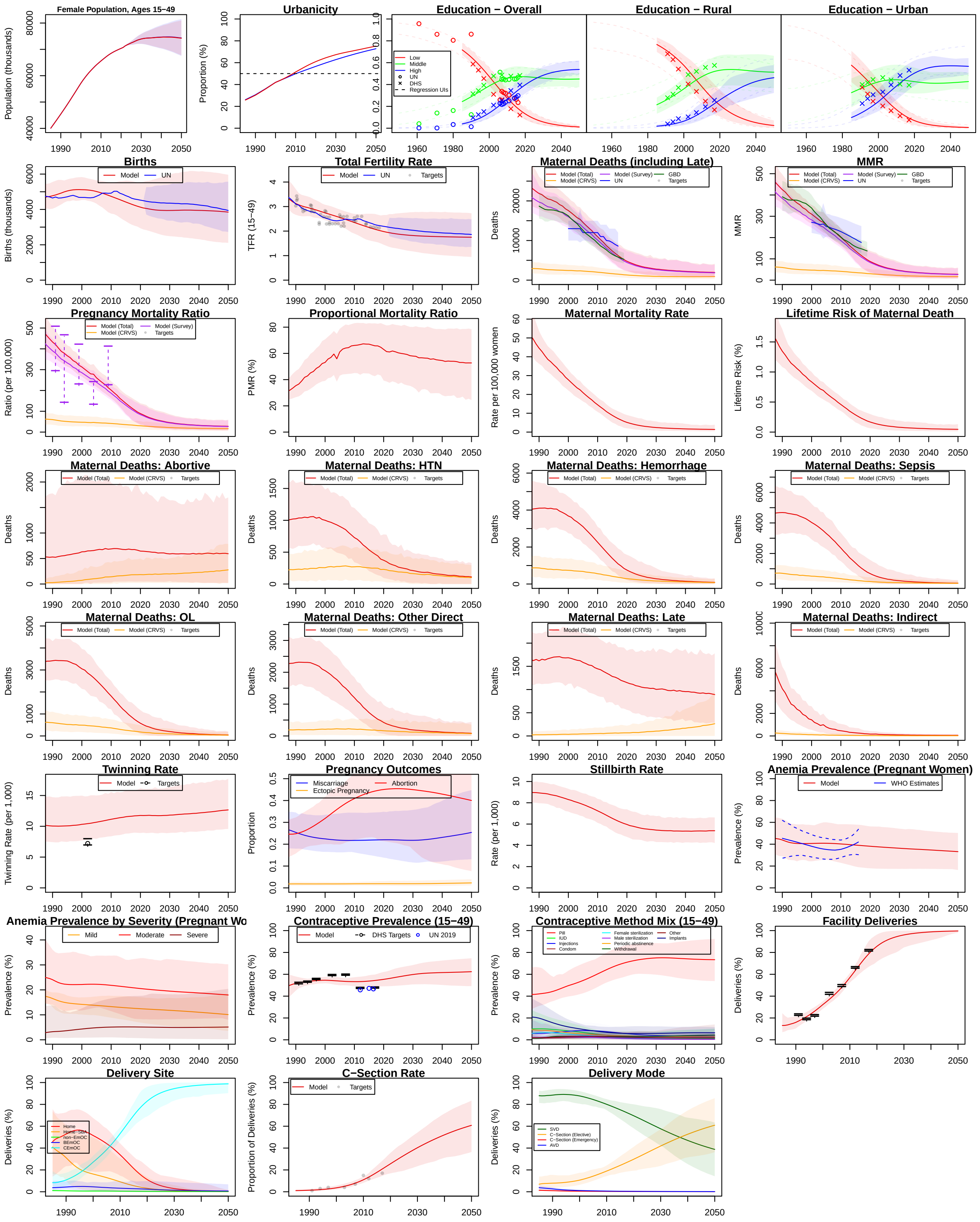

Iran (Islamic Republic of)

ISO Code	Region	Area	Income Group
IRN	Southern Asia	Asia	Upper middle income

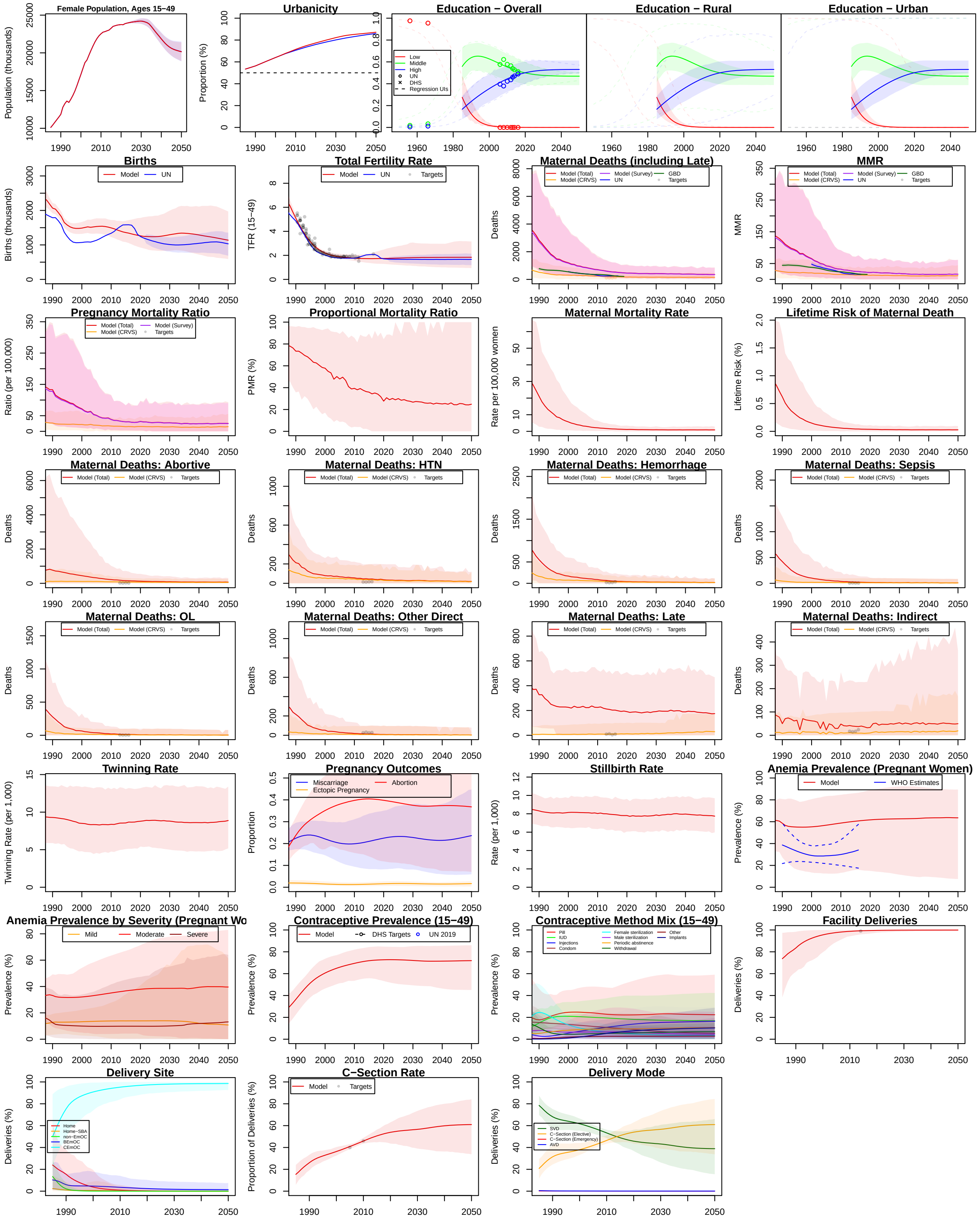

Iraq

ISO Code	Region	Area	Income Group
IRQ	Western Asia	Asia	Upper middle income

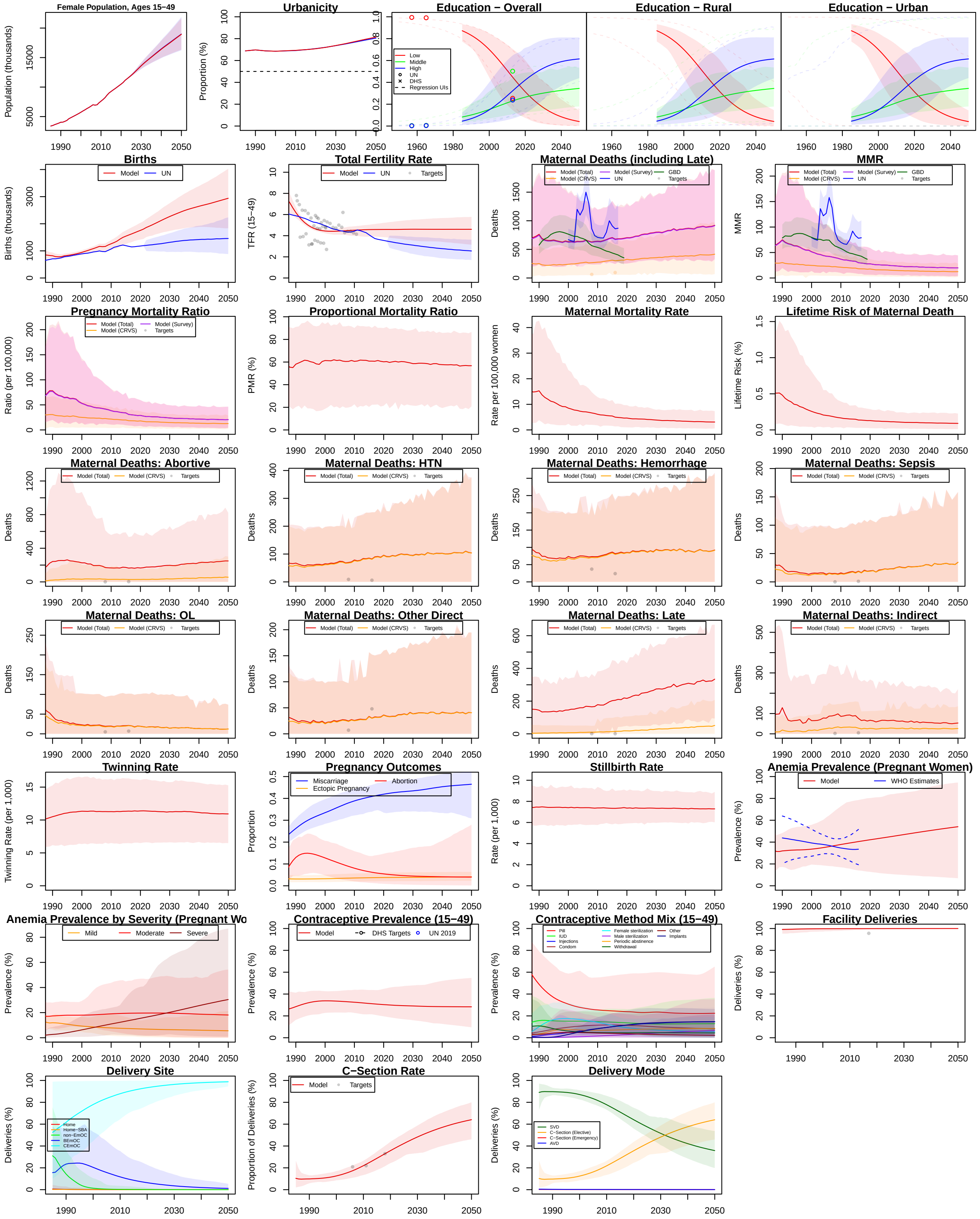

Ireland

ISO Code	Region	Area	Income Group
IRL	Northern Europe	Europe	High income

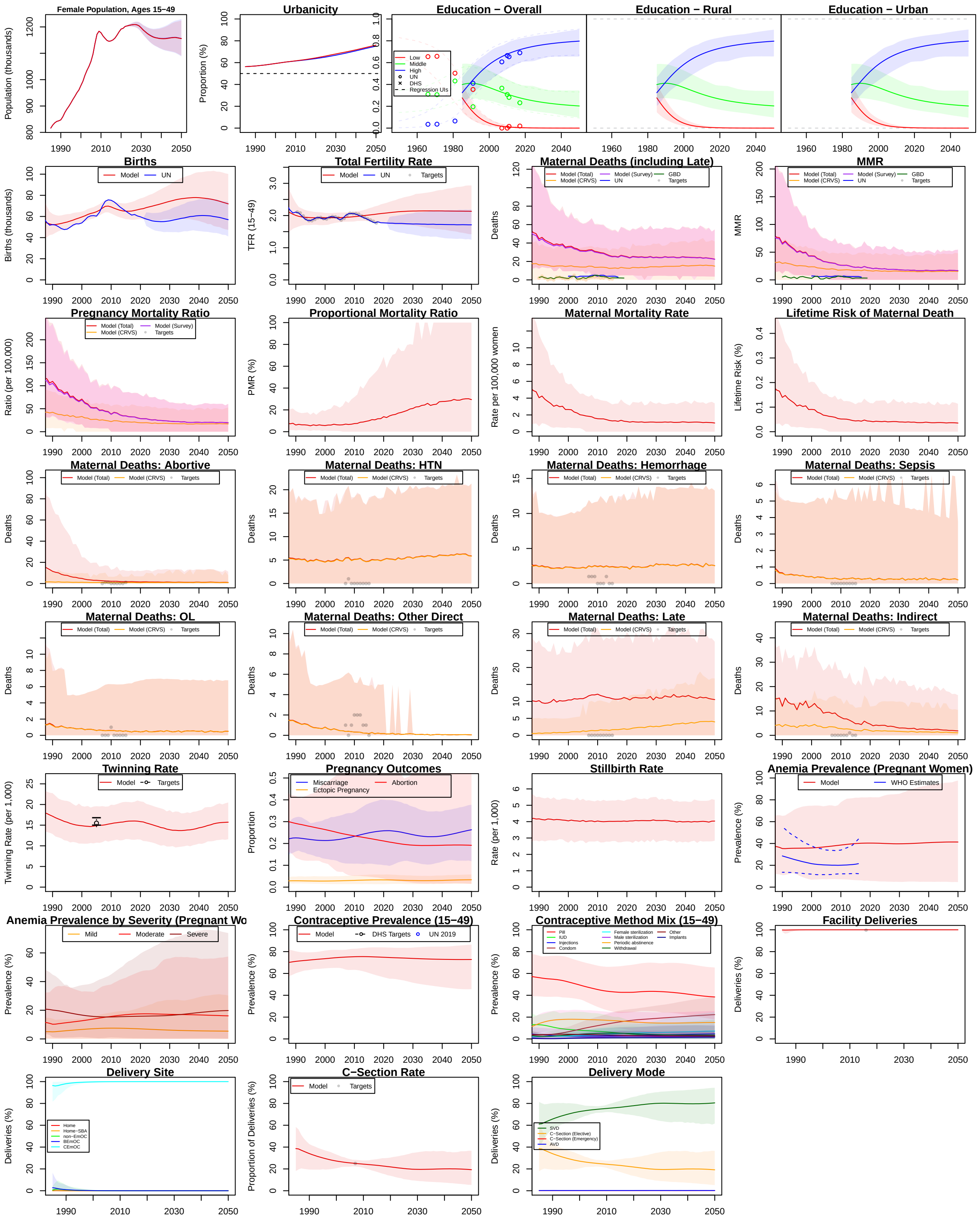

Israel

ISO Code	Region	Area	Income Group
ISR	Western Asia	Asia	High income

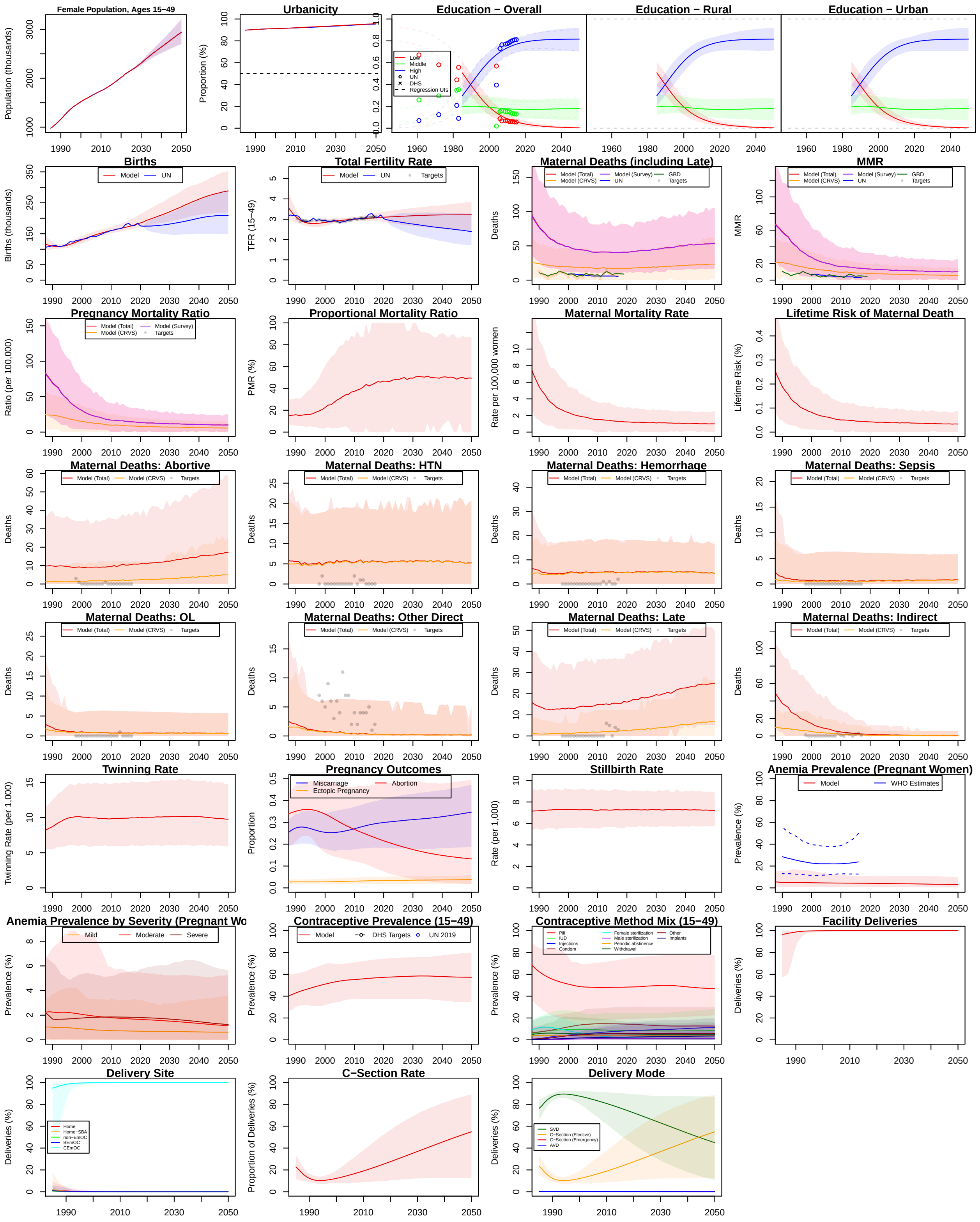

Italy

ISO Code	Region	Area	Income Group
ITA	Southern Europe	Europe	High income

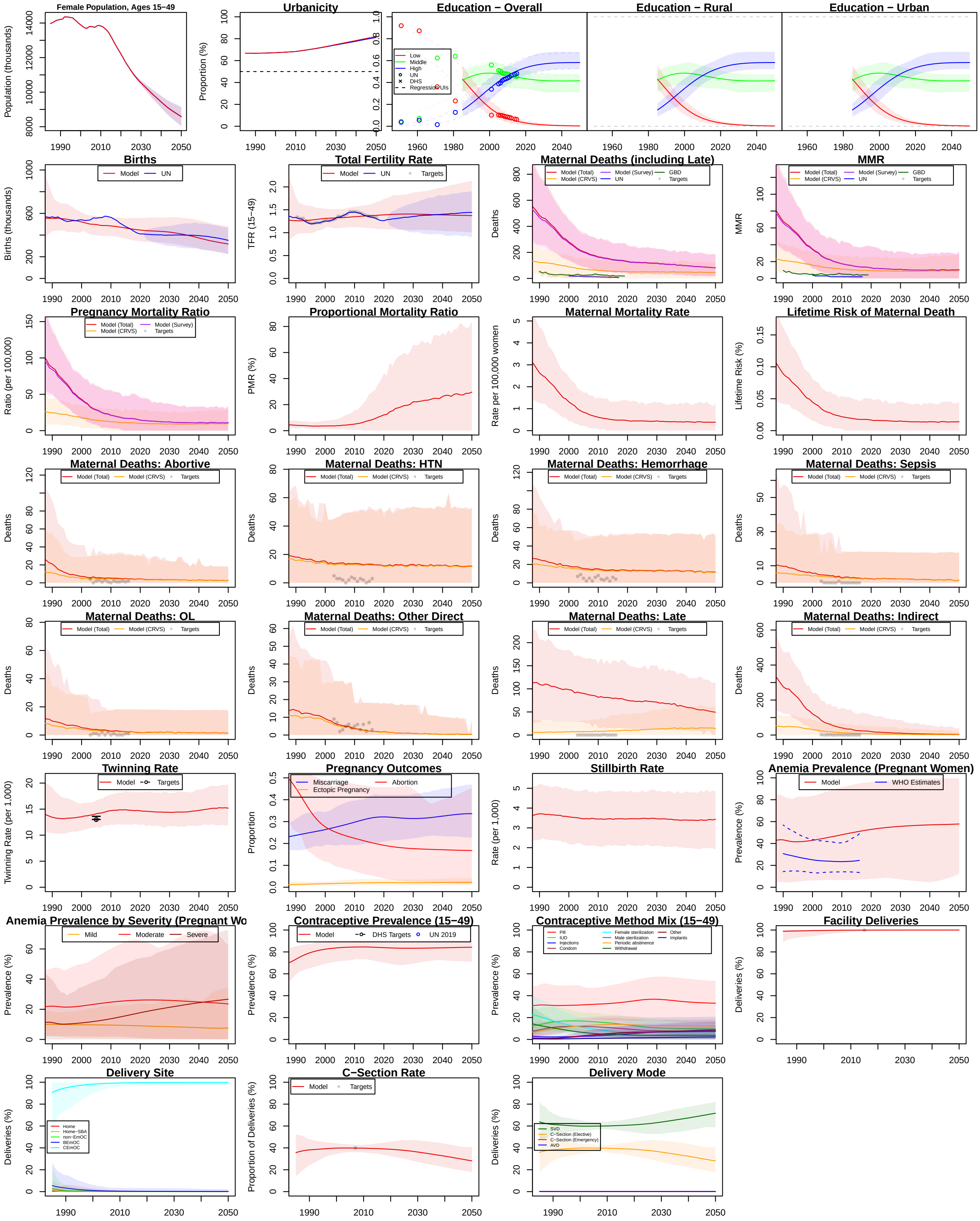

Jamaica

ISO Code	Region	Area	Income Group
JAM	Caribbean	Latin America and the Caribbean	Upper middle income

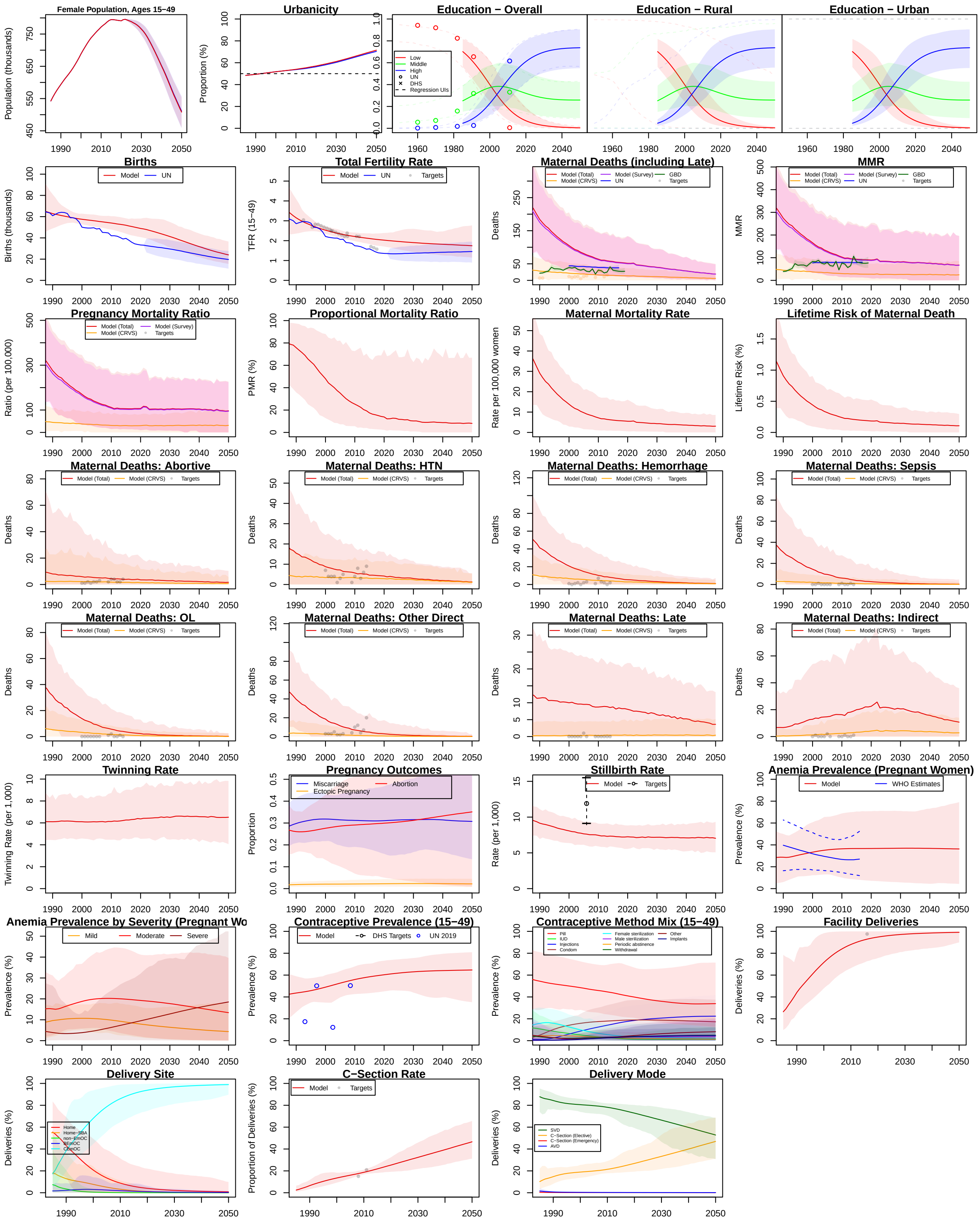

Japan

ISO Code	Region	Area	Income Group
JPN	Eastern Asia	Asia	High income

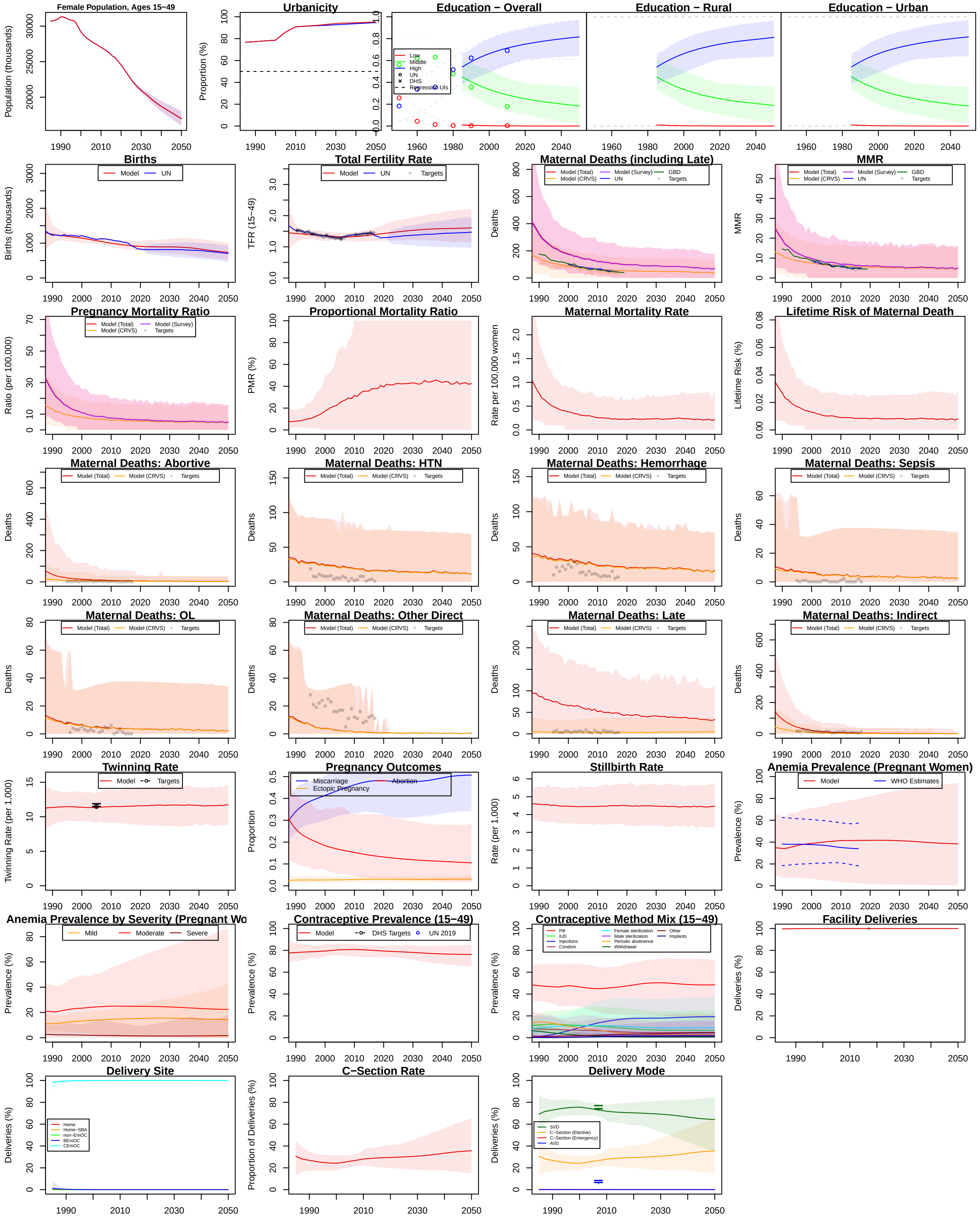

Jordan

ISO Code	Region	Area	Income Group
JOR	Western Asia	Asia	Upper middle income

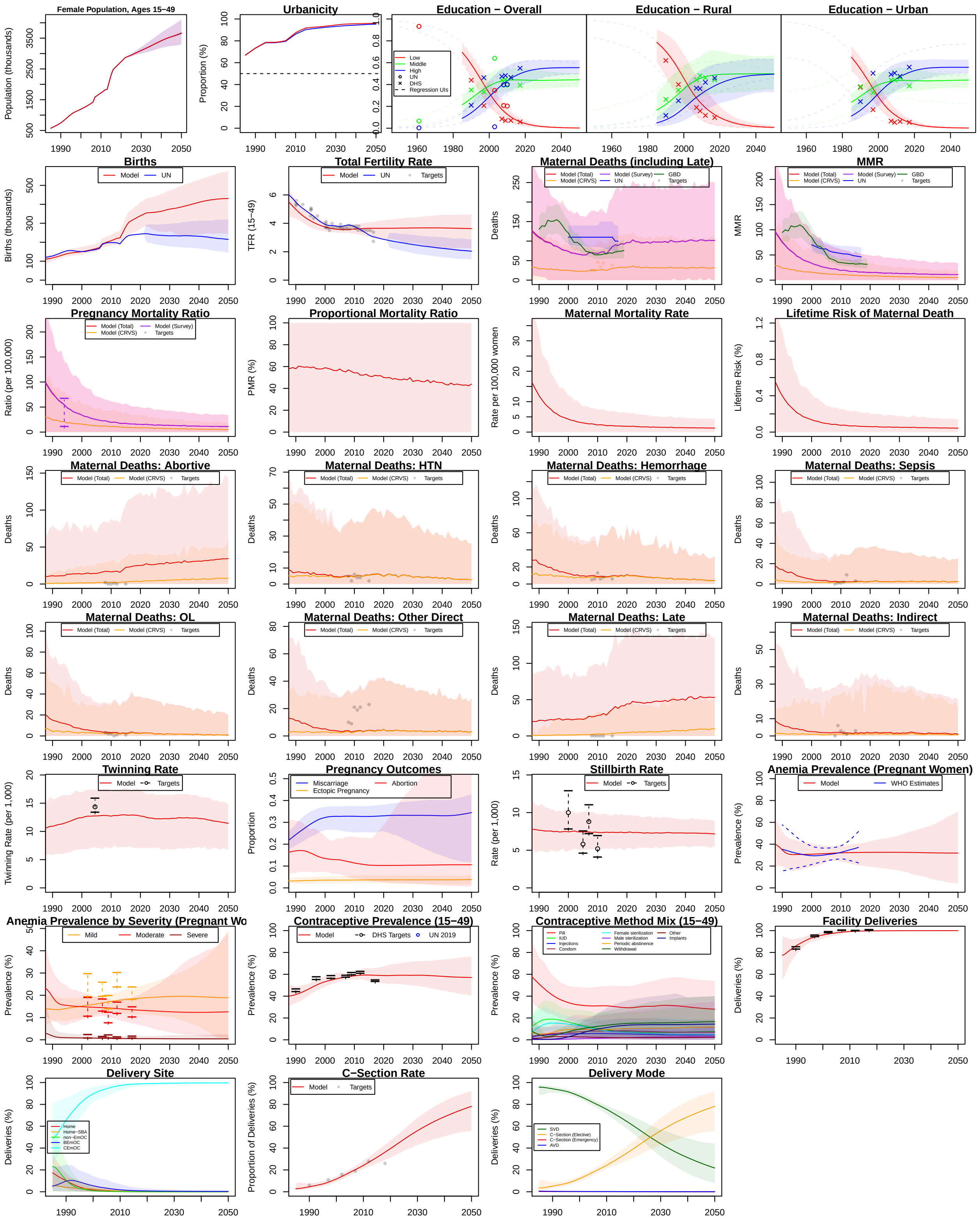

Kazakhstan

ISO Code	Region	Area	Income Group
KAZ	Central Asia	Asia	Upper middle income

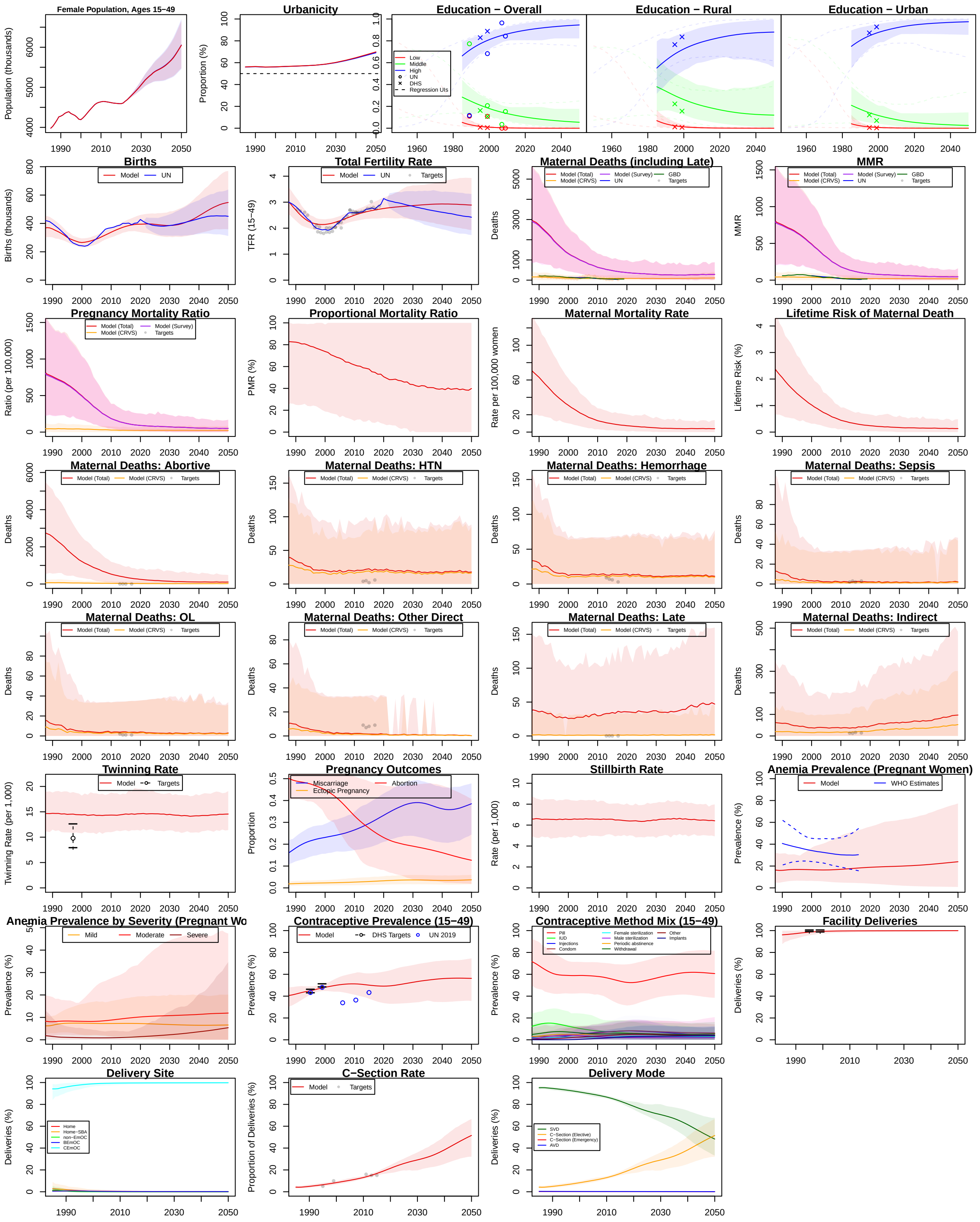

Kenya

ISO Code	Region	Area	Income Group
KEN	Eastern Africa	Africa	Lower middle income

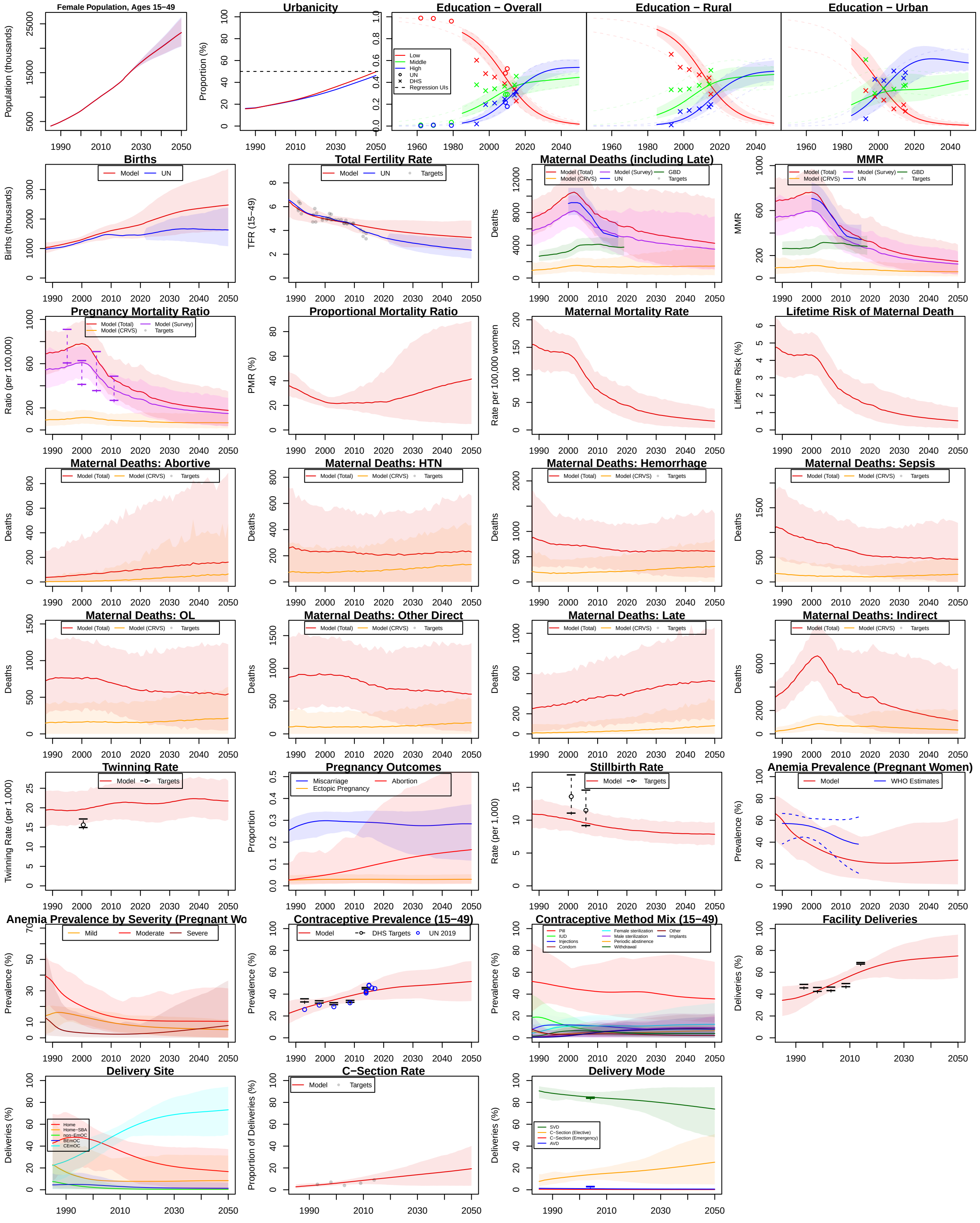

Kiribati

ISO Code	Region	Area	Income Group
KIR	Micronesia	Oceania	Lower middle income

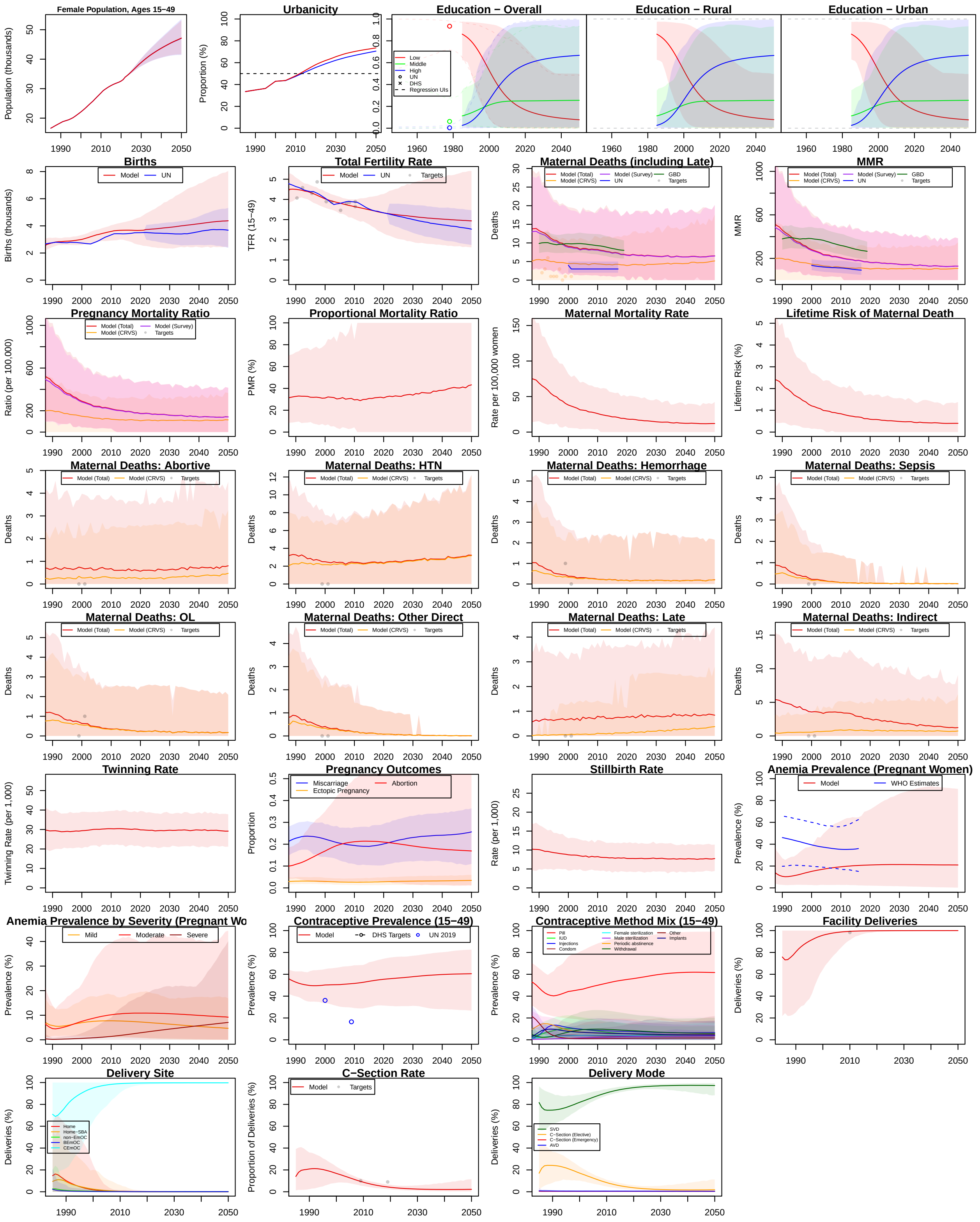

Kuwait

ISO Code	Region	Area	Income Group
KWT	Western Asia	Asia	High income

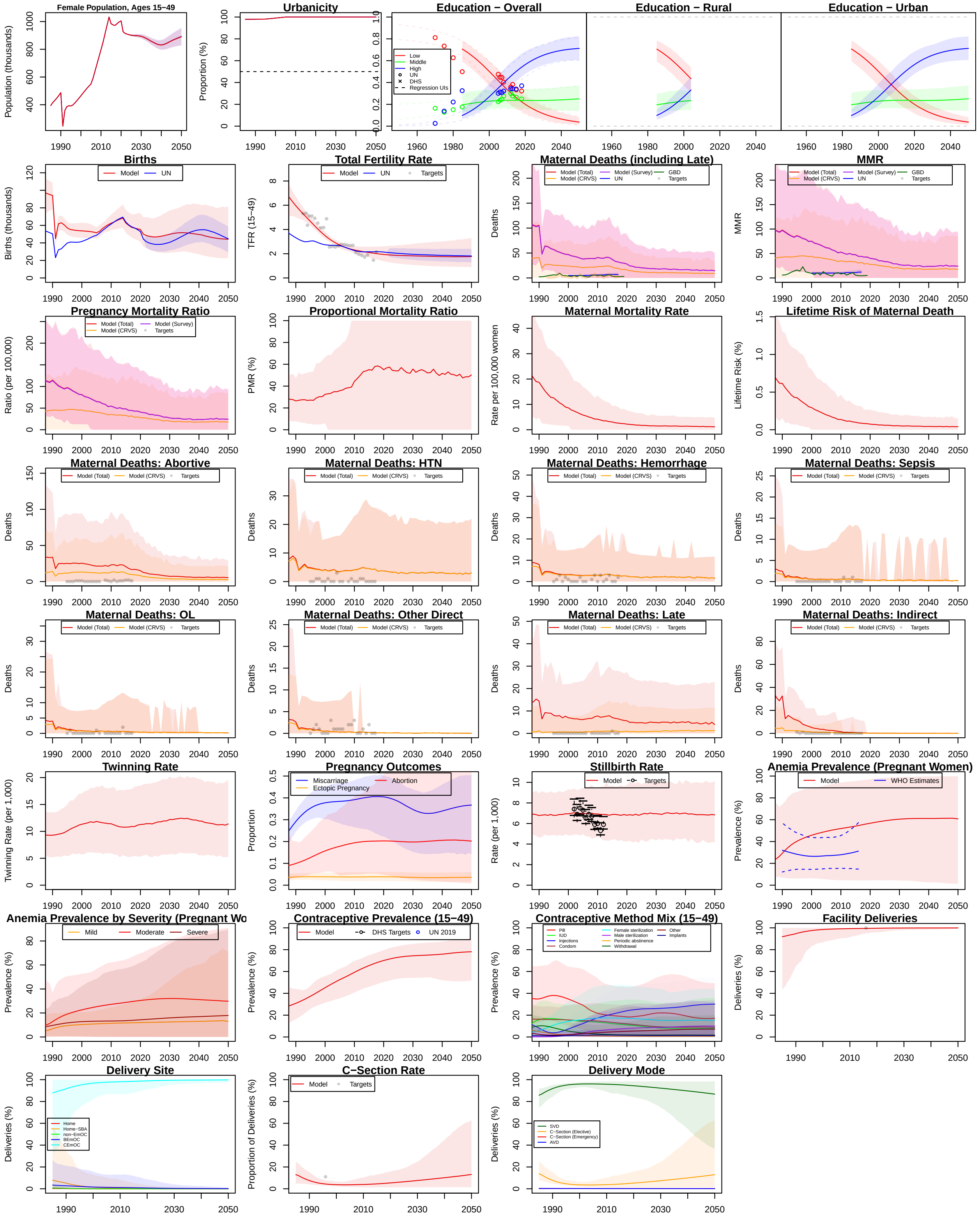

Kyrgyzstan

ISO Code	Region	Area	Income Group
KGZ	Central Asia	Asia	Lower middle income

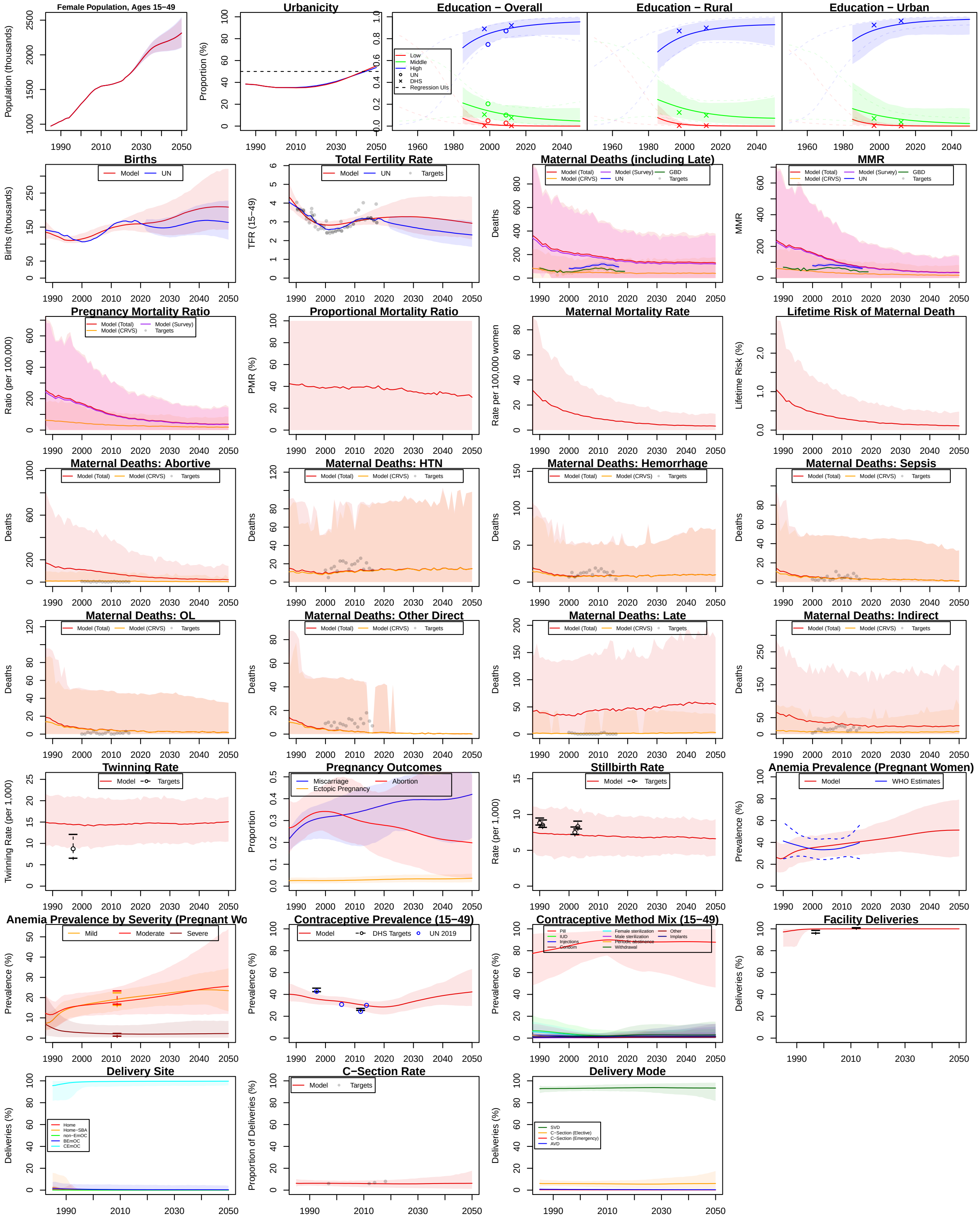

Lao People's Democratic Republic

ISO Code	Region	Area	Income Group
LAO	South-Eastern Asia	Asia	Lower middle income

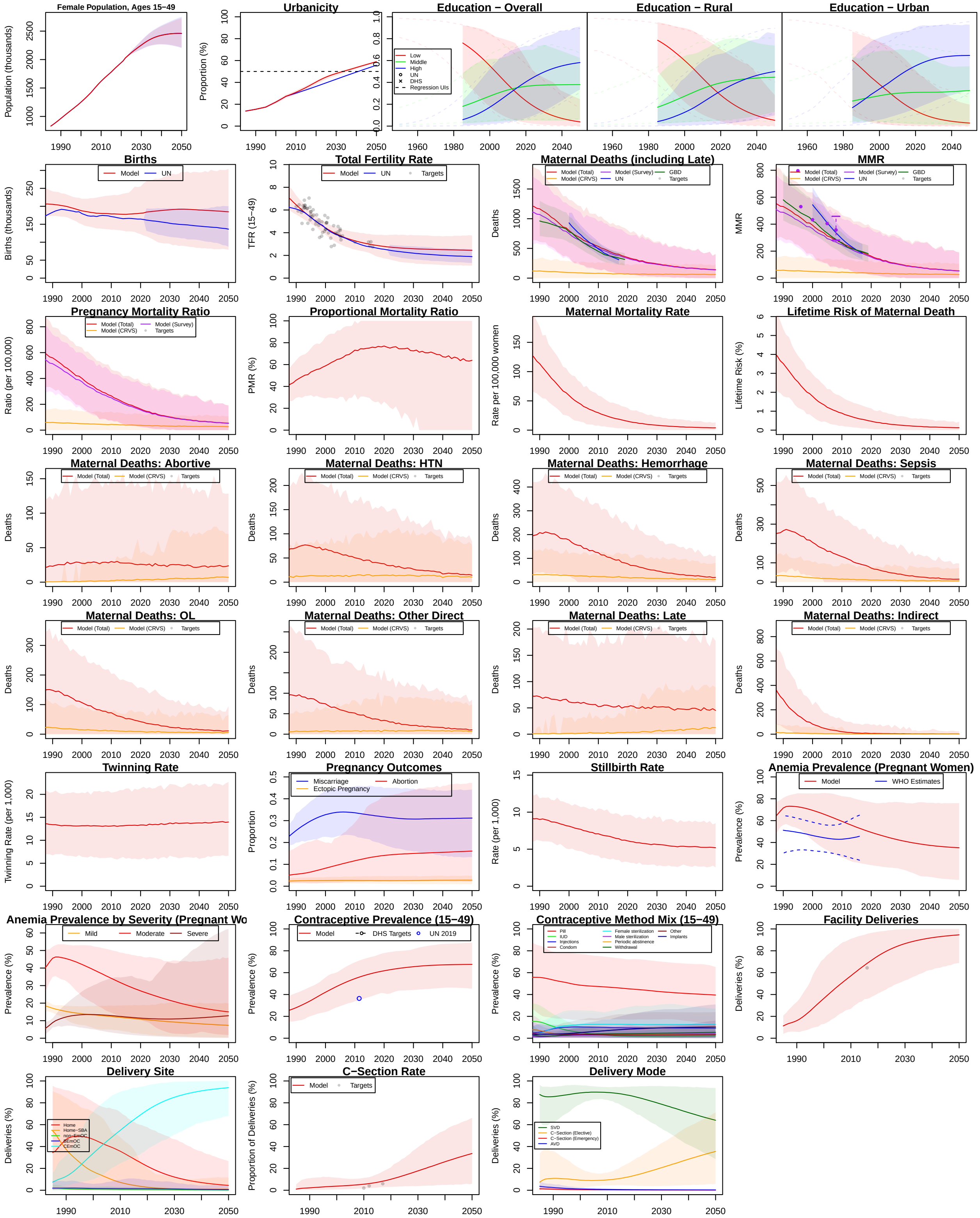

Latvia

ISO Code	Region	Area	Income Group
LVA	Northern Europe	Europe	High income

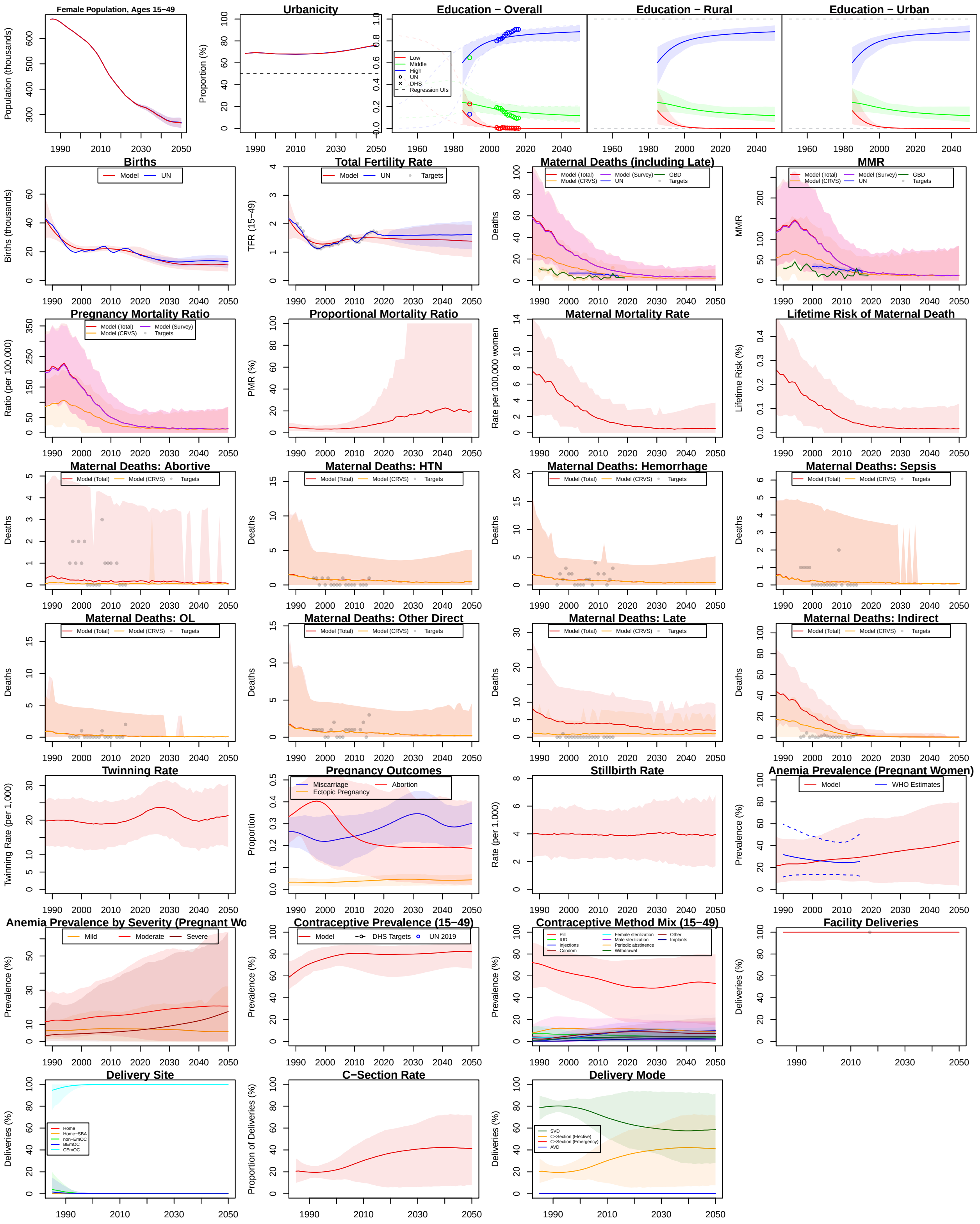

Lebanon

ISO Code	Region	Area	Income Group
LBN	Western Asia	Asia	Upper middle income

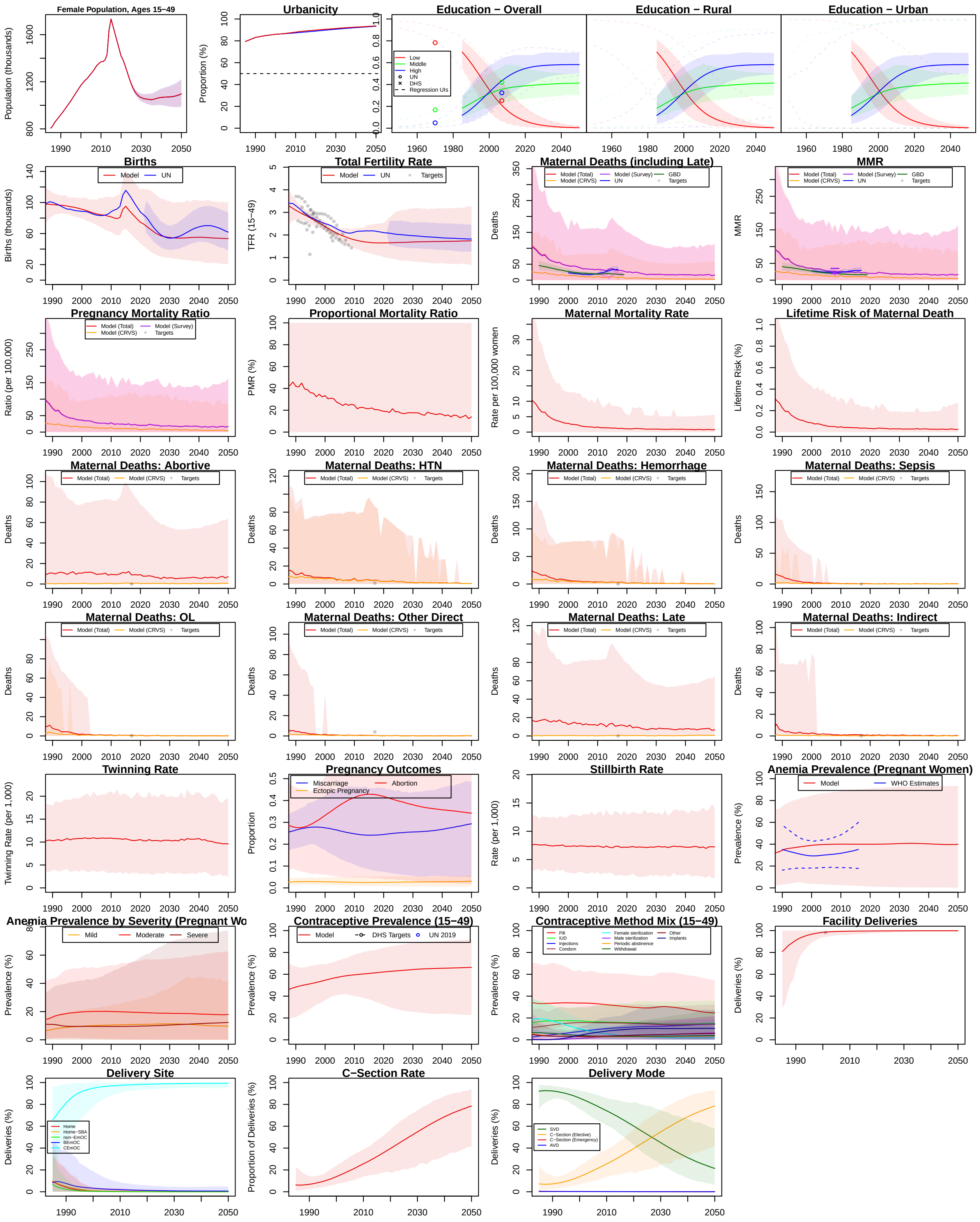

Lesotho

ISO Code	Region	Area	Income Group
LSO	Southern Africa	Africa	Lower middle income

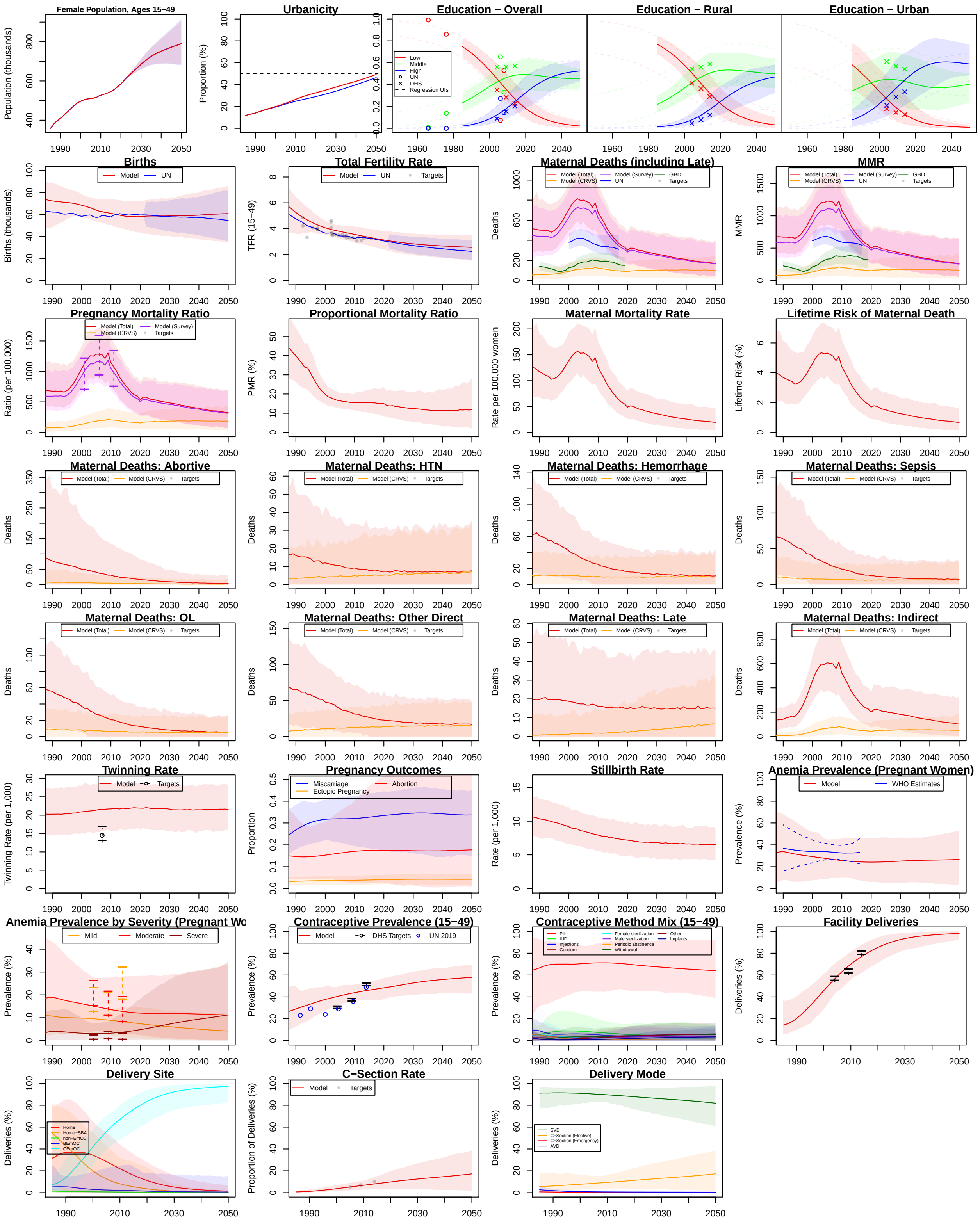

Liberia

ISO Code	Region	Area	Income Group
LBR	Western Africa	Africa	Low income

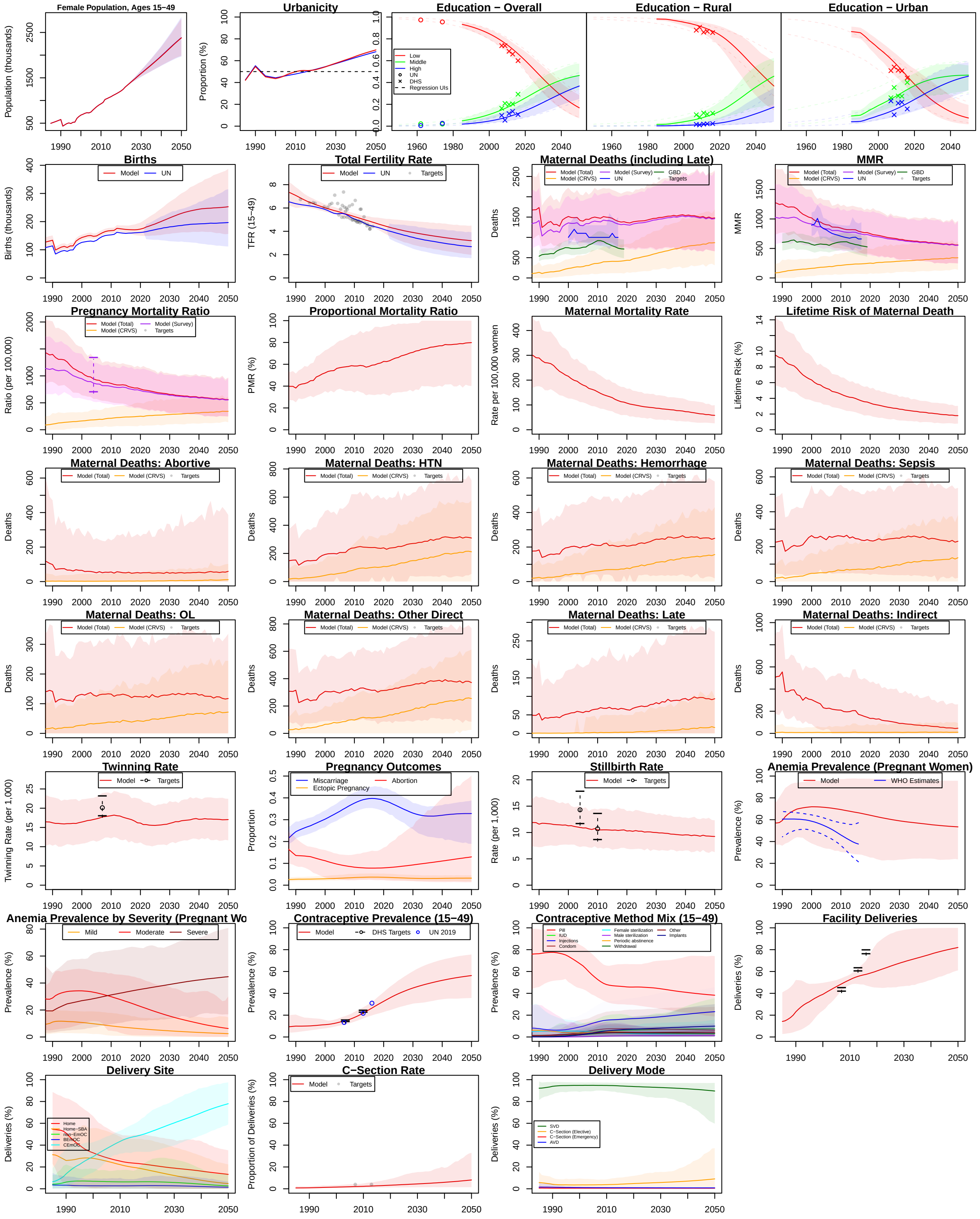

Libya

ISO Code	Region	Area	Income Group
LBY	Northern Africa	Africa	Upper middle income

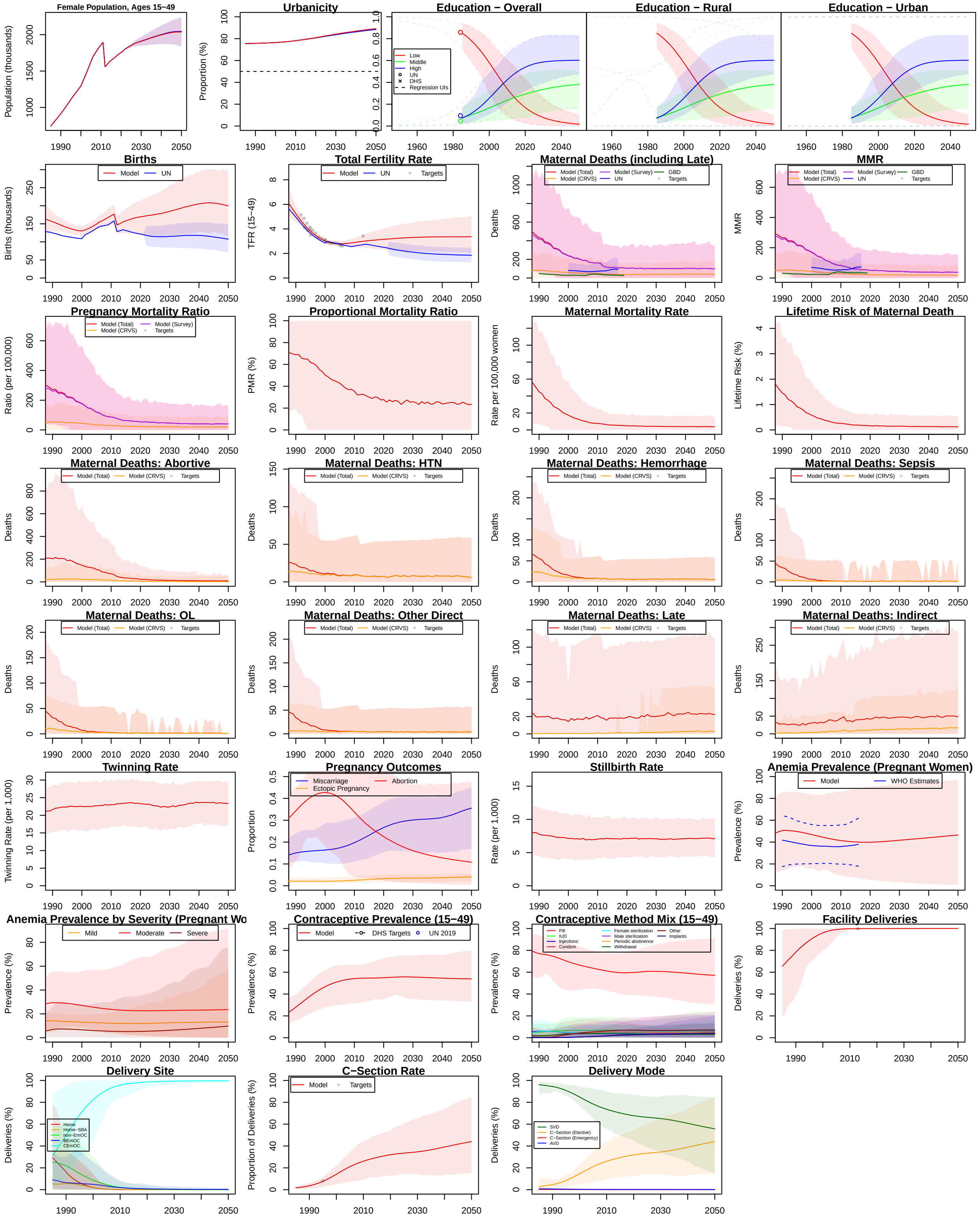

Liechtenstein

ISO Code	Region	Area	Income Group
LIE	Western Europe	Europe	High income

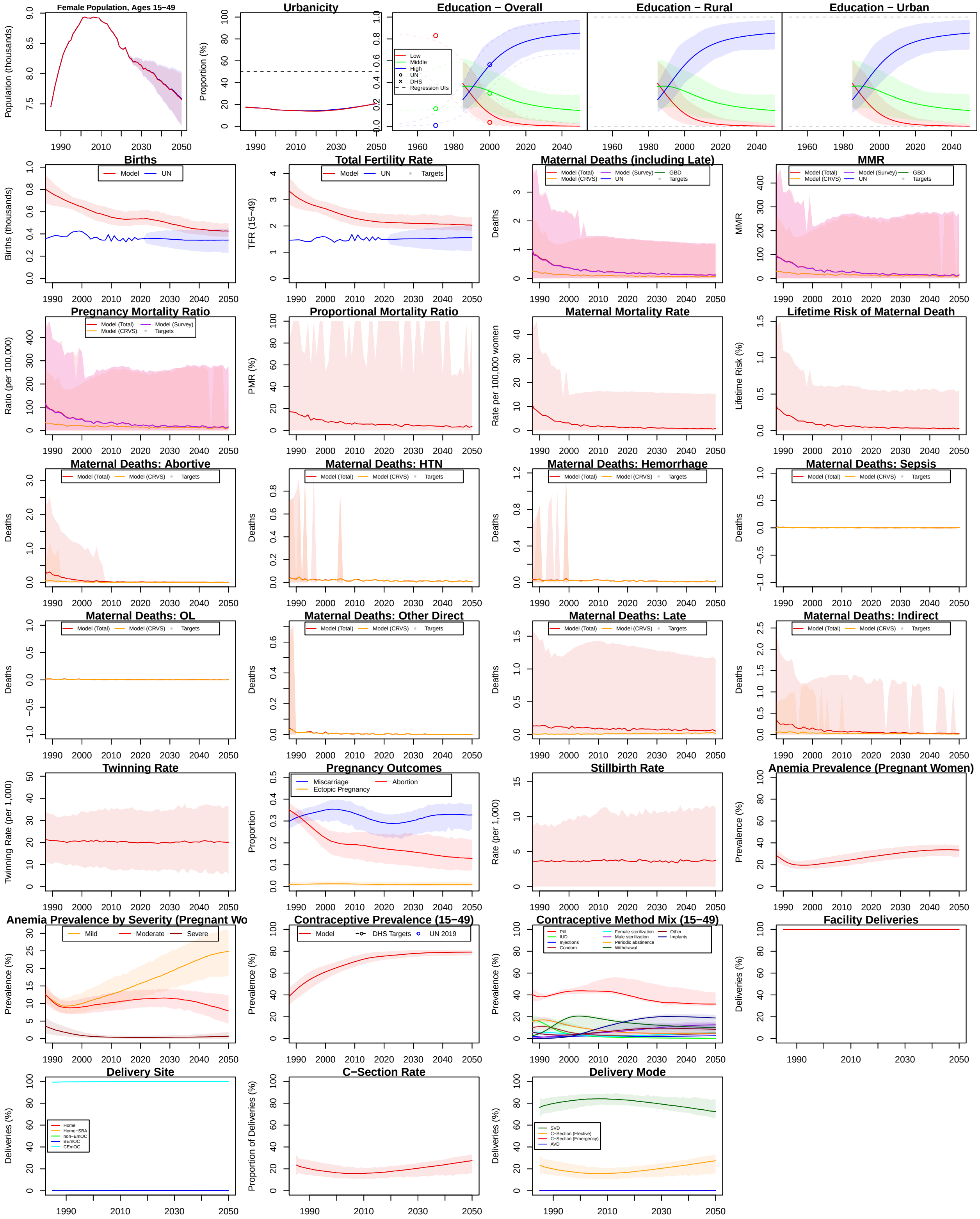

Lithuania

ISO Code	Region	Area	Income Group
LTU	Northern Europe	Europe	High income

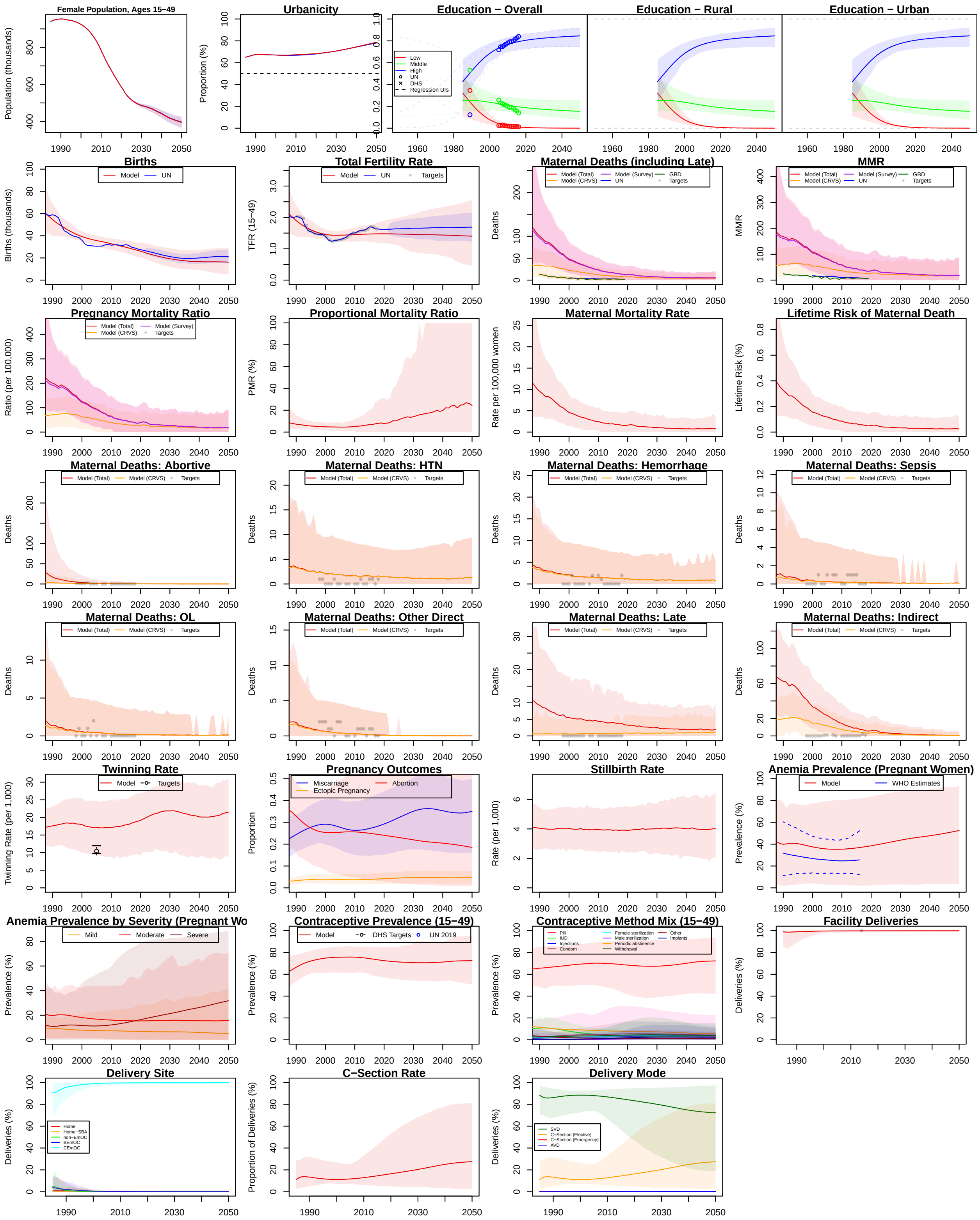

Luxembourg

ISO Code	Region	Area	Income Group
LUX	Western Europe	Europe	High income

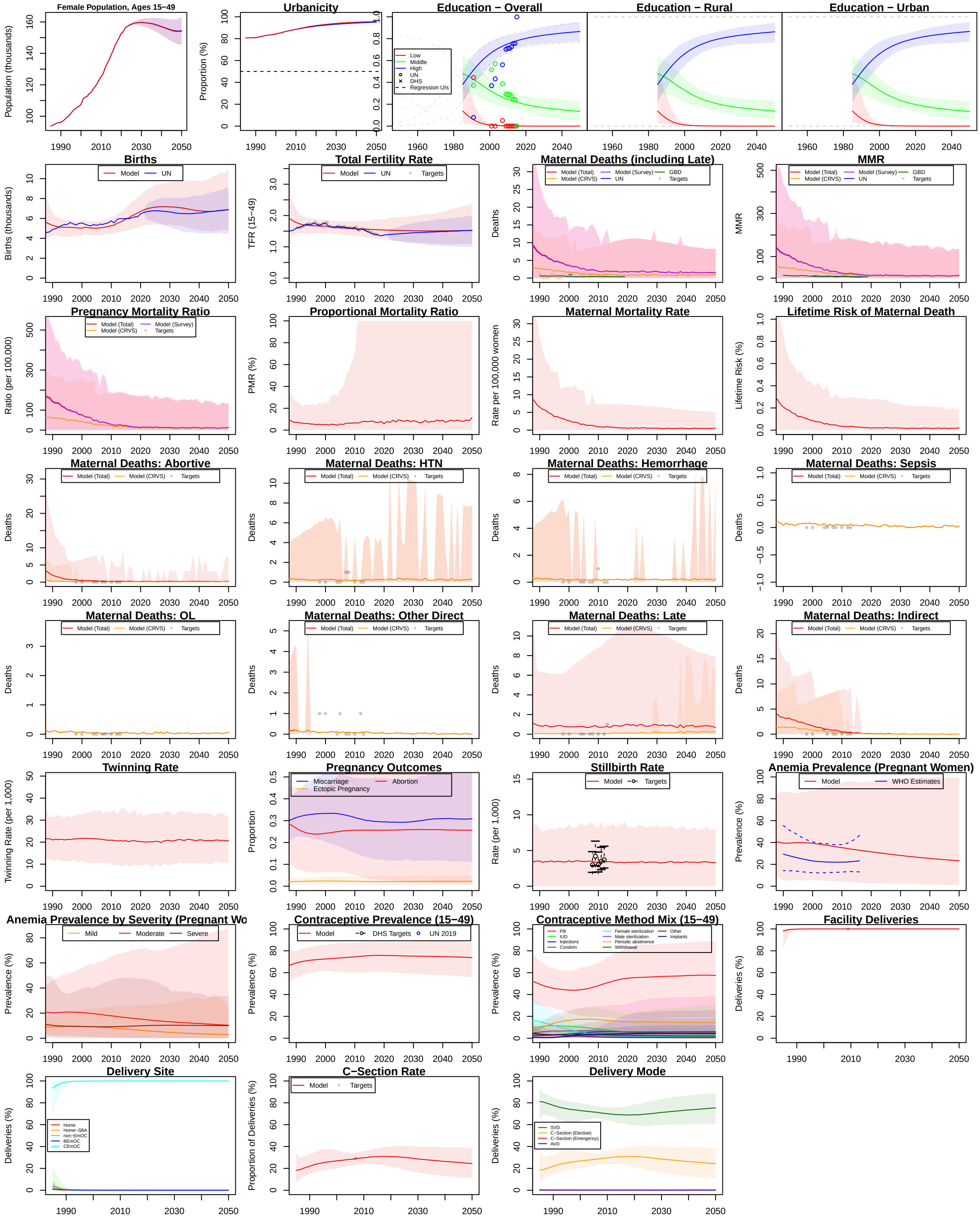

Madagascar

ISO Code	Region	Area	Income Group
MDG	Eastern Africa	Africa	Low income

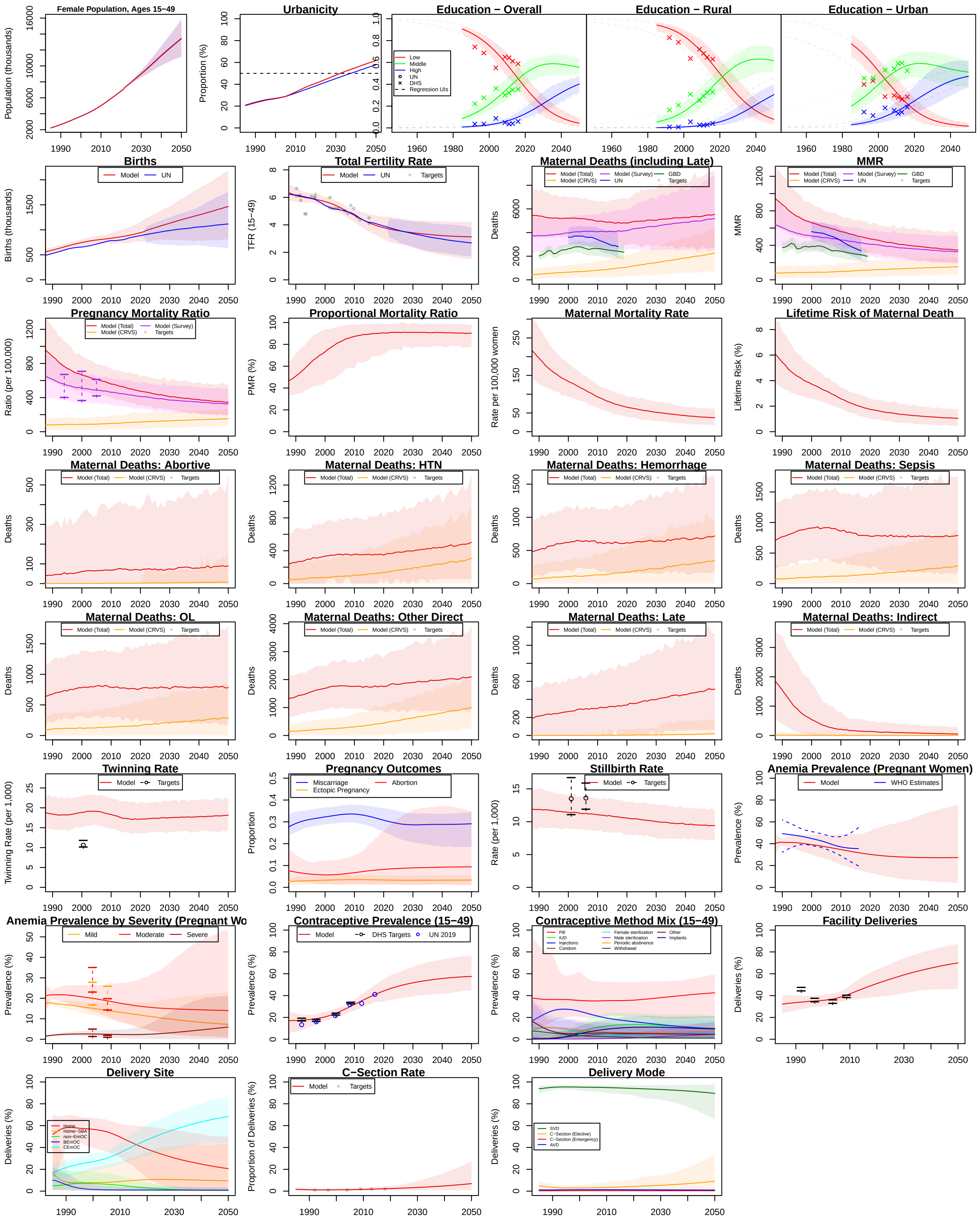

Malawi

ISO Code	Region	Area	Income Group
MWI	Eastern Africa	Africa	Low income

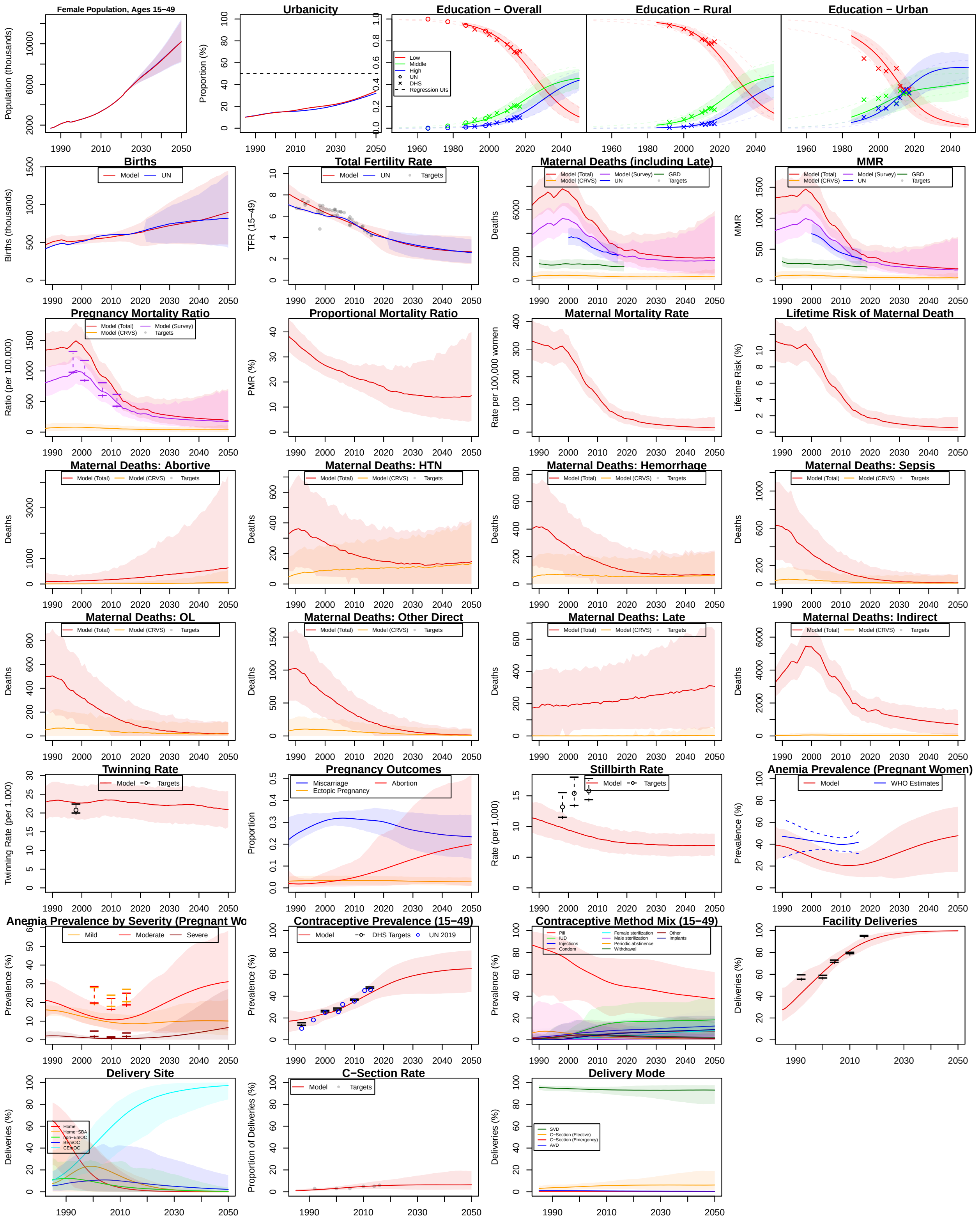

Malaysia

ISO Code	Region	Area	Income Group
MYS	South-Eastern Asia	Asia	Upper middle income

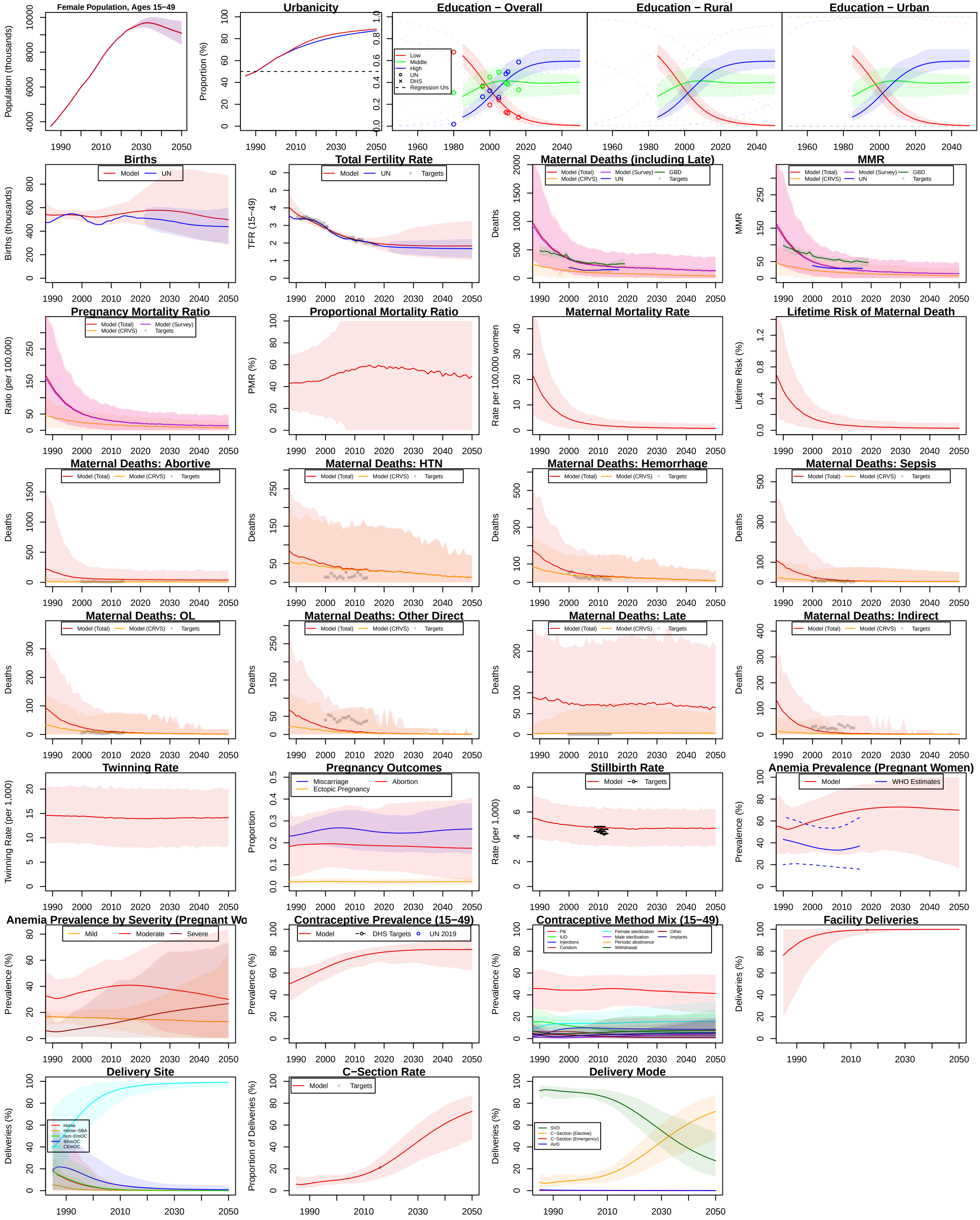

Maldives

ISO Code	Region	Area	Income Group
MDV	Southern Asia	Asia	Upper middle income

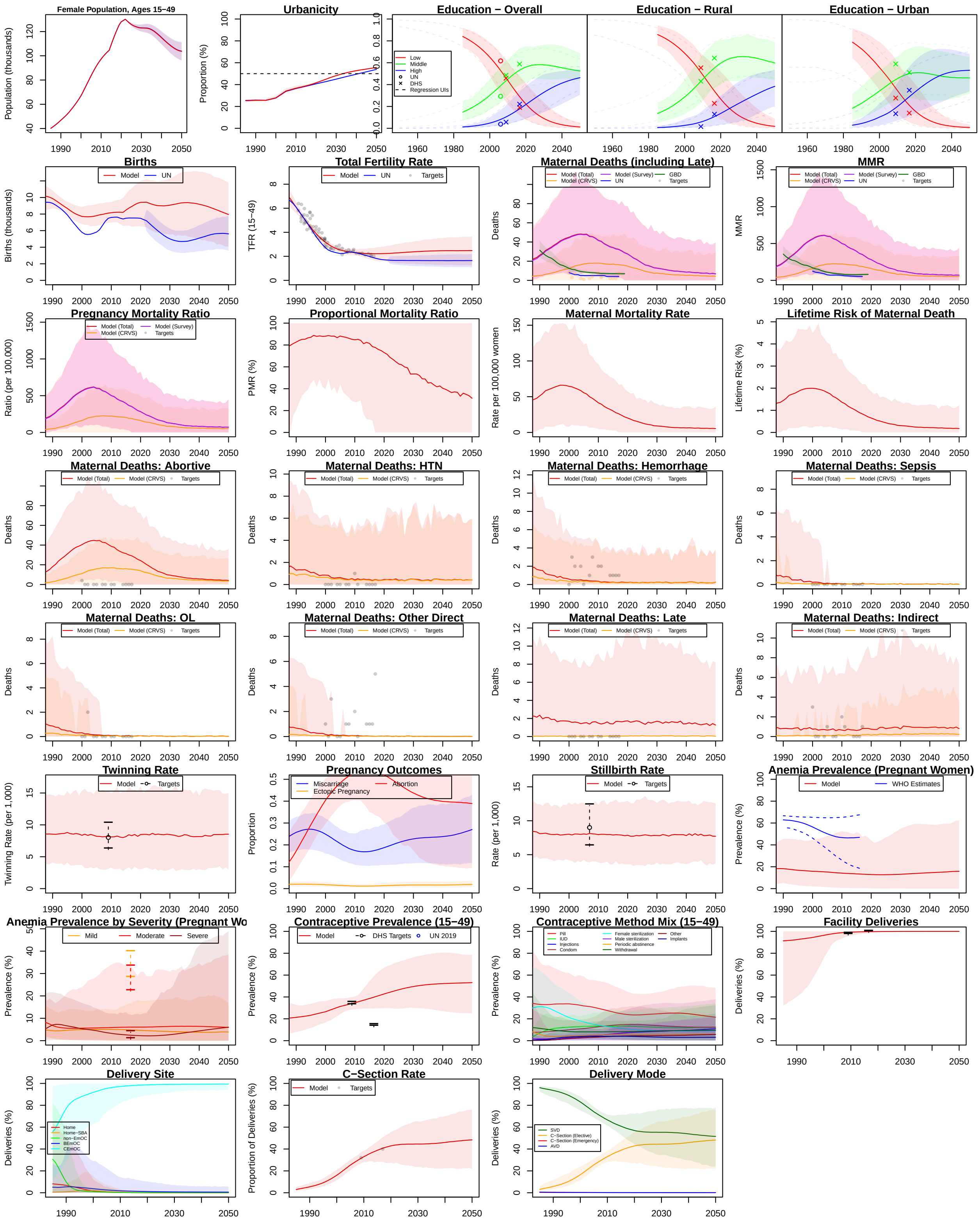

Mali

ISO Code	Region	Area	Income Group
MLI	Western Africa	Africa	Low income

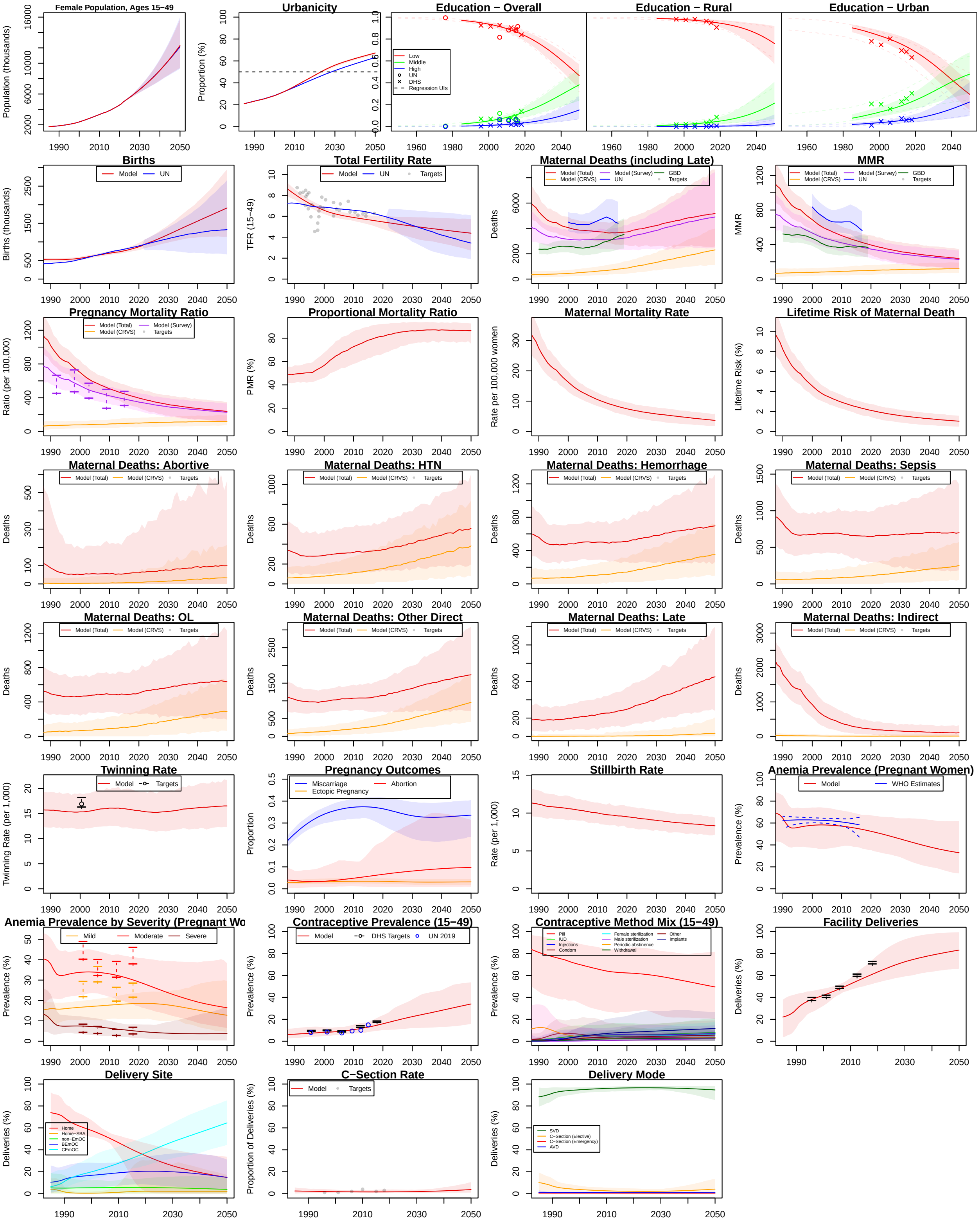

Malta

ISO Code	Region	Area	Income Group
MLT	Southern Europe	Europe	High income

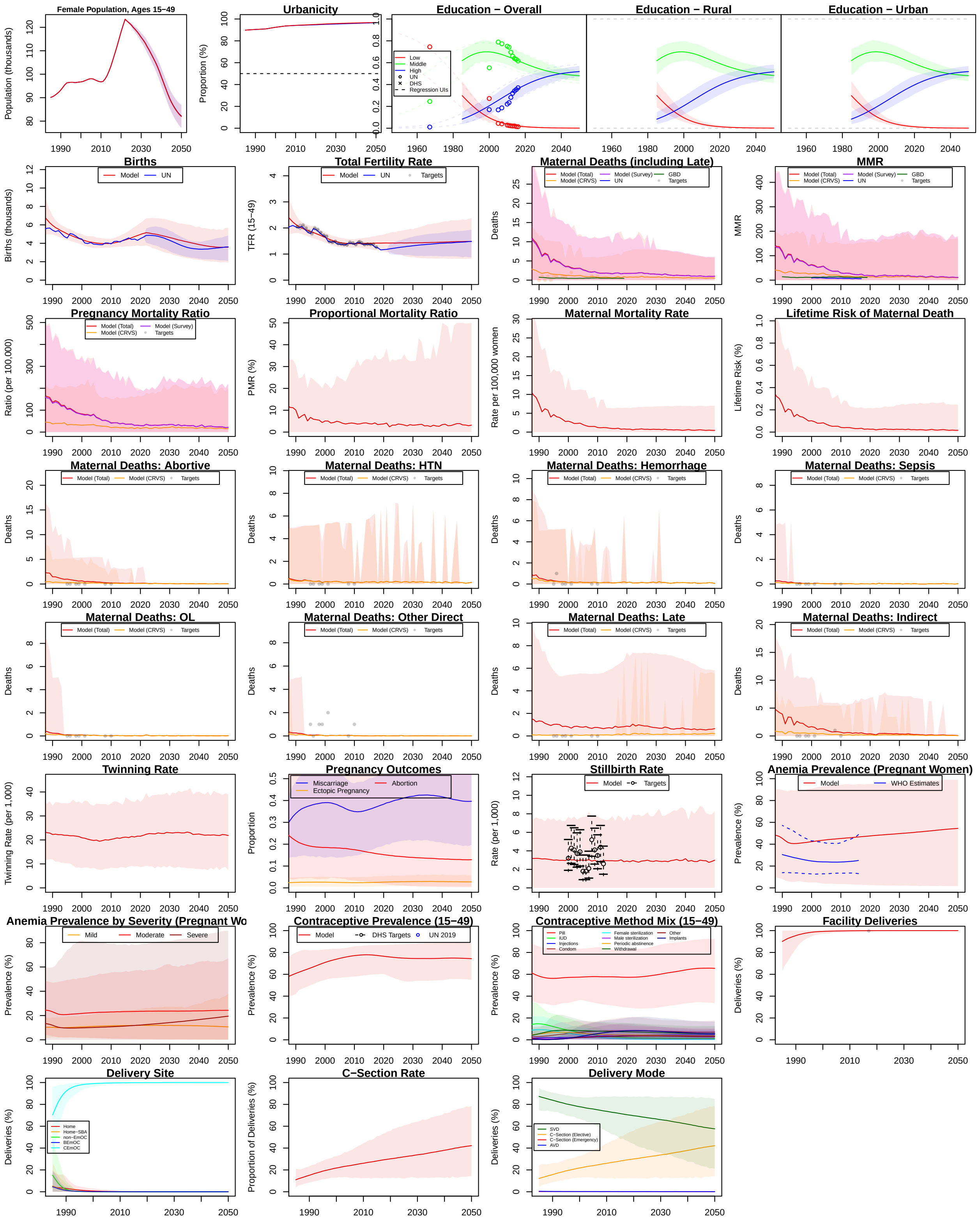

Marshall Islands

ISO Code	Region	Area	Income Group
MHL	Micronesia	Oceania	Upper middle income

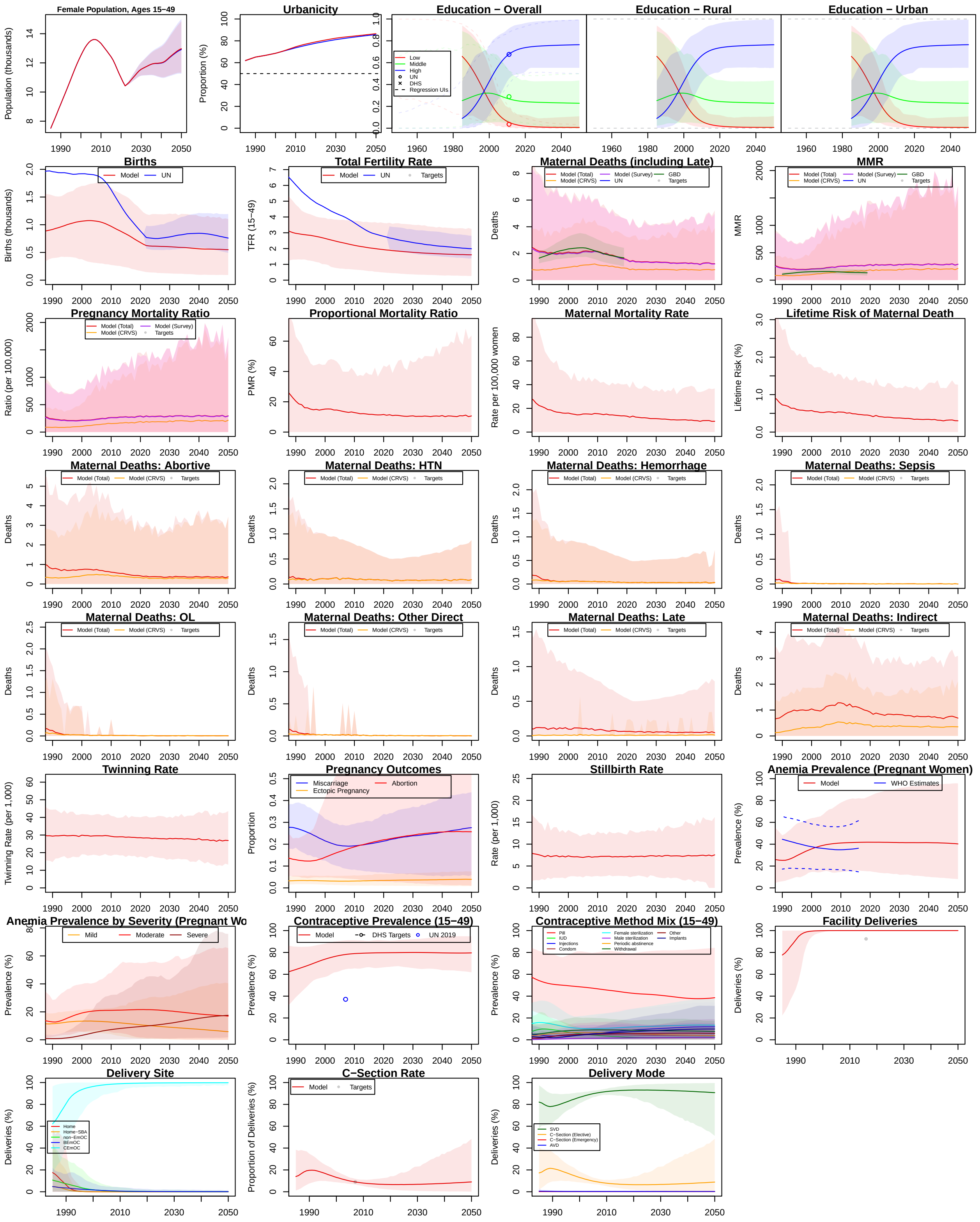

Mauritania

ISO Code	Region	Area	Income Group
MRT	Western Africa	Africa	Lower middle income

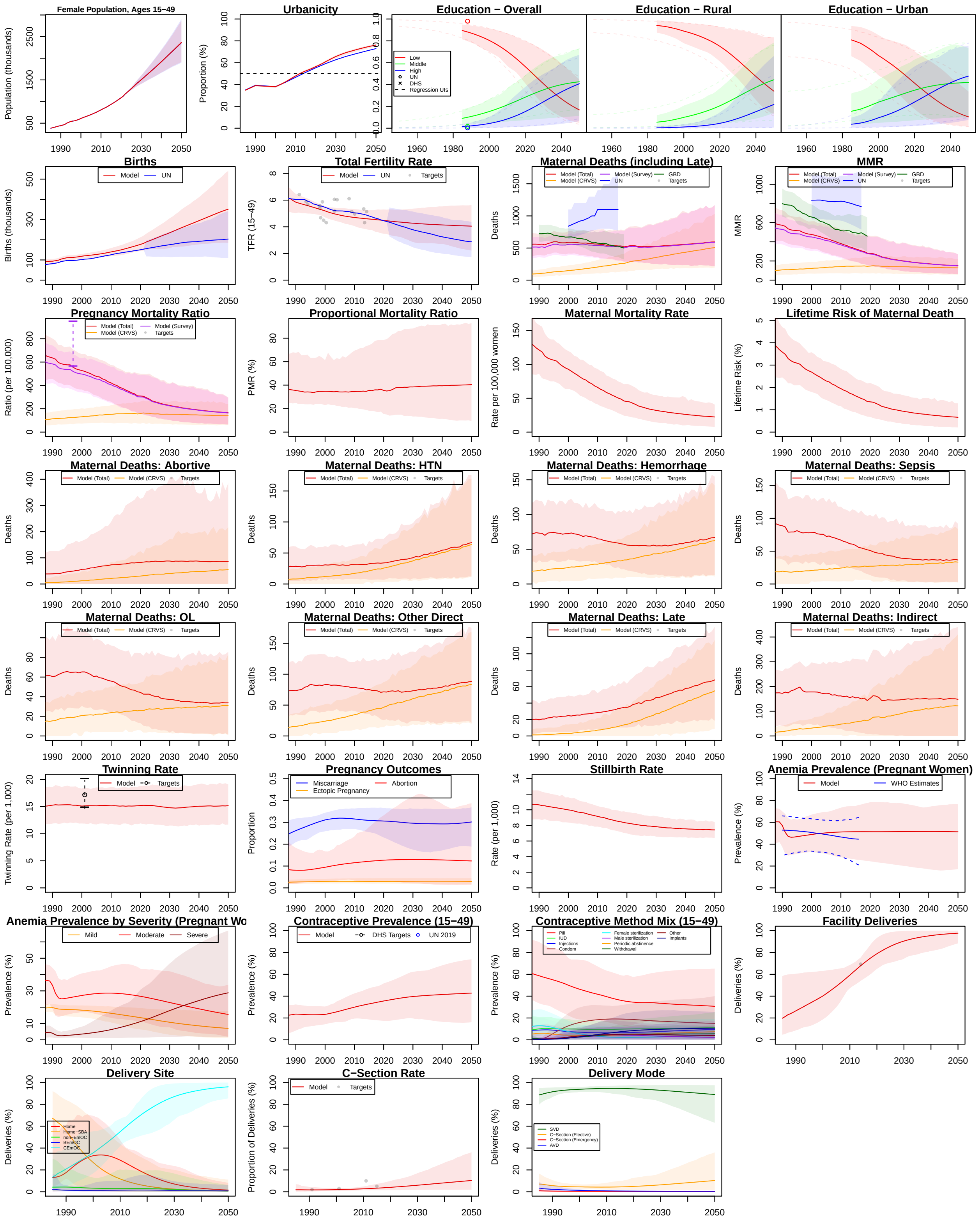

Mauritius

ISO Code	Region	Area	Income Group
MUS	Eastern Africa	Africa	Upper middle income

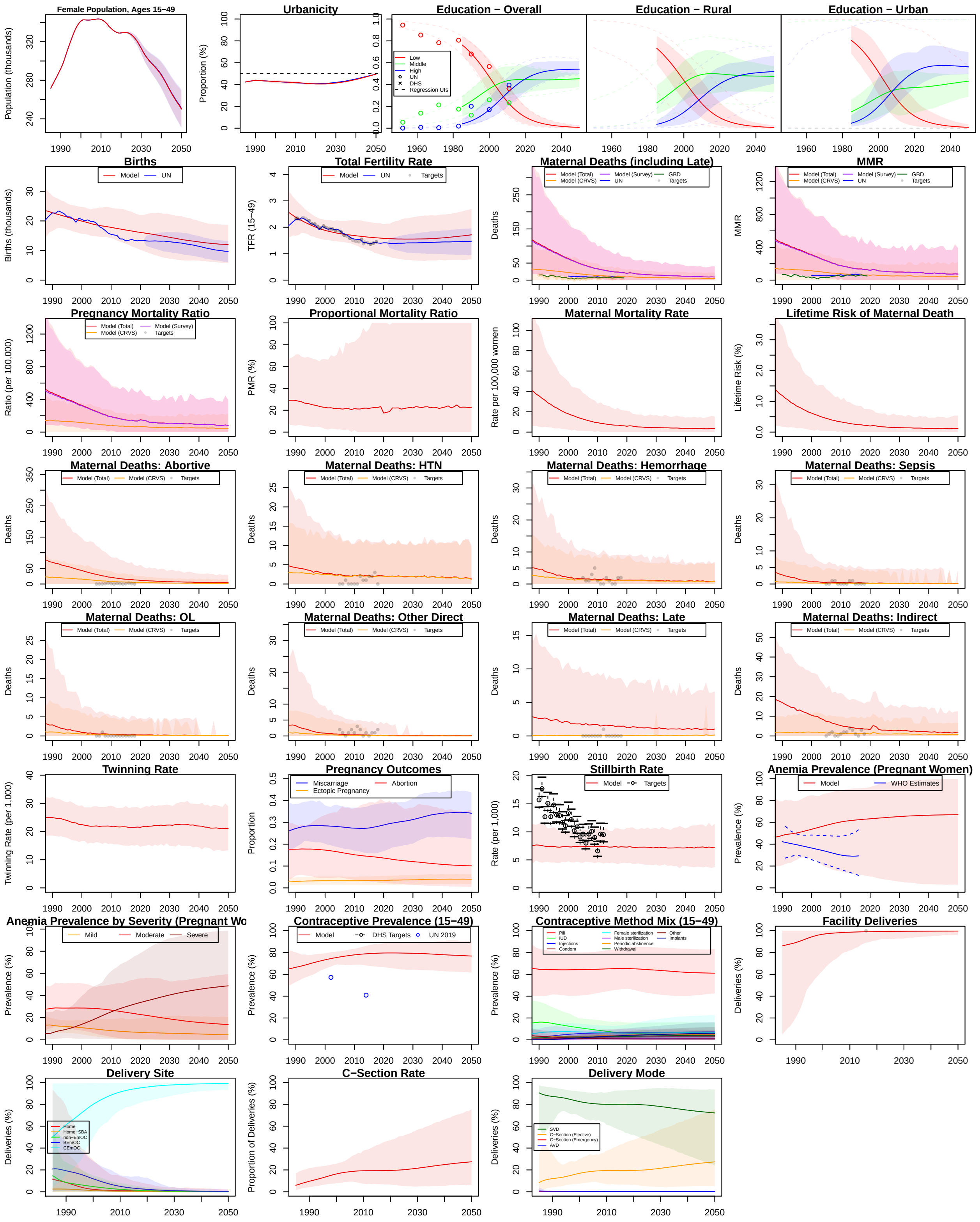

Mexico

ISO Code	Region	Area	Income Group
MEX	Central America	Latin America and the Caribbean	Upper middle income

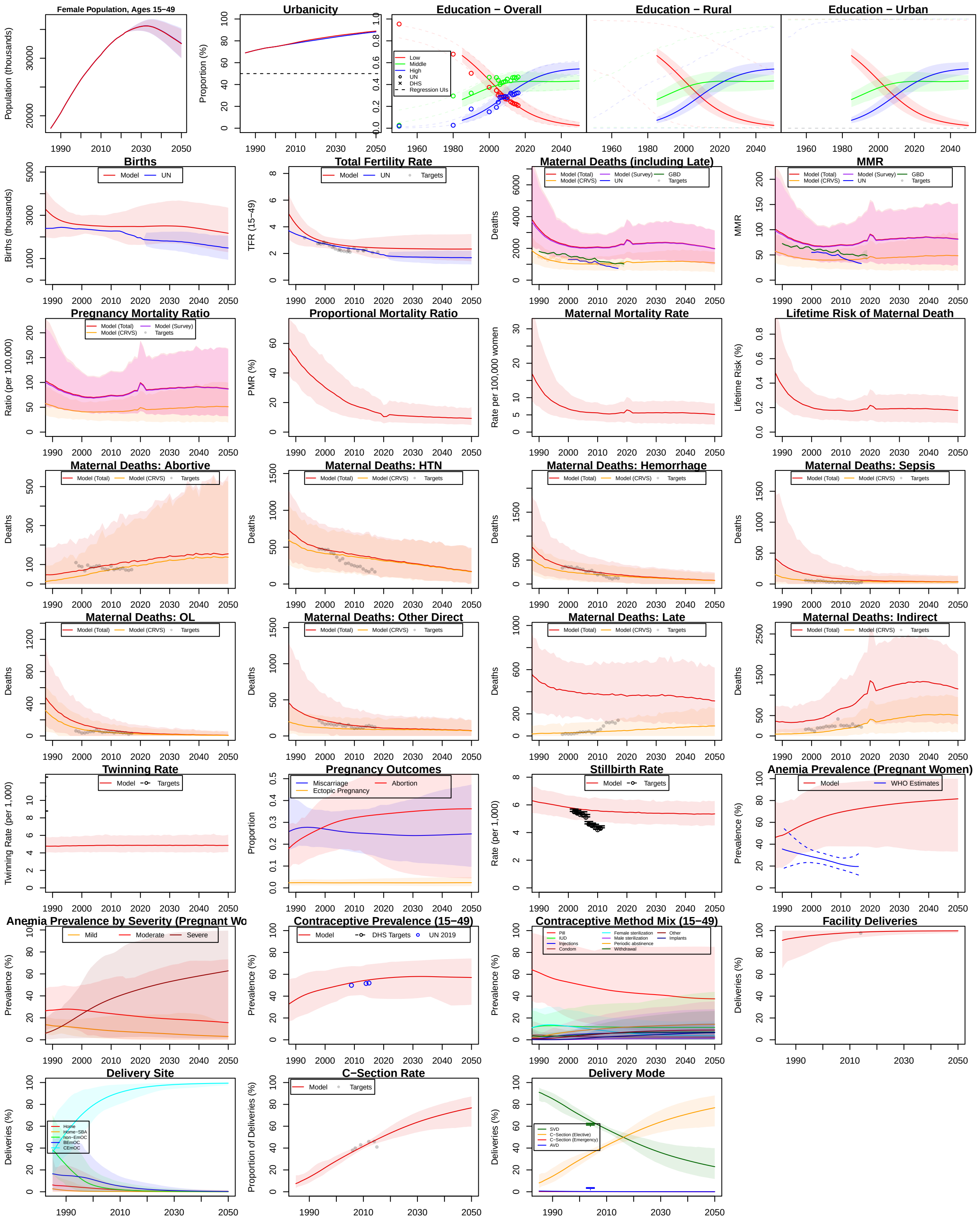

Micronesia (Fed. States of)

ISO Code	Region	Area	Income Group
FSM	Micronesia	Oceania	Lower middle income

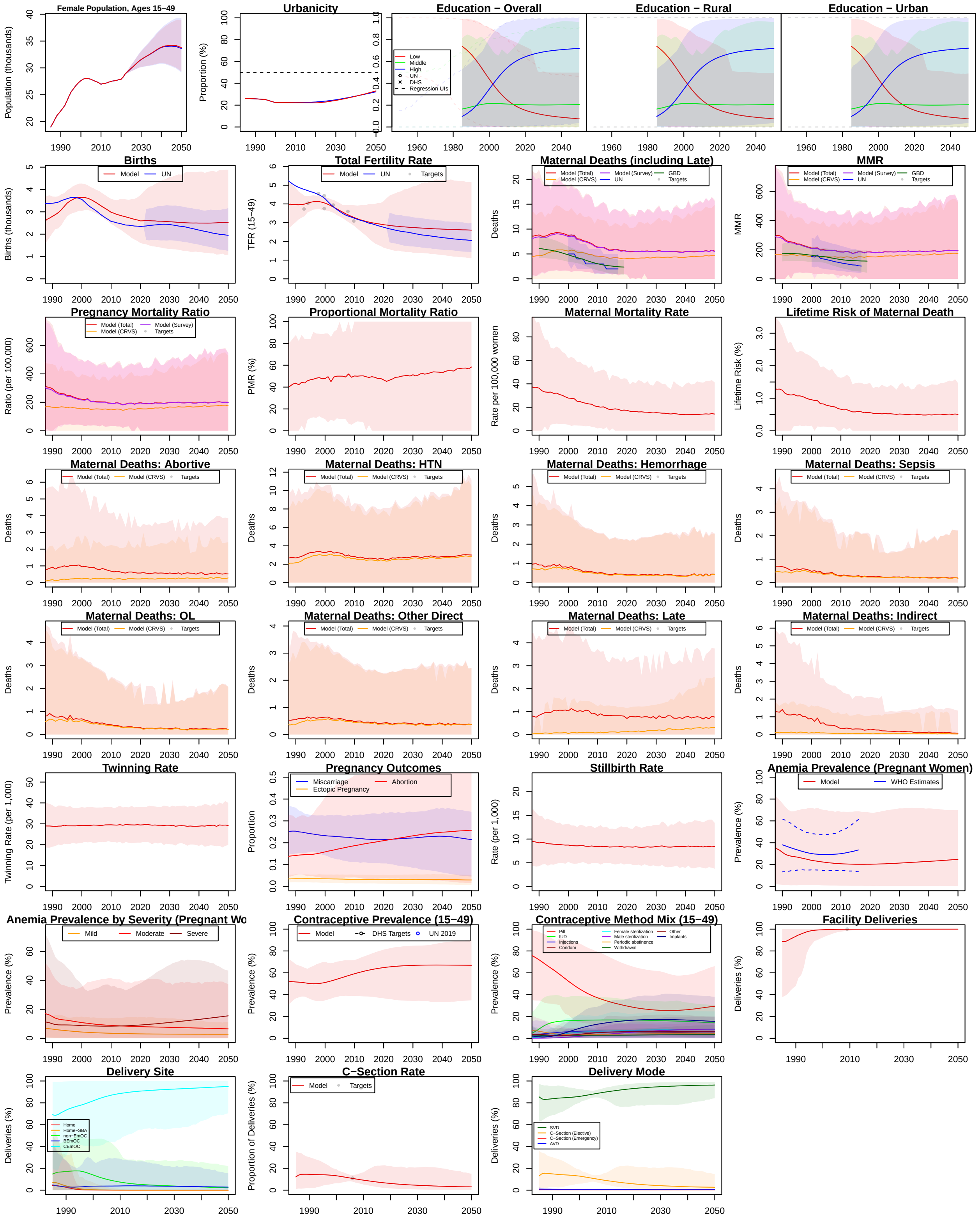

Monaco

ISO Code	Region	Area	Income Group
MCO	Western Europe	Europe	High income

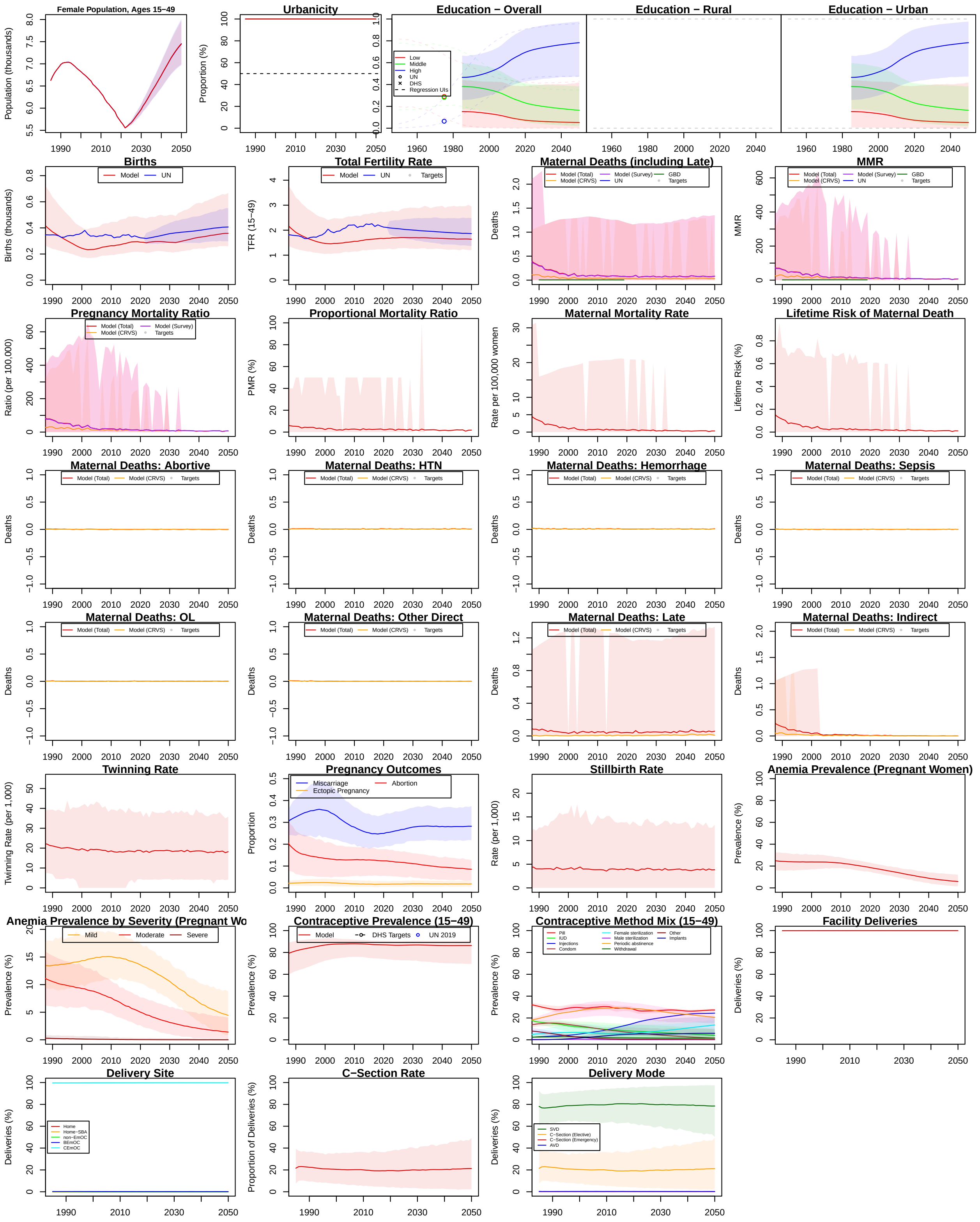

Mongolia

ISO Code	Region	Area	Income Group
MNG	Eastern Asia	Asia	Lower middle income

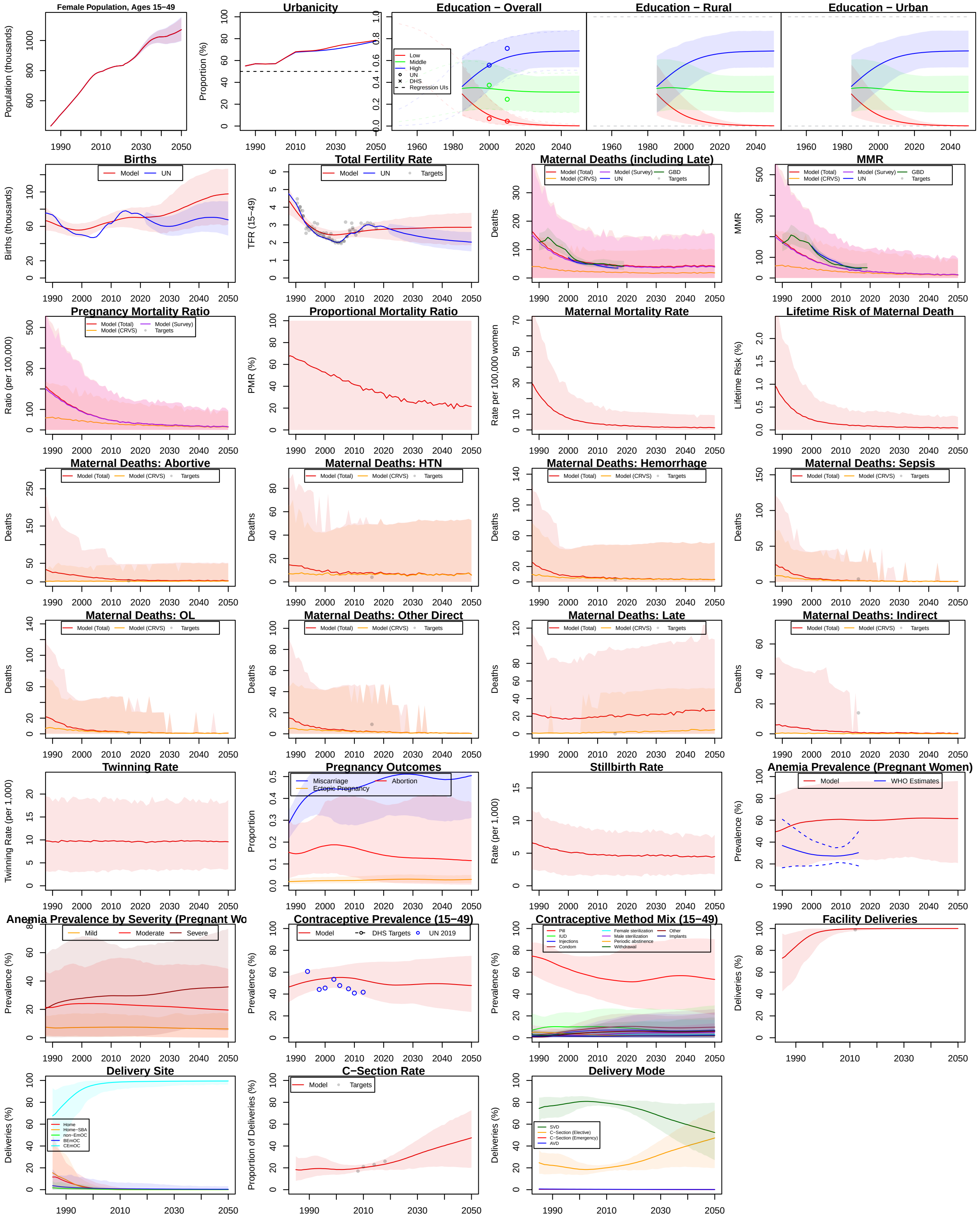

Montenegro

ISO Code	Region	Area	Income Group
MNE	Southern Europe	Europe	Upper middle income

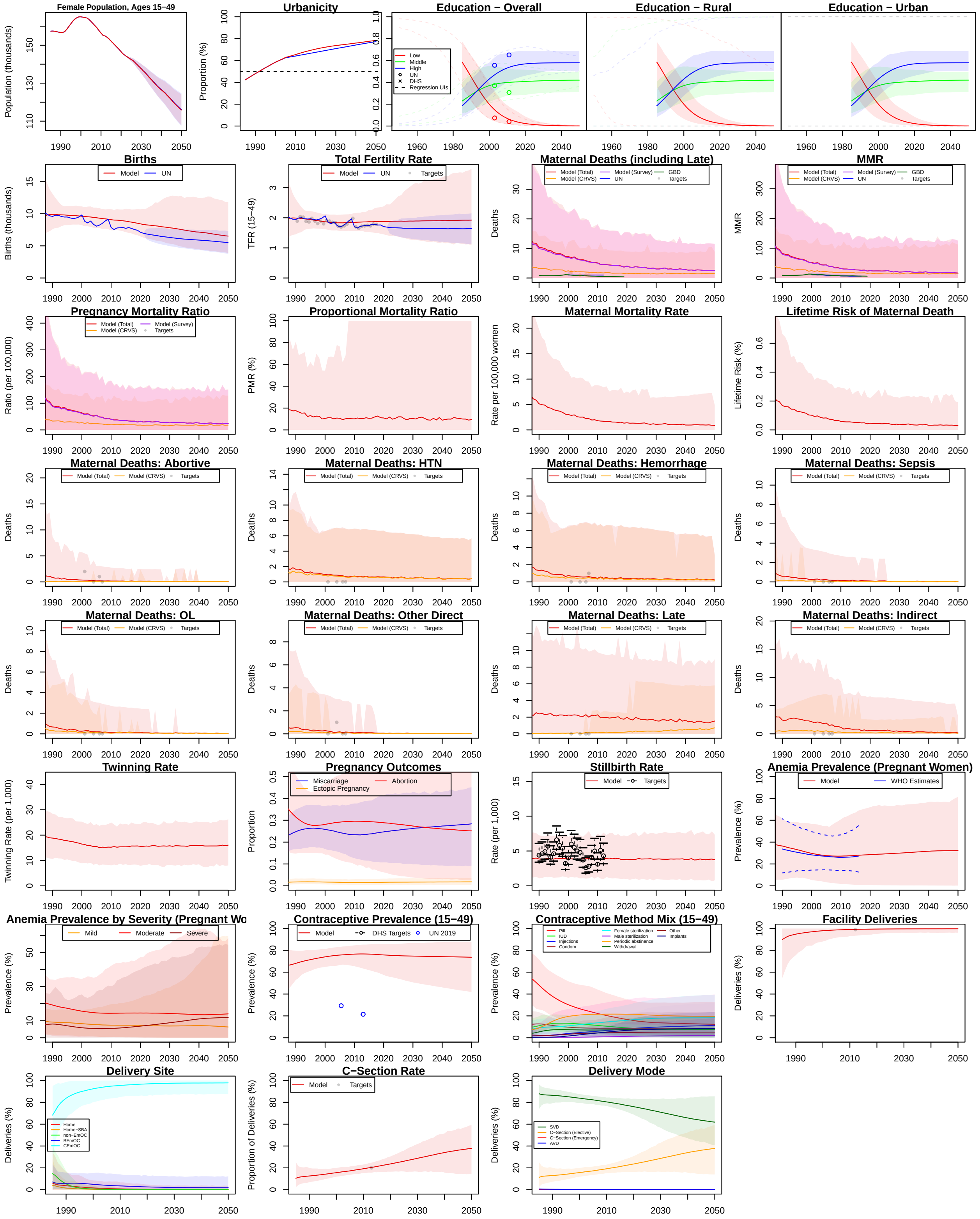

Morocco

ISO Code	Region	Area	Income Group
MAR	Northern Africa	Africa	Lower middle income

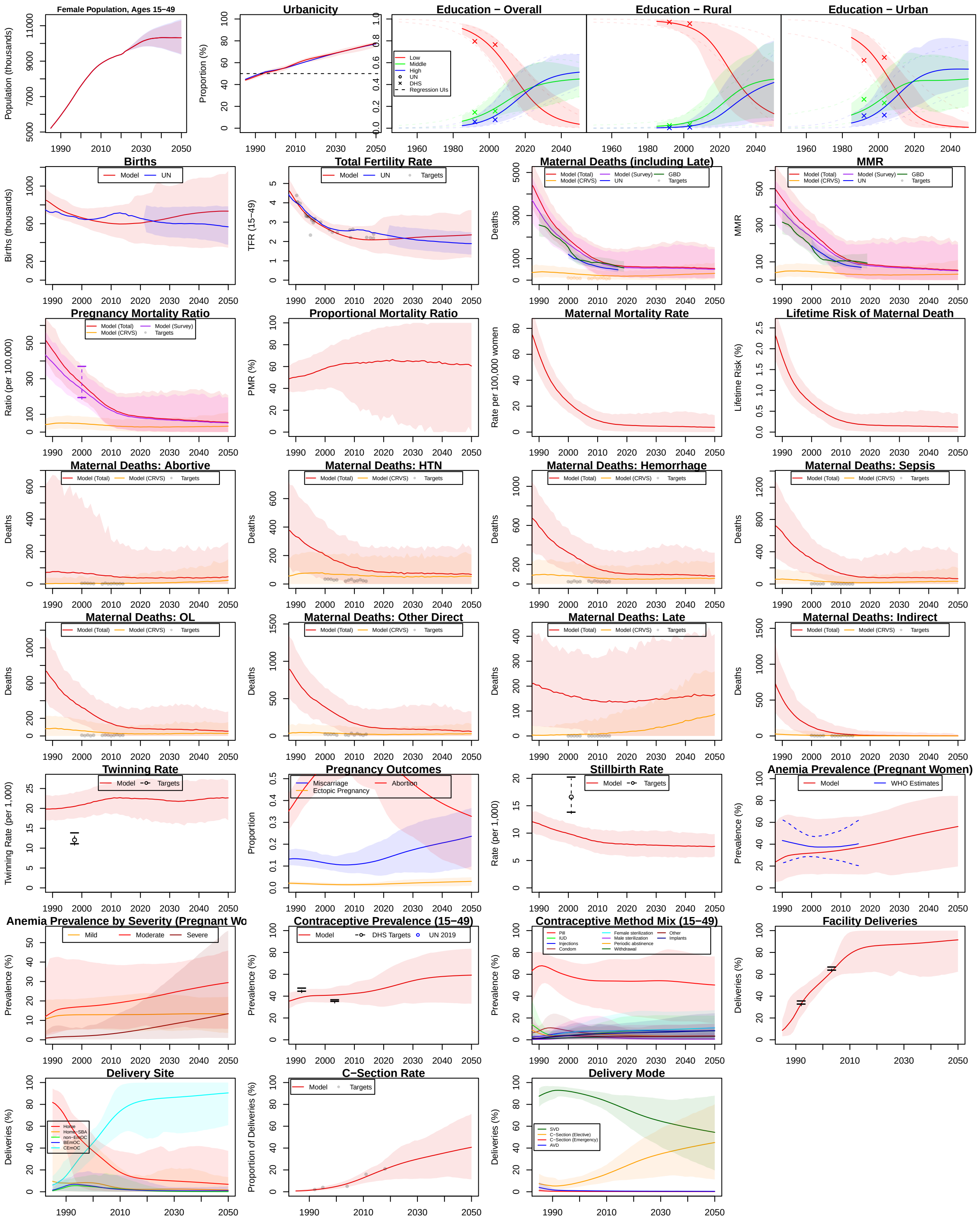

Mozambique

ISO Code	Region	Area	Income Group
MOZ	Eastern Africa	Africa	Low income

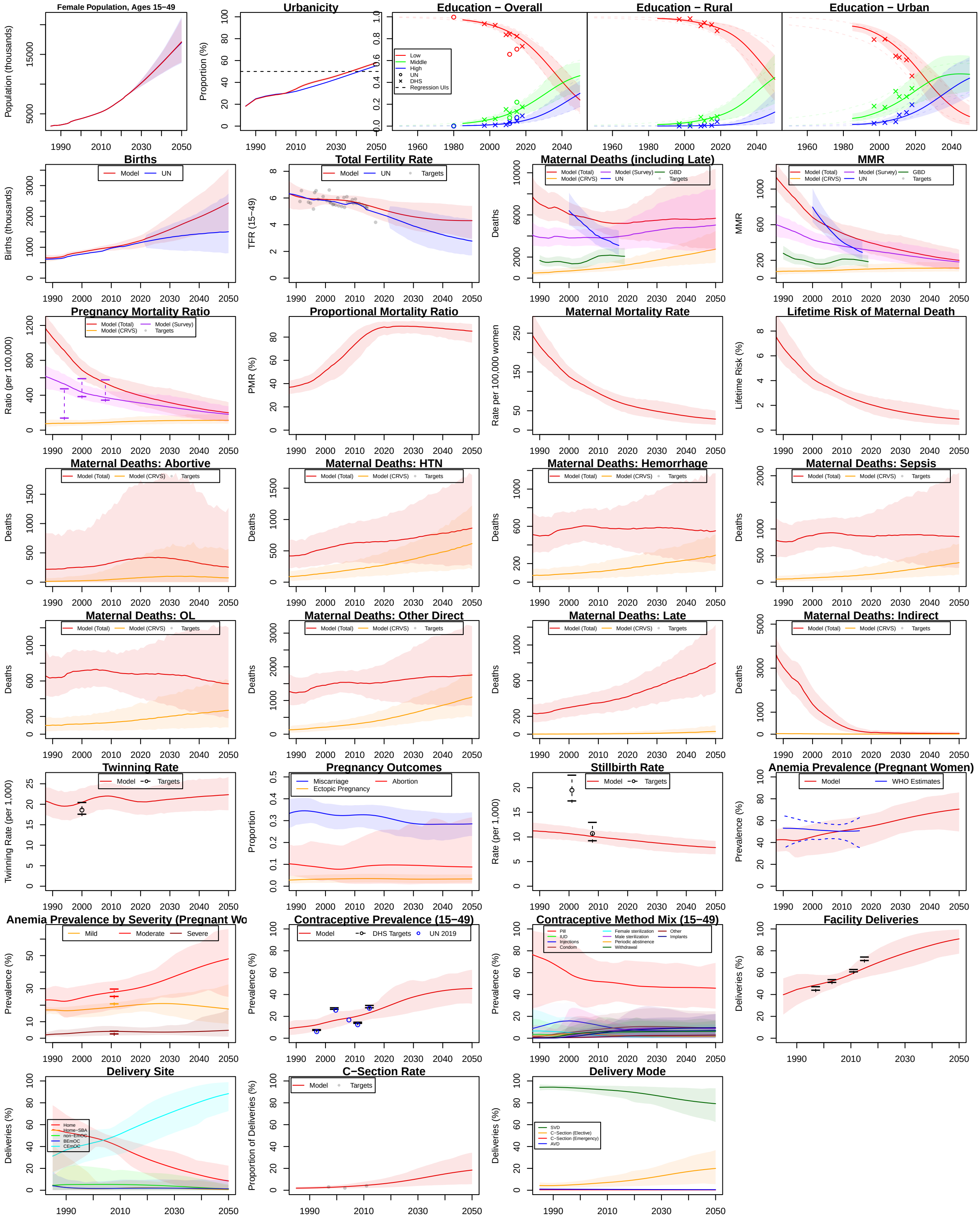

Myanmar

ISO Code	Region	Area	Income Group
MMR	South-Eastern Asia	Asia	Lower middle income

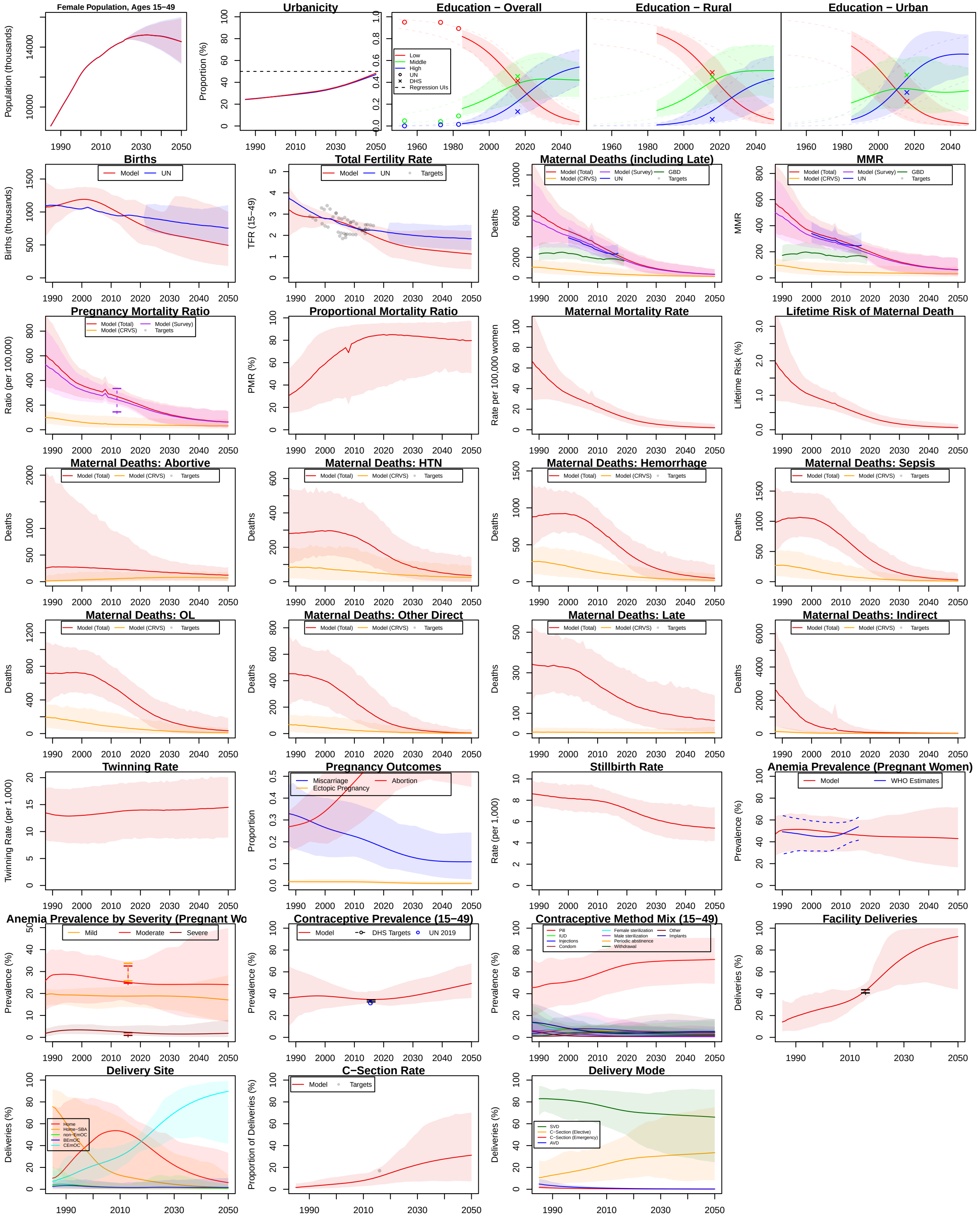

Namibia

ISO Code	Region	Area	Income Group
NAM	Southern Africa	Africa	Upper middle income

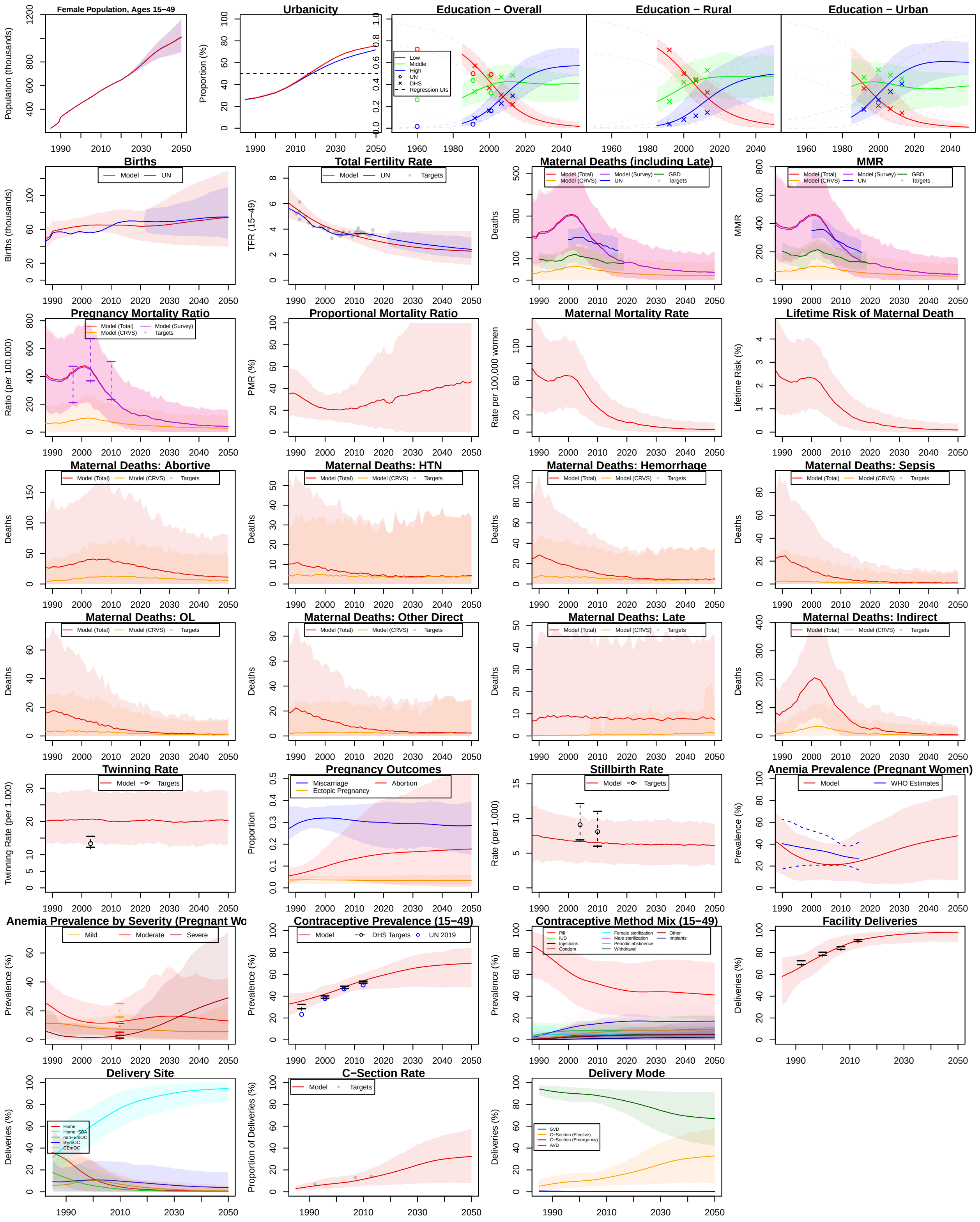

Nauru

ISO Code	Region	Area	Income Group
NRU	Micronesia	Oceania	Upper middle income

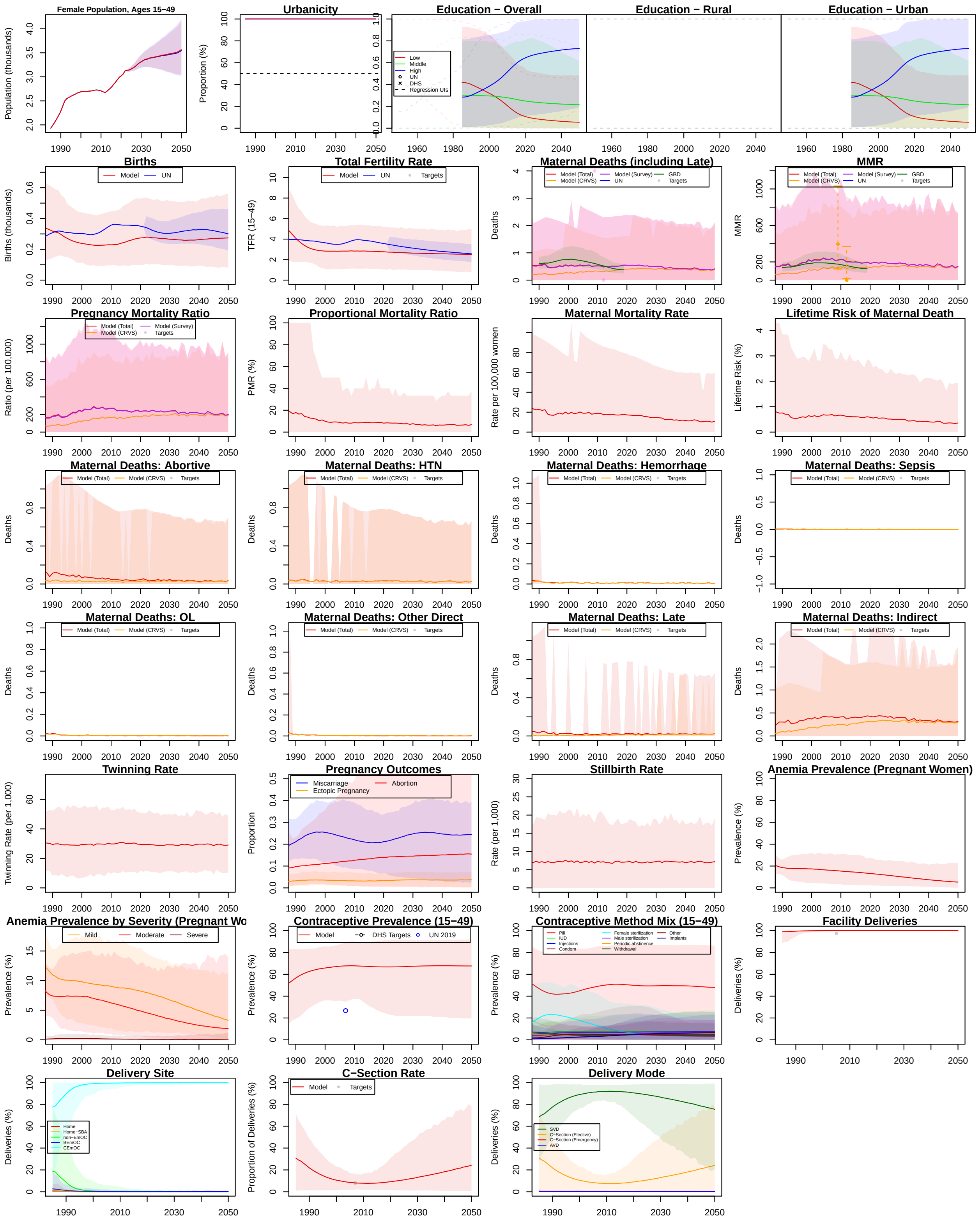

Nepal

ISO Code	Region	Area	Income Group
NPL	Southern Asia	Asia	Low income

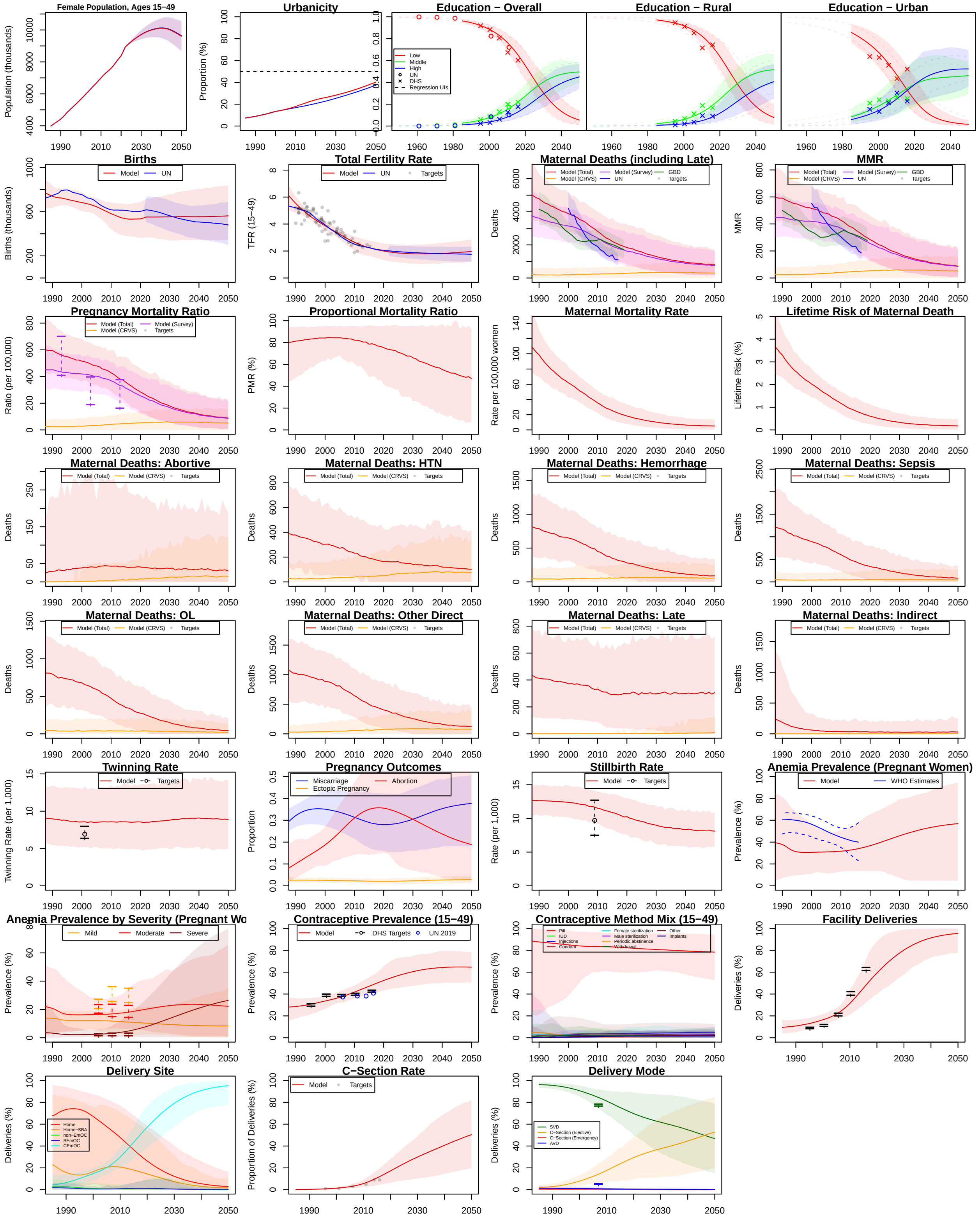

Netherlands

ISO Code	Region	Area	Income Group
NLD	Western Europe	Europe	High income

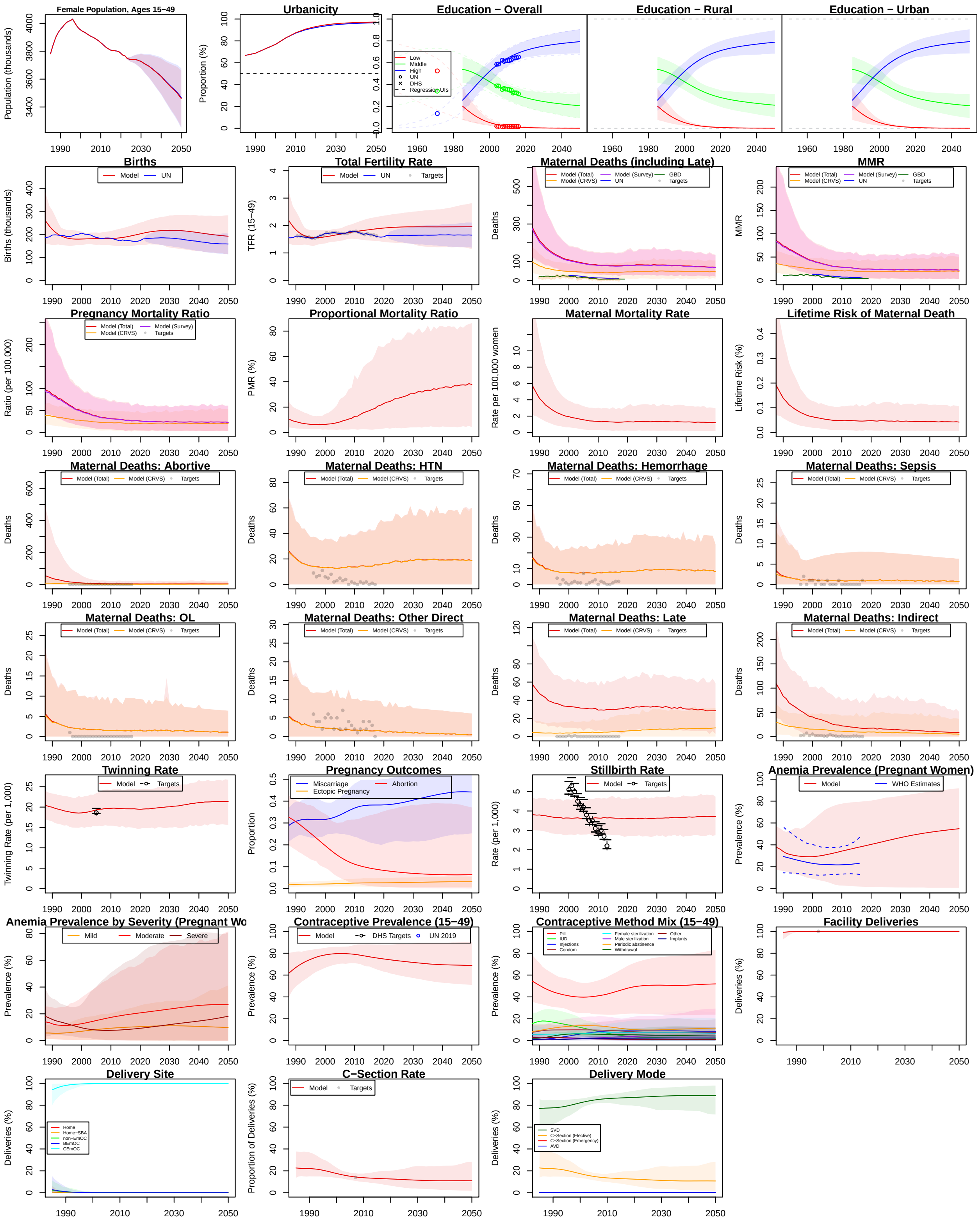

New Zealand

ISO Code	Region	Area	Income Group
NZL	Australia/New Zealand	Oceania	High income

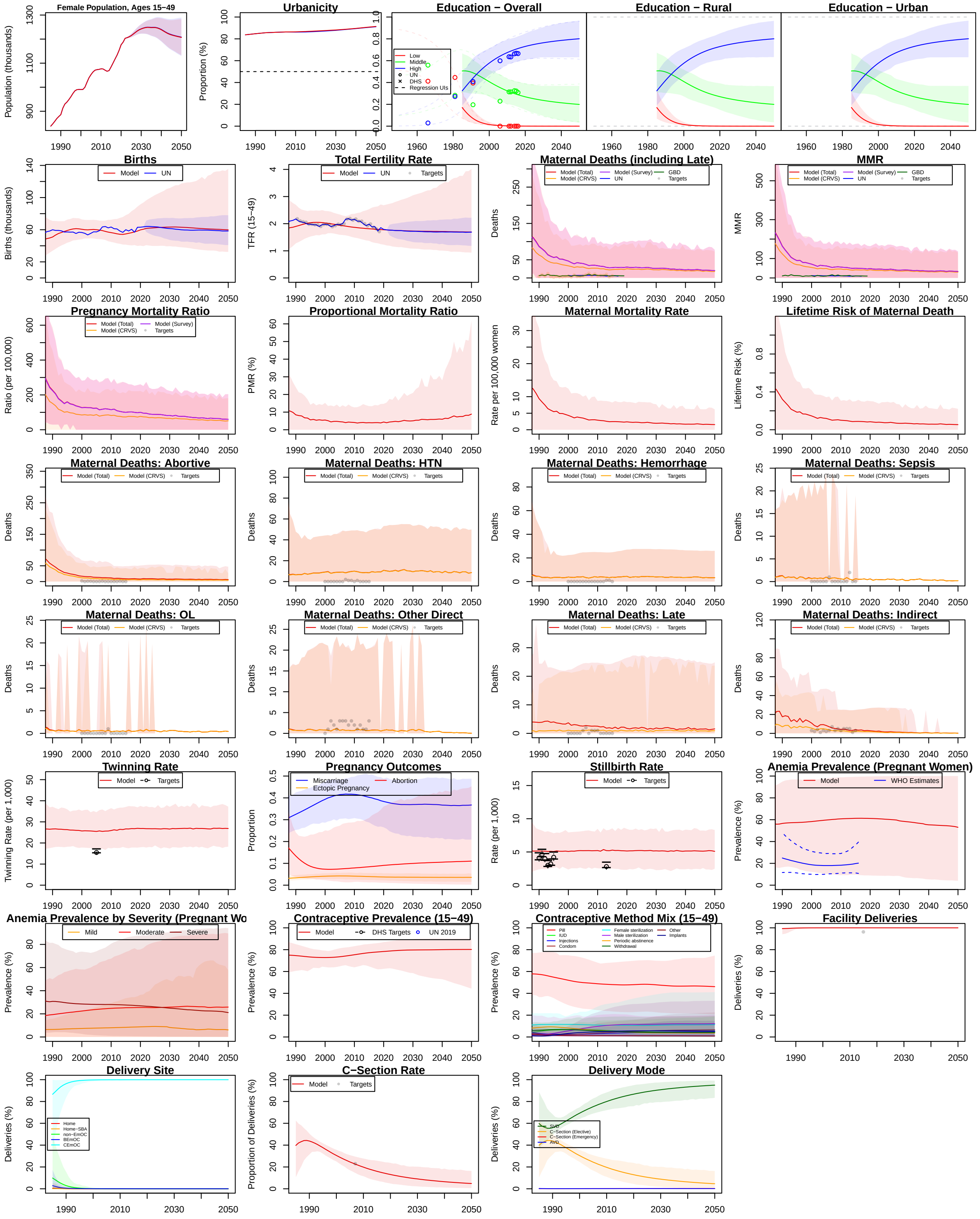

Nicaragua

ISO Code	Region	Area	Income Group
NIC	Central America	Latin America and the Caribbean	Lower middle income

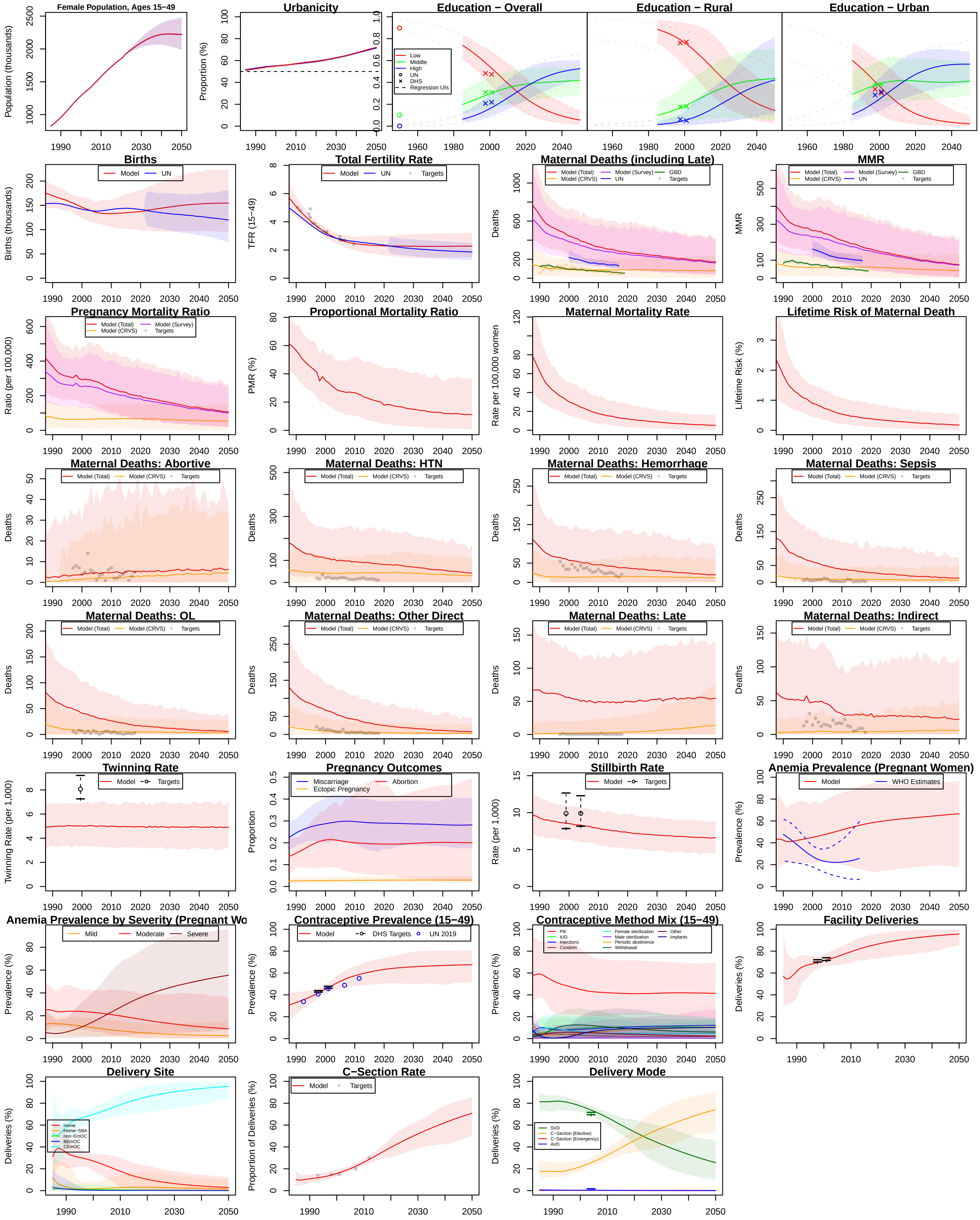

Niger

ISO Code	Region	Area	Income Group
NER	Western Africa	Africa	Low income

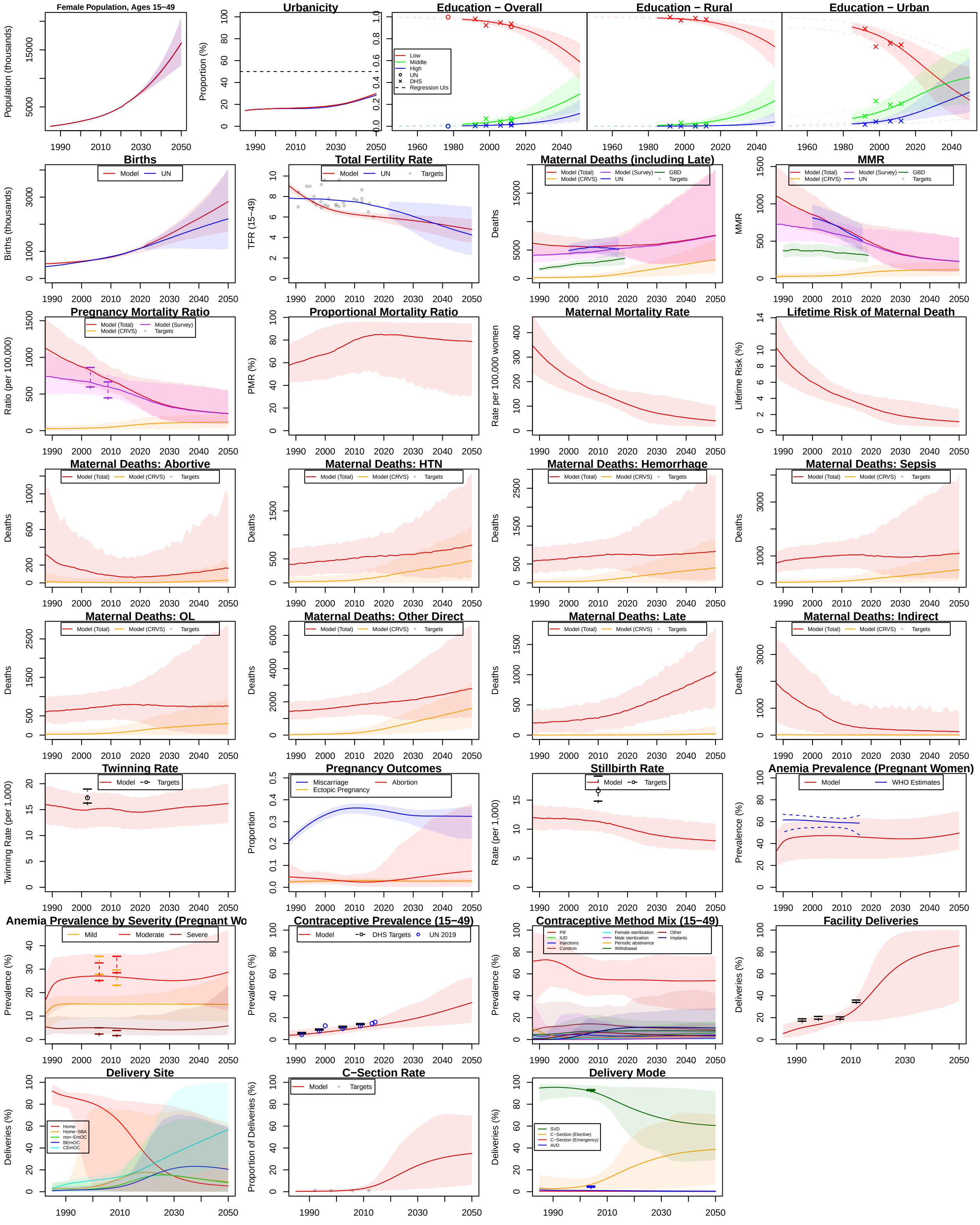

Nigeria

ISO Code	Region	Area	Income Group
NGA	Western Africa	Africa	Lower middle income

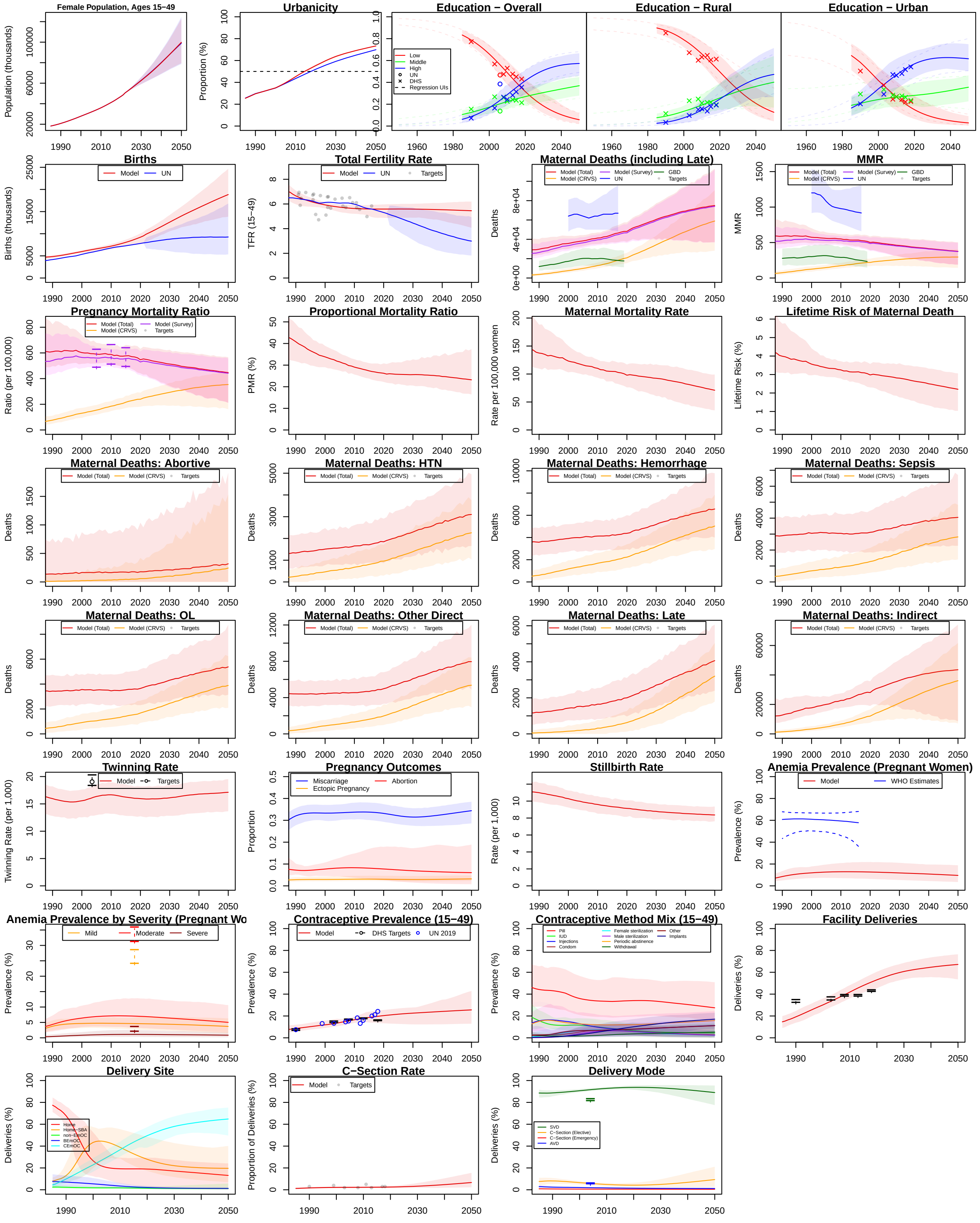

North Macedonia

ISO Code	Region	Area	Income Group
MKD	Southern Europe	Europe	Upper middle income

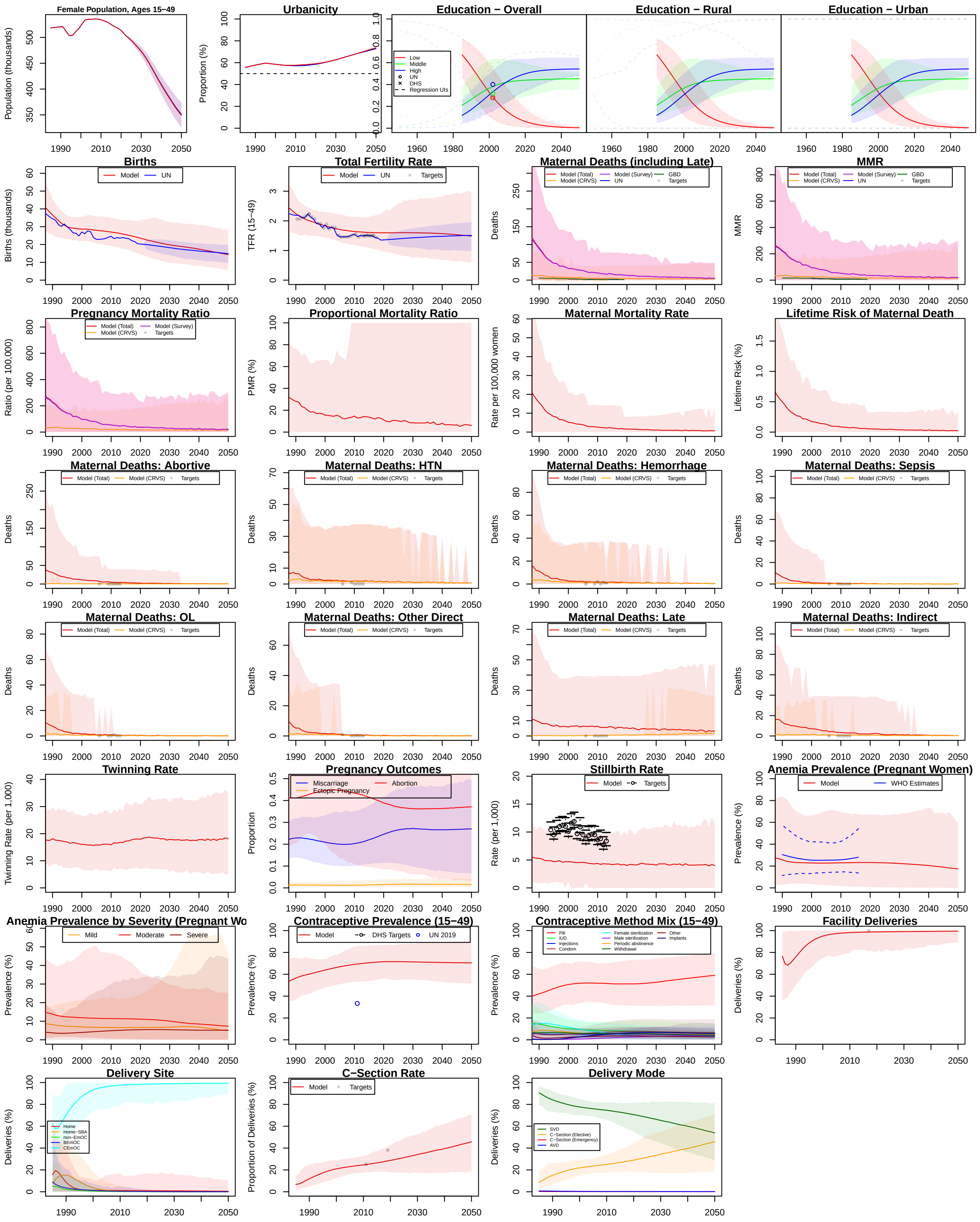

Norway

ISO Code	Region	Area	Income Group
NOR	Northern Europe	Europe	High income

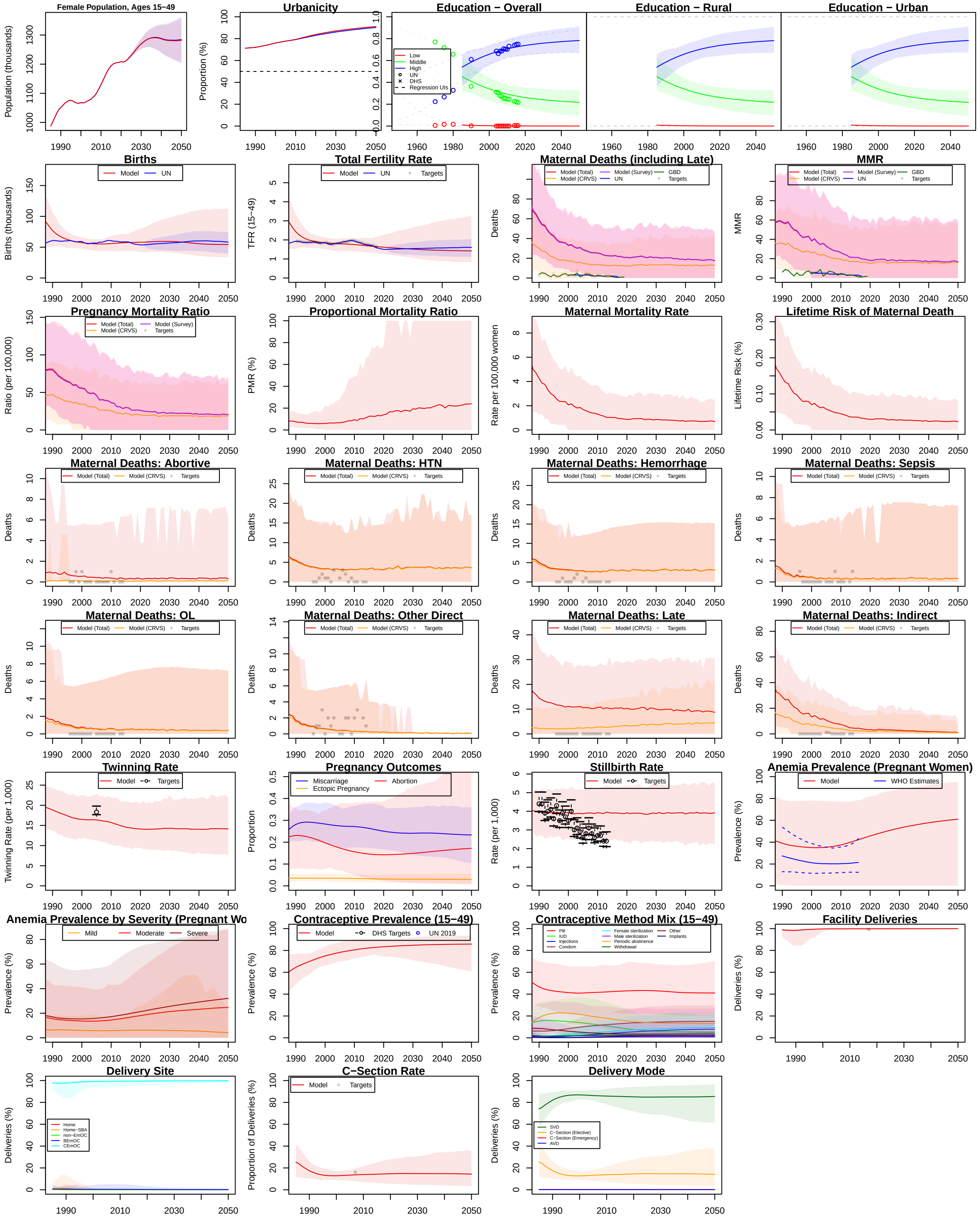

Oman

ISO Code	Region	Area	Income Group
OMN	Western Asia	Asia	High income

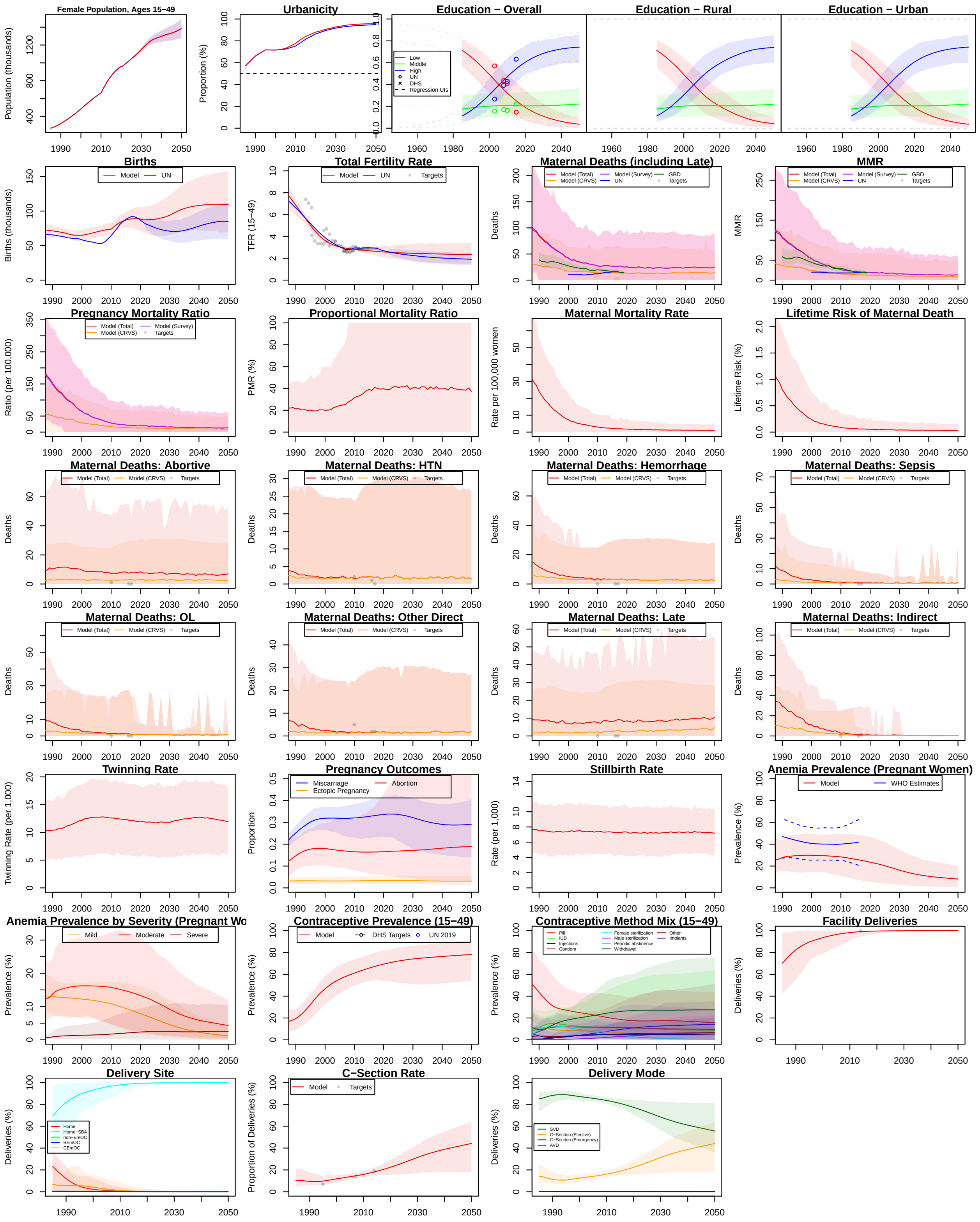

Pakistan

ISO Code	Region	Area	Income Group
PAK	Southern Asia	Asia	Lower middle income

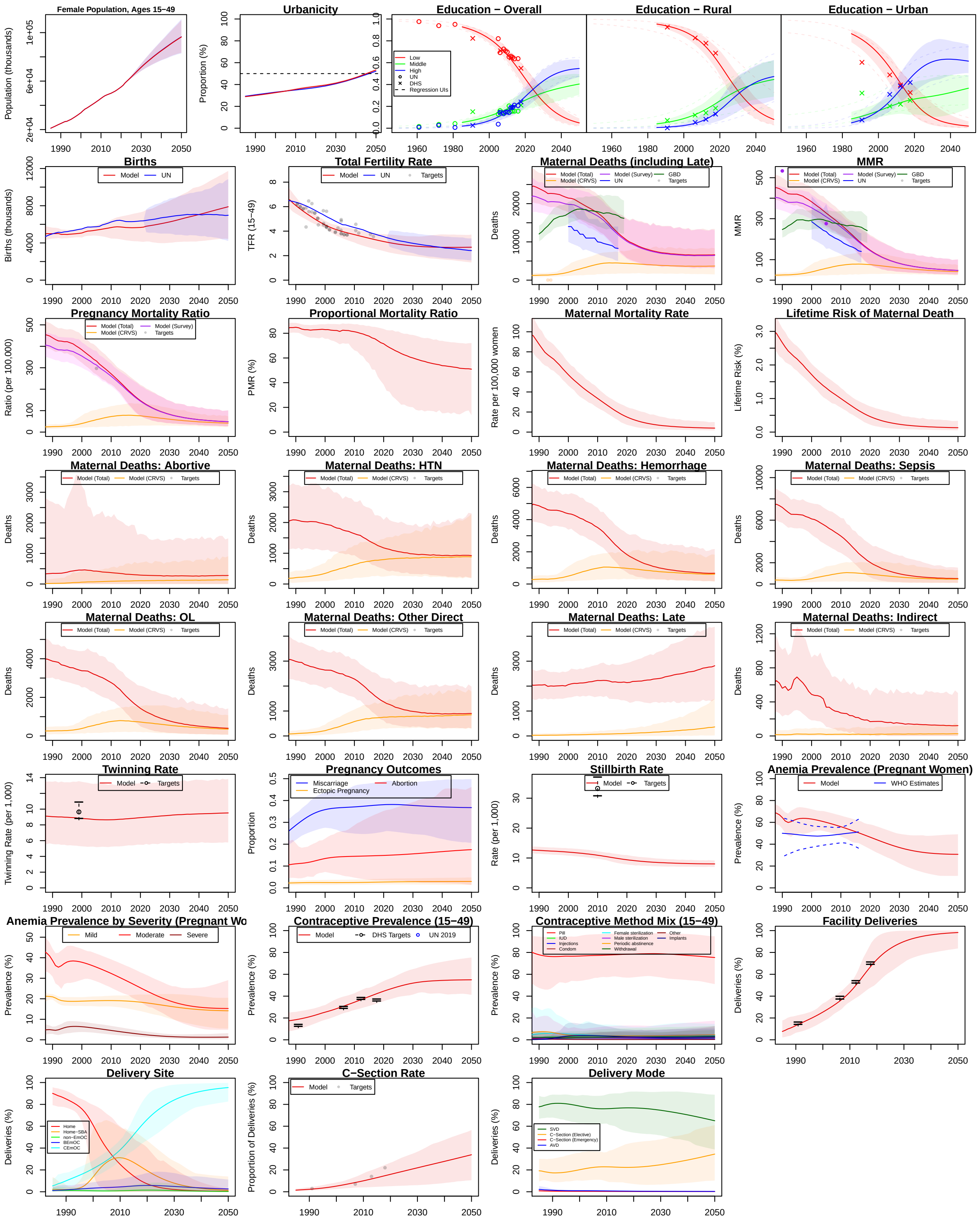

Palau

ISO Code	Region	Area	Income Group
PLW	Micronesia	Oceania	High income

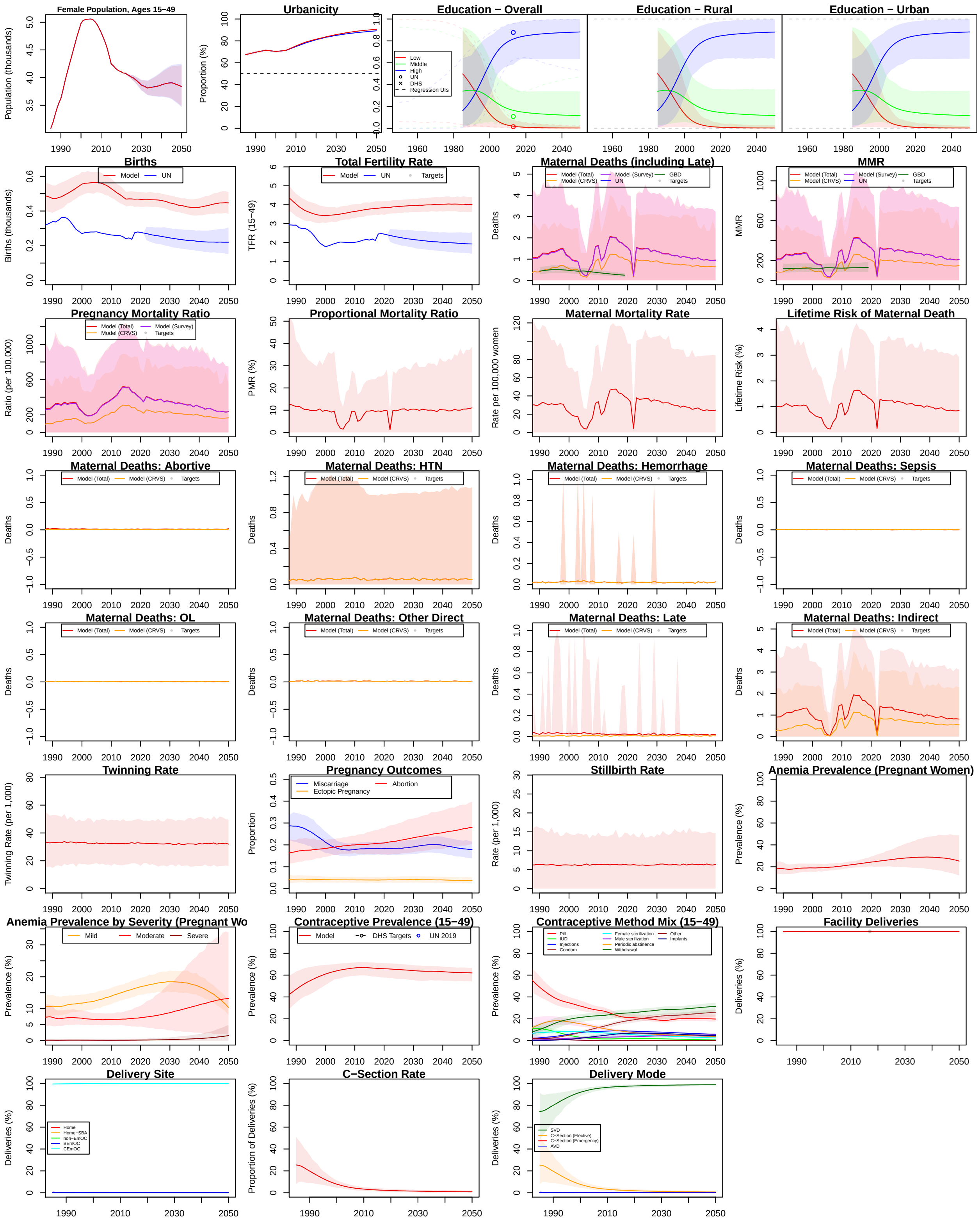

Panama

ISO Code	Region	Area	Income Group
PAN	Central America	Latin America and the Caribbean	High income

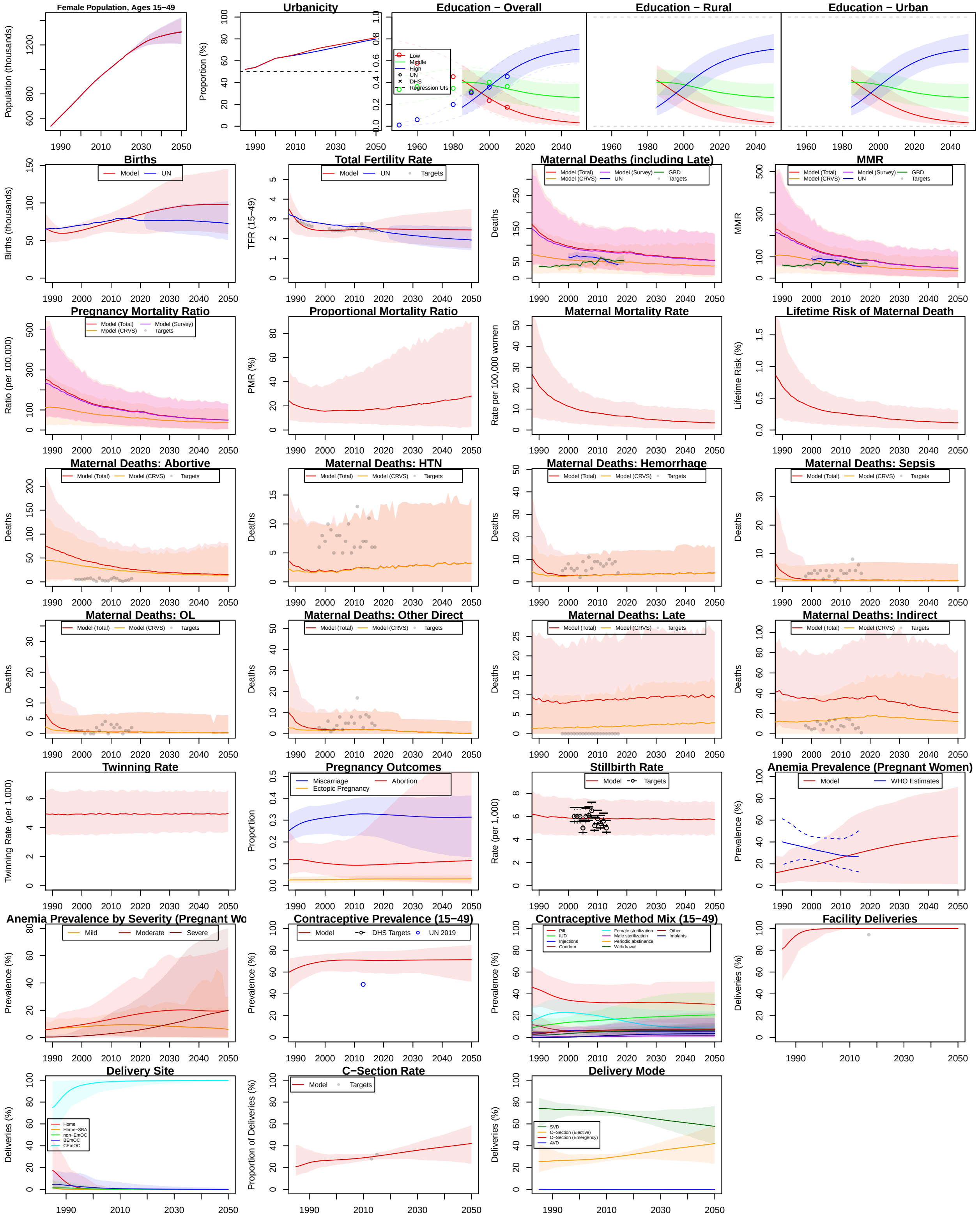

Papua New Guinea

ISO Code	Region	Area	Income Group
PNG	Melanesia	Oceania	Lower middle income

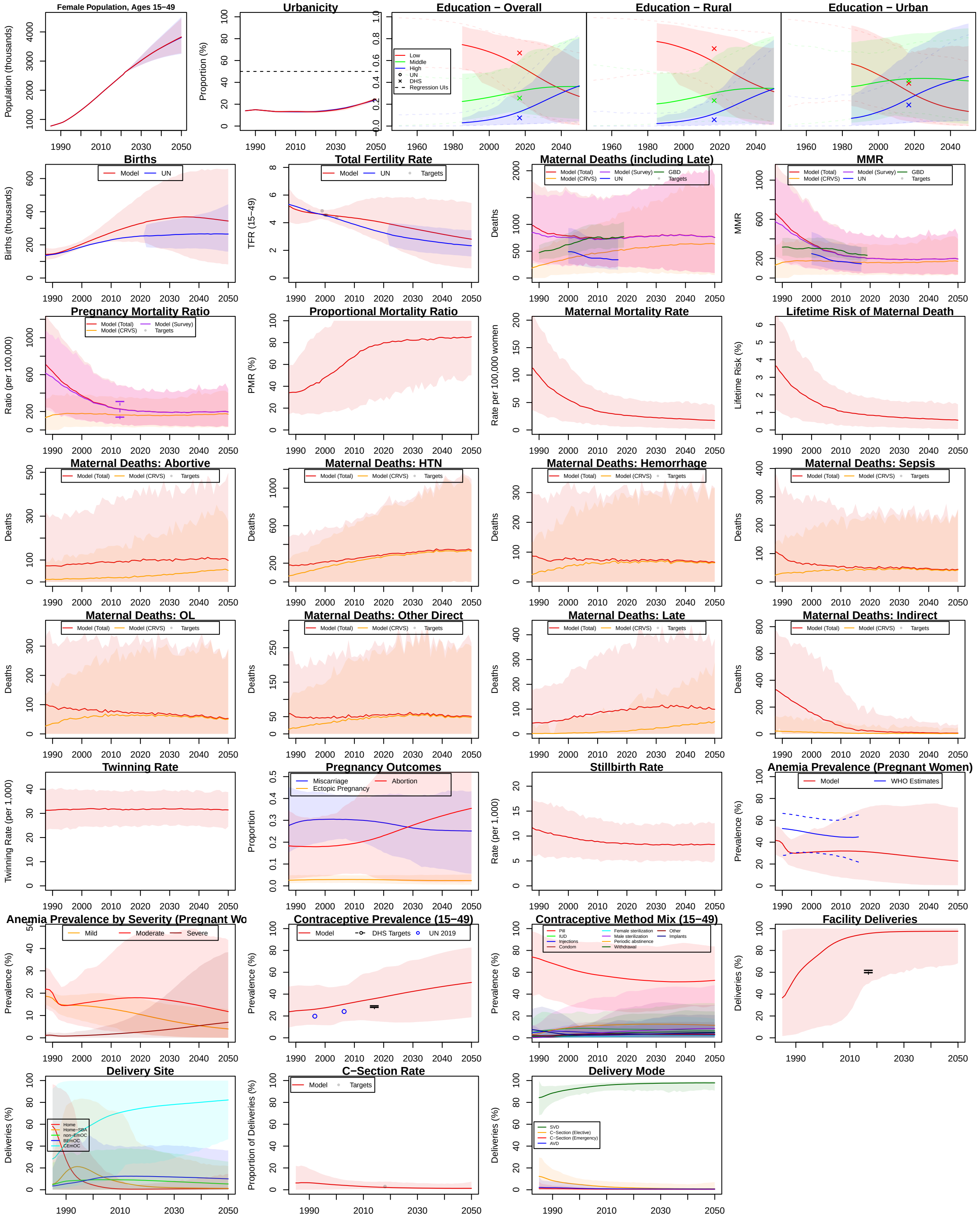

Paraguay

ISO Code	Region	Area	Income Group
PRY	South America	Latin America and the Caribbean	Upper middle income

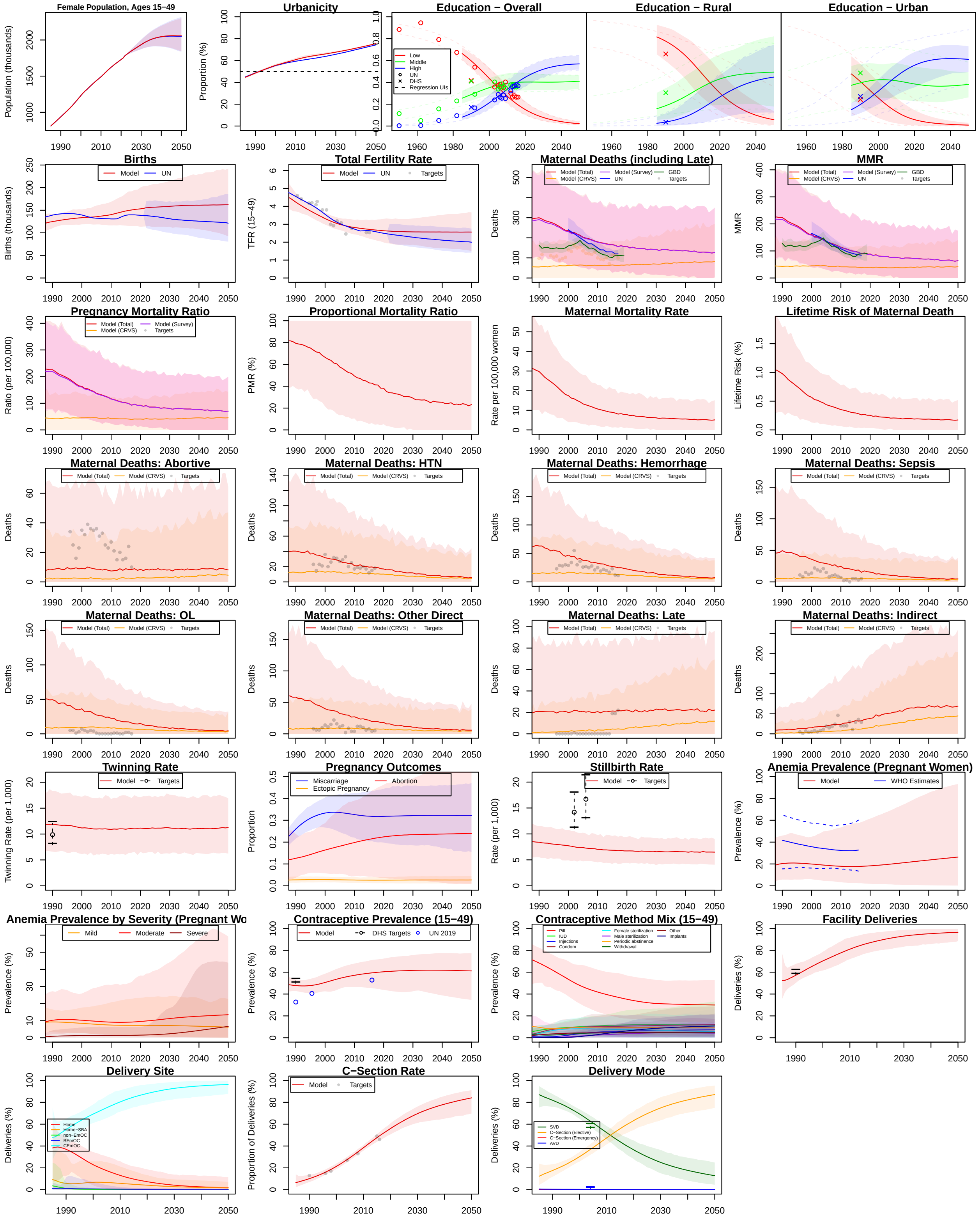

Peru

ISO Code	Region	Area	Income Group
PER	South America	Latin America and the Caribbean	Upper middle income

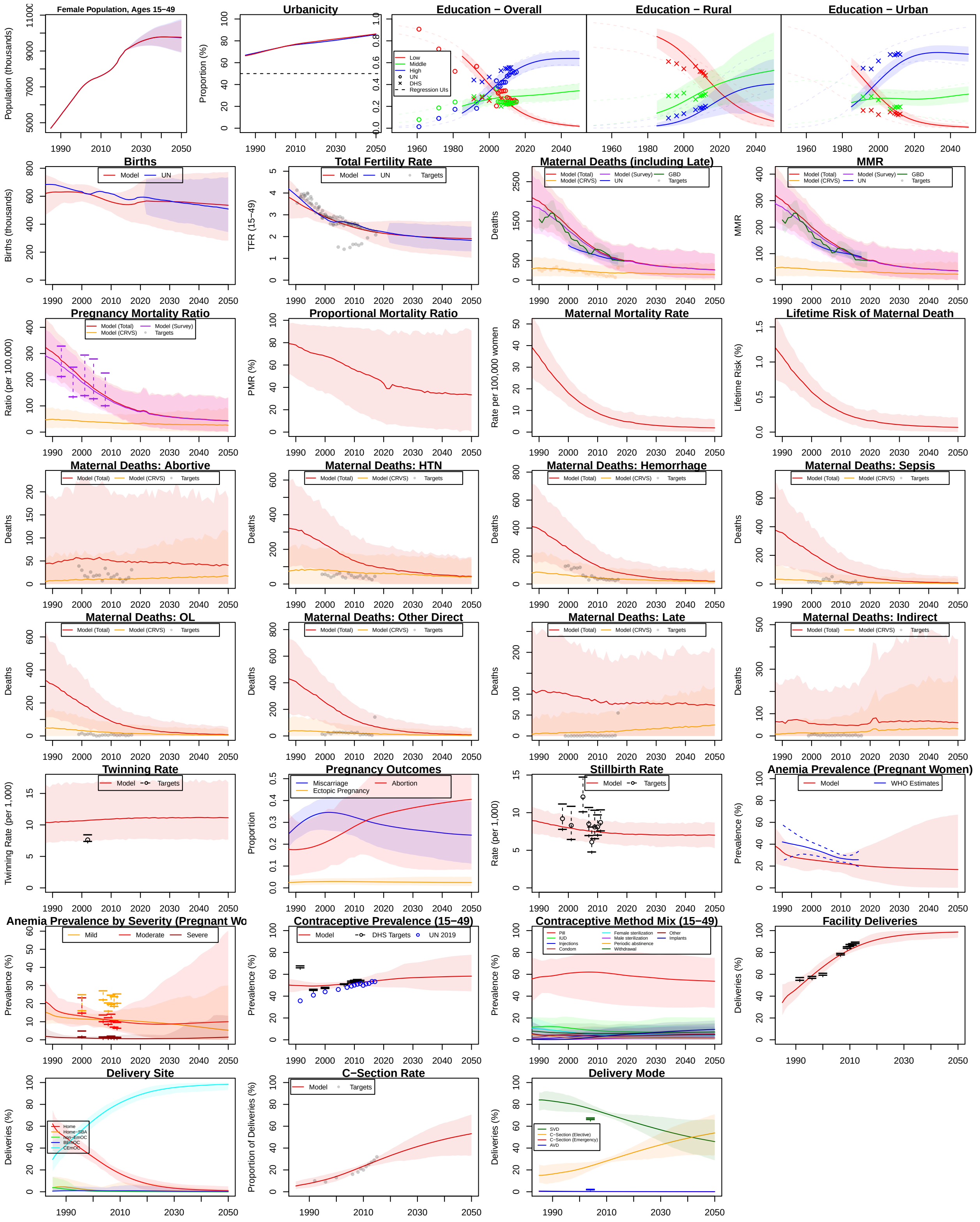

Philippines

ISO Code	Region	Area	Income Group
PHL	South-Eastern Asia	Asia	Lower middle income

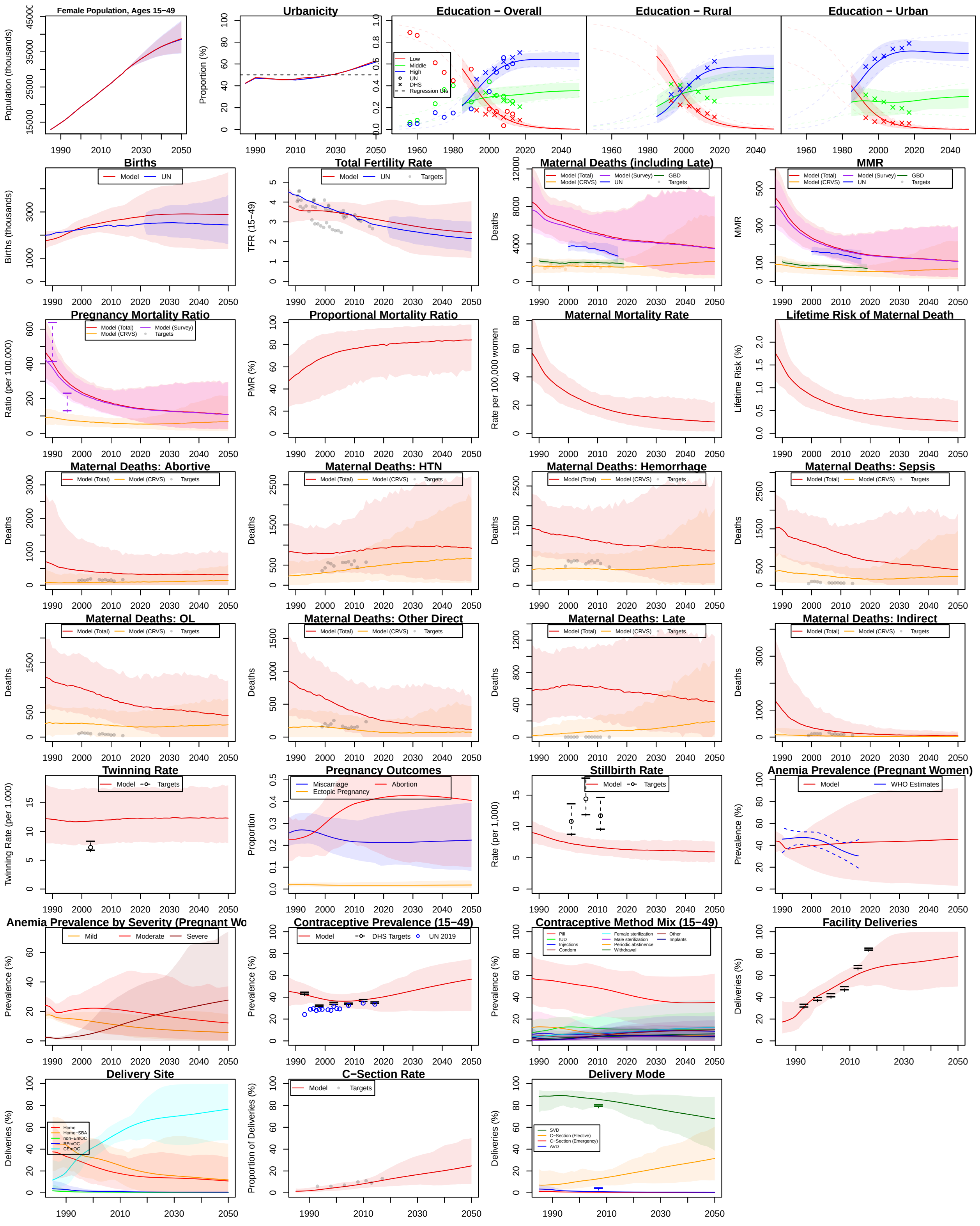

Poland

ISO Code	Region	Area	Income Group
POL	Eastern Europe	Europe	High income

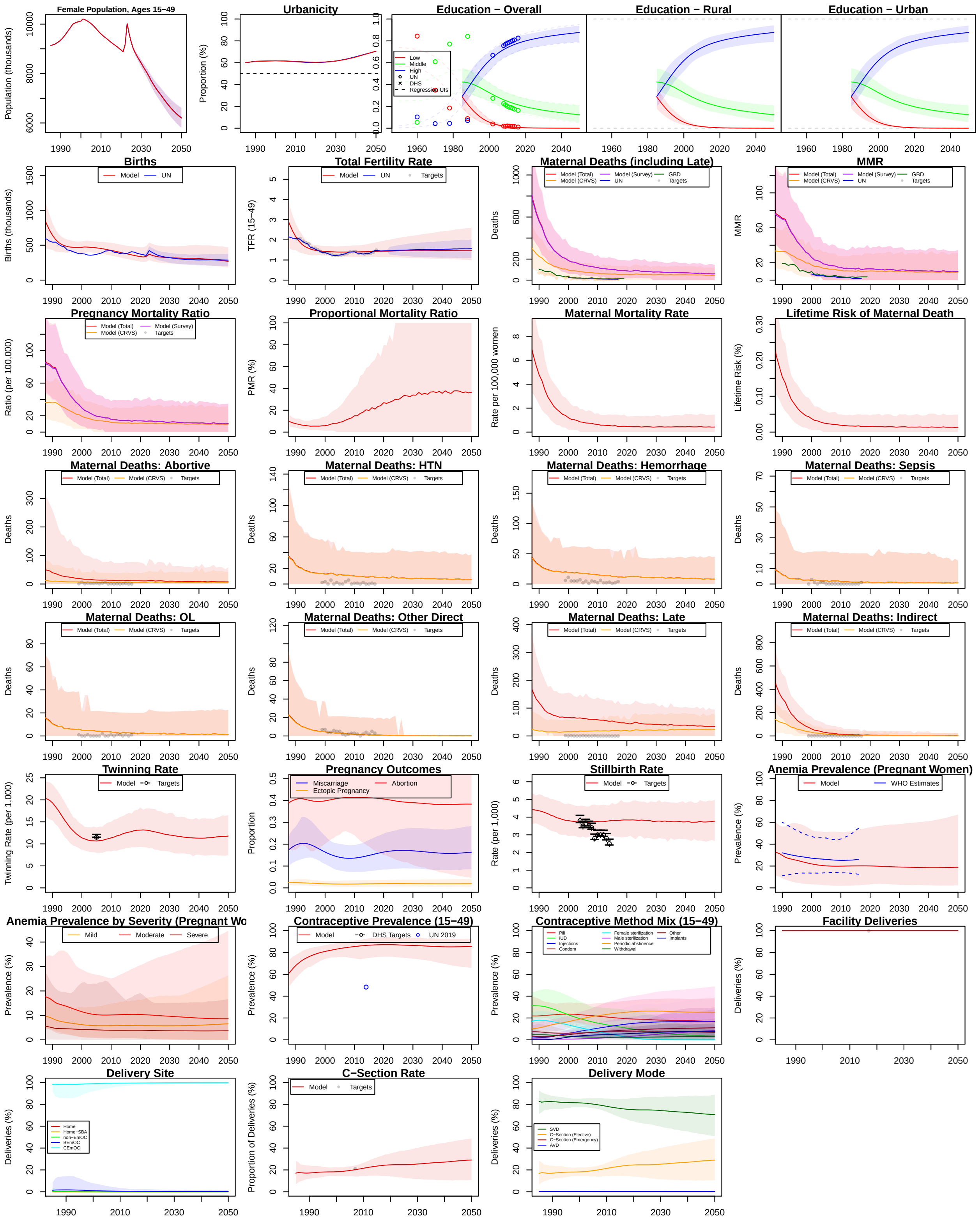

Portugal

ISO Code	Region	Area	Income Group
PRT	Southern Europe	Europe	High income

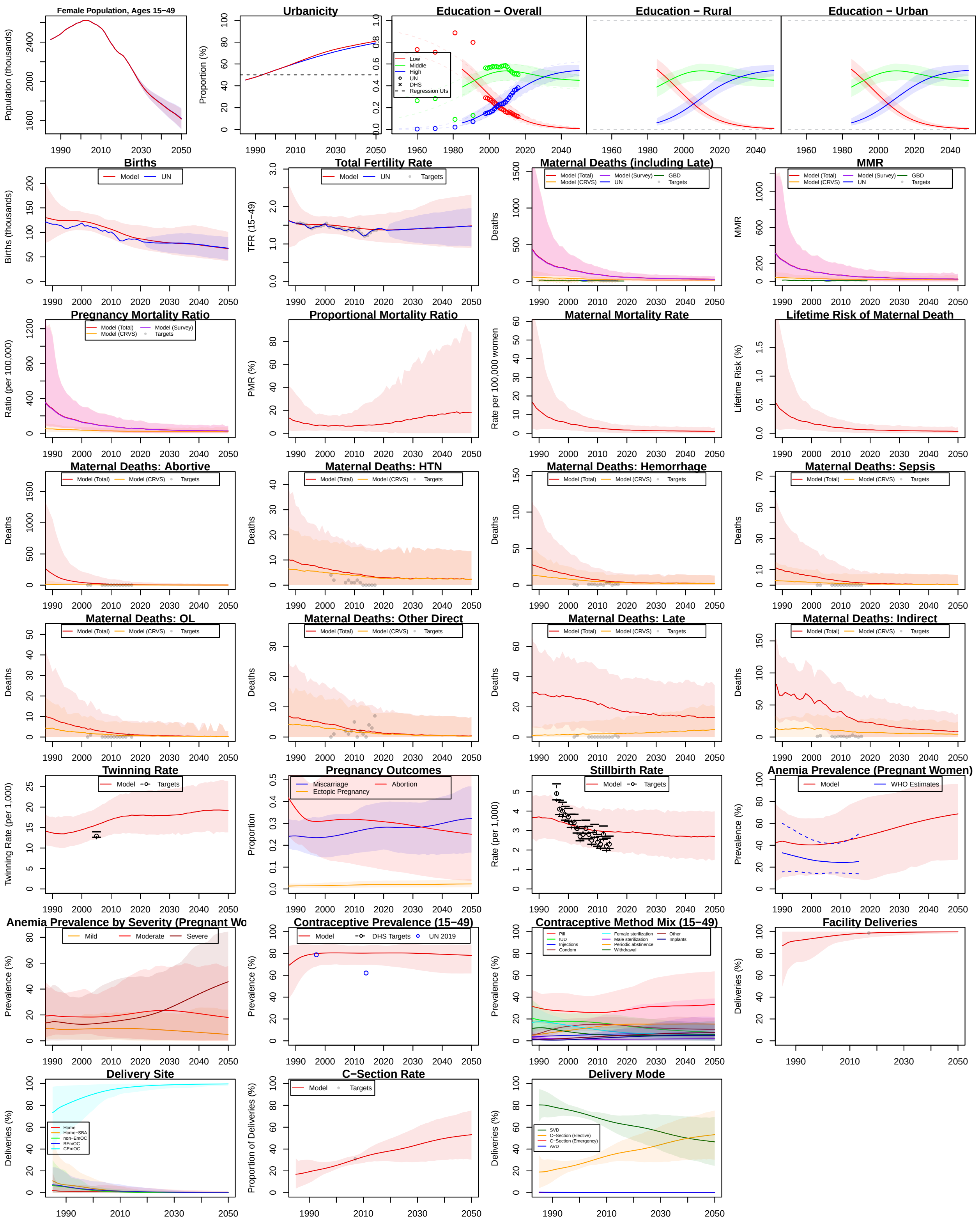

Puerto Rico

ISO Code	Region	Area	Income Group
PRI	Caribbean	Latin America and the Caribbean	High income

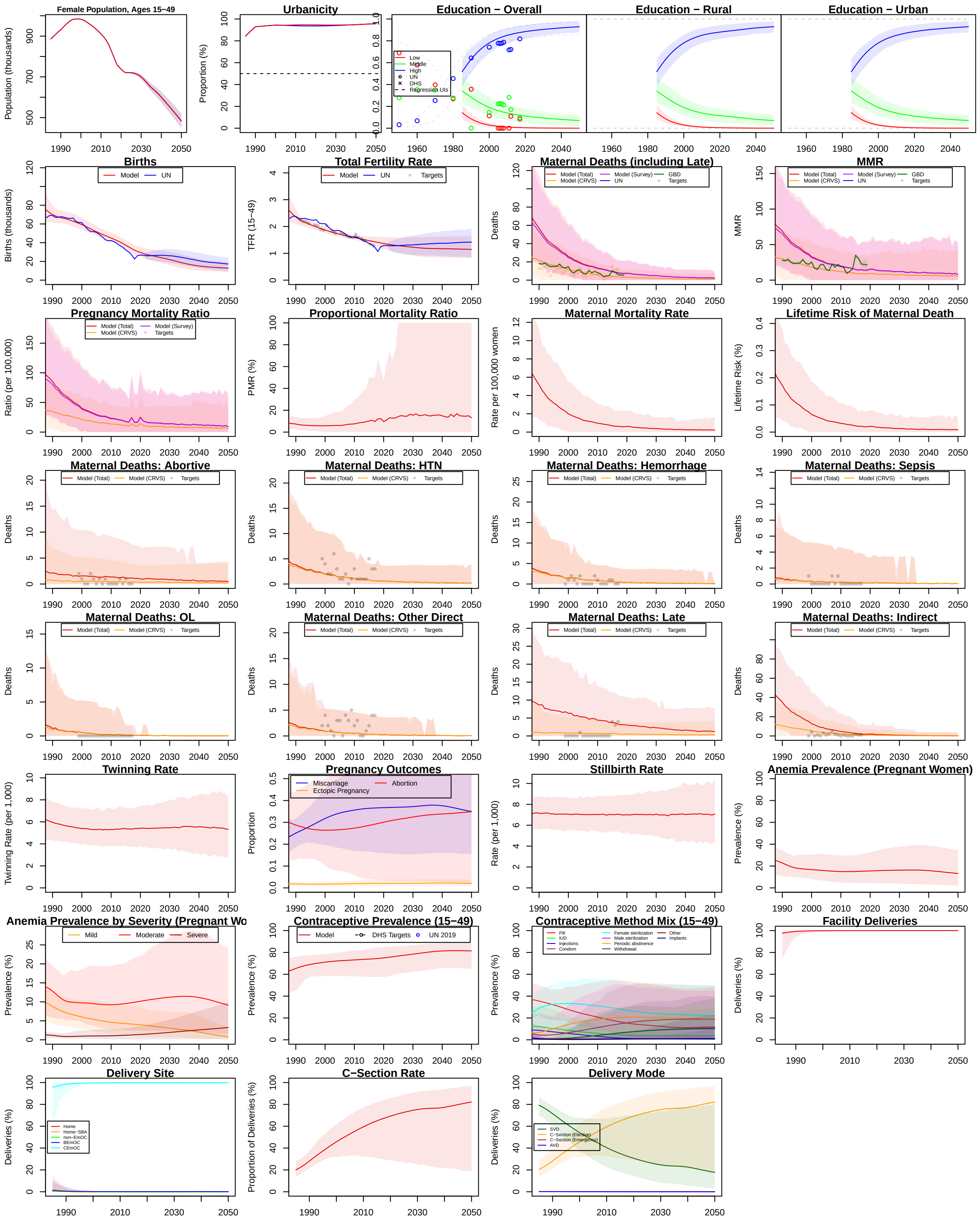

Qatar

ISO Code	Region	Area	Income Group
QAT	Western Asia	Asia	High income

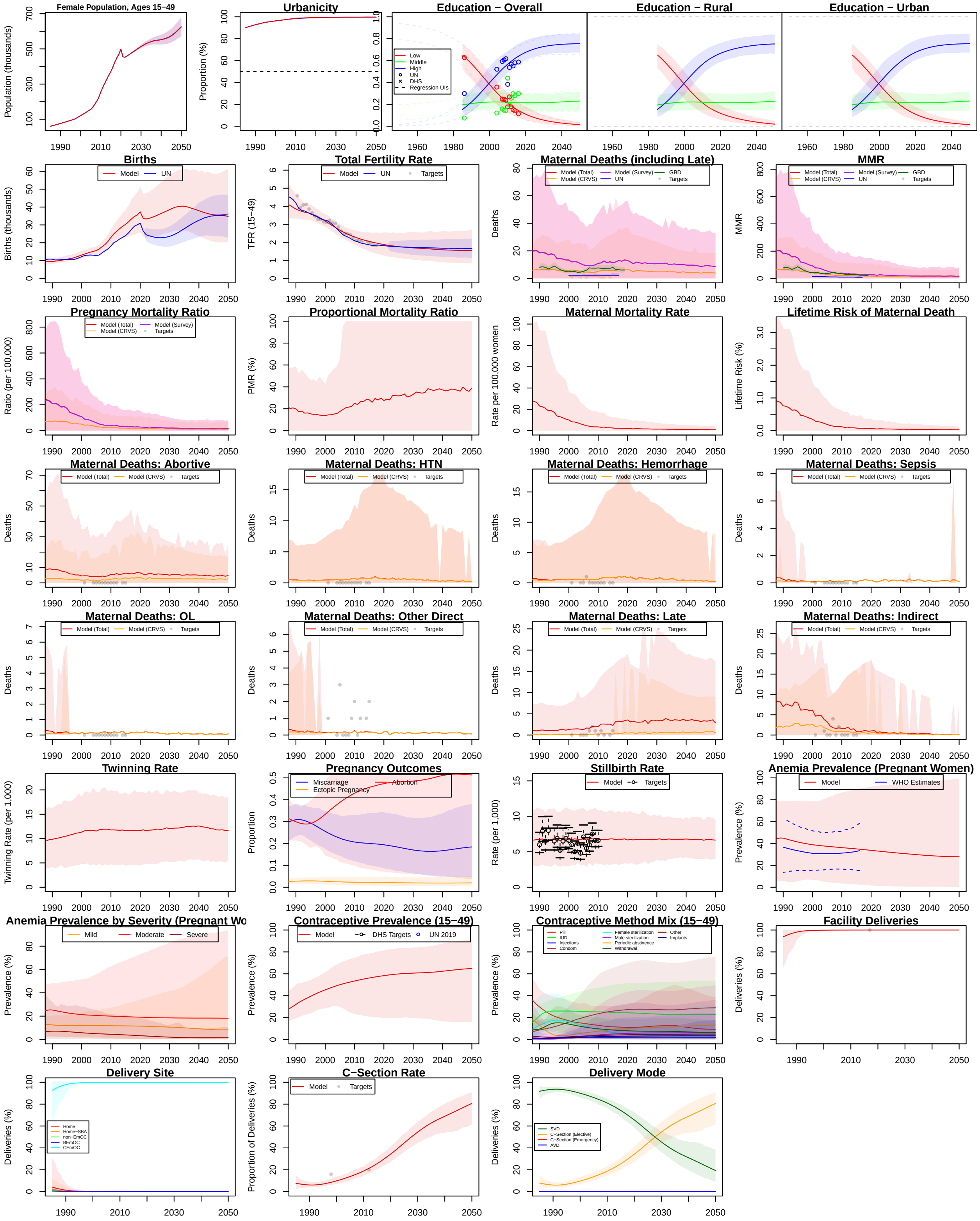

Republic of Korea

ISO Code	Region	Area	Income Group
KOR	Eastern Asia	Asia	High income

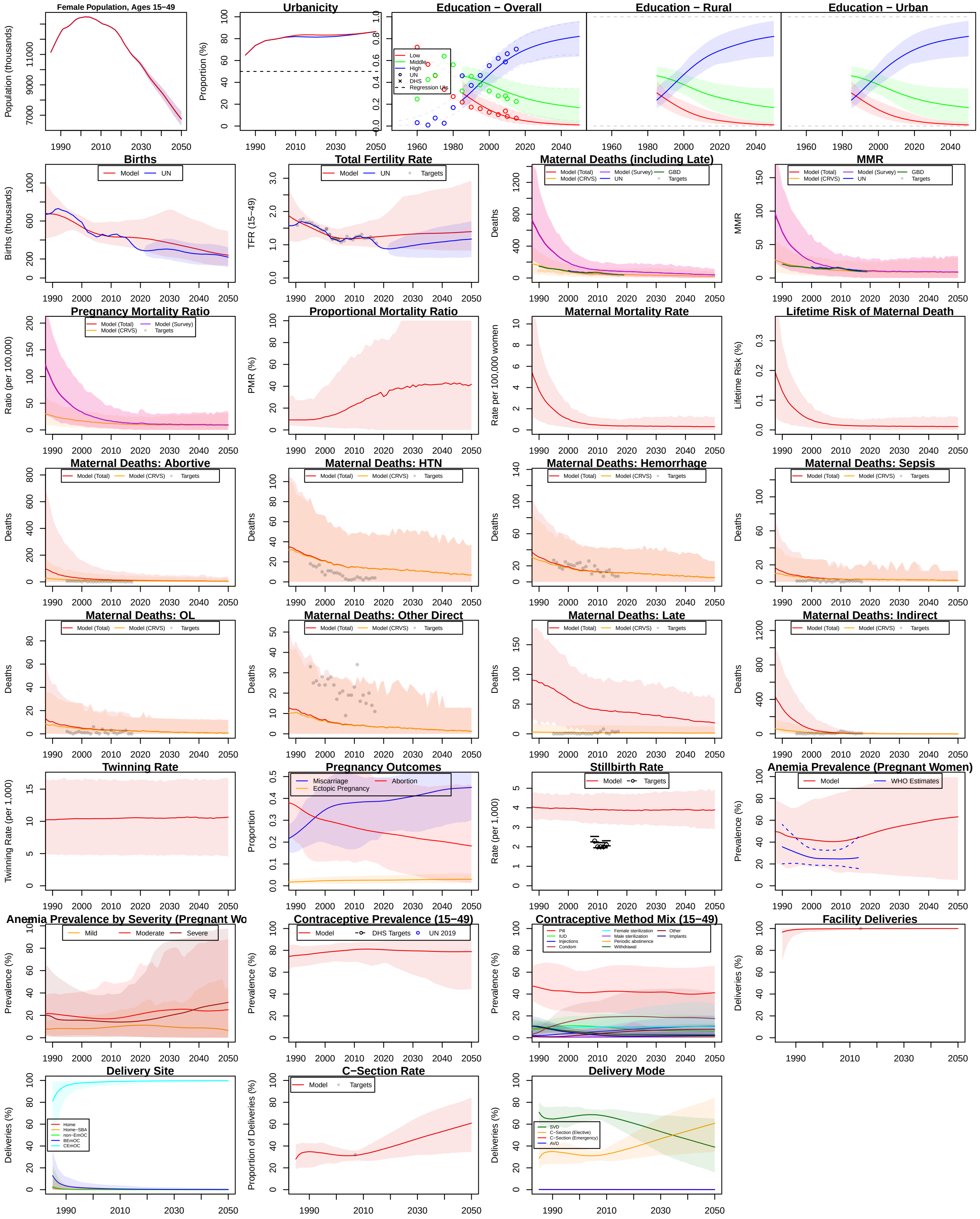

Republic of Moldova

ISO Code	Region	Area	Income Group
MDA	Eastern Europe	Europe	Lower middle income

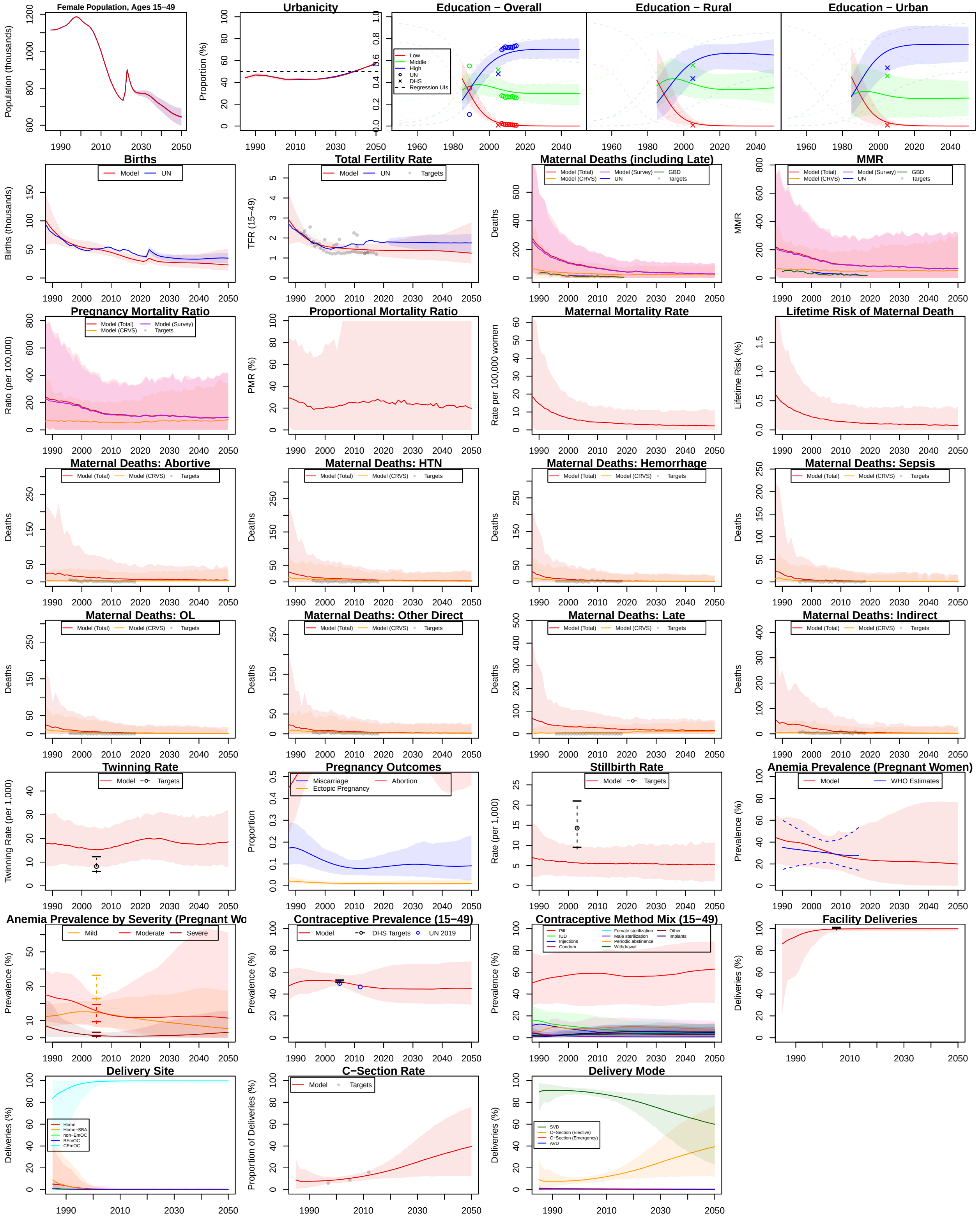

Romania

ISO Code	Region	Area	Income Group
ROU	Eastern Europe	Europe	Upper middle income

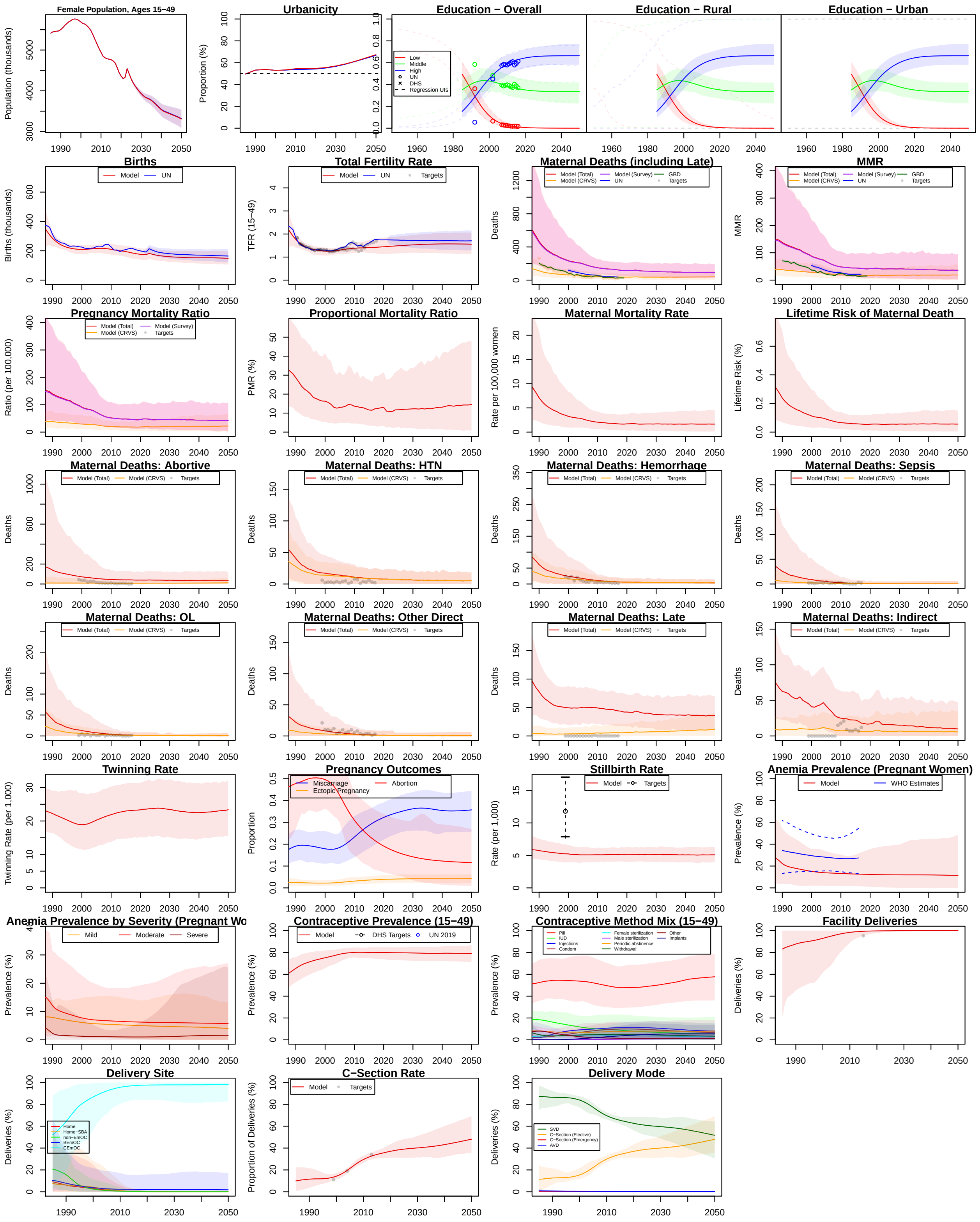

Russian Federation

ISO Code	Region	Area	Income Group
RUS	Eastern Europe	Europe	Upper middle income

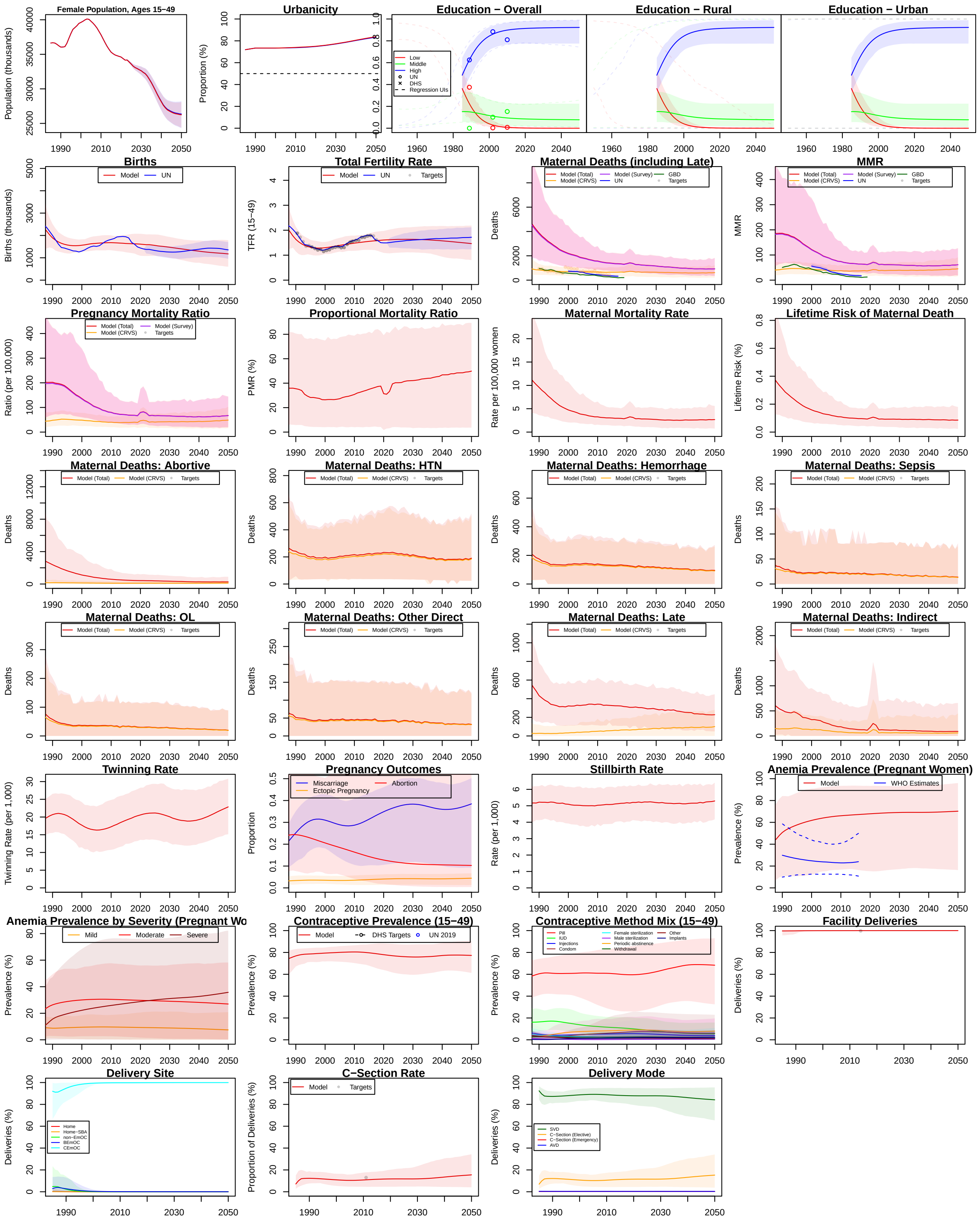

Rwanda

ISO Code	Region	Area	Income Group
RWA	Eastern Africa	Africa	Low income

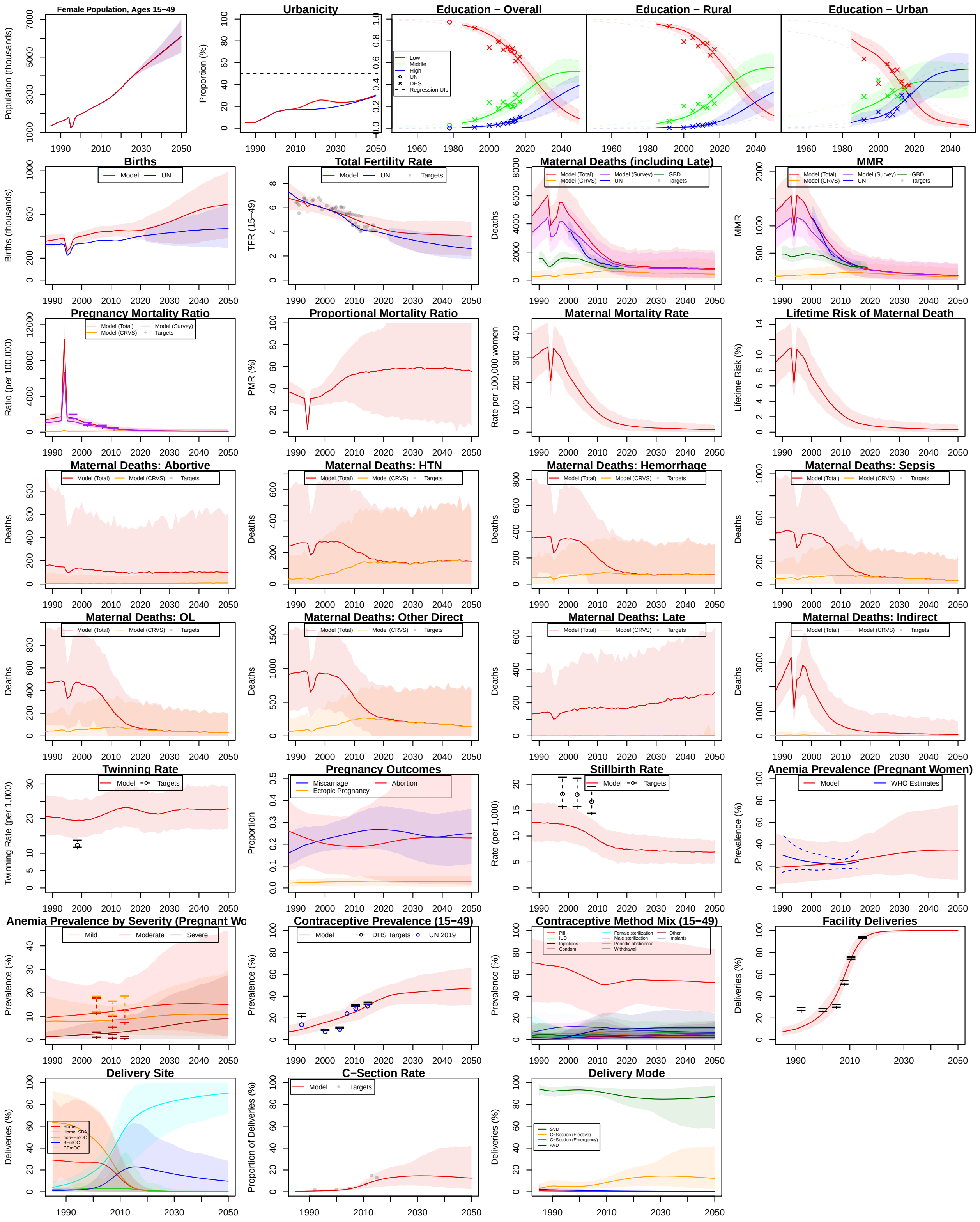

Saint Kitts and Nevis

ISO Code	Region	Area	Income Group
KNA	Caribbean	Latin America and the Caribbean	High income

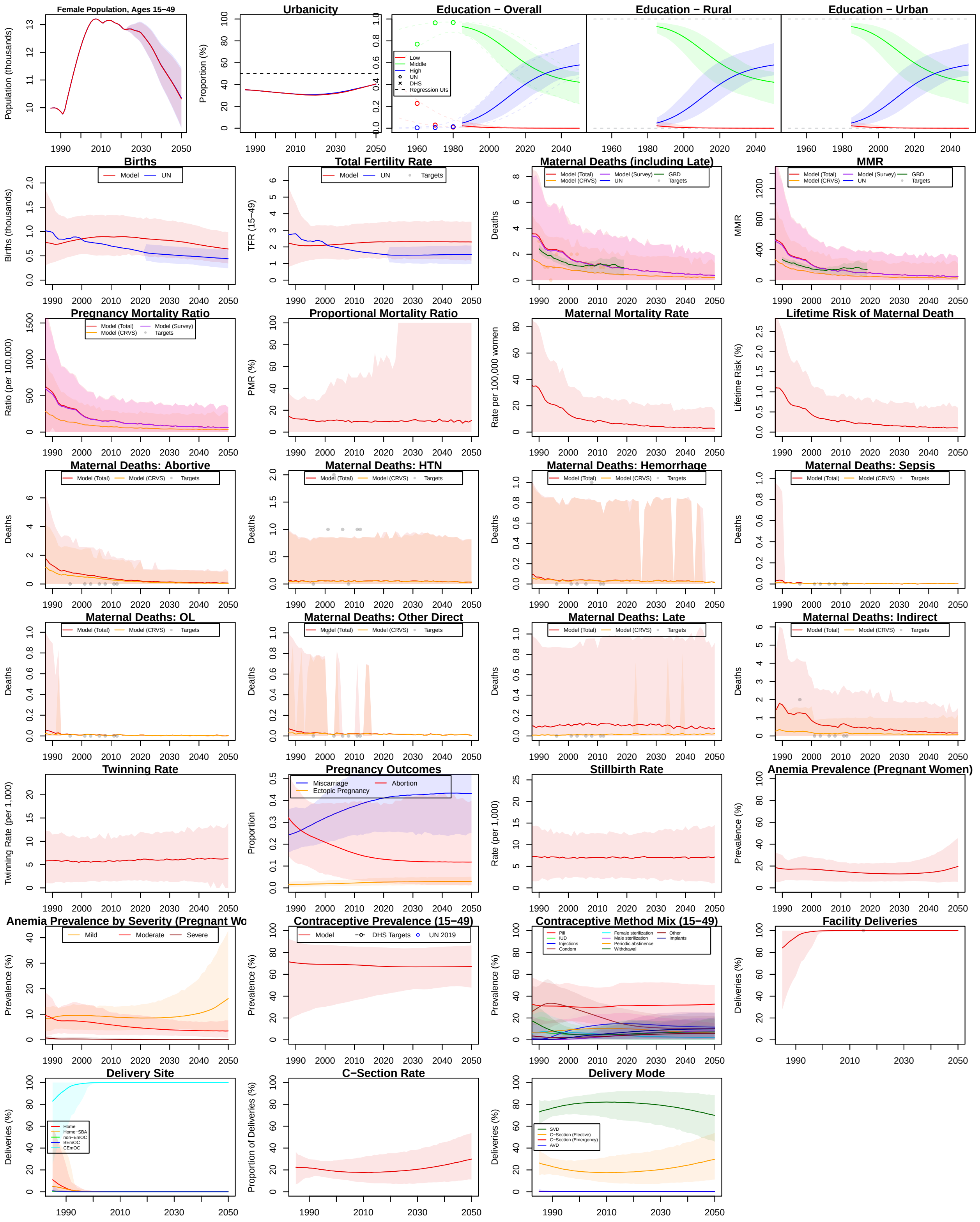

Saint Lucia

ISO Code	Region	Area	Income Group
LCA	Caribbean	Latin America and the Caribbean	Upper middle income

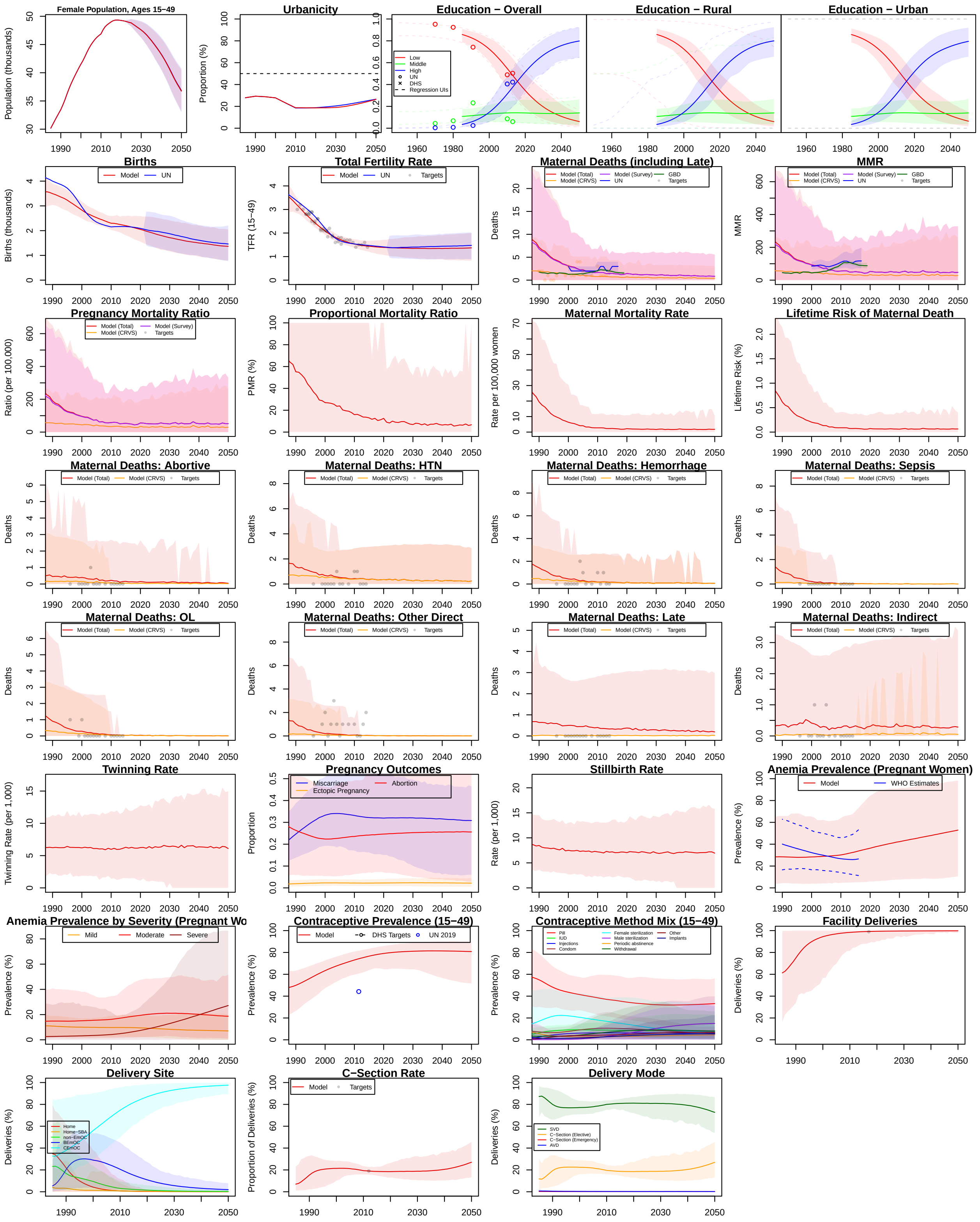

Saint Vincent and the Grenadines

ISO Code	Region	Area	Income Group
VCT	Caribbean	Latin America and the Caribbean	Upper middle income

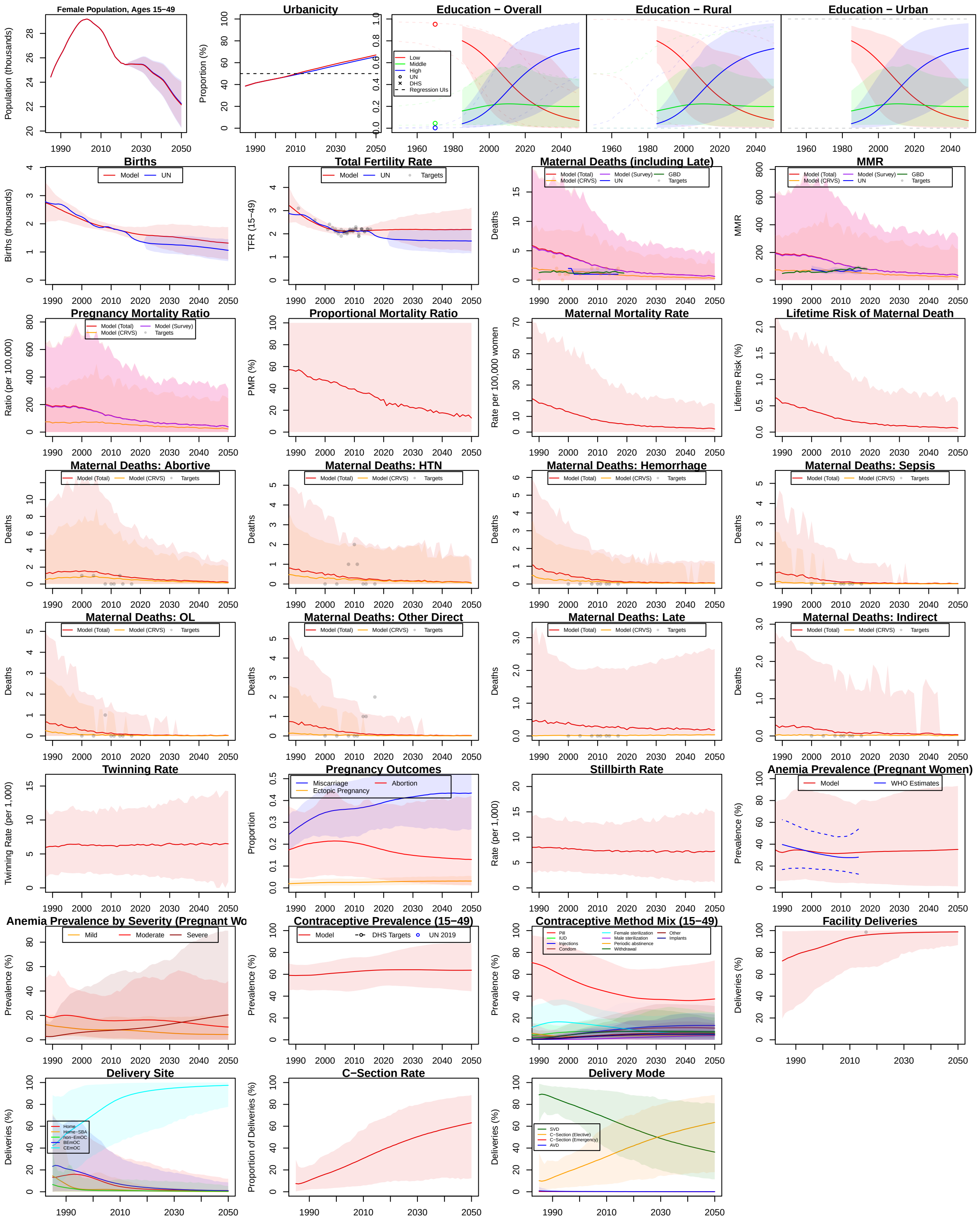

Samoa

ISO Code	Region	Area	Income Group
WSM	Polynesia	Oceania	Upper middle income

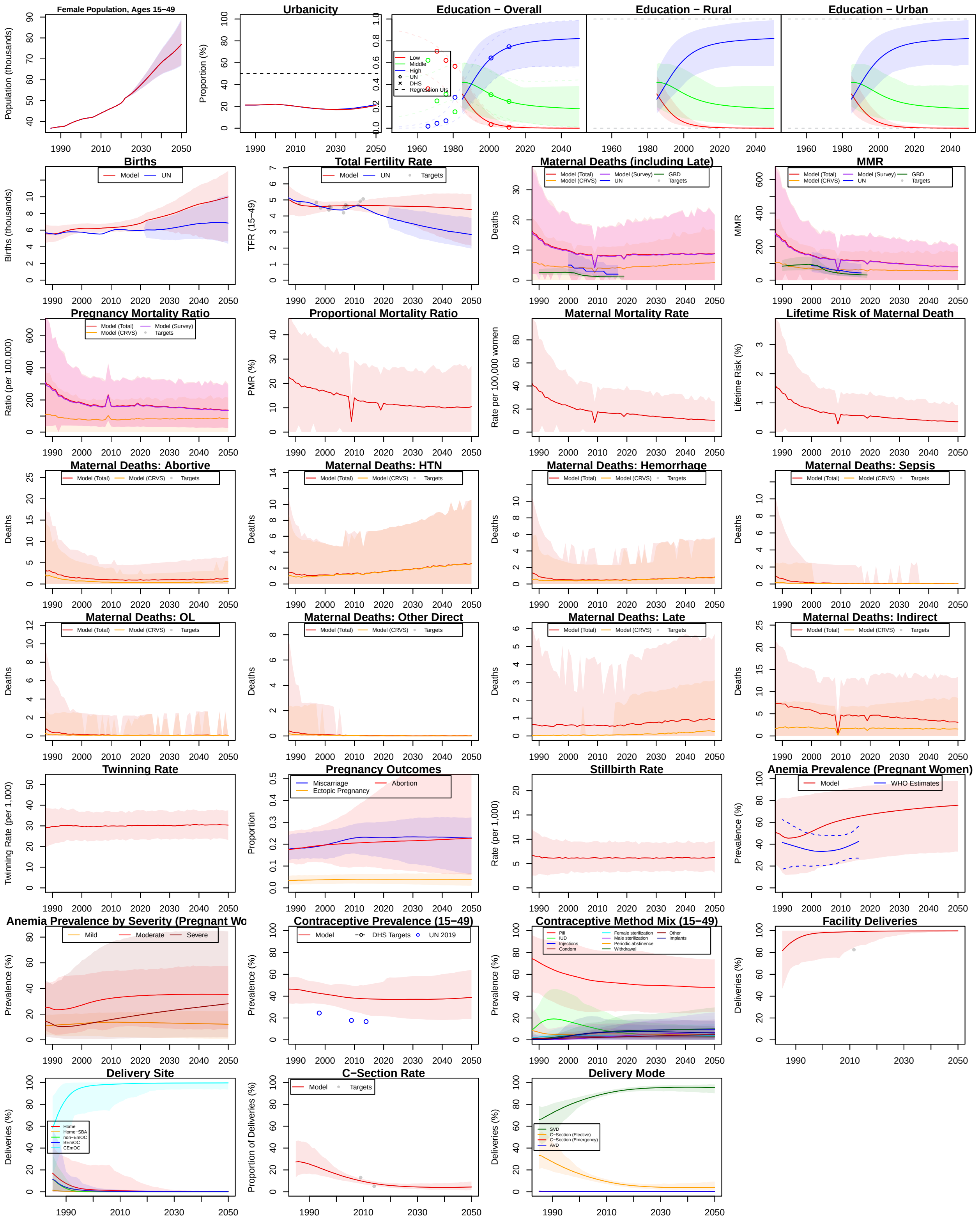

San Marino

ISO Code	Region	Area	Income Group
SMR	Southern Europe	Europe	High income

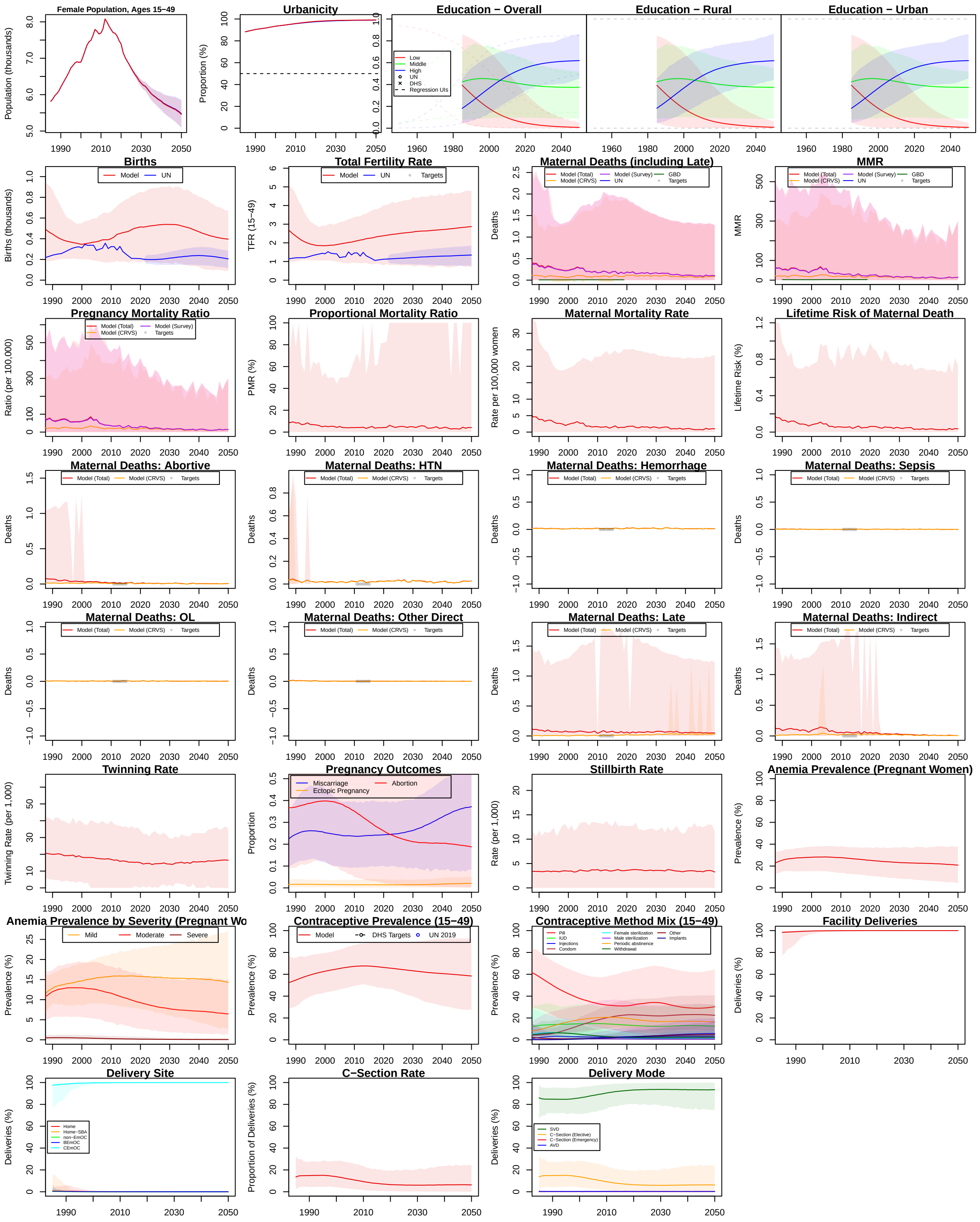

Sao Tome and Principe

ISO Code	Region	Area	Income Group
STP	Middle Africa	Africa	Lower middle income

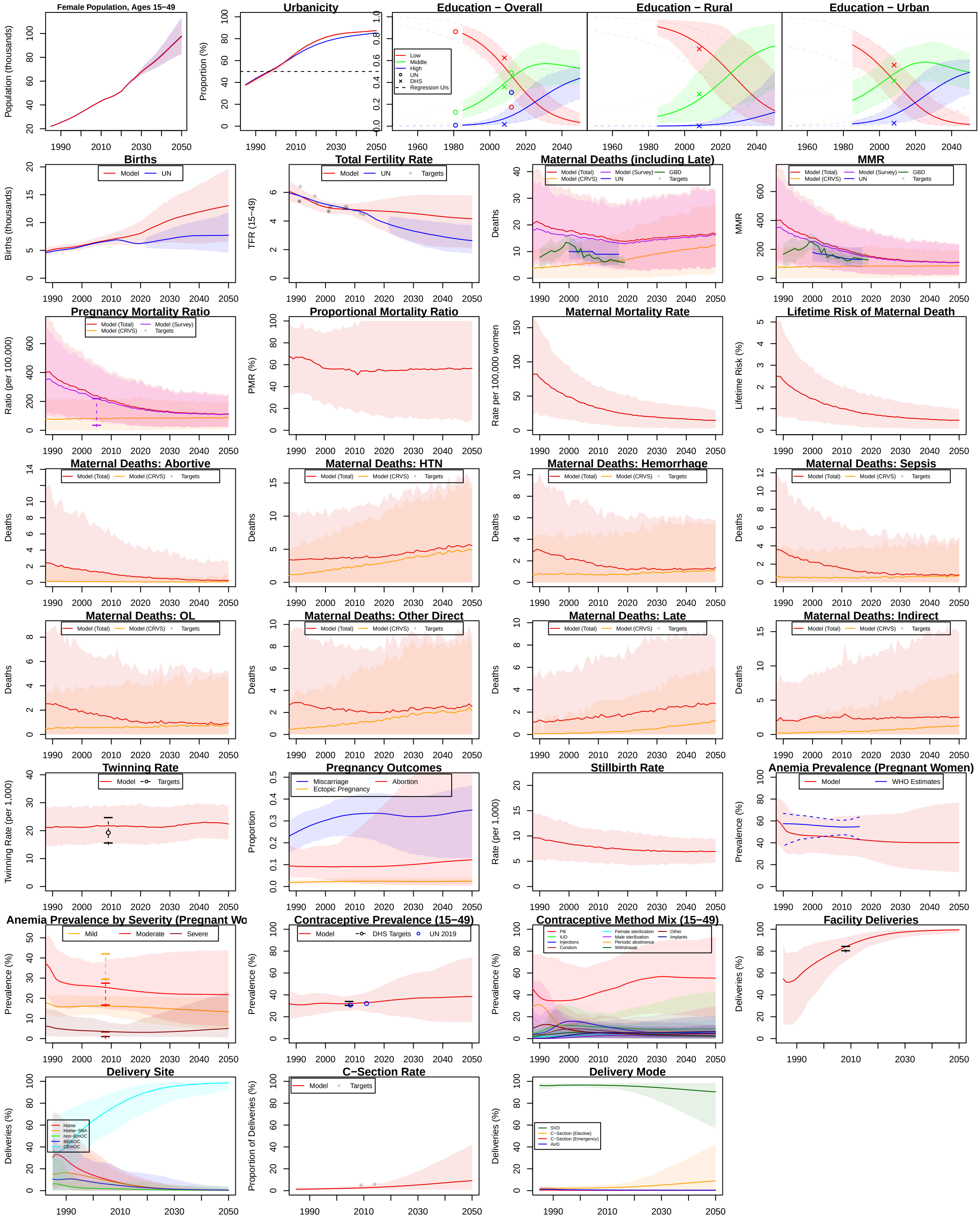

Saudi Arabia

ISO Code	Region	Area	Income Group
SAU	Western Asia	Asia	High income

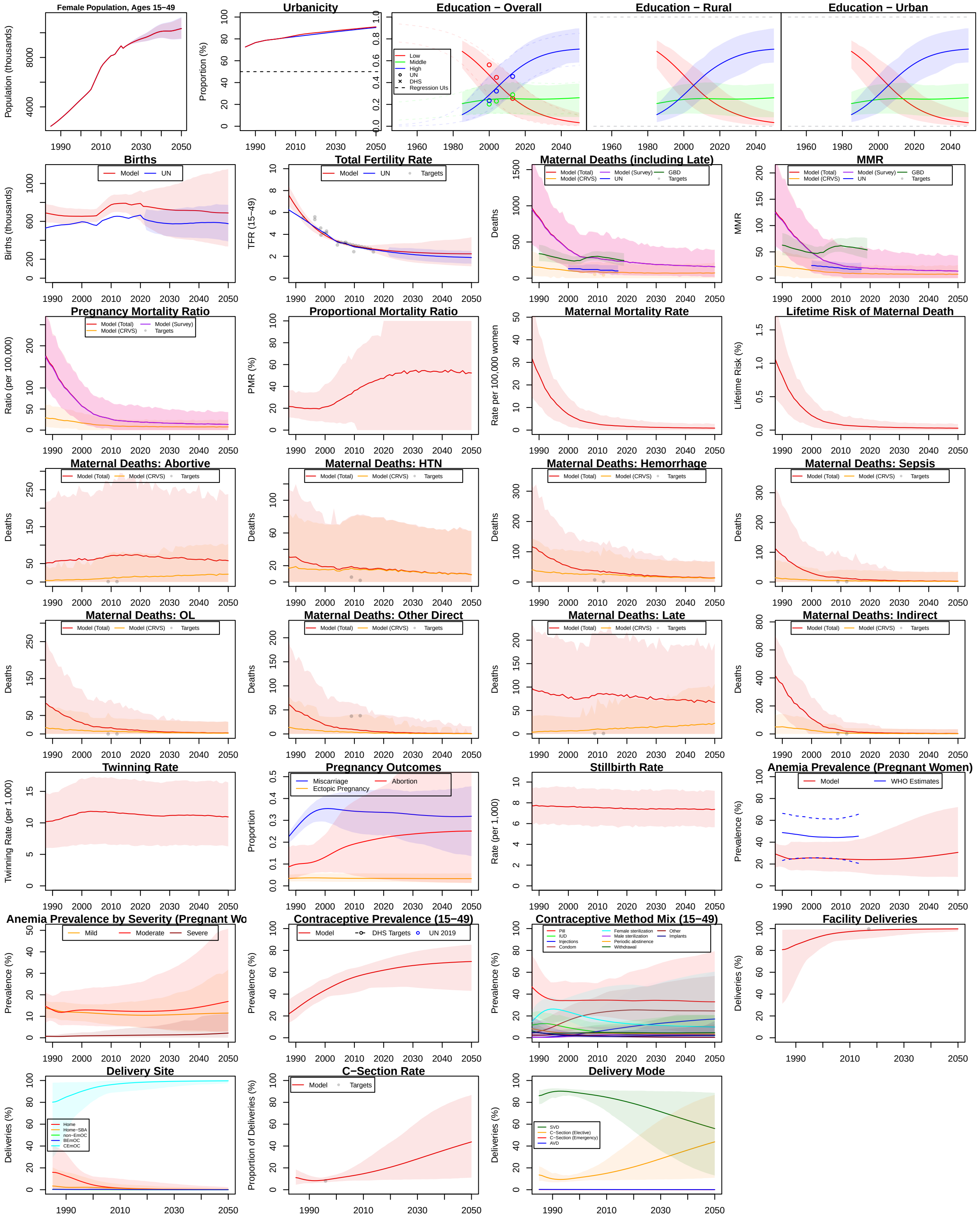

Senegal

ISO Code	Region	Area	Income Group
SEN	Western Africa	Africa	Low income

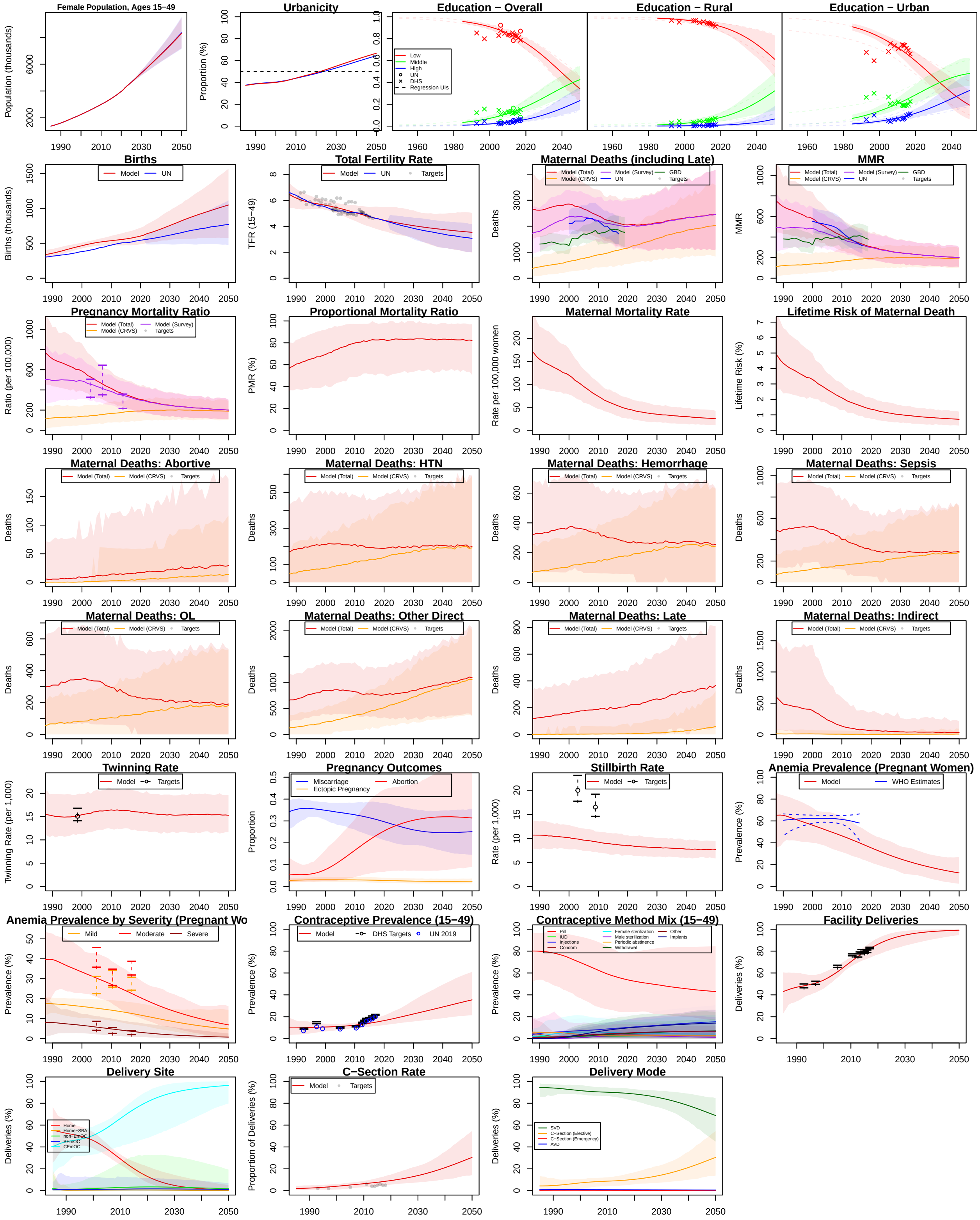

Serbia

ISO Code	Region	Area	Income Group
SRB	Southern Europe	Europe	Upper middle income

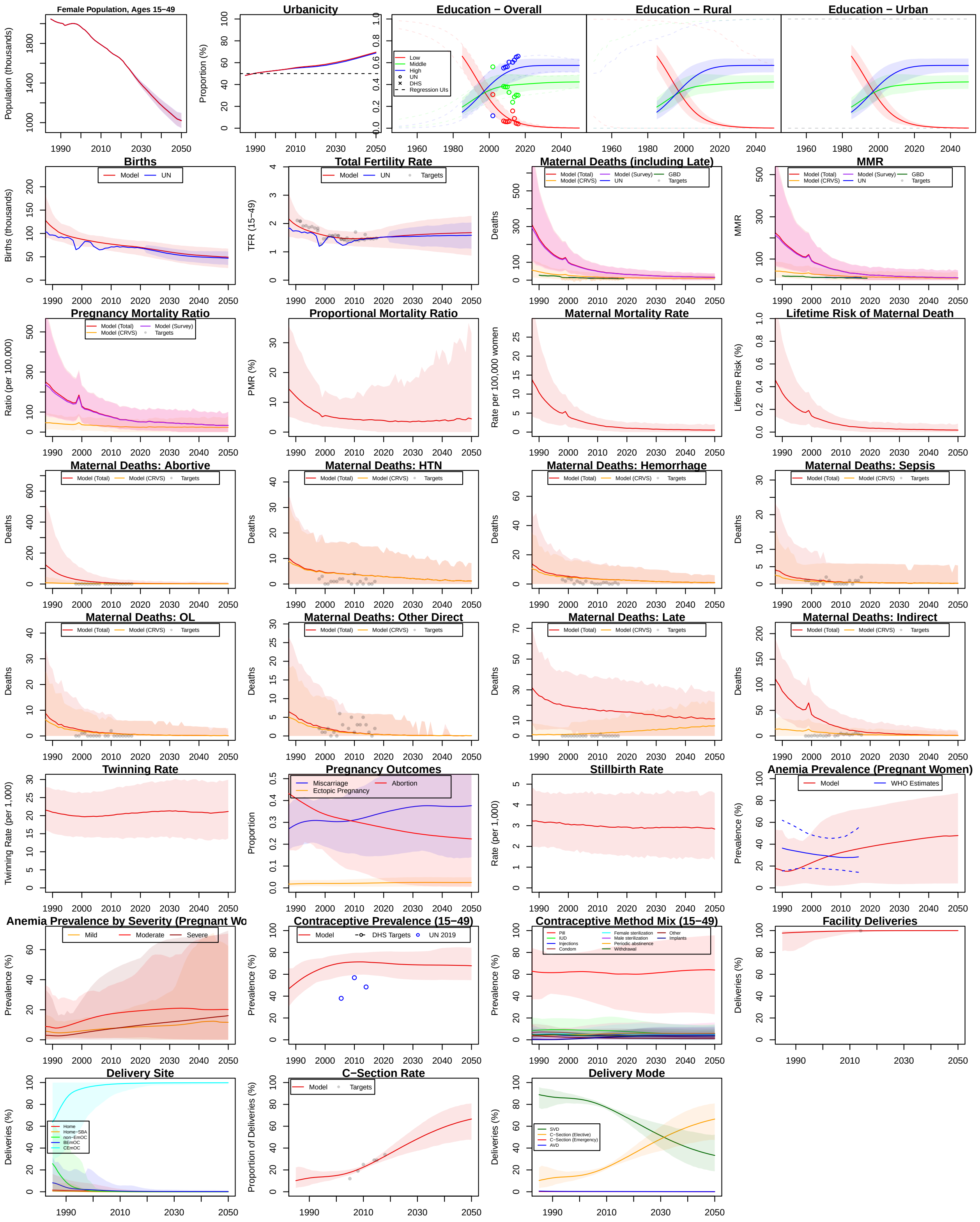

Seychelles

ISO Code	Region	Area	Income Group
SYC	Eastern Africa	Africa	High income

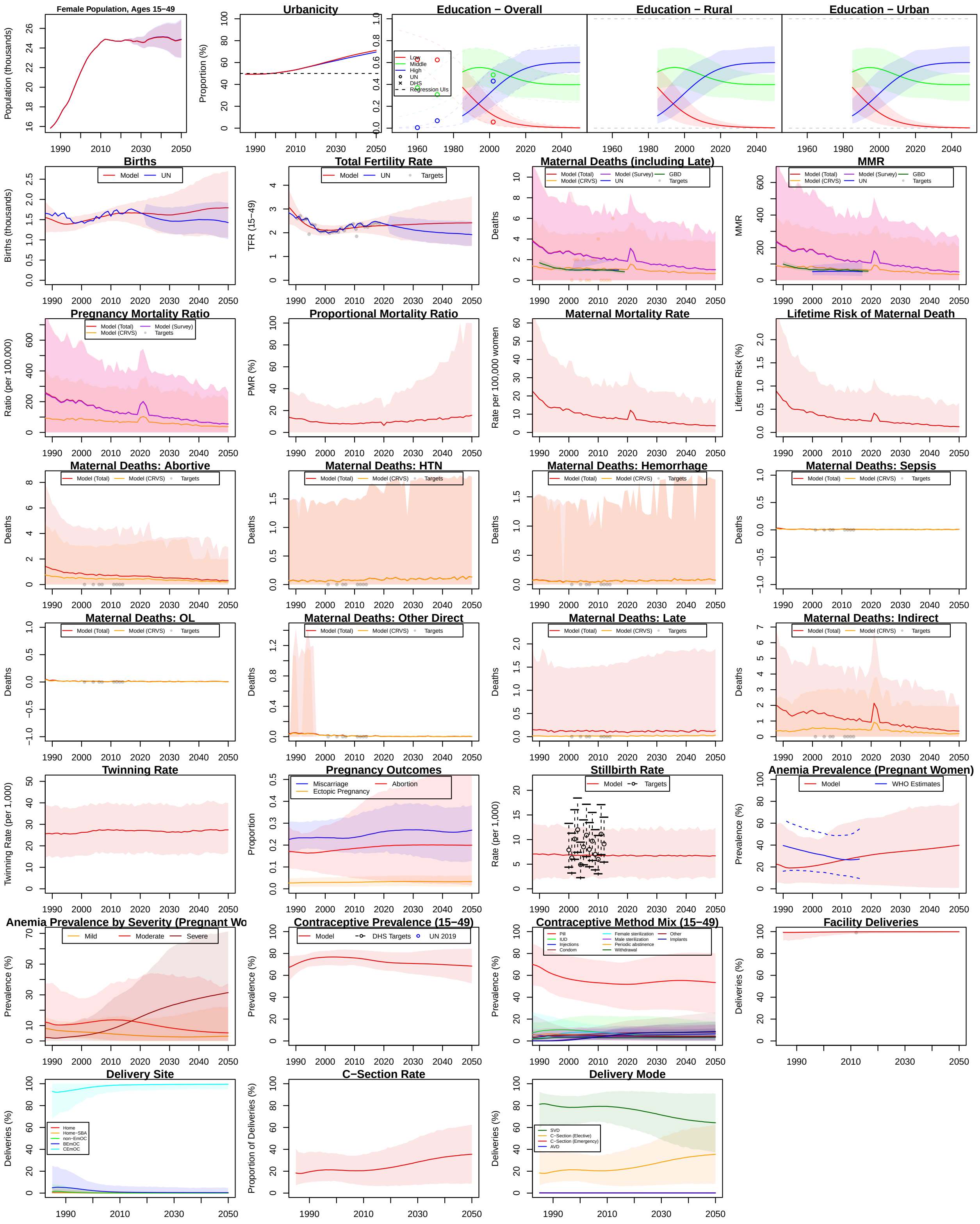

Sierra Leone

ISO Code	Region	Area	Income Group
SLE	Western Africa	Africa	Low income

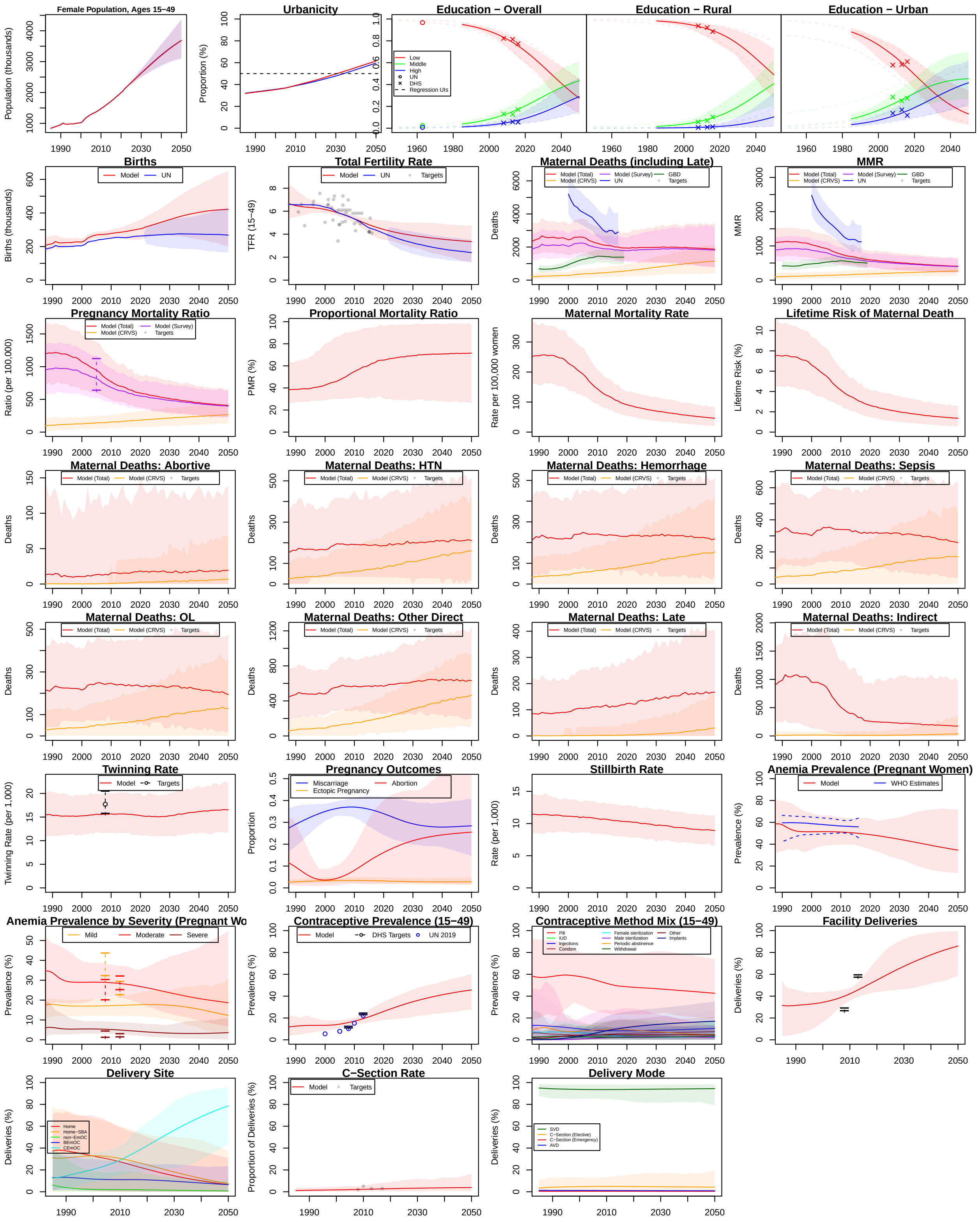

Singapore

ISO Code	Region	Area	Income Group
SGP	South-Eastern Asia	Asia	High income

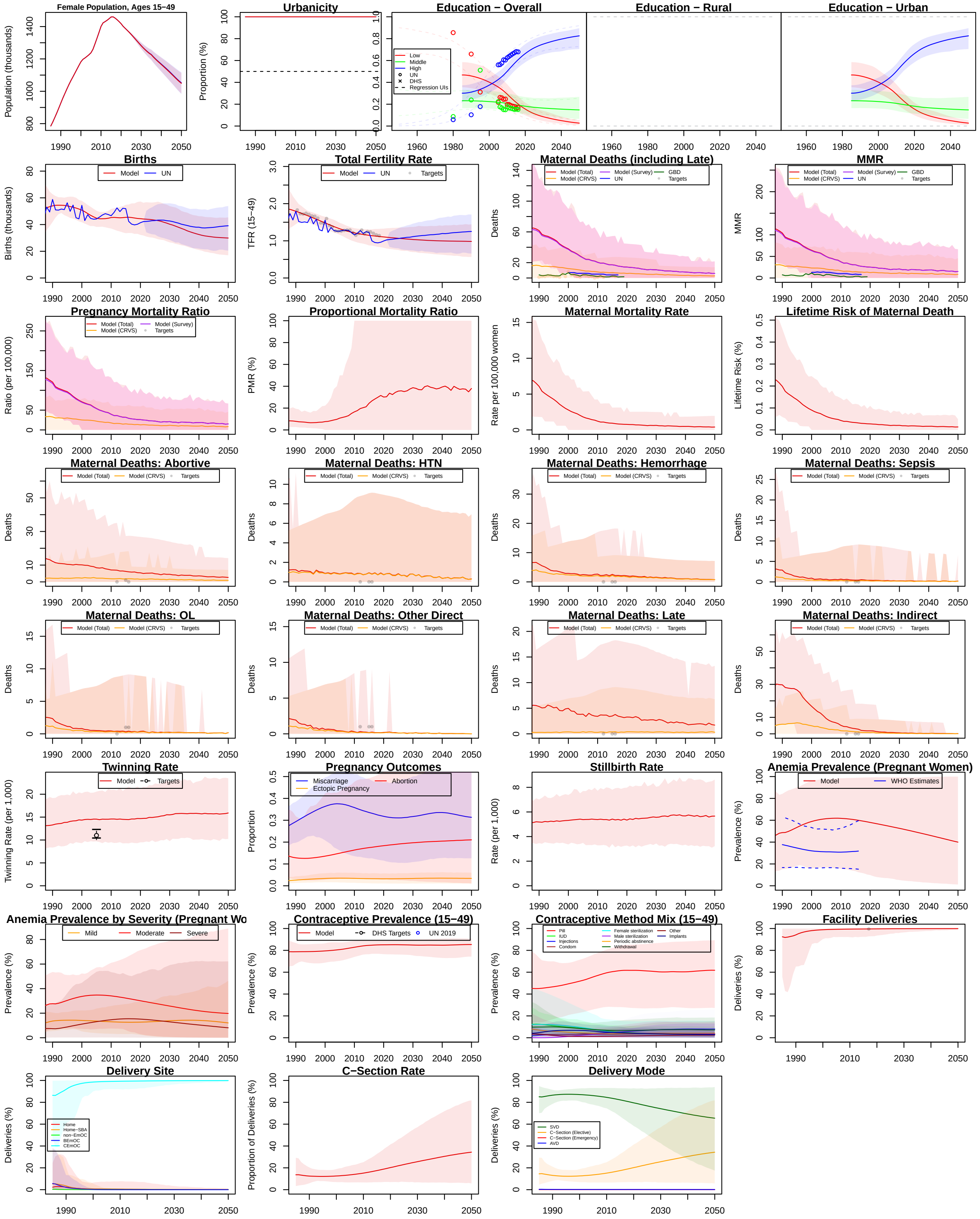

Slovakia

ISO Code	Region	Area	Income Group
SVK	Eastern Europe	Europe	High income

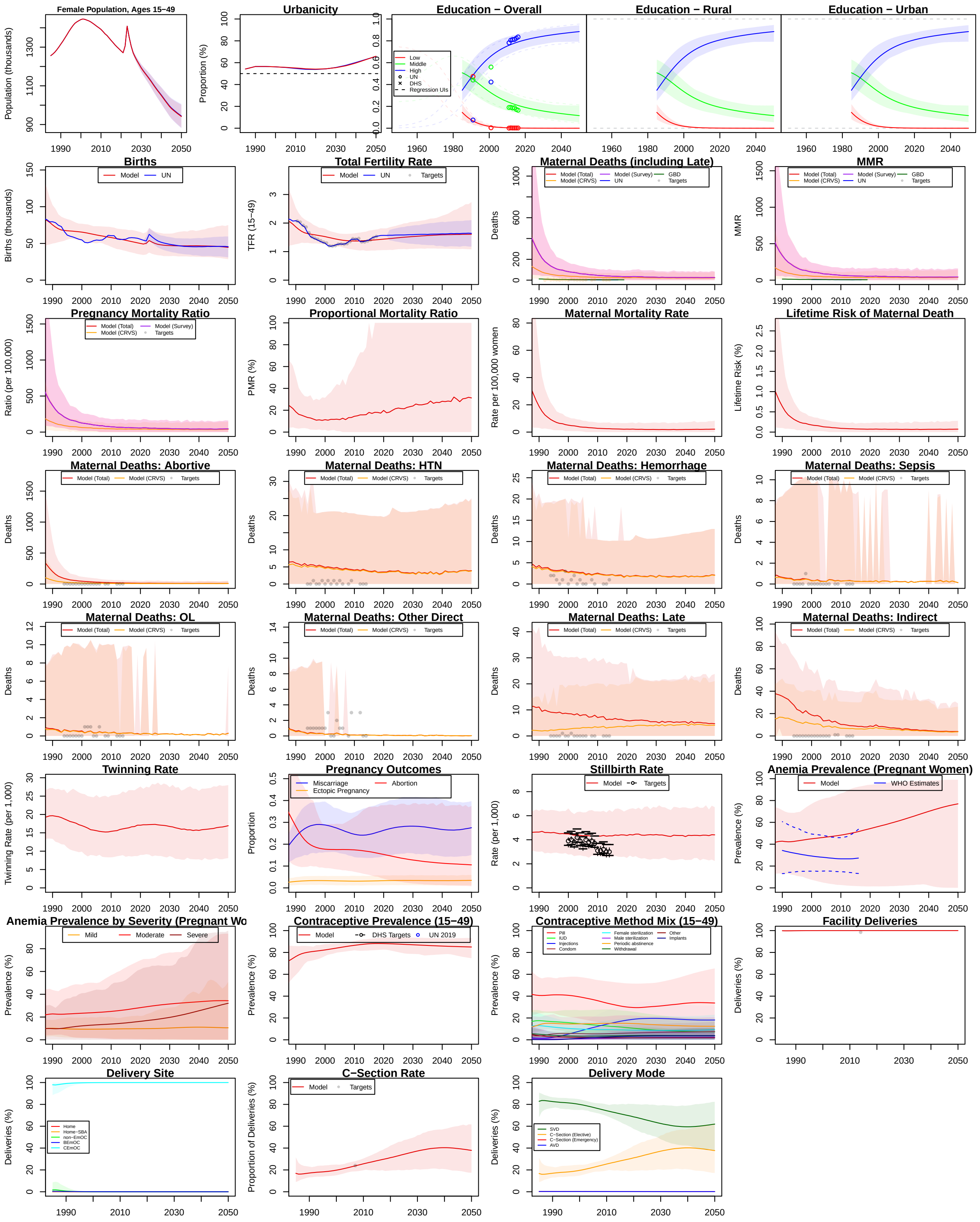

Slovenia

ISO Code	Region	Area	Income Group
SVN	Southern Europe	Europe	High income

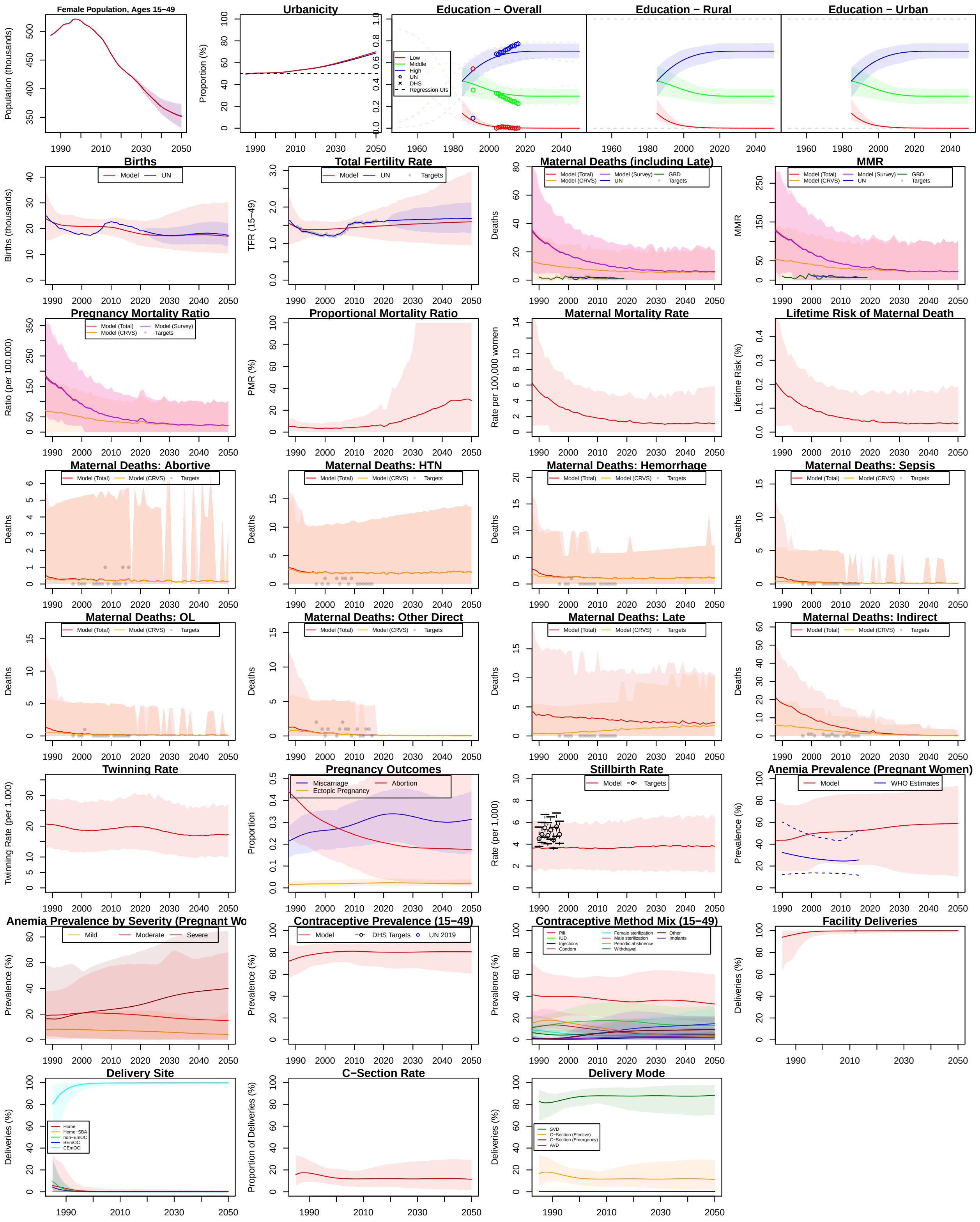

Solomon Islands

ISO Code	Region	Area	Income Group
SLB	Melanesia	Oceania	Lower middle income

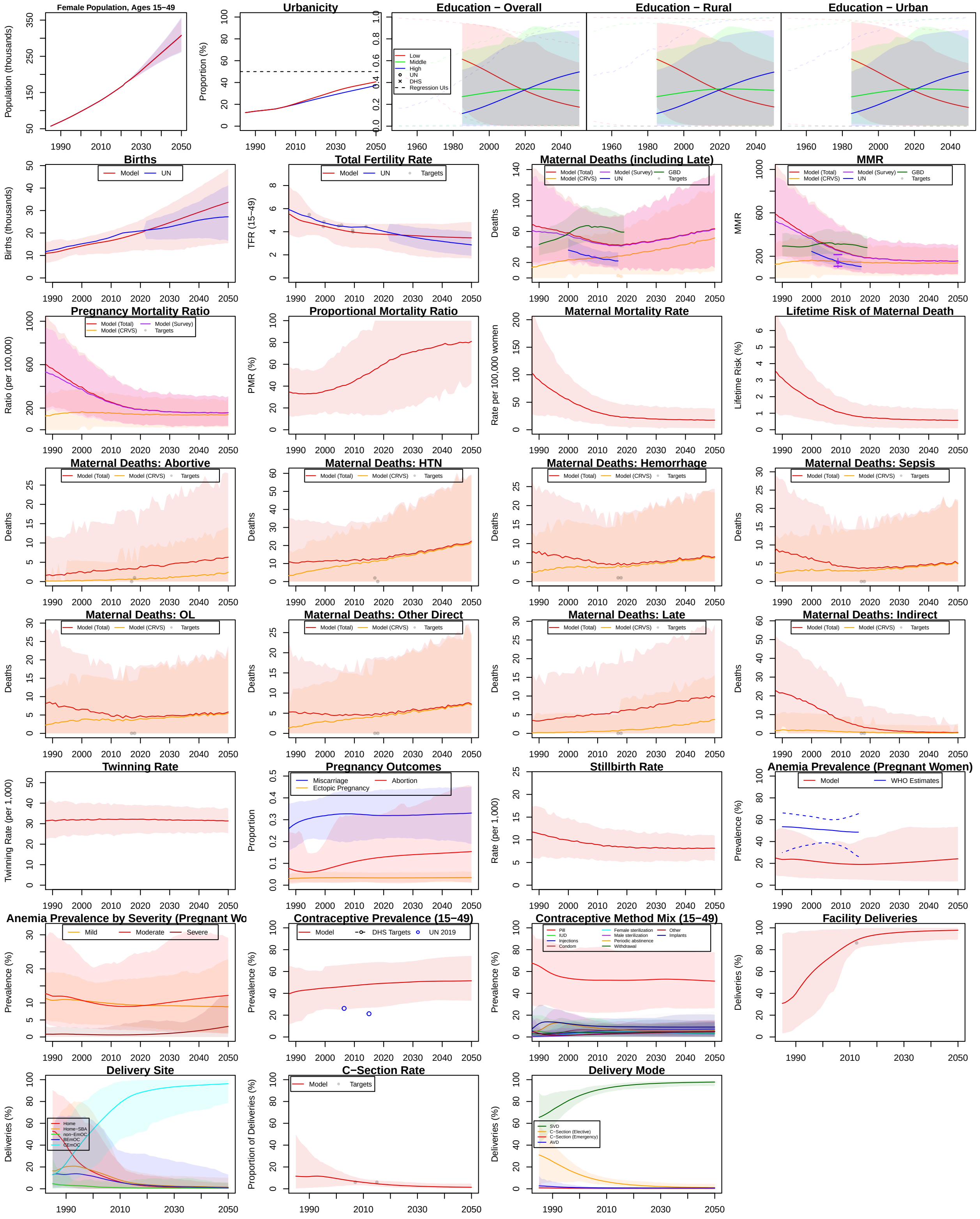

Somalia

ISO Code	Region	Area	Income Group
SOM	Eastern Africa	Africa	Low income

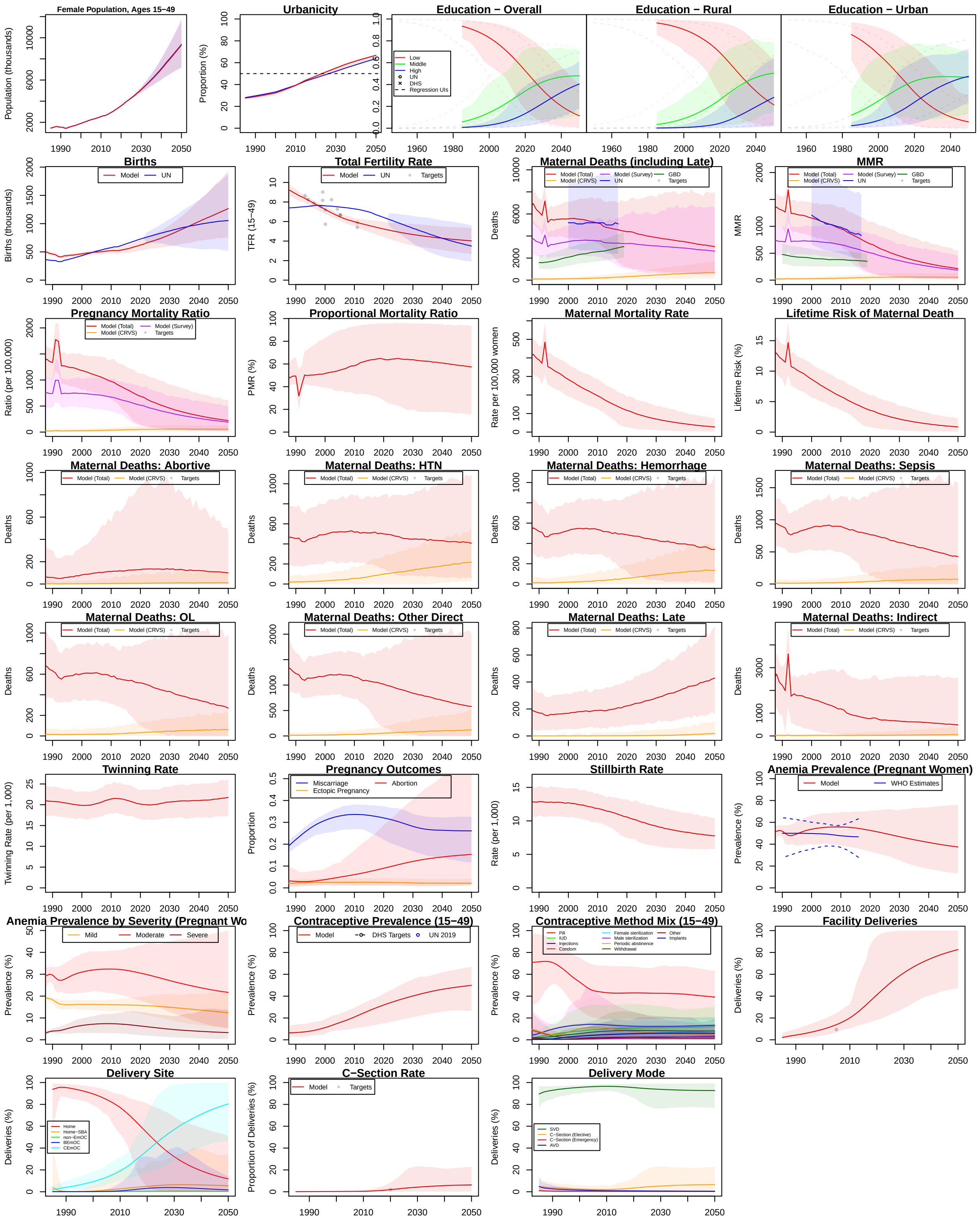

South Africa

ISO Code	Region	Area	Income Group
ZAF	Southern Africa	Africa	Upper middle income

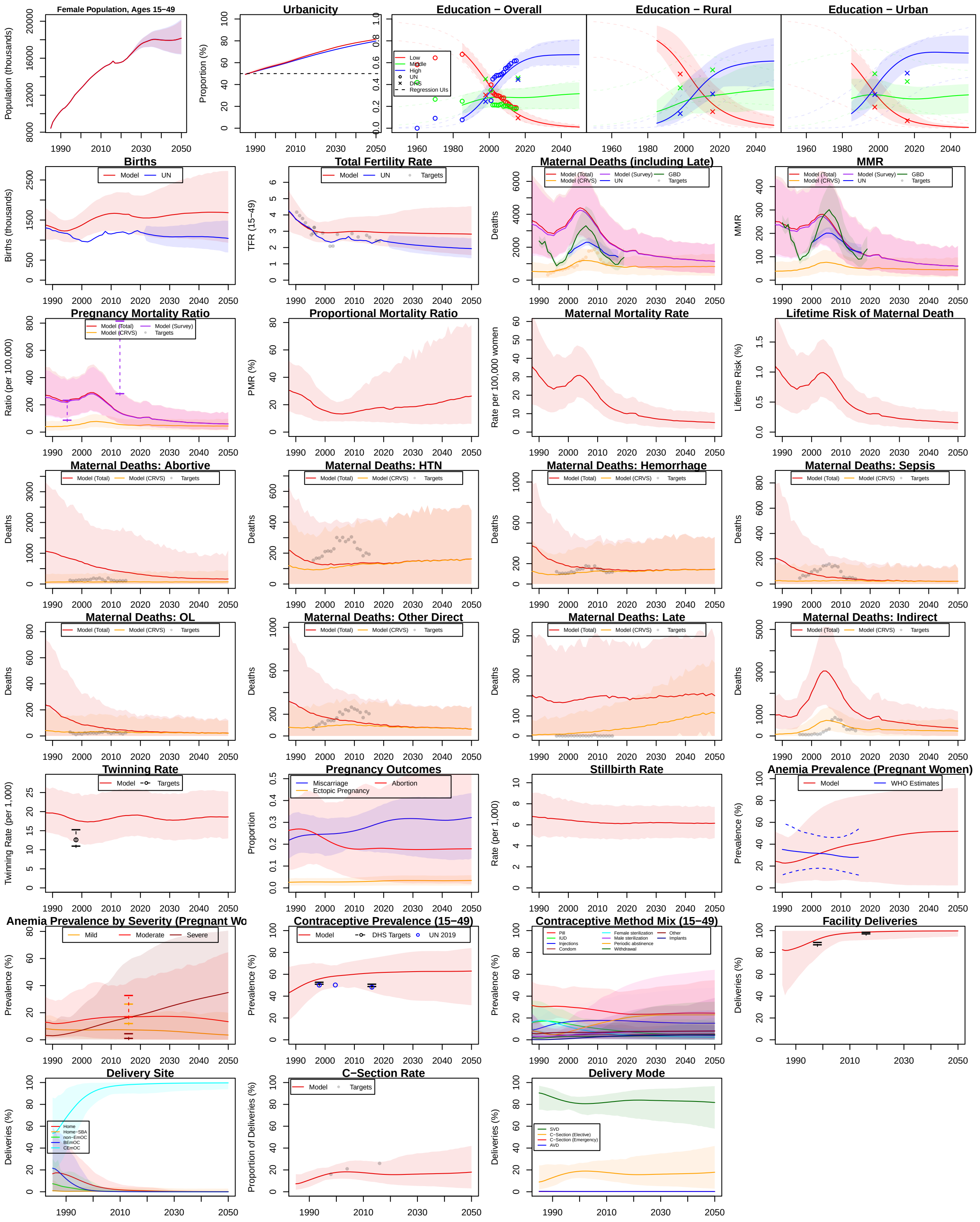

South Sudan

ISO Code	Region	Area	Income Group
SSD	Eastern Africa	Africa	Low income

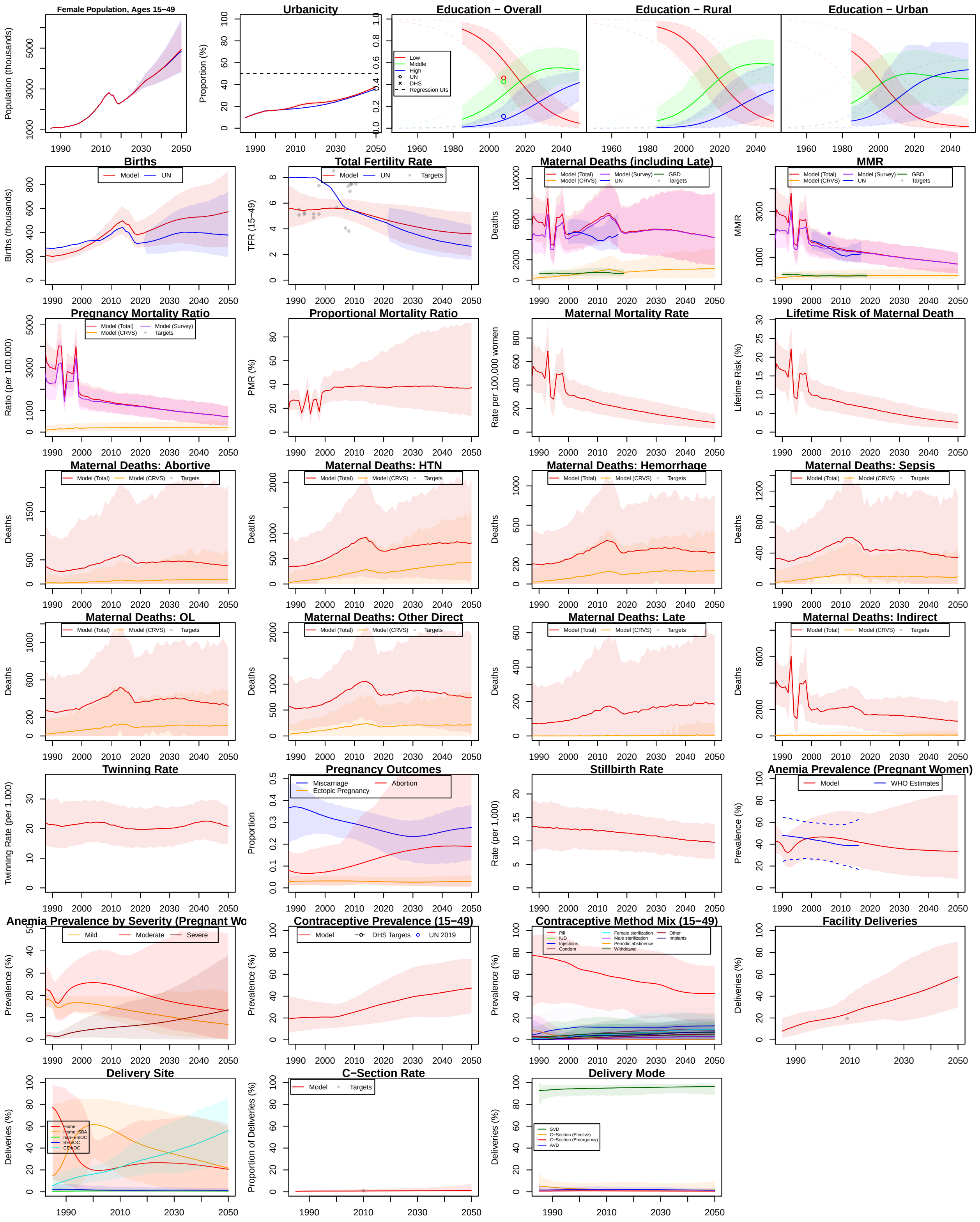

Spain

ISO Code	Region	Area	Income Group
ESP	Southern Europe	Europe	High income

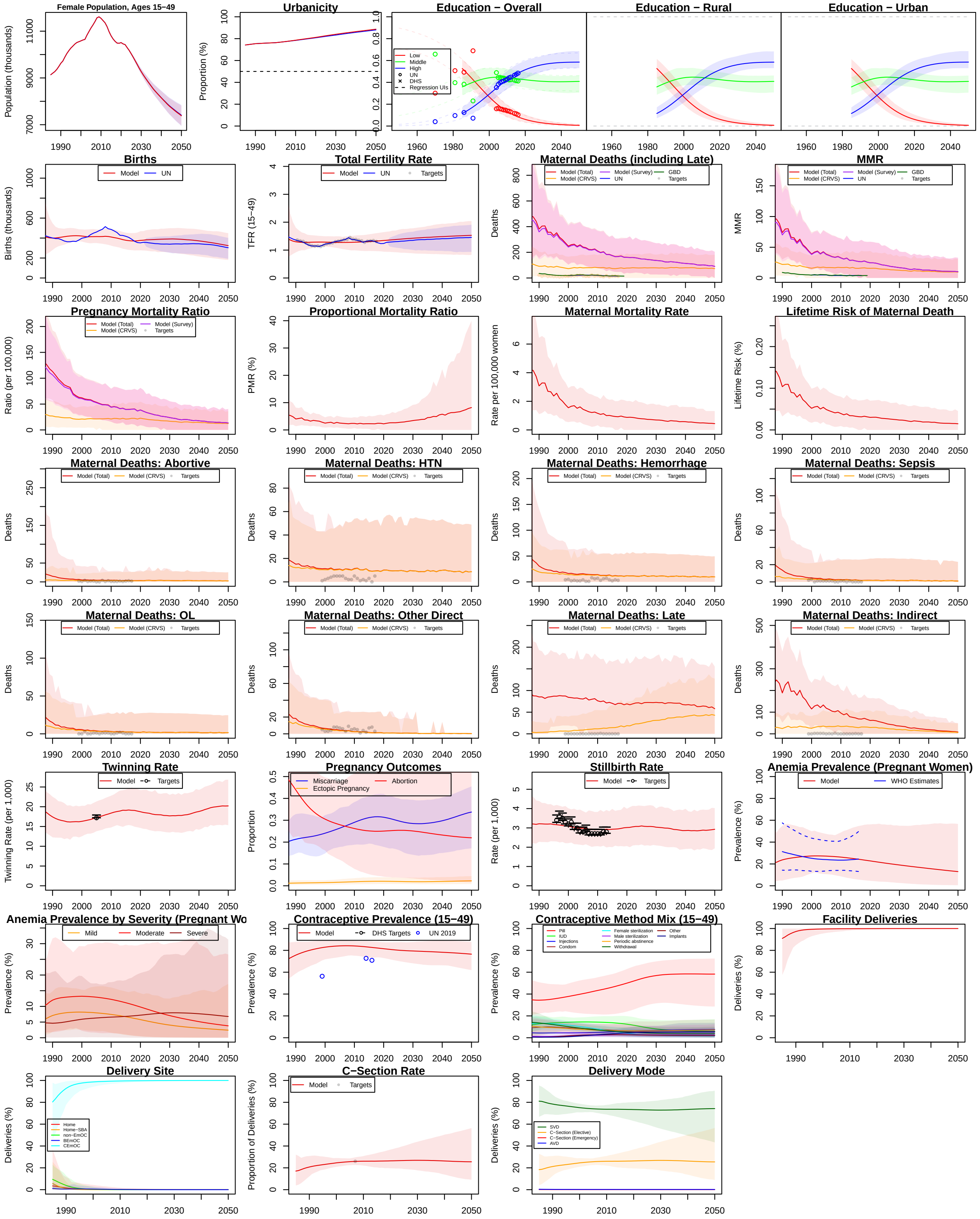

Sri Lanka

ISO Code	Region	Area	Income Group
LKA	Southern Asia	Asia	Lower middle income

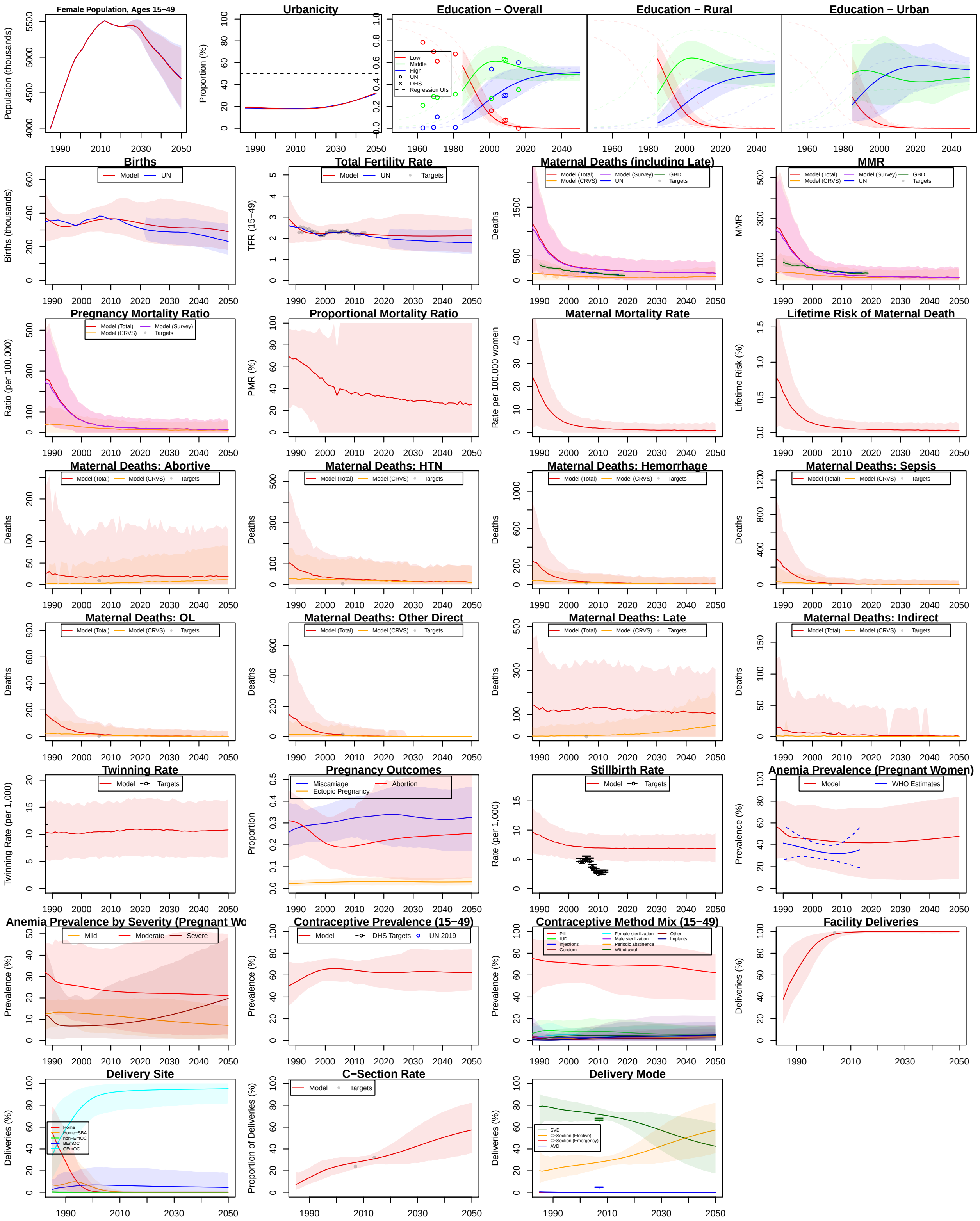

State of Palestine

ISO Code	Region	Area	Income Group
PSE	Western Asia	Asia	Lower middle income

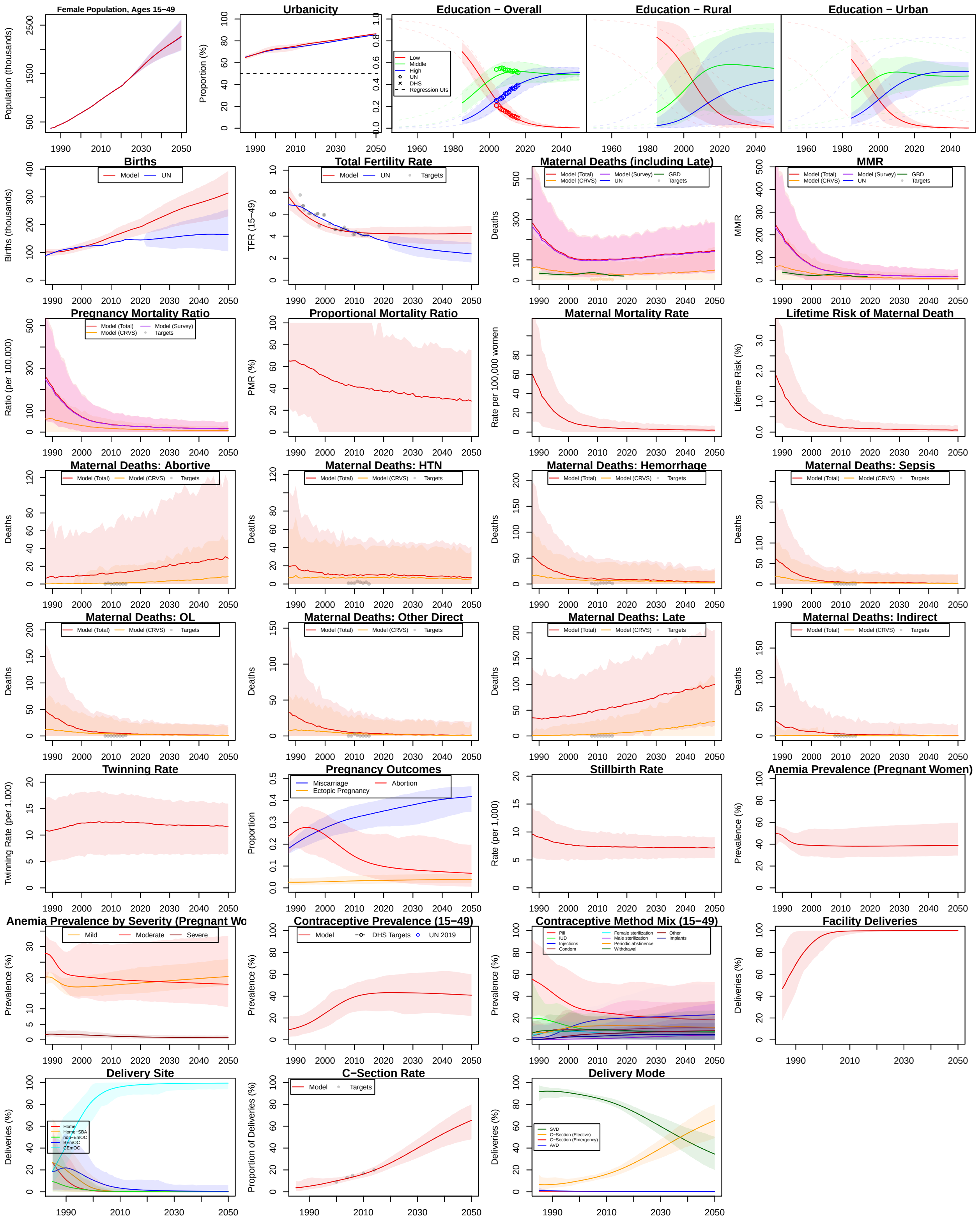

Sudan

ISO Code	Region	Area	Income Group
SDN	Northern Africa	Africa	Lower middle income

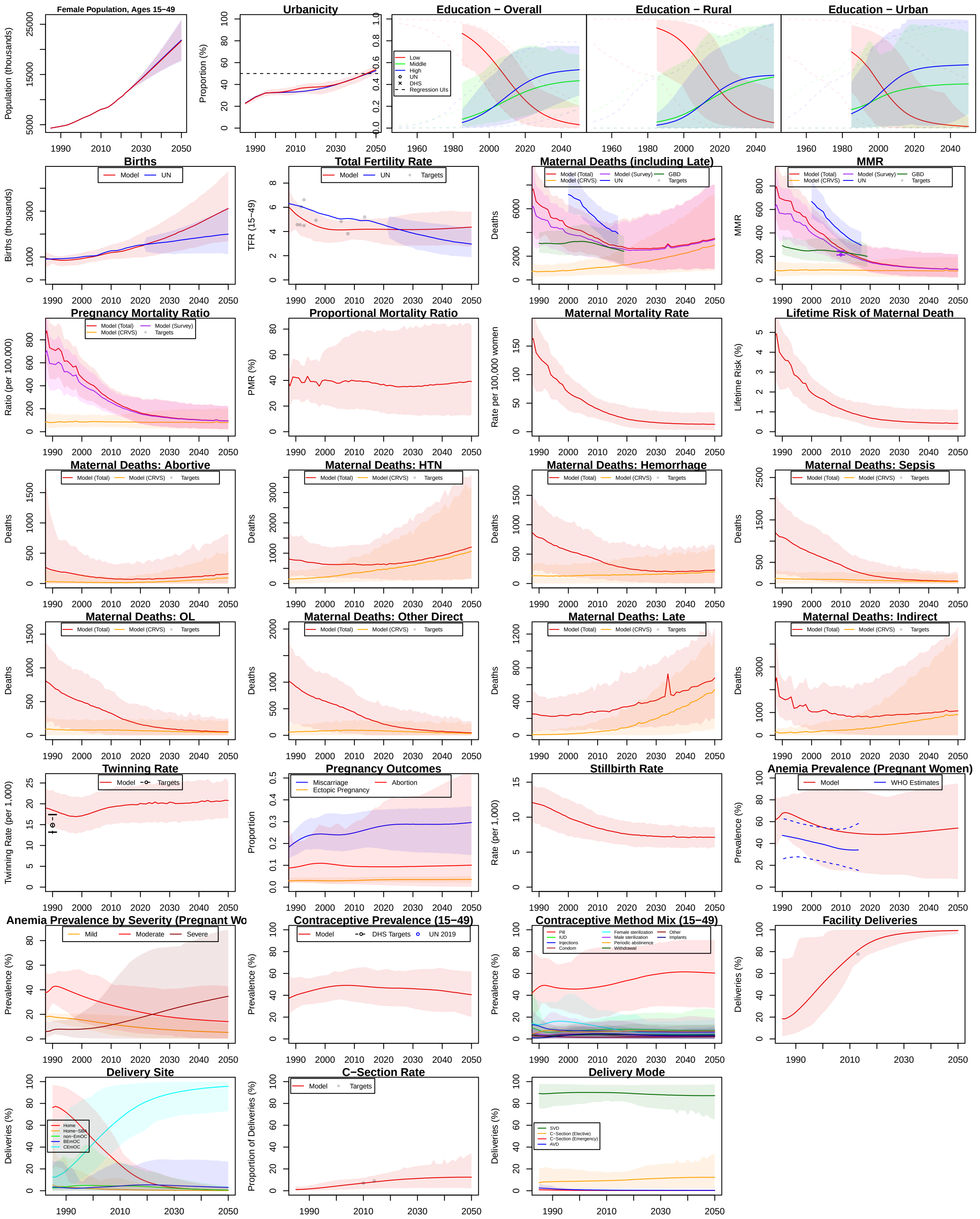

Suriname

ISO Code	Region	Area	Income Group
SUR	South America	Latin America and the Caribbean	Upper middle income

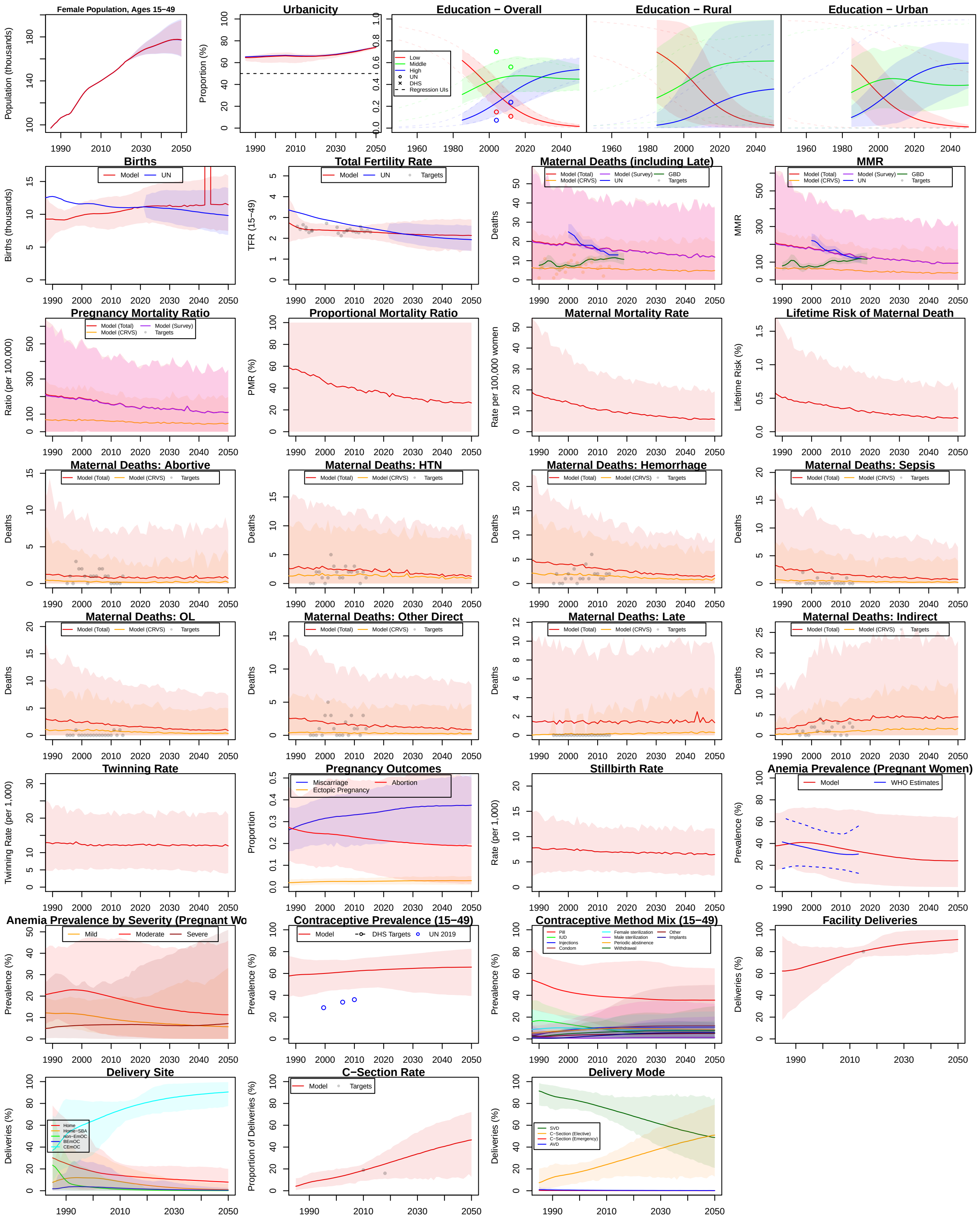

Sweden

ISO Code	Region	Area	Income Group
SWE	Northern Europe	Europe	High income

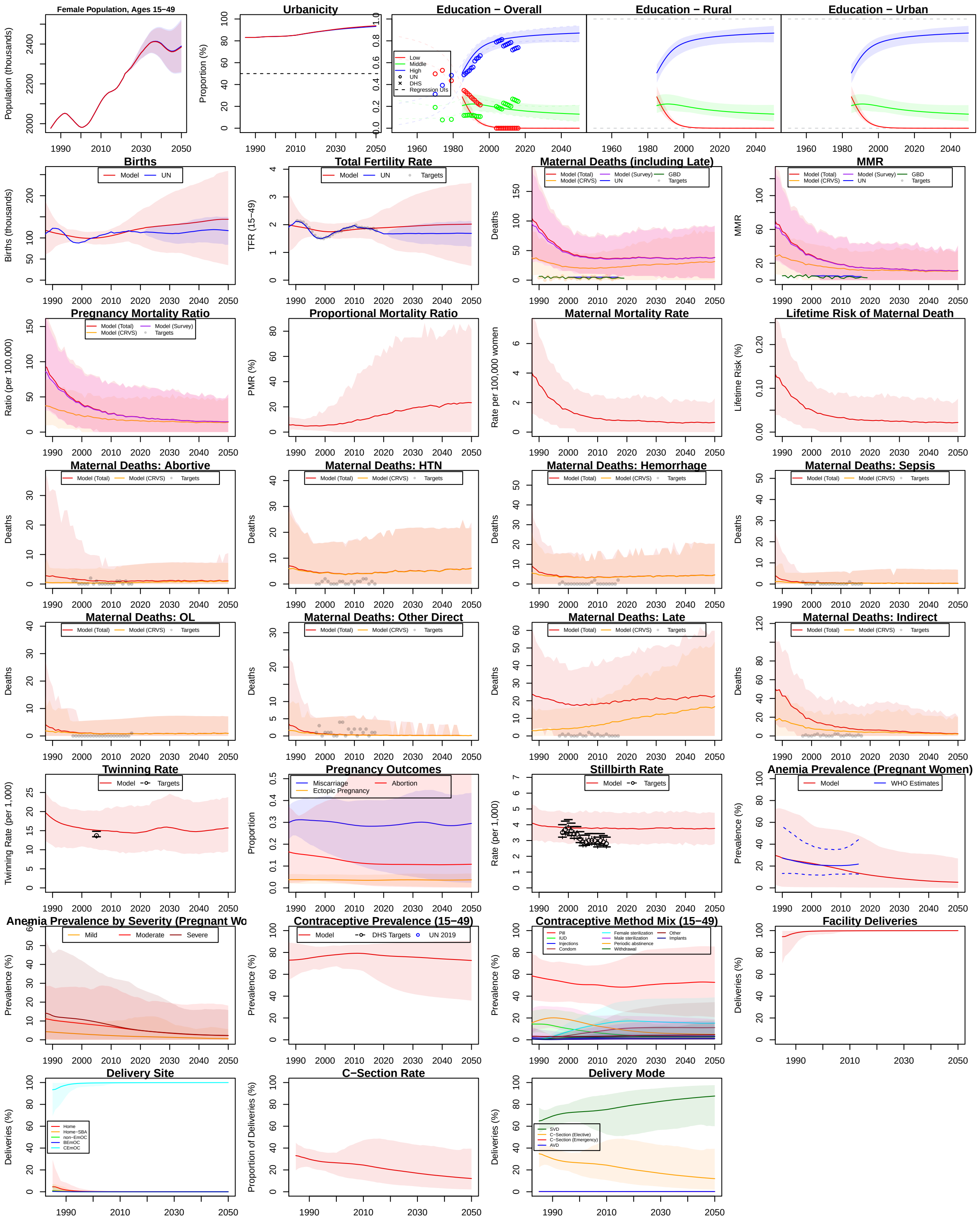

Switzerland

ISO Code	Region	Area	Income Group
CHE	Western Europe	Europe	High income

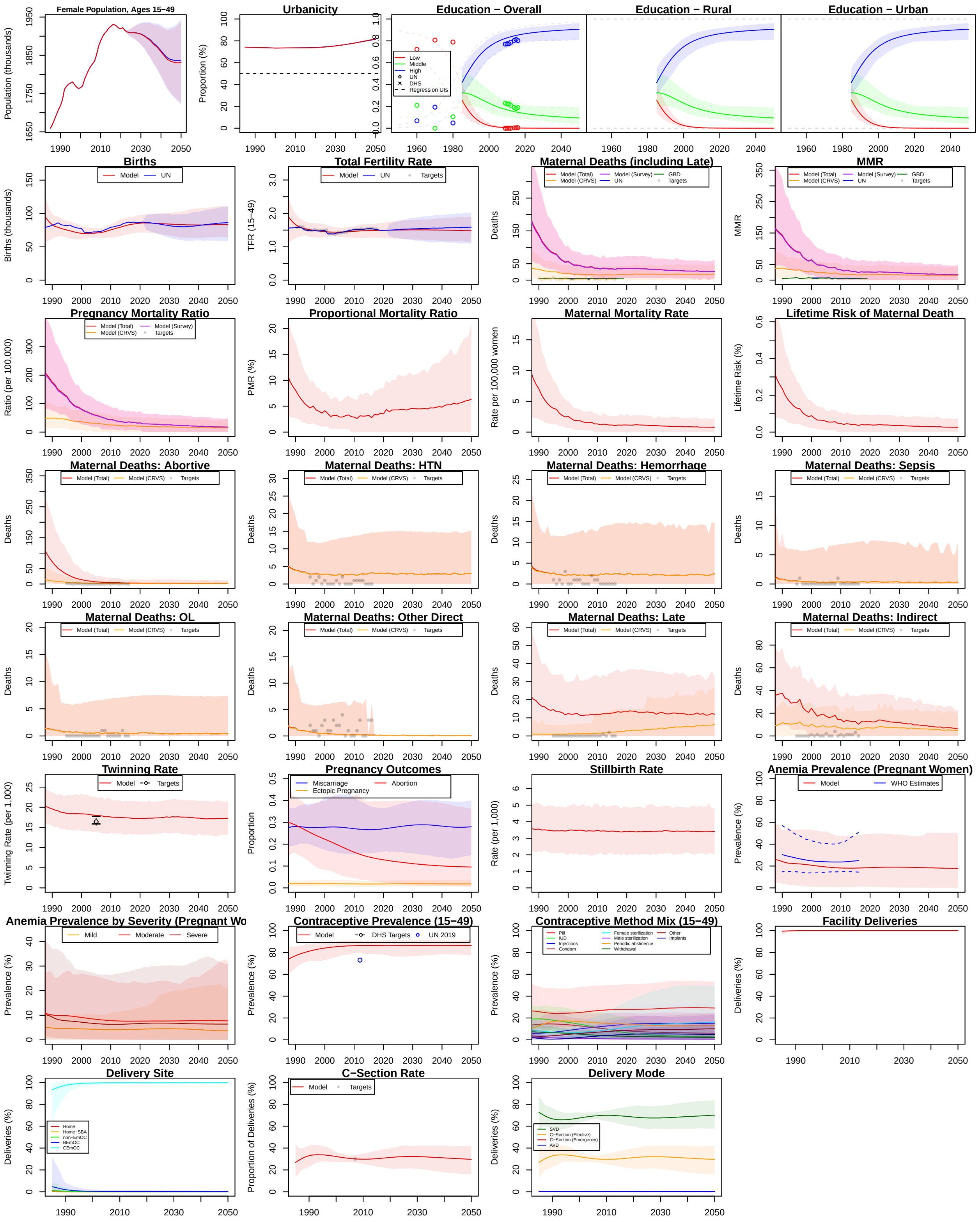

Syrian Arab Republic

ISO Code	Region	Area	Income Group
SYR	Western Asia	Asia	Low income

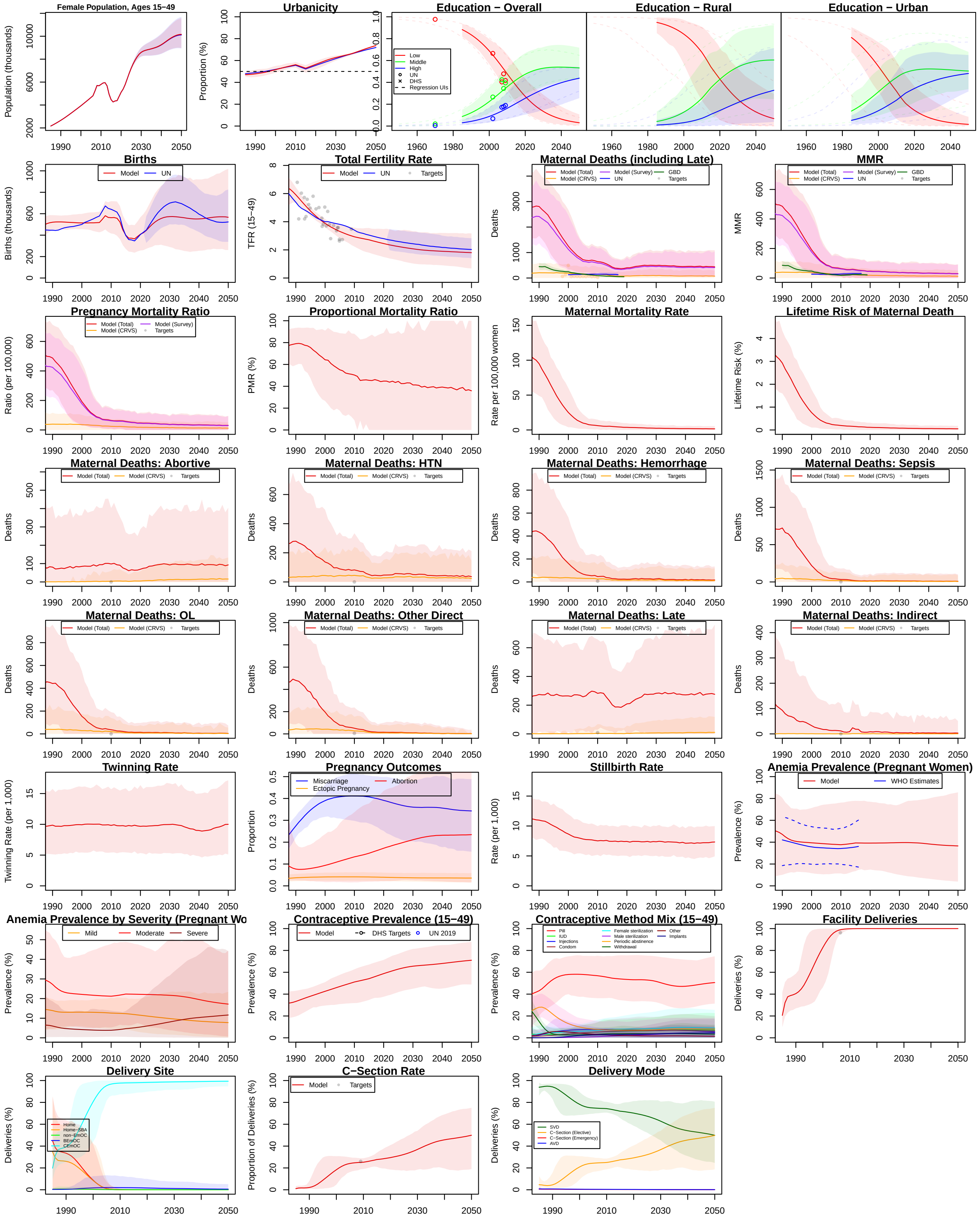

Taiwan

ISO Code	Region	Area	Income Group
TWN	Eastern Asia	Asia	High income

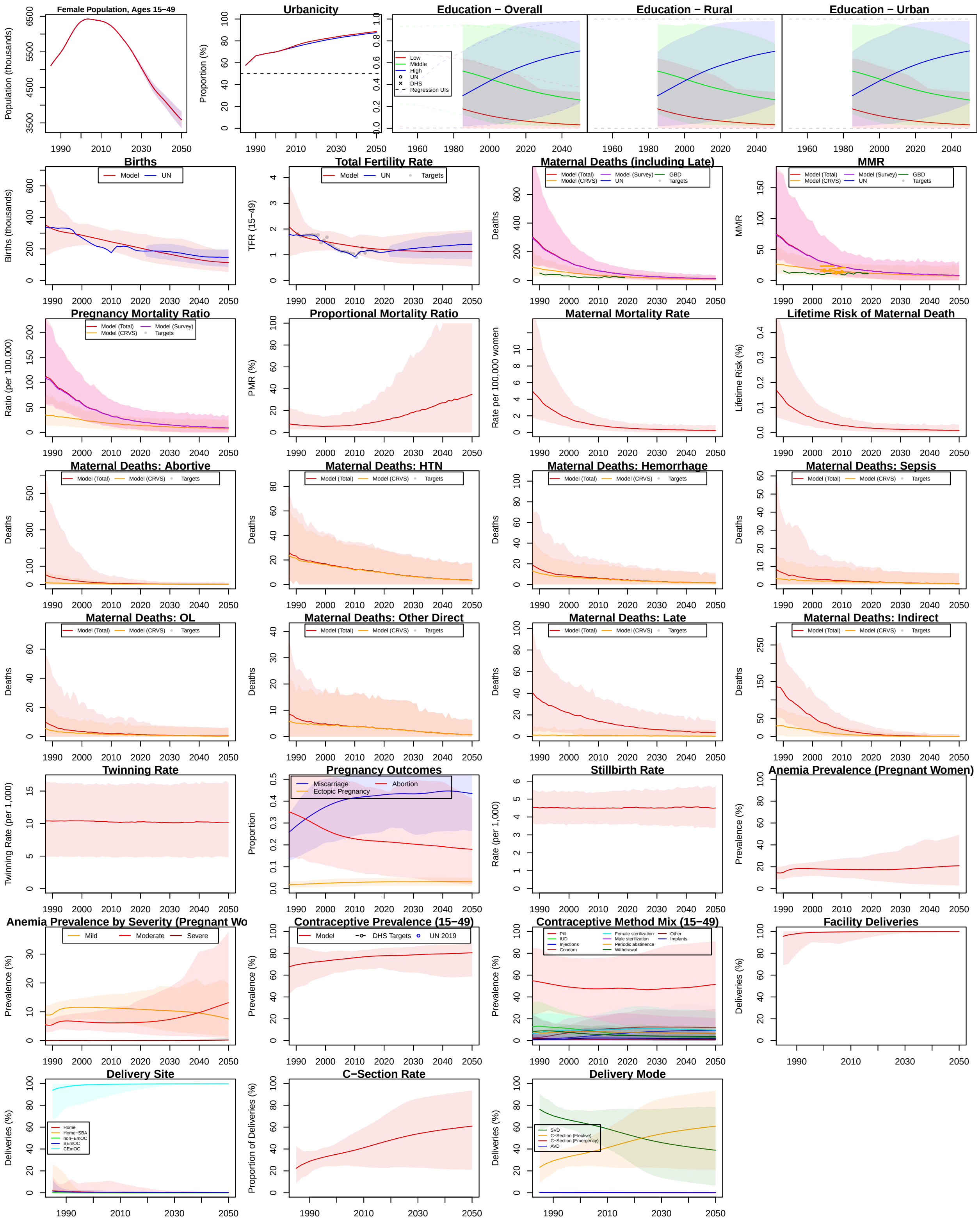

Tajikistan

ISO Code	Region	Area	Income Group
TJK	Central Asia	Asia	Low income

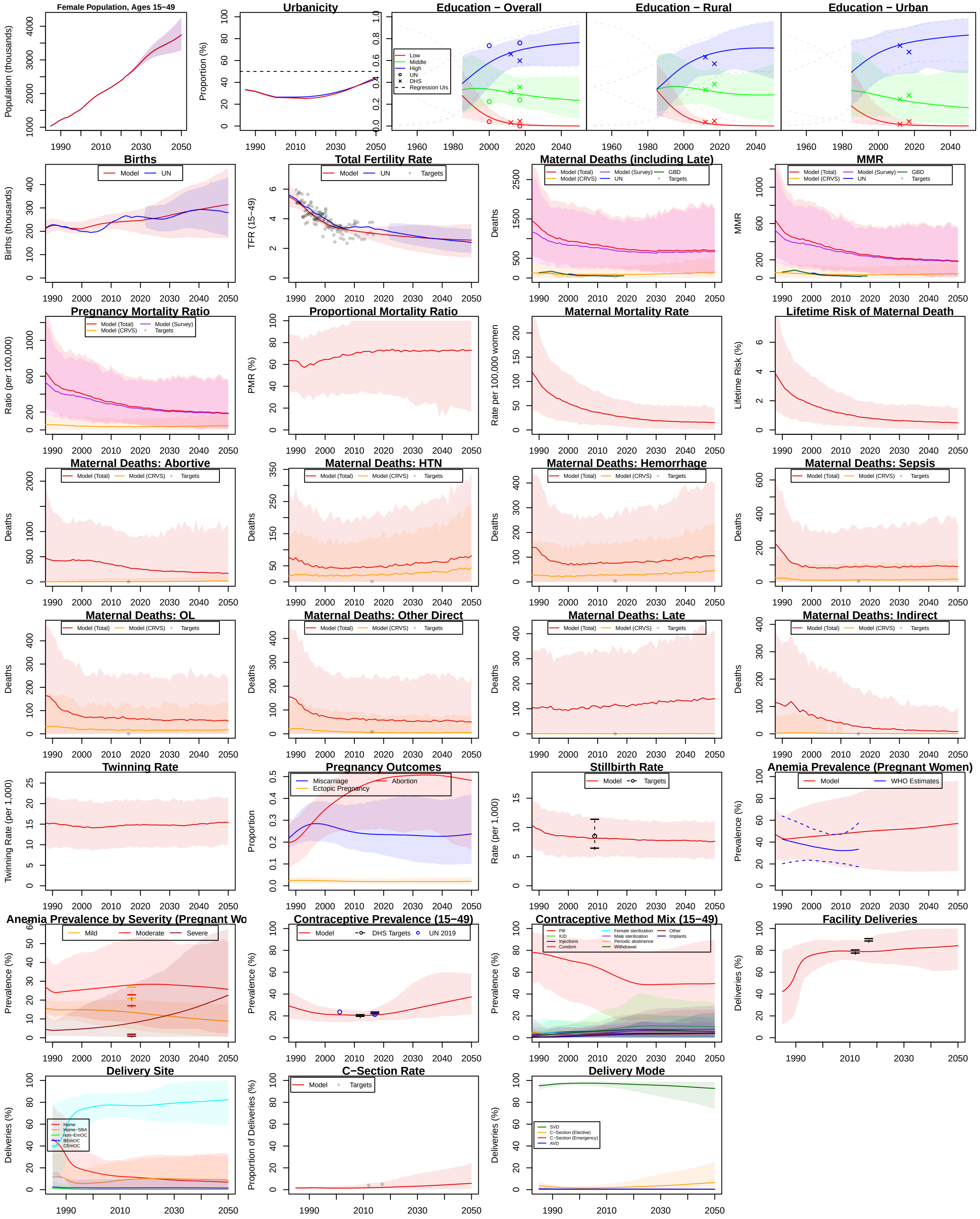

Thailand

ISO Code	Region	Area	Income Group
THA	South-Eastern Asia	Asia	Upper middle income

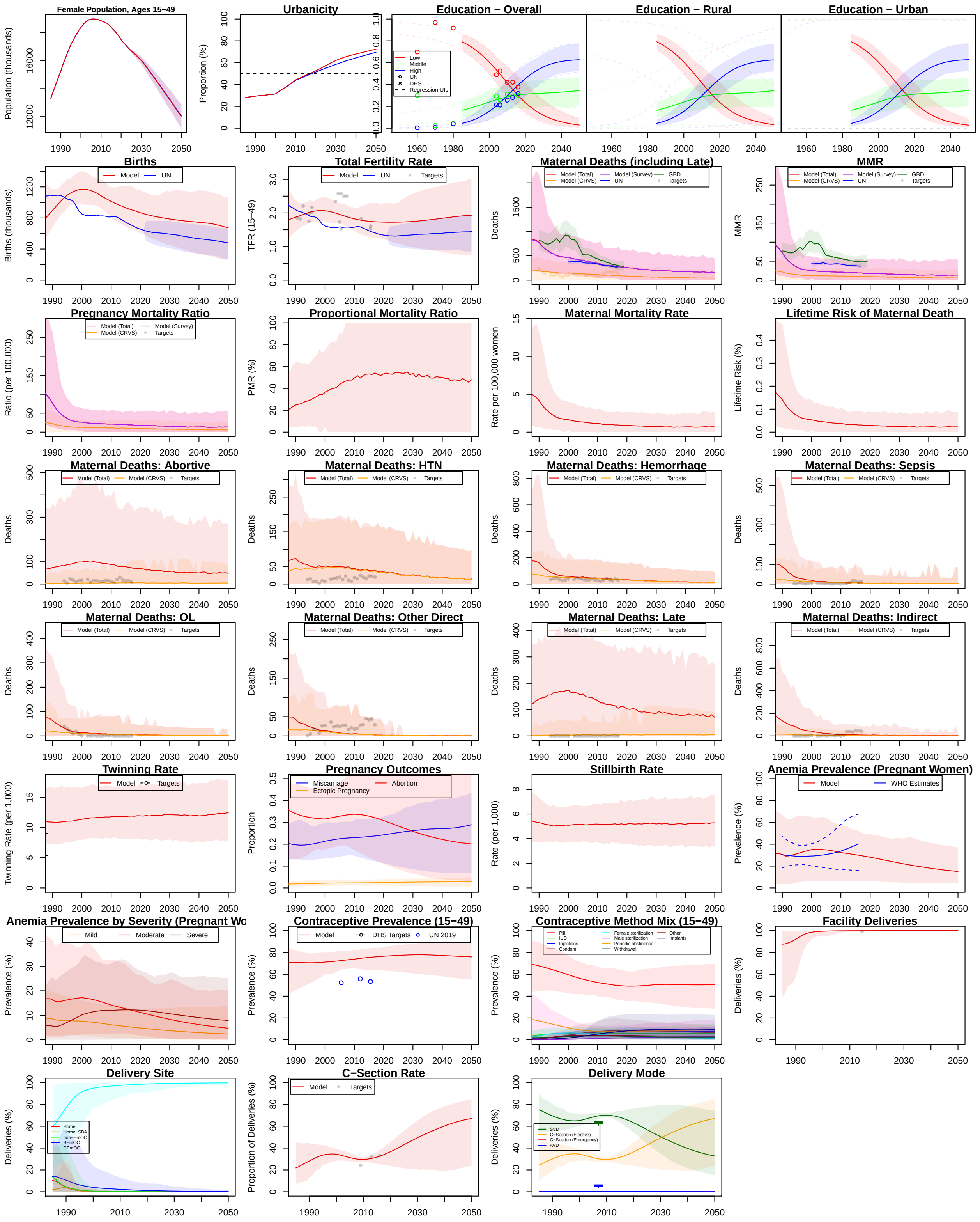

Timor-Leste

ISO Code	Region	Area	Income Group
TLS	South-Eastern Asia	Asia	Lower middle income

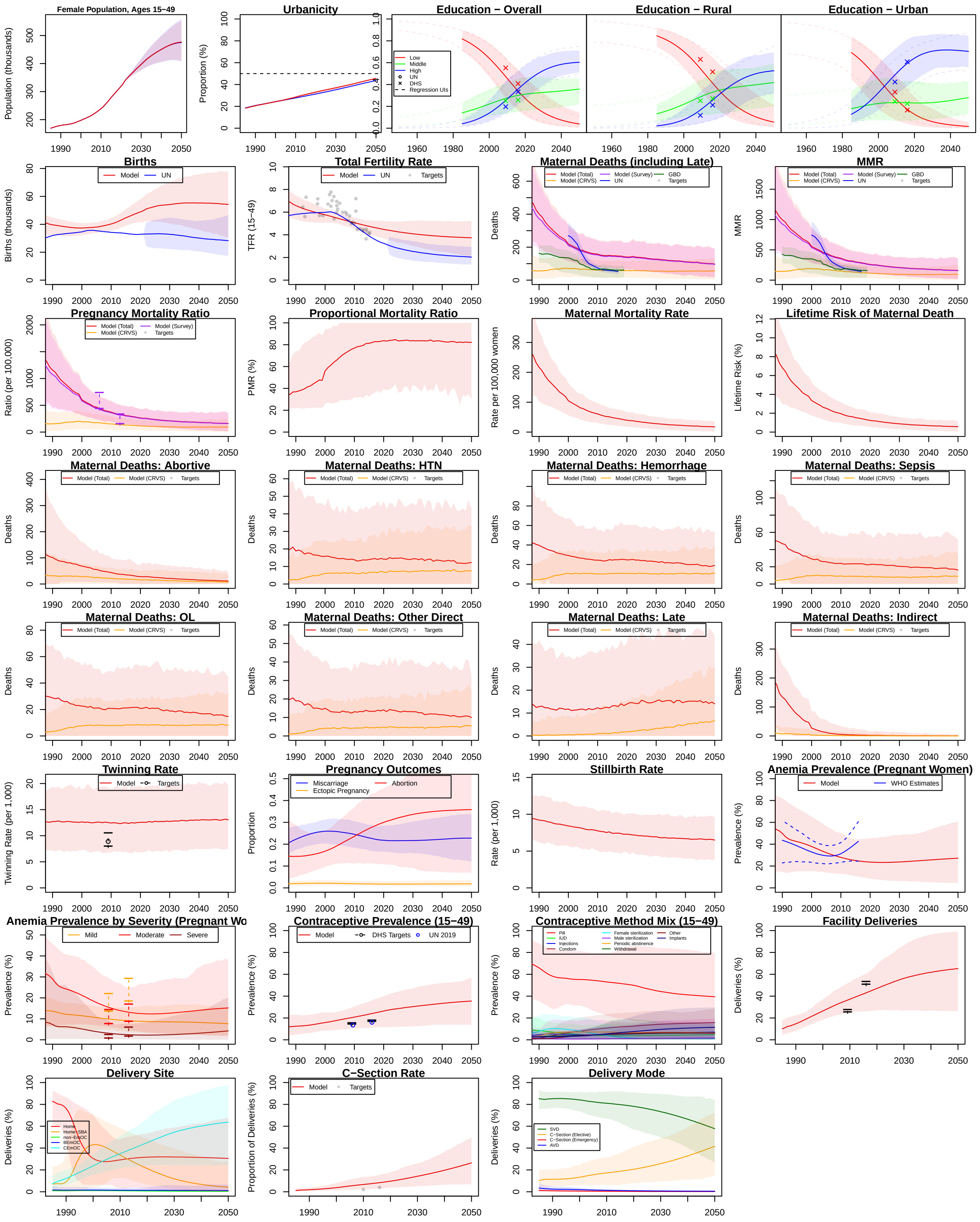

Togo

ISO Code	Region	Area	Income Group
TGO	Western Africa	Africa	Low income

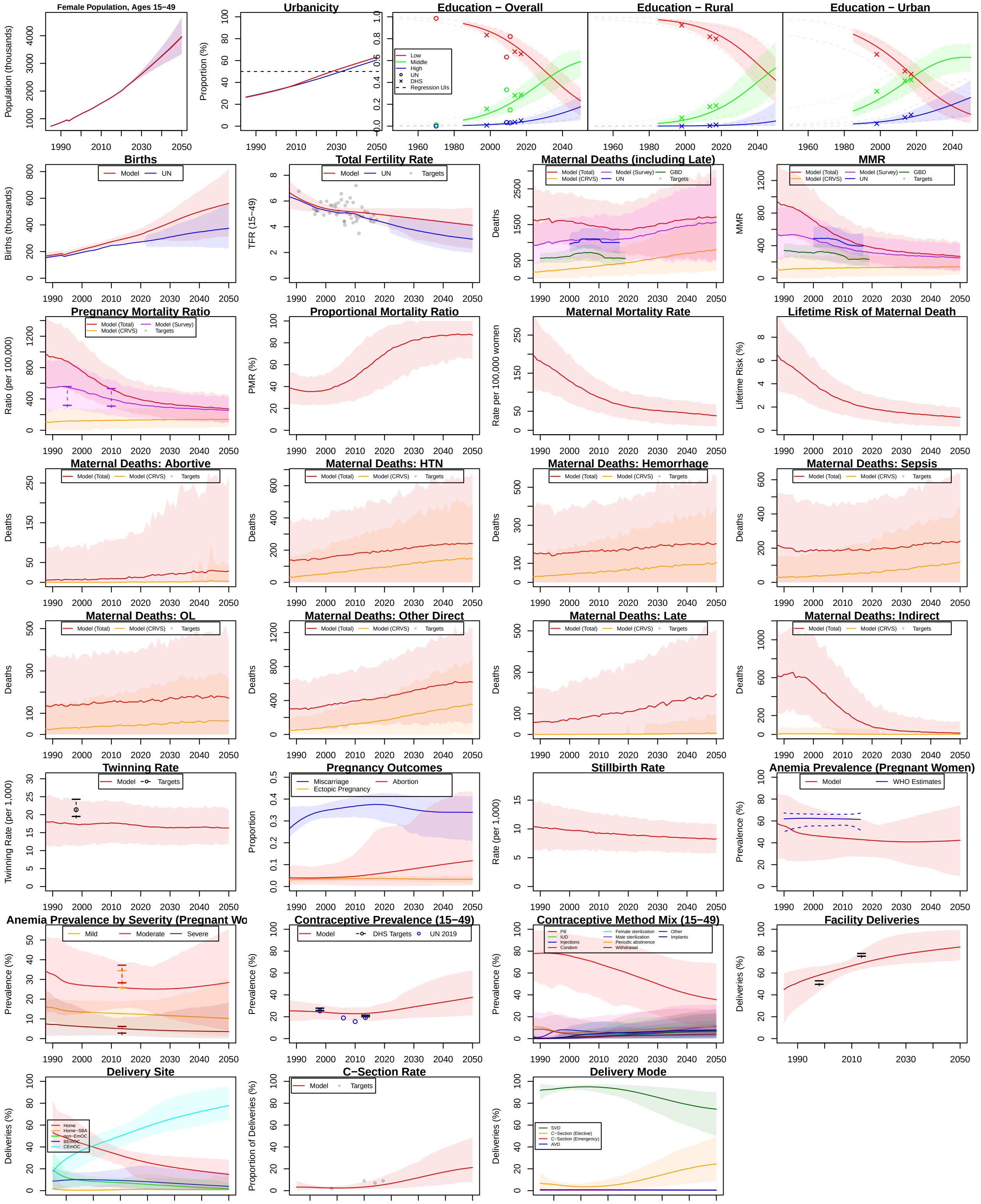

Tonga

ISO Code	Region	Area	Income Group
TON	Polynesia	Oceania	Upper middle income

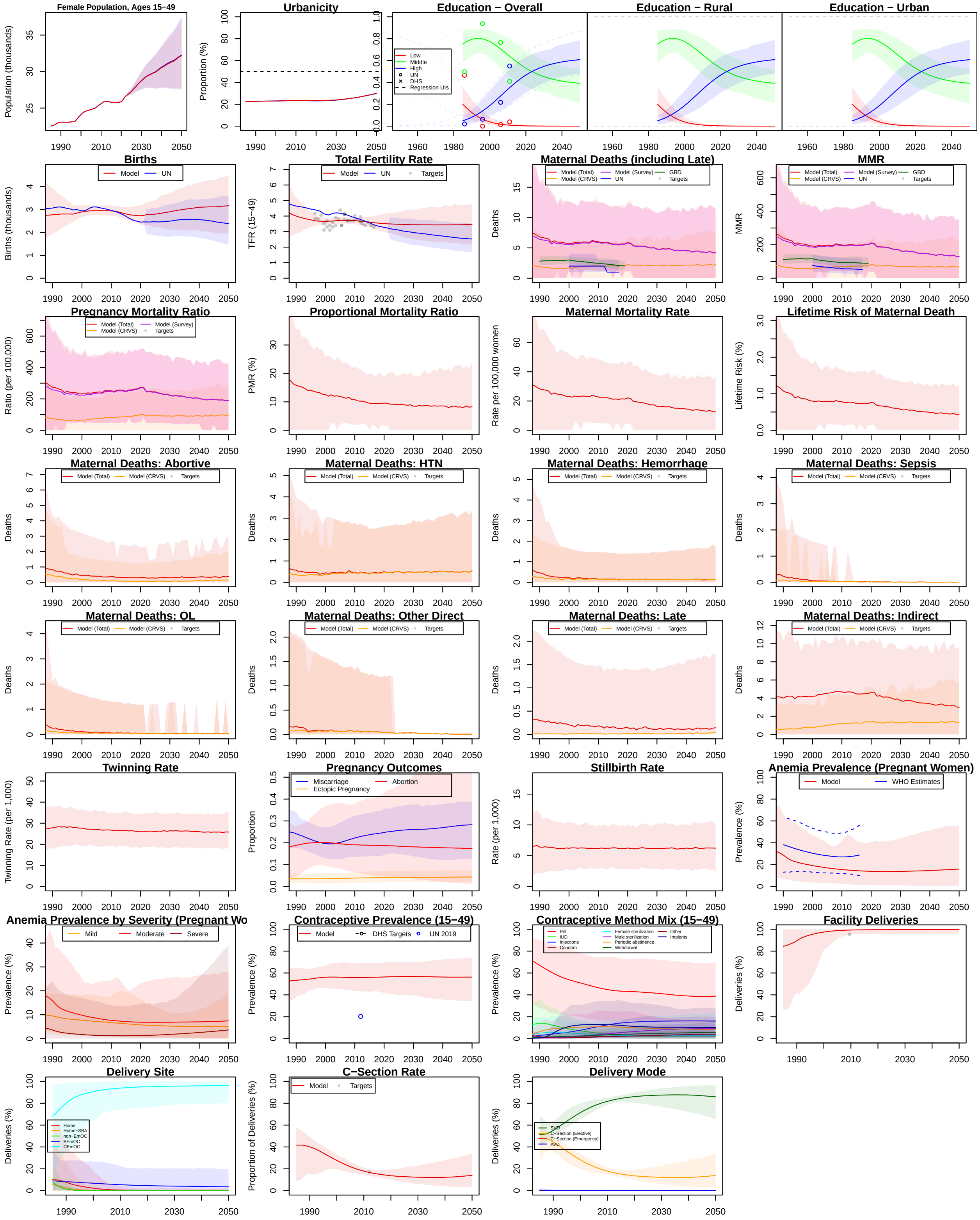

Trinidad and Tobago

ISO Code	Region	Area	Income Group
TTO	Caribbean	Latin America and the Caribbean	High income

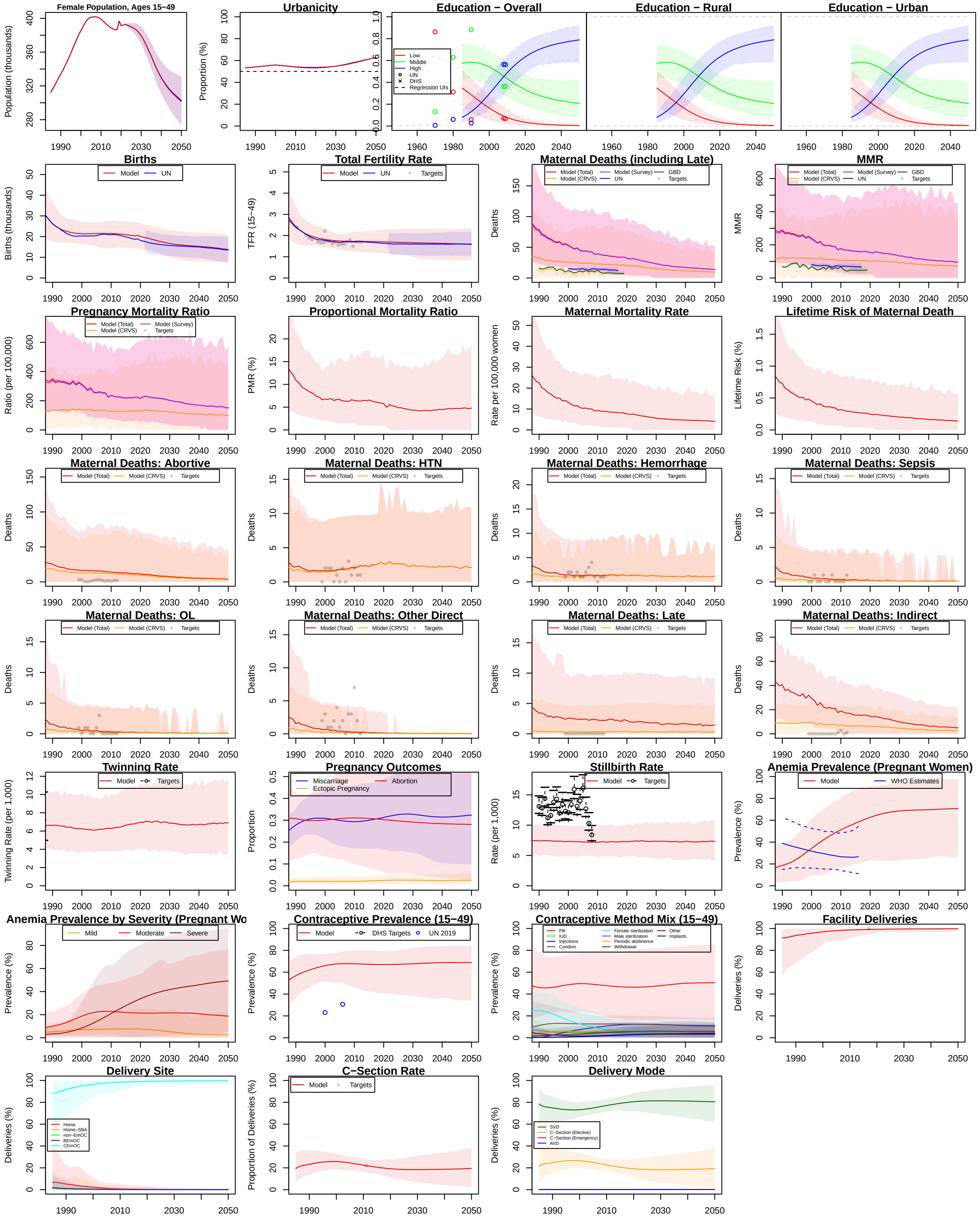

Tunisia

ISO Code	Region	Area	Income Group
TUN	Northern Africa	Africa	Lower middle income

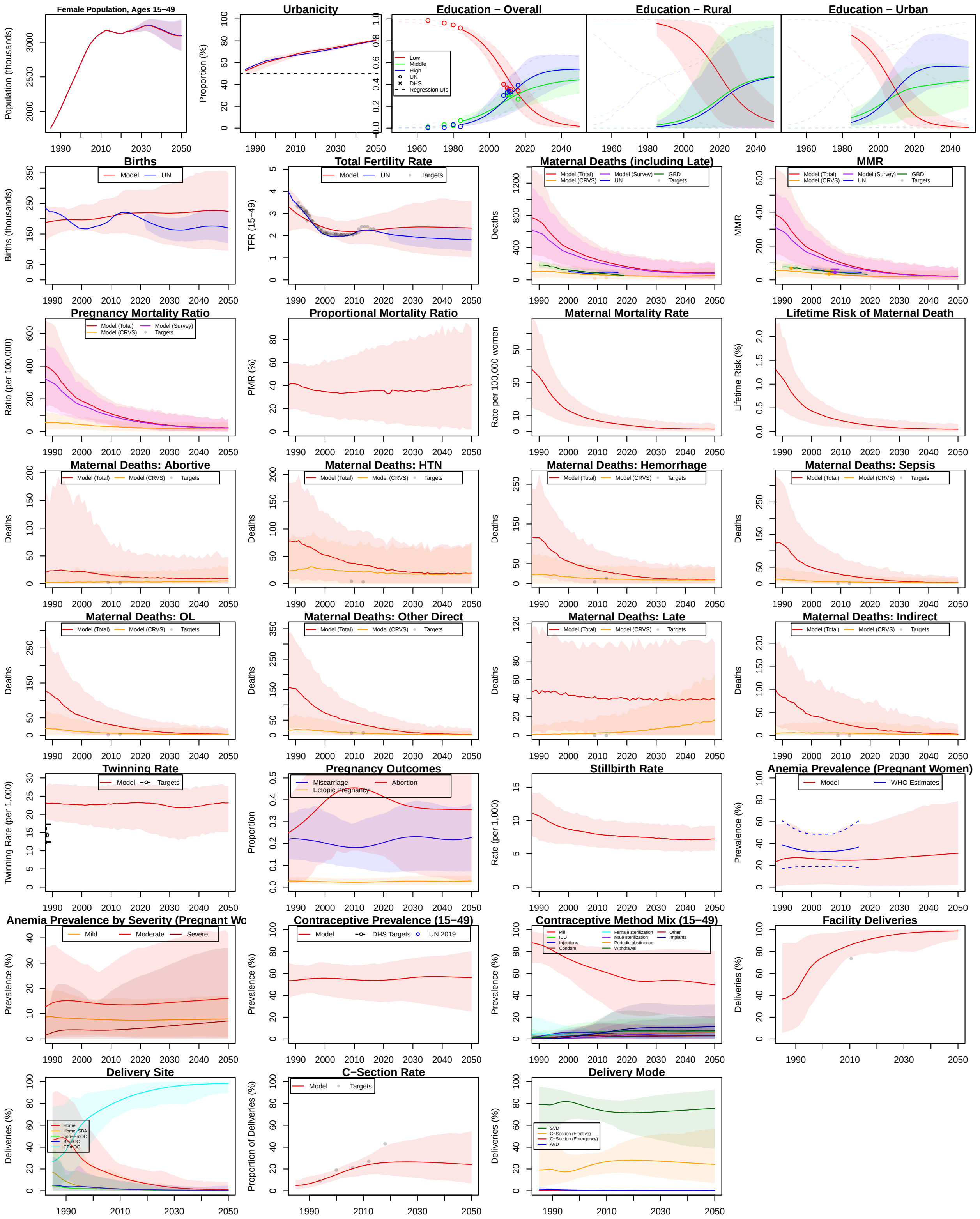

Türkiye

ISO Code	Region	Area	Income Group
TUR	Western Asia	Asia	Upper middle income

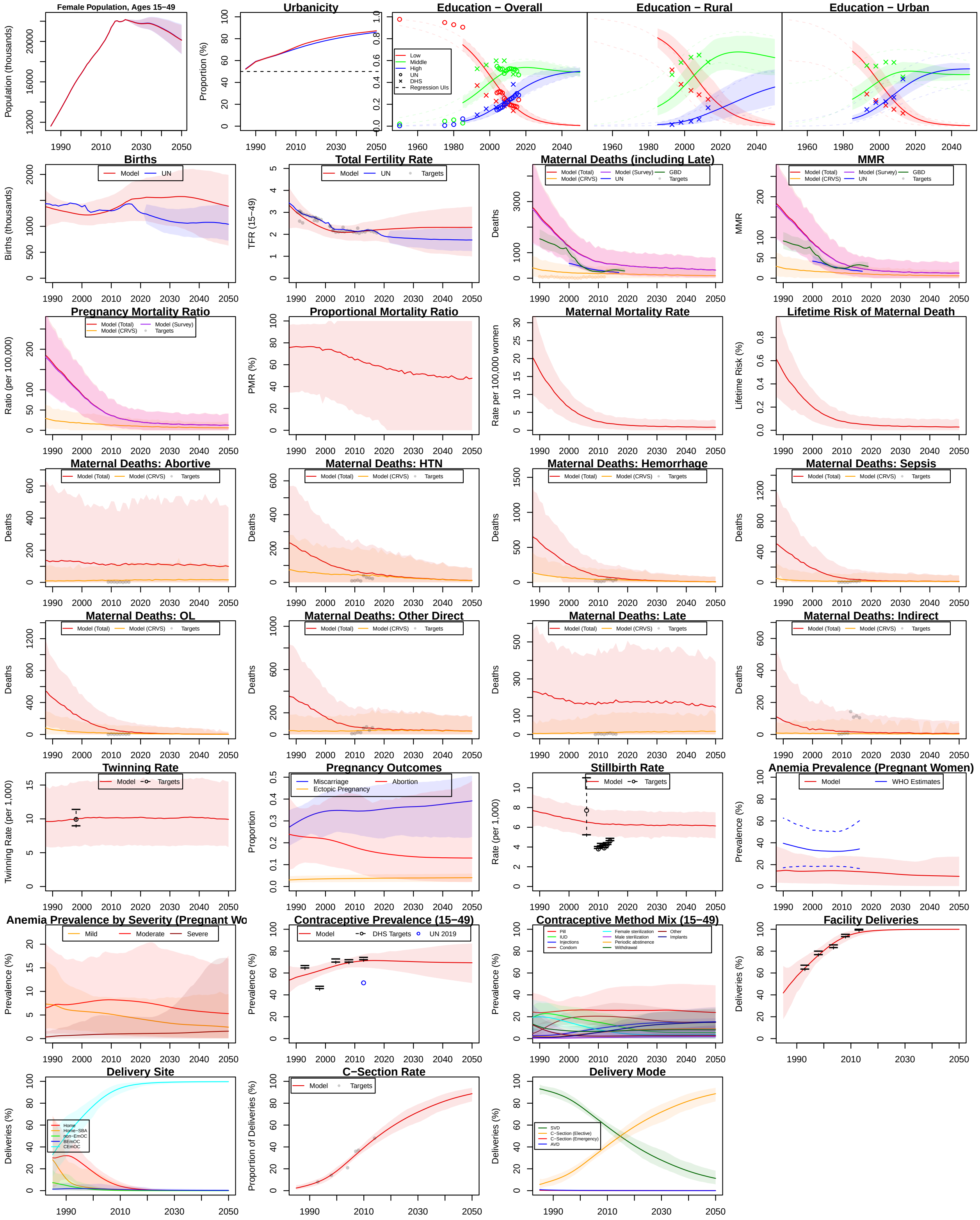

Turkmenistan

ISO Code	Region	Area	Income Group
TKM	Central Asia	Asia	Upper middle income

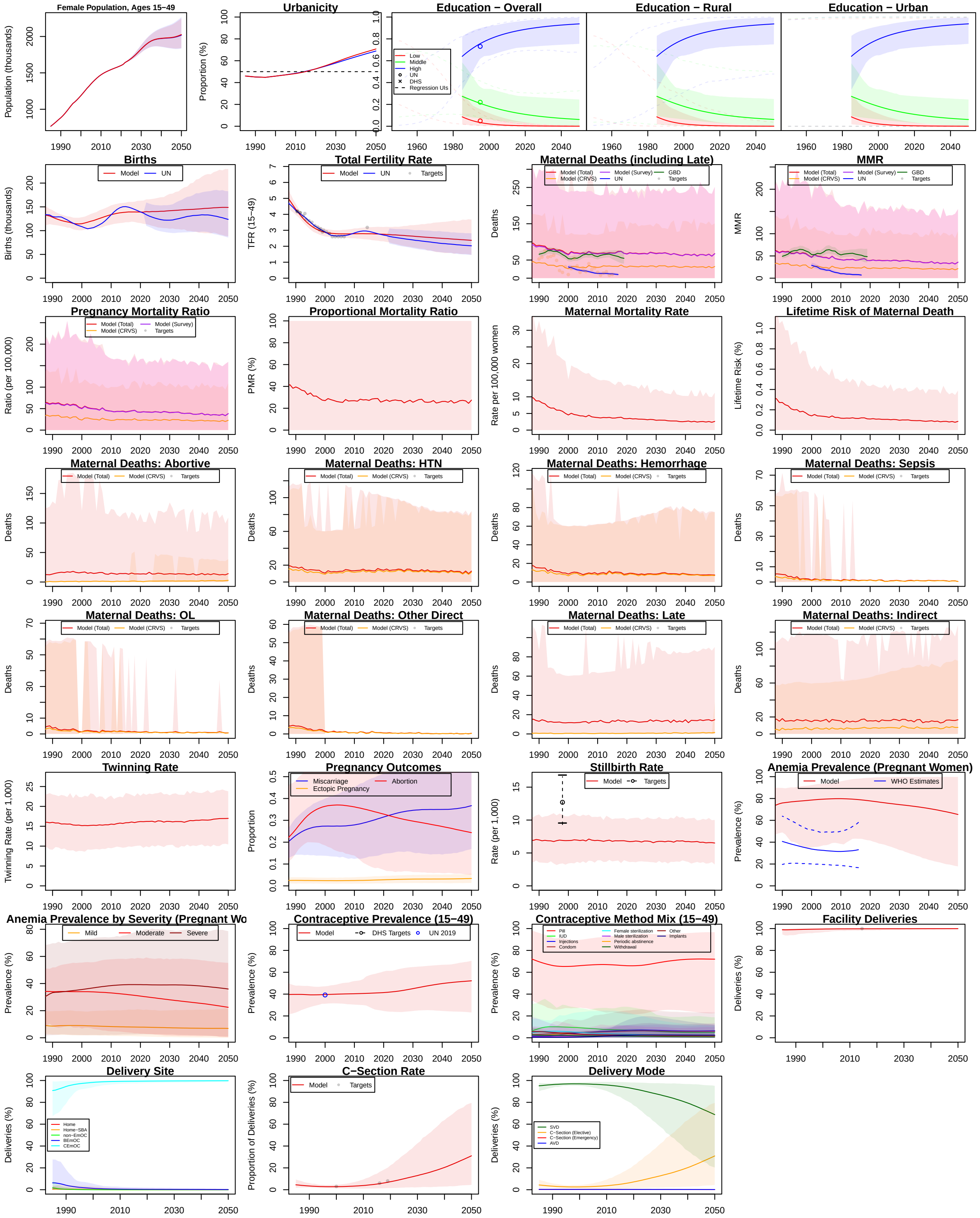

Tuvalu

ISO Code	Region	Area	Income Group
TUV	Polynesia	Oceania	Upper middle income

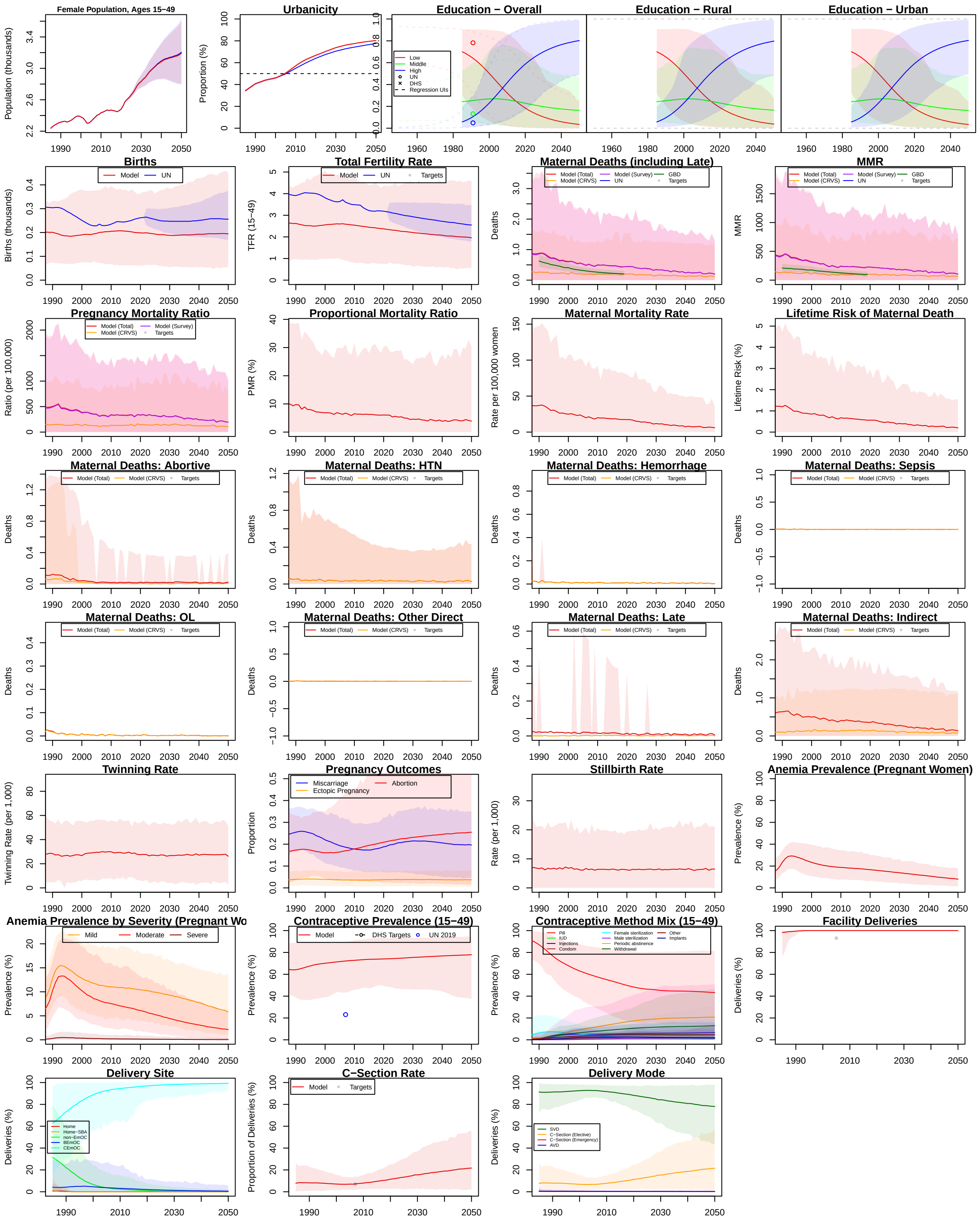

Uganda

ISO Code	Region	Area	Income Group
UGA	Eastern Africa	Africa	Low income

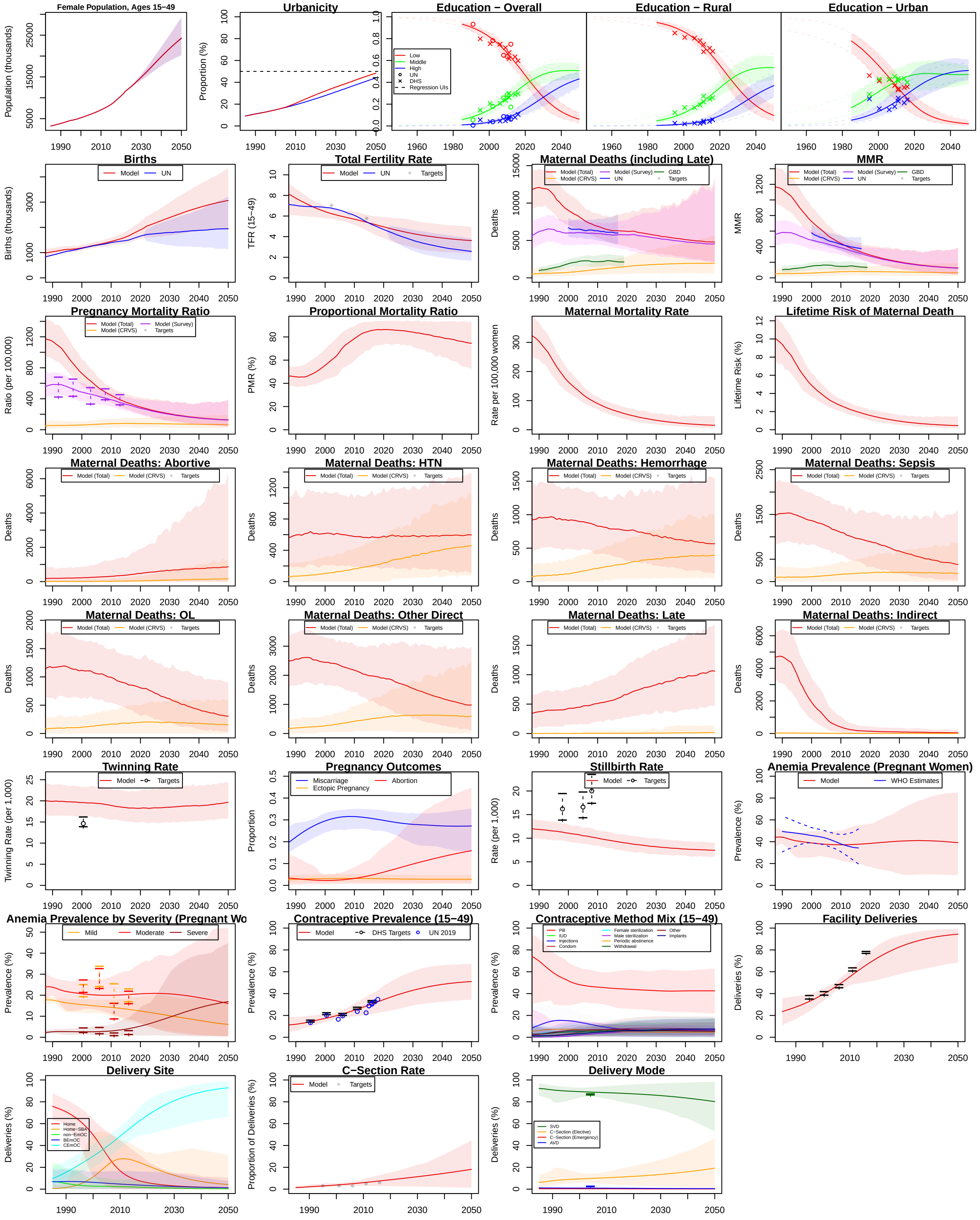

Ukraine

ISO Code	Region	Area	Income Group
UKR	Eastern Europe	Europe	Lower middle income

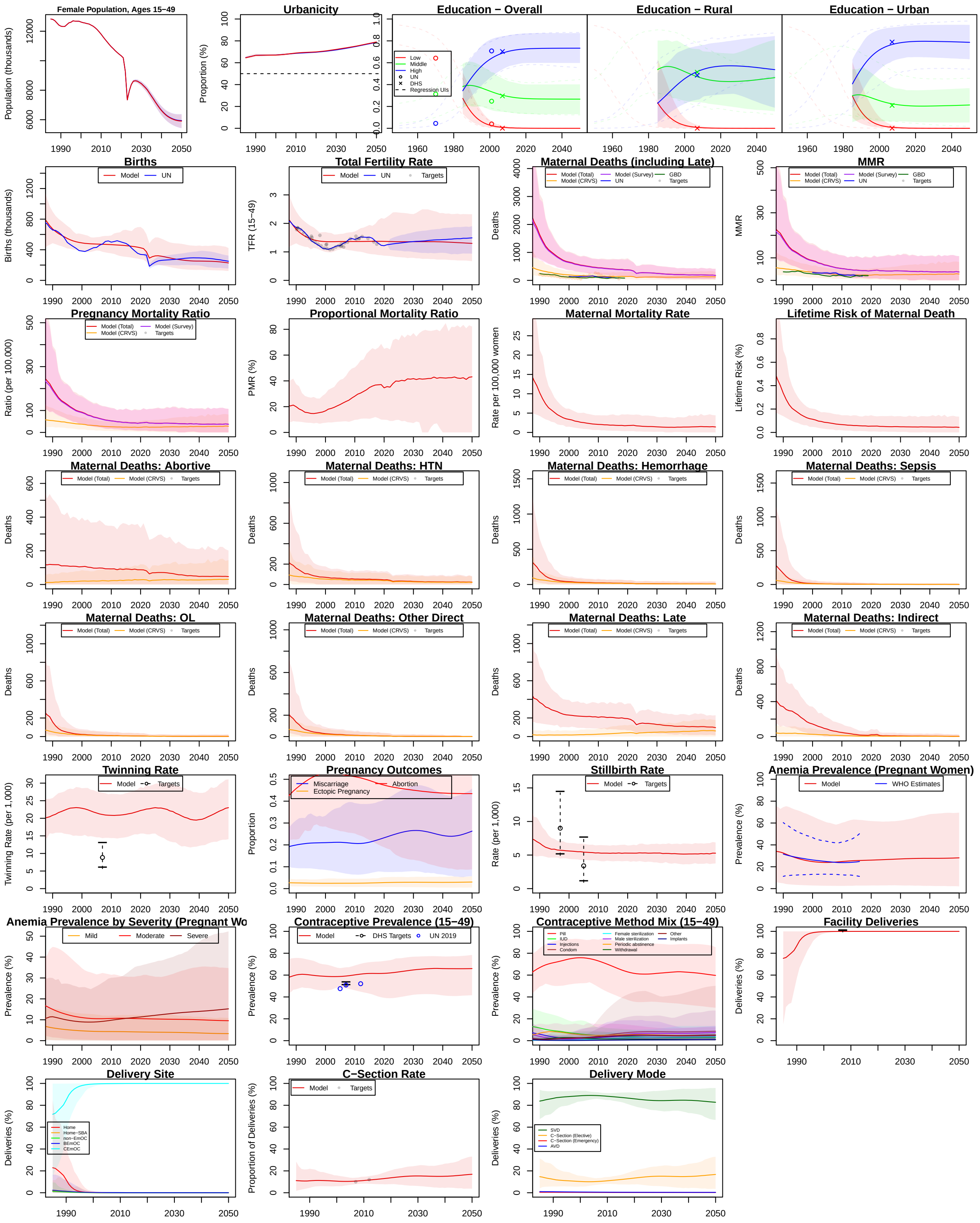

United Arab Emirates

ISO Code	Region	Area	Income Group
ARE	Western Asia	Asia	High income

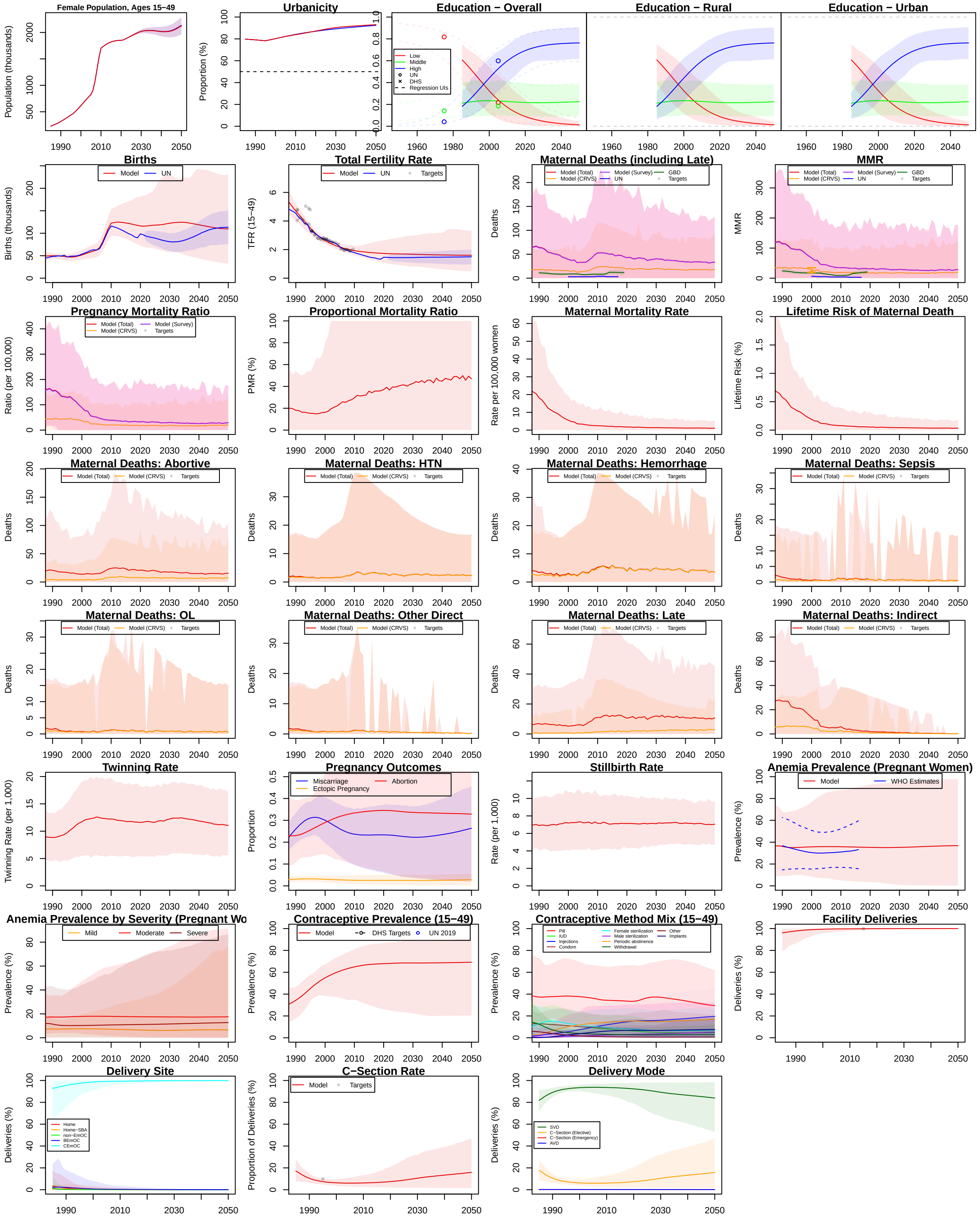

United Kingdom

ISO Code	Region	Area	Income Group
GBR	Northern Europe	Europe	High income

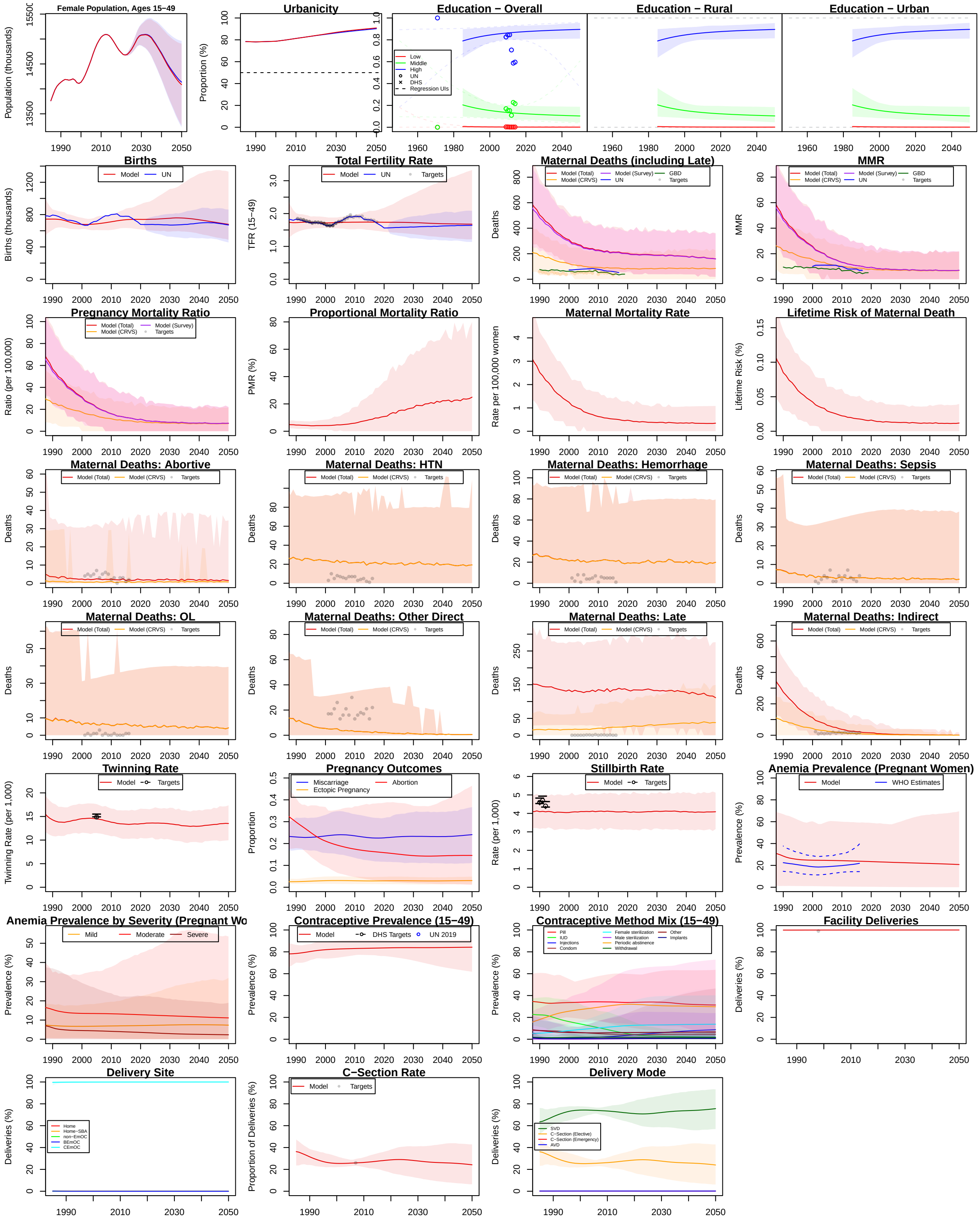

United Republic of Tanzania

ISO Code	Region	Area	Income Group
TZA	Eastern Africa	Africa	Low income

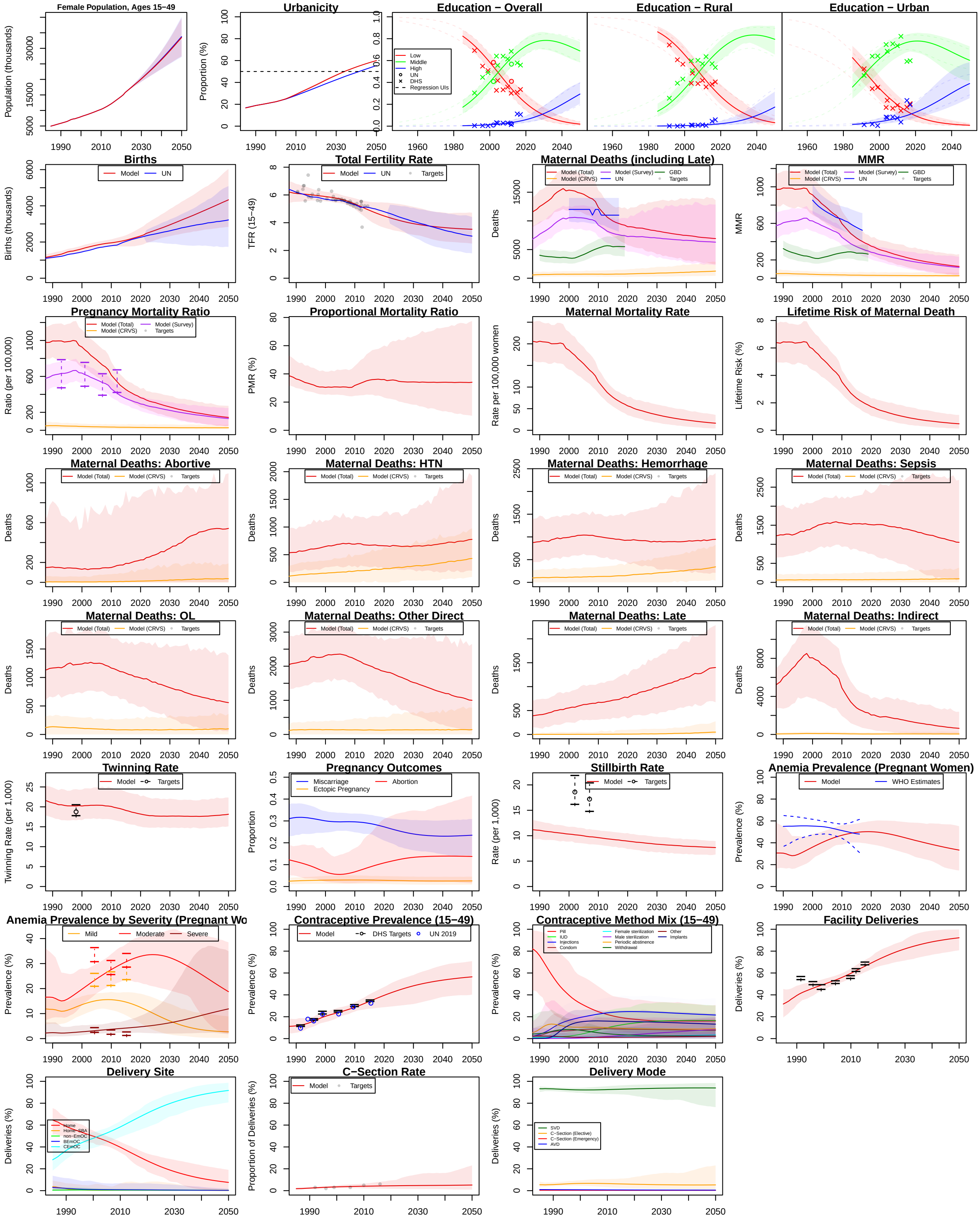

United States of America

ISO Code	Region	Area	Income Group
USA	Northern America	Northern America	High income

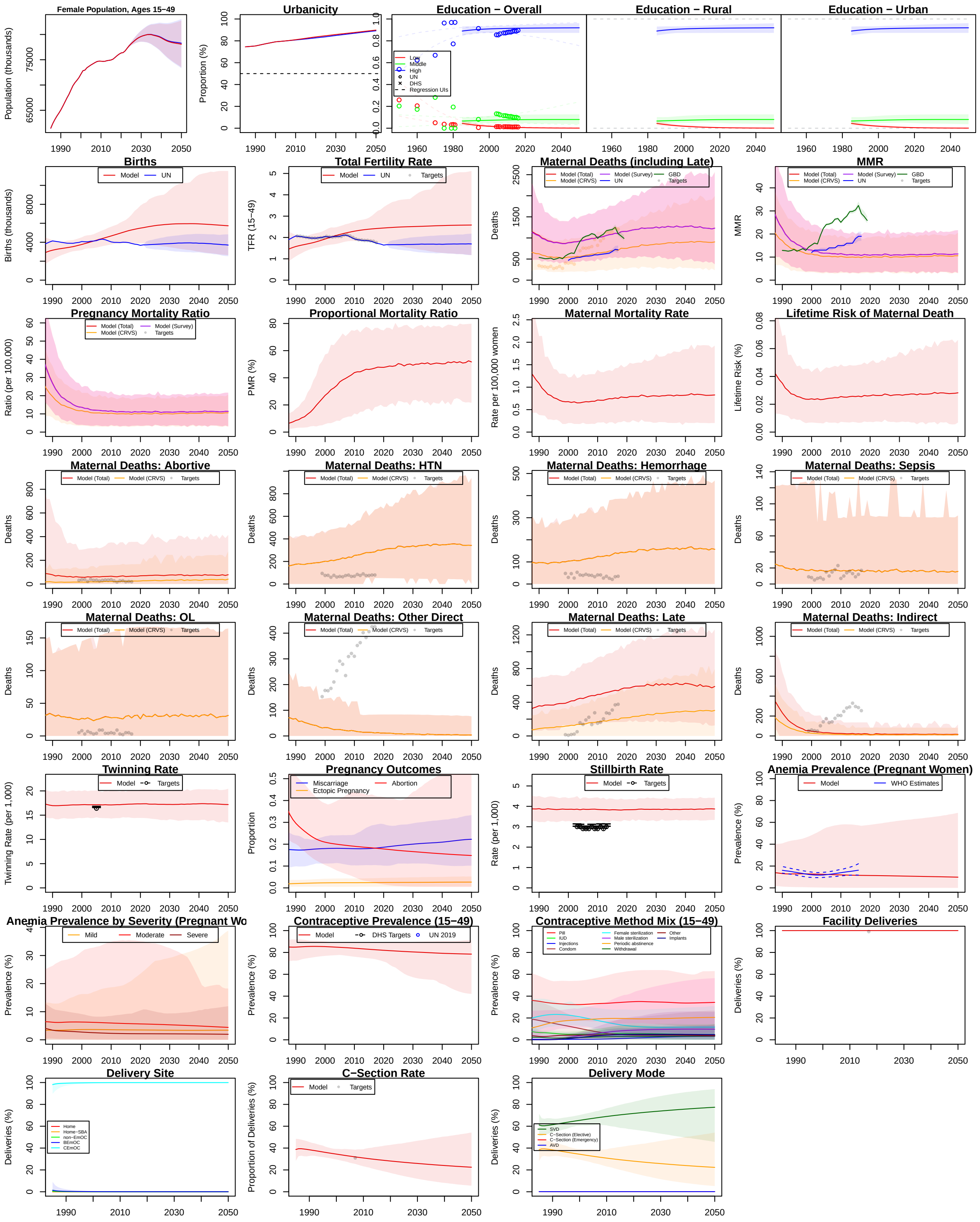

Uruguay

ISO Code	Region	Area	Income Group
URY	South America	Latin America and the Caribbean	High income

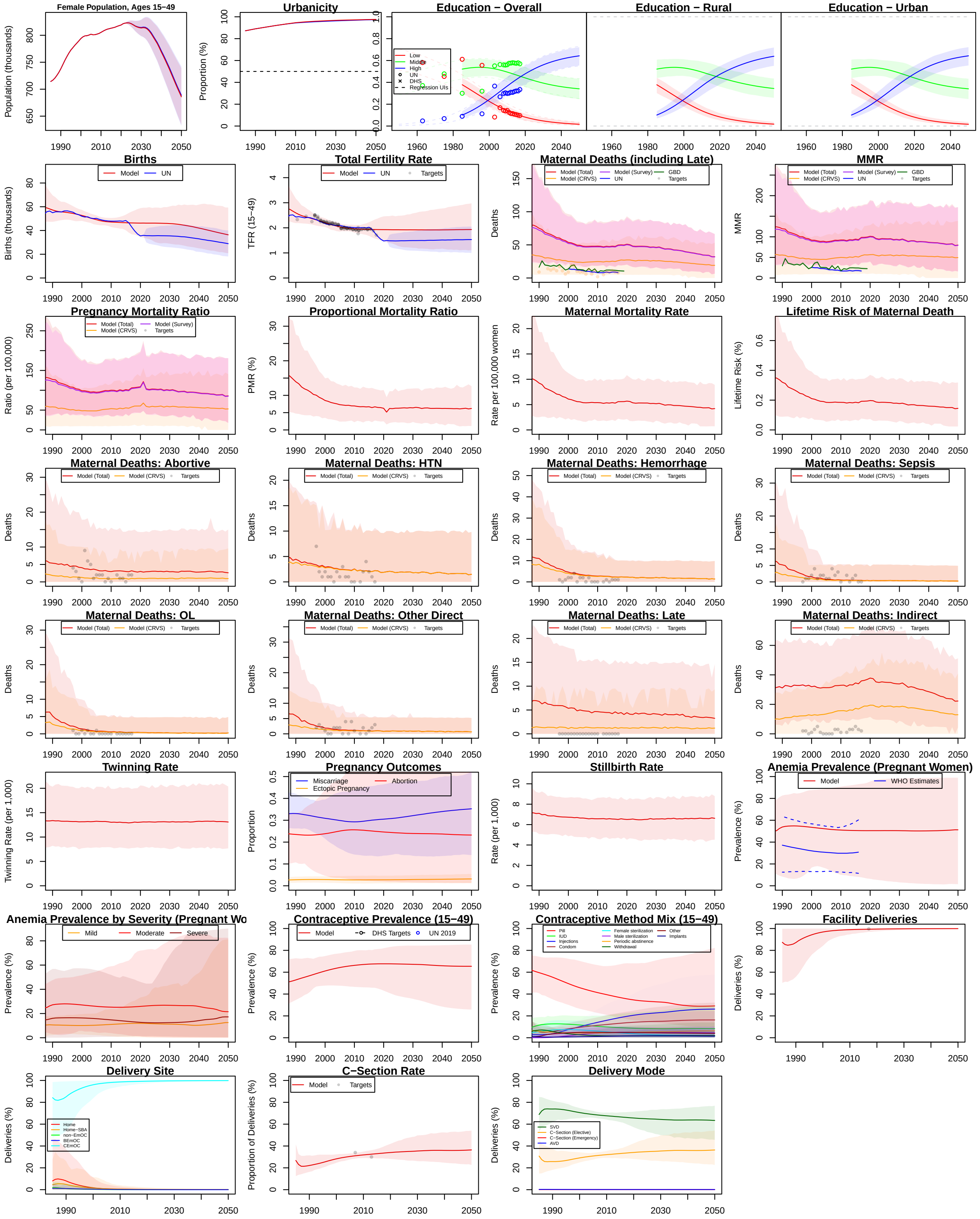

Uzbekistan

ISO Code	Region	Area	Income Group
UZB	Central Asia	Asia	Lower middle income

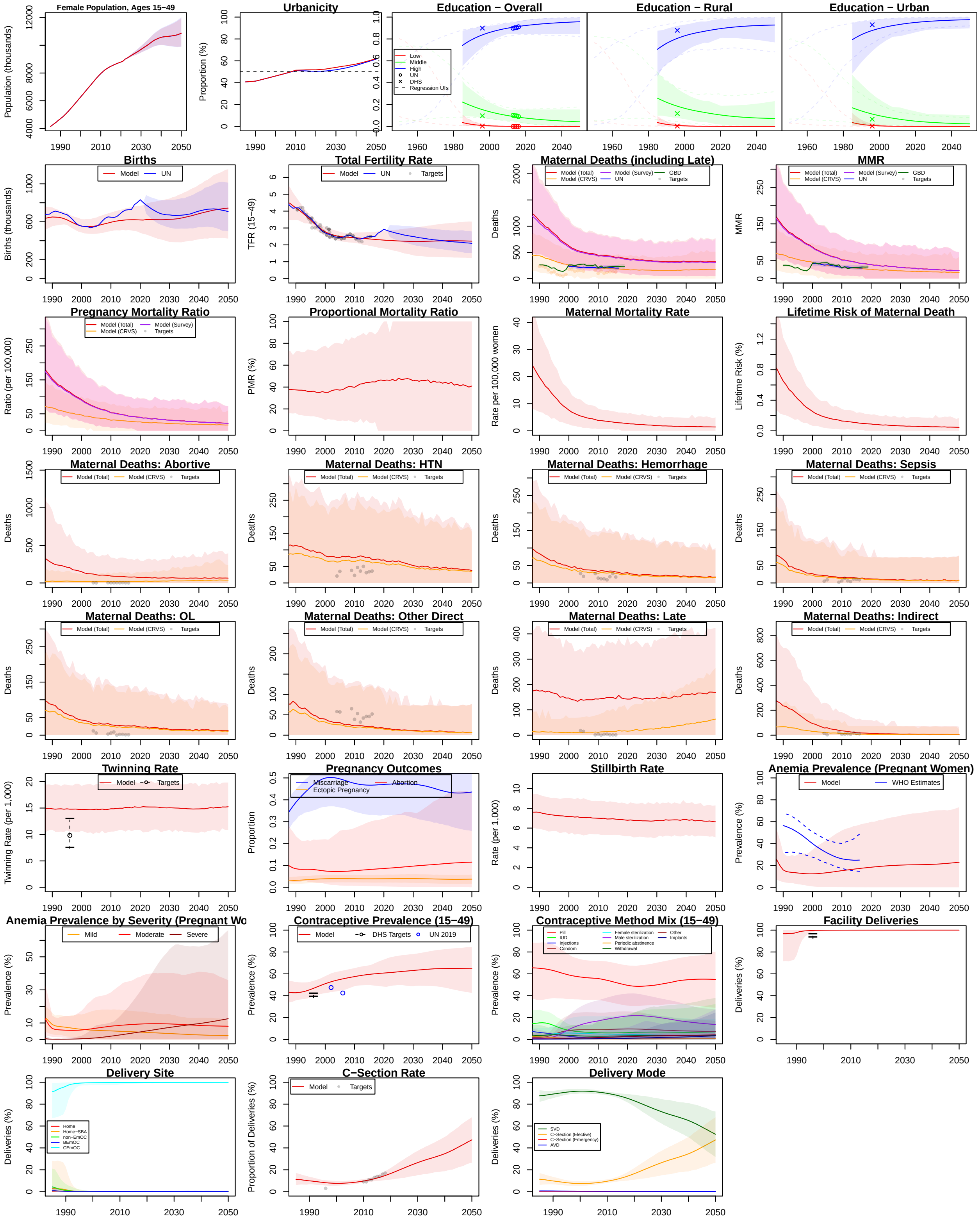

Vanuatu

ISO Code	Region	Area	Income Group
VUT	Melanesia	Oceania	Lower middle income

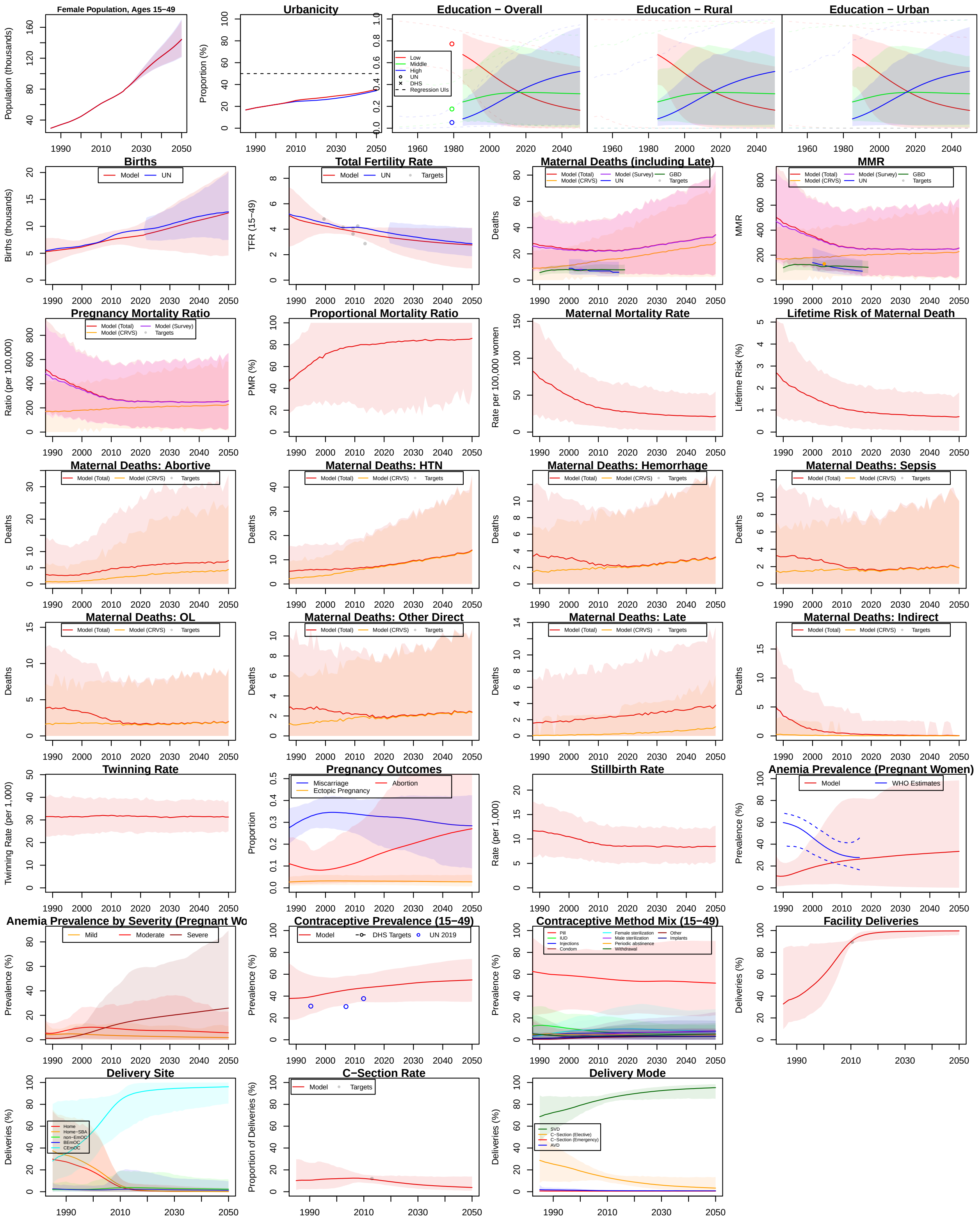

Venezuela (Bolivarian Republic of)

ISO Code	Region	Area	Income Group
VEN	South America	Latin America and the Caribbean	Upper middle income

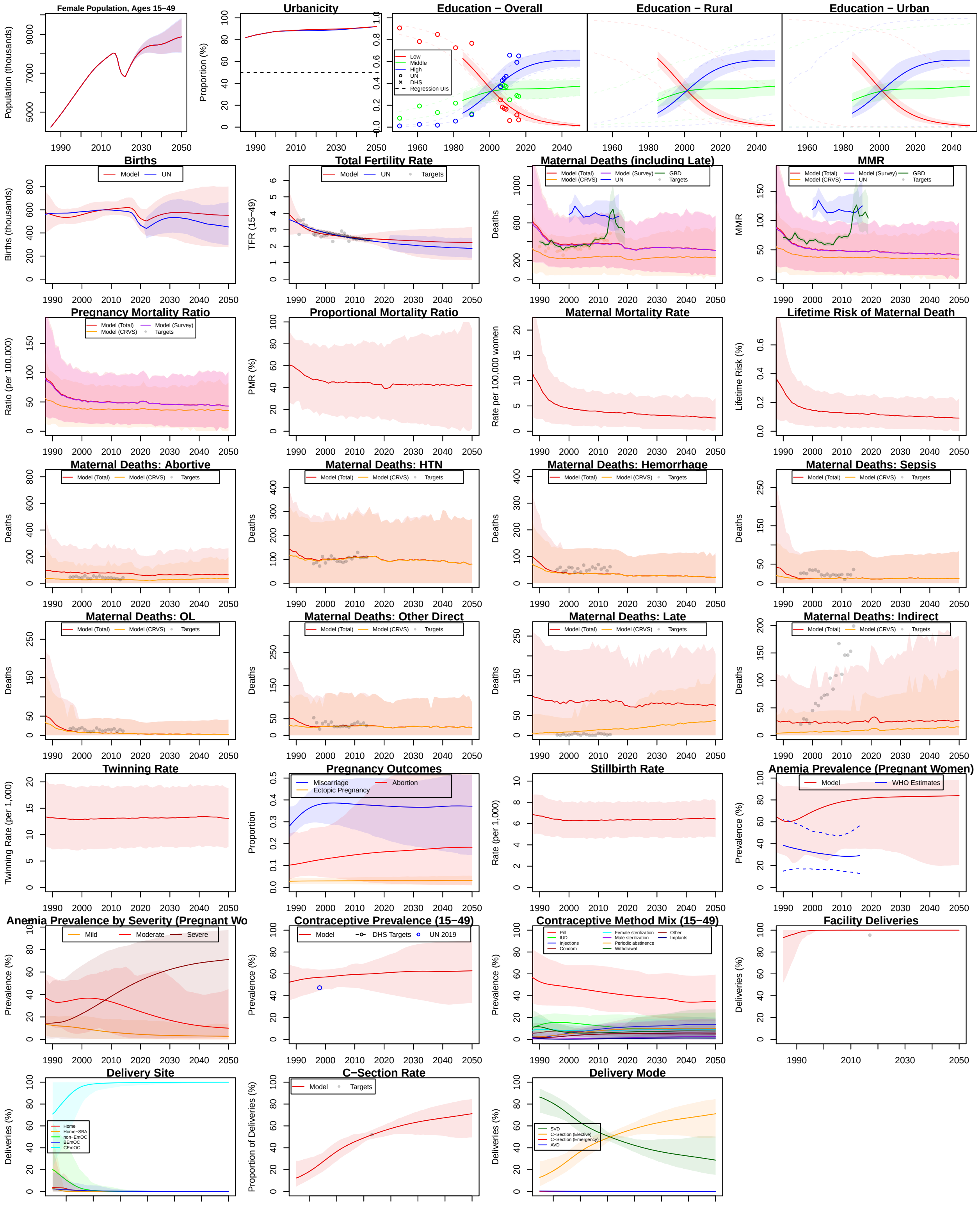

Viet Nam

ISO Code	Region	Area	Income Group
VNM	South-Eastern Asia	Asia	Lower middle income

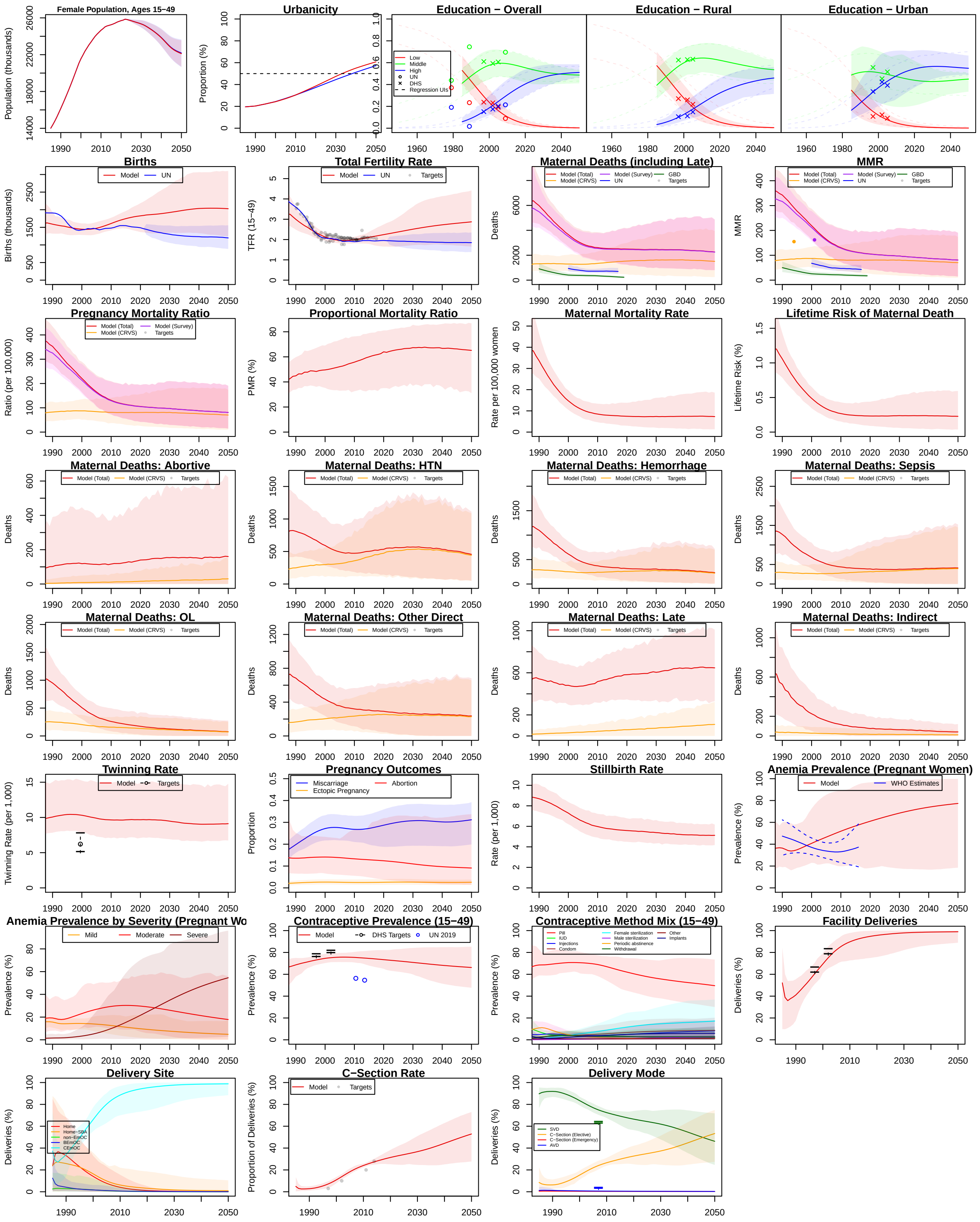

Yemen

ISO Code	Region	Area	Income Group
YEM	Western Asia	Asia	Low income

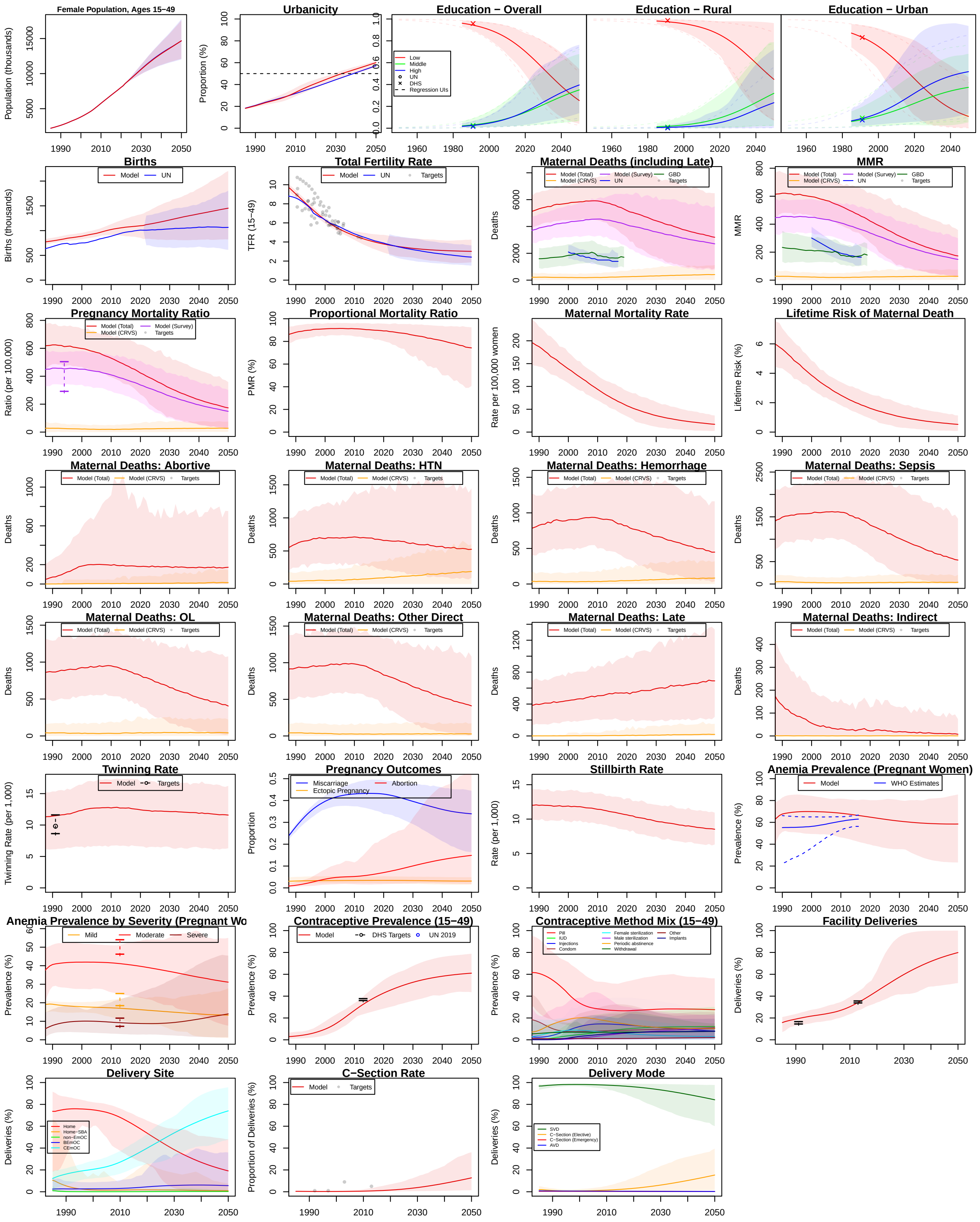

Zambia

ISO Code	Region	Area	Income Group
ZMB	Eastern Africa	Africa	Lower middle income

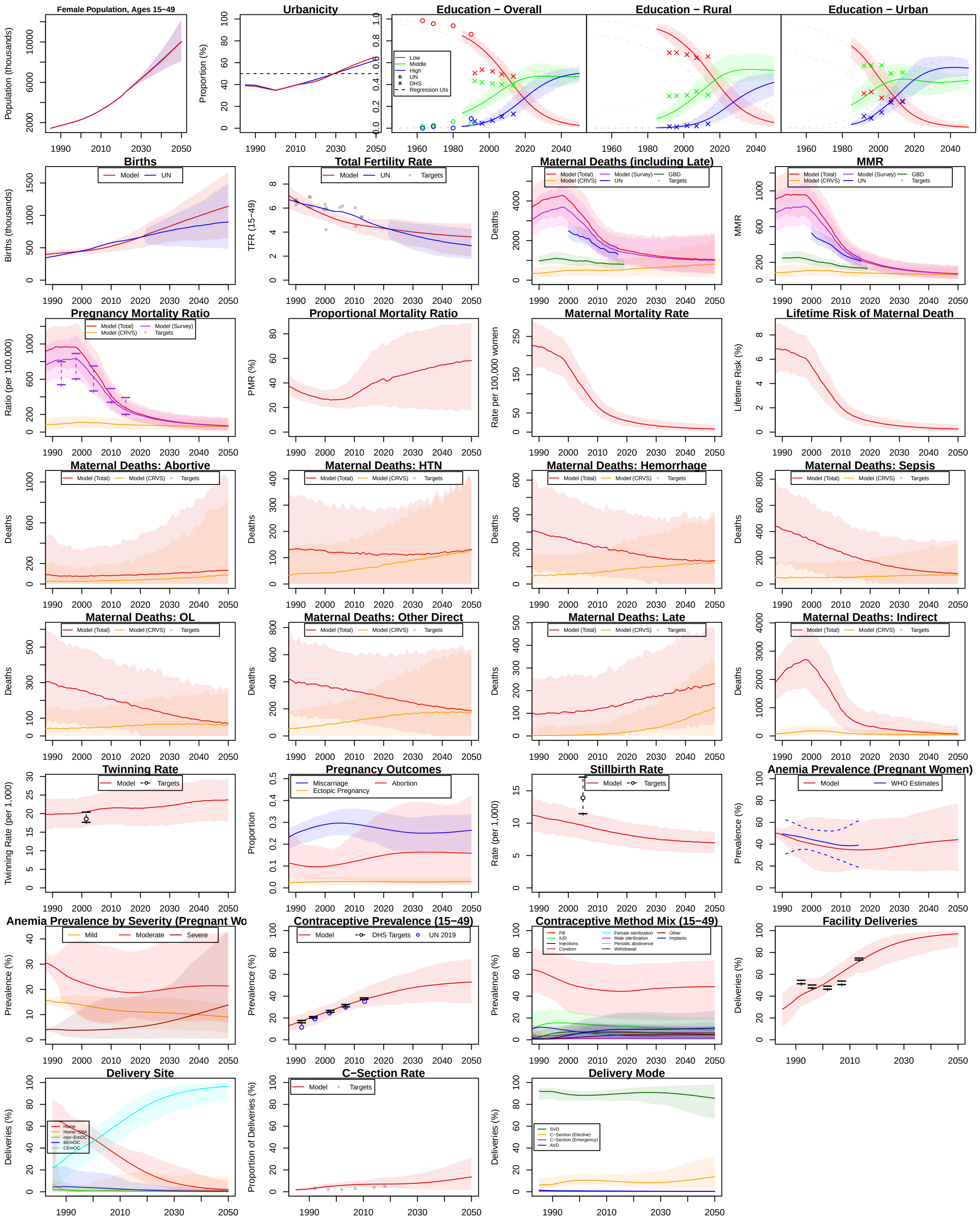

Zimbabwe

ISO Code	Region	Area	Income Group
ZWE	Eastern Africa	Africa	Low income

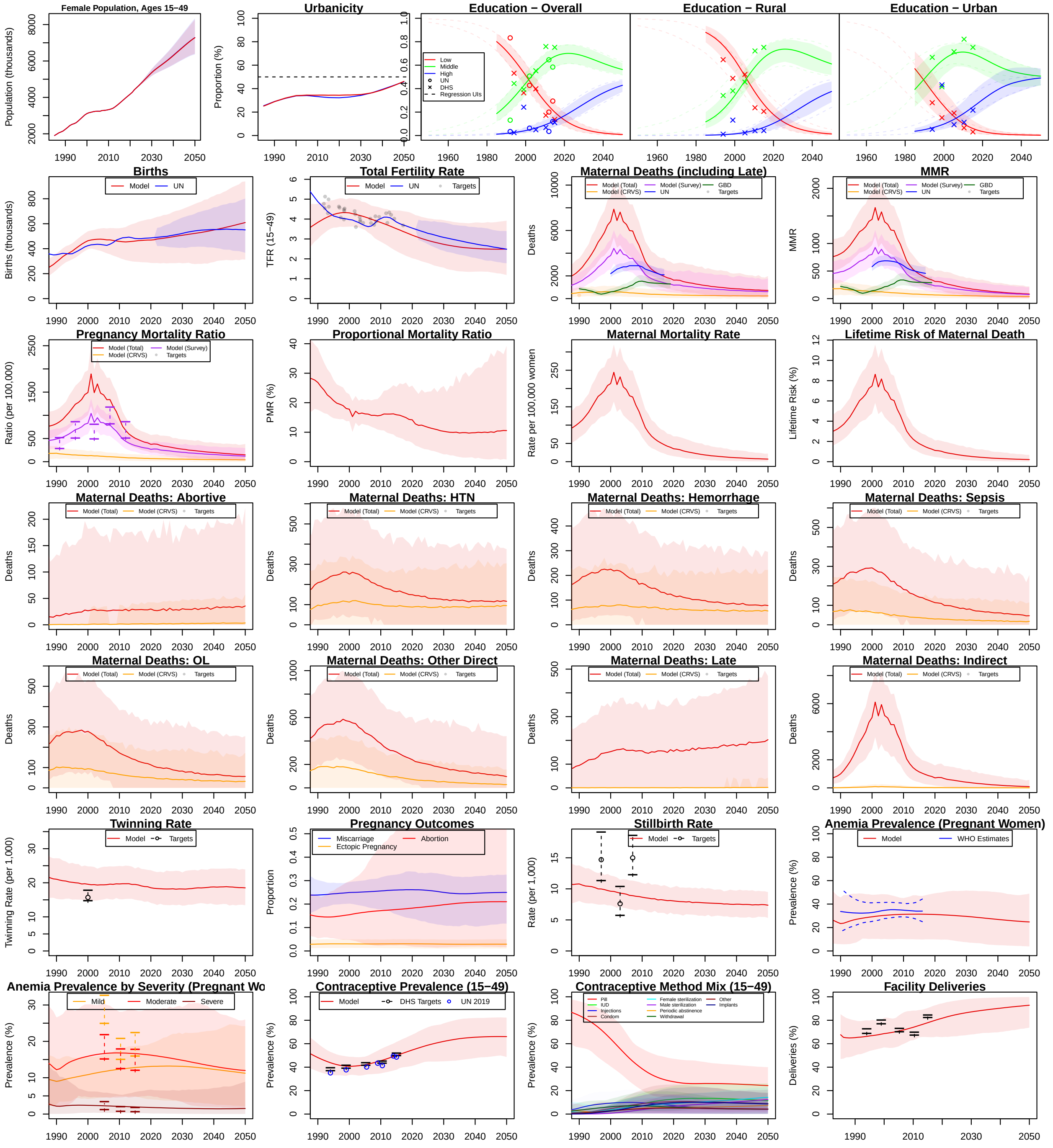

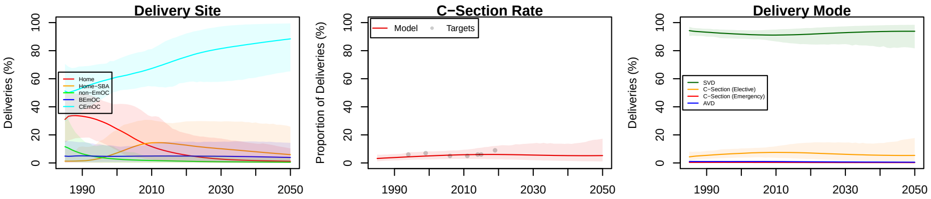
Delivery Site
Deliveries (%)
Home
Home−SBA
non−EmOC
BEmOC
CEmOC
C−Section Rate
Proportion of Deliveries (%)
Model
Targets
Delivery Mode
Deliveries (%)
SVD
C−Section (Elective)
C−Section (Emergency)
AVD
0
20
40
60
80
100
1990
2010
2030
2050

C Appendix C: Calibrated Parameters

C.1 Demographic Parameters

C.1.1 Proportion of Deaths Due to Injury

Hyperparameters

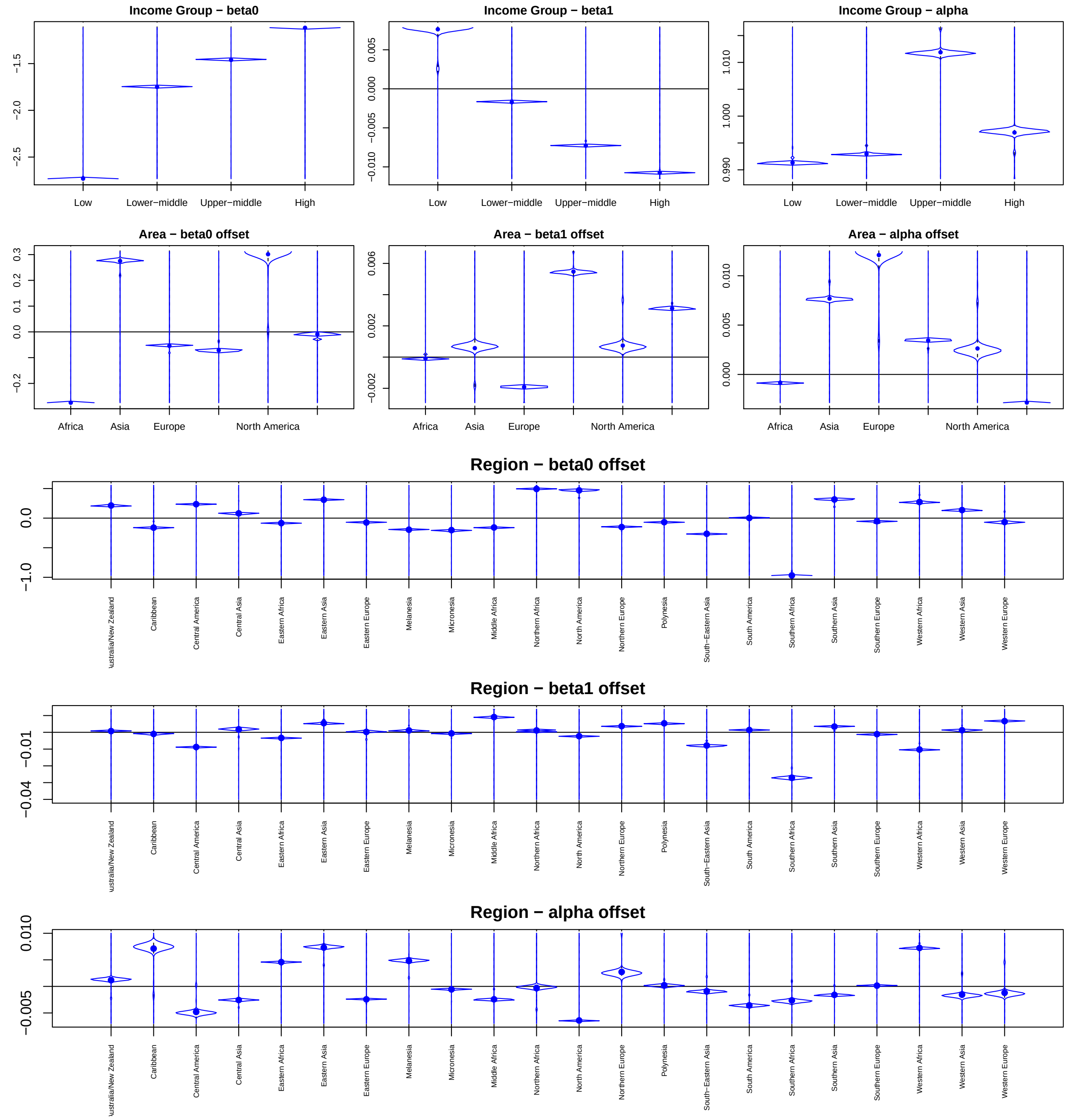

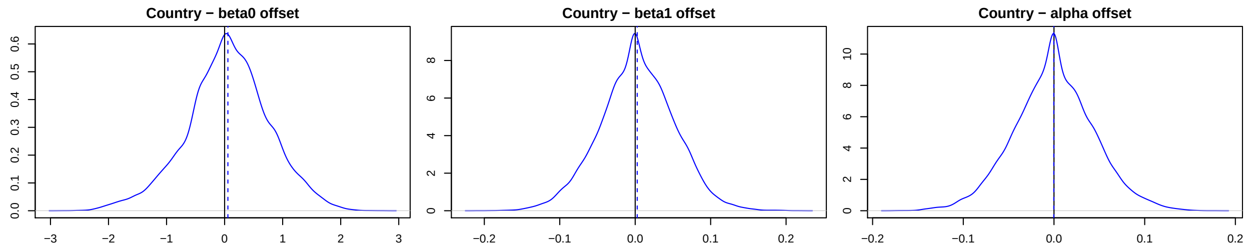
Country − beta0 offset
Country − beta1 offset
Country − alpha offset

Parameters

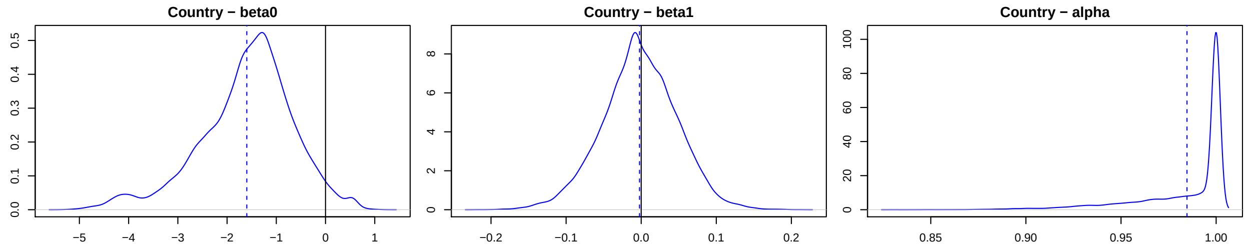
Country − beta0
Country − beta1
Country − alpha

C.2 Biological Parameters

C.2.1 Natural Fecundity/Fertility

Hyperparameters

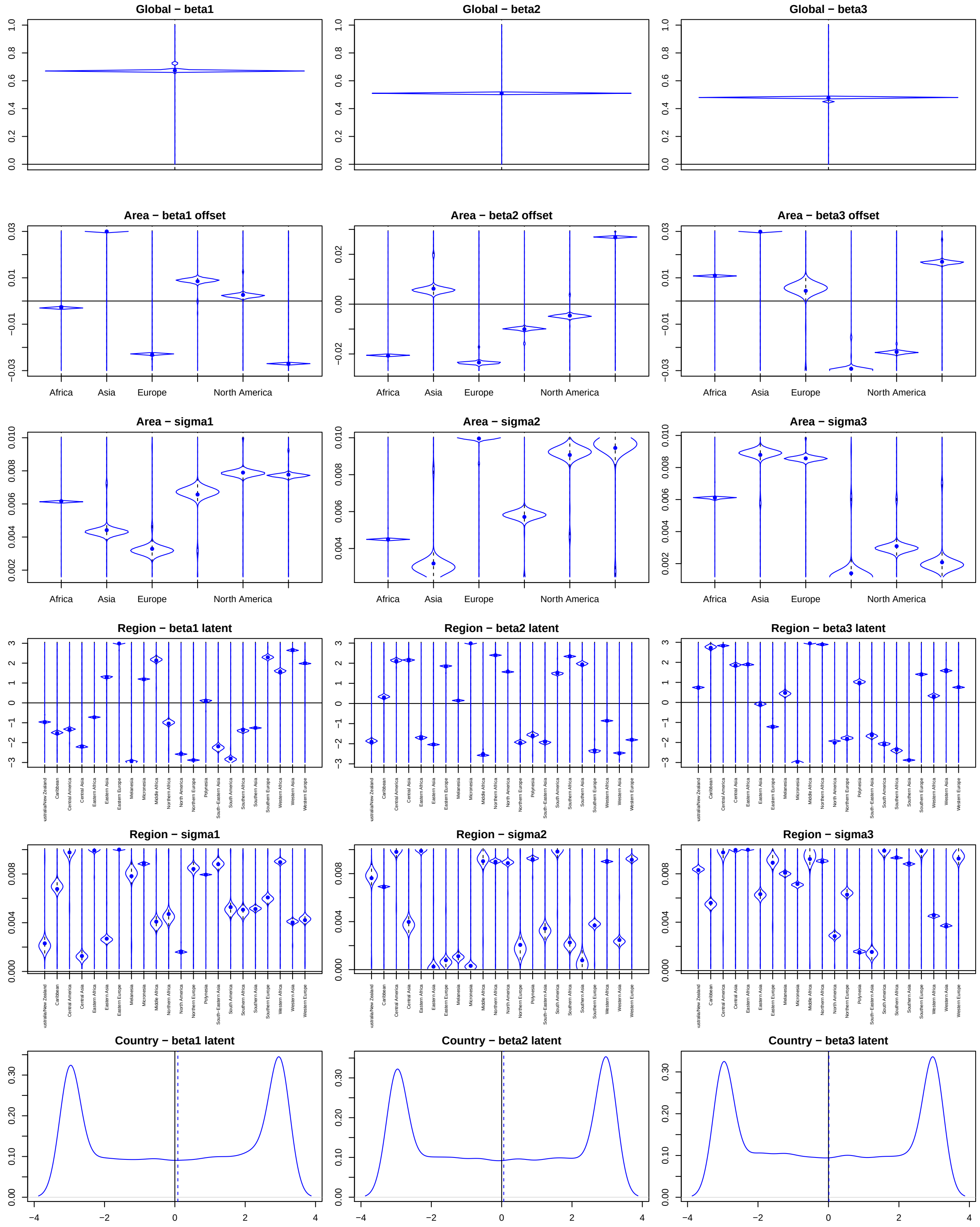

Parameters

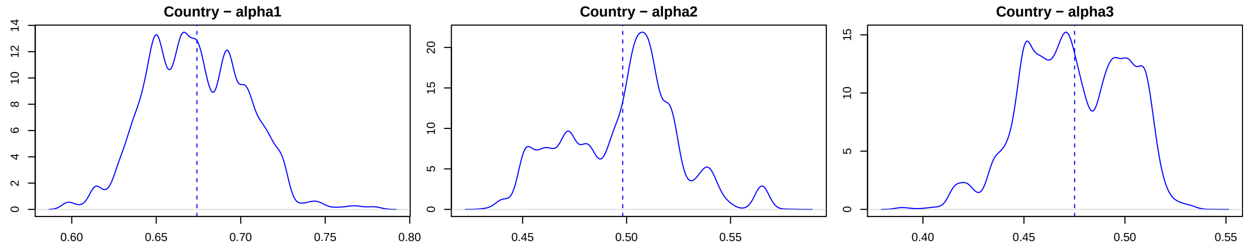
Country − alpha1
14
12
10
8
6
4
2
0
0.60
0.65
0.70
0.75
0.80
Country − alpha2
20
15
10
5
0
0.45
0.50
0.55
Country − alpha3
15
10
5
0
0.40
0.45
0.50
0.55

C.2.2 Sex Ratio - Primary

Hyperparameters

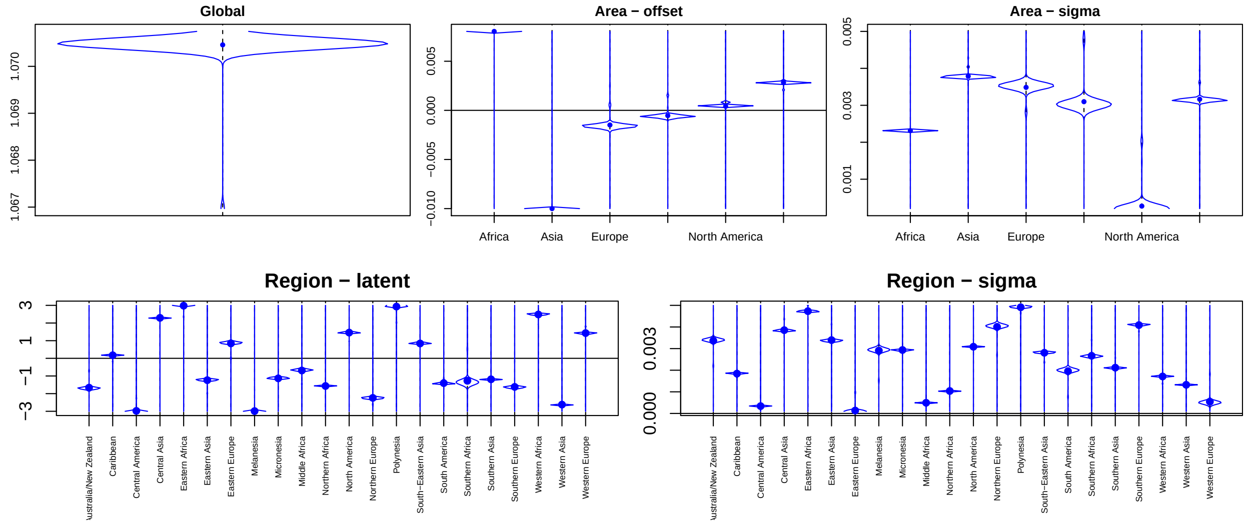

Parameters

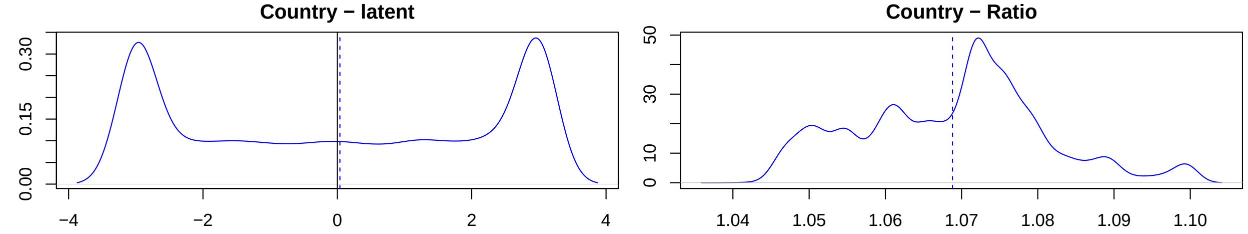

C.2.3 Twinning Rates

C.2.3.1 Monozygotic

Hyperparameters

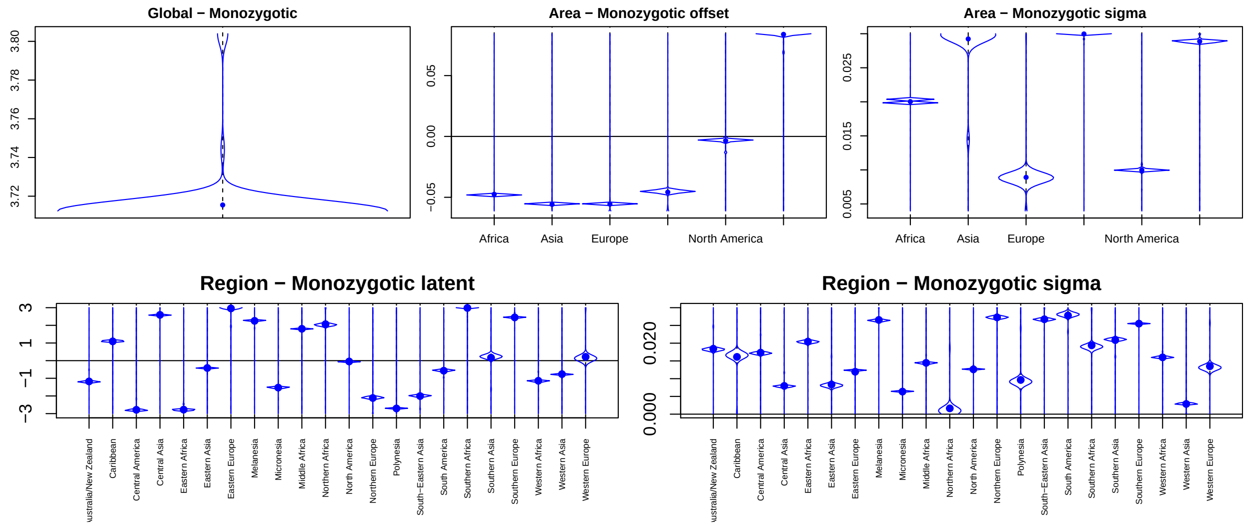

Parameters

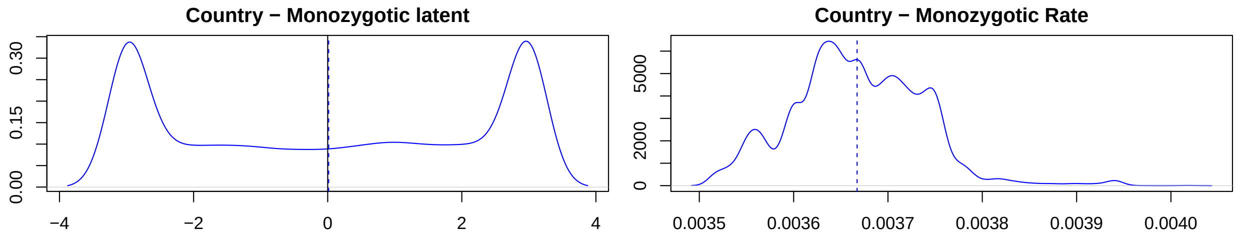

C.2.3.2 Dizygotic

Hyperparameters

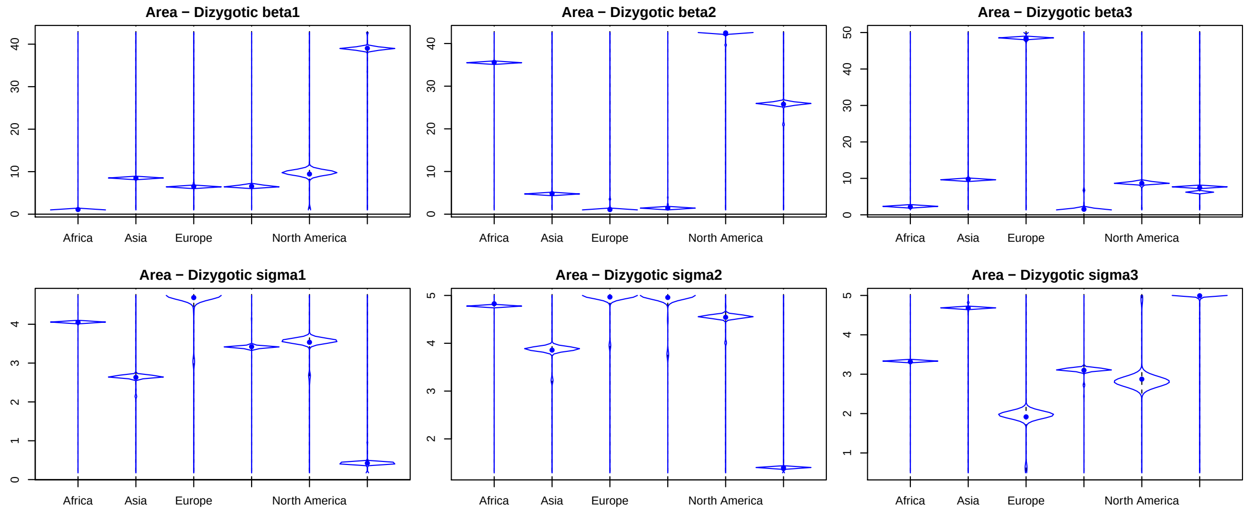

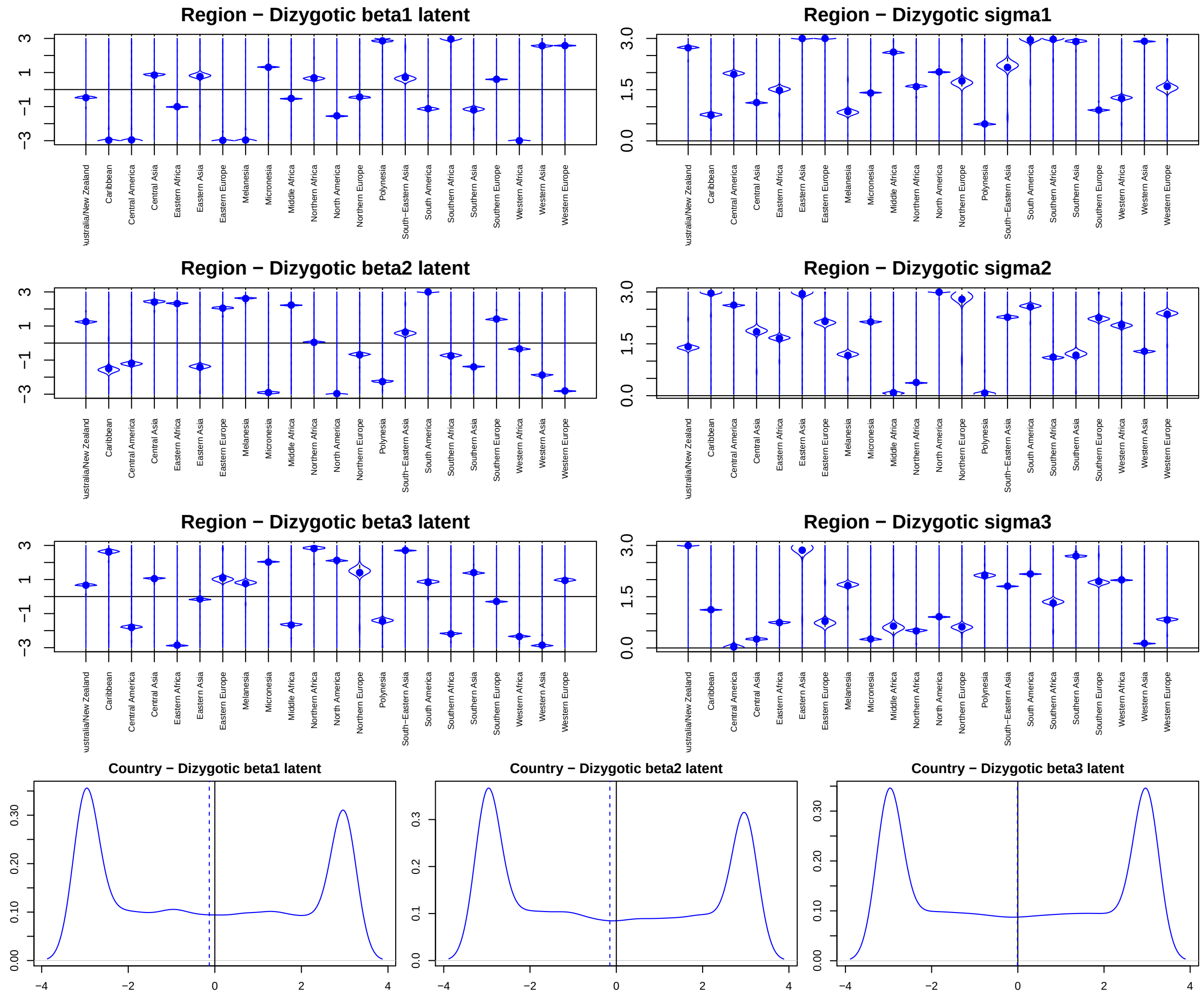
Region − Dizygotic beta1 latent
Region − Dizygotic sigma1
Region − Dizygotic beta2 latent
Region − Dizygotic sigma2
Region − Dizygotic beta3 latent
Region − Dizygotic sigma3
Country − Dizygotic beta1 latent
Country − Dizygotic beta2 latent
Country − Dizygotic beta3 latent

Parameters

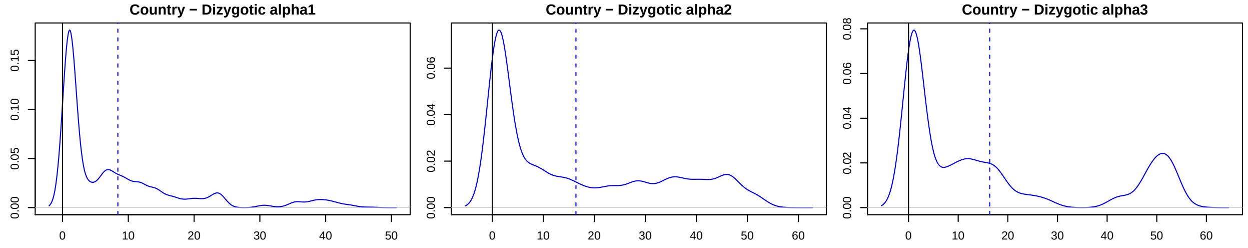
Country − Dizygotic alpha1
Country − Dizygotic alpha2
Country − Dizygotic alpha3

C.2.4 Miscarriage

C.2.4.1 Incidence

Hyperparameters

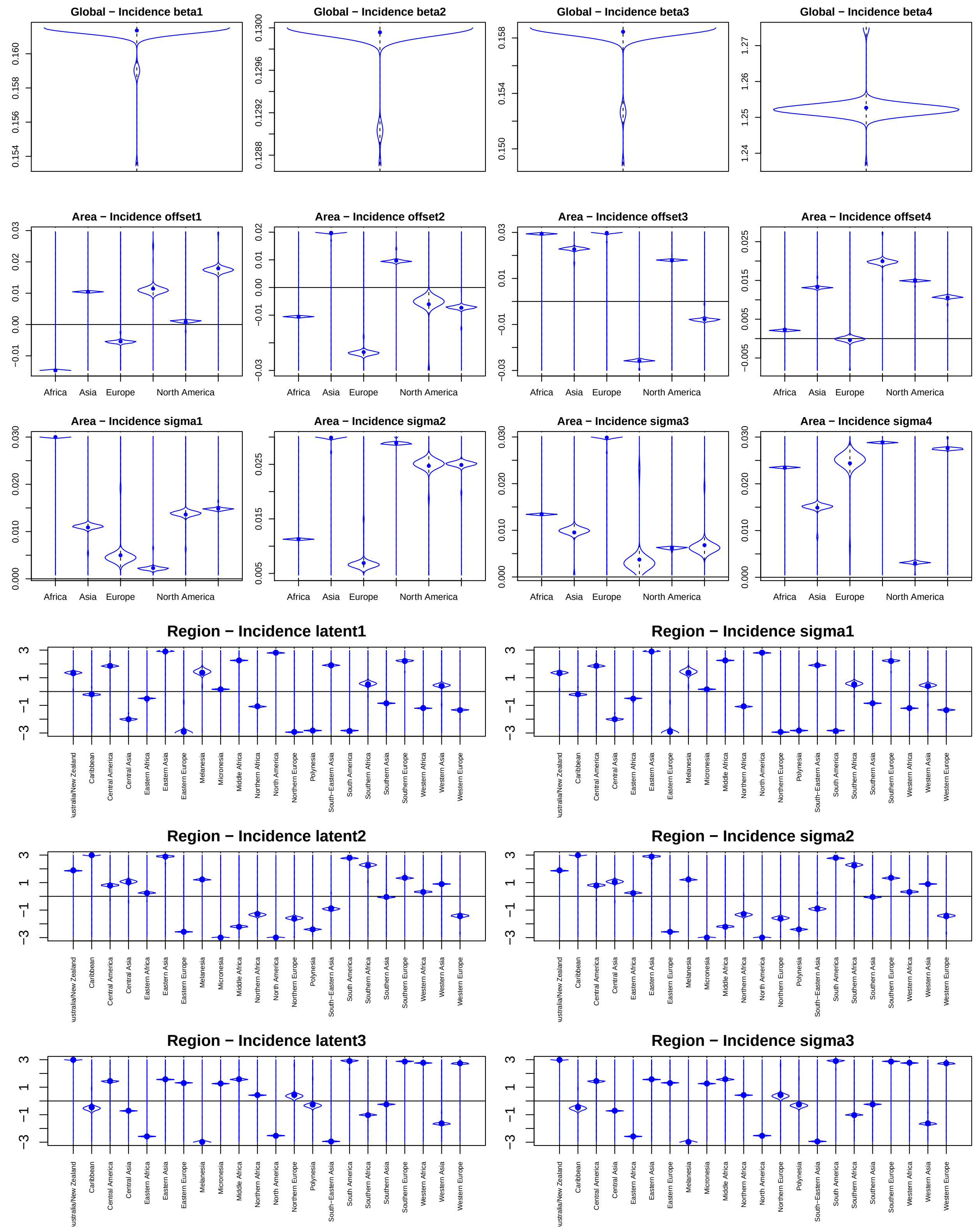

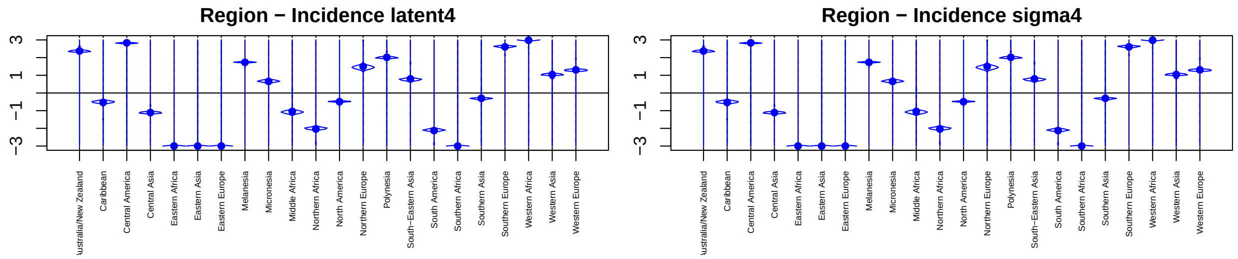

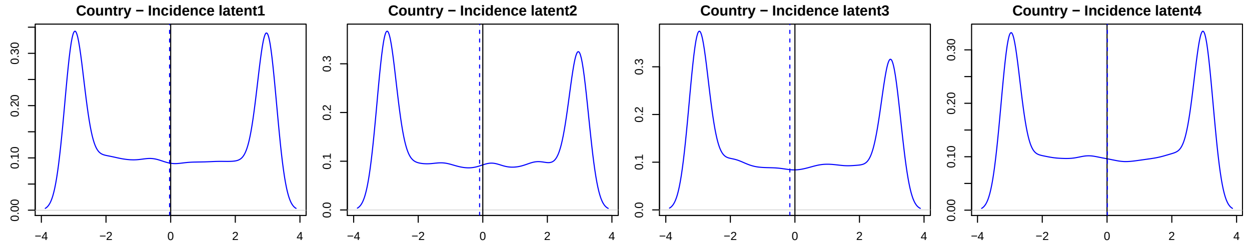

Parameters

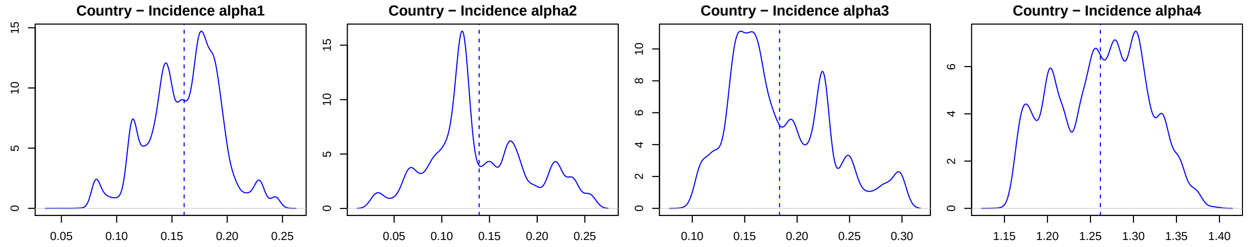

C.2.4.2 Relative Risk | Previous Miscarriage

Hyperparameters

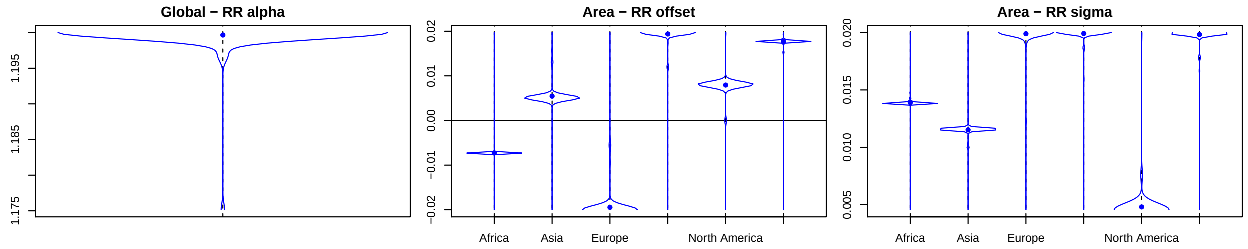

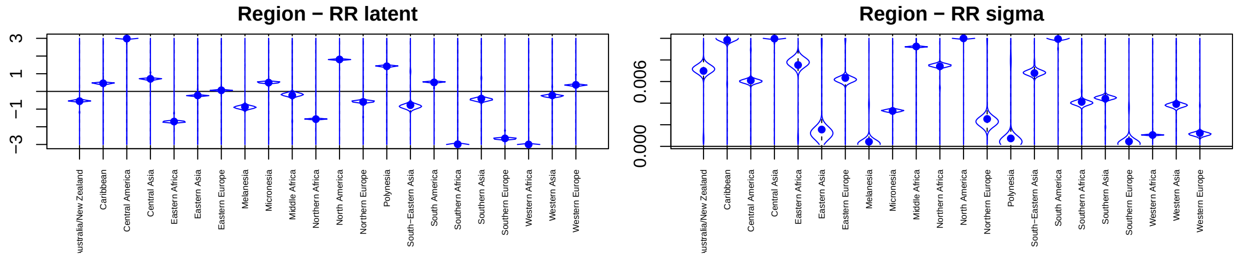

Parameters

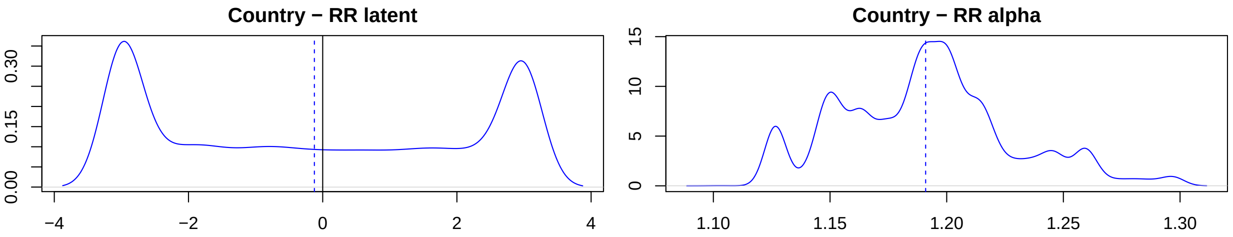
Country − RR latent
0.00
0.15
0.30
−4
−2
0
2
4
Country − RR alpha
0
5
10
15
1.10
1.15
1.20
1.25
1.30

C.2.5 Ectopic Pregnancy

C.2.5.1 Incidence

Hyperparameters

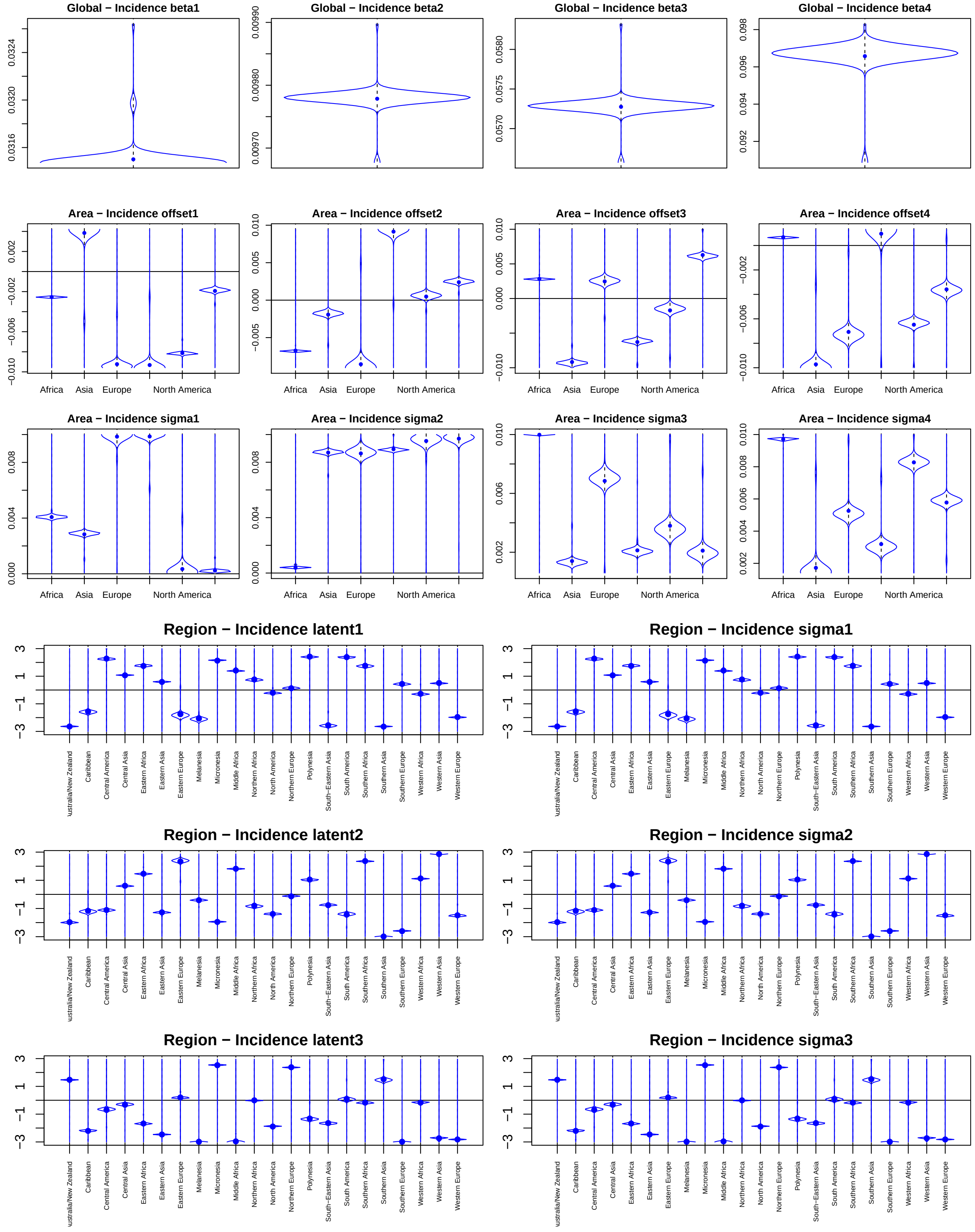

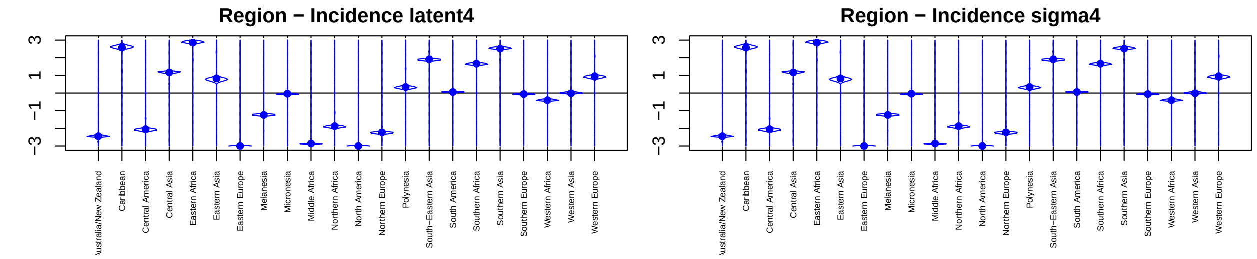

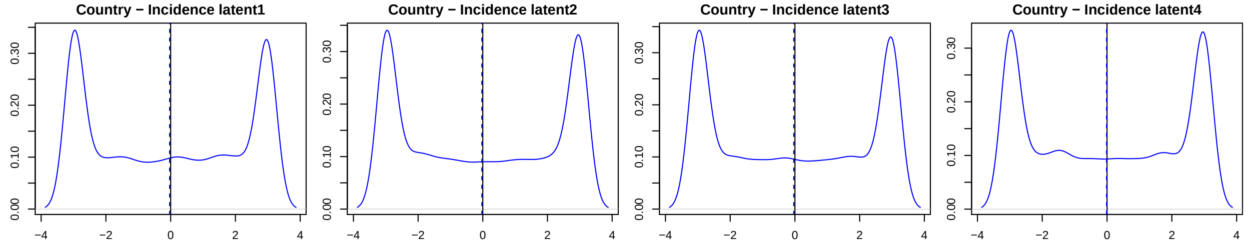

Parameters

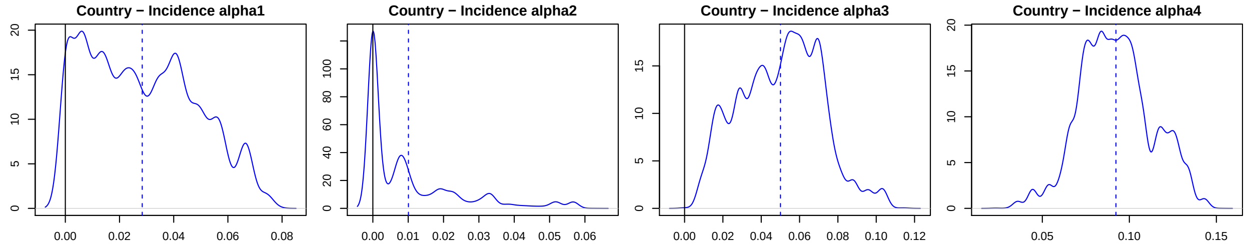

C.2.5.2 Recurrence

Hyperparameters

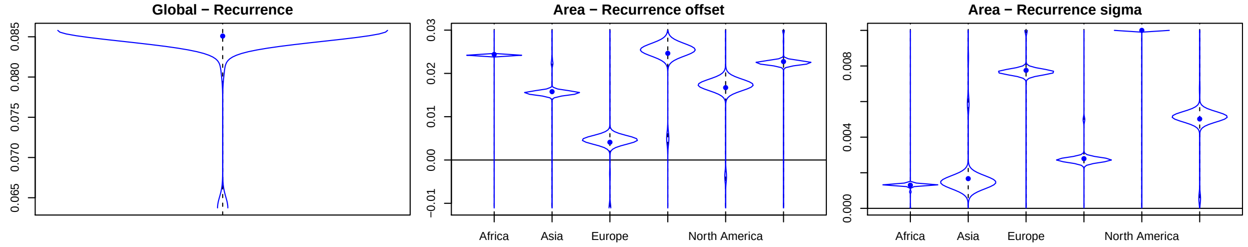

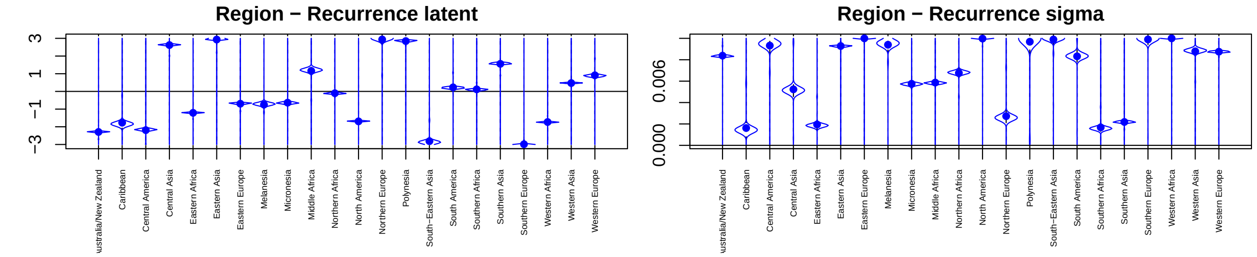

Parameters

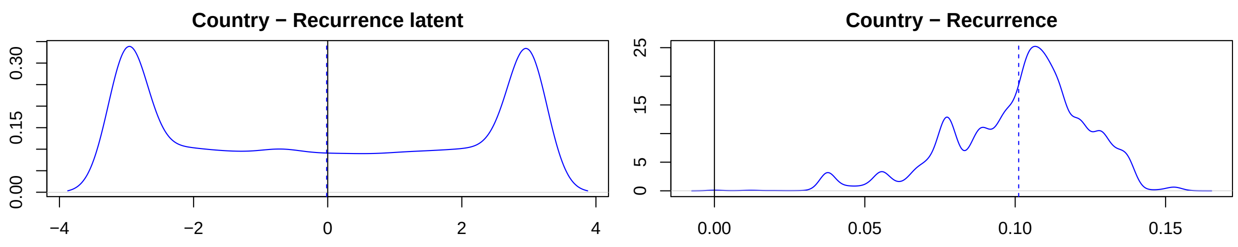

C.2.5.3 Rupture

Hyperparameters

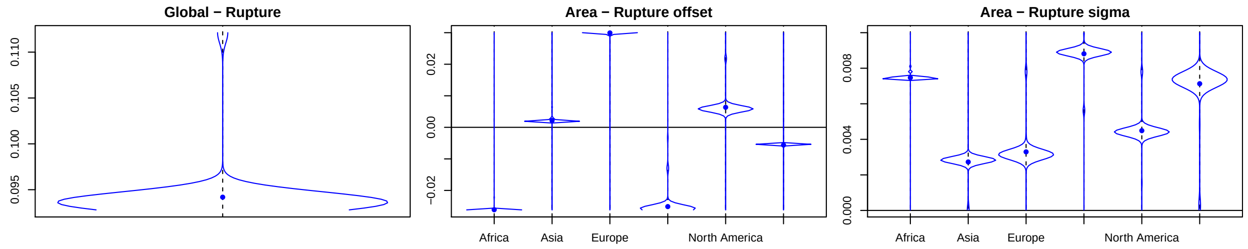

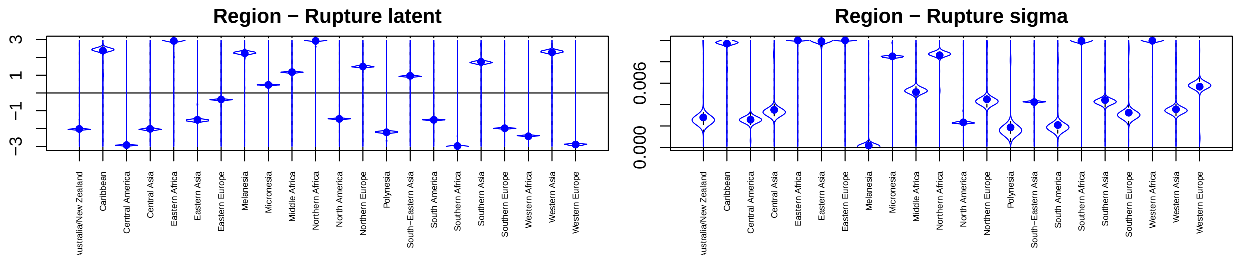

Parameters

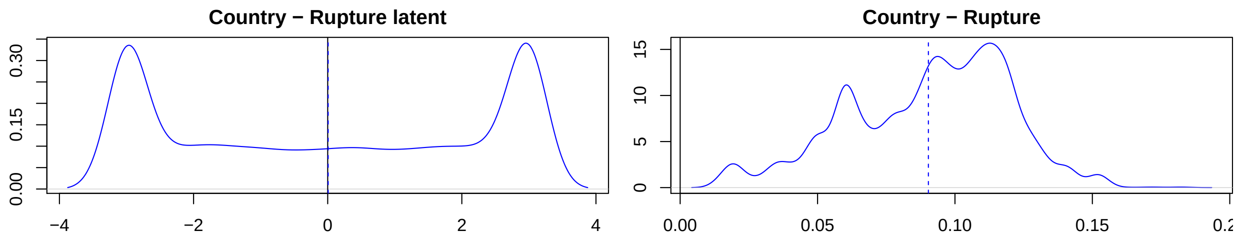

C.2.5.4 CFR

Hyperparameters

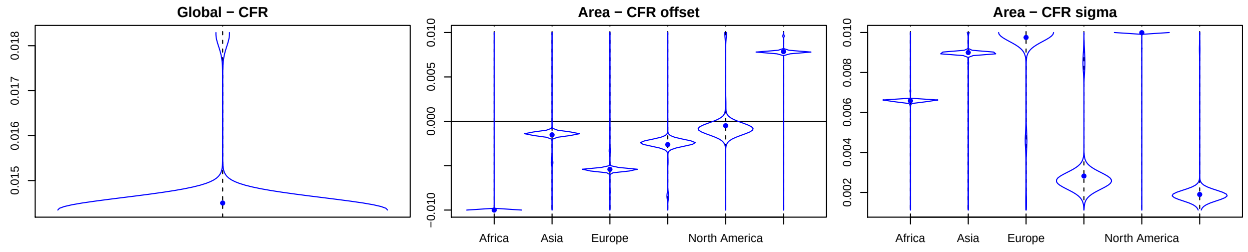

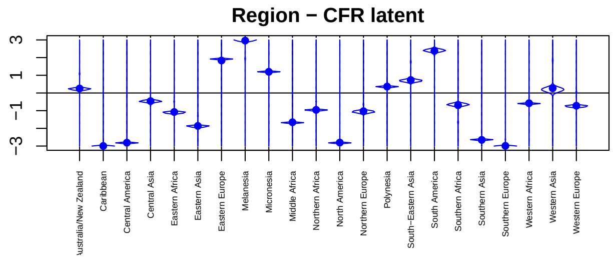
Region − CFR latent
3
1
−1
−3
ustralia/New Zealand
Caribbean
Central America
Central Asia
Eastern Africa
Eastern Asia
Eastern Europe
Melanesia
Micronesia
Middle Africa
Northern Africa
North America
Northern Europe
Polynesia
South−Eastern Asia
South America
Southern Africa
Southern Asia
Southern Europe
Western Africa
Western Asia
Western Europe

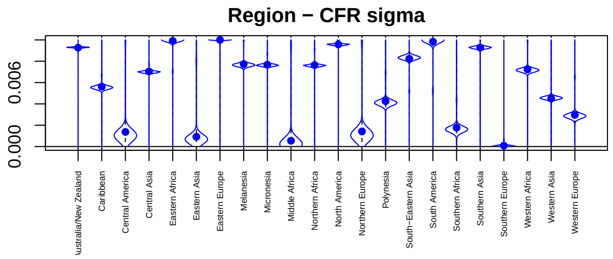
Region − CFR sigma
0.000
0.006
ustralia/New Zealand
Caribbean
Central America
Central Asia
Eastern Africa
Eastern Asia
Eastern Europe
Melanesia
Micronesia
Middle Africa
Northern Africa
North America
Northern Europe
Polynesia
South−Eastern Asia
South America
Southern Africa
Southern Asia
Southern Europe
Western Africa
Western Asia
Western Europe

Parameters

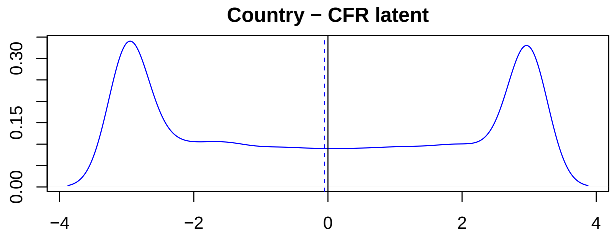
Country − CFR latent
0.00
0.15
0.30
−4
−2
0
2
4

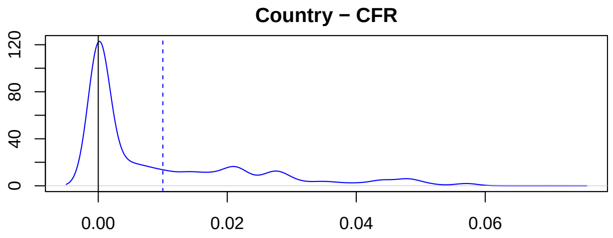
Country − CFR
0
40
80
120
0.00
0.02
0.04
0.06

C.2.6 Stillbirths

C.2.6.1 Incidence

Hyperparameters

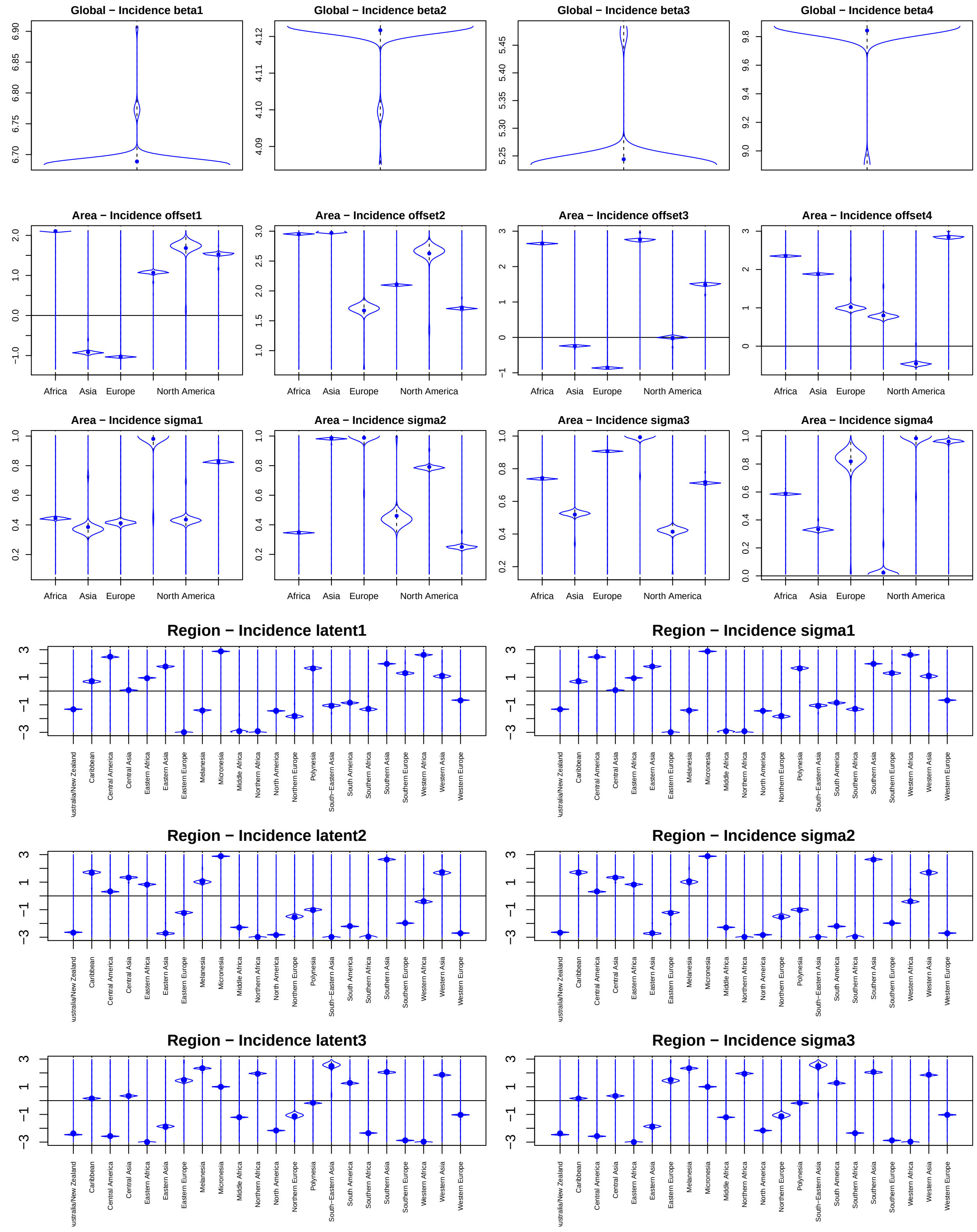

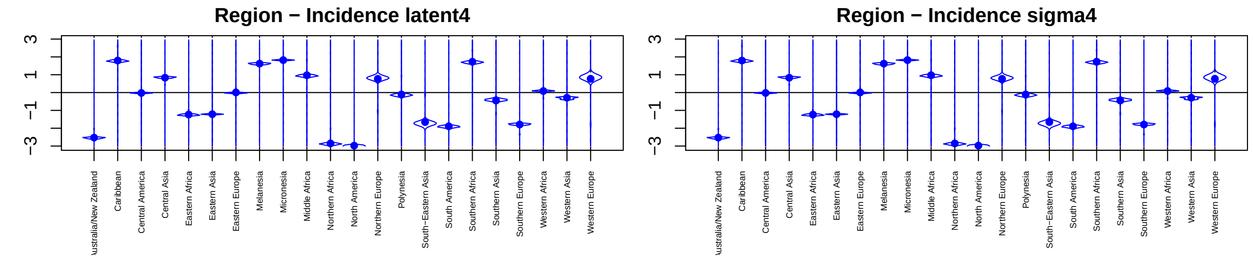

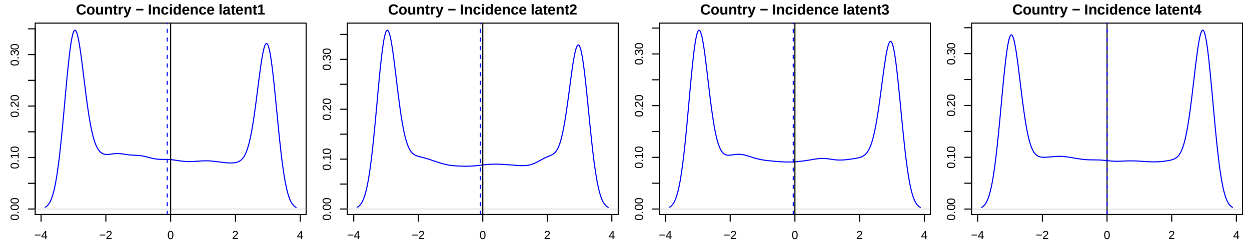

Parameters

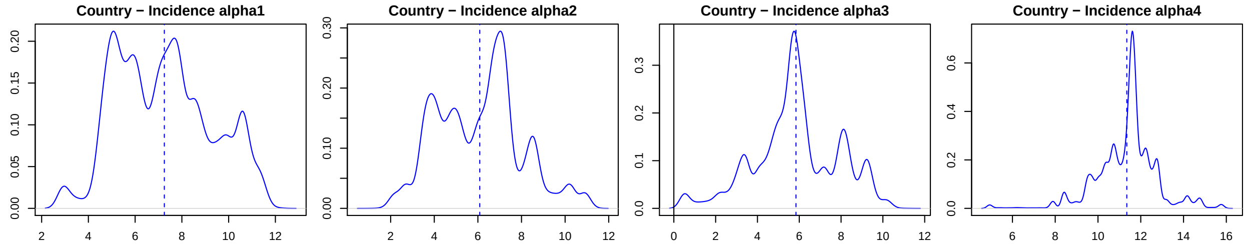

C.2.6.2 Male RR

Hyperparameters

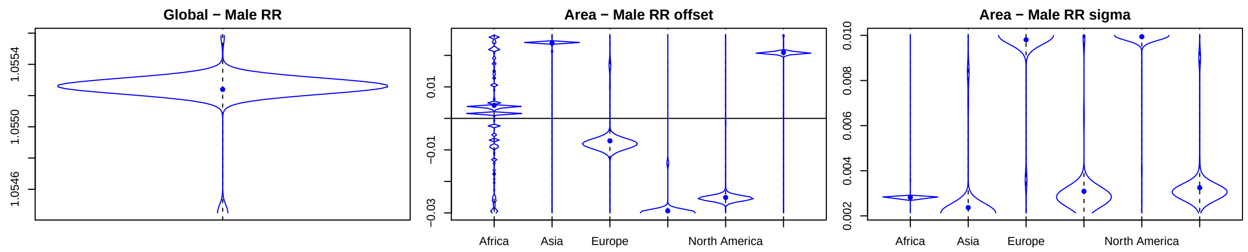

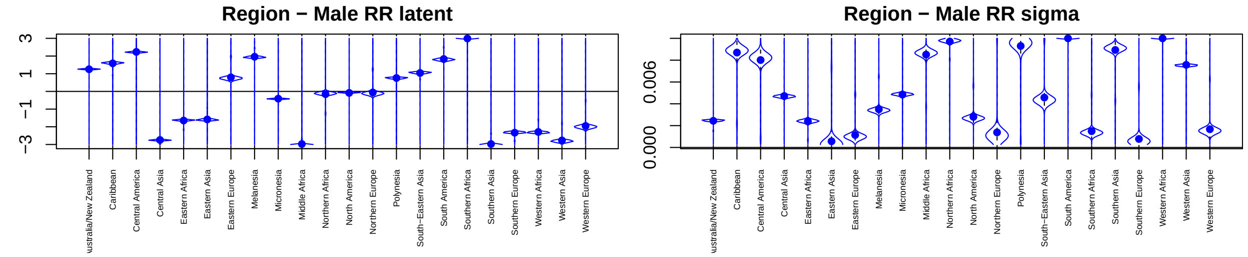

Parameters

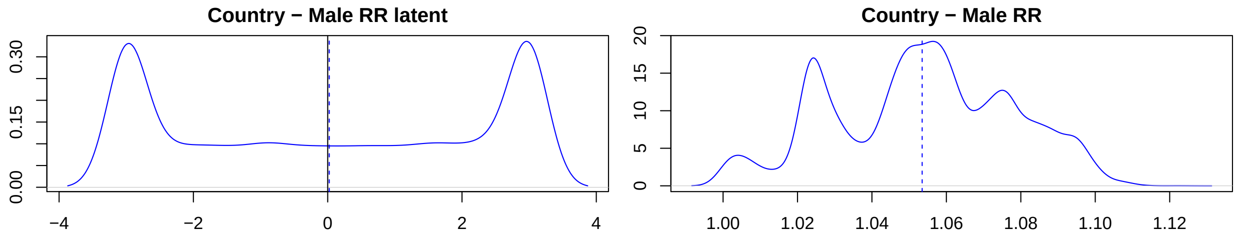
Country − Male RR latent
0.00
0.15
0.30
−4
−2
0
2
4
Country − Male RR
0
5
10
15
20
1.00
1.02
1.04
1.06
1.08
1.10
1.12

C.2.7 Lactational Amenorrhea

Hyperparameters

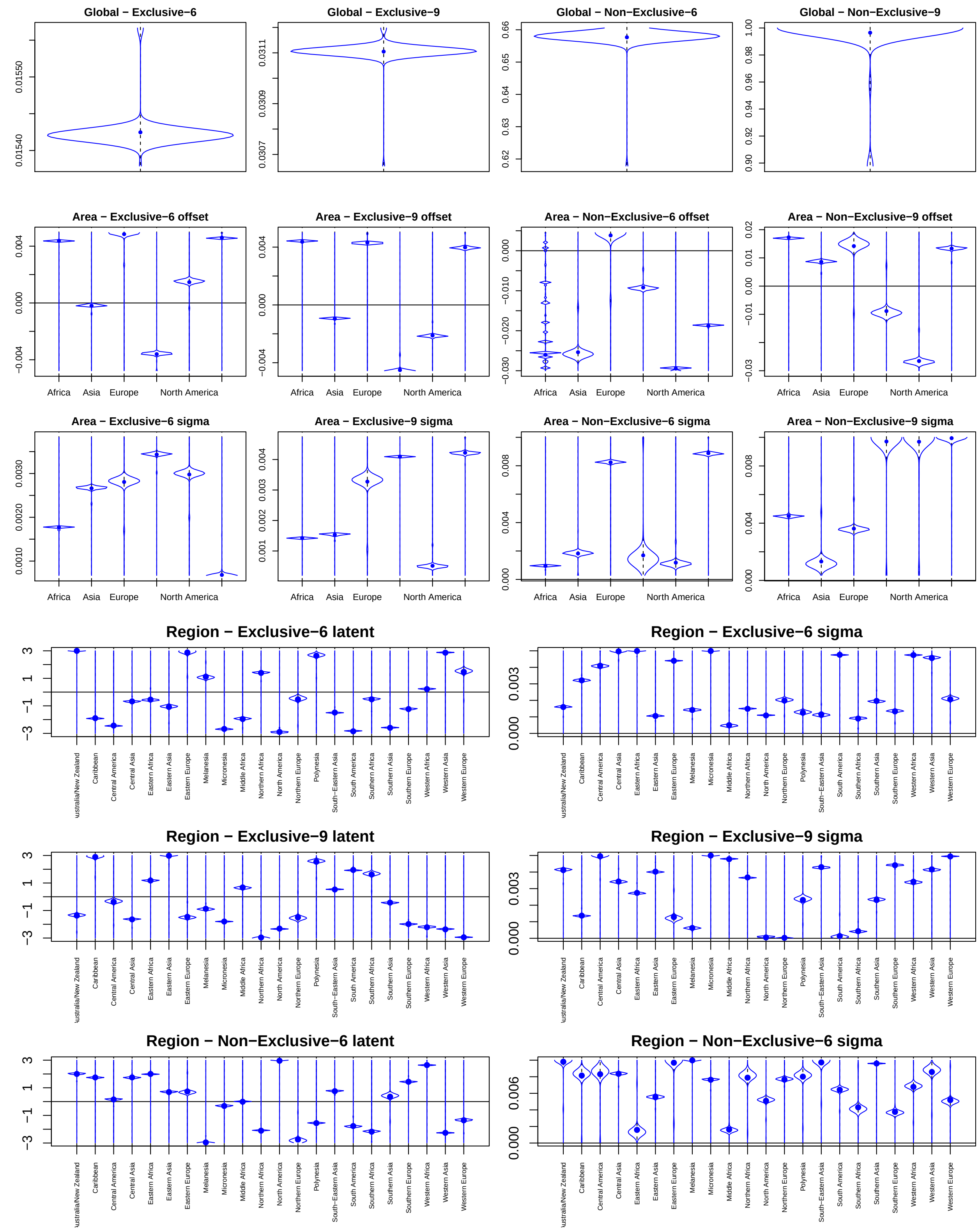

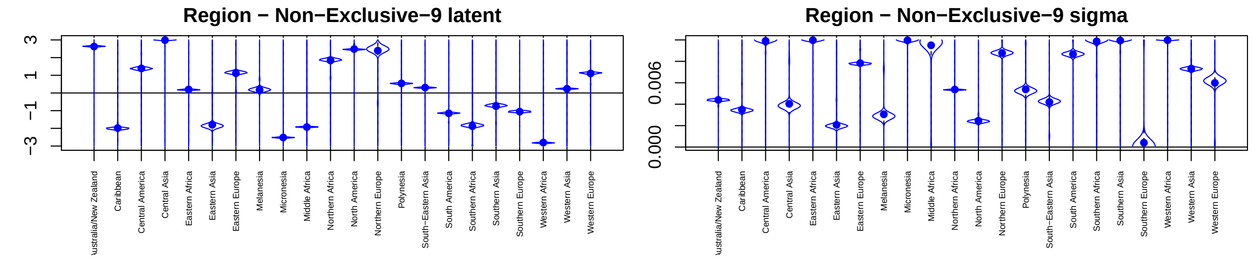
Region − Non−Exclusive−9 latent
Region − Non−Exclusive−9 sigma
Australia/New Zealand
Caribbean
Central America
Central Asia
Eastern Africa
Eastern Asia
Eastern Europe
Melanesia
Micronesia
Middle Africa
Northern Africa
North America
Northern Europe
Polynesia
South−Eastern Asia
South America
Southern Africa
Southern Asia
Southern Europe
Western Africa
Western Asia
Western Europe

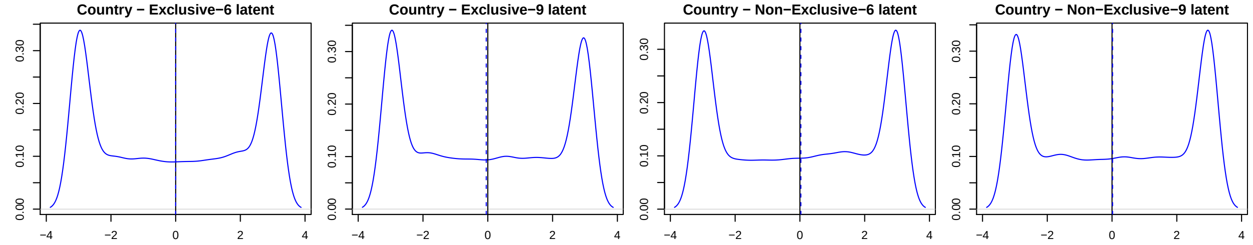
Country − Exclusive−6 latent
Country − Exclusive−9 latent
Country − Non−Exclusive−6 latent
Country − Non−Exclusive−9 latent

Parameters

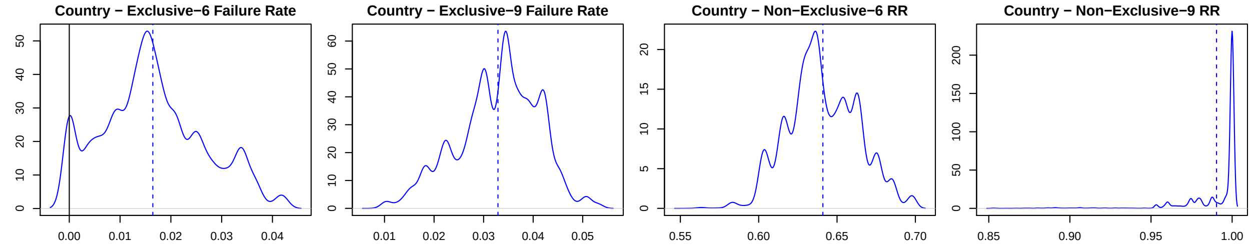
Country − Exclusive−6 Failure Rate
Country − Exclusive−9 Failure Rate
Country − Non−Exclusive−6 RR
Country − Non−Exclusive−9 RR

C.2.8 Menopause

Hyperparameters

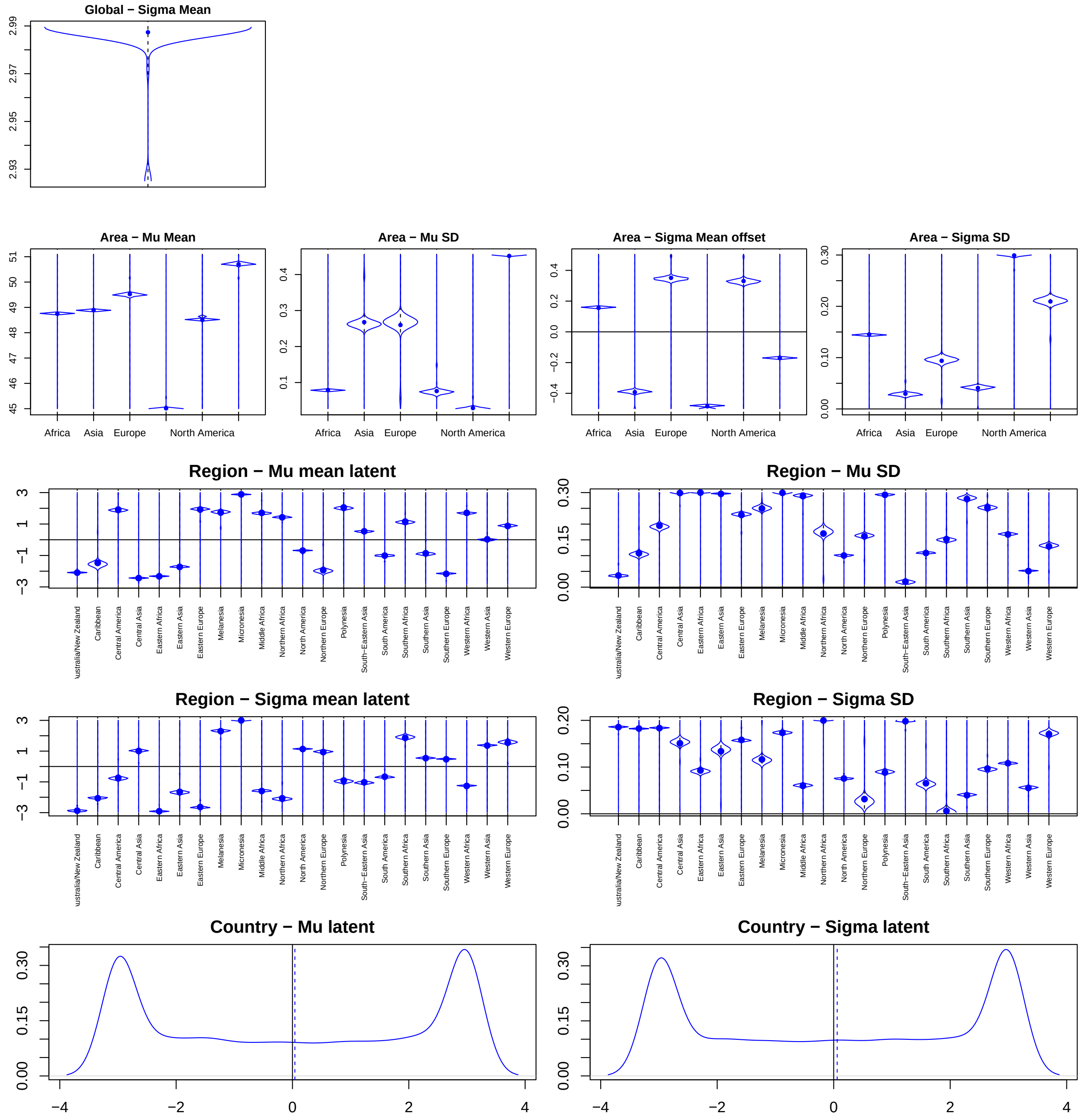

Parameters

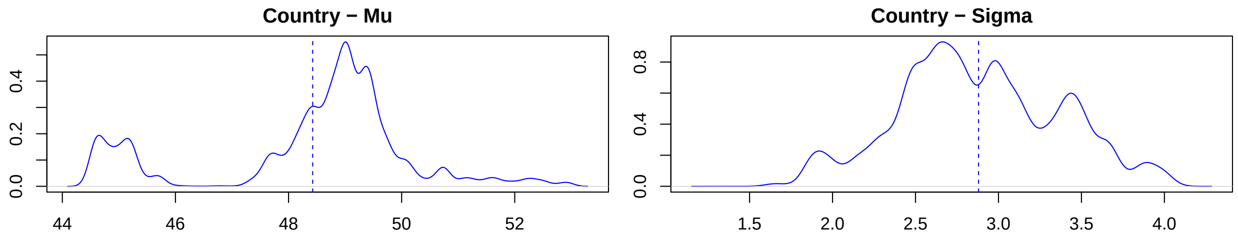

C.2.9 Anemia

Hyperparameters

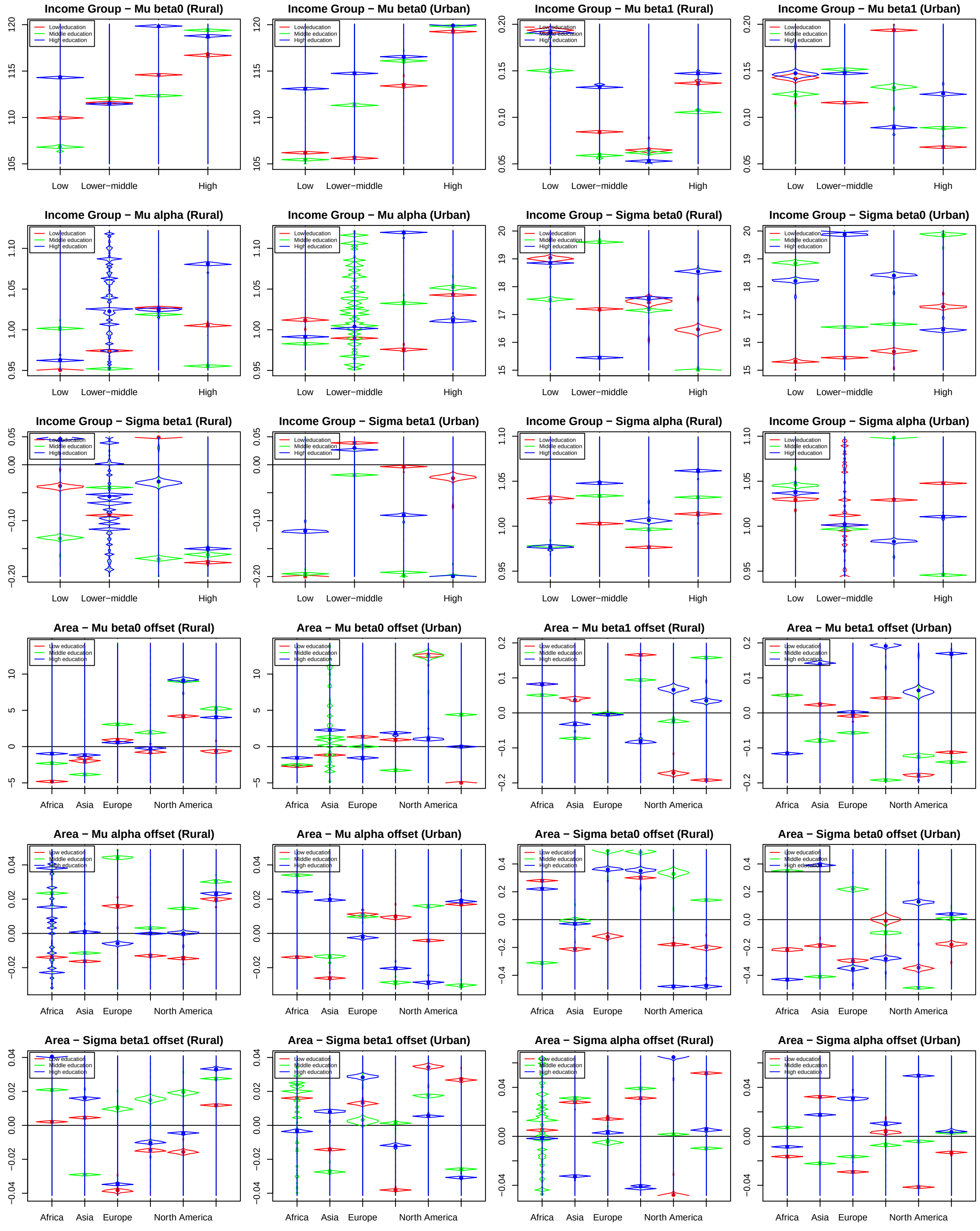

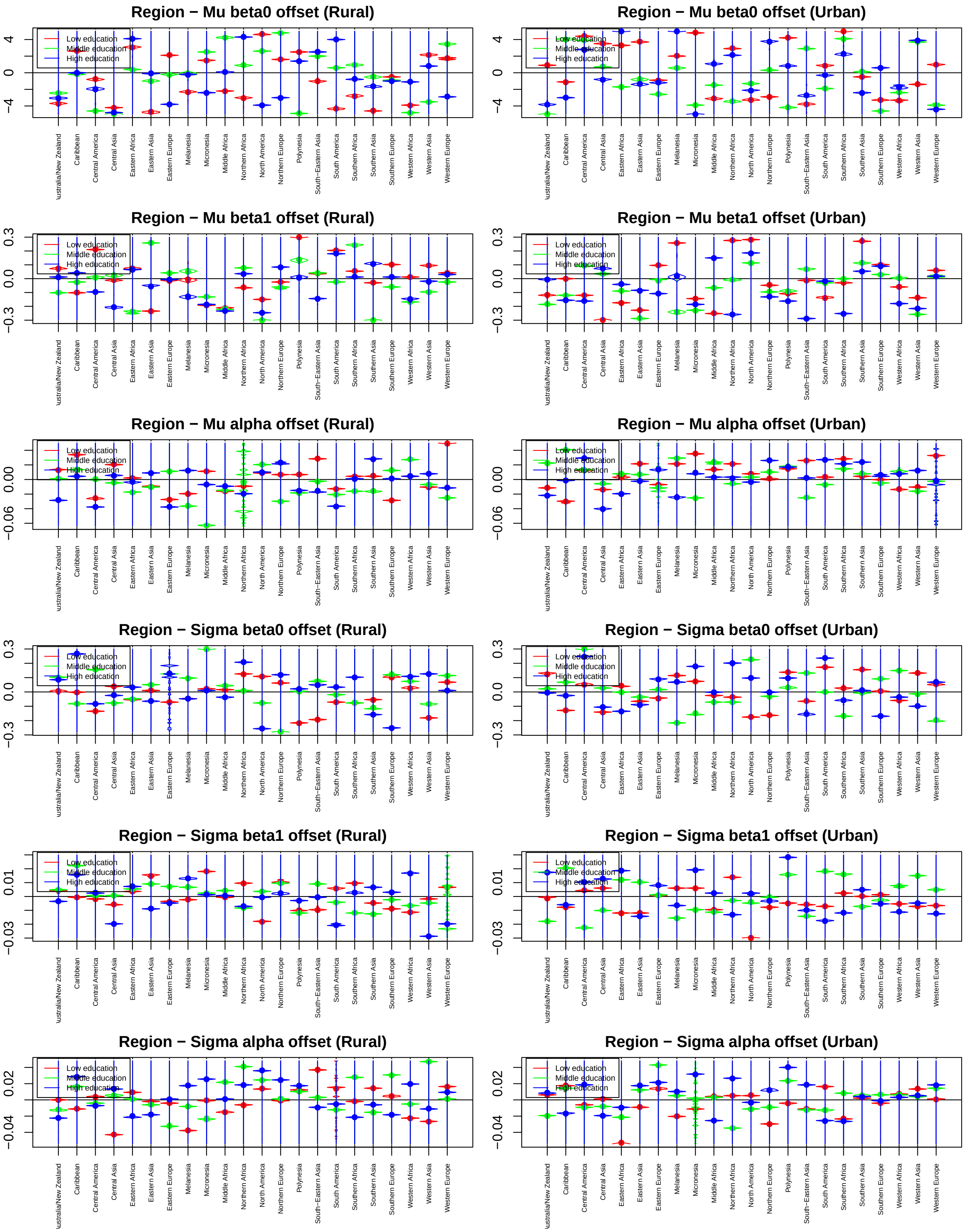
Region − Mu beta0 offset (Rural)
Region − Mu beta0 offset (Urban)
Region − Mu beta1 offset (Rural)
Region − Mu beta1 offset (Urban)
Region − Mu alpha offset (Rural)
Region − Mu alpha offset (Urban)
Region − Sigma beta0 offset (Rural)
Region − Sigma beta0 offset (Urban)
Region − Sigma beta1 offset (Rural)
Region − Sigma beta1 offset (Urban)
Region − Sigma alpha offset (Rural)
Region − Sigma alpha offset (Urban)
Low education
Middle education
High education
4
0
−4
0.3
0.0
−0.3
0.00
−0.06
0.01
−0.03
0.02
−0.04
ustralia/New Zealand
Caribbean
Central America
Central Asia
Eastern Africa
Eastern Asia
Eastern Europe
Melanesia
Micronesia
Middle Africa
Northern Africa
North America
Northern Europe
Polynesia
South−Eastern Asia
South America
Southern Africa
Southern Asia
Southern Europe
Western Africa
Western Asia
Western Europe

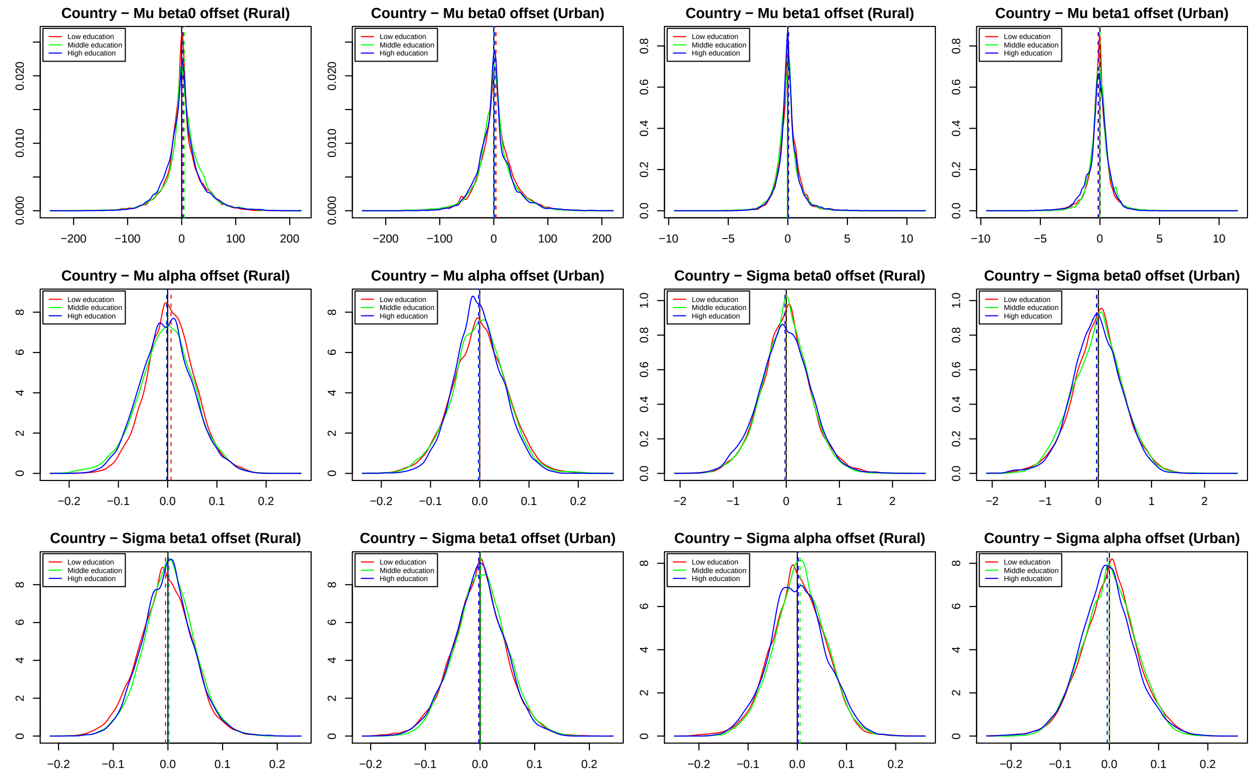
Country − Mu beta0 offset (Rural)
Country − Mu beta0 offset (Urban)
Country − Mu beta1 offset (Rural)
Country − Mu beta1 offset (Urban)
Country − Mu alpha offset (Rural)
Country − Mu alpha offset (Urban)
Country − Sigma beta0 offset (Rural)
Country − Sigma beta0 offset (Urban)
Country − Sigma beta1 offset (Rural)
Country − Sigma beta1 offset (Urban)
Country − Sigma alpha offset (Rural)
Country − Sigma alpha offset (Urban)
Low education
Middle education
High education

Parameters

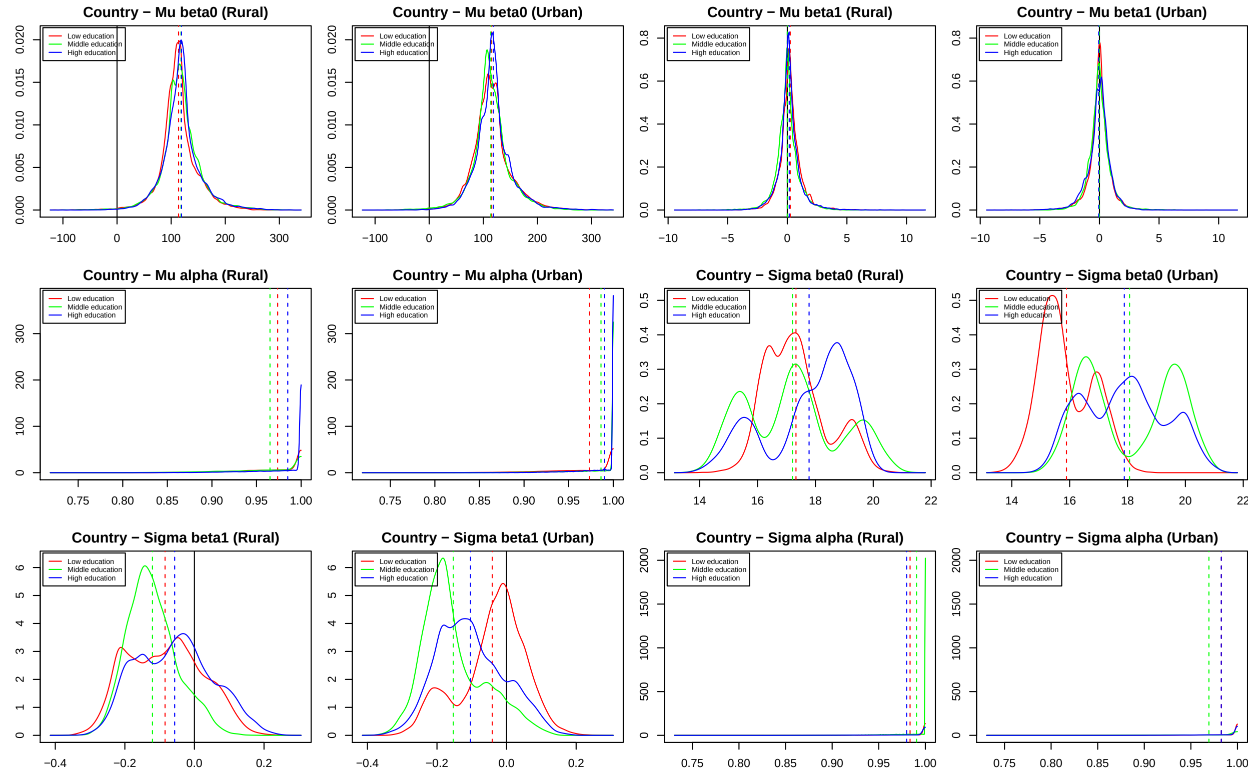
Country − Mu beta0 (Rural)
Country − Mu beta0 (Urban)
Country − Mu beta1 (Rural)
Country − Mu beta1 (Urban)
Country − Mu alpha (Rural)
Country − Mu alpha (Urban)
Country − Sigma beta0 (Rural)
Country − Sigma beta0 (Urban)
Country − Sigma beta1 (Rural)
Country − Sigma beta1 (Urban)
Country − Sigma alpha (Rural)
Country − Sigma alpha (Urban)
Low education
Middle education
High education

C.3 Family Planning

C.3.1 Age of Sexual Debut

Hyperparameters

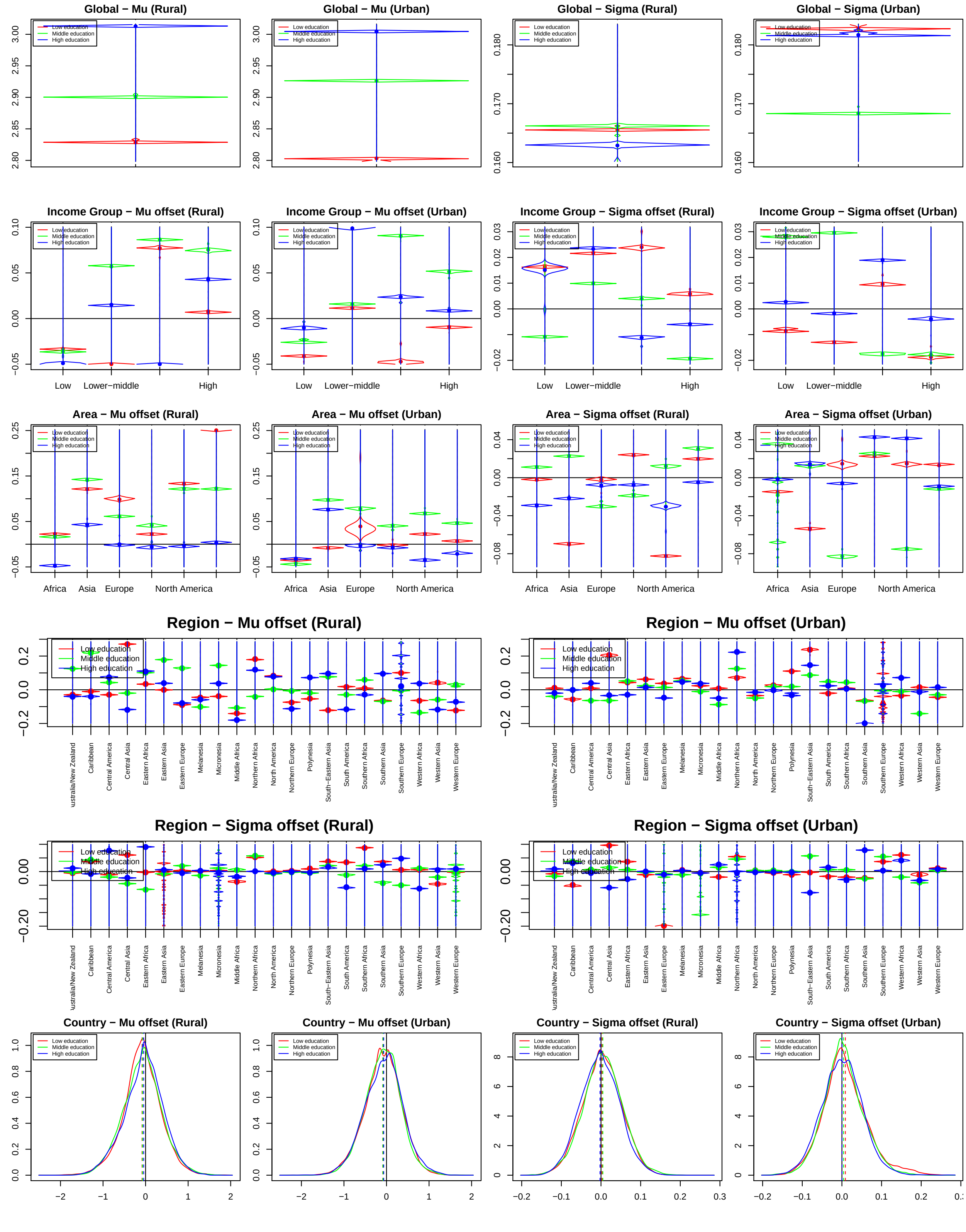

Parameters

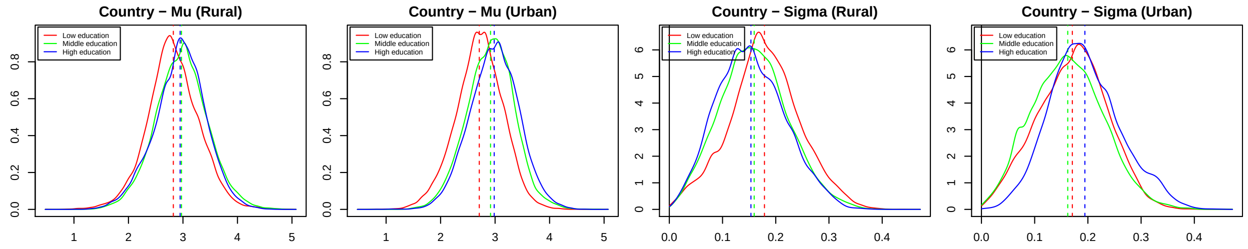
Country − Mu (Rural)
Country − Mu (Urban)
Country − Sigma (Rural)
Country − Sigma (Urban)
Low education
Middle education
High education

C.3.2 Number of Living Children

Hyperparameters

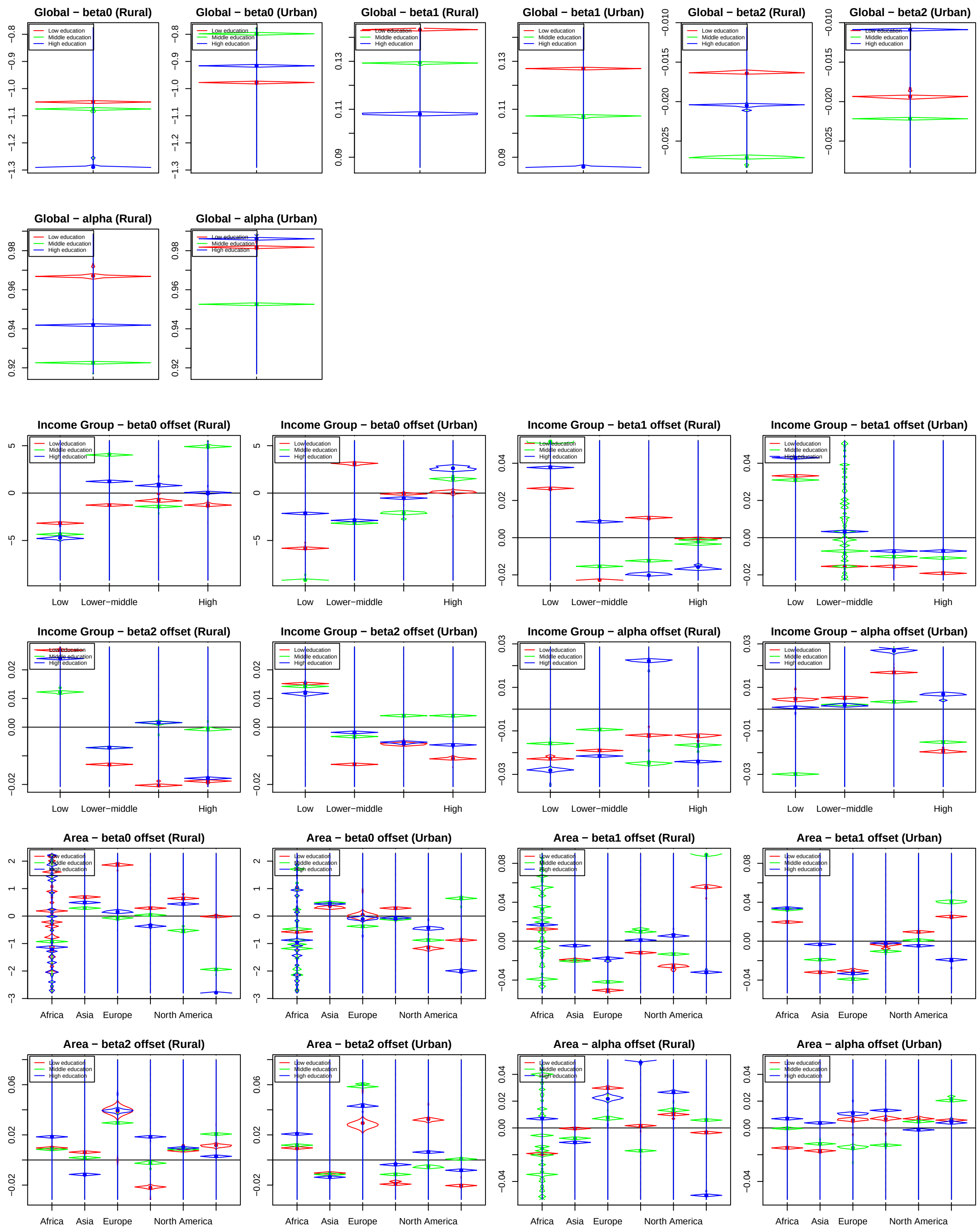

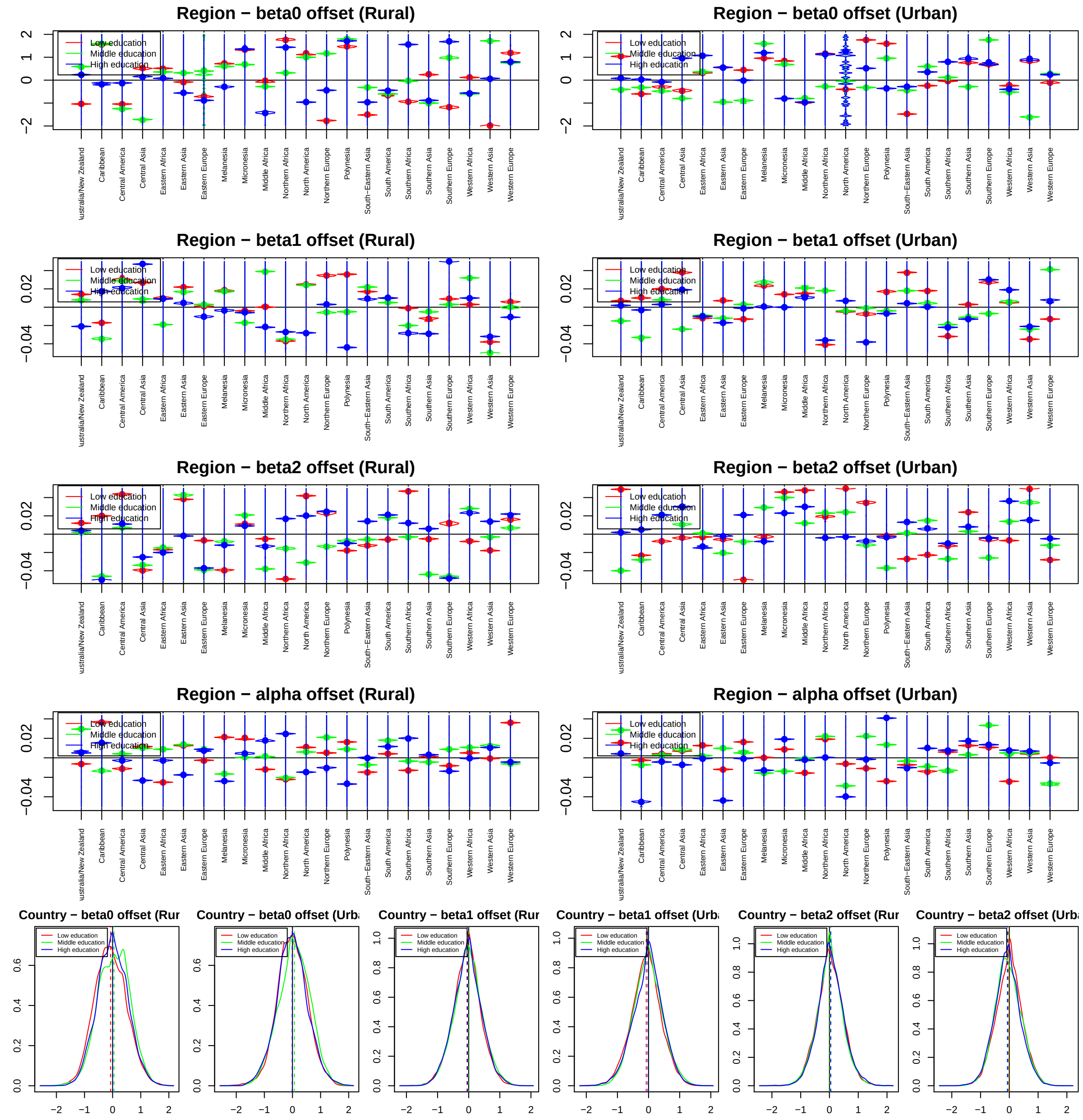
Region − beta0 offset (Rural)
Region − beta0 offset (Urban)
Region − beta1 offset (Rural)
Region − beta1 offset (Urban)
Region − beta2 offset (Rural)
Region − beta2 offset (Urban)
Region − alpha offset (Rural)
Region − alpha offset (Urban)
Low education
Middle education
High education
Country − beta0 offset (Rur
Country − beta0 offset (Urb
Country − beta1 offset (Rur
Country − beta1 offset (Urb
Country − beta2 offset (Rur
Country − beta2 offset (Urb

Parameters

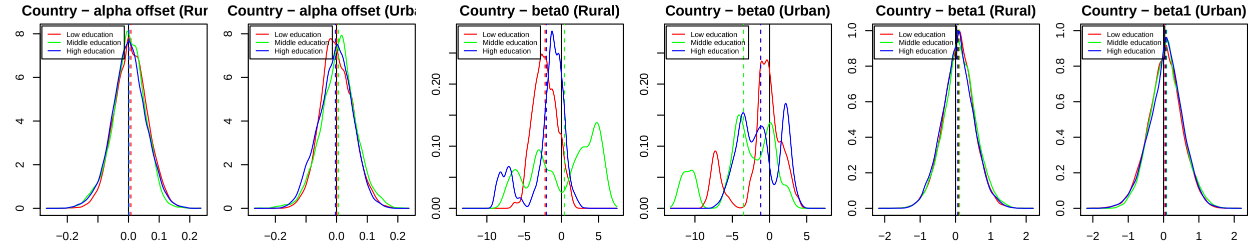
Country − alpha offset (Rur
Country − alpha offset (Urb
Country − beta0 (Rural)
Country − beta0 (Urban)
Country − beta1 (Rural)
Country − beta1 (Urban)
Low education
Middle education
High education

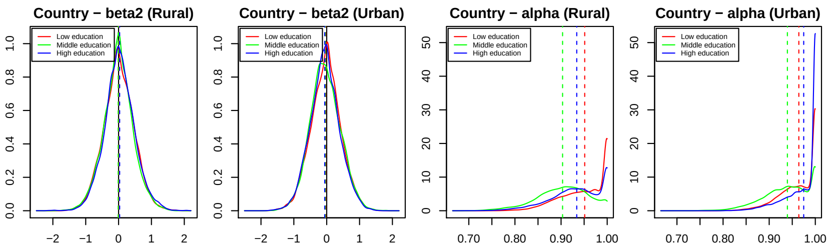
Country − beta2 (Rural)
Country − beta2 (Urban)
Country − alpha (Rural)
Country − alpha (Urban)
Low education
Middle education
High education

C.3.3 Desired Number of Children

Hyperparameters

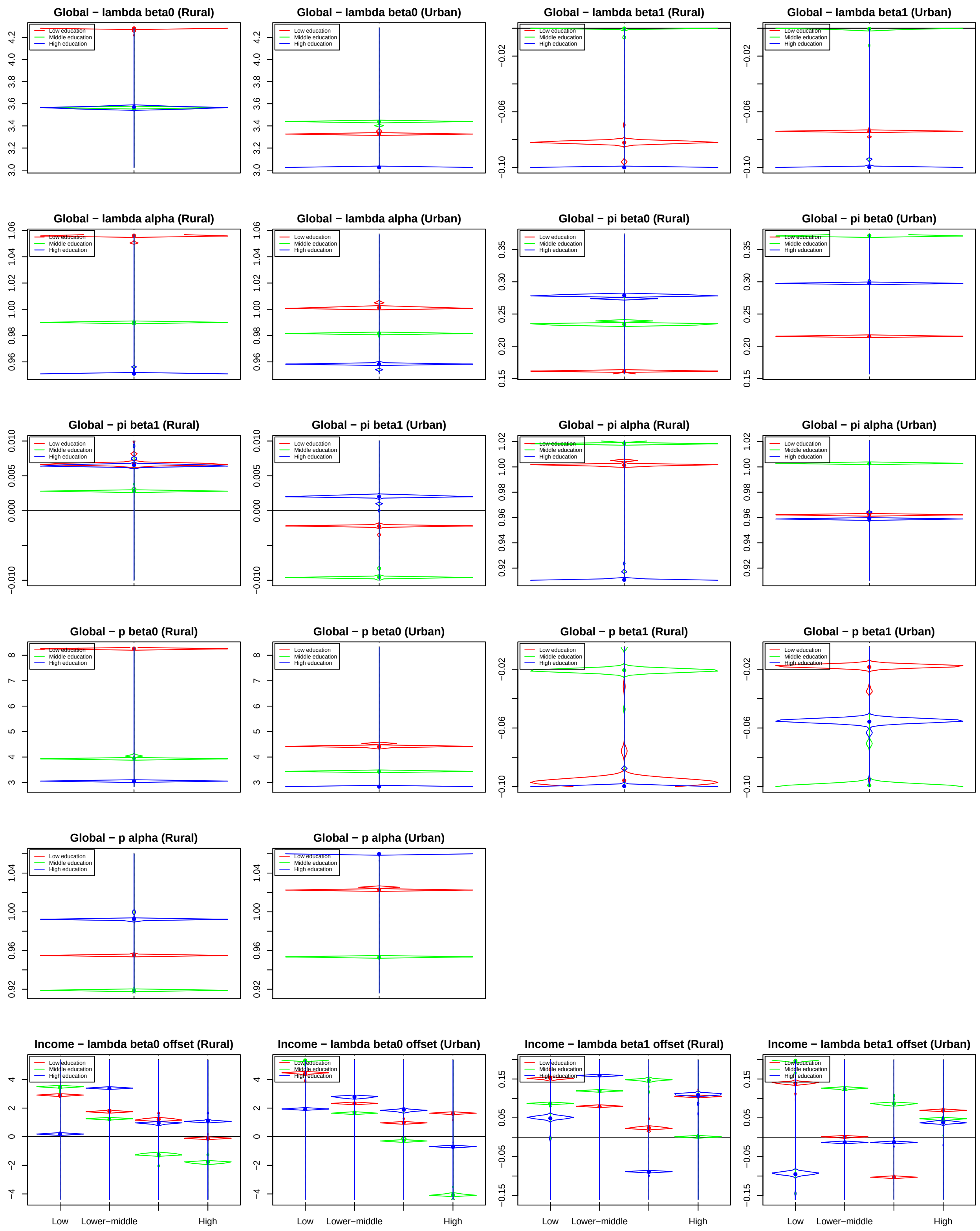

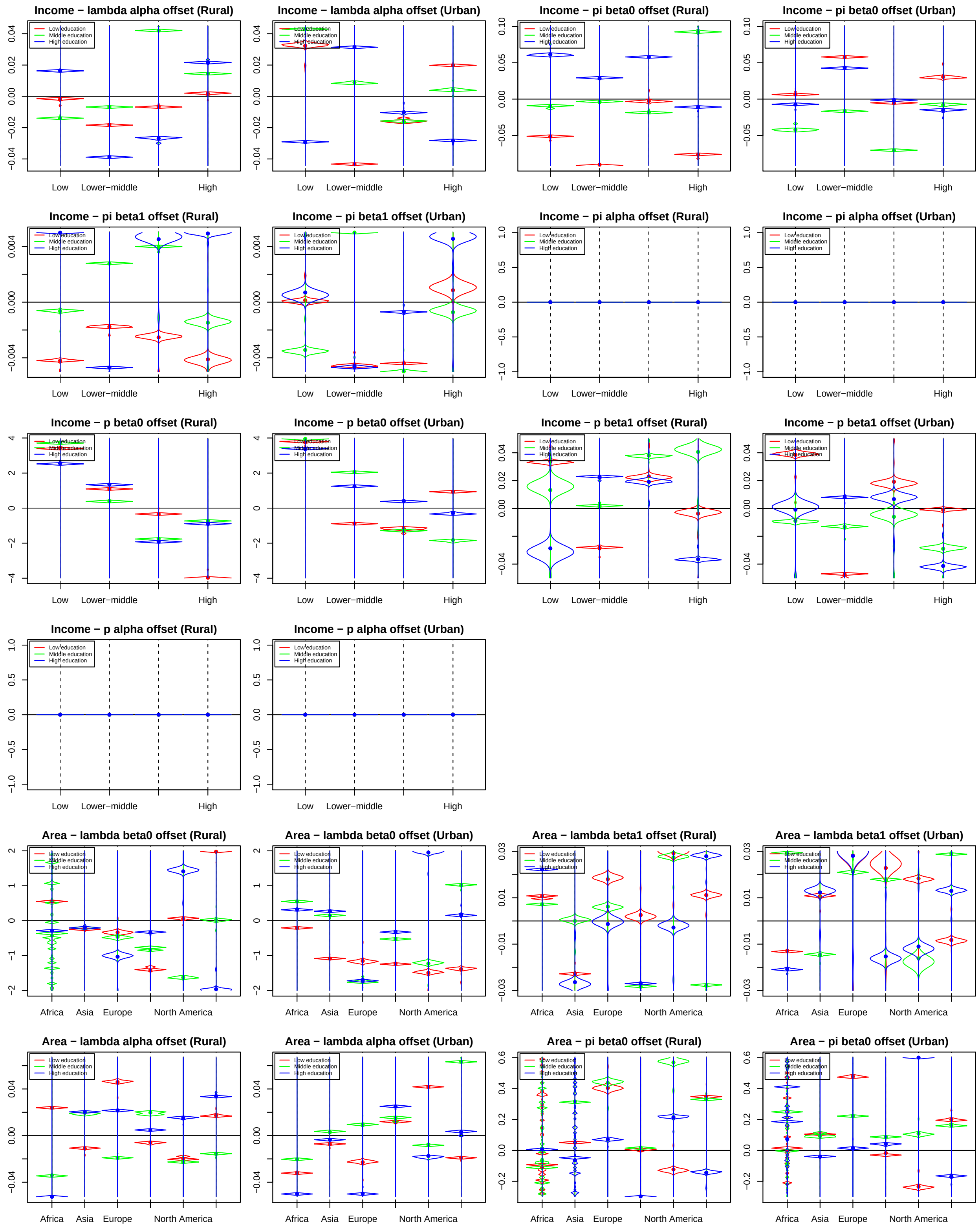
Income − lambda alpha offset (Rural)
Income − lambda alpha offset (Urban)
Income − pi beta0 offset (Rural)
Income − pi beta0 offset (Urban)
Income − pi beta1 offset (Rural)
Income − pi beta1 offset (Urban)
Income − pi alpha offset (Rural)
Income − pi alpha offset (Urban)
Income − p beta0 offset (Rural)
Income − p beta0 offset (Urban)
Income − p beta1 offset (Rural)
Income − p beta1 offset (Urban)
Income − p alpha offset (Rural)
Income − p alpha offset (Urban)
Area − lambda beta0 offset (Rural)
Area − lambda beta0 offset (Urban)
Area − lambda beta1 offset (Rural)
Area − lambda beta1 offset (Urban)
Area − lambda alpha offset (Rural)
Area − lambda alpha offset (Urban)
Area − pi beta0 offset (Rural)
Area − pi beta0 offset (Urban)
Low education
Middle education
High education
Low
Lower−middle
High
Africa
Asia
Europe
North America

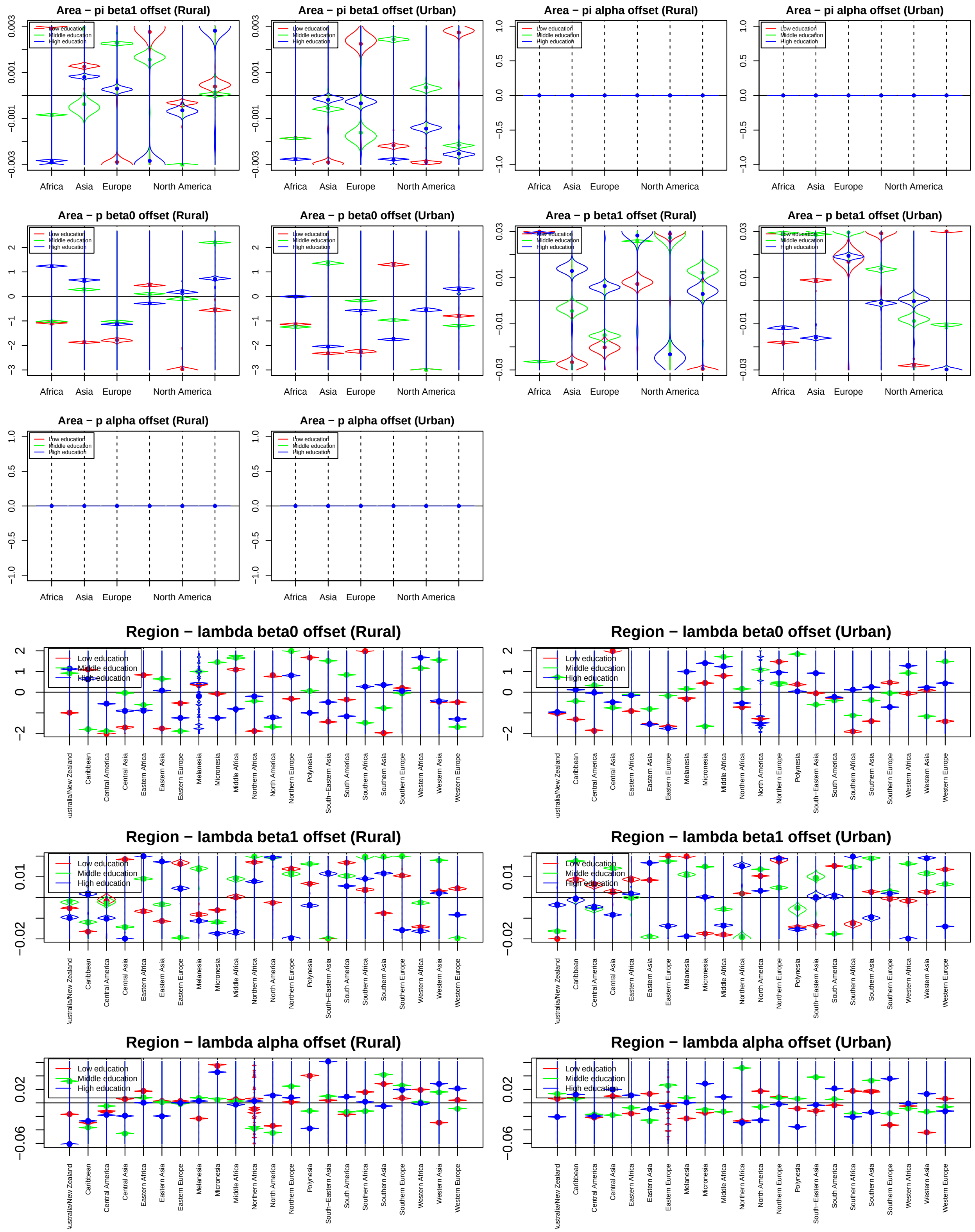
Area − pi beta1 offset (Rural)
Area − pi beta1 offset (Urban)
Area − pi alpha offset (Rural)
Area − pi alpha offset (Urban)
Area − p beta0 offset (Rural)
Area − p beta0 offset (Urban)
Area − p beta1 offset (Rural)
Area − p beta1 offset (Urban)
Area − p alpha offset (Rural)
Area − p alpha offset (Urban)
Low education
Middle education
High education
Africa
Asia
Europe
North America
Region − lambda beta0 offset (Rural)
Region − lambda beta0 offset (Urban)
Region − lambda beta1 offset (Rural)
Region − lambda beta1 offset (Urban)
Region − lambda alpha offset (Rural)
Region − lambda alpha offset (Urban)
Caribbean
Central America
Central Asia
Eastern Africa
Eastern Asia
Eastern Europe
Melanesia
Micronesia
Middle Africa
Northern Africa
North America
Northern Europe
Polynesia
South−Eastern Asia
South America
Southern Africa
Southern Asia
Southern Europe
Western Africa
Western Asia
Western Europe

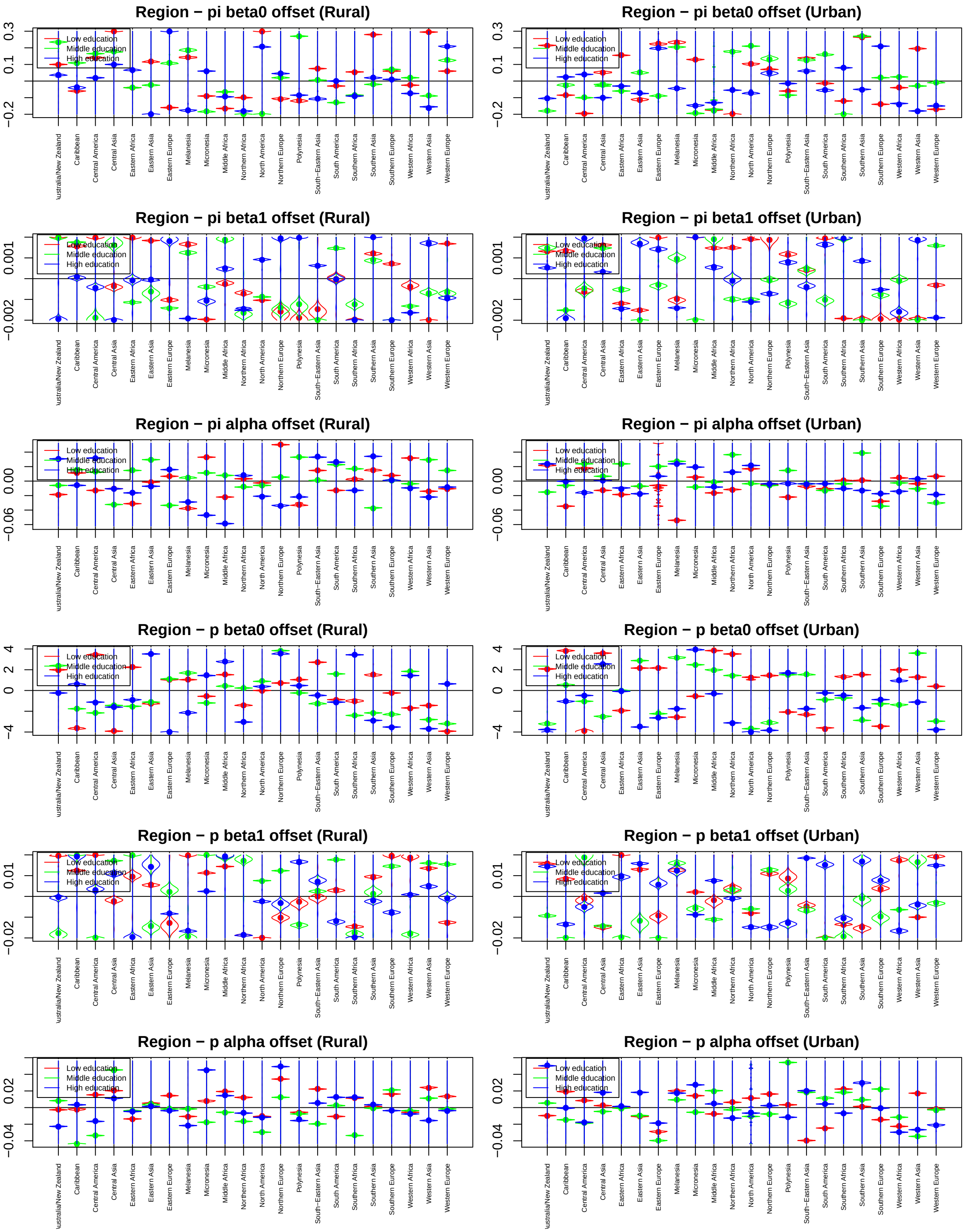
Region − pi beta0 offset (Rural)
Region − pi beta0 offset (Urban)
Region − pi beta1 offset (Rural)
Region − pi beta1 offset (Urban)
Region − pi alpha offset (Rural)
Region − pi alpha offset (Urban)
Region − p beta0 offset (Rural)
Region − p beta0 offset (Urban)
Region − p beta1 offset (Rural)
Region − p beta1 offset (Urban)
Region − p alpha offset (Rural)
Region − p alpha offset (Urban)
Low education
Middle education
High education
Caribbean
Central America
Central Asia
Eastern Africa
Eastern Asia
Eastern Europe
Melanesia
Micronesia
Middle Africa
Northern Africa
North America
Northern Europe
Polynesia
South−Eastern Asia
South America
Southern Africa
Southern Asia
Southern Europe
Western Africa
Western Asia
Western Europe

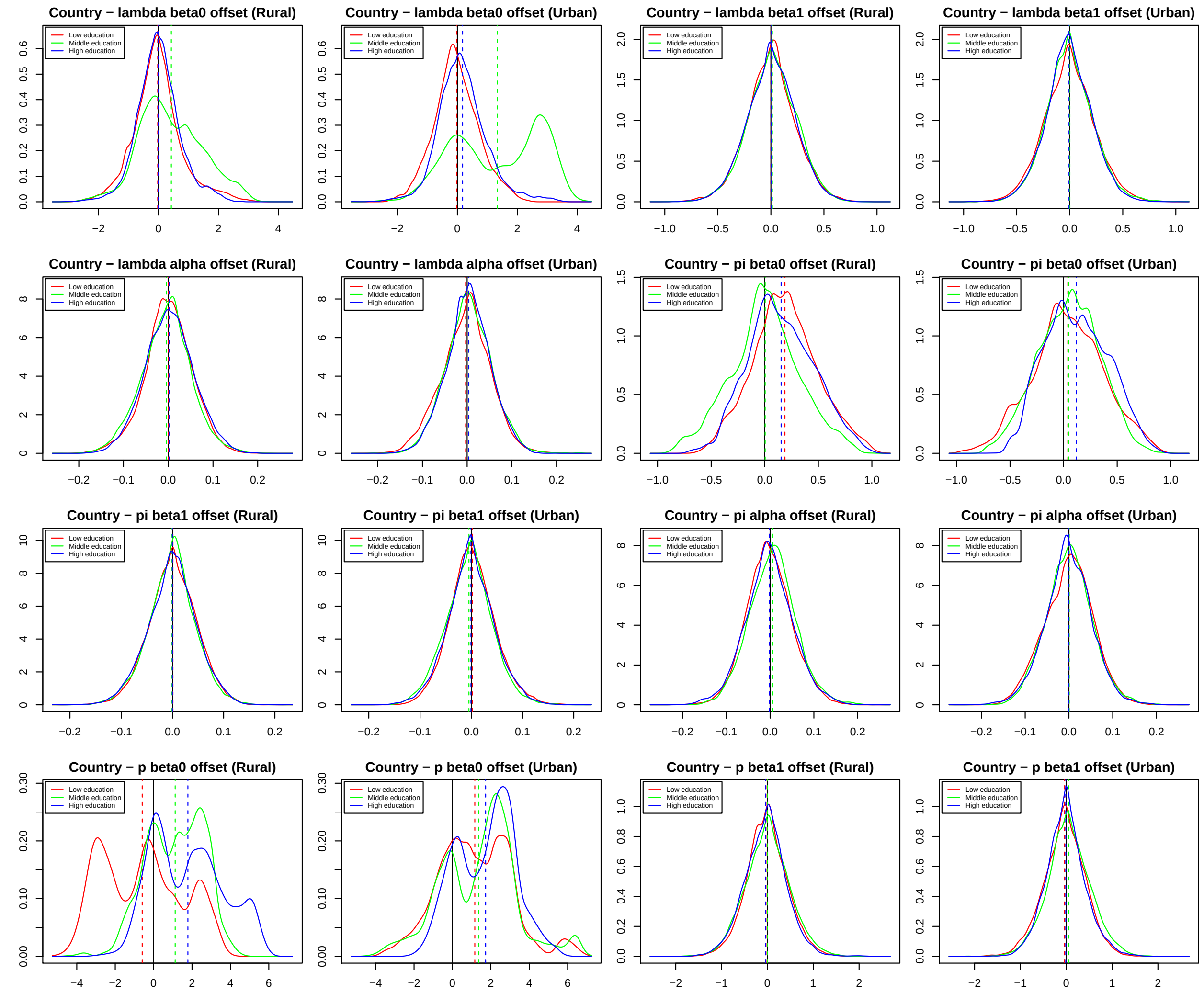
Country − lambda beta0 offset (Rural)
Country − lambda beta0 offset (Urban)
Country − lambda beta1 offset (Rural)
Country − lambda beta1 offset (Urban)
Country − lambda alpha offset (Rural)
Country − lambda alpha offset (Urban)
Country − pi beta0 offset (Rural)
Country − pi beta0 offset (Urban)
Country − pi beta1 offset (Rural)
Country − pi beta1 offset (Urban)
Country − pi alpha offset (Rural)
Country − pi alpha offset (Urban)
Country − p beta0 offset (Rural)
Country − p beta0 offset (Urban)
Country − p beta1 offset (Rural)
Country − p beta1 offset (Urban)
Low education
Middle education
High education

Parameters

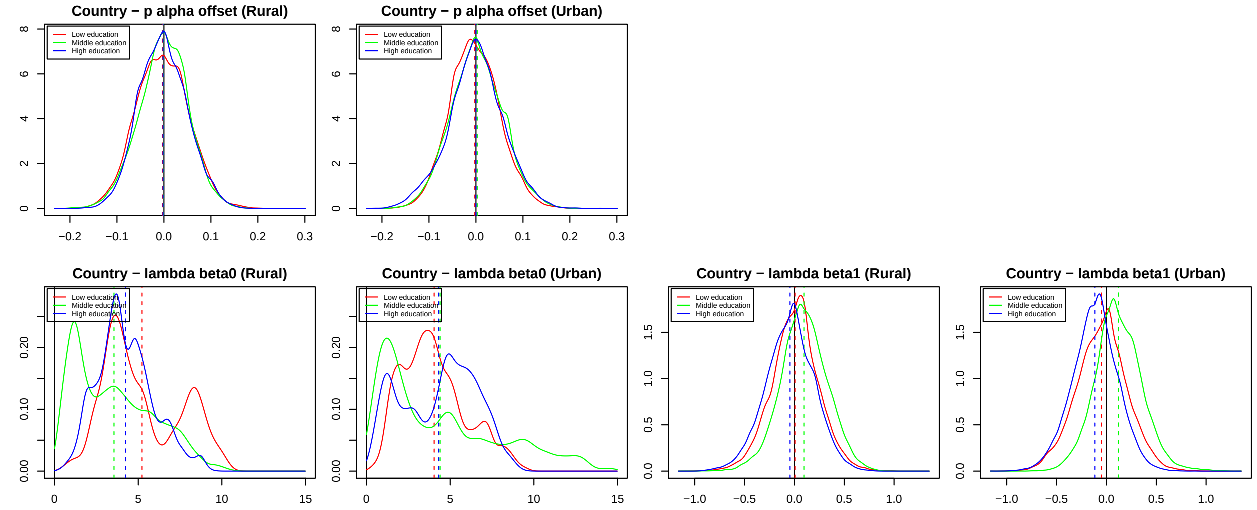
Country − p alpha offset (Rural)
Country − p alpha offset (Urban)
Country − lambda beta0 (Rural)
Country − lambda beta0 (Urban)
Country − lambda beta1 (Rural)
Country − lambda beta1 (Urban)
Low education
Middle education
High education

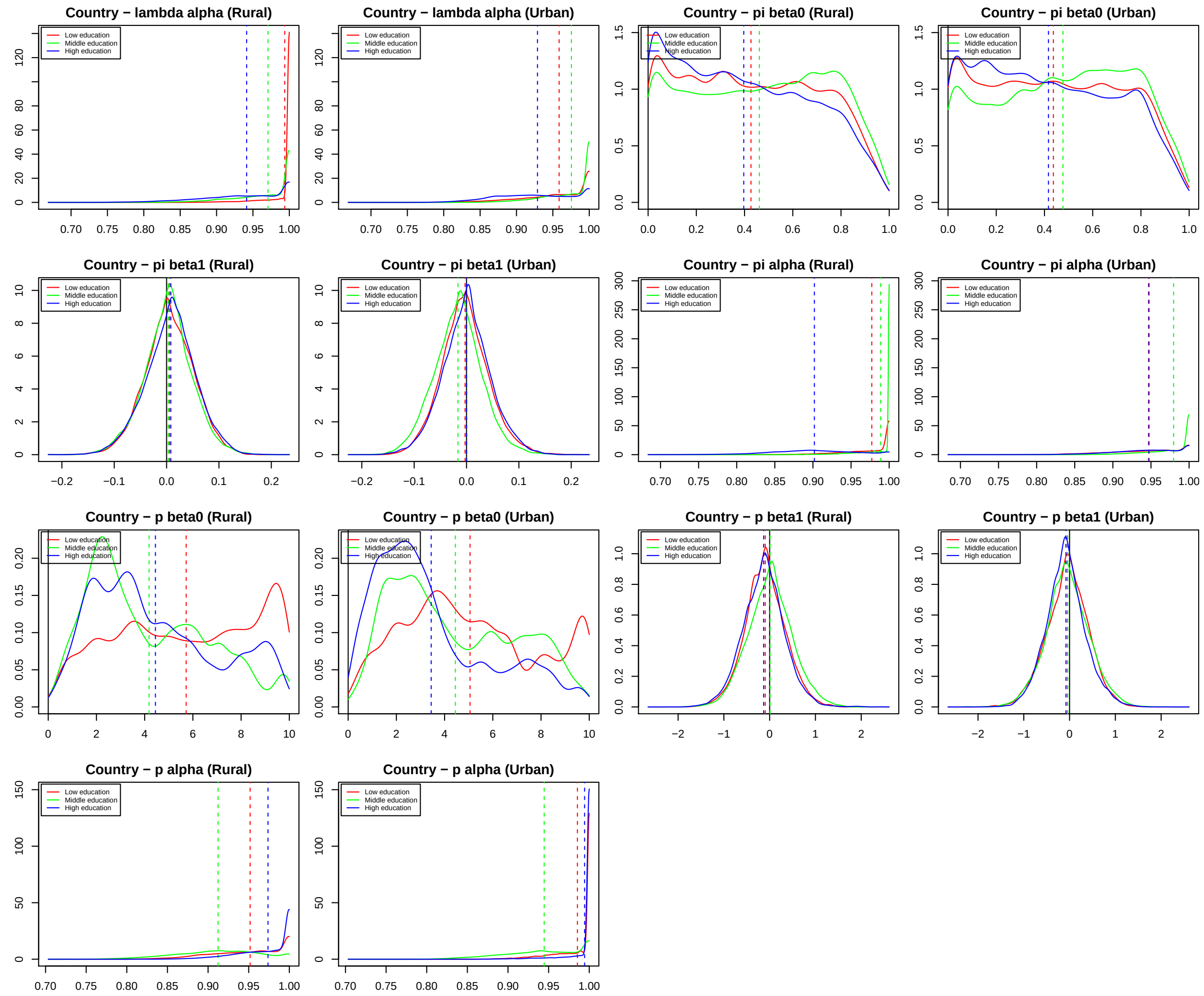
Country − lambda alpha (Rural)
Country − lambda alpha (Urban)
Country − pi beta0 (Rural)
Country − pi beta0 (Urban)
Country − pi beta1 (Rural)
Country − pi beta1 (Urban)
Country − pi alpha (Rural)
Country − pi alpha (Urban)
Country − p beta0 (Rural)
Country − p beta0 (Urban)
Country − p beta1 (Rural)
Country − p beta1 (Urban)
Country − p alpha (Rural)
Country − p alpha (Urban)
Low education
Middle education
High education

C.3.4 Unmet Need

C.3.4.1 No Need

Hyperparameters

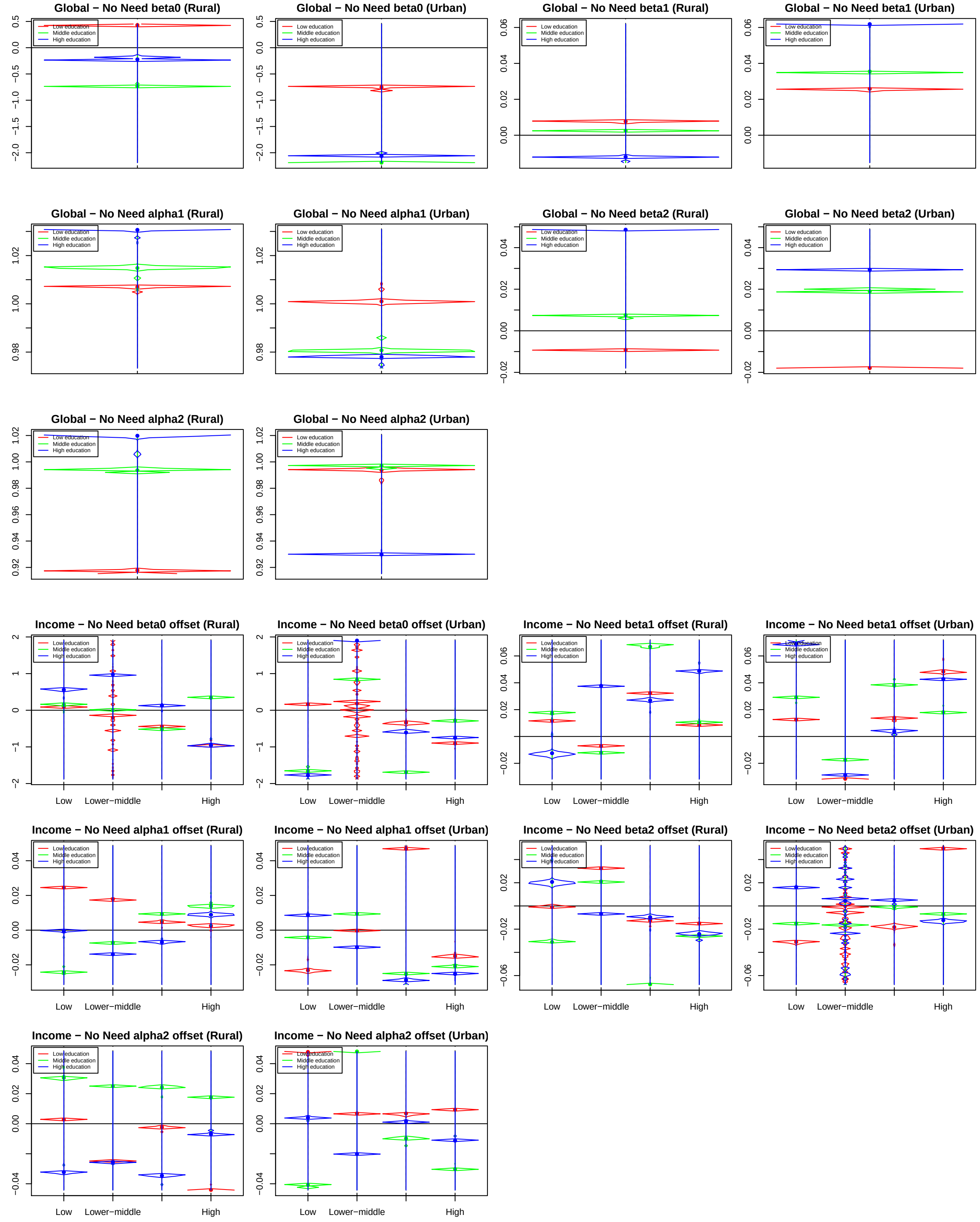

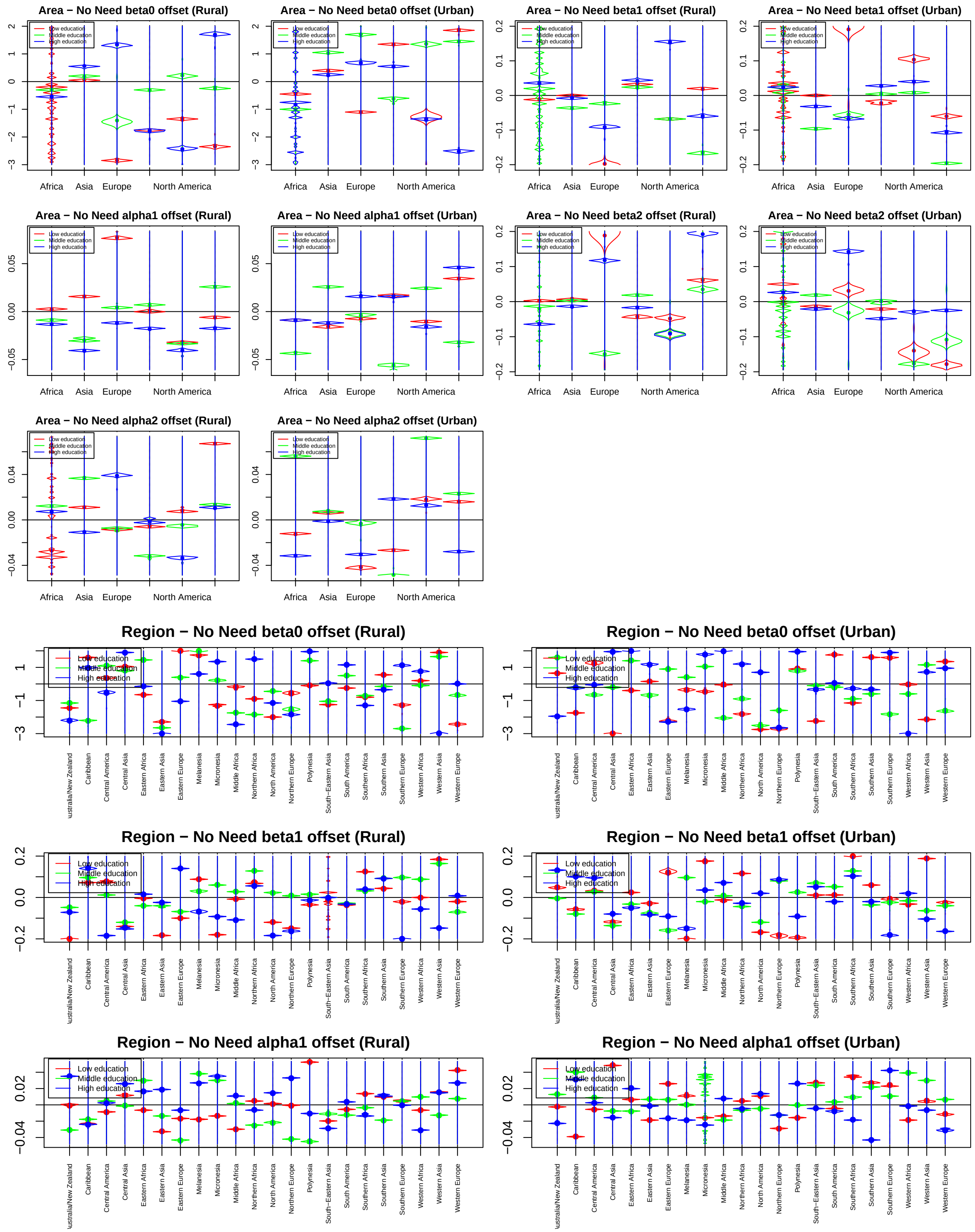
Area − No Need beta0 offset (Rural)
Area − No Need beta0 offset (Urban)
Area − No Need beta1 offset (Rural)
Area − No Need beta1 offset (Urban)
Area − No Need alpha1 offset (Rural)
Area − No Need alpha1 offset (Urban)
Area − No Need beta2 offset (Rural)
Area − No Need beta2 offset (Urban)
Area − No Need alpha2 offset (Rural)
Area − No Need alpha2 offset (Urban)
Low education
Middle education
High education
Africa
Asia
Europe
North America
Region − No Need beta0 offset (Rural)
Region − No Need beta0 offset (Urban)
Region − No Need beta1 offset (Rural)
Region − No Need beta1 offset (Urban)
Region − No Need alpha1 offset (Rural)
Region − No Need alpha1 offset (Urban)
Caribbean
Central America
Central Asia
Eastern Africa
Eastern Asia
Eastern Europe
Melanesia
Micronesia
Middle Africa
Northern Africa
North America
Northern Europe
Polynesia
South−Eastern Asia
South America
Southern Africa
Southern Asia
Southern Europe
Western Africa
Western Asia
Western Europe

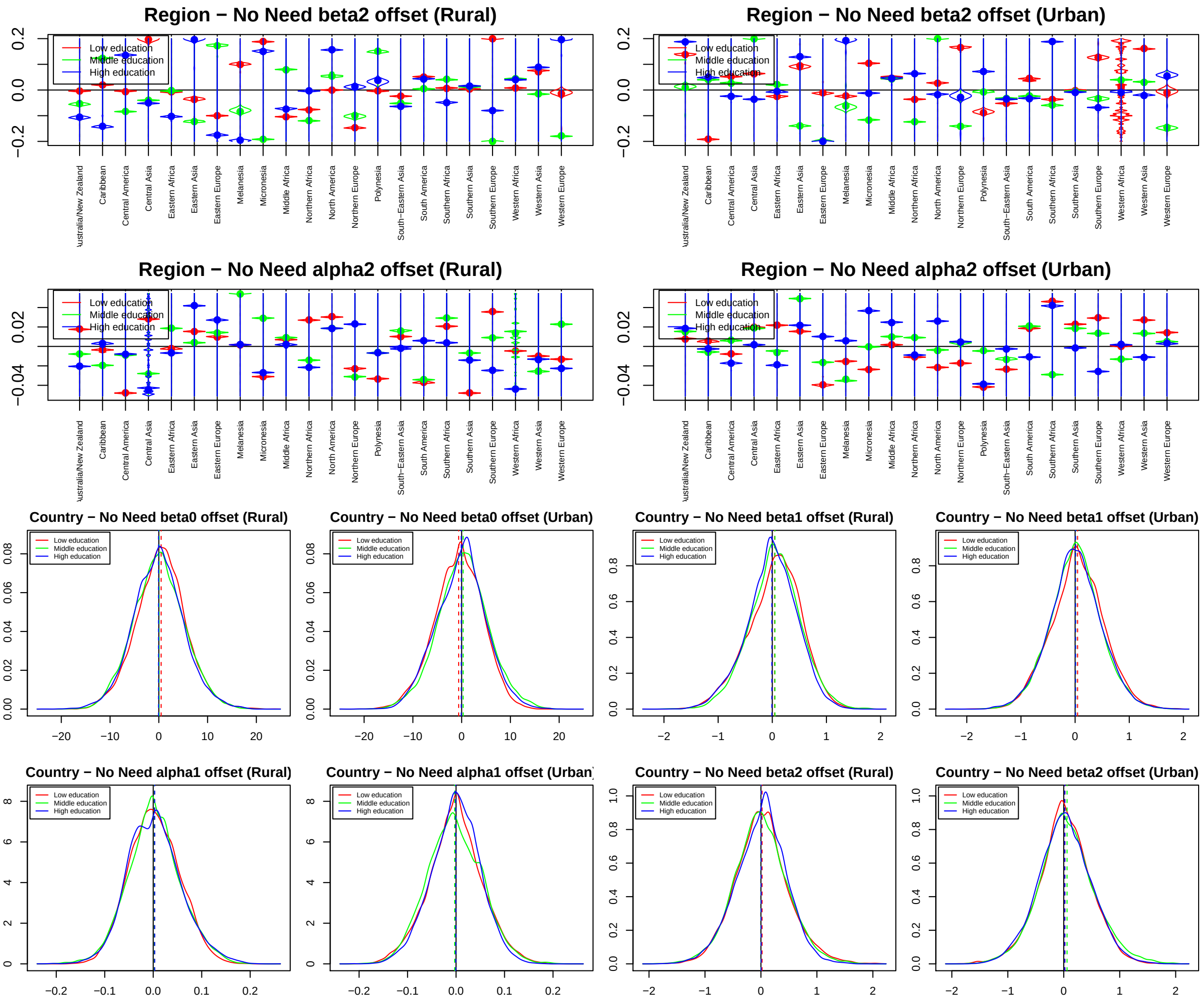
Region − No Need beta2 offset (Rural)
Region − No Need beta2 offset (Urban)
Region − No Need alpha2 offset (Rural)
Region − No Need alpha2 offset (Urban)
Country − No Need beta0 offset (Rural)
Country − No Need beta0 offset (Urban)
Country − No Need beta1 offset (Rural)
Country − No Need beta1 offset (Urban)
Country − No Need alpha1 offset (Rural)
Country − No Need alpha1 offset (Urban)
Country − No Need beta2 offset (Rural)
Country − No Need beta2 offset (Urban)
Low education
Middle education
High education

Parameters

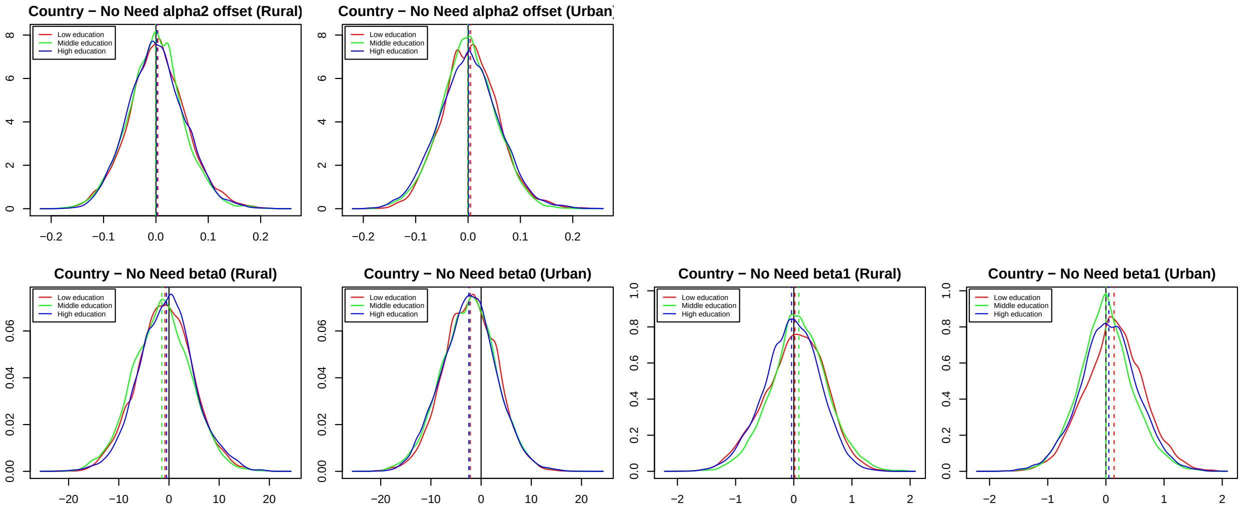
Country − No Need alpha2 offset (Rural)
Country − No Need alpha2 offset (Urban)
Country − No Need beta0 (Rural)
Country − No Need beta0 (Urban)
Country − No Need beta1 (Rural)
Country − No Need beta1 (Urban)
Low education
Middle education
High education

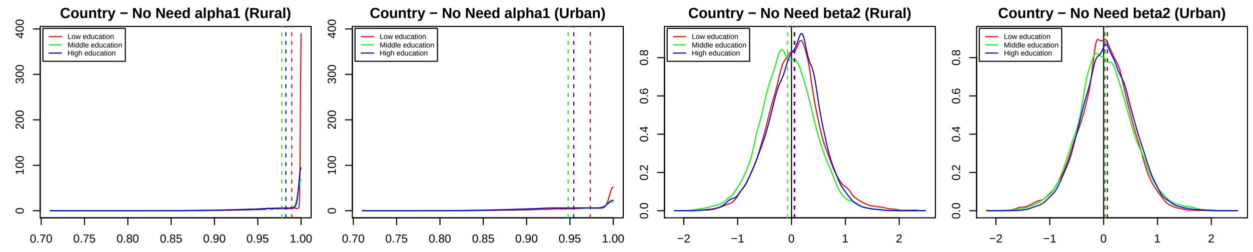

C.3.4.2 Spacing

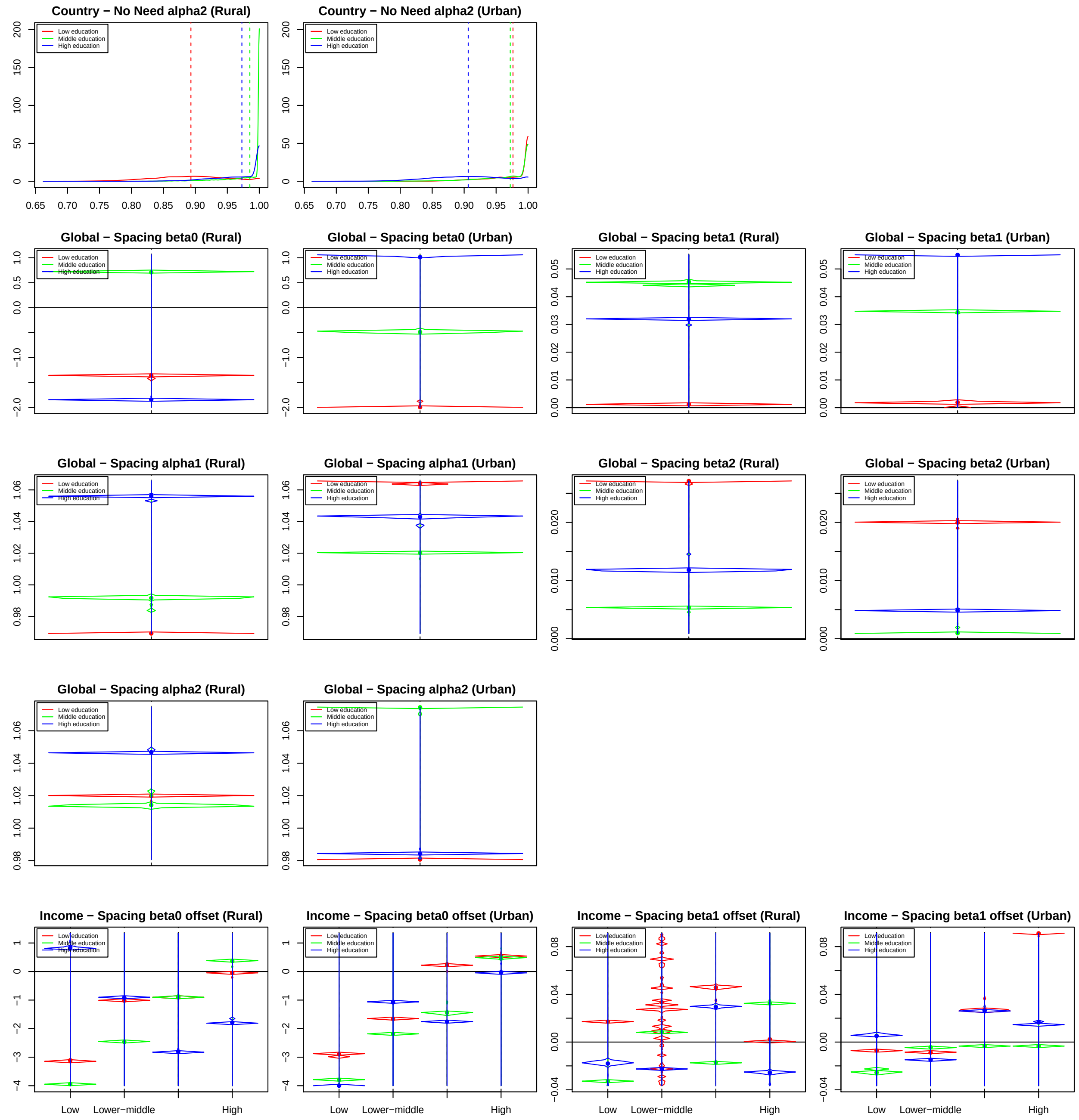

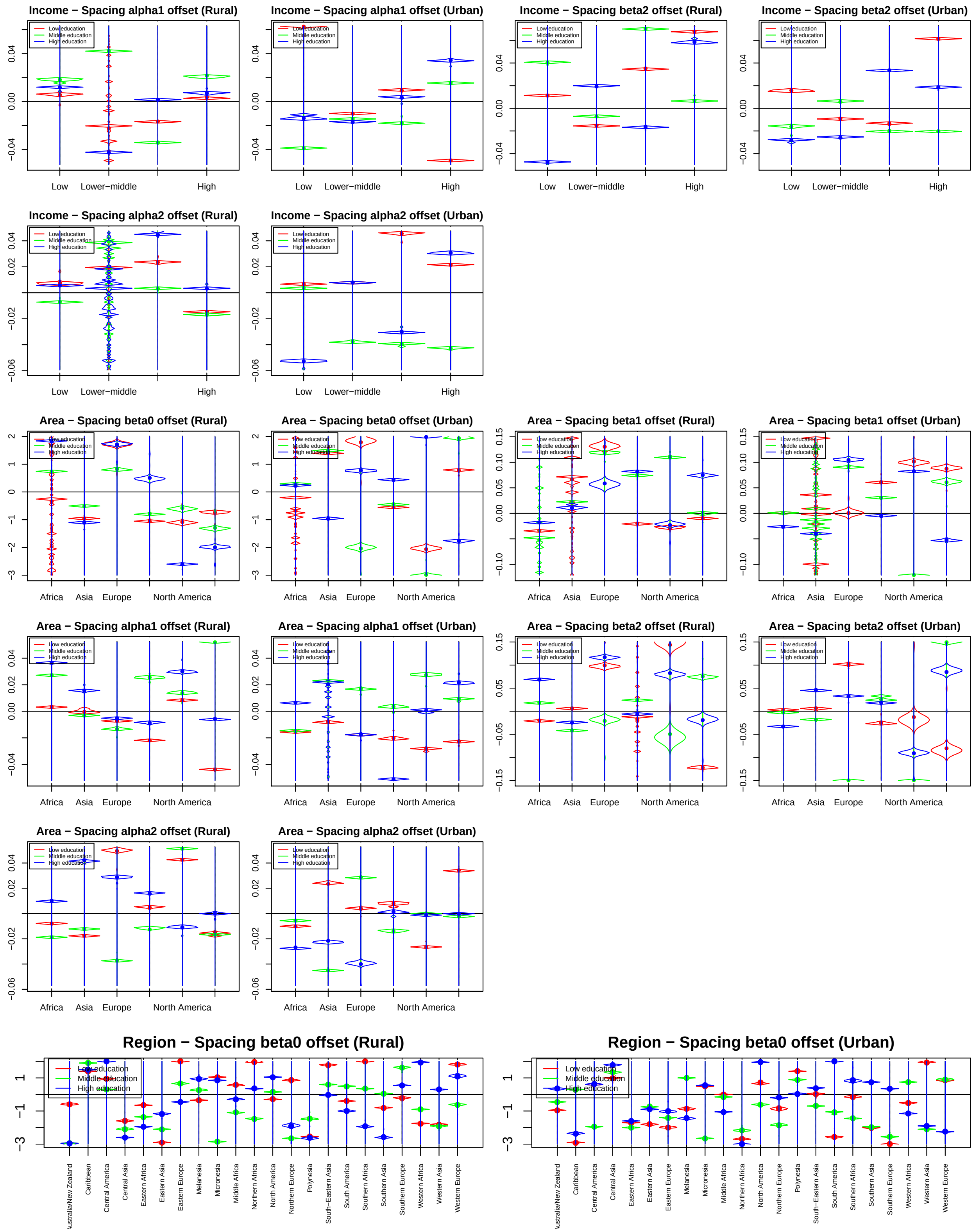
Income − Spacing alpha1 offset (Rural)
Income − Spacing alpha1 offset (Urban)
Income − Spacing beta2 offset (Rural)
Income − Spacing beta2 offset (Urban)
Income − Spacing alpha2 offset (Rural)
Income − Spacing alpha2 offset (Urban)
Area − Spacing beta0 offset (Rural)
Area − Spacing beta0 offset (Urban)
Area − Spacing beta1 offset (Rural)
Area − Spacing beta1 offset (Urban)
Area − Spacing alpha1 offset (Rural)
Area − Spacing alpha1 offset (Urban)
Area − Spacing beta2 offset (Rural)
Area − Spacing beta2 offset (Urban)
Area − Spacing alpha2 offset (Rural)
Area − Spacing alpha2 offset (Urban)
Region − Spacing beta0 offset (Rural)
Region − Spacing beta0 offset (Urban)
Low education
Middle education
High education
Low
Lower−middle
High
Africa
Asia
Europe
North America
Australia/New Zealand
Caribbean
Central America
Central Asia
Eastern Africa
Eastern Asia
Eastern Europe
Melanesia
Micronesia
Middle Africa
Northern Africa
North America
Northern Europe
Polynesia
South−Eastern Asia
South America
Southern Africa
Southern Asia
Southern Europe
Western Africa
Western Asia
Western Europe

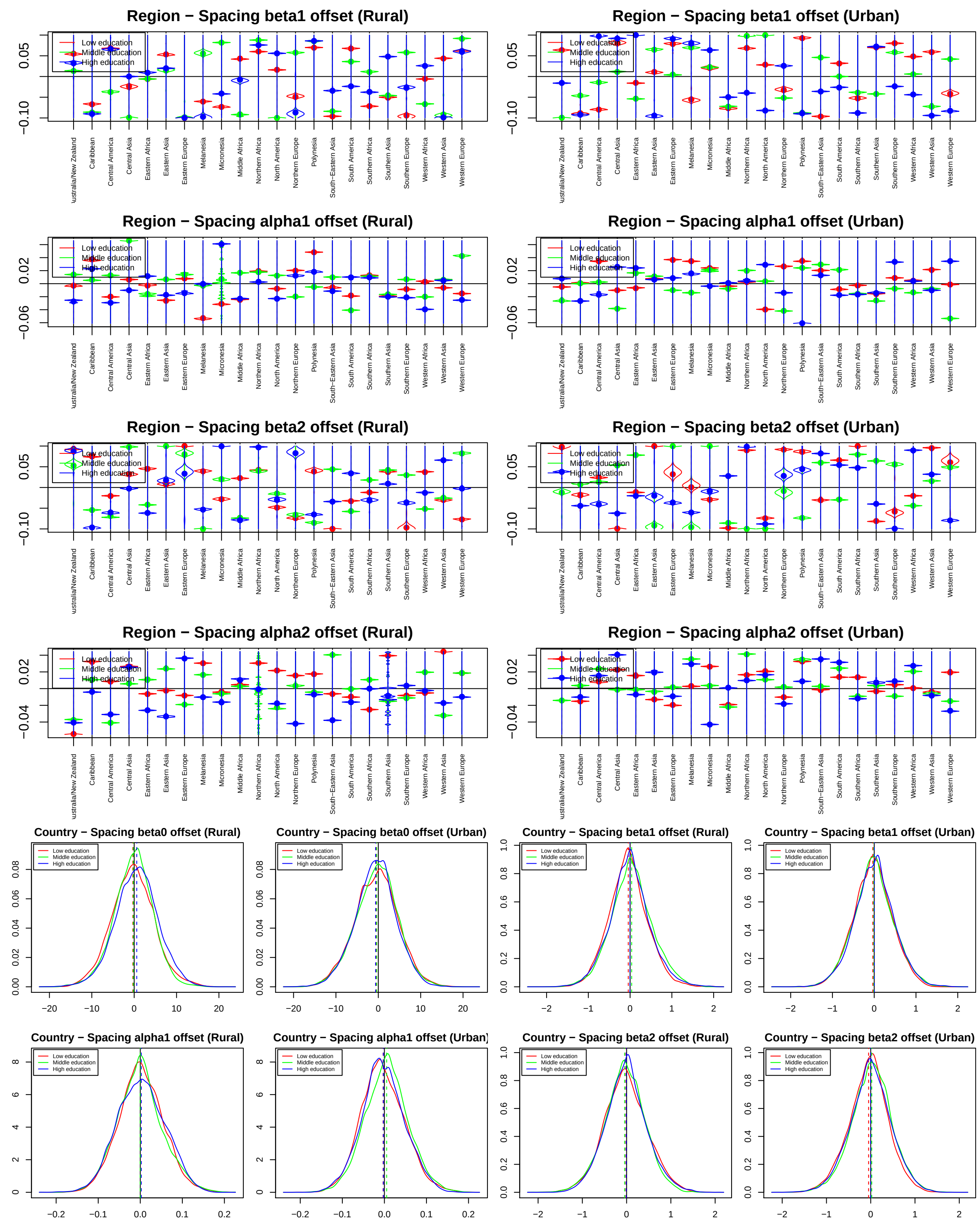
Region − Spacing beta1 offset (Rural)
Region − Spacing beta1 offset (Urban)
Region − Spacing alpha1 offset (Rural)
Region − Spacing alpha1 offset (Urban)
Region − Spacing beta2 offset (Rural)
Region − Spacing beta2 offset (Urban)
Region − Spacing alpha2 offset (Rural)
Region − Spacing alpha2 offset (Urban)
Low education
Middle education
High education
Australia/New Zealand
Caribbean
Central America
Central Asia
Eastern Africa
Eastern Asia
Eastern Europe
Melanesia
Micronesia
Middle Africa
Northern Africa
North America
Northern Europe
Polynesia
South−Eastern Asia
South America
Southern Africa
Southern Asia
Southern Europe
Western Africa
Western Asia
Western Europe
Country − Spacing beta0 offset (Rural)
Country − Spacing beta0 offset (Urban)
Country − Spacing beta1 offset (Rural)
Country − Spacing beta1 offset (Urban)
Country − Spacing alpha1 offset (Rural)
Country − Spacing alpha1 offset (Urban)
Country − Spacing beta2 offset (Rural)
Country − Spacing beta2 offset (Urban)

Parameters

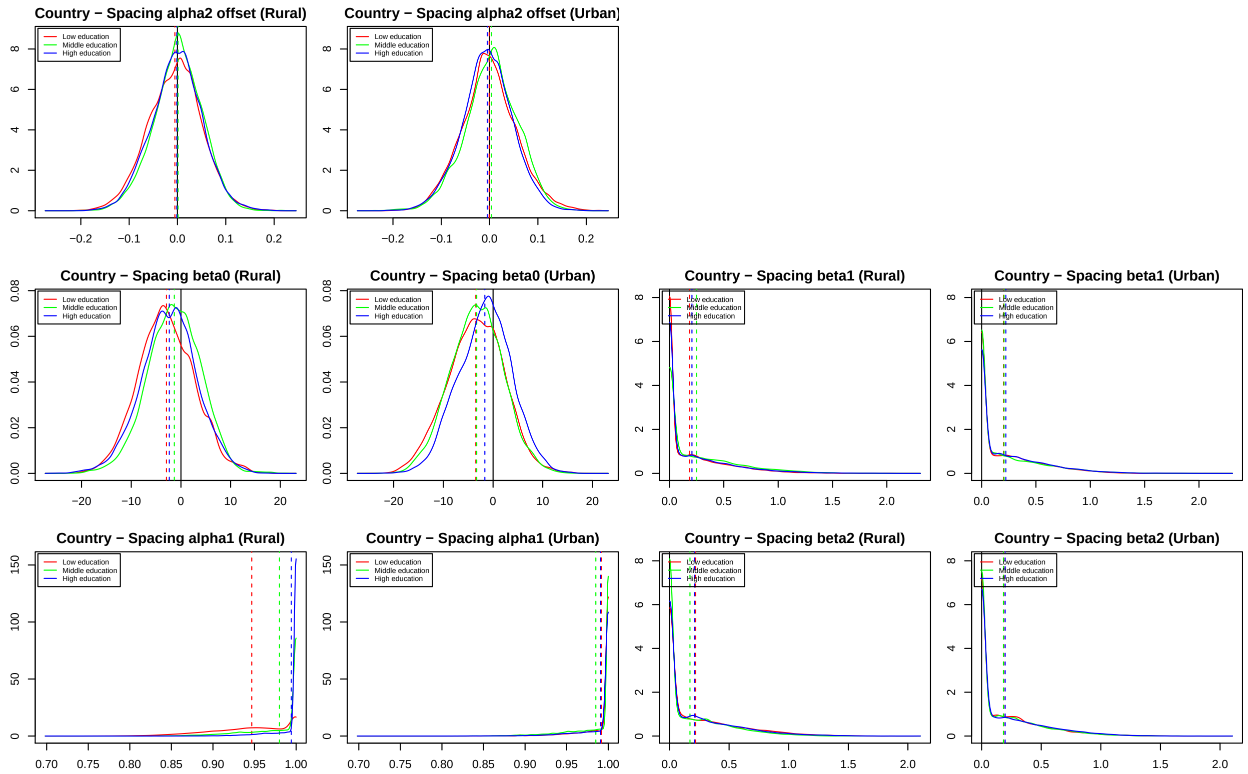

C.3.4.3 Limiting

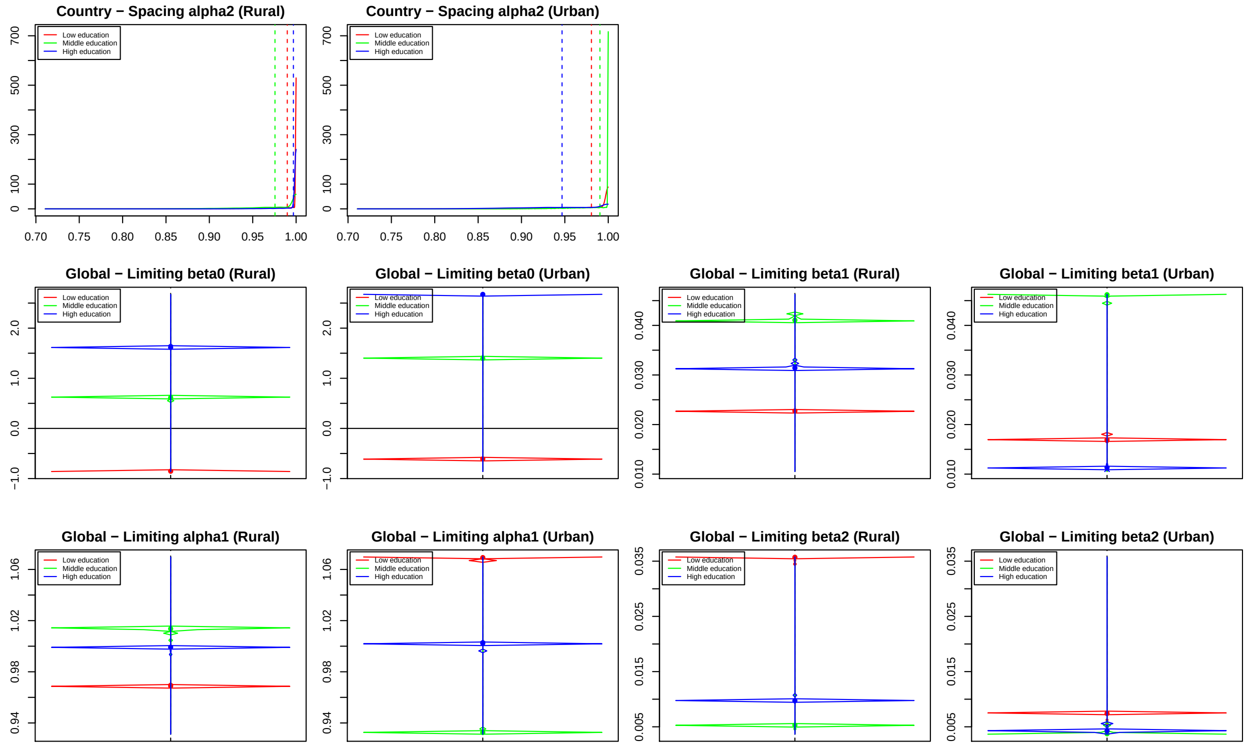

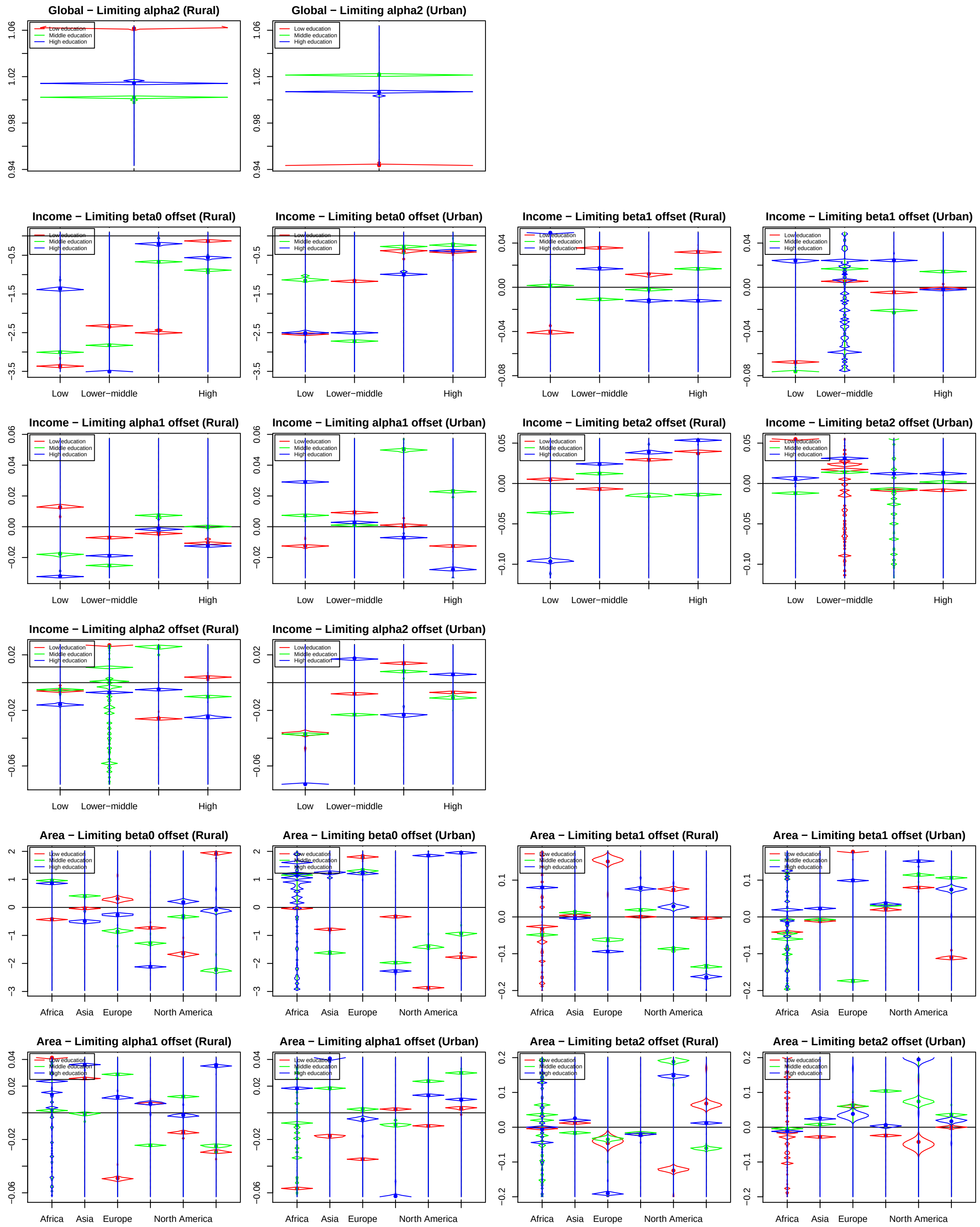
Global − Limiting alpha2 (Rural)
Global − Limiting alpha2 (Urban)
Income − Limiting beta0 offset (Rural)
Income − Limiting beta0 offset (Urban)
Income − Limiting beta1 offset (Rural)
Income − Limiting beta1 offset (Urban)
Income − Limiting alpha1 offset (Rural)
Income − Limiting alpha1 offset (Urban)
Income − Limiting beta2 offset (Rural)
Income − Limiting beta2 offset (Urban)
Income − Limiting alpha2 offset (Rural)
Income − Limiting alpha2 offset (Urban)
Area − Limiting beta0 offset (Rural)
Area − Limiting beta0 offset (Urban)
Area − Limiting beta1 offset (Rural)
Area − Limiting beta1 offset (Urban)
Area − Limiting alpha1 offset (Rural)
Area − Limiting alpha1 offset (Urban)
Area − Limiting beta2 offset (Rural)
Area − Limiting beta2 offset (Urban)
Low education
Middle education
High education
Low
Lower−middle
High
Africa
Asia
Europe
North America

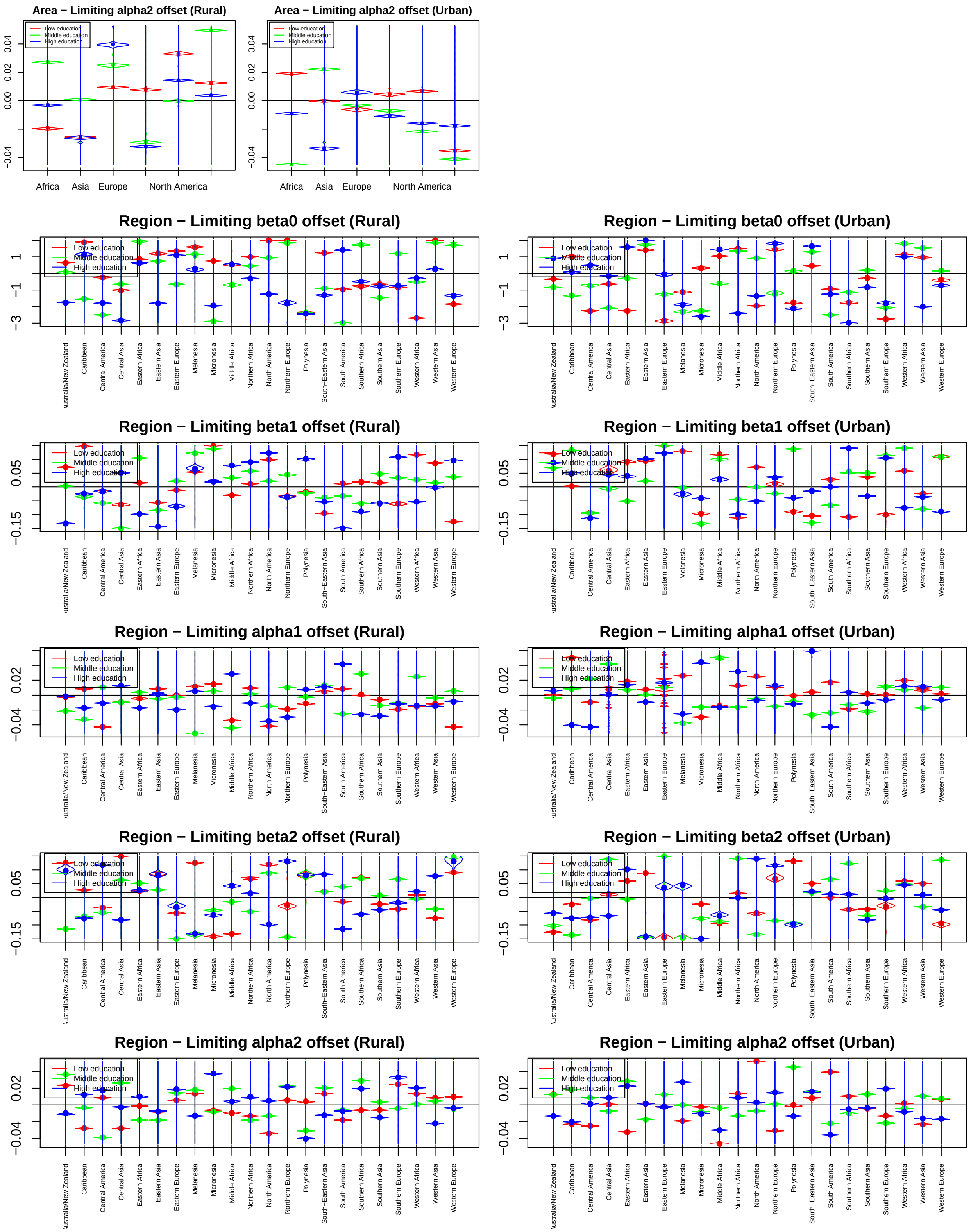
Area − Limiting alpha2 offset (Rural)
Area − Limiting alpha2 offset (Urban)
Low education
Middle education
High education
Africa
Asia
Europe
North America
Region − Limiting beta0 offset (Rural)
Region − Limiting beta0 offset (Urban)
Region − Limiting beta1 offset (Rural)
Region − Limiting beta1 offset (Urban)
Region − Limiting alpha1 offset (Rural)
Region − Limiting alpha1 offset (Urban)
Region − Limiting beta2 offset (Rural)
Region − Limiting beta2 offset (Urban)
Region − Limiting alpha2 offset (Rural)
Region − Limiting alpha2 offset (Urban)
Caribbean
Central America
Central Asia
Eastern Africa
Eastern Asia
Eastern Europe
Melanesia
Micronesia
Middle Africa
Northern Africa
North America
Northern Europe
Polynesia
South−Eastern Asia
South America
Southern Africa
Southern Asia
Southern Europe
Western Africa
Western Asia
Western Europe

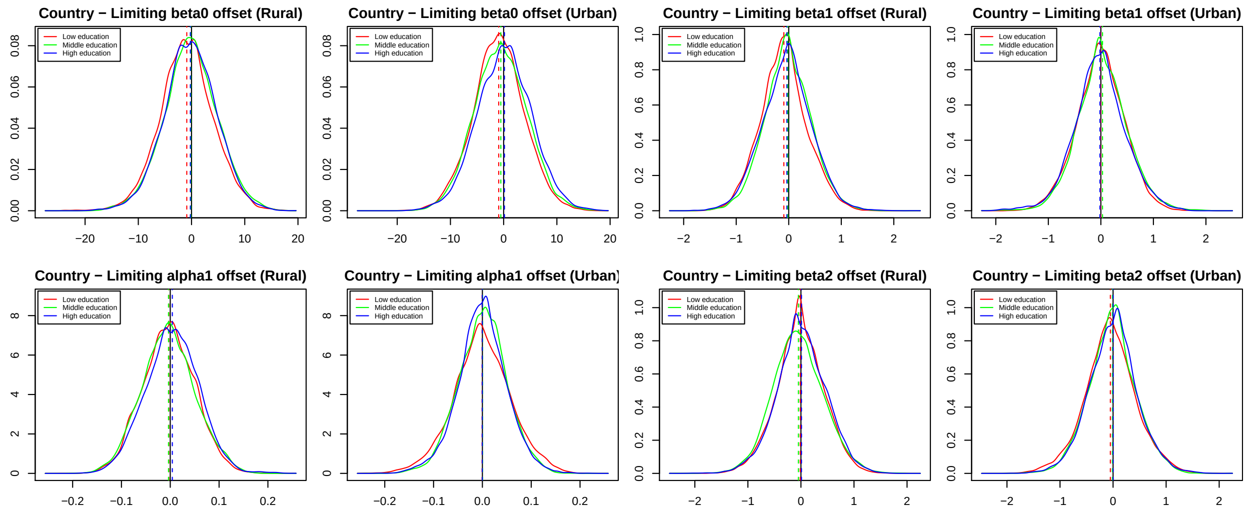
Country − Limiting beta0 offset (Rural)
Country − Limiting beta0 offset (Urban)
Country − Limiting beta1 offset (Rural)
Country − Limiting beta1 offset (Urban)
Country − Limiting alpha1 offset (Rural)
Country − Limiting alpha1 offset (Urban)
Country − Limiting beta2 offset (Rural)
Country − Limiting beta2 offset (Urban)
Low education
Middle education
High education

Parameters

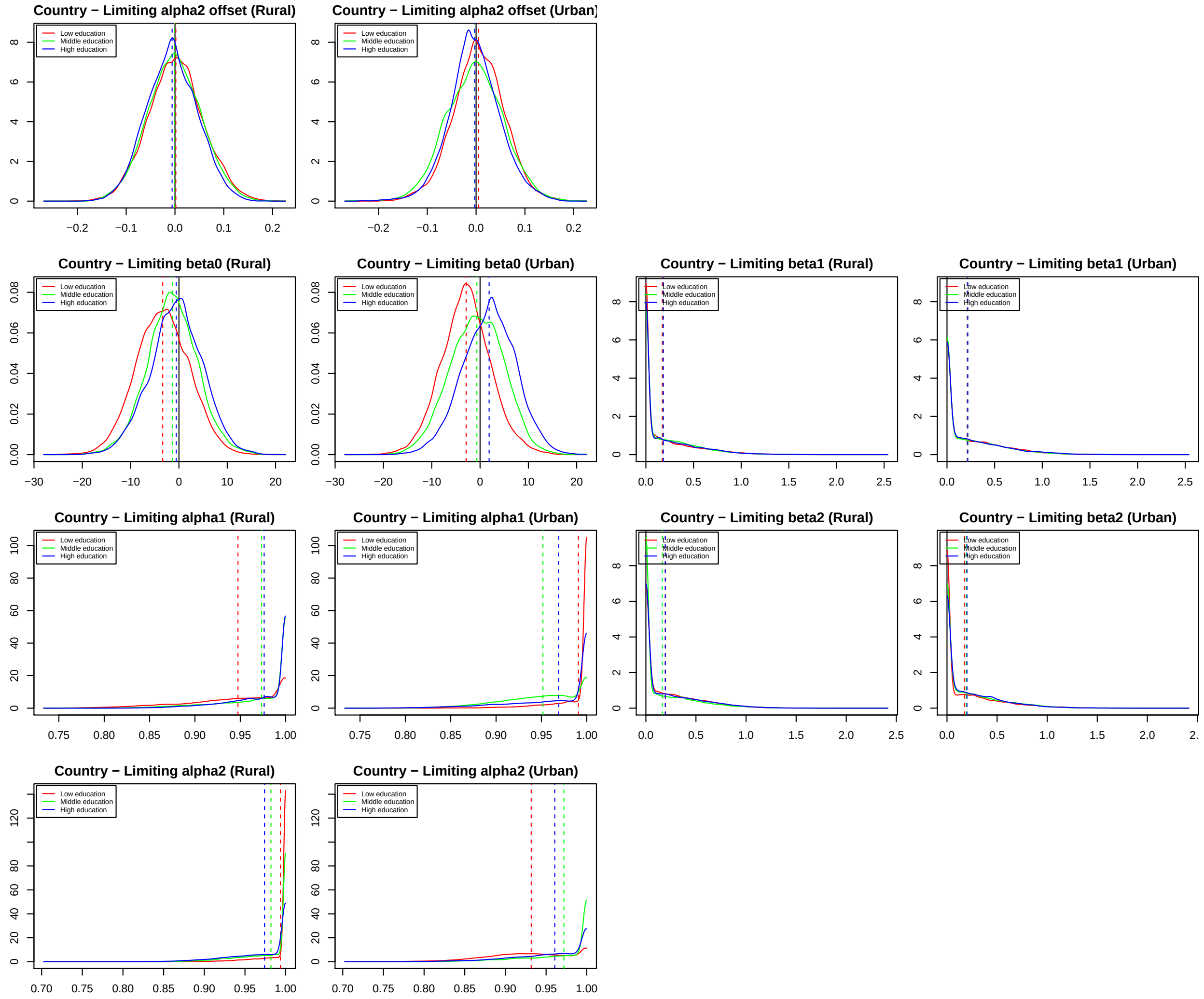
Country − Limiting alpha2 offset (Rural)
Country − Limiting alpha2 offset (Urban)
Country − Limiting beta0 (Rural)
Country − Limiting beta0 (Urban)
Country − Limiting beta1 (Rural)
Country − Limiting beta1 (Urban)
Country − Limiting alpha1 (Rural)
Country − Limiting alpha1 (Urban)
Country − Limiting beta2 (Rural)
Country − Limiting beta2 (Urban)
Country − Limiting alpha2 (Rural)
Country − Limiting alpha2 (Urban)
Low education
Middle education
High education

C.3.5 Contraception Method Mix

C.3.5.1 Pill

Hyperparameters

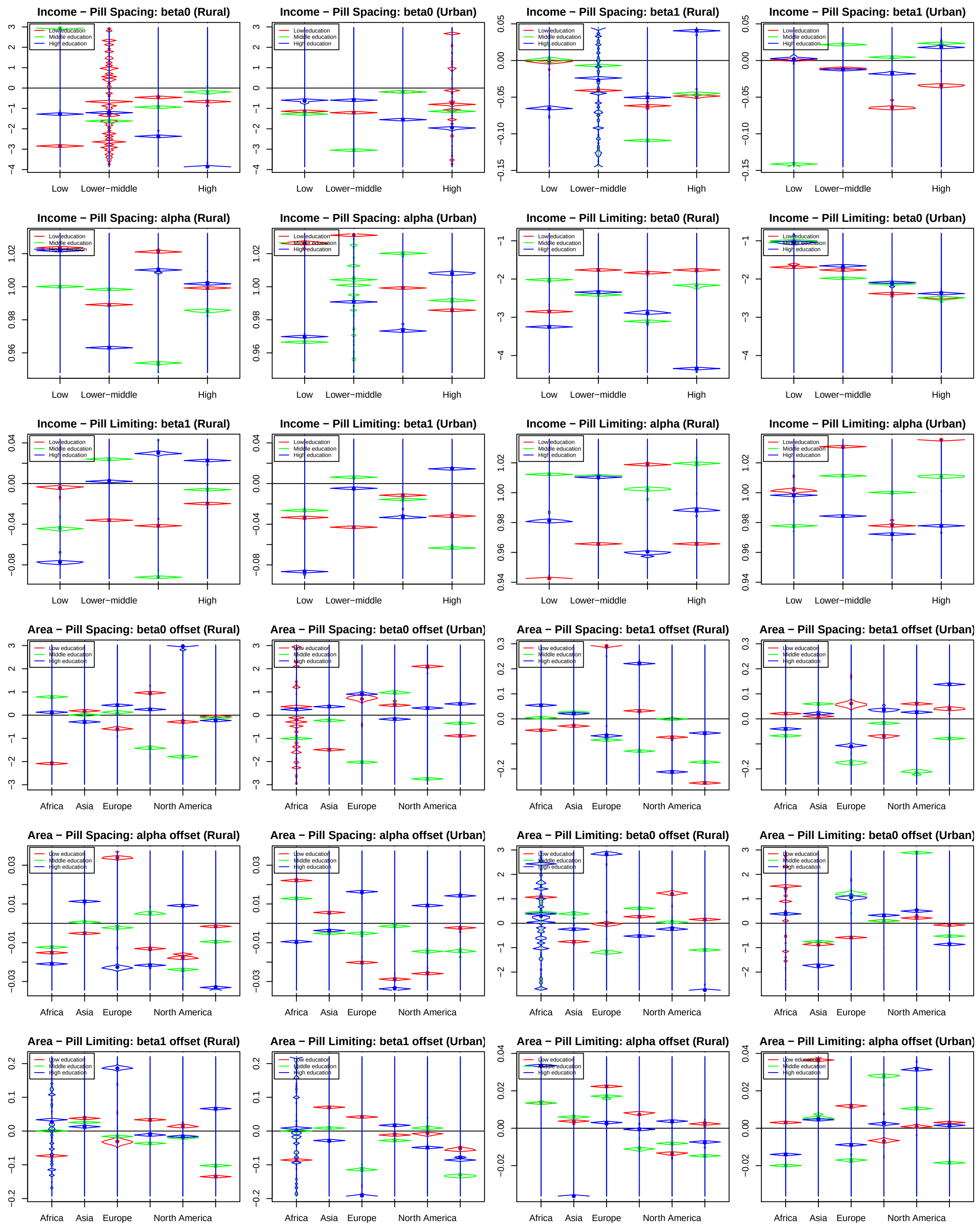

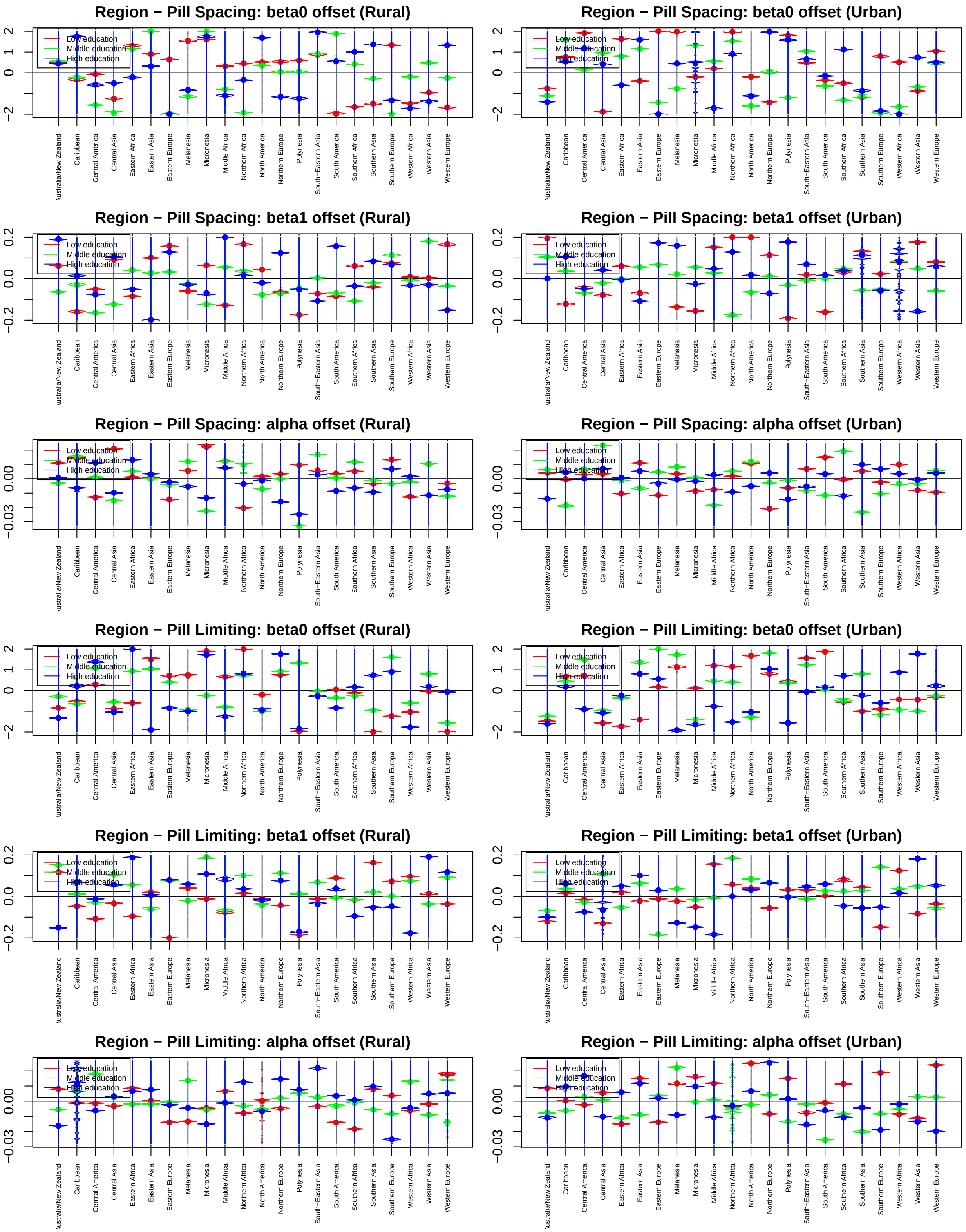
Region − Pill Spacing: beta0 offset (Rural)
Region − Pill Spacing: beta0 offset (Urban)
Region − Pill Spacing: beta1 offset (Rural)
Region − Pill Spacing: beta1 offset (Urban)
Region − Pill Spacing: alpha offset (Rural)
Region − Pill Spacing: alpha offset (Urban)
Region − Pill Limiting: beta0 offset (Rural)
Region − Pill Limiting: beta0 offset (Urban)
Region − Pill Limiting: beta1 offset (Rural)
Region − Pill Limiting: beta1 offset (Urban)
Region − Pill Limiting: alpha offset (Rural)
Region − Pill Limiting: alpha offset (Urban)
Low education
Middle education
High education
Australia/New Zealand
Caribbean
Central America
Central Asia
Eastern Africa
Eastern Asia
Eastern Europe
Melanesia
Micronesia
Middle Africa
Northern Africa
North America
Northern Europe
Polynesia
South−Eastern Asia
South America
Southern Africa
Southern Asia
Southern Europe
Western Africa
Western Asia
Western Europe

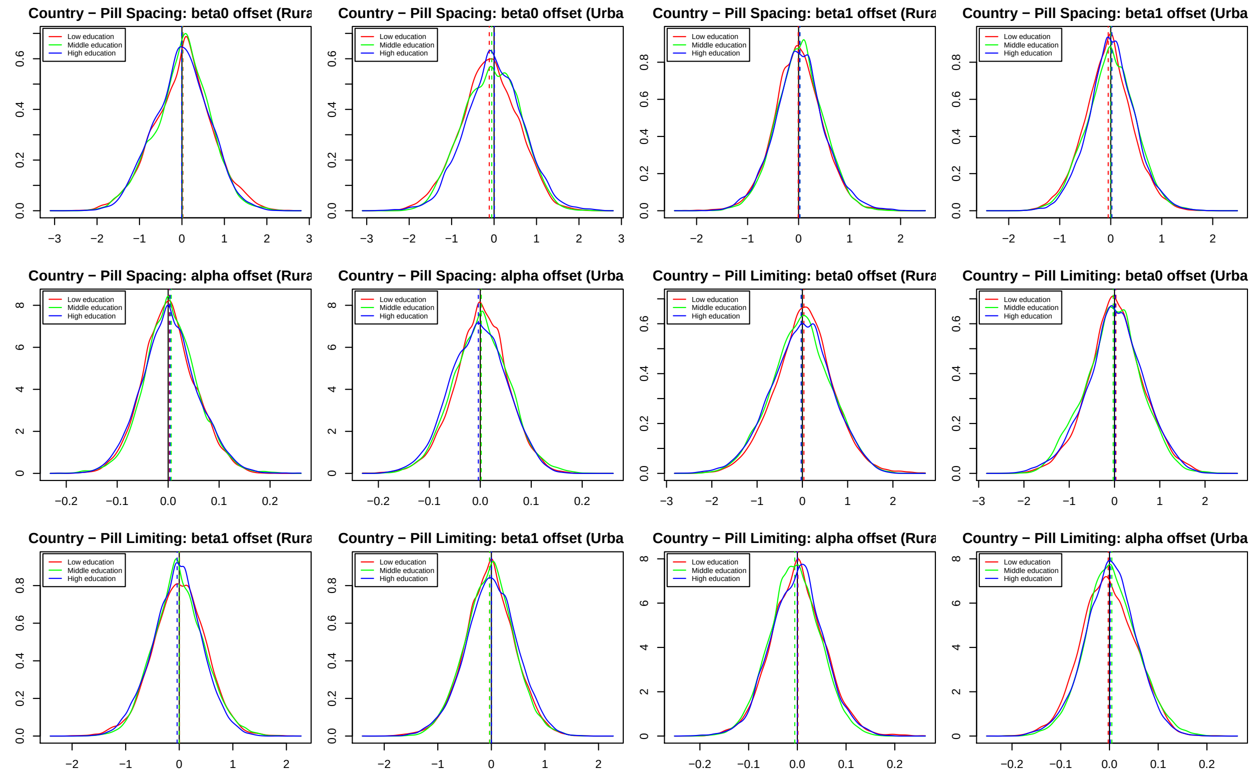

Parameters

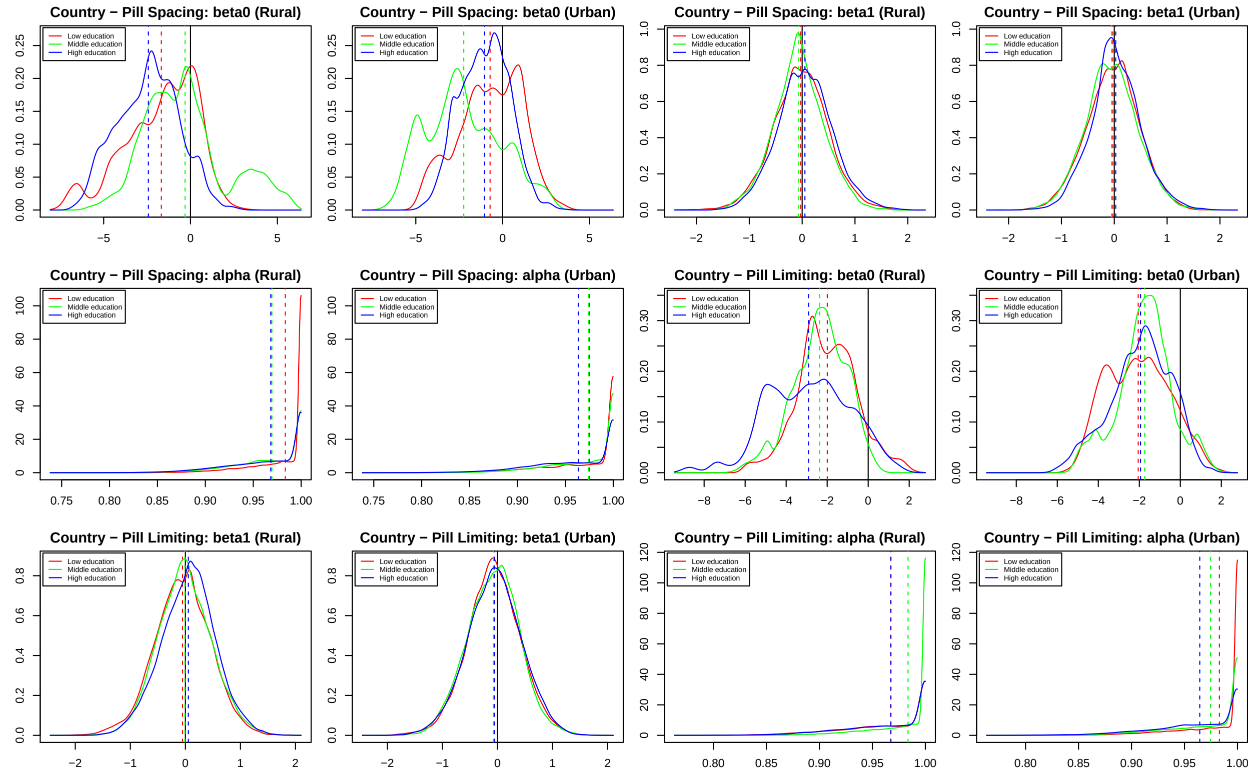

C.3.5.2 IUD

Hyperparameters

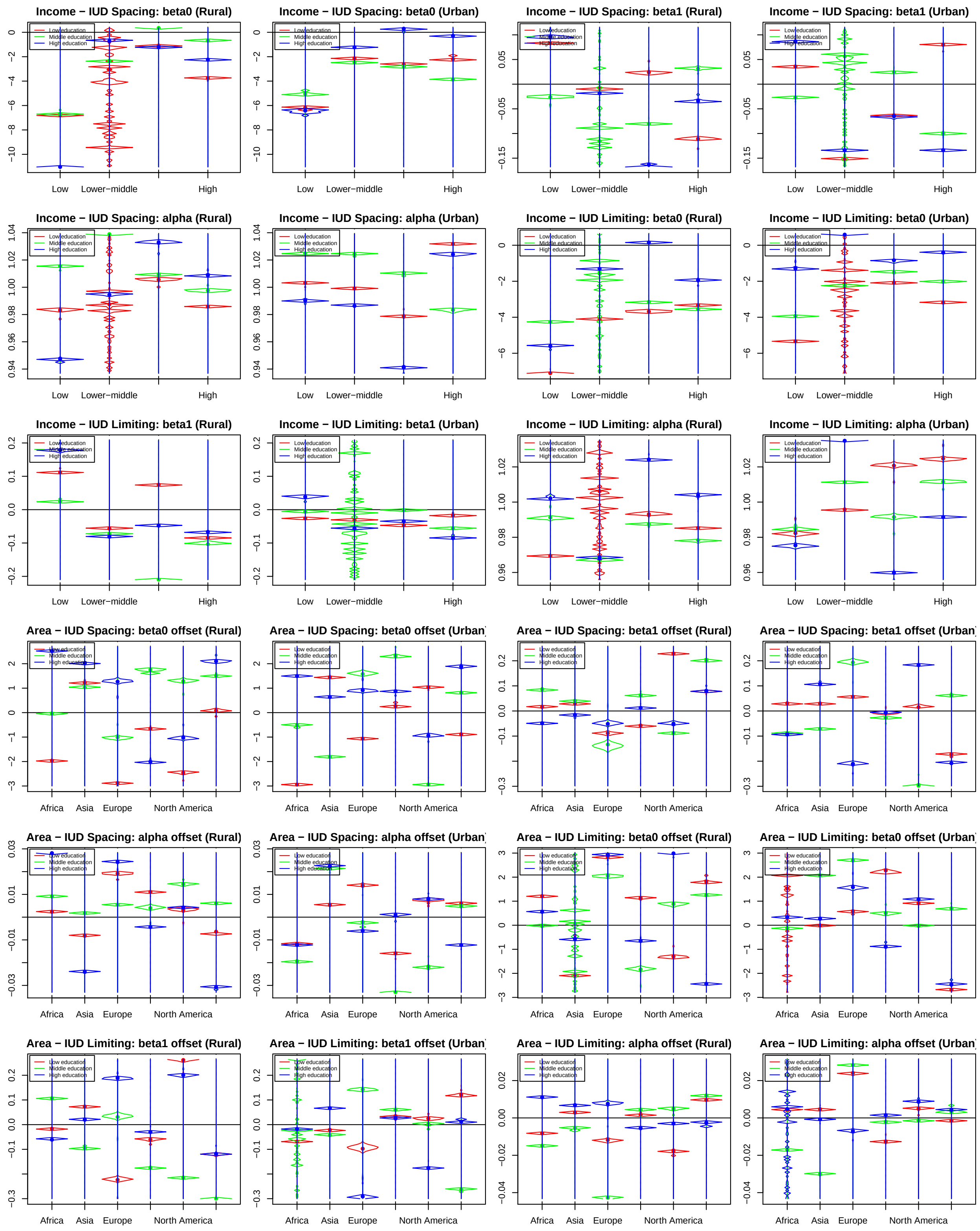

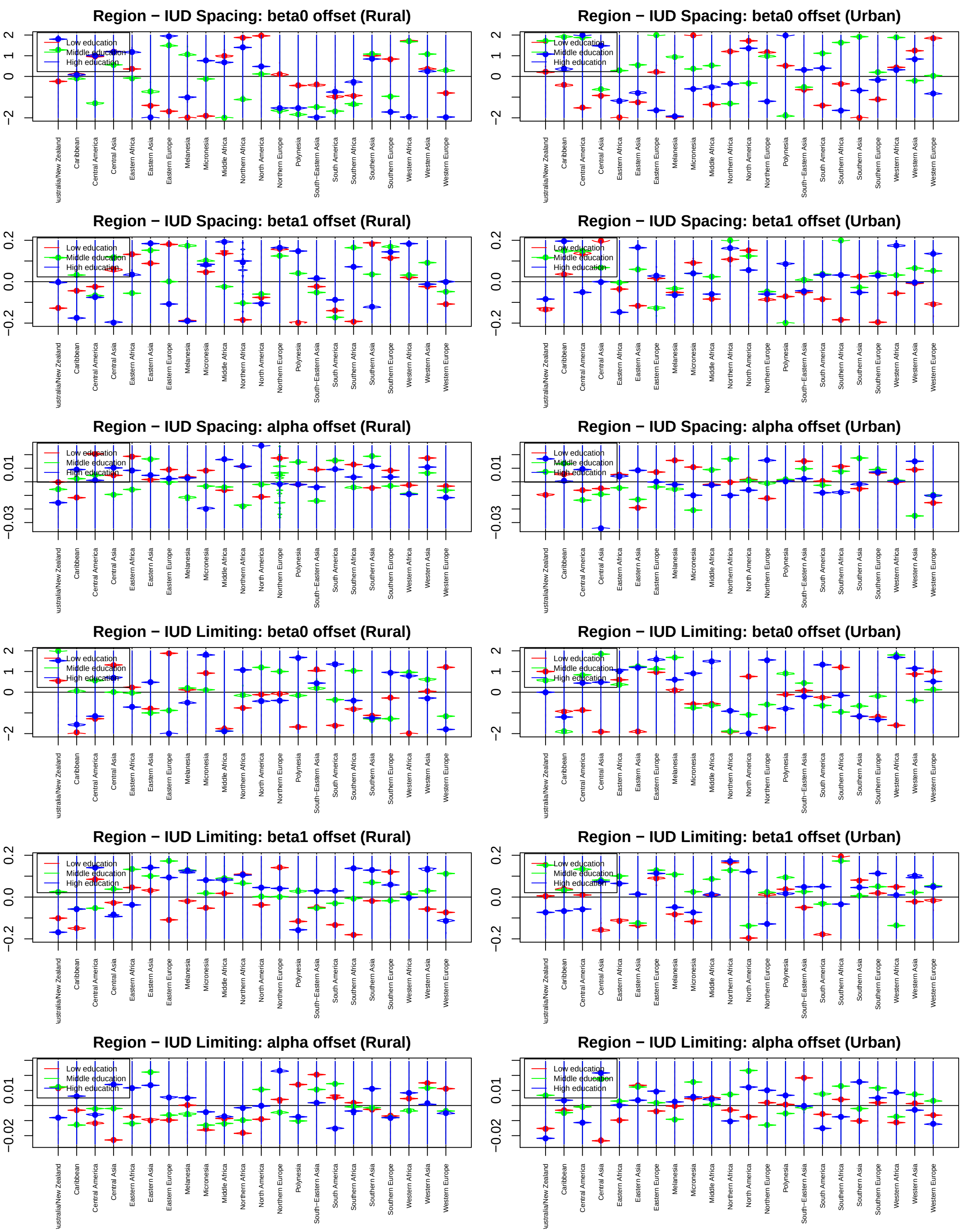
Region − IUD Spacing: beta0 offset (Rural)
Region − IUD Spacing: beta0 offset (Urban)
Region − IUD Spacing: beta1 offset (Rural)
Region − IUD Spacing: beta1 offset (Urban)
Region − IUD Spacing: alpha offset (Rural)
Region − IUD Spacing: alpha offset (Urban)
Region − IUD Limiting: beta0 offset (Rural)
Region − IUD Limiting: beta0 offset (Urban)
Region − IUD Limiting: beta1 offset (Rural)
Region − IUD Limiting: beta1 offset (Urban)
Region − IUD Limiting: alpha offset (Rural)
Region − IUD Limiting: alpha offset (Urban)
Low education
Middle education
High education
Australia/New Zealand
Caribbean
Central America
Central Asia
Eastern Africa
Eastern Asia
Eastern Europe
Melanesia
Micronesia
Middle Africa
Northern Africa
North America
Northern Europe
Polynesia
South−Eastern Asia
South America
Southern Africa
Southern Asia
Southern Europe
Western Africa
Western Asia
Western Europe

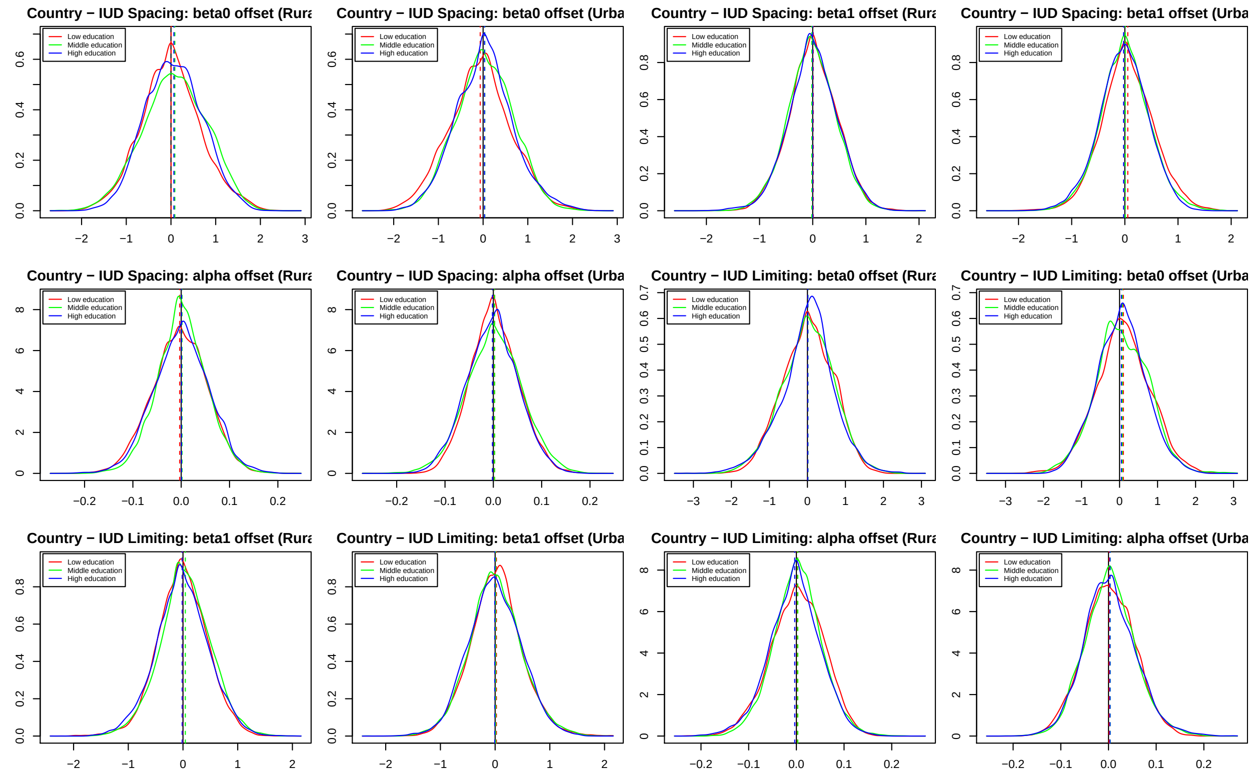

Parameters

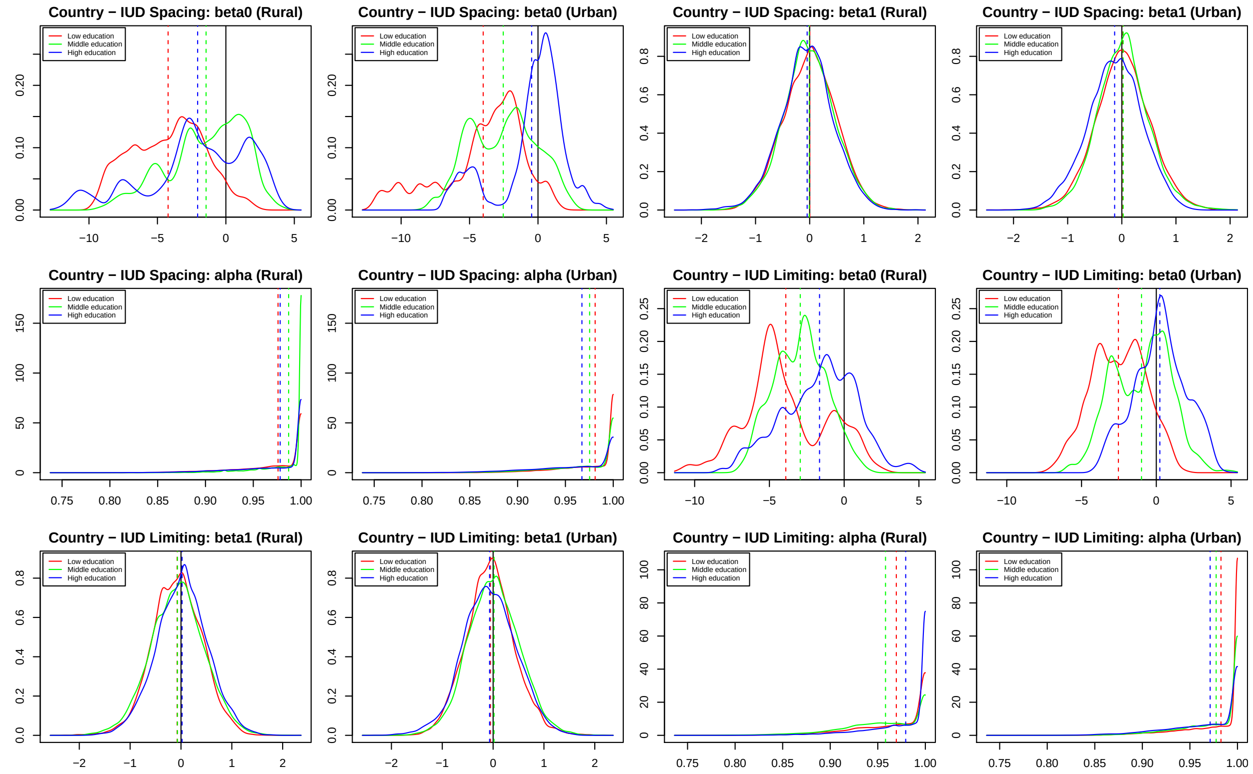

C.3.5.3 Injections

Hyperparameters

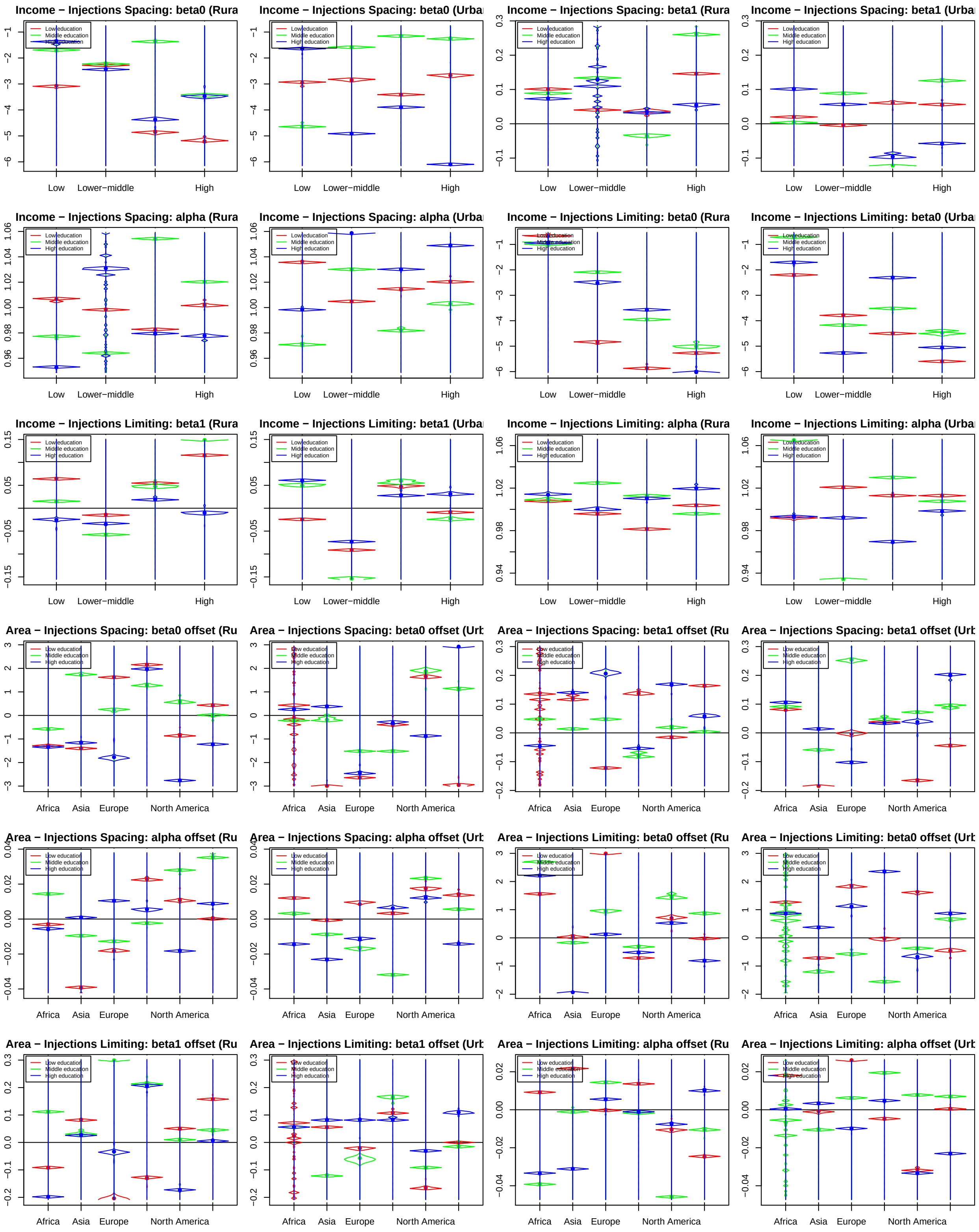

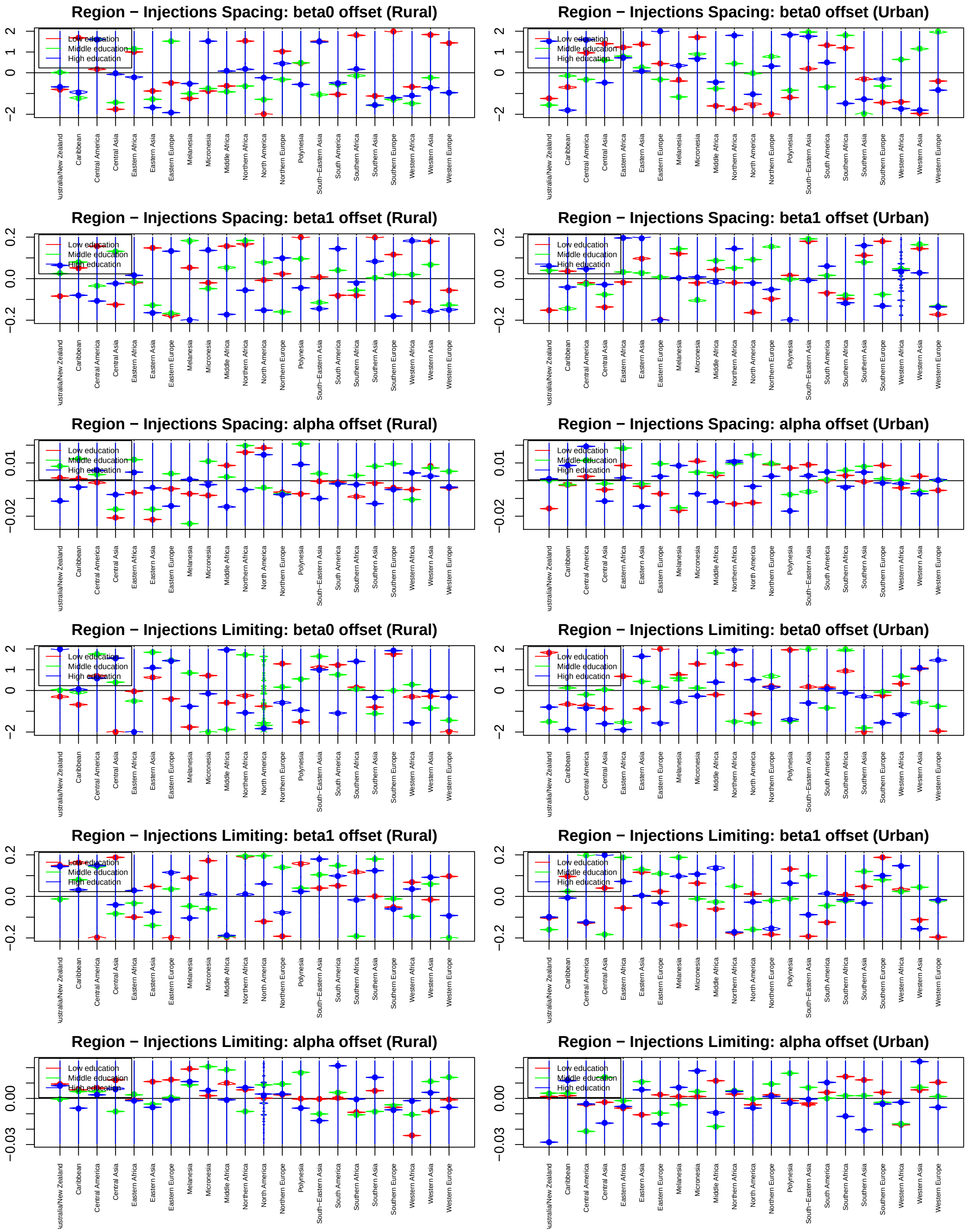

Region − Injections Spacing: beta0 offset (Rural)
Region − Injections Spacing: beta0 offset (Urban)
Region − Injections Spacing: beta1 offset (Rural)
Region − Injections Spacing: beta1 offset (Urban)
Region − Injections Spacing: alpha offset (Rural)
Region − Injections Spacing: alpha offset (Urban)
Region − Injections Limiting: beta0 offset (Rural)
Region − Injections Limiting: beta0 offset (Urban)
Region − Injections Limiting: beta1 offset (Rural)
Region − Injections Limiting: beta1 offset (Urban)
Region − Injections Limiting: alpha offset (Rural)
Region − Injections Limiting: alpha offset (Urban)
Low education
Middle education
High education
ustralia/New Zealand
Caribbean
Central America
Central Asia
Eastern Africa
Eastern Asia
Eastern Europe
Melanesia
Micronesia
Middle Africa
Northern Africa
North America
Northern Europe
Polynesia
South−Eastern Asia
South America
Southern Africa
Southern Asia
Southern Europe
Western Africa
Western Asia
Western Europe

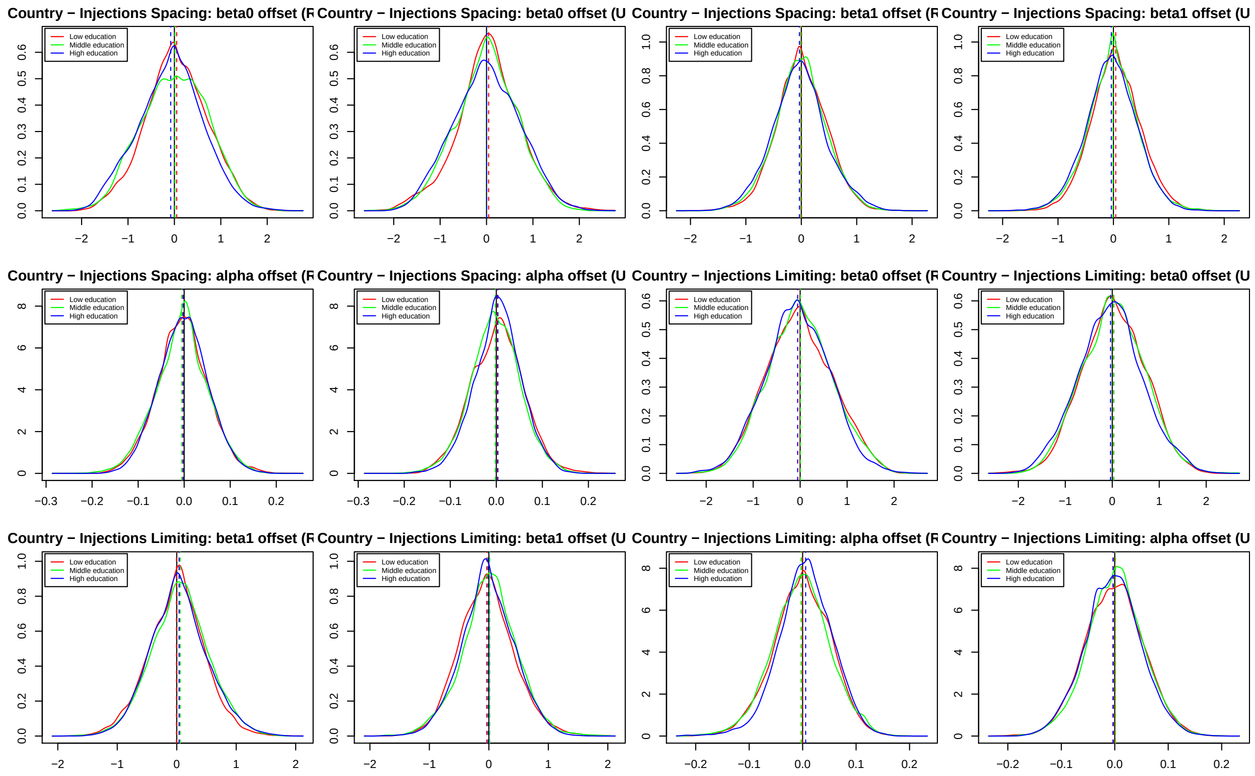

Parameters

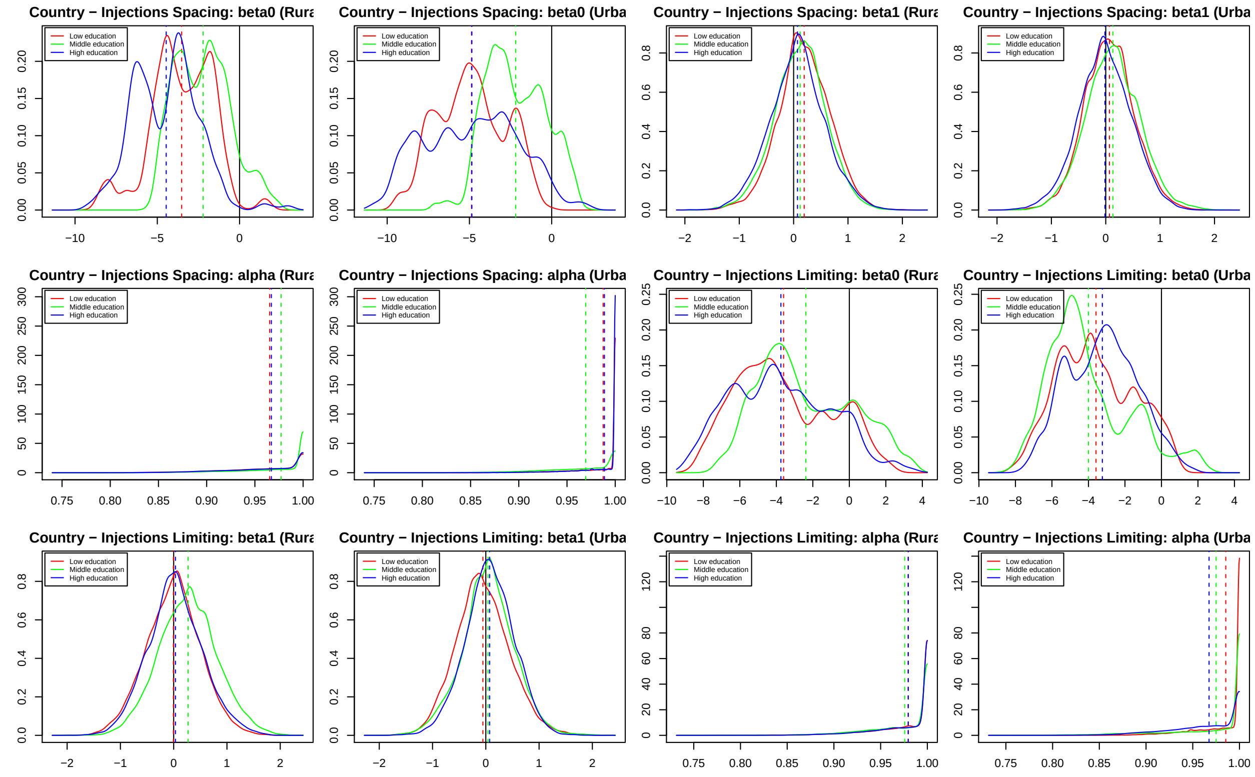

C.3.5.4 Condom

Hyperparameters

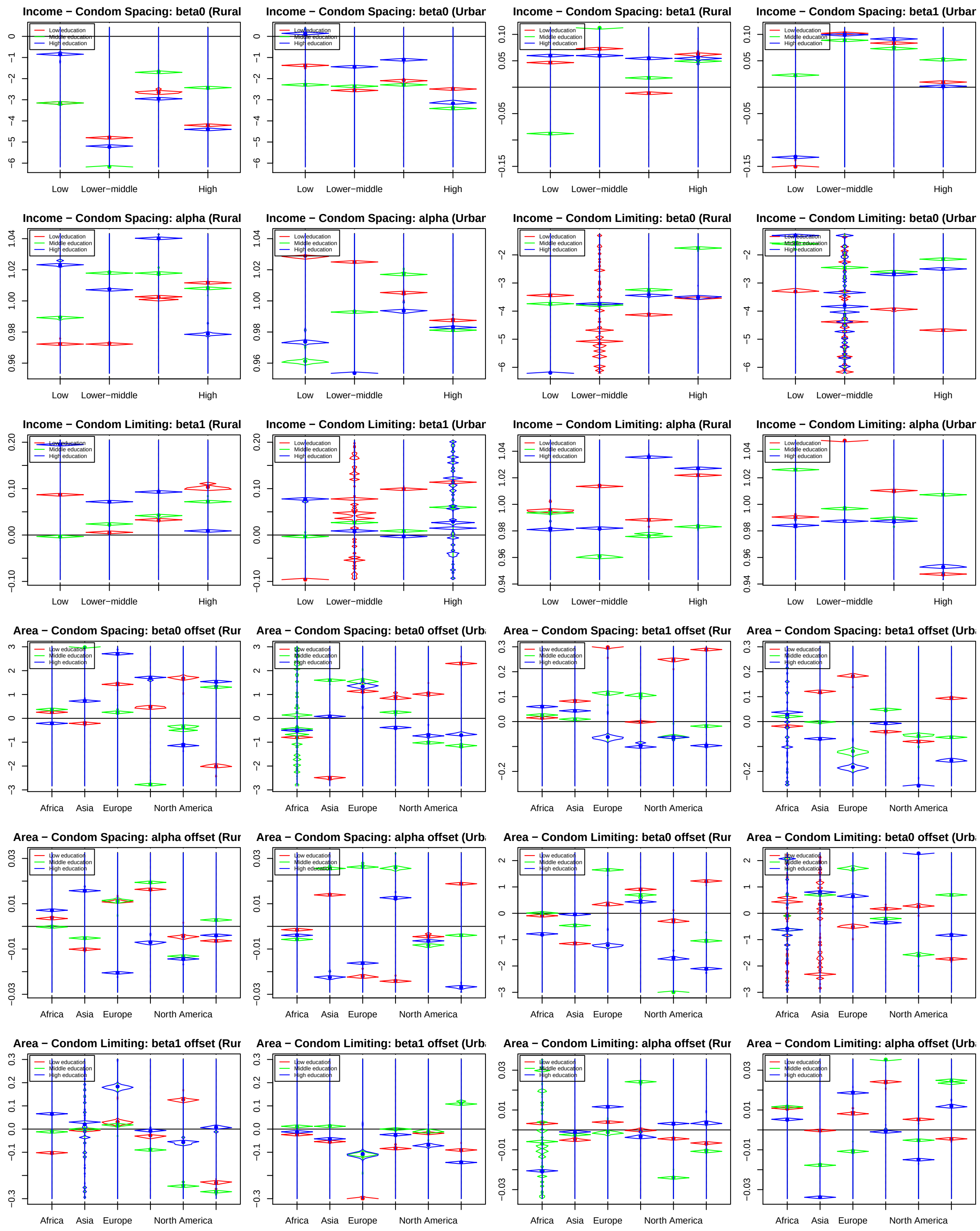

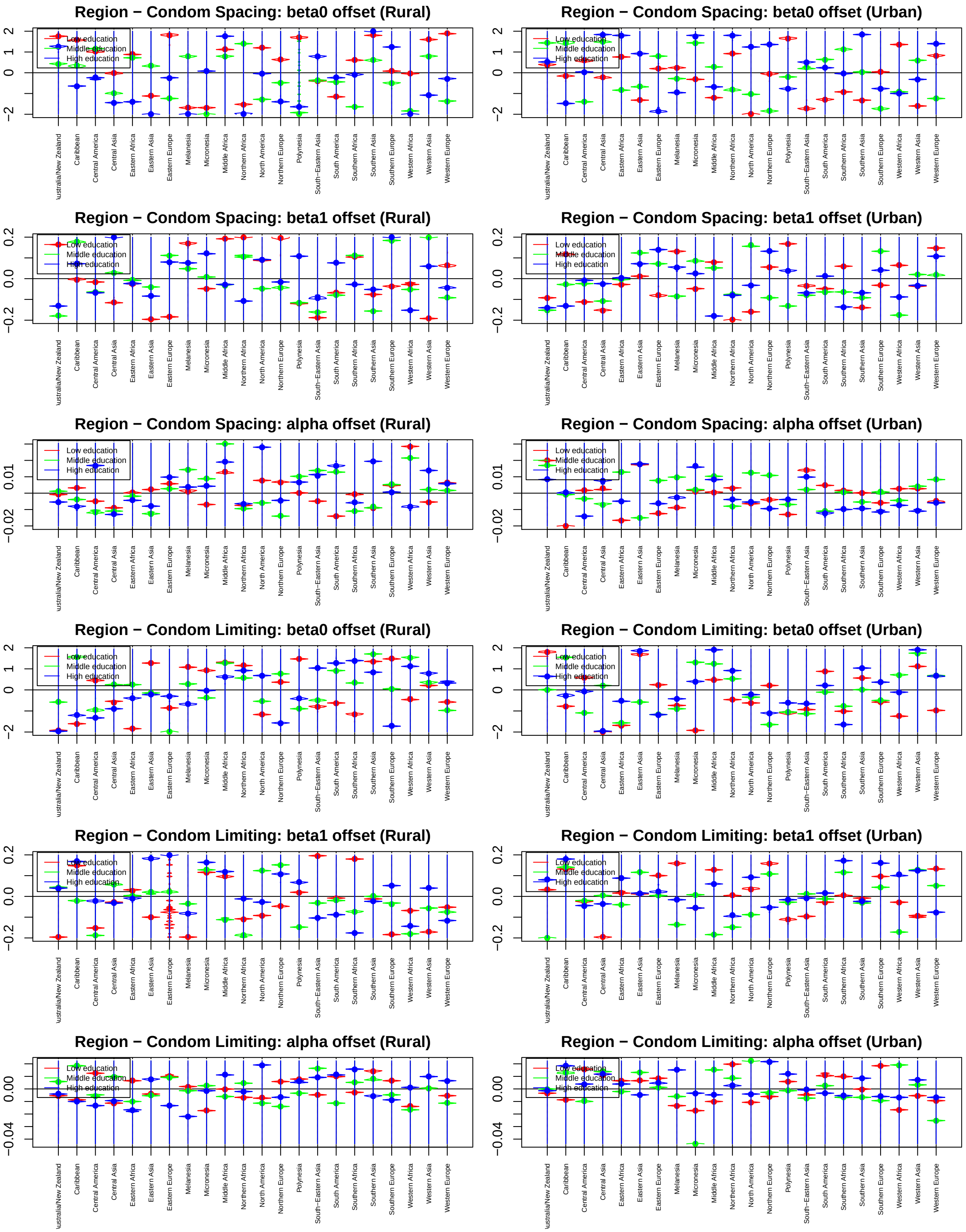
Region − Condom Spacing: beta0 offset (Rural)
Region − Condom Spacing: beta0 offset (Urban)
Region − Condom Spacing: beta1 offset (Rural)
Region − Condom Spacing: beta1 offset (Urban)
Region − Condom Spacing: alpha offset (Rural)
Region − Condom Spacing: alpha offset (Urban)
Region − Condom Limiting: beta0 offset (Rural)
Region − Condom Limiting: beta0 offset (Urban)
Region − Condom Limiting: beta1 offset (Rural)
Region − Condom Limiting: beta1 offset (Urban)
Region − Condom Limiting: alpha offset (Rural)
Region − Condom Limiting: alpha offset (Urban)
Low education
Middle education
High education
Australia/New Zealand
Caribbean
Central America
Central Asia
Eastern Africa
Eastern Asia
Eastern Europe
Melanesia
Micronesia
Middle Africa
Northern Africa
North America
Northern Europe
Polynesia
South−Eastern Asia
South America
Southern Africa
Southern Asia
Southern Europe
Western Africa
Western Asia
Western Europe

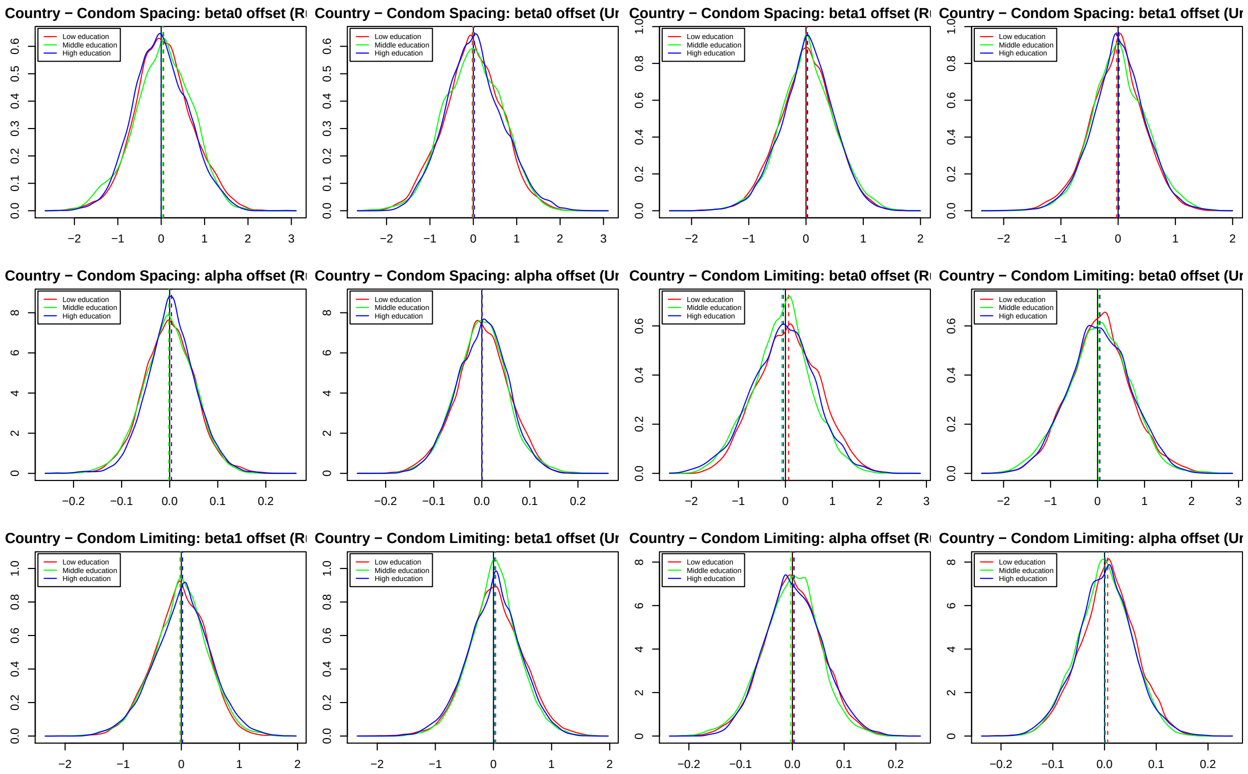

Parameters

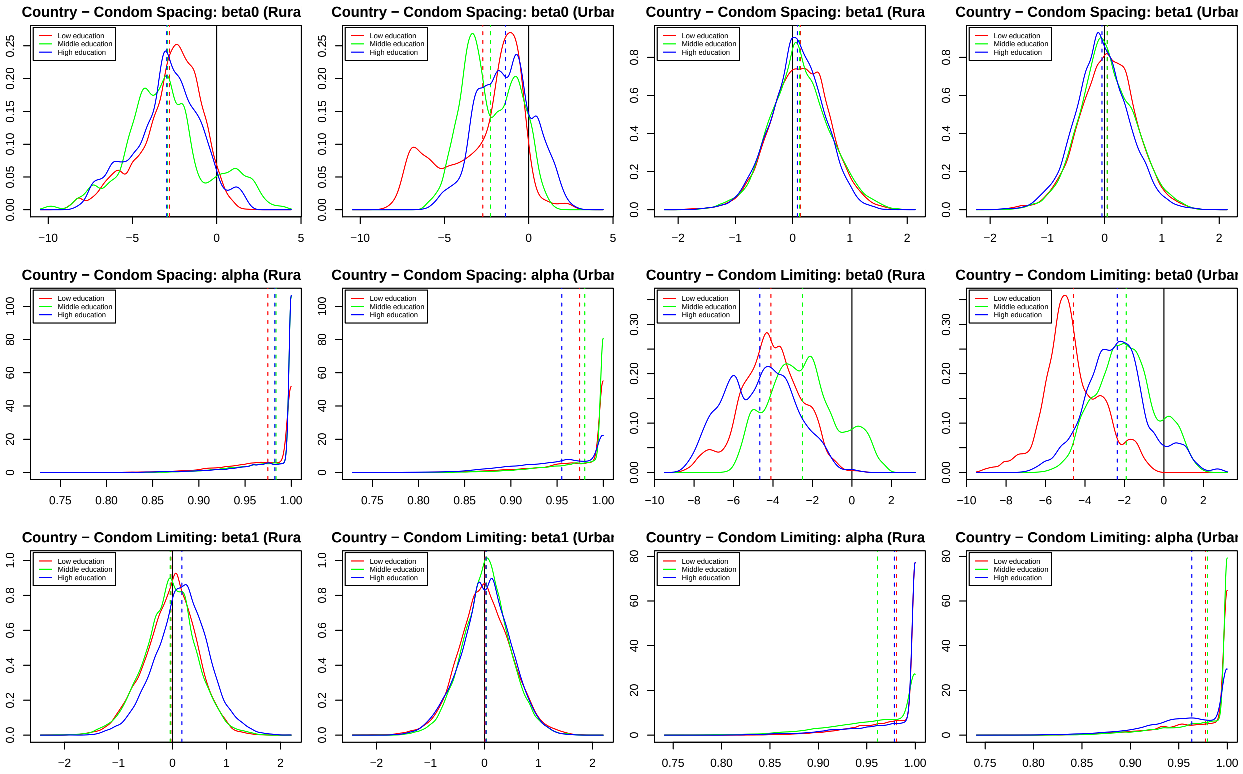

C.3.5.5 Female sterilization

Hyperparameters

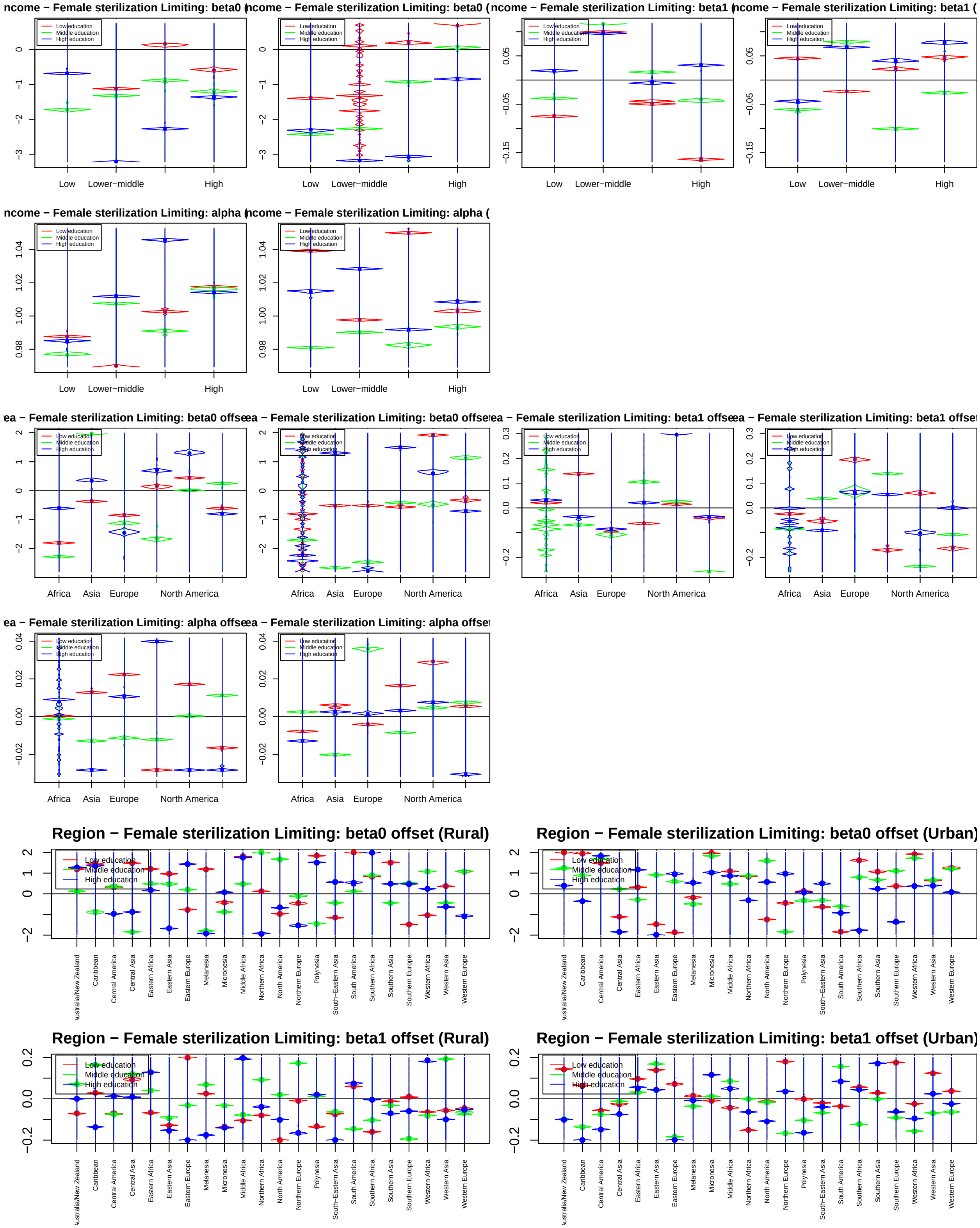

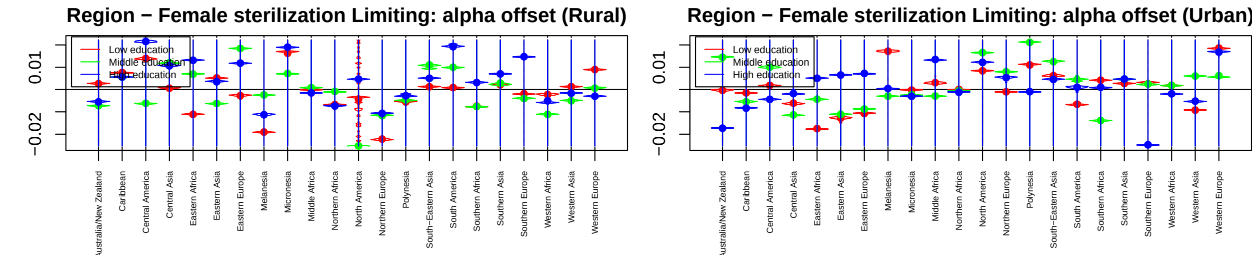

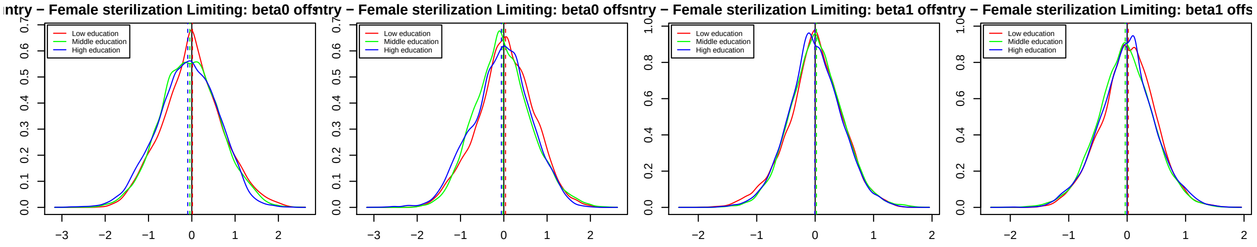

Parameters

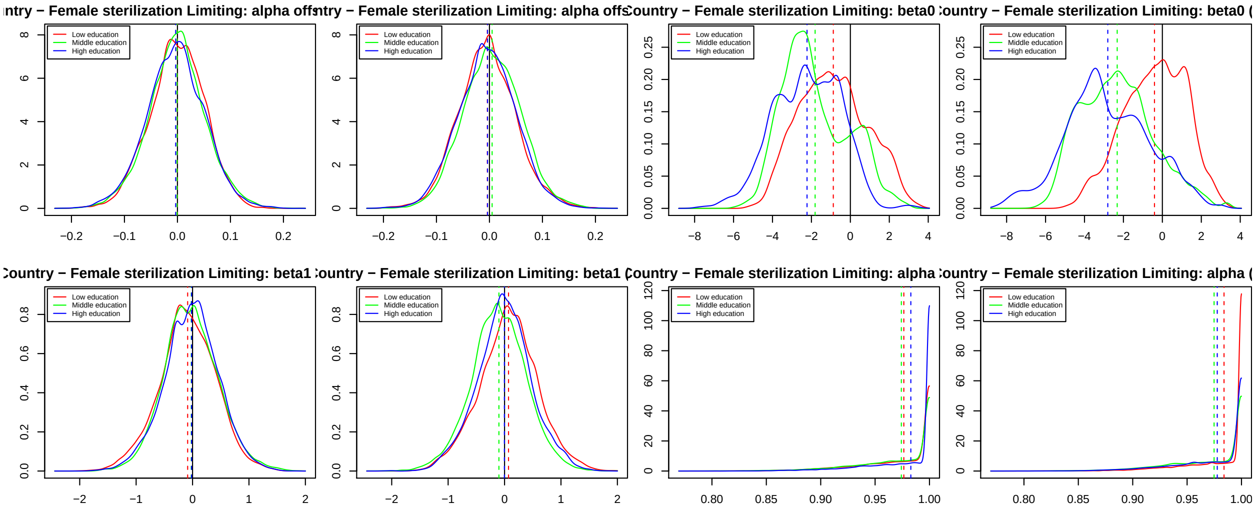

C.3.5.6 Male sterilization

Hyperparameters

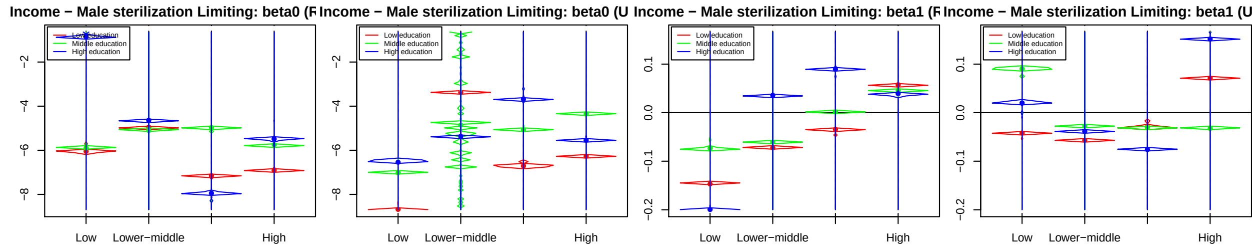

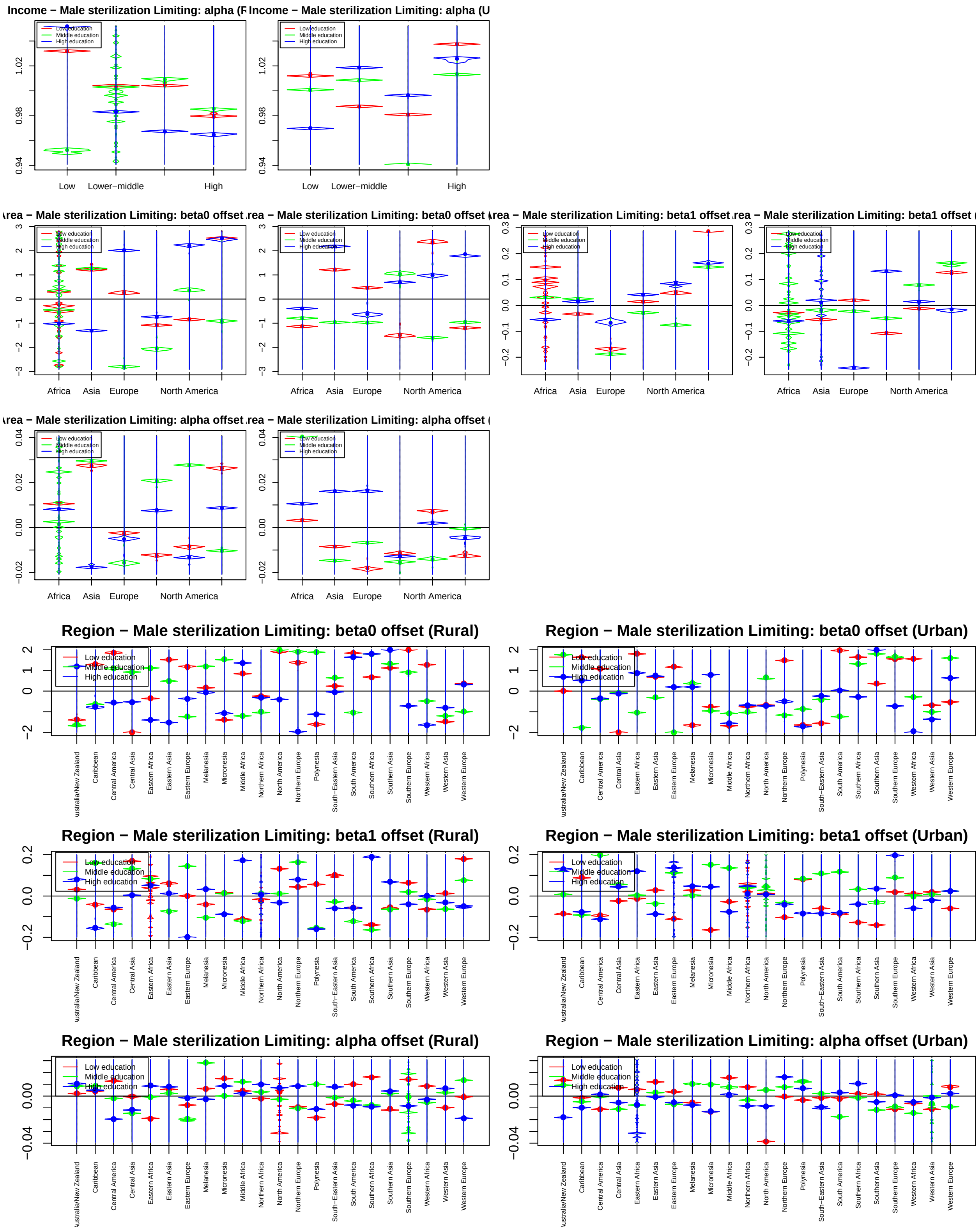
Income − Male sterilization Limiting: alpha (R
Income − Male sterilization Limiting: alpha (U
Low education
Middle education
High education
Low
Lower−middle
High
Area − Male sterilization Limiting: beta0 offset
Area − Male sterilization Limiting: beta1 offset
Area − Male sterilization Limiting: alpha offset
Africa
Asia
Europe
North America
Region − Male sterilization Limiting: beta0 offset (Rural)
Region − Male sterilization Limiting: beta0 offset (Urban)
Region − Male sterilization Limiting: beta1 offset (Rural)
Region − Male sterilization Limiting: beta1 offset (Urban)
Region − Male sterilization Limiting: alpha offset (Rural)
Region − Male sterilization Limiting: alpha offset (Urban)
Caribbean
Central America
Central Asia
Eastern Africa
Eastern Asia
Eastern Europe
Melanesia
Micronesia
Middle Africa
Northern Africa
North America
Northern Europe
Polynesia
South−Eastern Asia
South America
Southern Africa
Southern Asia
Southern Europe
Western Africa
Western Asia
Western Europe

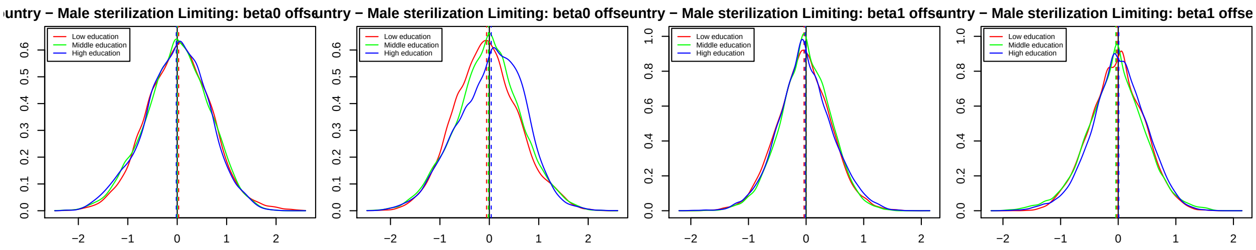

Parameters

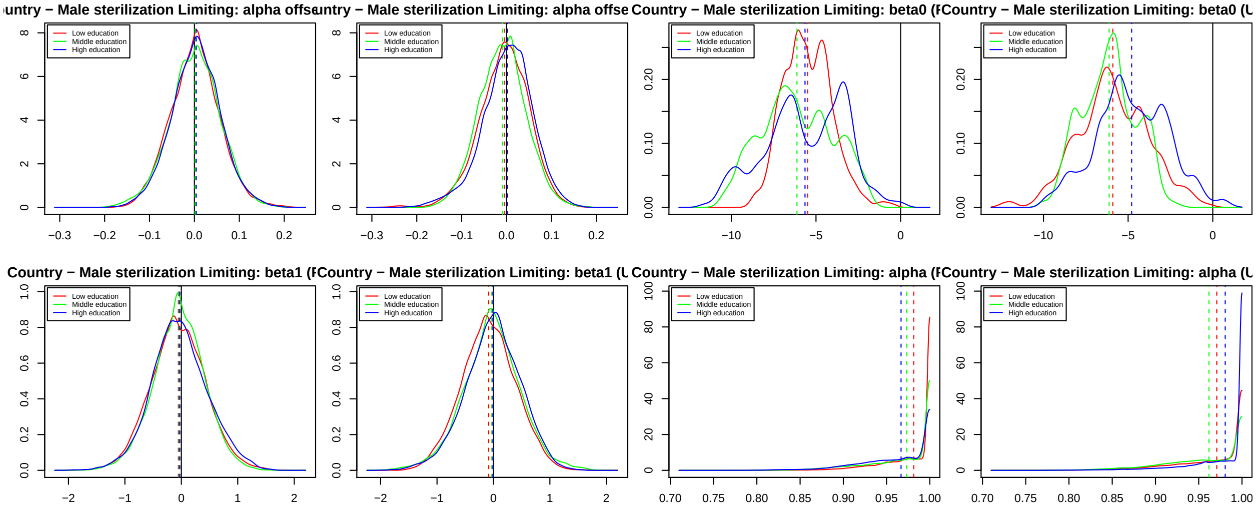

C.3.5.7 Periodic abstinence

Hyperparameters

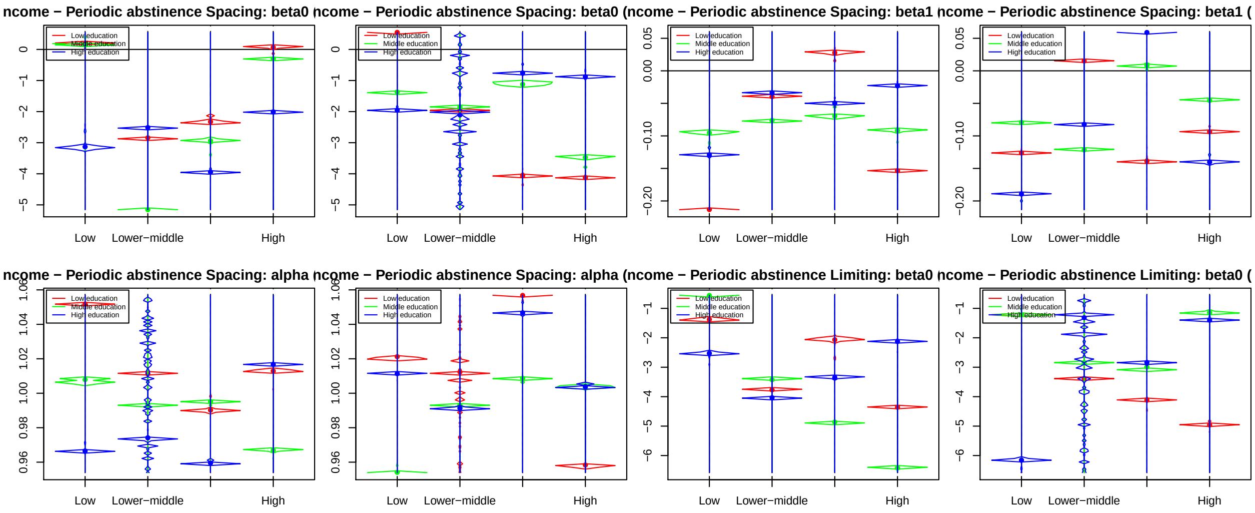

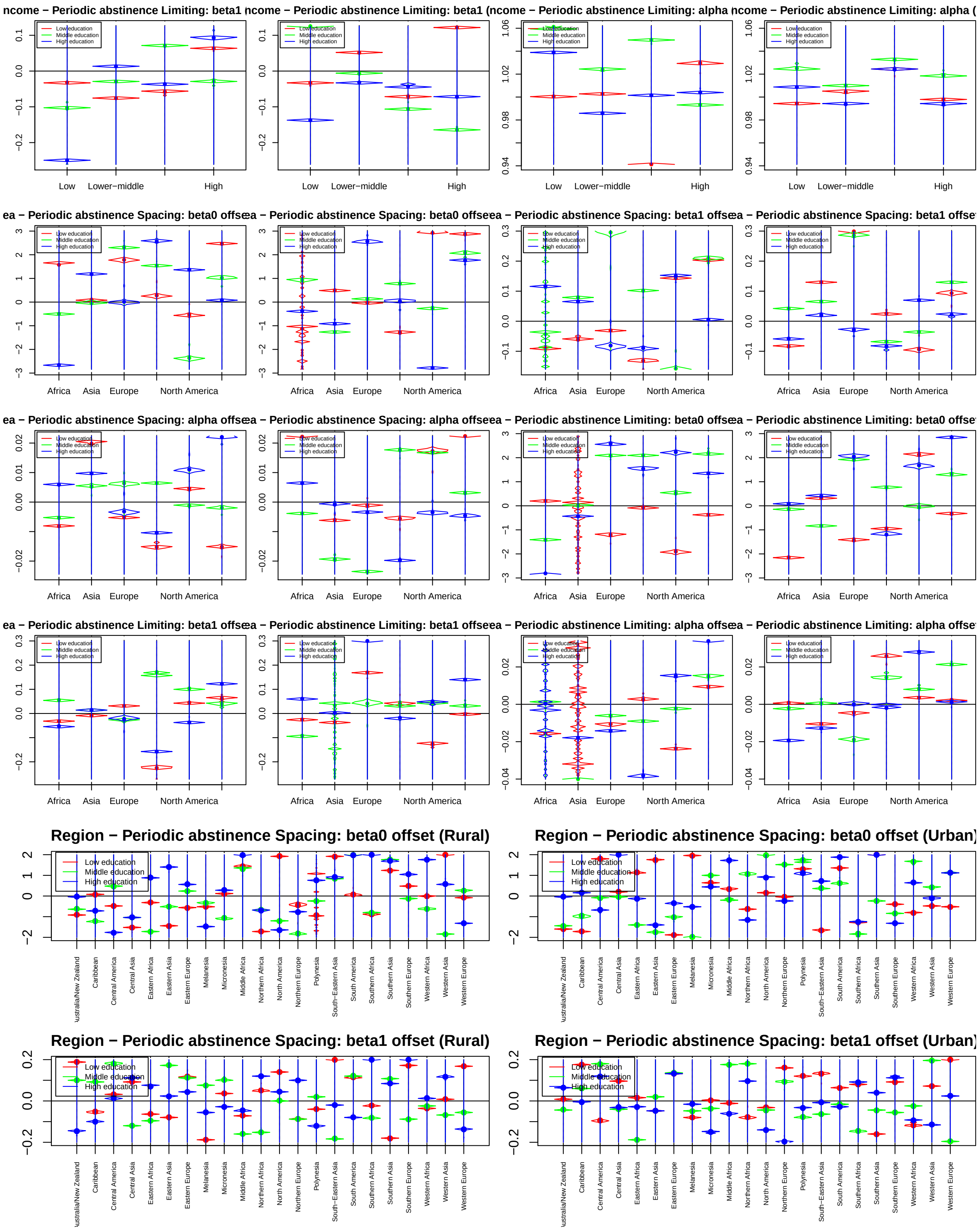
ncome − Periodic abstinence Limiting: beta1
ncome − Periodic abstinence Limiting: beta1 (
ncome − Periodic abstinence Limiting: alpha
ncome − Periodic abstinence Limiting: alpha (
Low education
Middle education
High education
Low
Lower−middle
High
ea − Periodic abstinence Spacing: beta0 offse
ea − Periodic abstinence Spacing: beta0 offse
ea − Periodic abstinence Spacing: beta1 offse
ea − Periodic abstinence Spacing: beta1 offse
Africa
Asia
Europe
North America
ea − Periodic abstinence Spacing: alpha offse
ea − Periodic abstinence Spacing: alpha offse
ea − Periodic abstinence Limiting: beta0 offse
ea − Periodic abstinence Limiting: beta0 offse
ea − Periodic abstinence Limiting: beta1 offse
ea − Periodic abstinence Limiting: beta1 offse
ea − Periodic abstinence Limiting: alpha offse
ea − Periodic abstinence Limiting: alpha offse
Region − Periodic abstinence Spacing: beta0 offset (Rural)
Region − Periodic abstinence Spacing: beta0 offset (Urban)
Region − Periodic abstinence Spacing: beta1 offset (Rural)
Region − Periodic abstinence Spacing: beta1 offset (Urban)
ustralia/New Zealand
Caribbean
Central America
Central Asia
Eastern Africa
Eastern Asia
Eastern Europe
Melanesia
Micronesia
Middle Africa
Northern Africa
North America
Northern Europe
Polynesia
South−Eastern Asia
South America
Southern Africa
Southern Asia
Southern Europe
Western Africa
Western Asia
Western Europe

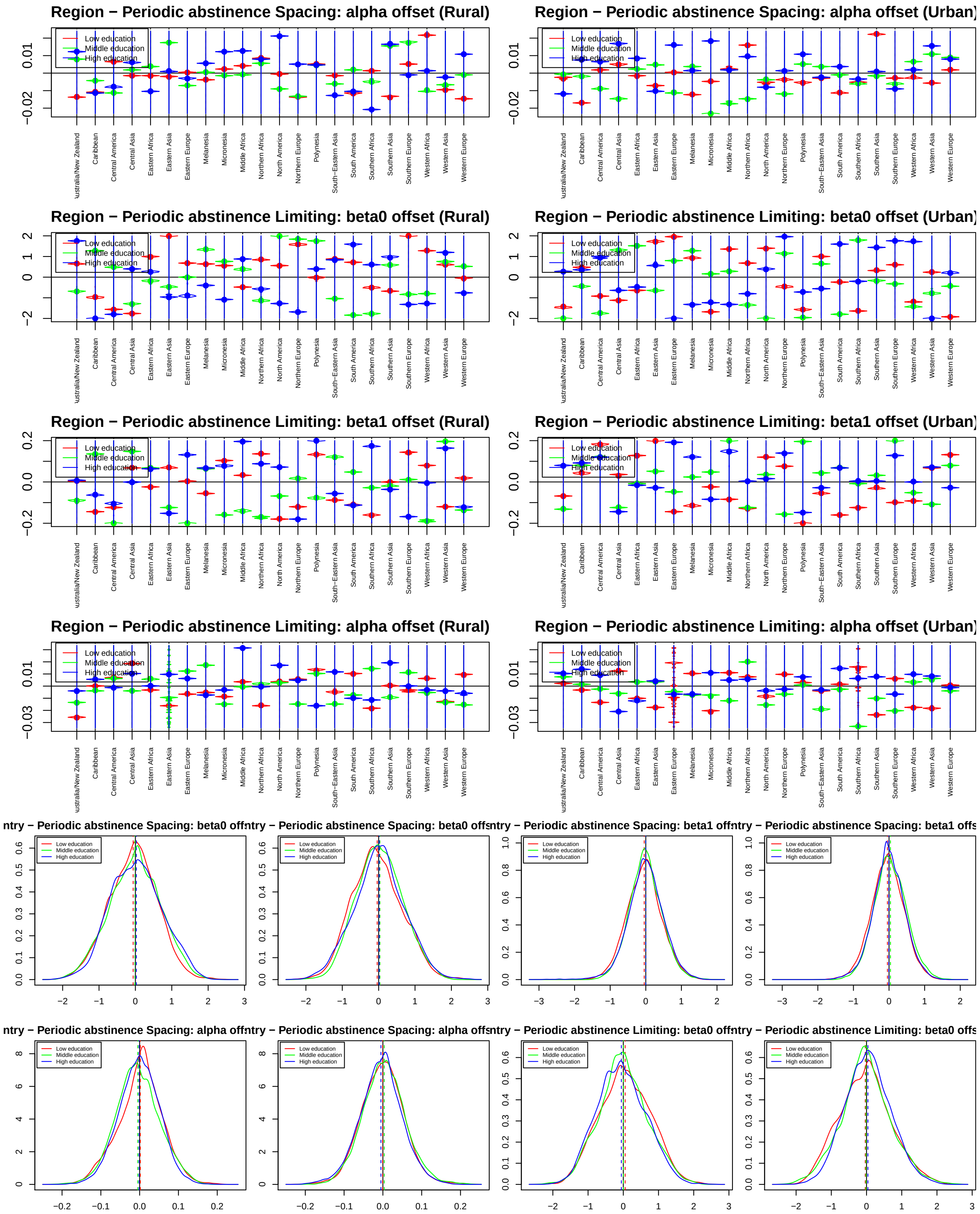

Region − Periodic abstinence Spacing: alpha offset (Rural)
Region − Periodic abstinence Spacing: alpha offset (Urban)
Region − Periodic abstinence Limiting: beta0 offset (Rural)
Region − Periodic abstinence Limiting: beta0 offset (Urban)
Region − Periodic abstinence Limiting: beta1 offset (Rural)
Region − Periodic abstinence Limiting: beta1 offset (Urban)
Region − Periodic abstinence Limiting: alpha offset (Rural)
Region − Periodic abstinence Limiting: alpha offset (Urban)
Low education
Middle education
High education
Australia/New Zealand
Caribbean
Central America
Central Asia
Eastern Africa
Eastern Asia
Eastern Europe
Melanesia
Micronesia
Middle Africa
Northern Africa
North America
Northern Europe
Polynesia
South−Eastern Asia
South America
Southern Africa
Southern Asia
Southern Europe
Western Africa
Western Asia
Western Europe
ntry − Periodic abstinence Spacing: beta0 off
ntry − Periodic abstinence Spacing: beta0 offs
ntry − Periodic abstinence Spacing: beta1 off
ntry − Periodic abstinence Spacing: beta1 offs
ntry − Periodic abstinence Spacing: alpha off
ntry − Periodic abstinence Spacing: alpha offs
ntry − Periodic abstinence Limiting: beta0 off
ntry − Periodic abstinence Limiting: beta0 offs

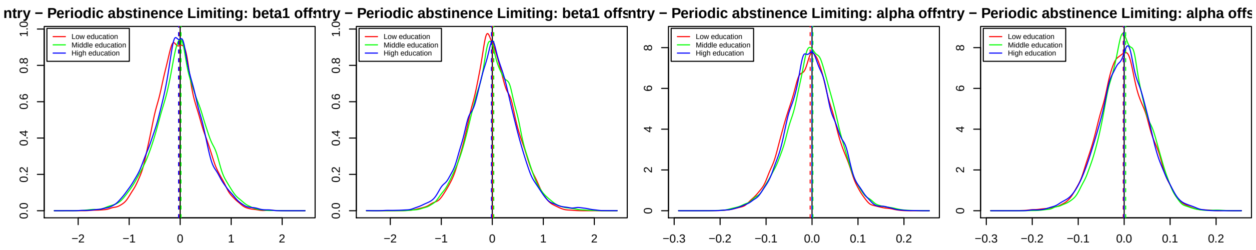

Parameters

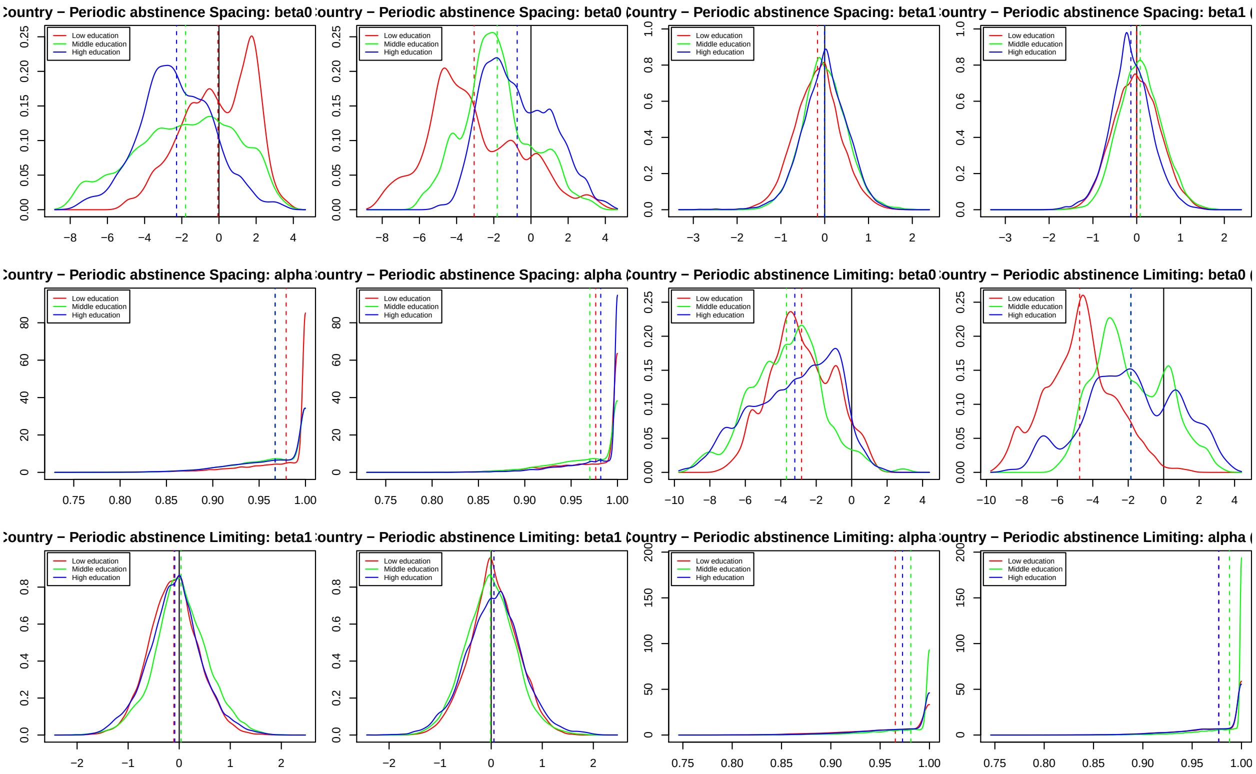

C.3.5.8 Withdrawal

Hyperparameters

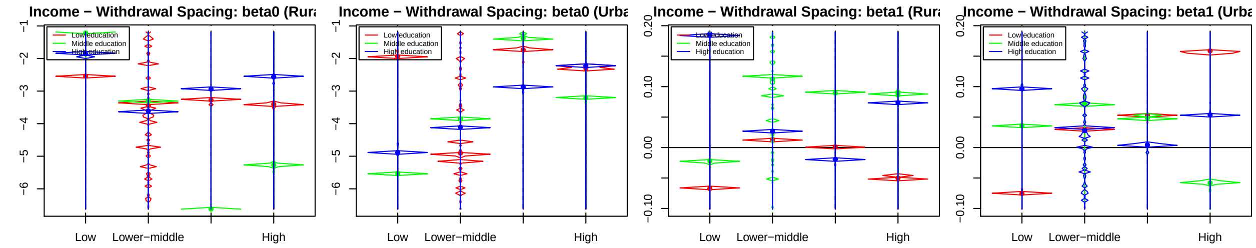

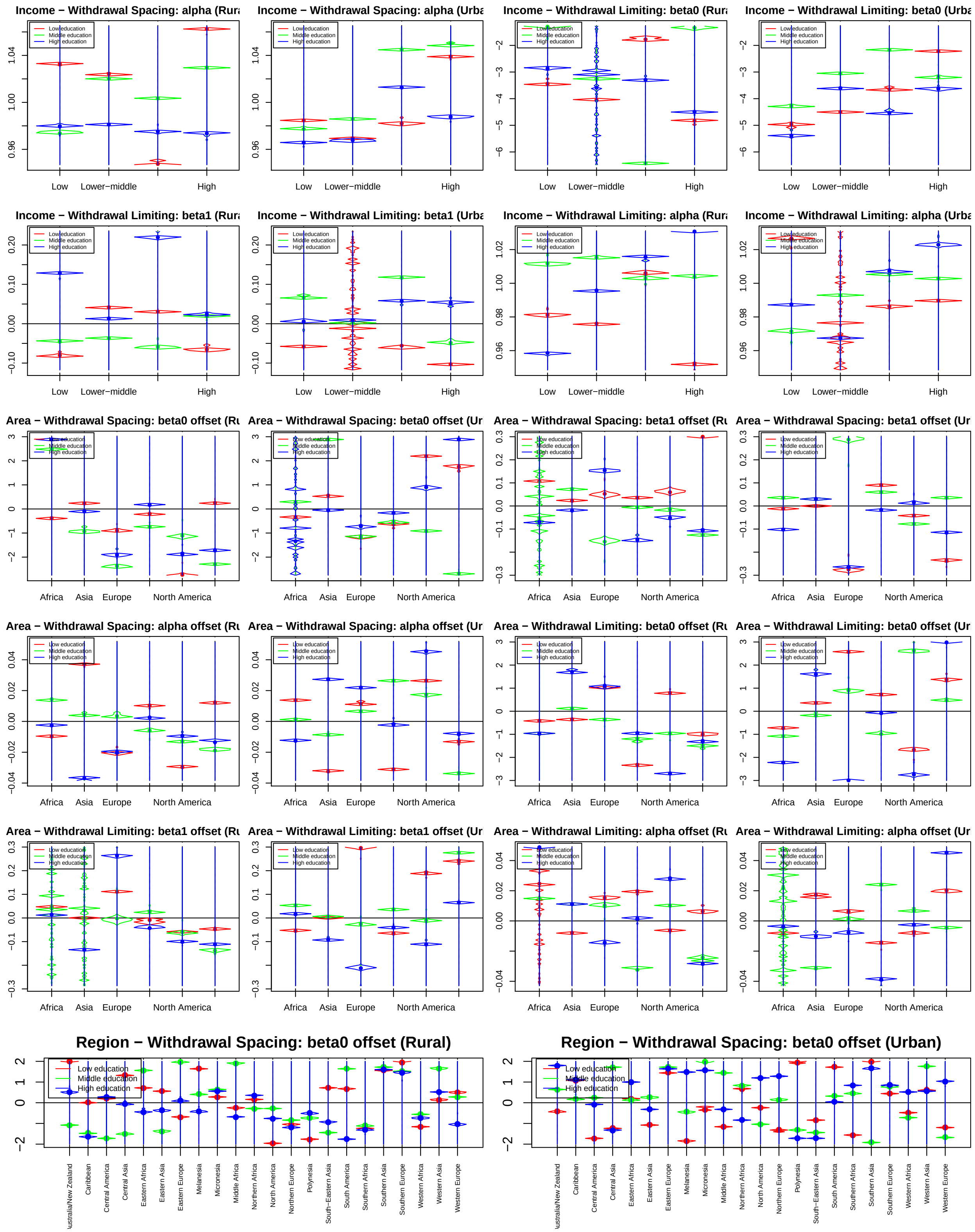
Income − Withdrawal Spacing: alpha (Rura
Income − Withdrawal Spacing: alpha (Urba
Income − Withdrawal Limiting: beta0 (Rura
Income − Withdrawal Limiting: beta0 (Urba
Income − Withdrawal Limiting: beta1 (Rura
Income − Withdrawal Limiting: beta1 (Urba
Income − Withdrawal Limiting: alpha (Rura
Income − Withdrawal Limiting: alpha (Urba
Area − Withdrawal Spacing: beta0 offset (Ru
Area − Withdrawal Spacing: beta0 offset (Ur
Area − Withdrawal Spacing: beta1 offset (Ru
Area − Withdrawal Spacing: beta1 offset (Ur
Area − Withdrawal Spacing: alpha offset (Ru
Area − Withdrawal Spacing: alpha offset (Ur
Area − Withdrawal Limiting: beta0 offset (Ru
Area − Withdrawal Limiting: beta0 offset (Ur
Area − Withdrawal Limiting: beta1 offset (Ru
Area − Withdrawal Limiting: beta1 offset (Ur
Area − Withdrawal Limiting: alpha offset (Ru
Area − Withdrawal Limiting: alpha offset (Ur
Region − Withdrawal Spacing: beta0 offset (Rural)
Region − Withdrawal Spacing: beta0 offset (Urban)
Low education
Middle education
High education
Low
Lower−middle
High
Africa
Asia
Europe
North America
Australia/New Zealand
Caribbean
Central America
Central Asia
Eastern Africa
Eastern Asia
Eastern Europe
Melanesia
Micronesia
Middle Africa
Northern Africa
North America
Northern Europe
Polynesia
South−Eastern Asia
South America
Southern Africa
Southern Asia
Southern Europe
Western Africa
Western Asia
Western Europe

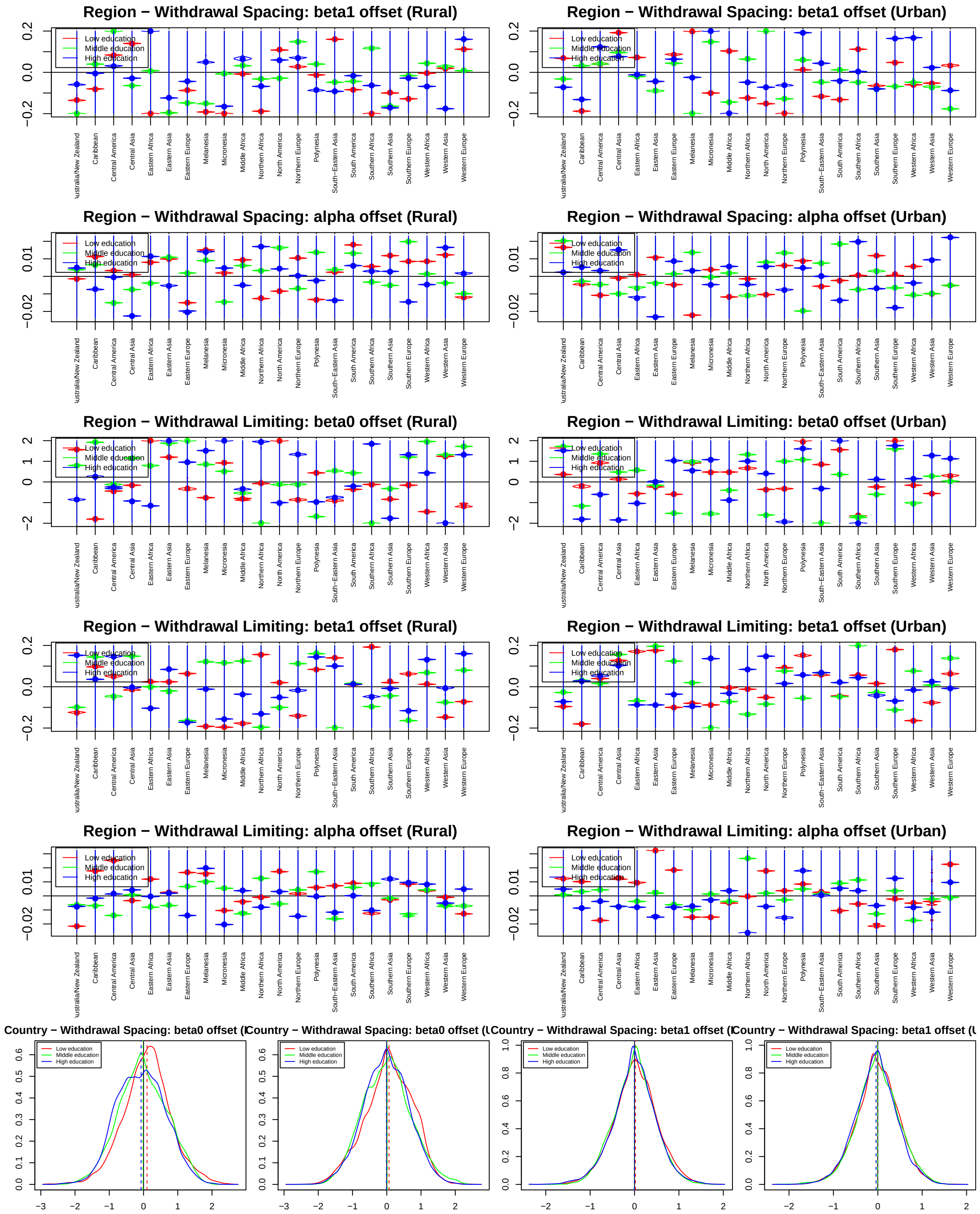
Region − Withdrawal Spacing: beta1 offset (Rural)
Region − Withdrawal Spacing: beta1 offset (Urban)
Region − Withdrawal Spacing: alpha offset (Rural)
Region − Withdrawal Spacing: alpha offset (Urban)
Region − Withdrawal Limiting: beta0 offset (Rural)
Region − Withdrawal Limiting: beta0 offset (Urban)
Region − Withdrawal Limiting: beta1 offset (Rural)
Region − Withdrawal Limiting: beta1 offset (Urban)
Region − Withdrawal Limiting: alpha offset (Rural)
Region − Withdrawal Limiting: alpha offset (Urban)
Low education
Middle education
High education
ustralia/New Zealand
Caribbean
Central America
Central Asia
Eastern Africa
Eastern Asia
Eastern Europe
Melanesia
Micronesia
Middle Africa
Northern Africa
North America
Northern Europe
Polynesia
South−Eastern Asia
South America
Southern Africa
Southern Asia
Southern Europe
Western Africa
Western Asia
Western Europe
Country − Withdrawal Spacing: beta0 offset (R
Country − Withdrawal Spacing: beta0 offset (U
Country − Withdrawal Spacing: beta1 offset (R
Country − Withdrawal Spacing: beta1 offset (U

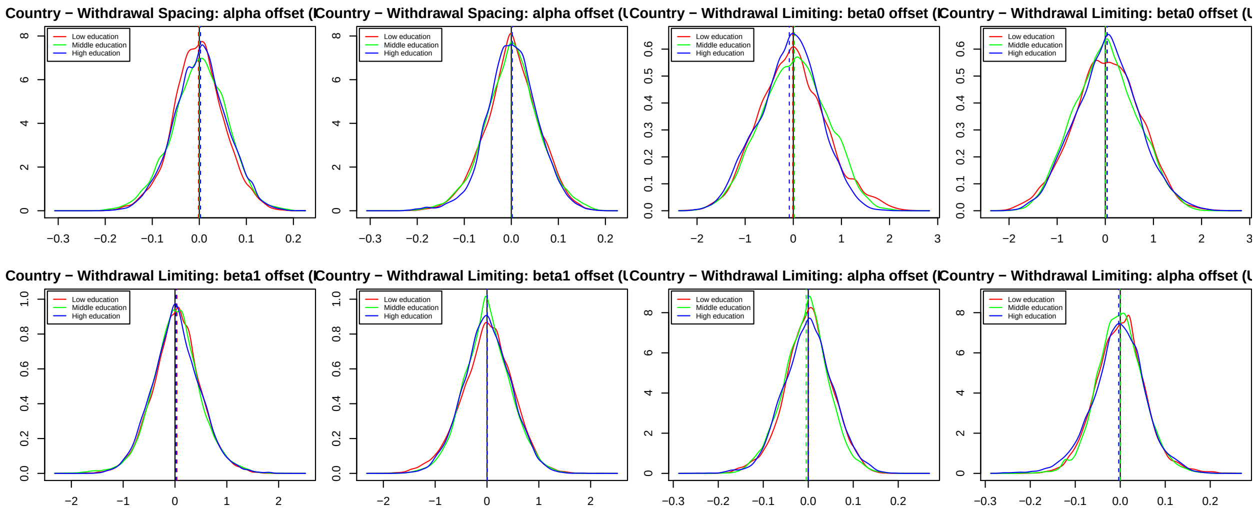

Parameters

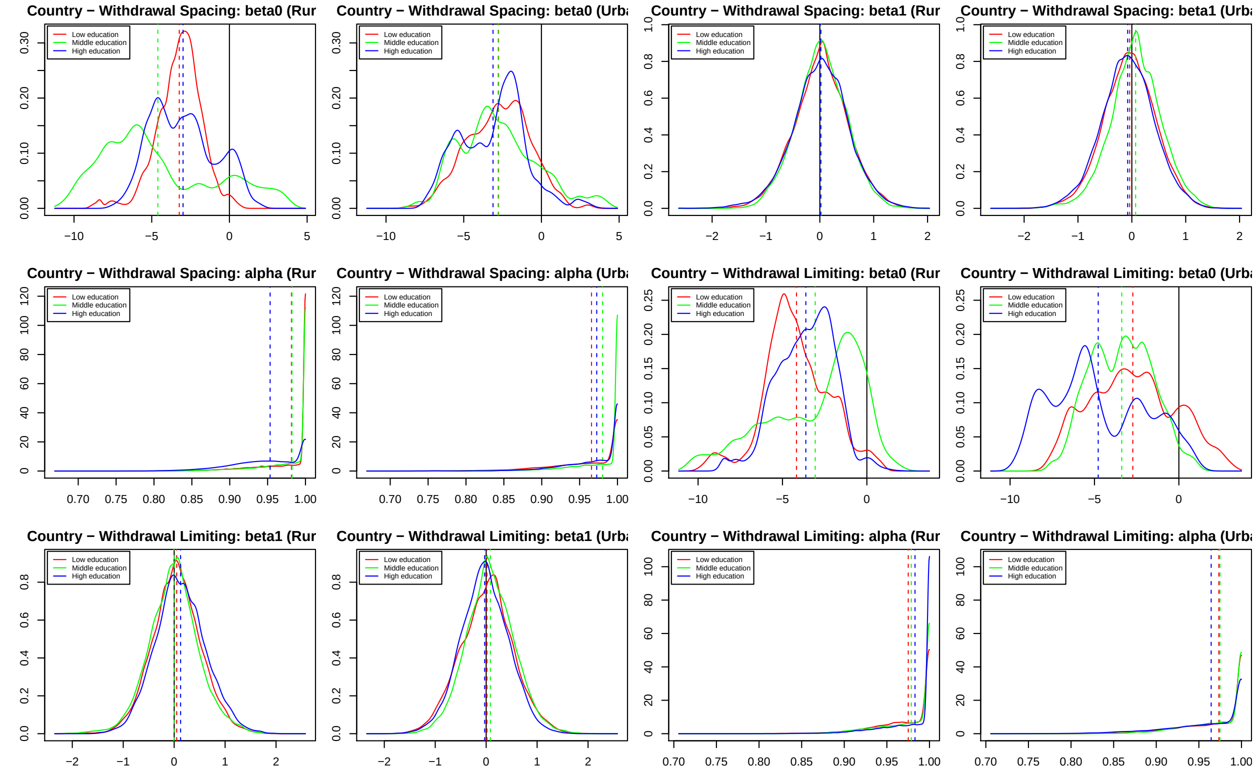

C.3.5.9 Other

Hyperparameters

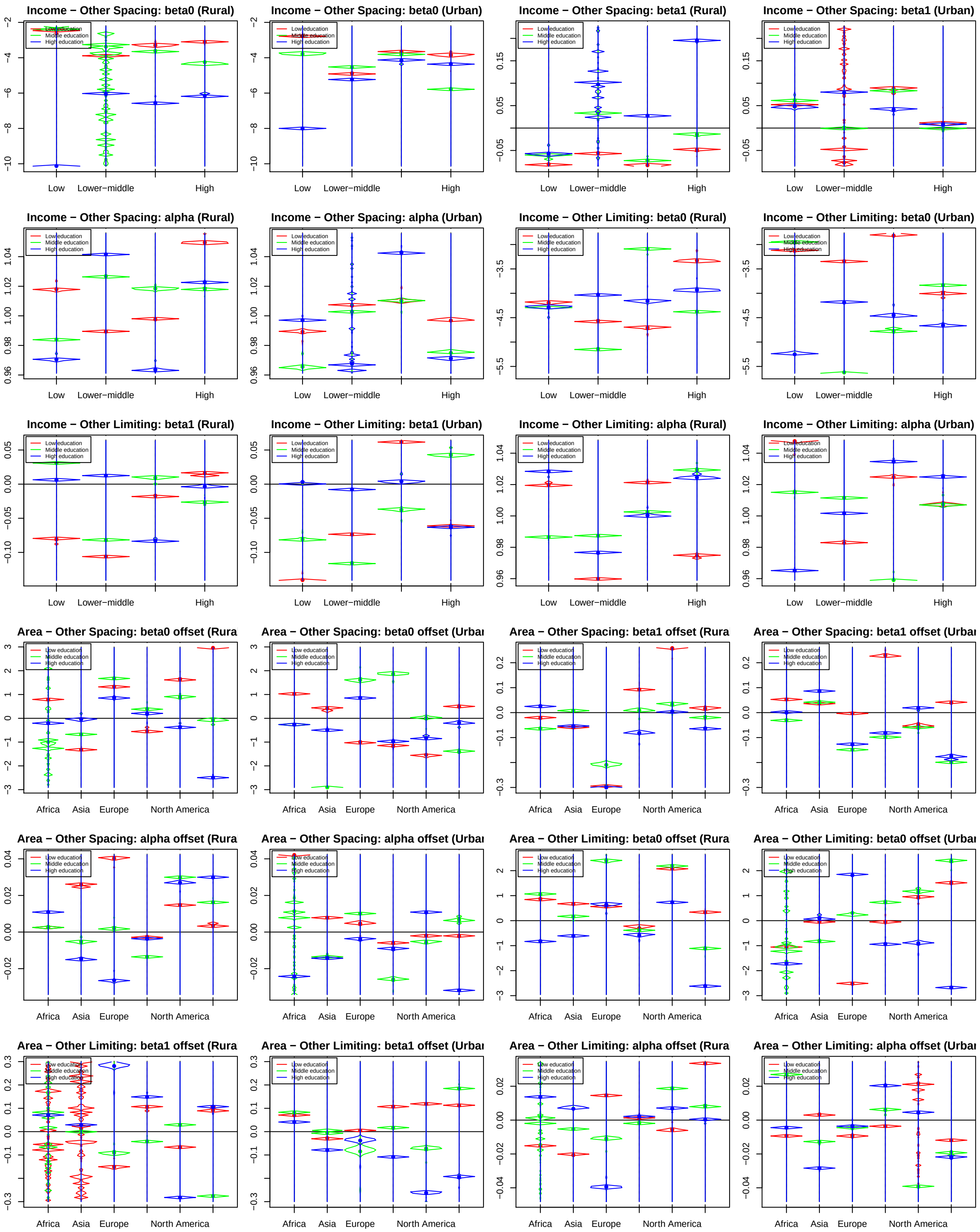
Income − Other Spacing: beta0 (Rural)
Income − Other Spacing: beta0 (Urban)
Income − Other Spacing: beta1 (Rural)
Income − Other Spacing: beta1 (Urban)
Income − Other Spacing: alpha (Rural)
Income − Other Spacing: alpha (Urban)
Income − Other Limiting: beta0 (Rural)
Income − Other Limiting: beta0 (Urban)
Income − Other Limiting: beta1 (Rural)
Income − Other Limiting: beta1 (Urban)
Income − Other Limiting: alpha (Rural)
Income − Other Limiting: alpha (Urban)
Area − Other Spacing: beta0 offset (Rura
Area − Other Spacing: beta0 offset (Urbai
Area − Other Spacing: beta1 offset (Rura
Area − Other Spacing: beta1 offset (Urbai
Area − Other Spacing: alpha offset (Rura
Area − Other Spacing: alpha offset (Urbai
Area − Other Limiting: beta0 offset (Rura
Area − Other Limiting: beta0 offset (Urbai
Area − Other Limiting: beta1 offset (Rura
Area − Other Limiting: beta1 offset (Urbai
Area − Other Limiting: alpha offset (Rura
Area − Other Limiting: alpha offset (Urbai
Low education
Middle education
High education
Low
Lower−middle
High
Africa
Asia
Europe
North America

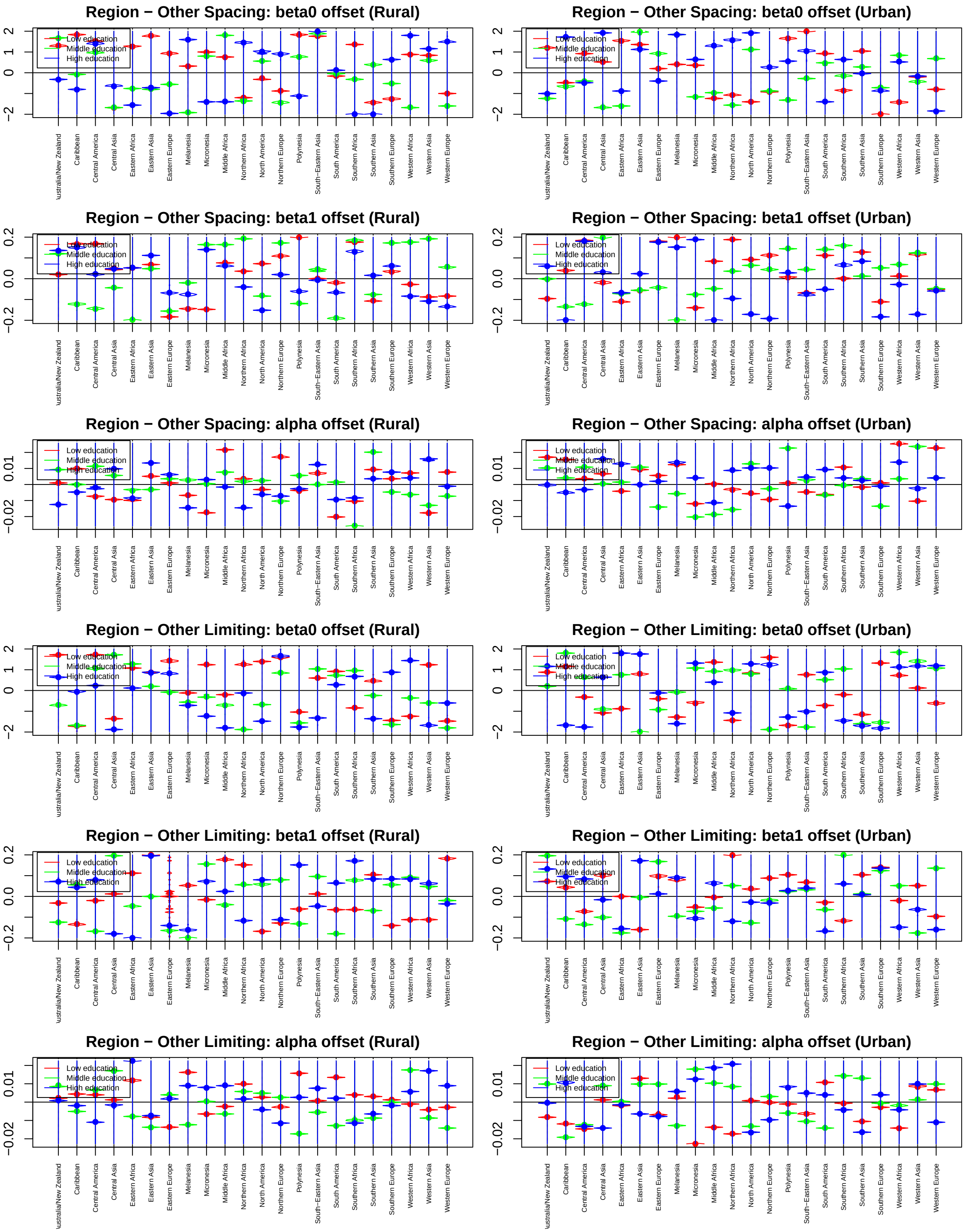
Region − Other Spacing: beta0 offset (Rural)
Region − Other Spacing: beta0 offset (Urban)
Region − Other Spacing: beta1 offset (Rural)
Region − Other Spacing: beta1 offset (Urban)
Region − Other Spacing: alpha offset (Rural)
Region − Other Spacing: alpha offset (Urban)
Region − Other Limiting: beta0 offset (Rural)
Region − Other Limiting: beta0 offset (Urban)
Region − Other Limiting: beta1 offset (Rural)
Region − Other Limiting: beta1 offset (Urban)
Region − Other Limiting: alpha offset (Rural)
Region − Other Limiting: alpha offset (Urban)
Low education
Middle education
High education
Australia/New Zealand
Caribbean
Central America
Central Asia
Eastern Africa
Eastern Asia
Eastern Europe
Melanesia
Micronesia
Middle Africa
Northern Africa
North America
Northern Europe
Polynesia
South−Eastern Asia
South America
Southern Africa
Southern Asia
Southern Europe
Western Africa
Western Asia
Western Europe

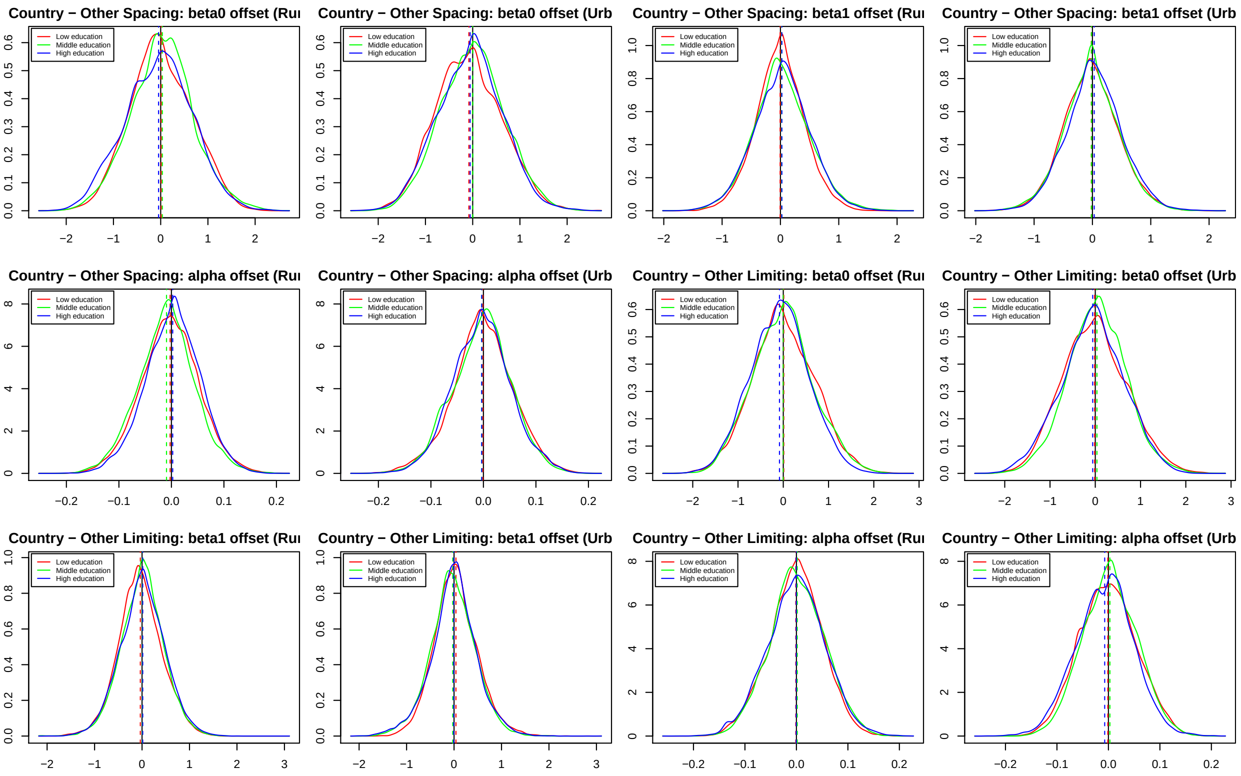

Parameters

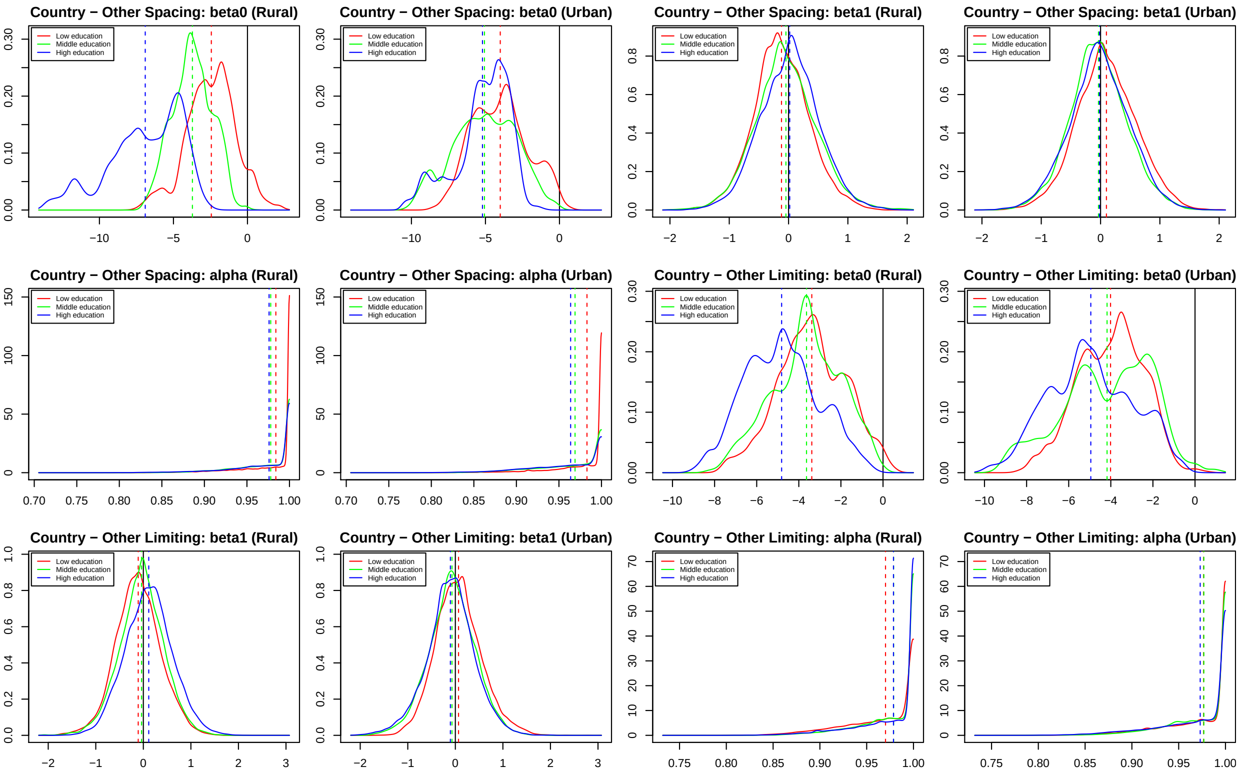

C.3.5.10 Implants

Hyperparameters

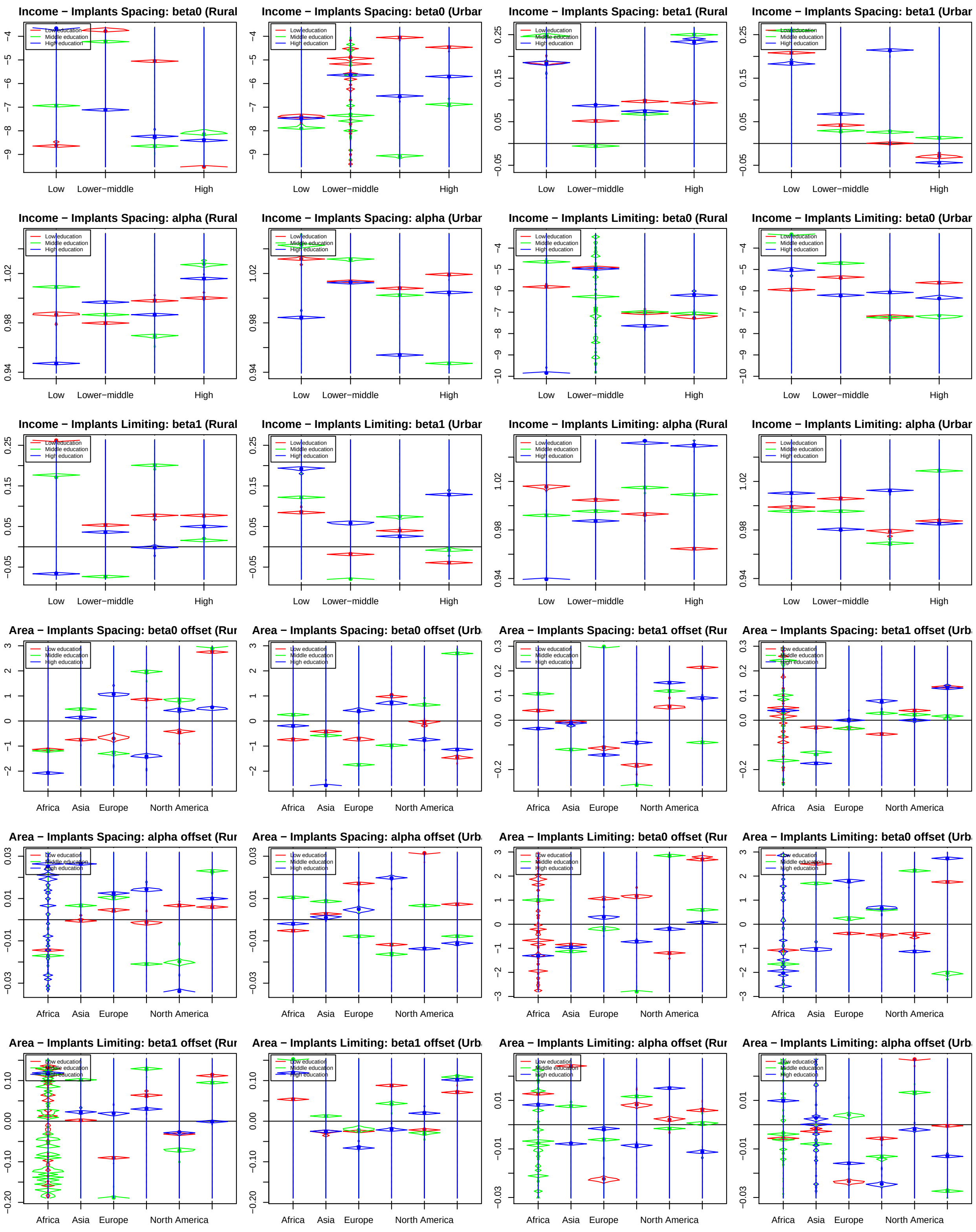

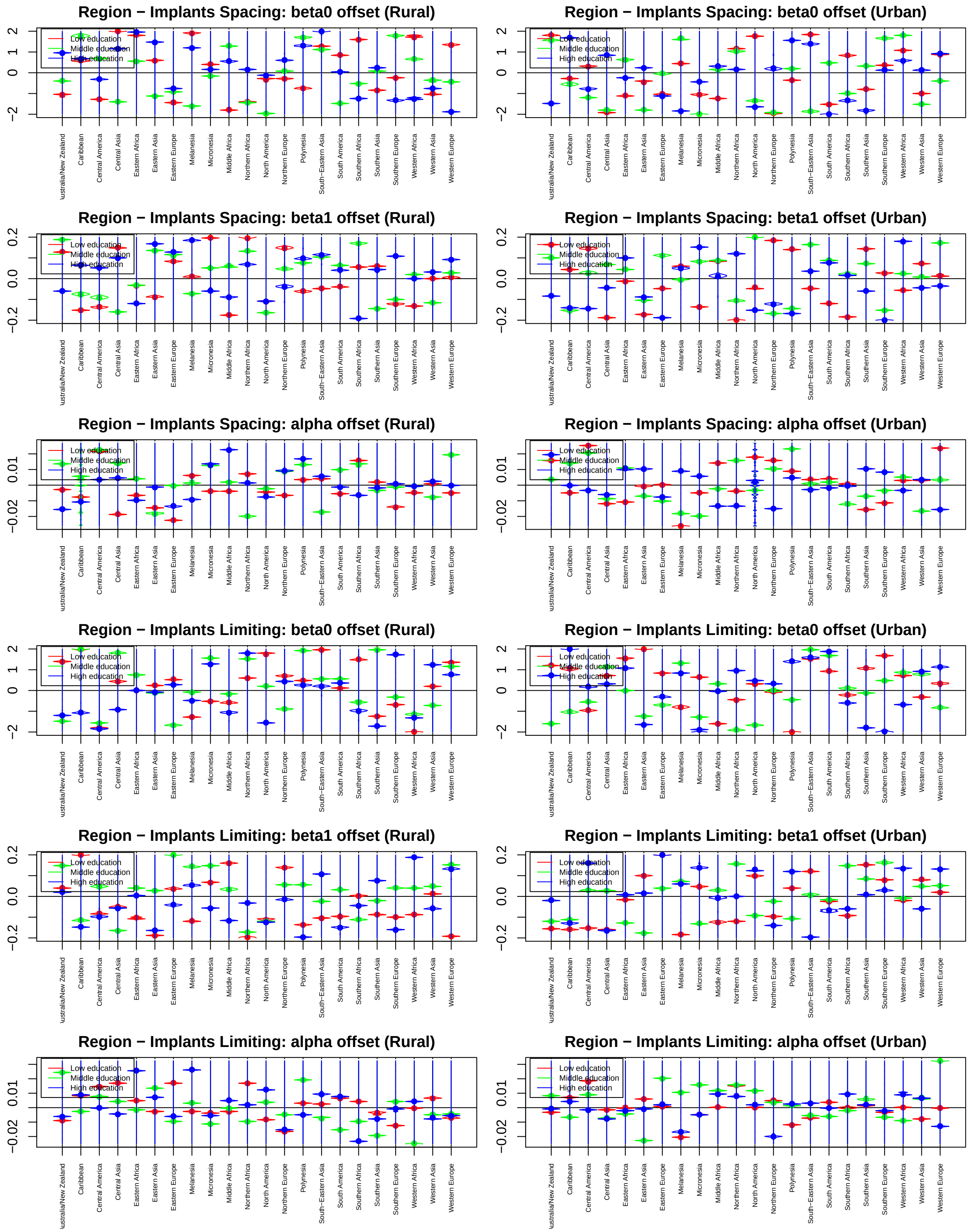
Region − Implants Spacing: beta0 offset (Rural)
Region − Implants Spacing: beta0 offset (Urban)
Region − Implants Spacing: beta1 offset (Rural)
Region − Implants Spacing: beta1 offset (Urban)
Region − Implants Spacing: alpha offset (Rural)
Region − Implants Spacing: alpha offset (Urban)
Region − Implants Limiting: beta0 offset (Rural)
Region − Implants Limiting: beta0 offset (Urban)
Region − Implants Limiting: beta1 offset (Rural)
Region − Implants Limiting: beta1 offset (Urban)
Region − Implants Limiting: alpha offset (Rural)
Region − Implants Limiting: alpha offset (Urban)
Low education
Middle education
High education
ustralia/New Zealand
Caribbean
Central America
Central Asia
Eastern Africa
Eastern Asia
Eastern Europe
Melanesia
Micronesia
Middle Africa
Northern Africa
North America
Northern Europe
Polynesia
South−Eastern Asia
South America
Southern Africa
Southern Asia
Southern Europe
Western Africa
Western Asia
Western Europe

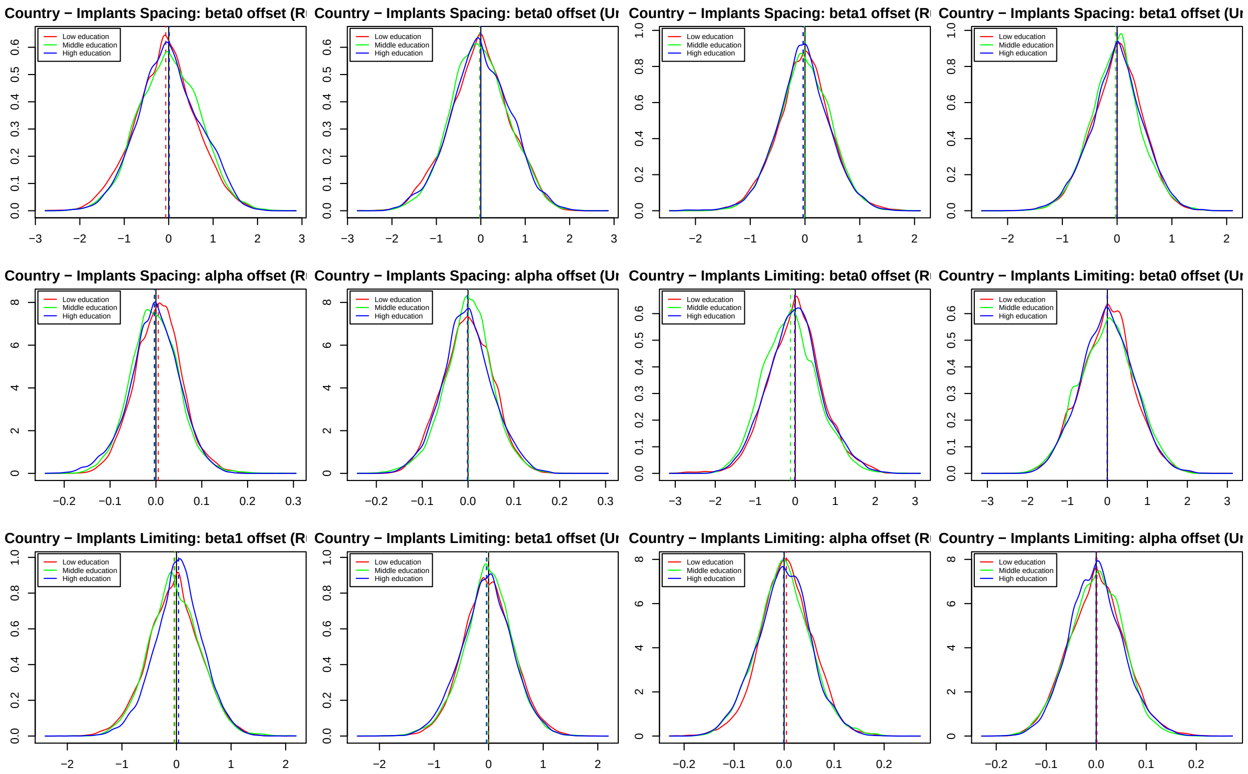
Country − Implants Spacing: beta0 offset (R
Country − Implants Spacing: beta0 offset (Ur
Country − Implants Spacing: beta1 offset (R
Country − Implants Spacing: beta1 offset (Ur
Country − Implants Spacing: alpha offset (R
Country − Implants Spacing: alpha offset (Ur
Country − Implants Limiting: beta0 offset (R
Country − Implants Limiting: beta0 offset (Ur
Country − Implants Limiting: beta1 offset (R
Country − Implants Limiting: beta1 offset (Ur
Country − Implants Limiting: alpha offset (R
Country − Implants Limiting: alpha offset (Ur
Low education
Middle education
High education

Parameters

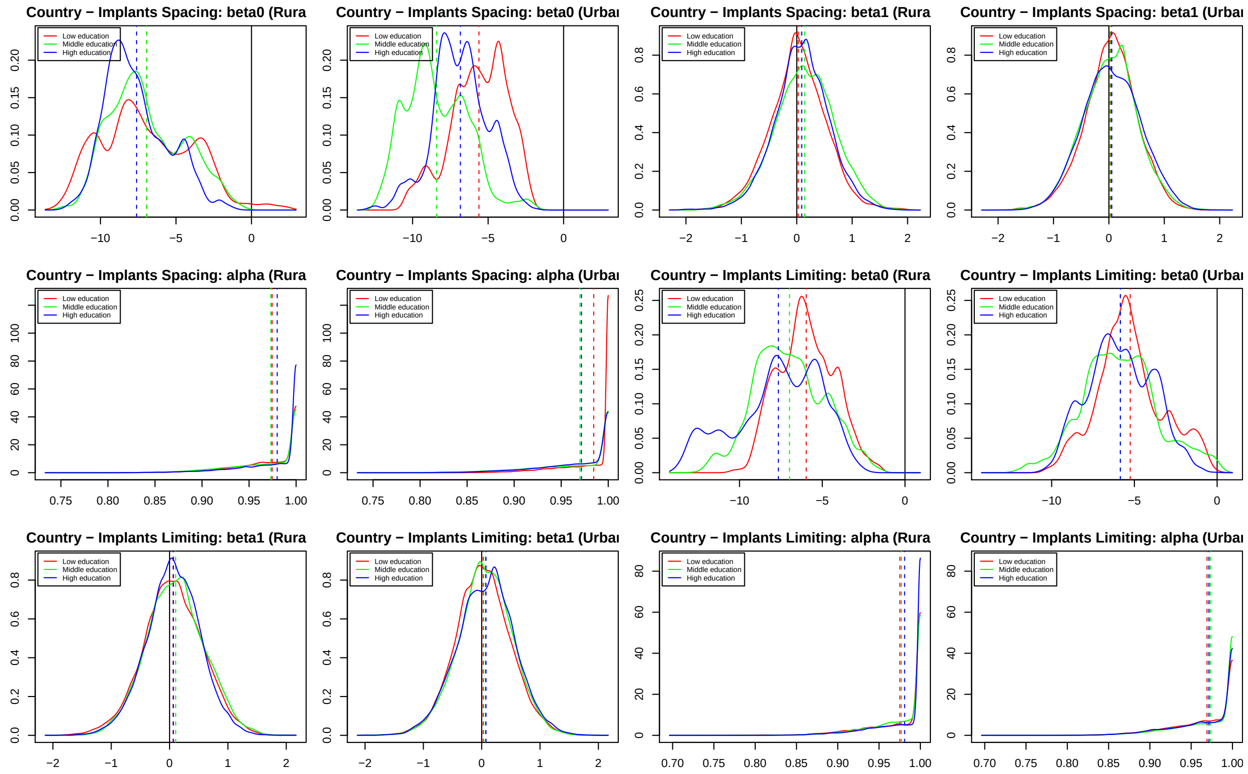
Country − Implants Spacing: beta0 (Rura
Country − Implants Spacing: beta0 (Urbai
Country − Implants Spacing: beta1 (Rura
Country − Implants Spacing: beta1 (Urbai
Country − Implants Spacing: alpha (Rura
Country − Implants Spacing: alpha (Urbai
Country − Implants Limiting: beta0 (Rura
Country − Implants Limiting: beta0 (Urbai
Country − Implants Limiting: beta1 (Rura
Country − Implants Limiting: beta1 (Urbai
Country − Implants Limiting: alpha (Rura
Country − Implants Limiting: alpha (Urbai
Low education
Middle education
High education

C.3.6 Contraception Failure Rates

C.3.6.1 Pill

Hyperparameters

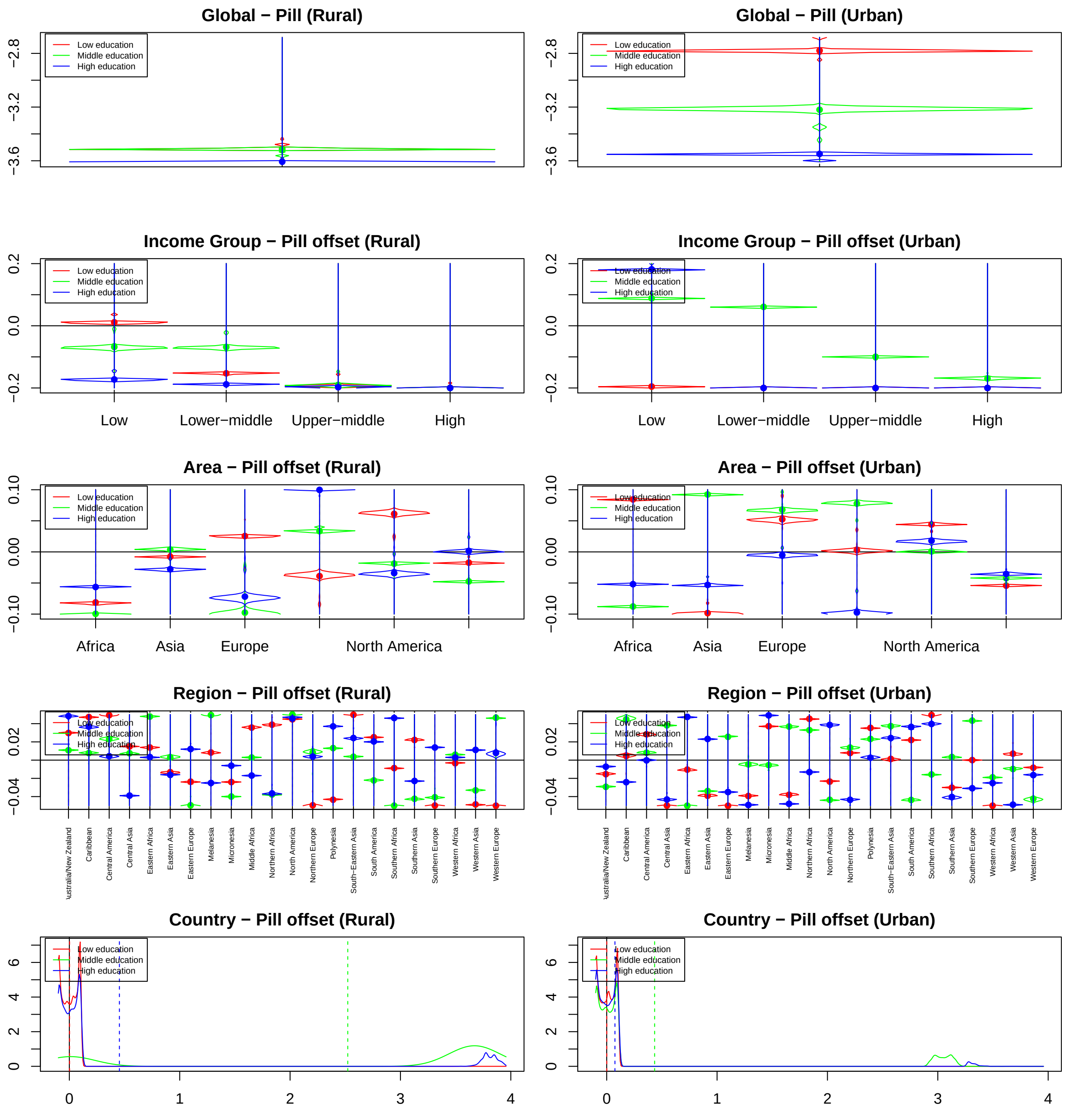

Parameters

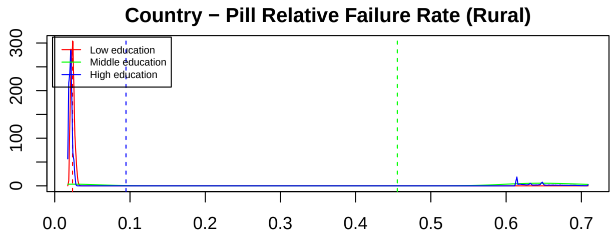

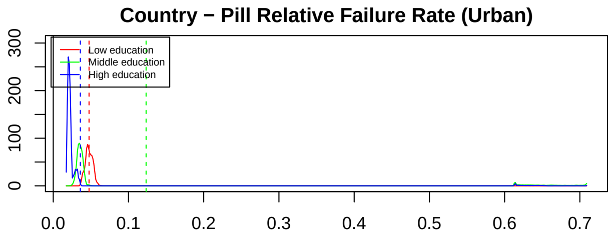

C.3.6.2 IUD

Hyperparameters

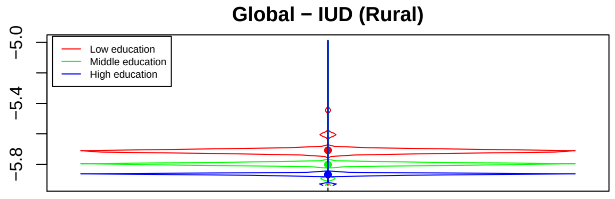

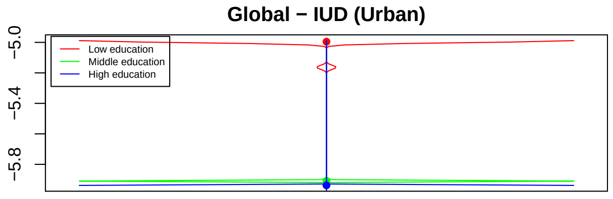

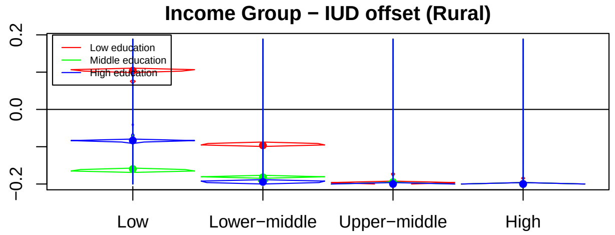

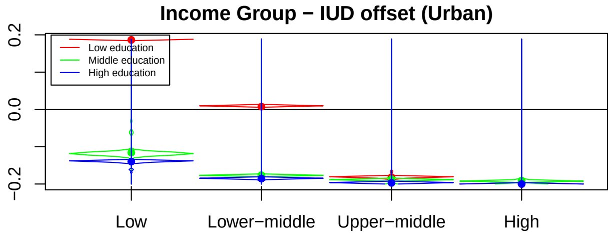

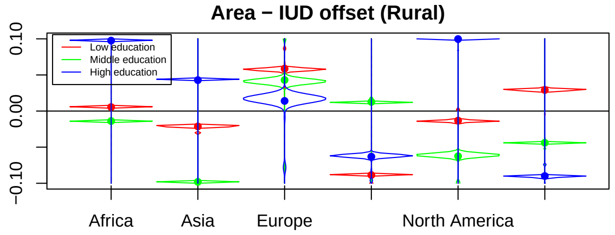

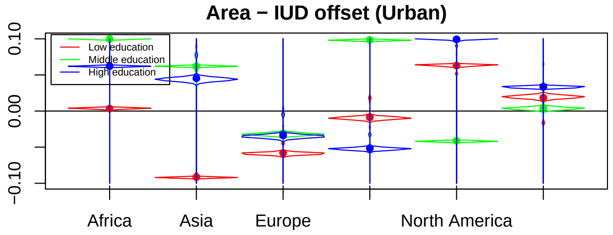

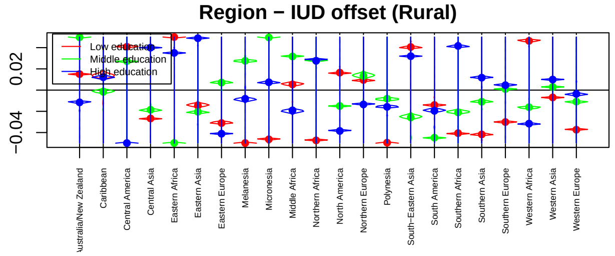

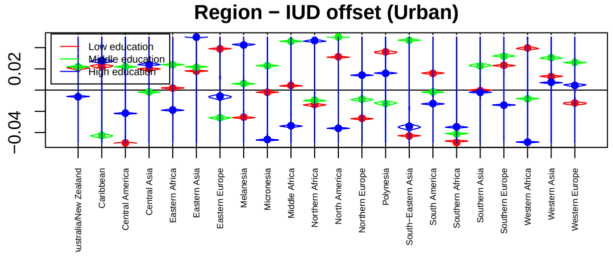

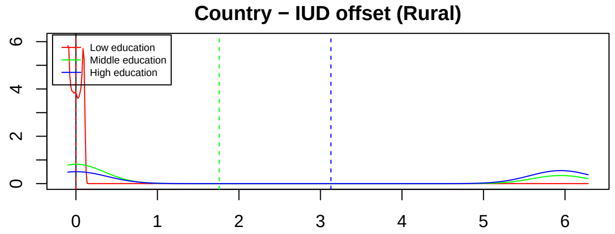

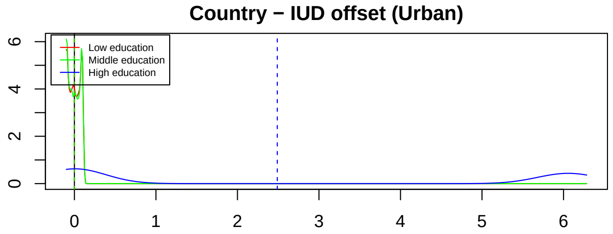

Parameters

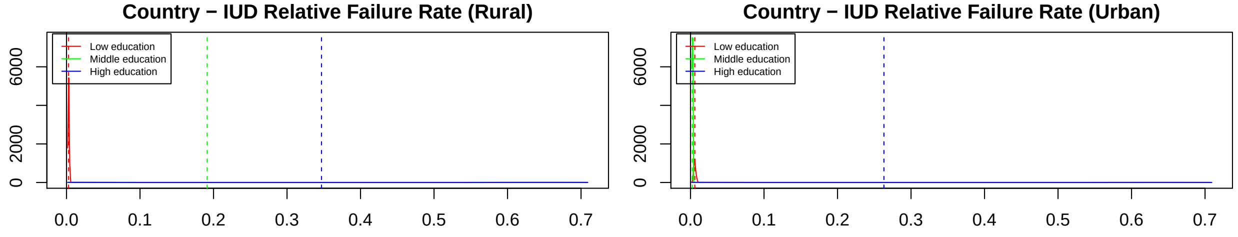

C.3.6.3 Injections

Hyperparameters

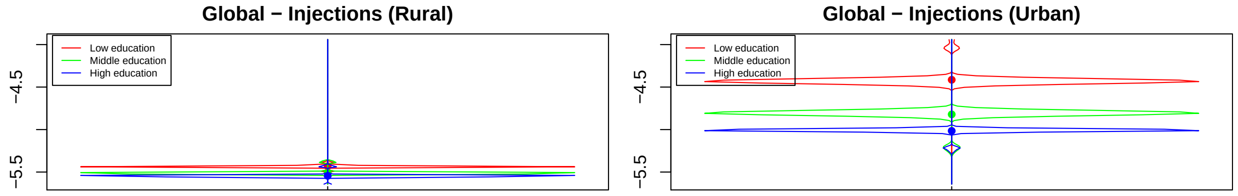

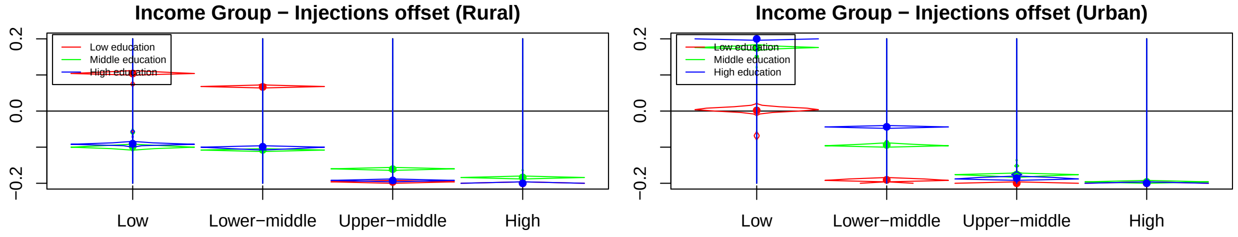

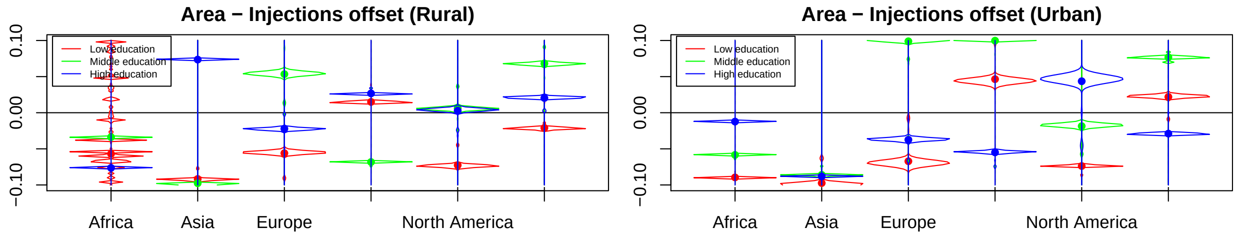

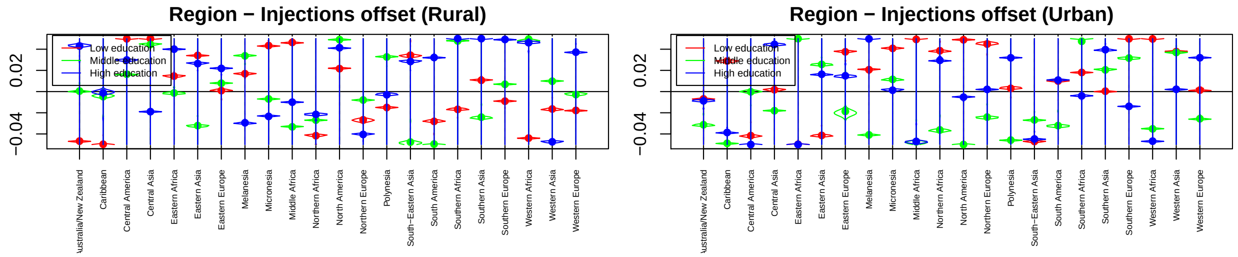

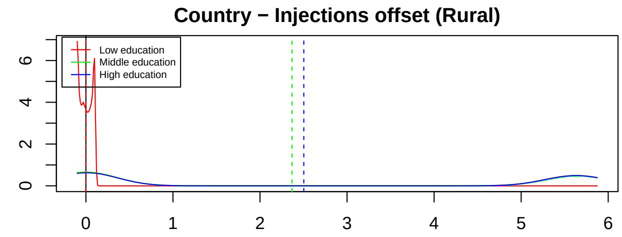

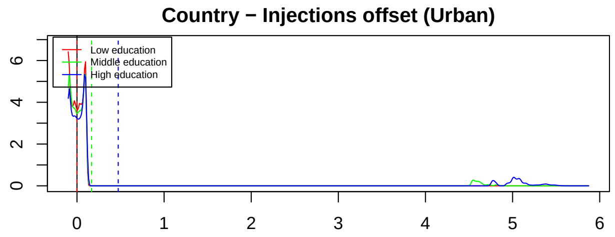

Parameters

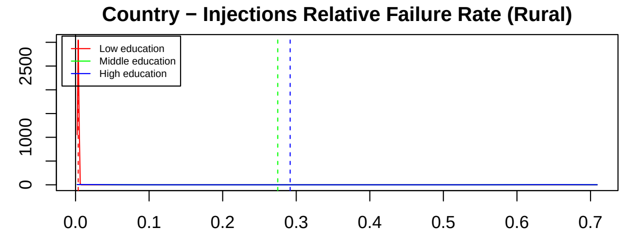

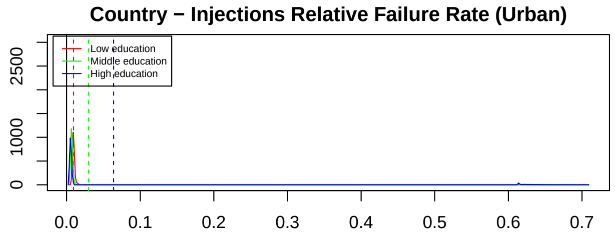

C.3.6.4 Condom

Hyperparameters

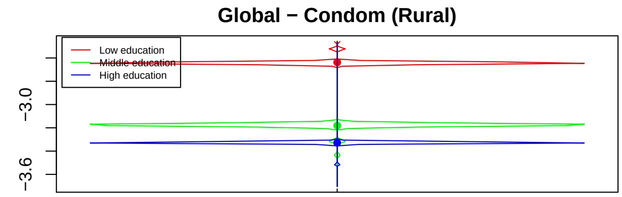

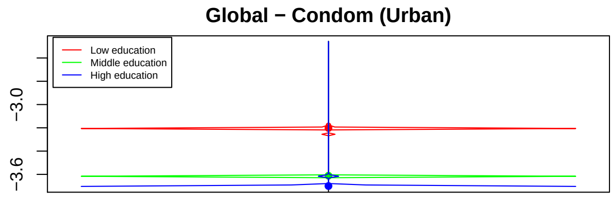

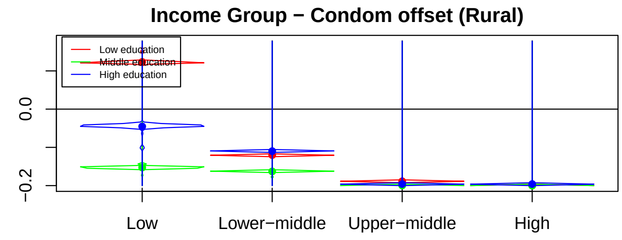

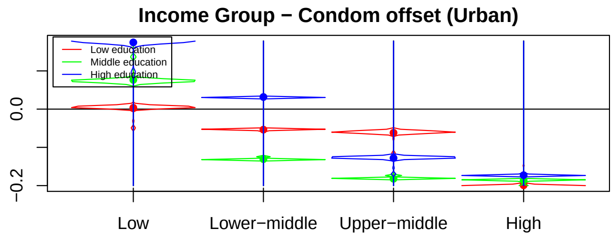

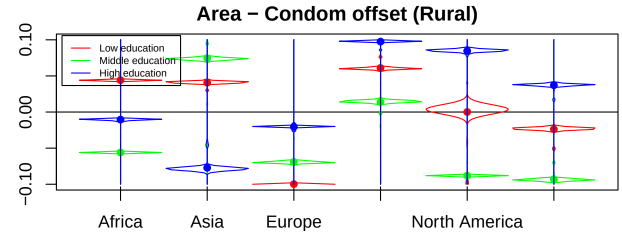

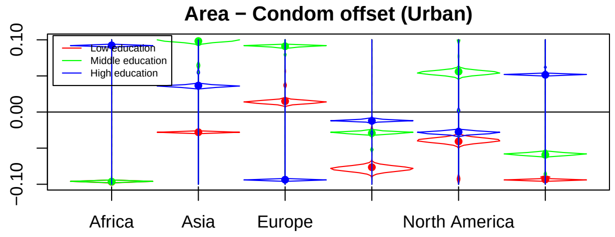

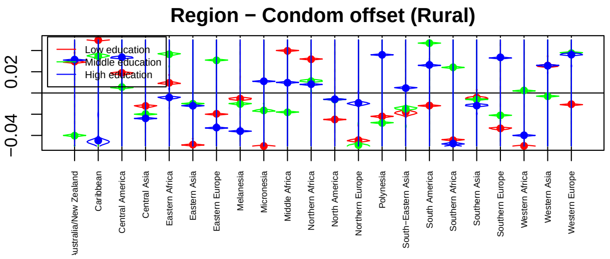

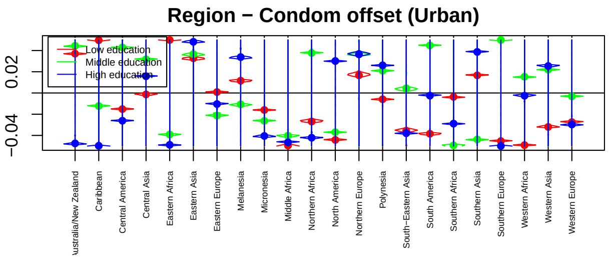

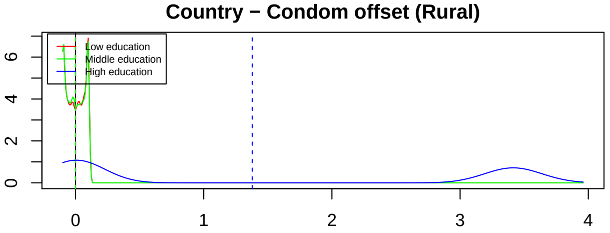

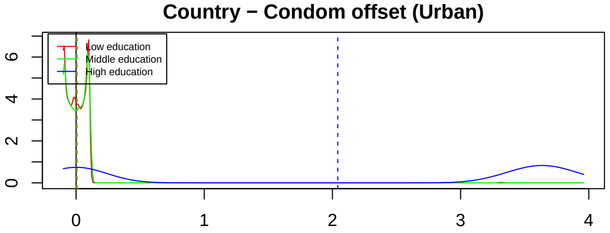

Parameters

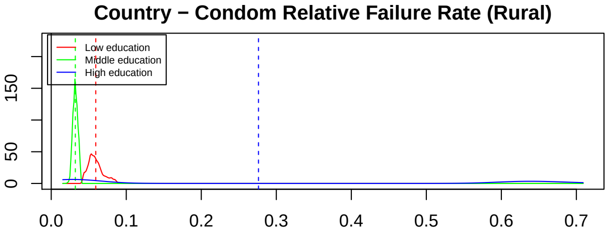

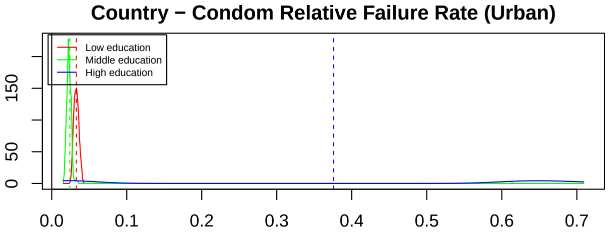

C.3.6.5 Female sterilization

Hyperparameters

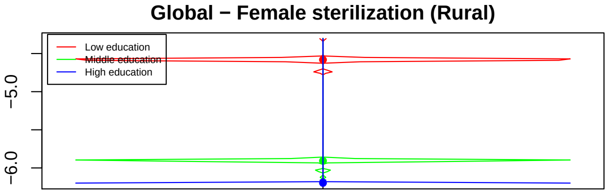

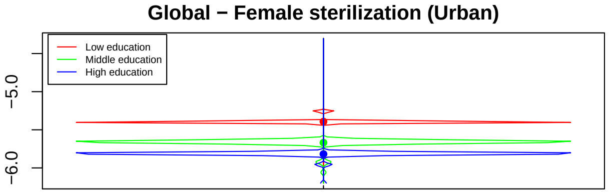

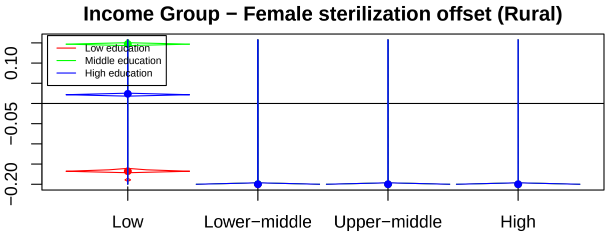

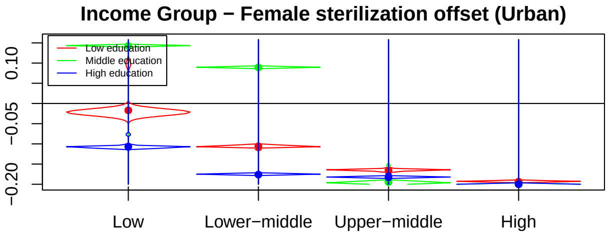

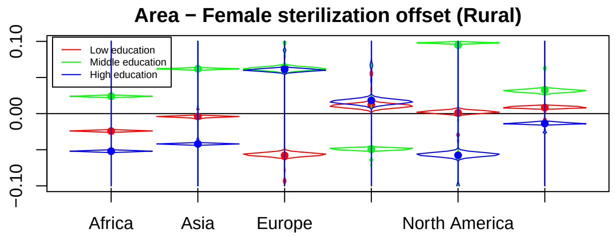

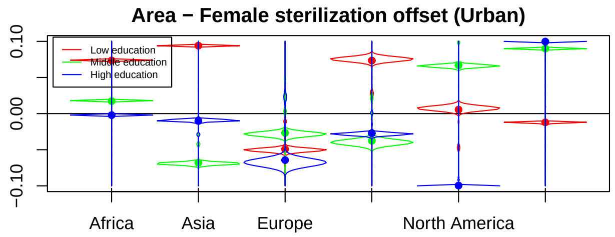

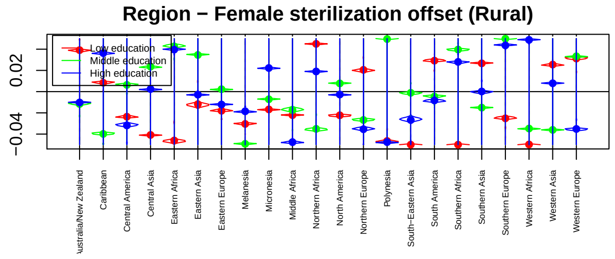

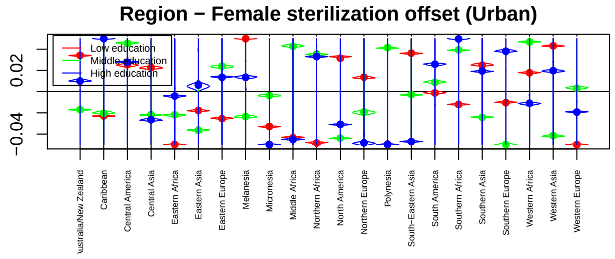

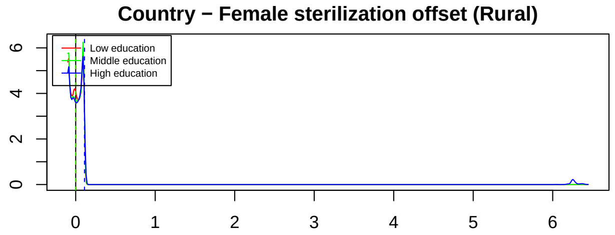

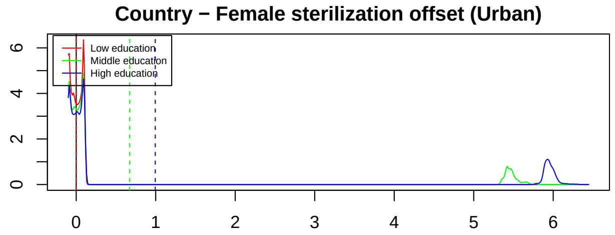

Parameters

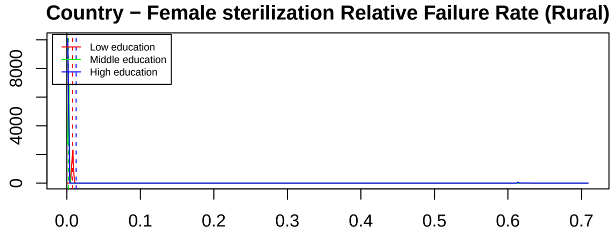

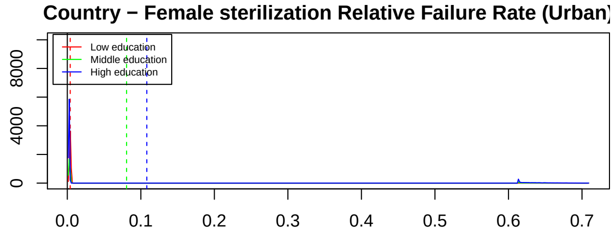

C.3.6.6 Male sterilization

Hyperparameters

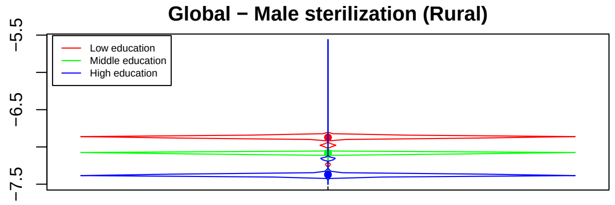

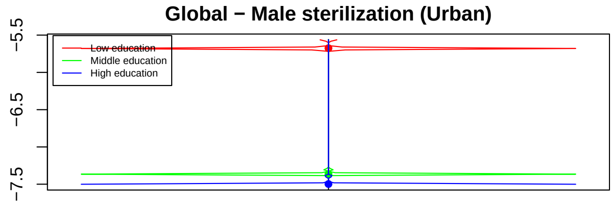

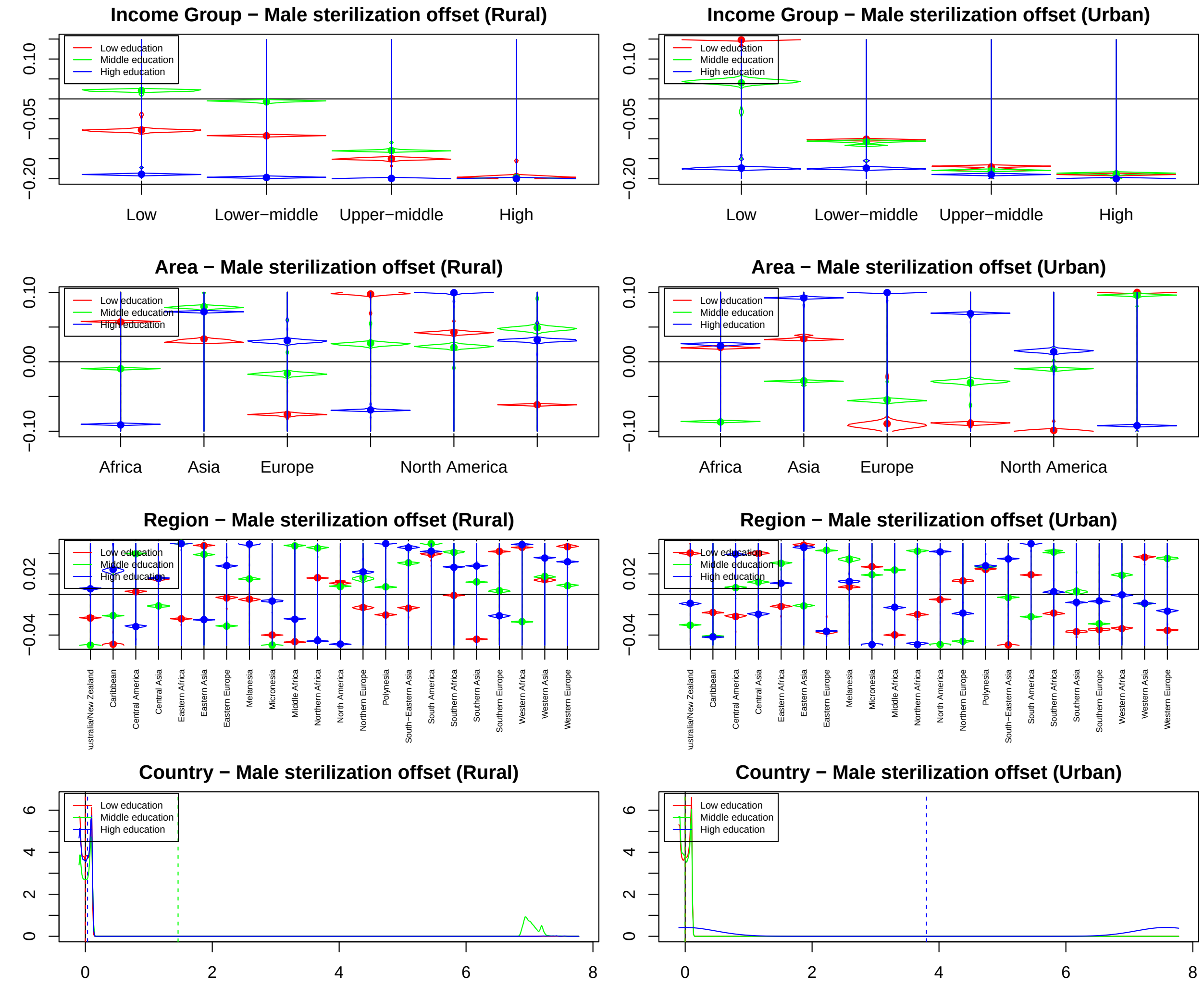

Parameters

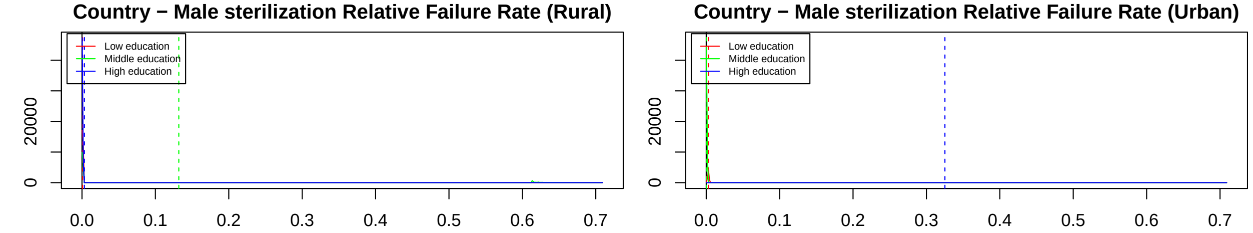

C.3.6.7 Periodic abstinence

Hyperparameters

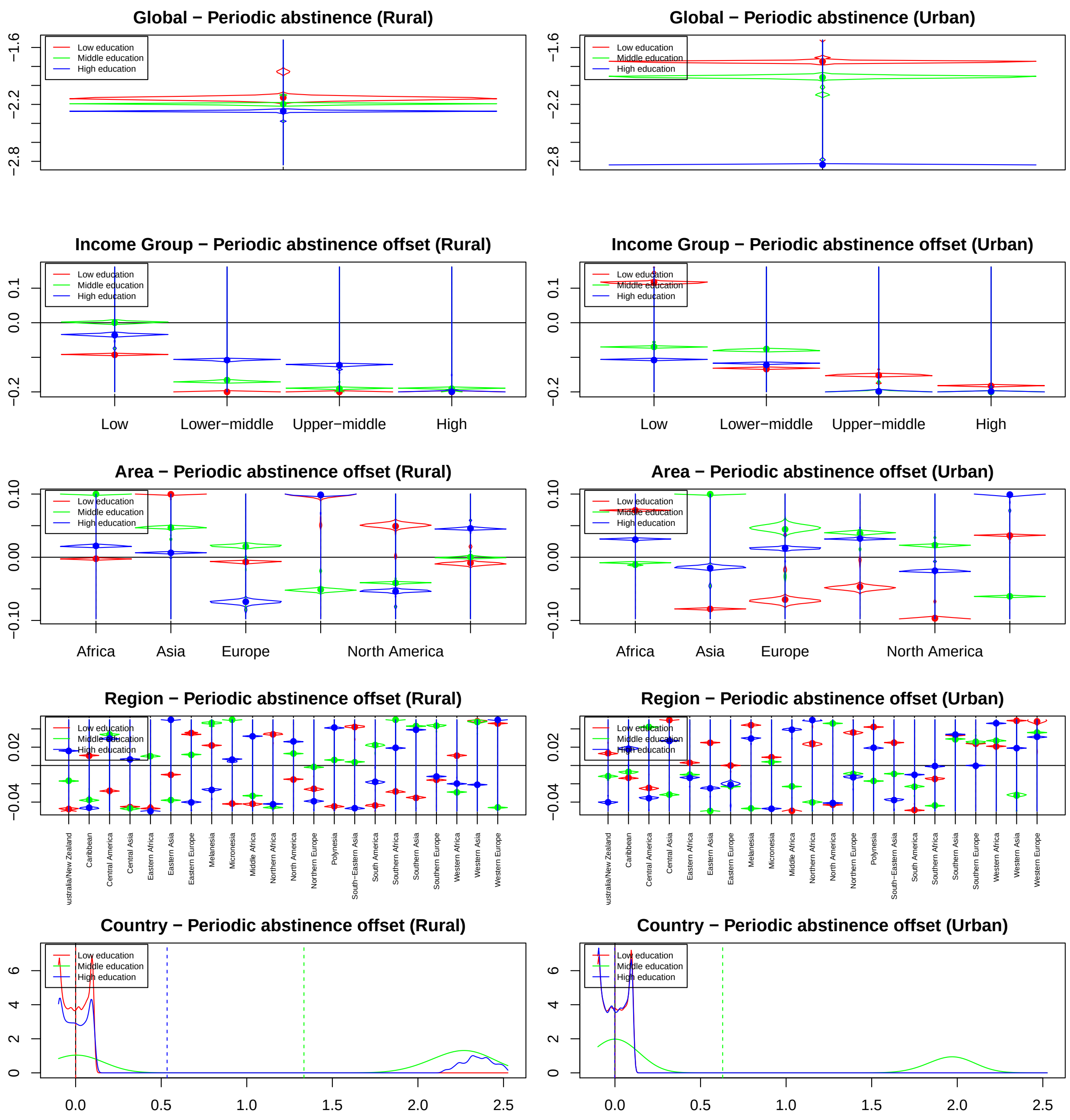

Parameters

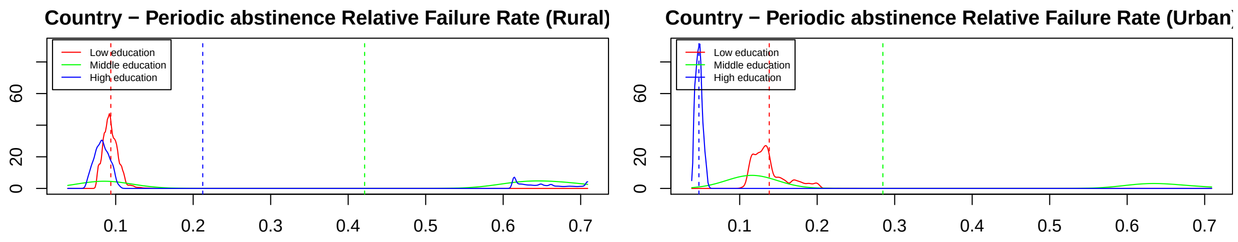

C.3.6.8 Withdrawal

Hyperparameters

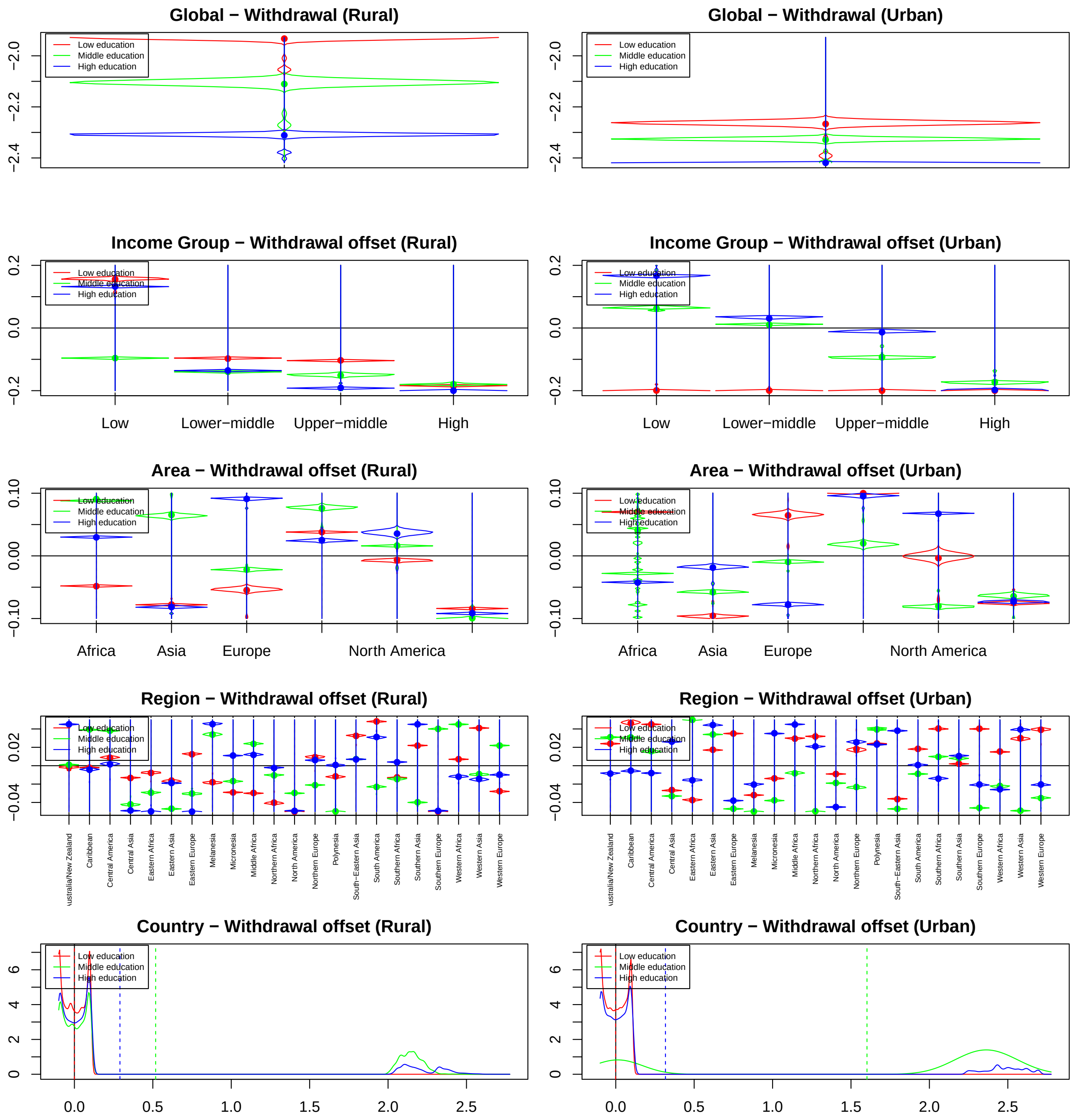

Parameters

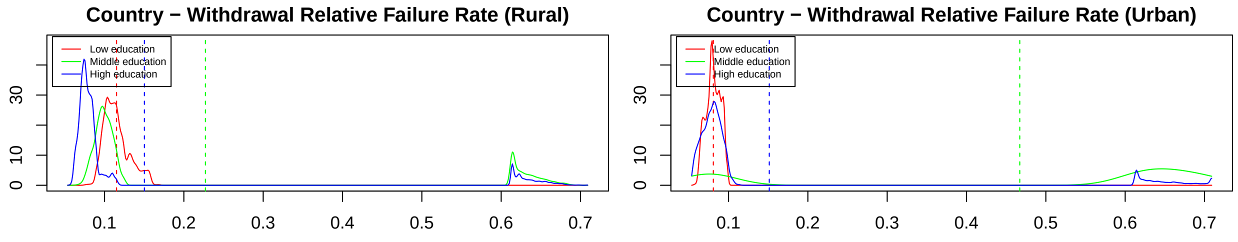

C.3.6.9 Other

Hyperparameters

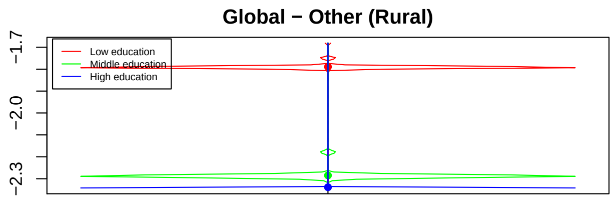

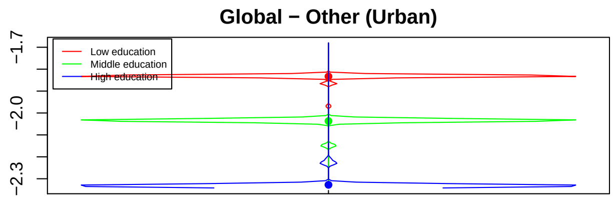

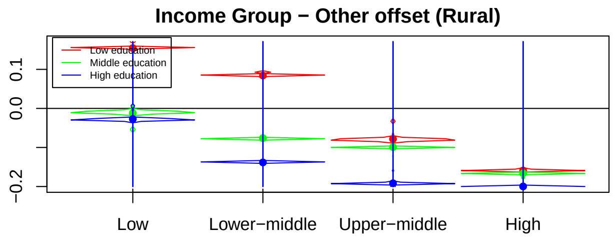

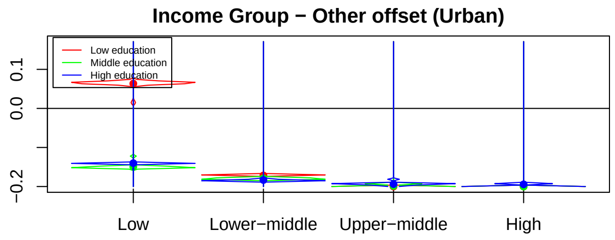

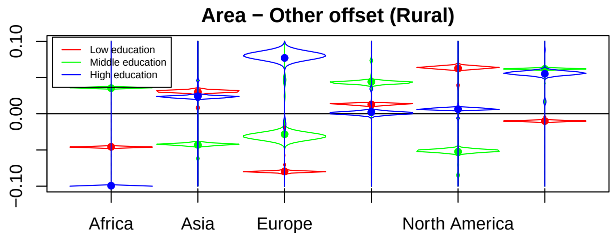

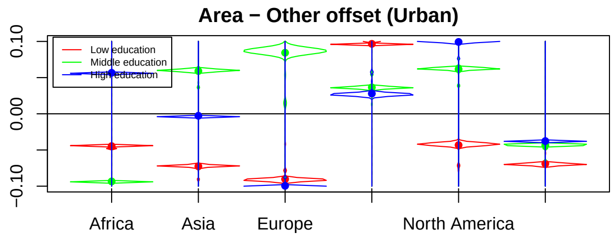

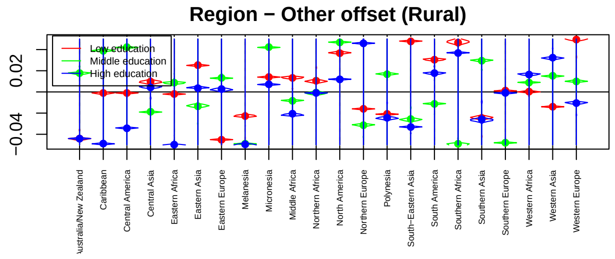

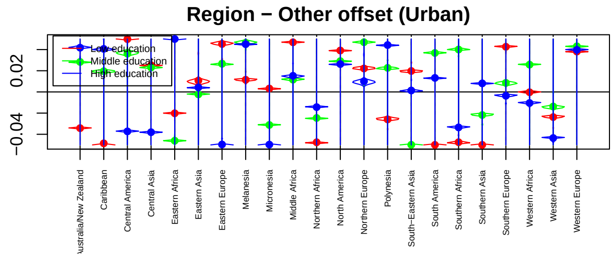

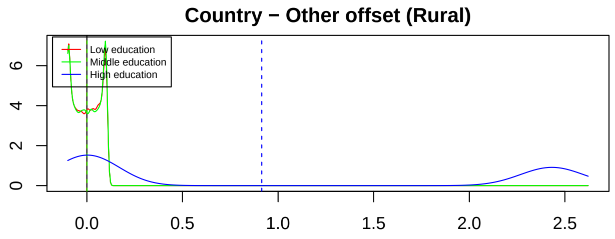

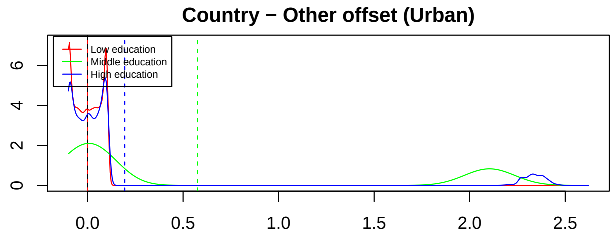

Parameters

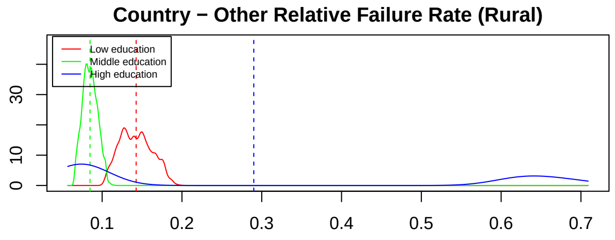

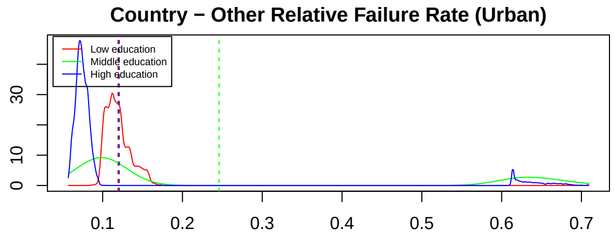

C.3.6.10 Implants

Hyperparameters

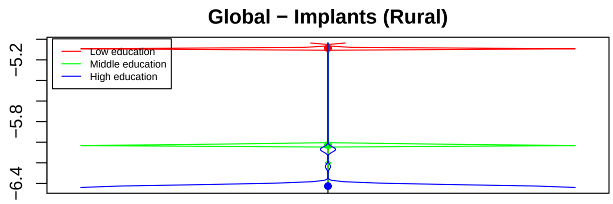

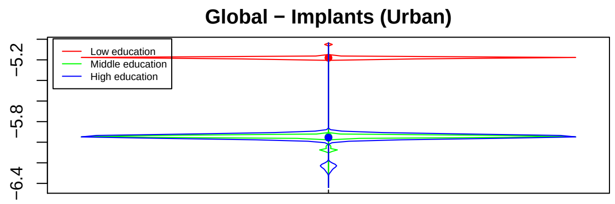

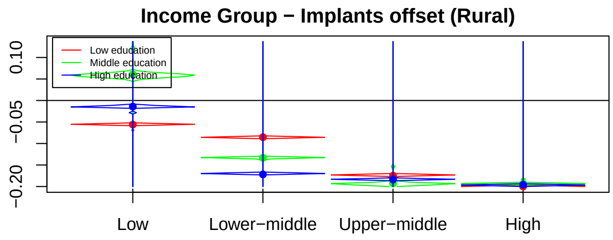

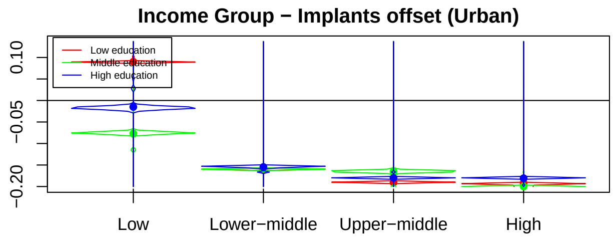

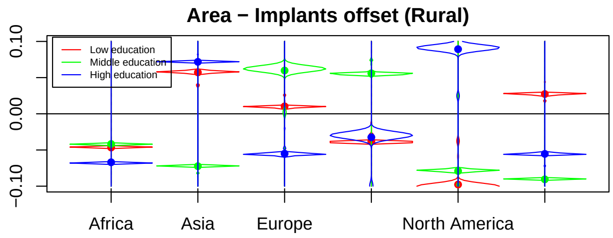

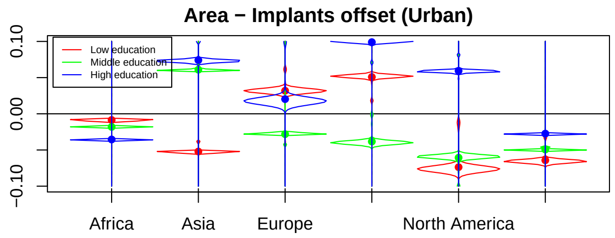

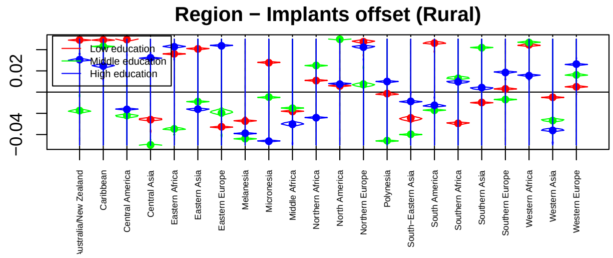

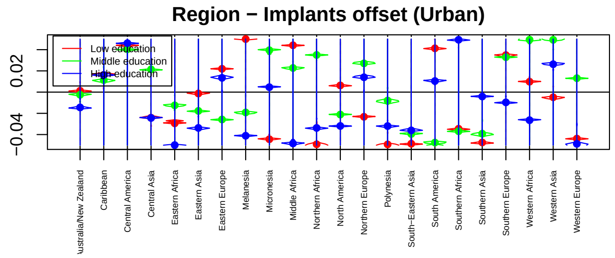

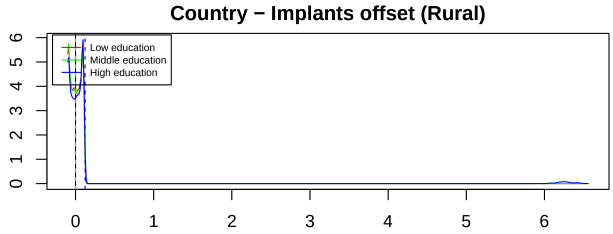

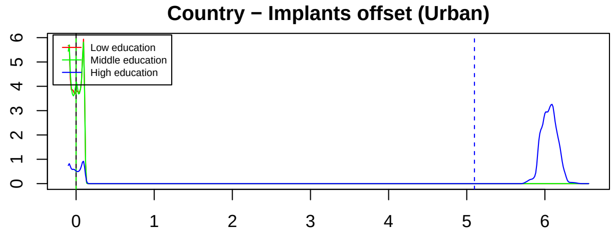

Parameters

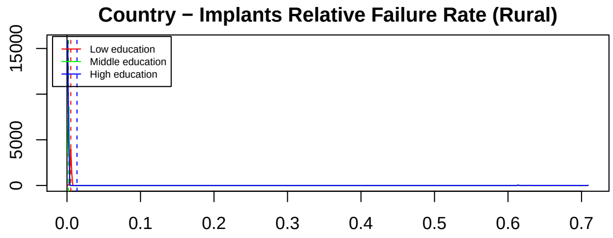

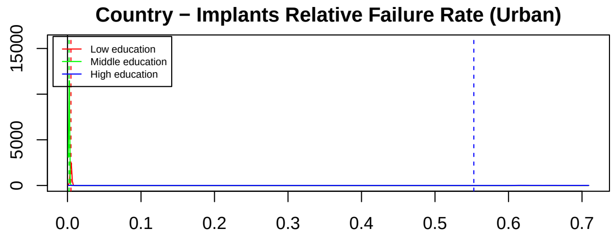

C.3.7 Method Duration of Use

C.3.7.1 Duration of Use

C.3.7.1.1 Pill

Hyperparameters

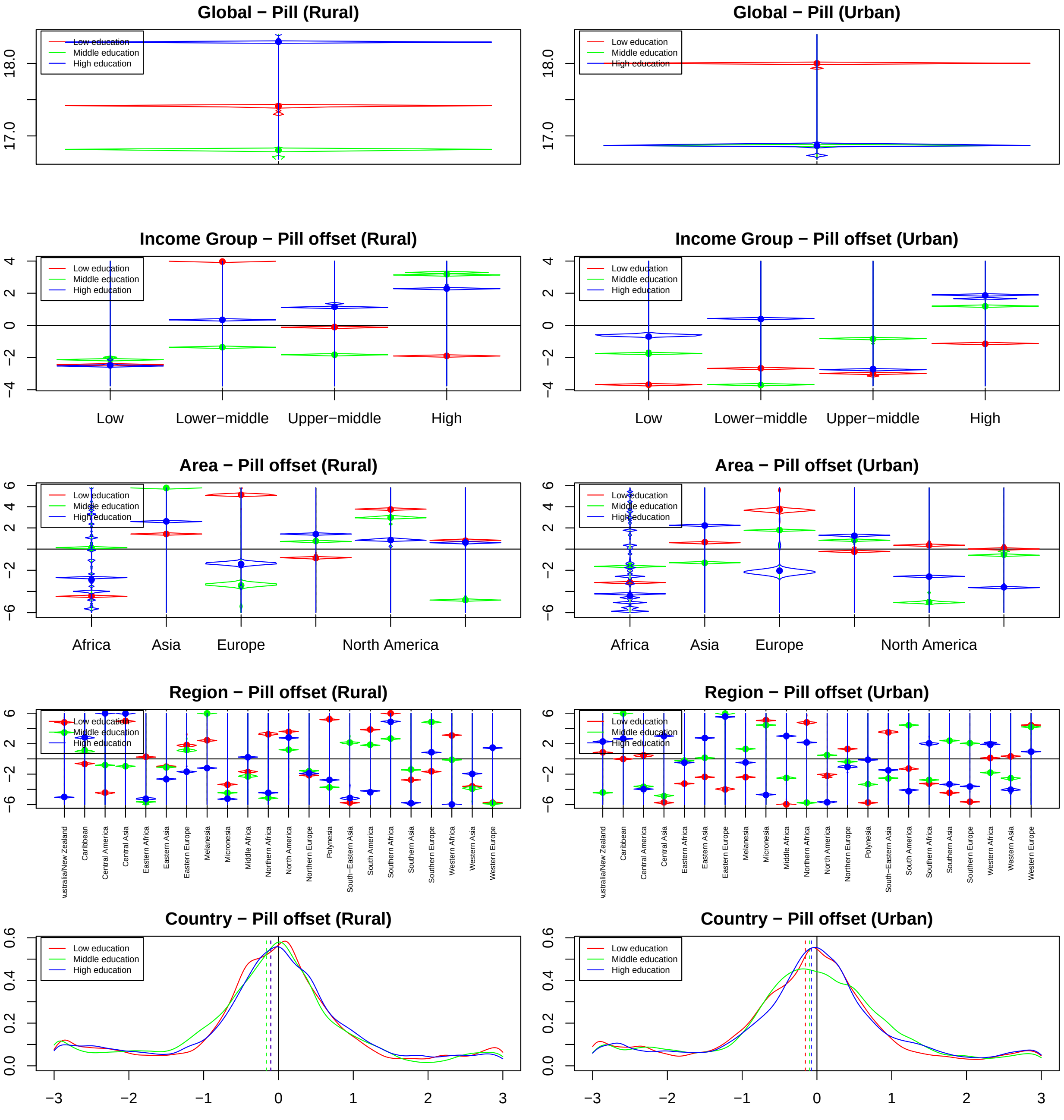

Parameters

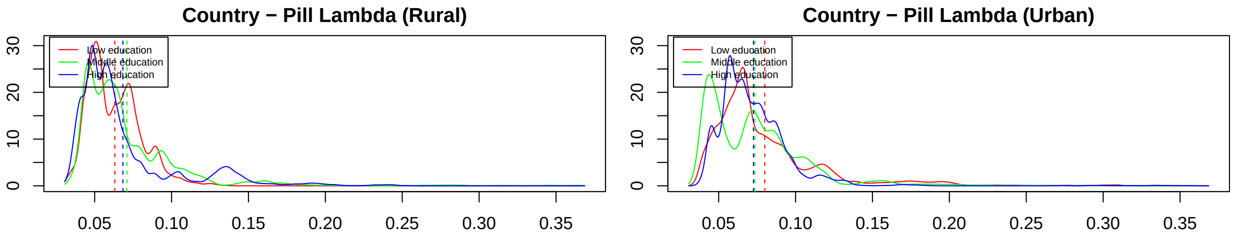

C.3.7.1.2 IUD

Hyperparameters

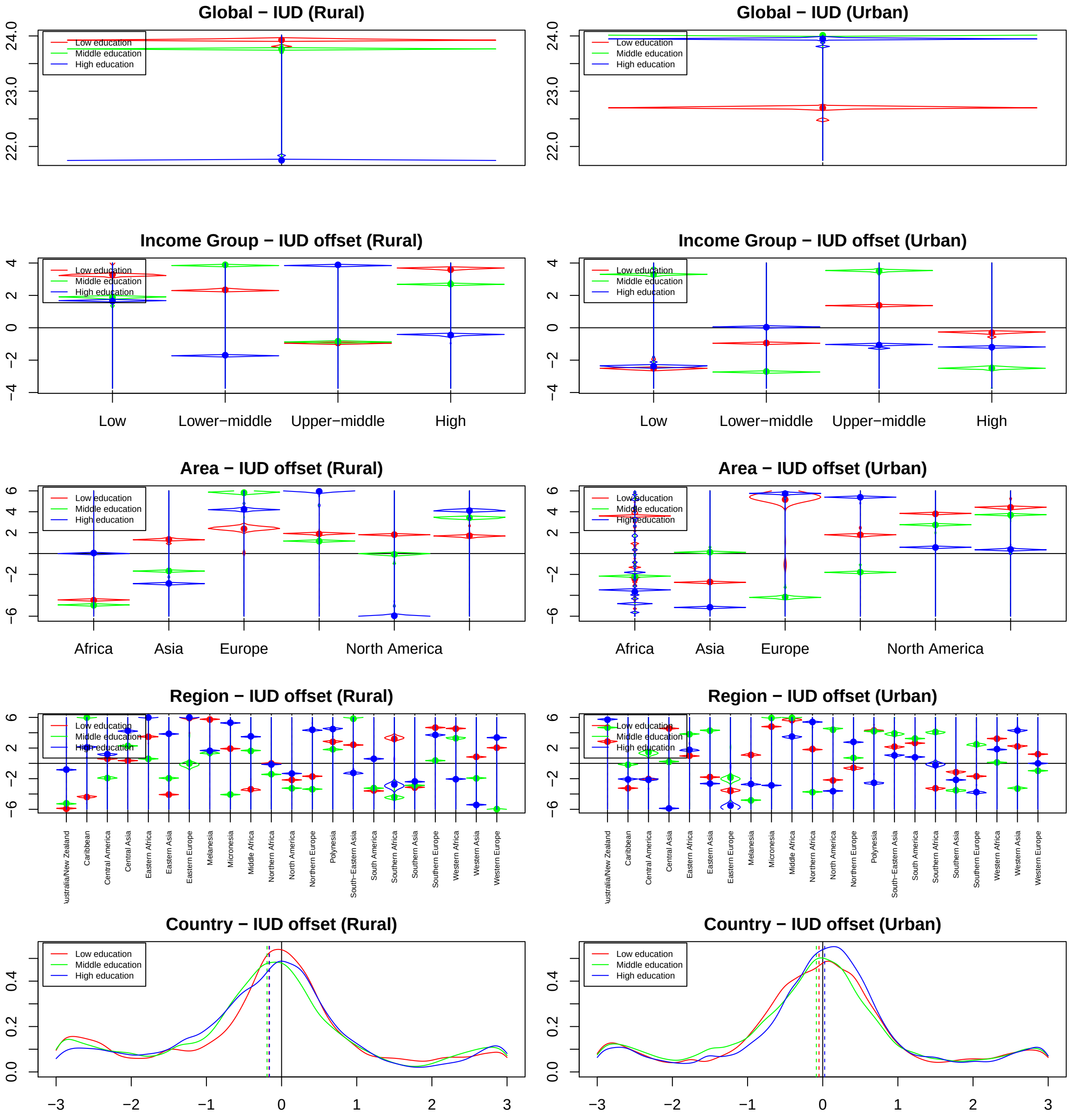

Parameters

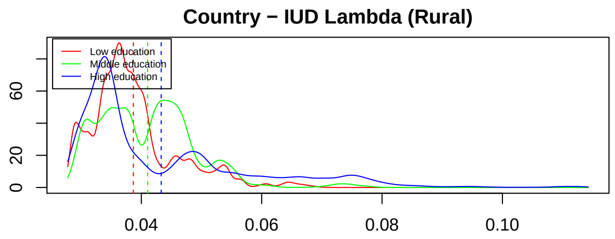

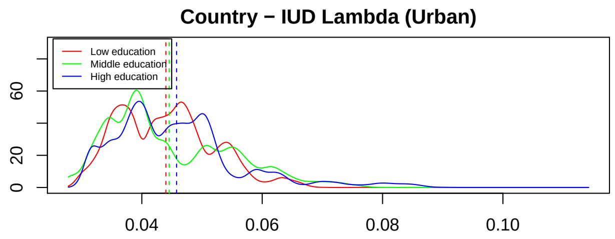

C.3.7.1.3 Injections

Hyperparameters

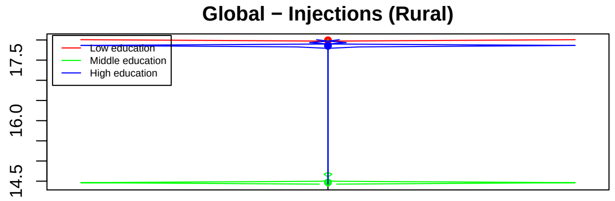

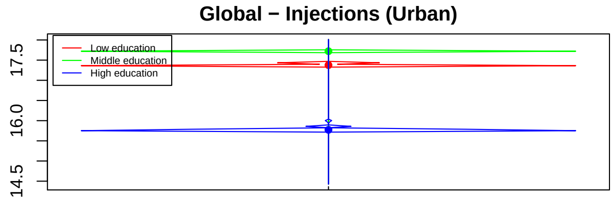

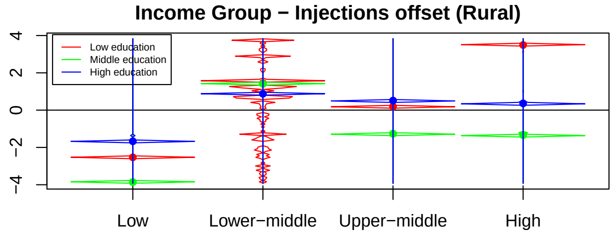

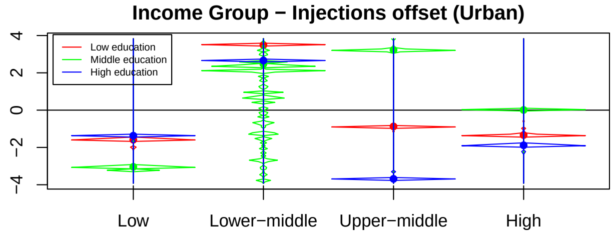

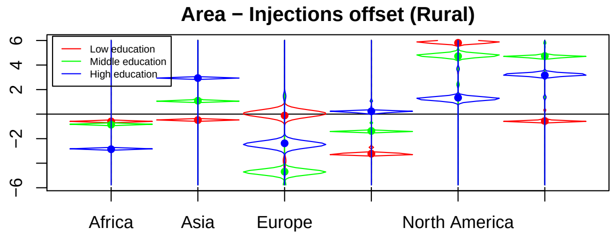

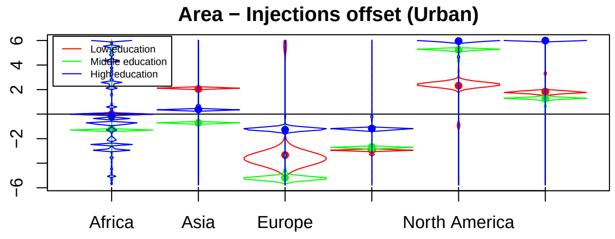

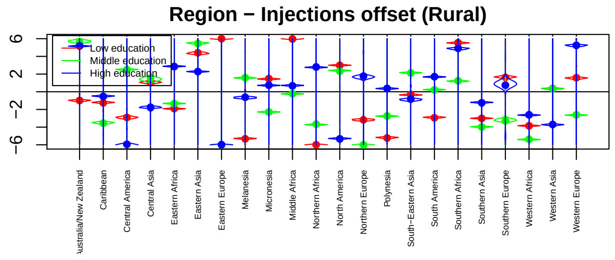

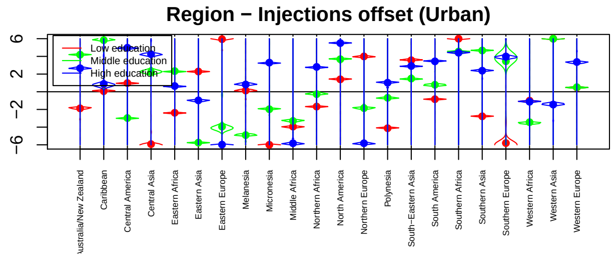

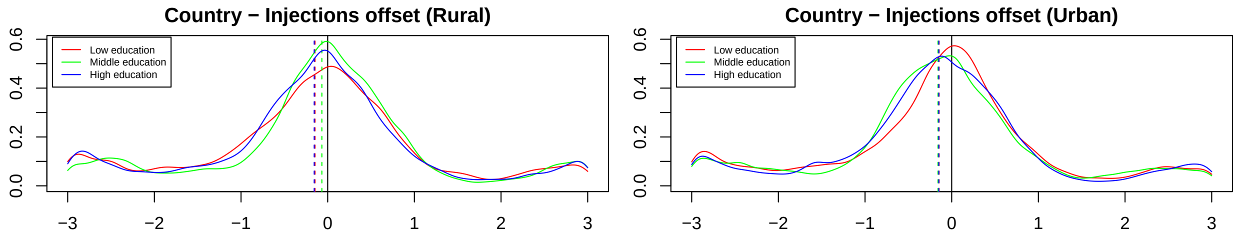

Parameters

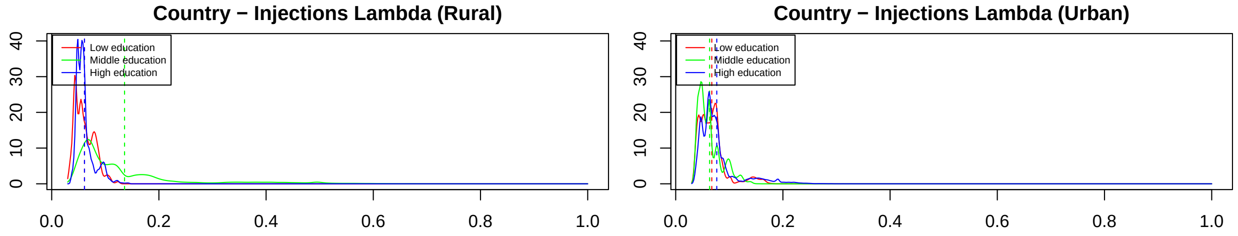

C.3.7.1.4 Condom

Hyperparameters

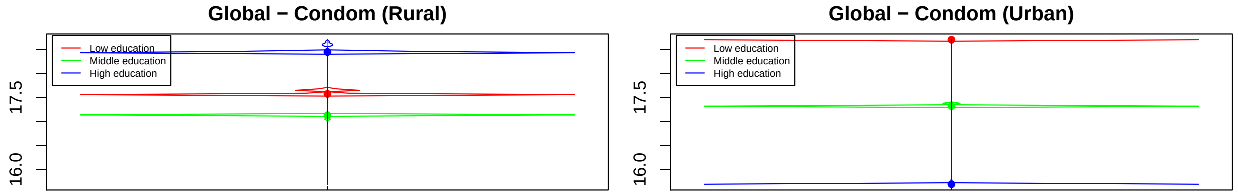

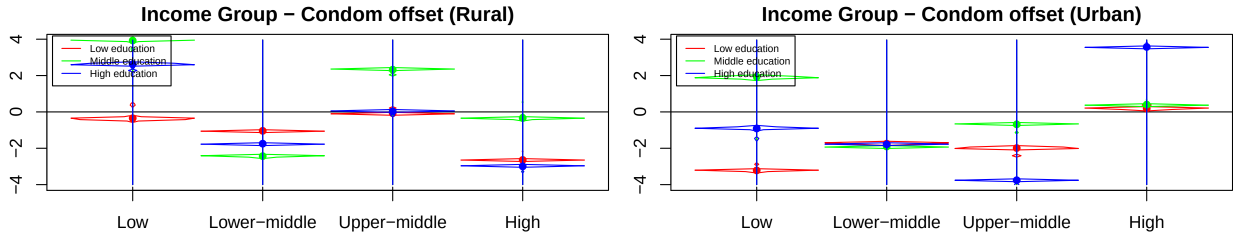

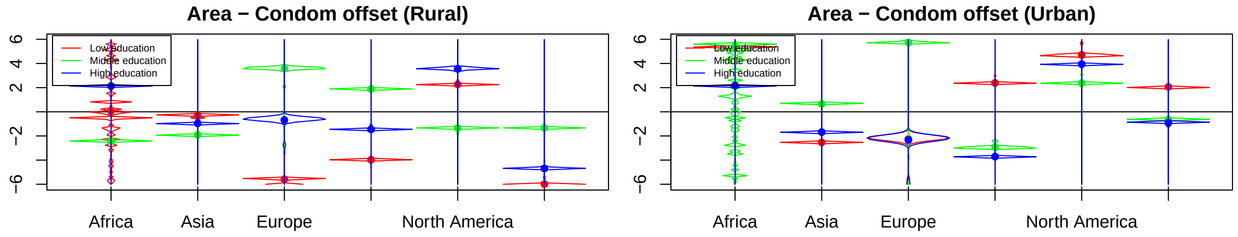

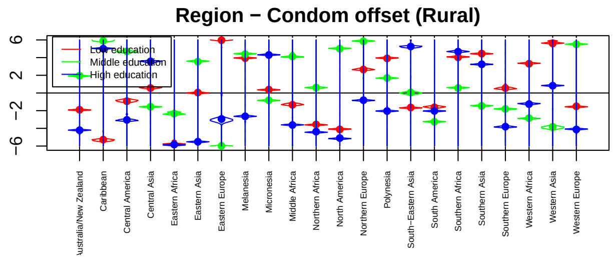

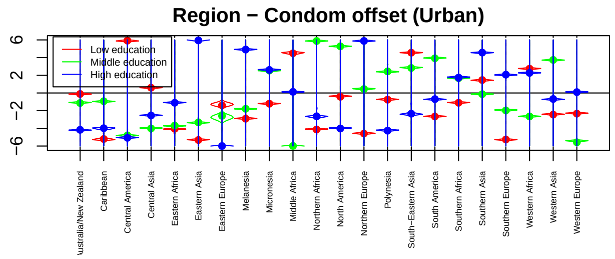

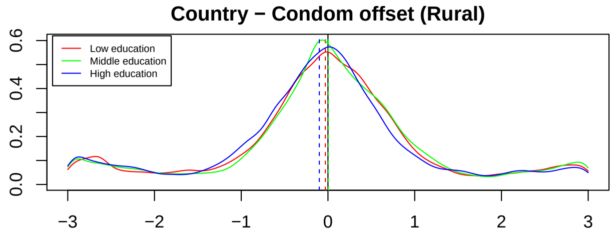

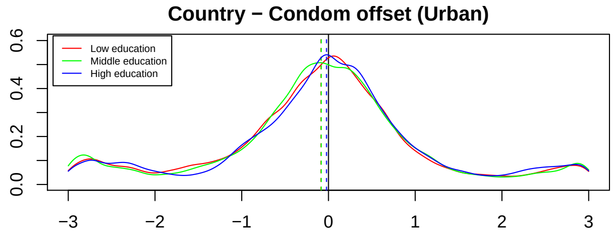

Parameters

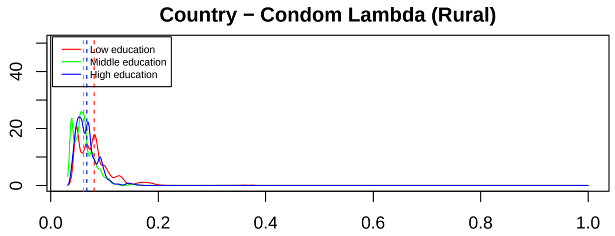

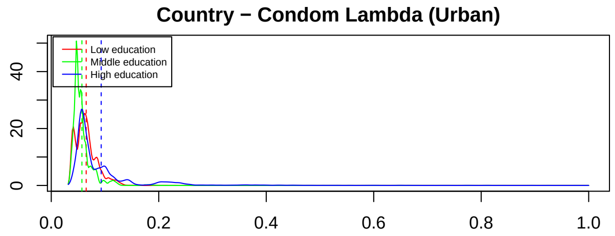

C.3.7.1.5 Periodic abstinence

Hyperparameters

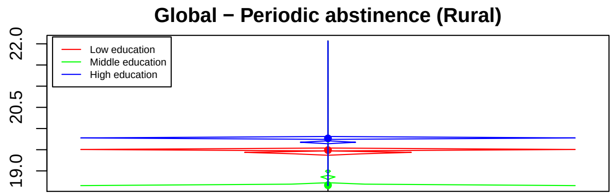

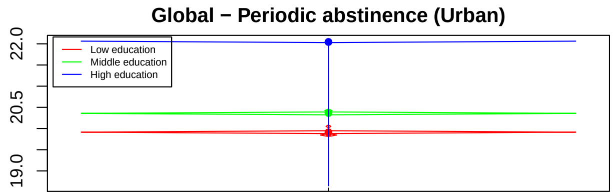

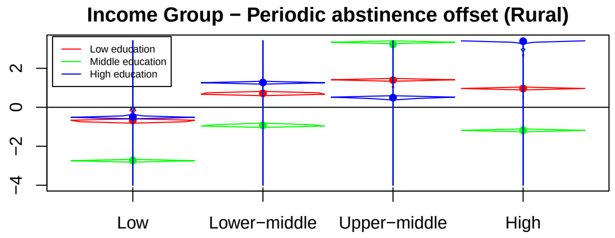

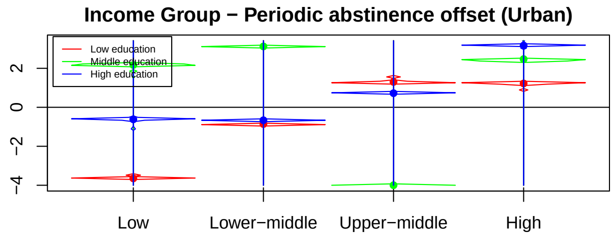

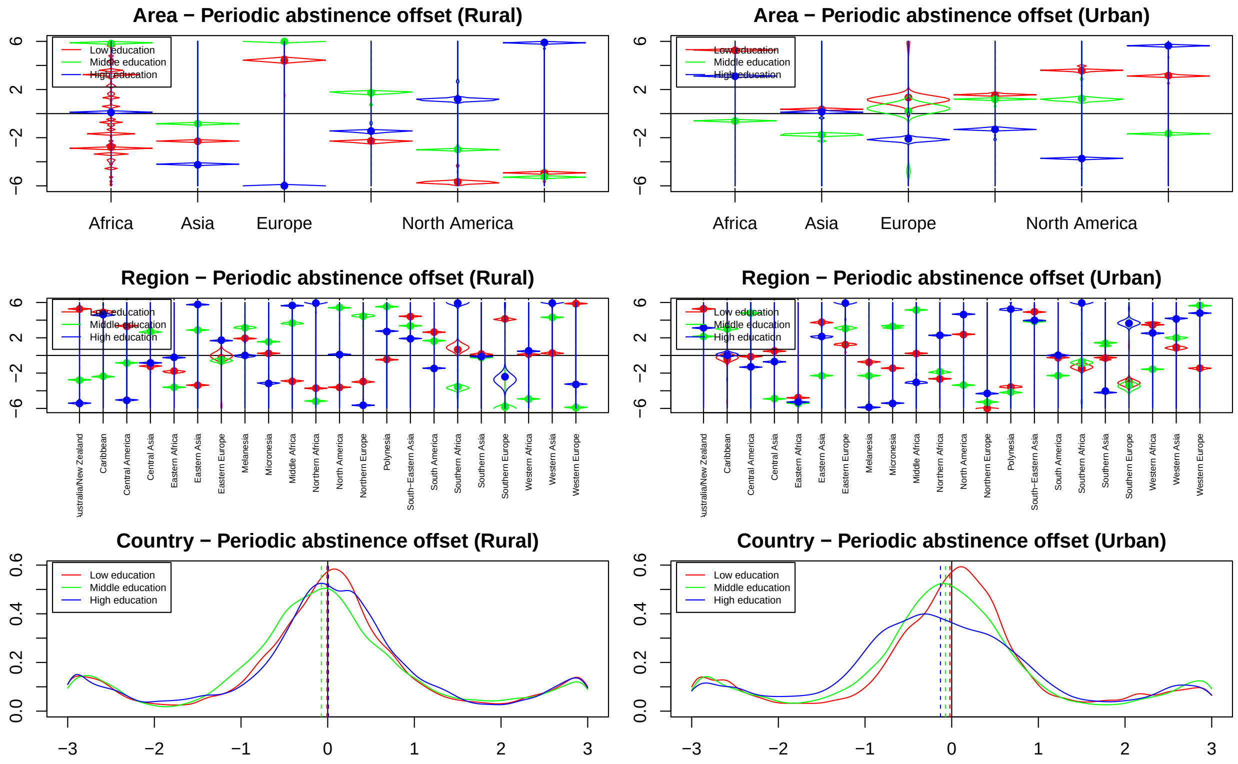

Parameters

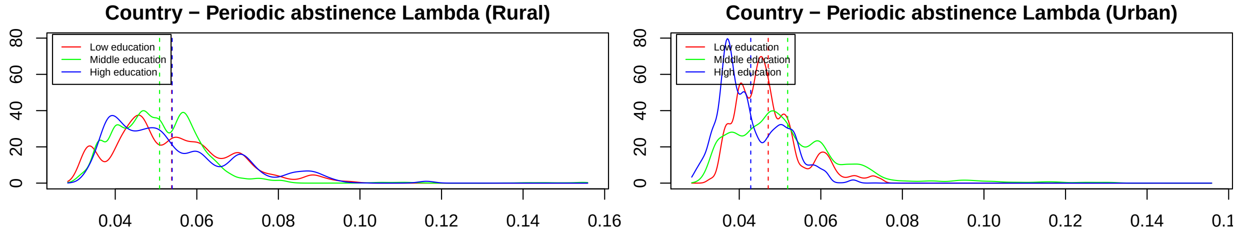

C.3.7.1.6 Withdrawal

Hyperparameters

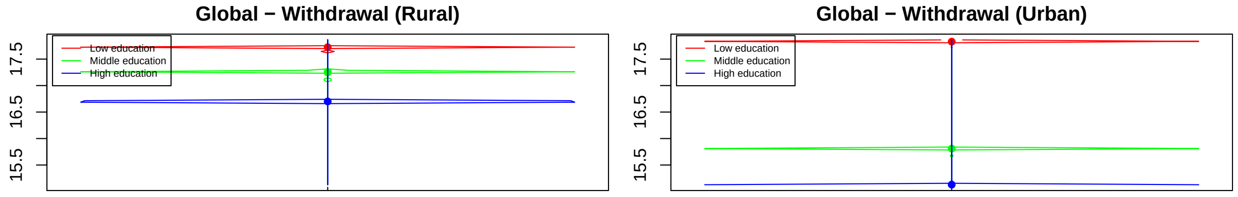

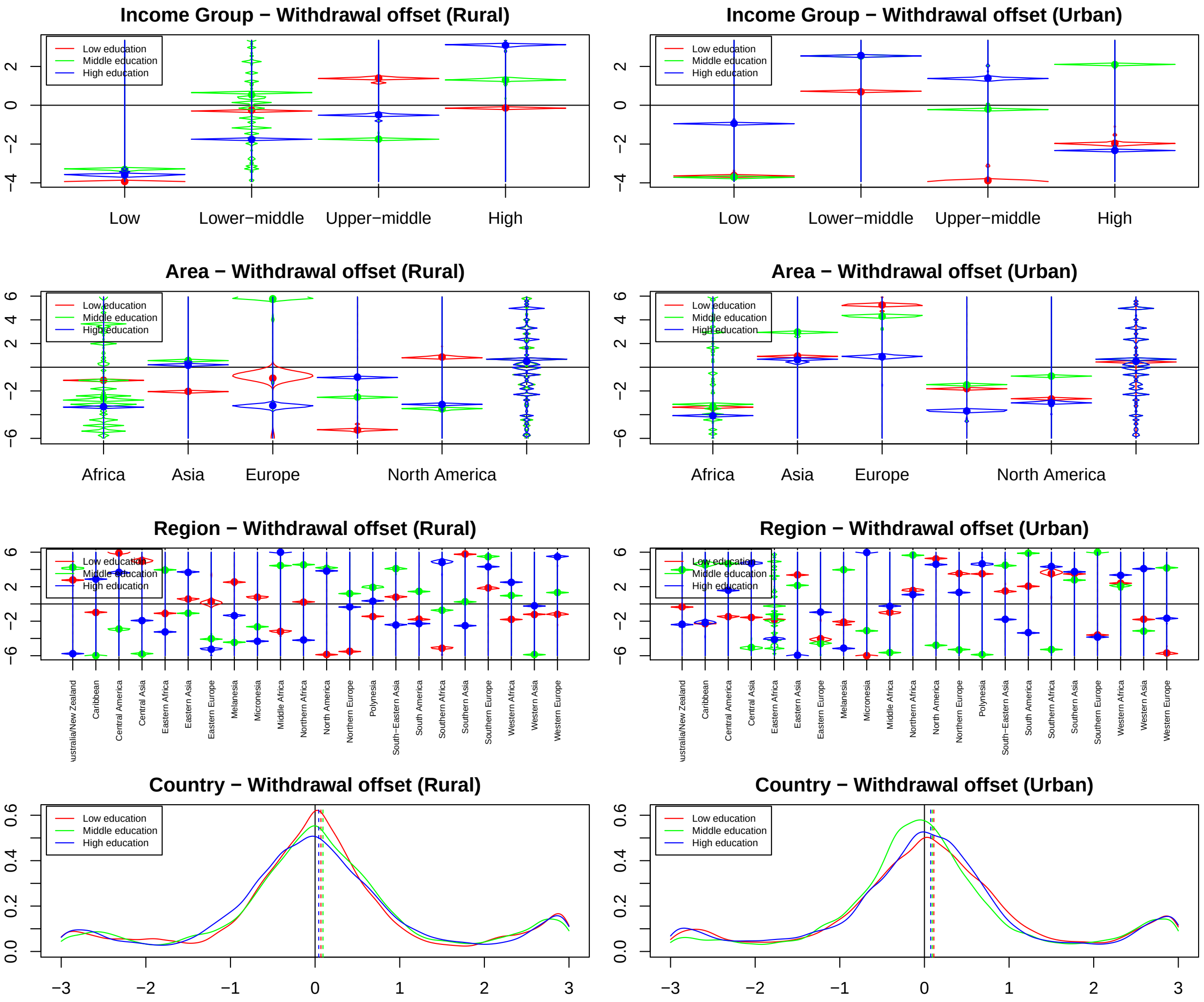

Parameters

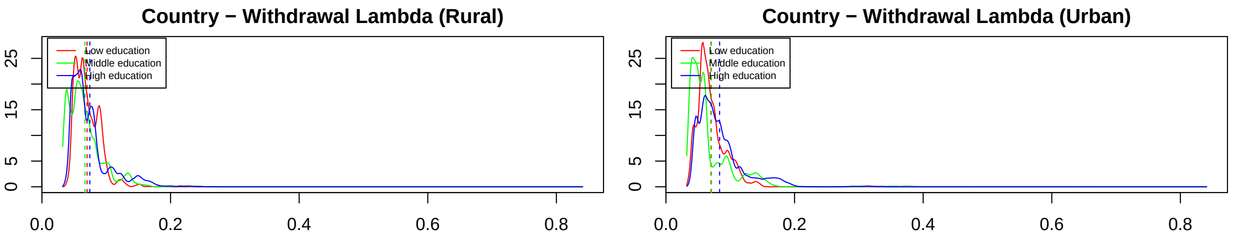

C.3.7.1.7 Other

Hyperparameters

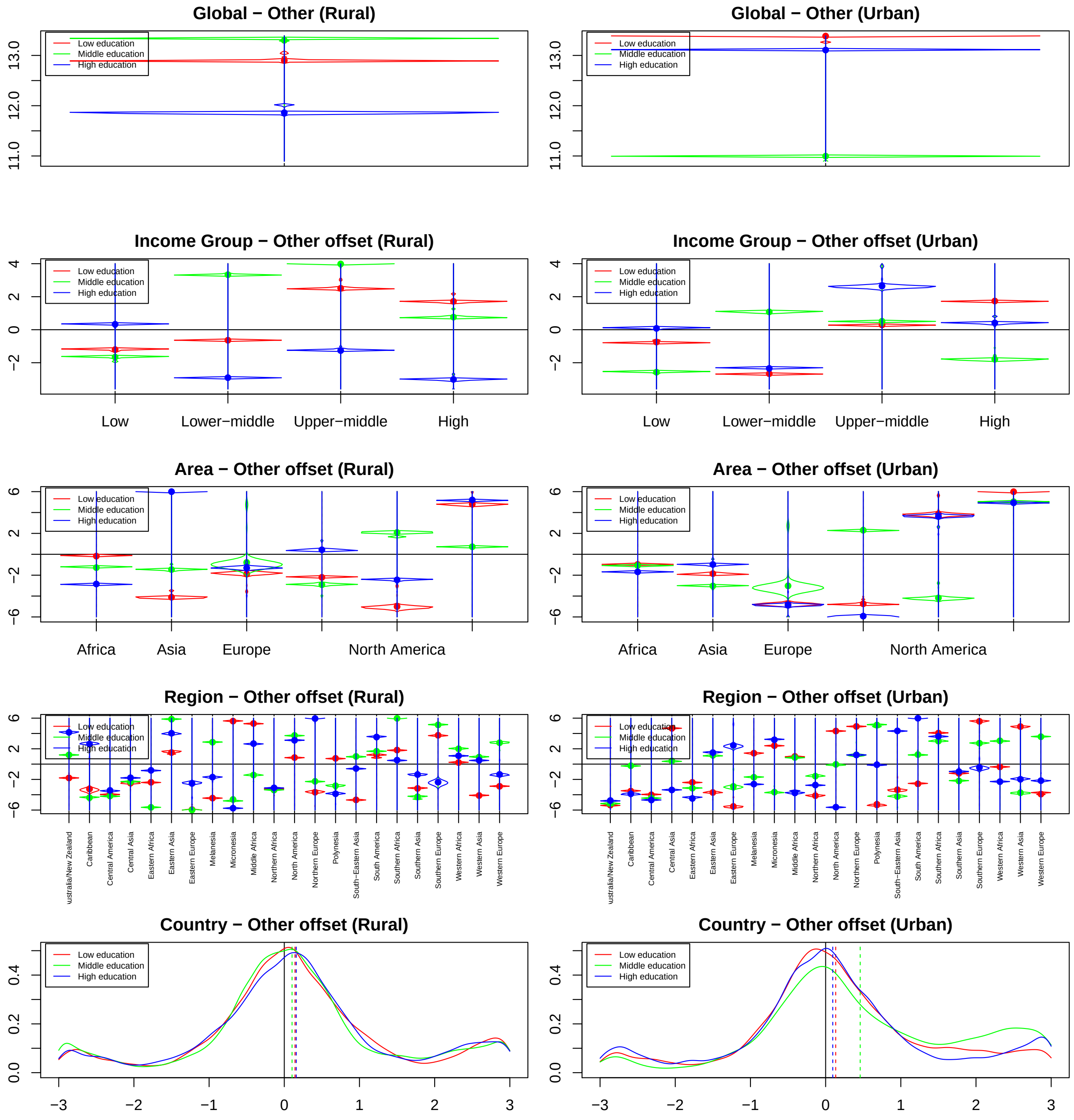

Parameters

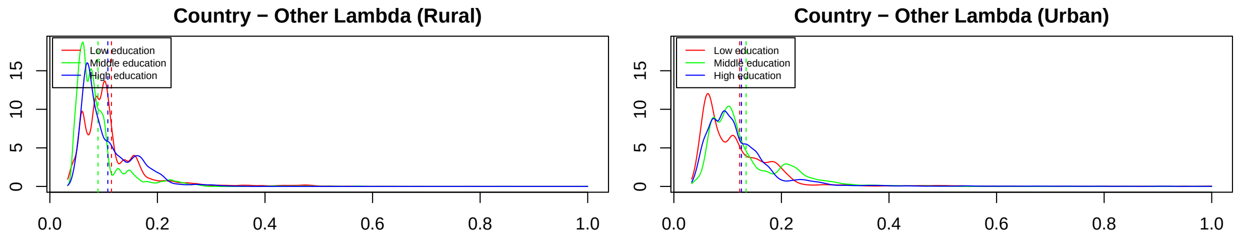

C.3.7.1.8 Implants

Hyperparameters

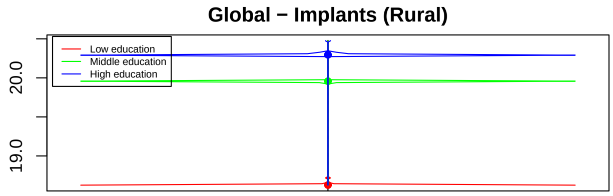

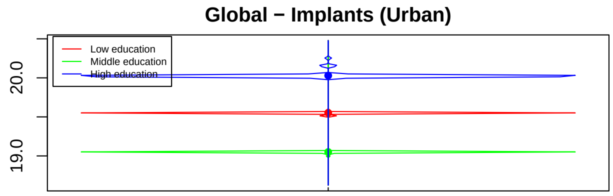

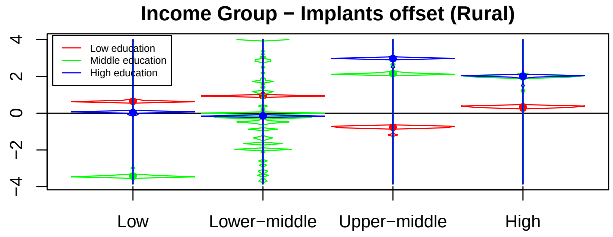

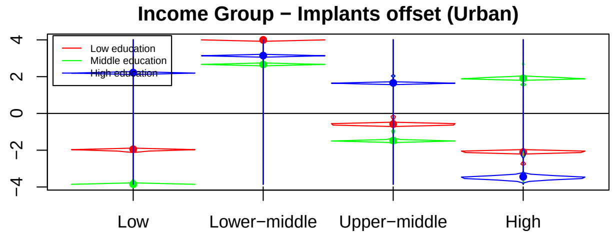

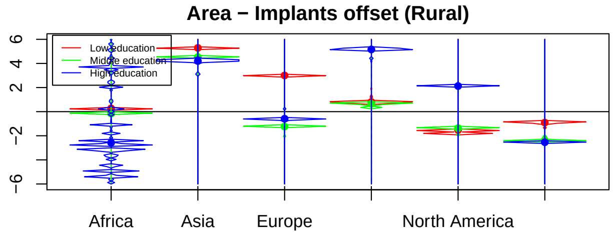

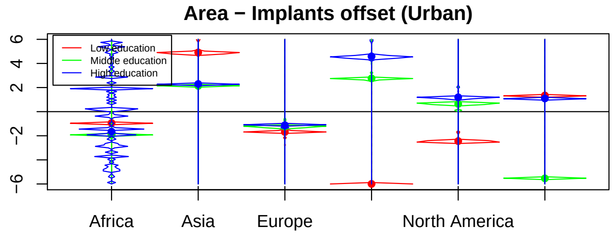

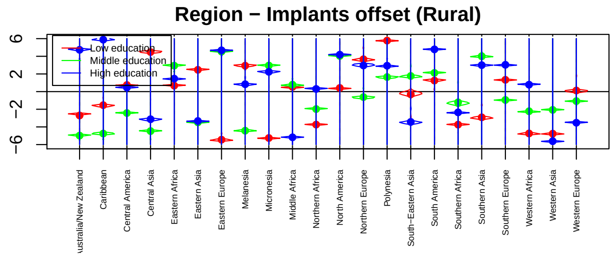

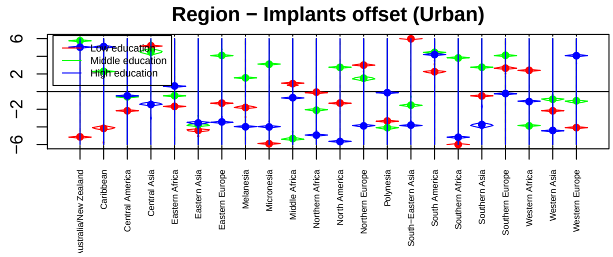

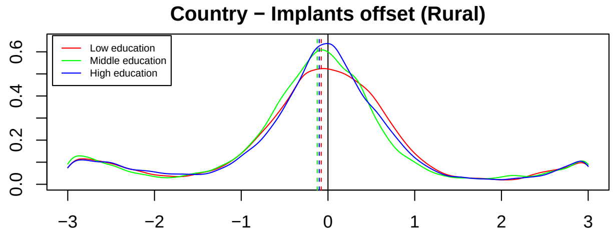

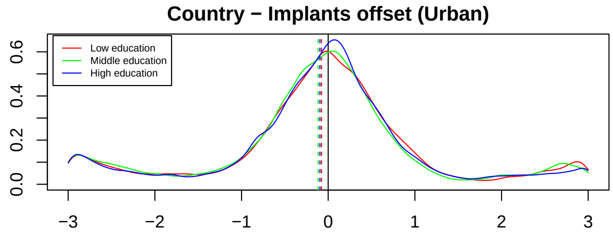

Parameters

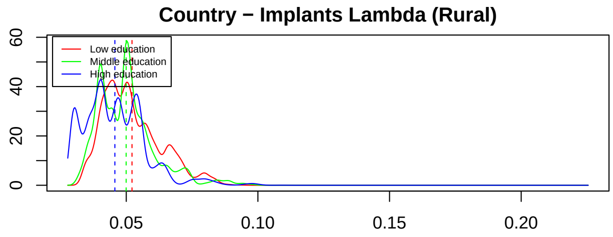

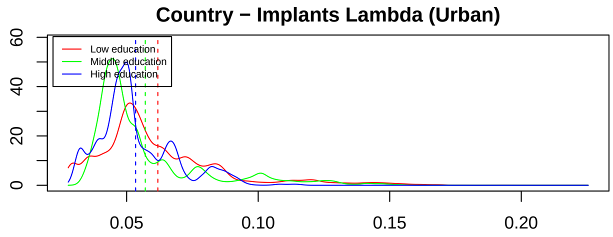

C.3.7.2 Discontinue All Methods

C.3.7.2.1 Pill

Hyperparameters

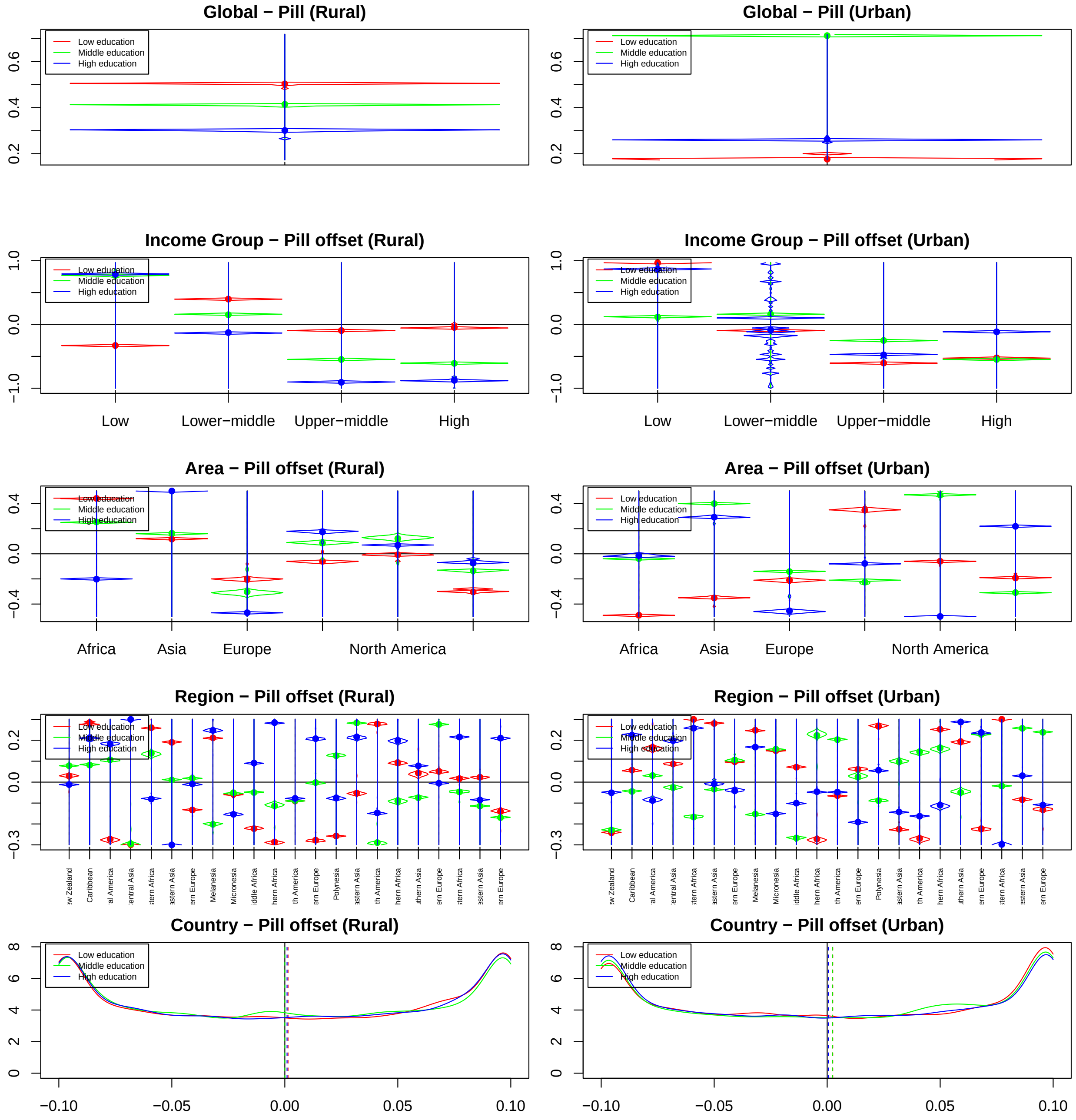

Parameters

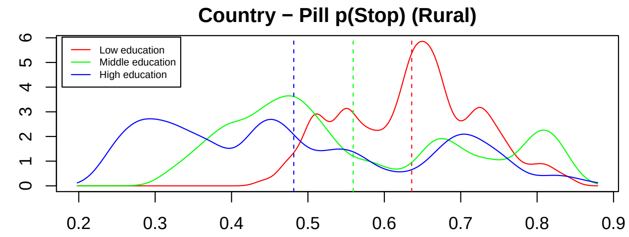

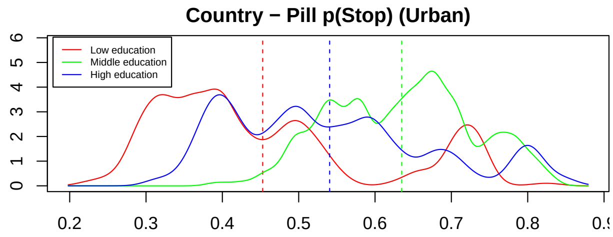

C.3.7.2.2 IUD

Hyperparameters

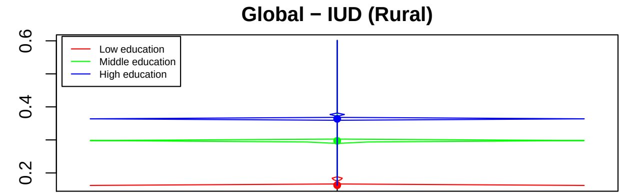

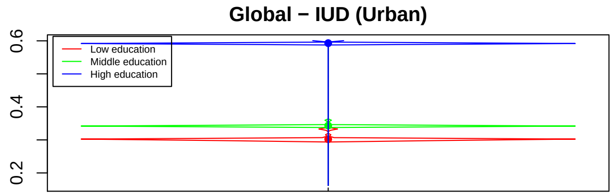

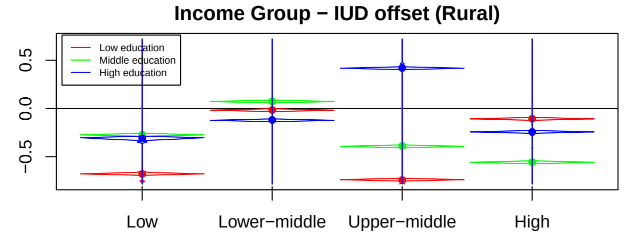

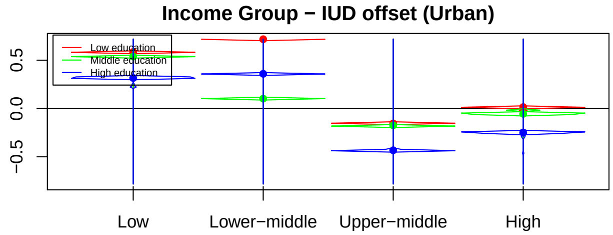

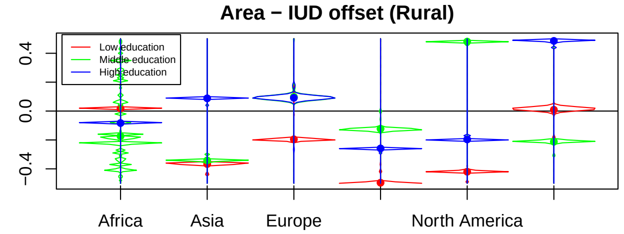

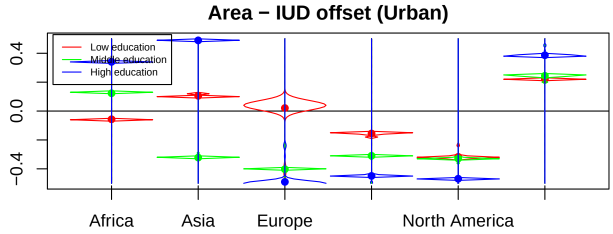

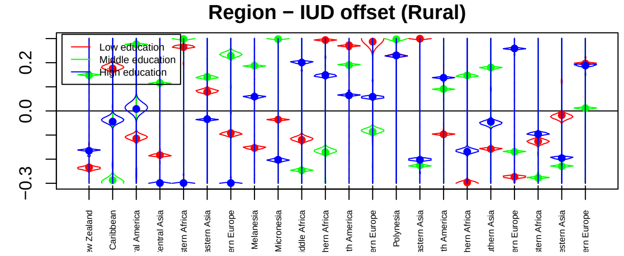

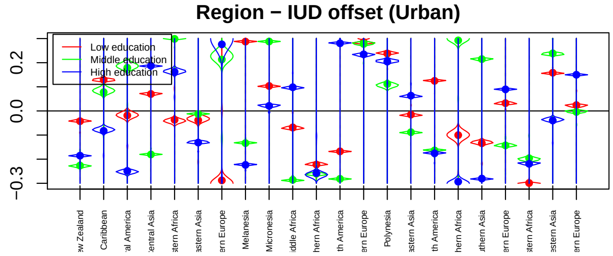

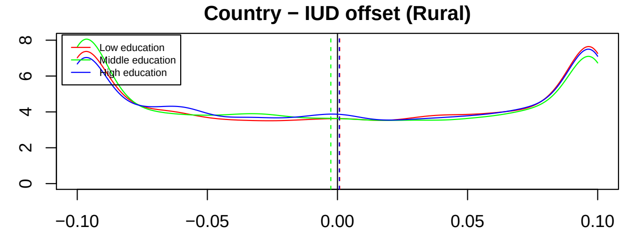

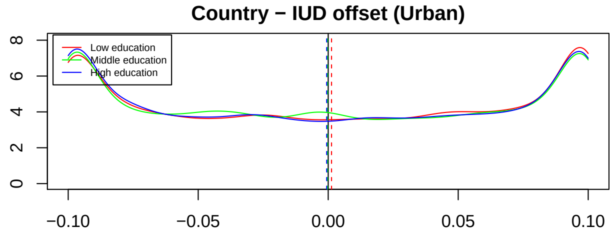

Parameters

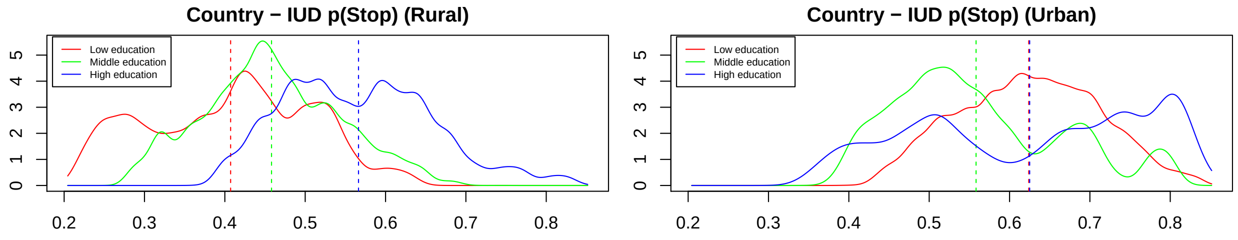

C.3.7.2.3 Injections

Hyperparameters

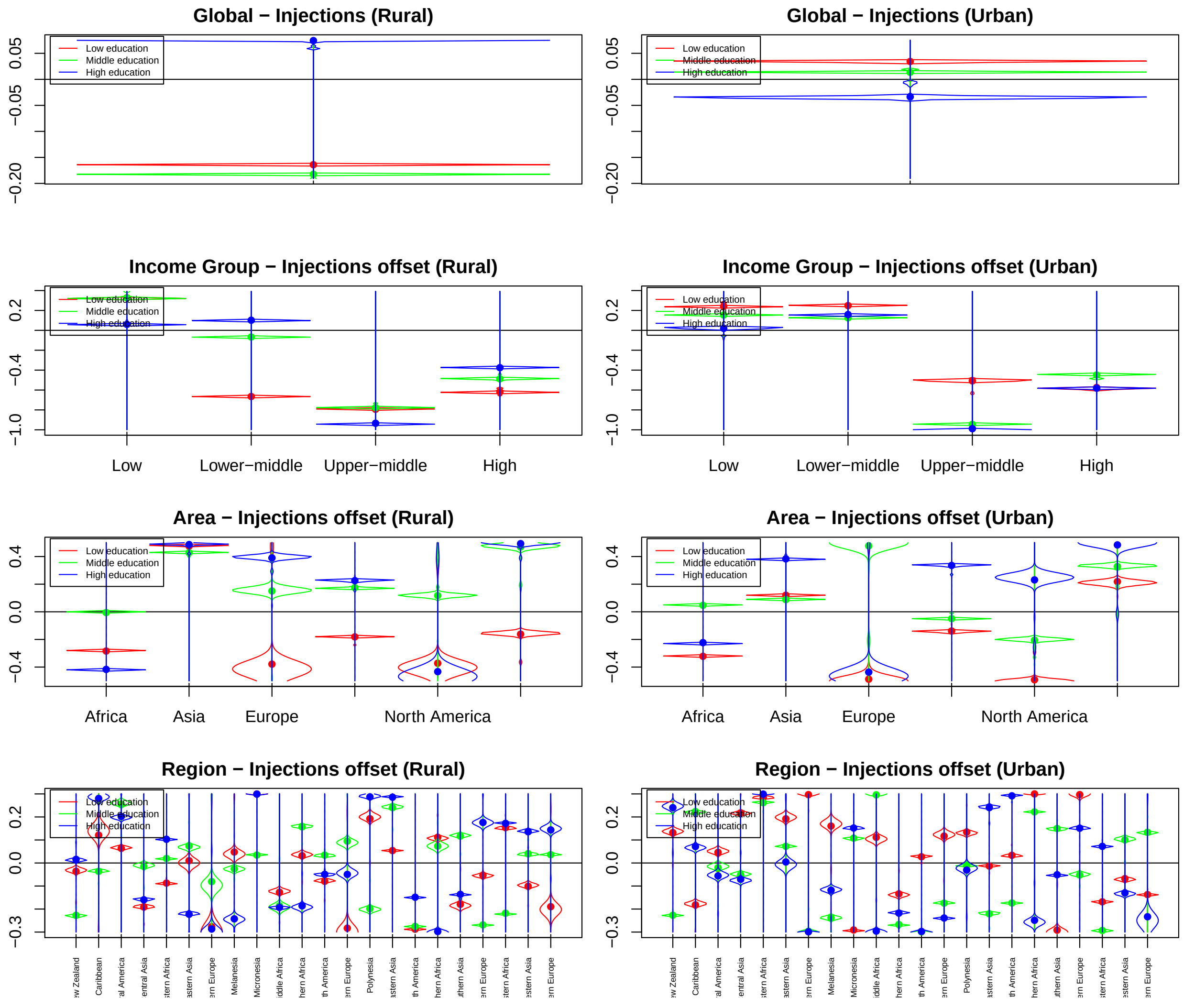

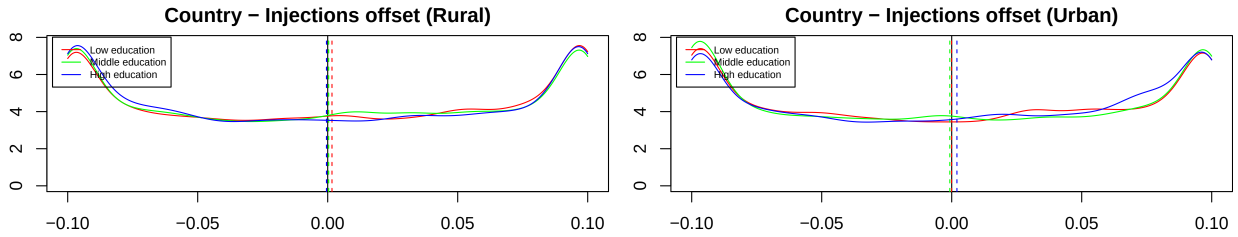

Parameters

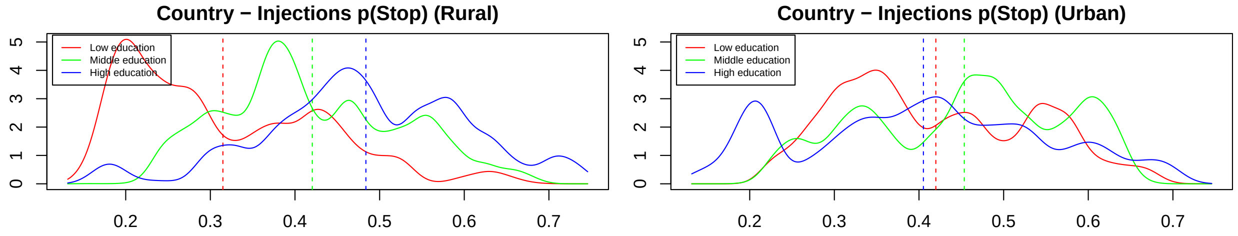

C.3.7.2.4 Condom

Hyperparameters

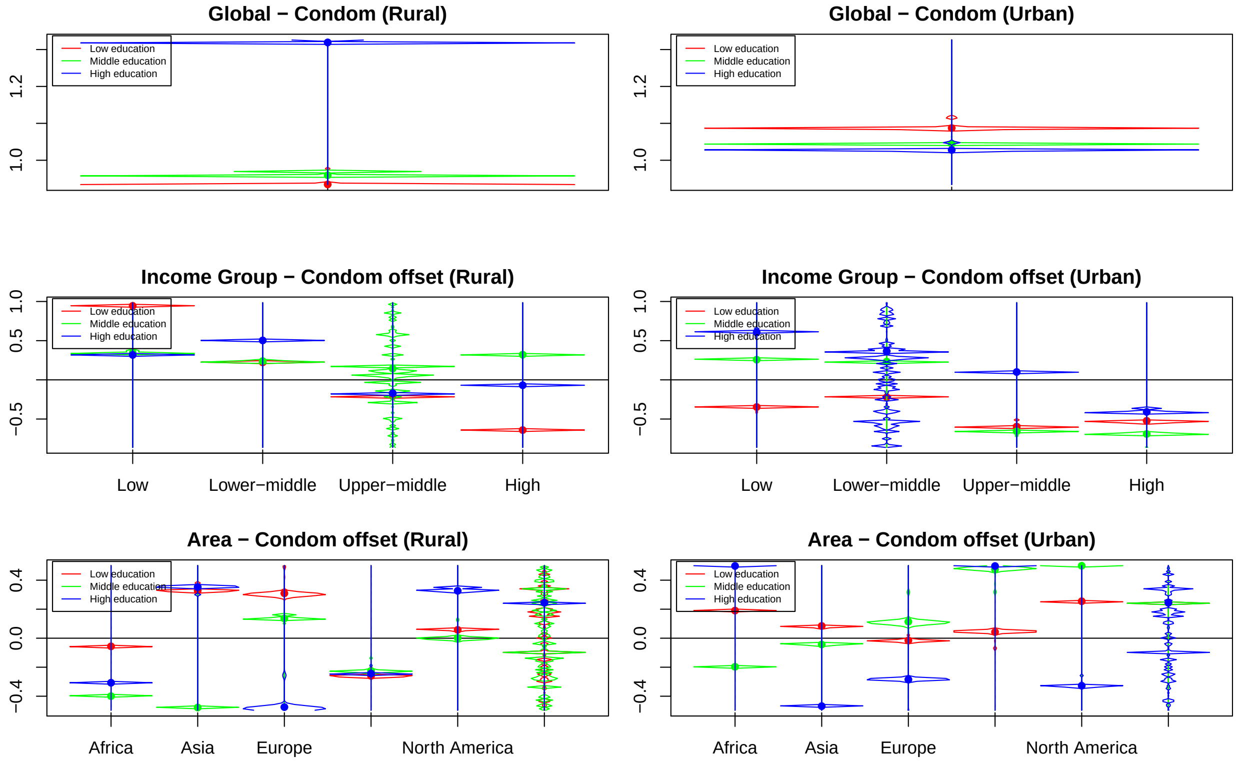

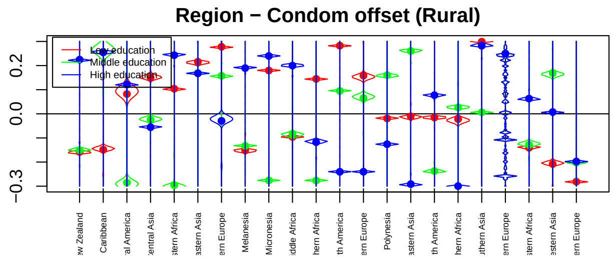

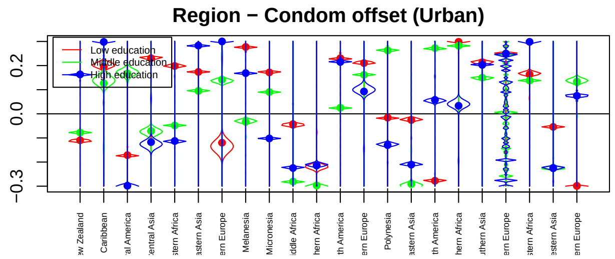

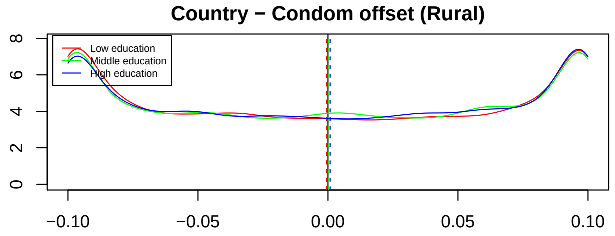

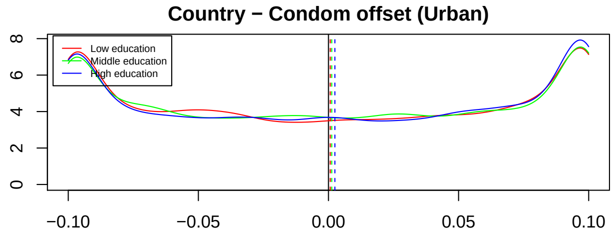

Parameters

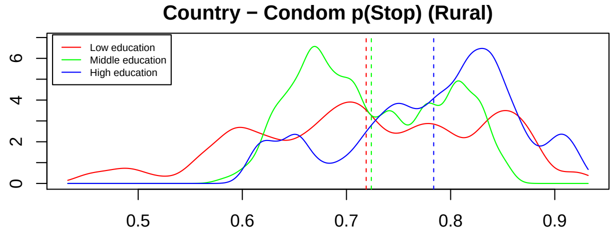

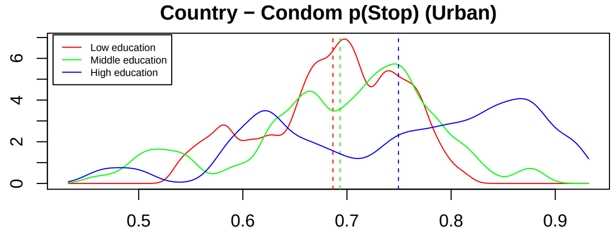

C.3.7.2.5 Periodic abstinence

Hyperparameters

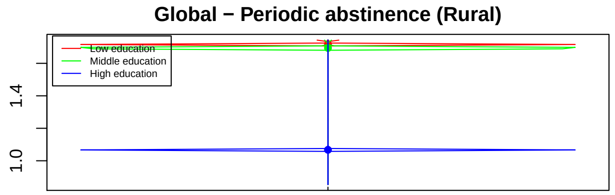

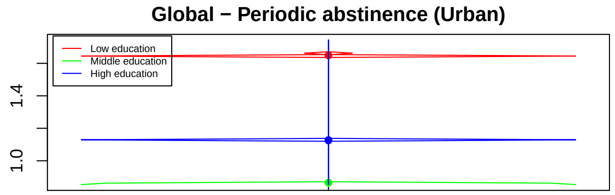

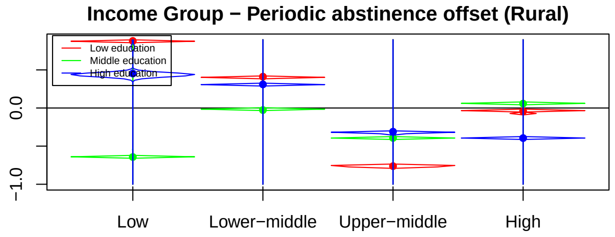

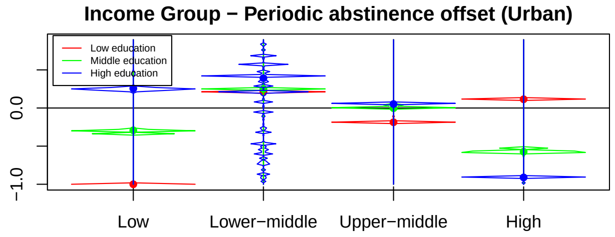

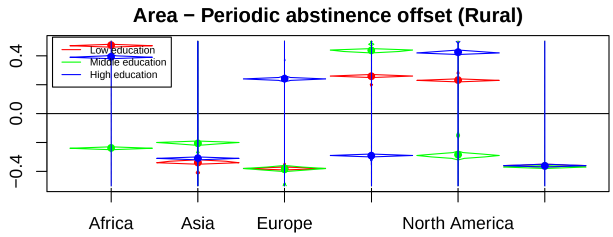

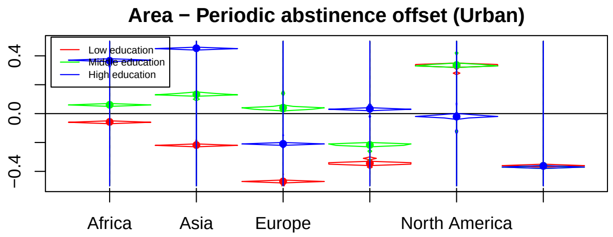

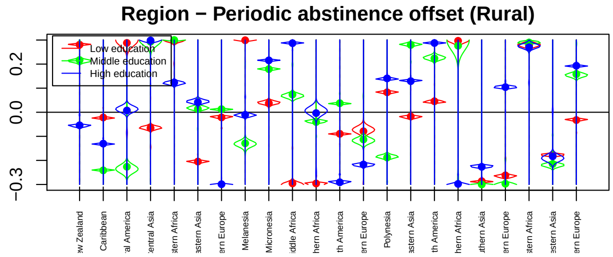

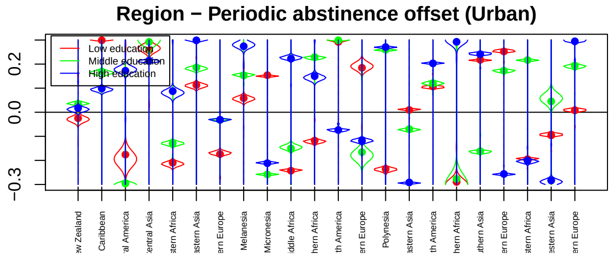

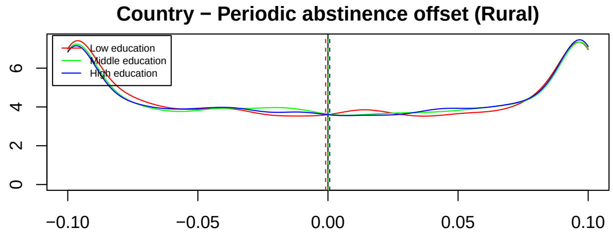

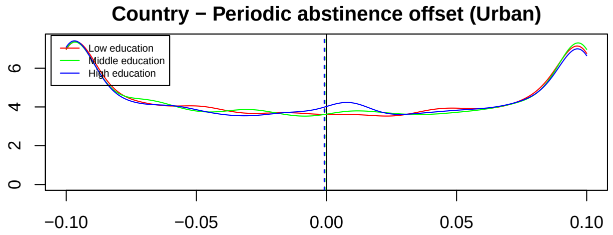

Parameters

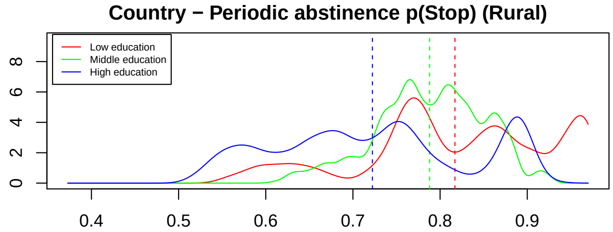

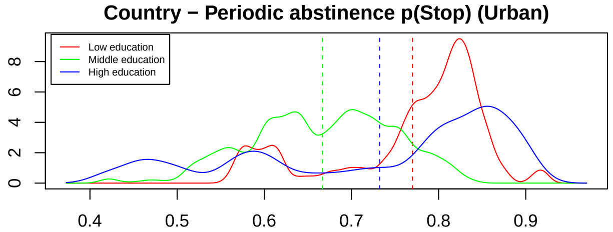

C.3.7.2.6 Withdrawal

Hyperparameters

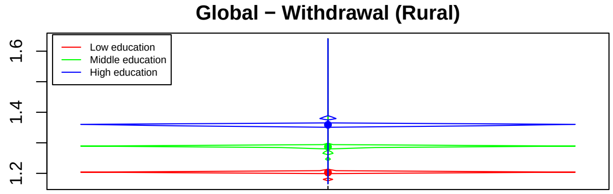

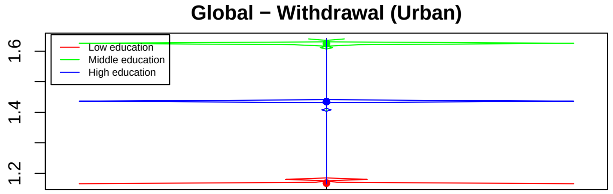

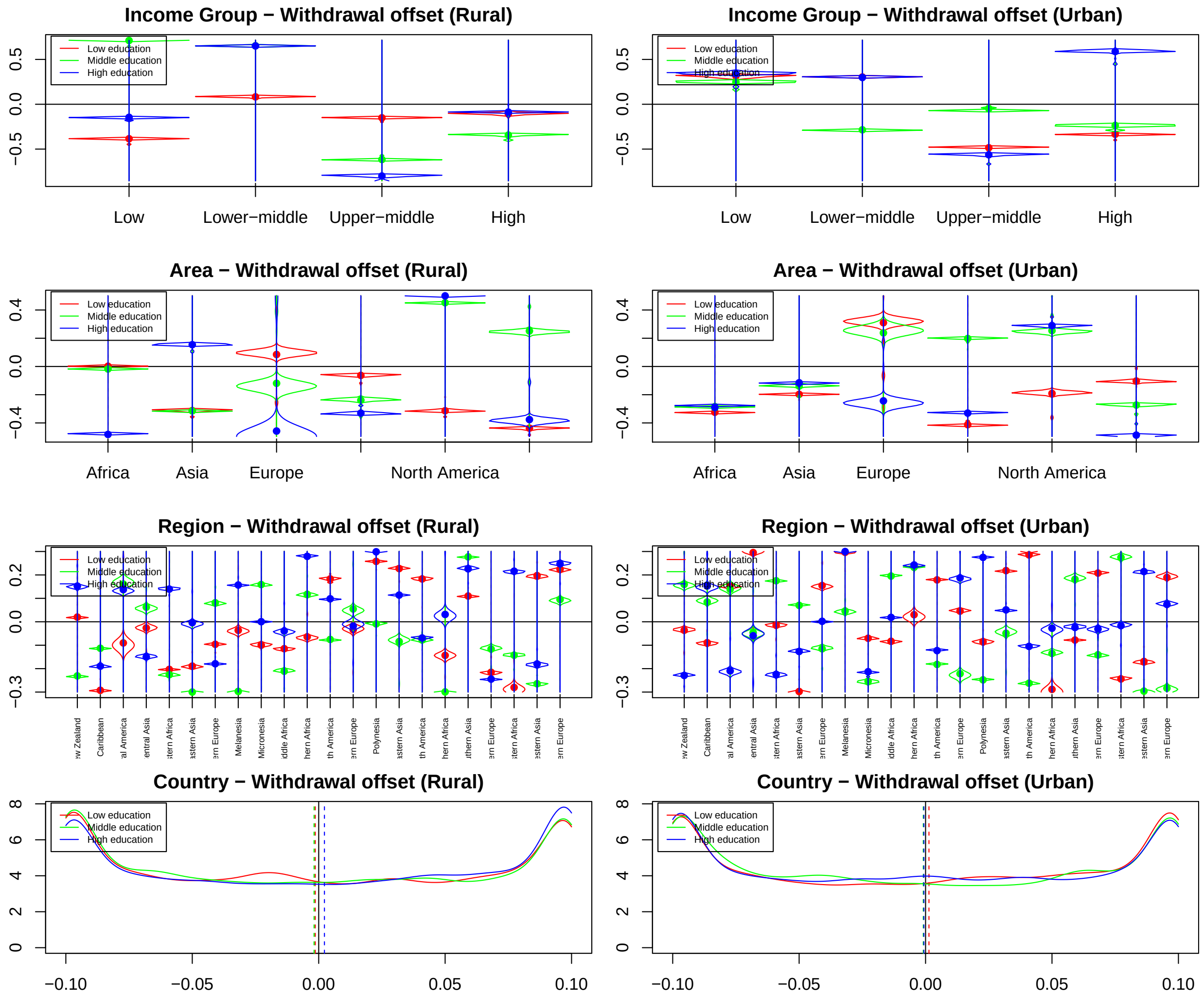

Parameters

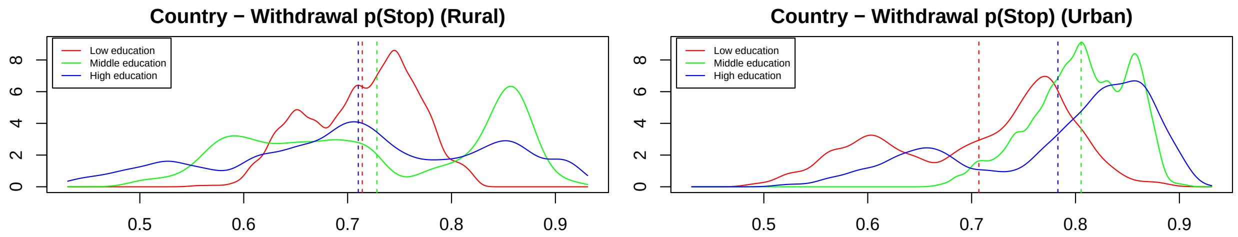

C.3.7.2.7 Other

Hyperparameters

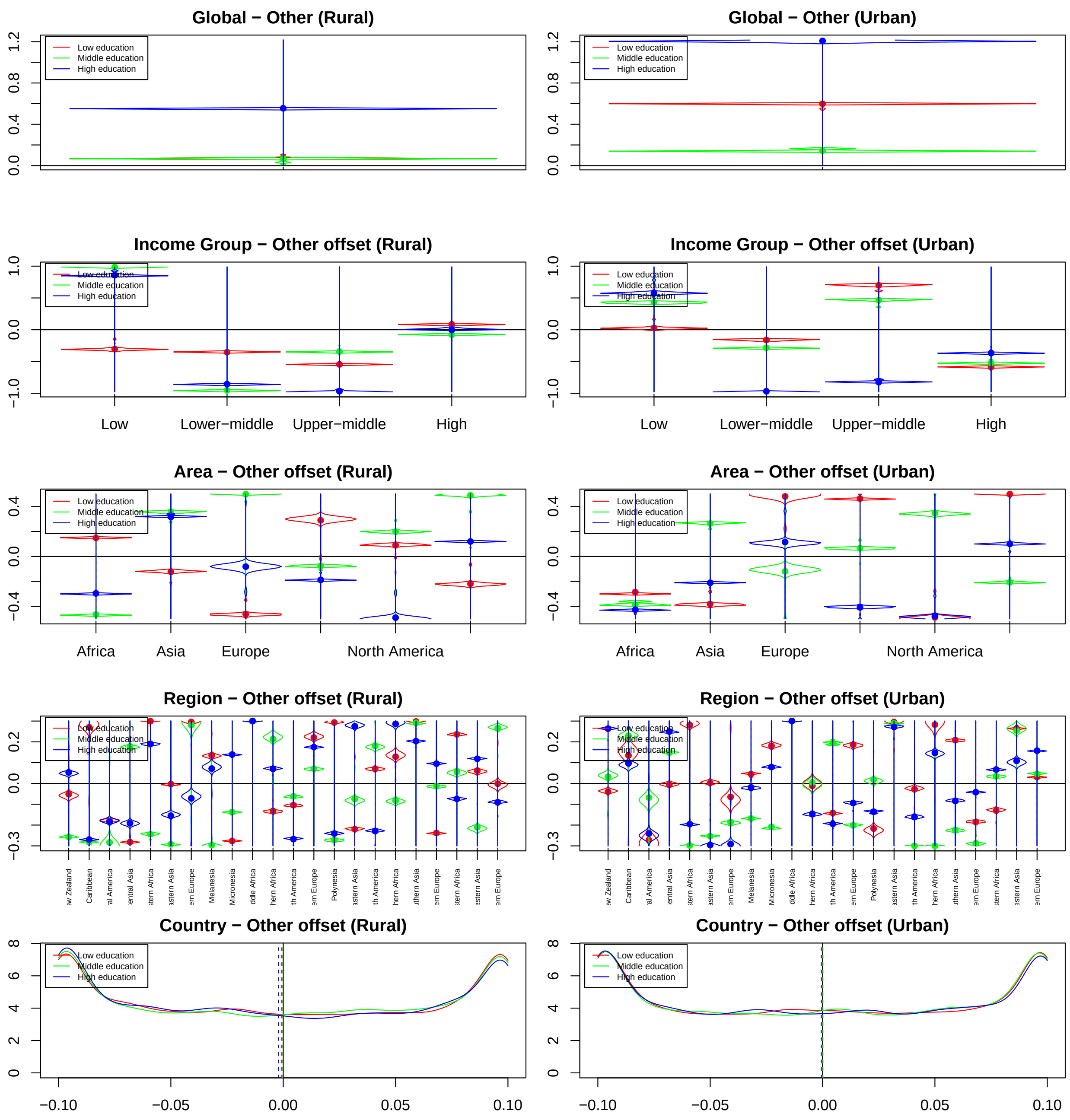

Parameters

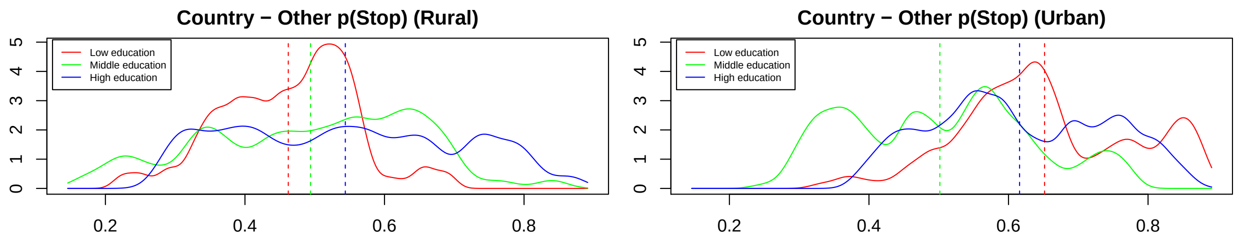

C.3.7.2.8 Implants

Hyperparameters

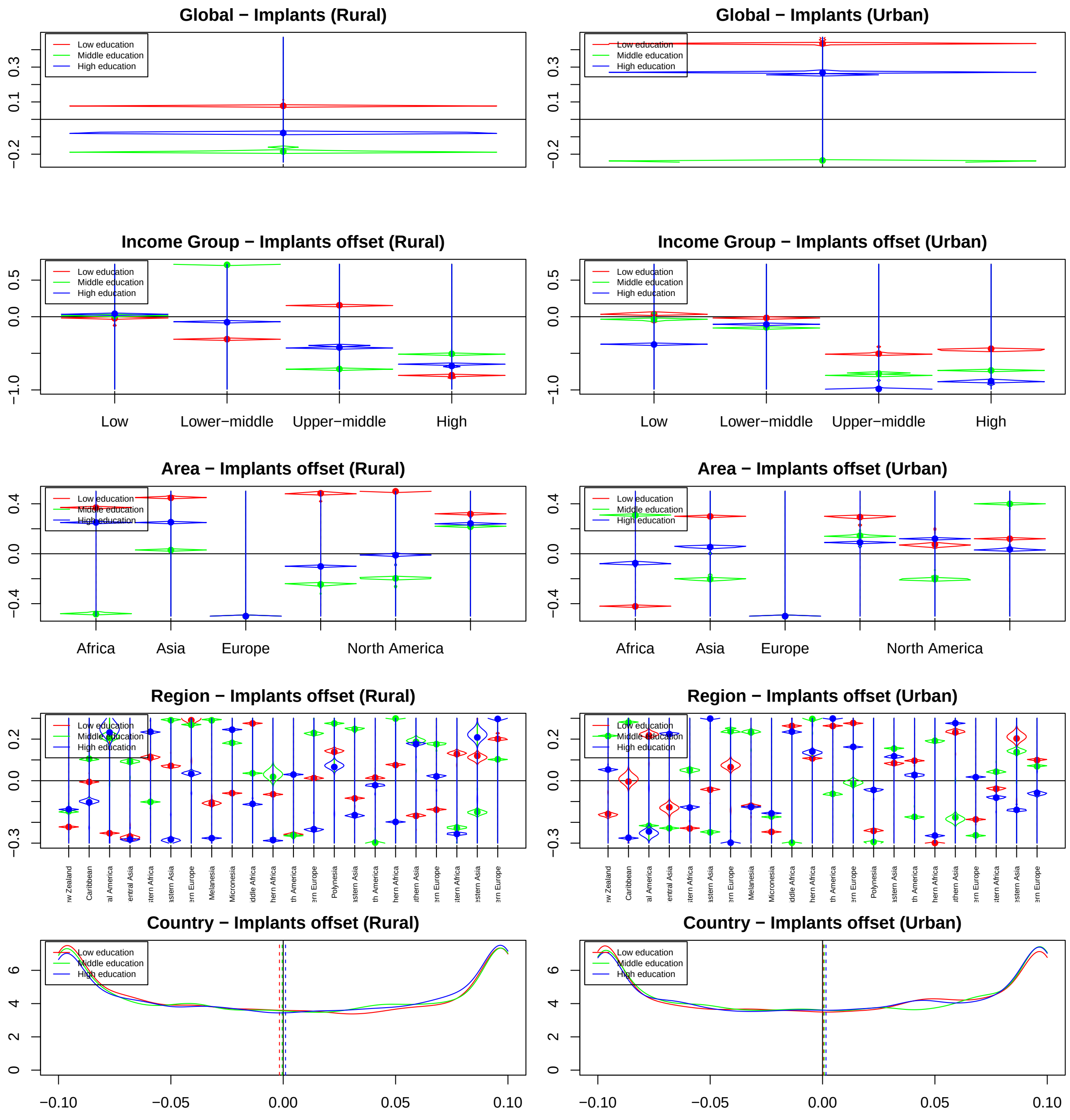

Parameters

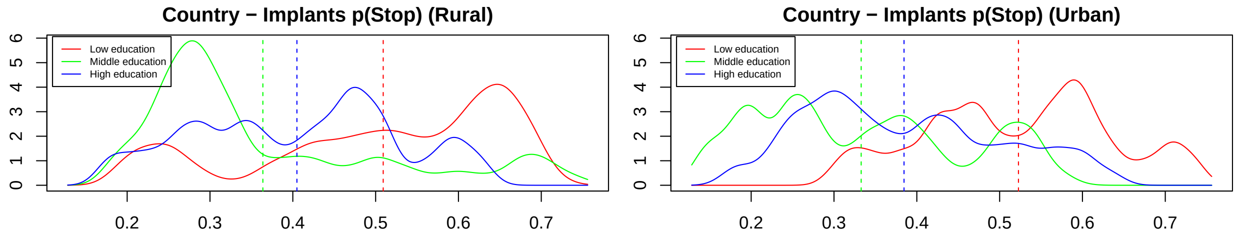

C.3.8 Abortion

C.3.8.1 p(Abortion|Unintended Pregnancy)

Hyperparameters

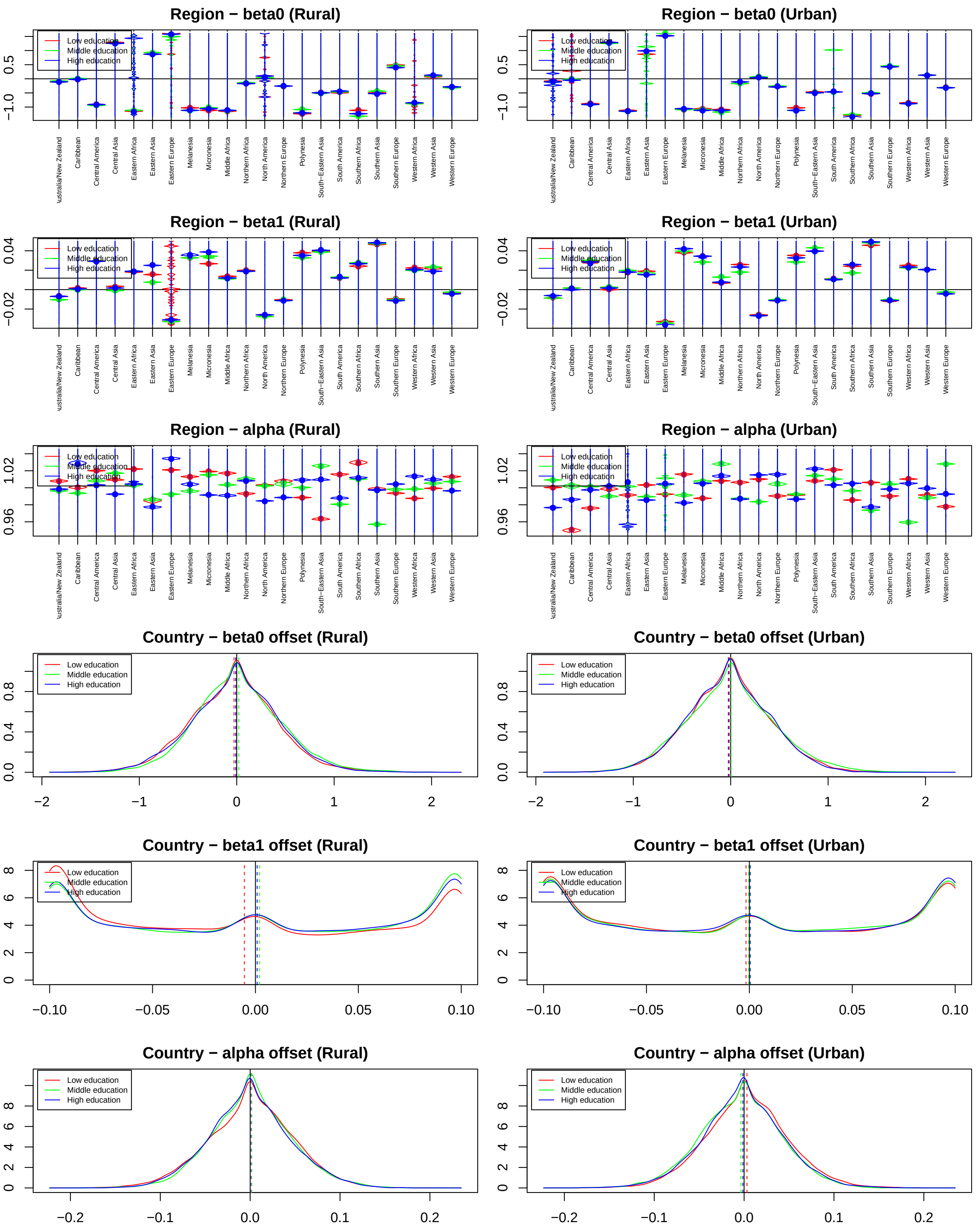

Parameters

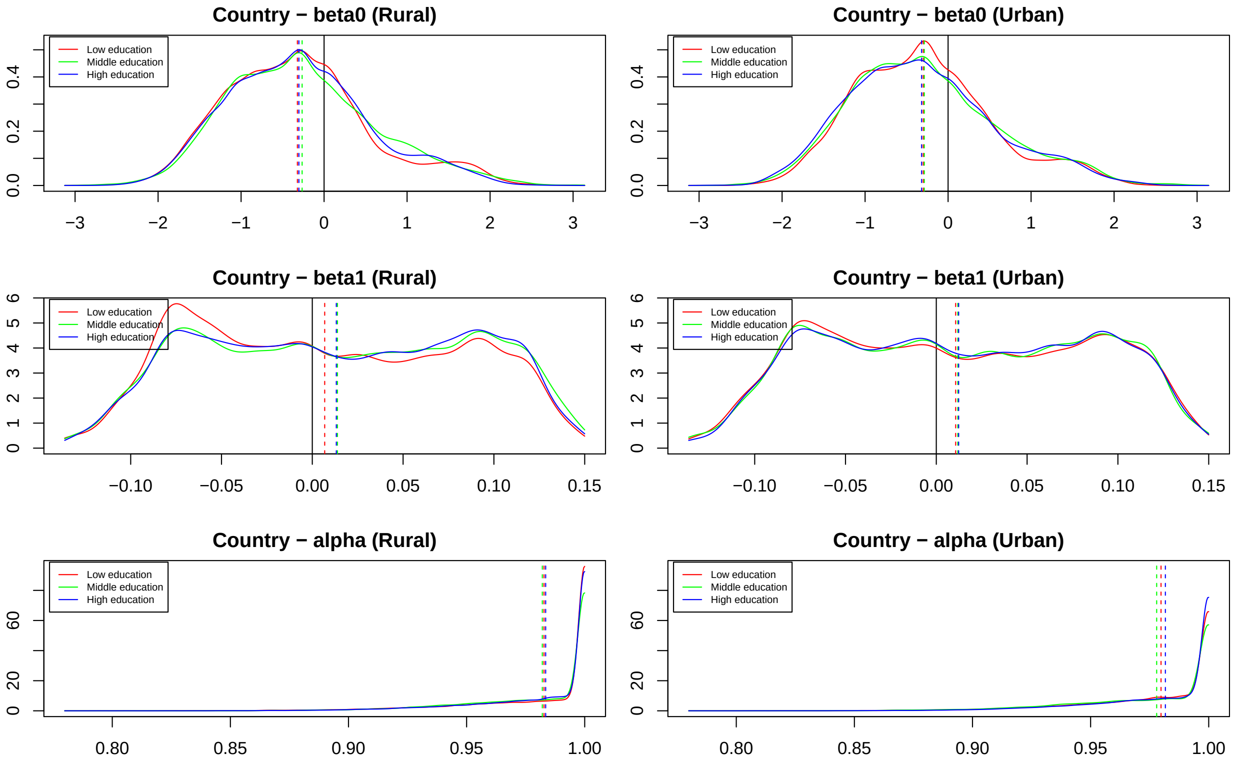

C.3.8.2 p(Safe Abortion|Abortion)

Hyperparameters

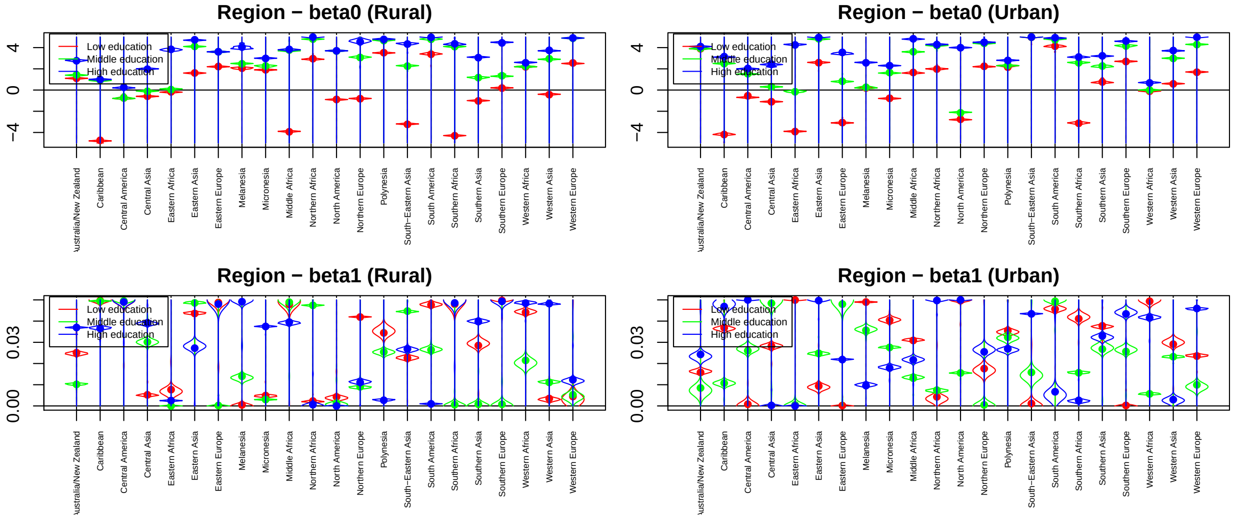

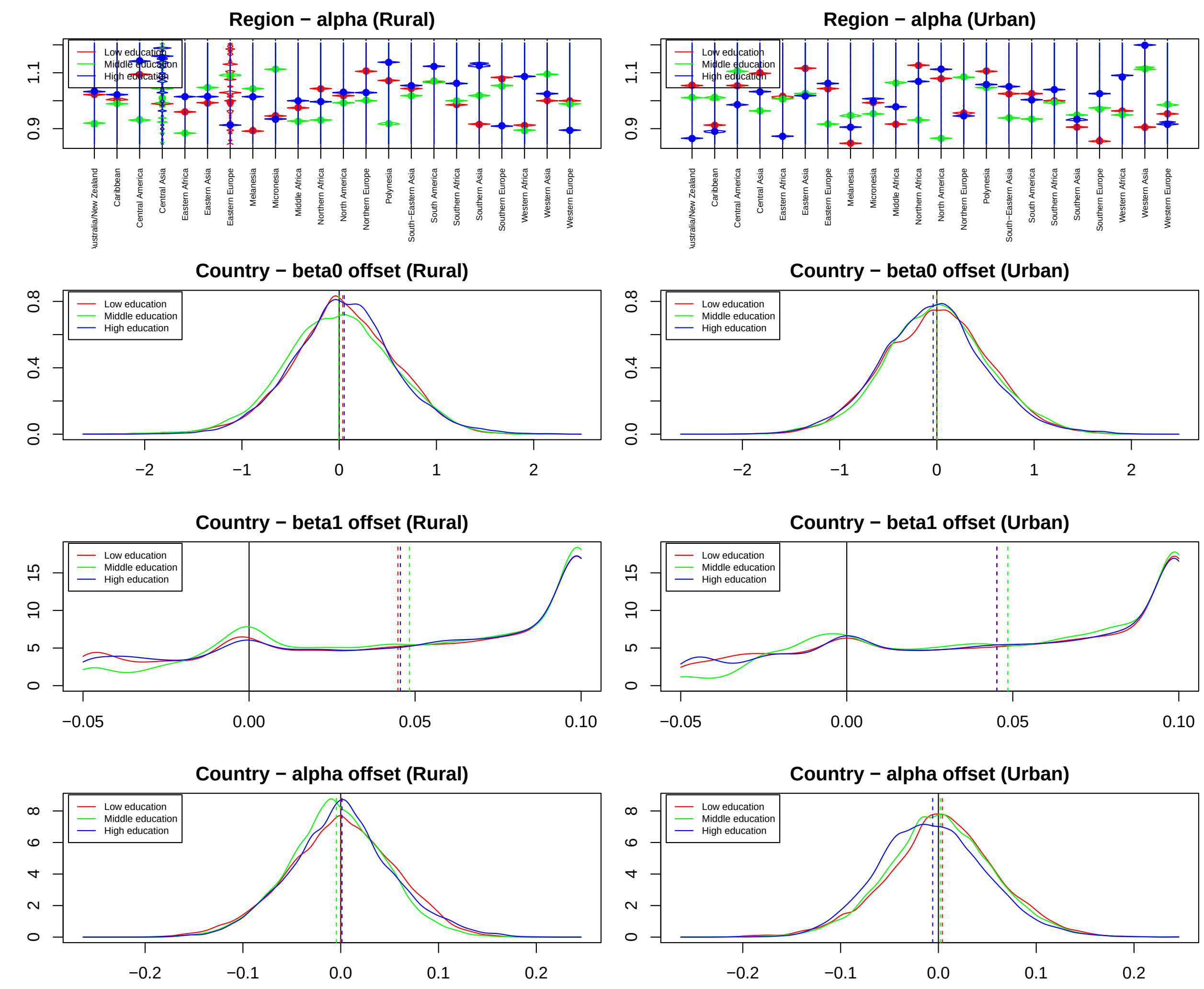
Region − alpha (Rural)
Region − alpha (Urban)
Country − beta0 offset (Rural)
Country − beta0 offset (Urban)
Country − beta1 offset (Rural)
Country − beta1 offset (Urban)
Country − alpha offset (Rural)
Country − alpha offset (Urban)
Low education
Middle education
High education

Parameters

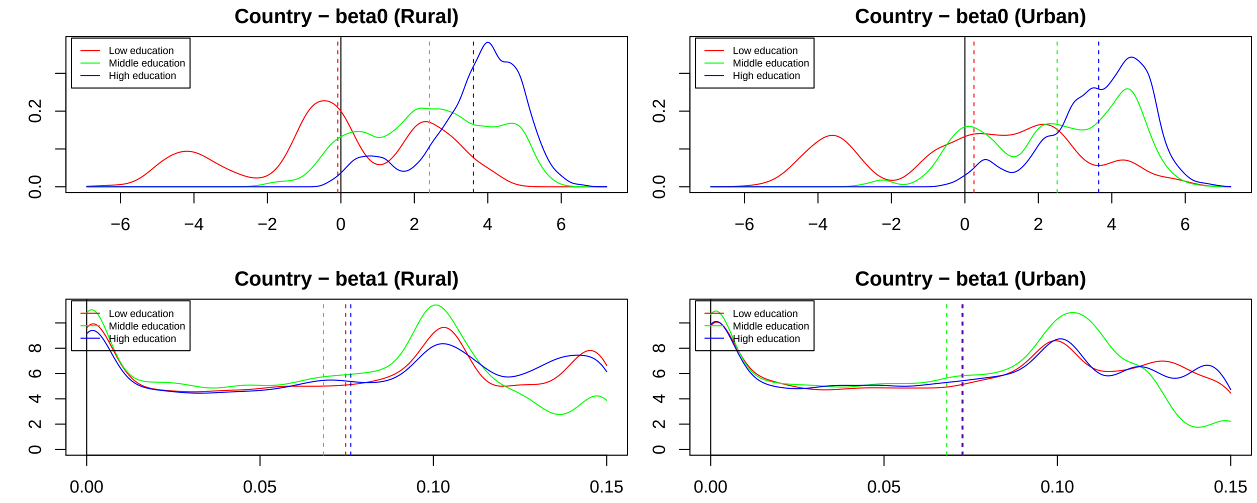
Country − beta0 (Rural)
Country − beta0 (Urban)
Country − beta1 (Rural)
Country − beta1 (Urban)
Low education
Middle education
High education

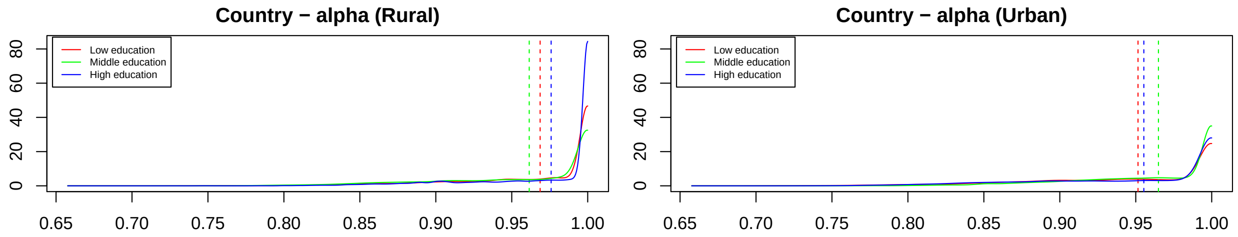

C.3.8.3 p(Mortality)

Hyperparameters

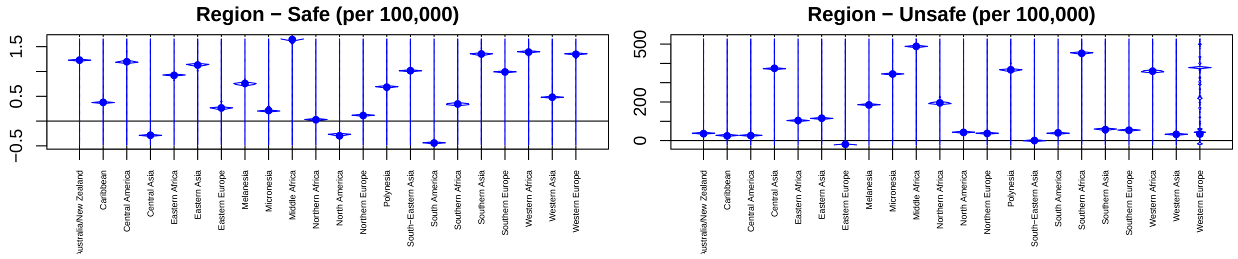

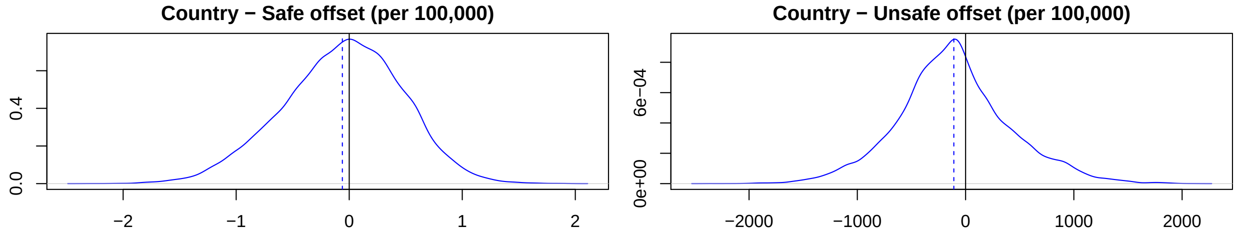

Parameters

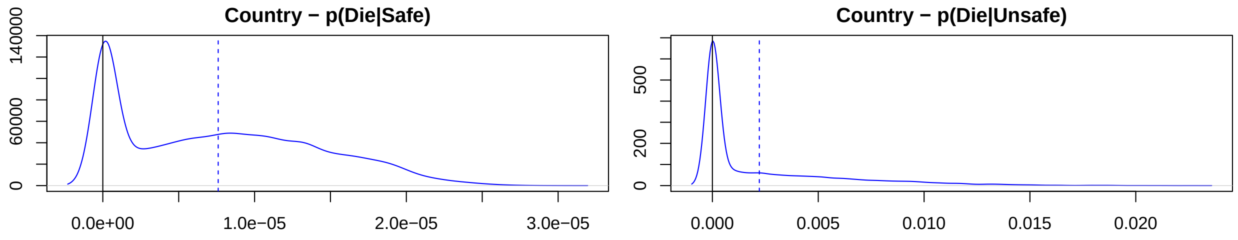

C.3.8.4 p(Infertility)

Hyperparameters

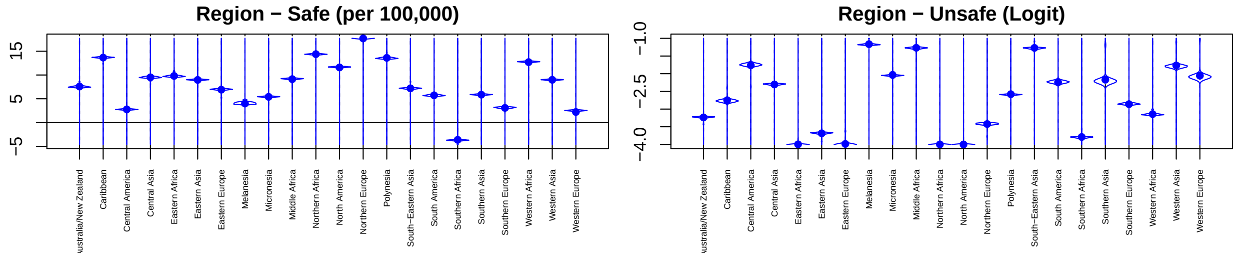

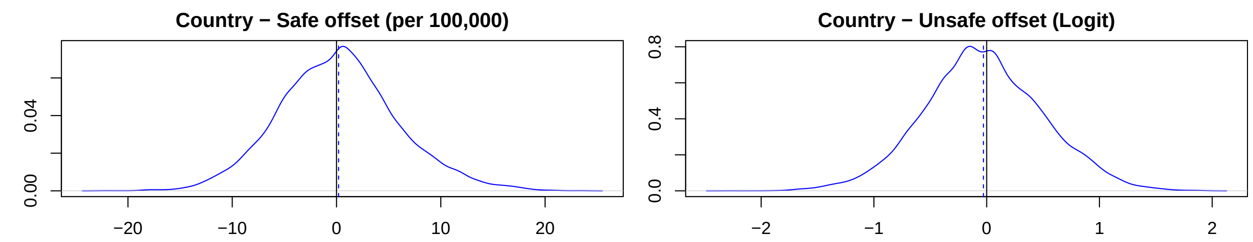

Parameters

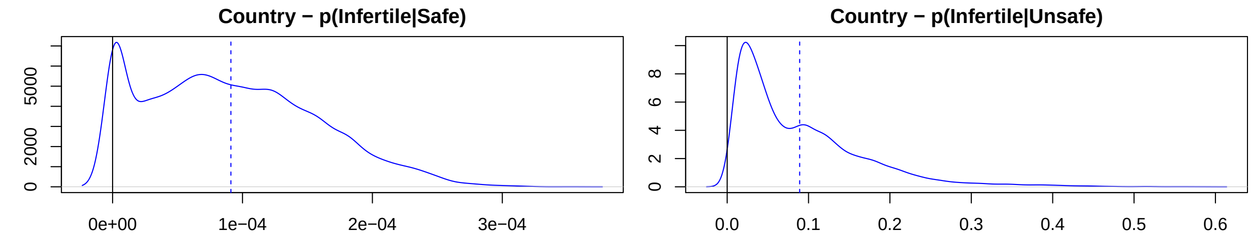

C.3.8.5 Anemia RR - Mortality

Hyperparameters

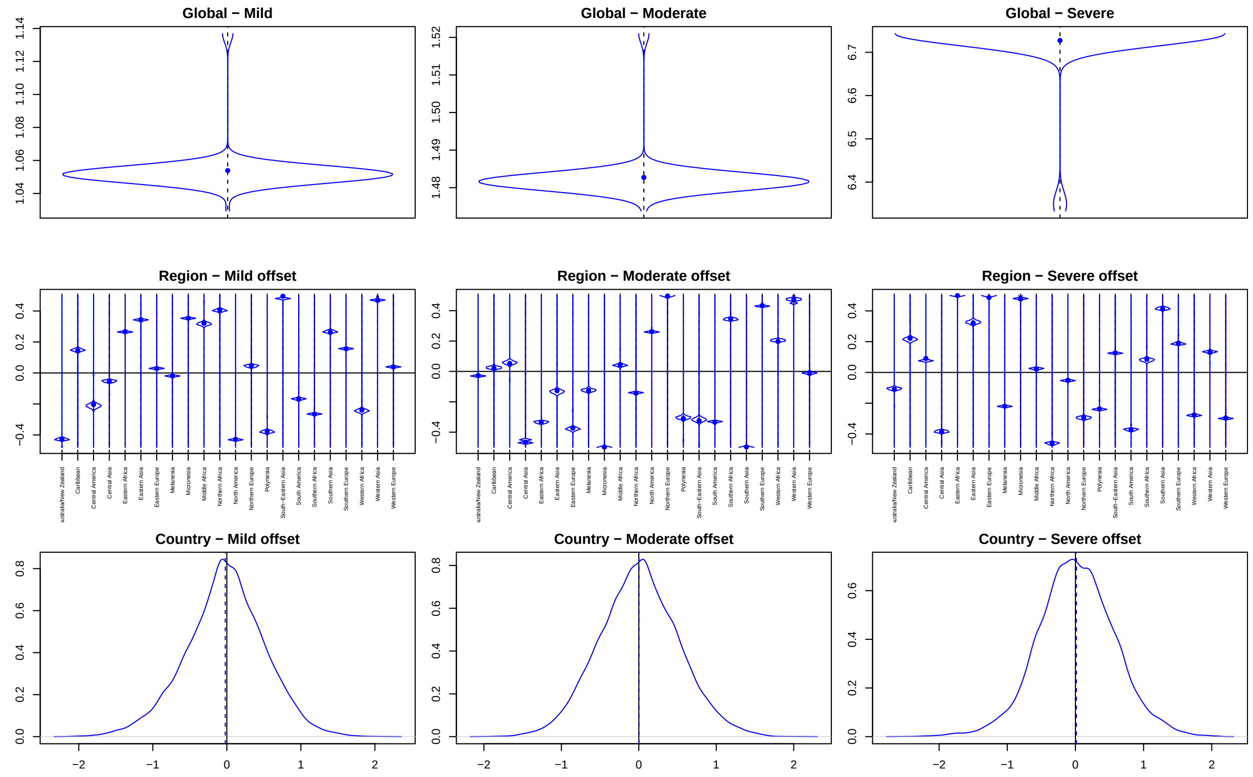

Parameters

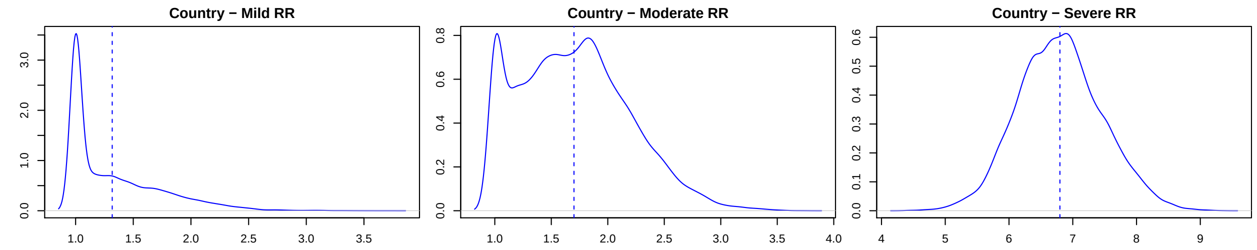
Country − Mild RR
Country − Moderate RR
Country − Severe RR

C.3.9 Sex Ratio - Secondary

Hyperparameters

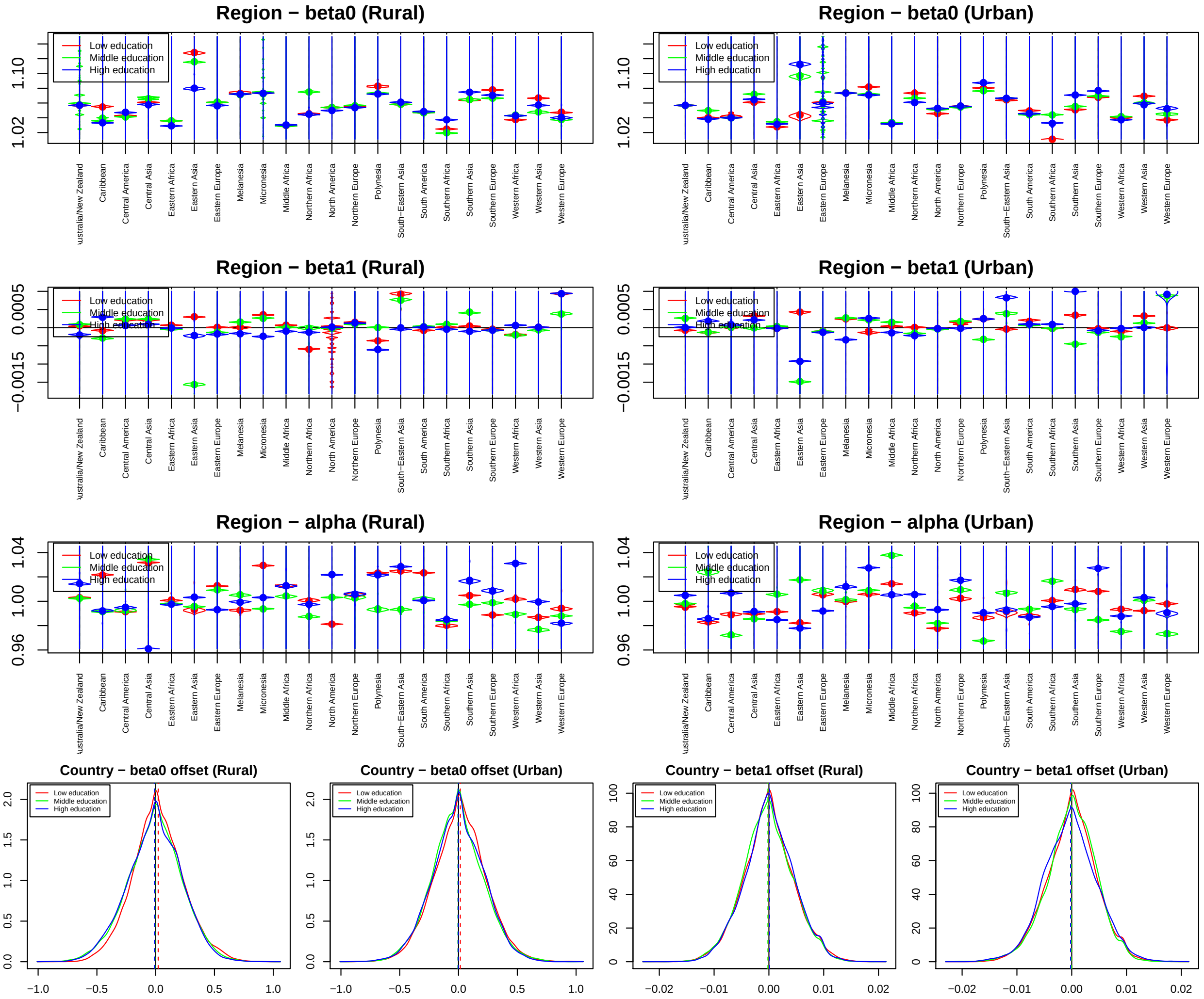

Parameters

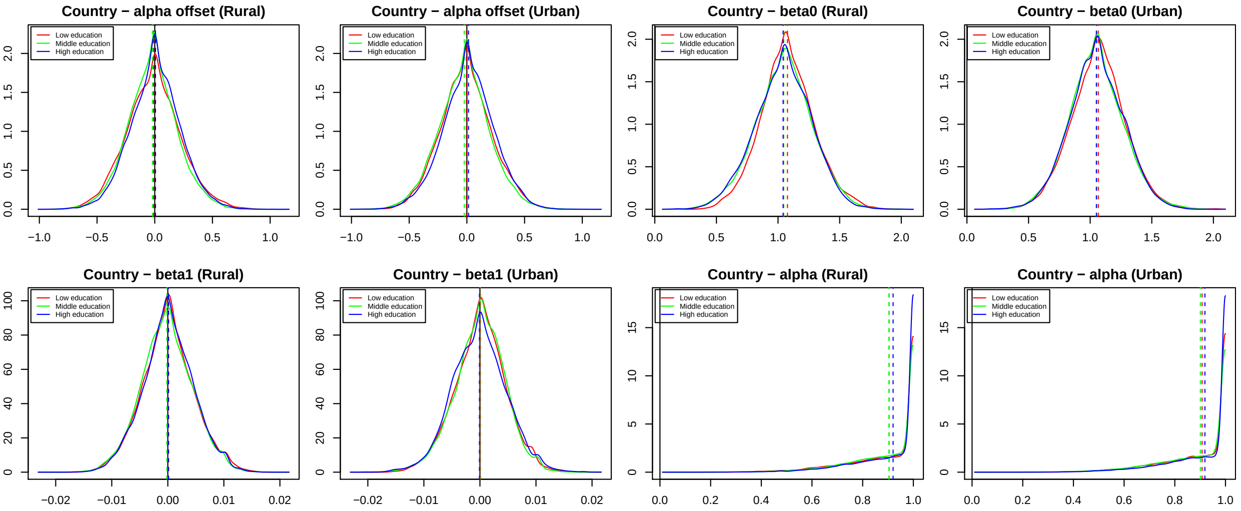

C.3.10 Breastfeeding

C.3.10.1 Any Breastfeeding

Hyperparameters

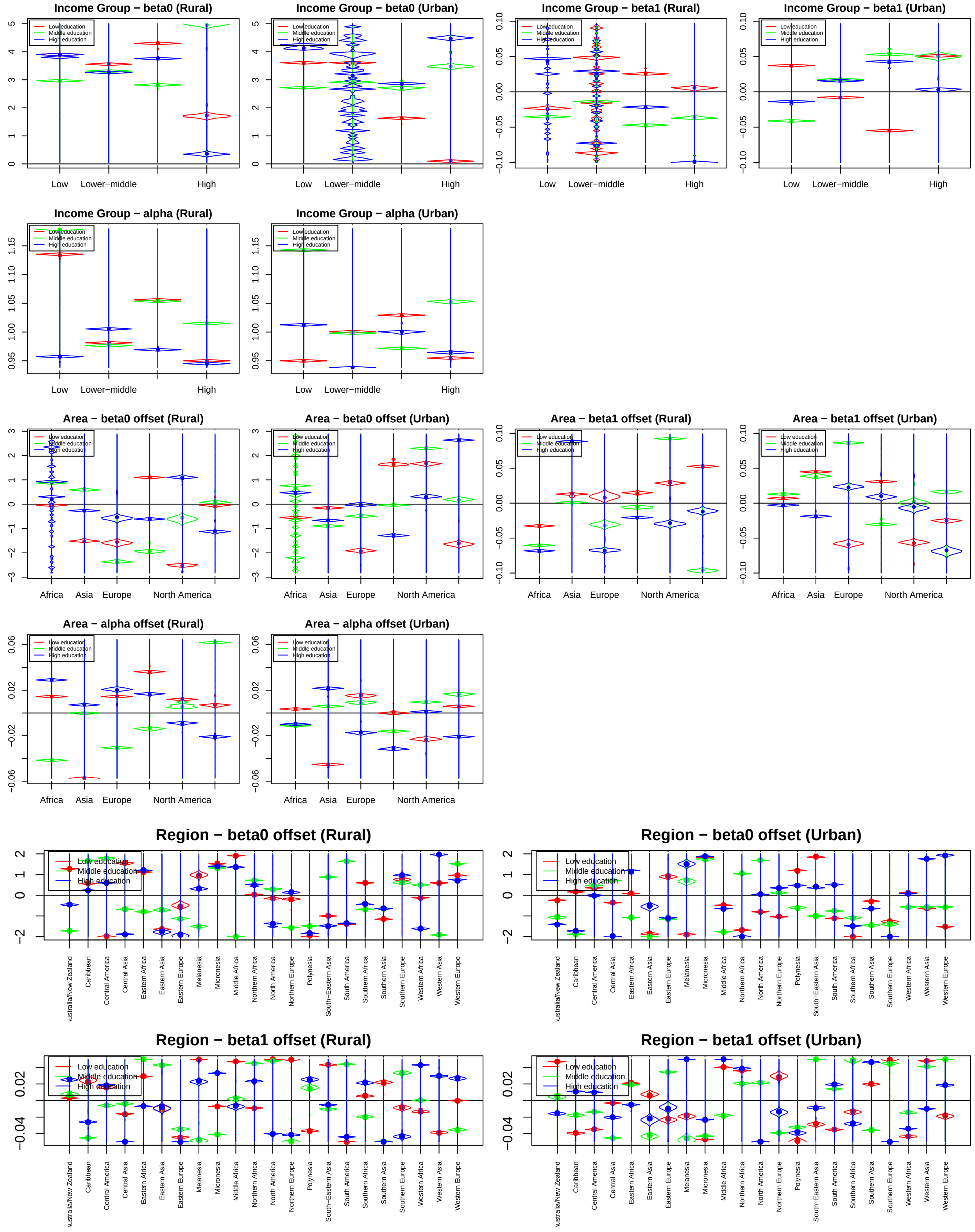

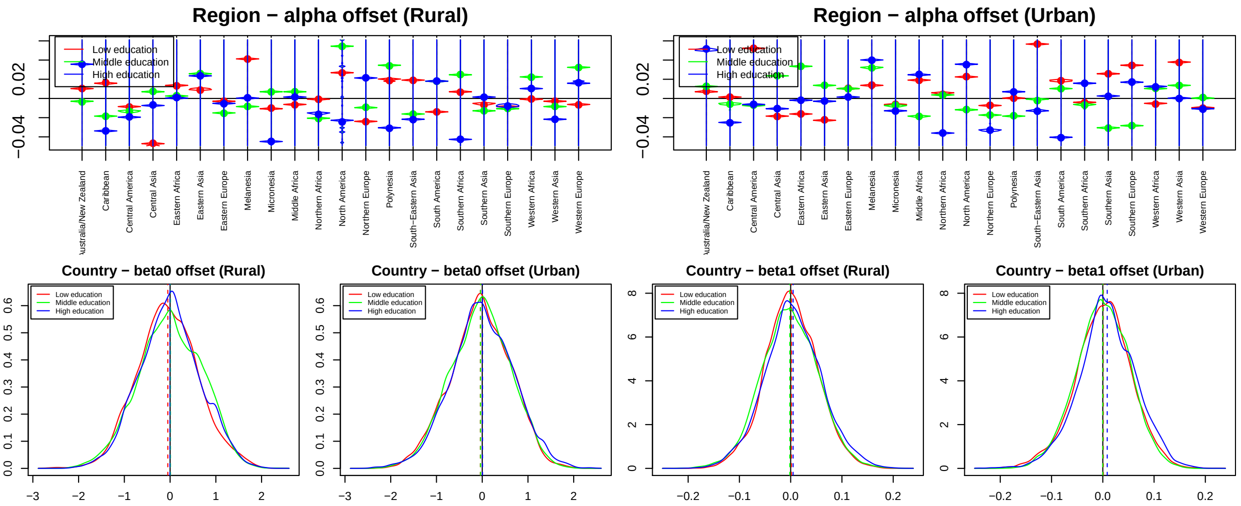

Parameters

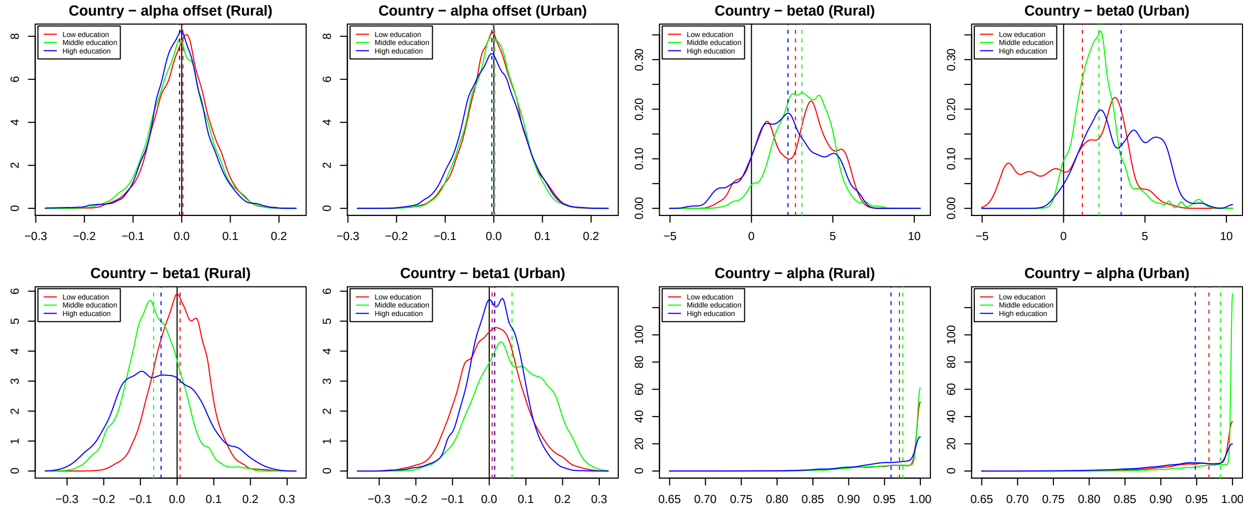

C.3.10.2 Exclusive Breastfeeding

Hyperparameters

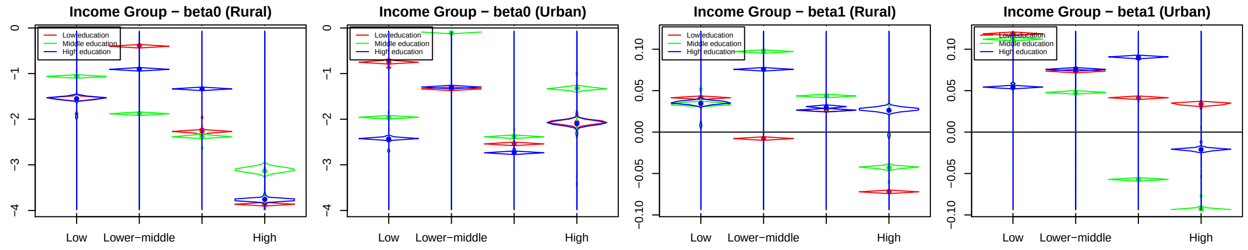

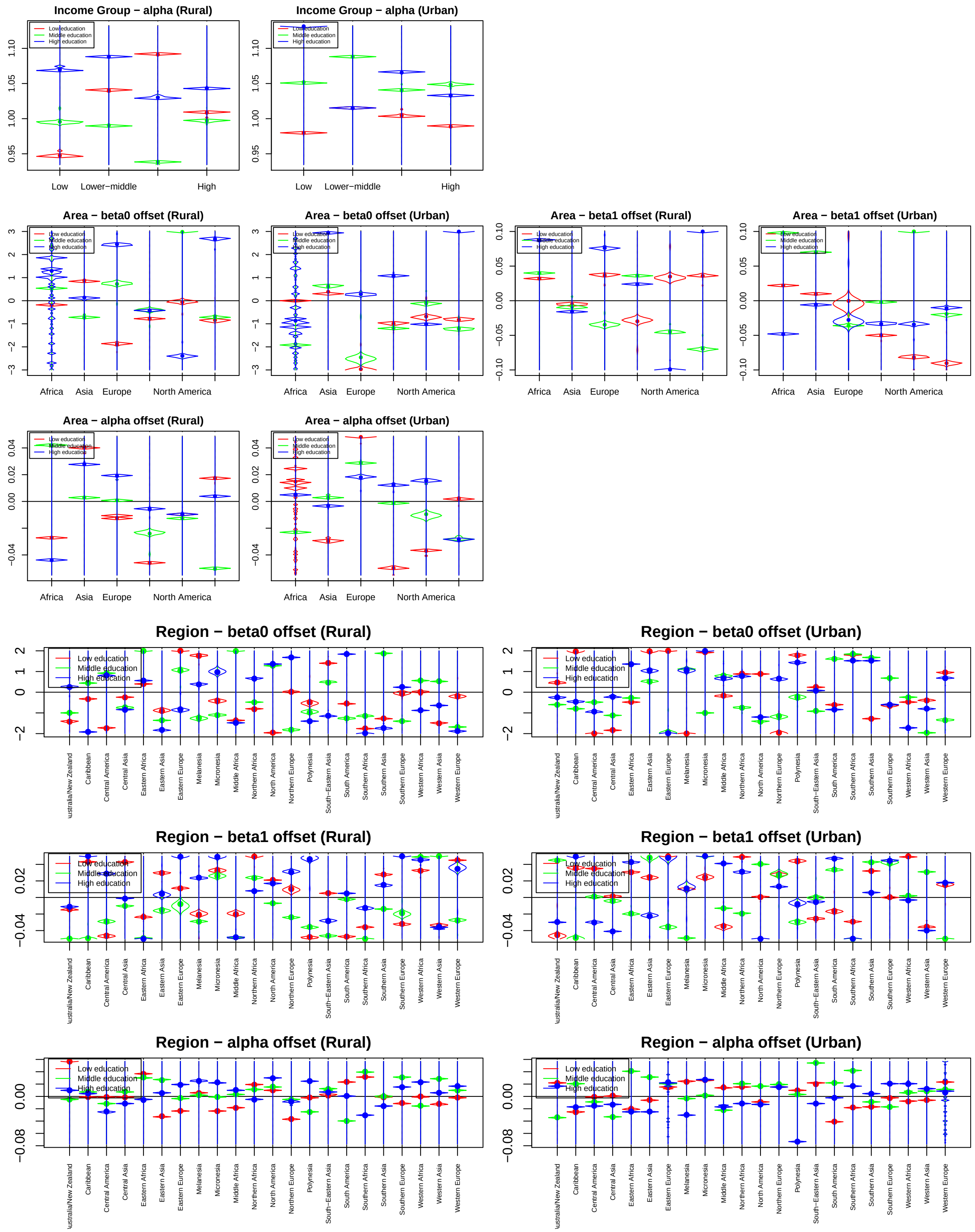
Income Group − alpha (Rural)
Income Group − alpha (Urban)
Low education
Middle education
High education
Low
Lower−middle
High
Area − beta0 offset (Rural)
Area − beta0 offset (Urban)
Area − beta1 offset (Rural)
Area − beta1 offset (Urban)
Area − alpha offset (Rural)
Area − alpha offset (Urban)
Africa
Asia
Europe
North America
Region − beta0 offset (Rural)
Region − beta0 offset (Urban)
Region − beta1 offset (Rural)
Region − beta1 offset (Urban)
Region − alpha offset (Rural)
Region − alpha offset (Urban)
Caribbean
Central America
Central Asia
Eastern Africa
Eastern Asia
Eastern Europe
Melanesia
Micronesia
Middle Africa
Northern Africa
North America
Northern Europe
Polynesia
South−Eastern Asia
South America
Southern Africa
Southern Asia
Southern Europe
Western Africa
Western Asia
Western Europe

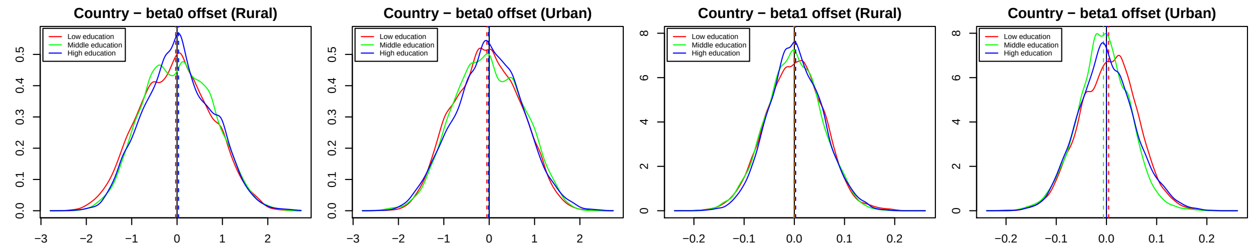

Parameters

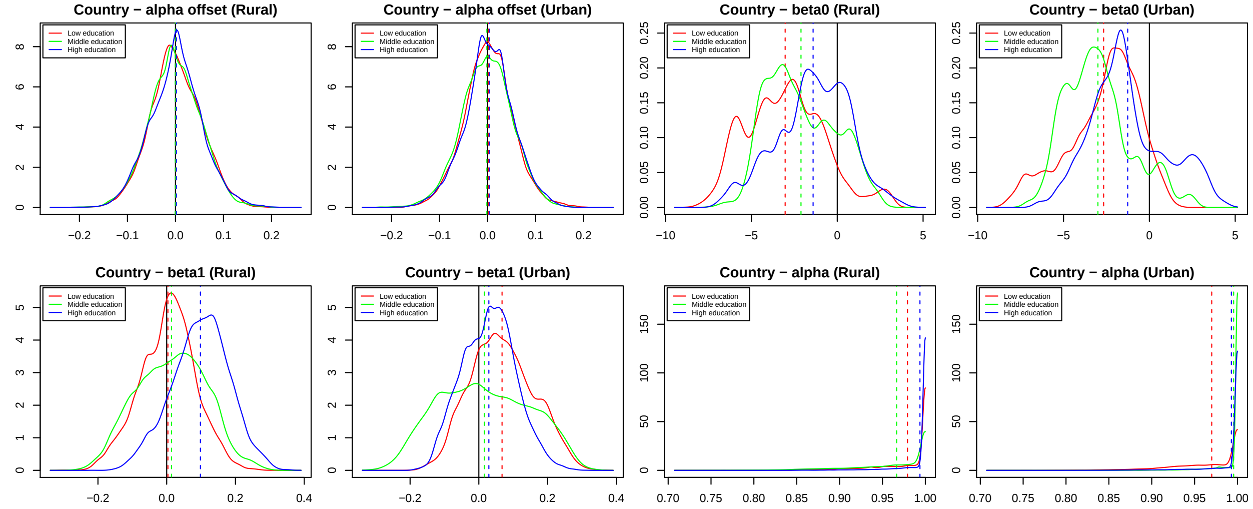

C.3.10.3 6 Months | Any Breastfeeding

Hyperparameters

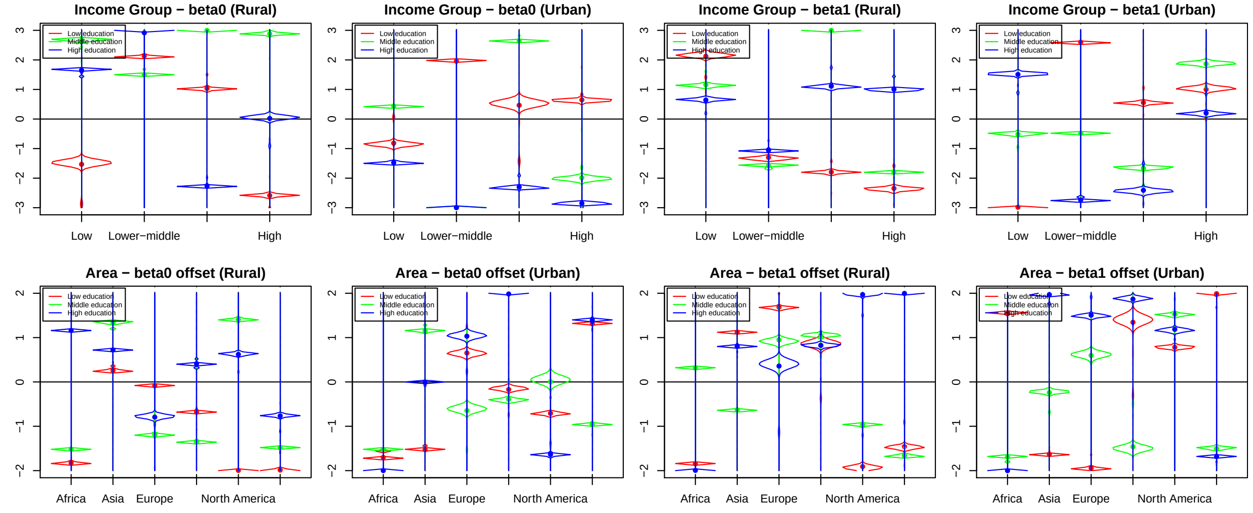

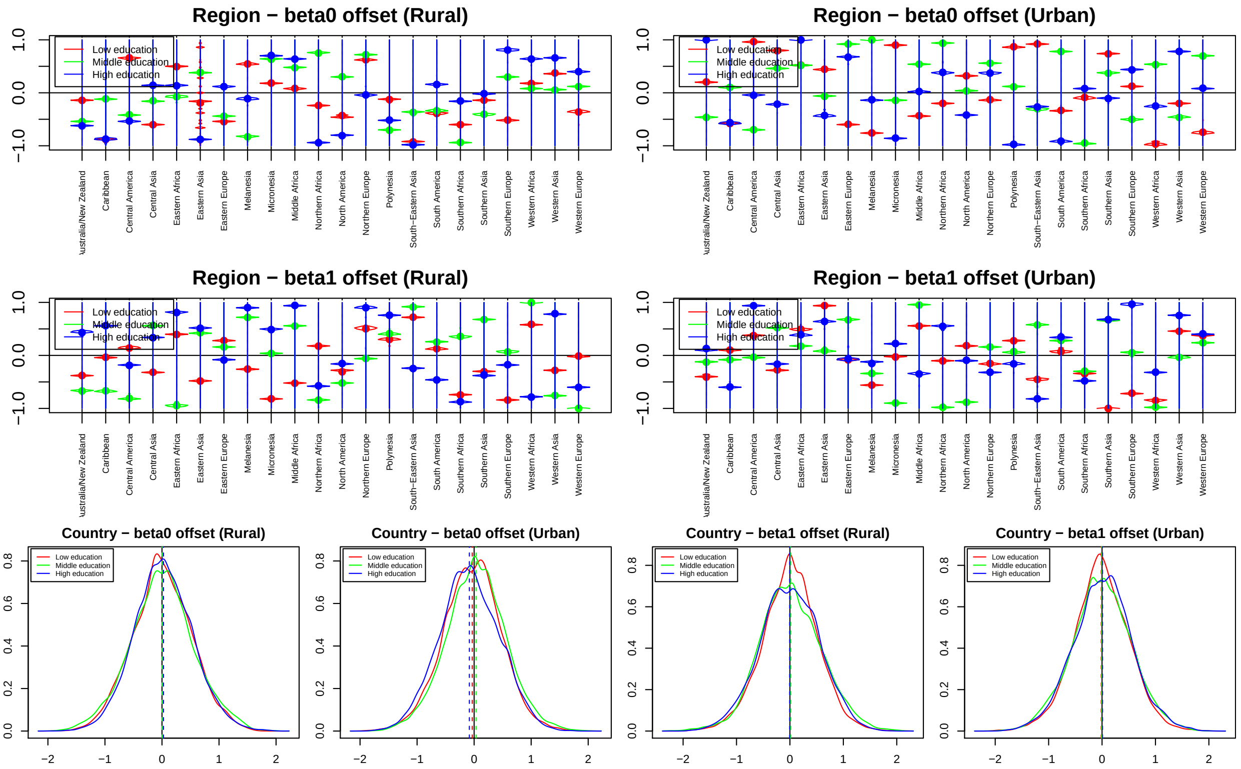
Region − beta0 offset (Rural)
Region − beta0 offset (Urban)
Region − beta1 offset (Rural)
Region − beta1 offset (Urban)
Country − beta0 offset (Rural)
Country − beta0 offset (Urban)
Country − beta1 offset (Rural)
Country − beta1 offset (Urban)
Low education
Middle education
High education

Parameters

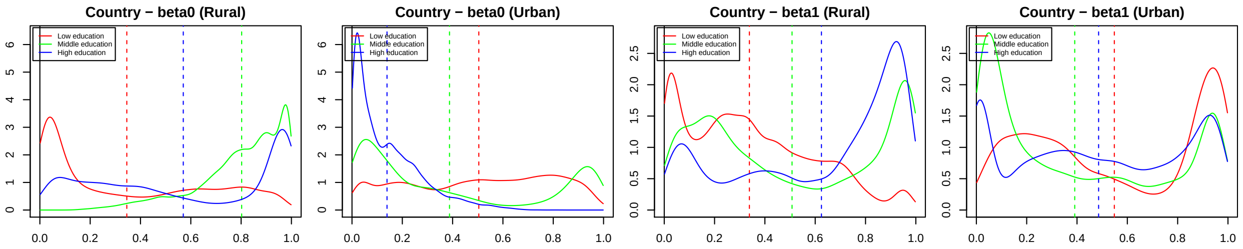
Country − beta0 (Rural)
Country − beta0 (Urban)
Country − beta1 (Rural)
Country − beta1 (Urban)
Low education
Middle education
High education

C.4 Health System Parameters

C.4.1 Antenatal Care

C.4.1.1 Any ANC

Hyperparameters

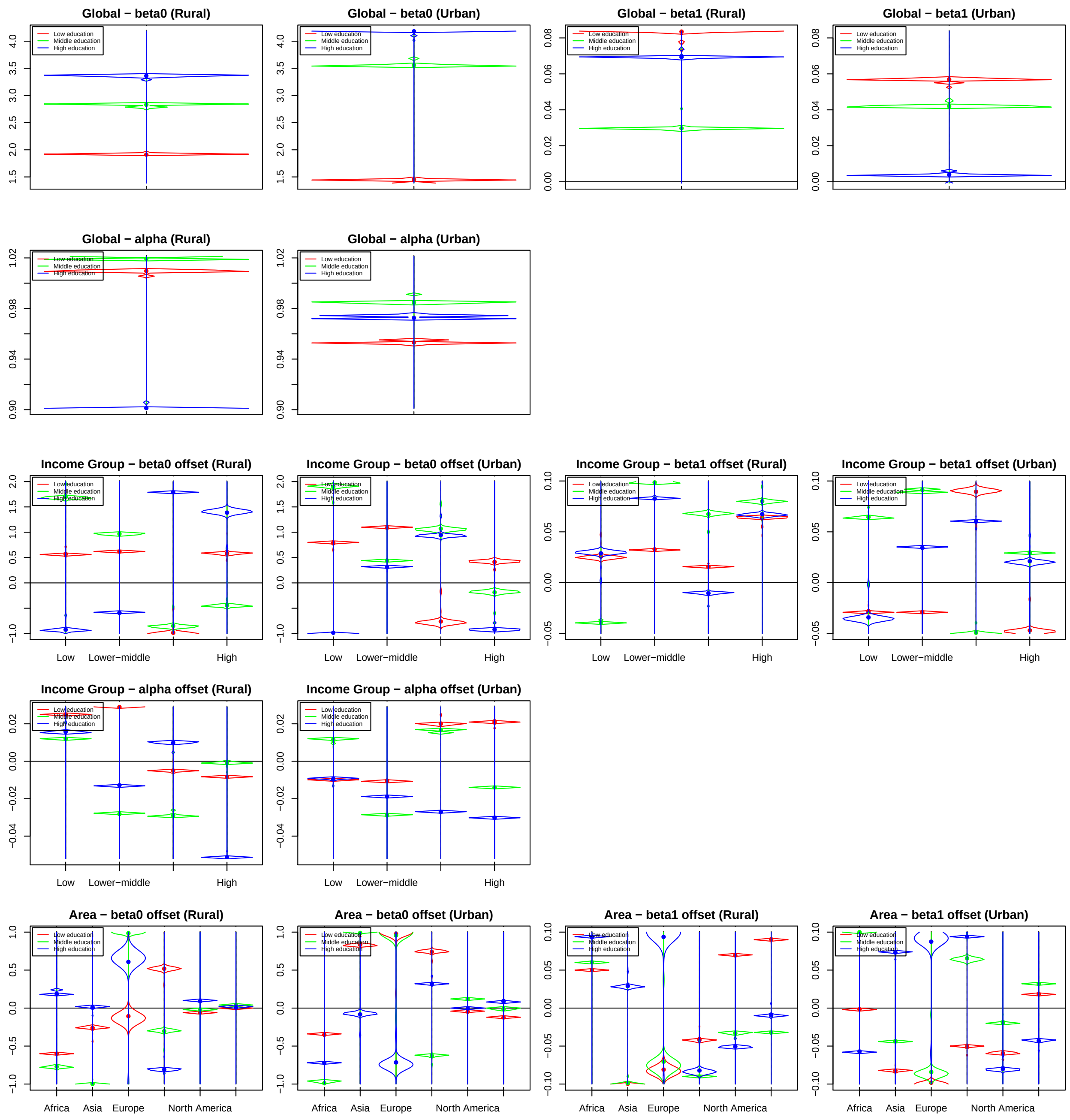

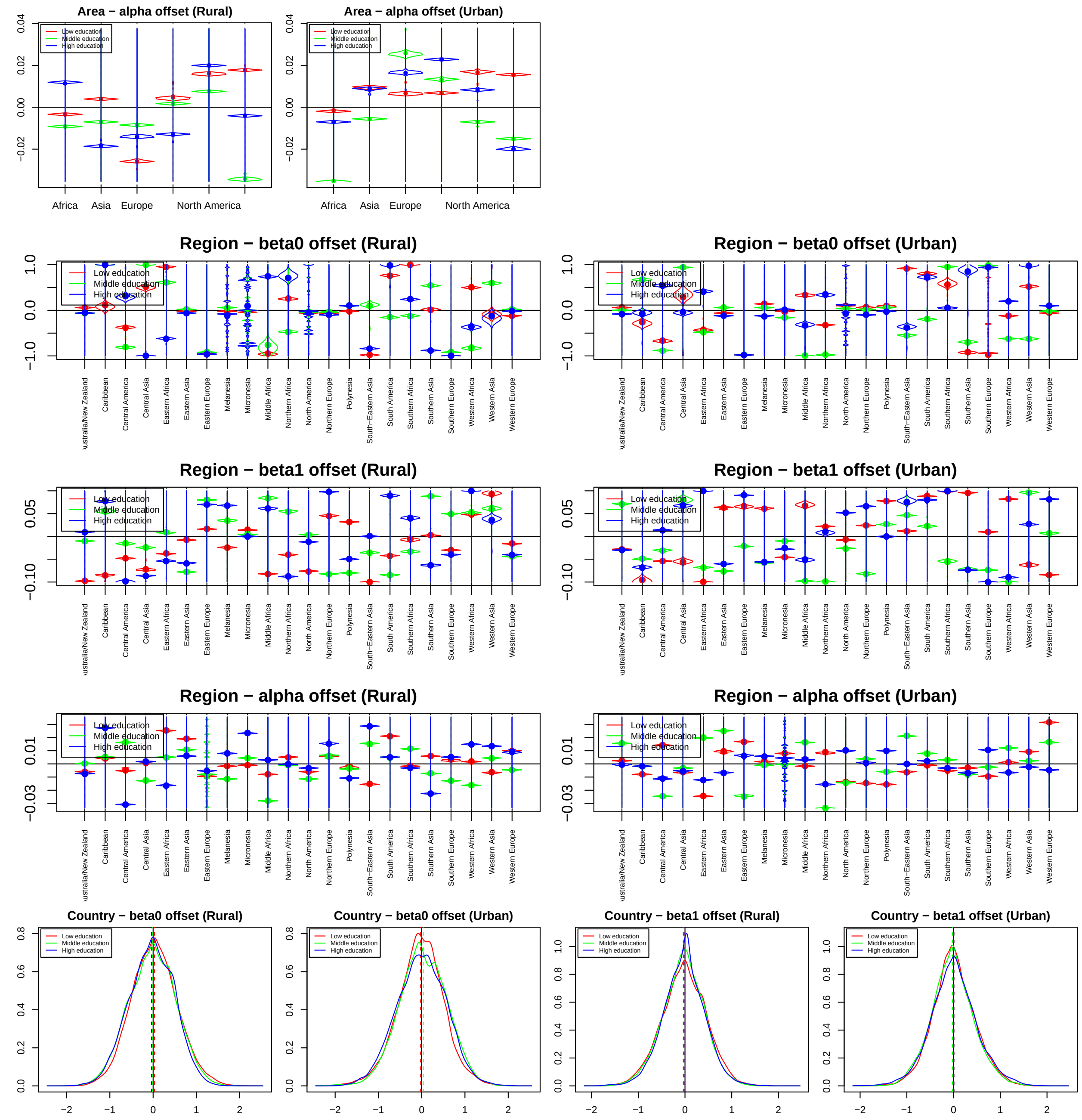
Area − alpha offset (Rural)
Area − alpha offset (Urban)
Low education
Middle education
High education
Africa
Asia
Europe
North America
Region − beta0 offset (Rural)
Region − beta0 offset (Urban)
Region − beta1 offset (Rural)
Region − beta1 offset (Urban)
Region − alpha offset (Rural)
Region − alpha offset (Urban)
Caribbean
Central America
Central Asia
Eastern Africa
Eastern Asia
Eastern Europe
Melanesia
Micronesia
Middle Africa
Northern Africa
North America
Northern Europe
Polynesia
South−Eastern Asia
South America
Southern Africa
Southern Asia
Southern Europe
Western Africa
Western Asia
Western Europe
Country − beta0 offset (Rural)
Country − beta0 offset (Urban)
Country − beta1 offset (Rural)
Country − beta1 offset (Urban)

Parameters

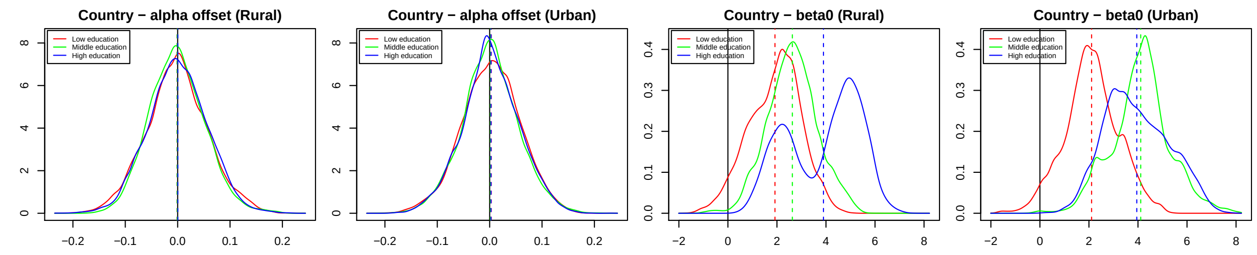
Country − alpha offset (Rural)
Country − alpha offset (Urban)
Country − beta0 (Rural)
Country − beta0 (Urban)
Low education
Middle education
High education

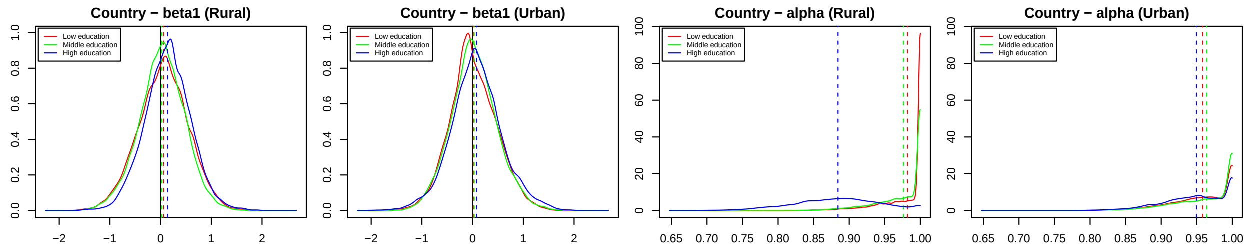

C.4.1.2 Number of Visits

Hyperparameters

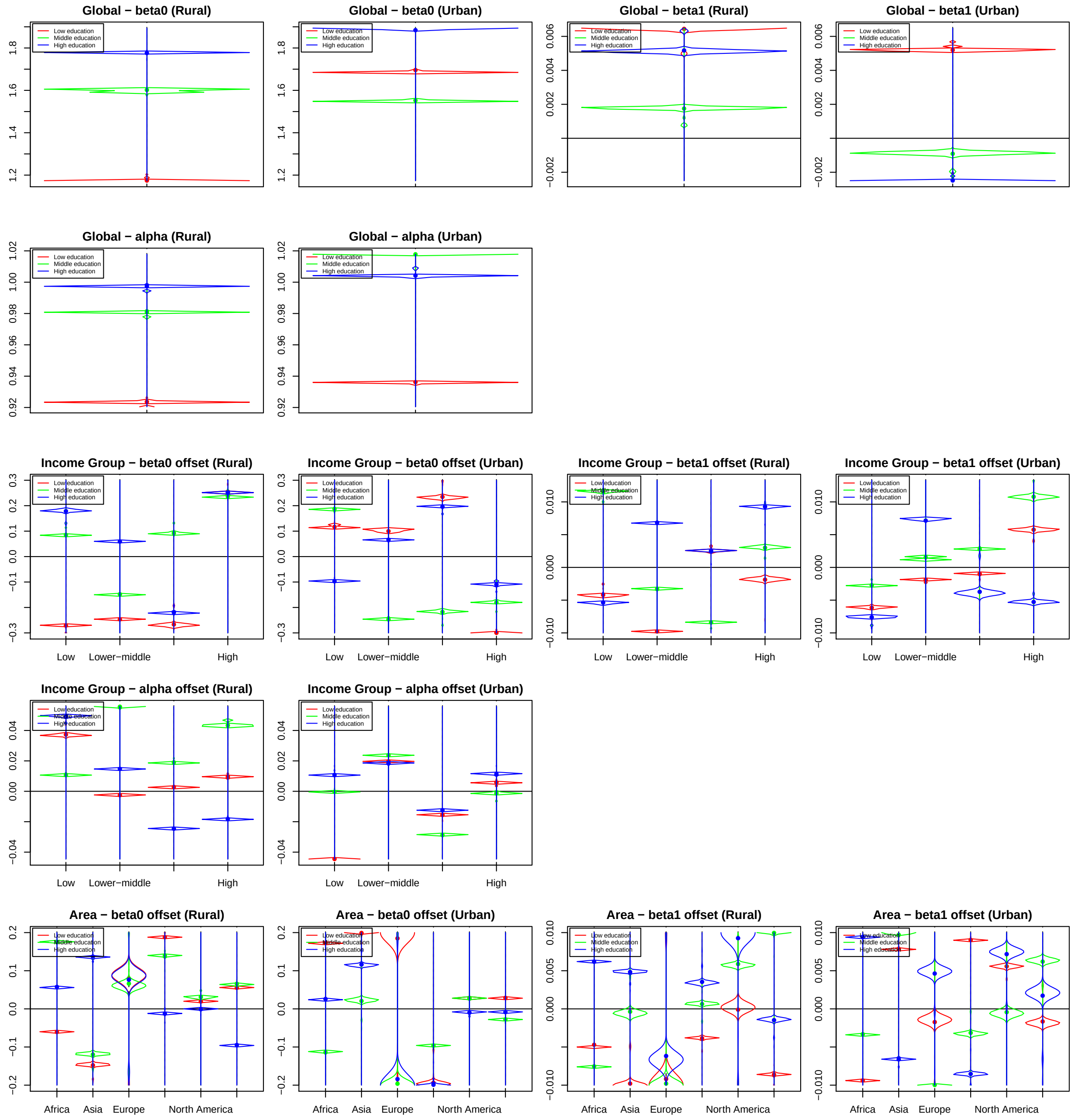

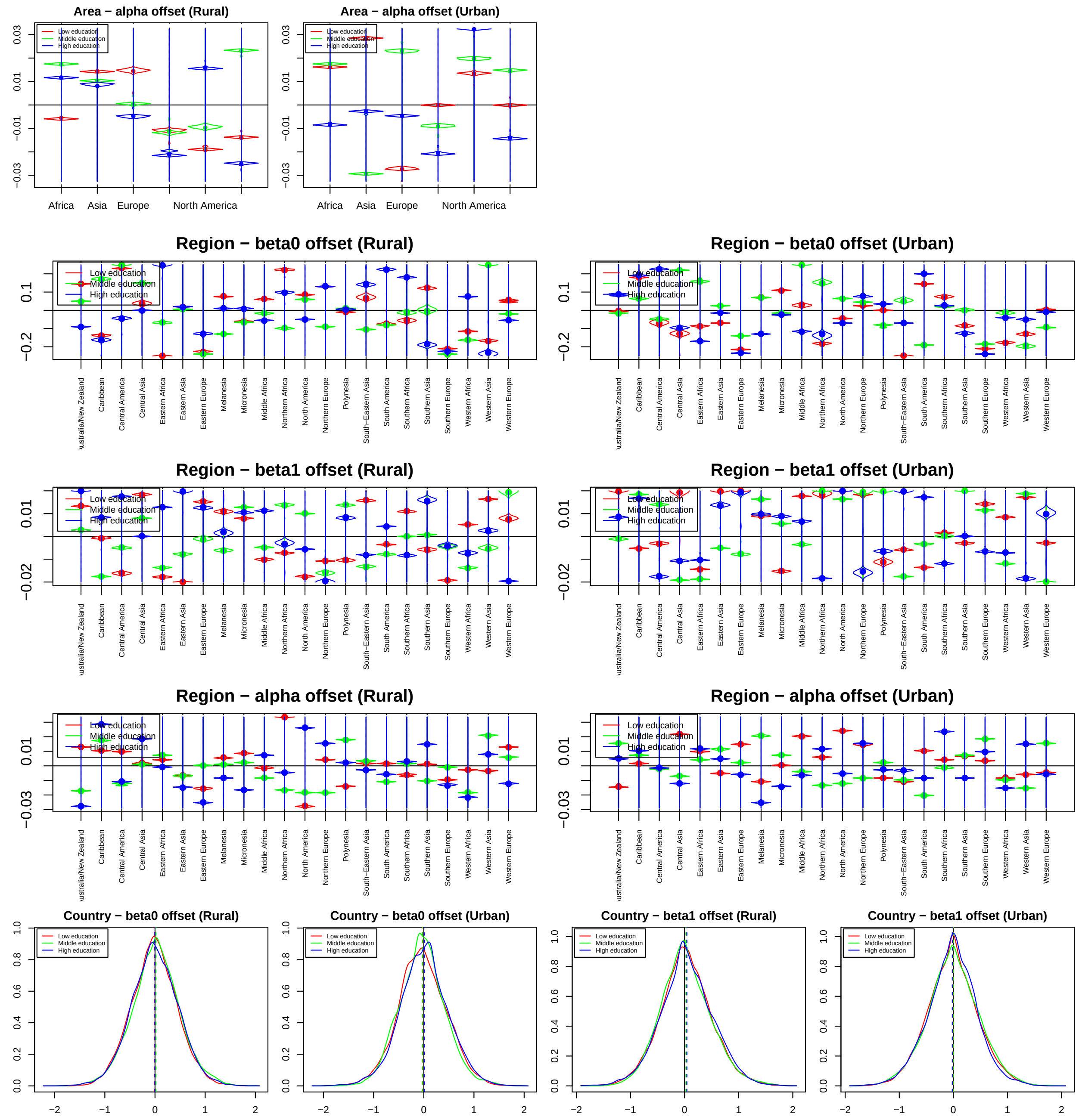
Area − alpha offset (Rural)
Area − alpha offset (Urban)
Low education
Middle education
High education
Africa
Asia
Europe
North America
Region − beta0 offset (Rural)
Region − beta0 offset (Urban)
Region − beta1 offset (Rural)
Region − beta1 offset (Urban)
Region − alpha offset (Rural)
Region − alpha offset (Urban)
Australia/New Zealand
Caribbean
Central America
Central Asia
Eastern Africa
Eastern Asia
Eastern Europe
Melanesia
Micronesia
Middle Africa
Northern Africa
North America
Northern Europe
Polynesia
South−Eastern Asia
South America
Southern Africa
Southern Asia
Southern Europe
Western Africa
Western Asia
Western Europe
Country − beta0 offset (Rural)
Country − beta0 offset (Urban)
Country − beta1 offset (Rural)
Country − beta1 offset (Urban)

Parameters

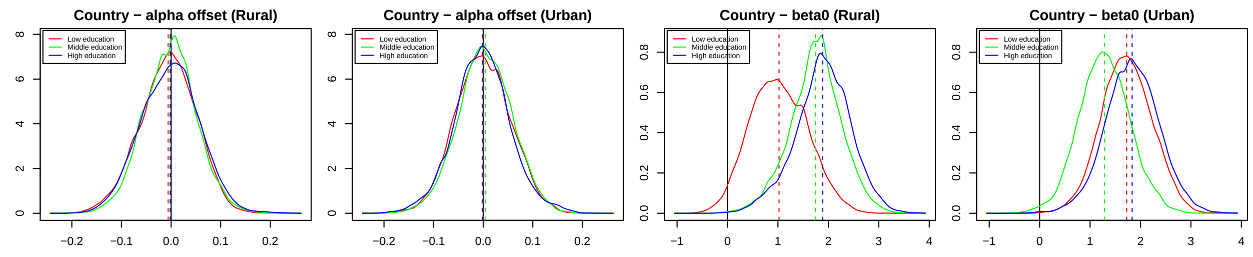
Country − alpha offset (Rural)
Country − alpha offset (Urban)
Country − beta0 (Rural)
Country − beta0 (Urban)
Low education
Middle education
High education

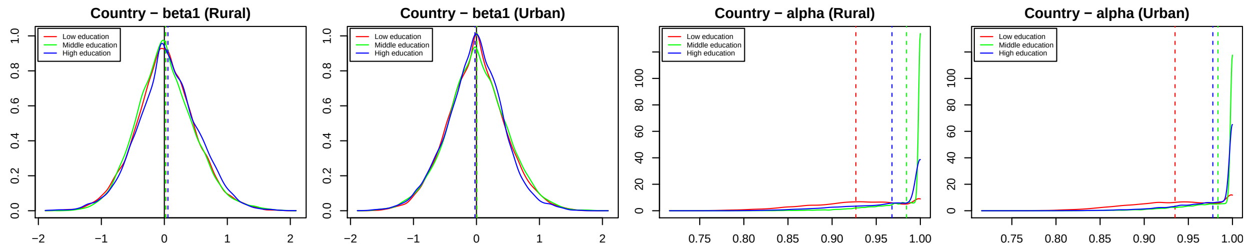

C.4.1.3 Hb Increase

Hyperparameters

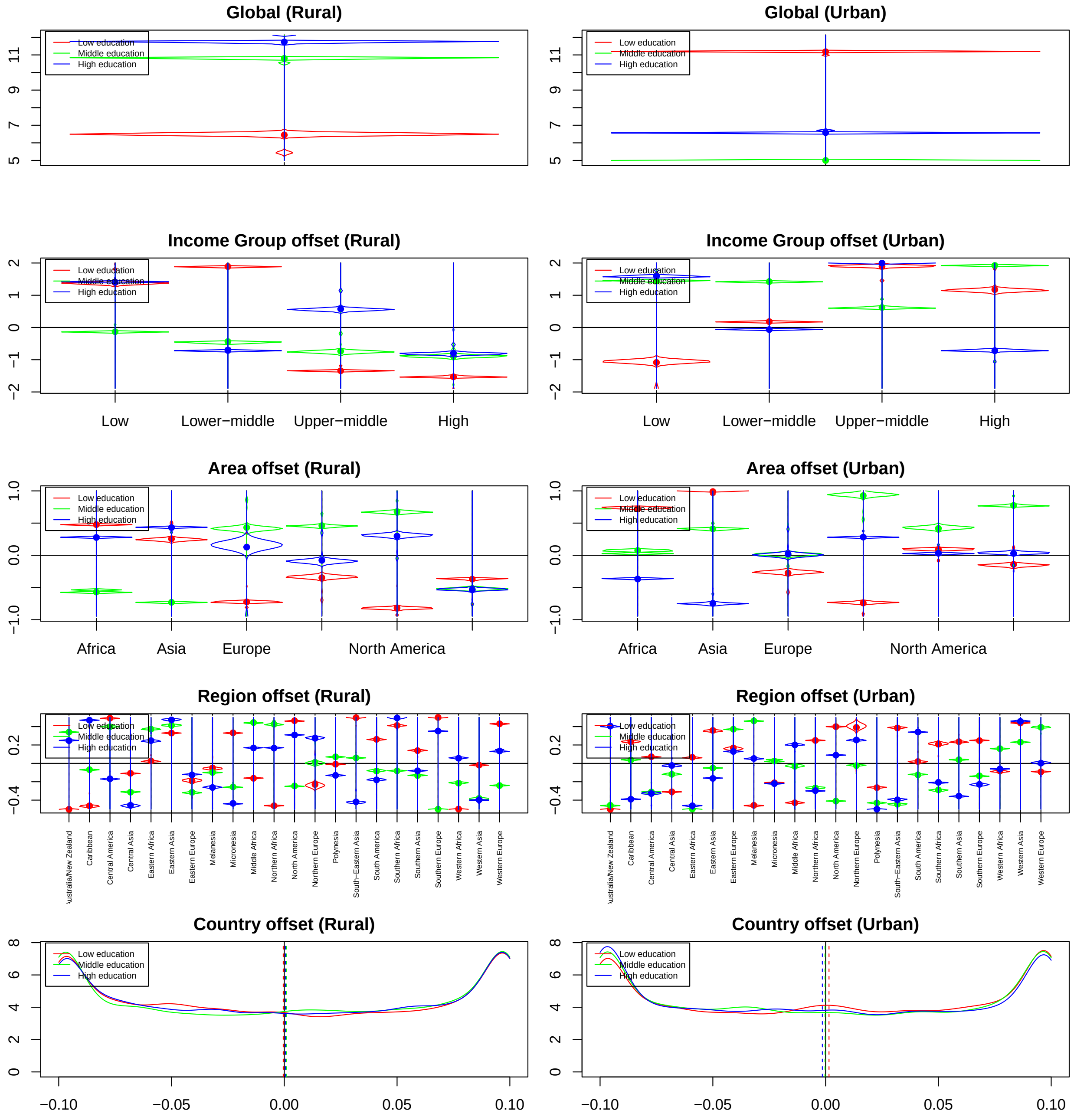

Parameters

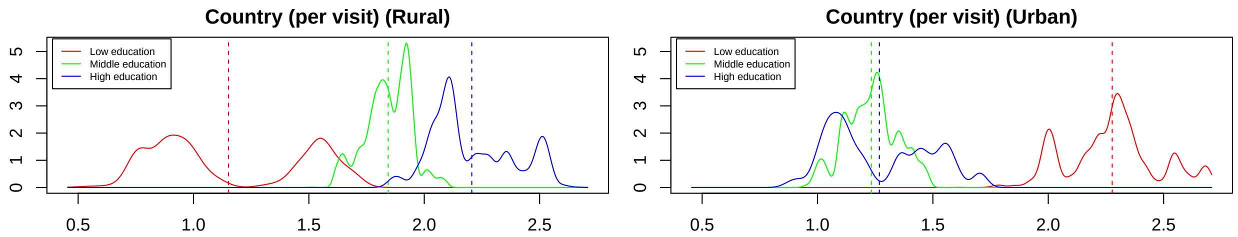

C.4.1.4 Recognition/Referral Increase

Hyperparameters

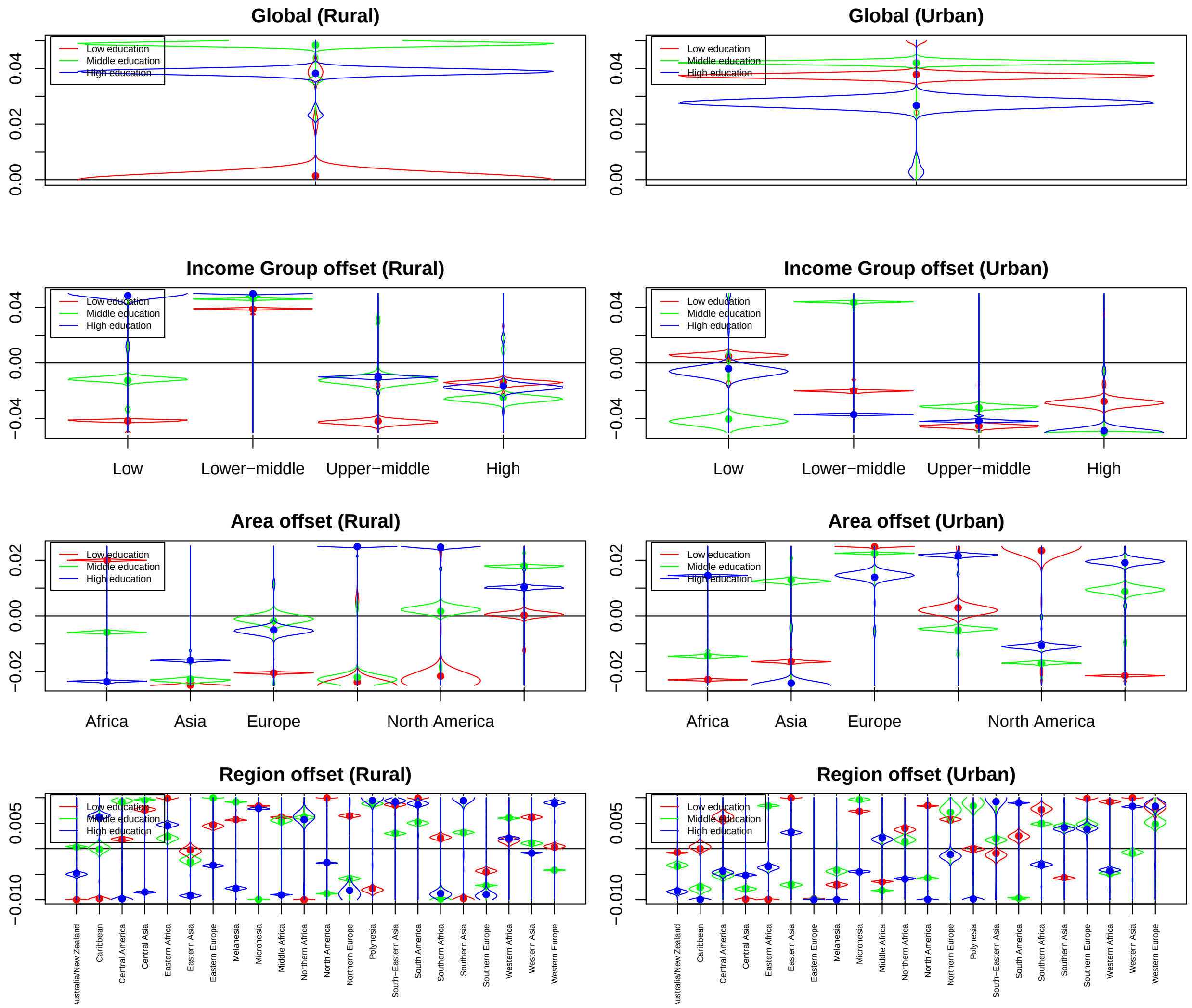

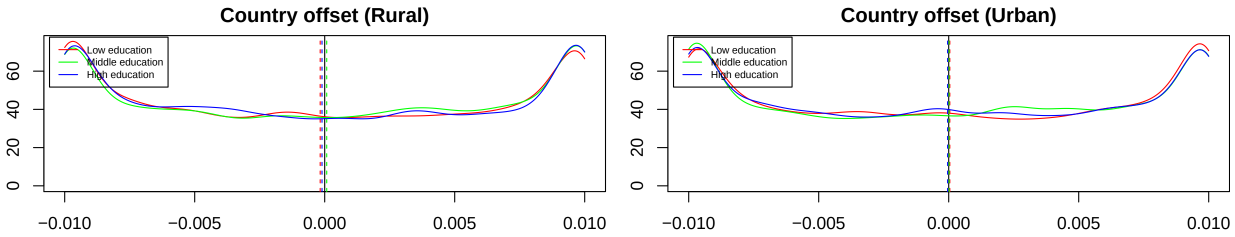
Country offset (Rural)
Low education
Middle education
High education
0
20
40
60
−0.010
−0.005
0.000
0.005
0.010
Country offset (Urban)
Low education
Middle education
High education
0
20
40
60
−0.010
−0.005
0.000
0.005
0.010

Parameters

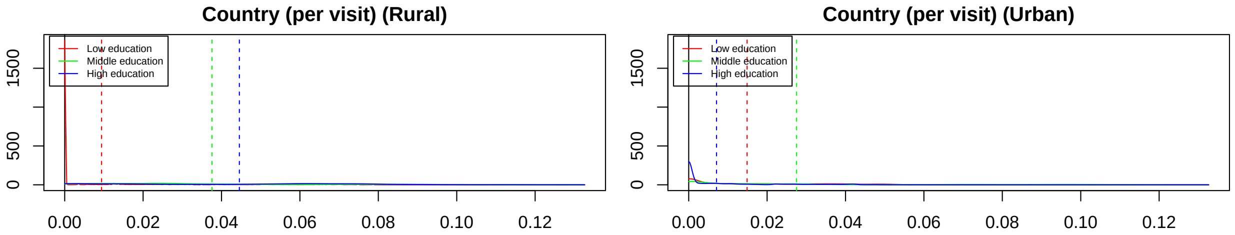
Country (per visit) (Rural)
Low education
Middle education
High education
0
500
1500
0.00
0.02
0.04
0.06
0.08
0.10
0.12
Country (per visit) (Urban)
Low education
Middle education
High education
0
500
1500
0.00
0.02
0.04
0.06
0.08
0.10
0.12

C.4.2 Starting Delivery Site

C.4.2.1 Facility Delivery

Hyperparameters

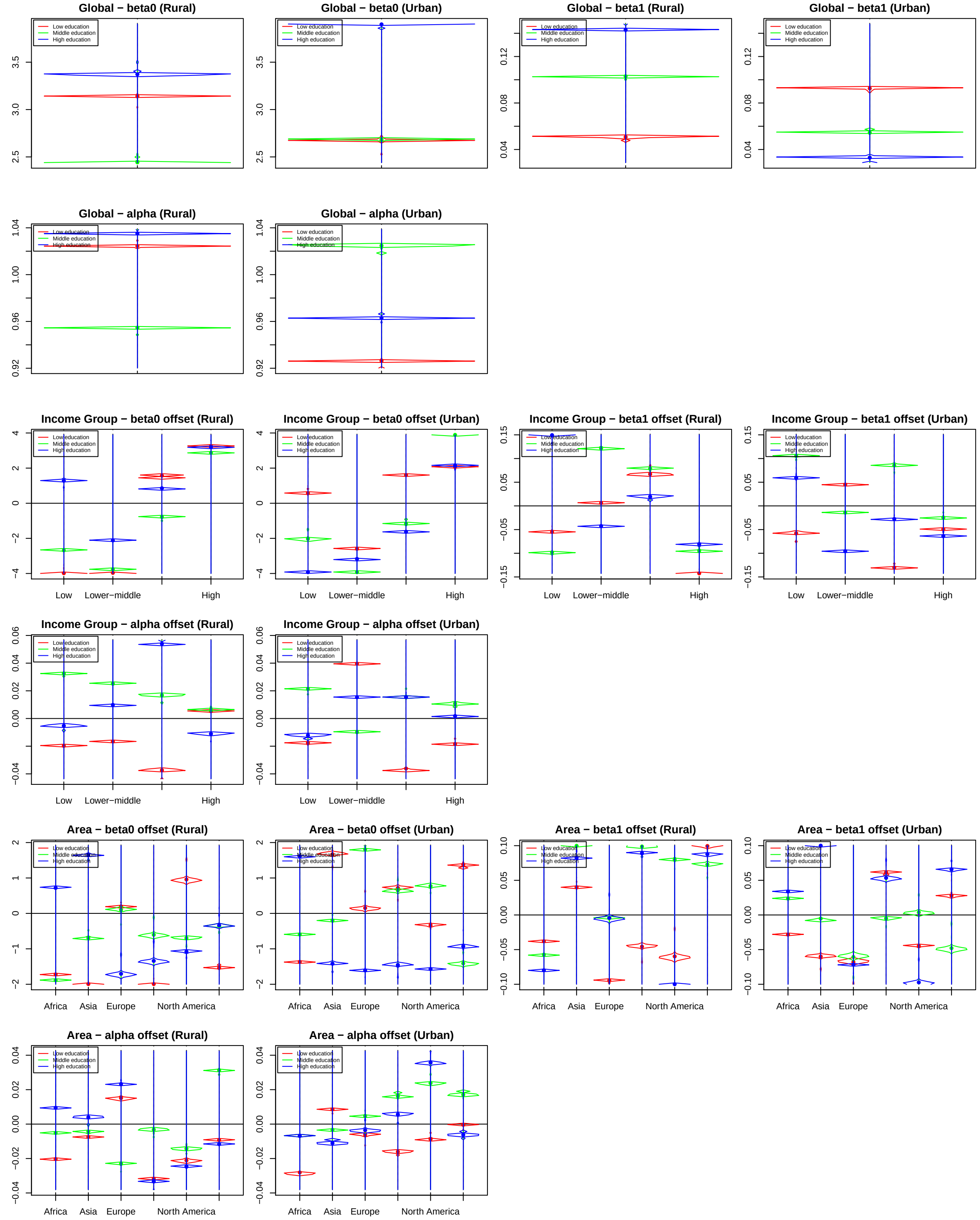

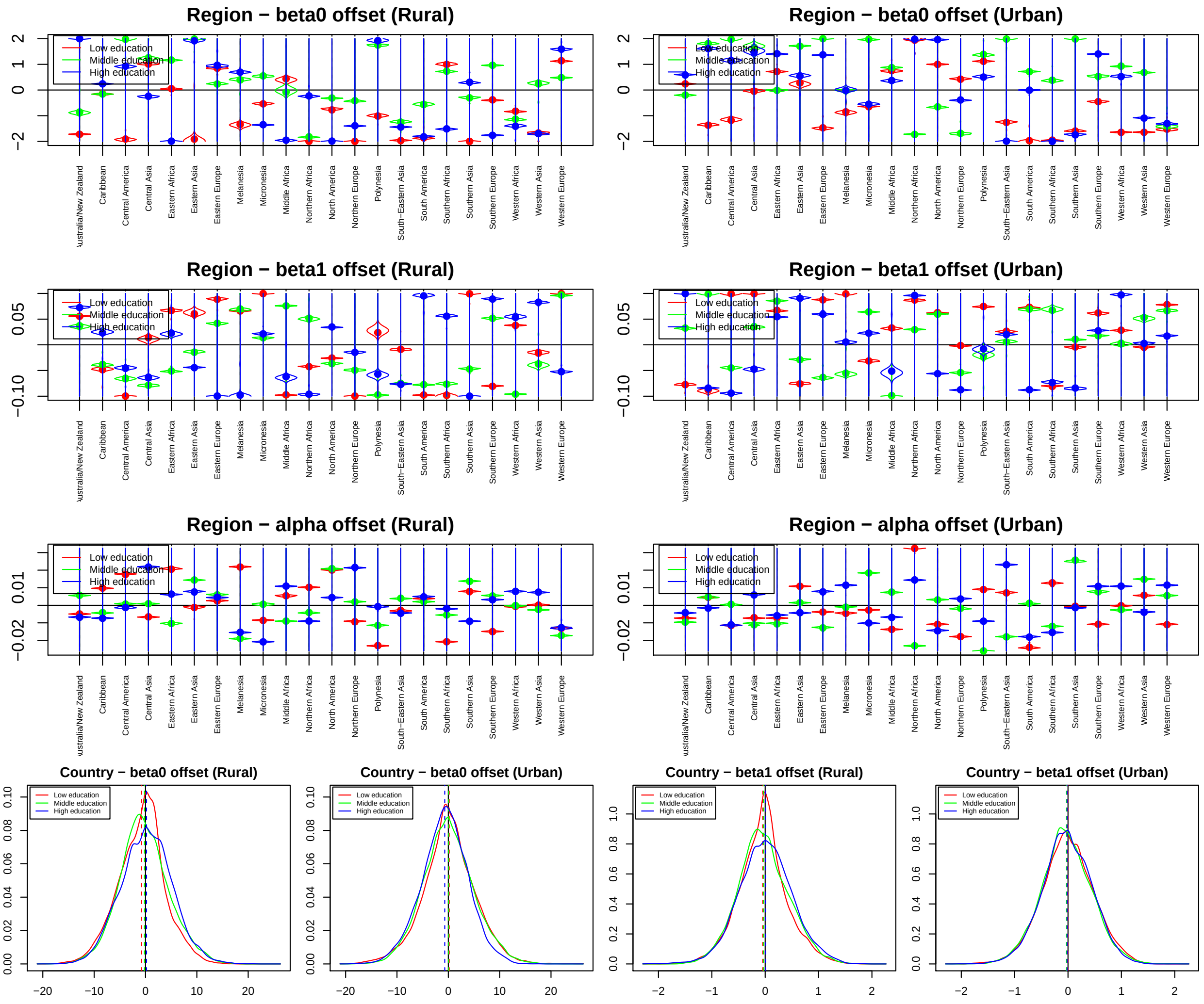

Parameters

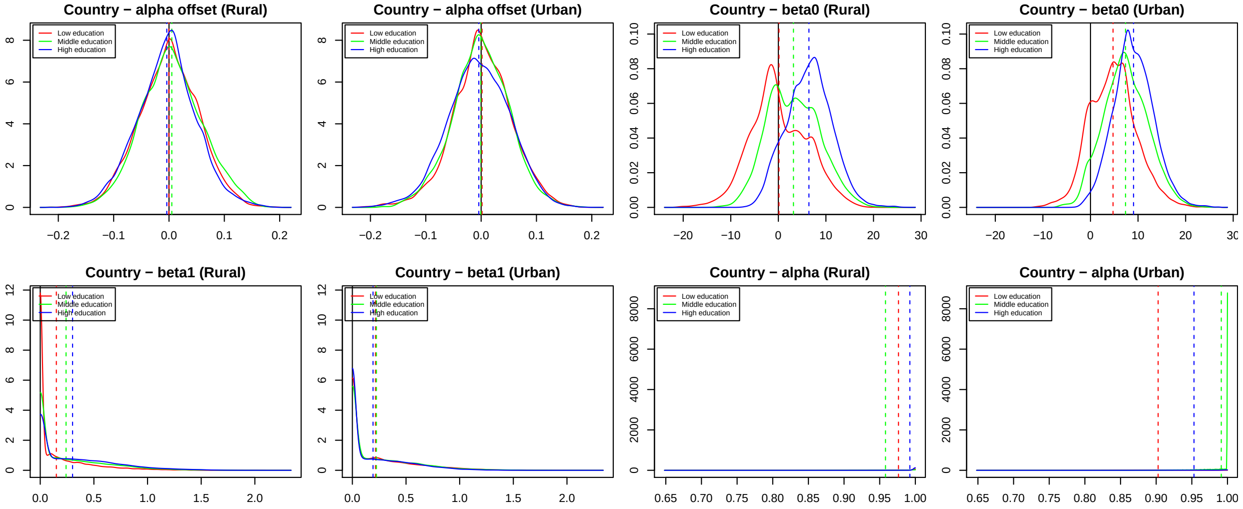

C.4.2.2 SBA|Home

Hyperparameters

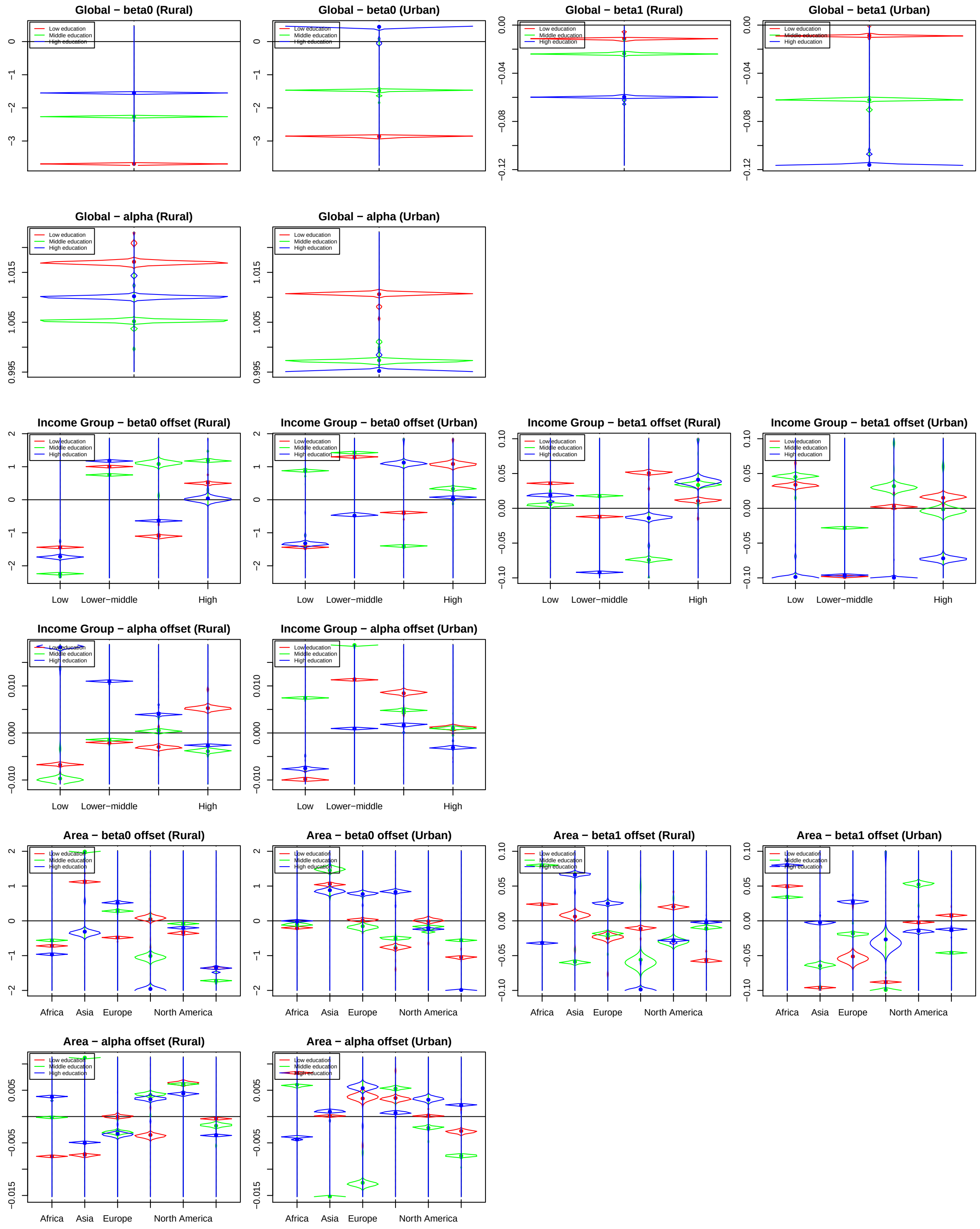

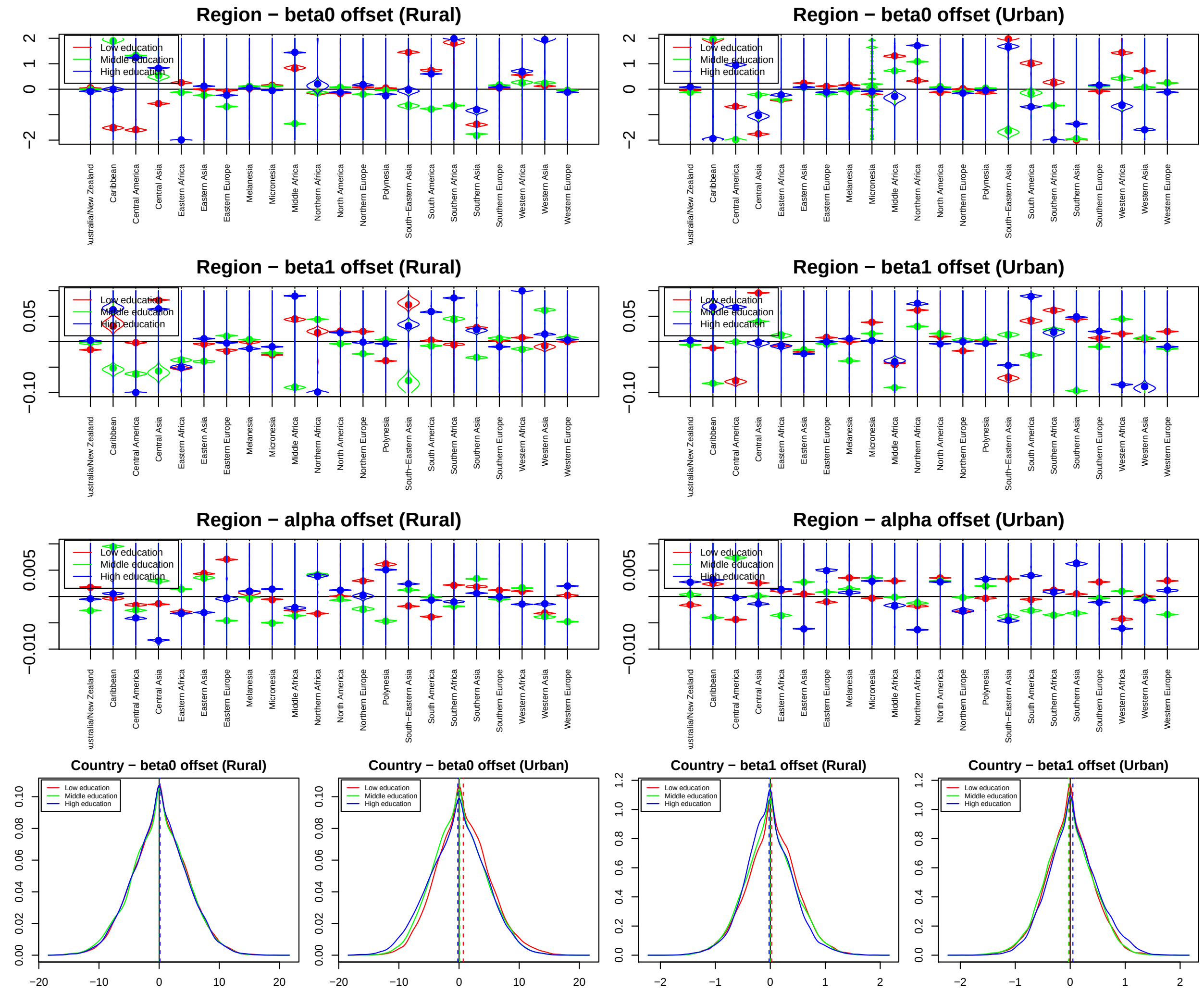

Parameters

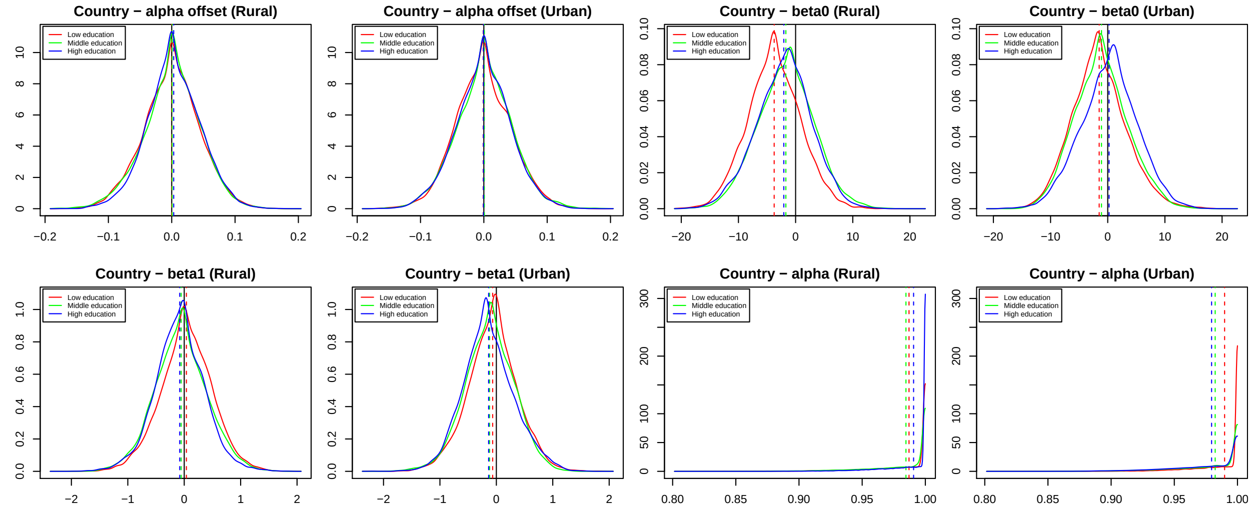

C.4.2.3 non-EmOC|Facility

Hyperparameters

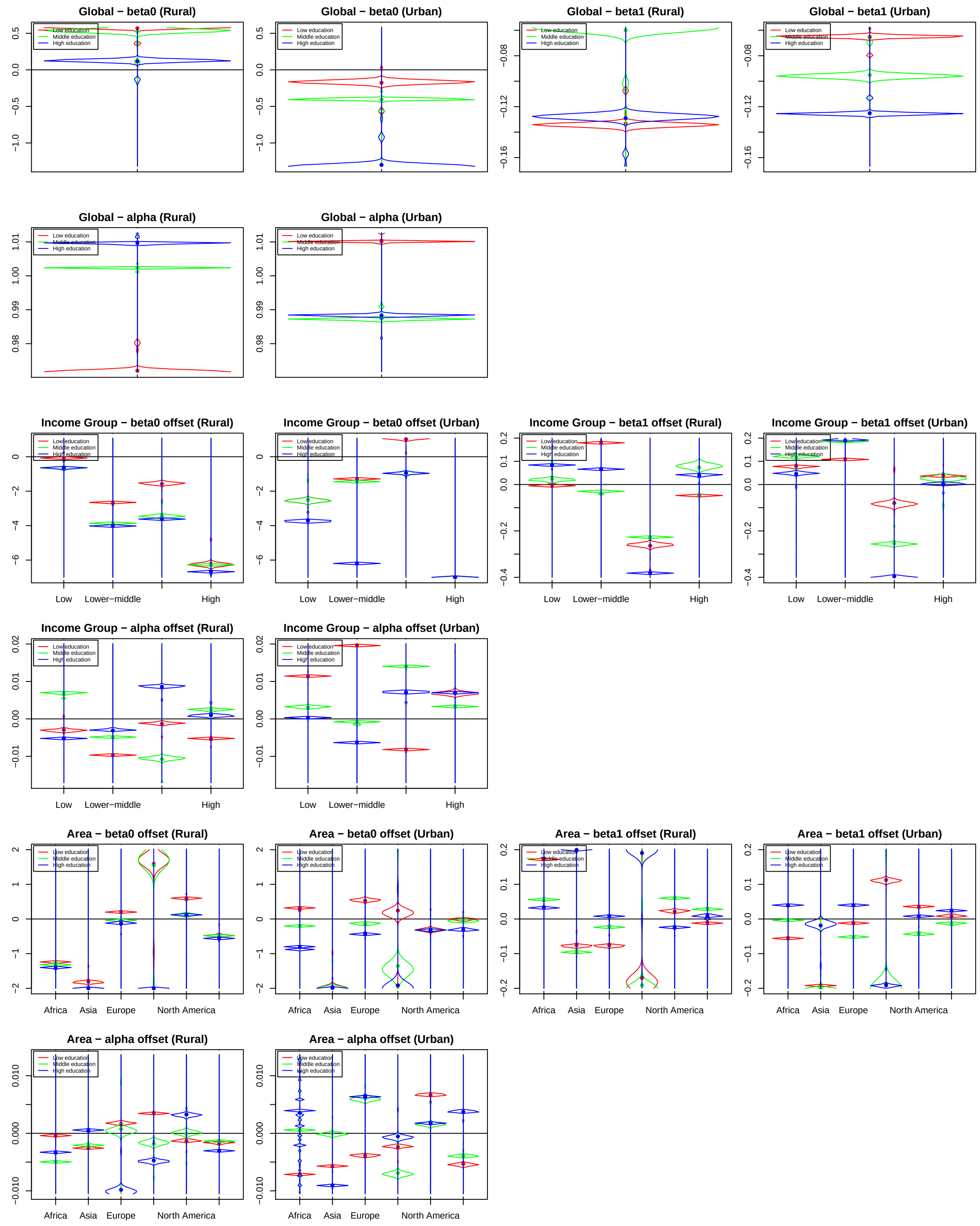

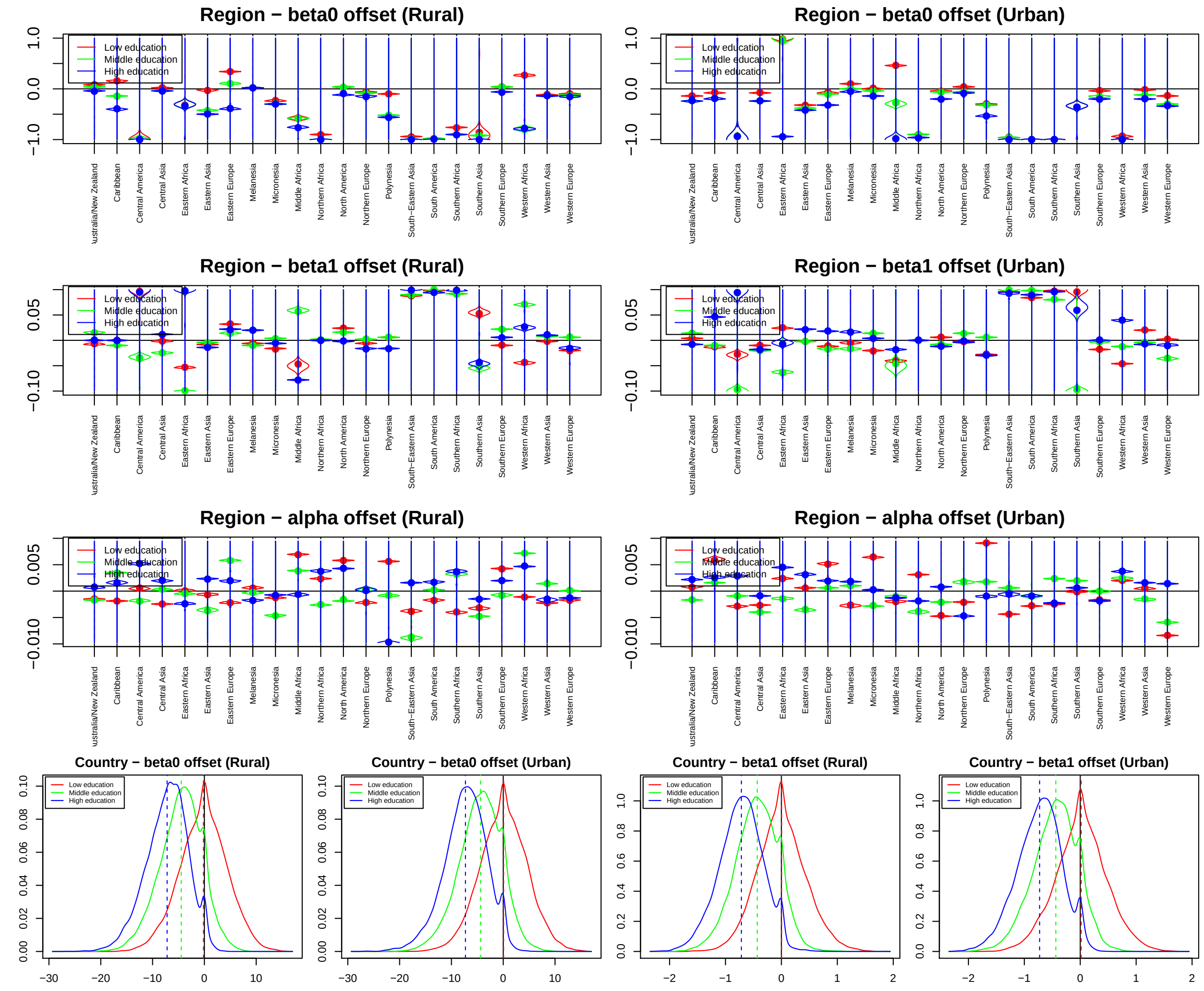

Parameters

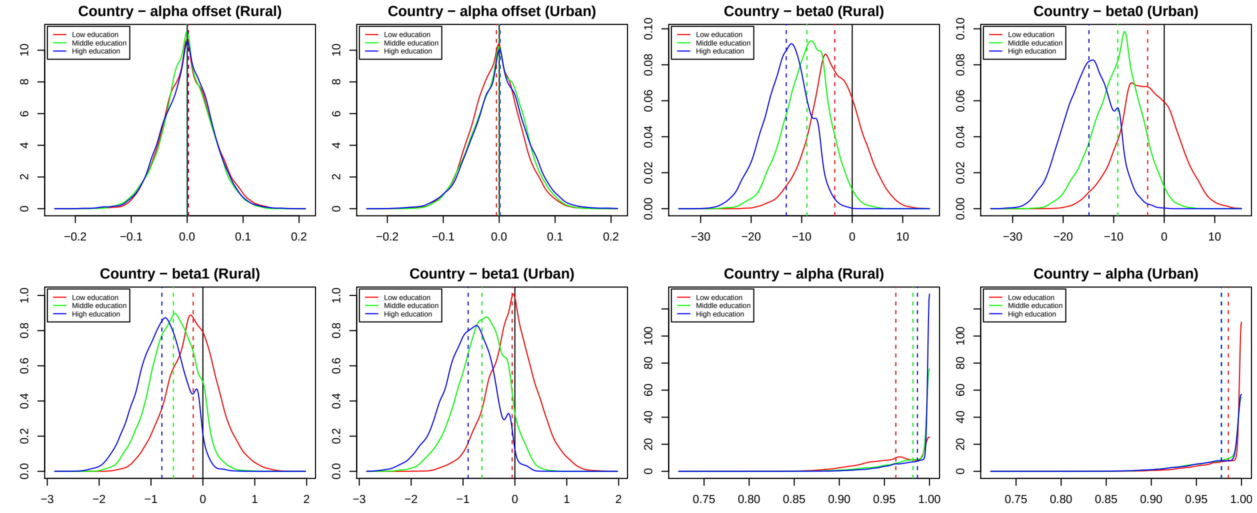

C.4.2.4 BEmOC|Facility

Hyperparameters

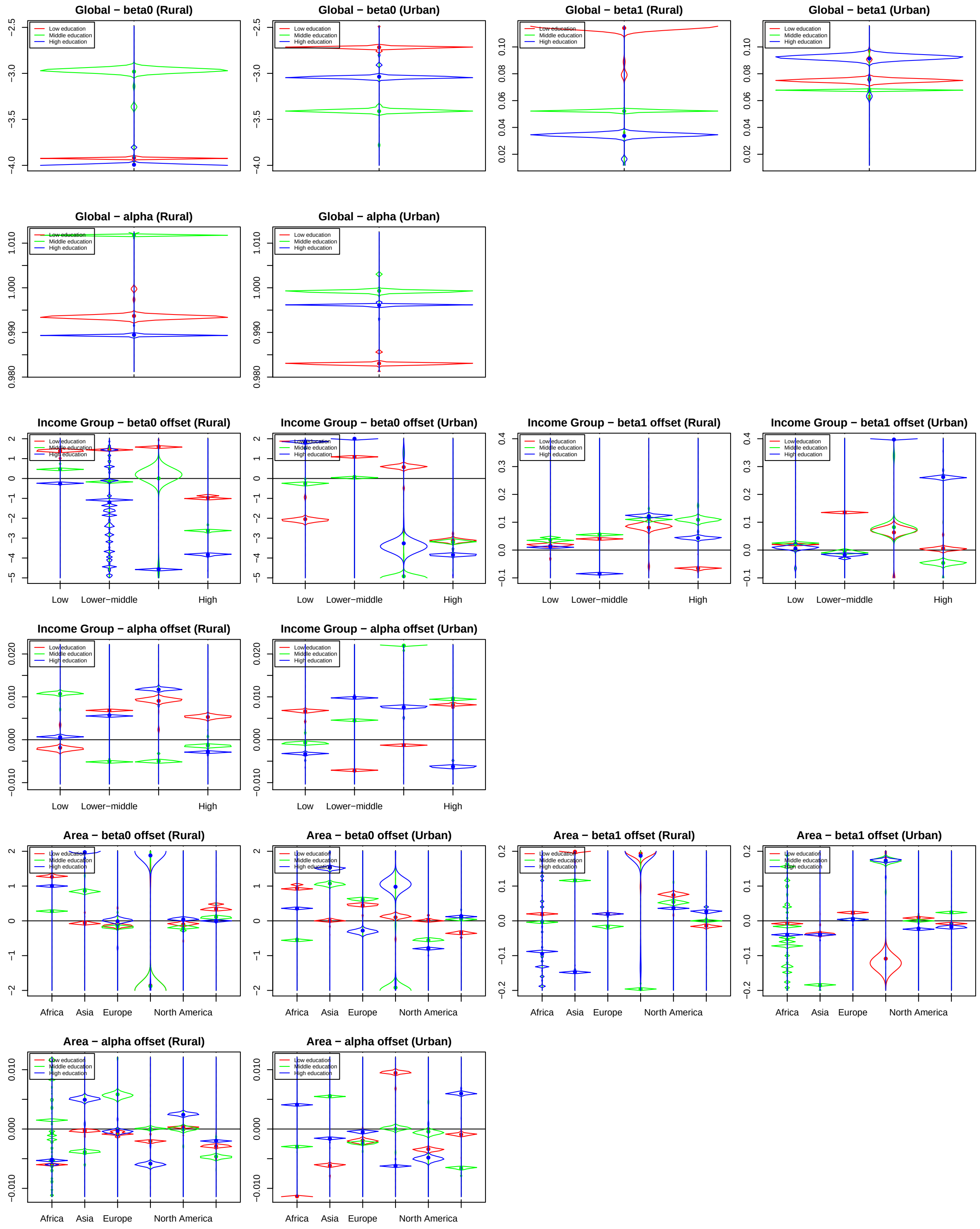

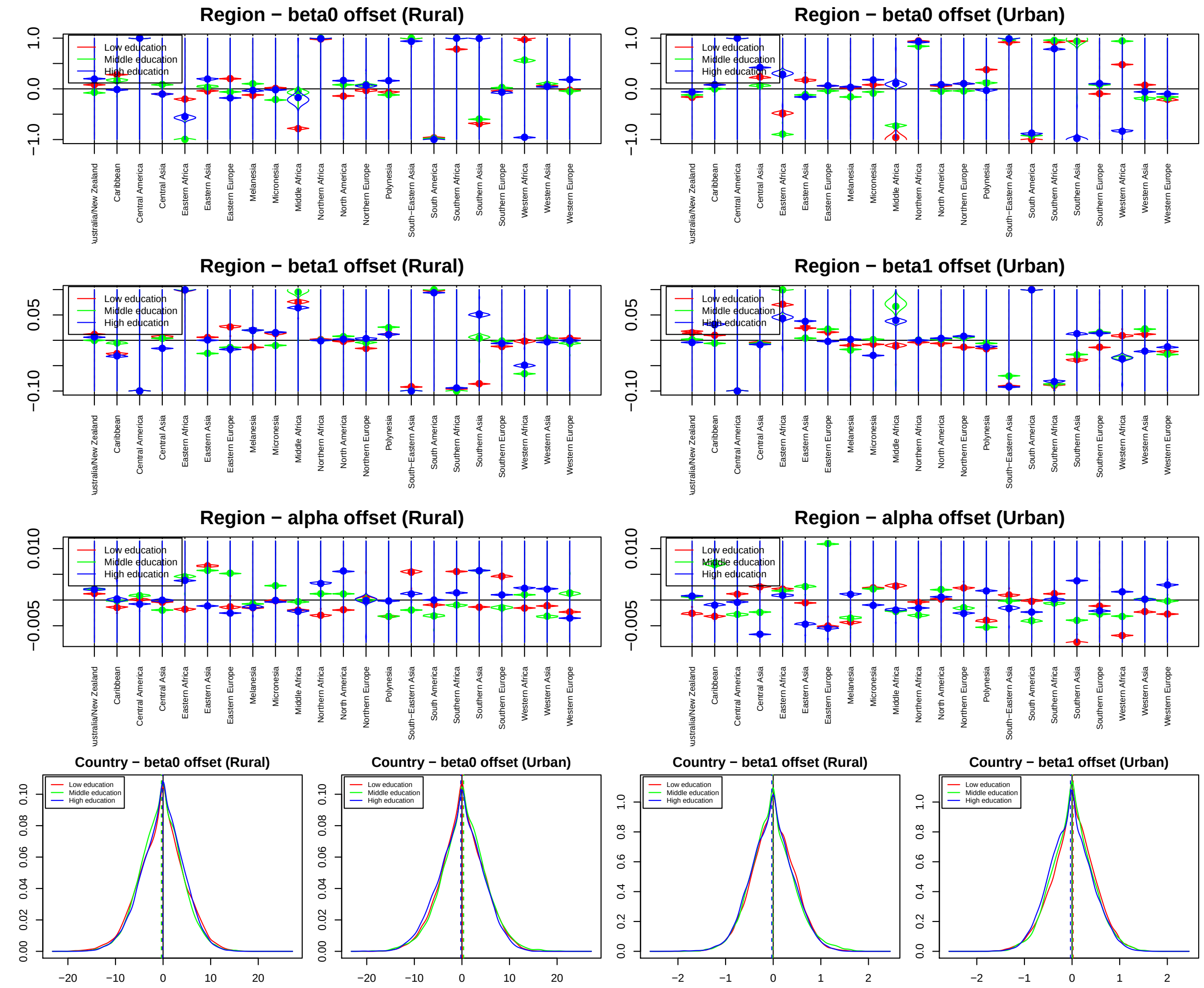

Parameters

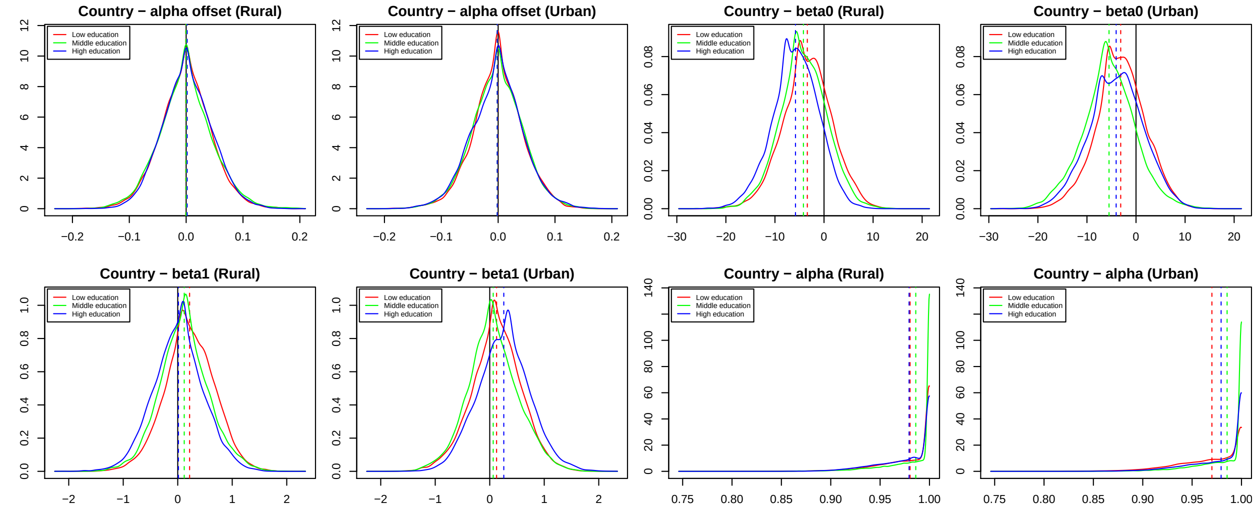

C.4.2.5 CEmOC|Facility

Hyperparameters

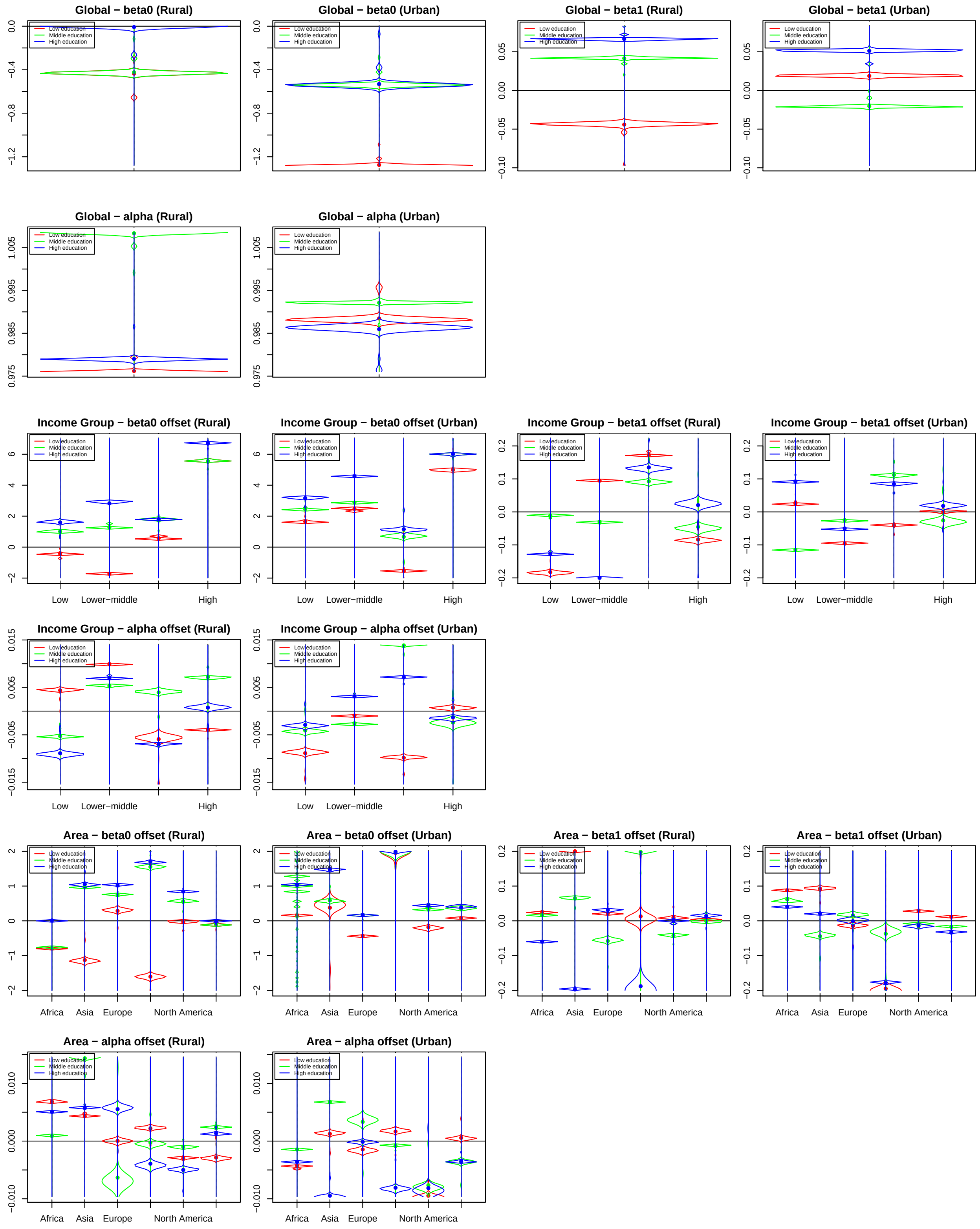

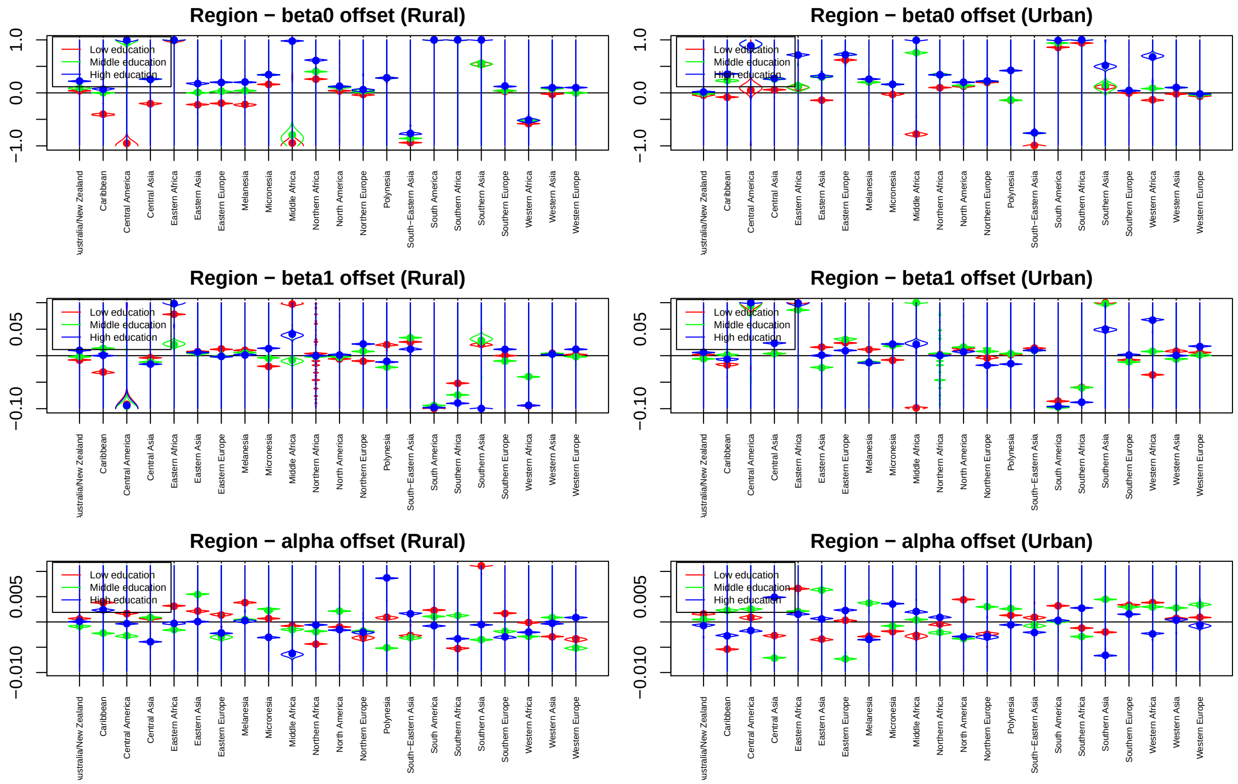
Region − beta0 offset (Rural)
Region − beta0 offset (Urban)
Region − beta1 offset (Rural)
Region − beta1 offset (Urban)
Region − alpha offset (Rural)
Region − alpha offset (Urban)
Low education
Middle education
High education
Caribbean
Central America
Central Asia
Eastern Africa
Eastern Asia
Eastern Europe
Melanesia
Micronesia
Middle Africa
Northern Africa
North America
Northern Europe
Polynesia
South−Eastern Asia
South America
Southern Africa
Southern Asia
Southern Europe
Western Africa
Western Asia
Western Europe

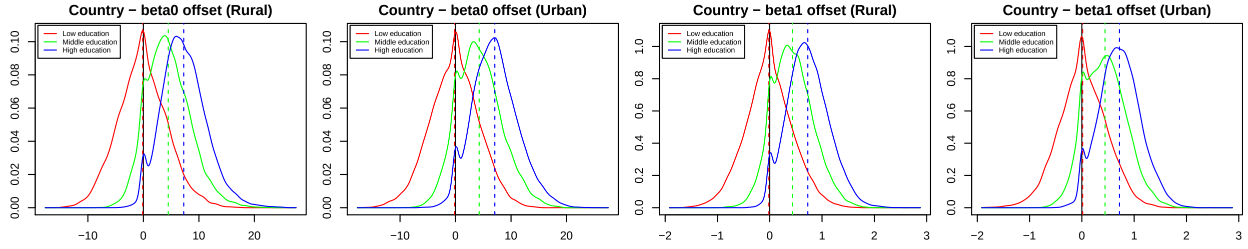
Country − beta0 offset (Rural)
Country − beta0 offset (Urban)
Country − beta1 offset (Rural)
Country − beta1 offset (Urban)
Low education
Middle education
High education

Parameters

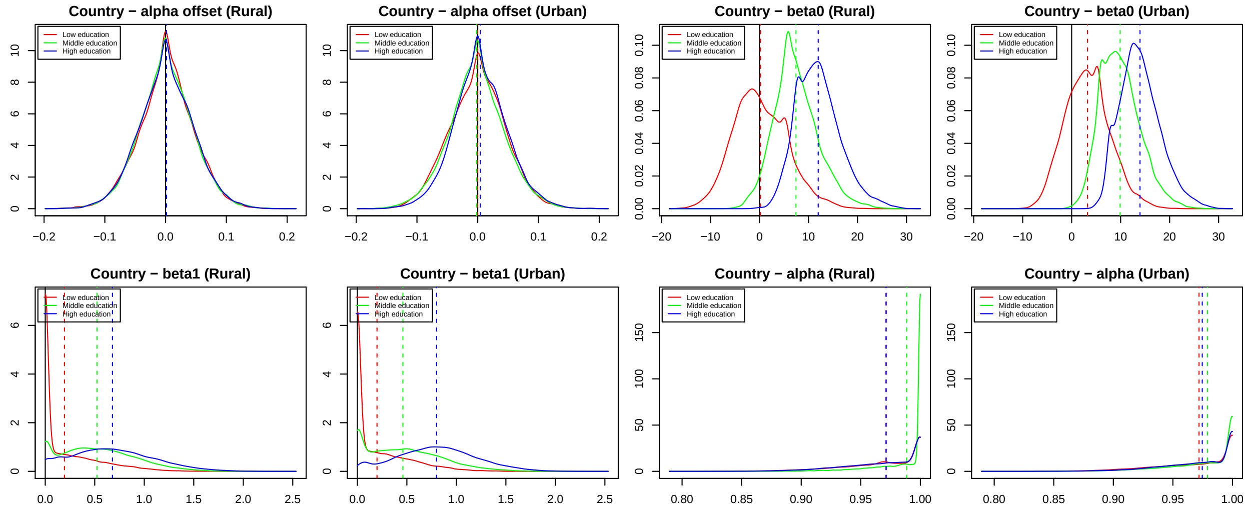
Country − alpha offset (Rural)
Country − alpha offset (Urban)
Country − beta0 (Rural)
Country − beta0 (Urban)
Country − beta1 (Rural)
Country − beta1 (Urban)
Country − alpha (Rural)
Country − alpha (Urban)
Low education
Middle education
High education

C.4.3 Recognition/Referral

C.4.3.1 Home

C.4.3.1.1 Pre-Eclampsia/Eclampsia

Hyperparameters

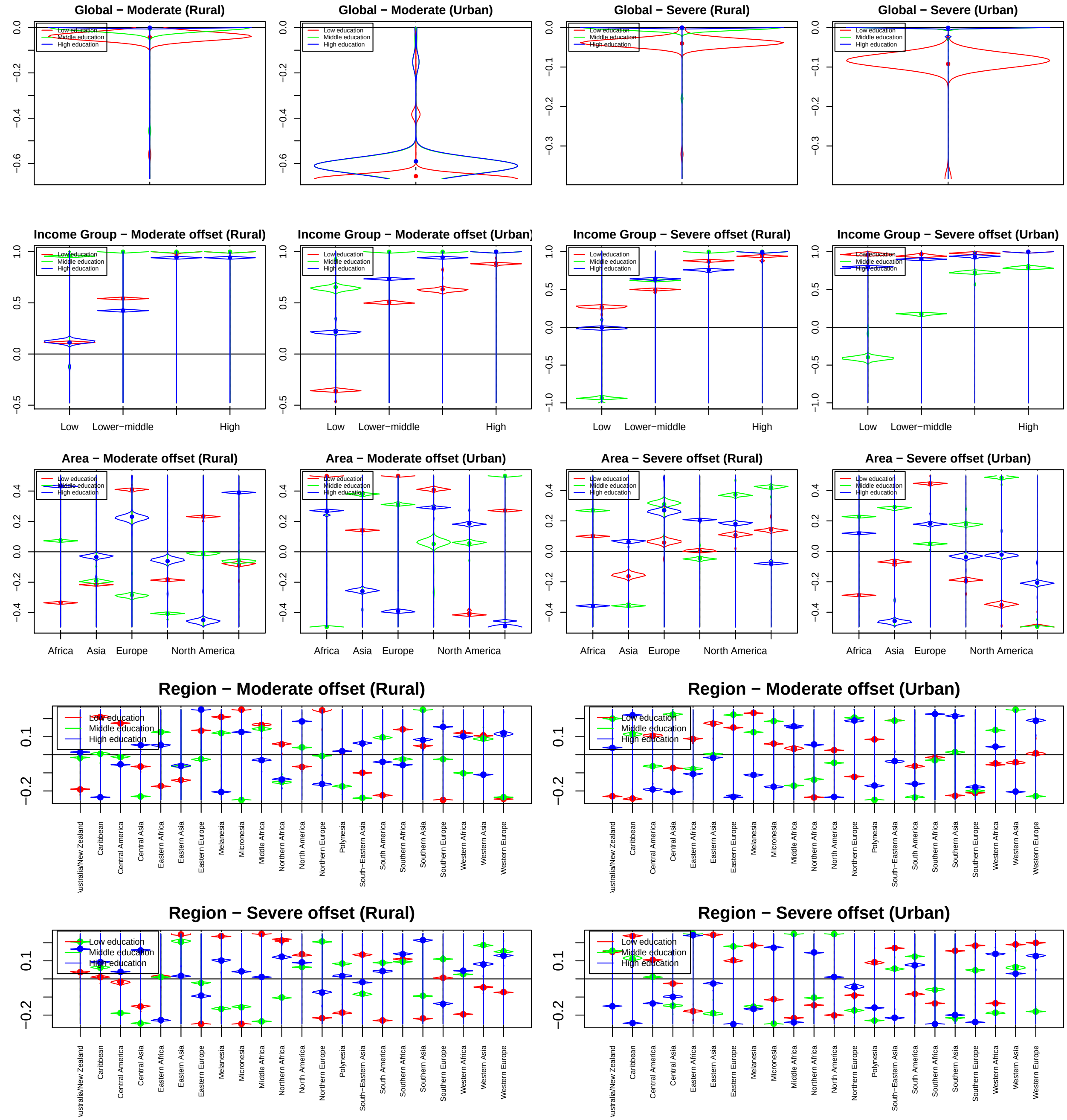

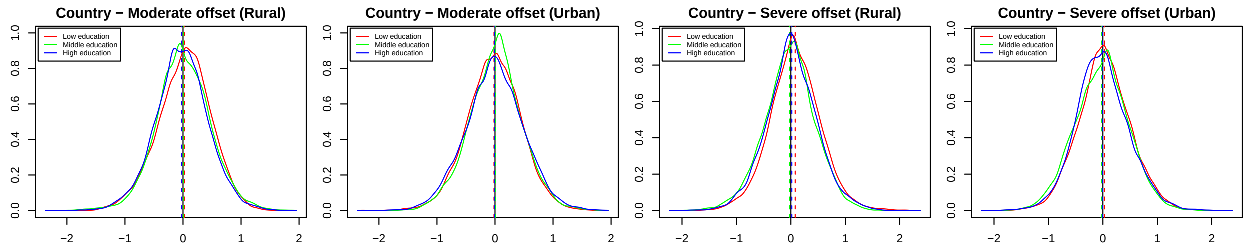

Parameters

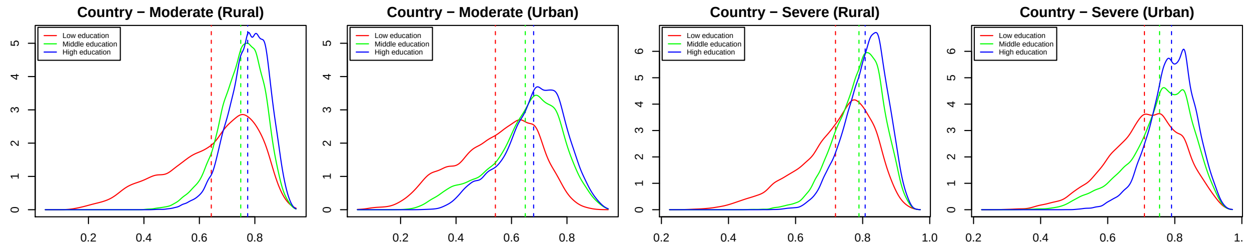

C.4.3.1.2 Obstructed Labour

Hyperparameters

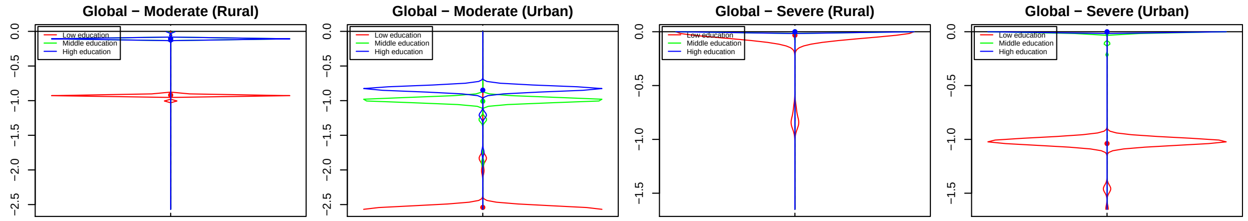

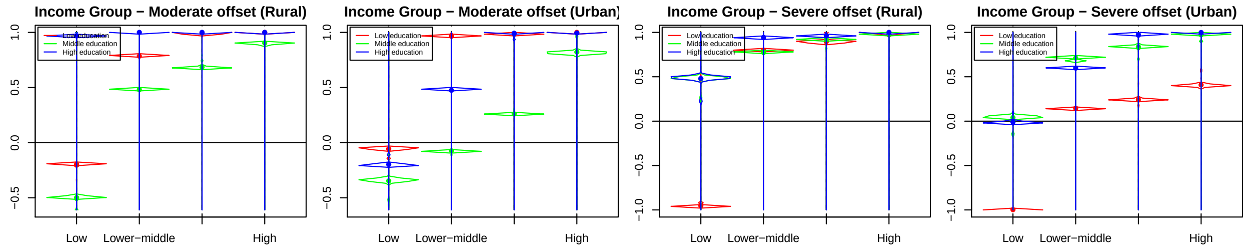

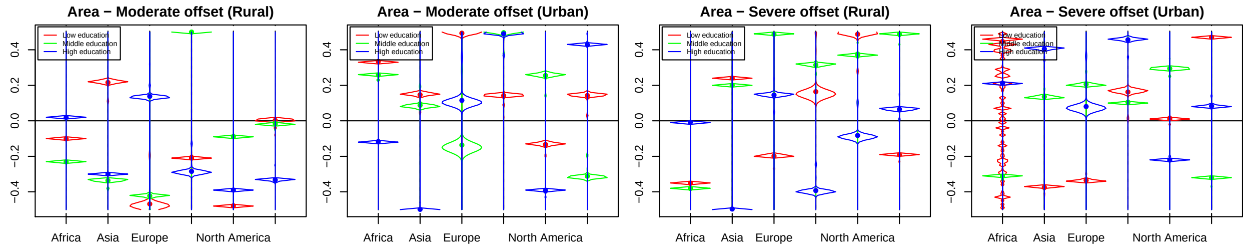

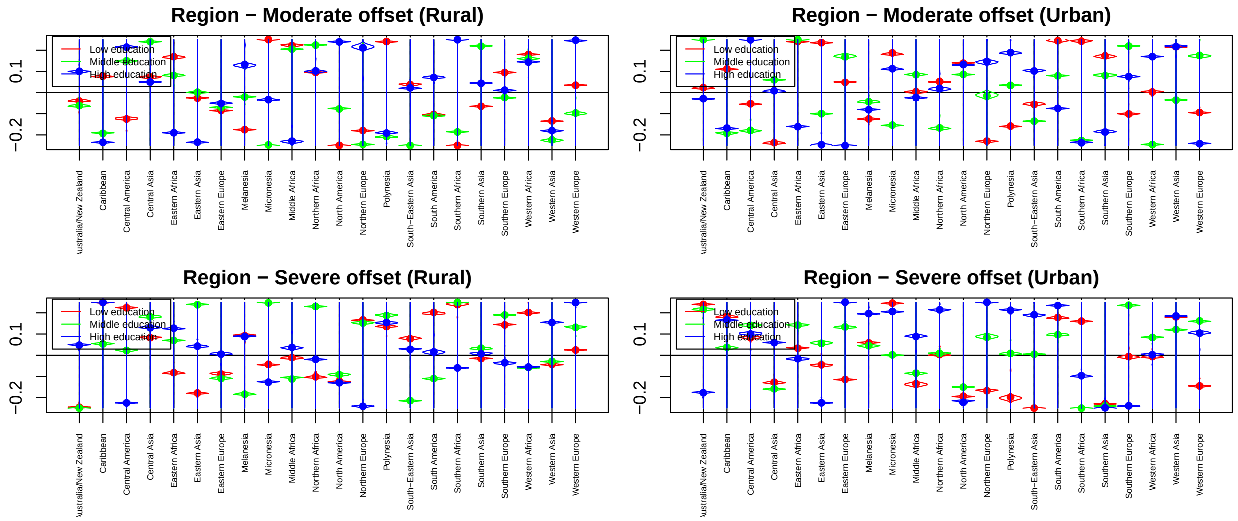

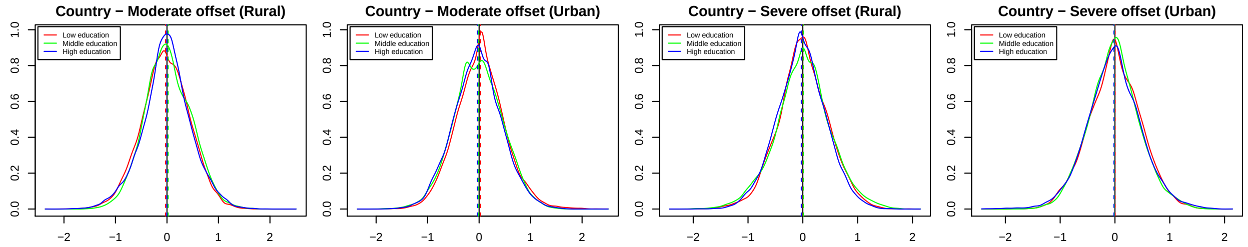

Parameters

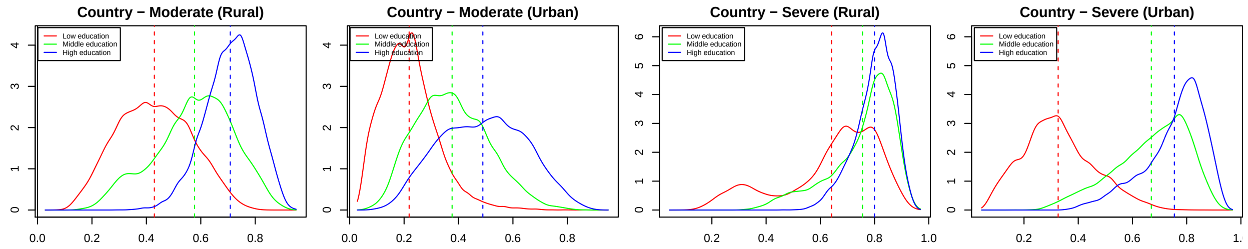

C.4.3.1.3 Postpartum Hemmorhage

Hyperparameters

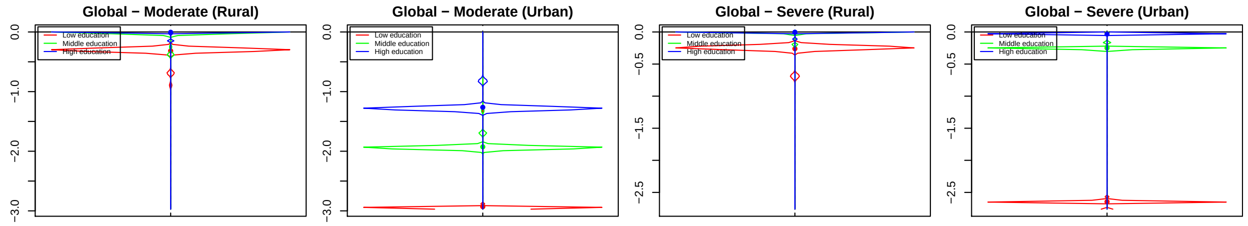

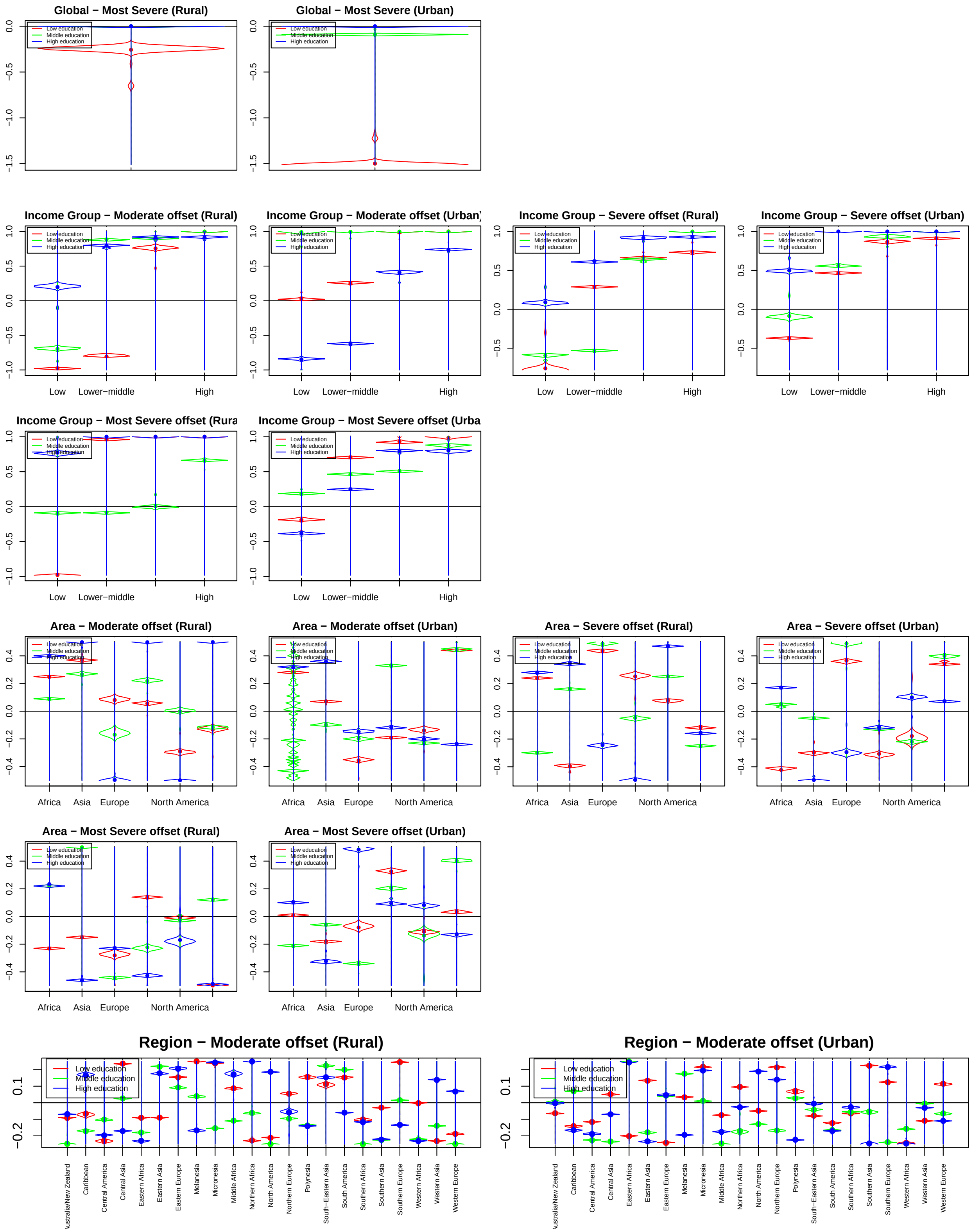
Global − Most Severe (Rural)
Global − Most Severe (Urban)
Low education
Middle education
High education
Income Group − Moderate offset (Rural)
Income Group − Moderate offset (Urban)
Income Group − Severe offset (Rural)
Income Group − Severe offset (Urban)
Income Group − Most Severe offset (Rura
Income Group − Most Severe offset (Urba
Low
Lower−middle
High
Area − Moderate offset (Rural)
Area − Moderate offset (Urban)
Area − Severe offset (Rural)
Area − Severe offset (Urban)
Area − Most Severe offset (Rural)
Area − Most Severe offset (Urban)
Africa
Asia
Europe
North America
Region − Moderate offset (Rural)
Region − Moderate offset (Urban)
Caribbean
Central America
Central Asia
Eastern Africa
Eastern Asia
Eastern Europe
Melanesia
Micronesia
Middle Africa
Northern Africa
North America
Northern Europe
Polynesia
South−Eastern Asia
South America
Southern Africa
Southern Asia
Southern Europe
Western Africa
Western Asia
Western Europe

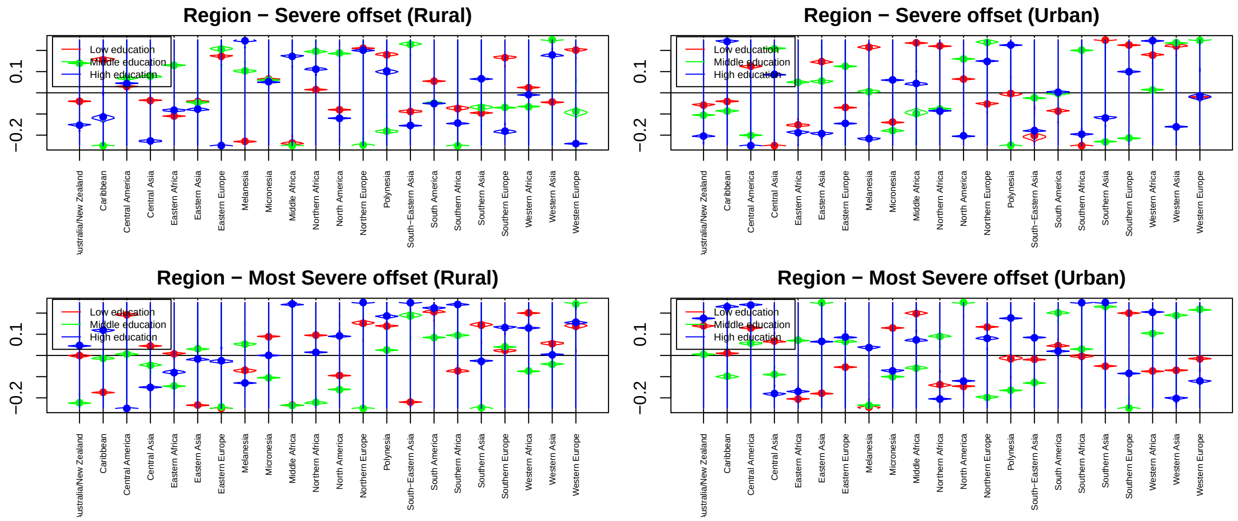

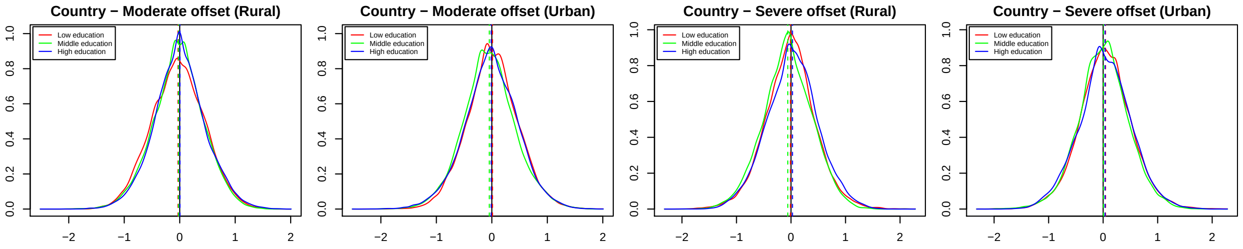

Parameters

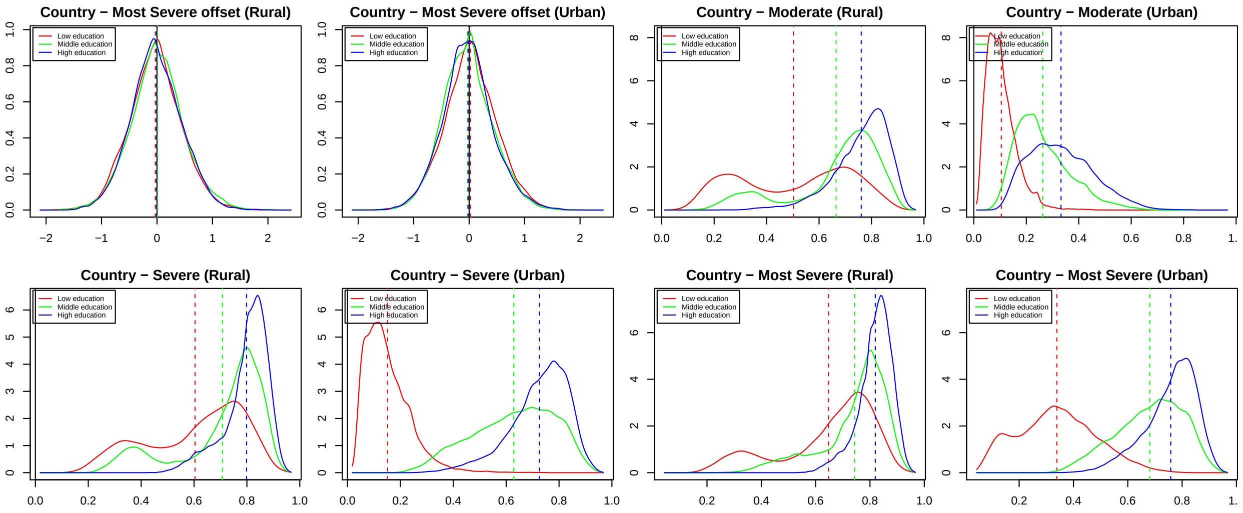

C.4.3.1.4 Sepsis

Hyperparameters

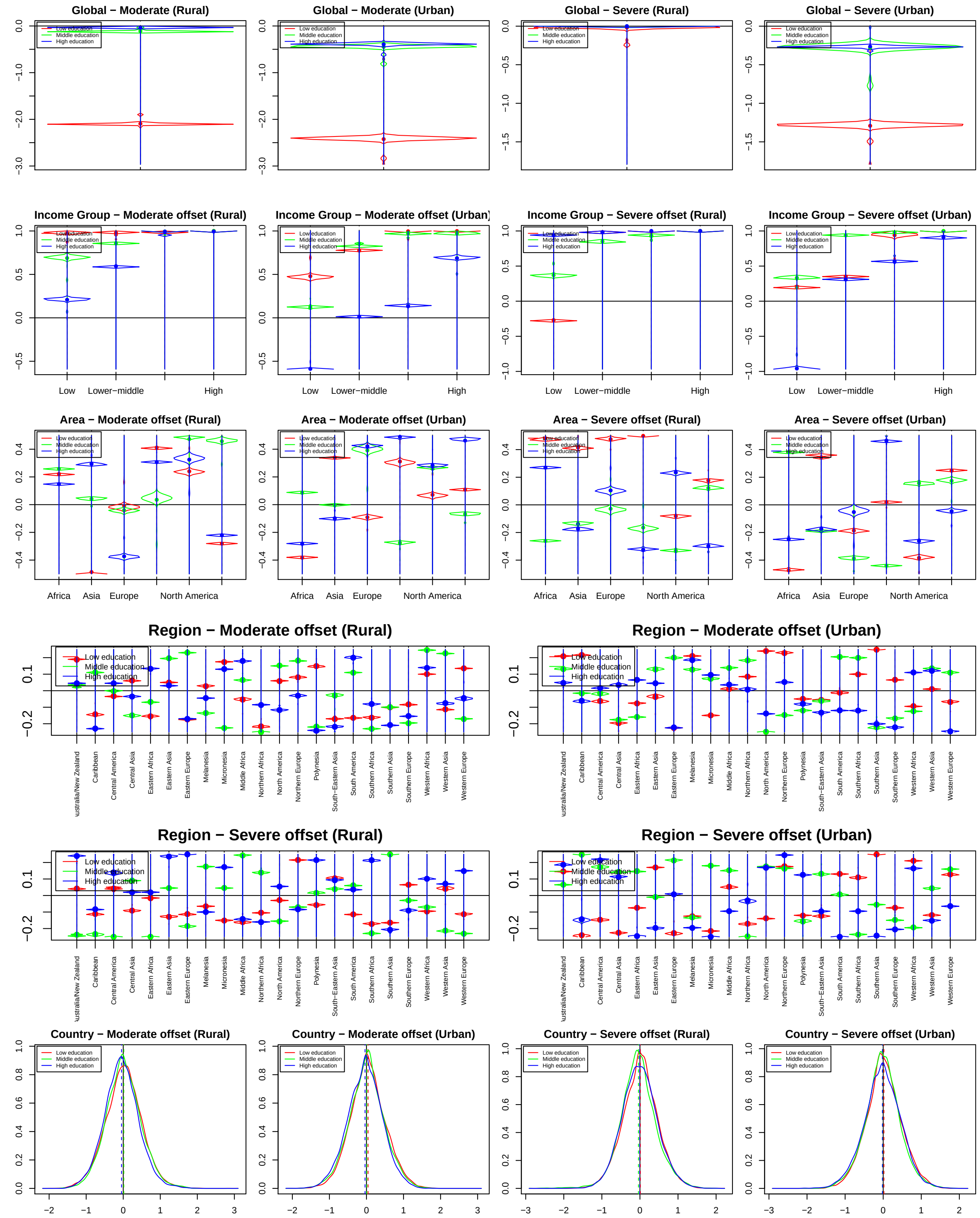
Global − Moderate (Rural)
Global − Moderate (Urban)
Global − Severe (Rural)
Global − Severe (Urban)
Income Group − Moderate offset (Rural)
Income Group − Moderate offset (Urban)
Income Group − Severe offset (Rural)
Income Group − Severe offset (Urban)
Low
Lower−middle
High
Area − Moderate offset (Rural)
Area − Moderate offset (Urban)
Area − Severe offset (Rural)
Area − Severe offset (Urban)
Africa
Asia
Europe
North America
Region − Moderate offset (Rural)
Region − Moderate offset (Urban)
Region − Severe offset (Rural)
Region − Severe offset (Urban)
ustralia/New Zealand
Caribbean
Central America
Central Asia
Eastern Africa
Eastern Asia
Eastern Europe
Melanesia
Micronesia
Middle Africa
Northern Africa
North America
Northern Europe
Polynesia
South−Eastern Asia
South America
Southern Africa
Southern Asia
Southern Europe
Western Africa
Western Asia
Western Europe
Country − Moderate offset (Rural)
Country − Moderate offset (Urban)
Country − Severe offset (Rural)
Country − Severe offset (Urban)
Low education
Middle education
High education

Parameters

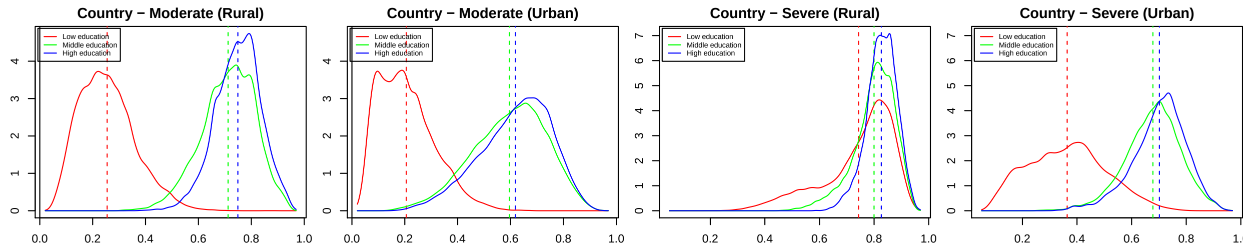

C.4.3.1.5 Other Hyperparameters

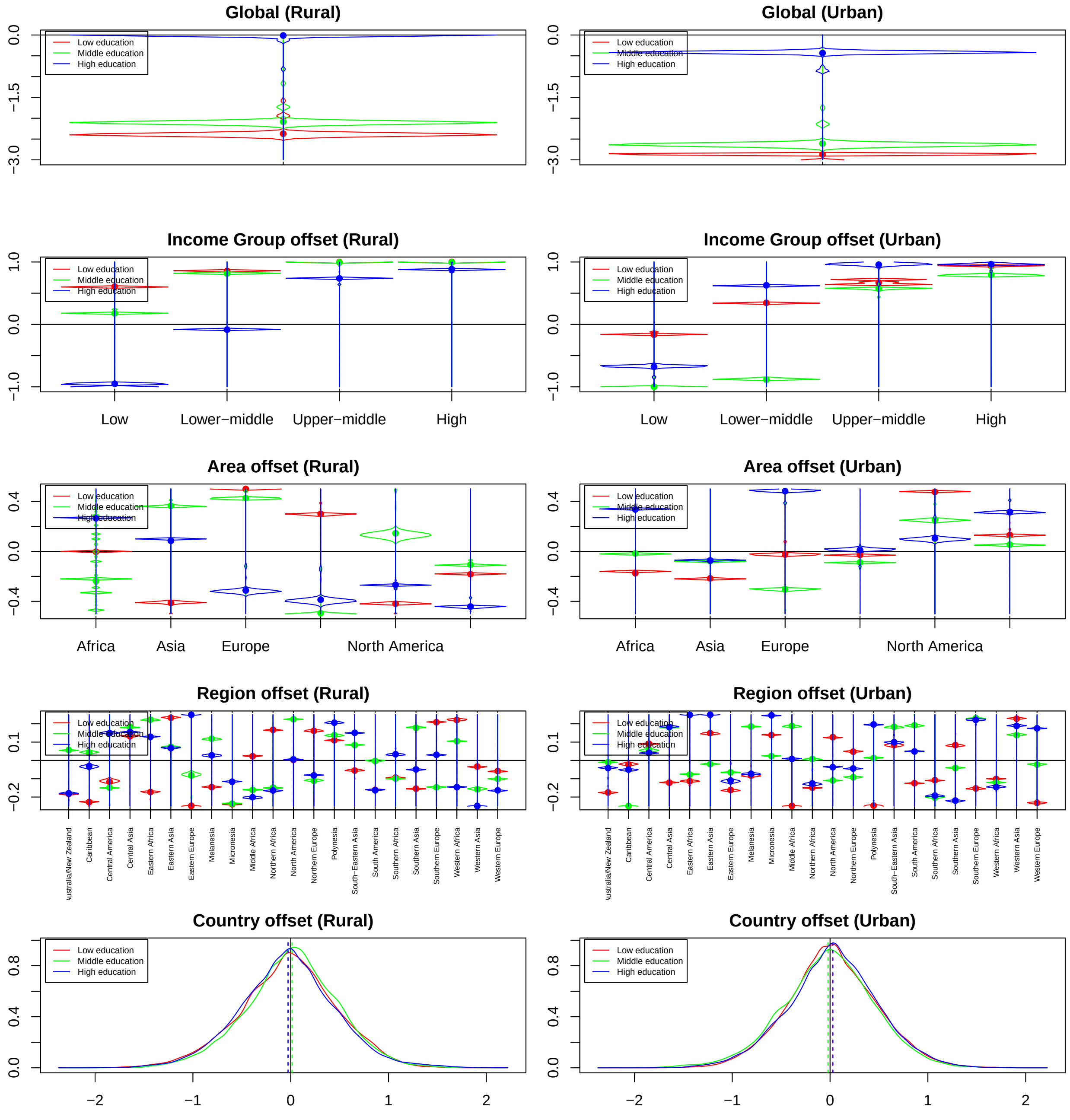

Parameters

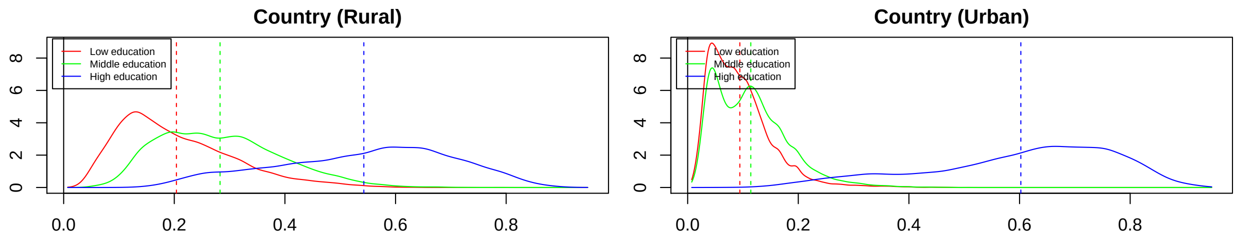

C.4.3.2 Home-SBA

C.4.3.2.1 Pre-Eclampsia/Eclampsia

Hyperparameters

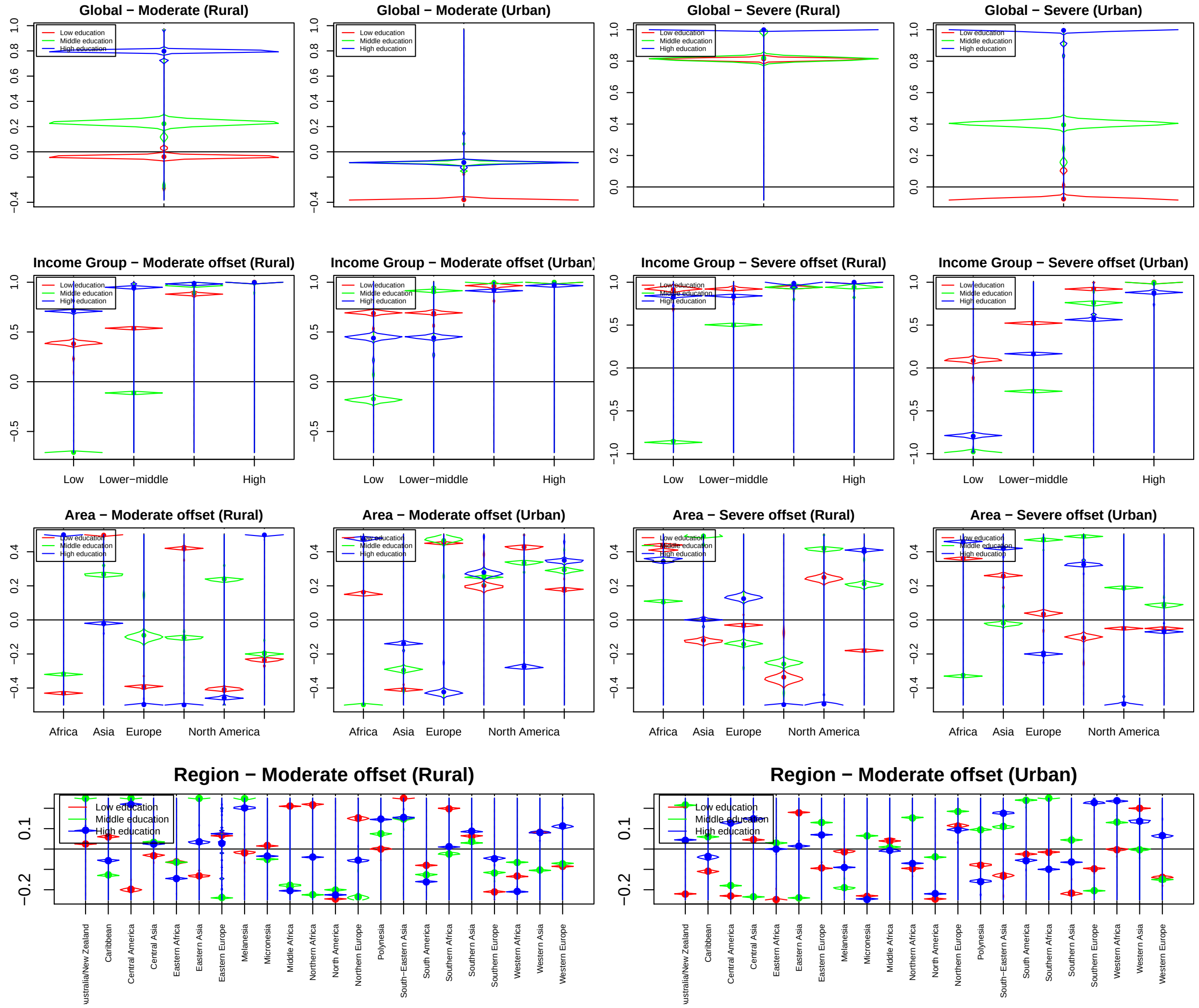

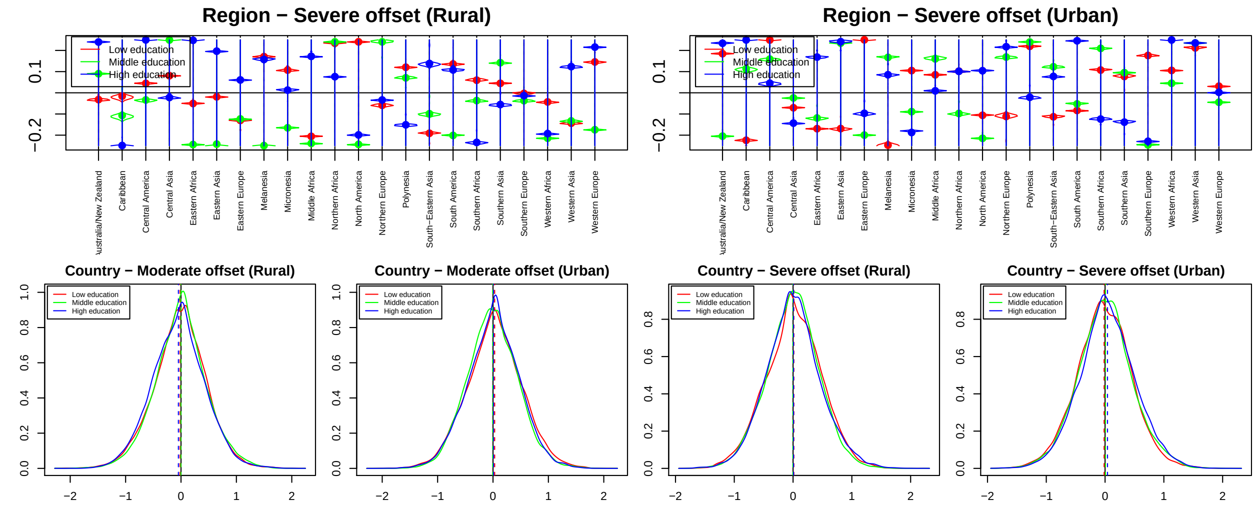

Parameters

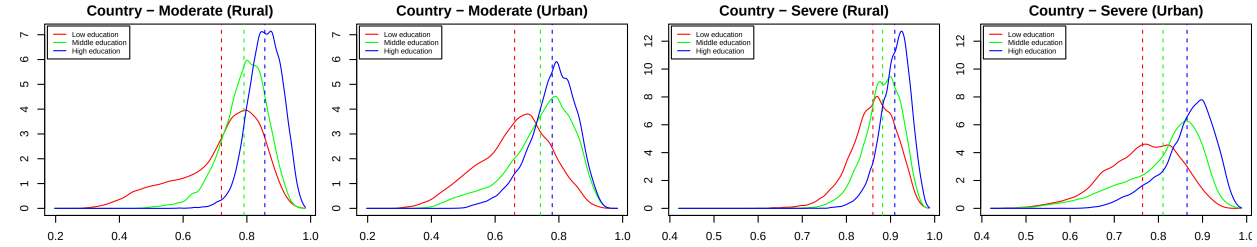

C.4.3.2.2 Obstructed Labour

Hyperparameters

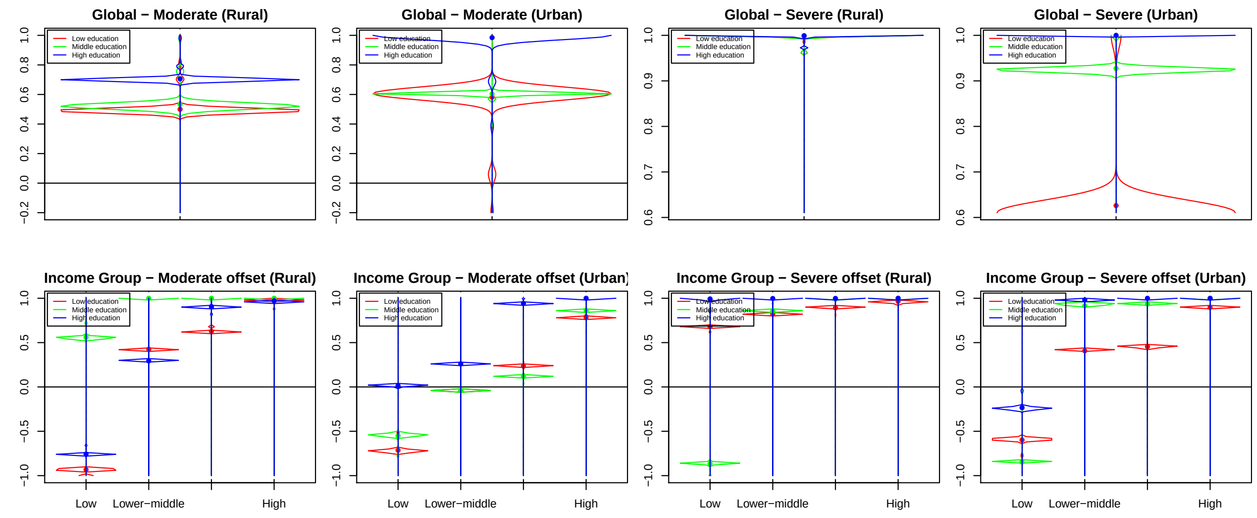

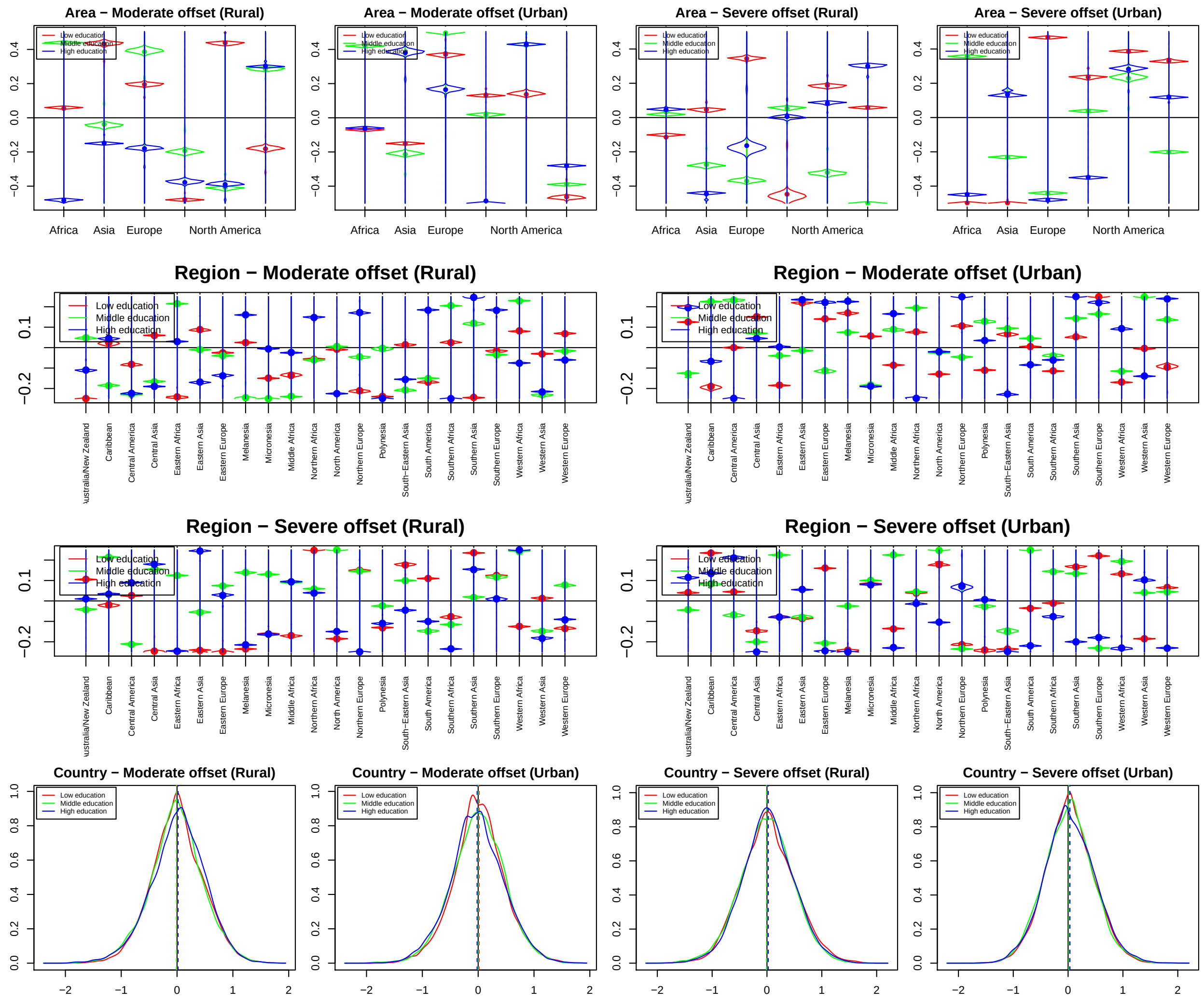

Parameters

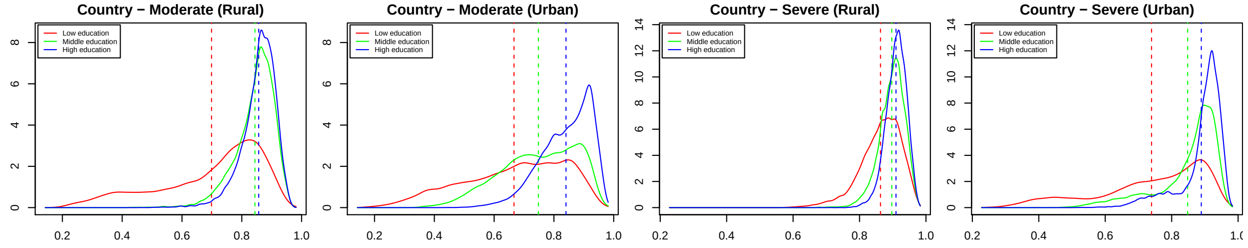

C.4.3.2.3 Postpartum Hemmorhage

Hyperparameters

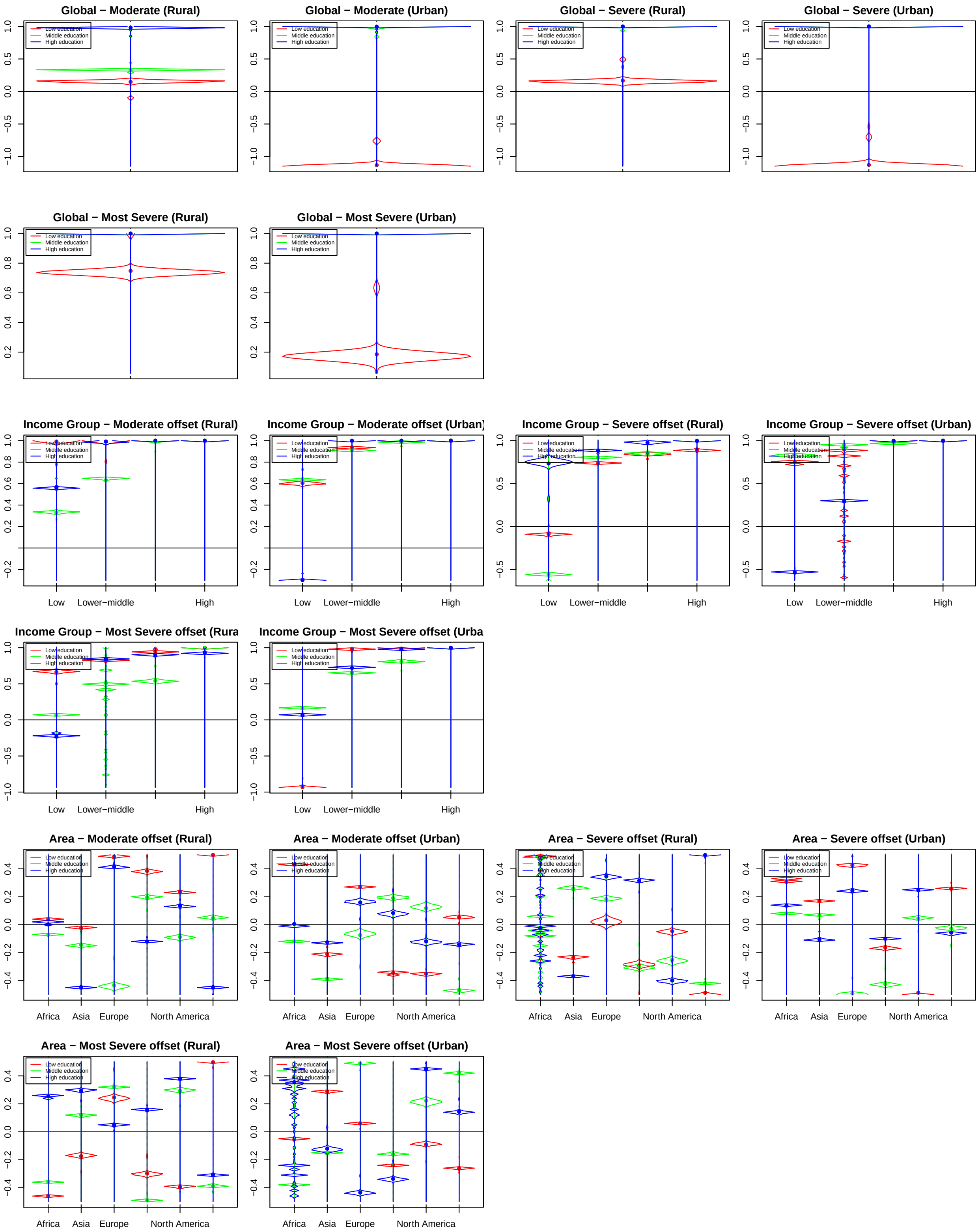
Global − Moderate (Rural)
Global − Moderate (Urban)
Global − Severe (Rural)
Global − Severe (Urban)
Global − Most Severe (Rural)
Global − Most Severe (Urban)
Income Group − Moderate offset (Rural)
Income Group − Moderate offset (Urban)
Income Group − Severe offset (Rural)
Income Group − Severe offset (Urban)
Income Group − Most Severe offset (Rura
Income Group − Most Severe offset (Urba
Area − Moderate offset (Rural)
Area − Moderate offset (Urban)
Area − Severe offset (Rural)
Area − Severe offset (Urban)
Area − Most Severe offset (Rural)
Area − Most Severe offset (Urban)
Low education
Middle education
High education
Low
Lower−middle
High
Africa
Asia
Europe
North America

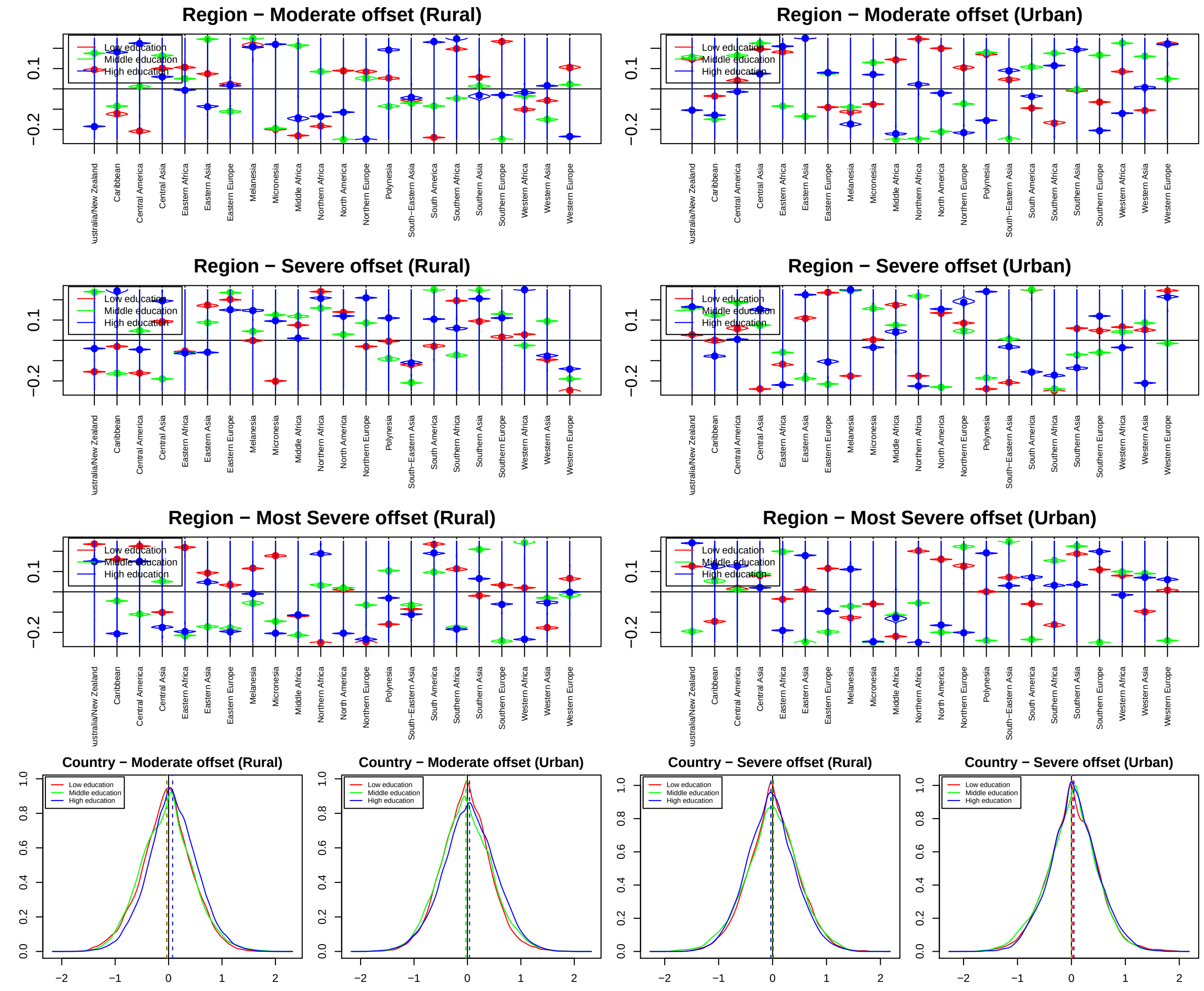

Parameters

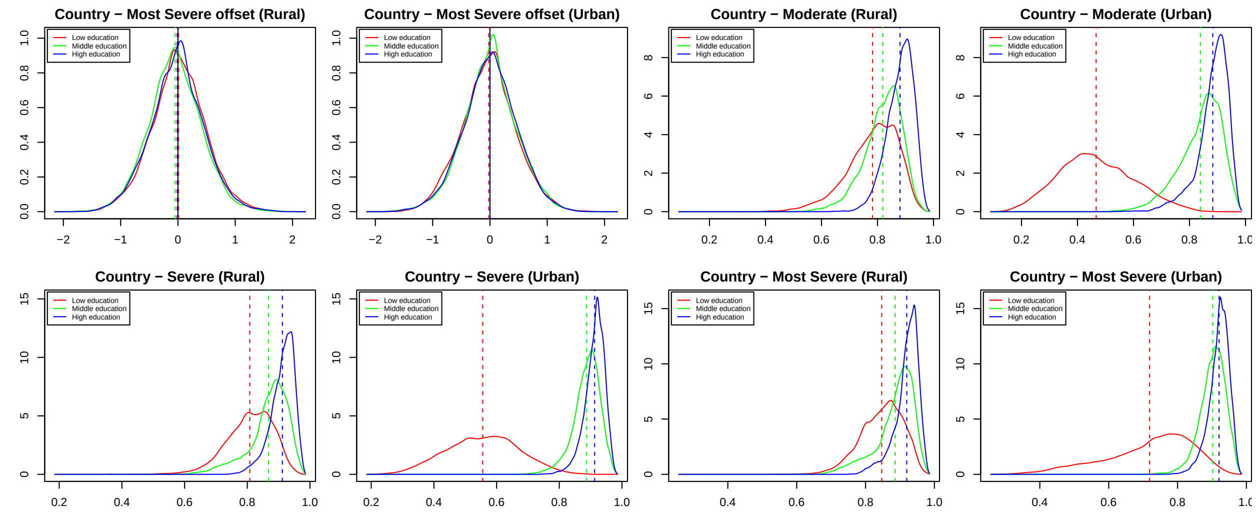

C.4.3.2.4 Sepsis

Hyperparameters

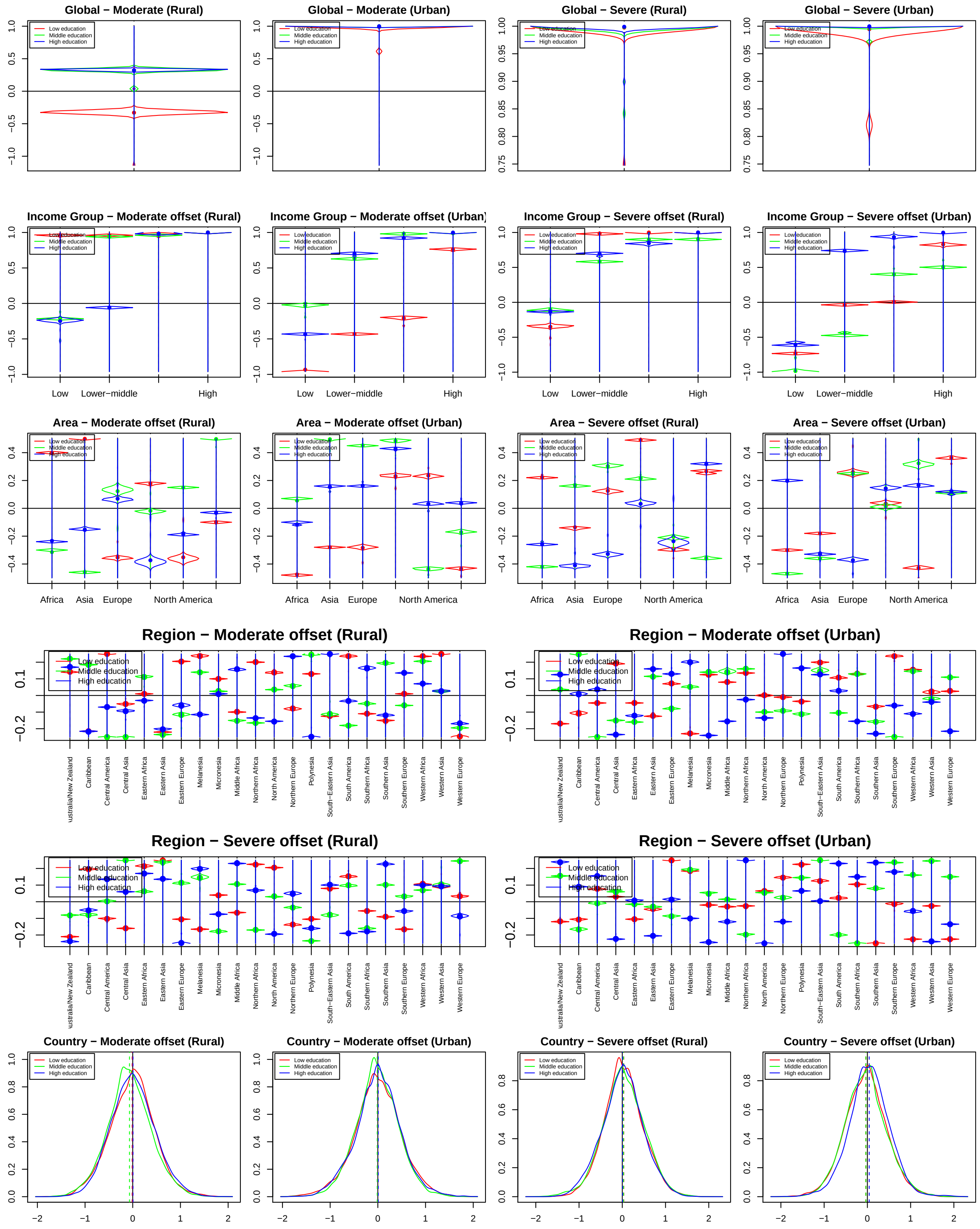

Parameters

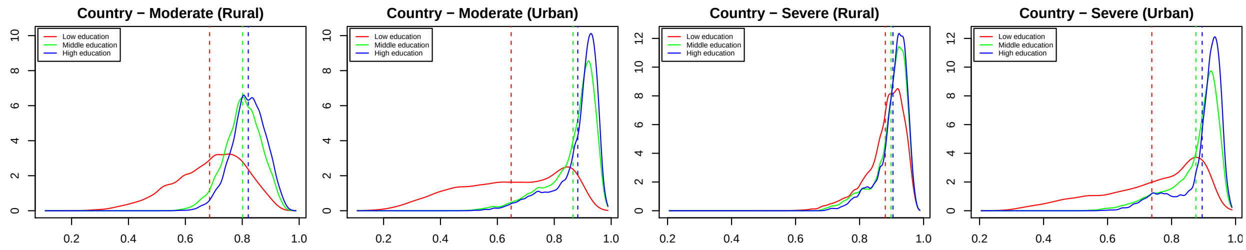

C.4.3.2.5 Other Hyperparameters

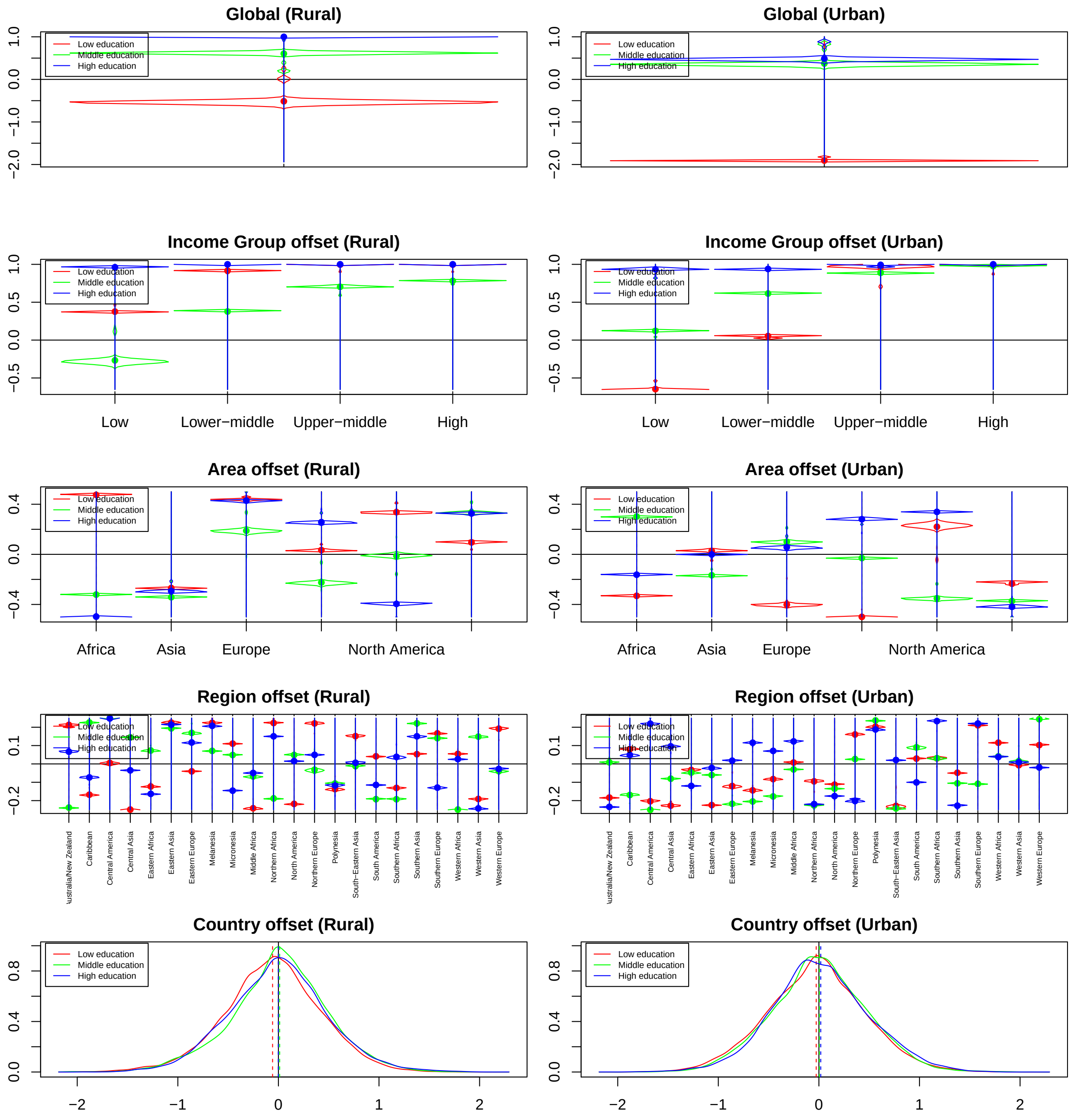

Parameters

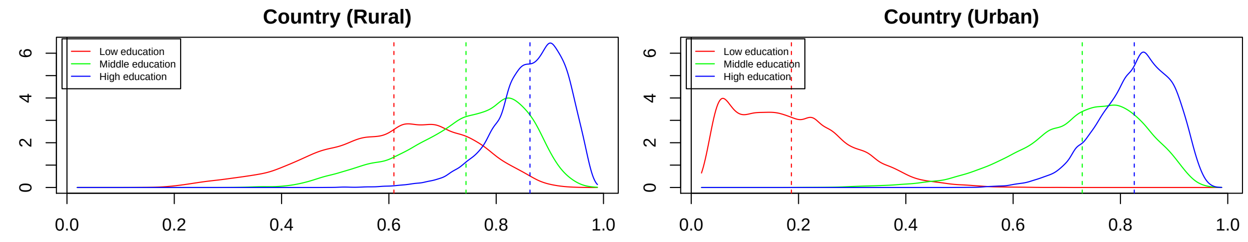

C.4.3.3 non-EmOC

C.4.3.3.1 Pre-Eclampsia/Eclampsia

Hyperparameters

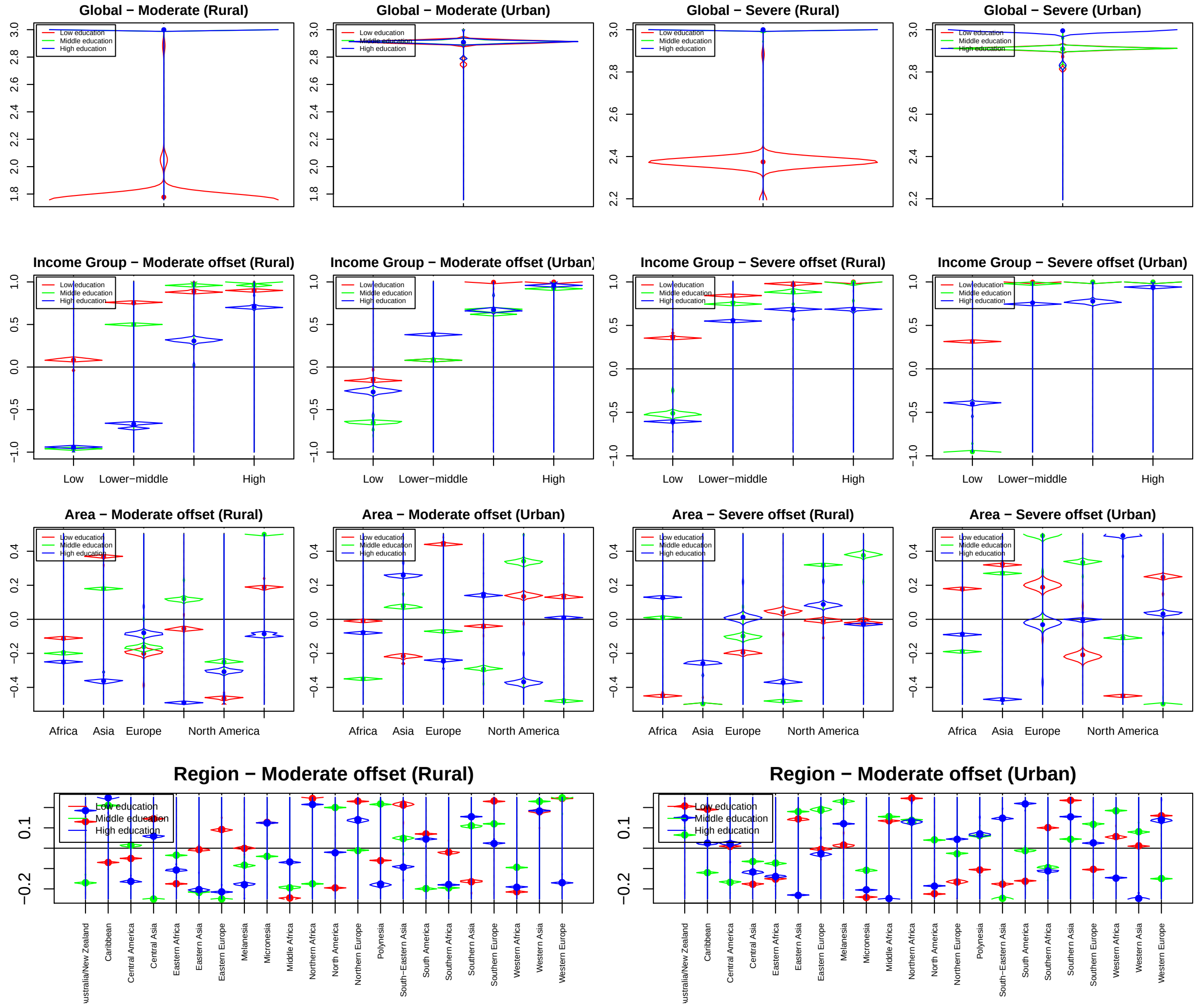

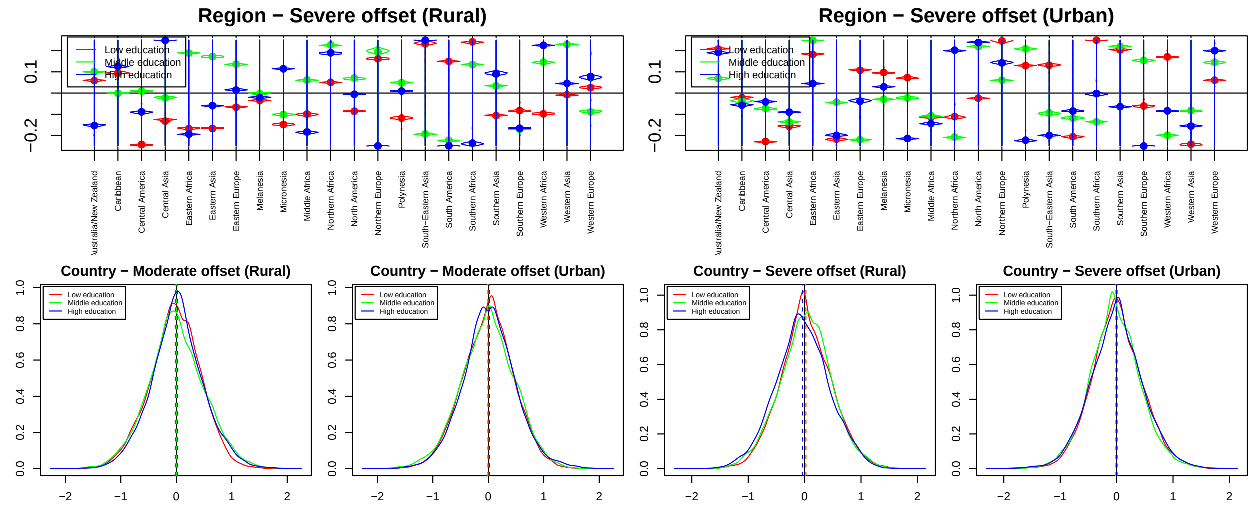

Parameters

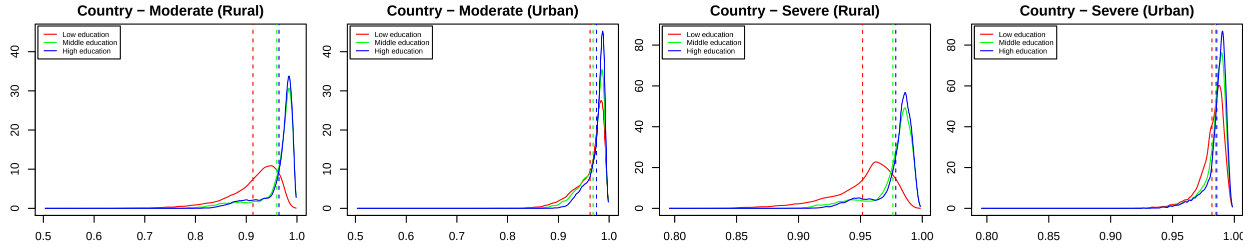

C.4.3.3.2 Obstructed Labour

Hyperparameters

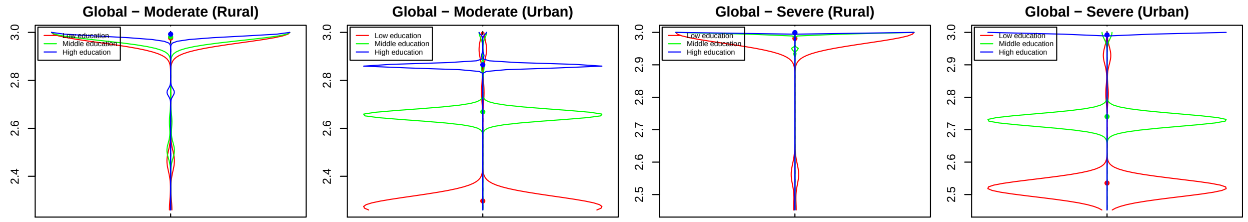

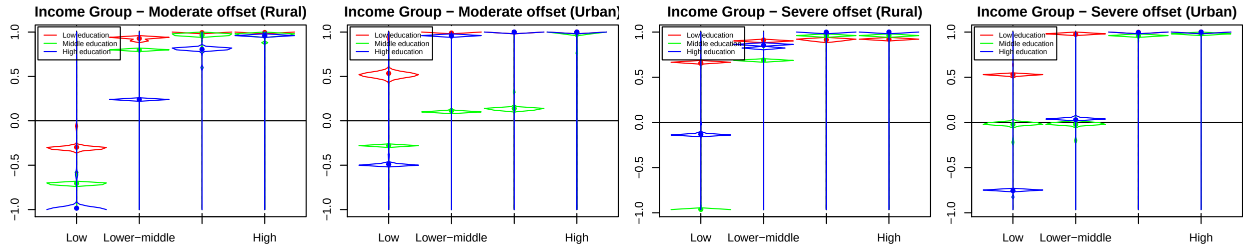

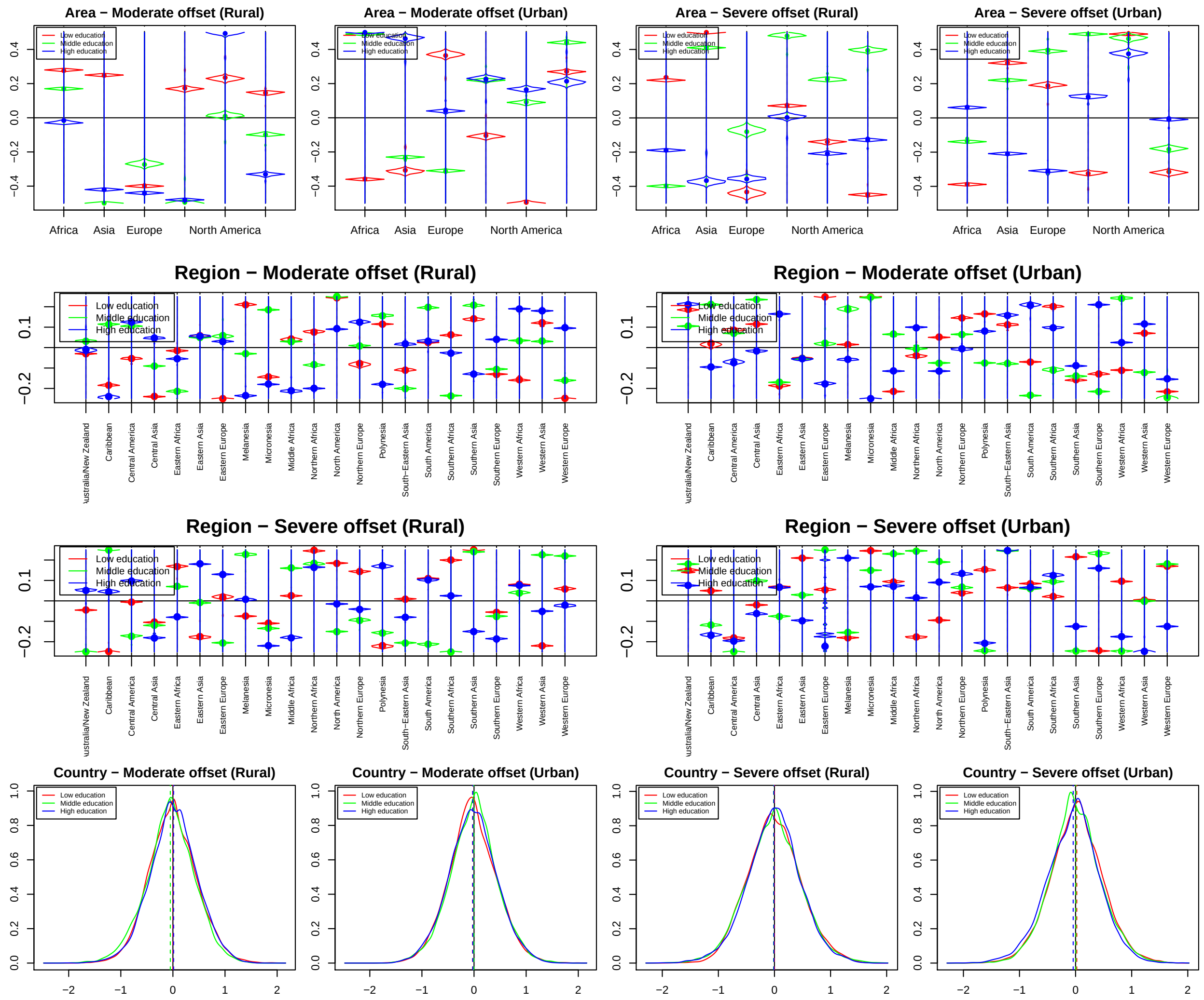

Parameters

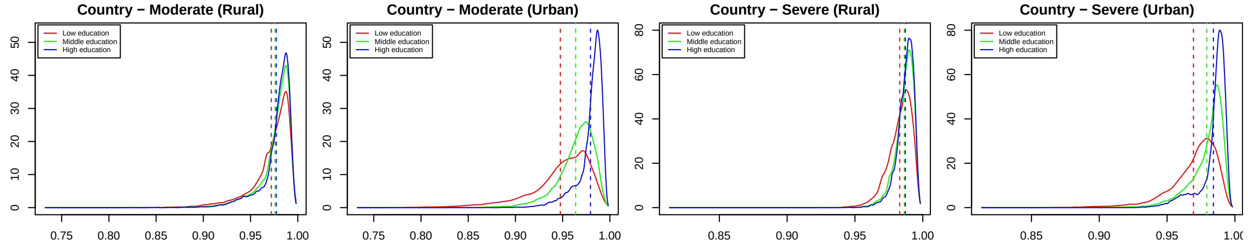

C.4.3.3.3 Postpartum Hemmorhage

Hyperparameters

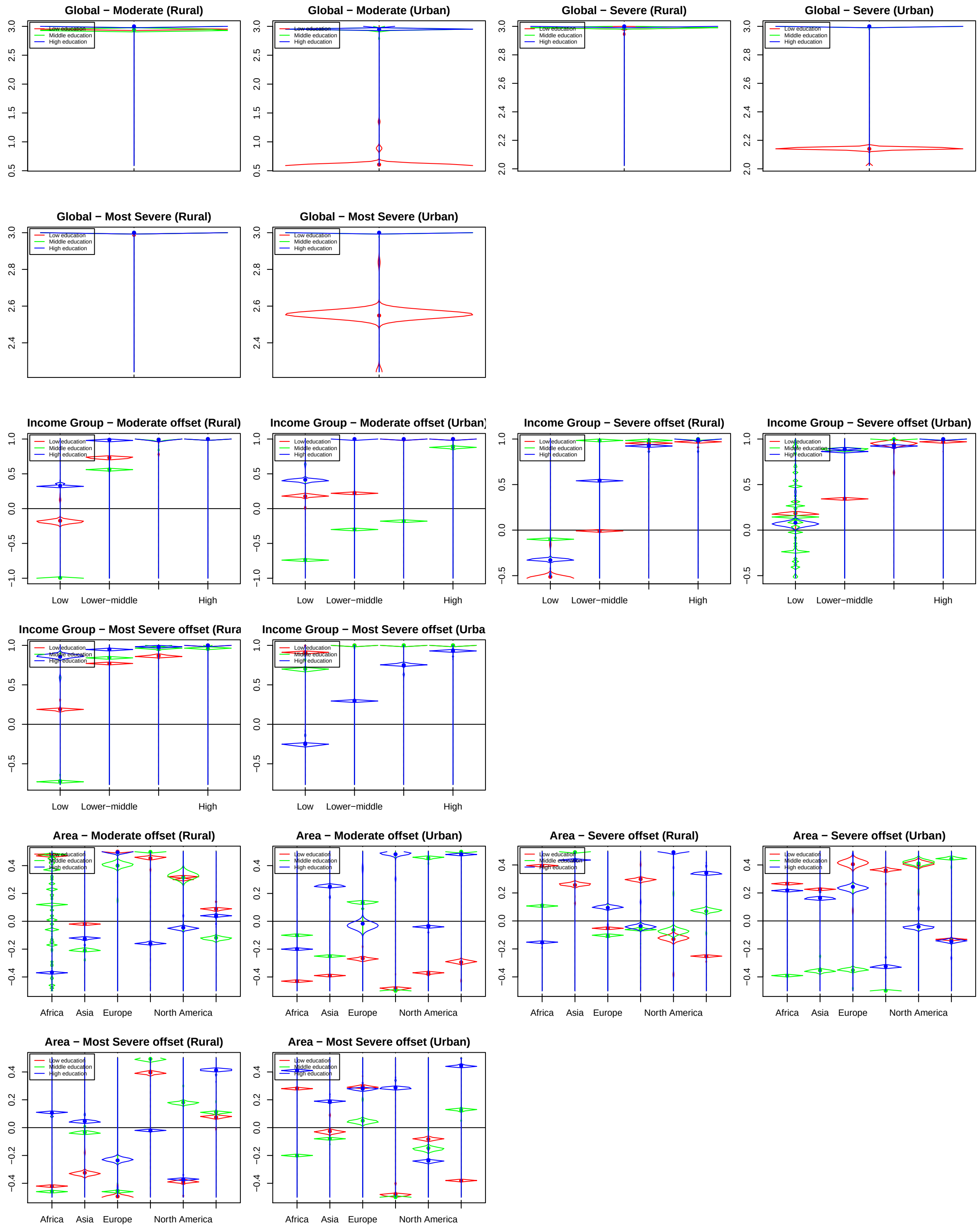
Global − Moderate (Rural)
Global − Moderate (Urban)
Global − Severe (Rural)
Global − Severe (Urban)
Global − Most Severe (Rural)
Global − Most Severe (Urban)
Income Group − Moderate offset (Rural)
Income Group − Moderate offset (Urban)
Income Group − Severe offset (Rural)
Income Group − Severe offset (Urban)
Income Group − Most Severe offset (Rura
Income Group − Most Severe offset (Urba
Area − Moderate offset (Rural)
Area − Moderate offset (Urban)
Area − Severe offset (Rural)
Area − Severe offset (Urban)
Area − Most Severe offset (Rural)
Area − Most Severe offset (Urban)
Low education
Middle education
High education
Low
Lower−middle
High
Africa
Asia
Europe
North America

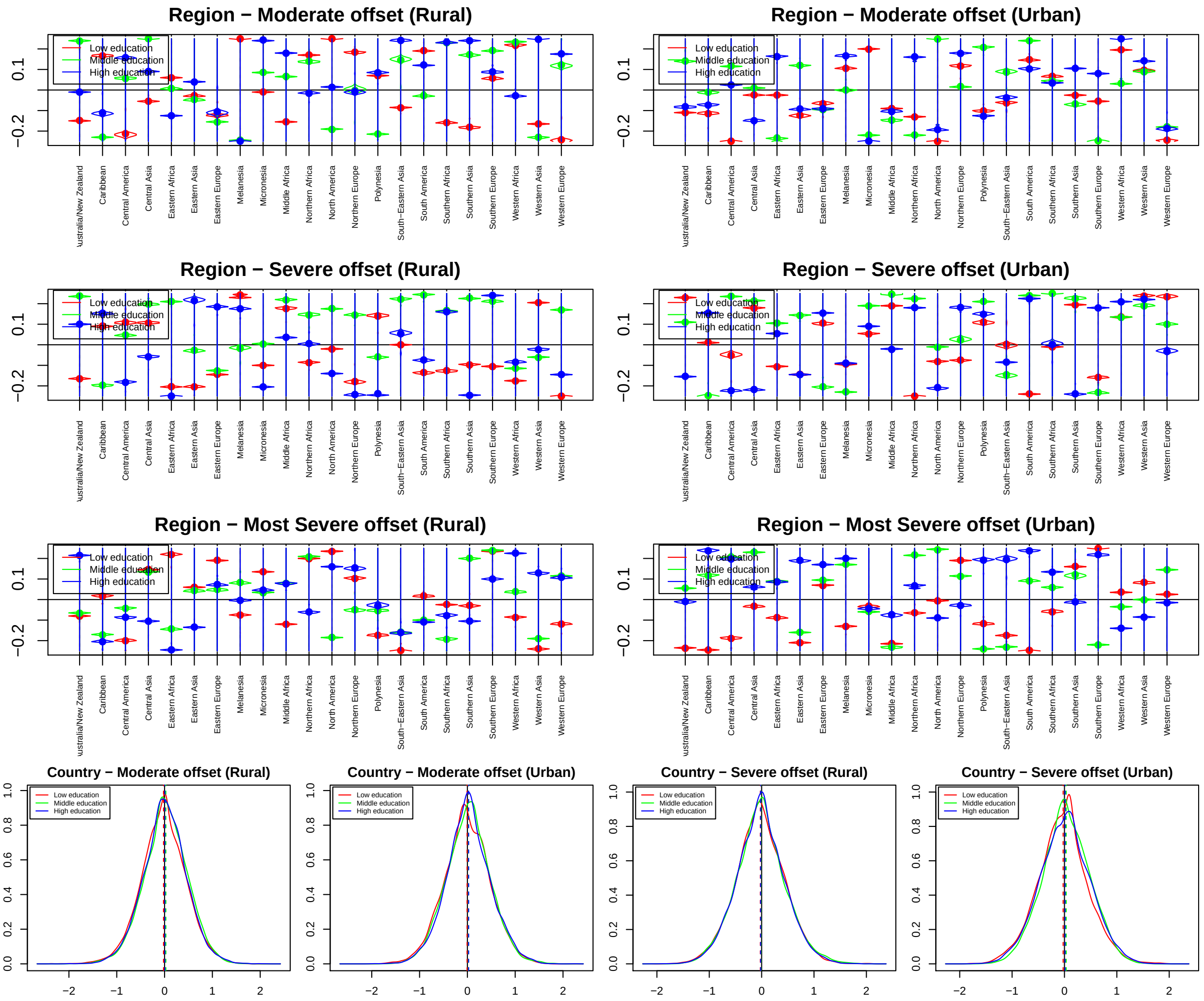

Parameters

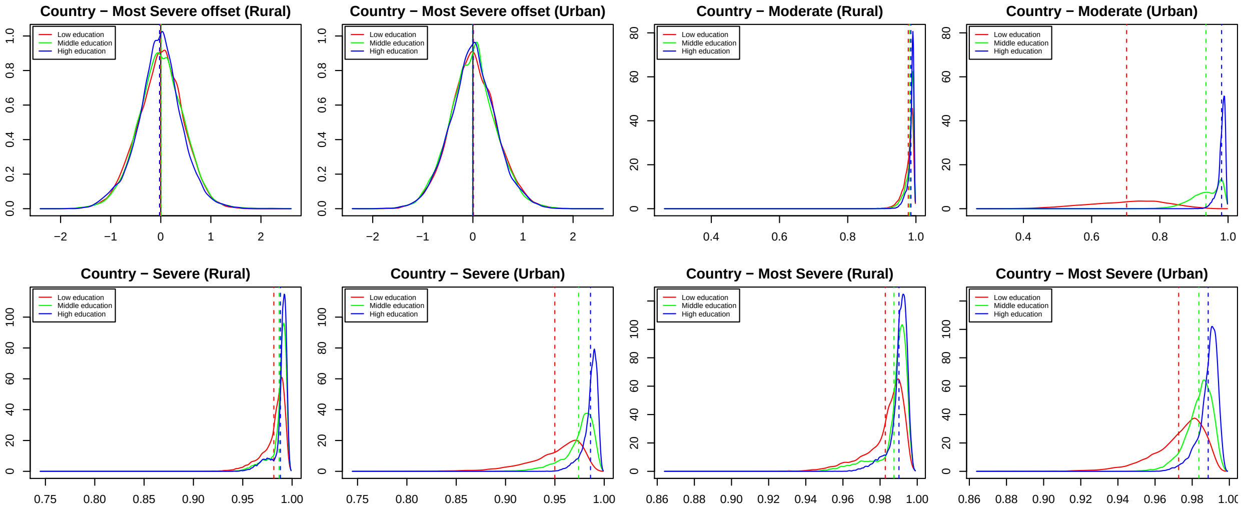

C.4.3.3.4 Sepsis

Hyperparameters

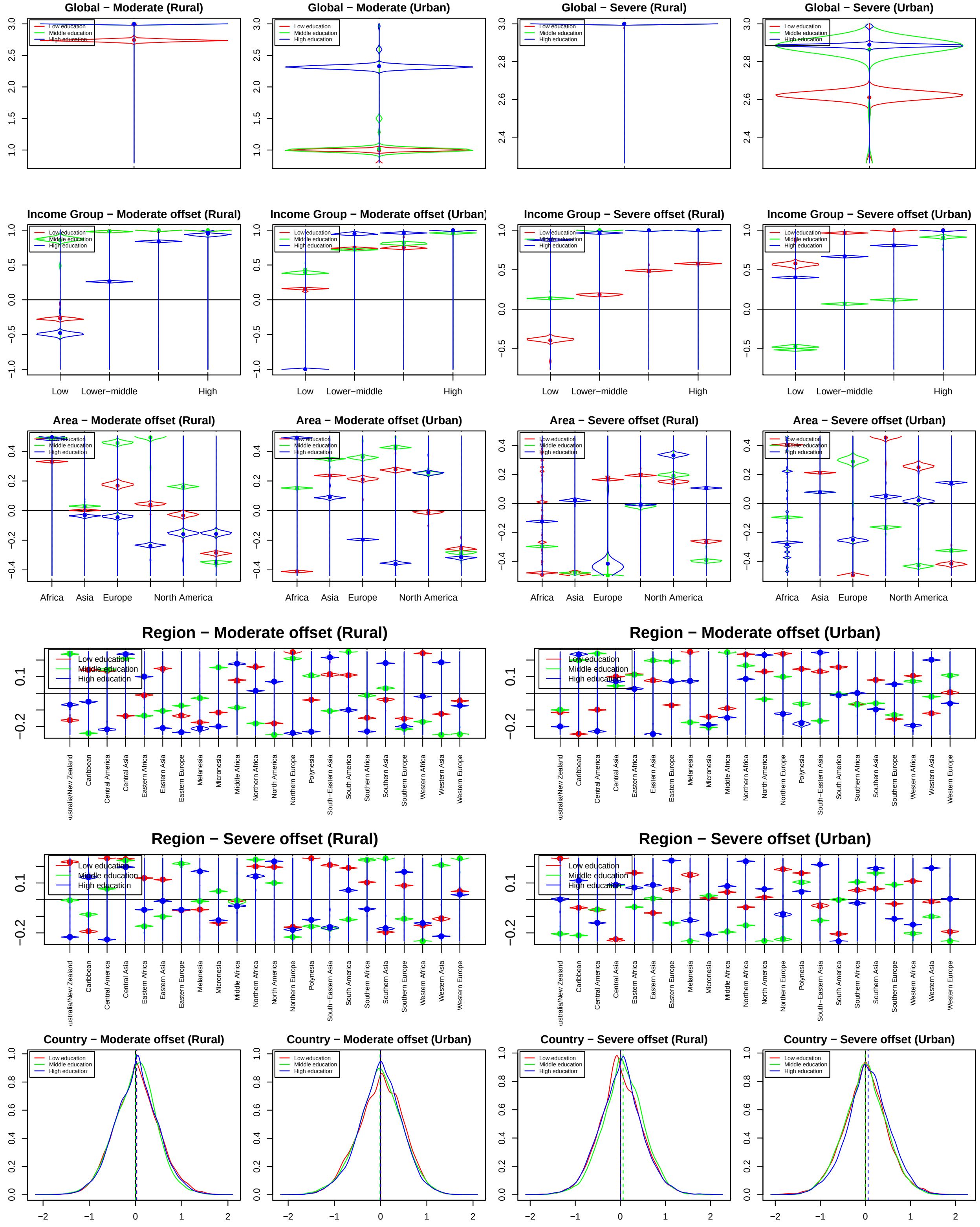

Parameters

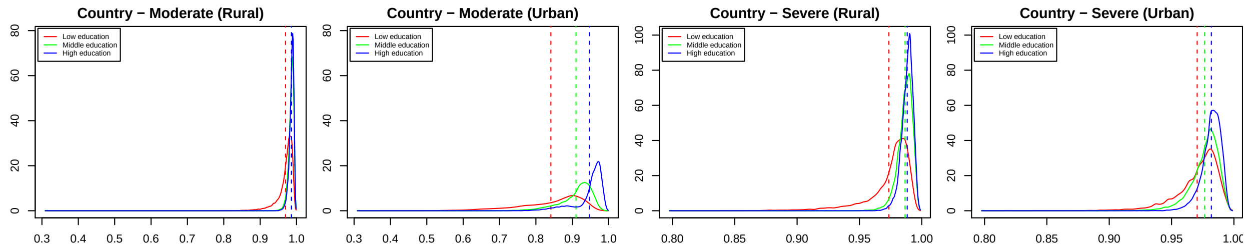

C.4.3.3.5 Other Hyperparameters

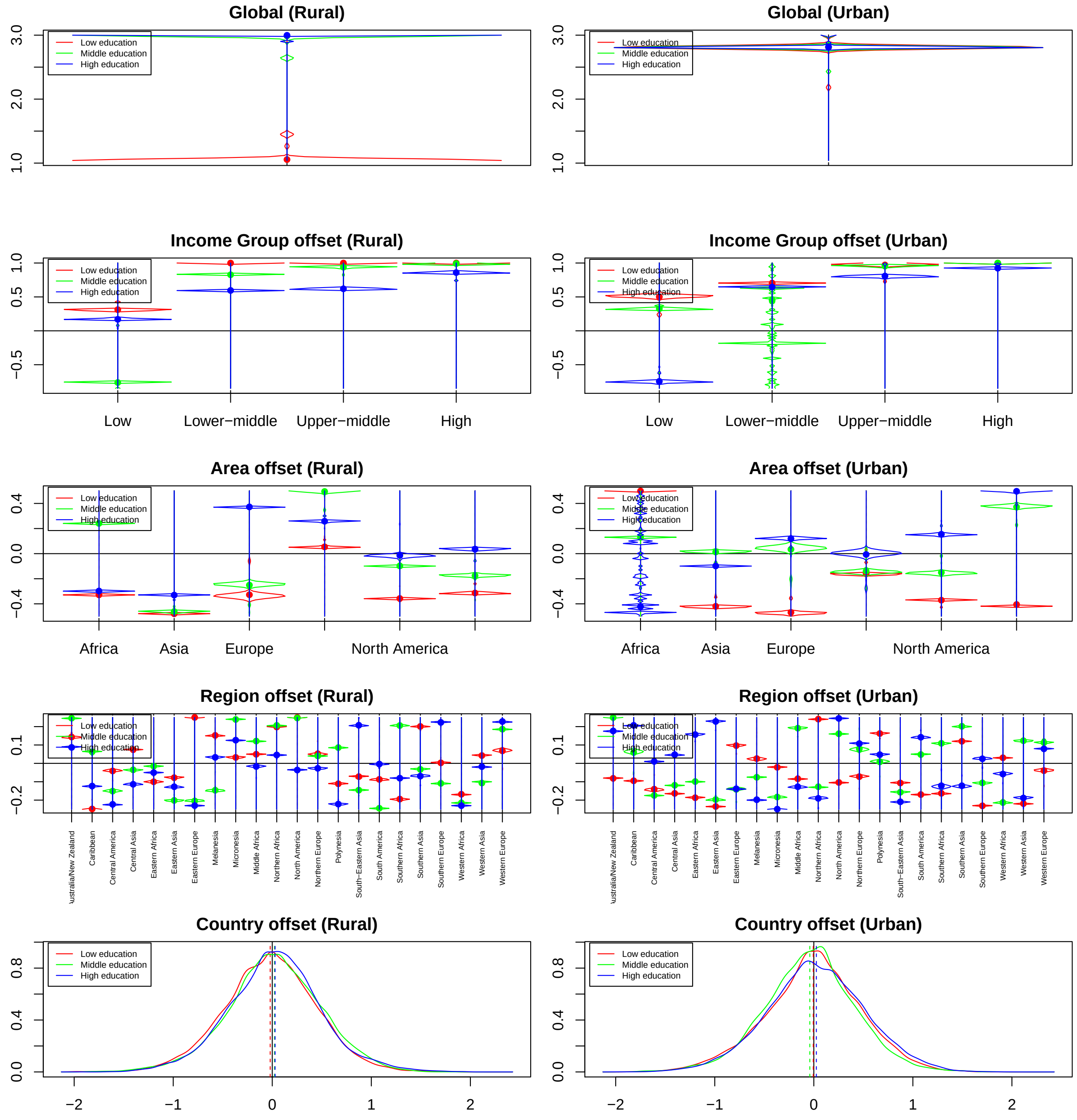

Parameters

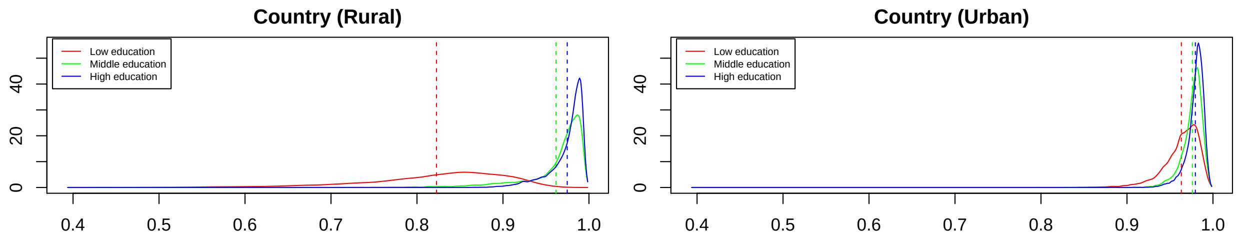

C.4.3.4 BEmOC

C.4.3.4.1 Pre-Eclampsia/Eclampsia

Hyperparameters

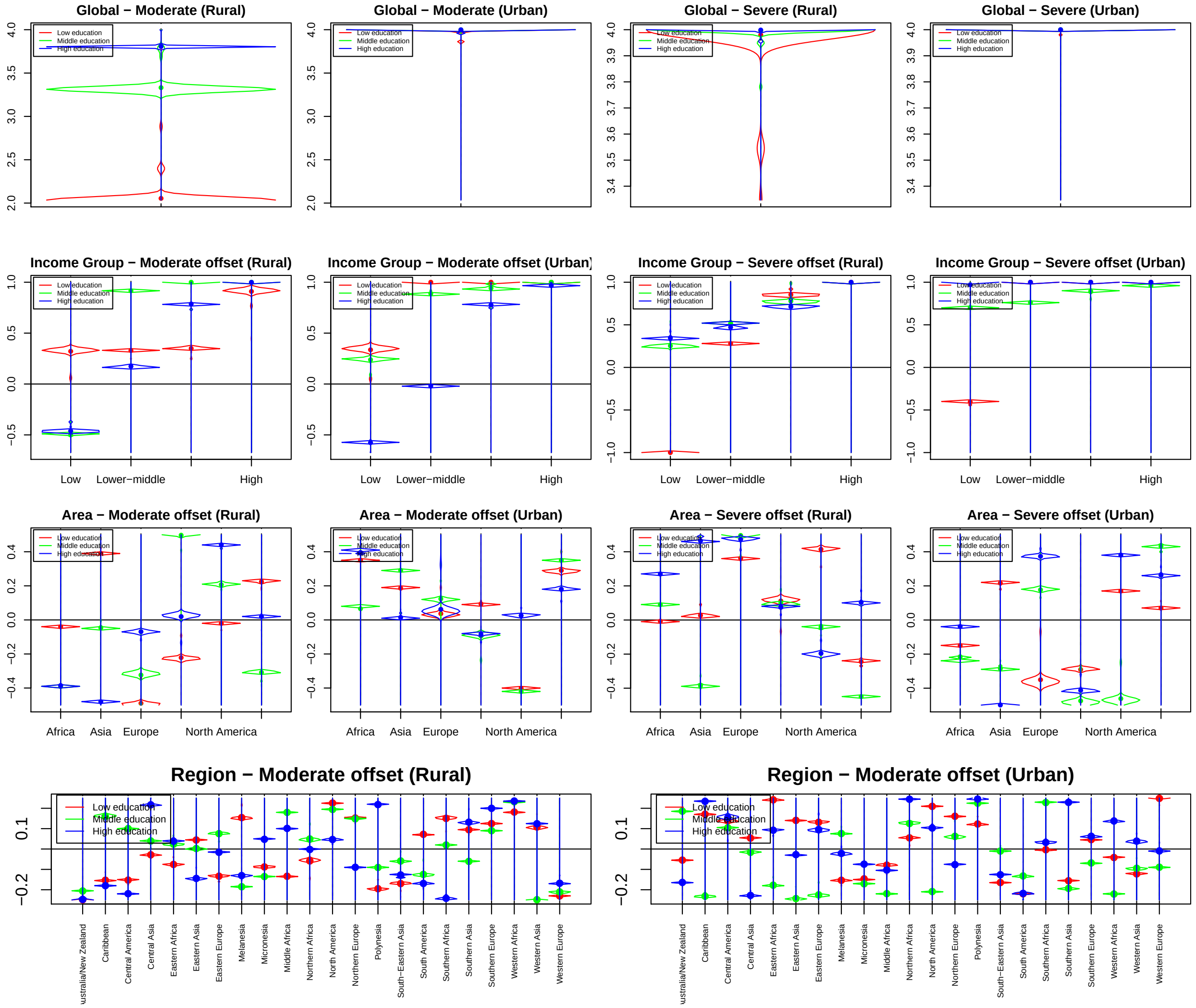

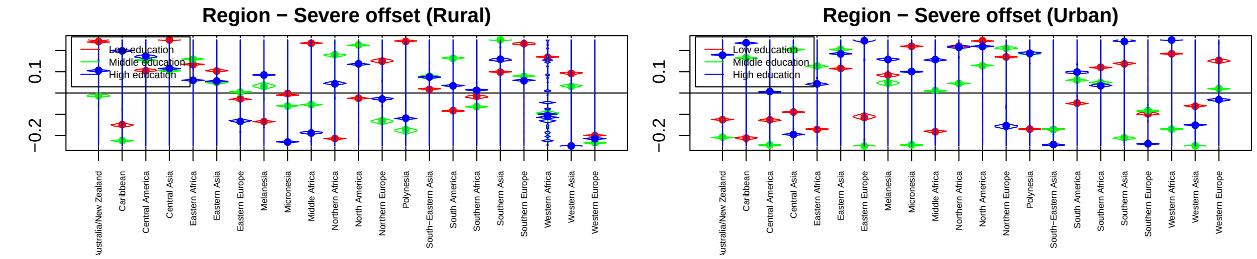

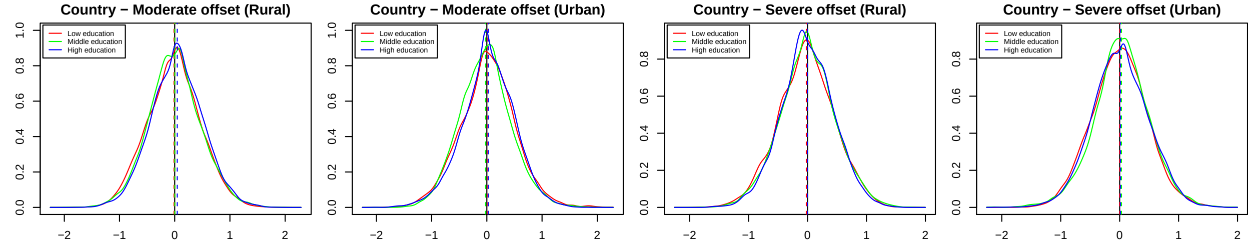

Parameters

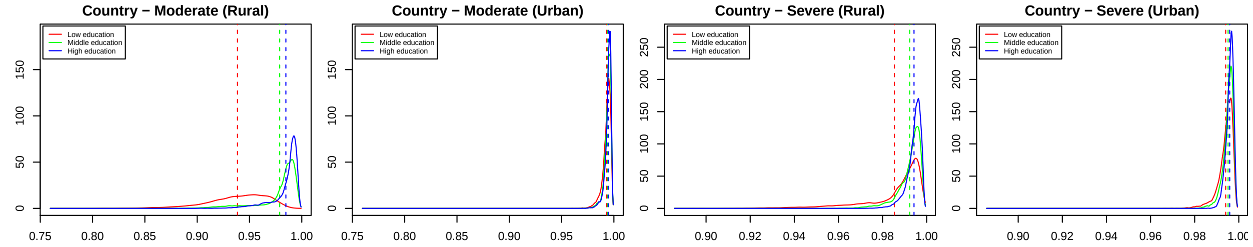

C.4.3.4.2 Obstructed Labour

Hyperparameters

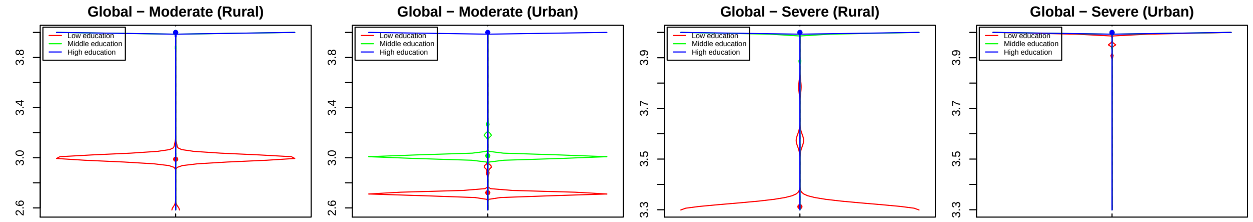

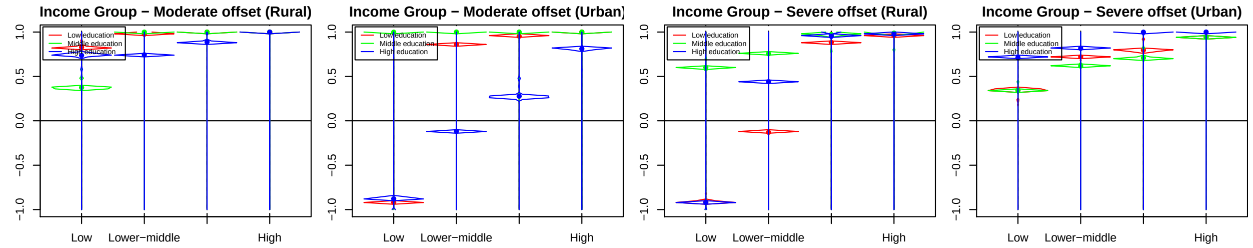

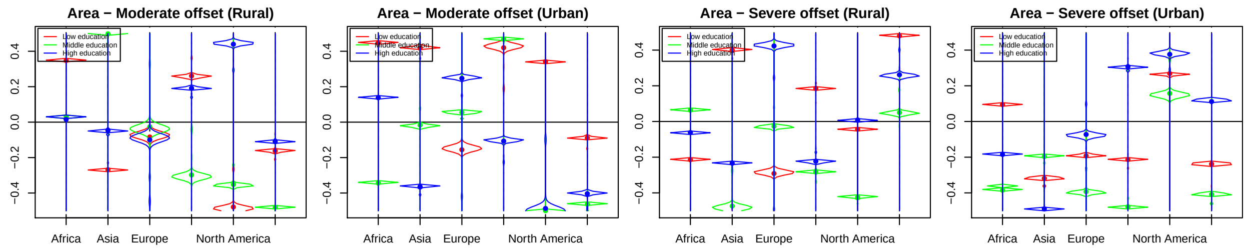

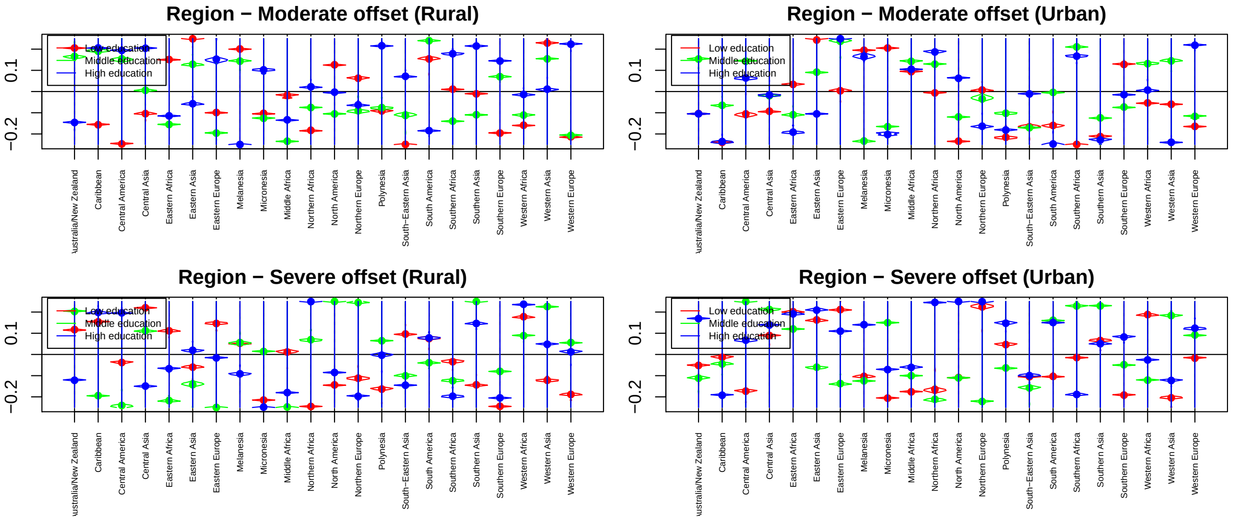

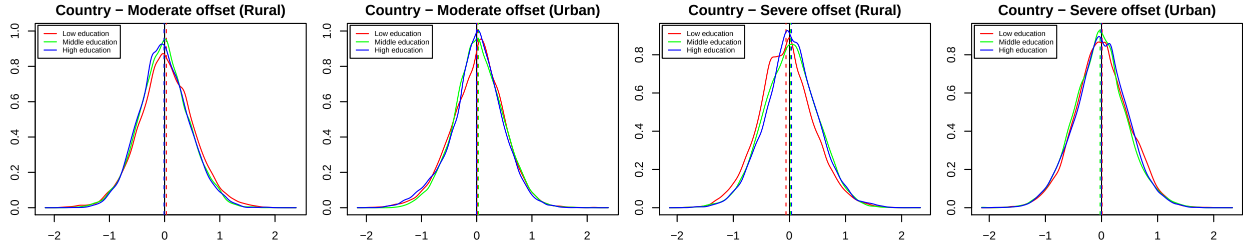

Parameters

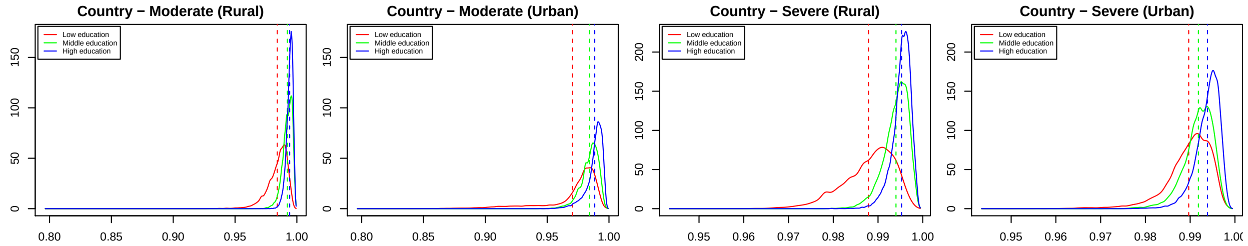

C.4.3.4.3 Postpartum Hemmorhage

Hyperparameters

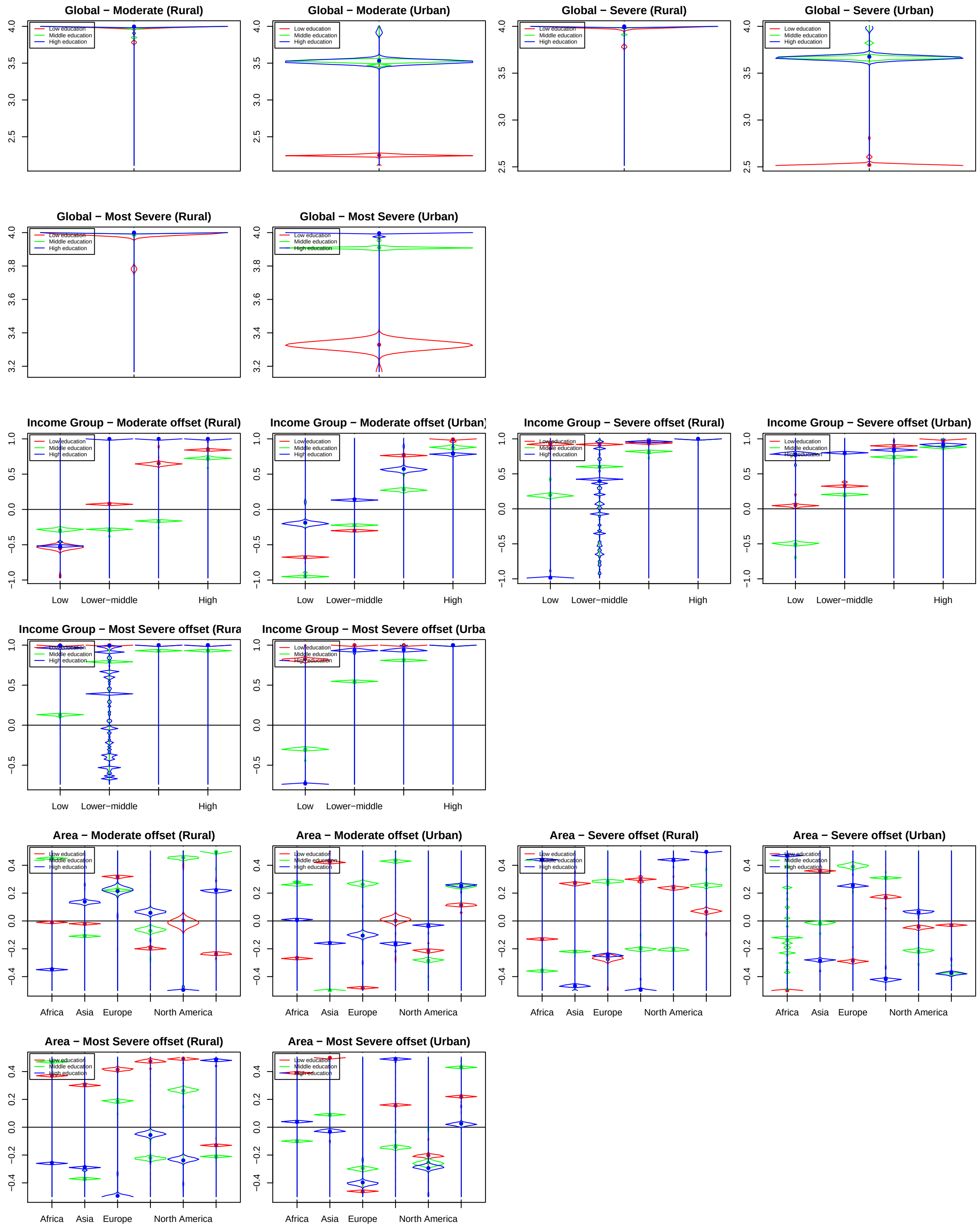
Global − Moderate (Rural)
Global − Moderate (Urban)
Global − Severe (Rural)
Global − Severe (Urban)
Global − Most Severe (Rural)
Global − Most Severe (Urban)
Income Group − Moderate offset (Rural)
Income Group − Moderate offset (Urban)
Income Group − Severe offset (Rural)
Income Group − Severe offset (Urban)
Income Group − Most Severe offset (Rura
Income Group − Most Severe offset (Urba
Area − Moderate offset (Rural)
Area − Moderate offset (Urban)
Area − Severe offset (Rural)
Area − Severe offset (Urban)
Area − Most Severe offset (Rural)
Area − Most Severe offset (Urban)
Low education
Middle education
High education
Low
Lower−middle
High
Africa
Asia
Europe
North America

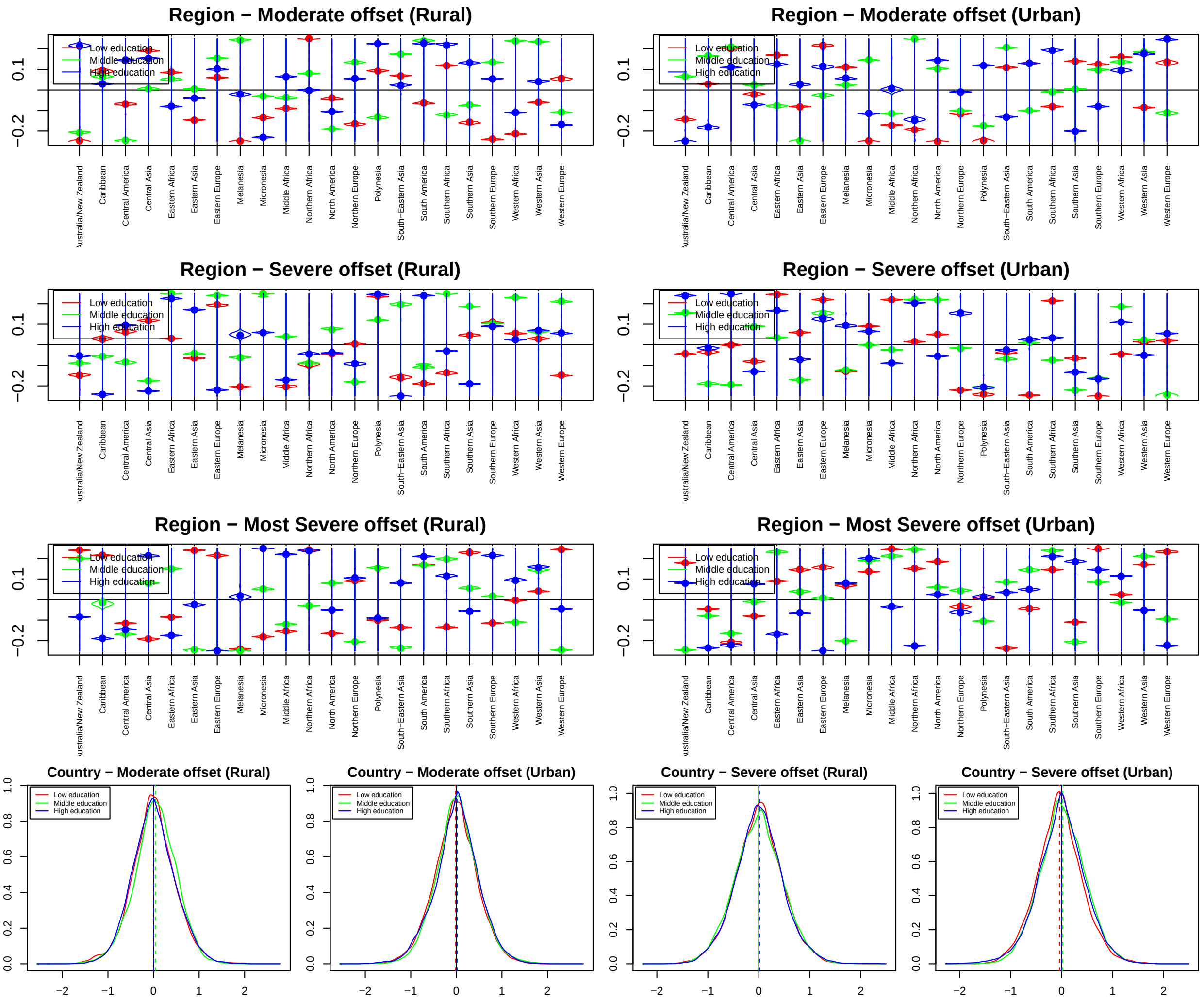

Parameters

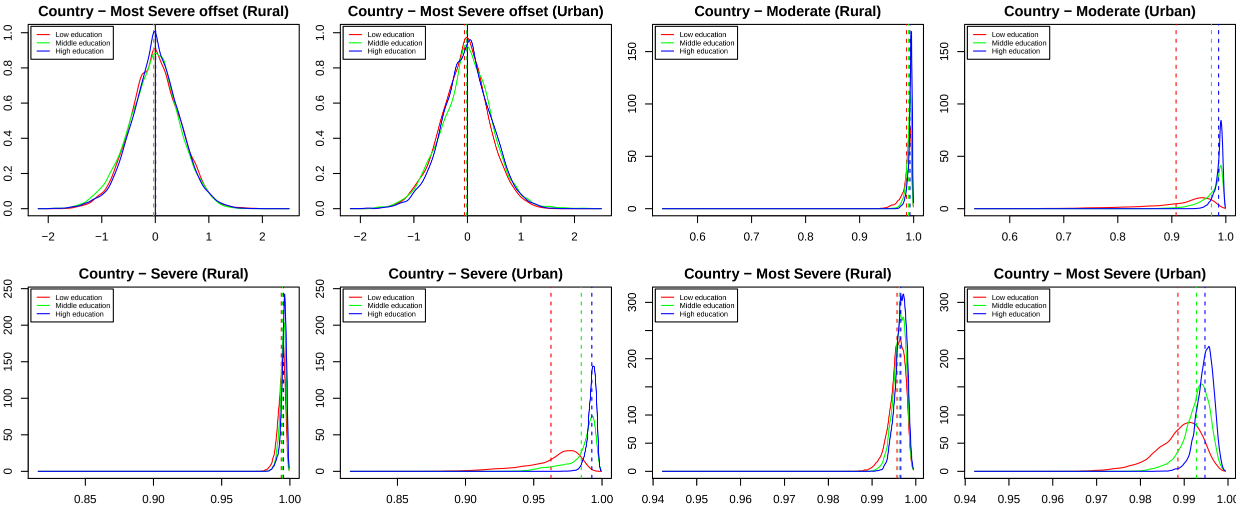

C.4.3.4.4 Sepsis

Hyperparameters

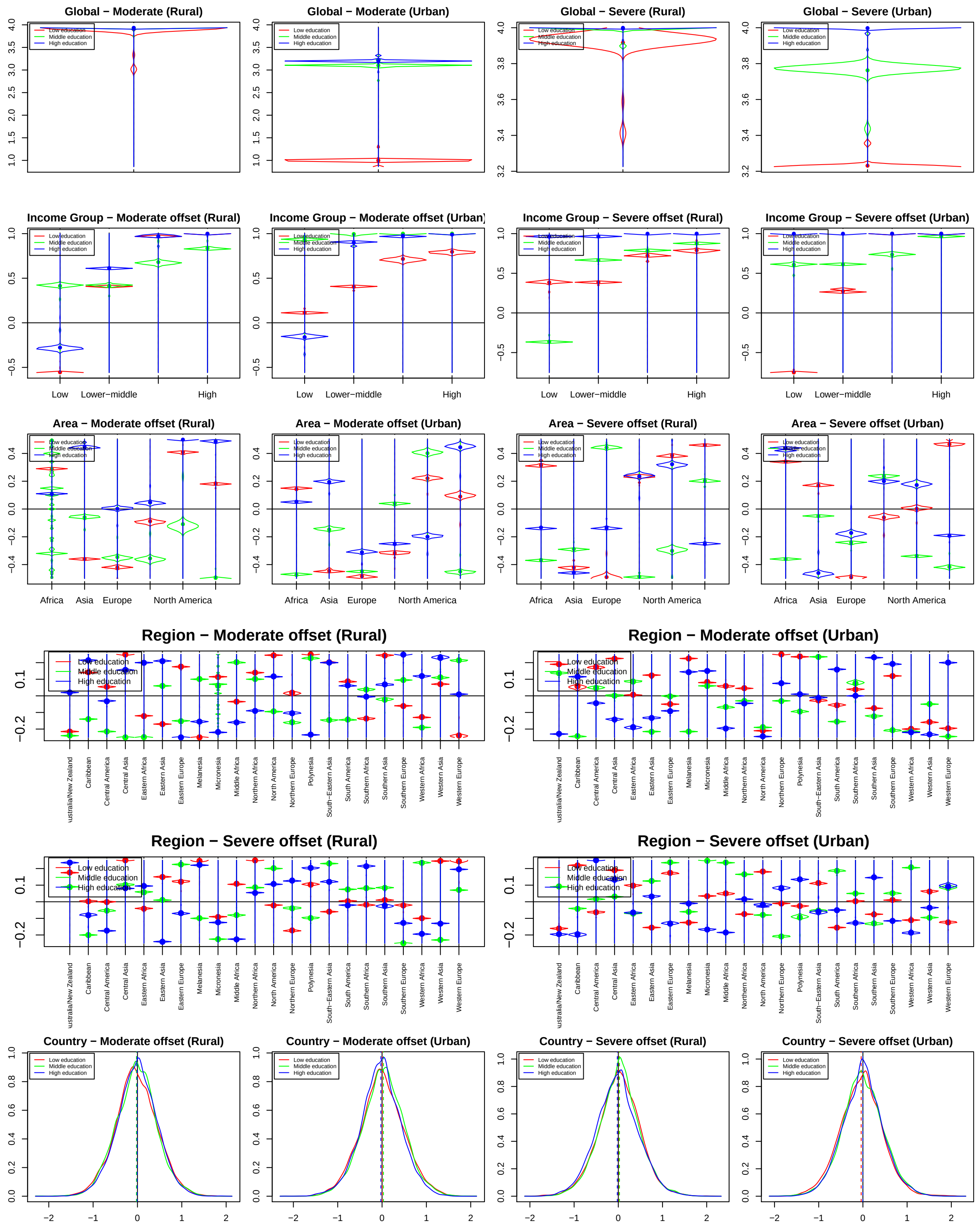

Parameters

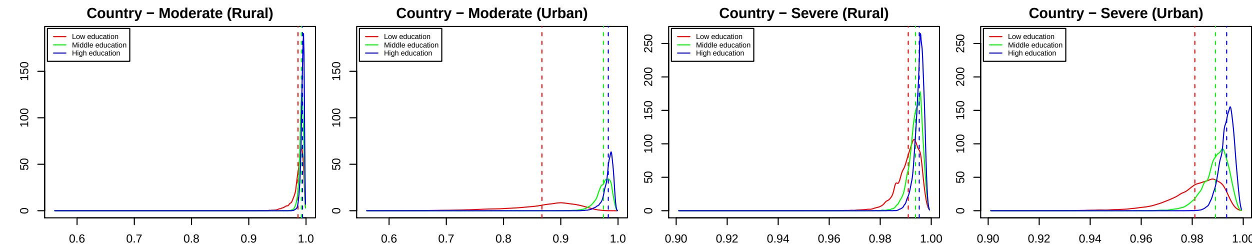

C.4.3.4.5 Other

Hyperparameters

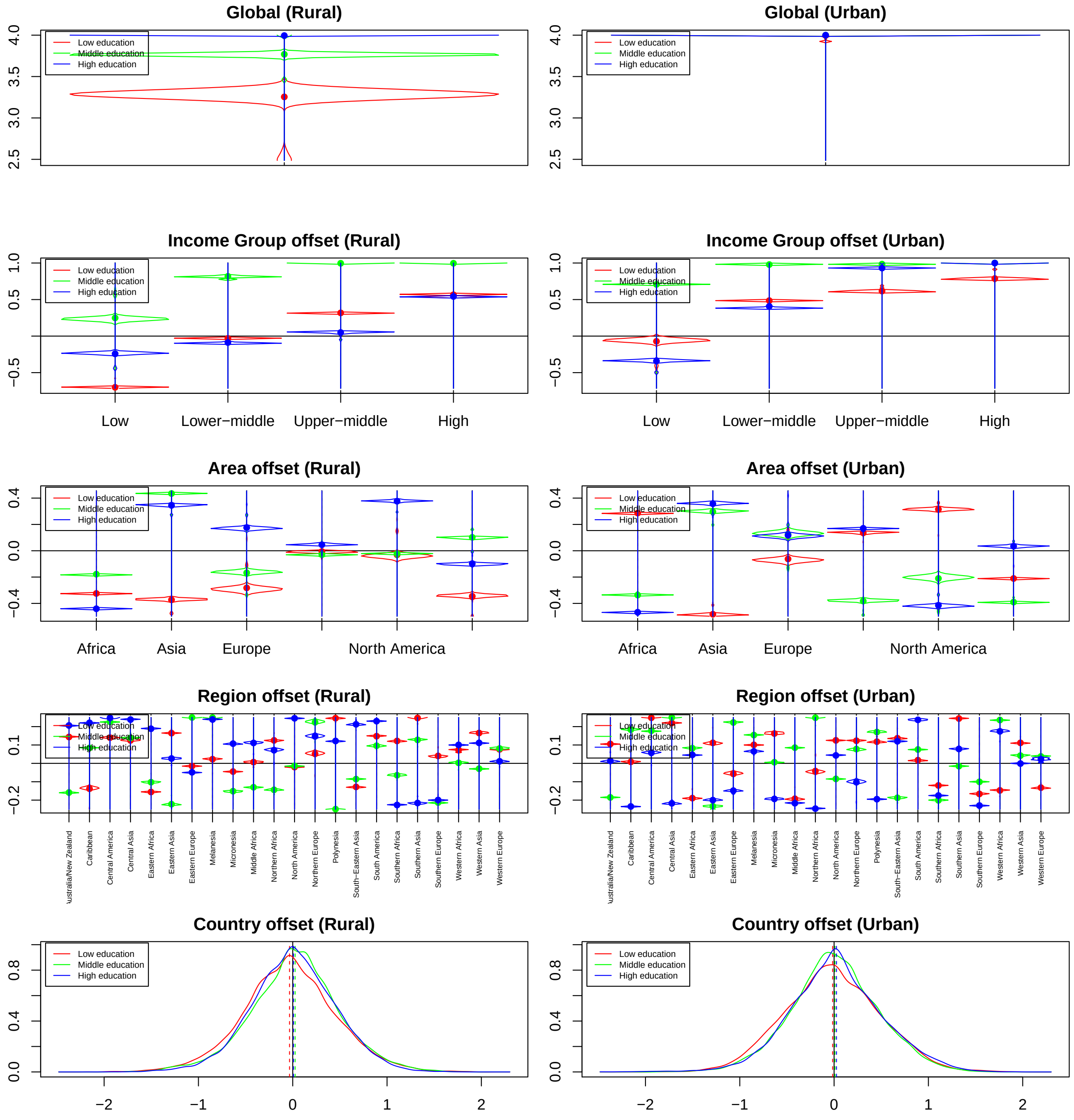

Parameters

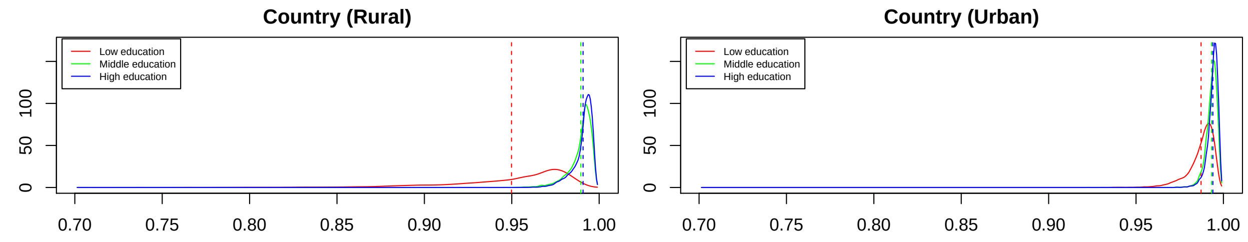

C.4.3.5 CEmOC

C.4.3.5.1 Pre-Eclampsia/Eclampsia

Hyperparameters

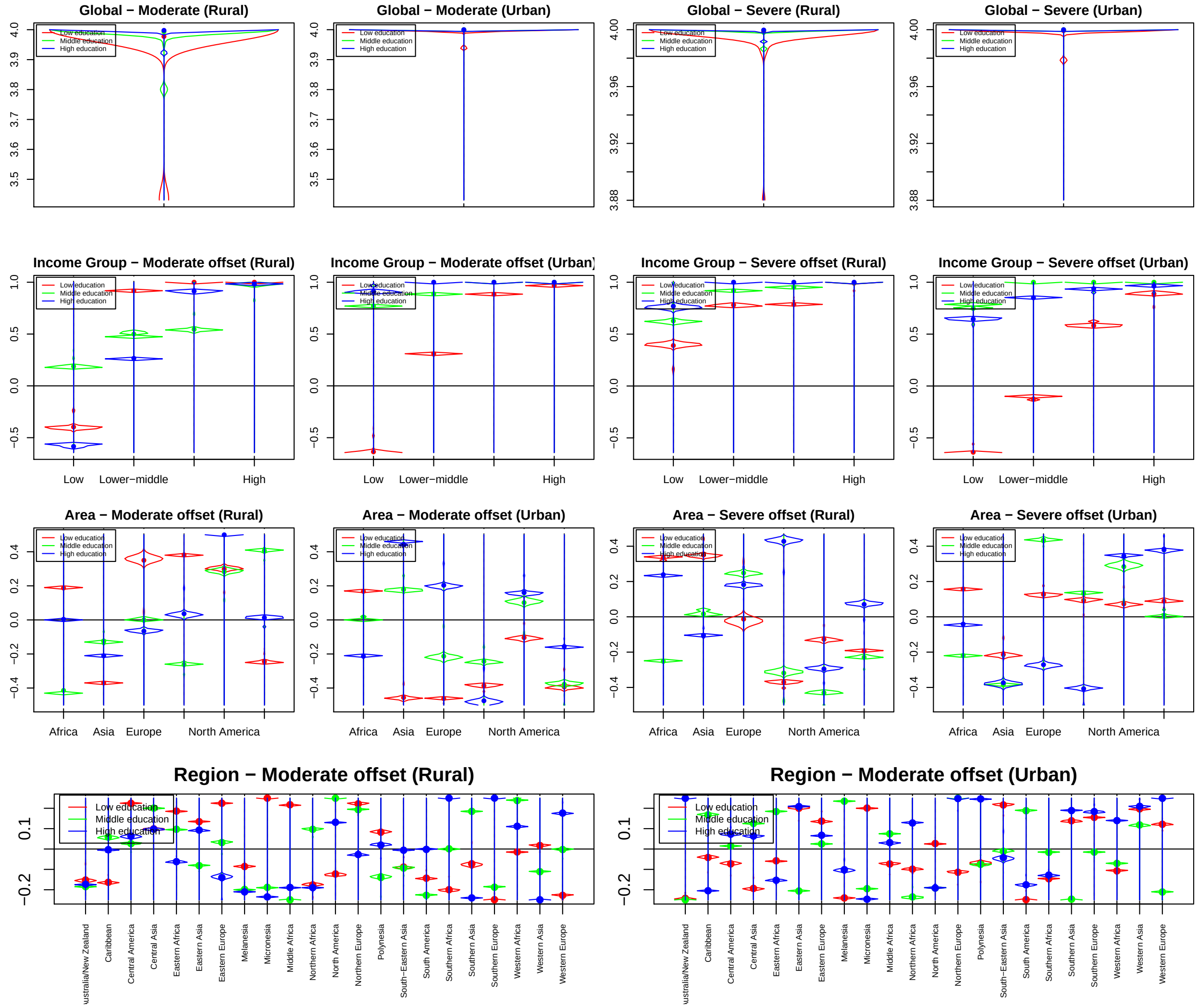

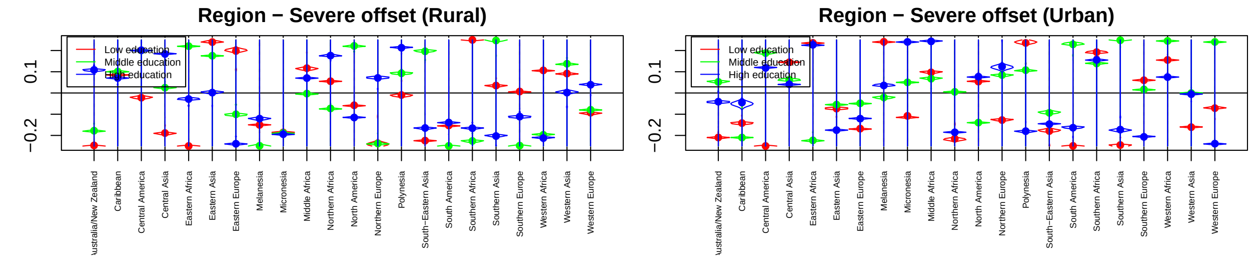

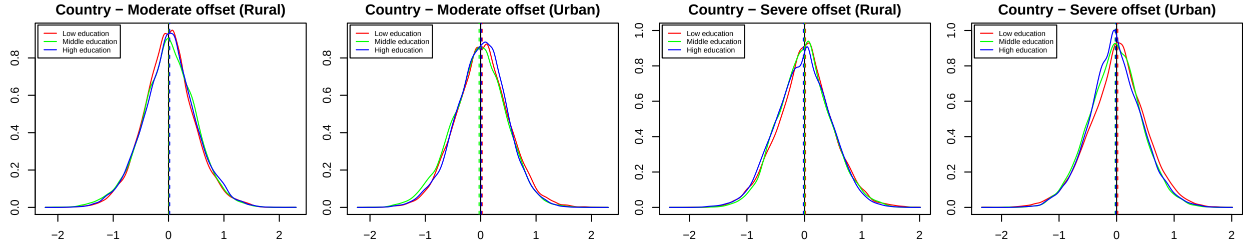

Parameters

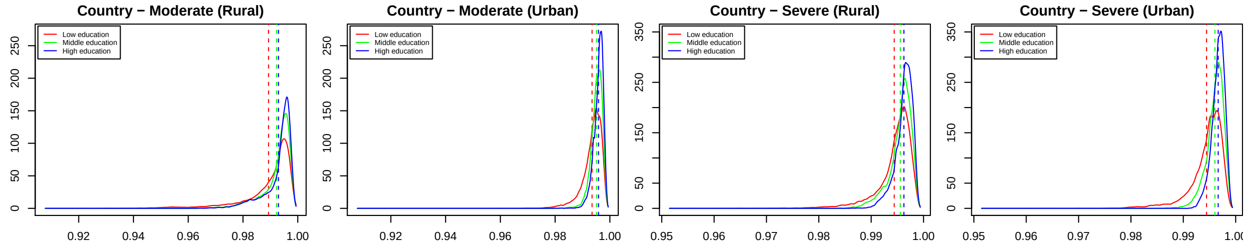

C.4.3.5.2 Obstructed Labour

Hyperparameters

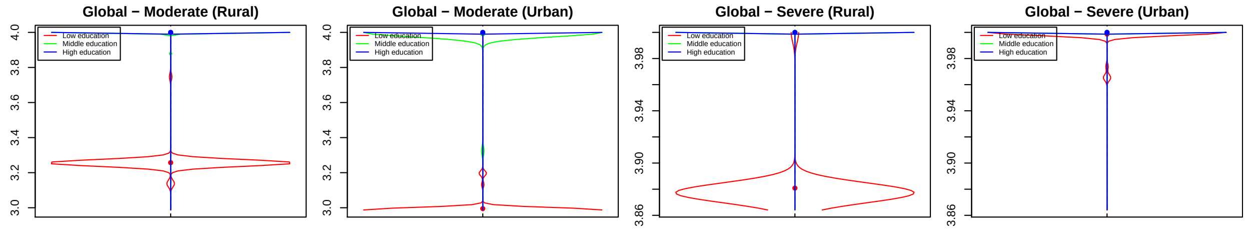

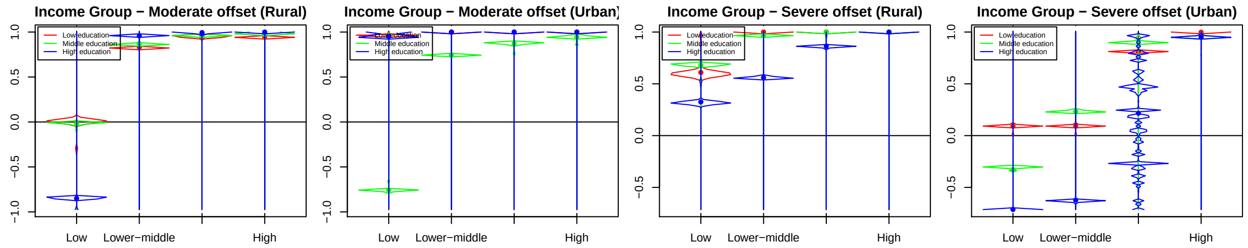

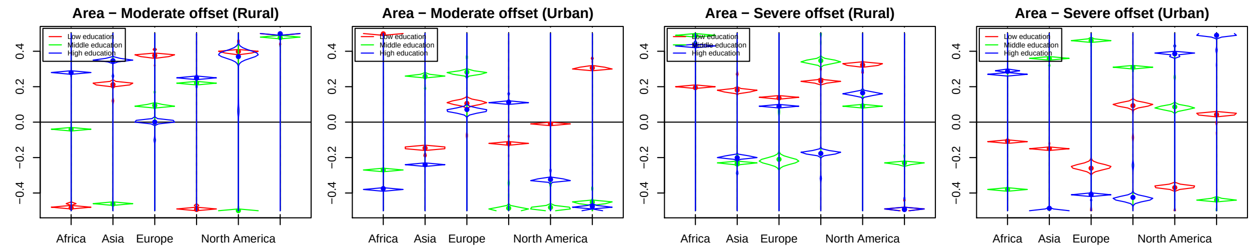

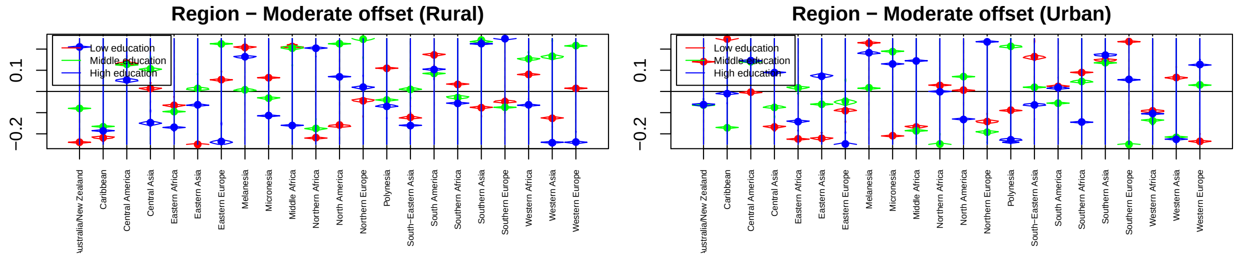

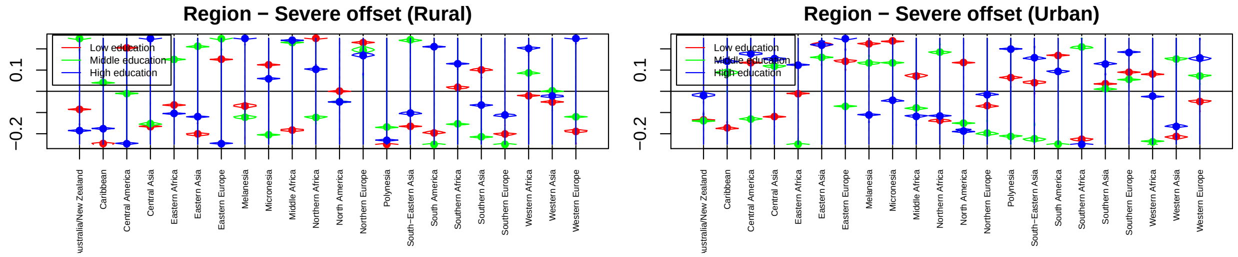

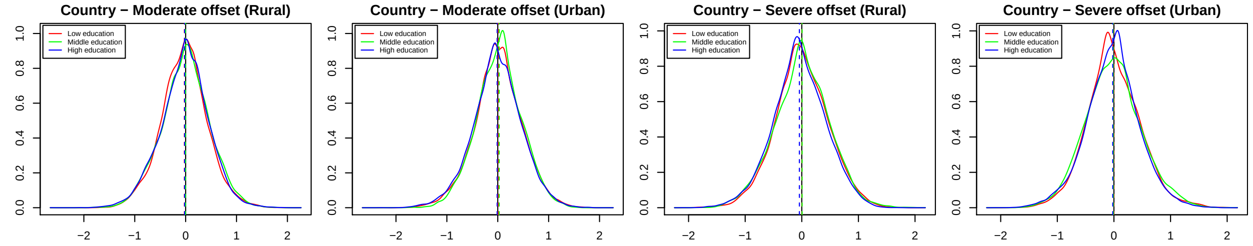

Parameters

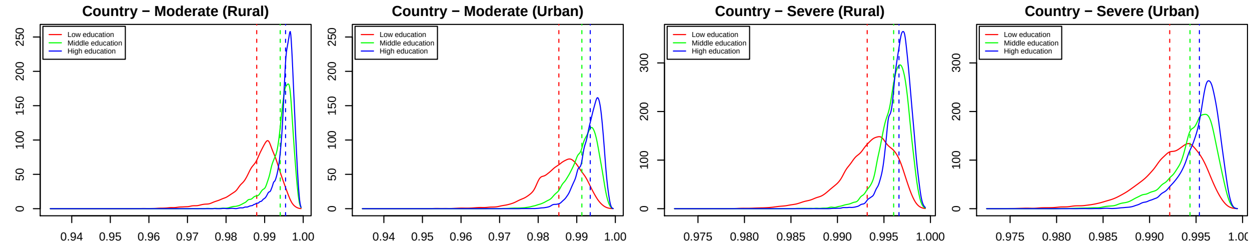

C.4.3.5.3 Postpartum Hemmorhage

Hyperparameters

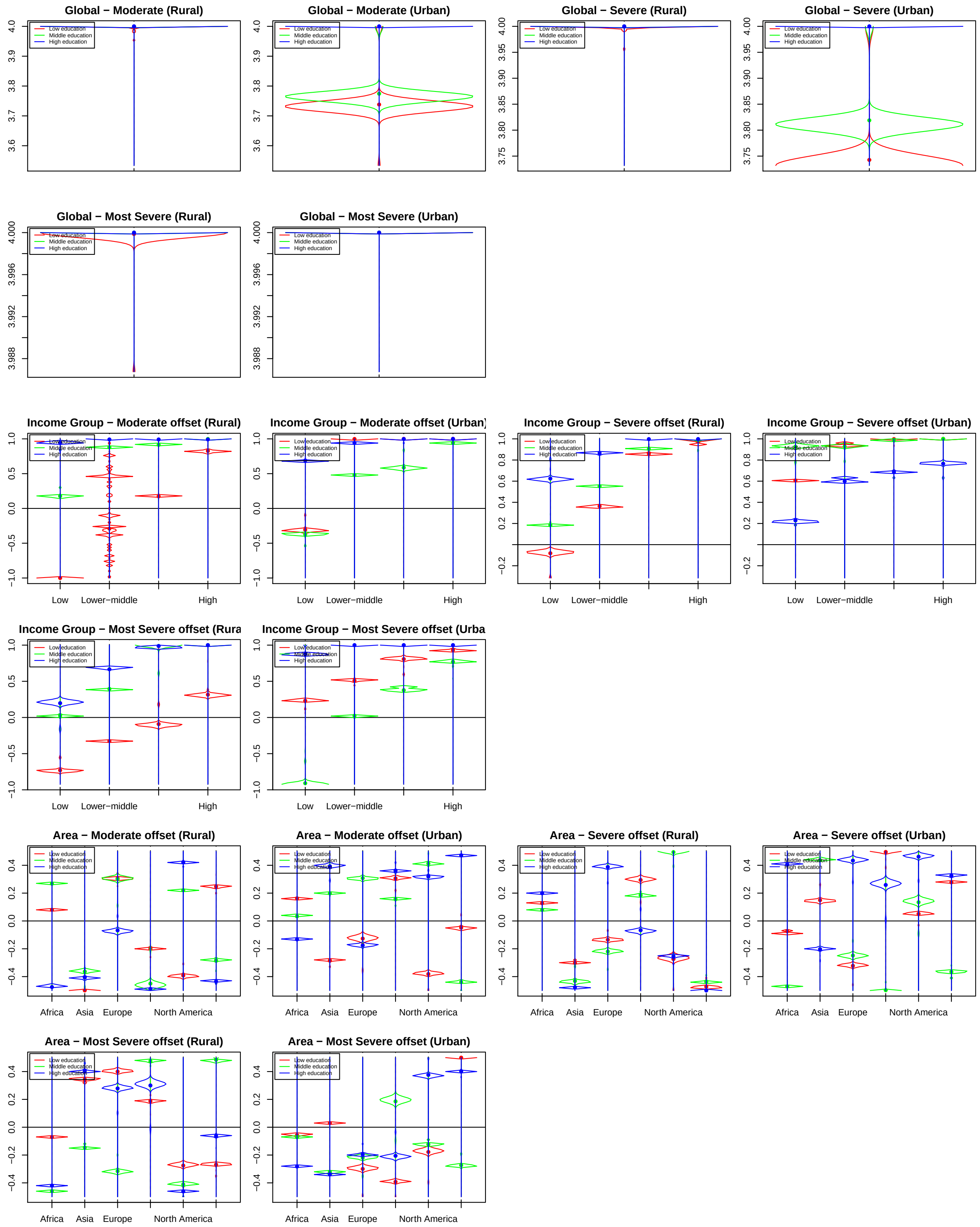
Global − Moderate (Rural)
Global − Moderate (Urban)
Global − Severe (Rural)
Global − Severe (Urban)
Global − Most Severe (Rural)
Global − Most Severe (Urban)
Income Group − Moderate offset (Rural)
Income Group − Moderate offset (Urban)
Income Group − Severe offset (Rural)
Income Group − Severe offset (Urban)
Income Group − Most Severe offset (Rura
Income Group − Most Severe offset (Urba
Area − Moderate offset (Rural)
Area − Moderate offset (Urban)
Area − Severe offset (Rural)
Area − Severe offset (Urban)
Area − Most Severe offset (Rural)
Area − Most Severe offset (Urban)
Low education
Middle education
High education
Low
Lower−middle
High
Africa
Asia
Europe
North America

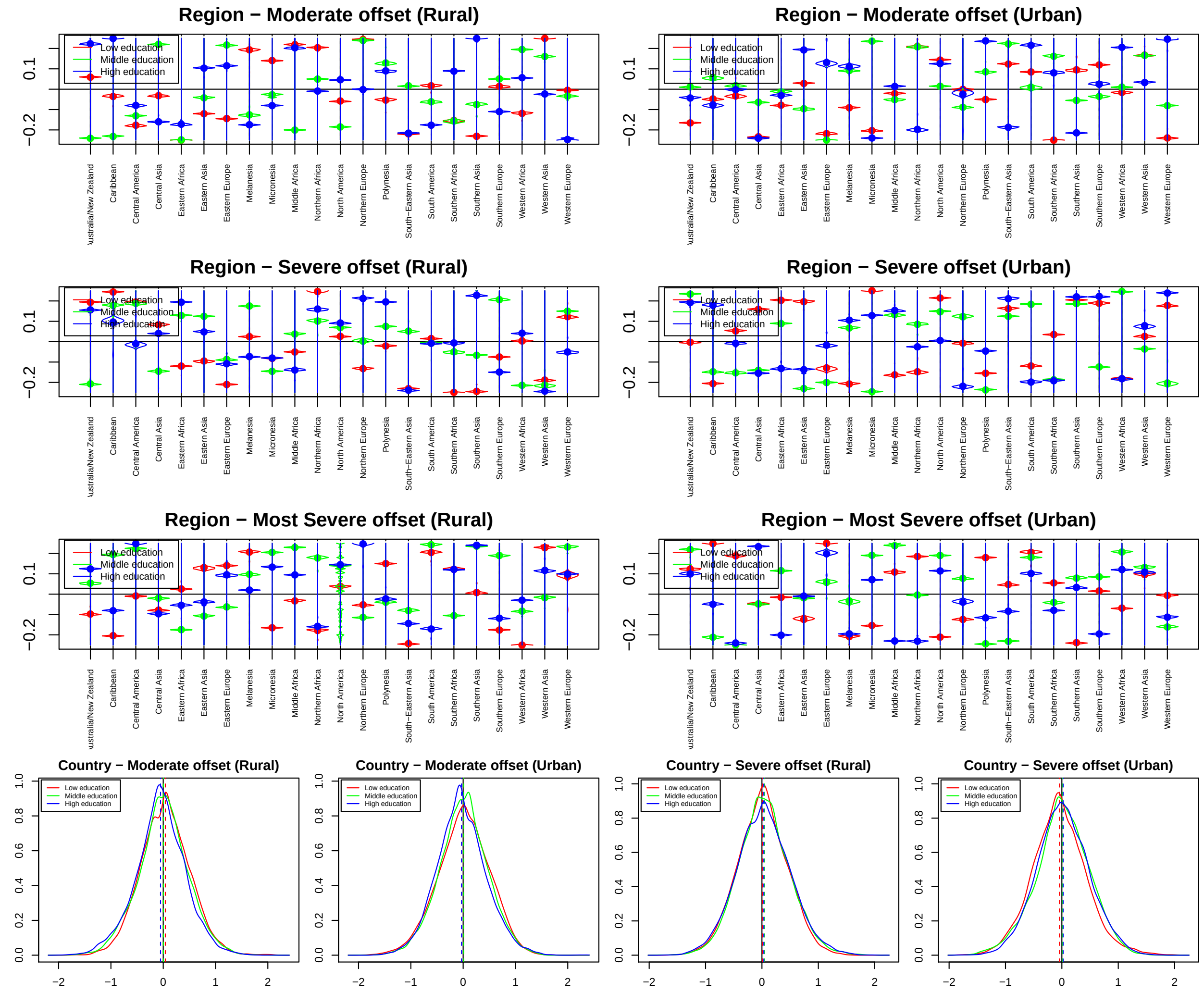

Parameters

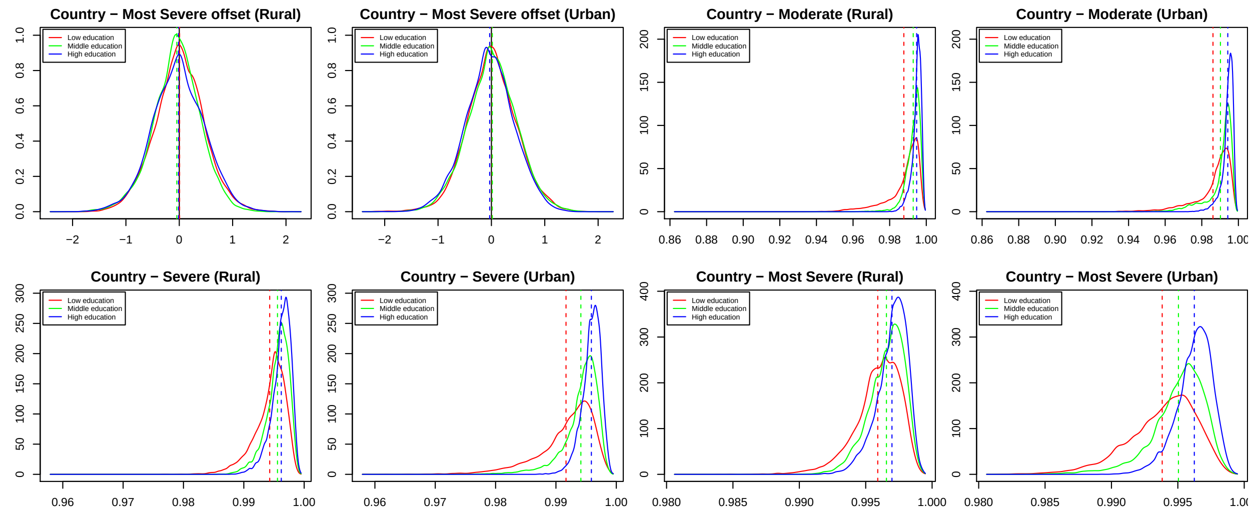

C.4.3.5.4 Sepsis

Hyperparameters

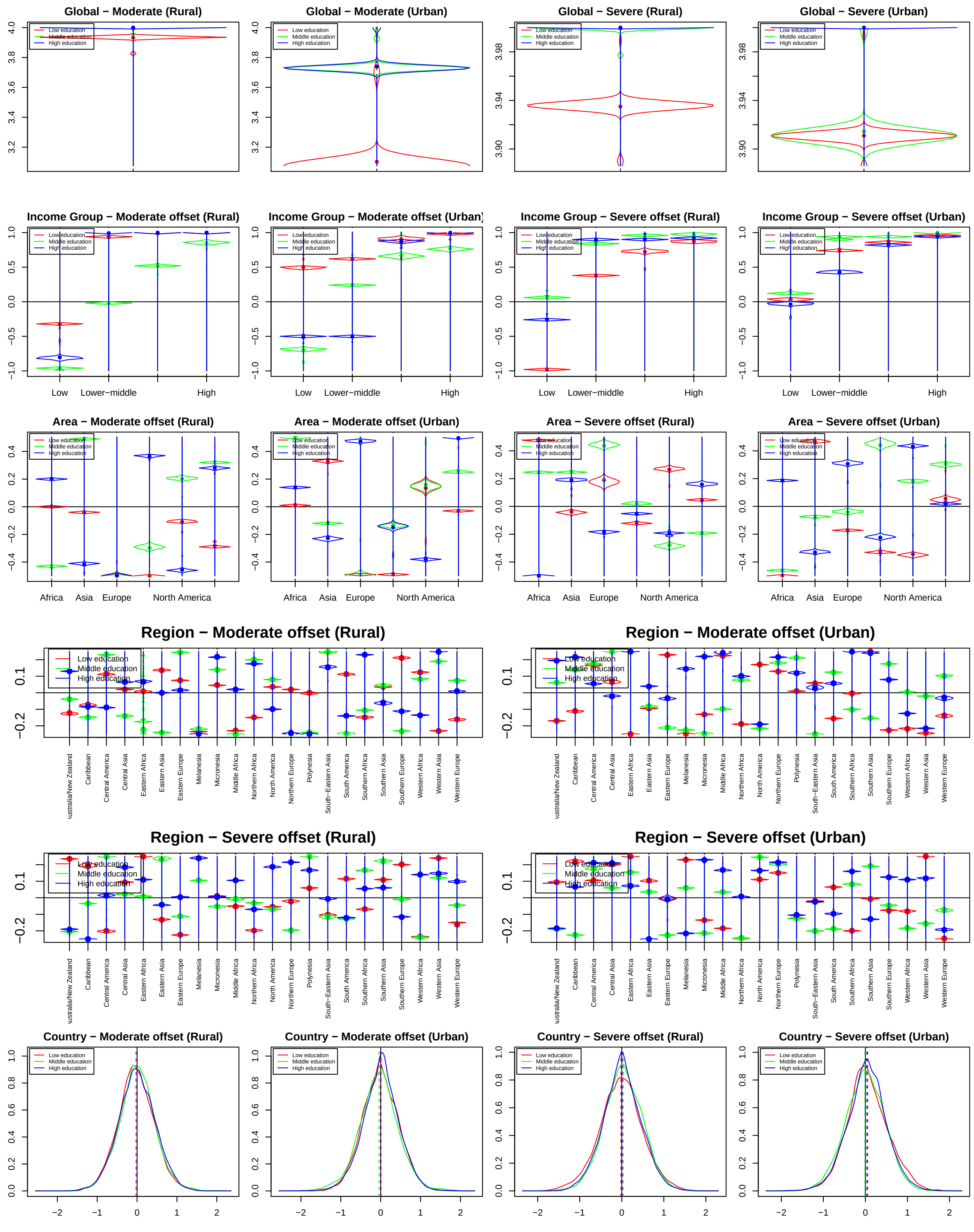

Parameters

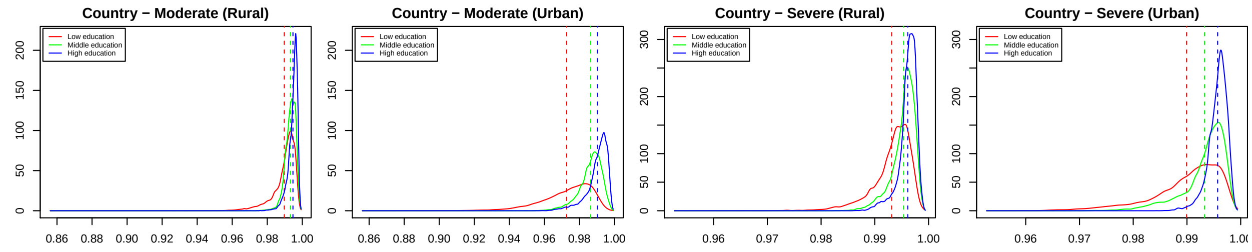

C.4.3.5.5 Other Hyperparameters

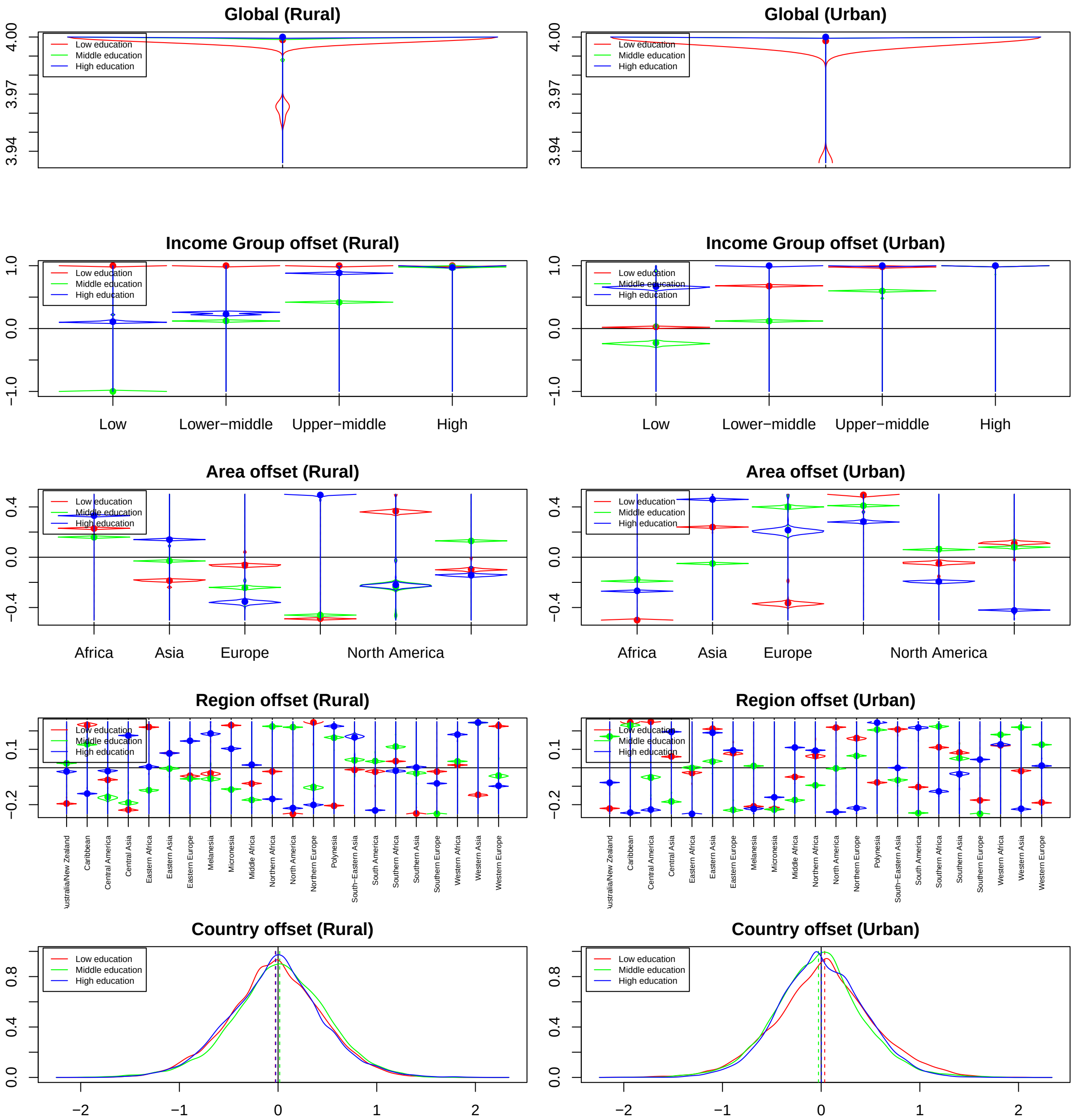

Parameters

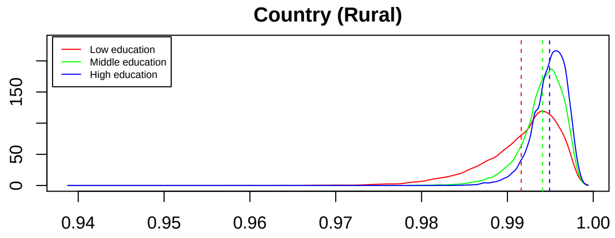
Country (Rural)
Low education
Middle education
High education
0
50
150
0.94
0.95
0.96
0.97
0.98
0.99
1.00

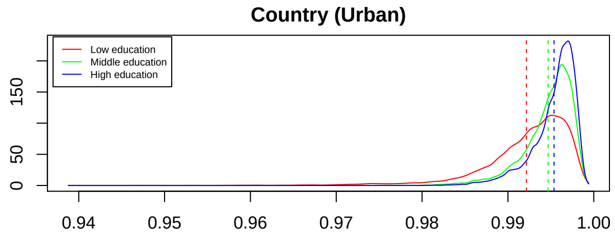
Country (Urban)
Low education
Middle education
High education
0
50
150
0.94
0.95
0.96
0.97
0.98
0.99
1.00

C.4.4 Transportation

C.4.4.1 Home

Hyperparameters

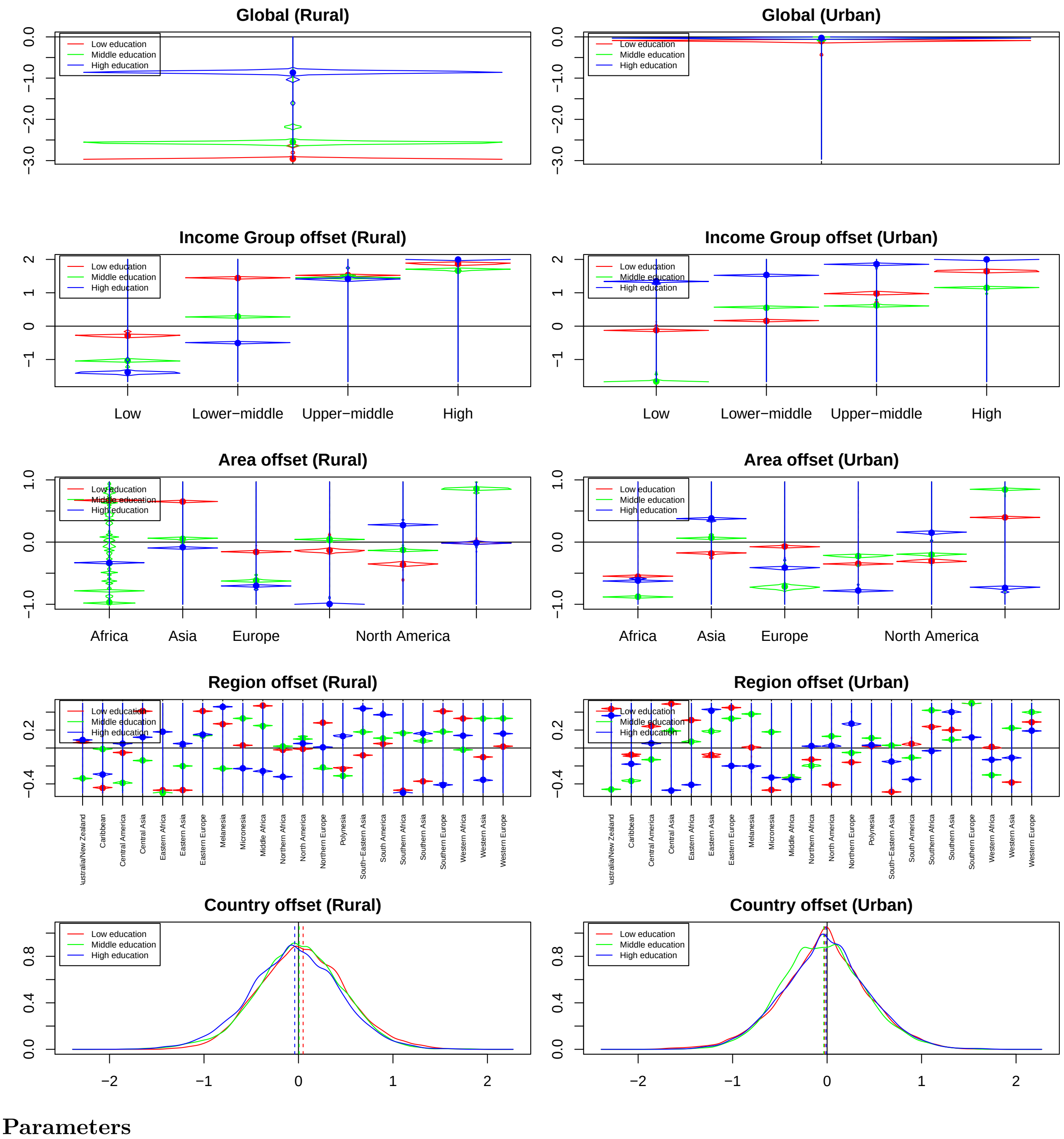

Parameters

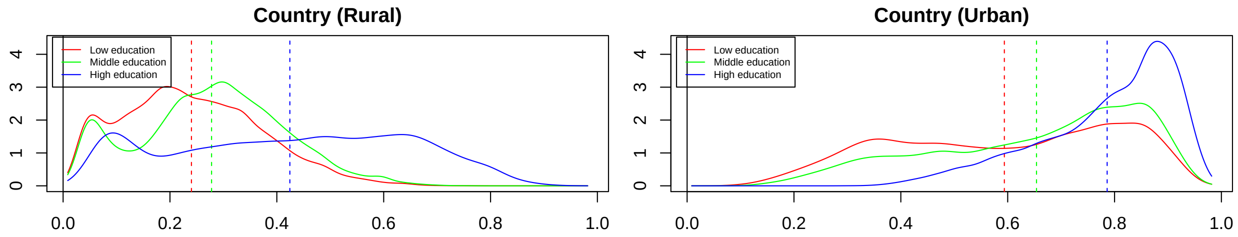

C.4.4.2 Home-SBA

Hyperparameters

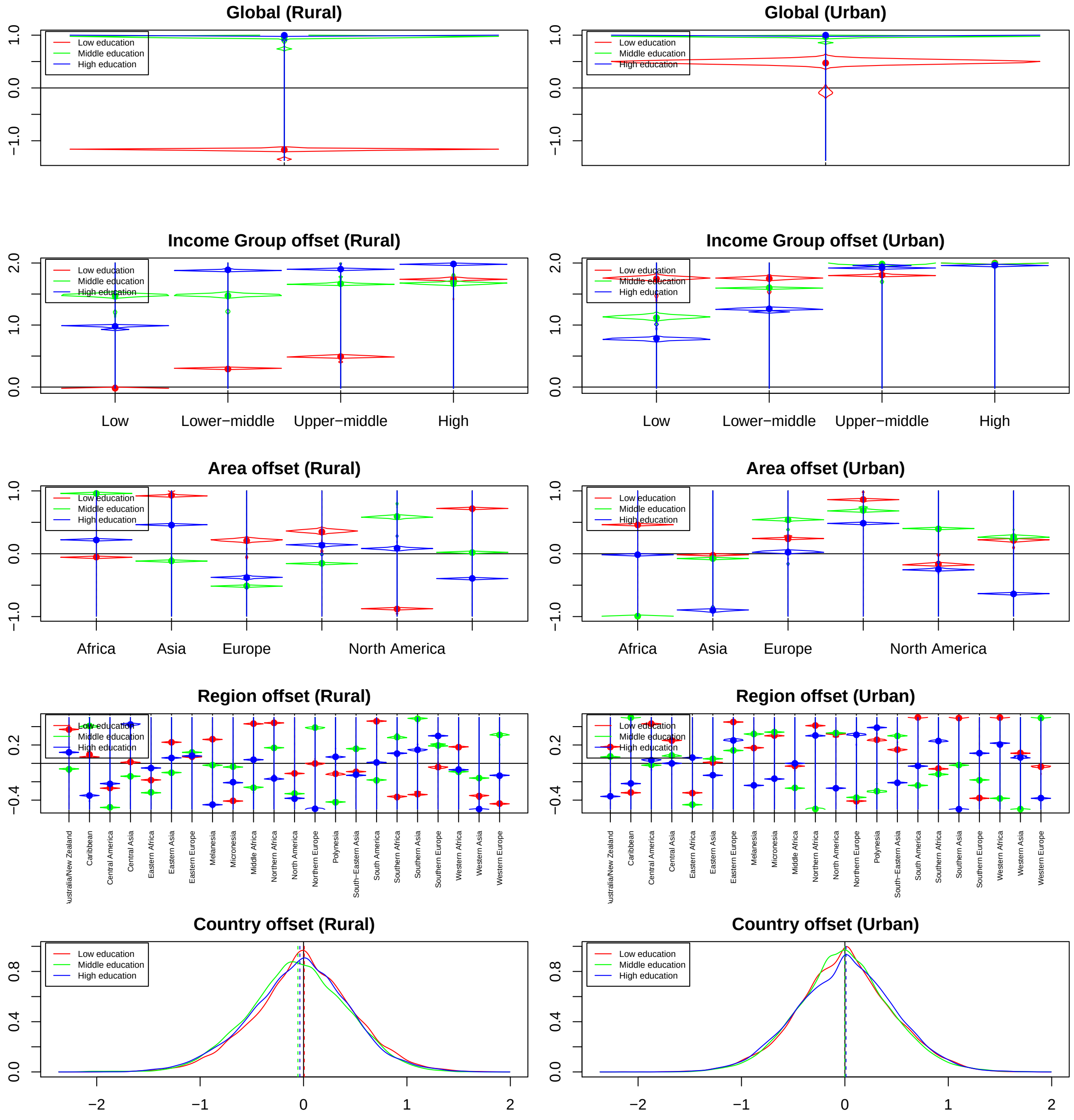

Parameters

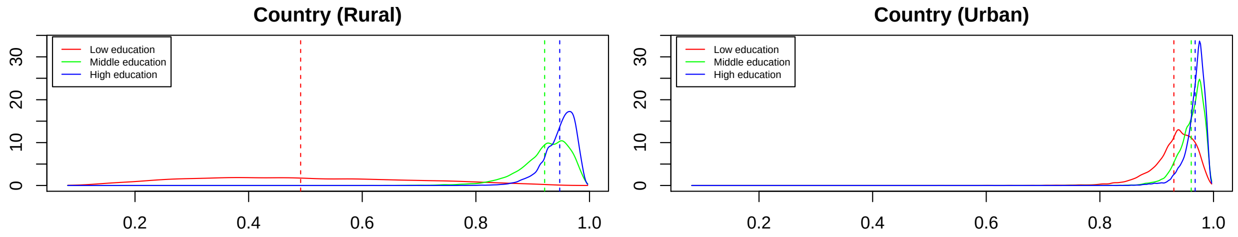

C.4.4.3 non-EmOC

Hyperparameters

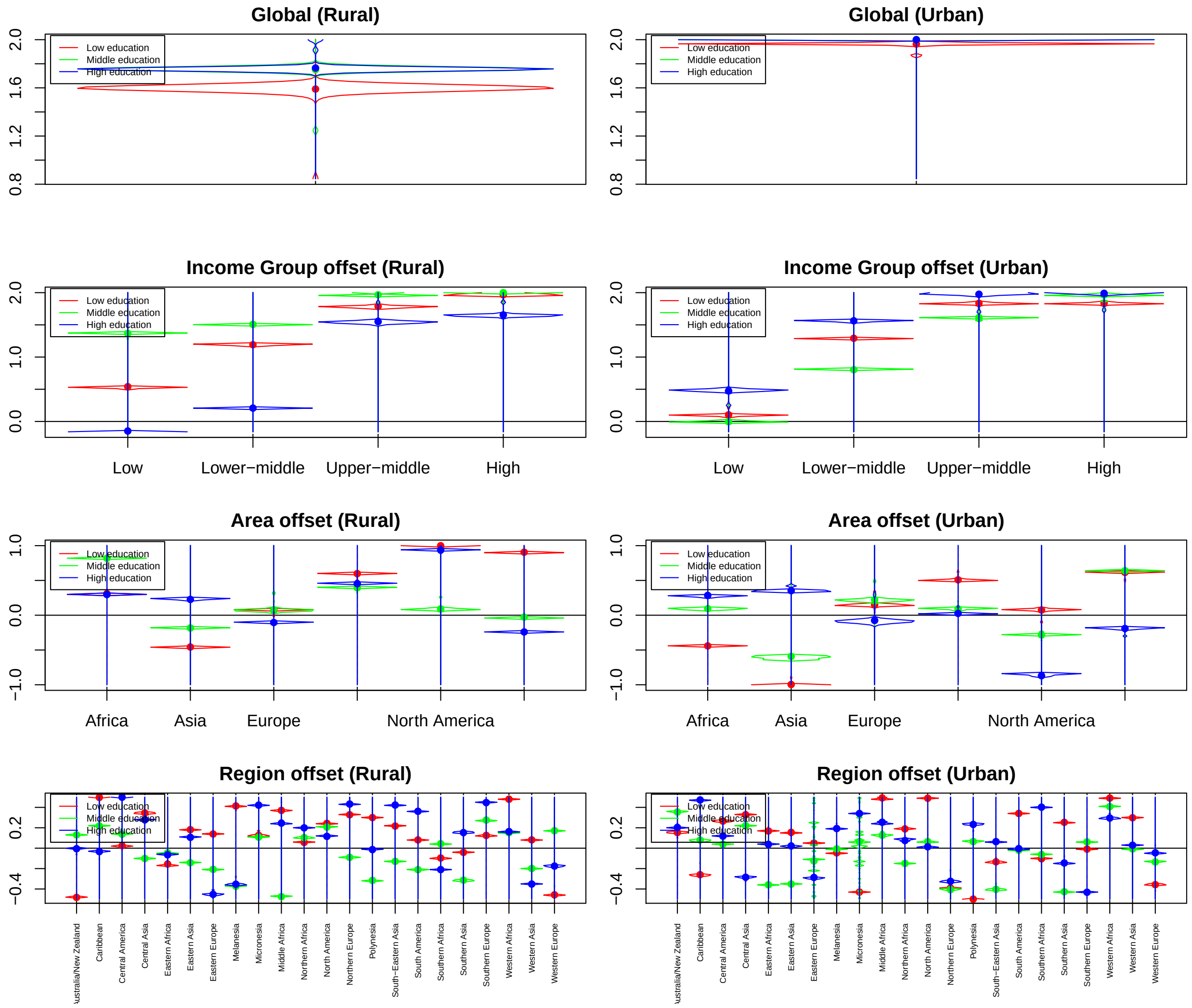

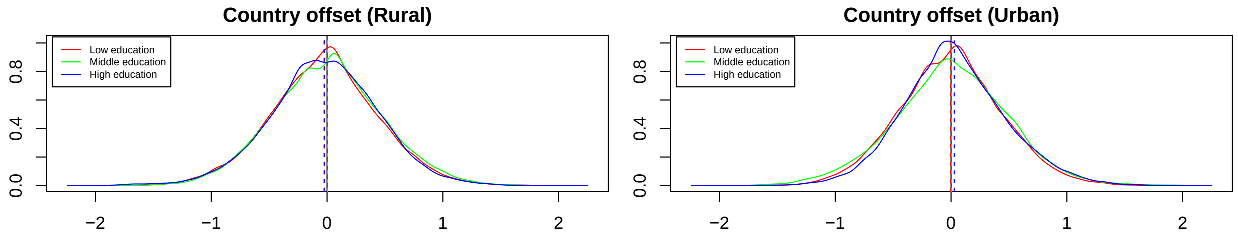

Parameters

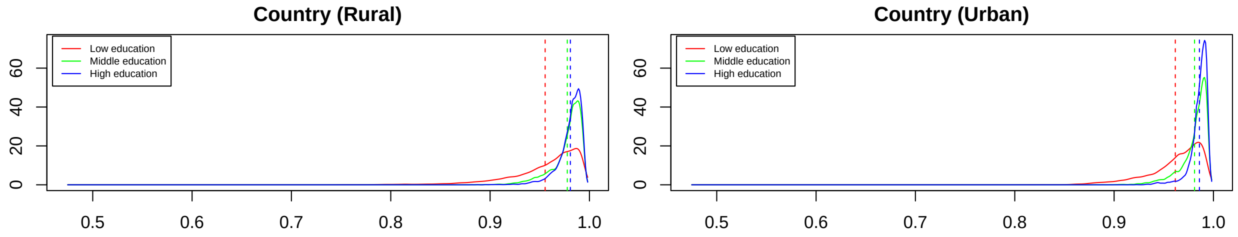

C.4.4.4 BEmOC

Hyperparameters

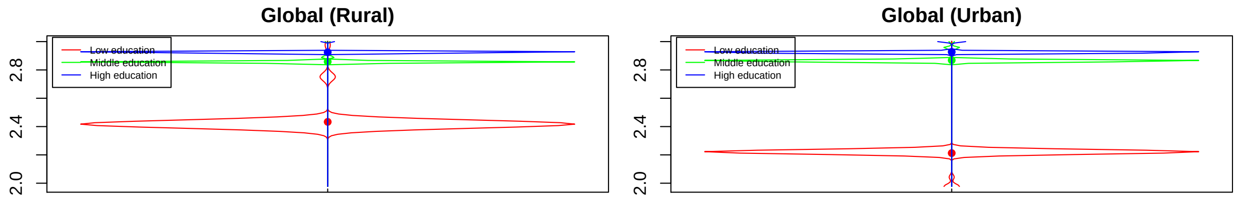

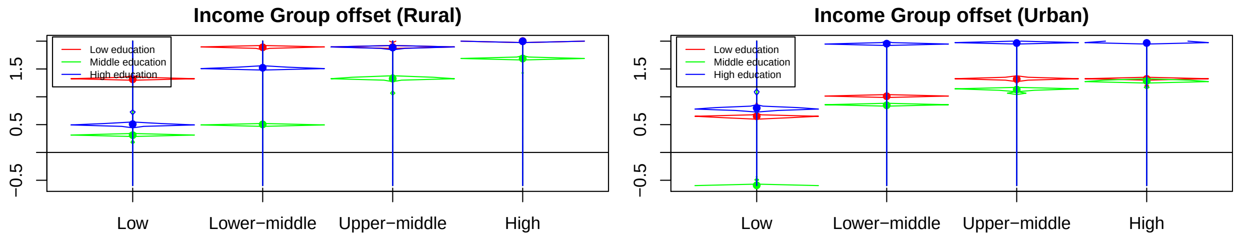

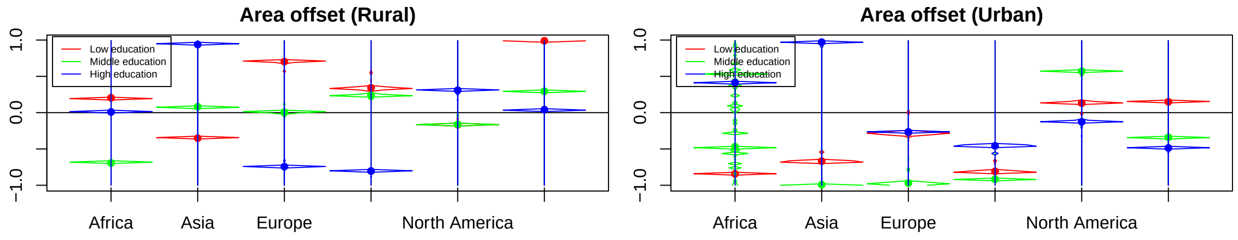

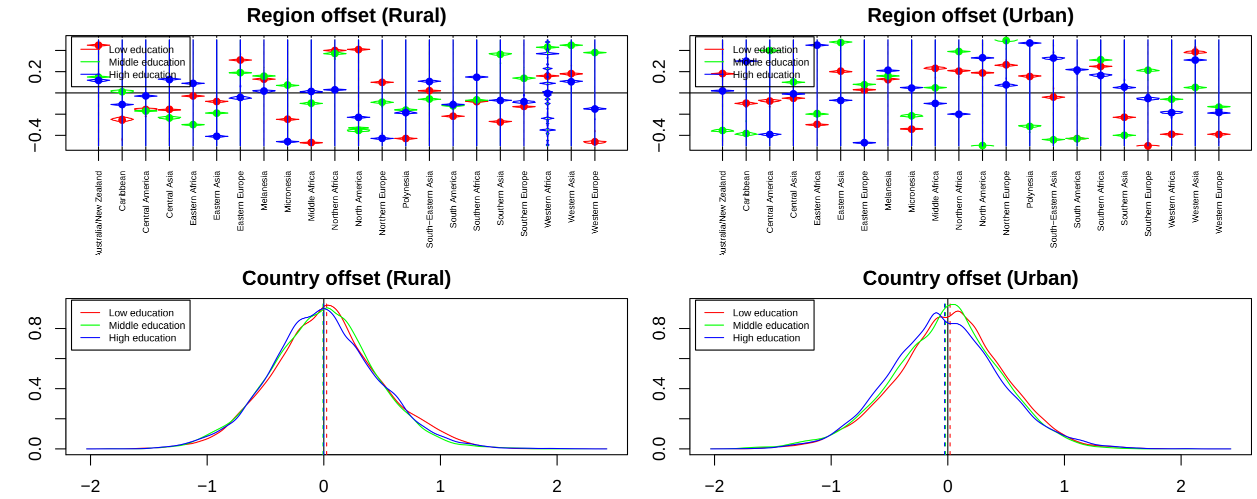
Region offset (Rural)
Region offset (Urban)
Low education
Middle education
High education
0.2
−0.4
Australia/New Zealand
Caribbean
Central America
Central Asia
Eastern Africa
Eastern Asia
Eastern Europe
Melanesia
Micronesia
Middle Africa
Northern Africa
North America
Northern Europe
Polynesia
South−Eastern Asia
South America
Southern Africa
Southern Asia
Southern Europe
Western Africa
Western Asia
Western Europe
Country offset (Rural)
Country offset (Urban)
0.0
0.4
0.8
−2
−1
0
1
2

Parameters

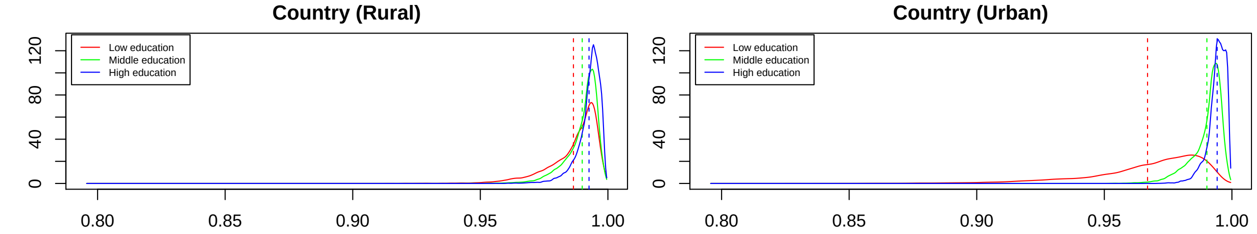
Country (Rural)
Country (Urban)
Low education
Middle education
High education
0
40
80
120
0.80
0.85
0.90
0.95
1.00

C.4.5 Referral Facility

C.4.5.1 Home

Hyperparameters

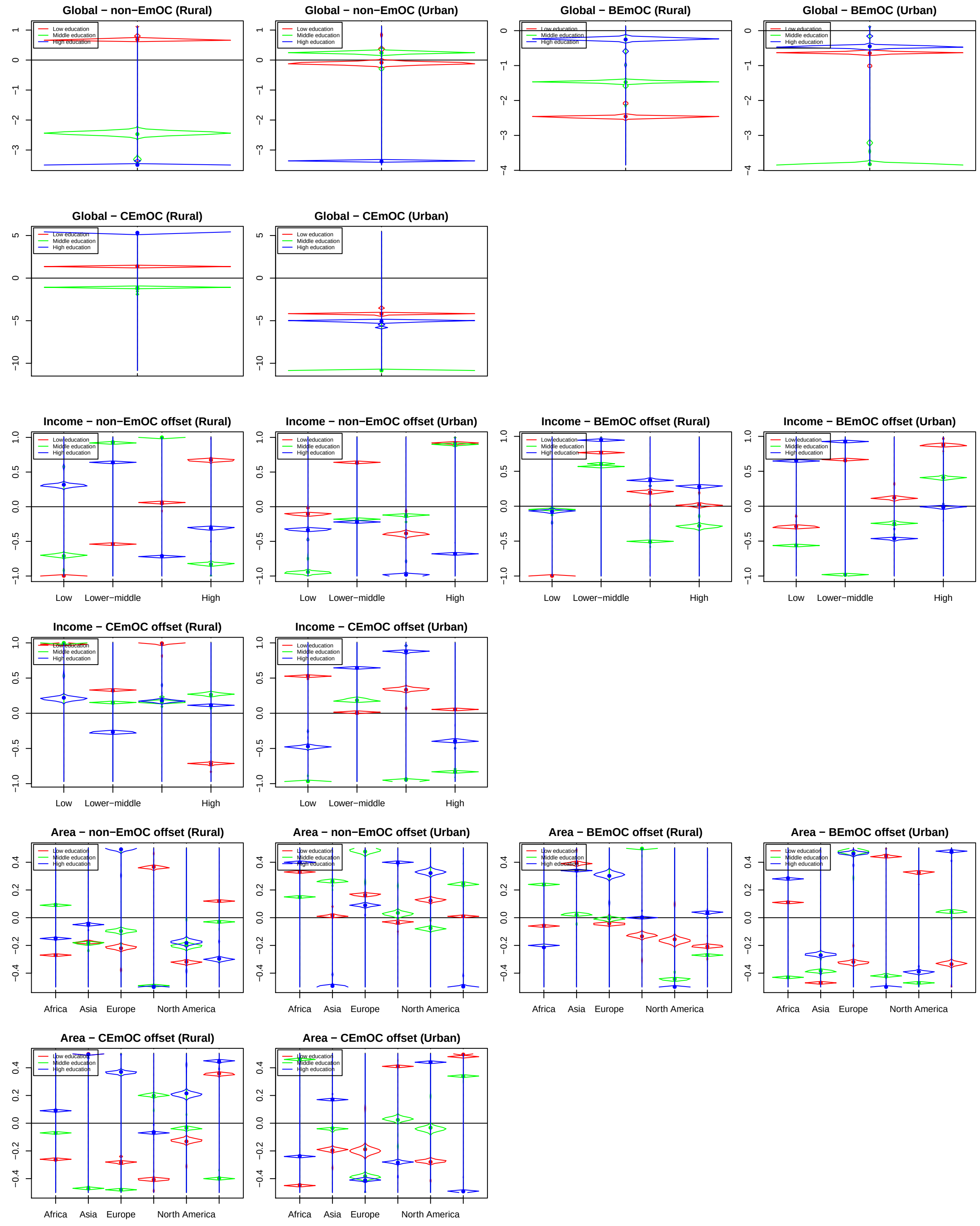

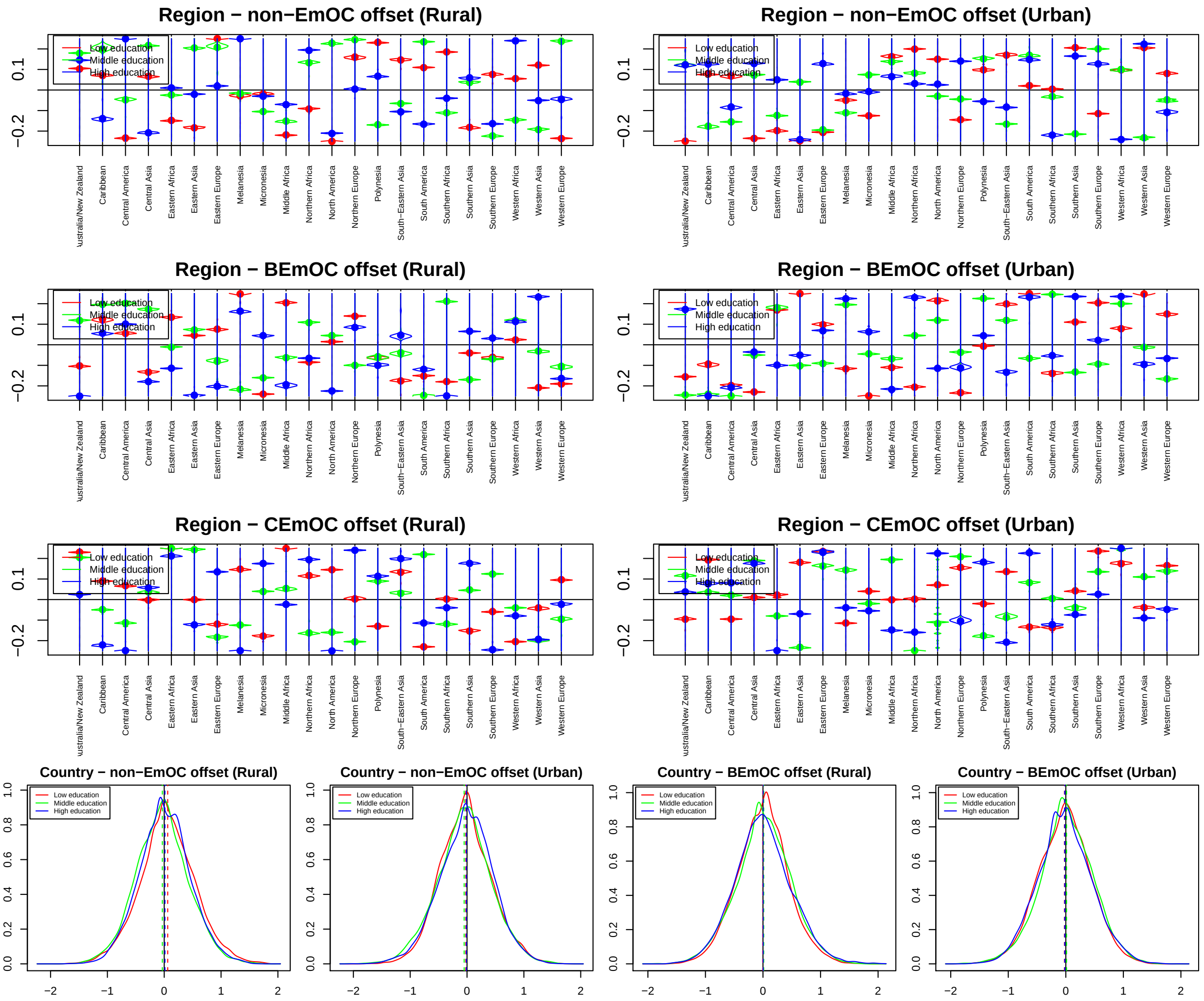

Parameters

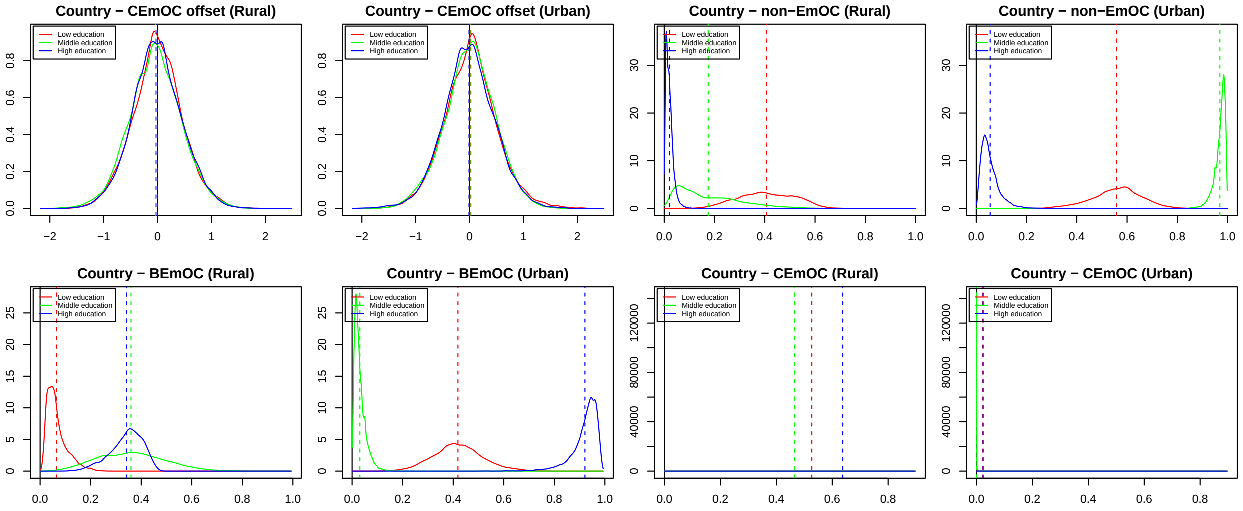

C.4.5.2 Home-SBA

Hyperparameters

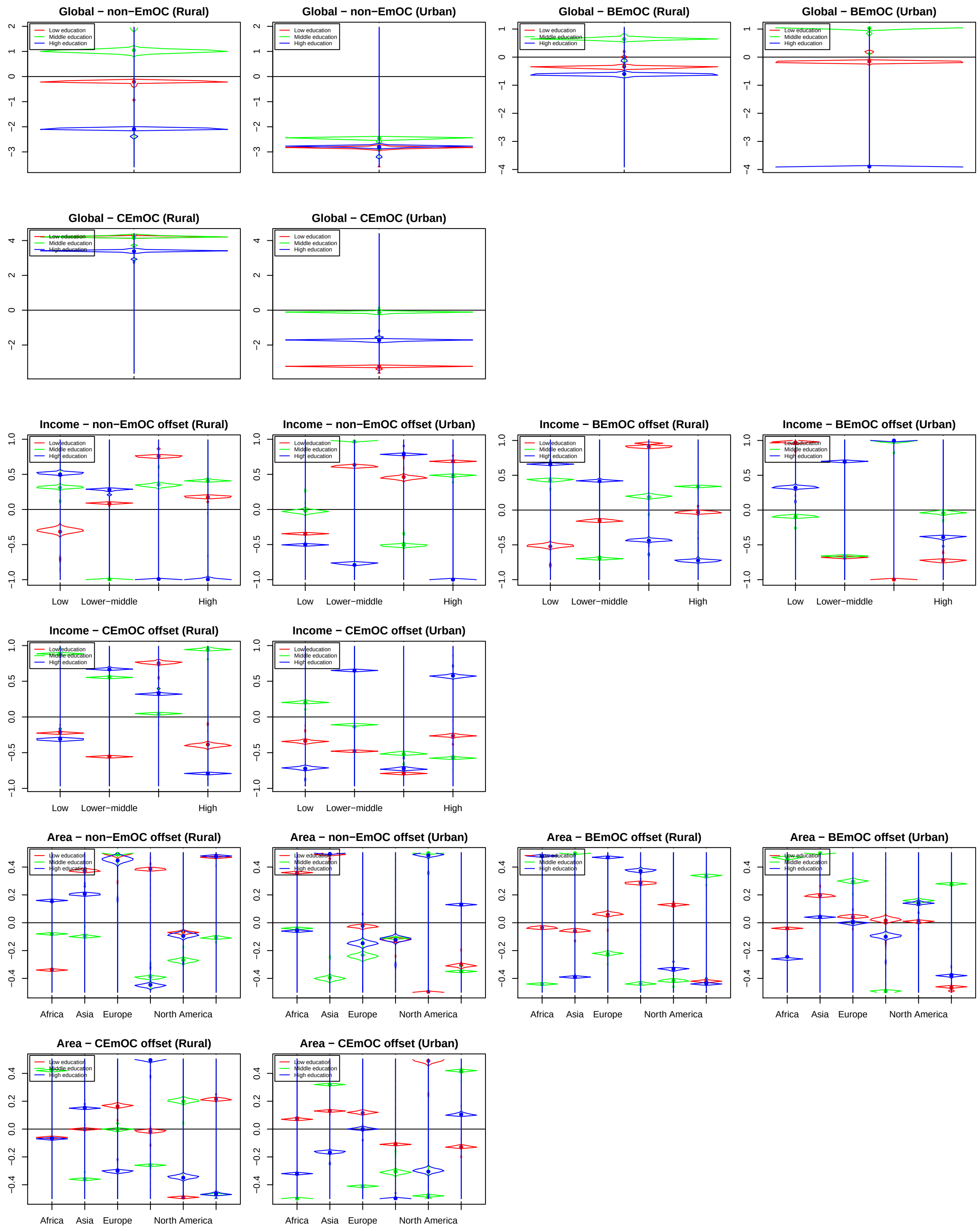

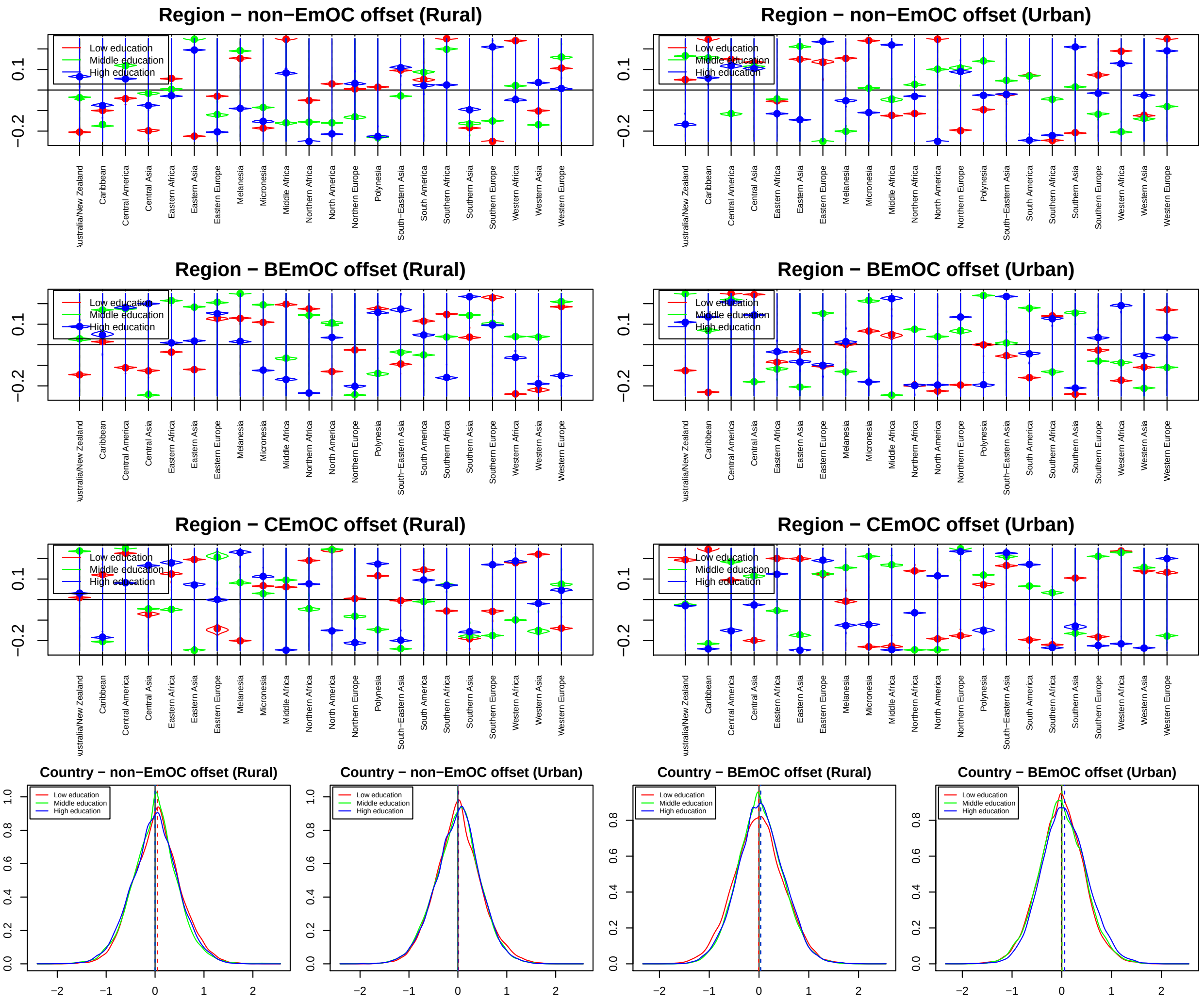

Parameters

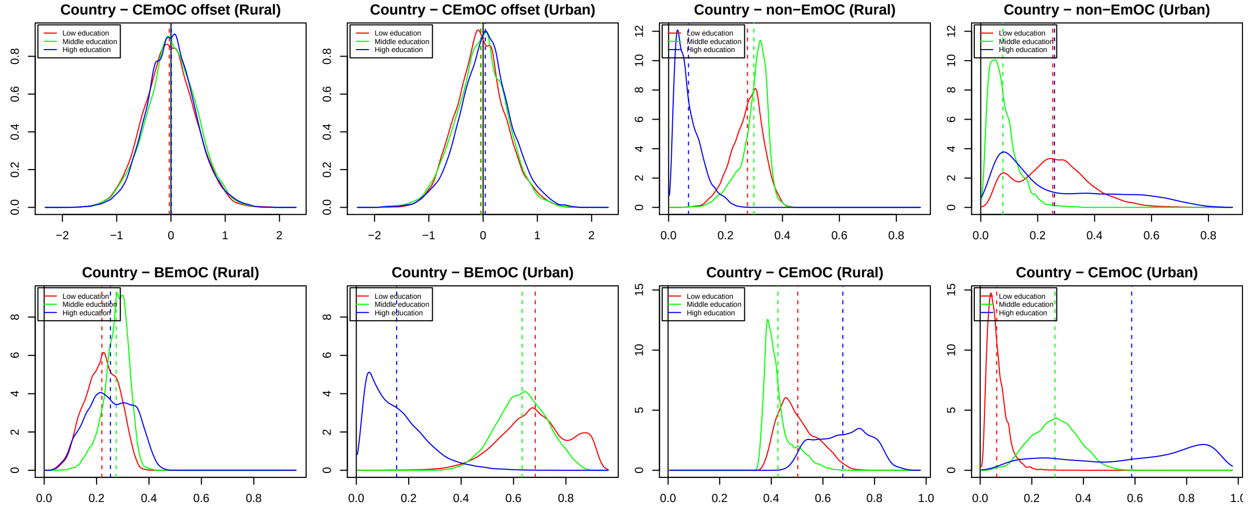

C.4.5.3 non-EmOC

Hyperparameters

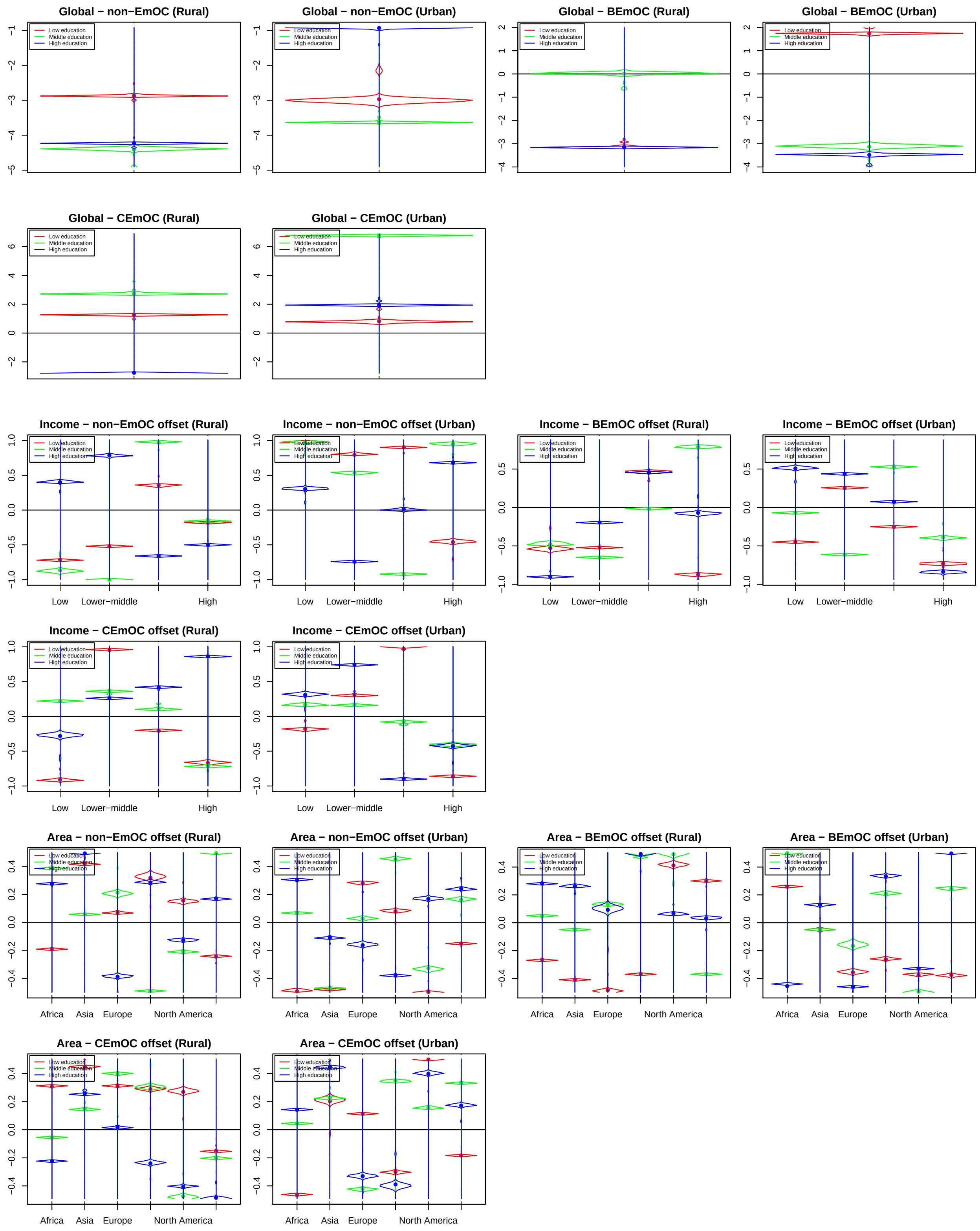

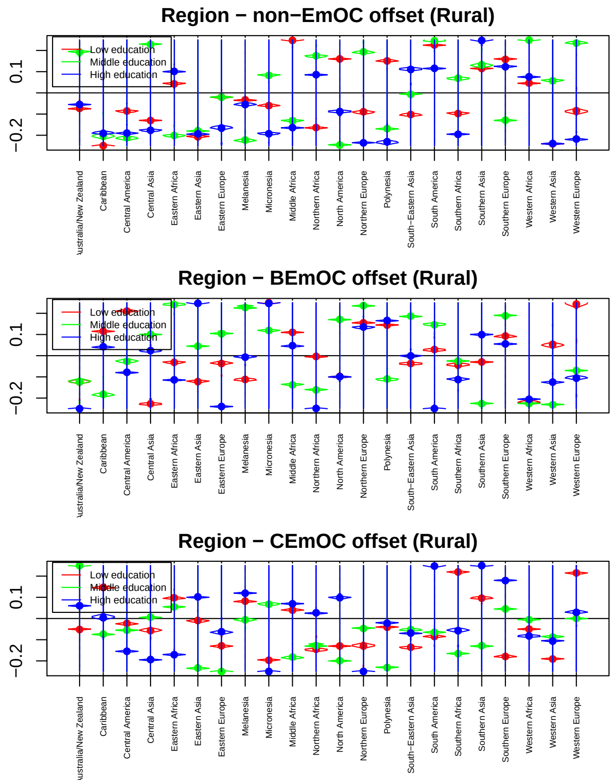

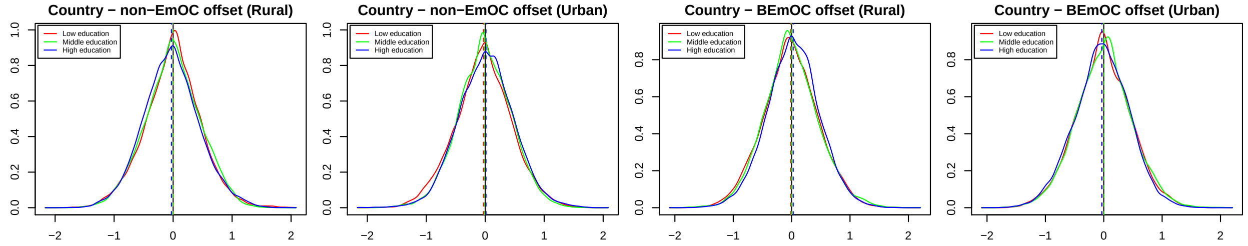

Parameters

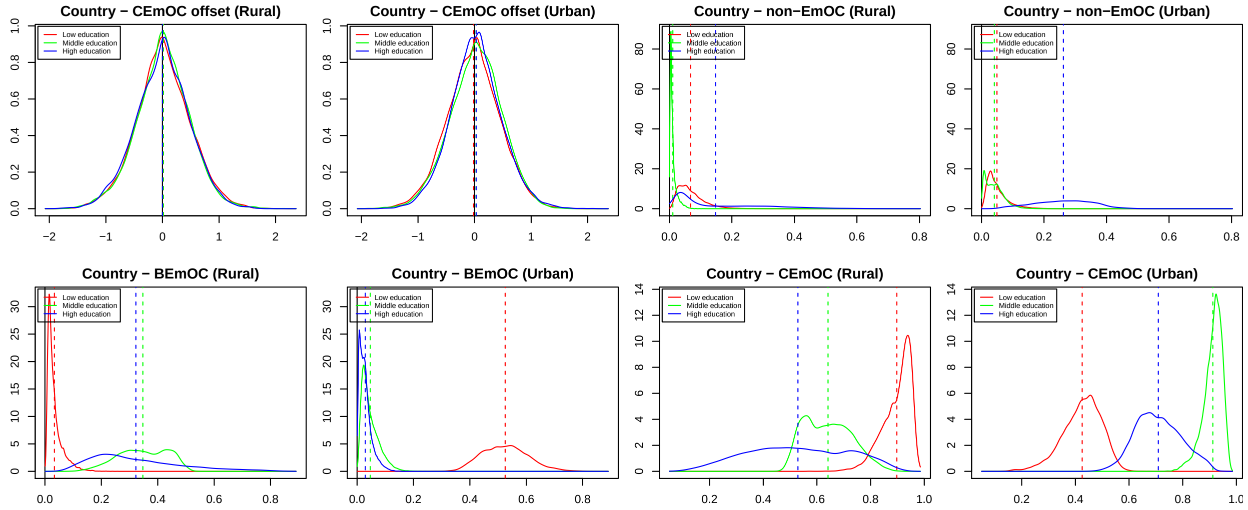

C.4.5.4 BEmOC

Hyperparameters

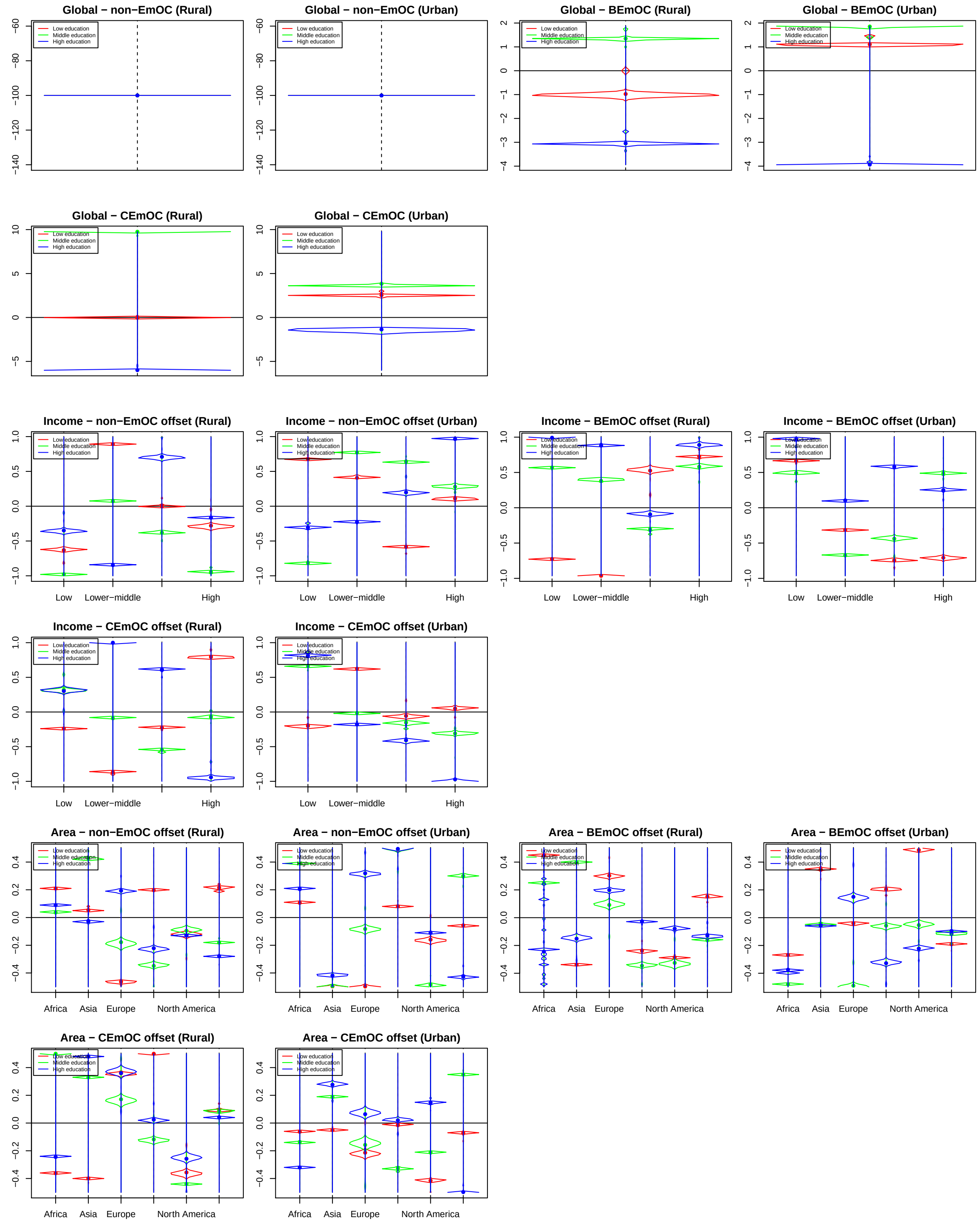

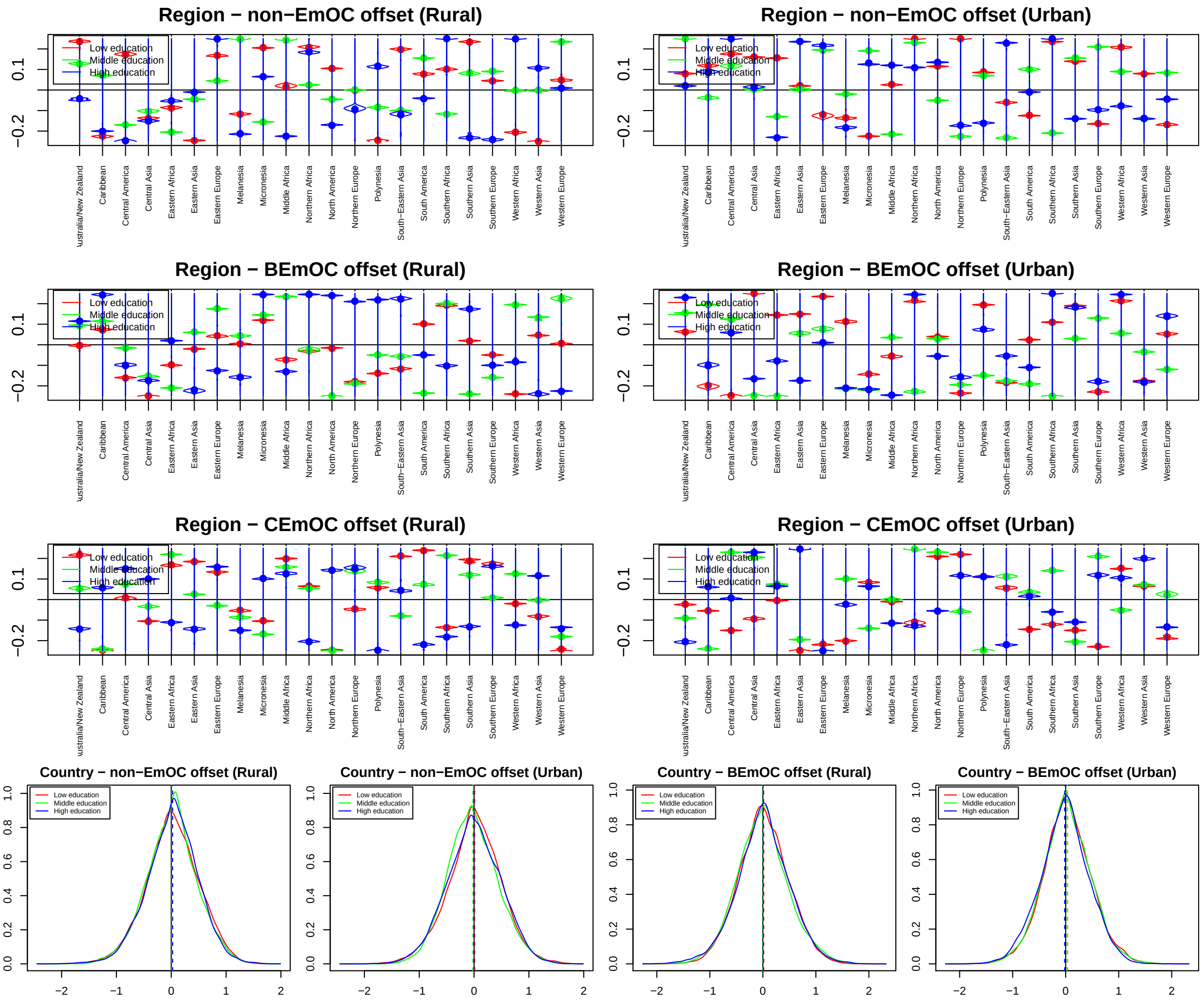

Region − non−EmOC offset (Rural)
Region − non−EmOC offset (Urban)
Region − BEmOC offset (Rural)
Region − BEmOC offset (Urban)
Region − CEmOC offset (Rural)
Region − CEmOC offset (Urban)
Low education
Middle education
High education
Australia/New Zealand
Caribbean
Central America
Central Asia
Eastern Africa
Eastern Asia
Eastern Europe
Melanesia
Micronesia
Middle Africa
Northern Africa
North America
Northern Europe
Polynesia
South−Eastern Asia
South America
Southern Africa
Southern Asia
Southern Europe
Western Africa
Western Asia
Western Europe
Country − non−EmOC offset (Rural)
Country − non−EmOC offset (Urban)
Country − BEmOC offset (Rural)
Country − BEmOC offset (Urban)

Parameters

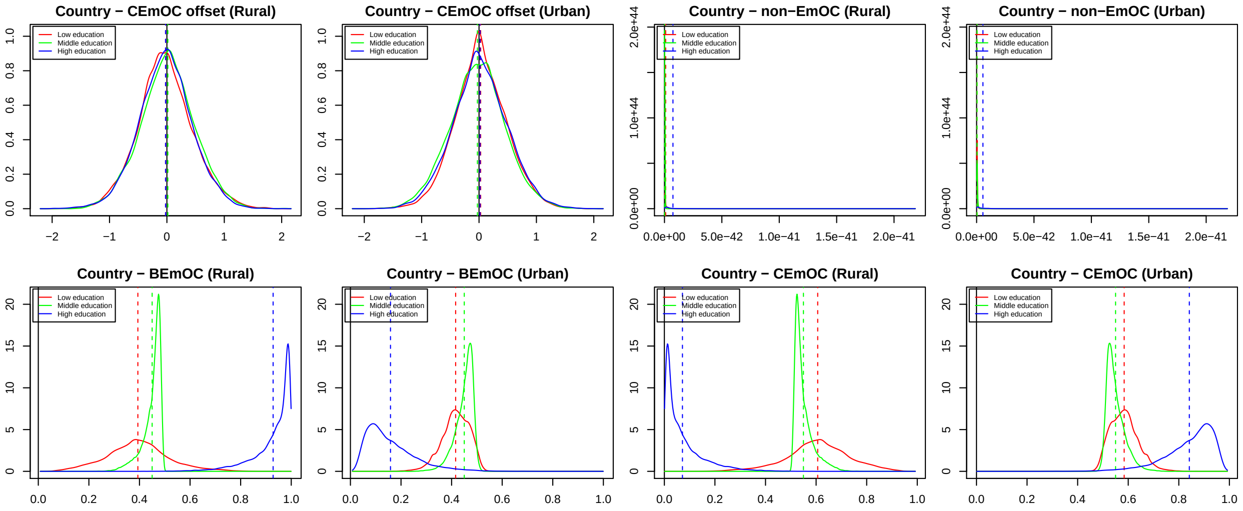

Country − CEmOC offset (Rural)
Country − CEmOC offset (Urban)
Country − non−EmOC (Rural)
Country − non−EmOC (Urban)
Country − BEmOC (Rural)
Country − BEmOC (Urban)
Country − CEmOC (Rural)
Country − CEmOC (Urban)
Low education
Middle education
High education

C.4.6 Under-reporting

C.4.6.1 CRVS

Hyperparameters

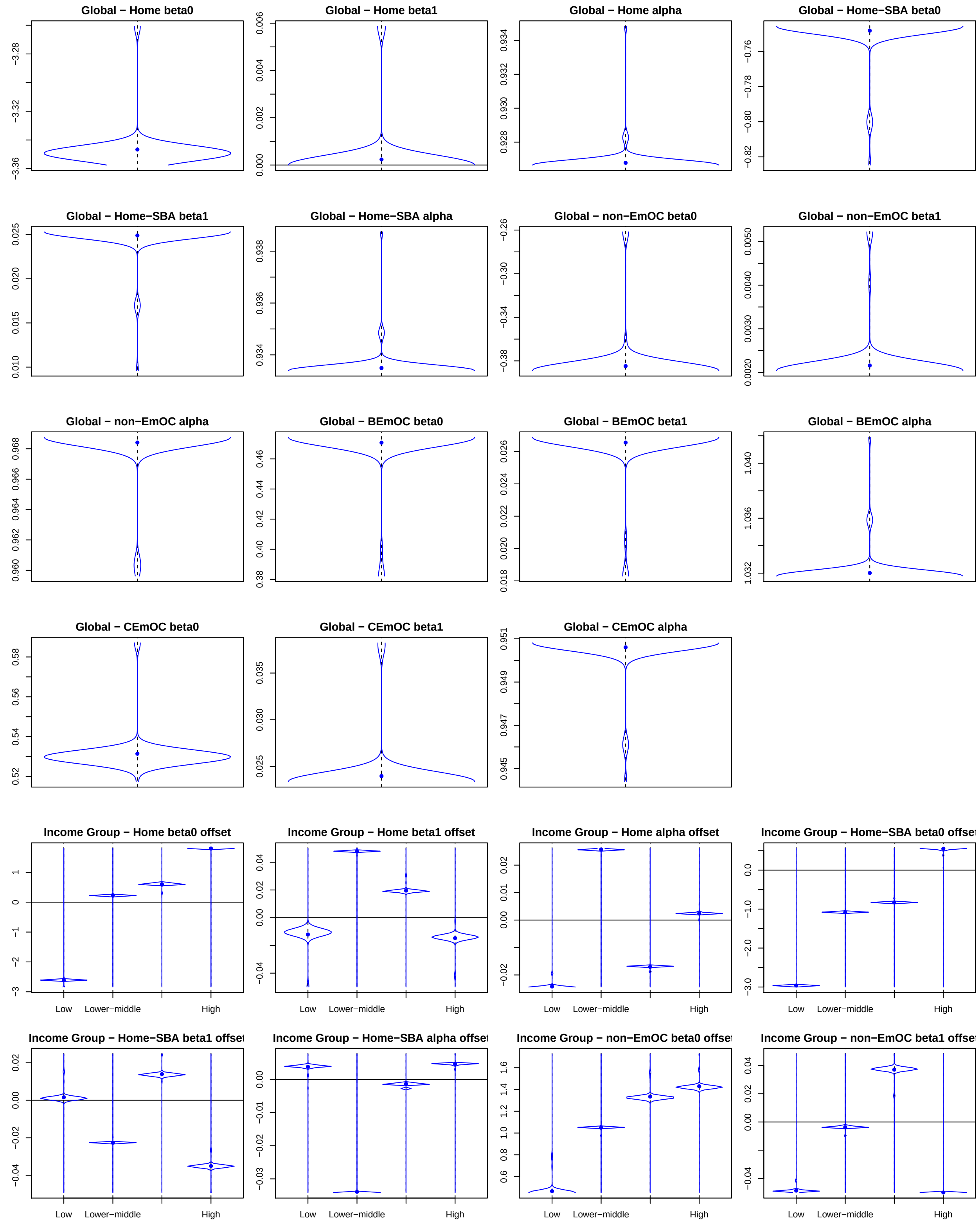

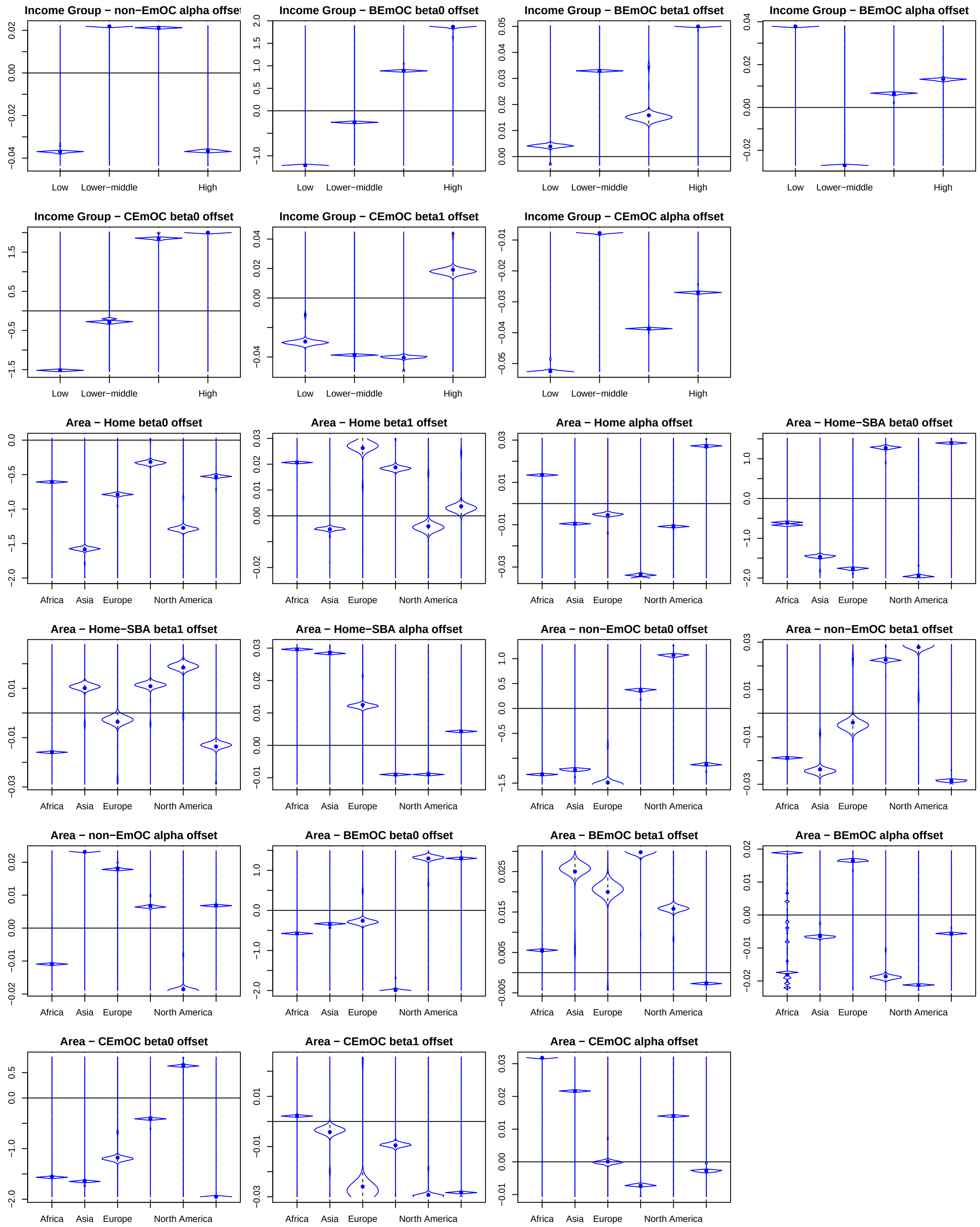
Income Group − non−EmOC alpha offset
Income Group − BEmOC beta0 offset
Income Group − BEmOC beta1 offset
Income Group − BEmOC alpha offset
Income Group − CEmOC beta0 offset
Income Group − CEmOC beta1 offset
Income Group − CEmOC alpha offset
Low
Lower−middle
High
Area − Home beta0 offset
Area − Home beta1 offset
Area − Home alpha offset
Area − Home−SBA beta0 offset
Area − Home−SBA beta1 offset
Area − Home−SBA alpha offset
Area − non−EmOC beta0 offset
Area − non−EmOC beta1 offset
Area − non−EmOC alpha offset
Area − BEmOC beta0 offset
Area − BEmOC beta1 offset
Area − BEmOC alpha offset
Area − CEmOC beta0 offset
Area − CEmOC beta1 offset
Area − CEmOC alpha offset
Africa
Asia
Europe
North America

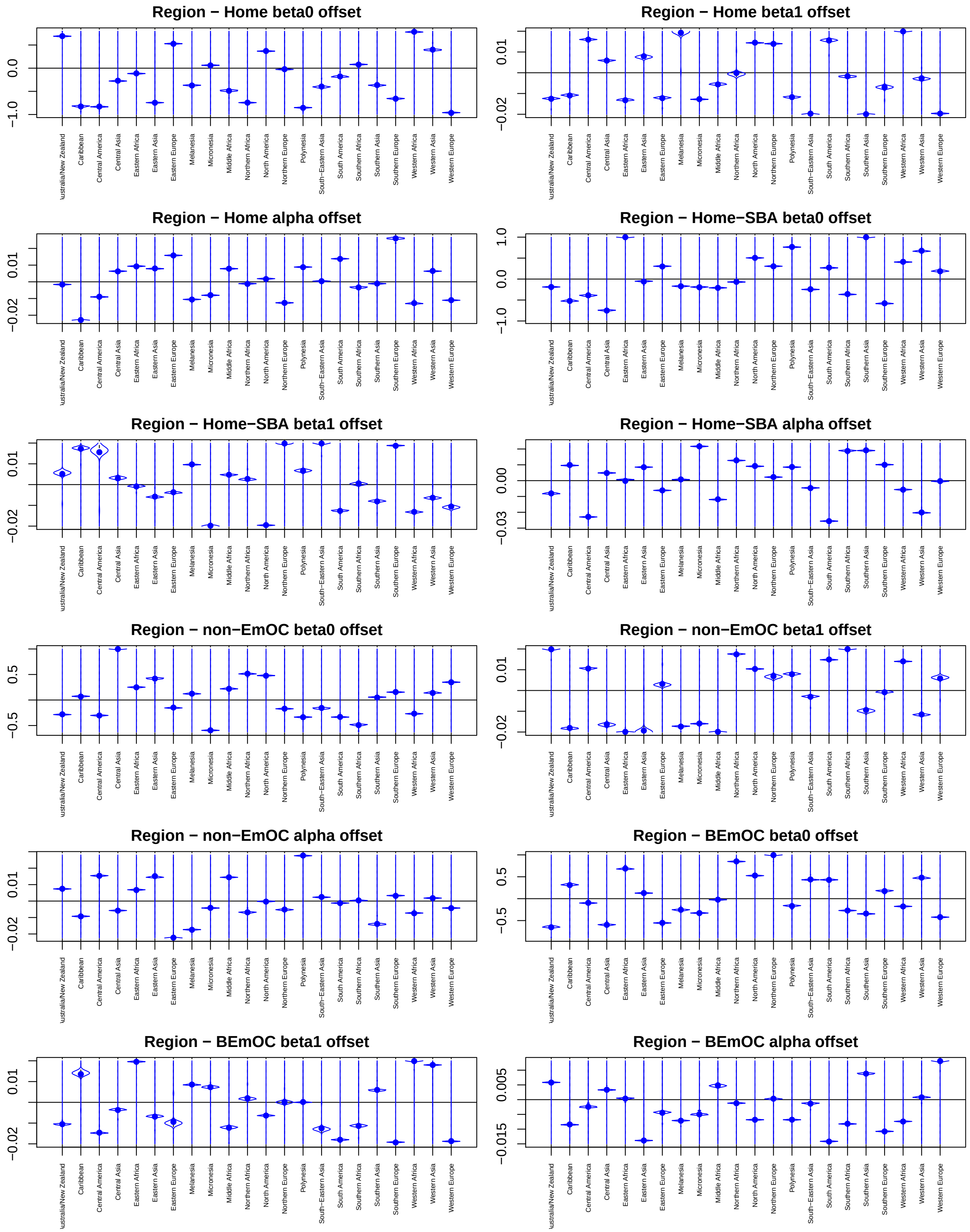
Region − Home beta0 offset
Region − Home beta1 offset
Region − Home alpha offset
Region − Home−SBA beta0 offset
Region − Home−SBA beta1 offset
Region − Home−SBA alpha offset
Region − non−EmOC beta0 offset
Region − non−EmOC beta1 offset
Region − non−EmOC alpha offset
Region − BEmOC beta0 offset
Region − BEmOC beta1 offset
Region − BEmOC alpha offset
ustralia/New Zealand
Caribbean
Central America
Central Asia
Eastern Africa
Eastern Asia
Eastern Europe
Melanesia
Micronesia
Middle Africa
Northern Africa
North America
Northern Europe
Polynesia
South−Eastern Asia
South America
Southern Africa
Southern Asia
Southern Europe
Western Africa
Western Asia
Western Europe

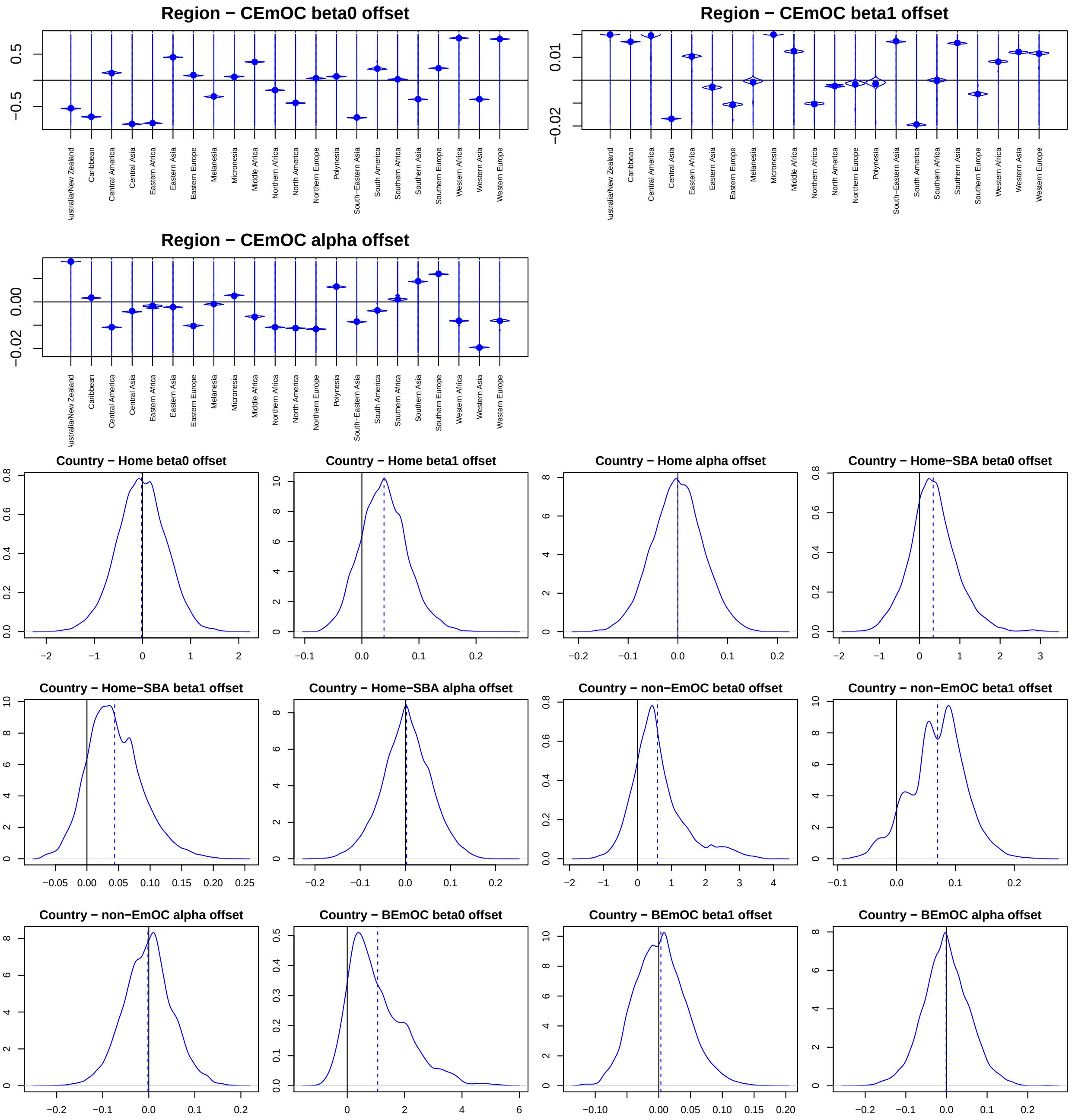
Region − CEmOC beta0 offset
Region − CEmOC beta1 offset
Region − CEmOC alpha offset
Country − Home beta0 offset
Country − Home beta1 offset
Country − Home alpha offset
Country − Home−SBA beta0 offset
Country − Home−SBA beta1 offset
Country − Home−SBA alpha offset
Country − non−EmOC beta0 offset
Country − non−EmOC beta1 offset
Country − non−EmOC alpha offset
Country − BEmOC beta0 offset
Country − BEmOC beta1 offset
Country − BEmOC alpha offset

Parameters

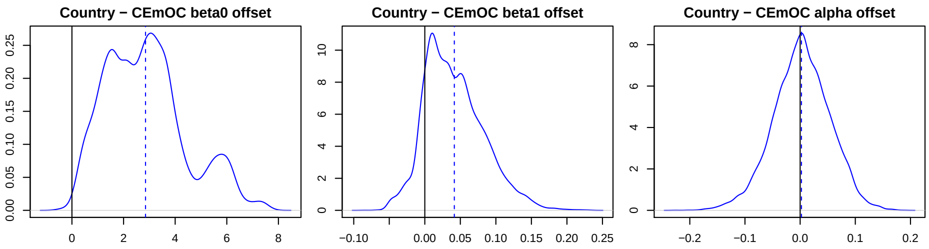
Country − CEmOC beta0 offset
Country − CEmOC beta1 offset
Country − CEmOC alpha offset

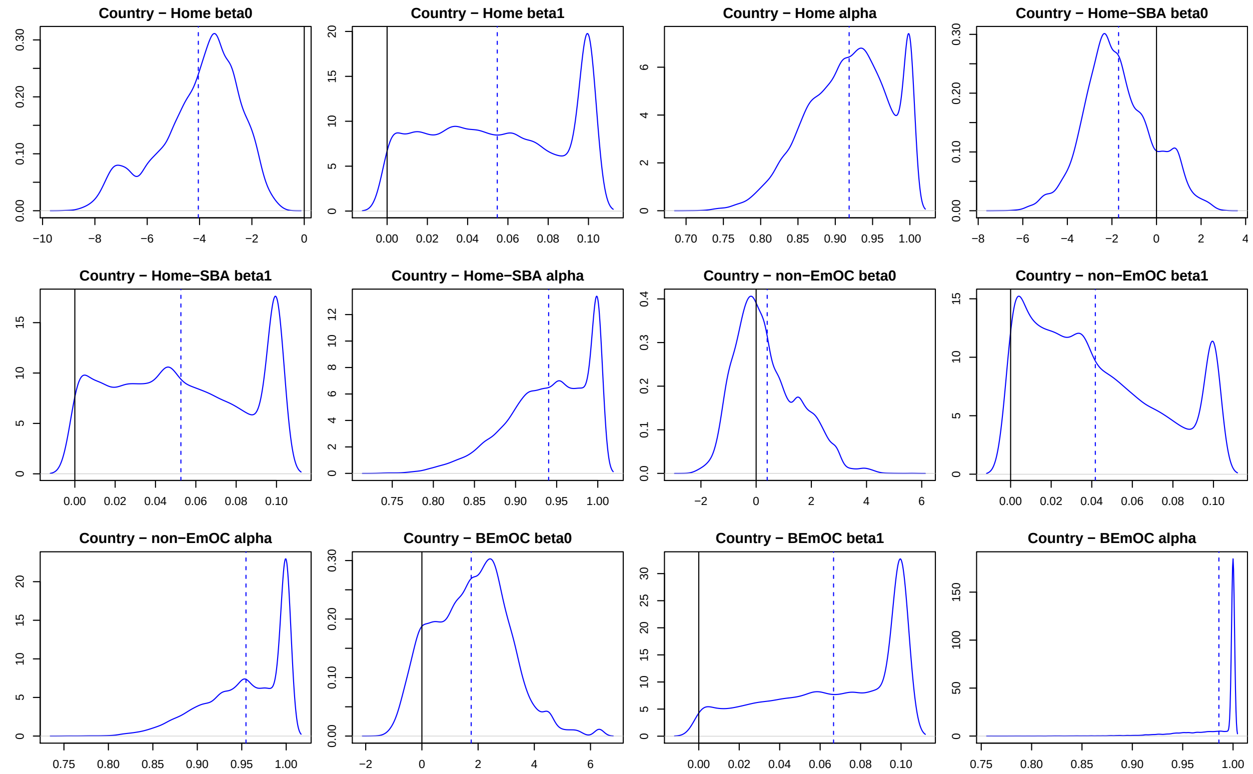

C.4.6.2 Survey

Hyperparameters

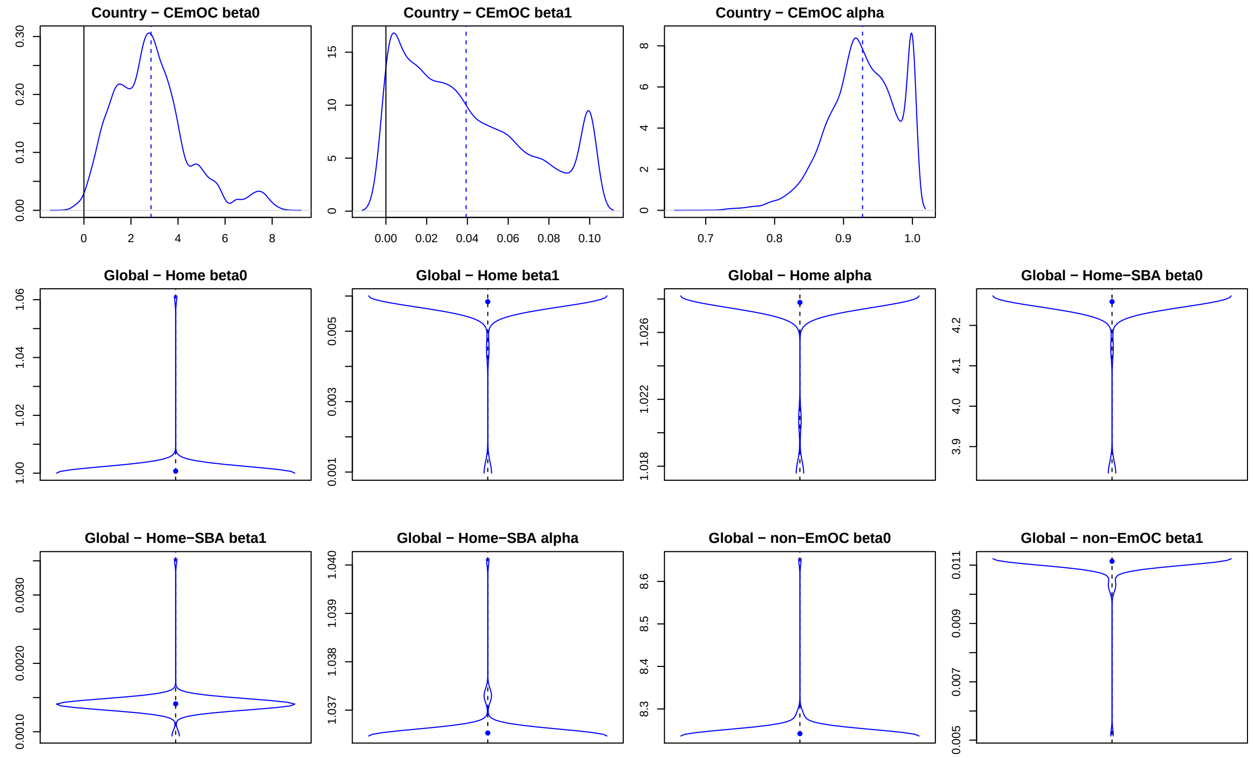

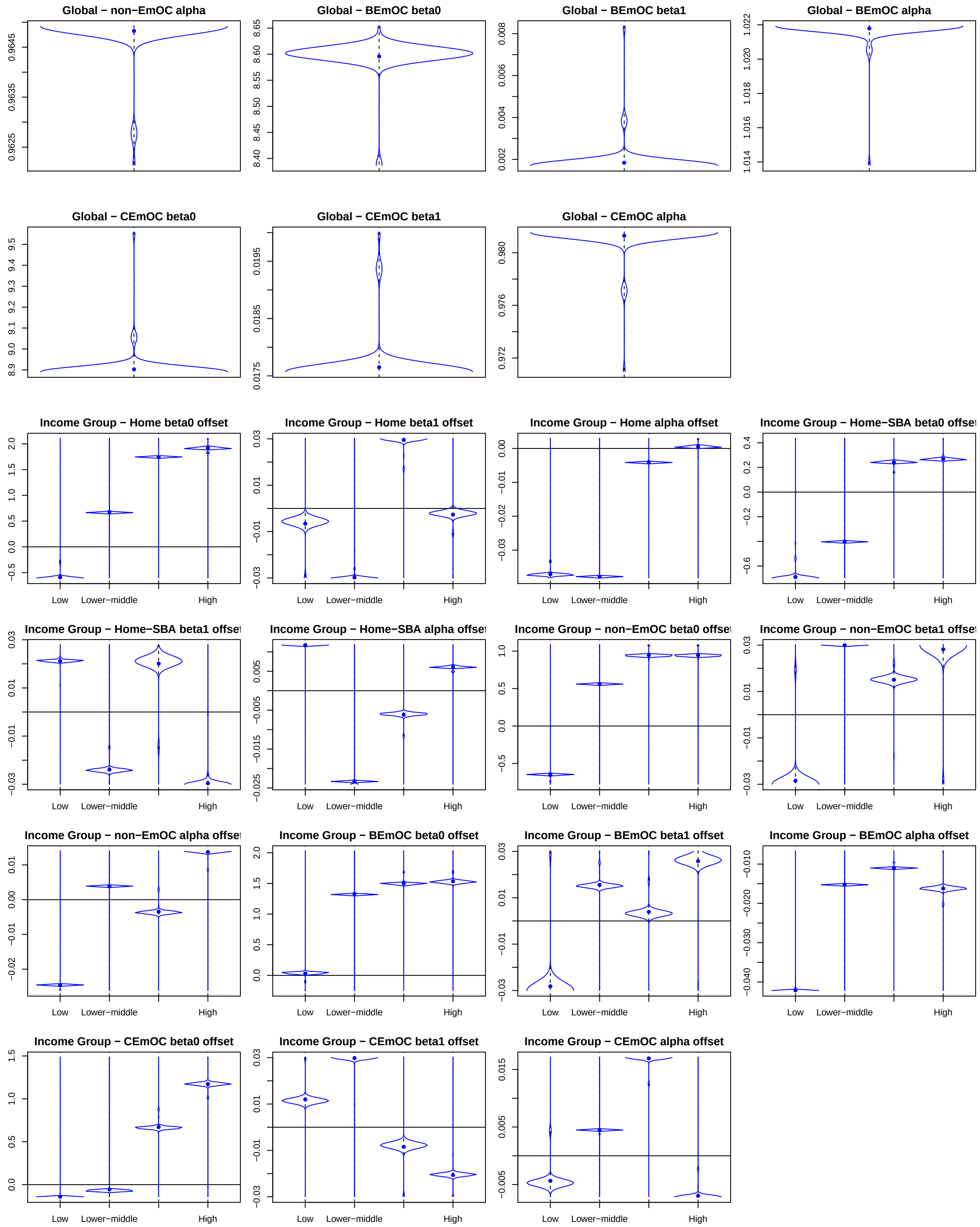
Global − non−EmOC alpha
Global − BEmOC beta0
Global − BEmOC beta1
Global − BEmOC alpha
Global − CEmOC beta0
Global − CEmOC beta1
Global − CEmOC alpha
Income Group − Home beta0 offset
Income Group − Home beta1 offset
Income Group − Home alpha offset
Income Group − Home−SBA beta0 offset
Income Group − Home−SBA beta1 offset
Income Group − Home−SBA alpha offset
Income Group − non−EmOC beta0 offset
Income Group − non−EmOC beta1 offset
Income Group − non−EmOC alpha offset
Income Group − BEmOC beta0 offset
Income Group − BEmOC beta1 offset
Income Group − BEmOC alpha offset
Income Group − CEmOC beta0 offset
Income Group − CEmOC beta1 offset
Income Group − CEmOC alpha offset
Low
Lower−middle
High

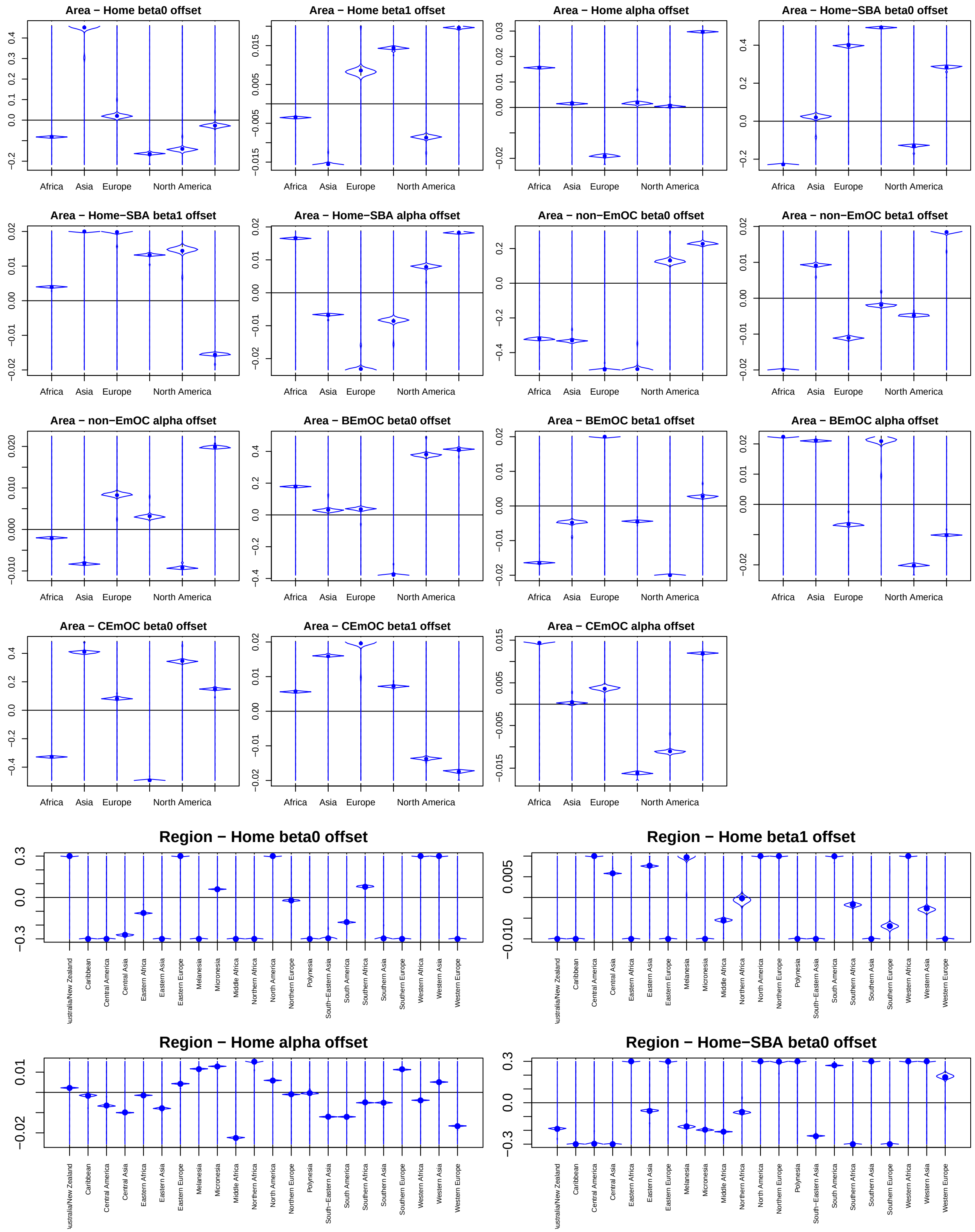
Area − Home beta0 offset
Area − Home beta1 offset
Area − Home alpha offset
Area − Home−SBA beta0 offset
Area − Home−SBA beta1 offset
Area − Home−SBA alpha offset
Area − non−EmOC beta0 offset
Area − non−EmOC beta1 offset
Area − non−EmOC alpha offset
Area − BEmOC beta0 offset
Area − BEmOC beta1 offset
Area − BEmOC alpha offset
Area − CEmOC beta0 offset
Area − CEmOC beta1 offset
Area − CEmOC alpha offset
Africa
Asia
Europe
North America
Region − Home beta0 offset
Region − Home beta1 offset
Region − Home alpha offset
Region − Home−SBA beta0 offset
Australia/New Zealand
Caribbean
Central America
Central Asia
Eastern Africa
Eastern Asia
Eastern Europe
Melanesia
Micronesia
Middle Africa
Northern Africa
North America
Northern Europe
Polynesia
South−Eastern Asia
South America
Southern Africa
Southern Asia
Southern Europe
Western Africa
Western Asia
Western Europe

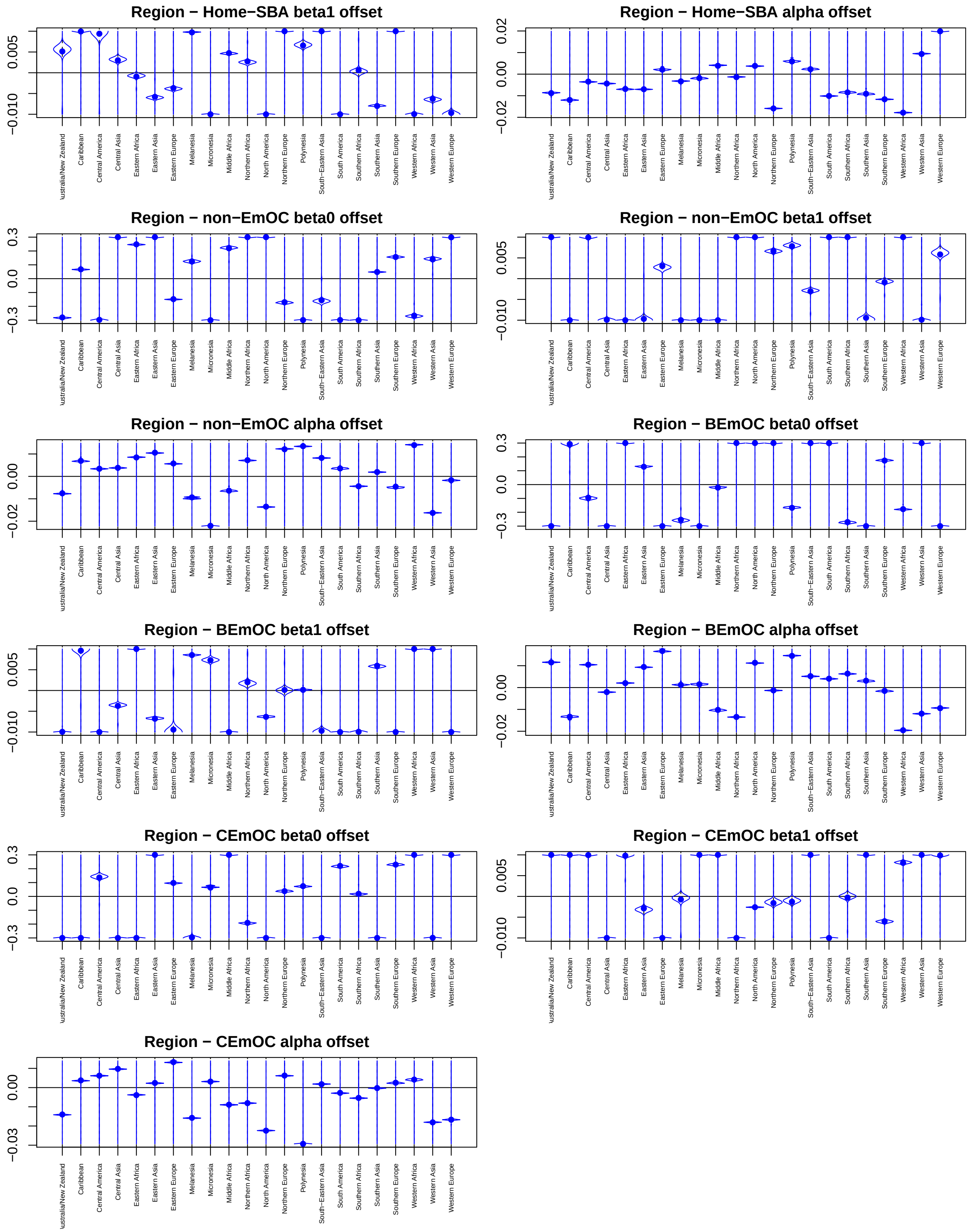

Region − Home−SBA beta1 offset
0.005
−0.010
Region − Home−SBA alpha offset
0.02
0.00
−0.02
Region − non−EmOC beta0 offset
0.3
0.0
−0.3
Region − non−EmOC beta1 offset
0.005
−0.010
Region − non−EmOC alpha offset
0.00
−0.02
Region − BEmOC beta0 offset
0.3
0.0
−0.3
Region − BEmOC beta1 offset
0.005
−0.010
Region − BEmOC alpha offset
0.00
−0.02
Region − CEmOC beta0 offset
0.3
0.0
−0.3
Region − CEmOC beta1 offset
0.005
−0.010
Region − CEmOC alpha offset
0.00
−0.03
ustralia/New Zealand
Caribbean
Central America
Central Asia
Eastern Africa
Eastern Asia
Eastern Europe
Melanesia
Micronesia
Middle Africa
Northern Africa
North America
Northern Europe
Polynesia
South−Eastern Asia
South America
Southern Africa
Southern Asia
Southern Europe
Western Africa
Western Asia
Western Europe

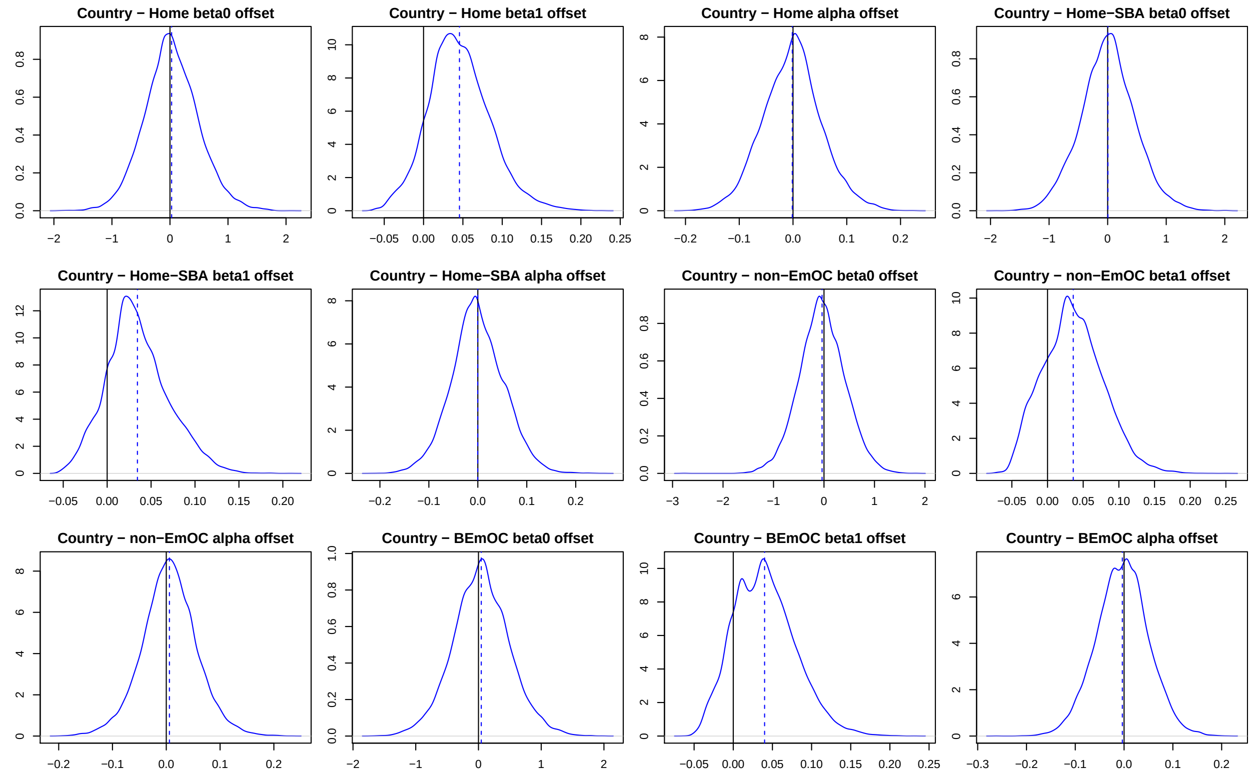
Country − Home beta0 offset
Country − Home beta1 offset
Country − Home alpha offset
Country − Home−SBA beta0 offset
Country − Home−SBA beta1 offset
Country − Home−SBA alpha offset
Country − non−EmOC beta0 offset
Country − non−EmOC beta1 offset
Country − non−EmOC alpha offset
Country − BEmOC beta0 offset
Country − BEmOC beta1 offset
Country − BEmOC alpha offset

Parameters

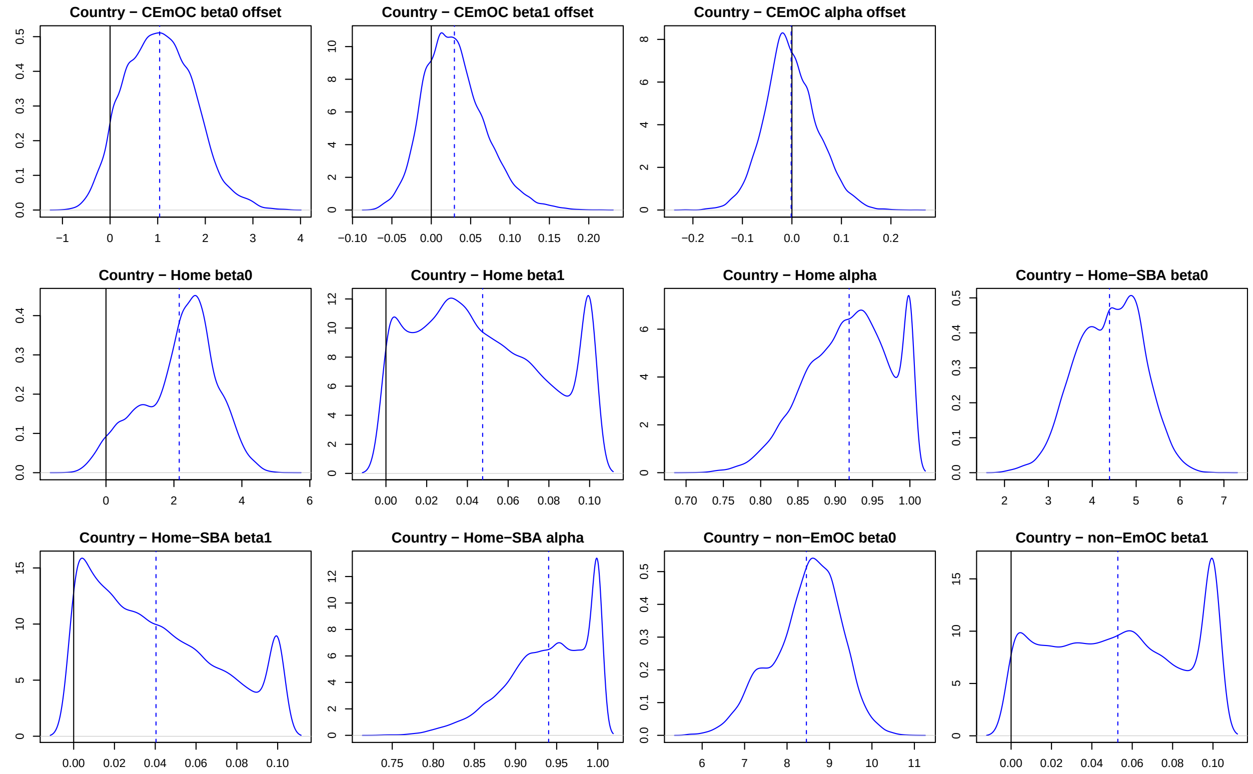
Country − CEmOC beta0 offset
Country − CEmOC beta1 offset
Country − CEmOC alpha offset
Country − Home beta0
Country − Home beta1
Country − Home alpha
Country − Home−SBA beta0
Country − Home−SBA beta1
Country − Home−SBA alpha
Country − non−EmOC beta0
Country − non−EmOC beta1

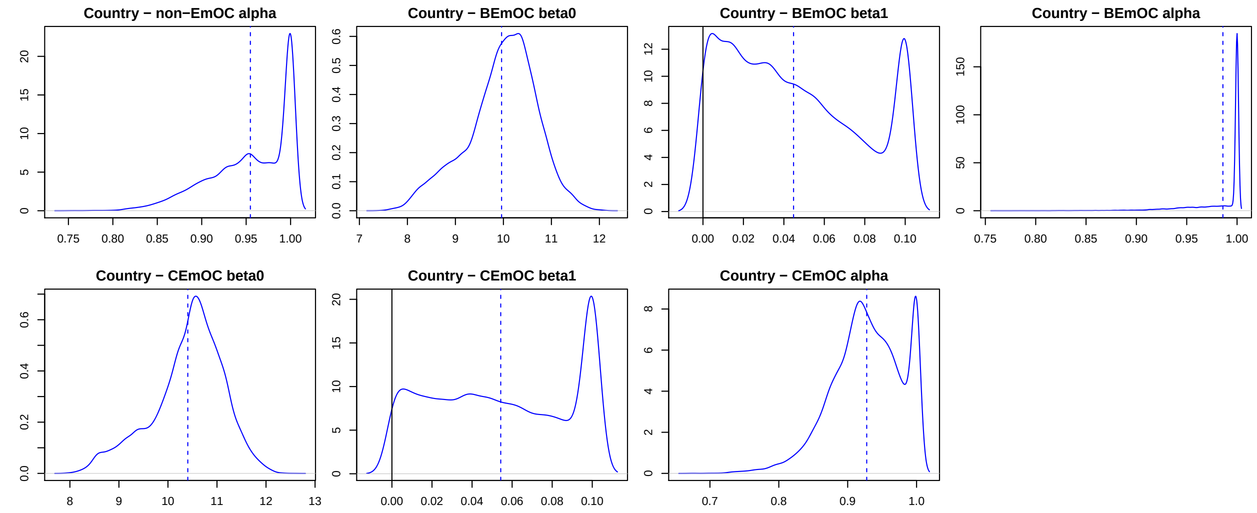

Country − non−EmOC alpha
Country − BEmOC beta0
Country − BEmOC beta1
Country − BEmOC alpha
Country − CEmOC beta0
Country − CEmOC beta1
Country − CEmOC alpha

C.4.7 Quality of Care

Hyperparameters

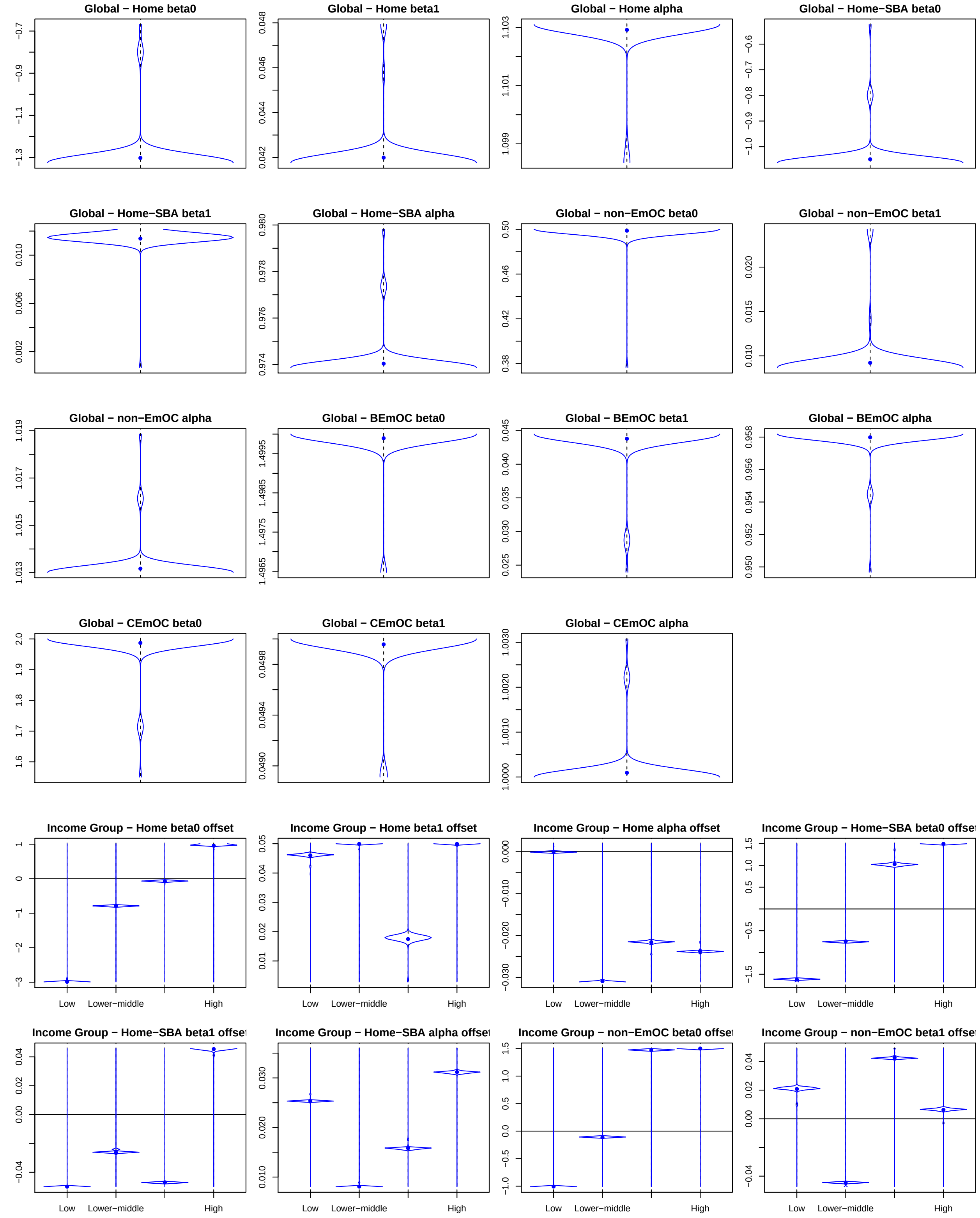

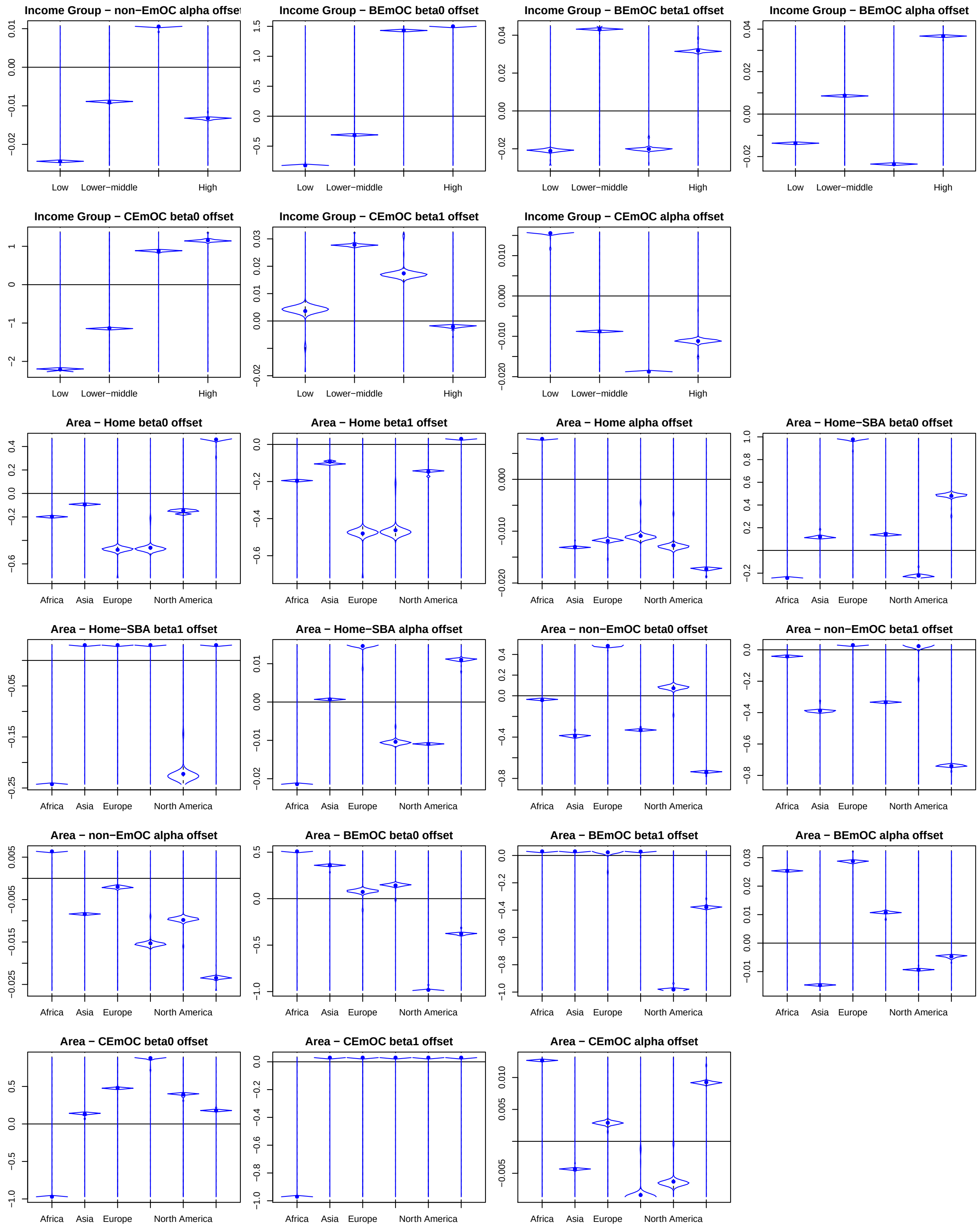
Income Group − non−EmOC alpha offset
Income Group − BEmOC beta0 offset
Income Group − BEmOC beta1 offset
Income Group − BEmOC alpha offset
Income Group − CEmOC beta0 offset
Income Group − CEmOC beta1 offset
Income Group − CEmOC alpha offset
Low
Lower−middle
High
Area − Home beta0 offset
Area − Home beta1 offset
Area − Home alpha offset
Area − Home−SBA beta0 offset
Area − Home−SBA beta1 offset
Area − Home−SBA alpha offset
Area − non−EmOC beta0 offset
Area − non−EmOC beta1 offset
Area − non−EmOC alpha offset
Area − BEmOC beta0 offset
Area − BEmOC beta1 offset
Area − BEmOC alpha offset
Area − CEmOC beta0 offset
Area − CEmOC beta1 offset
Area − CEmOC alpha offset
Africa
Asia
Europe
North America

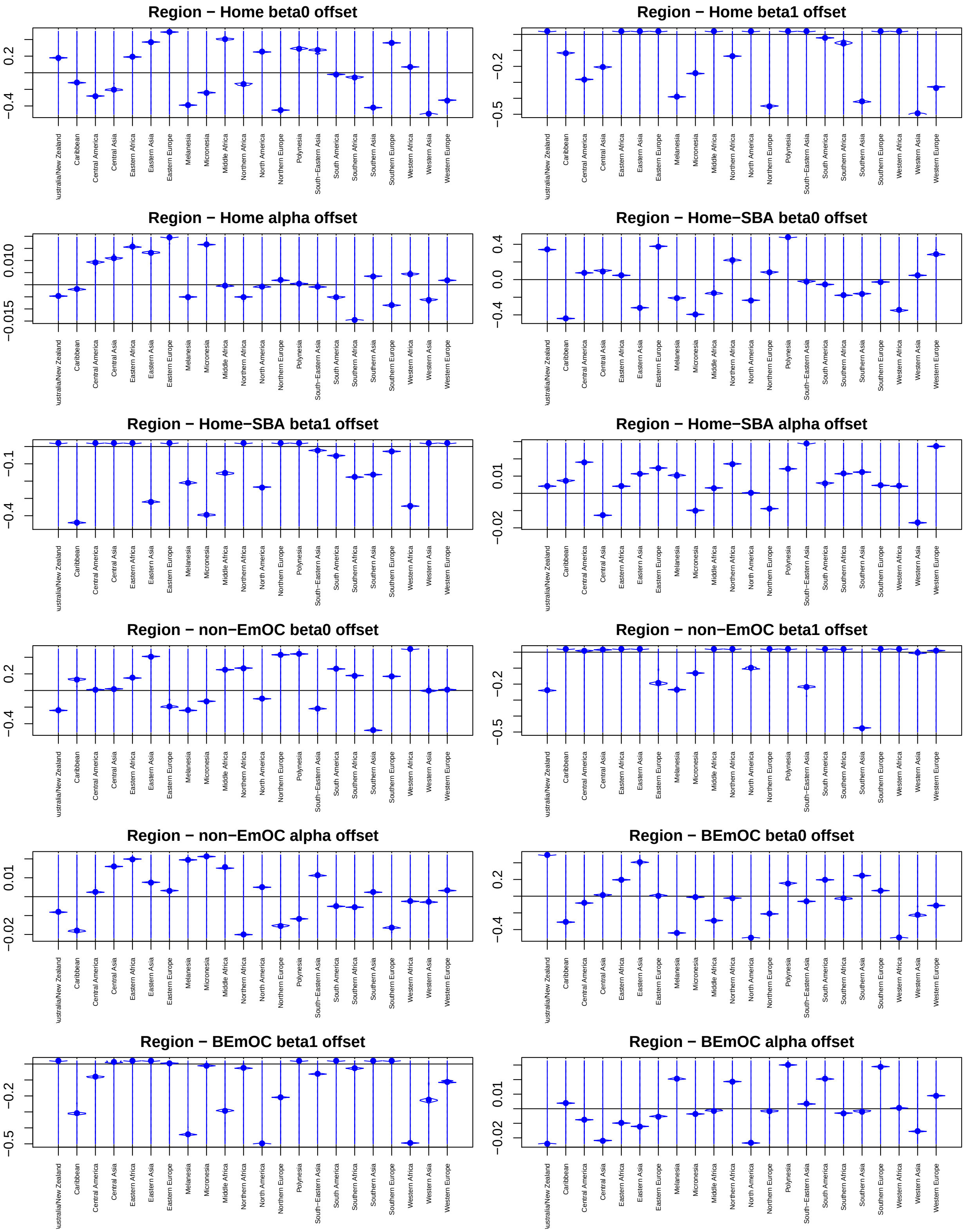
Region − Home beta0 offset
Region − Home beta1 offset
Region − Home alpha offset
Region − Home−SBA beta0 offset
Region − Home−SBA beta1 offset
Region − Home−SBA alpha offset
Region − non−EmOC beta0 offset
Region − non−EmOC beta1 offset
Region − non−EmOC alpha offset
Region − BEmOC beta0 offset
Region − BEmOC beta1 offset
Region − BEmOC alpha offset
ustralia/New Zealand
Caribbean
Central America
Central Asia
Eastern Africa
Eastern Asia
Eastern Europe
Melanesia
Micronesia
Middle Africa
Northern Africa
North America
Northern Europe
Polynesia
South−Eastern Asia
South America
Southern Africa
Southern Asia
Southern Europe
Western Africa
Western Asia
Western Europe

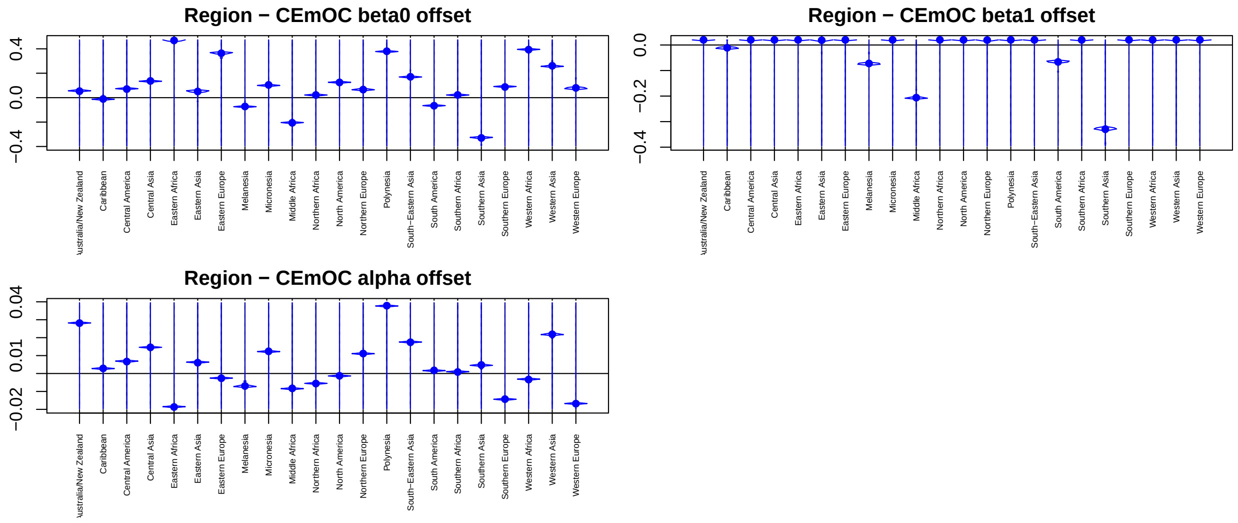
Region − CEmOC beta0 offset
Region − CEmOC beta1 offset
Region − CEmOC alpha offset
Australia/New Zealand
Caribbean
Central America
Central Asia
Eastern Africa
Eastern Asia
Eastern Europe
Melanesia
Micronesia
Middle Africa
Northern Africa
North America
Northern Europe
Polynesia
South−Eastern Asia
South America
Southern Africa
Southern Asia
Southern Europe
Western Africa
Western Asia
Western Europe

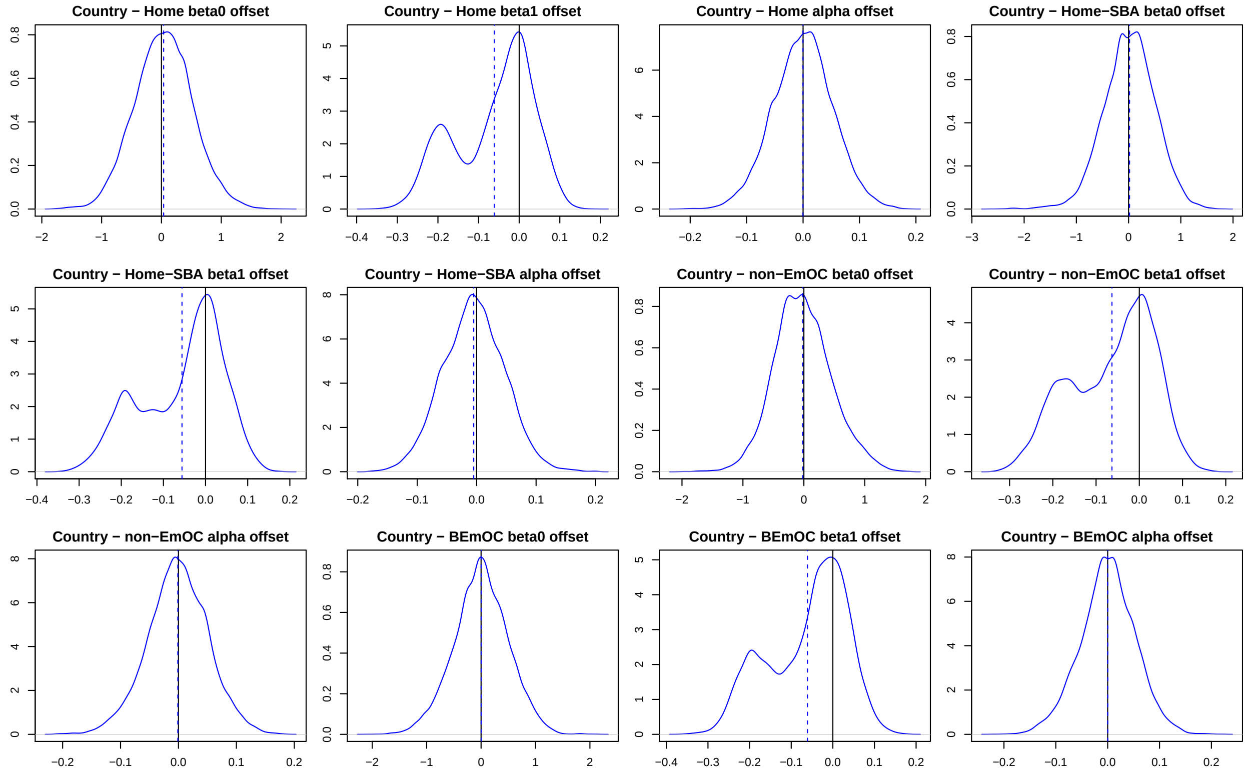
Country − Home beta0 offset
Country − Home beta1 offset
Country − Home alpha offset
Country − Home−SBA beta0 offset
Country − Home−SBA beta1 offset
Country − Home−SBA alpha offset
Country − non−EmOC beta0 offset
Country − non−EmOC beta1 offset
Country − non−EmOC alpha offset
Country − BEmOC beta0 offset
Country − BEmOC beta1 offset
Country − BEmOC alpha offset

Parameters

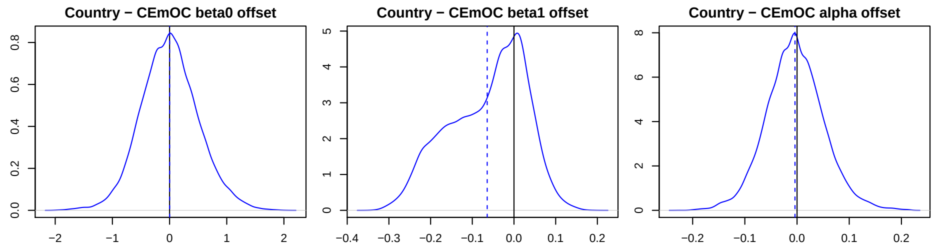
Country − CEmOC beta0 offset
Country − CEmOC beta1 offset
Country − CEmOC alpha offset

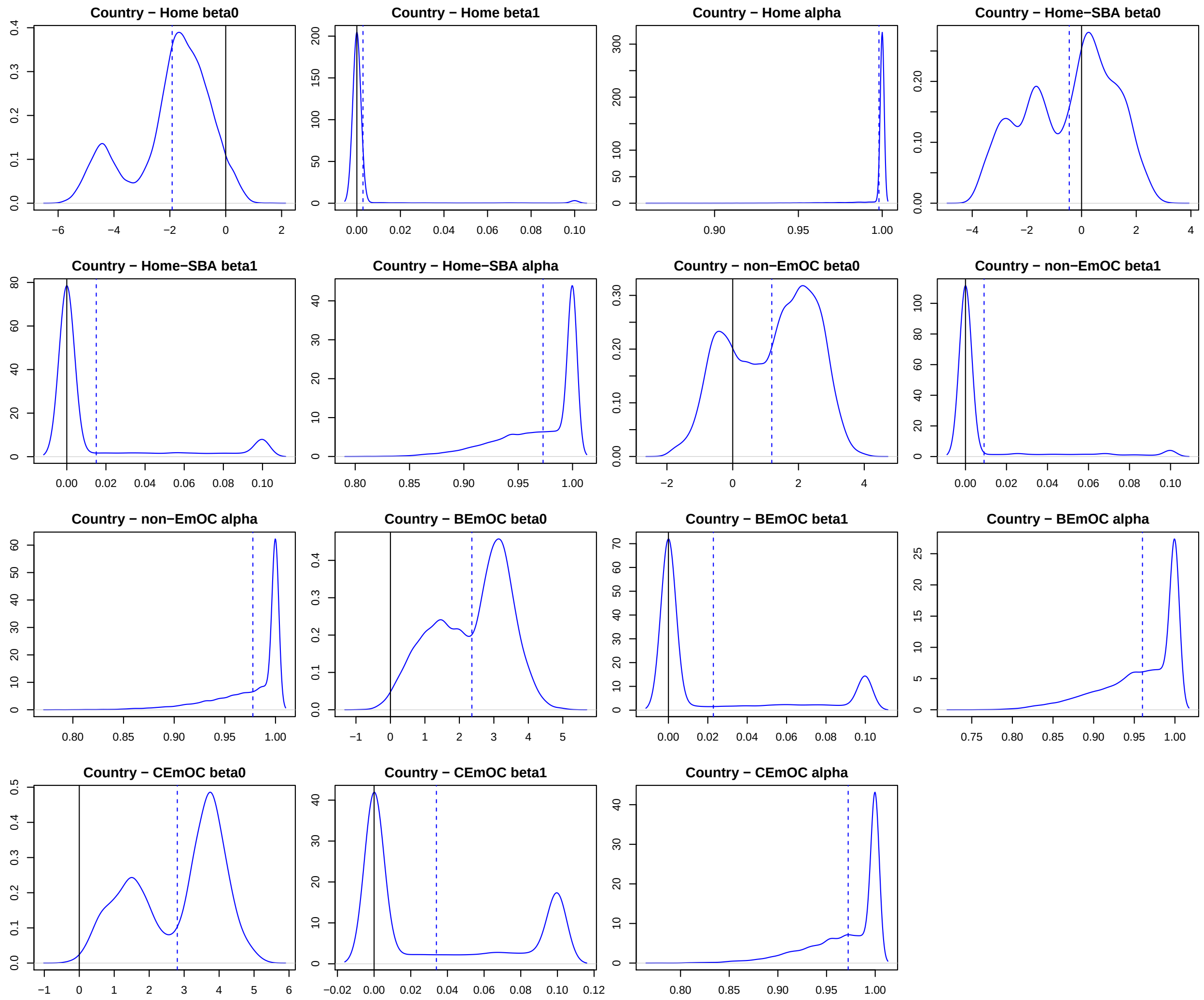
Country − Home beta0
Country − Home beta1
Country − Home alpha
Country − Home−SBA beta0
Country − Home−SBA beta1
Country − Home−SBA alpha
Country − non−EmOC beta0
Country − non−EmOC beta1
Country − non−EmOC alpha
Country − BEmOC beta0
Country − BEmOC beta1
Country − BEmOC alpha
Country − CEmOC beta0
Country − CEmOC beta1
Country − CEmOC alpha

C.5 Obstetric Complications

C.5.1 Pre-Eclampsia/Eclampsia

C.5.1.1 Incidence

Hyperparameters

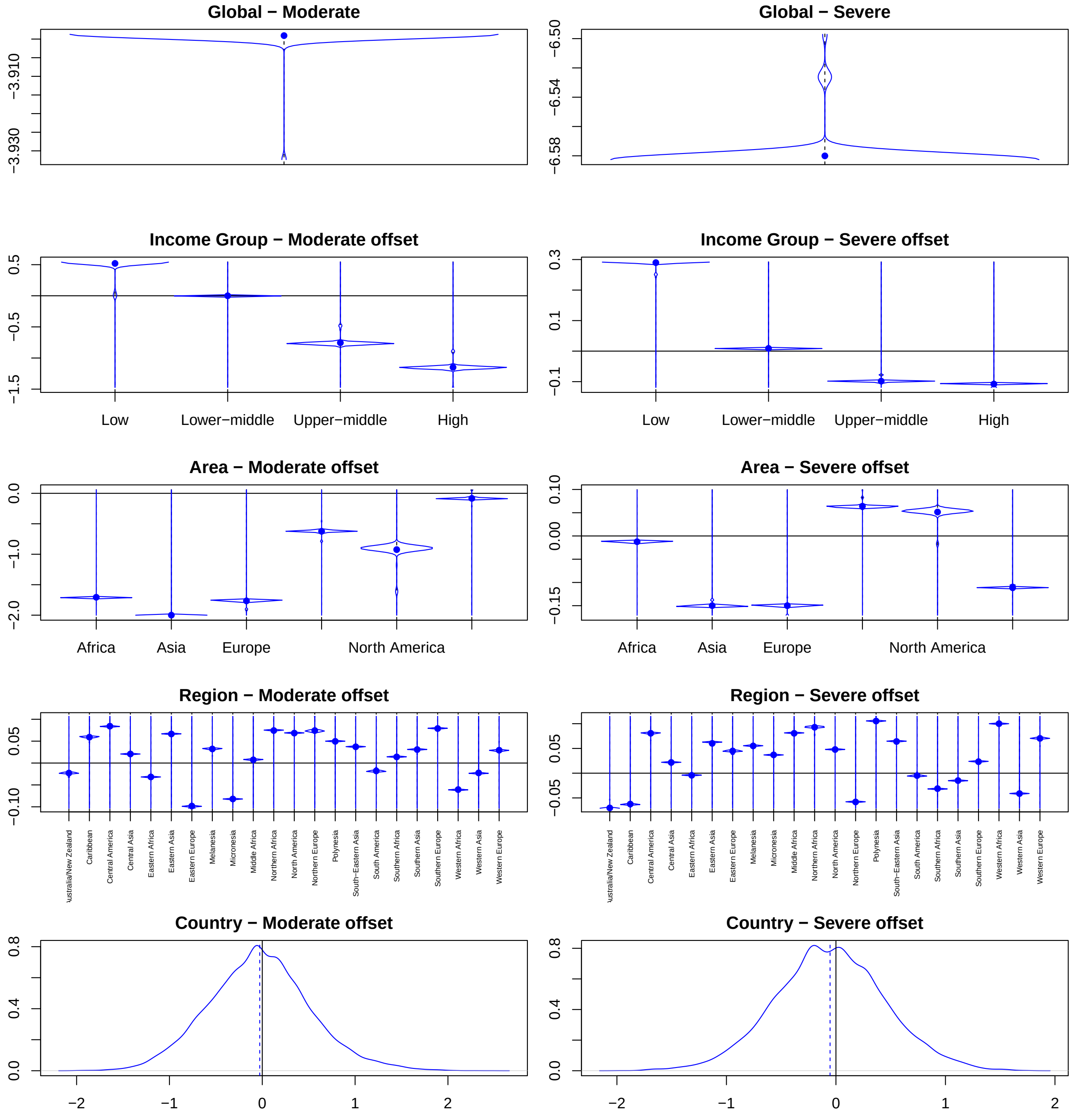

Parameters

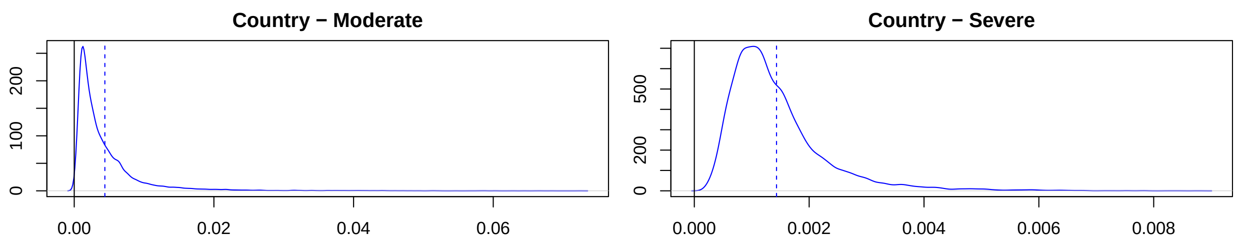

C.5.1.2 Risk Factors

Hyperparameters

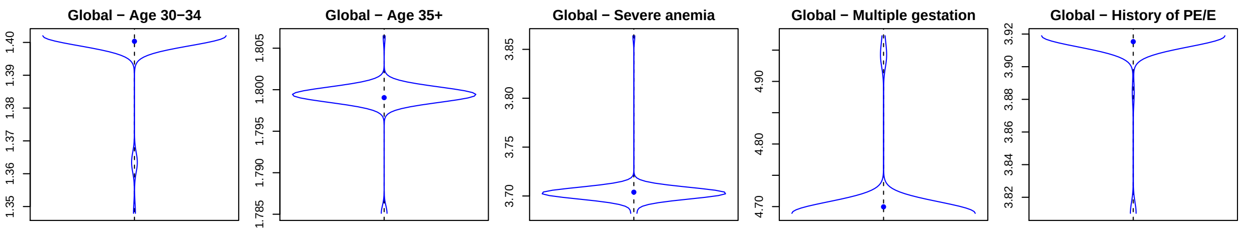

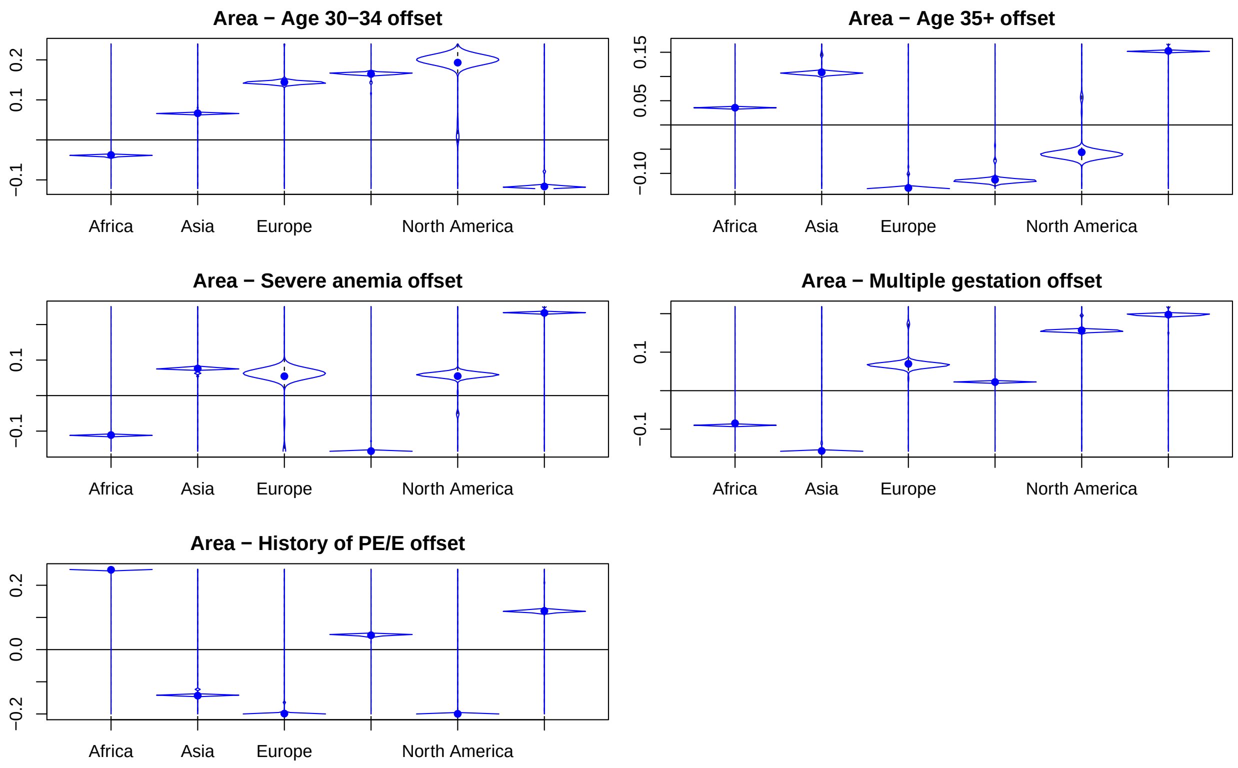

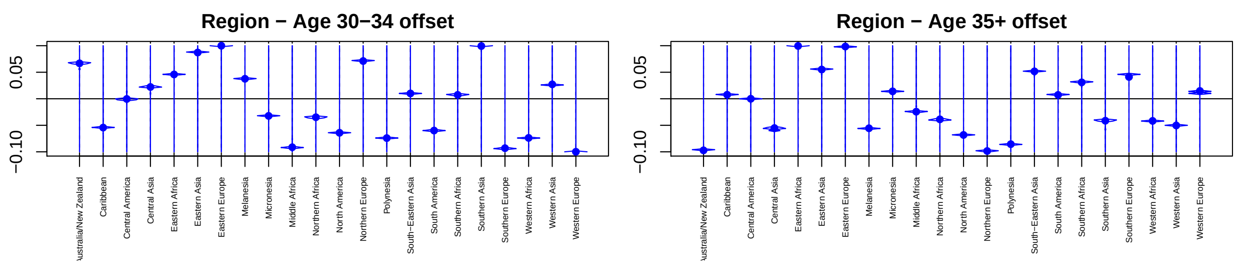

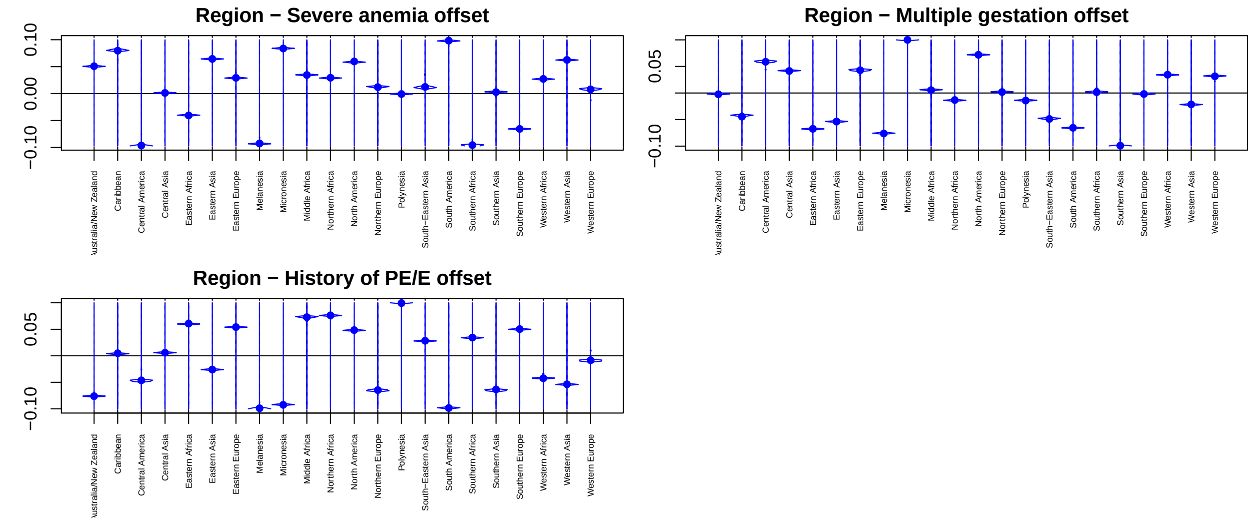

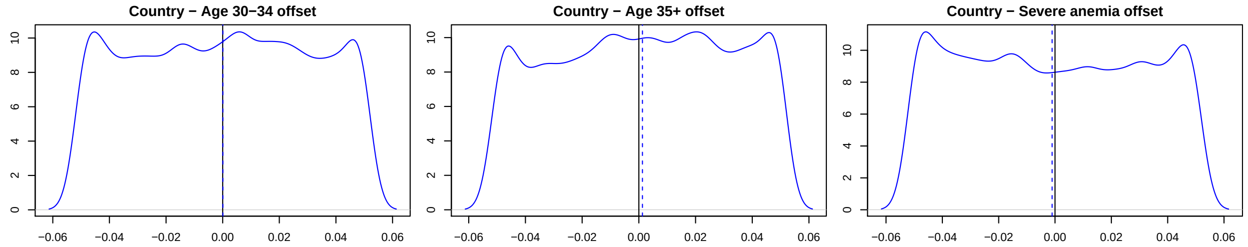

Parameters

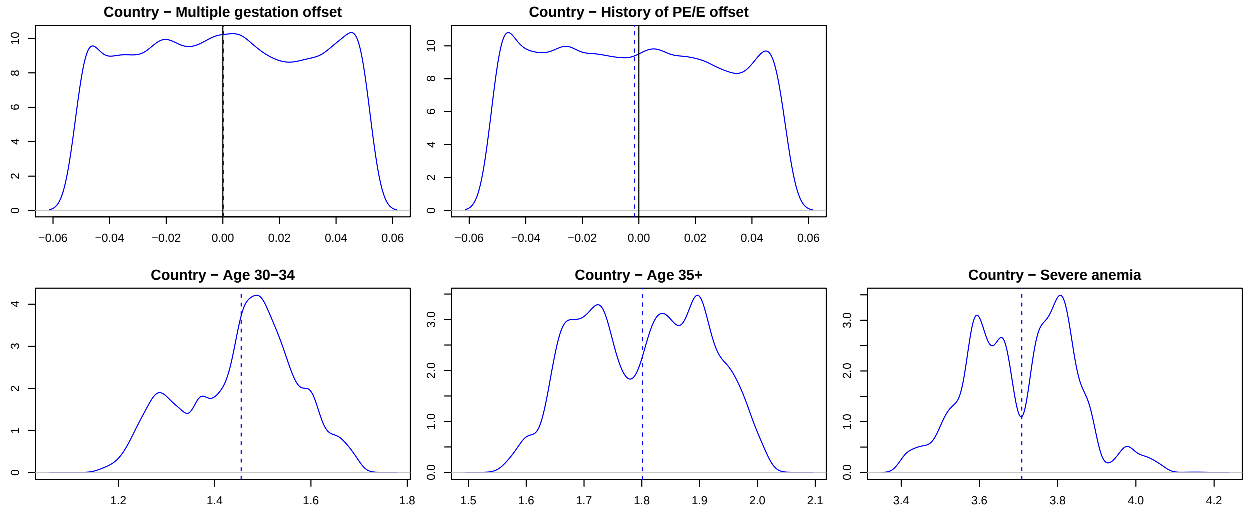

C.5.1.3 Outcomes

Hyperparameters

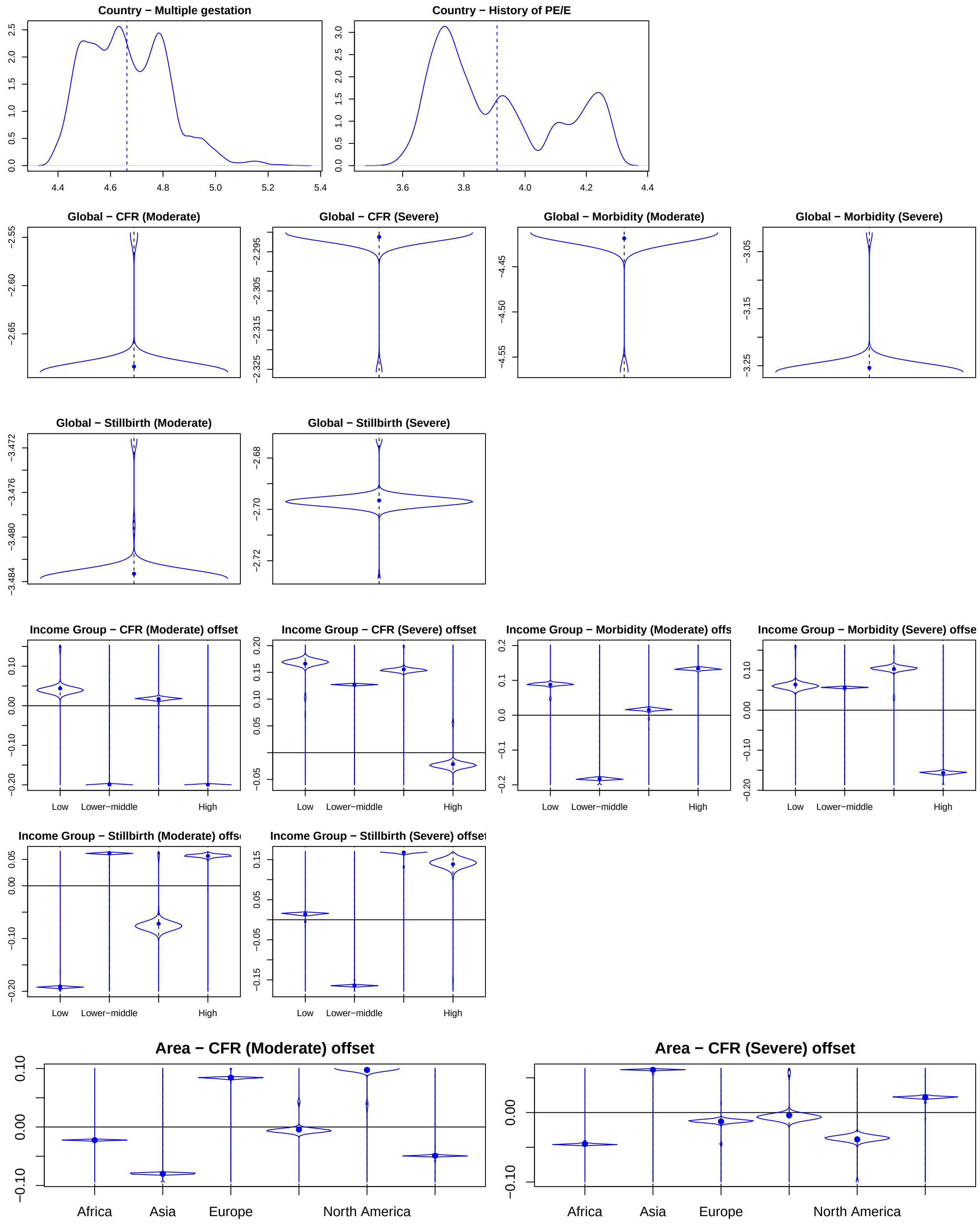
Country − Multiple gestation
Country − History of PE/E
Global − CFR (Moderate)
Global − CFR (Severe)
Global − Morbidity (Moderate)
Global − Morbidity (Severe)
Global − Stillbirth (Moderate)
Global − Stillbirth (Severe)
Income Group − CFR (Moderate) offset
Income Group − CFR (Severe) offset
Income Group − Morbidity (Moderate) offs
Income Group − Morbidity (Severe) offse
Income Group − Stillbirth (Moderate) offs
Income Group − Stillbirth (Severe) offset
Low
Lower−middle
High
Area − CFR (Moderate) offset
Area − CFR (Severe) offset
Africa
Asia
Europe
North America

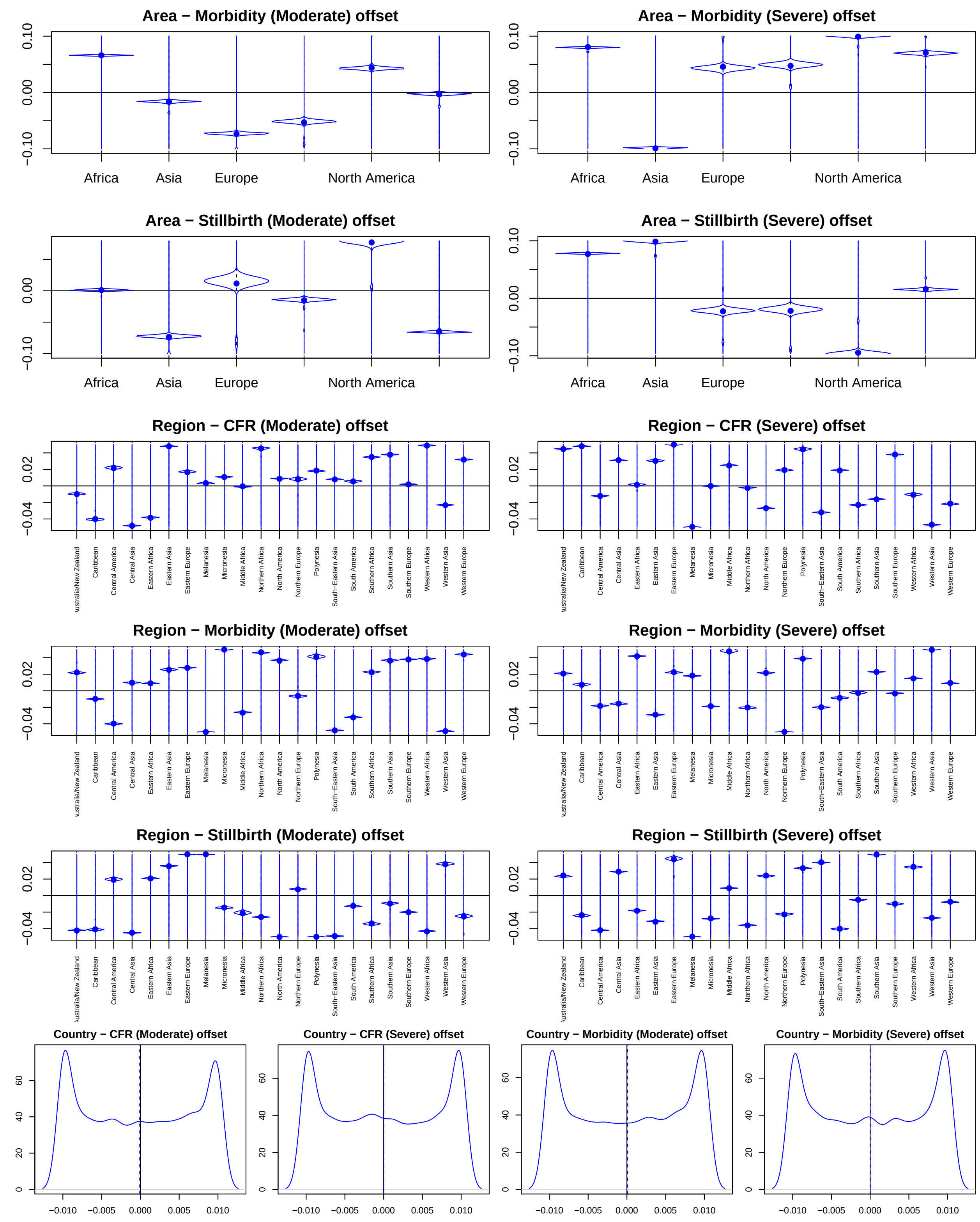
Area − Morbidity (Moderate) offset
Area − Morbidity (Severe) offset
Area − Stillbirth (Moderate) offset
Area − Stillbirth (Severe) offset
Africa
Asia
Europe
North America
Region − CFR (Moderate) offset
Region − CFR (Severe) offset
Region − Morbidity (Moderate) offset
Region − Morbidity (Severe) offset
Region − Stillbirth (Moderate) offset
Region − Stillbirth (Severe) offset
Caribbean
Central America
Central Asia
Eastern Africa
Eastern Asia
Eastern Europe
Melanesia
Micronesia
Middle Africa
Northern Africa
North America
Northern Europe
Polynesia
South−Eastern Asia
South America
Southern Africa
Southern Asia
Southern Europe
Western Africa
Western Asia
Western Europe
Country − CFR (Moderate) offset
Country − CFR (Severe) offset
Country − Morbidity (Moderate) offset
Country − Morbidity (Severe) offset

Parameters

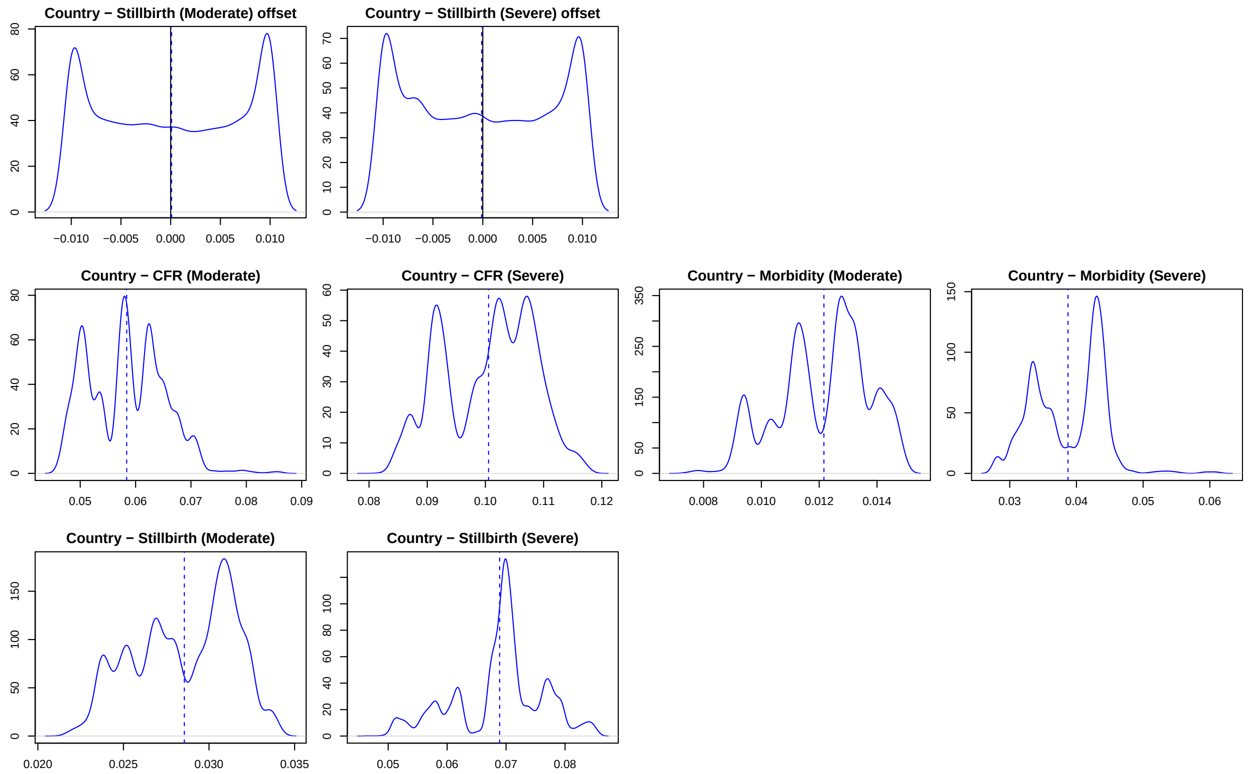
Country − Stillbirth (Moderate) offset
0 20 40 60 80
−0.010 −0.005 0.000 0.005 0.010
Country − Stillbirth (Severe) offset
0 10 20 30 40 50 60 70
−0.010 −0.005 0.000 0.005 0.010
Country − CFR (Moderate)
0 20 40 60 80
0.05 0.06 0.07 0.08 0.09
Country − CFR (Severe)
0 10 20 30 40 50 60
0.08 0.09 0.10 0.11 0.12
Country − Morbidity (Moderate)
0 50 150 250 350
0.008 0.010 0.012 0.014
Country − Morbidity (Severe)
0 50 100 150
0.03 0.04 0.05 0.06
Country − Stillbirth (Moderate)
0 50 100 150
0.020 0.025 0.030 0.035
Country − Stillbirth (Severe)
0 20 40 60 80 100
0.05 0.06 0.07 0.08

C.5.2 Obstructed Labour

C.5.2.1 Incidence

Hyperparameters

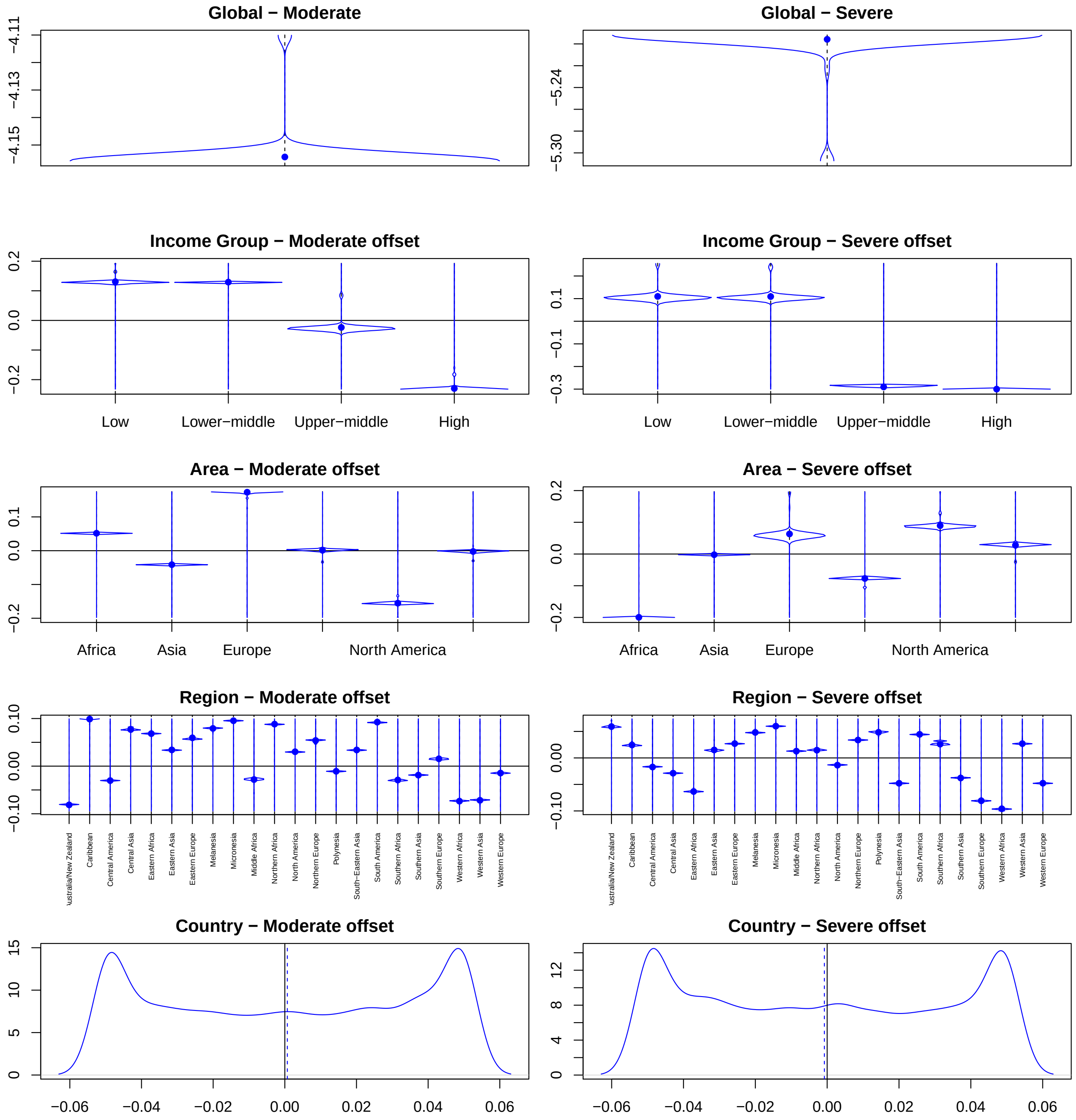

Parameters

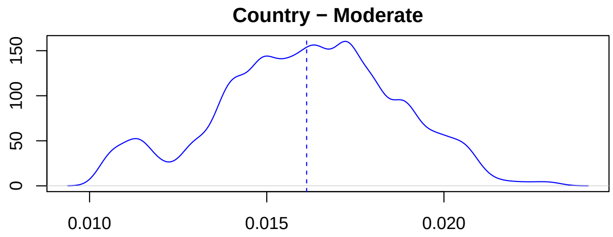

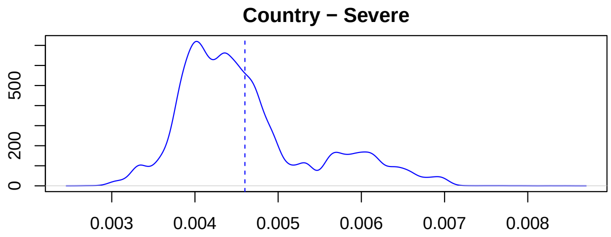

C.5.2.2 Risk Factors

Hyperparameters

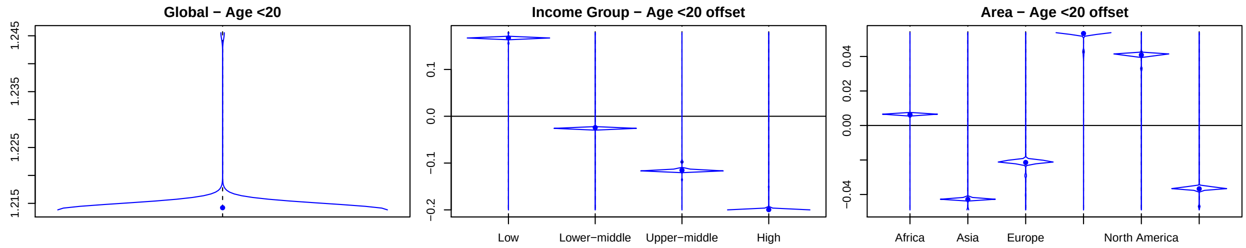

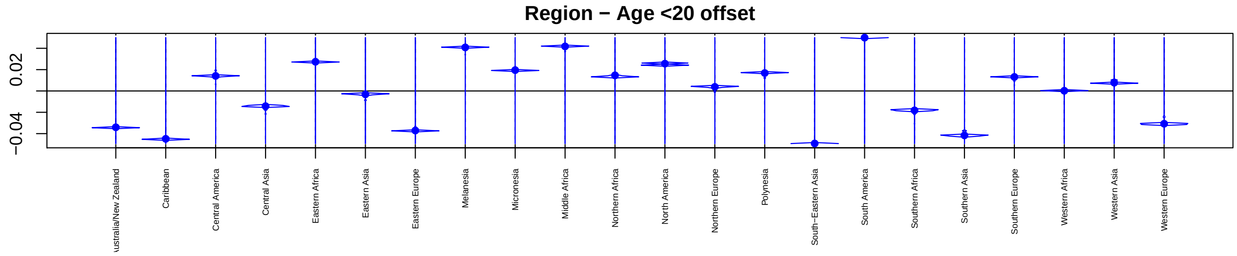

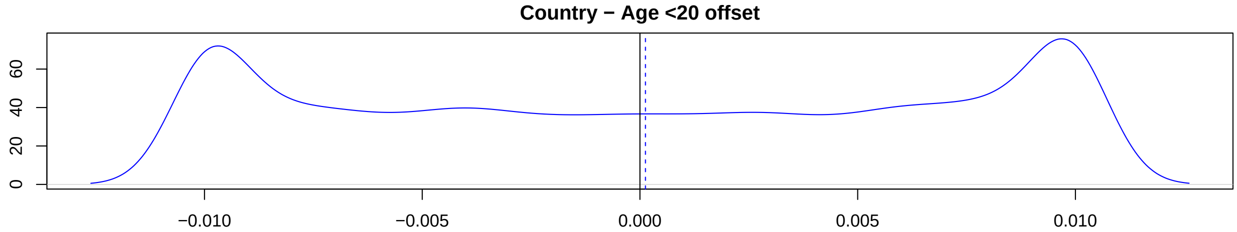

Parameters

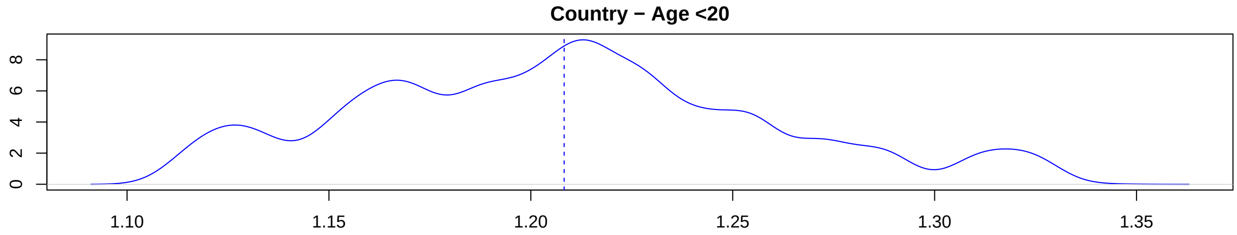

C.5.2.3 Outcomes

Hyperparameters

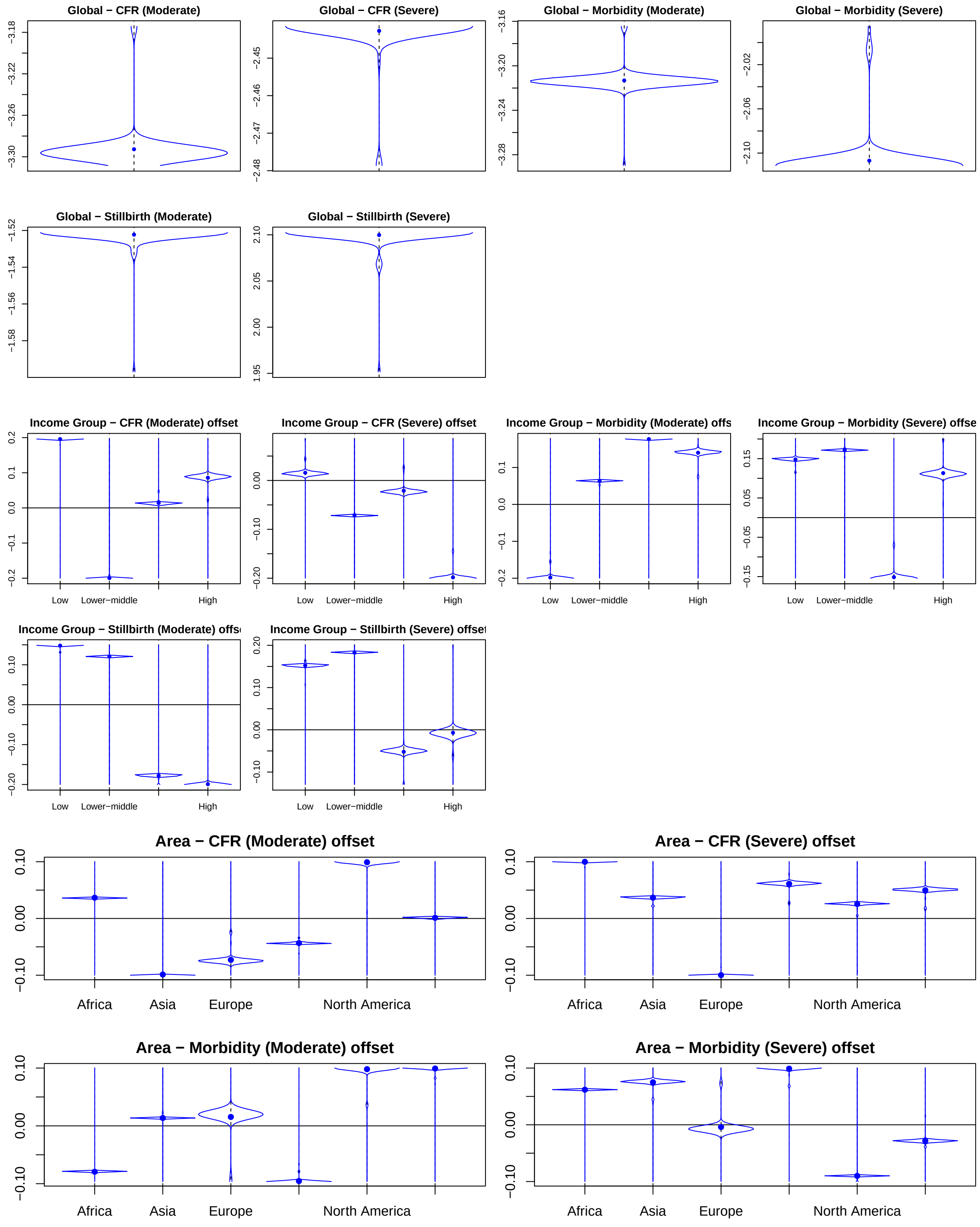
Global − CFR (Moderate)
Global − CFR (Severe)
Global − Morbidity (Moderate)
Global − Morbidity (Severe)
Global − Stillbirth (Moderate)
Global − Stillbirth (Severe)
Income Group − CFR (Moderate) offset
Income Group − CFR (Severe) offset
Income Group − Morbidity (Moderate) offs
Income Group − Morbidity (Severe) offse
Income Group − Stillbirth (Moderate) offs
Income Group − Stillbirth (Severe) offset
Low
Lower−middle
High
Area − CFR (Moderate) offset
Area − CFR (Severe) offset
Area − Morbidity (Moderate) offset
Area − Morbidity (Severe) offset
Africa
Asia
Europe
North America

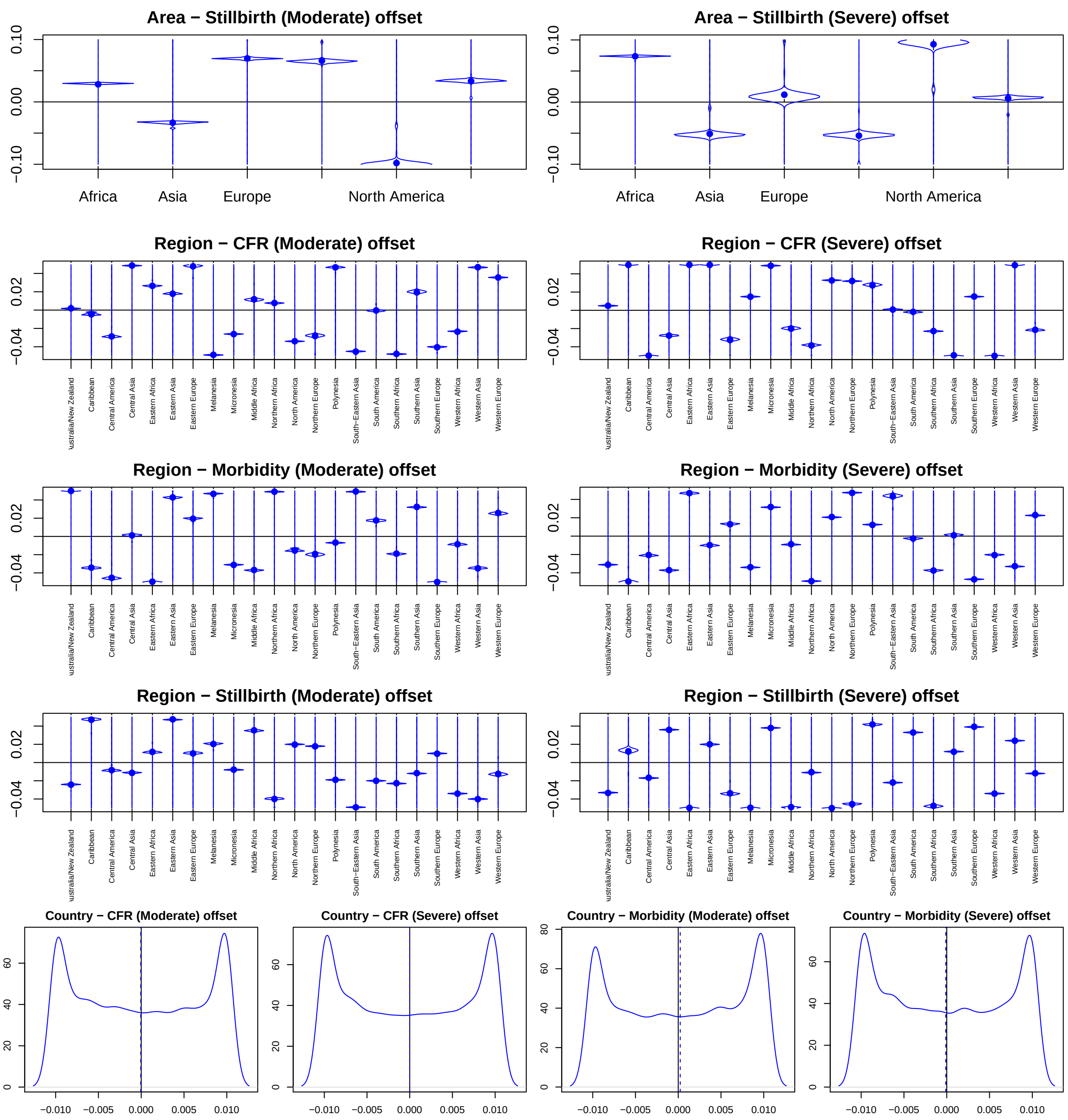
Area − Stillbirth (Moderate) offset
Area − Stillbirth (Severe) offset
Africa
Asia
Europe
North America
Region − CFR (Moderate) offset
Region − CFR (Severe) offset
Region − Morbidity (Moderate) offset
Region − Morbidity (Severe) offset
Region − Stillbirth (Moderate) offset
Region − Stillbirth (Severe) offset
Caribbean
Central America
Central Asia
Eastern Africa
Eastern Asia
Eastern Europe
Melanesia
Micronesia
Middle Africa
Northern Africa
North America
Northern Europe
Polynesia
South−Eastern Asia
South America
Southern Africa
Southern Asia
Southern Europe
Western Africa
Western Asia
Western Europe
Country − CFR (Moderate) offset
Country − CFR (Severe) offset
Country − Morbidity (Moderate) offset
Country − Morbidity (Severe) offset

Parameters

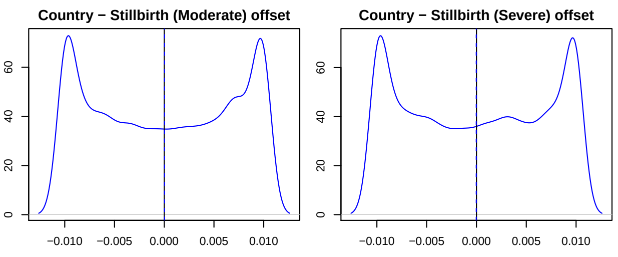
Country − Stillbirth (Moderate) offset
Country − Stillbirth (Severe) offset

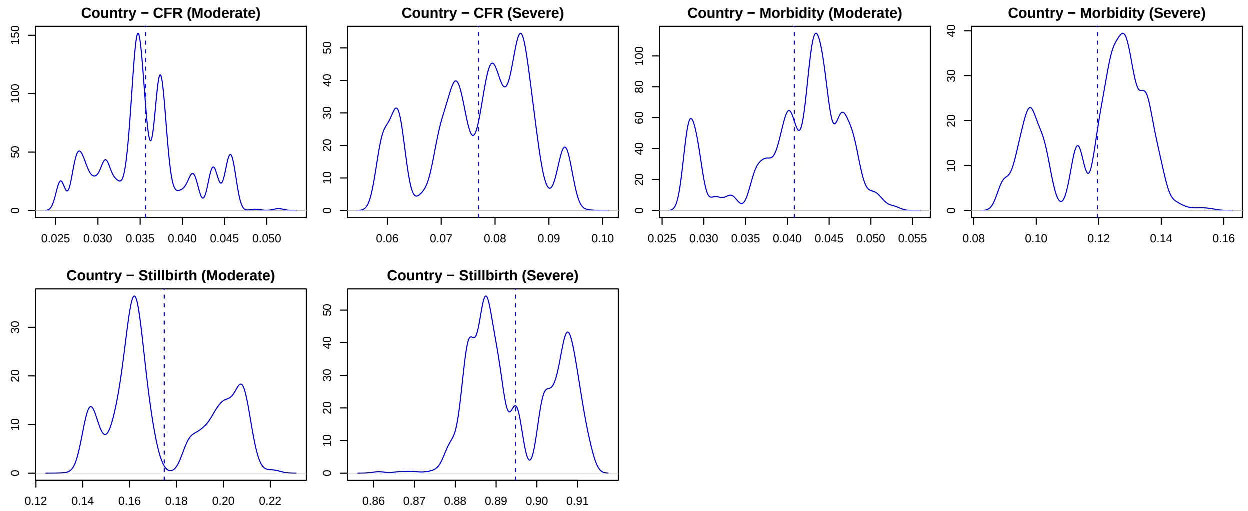
Country − CFR (Moderate)
0 50 100 150
0.025 0.030 0.035 0.040 0.045 0.050
Country − CFR (Severe)
0 10 20 30 40 50
0.06 0.07 0.08 0.09 0.10
Country − Morbidity (Moderate)
0 20 40 60 80 100
0.025 0.030 0.035 0.040 0.045 0.050 0.055
Country − Morbidity (Severe)
0 10 20 30 40
0.08 0.10 0.12 0.14 0.16
Country − Stillbirth (Moderate)
0 10 20 30
0.12 0.14 0.16 0.18 0.20 0.22
Country − Stillbirth (Severe)
0 10 20 30 40 50
0.86 0.87 0.88 0.89 0.90 0.91

C.5.3 Postpartum Hemorrhage

C.5.3.1 Incidence

Hyperparameters

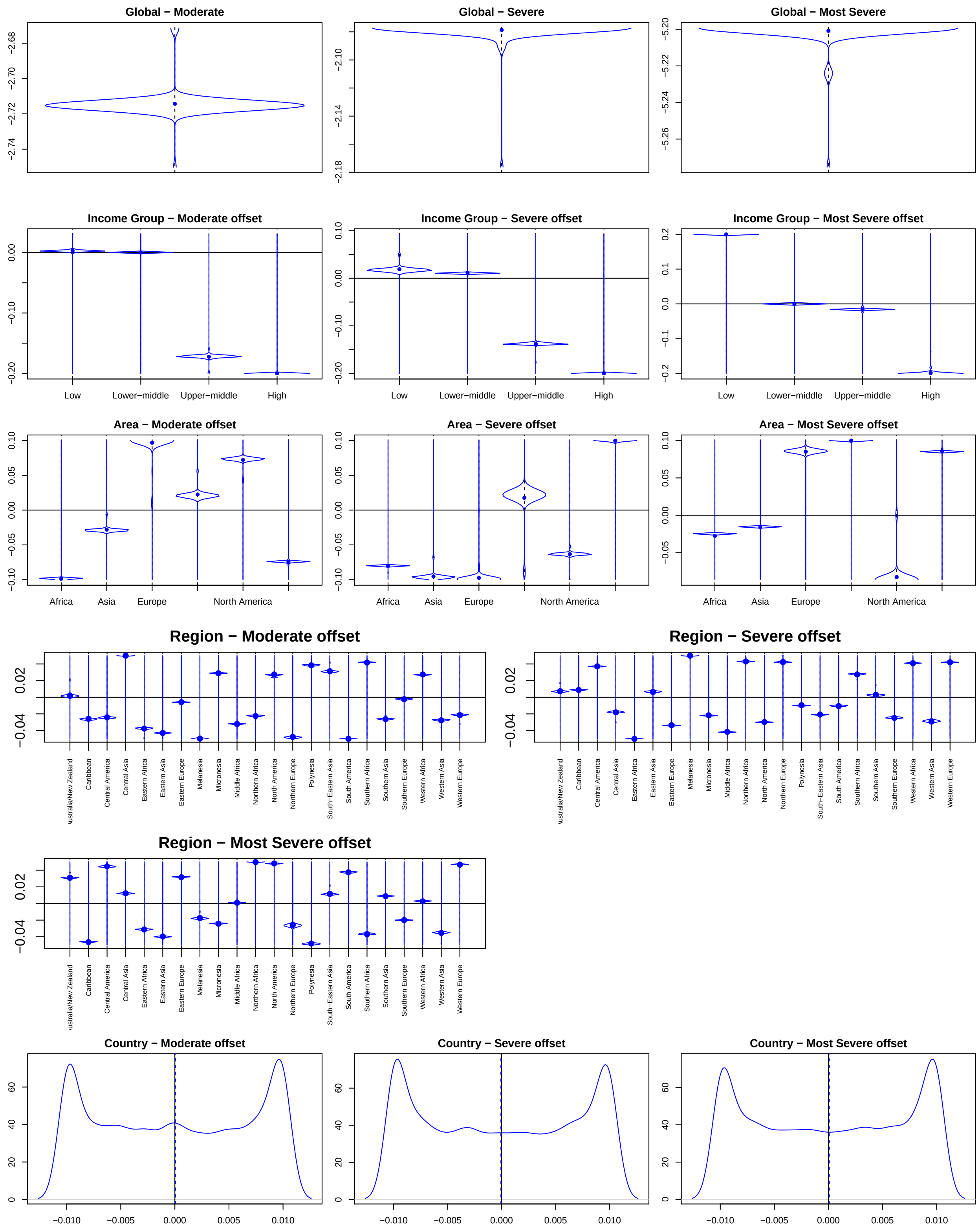

Parameters

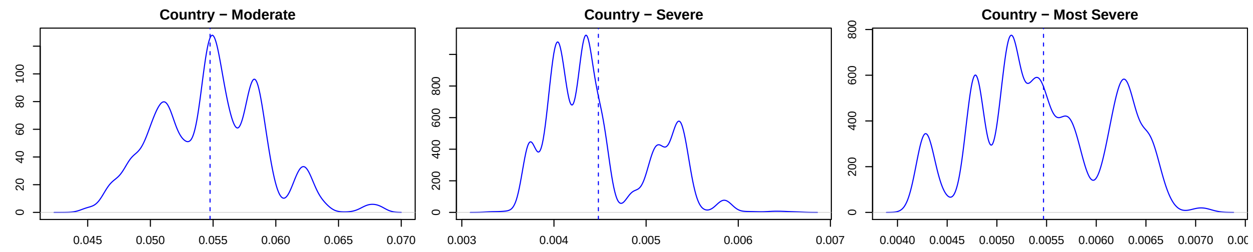

C.5.3.2 Morbidity/Mortality

Hyperparameters

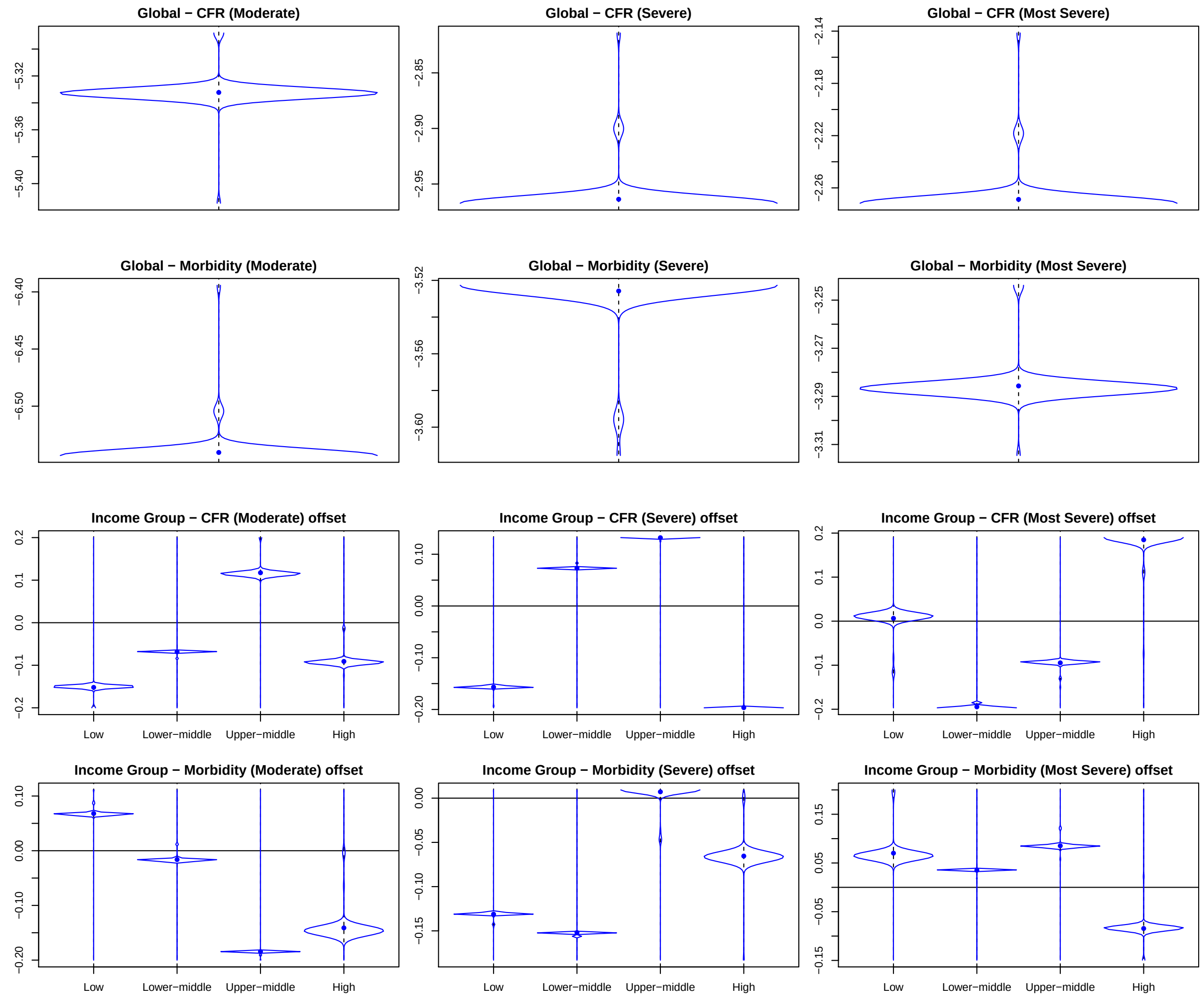

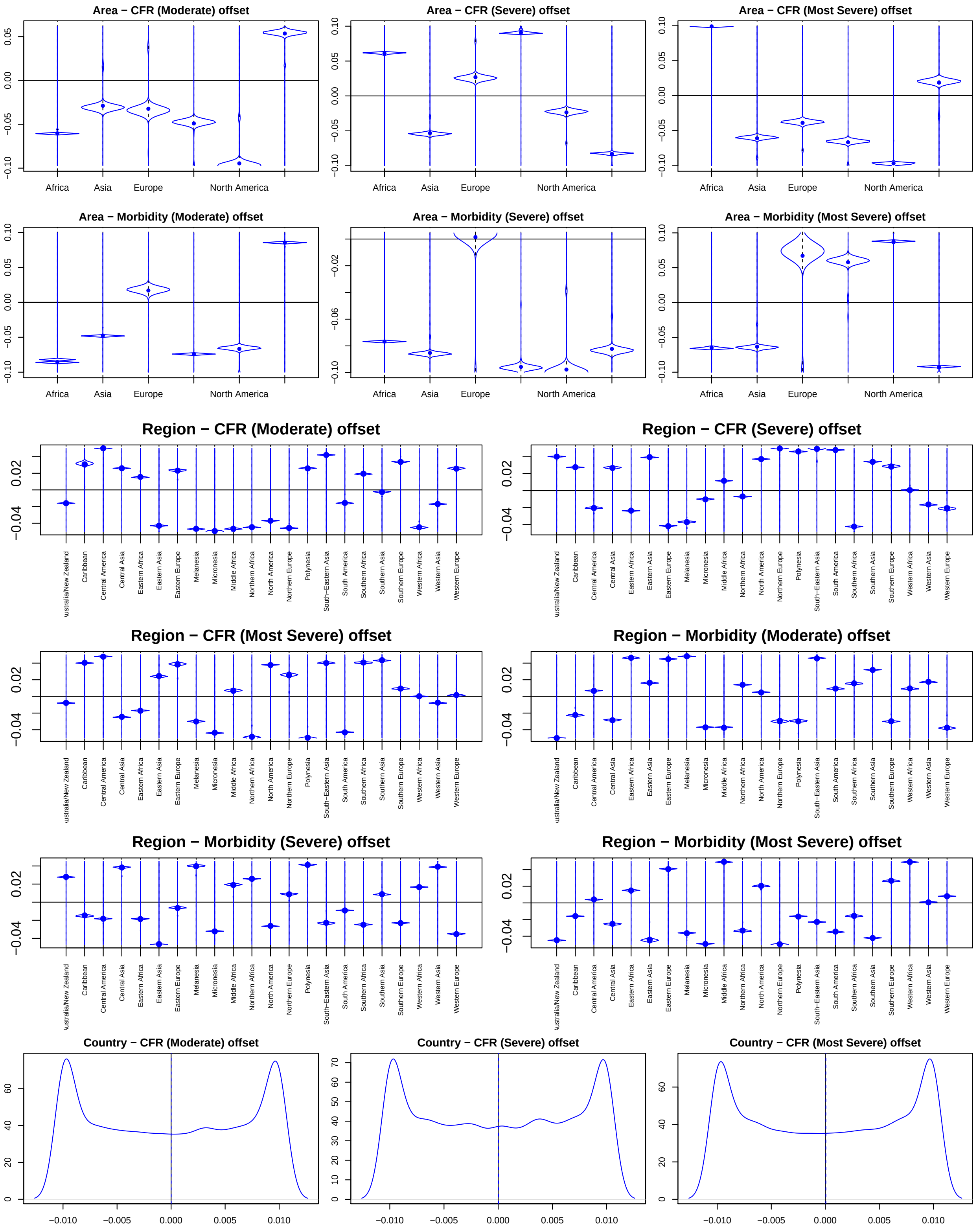

Area − CFR (Moderate) offset
Area − CFR (Severe) offset
Area − CFR (Most Severe) offset
Area − Morbidity (Moderate) offset
Area − Morbidity (Severe) offset
Area − Morbidity (Most Severe) offset
Africa
Asia
Europe
North America
Region − CFR (Moderate) offset
Region − CFR (Severe) offset
Region − CFR (Most Severe) offset
Region − Morbidity (Moderate) offset
Region − Morbidity (Severe) offset
Region − Morbidity (Most Severe) offset
ustralia/New Zealand
Caribbean
Central America
Central Asia
Eastern Africa
Eastern Asia
Eastern Europe
Melanesia
Micronesia
Middle Africa
Northern Africa
North America
Northern Europe
Polynesia
South−Eastern Asia
South America
Southern Africa
Southern Asia
Southern Europe
Western Africa
Western Asia
Western Europe
Country − CFR (Moderate) offset
Country − CFR (Severe) offset
Country − CFR (Most Severe) offset

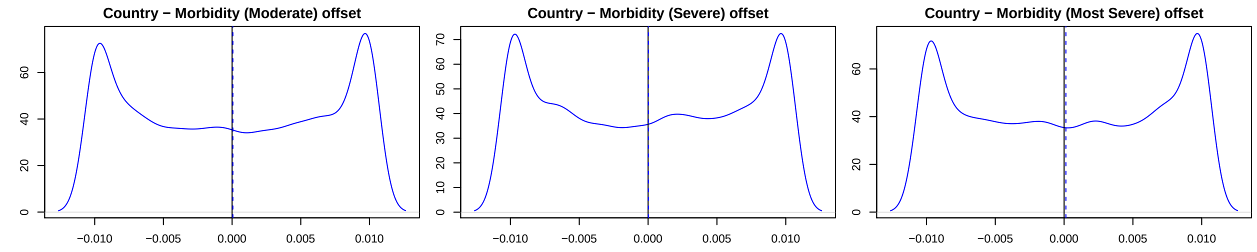

Parameters

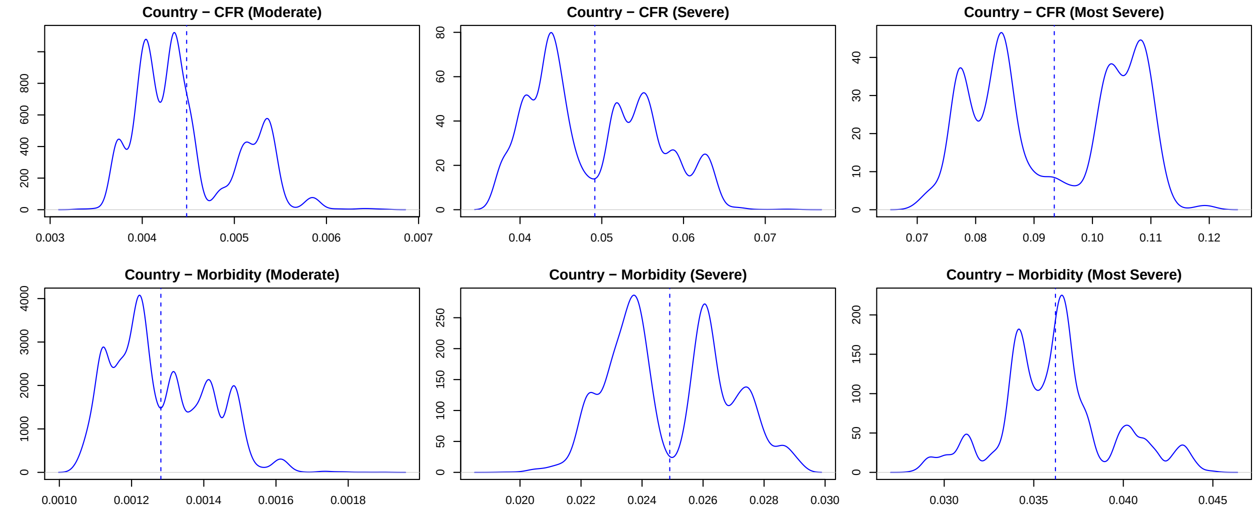

C.5.3.3 Anemia Relative Risks/Outcomes

Hyperparameters

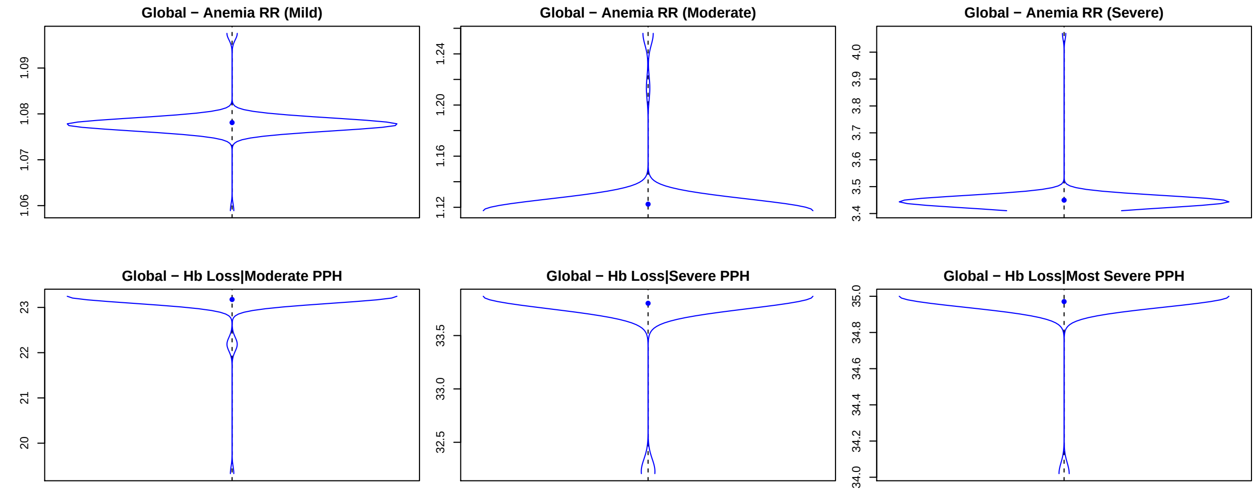

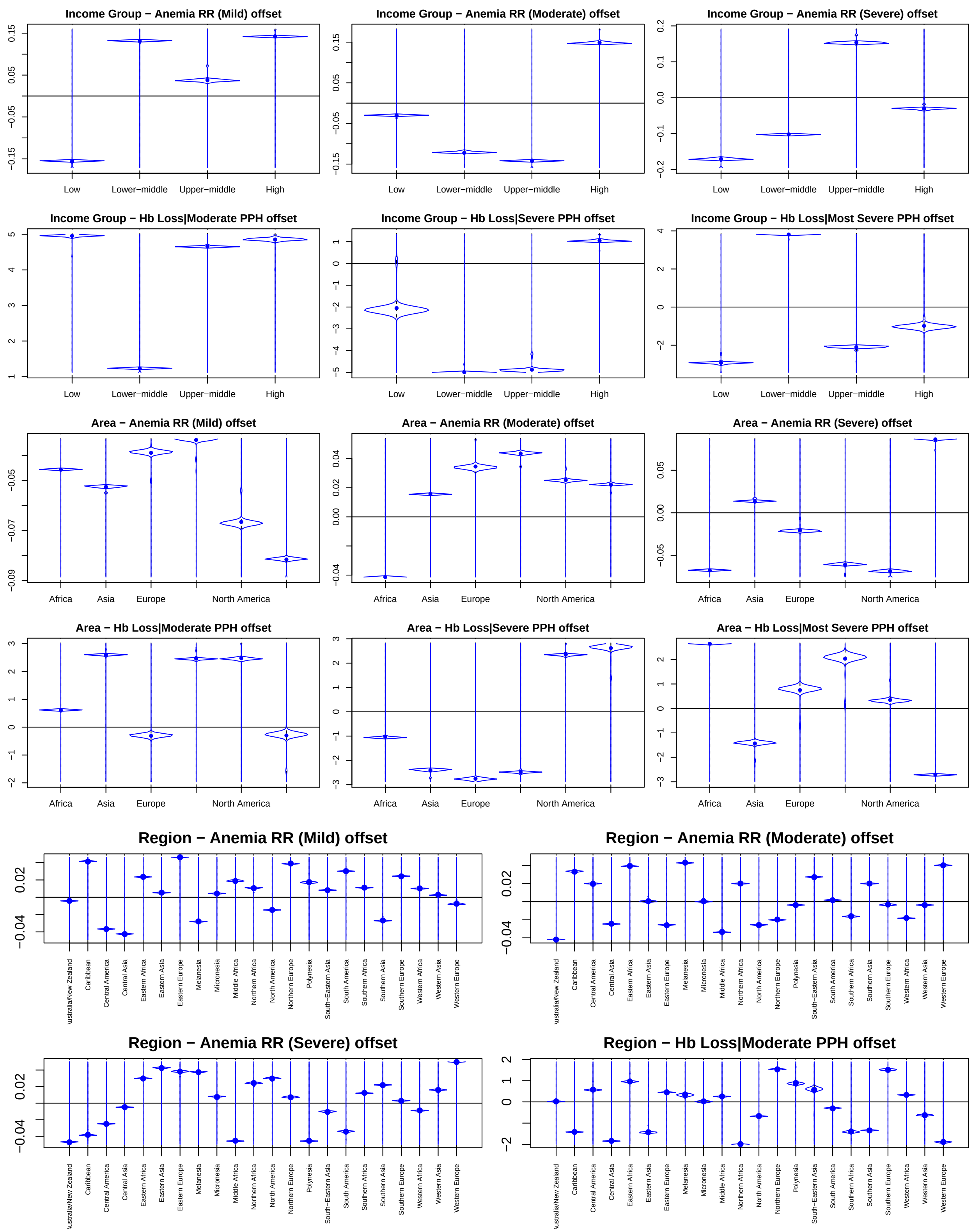
Income Group − Anemia RR (Mild) offset
Income Group − Anemia RR (Moderate) offset
Income Group − Anemia RR (Severe) offset
Low
Lower−middle
Upper−middle
High
Income Group − Hb Loss|Moderate PPH offset
Income Group − Hb Loss|Severe PPH offset
Income Group − Hb Loss|Most Severe PPH offset
Area − Anemia RR (Mild) offset
Area − Anemia RR (Moderate) offset
Area − Anemia RR (Severe) offset
Africa
Asia
Europe
North America
Area − Hb Loss|Moderate PPH offset
Area − Hb Loss|Severe PPH offset
Area − Hb Loss|Most Severe PPH offset
Region − Anemia RR (Mild) offset
Region − Anemia RR (Moderate) offset
Region − Anemia RR (Severe) offset
Region − Hb Loss|Moderate PPH offset
Caribbean
Central America
Central Asia
Eastern Africa
Eastern Asia
Eastern Europe
Melanesia
Micronesia
Middle Africa
Northern Africa
North America
Northern Europe
Polynesia
South−Eastern Asia
South America
Southern Africa
Southern Asia
Southern Europe
Western Africa
Western Asia
Western Europe

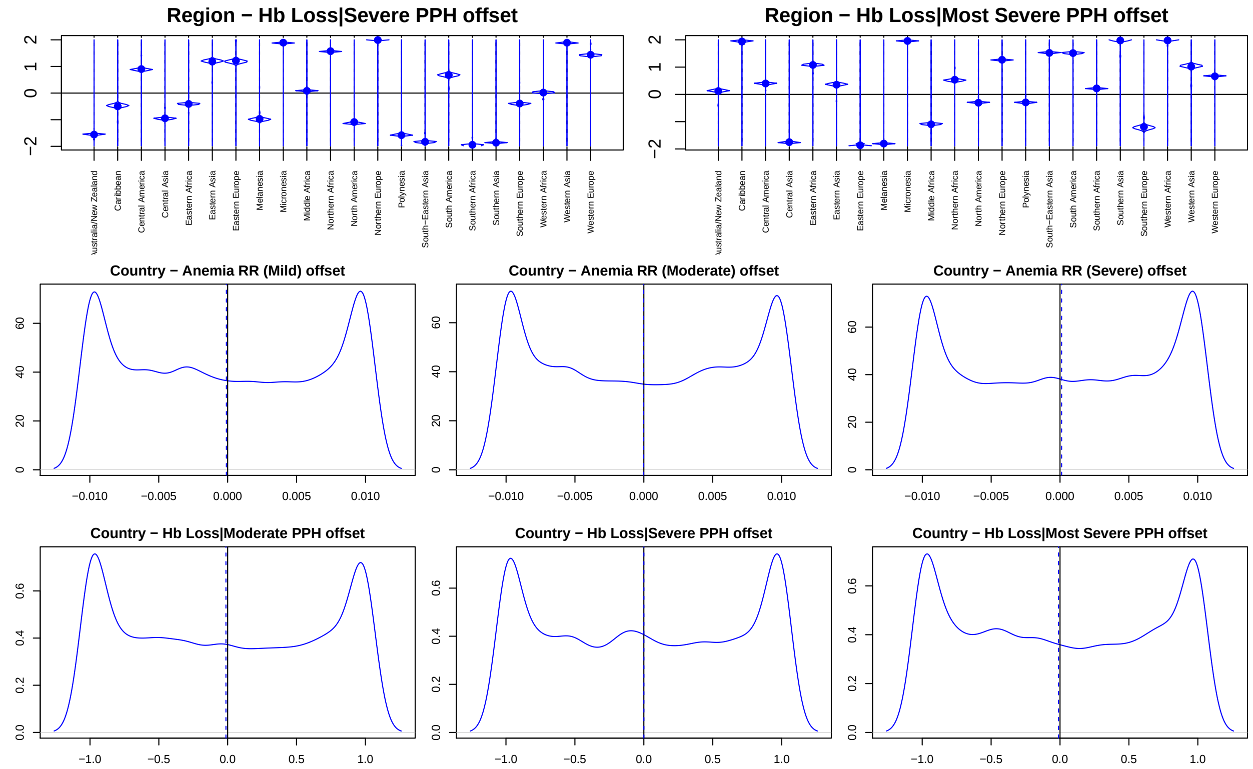
Region − Hb Loss|Severe PPH offset
Region − Hb Loss|Most Severe PPH offset
Australia/New Zealand
Caribbean
Central America
Central Asia
Eastern Africa
Eastern Asia
Eastern Europe
Melanesia
Micronesia
Middle Africa
Northern Africa
North America
Northern Europe
Polynesia
South−Eastern Asia
South America
Southern Africa
Southern Asia
Southern Europe
Western Africa
Western Asia
Western Europe
Country − Anemia RR (Mild) offset
Country − Anemia RR (Moderate) offset
Country − Anemia RR (Severe) offset
Country − Hb Loss|Moderate PPH offset
Country − Hb Loss|Severe PPH offset
Country − Hb Loss|Most Severe PPH offset

Parameters

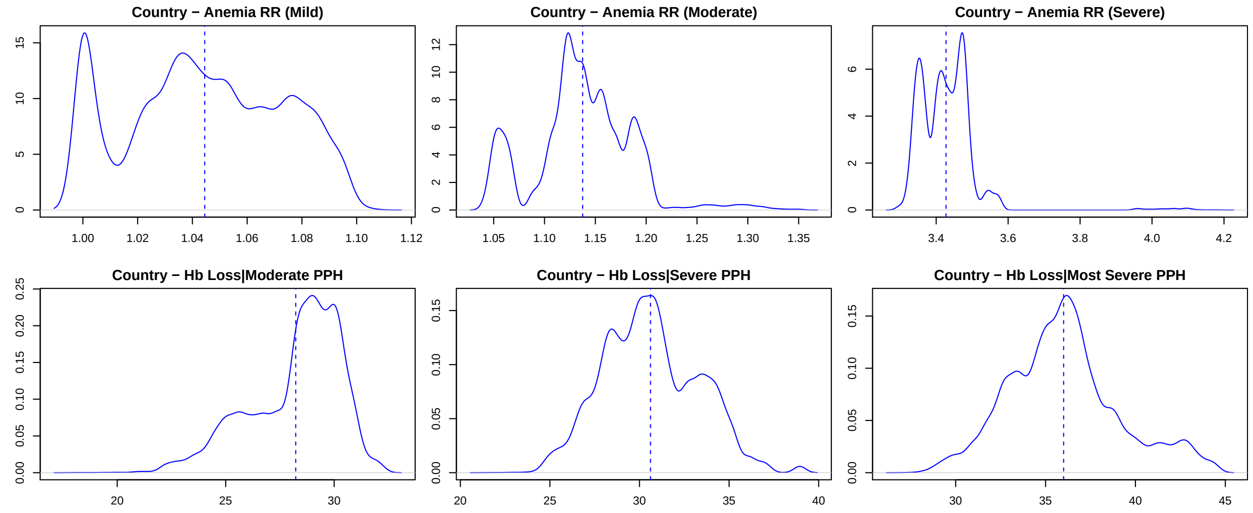
Country − Anemia RR (Mild)
Country − Anemia RR (Moderate)
Country − Anemia RR (Severe)
Country − Hb Loss|Moderate PPH
Country − Hb Loss|Severe PPH
Country − Hb Loss|Most Severe PPH

C.5.4 Sepsis

C.5.4.1 Incidence

Hyperparameters

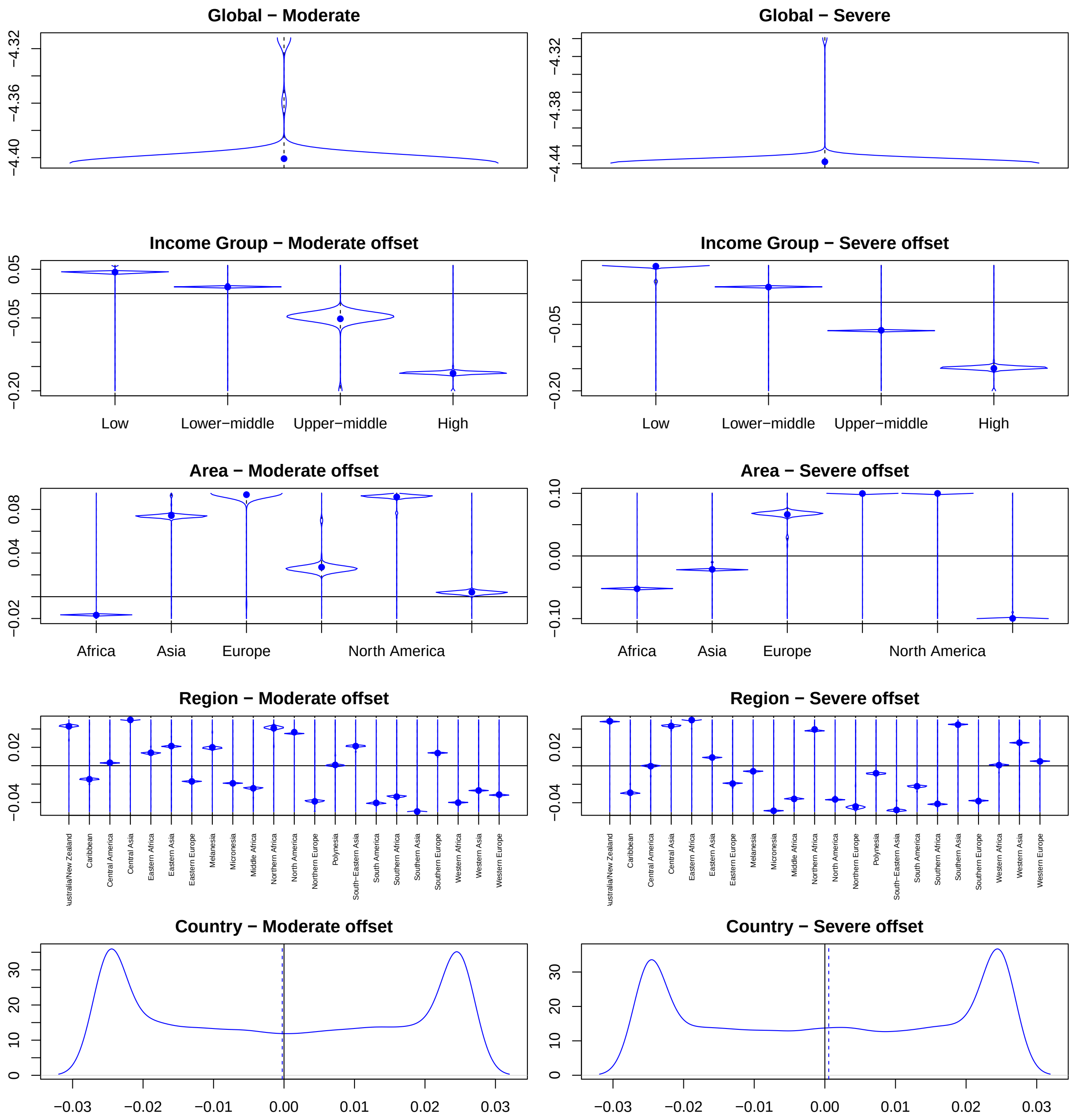

Parameters

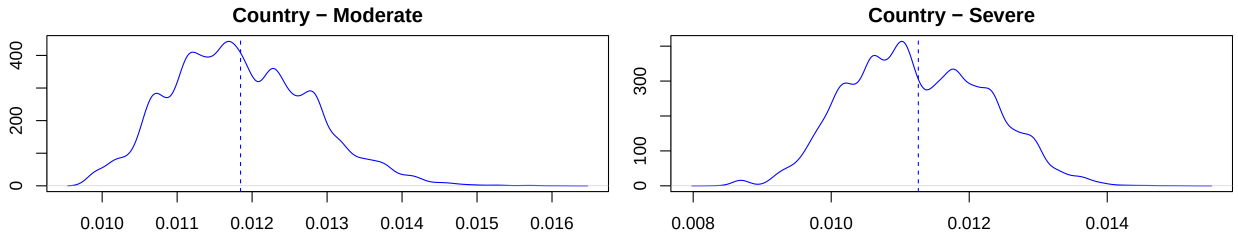

C.5.4.2 Risk Factors

Hyperparameters

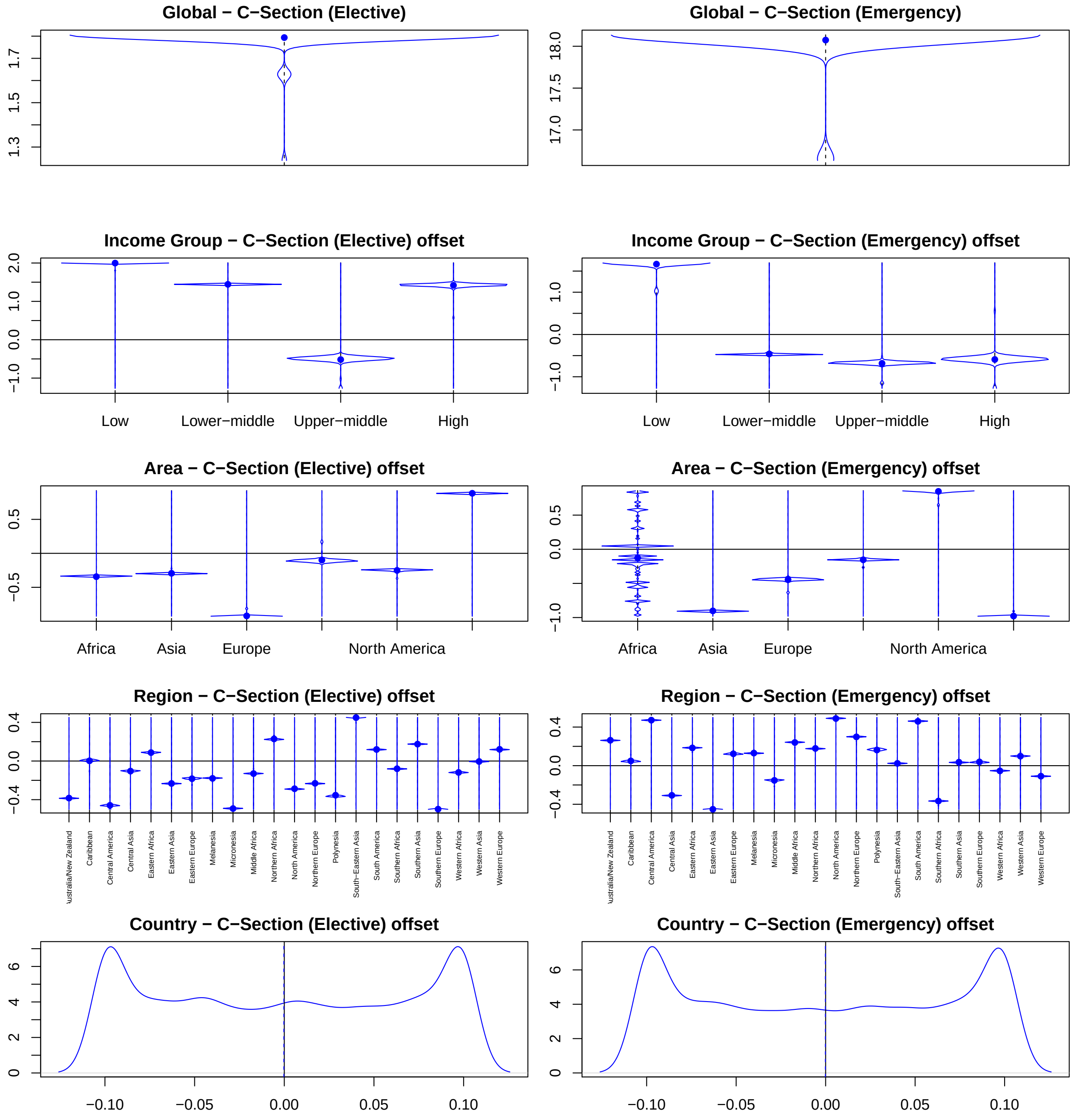

Parameters

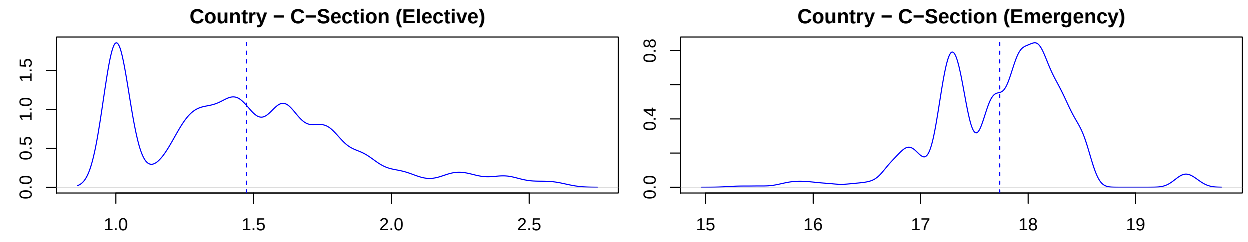

C.5.4.3 Mortality/Morbidity

Hyperparameters

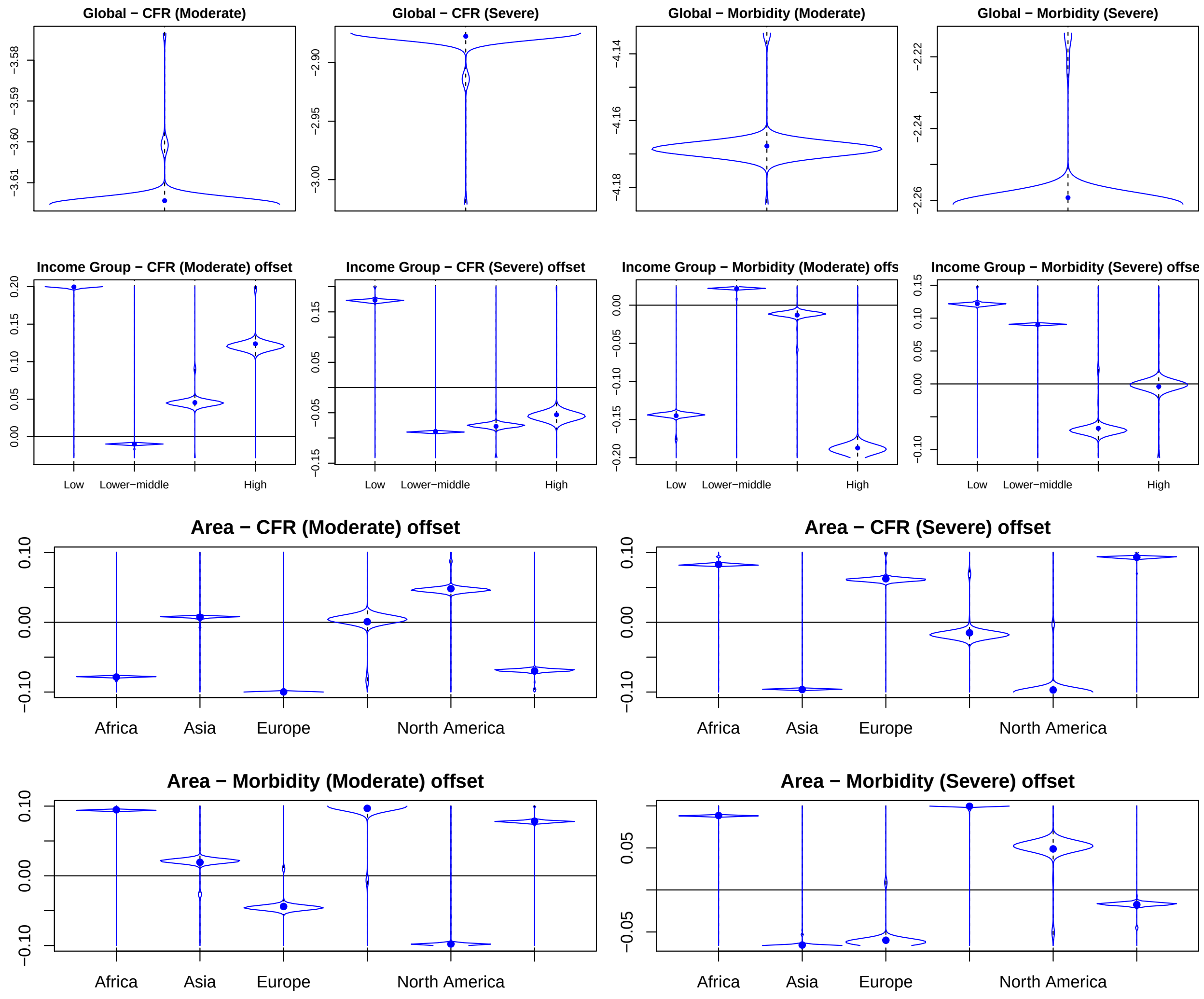

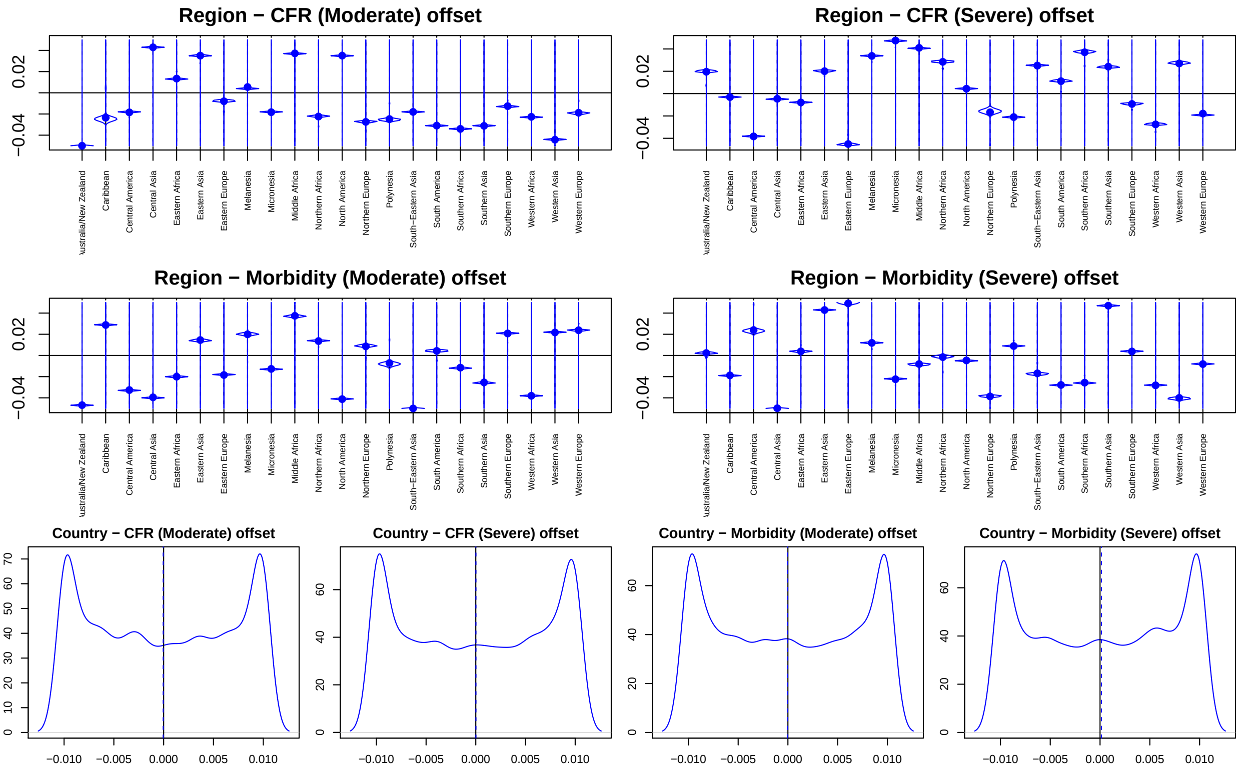

Parameters

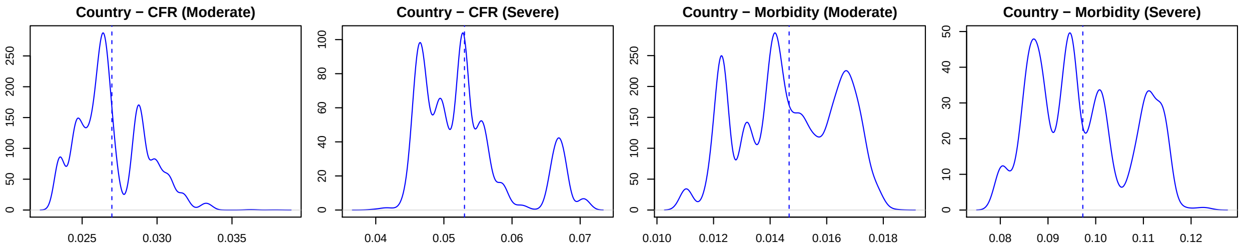

C.5.4.4 Anemia Relative Risks

Hyperparameters

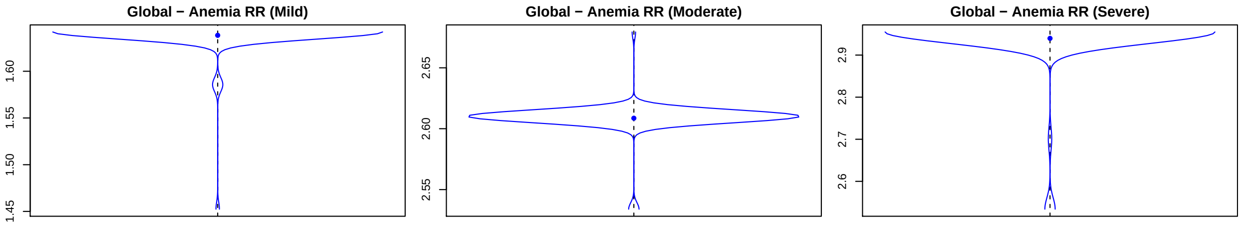

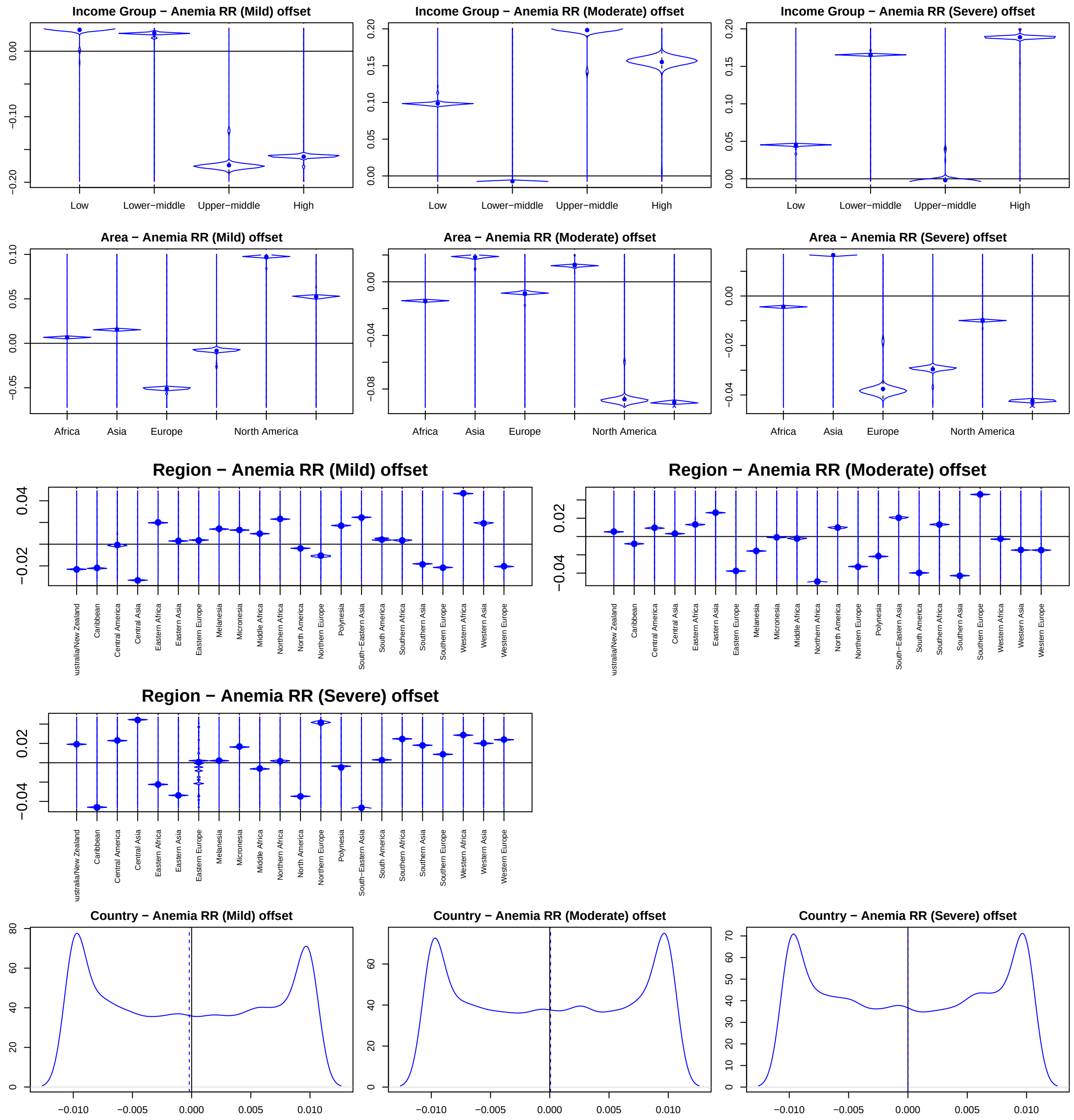

Income Group − Anemia RR (Mild) offset
Income Group − Anemia RR (Moderate) offset
Income Group − Anemia RR (Severe) offset
Low
Lower−middle
Upper−middle
High
Area − Anemia RR (Mild) offset
Area − Anemia RR (Moderate) offset
Area − Anemia RR (Severe) offset
Africa
Asia
Europe
North America
Region − Anemia RR (Mild) offset
Region − Anemia RR (Moderate) offset
Region − Anemia RR (Severe) offset
ustralia/New Zealand
Caribbean
Central America
Central Asia
Eastern Africa
Eastern Asia
Eastern Europe
Melanesia
Micronesia
Middle Africa
Northern Africa
North America
Northern Europe
Polynesia
South−Eastern Asia
South America
Southern Africa
Southern Asia
Southern Europe
Western Africa
Western Asia
Western Europe
Country − Anemia RR (Mild) offset
Country − Anemia RR (Moderate) offset
Country − Anemia RR (Severe) offset

Parameters

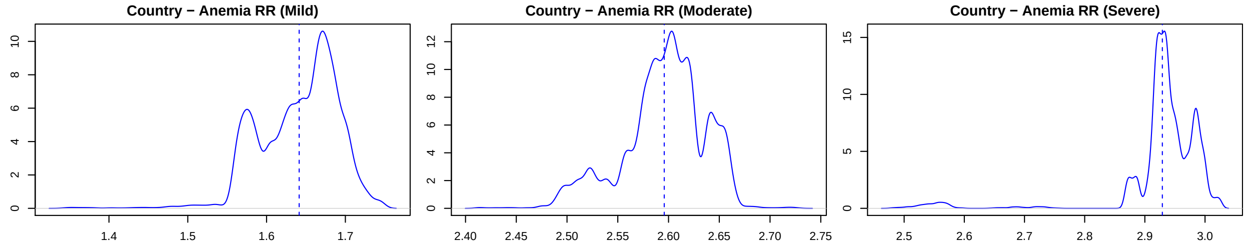

Country − Anemia RR (Mild)
Country − Anemia RR (Moderate)
Country − Anemia RR (Severe)

C.5.5 Other Direct

C.5.5.1 Incidence

Hyperparameters

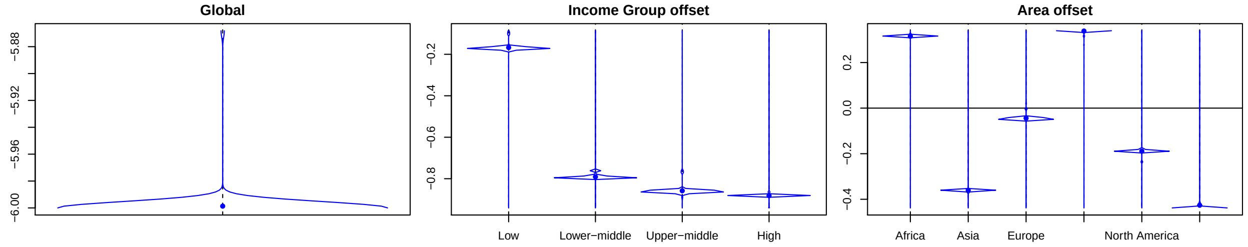

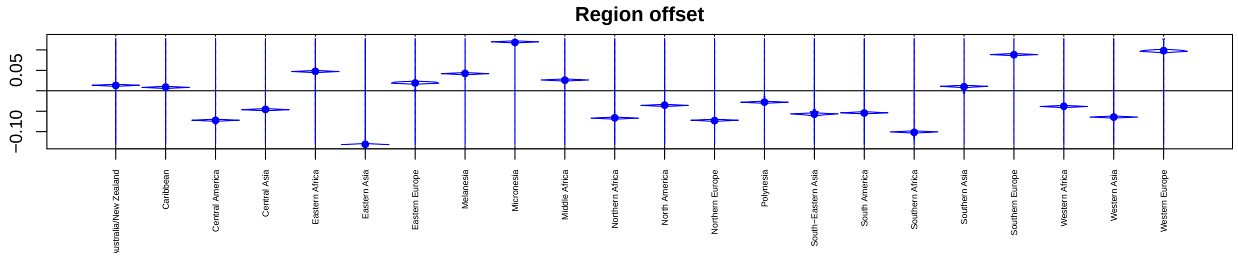

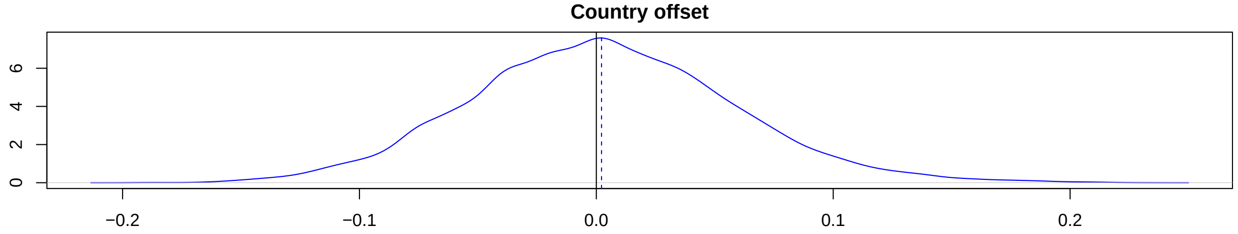

Parameters

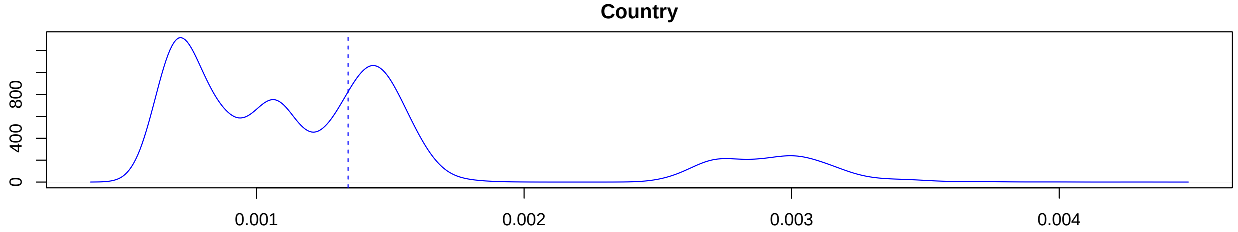

C.5.5.2 Mortality

Hyperparameters

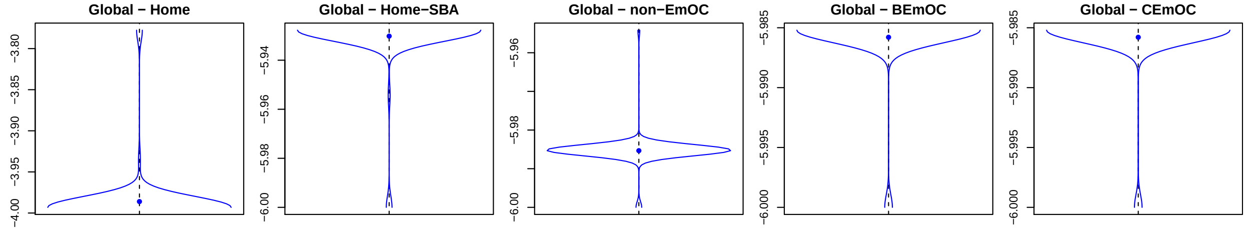

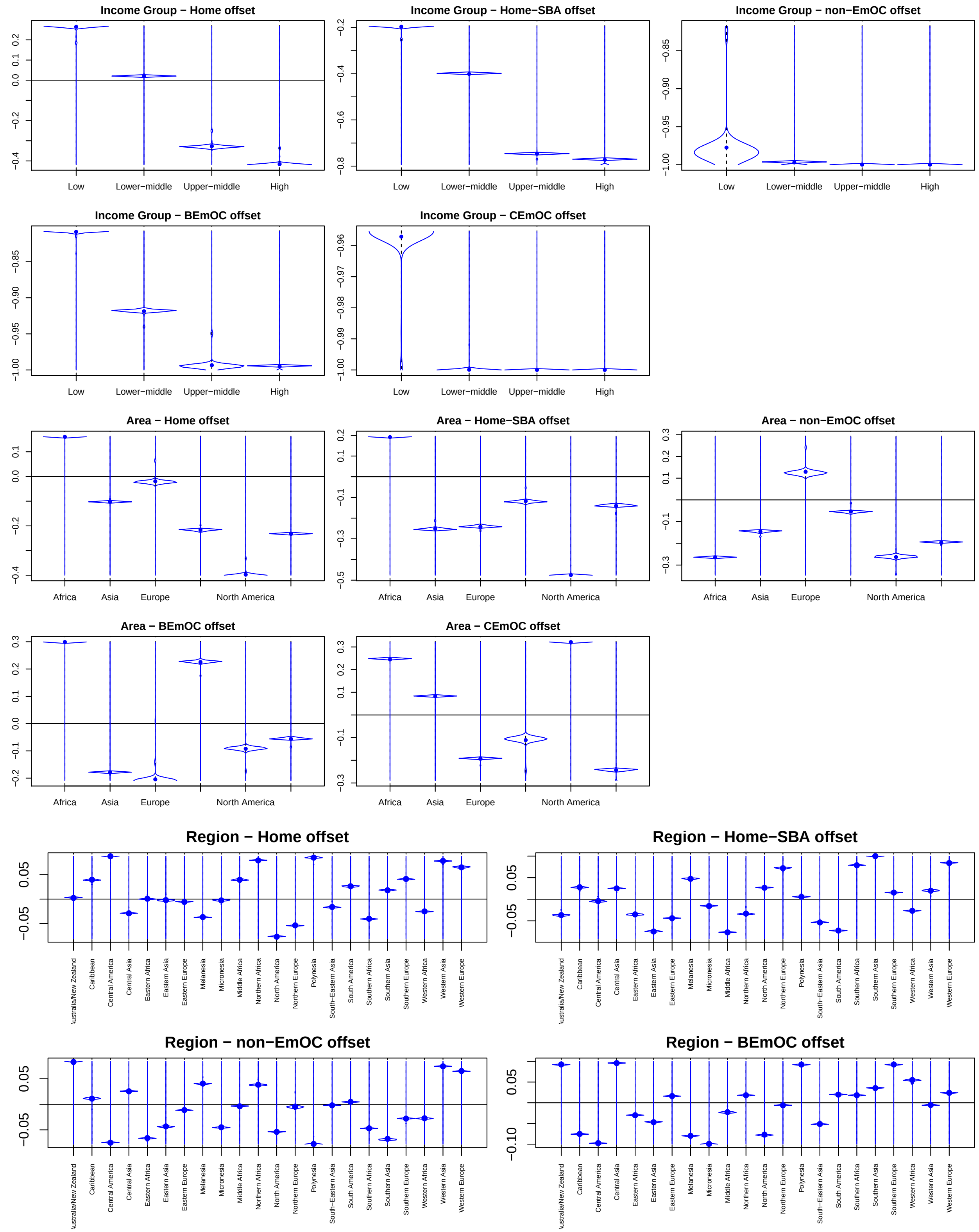
Income Group − Home offset
Income Group − Home−SBA offset
Income Group − non−EmOC offset
Income Group − BEmOC offset
Income Group − CEmOC offset
Low
Lower−middle
Upper−middle
High
Area − Home offset
Area − Home−SBA offset
Area − non−EmOC offset
Area − BEmOC offset
Area − CEmOC offset
Africa
Asia
Europe
North America
Region − Home offset
Region − Home−SBA offset
Region − non−EmOC offset
Region − BEmOC offset
Caribbean
Central America
Central Asia
Eastern Africa
Eastern Asia
Eastern Europe
Melanesia
Micronesia
Middle Africa
Northern Africa
North America
Northern Europe
Polynesia
South−Eastern Asia
South America
Southern Africa
Southern Asia
Southern Europe
Western Africa
Western Asia
Western Europe

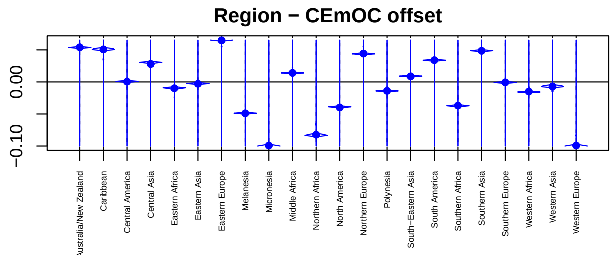
Region − CEmOC offset
0.00
−0.10
Australia/New Zealand
Caribbean
Central America
Central Asia
Eastern Africa
Eastern Asia
Eastern Europe
Melanesia
Micronesia
Middle Africa
Northern Africa
North America
Northern Europe
Polynesia
South−Eastern Asia
South America
Southern Africa
Southern Asia
Southern Europe
Western Africa
Western Asia
Western Europe

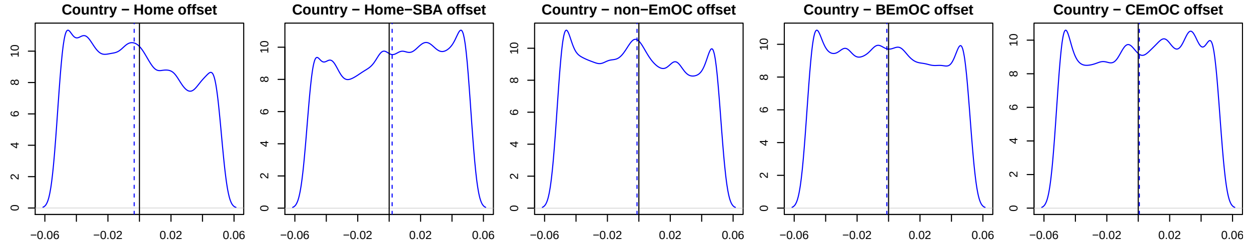
Country − Home offset
Country − Home−SBA offset
Country − non−EmOC offset
Country − BEmOC offset
Country − CEmOC offset
0
2
4
6
8
10
−0.06
−0.02
0.02
0.06

Parameters

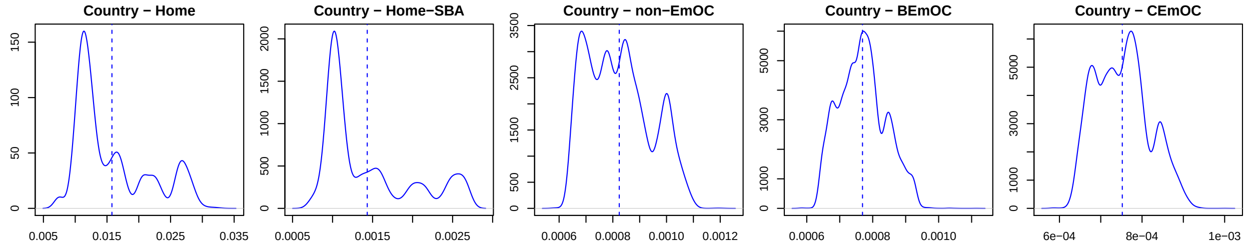
Country − Home
Country − Home−SBA
Country − non−EmOC
Country − BEmOC
Country − CEmOC
0
50
100
150
0.005
0.015
0.025
0.035
500
1000
1500
2000
0.0005
0.0015
0.0025
2500
3500
0.0006
0.0008
0.0010
0.0012
3000
5000
6e−04
8e−04
1e−03

C.5.6 Late Maternal Deaths

C.5.6.1 Incidence

Hyperparameters

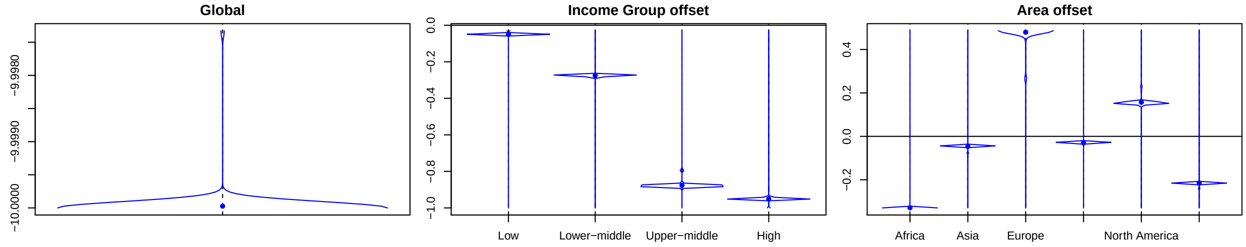

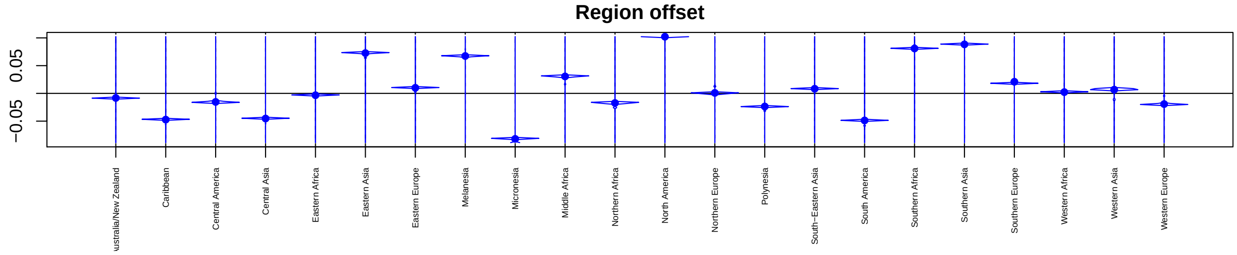

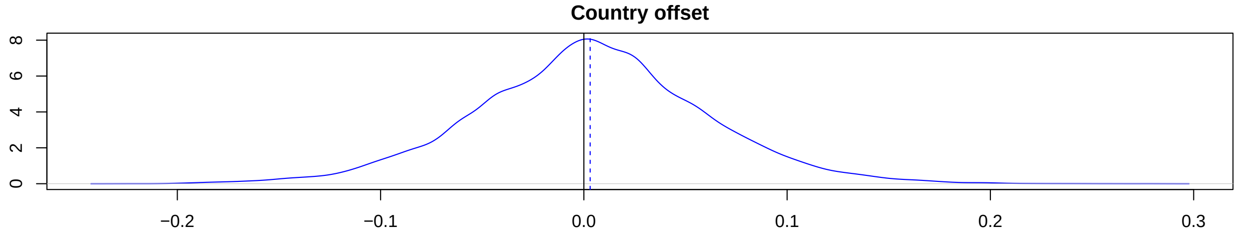

Parameters

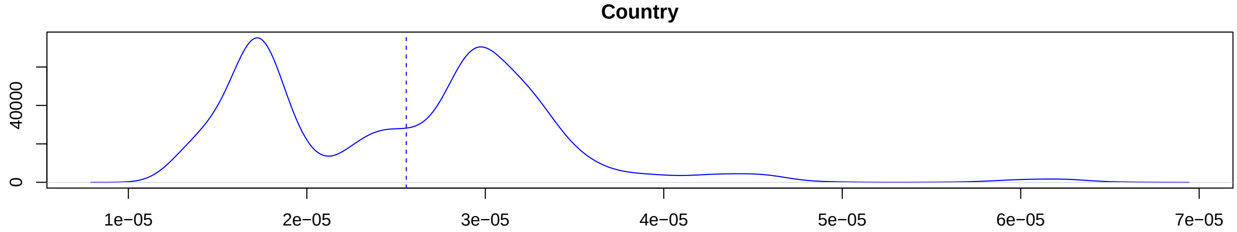

C.5.6.2 Mortality

Hyperparameters

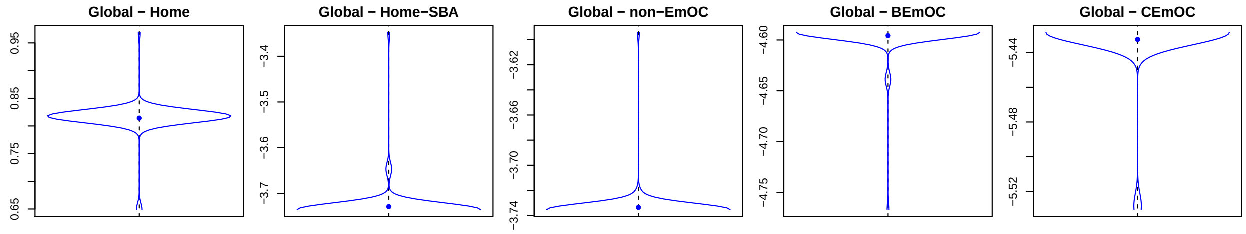

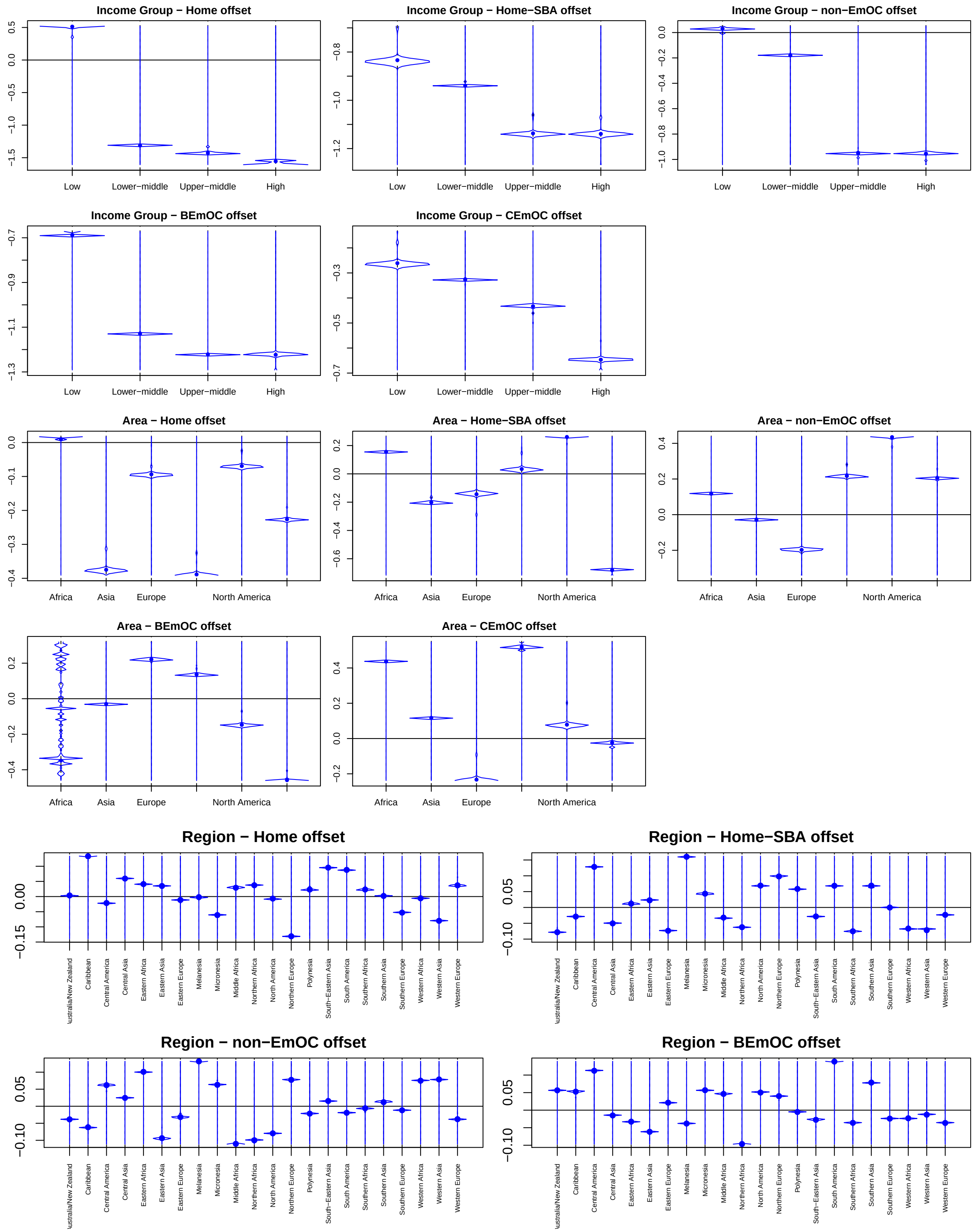
Income Group − Home offset
Income Group − Home−SBA offset
Income Group − non−EmOC offset
Income Group − BEmOC offset
Income Group − CEmOC offset
Low
Lower−middle
Upper−middle
High
Area − Home offset
Area − Home−SBA offset
Area − non−EmOC offset
Area − BEmOC offset
Area − CEmOC offset
Africa
Asia
Europe
North America
Region − Home offset
Region − Home−SBA offset
Region − non−EmOC offset
Region − BEmOC offset
Caribbean
Central America
Central Asia
Eastern Africa
Eastern Asia
Eastern Europe
Melanesia
Micronesia
Middle Africa
Northern Africa
North America
Northern Europe
Polynesia
South−Eastern Asia
South America
Southern Africa
Southern Asia
Southern Europe
Western Africa
Western Asia
Western Europe

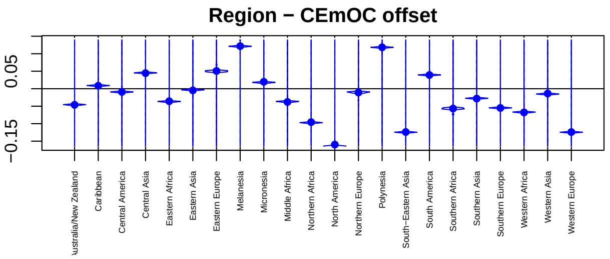
Region − CEmOC offset
0.05
−0.15
Australia/New Zealand
Caribbean
Central America
Central Asia
Eastern Africa
Eastern Asia
Eastern Europe
Melanesia
Micronesia
Middle Africa
Northern Africa
North America
Northern Europe
Polynesia
South−Eastern Asia
South America
Southern Africa
Southern Asia
Southern Europe
Western Africa
Western Asia
Western Europe

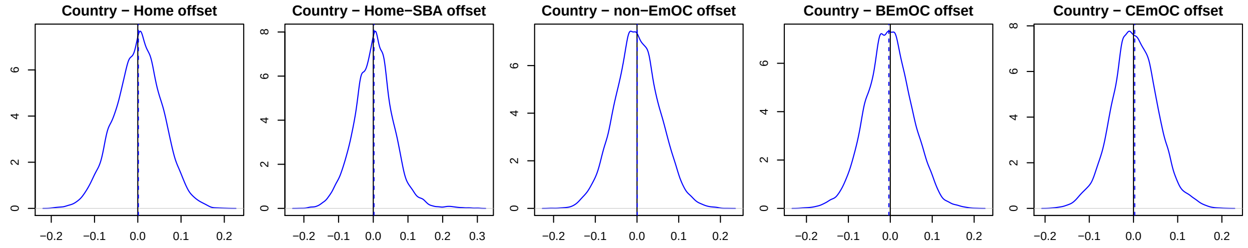
Country − Home offset
Country − Home−SBA offset
Country − non−EmOC offset
Country − BEmOC offset
Country − CEmOC offset

Parameters

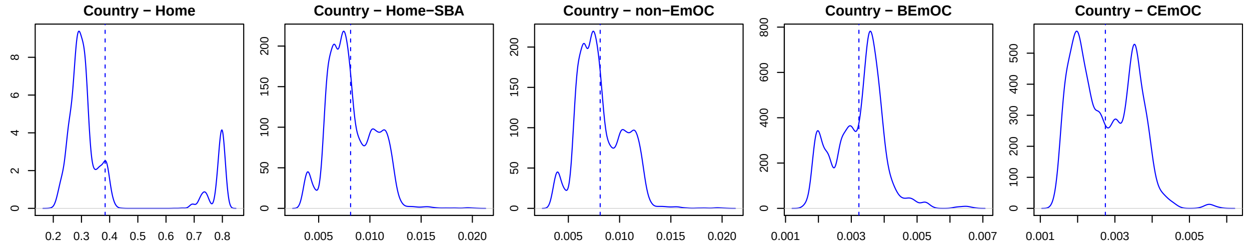
Country − Home
Country − Home−SBA
Country − non−EmOC
Country − BEmOC
Country − CEmOC

C.5.7 Indirect Maternal Deaths

Hyperparameters

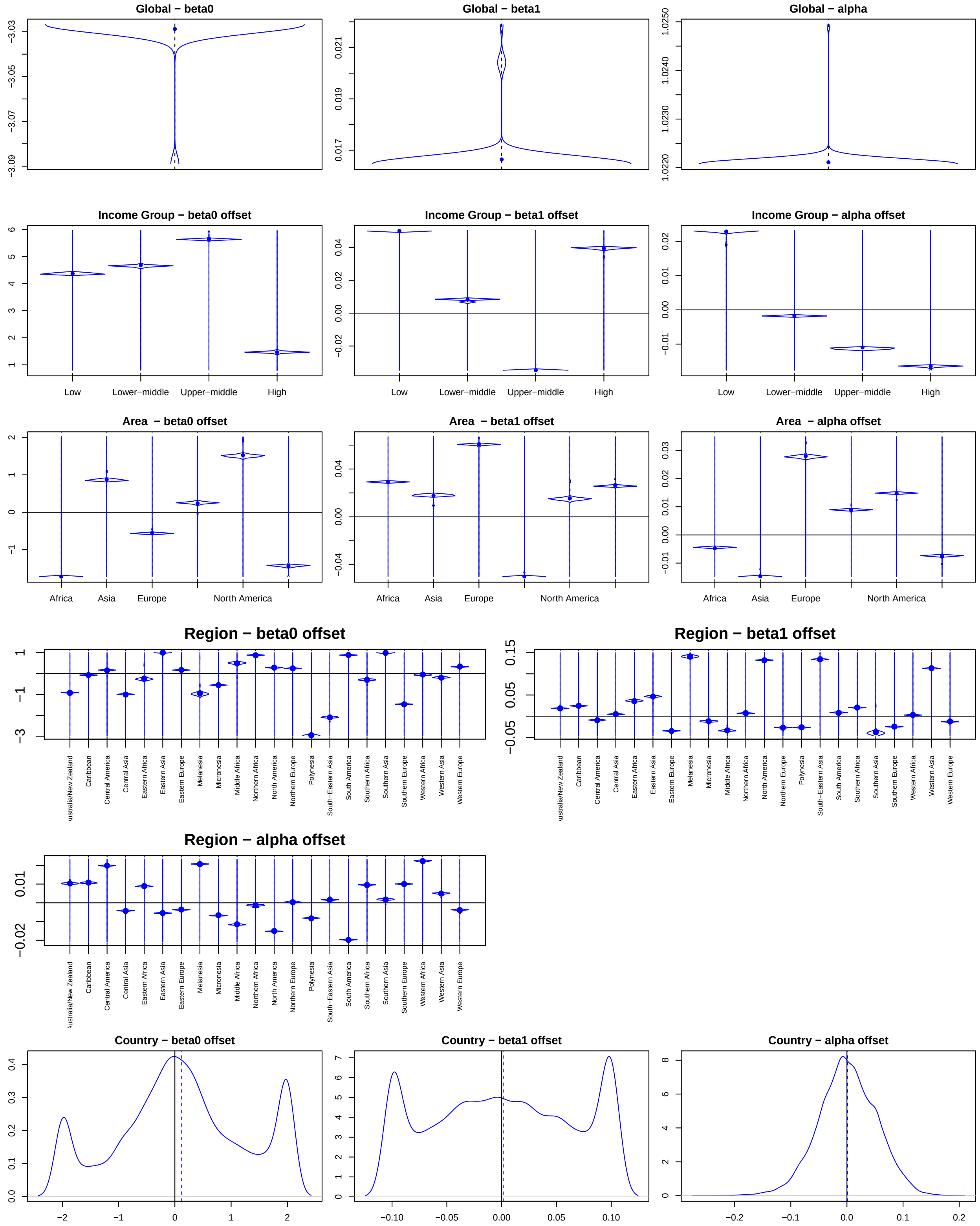

Parameters

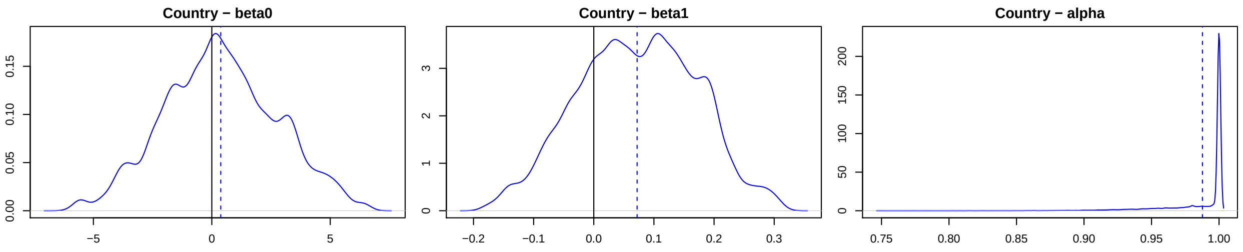

Country − beta0
0.00
0.05
0.10
0.15
−5
0
5
Country − beta1
0
1
2
3
−0.2
−0.1
0.0
0.1
0.2
0.3
Country − alpha
0
50
100
150
200
0.75
0.80
0.85
0.90
0.95
1.00

C.6 Clinical Interventions

C.6.1 Elective Caesarean Section

Hyperparameters

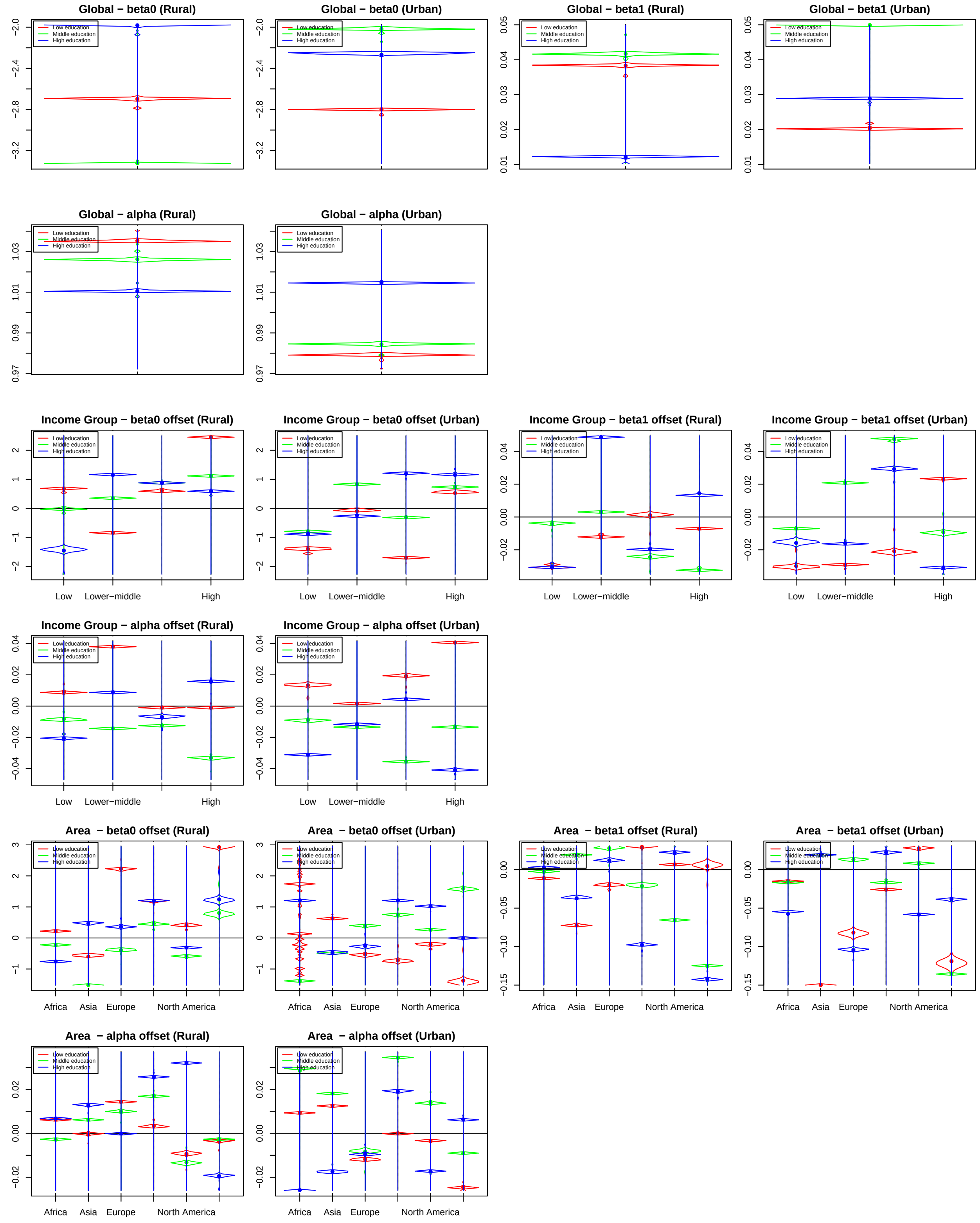

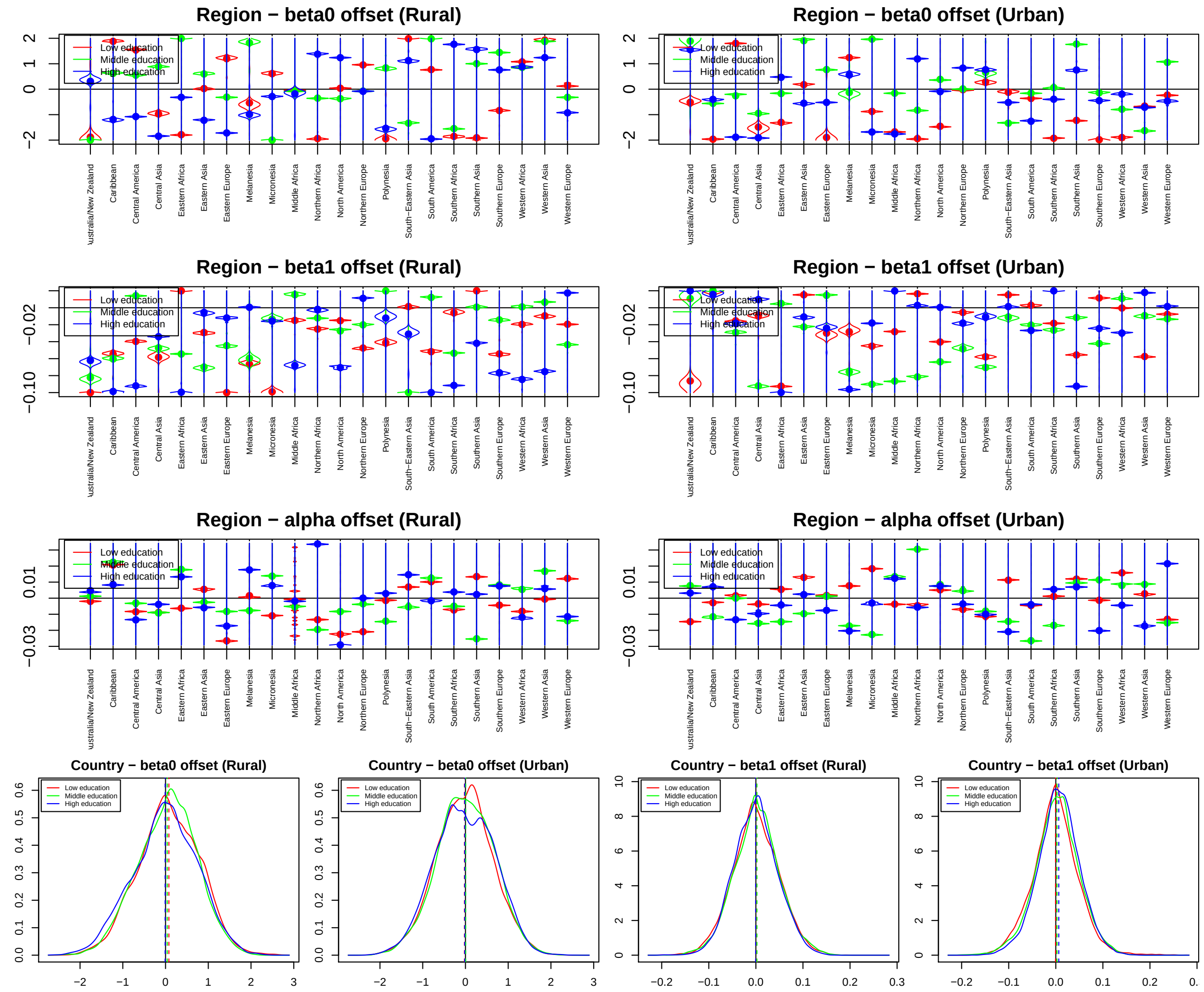
Region − beta0 offset (Rural)
Region − beta0 offset (Urban)
Region − beta1 offset (Rural)
Region − beta1 offset (Urban)
Region − alpha offset (Rural)
Region − alpha offset (Urban)
Low education
Middle education
High education
Caribbean
Central America
Central Asia
Eastern Africa
Eastern Asia
Eastern Europe
Melanesia
Micronesia
Middle Africa
Northern Africa
North America
Northern Europe
Polynesia
South−Eastern Asia
South America
Southern Africa
Southern Asia
Southern Europe
Western Africa
Western Asia
Western Europe
Country − beta0 offset (Rural)
Country − beta0 offset (Urban)
Country − beta1 offset (Rural)
Country − beta1 offset (Urban)

Parameters

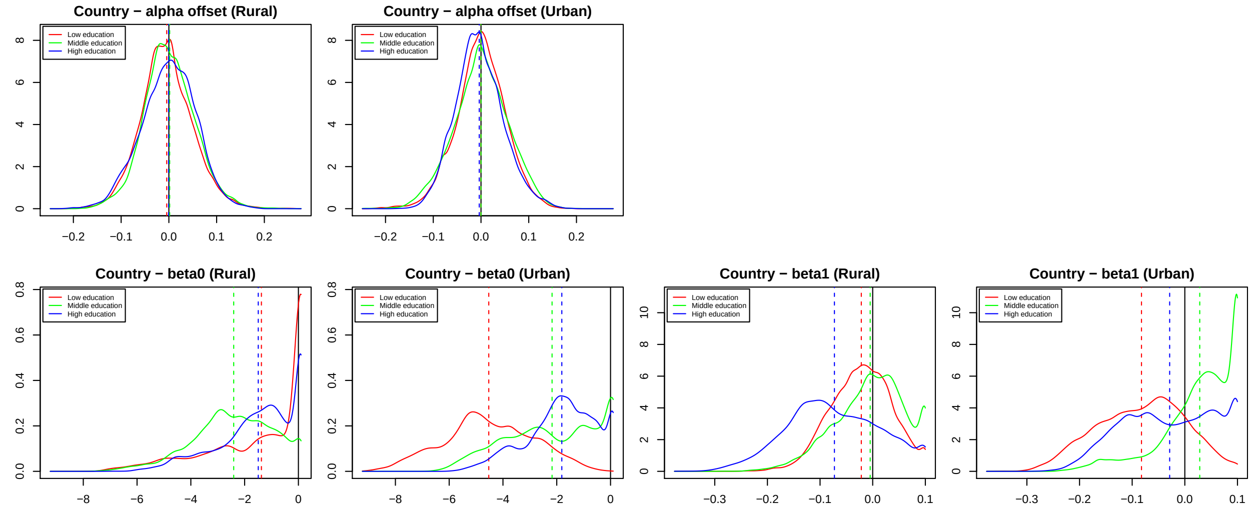
Country − alpha offset (Rural)
Country − alpha offset (Urban)
Country − beta0 (Rural)
Country − beta0 (Urban)
Country − beta1 (Rural)
Country − beta1 (Urban)
Low education
Middle education
High education

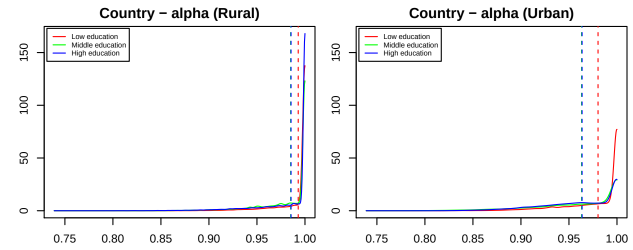

Country − alpha (Rural)
Low education
Middle education
High education
0
50
100
150
0.75
0.80
0.85
0.90
0.95
1.00
Country − alpha (Urban)
Low education
Middle education
High education
0
50
100
150
0.75
0.80
0.85
0.90
0.95
1.00

C.6.2 Active Management of Labour

C.6.2.1 Use

Hyperparameters

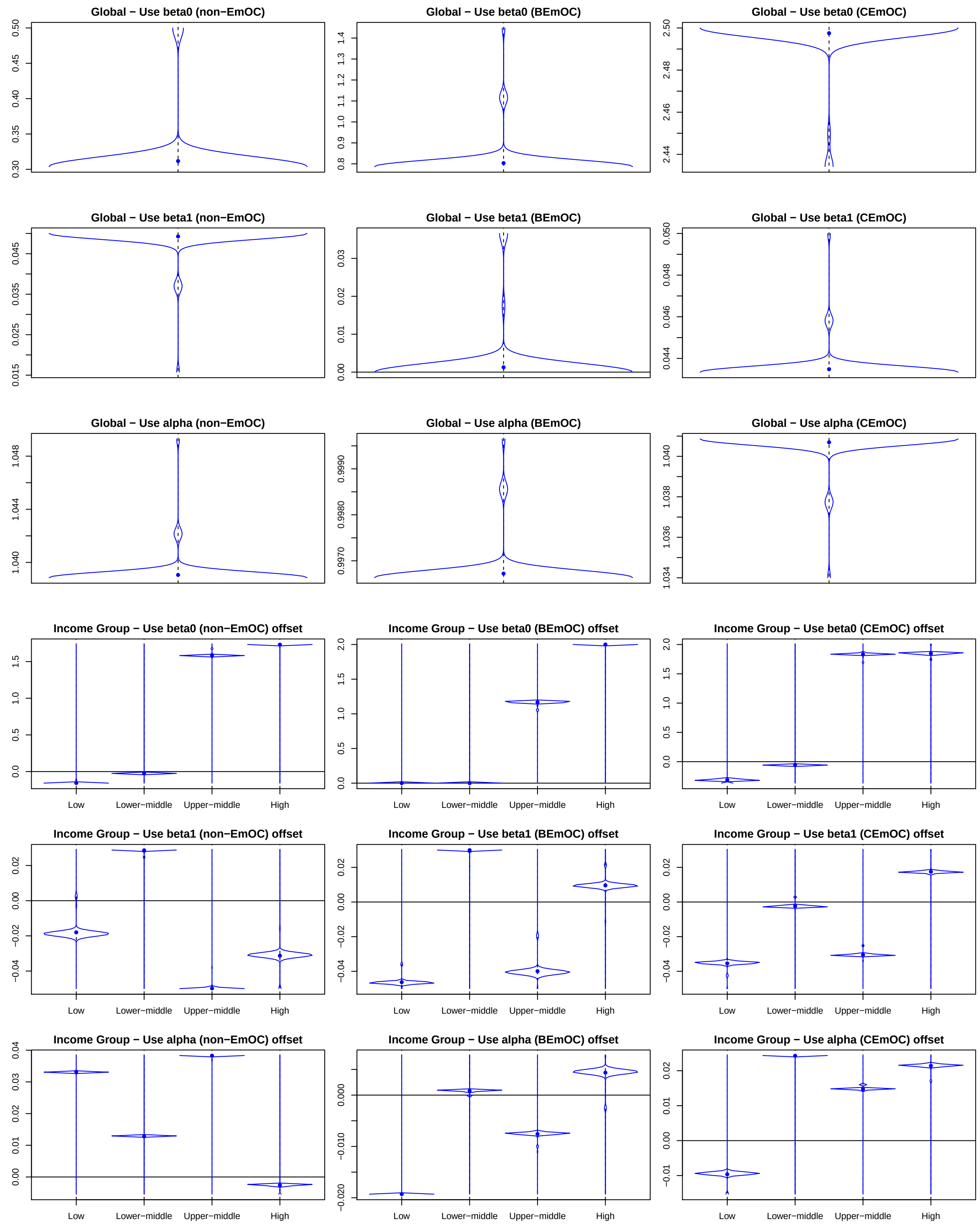

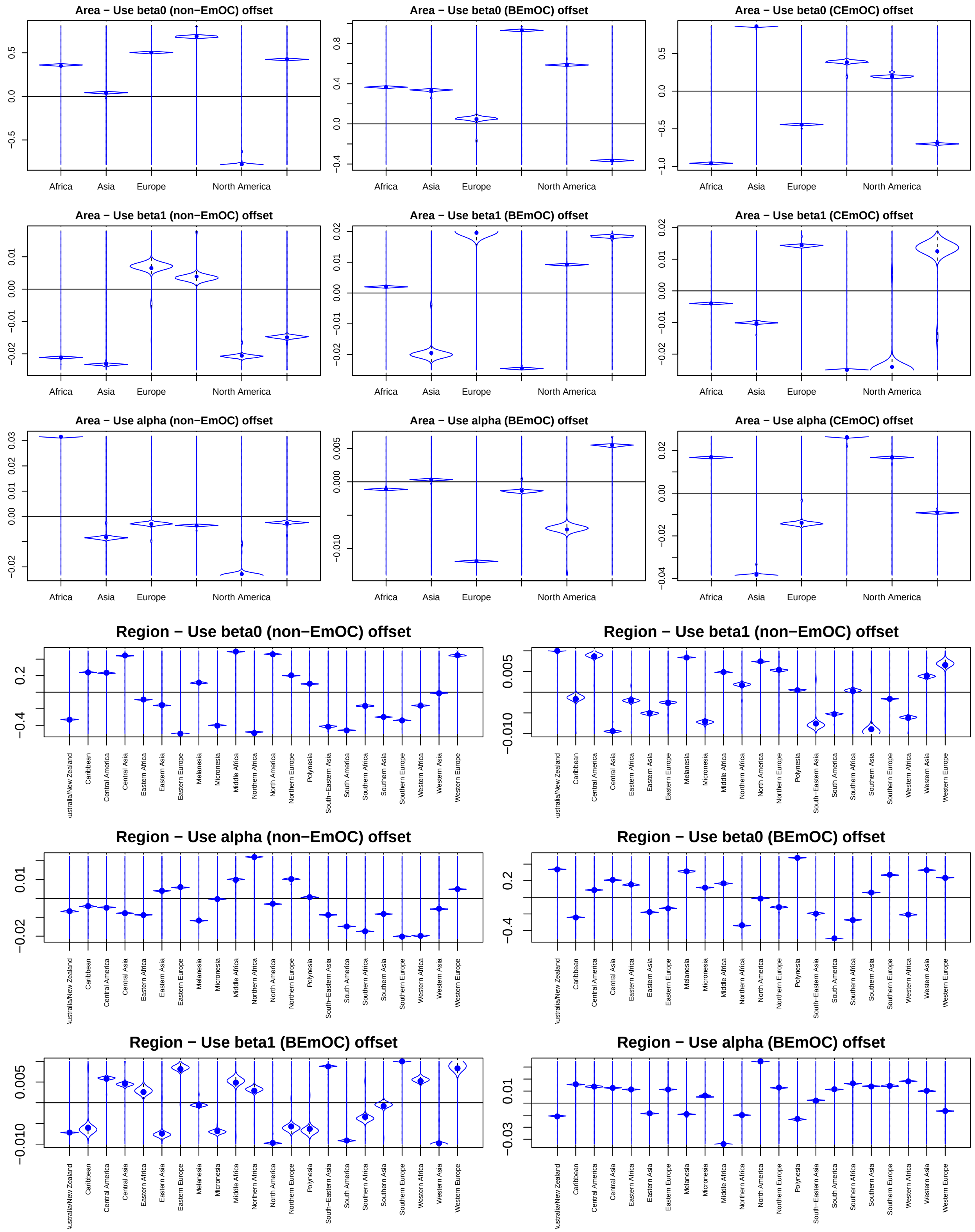
Area − Use beta0 (non−EmOC) offset
Area − Use beta0 (BEmOC) offset
Area − Use beta0 (CEmOC) offset
Area − Use beta1 (non−EmOC) offset
Area − Use beta1 (BEmOC) offset
Area − Use beta1 (CEmOC) offset
Area − Use alpha (non−EmOC) offset
Area − Use alpha (BEmOC) offset
Area − Use alpha (CEmOC) offset
Africa
Asia
Europe
North America
Region − Use beta0 (non−EmOC) offset
Region − Use beta1 (non−EmOC) offset
Region − Use alpha (non−EmOC) offset
Region − Use beta0 (BEmOC) offset
Region − Use beta1 (BEmOC) offset
Region − Use alpha (BEmOC) offset
Caribbean
Central America
Central Asia
Eastern Africa
Eastern Asia
Eastern Europe
Melanesia
Micronesia
Middle Africa
Northern Africa
North America
Northern Europe
Polynesia
South−Eastern Asia
South America
Southern Africa
Southern Asia
Southern Europe
Western Africa
Western Asia
Western Europe

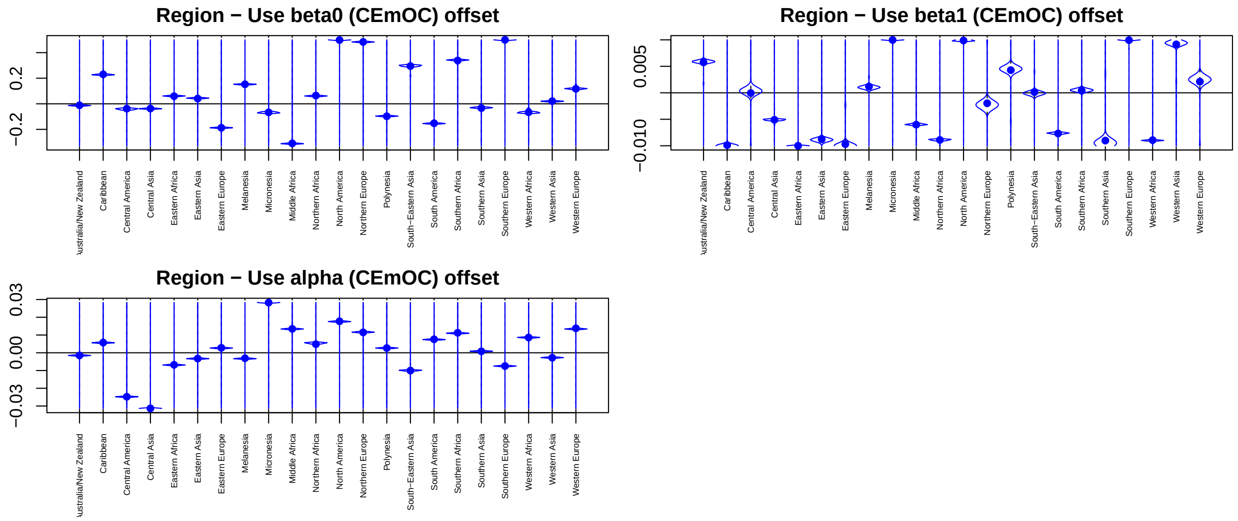
Region − Use beta0 (CEmOC) offset
Region − Use beta1 (CEmOC) offset
Region − Use alpha (CEmOC) offset
Australia/New Zealand
Caribbean
Central America
Central Asia
Eastern Africa
Eastern Asia
Eastern Europe
Melanesia
Micronesia
Middle Africa
Northern Africa
North America
Northern Europe
Polynesia
South−Eastern Asia
South America
Southern Africa
Southern Asia
Southern Europe
Western Africa
Western Asia
Western Europe

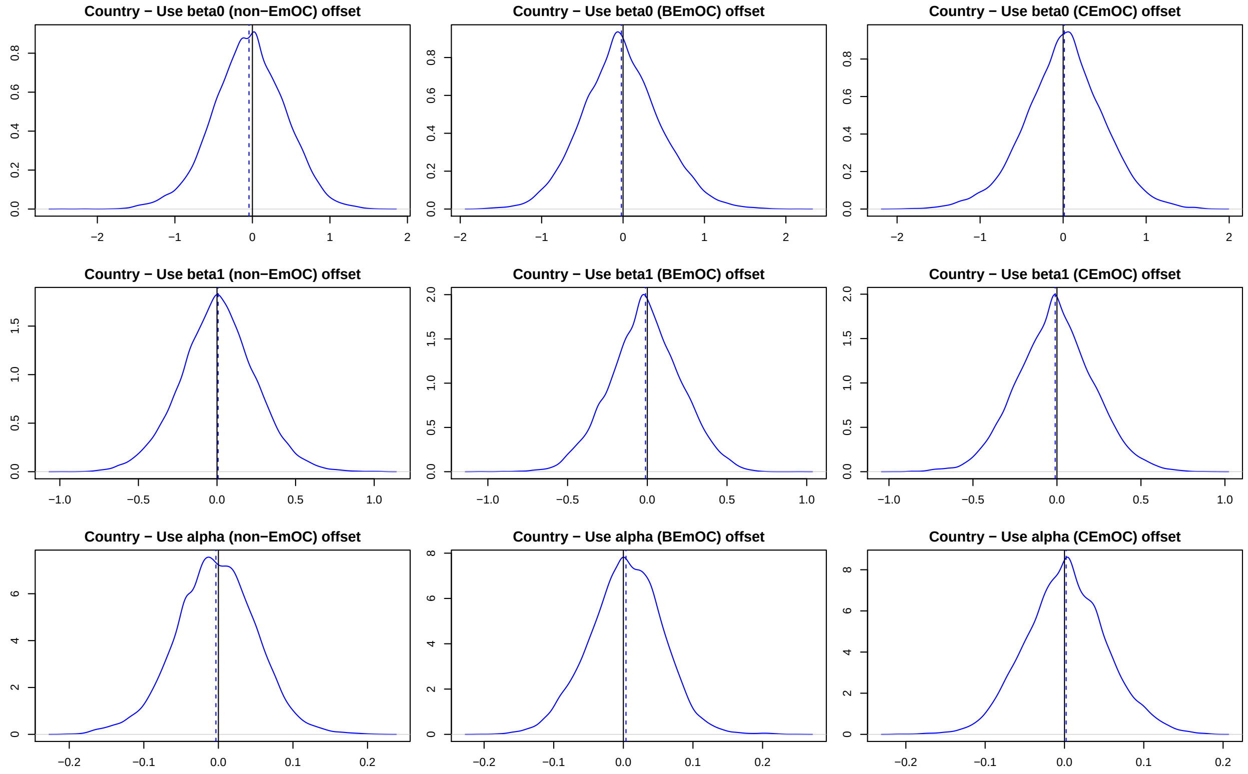
Country − Use beta0 (non−EmOC) offset
Country − Use beta0 (BEmOC) offset
Country − Use beta0 (CEmOC) offset
Country − Use beta1 (non−EmOC) offset
Country − Use beta1 (BEmOC) offset
Country − Use beta1 (CEmOC) offset
Country − Use alpha (non−EmOC) offset
Country − Use alpha (BEmOC) offset
Country − Use alpha (CEmOC) offset

Parameters

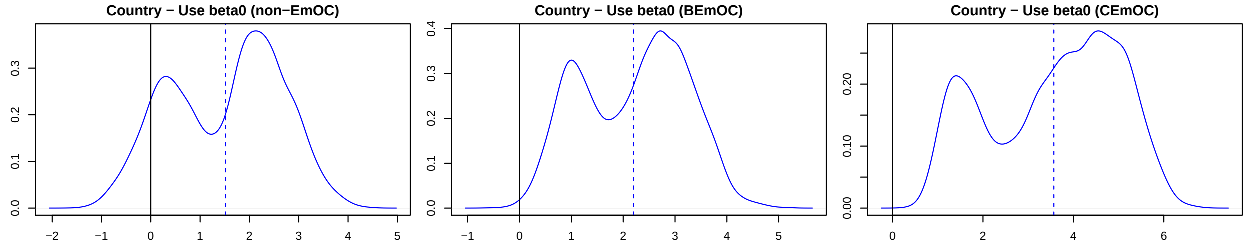
Country − Use beta0 (non−EmOC)
Country − Use beta0 (BEmOC)
Country − Use beta0 (CEmOC)

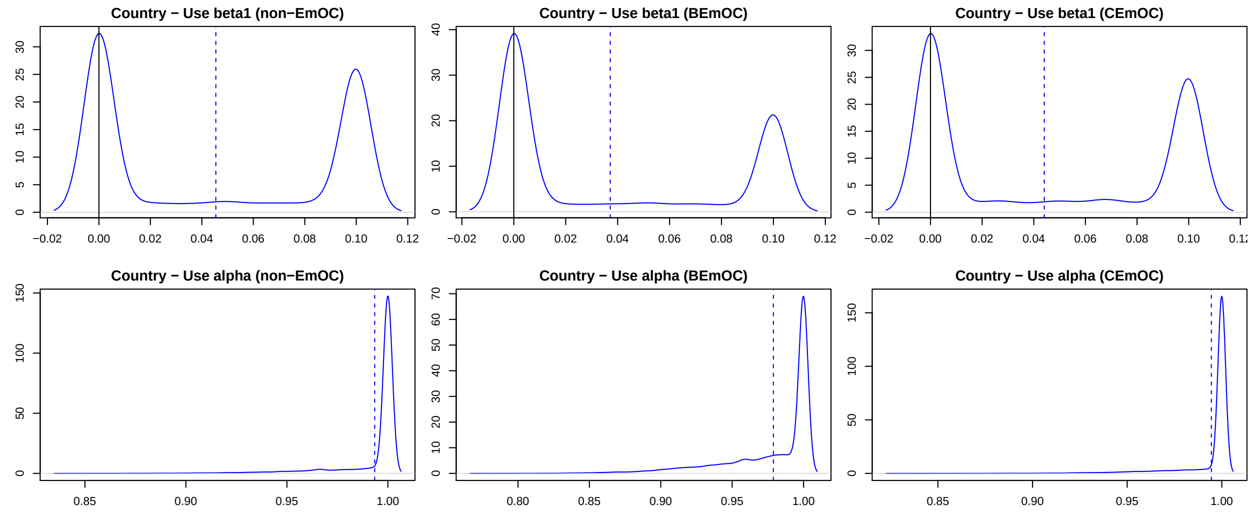

C.6.2.2 Efficacy

Hyperparameters

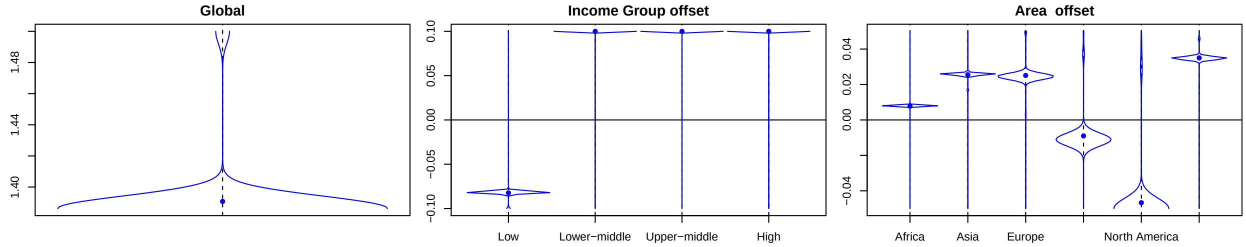

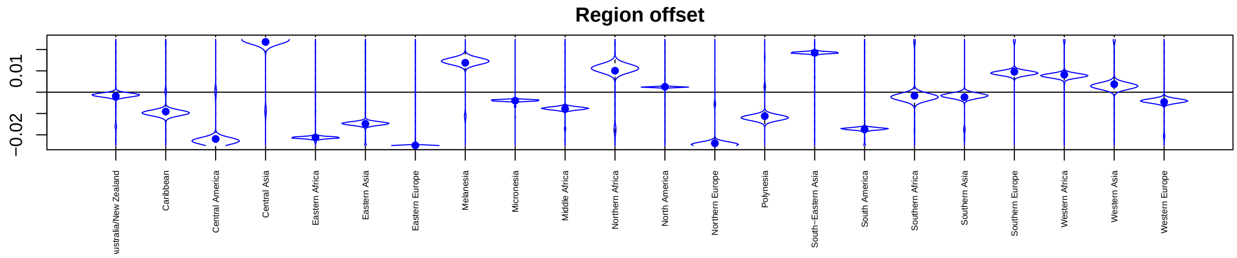

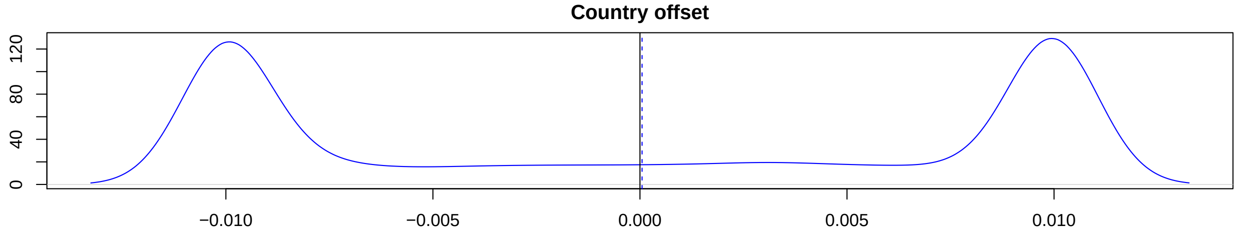

Parameters

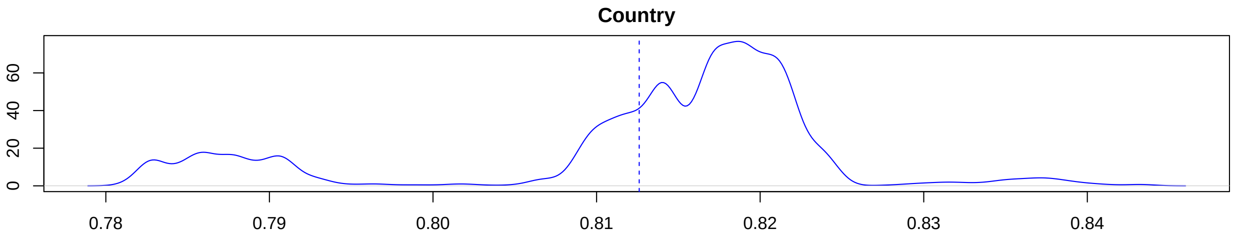
Country
0
20
40
60
0.78
0.79
0.80
0.81
0.82
0.83
0.84

C.6.3 Partograph

C.6.3.1 Use

Hyperparameters

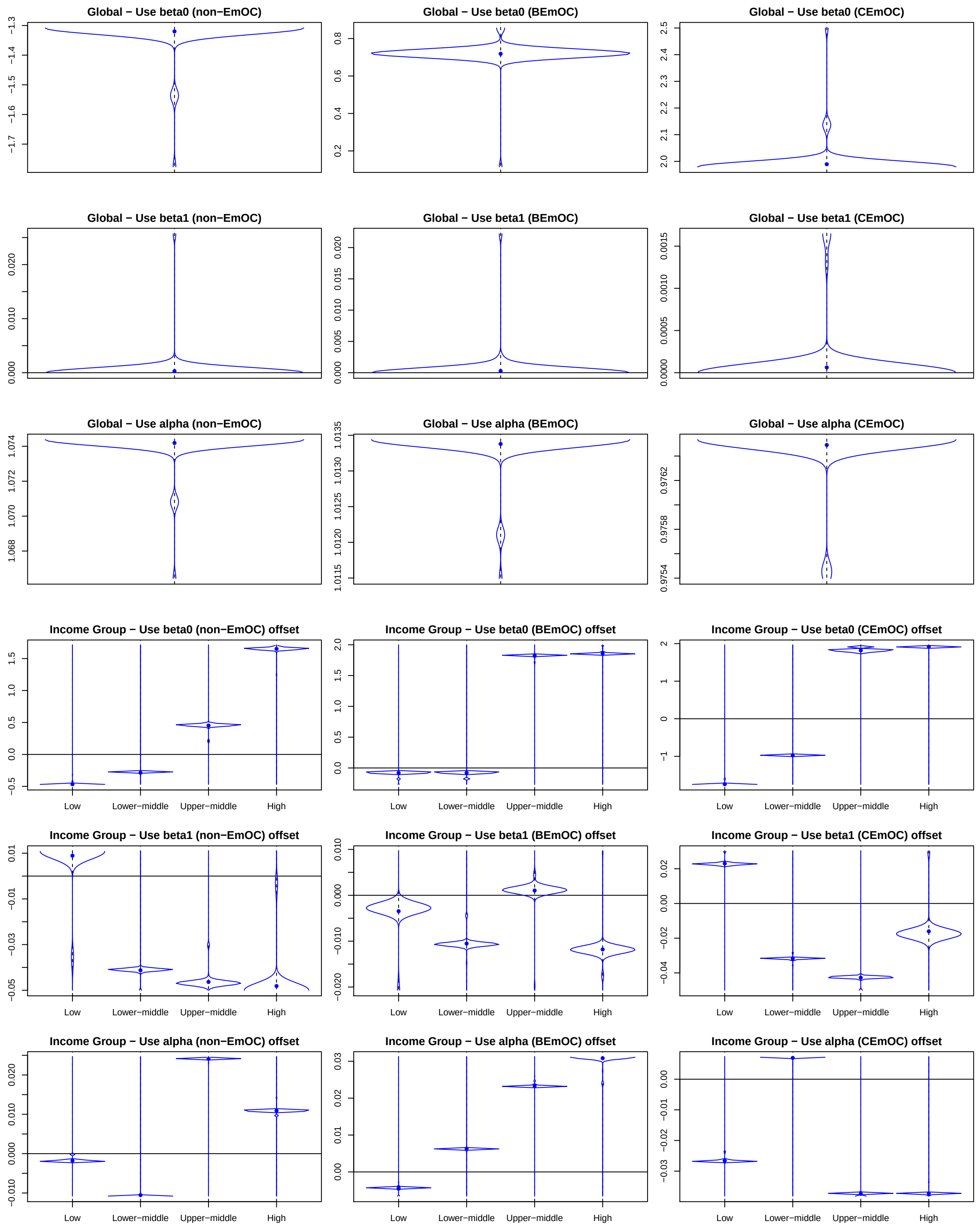

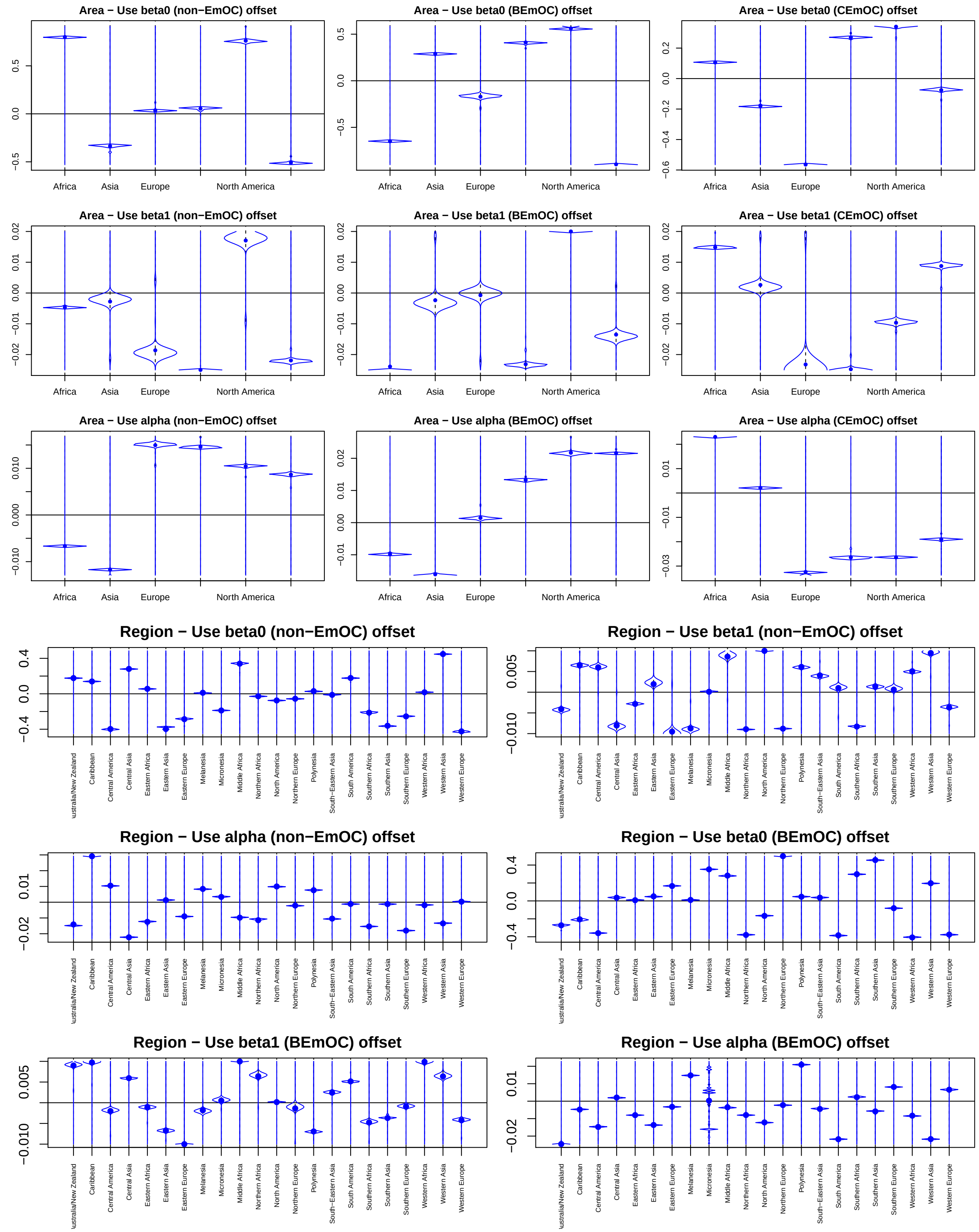
Area − Use beta0 (non−EmOC) offset
Area − Use beta0 (BEmOC) offset
Area − Use beta0 (CEmOC) offset
Area − Use beta1 (non−EmOC) offset
Area − Use beta1 (BEmOC) offset
Area − Use beta1 (CEmOC) offset
Area − Use alpha (non−EmOC) offset
Area − Use alpha (BEmOC) offset
Area − Use alpha (CEmOC) offset
Africa
Asia
Europe
North America
Region − Use beta0 (non−EmOC) offset
Region − Use beta1 (non−EmOC) offset
Region − Use alpha (non−EmOC) offset
Region − Use beta0 (BEmOC) offset
Region − Use beta1 (BEmOC) offset
Region − Use alpha (BEmOC) offset
ustralia/New Zealand
Caribbean
Central America
Central Asia
Eastern Africa
Eastern Asia
Eastern Europe
Melanesia
Micronesia
Middle Africa
Northern Africa
North America
Northern Europe
Polynesia
South−Eastern Asia
South America
Southern Africa
Southern Asia
Southern Europe
Western Africa
Western Asia
Western Europe

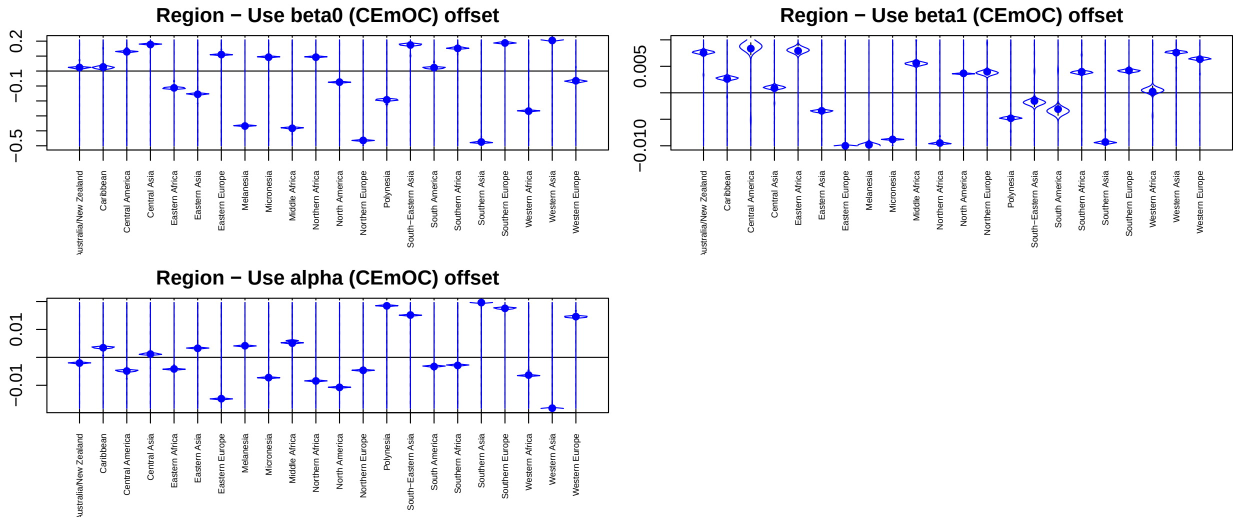
Region − Use beta0 (CEmOC) offset
Region − Use beta1 (CEmOC) offset
Region − Use alpha (CEmOC) offset

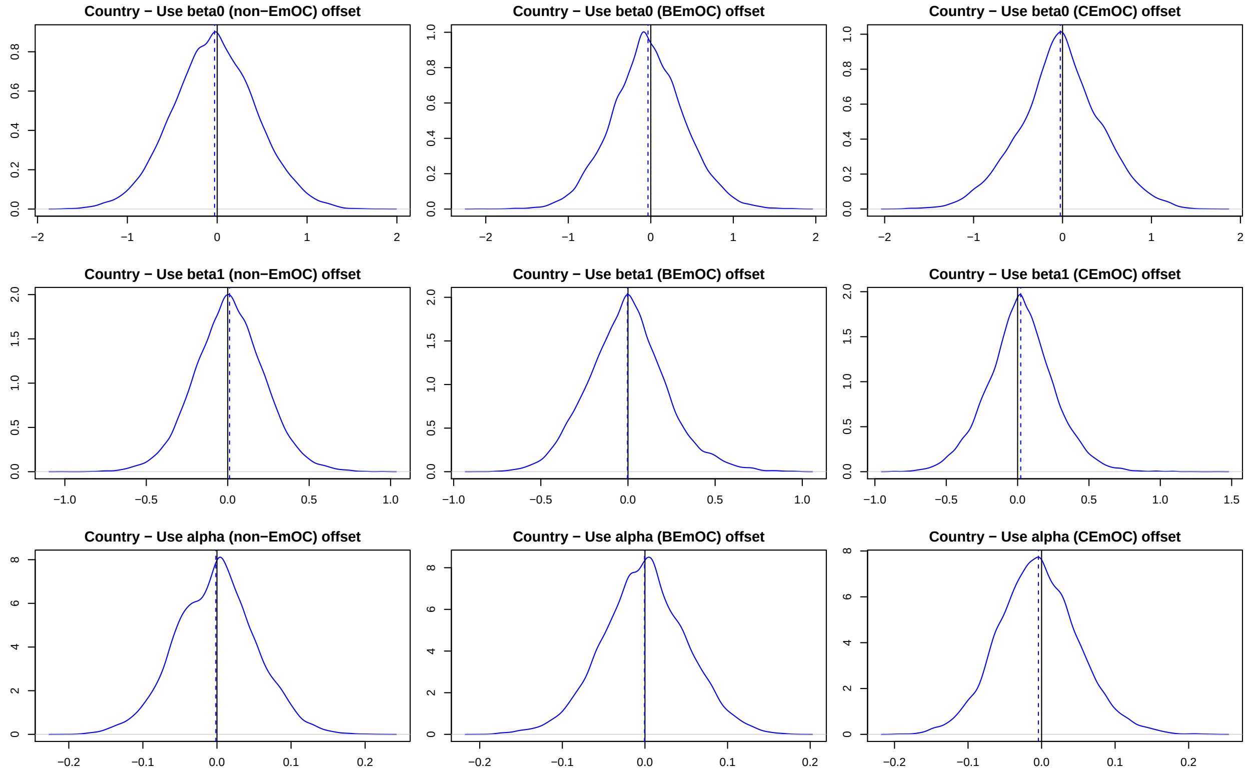
Country − Use beta0 (non−EmOC) offset
Country − Use beta0 (BEmOC) offset
Country − Use beta0 (CEmOC) offset
Country − Use beta1 (non−EmOC) offset
Country − Use beta1 (BEmOC) offset
Country − Use beta1 (CEmOC) offset
Country − Use alpha (non−EmOC) offset
Country − Use alpha (BEmOC) offset
Country − Use alpha (CEmOC) offset

Parameters

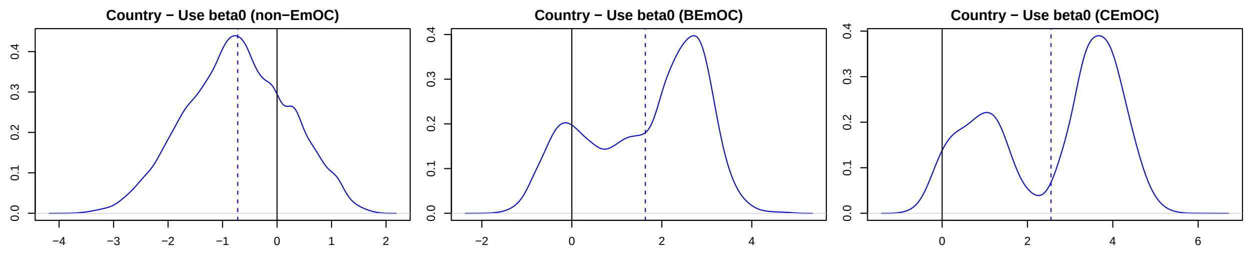
Country − Use beta0 (non−EmOC)
Country − Use beta0 (BEmOC)
Country − Use beta0 (CEmOC)

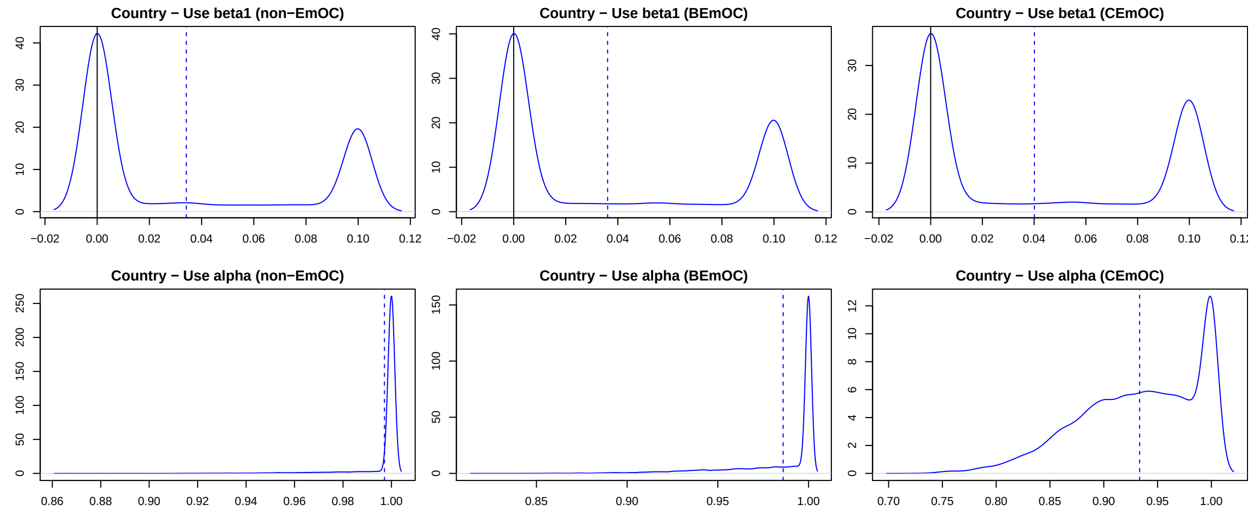

C.6.3.2 Efficacy

Hyperparameters

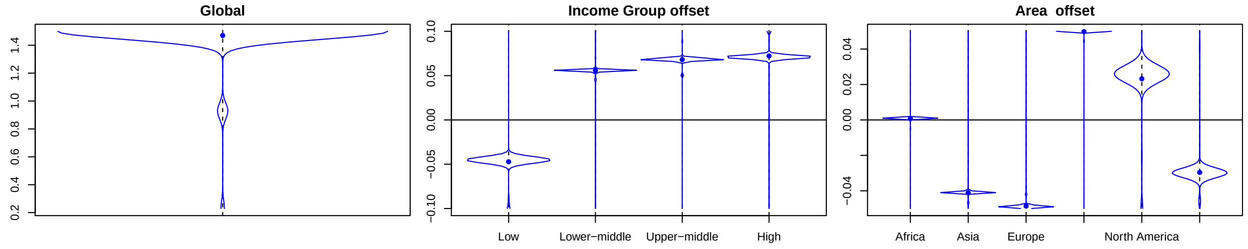

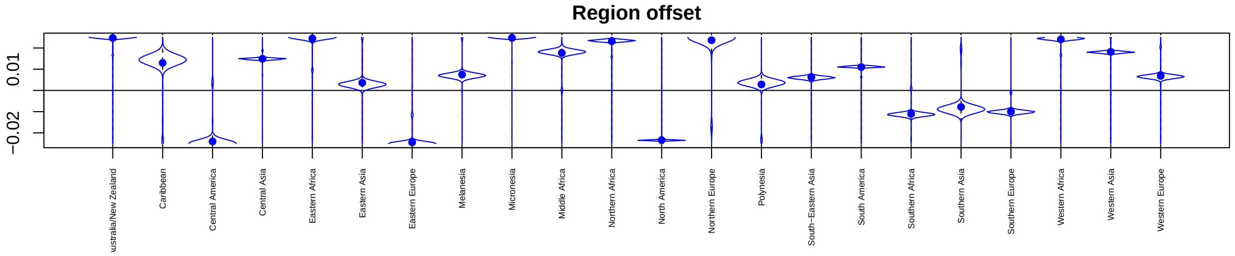

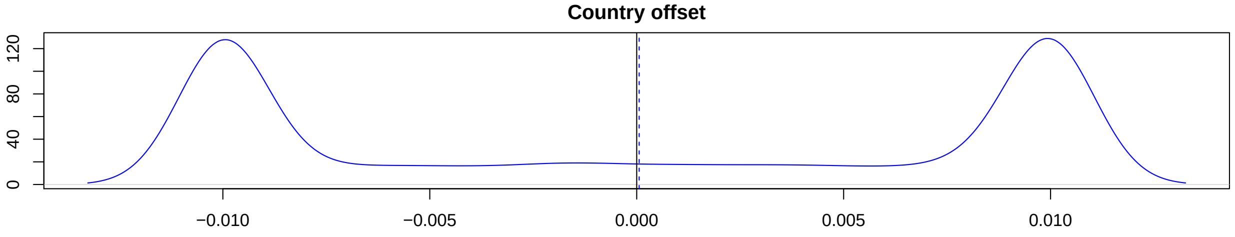

Parameters

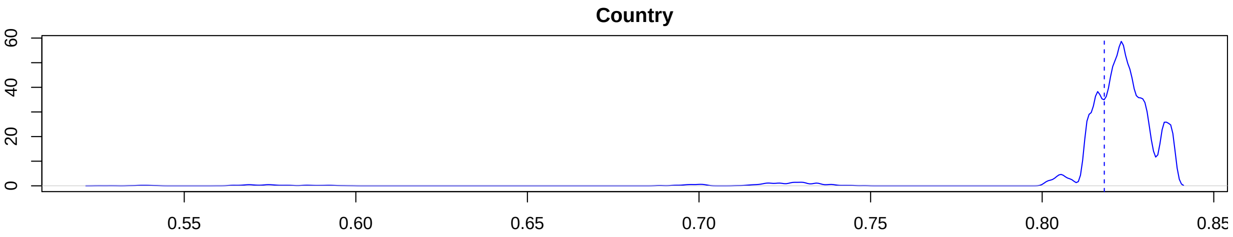
Country
0
20
40
60
0.55
0.60
0.65
0.70
0.75
0.80
0.85

C.6.4 Clean Delivery

C.6.4.1 Use

Hyperparameters

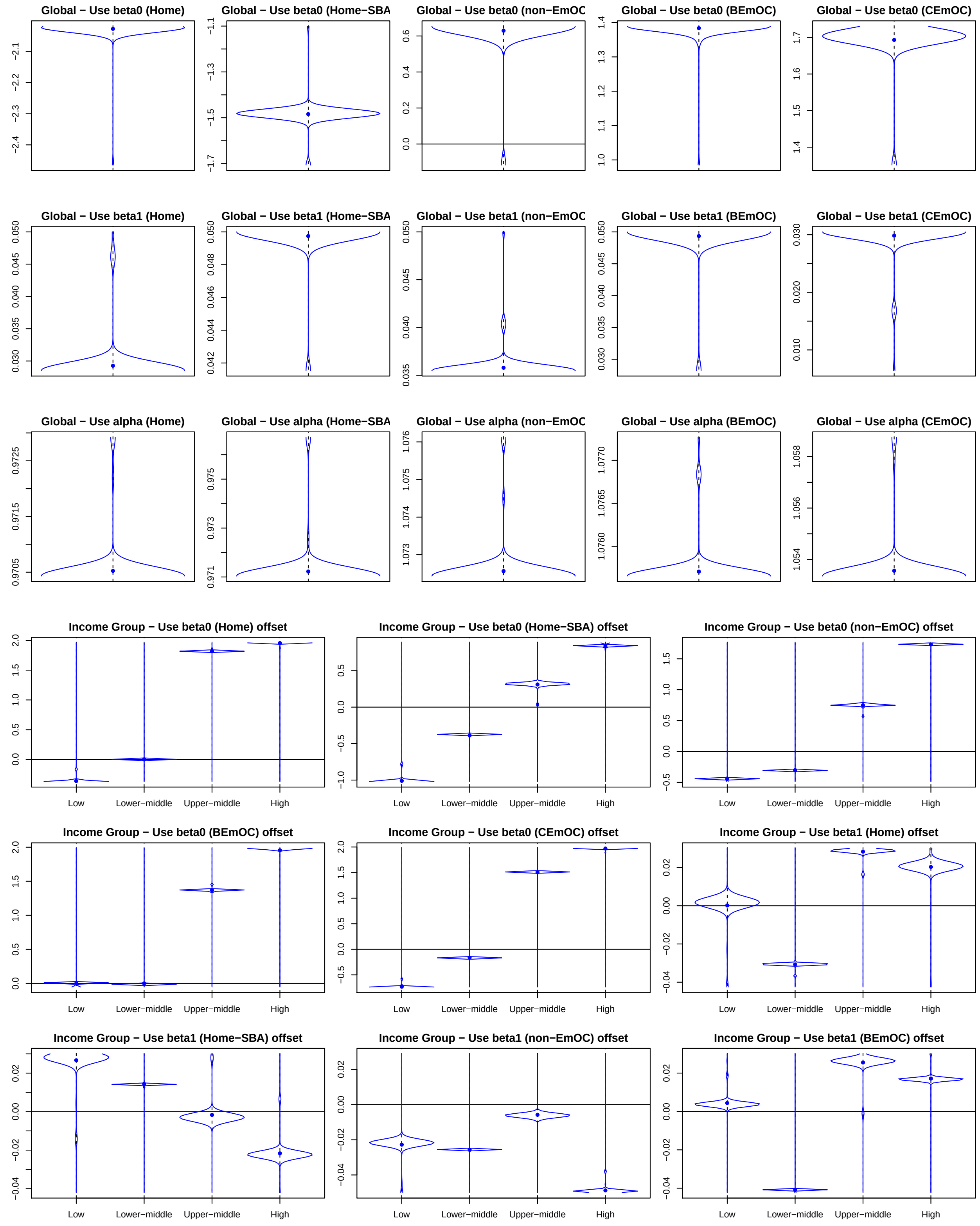

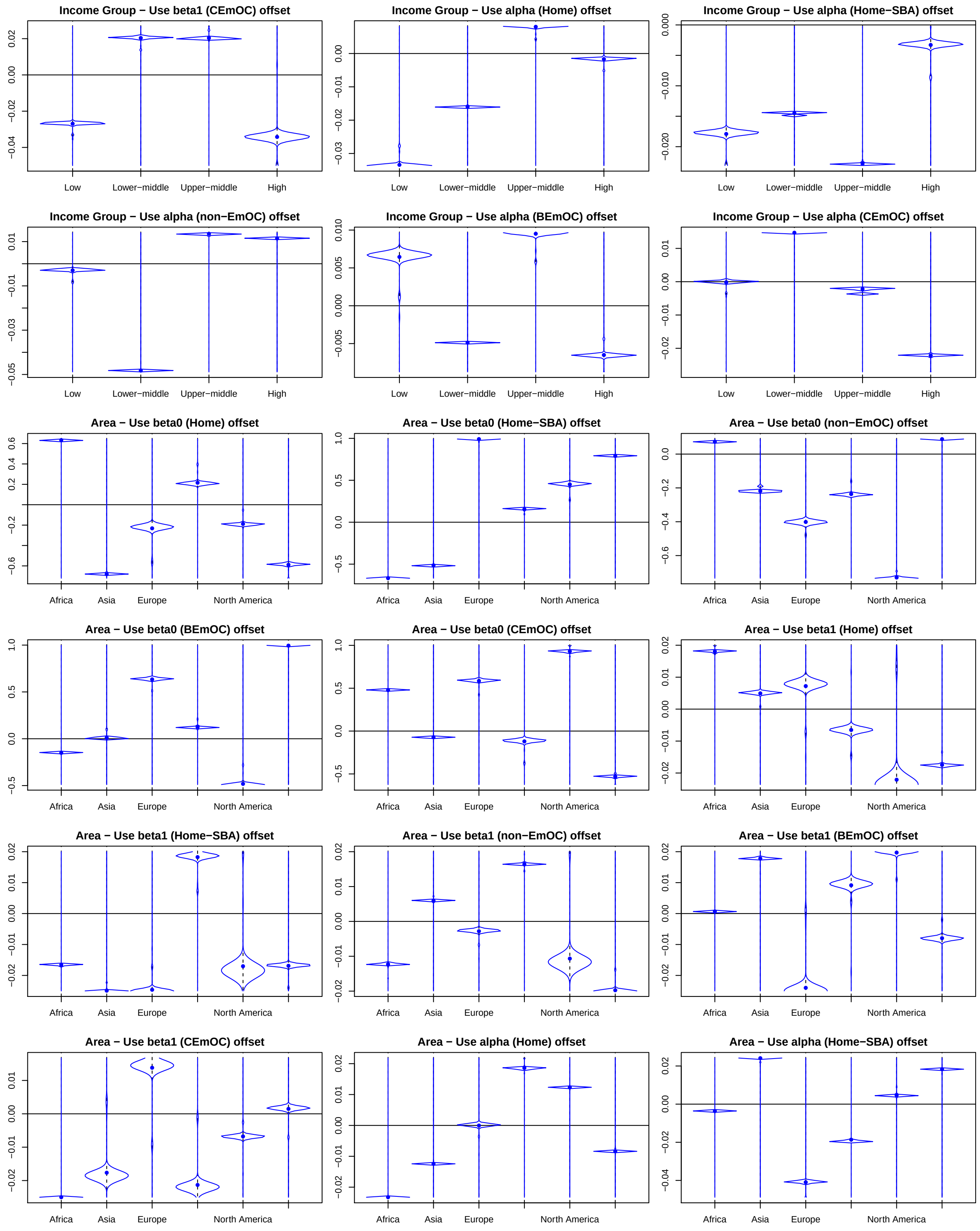
Income Group − Use beta1 (CEmOC) offset
Income Group − Use alpha (Home) offset
Income Group − Use alpha (Home−SBA) offset
Income Group − Use alpha (non−EmOC) offset
Income Group − Use alpha (BEmOC) offset
Income Group − Use alpha (CEmOC) offset
Low
Lower−middle
Upper−middle
High
Area − Use beta0 (Home) offset
Area − Use beta0 (Home−SBA) offset
Area − Use beta0 (non−EmOC) offset
Area − Use beta0 (BEmOC) offset
Area − Use beta0 (CEmOC) offset
Area − Use beta1 (Home) offset
Area − Use beta1 (Home−SBA) offset
Area − Use beta1 (non−EmOC) offset
Area − Use beta1 (BEmOC) offset
Area − Use beta1 (CEmOC) offset
Area − Use alpha (Home) offset
Area − Use alpha (Home−SBA) offset
Africa
Asia
Europe
North America

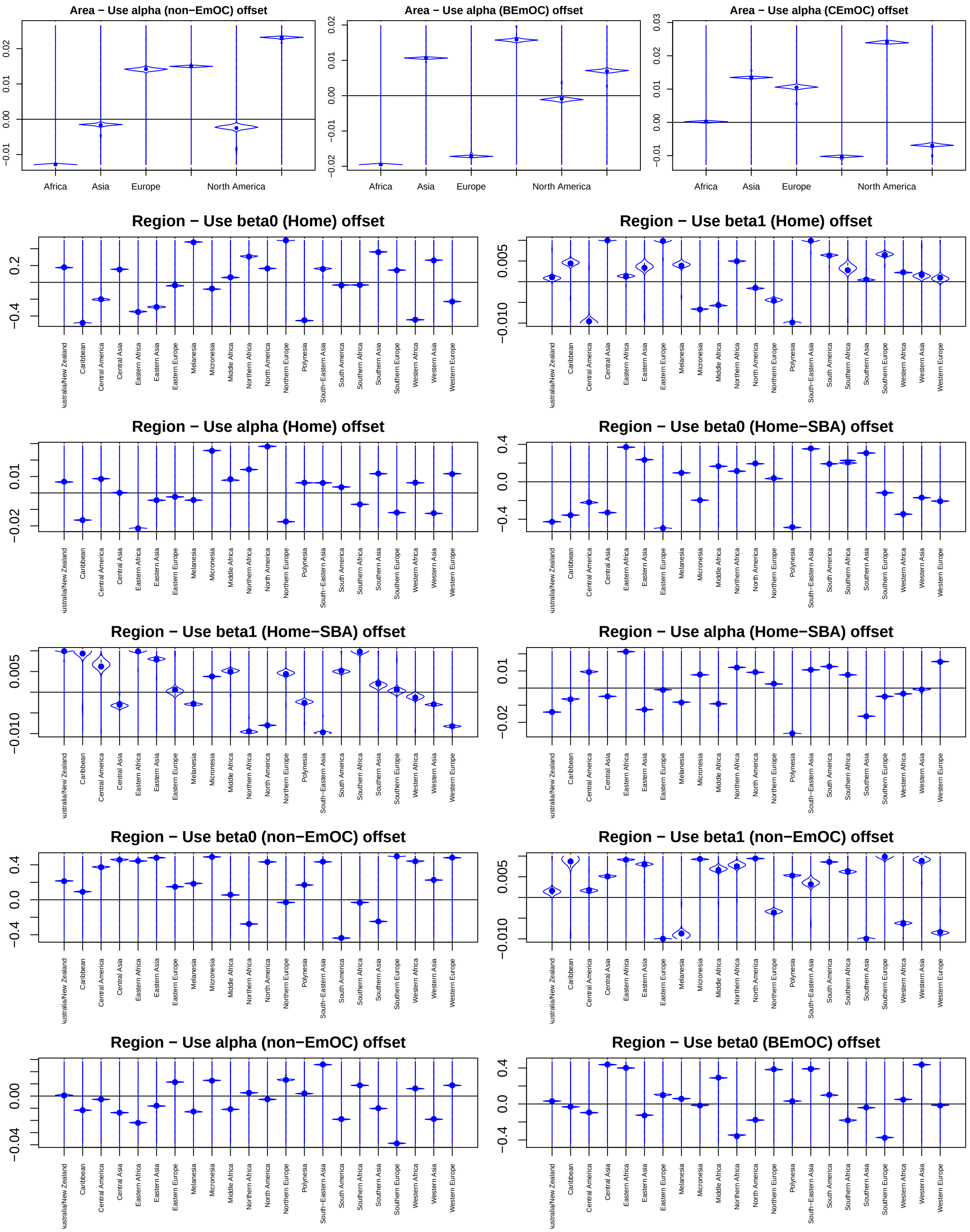
Area − Use alpha (non−EmOC) offset
Area − Use alpha (BEmOC) offset
Area − Use alpha (CEmOC) offset
Africa
Asia
Europe
North America
Region − Use beta0 (Home) offset
Region − Use beta1 (Home) offset
Region − Use alpha (Home) offset
Region − Use beta0 (Home−SBA) offset
Region − Use beta1 (Home−SBA) offset
Region − Use alpha (Home−SBA) offset
Region − Use beta0 (non−EmOC) offset
Region − Use beta1 (non−EmOC) offset
Region − Use alpha (non−EmOC) offset
Region − Use beta0 (BEmOC) offset
Australia/New Zealand
Caribbean
Central America
Central Asia
Eastern Africa
Eastern Asia
Eastern Europe
Melanesia
Micronesia
Middle Africa
Northern Africa
North America
Northern Europe
Polynesia
South−Eastern Asia
South America
Southern Africa
Southern Asia
Southern Europe
Western Africa
Western Asia
Western Europe

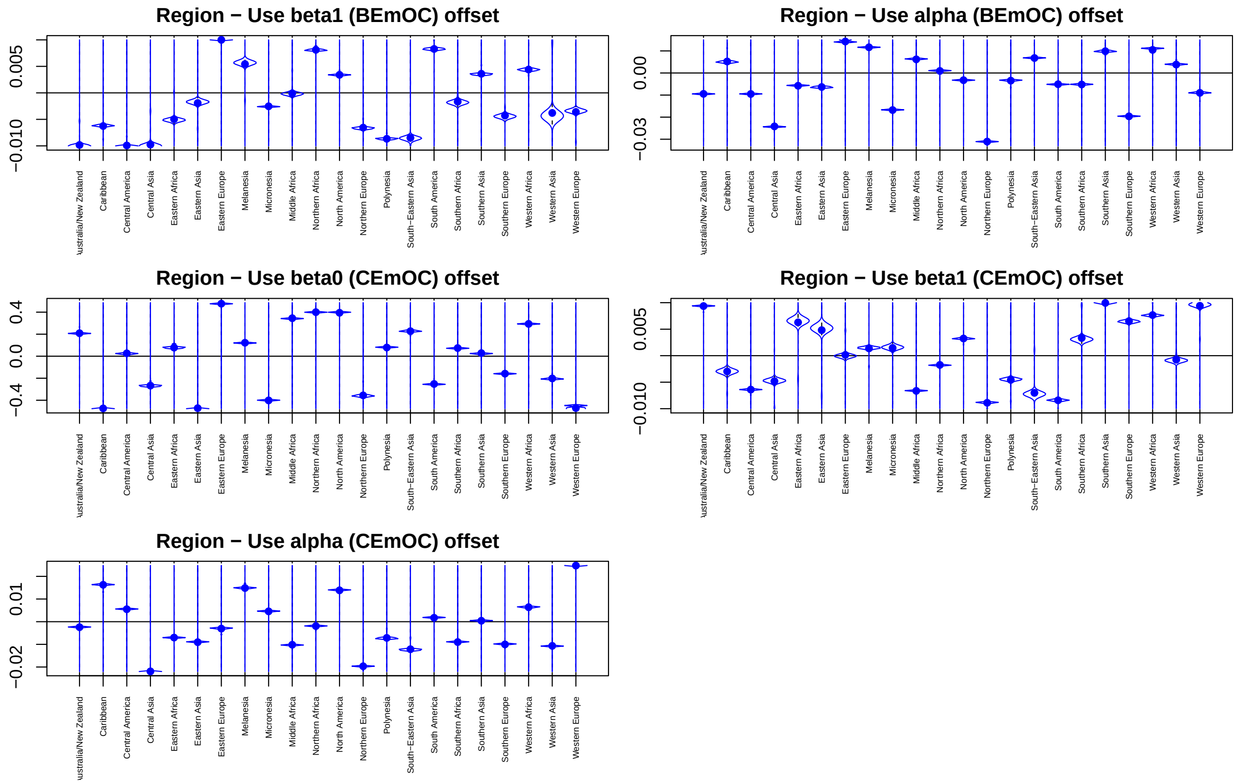
Region − Use beta1 (BEmOC) offset
Region − Use alpha (BEmOC) offset
Region − Use beta0 (CEmOC) offset
Region − Use beta1 (CEmOC) offset
Region − Use alpha (CEmOC) offset
Caribbean
Central America
Central Asia
Eastern Africa
Eastern Asia
Eastern Europe
Melanesia
Micronesia
Middle Africa
Northern Africa
North America
Northern Europe
Polynesia
South−Eastern Asia
South America
Southern Africa
Southern Asia
Southern Europe
Western Africa
Western Asia
Western Europe

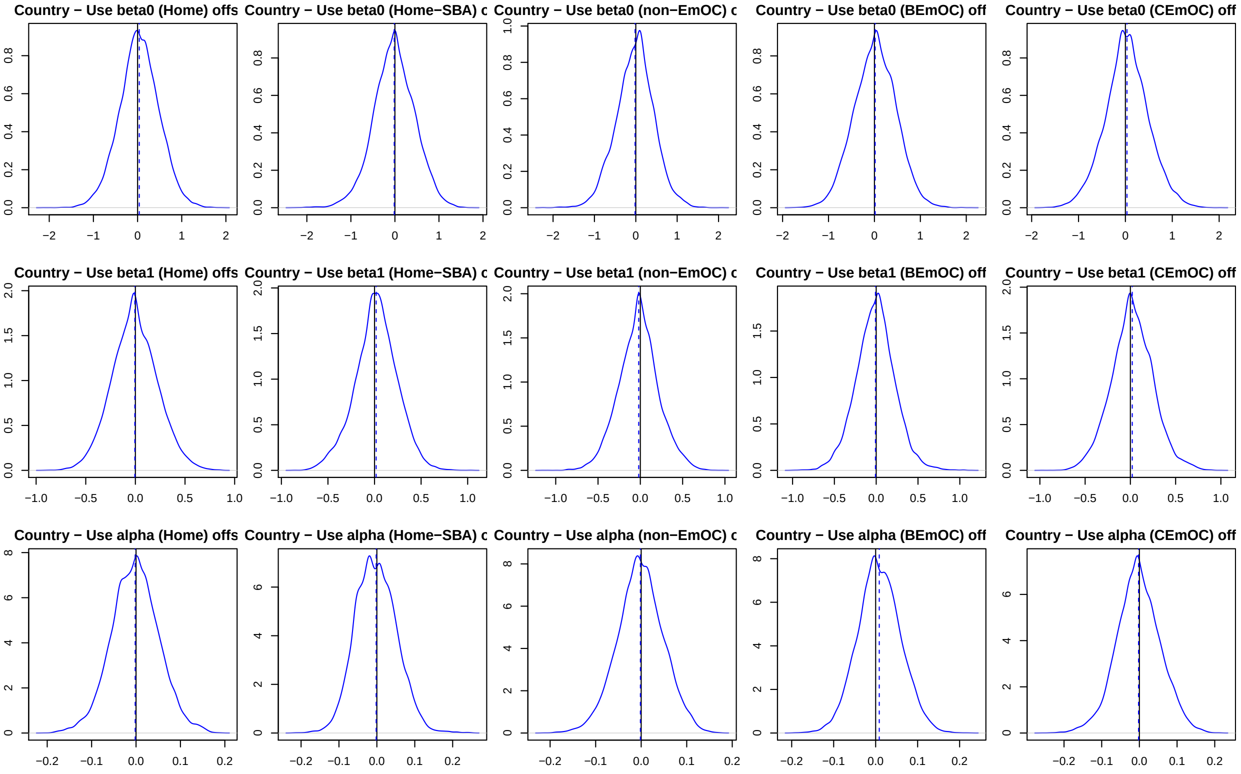
Country − Use beta0 (Home) offs
Country − Use beta0 (Home−SBA) c
Country − Use beta0 (non−EmOC) c
Country − Use beta0 (BEmOC) off
Country − Use beta0 (CEmOC) off
Country − Use beta1 (Home) offs
Country − Use beta1 (Home−SBA) c
Country − Use beta1 (non−EmOC) c
Country − Use beta1 (BEmOC) off
Country − Use beta1 (CEmOC) off
Country − Use alpha (Home) offs
Country − Use alpha (Home−SBA) c
Country − Use alpha (non−EmOC) c
Country − Use alpha (BEmOC) off
Country − Use alpha (CEmOC) off

Parameters

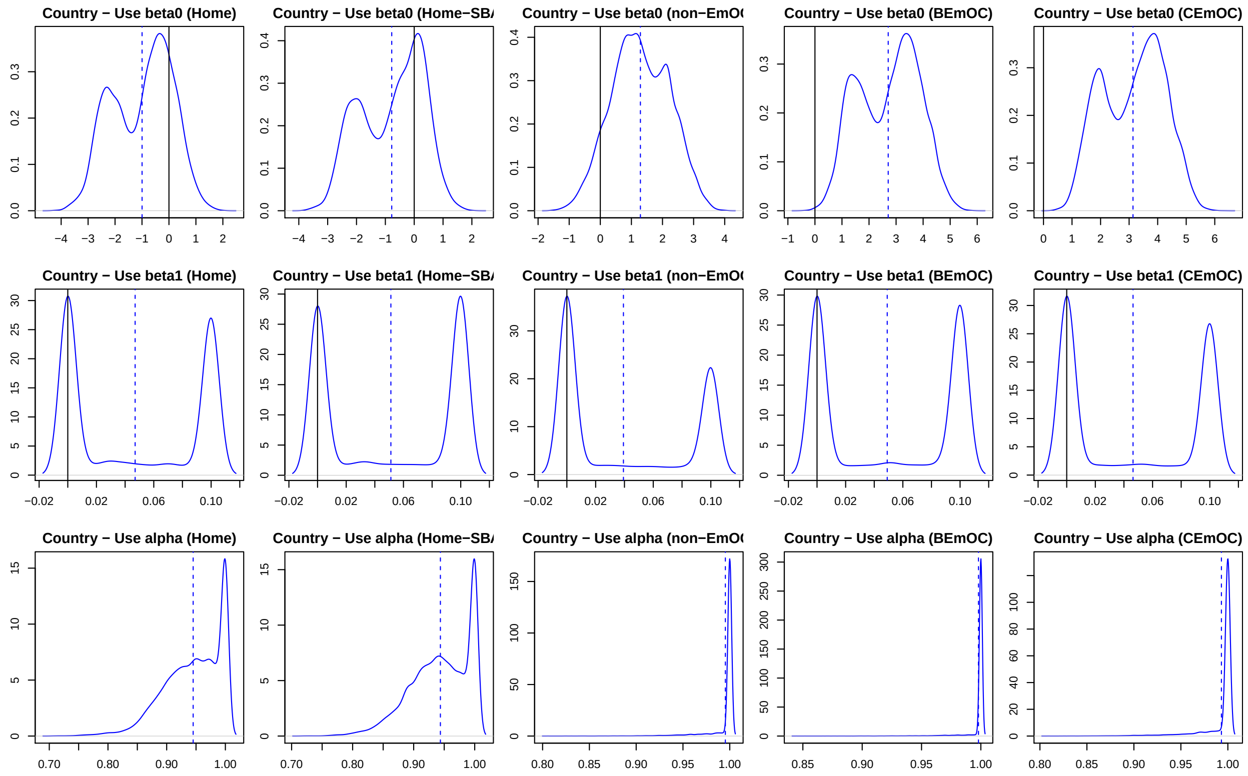

C.6.4.2 Efficacy

Hyperparameters

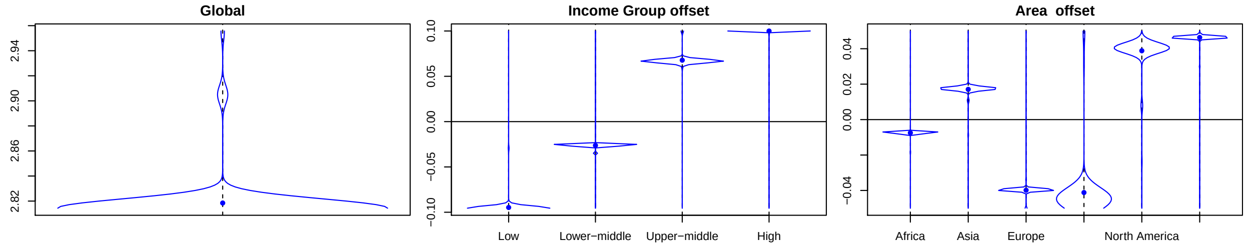

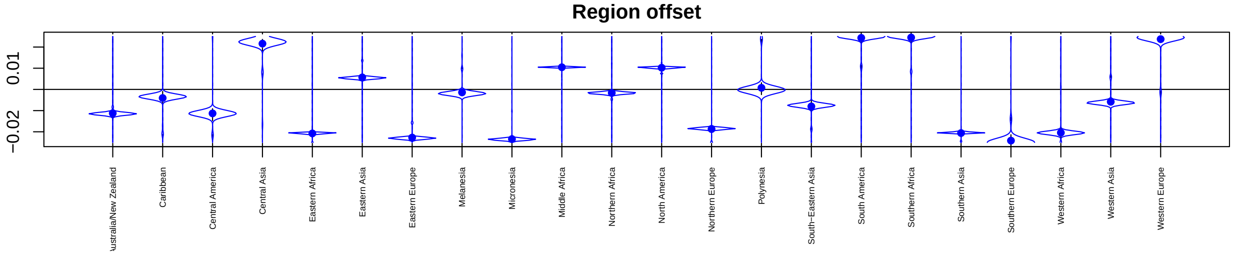

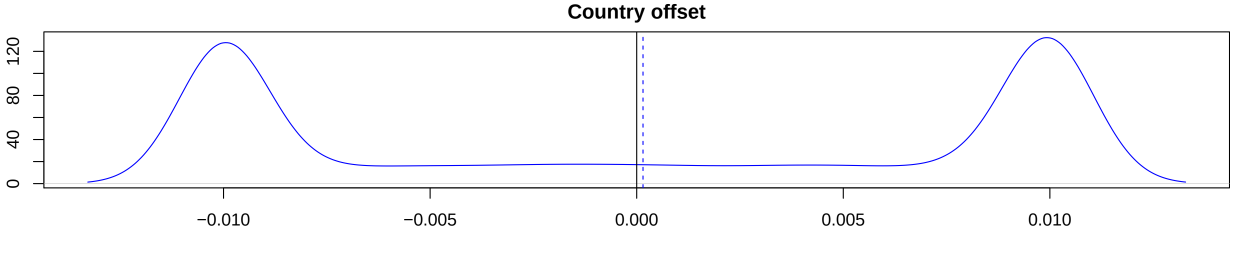

Parameters

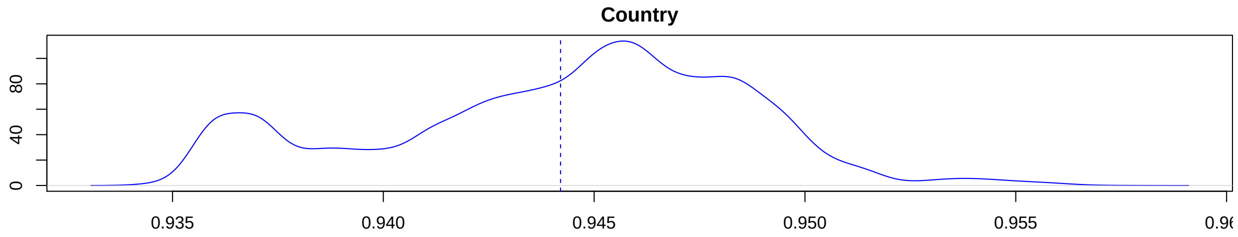

C.6.5 Management of Ectopic Pregnancy

C.6.5.1 Availability

Hyperparameters

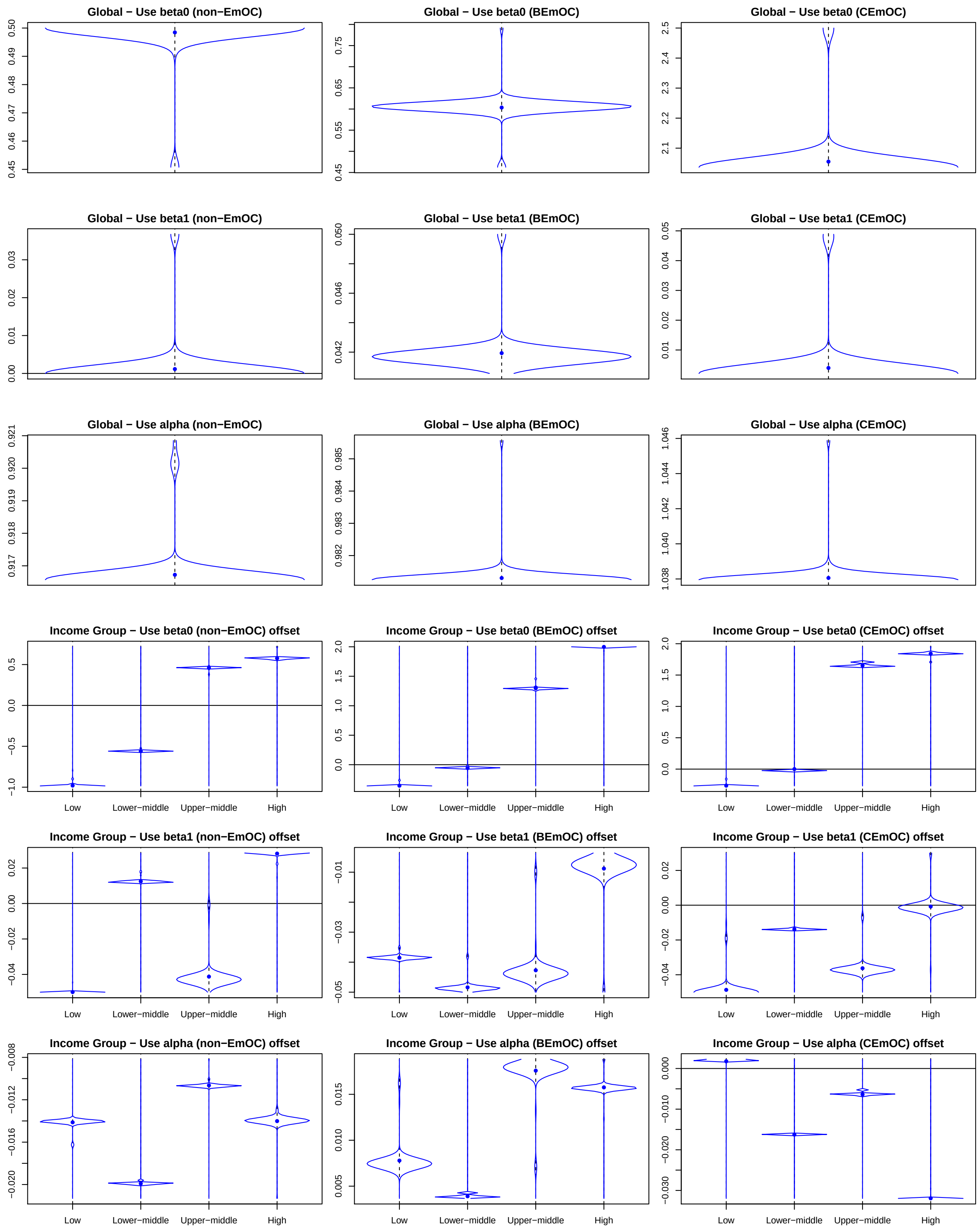

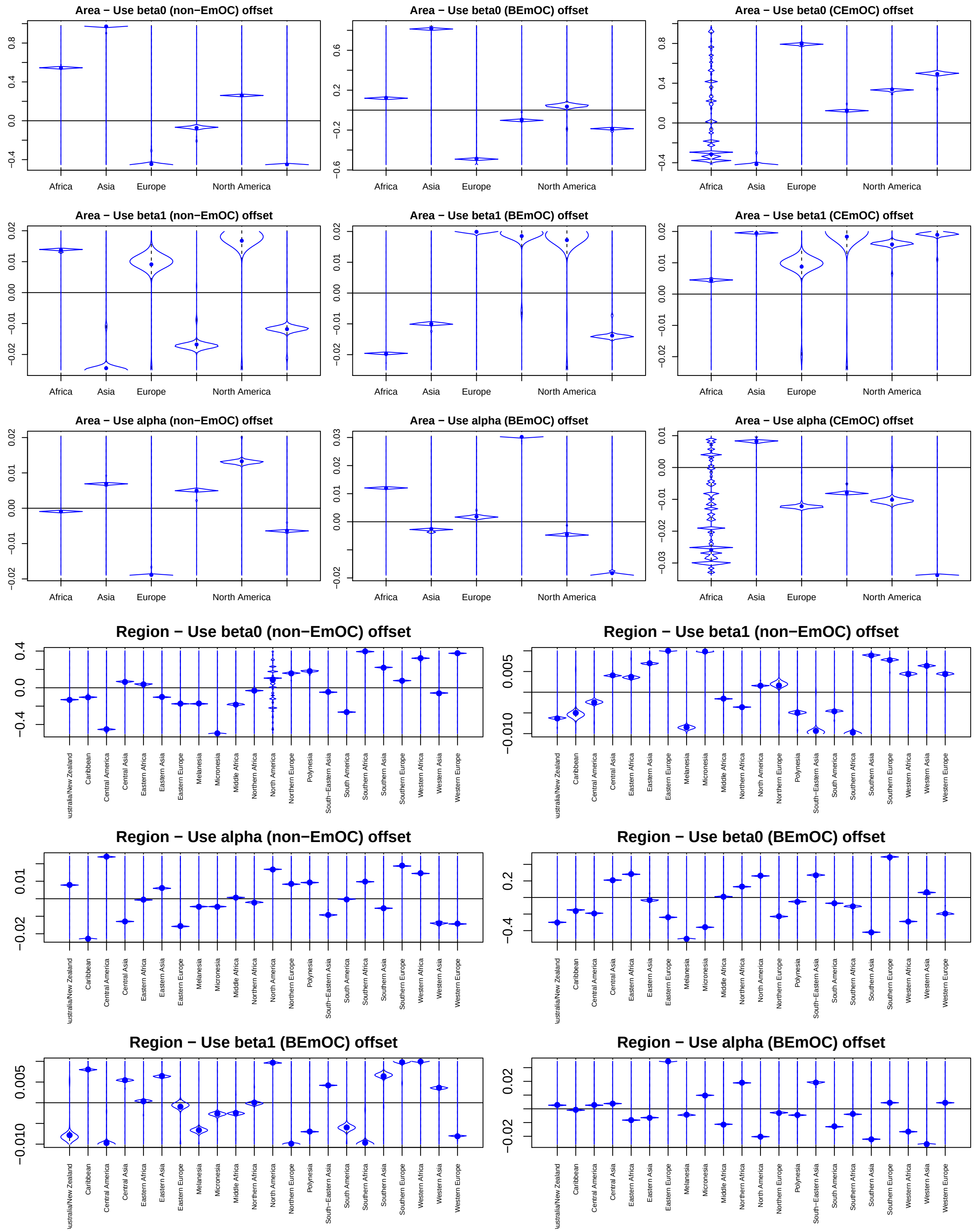
Area − Use beta0 (non−EmOC) offset
Area − Use beta0 (BEmOC) offset
Area − Use beta0 (CEmOC) offset
Area − Use beta1 (non−EmOC) offset
Area − Use beta1 (BEmOC) offset
Area − Use beta1 (CEmOC) offset
Area − Use alpha (non−EmOC) offset
Area − Use alpha (BEmOC) offset
Area − Use alpha (CEmOC) offset
Africa
Asia
Europe
North America
Region − Use beta0 (non−EmOC) offset
Region − Use beta1 (non−EmOC) offset
Region − Use alpha (non−EmOC) offset
Region − Use beta0 (BEmOC) offset
Region − Use beta1 (BEmOC) offset
Region − Use alpha (BEmOC) offset
Caribbean
Central America
Central Asia
Eastern Africa
Eastern Asia
Eastern Europe
Melanesia
Micronesia
Middle Africa
Northern Africa
North America
Northern Europe
Polynesia
South−Eastern Asia
South America
Southern Africa
Southern Asia
Southern Europe
Western Africa
Western Asia
Western Europe

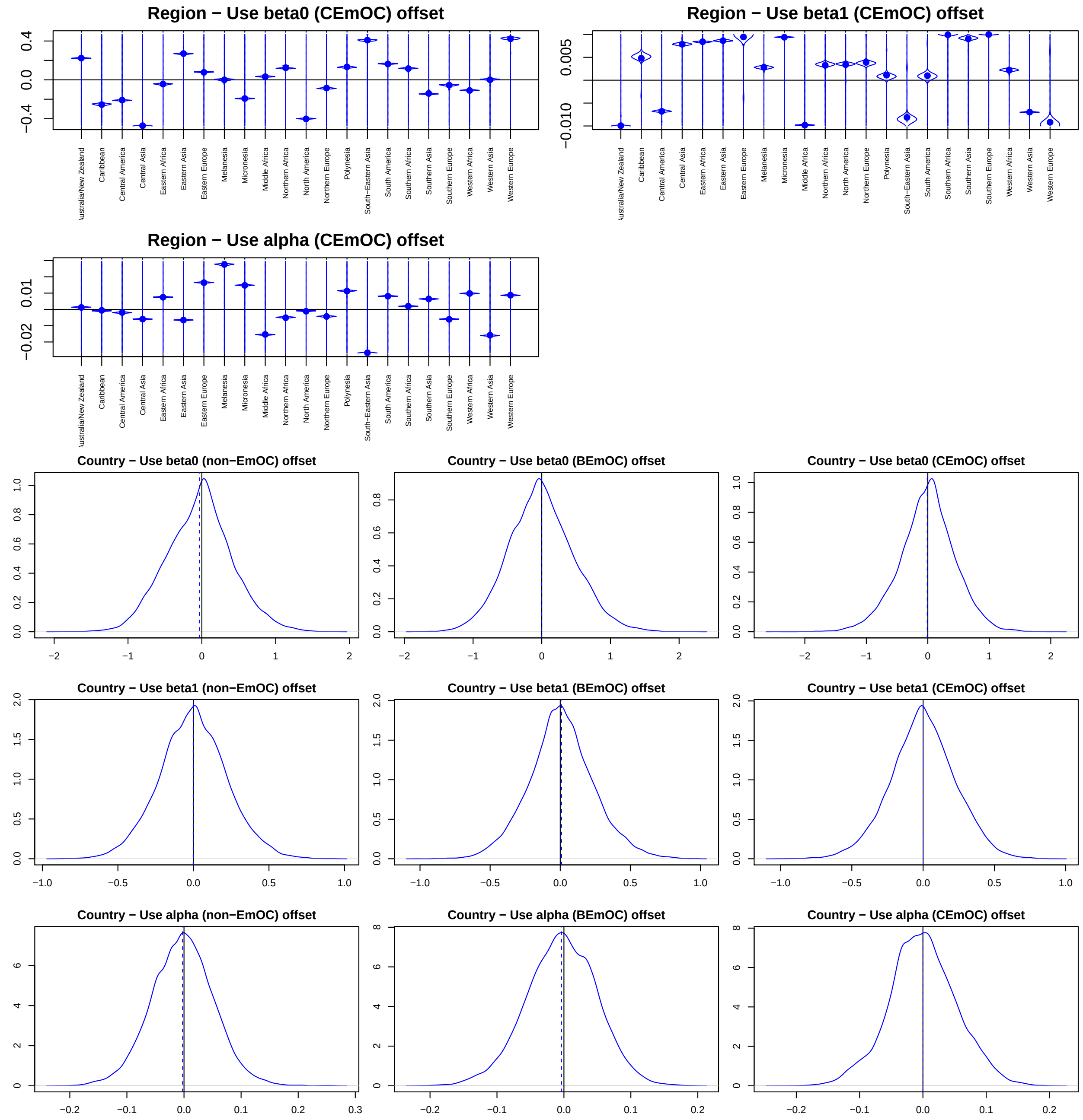
Region − Use beta0 (CEmOC) offset
Region − Use beta1 (CEmOC) offset
Region − Use alpha (CEmOC) offset
Australia/New Zealand
Caribbean
Central America
Central Asia
Eastern Africa
Eastern Asia
Eastern Europe
Melanesia
Micronesia
Middle Africa
Northern Africa
North America
Northern Europe
Polynesia
South−Eastern Asia
South America
Southern Africa
Southern Asia
Southern Europe
Western Africa
Western Asia
Western Europe
Country − Use beta0 (non−EmOC) offset
Country − Use beta0 (BEmOC) offset
Country − Use beta0 (CEmOC) offset
Country − Use beta1 (non−EmOC) offset
Country − Use beta1 (BEmOC) offset
Country − Use beta1 (CEmOC) offset
Country − Use alpha (non−EmOC) offset
Country − Use alpha (BEmOC) offset
Country − Use alpha (CEmOC) offset

Parameters

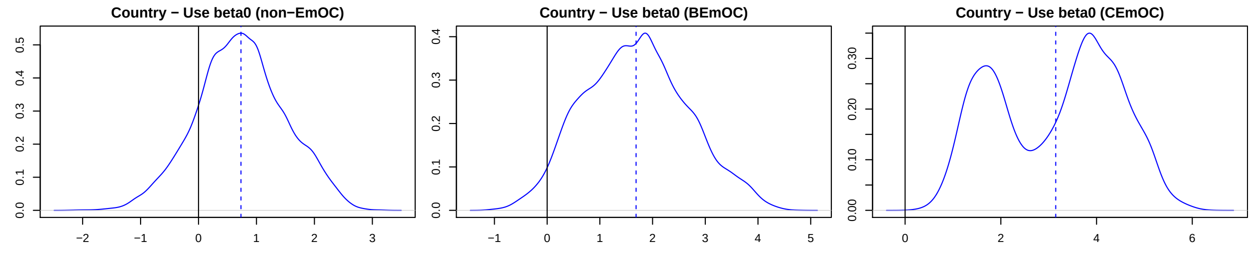
Country − Use beta0 (non−EmOC)
Country − Use beta0 (BEmOC)
Country − Use beta0 (CEmOC)

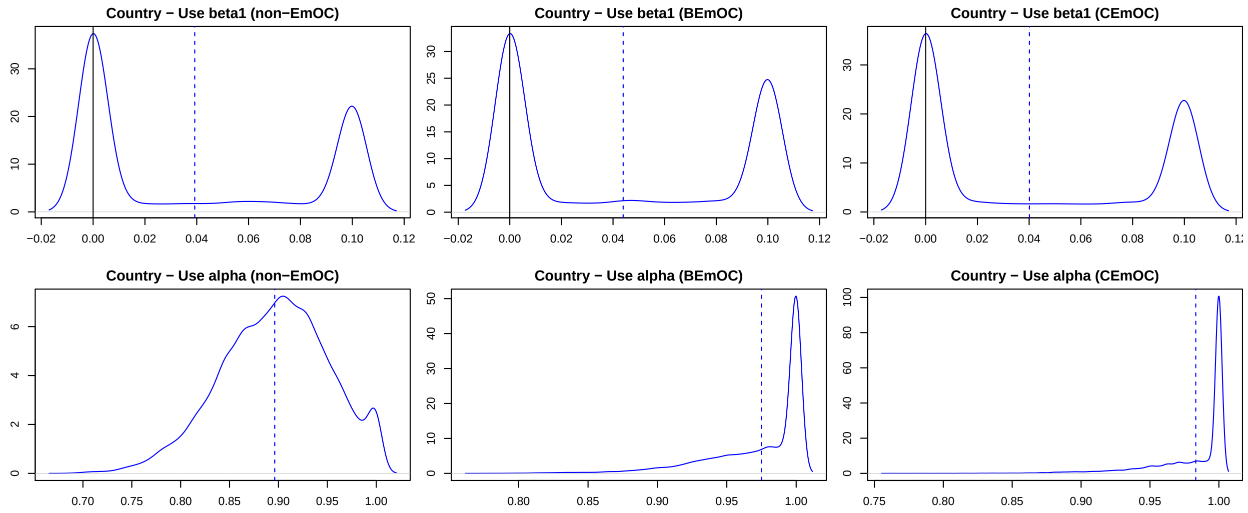

C.6.5.2 Efficacy

Hyperparameters

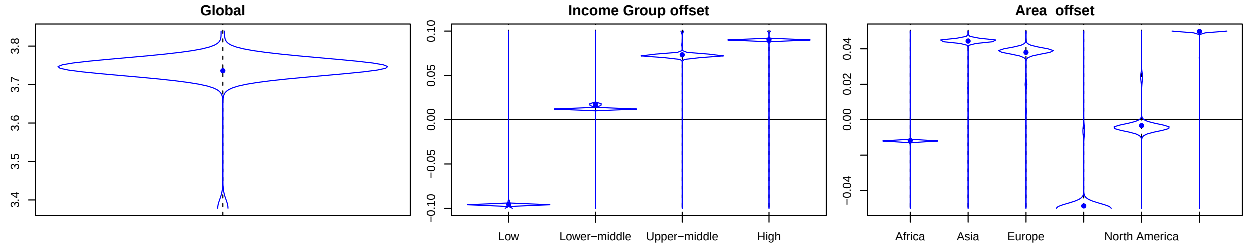

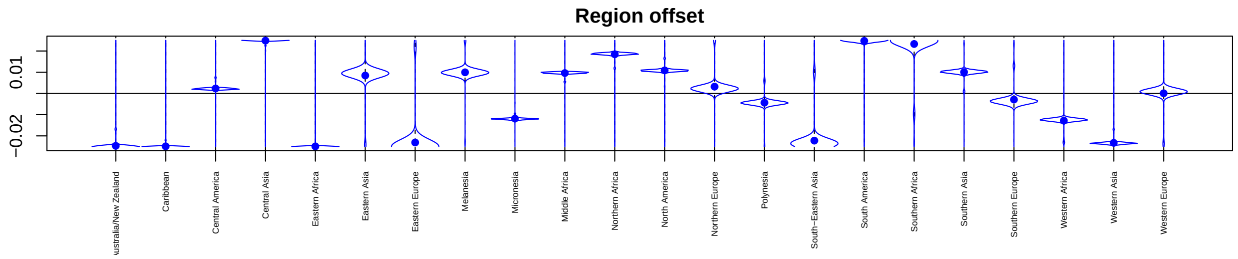

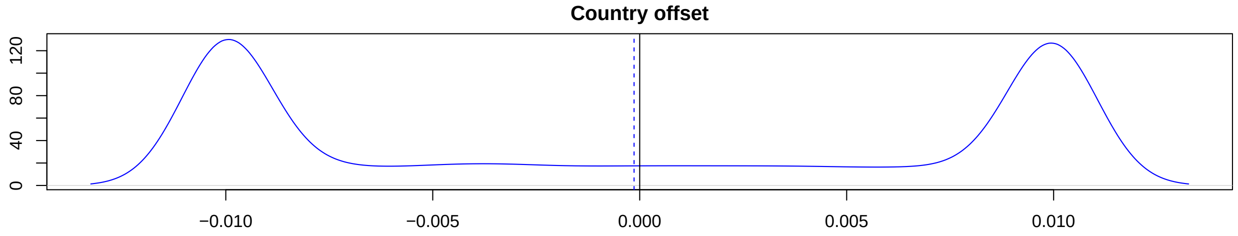

Parameters

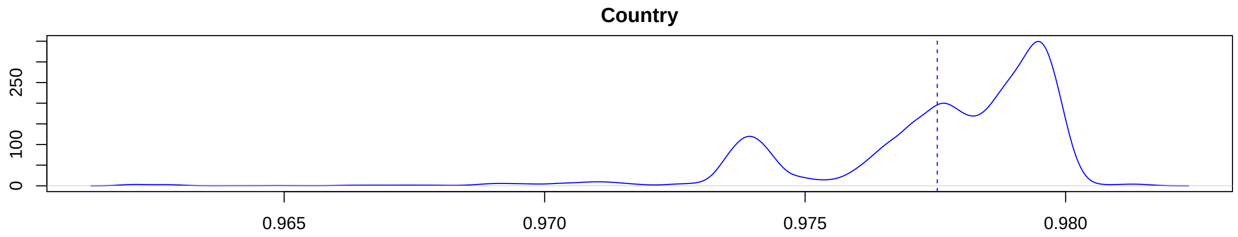
Country
0
100
250
0.965
0.970
0.975
0.980

C.6.6 Hypertension Management

C.6.6.1 Availability

Hyperparameters

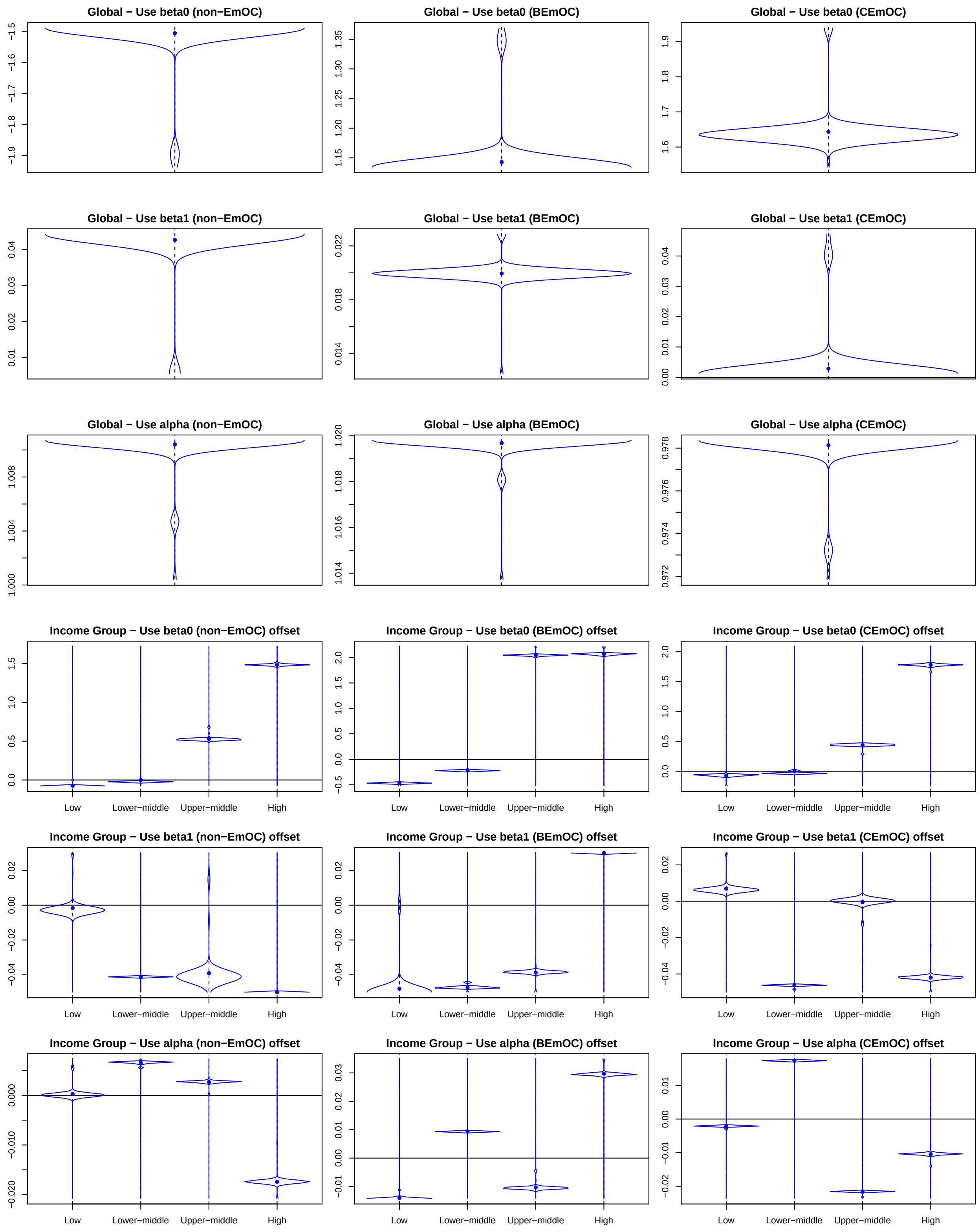

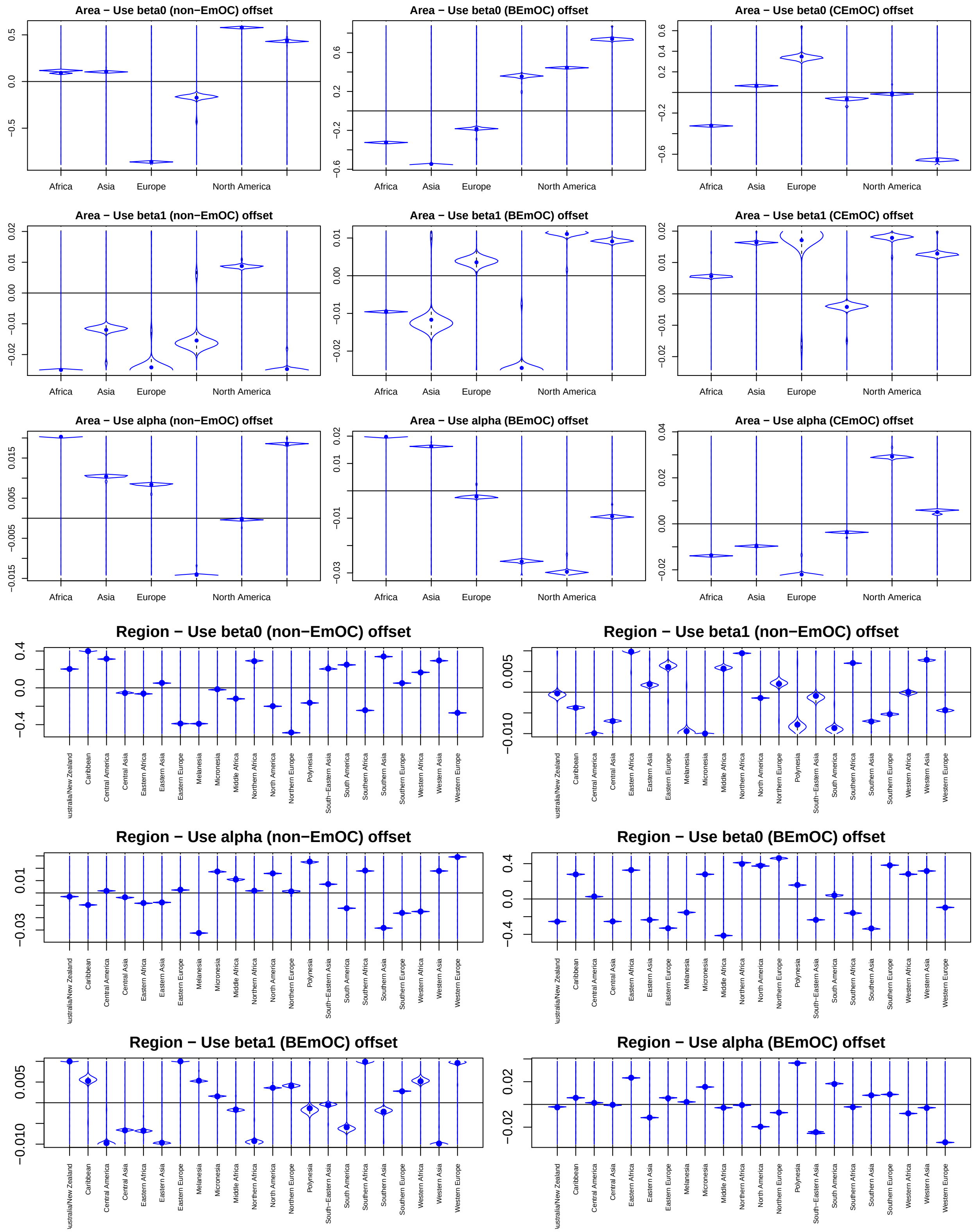

Area − Use beta0 (non−EmOC) offset
Area − Use beta0 (BEmOC) offset
Area − Use beta0 (CEmOC) offset
Area − Use beta1 (non−EmOC) offset
Area − Use beta1 (BEmOC) offset
Area − Use beta1 (CEmOC) offset
Area − Use alpha (non−EmOC) offset
Area − Use alpha (BEmOC) offset
Area − Use alpha (CEmOC) offset
Africa
Asia
Europe
North America
Region − Use beta0 (non−EmOC) offset
Region − Use beta1 (non−EmOC) offset
Region − Use alpha (non−EmOC) offset
Region − Use beta0 (BEmOC) offset
Region − Use beta1 (BEmOC) offset
Region − Use alpha (BEmOC) offset
ustralia/New Zealand
Caribbean
Central America
Central Asia
Eastern Africa
Eastern Asia
Eastern Europe
Melanesia
Micronesia
Middle Africa
Northern Africa
North America
Northern Europe
Polynesia
South−Eastern Asia
South America
Southern Africa
Southern Asia
Southern Europe
Western Africa
Western Asia
Western Europe

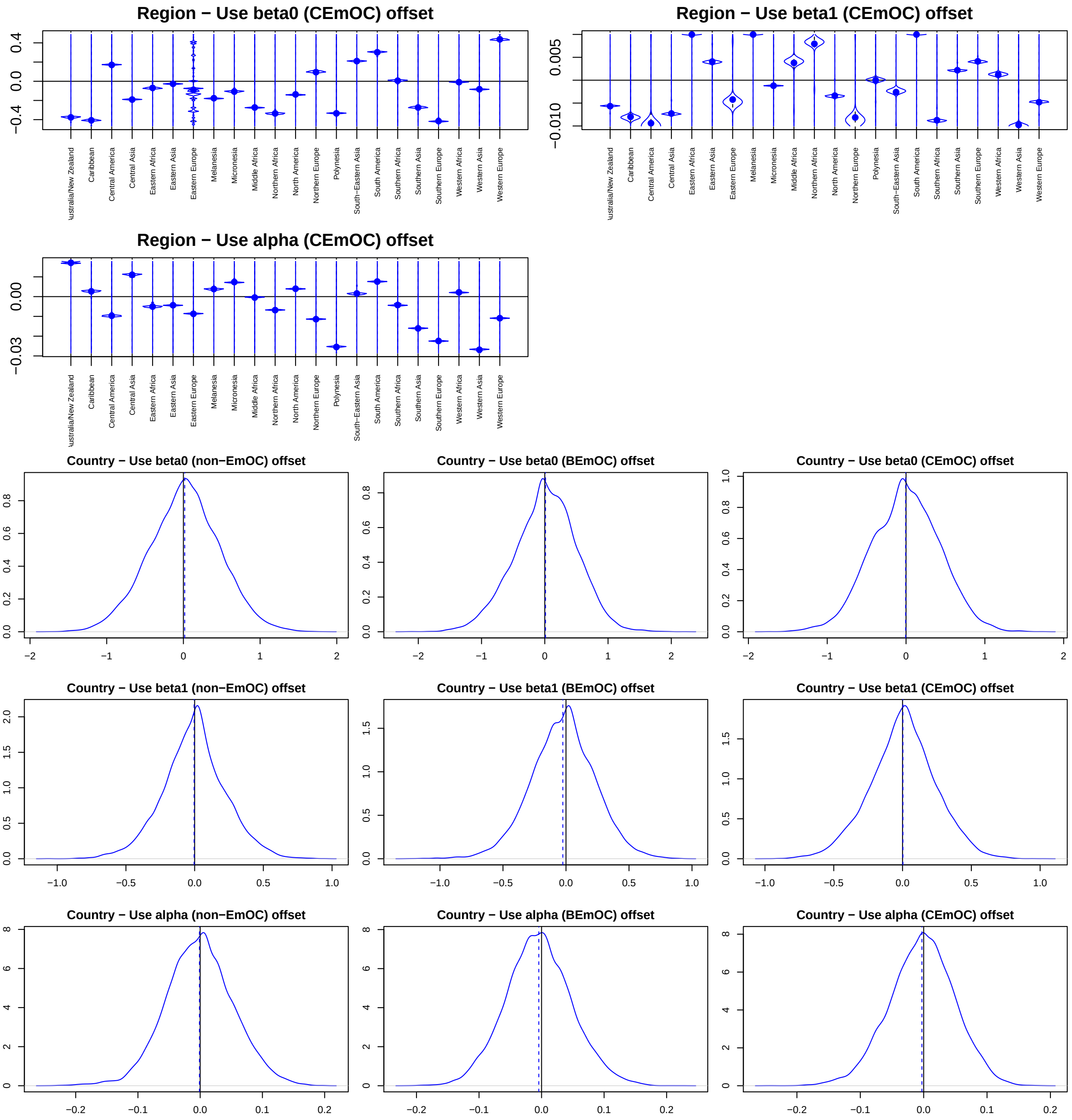
Region − Use beta0 (CEmOC) offset
Region − Use beta1 (CEmOC) offset
Region − Use alpha (CEmOC) offset
Country − Use beta0 (non−EmOC) offset
Country − Use beta0 (BEmOC) offset
Country − Use beta0 (CEmOC) offset
Country − Use beta1 (non−EmOC) offset
Country − Use beta1 (BEmOC) offset
Country − Use beta1 (CEmOC) offset
Country − Use alpha (non−EmOC) offset
Country − Use alpha (BEmOC) offset
Country − Use alpha (CEmOC) offset

Parameters

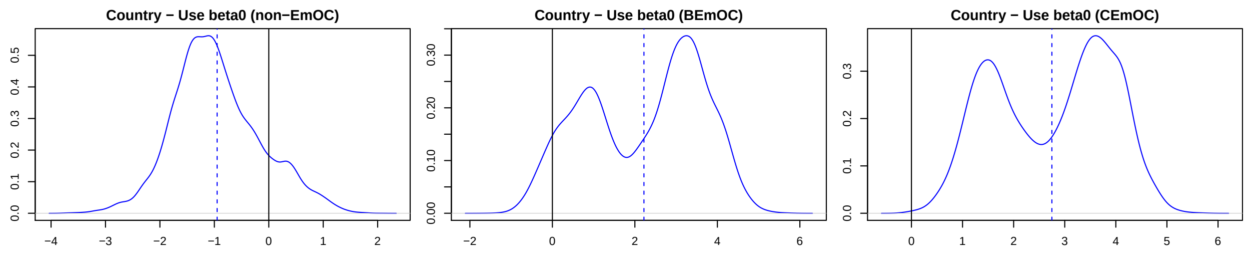
Country − Use beta0 (non−EmOC)
Country − Use beta0 (BEmOC)
Country − Use beta0 (CEmOC)

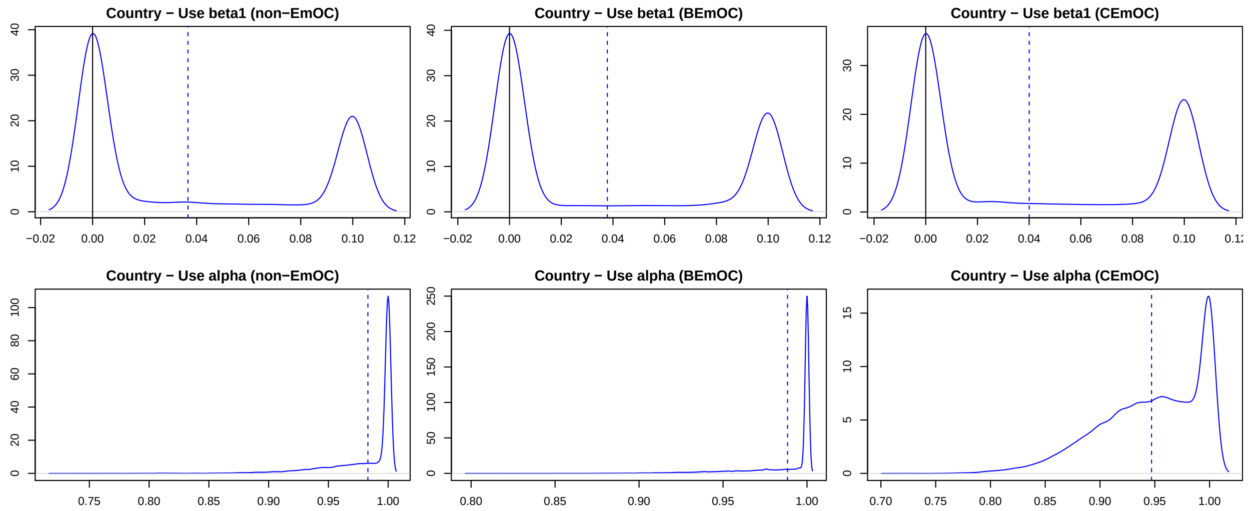

C.6.6.2 Efficacy

Hyperparameters

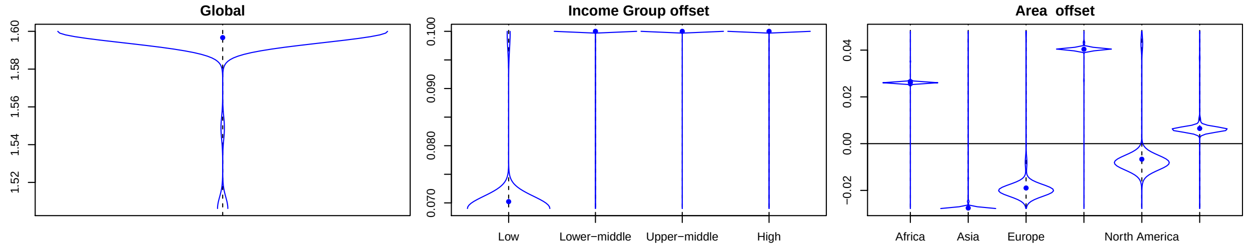

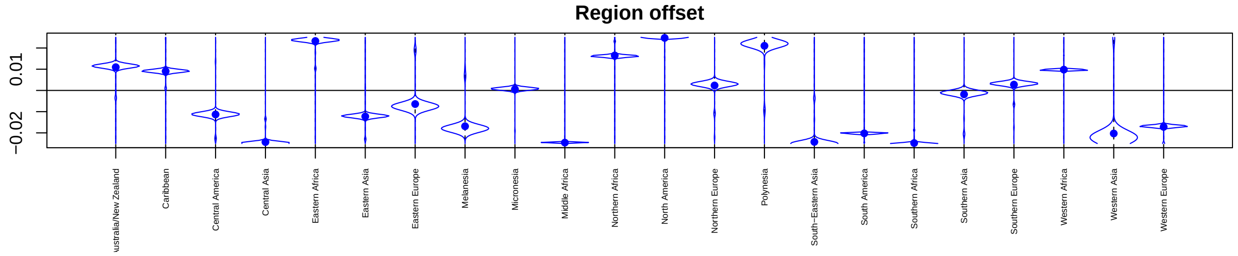

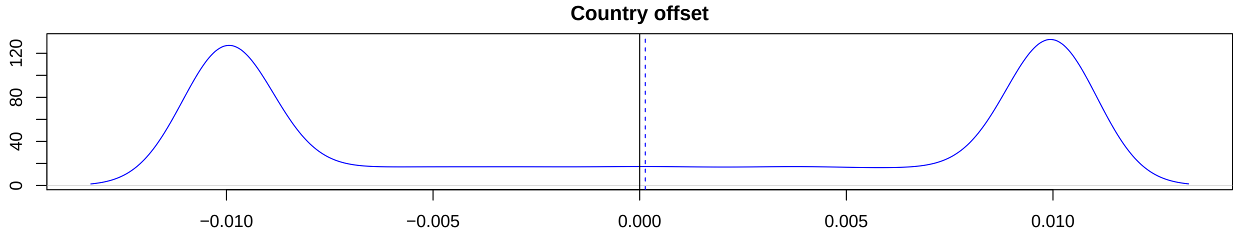

Parameters

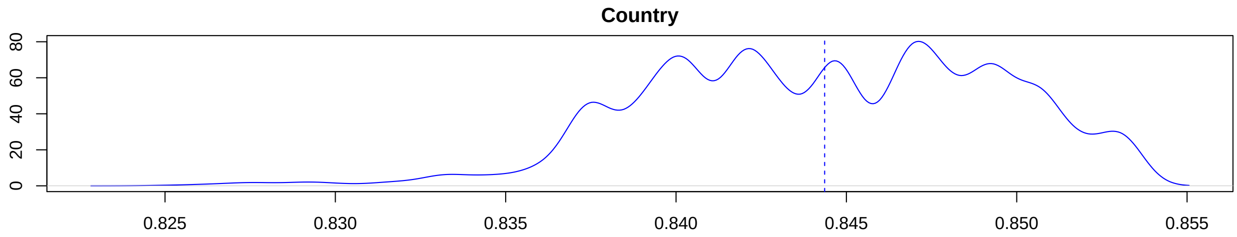

C.6.6.3 C-Section|EmOC

Hyperparameters

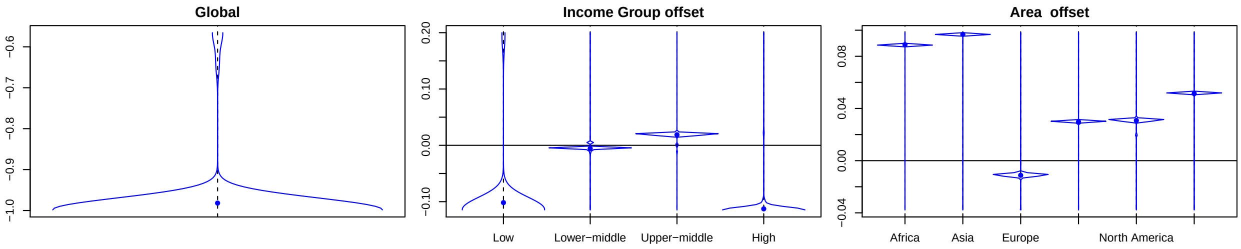

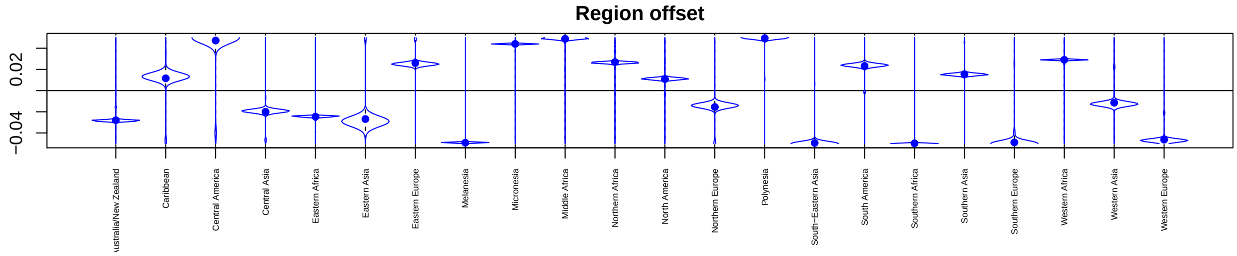

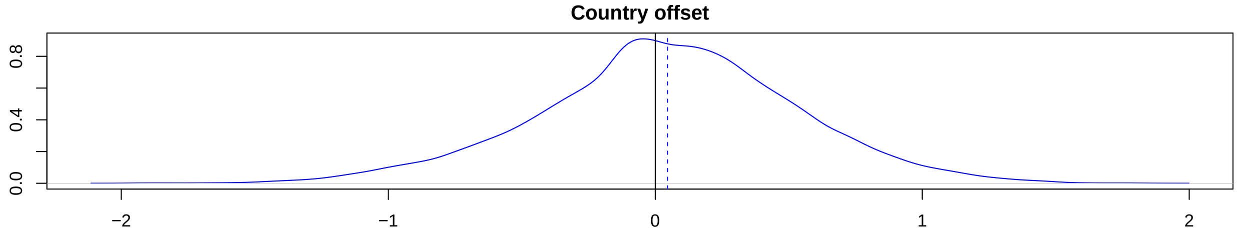

Parameters

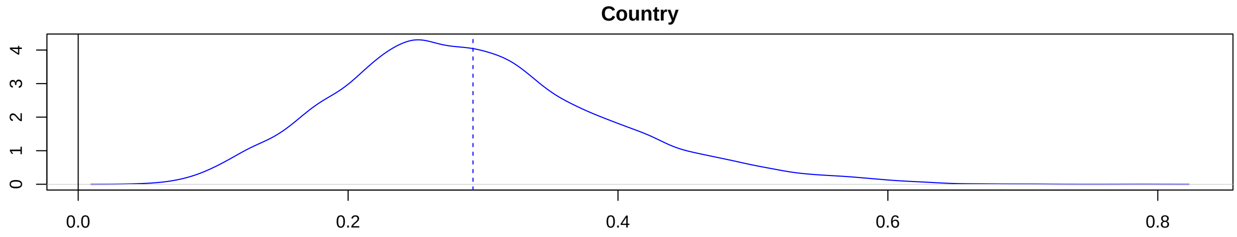

C.6.7 Assisted Delivery

C.6.7.1 Availability

Hyperparameters

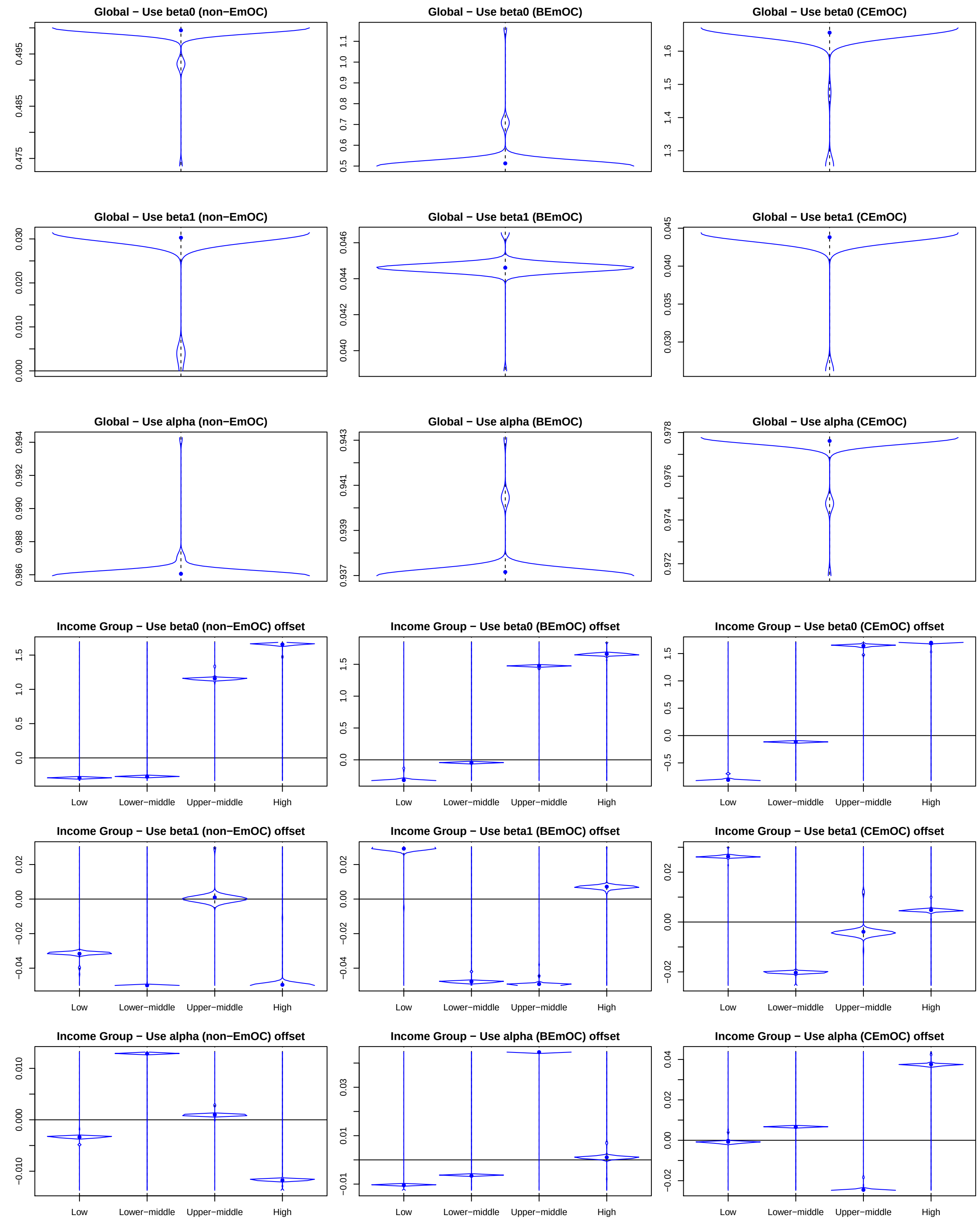

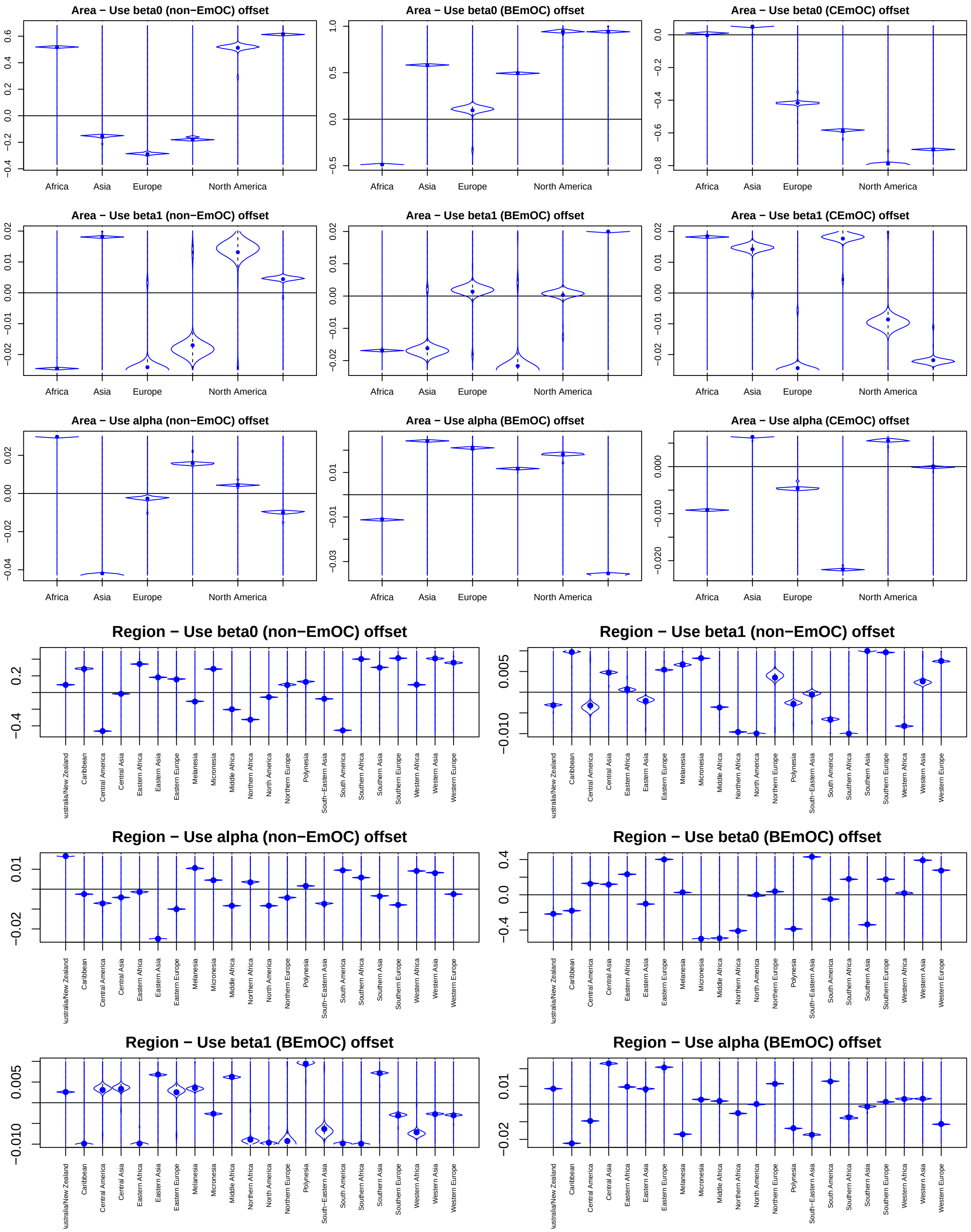

Area − Use beta0 (non−EmOC) offset
Area − Use beta0 (BEmOC) offset
Area − Use beta0 (CEmOC) offset
Area − Use beta1 (non−EmOC) offset
Area − Use beta1 (BEmOC) offset
Area − Use beta1 (CEmOC) offset
Area − Use alpha (non−EmOC) offset
Area − Use alpha (BEmOC) offset
Area − Use alpha (CEmOC) offset
Africa
Asia
Europe
North America
Region − Use beta0 (non−EmOC) offset
Region − Use beta1 (non−EmOC) offset
Region − Use alpha (non−EmOC) offset
Region − Use beta0 (BEmOC) offset
Region − Use beta1 (BEmOC) offset
Region − Use alpha (BEmOC) offset
Caribbean
Central America
Central Asia
Eastern Africa
Eastern Asia
Eastern Europe
Melanesia
Micronesia
Middle Africa
Northern Africa
North America
Northern Europe
Polynesia
South−Eastern Asia
South America
Southern Africa
Southern Asia
Southern Europe
Western Africa
Western Asia
Western Europe

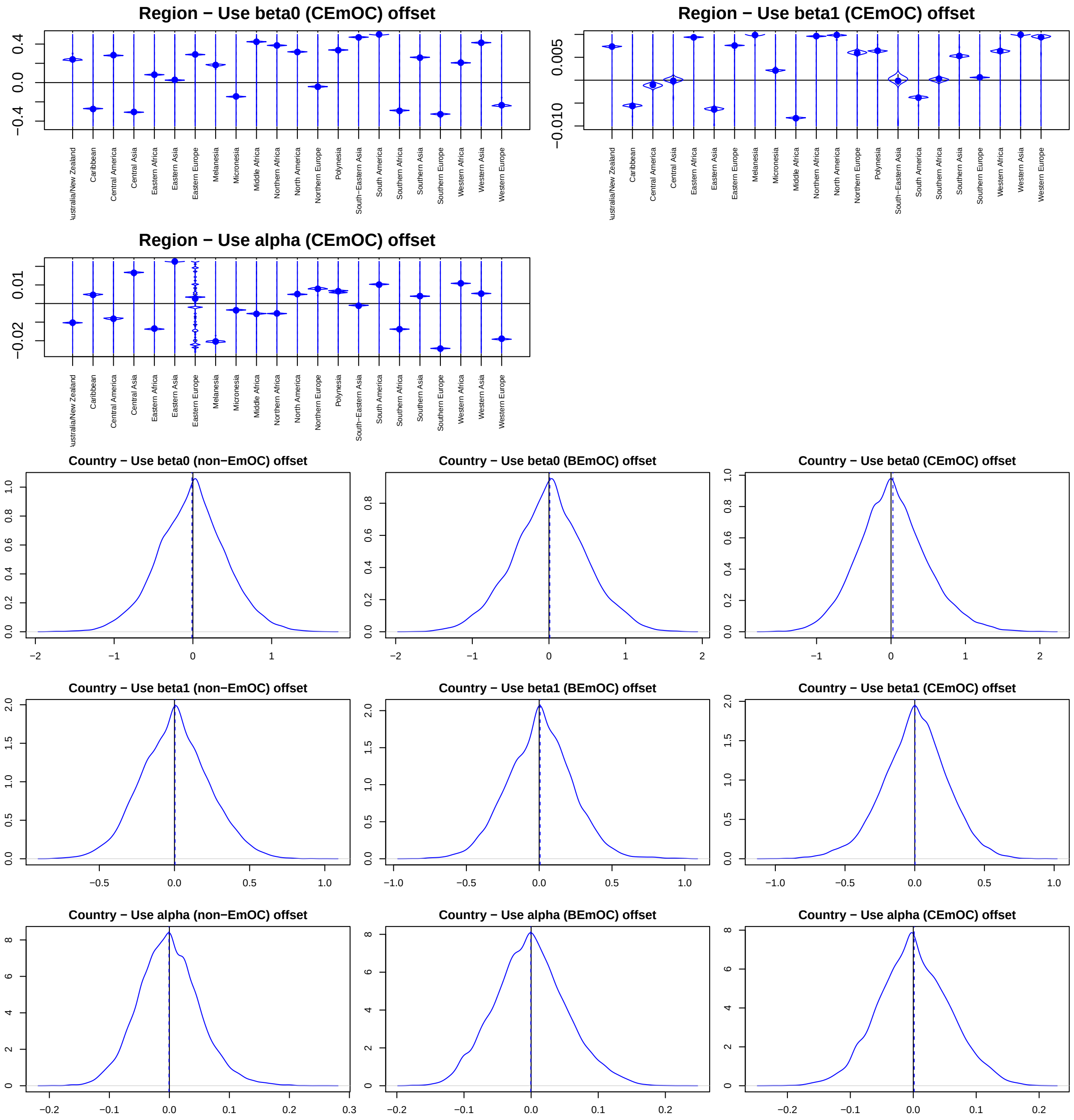
Region − Use beta0 (CEmOC) offset
Region − Use beta1 (CEmOC) offset
Region − Use alpha (CEmOC) offset
Australia/New Zealand
Caribbean
Central America
Central Asia
Eastern Africa
Eastern Asia
Eastern Europe
Melanesia
Micronesia
Middle Africa
Northern Africa
North America
Northern Europe
Polynesia
South−Eastern Asia
South America
Southern Africa
Southern Asia
Southern Europe
Western Africa
Western Asia
Western Europe
Country − Use beta0 (non−EmOC) offset
Country − Use beta0 (BEmOC) offset
Country − Use beta0 (CEmOC) offset
Country − Use beta1 (non−EmOC) offset
Country − Use beta1 (BEmOC) offset
Country − Use beta1 (CEmOC) offset
Country − Use alpha (non−EmOC) offset
Country − Use alpha (BEmOC) offset
Country − Use alpha (CEmOC) offset

Parameters

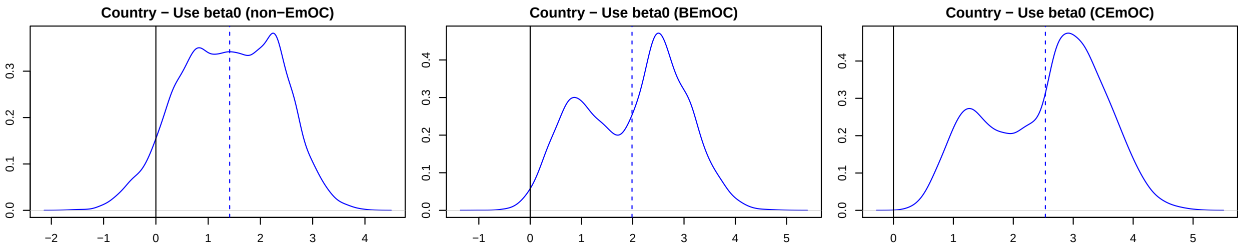
Country − Use beta0 (non−EmOC)
Country − Use beta0 (BEmOC)
Country − Use beta0 (CEmOC)

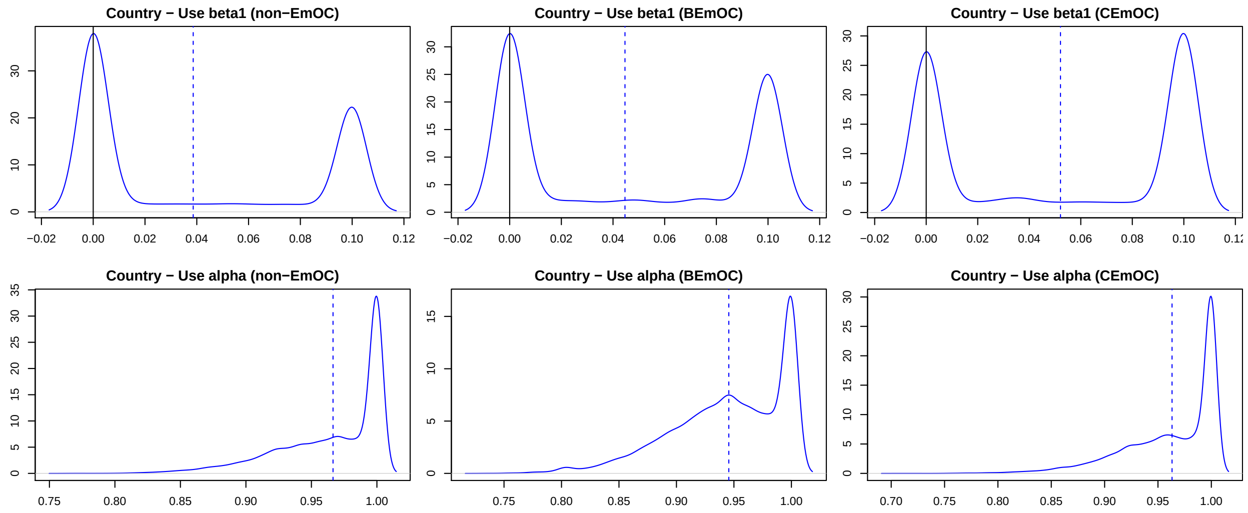

C.6.7.2 Efficacy

Hyperparameters

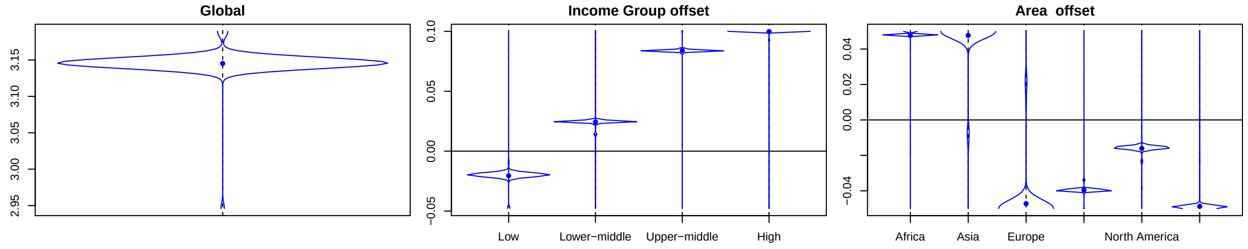

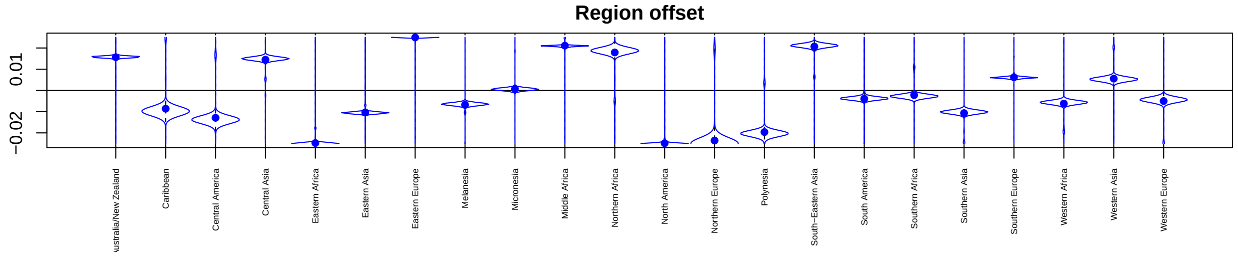

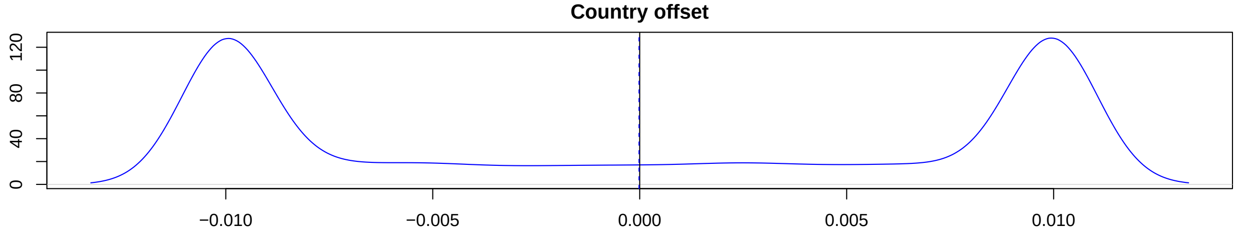

Parameters

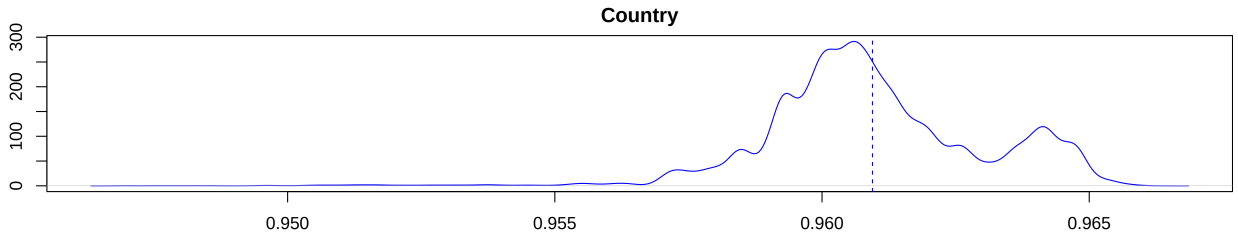

C.6.7.3 C-Section|EmOC, Moderate OL

Hyperparameters

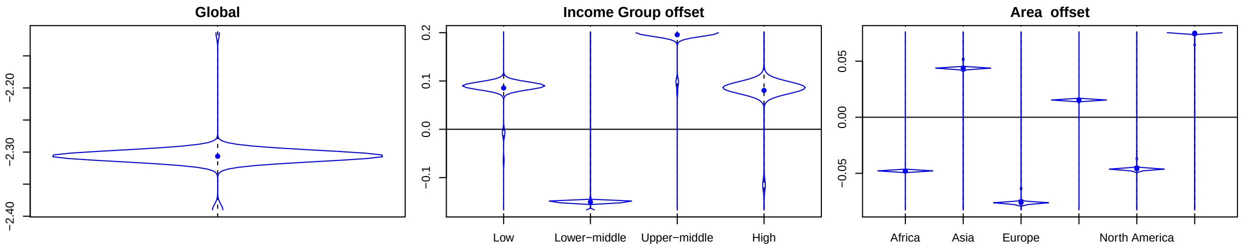

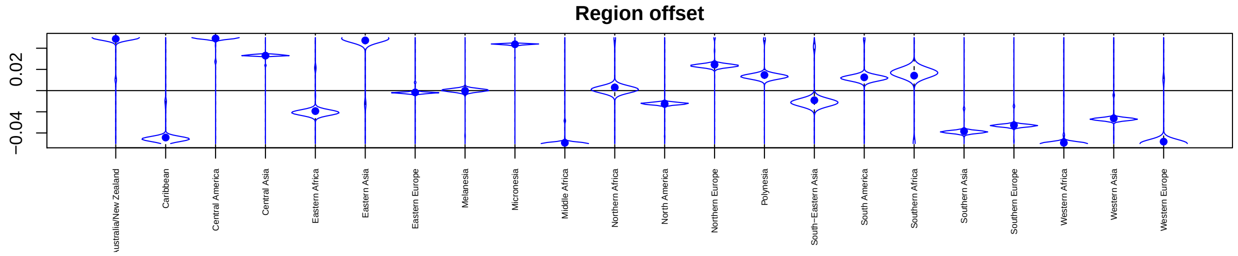

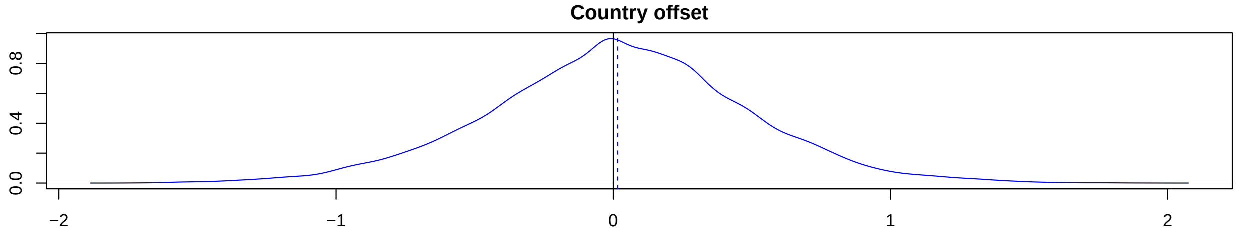

Parameters

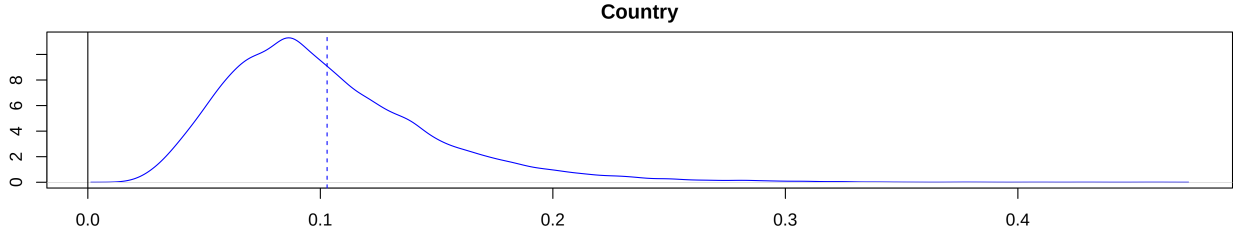

C.6.8 Hemorrhage Treatment

C.6.8.1 Availability

Hyperparameters

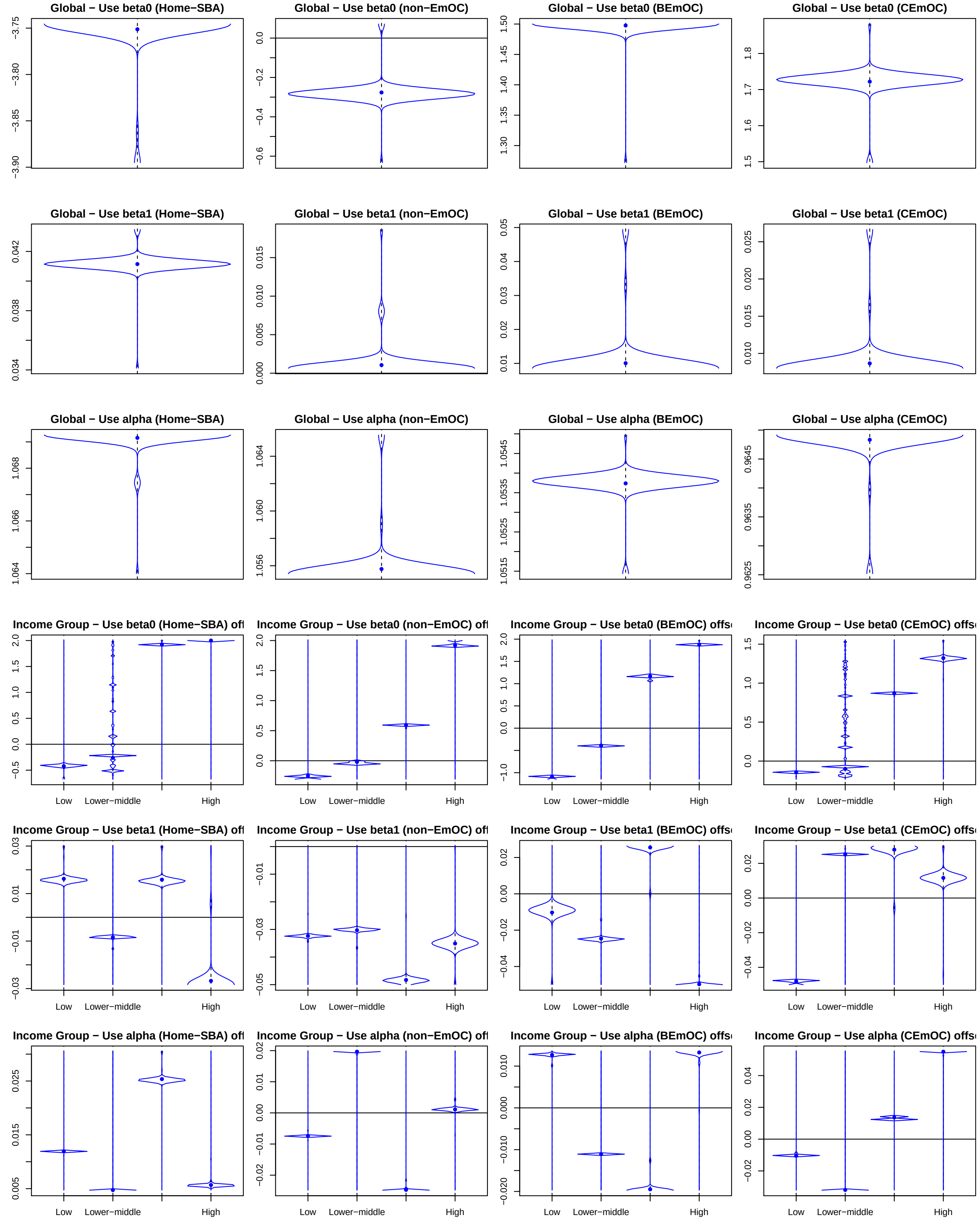

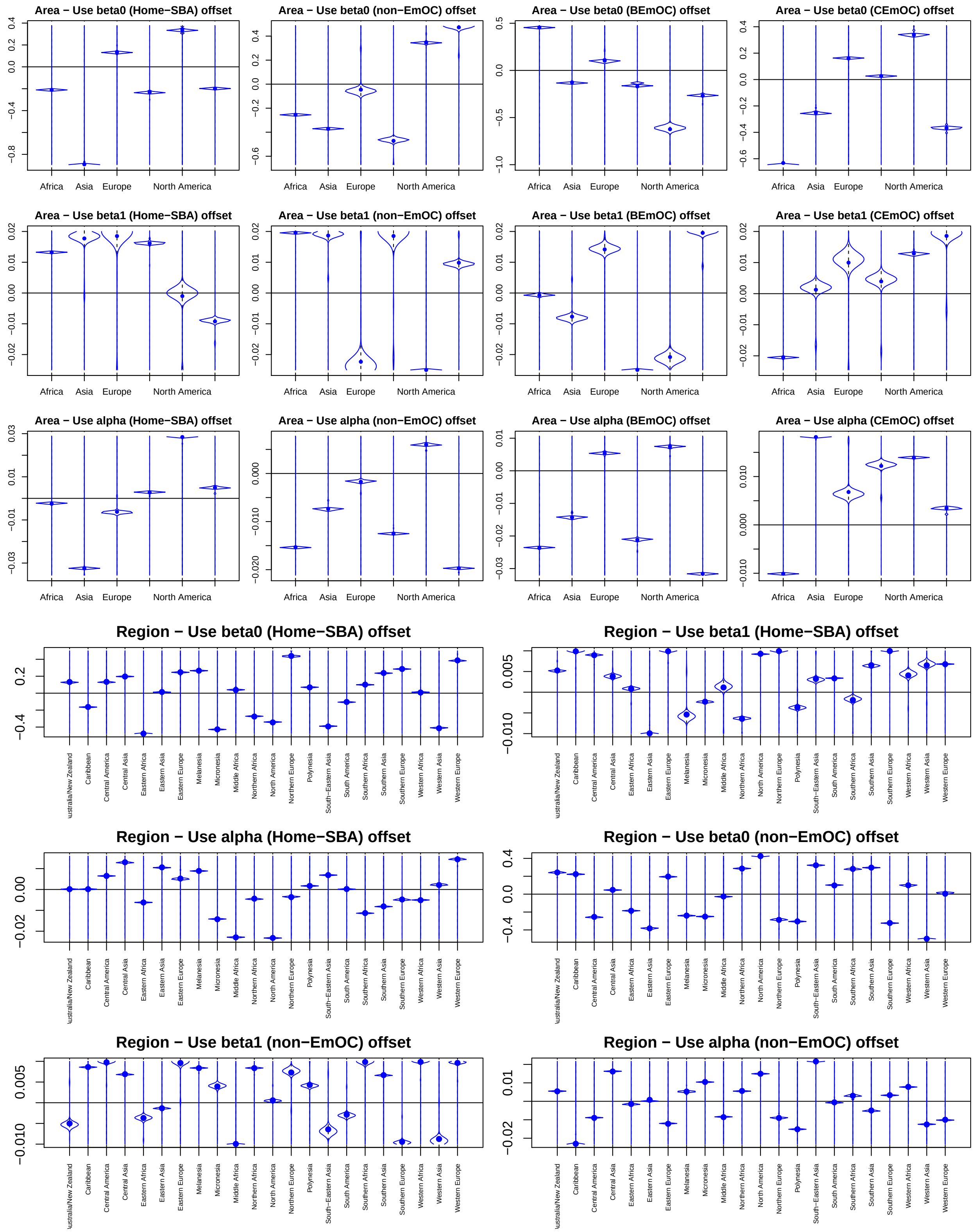
Area − Use beta0 (Home−SBA) offset
Area − Use beta0 (non−EmOC) offset
Area − Use beta0 (BEmOC) offset
Area − Use beta0 (CEmOC) offset
Area − Use beta1 (Home−SBA) offset
Area − Use beta1 (non−EmOC) offset
Area − Use beta1 (BEmOC) offset
Area − Use beta1 (CEmOC) offset
Area − Use alpha (Home−SBA) offset
Area − Use alpha (non−EmOC) offset
Area − Use alpha (BEmOC) offset
Area − Use alpha (CEmOC) offset
Africa
Asia
Europe
North America
Region − Use beta0 (Home−SBA) offset
Region − Use beta1 (Home−SBA) offset
Region − Use alpha (Home−SBA) offset
Region − Use beta0 (non−EmOC) offset
Region − Use beta1 (non−EmOC) offset
Region − Use alpha (non−EmOC) offset
Caribbean
Central America
Central Asia
Eastern Africa
Eastern Asia
Eastern Europe
Melanesia
Micronesia
Middle Africa
Northern Africa
North America
Northern Europe
Polynesia
South−Eastern Asia
South America
Southern Africa
Southern Asia
Southern Europe
Western Africa
Western Asia
Western Europe

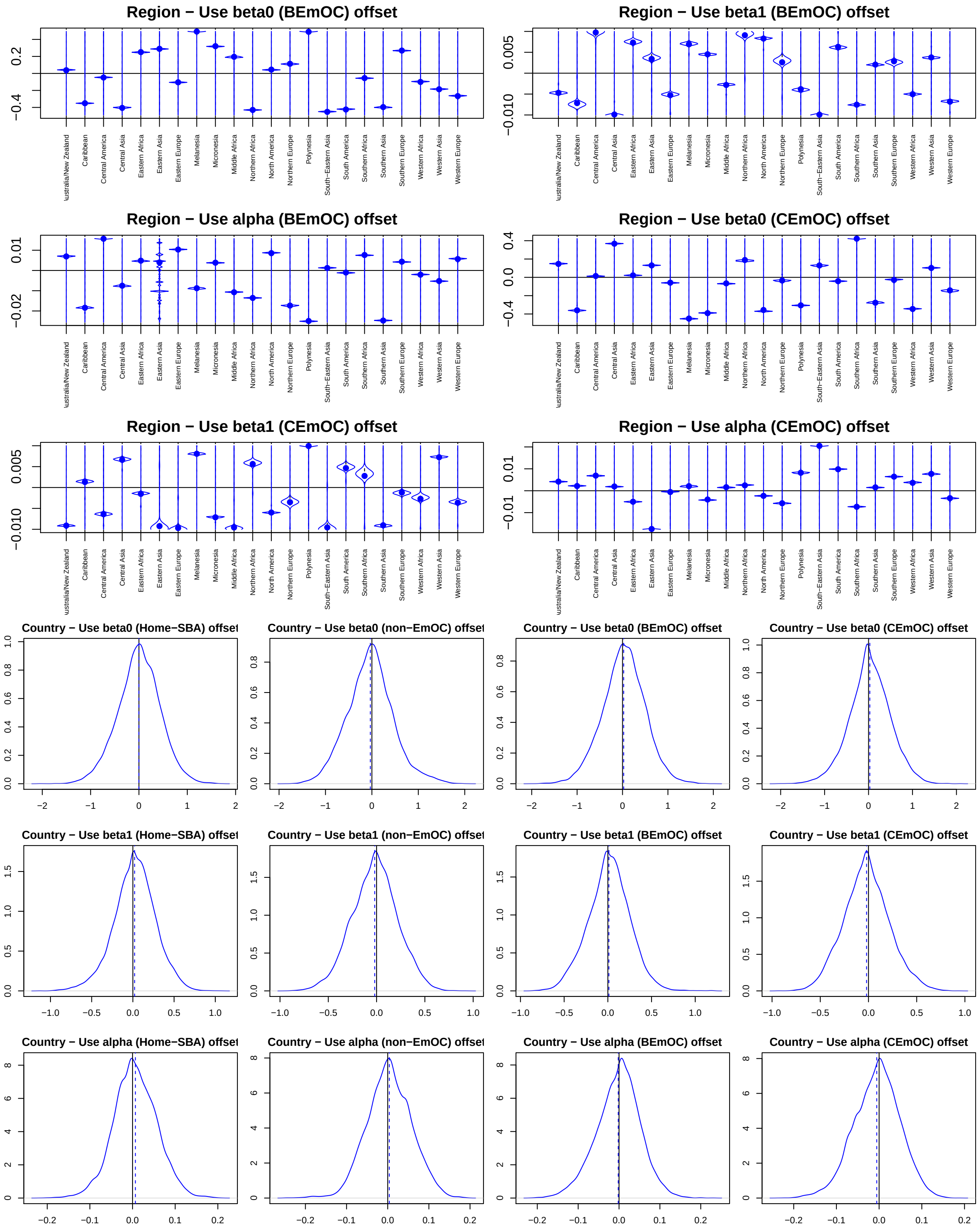
Region − Use beta0 (BEmOC) offset
Region − Use beta1 (BEmOC) offset
Region − Use alpha (BEmOC) offset
Region − Use beta0 (CEmOC) offset
Region − Use beta1 (CEmOC) offset
Region − Use alpha (CEmOC) offset
Country − Use beta0 (Home−SBA) offset
Country − Use beta0 (non−EmOC) offset
Country − Use beta0 (BEmOC) offset
Country − Use beta0 (CEmOC) offset
Country − Use beta1 (Home−SBA) offset
Country − Use beta1 (non−EmOC) offset
Country − Use beta1 (BEmOC) offset
Country − Use beta1 (CEmOC) offset
Country − Use alpha (Home−SBA) offset
Country − Use alpha (non−EmOC) offset
Country − Use alpha (BEmOC) offset
Country − Use alpha (CEmOC) offset

Parameters

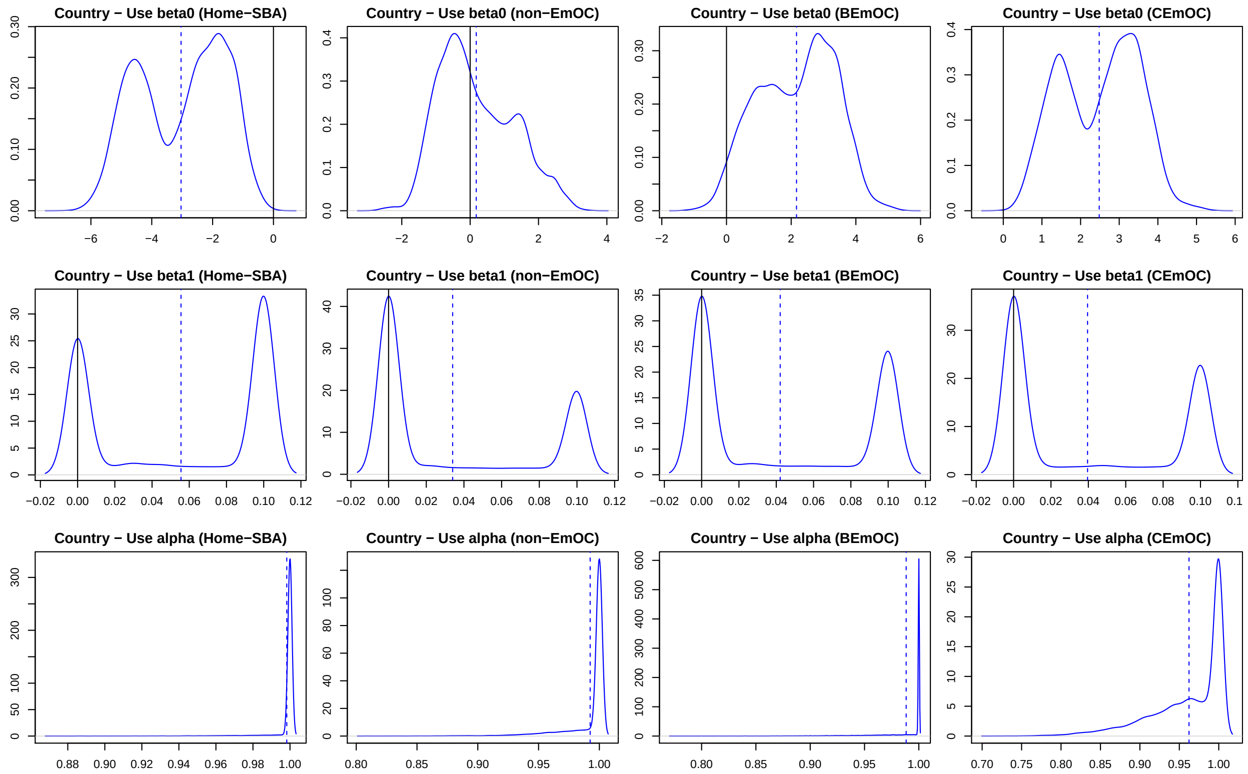

C.6.8.2 Efficacy

Hyperparameters

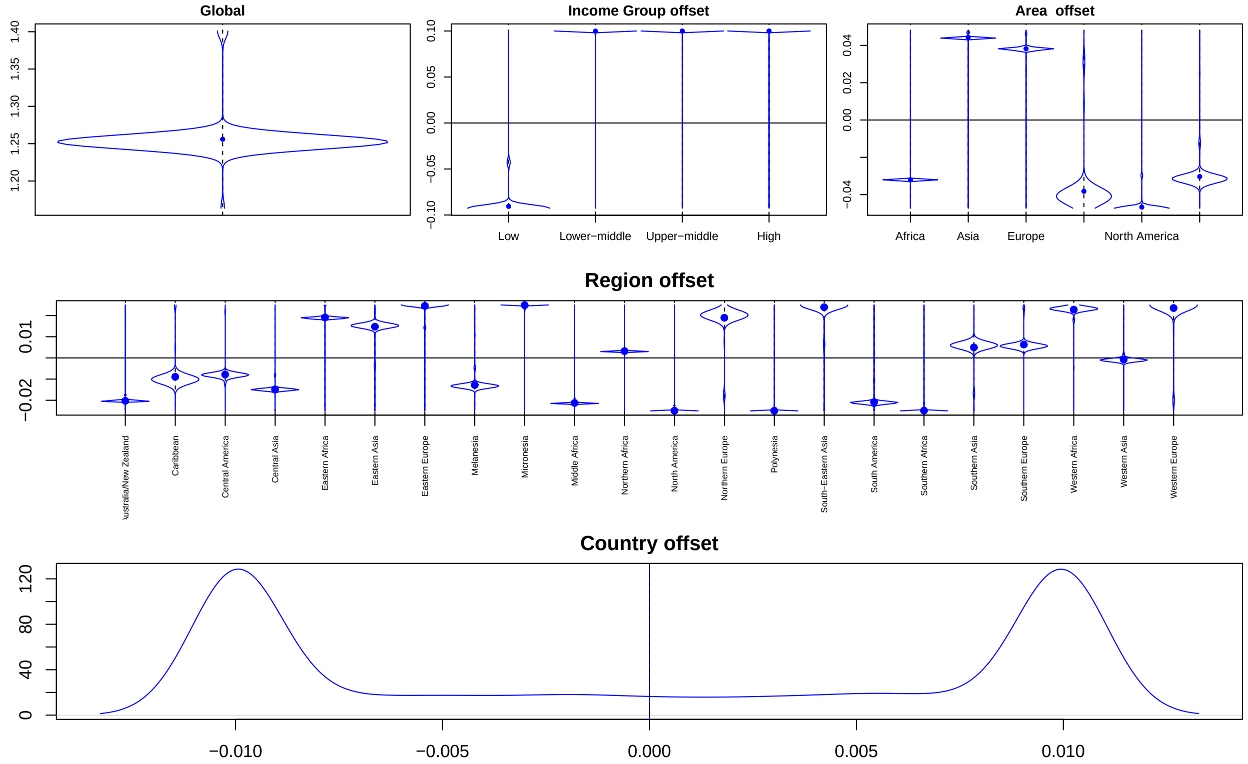

Parameters

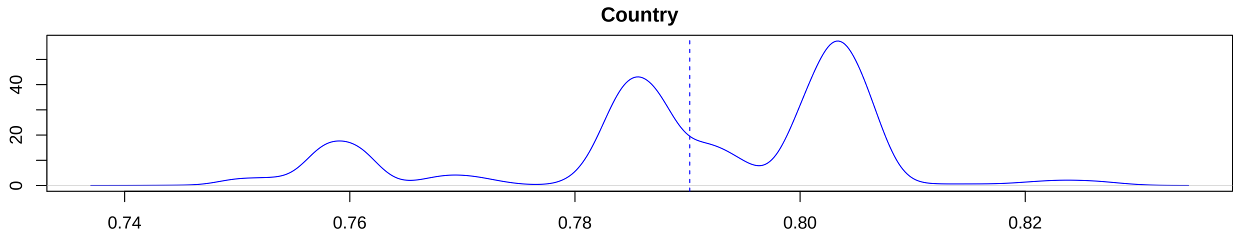

C.6.9 Antibiotics

C.6.9.1 Availability

Hyperparameters

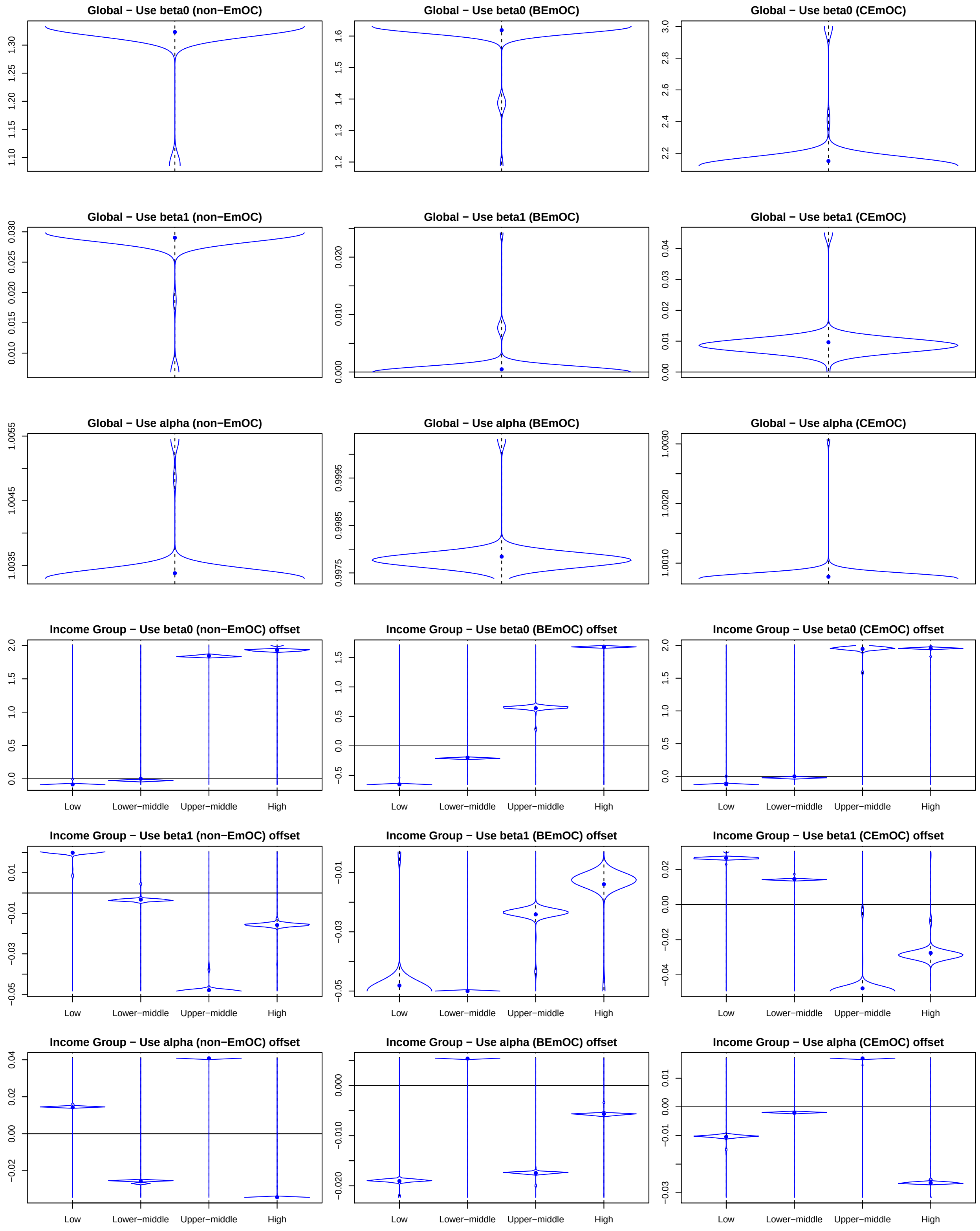

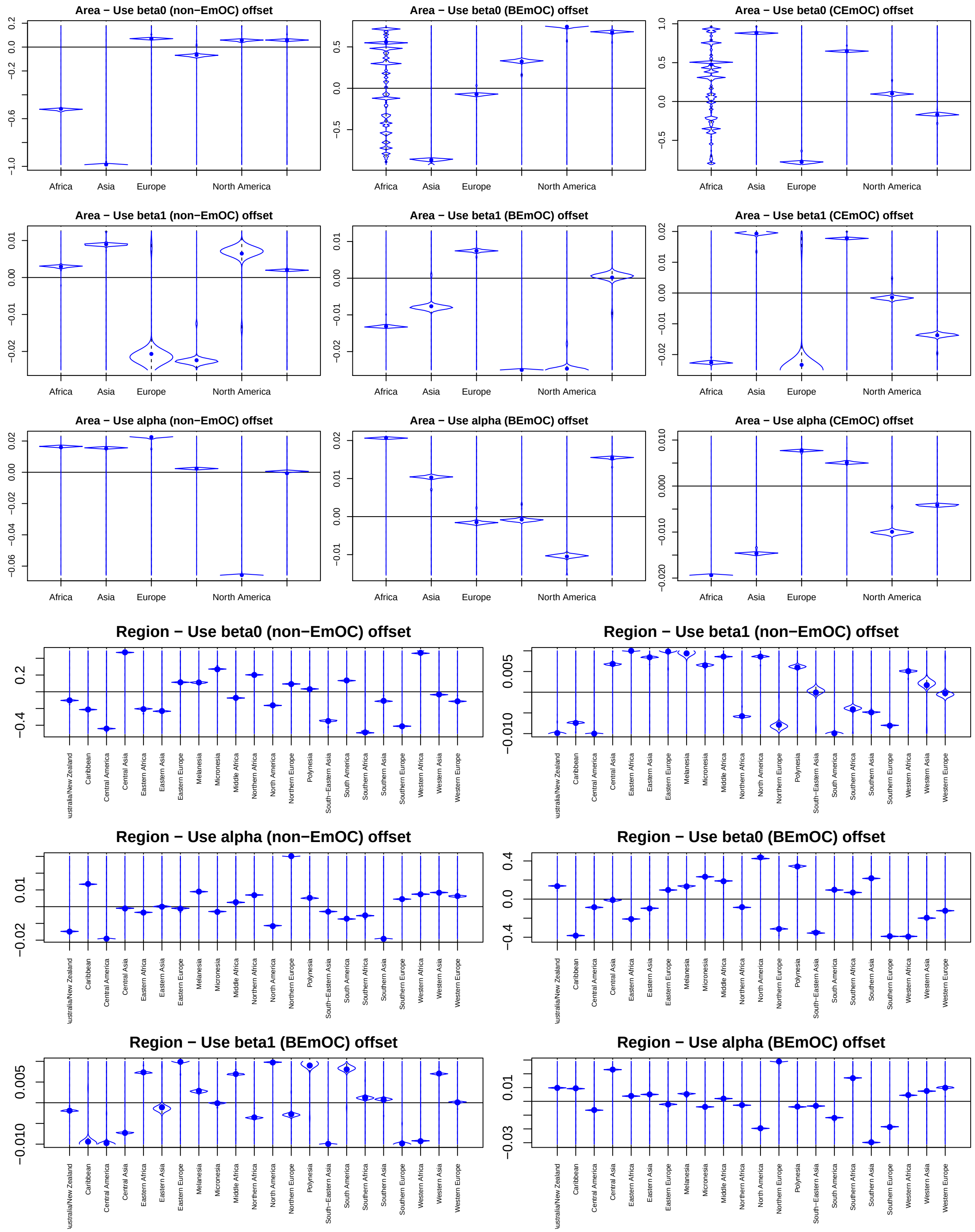
Area − Use beta0 (non−EmOC) offset
Area − Use beta0 (BEmOC) offset
Area − Use beta0 (CEmOC) offset
Area − Use beta1 (non−EmOC) offset
Area − Use beta1 (BEmOC) offset
Area − Use beta1 (CEmOC) offset
Area − Use alpha (non−EmOC) offset
Area − Use alpha (BEmOC) offset
Area − Use alpha (CEmOC) offset
Africa
Asia
Europe
North America
Region − Use beta0 (non−EmOC) offset
Region − Use beta1 (non−EmOC) offset
Region − Use alpha (non−EmOC) offset
Region − Use beta0 (BEmOC) offset
Region − Use beta1 (BEmOC) offset
Region − Use alpha (BEmOC) offset
Australia/New Zealand
Caribbean
Central America
Central Asia
Eastern Africa
Eastern Asia
Eastern Europe
Melanesia
Micronesia
Middle Africa
Northern Africa
North America
Northern Europe
Polynesia
South−Eastern Asia
South America
Southern Africa
Southern Asia
Southern Europe
Western Africa
Western Asia
Western Europe

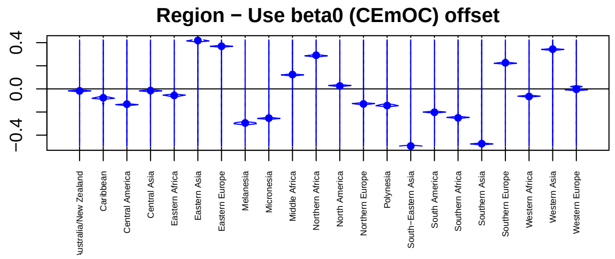
Region − Use beta0 (CEmOC) offset
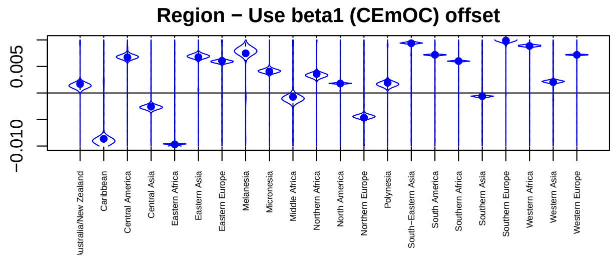
Region − Use beta1 (CEmOC) offset
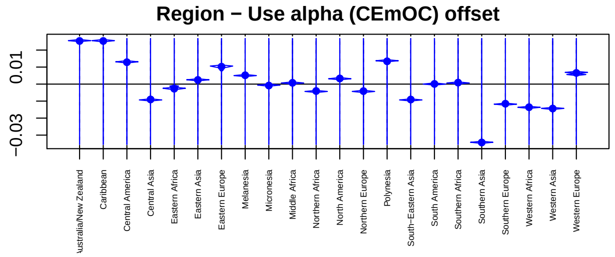
Region − Use alpha (CEmOC) offset
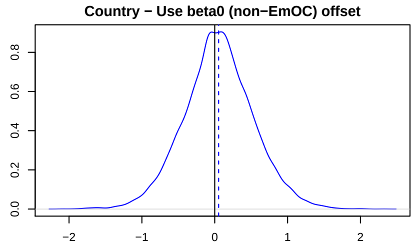
Country − Use beta0 (non−EmOC) offset
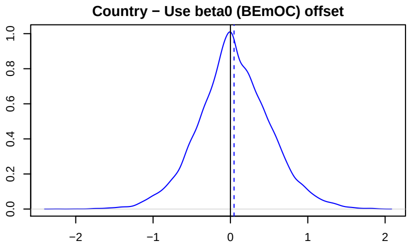
Country − Use beta0 (BEmOC) offset
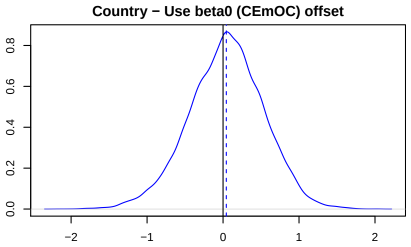
Country − Use beta0 (CEmOC) offset
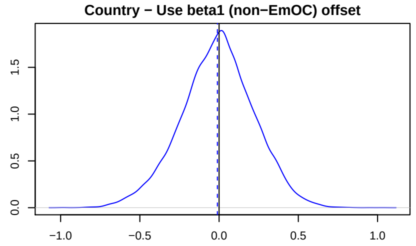
Country − Use beta1 (non−EmOC) offset
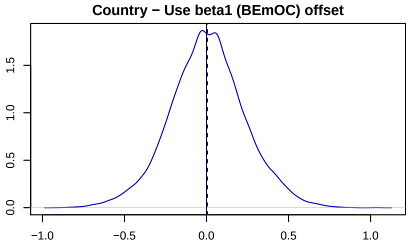
Country − Use beta1 (BEmOC) offset
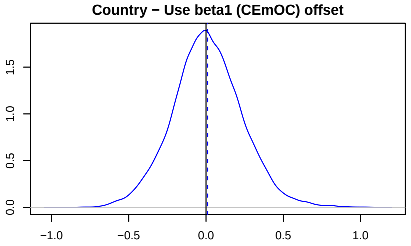
Country − Use beta1 (CEmOC) offset
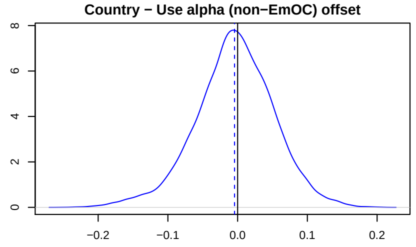
Country − Use alpha (non−EmOC) offset
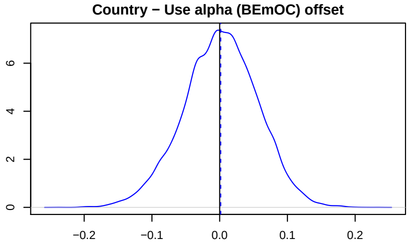
Country − Use alpha (BEmOC) offset
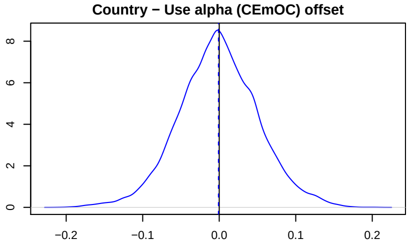
Country − Use alpha (CEmOC) offset

Parameters

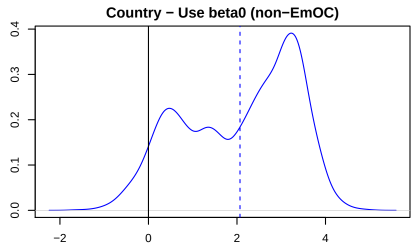
Country − Use beta0 (non−EmOC)
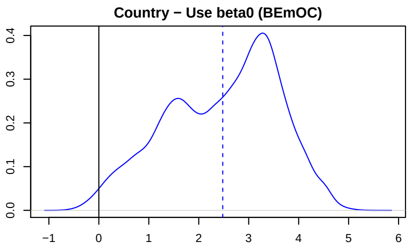
Country − Use beta0 (BEmOC)
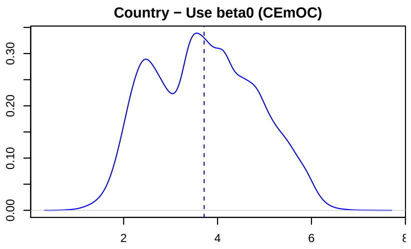
Country − Use beta0 (CEmOC)

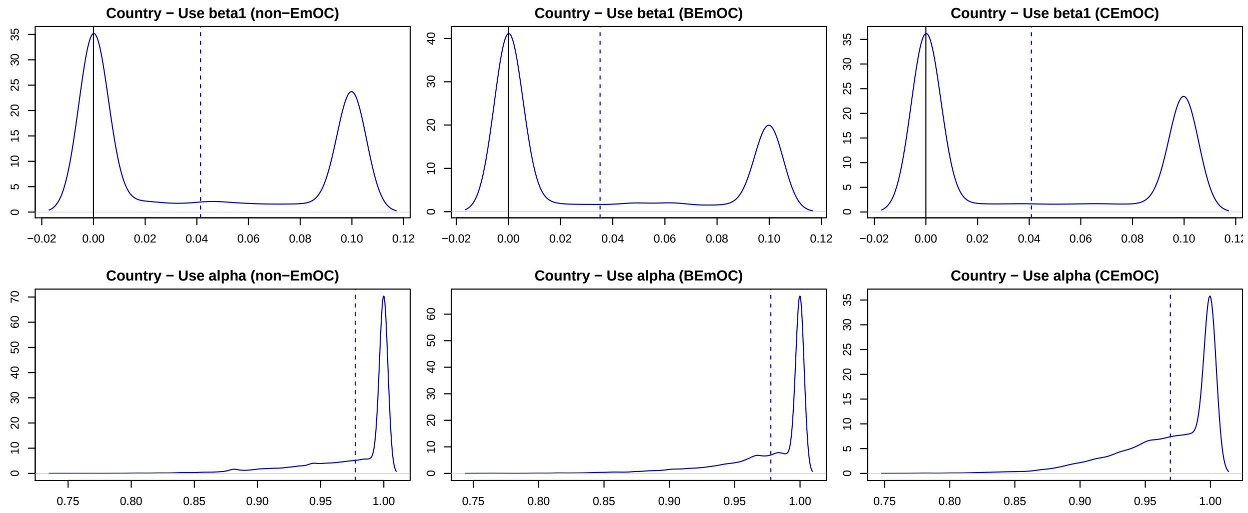

C.6.9.2 Efficacy

Hyperparameters

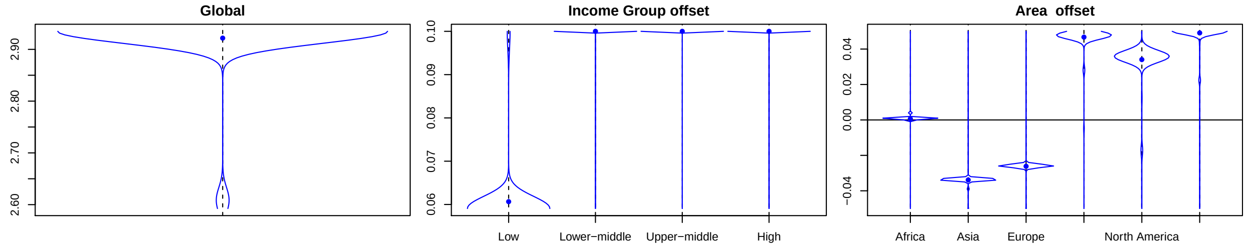

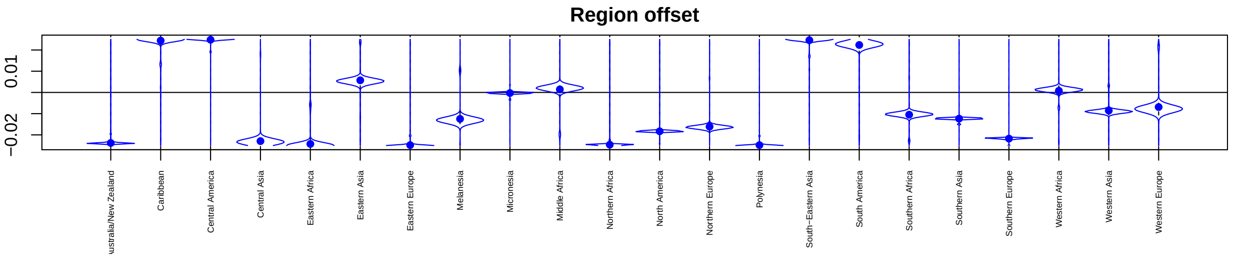

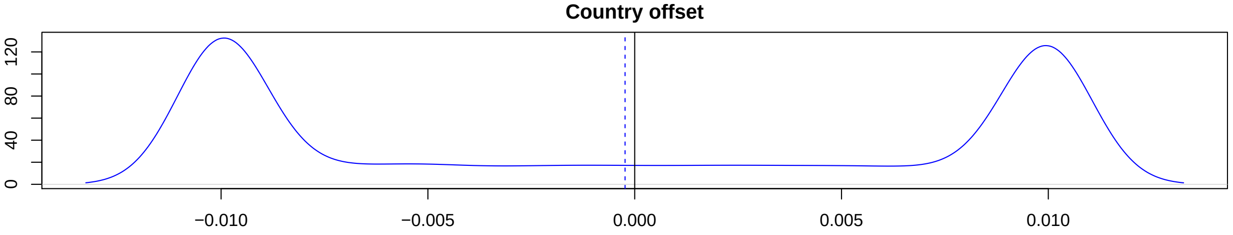

Parameters

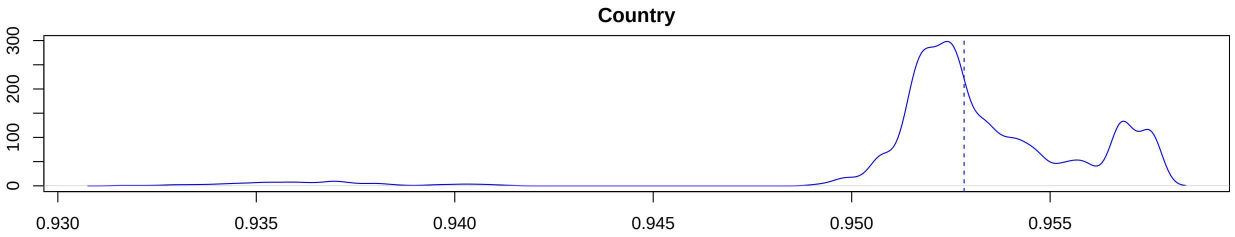
Country
0
100
200
300
0.930
0.935
0.940
0.945
0.950
0.955
